# 2R and remodeling of vertebrate signal transduction engine

**DOI:** 10.1186/1741-7007-8-146

**Published:** 2010-12-13

**Authors:** Lukasz Huminiecki, Carl Henrik Heldin

**Affiliations:** 1 Ludwig Institute for Cancer Research, Uppsala University, Box 595, SE-751 24 Uppsala, Sweden; 2 Evolutionary Systems Biology, Department of Cell and Molecular Biology (CMB), Karolinska Institutet, P.O. Box 285, SE-171 77 Stockholm, Sweden

## Abstract

****Background**:**

Whole genome duplication (WGD) is a special case of gene duplication, observed rarely in animals, whereby all genes duplicate simultaneously through polyploidisation. Two rounds of WGD (2R-WGD) occurred at the base of vertebrates, giving rise to an enormous wave of genetic novelty, but a systematic analysis of functional consequences of this event has not yet been performed.

****Results**:**

We show that 2R-WGD affected an overwhelming majority (74%) of signalling genes, in particular developmental pathways involving receptor tyrosine kinases, Wnt and transforming growth factor-β ligands, G protein-coupled receptors and the apoptosis pathway. 2R-retained genes, in contrast to tandem duplicates, were enriched in protein interaction domains and multifunctional signalling modules of Ras and mitogen-activated protein kinase cascades. 2R-WGD had a fundamental impact on the cell-cycle machinery, redefined molecular building blocks of the neuronal synapse, and was formative for vertebrate brains. We investigated 2R-associated nodes in the context of the human signalling network, as well as in an inferred ancestral pre-2R (AP2R) network, and found that hubs (particularly involving negative regulation) were preferentially retained, with high connectivity driving retention. Finally, microarrays and proteomics demonstrated a trend for gradual paralog expression divergence independent of the duplication mechanism, but inferred ancestral expression states suggested preferential subfunctionalisation among 2R-ohnologs (2ROs).

****Conclusions**:**

The 2R event left an indelible imprint on vertebrate signalling and the cell cycle. We show that 2R-WGD preferentially retained genes are associated with higher organismal complexity (for example, locomotion, nervous system, morphogenesis), while genes associated with basic cellular functions (for example, translation, replication, splicing, recombination; with the notable exception of cell cycle) tended to be excluded. 2R-WGD set the stage for the emergence of key vertebrate functional novelties (such as complex brains, circulatory system, heart, bone, cartilage, musculature and adipose tissue). A full explanation of the impact of 2R on evolution, function and the flow of information in vertebrate signalling networks is likely to have practical consequences for regenerative medicine, stem cell therapies and cancer treatment.

## Background

Most genes belong to gene families which are derived through consecutive cycles of gene duplication. In animals, in the absence of horizontal gene transfer, gene duplication is the most important source of evolutionary novelty. While most duplications are of single genes, predominantly in tandem arrangements, whole genome duplication (WGD) is a special case whereby all genes duplicate simultaneously through polyploidisation. A WGD is followed by the loss of the majority of duplicated genes in the process of rediploidisation. Over large evolutionary time scales, rediploidisation results in the formation of a paleopolyploid species such as *Saccharomyces cerevisiae. S. cerevisiae *was shown in a pioneering study to derive from a WGD which took place after the divergence of *Saccharomyces *from *Kluyveromyces *[1]. Pairs of genes derived from this WGD were shown to constitute about 13% of the yeast coding gene set [1].

Evidence has accumulated that single-gene duplications and WGDs result in preferential retention of different functional gene classes. In particular, WGDs may facilitate coevolution of interacting proteins, which are likely to resist single-gene duplication because of sensitivity to gene dosage [2,3]. Ohnologs, which are defined as paralogs derived from a WGD, were shown to be enriched in transcription factors (TFs) and signalling genes in animals, plants, yeast and Paramecium (reviewed in [4]). However, no detailed analysis of the consequences of these trends for functionality of signal transduction pathways and signalling networks has been undertaken for any of the known WGD events in plants, animals or protozoans.

The modern version of the two rounds (2R) hypothesis (recently reviewed in [5]) proposes that two WGDs occurred at the base of vertebrates after the divergence of urochordates and before the radiation of gnathostomes, most likely even before the cyclostome-gnathostome split [6]. Recent genomic studies have provided overwhelming support for the 2R hypothesis. In particular, strong evidence was derived through sequencing and analysis of genomes of the human [7], the fish *Tetraodon nigroviridis *[8] and the lancelet *Branchiostoma floridae *[9]. An important methodological advance was made by Dehal and Boore [10], who used an ingenious approach of mapping 2R paralogons by first identifying descendants of gene duplications mapping to the base of vertebrates by phylogenetic timing. Furthermore, successful attempts have been made recently at the reconstruction of the ancestral vertebrate genome before 2R-WGD [11].

Herein we present the first systematic analysis of the functional consequences of 2R-WGD using state-of-the-art methods for inference of orthology and duplications. We establish a gene retention percentage for each WGD, analyse preferences in types of retained genes and tissue expression signatures, contrast trends detected for 2R-WGD with those observed for tandem or segmental duplications and view gene family data through the lens of signalling network evolution.

## Results

### Phylogenetic timing links the emergence of vertebrates with the greatest wave of gene duplications in the history of the animal kingdom

We performed a comprehensive computational screen of duplication patterns in the TreeFam database of metazoan gene families (see Methods). Table 1 shows the distribution of inferred duplication events associated with different taxonomic units (sorted according to rank). The highest number of inferred duplications was associated with the emergence of vertebrates (7,701 duplication nodes), more than twice as many as for the second most abundant taxonomic unit, the Bilateria (3,313 nodes). Human 2R-ohnologs (2ROs) were mapped to 9,958 unique Entrez Genes (Additional file 1, Table S1), which were then placed on the genome defining a linear pattern of paralogons covering 83% of the length of human chromosomes (proving that they originated through whole genome duplication; see Methods, Identification of paralogons in the human genome).

**Table 1:** Taxonomic distribution of gene duplications, focusing on eight developmental pathways^a^

**Taxon**	**GPCRs**	**Hedgehog**	**JAK/STAT pathway**	**NHRs**	**NOTCH**	**RTKs**	**TGF-β**	**WNT**	**Sum for eight pathways**	**All genes**
***Kingdom and related***										
Metazoa	31	2	-	-	4	5	10	5	57	182
**Bilateria**	**503**	**31**	**12**	**16**	**54**	**187**	**59**	**86**	**948**	**3,313**
***Phyla and subphyla***										
Chordata	129	3	5	1	9	34	10	25	216	1,171
**Vertebrata**	**1,327**	**34**	**76**	**38**	**56**	**299**	**66**	**145**	**2,041**	**7,701**
***Superclass, class, subclass***										
Tetrapoda	85	-	4	1	2	6	1	1	100	500
Amniota	74	-	1	1	1	5	1	1	84	367
Mammalia	164	-	2	3	-	3	1	4	177	725
Theria	272	-	1	1	1	-	-	2	277	764
Eutheria	261	-	5	7	1	5	2	4	285	1,933
***Superorder, order, infraorder***										
Catarrhini	69	-	-	-	1	4	1	2	77	1,478
Clupeocephala	102	1	-	-	9	21	2	12	147	3,268
Laurasiatheria	23	-	-	-	-	-	-	-	23	111
Smegmamorpha	3	-	-	-	-	-	-	-	3	91
***Family, subfamily, genus***										
Ciona	21	-	-	-	-	2	-	-	23	1,242
Hominidae	62	-	-	-	-	3	1	1	67	628
Murinae	356	-	6	5	-	3	1	-	371	1,184
Percomorpha	15	-	-	-	-	2	-	1	18	1,479
Tetraodontidae	2	-	-	-	-	-	-	-	2	471
***Species***										
*Bos taurus*	15	-	-	-	1	1	-	-	17	2,472
*Canis familiaris*	222	-	-	-	-	-	-	-	222	1,122
*Ciona intestinalis*	1	-	-	-	-	-	-	-	1	2,041
*Ciona savignyi*	-	-	-	-	-	-	-	-	-	736
*Danio rerio*	96	3	-	-	3	5	-	2	109	4,076
*Fugu rubripes*	6	-	-	-	-	-	-	-	6	1425
*Gallus gallus*	8	-	-	-	-	-	-	-	8	1,075
*Gasterosteus aculeatus*	-	-	-	-	-	-	-	-	-	1,298
*Homo sapiens*	31	-	1	-	-	3	1	1	37	1,010
*Macaca mulatta*	5	-	-	-	-	-	-	-	5	1,504
*Monodelphis domestica*	5	-	-	-	-	-	-	-	5	2,363
*Mus musculus*	214	-	-	-	3	3	-	1	221	2,125
*Ornithorhynchus anatinus*	182	-	-	-	-	1	-	1	184	1,031
*Oryzias latipes*	7	-	-	-	-	-	-	-	7	1,097
*Pan troglodytes*	1	-	-	-	-	-	-	-	1	231
*Rattus norvegicus*	263	-	1	3	-	-	-	2	269	3,112
*Tetraodon nigroviridis*	-	-	-	-	-	-	-	-	-	1,749
*Xenopus tropicalis*	-	-	-	-	-	-	-	-	-	2,617

^a^Phylogenetic timing associates Vertebrata and Bilateria with the highest numbers of duplications (in bold). Total associated with a given taxon is given in the last column and underlined. Table rows are sorted according to taxonomic ranks (kingdom, phylum, class, order, family, genus, and species). Duplication numbers for all components of the eight selected pathways are given. GPCRs, G protein-coupled receptors; JAK/STAT, Janus kinase/signal transducer and activator of transcription; nuclear hormone receptors (NHRs); RTKs, receptor tyrosine kinases; TGF-β, transforming growth factor-β.

Next, we calculated the number of ancestral pre-2R (AP2R) chordate genes as 3,545 by analysing the topology of TreeFam trees. 2R-WGD was assumed to initially result in a fourfold increase minus genes lost later through rediploidisation. Thus, the overall retention percentage was estimated as 9,958 ÷ (3,545 × 4) × 100% = 70.2%. However, this was an overestimate, as 4,231 tandem or segmental duplications, identified as nodes younger than 2R-WGD, inflated the number of ohnologs. One also needs to take into account the number of gene families without ohnologs, estimated from the total of human genes in TreeFam minus 2ROs (14,892 - 9,958 = 4,934), or total of Ensembl-predicted human protein-coding genes minus 2ROs (23,438 - 9,958 = 13,480). The upper-bound estimate was therefore calculated as (9,958 - 4,231) ÷ (3,545 × 4 + 4,934) × 100% = 30% and the lower bound as (9,958 - 4,231) ÷ (3545 × 4 + 13,480) × 100% = 20.7%.

The number of duplication nodes which could be placed at the very base of vertebrates was 3,545. An additional 3,263 duplication nodes were children to these nodes, yet were still assigned to vertebrates by phylogenetic timing. These two waves of duplications, in close temporal succession, and of similar quantitative contributions, should be interpreted as differential signatures of two consecutive rounds of genome doubling of 2R-WGD, with similar retention rates (approximately 10%-15%, that is, half of the overall 2R-WGD retention rate).

### Gene duplication in the shared animal developmental toolkit

A key set of uniquely important conserved genes, known as the shared toolkit, control development in all animals. To better understand the evolution of the toolkit, we investigated duplication patterns of the eight key signalling pathways, namely, G protein-coupled receptors (GPCRs), receptor tyrosine kinases (RTKs), Wnt, Notch, transforming growth factor (TGF)-β, Janus kinase/signal transducer and activator of transcription (JAK/STAT), Hedgehog and nuclear hormone receptors. These duplication patterns are illustrated in Table 1 with two clear major waves of diversification: one at the emergence of Bilaterians and the other tied to the emergence of vertebrates (2R-WGD). Apart from these two waves, the toolkit was strongly conserved throughout vertebrates, although a few additional modifications were associated with teleosts (potentially reflecting a fish-specific genome duplication (FSGD)), tetrapods and mammals. GPCRs were perhaps most dynamic, particularly those involved in sensory information processing, which was likely a sign of environmental adaptation. For example, *Canis familiaris *(dog) was associated with 222 species-specific GPCR duplications (the majority of which map to families of olfactory receptors, such as TF344049, TF337111, TF337295, TF343679, TF337210 and TF336833).

### Gene families associated with the highest number of 2ROs

Gene families expanded during the course of 2R included predominantly TFs and signalling genes. Table 2 lists the top 20 expanded families. The highest number of 2R duplications was assigned to the T-box transcriptional factor family (19 gene duplication nodes); followed by integrin-α (13 nodes); GPCRs of the gonadotropin-releasing hormone/vasopressin/oxytocin family (11 nodes); and Cdc42, Wnt ligand, annexin and PDZ/LIM domain family (10 gene duplications each). The integrin-β repertoire (which pair with α-integrins in a combinatorial fashion) also underwent substantial expansion in the course of 2R (six duplication events).

**Table 2:** Top 20 gene families expanded in the course of 2R^a^

**TreeFam ID**	**Number of duplication nodes**	**Description**
TF106341	19	T-box TF
TF105391	13	Integrin, alpha
TF106499	11	Gonadotropin-releasing hormone receptor/arginine vasopressin receptor
TF101109	10	Cell division control protein 42 homolog
TF105310	10	Wingless-type MMTV integration site family
TF105452	10	Annexin
TF106408	10	PDZ and LIM domain protein
TF105128	8	Dual-specificity phosphatase 3/14/18/19/21/26
TF102004	7	Protein kinase A/C
TF102023	7	Caspase family, apoptosis-related cysteine protease
TF105094	7	Cytochrome P450, family 11/24/27
TF105122	7	Dual-specificity phosphatase 1/2/4-7/9/10
TF105049	6	Heat shock 27-kDa protein/crystallin, alpha
TF105100	6	Mitogen-activated protein kinase 8-14
TF105191	6	ATP-binding cassette, subfamily A (ABC1), member 1-4/7/12/13
TF105392	6	Integrin, beta
TF101079	5	Septin 1/2/4/5/7
TF102003	5	Tyrosine 3-monooxygenase/tryptophan 5-monooxygenase activation protein, epsilon polypeptide
TF102031	5	Phosphoinositide 3-kinase, class I/II
TF105272	5	B-cell translocation gene

^a^Families which underwent expansion consist predominantly of genes encoding TFs and signalling proteins. 2R, two rounds; MMTV, mouse mammary tumor virus.

### Functional enrichment associated with 2ROs

We searched for over- and underrepresented functional categories associated with 2ROs. Table 3 lists the top 20 overrepresented gene ontology (GO) biological process (BP) terms. Some terms were related, as the GO classification used was a mixture of all hierarchy levels. No ontology should be seen in isolation; instead, specific functions should always be viewed in the context of higher-level functions. Signal transduction (GO:0007165) was the top overrepresented term, with 853 of the total pool of 1,160 human-associated genes (that is, 74%) being 2ROs. The full list of overrepresented BP terms is given in Additional file 2, Table S2_bp, while overrepresented molecular function (MF) and cellular component (CC) terms are given in Additional file 3, TableS2_mf, and Additional file 4, TableS2_cc, respectively.

**Table 3:** Top 20 biological processes associated with 2ROs^a^

**ID**	***P *value**	**Description**
GO:0007165	2.12e-33	Signal transduction
GO:0007275	3.64e-26	Multicellular organismal development
GO:0007186	3.19e-19	G protein-coupled receptor protein signalling pathway
GO:0006468	1.13e-15	Protein amino acid phosphorylation
GO:0007154	1.10e-13	Cell communication
GO:0007268	7.50e-13	Synaptic transmission
GO:0022008	2.34e-12	Neurogenesis
GO:0030036	1.64e-11	Actin cytoskeleton organization and biogenesis
GO:0050877	2.07e-11	Neurological system process
GO:0006811	3.47e-11	Ion transport
GO:0051056	8.93e-11	Regulation of small GTPase-mediated signal transduction
GO:0006928	1.52e-10	Cell motility
GO:0009605	1.95e-10	Response to external stimulus
GO:0006796	2.60e-10	Phosphate metabolic process
GO:0050794	4.15e-10	Regulation of cellular process
GO:0007265	7.20e-10	Ras protein signal transduction
GO:0000904	8.59e-10	Cellular morphogenesis during differentiation
GO:0006813	1.04e-09	Potassium ion transport
GO:0051179	1.63e-09	Localization
GO:0048812	1.77e-09	Neurite morphogenesis

^a^Signal transduction is the top overrepresented term: Almost three quarters of human genes associated with this term are marked as 2R-ohnologs (2ROs). *P *values derive from the hypergeometric test with conditional correction taking into account the hierarchical structure of ontologies.

Specific GO terms revealed signalling pathways preferentially affected by 2R-WGD, that is, GPCRs, Ras and its regulators, Wnt pathway, and RTK-associated signalling (Additional file 2, Table S2_bp). Several terms also pointed to signalling associated with the cytoskeleton and cellular attachment (Additional file 2, Table S2_bp). Vertebrate evolutionary novelties could be associated with a high proportion of 2ROs. For example, a number of overrepresented terms could be linked with the muscular upgrade: muscle contraction, skeletal muscle development and myoblast differentiation (Additional file 2, Table S2_bp). Higher-level terms indicated a general trend towards greater organismal complexity, as well as expanded locomotory and sensory abilities characteristic of vertebrates (Additional file 2, Table S2_bp).

BP terms underrepresented among 2ROs (Additional file 5, Table S3_bp) were dominated by basic cellular functions strongly conserved throughout Eukaryota, such as translation, DNA repair, RNA splicing, DNA replication, protein folding, DNA recombination, cellular respiration, mRNA transport or ubiquitin-dependent protein catabolic process. Underrepresented MF and CC terms are given in Additional file 6, TableS3_mf, and Additional file 7, TableS3_cc, respectively.

It was also intriguing to directly compare over- and underrepresented CC terms. The former (Additional file 4, TableS2_cc) centered on the membrane and cellular skeleton, including plasma membrane, synapse, actin cytoskeleton, cell junction, postsynaptic membrane and contractile fiber. The latter (Additional file 7, TableS3_cc), in contrast, centered on organelles, cytoplasm and the nucleus, including mitochondrion, spliceosome, ribosomal subunit, proteasome complex, nucleolus, chromosome and cytoplasm.

Overall, these findings boldly underlined the conclusion that signal transduction genes were preferentially retained after 2R-WGD. In stark contrast, very different functional trends characterised duplications younger than 2R, corresponding to tandem or segmental duplications (mapped to 5,495 unique human Entrez Genes). Overrepresented BP terms associated with these genes (Additional file 8, Table S3_not2R-over) were strongly biased towards immune functions and DNA/nucleosome/chromatin packaging. Crucially, terms associated with cell communication, development and cell cycle were strongly underrepresented (Additional file 9, Table S3_not2R-under).

### Pathways overrepresented among 2ROs

Investigation of Kyoto Encyclopedia of Genes and Genomes (KEGG) pathways overrepresented in gene duplications associated with 2R (Additional file 10, Table S4) revealed four classes of pathways: (1) canonical signalling pathways (calcium signalling, mitogen-activated protein kinase (MAPK) signalling, Wnt signalling, insulin signalling, ErbB signalling, TGF-β signalling); (2) pathways associated with vertebrate novelties (neuroactive ligand-receptor interaction, axon guidance, melanogenesis, leukocyte transendothelial migration, adipocytokine signalling pathway, vascular endothelial growth factor signalling pathway, B cell receptor signalling pathway); (3) pathways associated with the cellular cytoskeleton, cell-cell and cell-extracellular matrix (ECM) interactions (regulation of actin cytoskeleton, focal adhesion, adherens junction, tight junction and ECM-receptor interaction); and (4) disease-associated pathways (renal cell carcinoma, chronic myeloid leukemia, long-term depression, colorectal cancer, type 2 diabetes mellitus, small cell lung cancer, glioma, pancreatic cancer).

### Protein domains overrepresented among 2ROs

All Pfam domains overrepresented among 2ROs were related to signal transduction (Table 4 and Additional file 11, Table S5). The most obvious were typical signalling domains, such as the protein tyrosine kinase domain, the serine/threonine kinase domain (harboured by cyclin-dependent kinases among others), Ras family signature, RhoGEF domain, RhoGAP domain, protein kinase C, protein-tyrosine phosphatase, neurotransmitter-gated ion channel domains and two types of 7TM domains (rhodopsin and secretin families). Several further domains were associated with transcriptional factors integrated with signalling pathways, that is, homeobox domain, helix-loop-helix, or ligand-binding domain of nuclear hormone receptor.

**Table 4:** Pfam domains overrepresented in gene duplications associated with 2R^a^

**Domain name**	**Pfam ID**	***P *value**
PH domain	PF00169	8.85e-23
Homeobox domain	PF00046	5.42e-12
7 transmembrane receptor (rhodopsin family)	PF00001	1.17e-11
Tyrosine kinase domain	PF07714	1.84e-10
Serine/threonine kinase domain	PF00069	3.18e-10
PDZ	PF00595	4.30e-10
Intermediate filament protein	PF00038	1.02e-09
EGF-like domain	PF00008	3.39e-08
EF hand	PF00036	5.20e-08
Helix-loop-helix DNA-binding domain	PF00010	8.32e-08
SH3 domain	PF00018	8.80e-08
Ras family	PF00071	9.14e-08
Ion transport protein	PF00520	1.27e-07
SH2 domain	PF00017	1.30e-07
C2 domain	PF00168	4.48e-07
Neurotransmitter-gated ion channel ligand binding domain	PF02931	7.02e-07
Neurotransmitter-gated ion channel transmembrane region	PF02932	7.02e-07
Protein tyrosine phosphatase	PF00102	1.24e-06
RhoGEF domain	PF00621	1.24e-06
Calponin homology (CH) domain	PF00307	2.32e-06
7 transmembrane receptor (Secretin family)	PF00002	3.11e-06
SAM domain (Sterile α motif)	PF00536	3.11e-06
Protein kinase C terminal domain	PF00433	5.10e-06
Hormone receptor domain	PF02793	5.68e-06
RhoGAP domain	PF00620	6.71e-06
Fibronectin type III domain	PF00041	7.08e-06
IPT/TIG domain	PF01833	9.61e-06
Ligand-binding domain of nuclear	PF00104	1.84e-05
hormone receptor		
Phosphotyrosine interaction domain (PTB/PID)	PF00640	2.75e-05
Phorbol esters/diacylglycerol binding domain (C1 domain)	PF00130	2.84e-05
Laminin G domain	PF02210	3.63e-05
E1-E2 ATPase	PF00122	7.87e-05

^a^All Pfam domains overrepresented in gene duplications associated with 2ROs at the *P *value cutoff of 0.0001 can be linked with signal transduction. *P *values derive from the hypergeometric test. EGF, epidermal growth factor; PH domain, pleckstrin homology domain (PH domain); SH3, Src homology 3; SH2, Src homology 2; ig-like, plexins, transcription factors (IPT/TIG); phosphotyrosine-binding domain/phosphotyrosine interaction domain (PTB/PID).

### Tissue expression signature associated with 2ROs

**Figure 1:**
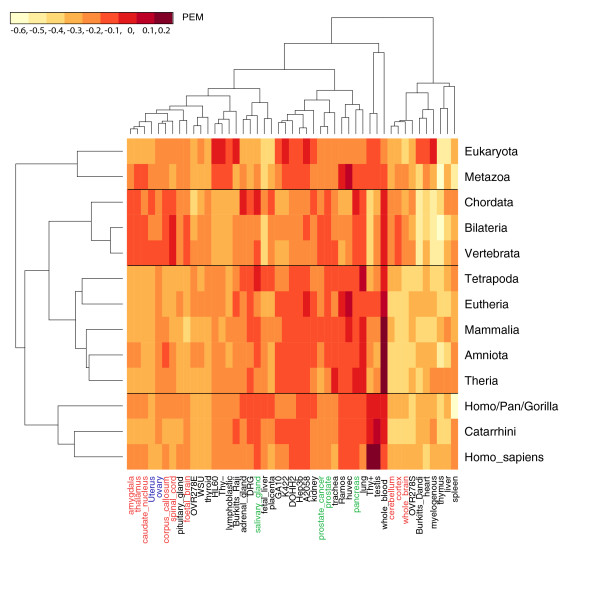
**Gene duplication timing and spatial expression domain of progeny genes**. The heatmap (false-color image) reveals the expression signature of vertebrate-specific gene duplications in the context of the broader evolutionary history of the animal kingdom. Tissues (horizontal axis) and taxons (vertical axis) were ordered using a simple hierarchical clustering algorithm (as visualized using dendrograms). The taxons are grouped into four clusters: **(a) **Eukaryota and Metazoa; **(b) **Bilateria, Chordata and Vertebrata; **(c) **Tetrapoda, Amniota, Mammalia, Eutheria and Theria; and **(d) **Catarrhini, Homo/Pan/Gorilla and *Homo sapiens*. Two round (2R) genes (labeled "Vertebrata") appear to be preferentially expressed in nervous tissues (amygdala, thalamus, caudate nucleus, corpus callosum, spinal cord, fetal brain, cerebellum, cortex and whole brain; highlighted in red), whole blood (in bold font), female reproductive track (uterus and ovary; highlighted in blue), several vertebrate-specific glands (salivary gland, prostate cancer, prostate, pancreas; highlighted in green) and the respiratory system (trachea and lung; in italics).

Figure 1 is a heatmap illustrating the relationship between the relative timing of gene duplications (as inferred by phylogenetic timing) and the spatial expression domain of progeny genes (as determined by mRNA levels). For example, a label "*Homo sapiens*" on the vertical axis signifies recent human-specific duplications. "Vertebrata" signifies duplications at the base of vertebrates linked with 2R-WGD. Dark red colour indicates preferential expression in a given tissue, while light yellow colour indicates a tendency for the exclusion of a given tissue from the expression domain of progeny genes. Taxons and tissues were ordered using the hierarchical clustering. It is striking and has never been previously reported that taxons comprise four chronological groups which can be aligned with major evolutionary transitions that have occurred in the course of animal evolution:

(a) Eukaryota and Metazoa: The emergence of the nucleated cell and establishment of multicellularity

(b) Bilateria, Chordata and Vertebrata: Bilateral symmetry and complex body plans

(c) Tetrapoda, Amniota, Mammalia, Eutheria and Theria: Diversification of vertebrates

(d) Homo/Pan/Gorilla, *Homo sapiens *and Catarrhini: The emergence of primates

The preferential expression of 2ROs in neuronal tissues demonstrated in Figure 1 is particularly exciting. Additional file 12, Table S6, lists 349 2ROs preferentially expressed in brain (preferential expression measure >0.4). GO terms (BP, CC and MF, respectively) preferentially associated with these genes are listed in Additional file 13, Table S7_bp; Additional file 14, Table S7_cc; and Additional file 15, Table S7_mf. The top three overrepresented terms in each category were as follows: synaptic transmission, neurological system process and cell communication (BP); synapse, cell junction and plasma membrane (CC); and calcium ion binding, transporter activity and calmodulin binding (MF).

**Figure 2:**
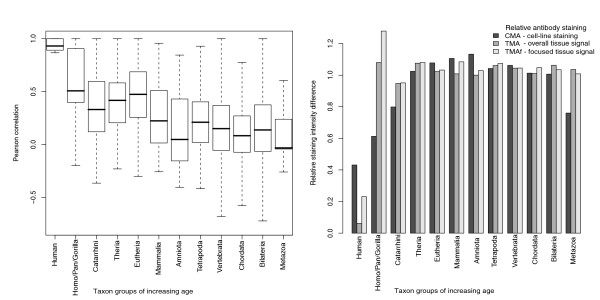
**Expression divergence in pairs of paralogs of different ages**. Expression similarity decays steadily over evolutionary time. (a) mRNA data and (b) proteomic data suggest similar trends. Proteomic data [12] are derived from relative antibody staining intensity examined either in tissue sections (tissue microarray (TMA), overall signal; tissue microarray focused (TMAf), focused signal in the staining cell subset) or through cell-line staining cell microarray (CMA). Figure 2a is a box-and-whisker plot, with thick black horizontal lines showing the median, a box drawn between the quartiles, dotted lines extending to the minimum and maximum and outliers illustrated as circles.

In the next step, we analysed expression divergence between paralogs and found that mRNA expression similarity decays steadily over evolutionary time. This is proven by the falling average expression correlation between pairs of paralogs grouped by increasing age, detected using a variety of measures of expression distance (Figure 2a) (Pearson product-moment correlation coefficient, Additional file 16, Figure S1; Kendall correlation coefficient, Additional file 17, Figure S2; Spearman correlation coefficient, Additional file 18, Figure S3; Manhattan distance, Additional file 19, Figure S4, simple difference in breadth of expression). No specific signature could be discerned for 2ROs: the overall rate of expression divergence was similar between tandem and WGD duplicates, at least over large evolutionary time scales.

Similar trends were inferred with relative protein concentration data derived from a large-scale antibody screen of human cell lines and tissues [12]. However, relative protein abundance between paralogs diverge with somewhat different temporal dynamics when tissue distribution (tissue microarray (TMA), overall; tissue microarray focused (TMAf), focused on positive regions) rather than cell line distribution (CMA) is considered (Figure 2b). CMA diverges gradually, reaching a plateau for duplications dated to mammals and older, similar to mRNA divergence (Figure 2a). In contrast, TMA and TMAf divergence is low for human-specific duplications, but subsequent divergence is extremely rapid. One possible explanation is that tissue protein levels are stabilised posttranscriptionally by a mRNA-level network of regulation, for example, through microRNA (miRNA), and this step is subtly influenced by the tissue microenvironment. The stabilising effect, however, decays rapidly over evolutionary time, as noncoding regions of paralogs, being under little selective constraint, rapidly diverge in sequence.

### Specific family examples

#### Ras system was greatly affected by 2R

Evolution of the Ras family (TreeFam family TF312796) was shaped predominantly by the 2R event. Three vertebrate co-orthologs of Ras, K-Ras, N-Ras and H-Ras, originated from 2R. Two further duplications in the Ras family could be mapped to 2R: (1) Ras-related protein Ral-A (RALA; located on chromosome 7) and Ras-related protein Ral-B (RALB; chromosome 2) and (2) Ras-related protein (RRAS; chromosome 19) and Ras-related protein 2 (RRAS2; chromosome 11).

RasGAPs are GTPase-activating proteins. There are two subfamilies of RasGAPs in TreeFam: TF105303 and TF105302. In TF105303, RAS protein activator-like 3 (located on chromosome 19), RAS protein activator-like 2 (chromosome 1), disabled homolog 2-interacting protein (DAB2IP; chromosome 9), and SYNGAP1 (chromosome 6) are 2ROs. DAB2IP acts as a tumor suppressor gene and is inactivated by methylation or polycomb Ezh2 complex and histone deacetylase in prostate cancer [13]. SynGAP is a synaptic-specific GTPase-activating protein [14]. There is only one RasGAP of that subfamily in fly (CG42270) and worm (gap-2). In the second subfamily of RasGAPs (TF105302), two duplications can be mapped to 2R: Ras GTPase-activating protein 4 (RASA4; chromosome 7) and RasGAP-activating-like protein 1 (RASAL1; chromosome 12), as well as Ras GTPase-activating protein 2 (RASA2; chromosome 3) and Ras GTPase-activating protein 3 (RASA3; chromosome 13).

#### Cell cycle machinery expanded dramatically in the course of 2R

Most cyclins, including key cell cycle-regulating groups A, B and D, underwent diversification at the base of vertebrates and are represented by two to four vertebrate-specific paralogs (Table 5). Analysis of the TreeFam database indicated that the following genes were also 2ROs: cyclin-dependent kinases CDK2 and CDK3 (TF300619), CDK4 and CDK6 (TF328559) and cyclin-dependent kinase inhibitors p21 and p27 (TF101038), as well as p18 and p19 (TF333311) and finally orthologs of the WEE1 (*S. pombe*) inactivator of the CDK/cyclin complex, namely, Wee1 and Wee2 (TF315075).

**Table 5:** Vertebrate-specific cyclin isoforms^a^

**Ancestral bilaterian gene**	**TreeFam ID**	**Vertebrate paralogs**
Cyclin A	TF101002	Cyclin A1, Cyclin A2
Cyclin B	TF101001	Cyclin B1, Cyclin B2
Cyclin D	TF101004	Cyclin D1, Cyclin D2, Cyclin D3
Cyclin E	TF101005	Cyclin E1, Cyclin E2
Cyclin G	TF101007	Cyclin G1, Cyclin G2
Cyclin I	TF101007	Cyclin I, Cyclin I2
Cyclin J	TF101009	Cyclin J, Cyclin J-like protein
Cyclin L	TF101011	Cyclin L1, Cyclin L2
Cyclin M	TF101012	Cyclin M1, Cyclin M2, Cyclin M3, Cyclin M4
Cyclin T	TF101014	Cyclin T2, Cyclin T2

^a^All cyclin subfamilies are represented by two to four vertebrate-specific paralogs. Cyclins A, B and D are key cell cycle regulators. Cyclin D provides the link between the cell cycle and the signal transduction machinery.

#### Neurotrophin family

**Figure 3:**
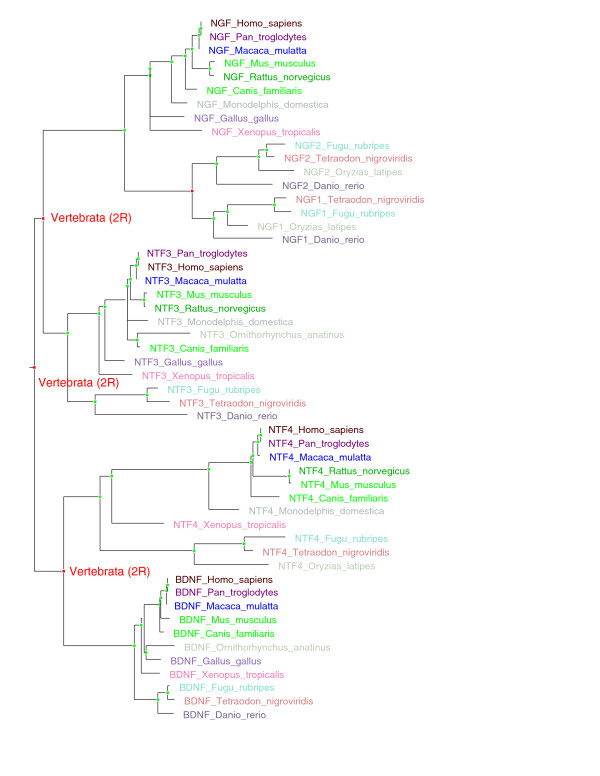
**Neurotrophins cluster into four ortholog groups which derive from two rounds of whole genome duplication (2R-WGD)**. Human neurotrophins (TF106463) include neurotrophin 4 (NTF4; located on chromosome 19), brain-derived neurotrophic factor (BDNF; chromosome 11), neurotrophin 3 (NTF3; chromosome 12) and nerve growth factor (NGF; chromosome 1). All other vertebrate neurotrophins can be clustered in clear ortholog groups with these four human genes. Phylogenetic timing places the duplications which gave rise to the four groups at the base of vertebrates. Red node dots signify duplication nodes, while green node dots signify speciation nodes. Labels "Vertebrata (2R)" signify nodes corresponding to 2R-WGD.

Neurotrophins are key growth factors influencing proliferation, differentiation, survival and death of neuronal cells. Human neurotrophins (TF106463) include NTF4 (located on chromosome 19), brain-derived neurotrophic factor (BDNF; chromosome 11), NTF3 (chromosome 12) and nerve growth factor (NGF; chromosome 1). NTF4, BDNF, NTF3 and NGF are evidently ohnologs deriving from the 2R-WGD (Figure 3). Interestingly, worm and fly lack orthologs of neurotrophins [15]. The expanded neurotrophin family is most likely involved in the sculpturing of characteristically complex vertebrate nervous systems, with a large, centralized and multicompartmental brain.

#### Histone deacetylases: Cell-specific regulators of chromatin structure

**Figure 4:**
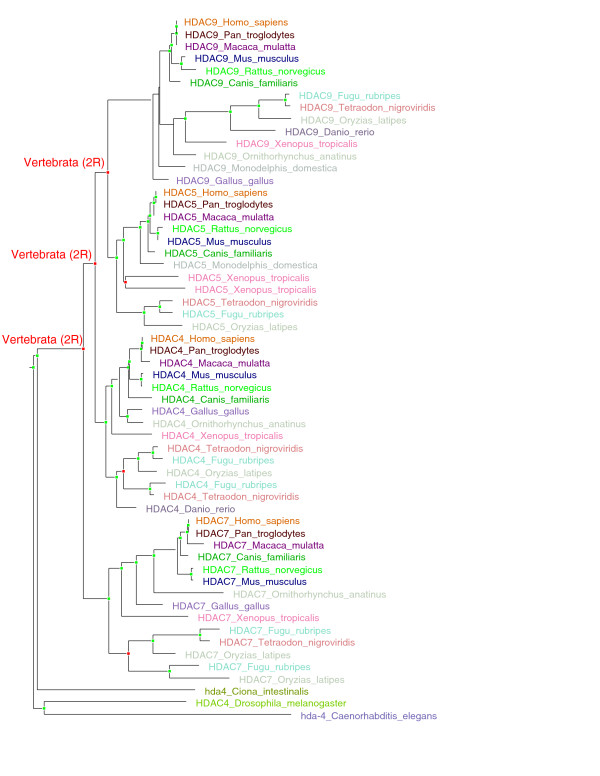
**Evolution of the family of class II histone deacetylases (HDACs) followed the 2R model**. The HDAC family (TF106174) is the family of class II histone deacetylases, which include four vertebrate genes: HDAC4 (located on chromosome 2), HDAC5 (located on chromosome 17), HDAC7 (chromosome 12) and HDAC9 (chromosome 7). There are only single genes for class II histone deacetylases in each *C. intestinalis *(hda4), fly (HDAC4) and worm (hda-4). Red node dots signify duplication nodes, while green node dots signify speciation nodes. Labels "Vertebrata (2R)" signify nodes corresponding to 2R-WGD.

The family of class II histone deacetylases (HDAC; TF106174) includes four vertebrate genes, that is, HDAC4 (located on chromosome 2), HDAC5 (chromosome 17), HDAC7 (chromosome 12) and HDAC9 (chromosome 7). There is only one HDAC in fly, worm and *C. intestinalis *(Figure 4). Vertebrate HDACs have cell type-specific expression patterns and link through an N-terminal extension to TFs from a number of signalling pathways [16]. There are links with vertebrate novelties in the skeletal, circulatory and muscular systems. HDAC4 is a corepressor controlling bone development [17]; HDAC5 and HDAC9 have been shown to suppress cardiac stress signals and control cardiac development [18]; and HDAC9 also couples neuronal activity to muscle chromatin acetylation and gene expression [19].

### 2R-WGD and the architecture of the human signalling network

First, we identified the total overlap between protein members of the 1,625-node human cancer signalling map (HCSM) of Cui *et al*. [20] and paralogous genes of different ages. (HCSM is not limited to cancer; it describes the entire human signalling network and is suitable for the evolutionary analyses of the type we have undertaken.) The overlap consisted of 237 nodes for duplications mapped to Tetrapoda and younger, 1,096 nodes for 2ROs, 194 nodes for duplications linked with the emergence of chordates and 620 for those linked with the emergence of bilaterians (Table 6). Second, separate overlaps were identified for subnetworks consisting of positive, negative and scaffolding edges alone. (Note that these categories are not mutually exclusive, as many nodes are linked to more than one edge type.)

**Table 6:** Paralogs and network connectivity: Outdegree, indegree and betweenness^a^

***Tetrapoda and younger*** ***(5,495 gene set linked)***			
*Subnetwork and overlap*	*Outdegree*	*Indegree*	*Betweenness*
All edges (237 nodes)	3.90 [4.07, 342]	3.61 [4.07, 99]	0.41 [0.47, 266]
Positive edges only (150 nodes)	2.27 [2.15, 676]	1.98 [2.16, 262]	0.40 [0.44, 365]
Negative edges only (71 nodes)	1.70 [1.34, 905]	1.22 [1.33, 338]	0.39 [0.42, 440]
Scaffolding only (144 nodes)	3.57 [3.71, 386]	3.57 [3.70, 385]	0.43 [0.47, 369]
***2ROs*** ***(9,958 gene set linked)***			
*Subnetwork and overlap*	*Outdegree*	*Indegree*	*Betweenness*
All edges (1,096 nodes)	**4.34 [4.06, 999]**	**4.29 [4.06, 984]**	2.33 [2.19, 916]
Positive edges only (771 nodes)	2.23 [2.16, 836]	2.19 [2.16, 666]	2.40 [2.25, 879]
Negative edges only (377 nodes)	**1.48 [1.33, 996]**	1.34 [1.33, 522]	**2.63 [2.30, 981]**
Scaffolds only (714 nodes)	3.85 [3.72, 898]	3.85 [3.72, 885]	2.40 [2.35, 626]
***Chordata*** ***(2,173 gene set linked)***			
*Subnetwork and overlap*	*Outdegree*	*Indegree*	*Betweenness*
All edges (194 nodes)	4.24 [4.04, 697]	4.26 [4.06, 697]	0.44 [0.38, 792]
Positive edges only (120 nodes)	2.59 [2.14, 932]	2.31 [2.15, 707]	0.47 [0.35, 909]
Negative edges only (61 nodes)	1.55 [1.33, 789]	**1.95 [1.33, 983]**	0.42 [0.36, 730]
Scaffolds only (141 nodes)	3.40 [3.70, 203]	3.40 [3.70, 216]	0.44 [0.47, 455]
***Bilateria*** ***(5,223 gene set linked)***			
*Subnetwork and overlap*	*Outdegree*	*Indegree*	*Betweenness*
All edges (620 nodes)	**4.87 [4.06, 1000]**	**4.72 [4.05, 1000]**	**1.60 [1.23, 1000]**
Positive edges only (428 nodes)	**2.61 [2.16, 999]**	**2.49 [2.15, 997]**	**1.65 [1.24, 999]**
Negative edges only (242 nodes)	**1.66 [1.33, 998]**	1.49 [1.34, 903]	**1.92 [1.48, 995]**
Scaffolds only (399 nodes)	**4.19 [3.73, 987]**	**4.19 [3.72, 990]**	1.45 [1.31, 801]

^a^Palogs linked with different taxons are mapped to the human cancer signalling map (HCSM). The entire network, as well as the subnetworks consisting of only positive, negative or scaffolding edges, are considered. The overlap between duplication sets of different ages and HCSM subnetworks is given in parentheses in the first column. In the remaining columns, following the value of the metric (outdegree, indegree or betweenness) calculated for the test subgraph, numbers in square brackets denote (1) the average value of the given metric calculated for 1,000 random subgraphs and (2) the number of 1,000 random subgraphs for which the given metric had lower value than for the test subgraph (the difference is significant at the *P *value cutoff of 0.05 if this number is lower than 50 or higher than 950, and in these cases the numbers are underlined

#### Network connectivity: Degree and betweenness

Table 6 illustrates the overall results of the analysis of network connectivity, suggesting that highly connected negative regulators were preferentially retained after 2R-WGD. All statistically significant differences corresponded to increases in connectivity and/or betweenness centrality. These statistically significant differences were mostly associated with either 2ROs or the emergence of Bilaterians. It should be noted that the degree of a node and its betweenness centrality were highly correlated (Pearson's *r *correlation, 0.91), and we cannot be certain whether the simple number of interacting partners (degree) or the amount of information flowing through the given node (betweenness centrality) correspond more closely to the biological properties of the network which were selected for in evolution.

#### Degree difference and edge conservation

We compared degree difference and edge conservation, following waves of duplications of different ages. Degree difference, that is, the absolute value of degree subtraction between two nodes, is a metric of paralog connectivity divergence. The edge conservation concept is considered in the Discussion section. Table 7 shows that these two metrics were inversely correlated, that is, the lower the average degree difference, the higher the percentage of conserved edges. Furthermore, edge conservation was higher and degree difference was lower between pairs of nodes linked with 2ROs than those linked with gene duplications mapping to the base of Chordates or Bilaterians. The differences in conservation of edges were highly statistically significant. For example, when the entire signalling network was considered, ratios of conserved edges to the total number of edges, calculated for each paralogous pair separately and then averaged, were (1) 0.281, (2) 0.083 and (3) 0.141, using Wilcoxon rank-sum test *P *values for pairwise comparisons 1 versus 2 and 1 versus 3 were 6.163e-08 and 1.235e-13, respectively. The differences were even more pronounced when distribution characteristics were considered: The percentages of paralogous pairs with more than one third of conserved edges were 39%, 9% and 17%, for (1), (2) and (3), respectively. The percentages of paralogous pairs which had at least one conserved edge were 65%, 35% and 47%, respectively.

**Table 7:** Paralogous gene pairs and conservation of signalling network connectivity: Shared edges and average degree difference^a^

***2ROs*** ***(9,958 gene set linked)***		
*Subnetwork and overlap*	*Shared edges*	*Average degree difference*
All edges (450 node pairs)	1,728/6,741 = 25.63%	7.17
Positive edges only (251 node pairs)	658/2,750 = 23.92%	4.87
Negative edges only (94 node pairs)	348/783 = 44.44%	3.05
Scaffolds only (254 node pairs)	652/2,445 = 26.66%	4.44
***Chordata*** ***(2,173 gene set linked)***		
*Subnetwork and overlap*	*Shared edges*	*Average degree difference*
All edges (67 node pairs)	130/1,149 = 11.31%	9.86
Positive edges only (37 node pairs)	66/483 = 13.66%	6.72
Negative edges only (11 node pairs)	24/102 = 23.52%	4.9
Scaffolds only (39 node pairs)	32/350 = 9.14%	4.66
***Bilateria*** ***(5,223 gene set linked)***		
*Subnetwork and overlap*	*Shared edges*	*Average degree difference*
All edges (452 node pairs)	1,198/8,356 = 14.33%	9.37
Positive edges only (271 node pairs)	440/3,686 = 11.93%	6.48
Negative edges only (126 node pairs)	178/1,120 = 15.89%	3.61
Scaffolds only (219 node pairs)	418/2,335 = 17.90%	4.90

^a^Paralogous gene pairs linked with different taxons are mapped to the human cancer signalling map (HCSM). The entire network, as well as the subnetworks consisting of only positive, negative or scaffolding edges, is considered. The overlap between pair sets of different ages and HCSM subnetworks is given in parentheses in the first column. In the second column, the number of shared edges versus the total is given (as the ratio and the resulting percentage value, calculated for the total set of paralogs). In the third column, the average difference in degree between such node pairs is given.

It should be noted that the number of random node pairs with shared edges is much lower than the values observed among paralogs. By random sampling, we approximate that the expected fraction of shared ages under the null hypothesis is only 0.6% (versus 25.63% for 2ROs), while only 4% of random node pairs have any shared edges (versus 65% for 2ROs). This strongly supports the conclusion that the presence of shared edges between duplicate nodes stems from their shared evolutionary ancestry.

Interestingly, for all age groups, conservation of regulatory edges with negative impact was higher than those with positive impact. This effect was strongest for pairs of paralogous nodes linked with 2ROs: 658 (23.92%) of 2,750 positive edges and 348 (44.44%) of 783 negative edges were conserved. Corresponding percentage figures for Chordates and Bilaterians were 13.66% versus 23.52% and 11.93% versus 15.89%, respectively (Table 7).

We further subdivided 2RO-linked regulatory conserved edges into those originating from the shared interaction node and directed towards the paralogous pair (conserved incoming edges, or CIEs) and those originating from the paralogous pair and directed towards the shared interaction node (conserved outgoing edges, or COEs), with similar percentage frequencies (22.28% versus 26.91% for positive edges only and 48.42% versus 45.54% for negative edges alone).

Finally, it should also be noted that the fraction of conserved edges for duplications mapping to Tetrapoda and younger is not significantly different from that calculated for 2ROs (62/275 = 22.54% versus 1,728/6,741 = 25.63%, respectively; Wilcoxon *P *value = 0.808), suggesting that there is no dominant linear correlation between duplicate age and edge conservation and that greater edge conservation among 2ROs than older single-gene duplications can be attributed in large measure to the inherent properties of genome duplication.

#### Paralogous nodes linked by a bridging edge

A bridging edge is an edge directly linking the paralogous node pair. Bridging interactions were preferentially of the scaffolding type. We identified 48 bridged 2RO pairs, with 35 scaffolding, 19 stimulatory and 5 inhibitory bridging edges (Additional file 20, Table 2ROs, bridged). The apparent excess of activatory links was simply a reflection of the general bias in network composition. Bridged pairs were also characterized by higher average number of edges, 26.7 per pair versus 15 per pair in the total set (Wilcoxon rank-sum test *P *value = 0.000196), and had a similar fraction of conserved edges (0.291 versus 0.281, *t*-test *P *value = 0.77).

#### Analysis of the inferred AP2R network and expression states

The fly ancestral ortholog set (FAOS) is a set of unique *Drosophila melanogaster *orthologs of human ohnolog pairs inferred from TreeFam with high confidence (bootstrap >75%). To investigate associated topology features, the FAOS was linked to the fly PPI network described by Giot *et al*. [21]. We found that FAOS nodes were characterised by increased degree (6.28 compared to the average of 5.52 for 1,000 randomly sampled subnetworks; *P *= 0.005). Nodes linked with mammalian and chordate duplications exhibited even higher-degree biases: 7.75 and 8.74 (*P *= 0.001 and 0.002, respectively).

We combined the FAOS with Fly Expression Atlas (FEA) [22] to infer AP2R expression states, as well as patterns of expression sub- and neofunctionalisation. The following FEA tissues were considered analogous to human tissues: brain, midgut, hindgut, heart, ovary, testis and fat. However, we found good correlation between the FEA and the GEA only for brain and not for the other six tissues (data not shown). On top of that, in the past we have found that brain had the highest number of uniquely expressed genes and was robust in comparative expression tests [23]. Therefore, we concentrated on brain expression.

To investigate the evolution of AP2R expression states, we identified gene triads consisting of a unique fly ortholog (member of the FAOS) and a pair of human paralogs, where the three genes could be linked to expression data in the FEA or the GEA. We then established whether these genes were preferentially expressed in brain. Preferential brain expression (PBE) was defined as brain signal higher than the average signal for all tissues. "b" denotes the PBE, while "nb" denotes the lack thereof. Triads in each of six possible configurations were then quantified and interpreted (Table 8). For example, "b: b & b" and "nb: nb & nb" correspond to straightforward conservation of brain expression status. More interestingly, "b: b & nb" signifies subfunctionalisation through loss of the PBE in one of the paralogs, while "nb: nb & b" is interpreted as neofunctionalisation through gain of the PBE.

**Table 8:** Duplication triads and patterns of expression subfunctionalisation and neofunctionalisation^a^

**Triad expression**	**Tetrapoda and younger**	**Chordata**	**2ROs**	**Evolutionary interpretation**
b: b &b	0	6	56	Ancestral expression conservation (b)
b: nb & b	5	11	123	Subfunctionalisation through loss of PBE in one duplicate
b: nb & nb	7	12	133	Gain or loss independent of gene duplication
Conservation/sub	0/5 = 0	6/11 = 0.55	56/123 = 0.46	Relative rate of subfunctionalisation
nb: b & b	1	3	18	Gain or loss independent of gene duplication
nb: nb & b	7	11	58	Neofunctionalisation through gain of PBE in one duplicate
nb: nb & nb	12	9	78	Ancestral expression conservation (nb)
Conservation/neo	12/7 = 1.71	9/11 = 0.82	78/58 = 1.34	Relative rate of neofunctionalisation
Sub/neo ratio	5/7 = 0.71	11/11 = 1	123/58 = 2.12	Ratio of sub- to neofunctionalisation

^a^We quantified triads depending on brain expression status of a pair of human duplicated genes mapped to unique fly ortholog, depending on timing of duplication ("b" denotes PBE, "nb" denotes lack thereof). PBE, preferential brain expression.

The first interesting observation was that subfunctionalisation was two to three times more common relative to neofunctionalisation among 2ROs than among other duplicates (Table 8). Clearly, subfunctionalisation, as a faster process, was well suited for network remodeling following 2R-WGD. The second important observation was that expression neofunctionalisation into brain was very rare among duplicates dating to Tetrapoda and younger, supporting the interpretation of 2R as the formative event for vertebrate brains.

## Discussion

2R-WGD occurred more than 450 million years ago, and most resulting gene duplicates were lost, leading to rediploidisation. Here we set out to functionally characterize retained 2ROs. First, we found that signal transduction was the most enriched GO term (in stark contrast to tandem or segmental duplications, where this term was underrepresented). In total, 74% of human signalling genes were descendants of 2ROs. Foreshadowing later findings, several GO terms were associated with the nervous system: neurogenesis, synaptic transmission, axon guidance, nervous system development and neuron differentiation (Additional file 2, Table S2_bp). Next, we searched for protein domains enriched among 2ROs and found many classic signalling domains, as well as well-known protein interaction (PI) domains, such as Src homology 2 (SH2), Src homology 3 (SH3), phosphotyrosine-binding domain (PTB) and PDZ (reviewed in [24]). The PI domains aid signalling by enabling dynamic formation of signalling protein complexes. For example, SH2 and PTB selectively recognise phosphorylated tyrosines, while SH3 binds proline-rich sequences with a characteristic motif Pro-X-X-Pro. SH2 proteins frequently form membrane-attached signal-processing complexes at autophosphorylated receptors and participate in positive and negative feedback loops of phosphorylation cascades. PTB-bearing proteins, in turn, are predominantly adaptors and docking stations, frequently anchored in the cell membrane (sometimes by means of a lipid-binding PH domain), and promoting assembly of large signalling complexes at autophosphorylated tyrosine kinases. Finally, PDZ domains recognise internal valine or leucine residues and are abundant in synapses, serving as scaffolds for the assembly of large signalling complexes involved in neurotransmission.

To better understand the evolutionary dynamics of 2R-WGD, we investigated the relationship between relative timing of gene duplication and spatial expression domain of progeny genes. The heatmap in Figure 1 revealed 2R's expression signature in the broader context of animal evolution. Significantly, a trend could be observed for brain and nervous tissue expression (amygdala, thalamus, caudate nucleus, corpus callosum, spinal cord, fetal brain, cerebellum, cortex and whole brain) to map to the taxonomic cluster (b), Bilateria, Chordata and Vertebrata, while being excluded from younger clusters (c) and (d). These expression patterns, taken together with the results of GO analysis, suggested that the molecular machinery of the vertebrate neuron was defined in the 2R event and strongly conserved thereafter. A previous focused study of fly and mouse noted that vertebrate synapses were far more complex than those of invertebrates [25], but the scale, the mechanism and the precise timing of this key evolutionary transition was hitherto unknown.

Development of large multicompartmentalised vertebrate brains is shaped by three layers of control [26]: (1) establishment of patterning centres that secrete diffusible signalling ligands, such as WNTs, BMPs and soluble bone morphogenetic protein (BMP) antagonists; (2) brain-specific transcriptional regulatory networks involving TFs such as paired box proteins (PAX) and forkhead box protein (FOXP); and (3) extensive neuronal apoptosis shaping the fine detail of brain structures and compartments. For example, in a direct mechanistic demonstration, mice deficient in cysteine-aspartic acid protease 3 (CASP3) exhibited decreased neuronal apoptosis and hyperplasia, resulting in gross brain abnormalities [27]. How important was 2R-WGD for the definition of this developmental toolkit? We found that multiple WNT ligands (TF105310), PAX2/5/8 and PAX1/9 (TF315397), FOXP1/2/3/4 (TF326978) and CASP3/7 (TF102023) were 2ROs. Previously, we showed that the evolution of the BMP/TGF-β pathway was guided almost entirely by 2R-WGD [28]. In conclusion, we identified most of the vertebrate brain developmental toolkit as 2ROs.

The exclusion of nervous tissue from the expression domain of newly formed mammalian and primate genes contradicts intuition. However, anatomical differences between vertebrate nervous systems can be sufficiently explained by changes in developmental expression patterns of existing regulatory and structural genes of the neuron. Higher complexity of mental functions in certain vertebrate lineages (for example, in primates, some birds, and dolphins) is likely to stem from these anatomical differences, as well as more complex ways in which neurons are connected, as demonstrated by the rising area of connectomics.

Uniquely in animal evolution, and in stark contrast to other basic cellular functions, 2R-WGD expanded the cell cycle machinery, in particular cyclins A and B, and the interface with signalling made up by cyclins D1-D3, CDK4/6, p21/p27 and p18/p19. Cyclin D levels (unlike cyclins A and B) do not correlate with cell-cycle phases but with extracellular mitogens, cytokines, hormones and juxtacrine ligands. Signalling pathways induce expression of cyclin D, which pairs with cyclin-dependent kinases (CDKs) of types 4 and 6, stimulating the cell to enter the cycle from G1. (This progression can be inhibited by cyclin-dependent kinase inhibitors p21/p27 and p18/p19.) We identify all four sets of genes involved (that is, cyclins D1-D3, CDK4/6, p21/p27 and p18/p19) as 2ROs.

Arguably, the cyclin/CDK engine might be a relatively late evolutionary invention, taking over from ancient kinases [29], and with the inherent tendency for redundancy characteristic of an integrating system [30]. However, cyclin/CDK signalling is very well documented in yeast. Regardless of the controversy regarding the nature of primordial cell-cycle regulators, the results presented here suggest that control over cell cycles became more important in large and long-lived animals and that expansion of the cyclin/CDK network, which occurred through genome duplication, facilitated fine-tuning of that control. No such regulatory upgrade was required for other basic cellular functions (such as translation, replication, splicing and recombination). We hope to open a new area of investigation into the differences of cell-cycle control between vertebrates and model species such as fly, worm and yeast, with important consequences for both basic and applied science. As cyclin D1-D3/CDK4/6 complexes have at least partially overlapping phosphorylation targets, the apparent functional redundancy serves to integrate multiple upstream signals. In other words, 2R-WGD most likely resulted in retention of duplicates with different signalling inputs but similar outputs. Kinetic modeling, protein interaction and target screens focused on differences between invertebrate and vertebrate cyclin/CDK networks should yield the first clues.

The next question we decided to ask was whether signalling network nodes linked with 2ROs exhibited some characteristic features, such as the degree (that is, the number of interaction partners) or betweenness centrality (that is, the amount of network traffic, or information, flowing through a given node). The degree of human 2RO nodes was significantly increased, with the strongest effect on outdegree of negative regulation (Table 6). This suggested that highly connected nodes, that is, network hubs, in particular those involving negative regulators, were preferentially retained. Enrichment of 2ROs in PI domains, as shown by PFAM analysis, also suggested higher interconnectedness of the post-2R network. The likely biological result of this trend towards greater network complexity was increased signalling robustness and cross-talk. Negative feedback loops, on the other hand, were likely to mediate inducible and temporary biological responses invoked by external stimuli or network oscillations facilitating spatiotemporal patterning during vertebrate development.

However, was high connectedness driving preferential retention, or was it merely a consequence of rediploidisation? If only we could sequence the genome of the AP2R animal! This is, of course, impossible, but some features can be inferred from extant species. To this end, we compared fly and human and found that hubs were already enriched in genes ancestral to 2ROs. High connectedness was therefore a factor contributing towards preferential retention. Interestingly, ancestral nodes associated with mammalian and chordate duplications exhibited even higher connectivity biases, but the progeny of these genes were not associated with human hubs. This could be explained by the evolutionary model in which all duplications preferentially target highly connected nodes but WGDs preserve their status as hubs, while tandem and segmental duplications remodel them towards reduced connectivity.

Do gene duplications conserve interactions or rewire duplicates with novel interaction partners? We must first define a few concepts which will help us approach network topology from the evolutionary perspective, with a focus on gene duplication. Let us define shared edges as a pair of edges extending between two nodes and an identical third node. A conserved edge, on the other hand, corresponds to an ancestral interaction in the ancestral network, which is still present in the extant network. We can see that shared edges between a pair of 2ROs are parsimoniously explained as conserved edges (derived from an ancestral interaction in the AP2R network), as the probability of gaining shared edges through convergent evolution is extremely low. Finally, a bridging edge is an edge directly linking the paralogous node pair, suggesting sophisticated forms of regulatory feedback and information processing between duplicates [31]. The bridging edge is an evolutionary novelty created as a consequence of duplication, possibly but not necessarily associated with ancestral proteins prone to homodimerisation.

**Figure 5:**
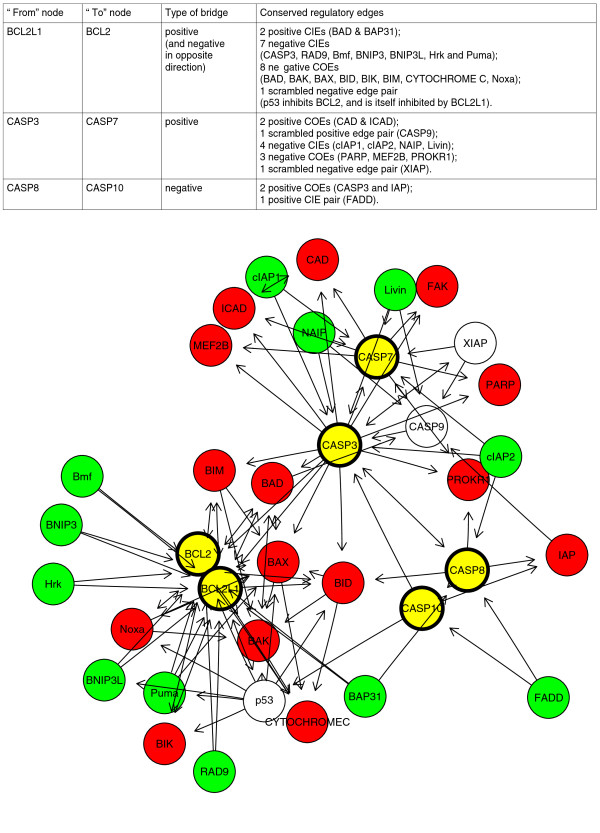
**Bridged pairs and shared edges between 2ROs in the apoptosis pathway**. Graphed representation of a subnetwork of the human cancer signalling map (HCSM) focusing on the apoptosis pathway is shown. There are three pairs of 2ROs in the subnetwork: BCL2-like 1 (BCL2L1) and BCL2, cysteine-aspartic acid protease 3 (CASP3) and CASP7, and CASP8 and CASP10. Nodes are colour-coded as follows: yellow signifies nodes mapping to 2ROs and green and red signify nodes mapping to conserved incoming edges (CIEs) and conserved outgoing edges (COEs), respectively. The edges shown are regulatory edges with the directionality indicated by arrows. CIEs suggest a common conserved regulator, located upstream in terms of information flow. In contrast, COEs indicate evolutionary conservation of a common regulatory target, located downstream. CASP8 and CASP10 are initiator caspases which locate to the death-inducing signalling complex (DISC). CASP3 and CASP7 are executioner caspases functioning downstream of initiator caspases. IAPs are inhibitors of apoptosis. The relative balance of antiapoptotic BCL2 and BCL2L1 versus proapoptotic BCL2-associated agonist of cell death, BCL2-antagonist/killer 1, BCL2-associated X protein, BH3 interacting domain death agonist, BCL2-interacting mediator of cell death, Puma and Noxa determines the activity of the intrinsic pathway of apoptosis.

When the concepts of shared, conserved and bridging edges are applied to HCSM (Table 7), a number of observations emerge: (1) the fraction of conserved edges is higher for 2ROs than for paralogs mapping to Chordates or Bilaterians, (2) the fraction of conserved regulatory edges with negative impact is higher than those with positive impact, and (3) complex novel network motifs are formed by bridged hubs (Figure 5). Figure 5 shows a graph representation of a HCSM subnetwork focusing on the apoptosis pathway featuring three bridged 2RO pairs. Overall, bridged pairs are extremely rich in signalling hubs, with twice the average number of interacting partners. In terms of the broader evolutionary impact, we propose that 450 million years ago, at the time of 2R, instantaneous doubling of the signalling network through WGD not only immediately expanded the available space of network states but also kick-started rapid coevolution of nodes into novel topologies. The cumulative effect was that of greatly increased phenotype space, enabling adaptation to an expanded range of physiological parameters, such as temperature, osmotic pressure, availability of nutrients and growth factors. Greater organismal adaptability facilitated, in turn, colonisation of novel environments or ecological niches. 2R-WGD was most likely an instantaneous speciation, in itself an extraordinary evolutionary event, somewhat contrary to the classic Darwinian view of gradual evolution. It probably took place under stress conditions on the fringes of the normal ecological range of the parental species. Few "hopeful monsters", with duplicated genomes, must have had an instant adaptability advantage to compete with AP2R parental populations, despite the increased costs of DNA replication, chromatin remodeling and chromosome segregation associated with polyploidy. For example, Conant and Wolfe [32] proposed that yeast WGD conferred an immediate selective advantage for growth in high-glucose environments through the increase of dosage of genes in the glycolytic pathway. In the longer term, as proven by our GO analysis, 2R-WGD likely also provided a drive for increased morphological complexity [33] and conferred greater evolvability, facilitating the emergence of vertebrate novelties.

## Conclusions

Herein we present the first global analysis of functional trends among 2R-WGD-retained genes using state-of-the-art methodology and a high-quality data set verified through manual curation. In a methodological advance, 2R ohnologs were identified using detailed phylogenetic trees on the basis of a tree-merging algorithm implemented in the TreeFam database. We found that 2R-WGD was the paramount source of novelty in vertebrate evolution, affecting an overwhelming majority (74%) of signalling genes, in particular developmental pathways involving receptor tyrosine kinases, Wnt and TGF-β ligands, GPCRs and apoptosis pathway. Moreover, 2R-WGD redefined vertebrate synapses and facilitated the formation of centralised brains. We show that 2R-WGD preferentially retained genes associated with higher organismal complexity (for example, locomotion, nervous system, morphogenesis), while genes associated with basic cellular functions (for example, translation, replication, splicing and recombination, with the notable exception of cell cycle) tended to be excluded. In conclusion, 2R-WGD left an indelible imprint on the vertebrate signalling network (including the interface with cell-cycle machinery) and set the stage for the emergence of key vertebrate functional novelties, facilitating the evolutionary success of this taxonomic group. Finally, we link observed functional trends to signalling network and expression evolution, investigating the human signalling network and the inferred AP2R network, a comparison that has never previously been performed.

## Methods

### Tree families database

TreeFam (Tree Families; http://www.treefam.org/) is a database of phylogenetic trees of animal genes [34]. TreeFam relies on a multistage computational pipeline and a tree-merging algorithm implemented in the TreeBeST phylogenetic engine. Several types of trees are utilized: (1) a maximum likelihood tree built using PHYML with the WAG model, (2) a maximum likelihood tree built using PHYML with the HKY model, (3) a neighbor-joining tree using *P *distance, (4) a neighbor-joining tree using *Ka *distance, and (5) a neighbor-joining tree using *Ks *distance. The merging procedure is implemented in the TreeBeST phylogenetic engine. In the first step of the merging procedure, the set of permitted branches and nodes is constructed, given the input set of trees. In the second step, the tree which optimizes the objective function is found. The objective function measures the similarity between a gene tree and the species tree (by minimizing the number of inferred gene duplications and losses), as well as the overall bootstrap support. TreeBeST has been tested extensively against knowledge of biologists, including manual curation, within the TreeFam and Ensembl databases [35].

TreeFam release 6 database was downloaded as a set of SQL instructions and data files, and reconstituted as a local MySQL database. The total data set extracted from TreeFam6 consisted of 11,635 trees containing 391,730 genes and assigned to 64 different taxonomic categories spanning the animal kingdom with *S. cerevisiae *and *A. thaliana *as outgroups. Perl scripts based on the provided TreeFam API http://treesoft.sourceforge.net/tf-perl-api.shtml were used to extract the data.

Figures 3 and 4 were produced using a locally installed version of the TreeBeST pipeline. Trees were rooted on time. A speciation and duplication inference (SDI) algorithm, based on the reconciliation of the gene tree with a trusted species tree [36], was used to infer orthology, paralogy, speciation nodes and gene duplication events. However, inferred duplication events with no species intersection support (SIS = 0) were attributed to locally incorrect gene tree topology.

### GO, KEGG and PFAM enrichment analysis

A hypergeometric test implemented in the Bioconductor package GOstats was used to detect enrichment in GO categories, KEGG pathways, PFAM domains or spatial clustering along human chromosomes. The test is implemented in function hyperGTest, which enables testing for both over- and underrepresentation of terms, and conditional correction taking into account the hierarchical structure of GO ontologies was used [37].

### Expression signatures of gene duplications throughout the animal kingdom

Expression data are derived from a Gene Expression Atlas [38], a collection of Affymetrix readings from 47 human tissues and cell lines (see Additional file 21, Table S8, and http://expression.gnf.org/human_annot.html). Mapping of Affymetrix clusters to Entrez Genes was derived from the R environment table hgu95aENTREZID. In case of multiple probes mapping to the same Entrez Gene, average values across the probes were taken. Original data from the Gene Expression Atlas were in the form of average difference (AD) values, representing the fluorescence assigned to a given gene probe after subtraction of background calculated from its mismatch control. The cutoff accepted in the GEA for a gene to be regarded as switched-on or expressed was AD = 200. The AD values were converted to preferential expression measures (PEMs). PEM is log_10_(S/A), where S is the Affymetrix signal for a given gene in a specific tissue and A is the arithmetic mean signal for the gene across all tissues. To avoid biasing PEM values by genes without detectable expression in any of the GEA tissues or by genes with very high average expression, data points with AD_avg _< 50 or AD_avg _> 999 were omitted (2,130 and 545 of the total 6,837 human genes mappable to the GEA data set). The actual number of genes with expression data corresponding to each taxonomic unit and average PEM values across 47 human GEA tissues are given in Additional file 22, Table S9. The heatmap in Figure 1 was generated using the standard R function and the brewer.pal palette from the RColorBrewer package. Tissues (horizontal axis) and taxons (vertical axis) were ordered using a simple hierarchical clustering algorithm and visualized using dendrograms.

For the analysis of tissue specificity of expression, two separate measures of tissue specificity were used: percentage breadth of expression and PEM_MAX_. Percentage breadth of expression was defined as the percentage of the 47 human tissues studied in which a given gene was expressed above the threshold level (AD = 200). PEM_MAX _is the maximal value of the PEM across the 47 tissues for a given gene.

### Identification of 2R paralogons in the human genome

We identify 2ROs in the human genome with high certainty by the combination of phylogenetic timing and paralogon detection. By phylogenetic timing, we mean the inference of relative evolutionary timing of gene duplications on the basis of the patterns of presence or absence of orthologs and paralogs in genomes of extant species, assuming a known and trusted species tree.

Paralogons (or multiplicons) are pairs of mutually paralogous chromosome regions in which gene content and gene order are conserved. Coverage of the majority of the genome by multiplicons is an unmistakable signature of a past WGD. It should be noted that in the case of ancient WGDs, such as the 2R event, a large number of positional rearrangements is bound to have occurred and the conservation of synteny is imperfect or sometimes even marginal. Various algorithms aiming at increased sensitivity have been developed, including the Automatic Detection of Homologous Regions (ADHoRe), which combines information across multiple homologous segments. In our analysis, multiplicon detection was performed using the i-ADHoRe 2.4 [39] implementation of the ADHoRe algorithm. The following parameter values were used: 30 intervening genes were allowed between anchor points, a minimum of five anchor points were required to define a multiplicon, a quality value of 0.8 and a probability cutoff of 0.00001. Multiplicon detection was used to verify gene duplications assigned to the 2R-WGD by phylogenetic timing. Positional mapping of human genes descending from the duplication node mapping the base of vertebrates yields a characteristic pattern of paralogous regions covering 83% of the length of the human genome, constituting a definitive signal of an ancient WGD (Additional file 23, Table S10, contains the list of genes with chromosomal location; Additional file 24, Table S11, defines the paralogy relationships between human 2ROs). Similarly to the analysis of Dehal and Boore [10], chromosomes 18, 21 and Y were found not to harbour any paralogous regions, suggesting that they originated after 2R-WGD (Additional file 25, Table S12, lists fractions contained in multiplicons by chromosome breakdown).

Additional confirmation can be derived from the fact that no simple spatial clustering in chromosomal gene order can be detected for the list of 2ROs, which would be expected if these paralogs arose in the standard fashion, that is, by tandem duplications. In contrast, such chromosomal clusters can be found for descendants of gene duplications mapped by phylogenetic timing to other taxons, such as Chordata (Additional file 26, Table S13), Amniota/Tetrapoda (Additional file 27, Table S14), Mammalia/Eutheria/Theria (Additional file 28, Table S15) or identified as human-specific (Additional file 29, Table S16).

### Analysis of the human signalling network

A manually curated signalling network consisting of 1,634 nodes, termed the human cancer signalling map (HCSM), was derived by Cui *et al*. [20] from the integration of several data sets: the BioCarta database http://www.biocarta.com/, the Cancer Cell Map http://cancer.cellmap.org/cellmap/ and the data set described by Ma'ayan *et al*. [40]. The integrated network consists of 5,059 edges divided into undirected 1,915 scaffolding links (neutral physical interactions) and directed 2,403 activator and 741 inhibitory links. The network was retrieved as a Microsoft Excel file (Microsoft Corp., Redmond, WA, USA) and processed into two tab-delimited files containing data on nodes and edges, respectively. These files were then processed into a format compatible with the R class graphNEL and analysed using the R package RBGL (an interface to the popular Boost C++ library of graph algorithms). Protein members of the signalling network were mapped to Entrez Gene Ids. Custom R functions were written to analyze the overlap between human gene sets mapped to gene duplications of different phylogenetic age and the protein nodes of the imported human signalling network. Nodes were compared using standard RBGL functions, with regard to degree (including indegree and outdegree), and betweenness centrality (function brandes.betweenness.centrality, method relative.betweenness.centrality.vertices) as shown in Table 6. *P *values were calculated by comparison with distributions of relevant function values calculated for random subgraphs of the same size as that obtained through mapping to a given test set. Random subgraphs were derived through permutation of node labels using sampling without replacement.

An analysis of conservation of network edges following gene duplications of different ages was also performed (Table 7). The number of identical edges was compared against the total number of edges linked to the pair of nodes identified as paralogs. The percentage of conserved edges was calculated and is shown in Table 7. An undirected graph representation of the human signalling network was used. The average difference in degree between paralogous nodes, and the number of paralogous nodes connected by a bridging edge, were also calculated (Table 7).

## Abbreviations

2R-WGD: two rounds of whole genome duplication at the base of vertebrates; 2ROs: 2R-ohnologs; AP2R: ancestral pre-2R; GPCRs: G protein-coupled receptors; RTKs: receptor tyrosine kinases; WGD: whole genome duplication.

## Authors' contributions

LH performed the analysis and wrote the manuscript. CHH contributed to writing the manuscript. LH and CHH together designed the study and interpreted the results.

## Authors' information

LH is a senior postdoctoral fellow in evolutionary systems biology at Ludwig Institute for Cancer Research, Uppsala Branch (LICR-UPP), funded by the ENFIN Network of Excellence for Systems Biology to conduct interdisciplinary research on the molecular evolution of animal signalling. CHH is a molecular biologist, director of LICR-UPP, and Swedish representative to the European Research Council.

## Supplementary Material

Additional file 1 **TableS1**. 2ROs mapped to Entrez Genes. Click here for file

Additional file 2 **TableS2_bp**. 2RO overrepresented BP terms. Click here for file

Additional file 3 **TableS2_mf**. 2RO overrepresented MF terms. Click here for file

Additional file 4 **TableS2_cc**. 2RO overrepresented CC terms. Click here for file

Additional file 5 **TableS3_bp**. 2RO underrepresented BP terms. Click here for file

Additional file 6 **TableS3_mf**. 2RO underrepresented MF terms. Click here for file

Additional file 7 **TableS3_cc**. 2RO underrepresented CC terms. Click here for file

Additional file 8 **TableS3_not2R-over**. Tandem/segmental duplication overrepresented BP terms. Click here for file

Additional file 9 **TableS3_not2R-under**. Tandem/segmental duplication underrepresented BP terms. Click here for file

Additional file 10 **TableS4**. 2RO overrepresented KEGG pathways. Click here for file

Additional file 11 **TableS5**. 2RO overrepresented PFAM domains. Click here for file

Additional file 12 **TableS6**. 349 2ROs preferentially expressed in brain. Click here for file

Additional file 13 **TableS7_bp**.html. 2ROs preferentially expressed in brain, overrepresented BP terms. Click here for file

Additional file 14 **TableS7_cc**.html. 2ROs preferentially expressed in brain, overrepresented CC terms. Click here for file

Additional file 15 **TableS7_mf**.html. 2ROs preferentially expressed in brain, overrepresented MF terms. Click here for file

Additional file 16 **FigureS1**. Duplication timing and expression divergence (Kendall correlation). Click here for file

Additional file 17 **FigureS2**. Duplication timing and expression divergence (Spearman's rank correlation). Click here for file

Additional file 18 **FigureS3**. Duplication timing and expression divergence (Manhattan distance). Click here for file

Additional file 19 **FigureS4**. Duplication timing and expression (difference in breadth of expression). Click here for file

Additional file 20 **2ROs-bridged**. Human 2RO bridged pairs. Click here for file

Additional file 21 **TableS8**. 47 human tissues and cell lines in Gene Expression Atlas. Click here for file

Additional file 22 **TableS9**. Matrix of average PEM values across tissues and taxons. Click here for file

Additional file 23 **TableS10**. Human genes with chromosomal location. Click here for file

Additional file 24 **TableS11**. Paralogy relationship between human 2ROs. Click here for file

Additional file 25 **TableS12**. Fractions contained in 2RO-defined multiplicons by chromosome breakdown, paralog regions cover 83% of the human genome proving a WGD. Click here for file

Additional file 26 **TableS13**. Chromosomal clusters for gene duplications mapped to Chordata. Click here for file

Additional file 27 **TableS14**. Chromosomal clusters for gene duplications mapped to Amniota/Tetrapoda. Click here for file

Additional file 28 **TableS15**. Chromosomal clusters for duplications mapped to Mammalia/Eutheria/Theria. Click here for file

Additional file 29 **TableS16**. Chromosomal clusters for human-specific duplications. Click here for file
