# Supplementary material for: 2R and remodeling of vertebrate signal transduction engine
Source: BMC Biol. 2010 Dec 13;8:146. doi: 10.1186/1741-7007-8-146 (PMC3238295; doi:10.1186/1741-7007-8-146)
Supplement: Additional file 1 — TableS1. 2ROs mapped to Entrez Genes. [file 1741-7007-8-146-S1.pdf]

Family	EntrezID	EnsemblID
TF329712	64102	ENSG00000000005
TF351634	2268	ENSG00000000938
TF326157	3075	ENSG00000000971
TF313214	57185	ENSG00000001461
TF330032	22875	ENSG00000001561
TF352628	6405	ENSG00000001617
TF350755	9957	ENSG00000002587
TF314750	26	ENSG00000002726
TF313938	23072	ENSG00000002746
TF319104	3927	ENSG00000002834
TF332117	29916	ENSG00000002919
TF328485	90293	ENSG00000003096
TF105093	56603	ENSG00000003137
TF317186	3382	ENSG00000003147
TF332997	79007	ENSG00000003249
TF331793	57679	ENSG00000003393
TF102023	843	ENSG00000003400
TF315349	7035	ENSG00000003436
TF315789	10181	ENSG00000003756
TF315197	9108	ENSG00000003987
TF315212	6542	ENSG00000003989
TF300808	381	ENSG00000004059
TF312962	23129	ENSG00000004399
TF332530	952	ENSG00000004468
TF105294	2288	ENSG00000004478
TF315789	10180	ENSG00000004534
TF313013	84254	ENSG00000004660
TF105049	126393	ENSG00000004776
TF351451	115703	ENSG00000004777
TF314918	5166	ENSG00000004799
TF105193	340273	ENSG00000004846
TF350743	170302	ENSG00000004848
TF313209	10165	ENSG00000004864
TF314162	7982	ENSG00000004866
TF313630	6521	ENSG00000004939
TF315710	799	ENSG00000004948
TF318198	1856	ENSG00000004975
TF351676	64063	ENSG00000005001
TF331055	8935	ENSG00000005020
TF300743	292	ENSG00000005022
TF350668	3207	ENSG00000005073
TF351603	4222	ENSG00000005102
TF329791	221981	ENSG00000005108
TF319186	56928	ENSG00000005206
TF325496	80256	ENSG00000005238
TF300262	51226	ENSG00000005243
TF314920	5577	ENSG00000005249
TF101097	1387	ENSG00000005339
TF316230	9256	ENSG00000005379
TF314316	4353	ENSG00000005381
TF315109	6936	ENSG00000005436
TF105193	5244	ENSG00000005471
TF106417	55904	ENSG00000005483
TF316183	30812	ENSG00000005513
TF352583	26224	ENSG00000005812
TF105391	3683	ENSG00000005844
TF314918	5164	ENSG00000005882
TF105391	3675	ENSG00000005884

TF335557	7543	ENSG00000005889
TF316339	3920	ENSG00000005893
TF105391	3674	ENSG00000005961
TF106501	9244	ENSG00000006016
TF320922	114881	ENSG00000006025
TF336444	26022	ENSG00000006042
TF317306	51087	ENSG00000006047
TF332742	3883	ENSG00000006059
TF105201	6833	ENSG00000006071
TF334888	6362	ENSG00000006074
TF334888	6348	ENSG00000006075
TF327980	10368	ENSG00000006116
TF314981	54972	ENSG00000006118
TF300318	163	ENSG00000006125
TF333405	6863	ENSG00000006128
TF313555	8913	ENSG00000006283
TF315720	1750	ENSG00000006377
TF105118	4293	ENSG00000006432
TF312796	5898	ENSG00000006451
TF325648	55971	ENSG00000006453
TF106480	80853	ENSG00000006459
TF316214	2115	ENSG00000006468
TF314264	221	ENSG00000006534
TF323570	57157	ENSG00000006576
TF334888	10344	ENSG00000006606
TF351276	9855	ENSG00000006607
TF325033	10083	ENSG00000006611
TF324982	6915	ENSG00000006638
TF313638	3475	ENSG00000006652
TF315551	56891	ENSG00000006659
TF316514	9912	ENSG00000006740
TF313468	85477	ENSG00000006747
TF314186	414	ENSG00000006756
TF314375	8735	ENSG00000006788
TF313640	79602	ENSG00000006831
TF101031	51265	ENSG00000006837
TF314310	151531	ENSG00000007001
TF351676	10942	ENSG00000007038
TF315213	57787	ENSG00000007047
TF324631	8842	ENSG00000007062
TF324410	4843	ENSG00000007171
TF312913	9058	ENSG00000007216
TF313010	79090	ENSG00000007255
TF314013	4145	ENSG00000007264
TF336032	974	ENSG00000007312
TF323985	6329	ENSG00000007314
TF314162	54879	ENSG00000007341
TF313097	8277	ENSG00000007350
TF320146	5080	ENSG00000007372
TF312988	64285	ENSG00000007384
TF317607	55692	ENSG00000007392
TF315824	9254	ENSG00000007402
TF315526	8938	ENSG00000007516
TF313443	7005	ENSG00000007866
TF326910	6401	ENSG00000007908
TF105354	27035	ENSG00000007952
TF105566	1870	ENSG00000007968
TF319919	6853	ENSG00000008056
TF314166	57172	ENSG00000008118

TF324076	65220	ENSG00000008130
TF313718	7021	ENSG00000008196
TF313718	83741	ENSG00000008197
TF352091	9265	ENSG00000008256
TF314733	53616	ENSG00000008277
TF315804	6856	ENSG00000008282
TF313096	9043	ENSG00000008294
TF323983	65010	ENSG00000008300
TF323983	1951	ENSG00000008300
TF316755	55200	ENSG00000008323
TF324811	84954	ENSG00000008382
TF323191	1407	ENSG00000008405
TF313889	4784	ENSG00000008441
TF354325	6482	ENSG00000008513
TF315428	64386	ENSG00000008516
TF316484	5310	ENSG00000008710
TF325073	23542	ENSG00000008735
TF323347	23221	ENSG00000008853
TF300706	54497	ENSG00000008869
TF300222	6224	ENSG00000008988
TF324707	7812	ENSG00000009307
TF316833	10178	ENSG00000009694
TF351610	5081	ENSG00000009709
TF330373	10747	ENSG00000009724
TF313644	199870	ENSG00000009780
TF313644	653740	ENSG00000009780
TF313644	654163	ENSG00000009780
TF324749	51085	ENSG00000009950
TF331658	10048	ENSG00000010017
TF318679	51513	ENSG00000010030
TF105032	7384	ENSG00000010256
TF352895	928	ENSG00000010278
TF331217	25900	ENSG00000010295
TF315710	2696	ENSG00000010310
TF325426	51533	ENSG00000010318
TF316102	56920	ENSG00000010319
TF331489	23166	ENSG00000010327
TF343812	6540	ENSG00000010379
TF331065	5646	ENSG00000010438
TF315363	695	ENSG00000010671
TF106488	22955	ENSG00000010803
TF351634	2534	ENSG00000010810
TF331837	3097	ENSG00000010818
TF317259	6924	ENSG00000011007
TF314619	11313	ENSG00000011009
TF314619	388499	ENSG00000011009
TF316663	9902	ENSG00000011028
TF106373	4831	ENSG00000011052
TF106373	654364	ENSG00000011052
TF106373	4830	ENSG00000011052
TF343812	6534	ENSG00000011083
TF352892	10867	ENSG00000011105
TF315245	9546	ENSG00000011132
TF313114	11185	ENSG00000011177
TF314196	51099	ENSG00000011198
TF318736	3730	ENSG00000011201
TF105407	26993	ENSG00000011243
TF316498	54799	ENSG00000011258
TF319824	5725	ENSG00000011304

TF318971	8193	ENSG00000011332
TF102031	5286	ENSG00000011405
TF106494	54443	ENSG00000011426
TF333705	58525	ENSG00000011451
TF317184	23637	ENSG00000011454
TF334562	1634	ENSG00000011465
TF315071	54814	ENSG00000011478
TF105121	8491	ENSG00000011566
TF350825	27033	ENSG00000011590
TF315453	2556	ENSG00000011677
TF334827	933	ENSG00000012124
TF316102	7869	ENSG00000012171
TF313265	4007	ENSG00000012211
TF324013	4057	ENSG00000012223
TF324013	728320	ENSG00000012223
TF323454	60481	ENSG00000012660
TF105320	240	ENSG00000012779
TF106476	8284	ENSG00000012817
TF329501	57658	ENSG00000012822
TF105121	11183	ENSG00000012983
TF314429	30845	ENSG00000013016
TF314720	51629	ENSG00000013306
TF101041	1195	ENSG00000013441
TF315041	10552	ENSG00000013455
TF316126	23357	ENSG00000013523
TF314401	9604	ENSG00000013561
TF329541	1774	ENSG00000013563
TF328887	50865	ENSG00000013583
TF321410	9052	ENSG00000013588
TF332922	10046	ENSG00000013619
TF332922	728030	ENSG00000013619
TF332922	730818	ENSG00000013619
TF329295	923	ENSG00000013725
TF333149	10460	ENSG00000013810
TF314748	823	ENSG00000014216
TF312893	55	ENSG00000014257
TF315197	10903	ENSG00000014914
TF320231	440193	ENSG00000015133
TF350501	10138	ENSG00000015153
TF316736	7454	ENSG00000015285
TF324523	1800	ENSG00000015413
TF333921	9782	ENSG00000015479
TF333921	401957	ENSG00000015479
TF333921	642536	ENSG00000015479
TF333921	648887	ENSG00000015479
TF333921	648893	ENSG00000015479
TF315534	64132	ENSG00000015532
TF326935	81551	ENSG00000015592
TF315442	3670	ENSG00000016082
TF332433	55830	ENSG00000016864
TF354251	27032	ENSG00000017260
TF332255	80725	ENSG00000017373
TF332820	3479	ENSG00000017427
TF328787	92745	ENSG00000017483
TF323904	22902	ENSG00000018189
TF351103	1272	ENSG00000018236
TF315185	6556	ENSG00000018280
TF326941	25937	ENSG00000018408
TF312838	477	ENSG00000018625

TF330875	23584	ENSG00000019102
TF329165	23187	ENSG00000019144
TF330855	8685	ENSG00000019169
TF105094	1591	ENSG00000019186
TF315515	6591	ENSG00000019549
TF317779	972	ENSG00000019582
TF329901	3082	ENSG00000019991
TF331206	25960	ENSG00000020181
TF333046	55734	ENSG00000020256
TF324165	23034	ENSG00000020577
TF321496	864	ENSG00000020633
TF331787	58473	ENSG00000021300
TF352619	1992	ENSG00000021355
TF105087	64816	ENSG00000021461
TF321302	9369	ENSG00000021645
TF312807	114879	ENSG00000021762
TF330498	732	ENSG00000021852
TF315453	2554	ENSG00000022355
TF330014	55655	ENSG00000022556
TF327695	57476	ENSG00000023171
TF330014	6050	ENSG00000023191
TF300724	211	ENSG00000023330
TF105356	330	ENSG00000023445
TF105199	1244	ENSG00000023839
TF333160	50619	ENSG00000023892
TF333115	51177	ENSG00000023902
TF323875	23304	ENSG00000024048
TF314429	30846	ENSG00000024422
TF328365	55635	ENSG00000024526
TF323549	25901	ENSG00000024862
TF300659	58528	ENSG00000025039
TF330401	3298	ENSG00000025156
TF106475	51230	ENSG00000025293
TF325617	8630	ENSG00000025423
TF352167	10062	ENSG00000025434
TF106202	10953	ENSG00000025772
TF354205	23633	ENSG00000025800
TF330122	7431	ENSG00000026025
TF333916	355	ENSG00000026103
TF315063	8635	ENSG00000026297
TF334173	960	ENSG00000026508
TF313103	3755	ENSG00000026559
TF314065	56895	ENSG00000026652
TF317532	11119	ENSG00000026950
TF351636	3645	ENSG00000027644
TF336893	9047	ENSG00000027869
TF106473	7444	ENSG00000028116
TF331157	7133	ENSG00000028137
TF316413	5452	ENSG00000028277
TF106439	65980	ENSG00000028310
TF313698	6642	ENSG00000028528
TF319983	56938	ENSG00000029153
TF335939	9774	ENSG00000029363
TF335939	728366	ENSG00000029363
TF335939	728372	ENSG00000029363
TF335939	731605	ENSG00000029363
TF315051	55334	ENSG00000029364
TF351263	286	ENSG00000029534
TF329365	9135	ENSG00000029725

TF105371	3149	ENSG00000029993
TF331189	22807	ENSG00000030419
TF328895	51306	ENSG00000031003
TF351451	57514	ENSG00000031081
TF313181	8498	ENSG00000031823
TF106427	5926	ENSG00000032219
TF300519	10908	ENSG00000032444
TF318303	54480	ENSG00000033100
TF351429	57554	ENSG00000033122
TF329487	9846	ENSG00000033327
TF300346	535	ENSG00000033627
TF323787	8554	ENSG00000033800
TF313630	9497	ENSG00000033867
TF315245	321	ENSG00000034053
TF106434	29128	ENSG00000034063
TF106434	728688	ENSG00000034063
TF324777	25897	ENSG00000034677
TF313686	7414	ENSG00000035403
TF328365	55789	ENSG00000035499
TF351123	23604	ENSG00000035664
TF300486	159	ENSG00000035687
TF332087	26228	ENSG00000035720
TF317409	7077	ENSG00000035862
TF331825	9172	ENSG00000036448
TF313494	6570	ENSG00000036565
TF106277	9099	ENSG00000036672
TF331269	846	ENSG00000036828
TF300477	27175	ENSG00000037042
TF325768	2324	ENSG00000037280
TF333205	4238	ENSG00000037749
TF316310	3224	ENSG00000037965
TF314740	55300	ENSG00000038210
TF314351	7092	ENSG00000038295
TF318080	7204	ENSG00000038382
TF332134	1462	ENSG00000038427
TF330855	4481	ENSG00000038945
TF324904	9765	ENSG00000039319
TF329332	79567	ENSG00000039523
TF330498	729	ENSG00000039537
TF331274	26064	ENSG00000039560
TF315803	54831	ENSG00000039987
TF315993	23035	ENSG00000040199
TF350296	27067	ENSG00000040341
TF313694	54896	ENSG00000040487
TF330080	65078	ENSG00000040608
TF331373	79142	ENSG00000040633
TF329887	1008	ENSG00000040731
TF325637	3631	ENSG00000040933
TF312895	5874	ENSG00000041353
TF329915	3371	ENSG00000041982
TF330127	90326	ENSG00000041988
TF329332	140876	ENSG00000042062
TF328375	54884	ENSG00000042445
TF313468	822	ENSG00000042493
TF314733	10863	ENSG00000042980
TF350735	8538	ENSG00000043039
TF313332	54165	ENSG00000043093
TF316118	23338	ENSG00000043143
TF351425	7546	ENSG00000043355

TF326567	3937	ENSG00000043462
TF316350	153	ENSG00000043591
TF314740	55361	ENSG00000043822
TF313686	1495	ENSG00000044115
TF313970	5256	ENSG00000044446
TF315608	2042	ENSG00000044524
TF315162	2824	ENSG00000046653
TF328639	80243	ENSG00000046889
TF300379	56474	ENSG00000047230
TF325496	56204	ENSG00000047346
TF105769	116984	ENSG00000047365
TF329807	1356	ENSG00000047457
TF332997	84062	ENSG00000047579
TF331465	7504	ENSG00000047597
TF314265	57101	ENSG00000047617
TF325918	6322	ENSG00000047634
TF324040	55841	ENSG00000047644
TF316710	395	ENSG00000047648
TF316006	27146	ENSG00000047662
TF316358	4134	ENSG00000047849
TF316358	729797	ENSG00000047849
TF316358	732227	ENSG00000047849
TF329035	57646	ENSG00000048028
TF106174	9734	ENSG00000048052
TF313002	26262	ENSG00000048140
TF324786	57545	ENSG00000048342
TF300465	50484	ENSG00000048392
TF351071	55885	ENSG00000048540
TF333971	2978	ENSG00000048545
TF314924	10659	ENSG00000048740
TF328642	23196	ENSG00000048828
TF315915	23518	ENSG00000048991
TF332900	1298	ENSG00000049089
TF313537	11174	ENSG00000049192
TF318445	8863	ENSG00000049246
TF313361	55040	ENSG00000049283
TF317514	4052	ENSG00000049323
TF314849	5954	ENSG00000049449
TF320364	57492	ENSG00000049618
TF320364	729446	ENSG00000049618
TF323658	23327	ENSG00000049759
TF313036	3074	ENSG00000049860
TF331442	63933	ENSG00000050393
TF313630	9498	ENSG00000050438
TF312903	10319	ENSG00000050555
TF324982	5733	ENSG00000050628
TF351138	79931	ENSG00000050730
TF105100	5601	ENSG00000050748
TF338175	91522	ENSG00000050767
TF328782	9564	ENSG00000050820
TF313941	84067	ENSG00000051009
TF324319	9709	ENSG00000051108
TF325627	9454	ENSG00000051128
TF102031	5291	ENSG00000051382
TF329090	54477	ENSG00000052126
TF351676	5652	ENSG00000052344
TF324337	80143	ENSG00000052723
TF350743	60529	ENSG00000052850
TF106409	23105	ENSG00000053108

TF105083	1112	ENSG00000053254
TF330614	728464	ENSG00000053328
TF330614	731200	ENSG00000053328
TF318080	23101	ENSG00000053524
TF333421	83714	ENSG00000053702
TF335359	3909	ENSG00000053747
TF315186	3784	ENSG00000053918
TF300091	27095	ENSG00000054116
TF335939	9967	ENSG00000054118
TF315158	29085	ENSG00000054148
TF332859	954	ENSG00000054179
TF316663	4065	ENSG00000054219
TF106427	51742	ENSG00000054267
TF324998	23596	ENSG00000054277
TF351976	5798	ENSG00000054356
TF105221	23095	ENSG00000054523
TF316127	2296	ENSG00000054598
TF314211	25771	ENSG00000054611
TF312866	57475	ENSG00000054690
TF300590	10079	ENSG00000054793
TF329591	140689	ENSG00000054803
TF106451	25884	ENSG00000054938
TF331128	23201	ENSG00000054965
TF332339	84957	ENSG00000054967
TF313130	3757	ENSG00000055118
TF324360	10827	ENSG00000055147
TF312925	26999	ENSG00000055163
TF332021	23118	ENSG00000055208
TF354317	58508	ENSG00000055609
TF354317	642678	ENSG00000055609
TF354317	645159	ENSG00000055609
TF317783	55283	ENSG00000055732
TF320243	114800	ENSG00000055813
TF318160	23369	ENSG00000055917
TF328982	3700	ENSG00000055955
TF328982	3697	ENSG00000055957
TF320194	728303	ENSG00000056097
TF320194	730732	ENSG00000056097
TF320194	51663	ENSG00000056097
TF331707	55609	ENSG00000056277
TF315303	10886	ENSG00000056291
TF105392	3690	ENSG00000056345
TF331518	112885	ENSG00000056487
TF321154	7185	ENSG00000056558
TF317698	54542	ENSG00000056586
TF324206	7703	ENSG00000056661
TF329644	55540	ENSG00000056736
TF312839	8908	ENSG00000056998
TF352619	6318	ENSG00000057149
TF321877	5318	ENSG00000057294
TF327329	2155	ENSG00000057593
TF300449	2665	ENSG00000057608
TF316545	639	ENSG00000057657
TF316292	57458	ENSG00000057704
TF106444	57504	ENSG00000057935
TF300576	8975	ENSG00000058056
TF326897	23200	ENSG00000058063
TF312903	3918	ENSG00000058085
TF106508	5218	ENSG00000058091

TF300348	29927	ENSG00000058262
TF105543	4659	ENSG00000058272
TF317296	5923	ENSG00000058335
TF315229	816	ENSG00000058404
TF101138	9696	ENSG00000058453
TF101138	652531	ENSG00000058453
TF300330	493	ENSG00000058668
TF313536	54432	ENSG00000058799
TF313104	1608	ENSG00000058866
TF314982	64718	ENSG00000059145
TF105087	6916	ENSG00000059377
TF338389	64761	ENSG00000059378
TF315654	4084	ENSG00000059728
TF106508	5128	ENSG00000059758
TF313762	6515	ENSG00000059804
TF319755	5662	ENSG00000059915
TF317306	8531	ENSG00000060138
TF315519	65125	ENSG00000060237
TF331377	11054	ENSG00000060491
TF316079	84699	ENSG00000060566
TF312900	10076	ENSG00000060656
TF316230	23504	ENSG00000060709
TF323987	1301	ENSG00000060718
TF333141	79832	ENSG00000060749
TF332308	30	ENSG00000060971
TF300882	586	ENSG00000060982
TF106174	51564	ENSG00000061273
TF331420	11178	ENSG00000061337
TF105310	7478	ENSG00000061492
TF323915	6676	ENSG00000061656
TF313683	10787	ENSG00000061676
TF314186	416	ENSG00000062096
TF314707	84649	ENSG00000062282
TF351636	4058	ENSG00000062524
TF312966	63916	ENSG00000062598
TF330037	124925	ENSG00000063015
TF352709	28968	ENSG00000063127
TF335495	29998	ENSG00000063169
TF354296	56848	ENSG00000063176
TF352926	770	ENSG00000063180
TF314111	11338	ENSG00000063244
TF313361	29924	ENSG00000063245
TF352074	10016	ENSG00000063438
TF352074	57491	ENSG00000063438
TF351613	2928	ENSG00000063515
TF315197	8776	ENSG00000063601
TF105317	2817	ENSG00000063660
TF105273	3029	ENSG00000063854
TF328387	6047	ENSG00000063978
TF328387	644006	ENSG00000063978
TF328387	650020	ENSG00000063978
TF102023	841	ENSG00000064012
TF332155	22998	ENSG00000064042
TF350606	1747	ENSG00000064195
TF332959	53340	ENSG00000064199
TF326070	8839	ENSG00000064205
TF354325	10402	ENSG00000064225
TF354251	9914	ENSG00000064270
TF106466	4804	ENSG00000064300

TF332268	50937	ENSG00000064309
TF105417	28996	ENSG00000064393
TF105417	653052	ENSG00000064393
TF314067	4207	ENSG00000064489
TF314067	729991	ENSG00000064489
TF314067	731041	ENSG00000064489
TF333112	8625	ENSG00000064490
TF314570	54929	ENSG00000064545
TF330052	9170	ENSG00000064547
TF323769	5476	ENSG00000064601
TF313191	6558	ENSG00000064651
TF332414	28966	ENSG00000064652
TF319337	2139	ENSG00000064655
TF313921	1265	ENSG00000064666
TF105191	10347	ENSG00000064687
TF106482	53339	ENSG00000064726
TF313011	55711	ENSG00000064763
TF315610	1117	ENSG00000064886
TF313526	22904	ENSG00000064932
TF106440	10362	ENSG00000064961
TF315710	10203	ENSG00000064989
TF320582	23294	ENSG00000064999
TF333498	7629	ENSG00000065029
TF314874	54887	ENSG00000065060
TF300673	2773	ENSG00000065135
TF102005	5586	ENSG00000065243
TF314319	9697	ENSG00000065308
TF352481	9423	ENSG00000065320
TF315710	9340	ENSG00000065325
TF313104	1606	ENSG00000065357
TF106002	2065	ENSG00000065361
TF105421	54763	ENSG00000065371
TF312824	91526	ENSG00000065413
TF314211	55633	ENSG00000065491
TF351123	4638	ENSG00000065534
TF314861	9892	ENSG00000065609
TF351445	9748	ENSG00000065613
TF300348	55176	ENSG00000065665
TF102004	5588	ENSG00000065675
TF314167	7089	ENSG00000065717
TF331945	51665	ENSG00000065802
TF325943	83641	ENSG00000065809
TF300537	4199	ENSG00000065833
TF317184	23216	ENSG00000065882
TF101060	8621	ENSG00000065883
TF323998	10797	ENSG00000065911
TF318755	84679	ENSG00000065923
TF333250	55810	ENSG00000065970
TF317306	4904	ENSG00000065978
TF314638	5141	ENSG00000065989
TF105556	5525	ENSG00000066027
TF313686	1496	ENSG00000066032
TF313377	1994	ENSG00000066044
TF317568	7075	ENSG00000066056
TF312871	57609	ENSG00000066084
TF106486	6602	ENSG00000066117
TF106449	9682	ENSG00000066135
TF317212	6550	ENSG00000066230
TF316357	25791	ENSG00000066248

TF323454	64834	ENSG00000066322
TF352494	6688	ENSG00000066336
TF314305	744	ENSG00000066382
TF316307	2263	ENSG00000066468
TF317832	2009	ENSG00000066629
TF330868	91283	ENSG00000066697
TF330868	8577	ENSG00000066697
TF105235	26153	ENSG00000066735
TF313482	55102	ENSG00000066739
TF300714	10565	ENSG00000066777
TF331404	9650	ENSG00000066855
TF314604	10734	ENSG00000066923
TF316834	4649	ENSG00000066933
TF300411	5214	ENSG00000067057
TF315506	1316	ENSG00000067082
TF316040	8611	ENSG00000067113
TF321506	4756	ENSG00000067141
TF314319	23471	ENSG00000067167
TF313970	5255	ENSG00000067177
TF313970	646780	ENSG00000067177
TF333916	7132	ENSG00000067182
TF316195	782	ENSG00000067191
TF317184	7813	ENSG00000067208
TF300390	5315	ENSG00000067225
TF300837	387	ENSG00000067560
TF300509	1659	ENSG00000067596
TF102004	5590	ENSG00000067606
TF335557	7544	ENSG00000067646
TF315600	6857	ENSG00000067715
TF329881	652725	ENSG00000067798
TF329881	89795	ENSG00000067798
TF315909	57595	ENSG00000067840
TF300330	492	ENSG00000067842
TF313551	6093	ENSG00000067900
TF313551	727758	ENSG00000067900
TF313551	732432	ENSG00000067900
TF314918	5165	ENSG00000067992
TF321598	8692	ENSG00000068001
TF106174	9759	ENSG00000068024
TF316307	2261	ENSG00000068078
TF332752	3430	ENSG00000068079
TF327852	79990	ENSG00000068137
TF314067	4205	ENSG00000068305
TF317174	7030	ENSG00000068323
TF314012	2182	ENSG00000068366
TF314246	3632	ENSG00000068383
TF314177	65055	ENSG00000068615
TF326897	23250	ENSG00000068650
TF330843	9741	ENSG00000068697
TF313783	57217	ENSG00000068724
TF312925	23191	ENSG00000068793
TF105222	3796	ENSG00000068796
TF312918	10235	ENSG00000068831
TF106237	23198	ENSG00000068878
TF105556	5526	ENSG00000068971
TF300309	5837	ENSG00000068976
TF351940	5307	ENSG00000069011
TF313147	57113	ENSG00000069018
TF313149	375449	ENSG00000069020

TF316380	221395	ENSG00000069122
TF316846	54549	ENSG00000069188
TF314733	8756	ENSG00000069206
TF332191	64710	ENSG00000069275
TF324563	8514	ENSG00000069424
TF105201	10060	ENSG00000069431
TF300280	139341	ENSG00000069509
TF313314	4129	ENSG00000069535
TF334382	1815	ENSG00000069696
TF337375	7049	ENSG00000069702
TF319283	8399	ENSG00000069764
TF314618	483	ENSG00000069849
TF323658	4734	ENSG00000069869
TF105098	5597	ENSG00000069956
TF312895	5873	ENSG00000069974
TF313681	27440	ENSG00000069998
TF315253	4040	ENSG00000070018
TF332183	57661	ENSG00000070047
TF323218	4925	ENSG00000070081
TF331744	5217	ENSG00000070087
TF315900	5774	ENSG00000070159
TF313446	6710	ENSG00000070182
TF317805	2255	ENSG00000070193
TF313325	23446	ENSG00000070214
TF327119	23293	ENSG00000070366
TF300059	8218	ENSG00000070371
TF317805	27006	ENSG00000070388
TF106409	10272	ENSG00000070404
TF317985	55658	ENSG00000070423
TF314879	55062	ENSG00000070540
TF316497	22844	ENSG00000070601
TF313193	3340	ENSG00000070614
TF315187	10947	ENSG00000070718
TF318250	1258	ENSG00000070729
TF300458	26986	ENSG00000070756
TF300458	652607	ENSG00000070756
TF318014	10420	ENSG00000070759
TF300483	1459	ENSG00000070770
TF315900	11099	ENSG00000070778
TF315229	815	ENSG00000070808
TF101109	998	ENSG00000070831
TF101109	643751	ENSG00000070831
TF320922	26031	ENSG00000070882
TF315608	2046	ENSG00000070886
TF313191	6559	ENSG00000070915
TF300330	490	ENSG00000070961
TF314204	29850	ENSG00000070985
TF351631	8440	ENSG00000071051
TF351631	729030	ENSG00000071051
TF105138	9448	ENSG00000071054
TF324570	11320	ENSG00000071073
TF335157	54860	ENSG00000071203
TF316851	79658	ENSG00000071205
TF313438	6196	ENSG00000071242
TF329370	22846	ENSG00000071246
TF313265	29995	ENSG00000071282
TF315257	6400	ENSG00000071537
TF325819	537	ENSG00000071553
TF321672	6929	ENSG00000071564

TF329785	28951	ENSG00000071575
TF325032	53615	ENSG00000071655
TF353414	60343	ENSG00000071889
TF326512	140469	ENSG00000071909
TF329887	28513	ENSG00000071991
TF352709	55117	ENSG00000072041
TF105429	51109	ENSG00000072042
TF313399	5566	ENSG00000072062
TF313399	730418	ENSG00000072062
TF351999	22859	ENSG00000072071
TF352676	87	ENSG00000072110
TF313438	27330	ENSG00000072133
TF313361	22905	ENSG00000072134
TF351977	26469	ENSG00000072135
TF314113	55679	ENSG00000072163
TF331962	10290	ENSG00000072195
TF331962	729871	ENSG00000072195
TF330709	84708	ENSG00000072201
TF314264	224	ENSG00000072210
TF312981	7037	ENSG00000072274
TF313894	6720	ENSG00000072310
TF313147	7224	ENSG00000072315
TF326216	27125	ENSG00000072364
TF101108	7321	ENSG00000072401
TF323347	9886	ENSG00000072422
TF101156	8243	ENSG00000072501
TF315213	2011	ENSG00000072518
TF330957	55743	ENSG00000072609
TF313393	8974	ENSG00000072682
TF335097	2213	ENSG00000072694
TF335097	9103	ENSG00000072694
TF326480	4775	ENSG00000072736
TF351445	6793	ENSG00000072786
TF105679	23291	ENSG00000072803
TF318315	9744	ENSG00000072818
TF314706	1400	ENSG00000072832
TF314715	51009	ENSG00000072849
TF313076	54847	ENSG00000072858
TF325693	54820	ENSG00000072864
TF331789	10335	ENSG00000072952
TF313483	79041	ENSG00000072954
TF300393	8907	ENSG00000072958
TF331051	5817	ENSG00000073008
TF326608	8517	ENSG00000073009
TF333142	56666	ENSG00000073150
TF106101	8626	ENSG00000073282
TF316085	80216	ENSG00000073331
TF314585	3993	ENSG00000073350
TF314585	652838	ENSG00000073350
TF314638	5151	ENSG00000073417
TF313867	1183	ENSG00000073464
TF106476	5927	ENSG00000073614
TF314733	4185	ENSG00000073670
TF105554	5523	ENSG00000073711
TF314677	10979	ENSG00000073712
TF105193	8647	ENSG00000073734
TF325617	10170	ENSG00000073737
TF329295	922	ENSG00000073754
TF329675	5743	ENSG00000073756

TF320229	10644	ENSG00000073792
TF105119	9175	ENSG00000073803
TF323961	6480	ENSG00000073849
TF106341	30009	ENSG00000073861
TF313568	10129	ENSG00000073910
TF314861	8301	ENSG00000073921
TF350216	2736	ENSG00000074047
TF101155	23332	ENSG00000074054
TF351641	4854	ENSG00000074181
TF105553	5522	ENSG00000074211
TF313443	8463	ENSG00000074219
TF332908	54825	ENSG00000074276
TF332776	6620	ENSG00000074317
TF332776	652730	ENSG00000074317
TF312917	7251	ENSG00000074319
TF300651	489	ENSG00000074370
TF316425	771	ENSG00000074410
TF312903	59277	ENSG00000074527
TF324572	9891	ENSG00000074590
TF324572	728670	ENSG00000074590
TF324572	732183	ENSG00000074590
TF313309	54878	ENSG00000074603
TF318759	9187	ENSG00000074621
TF329009	55205	ENSG00000074657
TF332598	8578	ENSG00000074660
TF313311	3998	ENSG00000074695
TF313326	51495	ENSG00000074696
TF313326	732402	ENSG00000074696
TF326495	26034	ENSG00000074706
TF105354	50508	ENSG00000074771
TF300391	2023	ENSG00000074800
TF313191	6557	ENSG00000074803
TF331430	55160	ENSG00000074964
TF315363	7294	ENSG00000074966
TF324060	9671	ENSG00000075035
TF315186	3785	ENSG00000075043
TF315303	6865	ENSG00000075073
TF314682	6717	ENSG00000075142
TF101527	8672	ENSG00000075151
TF316102	10371	ENSG00000075213
TF352628	10512	ENSG00000075223
TF323983	9620	ENSG00000075275
TF105310	7479	ENSG00000075290
TF333921	27332	ENSG00000075292
TF314720	55972	ENSG00000075303
TF313003	119	ENSG00000075340
TF317805	2249	ENSG00000075388
TF105303	9462	ENSG00000075391
TF315213	4140	ENSG00000075413
TF316401	64778	ENSG00000075420
TF326301	2355	ENSG00000075426
TF327980	27091	ENSG00000075429
TF327980	27092	ENSG00000075461
TF313568	285527	ENSG00000075539
TF321435	23505	ENSG00000075568
TF323992	6624	ENSG00000075618
TF300361	60	ENSG00000075624
TF300589	5337	ENSG00000075651
TF312838	479	ENSG00000075673

TF323254	284403	ENSG00000075702
TF323171	1739	ENSG00000075711
TF105605	7879	ENSG00000075785
TF315310	55973	ENSG00000075790
TF313842	25956	ENSG00000075826
TF329345	55843	ENSG00000075884
TF315397	5076	ENSG00000075891
TF315108	23609	ENSG00000075975
TF105646	4175	ENSG00000076003
TF323596	10179	ENSG00000076053
TF314644	5939	ENSG00000076067
TF329083	11176	ENSG00000076108
TF323502	25930	ENSG00000076201
TF351956	8786	ENSG00000076344
TF315701	113235	ENSG00000076351
TF312962	5362	ENSG00000076356
TF314176	88455	ENSG00000076513
TF317562	7163	ENSG00000076554
TF300061	32	ENSG00000076555
TF315266	22978	ENSG00000076685
TF330534	4162	ENSG00000076706
TF105317	2239	ENSG00000076716
TF321931	55796	ENSG00000076770
TF315529	57662	ENSG00000076826
TF318626	5909	ENSG00000076864
TF106495	9138	ENSG00000076928
TF313242	6813	ENSG00000076944
TF105395	27231	ENSG00000077009
TF313104	8527	ENSG00000077044
TF325130	83992	ENSG00000077063
TF312863	51412	ENSG00000077080
TF328382	5915	ENSG00000077092
TF105282	7155	ENSG00000077097
TF325632	4791	ENSG00000077150
TF105543	4660	ENSG00000077157
TF352179	23032	ENSG00000077254
TF105351	5063	ENSG00000077264
TF314748	827	ENSG00000077274
TF318770	1641	ENSG00000077279
TF300553	1781	ENSG00000077380
TF317511	54518	ENSG00000077420
TF318428	4034	ENSG00000077454
TF313644	143684	ENSG00000077458
TF352676	88	ENSG00000077522
TF329003	7107	ENSG00000077585
TF312981	10003	ENSG00000077616
TF316118	79960	ENSG00000077684
TF101128	7319	ENSG00000077721
TF316307	2260	ENSG00000077782
TF101156	27127	ENSG00000077935
TF317514	2192	ENSG00000077942
TF105391	8516	ENSG00000077943
TF319145	8530	ENSG00000077984
TF316358	4133	ENSG00000078018
TF323787	9063	ENSG00000078043
TF313542	273	ENSG00000078053
TF317006	369	ENSG00000078061
TF316339	27074	ENSG00000078081
TF319104	10529	ENSG00000078114

TF314076	7289	ENSG00000078246
TF354311	8871	ENSG00000078269
TF313845	108	ENSG00000078295
TF105556	5527	ENSG00000078304
TF315942	54715	ENSG00000078328
TF106149	2782	ENSG00000078369
TF317819	3205	ENSG00000078399
TF333184	1906	ENSG00000078401
TF316118	8028	ENSG00000078403
TF315710	117	ENSG00000078549
TF317805	26281	ENSG00000078579
TF350009	27334	ENSG00000078589
TF317770	9452	ENSG00000078596
TF315091	7419	ENSG00000078668
TF329702	57690	ENSG00000078687
TF106303	9139	ENSG00000078699
TF331600	1620	ENSG00000078725
TF323658	83737	ENSG00000078747
TF333017	58476	ENSG00000078804
TF314375	57644	ENSG00000078814
TF315617	80341	ENSG00000078898
TF106101	7161	ENSG00000078900
TF101108	51619	ENSG00000078967
TF333030	27098	ENSG00000079101
TF106303	862	ENSG00000079102
TF316817	1015	ENSG00000079112
TF105296	51661	ENSG00000079150
TF320922	114880	ENSG00000079156
TF315206	6507	ENSG00000079215
TF314050	8569	ENSG00000079277
TF315996	7145	ENSG00000079308
TF312890	56681	ENSG00000079332
TF312890	646260	ENSG00000079332
TF101053	8556	ENSG00000079335
TF313184	10411	ENSG00000079337
TF316289	29843	ENSG00000079387
TF105128	51207	ENSG00000079393
TF323955	5050	ENSG00000079462
TF316851	4983	ENSG00000079482
TF316381	55604	ENSG00000079691
TF300350	5236	ENSG00000079739
TF300362	1785	ENSG00000079805
TF351626	2037	ENSG00000079819
TF321703	22999	ENSG00000079841
TF315607	8417	ENSG00000079950
TF351926	5794	ENSG00000080031
TF315865	1638	ENSG00000080166
TF331078	131544	ENSG00000080200
TF315608	285220	ENSG00000080224
TF315710	6344	ENSG00000080293
TF321340	5991	ENSG00000080298
TF313630	8671	ENSG00000080493
TF300785	6595	ENSG00000080503
TF105451	50700	ENSG00000080511
TF314230	27244	ENSG00000080546
TF333654	11043	ENSG00000080561
TF323987	50509	ENSG00000080573
TF106424	10847	ENSG00000080603
TF317197	1361	ENSG00000080618

TF315605	1136	ENSG00000080644
TF315015	3781	ENSG00000080709
TF315040	5663	ENSG00000080815
TF300686	3320	ENSG00000080824
TF105568	5933	ENSG00000080839
TF321382	22839	ENSG00000080845
TF326157	3078	ENSG00000080910
TF323256	54665	ENSG00000081019
TF316816	260425	ENSG00000081026
TF333433	2920	ENSG00000081041
TF316865	1286	ENSG00000081052
TF318448	6932	ENSG00000081059
TF329887	1005	ENSG00000081138
TF331340	50939	ENSG00000081148
TF300034	384	ENSG00000081181
TF314067	4208	ENSG00000081189
TF312805	779	ENSG00000081248
TF321877	5317	ENSG00000081277
TF321877	652798	ENSG00000081277
TF314166	9262	ENSG00000081320
TF101053	8555	ENSG00000081377
TF315253	4036	ENSG00000081479
TF105356	4671	ENSG00000081770
TF105356	642009	ENSG00000081770
TF105356	643784	ENSG00000081770
TF105356	648984	ENSG00000081770
TF105356	651112	ENSG00000081770
TF105356	653406	ENSG00000081770
TF105356	728535	ENSG00000081770
TF312913	6561	ENSG00000081800
TF312963	93664	ENSG00000081803
TF332299	56131	ENSG00000081818
TF332299	56139	ENSG00000081842
TF332299	56136	ENSG00000081842
TF332299	56135	ENSG00000081842
TF332299	56134	ENSG00000081842
TF332299	26025	ENSG00000081853
TF332299	5098	ENSG00000081853
TF332299	56098	ENSG00000081853
TF332299	56097	ENSG00000081853
TF315993	23239	ENSG00000081913
TF338122	3595	ENSG00000081985
TF106486	6604	ENSG00000082014
TF337003	2533	ENSG00000082074
TF314263	58538	ENSG00000082126
TF319817	55437	ENSG00000082146
TF319817	644462	ENSG00000082146
TF319817	650168	ENSG00000082146
TF324313	9689	ENSG00000082153
TF106510	5241	ENSG00000082175
TF300537	4200	ENSG00000082212
TF101014	905	ENSG00000082258
TF314837	57579	ENSG00000082269
TF351626	23136	ENSG00000082397
TF333490	22837	ENSG00000082438
TF323171	1741	ENSG00000082458
TF313947	3776	ENSG00000082482
TF321154	7188	ENSG00000082512
TF315737	4986	ENSG00000082556

TF326681	4779	ENSG00000082641
TF329951	54437	ENSG00000082684
TF101104	2932	ENSG00000082701
TF105392	3693	ENSG00000082781
TF324969	23085	ENSG00000082805
TF317486	11342	ENSG00000082996
TF314204	80036	ENSG00000083067
TF316855	23033	ENSG00000083097
TF106483	7994	ENSG00000083168
TF315661	79670	ENSG00000083223
TF324551	9706	ENSG00000083290
TF314132	79977	ENSG00000083307
TF300825	3842	ENSG00000083312
TF313826	5351	ENSG00000083444
TF328633	5026	ENSG00000083454
TF105391	3682	ENSG00000083457
TF106415	23047	ENSG00000083642
TF351924	1833	ENSG00000083782
TF318734	1540	ENSG00000083799
TF313430	10998	ENSG00000083807
TF316403	2195	ENSG00000083857
TF313876	64744	ENSG00000084070
TF319444	54434	ENSG00000084112
TF317274	334	ENSG00000084234
TF327203	57613	ENSG00000084444
TF317540	6579	ENSG00000084453
TF321348	79570	ENSG00000084628
TF332900	1307	ENSG00000084636
TF318595	200081	ENSG00000084652
TF332652	8648	ENSG00000084676
TF314098	22979	ENSG00000084710
TF105223	3797	ENSG00000084731
TF313620	22924	ENSG00000084764
TF352892	3732	ENSG00000085117
TF333317	63035	ENSG00000085185
TF313172	546	ENSG00000085224
TF313172	642995	ENSG00000085224
TF313172	652458	ENSG00000085224
TF313172	728849	ENSG00000085224
TF315309	2122	ENSG00000085276
TF313797	9522	ENSG00000085365
TF300655	5550	ENSG00000085377
TF314470	57590	ENSG00000085449
TF315610	5016	ENSG00000085465
TF313492	29957	ENSG00000085491
TF326128	57549	ENSG00000085552
TF105193	5243	ENSG00000085563
TF316419	8895	ENSG00000085719
TF105310	7481	ENSG00000085741
TF314133	23259	ENSG00000085788
TF313761	22996	ENSG00000085831
TF324293	2060	ENSG00000085832
TF105328	4258	ENSG00000085871
TF315541	55054	ENSG00000085978
TF313149	23139	ENSG00000086015
TF105151	3301	ENSG00000086061
TF312834	2683	ENSG00000086062
TF312940	363	ENSG00000086159
TF312981	2346	ENSG00000086205

TF106374	51314	ENSG00000086288
TF328581	54749	ENSG00000086289
TF332117	29887	ENSG00000086300
TF313811	22929	ENSG00000086475
TF332328	3049	ENSG00000086506
TF318394	80271	ENSG00000086544
TF316403	2196	ENSG00000086570
TF313000	10959	ENSG00000086598
TF314471	56605	ENSG00000086619
TF313612	54469	ENSG00000086666
TF325617	3294	ENSG00000086696
TF318595	55787	ENSG00000086712
TF313342	5475	ENSG00000086717
TF315197	8898	ENSG00000087053
TF320310	7205	ENSG00000087077
TF315470	43	ENSG00000087085
TF313885	2512	ENSG00000087086
TF315834	581	ENSG00000087088
TF315210	51701	ENSG00000087095
TF313537	9509	ENSG00000087116
TF315428	4313	ENSG00000087245
TF336054	4504	ENSG00000087250
TF300673	2775	ENSG00000087258
TF313003	118	ENSG00000087274
TF105922	79944	ENSG00000087299
TF320666	22795	ENSG00000087303
TF316048	64395	ENSG00000087338
TF328485	57542	ENSG00000087448
TF300673	2778	ENSG00000087460
TF352031	10059	ENSG00000087470
TF316316	116154	ENSG00000087495
TF313718	7022	ENSG00000087510
TF105331	6790	ENSG00000087586
TF328782	57091	ENSG00000087589
TF321340	5990	ENSG00000087903
TF323232	339175	ENSG00000087995
TF321745	6820	ENSG00000088002
TF315900	5775	ENSG00000088179
TF313654	8570	ENSG00000088247
TF300673	2767	ENSG00000088256
TF325156	55616	ENSG00000088280
TF329039	1789	ENSG00000088305
TF314379	28954	ENSG00000088320
TF351626	2036	ENSG00000088367
TF330897	6564	ENSG00000088386
TF313629	23348	ENSG00000088387
TF300423	1795	ENSG00000088538
TF332764	51161	ENSG00000088543
TF314044	79822	ENSG00000088756
TF105545	23368	ENSG00000088808
TF321873	8455	ENSG00000088812
TF318348	54498	ENSG00000088826
TF334827	6614	ENSG00000088827
TF105291	2280	ENSG00000088832
TF312973	55968	ENSG00000088833
TF313391	57593	ENSG00000088881
TF315592	56265	ENSG00000088882
TF331420	9762	ENSG00000088899
TF300264	8655	ENSG00000088986

TF313698	27131	ENSG00000089006
TF328633	5027	ENSG00000089041
TF313272	9962	ENSG00000089057
TF329240	29058	ENSG00000089063
TF106480	84678	ENSG00000089094
TF315442	64211	ENSG00000089116
TF351844	22895	ENSG00000089169
TF105221	55614	ENSG00000089177
TF106341	6910	ENSG00000089225
TF324410	4842	ENSG00000089250
TF322599	2521	ENSG00000089280
TF327695	57655	ENSG00000089351
TF333443	5349	ENSG00000089356
TF329807	9843	ENSG00000089472
TF313294	29965	ENSG00000089486
TF313130	23415	ENSG00000089558
TF300337	23193	ENSG00000089597
TF351450	51291	ENSG00000089639
TF317561	8079	ENSG00000089693
TF314145	78990	ENSG00000089723
TF332229	7597	ENSG00000089775
TF314482	25977	ENSG00000089818
TF315892	393	ENSG00000089820
TF331274	170961	ENSG00000089847
TF105735	55760	ENSG00000089876
TF106450	23186	ENSG00000089902
TF331954	55668	ENSG00000089916
TF317514	8425	ENSG00000090006
TF317212	6548	ENSG00000090020
TF300842	10914	ENSG00000090060
TF318292	57060	ENSG00000090097
TF315837	5996	ENSG00000090104
TF313936	83719	ENSG00000090238
TF313387	29888	ENSG00000090372
TF328924	11213	ENSG00000090376
TF324882	4069	ENSG00000090382
TF325195	79594	ENSG00000090432
TF333729	26998	ENSG00000090512
TF320837	55214	ENSG00000090530
TF327221	9727	ENSG00000090565
TF300458	8761	ENSG00000090621
TF314319	79603	ENSG00000090661
TF317783	57192	ENSG00000090674
TF315495	1947	ENSG00000090776
TF329702	27327	ENSG00000090905
TF300299	8857	ENSG00000090920
TF300299	651441	ENSG00000090920
TF300299	652599	ENSG00000090920
TF328565	64857	ENSG00000090924
TF312967	57605	ENSG00000090975
TF314195	55763	ENSG00000090989
TF316413	5459	ENSG00000091010
TF312807	114882	ENSG00000091039
TF325526	113878	ENSG00000091073
TF321796	4897	ENSG00000091129
TF312903	3912	ENSG00000091136
TF313784	5172	ENSG00000091137
TF313784	1811	ENSG00000091138
TF313196	23335	ENSG00000091157

TF331549	3568	ENSG00000091181
TF105199	368	ENSG00000091262
TF317387	54918	ENSG00000091317
TF105391	3655	ENSG00000091409
TF313184	11069	ENSG00000091428
TF315257	23231	ENSG00000091490
TF324013	7018	ENSG00000091513
TF316834	51168	ENSG00000091536
TF334137	350	ENSG00000091583
TF312967	83394	ENSG00000091622
TF323288	79776	ENSG00000091656
TF313535	57084	ENSG00000091664
TF317197	1357	ENSG00000091704
TF323751	2099	ENSG00000091831
TF315837	26575	ENSG00000091844
TF334493	4345	ENSG00000091972
TF333630	1215	ENSG00000092009
TF106236	5720	ENSG00000092010
TF317210	84502	ENSG00000092051
TF314375	4625	ENSG00000092054
TF105008	1053	ENSG00000092067
TF313355	23428	ENSG00000092068
TF325426	55632	ENSG00000092140
TF330974	3183	ENSG00000092199
TF328883	57096	ENSG00000092200
TF106481	9878	ENSG00000092203
TF324278	7051	ENSG00000092295
TF324396	1618	ENSG00000092345
TF323190	90665	ENSG00000092377
TF316102	57556	ENSG00000092421
TF314204	54822	ENSG00000092439
TF317402	7301	ENSG00000092445
TF314748	825	ENSG00000092529
TF315125	8773	ENSG00000092531
TF106341	6913	ENSG00000092607
TF332900	1299	ENSG00000092758
TF313935	7430	ENSG00000092820
TF351553	4637	ENSG00000092841
TF101510	26523	ENSG00000092847
TF325195	117584	ENSG00000092871
TF315526	201294	ENSG00000092929
TF315284	79157	ENSG00000092931
TF314706	1808	ENSG00000092964
TF318514	7042	ENSG00000092969
TF331954	55105	ENSG00000092978
TF329140	1312	ENSG00000093010
TF323645	55350	ENSG00000093134
TF314109	9209	ENSG00000093167
TF105933	9117	ENSG00000093183
TF315453	2568	ENSG00000094755
TF332742	3881	ENSG00000094796
TF350503	23468	ENSG00000094916
TF320231	29911	ENSG00000095066
TF329555	120400	ENSG00000095110
TF319780	54566	ENSG00000095203
TF313483	55151	ENSG00000095209
TF329675	5742	ENSG00000095303
TF101106	23511	ENSG00000095319
TF313836	1384	ENSG00000095321

TF323756	10044	ENSG00000095370
TF317336	55357	ENSG00000095383
TF325033	25861	ENSG00000095397
TF316499	5146	ENSG00000095464
TF316499	730677	ENSG00000095464
TF316102	57715	ENSG00000095539
TF331860	64376	ENSG00000095574
TF326567	29760	ENSG00000095585
TF314351	7093	ENSG00000095587
TF105093	1592	ENSG00000095596
TF326896	56165	ENSG00000095627
TF320680	10580	ENSG00000095637
TF326512	53904	ENSG00000095777
TF328635	51322	ENSG00000095787
TF106464	1390	ENSG00000095794
TF351676	23430	ENSG00000095917
TF340686	284422	ENSG00000095932
TF331837	3096	ENSG00000095951
TF334441	54209	ENSG00000095970
TF313947	83795	ENSG00000095981
TF105294	2289	ENSG00000096060
TF105334	6732	ENSG00000096063
TF316118	27154	ENSG00000096070
TF314990	5225	ENSG00000096088
TF323345	28978	ENSG00000096092
TF334441	9436	ENSG00000096264
TF300686	3326	ENSG00000096384
TF300686	644816	ENSG00000096384
TF312815	3710	ENSG00000096433
TF331090	1832	ENSG00000096696
TF316157	3189	ENSG00000096746
TF327041	3717	ENSG00000096968
TF105081	25	ENSG00000097007
TF323458	84144	ENSG00000097096
TF314277	5125	ENSG00000099139
TF313000	11018	ENSG00000099203
TF318042	3983	ENSG00000099204
TF313448	22931	ENSG00000099246
TF330156	8829	ENSG00000099250
TF313367	56952	ENSG00000099256
TF105402	54873	ENSG00000099260
TF313002	23555	ENSG00000099282
TF332276	55506	ENSG00000099284
TF313149	23031	ENSG00000099308
TF316834	4650	ENSG00000099331
TF313947	9424	ENSG00000099337
TF338452	54620	ENSG00000099364
TF313763	112755	ENSG00000099365
TF106436	9739	ENSG00000099381
TF317441	9274	ENSG00000099385
TF315495	1943	ENSG00000099617
TF331130	255057	ENSG00000099625
TF320624	83259	ENSG00000099715
TF313406	4670	ENSG00000099783
TF319557	51257	ENSG00000099785
TF300908	9524	ENSG00000099797
TF101107	997	ENSG00000099804
TF318250	610	ENSG00000099822
TF318385	8045	ENSG00000099849

TF300196	4616	ENSG00000099860
TF105402	5064	ENSG00000099864
TF314050	2872	ENSG00000099875
TF324593	85358	ENSG00000099882
TF321877	421	ENSG00000099889
TF314569	27037	ENSG00000099899
TF354263	29801	ENSG00000099904
TF332598	91179	ENSG00000099910
TF343094	3053	ENSG00000099937
TF321436	1399	ENSG00000099942
TF328633	9127	ENSG00000099957
TF314715	91319	ENSG00000099958
TF313293	83874	ENSG00000099992
TF313608	2687	ENSG00000099998
TF316716	23384	ENSG00000100014
TF313936	29799	ENSG00000100027
TF105097	5594	ENSG00000100030
TF313608	2678	ENSG00000100031
TF317617	9647	ENSG00000100034
TF331401	1417	ENSG00000100053
TF331401	1415	ENSG00000100058
TF324207	4242	ENSG00000100060
TF351139	29775	ENSG00000100065
TF320843	91355	ENSG00000100068
TF313940	157	ENSG00000100077
TF324679	50487	ENSG00000100078
TF315551	3957	ENSG00000100079
TF318574	26088	ENSG00000100083
TF316514	23616	ENSG00000100092
TF316514	57026	ENSG00000100092
TF330037	23544	ENSG00000100095
TF315551	3956	ENSG00000100097
TF331319	113791	ENSG00000100100
TF331686	23598	ENSG00000100105
TF313608	91227	ENSG00000100121
TF313608	728226	ENSG00000100121
TF331401	1414	ENSG00000100122
TF330790	129138	ENSG00000100124
TF328311	85377	ENSG00000100139
TF313148	23761	ENSG00000100141
TF316183	6663	ENSG00000100146
TF313792	23539	ENSG00000100156
TF101078	55964	ENSG00000100167
TF352855	6523	ENSG00000100170
TF352855	6527	ENSG00000100191
TF314792	11015	ENSG00000100196
TF300332	10521	ENSG00000100201
TF331317	6942	ENSG00000100207
TF319837	7494	ENSG00000100219
TF313660	9929	ENSG00000100221
TF326626	9609	ENSG00000100228
TF317409	7078	ENSG00000100234
TF313227	9701	ENSG00000100239
TF318583	6305	ENSG00000100241
TF323915	25777	ENSG00000100242
TF314333	1727	ENSG00000100243
TF313985	26286	ENSG00000100262
TF313677	11252	ENSG00000100266
TF300318	162	ENSG00000100280

TF314105	10043	ENSG00000100284
TF330122	4744	ENSG00000100285
TF313549	1120	ENSG00000100288
TF314786	3162	ENSG00000100292
TF331356	164668	ENSG00000100298
TF342852	706	ENSG00000100300
TF316238	23551	ENSG00000100302
TF106456	23492	ENSG00000100307
TF319554	5155	ENSG00000100311
TF331025	164633	ENSG00000100314
TF300555	6122	ENSG00000100316
TF300555	653881	ENSG00000100316
TF315942	23543	ENSG00000100320
TF320995	9145	ENSG00000100321
TF315197	8897	ENSG00000100330
TF331032	54471	ENSG00000100335
TF334681	80832	ENSG00000100336
TF314272	150379	ENSG00000100341
TF334681	8542	ENSG00000100342
TF314272	80339	ENSG00000100344
TF333601	4627	ENSG00000100345
TF313555	8911	ENSG00000100346
TF354288	9402	ENSG00000100351
TF329702	23112	ENSG00000100354
TF332342	5816	ENSG00000100362
TF330850	4689	ENSG00000100365
TF324772	10478	ENSG00000100372
TF337874	3560	ENSG00000100385
TF101097	2033	ENSG00000100393
TF316498	83746	ENSG00000100395
TF329017	23264	ENSG00000100403
TF300874	5372	ENSG00000100417
TF328492	27351	ENSG00000100418
TF314514	64781	ENSG00000100422
TF316118	23774	ENSG00000100425
TF322818	9889	ENSG00000100426
TF313947	54207	ENSG00000100433
TF314196	63874	ENSG00000100439
TF315783	23351	ENSG00000100441
TF333630	1511	ENSG00000100448
TF333630	2999	ENSG00000100450
TF333630	3002	ENSG00000100453
TF320448	55147	ENSG00000100461
TF318242	1690	ENSG00000100473
TF317296	6655	ENSG00000100485
TF325139	51199	ENSG00000100503
TF300309	5836	ENSG00000100504
TF315216	114088	ENSG00000100505
TF300083	10175	ENSG00000100528
TF332246	26499	ENSG00000100558
TF329403	145497	ENSG00000100565
TF321667	283578	ENSG00000100580
TF313680	10598	ENSG00000100591
TF314602	23002	ENSG00000100592
TF331333	145501	ENSG00000100593
TF300452	9517	ENSG00000100596
TF331067	79890	ENSG00000100599
TF313403	5641	ENSG00000100600
TF329288	3705	ENSG00000100605

TF313590	5494	ENSG00000100614
TF315545	51804	ENSG00000100625
TF313267	57452	ENSG00000100626
TF314568	2079	ENSG00000100632
TF317772	3091	ENSG00000100644
TF332459	9766	ENSG00000100647
TF351335	6430	ENSG00000100650
TF343201	5267	ENSG00000100665
TF314308	6547	ENSG00000100678
TF329481	79038	ENSG00000100711
TF300623	4522	ENSG00000100714
TF313570	22990	ENSG00000100731
TF330024	623	ENSG00000100739
TF106473	7443	ENSG00000100749
TF316874	89932	ENSG00000100767
TF313438	9252	ENSG00000100784
TF315190	55671	ENSG00000100796
TF106223	5693	ENSG00000100804
TF106493	7528	ENSG00000100811
TF105537	90673	ENSG00000100829
TF105907	8106	ENSG00000100836
TF328782	10278	ENSG00000100842
TF324451	394	ENSG00000100852
TF316419	9362	ENSG00000100884
TF313572	57680	ENSG00000100888
TF314402	5106	ENSG00000100889
TF320166	4792	ENSG00000100906
TF313860	51016	ENSG00000100908
TF106236	5721	ENSG00000100911
TF323740	84312	ENSG00000100916
TF314269	29082	ENSG00000100931
TF300693	10484	ENSG00000100934
TF300378	51292	ENSG00000100938
TF326480	4776	ENSG00000100968
TF315617	5360	ENSG00000100979
TF315428	4318	ENSG00000100985
TF350743	30813	ENSG00000100987
TF300309	5834	ENSG00000100994
TF315122	26090	ENSG00000100997
TF325139	22981	ENSG00000101004
TF320906	10110	ENSG00000101049
TF326257	4605	ENSG00000101057
TF316148	140902	ENSG00000101074
TF352097	3172	ENSG00000101076
TF313168	57446	ENSG00000101079
TF354288	84174	ENSG00000101082
TF326480	4773	ENSG00000101096
TF315600	140730	ENSG00000101098
TF300458	80336	ENSG00000101104
TF354217	6789	ENSG00000101109
TF317003	57167	ENSG00000101115
TF328818	23394	ENSG00000101126
TF324994	55816	ENSG00000101134
TF316134	655	ENSG00000101144
TF317562	7165	ENSG00000101150
TF105164	80331	ENSG00000101152
TF313225	1522	ENSG00000101160
TF312873	51012	ENSG00000101166
TF312873	641796	ENSG00000101166

TF312873	646873	ENSG00000101166
TF351747	11255	ENSG00000101180
TF326170	4923	ENSG00000101188
TF350578	11083	ENSG00000101191
TF105356	79444	ENSG00000101197
TF321348	128414	ENSG00000101198
TF329914	57642	ENSG00000101203
TF315605	1137	ENSG00000101204
TF300304	1917	ENSG00000101210
TF314013	5753	ENSG00000101213
TF317090	26205	ENSG00000101216
TF323506	25876	ENSG00000101222
TF101056	994	ENSG00000101224
TF331333	140862	ENSG00000101230
TF315257	80343	ENSG00000101251
TF329785	57761	ENSG00000101255
TF319243	9770	ENSG00000101265
TF300483	1457	ENSG00000101266
TF314820	113278	ENSG00000101276
TF331799	343637	ENSG00000101282
TF313464	8760	ENSG00000101290
TF315303	128674	ENSG00000101292
TF332407	9751	ENSG00000101298
TF351123	85366	ENSG00000101306
TF300693	10483	ENSG00000101310
TF314677	55612	ENSG00000101311
TF316454	343702	ENSG00000101321
TF332620	5173	ENSG00000101327
TF328517	140706	ENSG00000101331
TF314218	10398	ENSG00000101335
TF351634	3055	ENSG00000101336
TF105352	57144	ENSG00000101349
TF105223	9371	ENSG00000101350
TF313620	22919	ENSG00000101367
TF351835	182	ENSG00000101384
TF317932	6640	ENSG00000101400
TF105566	1869	ENSG00000101412
TF320926	58490	ENSG00000101413
TF314269	128866	ENSG00000101421
TF315617	671	ENSG00000101425
TF319145	128821	ENSG00000101435
TF312818	140679	ENSG00000101438
TF319145	1471	ENSG00000101439
TF330729	434	ENSG00000101440
TF319145	1472	ENSG00000101441
TF316803	26051	ENSG00000101445
TF330777	81610	ENSG00000101447
TF312964	84557	ENSG00000101460
TF331357	79953	ENSG00000101463
TF318191	7125	ENSG00000101470
TF316475	57136	ENSG00000101474
TF314924	56853	ENSG00000101489
TF332241	9658	ENSG00000101493
TF329887	28316	ENSG00000101542
TF328818	22850	ENSG00000101544
TF317024	9218	ENSG00000101558
TF314095	9663	ENSG00000101577
TF331825	8736	ENSG00000101605
TF314218	10627	ENSG00000101608

TF323961	29906	ENSG00000101638
TF314923	4092	ENSG00000101665
TF324997	9388	ENSG00000101670
TF335359	284217	ENSG00000101680
TF331012	54941	ENSG00000101695
TF326440	23253	ENSG00000101745
TF325594	8715	ENSG00000101746
TF300212	286436	ENSG00000101812
TF326318	25878	ENSG00000101825
TF314847	115201	ENSG00000101844
TF314186	412	ENSG00000101846
TF323190	6907	ENSG00000101849
TF314562	10857	ENSG00000101856
TF333654	4281	ENSG00000101871
TF106338	2986	ENSG00000101890
TF314618	23439	ENSG00000101892
TF106366	5634	ENSG00000101911
TF351113	51311	ENSG00000101916
TF319778	56180	ENSG00000101928
TF106451	91851	ENSG00000101938
TF106452	6839	ENSG00000101945
TF336515	8406	ENSG00000101955
TF315453	2742	ENSG00000101958
TF105356	331	ENSG00000101966
TF314604	10735	ENSG00000101972
TF326897	286410	ENSG00000101974
TF318080	4168	ENSG00000101977
TF327329	2158	ENSG00000101981
TF105204	215	ENSG00000101986
TF105204	642762	ENSG00000101986
TF312805	778	ENSG00000102001
TF315804	6855	ENSG00000102003
TF317387	5355	ENSG00000102007
TF315363	660	ENSG00000102010
TF300680	5358	ENSG00000102024
TF318679	2000	ENSG00000102034
TF300674	6594	ENSG00000102038
TF315197	55613	ENSG00000102043
TF331945	140462	ENSG00000102048
TF105479	11230	ENSG00000102050
TF315783	340554	ENSG00000102053
TF106485	5931	ENSG00000102054
TF313103	3750	ENSG00000102057
TF324998	5956	ENSG00000102076
TF323211	9016	ENSG00000102078
TF316292	1527	ENSG00000102080
TF316292	653363	ENSG00000102080
TF316292	728447	ENSG00000102080
TF105427	2332	ENSG00000102081
TF106488	10389	ENSG00000102098
TF330156	6247	ENSG00000102104
TF323230	286526	ENSG00000102128
TF315391	2623	ENSG00000102145
TF314850	84061	ENSG00000102158
TF331693	9248	ENSG00000102195
TF316118	9767	ENSG00000102221
TF106508	5127	ENSG00000102225
TF106276	8237	ENSG00000102226
TF106336	9468	ENSG00000102230

TF331292	680	ENSG00000102239
TF326340	51442	ENSG00000102243
TF332169	959	ENSG00000102245
TF317409	7076	ENSG00000102265
TF315453	2564	ENSG00000102287
TF320624	27328	ENSG00000102290
TF320624	730420	ENSG00000102290
TF343077	2245	ENSG00000102302
TF328982	347365	ENSG00000102313
TF350556	11279	ENSG00000102349
TF336515	27286	ENSG00000102359
TF341184	94121	ENSG00000102362
TF316044	158866	ENSG00000102383
TF313044	54457	ENSG00000102387
TF312909	2717	ENSG00000102393
TF317067	80183	ENSG00000102445
TF317805	2259	ENSG00000102466
TF316350	3356	ENSG00000102468
TF324911	54602	ENSG00000102471
TF332331	10673	ENSG00000102524
TF316401	22862	ENSG00000102531
TF314910	81617	ENSG00000102547
TF315506	688	ENSG00000102554
TF105338	8428	ENSG00000102572
TF313175	54	ENSG00000102575
TF105162	5611	ENSG00000102580
TF300320	55757	ENSG00000102595
TF316105	8874	ENSG00000102606
TF317805	2254	ENSG00000102678
TF313538	6445	ENSG00000102683
TF314880	10166	ENSG00000102743
TF101178	3839	ENSG00000102753
TF325768	2321	ENSG00000102755
TF336312	28984	ENSG00000102760
TF313104	160851	ENSG00000102780
TF323170	84056	ENSG00000102781
TF323386	26512	ENSG00000102786
TF332296	730249	ENSG00000102794
TF332296	730803	ENSG00000102794
TF353029	79758	ENSG00000102796
TF328824	84935	ENSG00000102802
TF318837	8848	ENSG00000102804
TF315964	10562	ENSG00000102837
TF314969	23295	ENSG00000102858
TF330401	3299	ENSG00000102878
TF314280	11151	ENSG00000102879
TF105097	5595	ENSG00000102882
TF328545	79153	ENSG00000102886
TF312966	79767	ENSG00000102890
TF336054	84560	ENSG00000102891
TF314422	10204	ENSG00000102898
TF314422	729380	ENSG00000102898
TF314422	731535	ENSG00000102898
TF326480	10725	ENSG00000102908
TF315783	9683	ENSG00000102921
TF329591	869	ENSG00000102924
TF316174	51090	ENSG00000102934
TF331504	23090	ENSG00000102935
TF334888	6367	ENSG00000102962

TF334888	6361	ENSG00000102970
TF106430	10664	ENSG00000102974
TF312899	50855	ENSG00000102981
TF315428	4324	ENSG00000102996
TF314537	80777	ENSG00000103018
TF106373	4832	ENSG00000103024
TF313168	65009	ENSG00000103034
TF328787	55238	ENSG00000103042
TF332506	3038	ENSG00000103044
TF328678	55512	ENSG00000103056
TF313355	9057	ENSG00000103064
TF313258	23659	ENSG00000103066
TF316157	80004	ENSG00000103067
TF314665	22879	ENSG00000103111
TF315454	8312	ENSG00000103126
TF331029	54550	ENSG00000103154
TF313988	9717	ENSG00000103184
TF316148	83716	ENSG00000103196
TF106373	4833	ENSG00000103200
TF105199	4363	ENSG00000103222
TF351598	2294	ENSG00000103241
TF106273	64428	ENSG00000103245
TF105273	84264	ENSG00000103253
TF314984	65990	ENSG00000103254
TF313355	8140	ENSG00000103257
TF330918	79006	ENSG00000103260
TF313540	9028	ENSG00000103269
TF101122	7329	ENSG00000103275
TF332794	7783	ENSG00000103310
TF316085	29904	ENSG00000103319
TF322245	6650	ENSG00000103326
TF314295	9780	ENSG00000103335
TF351676	260429	ENSG00000103355
TF318574	23062	ENSG00000103365
TF312940	343	ENSG00000103375
TF106278	57478	ENSG00000103404
TF314786	3163	ENSG00000103415
TF105152	9093	ENSG00000103423
TF317003	6299	ENSG00000103449
TF106481	27324	ENSG00000103460
TF105568	5934	ENSG00000103479
TF315534	64131	ENSG00000103489
TF315534	283824	ENSG00000103489
TF328883	23322	ENSG00000103494
TF331686	4150	ENSG00000103495
TF337874	50615	ENSG00000103522
TF313462	79838	ENSG00000103534
TF343812	6530	ENSG00000103546
TF323183	9810	ENSG00000103549
TF313173	366	ENSG00000103569
TF314280	10391	ENSG00000103647
TF314013	1445	ENSG00000103653
TF314605	8120	ENSG00000103723
TF354286	23205	ENSG00000103740
TF321506	57722	ENSG00000103742
TF300099	8766	ENSG00000103769
TF332604	64927	ENSG00000103852
TF331083	80381	ENSG00000103855
TF316575	57214	ENSG00000103888

TF325627	9455	ENSG00000103942
TF314429	30844	ENSG00000103966
TF314452	25963	ENSG00000103978
TF300654	79895	ENSG00000104043
TF324278	9333	ENSG00000104055
TF332736	23359	ENSG00000104059
TF326036	2553	ENSG00000104064
TF315957	7082	ENSG00000104067
TF312896	23312	ENSG00000104093
TF321839	171177	ENSG00000104140
TF313406	50804	ENSG00000104177
TF325768	5157	ENSG00000104213
TF316044	51201	ENSG00000104219
TF318770	6101	ENSG00000104237
TF316425	760	ENSG00000104267
TF317907	7976	ENSG00000104290
TF106506	8767	ENSG00000104312
TF319337	2138	ENSG00000104313
TF325083	793	ENSG00000104327
TF314300	54928	ENSG00000104331
TF350133	6422	ENSG00000104332
TF330843	55353	ENSG00000104341
TF330843	729544	ENSG00000104341
TF313214	79815	ENSG00000104361
TF324269	3551	ENSG00000104365
TF329901	5327	ENSG00000104368
TF317210	56704	ENSG00000104369
TF330916	27121	ENSG00000104371
TF354217	6788	ENSG00000104375
TF327072	54332	ENSG00000104381
TF316157	54845	ENSG00000104413
TF326070	8840	ENSG00000104415
TF313168	10397	ENSG00000104419
TF319585	51101	ENSG00000104427
TF326935	11075	ENSG00000104435
TF316742	55156	ENSG00000104442
TF300009	83988	ENSG00000104490
TF336893	63898	ENSG00000104611
TF318470	23516	ENSG00000104635
TF315197	66036	ENSG00000104643
TF313689	23484	ENSG00000104660
TF324168	203069	ENSG00000104679
TF333916	8797	ENSG00000104689
TF105551	5516	ENSG00000104695
TF330122	4741	ENSG00000104722
TF314850	7991	ENSG00000104723
TF330122	4747	ENSG00000104725
TF331430	9639	ENSG00000104728
TF323290	54758	ENSG00000104731
TF314733	2515	ENSG00000104755
TF315424	665	ENSG00000104765
TF314076	7288	ENSG00000104804
TF323218	4924	ENSG00000104805
TF300306	2997	ENSG00000104812
TF105121	11184	ENSG00000104814
TF332940	114336	ENSG00000104818
TF354318	3191	ENSG00000104824
TF320166	4793	ENSG00000104825
TF332940	3972	ENSG00000104826

TF332940	1082	ENSG00000104827
TF332940	93659	ENSG00000104827
TF332940	94115	ENSG00000104827
TF313103	3743	ENSG00000104848
TF325632	5971	ENSG00000104856
TF316850	64130	ENSG00000104863
TF314214	1158	ENSG00000104879
TF325887	23370	ENSG00000104880
TF313535	57030	ENSG00000104888
TF314010	147700	ENSG00000104892
TF352892	951	ENSG00000104894
TF315153	4066	ENSG00000104903
TF314741	4946	ENSG00000104904
TF313254	8677	ENSG00000104915
TF105337	1760	ENSG00000104936
TF314296	79735	ENSG00000104946
TF313314	259307	ENSG00000104951
TF314167	166	ENSG00000104964
TF316981	4858	ENSG00000104967
TF313092	6449	ENSG00000104969
TF338122	9466	ENSG00000104998
TF106429	55723	ENSG00000105011
TF313321	7138	ENSG00000105048
TF106473	51231	ENSG00000105053
TF313227	22870	ENSG00000105063
TF315964	93145	ENSG00000105088
TF105303	64926	ENSG00000105122
TF105407	10270	ENSG00000105127
TF314403	79852	ENSG00000105131
TF323458	85360	ENSG00000105137
TF102023	23581	ENSG00000105141
TF315206	6511	ENSG00000105143
TF105331	6795	ENSG00000105146
TF101005	898	ENSG00000105173
TF315551	29124	ENSG00000105198
TF314624	9149	ENSG00000105204
TF315551	1178	ENSG00000105205
TF313514	26065	ENSG00000105216
TF102004	208	ENSG00000105221
TF313378	23646	ENSG00000105223
TF350595	57716	ENSG00000105227
TF323787	51588	ENSG00000105229
TF314159	9253	ENSG00000105245
TF331210	10148	ENSG00000105246
TF325799	56961	ENSG00000105251
TF333654	79187	ENSG00000105255
TF326096	25999	ENSG00000105270
TF320194	23217	ENSG00000105278
TF315206	6510	ENSG00000105281
TF314320	25865	ENSG00000105287
TF315606	27134	ENSG00000105289
TF317274	333	ENSG00000105290
TF336272	26093	ENSG00000105321
TF317301	11100	ENSG00000105323
TF318514	7040	ENSG00000105329
TF328397	10226	ENSG00000105355
TF333601	79784	ENSG00000105357
TF332441	27181	ENSG00000105366
TF336032	973	ENSG00000105369

TF330587	3982	ENSG00000105370
TF330587	4818	ENSG00000105374
TF332441	945	ENSG00000105383
TF351179	1406	ENSG00000105392
TF327041	7297	ENSG00000105397
TF321745	6822	ENSG00000105398
TF101059	11140	ENSG00000105401
TF316547	8775	ENSG00000105402
TF312838	478	ENSG00000105409
TF318093	56917	ENSG00000105419
TF312900	5802	ENSG00000105426
TF330308	84518	ENSG00000105427
TF317486	148066	ENSG00000105428
TF314792	10945	ENSG00000105438
TF352091	9266	ENSG00000105443
TF314731	2906	ENSG00000105464
TF320995	23546	ENSG00000105467
TF330481	6320	ENSG00000105472
TF332441	946	ENSG00000105492
TF325228	8605	ENSG00000105499
TF332441	8778	ENSG00000105501
TF334804	56344	ENSG00000105507
TF332506	3036	ENSG00000105509
TF313199	9545	ENSG00000105514
TF315869	1628	ENSG00000105516
TF323924	828	ENSG00000105519
TF316040	64748	ENSG00000105520
TF330777	54854	ENSG00000105523
TF350641	54922	ENSG00000105538
TF335872	26291	ENSG00000105550
TF300882	587	ENSG00000105552
TF106453	54531	ENSG00000105556
TF329090	57664	ENSG00000105559
TF105552	5518	ENSG00000105568
TF327980	59284	ENSG00000105605
TF315506	10661	ENSG00000105610
TF314536	1777	ENSG00000105612
TF313149	22983	ENSG00000105613
TF327041	3640	ENSG00000105639
TF327041	3718	ENSG00000105639
TF315015	3780	ENSG00000105642
TF313650	27106	ENSG00000105643
TF102033	5296	ENSG00000105647
TF313199	5864	ENSG00000105649
TF314638	5143	ENSG00000105650
TF326161	8178	ENSG00000105656
TF321571	23373	ENSG00000105662
TF319820	9757	ENSG00000105663
TF324917	1311	ENSG00000105664
TF312838	495	ENSG00000105675
TF332441	4099	ENSG00000105695
TF331226	25789	ENSG00000105696
TF323338	7392	ENSG00000105698
TF330877	51599	ENSG00000105699
TF105295	23770	ENSG00000105701
TF351678	3249	ENSG00000105707
TF332097	6324	ENSG00000105711
TF314340	80714	ENSG00000105717
TF351065	2077	ENSG00000105722

TF101104	2931	ENSG00000105723
TF334668	2901	ENSG00000105737
TF318626	23094	ENSG00000105738
TF323904	154661	ENSG00000105784
TF105302	10156	ENSG00000105808
TF105302	401331	ENSG00000105808
TF105302	648426	ENSG00000105808
TF105302	652668	ENSG00000105808
TF101022	1021	ENSG00000105810
TF105032	9512	ENSG00000105819
TF315349	7980	ENSG00000105825
TF333530	10135	ENSG00000105835
TF102031	5294	ENSG00000105851
TF105392	3696	ENSG00000105855
TF350150	6671	ENSG00000105866
TF316836	8701	ENSG00000105877
TF316836	728563	ENSG00000105877
TF316836	732169	ENSG00000105877
TF350606	1749	ENSG00000105880
TF332031	256227	ENSG00000105889
TF332376	5764	ENSG00000105894
TF314263	51678	ENSG00000105926
TF352821	1687	ENSG00000105928
TF300346	50617	ENSG00000105929
TF338389	56829	ENSG00000105939
TF300695	4967	ENSG00000105953
TF324540	11033	ENSG00000105963
TF317174	22797	ENSG00000105967
TF354232	94239	ENSG00000105968
TF315736	858	ENSG00000105971
TF315736	857	ENSG00000105974
TF317402	4233	ENSG00000105976
TF313485	64327	ENSG00000105983
TF105310	7472	ENSG00000105989
TF317730	3198	ENSG00000105991
TF105142	10049	ENSG00000105993
TF317730	3199	ENSG00000105996
TF315938	3200	ENSG00000105997
TF324207	3955	ENSG00000106003
TF316310	3202	ENSG00000106004
TF316310	3203	ENSG00000106006
TF315710	7434	ENSG00000106018
TF314629	6742	ENSG00000106028
TF330813	3209	ENSG00000106031
TF315938	2128	ENSG00000106038
TF329501	8887	ENSG00000106052
TF319651	1124	ENSG00000106069
TF317511	2887	ENSG00000106070
TF333490	392529	ENSG00000106078
TF333490	23242	ENSG00000106078
TF105296	55033	ENSG00000106080
TF313763	6804	ENSG00000106089
TF352118	10392	ENSG00000106100
TF343504	2617	ENSG00000106105
TF315710	1395	ENSG00000106113
TF315608	2051	ENSG00000106123
TF312940	358	ENSG00000106125
TF315710	2692	ENSG00000106128
TF318060	51142	ENSG00000106153

TF318060	645317	ENSG00000106153
TF334888	6369	ENSG00000106178
TF105049	3315	ENSG00000106211
TF330208	4885	ENSG00000106236
TF324338	11333	ENSG00000106244
TF105087	1577	ENSG00000106258
TF106348	4521	ENSG00000106268
TF351978	5803	ENSG00000106278
TF316736	8976	ENSG00000106299
TF321598	23553	ENSG00000106302
TF321598	6677	ENSG00000106304
TF312981	7036	ENSG00000106327
TF323992	29999	ENSG00000106328
TF319778	64598	ENSG00000106330
TF320146	5078	ENSG00000106331
TF314351	5118	ENSG00000106333
TF300378	3614	ENSG00000106348
TF325357	3268	ENSG00000106351
TF352620	5054	ENSG00000106366
TF312921	1174	ENSG00000106367
TF314707	346606	ENSG00000106384
TF317293	56913	ENSG00000106392
TF313826	8985	ENSG00000106397
TF331936	24146	ENSG00000106404
TF333159	113263	ENSG00000106415
TF314218	93408	ENSG00000106436
TF328907	54664	ENSG00000106460
TF314509	2146	ENSG00000106462
TF317907	6424	ENSG00000106483
TF300644	653857	ENSG00000106526
TF350705	11281	ENSG00000106536
TF323367	27075	ENSG00000106537
TF321449	10551	ENSG00000106541
TF352074	196	ENSG00000106546
TF326279	54927	ENSG00000106554
TF330845	26157	ENSG00000106560
TF350216	2737	ENSG00000106571
TF335271	55665	ENSG00000106608
TF314986	6009	ENSG00000106615
TF313247	51422	ENSG00000106617
TF315592	165	ENSG00000106624
TF314218	58498	ENSG00000106631
TF314238	2645	ENSG00000106633
TF317441	9275	ENSG00000106635
TF313267	168391	ENSG00000106648
TF326096	7461	ENSG00000106665
TF318014	3984	ENSG00000106683
TF315206	6505	ENSG00000106688
TF315442	9355	ENSG00000106689
TF333654	83856	ENSG00000106701
TF321823	79937	ENSG00000106714
TF321823	727745	ENSG00000106714
TF332665	10927	ENSG00000106723
TF105395	54981	ENSG00000106733
TF312903	1955	ENSG00000106780
TF314280	7464	ENSG00000106789
TF314724	7046	ENSG00000106799
TF313285	727	ENSG00000106804
TF351924	4969	ENSG00000106809

TF334562	54829	ENSG00000106819
TF314167	7091	ENSG00000106829
TF315442	26468	ENSG00000106852
TF300362	1759	ENSG00000106976
TF337375	2022	ENSG00000106991
TF313747	203	ENSG00000106992
TF101059	55664	ENSG00000106993
TF333404	6019	ENSG00000107014
TF333404	6013	ENSG00000107018
TF106449	23081	ENSG00000107077
TF313629	81704	ENSG00000107099
TF324499	23189	ENSG00000107104
TF313377	1993	ENSG00000107105
TF318014	7016	ENSG00000107140
TF314283	57582	ENSG00000107147
TF316425	768	ENSG00000107159
TF313654	8939	ENSG00000107164
TF315865	7306	ENSG00000107165
TF316079	10488	ENSG00000107175
TF330709	8777	ENSG00000107186
TF315442	8022	ENSG00000107187
TF330258	23586	ENSG00000107201
TF319618	8395	ENSG00000107242
TF350216	169792	ENSG00000107249
TF317296	2889	ENSG00000107263
TF315245	320	ENSG00000107282
TF313281	6456	ENSG00000107295
TF336103	5730	ENSG00000107317
TF105191	20	ENSG00000107331
TF325799	6461	ENSG00000107338
TF101107	54926	ENSG00000107341
TF314365	51104	ENSG00000107362
TF313612	7763	ENSG00000107372
TF318198	1855	ENSG00000107404
TF318198	652788	ENSG00000107404
TF106408	9124	ENSG00000107438
TF101009	54619	ENSG00000107443
TF103012	1791	ENSG00000107447
TF315391	2625	ENSG00000107485
TF321873	26033	ENSG00000107518
TF319243	83937	ENSG00000107551
TF330015	23268	ENSG00000107554
TF326172	22841	ENSG00000107560
TF313059	10613	ENSG00000107566
TF314351	8029	ENSG00000107611
TF316134	2662	ENSG00000107623
TF105100	5599	ENSG00000107643
TF314133	11196	ENSG00000107651
TF329516	59338	ENSG00000107679
TF316767	219699	ENSG00000107731
TF317779	9806	ENSG00000107742
TF105557	5532	ENSG00000107758
TF331021	54462	ENSG00000107771
TF314724	657	ENSG00000107779
TF300361	59	ENSG00000107796
TF325347	3195	ENSG00000107807
TF331420	84445	ENSG00000107816
TF313205	81855	ENSG00000107819
TF331645	81621	ENSG00000107821

TF331233	2253	ENSG00000107831
TF327704	10360	ENSG00000107833
TF326036	80351	ENSG00000107854
TF351940	5309	ENSG00000107859
TF329345	57584	ENSG00000107863
TF317658	22849	ENSG00000107864
TF319446	91452	ENSG00000107897
TF321960	23185	ENSG00000107929
TF314368	9148	ENSG00000107954
TF329347	9644	ENSG00000107957
TF330916	22943	ENSG00000107984
TF313391	253738	ENSG00000108001
TF324918	114815	ENSG00000108018
TF314464	219771	ENSG00000108100
TF316952	57178	ENSG00000108175
TF312801	10105	ENSG00000108179
TF313002	81619	ENSG00000108219
TF333155	9211	ENSG00000108231
TF313318	23232	ENSG00000108239
TF332742	25984	ENSG00000108244
TF331401	1411	ENSG00000108255
TF332832	57532	ENSG00000108256
TF317762	28964	ENSG00000108262
TF324174	79154	ENSG00000108272
TF316118	4302	ENSG00000108292
TF323904	10900	ENSG00000108309
TF328989	7343	ENSG00000108312
TF313184	51195	ENSG00000108352
TF351956	8787	ENSG00000108370
TF317074	54894	ENSG00000108375
TF105310	7473	ENSG00000108379
TF328708	443	ENSG00000108381
TF101079	5414	ENSG00000108387
TF315197	9110	ENSG00000108389
TF328633	5023	ENSG00000108405
TF332742	8688	ENSG00000108417
TF329833	51174	ENSG00000108423
TF312906	201158	ENSG00000108442
TF313438	6198	ENSG00000108443
TF350503	10951	ENSG00000108468
TF101021	1018	ENSG00000108504
TF323452	23125	ENSG00000108509
TF316867	9969	ENSG00000108510
TF316310	3216	ENSG00000108511
TF300391	2027	ENSG00000108515
TF331744	5216	ENSG00000108518
TF317486	26001	ENSG00000108523
TF316238	51655	ENSG00000108551
TF315605	1145	ENSG00000108556
TF331317	10743	ENSG00000108557
TF343812	6532	ENSG00000108576
TF314264	218	ENSG00000108602
TF106486	6603	ENSG00000108604
TF320995	9144	ENSG00000108639
TF300332	1655	ENSG00000108654
TF352091	9267	ENSG00000108669
TF331368	3959	ENSG00000108679
TF330663	40	ENSG00000108684
TF334888	6354	ENSG00000108688

TF334888	6347	ENSG00000108691
TF334888	6355	ENSG00000108700
TF334888	6346	ENSG00000108702
TF320327	6928	ENSG00000108753
TF332742	3882	ENSG00000108759
TF330258	79132	ENSG00000108771
TF105399	2648	ENSG00000108773
TF300199	5878	ENSG00000108774
TF105451	3292	ENSG00000108786
TF321823	8506	ENSG00000108797
TF314303	51225	ENSG00000108798
TF314509	2145	ENSG00000108799
TF315720	1748	ENSG00000108813
TF105540	84687	ENSG00000108819
TF323987	1277	ENSG00000108821
TF314655	6442	ENSG00000108823
TF330887	8153	ENSG00000108830
TF105320	239	ENSG00000108839
TF106174	10014	ENSG00000108840
TF105199	8714	ENSG00000108846
TF332778	5539	ENSG00000108849
TF314263	4355	ENSG00000108852
TF323658	64750	ENSG00000108854
TF105128	1845	ENSG00000108861
TF331651	786	ENSG00000108878
TF315869	3131	ENSG00000108924
TF313792	9120	ENSG00000108932
TF314920	5573	ENSG00000108946
TF315495	1949	ENSG00000108947
TF313276	54757	ENSG00000108950
TF313370	23531	ENSG00000108960
TF313474	25979	ENSG00000109016
TF329216	26118	ENSG00000109046
TF300009	5957	ENSG00000109047
TF314375	4619	ENSG00000109061
TF350449	9368	ENSG00000109062
TF314375	4621	ENSG00000109063
TF332780	7448	ENSG00000109072
TF315649	7126	ENSG00000109079
TF326183	30850	ENSG00000109089
TF330414	5376	ENSG00000109099
TF329867	8456	ENSG00000109101
TF314474	9094	ENSG00000109103
TF314203	230	ENSG00000109107
TF326626	83871	ENSG00000109113
TF351612	8929	ENSG00000109132
TF315453	2557	ENSG00000109158
TF106499	2798	ENSG00000109163
TF331616	57606	ENSG00000109171
TF327106	54940	ENSG00000109180
TF315472	7365	ENSG00000109181
TF354270	23142	ENSG00000109184
TF314144	64854	ENSG00000109189
TF321745	6783	ENSG00000109193
TF314908	26511	ENSG00000109220
TF338319	10874	ENSG00000109255
TF333433	5197	ENSG00000109272
TF325632	4790	ENSG00000109320
TF332773	374	ENSG00000109321

TF332773	727738	ENSG00000109321
TF101108	7323	ENSG00000109332
TF105100	5602	ENSG00000109339
TF318679	644935	ENSG00000109381
TF318679	1998	ENSG00000109381
TF323211	7350	ENSG00000109424
TF313145	23158	ENSG00000109436
TF325637	8821	ENSG00000109452
TF329487	2549	ENSG00000109458
TF105452	11199	ENSG00000109511
TF105284	80273	ENSG00000109519
TF313867	1182	ENSG00000109572
TF331018	23321	ENSG00000109654
TF313762	56606	ENSG00000109667
TF101074	55294	ENSG00000109670
TF326567	116449	ENSG00000109684
TF329088	7468	ENSG00000109685
TF330850	152503	ENSG00000109686
TF313487	57620	ENSG00000109689
TF351204	579	ENSG00000109705
TF315453	2743	ENSG00000109738
TF332530	683	ENSG00000109743
TF313184	9693	ENSG00000109756
TF329901	3083	ENSG00000109758
TF350556	51274	ENSG00000109787
TF330725	25854	ENSG00000109794
TF105671	7358	ENSG00000109814
TF343068	10891	ENSG00000109819
TF314957	29118	ENSG00000109832
TF105049	1410	ENSG00000109846
TF350825	7704	ENSG00000109906
TF313721	23788	ENSG00000109919
TF300299	7007	ENSG00000109927
TF326804	56253	ENSG00000109943
TF313522	27087	ENSG00000109956
TF105042	3312	ENSG00000109971
TF328633	5024	ENSG00000109991
TF328633	732475	ENSG00000109991
TF329720	4013	ENSG00000110002
TF323964	29907	ENSG00000110025
TF314113	9404	ENSG00000110031
TF325526	23220	ENSG00000110042
TF313482	23130	ENSG00000110046
TF314429	10938	ENSG00000110047
TF354320	5007	ENSG00000110048
TF314905	81622	ENSG00000110057
TF314905	255620	ENSG00000110057
TF314905	402457	ENSG00000110057
TF314905	641922	ENSG00000110057
TF314905	650562	ENSG00000110057
TF106433	51111	ENSG00000110066
TF313227	55291	ENSG00000110075
TF321302	9379	ENSG00000110076
TF335157	64231	ENSG00000110077
TF335157	51338	ENSG00000110079
TF354325	6484	ENSG00000110080
TF313836	1374	ENSG00000110090
TF101004	595	ENSG00000110092
TF332238	79073	ENSG00000110108

TF315303	887	ENSG00000110148
TF331018	10612	ENSG00000110171
TF105394	26973	ENSG00000110172
TF333142	24145	ENSG00000110218
TF334458	116519	ENSG00000110243
TF334458	337	ENSG00000110244
TF313267	374378	ENSG00000110328
TF105356	329	ENSG00000110330
TF315428	4321	ENSG00000110347
TF300440	1656	ENSG00000110367
TF314210	867	ENSG00000110395
TF331051	5818	ENSG00000110400
TF105417	10114	ENSG00000110422
TF332690	25758	ENSG00000110427
TF315206	6506	ENSG00000110436
TF330897	51296	ENSG00000110446
TF329295	921	ENSG00000110448
TF332376	4192	ENSG00000110492
TF352895	975	ENSG00000110651
TF313645	54733	ENSG00000110660
TF323472	55531	ENSG00000110675
TF320471	55553	ENSG00000110693
TF312967	9600	ENSG00000110697
TF314507	9049	ENSG00000110711
TF300346	10312	ENSG00000110719
TF313549	1119	ENSG00000110721
TF331016	84867	ENSG00000110786
TF320837	27239	ENSG00000110811
TF320837	10536	ENSG00000110811
TF314207	8496	ENSG00000110841
TF318732	25766	ENSG00000110844
TF314280	23603	ENSG00000110880
TF330663	41	ENSG00000110881
TF313887	1610	ENSG00000110887
TF329471	65981	ENSG00000110888
TF352892	441631	ENSG00000110900
TF315649	83892	ENSG00000110906
TF315185	4891	ENSG00000110911
TF323969	81566	ENSG00000110925
TF313013	10645	ENSG00000110931
TF313542	51411	ENSG00000110934
TF315077	10728	ENSG00000110958
TF315077	284672	ENSG00000110958
TF315077	441050	ENSG00000110958
TF315600	341359	ENSG00000110975
TF317441	605	ENSG00000110987
TF105094	1594	ENSG00000111012
TF316344	4618	ENSG00000111046
TF316344	4617	ENSG00000111049
TF316850	8825	ENSG00000111052
TF332742	3875	ENSG00000111057
TF315996	23371	ENSG00000111077
TF350216	2735	ENSG00000111087
TF314700	57460	ENSG00000111110
TF317732	2004	ENSG00000111145
TF343812	6539	ENSG00000111181
TF105310	81029	ENSG00000111186
TF314711	59341	ENSG00000111199
TF300608	56341	ENSG00000111218

TF338389	57097	ENSG00000111224
TF317805	2251	ENSG00000111241
TF314218	4633	ENSG00000111245
TF318206	23316	ENSG00000111249
TF323184	10019	ENSG00000111252
TF313103	3736	ENSG00000111262
TF105122	80824	ENSG00000111266
TF300455	217	ENSG00000111275
TF101038	1027	ENSG00000111276
TF321410	55507	ENSG00000111291
TF331388	83445	ENSG00000111305
TF331157	4055	ENSG00000111321
TF101037	8099	ENSG00000111328
TF300137	55766	ENSG00000111332
TF330920	4256	ENSG00000111341
TF105302	8437	ENSG00000111344
TF105387	397	ENSG00000111348
TF328787	81539	ENSG00000111371
TF318030	79785	ENSG00000111404
TF319848	8909	ENSG00000111405
TF316304	7421	ENSG00000111424
TF317907	11211	ENSG00000111432
TF313763	2054	ENSG00000111450
TF351999	283383	ENSG00000111452
TF300262	22818	ENSG00000111481
TF300355	55832	ENSG00000111530
TF333253	55801	ENSG00000111536
TF300199	5869	ENSG00000111540
TF316430	11052	ENSG00000111605
TF106448	1108	ENSG00000111642
TF314874	23074	ENSG00000111647
TF352014	51147	ENSG00000111653
TF313270	56994	ENSG00000111666
TF300576	8078	ENSG00000111667
TF312822	84727	ENSG00000111671
TF300391	2026	ENSG00000111674
TF328554	1822	ENSG00000111676
TF351632	5777	ENSG00000111679
TF323990	51559	ENSG00000111696
TF317540	28234	ENSG00000111700
TF331356	339	ENSG00000111701
TF300267	51026	ENSG00000111711
TF300306	2998	ENSG00000111713
TF314963	3945	ENSG00000111716
TF313827	5564	ENSG00000111725
TF314757	29915	ENSG00000111727
TF323961	6489	ENSG00000111728
TF331356	57379	ENSG00000111732
TF105954	11021	ENSG00000111737
TF331299	1911	ENSG00000111752
TF331299	653441	ENSG00000111752
TF105064	1337	ENSG00000111775
TF321340	5992	ENSG00000111783
TF314907	55188	ENSG00000111785
TF106261	8683	ENSG00000111786
TF324337	26127	ENSG00000111790
TF329914	1303	ENSG00000111799
TF317532	10384	ENSG00000111801
TF334118	29940	ENSG00000111817

TF328769	4117	ENSG00000111837
TF323345	51522	ENSG00000111843
TF315534	2651	ENSG00000111846
TF328782	4739	ENSG00000111859
TF331041	387119	ENSG00000111860
TF318170	84830	ENSG00000111863
TF106429	25842	ENSG00000111875
TF316006	79632	ENSG00000111879
TF313420	4121	ENSG00000111885
TF315453	2570	ENSG00000111886
TF312881	57515	ENSG00000111897
TF317562	7164	ENSG00000111907
TF353069	135114	ENSG00000111911
TF313530	135112	ENSG00000111912
TF329332	9750	ENSG00000111913
TF350709	23328	ENSG00000111961
TF101170	26271	ENSG00000112029
TF313720	54516	ENSG00000112031
TF316304	5467	ENSG00000112033
TF315737	4988	ENSG00000112038
TF314076	7287	ENSG00000112041
TF105100	1432	ENSG00000112062
TF314450	6005	ENSG00000112077
TF314369	222658	ENSG00000112078
TF105337	11329	ENSG00000112079
TF106263	6428	ENSG00000112081
TF314701	3605	ENSG00000112115
TF314701	112744	ENSG00000112116
TF106459	4172	ENSG00000112118
TF330957	9025	ENSG00000112130
TF316316	221692	ENSG00000112137
TF330345	266727	ENSG00000112139
TF328769	22858	ENSG00000112144
TF315710	2740	ENSG00000112164
TF316134	653	ENSG00000112175
TF326681	60468	ENSG00000112182
TF314235	221662	ENSG00000112183
TF313791	10486	ENSG00000112186
TF342852	222642	ENSG00000112212
TF314113	9457	ENSG00000112214
TF314878	202559	ENSG00000112232
TF105566	1871	ENSG00000112242
TF313384	7803	ENSG00000112245
TF317772	6492	ENSG00000112246
TF105385	154150	ENSG00000112273
TF332900	1297	ENSG00000112280
TF315031	8936	ENSG00000112290
TF331078	202	ENSG00000112297
TF323645	8876	ENSG00000112299
TF323645	8875	ENSG00000112303
TF313876	60682	ENSG00000112305
TF313522	135152	ENSG00000112309
TF101171	51053	ENSG00000112312
TF319337	2070	ENSG00000112319
TF314980	8724	ENSG00000112335
TF313535	10246	ENSG00000112337
TF312855	64065	ENSG00000112378
TF313792	117247	ENSG00000112394
TF321769	57211	ENSG00000112414

TF316316	9749	ENSG00000112419
TF330966	1235	ENSG00000112486
TF334274	6892	ENSG00000112493
TF106420	5252	ENSG00000112511
TF313269	51596	ENSG00000112514
TF314878	9444	ENSG00000112531
TF332113	4188	ENSG00000112559
TF317174	7942	ENSG00000112561
TF320666	64094	ENSG00000112562
TF101004	896	ENSG00000112576
TF300102	6908	ENSG00000112592
TF333971	2979	ENSG00000112599
TF331684	5961	ENSG00000112619
TF335495	23506	ENSG00000112624
TF105556	5528	ENSG00000112640
TF106349	11165	ENSG00000112664
TF105126	56940	ENSG00000112679
TF105067	1347	ENSG00000112695
TF300873	55754	ENSG00000112697
TF350136	26054	ENSG00000112701
TF331340	3617	ENSG00000112706
TF319554	7422	ENSG00000112715
TF313950	2030	ENSG00000112759
TF326070	8838	ENSG00000112761
TF317532	11120	ENSG00000112763
TF335359	3910	ENSG00000112769
TF315239	55603	ENSG00000112773
TF315438	53405	ENSG00000112782
TF331929	57666	ENSG00000112787
TF330032	59084	ENSG00000112796
TF315280	4224	ENSG00000112818
TF106341	9096	ENSG00000112837
TF351429	55914	ENSG00000112851
TF332299	56133	ENSG00000112852
TF300652	23438	ENSG00000112855
TF313152	4124	ENSG00000112893
TF329951	9037	ENSG00000112902
TF330498	730	ENSG00000112936
TF313939	11044	ENSG00000112941
TF330851	2690	ENSG00000112964
TF105361	3157	ENSG00000112972
TF105232	10112	ENSG00000112984
TF300636	23530	ENSG00000112992
TF332773	1839	ENSG00000113070
TF313630	83697	ENSG00000113073
TF326061	4015	ENSG00000113083
TF333630	3003	ENSG00000113088
TF329887	1007	ENSG00000113100
TF314331	10307	ENSG00000113108
TF319356	6678	ENSG00000113140
TF315153	9421	ENSG00000113196
TF332299	56132	ENSG00000113205
TF332299	26167	ENSG00000113209
TF332299	56130	ENSG00000113211
TF332299	56129	ENSG00000113212
TF314638	8622	ENSG00000113231
TF101041	57396	ENSG00000113240
TF332299	56121	ENSG00000113248
TF336163	26762	ENSG00000113249

TF313240	2916	ENSG00000113262
TF315363	3702	ENSG00000113263
TF317486	55819	ENSG00000113269
TF314186	411	ENSG00000113273
TF324917	7060	ENSG00000113296
TF323175	57472	ENSG00000113300
TF317532	79908	ENSG00000113303
TF317532	653117	ENSG00000113303
TF317296	5924	ENSG00000113319
TF315453	2566	ENSG00000113327
TF101007	900	ENSG00000113328
TF103052	10622	ENSG00000113356
TF329887	1004	ENSG00000113361
TF101181	4001	ENSG00000113368
TF313650	57561	ENSG00000113369
TF314360	64083	ENSG00000113384
TF106339	4883	ENSG00000113389
TF315960	83989	ENSG00000113391
TF313430	28965	ENSG00000113396
TF300858	6897	ENSG00000113407
TF319371	50805	ENSG00000113430
TF300395	4012	ENSG00000113441
TF314638	5144	ENSG00000113448
TF330851	5618	ENSG00000113494
TF313657	10723	ENSG00000113504
TF352820	7903	ENSG00000113532
TF300841	10007	ENSG00000113552
TF105551	5515	ENSG00000113575
TF317805	2246	ENSG00000113578
TF106510	2908	ENSG00000113580
TF338122	3977	ENSG00000113594
TF330498	735	ENSG00000113600
TF324040	23286	ENSG00000113645
TF332276	9555	ENSG00000113648
TF314706	1809	ENSG00000113657
TF314923	4090	ENSG00000113658
TF354246	1452	ENSG00000113712
TF325768	5159	ENSG00000113721
TF351605	1044	ENSG00000113722
TF300290	8992	ENSG00000113732
TF324693	8614	ENSG00000113739
TF317658	80315	ENSG00000113742
TF316350	3274	ENSG00000113749
TF318935	1627	ENSG00000113758
TF350019	23567	ENSG00000113761
TF316767	90249	ENSG00000113763
TF351103	5067	ENSG00000113805
TF328380	58515	ENSG00000113811
TF333729	3273	ENSG00000113905
TF330912	604	ENSG00000113916
TF105466	84100	ENSG00000113966
TF331083	942	ENSG00000114013
TF333368	51421	ENSG00000114019
TF323771	26355	ENSG00000114023
TF354205	3836	ENSG00000114030
TF315189	7337	ENSG00000114062
TF316894	5948	ENSG00000114113
TF316894	5947	ENSG00000114115
TF314220	55186	ENSG00000114120

TF313940	131890	ENSG00000114124
TF314396	7029	ENSG00000114126
TF105399	8850	ENSG00000114166
TF315470	590	ENSG00000114200
TF105310	7474	ENSG00000114251
TF313541	5210	ENSG00000114268
TF317805	2257	ENSG00000114279
TF314920	5576	ENSG00000114302
TF351373	3280	ENSG00000114315
TF106276	7375	ENSG00000114316
TF318315	23527	ENSG00000114331
TF300673	2779	ENSG00000114349
TF300673	2771	ENSG00000114353
TF321598	3373	ENSG00000114378
TF323584	11068	ENSG00000114395
TF105427	8087	ENSG00000114416
TF314210	868	ENSG00000114423
TF106149	59345	ENSG00000114450
TF331083	11148	ENSG00000114455
TF329118	27136	ENSG00000114487
TF328984	23150	ENSG00000114541
TF313647	54946	ENSG00000114544
TF105421	152015	ENSG00000114547
TF312962	5361	ENSG00000114554
TF300811	523	ENSG00000114573
TF333564	50512	ENSG00000114631
TF314939	57088	ENSG00000114698
TF321368	1154	ENSG00000114737
TF312891	7867	ENSG00000114738
TF352876	93	ENSG00000114739
TF315044	51555	ENSG00000114757
TF314978	13	ENSG00000114771
TF314417	10289	ENSG00000114784
TF316357	26084	ENSG00000114790
TF351654	54800	ENSG00000114796
TF313216	23007	ENSG00000114805
TF315710	7433	ENSG00000114812
TF318191	7134	ENSG00000114854
TF318563	4820	ENSG00000114857
TF300522	1181	ENSG00000114859
TF326978	27086	ENSG00000114861
TF101527	1981	ENSG00000114867
TF313630	6508	ENSG00000114923
TF314733	8745	ENSG00000114948
TF324413	1716	ENSG00000114956
TF300789	55233	ENSG00000114978
TF313311	81562	ENSG00000114988
TF331476	6242	ENSG00000114993
TF334888	6364	ENSG00000115009
TF318560	30818	ENSG00000115041
TF300420	10120	ENSG00000115073
TF351629	7535	ENSG00000115085
TF300644	10096	ENSG00000115091
TF332031	55240	ENSG00000115107
TF319780	57669	ENSG00000115109
TF314132	29842	ENSG00000115112
TF315007	10254	ENSG00000115145
TF316871	9381	ENSG00000115155
TF316315	9595	ENSG00000115165

TF314724	90	ENSG00000115170
TF323159	85461	ENSG00000115183
TF313382	7781	ENSG00000115194
TF315519	29959	ENSG00000115216
TF105392	3694	ENSG00000115221
TF325415	64838	ENSG00000115226
TF105391	3676	ENSG00000115232
TF318398	9784	ENSG00000115234
TF314638	5136	ENSG00000115252
TF314913	92840	ENSG00000115255
TF314277	54760	ENSG00000115257
TF332333	2641	ENSG00000115263
TF106496	10297	ENSG00000115266
TF330258	64135	ENSG00000115267
TF314682	25801	ENSG00000115271
TF317511	2888	ENSG00000115290
TF326096	79745	ENSG00000115295
TF325347	3196	ENSG00000115297
TF313446	6711	ENSG00000115306
TF105431	57142	ENSG00000115310
TF323480	27429	ENSG00000115317
TF326061	84695	ENSG00000115318
TF324994	1796	ENSG00000115325
TF324994	730244	ENSG00000115325
TF324994	732407	ENSG00000115325
TF313267	2591	ENSG00000115339
TF315303	6869	ENSG00000115353
TF320231	55704	ENSG00000115355
TF352986	84141	ENSG00000115363
TF300068	10314	ENSG00000115365
TF317514	2202	ENSG00000115380
TF329915	2335	ENSG00000115414
TF318648	6772	ENSG00000115415
TF313359	2744	ENSG00000115419
TF300842	64895	ENSG00000115421
TF331211	3485	ENSG00000115457
TF331211	3488	ENSG00000115461
TF320736	80303	ENSG00000115468
TF313676	3769	ENSG00000115474
TF331063	4759	ENSG00000115488
TF105382	23301	ENSG00000115504
TF351179	5013	ENSG00000115507
TF314447	80219	ENSG00000115520
TF315179	79031	ENSG00000115539
TF324723	55818	ENSG00000115548
TF313216	84812	ENSG00000115556
TF325519	7850	ENSG00000115590
TF313247	53632	ENSG00000115592
TF106487	150572	ENSG00000115593
TF325519	3554	ENSG00000115594
TF325519	8808	ENSG00000115598
TF325519	9173	ENSG00000115602
TF325519	8809	ENSG00000115604
TF325519	8807	ENSG00000115607
TF317212	6549	ENSG00000115616
TF331599	79083	ENSG00000115648
TF314588	60482	ENSG00000115665
TF323767	3069	ENSG00000115677
TF105338	10494	ENSG00000115694

TF327329	5624	ENSG00000115718
TF326217	3398	ENSG00000115738
TF300009	3241	ENSG00000115756
TF300760	4953	ENSG00000115758
TF331787	55041	ENSG00000115762
TF313387	6801	ENSG00000115808
TF314320	23683	ENSG00000115825
TF315071	25797	ENSG00000115828
TF313209	8604	ENSG00000115840
TF315854	151393	ENSG00000115841
TF350606	1746	ENSG00000115844
TF320463	6382	ENSG00000115884
TF313216	5334	ENSG00000115896
TF315206	6509	ENSG00000115902
TF317296	6654	ENSG00000115904
TF332135	7456	ENSG00000115935
TF105067	9167	ENSG00000115944
TF332246	5341	ENSG00000115956
TF330887	390	ENSG00000115963
TF323952	1386	ENSG00000115966
TF317300	22848	ENSG00000115977
TF323495	66008	ENSG00000115993
TF312915	7072	ENSG00000116001
TF329001	51449	ENSG00000116005
TF315737	84634	ENSG00000116014
TF317772	2034	ENSG00000116016
TF320364	1820	ENSG00000116017
TF314731	116444	ENSG00000116032
TF319504	25806	ENSG00000116035
TF300313	525	ENSG00000116039
TF326681	4780	ENSG00000116044
TF315608	2043	ENSG00000116106
TF323729	117583	ENSG00000116117
TF331144	607	ENSG00000116128
TF351612	5396	ENSG00000116132
TF315213	4139	ENSG00000116141
TF329915	7143	ENSG00000116147
TF101011	81669	ENSG00000116148
TF101011	727877	ENSG00000116148
TF331942	2882	ENSG00000116157
TF351676	25823	ENSG00000116176
TF331636	60676	ENSG00000116183
TF352150	55103	ENSG00000116191
TF331226	9528	ENSG00000116209
TF106448	26038	ENSG00000116254
TF316749	5768	ENSG00000116260
TF313242	6814	ENSG00000116266
TF331373	148479	ENSG00000116273
TF315906	57535	ENSG00000116299
TF315737	4985	ENSG00000116329
TF351335	6429	ENSG00000116350
TF312886	51102	ENSG00000116353
TF352511	3749	ENSG00000116396
TF313014	5906	ENSG00000116473
TF106171	3065	ENSG00000116478
TF314822	829	ENSG00000116489
TF324777	127544	ENSG00000116514
TF313797	10067	ENSG00000116521
TF342569	55223	ENSG00000116525

TF106416	55870	ENSG00000116539
TF321382	58512	ENSG00000116544
TF315795	6421	ENSG00000116560
TF321839	58480	ENSG00000116574
TF325887	9181	ENSG00000116584
TF314067	4209	ENSG00000116604
TF313629	85440	ENSG00000116641
TF320527	26232	ENSG00000116661
TF320527	26270	ENSG00000116663
TF105165	9829	ENSG00000116675
TF106501	3953	ENSG00000116678
TF314289	9927	ENSG00000116688
TF332780	10216	ENSG00000116690
TF329087	4688	ENSG00000116701
TF315179	5132	ENSG00000116703
TF313307	23169	ENSG00000116704
TF332708	400735	ENSG00000116708
TF332708	645359	ENSG00000116708
TF300196	1647	ENSG00000116717
TF332708	65121	ENSG00000116721
TF332708	390999	ENSG00000116726
TF315837	5997	ENSG00000116741
TF314019	6121	ENSG00000116745
TF300439	270	ENSG00000116748
TF106266	9295	ENSG00000116754
TF326157	10878	ENSG00000116785
TF323570	10745	ENSG00000116793
TF313718	339488	ENSG00000116819
TF350737	2494	ENSG00000116833
TF105224	23046	ENSG00000116852
TF315146	252839	ENSG00000116857
TF332273	55700	ENSG00000116871
TF105789	127700	ENSG00000116885
TF313360	8443	ENSG00000116906
TF300659	64121	ENSG00000116954
TF320666	4811	ENSG00000116962
TF315551	3964	ENSG00000116977
TF329831	84618	ENSG00000116981
TF300009	51440	ENSG00000116983
TF316134	656	ENSG00000116985
TF106001	4610	ENSG00000116990
TF318626	57568	ENSG00000116991
TF332794	57829	ENSG00000116996
TF350813	6018	ENSG00000117000
TF315186	9132	ENSG00000117013
TF321703	9783	ENSG00000117016
TF102004	10000	ENSG00000117020
TF323961	81849	ENSG00000117069
TF351999	23266	ENSG00000117114
TF333418	4237	ENSG00000117122
TF106476	10765	ENSG00000117139
TF300611	6675	ENSG00000117143
TF315837	5999	ENSG00000117152
TF319283	26279	ENSG00000117215
TF331602	2635	ENSG00000117226
TF331602	2633	ENSG00000117228
TF106508	5129	ENSG00000117266
TF324491	8934	ENSG00000117280
TF313650	10628	ENSG00000117289

TF315192	1889	ENSG00000117298
TF105363	3155	ENSG00000117305
TF326217	3399	ENSG00000117318
TF334137	4179	ENSG00000117335
TF314362	51107	ENSG00000117362
TF320837	64175	ENSG00000117385
TF313762	6513	ENSG00000117394
TF336573	4352	ENSG00000117400
TF332366	9048	ENSG00000117407
TF314539	9670	ENSG00000117408
TF312834	8704	ENSG00000117411
TF106489	8643	ENSG00000117425
TF106492	10327	ENSG00000117448
TF105181	5052	ENSG00000117450
TF102033	8503	ENSG00000117461
TF352892	10103	ENSG00000117472
TF313684	10560	ENSG00000117479
TF314455	2166	ENSG00000117480
TF321304	387338	ENSG00000117481
TF313000	50999	ENSG00000117500
TF313921	1266	ENSG00000117519
TF328738	23215	ENSG00000117523
TF332169	356	ENSG00000117560
TF319824	58155	ENSG00000117569
TF329114	3290	ENSG00000117594
TF328512	3664	ENSG00000117595
TF316040	163404	ENSG00000117598
TF316040	9890	ENSG00000117600
TF313579	11123	ENSG00000117602
TF313041	25949	ENSG00000117614
TF106450	55758	ENSG00000117625
TF326935	3925	ENSG00000117632
TF331404	56181	ENSG00000117640
TF313420	57134	ENSG00000117643
TF313438	6195	ENSG00000117676
TF316638	5629	ENSG00000117707
TF320364	8289	ENSG00000117713
TF105242	6118	ENSG00000117748
TF315607	23673	ENSG00000117758
TF316807	54996	ENSG00000117791
TF352855	200010	ENSG00000117834
TF321449	51060	ENSG00000117862
TF324255	57488	ENSG00000117868
TF315605	1143	ENSG00000117971
TF300299	727897	ENSG00000117983
TF314990	1509	ENSG00000117984
TF330481	78989	ENSG00000118004
TF314604	10274	ENSG00000118007
TF319820	4297	ENSG00000118058
TF315428	4317	ENSG00000118113
TF334458	335	ENSG00000118137
TF106431	645730	ENSG00000118156
TF314308	6543	ENSG00000118160
TF314909	6230	ENSG00000118181
TF313321	7139	ENSG00000118194
TF315529	23271	ENSG00000118200
TF316079	22926	ENSG00000118217
TF331401	1421	ENSG00000118231
TF334733	55686	ENSG00000118242

TF330156	8828	ENSG00000118257
TF106464	1385	ENSG00000118260
TF300210	7276	ENSG00000118271
TF312834	9331	ENSG00000118276
TF316425	23632	ENSG00000118298
TF331789	4033	ENSG00000118308
TF354252	23120	ENSG00000118322
TF324529	57558	ENSG00000118369
TF331399	27145	ENSG00000118407
TF105375	9324	ENSG00000118418
TF330052	1268	ENSG00000118432
TF328986	84251	ENSG00000118473
TF343620	727966	ENSG00000118479
TF343620	730504	ENSG00000118479
TF350578	23469	ENSG00000118482
TF332024	5325	ENSG00000118495
TF343227	84085	ENSG00000118496
TF324491	10981	ENSG00000118508
TF326257	4602	ENSG00000118513
TF320906	6446	ENSG00000118515
TF300034	383	ENSG00000118520
TF326070	1490	ENSG00000118523
TF350742	6943	ENSG00000118526
TF331105	26234	ENSG00000118564
TF313792	9194	ENSG00000118596
TF314218	103910	ENSG00000118680
TF314218	642045	ENSG00000118680
TF314218	642076	ENSG00000118680
TF315583	2309	ENSG00000118689
TF318093	60436	ENSG00000118707
TF313796	845	ENSG00000118729
TF315964	118427	ENSG00000118733
TF101007	10983	ENSG00000118816
TF331090	5493	ENSG00000118898
TF326088	29855	ENSG00000118900
TF350556	11278	ENSG00000118922
TF316166	7347	ENSG00000118939
TF352008	27253	ENSG00000118946
TF101004	894	ENSG00000118971
TF335872	8074	ENSG00000118972
TF314116	341511	ENSG00000118975
TF326161	22936	ENSG00000118985
TF332714	23314	ENSG00000119042
TF101128	7320	ENSG00000119048
TF314204	140803	ENSG00000119121
TF351003	687	ENSG00000119138
TF315957	9414	ENSG00000119139
TF316289	205564	ENSG00000119231
TF329066	80212	ENSG00000119242
TF315216	440730	ENSG00000119283
TF319824	9991	ENSG00000119314
TF101216	5887	ENSG00000119318
TF313386	6418	ENSG00000119335
TF300032	51552	ENSG00000119396
TF331018	22954	ENSG00000119401
TF106420	26147	ENSG00000119403
TF315701	57864	ENSG00000119457
TF315430	8013	ENSG00000119508
TF312824	27130	ENSG00000119509

TF352660	79695	ENSG00000119514
TF320336	57706	ENSG00000119522
TF338122	1441	ENSG00000119535
TF105012	9525	ENSG00000119541
TF318206	9480	ENSG00000119547
TF335684	84878	ENSG00000119574
TF316638	283571	ENSG00000119608
TF350743	338917	ENSG00000119614
TF319554	5228	ENSG00000119630
TF300288	97	ENSG00000119640
TF317075	64207	ENSG00000119669
TF317514	4053	ENSG00000119681
TF314292	55640	ENSG00000119686
TF331803	8111	ENSG00000119714
TF323751	2103	ENSG00000119715
TF333498	57862	ENSG00000119725
TF101109	23433	ENSG00000119729
TF351654	114818	ENSG00000119771
TF329039	1788	ENSG00000119772
TF105291	2281	ENSG00000119782
TF105251	64225	ENSG00000119787
TF320308	25940	ENSG00000119812
TF315551	29094	ENSG00000119862
TF332485	25927	ENSG00000119865
TF318131	53335	ENSG00000119866
TF313535	26503	ENSG00000119899
TF331377	79627	ENSG00000119900
TF330284	6975	ENSG00000119913
TF342671	3437	ENSG00000119917
TF351204	159296	ENSG00000119919
TF342671	3433	ENSG00000119922
TF313360	57678	ENSG00000119927
TF105537	5507	ENSG00000119938
TF101012	26507	ENSG00000119946
TF315654	4601	ENSG00000119950
TF315303	2834	ENSG00000119973
TF329169	26123	ENSG00000119977
TF318560	30819	ENSG00000120049
TF314089	2805	ENSG00000120053
TF350133	6425	ENSG00000120057
TF300673	10672	ENSG00000120063
TF316310	3218	ENSG00000120068
TF336511	284058	ENSG00000120071
TF316310	3215	ENSG00000120075
TF316310	3217	ENSG00000120087
TF315710	1394	ENSG00000120088
TF315938	3213	ENSG00000120093
TF317730	3211	ENSG00000120094
TF105122	1843	ENSG00000120129
TF314866	79646	ENSG00000120137
TF350699	4488	ENSG00000120149
TF317568	7010	ENSG00000120156
TF300789	79817	ENSG00000120162
TF333404	3641	ENSG00000120211
TF331083	29126	ENSG00000120217
TF315232	2891	ENSG00000120251
TF300623	25902	ENSG00000120254
TF325867	84918	ENSG00000120256
TF314431	5110	ENSG00000120265

TF328565	57480	ENSG00000120278
TF105769	64411	ENSG00000120318
TF332299	56128	ENSG00000120322
TF332299	56126	ENSG00000120324
TF332299	56127	ENSG00000120324
TF332299	56122	ENSG00000120327
TF332299	56124	ENSG00000120328
TF314880	83884	ENSG00000120329
TF329915	63923	ENSG00000120332
TF316276	89866	ENSG00000120341
TF313676	3762	ENSG00000120457
TF314260	407	ENSG00000120500
TF332255	56243	ENSG00000120549
TF324882	84569	ENSG00000120563
TF316663	4360	ENSG00000120586
TF314400	84898	ENSG00000120594
TF106438	80314	ENSG00000120616
TF323811	440073	ENSG00000120645
TF323811	728056	ENSG00000120645
TF323811	731035	ENSG00000120645
TF323802	55068	ENSG00000120658
TF332169	8600	ENSG00000120659
TF313720	9617	ENSG00000120662
TF320584	29103	ENSG00000120675
TF318679	1997	ENSG00000120690
TF314923	4093	ENSG00000120693
TF105043	10808	ENSG00000120694
TF332672	84078	ENSG00000120696
TF316269	7045	ENSG00000120708
TF332095	51307	ENSG00000120709
TF326855	51247	ENSG00000120727
TF343193	9499	ENSG00000120729
TF324723	51780	ENSG00000120733
TF318980	1958	ENSG00000120738
TF313229	27230	ENSG00000120742
TF300680	5357	ENSG00000120756
TF316650	7181	ENSG00000120798
TF328426	7112	ENSG00000120802
TF332433	83468	ENSG00000120820
TF330821	80298	ENSG00000120832
TF321368	8835	ENSG00000120833
TF105122	1846	ENSG00000120875
TF333030	1191	ENSG00000120885
TF333916	8795	ENSG00000120889
TF320680	10174	ENSG00000120896
TF316643	2185	ENSG00000120899
TF315605	1135	ENSG00000120903
TF331895	148	ENSG00000120907
TF105557	5533	ENSG00000120910
TF106408	64236	ENSG00000120913
TF328342	81790	ENSG00000120925
TF106304	4879	ENSG00000120937
TF331157	943	ENSG00000120949
TF332708	65122	ENSG00000120952
TF315171	51123	ENSG00000120963
TF315171	728713	ENSG00000120963
TF315171	731044	ENSG00000120963
TF314619	10434	ENSG00000120992
TF316148	83690	ENSG00000121005

TF312837	157506	ENSG00000121039
TF314316	8288	ENSG00000121053
TF351086	7706	ENSG00000121060
TF313419	8405	ENSG00000121067
TF106341	6909	ENSG00000121068
TF106341	9496	ENSG00000121075
TF300264	140735	ENSG00000121083
TF313348	389240	ENSG00000121089
TF333159	81558	ENSG00000121104
TF330836	9227	ENSG00000121207
TF321435	23240	ENSG00000121210
TF317532	53840	ENSG00000121236
TF317532	445372	ENSG00000121236
TF317532	117854	ENSG00000121236
TF313939	64282	ENSG00000121274
TF313845	113	ENSG00000121281
TF328447	57616	ENSG00000121297
TF313676	3764	ENSG00000121361
TF315795	55269	ENSG00000121390
TF315909	23024	ENSG00000121440
TF315442	89884	ENSG00000121454
TF105501	6045	ENSG00000121481
TF105933	26984	ENSG00000121542
TF326644	64091	ENSG00000121577
TF312834	8702	ENSG00000121578
TF105231	81930	ENSG00000121621
TF329606	2703	ENSG00000121634
TF313188	51029	ENSG00000121644
TF313188	731151	ENSG00000121644
TF325073	9479	ENSG00000121653
TF323191	1408	ENSG00000121671
TF324139	9409	ENSG00000121680
TF328365	91614	ENSG00000121690
TF336988	7750	ENSG00000121741
TF329606	10804	ENSG00000121742
TF329606	2700	ENSG00000121743
TF314296	64786	ENSG00000121749
TF331634	576	ENSG00000121753
TF315303	3061	ENSG00000121764
TF316894	2170	ENSG00000121769
TF314878	10657	ENSG00000121774
TF321110	55116	ENSG00000121775
TF330966	9034	ENSG00000121797
TF330966	1231	ENSG00000121807
TF330966	729230	ENSG00000121807
TF317985	27246	ENSG00000121848
TF103052	84265	ENSG00000121851
TF332184	2693	ENSG00000121853
TF332169	8743	ENSG00000121858
TF335557	51193	ENSG00000121864
TF351826	22865	ENSG00000121871
TF102031	5290	ENSG00000121879
TF106415	23244	ENSG00000121892
TF315592	119587	ENSG00000121898
TF332771	113452	ENSG00000121900
TF316872	114784	ENSG00000121904
TF300009	3208	ENSG00000121905
TF325296	140	ENSG00000121933
TF328344	29899	ENSG00000121957

TF330966	7852	ENSG00000121966
TF352876	92	ENSG00000121989
TF324824	22987	ENSG00000122012
TF325768	2322	ENSG00000122025
TF318030	387496	ENSG00000122035
TF314489	5412	ENSG00000122042
TF350709	54440	ENSG00000122122
TF317034	4952	ENSG00000122126
TF106341	50945	ENSG00000122145
TF319326	2331	ENSG00000122176
TF316344	4656	ENSG00000122180
TF329901	5340	ENSG00000122194
TF350755	9956	ENSG00000122254
TF350543	5930	ENSG00000122257
TF329017	29066	ENSG00000122299
TF105452	311	ENSG00000122359
TF106408	11155	ENSG00000122367
TF324998	94233	ENSG00000122375
TF328605	57489	ENSG00000122417
TF324982	5737	ENSG00000122420
TF333627	127495	ENSG00000122477
TF333705	84146	ENSG00000122482
TF313072	80148	ENSG00000122490
TF316952	83637	ENSG00000122515
TF332342	654231	ENSG00000122543
TF332342	730297	ENSG00000122543
TF101079	989	ENSG00000122545
TF351653	55975	ENSG00000122550
TF324319	64224	ENSG00000122557
TF350503	11335	ENSG00000122565
TF350503	653972	ENSG00000122565
TF351342	3181	ENSG00000122566
TF332135	644150	ENSG00000122574
TF333047	30010	ENSG00000122584
TF332778	4852	ENSG00000122585
TF317153	84668	ENSG00000122591
TF316310	3204	ENSG00000122592
TF351791	3624	ENSG00000122641
TF105296	11328	ENSG00000122642
TF314663	51251	ENSG00000122643
TF105464	10124	ENSG00000122644
TF103012	27434	ENSG00000122678
TF333286	10268	ENSG00000122679
TF315153	7291	ENSG00000122691
TF313138	152007	ENSG00000122694
TF314192	92014	ENSG00000122696
TF313162	1211	ENSG00000122705
TF313573	138474	ENSG00000122728
TF313476	48	ENSG00000122729
TF331210	1271	ENSG00000122756
TF332690	57670	ENSG00000122778
TF106455	8805	ENSG00000122779
TF106492	6718	ENSG00000122787
TF106349	170685	ENSG00000122824
TF315153	50674	ENSG00000122859
TF329901	5328	ENSG00000122861
TF342871	9469	ENSG00000122863
TF323767	80114	ENSG00000122870
TF324661	55847	ENSG00000122873

TF318980	1959	ENSG00000122877
TF313393	5033	ENSG00000122884
TF316867	23389	ENSG00000123066
TF333311	1031	ENSG00000123080
TF318022	26994	ENSG00000123091
TF318385	11228	ENSG00000123094
TF330859	79365	ENSG00000123095
TF312815	3709	ENSG00000123104
TF331029	64168	ENSG00000123119
TF323658	11059	ENSG00000123124
TF300442	10212	ENSG00000123136
TF102005	5585	ENSG00000123143
TF316380	976	ENSG00000123146
TF313878	10755	ENSG00000123159
TF314716	84650	ENSG00000123179
TF300460	540	ENSG00000123191
TF300459	57486	ENSG00000123213
TF326608	10133	ENSG00000123240
TF328982	80760	ENSG00000123243
TF106464	466	ENSG00000123268
TF315153	58158	ENSG00000123307
TF329345	64333	ENSG00000123329
TF313683	3071	ENSG00000123338
TF315428	4327	ENSG00000123342
TF315428	732415	ENSG00000123342
TF320553	65244	ENSG00000123352
TF323369	29095	ENSG00000123353
TF315430	3164	ENSG00000123358
TF314638	5153	ENSG00000123360
TF330813	3229	ENSG00000123364
TF101021	1017	ENSG00000123374
TF315253	4035	ENSG00000123384
TF350668	3227	ENSG00000123388
TF326681	4778	ENSG00000123405
TF351604	3228	ENSG00000123407
TF331189	64375	ENSG00000123411
TF300314	10376	ENSG00000123416
TF313206	25895	ENSG00000123427
TF331549	3598	ENSG00000123496
TF332902	1300	ENSG00000123500
TF326075	85015	ENSG00000123552
TF315162	5354	ENSG00000123560
TF343201	6906	ENSG00000123561
TF323400	9643	ENSG00000123562
TF300212	158983	ENSG00000123569
TF326442	51209	ENSG00000123570
TF105138	203447	ENSG00000123572
TF326442	9367	ENSG00000123595
TF323232	79828	ENSG00000123600
TF314664	79809	ENSG00000123607
TF332752	9111	ENSG00000123609
TF334173	7130	ENSG00000123610
TF314724	130399	ENSG00000123612
TF329083	29994	ENSG00000123636
TF314873	206358	ENSG00000123643
TF332340	55509	ENSG00000123685
TF313676	3759	ENSG00000123700
TF313014	57826	ENSG00000123728
TF323302	81579	ENSG00000123739

TF300630	79934	ENSG00000123815
TF313541	5208	ENSG00000123836
TF334137	722	ENSG00000123838
TF101510	27161	ENSG00000123908
TF315654	10608	ENSG00000123933
TF101142	1164	ENSG00000123975
TF314012	2181	ENSG00000123983
TF318303	79586	ENSG00000123989
TF314707	116255	ENSG00000124003
TF328699	79843	ENSG00000124019
TF313657	6560	ENSG00000124067
TF332646	4159	ENSG00000124089
TF106430	140690	ENSG00000124092
TF105371	10357	ENSG00000124097
TF326807	90203	ENSG00000124104
TF323436	79183	ENSG00000124120
TF328639	57580	ENSG00000124126
TF313103	3787	ENSG00000124134
TF313657	57468	ENSG00000124140
TF314044	343578	ENSG00000124143
TF320463	6385	ENSG00000124145
TF332652	8202	ENSG00000124151
TF330078	8785	ENSG00000124159
TF324704	57727	ENSG00000124160
TF317024	9217	ENSG00000124164
TF312899	84612	ENSG00000124171
TF313572	84181	ENSG00000124177
TF313216	5335	ENSG00000124181
TF106481	84969	ENSG00000124191
TF351335	6431	ENSG00000124193
TF327072	78997	ENSG00000124194
TF300714	10564	ENSG00000124198
TF323611	57169	ENSG00000124201
TF333184	1908	ENSG00000124205
TF106147	387522	ENSG00000124208
TF106147	7335	ENSG00000124208
TF106147	387521	ENSG00000124208
TF331262	57403	ENSG00000124209
TF105090	5740	ENSG00000124212
TF350296	6780	ENSG00000124214
TF316817	60437	ENSG00000124215
TF315515	6615	ENSG00000124216
TF331681	56937	ENSG00000124225
TF331012	55905	ENSG00000124226
TF326629	55653	ENSG00000124243
TF313947	60598	ENSG00000124249
TF314402	5105	ENSG00000124253
TF325581	64377	ENSG00000124302
TF323811	23096	ENSG00000124313
TF337874	3581	ENSG00000124334
TF323215	10617	ENSG00000124356
TF326855	400961	ENSG00000124374
TF314701	27189	ENSG00000124391
TF300654	10396	ENSG00000124406
TF323554	23326	ENSG00000124422
TF317772	64344	ENSG00000124440
TF331897	56269	ENSG00000124449
TF324278	2162	ENSG00000124491
TF313240	2914	ENSG00000124493

TF106431	55809	ENSG00000124496
TF313677	29993	ENSG00000124507
TF317532	10385	ENSG00000124508
TF300174	8294	ENSG00000124529
TF300174	8359	ENSG00000124529
TF300174	8360	ENSG00000124529
TF300174	8361	ENSG00000124529
TF300174	8362	ENSG00000124529
TF300174	8363	ENSG00000124529
TF300174	8364	ENSG00000124529
TF300174	8365	ENSG00000124529
TF300174	8366	ENSG00000124529
TF300174	8367	ENSG00000124529
TF300174	8368	ENSG00000124529
TF300174	8370	ENSG00000124529
TF300174	121504	ENSG00000124529
TF300174	554313	ENSG00000124529
TF317532	54718	ENSG00000124549
TF317532	696	ENSG00000124557
TF313535	10786	ENSG00000124564
TF313535	6568	ENSG00000124568
TF352619	5269	ENSG00000124570
TF313664	3007	ENSG00000124575
TF300174	8369	ENSG00000124578
TF300296	4835	ENSG00000124588
TF313265	29964	ENSG00000124593
TF316767	222643	ENSG00000124602
TF313664	3024	ENSG00000124610
TF319100	6204	ENSG00000124614
TF319100	730187	ENSG00000124614
TF319100	732348	ENSG00000124614
TF300137	3013	ENSG00000124642
TF313385	6954	ENSG00000124678
TF331356	10930	ENSG00000124701
TF328485	401265	ENSG00000124743
TF332934	81578	ENSG00000124749
TF101038	1026	ENSG00000124762
TF316183	6659	ENSG00000124766
TF316419	57699	ENSG00000124772
TF313947	89822	ENSG00000124780
TF321074	6745	ENSG00000124783
TF332589	51299	ENSG00000124785
TF350643	6310	ENSG00000124788
TF321496	860	ENSG00000124813
TF331806	442213	ENSG00000124814
TF324998	221391	ENSG00000124818
TF324146	9247	ENSG00000124827
TF314109	9208	ENSG00000124831
TF333433	6372	ENSG00000124875
TF312888	745	ENSG00000124920
TF350595	79026	ENSG00000124942
TF105806	55831	ENSG00000125037
TF333167	54436	ENSG00000125089
TF336054	4495	ENSG00000125144
TF336054	4502	ENSG00000125148
TF336054	441019	ENSG00000125148
TF324994	55715	ENSG00000125170
TF354206	9271	ENSG00000125207
TF330775	2841	ENSG00000125245

TF313014	5911	ENSG00000125249
TF315811	6555	ENSG00000125255
TF315495	1948	ENSG00000125266
TF351735	11166	ENSG00000125285
TF300394	9375	ENSG00000125304
TF105234	3834	ENSG00000125337
TF328512	3659	ENSG00000125347
TF316034	65109	ENSG00000125351
TF101080	23157	ENSG00000125354
TF331034	55026	ENSG00000125355
TF351789	652	ENSG00000125378
TF324982	5732	ENSG00000125384
TF330223	8603	ENSG00000125386
TF313940	2868	ENSG00000125388
TF316183	6662	ENSG00000125398
TF314375	4620	ENSG00000125414
TF350755	9953	ENSG00000125430
TF324506	399512	ENSG00000125434
TF318574	23163	ENSG00000125447
TF328854	57409	ENSG00000125457
TF333537	7270	ENSG00000125482
TF316128	56751	ENSG00000125492
TF105543	54776	ENSG00000125503
TF314013	6725	ENSG00000125508
TF315737	652222	ENSG00000125510
TF315737	4987	ENSG00000125510
TF326610	56731	ENSG00000125520
TF315737	2832	ENSG00000125522
TF322733	128408	ENSG00000125533
TF315397	7849	ENSG00000125618
TF331013	51141	ENSG00000125629
TF319755	23550	ENSG00000125637
TF313492	79085	ENSG00000125648
TF332366	5623	ENSG00000125650
TF315232	2892	ENSG00000125675
TF324954	5469	ENSG00000125686
TF300913	9349	ENSG00000125691
TF319817	92335	ENSG00000125695
TF314847	84938	ENSG00000125703
TF313285	718	ENSG00000125730
TF313285	653879	ENSG00000125730
TF323756	10045	ENSG00000125731
TF351162	9322	ENSG00000125733
TF314804	56927	ENSG00000125734
TF332169	8740	ENSG00000125735
TF326301	2354	ENSG00000125740
TF317832	24139	ENSG00000125746
TF321411	7408	ENSG00000125753
TF327131	27111	ENSG00000125775
TF314866	80025	ENSG00000125779
TF324278	7053	ENSG00000125780
TF316127	3170	ENSG00000125798
TF330714	22918	ENSG00000125810
TF315397	5075	ENSG00000125813
TF316547	63908	ENSG00000125814
TF319145	10047	ENSG00000125815
TF351204	644524	ENSG00000125816
TF351204	651062	ENSG00000125816
TF351204	4821	ENSG00000125820

TF319145	128817	ENSG00000125823
TF106376	56255	ENSG00000125827
TF319145	140880	ENSG00000125831
TF105336	140901	ENSG00000125834
TF314232	6628	ENSG00000125835
TF332090	80023	ENSG00000125841
TF333579	6238	ENSG00000125844
TF351789	650	ENSG00000125845
TF331598	23767	ENSG00000125848
TF315489	58495	ENSG00000125850
TF331647	64096	ENSG00000125861
TF328601	11034	ENSG00000125868
TF105942	128637	ENSG00000125875
TF315153	6939	ENSG00000125878
TF332724	56914	ENSG00000125879
TF315060	140836	ENSG00000125888
TF335735	55321	ENSG00000125895
TF330964	83541	ENSG00000125898
TF330052	8698	ENSG00000125910
TF105849	56926	ENSG00000125912
TF331915	4435	ENSG00000125931
TF314932	10236	ENSG00000125944
TF316134	8200	ENSG00000125965
TF315428	10893	ENSG00000125966
TF331029	63941	ENSG00000125967
TF326217	3397	ENSG00000125968
TF330974	22913	ENSG00000125970
TF331899	10137	ENSG00000125976
TF330777	128876	ENSG00000125998
TF332024	5326	ENSG00000126003
TF331292	2925	ENSG00000126010
TF106476	8242	ENSG00000126012
TF333368	154796	ENSG00000126016
TF101510	192669	ENSG00000126070
TF354325	6487	ENSG00000126091
TF314010	3831	ENSG00000126214
TF318080	23263	ENSG00000126217
TF327329	2159	ENSG00000126218
TF327329	8858	ENSG00000126231
TF350185	79414	ENSG00000126243
TF314682	826	ENSG00000126247
TF330775	2866	ENSG00000126251
TF333443	5348	ENSG00000126258
TF333443	53822	ENSG00000126258
TF327139	84063	ENSG00000126259
TF330775	2867	ENSG00000126262
TF105065	1340	ENSG00000126267
TF332742	8689	ENSG00000126337
TF328382	7067	ENSG00000126351
TF330966	1236	ENSG00000126353
TF328382	9572	ENSG00000126368
TF328512	3661	ENSG00000126456
TF300608	3276	ENSG00000126457
TF312796	6237	ENSG00000126458
TF332123	5639	ENSG00000126460
TF332183	58506	ENSG00000126461
TF333141	57479	ENSG00000126464
TF331598	23769	ENSG00000126500
TF315519	65266	ENSG00000126562

TF102004	5582	ENSG00000126583
TF315976	645832	ENSG00000126656
TF316697	117154	ENSG00000126733
TF317732	2002	ENSG00000126767
TF106195	10245	ENSG00000126768
TF333579	3895	ENSG00000126777
TF315545	6495	ENSG00000126778
TF101109	57381	ENSG00000126785
TF321382	9787	ENSG00000126787
TF105042	3306	ENSG00000126803
TF332229	22890	ENSG00000126804
TF328565	26030	ENSG00000126822
TF337915	11105	ENSG00000126856
TF300814	55288	ENSG00000126858
TF320736	83543	ENSG00000126878
TF329533	286336	ENSG00000126882
TF106499	554	ENSG00000126895
TF315811	8273	ENSG00000126903
TF105137	5605	ENSG00000126934
TF316157	3188	ENSG00000126945
TF314566	55998	ENSG00000126952
TF106191	1678	ENSG00000126953
TF300618	821	ENSG00000127022
TF315837	6003	ENSG00000127074
TF319326	4958	ENSG00000127083
TF316247	89846	ENSG00000127084
TF331837	59269	ENSG00000127124
TF333184	1907	ENSG00000127129
TF318131	64919	ENSG00000127152
TF321154	7186	ENSG00000127191
TF330373	5648	ENSG00000127241
TF300331	84239	ENSG00000127249
TF330836	57110	ENSG00000127252
TF313014	5908	ENSG00000127314
TF333253	50616	ENSG00000127318
TF315803	144453	ENSG00000127325
TF313748	117177	ENSG00000127328
TF351926	5787	ENSG00000127329
TF314624	8445	ENSG00000127334
TF331401	155051	ENSG00000127377
TF314711	56302	ENSG00000127412
TF334735	53834	ENSG00000127418
TF319283	5322	ENSG00000127472
TF316380	30817	ENSG00000127507
TF106187	23309	ENSG00000127511
TF324293	58513	ENSG00000127527
TF315506	10365	ENSG00000127528
TF320995	9143	ENSG00000127561
TF315349	117166	ENSG00000127578
TF300298	56604	ENSG00000127589
TF331090	23499	ENSG00000127603
TF300785	6597	ENSG00000127616
TF106449	23030	ENSG00000127663
TF300314	7277	ENSG00000127824
TF313468	7429	ENSG00000127831
TF331385	55504	ENSG00000127863
TF325756	6049	ENSG00000127870
TF319909	2791	ENSG00000127920
TF319909	2792	ENSG00000127928

TF316860	3092	ENSG00000127946
TF351977	5782	ENSG00000127947
TF332031	79689	ENSG00000127954
TF300673	2770	ENSG00000127955
TF330821	7978	ENSG00000127989
TF314655	8910	ENSG00000127990
TF350185	57622	ENSG00000128011
TF315463	7538	ENSG00000128016
TF106457	6691	ENSG00000128040
TF318030	65997	ENSG00000128045
TF325768	3791	ENSG00000128052
TF312799	57168	ENSG00000128203
TF314557	23753	ENSG00000128228
TF314802	9514	ENSG00000128242
TF102003	7533	ENSG00000128245
TF317532	5988	ENSG00000128250
TF317532	10739	ENSG00000128253
TF325296	135	ENSG00000128271
TF316136	468	ENSG00000128272
TF316136	643159	ENSG00000128272
TF316136	730136	ENSG00000128272
TF317532	10738	ENSG00000128276
TF317532	10739	ENSG00000128276
TF331725	11135	ENSG00000128283
TF334681	80833	ENSG00000128284
TF315737	2847	ENSG00000128285
TF312910	8459	ENSG00000128294
TF325648	80115	ENSG00000128298
TF315737	8484	ENSG00000128310
TF334681	80831	ENSG00000128313
TF335549	3543	ENSG00000128322
TF334681	23780	ENSG00000128335
TF101109	5880	ENSG00000128340
TF331356	200315	ENSG00000128383
TF331356	200316	ENSG00000128394
TF331356	60489	ENSG00000128394
TF324120	26150	ENSG00000128408
TF318099	96597	ENSG00000128438
TF316716	92521	ENSG00000128487
TF317197	51200	ENSG00000128510
TF300423	9732	ENSG00000128512
TF333564	5420	ENSG00000128567
TF326978	93986	ENSG00000128573
TF314205	57464	ENSG00000128578
TF105143	4189	ENSG00000128590
TF313685	2318	ENSG00000128591
TF334360	64101	ENSG00000128594
TF314849	813	ENSG00000128595
TF328512	3663	ENSG00000128604
TF332887	10234	ENSG00000128606
TF316780	389549	ENSG00000128610
TF324998	611	ENSG00000128617
TF312960	4430	ENSG00000128641
TF317730	3231	ENSG00000128645
TF315938	3232	ENSG00000128652
TF316499	50940	ENSG00000128655
TF342052	1123	ENSG00000128656
TF314688	2571	ENSG00000128683
TF323369	94101	ENSG00000128699

TF317819	3235	ENSG00000128709
TF317819	3236	ENSG00000128710
TF350668	3237	ENSG00000128713
TF330813	3239	ENSG00000128714
TF314232	6638	ENSG00000128739
TF323922	57045	ENSG00000128791
TF316134	2658	ENSG00000128802
TF323577	58504	ENSG00000128805
TF313658	159491	ENSG00000128815
TF328771	55930	ENSG00000128833
TF332247	84952	ENSG00000128849
TF315841	29767	ENSG00000128872
TF315292	146057	ENSG00000128881
TF351835	54567	ENSG00000128917
TF300455	8854	ENSG00000128918
TF300455	729593	ENSG00000128918
TF300455	731752	ENSG00000128918
TF314589	54629	ENSG00000128923
TF314718	10776	ENSG00000128989
TF314718	646227	ENSG00000128989
TF300316	54832	ENSG00000129003
TF326061	4016	ENSG00000129038
TF330966	51554	ENSG00000129048
TF330966	651872	ENSG00000129048
TF343193	23022	ENSG00000129116
TF316344	4654	ENSG00000129152
TF352511	3746	ENSG00000129159
TF313327	7166	ENSG00000129167
TF313758	8048	ENSG00000129170
TF105567	79733	ENSG00000129173
TF316183	6665	ENSG00000129194
TF300589	5338	ENSG00000129219
TF314507	23746	ENSG00000129221
TF316339	968	ENSG00000129226
TF314618	482	ENSG00000129244
TF105427	9513	ENSG00000129245
TF315428	79148	ENSG00000129270
TF334888	6351	ENSG00000129277
TF106475	51105	ENSG00000129292
TF101014	904	ENSG00000129315
TF320194	3609	ENSG00000129351
TF313325	57153	ENSG00000129353
TF300393	10053	ENSG00000129354
TF333311	1032	ENSG00000129355
TF333416	57509	ENSG00000129422
TF332441	27180	ENSG00000129450
TF106506	11035	ENSG00000129465
TF313845	196883	ENSG00000129467
TF315834	599	ENSG00000129473
TF320310	84962	ENSG00000129474
TF300706	25938	ENSG00000129493
TF316127	3169	ENSG00000129514
TF313698	58533	ENSG00000129515
TF314595	112399	ENSG00000129521
TF325689	4901	ENSG00000129535
TF106223	122706	ENSG00000129543
TF314913	7905	ENSG00000129625
TF333250	2302	ENSG00000129654
TF313988	6397	ENSG00000129657

TF312988	79651	ENSG00000129667
TF316105	9459	ENSG00000129675
TF332273	79649	ENSG00000129680
TF317805	2258	ENSG00000129682
TF315605	57053	ENSG00000129749
TF101111	1028	ENSG00000129757
TF313375	9426	ENSG00000129873
TF313375	203611	ENSG00000129873
TF316817	1013	ENSG00000129910
TF331003	58986	ENSG00000129925
TF315807	25759	ENSG00000129946
TF316040	79948	ENSG00000129951
TF332820	723961	ENSG00000129965
TF332820	3630	ENSG00000129965
TF314365	81926	ENSG00000129968
TF314365	648359	ENSG00000129968
TF315617	3929	ENSG00000129988
TF315600	6861	ENSG00000129990
TF313374	7137	ENSG00000129991
TF106303	863	ENSG00000129993
TF332123	79057	ENSG00000130032
TF313103	3741	ENSG00000130037
TF329556	84766	ENSG00000130038
TF300265	6232	ENSG00000130041
TF331873	158046	ENSG00000130045
TF314044	9754	ENSG00000130052
TF331752	27112	ENSG00000130054
TF313692	54857	ENSG00000130055
TF319736	6303	ENSG00000130066
TF313085	54552	ENSG00000130119
TF105572	23677	ENSG00000130147
TF313629	57572	ENSG00000130158
TF351700	3949	ENSG00000130164
TF313921	1264	ENSG00000130176
TF314979	90332	ENSG00000130201
TF331051	5819	ENSG00000130202
TF334458	348	ENSG00000130203
TF106204	10452	ENSG00000130204
TF300196	10912	ENSG00000130222
TF318428	57631	ENSG00000130224
TF313309	1804	ENSG00000130226
TF314248	23039	ENSG00000130227
TF326300	84800	ENSG00000130235
TF320308	147965	ENSG00000130244
TF325240	9667	ENSG00000130254
TF332134	1463	ENSG00000130287
TF105221	547	ENSG00000130294
TF313430	376497	ENSG00000130304
TF313826	79709	ENSG00000130309
TF354286	81616	ENSG00000130377
TF314586	4298	ENSG00000130382
TF316348	2527	ENSG00000130383
TF316134	9210	ENSG00000130385
TF350731	4301	ENSG00000130396
TF352676	81	ENSG00000130402
TF315041	10095	ENSG00000130429
TF315041	653888	ENSG00000130429
TF331651	59285	ENSG00000130433
TF324881	57688	ENSG00000130449

TF328986	23149	ENSG00000130475
TF312844	23025	ENSG00000130477
TF350229	55201	ENSG00000130479
TF328485	113730	ENSG00000130487
TF313752	9997	ENSG00000130489
TF314316	7837	ENSG00000130508
TF318961	170463	ENSG00000130511
TF313278	54858	ENSG00000130517
TF323952	3727	ENSG00000130522
TF314204	54795	ENSG00000130529
TF321745	25830	ENSG00000130540
TF315964	10439	ENSG00000130558
TF315529	157922	ENSG00000130559
TF314260	6295	ENSG00000130561
TF325918	140700	ENSG00000130590
TF313321	7140	ENSG00000130595
TF313374	7136	ENSG00000130598
TF323987	1289	ENSG00000130635
TF332232	50632	ENSG00000130643
TF300519	375775	ENSG00000130653
TF332328	3050	ENSG00000130656
TF105352	10298	ENSG00000130669
TF351530	3110	ENSG00000130675
TF331041	64793	ENSG00000130695
TF316520	6874	ENSG00000130699
TF315391	140628	ENSG00000130700
TF335359	3911	ENSG00000130702
TF328738	84726	ENSG00000130723
TF106455	10155	ENSG00000130726
TF313536	78992	ENSG00000130733
TF314847	84971	ENSG00000130734
TF321641	23211	ENSG00000130749
TF317772	4861	ENSG00000130751
TF315147	9535	ENSG00000130755
TF105118	4294	ENSG00000130758
TF316357	27237	ENSG00000130762
TF314230	83667	ENSG00000130766
TF333479	9473	ENSG00000130775
TF326096	6249	ENSG00000130779
TF316860	9026	ENSG00000130787
TF343812	6535	ENSG00000130821
TF343812	731026	ENSG00000130821
TF314166	139728	ENSG00000130822
TF312962	55558	ENSG00000130827
TF105122	1852	ENSG00000130829
TF313355	56301	ENSG00000130876
TF332149	4037	ENSG00000130881
TF316484	10343	ENSG00000130943
TF314591	3293	ENSG00000130948
TF318374	22927	ENSG00000130956
TF314824	8789	ENSG00000130957
TF313307	11046	ENSG00000130958
TF332123	5638	ENSG00000130962
TF300586	7317	ENSG00000130985
TF351549	9113	ENSG00000131023
TF313069	54869	ENSG00000131037
TF320448	9584	ENSG00000131051
TF105061	84701	ENSG00000131055
TF333329	2686	ENSG00000131067

TF300417	55902	ENSG00000131069
TF331385	60401	ENSG00000131080
TF316832	23229	ENSG00000131089
TF329591	10882	ENSG00000131094
TF330122	2670	ENSG00000131095
TF332778	5697	ENSG00000131096
TF314628	51751	ENSG00000131097
TF105061	1327	ENSG00000131143
TF313860	10328	ENSG00000131148
TF105574	6451	ENSG00000131171
TF313981	6569	ENSG00000131183
TF329901	2161	ENSG00000131187
TF326480	4772	ENSG00000131196
TF330978	3620	ENSG00000131203
TF329606	81025	ENSG00000131233
TF313791	10487	ENSG00000131236
TF327221	84440	ENSG00000131242
TF325756	51132	ENSG00000131263
TF351605	1046	ENSG00000131264
TF321154	7187	ENSG00000131323
TF316380	84658	ENSG00000131355
TF105573	9467	ENSG00000131370
TF333285	23180	ENSG00000131378
TF352511	3748	ENSG00000131398
TF314990	9476	ENSG00000131400
TF352167	7376	ENSG00000131408
TF324303	94030	ENSG00000131409
TF106408	8572	ENSG00000131435
TF300864	9945	ENSG00000131459
TF300477	7283	ENSG00000131462
TF106236	10197	ENSG00000131467
TF314750	8639	ENSG00000131471
TF333286	10266	ENSG00000131477
TF314750	314	ENSG00000131480
TF324388	2538	ENSG00000131482
TF328552	404734	ENSG00000131503
TF328552	54882	ENSG00000131503
TF328552	8637	ENSG00000131503
TF315383	1729	ENSG00000131504
TF324911	80762	ENSG00000131507
TF101108	7322	ENSG00000131508
TF318315	116983	ENSG00000131584
TF314265	55107	ENSG00000131620
TF314207	8500	ENSG00000131626
TF331319	79412	ENSG00000131650
TF350735	56033	ENSG00000131668
TF323538	4814	ENSG00000131669
TF316425	765	ENSG00000131686
TF350229	4131	ENSG00000131711
TF331549	3597	ENSG00000131724
TF314214	1160	ENSG00000131730
TF332742	3885	ENSG00000131737
TF332742	3884	ENSG00000131738
TF315996	84951	ENSG00000131746
TF313869	10948	ENSG00000131748
TF328382	5914	ENSG00000131759
TF332576	84152	ENSG00000131771
TF314878	10656	ENSG00000131773
TF325704	8799	ENSG00000131779

TF323787	10401	ENSG00000131788
TF313827	5565	ENSG00000131791
TF332940	2488	ENSG00000131808
TF323032	57663	ENSG00000131864
TF318303	22856	ENSG00000131873
TF318303	644691	ENSG00000131873
TF318303	651391	ENSG00000131873
TF314585	3996	ENSG00000131899
TF332386	8431	ENSG00000131910
TF316240	79727	ENSG00000131914
TF330127	55145	ENSG00000131931
TF323502	85415	ENSG00000131941
TF315122	145447	ENSG00000131969
TF105616	2643	ENSG00000131979
TF315551	3958	ENSG00000131981
TF313043	7332	ENSG00000131982
TF336377	79883	ENSG00000132000
TF105141	3337	ENSG00000132002
TF324881	65249	ENSG00000132003
TF321340	5989	ENSG00000132005
TF314229	54862	ENSG00000132024
TF330078	4148	ENSG00000132031
TF315442	3975	ENSG00000132130
TF323754	246176	ENSG00000132139
TF300061	31	ENSG00000132142
TF317006	5894	ENSG00000132155
TF343812	6538	ENSG00000132164
TF316304	5468	ENSG00000132170
TF335097	84824	ENSG00000132185
TF331033	84034	ENSG00000132205
TF314945	23647	ENSG00000132254
TF317532	85363	ENSG00000132256
TF318250	1262	ENSG00000132259
TF317532	10346	ENSG00000132274
TF314098	23167	ENSG00000132294
TF324350	79781	ENSG00000132321
TF318445	8864	ENSG00000132326
TF333286	10267	ENSG00000132329
TF351829	5791	ENSG00000132334
TF314032	5562	ENSG00000132356
TF335271	84674	ENSG00000132357
TF318626	23108	ENSG00000132359
TF317034	51763	ENSG00000132376
TF317350	5176	ENSG00000132386
TF101118	7326	ENSG00000132388
TF313318	57533	ENSG00000132405
TF326644	64208	ENSG00000132429
TF300068	55915	ENSG00000132434
TF313885	53940	ENSG00000132446
TF316157	2926	ENSG00000132463
TF328552	26057	ENSG00000132466
TF314141	23558	ENSG00000132471
TF314982	85451	ENSG00000132478
TF351086	91107	ENSG00000132481
TF101534	1984	ENSG00000132507
TF317405	23135	ENSG00000132510
TF314820	55065	ENSG00000132517
TF106338	3000	ENSG00000132518
TF331416	54739	ENSG00000132530

TF323171	1742	ENSG00000132535
TF330078	4147	ENSG00000132561
TF314177	51308	ENSG00000132563
TF300188	84105	ENSG00000132570
TF314557	6388	ENSG00000132581
TF333209	7014	ENSG00000132604
TF105012	27183	ENSG00000132612
TF320619	92154	ENSG00000132613
TF329492	116835	ENSG00000132622
TF315125	6616	ENSG00000132639
TF106482	22903	ENSG00000132640
TF331067	54453	ENSG00000132669
TF351829	5786	ENSG00000132670
TF315737	6754	ENSG00000132671
TF314450	57127	ENSG00000132677
TF312838	480	ENSG00000132681
TF332134	63827	ENSG00000132692
TF330208	1401	ENSG00000132693
TF106495	9826	ENSG00000132694
TF332134	60484	ENSG00000132702
TF330208	325	ENSG00000132703
TF315600	23208	ENSG00000132718
TF328708	91703	ENSG00000132744
TF314264	222	ENSG00000132746
TF314095	64900	ENSG00000132793
TF314235	55544	ENSG00000132819
TF331739	653968	ENSG00000132821
TF331739	128434	ENSG00000132821
TF312881	10955	ENSG00000132824
TF105537	5509	ENSG00000132825
TF314605	8546	ENSG00000132842
TF330709	10207	ENSG00000132849
TF324499	163782	ENSG00000132854
TF315600	6860	ENSG00000132872
TF320527	93611	ENSG00000132879
TF326170	56923	ENSG00000132911
TF316499	5145	ENSG00000132915
TF300654	51761	ENSG00000132932
TF333416	23281	ENSG00000132938
TF336988	9205	ENSG00000132950
TF101025	1024	ENSG00000132964
TF105328	241	ENSG00000132965
TF315031	10810	ENSG00000132970
TF326896	56163	ENSG00000132972
TF330052	2835	ENSG00000132975
TF320495	1131	ENSG00000133019
TF320495	730413	ENSG00000133019
TF314375	4626	ENSG00000133020
TF333601	4628	ENSG00000133026
TF313752	6341	ENSG00000133028
TF329258	23164	ENSG00000133030
TF315610	1116	ENSG00000133048
TF102031	5287	ENSG00000133056
TF315610	1118	ENSG00000133063
TF313647	254428	ENSG00000133065
TF316814	59352	ENSG00000133067
TF316292	9911	ENSG00000133069
TF318770	9201	ENSG00000133083
TF101002	8900	ENSG00000133101

TF326185	122042	ENSG00000133105
TF313147	7223	ENSG00000133107
TF316269	10631	ENSG00000133110
TF105750	161003	ENSG00000133115
TF314803	9365	ENSG00000133116
TF314044	90627	ENSG00000133121
TF325994	8471	ENSG00000133124
TF329118	79710	ENSG00000133131
TF317486	79589	ENSG00000133135
TF313145	54885	ENSG00000133138
TF315608	2048	ENSG00000133216
TF106482	55643	ENSG00000133243
TF337003	84106	ENSG00000133246
TF106433	84787	ENSG00000133247
TF316499	5158	ENSG00000133256
TF313349	1455	ENSG00000133275
TF341440	28992	ENSG00000133315
TF315551	85329	ENSG00000133317
TF105431	10313	ENSG00000133318
TF330836	5920	ENSG00000133321
TF330836	54979	ENSG00000133328
TF333601	4629	ENSG00000133392
TF326303	23037	ENSG00000133401
TF329118	22880	ENSG00000133422
TF319168	9215	ENSG00000133424
TF325759	2953	ENSG00000133433
TF325759	653689	ENSG00000133433
TF339614	84700	ENSG00000133454
TF313762	66035	ENSG00000133460
TF329591	114904	ENSG00000133466
TF330777	113828	ENSG00000133477
TF330845	474344	ENSG00000133561
TF330845	55303	ENSG00000133574
TF315108	23608	ENSG00000133606
TF317762	116988	ENSG00000133612
TF300644	57180	ENSG00000133627
TF105272	694	ENSG00000133639
TF300331	79572	ENSG00000133657
TF312796	3845	ENSG00000133703
TF300634	10526	ENSG00000133704
TF313194	3612	ENSG00000133731
TF316425	759	ENSG00000133742
TF333160	23075	ENSG00000133789
TF319983	406	ENSG00000133794
TF334173	10894	ENSG00000133800
TF300439	272	ENSG00000133805
TF318583	81846	ENSG00000133812
TF324129	9645	ENSG00000133816
TF312796	22800	ENSG00000133818
TF105128	78986	ENSG00000133878
TF318971	5977	ENSG00000133884
TF351613	145258	ENSG00000133937
TF314159	8650	ENSG00000133961
TF331917	23508	ENSG00000133985
TF314733	8748	ENSG00000134007
TF326061	4017	ENSG00000134013
TF313391	64641	ENSG00000134025
TF314733	27299	ENSG00000134028
TF325032	8932	ENSG00000134046

TF101001	891	ENSG00000134057
TF351113	4064	ENSG00000134061
TF328924	3656	ENSG00000134070
TF314166	8536	ENSG00000134072
TF313093	25917	ENSG00000134077
TF330859	8553	ENSG00000134107
TF105470	55207	ENSG00000134108
TF351103	27255	ENSG00000134115
TF321796	10752	ENSG00000134121
TF318093	4212	ENSG00000134138
TF314204	4308	ENSG00000134160
TF300673	2780	ENSG00000134183
TF352895	10100	ENSG00000134198
TF332940	7252	ENSG00000134200
TF315600	148281	ENSG00000134207
TF316171	10451	ENSG00000134215
TF315610	27159	ENSG00000134216
TF105361	3158	ENSG00000134240
TF351977	26191	ENSG00000134242
TF324918	6272	ENSG00000134243
TF105310	7482	ENSG00000134245
TF332702	5738	ENSG00000134247
TF314733	11085	ENSG00000134249
TF351641	4853	ENSG00000134250
TF313270	10390	ENSG00000134255
TF332702	9398	ENSG00000134256
TF331083	79679	ENSG00000134258
TF106463	4803	ENSG00000134259
TF326239	56907	ENSG00000134278
TF300808	377	ENSG00000134287
TF328907	79022	ENSG00000134291
TF328787	54407	ENSG00000134294
TF102003	10971	ENSG00000134308
TF314132	29841	ENSG00000134317
TF313551	9475	ENSG00000134318
TF106001	4613	ENSG00000134323
TF314095	23175	ENSG00000134324
TF314963	3939	ENSG00000134333
TF314265	63982	ENSG00000134343
TF338122	3572	ENSG00000134352
TF338122	649891	ENSG00000134352
TF106409	10468	ENSG00000134363
TF326157	10877	ENSG00000134365
TF329881	89796	ENSG00000134369
TF106195	10440	ENSG00000134375
TF326157	81494	ENSG00000134389
TF326157	3080	ENSG00000134391
TF313986	10595	ENSG00000134398
TF315976	30062	ENSG00000134438
TF351747	59340	ENSG00000134489
TF315332	284252	ENSG00000134504
TF323936	91768	ENSG00000134508
TF300423	1794	ENSG00000134516
TF330414	2012	ENSG00000134531
TF320471	6660	ENSG00000134532
TF317540	10599	ENSG00000134538
TF312893	53	ENSG00000134575
TF323032	83844	ENSG00000134588
TF300097	9363	ENSG00000134594

TF316183	6658	ENSG00000134595
TF105338	51765	ENSG00000134602
TF312981	219595	ENSG00000134612
TF354206	143689	ENSG00000134627
TF331693	4544	ENSG00000134640
TF318160	9698	ENSG00000134644
TF350578	90853	ENSG00000134668
TF331299	1912	ENSG00000134686
TF101077	55143	ENSG00000134690
TF101510	192670	ENSG00000134698
TF320231	51361	ENSG00000134709
TF317546	653189	ENSG00000134717
TF317546	91408	ENSG00000134717
TF315661	23318	ENSG00000134744
TF316817	1824	ENSG00000134755
TF331012	51444	ENSG00000134758
TF316817	1825	ENSG00000134762
TF316817	1823	ENSG00000134765
TF343849	1837	ENSG00000134769
TF316268	80206	ENSG00000134775
TF330024	187	ENSG00000134817
TF313604	9415	ENSG00000134824
TF330976	27202	ENSG00000134830
TF324568	9575	ENSG00000134852
TF325768	5156	ENSG00000134853
TF316865	1284	ENSG00000134871
TF331936	9071	ENSG00000134873
TF330044	22873	ENSG00000134874
TF323280	79070	ENSG00000134901
TF351451	9743	ENSG00000134909
TF331949	11095	ENSG00000134917
TF316214	2113	ENSG00000134954
TF314991	219855	ENSG00000134955
TF314803	152831	ENSG00000134962
TF313000	353376	ENSG00000134970
TF313000	51014	ENSG00000134970
TF106496	324	ENSG00000134982
TF314412	29979	ENSG00000135018
TF105452	301	ENSG00000135046
TF313739	1514	ENSG00000135047
TF316575	23670	ENSG00000135048
TF313794	23287	ENSG00000135049
TF331127	51280	ENSG00000135052
TF332736	9413	ENSG00000135063
TF314733	8728	ENSG00000135074
TF336163	84868	ENSG00000135077
TF101009	79616	ENSG00000135083
TF351444	51347	ENSG00000135090
TF329014	10993	ENSG00000135094
TF325419	4440	ENSG00000135097
TF320327	6927	ENSG00000135100
TF106341	6926	ENSG00000135111
TF331930	84900	ENSG00000135119
TF328633	5025	ENSG00000135124
TF326671	92558	ENSG00000135127
TF325526	1840	ENSG00000135144
TF331416	10906	ENSG00000135148
TF333537	9988	ENSG00000135164
TF332342	4951	ENSG00000135175

TF315472	79799	ENSG00000135220
TF315472	54490	ENSG00000135226
TF319230	50640	ENSG00000135241
TF105334	6733	ENSG00000135250
TF313265	26136	ENSG00000135269
TF332113	29969	ENSG00000135272
TF331634	577	ENSG00000135298
TF316350	3351	ENSG00000135312
TF314932	10492	ENSG00000135316
TF315608	2045	ENSG00000135333
TF317123	55122	ENSG00000135334
TF105116	6885	ENSG00000135341
TF329606	84694	ENSG00000135355
TF314826	79899	ENSG00000135362
TF351071	4005	ENSG00000135363
TF331518	51317	ENSG00000135365
TF318679	26298	ENSG00000135373
TF318679	2001	ENSG00000135374
TF332123	79056	ENSG00000135378
TF329471	4076	ENSG00000135387
TF300140	517	ENSG00000135390
TF330122	5630	ENSG00000135406
TF313468	10677	ENSG00000135407
TF318514	10220	ENSG00000135414
TF313359	27165	ENSG00000135423
TF105391	3679	ENSG00000135424
TF331566	9840	ENSG00000135426
TF325617	5959	ENSG00000135437
TF317762	116986	ENSG00000135439
TF317854	3891	ENSG00000135443
TF332576	5502	ENSG00000135447
TF323367	6302	ENSG00000135452
TF332297	2583	ENSG00000135454
TF314132	7024	ENSG00000135457
TF314447	93058	ENSG00000135469
TF319996	23017	ENSG00000135472
TF317854	3855	ENSG00000135480
TF351342	3178	ENSG00000135486
TF351342	389674	ENSG00000135486
TF351342	402562	ENSG00000135486
TF351342	645691	ENSG00000135486
TF313784	115557	ENSG00000135502
TF313784	65012	ENSG00000135502
TF314724	91	ENSG00000135503
TF312940	4284	ENSG00000135517
TF313130	23416	ENSG00000135519
TF332273	9053	ENSG00000135525
TF333380	8763	ENSG00000135535
TF333323	57224	ENSG00000135540
TF323617	23493	ENSG00000135547
TF330809	5570	ENSG00000135549
TF343107	9038	ENSG00000135569
TF331292	4829	ENSG00000135577
TF324129	64780	ENSG00000135596
TF316546	85021	ENSG00000135597
TF313763	8676	ENSG00000135604
TF315363	7006	ENSG00000135605
TF352903	10505	ENSG00000135622
TF318980	1961	ENSG00000135625

TF326172	26056	ENSG00000135631
TF316871	8291	ENSG00000135636
TF317015	2016	ENSG00000135638
TF328589	27345	ENSG00000135643
TF106276	9958	ENSG00000135655
TF313545	2799	ENSG00000135677
TF105306	4193	ENSG00000135679
TF328485	79786	ENSG00000135686
TF314019	53630	ENSG00000135697
TF342871	23563	ENSG00000135702
TF316268	29109	ENSG00000135723
TF317212	6553	ENSG00000135740
TF343201	183	ENSG00000135744
TF313570	80003	ENSG00000135749
TF313947	3775	ENSG00000135750
TF313947	728987	ENSG00000135750
TF313947	731259	ENSG00000135750
TF314595	54583	ENSG00000135766
TF314748	10753	ENSG00000135773
TF300491	2752	ENSG00000135821
TF313254	10228	ENSG00000135823
TF315837	85397	ENSG00000135824
TF333351	116496	ENSG00000135842
TF312903	3915	ENSG00000135862
TF317698	149041	ENSG00000135870
TF315605	1144	ENSG00000135902
TF351610	5077	ENSG00000135903
TF313629	55619	ENSG00000135905
TF323032	57695	ENSG00000135913
TF316350	3357	ENSG00000135914
TF317770	81618	ENSG00000135916
TF313684	80704	ENSG00000135917
TF352620	5270	ENSG00000135919
TF105310	80326	ENSG00000135925
TF319996	64114	ENSG00000135926
TF105094	1593	ENSG00000135929
TF101529	9470	ENSG00000135930
TF314910	51719	ENSG00000135932
TF326518	80705	ENSG00000135951
TF331385	10913	ENSG00000135960
TF328650	9392	ENSG00000135966
TF106438	26122	ENSG00000135999
TF316832	50649	ENSG00000136002
TF105422	23479	ENSG00000136003
TF354242	160428	ENSG00000136010
TF331489	55576	ENSG00000136011
TF315281	84101	ENSG00000136014
TF312962	10154	ENSG00000136040
TF328669	55198	ENSG00000136044
TF313647	84102	ENSG00000136052
TF313468	50853	ENSG00000136059
TF313685	2317	ENSG00000136068
TF106472	4752	ENSG00000136098
TF352008	5100	ENSG00000136099
TF333003	26586	ENSG00000136108
TF317184	9882	ENSG00000136111
TF318428	23143	ENSG00000136141
TF329478	55213	ENSG00000136144
TF325426	51131	ENSG00000136147

TF332155	4008	ENSG00000136153
TF335114	8796	ENSG00000136155
TF317770	9445	ENSG00000136156
TF325070	10253	ENSG00000136158
TF331292	1910	ENSG00000136160
TF329478	1102	ENSG00000136161
TF300680	3936	ENSG00000136167
TF106411	83852	ENSG00000136169
TF323890	9805	ENSG00000136193
TF315996	64759	ENSG00000136205
TF329827	285955	ENSG00000136206
TF329827	442609	ENSG00000136206
TF329827	653629	ENSG00000136206
TF329827	729597	ENSG00000136206
TF325581	55501	ENSG00000136213
TF320229	10643	ENSG00000136231
TF334865	10457	ENSG00000136235
TF313184	9771	ENSG00000136237
TF101109	5879	ENSG00000136238
TF314792	11014	ENSG00000136240
TF324313	28969	ENSG00000136261
TF313104	1607	ENSG00000136267
TF313348	23148	ENSG00000136274
TF318935	28988	ENSG00000136279
TF328517	83605	ENSG00000136280
TF312960	64005	ENSG00000136286
TF319025	80727	ENSG00000136295
TF313370	221938	ENSG00000136297
TF334321	27141	ENSG00000136305
TF351204	26257	ENSG00000136327
TF351204	7080	ENSG00000136352
TF313537	11173	ENSG00000136378
TF314365	58489	ENSG00000136379
TF313476	3658	ENSG00000136381
TF333088	53346	ENSG00000136404
TF313865	10518	ENSG00000136425
TF329501	10241	ENSG00000136436
TF332416	55316	ENSG00000136444
TF300701	4836	ENSG00000136448
TF106261	6426	ENSG00000136450
TF331686	7716	ENSG00000136451
TF332659	1101	ENSG00000136457
TF314900	55852	ENSG00000136478
TF332592	2689	ENSG00000136487
TF332592	1442	ENSG00000136488
TF313758	80774	ENSG00000136490
TF312863	86	ENSG00000136518
TF106265	6434	ENSG00000136527
TF323985	6326	ENSG00000136531
TF106341	10716	ENSG00000136535
TF323985	6332	ENSG00000136546
TF351634	640	ENSG00000136573
TF315391	2626	ENSG00000136574
TF324133	6498	ENSG00000136603
TF333253	3586	ENSG00000136634
TF323964	26750	ENSG00000136643
TF319243	83593	ENSG00000136653
TF313542	274	ENSG00000136717
TF312835	9394	ENSG00000136720

TF312835	728969	ENSG00000136720
TF300320	56886	ENSG00000136731
TF337016	2995	ENSG00000136732
TF315007	8027	ENSG00000136738
TF314688	2572	ENSG00000136750
TF314303	10006	ENSG00000136754
TF331443	56262	ENSG00000136802
TF318932	7295	ENSG00000136810
TF328605	4957	ENSG00000136811
TF314941	27348	ENSG00000136816
TF315506	9314	ENSG00000136826
TF314941	1861	ENSG00000136827
TF352150	9649	ENSG00000136828
TF333351	64855	ENSG00000136830
TF352818	27090	ENSG00000136840
TF315841	7111	ENSG00000136842
TF105303	153090	ENSG00000136848
TF313242	6812	ENSG00000136854
TF325324	29988	ENSG00000136856
TF329233	55755	ENSG00000136861
TF351113	7099	ENSG00000136869
TF314203	229	ENSG00000136872
TF352179	10868	ENSG00000136878
TF313965	9568	ENSG00000136928
TF106222	5695	ENSG00000136930
TF350737	2516	ENSG00000136931
TF329153	10244	ENSG00000136933
TF317206	10541	ENSG00000136938
TF315179	5082	ENSG00000136940
TF313739	1515	ENSG00000136943
TF315442	4010	ENSG00000136944
TF319716	81873	ENSG00000136950
TF330032	5168	ENSG00000136960
TF106001	4609	ENSG00000136997
TF106001	731404	ENSG00000136997
TF326070	4856	ENSG00000136999
TF331210	3590	ENSG00000137070
TF328468	55833	ENSG00000137073
TF325756	152006	ENSG00000137075
TF314677	7094	ENSG00000137076
TF317837	1761	ENSG00000137090
TF105141	25822	ENSG00000137094
TF331003	51754	ENSG00000137103
TF324791	9380	ENSG00000137106
TF300455	219	ENSG00000137124
TF331645	347252	ENSG00000137142
TF313237	55667	ENSG00000137145
TF300035	6194	ENSG00000137154
TF326978	116113	ENSG00000137166
TF314010	89953	ENSG00000137171
TF105221	63971	ENSG00000137177
TF320810	5292	ENSG00000137193
TF300378	2766	ENSG00000137198
TF313718	7020	ENSG00000137203
TF323345	81853	ENSG00000137210
TF323345	645203	ENSG00000137210
TF324300	55362	ENSG00000137216
TF324994	10817	ENSG00000137218
TF331612	93643	ENSG00000137221

TF314748	11131	ENSG00000137225
TF313765	27283	ENSG00000137251
TF315303	3062	ENSG00000137252
TF300137	3012	ENSG00000137259
TF300137	8335	ENSG00000137259
TF323356	9856	ENSG00000137261
TF328512	3662	ENSG00000137265
TF300298	7280	ENSG00000137267
TF351429	55227	ENSG00000137269
TF324146	8521	ENSG00000137270
TF351598	2295	ENSG00000137273
TF106506	8737	ENSG00000137275
TF300298	347733	ENSG00000137285
TF351623	3159	ENSG00000137309
TF324777	255488	ENSG00000137393
TF313721	23787	ENSG00000137409
TF335877	9982	ENSG00000137440
TF335877	83888	ENSG00000137441
TF317658	132864	ENSG00000137449
TF324020	85462	ENSG00000137460
TF351113	7097	ENSG00000137462
TF324557	9873	ENSG00000137478
TF314260	408	ENSG00000137486
TF317540	11309	ENSG00000137491
TF330114	5612	ENSG00000137492
TF330114	728748	ENSG00000137492
TF331442	60492	ENSG00000137500
TF341184	54843	ENSG00000137501
TF317167	2615	ENSG00000137507
TF314357	55298	ENSG00000137522
TF316148	51050	ENSG00000137558
TF323436	7274	ENSG00000137561
TF323437	8836	ENSG00000137563
TF313545	23213	ENSG00000137573
TF327131	6386	ENSG00000137575
TF329555	54827	ENSG00000137634
TF351678	56649	ENSG00000137648
TF313147	7225	ENSG00000137672
TF313147	730221	ENSG00000137672
TF313147	730335	ENSG00000137672
TF313147	731423	ENSG00000137672
TF315428	4316	ENSG00000137673
TF315428	9313	ENSG00000137674
TF315428	64066	ENSG00000137675
TF354270	84259	ENSG00000137692
TF326941	10413	ENSG00000137693
TF351086	23650	ENSG00000137699
TF105272	54766	ENSG00000137707
TF313935	5962	ENSG00000137710
TF105552	5519	ENSG00000137713
TF319845	2230	ENSG00000137714
TF333443	53826	ENSG00000137726
TF331062	57569	ENSG00000137727
TF333443	486	ENSG00000137731
TF315428	4322	ENSG00000137745
TF351678	84000	ENSG00000137747
TF102023	834	ENSG00000137752
TF102023	440068	ENSG00000137752
TF102023	838	ENSG00000137757

TF312844	440279	ENSG00000137766
TF325240	79811	ENSG00000137776
TF324917	7057	ENSG00000137801
TF323254	23005	ENSG00000137802
TF105391	22801	ENSG00000137809
TF323413	56965	ENSG00000137817
TF319738	54852	ENSG00000137819
TF315854	55177	ENSG00000137824
TF318394	3706	ENSG00000137825
TF331274	55075	ENSG00000137831
TF314923	4091	ENSG00000137834
TF313216	5330	ENSG00000137841
TF105352	56924	ENSG00000137843
TF352021	102	ENSG00000137845
TF314131	9153	ENSG00000137860
TF331851	64220	ENSG00000137868
TF331707	54816	ENSG00000137871
TF316102	80031	ENSG00000137872
TF323756	8412	ENSG00000137936
TF351162	54874	ENSG00000137942
TF317345	676	ENSG00000137948
TF313878	54810	ENSG00000137960
TF351450	9411	ENSG00000137962
TF313325	204962	ENSG00000137968
TF300740	653702	ENSG00000137970
TF300740	653949	ENSG00000137970
TF314536	58511	ENSG00000137976
TF313270	85465	ENSG00000138018
TF313845	109	ENSG00000138031
TF313590	5495	ENSG00000138032
TF316814	3973	ENSG00000138039
TF315190	57223	ENSG00000138041
TF313093	80745	ENSG00000138050
TF321745	391365	ENSG00000138068
TF300097	5861	ENSG00000138069
TF300467	10097	ENSG00000138071
TF105212	64240	ENSG00000138075
TF331033	11117	ENSG00000138080
TF313602	80204	ENSG00000138081
TF315545	6496	ENSG00000138083
TF331669	57159	ENSG00000138100
TF314232	1838	ENSG00000138101
TF300420	10121	ENSG00000138107
TF316871	26509	ENSG00000138119
TF326061	84171	ENSG00000138131
TF323215	57559	ENSG00000138134
TF325047	10660	ENSG00000138136
TF105016	84896	ENSG00000138138
TF333149	10579	ENSG00000138162
TF105122	1847	ENSG00000138166
TF329085	51063	ENSG00000138172
TF105463	403	ENSG00000138175
TF105232	9585	ENSG00000138182
TF332859	953	ENSG00000138185
TF315199	54536	ENSG00000138190
TF331445	5950	ENSG00000138207
TF330969	53836	ENSG00000138271
TF323302	84647	ENSG00000138308
TF329439	22891	ENSG00000138311

TF330284	170392	ENSG00000138315
TF313537	140766	ENSG00000138316
TF324004	80312	ENSG00000138336
TF343193	84665	ENSG00000138347
TF353036	316	ENSG00000138356
TF326440	580	ENSG00000138376
TF318648	6775	ENSG00000138378
TF318514	2660	ENSG00000138379
TF315501	4664	ENSG00000138386
TF106508	65061	ENSG00000138395
TF318563	9360	ENSG00000138398
TF313938	57520	ENSG00000138411
TF331566	6744	ENSG00000138434
TF333159	150864	ENSG00000138439
TF314303	10152	ENSG00000138443
TF105391	3685	ENSG00000138448
TF313463	30061	ENSG00000138449
TF314292	84925	ENSG00000138463
TF350136	57337	ENSG00000138468
TF333971	9626	ENSG00000138472
TF328965	83666	ENSG00000138496
TF328821	9728	ENSG00000138593
TF315841	29766	ENSG00000138594
TF319186	84888	ENSG00000138600
TF325799	90525	ENSG00000138606
TF314362	83464	ENSG00000138613
TF330132	8483	ENSG00000138615
TF318250	10021	ENSG00000138622
TF323577	83478	ENSG00000138639
TF328895	10144	ENSG00000138640
TF315189	8916	ENSG00000138641
TF315189	55008	ENSG00000138642
TF315189	51191	ENSG00000138646
TF352008	57575	ENSG00000138650
TF313193	64579	ENSG00000138653
TF314808	3184	ENSG00000138668
TF313261	5593	ENSG00000138669
TF313379	153020	ENSG00000138670
TF313842	22872	ENSG00000138674
TF315039	84803	ENSG00000138678
TF317805	2247	ENSG00000138685
TF314724	658	ENSG00000138696
TF323666	5910	ENSG00000138698
TF314516	55132	ENSG00000138709
TF314516	644578	ENSG00000138709
TF314516	648136	ENSG00000138709
TF316499	8654	ENSG00000138735
TF313147	7222	ENSG00000138741
TF333433	4283	ENSG00000138755
TF317300	55589	ENSG00000138756
TF325464	9908	ENSG00000138757
TF101080	55752	ENSG00000138758
TF317925	950	ENSG00000138760
TF101007	901	ENSG00000138764
TF323175	246175	ENSG00000138767
TF101031	8999	ENSG00000138769
TF333370	57619	ENSG00000138771
TF105452	306	ENSG00000138772
TF300887	27068	ENSG00000138777

TF352114	54848	ENSG00000138784
TF318448	51176	ENSG00000138795
TF313143	9061	ENSG00000138801
TF105557	5530	ENSG00000138814
TF318470	64116	ENSG00000138821
TF328754	4547	ENSG00000138823
TF316849	2201	ENSG00000138829
TF313096	23162	ENSG00000138834
TF318060	400916	ENSG00000138869
TF318060	646106	ENSG00000138869
TF313087	164714	ENSG00000138892
TF317334	91445	ENSG00000138942
TF318303	283358	ENSG00000139044
TF333297	5149	ENSG00000139053
TF106381	121506	ENSG00000139055
TF318679	2120	ENSG00000139083
TF352568	731158	ENSG00000139088
TF105224	55605	ENSG00000139116
TF316419	144402	ENSG00000139117
TF316247	121512	ENSG00000139132
TF102031	5288	ENSG00000139144
TF323911	58516	ENSG00000139146
TF313216	89869	ENSG00000139151
TF317540	53919	ENSG00000139155
TF313549	55500	ENSG00000139163
TF313265	144165	ENSG00000139174
TF330373	51279	ENSG00000139178
TF315946	9746	ENSG00000139182
TF313666	6843	ENSG00000139190
TF334274	55080	ENSG00000139192
TF316894	83758	ENSG00000139194
TF315044	5830	ENSG00000139197
TF328787	55089	ENSG00000139209
TF326838	347902	ENSG00000139211
TF332183	9169	ENSG00000139218
TF323987	1280	ENSG00000139219
TF314207	8499	ENSG00000139220
TF317206	23519	ENSG00000139223
TF325380	121227	ENSG00000139263
TF319557	92979	ENSG00000139266
TF316148	11010	ENSG00000139278
TF313327	121278	ENSG00000139287
TF332320	22822	ENSG00000139289
TF316814	8549	ENSG00000139292
TF105122	1848	ENSG00000139318
TF334562	4060	ENSG00000139329
TF319326	11081	ENSG00000139330
TF328876	50511	ENSG00000139351
TF322889	429	ENSG00000139352
TF323754	283431	ENSG00000139354
TF314981	114795	ENSG00000139364
TF330897	121260	ENSG00000139370
TF329014	113675	ENSG00000139410
TF317467	51228	ENSG00000139433
TF317467	645312	ENSG00000139433
TF317762	9815	ENSG00000139436
TF331508	84915	ENSG00000139438
TF329867	121643	ENSG00000139445
TF315197	9107	ENSG00000139505

TF315701	283537	ENSG00000139508
TF315212	6541	ENSG00000139514
TF330709	222484	ENSG00000139517
TF318470	283375	ENSG00000139540
TF315953	6895	ENSG00000139546
TF325617	8608	ENSG00000139547
TF106458	50846	ENSG00000139549
TF314724	94	ENSG00000139567
TF313902	79035	ENSG00000139579
TF105395	90634	ENSG00000139597
TF330455	1990	ENSG00000139610
TF314710	6601	ENSG00000139613
TF314319	91012	ENSG00000139624
TF105119	7786	ENSG00000139625
TF105392	3695	ENSG00000139626
TF313267	11226	ENSG00000139629
TF314688	51380	ENSG00000139631
TF313485	55716	ENSG00000139636
TF324255	23344	ENSG00000139641
TF317854	112802	ENSG00000139648
TF315506	283337	ENSG00000139651
TF314470	115825	ENSG00000139668
TF351342	144983	ENSG00000139675
TF350009	10161	ENSG00000139679
TF313526	55206	ENSG00000139697
TF106436	23067	ENSG00000139718
TF321840	79720	ENSG00000139722
TF315383	81624	ENSG00000139734
TF331616	122060	ENSG00000139737
TF319253	64062	ENSG00000139746
TF335721	84530	ENSG00000139767
TF354267	196541	ENSG00000139780
TF321931	10150	ENSG00000139793
TF351425	85416	ENSG00000139800
TF101153	8451	ENSG00000139842
TF332283	115669	ENSG00000139865
TF315737	6751	ENSG00000139874
TF329887	64403	ENSG00000139880
TF314379	161253	ENSG00000139890
TF329591	643866	ENSG00000139899
TF316981	4857	ENSG00000139910
TF324599	161247	ENSG00000139914
TF330345	161357	ENSG00000139915
TF106376	81542	ENSG00000139921
TF315388	122786	ENSG00000139926
TF314338	57161	ENSG00000139946
TF105431	6252	ENSG00000139970
TF314733	8747	ENSG00000139985
TF105429	145226	ENSG00000139988
TF314097	376267	ENSG00000139998
TF323751	2100	ENSG00000140009
TF313130	27133	ENSG00000140015
TF300393	85439	ENSG00000140022
TF331803	8477	ENSG00000140030
TF326301	122953	ENSG00000140044
TF333276	90050	ENSG00000140067
TF318759	123041	ENSG00000140090
TF317514	10516	ENSG00000140092
TF343094	51156	ENSG00000140093

TF351739	283600	ENSG00000140107
TF314961	91833	ENSG00000140153
TF313214	81614	ENSG00000140157
TF313657	9990	ENSG00000140199
TF321672	6938	ENSG00000140262
TF318444	256586	ENSG00000140280
TF313430	11001	ENSG00000140284
TF317805	2252	ENSG00000140285
TF315534	9245	ENSG00000140297
TF324164	663	ENSG00000140299
TF324144	85455	ENSG00000140323
TF314167	7090	ENSG00000140332
TF317206	8125	ENSG00000140350
TF313338	92912	ENSG00000140367
TF313677	9051	ENSG00000140368
TF315834	597	ENSG00000140379
TF106440	10363	ENSG00000140382
TF316345	10099	ENSG00000140391
TF332652	10499	ENSG00000140396
TF105151	55466	ENSG00000140403
TF351519	7168	ENSG00000140416
TF351636	3480	ENSG00000140443
TF313650	91947	ENSG00000140450
TF105094	1583	ENSG00000140459
TF313537	170691	ENSG00000140470
TF316990	55889	ENSG00000140478
TF314277	5046	ENSG00000140479
TF314924	60677	ENSG00000140488
TF313797	10066	ENSG00000140497
TF313311	79748	ENSG00000140506
TF332134	145864	ENSG00000140511
TF314450	51458	ENSG00000140519
TF313195	11057	ENSG00000140526
TF106465	4916	ENSG00000140538
TF330156	4240	ENSG00000140545
TF331510	374655	ENSG00000140548
TF314096	55898	ENSG00000140553
TF352820	8128	ENSG00000140557
TF323373	55784	ENSG00000140563
TF314277	5045	ENSG00000140564
TF313078	8826	ENSG00000140575
TF321571	64784	ENSG00000140577
TF313281	6457	ENSG00000140600
TF313648	23478	ENSG00000140612
TF101078	124404	ENSG00000140623
TF300874	5373	ENSG00000140650
TF352855	6524	ENSG00000140675
TF105391	3687	ENSG00000140678
TF314113	7041	ENSG00000140682
TF326183	1039	ENSG00000140743
TF351123	91807	ENSG00000140795
TF328786	85407	ENSG00000140807
TF332526	91862	ENSG00000140832
TF342871	10164	ENSG00000140835
TF323288	463	ENSG00000140836
TF316419	221184	ENSG00000140848
TF330014	84166	ENSG00000140853
TF105238	3801	ENSG00000140859
TF313537	170692	ENSG00000140873

TF317387	123920	ENSG00000140931
TF329887	1009	ENSG00000140937
TF312964	81631	ENSG00000140941
TF335574	23174	ENSG00000140948
TF315806	161931	ENSG00000140955
TF313502	29948	ENSG00000140961
TF328512	3394	ENSG00000140968
TF300814	89941	ENSG00000140983
TF300555	6123	ENSG00000140986
TF106423	9611	ENSG00000141027
TF314375	4622	ENSG00000141048
TF326024	93649	ENSG00000141052
TF317006	8844	ENSG00000141068
TF331658	57610	ENSG00000141084
TF330455	1506	ENSG00000141086
TF324523	64180	ENSG00000141096
TF323246	81577	ENSG00000141098
TF106367	5636	ENSG00000141127
TF314096	146862	ENSG00000141161
TF105222	84643	ENSG00000141200
TF105274	10140	ENSG00000141232
TF318216	9905	ENSG00000141258
TF331055	8631	ENSG00000141293
TF323890	90507	ENSG00000141295
TF319444	85464	ENSG00000141298
TF313540	162494	ENSG00000141314
TF324882	124912	ENSG00000141316
TF317067	9842	ENSG00000141342
TF324388	92579	ENSG00000141349
TF300059	1213	ENSG00000141367
TF335849	124773	ENSG00000141371
TF324583	51651	ENSG00000141378
TF330999	6760	ENSG00000141380
TF316520	6875	ENSG00000141384
TF105004	10939	ENSG00000141385
TF312873	10650	ENSG00000141391
TF313194	3613	ENSG00000141401
TF300673	2774	ENSG00000141404
TF318470	25800	ENSG00000141424
TF320926	55197	ENSG00000141425
TF313267	2589	ENSG00000141429
TF332804	116	ENSG00000141433
TF315280	4225	ENSG00000141434
TF314192	147407	ENSG00000141437
TF329726	64762	ENSG00000141441
TF314027	114799	ENSG00000141446
TF315391	2627	ENSG00000141448
TF314260	409	ENSG00000141480
TF312913	284111	ENSG00000141485
TF105138	50488	ENSG00000141503
TF319736	112483	ENSG00000141504
TF102035	23533	ENSG00000141506
TF106101	7157	ENSG00000141510
TF105387	396	ENSG00000141522
TF313462	11322	ENSG00000141524
TF313462	644590	ENSG00000141524
TF313462	652027	ENSG00000141524
TF313792	9123	ENSG00000141526
TF315606	79092	ENSG00000141527

TF319025	94015	ENSG00000141540
TF323230	10966	ENSG00000141542
TF300544	1453	ENSG00000141551
TF106273	26502	ENSG00000141562
TF325718	3607	ENSG00000141568
TF106456	57332	ENSG00000141570
TF314969	114804	ENSG00000141576
TF331381	79755	ENSG00000141579
TF106456	8535	ENSG00000141582
TF317681	494470	ENSG00000141622
TF105098	5596	ENSG00000141639
TF314923	4089	ENSG00000141646
TF335574	54877	ENSG00000141664
TF329591	147381	ENSG00000141668
TF320837	10609	ENSG00000141696
TF314663	115024	ENSG00000141698
TF329111	162427	ENSG00000141699
TF354315	8396	ENSG00000141720
TF106002	2064	ENSG00000141736
TF317511	2886	ENSG00000141738
TF313114	5409	ENSG00000141744
TF332878	342667	ENSG00000141750
TF331211	3487	ENSG00000141753
TF105296	60681	ENSG00000141756
TF312805	773	ENSG00000141837
TF317345	23476	ENSG00000141867
TF313889	4782	ENSG00000141905
TF316040	8612	ENSG00000141934
TF300411	5211	ENSG00000141959
TF351376	55527	ENSG00000141965
TF316171	7409	ENSG00000141968
TF314477	93343	ENSG00000141971
TF313865	117286	ENSG00000141977
TF338438	125972	ENSG00000141979
TF313281	6455	ENSG00000141985
TF106181	23410	ENSG00000142082
TF334894	10410	ENSG00000142089
TF334894	144383	ENSG00000142089
TF331207	1291	ENSG00000142156
TF332537	3454	ENSG00000142166
TF331207	1292	ENSG00000142173
TF315213	150094	ENSG00000142178
TF329039	29947	ENSG00000142182
TF314204	7226	ENSG00000142185
TF300282	757	ENSG00000142188
TF317274	351	ENSG00000142192
TF316855	9980	ENSG00000142197
TF102004	207	ENSG00000142208
TF333253	29949	ENSG00000142224
TF330414	2014	ENSG00000142227
TF352481	126147	ENSG00000142233
TF332280	114783	ENSG00000142235
TF314210	23624	ENSG00000142273
TF320310	126374	ENSG00000142279
TF313537	81794	ENSG00000142303
TF343812	6531	ENSG00000142319
TF300758	57140	ENSG00000142327
TF312960	4542	ENSG00000142347
TF330014	91662	ENSG00000142405

TF327980	59283	ENSG00000142408
TF316849	84467	ENSG00000142449
TF323332	10498	ENSG00000142453
TF317184	115704	ENSG00000142459
TF331371	9032	ENSG00000142484
TF324441	55244	ENSG00000142494
TF106221	5694	ENSG00000142507
TF332441	89790	ENSG00000142512
TF312893	93650	ENSG00000142513
TF352494	6689	ENSG00000142539
TF325565	402665	ENSG00000142549
TF314849	57333	ENSG00000142552
TF328554	473	ENSG00000142599
TF315192	79258	ENSG00000142606
TF315309	63976	ENSG00000142611
TF315309	647868	ENSG00000142611
TF330455	63036	ENSG00000142615
TF315608	1969	ENSG00000142627
TF316357	128272	ENSG00000142632
TF320736	79180	ENSG00000142634
TF331825	127294	ENSG00000142661
TF105574	83442	ENSG00000142669
TF326495	10256	ENSG00000142675
TF323356	79932	ENSG00000142687
TF352986	55194	ENSG00000142694
TF105115	9064	ENSG00000142733
TF341184	84958	ENSG00000142765
TF330455	23436	ENSG00000142789
TF330455	10136	ENSG00000142789
TF326548	3339	ENSG00000142798
TF318374	26135	ENSG00000142864
TF326070	3491	ENSG00000142871
TF313399	5567	ENSG00000142875
TF313765	64129	ENSG00000142910
TF300760	113451	ENSG00000142920
TF105222	11004	ENSG00000142945
TF312900	5792	ENSG00000142949
TF315803	266675	ENSG00000142959
TF300789	148932	ENSG00000142961
TF105088	1580	ENSG00000142973
TF315442	8543	ENSG00000143013
TF315804	284612	ENSG00000143028
TF316128	343472	ENSG00000143032
TF106420	22823	ENSG00000143033
TF313325	126969	ENSG00000143036
TF332702	3321	ENSG00000143061
TF332702	649698	ENSG00000143061
TF325130	55917	ENSG00000143079
TF314205	85369	ENSG00000143093
TF313103	3744	ENSG00000143105
TF352892	963	ENSG00000143119
TF332732	84432	ENSG00000143125
TF323983	1952	ENSG00000143126
TF105391	8515	ENSG00000143127
TF329606	2702	ENSG00000143140
TF331895	23432	ENSG00000143147
TF331895	730012	ENSG00000143147
TF331895	731581	ENSG00000143147
TF314618	481	ENSG00000143153

TF332951	57645	ENSG00000143157
TF324680	8804	ENSG00000143162
TF330875	10223	ENSG00000143167
TF352097	6258	ENSG00000143171
TF106341	9095	ENSG00000143178
TF315045	54499	ENSG00000143183
TF334888	6375	ENSG00000143184
TF334888	6846	ENSG00000143185
TF316413	5451	ENSG00000143190
TF330877	387597	ENSG00000143195
TF328602	1805	ENSG00000143196
TF335097	2212	ENSG00000143226
TF315837	8490	ENSG00000143248
TF316304	9970	ENSG00000143257
TF106277	27005	ENSG00000143258
TF326157	2165	ENSG00000143278
TF313796	844	ENSG00000143318
TF354340	81875	ENSG00000143319
TF316894	1382	ENSG00000143320
TF105385	3068	ENSG00000143321
TF105081	27	ENSG00000143322
TF314643	9213	ENSG00000143324
TF315837	6004	ENSG00000143333
TF333084	148753	ENSG00000143340
TF326318	83872	ENSG00000143341
TF315204	23179	ENSG00000143344
TF315442	56956	ENSG00000143355
TF323914	58497	ENSG00000143363
TF319910	6097	ENSG00000143365
TF331627	7286	ENSG00000143367
TF329009	57592	ENSG00000143373
TF300858	80222	ENSG00000143374
TF332247	57530	ENSG00000143375
TF106411	9869	ENSG00000143379
TF316874	54507	ENSG00000143382
TF315834	4170	ENSG00000143384
TF313739	1513	ENSG00000143387
TF321340	5993	ENSG00000143390
TF319618	8394	ENSG00000143398
TF317206	81611	ENSG00000143401
TF314589	55793	ENSG00000143409
TF105452	8416	ENSG00000143412
TF314319	29956	ENSG00000143418
TF314718	2029	ENSG00000143420
TF316102	10500	ENSG00000143434
TF319983	405	ENSG00000143437
TF331707	23126	ENSG00000143442
TF314741	51686	ENSG00000143450
TF313989	84072	ENSG00000143452
TF314360	55204	ENSG00000143457
TF326036	126626	ENSG00000143458
TF324269	9641	ENSG00000143466
TF351132	255928	ENSG00000143469
TF313130	3756	ENSG00000143473
TF314624	8444	ENSG00000143479
TF329370	79805	ENSG00000143494
TF106487	56950	ENSG00000143499
TF332459	55061	ENSG00000143502
TF105122	11221	ENSG00000143507

TF329059	79802	ENSG00000143512
TF105545	7159	ENSG00000143514
TF300654	57198	ENSG00000143515
TF314733	8751	ENSG00000143537
TF314097	5872	ENSG00000143545
TF332727	6279	ENSG00000143546
TF351519	7170	ENSG00000143549
TF313430	11000	ENSG00000143554
TF328468	9898	ENSG00000143569
TF317098	27173	ENSG00000143570
TF316079	148327	ENSG00000143578
TF315495	1944	ENSG00000143590
TF313173	89872	ENSG00000143595
TF315015	3782	ENSG00000143603
TF321369	57459	ENSG00000143614
TF315495	1945	ENSG00000143620
TF315072	6016	ENSG00000143622
TF300390	5313	ENSG00000143627
TF318250	57657	ENSG00000143630
TF300361	58	ENSG00000143632
TF332283	79573	ENSG00000143643
TF105118	84451	ENSG00000143674
TF328469	9859	ENSG00000143702
TF328469	642108	ENSG00000143702
TF328469	652766	ENSG00000143702
TF313582	8560	ENSG00000143753
TF300808	375	ENSG00000143761
TF318394	3707	ENSG00000143772
TF314473	2987	ENSG00000143774
TF313551	8476	ENSG00000143776
TF300083	149111	ENSG00000143786
TF314906	129642	ENSG00000143797
TF315040	5664	ENSG00000143801
TF101179	3930	ENSG00000143815
TF105310	7483	ENSG00000143816
TF313813	2052	ENSG00000143819
TF314990	5972	ENSG00000143839
TF320471	9580	ENSG00000143842
TF313549	55224	ENSG00000143845
TF314207	8497	ENSG00000143847
TF329090	22874	ENSG00000143850
TF331016	5778	ENSG00000143851
TF315600	127833	ENSG00000143858
TF350876	130497	ENSG00000143867
TF316134	151449	ENSG00000143869
TF300837	388	ENSG00000143878
TF314912	245973	ENSG00000143882
TF354318	92906	ENSG00000143889
TF105212	64241	ENSG00000143921
TF317832	27436	ENSG00000143924
TF300912	801	ENSG00000143933
TF300912	805	ENSG00000143933
TF300912	808	ENSG00000143933
TF328464	55252	ENSG00000143970
TF313195	84696	ENSG00000143994
TF324687	9027	ENSG00000144035
TF315199	23233	ENSG00000144036
TF313205	94097	ENSG00000144040
TF105735	165545	ENSG00000144045

TF323961	84620	ENSG00000144057
TF316174	7851	ENSG00000144063
TF312796	5899	ENSG00000144118
TF329591	165257	ENSG00000144119
TF314426	6574	ENSG00000144136
TF330076	129804	ENSG00000144152
TF318250	1261	ENSG00000144191
TF329826	129530	ENSG00000144214
TF326216	3899	ENSG00000144218
TF333047	11249	ENSG00000144227
TF313419	339745	ENSG00000144228
TF313419	642381	ENSG00000144228
TF313419	648489	ENSG00000144228
TF329791	80731	ENSG00000144229
TF330775	2840	ENSG00000144230
TF313267	114805	ENSG00000144278
TF321877	8502	ENSG00000144283
TF323985	6323	ENSG00000144285
TF313630	57282	ENSG00000144290
TF323890	79634	ENSG00000144306
TF326622	151126	ENSG00000144331
TF330868	23671	ENSG00000144339
TF101076	83879	ENSG00000144354
TF315720	1745	ENSG00000144355
TF300112	493911	ENSG00000144362
TF314159	51454	ENSG00000144366
TF331338	165215	ENSG00000144369
TF313206	151194	ENSG00000144401
TF315710	5746	ENSG00000144407
TF317197	130749	ENSG00000144410
TF323165	65065	ENSG00000144426
TF336511	151050	ENSG00000144445
TF105191	26154	ENSG00000144452
TF333489	57007	ENSG00000144476
TF314204	79054	ENSG00000144481
TF351373	55502	ENSG00000144485
TF326392	339768	ENSG00000144488
TF316419	151835	ENSG00000144550
TF101106	2177	ENSG00000144554
TF330846	9686	ENSG00000144560
TF300199	5868	ENSG00000144566
TF329111	79137	ENSG00000144567
TF313556	58190	ENSG00000144579
TF319557	57574	ENSG00000144583
TF326909	80852	ENSG00000144596
TF320864	85403	ENSG00000144597
TF351103	152330	ENSG00000144619
TF314138	51143	ENSG00000144635
TF314644	27303	ENSG00000144642
TF314688	339896	ENSG00000144644
TF312807	114884	ENSG00000144645
TF330966	1238	ENSG00000144648
TF330994	729085	ENSG00000144649
TF323969	64651	ENSG00000144655
TF105391	3680	ENSG00000144668
TF325082	2803	ENSG00000144674
TF313556	10217	ENSG00000144677
TF332878	6769	ENSG00000144681
TF323811	9922	ENSG00000144711

TF300355	23066	ENSG00000144712
TF351978	5793	ENSG00000144724
TF329644	54756	ENSG00000144730
TF105479	10550	ENSG00000144746
TF325380	26018	ENSG00000144749
TF351114	57408	ENSG00000144771
TF318578	10330	ENSG00000144785
TF320310	8994	ENSG00000144791
TF330224	64332	ENSG00000144802
TF332902	1295	ENSG00000144810
TF329555	91775	ENSG00000144815
TF314375	22989	ENSG00000144821
TF329165	257068	ENSG00000144824
TF329165	90102	ENSG00000144824
TF313921	29114	ENSG00000144834
TF324997	51365	ENSG00000144837
TF329417	141	ENSG00000144843
TF330875	152404	ENSG00000144847
TF316304	8856	ENSG00000144852
TF332268	91653	ENSG00000144857
TF330024	185	ENSG00000144891
TF324178	116931	ENSG00000144893
TF354242	10840	ENSG00000144908
TF312807	114885	ENSG00000144909
TF313147	7220	ENSG00000144935
TF314978	57552	ENSG00000144959
TF320310	4026	ENSG00000145012
TF317067	9711	ENSG00000145016
TF314252	7873	ENSG00000145050
TF314585	9515	ENSG00000145087
TF320864	55840	ENSG00000145088
TF330877	286676	ENSG00000145103
TF331371	116211	ENSG00000145107
TF332887	9353	ENSG00000145147
TF333729	197	ENSG00000145192
TF315192	9718	ENSG00000145194
TF329720	90113	ENSG00000145198
TF313784	10861	ENSG00000145217
TF315608	2044	ENSG00000145242
TF354252	57205	ENSG00000145246
TF327106	132299	ENSG00000145247
TF315811	345274	ENSG00000145283
TF330308	51316	ENSG00000145287
TF332776	6622	ENSG00000145335
TF315229	817	ENSG00000145349
TF324661	493856	ENSG00000145354
TF105007	115265	ENSG00000145358
TF351263	287	ENSG00000145362
TF101002	890	ENSG00000145386
TF323194	54532	ENSG00000145390
TF319557	55016	ENSG00000145416
TF350133	6423	ENSG00000145423
TF300037	6189	ENSG00000145425
TF314357	285533	ENSG00000145428
TF332130	56034	ENSG00000145431
TF315453	8001	ENSG00000145451
TF105421	83853	ENSG00000145491
TF328786	85409	ENSG00000145506
TF329887	1016	ENSG00000145526

TF313537	170690	ENSG00000145536
TF316834	4651	ENSG00000145555
TF338122	9180	ENSG00000145623
TF315472	133688	ENSG00000145626
TF101089	10769	ENSG00000145632
TF333630	3001	ENSG00000145649
TF102033	5295	ENSG00000145675
TF332134	1404	ENSG00000145681
TF321143	10184	ENSG00000145685
TF318961	23635	ENSG00000145687
TF313078	10788	ENSG00000145703
TF324035	167410	ENSG00000145721
TF352301	54826	ENSG00000145723
TF313594	23262	ENSG00000145725
TF317546	689	ENSG00000145741
TF317546	643412	ENSG00000145741
TF317546	647969	ENSG00000145741
TF323415	25816	ENSG00000145779
TF351376	56929	ENSG00000145780
TF332598	84466	ENSG00000145794
TF313537	171019	ENSG00000145808
TF313100	81555	ENSG00000145817
TF316851	23092	ENSG00000145819
TF333807	91368	ENSG00000145835
TF336163	91937	ENSG00000145850
TF318635	153830	ENSG00000145860
TF329591	114898	ENSG00000145861
TF315453	2559	ENSG00000145863
TF315453	2561	ENSG00000145864
TF329001	78991	ENSG00000145882
TF315453	2741	ENSG00000145888
TF351138	10318	ENSG00000145901
TF325464	10146	ENSG00000145907
TF331420	23138	ENSG00000145911
TF315176	64777	ENSG00000145916
TF325311	91272	ENSG00000145919
TF315172	10814	ENSG00000145920
TF316833	57451	ENSG00000145934
TF328589	3779	ENSG00000145936
TF351123	340156	ENSG00000145949
TF323246	54438	ENSG00000145990
TF319755	84249	ENSG00000146005
TF332659	26045	ENSG00000146006
TF331647	2676	ENSG00000146013
TF318770	51473	ENSG00000146038
TF313535	10050	ENSG00000146039
TF330223	54540	ENSG00000146067
TF313831	7941	ENSG00000146070
TF331157	27242	ENSG00000146072
TF325756	22838	ENSG00000146083
TF313379	255426	ENSG00000146090
TF324994	79930	ENSG00000146094
TF314602	23500	ENSG00000146122
TF105363	54511	ENSG00000146151
TF316247	221472	ENSG00000146192
TF351672	222663	ENSG00000146197
TF314265	50636	ENSG00000146205
TF313758	401262	ENSG00000146215
TF300740	285855	ENSG00000146223

TF300740	390800	ENSG00000146223
TF300740	642451	ENSG00000146223
TF320166	4794	ENSG00000146232
TF105090	51302	ENSG00000146233
TF351115	7162	ENSG00000146242
TF324197	55023	ENSG00000146247
TF329011	167681	ENSG00000146250
TF315453	2569	ENSG00000146276
TF333211	10957	ENSG00000146278
TF106488	256380	ENSG00000146285
TF323436	134829	ENSG00000146352
TF330052	2830	ENSG00000146360
TF314044	93663	ENSG00000146376
TF343107	9287	ENSG00000146378
TF343107	319100	ENSG00000146383
TF343107	83551	ENSG00000146385
TF343107	134864	ENSG00000146399
TF331404	113115	ENSG00000146410
TF332408	154091	ENSG00000146411
TF318170	51390	ENSG00000146416
TF319686	26230	ENSG00000146426
TF336988	9202	ENSG00000146463
TF332804	7432	ENSG00000146469
TF351702	221806	ENSG00000146530
TF300673	2768	ENSG00000146535
TF316846	730351	ENSG00000146555
TF316846	221935	ENSG00000146555
TF323952	9586	ENSG00000146592
TF106002	1956	ENSG00000146648
TF331211	3486	ENSG00000146674
TF313701	5814	ENSG00000146676
TF331211	3484	ENSG00000146678
TF329295	136853	ENSG00000146700
TF331369	22932	ENSG00000146707
TF314501	2631	ENSG00000146729
TF342569	135892	ENSG00000146755
TF331337	222255	ENSG00000146776
TF315127	142685	ENSG00000146809
TF313191	56996	ENSG00000146828
TF325513	64599	ENSG00000146830
TF300299	7455	ENSG00000146839
TF338389	92092	ENSG00000146858
TF315233	11011	ENSG00000146872
TF315608	2041	ENSG00000146904
TF318578	285888	ENSG00000146910
TF323921	136371	ENSG00000146926
TF326187	57502	ENSG00000146938
TF333370	357	ENSG00000146950
TF300097	401409	ENSG00000146955
TF317607	51631	ENSG00000146963
TF320336	27147	ENSG00000146966
TF350191	30011	ENSG00000147010
TF312855	83604	ENSG00000147027
TF341184	94122	ENSG00000147041
TF317405	7403	ENSG00000147050
TF332665	54466	ENSG00000147059
TF313935	4478	ENSG00000147065
TF313792	6567	ENSG00000147100
TF342871	56548	ENSG00000147119

TF300803	347517	ENSG00000147127
TF336988	9203	ENSG00000147130
TF313573	6872	ENSG00000147133
TF330775	84636	ENSG00000147138
TF315795	4841	ENSG00000147140
TF328984	90060	ENSG00000147144
TF350009	2846	ENSG00000147145
TF314716	10682	ENSG00000147155
TF314707	158835	ENSG00000147160
TF314980	29934	ENSG00000147164
TF105394	26548	ENSG00000147166
TF335557	7552	ENSG00000147180
TF315383	1730	ENSG00000147202
TF314566	56000	ENSG00000147206
TF106366	5631	ENSG00000147224
TF106366	221823	ENSG00000147224
TF316497	84443	ENSG00000147234
TF316350	3358	ENSG00000147246
TF313629	139818	ENSG00000147251
TF316710	158763	ENSG00000147256
TF105317	2719	ENSG00000147257
TF325411	139760	ENSG00000147262
TF331833	27316	ENSG00000147274
TF351429	9258	ENSG00000147324
TF313070	26260	ENSG00000147364
TF324998	2652	ENSG00000147380
TF324998	728458	ENSG00000147380
TF315453	55879	ENSG00000147402
TF318303	55790	ENSG00000147408
TF300313	526	ENSG00000147416
TF320327	79618	ENSG00000147421
TF315605	1142	ENSG00000147432
TF315605	8973	ENSG00000147434
TF324994	9046	ENSG00000147443
TF300423	80005	ENSG00000147459
TF313869	6770	ENSG00000147465
TF313059	11160	ENSG00000147475
TF317932	54212	ENSG00000147481
TF317299	9705	ENSG00000147488
TF351634	4067	ENSG00000147507
TF315837	8601	ENSG00000147509
TF333149	6867	ENSG00000147526
TF313115	51125	ENSG00000147533
TF323722	84513	ENSG00000147535
TF329088	54904	ENSG00000147548
TF105164	85479	ENSG00000147570
TF332956	1392	ENSG00000147571
TF331669	84675	ENSG00000147573
TF316894	5375	ENSG00000147588
TF314297	51110	ENSG00000147592
TF333209	7013	ENSG00000147601
TF300740	6129	ENSG00000147604
TF313784	115111	ENSG00000147606
TF314166	85481	ENSG00000147613
TF300857	245972	ENSG00000147614
TF332407	55638	ENSG00000147642
TF332149	29967	ENSG00000147650
TF331799	340419	ENSG00000147655
TF316174	114569	ENSG00000147676

TF330777	84985	ENSG00000147689
TF314837	51059	ENSG00000147724
TF323577	80728	ENSG00000147799
TF318470	55630	ENSG00000147804
TF314732	93100	ENSG00000147813
TF351700	7436	ENSG00000147852
TF312916	50808	ENSG00000147853
TF106434	115426	ENSG00000147854
TF313889	4781	ENSG00000147862
TF106445	9350	ENSG00000147869
TF328397	123	ENSG00000147872
TF106465	4915	ENSG00000148053
TF315136	414328	ENSG00000148057
TF315807	53358	ENSG00000148082
TF313511	84641	ENSG00000148110
TF332004	84909	ENSG00000148120
TF316040	54886	ENSG00000148123
TF325534	58499	ENSG00000148143
TF328543	401548	ENSG00000148158
TF105750	2040	ENSG00000148175
TF313468	2934	ENSG00000148180
TF350737	2649	ENSG00000148200
TF316224	286204	ENSG00000148204
TF332034	23245	ENSG00000148219
TF300001	6836	ENSG00000148248
TF330991	26301	ENSG00000148288
TF314174	28989	ENSG00000148335
TF313492	114789	ENSG00000148339
TF313896	84895	ENSG00000148343
TF336103	3934	ENSG00000148346
TF314804	57720	ENSG00000148358
TF323475	56623	ENSG00000148384
TF316276	9919	ENSG00000148396
TF351641	4851	ENSG00000148400
TF312805	774	ENSG00000148408
TF331184	138151	ENSG00000148411
TF318099	9712	ENSG00000148429
TF331338	221061	ENSG00000148468
TF318470	221074	ENSG00000148482
TF323961	338596	ENSG00000148488
TF323729	56288	ENSG00000148498
TF331759	6935	ENSG00000148516
TF328895	220965	ENSG00000148541
TF328627	29982	ENSG00000148572
TF328627	728381	ENSG00000148572
TF328627	730820	ENSG00000148572
TF332908	92211	ENSG00000148600
TF330861	26103	ENSG00000148602
TF324998	5995	ENSG00000148604
TF315189	26091	ENSG00000148634
TF315229	818	ENSG00000148660
TF331650	27063	ENSG00000148677
TF313003	120	ENSG00000148700
TF329901	3026	ENSG00000148702
TF319504	11023	ENSG00000148704
TF105145	54788	ENSG00000148719
TF101530	1979	ENSG00000148730
TF315303	64106	ENSG00000148734
TF318448	6934	ENSG00000148737

TF105095	1586	ENSG00000148795
TF330122	9118	ENSG00000148798
TF327063	84504	ENSG00000148826
TF318348	196743	ENSG00000148832
TF343068	23082	ENSG00000148840
TF332277	85450	ENSG00000148841
TF101012	54805	ENSG00000148842
TF314733	8038	ENSG00000148848
TF315837	6001	ENSG00000148908
TF314369	84280	ENSG00000148925
TF323754	2620	ENSG00000148935
TF316850	55327	ENSG00000148943
TF324303	57689	ENSG00000148948
TF312817	8525	ENSG00000149091
TF317350	710	ENSG00000149131
TF328358	8501	ENSG00000149150
TF351926	5795	ENSG00000149177
TF313985	84364	ENSG00000149182
TF314924	10658	ENSG00000149187
TF314230	143686	ENSG00000149212
TF333322	23052	ENSG00000149218
TF351654	283212	ENSG00000149243
TF316833	26011	ENSG00000149256
TF316833	729746	ENSG00000149256
TF314748	726	ENSG00000149260
TF105351	5058	ENSG00000149269
TF315783	85463	ENSG00000149289
TF326195	4684	ENSG00000149294
TF334382	1813	ENSG00000149295
TF315605	9177	ENSG00000149305
TF315232	2900	ENSG00000149403
TF330647	6768	ENSG00000149418
TF313608	92086	ENSG00000149435
TF314733	80332	ENSG00000149451
TF106444	9219	ENSG00000149480
TF313604	3992	ENSG00000149485
TF313604	3995	ENSG00000149485
TF313462	117532	ENSG00000149488
TF331684	6094	ENSG00000149489
TF317832	256364	ENSG00000149499
TF332794	22917	ENSG00000149506
TF335157	932	ENSG00000149516
TF313216	9651	ENSG00000149527
TF316430	79869	ENSG00000149532
TF335157	2206	ENSG00000149534
TF313522	26229	ENSG00000149541
TF313128	9638	ENSG00000149557
TF330875	90952	ENSG00000149564
TF327139	84623	ENSG00000149571
TF331728	10205	ENSG00000149573
TF331728	6327	ENSG00000149575
TF313076	51092	ENSG00000149577
TF313921	6876	ENSG00000149591
TF317210	57158	ENSG00000149596
TF105126	128853	ENSG00000149599
TF334329	85449	ENSG00000149633
TF331853	140710	ENSG00000149639
TF329887	64405	ENSG00000149654
TF323736	54915	ENSG00000149658

TF323936	81928	ENSG00000149679
TF314677	83706	ENSG00000149781
TF313216	5331	ENSG00000149782
TF331725	10435	ENSG00000149798
TF101179	7108	ENSG00000149809
TF106341	6911	ENSG00000149922
TF314203	226	ENSG00000149925
TF324847	83723	ENSG00000149926
TF315600	8448	ENSG00000149927
TF351444	9344	ENSG00000149930
TF351623	8091	ENSG00000149948
TF315428	4314	ENSG00000149968
TF326495	22866	ENSG00000149970
TF321796	53942	ENSG00000149972
TF319371	283078	ENSG00000150051
TF314263	143098	ENSG00000150054
TF314731	2904	ENSG00000150086
TF105392	3688	ENSG00000150093
TF105452	652846	ENSG00000150165
TF105452	728113	ENSG00000150165
TF333443	53828	ENSG00000150201
TF335097	2209	ENSG00000150337
TF324725	84159	ENSG00000150347
TF329887	1006	ENSG00000150394
TF313332	55208	ENSG00000150401
TF351549	26524	ENSG00000150457
TF351999	23284	ENSG00000150471
TF328699	220108	ENSG00000150510
TF332724	117153	ENSG00000150526
TF333137	4253	ENSG00000150527
TF332443	130576	ENSG00000150556
TF323207	27250	ENSG00000150593
TF316350	150	ENSG00000150594
TF315162	2823	ENSG00000150625
TF319554	7424	ENSG00000150630
TF323171	1740	ENSG00000150672
TF315837	64407	ENSG00000150681
TF329011	11098	ENSG00000150687
TF315197	54545	ENSG00000150712
TF332576	151242	ENSG00000150722
TF314984	134145	ENSG00000150756
TF300423	1793	ENSG00000150760
TF106191	26521	ENSG00000150779
TF354315	5305	ENSG00000150867
TF316876	341640	ENSG00000150893
TF315583	2308	ENSG00000150907
TF300464	9871	ENSG00000150961
TF105197	23457	ENSG00000150967
TF313489	196383	ENSG00000150977
TF312815	3708	ENSG00000150995
TF313097	84076	ENSG00000151005
TF313355	23657	ENSG00000151012
TF319114	57512	ENSG00000151025
TF324882	119180	ENSG00000151033
TF315824	93589	ENSG00000151062
TF320504	196513	ENSG00000151065
TF312805	775	ENSG00000151067
TF312805	652710	ENSG00000151067
TF313103	3742	ENSG00000151079

TF328382	7068	ENSG00000151090
TF300549	54995	ENSG00000151093
TF324663	144110	ENSG00000151117
TF106437	121551	ENSG00000151136
TF351263	288	ENSG00000151150
TF101212	144715	ENSG00000151164
TF300511	4143	ENSG00000151224
TF323210	283464	ENSG00000151233
TF352598	5756	ENSG00000151239
TF312871	22982	ENSG00000151240
TF101526	1977	ENSG00000151247
TF316816	9223	ENSG00000151276
TF313349	1456	ENSG00000151292
TF317762	119385	ENSG00000151303
TF317772	64067	ENSG00000151322
TF326916	283635	ENSG00000151327
TF326826	7069	ENSG00000151365
TF300537	10873	ENSG00000151376
TF325707	343930	ENSG00000151379
TF313537	81792	ENSG00000151388
TF315363	2241	ENSG00000151422
TF323159	57182	ENSG00000151458
TF331410	83643	ENSG00000151468
TF328984	55691	ENSG00000151474
TF351926	5800	ENSG00000151490
TF313069	2059	ENSG00000151491
TF317003	27164	ENSG00000151514
TF105932	5860	ENSG00000151552
TF313941	57700	ENSG00000151553
TF314265	121601	ENSG00000151572
TF334382	1814	ENSG00000151577
TF313243	166785	ENSG00000151611
TF333046	152485	ENSG00000151612
TF316413	5458	ENSG00000151615
TF331292	1909	ENSG00000151617
TF106510	4306	ENSG00000151623
TF106492	1646	ENSG00000151632
TF314706	10570	ENSG00000151640
TF351607	27287	ENSG00000151650
TF314733	101	ENSG00000151651
TF328982	3698	ENSG00000151655
TF324777	9781	ENSG00000151692
TF325156	8853	ENSG00000151693
TF350537	2313	ENSG00000151702
TF313676	3758	ENSG00000151704
TF328673	120224	ENSG00000151715
TF324040	80014	ENSG00000151718
TF313877	2180	ENSG00000151726
TF300743	291	ENSG00000151729
TF323833	636	ENSG00000151746
TF313229	387923	ENSG00000151778
TF326622	79750	ENSG00000151789
TF313798	341880	ENSG00000151812
TF315453	2555	ENSG00000151834
TF331145	26278	ENSG00000151835
TF300458	5042	ENSG00000151846
TF343156	55835	ENSG00000151849
TF331105	26272	ENSG00000151876
TF323413	79668	ENSG00000151883

TF331647	2674	ENSG00000151892
TF331090	667	ENSG00000151914
TF312915	7073	ENSG00000151923
TF102013	9531	ENSG00000151929
TF314981	121256	ENSG00000151952
TF323326	29970	ENSG00000151967
TF324035	128077	ENSG00000152022
TF315737	84539	ENSG00000152034
TF312921	130340	ENSG00000152056
TF317184	9910	ENSG00000152061
TF324847	148534	ENSG00000152078
TF332034	460	ENSG00000152092
TF331128	130074	ENSG00000152102
TF315900	5784	ENSG00000152104
TF313714	4249	ENSG00000152127
TF105049	26353	ENSG00000152137
TF331307	130733	ENSG00000152154
TF316413	5457	ENSG00000152192
TF350009	57105	ENSG00000152207
TF352434	2895	ENSG00000152208
TF315072	6014	ENSG00000152214
TF106416	26040	ENSG00000152217
TF313677	9050	ENSG00000152229
TF324388	57818	ENSG00000152254
TF314918	5163	ENSG00000152256
TF329631	5140	ENSG00000152270
TF318448	83439	ENSG00000152284
TF326567	284948	ENSG00000152292
TF313947	56659	ENSG00000152315
TF317779	6695	ENSG00000152377
TF315079	167555	ENSG00000152380
TF351403	2977	ENSG00000152402
TF331023	133746	ENSG00000152409
TF325627	9456	ENSG00000152413
TF324396	66037	ENSG00000152430
TF106452	79723	ENSG00000152455
TF300701	9397	ENSG00000152465
TF314144	219333	ENSG00000152484
TF325391	152137	ENSG00000152492
TF351230	814	ENSG00000152495
TF315216	55521	ENSG00000152503
TF315463	678	ENSG00000152518
TF312866	130271	ENSG00000152527
TF300411	5213	ENSG00000152556
TF315232	2893	ENSG00000152578
TF326318	285313	ENSG00000152580
TF319356	8404	ENSG00000152583
TF318563	1834	ENSG00000152591
TF321931	4154	ENSG00000152601
TF323924	133690	ENSG00000152611
TF300836	23171	ENSG00000152642
TF329606	2697	ENSG00000152661
TF312918	25780	ENSG00000152689
TF312890	51128	ENSG00000152700
TF343841	347732	ENSG00000152705
TF351276	10160	ENSG00000152767
TF342671	24138	ENSG00000152778
TF314866	53354	ENSG00000152782
TF327090	56978	ENSG00000152784

TF316134	651	ENSG00000152785
TF314808	9987	ENSG00000152795
TF320178	7402	ENSG00000152818
TF313240	2911	ENSG00000152822
TF312900	5796	ENSG00000152894
TF321823	85445	ENSG00000152910
TF313199	115827	ENSG00000152932
TF326161	153562	ENSG00000152939
TF313826	5352	ENSG00000152952
TF313395	55351	ENSG00000152953
TF332090	140767	ENSG00000152954
TF331900	152789	ENSG00000152969
TF351425	7545	ENSG00000152977
TF331206	166647	ENSG00000152990
TF317197	1360	ENSG00000153002
TF333155	55203	ENSG00000153012
TF313375	9425	ENSG00000153046
TF324381	23589	ENSG00000153048
TF328570	55024	ENSG00000153064
TF316724	1601	ENSG00000153071
TF300618	1047	ENSG00000153132
TF300674	8467	ENSG00000153147
TF316134	654	ENSG00000153162
TF319243	283349	ENSG00000153179
TF317301	3192	ENSG00000153187
TF317402	10461	ENSG00000153208
TF314452	84910	ENSG00000153214
TF331016	5801	ENSG00000153233
TF315430	4929	ENSG00000153234
TF316663	22925	ENSG00000153246
TF314644	5937	ENSG00000153250
TF323985	6328	ENSG00000153253
TF316780	55079	ENSG00000153266
TF316380	266977	ENSG00000153292
TF316380	221393	ENSG00000153294
TF319780	79981	ENSG00000153303
TF314541	51571	ENSG00000153310
TF325156	50807	ENSG00000153317
TF335682	153643	ENSG00000153347
TF334329	153478	ENSG00000153404
TF329433	124402	ENSG00000153443
TF352014	3621	ENSG00000153487
TF329417	113622	ENSG00000153531
TF314132	7342	ENSG00000153560
TF315176	64795	ENSG00000153561
TF316990	643699	ENSG00000153666
TF316990	727909	ENSG00000153666
TF316990	728047	ENSG00000153666
TF316990	728080	ENSG00000153666
TF316990	653073	ENSG00000153666
TF316990	653075	ENSG00000153666
TF316990	653125	ENSG00000153666
TF316990	653720	ENSG00000153666
TF312900	5789	ENSG00000153707
TF332089	286343	ENSG00000153714
TF326495	154043	ENSG00000153721
TF313429	2960	ENSG00000153767
TF318093	90316	ENSG00000153779
TF318093	730419	ENSG00000153779

TF319798	55625	ENSG00000153786
TF324316	339145	ENSG00000153789
TF317819	3206	ENSG00000153807
TF313676	3773	ENSG00000153822
TF329337	130888	ENSG00000153832
TF315332	79047	ENSG00000153885
TF317783	255231	ENSG00000153898
TF333155	163175	ENSG00000153902
TF314737	23576	ENSG00000153904
TF106266	140890	ENSG00000153914
TF313461	1105	ENSG00000153922
TF314968	162282	ENSG00000153930
TF325419	124540	ENSG00000153944
TF315824	781	ENSG00000153956
TF350755	9955	ENSG00000153976
TF328545	284161	ENSG00000153982
TF316102	223117	ENSG00000153993
TF105556	5529	ENSG00000154001
TF354288	10750	ENSG00000154016
TF352855	125206	ENSG00000154025
TF313747	26289	ENSG00000154027
TF332959	26256	ENSG00000154040
TF351375	147463	ENSG00000154065
TF325581	83539	ENSG00000154080
TF317210	57338	ENSG00000154118
TF313334	84959	ENSG00000154127
TF351053	54538	ENSG00000154133
TF351053	64221	ENSG00000154134
TF333142	116337	ENSG00000154143
TF329111	54463	ENSG00000154153
TF329887	1010	ENSG00000154162
TF330024	2838	ENSG00000154165
TF331037	25890	ENSG00000154175
TF313279	26207	ENSG00000154217
TF314229	200014	ENSG00000154222
TF314319	204219	ENSG00000154227
TF102004	5578	ENSG00000154229
TF313679	79705	ENSG00000154237
TF314802	64090	ENSG00000154252
TF330032	5169	ENSG00000154269
TF316166	7345	ENSG00000154277
TF333137	375056	ENSG00000154305
TF324144	84976	ENSG00000154309
TF105138	23043	ENSG00000154310
TF330468	83648	ENSG00000154319
TF300350	5239	ENSG00000154330
TF105310	89780	ENSG00000154342
TF331962	84033	ENSG00000154358
TF327043	91694	ENSG00000154359
TF321411	55740	ENSG00000154380
TF105537	5506	ENSG00000154415
TF105571	57630	ENSG00000154447
TF331602	115362	ENSG00000154451
TF351653	10324	ENSG00000154474
TF332434	2849	ENSG00000154478
TF313319	388650	ENSG00000154511
TF300140	518	ENSG00000154518
TF321823	728577	ENSG00000154529
TF106408	27295	ENSG00000154553

TF320680	8470	ENSG00000154556
TF300233	6921	ENSG00000154582
TF300233	644540	ENSG00000154582
TF300233	649647	ENSG00000154582
TF330875	1525	ENSG00000154639
TF105272	10950	ENSG00000154640
TF330715	140578	ENSG00000154645
TF326195	4685	ENSG00000154654
TF316498	91133	ENSG00000154655
TF314638	5137	ENSG00000154678
TF321331	154881	ENSG00000154710
TF321331	27342	ENSG00000154710
TF331459	58494	ENSG00000154721
TF329827	729597	ENSG00000154732
TF331949	9510	ENSG00000154734
TF331949	11096	ENSG00000154736
TF105310	7476	ENSG00000154764
TF343077	152273	ENSG00000154783
TF313216	23228	ENSG00000154822
TF105560	9989	ENSG00000154845
TF329491	147495	ENSG00000154856
TF314295	63895	ENSG00000154864
TF106278	124739	ENSG00000154914
TF300803	51560	ENSG00000154917
TF325310	146956	ENSG00000154920
TF315608	2047	ENSG00000154928
TF354241	84532	ENSG00000154930
TF352926	56934	ENSG00000154975
TF101080	346288	ENSG00000154997
TF315313	139322	ENSG00000155008
TF330916	27123	ENSG00000155011
TF321823	129684	ENSG00000155052
TF324631	150696	ENSG00000155066
TF333305	221264	ENSG00000155085
TF315506	7071	ENSG00000155090
TF351976	5799	ENSG00000155093
TF300760	51582	ENSG00000155096
TF314912	528	ENSG00000155097
TF316367	55529	ENSG00000155099
TF101025	23097	ENSG00000155111
TF332815	4082	ENSG00000155130
TF313761	158219	ENSG00000155158
TF313115	401647	ENSG00000155265
TF332434	27201	ENSG00000155269
TF314118	81894	ENSG00000155287
TF350709	64092	ENSG00000155307
TF329035	29761	ENSG00000155313
TF332065	65983	ENSG00000155324
TF300837	389	ENSG00000155366
TF314700	333926	ENSG00000155367
TF335802	1622	ENSG00000155368
TF313792	6566	ENSG00000155380
TF342569	378108	ENSG00000155428
TF313355	9056	ENSG00000155465
TF314516	23367	ENSG00000155506
TF314185	9337	ENSG00000155508
TF315232	2890	ENSG00000155511
TF106453	166968	ENSG00000155545
TF328570	118788	ENSG00000155629

TF326318	7273	ENSG00000155657
TF317153	285172	ENSG00000155744
TF317907	8324	ENSG00000155760
TF324034	64798	ENSG00000155792
TF326072	56776	ENSG00000155816
TF323183	56254	ENSG00000155827
TF343068	133522	ENSG00000155846
TF312966	9844	ENSG00000155849
TF313784	1836	ENSG00000155850
TF319394	158297	ENSG00000155875
TF318759	25769	ENSG00000155886
TF313845	114	ENSG00000155897
TF105302	5922	ENSG00000155903
TF354288	6503	ENSG00000155926
TF300032	116442	ENSG00000155961
TF315438	1193	ENSG00000155962
TF326216	2334	ENSG00000155966
TF320374	286097	ENSG00000155970
TF326909	23426	ENSG00000155974
TF105225	3798	ENSG00000155980
TF106311	10	ENSG00000156006
TF319755	23362	ENSG00000156011
TF106431	91748	ENSG00000156030
TF323914	158471	ENSG00000156035
TF300673	9630	ENSG00000156049
TF321199	145483	ENSG00000156050
TF300673	2776	ENSG00000156052
TF315472	7363	ENSG00000156096
TF332667	83873	ENSG00000156097
TF315428	4325	ENSG00000156103
TF300745	132	ENSG00000156110
TF314283	3778	ENSG00000156113
TF332340	10538	ENSG00000156127
TF324413	1633	ENSG00000156136
TF313537	9508	ENSG00000156140
TF350743	257	ENSG00000156150
TF313376	286148	ENSG00000156162
TF313342	5470	ENSG00000156194
TF351125	57188	ENSG00000156218
TF314131	9154	ENSG00000156222
TF331023	123720	ENSG00000156232
TF331023	647851	ENSG00000156232
TF331023	652637	ENSG00000156232
TF326075	10600	ENSG00000156256
TF326681	571	ENSG00000156273
TF331936	26285	ENSG00000156282
TF331936	9073	ENSG00000156284
TF316345	7102	ENSG00000156298
TF319686	7074	ENSG00000156299
TF324527	57466	ENSG00000156304
TF316990	728648	ENSG00000156363
TF331214	122416	ENSG00000156381
TF324918	22986	ENSG00000156395
TF316348	2528	ENSG00000156413
TF331233	8817	ENSG00000156427
TF320624	5097	ENSG00000156453
TF105571	153769	ENSG00000156463
TF316134	392255	ENSG00000156466
TF105553	5521	ENSG00000156475

TF300252	6156	ENSG00000156482
TF313103	3788	ENSG00000156486
TF300304	1915	ENSG00000156508
TF101170	286151	ENSG00000156509
TF314238	80201	ENSG00000156510
TF314238	3098	ENSG00000156515
TF325426	84295	ENSG00000156531
TF350185	57497	ENSG00000156564
TF354263	25921	ENSG00000156599
TF326759	27020	ENSG00000156642
TF106483	23522	ENSG00000156650
TF326172	80223	ENSG00000156675
TF316767	137970	ENSG00000156687
TF105100	5603	ENSG00000156711
TF102013	9530	ENSG00000156735
TF335157	931	ENSG00000156738
TF314783	29028	ENSG00000156802
TF313070	114907	ENSG00000156804
TF316169	391059	ENSG00000156869
TF320349	5261	ENSG00000156873
TF320349	650556	ENSG00000156873
TF313511	64645	ENSG00000156875
TF326199	163786	ENSG00000156876
TF105064	1339	ENSG00000156885
TF105391	3681	ENSG00000156886
TF321769	139378	ENSG00000156920
TF351425	7547	ENSG00000156925
TF321143	375323	ENSG00000156959
TF318639	93010	ENSG00000156966
TF324392	255027	ENSG00000156968
TF101524	1974	ENSG00000156976
TF316118	7862	ENSG00000156983
TF337899	285367	ENSG00000156990
TF333185	6750	ENSG00000157005
TF324316	137392	ENSG00000157021
TF324316	729073	ENSG00000157021
TF324904	9372	ENSG00000157077
TF300330	491	ENSG00000157087
TF324882	131375	ENSG00000157093
TF343812	6529	ENSG00000157103
TF328986	115548	ENSG00000157107
TF351070	11030	ENSG00000157110
TF351653	131377	ENSG00000157119
TF330498	731	ENSG00000157131
TF317409	7079	ENSG00000157150
TF319919	6854	ENSG00000157152
TF332469	3084	ENSG00000157168
TF315202	1376	ENSG00000157184
TF314482	55707	ENSG00000157191
TF351700	7804	ENSG00000157193
TF332031	261729	ENSG00000157214
TF318961	23648	ENSG00000157216
TF315428	4323	ENSG00000157227
TF317907	8321	ENSG00000157240
TF314957	11269	ENSG00000157349
TF354325	6483	ENSG00000157350
TF332708	343070	ENSG00000157358
TF332708	653619	ENSG00000157358
TF312805	776	ENSG00000157388

TF314186	415	ENSG00000157399
TF325768	3815	ENSG00000157404
TF325768	653882	ENSG00000157404
TF315824	55799	ENSG00000157445
TF101001	9133	ENSG00000157456
TF335682	145773	ENSG00000157470
TF312960	4643	ENSG00000157483
TF328669	26060	ENSG00000157500
TF332622	134265	ENSG00000157510
TF329224	1831	ENSG00000157514
TF314624	1859	ENSG00000157540
TF313676	3763	ENSG00000157542
TF313676	3772	ENSG00000157551
TF350537	2078	ENSG00000157554
TF316214	2114	ENSG00000157557
TF352892	90139	ENSG00000157570
TF105374	643790	ENSG00000157576
TF331484	4599	ENSG00000157601
TF316079	90993	ENSG00000157613
TF331604	25966	ENSG00000157617
TF332021	257397	ENSG00000157625
TF105402	114299	ENSG00000157654
TF105402	445815	ENSG00000157654
TF105402	11217	ENSG00000157654
TF312817	9162	ENSG00000157680
TF332414	79856	ENSG00000157734
TF326088	254048	ENSG00000157741
TF317006	673	ENSG00000157764
TF313981	10568	ENSG00000157765
TF332134	176	ENSG00000157766
TF334804	9478	ENSG00000157782
TF300189	10239	ENSG00000157823
TF325155	114793	ENSG00000157827
TF329726	150946	ENSG00000157833
TF331157	8764	ENSG00000157873
TF313865	130106	ENSG00000157884
TF332598	84465	ENSG00000157890
TF350641	55698	ENSG00000157927
TF324133	6497	ENSG00000157933
TF314879	26100	ENSG00000157954
TF314159	26119	ENSG00000157978
TF317762	116987	ENSG00000157985
TF332771	200634	ENSG00000157992
TF313831	5051	ENSG00000158006
TF314231	2134	ENSG00000158008
TF313382	7780	ENSG00000158014
TF331669	84676	ENSG00000158022
TF105122	1844	ENSG00000158050
TF314132	57822	ENSG00000158055
TF330014	338323	ENSG00000158077
TF313267	79623	ENSG00000158089
TF351631	4690	ENSG00000158092
TF300622	3242	ENSG00000158104
TF333472	127262	ENSG00000158109
TF353036	7498	ENSG00000158125
TF316454	55113	ENSG00000158156
TF101012	26504	ENSG00000158158
TF319337	2140	ENSG00000158161
TF330044	199221	ENSG00000158163

TF315031	10163	ENSG00000158195
TF313195	171586	ENSG00000158201
TF324255	83850	ENSG00000158220
TF315239	115572	ENSG00000158246
TF315946	64084	ENSG00000158258
TF332426	81035	ENSG00000158270
TF101153	8450	ENSG00000158290
TF331929	26053	ENSG00000158321
TF333370	57477	ENSG00000158352
TF101056	995	ENSG00000158402
TF300174	8294	ENSG00000158406
TF300174	8359	ENSG00000158406
TF300174	8360	ENSG00000158406
TF300174	8361	ENSG00000158406
TF300174	8362	ENSG00000158406
TF300174	8363	ENSG00000158406
TF300174	8364	ENSG00000158406
TF300174	8365	ENSG00000158406
TF300174	8366	ENSG00000158406
TF300174	8367	ENSG00000158406
TF300174	8368	ENSG00000158406
TF300174	8370	ENSG00000158406
TF300174	121504	ENSG00000158406
TF300174	554313	ENSG00000158406
TF324120	158787	ENSG00000158423
TF313002	340348	ENSG00000158457
TF332469	9542	ENSG00000158458
TF300415	23382	ENSG00000158467
TF312834	9334	ENSG00000158470
TF328840	9825	ENSG00000158480
TF317197	1358	ENSG00000158516
TF329347	648998	ENSG00000158517
TF329347	653361	ENSG00000158517
TF317197	93979	ENSG00000158525
TF105540	55607	ENSG00000158528
TF313692	81544	ENSG00000158555
TF300553	1780	ENSG00000158560
TF313541	5207	ENSG00000158571
TF300724	212	ENSG00000158578
TF314123	222068	ENSG00000158604
TF315039	137964	ENSG00000158669
TF316484	168507	ENSG00000158683
TF313921	8407	ENSG00000158710
TF317732	2005	ENSG00000158711
TF325412	85414	ENSG00000158715
TF105125	54935	ENSG00000158716
TF331012	115992	ENSG00000158717
TF323338	7391	ENSG00000158773
TF319283	64600	ENSG00000158786
TF328840	124044	ENSG00000158792
TF331807	9191	ENSG00000158796
TF332664	92822	ENSG00000158805
TF327704	10361	ENSG00000158806
TF331233	8822	ENSG00000158815
TF314486	978	ENSG00000158825
TF312834	8703	ENSG00000158850
TF318042	2039	ENSG00000158856
TF331949	9507	ENSG00000158859
TF313941	64760	ENSG00000158863

TF330937	2207	ENSG00000158869
TF106204	84134	ENSG00000158882
TF331728	4359	ENSG00000158887
TF105310	7484	ENSG00000158955
TF323815	56990	ENSG00000158985
TF324090	51735	ENSG00000158987
TF324090	96459	ENSG00000158987
TF351626	2035	ENSG00000159023
TF354311	8867	ENSG00000159082
TF315109	94104	ENSG00000159086
TF332537	3588	ENSG00000159113
TF324824	9900	ENSG00000159164
TF324693	6781	ENSG00000159167
TF313374	7135	ENSG00000159173
TF313758	1465	ENSG00000159176
TF330813	10481	ENSG00000159184
TF329591	714	ENSG00000159189
TF300140	516	ENSG00000159199
TF313579	1827	ENSG00000159200
TF354204	65264	ENSG00000159202
TF315438	54102	ENSG00000159212
TF321496	861	ENSG00000159216
TF320229	10642	ENSG00000159217
TF332333	2695	ENSG00000159224
TF329359	873	ENSG00000159228
TF329359	874	ENSG00000159231
TF300298	643224	ENSG00000159247
TF329606	57369	ENSG00000159248
TF300361	70	ENSG00000159251
TF329118	23515	ENSG00000159256
TF331936	23562	ENSG00000159261
TF317772	6493	ENSG00000159263
TF317067	440456	ENSG00000159266
TF316990	342096	ENSG00000159289
TF351672	80274	ENSG00000159307
TF337296	201175	ENSG00000159314
TF325228	283748	ENSG00000159337
TF313640	51094	ENSG00000159346
TF314333	51706	ENSG00000159348
TF300331	23400	ENSG00000159363
TF319371	79190	ENSG00000159387
TF105272	7832	ENSG00000159388
TF314238	3099	ENSG00000159399
TF330373	727728	ENSG00000159403
TF314924	11189	ENSG00000159409
TF323875	197131	ENSG00000159459
TF324278	116179	ENSG00000159495
TF315442	64843	ENSG00000159556
TF312861	1636	ENSG00000159640
TF326913	10417	ENSG00000159674
TF326279	84303	ENSG00000159685
TF313593	1487	ENSG00000159692
TF314440	51673	ENSG00000159713
TF317498	29800	ENSG00000159714
TF300857	9114	ENSG00000159720
TF330729	181	ENSG00000159723
TF320752	57732	ENSG00000159733
TF316381	146206	ENSG00000159753
TF331537	9715	ENSG00000159784

TF328814	6002	ENSG00000159788
TF314166	5681	ENSG00000159792
TF320310	7791	ENSG00000159840
TF105082	29	ENSG00000159842
TF106338	4882	ENSG00000159899
TF324451	2909	ENSG00000160007
TF324982	5739	ENSG00000160013
TF300912	801	ENSG00000160014
TF300912	805	ENSG00000160014
TF300912	808	ENSG00000160014
TF323549	79140	ENSG00000160050
TF330979	653121	ENSG00000160062
TF330979	730411	ENSG00000160062
TF300194	29101	ENSG00000160075
TF325415	252995	ENSG00000160097
TF318080	8997	ENSG00000160145
TF330132	148113	ENSG00000160161
TF105210	9619	ENSG00000160179
TF351678	64699	ENSG00000160183
TF313334	53347	ENSG00000160185
TF314991	54020	ENSG00000160190
TF318093	5316	ENSG00000160199
TF300143	7307	ENSG00000160201
TF105049	1409	ENSG00000160202
TF314065	56894	ENSG00000160216
TF329487	139716	ENSG00000160219
TF329408	8209	ENSG00000160221
TF331083	23308	ENSG00000160223
TF327070	81543	ENSG00000160233
TF105392	3689	ENSG00000160255
TF315204	5900	ENSG00000160271
TF333892	10841	ENSG00000160282
TF316171	7410	ENSG00000160293
TF332441	89858	ENSG00000160296
TF312871	23181	ENSG00000160305
TF332727	6285	ENSG00000160307
TF330587	125875	ENSG00000160318
TF313537	11093	ENSG00000160323
TF325324	11182	ENSG00000160326
TF328344	652251	ENSG00000160360
TF328344	26086	ENSG00000160360
TF105417	147746	ENSG00000160396
TF314941	27433	ENSG00000160404
TF323961	30815	ENSG00000160408
TF105429	112724	ENSG00000160439
TF102005	29941	ENSG00000160447
TF313446	57731	ENSG00000160460
TF313967	84446	ENSG00000160469
TF105065	125965	ENSG00000160471
TF330014	147945	ENSG00000160505
TF300136	728453	ENSG00000160516
TF300136	730288	ENSG00000160516
TF300136	730819	ENSG00000160516
TF323272	84814	ENSG00000160539
TF351444	57551	ENSG00000160551
TF331807	162989	ENSG00000160570
TF331728	196264	ENSG00000160588
TF331728	120425	ENSG00000160593
TF325240	6294	ENSG00000160633

TF335892	917	ENSG00000160654
TF332727	6271	ENSG00000160678
TF331447	26097	ENSG00000160679
TF330966	643	ENSG00000160683
TF331824	51043	ENSG00000160685
TF315807	6464	ENSG00000160691
TF315806	103	ENSG00000160710
TF331210	3570	ENSG00000160712
TF313338	55585	ENSG00000160714
TF315605	1141	ENSG00000160716
TF321571	200186	ENSG00000160741
TF314265	55129	ENSG00000160746
TF332736	10712	ENSG00000160767
TF319738	79957	ENSG00000160781
TF101181	4000	ENSG00000160789
TF330966	1234	ENSG00000160791
TF315710	5745	ENSG00000160801
TF314412	56893	ENSG00000160803
TF351553	4634	ENSG00000160808
TF331648	352954	ENSG00000160844
TF316307	2264	ENSG00000160867
TF105087	1576	ENSG00000160868
TF105087	1551	ENSG00000160870
TF331184	112939	ENSG00000160877
TF105094	1584	ENSG00000160882
TF314238	3101	ENSG00000160883
TF331376	9592	ENSG00000160888
TF342865	9551	ENSG00000160916
TF324982	5731	ENSG00000160951
TF332708	9684	ENSG00000160959
TF336589	136227	ENSG00000160963
TF318206	1523	ENSG00000160967
TF316803	84988	ENSG00000160972
TF313576	80228	ENSG00000160991
TF313576	729969	ENSG00000160991
TF313576	730276	ENSG00000160991
TF313576	730526	ENSG00000160991
TF323184	10603	ENSG00000160999
TF324570	11282	ENSG00000161013
TF332922	9794	ENSG00000161021
TF314924	60680	ENSG00000161082
TF318198	1857	ENSG00000161202
TF106336	5130	ENSG00000161217
TF320527	115290	ENSG00000161241
TF320527	126433	ENSG00000161243
TF300143	199746	ENSG00000161265
TF330127	199745	ENSG00000161277
TF105067	1346	ENSG00000161281
TF316009	11072	ENSG00000161326
TF314400	57125	ENSG00000161381
TF331189	22806	ENSG00000161405
TF314731	2905	ENSG00000161509
TF106367	5635	ENSG00000161542
TF332967	114757	ENSG00000161544
TF106262	6427	ENSG00000161547
TF334888	6352	ENSG00000161570
TF324882	57151	ENSG00000161572
TF334888	6360	ENSG00000161573
TF334888	6359	ENSG00000161574

TF318099	414060	ENSG00000161583
TF318099	653380	ENSG00000161583
TF318099	654341	ENSG00000161583
TF318099	729837	ENSG00000161583
TF105391	3678	ENSG00000161638
TF332441	114132	ENSG00000161640
TF326622	25946	ENSG00000161642
TF314263	4356	ENSG00000161647
TF334441	146894	ENSG00000161649
TF313660	126119	ENSG00000161677
TF324593	50944	ENSG00000161681
TF331338	284069	ENSG00000161682
TF313216	113026	ENSG00000161714
TF325155	91010	ENSG00000161791
TF312940	362	ENSG00000161798
TF318102	29127	ENSG00000161800
TF321960	113251	ENSG00000161813
TF316315	160622	ENSG00000161835
TF331660	125950	ENSG00000161847
TF317854	3890	ENSG00000161849
TF317854	3888	ENSG00000161850
TF314066	117283	ENSG00000161896
TF105320	246	ENSG00000161905
TF332664	115950	ENSG00000161914
TF330912	255877	ENSG00000161940
TF332331	407977	ENSG00000161955
TF332331	8742	ENSG00000161955
TF332331	8741	ENSG00000161955
TF316289	26168	ENSG00000161956
TF317805	2256	ENSG00000161958
TF101524	1973	ENSG00000161960
TF331381	146325	ENSG00000161992
TF331381	283948	ENSG00000161992
TF313408	339123	ENSG00000161999
TF315737	6755	ENSG00000162009
TF312822	90864	ENSG00000162032
TF350755	64711	ENSG00000162040
TF315420	57465	ENSG00000162065
TF352481	4917	ENSG00000162068
TF326671	146439	ENSG00000162069
TF313845	115	ENSG00000162104
TF324593	22941	ENSG00000162105
TF331063	10825	ENSG00000162139
TF323960	80150	ENSG00000162174
TF319909	2785	ENSG00000162188
TF332770	79081	ENSG00000162194
TF331917	283237	ENSG00000162222
TF314566	10482	ENSG00000162231
TF351739	283130	ENSG00000162241
TF328982	3699	ENSG00000162267
TF320504	55802	ENSG00000162290
TF313438	8986	ENSG00000162302
TF315253	4041	ENSG00000162337
TF335872	9965	ENSG00000162344
TF105088	284541	ENSG00000162365
TF105088	654164	ENSG00000162365
TF315153	6886	ENSG00000162367
TF313377	1996	ENSG00000162374
TF315206	6512	ENSG00000162383

TF328368	26027	ENSG00000162390
TF315079	338094	ENSG00000162391
TF316040	8613	ENSG00000162407
TF314032	5563	ENSG00000162409
TF324881	57643	ENSG00000162415
TF317090	10691	ENSG00000162419
TF312916	205	ENSG00000162433
TF312916	387851	ENSG00000162433
TF312916	645619	ENSG00000162433
TF312916	731007	ENSG00000162433
TF327041	3716	ENSG00000162434
TF331660	55225	ENSG00000162437
TF330455	11330	ENSG00000162438
TF314533	84328	ENSG00000162441
TF316894	116362	ENSG00000162444
TF320310	54751	ENSG00000162458
TF324506	284723	ENSG00000162461
TF334689	126755	ENSG00000162494
TF330078	4146	ENSG00000162510
TF330843	7805	ENSG00000162511
TF320463	9672	ENSG00000162512
TF106485	642954	ENSG00000162521
TF106485	648695	ENSG00000162521
TF106485	5928	ENSG00000162521
TF333323	57648	ENSG00000162522
TF333175	55450	ENSG00000162545
TF323513	249	ENSG00000162551
TF105310	54361	ENSG00000162552
TF332598	1953	ENSG00000162591
TF313014	9077	ENSG00000162595
TF343364	149224	ENSG00000162596
TF313889	4774	ENSG00000162599
TF324811	114803	ENSG00000162601
TF313654	8880	ENSG00000162613
TF105141	11080	ENSG00000162616
TF316380	64123	ENSG00000162618
TF315442	431707	ENSG00000162624
TF328543	51375	ENSG00000162627
TF318639	8707	ENSG00000162630
TF333945	22854	ENSG00000162631
TF320966	284611	ENSG00000162636
TF331602	2634	ENSG00000162645
TF300138	56900	ENSG00000162647
TF300138	727778	ENSG00000162647
TF331337	127002	ENSG00000162650
TF331602	115361	ENSG00000162654
TF105407	284695	ENSG00000162664
TF331600	339479	ENSG00000162670
TF350784	2672	ENSG00000162676
TF314283	343450	ENSG00000162687
TF320336	163486	ENSG00000162701
TF331779	23528	ENSG00000162702
TF319716	10092	ENSG00000162704
TF326804	57863	ENSG00000162706
TF330014	114548	ENSG00000162711
TF313676	3765	ENSG00000162728
TF332702	93185	ENSG00000162729
TF317840	4921	ENSG00000162733
TF313467	57216	ENSG00000162738

TF351220	25903	ENSG00000162745
TF335097	2215	ENSG00000162747
TF315442	4009	ENSG00000162761
TF334689	440699	ENSG00000162763
TF314292	28982	ENSG00000162769
TF326301	467	ENSG00000162772
TF315637	64783	ENSG00000162775
TF320336	79961	ENSG00000162777
TF331376	51278	ENSG00000162783
TF105235	55083	ENSG00000162849
TF329248	91461	ENSG00000162878
TF330775	165140	ENSG00000162881
TF312891	9261	ENSG00000162889
TF333253	50604	ENSG00000162891
TF333253	11009	ENSG00000162892
TF334441	5284	ENSG00000162896
TF334441	83953	ENSG00000162897
TF314748	824	ENSG00000162909
TF325632	5966	ENSG00000162924
TF333285	130132	ENSG00000162944
TF314748	92291	ENSG00000162949
TF332659	347730	ENSG00000162951
TF323801	79568	ENSG00000162972
TF105465	26225	ENSG00000162980
TF330836	151354	ENSG00000162981
TF313676	3760	ENSG00000162989
TF315153	4760	ENSG00000162992
TF317907	2487	ENSG00000162998
TF329439	150726	ENSG00000163013
TF300361	72	ENSG00000163017
TF314520	79677	ENSG00000163029
TF300009	7447	ENSG00000163032
TF314241	3020	ENSG00000163041
TF314241	3021	ENSG00000163041
TF314241	347376	ENSG00000163041
TF314241	644914	ENSG00000163041
TF314241	730740	ENSG00000163041
TF300630	56997	ENSG00000163050
TF313792	151473	ENSG00000163053
TF106461	2019	ENSG00000163064
TF318059	115677	ENSG00000163072
TF351791	3625	ENSG00000163083
TF330745	129446	ENSG00000163092
TF106408	10611	ENSG00000163110
TF323312	56957	ENSG00000163113
TF331650	200539	ENSG00000163126
TF313739	1520	ENSG00000163131
TF350699	4487	ENSG00000163132
TF324164	651044	ENSG00000163141
TF324164	149428	ENSG00000163141
TF329591	114905	ENSG00000163145
TF323415	79626	ENSG00000163154
TF318444	388695	ENSG00000163155
TF315841	29765	ENSG00000163157
TF317486	284996	ENSG00000163162
TF315504	55677	ENSG00000163166
TF331725	10602	ENSG00000163171
TF332727	6282	ENSG00000163191
TF332727	729659	ENSG00000163191

TF332727	730278	ENSG00000163191
TF332727	730558	ENSG00000163191
TF316134	27302	ENSG00000163217
TF323577	9938	ENSG00000163219
TF332727	6280	ENSG00000163220
TF332727	6283	ENSG00000163221
TF332938	7039	ENSG00000163235
TF314464	151195	ENSG00000163249
TF317907	7855	ENSG00000163251
TF331401	1420	ENSG00000163254
TF106305	4880	ENSG00000163273
TF300841	132789	ENSG00000163281
TF323513	250	ENSG00000163283
TF315453	2565	ENSG00000163285
TF323513	251	ENSG00000163286
TF315453	2560	ENSG00000163288
TF313214	152519	ENSG00000163293
TF323513	248	ENSG00000163295
TF328943	118429	ENSG00000163297
TF331751	84142	ENSG00000163322
TF324034	151556	ENSG00000163328
TF333202	57326	ENSG00000163346
TF331936	9076	ENSG00000163347
TF333020	90780	ENSG00000163348
TF105417	204851	ENSG00000163349
TF318242	1293	ENSG00000163359
TF328984	55765	ENSG00000163362
TF332672	84541	ENSG00000163376
TF331749	151647	ENSG00000163377
TF315841	56203	ENSG00000163380
TF300197	128240	ENSG00000163382
TF315303	886	ENSG00000163394
TF312838	476	ENSG00000163399
TF330897	6565	ENSG00000163406
TF332732	60675	ENSG00000163421
TF106409	11167	ENSG00000163430
TF315841	25802	ENSG00000163431
TF318679	1999	ENSG00000163435
TF315179	132954	ENSG00000163440
TF331645	3490	ENSG00000163453
TF315216	80128	ENSG00000163462
TF330966	3577	ENSG00000163464
TF325296	134	ENSG00000163485
TF315892	23380	ENSG00000163486
TF331401	1412	ENSG00000163499
TF106458	3549	ENSG00000163501
TF106341	8320	ENSG00000163508
TF331289	64343	ENSG00000163512
TF314724	7048	ENSG00000163513
TF106176	79885	ENSG00000163517
TF317514	2199	ENSG00000163520
TF314816	79411	ENSG00000163521
TF321796	23114	ENSG00000163531
TF352620	5274	ENSG00000163536
TF101155	23122	ENSG00000163539
TF324572	81788	ENSG00000163545
TF102004	5584	ENSG00000163558
TF101534	56648	ENSG00000163577
TF313762	6514	ENSG00000163581

TF330348	2168	ENSG00000163586
TF317186	130026	ENSG00000163596
TF317186	442064	ENSG00000163596
TF350501	23429	ENSG00000163602
TF312963	8618	ENSG00000163618
TF327063	4825	ENSG00000163623
TF313464	1040	ENSG00000163624
TF313658	23001	ENSG00000163625
TF315388	5783	ENSG00000163629
TF315804	132204	ENSG00000163630
TF331337	6314	ENSG00000163635
TF313265	166336	ENSG00000163637
TF313537	56999	ENSG00000163638
TF331875	7401	ENSG00000163646
TF328965	25976	ENSG00000163659
TF101011	57018	ENSG00000163660
TF330208	5806	ENSG00000163661
TF329541	1776	ENSG00000163687
TF314331	323	ENSG00000163697
TF316507	78987	ENSG00000163703
TF314351	26577	ENSG00000163710
TF318729	23350	ENSG00000163714
TF333433	2921	ENSG00000163734
TF333433	6374	ENSG00000163735
TF333433	5473	ENSG00000163736
TF333433	5196	ENSG00000163737
TF323998	441024	ENSG00000163738
TF333433	2919	ENSG00000163739
TF314939	57047	ENSG00000163746
TF317197	1359	ENSG00000163751
TF312839	2992	ENSG00000163754
TF331371	116441	ENSG00000163762
TF351991	54861	ENSG00000163788
TF350742	150921	ENSG00000163792
TF332956	7349	ENSG00000163794
TF350015	130557	ENSG00000163795
TF314942	151056	ENSG00000163803
TF329827	245711	ENSG00000163806
TF324278	7047	ENSG00000163810
TF319798	51304	ENSG00000163812
TF330481	7123	ENSG00000163815
TF343812	54716	ENSG00000163817
TF341788	79443	ENSG00000163820
TF330966	1230	ENSG00000163823
TF333627	79442	ENSG00000163827
TF343227	51725	ENSG00000163833
TF331779	84561	ENSG00000163848
TF331779	7707	ENSG00000163848
TF315035	349565	ENSG00000163864
TF336988	9204	ENSG00000163867
TF315232	2899	ENSG00000163873
TF315783	80149	ENSG00000163874
TF350556	28999	ENSG00000163884
TF333175	94032	ENSG00000163888
TF324997	200879	ENSG00000163898
TF316289	59343	ENSG00000163904
TF323617	26508	ENSG00000163909
TF324998	6010	ENSG00000163914
TF300223	116832	ENSG00000163923

TF313097	7086	ENSG00000163931
TF102004	5580	ENSG00000163932
TF316498	51460	ENSG00000163935
TF313085	26354	ENSG00000163938
TF336055	23272	ENSG00000163946
TF328974	50650	ENSG00000163947
TF316521	7884	ENSG00000163950
TF354263	131540	ENSG00000163958
TF316050	200931	ENSG00000163959
TF332796	165918	ENSG00000163961
TF313428	133060	ENSG00000163982
TF332727	6286	ENSG00000163993
TF331936	149461	ENSG00000164007
TF317532	114625	ENSG00000164010
TF324775	9255	ENSG00000164022
TF314547	166929	ENSG00000164023
TF105145	79982	ENSG00000164031
TF105145	646358	ENSG00000164031
TF105145	651395	ENSG00000164031
TF354232	3015	ENSG00000164032
TF328795	56898	ENSG00000164039
TF314562	10424	ENSG00000164040
TF101056	993	ENSG00000164045
TF312962	5364	ENSG00000164050
TF332572	51246	ENSG00000164054
TF325070	10252	ENSG00000164056
TF326082	8927	ENSG00000164061
TF312937	327	ENSG00000164062
TF105043	22824	ENSG00000164070
TF314166	79012	ENSG00000164076
TF314665	84315	ENSG00000164077
TF317402	4486	ENSG00000164078
TF328465	51368	ENSG00000164081
TF313240	2912	ENSG00000164082
TF105122	1849	ENSG00000164086
TF314700	132160	ENSG00000164088
TF320468	64850	ENSG00000164089
TF313497	80335	ENSG00000164091
TF313497	728505	ENSG00000164091
TF313497	730735	ENSG00000164091
TF351940	5308	ENSG00000164093
TF329295	8492	ENSG00000164099
TF313193	9348	ENSG00000164100
TF105371	3148	ENSG00000164104
TF315153	9464	ENSG00000164107
TF105452	308	ENSG00000164111
TF315806	132612	ENSG00000164113
TF106338	2982	ENSG00000164116
TF324093	3248	ENSG00000164120
TF331945	140458	ENSG00000164122
TF330994	51313	ENSG00000164125
TF315303	4886	ENSG00000164128
TF315303	4889	ENSG00000164129
TF106301	80155	ENSG00000164134
TF336199	3600	ENSG00000164136
TF314945	27236	ENSG00000164144
TF326812	54726	ENSG00000164164
TF105391	3673	ENSG00000164171
TF330156	10085	ENSG00000164176

TF314570	153396	ENSG00000164180
TF323454	79993	ENSG00000164181
TF313181	202151	ENSG00000164188
TF330775	2151	ENSG00000164220
TF332022	651746	ENSG00000164236
TF330775	2150	ENSG00000164251
TF106457	6690	ENSG00000164266
TF316350	3360	ENSG00000164270
TF105284	134266	ENSG00000164284
TF331942	493869	ENSG00000164294
TF312881	256987	ENSG00000164300
TF102023	836	ENSG00000164305
TF300395	51752	ENSG00000164307
TF300395	64167	ENSG00000164308
TF332948	9607	ENSG00000164326
TF313391	1879	ENSG00000164330
TF333112	57763	ENSG00000164331
TF325595	7098	ENSG00000164342
TF343812	348932	ENSG00000164363
TF316380	222611	ENSG00000164393
TF313877	23305	ENSG00000164398
TF101080	23176	ENSG00000164402
TF329606	375519	ENSG00000164411
TF315232	2898	ENSG00000164418
TF316894	2173	ENSG00000164434
TF325347	30012	ENSG00000164438
TF318595	167838	ENSG00000164440
TF331915	10370	ENSG00000164442
TF329085	221301	ENSG00000164451
TF106341	6862	ENSG00000164458
TF330156	285761	ENSG00000164465
TF313205	94081	ENSG00000164466
TF332635	114801	ENSG00000164484
TF331300	168002	ENSG00000164488
TF314585	134957	ENSG00000164506
TF300137	221613	ENSG00000164508
TF338122	133396	ENSG00000164509
TF329621	23366	ENSG00000164542
TF314166	9263	ENSG00000164543
TF106265	29896	ENSG00000164548
TF318250	348980	ENSG00000164588
TF331748	91977	ENSG00000164591
TF315153	63974	ENSG00000164600
TF331163	54329	ENSG00000164604
TF352583	26223	ENSG00000164616
TF343473	168667	ENSG00000164619
TF332339	285613	ENSG00000164620
TF313947	8645	ENSG00000164626
TF332031	26872	ENSG00000164647
TF101076	55536	ENSG00000164649
TF101076	728563	ENSG00000164649
TF101076	732169	ENSG00000164649
TF315506	221833	ENSG00000164651
TF315906	222223	ENSG00000164659
TF315281	25862	ENSG00000164663
TF323617	23462	ENSG00000164683
TF326610	619279	ENSG00000164684
TF316894	2171	ENSG00000164687
TF316894	387934	ENSG00000164687

TF316894	642956	ENSG00000164687
TF316894	728641	ENSG00000164687
TF316894	729163	ENSG00000164687
TF316894	731043	ENSG00000164687
TF316894	732031	ENSG00000164687
TF106458	6469	ENSG00000164690
TF331062	117289	ENSG00000164691
TF323987	1278	ENSG00000164692
TF314269	92421	ENSG00000164695
TF312913	26266	ENSG00000164707
TF300007	5224	ENSG00000164708
TF332280	22853	ENSG00000164715
TF331838	146861	ENSG00000164729
TF331838	731108	ENSG00000164729
TF316183	64321	ENSG00000164736
TF314044	10395	ENSG00000164741
TF313845	107	ENSG00000164742
TF323915	256979	ENSG00000164744
TF352097	3174	ENSG00000164749
TF101215	5885	ENSG00000164754
TF313382	169026	ENSG00000164756
TF324588	90390	ENSG00000164758
TF331157	4982	ENSG00000164761
TF320349	5260	ENSG00000164776
TF106461	2020	ENSG00000164778
TF313103	27012	ENSG00000164794
TF316872	114788	ENSG00000164796
TF313502	734	ENSG00000164823
TF323915	23353	ENSG00000164828
TF313530	55074	ENSG00000164830
TF335735	157753	ENSG00000164841
TF333506	115330	ENSG00000164849
TF333506	2852	ENSG00000164850
TF324410	4846	ENSG00000164867
TF328311	79778	ENSG00000164877
TF316425	761	ENSG00000164879
TF313630	6522	ENSG00000164889
TF331796	10922	ENSG00000164896
TF329265	83590	ENSG00000164897
TF351530	2636	ENSG00000164900
TF325718	221937	ENSG00000164916
TF350876	116039	ENSG00000164920
TF102003	7534	ENSG00000164924
TF317907	8323	ENSG00000164930
TF333017	94241	ENSG00000164938
TF316876	158326	ENSG00000164946
TF314379	2669	ENSG00000164949
TF313505	54704	ENSG00000164951
TF317053	91147	ENSG00000164953
TF325688	138716	ENSG00000164967
TF331928	203259	ENSG00000164970
TF105385	11168	ENSG00000164985
TF329247	51271	ENSG00000165006
TF313014	54769	ENSG00000165023
TF351629	6850	ENSG00000165025
TF105191	19	ENSG00000165029
TF328374	4783	ENSG00000165030
TF316321	137994	ENSG00000165046
TF323232	55798	ENSG00000165055

TF313399	5568	ENSG00000165059
TF350019	79698	ENSG00000165061
TF327063	157848	ENSG00000165066
TF331065	136242	ENSG00000165076
TF317197	57094	ENSG00000165078
TF313462	117531	ENSG00000165091
TF300455	216	ENSG00000165092
TF324790	138050	ENSG00000165102
TF313106	158158	ENSG00000165105
TF325946	55582	ENSG00000165115
TF314711	55503	ENSG00000165125
TF314824	2203	ENSG00000165140
TF333363	11244	ENSG00000165156
TF105354	1536	ENSG00000165168
TF326826	58526	ENSG00000165175
TF329347	648998	ENSG00000165178
TF331806	139411	ENSG00000165186
TF331945	140456	ENSG00000165192
TF352008	57526	ENSG00000165194
TF319554	2277	ENSG00000165197
TF320194	55342	ENSG00000165209
TF331936	1365	ENSG00000165215
TF315519	65268	ENSG00000165238
TF300460	538	ENSG00000165240
TF300460	644732	ENSG00000165240
TF326187	22829	ENSG00000165246
TF330998	139324	ENSG00000165259
TF313173	364	ENSG00000165269
TF313173	360	ENSG00000165272
TF300542	7415	ENSG00000165280
TF300542	2189	ENSG00000165280
TF324197	254065	ENSG00000165288
TF326378	26050	ENSG00000165300
TF329345	94134	ENSG00000165322
TF330595	159989	ENSG00000165325
TF315212	84889	ENSG00000165349
TF323386	203522	ENSG00000165359
TF331895	83550	ENSG00000165370
TF331936	9075	ENSG00000165376
TF350185	145581	ENSG00000165379
TF327090	118738	ENSG00000165388
TF328418	171546	ENSG00000165389
TF105452	652846	ENSG00000165390
TF105452	653145	ENSG00000165390
TF319557	220972	ENSG00000165406
TF316814	7253	ENSG00000165409
TF328601	1073	ENSG00000165410
TF350445	2957	ENSG00000165417
TF300692	283209	ENSG00000165434
TF314485	84457	ENSG00000165443
TF313792	220963	ENSG00000165449
TF323475	3636	ENSG00000165458
TF351612	401	ENSG00000165462
TF329606	2706	ENSG00000165474
TF314177	221035	ENSG00000165476
TF331199	220296	ENSG00000165478
TF320374	221154	ENSG00000165487
TF318093	63876	ENSG00000165495
TF314081	23588	ENSG00000165516

TF317832	161436	ENSG00000165521
TF337899	84881	ENSG00000165526
TF300808	382	ENSG00000165527
TF324300	57156	ENSG00000165548
TF351605	1045	ENSG00000165556
TF333006	219287	ENSG00000165566
TF332672	89890	ENSG00000165572
TF351179	5015	ENSG00000165588
TF331300	51339	ENSG00000165617
TF330775	27199	ENSG00000165621
TF315091	7417	ENSG00000165637
TF313494	6571	ENSG00000165646
TF324968	84858	ENSG00000165655
TF316697	1602	ENSG00000165659
TF331751	23172	ENSG00000165660
TF316749	169714	ENSG00000165661
TF329088	64324	ENSG00000165671
TF323802	10495	ENSG00000165675
TF317513	90167	ENSG00000165694
TF350784	8328	ENSG00000165702
TF313367	3251	ENSG00000165704
TF313319	138311	ENSG00000165716
TF313395	282974	ENSG00000165752
TF300280	65991	ENSG00000165775
TF316367	90809	ENSG00000165782
TF317098	29986	ENSG00000165794
TF313168	57447	ENSG00000165795
TF334329	55701	ENSG00000165801
TF332241	51222	ENSG00000165804
TF102023	840	ENSG00000165806
TF317532	153579	ENSG00000165810
TF318242	340706	ENSG00000165816
TF317003	6297	ENSG00000165821
TF323237	53349	ENSG00000165861
TF329492	259217	ENSG00000165868
TF323925	80019	ENSG00000165886
TF331650	26287	ENSG00000165887
TF105567	144455	ENSG00000165891
TF319168	120071	ENSG00000165905
TF313677	29763	ENSG00000165912
TF313783	145567	ENSG00000165914
TF313794	79841	ENSG00000165923
TF343201	390502	ENSG00000165951
TF343201	145264	ENSG00000165953
TF335163	79789	ENSG00000165959
TF315909	29951	ENSG00000165966
TF323325	4745	ENSG00000165973
TF329591	389941	ENSG00000165985
TF316195	783	ENSG00000165995
TF313326	9200	ENSG00000165996
TF105465	221079	ENSG00000165997
TF352511	3747	ENSG00000166006
TF106437	25841	ENSG00000166016
TF324168	27291	ENSG00000166024
TF333368	154810	ENSG00000166025
TF323480	5654	ENSG00000166033
TF324997	3990	ENSG00000166035
TF329178	9702	ENSG00000166037
TF313385	255394	ENSG00000166046

TF324568	139135	ENSG00000166049
TF321411	161742	ENSG00000166068
TF331459	83700	ENSG00000166086
TF314701	64806	ENSG00000166090
TF317387	116173	ENSG00000166091
TF331949	170689	ENSG00000166106
TF313465	55530	ENSG00000166111
TF300839	84706	ENSG00000166123
TF314097	51762	ENSG00000166128
TF105546	54866	ENSG00000166143
TF325867	6692	ENSG00000166145
TF316849	2200	ENSG00000166147
TF106499	552	ENSG00000166148
TF328365	120863	ENSG00000166153
TF351114	654429	ENSG00000166159
TF324998	2652	ENSG00000166160
TF324998	728458	ENSG00000166160
TF106439	29117	ENSG00000166164
TF314214	1152	ENSG00000166165
TF105679	8945	ENSG00000166167
TF326594	55323	ENSG00000166173
TF315453	2562	ENSG00000166206
TF318679	121599	ENSG00000166211
TF324994	10818	ENSG00000166225
TF300188	5092	ENSG00000166228
TF330875	79827	ENSG00000166250
TF332097	55800	ENSG00000166257
TF331084	252983	ENSG00000166263
TF315235	79156	ENSG00000166289
TF332068	55273	ENSG00000166292
TF314331	322	ENSG00000166313
TF330867	79933	ENSG00000166317
TF316403	8642	ENSG00000166341
TF314351	81832	ENSG00000166342
TF314537	1528	ENSG00000166347
TF300590	374868	ENSG00000166377
TF314207	8495	ENSG00000166387
TF314707	80168	ENSG00000166391
TF314333	51700	ENSG00000166394
TF352619	8710	ENSG00000166396
TF352619	5271	ENSG00000166401
TF314076	7275	ENSG00000166402
TF352619	5273	ENSG00000166404
TF351071	4004	ENSG00000166407
TF313196	256764	ENSG00000166415
TF316894	1381	ENSG00000166426
TF313378	122618	ENSG00000166428
TF332796	254225	ENSG00000166439
TF320336	6764	ENSG00000166444
TF313375	124359	ENSG00000166446
TF321506	283659	ENSG00000166450
TF316484	114780	ENSG00000166473
TF333498	7702	ENSG00000166478
TF313807	54495	ENSG00000166479
TF101088	7465	ENSG00000166483
TF102004	5579	ENSG00000166501
TF105385	50810	ENSG00000166503
TF313193	8509	ENSG00000166507
TF330481	10143	ENSG00000166509

TF331627	80323	ENSG00000166510
TF314005	3281	ENSG00000166530
TF313000	23423	ENSG00000166557
TF328787	146167	ENSG00000166558
TF313648	90701	ENSG00000166562
TF331867	339302	ENSG00000166569
TF315737	2587	ENSG00000166573
TF325693	81565	ENSG00000166579
TF316817	1014	ENSG00000166589
TF314379	6236	ENSG00000166592
TF332646	4160	ENSG00000166603
TF352619	89777	ENSG00000166634
TF315605	89832	ENSG00000166664
TF315605	732445	ENSG00000166664
TF329827	441273	ENSG00000166667
TF329427	80063	ENSG00000166669
TF315428	4319	ENSG00000166670
TF312906	780776	ENSG00000166676
TF351678	80975	ENSG00000166682
TF329090	144100	ENSG00000166689
TF334167	567	ENSG00000166710
TF329009	9640	ENSG00000166716
TF331127	113201	ENSG00000166734
TF315605	3359	ENSG00000166736
TF330800	440338	ENSG00000166737
TF313114	4837	ENSG00000166741
TF300367	164	ENSG00000166747
TF313794	123624	ENSG00000166748
TF313794	731136	ENSG00000166748
TF331543	89927	ENSG00000166780
TF313936	219539	ENSG00000166793
TF314963	3948	ENSG00000166796
TF314963	160287	ENSG00000166800
TF325946	374654	ENSG00000166813
TF325901	5346	ENSG00000166819
TF325704	8800	ENSG00000166821
TF314615	124491	ENSG00000166822
TF325707	55897	ENSG00000166823
TF300395	290	ENSG00000166825
TF351070	348093	ENSG00000166831
TF329881	89797	ENSG00000166833
TF329881	652800	ENSG00000166833
TF333615	348094	ENSG00000166839
TF328485	390594	ENSG00000166853
TF333489	11318	ENSG00000166856
TF350825	9880	ENSG00000166860
TF327980	10369	ENSG00000166862
TF312960	4640	ENSG00000166866
TF354284	63928	ENSG00000166869
TF314831	23306	ENSG00000166881
TF315501	4665	ENSG00000166886
TF323322	219988	ENSG00000166889
TF332887	114794	ENSG00000166897
TF313763	6809	ENSG00000166900
TF354315	79837	ENSG00000166908
TF315197	54893	ENSG00000166912
TF102003	7529	ENSG00000166913
TF106445	26585	ENSG00000166923
TF318837	81628	ENSG00000166925

TF335157	245802	ENSG00000166926
TF335157	58475	ENSG00000166927
TF335157	64232	ENSG00000166930
TF336444	23582	ENSG00000166946
TF324278	2038	ENSG00000166947
TF324278	343641	ENSG00000166948
TF314923	4088	ENSG00000166949
TF335157	83661	ENSG00000166959
TF335157	219995	ENSG00000166961
TF350229	4130	ENSG00000166963
TF313620	10982	ENSG00000166974
TF328177	59271	ENSG00000166979
TF314214	1159	ENSG00000166998
TF314214	548596	ENSG00000166998
TF106382	2923	ENSG00000167004
TF315720	4824	ENSG00000167034
TF318216	129049	ENSG00000167037
TF316009	150290	ENSG00000167065
TF315869	7008	ENSG00000167074
TF332297	124872	ENSG00000167080
TF314340	5090	ENSG00000167081
TF319909	2793	ENSG00000167083
TF323915	140732	ENSG00000167098
TF331870	201191	ENSG00000167100
TF315617	128859	ENSG00000167104
TF320966	399665	ENSG00000167106
TF316990	2801	ENSG00000167110
TF313430	10999	ENSG00000167114
TF313826	51148	ENSG00000167123
TF351612	51450	ENSG00000167157
TF315472	54576	ENSG00000167165
TF315472	54575	ENSG00000167165
TF315472	54600	ENSG00000167165
TF315472	54577	ENSG00000167165
TF315472	54578	ENSG00000167165
TF315472	54579	ENSG00000167165
TF315472	54657	ENSG00000167165
TF315472	54659	ENSG00000167165
TF315472	54658	ENSG00000167165
TF333189	79170	ENSG00000167183
TF321410	51704	ENSG00000167191
TF321436	1398	ENSG00000167193
TF316990	645752	ENSG00000167195
TF316990	653641	ENSG00000167195
TF316990	653643	ENSG00000167195
TF317336	23102	ENSG00000167202
TF330014	64127	ENSG00000167207
TF326807	124460	ENSG00000167208
TF334888	6368	ENSG00000167236
TF332820	3481	ENSG00000167244
TF101060	51755	ENSG00000167258
TF324523	64174	ENSG00000167261
TF315942	146713	ENSG00000167281
TF335892	915	ENSG00000167286
TF328771	4645	ENSG00000167306
TF313487	6786	ENSG00000167323
TF331357	112476	ENSG00000167371
TF331897	126298	ENSG00000167378
TF105554	28227	ENSG00000167393

TF319909	94235	ENSG00000167414
TF314316	4025	ENSG00000167419
TF329345	57636	ENSG00000167433
TF316425	762	ENSG00000167434
TF351519	7171	ENSG00000167460
TF314097	4218	ENSG00000167461
TF329735	90007	ENSG00000167470
TF333351	199786	ENSG00000167483
TF321369	54815	ENSG00000167491
TF326440	653103	ENSG00000167522
TF326440	29123	ENSG00000167522
TF324882	3906	ENSG00000167531
TF316195	784	ENSG00000167535
TF105429	147015	ENSG00000167536
TF314280	84940	ENSG00000167549
TF314986	121268	ENSG00000167550
TF101069	29946	ENSG00000167565
TF331208	57701	ENSG00000167566
TF300032	53916	ENSG00000167578
TF312940	359	ENSG00000167580
TF300836	2819	ENSG00000167588
TF317402	558	ENSG00000167601
TF330224	84807	ENSG00000167604
TF313462	147798	ENSG00000167608
TF332022	341405	ENSG00000167612
TF319025	57348	ENSG00000167614
TF331725	148170	ENSG00000167617
TF330014	199713	ENSG00000167634
TF105546	94274	ENSG00000167641
TF314528	90522	ENSG00000167645
TF324164	85300	ENSG00000167654
TF351123	1613	ENSG00000167657
TF300575	1938	ENSG00000167658
TF105385	84717	ENSG00000167674
TF328397	729359	ENSG00000167676
TF329951	10501	ENSG00000167680
TF324847	79850	ENSG00000167695
TF300839	2875	ENSG00000167701
TF105238	90990	ENSG00000167702
TF328358	124935	ENSG00000167703
TF313489	83547	ENSG00000167705
TF317350	5345	ENSG00000167711
TF314711	162514	ENSG00000167723
TF329114	374875	ENSG00000167733
TF106463	4909	ENSG00000167744
TF317854	144501	ENSG00000167767
TF317854	3848	ENSG00000167768
TF314145	55611	ENSG00000167770
TF106450	283248	ENSG00000167771
TF331211	3489	ENSG00000167779
TF334804	51475	ENSG00000167791
TF101037	10263	ENSG00000167797
TF106341	347853	ENSG00000167800
TF105181	7001	ENSG00000167815
TF334441	10871	ENSG00000167850
TF334441	11314	ENSG00000167851
TF332743	92162	ENSG00000167874
TF331090	2125	ENSG00000167880
TF313714	146664	ENSG00000167889

TF313462	147138	ENSG00000167895
TF105090	1581	ENSG00000167910
TF332742	192666	ENSG00000167916
TF327203	83986	ENSG00000167930
TF353019	50964	ENSG00000167941
TF330208	390667	ENSG00000167957
TF323428	25837	ENSG00000167964
TF329541	1775	ENSG00000167968
TF320582	57524	ENSG00000167971
TF105191	21	ENSG00000167972
TF313754	54442	ENSG00000167977
TF335721	23524	ENSG00000167978
TF330014	197358	ENSG00000167984
TF321840	55048	ENSG00000167987
TF330819	220001	ENSG00000167992
TF313748	5866	ENSG00000167994
TF315803	7439	ENSG00000167995
TF313885	2495	ENSG00000167996
TF314000	26580	ENSG00000168000
TF314498	6520	ENSG00000168003
TF330836	117245	ENSG00000168004
TF315541	89849	ENSG00000168010
TF314664	199223	ENSG00000168026
TF332859	956	ENSG00000168032
TF317997	1499	ENSG00000168036
TF332678	54986	ENSG00000168038
TF317514	4054	ENSG00000168056
TF312981	10004	ENSG00000168060
TF332340	116071	ENSG00000168062
TF105121	5871	ENSG00000168067
TF332426	51435	ENSG00000168077
TF330855	286133	ENSG00000168079
TF332620	5368	ENSG00000168081
TF323955	5049	ENSG00000168092
TF300032	5867	ENSG00000168118
TF313676	3761	ENSG00000168135
TF106417	55209	ENSG00000168137
TF330777	222584	ENSG00000168143
TF320231	84376	ENSG00000168172
TF105007	54541	ENSG00000168209
TF314117	3516	ENSG00000168214
TF313872	29063	ENSG00000168228
TF324982	5729	ENSG00000168229
TF319909	2786	ENSG00000168243
TF323925	92181	ENSG00000168246
TF314483	28511	ENSG00000168256
TF313103	169522	ENSG00000168263
TF317075	359948	ENSG00000168264
TF323990	64943	ENSG00000168268
TF316127	2299	ENSG00000168269
TF300137	3012	ENSG00000168274
TF300137	8335	ENSG00000168274
TF105225	3800	ENSG00000168280
TF105225	727742	ENSG00000168280
TF314772	4247	ENSG00000168282
TF324206	648	ENSG00000168283
TF313664	3008	ENSG00000168298
TF329329	115294	ENSG00000168300
TF329329	647621	ENSG00000168300

TF329329	649181	ENSG00000168300
TF329329	652095	ENSG00000168300
TF315332	200845	ENSG00000168301
TF325943	11170	ENSG00000168309
TF328512	3660	ENSG00000168310
TF330966	1524	ENSG00000168329
TF330745	165904	ENSG00000168334
TF320538	84684	ENSG00000168348
TF313582	123099	ENSG00000168350
TF323985	11280	ENSG00000168356
TF300808	378	ENSG00000168374
TF101079	4735	ENSG00000168385
TF331399	11259	ENSG00000168386
TF315127	142686	ENSG00000168387
TF331194	84879	ENSG00000168389
TF105197	6890	ENSG00000168394
TF352014	84289	ENSG00000168395
TF352014	727773	ENSG00000168395
TF314847	23192	ENSG00000168397
TF314847	727737	ENSG00000168397
TF330024	624	ENSG00000168398
TF331693	4543	ENSG00000168412
TF313103	93107	ENSG00000168418
TF329218	377007	ENSG00000168427
TF323926	80864	ENSG00000168452
TF324723	55806	ENSG00000168453
TF331262	11031	ENSG00000168461
TF314177	80346	ENSG00000168476
TF329915	7148	ENSG00000168477
TF333155	203190	ENSG00000168481
TF314351	649	ENSG00000168487
TF326591	11273	ENSG00000168488
TF314485	9796	ENSG00000168490
TF103049	661	ENSG00000168495
TF331031	8436	ENSG00000168497
TF331853	23255	ENSG00000168502
TF351530	2637	ENSG00000168505
TF329836	148738	ENSG00000168509
TF336851	124790	ENSG00000168517
TF312881	347735	ENSG00000168528
TF320495	1128	ENSG00000168539
TF323987	1281	ENSG00000168542
TF331647	2675	ENSG00000168546
TF352014	3622	ENSG00000168556
TF333807	55602	ENSG00000168564
TF314426	6575	ENSG00000168575
TF331401	1418	ENSG00000168582
TF329265	79089	ENSG00000168591
TF314733	11086	ENSG00000168594
TF318648	6774	ENSG00000168610
TF314733	8754	ENSG00000168615
TF314733	8749	ENSG00000168619
TF332366	2668	ENSG00000168621
TF315454	8313	ENSG00000168646
TF315472	167127	ENSG00000168671
TF330836	157638	ENSG00000168672
TF331681	753	ENSG00000168675
TF336573	3575	ENSG00000168685
TF315253	53353	ENSG00000168702

TF300415	10768	ENSG00000168710
TF330809	11142	ENSG00000168734
TF330819	255743	ENSG00000168743
TF316425	766	ENSG00000168748
TF316102	54910	ENSG00000168758
TF101012	26505	ENSG00000168763
TF326617	80319	ENSG00000168772
TF314214	1159	ENSG00000168775
TF314214	548596	ENSG00000168775
TF350757	6474	ENSG00000168779
TF313594	647471	ENSG00000168781
TF313594	9677	ENSG00000168781
TF313594	652849	ENSG00000168781
TF313002	10098	ENSG00000168785
TF330979	9925	ENSG00000168795
TF317932	6645	ENSG00000168807
TF332232	27065	ENSG00000168824
TF316350	3354	ENSG00000168830
TF314957	55308	ENSG00000168872
TF351735	8403	ENSG00000168875
TF314508	129303	ENSG00000168890
TF332127	51255	ENSG00000168894
TF317532	10917	ENSG00000168903
TF300511	4144	ENSG00000168906
TF325228	255189	ENSG00000168907
TF329775	57507	ENSG00000168916
TF331838	80723	ENSG00000168917
TF316321	3954	ENSG00000168924
TF330455	1504	ENSG00000168925
TF330455	440387	ENSG00000168928
TF325070	10251	ENSG00000168939
TF314195	29091	ENSG00000168952
TF331371	79853	ENSG00000168955
TF313240	2915	ENSG00000168959
TF315551	3965	ENSG00000168961
TF325228	8681	ENSG00000168970
TF332441	27036	ENSG00000168995
TF105566	1876	ENSG00000169016
TF105566	652869	ENSG00000169016
TF316865	1285	ENSG00000169031
TF105137	5604	ENSG00000169032
TF316157	3187	ENSG00000169045
TF325994	3667	ENSG00000169047
TF316034	65110	ENSG00000169062
TF106465	4920	ENSG00000169071
TF314574	8623	ENSG00000169093
TF333115	80301	ENSG00000169094
TF300743	293	ENSG00000169100
TF313349	53944	ENSG00000169118
TF330964	90362	ENSG00000169122
TF332622	84632	ENSG00000169129
TF316136	22809	ENSG00000169136
TF316971	7336	ENSG00000169139
TF314089	137362	ENSG00000169154
TF332666	23099	ENSG00000169155
TF313836	126129	ENSG00000169169
TF331388	146395	ENSG00000169181
TF313199	5865	ENSG00000169213
TF300740	648000	ENSG00000169216

TF331799	284654	ENSG00000169218
TF328814	10636	ENSG00000169220
TF313293	26000	ENSG00000169221
TF313311	10960	ENSG00000169223
TF313119	27166	ENSG00000169230
TF313119	649930	ENSG00000169230
TF324917	7059	ENSG00000169231
TF316425	11238	ENSG00000169239
TF315495	1942	ENSG00000169242
TF333433	3627	ENSG00000169245
TF333167	79628	ENSG00000169247
TF333433	6373	ENSG00000169248
TF316350	154	ENSG00000169252
TF318639	8706	ENSG00000169255
TF337047	114787	ENSG00000169258
TF105049	8988	ENSG00000169271
TF324563	7881	ENSG00000169282
TF325799	126669	ENSG00000169291
TF332386	190	ENSG00000169297
TF300692	55276	ENSG00000169299
TF313395	202374	ENSG00000169302
TF333913	11141	ENSG00000169306
TF330969	64805	ENSG00000169313
TF106381	204474	ENSG00000169340
TF330284	7369	ENSG00000169344
TF330284	2813	ENSG00000169347
TF333055	8738	ENSG00000169372
TF106187	25942	ENSG00000169375
TF105476	200894	ENSG00000169379
TF316643	5747	ENSG00000169398
TF350009	5724	ENSG00000169403
TF315897	5780	ENSG00000169410
TF106338	4881	ENSG00000169418
TF313947	51305	ENSG00000169427
TF333433	3576	ENSG00000169429
TF319243	166824	ENSG00000169435
TF332934	169044	ENSG00000169436
TF320463	6383	ENSG00000169439
TF323480	203100	ENSG00000169495
TF329516	59339	ENSG00000169499
TF315438	25932	ENSG00000169504
TF350009	1880	ENSG00000169508
TF331707	129025	ENSG00000169548
TF331759	9839	ENSG00000169554
TF329606	2705	ENSG00000169562
TF318292	5093	ENSG00000169564
TF315438	9022	ENSG00000169583
TF350399	646	ENSG00000169594
TF350399	652688	ENSG00000169594
TF328943	84168	ENSG00000169604
TF333003	150468	ENSG00000169607
TF315303	10887	ENSG00000169618
TF333488	23119	ENSG00000169635
TF331399	7798	ENSG00000169641
TF314313	284004	ENSG00000169660
TF314395	83985	ENSG00000169682
TF315605	1138	ENSG00000169684
TF336054	4490	ENSG00000169688
TF314867	10555	ENSG00000169692

TF331395	2815	ENSG00000169704
TF336054	4493	ENSG00000169715
TF324207	5986	ENSG00000169733
TF340612	7580	ENSG00000169740
TF319923	9079	ENSG00000169744
TF101109	5881	ENSG00000169750
TF314113	652842	ENSG00000169756
TF314113	3987	ENSG00000169756
TF314113	729260	ENSG00000169756
TF326187	22871	ENSG00000169760
TF334360	84894	ENSG00000169783
TF331833	159163	ENSG00000169800
TF331833	378951	ENSG00000169800
TF316157	3185	ENSG00000169813
TF323645	686	ENSG00000169814
TF318303	55454	ENSG00000169826
TF318303	644504	ENSG00000169826
TF315303	6870	ENSG00000169836
TF315938	219409	ENSG00000169840
TF320624	5099	ENSG00000169851
TF351053	6091	ENSG00000169855
TF318206	3175	ENSG00000169856
TF350009	5028	ENSG00000169860
TF321877	1501	ENSG00000169862
TF105310	7480	ENSG00000169884
TF316546	9185	ENSG00000169891
TF105391	3684	ENSG00000169896
TF312910	8460	ENSG00000169902
TF331371	7104	ENSG00000169903
TF332727	795	ENSG00000169906
TF331371	4071	ENSG00000169908
TF323312	161725	ENSG00000169918
TF317345	8019	ENSG00000169925
TF351003	51621	ENSG00000169926
TF316497	9758	ENSG00000169933
TF331342	23414	ENSG00000169946
TF331269	83756	ENSG00000169962
TF105113	10746	ENSG00000169967
TF326187	57555	ENSG00000169992
TF335306	4648	ENSG00000169994
TF106448	1107	ENSG00000170004
TF331599	25924	ENSG00000170011
TF102003	7532	ENSG00000170027
TF101117	10477	ENSG00000170035
TF329882	131368	ENSG00000170044
TF324563	9196	ENSG00000170049
TF343201	327657	ENSG00000170054
TF331292	9283	ENSG00000170075
TF332232	51617	ENSG00000170091
TF329827	285955	ENSG00000170092
TF329827	442572	ENSG00000170092
TF329827	442590	ENSG00000170092
TF343094	866	ENSG00000170099
TF316127	2298	ENSG00000170122
TF330024	2848	ENSG00000170128
TF101117	7324	ENSG00000170142
TF351342	220988	ENSG00000170144
TF315213	23235	ENSG00000170145
TF317486	57484	ENSG00000170153

TF326340	245806	ENSG00000170162
TF316310	3233	ENSG00000170166
TF315605	1140	ENSG00000170175
TF351604	3238	ENSG00000170178
TF324529	84640	ENSG00000170185
TF313792	9121	ENSG00000170190
TF106506	255239	ENSG00000170209
TF331895	147	ENSG00000170214
TF330348	2172	ENSG00000170231
TF331271	114825	ENSG00000170234
TF321199	84140	ENSG00000170264
TF314816	2720	ENSG00000170266
TF320837	10491	ENSG00000170275
TF105049	3316	ENSG00000170276
TF318250	54714	ENSG00000170289
TF316174	152189	ENSG00000170293
TF315551	284194	ENSG00000170298
TF316894	2167	ENSG00000170323
TF315388	143162	ENSG00000170324
TF318639	10678	ENSG00000170340
TF326301	2353	ENSG00000170345
TF313729	10972	ENSG00000170348
TF314923	4086	ENSG00000170365
TF319145	1473	ENSG00000170367
TF319145	1470	ENSG00000170369
TF317015	2018	ENSG00000170370
TF319145	1469	ENSG00000170373
TF315506	121340	ENSG00000170374
TF316102	9723	ENSG00000170381
TF334360	10446	ENSG00000170382
TF313924	7779	ENSG00000170385
TF318770	166614	ENSG00000170390
TF321410	55890	ENSG00000170412
TF331344	130827	ENSG00000170417
TF317854	3856	ENSG00000170421
TF317854	728638	ENSG00000170421
TF317854	730680	ENSG00000170421
TF317854	196374	ENSG00000170423
TF325296	136	ENSG00000170425
TF325617	121214	ENSG00000170426
TF314064	4255	ENSG00000170430
TF317854	3892	ENSG00000170442
TF317854	650428	ENSG00000170442
TF300652	3035	ENSG00000170445
TF317854	9119	ENSG00000170454
TF313237	160518	ENSG00000170456
TF105145	202052	ENSG00000170464
TF317854	286887	ENSG00000170465
TF324460	57148	ENSG00000170471
TF317854	3851	ENSG00000170477
TF313272	9963	ENSG00000170482
TF317854	121391	ENSG00000170484
TF324568	4862	ENSG00000170485
TF317854	140807	ENSG00000170486
TF327043	164832	ENSG00000170500
TF106467	79071	ENSG00000170522
TF317854	3889	ENSG00000170523
TF317854	652010	ENSG00000170523
TF313541	5209	ENSG00000170525

TF313462	79905	ENSG00000170537
TF352619	5272	ENSG00000170542
TF319371	79192	ENSG00000170549
TF319371	153572	ENSG00000170561
TF326759	133418	ENSG00000170571
TF315545	10736	ENSG00000170577
TF321382	9229	ENSG00000170579
TF318648	6773	ENSG00000170581
TF317075	26145	ENSG00000170604
TF105043	3308	ENSG00000170606
TF316127	3171	ENSG00000170608
TF313784	375611	ENSG00000170615
TF313538	6444	ENSG00000170624
TF325195	80196	ENSG00000170633
TF300288	98	ENSG00000170634
TF323952	11016	ENSG00000170653
TF105302	648426	ENSG00000170667
TF105302	652668	ENSG00000170667
TF331031	347273	ENSG00000170681
TF318131	162979	ENSG00000170684
TF317819	3219	ENSG00000170689
TF313087	284076	ENSG00000170703
TF315600	143425	ENSG00000170743
TF313103	3790	ENSG00000170745
TF331833	27288	ENSG00000170748
TF105225	3799	ENSG00000170759
TF331292	2861	ENSG00000170775
TF325887	11214	ENSG00000170776
TF317562	89882	ENSG00000170777
TF101069	55038	ENSG00000170779
TF312837	195814	ENSG00000170786
TF323480	94031	ENSG00000170801
TF105083	3344	ENSG00000170802
TF332742	8419	ENSG00000170819
TF316814	2492	ENSG00000170820
TF331163	2850	ENSG00000170837
TF332672	25948	ENSG00000170852
TF320619	9788	ENSG00000170873
TF330965	84437	ENSG00000170903
TF319738	85315	ENSG00000170915
TF329582	5314	ENSG00000170927
TF325032	85509	ENSG00000170948
TF331031	112464	ENSG00000170955
TF332506	3037	ENSG00000170961
TF332130	80310	ENSG00000170962
TF330052	1901	ENSG00000170989
TF312835	90161	ENSG00000171004
TF333020	26108	ENSG00000171016
TF331443	80131	ENSG00000171017
TF330809	5569	ENSG00000171033
TF316454	286046	ENSG00000171044
TF330976	2358	ENSG00000171049
TF330976	2357	ENSG00000171051
TF313128	9637	ENSG00000171055
TF316183	83595	ENSG00000171056
TF351636	238	ENSG00000171094
TF105482	883	ENSG00000171097
TF315197	4534	ENSG00000171100
TF351636	3643	ENSG00000171105

TF314289	55669	ENSG00000171109
TF314289	441511	ENSG00000171109
TF330845	155038	ENSG00000171115
TF332366	4902	ENSG00000171119
TF328589	27094	ENSG00000171121
TF316348	2525	ENSG00000171124
TF313103	170850	ENSG00000171126
TF351133	5581	ENSG00000171132
TF333404	117579	ENSG00000171136
TF331065	136541	ENSG00000171147
TF321368	9655	ENSG00000171150
TF321368	643884	ENSG00000171150
TF321368	651067	ENSG00000171150
TF332664	55657	ENSG00000171163
TF315232	2897	ENSG00000171189
TF333491	81603	ENSG00000171206
TF314351	81831	ENSG00000171208
TF331936	49861	ENSG00000171217
TF313551	55561	ENSG00000171219
TF323952	3726	ENSG00000171223
TF335755	219738	ENSG00000171224
TF315472	7364	ENSG00000171234
TF353019	25928	ENSG00000171243
TF330208	4884	ENSG00000171246
TF320308	283742	ENSG00000171262
TF336007	57597	ENSG00000171282
TF314577	2548	ENSG00000171298
TF313947	3777	ENSG00000171303
TF325581	50515	ENSG00000171310
TF300007	5223	ENSG00000171314
TF300007	642969	ENSG00000171314
TF313572	55636	ENSG00000171316
TF314027	157570	ENSG00000171320
TF332742	3880	ENSG00000171345
TF332742	3866	ENSG00000171346
TF332089	541468	ENSG00000171357
TF332742	8687	ENSG00000171360
TF313867	1184	ENSG00000171365
TF314440	11076	ENSG00000171368
TF313103	3752	ENSG00000171385
TF332742	3860	ENSG00000171401
TF332742	3857	ENSG00000171403
TF314638	27115	ENSG00000171408
TF106311	9	ENSG00000171428
TF332742	54474	ENSG00000171431
TF317006	283455	ENSG00000171435
TF332742	342574	ENSG00000171446
TF333162	57684	ENSG00000171448
TF101036	8941	ENSG00000171450
TF334118	92126	ENSG00000171451
TF328464	171023	ENSG00000171456
TF351835	65989	ENSG00000171462
TF312964	643246	ENSG00000171471
TF332135	147179	ENSG00000171475
TF324882	389852	ENSG00000171478
TF324882	729201	ENSG00000171478
TF330014	126206	ENSG00000171487
TF331443	84230	ENSG00000171488
TF324882	389852	ENSG00000171489

TF324882	729201	ENSG00000171489
TF331443	55144	ENSG00000171492
TF315201	133558	ENSG00000171495
TF344135	255631	ENSG00000171502
TF326185	59350	ENSG00000171509
TF330052	23566	ENSG00000171517
TF324982	5734	ENSG00000171522
TF324982	730002	ENSG00000171522
TF324982	730882	ENSG00000171522
TF315153	4761	ENSG00000171532
TF338320	4135	ENSG00000171533
TF351614	23440	ENSG00000171540
TF315834	598	ENSG00000171552
TF314595	112398	ENSG00000171570
TF316846	1826	ENSG00000171587
TF318522	10316	ENSG00000171596
TF315946	22883	ENSG00000171603
TF326617	51523	ENSG00000171604
TF102031	5293	ENSG00000171608
TF314220	84275	ENSG00000171612
TF314220	653698	ENSG00000171612
TF314220	654244	ENSG00000171612
TF329218	8507	ENSG00000171617
TF312822	80176	ENSG00000171621
TF330775	5031	ENSG00000171631
TF316840	2186	ENSG00000171634
TF332727	170591	ENSG00000171643
TF316214	2119	ENSG00000171656
TF330969	2857	ENSG00000171659
TF316755	57449	ENSG00000171680
TF329427	55729	ENSG00000171681
TF315837	10287	ENSG00000171700
TF314970	6919	ENSG00000171703
TF314265	203859	ENSG00000171714
TF323452	23261	ENSG00000171735
TF315551	3960	ENSG00000171747
TF331873	115861	ENSG00000171773
TF312918	115727	ENSG00000171777
TF315153	4807	ENSG00000171786
TF315834	596	ENSG00000171791
TF300379	1503	ENSG00000171793
TF332902	1296	ENSG00000171812
TF331271	170394	ENSG00000171813
TF332299	29930	ENSG00000171815
TF323538	4815	ENSG00000171840
TF314586	4300	ENSG00000171843
TF300465	6241	ENSG00000171848
TF330976	719	ENSG00000171860
TF343364	6201	ENSG00000171863
TF331895	146	ENSG00000171873
TF343477	84978	ENSG00000171877
TF312940	361	ENSG00000171885
TF105088	57834	ENSG00000171903
TF314677	83660	ENSG00000171914
TF315551	654346	ENSG00000171916
TF312906	51030	ENSG00000171928
TF332241	7764	ENSG00000171940
TF105088	126410	ENSG00000171954
TF324723	221037	ENSG00000171988

TF314963	92483	ENSG00000171989
TF330867	11346	ENSG00000171992
TF316174	4118	ENSG00000172005
TF300097	83452	ENSG00000172007
TF300459	7064	ENSG00000172009
TF351065	440695	ENSG00000172018
TF333213	2596	ENSG00000172020
TF314403	253152	ENSG00000172031
TF312903	3913	ENSG00000172037
TF323369	94103	ENSG00000172057
TF315506	8462	ENSG00000172059
TF351124	131578	ENSG00000172061
TF318390	6606	ENSG00000172062
TF318390	6607	ENSG00000172062
TF300789	126308	ENSG00000172081
TF300226	54205	ENSG00000172115
TF325083	794	ENSG00000172137
TF334888	6356	ENSG00000172156
TF343477	257019	ENSG00000172159
TF317932	6641	ENSG00000172164
TF319744	10892	ENSG00000172175
TF332592	5617	ENSG00000172179
TF354340	3669	ENSG00000172183
TF314906	154141	ENSG00000172197
TF326217	3400	ENSG00000172201
TF318505	2845	ENSG00000172209
TF330966	10663	ENSG00000172215
TF105008	1051	ENSG00000172216
TF351676	7177	ENSG00000172236
TF325565	257194	ENSG00000172260
TF341440	140733	ENSG00000172264
TF326759	682	ENSG00000172270
TF350923	25988	ENSG00000172273
TF313375	9085	ENSG00000172288
TF313375	253175	ENSG00000172288
TF314319	253782	ENSG00000172292
TF300452	55304	ENSG00000172296
TF318639	8708	ENSG00000172318
TF324381	27254	ENSG00000172346
TF313579	10231	ENSG00000172348
TF326303	3603	ENSG00000172349
TF105210	64137	ENSG00000172350
TF313375	9085	ENSG00000172352
TF313375	253175	ENSG00000172352
TF106149	2783	ENSG00000172354
TF326620	84331	ENSG00000172366
TF350449	79849	ENSG00000172367
TF331604	9854	ENSG00000172375
TF319983	9915	ENSG00000172379
TF319909	55970	ENSG00000172380
TF351676	83886	ENSG00000172382
TF331748	51778	ENSG00000172399
TF330867	171024	ENSG00000172403
TF105142	150353	ENSG00000172404
TF333404	10022	ENSG00000172410
TF300212	442461	ENSG00000172450
TF316348	10690	ENSG00000172461
TF324051	79694	ENSG00000172469
TF323230	142684	ENSG00000172476

TF326216	653886	ENSG00000172493
TF326216	4299	ENSG00000172493
TF328368	134526	ENSG00000172497
TF314757	3054	ENSG00000172534
TF313214	348938	ENSG00000172548
TF317932	54221	ENSG00000172554
TF317932	728185	ENSG00000172554
TF335961	408263	ENSG00000172568
TF329631	5139	ENSG00000172572
TF312918	10125	ENSG00000172575
TF351654	89857	ENSG00000172578
TF330887	27289	ENSG00000172602
TF101212	5883	ENSG00000172613
TF317514	30008	ENSG00000172638
TF314990	5222	ENSG00000172647
TF317762	729092	ENSG00000172650
TF317762	650155	ENSG00000172650
TF322599	8148	ENSG00000172660
TF350019	64393	ENSG00000172667
TF333479	387357	ENSG00000172673
TF334888	6363	ENSG00000172724
TF314280	57175	ENSG00000172725
TF313701	29942	ENSG00000172733
TF328601	1072	ENSG00000172757
TF316292	23023	ENSG00000172765
TF106301	79612	ENSG00000172766
TF316310	3222	ENSG00000172789
TF323428	326624	ENSG00000172794
TF313698	254122	ENSG00000172803
TF300215	6169	ENSG00000172809
TF105090	9420	ENSG00000172817
TF315489	5017	ENSG00000172818
TF328382	5916	ENSG00000172819
TF328382	731106	ENSG00000172819
TF319444	54961	ENSG00000172830
TF313505	57546	ENSG00000172840
TF350150	6670	ENSG00000172845
TF317854	3849	ENSG00000172867
TF312896	1657	ENSG00000172869
TF331360	51162	ENSG00000172889
TF101180	1717	ENSG00000172893
TF313490	26960	ENSG00000172915
TF313490	730471	ENSG00000172915
TF314176	338692	ENSG00000172932
TF105339	9943	ENSG00000172939
TF106480	23133	ENSG00000172943
TF106480	731069	ENSG00000172943
TF300429	130	ENSG00000172955
TF331465	150165	ENSG00000172967
TF315091	647153	ENSG00000172974
TF315091	729317	ENSG00000172974
TF323210	727936	ENSG00000172986
TF323210	732216	ENSG00000172986
TF328999	60495	ENSG00000172987
TF315915	10777	ENSG00000172995
TF313940	156	ENSG00000173020
TF325632	5970	ENSG00000173039
TF331508	55731	ENSG00000173065
TF350399	54796	ENSG00000173068

TF330024	339403	ENSG00000173080
TF328999	10855	ENSG00000173083
TF105042	3310	ENSG00000173110
TF334360	54674	ENSG00000173114
TF106480	22992	ENSG00000173120
TF336068	654790	ENSG00000173126
TF323751	2101	ENSG00000173153
TF313537	80070	ENSG00000173157
TF317511	65059	ENSG00000173166
TF313422	4580	ENSG00000173171
TF313845	111	ENSG00000173175
TF328965	54625	ENSG00000173193
TF350009	10800	ENSG00000173198
TF316072	165631	ENSG00000173200
TF101142	1163	ENSG00000173207
TF101142	729964	ENSG00000173207
TF101142	732142	ENSG00000173207
TF105204	225	ENSG00000173208
TF313680	130872	ENSG00000173209
TF318042	22885	ENSG00000173210
TF313467	81839	ENSG00000173218
TF325082	2804	ENSG00000173230
TF332591	134391	ENSG00000173250
TF317837	10655	ENSG00000173253
TF330308	153770	ENSG00000173261
TF313762	144195	ENSG00000173262
TF329003	56834	ENSG00000173264
TF332776	6623	ENSG00000173267
TF326036	8658	ENSG00000173273
TF331184	49854	ENSG00000173276
TF333617	344561	ENSG00000173302
TF331863	56977	ENSG00000173320
TF105118	4296	ENSG00000173327
TF329785	10221	ENSG00000173334
TF319145	128822	ENSG00000173335
TF313947	10089	ENSG00000173338
TF325595	54106	ENSG00000173366
TF325595	11344	ENSG00000173366
TF329591	713	ENSG00000173369
TF329591	712	ENSG00000173372
TF313245	79625	ENSG00000173376
TF312890	344988	ENSG00000173386
TF337908	132141	ENSG00000173389
TF316148	256710	ENSG00000173401
TF320538	3642	ENSG00000173404
TF316724	1600	ENSG00000173406
TF300259	440574	ENSG00000173436
TF105382	254102	ENSG00000173442
TF330127	83591	ENSG00000173451
TF105546	26472	ENSG00000173457
TF321449	155465	ENSG00000173467
TF314710	6599	ENSG00000173473
TF312900	5797	ENSG00000173482
TF319554	7423	ENSG00000173511
TF333916	8793	ENSG00000173530
TF329901	4485	ENSG00000173531
TF333916	8794	ENSG00000173535
TF300789	92597	ENSG00000173542
TF316876	1464	ENSG00000173546

TF314082	257364	ENSG00000173548
TF313902	64859	ENSG00000173559
TF316380	165082	ENSG00000173567
TF330014	126204	ENSG00000173572
TF313461	1106	ENSG00000173575
TF330966	2829	ENSG00000173578
TF330966	10803	ENSG00000173585
TF321745	27284	ENSG00000173597
TF106349	11163	ENSG00000173598
TF315472	10941	ENSG00000173610
TF331269	222545	ENSG00000173612
TF315035	64802	ENSG00000173614
TF330861	78999	ENSG00000173621
TF331065	5644	ENSG00000173636
TF313684	6573	ENSG00000173638
TF313800	9986	ENSG00000173653
TF331269	80835	ENSG00000173662
TF351373	390992	ENSG00000173673
TF335939	256643	ENSG00000173681
TF321769	10149	ENSG00000173698
TF315349	124857	ENSG00000173714
TF106200	9804	ENSG00000173726
TF325357	3267	ENSG00000173744
TF317997	3728	ENSG00000173801
TF323495	9001	ENSG00000173805
TF314417	10209	ENSG00000173812
TF314417	730144	ENSG00000173812
TF314417	731937	ENSG00000173812
TF329577	57674	ENSG00000173821
TF313130	81033	ENSG00000173826
TF101089	1263	ENSG00000173846
TF328974	10276	ENSG00000173848
TF300112	162466	ENSG00000173868
TF300298	347688	ENSG00000173876
TF331299	80012	ENSG00000173889
TF106456	84733	ENSG00000173894
TF313446	6712	ENSG00000173898
TF332742	162605	ENSG00000173908
TF320661	83759	ENSG00000173914
TF317730	3212	ENSG00000173917
TF329591	114897	ENSG00000173918
TF319557	115123	ENSG00000173926
TF319557	648089	ENSG00000173926
TF320661	5936	ENSG00000173933
TF320661	10432	ENSG00000173959
TF312973	165324	ENSG00000173960
TF105371	646993	ENSG00000173961
TF315976	84839	ENSG00000173976
TF317167	375387	ENSG00000174004
TF328485	80311	ENSG00000174010
TF315239	169966	ENSG00000174016
TF319909	2787	ENSG00000174021
TF353414	10447	ENSG00000174028
TF323211	253512	ENSG00000174032
TF102035	146850	ENSG00000174083
TF318099	727735	ENSG00000174115
TF351113	81793	ENSG00000174123
TF351113	7096	ENSG00000174125
TF351113	10333	ENSG00000174130

TF329836	285704	ENSG00000174136
TF332095	152877	ENSG00000174137
TF332647	57495	ENSG00000174145
TF323584	284613	ENSG00000174151
TF317762	728404	ENSG00000174194
TF106341	23269	ENSG00000174197
TF318398	169166	ENSG00000174226
TF313845	112	ENSG00000174233
TF313279	5306	ENSG00000174238
TF340612	7634	ENSG00000174255
TF315938	344191	ENSG00000174279
TF333100	57659	ENSG00000174282
TF333363	23051	ENSG00000174306
TF332320	23612	ENSG00000174307
TF313792	162515	ENSG00000174326
TF313792	201232	ENSG00000174327
TF350216	148979	ENSG00000174332
TF315605	55584	ENSG00000174343
TF336377	127435	ENSG00000174348
TF343812	340024	ENSG00000174358
TF324484	253959	ENSG00000174373
TF326170	7201	ENSG00000174417
TF328879	137735	ENSG00000174429
TF300651	488	ENSG00000174437
TF329913	402117	ENSG00000174453
TF321823	26047	ENSG00000174469
TF334360	158038	ENSG00000174482
TF313237	10260	ENSG00000174485
TF321506	9543	ENSG00000174498
TF313784	115019	ENSG00000174502
TF314338	246330	ENSG00000174516
TF331917	148014	ENSG00000174521
TF332537	53833	ENSG00000174564
TF300267	127845	ENSG00000174567
TF317123	79647	ENSG00000174574
TF319684	266743	ENSG00000174576
TF314319	133022	ENSG00000174599
TF330976	1240	ENSG00000174600
TF316126	90806	ENSG00000174606
TF315472	7368	ENSG00000174607
TF331833	378948	ENSG00000174622
TF331833	5940	ENSG00000174622
TF317540	6578	ENSG00000174640
TF313950	3177	ENSG00000174669
TF313967	9024	ENSG00000174672
TF329347	285590	ENSG00000174705
TF335877	143282	ENSG00000174721
TF328382	9975	ENSG00000174738
TF300458	140886	ENSG00000174740
TF323740	25855	ENSG00000174744
TF335755	132720	ENSG00000174749
TF312796	3265	ENSG00000174775
TF331067	9610	ENSG00000174791
TF342373	152816	ENSG00000174792
TF335838	152815	ENSG00000174796
TF326518	9662	ENSG00000174799
TF330714	57124	ENSG00000174807
TF350449	5174	ENSG00000174827
TF316380	2015	ENSG00000174837

TF320228	201627	ENSG00000174839
TF333463	375347	ENSG00000174844
TF314528	10897	ENSG00000174851
TF300083	254263	ENSG00000174871
TF330014	171389	ENSG00000174885
TF300097	81876	ENSG00000174903
TF331269	344760	ENSG00000174930
TF330037	26470	ENSG00000174938
TF330037	648541	ENSG00000174938
TF330037	652900	ENSG00000174938
TF312799	253982	ENSG00000174939
TF315649	253980	ENSG00000174943
TF330969	9934	ENSG00000174944
TF328603	155185	ENSG00000174945
TF330969	29909	ENSG00000174946
TF333380	388611	ENSG00000174950
TF351425	84107	ENSG00000174963
TF316425	763	ENSG00000174990
TF314010	64837	ENSG00000174996
TF313593	1488	ENSG00000175029
TF313593	645508	ENSG00000175029
TF342871	9435	ENSG00000175040
TF312923	79683	ENSG00000175048
TF312923	730077	ENSG00000175048
TF330122	1674	ENSG00000175084
TF105336	149420	ENSG00000175087
TF312822	92369	ENSG00000175093
TF314240	55690	ENSG00000175115
TF332815	65108	ENSG00000175130
TF313936	388403	ENSG00000175155
TF326804	253559	ENSG00000175161
TF330991	28	ENSG00000175164
TF317617	22843	ENSG00000175175
TF331537	131408	ENSG00000175182
TF313758	1466	ENSG00000175183
TF351791	3626	ENSG00000175189
TF106304	4878	ENSG00000175206
TF313556	10106	ENSG00000175215
TF324164	392	ENSG00000175220
TF314802	89792	ENSG00000175229
TF342871	8534	ENSG00000175264
TF316990	23015	ENSG00000175265
TF101005	9134	ENSG00000175305
TF320468	85007	ENSG00000175309
TF324946	257629	ENSG00000175311
TF319145	1474	ENSG00000175315
TF315060	8815	ENSG00000175334
TF315060	645870	ENSG00000175334
TF315605	1139	ENSG00000175344
TF315605	89832	ENSG00000175344
TF315605	732445	ENSG00000175344
TF315146	56674	ENSG00000175348
TF333421	56675	ENSG00000175352
TF315897	5771	ENSG00000175354
TF315897	646981	ENSG00000175354
TF351672	57758	ENSG00000175356
TF314923	4087	ENSG00000175387
TF105469	285598	ENSG00000175414
TF313162	1212	ENSG00000175416

TF324997	4023	ENSG00000175445
TF333320	317671	ENSG00000175449
TF313293	374403	ENSG00000175463
TF105553	55844	ENSG00000175470
TF323373	79772	ENSG00000175471
TF313309	57628	ENSG00000175497
TF321199	254187	ENSG00000175513
TF314412	143630	ENSG00000175518
TF314412	50613	ENSG00000175520
TF334804	57010	ENSG00000175544
TF327043	79836	ENSG00000175556
TF323211	7352	ENSG00000175564
TF323211	7351	ENSG00000175567
TF300803	5870	ENSG00000175582
TF300803	84084	ENSG00000175582
TF300803	150786	ENSG00000175582
TF350009	5029	ENSG00000175591
TF326301	8061	ENSG00000175592
TF320243	11007	ENSG00000175602
TF313438	6199	ENSG00000175634
TF314105	146691	ENSG00000175662
TF313965	165829	ENSG00000175697
TF324749	22877	ENSG00000175727
TF352097	7025	ENSG00000175745
TF102003	2810	ENSG00000175793
TF353884	4482	ENSG00000175806
TF350755	222537	ENSG00000175818
TF316214	2118	ENSG00000175832
TF315960	131909	ENSG00000175841
TF325648	10458	ENSG00000175866
TF324680	200407	ENSG00000175874
TF316310	3234	ENSG00000175879
TF315235	79666	ENSG00000175895
TF330052	9294	ENSG00000175898
TF105464	379	ENSG00000175906
TF334360	57633	ENSG00000175928
TF313576	93129	ENSG00000175938
TF329218	340359	ENSG00000175946
TF314474	84747	ENSG00000175970
TF320336	163259	ENSG00000175984
TF322889	56676	ENSG00000176009
TF300298	84617	ENSG00000176014
TF326271	116068	ENSG00000176018
TF326838	386724	ENSG00000176020
TF330647	344805	ENSG00000176040
TF331900	9832	ENSG00000176049
TF316545	257101	ENSG00000176083
TF331078	55057	ENSG00000176092
TF314066	9807	ENSG00000176095
TF105867	1479	ENSG00000176102
TF351634	7525	ENSG00000176105
TF332646	4161	ENSG00000176136
TF321110	55254	ENSG00000176142
TF313385	55346	ENSG00000176148
TF105318	2877	ENSG00000176153
TF316127	2290	ENSG00000176165
TF354296	8877	ENSG00000176170
TF315424	664	ENSG00000176171
TF334321	1149	ENSG00000176194

TF332659	80059	ENSG00000176204
TF329112	79915	ENSG00000176208
TF335802	414149	ENSG00000176244
TF314192	401612	ENSG00000176274
TF313014	643752	ENSG00000176276
TF329867	283150	ENSG00000176302
TF105070	1351	ENSG00000176340
TF313068	644338	ENSG00000176343
TF318639	79369	ENSG00000176383
TF325617	3291	ENSG00000176387
TF300758	6051	ENSG00000176393
TF317837	63951	ENSG00000176399
TF329606	349149	ENSG00000176402
TF321703	9699	ENSG00000176406
TF317532	283377	ENSG00000176422
TF321840	155382	ENSG00000176428
TF330714	161198	ENSG00000176435
TF101041	1196	ENSG00000176444
TF317540	28232	ENSG00000176463
TF329325	79446	ENSG00000176473
TF330836	11145	ENSG00000176485
TF313014	148252	ENSG00000176490
TF333189	222171	ENSG00000176532
TF328485	9920	ENSG00000176595
TF101181	84823	ENSG00000176619
TF315107	51320	ENSG00000176624
TF313989	150280	ENSG00000176635
TF331690	220441	ENSG00000176641
TF312960	4642	ENSG00000176658
TF318093	90655	ENSG00000176679
TF316127	2303	ENSG00000176692
TF106463	627	ENSG00000176697
TF101036	8851	ENSG00000176749
TF331208	344148	ENSG00000176771
TF323904	80230	ENSG00000176783
TF105296	360132	ENSG00000176826
TF319371	10265	ENSG00000176842
TF330918	284207	ENSG00000176845
TF329216	55884	ENSG00000176871
TF316183	6664	ENSG00000176887
TF338287	56892	ENSG00000176907
TF326024	284358	ENSG00000176909
TF317729	23141	ENSG00000176915
TF336103	733	ENSG00000176919
TF300194	286528	ENSG00000176960
TF105375	728234	ENSG00000176982
TF105375	730972	ENSG00000176982
TF300464	9632	ENSG00000176986
TF313422	345778	ENSG00000177034
TF323824	283232	ENSG00000177042
TF315545	147912	ENSG00000177045
TF317342	54503	ENSG00000177054
TF326731	150368	ENSG00000177096
TF331728	6330	ENSG00000177098
TF316846	57453	ENSG00000177103
TF101109	391	ENSG00000177105
TF313069	64787	ENSG00000177106
TF314265	196527	ENSG00000177119
TF331184	403341	ENSG00000177125

TF328876	171483	ENSG00000177138
TF331838	646000	ENSG00000177164
TF331838	652866	ENSG00000177164
TF324551	8408	ENSG00000177169
TF323436	157807	ENSG00000177182
TF313438	6197	ENSG00000177189
TF318639	374907	ENSG00000177191
TF342569	493829	ENSG00000177238
TF313103	3738	ENSG00000177272
TF317907	8325	ENSG00000177283
TF329606	219770	ENSG00000177291
TF331936	53842	ENSG00000177300
TF313103	3737	ENSG00000177301
TF320582	57513	ENSG00000177303
TF333100	253461	ENSG00000177311
TF333488	3090	ENSG00000177374
TF314207	8541	ENSG00000177380
TF329882	89766	ENSG00000177398
TF331842	219285	ENSG00000177409
TF318093	7050	ENSG00000177426
TF331032	125170	ENSG00000177427
TF314349	266812	ENSG00000177432
TF316650	7182	ENSG00000177463
TF331803	2828	ENSG00000177464
TF322733	167826	ENSG00000177468
TF331031	284119	ENSG00000177469
TF333100	10009	ENSG00000177485
TF319371	79191	ENSG00000177508
TF323961	51046	ENSG00000177511
TF332720	56475	ENSG00000177519
TF313209	79751	ENSG00000177542
TF329365	79874	ENSG00000177548
TF315153	4808	ENSG00000177551
TF332178	148109	ENSG00000177558
TF323190	79718	ENSG00000177565
TF325918	401474	ENSG00000177570
TF329295	9332	ENSG00000177575
TF320650	6181	ENSG00000177600
TF323952	3725	ENSG00000177606
TF329644	23765	ENSG00000177663
TF314272	57104	ENSG00000177666
TF314906	619373	ENSG00000177669
TF329295	283316	ENSG00000177675
TF335721	222183	ENSG00000177679
TF335838	168451	ENSG00000177683
TF329556	283229	ENSG00000177685
TF315116	387082	ENSG00000177688
TF312981	254827	ENSG00000177694
TF352892	977	ENSG00000177697
TF103046	5441	ENSG00000177700
TF331051	25945	ENSG00000177707
TF331838	83650	ENSG00000177710
TF316183	6666	ENSG00000177732
TF334042	85364	ENSG00000177764
TF331748	58529	ENSG00000177791
TF313676	3766	ENSG00000177807
TF332842	9849	ENSG00000177853
TF300189	1176	ENSG00000177879
TF300189	730730	ENSG00000177879

TF314822	93661	ENSG00000177938
TF300265	6232	ENSG00000177954
TF314907	60626	ENSG00000177963
TF336103	389812	ENSG00000177984
TF106499	285601	ENSG00000178015
TF332831	388886	ENSG00000178026
TF351125	92949	ENSG00000178031
TF329085	254228	ENSG00000178033
TF300378	3615	ENSG00000178035
TF331793	259173	ENSG00000178038
TF317561	4291	ENSG00000178053
TF351676	339906	ENSG00000178055
TF327695	54762	ENSG00000178075
TF332087	55620	ENSG00000178078
TF315605	170572	ENSG00000178084
TF105333	83983	ENSG00000178093
TF105333	730000	ENSG00000178093
TF105333	731055	ENSG00000178093
TF329233	9659	ENSG00000178104
TF329233	652164	ENSG00000178104
TF329233	652526	ENSG00000178104
TF329233	652879	ENSG00000178104
TF329233	727893	ENSG00000178104
TF329233	728802	ENSG00000178104
TF329233	728920	ENSG00000178104
TF329233	649752	ENSG00000178104
TF329233	727948	ENSG00000178104
TF332842	85460	ENSG00000178163
TF333006	205147	ENSG00000178171
TF331510	167465	ENSG00000178175
TF319589	254251	ENSG00000178177
TF312899	84552	ENSG00000178184
TF323184	25970	ENSG00000178188
TF315783	340152	ENSG00000178199
TF323280	143888	ENSG00000178202
TF335163	5339	ENSG00000178209
TF335163	652460	ENSG00000178209
TF336893	387694	ENSG00000178217
TF313267	63917	ENSG00000178234
TF351826	114798	ENSG00000178235
TF320251	282679	ENSG00000178301
TF313103	26251	ENSG00000178342
TF330800	152573	ENSG00000178343
TF300912	810	ENSG00000178363
TF300912	51806	ENSG00000178372
TF317067	389072	ENSG00000178385
TF316350	3350	ENSG00000178394
TF315153	63973	ENSG00000178403
TF300037	439992	ENSG00000178429
TF331981	55175	ENSG00000178502
TF333403	404217	ENSG00000178531
TF300894	788	ENSG00000178537
TF101142	652904	ENSG00000178556
TF106002	2066	ENSG00000178568
TF325689	4094	ENSG00000178573
TF314533	56998	ENSG00000178585
TF313986	2081	ENSG00000178607
TF323969	80034	ENSG00000178662
TF328965	84875	ENSG00000178685

TF321304	63899	ENSG00000178694
TF315332	115207	ENSG00000178695
TF317636	200895	ENSG00000178700
TF325688	54913	ENSG00000178718
TF319996	2907	ENSG00000178719
TF330714	7056	ENSG00000178726
TF351124	2814	ENSG00000178732
TF313763	415117	ENSG00000178750
TF331282	151176	ENSG00000178752
TF331928	57184	ENSG00000178761
TF333363	22882	ENSG00000178764
TF351124	1370	ENSG00000178772
TF316419	27132	ENSG00000178773
TF334441	124599	ENSG00000178789
TF313692	220032	ENSG00000178795
TF333386	132243	ENSG00000178804
TF342569	375593	ENSG00000178809
TF342569	378125	ENSG00000178809
TF331690	54546	ENSG00000178828
TF350742	9242	ENSG00000178860
TF334681	81575	ENSG00000178878
TF332387	144347	ENSG00000178882
TF313376	147991	ENSG00000178904
TF313376	147990	ENSG00000178904
TF313044	6879	ENSG00000178913
TF315551	3963	ENSG00000178934
TF315551	653499	ENSG00000178934
TF315551	728910	ENSG00000178934
TF315551	732032	ENSG00000178934
TF105165	2580	ENSG00000178950
TF331824	51341	ENSG00000178951
TF300432	7284	ENSG00000178952
TF331673	55030	ENSG00000178974
TF314082	112574	ENSG00000178996
TF331269	80834	ENSG00000179002
TF328485	127707	ENSG00000179023
TF313666	6844	ENSG00000179036
TF314979	283849	ENSG00000179044
TF317532	205860	ENSG00000179046
TF106493	132625	ENSG00000179059
TF333232	220388	ENSG00000179071
TF318445	5187	ENSG00000179094
TF316350	3355	ENSG00000179097
TF351373	84667	ENSG00000179111
TF324165	55095	ENSG00000179134
TF105094	1585	ENSG00000179142
TF330845	168537	ENSG00000179144
TF105320	59344	ENSG00000179148
TF330974	3183	ENSG00000179172
TF330974	343069	ENSG00000179172
TF330974	649330	ENSG00000179172
TF340612	144348	ENSG00000179195
TF338438	811	ENSG00000179218
TF101216	5886	ENSG00000179262
TF106445	199699	ENSG00000179284
TF315223	256472	ENSG00000179292
TF351632	5781	ENSG00000179295
TF351632	391771	ENSG00000179295
TF351632	647552	ENSG00000179295

TF335133	203430	ENSG00000179300
TF324060	23302	ENSG00000179314
TF300143	441722	ENSG00000179315
TF300143	649163	ENSG00000179315
TF300032	54734	ENSG00000179331
TF101041	1198	ENSG00000179335
TF101041	646505	ENSG00000179335
TF101041	649407	ENSG00000179335
TF315391	2624	ENSG00000179348
TF320364	10620	ENSG00000179361
TF314240	23241	ENSG00000179364
TF323472	255520	ENSG00000179387
TF318980	1960	ENSG00000179388
TF105317	2262	ENSG00000179399
TF105142	165721	ENSG00000179407
TF315108	7681	ENSG00000179455
TF337437	10472	ENSG00000179456
TF105320	242	ENSG00000179477
TF313535	246213	ENSG00000179520
TF325047	85474	ENSG00000179528
TF326378	139065	ENSG00000179542
TF316350	3352	ENSG00000179546
TF351947	146310	ENSG00000179580
TF352118	4261	ENSG00000179583
TF331342	161882	ENSG00000179588
TF105320	247	ENSG00000179593
TF313240	2918	ENSG00000179603
TF313240	652292	ENSG00000179603
TF331725	23580	ENSG00000179604
TF315149	84232	ENSG00000179632
TF314440	122664	ENSG00000179636
TF335097	2205	ENSG00000179639
TF332720	388394	ENSG00000179673
TF330014	126205	ENSG00000179709
TF331356	9582	ENSG00000179750
TF316127	2307	ENSG00000179772
TF329887	1003	ENSG00000179776
TF327070	116135	ENSG00000179796
TF331088	91663	ENSG00000179820
TF315201	727957	ENSG00000179832
TF315201	377711	ENSG00000179832
TF315637	29890	ENSG00000179837
TF313878	126326	ENSG00000179855
TF331915	163732	ENSG00000179862
TF105191	154664	ENSG00000179869
TF330014	204801	ENSG00000179873
TF331299	653441	ENSG00000179899
TF315915	22864	ENSG00000179912
TF318639	10331	ENSG00000179913
TF321302	9378	ENSG00000179915
TF313811	22928	ENSG00000179918
TF330966	1237	ENSG00000179934
TF340612	51333	ENSG00000179965
TF105356	642009	ENSG00000179978
TF105356	643784	ENSG00000179978
TF105356	648984	ENSG00000179978
TF105356	651112	ENSG00000179978
TF105356	652755	ENSG00000179978
TF105356	728519	ENSG00000179978

TF105356	728535	ENSG00000179978
TF328447	10194	ENSG00000179981
TF321368	122809	ENSG00000180008
TF351204	137814	ENSG00000180053
TF314508	284417	ENSG00000180061
TF324663	255043	ENSG00000180089
TF101079	1731	ENSG00000180096
TF313275	54952	ENSG00000180098
TF314979	11336	ENSG00000180104
TF326896	221400	ENSG00000180113
TF354246	122011	ENSG00000180138
TF105356	112401	ENSG00000180152
TF313327	7054	ENSG00000180176
TF314218	29895	ENSG00000180209
TF327329	2147	ENSG00000180210
TF315953	8575	ENSG00000180228
TF317681	223082	ENSG00000180233
TF324998	10692	ENSG00000180245
TF317212	389015	ENSG00000180251
TF343077	55785	ENSG00000180263
TF334275	124274	ENSG00000180269
TF313378	200150	ENSG00000180287
TF314741	4947	ENSG00000180304
TF314272	285848	ENSG00000180316
TF350743	8092	ENSG00000180318
TF315332	386618	ENSG00000180332
TF317907	2535	ENSG00000180340
TF329775	23060	ENSG00000180357
TF105351	5062	ENSG00000180370
TF327972	283254	ENSG00000180423
TF105090	1582	ENSG00000180432
TF329660	2619	ENSG00000180447
TF351450	23526	ENSG00000180448
TF316148	144321	ENSG00000180481
TF313896	374986	ENSG00000180488
TF331690	221687	ENSG00000180537
TF316348	2529	ENSG00000180549
TF318639	84752	ENSG00000180561
TF300137	8334	ENSG00000180573
TF329089	151963	ENSG00000180611
TF315938	170825	ENSG00000180613
TF315737	6752	ENSG00000180616
TF324206	84333	ENSG00000180628
TF324441	146802	ENSG00000180638
TF330498	5551	ENSG00000180644
TF315012	4081	ENSG00000180660
TF330800	387914	ENSG00000180730
TF105008	1052	ENSG00000180733
TF330052	53637	ENSG00000180739
TF331875	119467	ENSG00000180745
TF325581	166012	ENSG00000180767
TF330024	186	ENSG00000180772
TF314873	120103	ENSG00000180773
TF316044	253832	ENSG00000180776
TF314186	79642	ENSG00000180801
TF317819	3225	ENSG00000180806
TF105115	389840	ENSG00000180815
TF300887	5464	ENSG00000180817
TF317819	3226	ENSG00000180818

TF322733	27319	ENSG00000180828
TF338320	79929	ENSG00000180834
TF314990	643834	ENSG00000180860
TF314990	649034	ENSG00000180860
TF330966	3579	ENSG00000180871
TF106445	64388	ENSG00000180875
TF351429	23513	ENSG00000180900
TF313754	23510	ENSG00000180901
TF106499	5021	ENSG00000180914
TF330777	286077	ENSG00000180921
TF332667	118442	ENSG00000180929
TF313279	23760	ENSG00000180957
TF300296	1728	ENSG00000181019
TF354340	64782	ENSG00000181026
TF342971	9501	ENSG00000181031
TF335097	343413	ENSG00000181036
TF331155	284615	ENSG00000181039
TF314628	25994	ENSG00000181061
TF320495	1129	ENSG00000181072
TF106443	79813	ENSG00000181090
TF106443	652182	ENSG00000181090
TF329591	9370	ENSG00000181092
TF330775	2149	ENSG00000181104
TF327704	4869	ENSG00000181163
TF327704	440577	ENSG00000181163
TF332620	5179	ENSG00000181195
TF316350	151	ENSG00000181210
TF300137	92815	ENSG00000181218
TF314981	92293	ENSG00000181234
TF313492	284427	ENSG00000181240
TF329089	284114	ENSG00000181284
TF314981	124842	ENSG00000181291
TF106374	347736	ENSG00000181322
TF329807	341208	ENSG00000181333
TF332831	388341	ENSG00000181350
TF334888	6357	ENSG00000181374
TF334200	2837	ENSG00000181408
TF332280	9625	ENSG00000181409
TF332280	651771	ENSG00000181409
TF323386	55511	ENSG00000181433
TF316183	6657	ENSG00000181449
TF328673	55076	ENSG00000181458
TF313014	5912	ENSG00000181467
TF332229	57621	ENSG00000181472
TF319446	79777	ENSG00000181513
TF315012	10586	ENSG00000181541
TF315107	399664	ENSG00000181588
TF330969	53829	ENSG00000181631
TF332169	9966	ENSG00000181634
TF332320	7262	ENSG00000181649
TF313665	285973	ENSG00000181652
TF332024	5324	ENSG00000181690
TF335684	26137	ENSG00000181722
TF326838	57463	ENSG00000181754
TF330052	2827	ENSG00000181773
TF325804	284451	ENSG00000181781
TF312976	6478	ENSG00000181788
TF312976	730030	ENSG00000181788
TF312976	732092	ENSG00000181788

TF300324	22820	ENSG00000181789
TF331634	575	ENSG00000181790
TF318755	285195	ENSG00000181804
TF332339	768211	ENSG00000181826
TF321340	64864	ENSG00000181827
TF313762	6517	ENSG00000181856
TF313885	94033	ENSG00000181867
TF331936	1366	ENSG00000181885
TF314673	84890	ENSG00000181915
TF313247	5571	ENSG00000181929
TF315153	4762	ENSG00000181965
TF354231	64963	ENSG00000181991
TF343356	646976	ENSG00000181996
TF331476	219790	ENSG00000182010
TF324946	124590	ENSG00000182040
TF324570	25834	ENSG00000182050
TF333276	220382	ENSG00000182103
TF300873	161291	ENSG00000182107
TF318560	30820	ENSG00000182132
TF330032	339221	ENSG00000182156
TF316079	64764	ENSG00000182158
TF330775	286530	ENSG00000182162
TF316767	8633	ENSG00000182168
TF329836	56963	ENSG00000182175
TF300586	7318	ENSG00000182179
TF105371	441795	ENSG00000182181
TF331401	1419	ENSG00000182187
TF314231	2131	ENSG00000182197
TF300789	81532	ENSG00000182208
TF300174	8294	ENSG00000182217
TF300174	8359	ENSG00000182217
TF300174	8360	ENSG00000182217
TF300174	8361	ENSG00000182217
TF300174	8362	ENSG00000182217
TF300174	8363	ENSG00000182217
TF300174	8364	ENSG00000182217
TF300174	8365	ENSG00000182217
TF300174	8366	ENSG00000182217
TF300174	8367	ENSG00000182217
TF300174	8368	ENSG00000182217
TF300174	8370	ENSG00000182217
TF300174	121504	ENSG00000182217
TF300174	554313	ENSG00000182217
TF329059	84439	ENSG00000182218
TF331383	326340	ENSG00000182223
TF329595	25825	ENSG00000182240
TF316292	1527	ENSG00000182242
TF316292	653363	ENSG00000182242
TF316292	728447	ENSG00000182242
TF101117	7325	ENSG00000182247
TF330122	23336	ENSG00000182253
TF313103	3739	ENSG00000182255
TF315453	2567	ENSG00000182256
TF341481	55267	ENSG00000182257
TF330014	338322	ENSG00000182261
TF105015	55137	ENSG00000182263
TF318303	338707	ENSG00000182272
TF312921	8905	ENSG00000182287
TF312921	653653	ENSG00000182287

TF312921	654127	ENSG00000182287
TF325032	125997	ENSG00000182315
TF325032	653657	ENSG00000182315
TF331193	157285	ENSG00000182319
TF313676	3770	ENSG00000182324
TF330373	716	ENSG00000182326
TF316097	388323	ENSG00000182327
TF332708	391002	ENSG00000182330
TF329218	143879	ENSG00000182359
TF314457	55344	ENSG00000182378
TF333047	11247	ENSG00000182379
TF316195	785	ENSG00000182389
TF313010	122553	ENSG00000182400
TF313375	9426	ENSG00000182415
TF313375	203611	ENSG00000182415
TF334029	131149	ENSG00000182447
TF313947	50801	ENSG00000182450
TF328447	128553	ENSG00000182463
TF314748	147968	ENSG00000182472
TF101178	3838	ENSG00000182481
TF331648	652968	ENSG00000182482
TF331465	402415	ENSG00000182489
TF334562	633	ENSG00000182492
TF313576	84876	ENSG00000182500
TF321143	340596	ENSG00000182508
TF315363	2242	ENSG00000182511
TF300102	387332	ENSG00000182521
TF315736	859	ENSG00000182533
TF318014	3985	ENSG00000182541
TF314395	201305	ENSG00000182557
TF332714	6304	ENSG00000182568
TF333047	11248	ENSG00000182575
TF325768	1436	ENSG00000182578
TF315608	2049	ENSG00000182580
TF350755	9951	ENSG00000182601
TF323495	22906	ENSG00000182606
TF300137	8331	ENSG00000182611
TF313002	83882	ENSG00000182612
TF313216	23236	ENSG00000182621
TF330024	51289	ENSG00000182631
TF325565	50863	ENSG00000182667
TF315737	8811	ENSG00000182687
TF318060	392221	ENSG00000182699
TF333443	406875	ENSG00000182707
TF105371	729595	ENSG00000182711
TF105371	730702	ENSG00000182711
TF105452	302	ENSG00000182718
TF351956	9628	ENSG00000182732
TF316310	3214	ENSG00000182742
TF332887	392617	ENSG00000182743
TF319738	164091	ENSG00000182749
TF331636	5069	ENSG00000182752
TF325689	389692	ENSG00000182759
TF105374	728111	ENSG00000182765
TF105374	731576	ENSG00000182765
TF352434	2894	ENSG00000182771
TF330775	8843	ENSG00000182782
TF330775	338442	ENSG00000182782
TF317532	442247	ENSG00000182805

TF313758	1397	ENSG00000182809
TF321667	64746	ENSG00000182827
TF314457	345557	ENSG00000182836
TF315374	284013	ENSG00000182853
TF351634	3932	ENSG00000182866
TF313267	50614	ENSG00000182870
TF313267	729185	ENSG00000182870
TF315821	80781	ENSG00000182871
TF315789	8241	ENSG00000182872
TF351956	6000	ENSG00000182901
TF313209	83733	ENSG00000182902
TF330595	80254	ENSG00000182923
TF313428	347741	ENSG00000182938
TF322599	2130	ENSG00000182944
TF325804	161753	ENSG00000182950
TF105375	10473	ENSG00000182952
TF300136	650788	ENSG00000182953
TF316832	221178	ENSG00000182957
TF329606	10052	ENSG00000182963
TF316183	6656	ENSG00000182968
TF106444	9112	ENSG00000182979
TF334360	645191	ENSG00000183014
TF300308	161	ENSG00000183020
TF314308	6546	ENSG00000183023
TF313428	92736	ENSG00000183034
TF336068	5121	ENSG00000183036
TF313608	728226	ENSG00000183038
TF312920	1468	ENSG00000183048
TF314166	57118	ENSG00000183049
TF326271	145748	ENSG00000183060
TF314141	164684	ENSG00000183066
TF351204	1482	ENSG00000183072
TF331648	389523	ENSG00000183086
TF352157	652612	ENSG00000183087
TF352157	2621	ENSG00000183087
TF316876	166752	ENSG00000183090
TF319104	4703	ENSG00000183091
TF331612	57596	ENSG00000183092
TF105317	10082	ENSG00000183098
TF330015	389337	ENSG00000183111
TF314159	163933	ENSG00000183114
TF316872	64478	ENSG00000183117
TF330976	11251	ENSG00000183134
TF329178	285753	ENSG00000183137
TF336045	53820	ENSG00000183145
TF331025	83698	ENSG00000183166
TF330989	126567	ENSG00000183186
TF342871	4166	ENSG00000183196
TF313686	29119	ENSG00000183230
TF316230	150221	ENSG00000183246
TF329310	754	ENSG00000183255
TF333138	147372	ENSG00000183287
TF328876	171482	ENSG00000183304
TF315608	284656	ENSG00000183317
TF329827	388333	ENSG00000183318
TF333317	54880	ENSG00000183337
TF331602	163351	ENSG00000183347
TF331357	646658	ENSG00000183379
TF331357	649434	ENSG00000183379

TF314113	2275	ENSG00000183386
TF328989	120126	ENSG00000183417
TF328989	730436	ENSG00000183417
TF106506	54101	ENSG00000183421
TF330861	345193	ENSG00000183423
TF317532	391712	ENSG00000183439
TF314731	2903	ENSG00000183454
TF315737	6753	ENSG00000183473
TF331803	29933	ENSG00000183484
TF331484	4600	ENSG00000183486
TF106424	57634	ENSG00000183495
TF315107	84206	ENSG00000183496
TF315239	54855	ENSG00000183508
TF300137	8337	ENSG00000183558
TF300137	723790	ENSG00000183558
TF318292	54039	ENSG00000183570
TF323415	388121	ENSG00000183578
TF317074	84133	ENSG00000183579
TF314241	126961	ENSG00000183598
TF314241	333932	ENSG00000183598
TF314241	653604	ENSG00000183598
TF330468	84734	ENSG00000183615
TF330966	1232	ENSG00000183625
TF318770	94137	ENSG00000183638
TF329218	64410	ENSG00000183655
TF331749	407738	ENSG00000183662
TF330976	2825	ENSG00000183671
TF316134	353500	ENSG00000183682
TF332387	359845	ENSG00000183688
TF353745	9241	ENSG00000183691
TF314310	7378	ENSG00000183696
TF319100	654029	ENSG00000183701
TF325565	4978	ENSG00000183715
TF321143	10186	ENSG00000183722
TF317387	146223	ENSG00000183723
TF300282	23585	ENSG00000183726
TF315737	2831	ENSG00000183729
TF322889	430	ENSG00000183734
TF324269	29110	ENSG00000183735
TF106456	23467	ENSG00000183741
TF106456	23466	ENSG00000183741
TF105572	346389	ENSG00000183742
TF316663	414308	ENSG00000183748
TF331319	83999	ENSG00000183762
TF316127	668	ENSG00000183770
TF314028	150209	ENSG00000183773
TF315332	57528	ENSG00000183775
TF318639	10317	ENSG00000183778
TF324968	80139	ENSG00000183779
TF313798	148641	ENSG00000183780
TF315332	386617	ENSG00000183783
TF317259	162699	ENSG00000183791
TF331033	90187	ENSG00000183798
TF323771	221303	ENSG00000183807
TF331899	389677	ENSG00000183808
TF341248	727958	ENSG00000183812
TF330966	1233	ENSG00000183813
TF353414	54097	ENSG00000183844
TF327139	55243	ENSG00000183853

TF313078	128239	ENSG00000183856
TF318250	1260	ENSG00000183862
TF105274	10766	ENSG00000183864
TF323985	6331	ENSG00000183873
TF323985	650400	ENSG00000183873
TF323985	731231	ENSG00000183873
TF314186	340075	ENSG00000183876
TF317405	7404	ENSG00000183878
TF334689	219527	ENSG00000183908
TF343096	4068	ENSG00000183918
TF300174	8294	ENSG00000183941
TF300174	8359	ENSG00000183941
TF300174	8360	ENSG00000183941
TF300174	8361	ENSG00000183941
TF300174	8362	ENSG00000183941
TF300174	8363	ENSG00000183941
TF300174	8364	ENSG00000183941
TF300174	8365	ENSG00000183941
TF300174	8366	ENSG00000183941
TF300174	8367	ENSG00000183941
TF300174	8368	ENSG00000183941
TF300174	8370	ENSG00000183941
TF300174	121504	ENSG00000183941
TF300174	554313	ENSG00000183941
TF313130	131096	ENSG00000183960
TF316716	6525	ENSG00000183963
TF352818	256435	ENSG00000184005
TF313384	8073	ENSG00000184007
TF300361	71	ENSG00000184009
TF351678	7113	ENSG00000184012
TF313237	23258	ENSG00000184014
TF106341	6899	ENSG00000184058
TF324540	55803	ENSG00000184060
TF328642	54954	ENSG00000184083
TF331936	7122	ENSG00000184113
TF314501	8508	ENSG00000184117
TF321796	6900	ENSG00000184144
TF315186	3786	ENSG00000184156
TF331282	388581	ENSG00000184163
TF316507	79174	ENSG00000184164
TF313676	3768	ENSG00000184185
TF331163	54328	ENSG00000184194
TF105536	5504	ENSG00000184203
TF316990	161527	ENSG00000184206
TF314344	283871	ENSG00000184207
TF314707	347516	ENSG00000184210
TF328924	3654	ENSG00000184216
TF322733	116448	ENSG00000184221
TF320624	5101	ENSG00000184226
TF300455	220	ENSG00000184254
TF300137	8338	ENSG00000184260
TF313947	56660	ENSG00000184261
TF300137	317772	ENSG00000184270
TF350705	5463	ENSG00000184271
TF325032	284428	ENSG00000184301
TF315545	4990	ENSG00000184302
TF314320	5587	ENSG00000184304
TF331021	401145	ENSG00000184305
TF314116	284942	ENSG00000184319

TF105334	26576	ENSG00000184343
TF337908	389123	ENSG00000184345
TF332887	6586	ENSG00000184347
TF300137	8329	ENSG00000184348
TF300137	8330	ENSG00000184348
TF300137	8332	ENSG00000184348
TF300137	8336	ENSG00000184348
TF300137	8969	ENSG00000184348
TF300137	85235	ENSG00000184348
TF315495	1946	ENSG00000184349
TF313664	3009	ENSG00000184357
TF321877	11187	ENSG00000184363
TF332273	256714	ENSG00000184368
TF330481	10584	ENSG00000184374
TF332922	84441	ENSG00000184384
TF326233	340529	ENSG00000184388
TF326233	645974	ENSG00000184388
TF330991	127550	ENSG00000184389
TF330999	26039	ENSG00000184402
TF343356	81787	ENSG00000184405
TF313103	3751	ENSG00000184408
TF105281	116447	ENSG00000184428
TF335838	80764	ENSG00000184436
TF330966	2826	ENSG00000184451
TF336902	400746	ENSG00000184454
TF315617	254240	ENSG00000184459
TF329591	390664	ENSG00000184471
TF315583	4303	ENSG00000184481
TF316413	5454	ENSG00000184486
TF313384	11156	ENSG00000184489
TF316127	200350	ENSG00000184492
TF331034	348013	ENSG00000184497
TF352157	5627	ENSG00000184500
TF313474	201140	ENSG00000184544
TF105122	1850	ENSG00000184545
TF321368	9021	ENSG00000184557
TF326378	84189	ENSG00000184564
TF354206	440822	ENSG00000184571
TF350009	57121	ENSG00000184574
TF314638	5142	ENSG00000184588
TF331749	284467	ENSG00000184599
TF313130	90134	ENSG00000184611
TF323325	4753	ENSG00000184613
TF324178	9968	ENSG00000184634
TF101078	10801	ENSG00000184640
TF316127	349334	ENSG00000184659
TF316127	100036519	ENSG00000184659
TF330974	138046	ENSG00000184672
TF325759	2952	ENSG00000184674
TF333006	139285	ENSG00000184675
TF331936	9074	ENSG00000184697
TF317206	23520	ENSG00000184701
TF101079	5413	ENSG00000184702
TF334689	389816	ENSG00000184709
TF312881	619189	ENSG00000184716
TF330964	642273	ENSG00000184731
TF105251	25923	ENSG00000184743
TF315116	652489	ENSG00000184763
TF324570	641515	ENSG00000184774

TF319736	340562	ENSG00000184788
TF354320	23762	ENSG00000184792
TF300137	85235	ENSG00000184825
TF331824	201501	ENSG00000184828
TF315313	79135	ENSG00000184831
TF332746	51334	ENSG00000184838
TF314123	54732	ENSG00000184840
TF316350	1812	ENSG00000184845
TF313574	93517	ENSG00000184860
TF333209	283523	ENSG00000184862
TF333209	646316	ENSG00000184862
TF333209	648377	ENSG00000184862
TF333209	650291	ENSG00000184862
TF106482	90135	ENSG00000184887
TF313664	8971	ENSG00000184897
TF315116	6612	ENSG00000184900
TF300522	1188	ENSG00000184908
TF351835	3714	ENSG00000184916
TF325155	752	ENSG00000184922
TF336103	286256	ENSG00000184925
TF320251	375318	ENSG00000184945
TF300299	4588	ENSG00000184956
TF320495	1133	ENSG00000184984
TF324918	57537	ENSG00000184985
TF328726	80757	ENSG00000184986
TF328907	113277	ENSG00000184988
TF328907	728772	ENSG00000184988
TF332238	140707	ENSG00000184992
TF321340	222546	ENSG00000185002
TF351053	6092	ENSG00000185008
TF315187	26985	ENSG00000185009
TF329807	2157	ENSG00000185010
TF329831	57665	ENSG00000185013
TF329831	93034	ENSG00000185013
TF316425	377677	ENSG00000185015
TF325689	23764	ENSG00000185022
TF332708	389257	ENSG00000185028
TF316102	10509	ENSG00000185033
TF313865	10519	ENSG00000185043
TF320582	56899	ENSG00000185046
TF318759	57419	ENSG00000185052
TF313538	137868	ENSG00000185053
TF317854	51350	ENSG00000185069
TF332578	283777	ENSG00000185087
TF300265	51065	ENSG00000185088
TF324051	149175	ENSG00000185090
TF300486	122622	ENSG00000185100
TF314265	338440	ENSG00000185101
TF331088	255275	ENSG00000185105
TF314159	131583	ENSG00000185112
TF330401	3297	ENSG00000185122
TF330401	642255	ENSG00000185122
TF318099	729873	ENSG00000185128
TF318099	729877	ENSG00000185128
TF313701	5813	ENSG00000185129
TF317034	27124	ENSG00000185133
TF315303	4887	ENSG00000185149
TF325519	59307	ENSG00000185187
TF333630	400668	ENSG00000185198

TF334894	10581	ENSG00000185201
TF328485	84861	ENSG00000185214
TF314979	7127	ENSG00000185215
TF332646	4158	ENSG00000185231
TF300099	9230	ENSG00000185236
TF351114	2811	ENSG00000185245
TF314746	55015	ENSG00000185246
TF343694	645627	ENSG00000185251
TF343694	646354	ENSG00000185251
TF343694	649294	ENSG00000185251
TF343694	649396	ENSG00000185251
TF343694	729469	ENSG00000185251
TF343694	730919	ENSG00000185251
TF316292	1527	ENSG00000185254
TF316292	653363	ENSG00000185254
TF316292	728447	ENSG00000185254
TF329433	283991	ENSG00000185262
TF314252	441549	ENSG00000185267
TF324830	147111	ENSG00000185269
TF324830	388444	ENSG00000185269
TF324830	653520	ENSG00000185269
TF323596	54033	ENSG00000185272
TF313267	64409	ENSG00000185274
TF332666	84614	ENSG00000185278
TF331549	3563	ENSG00000185291
TF319186	162540	ENSG00000185294
TF323554	158880	ENSG00000185295
TF323985	6336	ENSG00000185313
TF321368	8651	ENSG00000185338
TF323754	10634	ENSG00000185340
TF300346	23545	ENSG00000185344
TF312835	266722	ENSG00000185352
TF323415	126282	ENSG00000185361
TF105100	5600	ENSG00000185386
TF317805	653449	ENSG00000185390
TF300858	123283	ENSG00000185418
TF105574	6450	ENSG00000185437
TF337047	285513	ENSG00000185477
TF317854	3854	ENSG00000185479
TF332878	246329	ENSG00000185482
TF106465	4919	ENSG00000185483
TF328512	3665	ENSG00000185507
TF316498	26013	ENSG00000185513
TF324824	9899	ENSG00000185518
TF331537	348487	ENSG00000185519
TF333297	5148	ENSG00000185527
TF313261	5592	ENSG00000185532
TF352097	7026	ENSG00000185551
TF314566	56001	ENSG00000185554
TF314566	650686	ENSG00000185554
TF314566	728343	ENSG00000185554
TF351835	8788	ENSG00000185559
TF325565	4045	ENSG00000185565
TF351220	169611	ENSG00000185585
TF350150	6667	ENSG00000185591
TF329345	201176	ENSG00000185602
TF332839	389119	ENSG00000185614
TF106381	64714	ENSG00000185615
TF324206	10336	ENSG00000185619

TF106381	5034	ENSG00000185624
TF314340	5087	ENSG00000185630
TF106383	56901	ENSG00000185633
TF315807	399694	ENSG00000185634
TF317854	338785	ENSG00000185640
TF315463	677	ENSG00000185650
TF313043	7332	ENSG00000185651
TF106463	4908	ENSG00000185652
TF324197	54014	ENSG00000185658
TF334865	6490	ENSG00000185664
TF319919	8224	ENSG00000185666
TF316413	5453	ENSG00000185668
TF315515	333929	ENSG00000185669
TF330979	79842	ENSG00000185670
TF329826	254773	ENSG00000185674
TF332708	23532	ENSG00000185686
TF326257	4603	ENSG00000185697
TF323736	253943	ENSG00000185728
TF315806	105	ENSG00000185736
TF342671	3434	ENSG00000185745
TF315186	56479	ENSG00000185760
TF318560	80333	ENSG00000185774
TF323400	10933	ENSG00000185787
TF323400	10934	ENSG00000185787
TF330014	338321	ENSG00000185792
TF314961	1762	ENSG00000185800
TF314820	79581	ENSG00000185803
TF331189	10320	ENSG00000185811
TF324687	339983	ENSG00000185818
TF314116	391282	ENSG00000185822
TF314116	652167	ENSG00000185822
TF315310	10134	ENSG00000185825
TF342569	140691	ENSG00000185880
TF334894	8519	ENSG00000185885
TF351676	339501	ENSG00000185888
TF316339	3916	ENSG00000185896
TF330775	2865	ENSG00000185897
TF330775	731823	ENSG00000185897
TF328485	257240	ENSG00000185915
TF106489	5727	ENSG00000185920
TF330080	146760	ENSG00000185924
TF329085	255022	ENSG00000185933
TF321348	286183	ENSG00000185942
TF314566	56001	ENSG00000185945
TF314566	650686	ENSG00000185945
TF314566	728343	ENSG00000185945
TF325994	8660	ENSG00000185950
TF350757	6473	ENSG00000185960
TF323833	23299	ENSG00000185963
TF331981	881	ENSG00000185972
TF313940	6011	ENSG00000185974
TF300137	83740	ENSG00000185978
TF300137	474381	ENSG00000185978
TF351826	84631	ENSG00000185985
TF105302	22821	ENSG00000185989
TF318428	84859	ENSG00000186001
TF314618	496	ENSG00000186009
TF300197	374887	ENSG00000186010
TF326072	342184	ENSG00000186031

TF315605	285242	ENSG00000186038
TF317854	319101	ENSG00000186049
TF315153	6887	ENSG00000186051
TF332213	147166	ENSG00000186060
TF334441	146722	ENSG00000186074
TF300007	441531	ENSG00000186076
TF300007	728188	ENSG00000186076
TF317854	3852	ENSG00000186081
TF315605	200909	ENSG00000186090
TF351179	503582	ENSG00000186103
TF330790	157567	ENSG00000186106
TF319618	23396	ENSG00000186111
TF105088	8529	ENSG00000186115
TF333162	10773	ENSG00000186130
TF105088	199974	ENSG00000186160
TF331144	283149	ENSG00000186174
TF317681	84937	ENSG00000186187
TF315617	359710	ENSG00000186190
TF315617	149954	ENSG00000186191
TF337010	123264	ENSG00000186198
TF105088	66002	ENSG00000186204
TF316807	64757	ENSG00000186205
TF331362	345079	ENSG00000186212
TF300194	136157	ENSG00000186217
TF300194	392982	ENSG00000186217
TF326629	55330	ENSG00000186222
TF300194	390031	ENSG00000186223
TF300194	390033	ENSG00000186223
TF300194	441584	ENSG00000186223
TF300194	651313	ENSG00000186223
TF328485	114792	ENSG00000186231
TF300194	143506	ENSG00000186232
TF300194	196120	ENSG00000186232
TF300194	650533	ENSG00000186232
TF300194	651308	ENSG00000186232
TF300194	650536	ENSG00000186232
TF326024	57496	ENSG00000186260
TF106449	55693	ENSG00000186280
TF313360	150763	ENSG00000186281
TF313360	653924	ENSG00000186281
TF314941	64222	ENSG00000186283
TF326233	340529	ENSG00000186288
TF326233	645974	ENSG00000186288
TF315453	2558	ENSG00000186297
TF315453	727729	ENSG00000186297
TF314349	4675	ENSG00000186310
TF329595	23621	ENSG00000186318
TF331562	388531	ENSG00000186326
TF314873	285641	ENSG00000186334
TF314873	153201	ENSG00000186335
TF324917	7058	ENSG00000186340
TF352097	6256	ENSG00000186350
TF338463	353322	ENSG00000186352
TF105088	260293	ENSG00000186377
TF342865	645225	ENSG00000186390
TF332742	353288	ENSG00000186393
TF332742	3858	ENSG00000186395
TF334441	342510	ENSG00000186407
TF315964	342035	ENSG00000186417

TF101178	3840	ENSG00000186432
TF314141	147468	ENSG00000186433
TF317854	3850	ENSG00000186442
TF340612	115560	ENSG00000186446
TF314349	4674	ENSG00000186462
TF313173	730908	ENSG00000186466
TF319909	54331	ENSG00000186469
TF331083	11118	ENSG00000186470
TF326082	27445	ENSG00000186472
TF331013	3638	ENSG00000186480
TF317299	23040	ENSG00000186487
TF300522	1187	ENSG00000186510
TF351451	257106	ENSG00000186517
TF101080	151011	ENSG00000186522
TF105088	11283	ENSG00000186526
TF105088	4051	ENSG00000186529
TF316127	2306	ENSG00000186564
TF313935	4771	ENSG00000186575
TF323170	11104	ENSG00000186625
TF315216	123722	ENSG00000186628
TF316380	326342	ENSG00000186629
TF105769	116985	ENSG00000186635
TF329827	441258	ENSG00000186645
TF329827	441272	ENSG00000186645
TF329827	641776	ENSG00000186645
TF329827	643862	ENSG00000186645
TF329827	643909	ENSG00000186645
TF329827	728524	ENSG00000186645
TF329827	730294	ENSG00000186645
TF329827	730304	ENSG00000186645
TF329827	730307	ENSG00000186645
TF316381	90668	ENSG00000186648
TF314826	23779	ENSG00000186654
TF314826	55615	ENSG00000186654
TF314826	553158	ENSG00000186654
TF332664	386607	ENSG00000186660
TF332664	80829	ENSG00000186660
TF332664	1270	ENSG00000186660
TF324061	144233	ENSG00000186666
TF105094	339761	ENSG00000186684
TF314116	130773	ENSG00000186698
TF105082	613	ENSG00000186716
TF105082	732425	ENSG00000186716
TF314305	727731	ENSG00000186732
TF323992	25794	ENSG00000186765
TF316127	399823	ENSG00000186766
TF332665	139886	ENSG00000186767
TF332665	474343	ENSG00000186787
TF321598	8372	ENSG00000186792
TF105452	244	ENSG00000186807
TF105452	652846	ENSG00000186807
TF330966	2833	ENSG00000186810
TF328550	53373	ENSG00000186815
TF332742	3872	ENSG00000186831
TF332742	3868	ENSG00000186832
TF336851	10614	ENSG00000186834
TF332742	3861	ENSG00000186847
TF325033	79955	ENSG00000186862
TF315303	84109	ENSG00000186867

TF316358	4137	ENSG00000186868
TF323824	200728	ENSG00000186889
TF317805	2248	ENSG00000186895
TF329591	338761	ENSG00000186897
TF330080	349667	ENSG00000186907
TF317342	23390	ENSG00000186908
TF343201	256394	ENSG00000186910
TF350009	5030	ENSG00000186912
TF326610	55893	ENSG00000186918
TF318060	645345	ENSG00000186940
TF316304	5465	ENSG00000186951
TF324499	256949	ENSG00000186994
TF336589	129080	ENSG00000186998
TF316484	342372	ENSG00000187008
TF314450	6007	ENSG00000187010
TF326392	83715	ENSG00000187017
TF332601	138428	ENSG00000187024
TF331299	344658	ENSG00000187033
TF330647	164656	ENSG00000187045
TF105088	1579	ENSG00000187048
TF323824	51259	ENSG00000187049
TF313443	7003	ENSG00000187079
TF313216	5333	ENSG00000187091
TF333419	885	ENSG00000187094
TF315029	957	ENSG00000187097
TF317174	4286	ENSG00000187098
TF314349	4673	ENSG00000187109
TF332887	6585	ENSG00000187122
TF332443	130574	ENSG00000187123
TF106492	1645	ENSG00000187134
TF106492	648517	ENSG00000187134
TF331739	342865	ENSG00000187135
TF316127	27022	ENSG00000187140
TF324716	55182	ENSG00000187147
TF326250	57698	ENSG00000187164
TF324396	57054	ENSG00000187191
TF324396	57055	ENSG00000187191
TF336054	4501	ENSG00000187193
TF315534	2650	ENSG00000187210
TF351162	23048	ENSG00000187239
TF332742	3859	ENSG00000187242
TF330534	4059	ENSG00000187244
TF323256	222194	ENSG00000187257
TF336573	2057	ENSG00000187266
TF328876	171484	ENSG00000187268
TF334321	63924	ENSG00000187288
TF321506	1630	ENSG00000187323
TF332299	56123	ENSG00000187372
TF316816	9863	ENSG00000187391
TF331644	338645	ENSG00000187398
TF321143	375612	ENSG00000187416
TF354284	11261	ENSG00000187446
TF330976	2359	ENSG00000187474
TF313664	3010	ENSG00000187475
TF343201	388007	ENSG00000187483
TF313676	3767	ENSG00000187486
TF316865	1282	ENSG00000187498
TF329606	2701	ENSG00000187513
TF300331	344905	ENSG00000187527

TF332708	343071	ENSG00000187545
TF332708	441872	ENSG00000187545
TF326736	646643	ENSG00000187550
TF105093	340665	ENSG00000187553
TF351113	7100	ENSG00000187554
TF326882	342977	ENSG00000187556
TF316127	286380	ENSG00000187559
TF316127	387054	ENSG00000187559
TF331018	378884	ENSG00000187566
TF317532	729974	ENSG00000187574
TF105070	341947	ENSG00000187581
TF316105	84069	ENSG00000187583
TF333209	646359	ENSG00000187589
TF326622	201181	ENSG00000187595
TF342373	200424	ENSG00000187605
TF331299	148398	ENSG00000187634
TF332134	404037	ENSG00000187664
TF324969	26059	ENSG00000187672
TF325070	81848	ENSG00000187678
TF312796	3266	ENSG00000187682
TF314711	51393	ENSG00000187688
TF316874	79875	ENSG00000187720
TF315453	2563	ENSG00000187730
TF314970	6917	ENSG00000187735
TF328821	79048	ENSG00000187742
TF300429	124	ENSG00000187758
TF316102	10507	ENSG00000187764
TF316240	389421	ENSG00000187772
TF315153	642658	ENSG00000187786
TF351139	64170	ENSG00000187796
TF332598	375033	ENSG00000187800
TF331362	347454	ENSG00000187808
TF335133	340595	ENSG00000187823
TF313664	3006	ENSG00000187837
TF314939	57048	ENSG00000187838
TF101530	1978	ENSG00000187840
TF328633	22953	ENSG00000187848
TF322889	121549	ENSG00000187855
TF331647	389400	ENSG00000187871
TF337003	199920	ENSG00000187889
TF330800	729956	ENSG00000187902
TF329295	1755	ENSG00000187908
TF329914	7373	ENSG00000187955
TF351322	92737	ENSG00000187957
TF332635	399474	ENSG00000187975
TF319283	391013	ENSG00000187980
TF331067	126432	ENSG00000187994
TF333472	285386	ENSG00000188001
TF330875	391123	ENSG00000188004
TF318093	56917	ENSG00000188013
TF332727	6274	ENSG00000188015
TF314412	29978	ENSG00000188021
TF313489	353116	ENSG00000188026
TF313739	392360	ENSG00000188029
TF332589	123904	ENSG00000188038
TF105464	10123	ENSG00000188042
TF317486	168433	ENSG00000188050
TF300032	115273	ENSG00000188060
TF105310	7477	ENSG00000188064

TF105310	649279	ENSG00000188064
TF325228	123745	ENSG00000188089
TF325707	145873	ENSG00000188095
TF351322	346007	ENSG00000188107
TF322889	647219	ENSG00000188108
TF105100	6300	ENSG00000188130
TF331155	390616	ENSG00000188134
TF316865	1287	ENSG00000188153
TF333323	4810	ENSG00000188158
TF300299	340990	ENSG00000188162
TF331199	253012	ENSG00000188175
TF316716	342527	ENSG00000188176
TF321641	376940	ENSG00000188177
TF314920	5575	ENSG00000188191
TF314920	645590	ENSG00000188191
TF317762	119016	ENSG00000188234
TF317762	728005	ENSG00000188234
TF319283	5320	ENSG00000188257
TF341788	285180	ENSG00000188282
TF351373	57801	ENSG00000188290
TF351429	344657	ENSG00000188306
TF351429	652189	ENSG00000188306
TF314939	5359	ENSG00000188313
TF326736	388228	ENSG00000188322
TF328787	10991	ENSG00000188338
TF314290	2963	ENSG00000188342
TF331369	7784	ENSG00000188372
TF105558	5535	ENSG00000188386
TF332372	2844	ENSG00000188394
TF326910	6402	ENSG00000188404
TF326882	339345	ENSG00000188425
TF315031	647909	ENSG00000188459
TF331376	389792	ENSG00000188483
TF300137	3014	ENSG00000188486
TF343201	5104	ENSG00000188488
TF320527	342897	ENSG00000188505
TF338175	84570	ENSG00000188517
TF330777	644815	ENSG00000188522
TF332328	3039	ENSG00000188536
TF332328	3040	ENSG00000188536
TF336272	388115	ENSG00000188549
TF324484	57186	ENSG00000188559
TF321348	154215	ENSG00000188580
TF315116	6613	ENSG00000188612
TF315116	728825	ENSG00000188612
TF315116	730776	ENSG00000188612
TF320562	340784	ENSG00000188620
TF332727	140576	ENSG00000188643
TF319394	283726	ENSG00000188659
TF314450	6006	ENSG00000188672
TF330978	169355	ENSG00000188676
TF314025	29780	ENSG00000188677
TF317532	342931	ENSG00000188683
TF317532	649055	ENSG00000188683
TF315153	642658	ENSG00000188686
TF313630	57835	ENSG00000188687
TF312923	51114	ENSG00000188706
TF105128	338599	ENSG00000188716
TF329913	375567	ENSG00000188730

TF313552	144404	ENSG00000188735
TF329087	10811	ENSG00000188747
TF323324	130612	ENSG00000188760
TF317907	8326	ENSG00000188763
TF321411	399473	ENSG00000188766
TF351924	26254	ENSG00000188770
TF316350	155	ENSG00000188778
TF324133	390598	ENSG00000188779
TF343841	378807	ENSG00000188782
TF319326	5549	ENSG00000188783
TF319283	30814	ENSG00000188784
TF320562	3167	ENSG00000188816
TF317498	79844	ENSG00000188818
TF317498	649917	ENSG00000188818
TF317498	652290	ENSG00000188818
TF329085	441168	ENSG00000188820
TF330052	1269	ENSG00000188822
TF332859	377841	ENSG00000188833
TF105566	139542	ENSG00000188841
TF318099	653380	ENSG00000188849
TF318099	729837	ENSG00000188849
TF329533	149297	ENSG00000188859
TF319114	440435	ENSG00000188888
TF313679	120892	ENSG00000188906
TF329606	2707	ENSG00000188910
TF333465	642938	ENSG00000188916
TF313326	401494	ENSG00000188921
TF337463	60506	ENSG00000188937
TF314978	126767	ENSG00000188984
TF317636	1719	ENSG00000188985
TF317636	643509	ENSG00000188985
TF300174	8294	ENSG00000188987
TF300174	8359	ENSG00000188987
TF300174	8360	ENSG00000188987
TF300174	8361	ENSG00000188987
TF300174	8362	ENSG00000188987
TF300174	8363	ENSG00000188987
TF300174	8364	ENSG00000188987
TF300174	8365	ENSG00000188987
TF300174	8366	ENSG00000188987
TF300174	8367	ENSG00000188987
TF300174	8368	ENSG00000188987
TF300174	8370	ENSG00000188987
TF300174	121504	ENSG00000188987
TF300174	554313	ENSG00000188987
TF324997	149998	ENSG00000188992
TF350813	23036	ENSG00000188994
TF315332	283219	ENSG00000188997
TF316009	63904	ENSG00000189037
TF106383	4697	ENSG00000189043
TF331930	51136	ENSG00000189050
TF332940	1082	ENSG00000189052
TF332940	93659	ENSG00000189052
TF332940	94115	ENSG00000189052
TF313294	9516	ENSG00000189067
TF313552	83862	ENSG00000189077
TF351676	345062	ENSG00000189099
TF333913	26280	ENSG00000189108
TF315506	80320	ENSG00000189120

TF331155	340120	ENSG00000189127
TF331936	1364	ENSG00000189143
TF323345	645843	ENSG00000189156
TF327169	51155	ENSG00000189159
TF332592	2688	ENSG00000189162
TF331383	646799	ENSG00000189167
TF332727	6284	ENSG00000189171
TF317854	374454	ENSG00000189182
TF352008	54510	ENSG00000189184
TF324841	388021	ENSG00000189203
TF313314	4128	ENSG00000189221
TF333211	55629	ENSG00000189266
TF329606	2709	ENSG00000189280
TF105432	2272	ENSG00000189283
TF105231	146909	ENSG00000189285
TF329867	139628	ENSG00000189299
TF318099	414059	ENSG00000189309
TF318099	643947	ENSG00000189309
TF332095	9679	ENSG00000189319
TF333387	389558	ENSG00000189320
TF332727	57402	ENSG00000189334
TF315518	165186	ENSG00000189350
TF105371	3146	ENSG00000189403
TF315428	8510	ENSG00000189409
TF329606	127534	ENSG00000189433
TF312962	91584	ENSG00000189437
TF325519	3556	ENSG00000196083
TF312900	11122	ENSG00000196090
TF315397	5079	ENSG00000196092
TF317779	50859	ENSG00000196104
TF329621	653319	ENSG00000196123
TF317299	4661	ENSG00000196132
TF343201	12	ENSG00000196136
TF106492	8644	ENSG00000196139
TF320553	26010	ENSG00000196141
TF328991	151525	ENSG00000196151
TF332727	6275	ENSG00000196154
TF334329	25894	ENSG00000196155
TF331721	124602	ENSG00000196169
TF331721	350383	ENSG00000196169
TF300174	8294	ENSG00000196176
TF300174	8359	ENSG00000196176
TF300174	8360	ENSG00000196176
TF300174	8361	ENSG00000196176
TF300174	8362	ENSG00000196176
TF300174	8363	ENSG00000196176
TF300174	8364	ENSG00000196176
TF300174	8365	ENSG00000196176
TF300174	8366	ENSG00000196176
TF300174	8367	ENSG00000196176
TF300174	8368	ENSG00000196176
TF300174	8370	ENSG00000196176
TF300174	121504	ENSG00000196176
TF300174	554313	ENSG00000196176
TF324300	9725	ENSG00000196187
TF314990	1510	ENSG00000196188
TF316102	64218	ENSG00000196189
TF329531	9687	ENSG00000196208
TF315244	6261	ENSG00000196218

TF315892	9901	ENSG00000196220
TF321745	442038	ENSG00000196228
TF319589	84458	ENSG00000196233
TF312801	5478	ENSG00000196262
TF312801	128192	ENSG00000196262
TF312801	131691	ENSG00000196262
TF312801	341457	ENSG00000196262
TF312801	643997	ENSG00000196262
TF312801	653214	ENSG00000196262
TF312801	654188	ENSG00000196262
TF312801	650332	ENSG00000196262
TF313240	2917	ENSG00000196277
TF314241	3020	ENSG00000196285
TF314241	3021	ENSG00000196285
TF314241	347376	ENSG00000196285
TF314241	644914	ENSG00000196285
TF314241	730740	ENSG00000196285
TF300651	487	ENSG00000196296
TF106492	340811	ENSG00000196326
TF330845	55340	ENSG00000196329
TF332940	94027	ENSG00000196337
TF326187	54413	ENSG00000196338
TF300429	131	ENSG00000196344
TF101059	390688	ENSG00000196347
TF334137	1604	ENSG00000196352
TF316419	131034	ENSG00000196353
TF333945	84628	ENSG00000196358
TF313377	1995	ENSG00000196361
TF106349	55190	ENSG00000196368
TF316348	2526	ENSG00000196371
TF331945	79754	ENSG00000196372
TF313645	222553	ENSG00000196376
TF315897	5770	ENSG00000196396
TF321411	51466	ENSG00000196405
TF329347	124056	ENSG00000196408
TF315608	2050	ENSG00000196411
TF332727	6276	ENSG00000196420
TF318837	9819	ENSG00000196428
TF331401	1413	ENSG00000196431
TF314574	438	ENSG00000196433
TF331869	727800	ENSG00000196435
TF351553	140465	ENSG00000196465
TF312976	6477	ENSG00000196470
TF323751	2104	ENSG00000196482
TF106423	9612	ENSG00000196498
TF321745	6817	ENSG00000196502
TF105469	132946	ENSG00000196503
TF318732	55660	ENSG00000196504
TF101534	143244	ENSG00000196513
TF101534	642592	ENSG00000196513
TF332622	60312	ENSG00000196526
TF313348	4666	ENSG00000196531
TF339614	399687	ENSG00000196535
TF328418	165679	ENSG00000196542
TF313152	4122	ENSG00000196547
TF315192	4311	ENSG00000196549
TF313555	8912	ENSG00000196557
TF313545	55959	ENSG00000196562
TF335359	3908	ENSG00000196569

TF331744	345456	ENSG00000196570
TF312962	23654	ENSG00000196576
TF326024	57591	ENSG00000196588
TF325032	729458	ENSG00000196589
TF106171	3066	ENSG00000196591
TF315428	4312	ENSG00000196611
TF300429	125	ENSG00000196616
TF300429	126	ENSG00000196616
TF315472	7366	ENSG00000196620
TF315472	728160	ENSG00000196620
TF316230	85376	ENSG00000196622
TF321672	6925	ENSG00000196628
TF315519	65267	ENSG00000196632
TF325595	51284	ENSG00000196664
TF333387	399888	ENSG00000196666
TF336893	84941	ENSG00000196684
TF314711	7442	ENSG00000196689
TF331185	57473	ENSG00000196700
TF328603	51321	ENSG00000196704
TF333390	389658	ENSG00000196711
TF351123	1612	ENSG00000196730
TF344135	85301	ENSG00000196739
TF300137	8329	ENSG00000196747
TF300137	8330	ENSG00000196747
TF300137	8332	ENSG00000196747
TF300137	8336	ENSG00000196747
TF300137	8969	ENSG00000196747
TF300137	85235	ENSG00000196747
TF332727	6273	ENSG00000196754
TF316413	5456	ENSG00000196767
TF314167	7088	ENSG00000196781
TF332922	55534	ENSG00000196782
TF334681	80830	ENSG00000196785
TF300137	8329	ENSG00000196787
TF300137	8330	ENSG00000196787
TF300137	8332	ENSG00000196787
TF300137	8336	ENSG00000196787
TF300137	8969	ENSG00000196787
TF300137	85235	ENSG00000196787
TF313387	29966	ENSG00000196792
TF315605	1146	ENSG00000196811
TF314477	89853	ENSG00000196814
TF324725	10865	ENSG00000196843
TF332742	390792	ENSG00000196859
TF106200	387990	ENSG00000196860
TF313348	342538	ENSG00000196861
TF300137	8329	ENSG00000196866
TF300137	8330	ENSG00000196866
TF300137	8332	ENSG00000196866
TF300137	8336	ENSG00000196866
TF300137	8969	ENSG00000196866
TF300137	85235	ENSG00000196866
TF323985	6334	ENSG00000196876
TF354205	3841	ENSG00000196911
TF106495	23365	ENSG00000196914
TF330775	27198	ENSG00000196917
TF106408	9260	ENSG00000196923
TF313685	2316	ENSG00000196924
TF316230	440804	ENSG00000196934

TF315892	57522	ENSG00000196935
TF353414	10447	ENSG00000196937
TF318470	57181	ENSG00000196950
TF102023	837	ENSG00000196954
TF300308	160	ENSG00000196961
TF332299	57717	ENSG00000196963
TF105452	307	ENSG00000196975
TF333084	642968	ENSG00000196990
TF340612	7697	ENSG00000197008
TF101069	29950	ENSG00000197019
TF105452	309	ENSG00000197043
TF315147	2764	ENSG00000197045
TF300174	8294	ENSG00000197061
TF300174	8359	ENSG00000197061
TF300174	8360	ENSG00000197061
TF300174	8361	ENSG00000197061
TF300174	8362	ENSG00000197061
TF300174	8363	ENSG00000197061
TF300174	8364	ENSG00000197061
TF300174	8365	ENSG00000197061
TF300174	8366	ENSG00000197061
TF300174	8367	ENSG00000197061
TF300174	8368	ENSG00000197061
TF300174	8370	ENSG00000197061
TF300174	121504	ENSG00000197061
TF300174	554313	ENSG00000197061
TF340612	7741	ENSG00000197062
TF325689	4097	ENSG00000197063
TF313650	92714	ENSG00000197070
TF332742	3886	ENSG00000197079
TF316990	440295	ENSG00000197092
TF314802	79690	ENSG00000197093
TF352709	388662	ENSG00000197106
TF318292	5094	ENSG00000197111
TF351634	6714	ENSG00000197122
TF313570	399909	ENSG00000197136
TF314733	203102	ENSG00000197140
TF313877	51703	ENSG00000197142
TF331443	23507	ENSG00000197147
TF321745	6799	ENSG00000197165
TF106472	341676	ENSG00000197168
TF331206	84435	ENSG00000197177
TF325594	140688	ENSG00000197183
TF354343	9583	ENSG00000197217
TF313145	23061	ENSG00000197226
TF300174	8294	ENSG00000197238
TF300174	8359	ENSG00000197238
TF300174	8360	ENSG00000197238
TF300174	8361	ENSG00000197238
TF300174	8362	ENSG00000197238
TF300174	8363	ENSG00000197238
TF300174	8364	ENSG00000197238
TF300174	8365	ENSG00000197238
TF300174	8366	ENSG00000197238
TF300174	8367	ENSG00000197238
TF300174	8368	ENSG00000197238
TF300174	8370	ENSG00000197238
TF300174	121504	ENSG00000197238
TF300174	554313	ENSG00000197238

TF330964	79927	ENSG00000197245
TF343201	5265	ENSG00000197249
TF351676	64499	ENSG00000197253
TF324499	25959	ENSG00000197256
TF334888	728835	ENSG00000197262
TF334888	730424	ENSG00000197262
TF334888	9560	ENSG00000197262
TF334888	388372	ENSG00000197262
TF105303	8831	ENSG00000197283
TF324599	128486	ENSG00000197296
TF300136	6234	ENSG00000197303
TF300136	645899	ENSG00000197303
TF300136	646195	ENSG00000197303
TF300136	650788	ENSG00000197303
TF316081	6840	ENSG00000197321
TF106455	51592	ENSG00000197323
TF332149	26020	ENSG00000197324
TF314338	57162	ENSG00000197329
TF340612	401898	ENSG00000197332
TF340612	730571	ENSG00000197332
TF300611	91373	ENSG00000197355
TF331300	147906	ENSG00000197380
TF315806	104	ENSG00000197381
TF330976	728	ENSG00000197405
TF329721	1735	ENSG00000197406
TF342343	641971	ENSG00000197412
TF342343	646999	ENSG00000197412
TF316990	283767	ENSG00000197414
TF316990	338999	ENSG00000197414
TF316990	440233	ENSG00000197414
TF316990	440243	ENSG00000197414
TF316990	729272	ENSG00000197414
TF316894	646486	ENSG00000197416
TF316894	650722	ENSG00000197416
TF313608	728441	ENSG00000197421
TF105115	4217	ENSG00000197442
TF300695	55753	ENSG00000197444
TF314808	3182	ENSG00000197451
TF333305	401271	ENSG00000197453
TF319554	5154	ENSG00000197461
TF338175	1305	ENSG00000197467
TF332299	56125	ENSG00000197479
TF332408	81031	ENSG00000197496
TF314131	64078	ENSG00000197506
TF313885	389844	ENSG00000197518
TF313885	441490	ENSG00000197518
TF326916	400823	ENSG00000197520
TF328771	4644	ENSG00000197535
TF332708	649179	ENSG00000197549
TF318626	26037	ENSG00000197555
TF300299	729997	ENSG00000197558
TF323230	57799	ENSG00000197562
TF316865	1288	ENSG00000197565
TF316310	3201	ENSG00000197576
TF314019	83875	ENSG00000197580
TF105318	2876	ENSG00000197582
TF328589	10242	ENSG00000197584
TF315029	955	ENSG00000197586
TF351609	127343	ENSG00000197587

TF330032	5167	ENSG00000197594
TF313011	84188	ENSG00000197601
TF332233	30816	ENSG00000197604
TF333418	8076	ENSG00000197614
TF314375	4624	ENSG00000197616
TF323815	56882	ENSG00000197622
TF352619	5055	ENSG00000197632
TF352619	5275	ENSG00000197641
TF331083	80380	ENSG00000197646
TF318099	729873	ENSG00000197681
TF318099	729877	ENSG00000197681
TF314025	55742	ENSG00000197702
TF328485	57565	ENSG00000197705
TF324360	92689	ENSG00000197712
TF334137	1379	ENSG00000197721
TF106480	5253	ENSG00000197724
TF332727	6281	ENSG00000197747
TF321143	222662	ENSG00000197753
TF313068	6168	ENSG00000197756
TF316310	3223	ENSG00000197757
TF314782	114112	ENSG00000197763
TF333630	1675	ENSG00000197766
TF325310	197342	ENSG00000197774
TF314081	122773	ENSG00000197776
TF326161	4950	ENSG00000197822
TF326161	647859	ENSG00000197822
TF341248	724094	ENSG00000197833
TF300174	8294	ENSG00000197837
TF300174	8359	ENSG00000197837
TF300174	8360	ENSG00000197837
TF300174	8361	ENSG00000197837
TF300174	8362	ENSG00000197837
TF300174	8363	ENSG00000197837
TF300174	8364	ENSG00000197837
TF300174	8365	ENSG00000197837
TF300174	8366	ENSG00000197837
TF300174	8367	ENSG00000197837
TF300174	8368	ENSG00000197837
TF300174	8370	ENSG00000197837
TF300174	121504	ENSG00000197837
TF300174	554313	ENSG00000197837
TF332839	55924	ENSG00000197852
TF316874	9719	ENSG00000197859
TF316874	653348	ENSG00000197859
TF313092	54557	ENSG00000197860
TF332441	100049587	ENSG00000197865
TF314541	81553	ENSG00000197872
TF312960	4641	ENSG00000197879
TF314483	28512	ENSG00000197885
TF315472	7367	ENSG00000197888
TF105221	23303	ENSG00000197892
TF313758	4892	ENSG00000197893
TF300429	128	ENSG00000197894
TF300429	642443	ENSG00000197894
TF300429	647346	ENSG00000197894
TF300174	8294	ENSG00000197914
TF300174	8359	ENSG00000197914
TF300174	8360	ENSG00000197914
TF300174	8361	ENSG00000197914

TF300174	8362	ENSG00000197914
TF300174	8363	ENSG00000197914
TF300174	8364	ENSG00000197914
TF300174	8365	ENSG00000197914
TF300174	8366	ENSG00000197914
TF300174	8367	ENSG00000197914
TF300174	8368	ENSG00000197914
TF300174	8370	ENSG00000197914
TF300174	121504	ENSG00000197914
TF300174	554313	ENSG00000197914
TF351373	388585	ENSG00000197921
TF314471	30001	ENSG00000197930
TF313216	5336	ENSG00000197943
TF324557	89848	ENSG00000197948
TF314978	344752	ENSG00000197953
TF332727	6277	ENSG00000197956
TF300362	26052	ENSG00000197959
TF331728	9019	ENSG00000197965
TF300316	23230	ENSG00000197969
TF320443	8227	ENSG00000197976
TF323454	54898	ENSG00000197977
TF320624	64881	ENSG00000197991
TF315192	3792	ENSG00000197993
TF321382	9228	ENSG00000198010
TF354343	57089	ENSG00000198018
TF335097	2210	ENSG00000198019
TF317762	728005	ENSG00000198035
TF106499	553	ENSG00000198049
TF313940	2870	ENSG00000198055
TF316219	54708	ENSG00000198060
TF321745	27233	ENSG00000198075
TF333100	7541	ENSG00000198081
TF300137	474382	ENSG00000198082
TF350191	23607	ENSG00000198087
TF300429	127	ENSG00000198099
TF318303	337876	ENSG00000198108
TF314941	54863	ENSG00000198113
TF330052	1902	ENSG00000198121
TF332967	4151	ENSG00000198125
TF336481	161145	ENSG00000198133
TF331362	65124	ENSG00000198142
TF105374	79366	ENSG00000198157
TF106453	57708	ENSG00000198160
TF312801	164022	ENSG00000198161
TF312801	653505	ENSG00000198161
TF312801	653598	ENSG00000198161
TF313420	10905	ENSG00000198162
TF314396	7027	ENSG00000198176
TF314396	441488	ENSG00000198176
TF321745	6819	ENSG00000198203
TF323964	83694	ENSG00000198208
TF300298	4157	ENSG00000198211
TF300298	10381	ENSG00000198211
TF312805	777	ENSG00000198216
TF336988	54870	ENSG00000198218
TF331549	1438	ENSG00000198223
TF314116	6147	ENSG00000198242
TF314116	401904	ENSG00000198242
TF314116	647099	ENSG00000198242

TF314116	728134	ENSG00000198242
TF314116	729617	ENSG00000198242
TF314116	729798	ENSG00000198242
TF314116	732089	ENSG00000198242
TF314116	730663	ENSG00000198242
TF314116	652071	ENSG00000198242
TF105902	54963	ENSG00000198276
TF351139	84433	ENSG00000198286
TF329827	441258	ENSG00000198305
TF329827	441272	ENSG00000198305
TF329827	641776	ENSG00000198305
TF329827	643862	ENSG00000198305
TF329827	643909	ENSG00000198305
TF329827	728524	ENSG00000198305
TF329827	730294	ENSG00000198305
TF329827	730304	ENSG00000198305
TF329827	730307	ENSG00000198305
TF300137	83740	ENSG00000198307
TF300137	474381	ENSG00000198307
TF326731	144717	ENSG00000198324
TF300174	8294	ENSG00000198327
TF300174	8359	ENSG00000198327
TF300174	8360	ENSG00000198327
TF300174	8361	ENSG00000198327
TF300174	8362	ENSG00000198327
TF300174	8363	ENSG00000198327
TF300174	8364	ENSG00000198327
TF300174	8365	ENSG00000198327
TF300174	8366	ENSG00000198327
TF300174	8367	ENSG00000198327
TF300174	8368	ENSG00000198327
TF300174	8370	ENSG00000198327
TF300174	121504	ENSG00000198327
TF300174	554313	ENSG00000198327
TF351553	4635	ENSG00000198336
TF300174	8294	ENSG00000198339
TF300174	8359	ENSG00000198339
TF300174	8360	ENSG00000198339
TF300174	8361	ENSG00000198339
TF300174	8362	ENSG00000198339
TF300174	8363	ENSG00000198339
TF300174	8364	ENSG00000198339
TF300174	8365	ENSG00000198339
TF300174	8366	ENSG00000198339
TF300174	8367	ENSG00000198339
TF300174	8368	ENSG00000198339
TF300174	8370	ENSG00000198339
TF300174	121504	ENSG00000198339
TF300174	554313	ENSG00000198339
TF316310	3221	ENSG00000198353
TF323731	340578	ENSG00000198354
TF320810	415116	ENSG00000198355
TF312801	164022	ENSG00000198360
TF312801	653505	ENSG00000198360
TF312801	653598	ENSG00000198360
TF321411	200734	ENSG00000198369
TF323658	11060	ENSG00000198373
TF300137	8329	ENSG00000198374
TF300137	8330	ENSG00000198374

TF300137	8332	ENSG00000198374
TF300137	8336	ENSG00000198374
TF300137	8969	ENSG00000198374
TF300137	85235	ENSG00000198374
TF300864	2673	ENSG00000198380
TF324293	50618	ENSG00000198399
TF106465	4914	ENSG00000198400
TF313732	10724	ENSG00000198408
TF336054	4494	ENSG00000198417
TF314782	7296	ENSG00000198431
TF333311	441478	ENSG00000198435
TF105095	1589	ENSG00000198457
TF351519	7169	ENSG00000198467
TF331707	140883	ENSG00000198477
TF105574	83699	ENSG00000198478
TF331274	148741	ENSG00000198483
TF318639	192134	ENSG00000198488
TF323736	51441	ENSG00000198492
TF105251	51062	ENSG00000198513
TF325689	7975	ENSG00000198517
TF300174	8294	ENSG00000198518
TF300174	8359	ENSG00000198518
TF300174	8360	ENSG00000198518
TF300174	8361	ENSG00000198518
TF300174	8362	ENSG00000198518
TF300174	8363	ENSG00000198518
TF300174	8364	ENSG00000198518
TF300174	8365	ENSG00000198518
TF300174	8366	ENSG00000198518
TF300174	8367	ENSG00000198518
TF300174	8368	ENSG00000198518
TF300174	8370	ENSG00000198518
TF300174	121504	ENSG00000198518
TF300174	554313	ENSG00000198518
TF331185	84450	ENSG00000198522
TF330989	145741	ENSG00000198535
TF300174	8294	ENSG00000198558
TF300174	8359	ENSG00000198558
TF300174	8360	ENSG00000198558
TF300174	8361	ENSG00000198558
TF300174	8362	ENSG00000198558
TF300174	8363	ENSG00000198558
TF300174	8364	ENSG00000198558
TF300174	8365	ENSG00000198558
TF300174	8366	ENSG00000198558
TF300174	8367	ENSG00000198558
TF300174	8368	ENSG00000198558
TF300174	8370	ENSG00000198558
TF300174	121504	ENSG00000198558
TF300174	554313	ENSG00000198558
TF321877	1500	ENSG00000198561
TF300442	7919	ENSG00000198563
TF313981	142680	ENSG00000198569
TF313981	652661	ENSG00000198569
TF331573	343035	ENSG00000198570
TF343096	117157	ENSG00000198574
TF315233	9874	ENSG00000198586
TF313490	987	ENSG00000198589
TF332241	9745	ENSG00000198597

TF315428	4326	ENSG00000198598
TF106492	1109	ENSG00000198610
TF312801	5478	ENSG00000198618
TF312801	128192	ENSG00000198618
TF312801	653214	ENSG00000198618
TF312801	654188	ENSG00000198618
TF333416	26112	ENSG00000198624
TF105306	4194	ENSG00000198625
TF315244	6262	ENSG00000198626
TF328485	55958	ENSG00000198642
TF353414	131177	ENSG00000198643
TF105339	27347	ENSG00000198648
TF314365	653401	ENSG00000198658
TF300912	801	ENSG00000198668
TF300912	805	ENSG00000198668
TF300912	808	ENSG00000198668
TF316454	389668	ENSG00000198669
TF329901	4018	ENSG00000198670
TF331749	338811	ENSG00000198673
TF313143	9060	ENSG00000198682
TF318755	10479	ENSG00000198689
TF105191	24	ENSG00000198691
TF315518	23116	ENSG00000198718
TF351835	28514	ENSG00000198719
TF314176	124930	ENSG00000198720
TF312844	10497	ENSG00000198722
TF319923	8861	ENSG00000198728
TF105546	81706	ENSG00000198729
TF320666	64093	ENSG00000198732
TF329807	2153	ENSG00000198734
TF332659	347731	ENSG00000198739
TF332655	22834	ENSG00000198740
TF323658	57154	ENSG00000198742
TF323658	731429	ENSG00000198742
TF331648	729438	ENSG00000198750
TF331648	730322	ENSG00000198750
TF313551	9578	ENSG00000198752
TF312962	5365	ENSG00000198753
TF313826	23127	ENSG00000198756
TF313069	79574	ENSG00000198758
TF330819	25975	ENSG00000198759
TF106493	404281	ENSG00000198767
TF329491	164284	ENSG00000198768
TF326923	9182	ENSG00000198774
TF332578	26049	ENSG00000198780
TF314731	116443	ENSG00000198785
TF300299	4583	ENSG00000198788
TF300299	652741	ENSG00000198788
TF314185	29883	ENSG00000198791
TF314160	25829	ENSG00000198792
TF313797	192683	ENSG00000198794
TF331504	25925	ENSG00000198795
TF331600	57795	ENSG00000198797
TF325380	9860	ENSG00000198799
TF300049	4860	ENSG00000198805
TF315397	5083	ENSG00000198807
TF332853	376132	ENSG00000198812
TF333250	22887	ENSG00000198815
TF315157	113402	ENSG00000198818

TF330937	919	ENSG00000198821
TF313240	2913	ENSG00000198822
TF350009	56670	ENSG00000198829
TF105375	3151	ENSG00000198830
TF105375	643872	ENSG00000198830
TF105375	644498	ENSG00000198830
TF105375	648822	ENSG00000198830
TF105375	649445	ENSG00000198830
TF105375	727795	ENSG00000198830
TF105375	728632	ENSG00000198830
TF105375	729505	ENSG00000198830
TF105375	729687	ENSG00000198830
TF105375	730562	ENSG00000198830
TF105375	731740	ENSG00000198830
TF105375	732081	ENSG00000198830
TF329606	57165	ENSG00000198835
TF315244	6263	ENSG00000198838
TF321235	51714	ENSG00000198843
TF316357	22899	ENSG00000198844
TF106481	9760	ENSG00000198846
TF335892	916	ENSG00000198851
TF313940	2869	ENSG00000198873
TF105374	728851	ENSG00000198875
TF105374	731104	ENSG00000198875
TF323731	25853	ENSG00000198876
TF316498	57713	ENSG00000198879
TF331945	142689	ENSG00000198881
TF332277	150771	ENSG00000198885
TF323731	139170	ENSG00000198889
TF332572	149345	ENSG00000198892
TF314822	830	ENSG00000198898
TF105281	7150	ENSG00000198900
TF323976	9055	ENSG00000198901
TF331356	27350	ENSG00000198904
TF331356	140564	ENSG00000198904
TF105113	4215	ENSG00000198909
TF313894	6721	ENSG00000198911
TF316413	5455	ENSG00000198914
TF313379	221002	ENSG00000198915
TF300223	6170	ENSG00000198918
TF313665	79065	ENSG00000198925
TF317226	9722	ENSG00000198929
TF331289	9755	ENSG00000198933
TF312801	164022	ENSG00000198936
TF312801	653505	ENSG00000198936
TF312801	653598	ENSG00000198936
TF331362	134548	ENSG00000198944
TF316498	84456	ENSG00000198945
TF320178	1756	ENSG00000198947
TF333205	9848	ENSG00000198948
TF312909	4668	ENSG00000198951
TF327119	23381	ENSG00000198952
TF324278	7052	ENSG00000198959
TF319910	6096	ENSG00000198963
TF314547	259230	ENSG00000198964
TF331395	2812	ENSG00000203618
TF314748	388743	ENSG00000203697
TF314175	128387	ENSG00000203705
TF332372	9293	ENSG00000203737

TF314174	149281	ENSG00000203740
TF335097	2214	ENSG00000203747
TF335097	652578	ENSG00000203747
TF344263	399829	ENSG00000203774
TF313887	8528	ENSG00000203797
TF323722	196051	ENSG00000203805
TF314241	126961	ENSG00000203811
TF314241	333932	ENSG00000203811
TF314241	653604	ENSG00000203811
TF314241	731820	ENSG00000203811
TF300137	8337	ENSG00000203812
TF300137	723790	ENSG00000203812
TF335097	644410	ENSG00000203820
TF332098	81552	ENSG00000203833
TF332098	653930	ENSG00000203833
TF332098	729086	ENSG00000203833
TF314365	653401	ENSG00000203835
TF312801	644591	ENSG00000203847
TF312801	728945	ENSG00000203847
TF312801	730262	ENSG00000203847
TF314241	126961	ENSG00000203852
TF314241	333932	ENSG00000203852
TF314241	653604	ENSG00000203852
TF333921	282996	ENSG00000203867
TF336045	134701	ENSG00000203877
TF300449	2664	ENSG00000203879
TF329329	55251	ENSG00000203880
TF316183	54345	ENSG00000203883
TF313386	646817	ENSG00000203911
TF325918	148418	ENSG00000203943
TF330991	360203	ENSG00000204007
TF342671	439996	ENSG00000204010
TF313392	388630	ENSG00000204018
TF330861	340745	ENSG00000204033
TF332997	55861	ENSG00000204070
TF317034	3633	ENSG00000204084
TF105242	29935	ENSG00000204086
TF331063	129807	ENSG00000204099
TF325689	9935	ENSG00000204103
TF314908	53344	ENSG00000204116
TF325513	26058	ENSG00000204120
TF323904	55680	ENSG00000204130
TF333323	340527	ENSG00000204131
TF316316	65979	ENSG00000204138
TF317762	414189	ENSG00000204149
TF323527	8505	ENSG00000204155
TF323527	727726	ENSG00000204155
TF312923	84243	ENSG00000204160
TF317762	653268	ENSG00000204169
TF315303	5540	ENSG00000204174
TF337047	9721	ENSG00000204175
TF324023	55219	ENSG00000204178
TF314707	158833	ENSG00000204195
TF314724	659	ENSG00000204217
TF315204	5863	ENSG00000204218
TF314970	6920	ENSG00000204219
TF318639	8705	ENSG00000204222
TF105501	6015	ENSG00000204227
TF352097	6257	ENSG00000204231

TF323987	1302	ENSG00000204248
TF317345	6046	ENSG00000204256
TF106221	5698	ENSG00000204261
TF323987	1290	ENSG00000204262
TF106223	5696	ENSG00000204264
TF105197	6891	ENSG00000204267
TF332665	169981	ENSG00000204271
TF331083	56244	ENSG00000204290
TF315821	1306	ENSG00000204291
TF314340	5089	ENSG00000204304
TF317334	6048	ENSG00000204308
TF314867	10554	ENSG00000204310
TF352821	494513	ENSG00000204311
TF313285	720	ENSG00000204319
TF315506	389058	ENSG00000204335
TF313285	721	ENSG00000204342
TF331368	388419	ENSG00000204347
TF330194	629	ENSG00000204359
TF330194	717	ENSG00000204364
TF332666	221527	ENSG00000204366
TF106443	10919	ENSG00000204371
TF330715	143903	ENSG00000204381
TF313325	80736	ENSG00000204385
TF331063	4758	ENSG00000204386
TF105042	3304	ENSG00000204388
TF105042	3304	ENSG00000204389
TF105042	3305	ENSG00000204390
TF329905	80737	ENSG00000204396
TF332592	1444	ENSG00000204414
TF300361	641455	ENSG00000204434
TF300361	727848	ENSG00000204434
TF314462	1460	ENSG00000204435
TF314462	58496	ENSG00000204435
TF331752	728215	ENSG00000204442
TF331752	731895	ENSG00000204442
TF328989	642623	ENSG00000204448
TF328738	7916	ENSG00000204469
TF320736	199	ENSG00000204472
TF332778	644059	ENSG00000204474
TF332708	391001	ENSG00000204478
TF332708	645425	ENSG00000204478
TF332708	649345	ENSG00000204479
TF332708	645414	ENSG00000204480
TF332708	729528	ENSG00000204481
TF332708	729516	ENSG00000204485
TF332708	391001	ENSG00000204486
TF332708	645425	ENSG00000204486
TF332708	654348	ENSG00000204488
TF332169	7124	ENSG00000204490
TF332708	391003	ENSG00000204491
TF332708	401940	ENSG00000204491
TF332169	4049	ENSG00000204496
TF332708	343070	ENSG00000204501
TF332708	653619	ENSG00000204501
TF332708	343068	ENSG00000204502
TF332708	401940	ENSG00000204503
TF332708	441873	ENSG00000204505
TF332708	729368	ENSG00000204507
TF332708	653606	ENSG00000204508

TF332708	440561	ENSG00000204509
TF332708	441871	ENSG00000204510
TF314978	343066	ENSG00000204518
TF331395	441617	ENSG00000204545
TF317840	780	ENSG00000204580
TF317532	135644	ENSG00000204614
TF317532	11074	ENSG00000204616
TF317532	80352	ENSG00000204618
TF313145	11138	ENSG00000204634
TF338319	129521	ENSG00000204640
TF331083	4340	ENSG00000204655
TF328989	643615	ENSG00000204664
TF328989	648213	ENSG00000204664
TF328989	129870	ENSG00000204699
TF328989	728231	ENSG00000204699
TF328989	129870	ENSG00000204705
TF328989	728231	ENSG00000204705
TF329827	387778	ENSG00000204710
TF332940	114335	ENSG00000204748
TF314248	64901	ENSG00000204764
TF316127	653404	ENSG00000204793
TF317805	728195	ENSG00000204801
TF317805	728422	ENSG00000204801
TF317805	728433	ENSG00000204801
TF317805	728195	ENSG00000204816
TF317805	728422	ENSG00000204816
TF317805	728433	ENSG00000204816
TF317805	728195	ENSG00000204817
TF317805	728422	ENSG00000204817
TF317805	728433	ENSG00000204817
TF316127	349334	ENSG00000204828
TF316127	100036519	ENSG00000204828
TF326591	6311	ENSG00000204842
TF329169	79600	ENSG00000204852
TF332742	125115	ENSG00000204889
TF332742	728760	ENSG00000204889
TF332742	147183	ENSG00000204897
TF315372	643226	ENSG00000204928
TF332853	390205	ENSG00000204950
TF332299	56113	ENSG00000204955
TF332299	56114	ENSG00000204956
TF332299	9752	ENSG00000204961
TF332299	56140	ENSG00000204962
TF332299	56141	ENSG00000204963
TF332299	56142	ENSG00000204964
TF332299	56143	ENSG00000204965
TF332299	56144	ENSG00000204967
TF332299	56145	ENSG00000204968
TF332299	56146	ENSG00000204969
TF332299	56147	ENSG00000204970
TF314990	643847	ENSG00000204973
TF314990	649034	ENSG00000204973
TF331669	10206	ENSG00000204977
TF331065	5645	ENSG00000204982
TF326239	84501	ENSG00000204991
TF343894	389333	ENSG00000205012
TF334888	6349	ENSG00000205017
TF334888	414062	ENSG00000205017
TF334888	728830	ENSG00000205017

TF334888	730422	ENSG00000205017
TF334888	728835	ENSG00000205020
TF334888	730424	ENSG00000205020
TF334888	9560	ENSG00000205020
TF334888	388372	ENSG00000205020
TF334888	6349	ENSG00000205021
TF334888	414062	ENSG00000205021
TF334888	728830	ENSG00000205021
TF334888	730422	ENSG00000205021
TF105907	390748	ENSG00000205022
TF329582	93035	ENSG00000205038
TF328989	646759	ENSG00000205042
TF328989	652438	ENSG00000205042
TF315551	3963	ENSG00000205076
TF315551	653499	ENSG00000205076
TF315551	728910	ENSG00000205076
TF315551	732032	ENSG00000205076
TF300137	646584	ENSG00000205079
TF101007	645121	ENSG00000205089
TF332743	643965	ENSG00000205116
TF315811	347051	ENSG00000205184
TF316894	646480	ENSG00000205186
TF330979	65986	ENSG00000205189
TF316814	55366	ENSG00000205213
TF106222	5699	ENSG00000205220
TF318242	5212	ENSG00000205221
TF329827	441273	ENSG00000205238
TF314707	646409	ENSG00000205267
TF314638	5150	ENSG00000205268
TF314615	100113407	ENSG00000205269
TF333403	613212	ENSG00000205279
TF313698	6643	ENSG00000205302
TF315534	644378	ENSG00000205318
TF315534	649414	ENSG00000205318
TF300634	10527	ENSG00000205339
TF336054	4496	ENSG00000205358
TF336054	645745	ENSG00000205358
TF336054	441771	ENSG00000205360
TF336054	4489	ENSG00000205362
TF336054	4499	ENSG00000205364
TF330647	3426	ENSG00000205403
TF313386	389217	ENSG00000205405
TF313386	650865	ENSG00000205405
TF331842	54809	ENSG00000205413
TF317854	3853	ENSG00000205420
TF317854	3887	ENSG00000205426
TF314979	91828	ENSG00000205436
TF325819	92270	ENSG00000205464
TF330989	388125	ENSG00000205502
TF329827	441258	ENSG00000205505
TF329827	441272	ENSG00000205505
TF329827	641776	ENSG00000205505
TF329827	643862	ENSG00000205505
TF329827	643909	ENSG00000205505
TF329827	728524	ENSG00000205505
TF329827	730294	ENSG00000205505
TF329827	730304	ENSG00000205505
TF329827	730307	ENSG00000205505
TF329827	441258	ENSG00000205506

TF329827	441272	ENSG00000205506
TF329827	641776	ENSG00000205506
TF329827	643862	ENSG00000205506
TF329827	643909	ENSG00000205506
TF329827	728524	ENSG00000205506
TF329827	730294	ENSG00000205506
TF329827	730304	ENSG00000205506
TF329827	730307	ENSG00000205506
TF329827	441258	ENSG00000205509
TF329827	441272	ENSG00000205509
TF329827	641776	ENSG00000205509
TF329827	643862	ENSG00000205509
TF329827	643909	ENSG00000205509
TF329827	728524	ENSG00000205509
TF329827	730294	ENSG00000205509
TF329827	730304	ENSG00000205509
TF329827	730307	ENSG00000205509
TF315204	57139	ENSG00000205517
TF314349	4676	ENSG00000205531
TF313836	1375	ENSG00000205560
TF318390	6606	ENSG00000205571
TF318390	6607	ENSG00000205571
TF329827	441258	ENSG00000205580
TF329827	441272	ENSG00000205580
TF329827	641776	ENSG00000205580
TF329827	643862	ENSG00000205580
TF329827	643909	ENSG00000205580
TF329827	728524	ENSG00000205580
TF329827	730294	ENSG00000205580
TF329827	730304	ENSG00000205580
TF329827	730307	ENSG00000205580
TF320228	414918	ENSG00000205593
TF332773	374	ENSG00000205595
TF332773	727738	ENSG00000205595
TF313115	641763	ENSG00000205596
TF313115	644667	ENSG00000205596
TF313115	650136	ENSG00000205596
TF331194	388931	ENSG00000205639
TF343356	389906	ENSG00000205664
TF343356	728687	ENSG00000205664
TF314186	347527	ENSG00000205667
TF300908	253017	ENSG00000205678
TF318971	8110	ENSG00000205683
TF325032	729458	ENSG00000205717
TF325032	653656	ENSG00000205718
TF324293	6453	ENSG00000205726
TF332277	162073	ENSG00000205730
TF343600	727902	ENSG00000205737
TF320336	79958	ENSG00000205744
TF317540	338821	ENSG00000205754
TF339123	192668	ENSG00000205795
TF323272	403313	ENSG00000205808
TF332604	153657	ENSG00000205838
TF334029	338872	ENSG00000205850
TF334029	387911	ENSG00000205863
TF324396	1617	ENSG00000205916
TF324396	57135	ENSG00000205916
TF318206	390874	ENSG00000205922
TF322733	10215	ENSG00000205927

TF324396	57055	ENSG00000205944
TF320584	131118	ENSG00000205981
TF320584	644589	ENSG00000205981
TF331833	159163	ENSG00000206011
TF331833	378951	ENSG00000206011
TF334894	387733	ENSG00000206013
TF331833	5940	ENSG00000206046
TF331833	378949	ENSG00000206046
TF331833	378950	ENSG00000206046
TF324994	220164	ENSG00000206052
TF327169	90861	ENSG00000206053
TF335549	91353	ENSG00000206066
TF352619	6317	ENSG00000206073
TF352619	5268	ENSG00000206075
TF325759	284890	ENSG00000206090
TF337915	4197	ENSG00000206115
TF314116	729480	ENSG00000206147
TF314116	731167	ENSG00000206147
TF316128	54729	ENSG00000206148
TF316128	390010	ENSG00000206148
TF105387	398	ENSG00000206156
TF332328	3039	ENSG00000206172
TF332328	3040	ENSG00000206172
TF332328	3042	ENSG00000206177
TF317259	51224	ENSG00000206181
TF317259	728929	ENSG00000206182
TF317259	653420	ENSG00000206183
TF354252	57194	ENSG00000206190
TF313326	201562	ENSG00000206527
TF326340	389136	ENSG00000206538
TF351676	29122	ENSG00000206549
TF312824	23243	ENSG00000206560
TF316454	114786	ENSG00000206579
TF333425	6414	ENSG00000211446
TF329721	1734	ENSG00000211448
TF329721	1733	ENSG00000211452
TF105337	23012	ENSG00000211455
TF336708	650751	ENSG00000211774
TF336708	650780	ENSG00000211776
TF332233	405754	ENSG00000212121
TF105375	3151	ENSG00000212769
TF105375	643872	ENSG00000212769
TF105375	644498	ENSG00000212769
TF105375	644992	ENSG00000212769
TF105375	648822	ENSG00000212769
TF105375	649445	ENSG00000212769
TF105375	727795	ENSG00000212769
TF105375	728632	ENSG00000212769
TF105375	729505	ENSG00000212769
TF105375	729687	ENSG00000212769
TF105375	730562	ENSG00000212769
TF105375	731740	ENSG00000212769
TF105375	732081	ENSG00000212769
TF331869	727800	ENSG00000212864
TF331871	254958	ENSG00000212867
TF331871	392242	ENSG00000212867
TF331871	401467	ENSG00000212867
TF331871	441359	ENSG00000212867
TF331871	441361	ENSG00000212867

TF331871	441363	ENSG00000212867
TF331871	650913	ENSG00000212867
TF331871	653042	ENSG00000212867
TF331871	653185	ENSG00000212867
TF315600	84258	ENSG00000213023
TF332940	1082	ENSG00000213030
TF332940	93659	ENSG00000213030
TF332940	94115	ENSG00000213030
TF332724	8190	ENSG00000213054
TF315157	375035	ENSG00000213064
TF324527	22828	ENSG00000213079
TF331401	1427	ENSG00000213139
TF329218	151230	ENSG00000213160
TF334360	339398	ENSG00000213171
TF331669	286827	ENSG00000213186
TF329591	114902	ENSG00000213192
TF330663	9311	ENSG00000213199
TF330845	170575	ENSG00000213203
TF332592	1443	ENSG00000213218
TF343387	388677	ENSG00000213240
TF312796	4893	ENSG00000213281
TF105328	4056	ENSG00000213316
TF324269	1147	ENSG00000213341
TF315654	83463	ENSG00000213347
TF313258	3931	ENSG00000213398
TF105317	221914	ENSG00000213420
TF318626	6494	ENSG00000213445
TF313087	440307	ENSG00000213471
TF318099	84218	ENSG00000213474
TF334888	6358	ENSG00000213494
TF331602	388646	ENSG00000213512
TF331833	56267	ENSG00000213516
TF324457	389125	ENSG00000213533
TF324457	375346	ENSG00000213533
TF331867	594855	ENSG00000213578
TF315091	7416	ENSG00000213585
TF332666	221504	ENSG00000213588
TF321745	6818	ENSG00000213599
TF321745	445329	ENSG00000213599
TF326935	50861	ENSG00000213603
TF319909	2788	ENSG00000213611
TF313036	3073	ENSG00000213614
TF313689	54741	ENSG00000213625
TF332770	81606	ENSG00000213626
TF321745	6818	ENSG00000213648
TF321745	445329	ENSG00000213648
TF316079	1388	ENSG00000213676
TF330052	1903	ENSG00000213694
TF315438	1192	ENSG00000213719
TF314737	23564	ENSG00000213722
TF300217	6235	ENSG00000213741
TF300217	642892	ENSG00000213741
TF300217	647361	ENSG00000213741
TF314116	653789	ENSG00000213753
TF315472	10720	ENSG00000213759
TF330414	2013	ENSG00000213853
TF315332	147040	ENSG00000213859
TF330976	1241	ENSG00000213903
TF330976	56413	ENSG00000213906

TF329541	1773	ENSG00000213918
TF300544	1454	ENSG00000213923
TF328512	10379	ENSG00000213928
TF331936	9080	ENSG00000213937
TF105391	3672	ENSG00000213949
TF300367	8906	ENSG00000213983
TF300467	649125	ENSG00000213985
TF333088	53345	ENSG00000213996
TF300337	2595	ENSG00000214013
TF313087	10093	ENSG00000214021
TF313087	26140	ENSG00000214021
TF101178	402715	ENSG00000214035
TF352892	7106	ENSG00000214063
TF316419	8904	ENSG00000214078
TF105553	5520	ENSG00000214122
TF316871	90342	ENSG00000214272
TF329827	441272	ENSG00000214300
TF330736	729581	ENSG00000214316
TF330736	731583	ENSG00000214316
TF316127	344167	ENSG00000214336
TF331853	387104	ENSG00000214338
TF336103	401562	ENSG00000214402
TF300673	346562	ENSG00000214415
TF344181	730269	ENSG00000214421
TF343797	57412	ENSG00000214435
TF300189	348110	ENSG00000214446
TF328397	440503	ENSG00000214456
TF314697	51400	ENSG00000214517
TF354285	10809	ENSG00000214530
TF319394	642677	ENSG00000214545
TF319394	648819	ENSG00000214545
TF343620	389405	ENSG00000214559
TF332299	56105	ENSG00000214567
TF332299	56099	ENSG00000214570
TF332299	9708	ENSG00000214574
TF317658	64506	ENSG00000214575
TF332299	56109	ENSG00000214580
TF332299	56102	ENSG00000214583
TF332299	56112	ENSG00000214594
TF332299	56137	ENSG00000214600
TF332299	56138	ENSG00000214608
TF337908	389124	ENSG00000214681
TF337908	440956	ENSG00000214686
TF337908	652088	ENSG00000214686
TF313638	7866	ENSG00000214706
TF322818	9189	ENSG00000214717
TF317301	221092	ENSG00000214753
TF101079	641977	ENSG00000214765
TF316871	654463	ENSG00000214814
TF333202	9236	ENSG00000214882
TF300740	728962	ENSG00000214932
TF300740	730452	ENSG00000214932
TF312801	390299	ENSG00000214975
TF331388	644070	ENSG00000214978
TF319230	643773	ENSG00000214979
TF331207	340267	ENSG00000215018
TF314368	84461	ENSG00000215041
TF312973	137886	ENSG00000215114
TF315551	402635	ENSG00000215132

TF314160	202915	ENSG00000215155
TF340612	728587	ENSG00000215180
TF340612	730442	ENSG00000215180
TF336561	4586	ENSG00000215182
TF336561	730855	ENSG00000215182
TF315372	389207	ENSG00000215203
TF316990	440270	ENSG00000215252
TF333363	57594	ENSG00000215271
TF320995	648629	ENSG00000215315
TF314218	4636	ENSG00000215375
TF314218	649851	ENSG00000215375
TF316990	727832	ENSG00000215405
TF323502	440368	ENSG00000215407
TF323502	654253	ENSG00000215407
TF105536	728970	ENSG00000215471
TF105536	731930	ENSG00000215471
TF312976	283514	ENSG00000215475
TF328464	80816	ENSG00000215491
TF329487	128954	ENSG00000215568
TF333084	642968	ENSG00000215611
TF333084	643750	ENSG00000215611
TF320562	3166	ENSG00000215612
TF334888	728835	ENSG00000215627
TF334888	730424	ENSG00000215627
TF334888	9560	ENSG00000215627
TF334888	388372	ENSG00000215627
TF334888	6349	ENSG00000215629
TF334888	414062	ENSG00000215629
TF334888	728830	ENSG00000215629
TF334888	730422	ENSG00000215629
TF331083	79908	ENSG00000215631
TF331083	653117	ENSG00000215631
TF315710	2642	ENSG00000215644
TF319554	729619	ENSG00000215682
TF328907	113277	ENSG00000215684
TF328907	728772	ENSG00000215684
TF328428	4077	ENSG00000215691
TF328428	727732	ENSG00000215691
TF352014	84289	ENSG00000215693
TF352014	727773	ENSG00000215693
TF314847	23192	ENSG00000215697
TF314847	727737	ENSG00000215697
TF333211	55629	ENSG00000215700
TF330455	51032	ENSG00000215704
TF300138	727778	ENSG00000215717
TF318099	414060	ENSG00000215748
TF318099	653380	ENSG00000215748
TF318099	654341	ENSG00000215748
TF318099	729837	ENSG00000215748
TF334458	335	ENSG00000215756
TF314850	728866	ENSG00000215762
TF313172	642995	ENSG00000215766
TF313172	652458	ENSG00000215766
TF313172	728849	ENSG00000215766
TF101178	3838	ENSG00000215769
TF351844	8447	ENSG00000215770
TF332742	390792	ENSG00000215772
TF332742	125115	ENSG00000215773
TF332742	728760	ENSG00000215773

TF330966	727811	ENSG00000215775
TF330966	1234	ENSG00000215778
TF330966	1231	ENSG00000215782
TF330966	729230	ENSG00000215782
TF333916	8718	ENSG00000215788
TF316097	80772	ENSG00000215792
TF315837	431704	ENSG00000215825
TF350449	652793	ENSG00000215859
TF350449	652793	ENSG00000215860
TF331083	391037	ENSG00000215889
TF330979	653121	ENSG00000215897
TF330979	728116	ENSG00000215897
