# Supplementary material for: 2R and remodeling of vertebrate signal transduction engine
Source: BMC Biol. 2010 Dec 13;8:146. doi: 10.1186/1741-7007-8-146 (PMC3238295; doi:10.1186/1741-7007-8-146)
Supplement: Additional file 2 — TableS2_bp. 2RO overrepresented BP terms. [file 1741-7007-8-146-S2.pdf]

GOBPID	Pvalue	OddsRatio	ExpCount	Count	Size	Term
GO:0007165	2.12253497811915e-33	2.29068000721493	673.077947049253	853	1160	signal transduction
GO:0007275	3.64407333691957e-26	1.94516892303586	835.616993292121	1007	1396	multicellular organismal development
GO:0007186	3.19748000356857e-19	3.01861330851559	220.848041775457	299	368	G-protein coupled receptor protein signaling pathway
GO:0006468	1.13961692402044e-15	2.32444322840664	282.696658431933	362	470	protein amino acid phosphorylation
GO:0007154	1.10624738535357e-13	3.06192943498643	122.778075343689	175	224	cell communication
GO:0007268	7.50673471160711e-13	3.26881378346683	125.709790664413	173	209	synaptic transmission
GO:0022008	2.34057420509259e-12	3.48382528324389	110.672734364842	154	184	neurogenesis
GO:0030036	1.64073992060226e-11	3.99246895559645	87.2149265375114	124	145	actin cytoskeleton organization and biogenesis
GO:0050877	2.07828989473169e-11	1.977404245675	288.711480951762	356	480	neurological system process
GO:0006811	3.47726420663926e-11	3.54105314130961	94.7954698665229	133	159	ion transport
GO:0051056	8.93113129775685e-11	4.2859151628779	75.1852814978546	108	125	regulation of small GTPase mediated signal transduction
GO:0006928	1.52367204259587e-10	2.27706911324406	185.256533610714	237	308	cell motility
GO:0009605	1.95947372831575e-10	1.91736885740127	282.696658431933	346	470	response to external stimulus
GO:0006796	2.60722353281314e-10	1.71481105820397	403.594591080484	478	671	phosphate metabolic process
GO:0050794	4.15193134883487e-10	1.36317338383094	1497.69080743726	1621	2490	regulation of cellular process
GO:0007265	7.20715281094679e-10	3.3492669924478	89.6208555454427	124	149	Ras protein signal transduction
GO:0000904	8.5905530823506e-10	5.44455445544554	54.7348849304382	81	91	cellular morphogenesis during differentiation
GO:0006813	1.04815274917313e-09	6.21204964850615	49.3215446625926	74	82	potassium ion transport
GO:0051179	1.63336460531214e-09	2.25889250698411	159.932280644636	206	272	localization
GO:0048812	1.77238803398555e-09	5.67169719169719	51.1259914185412	76	85	neurite morphogenesis
GO:0030030	2.92008332493392e-09	3.43190880895799	80.5986217657002	112	134	cell projection organization and biogenesis
GO:0032990	2.92008332493392e-09	3.43190880895799	80.5986217657002	112	134	cell part morphogenesis
GO:0065008	3.87207154205869e-09	1.63002186856403	422.240540891952	493	702	regulation of biological quality
GO:0007155	4.9439754969163e-09	1.77373693379791	301.944090495384	362	502	cell adhesion
GO:0019935	6.29365390832272e-09	4.39449121677706	58.945260694318	85	98	cyclic-nucleotide-mediated signaling
GO:0007399	7.7750470257911e-09	2.09088768542501	178.713201012388	225	300	nervous system development
GO:0009653	9.27052661145663e-09	2.06811727194961	181.573147647377	228	305	anatomical structure morphogenesis
GO:0048666	1.10240758084974e-08	3.86672915747684	64.9600832141464	92	108	neuron development
GO:0048513	1.31803566490544e-08	1.69069233293230	331.581656085371	393	557	organ development

GO:0007169	1.40476316580050e-08	2.99649849583272	88.417891041477	120	147	transmembrane receptor protein tyrosine kinase signaling pathway
GO:0007242	5.60912425093736e-08	1.79912888317503	235.661686679698	286	401	intracellular signaling cascade
GO:0009887	9.21070478554821e-08	2.03394433129667	166.009101547263	207	276	organ morphogenesis
GO:0045165	1.17494176989176e-07	8.19253878086083	31.8785593550904	49	53	cell fate commitment
GO:0006936	1.48339449444086e-07	2.84705661920011	81.8015862696658	110	136	muscle contraction
GO:0006816	1.73403794617357e-07	6.13450740173558	36.6904173709531	55	61	calcium ion transport
GO:0007423	1.81729268473820e-07	8.02359108781127	31.2770771031075	48	52	sensory organ development
GO:0007389	2.48078606493662e-07	3.86897216165509	52.9304381744897	75	88	pattern specification process
GO:0006812	2.97124869998219e-07	1.97383213801124	164.806137043297	204	274	cation transport
GO:0048856	3.43655325147134e-07	2.06206458667701	135.564967478445	172	234	anatomical structure development
GO:0007243	5.84686677875996e-07	1.94348668280872	163.001690287349	201	271	protein kinase cascade
GO:0051234	7.29141248515552e-07	1.33058033770080	931.094526069432	1014	1548	establishment of localization
GO:0007264	1.00322641392348e-06	3.22961100784938	57.8127744510978	80	97	small GTPase mediated signal transduction
GO:0007188	1.37872925664262e-06	4.38663844488237	40.9007931348329	59	68	G-protein signaling, coupled to cAMP nucleotide second messenger
GO:0035023	1.56987388391459e-06	7.17995854243945	28.2696658431933	43	47	regulation of Rho protein signal transduction
GO:0007010	1.62051511500307e-06	1.77701847633511	196.083214146405	236	326	cytoskeleton organization and biogenesis
GO:0007269	1.76847127703082e-06	Inf	15.6385385515538	26	26	neurotransmitter secretion
GO:0015698	2.33220368175011e-06	3.11507264000828	57.7422961903524	79	96	inorganic anion transport
GO:0030182	2.70665837439892e-06	11.5108888888889	21.5249901094554	34	36	neuron differentiation
GO:0030154	3.26696966947964e-06	1.38158593490006	583.399706979222	648	981	cell differentiation
GO:0050789	3.65631195774205e-06	2.7378640776699	61.6293421307976	84	107	regulation of biological process
GO:0006954	3.9827477953863e-06	1.94375133128442	137.739435704070	170	229	inflammatory response
GO:0050790	5.34034454977951e-06	1.73349479589803	190.669873878559	228	317	regulation of catalytic activity
GO:0007519	6.59456449423561e-06	5.12059679767103	31.2770771031075	46	52	skeletal muscle development
GO:0007626	7.55569638474955e-06	2.33954504562396	83.6060330256144	108	139	locomotory behavior
GO:0006470	8.39377459573641e-06	2.95474814843538	55.336367182421	75	92	protein amino acid dephosphorylation
GO:0007411	8.52186282088984e-06	6.50637671680837	25.863736835262	39	43	axon guidance
GO:0016055	1.12883455862095e-05	3.29356220281253	46.3141334026785	64	77	Wnt receptor signaling pathway
GO:0045445	1.36219095815813e-05	Inf	13.2326095436224	22	22	myoblast differentiation
GO:0048518	1.46190106521720e-05	1.38784719834601	485.997659602132	541	808	

positive regulation of biological process

GO:0048878 1.48707400507948e-05 1.87262685274522 134.130542192173 164 223

chemical homeostasis

GO:0003008 1.56467783462815e-05 1.86926196269262 130.273193731799 160 220

system process

GO:0040012 3.02206069163703e-05 4.29132753297783 31.2770771031075 45 52

regulation of locomotion

GO:0008277 3.14104058877612e-05 9.66140961496628 18.6459498114680 29 31

regulation of G-protein coupled receptor protein signaling pathway

GO:0032012 3.23986551645739e-05 16.64855466203 15.6385385515538 25 26

regulation of ARF protein signal transduction

GO:0051216 3.23986551645739e-05 16.64855466203 15.6385385515538 25 26

cartilage development

GO:0006935 4.49780338790442e-05 2.35804319083297 67.9674944740606 88 113

chemotaxis

GO:0030334 4.65092854006211e-05 5.06887532693984 25.863736835262 38 43

regulation of cell migration

GO:0048747 6.08120813018423e-05 6.88589046064563 20.4503965674165 31 34

muscle fiber development

GO:0007167 6.94898469779312e-05 2.91497975708502 44.1998939554613 60 74

enzyme linked receptor protein signaling pathway

GO:0048598 7.45421626291458e-05 3.56021552351828 34.2844883630217 48 57

embryonic morphogenesis

GO:0007610 8.41354697929398e-05 2.59863639769237 52.0479343220339 69 87

behavior
