# Supplementary material for: 2R and remodeling of vertebrate signal transduction engine
Source: BMC Biol. 2010 Dec 13;8:146. doi: 10.1186/1741-7007-8-146 (PMC3238295; doi:10.1186/1741-7007-8-146)
Supplement: Additional file 3 — TableS2_mf. 2RO overrepresented MF terms. [file 1741-7007-8-146-S3.pdf]

GOBPID	Pvalue	OddsRatio	ExpCount	Count	Size	Term
GO:0005509	3.24605284709689e-27	3.0802039415629	315.420736288505	427	525	calcium ion binding
GO:0022803	3.36248278003361e-17	4.37355024278601	124.365890308039	179	207	passive transmembrane transporter activity
GO:0022836	7.31192216344499e-17	5.21748116254037	103.938642624593	153	173	gated channel activity
GO:0022838	3.19774574720901e-16	4.22146718146718	120.761081893313	173	201	substrate specific channel activity
GO:0043565	3.87923118500125e-16	2.87665599131972	195.8612572001	263	326	sequence-specific DNA binding
GO:0003700	1.23279817168165e-14	2.02140488748864	365.287252692211	452	608	transcription factor activity
GO:0003779	1.06082533320199e-11	3.23424941072	114.152266466316	157	190	actin binding
GO:0030234	5.5619418669838e-11	1.93339575354810	292.590282995242	359	487	enzyme regulator activity
GO:0008528	7.58193975701377e-11	8.19350970017637	47.4633107938893	73	79	peptide receptor activity, G-protein coupled
GO:0004672	5.45344259136217e-10	4.10302974636207	70.2756918279849	101	118	protein kinase activity
GO:0030955	7.76298632946176e-10	8.6063371356147	41.4552967693464	64	69	potassium ion binding
GO:0030594	8.3790839920481e-10	10.0802026171380	38.4512897570749	60	64	neurotransmitter receptor activity
GO:0005244	8.77058461064348e-10	4.6688124959153	61.8825444527924	90	103	voltage-gated ion channel activity
GO:0022892	1.47917151912404e-09	1.73090539633225	352.069621838217	419	586	substrate-specific transporter activity
GO:0005083	1.47958921714073e-09	3.35288879390183	85.9146005509642	119	143	small GTPase regulator activity
GO:0043167	2.60107787189115e-09	1.36152458458746	1258.67893814175	1371	2095	ion binding
GO:0004871	3.00361868664367e-09	2.32234333256338	140.827973819010	184	243	signal transducer activity
GO:0005516	8.18073746072423e-09	4.1868271825818	60.6809416478838	87	101	calmodulin binding
GO:0016791	1.65050198508049e-08	2.81370605020071	96.1282243926872	129	160	phosphoric monoester hydrolase activity
GO:0005261	2.14634624625190e-08	4.06191289496157	58.7531749025525	84	98	cation channel activity
GO:0004725	3.88665396779243e-08	5.45901119797169	43.8585023791635	65	73	protein tyrosine phosphatase activity
GO:0004888	3.93422094534357e-08	1.98909007563603	181.402144425785	226	306	transmembrane receptor activity
GO:0005096	4.53768499620976e-08	3.24287032249562	73.297771099424	101	122	GTPase activator activity
GO:0015276	1.28765676317365e-07	5.20375844594595	42.0560981718006	62	70	ligand-gated ion channel activity
GO:0004674	2.80195077144192e-07	1.93770215241419	173.631605309291	214	289	protein serine/threonine kinase activity
GO:0015075	6.12104246384114e-07	1.81658249158249	196.243575136093	238	328	ion transmembrane transporter activity
GO:0005089	7.92703225048938e-07	12.3799621928166	23.4312546957175	37	39	Rho guanyl-nucleotide exchange factor activity
GO:0005085	1.94952033276504e-06	3.56352782341363	49.2657150012522	69	82	guanyl-nucleotide exchange factor activity
GO:0008509	4.13697880814303e-06	3.22268303004655	52.2697220135237	72	87	anion transmembrane transporter activity

GO:0016772 4.85958865140224e-06   1.49053867886517 361.081642875031 412   601
transferase activity, transferring phosphorus-containing groups
GO:0001584 6.52353838216045e-06   2.63674644558653 66.9280384469457 89   112
rhodopsin-like receptor activity
GO:0005057 6.52642435745579e-06   2.50940061852634 73.8985725018783 97   123
receptor signaling protein activity
GO:0005231 7.85984078217498e-06   10.6957616449853 20.4272476834460 32   34
excitatory extracellular ligand-gated ion channel activity
GO:0004714 8.96736531788913e-06   4.2705919528123 35.4472827448034 51   59
transmembrane receptor protein tyrosine kinase activity
GO:0004930 9.33735553447448e-06   4.61177349562619 32.11597177678 47   54
G-protein coupled receptor activity
GO:0005200 1.23296070146966e-05   3.40820491168813 43.8585023791635 61   73
structural constituent of cytoskeleton
GO:0005249 1.36674427933942e-05   4.49550470577623 32.4432757325319 47   54
voltage-gated potassium channel activity
GO:0005201 1.57023460597352e-05   3.68522032468902 39.0520911595292 55   65
extracellular matrix structural constituent
GO:0003707 2.05347376846379e-05   4.79586400280407 29.4392687202605 43   49
steroid hormone receptor activity
GO:0060090 2.05347376846379e-05   4.79586400280407 29.4392687202605 43   49
molecular adaptor activity
GO:0004872 2.59937909386231e-05   1.64734299516908 185.382131324004 220   316
receptor activity
GO:0042277 2.96629127445873e-05   2.30418666828705 74.4993739043326 96   124
peptide binding
GO:0004715 3.85464865290736e-05   7.12826968806826 21.0280490859003 32   35
non-membrane spanning protein tyrosine kinase activity
GO:0019887 4.22686607425392e-05   4.20590179698299 30.6408715251690 44   51
protein kinase regulator activity
GO:0015662 4.32347543327539e-05   5.84925467142557 23.4312546957175 35   39
ATPase activity, coupled to transmembrane movement of ions, phosphorylative mechanism
GO:0019992 4.49634028912984e-05   5.08210084033613 25.8344603055347 38   43
diacylglycerol binding
GO:0003924 7.3552995984775e-05   2.14556975059896 78.1041823190584 99   130
GTPase activity
GO:0015291 9.0179781699815e-05   2.32071403322729 64.2857500626096 83   107
secondary active transmembrane transporter activity
GO:0005262 9.02264754053005e-05   6.67994966442953 19.8264462809917 30   33
calcium channel activity
GO:0005515 9.07558205567014e-05   1.1900072003774 2620.47507926443 2702   4386
protein binding
GO:0031404 9.49570979107712e-05   4.34608110947678 27.0360631104433 39   45
chloride ion binding
