# Supplementary material for: 2R and remodeling of vertebrate signal transduction engine
Source: BMC Biol. 2010 Dec 13;8:146. doi: 10.1186/1741-7007-8-146 (PMC3238295; doi:10.1186/1741-7007-8-146)
Supplement: Additional file 4 — TableS2_cc. 2RO overrepresented CC terms. [file 1741-7007-8-146-S4.pdf]

GOBPID	Pvalue	OddsRatio	ExpCount	Count	Size	Term
GO:0005887	4.22736057161309e-26	2.27676323399539	528.308357715303	669	884	integral to plasma membrane
GO:0044459	7.41535513324731e-22	2.03750923378609	558.241706161137	691	948	plasma membrane part
GO:0031224	1.69185294399172e-18	1.5702627535146	1353.62868344505	1524	2259	intrinsic to membrane
GO:0005886	2.41314295363781e-18	2.40641784037559	283.877557160048	374	499	plasma membrane
GO:0045202	2.67495464360311e-14	5.3398683069586	84.489439736942	125	141	synapse
GO:0015629	7.0461486871078e-12	3.46690301113873	105.461995700013	147	176	actin cytoskeleton
GO:0030054	1.23317304007120e-11	3.76395314680179	92.5166155419223	131	155	cell junction
GO:0045211	8.90765839925645e-09	6.28320748372107	43.1435436954597	65	72	postsynaptic membrane
GO:0043292	2.23768896757161e-08	7.56873131140538	36.5521689642089	56	61	contractile fiber
GO:0005578	1.23354280598778e-06	2.27213177447552	103.435373285932	133	173	proteinaceous extracellular matrix
GO:0030017	1.58287350731992e-06	6.20392156862745	30.5600101176173	46	51	sarcomere
GO:0005882	8.0641777482977e-06	4.29941860465116	35.3537371948906	51	59	intermediate filament
GO:0005911	1.34724341905316e-05	3.06169878658101	49.7349184267105	68	83	intercellular junction
GO:0005581	2.84606243207579e-05	9.75185814397961	18.5756924244340	29	31	collagen
GO:0043235	4.20714064949375e-05	2.98805450323842	45.5404072340964	62	76	receptor complex
GO:0016323	5.12005201884465e-05	3.87377450980392	32.3576577715948	46	54	basolateral plasma membrane
GO:0008076	5.97718502165824e-05	4.48843479494820	27.5639306943215	40	46	voltage-gated potassium channel complex
