# Supplementary material for: 2R and remodeling of vertebrate signal transduction engine
Source: BMC Biol. 2010 Dec 13;8:146. doi: 10.1186/1741-7007-8-146 (PMC3238295; doi:10.1186/1741-7007-8-146)
Supplement: Additional file 5 — TableS3_bp. 2RO underrepresented BP terms. [file 1741-7007-8-146-S5.pdf]

GOBPID	Pvalue	OddsRatio	ExpCount	Count	Size	Term
GO:0009719	1.89187019845306e-21	0.308709949029976	170.820959563126	93	284	response to endogenous stimulus
GO:0006412	3.08426690393565e-18	0.329661558109834	158.791314523469	90	264	translation
GO:0022613	2.52697191398225e-16	0.0286660382374979	28.4075436982521	2	47	ribonucleoprotein complex biogenesis and assembly
GO:0006364	3.37289865730235e-14	0.0176946676406136	22.8563255753478	1	38	rRNA processing
GO:0009058	2.30696907166646e-13	0.576103787112961	470.359121050579	375	782	biosynthetic process
GO:0006281	1.59296748144161e-12	0.209403412629219	56.3869822485207	23	93	DNA repair
GO:0042773	6.02814238411843e-12	0	16.8415030555194	0	28	ATP synthesis coupled electron transport
GO:0000279	3.92391126348810e-11	0.365575817660124	108.266805356911	65	180	M phase
GO:0044248	6.98081597604716e-11	0.510941566668616	236.984007281238	175	394	cellular catabolic process
GO:0006139	7.88180226274542e-11	0.713873701351174	1218.00156026525	1096	2025	nucleobase, nucleoside, nucleotide and nucleic acid metabolic process
GO:0006120	2.43857974291836e-10	0	14.4355740475881	0	24	mitochondrial electron transport, NADH to ubiquinone
GO:0008033	1.54916130893794e-09	0	13.2326095436224	0	22	tRNA processing
GO:0008380	2.53675782724229e-09	0.377698420142844	93.2297490573397	57	155	RNA splicing
GO:0006261	1.08915318758406e-08	0.263687678897439	48.118580158627	23	80	DNA-dependent DNA replication
GO:0019882	4.10355131544594e-08	0.164013428633312	27.0667013392277	9	45	antigen processing and presentation
GO:0043170	4.88118770176965e-08	0.778879587894398	2170.74944740606	2056	3609	macromolecule metabolic process
GO:0006082	5.35386937351259e-08	0.542515936497625	194.880249642439	148	324	organic acid metabolic process
GO:0006397	6.2560285136738e-08	0.443240669087446	108.266805356911	73	180	mRNA processing
GO:0006732	7.07242594639376e-08	0.188672570411284	29.5457154036781	11	49	coenzyme metabolic process
GO:0000278	1.2899604595498e-07	0.489055991704414	132.927577688207	95	221	mitotic cell cycle
GO:0006084	3.81319667789228e-07	0.0626956665018949	13.8340917956053	2	23	acetyl-CoA metabolic process
GO:0007067	3.99649800386421e-07	0.419150731158605	83.0045507736315	54	138	mitosis
GO:0022618	5.05256080335086e-07	0.321295143212951	49.3215446625926	27	82	protein-RNA complex assembly
GO:0022402	5.54261453782072e-07	0.545872239513858	166.921824104235	127	277	cell cycle process
GO:0006260	1.64320220039472e-06	0.323066841415465	45.3586913677572	25	75	DNA replication
GO:0006099	2.01932901577923e-06	0.0693407393917319	12.6311272916396	2	21	tricarboxylic acid cycle
GO:0043285	2.92800434218538e-06	0.53208024579794	132.326095436224	99	220	biopolymer catabolic process
GO:0006520	3.18954854677354e-06	0.433559297324878	75.3226016684046	50	125	amino acid metabolic process
GO:0006302	4.02550258087706e-06	0.157922077922078	18.6459498114680	6	31	double-strand break repair

GO:0006298	4.19970463008768e-06	0.0987994808565866	13.8340917956053	3	23	mismatch repair
GO:0044237	4.78397364111401e-06	0.764481208098143	1133.73963133641	1060	1786	cellular metabolic process
GO:0006091	5.62688830541663e-06	0.489302172481896	96.8386425692368	69	161	generation of precursor metabolites and energy
GO:0006457	5.7106845841347e-06	0.412717053938428	63.7571187101807	41	106	protein folding
GO:0006310	6.50620487733581e-06	0.302738739502028	36.1563517915309	19	60	DNA recombination
GO:0045333	7.68860800199753e-06	0.194921643167637	21.0518788193993	8	35	cellular respiration
GO:0009109	9.23907020738242e-06	0.104033607705181	13.2326095436224	3	22	coenzyme catabolic process
GO:0007131	9.98790155873847e-06	0.043963963963964	9.6237160317254	1	16	meiotic recombination
GO:0051028	1.12838905408775e-05	0.303084935897436	34.2844883630217	18	57	mRNA transport
GO:0006289	1.57101334777048e-05	0.131761142362614	14.4355740475881	4	24	nucleotide-excision repair
GO:0006506	1.58393434529639e-05	0	7.21778702379405	0	12	GPI anchor biosynthetic process
GO:0007126	2.04606385487712e-05	0.267105191849122	27.0667013392277	13	45	meiosis
GO:0044238	2.14061437642060e-05	0.794883562208023	1424.83409812919	1351	2287	primary metabolic process
GO:0051321	2.32693541679138e-05	0.278903519146364	28.2696658431933	14	47	meiotic cell cycle
GO:0050657	2.55624428578286e-05	0.343934880642092	38.4948641269016	22	64	nucleic acid transport
GO:0051236	2.55624428578286e-05	0.343934880642092	38.4948641269016	22	64	establishment of RNA localization
GO:0043632	3.05160850065138e-05	0.459286880730656	68.5689767260434	47	114	modification-dependent macromolecule catabolic process
GO:0051603	3.05160850065138e-05	0.459286880730656	68.5689767260434	47	114	proteolysis involved in cellular protein catabolic process
GO:0009411	3.36390621479468e-05	0.179634002361275	16.8415030555194	6	28	response to UV
GO:0006284	4.38199751722783e-05	0.116349200290110	12.0296450396567	3	20	base-excision repair
GO:0006511	4.58397483309212e-05	0.466401291799850	67.9674944740606	47	113	ubiquitin-dependent protein catabolic process
GO:0007093	4.90172640687698e-05	0.164737069898290	15.0370562995709	5	25	mitotic cell cycle checkpoint
GO:0051188	9.39355593240936e-05	0.268191485092894	22.8563255753478	11	38	cofactor biosynthetic process
GO:0009069	9.43465855882152e-05	0.123661583387411	11.4281627876739	3	19	serine family amino acid metabolic process
