# Supplementary material for: 2R and remodeling of vertebrate signal transduction engine
Source: BMC Biol. 2010 Dec 13;8:146. doi: 10.1186/1741-7007-8-146 (PMC3238295; doi:10.1186/1741-7007-8-146)
Supplement: Additional file 6 — TableS3_mf. 2RO underrepresented MF terms. [file 1741-7007-8-146-S6.pdf]

GOBPID	Pvalue	OddsRatio	ExpCount	Count	Size	Term
GO:0003735	1.05927211700559e-13	0.183764790501064	54.6729276233408	20	91	structural constituent of ribosome
GO:0004540	7.94174818393611e-11	0.0424681858538029	19.8264462809917	2	33	ribonuclease activity
GO:0003723	2.18792120646068e-10	0.534844209485358	258.842290306379	196	430	RNA binding
GO:0003954	6.4057755025621e-10	0	13.8184322564488	0	23	NADH dehydrogenase activity
GO:0008137	6.4057755025621e-10	0	13.8184322564488	0	23	NADH dehydrogenase (ubiquinone) activity
GO:0016655	1.62563591221903e-09	0.0263748175943298	15.6208364638117	1	26	oxidoreductase activity, acting on NADH or NADPH, quinone or similar compound as acceptor
GO:0016779	5.90361989011617e-09	0.214341194445156	39.0520911595292	16	65	nucleotidyltransferase activity
GO:0004519	1.07402366243746e-08	0.176377078163930	31.2416729276233	11	52	endonuclease activity
GO:0003684	7.30168647750418e-08	0.097683833186545	18.6248434760831	4	31	damaged DNA binding
GO:0016874	2.03680149319485e-07	0.473158593978698	117.156273478588	82	195	ligase activity
GO:0008026	3.1555119537859e-07	0.254073890969938	36.648885549712	17	61	ATP-dependent helicase activity
GO:0016887	3.57986983325186e-07	0.510935657187787	139.385925369396	102	232	ATPase activity
GO:0003899	8.83912121843695e-07	0.0900369703289411	15.0200350613574	3	25	DNA-directed RNA polymerase activity
GO:0016491	1.03799528115650e-06	0.608404369243949	237.973346743777	192	395	oxidoreductase activity
GO:0045182	1.36975611725243e-06	0.322175800971971	45.6609065865264	25	76	translation regulator activity
GO:0016627	2.03378856446631e-06	0.152273019134455	19.2256448785374	6	32	oxidoreductase activity, acting on the CH-CH group of donors
GO:0008094	3.52881983146429e-06	0.212695717789096	24.6328575006261	10	41	DNA-dependent ATPase activity
GO:0008168	3.83327894484249e-06	0.28903969792836	35.4472827448034	18	59	methyltransferase activity
GO:0030983	4.32314854031895e-06	0.0413279132791328	10.2136238417230	1	17	mismatched DNA binding
GO:0005126	5.3358369852712e-06	0.204714827802219	22.8304532932632	9	38	hematopoietin/interferon-class (D200-domain) cytokine receptor binding
GO:0003743	5.36513484304688e-06	0.239714318276792	27.0360631104433	12	45	translation initiation factor activity
GO:0016891	1.02442407030495e-05	0.0440970050726148	9.61282243926872	1	16	endoribonuclease activity, producing 5'-phosphomonoesters
GO:0050660	1.03247548731451e-05	0.212093189750321	22.2296518908089	9	37	FAD binding
GO:0003823	1.56414135346619e-05	0.203115464910872	20.4272476834460	8	34	antigen binding
GO:0004659	1.61754788600059e-05	0	7.20961682945154	0	12	prenyltransferase activity
GO:0004527	2.46948172005448e-05	0.157322682880788	15.6208364638117	5	26	exonuclease activity
GO:0003676	2.77292975989932e-05	0.777000799268876	786.066365808335	721	1288	nucleic acid binding
GO:0032395	4.06041686838361e-05	0	6.60881542699725	0	11	MHC class II receptor activity
GO:0031202	7.14242482498836e-05	0.146942937931674	13.2176308539945	4	22	

RNA splicing factor activity, transesterification mechanism

GO:0050662	8.83613429911064e-05	0.327535624476111	30.7257516668763	17	51

coenzyme binding
