# Supplementary material for: 2R and remodeling of vertebrate signal transduction engine
Source: BMC Biol. 2010 Dec 13;8:146. doi: 10.1186/1741-7007-8-146 (PMC3238295; doi:10.1186/1741-7007-8-146)
Supplement: Additional file 7 — TableS3_cc. 2RO underrepresented CC terms. [file 1741-7007-8-146-S7.pdf]

GOBPID	Pvalue	OddsRatio	ExpCount	Count	Size	Term
GO:0005739	1.37517571945315e-28	0.346555385724095	287.623624636398	171	480	mitochondrion
GO:0043233	1.90401401658214e-25	0.408486573791412	355.934235487543	235	594	organelle lumen
GO:0043226	6.70028226764536e-23	0.6331274910546	2665.31225496396	2454	4448	organelle
GO:0043231	4.90111516865438e-19	0.654340799674525	2115.31977918406	1930	3440	intracellular membrane-bound organelle
GO:0005759	5.80458781361221e-15	0.188353658536585	61.7192361198938	23	103	mitochondrial matrix
GO:0005743	1.01049080406260e-13	0.290073442312248	93.4776780068294	48	156	mitochondrial inner membrane
GO:0031975	3.99567772759824e-12	0.462729500258942	202.534969014797	141	338	envelope
GO:0005681	5.48196158268954e-11	0.244282076158137	57.5247249272796	26	96	spliceosome
GO:0033279	8.98027009001788e-11	0.196598252674686	44.341975464778	17	74	ribosomal subunit
GO:0044446	1.05577449985545e-10	0.671604158675245	802.678711484594	701	1294	intracellular organelle part
GO:0005740	2.11428691493619e-10	0.416999946675199	130.629062855697	85	218	mitochondrial envelope
GO:0005747	2.79967919936240e-10	0	14.3811812318199	0	24	mitochondrial respiratory chain complex I
GO:0030964	2.79967919936240e-10	0	14.3811812318199	0	24	NADH dehydrogenase complex
GO:0032991	9.30892412062197e-10	0.679469992428975	721.834424941053	628	1182	macromolecular complex
GO:0030529	9.29897189583677e-09	0.309372397607692	61.3051066580478	33	101	ribonucleoprotein complex
GO:0044428	2.44138038306306e-07	0.49245406824147	131.461279461279	95	215	nuclear part
GO:0000502	2.72789558776555e-07	0.189388445733623	26.9647148096623	10	45	proteasome complex
GO:0016591	4.20898023973944e-07	0.264745762711864	37.7506007335273	18	63	DNA-directed RNA polymerase II, holoenzyme
GO:0030532	4.27142830521822e-07	0.0633043758043758	13.7819653471607	2	23	small nuclear ribonucleoprotein complex
GO:0044452	4.27142830521822e-07	0.0633043758043758	13.7819653471607	2	23	nucleolar part
GO:0005654	5.73112486275947e-07	0.575516575546168	199.538889591501	156	333	nucleoplasm
GO:0005730	1.57781699890681e-06	0.334826526482809	48.0568239472349	27	80	nucleolus
GO:0031090	5.4842849892141e-06	0.721029495480131	499.746047805742	440	834	organelle membrane
GO:0005694	6.14589241230648e-06	0.552673602544069	139.018085240926	106	232	chromosome
GO:0044424	6.53199085803424e-06	0.72003417433128	724.54535428729	666	1114	intracellular part
GO:0005737	6.97656139144334e-06	0.81795811693556	2194.92778550651	2100	3663	cytoplasm
GO:0005777	7.58379467928097e-06	0.298315677966102	34.7545213102314	18	58	peroxisome
GO:0042613	1.69627759887077e-05	0	7.19059061590995	0	12	MHC class II protein complex
GO:0022625	1.70020318262157e-05	0.204456009107172	20.3733400784115	8	34	cytosolic large ribosomal subunit

GO:0005819	3.64250527500969e-05	0.313381110405046	31.7584418869356	17	53	spindle
GO:0005815	4.1247080463809e-05	0.429710737170516	56.3262931579613	37	94	microtubule organizing center
GO:0005852	4.24123113384533e-05	0	6.59137473125079	0	11	eukaryotic translation initiation factor 3 complex
GO:0042612	5.97079367811079e-05	0.0512495737321576	8.38902238522828	1	14	MHC class I protein complex
GO:0022627	6.28977268924607e-05	0.221634954193094	19.1749083090932	8	32	cytosolic small ribosomal subunit
GO:0016604	6.35901479649218e-05	0.420424262241478	50.9333501960288	33	85	nuclear body
GO:0000775	6.67770625807084e-05	0.274928130517898	24.5678512710257	12	41	chromosome, pericentric region
