# Supplementary material for: 2R and remodeling of vertebrate signal transduction engine
Source: BMC Biol. 2010 Dec 13;8:146. doi: 10.1186/1741-7007-8-146 (PMC3238295; doi:10.1186/1741-7007-8-146)
Supplement: Additional file 8 — TableS3_not2R-over. Tandem/segmental duplication overrepresented BP terms. [file 1741-7007-8-146-S8.html]

Gene to GO BP Conditional test for over-representation

| GOBPID | Pvalue | OddsRatio | ExpCount | Count | Size | Term |
| GO:0006955 | 0.000 | 2.568 | 79 | 147 | 372 | immune response |
| GO:0006412 | 0.000 | 2.965 | 57 | 115 | 264 | translation |
| GO:0042742 | 0.000 | 7.708 | 9 | 29 | 43 | defense response to bacterium |
| GO:0006954 | 0.000 | 2.499 | 49 | 91 | 229 | inflammatory response |
| GO:0006935 | 0.000 | 3.433 | 24 | 54 | 113 | chemotaxis |
| GO:0006334 | 0.000 | 6.567 | 10 | 30 | 47 | nucleosome assembly |
| GO:0051707 | 0.000 | 5.201 | 11 | 29 | 50 | response to other organism |
| GO:0006968 | 0.000 | 3.954 | 14 | 34 | 66 | cellular defense response |
| GO:0006333 | 0.000 | 3.452 | 17 | 38 | 79 | chromatin assembly or disassembly |
| GO:0006323 | 0.000 | 3.505 | 15 | 33 | 68 | DNA packaging |
| GO:0007626 | 0.000 | 2.447 | 30 | 55 | 139 | locomotory behavior |
| GO:0006952 | 0.000 | 3.117 | 15 | 33 | 75 | defense response |
| GO:0045087 | 0.000 | 3.046 | 15 | 32 | 71 | innate immune response |
| GO:0009605 | 0.000 | 1.588 | 101 | 139 | 470 | response to external stimulus |
| GO:0009615 | 0.000 | 2.784 | 17 | 33 | 77 | response to virus |
| GO:0006805 | 0.000 | 6.851 | 4 | 13 | 20 | xenobiotic metabolic process |
| GO:0006953 | 0.000 | 5.740 | 5 | 14 | 23 | acute-phase response |
| GO:0000003 | 0.000 | 1.639 | 68 | 97 | 319 | reproduction |
| GO:0002504 | 0.000 | 9.210 | 3 | 10 | 14 | antigen processing and presentation of peptide or polysaccharide antigen via MHC class II |
