# Supplementary material for: 2R and remodeling of vertebrate signal transduction engine
Source: BMC Biol. 2010 Dec 13;8:146. doi: 10.1186/1741-7007-8-146 (PMC3238295; doi:10.1186/1741-7007-8-146)
Supplement: Additional file 9 — TableS3_not2R-under. Tandem/segmental duplication underrepresented BP terms. [file 1741-7007-8-146-S9.html]

Gene to GO BP Conditional test for under-representation

| GOBPID | Pvalue | OddsRatio | ExpCount | Count | Size | Term |
| GO:0007154 | 0.000 | 0.589 | 496 | 362 | 2314 | cell communication |
| GO:0006468 | 0.000 | 0.343 | 101 | 42 | 470 | protein amino acid phosphorylation |
| GO:0006464 | 0.000 | 0.529 | 222 | 139 | 1035 | protein modification process |
| GO:0006796 | 0.000 | 0.456 | 144 | 78 | 671 | phosphate metabolic process |
| GO:0007242 | 0.000 | 0.523 | 199 | 123 | 930 | intracellular signaling cascade |
| GO:0007399 | 0.000 | 0.451 | 104 | 55 | 484 | nervous system development |
| GO:0043283 | 0.000 | 0.716 | 595 | 498 | 2774 | biopolymer metabolic process |
| GO:0006366 | 0.000 | 0.535 | 109 | 67 | 510 | transcription from RNA polymerase II promoter |
| GO:0016192 | 0.000 | 0.457 | 70 | 37 | 326 | vesicle-mediated transport |
| GO:0007275 | 0.000 | 0.688 | 281 | 218 | 1289 | multicellular organismal development |
| GO:0065007 | 0.000 | 0.759 | 640 | 557 | 2985 | biological regulation |
| GO:0009790 | 0.000 | 0.283 | 32 | 11 | 151 | embryonic development |
| GO:0045184 | 0.000 | 0.539 | 95 | 58 | 441 | establishment of protein localization |
| GO:0003008 | 0.000 | 0.623 | 150 | 105 | 700 | system process |
| GO:0048856 | 0.000 | 0.634 | 157 | 112 | 714 | anatomical structure development |
| GO:0016043 | 0.000 | 0.734 | 342 | 280 | 1597 | cellular component organization and biogenesis |
| GO:0007389 | 0.000 | 0.172 | 19 | 4 | 88 | pattern specification process |
| GO:0048699 | 0.000 | 0.351 | 36 | 15 | 169 | generation of neurons |
| GO:0006461 | 0.000 | 0.427 | 48 | 24 | 226 | protein complex assembly |
| GO:0033036 | 0.000 | 0.593 | 108 | 72 | 504 | macromolecule localization |
| GO:0048666 | 0.000 | 0.251 | 23 | 7 | 108 | neuron development |
| GO:0043009 | 0.000 | 0.113 | 14 | 2 | 66 | chordate embryonic development |
| GO:0007167 | 0.000 | 0.151 | 16 | 3 | 74 | enzyme linked receptor protein signaling pathway |
| GO:0019226 | 0.000 | 0.449 | 50 | 26 | 234 | transmission of nerve impulse |
| GO:0030030 | 0.000 | 0.323 | 29 | 11 | 134 | cell projection organization and biogenesis |
| GO:0032990 | 0.000 | 0.323 | 29 | 11 | 134 | cell part morphogenesis |
| GO:0007050 | 0.000 | 0.117 | 14 | 2 | 64 | cell cycle arrest |
| GO:0009653 | 0.000 | 0.635 | 129 | 91 | 600 | anatomical structure morphogenesis |
| GO:0000165 | 0.000 | 0.302 | 25 | 9 | 117 | MAPKKK cascade |
| GO:0009966 | 0.000 | 0.554 | 75 | 47 | 351 | regulation of signal transduction |
| GO:0007420 | 0.000 | 0.270 | 22 | 7 | 101 | brain development |
| GO:0001501 | 0.000 | 0.384 | 33 | 15 | 156 | skeletal development |
| GO:0007265 | 0.000 | 0.375 | 32 | 14 | 149 | Ras protein signal transduction |
| GO:0016055 | 0.000 | 0.199 | 17 | 4 | 77 | Wnt receptor signaling pathway |
| GO:0006886 | 0.000 | 0.516 | 58 | 34 | 271 | intracellular protein transport |
| GO:0007169 | 0.000 | 0.380 | 32 | 14 | 147 | transmembrane receptor protein tyrosine kinase signaling pathway |
