# Supplementary material for: 2R and remodeling of vertebrate signal transduction engine
Source: BMC Biol. 2010 Dec 13;8:146. doi: 10.1186/1741-7007-8-146 (PMC3238295; doi:10.1186/1741-7007-8-146)
Supplement: Additional file 10 — TableS4. 2RO overrepresented KEGG pathways. [file 1741-7007-8-146-S10.pdf]

KEGGID	Pvalue	OddsRatio	ExpCount	Count	Size	Term
04080	3.48310598994490e-21	8.47022502744237	118.108484455959	171	183	Neuroactive ligand-receptor interaction
04810	2.78919101525287e-17	6.2847790507365	112.945272020725	160	175	Regulation of actin cytoskeleton
04510	5.69427207139847e-16	5.32941712204007	116.817681347150	163	181	Focal adhesion
04020	6.91695334227909e-14	5.61029524834692	96.1648316062176	135	149	Calcium signaling pathway
04010	3.12984116315739e-11	3.11714387741852	138.761334196891	181	215	MAPK signaling pathway
04360	7.01088823739418e-11	6.65072368421053	65.1855569948187	93	101	Axon guidance
04310	4.73124102280156e-09	4.56721138639958	69.7033678756477	96	108	Wnt signaling pathway
04520	7.304734036533e-09	9.04924831518922	43.8873056994819	64	68	Adherens junction
01430	2.17197052805683e-08	4.55223097112861	63.8947538860104	88	99	Cell Communication
04910	1.31046474386026e-07	3.32759533898305	79.3843911917098	105	123	Insulin signaling pathway
04916	1.9132884874294e-07	5.01931581685744	50.9867227979275	71	79	Melanogenesis
04670	2.45184150397198e-07	3.97118910424306	61.958549222798	84	96	Leukocyte transendothelial migration
04530	3.86962209449371e-07	3.60928121248499	66.476360103627	89	103	Tight junction
04920	5.03425264513237e-07	6.41838842975207	40.014896373057	57	62	Adipocytokine signaling pathway
05211	1.97197948240977e-06	5.34375	40.6602979274611	57	63	Renal cell carcinoma
05220	2.06451918685224e-06	4.50803525142561	46.4689119170984	64	72	Chronic myeloid leukemia
04730	2.44024851894935e-06	4.82447712659818	43.2419041450777	60	67	Long-term depression
04012	2.62785464598194e-06	3.94955798231929	51.6321243523316	70	80	ErbB signaling pathway
05210	4.52565975062284e-06	4.06811894882434	47.7597150259067	65	74	Colorectal cancer
04930	8.55061061737476e-06	10.3376789366053	25.1706606217617	37	39	Type II diabetes mellitus
05222	1.01290645925530e-05	3.38261322228006	54.2137305699482	72	84	Small cell lung cancer
04720	2.04254121975782e-05	4.00045196280992	41.9511010362694	57	65	Long-term potentiation
04370	2.53350517540441e-05	4.24624447717231	38.7240932642487	53	60	VEGF signaling pathway
05214	2.53350517540441e-05	4.24624447717231	38.7240932642487	53	60	Glioma
04662	3.51061873192778e-05	4.16398027526312	38.0786917098446	52	59	B cell receptor signaling pathway
05212	4.29080416487466e-05	3.42572463768116	45.8235103626943	61	71	Pancreatic cancer
04350	4.40475369628467e-05	3.13953790238837	50.9867227979275	67	79	TGF-beta signaling pathway
04512	5.87202429118027e-05	3.09107420861443	50.3413212435233	66	78	ECM-receptor interaction
