# Supplementary material for: 2R and remodeling of vertebrate signal transduction engine
Source: BMC Biol. 2010 Dec 13;8:146. doi: 10.1186/1741-7007-8-146 (PMC3238295; doi:10.1186/1741-7007-8-146)
Supplement: Additional file 11 — TableS5. 2RO overrepresented PFAM domains. [file 1741-7007-8-146-S11.pdf]

Domain	PFAMID	Pvalue	OddsRatio	ExpCount	Count	Size	Term
PH domain	PF00169	8.85861523258948e-23	15.1736915701159	79.91987548997	129	135	PF00169
Homeobox domain	PF00046	5.42693389828116e-12	5.15380138541903	69.2638920913074	103	117	PF00046
7 transmembrane receptor (rhodopsin family)	PF00001	1.17047204711829e-11	3.44219838808815	101.231842287295	142	171	PF00001
Protein tyrosine kinase	PF07714	1.84765685025853e-10	6.12847222222222	52.0959188379064	79	88	PF07714
Protein kinase domain	PF00069	3.18724060421142e-10	2.40283608032813	153.919760202905	201	260	PF00069
PDZ	PF00595	4.30787441344952e-10	5.9709590967005	50.9119206824994	77	86	PF00595
Intermediate filament protein	PF00038	1.02135213286146e-09	31.2789783889980	27.2319575743602	45	46	PF00038
EGF-like domain	PF00008	3.39265215476029e-08	3.75047039017627	60.3839059257551	86	102	PF00008
EF hand	PF00036	5.20144301326466e-08	3.48959365708622	63.935900391976	90	108	PF00036
Helix-loop-helix DNA-binding domain	PF00010	8.3232318722042e-08	6.37516404199475	36.1119437399124	55	61	PF00010
SH3 domain	PF00018	8.80352981284544e-08	3.19156295183285	69.2638920913074	96	117	PF00018
Ras family	PF00071	9.14429124865228e-08	4.95015228203948	43.2159326723542	64	73	PF00071
Ion transport protein	PF00520	1.27623701360423e-07	6.25802007478843	35.5199446622089	54	60	PF00520
SH2 domain	PF00017	1.30723680114440e-07	4.59487078319195	44.9919299054646	66	76	PF00017
C2 domain	PF00168	4.48262361962342e-07	4.63711001642036	40.8479363615402	60	69	PF00168
Neurotransmitter-gated ion-channel ligand binding domain	PF02931	7.02191338811733e-07	22.1861650009798	19.5359695642149	32	33	PF02931
Neurotransmitter-gated ion-channel transmembrane region	PF02932	7.02191338811733e-07	22.1861650009798	19.5359695642149	32	33	PF02932
Protein-tyrosine phosphatase	PF00102	1.24044108942520e-06	12.1367647058824	21.9039658750288	35	37	PF00102
RhoGEF domain	PF00621	1.24044108942520e-06	12.1367647058824	21.9039658750288	35	37	PF00621
Calponin homology (CH) domain	PF00307	2.3222085263921e-06	6.10866234531526	29.0079548074706	44	49	PF00307
7 transmembrane receptor (Secretin family)	PF00002	3.11096559185978e-06	20.0943987465727	17.7599723311044	29	30	PF00002
SAM domain (Sterile alpha motif)	PF00536	3.11096559185978e-06	20.0943987465727	17.7599723311044	29	30	PF00536
Protein kinase C terminal domain	PF00433	5.10972563064141e-06	11.0899470899471	20.1279686419184	32	34	PF00433
Hormone receptor domain	PF02793	5.68372571814912e-06	Inf	13.6159787871801	23	23	PF02793
RhoGAP domain	PF00620	6.71168235547895e-06	8.08888888888889	22.4959649527323	35	38	PF00620
Fibronectin type III domain	PF00041	7.08646743132626e-06	2.63463791083856	65.119898547383	87	110	PF00041
IPT/TIG domain	PF01833	9.61775765957877e-06	Inf	13.0239797094766	22	22	PF01833
Ligand-binding domain of nuclear hormone receptor Ligand-binding domain of nuclear hormone receptor	PF00104	1.84083733963050e-05	4.85588062045945				

28.4159557297671 42 48 PF00104
Phosphotyrosine interaction domain (PTB/PID) PF00640 2.75328432797022e-05
Inf 11.8399815540696 20 20 PF00640
Phorbol esters/diacylglycerol binding domain (C1 domain) PF00130
2.84318242650498e-05 5.26945261918776 25.4559603412497 38 43
PF00130
Laminin G domain PF02210 3.63372806199034e-05 16.6135785560556
14.7999769425870 24 25 PF02210
E1-E2 ATPase PF00122 7.8793325408234e-05 Inf 10.6559833986627 18
18 PF00122
