# Supplementary material for: 2R and remodeling of vertebrate signal transduction engine
Source: BMC Biol. 2010 Dec 13;8:146. doi: 10.1186/1741-7007-8-146 (PMC3238295; doi:10.1186/1741-7007-8-146)
Supplement: Additional file 12 — TableS6. 349 2ROs preferentially expressed in brain. [file 1741-7007-8-146-S12.pdf]

Family EnsemblID Description Symbol
TF101079 ENSG00000108387 Septin-4 (Peanut-like protein 2) (Brain protein H5) (Cell division control-related protein 2) (hCDCREL-2) (Bradeion beta) (CE5B3 beta) (Cerebral protein 7). [Source:Uniprot/SWISSPROT;Acc:O43236]SEPT4
TF102004 ENSG00000166501 Protein kinase C beta type (EC 2.7.11.13) (PKC-beta) (PKC-B). [Source:Uniprot/SWISSPROT;Acc:P05771] PRKCB1
TF102004 ENSG00000067606 Protein kinase C zeta type (EC 2.7.11.13) (nPKC-zeta). [Source:Uniprot/SWISSPROT;Acc:Q05513] PRKCZ
TF105042 ENSG00000126803 Heat shock-related 70 kDa protein 2 (Heat shock 70 kDa protein 2). [Source:Uniprot/SWISSPROT;Acc:P54652]HSPA2
TF105082 ENSG00000159842 Active breakpoint cluster region-related protein. [Source:Uniprot/SWISSPROT;Acc:Q12979] ABR
TF105100 ENSG00000109339 Mitogen-activated protein kinase 10 (EC 2.7.11.24) (Stress-activated protein kinase JNK3) (c-Jun N-terminal kinase 3) (MAP kinase p49 3F12). [Source:Uniprot/SWISSPROT;Acc:P53779] MAPK10
TF105118 ENSG00000006432 Mitogen-activated protein kinase kinase kinase 9 (EC 2.7.11.25) (Mixed lineage kinase 1). [Source:Uniprot/SWISSPROT;Acc:P80192] MAP3K9
TF105122 ENSG00000184545 Dual specificity protein phosphatase 8 (EC 3.1.3.48) (EC 3.1.3.16) (Dual specificity protein phosphatase hVH-5). [Source:Uniprot/SWISSPROT;Acc:Q13202] DUSP8
TF105137 ENSG00000169032 Dual specificity mitogen-activated protein kinase kinase 1 (EC 2.7.12.2) (MAP kinase kinase 1) (MAPKK 1) (ERK activator kinase 1) (MAPK/ERK kinase 1) (MEK1). [Source:Uniprot/SWISSPROT;Acc:Q02750] MAP2K1
TF105138 ENSG00000141503 Misshapen-like kinase 1 (EC 2.7.11.1) (Mitogen-activated protein kinase kinase kinase kinase 6) (MAPK/ERK kinase kinase kinase 6) (MEK kinase kinase 6) (MEKKK 6) (Misshapen/NIK-related kinase) (GCK family kinase MiNK). [Source:Uniprot/SWISSPROT;Acc:Q8N4C8] MINK1
TF105191 ENSG00000107331 ATP-binding cassette sub-family A member 2 (ATP-binding cassette transporter 2) (ATP-binding cassette 2). [Source:Uniprot/SWISSPROT;Acc:Q9BZC7] ABCA2
TF105191 ENSG00000167972 ATP-binding cassette sub-family A member 3 (ATP-binding cassette transporter 3) (ATP-binding cassette 3) (ABC-C transporter). [Source:Uniprot/SWISSPROT;Acc:Q99758] ABCA3
TF105221 ENSG00000054523 Kinesin-like protein KIF1B (Klp). [Source:Uniprot/SWISSPROT;Acc:O60333] KIF1B
TF105223 ENSG00000084731 Kinesin-like protein KIF3C. [Source:Uniprot/SWISSPROT;Acc:O14782] KIF3C
TF105225 ENSG00000168280 Kinesin heavy chain isoform 5C (Kinesin heavy chain neuron-specific 2). [Source:Uniprot/SWISSPROT;Acc:O60282] KIF5C
TF105291 ENSG00000119782 FK506-binding protein 1B (EC 5.2.1.8) (Peptidyl-prolyl cis-trans isomerase 1B) (PPIase 1B) (Rotamase 1B) (12.6 kDa FKBP) (FKBP-12.6) (Immunophilin FKBP12.6) (h-FKBP-12). [Source:Uniprot/SWISSPROT;Acc:P68106] FKBP1B
TF105295 ENSG00000105701 FK506-binding protein 8 (EC 5.2.1.8) (Peptidyl-prolyl cis-trans isomerase) (PPIase) (Rotamase) (38 kDa FK506-binding protein) (FKBPR38) (hFKBP38). [Source:Uniprot/SWISSPROT;Acc:Q14318] FKBP8
TF105338 ENSG00000115694 Serine/threonine-protein kinase 25 (EC 2.7.11.1) (Sterile 20/oxidant stress-response kinase 1) (Ste20/oxidant stress response kinase 1) (SOK-1) (Ste20-like kinase). [Source:Uniprot/SWISSPROT;Acc:O00506] STK25
TF105351 ENSG00000149269 Serine/threonine-protein kinase PAK 1 (EC 2.7.11.1) (p21-activated kinase 1) (PAK-1) (p65-PAK) (Alpha-PAK). [Source:Uniprot/SWISSPROT;Acc:Q13153] PAK1
TF105402 ENSG00000099864 Paralemmin. [Source:Uniprot/SWISSPROT;Acc:O75781] PALM
TF105431 ENSG00000139970 Reticulon-1 (Neuroendocrine-specific protein). [Source:Uniprot/SWISSPROT;Acc:Q16799] RTN1
TF105463 ENSG00000138175 ADP-ribosylation factor-like protein 3. [Source:Uniprot/SWISSPROT;Acc:P36405] ARL3
TF105556 ENSG00000068971 Serine/threonine-protein phosphatase 2A 56 kDa

regulatory subunit beta isoform (PP2A, B subunit, B' beta isoform) (PP2A, B subunit, B56 beta isoform) (PP2A, B subunit, PR61 beta isoform) (PP2A, B subunit, R5 beta isoform). [Source:Uniprot/SWISSPROT;Acc:Q15173] PPP2R5B
TF105557 ENSG00000107758 Serine/threonine-protein phosphatase 2B catalytic subunit beta isoform (EC 3.1.3.16) (Calmodulin-dependent calcineurin A subunit beta isoform) (CAM-PRP catalytic subunit). [Source:Uniprot/SWISSPROT;Acc:P16298] PPP3CB
TF105932 ENSG00000151552 Dihydropteridine reductase (EC 1.5.1.34) (HDHPR) (Quinoid dihydropteridine reductase). [Source:Uniprot/SWISSPROT;Acc:P09417] QDPR
TF106341 ENSG00000136535 T-brain-1 protein (T-box brain protein 1) (TBR-1) (TES-56). [Source:Uniprot/SWISSPROT;Acc:Q16650] TBR1
TF106443 ENSG00000204371 Histone-lysine N-methyltransferase, H3 lysine-9 specific 3 (EC 2.1.1.43) (Histone H3-K9 methyltransferase 3) (H3-K9-HMTase 3) (Euchromatic histone-lysine N-methyltransferase 2) (HLA-B-associated transcript 8) (Protein G9a). [Source:Uniprot/SWISSPROT;Acc:Q96KQ7] EHMT2
TF106448 ENSG00000116254 Chromodomain helicase-DNA-binding protein 5 (EC 3.6.1.-) (ATP- dependent helicase CHD5) (CHD-5). [Source:Uniprot/SWISSPROT;Acc:Q8TDI0] CHD5
TF106456 ENSG00000100307 Chromobox protein homolog 7. [Source:Uniprot/SWISSPROT;Acc:O95931] CBX7
TF106456 ENSG00000183741 Chromobox protein homolog 6. [Source:Uniprot/SWISSPROT;Acc:O95503] NPTXR
TF106465 ENSG00000148053 BDNF/NT-3 growth factors receptor precursor (EC 2.7.10.1) (Neurotrophic tyrosine kinase receptor type 2) (TrkB tyrosine kinase) (GP145-TrkB) (Trk-B). [Source:Uniprot/SWISSPROT;Acc:Q16620]NTRK2
TF106482 ENSG00000132640 BTB/POZ domain-containing protein 3. [Source:Uniprot/SWISSPROT;Acc:Q9Y2F9] BTBD3
TF106486 ENSG00000082014 SWI/SNF-related matrix-associated actin-dependent regulator of chromatin subfamily D member 3 (60 kDa BRG-1/Brm-associated factor subunit C) (BRG1-associated factor 60C). [Source:Uniprot/SWISSPROT;Acc:Q6STE5] SMARCD3
TF106496 ENSG00000115266 adenomatosis polyposis coli 2 [Source:RefSeq_peptide;Acc:NP_005874] APC2
TF106508 ENSG00000102225 Serine/threonine-protein kinase PCTAIRE-1 (EC 2.7.11.22) (PCTAIRE- motif protein kinase 1). [Source:Uniprot/SWISSPROT;Acc:Q00536] PCTK1
TF300009 ENSG00000163032 Visinin-like protein 1 (VILIP) (Hippocalcin-like protein 3) (HLP3). [Source:Uniprot/SWISSPROT;Acc:P62760] VSNL1
TF300009 ENSG00000121905 Neuron-specific calcium-binding protein hippocalcin (Calcium-binding protein BDR-2). [Source:Uniprot/SWISSPROT;Acc:P84074] HPCA
TF300009 ENSG00000104490 Neurocalcin-delta. [Source:Uniprot/SWISSPROT;Acc:P61601] NCALD
TF300097 ENSG00000134594 Ras-related protein Rab-33A (Small GTP-binding protein S10). [Source:Uniprot/SWISSPROT;Acc:Q14088] RAB33A
TF300189 ENSG00000157823 AP-3 complex subunit sigma-2 (Adapter-related protein complex 3 sigma- 2 subunit) (Sigma-adaptin 3b) (AP-3 complex sigma-3B subunit) (Sigma- 3B-adaptin). [Source:Uniprot/SWISSPROT;Acc:P59780] AP3S2
TF300288 ENSG00000170634 Acylphosphatase-2 (EC 3.6.1.7) (Acylphosphate phosphohydrolase 2) (Acylphosphatase, muscle type isozyme). [Source:Uniprot/SWISSPROT;Acc:P14621] ACYP2
TF300304 ENSG00000101210 Elongation factor 1-alpha 2 (EF-1-alpha-2) (Elongation factor 1 A-2) (eEF1A-2) (Statin S1). [Source:Uniprot/SWISSPROT;Acc:Q05639] EEF1A2
TF300314 ENSG00000127824 Tubulin alpha-4A chain (Tubulin alpha-1 chain) (Alpha-tubulin 1) (Testis-specific alpha-tubulin) (Tubulin H2-alpha). [Source:Uniprot/SWISSPROT;Acc:P68366] TUBA4A
TF300330 ENSG00000157087 Plasma membrane calcium-transporting ATPase 2 (EC 3.6.3.8) (PMCA2) (Plasma membrane calcium pump isoform 2) (Plasma membrane calcium ATPase isoform 2). [Source:Uniprot/SWISSPROT;Acc:Q01814] ATP2B2
TF300346 ENSG00000033627 Vacuolar proton translocating ATPase 116 kDa subunit

a isoform 1 (V- ATPase 116 kDa isoform a1) (Clathrin-coated vesicle/synaptic vesicle proton pump 116 kDa subunit) (Vacuolar proton pump subunit 1) (Vacuolar adenosine triphosphatase subunit Ac116). [Source:Uniprot/SWISSPROT;Acc:Q93050] ATP6V0A1
TF300362 ENSG00000106976 Dynamin-1 (EC 3.6.5.5). [Source:Uniprot/SWISSPROT;Acc:Q05193] DNM1
TF300391 ENSG00000111674 Gamma-enolase (EC 4.2.1.11) (2-phospho-D-glycerate hydro-lyase) (Neural enolase) (Neuron-specific enolase) (NSE) (Enolase 2). [Source:Uniprot/SWISSPROT;Acc:P09104] ENO2
TF300411 ENSG00000067057 6-phosphofructokinase type C (EC 2.7.1.11) (Phosphofructokinase 1) (Phosphohexokinase) (Phosphofructo-1-kinase isozyme C) (PFK-C) (6- phosphofructokinase, platelet type). [Source:Uniprot/SWISSPROT;Acc:Q01813] PFKP
TF300415 ENSG00000168710 Putative adenosylhomocysteinase 2 (EC 3.3.1.1) (S-adenosyl-L- homocysteine hydrolase 2) (AdoHcyase 2) (S-adenosylhomocysteine hydrolase-like 1) (DC-expressed AHCY-like molecule). [Source:Uniprot/SWISSPROT;Acc:O43865] AHCYL1
TF300420 ENSG00000115073 Beta-centractin (Actin-related protein 1B) (ARP1B). [Source:Uniprot/SWISSPROT;Acc:P42025] ACTR1B
TF300423 ENSG00000088538 Dedicator of cytokinesis protein 3 (Modifier of cell adhesion) (Presenilin-binding protein) (PBP). [Source:Uniprot/SWISSPROT;Acc:Q8IZD9] DOCK3
TF300537 ENSG00000151376 NADP-dependent malic enzyme, mitochondrial precursor (EC 1.1.1.40) (NADP-ME) (Malic enzyme 3). [Source:Uniprot/SWISSPROT;Acc:Q16798] ME3
TF300553 ENSG00000158560 Cytoplasmic dynein 1 intermediate chain 1 (Dynein intermediate chain 1, cytosolic) (DH IC-1) (Cytoplasmic dynein intermediate chain 1). [Source:Uniprot/SWISSPROT;Acc:O14576] DYNC1I1
TF300590 ENSG00000054793 Probable phospholipid-transporting ATPase IIA (EC 3.6.3.1) (ATPase class II type 9A) (ATPase IIA). [Source:Uniprot/SWISSPROT;Acc:O75110] ATP9A
TF300651 ENSG00000174437 Sarcoplasmic/endoplasmic reticulum calcium ATPase 2 (EC 3.6.3.8) (Calcium pump 2) (SERCA2) (SR Ca(2+)-ATPase 2) (Calcium-transporting ATPase sarcoplasmic reticulum type, slow twitch skeletal muscle isoform) (Endoplasmic reticulum class 1/2 Ca(2+) ATPase). [Source:Uniprot/SWISSPROT;Acc:P16615] ATP2A2
TF300673 ENSG00000087258 Guanine nucleotide-binding protein G(o) subunit alpha 1. [Source:Uniprot/SWISSPROT;Acc:P09471] GNAO1
TF300803 ENSG00000175582 Ras-related protein Rab-6A (Rab-6). [Source:Uniprot/SWISSPROT;Acc:P20340] RAB6A
TF300912 ENSG00000143933 Calmodulin (CaM). [Source:Uniprot/SWISSPROT;Acc:P62158] CALM1
TF300912 ENSG00000160014 Calmodulin (CaM). [Source:Uniprot/SWISSPROT;Acc:P62158] CALM1
TF312796 ENSG00000174775 GTPase HRas precursor (Transforming protein p21) (p21ras) (H-Ras-1) (c-H-ras) (Ha-Ras). [Source:Uniprot/SWISSPROT;Acc:P01112] HRAS
TF312817 ENSG00000149091 Diacylglycerol kinase zeta (EC 2.7.1.107) (Diglyceride kinase zeta) (DGK-zeta) (DAG kinase zeta). [Source:Uniprot/SWISSPROT;Acc:Q13574] DGKZ
TF312838 ENSG00000018625 Sodium/potassium-transporting ATPase subunit alpha-2 precursor (EC 3.6.3.9) (Sodium pump subunit alpha-2) (Na(+)/K(+) ATPase alpha-2 subunit). [Source:Uniprot/SWISSPROT;Acc:P50993] ATP1A2
TF312863 ENSG00000077080 Actin-like protein 6B (53 kDa BRG1-associated factor B) (Actin-related protein Baf53b) (ArpNalpha). [Source:Uniprot/SWISSPROT;Acc:O94805] ACTL6B
TF312888 ENSG00000124920 Uncharacterized protein C11orf9 (Best macular dystrophy-related protein) (Fragment). [Source:Uniprot/SPTREMBL;Acc:Q9Y2G1] C11orf9
TF312899 ENSG00000102981 Partitioning defective 6 homolog alpha (PAR-6 alpha)

(PAR-6A) (PAR-6) (PAR6C) (Tax interaction protein 40) (TIP-40).
[Source:Uniprot/SWISSPROT;Acc:Q9NPB6] PARD6A
TF312962 ENSG00000164050 Plexin-B1 precursor (Semaphorin receptor SEP).
[Source:Uniprot/SWISSPROT;Acc:O43157] PLXNB1
TF312963 ENSG00000163618 Calcium-dependent secretion activator 1 (Calcium-dependent activator protein for secretion 1) (CAPS-1).
[Source:Uniprot/SWISSPROT;Acc:Q9ULU8] CADPS
TF312966 ENSG00000155849 Engulfment and cell motility protein 1 (CED-12 homolog). [Source:Uniprot/SWISSPROT;Acc:Q92556] ELMO1
TF313002 ENSG00000168785 Tetraspanin-5 (Tspan-5) (Transmembrane 4 superfamily member 9) (Tetraspan NET-4). [Source:Uniprot/SWISSPROT;Acc:P62079] TSPAN5
TF313013 ENSG00000110931 Calcium/calmodulin-dependent protein kinase kinase 2 (EC 2.7.11.17) (Calcium/calmodulin-dependent protein kinase kinase beta) (CaM-kinase kinase beta) (CaM-KK beta) (CaMKK beta).
[Source:Uniprot/SWISSPROT;Acc:Q96RR4] CAMKK2
TF313043 ENSG00000131982 ubiquitin-conjugating enzyme E2L 3 isoform 2
[Source:RefSeq_peptide;Acc:NP_937800] UBE2L3
TF313096 ENSG00000138834 C-jun-amino-terminal kinase-interacting protein 3 (JNK-interacting protein 3) (JIP-3) (JNK MAP kinase scaffold protein 3) (Mitogen-activated protein kinase 8-interacting protein 3).
[Source:Uniprot/SWISSPROT;Acc:Q9UPT6] MAPK8IP3
TF313103 ENSG00000026559 Potassium voltage-gated channel subfamily G member 1 (Voltage-gated potassium channel subunit Kv6.1) (kH2).
[Source:Uniprot/SWISSPROT;Acc:Q9UIX4] KCNG1
TF313128 ENSG00000149557 Fasciculation and elongation protein zeta 1 (Zygin-1) (Zygin I). [Source:Uniprot/SWISSPROT;Acc:Q99689] FEZ1
TF313149 ENSG00000105613 Microtubule-associated serine/threonine-protein kinase 1 (EC 2.7.11.1) (Syntrophin-associated serine/threonine-protein kinase).
[Source:Uniprot/SWISSPROT;Acc:Q9Y2H9] MAST1
TF313149 ENSG00000099308 Microtubule-associated serine/threonine-protein kinase 3 (EC 2.7.11.1). [Source:Uniprot/SWISSPROT;Acc:O60307] MAST3
TF313162 ENSG00000175416 Clathrin light chain B (Lcb).
[Source:Uniprot/SWISSPROT;Acc:P09497] CLTB
TF313168 ENSG00000103034 Protein NDRG4 (Brain development-related molecule 1) (Vascular smooth muscle cell-associated protein 8) (SMAP-8).
[Source:Uniprot/SWISSPROT;Acc:Q9ULP0] NDRG4
TF313209 ENSG00000115840 Calcium-binding mitochondrial carrier protein Aralar1 (Mitochondrial aspartate glutamate carrier 1) (Solute carrier family 25 member 12).
[Source:Uniprot/SWISSPROT;Acc:O75746] SLC25A12
TF313216 ENSG00000182621 1-phosphatidylinositol-4,5-bisphosphate phosphodiesterase beta 1 (EC 3.1.4.11) (Phosphoinositide phospholipase C) (Phospholipase C- beta-1) (PLC-beta-1) (PLC-I) (PLC-154).
[Source:Uniprot/SWISSPROT;Acc:Q9NQ66] PLCB1
TF313237 ENSG00000184014 Rab6-interacting protein 1 (Rab6IP1).
[Source:Uniprot/SWISSPROT;Acc:Q6IQ26] RAB6IP1
TF313240 ENSG00000168959 Metabotropic glutamate receptor 5 precursor (mGluR5).
[Source:Uniprot/SWISSPROT;Acc:P41594] GRM5
TF313240 ENSG00000198822 Metabotropic glutamate receptor 3 precursor (mGluR3).
[Source:Uniprot/SWISSPROT;Acc:Q14832] GRM3
TF313242 ENSG00000136854 Syntaxin-binding protein 1 (Unc-18 homolog) (Unc-18A) (Unc-18-1) (N- Sec1) (p67). [Source:Uniprot/SWISSPROT;Acc:P61764] STXBP1
TF313281 ENSG00000107295 SH3-containing GRB2-like protein 2 (EC 2.3.1.-) (Endophilin-1) (Endophilin-A1) (SH3 domain protein 2A) (EEN-B1).
[Source:Uniprot/SWISSPROT;Acc:Q99962] SH3GL2
TF313309 ENSG00000130226 Dipeptidyl aminopeptidase-like protein 6 (Dipeptidylpeptidase VI) (Dipeptidylpeptidase 6) (Dipeptidyl peptidase IV-like protein) (Dipeptidyl aminopeptidase-related protein) (DPPX).
[Source:Uniprot/SWISSPROT;Acc:P42658] DPP6
TF313348 ENSG00000136274 NAC-alpha domain-containing protein 1.

[Source:Uniprot/SWISSPROT;Acc:O15069] NACAD
TF313359 ENSG00000135423 Glutaminase liver isoform, mitochondrial precursor (EC 3.5.1.2) (GLS) (L-glutamine amidohydrolase) (L-glutaminase). [Source:Uniprot/SWISSPROT;Acc:Q9UI32] GLS2
TF313361 ENSG00000072134 Epsin-2 (EPS-15-interacting protein 2). [Source:Uniprot/SWISSPROT;Acc:O95208] EPN2
TF313378 ENSG00000105223 Phospholipase D3 (EC 3.1.4.4) (PLD 3) (Choline phosphatase 3) (Phosphatidylcholine-hydrolyzing phospholipase D3) (HindIII K4L homolog) (Hu-K4). [Source:Uniprot/SWISSPROT;Acc:Q8IV08] PLD3
TF313438 ENSG00000071242 Ribosomal protein S6 kinase alpha-2 (EC 2.7.11.1) (S6K-alpha 2) (90 kDa ribosomal protein S6 kinase 2) (p90-RSK 2) (Ribosomal S6 kinase 3) (RSK-3) (pp90RSK3) (MAP kinase-activated protein kinase 1c) (MAPKAPK1C). [Source:Uniprot/SWISSPROT;Acc:Q15349] RPS6KA2
TF313446 ENSG00000173898 Spectrin beta chain, brain 2 (Spectrin, non-erythroid beta chain 2) (Beta-III spectrin). [Source:Uniprot/SWISSPROT;Acc:O15020] SPTBN2
TF313535 ENSG00000104888 solute carrier family 17, member 7 [Source:RefSeq_peptide;Acc:NP_064705] SLC17A7
TF313542 ENSG00000078053 Amphiphysin. [Source:Uniprot/SWISSPROT;Acc:P49418] AMPH
TF313579 ENSG00000172348 Calcipressin-2 (Thyroid hormone-responsive protein ZAKI-4) (Down syndrome candidate region 1-like 1) (Myocyte-enriched calcineurin-interacting protein 2) (MCIP2). [Source:Uniprot/SWISSPROT;Acc:Q14206] RCAN2
TF313620 ENSG00000166974 Microtubule-associated protein RP/EB family member 2 (APC-binding protein EB2) (End-binding protein 2) (EB2). [Source:Uniprot/SWISSPROT;Acc:Q15555] MAPRE2
TF313630 ENSG00000033867 Sodium bicarbonate cotransporter 3 (Sodium bicarbonate cotransporter 2) (Sodium bicarbonate cotransporter 2b) (Bicarbonate transporter) (Solute carrier family 4 member 7). [Source:Uniprot/SWISSPROT;Acc:Q9Y6M7] SLC4A7
TF313657 ENSG00000124140 Solute carrier family 12 member 5 (Electroneutral potassium-chloride cotransporter 2) (Erythroid K-Cl cotransporter 2) (Neuronal K-Cl cotransporter) (hKCC2). [Source:Uniprot/SWISSPROT;Acc:Q9H2X9] SLC12A5
TF313676 ENSG00000168135 Inward rectifier potassium channel 4 (Potassium channel, inwardly rectifying subfamily J member 4) (Inward rectifier K(+) channel Kir2.3) (Hippocampal inward rectifier) (HIR) (HRK1) (HIRK2). [Source:Uniprot/SWISSPROT;Acc:P48050] KCNJ4
TF313686 ENSG00000066032 catenin, alpha 2 [Source:RefSeq_peptide;Acc:NP_004380] CTNNA2
TF313747 ENSG00000106992 Adenylate kinase isoenzyme 1 (EC 2.7.4.3) (ATP-AMP transphosphorylase) (AK1) (Myokinase). [Source:Uniprot/SWISSPROT;Acc:P00568] AK1
TF313754 ENSG00000180901 BTB/POZ domain-containing protein KCTD2. [Source:Uniprot/SWISSPROT;Acc:Q14681] KCTD2
TF313763 ENSG00000106089 Syntaxin-1A (Neuron-specific antigen HPC-1). [Source:Uniprot/SWISSPROT;Acc:Q16623] STX1A
TF313791 ENSG00000112186 Adenylyl cyclase-associated protein 2 (CAP 2). [Source:Uniprot/SWISSPROT;Acc:P40123] CAP2
TF313845 ENSG00000164742 Adenylate cyclase type 1 (EC 4.6.1.1) (Adenylate cyclase type I) (ATP pyrophosphate-lyase 1) (Adenylyl cyclase 1) (Ca(2+)/calmodulin- activated adenylyl cyclase). [Source:Uniprot/SWISSPROT;Acc:Q08828] ADCY1
TF313867 ENSG00000073464 Chloride channel protein 4 (ClC-4). [Source:Uniprot/SWISSPROT;Acc:P51793] CLCN4
TF313877 ENSG00000164398 Long-chain-fatty-acid--CoA ligase 6 (EC 6.2.1.3) (Long-chain acyl-CoA synthetase 6) (LACS 6). [Source:Uniprot/SWISSPROT;Acc:Q9UKU0] ACSL6
TF313921 ENSG00000144834 Transgelin-3 (Neuronal protein NP25) (Neuronal protein 22) (NP22). [Source:Uniprot/SWISSPROT;Acc:Q9UI15] TAGLN3
TF313965 ENSG00000136928 Gamma-aminobutyric acid type B receptor, subunit 2 precursor (GABA-B receptor 2) (GABA-B-R2) (Gb2) (GABABR2) (G-protein coupled

receptor 51) (HG20). [Source:Uniprot/SWISSPROT;Acc:O75899] GABBR2
TF313988 ENSG00000103184 SEC14-like 5 [Source:RefSeq_peptide;Acc:NP_055507] SEC14L5
TF314000 ENSG00000168000 Seipin (Bernardinelli-Seip congenital lipodystrophy type 2 protein). [Source:Uniprot/SWISSPROT;Acc:Q96G97] BSCL2
TF314013 ENSG00000007264 Megakaryocyte-associated tyrosine-protein kinase (EC 2.7.10.2) (Tyrosine-protein kinase CTK) (Protein kinase HYL) (Hematopoietic consensus tyrosine-lacking kinase). [Source:Uniprot/SWISSPROT;Acc:P42679] MATK
TF314066 ENSG00000176095 Inositol hexaphosphate kinase 1 (EC 2.7.4.21) (InsP6 kinase 1) (Inositol hexakisphosphate kinase 1). [Source:Uniprot/SWISSPROT;Acc:Q92551] IHPK1
TF314067 ENSG00000081189 Myocyte-specific enhancer factor 2C. [Source:Uniprot/SWISSPROT;Acc:Q06413] MEF2C
TF314089 ENSG00000120053 Aspartate aminotransferase, cytoplasmic (EC 2.6.1.1) (Transaminase A) (Glutamate oxaloacetate transaminase 1). [Source:Uniprot/SWISSPROT;Acc:P17174] GOT1
TF314160 ENSG00000198792 Putative MAP kinase-activating protein C22orf5 (Putative MAPK- activating protein FM08). [Source:Uniprot/SWISSPROT;Acc:Q9Y519] C22orf5
TF314166 ENSG00000008118 Calcium/calmodulin-dependent protein kinase type 1G (EC 2.7.11.17) (CaM kinase IG) (CaM kinase I gamma) (CaMKI gamma) (CaMKI-gamma) (CaM- KI gamma) (CaMKIG) (CaMK-like CREB kinase III) (CLICK III). [Source:Uniprot/SWISSPROT;Acc:Q96NX5] CAMK1G
TF314177 ENSG00000132563 Receptor expression-enhancing protein 2. [Source:Uniprot/SWISSPROT;Acc:Q9BRK0] REEP2
TF314207 ENSG00000143847 Liprin-alpha-4 (Protein tyrosine phosphatase receptor type f polypeptide-interacting protein alpha-4) (PTPRF-interacting protein alpha-4). [Source:Uniprot/SWISSPROT;Acc:O75335] PPFIA4
TF314214 ENSG00000168775 Creatine kinase, ubiquitous mitochondrial precursor (EC 2.7.3.2) (U- MtCK) (Mia-CK) (Acidic-type mitochondrial creatine kinase). [Source:Uniprot/SWISSPROT;Acc:P12532] CKMT1B
TF314232 ENSG00000138101 Dystrobrevin beta (Beta-dystrobrevin) (DTN-B). [Source:Uniprot/SWISSPROT;Acc:O60941] DTNB
TF314280 ENSG00000103647 Coronin-2B (Coronin-like protein C) (Clipin-C) (Protein FC96). [Source:Uniprot/SWISSPROT;Acc:Q9UQ03] CORO2B
TF314308 ENSG00000118160 Sodium/calcium exchanger 2 precursor (Na(+)/Ca(2+)-exchange protein 2). [Source:Uniprot/SWISSPROT;Acc:Q9UPR5] SLC8A2
TF314331 ENSG00000166313 Amyloid beta A4 precursor protein-binding family B member 1 (Fe65 protein). [Source:Uniprot/SWISSPROT;Acc:O00213] APBB1
TF314349 ENSG00000186310 Nucleosome assembly protein 1-like 3. [Source:Uniprot/SWISSPROT;Acc:Q99457] NAP1L3
TF314368 ENSG00000107954 Neuralized-like protein 1 (h-neuralized 1) (h-neu) (RING finger protein 67). [Source:Uniprot/SWISSPROT;Acc:O76050] NEURL
TF314440 ENSG00000171368 Tubulin polymerization-promoting protein (TPPP) (25 kDa brain-specific protein) (p25-alpha) (p24) (p25). [Source:Uniprot/SWISSPROT;Acc:O94811] TPPP
TF314482 ENSG00000089818 Adaptin ear-binding coat-associated protein 1 (NECAP-1). [Source:Uniprot/SWISSPROT;Acc:Q8NC96] NECAP1
TF314485 ENSG00000168490 Phytanoyl-CoA hydroxylase-interacting protein (Phytanoyl-CoA hydroxylase-associated protein 1) (PAHXAP1) (PAHX-AP1). [Source:Uniprot/SWISSPROT;Acc:Q92561] PHYHIP
TF314539 ENSG00000117408 Importin-13 (Imp13) (Ran-binding protein 13) (RanBP13) (Karyopherin 13) (Kap13). [Source:Uniprot/SWISSPROT;Acc:O94829] IPO13
TF314602 ENSG00000146122 Disheveled-associated activator of morphogenesis 2. [Source:Uniprot/SWISSPROT;Acc:Q86T65] DAAM2
TF314605 ENSG00000103723 AP-3 complex subunit beta-2 (Adapter-related protein complex 3 beta-2 subunit) (Beta3B-adaptin) (Adaptor protein complex AP-3 beta-2 subunit) (AP-3 complex beta-2 subunit) (Clathrin assembly protein complex 3 beta-2 large chain) (Neuron-specific vesicle c [Source:Uniprot/SWISSPROT;Acc:Q13367]

AP3B2
TF314618 ENSG00000129244 Sodium/potassium-transporting ATPase subunit beta-2 (Sodium/potassium- dependent ATPase beta-2 subunit). [Source:Uniprot/SWISSPROT;Acc:P14415] ATP1B2
TF314677 ENSG00000171914 Talin-2. [Source:Uniprot/SWISSPROT;Acc:Q9Y4G6] TLN2
TF314688 ENSG00000136750 Glutamate decarboxylase 2 (EC 4.1.1.15) (Glutamate decarboxylase 65 kDa isoform) (GAD-65) (65 kDa glutamic acid decarboxylase). [Source:Uniprot/SWISSPROT;Acc:Q05329] GAD2
TF314700 ENSG00000111110 Protein phosphatase 1H (EC 3.1.3.16). [Source:Uniprot/SWISSPROT;Acc:Q9ULR3] PPM1H
TF314706 ENSG00000072832 Dihydropyrimidinase-related protein 1 (DRP-1) (Collapsin response mediator protein 1) (CRMP-1). [Source:Uniprot/SWISSPROT;Acc:Q14194] CRMP1
TF314710 ENSG00000139613 SWI/SNF-related matrix-associated actin-dependent regulator of chromatin subfamily C member 2 (SWI/SNF complex 170 kDa subunit) (BRG1-associated factor 170). [Source:Uniprot/SWISSPROT;Acc:Q8TAQ2] SMARCC2
TF314718 ENSG00000128989 cAMP-regulated phosphoprotein 19 (ARPP-19). [Source:Uniprot/SWISSPROT;Acc:P56211] ARPP-19
TF314724 ENSG00000135503 Activin receptor type 1B precursor (EC 2.7.11.30) (ACTR-IB) (Serine/threonine-protein kinase receptor R2) (SKR2) (Activin receptor-like kinase 4) (ALK-4). [Source:Uniprot/SWISSPROT;Acc:P36896] ACVR1B
TF314861 ENSG00000065609 Clathrin coat assembly protein AP180 (Clathrin coat-associated protein AP180) (91 kDa synaptosomal-associated protein). [Source:Uniprot/SWISSPROT;Acc:O60641] SNAP91
TF314878 ENSG00000112531 Quaking protein (Hqk). [Source:Uniprot/SWISSPROT;Acc:Q96PU8] QKI
TF314920 ENSG00000188191 cAMP-dependent protein kinase type I-beta regulatory subunit. [Source:Uniprot/SWISSPROT;Acc:P31321] PRKAR1B
TF314970 ENSG00000171703 Transcription elongation factor A protein 2 (Transcription elongation factor S-II protein 2) (Testis-specific S-II) (Transcription elongation factor TFIIS.l). [Source:Uniprot/SWISSPROT;Acc:Q15560] TCEA2
TF315029 ENSG00000197586 Ectonucleoside triphosphate diphosphohydrolase 6 (EC 3.6.1.6) (NTPDase 6) (CD39 antigen-like 2). [Source:Uniprot/SWISSPROT;Acc:O75354] ENTPD6
TF315031 ENSG00000132970 Wiskott-Aldrich syndrome protein family member 3 (WASP-family protein member 3) (Protein WAVE-3) (Verprolin homology domain-containing protein 3). [Source:Uniprot/SWISSPROT;Acc:Q9UPY6] WASF3
TF315044 ENSG00000139197 Peroxisomal targeting signal 1 receptor (Peroxisome receptor 1) (Peroxisomal C-terminal targeting signal import receptor) (PTS1-BP) (Peroxin-5) (PTS1 receptor). [Source:Uniprot/SWISSPROT;Acc:P50542] PEX5
TF315125 ENSG00000132639 Synaptosomal-associated protein 25 (SNAP-25) (Synaptosomal-associated 25 kDa protein) (Super protein) (SUP). [Source:Uniprot/SWISSPROT;Acc:P60880] SNAP25
TF315162 ENSG00000046653 Neuronal membrane glycoprotein M6-b (M6b). [Source:Uniprot/SWISSPROT;Acc:Q13491] GPM6B
TF315186 ENSG00000075043 Potassium voltage-gated channel subfamily KQT member 2 (Voltage-gated potassium channel subunit Kv7.2) (Neuroblastoma-specific potassium channel subunit alpha KvLQT2) (KQT-like 2). [Source:Uniprot/SWISSPROT;Acc:O43526] KCNQ2
TF315206 ENSG00000079215 Excitatory amino acid transporter 1 (Solute carrier family 1 member 3) (Sodium-dependent glutamate/aspartate transporter 1) (GLAST-1). [Source:Uniprot/SWISSPROT;Acc:P43003] SLC1A3
TF315206 ENSG00000110436 Excitatory amino acid transporter 2 (Solute carrier family 1 member 2) (Sodium-dependent glutamate/aspartate transporter 2) (Glutamate/aspartate transporter II). [Source:Uniprot/SWISSPROT;Acc:P43004] SLC1A2
TF315213 ENSG00000170145 Serine/threonine-protein kinase SNF1-like kinase 2 (EC 2.7.11.1) (Qin- induced kinase) (SIK2). [Source:Uniprot/SWISSPROT;Acc:Q9H0K1] SNF1LK2

TF315229 ENSG00000070808 Calcium/calmodulin-dependent protein kinase type II alpha chain (EC 2.7.11.17) (CaM-kinase II alpha chain) (CaM kinase II alpha subunit) (CaMK-II subunit alpha). [Source:Uniprot/SWISSPROT;Acc:Q9UQM7] CAMK2A
TF315229 ENSG00000058404 Calcium/calmodulin-dependent protein kinase type II beta chain (EC 2.7.11.17) (CaM-kinase II beta chain) (CaM kinase II subunit beta) (CaMK-II subunit beta). [Source:Uniprot/SWISSPROT;Acc:Q13554] CAMK2B
TF315232 ENSG00000120251 Glutamate receptor 2 precursor (GluR-2) (GluR-B) (GluR-K2) (Glutamate receptor ionotropic, AMPA 2) (AMPA-selective glutamate receptor 2). [Source:Uniprot/SWISSPROT;Acc:P42262] GRIA2
TF315245 ENSG00000034053 Amyloid beta A4 precursor protein-binding family A member 2 (Neuron- specific X11L protein) (Neuronal Munc18-1-interacting protein 2) (Mint-2) (Adapter protein X11beta). [Source:Uniprot/SWISSPROT;Acc:Q99767] APBA2
TF315442 ENSG00000106689 LIM/homeobox protein Lhx2 (Homeobox protein LH-2). [Source:Uniprot/SWISSPROT;Acc:P50458] LHX2
TF315442 ENSG00000143013 LIM domain transcription factor LMO4 (LIM domain only protein 4) (LMO- 4) (Breast tumor autoantigen). [Source:Uniprot/SWISSPROT;Acc:P61968] LMO4
TF315453 ENSG00000113327 Gamma-aminobutyric-acid receptor subunit gamma-2 precursor (GABA(A) receptor subunit gamma-2). [Source:Uniprot/SWISSPROT;Acc:P18507] GABRG2
TF315453 ENSG00000186297 Gamma-aminobutyric-acid receptor subunit alpha-5 precursor (GABA(A) receptor subunit alpha-5). [Source:Uniprot/SWISSPROT;Acc:P31644] GABRA5
TF315453 ENSG00000151834 Gamma-aminobutyric-acid receptor subunit alpha-2 precursor (GABA(A) receptor subunit alpha-2). [Source:Uniprot/SWISSPROT;Acc:P47869] GABRA2
TF315453 ENSG00000163288 Gamma-aminobutyric-acid receptor subunit beta-1 precursor (GABA(A) receptor subunit beta-1). [Source:Uniprot/SWISSPROT;Acc:P18505] GABRB1
TF315453 ENSG00000187730 Gamma-aminobutyric-acid receptor subunit delta precursor (GABA(A) receptor subunit delta). [Source:Uniprot/SWISSPROT;Acc:O14764] GABRD
TF315453 ENSG00000109738 Glycine receptor subunit beta precursor (Glycine receptor 58 kDa subunit). [Source:Uniprot/SWISSPROT;Acc:P48167] GLRB
TF315495 ENSG00000108947 Ephrin-B3 precursor (EPH-related receptor tyrosine kinase ligand 8) (LERK-8) (EPH-related receptor transmembrane ligand ELK-L3). [Source:Uniprot/SWISSPROT;Acc:Q15768] EFNB3
TF315506 ENSG00000136826 Krueppel-like factor 4 (Epithelial zinc finger protein EZF) (Gut- enriched krueppel-like factor). [Source:Uniprot/SWISSPROT;Acc:O43474] KLF4
TF315526 ENSG00000007516 BAI1-associated protein 3 (BAP3). [Source:Uniprot/SWISSPROT;Acc:O94812] BAIAP3
TF315600 ENSG00000132718 Synaptotagmin-11 (Synaptotagmin XI) (SytXI). [Source:Uniprot/SWISSPROT;Acc:Q9BT88] SYT11
TF315600 ENSG00000129990 Synaptotagmin-5 (Synaptotagmin V) (SytV). [Source:Uniprot/SWISSPROT;Acc:O00445] SYT5
TF315600 ENSG00000067715 Synaptotagmin-1 (Synaptotagmin I) (SytI) (p65). [Source:Uniprot/SWISSPROT;Acc:P21579] SYT1
TF315605 ENSG00000175344 Neuronal acetylcholine receptor subunit alpha-7 precursor. [Source:Uniprot/SWISSPROT;Acc:P36544] CHRNA7
TF315608 ENSG00000116106 Ephrin type-A receptor 4 precursor (EC 2.7.10.1) (Tyrosine-protein kinase receptor SEK) (Receptor protein-tyrosine kinase HEK8) (Tyrosine-protein kinase TYRO1). [Source:Uniprot/SWISSPROT;Acc:P54764] EPHA4
TF315608 ENSG00000106123 Ephrin type-B receptor 6 precursor (Tyrosine-protein kinase-defective receptor EPH-6) (HEP). [Source:Uniprot/SWISSPROT;Acc:O15197] EPHB6
TF315654 ENSG00000123933 Max-interacting transcriptional repressor MAD4 (Max-associated protein 4) (MAX dimerization protein 4). [Source:Uniprot/SWISSPROT;Acc:Q14582] MXD4

TF315804 ENSG00000102003 Synaptophysin (Major synaptic vesicle protein p38). [Source:Uniprot/SWISSPROT;Acc:P08247] SYP
TF315834 ENSG00000129473 Apoptosis regulator Bcl-W (Bcl-2-like 2 protein). [Source:Uniprot/SWISSPROT;Acc:Q92843] BCL2L2
TF315837 ENSG00000117152 Regulator of G-protein signaling 4 (RGS4) (RGP4). [Source:Uniprot/SWISSPROT;Acc:P49798] RGS4
TF315869 ENSG00000105516 D site-binding protein (Albumin D box-binding protein) (Albumin D- element-binding protein) (TAXREB302). [Source:Uniprot/SWISSPROT;Acc:Q10586] DBP
TF315892 ENSG00000196220 SLIT-ROBO Rho GTPase-activating protein 3 (srGAP3) (srGAP2) (WAVE- associated Rac GTPase-activating protein) (WRP) (Mental disorder-associated GAP) (Rho GTPase-activating protein 14). [Source:Uniprot/SWISSPROT;Acc:O43295] SRGAP3
TF315946 ENSG00000139182 Calsyntenin-3 precursor. [Source:Uniprot/SWISSPROT;Acc:Q9BQT9] CLSTN3
TF315964 ENSG00000130558 Noelin precursor (Neuronal olfactomedin-related ER localized protein) (Olfactomedin-1). [Source:Uniprot/SWISSPROT;Acc:Q99784] OLFM1
TF315993 ENSG00000081913 PH domain leucine-rich repeat-containing protein phosphatase (EC 3.1.3.16) (PH domain leucine-rich repeat protein phosphatase) (Pleckstrin homology domain-containing family E protein 1) (Suprachiasmatic nucleus circadian oscillatory protein) (hSCOP). [Source:Uniprot/SWISSPROT;Acc:O60346] PHLPP
TF316040 ENSG00000117600 plasticity related gene 1 [Source:RefSeq_peptide;Acc:NP_055654] LPPR4
TF316118 ENSG00000043143 Protein Jade-2 (PHD finger protein 15). [Source:Uniprot/SWISSPROT;Acc:Q9NQC1] PHF15
TF316127 ENSG00000176165 Forkhead box protein G1B (Forkhead-related protein FKHL1) (Transcription factor BF-1) (Brain factor 1) (BF1) (HFK1). [Source:Uniprot/SWISSPROT;Acc:P55315] FOXG1
TF316166 ENSG00000154277 Ubiquitin carboxyl-terminal hydrolase isozyme L1 (EC 3.4.19.12) (EC 6.-.-.-) (UCH-L1) (Ubiquitin thioesterase L1) (Neuron cytoplasmic protein 9.5) (PGP 9.5) (PGP9.5). [Source:Uniprot/SWISSPROT;Acc:P09936] UCHL1
TF316174 ENSG00000102934 Plasmolipin (Plasma membrane proteolipid). [Source:Uniprot/SWISSPROT;Acc:Q9Y342] PLLP
TF316174 ENSG00000172005 Myelin and lymphocyte protein (T-lymphocyte maturation-associated protein). [Source:Uniprot/SWISSPROT;Acc:P21145] MAL
TF316195 ENSG00000067191 Voltage-dependent L-type calcium channel subunit beta-1 (CAB1) (Calcium channel voltage-dependent subunit beta 1). [Source:Uniprot/SWISSPROT;Acc:Q02641] CACNB1
TF316195 ENSG00000167535 Voltage-dependent L-type calcium channel subunit beta-3 (CAB3) (Calcium channel voltage-dependent subunit beta 3). [Source:Uniprot/SWISSPROT;Acc:P54284] CACNB3
TF316214 ENSG00000157557 Protein C-ets-2. [Source:Uniprot/SWISSPROT;Acc:P15036] ETS2
TF316214 ENSG00000171656 ETS translocation variant 5 (Ets-related protein ERM). [Source:Uniprot/SWISSPROT;Acc:P41161] ETV5
TF316230 ENSG00000060709 RIM-binding protein 2 (RIM-BP2). [Source:Uniprot/SWISSPROT;Acc:O15034] RIMBP2
TF316304 ENSG00000112033 Peroxisome proliferator-activated receptor delta (PPAR-delta) (PPAR- beta) (Nuclear hormone receptor 1) (NUC1) (NUCI). [Source:Uniprot/SWISSPROT;Acc:Q03181] PPARD
TF316307 ENSG00000068078 Fibroblast growth factor receptor 3 precursor (EC 2.7.10.1) (FGFR-3) (CD333 antigen). [Source:Uniprot/SWISSPROT;Acc:P22607] FGFR3
TF316345 ENSG00000156298 Tetraspanin-7 (Tspan-7) (Transmembrane 4 superfamily member 2) (Cell surface glycoprotein A15) (T-cell acute lymphoblastic leukemia-associated antigen 1) (TALLA-1) (Membrane component, X chromosome, surface marker 1) (CD231 antigen). [Source:Uniprot/SWISSPROT;Acc:P41732] TSPAN7
TF316348 ENSG00000172461 Alpha-(1,3)-fucosyltransferase (EC 2.4.1.-) (Galactoside 3-L- fucosyltransferase) (Fucosyltransferase 9) (FucT-IX).

[Source:Uniprot/SWISSPROT;Acc:Q9Y231]	FUT9
TF316358	ENSG00000186868	Microtubule-associated protein tau (Neurofibrillary tangle protein) (Paired helical filament-tau) (PHF-tau). [Source:Uniprot/SWISSPROT;Acc:P10636]	MAPT
TF316803	ENSG00000101445	Protein phosphatase 1 regulatory inhibitor subunit 16B (TGF-beta- inhibited membrane-associated protein) (hTIMAP) (CAAX box protein TIMAP) (Ankyrin repeat domain protein 4). [Source:Uniprot/SWISSPROT;Acc:Q96T49]	PPP1R16B
TF316832	ENSG00000131089	Rho guanine nucleotide exchange factor 9 (Rac/Cdc42 guanine nucleotide exchange factor 9) (Collybistin) (PEM-2 homolog). [Source:Uniprot/SWISSPROT;Acc:O43307]	ARHGEF9
TF316981	ENSG00000139910	RNA-binding protein Nova-1 (Neuro-oncological ventral antigen 1) (Onconeural ventral antigen 1) (Paraneoplastic Ri antigen) (Ventral neuron-specific protein 1). [Source:Uniprot/SWISSPROT;Acc:P51513]	NOVA1
TF317226	ENSG00000198929	Carboxyl-terminal PDZ ligand of neuronal nitric oxide synthase protein (C--terminal PDZ ligand of neuronal nitric oxide synthase protein) (Nitric oxide synthase 1 adaptor protein). [Source:Uniprot/SWISSPROT;Acc:O75052]	NOS1AP
TF317274	ENSG00000105290	Amyloid-like protein 1 precursor (APLP) (APLP-1) [Contains: C30]. [Source:Uniprot/SWISSPROT;Acc:P51693]	APLP1
TF317299	ENSG00000186487	Myelin transcription factor 1-like protein (MyT1L protein) (MyT1-L). [Source:Uniprot/SWISSPROT;Acc:Q9UL68]	MYT1L
TF317402	ENSG00000092445	Tyrosine-protein kinase receptor TYRO3 precursor (EC 2.7.10.1) (Tyrosine-protein kinase RSE) (Tyrosine-protein kinase SKY) (Tyrosine-protein kinase DTK) (Protein-tyrosine kinase byk). [Source:Uniprot/SWISSPROT;Acc:Q06418]	TYRO3
TF317561	ENSG00000089693	Myeloid leukemia factor 2 (Myelodysplasia-myeloid leukemia factor 2). [Source:Uniprot/SWISSPROT;Acc:Q15773]	MLF2
TF317762	ENSG00000157985	Centaurin-gamma 2 (ARF-GAP with GTP-binding protein-like, ankyrin repeat and pleckstrin homology domains 1) (AGAP-1) (GTP-binding and GTPase-activating protein 1) (GGAP1). [Source:Uniprot/SWISSPROT;Acc:Q9UPQ3]	CENTG2
TF317762	ENSG00000135439	Centaurin-gamma 1 (ARF-GAP with GTP-binding protein-like, ankyrin repeat and pleckstrin homology domains 2) (AGAP-2) (Phosphatidylinositol-3-kinase enhancer) (PIKE) (GTP-binding and GTPase-activating protein 2) (GGAP2). [Source:Uniprot/SWISSPROT;Acc:Q99490]	CENTG1
TF317805	ENSG00000113578	Heparin-binding growth factor 1 precursor (HBGF-1) (Acidic fibroblast growth factor) (aFGF) (Beta-endothelial cell growth factor) (ECGF- beta). [Source:Uniprot/SWISSPROT;Acc:P05230]	FGF1
TF317932	ENSG00000101400	Alpha-1-syntrophin (59 kDa dystrophin-associated protein A1 acidic component 1) (Pro-TGF-alpha cytoplasmic domain-interacting protein 1) (TACIP1) (Syntrophin 1). [Source:Uniprot/SWISSPROT;Acc:Q13424]	SNTA1
TF318042	ENSG00000158856	Dematin (Erythrocyte membrane protein band 4.9). [Source:Uniprot/SWISSPROT;Acc:Q08495]	EPB49
TF318080	ENSG00000126217	Guanine nucleotide exchange factor DBS (DBL's big sister) (MCF2- transforming sequence-like protein). [Source:Uniprot/SWISSPROT;Acc:O15068]	MCF2L
TF318080	ENSG00000160145	Kalirin (Huntingtin-associated protein-interacting protein) (Protein Duo). [Source:Uniprot/SWISSPROT;Acc:O60229]	KALRN
TF318131	ENSG00000119866	B-cell lymphoma/leukemia 11A (B-cell CLL/lymphoma 11A) (COUP-TF- interacting protein 1) (Ecotropic viral integration site 9 protein homolog) (EVI-9). [Source:Uniprot/SWISSPROT;Acc:Q9H165]	BCL11A
TF318206	ENSG00000111249	Homeobox protein cut-like 2 (Homeobox protein Cux-2) (Cut-like 2). [Source:Uniprot/SWISSPROT;Acc:O14529]	CUTL2
TF318374	ENSG00000130956	Intracellular hyaluronan-binding protein 4 (IHABP4) (Ki-1/57 intracellular antigen). [Source:Uniprot/SWISSPROT;Acc:Q5JVS0] HABP4
TF318583	ENSG00000100241	Myotubularin-related protein 5 (SET-binding factor 1) (Sbf1). [Source:Uniprot/SWISSPROT;Acc:O95248]	SBF1
TF318626	ENSG00000197555	Signal-induced proliferation-associated 1-like protein 1 (High-risk human papilloma viruses E6 oncoproteins targeted protein 1)

(E6- targeted protein 1). [Source:Uniprot/SWISSPROT;Acc:O43166] SIPA1L1
TF318626 ENSG00000076864 Rap1 GTPase-activating protein 1 (Rap1GAP). [Source:Uniprot/SWISSPROT;Acc:P47736] RAP1GAP
TF318755 ENSG00000198689 Sodium/hydrogen exchanger 6 (Na(+)/H(+) exchanger 6) (NHE-6) (Solute carrier family 9 member 6). [Source:Uniprot/SWISSPROT;Acc:Q92581] SLC9A6
TF318770 ENSG00000133083 Serine/threonine-protein kinase DCLK1 (EC 2.7.11.1) (Doublecortin-like and CAM kinase-like 1) (Doublecortin-like kinase 1). [Source:Uniprot/SWISSPROT;Acc:O15075] DCLK1
TF318837 ENSG00000166925 TSC22 domain family protein 4 (TSC22-related-inducible leucine zipper protein 2) (Tsc-22-like protein THG-1). [Source:Uniprot/SWISSPROT;Acc:Q9Y3Q8] TSC22D4
TF318971 ENSG00000011332 Zinc finger protein neuro-d4 (D4, zinc and double PHD fingers family 1). [Source:Uniprot/SWISSPROT;Acc:Q92782] DPF1
TF318980 ENSG00000179388 Early growth response protein 3 (EGR-3) (Zinc finger protein pilot). [Source:Uniprot/SWISSPROT;Acc:Q06889] EGR3
TF319168 ENSG00000133424 Glycosyltransferase-like protein LARGE1 (EC 2.4.-.-) (Acetylglucosaminyltransferase-like 1A). [Source:Uniprot/SWISSPROT;Acc:O95461] LARGE
TF319618 ENSG00000186111 Phosphatidylinositol-4-phosphate 5-kinase type-1 gamma (EC 2.7.1.68) (Phosphatidylinositol-4-phosphate 5-kinase type I gamma) (PtdIns(4)P- 5-kinase gamma) (PtdInsPKIgamma) (PIP5KIgamma). [Source:Uniprot/SWISSPROT;Acc:O60331] PIP5K1C
TF319755 ENSG00000059915 pleckstrin and Sec7 domain containing [Source:RefSeq_peptide;Acc:NP_002770] PSD
TF319919 ENSG00000157152 Synapsin-2 (Synapsin II). [Source:Uniprot/SWISSPROT;Acc:Q92777] SYN2
TF319923 ENSG00000169744 LIM domain-binding protein 2 (Carboxyl-terminal LIM domain-binding protein 1) (CLIM-1) (LIM domain-binding factor CLIM1). [Source:Uniprot/SWISSPROT;Acc:O43679] LDB2
TF319983 ENSG00000172379 Aryl hydrocarbon receptor nuclear translocator 2 (ARNT protein 2). [Source:Uniprot/SWISSPROT;Acc:Q9HBZ2] ARNT2
TF319996 ENSG00000135472 Fas apoptotic inhibitory molecule 2 (Lifeguard protein) (Transmembrane BAX inhibitor motif-containing protein 2). [Source:Uniprot/SWISSPROT;Acc:Q9BWQ8] FAIM2
TF320243 ENSG00000175602 Delta-interacting protein A (Hepatitis delta antigen-interacting protein A) (Coiled-coil domain-containing protein 85B). [Source:Uniprot/SWISSPROT;Acc:Q15834] CCDC85B
TF320619 ENSG00000132613 Actin-bundling protein with BAIAP2 homology. [Source:Uniprot/SPTREMBL;Acc:Q765P7] ABBA-1
TF320736 ENSG00000115468 EF-hand domain-containing protein 1 (Swiprosin-2). [Source:Uniprot/SWISSPROT;Acc:Q9BUP0] EFHD1
TF320995 ENSG00000127561 Synaptogyrin-3. [Source:Uniprot/SWISSPROT;Acc:O43761] SYNGR3
TF321302 ENSG00000110076 Neurexin-2-alpha precursor (Neurexin II-alpha). [Source:Uniprot/SWISSPROT;Acc:Q9P2S2] NRXN2
TF321410 ENSG00000167191 G-protein coupled receptor family C group 5 member B precursor (Retinoic acid-induced gene 2 protein) (RAIG-2) (A-69G12.1). [Source:Uniprot/SWISSPROT;Acc:Q9NZH0] GPRC5B
TF321703 ENSG00000117016 Regulating synaptic membrane exocytosis protein 3 (Nim3) (Rab-3- interacting molecule 3) (RIM 3) (RIM3 gamma). [Source:Uniprot/SWISSPROT;Acc:Q9UJD0] RIMS3
TF321745 ENSG00000130540 Sulfotransferase 4A1 (EC 2.8.2.-) (Brain sulfotransferase-like protein) (hBR-STL) (hBR-STL-1) (Nervous system sulfotransferase) (NST). [Source:Uniprot/SWISSPROT;Acc:Q9BR01] SULT4A1
TF321796 ENSG00000184144 Contactin-2 precursor (Axonin-1) (Axonal glycoprotein TAG-1) (Transient axonal glycoprotein 1) (TAX-1). [Source:Uniprot/SWISSPROT;Acc:Q02246] CNTN2
TF321796 ENSG00000163531 Neurofascin precursor.

[Source:Uniprot/SWISSPROT;Acc:O94856] NFASC
TF321823 ENSG00000174469 Contactin-associated protein-like 2 precursor (Cell recognition molecule Caspr2). [Source:Uniprot/SWISSPROT;Acc:Q9UHC6] CNTNAP2
TF321877 ENSG00000169862 Catenin delta-2 (Delta-catenin) (Neural plakophilin-related ARM-repeat protein) (NPRAP) (Neurojungin) (GT24). [Source:Uniprot/SWISSPROT;Acc:Q9UQB3] CTNND2
TF322733 ENSG00000205927 Oligodendrocyte transcription factor 2 (Oligo2) (Class B basic helix- loop-helix protein 1) (bHLHB1) (Protein kinase C-binding protein RACK17) (Protein kinase C-binding protein 2). [Source:Uniprot/SWISSPROT;Acc:Q13516] OLIG2
TF323183 ENSG00000155827 E3 ubiquitin-protein ligase BRE1A (EC 6.3.2.-) (BRE1-A) (hBRE1) (RING finger protein 20). [Source:Uniprot/SWISSPROT;Acc:Q5VTR2] RNF20
TF323230 ENSG00000141542 Ras-related protein Rab-40B (SOCS box-containing protein RAR) (Rar protein). [Source:Uniprot/SWISSPROT;Acc:Q12829] RAB40B
TF323325 ENSG00000165973 Protein kinase C-binding protein NELL1 precursor (NEL-like protein 1) (Nel-related protein 1). [Source:Uniprot/SWISSPROT;Acc:Q92832] NELL1
TF323326 ENSG00000151967 Schwannomin-interacting protein 1 (SCHIP-1). [Source:Uniprot/SWISSPROT;Acc:Q9P0W5] SCHIP1
TF323452 ENSG00000171735 Calmodulin-binding transcription activator 1. [Source:Uniprot/SWISSPROT;Acc:Q9Y6Y1] CAMTA1
TF323754 ENSG00000185340 GAS2-like protein 1 (Growth arrest-specific 2-like 1) (GAS2-related protein on chromosome 22) (GAR22 protein). [Source:Uniprot/SWISSPROT;Acc:Q99501] GAS2L1
TF323811 ENSG00000144711 IQ motif and Sec7 domain-containing protein 1 (ADP-ribosylation factors guanine nucleotide-exchange protein 100) (ADP-ribosylation factors guanine nucleotide-exchange protein 2) (Brefeldin-resistant Arf-GEF 2 protein). [Source:Uniprot/SWISSPROT;Acc:Q6DN90] IQSEC1
TF323811 ENSG00000120645 IQ motif and Sec7 domain-containing protein 3. [Source:Uniprot/SWISSPROT;Acc:Q9UPP2] IQSEC3
TF323890 ENSG00000136193 Secernin-1. [Source:Uniprot/SWISSPROT;Acc:Q12765] SCRN1
TF323904 ENSG00000108309 RUN domain containing 3A [Source:RefSeq_peptide;Acc:NP_006686] RPIP8
TF323983 ENSG00000143126 Cadherin EGF LAG seven-pass G-type receptor 2 precursor (Epidermal growth factor-like 2) (Multiple epidermal growth factor-like domains 3) (Flamingo 1). [Source:Uniprot/SWISSPROT;Acc:Q9HCU4] CELSR2
TF323983 ENSG00000008300 Solute carrier family 26 member 6 (Pendrin-like protein 1) (Pendrin L1). [Source:Uniprot/SWISSPROT;Acc:Q9BXS9] SLC26A6
TF324129 ENSG00000133816 Protein MICAL-2. [Source:Uniprot/SWISSPROT;Acc:O94851] MICAL2
TF324563 ENSG00000069424 Voltage-gated potassium channel subunit beta-2 (K(+) channel subunit beta-2) (Kv-beta-2) (HKvbeta2). [Source:Uniprot/SWISSPROT;Acc:Q13303] KCNAB2
TF324572 ENSG00000074590 NUAK family SNF1-like kinase 1 (EC 2.7.11.1) (AMPK-related protein kinase 5). [Source:Uniprot/SWISSPROT;Acc:O60285] NUAK1
TF324824 ENSG00000159164 Synaptic vesicle glycoprotein 2A. [Source:Uniprot/SWISSPROT;Acc:Q7L0J3] SV2A
TF324824 ENSG00000185518 Synaptic vesicle glycoprotein 2B. [Source:Uniprot/SWISSPROT;Acc:Q7L1I2] SV2B
TF324994 ENSG00000101134 Docking protein 5 (Downstream of tyrosine kinase 5) (IRS6) (Protein dok-5). [Source:Uniprot/SWISSPROT;Acc:Q9P104] DOK5
TF325073 ENSG00000008735 C-jun-amino-terminal kinase-interacting protein 2 (JNK-interacting protein 2) (JIP-2) (JNK MAP kinase scaffold protein 2) (Islet-brain-2) (IB-2) (Mitogen-activated protein kinase 8-interacting protein 2). [Source:Uniprot/SWISSPROT;Acc:Q13387] MAPK8IP2
TF325296 ENSG00000163485 Adenosine A1 receptor. [Source:Uniprot/SWISSPROT;Acc:P30542] ADORA1
TF325627 ENSG00000152413 Homer protein homolog 1.

[Source:Uniprot/SWISSPROT;Acc:Q86YM7] HOMER1
TF325648 ENSG00000175866 Brain-specific angiogenesis inhibitor 1-associated protein 2 (BAI1- associated protein 2) (BAI-associated protein 2) (Protein BAP2) (Insulin receptor substrate p53) (IRSp53) (Insulin receptor substrate protein of 53 kDa) (Insulin receptor substrate p53/p5 [Source:Uniprot/SWISSPROT;Acc:Q9UQB8] BAIAP2
TF325943 ENSG00000168309 Protein FAM107A (Down-regulated in renal cell carcinoma 1) (Protein TU3A). [Source:Uniprot/SWISSPROT;Acc:O95990] FAM107A
TF326082 ENSG00000186472 Protein piccolo (Aczonin). [Source:Uniprot/SWISSPROT;Acc:Q9Y6V0] PCLO
TF326096 ENSG00000105270 CAP-Gly domain-containing linker protein 3 (Cytoplasmic linker protein 170-related 59 kDa protein) (CLIPR-59) (CLIP-170-related 59 kDa protein). [Source:Uniprot/SWISSPROT;Acc:Q96DZ5] CLIP3
TF326378 ENSG00000165300 SLIT and NTRK-like protein 5 precursor (Leucine-rich repeat-containing protein 11). [Source:Uniprot/SWISSPROT;Acc:O94991] SLITRK5
TF326495 ENSG00000074706 phosphoinositide-binding protein PIP3-E [Source:RefSeq_peptide;Acc:NP_056368] PIP3-E
TF326804 ENSG00000162706 Cell adhesion molecule 3 precursor (Immunoglobulin superfamily member 4B) (Nectin-like protein 1) (TSLC1-like protein 1) (Synaptic cell adhesion molecule 3) (Brain immunoglobulin receptor). [Source:Uniprot/SWISSPROT;Acc:Q8N126] CADM3
TF326935 ENSG00000104435 Stathmin-2 (SCG10 protein) (Superior cervical ganglion-10 protein). [Source:Uniprot/SWISSPROT;Acc:Q93045]STMN2
TF327695 ENSG00000023171 GRAM domain-containing protein 1B. [Source:Uniprot/SWISSPROT;Acc:Q3KR37] GRAMD1B
TF327980 ENSG00000006116 Voltage-dependent calcium channel gamma-3 subunit (Neuronal voltage- gated calcium channel gamma-3 subunit). [Source:Uniprot/SWISSPROT;Acc:O60359] CACNG3
TF328382 ENSG00000126351 Thyroid hormone receptor alpha (C-erbA-alpha) (c-erbA-1) (EAR-7) (EAR7). [Source:Uniprot/SWISSPROT;Acc:P10827] THRA
TF328485 ENSG00000176595 Kelch repeat and BTB domain-containing protein 11 (Kelch domain- containing protein 7B). [Source:Uniprot/SWISSPROT;Acc:O94819] KBTBD11
TF328924 ENSG00000184216 Interleukin-1 receptor-associated kinase 1 (EC 2.7.11.1) (IRAK-1). [Source:Uniprot/SWISSPROT;Acc:P51617] IRAK1
TF329288 ENSG00000100605 Inositol-tetrakisphosphate 1-kinase (EC 2.7.1.134) (Inositol- triphosphate 5/6-kinase) (EC 2.7.1.159) (Inositol 1,3,4-trisphosphate 5/6-kinase) (Ins(1,3,4)P(3) 5/6-kinase). [Source:Uniprot/SWISSPROT;Acc:Q13572] ITPK1
TF329439 ENSG00000138311 Talanin. [Source:Uniprot/SWISSPROT;Acc:Q70YC4] ZNF365
TF329492 ENSG00000165868 Heat shock 70 kDa protein 12A. [Source:Uniprot/SWISSPROT;Acc:O43301] HSPA12A
TF329721 ENSG00000211448 Type II iodothyronine deiodinase (EC 1.97.1.10) (Type-II 5' deiodinase) (DIOII) (Type 2 DI) (5DII). [Source:Uniprot/SWISSPROT;Acc:Q92813] DIO2
TF329881 ENSG00000067798 Neuron navigator 3 (Steerin-3) (Pore membrane and/or filament- interacting-like protein 1) (Unc-53 homolog 3) (unc53H3). [Source:Uniprot/SWISSPROT;Acc:Q8IVL0] LOC652725
TF330024 ENSG00000168398 B2 bradykinin receptor (BK-2 receptor) (B2R). [Source:Uniprot/SWISSPROT;Acc:P30411] BDKRB2
TF330032 ENSG00000136960 Ectonucleotide pyrophosphatase/phosphodiesterase family member 2 precursor (EC 3.1.4.39) (E-NPP 2) (Extracellular lysophospholipase D) (LysoPLD) (Autotaxin). [Source:Uniprot/SWISSPROT;Acc:Q13822] ENPP2
TF330122 ENSG00000104722 Neurofilament medium polypeptide (NF-M) (Neurofilament triplet M protein) (160 kDa neurofilament protein) (Neurofilament 3). [Source:Uniprot/SWISSPROT;Acc:P07197] NEFM
TF330122 ENSG00000104725 Neurofilament light polypeptide (NF-L) (Neurofilament triplet L protein) (68 kDa neurofilament protein). [Source:Uniprot/SWISSPROT;Acc:P07196] NEFL

TF330208 ENSG00000171246 Neuronal pentraxin-1 precursor (NP1) (Neuronal pentraxin I) (NP-I). [Source:Uniprot/SWISSPROT;Acc:Q15818] NPTX1
TF330208 ENSG00000106236 Neuronal pentraxin-2 precursor (NP2) (Neuronal pentraxin II) (NP-II). [Source:Uniprot/SWISSPROT;Acc:P47972] NPTX2
TF330663 ENSG00000108684 Amiloride-sensitive cation channel 1, neuronal (Amiloride-sensitive brain sodium channel) (Amiloride-sensitive cation channel neuronal 1) (Acid-sensing ion channel 2) (ASIC2) (Brain sodium channel 1) (BNaC1) (BNC1) (Mammalian degenerin homolog). [Source:Uniprot/SWISSPROT;Acc:Q16515] ACCN1
TF330974 ENSG00000184672 RALY RNA binding protein-like isoform 2 [Source:RefSeq_peptide;Acc:NP_001093863] LOC138046
TF331083 ENSG00000204655 myelin oligodendrocyte glycoprotein isoform alpha3 precursor [Source:RefSeq_peptide;Acc:NP_001008229] MOG
TF331128 ENSG00000054965 Uncharacterized protein KIAA0280. [Source:Uniprot/SWISSPROT;Acc:Q92567] KIAA0280
TF331292 ENSG00000170775 Probable G-protein coupled receptor 37 precursor (Endothelin B receptor-like protein 1) (ETBR-LP-1) (Parkin-associated endothelin receptor-like receptor) (PAELR). [Source:Uniprot/SWISSPROT;Acc:O15354] GPR37
TF331395 ENSG00000203618 Platelet glycoprotein Ib beta chain precursor (GP-Ib beta) (GPIbB) (GPIb-beta) (Antigen CD42b-beta) (CD42c antigen). [Source:Uniprot/SWISSPROT;Acc:P13224] GP1BB
TF331420 ENSG00000088899 Uncharacterized protein KIAA0552. [Source:Uniprot/SWISSPROT;Acc:O60299] ProSAPiP1
TF331510 ENSG00000140548 Zinc finger protein 710. [Source:Uniprot/SWISSPROT;Acc:Q8N1W2] ZNF710
TF331537 ENSG00000175182 Protein FAM131A precursor. [Source:Uniprot/SWISSPROT;Acc:Q6UXB0] FAM131A
TF331537 ENSG00000159784 Protein FAM131B. [Source:Uniprot/SWISSPROT;Acc:Q86XD5] FAM131B
TF331543 ENSG00000166780 Uncharacterized protein C16orf45. [Source:Uniprot/SWISSPROT;Acc:Q96MC5] C16orf45
TF331598 ENSG00000126500 Leucine-rich repeat transmembrane protein FLRT1 precursor (Fibronectin-like domain-containing leucine-rich transmembrane protein 1). [Source:Uniprot/SWISSPROT;Acc:Q9NZU1] FLRT1
TF331600 ENSG00000078725 Deleted in bladder cancer protein 1 precursor (Protein FAM5A). [Source:Uniprot/SWISSPROT;Acc:O60477] DBC1
TF331634 ENSG00000121753 Brain-specific angiogenesis inhibitor 2 precursor. [Source:Uniprot/SWISSPROT;Acc:O60241] BAI2
TF331647 ENSG00000168546 GDNF family receptor alpha-2 precursor (GFR-alpha-2) (Neurturin receptor alpha) (NTNR-alpha) (NRTNR-alpha) (TGF-beta-related neurotrophic factor receptor 2) (GDNF receptor beta) (GDNFR-beta) (RET ligand 2). [Source:Uniprot/SWISSPROT;Acc:O00451] GFRA2
TF331744 ENSG00000070087 Profilin-2 (Profilin II). [Source:Uniprot/SWISSPROT;Acc:P35080] PFN2
TF331824 ENSG00000178951 Zinc finger and BTB domain-containing protein 7A (Leukemia/lymphoma- related factor) (Factor that binds to inducer of short transcripts protein 1) (Factor binding IST protein 1) (FBI-1) (HIV-1 1st-binding protein 1) (TTF-I-interacting peptide 21) (TIP21). [Source:Uniprot/SWISSPROT;Acc:O95365] ZBTB7A
TF331936 ENSG00000134873 Claudin-10 (OSP-like protein). [Source:Uniprot/SWISSPROT;Acc:P78369] CLDN10
TF332034 ENSG00000152092 Astrotactin-1 precursor. [Source:Uniprot/SWISSPROT;Acc:O14525] ASTN1
TF332097 ENSG00000105711 Sodium channel subunit beta-1 precursor. [Source:Uniprot/SWISSPROT;Acc:Q07699] SCN1B
TF332134 ENSG00000130287 Neurocan core protein precursor (Chondroitin sulfate proteoglycan 3). [Source:Uniprot/SWISSPROT;Acc:O14594] NCAN
TF332149 ENSG00000130881 Low-density lipoprotein receptor-related protein 3 precursor (hLRp105). [Source:Uniprot/SWISSPROT;Acc:O75074] LRP3
TF332299 ENSG00000081853 Protocadherin gamma A12 precursor (PCDH-gamma-A12)

(Cadherin-21) (Fibroblast cadherin 3). [Source:Uniprot/SWISSPROT;Acc:O60330]
PCDHGA12
TF332342 ENSG00000100362 Parvalbumin alpha.
[Source:Uniprot/SWISSPROT;Acc:P20472] PVALB
TF332376 ENSG00000105894 Pleiotrophin precursor (PTN) (Heparin-binding growth-associated molecule) (HB-GAM) (Heparin-binding growth factor 8) (HBGF-8) (Osteoblast-specific factor 1) (OSF-1) (Heparin-binding neurite outgrowth-promoting factor 1) (HBNF-1) (Heparin-binding brain mit [Source:Uniprot/SWISSPROT;Acc:P21246]
PTN
TF332407 ENSG00000101298 Syntaphilin. [Source:Uniprot/SWISSPROT;Acc:O15079]
SNPH
TF332659 ENSG00000146006 Leucine-rich repeat transmembrane neuronal protein 2 precursor (Leucine-rich repeat neuronal 2 protein).
[Source:Uniprot/SWISSPROT;Acc:O43300] LRRTM2
TF332666 ENSG00000169155 Zinc finger and BTB domain-containing protein 43 (Zinc finger protein 297B) (ZnF-x) (Zinc finger and BTB domain-containing protein 22B). [Source:Uniprot/SWISSPROT;Acc:O43298] ZBTB43
TF332727 ENSG00000160307 Protein S100-B (S100 calcium-binding protein B) (S-100 protein beta subunit) (S-100 protein beta chain).
[Source:Uniprot/SWISSPROT;Acc:P04271] S100B
TF332776 ENSG00000173267 Gamma-synuclein (Persyn) (Breast cancer-specific gene 1 protein) (Synoretin) (SR). [Source:Uniprot/SWISSPROT;Acc:O76070] SNCG
TF332778 ENSG00000122585 Neuropeptide Y precursor [Contains: Neuropeptide Y (Neuropeptide tyrosine) (NPY); C-flanking peptide of NPY (CPON)].
[Source:Uniprot/SWISSPROT;Acc:P01303] NPY
TF332887 ENSG00000187122 Slit homolog 1 protein precursor (Slit-1) (Multiple epidermal growth factor-like domains 4). [Source:Uniprot/SWISSPROT;Acc:O75093]
SLIT1
TF333185 ENSG00000157005 Somatostatin precursor (Growth hormone release-inhibiting factor) [Contains: Somatostatin-28; Somatostatin-14].
[Source:Uniprot/SWISSPROT;Acc:P61278] SST
TF333205 ENSG00000198948 Microfibrillar-associated protein 3-like precursor (Testis development protein NYD-SP9). [Source:Uniprot/SWISSPROT;Acc:O75121] MFAP3L
TF333213 ENSG00000172020 Neuromodulin (Axonal membrane protein GAP-43) (Growth-associated protein 43) (PP46) (Neural phosphoprotein B-50).
[Source:Uniprot/SWISSPROT;Acc:P17677] GAP43
TF333405 ENSG00000006128 Protachykinin 1 precursor (PPT) [Contains: Substance P; Neurokinin A (NKA) (Substance K) (Neuromedin L); Neuropeptide K (NPK); Neuropeptide gamma; C-terminal-flanking peptide].
[Source:Uniprot/SWISSPROT;Acc:P20366] TAC1
TF333419 ENSG00000187094 Cholecystokinins precursor (CCK) [Contains: Cholecystokinin 58 (CCK58); Cholecystokinin 39 (CCK39); Cholecystokinin 33 (CCK33); Cholecystokinin 12 (CCK12); Cholecystokinin 8 (CCK8)].
[Source:Uniprot/SWISSPROT;Acc:P06307] CCK
TF333490 ENSG00000106078 Protein cordon-bleu.
[Source:Uniprot/SWISSPROT;Acc:O75128] LOC392529
TF334360 ENSG00000170382 Leucine-rich repeat neuronal protein 5 precursor (Glioma amplified on chromosome 1 protein). [Source:Uniprot/SWISSPROT;Acc:O75325]
LRRN2
TF334493 ENSG00000091972 OX-2 membrane glycoprotein precursor (CD200 antigen).
[Source:Uniprot/SWISSPROT;Acc:P41217] CD200
TF334668 ENSG00000105737 Glutamate receptor, ionotropic kainate 5 precursor (Glutamate receptor KA-2) (KA2) (Excitatory amino acid receptor 2) (EAA2).
[Source:Uniprot/SWISSPROT;Acc:Q16478] GRIK5
TF334804 ENSG00000157782 Calcium-binding protein 1 (CaBP1) (Calbrain).
[Source:Uniprot/SWISSPROT;Acc:Q9NZU7] CABP1
TF342052 ENSG00000128656 N-chimaerin (NC) (N-chimerin) (Alpha chimerin) (A-chimaerin) (Rho GTPase-activating protein 2). [Source:Uniprot/SWISSPROT;Acc:P15882]
CHN1

TF342871 ENSG00000175264 Carbohydrate sulfotransferase 1 (EC 2.8.2.21) (Keratan sulfate Gal-6 sulfotransferase) (KSST) (KSGal6ST) (KS6ST) (Galactose/N-acetylglucosamine/N-acetylglucosamine 6-O-sulfotransferase 1) (GST-1). [Source:Uniprot/SWISSPROT;Acc:O43916] CHST1
TF343812 ENSG00000130821 Sodium- and chloride-dependent creatine transporter 1 (CT1) (Creatine transporter 1) (Solute carrier family 6 member 8). [Source:Uniprot/SWISSPROT;Acc:P48029] SLC6A8
TF350229 ENSG00000166963 Microtubule-associated protein 1A (MAP 1A) (Proliferation-related protein p80) [Contains: MAP1 light chain LC2]. [Source:Uniprot/SWISSPROT;Acc:P78559] MAP1A
TF351071 ENSG00000048540 LIM domain only protein 3 (Neuronal-specific transcription factor DAT1) (Rhombotin-3). [Source:Uniprot/SWISSPROT;Acc:Q8TAP4] LMO3
TF351204 ENSG00000125820 Homeobox protein Nkx-2.2 (Homeobox protein NK-2 homolog B). [Source:Uniprot/SWISSPROT;Acc:O95096] NKX2-2
TF351553 ENSG00000196465 Myosin light polypeptide 6B (Smooth muscle and nonmuscle myosin light chain alkali 6B) (Myosin light chain 1 slow-twitch muscle A isoform) (MLC1sa). [Source:Uniprot/SWISSPROT;Acc:P14649] MYL6B
TF351626 ENSG00000088367 Band 4.1-like protein 1 (Neuronal protein 4.1) (4.1N). [Source:Uniprot/SWISSPROT;Acc:Q9H4G0] EPB41L1
TF351700 ENSG00000157193 Low-density lipoprotein receptor-related protein 8 precursor (Apolipoprotein E receptor 2). [Source:Uniprot/SWISSPROT;Acc:Q14114] LRP8
TF351844 ENSG00000089169 Rabphilin-3A (Exophilin-1). [Source:Uniprot/SWISSPROT;Acc:Q9Y2J0] RPH3A
TF351956 ENSG00000182901 Regulator of G-protein signaling 7 (RGS7). [Source:Uniprot/SWISSPROT;Acc:P49802] RGS7
TF351976 ENSG00000155093 Receptor-type tyrosine-protein phosphatase N2 precursor (EC 3.1.3.48) (R-PTP-N2) (Islet cell autoantigen-related protein) (ICAAR) (IAR) (Phogrin). [Source:Uniprot/SWISSPROT;Acc:Q92932] PTPRN2
TF351999 ENSG00000072071 Latrophilin-1 precursor (Calcium-independent alpha-latrotoxin receptor 1) (Lectomedin-2). [Source:Uniprot/SWISSPROT;Acc:O94910] LPHN1
TF352620 ENSG00000163536 Neuroserpin precursor (Serpin I1) (Protease inhibitor 12). [Source:Uniprot/SWISSPROT;Acc:Q99574] SERPINI1
TF352926 ENSG00000063180 Carbonic anhydrase-related protein 11 precursor (CA-XI) (CARP XI) (CA- RP XI) (Carbonic anhydrase-related protein 2) (CARP-2) (CA-RP II). [Source:Uniprot/SWISSPROT;Acc:O75493] CA11
TF354311 ENSG00000159082 Synaptojanin-1 (EC 3.1.3.36) (Synaptic inositol-1,4,5-trisphosphate 5- phosphatase 1). [Source:Uniprot/SWISSPROT;Acc:O43426] SYNJ1
TF354315 ENSG00000141720 Phosphatidylinositol-4-phosphate 5-kinase type-2 beta (EC 2.7.1.68) (Phosphatidylinositol-4-phosphate 5-kinase type II beta) (1-phosphatidylinositol-4-phosphate 5-kinase 2-beta) (PtdIns(4)P-5-kinase isoform 2-beta) (PIP5KII-beta) (Diphosphoinositide kina [Source:Uniprot/SWISSPROT;Acc:P78356] PIP5K2B
