# Supplementary material for: 2R and remodeling of vertebrate signal transduction engine
Source: BMC Biol. 2010 Dec 13;8:146. doi: 10.1186/1741-7007-8-146 (PMC3238295; doi:10.1186/1741-7007-8-146)
Supplement: Additional file 13 — TableS7_bp.html. 2ROs preferentially expressed in brain, overrepresented BP terms. [file 1741-7007-8-146-S13.html]

Gene to GO BP Conditional test for over-representation

| GOBPID | Pvalue | OddsRatio | ExpCount | Count | Size | Term |
| GO:0007268 | 0.000 | 5.327 | 6 | 27 | 155 | synaptic transmission |
| GO:0050877 | 0.000 | 2.846 | 20 | 49 | 480 | neurological system process |
| GO:0007154 | 0.000 | 1.944 | 98 | 146 | 2314 | cell communication |
| GO:0051179 | 0.000 | 1.996 | 77 | 122 | 1820 | localization |
| GO:0001505 | 0.000 | 8.217 | 2 | 14 | 54 | regulation of neurotransmitter levels |
| GO:0030182 | 0.000 | 3.585 | 6 | 19 | 144 | neuron differentiation |
| GO:0007399 | 0.000 | 3.205 | 7 | 21 | 190 | nervous system development |
| GO:0022008 | 0.000 | 3.042 | 8 | 21 | 184 | neurogenesis |
| GO:0003001 | 0.000 | 5.795 | 2 | 10 | 50 | generation of a signal involved in cell-cell signaling |
| GO:0032501 | 0.000 | 1.576 | 91 | 122 | 2148 | multicellular organismal process |
| GO:0007417 | 0.000 | 2.860 | 7 | 19 | 175 | central nervous system development |
| GO:0031175 | 0.000 | 3.735 | 4 | 13 | 94 | neurite development |
| GO:0006816 | 0.000 | 4.538 | 3 | 10 | 61 | calcium ion transport |
| GO:0006810 | 0.000 | 1.707 | 44 | 67 | 1131 | transport |
| GO:0006812 | 0.000 | 2.374 | 12 | 25 | 274 | cation transport |
| GO:0016192 | 0.000 | 2.707 | 8 | 19 | 189 | vesicle-mediated transport |
| GO:0060052 | 0.000 | 68.396 | 0 | 3 | 4 | neurofilament cytoskeleton organization and biogenesis |
| GO:0006836 | 0.000 | 6.443 | 1 | 7 | 32 | neurotransmitter transport |
| GO:0007409 | 0.001 | 3.639 | 3 | 11 | 81 | axonogenesis |
| GO:0006897 | 0.001 | 2.863 | 6 | 15 | 137 | endocytosis |
| GO:0007018 | 0.001 | 4.726 | 2 | 8 | 47 | microtubule-based movement |
| GO:0007158 | 0.001 | 34.193 | 0 | 3 | 5 | neuron adhesion |
| GO:0016079 | 0.001 | 34.193 | 0 | 3 | 5 | synaptic vesicle exocytosis |
| GO:0006814 | 0.001 | 3.789 | 3 | 10 | 71 | sodium ion transport |
| GO:0048667 | 0.001 | 3.441 | 4 | 11 | 85 | neuron morphogenesis during differentiation |
