# Supplementary material for: 2R and remodeling of vertebrate signal transduction engine
Source: BMC Biol. 2010 Dec 13;8:146. doi: 10.1186/1741-7007-8-146 (PMC3238295; doi:10.1186/1741-7007-8-146)
Supplement: Additional file 14 — TableS7_cc.html. 2ROs preferentially expressed in brain, overrepresented CC terms. [file 1741-7007-8-146-S14.html]

Gene to GO CC Conditional test for over-representation

| GOCCID | Pvalue | OddsRatio | ExpCount | Count | Size | Term |
| GO:0045202 | 0.000 | 8.474 | 6 | 36 | 141 | synapse |
| GO:0030054 | 0.000 | 4.997 | 10 | 40 | 238 | cell junction |
| GO:0005886 | 0.000 | 2.162 | 75 | 126 | 1758 | plasma membrane |
| GO:0044425 | 0.000 | 1.722 | 109 | 149 | 2605 | membrane part |
| GO:0008021 | 0.000 | 8.600 | 1 | 9 | 33 | synaptic vesicle |
| GO:0030135 | 0.000 | 4.843 | 3 | 13 | 75 | coated vesicle |
| GO:0045211 | 0.000 | 4.607 | 3 | 12 | 72 | postsynaptic membrane |
| GO:0005954 | 0.000 | Inf | 0 | 3 | 3 | calcium- and calmodulin-dependent protein kinase complex |
| GO:0031982 | 0.000 | 2.450 | 12 | 27 | 286 | vesicle |
| GO:0030424 | 0.000 | 7.982 | 1 | 7 | 27 | axon |
| GO:0012506 | 0.000 | 4.073 | 3 | 11 | 73 | vesicle membrane |
| GO:0044433 | 0.001 | 4.018 | 3 | 10 | 67 | cytoplasmic vesicle part |
| GO:0016021 | 0.001 | 1.485 | 94 | 122 | 2208 | integral to membrane |
| GO:0005623 | 0.001 | 2.273 | 308 | 324 | 7216 | cell |
| GO:0005856 | 0.001 | 1.787 | 27 | 44 | 629 | cytoskeleton |
