# Supplementary material for: 2R and remodeling of vertebrate signal transduction engine
Source: BMC Biol. 2010 Dec 13;8:146. doi: 10.1186/1741-7007-8-146 (PMC3238295; doi:10.1186/1741-7007-8-146)
Supplement: Additional file 15 — TableS7_mf.html. 2ROs preferentially expressed in brain, overrepresented MF terms. [file 1741-7007-8-146-S15.html]

Gene to GO MF Conditional test for over-representation

| GOMFID | Pvalue | OddsRatio | ExpCount | Count | Size | Term |
| GO:0005509 | 0.000 | 2.453 | 22 | 48 | 525 | calcium ion binding |
| GO:0005215 | 0.000 | 2.048 | 30 | 55 | 709 | transporter activity |
| GO:0005516 | 0.000 | 4.031 | 4 | 15 | 101 | calmodulin binding |
| GO:0022891 | 0.000 | 2.208 | 20 | 39 | 460 | substrate-specific transmembrane transporter activity |
| GO:0015267 | 0.000 | 2.772 | 9 | 22 | 207 | channel activity |
| GO:0005216 | 0.000 | 2.842 | 8 | 21 | 193 | ion channel activity |
| GO:0005078 | 0.000 | Inf | 0 | 3 | 3 | MAP-kinase scaffold activity |
| GO:0019894 | 0.000 | 30.142 | 0 | 4 | 7 | kinesin binding |
| GO:0004683 | 0.000 | 14.162 | 1 | 5 | 13 | calmodulin-dependent protein kinase activity |
| GO:0005230 | 0.000 | 5.138 | 2 | 9 | 49 | extracellular ligand-gated ion channel activity |
| GO:0008509 | 0.000 | 3.670 | 4 | 12 | 87 | anion transmembrane transporter activity |
| GO:0016301 | 0.000 | 1.921 | 23 | 40 | 533 | kinase activity |
| GO:0005388 | 0.000 | 18.080 | 0 | 4 | 9 | calcium-transporting ATPase activity |
| GO:0008066 | 0.000 | 7.565 | 1 | 6 | 24 | glutamate receptor activity |
| GO:0015631 | 0.000 | 5.062 | 2 | 8 | 44 | tubulin binding |
| GO:0016773 | 0.001 | 1.931 | 20 | 36 | 475 | phosphotransferase activity, alcohol group as acceptor |
| GO:0032555 | 0.001 | 1.621 | 46 | 67 | 1066 | purine ribonucleotide binding |
| GO:0022834 | 0.001 | 3.807 | 3 | 10 | 70 | ligand-gated channel activity |
| GO:0004890 | 0.001 | 8.709 | 1 | 5 | 18 | GABA-A receptor activity |
| GO:0043167 | 0.001 | 1.469 | 90 | 116 | 2095 | ion binding |
| GO:0008324 | 0.001 | 2.109 | 13 | 26 | 313 | cation transmembrane transporter activity |
| GO:0031402 | 0.001 | 4.024 | 3 | 9 | 60 | sodium ion binding |
| GO:0000287 | 0.001 | 2.246 | 11 | 22 | 249 | magnesium ion binding |
