# Supplementary figures and images for: 2R and remodeling of vertebrate signal transduction engine
Source: BMC Biol. 2010 Dec 13;8:146. doi: 10.1186/1741-7007-8-146 (PMC3238295; doi:10.1186/1741-7007-8-146)

**Figure S1**

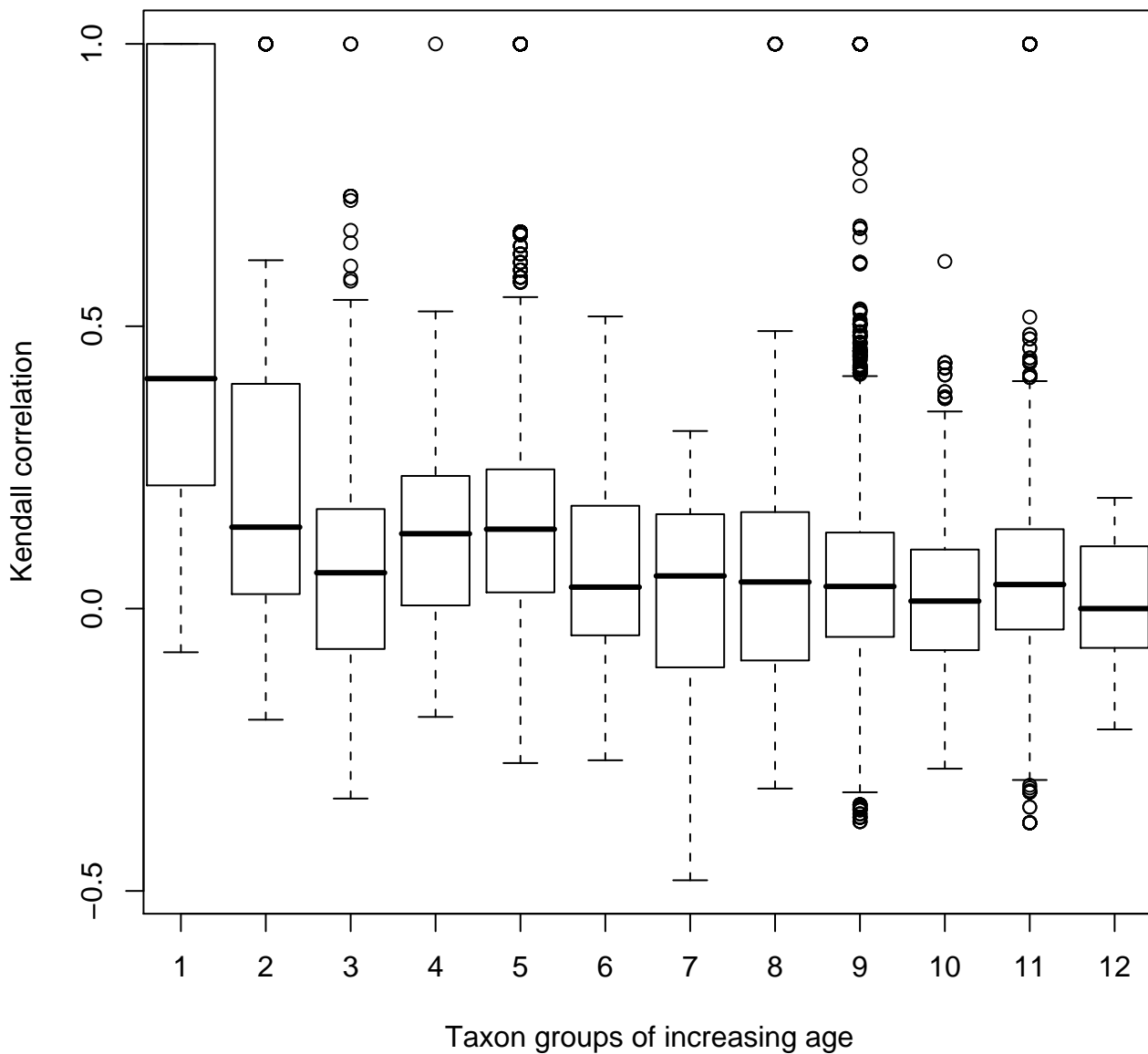

Supplement: Additional file 16 — FigureS1. Duplication timing and expression divergence (Kendall correlation). [file 1741-7007-8-146-S16.pdf]

**Figure S2**

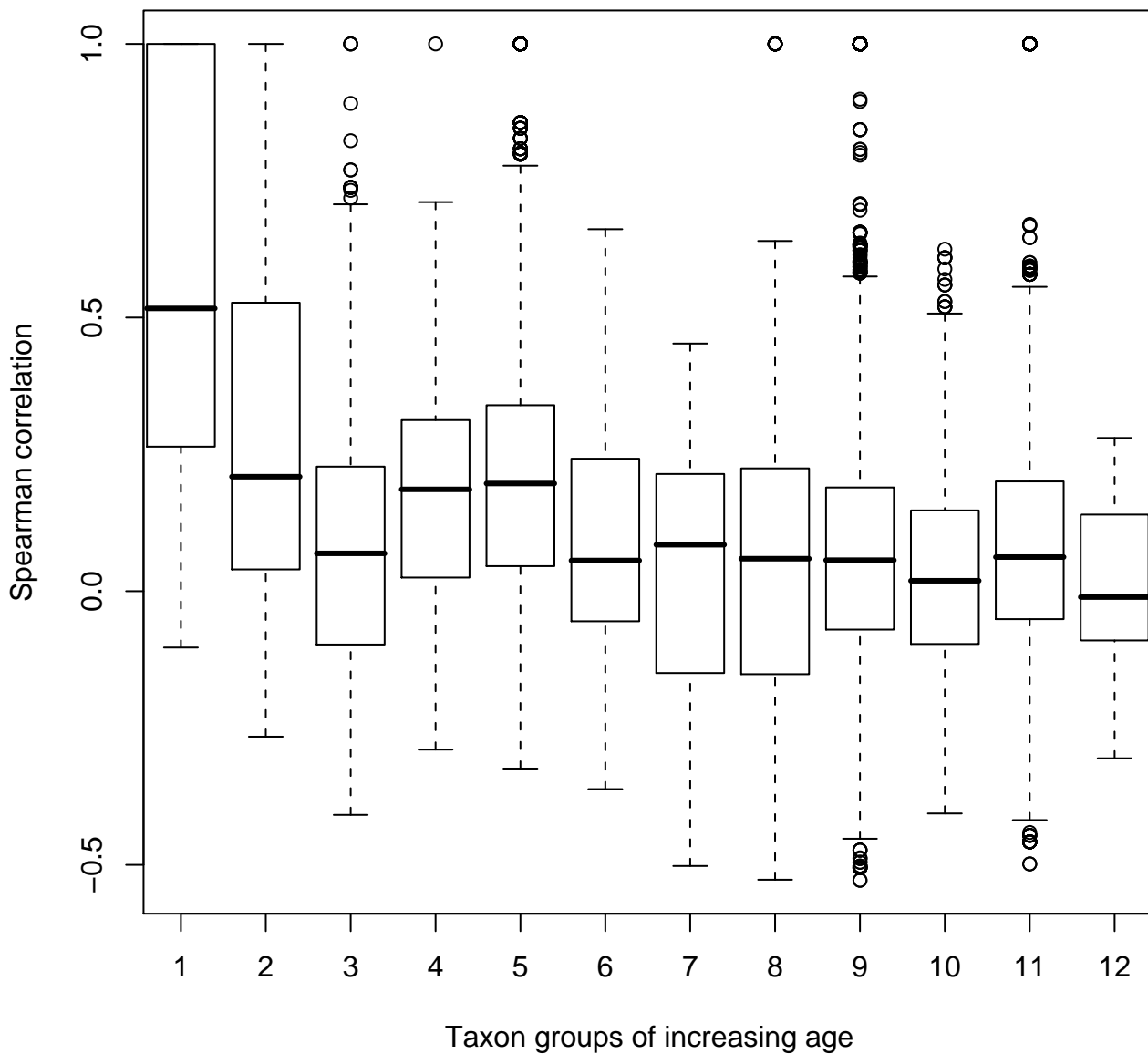

Supplement: Additional file 17 — FigureS2. Duplication timing and expression divergence (Spearman's rank correlation). [file 1741-7007-8-146-S17.pdf]

**Figure S3**

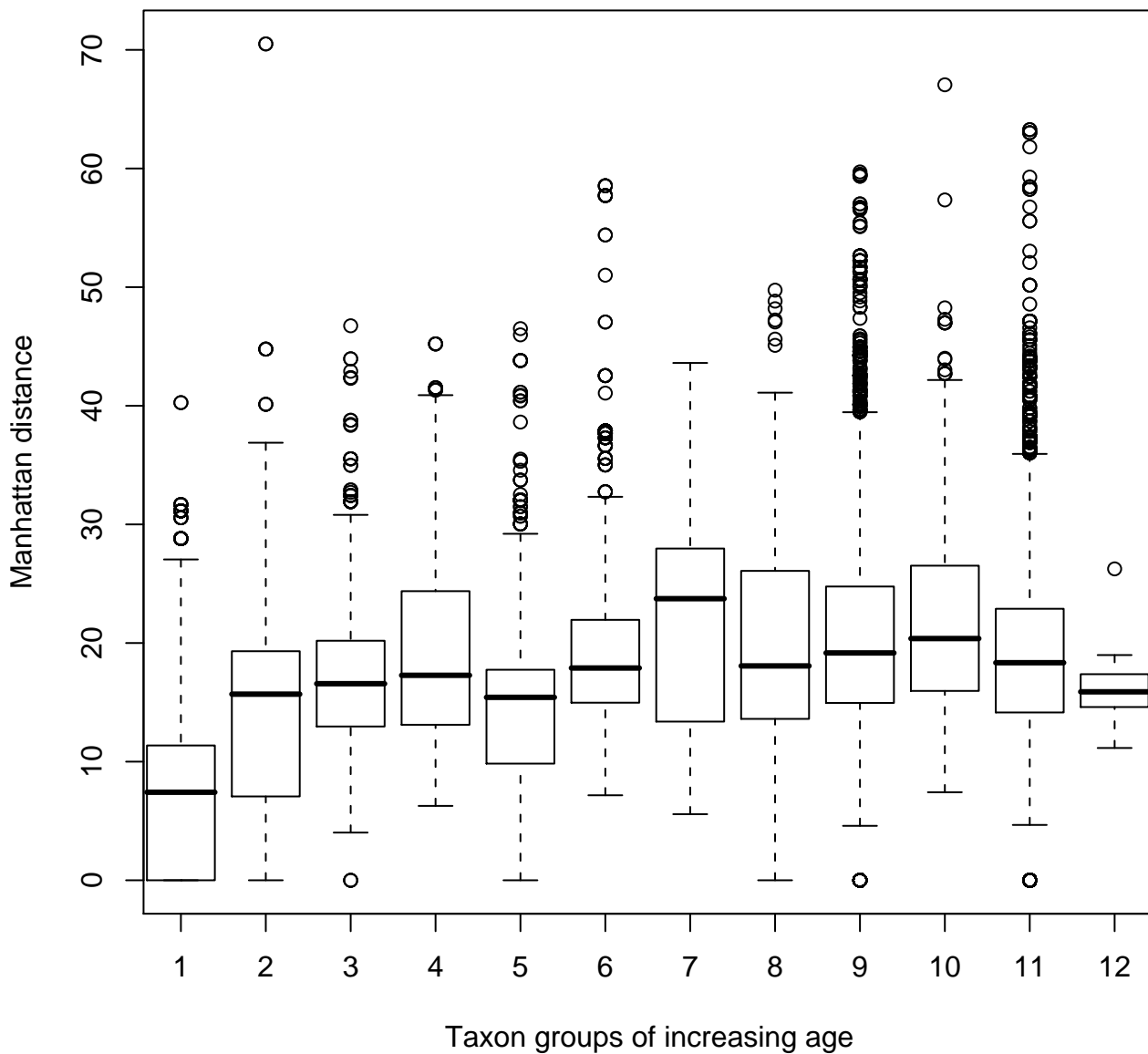

Supplement: Additional file 18 — FigureS3. Duplication timing and expression divergence (Manhattan distance). [file 1741-7007-8-146-S18.pdf]

**Figure S4**

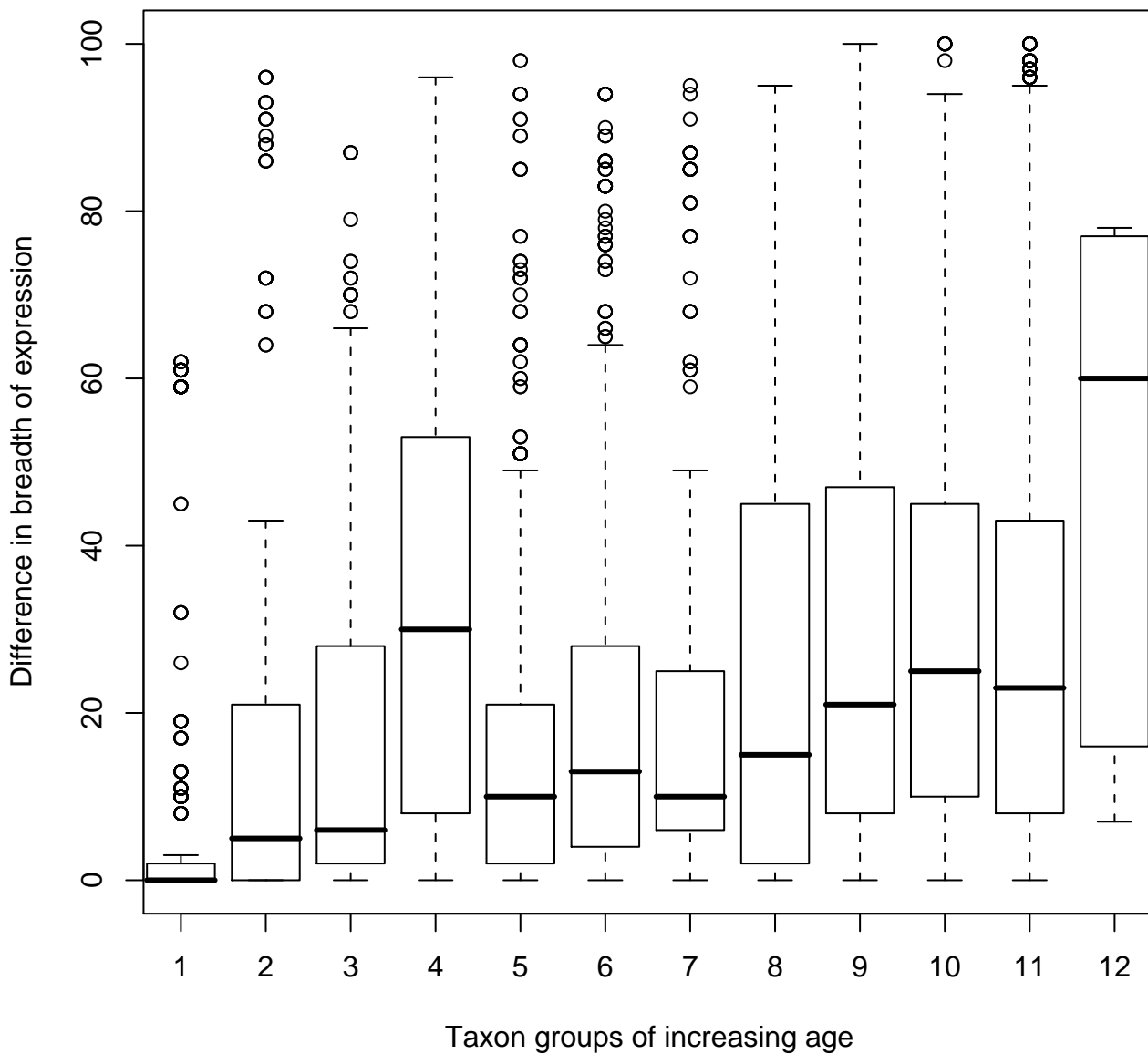

Supplement: Additional file 19 — FigureS4. Duplication timing and expression (difference in breadth of expression). [file 1741-7007-8-146-S19.pdf]
