# Supplementary material for: 2R and remodeling of vertebrate signal transduction engine
Source: BMC Biol. 2010 Dec 13;8:146. doi: 10.1186/1741-7007-8-146 (PMC3238295; doi:10.1186/1741-7007-8-146)
Supplement: Additional file 20 — 2ROs-bridged. Human 2RO bridged pairs. [file 1741-7007-8-146-S20.pdf]

Node1Name	Node1ENTREZID	Node2Name	Node2ENTREZID
cIAP1	329	XIAP	331
CASP3	836	CASP7	840
CASP10	843	CASP8	841
EGFR	1956	ERBB2	2064
EGR2	1959	EGR3	1960
ETS1	2113	ETS2	2114
F7	2155	F9	2158
HDAC1	3065	HDAC2	3066
ID2	3398	ID3	3399
LMNA	4000	LMNB1	4001
LMNA	4000	LMNB2	84823
SMAD2	4087	SMAD3	4088
PAX2	5076	PAX5	5079
RIIa	5576	RIIb	5577
FAK	5747	PTK	2185
SHP1	5777	SHP2	5781
STAT1	6772	STAT3	6774
TLR2	7097	TLR6	10333
TLR2	7097	TLR1	7096
SMRT	9612	NCOR	9611
BCL2	596	BCL2L1	598
ALK3	657	BMPR1	658
CBP	1387	p300	2033
DISHEVELED	1855	DVL3	1857
F10	2159	F7	2155
HMG1	3146	HMG2	3148
c-JUN	3725	JUND	3727
LMNB1	4001	LMNB2	84823
PDE6A	5145	PDE6B	5158
MEK1	5604	MEK2	5605
p107	5933	P130	5934
SP1	6667	SP3	6670
TRAF1	7185	TRAF2	7186
MNK1	8569	MNK2	2872
HAND1	9421	HAND2	9464
