# Supplementary material for: 2R and remodeling of vertebrate signal transduction engine
Source: BMC Biol. 2010 Dec 13;8:146. doi: 10.1186/1741-7007-8-146 (PMC3238295; doi:10.1186/1741-7007-8-146)
Supplement: Additional file 21 — TableS8. 47 human tissues and cell lines in Gene Expression Atlas. [file 1741-7007-8-146-S21.pdf]

ID	Description
1	foetal_brain
2	cerebellum
3	whole_brain
4	cortex
5	caudate_nucleus
6	amygdala
7	thalamus
8	corpus_callosum
9	spinal_cord
10	whole blood
11	testis
12	pancreas
13	placenta
14	pituitary gland
15	thyroid
16	prostate cancer
17	prostate
18	ovary
19	OVR278E
20	OVR278S
21	Uterus
22	DRG
23	salivary_gland
24	trachea
25	lung
26	thymus
27	spleen
28	adrenal gland
29	kidney
30	fetal liver
31	liver
32	heart
33	huvec
34	Thy+
35	Thy-
36	myelogenous
37	lymphoblastic
38	Burkitts_Dandi
39	Burkitts_Raji
40	Hep3b
41	A2058
42	DOHH2
43	GA10
44	HL60
45	K422
46	Ramos
47	WSU
