# Supplementary material for: 2R and remodeling of vertebrate signal transduction engine
Source: BMC Biol. 2010 Dec 13;8:146. doi: 10.1186/1741-7007-8-146 (PMC3238295; doi:10.1186/1741-7007-8-146)
Supplement: Additional file 22 — TableS9. Matrix of average PEM values across tissues and taxons. [file 1741-7007-8-146-S22.pdf]

HUMAN 181   -0.2603650 -0.3804321 -0.4073224 -0.4182602 -0.3286219 -0.3264714
      -0.3223601 -0.3297349 -0.3778511 -0.1666399 0.2817169   -0.1210589
      -0.3597430 -0.2463699 -0.3565859 -0.3264141 -0.3090213 -0.3531424 -0.396
3568 -0.4433977 -0.3599885 -0.2448773 -0.1308396 -0.3911948 -0.2660835 -0.454
5106 -0.3173470 -0.2688576 -0.2849280 -0.3062897 -0.3682242 -0.4976498 -0.133
7774 0.2314681   -0.2806972 -0.3432177 -0.2689231 -0.4515990 -0.2642913
      -0.2018936 -0.2019083 -0.1738183 -0.2139051 -0.3210622 -0.2389722 -0.201
9640 -0.4425532
Homo/Pan/Gorilla 181   -0.3192602 -0.3690323 -0.4244086 -0.4084796 -0.3405118
      -0.3356968 -0.3183957 -0.2999613 -0.3416710 -0.1066860 -0.1002925 -0.189
9054 -0.2031376 -0.2771892 -0.4071140 -0.3385290 -0.3048581 -0.3348323 -0.410
2301 -0.4596258 -0.4051957 -0.2076925 -0.1974667 -0.3491742 -0.2502366 -0.475
5247 -0.7156946 -0.2395355 -0.1819699 -0.1998172 -0.3984516 -0.4287527 -0.201
0559 -0.1509548 -0.3418581 -0.3941806 -0.3100624 -0.4247634 -0.2969527 -0.240
5935 -0.2233634 -0.2806774 -0.3343398 -0.3154258 -0.2698344 -0.2555161 -0.378
9075
Catarrhini 328   -0.2590191 -0.3983655 -0.4553155 -0.4095273 -0.3703909
      -0.3474255 -0.3442273 -0.3097173 -0.3235409 -0.0910418 -0.0252555 -0.147
0518 -0.2615982 -0.3200509 -0.3125336 -0.2844409 -0.2520000 -0.2905209 -0.317
5636 -0.4118927 -0.3564527 -0.2016345 -0.1704300 -0.2788036 -0.1928036 -0.461
0218 -0.2926564 -0.2362764 -0.2604255 -0.2621655 -0.4090318 -0.3504827 -0.159
3264 -0.0578273 -0.2412473 -0.2738545 -0.2625427 -0.3442245 -0.1816500 -0.146
6509 -0.1562173 -0.1352218 -0.1517718 -0.2589282 -0.2146100 -0.1928545 -0.342
4555
Theria   207   -0.3628306 -0.3936290 -0.4180161 -0.3804500 -0.3426968
      -0.3243032 -0.3101710 -0.2563355 -0.2863935 0.0701903   -0.2545629
      -0.1236226 -0.3459032 -0.3359500 -0.3177855 -0.2946129 -0.2747613 -0.270
2129 -0.3164403 -0.3666048 -0.3051790 -0.1792774 -0.2778613 -0.1160903 -0.076
9468 -0.2502032 -0.2882226 -0.2706177 -0.2353758 -0.2806242 -0.2676097 -0.365
7516 -0.1762903 -0.2959871 -0.2580065 -0.2633177 -0.2325500 -0.3779452 -0.288
7887 -0.1534919 -0.1475887 -0.1992565 -0.1958710 -0.2319210 -0.1770419 -0.179
1806 -0.3152435
Eutheria   472   -0.2591395 -0.3648808 -0.3847364 -0.3584931 -0.3191444
      -0.2855195 -0.2910390 -0.2653443 -0.2791082 0.0038446   -0.1434756
      -0.1555964 -0.2545876 -0.2466914 -0.2481631 -0.1813943 -0.1631208 -0.220
7891 -0.2300286 -0.3332048 -0.2744525 -0.1659077 -0.2139819 -0.2146831 -0.098
3311 -0.3841280 -0.3303493 -0.2288386 -0.1618885 -0.2675953 -0.3401631 -0.302
0242 0.0289841   -0.1035799 -0.2020840 -0.2851538 -0.2516271 -0.3188429
      -0.1329259 -0.1040417 -0.0748122 -0.1288523 -0.1020588 -0.2475557 -0.101
5892 -0.0355288 -0.2351884
Mammalia   333   -0.4146730 -0.4050428 -0.4261367 -0.4234828 -0.3760619
      -0.3594599 -0.3618793 -0.2804095 -0.3141291 0.0798365   -0.2909687
      -0.1434545 -0.2252139 -0.3599908 -0.3646196 -0.2094217 -0.1886188 -0.303
1276 -0.3433231 -0.3978824 -0.3483866 -0.1665477 -0.2098770 -0.1616769 -0.079
8701 -0.3476860 -0.3314607 -0.2224775 -0.1594293 -0.2445699 -0.2958594 -0.426
5050 -0.0345592 -0.2160978 -0.3452093 -0.4341039 -0.3098976 -0.4287922 -0.290
3911 -0.1840321 -0.1530626 -0.1829037 -0.2283529 -0.3027869 -0.1854859 -0.118
9045 -0.3125187
Amniota   207   -0.3346677 -0.3841088 -0.4011903 -0.3887840 -0.2945378
      -0.2689743 -0.2699350 -0.1928746 -0.2654184 0.0893565   -0.2937704
      -0.1712689 -0.3086360 -0.3265423 -0.3500574 -0.2378822 -0.1989985 -0.235
6299 -0.2553776 -0.3830378 -0.2930272 -0.2567356 -0.1951586 -0.1611299 -0.101
6677 -0.4649592 -0.2509215 -0.2782689 -0.1946480 -0.2818520 -0.4342598 -0.311
8505 -0.1425151 -0.2817130 -0.2661103 -0.3724094 -0.2988610 -0.3689426 -0.238
5483 -0.1469381 -0.1271934 -0.1593882 -0.1945181 -0.2509517 -0.1775861 -0.203
0332 -0.2434184
Tetrapoda   249   -0.3065975 -0.3040568 -0.3647204 -0.3164716 -0.3164516
      -0.2932181 -0.2874101 -0.2699862 -0.2680568 -0.0558510 -0.2059309 -0.128
9601 -0.1541183 -0.2995637 -0.2281551 -0.1900922 -0.1851659 -0.2190092 -0.252

7404 -0.3409432 -0.3019017 -0.1337773 -0.1121736 -0.1754316 -0.0024731 -0.338
7035 -0.2502458 -0.1517435 -0.2170276 -0.1315392 -0.2629616 -0.2865253 -0.147
3932 -0.3134516 -0.2499662 -0.3321859 -0.2844286 -0.3300737 -0.2192074 -0.142
8786 -0.1264132 -0.1701260 -0.2019816 -0.2258356 -0.1885038 -0.1792550 -0.265
1935
Euteleostomi 3831 -0.1511665 -0.1992902 -0.2177880 -0.1782080 -0.1628838
-0.1482232 -0.1513408 -0.1481344 -0.1284573 -0.1273487 -0.2307786 -0.172
3530 -0.2262009 -0.2090459 -0.2195895 -0.1796110 -0.1682968 -0.1703489 -0.228
9476 -0.2328910 -0.1850581 -0.1983432 -0.1807743 -0.1889303 -0.1580656 -0.356
9823 -0.2063423 -0.1992088 -0.2288586 -0.3111837 -0.2753267 -0.2789502 -0.229
9913 -0.3170206 -0.2631215 -0.3495286 -0.2858210 -0.3515679 -0.2190547 -0.214
9552 -0.2065806 -0.2265624 -0.2629741 -0.2676943 -0.2457447 -0.2547663 -0.214
6922
Chordata 759 -0.2447150 -0.2481974 -0.2832364 -0.2320477 -0.2226240
-0.2110260 -0.2051998 -0.2097419 -0.1769860 -0.1536577 -0.2707699 -0.155
5311 -0.2051281 -0.2289442 -0.2258069 -0.2227121 -0.2194367 -0.2301184 -0.312
9533 -0.2580997 -0.2028262 -0.1800881 -0.1667492 -0.2247029 -0.2079913 -0.337
0178 -0.2486100 -0.1574333 -0.2095542 -0.2449413 -0.2430953 -0.3160477 -0.276
6813 -0.3456880 -0.2922682 -0.3709182 -0.2941131 -0.3695912 -0.2747805 -0.229
4723 -0.2399745 -0.2479374 -0.2734733 -0.2833115 -0.2789562 -0.2978334 -0.285
3659
Bilateria 2040 -0.1376831 -0.1981130 -0.2045502 -0.1728228 -0.1576192
-0.1451168 -0.1486780 -0.1449278 -0.1314568 -0.1296413 -0.2057975 -0.153
5763 -0.2161805 -0.2107501 -0.2019232 -0.1594488 -0.1458532 -0.1854910 -0.223
2246 -0.2413994 -0.1911388 -0.1846162 -0.1800615 -0.1911638 -0.1603022 -0.354
8826 -0.2261801 -0.1799707 -0.2134363 -0.2966444 -0.2739892 -0.2680430 -0.189
5776 -0.2662469 -0.2362634 -0.3225941 -0.2514250 -0.3246345 -0.1928343 -0.185
7938 -0.1681989 -0.1960380 -0.2191569 -0.2366251 -0.2210745 -0.2175619 -0.213
0347
Eukaryota 178 -0.1702547 -0.2628421 -0.2908674 -0.2363368 -0.2250926
-0.2362653 -0.2146379 -0.1738589 -0.1930484 -0.2018695 -0.0850042 -0.186
6358 -0.3020379 -0.1890884 -0.2088737 -0.1851600 -0.1740274 -0.1787284 -0.207
9284 -0.2490884 -0.2179937 -0.2455747 -0.2114274 -0.1965032 -0.1584568 -0.444
0232 -0.2801137 -0.2169474 -0.1047368 -0.3174379 -0.3880505 -0.0860989 -0.176
6274 -0.1152484 0.0023305 -0.0368211 -0.1115116 -0.0946063 -0.0273937
-0.0541768 -0.0391011 -0.1032042 -0.0561726 -0.0341558 -0.0442905 -0.144
4568 -0.1838758
Fungi/Metazoa 201 -0.1517709 -0.2091727 -0.1869218 -0.1865473 -0.1383036
-0.1651055 -0.1267000 -0.1717818 -0.1979055 -0.1311200 -0.1454473 -0.144
6655 -0.2355600 -0.1601164 -0.2177800 -0.1625164 -0.1765400 -0.1736873 -0.228
5036 -0.2313000 -0.1720691 -0.2174491 -0.1851236 -0.1715109 -0.1513345 -0.358
2345 -0.3284927 -0.1772636 -0.1828564 -0.2424182 -0.2318473 -0.1709164 -0.058
6382 -0.1473691 -0.1110618 -0.2024618 -0.1378873 -0.2525564 -0.2188691 -0.142
0255 -0.1334618 -0.1289291 -0.1754764 -0.1469018 -0.1370255 -0.0835818 -0.209
4345
