# Supplementary material for: 2R and remodeling of vertebrate signal transduction engine
Source: BMC Biol. 2010 Dec 13;8:146. doi: 10.1186/1741-7007-8-146 (PMC3238295; doi:10.1186/1741-7007-8-146)
Supplement: Additional file 23 — TableS10. Human genes with chromosomal location. [file 1741-7007-8-146-S23.pdf]

Gene	Chromosome	Position
ENSG00000157456	chromosome15	57184760
ENSG00000134057	chromosome5	68498846
ENSG00000145386	chromosome4	122964234
ENSG00000133101	chromosome13	35904759
ENSG00000110092	chromosome11	69165263
ENSG00000110092	chromosome11	69165263
ENSG00000112576	chromosome6	42124403
ENSG00000118971	chromosome12	4253468
ENSG00000118971	chromosome12	4253468
ENSG00000112576	chromosome6	42124403
ENSG00000105173	chromosome19	34995303
ENSG00000175305	chromosome8	95975626
ENSG00000205089	chromosome5	132111087
ENSG00000118816	chromosome4	78206540
ENSG00000138764	chromosome4	78298710
ENSG00000113328	chromosome5	162798841
ENSG00000107443	chromosome10	97794141
ENSG00000135083	chromosome5	159699107
ENSG00000163660	chromosome3	158360578
ENSG00000116148	chromosome1	1324550
ENSG00000215726	supercontigNT_113871	172638
ENSG00000168763	chromosome2	96845742
ENSG00000119946	chromosome10	101080074
ENSG00000158158	chromosome2	96790464
ENSG00000158158	chromosome2	96790464
ENSG00000168763	chromosome2	96845742
ENSG00000119946	chromosome10	101080074
ENSG00000148842	chromosome10	104668228
ENSG00000119946	chromosome10	101080074
ENSG00000168763	chromosome2	96845742
ENSG00000082258	chromosome2	135392895
ENSG00000129315	chromosome12	47396726
ENSG00000082258	chromosome2	135392895
ENSG00000123374	chromosome12	54647060
ENSG00000108504	chromosome17	71509102
ENSG00000105810	chromosome7	92300574
ENSG00000132964	chromosome13	25726779
ENSG00000155111	chromosome6	111243033
ENSG00000138769	chromosome4	76770197
ENSG00000006837	chromosome5	133730114
ENSG00000176749	chromosome17	27838752
ENSG00000171450	chromosome2	219532787
ENSG00000176749	chromosome17	27838752
ENSG00000167797	chromosome11	67032130
ENSG00000111328	chromosome12	122322119
ENSG00000124762	chromosome6	36759857
ENSG00000111276	chromosome12	12762041
ENSG00000113240	chromosome5	177983024
ENSG00000013441	chromosome2	201434831
ENSG00000178340	chromosome7	23592685
ENSG00000176444	chromosome1	153507393
ENSG00000215793	chromosome1	246167234
ENSG00000179335	chromosome15	72698591
ENSG00000178340	chromosome7	23592685
ENSG00000176444	chromosome1	153507393
ENSG00000113240	chromosome5	177983024
ENSG00000013441	chromosome2	201434831
ENSG00000079335	chromosome1	100591099

ENSG00000081377	chromosome9	98421482
ENSG00000101224	chromosome20	3725179
ENSG00000164045	chromosome3	48204442
ENSG00000158402	chromosome5	137694769
ENSG00000106993	chromosome9	4669768
ENSG00000196347	chromosome16	28620434
ENSG00000196585	chromosome16	28332525
ENSG00000105401	chromosome19	10375156
ENSG00000167258	chromosome17	34871851
ENSG00000065883	chromosome7	39956766
ENSG00000167565	chromosome19	45639828
ENSG00000197019	chromosome19	45621294
ENSG00000170779	chromosome14	104549312
ENSG00000167565	chromosome19	45639828
ENSG00000197019	chromosome19	45621294
ENSG00000109670	chromosome4	153552406
ENSG00000109670	chromosome4	153552406
ENSG00000144354	chromosome2	173927938
ENSG00000164649	chromosome7	21951948
ENSG00000164649	chromosome7	21951948
ENSG00000144354	chromosome2	173927938
ENSG00000134690	chromosome1	37930950
ENSG00000184640	chromosome17	72827866
ENSG00000140623	chromosome16	4778320
ENSG00000140623	chromosome16	4778320
ENSG00000184640	chromosome17	72827866
ENSG00000100167	chromosome22	40703015
ENSG00000184640	chromosome17	72827866
ENSG00000184702	chromosome22	18082112
ENSG00000108387	chromosome17	53964324
ENSG00000180096	chromosome16	30301350
ENSG00000168385	chromosome2	241912321
ENSG00000214005	chromosome7	151695537
ENSG00000122545	chromosome7	35807187
ENSG00000214765	chromosome7	45771956
ENSG00000214834	chromosome9	100408925
ENSG00000122545	chromosome7	35807187
ENSG00000214765	chromosome7	45771956
ENSG00000214834	chromosome9	100408925
ENSG00000214005	chromosome7	151695537
ENSG00000108387	chromosome17	53964324
ENSG00000184702	chromosome22	18082112
ENSG00000164402	chromosome5	132140729
ENSG00000186522	chromosome2	109728694
ENSG00000154997	chromosome7	55881828
ENSG00000125354	chromosomeX	118711367
ENSG00000138758	chromosome4	78090081
ENSG00000186522	chromosome2	109728694
ENSG00000154997	chromosome7	55881828
ENSG00000164402	chromosome5	132140729
ENSG00000138758	chromosome4	78090081
ENSG00000125354	chromosomeX	118711367
ENSG00000214102	chromosome7	141055028
ENSG00000166483	chromosome11	9552057
ENSG00000173846	chromosome1	45038723
ENSG00000145632	chromosome5	57791544
ENSG00000145632	chromosome5	57791544
ENSG00000173846	chromosome1	45038723
ENSG00000185988	chromosome19	1477996

ENSG00000100393	chromosome22	39818955
ENSG00000005339	chromosome16	3870123
ENSG00000082701	chromosome3	121294972
ENSG00000105723	chromosome19	47438458
ENSG00000144554	chromosome3	10045342
ENSG00000095319	chromosome9	130749817
ENSG00000099804	chromosome19	482932
ENSG00000107341	chromosome9	33807756
ENSG00000131508	chromosome5	138921371
ENSG00000109332	chromosome4	104009343
ENSG00000184888	chromosomeX	84076202
ENSG00000072401	chromosome10	59764968
ENSG00000131508	chromosome5	138921371
ENSG00000072401	chromosome10	59764968
ENSG00000078967	chromosome7	43932657
ENSG00000119729	chromosome2	46623690
ENSG00000126785	chromosome14	62741341
ENSG00000136238	chromosome7	6380892
ENSG00000128340	chromosome22	35970135
ENSG00000169750	chromosome17	77582927
ENSG00000070831	chromosome1	22277559
ENSG00000070831	chromosome1	22277559
ENSG00000128340	chromosome22	35970135
ENSG00000136238	chromosome7	6380892
ENSG00000070831	chromosome1	22277559
ENSG00000070831	chromosome1	22277559
ENSG00000177105	chromosome11	3805945
ENSG00000129757	chromosome11	2863296
ENSG00000170035	chromosome2	181555015
ENSG00000182247	chromosome3	23225195
ENSG00000170142	chromosome3	23823765
ENSG00000170142	chromosome3	23823765
ENSG00000182247	chromosome3	23225195
ENSG00000170142	chromosome3	23823765
ENSG00000170035	chromosome2	181555015
ENSG00000170142	chromosome3	23823765
ENSG00000182247	chromosome3	23225195
ENSG00000132388	chromosome17	4216562
ENSG00000103275	chromosome16	1304033
ENSG00000077721	chromosomeX	118592703
ENSG00000077721	chromosomeX	118592703
ENSG00000119048	chromosome5	133735186
ENSG00000058453	chromosome1	17121101
ENSG00000123975	chromosome9	91116028
ENSG00000123975	chromosome9	91116028
ENSG00000173207	chromosome1	153213846
ENSG00000178556	chromosomeX	30545492
ENSG00000139842	chromosome13	112911943
ENSG00000158290	chromosomeX	119592501
ENSG00000074054	chromosome2	122079942
ENSG00000163539	chromosome3	33734499
ENSG00000077935	chromosome22	44188113
ENSG00000072501	chromosomeX	53466275
ENSG00000156509	chromosome8	101223556
ENSG00000112029	chromosome6	153345788
ENSG00000112312	chromosome6	24885454
ENSG00000102753	chromosome13	49264644
ENSG00000186432	chromosome3	161765765
ENSG00000185467	chromosome7	98643026

ENSG00000182481	chromosome17	63463711
ENSG00000215769	supercontigNT_113939	112979
ENSG00000214035	chromosome7	144333895
ENSG00000214180	chromosome17	60185184
ENSG00000143815	chromosome1	223678401
ENSG00000149809	chromosome11	64636064
ENSG00000172893	chromosome11	70833647
ENSG00000160789	chromosome1	154351334
ENSG00000160789	chromosome1	154351334
ENSG00000113368	chromosome5	126141100
ENSG00000176619	chromosome19	2407872
ENSG00000176619	chromosome19	2407872
ENSG00000113368	chromosome5	126141100
ENSG00000172613	chromosome11	66916092
ENSG00000151164	chromosome12	109424535
ENSG00000164754	chromosome8	117948150
ENSG00000119318	chromosome9	109085732
ENSG00000179262	chromosome19	12917763
ENSG00000119318	chromosome9	109085732
ENSG00000126070	chromosome1	36169615
ENSG00000092847	chromosome1	36121610
ENSG00000134698	chromosome1	36046605
ENSG00000092847	chromosome1	36121610
ENSG00000126070	chromosome1	36169615
ENSG00000123908	chromosome8	141714788
ENSG00000092847	chromosome1	36121610
ENSG00000126070	chromosome1	36169615
ENSG00000134698	chromosome1	36046605
ENSG00000161960	chromosome17	7403807
ENSG00000156976	chromosome3	187984094
ENSG00000175766	chromosome5	176001486
ENSG00000151247	chromosome4	100069287
ENSG00000151247	chromosome4	100069287
ENSG00000175766	chromosome5	176001486
ENSG00000114867	chromosome3	185515809
ENSG00000075151	chromosome1	21367132
ENSG00000135930	chromosome2	233123652
ENSG00000187840	chromosome8	38007249
ENSG00000148730	chromosome10	71834164
ENSG00000148730	chromosome10	71834164
ENSG00000187840	chromosome8	38007249
ENSG00000163577	chromosome3	172108290
ENSG00000132507	chromosome17	7151090
ENSG00000196513	chromosome10	80942364
ENSG00000163577	chromosome3	172108290
ENSG00000132507	chromosome17	7151090
ENSG00000196513	chromosome10	80942364
ENSG00000132507	chromosome17	7151090
ENSG00000196513	chromosome10	80942364
ENSG00000163577	chromosome3	172108290
ENSG00000215108	chromosomeX	72411340
ENSG00000164924	chromosome8	102030294
ENSG00000166913	chromosome20	42963589
ENSG00000170027	chromosome7	75826062
ENSG00000128245	chromosome22	30670720
ENSG00000215108	chromosomeX	72411340
ENSG00000164924	chromosome8	102030294
ENSG00000166913	chromosome20	42963589
ENSG00000175793	chromosome1	27062291

ENSG00000134308	chromosome2	9688033
ENSG00000166913	chromosome20	42963589
ENSG00000215108	chromosomeX	72411340
ENSG00000164924	chromosome8	102030294
ENSG00000175793	chromosome1	27062291
ENSG00000134308	chromosome2	9688033
ENSG00000215108	chromosomeX	72411340
ENSG00000164924	chromosome8	102030294
ENSG00000166913	chromosome20	42963589
ENSG00000128245	chromosome22	30670720
ENSG00000170027	chromosome7	75826062
ENSG00000154229	chromosome17	61729432
ENSG00000166501	chromosome16	23754998
ENSG00000126583	chromosome19	59077561
ENSG00000163932	chromosome3	53187479
ENSG00000065675	chromosome10	6597104
ENSG00000067606	chromosome1	1971930
ENSG00000163558	chromosome3	171423152
ENSG00000166501	chromosome16	23754998
ENSG00000154229	chromosome17	61729432
ENSG00000117020	chromosome1	242073180
ENSG00000142208	chromosome14	104330026
ENSG00000105221	chromosome19	45463015
ENSG00000117020	chromosome1	242073180
ENSG00000105221	chromosome19	45463015
ENSG00000142208	chromosome14	104330026
ENSG00000123143	chromosome19	14405334
ENSG00000065243	chromosome1	88922852
ENSG00000160447	chromosome9	130505016
ENSG00000065243	chromosome1	88922852
ENSG00000123143	chromosome19	14405334
ENSG00000151929	chromosome10	121401178
ENSG00000156735	chromosome8	38153545
ENSG00000003400	chromosome2	201758746
ENSG00000064012	chromosome2	201831200
ENSG00000164305	chromosome4	185801301
ENSG00000165806	chromosome10	115441721
ENSG00000064012	chromosome2	201831200
ENSG00000003400	chromosome2	201758746
ENSG00000064012	chromosome2	201831200
ENSG00000003400	chromosome2	201758746
ENSG00000137752	chromosome11	104477352
ENSG00000137757	chromosome11	104384958
ENSG00000196954	chromosome11	104344463
ENSG00000204403	chromosome11	104274352
ENSG00000105141	chromosome19	15024063
ENSG00000064012	chromosome2	201831200
ENSG00000003400	chromosome2	201758746
ENSG00000165806	chromosome10	115441721
ENSG00000164305	chromosome4	185801301
ENSG00000133056	chromosome1	202705554
ENSG00000011405	chromosome11	17147865
ENSG00000139144	chromosome12	18326283
ENSG00000105851	chromosome7	106295243
ENSG00000171608	chromosome1	9693101
ENSG00000051382	chromosome3	139960876
ENSG00000121879	chromosome3	180399308
ENSG00000011405	chromosome11	17147865
ENSG00000133056	chromosome1	202705554

ENSG00000145675	chromosome5	67558260
ENSG00000105647	chromosome19	18127690
ENSG00000117461	chromosome1	46370212
ENSG00000105647	chromosome19	18127690
ENSG00000145675	chromosome5	67558260
ENSG00000117461	chromosome1	46370212
ENSG00000105647	chromosome19	18127690
ENSG00000145675	chromosome5	67558260
ENSG00000105647	chromosome19	18127690
ENSG00000145675	chromosome5	67558260
ENSG00000117461	chromosome1	46370212
ENSG00000141506	chromosome17	8755537
ENSG00000174083	chromosome17	8711723
ENSG00000107447	chromosome10	98054245
ENSG00000122678	chromosome7	44088563
ENSG00000177700	chromosome11	832509
ENSG00000168495	chromosome8	22158908
ENSG00000121851	chromosome1	144171580
ENSG00000113356	chromosome5	89817141
ENSG00000121851	chromosome1	144171580
ENSG00000141385	chromosome18	12367082
ENSG00000145358	chromosome4	101330164
ENSG00000168209	chromosome10	73703981
ENSG00000168209	chromosome10	73703981
ENSG00000145358	chromosome4	101330164
ENSG00000180733	chromosome8	48813236
ENSG00000172216	chromosome20	48240978
ENSG00000092067	chromosome14	22658141
ENSG00000092067	chromosome14	22658141
ENSG00000172216	chromosome20	48240978
ENSG00000180733	chromosome8	48813236
ENSG00000180733	chromosome8	48813236
ENSG00000172216	chromosome20	48240978
ENSG00000119541	chromosome18	59240473
ENSG00000132612	chromosome16	67895081
ENSG00000182263	chromosome2	164299684
ENSG00000205432	chromosome12	50502483
ENSG00000205432	chromosome12	50502483
ENSG00000182263	chromosome2	164299684
ENSG00000138138	chromosome10	89564337
ENSG00000010256	chromosome3	48622058
ENSG00000105819	chromosome7	102725143
ENSG00000109971	chromosome11	122437243
ENSG00000126803	chromosome14	64077321
ENSG00000204388	chromosome6	31903707
ENSG00000204389	chromosome6	31891513
ENSG00000204390	chromosome6	31887729
ENSG00000173110	chromosome1	159761073
ENSG00000126803	chromosome14	64077321
ENSG00000204388	chromosome6	31903707
ENSG00000204389	chromosome6	31891513
ENSG00000204390	chromosome6	31887729
ENSG00000173110	chromosome1	159761073
ENSG00000109971	chromosome11	122437243
ENSG00000204388	chromosome6	31903707
ENSG00000204389	chromosome6	31891513
ENSG00000204390	chromosome6	31887729
ENSG00000173110	chromosome1	159761073
ENSG00000126803	chromosome14	64077321

ENSG00000164070	chromosome4	128923156
ENSG00000170606	chromosome5	132415842
ENSG00000120694	chromosome13	30633720
ENSG00000170606	chromosome5	132415842
ENSG00000164070	chromosome4	128923156
ENSG00000160202	chromosome21	43462279
ENSG00000004776	chromosome19	40939750
ENSG00000106211	chromosome7	75769966
ENSG00000152137	chromosome12	118101501
ENSG00000170276	chromosome11	111288764
ENSG00000169271	chromosome5	53787377
ENSG00000109846	chromosome11	111287659
ENSG00000004776	chromosome19	40939750
ENSG00000160202	chromosome21	43462279
ENSG00000004776	chromosome19	40939750
ENSG00000160202	chromosome21	43462279
ENSG00000109846	chromosome11	111287659
ENSG00000169271	chromosome5	53787377
ENSG00000170276	chromosome11	111288764
ENSG00000106211	chromosome7	75769966
ENSG00000131143	chromosome16	84392313
ENSG00000131055	chromosome20	29690482
ENSG00000131055	chromosome20	29690482
ENSG00000131143	chromosome16	84392313
ENSG00000156885	chromosome16	31347148
ENSG00000111775	chromosome12	119360313
ENSG00000164605	chromosome6	37120614
ENSG00000160471	chromosome19	60557702
ENSG00000126267	chromosome19	40831611
ENSG00000172912	chromosome22	39295238
ENSG00000115944	chromosome2	42441806
ENSG00000161281	chromosome19	41335150
ENSG00000112695	chromosome6	76023213
ENSG00000115944	chromosome2	42441806
ENSG00000112695	chromosome6	76023213
ENSG00000161281	chromosome19	41335150
ENSG00000176340	chromosome11	63498729
ENSG00000176340	chromosome11	63498729
ENSG00000187581	chromosome14	92883368
ENSG00000097007	chromosome9	132579528
ENSG00000143322	chromosome1	177465156
ENSG00000159842	chromosome17	1029772
ENSG00000186716	chromosome22	21853148
ENSG00000186716	chromosome22	21853148
ENSG00000159842	chromosome17	1029772
ENSG00000170802	chromosome2	48426858
ENSG00000053254	chromosome14	88948574
ENSG00000106258	chromosome7	99115456
ENSG00000160870	chromosome7	99170653
ENSG00000160868	chromosome7	99219641
ENSG00000021461	chromosome7	99263675
ENSG00000106258	chromosome7	99115456
ENSG00000160870	chromosome7	99170653
ENSG00000160868	chromosome7	99219641
ENSG00000021461	chromosome7	99263675
ENSG00000059377	chromosome7	139175656
ENSG00000187048	chromosome1	47179693
ENSG00000162365	chromosome1	47375745
ENSG00000154198	chromosome1	47138694

ENSG00000186160	chromosome1	47305750
ENSG00000186377	chromosome1	47262077
ENSG00000142973	chromosome1	47037341
ENSG00000186529	chromosome19	15613226
ENSG00000186115	chromosome19	15869422
ENSG00000171903	chromosome19	15906219
ENSG00000186204	chromosome19	15645340
ENSG00000186526	chromosome19	15587428
ENSG00000171954	chromosome19	15497148
ENSG00000180432	chromosome3	42892313
ENSG00000124212	chromosome20	47618061
ENSG00000167910	chromosome8	59575212
ENSG00000172817	chromosome8	65873699
ENSG00000167910	chromosome8	59575212
ENSG00000124212	chromosome20	47618061
ENSG00000180432	chromosome3	42892313
ENSG00000146233	chromosome6	46728279
ENSG00000187553	chromosome10	94811011
ENSG00000003137	chromosome2	72228472
ENSG00000003137	chromosome2	72228472
ENSG00000187553	chromosome10	94811011
ENSG00000095596	chromosome10	94823682
ENSG00000111012	chromosome12	56447092
ENSG00000135929	chromosome2	219355150
ENSG00000186684	chromosome2	127677596
ENSG00000135929	chromosome2	219355150
ENSG00000019186	chromosome20	52223526
ENSG00000140459	chromosome15	72446980
ENSG00000160882	chromosome8	143958232
ENSG00000179142	chromosome8	143996259
ENSG00000135929	chromosome2	219355150
ENSG00000111012	chromosome12	56447092
ENSG00000186684	chromosome2	127677596
ENSG00000111012	chromosome12	56447092
ENSG00000135929	chromosome2	219355150
ENSG00000019186	chromosome20	52223526
ENSG00000019186	chromosome20	52223526
ENSG00000186684	chromosome2	127677596
ENSG00000111012	chromosome12	56447092
ENSG00000135929	chromosome2	219355150
ENSG00000160882	chromosome8	143958232
ENSG00000179142	chromosome8	143996259
ENSG00000140459	chromosome15	72446980
ENSG00000148795	chromosome10	104587109
ENSG00000148795	chromosome10	104587109
ENSG00000198457	chromosome6	32114179
ENSG00000102882	chromosome16	30042032
ENSG00000100030	chromosome22	20551731
ENSG00000141639	chromosome18	46444327
ENSG00000069956	chromosome15	50125950
ENSG00000215542	chromosome21	22732534
ENSG00000109339	chromosome4	87334579
ENSG00000050748	chromosome5	179640168
ENSG00000156711	chromosome6	36206338
ENSG00000188130	chromosome22	49042066
ENSG00000185386	chromosome22	49050849
ENSG00000185386	chromosome22	49050849
ENSG00000156711	chromosome6	36206338
ENSG00000188130	chromosome22	49042066

ENSG00000112062	chromosome6	36103913
ENSG00000156711	chromosome6	36206338
ENSG00000188130	chromosome22	49042066
ENSG00000107643	chromosome10	49279710
ENSG00000050748	chromosome5	179640168
ENSG00000109339	chromosome4	87334579
ENSG00000188130	chromosome22	49042066
ENSG00000156711	chromosome6	36206338
ENSG00000198909	chromosome17	59053852
ENSG00000169967	chromosome2	127817224
ENSG00000198909	chromosome17	59053852
ENSG00000180815	chromosomeX	19443301
ENSG00000197442	chromosome6	137155229
ENSG00000142733	chromosome1	27565676
ENSG00000197442	chromosome6	137155229
ENSG00000180815	chromosomeX	19443301
ENSG00000135341	chromosome6	91353324
ENSG00000130758	chromosome19	45389779
ENSG00000006432	chromosome14	70345642
ENSG00000143674	chromosome1	231530398
ENSG00000143674	chromosome1	231530398
ENSG00000130758	chromosome19	45389779
ENSG00000006432	chromosome14	70345642
ENSG00000173327	chromosome11	65137804
ENSG00000006432	chromosome14	70345642
ENSG00000130758	chromosome19	45389779
ENSG00000139625	chromosome12	52167443
ENSG00000073803	chromosome3	186629064
ENSG00000168067	chromosome11	64327198
ENSG00000104814	chromosome19	43800376
ENSG00000011566	chromosome2	39517633
ENSG00000104814	chromosome19	43800376
ENSG00000168067	chromosome11	64327198
ENSG00000104814	chromosome19	43800376
ENSG00000168067	chromosome11	64327198
ENSG00000011566	chromosome2	39517633
ENSG00000012983	chromosome14	50068518
ENSG00000184545	chromosome11	1543633
ENSG00000111266	chromosome12	12565300
ENSG00000143507	chromosome1	219979710
ENSG00000120875	chromosome8	29263715
ENSG00000120129	chromosome5	172130562
ENSG00000139318	chromosome12	88269948
ENSG00000164086	chromosome3	52065270
ENSG00000130829	chromosomeX	152562128
ENSG00000138166	chromosome10	112247870
ENSG00000120129	chromosome5	172130562
ENSG00000120875	chromosome8	29263715
ENSG00000158050	chromosome2	96174821
ENSG00000120129	chromosome5	172130562
ENSG00000120875	chromosome8	29263715
ENSG00000138166	chromosome10	112247870
ENSG00000164086	chromosome3	52065270
ENSG00000139318	chromosome12	88269948
ENSG00000158716	chromosome1	158017515
ENSG00000158716	chromosome1	158017515
ENSG00000112679	chromosome6	237540
ENSG00000112679	chromosome6	237540
ENSG00000149599	chromosome20	29924484

ENSG00000079393	chromosome10	76538922
ENSG00000133878	chromosome8	33574576
ENSG00000079393	chromosome10	76538922
ENSG00000188716	chromosome10	76488279
ENSG00000188716	chromosome10	76488279
ENSG00000188716	chromosome10	76488279
ENSG00000188716	chromosome10	76488279
ENSG00000079393	chromosome10	76538922
ENSG00000079393	chromosome10	76538922
ENSG00000188716	chromosome10	76488279
ENSG00000133878	chromosome8	33574576
ENSG00000133878	chromosome8	33574576
ENSG00000079393	chromosome10	76538922
ENSG00000188716	chromosome10	76488279
ENSG00000133878	chromosome8	33574576
ENSG00000079393	chromosome10	76538922
ENSG00000188716	chromosome10	76488279
ENSG00000108861	chromosome17	39211819
ENSG00000212825	chromosome7	128553808
ENSG00000126934	chromosome19	4074873
ENSG00000169032	chromosome15	64466740
ENSG00000071054	chromosome2	101680975
ENSG00000123572	chromosomeX	104953495
ENSG00000141503	chromosome17	4728441
ENSG00000154310	chromosome3	172660547
ENSG00000123572	chromosomeX	104953495
ENSG00000071054	chromosome2	101680975
ENSG00000154310	chromosome3	172660547
ENSG00000141503	chromosome17	4728441
ENSG00000132002	chromosome19	14490162
ENSG00000162616	chromosome1	78243383
ENSG00000137094	chromosome9	34983231
ENSG00000162616	chromosome1	78243383
ENSG00000132002	chromosome19	14490162
ENSG00000105993	chromosome7	156844028
ENSG00000172404	chromosome22	39587945
ENSG00000179407	chromosome3	129664779
ENSG00000128590	chromosome7	107999407
ENSG00000170464	chromosome5	138803034
ENSG00000164031	chromosome4	101086752
ENSG00000148719	chromosome10	73784662
ENSG00000164031	chromosome4	101086752
ENSG00000170464	chromosome5	138803034
ENSG00000140403	chromosome15	76343711
ENSG00000086061	chromosome9	33016483
ENSG00000103423	chromosome16	4415884
ENSG00000102580	chromosome13	95127511
ENSG00000101152	chromosome20	62030143
ENSG00000147570	chromosome8	67126337
ENSG00000116675	chromosome1	65503182
ENSG00000178950	chromosome4	915976
ENSG00000117450	chromosome1	45757303
ENSG00000167815	chromosome19	12773076
ENSG00000198691	chromosome1	94359298
ENSG00000064687	chromosome19	992361
ENSG00000165029	chromosome9	106705782
ENSG00000064687	chromosome19	992361
ENSG00000165029	chromosome9	106705782
ENSG00000144452	chromosome2	215711177

ENSG00000179869	chromosome7	48208391
ENSG00000064687	chromosome19	992361
ENSG00000165029	chromosome9	106705782
ENSG00000198691	chromosome1	94359298
ENSG00000107331	chromosome9	139043146
ENSG00000167972	chromosome16	2318615
ENSG00000165029	chromosome9	106705782
ENSG00000064687	chromosome19	992361
ENSG00000004846	chromosome7	20657571
ENSG00000085563	chromosome7	87067437
ENSG00000005471	chromosome7	86942718
ENSG00000073734	chromosome2	169582882
ENSG00000085563	chromosome7	87067437
ENSG00000005471	chromosome7	86942718
ENSG00000004846	chromosome7	20657571
ENSG00000204267	chromosome6	32913989
ENSG00000168394	chromosome6	32929572
ENSG00000168394	chromosome6	32929572
ENSG00000204267	chromosome6	32913989
ENSG00000150967	chromosome12	122010736
ENSG00000091262	chromosome16	16224793
ENSG00000103222	chromosome16	15951110
ENSG00000023839	chromosome10	101532592
ENSG00000103222	chromosome16	15951110
ENSG00000091262	chromosome16	16224793
ENSG00000108846	chromosome17	46067297
ENSG00000006071	chromosome11	17454942
ENSG00000069431	chromosome12	21980876
ENSG00000173208	chromosome12	38299685
ENSG00000101986	chromosomeX	152643916
ENSG00000172350	chromosome11	118525886
ENSG00000160179	chromosome21	42494855
ENSG00000143921	chromosome2	43919697
ENSG00000138075	chromosome2	43919323
ENSG00000197892	chromosome8	29176471
ENSG00000137177	chromosome6	18095674
ENSG00000089177	chromosome20	16501921
ENSG00000130294	chromosome2	241385843
ENSG00000054523	chromosome1	10214974
ENSG00000141200	chromosome17	49255394
ENSG00000068796	chromosome5	61640092
ENSG00000142945	chromosome1	44978192
ENSG00000084731	chromosome2	26058291
ENSG00000101350	chromosome20	30361242
ENSG00000116852	chromosome1	199259135
ENSG00000116852	chromosome1	199259135
ENSG00000139116	chromosome12	38123190
ENSG00000155980	chromosome12	56230322
ENSG00000168280	chromosome2	149502044
ENSG00000170759	chromosome10	32384908
ENSG00000168280	chromosome2	149502044
ENSG00000155980	chromosome12	56230322
ENSG00000121621	chromosome11	28076071
ENSG00000189285	chromosome17	40369245
ENSG00000112984	chromosome5	137543269
ENSG00000138182	chromosome10	91455032
ENSG00000112984	chromosome5	137543269
ENSG00000125337	chromosome6	168173115
ENSG00000066735	chromosome14	103701972

ENSG00000162849	chromosome1	243741031
ENSG00000162849	chromosome1	243741031
ENSG00000066735	chromosome14	103701972
ENSG00000140859	chromosome16	56389657
ENSG00000140859	chromosome16	56389657
ENSG00000167702	chromosome8	145662611
ENSG00000204086	chromosomeX	96025966
ENSG00000117748	chromosome1	28113759
ENSG00000198513	chromosome14	50096734
ENSG00000119787	chromosome2	38457907
ENSG00000184743	chromosome11	63195764
ENSG00000119787	chromosome2	38457907
ENSG00000119787	chromosome2	38457907
ENSG00000198513	chromosome14	50096734
ENSG00000159388	chromosome1	201541358
ENSG00000159388	chromosome1	201541358
ENSG00000133639	chromosome12	91063443
ENSG00000154640	chromosome21	17903334
ENSG00000137707	chromosome11	110874712
ENSG00000154640	chromosome21	17903334
ENSG00000137707	chromosome11	110874712
ENSG00000133639	chromosome12	91063443
ENSG00000159388	chromosome1	201541358
ENSG00000137707	chromosome11	110874712
ENSG00000154640	chromosome21	17903334
ENSG00000063854	chromosome16	1816790
ENSG00000103253	chromosome16	717511
ENSG00000141232	chromosome17	46296378
ENSG00000183864	chromosome22	40163296
ENSG00000198900	chromosome20	39091122
ENSG00000184428	chromosome8	144488407
ENSG00000198900	chromosome20	39091122
ENSG00000131747	chromosome17	35827935
ENSG00000077097	chromosome3	25680793
ENSG00000109519	chromosome4	7120661
ENSG00000214621	chromosome7	65892237
ENSG00000164284	chromosome5	148705296
ENSG00000119782	chromosome2	24126224
ENSG00000198225	chromosome6	63979421
ENSG00000088832	chromosome20	1321754
ENSG00000198225	chromosome6	63979421
ENSG00000088832	chromosome20	1321754
ENSG00000119782	chromosome2	24126224
ENSG00000004478	chromosome12	2774567
ENSG00000096060	chromosome6	35718580
ENSG00000105701	chromosome19	18513781
ENSG00000141756	chromosome17	37222813
ENSG00000122642	chromosome7	32963711
ENSG00000176826	chromosome7	55723054
ENSG00000106080	chromosome7	30032650
ENSG00000079150	chromosome2	179051473
ENSG00000111344	chromosome12	112057651
ENSG00000105808	chromosome7	102044357
ENSG00000170667	chromosome7	101945163
ENSG00000105808	chromosome7	102044357
ENSG00000170667	chromosome7	101945163
ENSG00000111344	chromosome12	112057651
ENSG00000185989	chromosome13	113916067
ENSG00000155903	chromosome3	142688616

ENSG00000136848	chromosome9	123369217
ENSG00000075391	chromosome1	176330305
ENSG00000197283	chromosome6	33496020
ENSG00000136848	chromosome9	123369217
ENSG00000136848	chromosome9	123369217
ENSG00000197283	chromosome6	33496020
ENSG00000075391	chromosome1	176330305
ENSG00000197283	chromosome6	33496020
ENSG00000136848	chromosome9	123369217
ENSG00000105122	chromosome19	15436378
ENSG00000197283	chromosome6	33496020
ENSG00000136848	chromosome9	123369217
ENSG00000075391	chromosome1	176330305
ENSG00000198625	chromosome1	202761270
ENSG00000135679	chromosome12	67488525
ENSG00000154342	chromosome1	226261453
ENSG00000108379	chromosome17	42250963
ENSG00000085741	chromosome11	75595100
ENSG00000162552	chromosome1	22342003
ENSG00000169884	chromosome12	47650580
ENSG00000135925	chromosome2	219453962
ENSG00000158955	chromosome17	42265711
ENSG00000143816	chromosome1	226202212
ENSG00000061492	chromosome5	137447678
ENSG00000075290	chromosome10	102212916
ENSG00000188064	chromosome22	44751299
ENSG00000154764	chromosome3	13896315
ENSG00000154764	chromosome3	13896315
ENSG00000188064	chromosome22	44751299
ENSG00000111186	chromosome12	1610782
ENSG00000114251	chromosome3	55496333
ENSG00000134245	chromosome1	112811683
ENSG00000105989	chromosome7	116750280
ENSG00000063660	chromosome2	241024009
ENSG00000213420	chromosome7	99612759
ENSG00000147257	chromosomeX	132947143
ENSG00000179399	chromosome13	90849302
ENSG00000183098	chromosome13	92677711
ENSG00000076716	chromosomeX	132376660
ENSG00000213420	chromosome7	99612759
ENSG00000063660	chromosome2	241024009
ENSG00000076716	chromosomeX	132376660
ENSG00000183098	chromosome13	92677711
ENSG00000179399	chromosome13	90849302
ENSG00000147257	chromosomeX	132947143
ENSG00000176153	chromosome14	64479198
ENSG00000197582	chromosome3	49370716
ENSG00000176153	chromosome14	64479198
ENSG00000176153	chromosome14	64479198
ENSG00000197582	chromosome3	49370716
ENSG00000012779	chromosome10	45189734
ENSG00000012779	chromosome10	45189734
ENSG00000214859	chromosome9	94715369
ENSG00000161905	chromosome17	4491696
ENSG00000188101	chromosome17	6738409
ENSG00000108839	chromosome17	6840161
ENSG00000179148	chromosome17	7962502
ENSG00000179477	chromosome17	7931486
ENSG00000179593	chromosome17	7883199

ENSG00000179148	chromosome17	7962502
ENSG00000179477	chromosome17	7931486
ENSG00000179593	chromosome17	7883199
ENSG00000214859	chromosome9	94715369
ENSG00000161905	chromosome17	4491696
ENSG00000188101	chromosome17	6738409
ENSG00000108839	chromosome17	6840161
ENSG00000213316	chromosome5	179153688
ENSG00000085871	chromosome4	140806624
ENSG00000132965	chromosome13	30207743
ENSG00000085871	chromosome4	140806624
ENSG00000213316	chromosome5	179153688
ENSG00000087586	chromosome20	54396661
ENSG00000105146	chromosome19	62434429
ENSG00000178093	chromosome19	19487237
ENSG00000214601	chromosome8	145414584
ENSG00000214624	chromosome8	145270556
ENSG00000184343	chromosomeX	152699736
ENSG00000135250	chromosome7	104816583
ENSG00000184343	chromosomeX	152699736
ENSG00000096063	chromosome6	35996812
ENSG00000184343	chromosomeX	152699736
ENSG00000135250	chromosome7	104816583
ENSG00000175087	chromosome1	26313387
ENSG00000125834	chromosome20	2031519
ENSG00000125834	chromosome20	2031519
ENSG00000175087	chromosome1	26313387
ENSG00000104936	chromosome19	50977470
ENSG00000211455	chromosome12	27341921
ENSG00000112079	chromosome6	36615958
ENSG00000104936	chromosome19	50977470
ENSG00000115694	chromosome2	242096124
ENSG00000102572	chromosome13	98027201
ENSG00000134602	chromosomeX	130984987
ENSG00000102572	chromosome13	98027201
ENSG00000115694	chromosome2	242096124
ENSG00000172939	chromosome3	38182372
ENSG00000198648	chromosome2	168812192
ENSG00000180370	chromosome3	197993915
ENSG00000149269	chromosome11	76781214
ENSG00000077264	chromosomeX	110252988
ENSG00000077264	chromosomeX	110252988
ENSG00000149269	chromosome11	76781214
ENSG00000149269	chromosome11	76781214
ENSG00000077264	chromosomeX	110252988
ENSG00000180370	chromosome3	197993915
ENSG00000137843	chromosome15	38344279
ENSG00000130669	chromosome19	44352034
ENSG00000101349	chromosome20	9572977
ENSG00000101349	chromosome20	9572977
ENSG00000130669	chromosome19	44352034
ENSG00000007952	chromosomeX	100015785
ENSG00000165168	chromosomeX	37524275
ENSG00000074771	chromosome6	155818627
ENSG00000165168	chromosomeX	37524275
ENSG00000007952	chromosomeX	100015785
ENSG00000101197	chromosome20	61337894
ENSG00000110330	chromosome11	101725796
ENSG00000023445	chromosome11	101700451

ENSG00000110330	chromosome11	101725796
ENSG00000023445	chromosome11	101700451
ENSG00000101197	chromosome20	61337894
ENSG00000180152	chromosome19	58485440
ENSG00000101966	chromosomeX	122847194
ENSG00000180152	chromosome19	58485440
ENSG00000101966	chromosomeX	122847194
ENSG00000110330	chromosome11	101725796
ENSG00000023445	chromosome11	101700451
ENSG00000101197	chromosome20	61337894
ENSG00000081770	chromosome5	70355957
ENSG00000179978	chromosome5	69460020
ENSG00000112972	chromosome5	43334825
ENSG00000134240	chromosome1	120112991
ENSG00000117305	chromosome1	24024493
ENSG00000146151	chromosome6	55551813
ENSG00000164104	chromosome4	174491376
ENSG00000132967	chromosome3	22398314
ENSG00000189403	chromosome13	29935818
ENSG00000100118	chromosome22	25286492
ENSG00000124097	chromosome20	55497490
ENSG00000214852	chromosome2	9076480
ENSG00000215308	chromosomeX	36139152
ENSG00000182181	chromosome17	38053722
ENSG00000173961	chromosome9	36294928
ENSG00000029993	chromosomeX	149904731
ENSG00000182711	chromosome5	179053804
ENSG00000180212	chromosome20	32885432
ENSG00000214364	chromosome6	122221620
ENSG00000182181	chromosome17	38053722
ENSG00000173961	chromosome9	36294928
ENSG00000029993	chromosomeX	149904731
ENSG00000182711	chromosome5	179053804
ENSG00000180212	chromosome20	32885432
ENSG00000214364	chromosome6	122221620
ENSG00000132967	chromosome3	22398314
ENSG00000189403	chromosome13	29935818
ENSG00000100118	chromosome22	25286492
ENSG00000124097	chromosome20	55497490
ENSG00000214852	chromosome2	9076480
ENSG00000215308	chromosomeX	36139152
ENSG00000164104	chromosome4	174491376
ENSG00000198157	chromosomeX	80263705
ENSG00000197366	chromosome10	124643966
ENSG00000182765	chromosome18	56613130
ENSG00000172186	chromosome18	53837292
ENSG00000184409	chromosomeX	153698871
ENSG00000198875	chromosome2	97810143
ENSG00000157576	chromosome15	91056042
ENSG00000197366	chromosome10	124643966
ENSG00000182765	chromosome18	56613130
ENSG00000172186	chromosome18	53837292
ENSG00000184409	chromosomeX	153698871
ENSG00000198875	chromosome2	97810143
ENSG00000157576	chromosome15	91056042
ENSG00000198157	chromosomeX	80263705
ENSG00000214578	chromosome10	85831162
ENSG00000198830	chromosome1	26671691
ENSG00000212769	chromosome15	27810209

ENSG00000176982	chromosome9	15578353
ENSG00000180150	chromosome22	42529445
ENSG00000182952	chromosome6	26653414
ENSG00000118418	chromosome6	80000997
ENSG00000118418	chromosome6	80000997
ENSG00000214578	chromosome10	85831162
ENSG00000198830	chromosome1	26671691
ENSG00000212769	chromosome15	27810209
ENSG00000176982	chromosome9	15578353
ENSG00000180150	chromosome22	42529445
ENSG00000182952	chromosome6	26653414
ENSG00000173442	chromosome11	65100389
ENSG00000115504	chromosome2	62787831
ENSG00000167674	chromosome19	4423348
ENSG00000112273	chromosome6	22677784
ENSG00000143321	chromosome1	154988780
ENSG00000166503	chromosome15	81667171
ENSG00000164985	chromosome9	15500187
ENSG00000164985	chromosome9	15500187
ENSG00000112273	chromosome6	22677784
ENSG00000143321	chromosome1	154988780
ENSG00000166503	chromosome15	81667171
ENSG00000166503	chromosome15	81667171
ENSG00000112273	chromosome6	22677784
ENSG00000143321	chromosome1	154988780
ENSG00000141522	chromosome17	77421096
ENSG00000215642	supercontigNT_113944	124392
ENSG00000111348	chromosome12	14994914
ENSG00000206156	chromosome16	270682
ENSG00000206156	chromosome16	270682
ENSG00000111348	chromosome12	14994914
ENSG00000144668	chromosome3	37468870
ENSG00000115232	chromosome2	182030607
ENSG00000077943	chromosome10	15801777
ENSG00000005961	chromosome17	39822368
ENSG00000140678	chromosome16	31274077
ENSG00000156886	chromosome16	31312183
ENSG00000169896	chromosome16	31176008
ENSG00000005844	chromosome16	30391660
ENSG00000083457	chromosome17	3651188
ENSG00000083457	chromosome17	3651188
ENSG00000140678	chromosome16	31274077
ENSG00000156886	chromosome16	31312183
ENSG00000169896	chromosome16	31176008
ENSG00000005844	chromosome16	30391660
ENSG00000213949	chromosome5	52119945
ENSG00000164171	chromosome5	52321056
ENSG00000143127	chromosome1	144236423
ENSG00000137809	chromosome15	66511550
ENSG00000213949	chromosome5	52119945
ENSG00000164171	chromosome5	52321056
ENSG00000143127	chromosome1	144236423
ENSG00000137809	chromosome15	66511550
ENSG00000083457	chromosome17	3651188
ENSG00000140678	chromosome16	31274077
ENSG00000156886	chromosome16	31312183
ENSG00000169896	chromosome16	31176008
ENSG00000005844	chromosome16	30391660
ENSG00000091409	chromosome2	173000763

ENSG00000135424	chromosome12	54387734
ENSG00000164171	chromosome5	52321056
ENSG00000213949	chromosome5	52119945
ENSG00000005844	chromosome16	30391660
ENSG00000140678	chromosome16	31274077
ENSG00000156886	chromosome16	31312183
ENSG00000169896	chromosome16	31176008
ENSG00000138448	chromosome2	187163053
ENSG00000161638	chromosome12	53099250
ENSG00000005961	chromosome17	39822368
ENSG00000077943	chromosome10	15801777
ENSG00000137809	chromosome15	66511550
ENSG00000143127	chromosome1	144236423
ENSG00000135424	chromosome12	54387734
ENSG00000091409	chromosome2	173000763
ENSG00000005884	chromosome17	45488803
ENSG00000143127	chromosome1	144236423
ENSG00000137809	chromosome15	66511550
ENSG00000213949	chromosome5	52119945
ENSG00000164171	chromosome5	52321056
ENSG00000161638	chromosome12	53099250
ENSG00000138448	chromosome2	187163053
ENSG00000115221	chromosome2	160764821
ENSG00000105855	chromosome7	20337955
ENSG00000056345	chromosome17	42686227
ENSG00000082781	chromosome3	126088538
ENSG00000160255	chromosome21	45155126
ENSG00000139626	chromosome12	51880495
ENSG00000082781	chromosome3	126088538
ENSG00000082781	chromosome3	126088538
ENSG00000056345	chromosome17	42686227
ENSG00000105855	chromosome7	20337955
ENSG00000115221	chromosome2	160764821
ENSG00000150093	chromosome10	33264493
ENSG00000139626	chromosome12	51880495
ENSG00000160255	chromosome21	45155126
ENSG00000147166	chromosomeX	70438382
ENSG00000110172	chromosome11	89595771
ENSG00000077009	chromosome19	3884670
ENSG00000106733	chromosome9	76887852
ENSG00000077009	chromosome19	3884670
ENSG00000139597	chromosome13	31900220
ENSG00000077009	chromosome19	3884670
ENSG00000106733	chromosome9	76887852
ENSG00000114166	chromosome3	20056974
ENSG00000108773	chromosome17	37526849
ENSG00000099864	chromosome19	659949
ENSG00000099260	chromosome1	99884462
ENSG00000099864	chromosome19	659949
ENSG00000099260	chromosome1	99884462
ENSG00000099864	chromosome19	659949
ENSG00000157654	chromosome9	111442963
ENSG00000162664	chromosome1	90230310
ENSG00000105127	chromosome19	15351543
ENSG00000011243	chromosome19	15382488
ENSG00000110422	chromosome11	33264537
ENSG00000064393	chromosome7	139068328
ENSG00000163349	chromosome1	114284529
ENSG00000064393	chromosome7	139068328

ENSG00000163349	chromosome1	114284529
ENSG00000110422	chromosome11	33264537
ENSG00000160396	chromosome19	45587650
ENSG00000163349	chromosome1	114284529
ENSG00000064393	chromosome7	139068328
ENSG00000145491	chromosome5	10495280
ENSG00000065371	chromosome3	125182019
ENSG00000114547	chromosome3	127173588
ENSG00000136003	chromosome12	107480529
ENSG00000102081	chromosomeX	146801390
ENSG00000129245	chromosome17	7458576
ENSG00000114416	chromosome3	182113168
ENSG00000114416	chromosome3	182113168
ENSG00000129245	chromosome17	7458576
ENSG00000139988	chromosome14	67259113
ENSG00000072042	chromosome14	67232174
ENSG00000139988	chromosome14	67259113
ENSG00000072042	chromosome14	67232174
ENSG00000139988	chromosome14	67259113
ENSG00000072042	chromosome14	67232174
ENSG00000160439	chromosome19	60266213
ENSG00000167536	chromosome17	24254089
ENSG00000133318	chromosome11	63205685
ENSG00000139970	chromosome14	59407102
ENSG00000115310	chromosome2	55130941
ENSG00000139970	chromosome14	59407102
ENSG00000133318	chromosome11	63205685
ENSG00000189283	chromosome3	60497736
ENSG00000108785	chromosome17	37952308
ENSG00000108786	chromosome17	37958478
ENSG00000080511	chromosome19	9985174
ENSG00000080511	chromosome19	9985174
ENSG00000080511	chromosome19	9985174
ENSG00000108785	chromosome17	37952308
ENSG00000108786	chromosome17	37958478
ENSG00000164111	chromosome4	122837195
ENSG00000135046	chromosome9	74959060
ENSG00000143412	chromosome1	149222206
ENSG00000183059	chromosome9	33614272
ENSG00000182718	chromosome15	58477405
ENSG00000164111	chromosome4	122837195
ENSG00000165390	chromosome10	47875435
ENSG00000150165	chromosome10	46593925
ENSG00000186807	chromosome10	47217118
ENSG00000197043	chromosome5	150517565
ENSG00000109511	chromosome4	169250427
ENSG00000196975	chromosome2	69862202
ENSG00000165390	chromosome10	47875435
ENSG00000150165	chromosome10	46593925
ENSG00000186807	chromosome10	47217118
ENSG00000164111	chromosome4	122837195
ENSG00000138772	chromosome4	79694652
ENSG00000183059	chromosome9	33614272
ENSG00000182718	chromosome15	58477405
ENSG00000143412	chromosome1	149222206
ENSG00000143412	chromosome1	149222206
ENSG00000183059	chromosome9	33614272
ENSG00000182718	chromosome15	58477405
ENSG00000135046	chromosome9	74959060

ENSG00000109511	chromosome4	169250427
ENSG00000197043	chromosome5	150517565
ENSG00000165390	chromosome10	47875435
ENSG00000150165	chromosome10	46593925
ENSG00000186807	chromosome10	47217118
ENSG00000164111	chromosome4	122837195
ENSG00000138772	chromosome4	79694652
ENSG00000196975	chromosome2	69862202
ENSG00000165390	chromosome10	47875435
ENSG00000150165	chromosome10	46593925
ENSG00000186807	chromosome10	47217118
ENSG00000164111	chromosome4	122837195
ENSG00000138772	chromosome4	79694652
ENSG00000196975	chromosome2	69862202
ENSG00000109511	chromosome4	169250427
ENSG00000197043	chromosome5	150517565
ENSG00000138772	chromosome4	79694652
ENSG00000165390	chromosome10	47875435
ENSG00000150165	chromosome10	46593925
ENSG00000186807	chromosome10	47217118
ENSG00000164111	chromosome4	122837195
ENSG00000122359	chromosome10	81922598
ENSG00000109511	chromosome4	169250427
ENSG00000197043	chromosome5	150517565
ENSG00000165390	chromosome10	47875435
ENSG00000150165	chromosome10	46593925
ENSG00000186807	chromosome10	47217118
ENSG00000164111	chromosome4	122837195
ENSG00000138772	chromosome4	79694652
ENSG00000196975	chromosome2	69862202
ENSG00000143412	chromosome1	149222206
ENSG00000183059	chromosome9	33614272
ENSG00000182718	chromosome15	58477405
ENSG00000135046	chromosome9	74959060
ENSG00000138175	chromosome10	104464023
ENSG00000138175	chromosome10	104464023
ENSG00000188042	chromosome2	235069970
ENSG00000122644	chromosome7	12694405
ENSG00000122872	chromosome10	62114269
ENSG00000175906	chromosome17	38832627
ENSG00000122644	chromosome7	12694405
ENSG00000122872	chromosome10	62114269
ENSG00000188042	chromosome2	235069970
ENSG00000122644	chromosome7	12694405
ENSG00000122872	chromosome10	62114269
ENSG00000175906	chromosome17	38832627
ENSG00000141748	chromosome17	34575543
ENSG00000165997	chromosome10	18988573
ENSG00000165997	chromosome10	18988573
ENSG00000141748	chromosome17	34575543
ENSG00000215451	chromosome22	23411460
ENSG00000162980	chromosome2	152392937
ENSG00000113966	chromosome3	98969642
ENSG00000196503	chromosome4	57066355
ENSG00000175414	chromosome5	175725173
ENSG00000134108	chromosome3	5139151
ENSG00000169379	chromosome3	95181958
ENSG00000174225	chromosomeX	100115335
ENSG00000144746	chromosome3	69216889

ENSG00000102050	chromosomeX	48818591
ENSG00000171097	chromosome9	130647509
ENSG00000121481	chromosome1	183323309
ENSG00000204227	chromosome6	33284578
ENSG00000215471	chromosome13	45910125
ENSG00000184203	chromosome3	196751138
ENSG00000102055	chromosomeX	42522275
ENSG00000215031	chromosome17	7174495
ENSG00000119938	chromosome10	93382707
ENSG00000119938	chromosome10	93382707
ENSG00000119938	chromosome10	93382707
ENSG00000154415	chromosome7	113346288
ENSG00000100829	chromosome14	22841836
ENSG00000132825	chromosome20	57948382
ENSG00000154415	chromosome7	113346288
ENSG00000108819	chromosome17	45582874
ENSG00000158528	chromosome7	94377362
ENSG00000158528	chromosome7	94377362
ENSG00000108819	chromosome17	45582874
ENSG00000058272	chromosome12	78852843
ENSG00000077157	chromosome1	200584603
ENSG00000125503	chromosome19	60320724
ENSG00000125503	chromosome19	60320724
ENSG00000077157	chromosome1	200584603
ENSG00000058272	chromosome12	78852843
ENSG00000143514	chromosome1	222075778
ENSG00000088808	chromosome14	103383398
ENSG00000173457	chromosome11	63770722
ENSG00000198729	chromosome6	150506022
ENSG00000167641	chromosome19	43438820
ENSG00000166143	chromosome15	38908132
ENSG00000167641	chromosome19	43438820
ENSG00000173457	chromosome11	63770722
ENSG00000198729	chromosome6	150506022
ENSG00000198729	chromosome6	150506022
ENSG00000173457	chromosome11	63770722
ENSG00000104695	chromosome8	30789480
ENSG00000113575	chromosome5	133589452
ENSG00000113575	chromosome5	133589452
ENSG00000104695	chromosome8	30789480
ENSG00000113575	chromosome5	133589452
ENSG00000104695	chromosome8	30789480
ENSG00000137713	chromosome11	111142296
ENSG00000105568	chromosome19	57385162
ENSG00000156475	chromosome5	146415500
ENSG00000074211	chromosome4	6524858
ENSG00000214122	chromosome8	26205073
ENSG00000175470	chromosome10	133597951
ENSG00000214122	chromosome8	26205073
ENSG00000156475	chromosome5	146415500
ENSG00000074211	chromosome4	6524858
ENSG00000074211	chromosome4	6524858
ENSG00000156475	chromosome5	146415500
ENSG00000073711	chromosome3	137203031
ENSG00000167393	chromosomeX	267427
ENSG00000073711	chromosome3	137203031
ENSG00000066027	chromosome1	210526076
ENSG00000068971	chromosome11	64449702
ENSG00000078304	chromosome14	101297984

ENSG00000078304	chromosome14	101297984
ENSG00000078304	chromosome14	101297984
ENSG00000112640	chromosome6	43060394
ENSG00000154001	chromosome14	63076157
ENSG00000068971	chromosome11	64449702
ENSG00000066027	chromosome1	210526076
ENSG00000138814	chromosome4	102487109
ENSG00000107758	chromosome10	74925654
ENSG00000120910	chromosome8	22354769
ENSG00000120910	chromosome8	22354769
ENSG00000107758	chromosome10	74925654
ENSG00000188386	chromosome9	103397034
ENSG00000124224	chromosome20	56317888
ENSG00000215443	chromosome20	56254267
ENSG00000154845	chromosome18	9604606
ENSG00000112242	chromosome6	20510443
ENSG00000007968	chromosome1	23729873
ENSG00000101412	chromosome20	31737732
ENSG00000101412	chromosome20	31737732
ENSG00000112242	chromosome6	20510443
ENSG00000007968	chromosome1	23729873
ENSG00000169016	chromosome2	11523457
ENSG00000188841	chromosomeX	135008174
ENSG00000007968	chromosome1	23729873
ENSG00000112242	chromosome6	20510443
ENSG00000169016	chromosome2	11523457
ENSG00000188841	chromosomeX	135008174
ENSG00000165891	chromosome12	75982547
ENSG00000129173	chromosome11	19219966
ENSG00000103479	chromosome16	52025970
ENSG00000080839	chromosome20	35157746
ENSG00000172985	chromosome2	109381463
ENSG00000154447	chromosome4	170426939
ENSG00000172985	chromosome2	109381463
ENSG00000156463	chromosome5	145297685
ENSG00000172985	chromosome2	109381463
ENSG00000172985	chromosome2	109381463
ENSG00000154447	chromosome4	170426939
ENSG00000130147	chromosome2	235608386
ENSG00000183742	chromosome7	20168011
ENSG00000131370	chromosome3	15348920
ENSG00000185437	chromosome21	39745704
ENSG00000131171	chromosomeX	80344356
ENSG00000198478	chromosome6	80397898
ENSG00000131171	chromosomeX	80344356
ENSG00000185437	chromosome21	39745704
ENSG00000142669	chromosome1	26478833
ENSG00000214636	chromosome6	52630130
ENSG00000142669	chromosome1	26478833
ENSG00000214636	chromosome6	52630130
ENSG00000075785	chromosome3	129996901
ENSG00000075785	chromosome3	129996901
ENSG00000131979	chromosome14	54439132
ENSG00000076003	chromosome2	136350406
ENSG00000109814	chromosome4	39199528
ENSG00000072803	chromosome5	171366205
ENSG00000166167	chromosome10	103103928
ENSG00000089876	chromosome10	127559384
ENSG00000144045	chromosome2	74605721

ENSG00000133115	chromosome13	38462859
ENSG00000148175	chromosome9	123172287
ENSG00000148175	chromosome9	123172287
ENSG00000133115	chromosome13	38462859
ENSG00000120318	chromosome5	141040238
ENSG00000047365	chromosome4	35907504
ENSG00000186635	chromosome11	72115822
ENSG00000047365	chromosome4	35907504
ENSG00000120318	chromosome5	141040238
ENSG00000116885	chromosome1	36688558
ENSG00000125037	chromosome3	10003346
ENSG00000125912	chromosome19	3137029
ENSG00000176102	chromosome11	33139448
ENSG00000198276	chromosome20	62058170
ENSG00000100836	chromosome14	22860519
ENSG00000205022	chromosome16	87460516
ENSG00000087299	chromosome14	49848619
ENSG00000151552	chromosome4	17122776
ENSG00000093183	chromosome3	42585543
ENSG00000121542	chromosome3	124410755
ENSG00000125875	chromosome20	391145
ENSG00000111737	chromosome12	119038838
ENSG00000136997	chromosome8	128818022
ENSG00000134323	chromosome2	15999638
ENSG00000116990	chromosome1	40140148
ENSG00000134323	chromosome2	15999638
ENSG00000136997	chromosome8	128818022
ENSG00000065361	chromosome12	54760352
ENSG00000178568	chromosome2	213111500
ENSG00000141736	chromosome17	35110018
ENSG00000146648	chromosome7	55054465
ENSG00000178568	chromosome2	213111500
ENSG00000065361	chromosome12	54760352
ENSG00000146648	chromosome7	55054465
ENSG00000141736	chromosome17	35110018
ENSG00000073282	chromosome3	190831999
ENSG00000078900	chromosome1	3588790
ENSG00000141510	chromosome17	7520638
ENSG00000078900	chromosome1	3588790
ENSG00000073282	chromosome3	190831999
ENSG00000124208	chromosome20	48203582
ENSG00000078369	chromosome1	1746753
ENSG00000114450	chromosome3	180626683
ENSG00000114450	chromosome3	180626683
ENSG00000078369	chromosome1	1746753
ENSG00000172354	chromosome7	100111825
ENSG00000114450	chromosome3	180626683
ENSG00000078369	chromosome1	1746753
ENSG00000196591	chromosome6	114399048
ENSG00000116478	chromosome1	32530358
ENSG00000116478	chromosome1	32530358
ENSG00000196591	chromosome6	114399048
ENSG00000048052	chromosome7	18584322
ENSG00000108840	chromosome17	39550539
ENSG00000068024	chromosome2	239939332
ENSG00000048052	chromosome7	18584322
ENSG00000108840	chromosome17	39550539
ENSG00000068024	chromosome2	239939332
ENSG00000061273	chromosome12	46499836

ENSG00000108840	chromosome17	39550539
ENSG00000048052	chromosome7	18584322
ENSG00000108840	chromosome17	39550539
ENSG00000068024	chromosome2	239939332
ENSG00000048052	chromosome7	18584322
ENSG00000108840	chromosome17	39550539
ENSG00000163517	chromosome3	13496848
ENSG00000142082	chromosome11	226329
ENSG00000169375	chromosome15	73509770
ENSG00000127511	chromosome19	16801232
ENSG00000150779	chromosome11	111462658
ENSG00000126953	chromosomeX	100490309
ENSG00000134375	chromosome1	200191278
ENSG00000126768	chromosomeX	48639977
ENSG00000173726	chromosome1	233358654
ENSG00000196860	chromosome14	57932429
ENSG00000173726	chromosome1	233358654
ENSG00000025772	chromosome20	43022389
ENSG00000130204	chromosome19	50086513
ENSG00000158882	chromosome1	159462904
ENSG00000204261	chromosome6	32929985
ENSG00000204261	chromosome6	32929985
ENSG00000142507	chromosome17	4646448
ENSG00000205220	chromosome16	66528154
ENSG00000136930	chromosome9	126217526
ENSG00000205220	chromosome16	66528154
ENSG00000129543	chromosome14	22581275
ENSG00000204264	chromosome6	32920169
ENSG00000100804	chromosome14	22573940
ENSG00000204264	chromosome6	32920169
ENSG00000129543	chromosome14	22581275
ENSG00000131467	chromosome17	38239175
ENSG00000092010	chromosome14	23675312
ENSG00000174144	chromosome5	97574862
ENSG00000100911	chromosome14	23685631
ENSG00000068878	chromosome2	54051291
ENSG00000136450	chromosome17	53439498
ENSG00000111786	chromosome12	119391796
ENSG00000136450	chromosome17	53439498
ENSG00000161547	chromosome17	72244838
ENSG00000214754	chromosome7	47058916
ENSG00000112081	chromosome6	36672518
ENSG00000136527	chromosome3	187138454
ENSG00000164548	chromosome7	23537969
ENSG00000116754	chromosome1	70459908
ENSG00000153914	chromosome5	65475961
ENSG00000141562	chromosome17	78009969
ENSG00000103245	chromosome16	730987
ENSG00000114316	chromosome3	49352462
ENSG00000135655	chromosome12	60940463
ENSG00000135655	chromosome12	60940463
ENSG00000114316	chromosome3	49352462
ENSG00000102226	chromosomeX	46977258
ENSG00000036672	chromosome11	118749401
ENSG00000036672	chromosome11	118749401
ENSG00000143258	chromosome1	159397055
ENSG00000103404	chromosome16	23068093
ENSG00000154914	chromosome17	9489675
ENSG00000164134	chromosome4	140442382

ENSG00000172766	chromosome13	40783665
ENSG00000078699	chromosome20	31625668
ENSG00000129993	chromosome16	87570717
ENSG00000079102	chromosome8	93157457
ENSG00000079102	chromosome8	93157457
ENSG00000129993	chromosome16	87570717
ENSG00000120937	chromosome1	11841478
ENSG00000175206	chromosome1	11830428
ENSG00000163273	chromosome2	232499204
ENSG00000156006	chromosome8	18301794
ENSG00000171428	chromosome8	18123837
ENSG00000102230	chromosomeX	24600671
ENSG00000161217	chromosome3	197481800
ENSG00000169418	chromosome1	151918209
ENSG00000159899	chromosome9	35782406
ENSG00000132518	chromosome17	7847091
ENSG00000101890	chromosomeX	108605822
ENSG00000164116	chromosome4	156837470
ENSG00000101890	chromosomeX	108605822
ENSG00000132518	chromosome17	7847091
ENSG00000113389	chromosome5	32747640
ENSG00000163508	chromosome3	27738790
ENSG00000136535	chromosome2	161981168
ENSG00000089225	chromosome12	113326087
ENSG00000121075	chromosome17	56888634
ENSG00000112837	chromosome6	85530619
ENSG00000092607	chromosome1	119331942
ENSG00000149922	chromosome16	30010016
ENSG00000174197	chromosome15	39748385
ENSG00000143178	chromosome1	166516953
ENSG00000164458	chromosome6	166501070
ENSG00000135111	chromosome12	113605389
ENSG00000164458	chromosome6	166501070
ENSG00000143178	chromosome1	166516953
ENSG00000122145	chromosomeX	79164425
ENSG00000092607	chromosome1	119331942
ENSG00000112837	chromosome6	85530619
ENSG00000163508	chromosome3	27738790
ENSG00000174197	chromosome15	39748385
ENSG00000149922	chromosome16	30010016
ENSG00000121068	chromosome17	56832350
ENSG00000135111	chromosome12	113605389
ENSG00000149922	chromosome16	30010016
ENSG00000174197	chromosome15	39748385
ENSG00000121075	chromosome17	56888634
ENSG00000089225	chromosome12	113326087
ENSG00000136535	chromosome2	161981168
ENSG00000163508	chromosome3	27738790
ENSG00000073861	chromosome17	43165820
ENSG00000184058	chromosome22	18127167
ENSG00000167800	chromosome11	67163613
ENSG00000136535	chromosome2	161981168
ENSG00000163508	chromosome3	27738790
ENSG00000089225	chromosome12	113326087
ENSG00000106268	chromosome7	2249153
ENSG00000112664	chromosome6	34468118
ENSG00000196368	chromosomeX	51256037
ENSG00000122824	chromosomeX	51092558
ENSG00000173598	chromosome12	92296230

ENSG00000196368	chromosomeX	51256037
ENSG00000122824	chromosomeX	51092558
ENSG00000173598	chromosome12	92296230
ENSG00000112664	chromosome6	34468118
ENSG00000101911	chromosomeX	12719538
ENSG00000147224	chromosomeX	106758515
ENSG00000161542	chromosome17	71861293
ENSG00000141127	chromosome17	18709872
ENSG00000103200	chromosome16	387224
ENSG00000103024	chromosome16	1761537
ENSG00000123009	chromosome12	119204489
ENSG00000011052	chromosome17	46586734
ENSG00000103024	chromosome16	1761537
ENSG00000103200	chromosome16	387224
ENSG00000103024	chromosome16	1761537
ENSG00000103200	chromosome16	387224
ENSG00000123009	chromosome12	119204489
ENSG00000011052	chromosome17	46586734
ENSG00000181322	chromosome3	139530868
ENSG00000086288	chromosome7	37856394
ENSG00000139921	chromosome14	50776761
ENSG00000125827	chromosome20	7948261
ENSG00000185615	chromosome16	273171
ENSG00000139055	chromosome12	14982710
ENSG00000169340	chromosome16	20318124
ENSG00000169340	chromosome16	20318124
ENSG00000139055	chromosome12	14982710
ENSG00000185624	chromosome17	77411637
ENSG00000139055	chromosome12	14982710
ENSG00000169340	chromosome16	20318124
ENSG00000185615	chromosome16	273171
ENSG00000167004	chromosome15	41826030
ENSG00000185633	chromosome12	55917136
ENSG00000189043	chromosome7	10946210
ENSG00000212665	chromosome1	107849348
ENSG00000154553	chromosome4	186693583
ENSG00000107438	chromosome10	97040663
ENSG00000214895	chromosome14	49586836
ENSG00000131435	chromosome5	131621327
ENSG00000107438	chromosome10	97040663
ENSG00000214895	chromosome14	49586836
ENSG00000154553	chromosome4	186693583
ENSG00000122367	chromosome10	88418429
ENSG00000196923	chromosome5	176856120
ENSG00000163110	chromosome4	95595463
ENSG00000120913	chromosome8	22492238
ENSG00000107438	chromosome10	97040663
ENSG00000214895	chromosome14	49586836
ENSG00000154553	chromosome4	186693583
ENSG00000131435	chromosome5	131621327
ENSG00000196923	chromosome5	176856120
ENSG00000163110	chromosome4	95595463
ENSG00000122367	chromosome10	88418429
ENSG00000154553	chromosome4	186693583
ENSG00000122367	chromosome10	88418429
ENSG00000122367	chromosome10	88418429
ENSG00000163110	chromosome4	95595463
ENSG00000196923	chromosome5	176856120
ENSG00000070404	chromosome19	627424

ENSG00000134363	chromosome5	52812379
ENSG00000163430	chromosome3	121652284
ENSG00000134363	chromosome5	52812379
ENSG00000070404	chromosome19	627424
ENSG00000163430	chromosome3	121652284
ENSG00000053108	chromosome5	132967574
ENSG00000143379	chromosome1	149165878
ENSG00000143379	chromosome1	149165878
ENSG00000136169	chromosome13	48924031
ENSG00000083642	chromosome13	32120910
ENSG00000121892	chromosome4	39654593
ENSG00000152217	chromosome18	40535310
ENSG00000152217	chromosome18	40535310
ENSG00000116539	chromosome1	153757935
ENSG00000152217	chromosome18	40535310
ENSG00000168137	chromosome3	9441891
ENSG00000005483	chromosome7	104468636
ENSG00000119403	chromosome9	122676841
ENSG00000112511	chromosome6	33488019
ENSG00000143033	chromosome1	93317663
ENSG00000112511	chromosome6	33488019
ENSG00000119403	chromosome9	122676841
ENSG00000196498	chromosome12	123545751
ENSG00000141027	chromosome17	16038609
ENSG00000183495	chromosome12	131011118
ENSG00000080603	chromosome16	30619647
ENSG00000032219	chromosome14	57836326
ENSG00000054267	chromosome1	233556858
ENSG00000105011	chromosome19	14108269
ENSG00000111875	chromosome6	119263629
ENSG00000102974	chromosome16	66162569
ENSG00000124092	chromosome20	55532668
ENSG00000118156	chromosome19	52750926
ENSG00000156030	chromosome14	73276465
ENSG00000124496	chromosome6	42345307
ENSG00000156030	chromosome14	73276465
ENSG00000118156	chromosome19	52750926
ENSG00000110066	chromosome11	67714120
ENSG00000133247	chromosome19	60545117
ENSG00000034063	chromosome19	4861859
ENSG00000147854	chromosome9	6403491
ENSG00000099381	chromosome16	30877554
ENSG00000139718	chromosome12	120741761
ENSG00000139718	chromosome12	120741761
ENSG00000099381	chromosome16	30877554
ENSG00000151136	chromosome12	106236848
ENSG00000166016	chromosome11	34335149
ENSG00000120616	chromosome10	32707428
ENSG00000135999	chromosome2	149119057
ENSG00000028310	chromosome5	945902
ENSG00000214423	chromosome6	111857720
ENSG00000166164	chromosome16	48960187
ENSG00000215415	chromosome13	94209528
ENSG00000064961	chromosome19	3524308
ENSG00000140382	chromosome15	75537805
ENSG00000181090	chromosome9	139711440
ENSG00000204371	chromosome6	31973434
ENSG00000057935	chromosome2	42649390
ENSG00000182979	chromosome14	104957418

ENSG00000149480	chromosome11	62125499
ENSG00000182979	chromosome14	104957418
ENSG00000057935	chromosome2	42649390
ENSG00000166923	chromosome15	30810184
ENSG00000180875	chromosome1	238723399
ENSG00000179284	chromosome19	12936975
ENSG00000147869	chromosome9	14712671
ENSG00000170004	chromosome17	7728892
ENSG00000116254	chromosome1	6162671
ENSG00000111642	chromosome12	6585801
ENSG00000116254	chromosome1	6162671
ENSG00000170004	chromosome17	7728892
ENSG00000066135	chromosome1	43891434
ENSG00000186280	chromosome11	94370185
ENSG00000127663	chromosome19	4983902
ENSG00000107077	chromosome9	6782989
ENSG00000107077	chromosome9	6782989
ENSG00000186280	chromosome11	94370185
ENSG00000127663	chromosome19	4983902
ENSG00000127663	chromosome19	4983902
ENSG00000186280	chromosome11	94370185
ENSG00000117625	chromosome1	209499526
ENSG00000089902	chromosome14	102128986
ENSG00000089902	chromosome14	102128986
ENSG00000117625	chromosome1	209499526
ENSG00000167771	chromosome11	63440505
ENSG00000054938	chromosome11	74119582
ENSG00000101938	chromosomeX	109922066
ENSG00000152455	chromosome10	14960894
ENSG00000101945	chromosomeX	48440120
ENSG00000101945	chromosomeX	48440120
ENSG00000152455	chromosome10	14960894
ENSG00000101945	chromosomeX	48440120
ENSG00000155545	chromosome5	56292378
ENSG00000105556	chromosome19	295783
ENSG00000198160	chromosome1	67153227
ENSG00000198160	chromosome1	67153227
ENSG00000105556	chromosome19	295783
ENSG00000122779	chromosome7	137795834
ENSG00000197323	chromosome1	114855221
ENSG00000166436	chromosome11	8629932
ENSG00000197323	chromosome1	114855221
ENSG00000122779	chromosome7	137795834
ENSG00000130726	chromosome19	63747937
ENSG00000197323	chromosome1	114855221
ENSG00000122779	chromosome7	137795834
ENSG00000166436	chromosome11	8629932
ENSG00000100307	chromosome22	37878396
ENSG00000141582	chromosome17	75427646
ENSG00000173894	chromosome17	75366630
ENSG00000183741	chromosome22	37598142
ENSG00000141570	chromosome17	75385393
ENSG00000141582	chromosome17	75427646
ENSG00000100307	chromosome22	37878396
ENSG00000173894	chromosome17	75366630
ENSG00000100307	chromosome22	37878396
ENSG00000141582	chromosome17	75427646
ENSG00000141570	chromosome17	75385393
ENSG00000183741	chromosome22	37598142

ENSG00000100307	chromosome22	37878396
ENSG00000164266	chromosome5	147191334
ENSG00000128040	chromosome4	57382586
ENSG00000164266	chromosome5	147191334
ENSG00000164266	chromosome5	147191334
ENSG00000164690	chromosome7	155297578
ENSG00000163501	chromosome2	219633434
ENSG00000139549	chromosome12	47774563
ENSG00000163501	chromosome2	219633434
ENSG00000164690	chromosome7	155297578
ENSG00000112118	chromosome6	52257432
ENSG00000164778	chromosome7	154943834
ENSG00000163064	chromosome2	119321214
ENSG00000164778	chromosome7	154943834
ENSG00000176697	chromosome11	27699538
ENSG00000167744	chromosome19	54257067
ENSG00000212844	chromosome19	54234322
ENSG00000134259	chromosome1	115630940
ENSG00000185652	chromosome12	5473642
ENSG00000185652	chromosome12	5473642
ENSG00000134259	chromosome1	115630940
ENSG00000167744	chromosome19	54257067
ENSG00000212844	chromosome19	54234322
ENSG00000176697	chromosome11	27699538
ENSG00000095794	chromosome10	35466770
ENSG00000118260	chromosome2	208128605
ENSG00000123268	chromosome12	49460196
ENSG00000118260	chromosome2	208128605
ENSG00000095794	chromosome10	35466770
ENSG00000140538	chromosome15	86600389
ENSG00000148053	chromosome9	86475346
ENSG00000198400	chromosome1	155052246
ENSG00000148053	chromosome9	86475346
ENSG00000140538	chromosome15	86600389
ENSG00000185483	chromosome1	64012677
ENSG00000169071	chromosome9	93752067
ENSG00000064300	chromosome17	44927779
ENSG00000214778	chromosome3	47028036
ENSG00000170522	chromosome4	111338941
ENSG00000197168	chromosome13	51599620
ENSG00000136098	chromosome13	51628328
ENSG00000197168	chromosome13	51599620
ENSG00000028116	chromosome2	58129471
ENSG00000100749	chromosome14	96369562
ENSG00000100749	chromosome14	96369562
ENSG00000028116	chromosome2	58129471
ENSG00000105053	chromosome19	55211232
ENSG00000025293	chromosome20	33852859
ENSG00000129292	chromosome8	133859257
ENSG00000129292	chromosome8	133859257
ENSG00000073614	chromosome12	368644
ENSG00000117139	chromosome1	201044467
ENSG00000012817	chromosomeY	17656289
ENSG00000126012	chromosomeX	53270797
ENSG00000117139	chromosome1	201044467
ENSG00000073614	chromosome12	368644
ENSG00000006459	chromosome7	139523207
ENSG00000172943	chromosomeX	54088049
ENSG00000197724	chromosome9	95378877

ENSG00000197724	chromosome9	95378877
ENSG00000172943	chromosomeX	54088049
ENSG00000173120	chromosome11	66698221
ENSG00000089094	chromosome12	120503200
ENSG00000124191	chromosome20	42007967
ENSG00000198846	chromosome8	60194101
ENSG00000198846	chromosome8	60194101
ENSG00000124191	chromosome20	42007967
ENSG00000103460	chromosome16	51138137
ENSG00000092203	chromosome14	21015278
ENSG00000103460	chromosome16	51138137
ENSG00000198846	chromosome8	60194101
ENSG00000124191	chromosome20	42007967
ENSG00000184887	chromosome14	104786549
ENSG00000132640	chromosome20	11846924
ENSG00000133243	chromosome19	1966703
ENSG00000064726	chromosome15	81526908
ENSG00000156650	chromosome10	76272622
ENSG00000083168	chromosome8	42025653
ENSG00000102054	chromosomeX	16798098
ENSG00000162521	chromosome1	32889495
ENSG00000108604	chromosome17	59273662
ENSG00000082014	chromosome7	150603172
ENSG00000066117	chromosome12	48765420
ENSG00000082014	chromosome7	150603172
ENSG00000108604	chromosome17	59273662
ENSG00000143499	chromosome1	212521221
ENSG00000115593	chromosome2	88148499
ENSG00000143499	chromosome1	212521221
ENSG00000115593	chromosome2	88148499
ENSG00000102098	chromosomeX	18262113
ENSG00000010803	chromosome1	41424393
ENSG00000146285	chromosome6	108200225
ENSG00000146285	chromosome6	108200225
ENSG00000010803	chromosome1	41424393
ENSG00000185920	chromosome9	97318924
ENSG00000117425	chromosome1	45081192
ENSG00000215267	chromosome10	5320443
ENSG00000196326	chromosome10	5217068
ENSG00000151631	chromosome10	4924162
ENSG00000198610	chromosome10	5228831
ENSG00000196139	chromosome10	5126637
ENSG00000151632	chromosome10	5036067
ENSG00000187134	chromosome10	4995637
ENSG00000122787	chromosome7	137411805
ENSG00000117448	chromosome1	45800054
ENSG00000122787	chromosome7	137411805
ENSG00000215267	chromosome10	5320443
ENSG00000196326	chromosome10	5217068
ENSG00000151631	chromosome10	4924162
ENSG00000198610	chromosome10	5228831
ENSG00000196139	chromosome10	5126637
ENSG00000151632	chromosome10	5036067
ENSG00000187134	chromosome10	4995637
ENSG00000100811	chromosome14	99775335
ENSG00000198767	chromosomeX	21784524
ENSG00000100811	chromosome14	99775335
ENSG00000179059	chromosome4	189160956
ENSG00000100811	chromosome14	99775335

ENSG00000198767	chromosomeX	21784524
ENSG00000011426	chromosome7	36396161
ENSG00000132694	chromosome1	155280747
ENSG00000076928	chromosome19	47079108
ENSG00000196914	chromosome11	119713163
ENSG00000196914	chromosome11	119713163
ENSG00000076928	chromosome19	47079108
ENSG00000134982	chromosome5	112118487
ENSG00000115266	chromosome19	1404001
ENSG00000109163	chromosome4	68302649
ENSG00000211451	chromosome1	144227055
ENSG00000178015	chromosome5	94981736
ENSG00000211451	chromosome1	144227055
ENSG00000109163	chromosome4	68302649
ENSG00000211451	chromosome1	144227055
ENSG00000211451	chromosome1	144227055
ENSG00000126895	chromosomeX	152823794
ENSG00000198049	chromosome1	204391064
ENSG00000166148	chromosome12	61830884
ENSG00000180914	chromosome3	8784874
ENSG00000180914	chromosome3	8784874
ENSG00000198049	chromosome1	204391064
ENSG00000166148	chromosome12	61830884
ENSG00000126895	chromosomeX	152823794
ENSG00000166148	chromosome12	61830884
ENSG00000198049	chromosome1	204391064
ENSG00000126895	chromosomeX	152823794
ENSG00000116678	chromosome1	65803837
ENSG00000006016	chromosome19	18578632
ENSG00000137275	chromosome6	3022057
ENSG00000129465	chromosome14	23878873
ENSG00000104312	chromosome8	90839424
ENSG00000170209	chromosome11	112763817
ENSG00000183421	chromosome21	42060271
ENSG00000129465	chromosome14	23878873
ENSG00000137275	chromosome6	3022057
ENSG00000170209	chromosome11	112763817
ENSG00000183421	chromosome21	42060271
ENSG00000104312	chromosome8	90839424
ENSG00000183421	chromosome21	42060271
ENSG00000170209	chromosome11	112763817
ENSG00000059758	chromosome12	95252746
ENSG00000117266	chromosome1	203758919
ENSG00000117266	chromosome1	203758919
ENSG00000059758	chromosome12	95252746
ENSG00000117266	chromosome1	203758919
ENSG00000059758	chromosome12	95252746
ENSG00000102225	chromosomeX	46967901
ENSG00000138395	chromosome2	202379633
ENSG00000058091	chromosome7	90063878
ENSG00000082175	chromosome11	100505012
ENSG00000082175	chromosome11	100505012
ENSG00000151623	chromosome4	149577463
ENSG00000113580	chromosome5	142760598
ENSG00000113580	chromosome5	142760598
ENSG00000151623	chromosome4	149577463
ENSG00000148248	chromosome9	135232662
ENSG00000164708	chromosome7	44071654
ENSG00000215178	chromosome9	35933926

ENSG00000171314	chromosome10	99176055
ENSG00000197041	chromosome12	102948687
ENSG00000186076	chromosomeX	77111792
ENSG00000214206	chromosome12	94590212
ENSG00000214824	chromosome2	23949389
ENSG00000109047	chromosome17	9749223
ENSG00000163032	chromosome2	17636823
ENSG00000116983	chromosome1	39922863
ENSG00000121905	chromosome1	33127087
ENSG00000115756	chromosome2	10477335
ENSG00000109047	chromosome17	9749223
ENSG00000104490	chromosome8	102801049
ENSG00000115756	chromosome2	10477335
ENSG00000121905	chromosome1	33127087
ENSG00000104490	chromosome8	102801049
ENSG00000115756	chromosome2	10477335
ENSG00000119396	chromosome9	122995512
ENSG00000183940	chromosome14	89427888
ENSG00000188060	chromosome1	28791611
ENSG00000179331	chromosome11	107304505
ENSG00000155961	chromosomeX	154146768
ENSG00000168118	chromosome1	227473710
ENSG00000167578	chromosome19	45976121
ENSG00000183940	chromosome14	89427888
ENSG00000188060	chromosome1	28791611
ENSG00000155961	chromosomeX	154146768
ENSG00000179331	chromosome11	107304505
ENSG00000118520	chromosome6	131936116
ENSG00000081181	chromosome14	67156448
ENSG00000137154	chromosome9	19385743
ENSG00000214908	chromosome9	88180442
ENSG00000173322	chromosome12	12895183
ENSG00000178429	chromosome10	86310179
ENSG00000145425	chromosome4	152240255
ENSG00000187899	chromosome15	57847843
ENSG00000215836	chromosome1	164258076
ENSG00000198805	chromosome14	19992539
ENSG00000141367	chromosome17	55052275
ENSG00000070371	chromosome22	17659165
ENSG00000076555	chromosome12	108061594
ENSG00000132142	chromosome17	32840614
ENSG00000115365	chromosome2	211049366
ENSG00000132434	chromosome7	55401213
ENSG00000174871	chromosome11	65802504
ENSG00000143786	chromosome1	222871500
ENSG00000100528	chromosome14	53977796
ENSG00000143786	chromosome1	222871500
ENSG00000174871	chromosome11	65802504
ENSG00000054116	chromosome1	36387567
ENSG00000173626	chromosome6	116973371
ENSG00000174903	chromosome11	65777735
ENSG00000138069	chromosome2	65194788
ENSG00000138069	chromosome2	65194788
ENSG00000174903	chromosome11	65777735
ENSG00000146955	chromosome7	139753916
ENSG00000185051	chromosome16	45218655
ENSG00000134594	chromosomeX	129133718
ENSG00000172007	chromosome4	140594800
ENSG00000174903	chromosome11	65777735

ENSG00000138069	chromosome2	65194788
ENSG00000174903	chromosome11	65777735
ENSG00000138069	chromosome2	65194788
ENSG00000134594	chromosomeX	129133718
ENSG00000185236	chromosome19	8361301
ENSG00000103769	chromosome15	63948978
ENSG00000185236	chromosome19	8361301
ENSG00000112592	chromosome6	170708043
ENSG00000182521	chromosome14	54977017
ENSG00000112592	chromosome6	170708043
ENSG00000112592	chromosome6	170708043
ENSG00000182521	chromosome14	54977017
ENSG00000173868	chromosome17	44659053
ENSG00000144362	chromosome2	170265728
ENSG00000160516	chromosome7	71045180
ENSG00000182953	chromosome17	18416791
ENSG00000197303	chromosome11	82078249
ENSG00000188486	chromosome11	118471315
ENSG00000184270	chromosome1	148126091
ENSG00000164508	chromosome6	25834735
ENSG00000203812	chromosome1	148089305
ENSG00000183558	chromosome1	148080890
ENSG00000184260	chromosome1	148125149
ENSG00000111332	chromosome12	14818672
ENSG00000180573	chromosome6	26232440
ENSG00000181218	chromosome1	226712142
ENSG00000168274	chromosome6	26325182
ENSG00000137259	chromosome6	26141776
ENSG00000124642	chromosome6	26307451
ENSG00000182611	chromosome6	27890498
ENSG00000196787	chromosome6	27208830
ENSG00000184348	chromosome6	27914097
ENSG00000198374	chromosome6	27941112
ENSG00000196747	chromosome6	27883967
ENSG00000184825	chromosome6	27222887
ENSG00000196866	chromosome6	27968907
ENSG00000158403	chromosome6	26380748
ENSG00000198082	chromosomeX	153766519
ENSG00000198307	chromosomeX	154263630
ENSG00000185978	chromosomeX	154342783
ENSG00000205079	chromosomeX	36647927
ENSG00000205070	chromosomeX	37738541
ENSG00000184270	chromosome1	148126091
ENSG00000188486	chromosome11	118471315
ENSG00000198082	chromosomeX	153766519
ENSG00000198307	chromosomeX	154263630
ENSG00000185978	chromosomeX	154342783
ENSG00000205079	chromosomeX	36647927
ENSG00000205070	chromosomeX	37738541
ENSG00000184270	chromosome1	148126091
ENSG00000184270	chromosome1	148126091
ENSG00000198082	chromosomeX	153766519
ENSG00000198307	chromosomeX	154263630
ENSG00000185978	chromosomeX	154342783
ENSG00000205079	chromosomeX	36647927
ENSG00000205070	chromosomeX	37738541
ENSG00000164508	chromosome6	25834735
ENSG00000203812	chromosome1	148089305
ENSG00000183558	chromosome1	148080890

ENSG00000184260	chromosome1	148125149
ENSG00000111332	chromosome12	14818672
ENSG00000180573	chromosome6	26232440
ENSG00000181218	chromosome1	226712142
ENSG00000168274	chromosome6	26325182
ENSG00000137259	chromosome6	26141776
ENSG00000124642	chromosome6	26307451
ENSG00000182611	chromosome6	27890498
ENSG00000196787	chromosome6	27208830
ENSG00000184348	chromosome6	27914097
ENSG00000198374	chromosome6	27941112
ENSG00000196747	chromosome6	27883967
ENSG00000184825	chromosome6	27222887
ENSG00000196866	chromosome6	27968907
ENSG00000158403	chromosome6	26380748
ENSG00000215717	supercontigNT_113870	124215
ENSG00000162647	chromosome1	109432406
ENSG00000154518	chromosome2	175754368
ENSG00000135390	chromosome12	52356244
ENSG00000159199	chromosome17	44325779
ENSG00000135390	chromosome12	52356244
ENSG00000159199	chromosome17	44325779
ENSG00000179315	chromosome15	33171974
ENSG00000160201	chromosome21	43400674
ENSG00000160201	chromosome21	43400674
ENSG00000179315	chromosome15	33171974
ENSG00000161265	chromosome19	40928133
ENSG00000124578	chromosome6	26355185
ENSG00000188987	chromosome6	26297284
ENSG00000198558	chromosome6	27949268
ENSG00000124529	chromosome6	26135460
ENSG00000197061	chromosome6	26212155
ENSG00000197837	chromosome12	14815286
ENSG00000197238	chromosome6	27899882
ENSG00000196176	chromosome6	26129886
ENSG00000197914	chromosome6	27907285
ENSG00000183941	chromosome1	148070873
ENSG00000182217	chromosome1	148099322
ENSG00000158406	chromosome6	26393707
ENSG00000197238	chromosome6	27899882
ENSG00000197837	chromosome12	14815286
ENSG00000197238	chromosome6	27899882
ENSG00000198339	chromosome6	27215067
ENSG00000198339	chromosome6	27215067
ENSG00000198518	chromosome6	26312852
ENSG00000183941	chromosome1	148070873
ENSG00000196176	chromosome6	26129886
ENSG00000197914	chromosome6	27907285
ENSG00000188987	chromosome6	26297284
ENSG00000198558	chromosome6	27949268
ENSG00000124529	chromosome6	26135460
ENSG00000197061	chromosome6	26212155
ENSG00000197837	chromosome12	14815286
ENSG00000197238	chromosome6	27899882
ENSG00000182217	chromosome1	148099322
ENSG00000158406	chromosome6	26393707
ENSG00000196176	chromosome6	26129886
ENSG00000197914	chromosome6	27907285
ENSG00000183941	chromosome1	148070873

ENSG00000188987	chromosome6	26297284
ENSG00000198558	chromosome6	27949268
ENSG00000124529	chromosome6	26135460
ENSG00000197061	chromosome6	26212155
ENSG00000197837	chromosome12	14815286
ENSG00000197238	chromosome6	27899882
ENSG00000196176	chromosome6	26129886
ENSG00000197914	chromosome6	27907285
ENSG00000197238	chromosome6	27899882
ENSG00000197061	chromosome6	26212155
ENSG00000124529	chromosome6	26135460
ENSG00000124529	chromosome6	26135460
ENSG00000197061	chromosome6	26212155
ENSG00000188987	chromosome6	26297284
ENSG00000198558	chromosome6	27949268
ENSG00000198518	chromosome6	26312852
ENSG00000198339	chromosome6	27215067
ENSG00000197914	chromosome6	27907285
ENSG00000196176	chromosome6	26129886
ENSG00000197914	chromosome6	27907285
ENSG00000197837	chromosome12	14815286
ENSG00000197238	chromosome6	27899882
ENSG00000188987	chromosome6	26297284
ENSG00000198558	chromosome6	27949268
ENSG00000124529	chromosome6	26135460
ENSG00000197061	chromosome6	26212155
ENSG00000198327	chromosome6	26348633
ENSG00000198518	chromosome6	26312852
ENSG00000198339	chromosome6	27215067
ENSG00000197914	chromosome6	27907285
ENSG00000124578	chromosome6	26355185
ENSG00000197238	chromosome6	27899882
ENSG00000197238	chromosome6	27899882
ENSG00000124578	chromosome6	26355185
ENSG00000183941	chromosome1	148070873
ENSG00000197914	chromosome6	27907285
ENSG00000124529	chromosome6	26135460
ENSG00000197061	chromosome6	26212155
ENSG00000124578	chromosome6	26355185
ENSG00000197238	chromosome6	27899882
ENSG00000197837	chromosome12	14815286
ENSG00000132570	chromosome5	134268729
ENSG00000166228	chromosome10	72318297
ENSG00000157823	chromosome15	88238186
ENSG00000214446	chromosome15	88257072
ENSG00000177879	chromosome5	115205634
ENSG00000157823	chromosome15	88238186
ENSG00000214446	chromosome15	88257072
ENSG00000157823	chromosome15	88238186
ENSG00000214446	chromosome15	88257072
ENSG00000214446	chromosome15	88257072
ENSG00000157823	chromosome15	88238186
ENSG00000160075	chromosome1	1499801
ENSG00000176960	chromosomeX	113531383
ENSG00000186217	chromosome7	123903661
ENSG00000186223	chromosome11	4220447
ENSG00000186232	chromosome11	4307671
ENSG00000099860	chromosome19	2427357
ENSG00000130222	chromosome9	91409857

ENSG00000116717	chromosome1	67923766
ENSG00000130222	chromosome9	91409857
ENSG00000099860	chromosome19	2427357
ENSG00000163382	chromosome1	154828221
ENSG00000186010	chromosome19	19500721
ENSG00000111540	chromosome12	54667012
ENSG00000108774	chromosome17	37536047
ENSG00000144566	chromosome3	19967395
ENSG00000108774	chromosome17	37536047
ENSG00000144566	chromosome3	19967395
ENSG00000111540	chromosome12	54667012
ENSG00000144566	chromosome3	19967395
ENSG00000108774	chromosome17	37536047
ENSG00000118271	chromosome18	27425864
ENSG00000172450	chromosomeX	103046270
ENSG00000101812	chromosomeX	103154925
ENSG00000123569	chromosomeX	103196496
ENSG00000172809	chromosome17	69711690
ENSG00000172809	chromosome17	69711690
ENSG00000213741	chromosome14	49122815
ENSG00000008988	chromosome8	57149497
ENSG00000214289	chromosome3	135557939
ENSG00000163923	chromosome3	188321783
ENSG00000198918	chromosomeX	118809568
ENSG00000174440	chromosome2	100353440
ENSG00000188512	chromosome11	31259104
ENSG00000214810	chromosome6	34295196
ENSG00000172115	chromosome7	25130264
ENSG00000154582	chromosome8	75034558
ENSG00000105694	chromosome19	2489108
ENSG00000154582	chromosome8	75034558
ENSG00000105694	chromosome19	2489108
ENSG00000172786	chromosome1	172326234
ENSG00000156482	chromosome8	99129035
ENSG00000173180	chromosome6	114267159
ENSG00000173436	chromosome1	19796127
ENSG00000005243	chromosome17	43470139
ENSG00000111481	chromosome12	53005215
ENSG00000121083	chromosome17	53519451
ENSG00000088986	chromosome12	119418608
ENSG00000185088	chromosome15	61236658
ENSG00000130041	chromosome12	3191345
ENSG00000163144	chromosome4	22204917
ENSG00000177954	chromosome1	152229897
ENSG00000198731	chromosome1	37794435
ENSG00000111711	chromosome12	21546125
ENSG00000174567	chromosome1	202449658
ENSG00000069509	chromosomeX	44287023
ENSG00000182814	chromosome2	84371454
ENSG00000165775	chromosomeX	153908408
ENSG00000183726	chromosome1	25539565
ENSG00000142188	chromosome21	33763063
ENSG00000119640	chromosome14	74600010
ENSG00000170634	chromosome2	54196256
ENSG00000113732	chromosome5	172343470
ENSG00000124588	chromosome6	2939231
ENSG00000181019	chromosome16	68317844
ENSG00000176014	chromosome18	12298292
ENSG00000198211	chromosome16	88513168

ENSG00000137285	chromosome6	3172777
ENSG00000137267	chromosome6	3102697
ENSG00000215801	chromosome1	240287010
ENSG00000215813	chromosome1	225760506
ENSG00000215814	chromosome1	225749467
ENSG00000127589	chromosome4	191143018
ENSG00000159247	chromosome9	140189317
ENSG00000173213	chromosome18	39558
ENSG00000173876	chromosome10	85179
ENSG00000117983	chromosome11	1200930
ENSG00000196292	chromosome11	1141627
ENSG00000198788	chromosome11	1064902
ENSG00000184956	chromosome11	1026656
ENSG00000184956	chromosome11	1026656
ENSG00000117983	chromosome11	1200930
ENSG00000196292	chromosome11	1141627
ENSG00000198788	chromosome11	1064902
ENSG00000109927	chromosome11	120478585
ENSG00000146839	chromosome7	100169728
ENSG00000109927	chromosome11	120478585
ENSG00000184956	chromosome11	1026656
ENSG00000188162	chromosome11	17526303
ENSG00000197558	chromosome7	149115353
ENSG00000146839	chromosome7	100169728
ENSG00000117983	chromosome11	1200930
ENSG00000196292	chromosome11	1141627
ENSG00000198788	chromosome11	1064902
ENSG00000117983	chromosome11	1200930
ENSG00000196292	chromosome11	1141627
ENSG00000090920	chromosome19	45132366
ENSG00000109927	chromosome11	120478585
ENSG00000146839	chromosome7	100169728
ENSG00000156508	chromosome6	74286471
ENSG00000185637	chromosome9	134884694
ENSG00000214744	chromosome12	17034871
ENSG00000215093	chromosomeX	85303020
ENSG00000101210	chromosome20	61599561
ENSG00000156508	chromosome6	74286471
ENSG00000185637	chromosome9	134884694
ENSG00000214744	chromosome12	17034871
ENSG00000215093	chromosomeX	85303020
ENSG00000101210	chromosome20	61599561
ENSG00000101210	chromosome20	61599561
ENSG00000156508	chromosome6	74286471
ENSG00000185637	chromosome9	134884694
ENSG00000214744	chromosome12	17034871
ENSG00000215093	chromosomeX	85303020
ENSG00000156508	chromosome6	74286471
ENSG00000185637	chromosome9	134884694
ENSG00000214744	chromosome12	17034871
ENSG00000215093	chromosomeX	85303020
ENSG00000101210	chromosome20	61599561
ENSG00000156508	chromosome6	74286471
ENSG00000185637	chromosome9	134884694
ENSG00000214744	chromosome12	17034871
ENSG00000215093	chromosomeX	85303020
ENSG00000101210	chromosome20	61599561
ENSG00000104812	chromosome19	54188182
ENSG00000111713	chromosome12	21648794

ENSG00000196961	chromosome19	54962203
ENSG00000183020	chromosome11	916022
ENSG00000068976	chromosome11	64283947
ENSG00000100994	chromosome20	25176815
ENSG00000100994	chromosome20	25176815
ENSG00000068976	chromosome11	64283947
ENSG00000100504	chromosome14	50480872
ENSG00000147416	chromosome8	20099198
ENSG00000147416	chromosome8	20099198
ENSG00000116039	chromosome2	71016593
ENSG00000214391	chromosome11	89655474
ENSG00000163526	chromosome2	219839190
ENSG00000127824	chromosome2	219826825
ENSG00000127824	chromosome2	219826825
ENSG00000163526	chromosome2	219839190
ENSG00000123416	chromosome12	47811351
ENSG00000163526	chromosome2	219839190
ENSG00000127824	chromosome2	219826825
ENSG00000214391	chromosome11	89655474
ENSG00000129003	chromosome15	60139866
ENSG00000197969	chromosome9	78982441
ENSG00000100280	chromosome22	28093233
ENSG00000006125	chromosome17	30945163
ENSG00000136731	chromosome2	128565402
ENSG00000102595	chromosome13	95503568
ENSG00000181789	chromosome3	130451243
ENSG00000157087	chromosome3	10466228
ENSG00000067842	chromosomeX	152454900
ENSG00000058668	chromosome1	201918957
ENSG00000067842	chromosomeX	152454900
ENSG00000157087	chromosome3	10466228
ENSG00000070961	chromosome12	88573795
ENSG00000067842	chromosomeX	152454900
ENSG00000157087	chromosome3	10466228
ENSG00000058668	chromosome1	201918957
ENSG00000070961	chromosome12	88573795
ENSG00000058668	chromosome1	201918957
ENSG00000067842	chromosomeX	152454900
ENSG00000157087	chromosome3	10466228
ENSG00000159363	chromosome1	17210821
ENSG00000127249	chromosome3	194755283
ENSG00000187527	chromosome3	194579209
ENSG00000133657	chromosome3	195664209
ENSG00000133657	chromosome3	195664209
ENSG00000127249	chromosome3	194755283
ENSG00000187527	chromosome3	194579209
ENSG00000100201	chromosome22	37232189
ENSG00000108654	chromosome17	59932700
ENSG00000089597	chromosome11	62170648
ENSG00000214013	chromosome15	40353972
ENSG00000033627	chromosome17	37866439
ENSG00000105929	chromosome7	138106533
ENSG00000110719	chromosome11	67565315
ENSG00000185344	chromosome12	122763066
ENSG00000110719	chromosome11	67565315
ENSG00000185344	chromosome12	122763066
ENSG00000105929	chromosome7	138106533
ENSG00000185344	chromosome12	122763066
ENSG00000110719	chromosome11	67565315

ENSG00000110719	chromosome11	67565315
ENSG00000185344	chromosome12	122763066
ENSG00000058262	chromosome3	129254086
ENSG00000065665	chromosome10	12211701
ENSG00000079739	chromosome1	63831658
ENSG00000079739	chromosome1	63831658
ENSG00000204794	chromosome9	68199367
ENSG00000154330	chromosome9	70161864
ENSG00000111530	chromosome12	65949765
ENSG00000144712	chromosome3	12813220
ENSG00000163017	chromosome2	73981947
ENSG00000107796	chromosome10	90698668
ENSG00000143632	chromosome1	227635486
ENSG00000163017	chromosome2	73981947
ENSG00000107796	chromosome10	90698668
ENSG00000159251	chromosome15	32874302
ENSG00000143632	chromosome1	227635486
ENSG00000214555	chromosome15	81192887
ENSG00000215141	chromosome11	1781902
ENSG00000204434	chromosome2	132100182
ENSG00000075624	chromosome7	5535815
ENSG00000184009	chromosome17	77093976
ENSG00000143632	chromosome1	227635486
ENSG00000159251	chromosome15	32874302
ENSG00000163017	chromosome2	73981947
ENSG00000107796	chromosome10	90698668
ENSG00000143632	chromosome1	227635486
ENSG00000163017	chromosome2	73981947
ENSG00000107796	chromosome10	90698668
ENSG00000143632	chromosome1	227635486
ENSG00000159251	chromosome15	32874302
ENSG00000184009	chromosome17	77093976
ENSG00000163017	chromosome2	73981947
ENSG00000107796	chromosome10	90698668
ENSG00000184009	chromosome17	77093976
ENSG00000214555	chromosome15	81192887
ENSG00000215141	chromosome11	1781902
ENSG00000204434	chromosome2	132100182
ENSG00000075624	chromosome7	5535815
ENSG00000079805	chromosome19	10689919
ENSG00000197959	chromosome1	170077420
ENSG00000106976	chromosome9	130005571
ENSG00000197959	chromosome1	170077420
ENSG00000079805	chromosome19	10689919
ENSG00000166747	chromosome16	70380884
ENSG00000213983	chromosome14	23106364
ENSG00000106348	chromosome7	127837192
ENSG00000178035	chromosome3	49041788
ENSG00000100938	chromosome14	23772300
ENSG00000137198	chromosome6	16346904
ENSG00000171793	chromosome1	41221550
ENSG00000047230	chromosomeX	16630947
ENSG00000067225	chromosome15	70298493
ENSG00000143627	chromosome1	153537844
ENSG00000108515	chromosome17	4795870
ENSG00000111674	chromosome12	6895258
ENSG00000215809	chromosome1	234713088
ENSG00000074800	chromosome1	8857555
ENSG00000214431	chromosome15	90765134

ENSG00000108515	chromosome17	4795870
ENSG00000111674	chromosome12	6895258
ENSG00000111674	chromosome12	6895258
ENSG00000108515	chromosome17	4795870
ENSG00000140022	chromosome14	80934480
ENSG00000129354	chromosome19	10558908
ENSG00000072958	chromosome19	16169838
ENSG00000125304	chromosome13	98951862
ENSG00000164307	chromosome5	96165386
ENSG00000164308	chromosome5	96241146
ENSG00000166825	chromosome15	88150819
ENSG00000113441	chromosome5	96297153
ENSG00000164308	chromosome5	96241146
ENSG00000164307	chromosome5	96165386
ENSG00000067057	chromosome10	3099788
ENSG00000141959	chromosome21	44544417
ENSG00000152556	chromosome12	46787424
ENSG00000152556	chromosome12	46787424
ENSG00000141959	chromosome21	44544417
ENSG00000168710	chromosome1	110329198
ENSG00000158467	chromosome7	128652154
ENSG00000158467	chromosome7	128652154
ENSG00000168710	chromosome1	110329198
ENSG00000131069	chromosome20	32926564
ENSG00000115073	chromosome2	97646786
ENSG00000138107	chromosome10	104252395
ENSG00000134516	chromosome5	168996909
ENSG00000147459	chromosome8	25098341
ENSG00000150760	chromosome10	128584077
ENSG00000088538	chromosome3	50791106
ENSG00000128512	chromosome7	111431424
ENSG00000150760	chromosome10	128584077
ENSG00000147459	chromosome8	25098341
ENSG00000197894	chromosome4	100240091
ENSG00000198099	chromosome4	100284397
ENSG00000187758	chromosome4	100431095
ENSG00000196616	chromosome4	100461512
ENSG00000196344	chromosome4	100575450
ENSG00000172955	chromosome4	100359333
ENSG00000178952	chromosome16	28765092
ENSG00000133805	chromosome11	10429145
ENSG00000116748	chromosome1	115039715
ENSG00000110367	chromosome11	118162171
ENSG00000123136	chromosome19	14385033
ENSG00000198563	chromosome6	31617457
ENSG00000203879	chromosomeX	153318795
ENSG00000057608	chromosome10	5895228
ENSG00000203879	chromosomeX	153318795
ENSG00000057608	chromosome10	5895228
ENSG00000057608	chromosome10	5895228
ENSG00000203879	chromosomeX	153318795
ENSG00000100596	chromosome14	77152676
ENSG00000172296	chromosome20	12937916
ENSG00000184254	chromosome15	99237603
ENSG00000128918	chromosome15	56145141
ENSG00000165092	chromosome9	74757737
ENSG00000128918	chromosome15	56145141
ENSG00000184254	chromosome15	99237603
ENSG00000111275	chromosome12	110689170

ENSG00000137124	chromosome9	38385746
ENSG00000151846	chromosome13	24568337
ENSG00000215587	chromosomeY	9241811
ENSG00000070756	chromosome8	101802988
ENSG00000174740	chromosomeX	90577233
ENSG00000090621	chromosome1	39814211
ENSG00000101104	chromosome20	42972199
ENSG00000174740	chromosomeX	90577233
ENSG00000090621	chromosome1	39814211
ENSG00000151846	chromosome13	24568337
ENSG00000215587	chromosomeY	9241811
ENSG00000070756	chromosome8	101802988
ENSG00000123213	chromosome5	65053957
ENSG00000172009	chromosome19	2736661
ENSG00000123191	chromosome13	51483475
ENSG00000165240	chromosomeX	77104778
ENSG00000215763	supercontigNT_113965	504703
ENSG00000150961	chromosome4	119974300
ENSG00000176986	chromosome10	75176597
ENSG00000171848	chromosome2	10180197
ENSG00000048392	chromosome8	103320279
ENSG00000138071	chromosome2	65308548
ENSG00000213985	chromosome19	20229529
ENSG00000131462	chromosome17	38015282
ENSG00000037042	chromosome17	38065048
ENSG00000070770	chromosome16	56789142
ENSG00000101266	chromosome20	437196
ENSG00000070770	chromosome16	56789142
ENSG00000035687	chromosome1	242681743
ENSG00000185100	chromosome14	104261653
ENSG00000185100	chromosome14	104261653
ENSG00000035687	chromosome1	242681743
ENSG00000135821	chromosome1	180624496
ENSG00000067596	chromosome17	38916932
ENSG00000168906	chromosome2	85619922
ENSG00000151224	chromosome10	82039160
ENSG00000151224	chromosome10	82039160
ENSG00000168906	chromosome2	85619922
ENSG00000130653	chromosome9	139564471
ENSG00000032444	chromosome19	7506437
ENSG00000114859	chromosome3	185561962
ENSG00000114859	chromosome3	185561962
ENSG00000114859	chromosome3	185561962
ENSG00000186510	chromosome1	16221702
ENSG00000184908	chromosome1	16243575
ENSG00000151376	chromosome11	86060635
ENSG00000065833	chromosome6	84197393
ENSG00000065833	chromosome6	84197393
ENSG00000151376	chromosome11	86060635
ENSG00000082212	chromosome18	46676189
ENSG00000165280	chromosome9	35069522
ENSG00000213923	chromosome22	37040109
ENSG00000141551	chromosome17	77824547
ENSG00000213923	chromosome22	37040109
ENSG00000141551	chromosome17	77824547
ENSG00000151093	chromosome3	25807516
ENSG00000077380	chromosome2	172254912
ENSG00000158560	chromosome7	95271978
ENSG00000140986	chromosome16	1944634

ENSG00000100316	chromosome22	38045549
ENSG00000167658	chromosome19	3936379
ENSG00000111667	chromosome12	6831605
ENSG00000058056	chromosome3	180853708
ENSG00000130985	chromosomeX	46942657
ENSG00000182179	chromosome3	49826225
ENSG00000075651	chromosome3	172938536
ENSG00000075651	chromosome3	172938536
ENSG00000129219	chromosome17	4658032
ENSG00000054793	chromosome20	49818316
ENSG00000166377	chromosome18	74930399
ENSG00000111218	chromosome12	3471053
ENSG00000126457	chromosome19	54872350
ENSG00000197355	chromosome9	139092412
ENSG00000117143	chromosome1	160802483
ENSG00000153132	chromosome4	141553683
ENSG00000127022	chromosome5	179065289
ENSG00000127022	chromosome5	179065289
ENSG00000153132	chromosome4	141553683
ENSG00000158104	chromosome12	120781113
ENSG00000100714	chromosome14	63924899
ENSG00000120254	chromosome6	151228652
ENSG00000163050	chromosome1	225215710
ENSG00000123815	chromosome19	45912378
ENSG00000133704	chromosome12	30739849
ENSG00000205339	chromosome11	9362887
ENSG00000112992	chromosome5	43645055
ENSG00000115091	chromosome2	114364327
ENSG00000106526	chromosome7	149623319
ENSG00000133627	chromosome7	152087901
ENSG00000196296	chromosome16	28797305
ENSG00000074370	chromosome17	3814491
ENSG00000174437	chromosome12	109203978
ENSG00000074370	chromosome17	3814491
ENSG00000196296	chromosome16	28797305
ENSG00000174437	chromosome12	109203978
ENSG00000112855	chromosome5	140051418
ENSG00000170445	chromosome5	140051074
ENSG00000143515	chromosome1	152566900
ENSG00000104043	chromosome15	48186456
ENSG00000104043	chromosome15	48186456
ENSG00000143515	chromosome1	152566900
ENSG00000132932	chromosome13	24844351
ENSG00000124406	chromosome4	42353648
ENSG00000085377	chromosome6	105957460
ENSG00000116954	chromosome1	39097906
ENSG00000025039	chromosome6	90178432
ENSG00000065135	chromosome1	109892866
ENSG00000127955	chromosome7	79602413
ENSG00000214622	chromosome10	79499305
ENSG00000114353	chromosome3	50248772
ENSG00000146535	chromosome7	2850322
ENSG00000214622	chromosome10	79499305
ENSG00000114353	chromosome3	50248772
ENSG00000065135	chromosome1	109892866
ENSG00000127955	chromosome7	79602413
ENSG00000114349	chromosome3	50204163
ENSG00000134183	chromosome1	109957016
ENSG00000214415	chromosome7	79979179

ENSG00000065135	chromosome1	109892866
ENSG00000127955	chromosome7	79602413
ENSG00000141404	chromosome18	11679563
ENSG00000087460	chromosome20	56861716
ENSG00000141404	chromosome18	11679563
ENSG00000065135	chromosome1	109892866
ENSG00000127955	chromosome7	79602413
ENSG00000088256	chromosome19	3045650
ENSG00000156052	chromosome9	79835972
ENSG00000156049	chromosome9	79452530
ENSG00000120063	chromosome17	60483174
ENSG00000146535	chromosome7	2850322
ENSG00000087258	chromosome16	54783649
ENSG00000156052	chromosome9	79835972
ENSG00000088256	chromosome19	3045650
ENSG00000134183	chromosome1	109957016
ENSG00000214415	chromosome7	79979179
ENSG00000114349	chromosome3	50204163
ENSG00000153147	chromosome4	144654528
ENSG00000102038	chromosomeX	128485029
ENSG00000102024	chromosomeX	114750819
ENSG00000136167	chromosome13	45631799
ENSG00000136167	chromosome13	45631799
ENSG00000102024	chromosomeX	114750819
ENSG00000120756	chromosome3	143865770
ENSG00000205100	chromosome4	190631296
ENSG00000080824	chromosome14	101675495
ENSG00000096384	chromosome6	44324345
ENSG00000183199	chromosome4	89032019
ENSG00000205527	chromosome15	56772617
ENSG00000205940	chromosome4	12944136
ENSG00000169299	chromosome4	37504750
ENSG00000165434	chromosome11	73786855
ENSG00000101310	chromosome20	18439480
ENSG00000100934	chromosome14	38635074
ENSG00000105953	chromosome7	44630468
ENSG00000197444	chromosome10	50636645
ENSG00000136448	chromosome17	40494224
ENSG00000152465	chromosome10	15250618
ENSG00000008869	chromosome2	37164062
ENSG00000129493	chromosome14	30941933
ENSG00000124198	chromosome20	46971834
ENSG00000066777	chromosome8	68418077
ENSG00000023330	chromosome3	52208298
ENSG00000158578	chromosomeX	55070966
ENSG00000181416	chromosome2	203613076
ENSG00000215402	chromosome13	98400703
ENSG00000146223	chromosome6	42955654
ENSG00000215297	chromosome9	3356738
ENSG00000137970	chromosome1	96935140
ENSG00000169216	chromosome5	76913957
ENSG00000214485	chromosome5	149444095
ENSG00000147604	chromosome8	74375858
ENSG00000171549	chromosome7	107937913
ENSG00000214557	chromosome10	90368813
ENSG00000182520	chromosome12	19133352
ENSG00000214932	chromosomeX	135700866
ENSG00000169100	chromosomeX	1470903
ENSG00000005022	chromosomeX	118486507

ENSG00000005022	chromosomeX	118486507
ENSG00000169100	chromosomeX	1470903
ENSG00000151729	chromosome4	186301521
ENSG00000156110	chromosome10	75581043
ENSG00000142327	chromosome2	241161224
ENSG00000176393	chromosome1	200218418
ENSG00000115758	chromosome2	10502610
ENSG00000142920	chromosome1	33320438
ENSG00000155096	chromosome8	103925057
ENSG00000142920	chromosome1	33320438
ENSG00000155096	chromosome8	103925057
ENSG00000115758	chromosome2	10502610
ENSG00000155096	chromosome8	103925057
ENSG00000142920	chromosome1	33320438
ENSG00000127616	chromosome19	10955828
ENSG00000080503	chromosome9	2019023
ENSG00000142961	chromosome1	46853336
ENSG00000120162	chromosome9	27445549
ENSG00000172081	chromosome19	2029560
ENSG00000172081	chromosome19	2029560
ENSG00000120162	chromosome9	27445549
ENSG00000173542	chromosome4	71986965
ENSG00000114978	chromosome2	74268261
ENSG00000182208	chromosome11	1465805
ENSG00000175582	chromosome11	73149329
ENSG00000154917	chromosome3	135097001
ENSG00000147127	chromosomeX	69418793
ENSG00000154917	chromosome3	135097001
ENSG00000154917	chromosome3	135097001
ENSG00000175582	chromosome11	73149329
ENSG00000165527	chromosome14	49430205
ENSG00000143761	chromosome1	226351439
ENSG00000134287	chromosome12	47621146
ENSG00000134287	chromosome12	47621146
ENSG00000004059	chromosome7	127015789
ENSG00000168374	chromosome3	57557908
ENSG00000168374	chromosome3	57557908
ENSG00000004059	chromosome7	127015789
ENSG00000143761	chromosome1	226351439
ENSG00000134287	chromosome12	47621146
ENSG00000134287	chromosome12	47621146
ENSG00000143761	chromosome1	226351439
ENSG00000143761	chromosome1	226351439
ENSG00000134287	chromosome12	47621146
ENSG00000114573	chromosome3	114980291
ENSG00000126858	chromosome17	27493825
ENSG00000140983	chromosome16	658201
ENSG00000105576	chromosome19	12692792
ENSG00000083312	chromosome5	72148321
ENSG00000167588	chromosome12	48784101
ENSG00000152642	chromosome3	32123208
ENSG00000152642	chromosome3	32123208
ENSG00000067560	chromosome3	49388027
ENSG00000155366	chromosome1	113047945
ENSG00000143878	chromosome2	20510708
ENSG00000067560	chromosome3	49388027
ENSG00000155366	chromosome1	113047945
ENSG00000067560	chromosome3	49388027
ENSG00000155366	chromosome1	113047945

ENSG00000155366	chromosome1	113047945
ENSG00000067560	chromosome3	49388027
ENSG00000155366	chromosome1	113047945
ENSG00000155366	chromosome1	113047945
ENSG00000067560	chromosome3	49388027
ENSG00000167701	chromosome8	145700496
ENSG00000166123	chromosome16	45476129
ENSG00000166123	chromosome16	45476129
ENSG00000167701	chromosome8	145700496
ENSG00000113552	chromosome5	141371785
ENSG00000163281	chromosome4	44418982
ENSG00000115421	chromosome2	60837118
ENSG00000090060	chromosome14	96038683
ENSG00000159720	chromosome16	66072491
ENSG00000147614	chromosome8	87180324
ENSG00000185418	chromosome15	100082114
ENSG00000113407	chromosome5	33476950
ENSG00000143374	chromosome1	148726551
ENSG00000113407	chromosome5	33476950
ENSG00000185418	chromosome15	100082114
ENSG00000131459	chromosome5	179712824
ENSG00000198380	chromosome2	69467708
ENSG00000112697	chromosome6	76051075
ENSG00000182107	chromosome14	60817619
ENSG00000182107	chromosome14	60817619
ENSG00000112697	chromosome6	76051075
ENSG00000100417	chromosome22	40315756
ENSG00000140650	chromosome16	8799241
ENSG00000060982	chromosome12	24993335
ENSG00000105552	chromosome19	54006077
ENSG00000180817	chromosome10	71663090
ENSG00000138777	chromosome4	106614657
ENSG00000178537	chromosome3	48911232
ENSG00000099797	chromosome19	14501509
ENSG00000205678	chromosome4	64957665
ENSG00000160014	chromosome19	51796532
ENSG00000178372	chromosome10	5531402
ENSG00000178363	chromosome10	5557049
ENSG00000160014	chromosome19	51796532
ENSG00000178372	chromosome10	5531402
ENSG00000178363	chromosome10	5557049
ENSG00000160014	chromosome19	51796532
ENSG00000178372	chromosome10	5531402
ENSG00000178363	chromosome10	5557049
ENSG00000143933	chromosome2	47257087
ENSG00000198668	chromosome14	89933328
ENSG00000143933	chromosome2	47257087
ENSG00000198668	chromosome14	89933328
ENSG00000125691	chromosome17	34263490
ENSG00000125691	chromosome17	34263490
ENSG00000144118	chromosome2	120752711
ENSG00000006451	chromosome7	39692792
ENSG00000144118	chromosome2	120752711
ENSG00000133703	chromosome12	25289586
ENSG00000213281	chromosome1	115060305
ENSG00000187682	chromosomeX	48572478
ENSG00000213281	chromosome1	115060305
ENSG00000133703	chromosome12	25289586
ENSG00000174775	chromosome11	524323

ENSG00000174775	chromosome11	524323
ENSG00000213281	chromosome1	115060305
ENSG00000133703	chromosome12	25289586
ENSG00000213281	chromosome1	115060305
ENSG00000133703	chromosome12	25289586
ENSG00000133818	chromosome11	14336993
ENSG00000126458	chromosome19	54835168
ENSG00000128203	chromosome22	25159582
ENSG00000174939	chromosome16	29819794
ENSG00000198936	chromosome1	147819702
ENSG00000198161	chromosome1	146421969
ENSG00000198360	chromosome1	143075529
ENSG00000203847	chromosome1	142559372
ENSG00000215551	chromosome20	25297399
ENSG00000150276	chromosome13	52292633
ENSG00000196262	chromosome7	44802849
ENSG00000198618	chromosome21	19151968
ENSG00000214975	chromosome6	25084626
ENSG00000108179	chromosome10	80777311
ENSG00000198936	chromosome1	147819702
ENSG00000198161	chromosome1	146421969
ENSG00000198360	chromosome1	143075529
ENSG00000203847	chromosome1	142559372
ENSG00000215551	chromosome20	25297399
ENSG00000150276	chromosome13	52292633
ENSG00000196262	chromosome7	44802849
ENSG00000198618	chromosome21	19151968
ENSG00000214975	chromosome6	25084626
ENSG00000108179	chromosome10	80777311
ENSG00000108179	chromosome10	80777311
ENSG00000198936	chromosome1	147819702
ENSG00000198161	chromosome1	146421969
ENSG00000198360	chromosome1	143075529
ENSG00000203847	chromosome1	142559372
ENSG00000215551	chromosome20	25297399
ENSG00000150276	chromosome13	52292633
ENSG00000196262	chromosome7	44802849
ENSG00000198618	chromosome21	19151968
ENSG00000214975	chromosome6	25084626
ENSG00000198216	chromosome1	179719504
ENSG00000141837	chromosome19	13478039
ENSG00000148408	chromosome9	139892207
ENSG00000102001	chromosomeX	48976716
ENSG00000157388	chromosome3	53504234
ENSG00000151067	chromosome12	2032990
ENSG00000157388	chromosome3	53504234
ENSG00000102001	chromosomeX	48976716
ENSG00000148408	chromosome9	139892207
ENSG00000141837	chromosome19	13478039
ENSG00000081248	chromosome1	199348091
ENSG00000151067	chromosome12	2032990
ENSG00000102001	chromosomeX	48976716
ENSG00000157388	chromosome3	53504234
ENSG00000144645	chromosome3	31997676
ENSG00000144909	chromosome3	126796335
ENSG00000091039	chromosome12	75405463
ENSG00000021762	chromosome11	3106955
ENSG00000150995	chromosome3	4533176
ENSG00000123104	chromosome12	26876982

ENSG00000123104	chromosome12	26876982
ENSG00000150995	chromosome3	4533176
ENSG00000096433	chromosome6	33697358
ENSG00000149091	chromosome11	46311402
ENSG00000157680	chromosome7	137182149
ENSG00000101438	chromosome20	36786782
ENSG00000175093	chromosome3	142267637
ENSG00000111671	chromosome12	6852327
ENSG00000171621	chromosome1	9338538
ENSG00000162032	chromosome16	1771486
ENSG00000171621	chromosome1	9338538
ENSG00000111671	chromosome12	6852327
ENSG00000111671	chromosome12	6852327
ENSG00000171621	chromosome1	9338538
ENSG00000175093	chromosome3	142267637
ENSG00000139645	chromosome12	54938321
ENSG00000065413	chromosome2	197760028
ENSG00000206560	chromosome3	15813033
ENSG00000206560	chromosome3	15813033
ENSG00000065413	chromosome2	197760028
ENSG00000119509	chromosome9	101906625
ENSG00000158850	chromosome1	159412475
ENSG00000086062	chromosome9	33157168
ENSG00000086062	chromosome9	33157168
ENSG00000117411	chromosome1	44219420
ENSG00000117411	chromosome1	44219420
ENSG00000086062	chromosome9	33157168
ENSG00000121578	chromosome3	120431637
ENSG00000158850	chromosome1	159412475
ENSG00000118276	chromosome18	27518388
ENSG00000158470	chromosome20	47763635
ENSG00000158850	chromosome1	159412475
ENSG00000121578	chromosome3	120431637
ENSG00000185352	chromosome13	95541118
ENSG00000171004	chromosomeX	131920303
ENSG00000136720	chromosome2	128792608
ENSG00000171004	chromosomeX	131920303
ENSG00000185352	chromosome13	95541118
ENSG00000171004	chromosomeX	131920303
ENSG00000185352	chromosome13	95541118
ENSG00000136720	chromosome2	128792608
ENSG00000170786	chromosome8	57391461
ENSG00000170786	chromosome8	57391461
ENSG00000121039	chromosome8	74370079
ENSG00000105409	chromosome19	47190069
ENSG00000132681	chromosome1	158388455
ENSG00000018625	chromosome1	158349930
ENSG00000163399	chromosome1	116717657
ENSG00000132681	chromosome1	158388455
ENSG00000018625	chromosome1	158349930
ENSG00000105409	chromosome19	47190069
ENSG00000075673	chromosome13	24152882
ENSG00000105675	chromosome19	40746372
ENSG00000163754	chromosome3	150192118
ENSG00000056998	chromosomeX	2756860
ENSG00000056998	chromosomeX	2756860
ENSG00000163754	chromosome3	150192118
ENSG00000198722	chromosome9	35152281
ENSG00000130477	chromosome19	17647979

ENSG00000130477	chromosome19	17647979
ENSG00000198722	chromosome9	35152281
ENSG00000137766	chromosome15	52314521
ENSG00000147027	chromosomeX	34585068
ENSG00000112378	chromosome6	138470171
ENSG00000176711	chromosome15	88619365
ENSG00000147027	chromosomeX	34585068
ENSG00000159640	chromosome17	58908188
ENSG00000136518	chromosome3	180763575
ENSG00000077080	chromosome7	100091914
ENSG00000152527	chromosome2	43725317
ENSG00000054690	chromosome14	67069786
ENSG00000151240	chromosome10	725519
ENSG00000160305	chromosome21	46703473
ENSG00000066084	chromosome12	49185191
ENSG00000151240	chromosome10	725519
ENSG00000066084	chromosome12	49185191
ENSG00000160305	chromosome21	46703473
ENSG00000151240	chromosome10	725519
ENSG00000101166	chromosome20	57051181
ENSG00000141391	chromosome18	12397975
ENSG00000111897	chromosome6	122834582
ENSG00000132824	chromosome20	42584007
ENSG00000164300	chromosome5	79534811
ENSG00000184716	chromosome15	41888068
ENSG00000168528	chromosome1	31655272
ENSG00000132824	chromosome20	42584007
ENSG00000111897	chromosome6	122834582
ENSG00000116353	chromosome1	29430006
ENSG00000124920	chromosome11	61276697
ENSG00000152700	chromosome5	133987609
ENSG00000173386	chromosome4	121482662
ENSG00000079332	chromosome10	71591678
ENSG00000114738	chromosome3	50630001
ENSG00000162889	chromosome1	204925198
ENSG00000142513	chromosome19	55985484
ENSG00000134575	chromosome11	47226917
ENSG00000014257	chromosome3	133518991
ENSG00000134575	chromosome11	47226917
ENSG00000142513	chromosome19	55985484
ENSG00000041353	chromosome18	50695815
ENSG00000069974	chromosome15	53314425
ENSG00000172869	chromosome5	118435164
ENSG00000104093	chromosome15	49702035
ENSG00000178184	chromosome18	76106223
ENSG00000124171	chromosome20	48781731
ENSG00000102981	chromosome16	66252443
ENSG00000124171	chromosome20	48781731
ENSG00000178184	chromosome18	76106223
ENSG00000152894	chromosome6	128883197
ENSG00000173482	chromosome18	7557817
ENSG00000196090	chromosome20	41251788
ENSG00000196090	chromosome20	41251788
ENSG00000173482	chromosome18	7557817
ENSG00000153707	chromosome9	8723844
ENSG00000105426	chromosome19	5237152
ENSG00000142949	chromosome1	43770843
ENSG00000060656	chromosome1	29435744
ENSG00000173482	chromosome18	7557817

ENSG00000196090	chromosome20	41251788
ENSG00000152894	chromosome6	128883197
ENSG00000105426	chromosome19	5237152
ENSG00000153707	chromosome9	8723844
ENSG00000106780	chromosome9	122516458
ENSG00000106780	chromosome9	122516458
ENSG00000135862	chromosome1	181259475
ENSG00000058085	chromosome1	181422111
ENSG00000050555	chromosome9	132874423
ENSG00000091136	chromosome7	107430567
ENSG00000172037	chromosome3	49145305
ENSG00000050555	chromosome9	132874423
ENSG00000058085	chromosome1	181422111
ENSG00000074527	chromosome12	94708219
ENSG00000108442	chromosome17	15407488
ENSG00000171928	chromosome17	18625332
ENSG00000108442	chromosome17	15407488
ENSG00000171928	chromosome17	18625332
ENSG00000166676	chromosome16	10819996
ENSG00000198951	chromosome22	40796365
ENSG00000102393	chromosomeX	100549548
ENSG00000169902	chromosome7	65342848
ENSG00000128294	chromosome22	25267597
ENSG00000169902	chromosome7	65342848
ENSG00000081800	chromosome7	122627237
ENSG00000164707	chromosome7	135062785
ENSG00000007216	chromosome17	23824858
ENSG00000141485	chromosome17	6557377
ENSG00000081800	chromosome7	122627237
ENSG00000141485	chromosome17	6557377
ENSG00000007216	chromosome17	23824858
ENSG00000164707	chromosome7	135062785
ENSG00000081800	chromosome7	122627237
ENSG00000116001	chromosome2	70329067
ENSG00000151923	chromosome10	121345968
ENSG00000162433	chromosome1	65386679
ENSG00000147853	chromosome9	4731088
ENSG00000147853	chromosome9	4731088
ENSG00000162433	chromosome1	65386679
ENSG00000074319	chromosome11	18504940
ENSG00000171777	chromosome19	43608572
ENSG00000172575	chromosome15	36644122
ENSG00000152689	chromosome2	33593708
ENSG00000068831	chromosome11	64269509
ENSG00000172575	chromosome15	36644122
ENSG00000171777	chromosome19	43608572
ENSG00000068831	chromosome11	64269509
ENSG00000152689	chromosome2	33593708
ENSG00000183048	chromosome17	77289862
ENSG00000182287	chromosomeX	15780569
ENSG00000106367	chromosome7	100586595
ENSG00000152056	chromosome2	224410412
ENSG00000106367	chromosome7	100586595
ENSG00000182287	chromosomeX	15780569
ENSG00000204160	chromosome1	27025883
ENSG00000175048	chromosome6	157723042
ENSG00000175048	chromosome6	157723042
ENSG00000204160	chromosome1	27025883
ENSG00000188706	chromosomeX	128803603

ENSG00000055163	chromosome5	156644935
ENSG00000068793	chromosome15	20477224
ENSG00000164062	chromosome3	49686839
ENSG00000135517	chromosome12	55134665
ENSG00000167580	chromosome12	48630881
ENSG00000086159	chromosome12	48653224
ENSG00000161798	chromosome12	48642068
ENSG00000106125	chromosome7	30918050
ENSG00000167580	chromosome12	48630881
ENSG00000086159	chromosome12	48653224
ENSG00000161798	chromosome12	48642068
ENSG00000135517	chromosome12	55134665
ENSG00000171885	chromosome18	22699673
ENSG00000167580	chromosome12	48630881
ENSG00000086159	chromosome12	48653224
ENSG00000161798	chromosome12	48642068
ENSG00000135517	chromosome12	55134665
ENSG00000106125	chromosome7	30918050
ENSG00000167580	chromosome12	48630881
ENSG00000086159	chromosome12	48653224
ENSG00000161798	chromosome12	48642068
ENSG00000135517	chromosome12	55134665
ENSG00000106125	chromosome7	30918050
ENSG00000103375	chromosome16	25135868
ENSG00000197879	chromosome17	1342532
ENSG00000157483	chromosome15	57451992
ENSG00000142347	chromosome19	8548329
ENSG00000128641	chromosome2	191849867
ENSG00000166866	chromosome12	55728375
ENSG00000176658	chromosome17	28228004
ENSG00000136286	chromosome7	44985086
ENSG00000157483	chromosome15	57451992
ENSG00000076356	chromosome1	206457936
ENSG00000189437	chromosome7	131843993
ENSG00000114554	chromosome3	128190127
ENSG00000130827	chromosomeX	153341718
ENSG00000196576	chromosome22	49071141
ENSG00000198753	chromosomeX	152684185
ENSG00000164050	chromosome3	48441025
ENSG00000136040	chromosome12	93066879
ENSG00000004399	chromosome3	130808173
ENSG00000164050	chromosome3	48441025
ENSG00000198753	chromosomeX	152684185
ENSG00000130827	chromosomeX	153341718
ENSG00000114554	chromosome3	128190127
ENSG00000189437	chromosome7	131843993
ENSG00000076356	chromosome1	206457936
ENSG00000163618	chromosome3	62835745
ENSG00000081803	chromosome7	122313628
ENSG00000101460	chromosome20	32601443
ENSG00000140941	chromosome16	85983535
ENSG00000171471	chromosome12	115498131
ENSG00000062598	chromosome20	44456529
ENSG00000155849	chromosome7	37348820
ENSG00000102890	chromosome16	65790572
ENSG00000155849	chromosome7	37348820
ENSG00000062598	chromosome20	44456529
ENSG00000090975	chromosome12	122085091
ENSG00000091622	chromosome17	6400451

ENSG00000091622	chromosome17	6400451
ENSG00000090975	chromosome12	122085091
ENSG00000110697	chromosome11	67028228
ENSG00000173960	chromosome2	24034689
ENSG00000215114	chromosome8	59486499
ENSG00000088833	chromosome20	1395470
ENSG00000088833	chromosome20	1395470
ENSG00000215114	chromosome8	59486499
ENSG00000181788	chromosome3	151963327
ENSG00000215475	chromosome13	45323766
ENSG00000196470	chromosome16	46976716
ENSG00000106327	chromosome7	100077069
ENSG00000072274	chromosome3	197288368
ENSG00000177694	chromosome3	176297273
ENSG00000168060	chromosome11	64582570
ENSG00000086205	chromosome11	49186538
ENSG00000134612	chromosome11	89011995
ENSG00000077616	chromosome11	89507575
ENSG00000072274	chromosome3	197288368
ENSG00000106327	chromosome7	100077069
ENSG00000007384	chromosome16	55014
ENSG00000129667	chromosome17	71989202
ENSG00000099203	chromosome19	10807868
ENSG00000117500	chromosome1	93418388
ENSG00000086598	chromosome12	122635137
ENSG00000134970	chromosome5	114989389
ENSG00000166557	chromosome15	77390647
ENSG00000099282	chromosome10	70881357
ENSG00000168785	chromosome4	99798401
ENSG00000048140	chromosome5	176007223
ENSG00000048140	chromosome5	176007223
ENSG00000168785	chromosome4	99798401
ENSG00000108219	chromosome10	82238970
ENSG00000158457	chromosome7	128572057
ENSG00000099282	chromosome10	70881357
ENSG00000182612	chromosome17	77219844
ENSG00000158457	chromosome7	128572057
ENSG00000148700	chromosome10	111850402
ENSG00000075340	chromosome2	70787049
ENSG00000075340	chromosome2	70787049
ENSG00000148700	chromosome10	111850402
ENSG00000087274	chromosome4	2847441
ENSG00000182400	chromosome14	38709051
ENSG00000007255	chromosome19	50373317
ENSG00000197601	chromosome11	13672889
ENSG00000064763	chromosome12	29314650
ENSG00000004660	chromosome17	3740774
ENSG00000110931	chromosome12	120219939
ENSG00000176490	chromosome19	2668805
ENSG00000165023	chromosome9	92415930
ENSG00000165023	chromosome9	92415930
ENSG00000214405	chromosome3	105264567
ENSG00000127314	chromosome12	67328772
ENSG00000176276	chromosome5	75505765
ENSG00000181467	chromosome3	154363173
ENSG00000125249	chromosome13	96884726
ENSG00000165023	chromosome9	92415930
ENSG00000176490	chromosome19	2668805
ENSG00000162595	chromosome1	68285569

ENSG00000116473	chromosome1	112035506
ENSG00000214405	chromosome3	105264567
ENSG00000127314	chromosome12	67328772
ENSG00000176276	chromosome5	75505765
ENSG00000125249	chromosome13	96884726
ENSG00000181467	chromosome3	154363173
ENSG00000214492	chromosome14	102815401
ENSG00000123728	chromosomeX	131178978
ENSG00000049860	chromosome5	74016842
ENSG00000213614	chromosome15	70455368
ENSG00000117614	chromosome1	25431546
ENSG00000131982	chromosome14	54765688
ENSG00000185651	chromosome22	20252034
ENSG00000178913	chromosome5	140679796
ENSG00000102387	chromosomeX	100434690
ENSG00000107566	chromosome10	101935368
ENSG00000147475	chromosome8	37714599
ENSG00000176343	chromosome11	111265726
ENSG00000197756	chromosome2	217071843
ENSG00000131037	chromosome19	60275378
ENSG00000177106	chromosome11	699408
ENSG00000131037	chromosome19	60275378
ENSG00000151491	chromosome12	15727153
ENSG00000131037	chromosome19	60275378
ENSG00000177106	chromosome11	699408
ENSG00000198758	chromosome1	110108093
ENSG00000151491	chromosome12	15727153
ENSG00000177106	chromosome11	699408
ENSG00000131037	chromosome19	60275378
ENSG00000131037	chromosome19	60275378
ENSG00000147364	chromosome8	353080
ENSG00000156804	chromosome8	124622436
ENSG00000122490	chromosome18	75811915
ENSG00000149577	chromosome11	116555190
ENSG00000072858	chromosome3	114734559
ENSG00000145703	chromosome5	75735010
ENSG00000140575	chromosome15	88732578
ENSG00000140575	chromosome15	88732578
ENSG00000145703	chromosome5	75735010
ENSG00000183856	chromosome1	154808946
ENSG00000130119	chromosomeX	54570464
ENSG00000163938	chromosome3	52695149
ENSG00000214021	chromosome3	9809584
ENSG00000138892	chromosome22	48835874
ENSG00000170703	chromosome17	44249440
ENSG00000213471	chromosome15	88594867
ENSG00000197860	chromosome5	65052391
ENSG00000104969	chromosome19	2720067
ENSG00000138050	chromosome2	39859794
ENSG00000134077	chromosome3	9381753
ENSG00000138834	chromosome16	1696342
ENSG00000008294	chromosome17	46553017
ENSG00000008294	chromosome17	46553017
ENSG00000138834	chromosome16	1696342
ENSG00000163931	chromosome3	53264999
ENSG00000151005	chromosome4	164614337
ENSG00000007350	chromosomeX	153177407
ENSG00000145817	chromosome5	143529713
ENSG00000177752	chromosome4	44348399

ENSG00000164794	chromosome8	111055794
ENSG00000168263	chromosome9	2707740
ENSG00000178342	chromosome18	75724656
ENSG00000026559	chromosome20	49060283
ENSG00000168418	chromosome16	82828593
ENSG00000171126	chromosome2	42574146
ENSG00000026559	chromosome20	49060283
ENSG00000178342	chromosome18	75724656
ENSG00000111262	chromosome12	4890806
ENSG00000177301	chromosome1	110948928
ENSG00000168263	chromosome9	2707740
ENSG00000164794	chromosome8	111055794
ENSG00000182255	chromosome11	29990802
ENSG00000130037	chromosome12	5023575
ENSG00000143105	chromosome1	110862933
ENSG00000177272	chromosome1	111018955
ENSG00000177301	chromosome1	110948928
ENSG00000111262	chromosome12	4890806
ENSG00000151079	chromosome12	4789469
ENSG00000104848	chromosome19	54267655
ENSG00000177301	chromosome1	110948928
ENSG00000111262	chromosome12	4890806
ENSG00000151079	chromosome12	4789469
ENSG00000177272	chromosome1	111018955
ENSG00000130037	chromosome12	5023575
ENSG00000143105	chromosome1	110862933
ENSG00000177272	chromosome1	111018955
ENSG00000177301	chromosome1	110948928
ENSG00000111262	chromosome12	4890806
ENSG00000151079	chromosome12	4789469
ENSG00000182255	chromosome11	29990802
ENSG00000151079	chromosome12	4789469
ENSG00000177301	chromosome1	110948928
ENSG00000111262	chromosome12	4890806
ENSG00000177272	chromosome1	111018955
ENSG00000177301	chromosome1	110948928
ENSG00000111262	chromosome12	4890806
ENSG00000151079	chromosome12	4789469
ENSG00000130037	chromosome12	5023575
ENSG00000143105	chromosome1	110862933
ENSG00000184408	chromosome7	119701923
ENSG00000171385	chromosome1	112326872
ENSG00000104848	chromosome19	54267655
ENSG00000182255	chromosome11	29990802
ENSG00000130037	chromosome12	5023575
ENSG00000143105	chromosome1	110862933
ENSG00000177272	chromosome1	111018955
ENSG00000177301	chromosome1	110948928
ENSG00000111262	chromosome12	4890806
ENSG00000151079	chromosome12	4789469
ENSG00000143105	chromosome1	110862933
ENSG00000130037	chromosome12	5023575
ENSG00000168418	chromosome16	82828593
ENSG00000178342	chromosome18	75724656
ENSG00000026559	chromosome20	49060283
ENSG00000170745	chromosome2	17975757
ENSG00000156486	chromosome8	99509384
ENSG00000124134	chromosome20	43161292
ENSG00000171126	chromosome2	42574146

ENSG00000178342	chromosome18	75724656
ENSG00000026559	chromosome20	49060283
ENSG00000168418	chromosome16	82828593
ENSG00000171385	chromosome1	112326872
ENSG00000184408	chromosome7	119701923
ENSG00000102057	chromosomeX	48711623
ENSG00000177272	chromosome1	111018955
ENSG00000177301	chromosome1	110948928
ENSG00000111262	chromosome12	4890806
ENSG00000151079	chromosome12	4789469
ENSG00000178342	chromosome18	75724656
ENSG00000026559	chromosome20	49060283
ENSG00000168418	chromosome16	82828593
ENSG00000171126	chromosome2	42574146
ENSG00000164794	chromosome8	111055794
ENSG00000168263	chromosome9	2707740
ENSG00000156486	chromosome8	99509384
ENSG00000124134	chromosome20	43161292
ENSG00000170745	chromosome2	17975757
ENSG00000124134	chromosome20	43161292
ENSG00000156486	chromosome8	99509384
ENSG00000168263	chromosome9	2707740
ENSG00000136267	chromosome7	14847414
ENSG00000058866	chromosome3	187520943
ENSG00000077044	chromosome2	233927904
ENSG00000102780	chromosome13	41520910
ENSG00000077044	chromosome2	233927904
ENSG00000102780	chromosome13	41520910
ENSG00000065357	chromosome12	54616555
ENSG00000058866	chromosome3	187520943
ENSG00000136267	chromosome7	14847414
ENSG00000165105	chromosome9	84867603
ENSG00000141744	chromosome17	35078255
ENSG00000011177	chromosome7	30758292
ENSG00000166741	chromosome11	113672489
ENSG00000155265	chromosome10	99600051
ENSG00000147533	chromosome8	41467531
ENSG00000205596	chromosome7	65173925
ENSG00000169230	chromosome5	176663593
ENSG00000171055	chromosome2	36678790
ENSG00000149557	chromosome11	124864884
ENSG00000184611	chromosome2	163403275
ENSG00000055118	chromosome7	150305935
ENSG00000173826	chromosome17	58954507
ENSG00000173826	chromosome17	58954507
ENSG00000055118	chromosome7	150305935
ENSG00000183960	chromosome3	19165216
ENSG00000089558	chromosome17	37586490
ENSG00000135519	chromosome12	48219467
ENSG00000173826	chromosome17	58954507
ENSG00000089558	chromosome17	37586490
ENSG00000183960	chromosome3	19165216
ENSG00000140015	chromosome14	62638335
ENSG00000143473	chromosome1	209373911
ENSG00000122694	chromosome9	36126032
ENSG00000198682	chromosome10	89409719
ENSG00000138801	chromosome4	108860785
ENSG00000197226	chromosome5	179267461
ENSG00000109436	chromosome4	141896650

ENSG00000204634	chromosome2	101033393
ENSG00000133138	chromosomeX	105932566
ENSG00000109436	chromosome4	141896650
ENSG00000197226	chromosome5	179267461
ENSG00000133138	chromosomeX	105932566
ENSG00000204634	chromosome2	101033393
ENSG00000072315	chromosomeX	111082305
ENSG00000133107	chromosome13	37255471
ENSG00000144935	chromosome3	143926092
ENSG00000069018	chromosome5	135720972
ENSG00000138741	chromosome4	123092286
ENSG00000133107	chromosome13	37255471
ENSG00000072315	chromosomeX	111082305
ENSG00000137672	chromosome11	100959445
ENSG00000138741	chromosome4	123092286
ENSG00000069018	chromosome5	135720972
ENSG00000100141	chromosome22	30388195
ENSG00000099308	chromosome19	18095430
ENSG00000069020	chromosome5	66290832
ENSG00000086015	chromosome1	46042155
ENSG00000105613	chromosome19	12810387
ENSG00000069020	chromosome5	66290832
ENSG00000099308	chromosome19	18095430
ENSG00000105613	chromosome19	12810387
ENSG00000086015	chromosome1	46042155
ENSG00000112893	chromosome5	109054018
ENSG00000196547	chromosome15	89248442
ENSG00000196547	chromosome15	89248442
ENSG00000112893	chromosome5	109054018
ENSG00000175416	chromosome5	175775971
ENSG00000122705	chromosome9	36181054
ENSG00000122705	chromosome9	36181054
ENSG00000175416	chromosome5	175775971
ENSG00000104419	chromosome8	134365737
ENSG00000101079	chromosome20	34783553
ENSG00000103034	chromosome16	57055836
ENSG00000165795	chromosome14	20561315
ENSG00000101079	chromosome20	34783553
ENSG00000104419	chromosome8	134365737
ENSG00000165795	chromosome14	20561315
ENSG00000103034	chromosome16	57055836
ENSG00000085224	chromosomeX	76928243
ENSG00000215766	supercontigNT_113965	386312
ENSG00000176115	chromosome9	66778517
ENSG00000181997	chromosome9	42849340
ENSG00000186466	chromosome9	66977884
ENSG00000165269	chromosome9	33391261
ENSG00000143595	chromosome1	152560256
ENSG00000176115	chromosome9	66778517
ENSG00000181997	chromosome9	42849340
ENSG00000186466	chromosome9	66977884
ENSG00000165269	chromosome9	33391261
ENSG00000143595	chromosome1	152560256
ENSG00000103569	chromosome15	56218057
ENSG00000165272	chromosome9	33437529
ENSG00000143595	chromosome1	152560256
ENSG00000176115	chromosome9	66778517
ENSG00000181997	chromosome9	42849340
ENSG00000186466	chromosome9	66977884

ENSG00000165269	chromosome9	33391261
ENSG00000103569	chromosome15	56218057
ENSG00000176115	chromosome9	66778517
ENSG00000181997	chromosome9	42849340
ENSG00000186466	chromosome9	66977884
ENSG00000165269	chromosome9	33391261
ENSG00000143595	chromosome1	152560256
ENSG00000102575	chromosome19	11549133
ENSG00000031823	chromosome19	5929094
ENSG00000164188	chromosome5	36337276
ENSG00000091428	chromosome2	173308958
ENSG00000108352	chromosome17	35594011
ENSG00000136237	chromosome7	22316165
ENSG00000091428	chromosome2	173308958
ENSG00000109756	chromosome4	160408758
ENSG00000108352	chromosome17	35594011
ENSG00000136237	chromosome7	22316165
ENSG00000091428	chromosome2	173308958
ENSG00000079337	chromosome12	46438607
ENSG00000136237	chromosome7	22316165
ENSG00000108352	chromosome17	35594011
ENSG00000109756	chromosome4	160408758
ENSG00000121644	chromosome1	242883239
ENSG00000064651	chromosome5	127447546
ENSG00000070915	chromosome16	55456649
ENSG00000074803	chromosome15	46287209
ENSG00000064651	chromosome5	127447546
ENSG00000146828	chromosome7	100289756
ENSG00000070915	chromosome16	55456649
ENSG00000064651	chromosome5	127447546
ENSG00000074803	chromosome15	46287209
ENSG00000166507	chromosome10	75238153
ENSG00000070614	chromosome5	149881010
ENSG00000138653	chromosome4	116217642
ENSG00000164100	chromosome4	119194514
ENSG00000166507	chromosome10	75238153
ENSG00000138653	chromosome4	116217642
ENSG00000164100	chromosome4	119194514
ENSG00000070614	chromosome5	149881010
ENSG00000133731	chromosome8	82756351
ENSG00000141401	chromosome18	11971669
ENSG00000140526	chromosome15	87460563
ENSG00000158201	chromosome18	17538724
ENSG00000143994	chromosome2	27200321
ENSG00000091157	chromosome18	52490745
ENSG00000166415	chromosome15	51812639
ENSG00000152932	chromosome5	57914793
ENSG00000105649	chromosome19	18174551
ENSG00000169213	chromosome1	52215378
ENSG00000105649	chromosome19	18174551
ENSG00000152932	chromosome5	57914793
ENSG00000105514	chromosome19	11309076
ENSG00000169213	chromosome1	52215378
ENSG00000152932	chromosome5	57914793
ENSG00000105649	chromosome19	18174551
ENSG00000144040	chromosome2	73152343
ENSG00000107819	chromosome10	102782221
ENSG00000164466	chromosome5	174851713
ENSG00000144401	chromosome2	208197345

ENSG00000123427	chromosome12	56452775
ENSG00000115840	chromosome2	172458971
ENSG00000004864	chromosome7	95789205
ENSG00000182902	chromosome22	16442711
ENSG00000177542	chromosome11	785007
ENSG00000177542	chromosome11	785007
ENSG00000182902	chromosome22	16442711
ENSG00000172548	chromosome5	156819907
ENSG00000163293	chromosome4	47713614
ENSG00000140157	chromosome15	20572778
ENSG00000001461	chromosome1	24618625
ENSG00000104361	chromosome8	99375542
ENSG00000140157	chromosome15	20572778
ENSG00000163293	chromosome4	47713614
ENSG00000140157	chromosome15	20572778
ENSG00000115896	chromosome2	198574975
ENSG00000154822	chromosome3	17026221
ENSG00000149782	chromosome11	63775629
ENSG00000182621	chromosome20	8061299
ENSG00000137841	chromosome15	38387166
ENSG00000154822	chromosome3	17026221
ENSG00000115896	chromosome2	198574975
ENSG00000149527	chromosome1	2397888
ENSG00000115556	chromosome2	219188707
ENSG00000161714	chromosome17	40565304
ENSG00000187091	chromosome3	38046035
ENSG00000139151	chromosome12	18781573
ENSG00000161714	chromosome17	40565304
ENSG00000115556	chromosome2	219188707
ENSG00000154822	chromosome3	17026221
ENSG00000115896	chromosome2	198574975
ENSG00000124181	chromosome20	39199696
ENSG00000124181	chromosome20	39199696
ENSG00000197943	chromosome16	80377096
ENSG00000182621	chromosome20	8061299
ENSG00000149782	chromosome11	63775629
ENSG00000187091	chromosome3	38046035
ENSG00000115556	chromosome2	219188707
ENSG00000161714	chromosome17	40565304
ENSG00000149527	chromosome1	2397888
ENSG00000114805	chromosome3	156876523
ENSG00000101160	chromosome20	57015579
ENSG00000100239	chromosome22	49179204
ENSG00000105063	chromosome19	60450470
ENSG00000110075	chromosome11	68061709
ENSG00000110075	chromosome11	68061709
ENSG00000105063	chromosome19	60450470
ENSG00000120742	chromosome3	151746613
ENSG00000151778	chromosome13	43846162
ENSG00000151778	chromosome13	43846162
ENSG00000120742	chromosome3	151746613
ENSG00000137145	chromosome9	19280782
ENSG00000198837	chromosome1	152183330
ENSG00000174485	chromosome15	63835843
ENSG00000174485	chromosome15	63835843
ENSG00000198837	chromosome1	152183330
ENSG00000174485	chromosome15	63835843
ENSG00000170456	chromosome12	31635034
ENSG00000184014	chromosome11	9243193

ENSG00000164082	chromosome3	51716148
ENSG00000198822	chromosome7	86232398
ENSG00000152822	chromosome6	146392347
ENSG00000168959	chromosome11	88420689
ENSG00000179603	chromosome7	126670495
ENSG00000124493	chromosome6	34231146
ENSG00000113262	chromosome5	178354552
ENSG00000124493	chromosome6	34231146
ENSG00000179603	chromosome7	126670495
ENSG00000196277	chromosome3	6878076
ENSG00000124493	chromosome6	34231146
ENSG00000179603	chromosome7	126670495
ENSG00000113262	chromosome5	178354552
ENSG00000136854	chromosome9	129414504
ENSG00000076944	chromosome19	7608036
ENSG00000076944	chromosome19	7608036
ENSG00000136854	chromosome9	129414504
ENSG00000116266	chromosome1	109090883
ENSG00000151611	chromosome4	146779742
ENSG00000173376	chromosome4	122186443
ENSG00000173376	chromosome4	122186443
ENSG00000115592	chromosome2	219404738
ENSG00000181929	chromosome12	47698791
ENSG00000106617	chromosome7	151204639
ENSG00000106617	chromosome7	151204639
ENSG00000106617	chromosome7	151204639
ENSG00000181929	chromosome12	47698791
ENSG00000104915	chromosome19	13121988
ENSG00000135823	chromosome1	179258433
ENSG00000103066	chromosome16	66836831
ENSG00000213398	chromosome16	66535506
ENSG00000138669	chromosome4	82345226
ENSG00000185532	chromosome10	52421181
ENSG00000139174	chromosome12	41152586
ENSG00000012211	chromosomeX	48929695
ENSG00000163637	chromosome3	64159644
ENSG00000012211	chromosomeX	48929695
ENSG00000163637	chromosome3	64159644
ENSG00000139174	chromosome12	41152586
ENSG00000124593	chromosome6	41859190
ENSG00000071282	chromosome3	8518508
ENSG00000135269	chromosome7	115637998
ENSG00000163637	chromosome3	64159644
ENSG00000012211	chromosomeX	48929695
ENSG00000110328	chromosome11	11599717
ENSG00000185274	chromosome7	70235725
ENSG00000182870	chromosome12	131415863
ENSG00000185274	chromosome7	70235725
ENSG00000182870	chromosome12	131415863
ENSG00000110328	chromosome11	11599717
ENSG00000139629	chromosome12	50059833
ENSG00000115339	chromosome2	166335457
ENSG00000185274	chromosome7	70235725
ENSG00000182870	chromosome12	131415863
ENSG00000158089	chromosome2	31214457
ENSG00000100626	chromosome14	68796761
ENSG00000178234	chromosome7	151422246
ENSG00000106648	chromosome7	151295265
ENSG00000144278	chromosome2	154509257

ENSG00000141429	chromosome18	31488625
ENSG00000182870	chromosome12	131415863
ENSG00000185274	chromosome7	70235725
ENSG00000112514	chromosome6	33493941
ENSG00000138018	chromosome2	26422605
ENSG00000134255	chromosome1	111491860
ENSG00000111666	chromosome12	100615771
ENSG00000089057	chromosome20	4861209
ENSG00000170482	chromosome5	138746842
ENSG00000180098	chromosome1	28752210
ENSG00000108950	chromosome17	64108403
ENSG00000108950	chromosome17	64108403
ENSG00000130517	chromosome19	18312492
ENSG00000183571	chromosome15	97330386
ENSG00000180957	chromosome22	26645180
ENSG00000174238	chromosome17	1412605
ENSG00000180957	chromosome22	26645180
ENSG00000154217	chromosome17	62804595
ENSG00000180957	chromosome22	26645180
ENSG00000174238	chromosome17	1412605
ENSG00000107295	chromosome9	17569241
ENSG00000141985	chromosome19	4351366
ENSG00000140600	chromosome15	81950590
ENSG00000141985	chromosome19	4351366
ENSG00000107295	chromosome9	17569241
ENSG00000204319	chromosome6	32090601
ENSG00000204342	chromosome6	32057864
ENSG00000125730	chromosome19	6671601
ENSG00000204319	chromosome6	32090601
ENSG00000204342	chromosome6	32057864
ENSG00000204319	chromosome6	32090601
ENSG00000204342	chromosome6	32057864
ENSG00000125730	chromosome19	6671601
ENSG00000106804	chromosome9	122852346
ENSG00000169221	chromosome16	30288181
ENSG00000099992	chromosome22	29052871
ENSG00000175463	chromosome11	66928250
ENSG00000099992	chromosome22	29052871
ENSG00000169221	chromosome16	30288181
ENSG00000215079	chromosome16	8883567
ENSG00000089486	chromosome16	4504133
ENSG00000189067	chromosome16	11558088
ENSG00000089486	chromosome16	4504133
ENSG00000089486	chromosome16	4504133
ENSG00000215079	chromosome16	8883567
ENSG00000116704	chromosome1	67292285
ENSG00000130958	chromosome9	98185738
ENSG00000116704	chromosome1	67292285
ENSG00000175497	chromosome2	115636004
ENSG00000130226	chromosome7	153380839
ENSG00000175497	chromosome2	115636004
ENSG00000142002	chromosome19	4674835
ENSG00000074603	chromosome15	63595084
ENSG00000074695	chromosome18	55177457
ENSG00000140506	chromosome15	72892249
ENSG00000114988	chromosome2	96769505
ENSG00000169223	chromosome5	176711255
ENSG00000189221	chromosomeX	43400534
ENSG00000069535	chromosomeX	43626631

ENSG00000104951	chromosome19	55096787
ENSG00000189221	chromosomeX	43400534
ENSG00000069535	chromosomeX	43626631
ENSG00000069535	chromosomeX	43626631
ENSG00000189221	chromosomeX	43400534
ENSG00000104951	chromosome19	55096787
ENSG00000108239	chromosome10	96152361
ENSG00000132405	chromosome4	6976063
ENSG00000154511	chromosome1	93199575
ENSG00000165716	chromosome9	138726939
ENSG00000118194	chromosome1	199609006
ENSG00000130595	chromosome11	1900681
ENSG00000105048	chromosome19	60350338
ENSG00000118194	chromosome1	199609006
ENSG00000105048	chromosome19	60350338
ENSG00000130595	chromosome11	1900681
ENSG00000105048	chromosome19	60350338
ENSG00000130595	chromosome11	1900681
ENSG00000118194	chromosome1	199609006
ENSG00000129353	chromosome19	10597310
ENSG00000137968	chromosome1	75779721
ENSG00000204385	chromosome6	31954737
ENSG00000137968	chromosome1	75779721
ENSG00000129353	chromosome19	10597310
ENSG00000070214	chromosome9	107046971
ENSG00000143036	chromosome1	95058596
ENSG00000074696	chromosome15	63610023
ENSG00000188921	chromosome9	21021590
ENSG00000165996	chromosome10	17699351
ENSG00000206527	chromosome3	124786565
ENSG00000129167	chromosome11	18018886
ENSG00000139287	chromosome12	70619034
ENSG00000180176	chromosome11	2149593
ENSG00000150401	chromosome13	113192986
ENSG00000043093	chromosome3	184180972
ENSG00000154127	chromosome11	122031968
ENSG00000160185	chromosome21	42697124
ENSG00000140367	chromosome15	73923063
ENSG00000189136	chromosome15	82913935
ENSG00000160714	chromosome1	152797654
ENSG00000156194	chromosome4	77036503
ENSG00000086717	chromosomeX	18635821
ENSG00000136274	chromosome7	45092184
ENSG00000121089	chromosome4	166083892
ENSG00000196861	chromosome17	57023324
ENSG00000196531	chromosome12	55404573
ENSG00000169118	chromosome15	62379752
ENSG00000151292	chromosome5	122909257
ENSG00000133275	chromosome19	1920772
ENSG00000133275	chromosome19	1920772
ENSG00000151292	chromosome5	122909257
ENSG00000155465	chromosome14	22354396
ENSG00000103064	chromosome16	66866131
ENSG00000130876	chromosome19	38408450
ENSG00000092068	chromosome14	22721964
ENSG00000151012	chromosome4	139382674
ENSG00000155465	chromosome14	22354396
ENSG00000103064	chromosome16	66866131
ENSG00000155465	chromosome14	22354396

ENSG00000103064	chromosome16	66866131
ENSG00000103257	chromosome16	86460530
ENSG00000115419	chromosome2	191454056
ENSG00000135423	chromosome12	55168170
ENSG00000116906	chromosome1	229443748
ENSG00000119927	chromosome10	113931533
ENSG00000186281	chromosome2	96061835
ENSG00000049283	chromosome17	45968917
ENSG00000063245	chromosome19	60881806
ENSG00000072134	chromosome17	19127026
ENSG00000072134	chromosome17	19127026
ENSG00000063245	chromosome19	60881806
ENSG00000113282	chromosome5	157177121
ENSG00000165704	chromosomeX	133422008
ENSG00000165704	chromosomeX	133422008
ENSG00000099256	chromosome10	25281511
ENSG00000108960	chromosome17	50854056
ENSG00000136297	chromosome7	4935435
ENSG00000130598	chromosome11	1817503
ENSG00000129991	chromosome19	60360770
ENSG00000129991	chromosome19	60360770
ENSG00000130598	chromosome11	1817503
ENSG00000159173	chromosome1	199653545
ENSG00000159173	chromosome1	199653545
ENSG00000159173	chromosome1	199653545
ENSG00000129991	chromosome19	60360770
ENSG00000130598	chromosome11	1817503
ENSG00000166446	chromosome16	79395680
ENSG00000153046	chromosome6	4661012
ENSG00000215563	chromosomeY	19500769
ENSG00000182415	chromosomeY	15937869
ENSG00000129873	chromosomeY	15791647
ENSG00000172288	chromosomeY	23468458
ENSG00000172352	chromosomeY	21893704
ENSG00000156162	chromosome8	95801403
ENSG00000178904	chromosome19	37591000
ENSG00000162374	chromosome1	50342251
ENSG00000196361	chromosome19	11452424
ENSG00000066044	chromosome19	7962700
ENSG00000196361	chromosome19	11452424
ENSG00000162374	chromosome1	50342251
ENSG00000107105	chromosome9	23755079
ENSG00000107105	chromosome9	23755079
ENSG00000196361	chromosome19	11452424
ENSG00000162374	chromosome1	50342251
ENSG00000196361	chromosome19	11452424
ENSG00000162374	chromosome1	50342251
ENSG00000107105	chromosome9	23755079
ENSG00000180287	chromosome1	240578074
ENSG00000105223	chromosome19	45564231
ENSG00000166428	chromosome14	104464523
ENSG00000166428	chromosome14	104464523
ENSG00000105223	chromosome19	45564231
ENSG00000180287	chromosome1	240578074
ENSG00000138670	chromosome4	82612023
ENSG00000146090	chromosome5	179568775
ENSG00000146090	chromosome5	179568775
ENSG00000138670	chromosome4	82612023
ENSG00000198915	chromosome10	43045109

ENSG00000115194	chromosome2	27339279
ENSG00000158014	chromosome1	26244975
ENSG00000115194	chromosome2	27339279
ENSG00000164756	chromosome8	118216748
ENSG00000158014	chromosome1	26244975
ENSG00000115194	chromosome2	27339279
ENSG00000184007	chromosome1	32157254
ENSG00000112245	chromosome6	64344745
ENSG00000184489	chromosome8	142501523
ENSG00000112245	chromosome6	64344745
ENSG00000184007	chromosome1	32157254
ENSG00000166046	chromosome12	105228984
ENSG00000176148	chromosome11	33021896
ENSG00000124678	chromosome6	35217082
ENSG00000215046	chromosomeX	101363215
ENSG00000215029	chromosomeX	101607122
ENSG00000176148	chromosome11	33021896
ENSG00000166046	chromosome12	105228984
ENSG00000203911	chromosome1	92313557
ENSG00000205405	chromosome4	110694700
ENSG00000215829	chromosome1	177419370
ENSG00000119335	chromosome9	130485996
ENSG00000214236	chromosome3	152469481
ENSG00000196792	chromosome14	30565143
ENSG00000115808	chromosome2	37047111
ENSG00000090372	chromosome19	51941528
ENSG00000115808	chromosome2	37047111
ENSG00000196792	chromosome14	30565143
ENSG00000164330	chromosome5	158459065
ENSG00000088881	chromosome20	2621792
ENSG00000088881	chromosome20	2621792
ENSG00000164330	chromosome5	158459065
ENSG00000108001	chromosome10	131652023
ENSG00000088881	chromosome20	2621792
ENSG00000164330	chromosome5	158459065
ENSG00000134025	chromosome8	25958293
ENSG00000108001	chromosome10	131652023
ENSG00000088881	chromosome20	2621792
ENSG00000164330	chromosome5	158459065
ENSG00000108001	chromosome10	131652023
ENSG00000088881	chromosome20	2621792
ENSG00000164330	chromosome5	158459065
ENSG00000134025	chromosome8	25958293
ENSG00000204018	chromosome1	48039882
ENSG00000186854	chromosome2	84961881
ENSG00000204018	chromosome1	48039882
ENSG00000204018	chromosome1	48039882
ENSG00000186854	chromosome2	84961881
ENSG00000072682	chromosome5	131582219
ENSG00000122884	chromosome10	74504648
ENSG00000122884	chromosome10	74504648
ENSG00000072682	chromosome5	131582219
ENSG00000169302	chromosome5	146599391
ENSG00000152953	chromosome4	5104492
ENSG00000165752	chromosome10	133995342
ENSG00000152953	chromosome4	5104492
ENSG00000169302	chromosome5	146599391
ENSG00000152953	chromosome4	5104492
ENSG00000169302	chromosome5	146599391

ENSG00000165752	chromosome10	133995342
ENSG00000142875	chromosome1	84316597
ENSG00000165059	chromosome9	70818829
ENSG00000072062	chromosome19	14089360
ENSG00000100600	chromosome14	92268885
ENSG00000104177	chromosome15	46257727
ENSG00000099783	chromosome19	8415883
ENSG00000215013	chromosome15	42528835
ENSG00000161999	chromosome16	674531
ENSG00000144228	chromosome2	139024257
ENSG00000121067	chromosome17	45055172
ENSG00000198162	chromosome1	117712329
ENSG00000117643	chromosome1	25816876
ENSG00000111885	chromosome6	119711930
ENSG00000111885	chromosome6	119711930
ENSG00000117643	chromosome1	25816876
ENSG00000173171	chromosome1	153445220
ENSG00000177034	chromosome5	79322818
ENSG00000183034	chromosome17	70432323
ENSG00000182938	chromosome17	70443492
ENSG00000182938	chromosome17	70443492
ENSG00000183034	chromosome17	70432323
ENSG00000214333	chromosome2	91141263
ENSG00000163982	chromosome4	4279493
ENSG00000153767	chromosome3	121952090
ENSG00000143554	chromosome1	152014214
ENSG00000113396	chromosome5	128329730
ENSG00000083807	chromosome19	63715135
ENSG00000130304	chromosome19	17442350
ENSG00000167114	chromosome9	130142863
ENSG00000140284	chromosome15	48261917
ENSG00000083807	chromosome19	63715135
ENSG00000143554	chromosome1	152014214
ENSG00000113396	chromosome5	128329730
ENSG00000113396	chromosome5	128329730
ENSG00000143554	chromosome1	152014214
ENSG00000108443	chromosome17	55325328
ENSG00000214817	chromosome17	20560388
ENSG00000175634	chromosome11	66952593
ENSG00000177189	chromosomeX	20194672
ENSG00000072133	chromosomeX	83329564
ENSG00000071242	chromosome6	167195649
ENSG00000117676	chromosome1	26728999
ENSG00000072133	chromosomeX	83329564
ENSG00000177189	chromosomeX	20194672
ENSG00000117676	chromosome1	26728999
ENSG00000071242	chromosome6	167195649
ENSG00000162302	chromosome11	63883284
ENSG00000100784	chromosome14	90596532
ENSG00000007866	chromosome6	35562466
ENSG00000074219	chromosome19	54555145
ENSG00000187079	chromosome11	12742356
ENSG00000187079	chromosome11	12742356
ENSG00000074219	chromosome19	54555145
ENSG00000187079	chromosome11	12742356
ENSG00000070182	chromosome14	64359578
ENSG00000173898	chromosome11	66245288
ENSG00000115306	chromosome2	54607021
ENSG00000160460	chromosome19	45670369

ENSG00000115306	chromosome2	54607021
ENSG00000173898	chromosome11	66245288
ENSG00000173898	chromosome11	66245288
ENSG00000115306	chromosome2	54607021
ENSG00000070182	chromosome14	64359578
ENSG00000099246	chromosome10	27833305
ENSG00000099246	chromosome10	27833305
ENSG00000173575	chromosome15	91245472
ENSG00000153922	chromosome5	98289991
ENSG00000149488	chromosome20	2465268
ENSG00000165091	chromosome9	74453385
ENSG00000170537	chromosome16	18902887
ENSG00000167608	chromosome19	59368625
ENSG00000167895	chromosome17	73639265
ENSG00000167895	chromosome17	73639265
ENSG00000167608	chromosome19	59368625
ENSG00000141524	chromosome17	73634509
ENSG00000103534	chromosome16	19358862
ENSG00000138449	chromosome2	190153432
ENSG00000101290	chromosome20	5055739
ENSG00000163624	chromosome4	85723579
ENSG00000166111	chromosome12	107856861
ENSG00000173218	chromosome1	115995558
ENSG00000162738	chromosome1	158652252
ENSG00000136059	chromosome3	38010168
ENSG00000127831	chromosome2	218996746
ENSG00000135407	chromosome12	56496091
ENSG00000148180	chromosome9	123101961
ENSG00000006747	chromosome7	12576938
ENSG00000135407	chromosome12	56496091
ENSG00000127831	chromosome2	218996746
ENSG00000006747	chromosome7	12576938
ENSG00000148180	chromosome9	123101961
ENSG00000042493	chromosome2	85482780
ENSG00000006747	chromosome7	12576938
ENSG00000184544	chromosome17	9635372
ENSG00000109016	chromosome17	20970877
ENSG00000122729	chromosome9	32395505
ENSG00000136381	chromosome15	76517735
ENSG00000066739	chromosome14	95899067
ENSG00000110046	chromosome11	64441184
ENSG00000072954	chromosome19	16633029
ENSG00000095209	chromosome9	107496763
ENSG00000105983	chromosome7	156378449
ENSG00000139636	chromosome12	47790606
ENSG00000167323	chromosome11	3834077
ENSG00000109689	chromosome4	26471678
ENSG00000167705	chromosome17	1499849
ENSG00000188026	chromosome12	122584457
ENSG00000150977	chromosome12	122486921
ENSG00000188026	chromosome12	122584457
ENSG00000167705	chromosome17	1499849
ENSG00000172915	chromosome13	34414958
ENSG00000198589	chromosome4	152155245
ENSG00000125648	chromosome19	6410640
ENSG00000181240	chromosome19	6384705
ENSG00000148339	chromosome9	129870420
ENSG00000085491	chromosome1	108544284
ENSG00000181240	chromosome19	6384705

ENSG00000125648	chromosome19	6410640
ENSG00000148339	chromosome9	129870420
ENSG00000181240	chromosome19	6384705
ENSG00000125648	chromosome19	6410640
ENSG00000085491	chromosome1	108544284
ENSG00000036565	chromosome8	20082756
ENSG00000165646	chromosome10	118991195
ENSG00000164091	chromosome3	52287418
ENSG00000140961	chromosome16	82540556
ENSG00000164823	chromosome8	90991057
ENSG00000164951	chromosome8	94998356
ENSG00000164951	chromosome8	94998356
ENSG00000172840	chromosome16	65475689
ENSG00000156875	chromosome1	100276377
ENSG00000148110	chromosome9	96176923
ENSG00000156875	chromosome1	100276377
ENSG00000149657	chromosome20	60131118
ENSG00000105216	chromosome19	39355388
ENSG00000109956	chromosome11	133762764
ENSG00000109956	chromosome11	133762764
ENSG00000109956	chromosome11	133762764
ENSG00000112309	chromosome6	71722854
ENSG00000112309	chromosome6	71722854
ENSG00000109956	chromosome11	133762764
ENSG00000149541	chromosome11	62145996
ENSG00000109956	chromosome11	133762764
ENSG00000109956	chromosome11	133762764
ENSG00000064932	chromosome19	1105351
ENSG00000139697	chromosome12	122400942
ENSG00000111912	chromosome6	126178194
ENSG00000111912	chromosome6	126178194
ENSG00000164830	chromosome8	107760564
ENSG00000104888	chromosome19	54636449
ENSG00000179520	chromosome12	99275301
ENSG00000091664	chromosome11	22316656
ENSG00000179520	chromosome12	99275301
ENSG00000104888	chromosome19	54636449
ENSG00000119899	chromosome6	74420331
ENSG00000112337	chromosome6	26034004
ENSG00000146039	chromosome6	25870170
ENSG00000124568	chromosome6	25938765
ENSG00000124564	chromosome6	25976595
ENSG00000130733	chromosome19	10900087
ENSG00000058799	chromosome1	54127199
ENSG00000151388	chromosome5	33927719
ENSG00000136378	chromosome15	76890618
ENSG00000214644	chromosome15	76055444
ENSG00000215743	supercontigNT_113927	47093
ENSG00000140873	chromosome16	76026094
ENSG00000145536	chromosome5	5193581
ENSG00000049192	chromosome5	64805257
ENSG00000142303	chromosome19	8576596
ENSG00000140470	chromosome15	98699628
ENSG00000145808	chromosome5	128824002
ENSG00000160323	chromosome9	135277385
ENSG00000156140	chromosome4	73653344
ENSG00000138316	chromosome10	72102565
ENSG00000087116	chromosome5	178704936
ENSG00000138316	chromosome10	72102565

ENSG00000156140	chromosome4	73653344
ENSG00000156140	chromosome4	73653344
ENSG00000138316	chromosome10	72102565
ENSG00000087116	chromosome5	178704936
ENSG00000160323	chromosome9	135277385
ENSG00000145808	chromosome5	128824002
ENSG00000140470	chromosome15	98699628
ENSG00000087116	chromosome5	178704936
ENSG00000156140	chromosome4	73653344
ENSG00000138316	chromosome10	72102565
ENSG00000163638	chromosome3	64648374
ENSG00000173157	chromosome12	42231992
ENSG00000102683	chromosome13	22675834
ENSG00000170624	chromosome5	155704077
ENSG00000185053	chromosome8	15139504
ENSG00000170624	chromosome5	155704077
ENSG00000185053	chromosome8	15139504
ENSG00000102683	chromosome13	22675834
ENSG00000185053	chromosome8	15139504
ENSG00000170624	chromosome5	155704077
ENSG00000141314	chromosome17	27617322
ENSG00000103269	chromosome16	665792
ENSG00000158571	chromosomeX	55037166
ENSG00000114268	chromosome3	48569215
ENSG00000123836	chromosome1	205294686
ENSG00000170525	chromosome10	6226967
ENSG00000114268	chromosome3	48569215
ENSG00000158571	chromosomeX	55037166
ENSG00000170525	chromosome10	6226967
ENSG00000123836	chromosome1	205294686
ENSG00000136717	chromosome2	127580990
ENSG00000078053	chromosome7	38637477
ENSG00000110934	chromosome12	50004154
ENSG00000078053	chromosome7	38637477
ENSG00000136717	chromosome2	127580990
ENSG00000078053	chromosome7	38637477
ENSG00000136717	chromosome2	127580990
ENSG00000110934	chromosome12	50004154
ENSG00000135677	chromosome12	63439324
ENSG00000196562	chromosome20	45819515
ENSG00000137573	chromosome8	70638765
ENSG00000137573	chromosome8	70638765
ENSG00000196562	chromosome20	45819515
ENSG00000100288	chromosome22	49368077
ENSG00000110721	chromosome11	67645221
ENSG00000143845	chromosome1	202387604
ENSG00000139163	chromosome12	22669365
ENSG00000139163	chromosome12	22669365
ENSG00000143845	chromosome1	202387604
ENSG00000134318	chromosome2	11401834
ENSG00000143776	chromosome1	225571507
ENSG00000198752	chromosome14	102593264
ENSG00000198752	chromosome14	102593264
ENSG00000143776	chromosome1	225571507
ENSG00000171219	chromosome11	64368618
ENSG00000171219	chromosome11	64368618
ENSG00000198752	chromosome14	102593264
ENSG00000143776	chromosome1	225571507
ENSG00000134318	chromosome2	11401834

ENSG00000067900	chromosome18	16944870
ENSG00000189077	chromosome7	75459457
ENSG00000188735	chromosome12	120635185
ENSG00000100346	chromosome22	38296704
ENSG00000196557	chromosome16	1143739
ENSG00000006283	chromosome17	45993820
ENSG00000196557	chromosome16	1143739
ENSG00000100346	chromosome22	38296704
ENSG00000144579	chromosome2	218973058
ENSG00000175215	chromosome12	56526486
ENSG00000144677	chromosome3	37878695
ENSG00000175215	chromosome12	56526486
ENSG00000144579	chromosome2	218973058
ENSG00000073910	chromosome13	31503933
ENSG00000075539	chromosome4	48350561
ENSG00000197136	chromosome11	65150911
ENSG00000100731	chromosome14	70444321
ENSG00000135749	chromosome1	231342211
ENSG00000100731	chromosome14	70444321
ENSG00000197136	chromosome11	65150911
ENSG00000171316	chromosome8	61816546
ENSG00000124177	chromosome20	39626202
ENSG00000177200	chromosome16	51747503
ENSG00000100888	chromosome14	20969238
ENSG00000100888	chromosome14	20969238
ENSG00000177200	chromosome16	51747503
ENSG00000124177	chromosome20	39626202
ENSG00000171316	chromosome8	61816546
ENSG00000122728	chromosome9	32625578
ENSG00000147133	chromosomeX	70502890
ENSG00000183921	chromosome16	22085092
ENSG00000184860	chromosome16	80591965
ENSG00000182500	chromosome12	120549031
ENSG00000175938	chromosome16	30868112
ENSG00000160991	chromosome7	101866409
ENSG00000160991	chromosome7	101866409
ENSG00000175938	chromosome16	30868112
ENSG00000117602	chromosome1	24713450
ENSG00000172348	chromosome6	46532673
ENSG00000159200	chromosome21	34909181
ENSG00000172348	chromosome6	46532673
ENSG00000117602	chromosome1	24713450
ENSG00000168350	chromosome14	99695678
ENSG00000168350	chromosome14	99695678
ENSG00000143753	chromosome1	222437662
ENSG00000138032	chromosome2	44281843
ENSG00000100614	chromosome14	59819175
ENSG00000177886	chromosome19	50693805
ENSG00000100614	chromosome14	59819175
ENSG00000138032	chromosome2	44281843
ENSG00000159692	chromosome4	1232892
ENSG00000175029	chromosome10	126717614
ENSG00000175029	chromosome10	126717614
ENSG00000159692	chromosome4	1232892
ENSG00000168781	chromosome15	41763830
ENSG00000145725	chromosome5	102493157
ENSG00000138081	chromosome2	47920393
ENSG00000134824	chromosome11	61340334
ENSG00000214876	chromosome11	56414887

ENSG00000149485	chromosome11	61415430
ENSG00000099998	chromosome22	22970694
ENSG00000133475	chromosome22	19893824
ENSG00000183038	chromosome22	18747798
ENSG00000215500	chromosome22	18941249
ENSG00000100031	chromosome22	23337049
ENSG00000197421	chromosome22	17158736
ENSG00000215610	supercontigNT_113923	99575
ENSG00000215466	chromosome22	22975435
ENSG00000100121	chromosome22	21318816
ENSG00000149435	chromosome20	23915249
ENSG00000107372	chromosome9	74169345
ENSG00000086666	chromosome15	78199742
ENSG00000107372	chromosome9	74169345
ENSG00000166974	chromosome18	30875502
ENSG00000084764	chromosome2	27098591
ENSG00000101367	chromosome20	30877395
ENSG00000084764	chromosome2	27098591
ENSG00000166974	chromosome18	30875502
ENSG00000147251	chromosomeX	117513963
ENSG00000088387	chromosome13	98536607
ENSG00000135905	chromosome2	225615336
ENSG00000088387	chromosome13	98536607
ENSG00000147251	chromosomeX	117513963
ENSG00000130158	chromosome19	11234117
ENSG00000116641	chromosome1	62926524
ENSG00000107099	chromosome9	276509
ENSG00000116641	chromosome1	62926524
ENSG00000130158	chromosome19	11234117
ENSG00000144290	chromosome2	162189302
ENSG00000050438	chromosome12	50071399
ENSG00000080493	chromosome4	72321158
ENSG00000188687	chromosome2	74395659
ENSG00000004939	chromosome17	39695761
ENSG00000114923	chromosome2	220200959
ENSG00000164889	chromosome7	150390008
ENSG00000188687	chromosome2	74395659
ENSG00000080493	chromosome4	72321158
ENSG00000113073	chromosome5	139720006
ENSG00000033867	chromosome3	27473179
ENSG00000050438	chromosome12	50071399
ENSG00000144290	chromosome2	162189302
ENSG00000113073	chromosome5	139720006
ENSG00000188687	chromosome2	74395659
ENSG00000080493	chromosome4	72321158
ENSG00000050438	chromosome12	50071399
ENSG00000144290	chromosome2	162189302
ENSG00000033867	chromosome3	27473179
ENSG00000164889	chromosome7	150390008
ENSG00000114923	chromosome2	220200959
ENSG00000214706	chromosome3	50305354
ENSG00000006652	chromosome7	111877980
ENSG00000006831	chromosome12	1733771
ENSG00000159346	chromosome1	201186822
ENSG00000006831	chromosome12	1733771
ENSG00000077458	chromosome11	95162291
ENSG00000009780	chromosome1	27925179
ENSG00000110660	chromosome11	107234704
ENSG00000196376	chromosome6	118335583

ENSG00000136052	chromosome12	103846436
ENSG00000114544	chromosome3	127269753
ENSG00000133065	chromosome1	204046193
ENSG00000114544	chromosome3	127269753
ENSG00000136052	chromosome12	103846436
ENSG00000140612	chromosome15	83060530
ENSG00000166562	chromosome18	54958161
ENSG00000197070	chromosome9	139619991
ENSG00000140450	chromosome15	96305096
ENSG00000117289	chromosome1	144150160
ENSG00000105643	chromosome19	17973124
ENSG00000113369	chromosome5	90714666
ENSG00000113369	chromosome5	90714666
ENSG00000113369	chromosome5	90714666
ENSG00000105643	chromosome19	17973124
ENSG00000117289	chromosome1	144150160
ENSG00000140450	chromosome15	96305096
ENSG00000105643	chromosome19	17973124
ENSG00000113369	chromosome5	90714666
ENSG00000140450	chromosome15	96305096
ENSG00000117289	chromosome1	144150160
ENSG00000107164	chromosome9	132444889
ENSG00000162613	chromosome1	78217277
ENSG00000088247	chromosome19	6375809
ENSG00000215224	chromosome9	21685172
ENSG00000088247	chromosome19	6375809
ENSG00000215224	chromosome9	21685172
ENSG00000162613	chromosome1	78217277
ENSG00000124067	chromosome16	66559958
ENSG00000140199	chromosome15	32416174
ENSG00000113504	chromosome5	1165107
ENSG00000124140	chromosome20	44091391
ENSG00000124140	chromosome20	44091391
ENSG00000113504	chromosome5	1165107
ENSG00000140199	chromosome15	32416174
ENSG00000124067	chromosome16	66559958
ENSG00000128815	chromosome10	49587784
ENSG00000163625	chromosome4	86000769
ENSG00000161677	chromosome19	55705501
ENSG00000100221	chromosome22	37425939
ENSG00000184897	chromosome3	130517436
ENSG00000187475	chromosome6	26216301
ENSG00000124610	chromosome6	26125940
ENSG00000184357	chromosome6	27943287
ENSG00000168298	chromosome6	26264598
ENSG00000187837	chromosome6	26164636
ENSG00000124575	chromosome6	26343141
ENSG00000196440	chromosomeX	100629261
ENSG00000187475	chromosome6	26216301
ENSG00000124610	chromosome6	26125940
ENSG00000184357	chromosome6	27943287
ENSG00000168298	chromosome6	26264598
ENSG00000187837	chromosome6	26164636
ENSG00000124575	chromosome6	26343141
ENSG00000187475	chromosome6	26216301
ENSG00000124610	chromosome6	26125940
ENSG00000184357	chromosome6	27943287
ENSG00000168298	chromosome6	26264598
ENSG00000187837	chromosome6	26164636

ENSG00000124575	chromosome6	26343141
ENSG00000184897	chromosome3	130517436
ENSG00000198925	chromosome2	219802609
ENSG00000181652	chromosome7	150352444
ENSG00000139190	chromosome12	6449959
ENSG00000179036	chromosome17	8006923
ENSG00000120457	chromosome11	128286379
ENSG00000157542	chromosome21	38134855
ENSG00000168135	chromosome22	37154084
ENSG00000184185	chromosome17	21259248
ENSG00000157542	chromosome21	38134855
ENSG00000120457	chromosome11	128286379
ENSG00000162728	chromosome1	158320445
ENSG00000187486	chromosome11	17366215
ENSG00000162989	chromosome2	155263534
ENSG00000151704	chromosome11	128217523
ENSG00000115474	chromosome2	233344317
ENSG00000157551	chromosome21	38593054
ENSG00000177807	chromosome1	158278947
ENSG00000157542	chromosome21	38134855
ENSG00000120457	chromosome11	128286379
ENSG00000177807	chromosome1	158278947
ENSG00000157551	chromosome21	38593054
ENSG00000187486	chromosome11	17366215
ENSG00000121361	chromosome12	21817818
ENSG00000184185	chromosome17	21259248
ENSG00000168135	chromosome22	37154084
ENSG00000182324	chromosome19	53656794
ENSG00000123700	chromosome17	65682776
ENSG00000182324	chromosome19	53656794
ENSG00000123700	chromosome17	65682776
ENSG00000184185	chromosome17	21259248
ENSG00000168135	chromosome22	37154084
ENSG00000153822	chromosome17	65639824
ENSG00000123700	chromosome17	65682776
ENSG00000182324	chromosome19	53656794
ENSG00000157551	chromosome21	38593054
ENSG00000177807	chromosome1	158278947
ENSG00000115474	chromosome2	233344317
ENSG00000140368	chromosome15	75074973
ENSG00000152229	chromosome18	41906153
ENSG00000100266	chromosome22	41638031
ENSG00000124507	chromosome6	34602061
ENSG00000165912	chromosome11	47160855
ENSG00000165912	chromosome11	47160855
ENSG00000100266	chromosome22	41638031
ENSG00000124507	chromosome6	34602061
ENSG00000124507	chromosome6	34602061
ENSG00000100266	chromosome22	41638031
ENSG00000188906	chromosome12	38905201
ENSG00000154237	chromosome15	99282361
ENSG00000100591	chromosome14	76994286
ENSG00000173209	chromosome2	61258178
ENSG00000069998	chromosome22	16026135
ENSG00000061676	chromosome2	183611073
ENSG00000123338	chromosome12	53177841
ENSG00000135917	chromosome2	228275279
ENSG00000117479	chromosome1	167721629
ENSG00000117479	chromosome1	167721629

ENSG00000135917	chromosome2	228275279
ENSG00000173638	chromosome21	45782302
ENSG00000196924	chromosomeX	153252808
ENSG00000136068	chromosome3	57969332
ENSG00000128591	chromosome7	128257928
ENSG00000136068	chromosome3	57969332
ENSG00000196924	chromosomeX	153252808
ENSG00000066032	chromosome2	79732191
ENSG00000044115	chromosome5	138145513
ENSG00000044115	chromosome5	138145513
ENSG00000066032	chromosome2	79732191
ENSG00000183230	chromosome10	69078535
ENSG00000035403	chromosome10	75427972
ENSG00000213625	chromosome1	65658903
ENSG00000104660	chromosome8	30072571
ENSG00000178795	chromosome11	76673831
ENSG00000158555	chromosome11	74866429
ENSG00000158555	chromosome11	74866429
ENSG00000178795	chromosome11	76673831
ENSG00000130055	chromosomeX	69561560
ENSG00000040487	chromosome1	19516759
ENSG00000129515	chromosome14	34169110
ENSG00000172803	chromosome11	65358113
ENSG00000089006	chromosome20	17897069
ENSG00000172803	chromosome11	65358113
ENSG00000129515	chromosome14	34169110
ENSG00000028528	chromosome15	62175266
ENSG00000205302	chromosome5	122138698
ENSG00000146676	chromosome7	44891473
ENSG00000185129	chromosome5	139473951
ENSG00000185129	chromosome5	139473951
ENSG00000146676	chromosome7	44891473
ENSG00000172733	chromosome8	31009841
ENSG00000167889	chromosome17	72376704
ENSG00000152127	chromosome2	134728445
ENSG00000167889	chromosome17	72376704
ENSG00000152127	chromosome2	134728445
ENSG00000087510	chromosome20	54638008
ENSG00000116819	chromosome1	35811766
ENSG00000008196	chromosome6	50894564
ENSG00000137203	chromosome6	10527670
ENSG00000116819	chromosome1	35811766
ENSG00000008196	chromosome6	50894564
ENSG00000137203	chromosome6	10527670
ENSG00000087510	chromosome20	54638008
ENSG00000008197	chromosome6	50789728
ENSG00000008196	chromosome6	50894564
ENSG00000116819	chromosome1	35811766
ENSG00000137203	chromosome6	10527670
ENSG00000116819	chromosome1	35811766
ENSG00000008196	chromosome6	50894564
ENSG00000120662	chromosome13	40733044
ENSG00000180015	chromosome4	189896500
ENSG00000112031	chromosome6	153365514
ENSG00000109919	chromosome11	47620594
ENSG00000137409	chromosome6	37061928
ENSG00000214315	chromosome6	139013804
ENSG00000170348	chromosome14	74713036
ENSG00000198408	chromosome10	103567770

ENSG00000198408	chromosome10	103567770
ENSG00000143387	chromosome1	149046525
ENSG00000163131	chromosome1	149003864
ENSG00000163131	chromosome1	149003864
ENSG00000143387	chromosome1	149046525
ENSG00000163131	chromosome1	149003864
ENSG00000143387	chromosome1	149046525
ENSG00000163131	chromosome1	149003864
ENSG00000135047	chromosome9	89532339
ENSG00000204437	chromosome10	81622863
ENSG00000136943	chromosome9	98840147
ENSG00000188029	chromosome9	89577650
ENSG00000106992	chromosome9	129676718
ENSG00000214347	chromosome19	6347382
ENSG00000154027	chromosome1	77520587
ENSG00000127328	chromosome12	68419894
ENSG00000167994	chromosome11	61444241
ENSG00000130489	chromosome22	49309707
ENSG00000133028	chromosome17	10541550
ENSG00000214586	chromosome12	46841145
ENSG00000167977	chromosome16	2672551
ENSG00000180901	chromosome17	70554876
ENSG00000197893	chromosome10	115413632
ENSG00000182809	chromosome14	105012245
ENSG00000146215	chromosome6	43384509
ENSG00000136490	chromosome17	59130391
ENSG00000197893	chromosome10	115413632
ENSG00000159176	chromosome1	199732055
ENSG00000129170	chromosome11	19170572
ENSG00000175183	chromosome12	75784172
ENSG00000136490	chromosome17	59130391
ENSG00000175183	chromosome12	75784172
ENSG00000129170	chromosome11	19170572
ENSG00000085831	chromosome1	51569587
ENSG00000085831	chromosome1	51569587
ENSG00000155158	chromosome9	15297250
ENSG00000059804	chromosome12	7982247
ENSG00000173262	chromosome12	7907319
ENSG00000117394	chromosome1	43196910
ENSG00000181856	chromosome17	7125978
ENSG00000163581	chromosome3	172227154
ENSG00000163581	chromosome3	172227154
ENSG00000181856	chromosome17	7125978
ENSG00000133460	chromosome22	22521912
ENSG00000109667	chromosome4	9636689
ENSG00000181856	chromosome17	7125978
ENSG00000163581	chromosome3	172227154
ENSG00000059804	chromosome12	7982247
ENSG00000173262	chromosome12	7907319
ENSG00000117394	chromosome1	43196910
ENSG00000117394	chromosome1	43196910
ENSG00000059804	chromosome12	7982247
ENSG00000173262	chromosome12	7907319
ENSG00000111450	chromosome12	129889598
ENSG00000166900	chromosome11	59279655
ENSG00000106089	chromosome7	72771898
ENSG00000099365	chromosome16	30929219
ENSG00000166900	chromosome11	59279655
ENSG00000135604	chromosome6	144549458

ENSG00000178750	chromosome3	95216804
ENSG00000142910	chromosome1	31815337
ENSG00000137251	chromosome6	54280823
ENSG00000165914	chromosome14	90352393
ENSG00000068724	chromosome2	47022185
ENSG00000145217	chromosome4	975492
ENSG00000155850	chromosome5	149337409
ENSG00000170615	chromosome7	102849198
ENSG00000170615	chromosome7	102849198
ENSG00000174502	chromosome1	204171572
ENSG00000091138	chromosome7	107222191
ENSG00000091137	chromosome7	107089323
ENSG00000135502	chromosome12	56290705
ENSG00000147606	chromosome8	92331056
ENSG00000112186	chromosome6	17529766
ENSG00000131236	chromosome1	40297606
ENSG00000118596	chromosome12	58384850
ENSG00000155380	chromosome1	113273454
ENSG00000112394	chromosome6	111515649
ENSG00000147100	chromosomeX	73557976
ENSG00000118596	chromosome12	58384850
ENSG00000155380	chromosome1	113273454
ENSG00000100156	chromosome22	36808827
ENSG00000141526	chromosome17	77787174
ENSG00000118596	chromosome12	58384850
ENSG00000155380	chromosome1	113273454
ENSG00000141526	chromosome17	77787174
ENSG00000100156	chromosome22	36808827
ENSG00000155380	chromosome1	113273454
ENSG00000118596	chromosome12	58384850
ENSG00000165449	chromosome10	61114056
ENSG00000163053	chromosome2	230632313
ENSG00000170190	chromosome17	70601327
ENSG00000100156	chromosome22	36808827
ENSG00000141526	chromosome17	77787174
ENSG00000118596	chromosome12	58384850
ENSG00000155380	chromosome1	113273454
ENSG00000108932	chromosome17	63786057
ENSG00000174327	chromosome17	6880426
ENSG00000174326	chromosome17	6887629
ENSG00000108932	chromosome17	63786057
ENSG00000166748	chromosome15	84487957
ENSG00000135049	chromosome9	87517269
ENSG00000165923	chromosome11	47692779
ENSG00000146856	chromosome7	134323225
ENSG00000143318	chromosome1	158427166
ENSG00000118729	chromosome1	116112686
ENSG00000118729	chromosome1	116112686
ENSG00000198794	chromosome15	73091234
ENSG00000140497	chromosome15	72952650
ENSG00000116521	chromosome1	153498567
ENSG00000085365	chromosome5	77692252
ENSG00000085365	chromosome5	77692252
ENSG00000116521	chromosome1	153498567
ENSG00000183780	chromosome1	232107447
ENSG00000151812	chromosome14	57130594
ENSG00000173653	chromosome11	66367503
ENSG00000166479	chromosome18	64533199
ENSG00000179918	chromosome16	30364550

ENSG00000086475	chromosome10	13426957
ENSG00000143819	chromosome1	224083054
ENSG00000152952	chromosome3	147361467
ENSG00000083444	chromosome1	11917424
ENSG00000198756	chromosome1	182273451
ENSG00000130309	chromosome19	17527523
ENSG00000167123	chromosome9	130222978
ENSG00000130309	chromosome19	17527523
ENSG00000198756	chromosome1	182273451
ENSG00000083444	chromosome1	11917424
ENSG00000152952	chromosome3	147361467
ENSG00000106397	chromosome7	100647276
ENSG00000111725	chromosome12	118590433
ENSG00000131791	chromosome1	145110348
ENSG00000146070	chromosome6	46798588
ENSG00000158006	chromosome1	26189895
ENSG00000110090	chromosome11	68339519
ENSG00000110090	chromosome11	68339519
ENSG00000169169	chromosome19	54887322
ENSG00000205560	chromosome22	49363211
ENSG00000095321	chromosome9	130912610
ENSG00000138674	chromosome4	84022114
ENSG00000075826	chromosome10	102266700
ENSG00000121281	chromosome16	48879592
ENSG00000129467	chromosome14	23873699
ENSG00000078295	chromosome5	7449410
ENSG00000155897	chromosome8	132121762
ENSG00000164742	chromosome7	45580668
ENSG00000162104	chromosome16	4105445
ENSG00000138031	chromosome2	24995361
ENSG00000174233	chromosome12	47463485
ENSG00000173175	chromosome3	124650083
ENSG00000129467	chromosome14	23873699
ENSG00000078295	chromosome5	7449410
ENSG00000121281	chromosome16	48879592
ENSG00000174233	chromosome12	47463485
ENSG00000078295	chromosome5	7449410
ENSG00000129467	chromosome14	23873699
ENSG00000131148	chromosome16	84390403
ENSG00000100908	chromosome14	23680354
ENSG00000136425	chromosome15	76210613
ENSG00000141977	chromosome19	16145287
ENSG00000185043	chromosome15	88578122
ENSG00000157884	chromosome2	26717687
ENSG00000171365	chromosomeX	49721321
ENSG00000073464	chromosomeX	10113073
ENSG00000109572	chromosome4	170793655
ENSG00000073464	chromosomeX	10113073
ENSG00000171365	chromosomeX	49721321
ENSG00000147465	chromosome8	38127494
ENSG00000147465	chromosome8	38127494
ENSG00000131748	chromosome17	35063311
ENSG00000168228	chromosome4	24923530
ENSG00000112305	chromosome6	71434448
ENSG00000084070	chromosome1	40612380
ENSG00000112305	chromosome6	71434448
ENSG00000164398	chromosome5	131375513
ENSG00000151726	chromosome4	185961663
ENSG00000197142	chromosome10	114126058

ENSG00000197142	chromosome10	114126058
ENSG00000164398	chromosome5	131375513
ENSG00000151726	chromosome4	185961663
ENSG00000151726	chromosome4	185961663
ENSG00000164398	chromosome5	131375513
ENSG00000123159	chromosome19	14454789
ENSG00000137960	chromosome1	78284367
ENSG00000179855	chromosome19	3536596
ENSG00000137960	chromosome1	78284367
ENSG00000123159	chromosome19	14454789
ENSG00000197518	chromosomeX	37236169
ENSG00000132446	chromosomeX	30999992
ENSG00000167996	chromosome11	61491474
ENSG00000173477	chromosome1	226889787
ENSG00000176778	chromosome5	17409396
ENSG00000215234	chromosome9	15517874
ENSG00000180795	chromosomeX	146941343
ENSG00000181867	chromosome5	121215558
ENSG00000197518	chromosomeX	37236169
ENSG00000132446	chromosomeX	30999992
ENSG00000167996	chromosome11	61491474
ENSG00000173477	chromosome1	226889787
ENSG00000176778	chromosome5	17409396
ENSG00000215234	chromosome9	15517874
ENSG00000180795	chromosomeX	146941343
ENSG00000181867	chromosome5	121215558
ENSG00000171878	chromosome20	3952564
ENSG00000087086	chromosome19	54160577
ENSG00000171878	chromosome20	3952564
ENSG00000087086	chromosome19	54160577
ENSG00000171878	chromosome20	3952564
ENSG00000087086	chromosome19	54160577
ENSG00000197518	chromosomeX	37236169
ENSG00000132446	chromosomeX	30999992
ENSG00000167996	chromosome11	61491474
ENSG00000173477	chromosome1	226889787
ENSG00000176778	chromosome5	17409396
ENSG00000215234	chromosome9	15517874
ENSG00000180795	chromosomeX	146941343
ENSG00000181867	chromosome5	121215558
ENSG00000110887	chromosome12	107802912
ENSG00000203797	chromosome6	110843443
ENSG00000008441	chromosome19	12967652
ENSG00000162599	chromosome1	61103631
ENSG00000162599	chromosome1	61103631
ENSG00000008441	chromosome19	12967652
ENSG00000141905	chromosome19	3310681
ENSG00000147862	chromosome9	14388631
ENSG00000147862	chromosome9	14388631
ENSG00000141905	chromosome19	3310681
ENSG00000141905	chromosome19	3310681
ENSG00000147862	chromosome9	14388631
ENSG00000141905	chromosome19	3310681
ENSG00000147862	chromosome9	14388631
ENSG00000198911	chromosome22	40559221
ENSG00000072310	chromosome17	17680857
ENSG00000148343	chromosome9	130838745
ENSG00000180488	chromosome1	78017929
ENSG00000173559	chromosome2	192251586

ENSG00000139579	chromosome12	54904908
ENSG00000144834	chromosome3	113200960
ENSG00000158710	chromosome1	158159885
ENSG00000130176	chromosome19	11510743
ENSG00000064666	chromosome19	977661
ENSG00000215433	chromosome22	28773183
ENSG00000204826	chromosome9	42999838
ENSG00000204782	chromosome9	68789308
ENSG00000117519	chromosome1	95165294
ENSG00000149591	chromosome11	116578940
ENSG00000158710	chromosome1	158159885
ENSG00000144834	chromosome3	113200960
ENSG00000117519	chromosome1	95165294
ENSG00000064666	chromosome19	977661
ENSG00000215433	chromosome22	28773183
ENSG00000204826	chromosome9	42999838
ENSG00000204782	chromosome9	68789308
ENSG00000170385	chromosome1	209818578
ENSG00000186575	chromosome22	28329904
ENSG00000137710	chromosome11	109655628
ENSG00000147065	chromosomeX	64804434
ENSG00000092820	chromosome6	159159114
ENSG00000147065	chromosomeX	64804434
ENSG00000137710	chromosome11	109655628
ENSG00000100027	chromosome22	20395034
ENSG00000175155	chromosome17	54785553
ENSG00000090238	chromosome16	30014438
ENSG00000166793	chromosome11	57171157
ENSG00000175155	chromosome17	54785553
ENSG00000100027	chromosome22	20395034
ENSG00000090238	chromosome16	30014438
ENSG00000100027	chromosome22	20395034
ENSG00000175155	chromosome17	54785553
ENSG00000138411	chromosome2	197006393
ENSG00000002746	chromosome7	43250030
ENSG00000112941	chromosome5	6790657
ENSG00000121274	chromosome16	48745280
ENSG00000112941	chromosome5	6790657
ENSG00000100077	chromosome22	24291008
ENSG00000173020	chromosome11	66790747
ENSG00000185974	chromosome13	113369703
ENSG00000125388	chromosome4	2935137
ENSG00000198873	chromosome10	120957282
ENSG00000198055	chromosome5	176786453
ENSG00000198873	chromosome10	120957282
ENSG00000114124	chromosome3	142979817
ENSG00000185974	chromosome13	113369703
ENSG00000198873	chromosome10	120957282
ENSG00000125388	chromosome4	2935137
ENSG00000151553	chromosome10	116571828
ENSG00000158863	chromosome8	22002711
ENSG00000164142	chromosome4	152706862
ENSG00000051009	chromosome11	6202323
ENSG00000124780	chromosome6	39390075
ENSG00000095981	chromosome6	39398295
ENSG00000095981	chromosome6	39398295
ENSG00000124780	chromosome6	39390075
ENSG00000173338	chromosome11	65119820
ENSG00000135750	chromosome1	231816541

ENSG00000099337	chromosome19	43502431
ENSG00000124249	chromosome20	42807966
ENSG00000169427	chromosome8	140784418
ENSG00000164626	chromosome6	39304866
ENSG00000124780	chromosome6	39390075
ENSG00000095981	chromosome6	39398295
ENSG00000100433	chromosome14	87862553
ENSG00000082482	chromosome1	213245888
ENSG00000182450	chromosome11	63817067
ENSG00000171303	chromosome2	26769248
ENSG00000169427	chromosome8	140784418
ENSG00000124249	chromosome20	42807966
ENSG00000135750	chromosome1	231816541
ENSG00000173338	chromosome11	65119820
ENSG00000135750	chromosome1	231816541
ENSG00000152315	chromosome14	89598303
ENSG00000184261	chromosome2	47650975
ENSG00000164626	chromosome6	39304866
ENSG00000171303	chromosome2	26769248
ENSG00000184261	chromosome2	47650975
ENSG00000152315	chromosome14	89598303
ENSG00000082482	chromosome1	213245888
ENSG00000100433	chromosome14	87862553
ENSG00000112759	chromosome6	44303029
ENSG00000174669	chromosome11	65895639
ENSG00000136928	chromosome9	100510841
ENSG00000175697	chromosome3	121445636
ENSG00000136928	chromosome9	100510841
ENSG00000174672	chromosome11	1368091
ENSG00000160469	chromosome19	60487139
ENSG00000044446	chromosomeX	18911972
ENSG00000067177	chromosomeX	71850454
ENSG00000131183	chromosome5	176745349
ENSG00000157765	chromosome4	25273221
ENSG00000198569	chromosome9	139245976
ENSG00000157765	chromosome4	25273221
ENSG00000131183	chromosome5	176745349
ENSG00000100262	chromosome22	41583133
ENSG00000149182	chromosome11	47154981
ENSG00000178607	chromosome17	59529655
ENSG00000134398	chromosome16	23632154
ENSG00000129657	chromosome17	72651274
ENSG00000103184	chromosome16	4948319
ENSG00000176635	chromosome22	28819942
ENSG00000143452	chromosome1	148958606
ENSG00000168000	chromosome11	62229561
ENSG00000168999	chromosome4	111392705
ENSG00000166530	chromosome16	82399183
ENSG00000168999	chromosome4	111392705
ENSG00000166530	chromosome16	82399183
ENSG00000174996	chromosome11	65782642
ENSG00000104892	chromosome19	50540598
ENSG00000137171	chromosome6	43135926
ENSG00000126214	chromosome14	103190655
ENSG00000104892	chromosome19	50540598
ENSG00000137171	chromosome6	43135926
ENSG00000174996	chromosome11	65782642
ENSG00000104892	chromosome19	50540598
ENSG00000137171	chromosome6	43135926

ENSG00000174996	chromosome11	65782642
ENSG00000123983	chromosome2	223481735
ENSG00000068366	chromosomeX	108813372
ENSG00000103653	chromosome15	72877692
ENSG00000007264	chromosome19	3740346
ENSG00000101213	chromosome20	61639112
ENSG00000125508	chromosome20	61649261
ENSG00000101213	chromosome20	61639112
ENSG00000125508	chromosome20	61649261
ENSG00000135697	chromosome16	79830015
ENSG00000197580	chromosome11	111551535
ENSG00000116745	chromosome1	68688177
ENSG00000188677	chromosome22	42726554
ENSG00000197702	chromosome11	12355771
ENSG00000171320	chromosome8	27688951
ENSG00000141446	chromosome18	17408803
ENSG00000183773	chromosome22	19652233
ENSG00000132356	chromosome5	40834049
ENSG00000162409	chromosome1	56883649
ENSG00000088756	chromosome18	6814762
ENSG00000124143	chromosome20	36696740
ENSG00000130052	chromosomeX	67784450
ENSG00000133121	chromosome13	32757776
ENSG00000164741	chromosome8	13401952
ENSG00000164741	chromosome8	13401952
ENSG00000130052	chromosomeX	67784450
ENSG00000133121	chromosome13	32757776
ENSG00000146376	chromosome6	130072975
ENSG00000124143	chromosome20	36696740
ENSG00000088756	chromosome18	6814762
ENSG00000133121	chromosome13	32757776
ENSG00000130052	chromosomeX	67784450
ENSG00000099875	chromosome19	2001851
ENSG00000079277	chromosome1	46832406
ENSG00000170430	chromosome10	131224507
ENSG00000160216	chromosome21	44203991
ENSG00000026652	chromosome6	161573236
ENSG00000176095	chromosome3	49760478
ENSG00000161896	chromosome6	33811232
ENSG00000064489	chromosome19	19122545
ENSG00000116604	chromosome1	154719709
ENSG00000116604	chromosome1	154719709
ENSG00000081189	chromosome5	88155362
ENSG00000068305	chromosome15	97990848
ENSG00000068305	chromosome15	97990848
ENSG00000081189	chromosome5	88155362
ENSG00000081189	chromosome5	88155362
ENSG00000068305	chromosome15	97990848
ENSG00000116604	chromosome1	154719709
ENSG00000064489	chromosome19	19122545
ENSG00000166402	chromosome11	8016997
ENSG00000078246	chromosome12	2870375
ENSG00000078246	chromosome12	2870375
ENSG00000166402	chromosome11	8016997
ENSG00000104804	chromosome19	54092938
ENSG00000112041	chromosome6	35588614
ENSG00000104804	chromosome19	54092938
ENSG00000078246	chromosome12	2870375
ENSG00000166402	chromosome11	8016997

ENSG00000197776	chromosome14	49229663
ENSG00000165516	chromosome14	49304937
ENSG00000178996	chromosome5	53849540
ENSG00000173548	chromosome15	73728499
ENSG00000120053	chromosome10	101180313
ENSG00000169154	chromosome8	37916705
ENSG00000215618	supercontigNT_113903	9705
ENSG00000101577	chromosome18	2950839
ENSG00000134324	chromosome2	11798997
ENSG00000132793	chromosome20	39407858
ENSG00000134324	chromosome2	11798997
ENSG00000101577	chromosome18	2950839
ENSG00000134324	chromosome2	11798997
ENSG00000101577	chromosome18	2950839
ENSG00000132793	chromosome20	39407858
ENSG00000141161	chromosome17	30499396
ENSG00000140553	chromosome15	89279559
ENSG00000166128	chromosome15	61268877
ENSG00000139998	chromosome14	64508548
ENSG00000143545	chromosome1	152225337
ENSG00000166128	chromosome15	61268877
ENSG00000167461	chromosome19	16083712
ENSG00000084710	chromosome2	25118503
ENSG00000132294	chromosome8	133026195
ENSG00000175662	chromosome17	17816455
ENSG00000100284	chromosome22	34025922
ENSG00000124831	chromosome2	238201027
ENSG00000093167	chromosome3	37165479
ENSG00000093167	chromosome3	37165479
ENSG00000124831	chromosome2	238201027
ENSG00000063244	chromosome19	60858283
ENSG00000063244	chromosome19	60858283
ENSG00000112214	chromosome6	97158211
ENSG00000112214	chromosome6	97158211
ENSG00000183386	chromosome1	38237672
ENSG00000089159	chromosome12	119187816
ENSG00000110031	chromosome11	58099822
ENSG00000140682	chromosome16	31391011
ENSG00000089159	chromosome12	119187816
ENSG00000169756	chromosome2	108637963
ENSG00000072163	chromosome2	128149069
ENSG00000140682	chromosome16	31391011
ENSG00000110031	chromosome11	58099822
ENSG00000198242	chromosome17	24071151
ENSG00000185822	chromosome21	39421366
ENSG00000215387	chromosome8	5321439
ENSG00000186698	chromosome2	64428378
ENSG00000215140	chromosome16	2913232
ENSG00000206147	chromosome16	376764
ENSG00000186239	chromosome14	34024936
ENSG00000215001	chromosome7	7748719
ENSG00000213753	chromosome19	63802268
ENSG00000212932	chromosome21	46935223
ENSG00000184319	chromosome22	49584071
ENSG00000214230	chromosome7	102580029
ENSG00000118975	chromosome12	2761331
ENSG00000215133	chromosome9	41876122
ENSG00000168214	chromosome4	25930595
ENSG00000155287	chromosome10	101370083

ENSG00000147454	chromosome8	23442461
ENSG00000155287	chromosome10	101370083
ENSG00000184840	chromosome5	176951822
ENSG00000158604	chromosome7	44588331
ENSG00000197506	chromosome9	86145369
ENSG00000156222	chromosome15	83231996
ENSG00000137860	chromosome15	43332706
ENSG00000153560	chromosome3	33456345
ENSG00000115112	chromosome2	121759156
ENSG00000214547	chromosome17	34647610
ENSG00000135457	chromosome12	49852473
ENSG00000135457	chromosome12	49852473
ENSG00000115112	chromosome2	121759156
ENSG00000214547	chromosome17	34647610
ENSG00000214547	chromosome17	34647610
ENSG00000115112	chromosome2	121759156
ENSG00000134317	chromosome2	10001257
ENSG00000158055	chromosome1	24513251
ENSG00000083307	chromosome8	102574174
ENSG00000158055	chromosome1	24513251
ENSG00000134317	chromosome2	10001257
ENSG00000107651	chromosome10	121642285
ENSG00000085788	chromosome8	38209670
ENSG00000144635	chromosome3	32587267
ENSG00000183066	chromosome22	40724769
ENSG00000132471	chromosome17	71362974
ENSG00000186433	chromosome18	20851167
ENSG00000152484	chromosome13	26643777
ENSG00000152484	chromosome13	26643777
ENSG00000109189	chromosome4	53203748
ENSG00000167770	chromosome11	63510505
ENSG00000089723	chromosome14	93562637
ENSG00000157978	chromosome1	25742777
ENSG00000157978	chromosome1	25742777
ENSG00000133961	chromosome14	72892213
ENSG00000105245	chromosome19	45888229
ENSG00000183114	chromosome1	20752054
ENSG00000185112	chromosome3	195888845
ENSG00000144366	chromosome2	189050675
ENSG00000198792	chromosome22	36973914
ENSG00000215155	chromosome7	1561647
ENSG00000004866	chromosome7	116380831
ENSG00000007341	chromosome1	112963259
ENSG00000164076	chromosome3	49874826
ENSG00000183049	chromosome10	12431824
ENSG00000134072	chromosome3	9784434
ENSG00000008118	chromosome1	207834952
ENSG00000130822	chromosomeX	152591726
ENSG00000081320	chromosome2	196736353
ENSG00000164543	chromosome7	43589368
ENSG00000164076	chromosome3	49874826
ENSG00000008118	chromosome1	207834952
ENSG00000183049	chromosome10	12431824
ENSG00000134072	chromosome3	9784434
ENSG00000134072	chromosome3	9784434
ENSG00000183049	chromosome10	12431824
ENSG00000164076	chromosome3	49874826
ENSG00000081320	chromosome2	196736353
ENSG00000159792	chromosome16	66500154

ENSG00000147613	chromosome8	87150968
ENSG00000065717	chromosome19	2979903
ENSG00000104964	chromosome19	3013926
ENSG00000140332	chromosome15	68176192
ENSG00000106829	chromosome9	81377526
ENSG00000196781	chromosome9	83492976
ENSG00000140332	chromosome15	68176192
ENSG00000065717	chromosome19	2979903
ENSG00000104964	chromosome19	3013926
ENSG00000196781	chromosome9	83492976
ENSG00000106829	chromosome9	81377526
ENSG00000104964	chromosome19	3013926
ENSG00000104964	chromosome19	3013926
ENSG00000065717	chromosome19	2979903
ENSG00000065717	chromosome19	2979903
ENSG00000104964	chromosome19	3013926
ENSG00000203740	chromosome1	168381873
ENSG00000148335	chromosome9	131434804
ENSG00000203705	chromosome1	211031887
ENSG00000198720	chromosome17	24944766
ENSG00000172932	chromosome11	66814404
ENSG00000172932	chromosome11	66814404
ENSG00000198720	chromosome17	24944766
ENSG00000076513	chromosome12	108921877
ENSG00000068615	chromosome2	86418145
ENSG00000132563	chromosome5	137802797
ENSG00000165476	chromosome10	64992338
ENSG00000168476	chromosome8	22054941
ENSG00000165476	chromosome10	64992338
ENSG00000068615	chromosome2	86418145
ENSG00000132563	chromosome5	137802797
ENSG00000132563	chromosome5	137802797
ENSG00000068615	chromosome2	86418145
ENSG00000155508	chromosome5	154223032
ENSG00000198791	chromosome8	17147033
ENSG00000180801	chromosome4	115119440
ENSG00000183876	chromosome5	149662130
ENSG00000113273	chromosome5	78316828
ENSG00000183876	chromosome5	149662130
ENSG00000180801	chromosome4	115119440
ENSG00000180801	chromosome4	115119440
ENSG00000101846	chromosomeX	7147717
ENSG00000157399	chromosomeX	2888442
ENSG00000062096	chromosomeX	2986652
ENSG00000205667	chromosomeX	2934654
ENSG00000006756	chromosomeX	2857317
ENSG00000176274	chromosomeX	103236597
ENSG00000122696	chromosome9	37878548
ENSG00000141437	chromosome18	27594623
ENSG00000189332	chromosome11	18187791
ENSG00000090989	chromosome4	56419247
ENSG00000090989	chromosome4	56419247
ENSG00000168952	chromosome14	24513865
ENSG00000011198	chromosome3	43707489
ENSG00000100439	chromosome14	22137056
ENSG00000149925	chromosome16	29985720
ENSG00000109107	chromosome17	23926678
ENSG00000136872	chromosome9	103232991
ENSG00000109107	chromosome17	23926678

ENSG00000149925	chromosome16	29985720
ENSG00000070985	chromosome11	2400843
ENSG00000130529	chromosome19	54352861
ENSG00000142185	chromosome21	44598012
ENSG00000142185	chromosome21	44598012
ENSG00000070985	chromosome11	2400843
ENSG00000130529	chromosome19	54352861
ENSG00000144481	chromosome2	234499922
ENSG00000130529	chromosome19	54352861
ENSG00000119121	chromosome9	76692593
ENSG00000092439	chromosome15	48766023
ENSG00000134160	chromosome15	29156417
ENSG00000083067	chromosome9	73251572
ENSG00000083067	chromosome9	73251572
ENSG00000134160	chromosome15	29156417
ENSG00000092439	chromosome15	48766023
ENSG00000119121	chromosome9	76692593
ENSG00000130529	chromosome19	54352861
ENSG00000070985	chromosome11	2400843
ENSG00000143093	chromosome1	110378783
ENSG00000128578	chromosome7	128861550
ENSG00000143847	chromosome1	201274760
ENSG00000139220	chromosome12	80672132
ENSG00000131626	chromosome11	69795927
ENSG00000139220	chromosome12	80672132
ENSG00000143847	chromosome1	201274760
ENSG00000177380	chromosome19	54322943
ENSG00000131626	chromosome11	69795927
ENSG00000143847	chromosome1	201274760
ENSG00000139220	chromosome12	80672132
ENSG00000110841	chromosome12	27677597
ENSG00000166387	chromosome11	7527283
ENSG00000142273	chromosome19	49973029
ENSG00000110395	chromosome11	118582338
ENSG00000114423	chromosome3	107069112
ENSG00000110395	chromosome11	118582338
ENSG00000142273	chromosome19	49973029
ENSG00000065491	chromosome6	37333522
ENSG00000054611	chromosome22	45537348
ENSG00000104879	chromosome19	50514812
ENSG00000131730	chromosome5	80582708
ENSG00000166998	chromosome15	41773541
ENSG00000168775	chromosome15	41673709
ENSG00000104879	chromosome19	50514812
ENSG00000166165	chromosome14	103058584
ENSG00000111245	chromosome12	109842717
ENSG00000180209	chromosome16	30293680
ENSG00000106436	chromosome7	101059119
ENSG00000106436	chromosome7	101059119
ENSG00000111245	chromosome12	109842717
ENSG00000180209	chromosome16	30293680
ENSG00000215375	chromosome4	661816
ENSG00000106631	chromosome7	44147430
ENSG00000180209	chromosome16	30293680
ENSG00000111245	chromosome12	109842717
ENSG00000106631	chromosome7	44147430
ENSG00000215375	chromosome4	661816
ENSG00000101335	chromosome20	34606702
ENSG00000101608	chromosome18	3243246

ENSG00000118680	chromosome18	3262897
ENSG00000106436	chromosome7	101059119
ENSG00000111245	chromosome12	109842717
ENSG00000180209	chromosome16	30293680
ENSG00000114120	chromosome3	142143587
ENSG00000171612	chromosome1	9522338
ENSG00000171612	chromosome1	9522338
ENSG00000154222	chromosome1	52602881
ENSG00000132024	chromosome19	13878255
ENSG00000080546	chromosome6	109521970
ENSG00000149212	chromosome11	94603673
ENSG00000130766	chromosome1	28458946
ENSG00000149212	chromosome11	94603673
ENSG00000080546	chromosome6	109521970
ENSG00000149212	chromosome11	94603673
ENSG00000080546	chromosome6	109521970
ENSG00000130766	chromosome1	28458946
ENSG00000182197	chromosome8	119192467
ENSG00000158008	chromosome1	26221725
ENSG00000182197	chromosome8	119192467
ENSG00000138101	chromosome2	25729034
ENSG00000125835	chromosome20	2399337
ENSG00000128739	chromosome15	22770598
ENSG00000132819	chromosome20	55400045
ENSG00000112183	chromosome6	17389792
ENSG00000132819	chromosome20	55400045
ENSG00000156515	chromosome10	70718506
ENSG00000156510	chromosome10	70650198
ENSG00000156510	chromosome10	70650198
ENSG00000156515	chromosome10	70718506
ENSG00000159399	chromosome2	74915216
ENSG00000160883	chromosome5	176255767
ENSG00000159399	chromosome2	74915216
ENSG00000156510	chromosome10	70650198
ENSG00000156515	chromosome10	70718506
ENSG00000106633	chromosome7	44195078
ENSG00000159399	chromosome2	74915216
ENSG00000156510	chromosome10	70650198
ENSG00000156515	chromosome10	70718506
ENSG00000160883	chromosome5	176255767
ENSG00000106633	chromosome7	44195078
ENSG00000160883	chromosome5	176255767
ENSG00000159399	chromosome2	74915216
ENSG00000156510	chromosome10	70650198
ENSG00000156515	chromosome10	70718506
ENSG00000175115	chromosome11	65594534
ENSG00000179364	chromosome14	104852301
ENSG00000175115	chromosome11	65594534
ENSG00000203811	chromosome1	148079354
ENSG00000183598	chromosome1	148051861
ENSG00000203811	chromosome1	148079354
ENSG00000203852	chromosome1	148090841
ENSG00000163041	chromosome1	224318676
ENSG00000196285	chromosome2	175292882
ENSG00000068383	chromosome10	134201591
ENSG00000130227	chromosome8	21833123
ENSG00000204764	chromosome5	170221616
ENSG00000145050	chromosome3	51397808
ENSG00000185267	chromosome10	14919952

ENSG00000120500	chromosomeX	69405961
ENSG00000130561	chromosome2	233882575
ENSG00000141480	chromosome17	4560766
ENSG00000130561	chromosome2	233882575
ENSG00000120500	chromosomeX	69405961
ENSG00000137486	chromosome11	74740525
ENSG00000137486	chromosome11	74740525
ENSG00000130561	chromosome2	233882575
ENSG00000120500	chromosomeX	69405961
ENSG00000141480	chromosome17	4560766
ENSG00000161647	chromosome17	39264876
ENSG00000150054	chromosome10	28567540
ENSG00000082126	chromosome2	202266923
ENSG00000150054	chromosome10	28567540
ENSG00000161647	chromosome17	39264876
ENSG00000150054	chromosome10	28567540
ENSG00000161647	chromosome17	39264876
ENSG00000082126	chromosome2	202266923
ENSG00000108852	chromosome17	39337355
ENSG00000105926	chromosome7	24629812
ENSG00000132746	chromosome11	67190983
ENSG00000006534	chromosome11	67539344
ENSG00000072210	chromosome17	19492877
ENSG00000108602	chromosome17	19592337
ENSG00000132746	chromosome11	67190983
ENSG00000006534	chromosome11	67539344
ENSG00000146205	chromosome2	241776700
ENSG00000160746	chromosome3	43622349
ENSG00000185101	chromosome11	431927
ENSG00000151572	chromosome12	99712894
ENSG00000134343	chromosome11	26310204
ENSG00000047617	chromosome12	5925588
ENSG00000131620	chromosome11	69602361
ENSG00000171714	chromosome11	22171615
ENSG00000177119	chromosome12	43896372
ENSG00000134343	chromosome11	26310204
ENSG00000151572	chromosome12	99712894
ENSG00000101421	chromosome20	31862936
ENSG00000164695	chromosome8	82807417
ENSG00000164695	chromosome8	82807417
ENSG00000101421	chromosome20	31862936
ENSG00000100931	chromosome14	23752615
ENSG00000100341	chromosome22	42619094
ENSG00000100344	chromosome22	42651125
ENSG00000177666	chromosome11	809719
ENSG00000180316	chromosome6	36346215
ENSG00000177666	chromosome11	809719
ENSG00000100341	chromosome22	42619094
ENSG00000100344	chromosome22	42651125
ENSG00000177666	chromosome11	809719
ENSG00000099139	chromosome9	77695918
ENSG00000099139	chromosome9	77695918
ENSG00000140479	chromosome15	99847397
ENSG00000140564	chromosome15	89219975
ENSG00000115257	chromosome19	1441346
ENSG00000106789	chromosome9	99959764
ENSG00000103647	chromosome15	66658656
ENSG00000110880	chromosome12	107619224
ENSG00000172725	chromosome11	66967530

ENSG00000167549	chromosome17	24974043
ENSG00000102879	chromosome16	30104032
ENSG00000102879	chromosome16	30104032
ENSG00000167549	chromosome17	24974043
ENSG00000172725	chromosome11	66967530
ENSG00000110880	chromosome12	107619224
ENSG00000172725	chromosome11	66967530
ENSG00000110880	chromosome12	107619224
ENSG00000102879	chromosome16	30104032
ENSG00000167549	chromosome17	24974043
ENSG00000162687	chromosome1	194844063
ENSG00000107147	chromosome9	137733926
ENSG00000107147	chromosome9	137733926
ENSG00000162687	chromosome1	194844063
ENSG00000156113	chromosome10	79067407
ENSG00000215262	chromosome8	36780592
ENSG00000171109	chromosome3	180549334
ENSG00000116688	chromosome1	11971813
ENSG00000188342	chromosome13	44592791
ENSG00000188342	chromosome13	44592791
ENSG00000119686	chromosome14	75115069
ENSG00000162769	chromosome1	211098418
ENSG00000138463	chromosome3	123996730
ENSG00000162769	chromosome1	211098418
ENSG00000154864	chromosome18	10687799
ENSG00000175388	chromosome18	10777141
ENSG00000103335	chromosome16	87330318
ENSG00000175388	chromosome18	10777141
ENSG00000154864	chromosome18	10687799
ENSG00000154864	chromosome18	10687799
ENSG00000175388	chromosome18	10777141
ENSG00000121749	chromosome12	70519829
ENSG00000104946	chromosome19	55072793
ENSG00000147592	chromosome8	71743910
ENSG00000104331	chromosome8	58068699
ENSG00000136754	chromosome10	27189799
ENSG00000138443	chromosome2	203901483
ENSG00000138443	chromosome2	203901483
ENSG00000136754	chromosome10	27189799
ENSG00000108798	chromosome17	44643086
ENSG00000066382	chromosome11	30558506
ENSG00000186732	chromosome22	42150936
ENSG00000183023	chromosome2	40510925
ENSG00000183023	chromosome2	40510925
ENSG00000100678	chromosome14	69704893
ENSG00000118160	chromosome19	52661473
ENSG00000118160	chromosome19	52661473
ENSG00000100678	chromosome14	69704893
ENSG00000183696	chromosome7	48100906
ENSG00000007001	chromosome2	158666822
ENSG00000169660	chromosome17	77970965
ENSG00000130508	chromosome2	1727286
ENSG00000147485	chromosome8	52484334
ENSG00000005381	chromosome17	53713119
ENSG00000121053	chromosome17	53625207
ENSG00000167419	chromosome17	53675340
ENSG00000065308	chromosome6	52549673
ENSG00000174599	chromosome4	118225998
ENSG00000067167	chromosome8	71682989

ENSG00000143418	chromosome1	149211008
ENSG00000154227	chromosome15	98861470
ENSG00000090661	chromosome19	8221961
ENSG00000154227	chromosome15	98861470
ENSG00000143418	chromosome1	149211008
ENSG00000139624	chromosome12	48847348
ENSG00000172292	chromosome2	169021205
ENSG00000105287	chromosome19	51911468
ENSG00000184304	chromosome14	29466470
ENSG00000115825	chromosome2	37397172
ENSG00000184304	chromosome14	29466470
ENSG00000115825	chromosome2	37397172
ENSG00000105287	chromosome19	51911468
ENSG00000115825	chromosome2	37397172
ENSG00000184304	chromosome14	29466470
ENSG00000163697	chromosome4	40730029
ENSG00000113108	chromosome5	139924015
ENSG00000163697	chromosome4	40730029
ENSG00000166313	chromosome11	6389154
ENSG00000163697	chromosome4	40730029
ENSG00000113108	chromosome5	139924015
ENSG00000100243	chromosome22	41375266
ENSG00000159348	chromosome1	201202957
ENSG00000166394	chromosome11	7650633
ENSG00000159348	chromosome1	201202957
ENSG00000100243	chromosome22	41375266
ENSG00000197329	chromosome2	64188949
ENSG00000174516	chromosome11	65992176
ENSG00000139946	chromosome14	55655132
ENSG00000174516	chromosome11	65992176
ENSG00000197329	chromosome2	64188949
ENSG00000167081	chromosome9	127549554
ENSG00000185630	chromosome1	162795684
ENSG00000105717	chromosome19	19590439
ENSG00000105717	chromosome19	19590439
ENSG00000167081	chromosome9	127549554
ENSG00000185630	chromosome1	162795684
ENSG00000204304	chromosome6	32265671
ENSG00000185630	chromosome1	162795684
ENSG00000167081	chromosome9	127549554
ENSG00000185630	chromosome1	162795684
ENSG00000184207	chromosome16	2204780
ENSG00000205531	chromosome11	2957027
ENSG00000177432	chromosome4	89837929
ENSG00000186462	chromosomeX	72351054
ENSG00000186310	chromosomeX	92814960
ENSG00000187109	chromosome12	74754267
ENSG00000163710	chromosome3	144090429
ENSG00000106333	chromosome7	100038016
ENSG00000171208	chromosome16	45735025
ENSG00000166342	chromosome18	68685507
ENSG00000107611	chromosome10	17211771
ENSG00000171208	chromosome16	45735025
ENSG00000166342	chromosome18	68685507
ENSG00000038295	chromosome4	167014507
ENSG00000095587	chromosome10	98263433
ENSG00000168487	chromosome8	22078864
ENSG00000166342	chromosome18	68685507
ENSG00000171208	chromosome16	45735025

ENSG00000168487	chromosome8	22078864
ENSG00000095587	chromosome10	98263433
ENSG00000137522	chromosome11	71317754
ENSG00000145428	chromosome4	154900465
ENSG00000113384	chromosome5	32209898
ENSG00000143457	chromosome1	148933939
ENSG00000138613	chromosome15	61356876
ENSG00000117362	chromosome1	148507835
ENSG00000129968	chromosome19	1832566
ENSG00000198658	chromosome1	144788195
ENSG00000203835	chromosome1	146085298
ENSG00000182502	chromosome22	20802375
ENSG00000182556	chromosome22	19355375
ENSG00000107362	chromosome9	73679817
ENSG00000136379	chromosome15	78774739
ENSG00000107362	chromosome9	73679817
ENSG00000129968	chromosome19	1832566
ENSG00000198658	chromosome1	144788195
ENSG00000203835	chromosome1	146085298
ENSG00000182502	chromosome22	20802375
ENSG00000182556	chromosome22	19355375
ENSG00000215041	chromosome17	7173362
ENSG00000214357	chromosome5	172001015
ENSG00000107954	chromosome10	105244135
ENSG00000107954	chromosome10	105244135
ENSG00000214357	chromosome5	172001015
ENSG00000148925	chromosome11	13423248
ENSG00000112078	chromosome6	36545853
ENSG00000109063	chromosome17	10499107
ENSG00000109061	chromosome17	10360685
ENSG00000125414	chromosome17	10391963
ENSG00000133020	chromosome17	10264270
ENSG00000141048	chromosome17	10310788
ENSG00000006788	chromosome17	10208573
ENSG00000109063	chromosome17	10499107
ENSG00000109061	chromosome17	10360685
ENSG00000125414	chromosome17	10391963
ENSG00000133020	chromosome17	10264270
ENSG00000141048	chromosome17	10310788
ENSG00000006788	chromosome17	10208573
ENSG00000092054	chromosome14	22972782
ENSG00000197616	chromosome14	22946273
ENSG00000144821	chromosome3	109730803
ENSG00000078814	chromosome20	33026878
ENSG00000006788	chromosome17	10208573
ENSG00000109061	chromosome17	10360685
ENSG00000125414	chromosome17	10391963
ENSG00000133020	chromosome17	10264270
ENSG00000141048	chromosome17	10310788
ENSG00000006788	chromosome17	10208573
ENSG00000109063	chromosome17	10499107
ENSG00000078814	chromosome20	33026878
ENSG00000144821	chromosome3	109730803
ENSG00000006788	chromosome17	10208573
ENSG00000109061	chromosome17	10360685
ENSG00000125414	chromosome17	10391963
ENSG00000133020	chromosome17	10264270
ENSG00000141048	chromosome17	10310788
ENSG00000092054	chromosome14	22972782

ENSG00000197616	chromosome14	22946273
ENSG00000109063	chromosome17	10499107
ENSG00000109061	chromosome17	10360685
ENSG00000125414	chromosome17	10391963
ENSG00000133020	chromosome17	10264270
ENSG00000141048	chromosome17	10310788
ENSG00000006788	chromosome17	10208573
ENSG00000139890	chromosome14	22422367
ENSG00000164949	chromosome8	95341908
ENSG00000166592	chromosome16	65516584
ENSG00000164949	chromosome8	95341908
ENSG00000166592	chromosome16	65516584
ENSG00000139890	chromosome14	22422367
ENSG00000088320	chromosome20	29527910
ENSG00000166592	chromosome16	65516584
ENSG00000164949	chromosome8	95341908
ENSG00000088320	chromosome20	29527910
ENSG00000164949	chromosome8	95341908
ENSG00000166592	chromosome16	65516584
ENSG00000139890	chromosome14	22422367
ENSG00000130332	chromosome19	2279711
ENSG00000182557	chromosome17	4284012
ENSG00000169682	chromosome16	28893974
ENSG00000183018	chromosome17	4349092
ENSG00000169682	chromosome16	28893974
ENSG00000183018	chromosome17	4349092
ENSG00000182557	chromosome17	4284012
ENSG00000183018	chromosome17	4349092
ENSG00000169682	chromosome16	28893974
ENSG00000198176	chromosome13	113288154
ENSG00000114126	chromosome3	143295498
ENSG00000120594	chromosome10	20146015
ENSG00000161381	chromosome17	34561217
ENSG00000013561	chromosome5	141333338
ENSG00000124253	chromosome20	55569874
ENSG00000100889	chromosome14	23633455
ENSG00000172031	chromosome1	92268225
ENSG00000105131	chromosome19	15204023
ENSG00000160803	chromosome1	154290117
ENSG00000175520	chromosome11	5487365
ENSG00000175518	chromosome11	5494248
ENSG00000188021	chromosomeX	56607032
ENSG00000135018	chromosome9	85512415
ENSG00000114784	chromosome3	40326437
ENSG00000173812	chromosome17	37098817
ENSG00000102898	chromosome16	66456535
ENSG00000144136	chromosome2	113120877
ENSG00000168575	chromosome8	42449066
ENSG00000110047	chromosome11	64402513
ENSG00000013016	chromosome2	31310992
ENSG00000024422	chromosome19	52911682
ENSG00000013016	chromosome2	31310992
ENSG00000110047	chromosome11	64402513
ENSG00000013016	chromosome2	31310992
ENSG00000110047	chromosome11	64402513
ENSG00000024422	chromosome19	52911682
ENSG00000103966	chromosome15	40051985
ENSG00000013016	chromosome2	31310992
ENSG00000110047	chromosome11	64402513

ENSG00000120265	chromosome6	150112731
ENSG00000171368	chromosome5	731176
ENSG00000179636	chromosome14	20568581
ENSG00000159713	chromosome16	65982516
ENSG00000159713	chromosome16	65982516
ENSG00000179636	chromosome14	20568581
ENSG00000115520	chromosome2	198026530
ENSG00000135469	chromosome12	54947170
ENSG00000135469	chromosome12	54947170
ENSG00000115520	chromosome2	198026530
ENSG00000140519	chromosome15	87840780
ENSG00000132677	chromosome1	154605627
ENSG00000188672	chromosome1	25619865
ENSG00000187010	chromosome1	25471626
ENSG00000112077	chromosome6	49712485
ENSG00000132677	chromosome1	154605627
ENSG00000140519	chromosome15	87840780
ENSG00000132677	chromosome1	154605627
ENSG00000140519	chromosome15	87840780
ENSG00000188672	chromosome1	25619865
ENSG00000187010	chromosome1	25471626
ENSG00000112077	chromosome6	49712485
ENSG00000112077	chromosome6	49712485
ENSG00000188672	chromosome1	25619865
ENSG00000187010	chromosome1	25471626
ENSG00000103978	chromosome15	40352910
ENSG00000103978	chromosome15	40352910
ENSG00000153214	chromosome2	112529640
ENSG00000117480	chromosome1	46632608
ENSG00000117480	chromosome1	46632608
ENSG00000182378	chromosomeX	140855
ENSG00000182836	chromosome5	41546386
ENSG00000182378	chromosomeX	140855
ENSG00000182836	chromosome5	41546386
ENSG00000182378	chromosomeX	140855
ENSG00000204435	chromosome6	31741204
ENSG00000108100	chromosome10	35665988
ENSG00000184766	chromosome19	33138940
ENSG00000182632	chromosome10	42285660
ENSG00000179296	chromosome10	48909518
ENSG00000204150	chromosome10	47178564
ENSG00000163249	chromosome2	208284584
ENSG00000215169	chromosomeX	64958817
ENSG00000139668	chromosome13	51056825
ENSG00000085449	chromosome2	224518246
ENSG00000086619	chromosome1	234511707
ENSG00000197930	chromosome14	52231950
ENSG00000143774	chromosome1	226394627
ENSG00000109103	chromosome17	23903703
ENSG00000175970	chromosome12	119632661
ENSG00000175970	chromosome12	119632661
ENSG00000109103	chromosome17	23903703
ENSG00000196814	chromosome9	128129030
ENSG00000196814	chromosome9	128129030
ENSG00000141971	chromosome19	17391945
ENSG00000089818	chromosome12	8126152
ENSG00000157191	chromosome1	16639844
ENSG00000168256	chromosome17	37427122
ENSG00000197885	chromosome3	23927400

ENSG00000165443	chromosome10	60606620
ENSG00000168490	chromosome8	22141816
ENSG00000158825	chromosome1	20788210
ENSG00000122042	chromosome13	29321676
ENSG00000168003	chromosome11	62380318
ENSG00000146729	chromosome7	55999797
ENSG00000184117	chromosome22	28307072
ENSG00000129221	chromosome17	6279149
ENSG00000110711	chromosome11	67007206
ENSG00000129221	chromosome17	6279149
ENSG00000180061	chromosome19	60524217
ENSG00000168890	chromosome2	85682583
ENSG00000168890	chromosome2	85682583
ENSG00000168890	chromosome2	85682583
ENSG00000180061	chromosome19	60524217
ENSG00000106462	chromosome7	148175324
ENSG00000108799	chromosome17	38134486
ENSG00000100422	chromosome22	45512710
ENSG00000138709	chromosome4	129215557
ENSG00000155506	chromosome5	154072679
ENSG00000163029	chromosome2	17790695
ENSG00000174851	chromosome11	65813033
ENSG00000167645	chromosome19	43498398
ENSG00000178585	chromosome1	9892907
ENSG00000162441	chromosome1	9919265
ENSG00000137976	chromosome1	84636836
ENSG00000105612	chromosome19	12853190
ENSG00000105612	chromosome19	12853190
ENSG00000137976	chromosome1	84636836
ENSG00000103018	chromosome16	68016088
ENSG00000166347	chromosome18	70110091
ENSG00000117408	chromosome1	44185860
ENSG00000197872	chromosome2	16632869
ENSG00000197872	chromosome2	16632869
ENSG00000153310	chromosome8	130960890
ENSG00000153310	chromosome8	130960890
ENSG00000154316	chromosome8	11234597
ENSG00000164023	chromosome4	109036159
ENSG00000198964	chromosome10	51773899
ENSG00000128228	chromosome22	20326626
ENSG00000132581	chromosome17	24013022
ENSG00000164040	chromosome4	129428468
ENSG00000101856	chromosomeX	118254355
ENSG00000162231	chromosome11	62329405
ENSG00000185554	chromosomeX	101448697
ENSG00000185945	chromosomeX	101521614
ENSG00000126952	chromosomeX	100984421
ENSG00000196970	chromosomeX	101704810
ENSG00000147206	chromosomeX	102234683
ENSG00000100632	chromosome14	68934704
ENSG00000100632	chromosome14	68934704
ENSG00000100632	chromosome14	68934704
ENSG00000100632	chromosome14	68934704
ENSG00000099899	chromosome22	18484430
ENSG00000064545	chromosome19	19110238
ENSG00000164180	chromosome5	87600297
ENSG00000196433	chromosomeX	1694093
ENSG00000169093	chromosomeX	1531734
ENSG00000196433	chromosomeX	1694093

ENSG00000171298	chromosome17	75692981
ENSG00000073350	chromosome17	71033373
ENSG00000131899	chromosome17	18069722
ENSG00000164506	chromosome6	147567362
ENSG00000145087	chromosome3	122111116
ENSG00000130382	chromosome19	6230796
ENSG00000171843	chromosome9	20612256
ENSG00000115665	chromosome2	107971044
ENSG00000128923	chromosome15	56850887
ENSG00000143409	chromosome1	149241826
ENSG00000130948	chromosome9	98104208
ENSG00000129521	chromosome14	33490019
ENSG00000171570	chromosome19	45998318
ENSG00000135766	chromosome1	229624258
ENSG00000171570	chromosome19	45998318
ENSG00000129521	chromosome14	33490019
ENSG00000100592	chromosome14	58799949
ENSG00000146122	chromosome6	39932057
ENSG00000100592	chromosome14	58799949
ENSG00000118007	chromosome3	137832431
ENSG00000101972	chromosomeX	122984159
ENSG00000066923	chromosome7	99616116
ENSG00000118007	chromosome3	137832431
ENSG00000101972	chromosomeX	122984159
ENSG00000101972	chromosomeX	122984159
ENSG00000118007	chromosome3	137832431
ENSG00000103723	chromosome15	81175513
ENSG00000132842	chromosome5	77626160
ENSG00000166822	chromosome16	74056000
ENSG00000205269	chromosome6	11646497
ENSG00000129244	chromosome17	7495562
ENSG00000069849	chromosome3	143078334
ENSG00000143153	chromosome1	167342692
ENSG00000186009	chromosome13	113360461
ENSG00000101892	chromosomeX	119380052
ENSG00000069849	chromosome3	143078334
ENSG00000129244	chromosome17	7495562
ENSG00000069849	chromosome3	143078334
ENSG00000129244	chromosome17	7495562
ENSG00000101892	chromosomeX	119380052
ENSG00000101892	chromosomeX	119380052
ENSG00000069849	chromosome3	143078334
ENSG00000129244	chromosome17	7495562
ENSG00000186009	chromosome13	113360461
ENSG00000011009	chromosome1	23991760
ENSG00000120992	chromosome8	55176937
ENSG00000143479	chromosome1	204866731
ENSG00000127334	chromosome12	66329181
ENSG00000157540	chromosome21	37714547
ENSG00000105204	chromosome19	45014348
ENSG00000181061	chromosome3	42810750
ENSG00000212646	chromosome12	51822104
ENSG00000214511	chromosome12	49634049
ENSG00000131097	chromosome17	40281047
ENSG00000106028	chromosome7	141085436
ENSG00000065989	chromosome19	10388619
ENSG00000184588	chromosome1	66151586
ENSG00000184588	chromosome1	66151586
ENSG00000065989	chromosome19	10388619

ENSG00000113448	chromosome5	58688430
ENSG00000105650	chromosome19	18205002
ENSG00000115252	chromosome2	183095349
ENSG00000154678	chromosome7	32304873
ENSG00000123360	chromosome12	53229924
ENSG00000154678	chromosome7	32304873
ENSG00000115252	chromosome2	183095349
ENSG00000113231	chromosome5	76542507
ENSG00000073417	chromosome15	83326398
ENSG00000205268	chromosome8	66916298
ENSG00000171408	chromosome6	136214830
ENSG00000105650	chromosome19	18205002
ENSG00000113448	chromosome5	58688430
ENSG00000143324	chromosome1	178867961
ENSG00000076067	chromosome12	55202075
ENSG00000144642	chromosome3	29297943
ENSG00000153250	chromosome2	161058121
ENSG00000153250	chromosome2	161058121
ENSG00000076067	chromosome12	55202075
ENSG00000144642	chromosome3	29297943
ENSG00000153250	chromosome2	161058121
ENSG00000076067	chromosome12	55202075
ENSG00000144642	chromosome3	29297943
ENSG00000153250	chromosome2	161058121
ENSG00000076067	chromosome12	55202075
ENSG00000144642	chromosome3	29297943
ENSG00000144642	chromosome3	29297943
ENSG00000076067	chromosome12	55202075
ENSG00000127990	chromosome7	94123347
ENSG00000108823	chromosome17	45598401
ENSG00000141698	chromosome17	37245724
ENSG00000122643	chromosome7	33068858
ENSG00000123607	chromosome2	166524257
ENSG00000168026	chromosome3	39124333
ENSG00000164077	chromosome3	49925784
ENSG00000103111	chromosome16	75782620
ENSG00000181915	chromosome10	64234826
ENSG00000171914	chromosome15	60726802
ENSG00000137076	chromosome9	35715692
ENSG00000073712	chromosome14	52487037
ENSG00000101311	chromosome20	6048202
ENSG00000149781	chromosome11	63731413
ENSG00000101311	chromosome20	6048202
ENSG00000073712	chromosome14	52487037
ENSG00000126247	chromosome19	41323754
ENSG00000075142	chromosome7	87694250
ENSG00000115271	chromosome2	162909005
ENSG00000115271	chromosome2	162909005
ENSG00000075142	chromosome7	87694250
ENSG00000139631	chromosome12	51860960
ENSG00000144644	chromosome3	30866591
ENSG00000128683	chromosome2	171383348
ENSG00000136750	chromosome10	26545745
ENSG00000215438	chromosome18	56441148
ENSG00000136750	chromosome10	26545745
ENSG00000128683	chromosome2	171383348
ENSG00000214517	chromosome11	73560115
ENSG00000111110	chromosome12	61614736
ENSG00000155367	chromosome1	113059447

ENSG00000155367	chromosome1	113059447
ENSG00000111110	chromosome12	61614736
ENSG00000164088	chromosome3	52256171
ENSG00000112116	chromosome6	52217187
ENSG00000112115	chromosome6	52159189
ENSG00000166090	chromosome14	22911941
ENSG00000124391	chromosome16	87232551
ENSG00000092964	chromosome8	26491688
ENSG00000151640	chromosome10	133850558
ENSG00000113657	chromosome5	146869615
ENSG00000072832	chromosome4	5945598
ENSG00000072832	chromosome4	5945598
ENSG00000113657	chromosome5	146869615
ENSG00000151640	chromosome10	133850558
ENSG00000092964	chromosome8	26491688
ENSG00000124003	chromosome2	223244749
ENSG00000124003	chromosome2	223244749
ENSG00000205267	chromosome7	100633277
ENSG00000166391	chromosome11	75106582
ENSG00000205267	chromosome7	100633277
ENSG00000124003	chromosome2	223244749
ENSG00000106384	chromosome7	100630856
ENSG00000147160	chromosomeX	69186508
ENSG00000184210	chromosomeX	69314158
ENSG00000204195	chromosomeX	69371271
ENSG00000062282	chromosome11	75157685
ENSG00000062282	chromosome11	75157685
ENSG00000147160	chromosomeX	69186508
ENSG00000184210	chromosomeX	69314158
ENSG00000204195	chromosomeX	69371271
ENSG00000205267	chromosome7	100633277
ENSG00000124003	chromosome2	223244749
ENSG00000166391	chromosome11	75106582
ENSG00000147160	chromosomeX	69186508
ENSG00000184210	chromosomeX	69314158
ENSG00000204195	chromosomeX	69371271
ENSG00000062282	chromosome11	75157685
ENSG00000106384	chromosome7	100630856
ENSG00000173473	chromosome3	47798292
ENSG00000139613	chromosome12	54869549
ENSG00000111199	chromosome12	108736985
ENSG00000196689	chromosome17	3442394
ENSG00000187688	chromosome17	16261708
ENSG00000196689	chromosome17	3442394
ENSG00000187688	chromosome17	16261708
ENSG00000111199	chromosome12	108736985
ENSG00000167723	chromosome17	3404895
ENSG00000167723	chromosome17	3404895
ENSG00000196689	chromosome17	3442394
ENSG00000187688	chromosome17	16261708
ENSG00000111199	chromosome12	108736985
ENSG00000127412	chromosome7	142340679
ENSG00000165125	chromosome7	142293384
ENSG00000167723	chromosome17	3404895
ENSG00000196689	chromosome17	3442394
ENSG00000187688	chromosome17	16261708
ENSG00000111199	chromosome12	108736985
ENSG00000127412	chromosome7	142340679
ENSG00000165125	chromosome7	142293384

ENSG00000072849	chromosome17	5330206
ENSG00000099958	chromosome22	22511175
ENSG00000147155	chromosomeX	48267104
ENSG00000123179	chromosome13	49163562
ENSG00000214469	chromosome16	62852646
ENSG00000128989	chromosome15	50648382
ENSG00000143420	chromosome1	148868673
ENSG00000075303	chromosome7	87325979
ENSG00000013306	chromosome17	39756457
ENSG00000106799	chromosome9	100907309
ENSG00000135503	chromosome12	50631795
ENSG00000204217	chromosome2	202950443
ENSG00000115170	chromosome2	158364252
ENSG00000163513	chromosome3	30623380
ENSG00000115170	chromosome2	158364252
ENSG00000139567	chromosome12	50592526
ENSG00000135503	chromosome12	50631795
ENSG00000106799	chromosome9	100907309
ENSG00000123612	chromosome2	158193403
ENSG00000204217	chromosome2	202950443
ENSG00000138696	chromosome4	96244599
ENSG00000107779	chromosome10	88625756
ENSG00000150086	chromosome12	13910410
ENSG00000183454	chromosome16	10181770
ENSG00000161509	chromosome17	70368254
ENSG00000105464	chromosome19	53593462
ENSG00000183454	chromosome16	10181770
ENSG00000150086	chromosome12	13910410
ENSG00000116032	chromosome19	951437
ENSG00000198785	chromosome9	103540083
ENSG00000105464	chromosome19	53593462
ENSG00000161509	chromosome17	70368254
ENSG00000147813	chromosome8	144731632
ENSG00000151651	chromosome10	134940312
ENSG00000069206	chromosome8	24354567
ENSG00000042980	chromosome8	24207608
ENSG00000134028	chromosome8	24297963
ENSG00000008277	chromosome7	87401717
ENSG00000073670	chromosome17	40192094
ENSG00000168615	chromosome8	38973740
ENSG00000168619	chromosome8	39561299
ENSG00000196115	chromosome8	39282846
ENSG00000197140	chromosome8	39084320
ENSG00000104755	chromosome8	39814862
ENSG00000134007	chromosome14	70061378
ENSG00000139985	chromosome14	69993970
ENSG00000168594	chromosome4	176133252
ENSG00000134249	chromosome1	120240483
ENSG00000214137	chromosome12	110821671
ENSG00000149451	chromosome20	3610652
ENSG00000135074	chromosome5	156935283
ENSG00000148848	chromosome10	128066705
ENSG00000143537	chromosome1	153290487
ENSG00000168615	chromosome8	38973740
ENSG00000143537	chromosome1	153290487
ENSG00000149451	chromosome20	3610652
ENSG00000135074	chromosome5	156935283
ENSG00000148848	chromosome10	128066705
ENSG00000069206	chromosome8	24354567

ENSG00000042980	chromosome8	24207608
ENSG00000134028	chromosome8	24297963
ENSG00000151651	chromosome10	134940312
ENSG00000073670	chromosome17	40192094
ENSG00000008277	chromosome7	87401717
ENSG00000114948	chromosome2	207016836
ENSG00000148848	chromosome10	128066705
ENSG00000149451	chromosome20	3610652
ENSG00000135074	chromosome5	156935283
ENSG00000135074	chromosome5	156935283
ENSG00000149451	chromosome20	3610652
ENSG00000069206	chromosome8	24354567
ENSG00000042980	chromosome8	24207608
ENSG00000134028	chromosome8	24297963
ENSG00000151651	chromosome10	134940312
ENSG00000143537	chromosome1	153290487
ENSG00000149451	chromosome20	3610652
ENSG00000135074	chromosome5	156935283
ENSG00000148848	chromosome10	128066705
ENSG00000168619	chromosome8	39561299
ENSG00000196115	chromosome8	39282846
ENSG00000197140	chromosome8	39084320
ENSG00000104755	chromosome8	39814862
ENSG00000134007	chromosome14	70061378
ENSG00000139985	chromosome14	69993970
ENSG00000168594	chromosome4	176133252
ENSG00000134249	chromosome1	120240483
ENSG00000214137	chromosome12	110821671
ENSG00000168615	chromosome8	38973740
ENSG00000213722	chromosome6	31804918
ENSG00000153904	chromosome1	85703317
ENSG00000043822	chromosome10	99390490
ENSG00000038210	chromosome4	24844884
ENSG00000104904	chromosome19	2220598
ENSG00000180304	chromosome15	62770861
ENSG00000180304	chromosome15	62770861
ENSG00000104904	chromosome19	2220598
ENSG00000143450	chromosome1	150002236
ENSG00000104904	chromosome19	2220598
ENSG00000185246	chromosome14	44635625
ENSG00000162909	chromosome1	221966966
ENSG00000203697	chromosome1	221919972
ENSG00000149260	chromosome11	76461747
ENSG00000077274	chromosomeX	110393821
ENSG00000162949	chromosome2	30863696
ENSG00000214711	chromosome2	31281818
ENSG00000182472	chromosome19	43926646
ENSG00000014216	chromosome11	64706749
ENSG00000137225	chromosome6	44234564
ENSG00000203697	chromosome1	221919972
ENSG00000162909	chromosome1	221966966
ENSG00000135773	chromosome1	228949866
ENSG00000092529	chromosome15	40433896
ENSG00000137225	chromosome6	44234564
ENSG00000014216	chromosome11	64706749
ENSG00000014216	chromosome11	64706749
ENSG00000137225	chromosome6	44234564
ENSG00000203697	chromosome1	221919972
ENSG00000162909	chromosome1	221966966

ENSG00000162949	chromosome2	30863696
ENSG00000214711	chromosome2	31281818
ENSG00000182472	chromosome19	43926646
ENSG00000077274	chromosomeX	110393821
ENSG00000149260	chromosome11	76461747
ENSG00000182472	chromosome19	43926646
ENSG00000162949	chromosome2	30863696
ENSG00000214711	chromosome2	31281818
ENSG00000203697	chromosome1	221919972
ENSG00000162909	chromosome1	221966966
ENSG00000014216	chromosome11	64706749
ENSG00000137225	chromosome6	44234564
ENSG00000092529	chromosome15	40433896
ENSG00000135773	chromosome1	228949866
ENSG00000002726	chromosome7	150184492
ENSG00000131480	chromosome17	38250170
ENSG00000131471	chromosome17	38256887
ENSG00000172534	chromosomeX	152889486
ENSG00000111727	chromosome12	102982469
ENSG00000168282	chromosome14	49157737
ENSG00000197763	chromosome3	127856329
ENSG00000198431	chromosome12	103206688
ENSG00000156802	chromosome8	124477779
ENSG00000100292	chromosome22	34107167
ENSG00000103415	chromosome16	4495527
ENSG00000100196	chromosome22	37194185
ENSG00000136240	chromosome7	6490214
ENSG00000105438	chromosome19	53586428
ENSG00000136240	chromosome7	6490214
ENSG00000100196	chromosome22	37194185
ENSG00000128242	chromosome22	29283380
ENSG00000154252	chromosome2	242365044
ENSG00000197093	chromosome7	99602655
ENSG00000197093	chromosome7	99602655
ENSG00000197093	chromosome7	99602655
ENSG00000154252	chromosome2	242365044
ENSG00000154252	chromosome2	242365044
ENSG00000197093	chromosome7	99602655
ENSG00000128242	chromosome22	29283380
ENSG00000175229	chromosome11	65569463
ENSG00000154252	chromosome2	242365044
ENSG00000197093	chromosome7	99602655
ENSG00000133116	chromosome13	32488579
ENSG00000134962	chromosome4	39084965
ENSG00000125734	chromosome19	6688588
ENSG00000148358	chromosome9	131856033
ENSG00000197451	chromosome5	177564445
ENSG00000152795	chromosome4	83569868
ENSG00000138668	chromosome4	83513856
ENSG00000215173	chromosomeX	63181265
ENSG00000138668	chromosome4	83513856
ENSG00000215173	chromosomeX	63181265
ENSG00000152795	chromosome4	83569868
ENSG00000170266	chromosome3	33113582
ENSG00000163521	chromosome2	219816540
ENSG00000101276	chromosome20	694419
ENSG00000132517	chromosome17	4878626
ENSG00000185803	chromosome8	145553762
ENSG00000101276	chromosome20	694419

ENSG00000116489	chromosome1	112963990
ENSG00000198898	chromosome7	116289902
ENSG00000198898	chromosome7	116289902
ENSG00000116489	chromosome1	112963990
ENSG00000177938	chromosome12	18782470
ENSG00000130957	chromosome9	96395830
ENSG00000130957	chromosome9	96395830
ENSG00000130957	chromosome9	96395830
ENSG00000165140	chromosome9	96441414
ENSG00000186654	chromosome22	43477019
ENSG00000135362	chromosome11	36379248
ENSG00000186654	chromosome22	43477019
ENSG00000166881	chromosome12	55758796
ENSG00000189362	chromosome2	191107627
ENSG00000082269	chromosome6	71194767
ENSG00000147724	chromosome8	139449409
ENSG00000130734	chromosome19	10515767
ENSG00000125703	chromosome1	63042046
ENSG00000168397	chromosome2	242225539
ENSG00000215697	supercontigNT_113880	54817
ENSG00000101844	chromosomeX	107221623
ENSG00000142552	chromosome19	54723542
ENSG00000049449	chromosome11	32069319
ENSG00000128595	chromosome7	128175874
ENSG00000049449	chromosome11	32069319
ENSG00000142552	chromosome19	54723542
ENSG00000128595	chromosome7	128175874
ENSG00000104723	chromosome8	15442311
ENSG00000102158	chromosomeX	77037564
ENSG00000215762	supercontigNT_113965	542775
ENSG00000073921	chromosome11	85457471
ENSG00000073921	chromosome11	85457471
ENSG00000065609	chromosome6	84474366
ENSG00000152782	chromosome10	91395040
ENSG00000120137	chromosome5	167938867
ENSG00000125779	chromosome20	3817748
ENSG00000169692	chromosome9	138701631
ENSG00000204310	chromosome6	32247252
ENSG00000180773	chromosome11	92570647
ENSG00000186335	chromosome5	150707215
ENSG00000123643	chromosome5	150818547
ENSG00000186334	chromosome5	150663109
ENSG00000111647	chromosome12	99060545
ENSG00000065060	chromosome6	34868006
ENSG00000121774	chromosome1	32252184
ENSG00000112232	chromosome6	63053813
ENSG00000131773	chromosome8	136539292
ENSG00000112531	chromosome6	163755783
ENSG00000131773	chromosome8	136539292
ENSG00000112232	chromosome6	63053813
ENSG00000070540	chromosome17	63965158
ENSG00000157954	chromosome7	5196577
ENSG00000120329	chromosome5	140663617
ENSG00000102743	chromosome13	40265363
ENSG00000136478	chromosome17	59645310
ENSG00000110057	chromosome11	67528090
ENSG00000155070	chromosome7	6865984
ENSG00000184795	chromosome4	9104481
ENSG00000172971	chromosome3	75755592

ENSG00000172197	chromosome6	20320445
ENSG00000143797	chromosome2	9061195
ENSG00000177669	chromosome8	30109991
ENSG00000143797	chromosome2	9061195
ENSG00000172197	chromosome6	20320445
ENSG00000177963	chromosome11	198855
ENSG00000177963	chromosome11	198855
ENSG00000111785	chromosome12	105692654
ENSG00000109220	chromosome4	54625225
ENSG00000204116	chromosomeX	72699765
ENSG00000188094	chromosome5	115415881
ENSG00000198940	chromosome1	209413451
ENSG00000118181	chromosome11	118394205
ENSG00000135932	chromosome2	231332961
ENSG00000102547	chromosome13	48855048
ENSG00000102547	chromosome13	48855048
ENSG00000135932	chromosome2	231332961
ENSG00000155097	chromosome8	104122241
ENSG00000143882	chromosome2	10780427
ENSG00000115255	chromosome19	1442067
ENSG00000129625	chromosome5	112285787
ENSG00000004799	chromosome7	95063542
ENSG00000005882	chromosome17	45527799
ENSG00000152256	chromosome2	173129125
ENSG00000067992	chromosomeX	24393494
ENSG00000152256	chromosome2	173129125
ENSG00000004799	chromosome7	95063542
ENSG00000005882	chromosome17	45527799
ENSG00000005882	chromosome17	45527799
ENSG00000004799	chromosome7	95063542
ENSG00000188191	chromosome7	717669
ENSG00000108946	chromosome17	64023136
ENSG00000114302	chromosome3	48860034
ENSG00000005249	chromosome7	106472589
ENSG00000141646	chromosome18	46827415
ENSG00000170365	chromosome4	146655216
ENSG00000113658	chromosome5	135517349
ENSG00000137834	chromosome15	64782651
ENSG00000101665	chromosome18	44730793
ENSG00000175387	chromosome18	43677126
ENSG00000166949	chromosome15	65145547
ENSG00000120693	chromosome13	36351827
ENSG00000113658	chromosome5	135517349
ENSG00000170365	chromosome4	146655216
ENSG00000141646	chromosome18	46827415
ENSG00000048740	chromosome10	11100098
ENSG00000149187	chromosome11	47467143
ENSG00000140488	chromosome15	70399270
ENSG00000159409	chromosome1	149955897
ENSG00000101489	chromosome18	33399603
ENSG00000161082	chromosome19	3175738
ENSG00000161082	chromosome19	3175738
ENSG00000101489	chromosome18	33399603
ENSG00000159409	chromosome1	149955897
ENSG00000140488	chromosome15	70399270
ENSG00000135316	chromosome6	86407877
ENSG00000125944	chromosome1	23540089
ENSG00000163746	chromosome3	147660382
ENSG00000188313	chromosome3	147737030

ENSG00000114698	chromosome3	147422549
ENSG00000163746	chromosome3	147660382
ENSG00000188313	chromosome3	147737030
ENSG00000187838	chromosome17	7238154
ENSG00000114698	chromosome3	147422549
ENSG00000163746	chromosome3	147660382
ENSG00000188313	chromosome3	147737030
ENSG00000136816	chromosome9	131605313
ENSG00000136827	chromosome9	131626186
ENSG00000186283	chromosome1	177317887
ENSG00000198113	chromosome9	139292963
ENSG00000160404	chromosome9	129537385
ENSG00000136816	chromosome9	131605313
ENSG00000136827	chromosome9	131626186
ENSG00000186283	chromosome1	177317887
ENSG00000186283	chromosome1	177317887
ENSG00000136816	chromosome9	131605313
ENSG00000136827	chromosome9	131626186
ENSG00000160404	chromosome9	129537385
ENSG00000198113	chromosome9	139292963
ENSG00000163803	chromosome2	28572486
ENSG00000164144	chromosome4	153970236
ENSG00000132254	chromosome11	6458228
ENSG00000157349	chromosome16	68890702
ENSG00000168872	chromosome16	68938396
ENSG00000109832	chromosome11	125279623
ENSG00000157349	chromosome16	68890702
ENSG00000168872	chromosome16	68938396
ENSG00000157349	chromosome16	68890702
ENSG00000168872	chromosome16	68938396
ENSG00000140153	chromosome14	101676014
ENSG00000185800	chromosome19	50987855
ENSG00000111716	chromosome12	21698873
ENSG00000171989	chromosome15	57286432
ENSG00000166800	chromosome11	18434804
ENSG00000166796	chromosome11	18390841
ENSG00000134333	chromosome11	18374966
ENSG00000153930	chromosome17	51585870
ENSG00000153930	chromosome17	51585870
ENSG00000141576	chromosome17	71747917
ENSG00000102858	chromosome16	4614963
ENSG00000171703	chromosome20	62165110
ENSG00000187735	chromosome8	55097239
ENSG00000204219	chromosome1	23623714
ENSG00000187735	chromosome8	55097239
ENSG00000171703	chromosome20	62165110
ENSG00000144959	chromosome3	173911565
ENSG00000114771	chromosome3	153014641
ENSG00000197953	chromosome3	152934580
ENSG00000114771	chromosome3	153014641
ENSG00000197953	chromosome3	152934580
ENSG00000144959	chromosome3	173911565
ENSG00000188984	chromosome1	12698931
ENSG00000204518	chromosome1	12627153
ENSG00000130201	chromosome19	50426951
ENSG00000205436	chromosome14	102636310
ENSG00000185215	chromosome14	102662548
ENSG00000180104	chromosome5	499291
ENSG00000179044	chromosome16	65781081

ENSG00000205436	chromosome14	102636310
ENSG00000185215	chromosome14	102662548
ENSG00000130201	chromosome19	50426951
ENSG00000185215	chromosome14	102662548
ENSG00000205436	chromosome14	102636310
ENSG00000205436	chromosome14	102636310
ENSG00000185215	chromosome14	102662548
ENSG00000130201	chromosome19	50426951
ENSG00000179044	chromosome16	65781081
ENSG00000112335	chromosome6	108688819
ENSG00000147164	chromosomeX	70204882
ENSG00000139364	chromosome12	124377123
ENSG00000181234	chromosome12	127666681
ENSG00000151952	chromosome12	128953838
ENSG00000181291	chromosome17	29932209
ENSG00000139364	chromosome12	124377123
ENSG00000181234	chromosome12	127666681
ENSG00000151952	chromosome12	128953838
ENSG00000181291	chromosome17	29932209
ENSG00000139364	chromosome12	124377123
ENSG00000181234	chromosome12	127666681
ENSG00000151952	chromosome12	128953838
ENSG00000006118	chromosome11	60448682
ENSG00000139364	chromosome12	124377123
ENSG00000181234	chromosome12	127666681
ENSG00000151952	chromosome12	128953838
ENSG00000059145	chromosome16	1404694
ENSG00000132478	chromosome17	71292557
ENSG00000103254	chromosome16	710605
ENSG00000103254	chromosome16	710605
ENSG00000150756	chromosome5	10302986
ENSG00000106615	chromosome7	150847531
ENSG00000167550	chromosome12	47749836
ENSG00000215868	chromosome1	110824911
ENSG00000172647	chromosome11	60765275
ENSG00000180860	chromosome11	60727613
ENSG00000204973	chromosome11	60746449
ENSG00000096088	chromosome6	41823051
ENSG00000184488	chromosome1	111735361
ENSG00000172647	chromosome11	60765275
ENSG00000180860	chromosome11	60727613
ENSG00000204973	chromosome11	60746449
ENSG00000215868	chromosome1	110824911
ENSG00000117984	chromosome11	1741666
ENSG00000131400	chromosome19	55560691
ENSG00000131401	chromosome19	55539825
ENSG00000143839	chromosome1	202402045
ENSG00000131400	chromosome19	55560691
ENSG00000131401	chromosome19	55539825
ENSG00000117984	chromosome11	1741666
ENSG00000196188	chromosome1	204484200
ENSG00000172647	chromosome11	60765275
ENSG00000180860	chromosome11	60727613
ENSG00000204973	chromosome11	60746449
ENSG00000215868	chromosome1	110824911
ENSG00000096088	chromosome6	41823051
ENSG00000184488	chromosome1	111735361
ENSG00000172647	chromosome11	60765275
ENSG00000180860	chromosome11	60727613

ENSG00000204973	chromosome11	60746449
ENSG00000215868	chromosome1	110824911
ENSG00000096088	chromosome6	41823051
ENSG00000184488	chromosome1	111735361
ENSG00000196188	chromosome1	204484200
ENSG00000160190	chromosome21	42811634
ENSG00000134955	chromosome11	124438441
ENSG00000136738	chromosome10	17726345
ENSG00000115145	chromosome2	152740403
ENSG00000181541	chromosome4	151723632
ENSG00000180660	chromosome13	34948276
ENSG00000080709	chromosome5	113725565
ENSG00000105642	chromosome19	17945647
ENSG00000143603	chromosome1	153109065
ENSG00000105642	chromosome19	17945647
ENSG00000080709	chromosome5	113725565
ENSG00000187097	chromosome14	73524559
ENSG00000197586	chromosome20	25124368
ENSG00000112290	chromosome6	110555498
ENSG00000188459	chromosomeX	47542269
ENSG00000158195	chromosome1	27627988
ENSG00000132970	chromosome13	26114408
ENSG00000132970	chromosome13	26114408
ENSG00000188459	chromosomeX	47542269
ENSG00000158195	chromosome1	27627988
ENSG00000112290	chromosome6	110555498
ENSG00000173614	chromosome1	9954719
ENSG00000163864	chromosome3	140829257
ENSG00000138678	chromosome4	84676800
ENSG00000158669	chromosome8	41575816
ENSG00000143801	chromosome1	225134970
ENSG00000080815	chromosome14	72684481
ENSG00000130429	chromosome7	98821274
ENSG00000013455	chromosome7	98768913
ENSG00000013455	chromosome7	98768913
ENSG00000130429	chromosome7	98821274
ENSG00000114757	chromosome3	181237082
ENSG00000139197	chromosome12	7234241
ENSG00000143183	chromosome1	164004613
ENSG00000029364	chromosome14	68935840
ENSG00000175334	chromosome11	65527298
ENSG00000175334	chromosome11	65527298
ENSG00000125888	chromosome20	17653671
ENSG00000026297	chromosome6	167289661
ENSG00000011478	chromosome19	50887802
ENSG00000115828	chromosome2	37425379
ENSG00000143622	chromosome1	154147301
ENSG00000152214	chromosome18	38949483
ENSG00000110958	chromosome12	55368051
ENSG00000110958	chromosome12	55368051
ENSG00000110958	chromosome12	55368051
ENSG00000110958	chromosome12	55368051
ENSG00000152380	chromosome5	79819699
ENSG00000162391	chromosome1	54861657
ENSG00000172974	chromosome2	65285747
ENSG00000165637	chromosome10	76640923
ENSG00000213585	chromosome5	133356603
ENSG00000215818	chromosome1	213616441
ENSG00000078668	chromosome8	42370887

ENSG00000213585	chromosome5	133356603
ENSG00000215818	chromosome1	213616441
ENSG00000172974	chromosome2	65285747
ENSG00000165637	chromosome10	76640923
ENSG00000181588	chromosome19	1518877
ENSG00000203759	chromosome1	154318414
ENSG00000183496	chromosome15	80125102
ENSG00000183496	chromosome15	80125102
ENSG00000203759	chromosome1	154318414
ENSG00000176624	chromosome18	46977104
ENSG00000203759	chromosome1	154318414
ENSG00000183496	chromosome15	80125102
ENSG00000181588	chromosome19	1518877
ENSG00000075975	chromosome3	12573650
ENSG00000075975	chromosome3	12573650
ENSG00000179455	chromosome15	21362023
ENSG00000133606	chromosome7	139825614
ENSG00000179455	chromosome15	21362023
ENSG00000133606	chromosome7	139825614
ENSG00000075975	chromosome3	12573650
ENSG00000005436	chromosome2	75791490
ENSG00000159086	chromosome21	33065851
ENSG00000184900	chromosome21	45062312
ENSG00000184900	chromosome21	45062312
ENSG00000177688	chromosome6	149763221
ENSG00000184763	chromosome7	55767331
ENSG00000188612	chromosomeX	114859806
ENSG00000184900	chromosome21	45062312
ENSG00000184900	chromosome21	45062312
ENSG00000184900	chromosome21	45062312
ENSG00000177688	chromosome6	149763221
ENSG00000184763	chromosome7	55767331
ENSG00000188612	chromosomeX	114859806
ENSG00000100997	chromosome20	25319340
ENSG00000131969	chromosome14	50402270
ENSG00000132639	chromosome20	10204140
ENSG00000092531	chromosome15	40591337
ENSG00000132639	chromosome20	10204140
ENSG00000146809	chromosome7	123041793
ENSG00000168387	chromosome3	57301102
ENSG00000148057	chromosome9	85427907
ENSG00000116857	chromosome1	199389675
ENSG00000175348	chromosome11	8942437
ENSG00000197045	chromosome14	54025396
ENSG00000130755	chromosome19	44518457
ENSG00000179632	chromosome8	145232575
ENSG00000122691	chromosome7	19123470
ENSG00000186051	chromosome9	107464599
ENSG00000104903	chromosome19	13072986
ENSG00000162367	chromosome1	47464148
ENSG00000171532	chromosome17	35016379
ENSG00000162992	chromosome2	182251833
ENSG00000123307	chromosome12	53706491
ENSG00000122859	chromosome10	71002806
ENSG00000181965	chromosome5	134899280
ENSG00000178403	chromosome4	113656081
ENSG00000162367	chromosome1	47464148
ENSG00000177551	chromosome1	116182517
ENSG00000171786	chromosome1	158607146

ENSG00000162367	chromosome1	47464148
ENSG00000104903	chromosome19	13072986
ENSG00000164600	chromosome7	31345408
ENSG00000162992	chromosome2	182251833
ENSG00000123307	chromosome12	53706491
ENSG00000171532	chromosome17	35016379
ENSG00000125878	chromosome20	538882
ENSG00000187786	chromosome8	145393505
ENSG00000188686	chromosome8	145461411
ENSG00000164107	chromosome4	174687016
ENSG00000113196	chromosome5	153837762
ENSG00000171786	chromosome1	158607146
ENSG00000177551	chromosome1	116182517
ENSG00000122691	chromosome7	19123470
ENSG00000162992	chromosome2	182251833
ENSG00000123307	chromosome12	53706491
ENSG00000171532	chromosome17	35016379
ENSG00000164600	chromosome7	31345408
ENSG00000125878	chromosome20	538882
ENSG00000187786	chromosome8	145393505
ENSG00000188686	chromosome8	145461411
ENSG00000125878	chromosome20	538882
ENSG00000123307	chromosome12	53706491
ENSG00000162992	chromosome2	182251833
ENSG00000213064	chromosome1	166461942
ENSG00000198818	chromosome6	166675960
ENSG00000054148	chromosome9	138863704
ENSG00000150625	chromosome4	176970373
ENSG00000046653	chromosomeX	13866758
ENSG00000123560	chromosomeX	102918544
ENSG00000123560	chromosomeX	102918544
ENSG00000046653	chromosomeX	13866758
ENSG00000120963	chromosome8	102283146
ENSG00000145920	chromosome5	175238349
ENSG00000145916	chromosome5	177497727
ENSG00000153561	chromosome2	86801302
ENSG00000115539	chromosome2	100534204
ENSG00000215109	chromosome8	61462375
ENSG00000163440	chromosome4	56143260
ENSG00000116703	chromosome1	184696862
ENSG00000136940	chromosome9	124628888
ENSG00000110911	chromosome12	49708276
ENSG00000018280	chromosome2	218955336
ENSG00000053918	chromosome11	2422905
ENSG00000185760	chromosome6	73388639
ENSG00000117013	chromosome1	41022353
ENSG00000117013	chromosome1	41022353
ENSG00000185760	chromosome6	73388639
ENSG00000184156	chromosome8	133561962
ENSG00000075043	chromosome20	61574261
ENSG00000075043	chromosome20	61574261
ENSG00000184156	chromosome8	133561962
ENSG00000184156	chromosome8	133561962
ENSG00000075043	chromosome20	61574261
ENSG00000117013	chromosome1	41022353
ENSG00000185760	chromosome6	73388639
ENSG00000185009	chromosome10	75568144
ENSG00000070718	chromosome8	42131363
ENSG00000148634	chromosome10	69502872

ENSG00000138641	chromosome4	89745998
ENSG00000138641	chromosome4	89745998
ENSG00000148634	chromosome10	69502872
ENSG00000138646	chromosome4	89597444
ENSG00000138642	chromosome4	89519098
ENSG00000114062	chromosome15	23204889
ENSG00000100796	chromosome14	91045745
ENSG00000182112	chromosomeX	27391335
ENSG00000138041	chromosome2	55697926
ENSG00000145194	chromosome3	185450177
ENSG00000117298	chromosome1	21544459
ENSG00000196549	chromosome3	156284651
ENSG00000142606	chromosome1	2550784
ENSG00000117298	chromosome1	21544459
ENSG00000145194	chromosome3	185450177
ENSG00000197993	chromosome7	142369416
ENSG00000003987	chromosome8	17315173
ENSG00000102043	chromosomeX	63531969
ENSG00000139505	chromosome13	24759386
ENSG00000150712	chromosome5	32348702
ENSG00000166912	chromosome15	29056326
ENSG00000014914	chromosome1	148176877
ENSG00000063601	chromosomeX	149612662
ENSG00000087053	chromosome11	95296767
ENSG00000184416	chromosome1	32479690
ENSG00000104643	chromosome8	11179808
ENSG00000166912	chromosome15	29056326
ENSG00000150712	chromosome5	32348702
ENSG00000087053	chromosome11	95296767
ENSG00000063601	chromosomeX	149612662
ENSG00000171100	chromosomeX	149511735
ENSG00000139505	chromosome13	24759386
ENSG00000102043	chromosomeX	63531969
ENSG00000108389	chromosome17	53948943
ENSG00000100330	chromosome22	28697049
ENSG00000138190	chromosome10	94584476
ENSG00000144036	chromosome2	72906548
ENSG00000179832	chromosome8	145274928
ENSG00000185038	chromosome2	234351791
ENSG00000171495	chromosome5	41113220
ENSG00000157184	chromosome1	53435204
ENSG00000160271	chromosome9	135014414
ENSG00000143344	chromosome1	181977916
ENSG00000143344	chromosome1	181977916
ENSG00000160271	chromosome9	135014414
ENSG00000204218	chromosome6	33374366
ENSG00000205517	chromosome19	11390955
ENSG00000205517	chromosome19	11390955
ENSG00000204218	chromosome6	33374366
ENSG00000105143	chromosome19	14944723
ENSG00000079215	chromosome5	36644283
ENSG00000162383	chromosome1	53380710
ENSG00000105143	chromosome19	14944723
ENSG00000079215	chromosome5	36644283
ENSG00000079215	chromosome5	36644283
ENSG00000105143	chromosome19	14944723
ENSG00000105281	chromosome19	51983063
ENSG00000115902	chromosome2	65070282
ENSG00000110436	chromosome11	35397090

ENSG00000162383	chromosome1	53380710
ENSG00000105143	chromosome19	14944723
ENSG00000079215	chromosome5	36644283
ENSG00000162383	chromosome1	53380710
ENSG00000115902	chromosome2	65070282
ENSG00000105281	chromosome19	51983063
ENSG00000106688	chromosome9	4480680
ENSG00000087095	chromosome17	23394063
ENSG00000003989	chromosome8	17440713
ENSG00000139514	chromosome13	29008326
ENSG00000165349	chromosomeX	70066573
ENSG00000003989	chromosome8	17440713
ENSG00000165349	chromosomeX	70066573
ENSG00000139514	chromosome13	29008326
ENSG00000075413	chromosome14	102922120
ENSG00000007047	chromosome19	50446687
ENSG00000116141	chromosome1	218768788
ENSG00000116141	chromosome1	218768788
ENSG00000007047	chromosome19	50446687
ENSG00000075413	chromosome14	102922120
ENSG00000007047	chromosome19	50446687
ENSG00000075413	chromosome14	102922120
ENSG00000116141	chromosome1	218768788
ENSG00000072518	chromosome11	63363555
ENSG00000170145	chromosome11	110978498
ENSG00000142178	chromosome21	43670487
ENSG00000163462	chromosome1	153413067
ENSG00000152503	chromosome5	114543634
ENSG00000186628	chromosome15	81253197
ENSG00000100505	chromosome14	50631408
ENSG00000119283	chromosome1	229365339
ENSG00000186628	chromosome15	81253197
ENSG00000152503	chromosome5	114543634
ENSG00000163462	chromosome1	153413067
ENSG00000178233	chromosome6	44346458
ENSG00000178233	chromosome6	44346458
ENSG00000179292	chromosome11	65816061
ENSG00000145349	chromosome4	114901674
ENSG00000070808	chromosome5	149649535
ENSG00000148660	chromosome10	75304226
ENSG00000058404	chromosome7	44331546
ENSG00000145349	chromosome4	114901674
ENSG00000058404	chromosome7	44331546
ENSG00000148660	chromosome10	75304226
ENSG00000070808	chromosome5	149649535
ENSG00000145349	chromosome4	114901674
ENSG00000171189	chromosome21	30233690
ENSG00000164418	chromosome6	101953875
ENSG00000163873	chromosome1	37272297
ENSG00000164418	chromosome6	101953875
ENSG00000171189	chromosome21	30233690
ENSG00000163873	chromosome1	37272297
ENSG00000171189	chromosome21	30233690
ENSG00000164418	chromosome6	101953875
ENSG00000149403	chromosome11	120036238
ENSG00000155511	chromosome5	152850642
ENSG00000120251	chromosome4	158361645
ENSG00000152578	chromosome11	104986935
ENSG00000125675	chromosomeX	122146069

ENSG00000125675	chromosomeX	122146069
ENSG00000152578	chromosome11	104986935
ENSG00000120251	chromosome4	158361645
ENSG00000155511	chromosome5	152850642
ENSG00000149403	chromosome11	120036238
ENSG00000198586	chromosome2	171725185
ENSG00000215170	chromosome10	38149926
ENSG00000146872	chromosome17	57912219
ENSG00000214705	chromosome17	29391005
ENSG00000166289	chromosome19	34856587
ENSG00000175895	chromosome8	96235449
ENSG00000174016	chromosomeX	79584695
ENSG00000183508	chromosome1	117967014
ENSG00000158246	chromosome1	27211746
ENSG00000112773	chromosome6	82519211
ENSG00000158246	chromosome1	27211746
ENSG00000112773	chromosome6	82519211
ENSG00000183508	chromosome1	117967014
ENSG00000112773	chromosome6	82519211
ENSG00000158246	chromosome1	27211746
ENSG00000196218	chromosome19	43616310
ENSG00000198838	chromosome15	31390539
ENSG00000198626	chromosome1	235272452
ENSG00000198626	chromosome1	235272452
ENSG00000196218	chromosome19	43616310
ENSG00000198838	chromosome15	31390539
ENSG00000198838	chromosome15	31390539
ENSG00000196218	chromosome19	43616310
ENSG00000034053	chromosome15	27133380
ENSG00000107282	chromosome9	71321947
ENSG00000107282	chromosome9	71321947
ENSG00000034053	chromosome15	27133380
ENSG00000011132	chromosome19	3711263
ENSG00000168702	chromosome2	142604880
ENSG00000123384	chromosome12	55809015
ENSG00000123384	chromosome12	55809015
ENSG00000168702	chromosome2	142604880
ENSG00000162337	chromosome11	67836759
ENSG00000070018	chromosome12	12310937
ENSG00000081479	chromosome2	169927156
ENSG00000071537	chromosome14	81069842
ENSG00000101251	chromosome20	13919181
ENSG00000091490	chromosome4	25474233
ENSG00000144130	chromosome2	113195534
ENSG00000076685	chromosome10	104924706
ENSG00000141434	chromosome18	28024032
ENSG00000112818	chromosome6	46869095
ENSG00000112818	chromosome6	46869095
ENSG00000141434	chromosome18	28024032
ENSG00000164663	chromosome6	41882700
ENSG00000136014	chromosome12	94452164
ENSG00000092931	chromosome17	72246030
ENSG00000128881	chromosome15	40958108
ENSG00000164128	chromosome4	164467157
ENSG00000159279	chromosome5	137171799
ENSG00000204174	chromosome10	46506790
ENSG00000164129	chromosome4	164490876
ENSG00000185149	chromosome4	156354542
ENSG00000204174	chromosome10	46506790

ENSG00000056291	chromosome4	73116483
ENSG00000148734	chromosome10	71696161
ENSG00000119973	chromosome10	120344747
ENSG00000101292	chromosome20	5243016
ENSG00000169618	chromosome2	68726458
ENSG00000164129	chromosome4	164490876
ENSG00000164128	chromosome4	164467157
ENSG00000159279	chromosome5	137171799
ENSG00000204174	chromosome10	46506790
ENSG00000101292	chromosome20	5243016
ENSG00000169618	chromosome2	68726458
ENSG00000186867	chromosome4	122521253
ENSG00000163394	chromosome4	26100988
ENSG00000110148	chromosome11	6237735
ENSG00000110148	chromosome11	6237735
ENSG00000163394	chromosome4	26100988
ENSG00000119973	chromosome10	120344747
ENSG00000185149	chromosome4	156354542
ENSG00000186867	chromosome4	122521253
ENSG00000148734	chromosome10	71696161
ENSG00000056291	chromosome4	73116483
ENSG00000148734	chromosome10	71696161
ENSG00000056291	chromosome4	73116483
ENSG00000186867	chromosome4	122521253
ENSG00000115353	chromosome2	75279569
ENSG00000169836	chromosome4	104860282
ENSG00000075073	chromosome10	70846086
ENSG00000204174	chromosome10	46506790
ENSG00000164128	chromosome4	164467157
ENSG00000159279	chromosome5	137171799
ENSG00000137252	chromosome6	55147345
ENSG00000121764	chromosome1	31857381
ENSG00000159279	chromosome5	137171799
ENSG00000164128	chromosome4	164467157
ENSG00000169836	chromosome4	104860282
ENSG00000115353	chromosome2	75279569
ENSG00000142611	chromosome1	2975684
ENSG00000085276	chromosome3	170347055
ENSG00000075790	chromosome7	107008454
ENSG00000185825	chromosomeX	152641894
ENSG00000184831	chromosomeX	23835741
ENSG00000155008	chromosomeX	84187963
ENSG00000178695	chromosome13	76358285
ENSG00000134504	chromosome18	22335198
ENSG00000153885	chromosome19	38983200
ENSG00000183775	chromosome5	143566471
ENSG00000178695	chromosome13	76358285
ENSG00000183783	chromosome4	44145298
ENSG00000178695	chromosome13	76358285
ENSG00000183775	chromosome5	143566471
ENSG00000213859	chromosome17	7196986
ENSG00000188997	chromosome11	77563249
ENSG00000134504	chromosome18	22335198
ENSG00000180332	chromosome13	44666703
ENSG00000188997	chromosome11	77563249
ENSG00000213859	chromosome17	7196986
ENSG00000168301	chromosome3	58459523
ENSG00000168301	chromosome3	58459523
ENSG00000188997	chromosome11	77563249

ENSG00000213859	chromosome17	7196986
ENSG00000003436	chromosome2	188086619
ENSG00000105825	chromosome7	93357927
ENSG00000105825	chromosome7	93357927
ENSG00000003436	chromosome2	188086619
ENSG00000173714	chromosome17	46268298
ENSG00000127578	chromosome16	621255
ENSG00000010671	chromosomeX	100516929
ENSG00000102010	chromosomeX	15436398
ENSG00000074966	chromosome4	47830945
ENSG00000135605	chromosome4	47925389
ENSG00000135605	chromosome4	47925389
ENSG00000074966	chromosome4	47830945
ENSG00000113263	chromosome5	156540567
ENSG00000102010	chromosomeX	15436398
ENSG00000010671	chromosomeX	100516929
ENSG00000182511	chromosome15	89229280
ENSG00000151422	chromosome5	108161783
ENSG00000113263	chromosome5	156540567
ENSG00000135605	chromosome4	47925389
ENSG00000074966	chromosome4	47830945
ENSG00000215203	chromosome4	42590041
ENSG00000204928	chromosome5	145232725
ENSG00000182853	chromosome17	4636388
ENSG00000139734	chromosome13	59636013
ENSG00000147202	chromosomeX	95826714
ENSG00000131504	chromosome5	140978747
ENSG00000147202	chromosomeX	95826714
ENSG00000139734	chromosome13	59636013
ENSG00000139926	chromosome14	51226305
ENSG00000163629	chromosome4	87775434
ENSG00000170324	chromosome10	49152617
ENSG00000170324	chromosome10	49152617
ENSG00000163629	chromosome4	87775434
ENSG00000141448	chromosome18	18005104
ENSG00000136574	chromosome8	11603231
ENSG00000107485	chromosome10	8137625
ENSG00000179348	chromosome3	129688565
ENSG00000102145	chromosomeX	48534461
ENSG00000130700	chromosome20	60483973
ENSG00000136574	chromosome8	11603231
ENSG00000141448	chromosome18	18005104
ENSG00000179348	chromosome3	129688565
ENSG00000107485	chromosome10	8137625
ENSG00000125618	chromosome2	113752442
ENSG00000196092	chromosome9	37024029
ENSG00000075891	chromosome10	102496008
ENSG00000198807	chromosome14	36201046
ENSG00000125813	chromosome20	21634360
ENSG00000075891	chromosome10	102496008
ENSG00000196092	chromosome9	37024029
ENSG00000196092	chromosome9	37024029
ENSG00000075891	chromosome10	102496008
ENSG00000125618	chromosome2	113752442
ENSG00000162065	chromosome16	2486151
ENSG00000176171	chromosome10	133645301
ENSG00000104765	chromosome8	26296564
ENSG00000176171	chromosome10	133645301
ENSG00000104765	chromosome8	26296564

ENSG00000104765	chromosome8	26296564
ENSG00000176171	chromosome10	133645301
ENSG00000137745	chromosome11	102331645
ENSG00000137674	chromosome11	102001261
ENSG00000008516	chromosome16	3036920
ENSG00000198598	chromosome12	130878993
ENSG00000156103	chromosome8	89408552
ENSG00000125966	chromosome20	33278117
ENSG00000123342	chromosome12	54522882
ENSG00000129270	chromosome17	31146495
ENSG00000215914	chromosome1	1621509
ENSG00000189409	chromosome1	1557461
ENSG00000102996	chromosome16	56617756
ENSG00000125966	chromosome20	33278117
ENSG00000156103	chromosome8	89408552
ENSG00000137674	chromosome11	102001261
ENSG00000166670	chromosome11	102156533
ENSG00000149968	chromosome11	102219488
ENSG00000137675	chromosome11	102081656
ENSG00000118113	chromosome11	102100797
ENSG00000196611	chromosome11	102174034
ENSG00000110347	chromosome11	102250878
ENSG00000137673	chromosome11	101906642
ENSG00000087245	chromosome16	54070893
ENSG00000100985	chromosome20	44070973
ENSG00000198598	chromosome12	130878993
ENSG00000137745	chromosome11	102331645
ENSG00000166670	chromosome11	102156533
ENSG00000149968	chromosome11	102219488
ENSG00000137675	chromosome11	102081656
ENSG00000118113	chromosome11	102100797
ENSG00000196611	chromosome11	102174034
ENSG00000110347	chromosome11	102250878
ENSG00000137673	chromosome11	101906642
ENSG00000137674	chromosome11	102001261
ENSG00000157227	chromosome14	22375867
ENSG00000125966	chromosome20	33278117
ENSG00000156103	chromosome8	89408552
ENSG00000102996	chromosome16	56617756
ENSG00000008516	chromosome16	3036920
ENSG00000123358	chromosome12	50718761
ENSG00000119508	chromosome9	101628874
ENSG00000153234	chromosome2	156894945
ENSG00000153234	chromosome2	156894945
ENSG00000119508	chromosome9	101628874
ENSG00000153234	chromosome2	156894945
ENSG00000213719	chromosome6	31812057
ENSG00000214258	chromosome16	85026992
ENSG00000169583	chromosome9	139010817
ENSG00000159212	chromosome21	34963558
ENSG00000169504	chromosome1	24944632
ENSG00000112782	chromosome6	46155939
ENSG00000155962	chromosomeX	154216931
ENSG00000112782	chromosome6	46155939
ENSG00000169504	chromosome1	24944632
ENSG00000159212	chromosome21	34963558
ENSG00000169504	chromosome1	24944632
ENSG00000112782	chromosome6	46155939
ENSG00000213719	chromosome6	31812057

ENSG00000214258	chromosome16	85026992
ENSG00000169583	chromosome9	139010817
ENSG00000169583	chromosome9	139010817
ENSG00000213719	chromosome6	31812057
ENSG00000214258	chromosome16	85026992
ENSG00000169504	chromosome1	24944632
ENSG00000112782	chromosome6	46155939
ENSG00000159212	chromosome21	34963558
ENSG00000121454	chromosome1	178466288
ENSG00000107187	chromosome9	138236680
ENSG00000143355	chromosome1	196148285
ENSG00000106689	chromosome9	125814449
ENSG00000159556	chromosome15	74416280
ENSG00000132130	chromosome17	32369608
ENSG00000106852	chromosome9	124030841
ENSG00000136944	chromosome9	128416550
ENSG00000162761	chromosome1	163591421
ENSG00000162761	chromosome1	163591421
ENSG00000136944	chromosome9	128416550
ENSG00000143013	chromosome1	87570287
ENSG00000106852	chromosome9	124030841
ENSG00000162624	chromosome1	75368988
ENSG00000016082	chromosome5	50715263
ENSG00000106689	chromosome9	125814449
ENSG00000143355	chromosome1	196148285
ENSG00000132130	chromosome17	32369608
ENSG00000089116	chromosome12	112393687
ENSG00000143355	chromosome1	196148285
ENSG00000143013	chromosome1	87570287
ENSG00000166206	chromosome15	24767330
ENSG00000145864	chromosome5	160906228
ENSG00000109738	chromosome4	158218627
ENSG00000186297	chromosome15	24665489
ENSG00000011677	chromosomeX	151283699
ENSG00000011677	chromosomeX	151283699
ENSG00000186297	chromosome15	24665489
ENSG00000022355	chromosome5	161210395
ENSG00000151834	chromosome4	46085481
ENSG00000109158	chromosome4	46690199
ENSG00000145863	chromosome5	161045574
ENSG00000163288	chromosome4	46728426
ENSG00000145864	chromosome5	160906228
ENSG00000166206	chromosome15	24767330
ENSG00000147402	chromosomeX	151557313
ENSG00000094755	chromosome5	170148198
ENSG00000187730	chromosome1	1940723
ENSG00000145888	chromosome5	151284304
ENSG00000145451	chromosome4	175986538
ENSG00000182256	chromosome15	24799429
ENSG00000102287	chromosomeX	150893754
ENSG00000094755	chromosome5	170148198
ENSG00000183185	chromosome3	99236522
ENSG00000111886	chromosome6	90081604
ENSG00000146276	chromosome6	89983743
ENSG00000022355	chromosome5	161210395
ENSG00000151834	chromosome4	46085481
ENSG00000011677	chromosomeX	151283699
ENSG00000186297	chromosome15	24665489
ENSG00000188828	chromosomeX	102866561

ENSG00000101958	chromosomeX	14458101
ENSG00000113327	chromosome5	161427584
ENSG00000163285	chromosome4	45820688
ENSG00000145863	chromosome5	161045574
ENSG00000109158	chromosome4	46690199
ENSG00000151834	chromosome4	46085481
ENSG00000022355	chromosome5	161210395
ENSG00000145864	chromosome5	160906228
ENSG00000166206	chromosome15	24767330
ENSG00000163288	chromosome4	46728426
ENSG00000163285	chromosome4	45820688
ENSG00000113327	chromosome5	161427584
ENSG00000102287	chromosomeX	150893754
ENSG00000182256	chromosome15	24799429
ENSG00000111886	chromosome6	90081604
ENSG00000146276	chromosome6	89983743
ENSG00000101958	chromosomeX	14458101
ENSG00000188828	chromosomeX	102866561
ENSG00000145451	chromosome4	175986538
ENSG00000145888	chromosome5	151284304
ENSG00000111886	chromosome6	90081604
ENSG00000146276	chromosome6	89983743
ENSG00000168646	chromosome17	60985201
ENSG00000103126	chromosome16	337027
ENSG00000103126	chromosome16	337027
ENSG00000168646	chromosome17	60985201
ENSG00000128016	chromosome19	44589385
ENSG00000185650	chromosome14	68329409
ENSG00000152518	chromosome2	43306959
ENSG00000128016	chromosome19	44589385
ENSG00000087085	chromosome7	100329790
ENSG00000114200	chromosome3	167031516
ENSG00000174607	chromosome4	115763486
ENSG00000145626	chromosome5	36027100
ENSG00000168671	chromosome5	36102649
ENSG00000167165	chromosome2	234191093
ENSG00000197592	chromosome4	69570966
ENSG00000196620	chromosome4	69218932
ENSG00000197888	chromosome4	69116798
ENSG00000213759	chromosome4	70115030
ENSG00000135226	chromosome4	70180808
ENSG00000198277	chromosome4	69909217
ENSG00000109181	chromosome4	69716312
ENSG00000171234	chromosome4	69996828
ENSG00000156096	chromosome4	70396169
ENSG00000135220	chromosome4	69852068
ENSG00000173610	chromosome4	70547952
ENSG00000167165	chromosome2	234191093
ENSG00000174607	chromosome4	115763486
ENSG00000197592	chromosome4	69570966
ENSG00000196620	chromosome4	69218932
ENSG00000197888	chromosome4	69116798
ENSG00000213759	chromosome4	70115030
ENSG00000135226	chromosome4	70180808
ENSG00000198277	chromosome4	69909217
ENSG00000109181	chromosome4	69716312
ENSG00000171234	chromosome4	69996828
ENSG00000156096	chromosome4	70396169
ENSG00000135220	chromosome4	69852068

ENSG00000173610	chromosome4	70547952
ENSG00000167165	chromosome2	234191093
ENSG00000197592	chromosome4	69570966
ENSG00000196620	chromosome4	69218932
ENSG00000197888	chromosome4	69116798
ENSG00000213759	chromosome4	70115030
ENSG00000135226	chromosome4	70180808
ENSG00000198277	chromosome4	69909217
ENSG00000109181	chromosome4	69716312
ENSG00000171234	chromosome4	69996828
ENSG00000156096	chromosome4	70396169
ENSG00000135220	chromosome4	69852068
ENSG00000173610	chromosome4	70547952
ENSG00000167165	chromosome2	234191093
ENSG00000145626	chromosome5	36027100
ENSG00000168671	chromosome5	36102649
ENSG00000174607	chromosome4	115763486
ENSG00000172818	chromosome11	65311421
ENSG00000125850	chromosome20	17986279
ENSG00000108947	chromosome17	7549642
ENSG00000090776	chromosomeX	67966345
ENSG00000125266	chromosome13	105985314
ENSG00000099617	chromosome19	1237168
ENSG00000184349	chromosome5	107034214
ENSG00000143590	chromosome1	153318042
ENSG00000143620	chromosome1	153302924
ENSG00000143590	chromosome1	153318042
ENSG00000099617	chromosome19	1237168
ENSG00000184349	chromosome5	107034214
ENSG00000169242	chromosome1	153367078
ENSG00000143620	chromosome1	153302924
ENSG00000099617	chromosome19	1237168
ENSG00000184349	chromosome5	107034214
ENSG00000143590	chromosome1	153318042
ENSG00000184349	chromosome5	107034214
ENSG00000099617	chromosome19	1237168
ENSG00000125266	chromosome13	105985314
ENSG00000090776	chromosomeX	67966345
ENSG00000138386	chromosome2	191232148
ENSG00000214916	chromosomeX	150146846
ENSG00000166886	chromosome12	55769322
ENSG00000206384	chromosome3	131788159
ENSG00000163166	chromosome2	128000255
ENSG00000139651	chromosome12	51864534
ENSG00000204335	chromosome2	171280269
ENSG00000189120	chromosome17	43280795
ENSG00000170374	chromosome12	52009490
ENSG00000127528	chromosome19	16296735
ENSG00000136826	chromosome9	109291131
ENSG00000102554	chromosome13	72531467
ENSG00000164651	chromosome7	20792943
ENSG00000105610	chromosome19	12858955
ENSG00000127528	chromosome19	16296735
ENSG00000136826	chromosome9	109291131
ENSG00000164651	chromosome7	20792943
ENSG00000067082	chromosome10	3817207
ENSG00000172059	chromosome2	10101295
ENSG00000155090	chromosome8	103737006
ENSG00000127528	chromosome19	16296735

ENSG00000136826	chromosome9	109291131
ENSG00000105610	chromosome19	12858955
ENSG00000164651	chromosome7	20792943
ENSG00000164651	chromosome7	20792943
ENSG00000170374	chromosome12	52009490
ENSG00000189120	chromosome17	43280795
ENSG00000067082	chromosome10	3817207
ENSG00000139651	chromosome12	51864534
ENSG00000197550	chromosome9	69137176
ENSG00000197294	chromosome2	132577907
ENSG00000124216	chromosome20	48033004
ENSG00000177835	chromosome2	210381773
ENSG00000019549	chromosome8	49996378
ENSG00000185669	chromosome16	87280316
ENSG00000019549	chromosome8	49996378
ENSG00000124216	chromosome20	48033004
ENSG00000177835	chromosome2	210381773
ENSG00000198718	chromosome14	44501375
ENSG00000189350	chromosome2	29098587
ENSG00000060237	chromosome12	732993
ENSG00000126562	chromosome17	38186243
ENSG00000196632	chromosomeX	54376832
ENSG00000165238	chromosome9	94987033
ENSG00000126562	chromosome17	38186243
ENSG00000060237	chromosome12	732993
ENSG00000165238	chromosome9	94987033
ENSG00000196632	chromosomeX	54376832
ENSG00000115216	chromosome2	27509645
ENSG00000007516	chromosome16	1324664
ENSG00000092929	chromosome17	71352014
ENSG00000118200	chromosome1	198975579
ENSG00000130559	chromosome9	137938827
ENSG00000076826	chromosome19	7566889
ENSG00000130559	chromosome9	137938827
ENSG00000118200	chromosome1	198975579
ENSG00000124091	chromosome20	54505829
ENSG00000205318	chromosome6	10742213
ENSG00000111846	chromosome6	10637131
ENSG00000140297	chromosome15	57697730
ENSG00000187210	chromosome9	78307118
ENSG00000103489	chromosome16	17472155
ENSG00000015532	chromosome17	45778501
ENSG00000017621	chromosomeX	48908043
ENSG00000085978	chromosome2	233825213
ENSG00000168010	chromosome11	72203139
ENSG00000138083	chromosome2	45022748
ENSG00000126778	chromosome14	60185661
ENSG00000177045	chromosome19	50963943
ENSG00000100625	chromosome14	60260730
ENSG00000138083	chromosome2	45022748
ENSG00000184302	chromosome14	60045693
ENSG00000170577	chromosome2	45089754
ENSG00000126778	chromosome14	60185661
ENSG00000184302	chromosome14	60045693
ENSG00000138083	chromosome2	45022748
ENSG00000171916	chromosome17	18320894
ENSG00000170298	chromosome17	20311376
ENSG00000168961	chromosome17	22982419
ENSG00000133317	chromosome11	63030441

ENSG00000116977	chromosome1	234755994
ENSG00000171747	chromosome19	43995367
ENSG00000119862	chromosome2	64535184
ENSG00000119862	chromosome2	64535184
ENSG00000131981	chromosome14	54673845
ENSG00000171747	chromosome19	43995367
ENSG00000171916	chromosome17	18320894
ENSG00000170298	chromosome17	20311376
ENSG00000168961	chromosome17	22982419
ENSG00000133317	chromosome11	63030441
ENSG00000116977	chromosome1	234755994
ENSG00000197778	chromosome19	44861900
ENSG00000105198	chromosome19	44784556
ENSG00000006659	chromosome19	44878400
ENSG00000105205	chromosome19	44921513
ENSG00000178934	chromosome19	43971707
ENSG00000205076	chromosome19	43955973
ENSG00000133317	chromosome11	63030441
ENSG00000116977	chromosome1	234755994
ENSG00000171916	chromosome17	18320894
ENSG00000170298	chromosome17	20311376
ENSG00000168961	chromosome17	22982419
ENSG00000171747	chromosome19	43995367
ENSG00000171916	chromosome17	18320894
ENSG00000170298	chromosome17	20311376
ENSG00000168961	chromosome17	22982419
ENSG00000133317	chromosome11	63030441
ENSG00000116977	chromosome1	234755994
ENSG00000119862	chromosome2	64535184
ENSG00000100079	chromosome22	36305859
ENSG00000100097	chromosome22	36401656
ENSG00000131981	chromosome14	54673845
ENSG00000119862	chromosome2	64535184
ENSG00000215132	chromosome7	2482544
ENSG00000131981	chromosome14	54673845
ENSG00000116977	chromosome1	234755994
ENSG00000133317	chromosome11	63030441
ENSG00000150907	chromosome13	40138350
ENSG00000184481	chromosomeX	70233104
ENSG00000204060	chromosome1	41600190
ENSG00000118689	chromosome6	108989105
ENSG00000118689	chromosome6	108989105
ENSG00000204060	chromosome1	41600190
ENSG00000184481	chromosomeX	70233104
ENSG00000150907	chromosome13	40138350
ENSG00000088882	chromosome20	2729219
ENSG00000121898	chromosome10	125641166
ENSG00000106624	chromosome7	44110790
ENSG00000106624	chromosome7	44110790
ENSG00000121898	chromosome10	125641166
ENSG00000149927	chromosome16	29929045
ENSG00000067715	chromosome12	78135431
ENSG00000134207	chromosome1	114497918
ENSG00000110975	chromosome12	33483725
ENSG00000170743	chromosome11	7229994
ENSG00000110975	chromosome12	33483725
ENSG00000134207	chromosome1	114497918
ENSG00000101098	chromosome20	42872327
ENSG00000149927	chromosome16	29929045

ENSG00000214569	chromosome11	67140296
ENSG00000143858	chromosome1	200841524
ENSG00000129990	chromosome19	60382222
ENSG00000170743	chromosome11	7229994
ENSG00000067715	chromosome12	78135431
ENSG00000132872	chromosome18	39111245
ENSG00000132718	chromosome1	154096177
ENSG00000214409	chromosome7	82228732
ENSG00000214408	chromosome7	82314452
ENSG00000214409	chromosome7	82228732
ENSG00000101098	chromosome20	42872327
ENSG00000110975	chromosome12	33483725
ENSG00000134207	chromosome1	114497918
ENSG00000170743	chromosome11	7229994
ENSG00000213023	chromosome19	55832481
ENSG00000117971	chromosome15	76720531
ENSG00000160716	chromosome1	152807145
ENSG00000147432	chromosome8	42671847
ENSG00000169684	chromosome15	76645117
ENSG00000108556	chromosome17	4747138
ENSG00000196811	chromosome2	233112702
ENSG00000149305	chromosome11	113280866
ENSG00000166736	chromosome11	113351240
ENSG00000166736	chromosome11	113351240
ENSG00000149305	chromosome11	113280866
ENSG00000186090	chromosome3	185233313
ENSG00000178084	chromosome3	185253563
ENSG00000186038	chromosome3	185300855
ENSG00000186090	chromosome3	185233313
ENSG00000178084	chromosome3	185253563
ENSG00000186038	chromosome3	185300855
ENSG00000166736	chromosome11	113351240
ENSG00000149305	chromosome11	113280866
ENSG00000135902	chromosome2	233099170
ENSG00000196811	chromosome2	233112702
ENSG00000108556	chromosome17	4747138
ENSG00000175344	chromosome15	30110090
ENSG00000166664	chromosome15	28462847
ENSG00000174343	chromosome4	40032242
ENSG00000160716	chromosome1	152807145
ENSG00000117971	chromosome15	76720531
ENSG00000101204	chromosome20	61462962
ENSG00000120903	chromosome8	27384493
ENSG00000147434	chromosome8	42742731
ENSG00000080644	chromosome15	76700192
ENSG00000186090	chromosome3	185233313
ENSG00000178084	chromosome3	185253563
ENSG00000186038	chromosome3	185300855
ENSG00000174343	chromosome4	40032242
ENSG00000129749	chromosome11	3649119
ENSG00000170175	chromosome17	7289171
ENSG00000196811	chromosome2	233112702
ENSG00000108556	chromosome17	4747138
ENSG00000135902	chromosome2	233099170
ENSG00000175344	chromosome15	30110090
ENSG00000166664	chromosome15	28462847
ENSG00000141527	chromosome17	75769833
ENSG00000141527	chromosome17	75769833
ENSG00000105289	chromosome19	3679374

ENSG00000117758	chromosome1	27972406
ENSG00000079950	chromosome6	132866363
ENSG00000116106	chromosome2	222145213
ENSG00000070886	chromosome1	22762716
ENSG00000183317	chromosome1	38003326
ENSG00000135333	chromosome6	94185781
ENSG00000145242	chromosome4	66218056
ENSG00000044524	chromosome3	89239589
ENSG00000080224	chromosome3	98427797
ENSG00000106123	chromosome7	142271108
ENSG00000196411	chromosome7	100262589
ENSG00000182580	chromosome3	185762718
ENSG00000133216	chromosome1	22910063
ENSG00000154928	chromosome3	135996945
ENSG00000146904	chromosome7	142816021
ENSG00000142627	chromosome1	16355015
ENSG00000070886	chromosome1	22762716
ENSG00000183317	chromosome1	38003326
ENSG00000135333	chromosome6	94185781
ENSG00000145242	chromosome4	66218056
ENSG00000044524	chromosome3	89239589
ENSG00000080224	chromosome3	98427797
ENSG00000116106	chromosome2	222145213
ENSG00000196411	chromosome7	100262589
ENSG00000106123	chromosome7	142271108
ENSG00000070886	chromosome1	22762716
ENSG00000183317	chromosome1	38003326
ENSG00000135333	chromosome6	94185781
ENSG00000145242	chromosome4	66218056
ENSG00000044524	chromosome3	89239589
ENSG00000080224	chromosome3	98427797
ENSG00000116106	chromosome2	222145213
ENSG00000146904	chromosome7	142816021
ENSG00000142627	chromosome1	16355015
ENSG00000133216	chromosome1	22910063
ENSG00000154928	chromosome3	135996945
ENSG00000182580	chromosome3	185762718
ENSG00000142627	chromosome1	16355015
ENSG00000146904	chromosome7	142816021
ENSG00000145242	chromosome4	66218056
ENSG00000044524	chromosome3	89239589
ENSG00000080224	chromosome3	98427797
ENSG00000070886	chromosome1	22762716
ENSG00000183317	chromosome1	38003326
ENSG00000135333	chromosome6	94185781
ENSG00000116106	chromosome2	222145213
ENSG00000080224	chromosome3	98427797
ENSG00000044524	chromosome3	89239589
ENSG00000154928	chromosome3	135996945
ENSG00000133216	chromosome1	22910063
ENSG00000044524	chromosome3	89239589
ENSG00000080224	chromosome3	98427797
ENSG00000145242	chromosome4	66218056
ENSG00000182580	chromosome3	185762718
ENSG00000133216	chromosome1	22910063
ENSG00000154928	chromosome3	135996945
ENSG00000106123	chromosome7	142271108
ENSG00000196411	chromosome7	100262589
ENSG00000135333	chromosome6	94185781

ENSG00000183317	chromosome1	38003326
ENSG00000183317	chromosome1	38003326
ENSG00000135333	chromosome6	94185781
ENSG00000070886	chromosome1	22762716
ENSG00000085465	chromosome1	111771867
ENSG00000134216	chromosome1	111635007
ENSG00000203878	chromosome1	111626726
ENSG00000134216	chromosome1	111635007
ENSG00000203878	chromosome1	111626726
ENSG00000134216	chromosome1	111635007
ENSG00000203878	chromosome1	111626726
ENSG00000085465	chromosome1	111771867
ENSG00000064886	chromosome1	111571875
ENSG00000133048	chromosome1	201422375
ENSG00000133063	chromosome1	201465418
ENSG00000134216	chromosome1	111635007
ENSG00000203878	chromosome1	111626726
ENSG00000078898	chromosome20	31060042
ENSG00000186190	chromosome20	31106891
ENSG00000186191	chromosome20	31134416
ENSG00000167104	chromosome20	31083115
ENSG00000184459	chromosome22	31183374
ENSG00000129988	chromosome20	36408334
ENSG00000101425	chromosome20	36366028
ENSG00000100979	chromosome20	43973499
ENSG00000129988	chromosome20	36408334
ENSG00000101425	chromosome20	36366028
ENSG00000078898	chromosome20	31060042
ENSG00000186190	chromosome20	31106891
ENSG00000186191	chromosome20	31134416
ENSG00000167104	chromosome20	31083115
ENSG00000184459	chromosome22	31183374
ENSG00000078898	chromosome20	31060042
ENSG00000186190	chromosome20	31106891
ENSG00000186191	chromosome20	31134416
ENSG00000167104	chromosome20	31083115
ENSG00000184459	chromosome22	31183374
ENSG00000162775	chromosome1	110678039
ENSG00000179837	chromosome3	51403871
ENSG00000109079	chromosome17	23690675
ENSG00000174943	chromosome16	29844856
ENSG00000110906	chromosome12	108399451
ENSG00000110906	chromosome12	108399451
ENSG00000174943	chromosome16	29844856
ENSG00000119950	chromosome10	111957557
ENSG00000059728	chromosome2	69995967
ENSG00000059728	chromosome2	69995967
ENSG00000119950	chromosome10	111957557
ENSG00000123933	chromosome4	2233506
ENSG00000213347	chromosome5	176671438
ENSG00000123933	chromosome4	2233506
ENSG00000059728	chromosome2	69995967
ENSG00000119950	chromosome10	111957557
ENSG00000134744	chromosome1	52764541
ENSG00000083223	chromosome9	88157935
ENSG00000076351	chromosome17	23757260
ENSG00000139508	chromosome13	28190136
ENSG00000119457	chromosome9	114692783
ENSG00000119457	chromosome9	114692783

ENSG00000139508	chromosome13	28190136
ENSG00000076351	chromosome17	23757260
ENSG00000064989	chromosome2	187958601
ENSG00000106018	chromosome7	158630225
ENSG00000114812	chromosome3	42519232
ENSG00000106128	chromosome7	30970209
ENSG00000078549	chromosome7	31069406
ENSG00000120088	chromosome17	41217674
ENSG00000106113	chromosome7	30706175
ENSG00000064989	chromosome2	187958601
ENSG00000004948	chromosome7	92954230
ENSG00000160801	chromosome3	46900054
ENSG00000144407	chromosome2	208980014
ENSG00000112164	chromosome6	39124595
ENSG00000010310	chromosome19	50864619
ENSG00000215644	supercontigNT_113944	62491
ENSG00000065325	chromosome17	9670106
ENSG00000010310	chromosome19	50864619
ENSG00000010310	chromosome19	50864619
ENSG00000215644	supercontigNT_113944	62491
ENSG00000112164	chromosome6	39124595
ENSG00000160801	chromosome3	46900054
ENSG00000106128	chromosome7	30970209
ENSG00000106018	chromosome7	158630225
ENSG00000114812	chromosome3	42519232
ENSG00000106128	chromosome7	30970209
ENSG00000114812	chromosome3	42519232
ENSG00000106018	chromosome7	158630225
ENSG00000078549	chromosome7	31069406
ENSG00000106018	chromosome7	158630225
ENSG00000114812	chromosome3	42519232
ENSG00000106128	chromosome7	30970209
ENSG00000080293	chromosome2	119998273
ENSG00000215644	supercontigNT_113944	62491
ENSG00000010310	chromosome19	50864619
ENSG00000108813	chromosome17	45401832
ENSG00000006377	chromosome7	96473226
ENSG00000167034	chromosome8	23596348
ENSG00000006377	chromosome7	96473226
ENSG00000108813	chromosome17	45401832
ENSG00000144355	chromosome2	172658652
ENSG00000105971	chromosome7	115926597
ENSG00000105974	chromosome7	115952353
ENSG00000182533	chromosome3	8750428
ENSG00000105971	chromosome7	115926597
ENSG00000182533	chromosome3	8750428
ENSG00000105974	chromosome7	115952353
ENSG00000182533	chromosome3	8750428
ENSG00000105974	chromosome7	115952353
ENSG00000082556	chromosome8	54326151
ENSG00000116329	chromosome1	29011483
ENSG00000112038	chromosome6	154402187
ENSG00000166573	chromosome18	73091493
ENSG00000152034	chromosome6	100510745
ENSG00000128285	chromosome22	39405396
ENSG00000128310	chromosome22	36549360
ENSG00000182687	chromosome17	71582560
ENSG00000152034	chromosome6	100510745
ENSG00000166573	chromosome18	73091493

ENSG00000128310	chromosome22	36549360
ENSG00000182687	chromosome17	71582560
ENSG00000183473	chromosome22	35933789
ENSG00000162009	chromosome16	1068870
ENSG00000116329	chromosome1	29011483
ENSG00000112038	chromosome6	154402187
ENSG00000082556	chromosome8	54326151
ENSG00000125510	chromosome20	62194518
ENSG00000125510	chromosome20	62194518
ENSG00000116329	chromosome1	29011483
ENSG00000112038	chromosome6	154402187
ENSG00000082556	chromosome8	54326151
ENSG00000125522	chromosome20	62208629
ENSG00000183729	chromosome8	54015021
ENSG00000112038	chromosome6	154402187
ENSG00000116329	chromosome1	29011483
ENSG00000180616	chromosome17	68677054
ENSG00000139874	chromosome14	37748346
ENSG00000132671	chromosome20	22964121
ENSG00000162009	chromosome16	1068870
ENSG00000183473	chromosome22	35933789
ENSG00000132671	chromosome20	22964121
ENSG00000139874	chromosome14	37748346
ENSG00000182687	chromosome17	71582560
ENSG00000128310	chromosome22	36549360
ENSG00000128285	chromosome22	39405396
ENSG00000152034	chromosome6	100510745
ENSG00000139874	chromosome14	37748346
ENSG00000132671	chromosome20	22964121
ENSG00000162009	chromosome16	1068870
ENSG00000183473	chromosome22	35933789
ENSG00000180616	chromosome17	68677054
ENSG00000125522	chromosome20	62208629
ENSG00000183729	chromosome8	54015021
ENSG00000125510	chromosome20	62194518
ENSG00000116329	chromosome1	29011483
ENSG00000112038	chromosome6	154402187
ENSG00000082556	chromosome8	54326151
ENSG00000183729	chromosome8	54015021
ENSG00000125522	chromosome20	62208629
ENSG00000180616	chromosome17	68677054
ENSG00000183729	chromosome8	54015021
ENSG00000162009	chromosome16	1068870
ENSG00000183473	chromosome22	35933789
ENSG00000139874	chromosome14	37748346
ENSG00000132671	chromosome20	22964121
ENSG00000125522	chromosome20	62208629
ENSG00000183729	chromosome8	54015021
ENSG00000125510	chromosome20	62194518
ENSG00000116329	chromosome1	29011483
ENSG00000112038	chromosome6	154402187
ENSG00000082556	chromosome8	54326151
ENSG00000139874	chromosome14	37748346
ENSG00000132671	chromosome20	22964121
ENSG00000162009	chromosome16	1068870
ENSG00000183473	chromosome22	35933789
ENSG00000180616	chromosome17	68677054
ENSG00000116014	chromosome19	868503
ENSG00000100441	chromosome14	23969777

ENSG00000102921	chromosome16	47201526
ENSG00000149289	chromosome11	109512670
ENSG00000102053	chromosomeX	64625440
ENSG00000102053	chromosomeX	64625440
ENSG00000149289	chromosome11	109512670
ENSG00000163874	chromosome1	37713685
ENSG00000178199	chromosome6	149837373
ENSG00000163874	chromosome1	37713685
ENSG00000102053	chromosomeX	64625440
ENSG00000149289	chromosome11	109512670
ENSG00000182872	chromosomeX	46891825
ENSG00000003756	chromosome3	50102872
ENSG00000004534	chromosome3	49975079
ENSG00000003756	chromosome3	50102872
ENSG00000182872	chromosomeX	46891825
ENSG00000116560	chromosome1	35431238
ENSG00000121390	chromosome13	19254898
ENSG00000147140	chromosomeX	70427213
ENSG00000121390	chromosome13	19254898
ENSG00000116560	chromosome1	35431238
ENSG00000142959	chromosome1	45025965
ENSG00000039987	chromosome19	12724407
ENSG00000167995	chromosome11	61474404
ENSG00000039987	chromosome19	12724407
ENSG00000142959	chromosome1	45025965
ENSG00000127325	chromosome12	68377846
ENSG00000039987	chromosome19	12724407
ENSG00000142959	chromosome1	45025965
ENSG00000167995	chromosome11	61474404
ENSG00000102003	chromosomeX	48943590
ENSG00000163630	chromosome3	63439181
ENSG00000143028	chromosome1	109810989
ENSG00000008282	chromosome7	105540212
ENSG00000163630	chromosome3	63439181
ENSG00000102003	chromosomeX	48943590
ENSG00000163630	chromosome3	63439181
ENSG00000102003	chromosomeX	48943590
ENSG00000008282	chromosome7	105540212
ENSG00000197381	chromosome21	45379069
ENSG00000185736	chromosome10	1769345
ENSG00000160710	chromosome1	152867099
ENSG00000140955	chromosome16	82782338
ENSG00000164113	chromosome4	123520675
ENSG00000164113	chromosome4	123520675
ENSG00000140955	chromosome16	82782338
ENSG00000148082	chromosome9	90983196
ENSG00000129946	chromosome19	411904
ENSG00000129946	chromosome19	411904
ENSG00000148082	chromosome9	90983196
ENSG00000160691	chromosome1	153209627
ENSG00000160691	chromosome1	153209627
ENSG00000129946	chromosome19	411904
ENSG00000148082	chromosome9	90983196
ENSG00000185634	chromosome15	47042505
ENSG00000126903	chromosomeX	153370474
ENSG00000205184	chromosome8	82769763
ENSG00000125255	chromosome13	102516601
ENSG00000145283	chromosome4	87989293
ENSG00000204291	chromosome9	100746165

ENSG00000182871	chromosome21	45649574
ENSG00000151062	chromosome12	1897901
ENSG00000157445	chromosome3	54131781
ENSG00000007402	chromosome3	50515859
ENSG00000153956	chromosome7	81910712
ENSG00000153956	chromosome7	81910712
ENSG00000007402	chromosome3	50515859
ENSG00000087088	chromosome19	54149998
ENSG00000143384	chromosome1	148818631
ENSG00000140379	chromosome15	78050517
ENSG00000171552	chromosome20	29773683
ENSG00000129473	chromosome14	22846817
ENSG00000171791	chromosome18	59136880
ENSG00000129473	chromosome14	22846817
ENSG00000171552	chromosome20	29773683
ENSG00000140379	chromosome15	78050517
ENSG00000143384	chromosome1	148818631
ENSG00000129473	chromosome14	22846817
ENSG00000171552	chromosome20	29773683
ENSG00000171791	chromosome18	59136880
ENSG00000150681	chromosome1	190394391
ENSG00000090104	chromosome1	190811546
ENSG00000127074	chromosome1	190880088
ENSG00000215825	chromosome1	190578790
ENSG00000116741	chromosome1	191044825
ENSG00000143248	chromosome1	161454009
ENSG00000135824	chromosome1	180911878
ENSG00000143333	chromosome1	180840018
ENSG00000090104	chromosome1	190811546
ENSG00000127074	chromosome1	190880088
ENSG00000215825	chromosome1	190578790
ENSG00000150681	chromosome1	190394391
ENSG00000147509	chromosome8	54927013
ENSG00000171700	chromosome20	62178684
ENSG00000143248	chromosome1	161454009
ENSG00000150681	chromosome1	190394391
ENSG00000090104	chromosome1	190811546
ENSG00000127074	chromosome1	190880088
ENSG00000215825	chromosome1	190578790
ENSG00000116741	chromosome1	191044825
ENSG00000143248	chromosome1	161454009
ENSG00000215825	chromosome1	190578790
ENSG00000090104	chromosome1	190811546
ENSG00000127074	chromosome1	190880088
ENSG00000171700	chromosome20	62178684
ENSG00000147509	chromosome8	54927013
ENSG00000091844	chromosome6	153406847
ENSG00000116741	chromosome1	191044825
ENSG00000150681	chromosome1	190394391
ENSG00000090104	chromosome1	190811546
ENSG00000127074	chromosome1	190880088
ENSG00000215825	chromosome1	190578790
ENSG00000135824	chromosome1	180911878
ENSG00000143333	chromosome1	180840018
ENSG00000150681	chromosome1	190394391
ENSG00000090104	chromosome1	190811546
ENSG00000127074	chromosome1	190880088
ENSG00000215825	chromosome1	190578790
ENSG00000116741	chromosome1	191044825

ENSG00000143248	chromosome1	161454009
ENSG00000117152	chromosome1	161305899
ENSG00000117152	chromosome1	161305899
ENSG00000135824	chromosome1	180911878
ENSG00000143333	chromosome1	180840018
ENSG00000150681	chromosome1	190394391
ENSG00000090104	chromosome1	190811546
ENSG00000127074	chromosome1	190880088
ENSG00000215825	chromosome1	190578790
ENSG00000116741	chromosome1	191044825
ENSG00000143248	chromosome1	161454009
ENSG00000117152	chromosome1	161305899
ENSG00000135824	chromosome1	180911878
ENSG00000143333	chromosome1	180840018
ENSG00000150681	chromosome1	190394391
ENSG00000090104	chromosome1	190811546
ENSG00000127074	chromosome1	190880088
ENSG00000215825	chromosome1	190578790
ENSG00000116741	chromosome1	191044825
ENSG00000143248	chromosome1	161454009
ENSG00000127074	chromosome1	190880088
ENSG00000090104	chromosome1	190811546
ENSG00000148908	chromosome10	121292141
ENSG00000128872	chromosome15	49845931
ENSG00000138594	chromosome15	49942374
ENSG00000163157	chromosome1	149413976
ENSG00000136842	chromosome9	99326292
ENSG00000163157	chromosome1	149413976
ENSG00000128872	chromosome15	49845931
ENSG00000138594	chromosome15	49942374
ENSG00000170807	chromosome7	123083155
ENSG00000163431	chromosome1	200182092
ENSG00000163431	chromosome1	200182092
ENSG00000170807	chromosome7	123083155
ENSG00000163380	chromosome3	69254228
ENSG00000137824	chromosome15	38834274
ENSG00000115841	chromosome2	38009925
ENSG00000107165	chromosome9	12683997
ENSG00000080166	chromosome13	93929511
ENSG00000167074	chromosome22	40107995
ENSG00000105516	chromosome19	53832064
ENSG00000108924	chromosome17	50697845
ENSG00000108924	chromosome17	50697845
ENSG00000105516	chromosome19	53832064
ENSG00000167074	chromosome22	40107995
ENSG00000196220	chromosome3	9265636
ENSG00000163486	chromosome1	204582821
ENSG00000196935	chromosome12	62524864
ENSG00000089820	chromosomeX	152844851
ENSG00000163486	chromosome1	204582821
ENSG00000196935	chromosome12	62524864
ENSG00000196220	chromosome3	9265636
ENSG00000196935	chromosome12	62524864
ENSG00000163486	chromosome1	204582821
ENSG00000169410	chromosome15	73658173
ENSG00000175354	chromosome18	12874309
ENSG00000196396	chromosome20	48560472
ENSG00000088179	chromosome2	120283900
ENSG00000070159	chromosome9	111265536

ENSG00000152104	chromosome1	212704770
ENSG00000070778	chromosome14	88086515
ENSG00000164659	chromosome7	86409333
ENSG00000116299	chromosome1	109458329
ENSG00000067840	chromosomeX	152748948
ENSG00000121440	chromosome3	73756667
ENSG00000165966	chromosome12	40118014
ENSG00000165966	chromosome12	40118014
ENSG00000121440	chromosome3	73756667
ENSG00000048991	chromosome2	136078886
ENSG00000179912	chromosome12	55990479
ENSG00000172995	chromosome3	35698248
ENSG00000179912	chromosome12	55990479
ENSG00000048991	chromosome2	136078886
ENSG00000172995	chromosome3	35698248
ENSG00000048991	chromosome2	136078886
ENSG00000179912	chromosome12	55990479
ENSG00000180613	chromosome4	54661269
ENSG00000169840	chromosome13	27264828
ENSG00000128652	chromosome2	176742089
ENSG00000105997	chromosome7	27116785
ENSG00000120093	chromosome17	43984836
ENSG00000105997	chromosome7	27116785
ENSG00000120093	chromosome17	43984836
ENSG00000128652	chromosome2	176742089
ENSG00000128652	chromosome2	176742089
ENSG00000105997	chromosome7	27116785
ENSG00000120093	chromosome17	43984836
ENSG00000106038	chromosome7	27249175
ENSG00000174279	chromosome2	176656751
ENSG00000120093	chromosome17	43984836
ENSG00000105997	chromosome7	27116785
ENSG00000128652	chromosome2	176742089
ENSG00000105997	chromosome7	27116785
ENSG00000120093	chromosome17	43984836
ENSG00000100320	chromosome22	34754420
ENSG00000078328	chromosome16	7323004
ENSG00000167281	chromosome17	74623429
ENSG00000167281	chromosome17	74623429
ENSG00000078328	chromosome16	7323004
ENSG00000139182	chromosome12	7174512
ENSG00000171603	chromosome1	9806379
ENSG00000158258	chromosome3	141136907
ENSG00000171603	chromosome1	9806379
ENSG00000139182	chromosome12	7174512
ENSG00000139546	chromosome12	52181460
ENSG00000180228	chromosome2	179024004
ENSG00000104067	chromosome15	27901524
ENSG00000119139	chromosome9	70979109
ENSG00000113391	chromosome5	93436213
ENSG00000175841	chromosome3	102723467
ENSG00000102837	chromosome13	52500973
ENSG00000215081	chromosome11	5500874
ENSG00000130558	chromosome9	137107374
ENSG00000118733	chromosome1	102234961
ENSG00000186417	chromosome15	49421174
ENSG00000102837	chromosome13	52500973
ENSG00000215081	chromosome11	5500874
ENSG00000105088	chromosome19	9908043

ENSG00000118733	chromosome1	102234961
ENSG00000130558	chromosome9	137107374
ENSG00000102837	chromosome13	52500973
ENSG00000215081	chromosome11	5500874
ENSG00000118733	chromosome1	102234961
ENSG00000130558	chromosome9	137107374
ENSG00000105088	chromosome19	9908043
ENSG00000126656	chromosome17	23716393
ENSG00000134438	chromosome18	55091419
ENSG00000173976	chromosome19	3722741
ENSG00000081913	chromosome18	58533897
ENSG00000040199	chromosome16	70306200
ENSG00000079308	chromosome2	218476610
ENSG00000136205	chromosome7	47445760
ENSG00000111077	chromosome12	51729195
ENSG00000136205	chromosome7	47445760
ENSG00000079308	chromosome2	218476610
ENSG00000111077	chromosome12	51729195
ENSG00000079308	chromosome2	218476610
ENSG00000136205	chromosome7	47445760
ENSG00000131746	chromosome17	35906204
ENSG00000136205	chromosome7	47445760
ENSG00000111879	chromosome6	119441164
ENSG00000047662	chromosome4	17392045
ENSG00000167065	chromosome22	29389991
ENSG00000189037	chromosomeX	44588323
ENSG00000161326	chromosome17	32946488
ENSG00000161326	chromosome17	32946488
ENSG00000167065	chromosome22	29389991
ENSG00000189037	chromosomeX	44588323
ENSG00000169062	chromosome13	114065217
ENSG00000125351	chromosomeX	118870920
ENSG00000162407	chromosome1	56817278
ENSG00000117598	chromosome1	99242993
ENSG00000148123	chromosome9	102987598
ENSG00000105520	chromosome19	11329350
ENSG00000148123	chromosome9	102987598
ENSG00000105520	chromosome19	11329350
ENSG00000117598	chromosome1	99242993
ENSG00000162407	chromosome1	56817278
ENSG00000117598	chromosome1	99242993
ENSG00000148123	chromosome9	102987598
ENSG00000105520	chromosome19	11329350
ENSG00000129951	chromosome19	772560
ENSG00000117600	chromosome1	99502594
ENSG00000117600	chromosome1	99502594
ENSG00000129951	chromosome19	772560
ENSG00000117598	chromosome1	99242993
ENSG00000141934	chromosome19	242337
ENSG00000067113	chromosome5	54866494
ENSG00000162407	chromosome1	56817278
ENSG00000067113	chromosome5	54866494
ENSG00000141934	chromosome19	242337
ENSG00000105520	chromosome19	11329350
ENSG00000148123	chromosome9	102987598
ENSG00000102383	chromosomeX	74659585
ENSG00000104219	chromosome8	17058412
ENSG00000180776	chromosome13	20931428
ENSG00000180776	chromosome13	20931428

ENSG00000104219	chromosome8	17058412
ENSG00000087338	chromosome2	69910529
ENSG00000163959	chromosome3	197427981
ENSG00000173200	chromosome3	123779271
ENSG00000182158	chromosome7	137336992
ENSG00000157613	chromosome11	46256239
ENSG00000118217	chromosome1	160002775
ENSG00000213676	chromosome6	32203963
ENSG00000143578	chromosome1	152207626
ENSG00000060566	chromosome19	4104745
ENSG00000107175	chromosome9	35722770
ENSG00000060566	chromosome19	4104745
ENSG00000143578	chromosome1	152207626
ENSG00000197321	chromosome10	29883994
ENSG00000103319	chromosome16	22144552
ENSG00000073331	chromosome4	113518383
ENSG00000182327	chromosome17	4639047
ENSG00000215792	chromosome1	1252154
ENSG00000215747	supercontigNT_113871	25736
ENSG00000153993	chromosome7	84589144
ENSG00000153993	chromosome7	84589144
ENSG00000170381	chromosome7	83115795
ENSG00000010319	chromosome3	52454084
ENSG00000012171	chromosome3	50281677
ENSG00000075213	chromosome7	83661839
ENSG00000170381	chromosome7	83115795
ENSG00000010319	chromosome3	52454084
ENSG00000012171	chromosome3	50281677
ENSG00000075213	chromosome7	83661839
ENSG00000153993	chromosome7	84589144
ENSG00000196189	chromosome1	154390994
ENSG00000185033	chromosome15	88545815
ENSG00000168758	chromosome2	96897351
ENSG00000095539	chromosome10	102722660
ENSG00000187764	chromosome9	91210192
ENSG00000092421	chromosome5	115868540
ENSG00000137872	chromosome15	45839288
ENSG00000143434	chromosome1	149381722
ENSG00000075213	chromosome7	83661839
ENSG00000012171	chromosome3	50281677
ENSG00000092421	chromosome5	115868540
ENSG00000095539	chromosome10	102722660
ENSG00000168758	chromosome2	96897351
ENSG00000187764	chromosome9	91210192
ENSG00000010319	chromosome3	52454084
ENSG00000170381	chromosome7	83115795
ENSG00000012171	chromosome3	50281677
ENSG00000075213	chromosome7	83661839
ENSG00000170381	chromosome7	83115795
ENSG00000010319	chromosome3	52454084
ENSG00000168758	chromosome2	96897351
ENSG00000095539	chromosome10	102722660
ENSG00000196189	chromosome1	154390994
ENSG00000185033	chromosome15	88545815
ENSG00000170381	chromosome7	83115795
ENSG00000010319	chromosome3	52454084
ENSG00000187764	chromosome9	91210192
ENSG00000196189	chromosome1	154390994
ENSG00000185033	chromosome15	88545815

ENSG00000168758	chromosome2	96897351
ENSG00000095539	chromosome10	102722660
ENSG00000185033	chromosome15	88545815
ENSG00000196189	chromosome1	154390994
ENSG00000143434	chromosome1	149381722
ENSG00000137872	chromosome15	45839288
ENSG00000102606	chromosome13	110565875
ENSG00000129675	chromosomeX	135690708
ENSG00000129675	chromosomeX	135690708
ENSG00000102606	chromosome13	110565875
ENSG00000187583	chromosome1	891775
ENSG00000108292	chromosome17	34115412
ENSG00000078403	chromosome10	21863580
ENSG00000100425	chromosome22	48603970
ENSG00000096070	chromosome6	36276078
ENSG00000102221	chromosomeX	46729240
ENSG00000077684	chromosome4	129972364
ENSG00000043143	chromosome5	133899447
ENSG00000096070	chromosome6	36276078
ENSG00000100425	chromosome22	48603970
ENSG00000156983	chromosome3	9750825
ENSG00000043143	chromosome5	133899447
ENSG00000077684	chromosome4	129972364
ENSG00000174606	chromosome1	211255637
ENSG00000013523	chromosome14	76348924
ENSG00000168269	chromosome5	169465540
ENSG00000125798	chromosome20	22512899
ENSG00000129514	chromosome14	37133929
ENSG00000170608	chromosome19	51059555
ENSG00000176692	chromosome16	85158443
ENSG00000054598	chromosome6	1555680
ENSG00000179772	chromosome20	29897007
ENSG00000176165	chromosome14	28306237
ENSG00000183770	chromosome3	140148255
ENSG00000184492	chromosome2	113973304
ENSG00000187559	chromosome9	70107688
ENSG00000204793	chromosome9	68491433
ENSG00000184659	chromosome9	69668720
ENSG00000204828	chromosome9	42708062
ENSG00000204779	chromosome9	69467804
ENSG00000170122	chromosome9	108120
ENSG00000186564	chromosome1	47676395
ENSG00000187140	chromosome1	63561318
ENSG00000176692	chromosome16	85158443
ENSG00000176692	chromosome16	85158443
ENSG00000176165	chromosome14	28306237
ENSG00000184492	chromosome2	113973304
ENSG00000187559	chromosome9	70107688
ENSG00000204793	chromosome9	68491433
ENSG00000184659	chromosome9	69668720
ENSG00000204828	chromosome9	42708062
ENSG00000204779	chromosome9	69467804
ENSG00000170122	chromosome9	108120
ENSG00000186564	chromosome1	47676395
ENSG00000187140	chromosome1	63561318
ENSG00000168269	chromosome5	169465540
ENSG00000214336	chromosome2	88533028
ENSG00000186766	chromosome10	129425504
ENSG00000206148	chromosome4	1387182

ENSG00000143032	chromosome1	90955341
ENSG00000143032	chromosome1	90955341
ENSG00000125492	chromosome9	134448006
ENSG00000143869	chromosome2	20730481
ENSG00000125965	chromosome20	33489123
ENSG00000156466	chromosome8	97242097
ENSG00000163217	chromosome2	68951995
ENSG00000128802	chromosome10	48036700
ENSG00000125965	chromosome20	33489123
ENSG00000143869	chromosome2	20730481
ENSG00000101144	chromosome20	55274586
ENSG00000153162	chromosome6	7672188
ENSG00000130385	chromosomeX	50670524
ENSG00000143869	chromosome2	20730481
ENSG00000125965	chromosome20	33489123
ENSG00000156466	chromosome8	97242097
ENSG00000153162	chromosome6	7672188
ENSG00000101144	chromosome20	55274586
ENSG00000112175	chromosome6	55847623
ENSG00000107623	chromosome10	48058717
ENSG00000152785	chromosome4	82171463
ENSG00000156466	chromosome8	97242097
ENSG00000143869	chromosome2	20730481
ENSG00000125965	chromosome20	33489123
ENSG00000112175	chromosome6	55847623
ENSG00000153162	chromosome6	7672188
ENSG00000101144	chromosome20	55274586
ENSG00000183682	chromosome1	39730251
ENSG00000116985	chromosome1	40026745
ENSG00000169136	chromosome19	55125920
ENSG00000128272	chromosome22	38247397
ENSG00000121005	chromosome8	76060778
ENSG00000103196	chromosome16	83429603
ENSG00000101074	chromosome20	42399212
ENSG00000137558	chromosome8	75900040
ENSG00000103196	chromosome16	83429603
ENSG00000121005	chromosome8	76060778
ENSG00000173401	chromosome12	74014776
ENSG00000180481	chromosome12	74071149
ENSG00000139278	chromosome12	74160928
ENSG00000137558	chromosome8	75900040
ENSG00000101074	chromosome20	42399212
ENSG00000103196	chromosome16	83429603
ENSG00000121005	chromosome8	76060778
ENSG00000101074	chromosome20	42399212
ENSG00000137558	chromosome8	75900040
ENSG00000104413	chromosome8	95722262
ENSG00000103067	chromosome16	66827450
ENSG00000132463	chromosome4	71924409
ENSG00000169813	chromosome10	43203339
ENSG00000169045	chromosome5	178982741
ENSG00000126945	chromosomeX	100553633
ENSG00000096746	chromosome10	69766985
ENSG00000096746	chromosome10	69766985
ENSG00000169813	chromosome10	43203339
ENSG00000169045	chromosome5	178982741
ENSG00000126945	chromosomeX	100553633
ENSG00000118939	chromosome13	75021958
ENSG00000154277	chromosome4	40953751

ENSG00000156869	chromosome1	99986913
ENSG00000134215	chromosome1	108309015
ENSG00000141968	chromosome19	6723819
ENSG00000141968	chromosome19	6723819
ENSG00000134215	chromosome1	108309015
ENSG00000160293	chromosome9	135847222
ENSG00000144063	chromosome2	110230659
ENSG00000147676	chromosome8	120289893
ENSG00000172005	chromosome2	95055265
ENSG00000170293	chromosome3	32255469
ENSG00000102934	chromosome16	55875954
ENSG00000170293	chromosome3	32255469
ENSG00000102934	chromosome16	55875954
ENSG00000144063	chromosome2	110230659
ENSG00000147676	chromosome8	120289893
ENSG00000172005	chromosome2	95055265
ENSG00000155254	chromosome10	99463624
ENSG00000147676	chromosome8	120289893
ENSG00000172005	chromosome2	95055265
ENSG00000144063	chromosome2	110230659
ENSG00000172005	chromosome2	95055265
ENSG00000147676	chromosome8	120289893
ENSG00000102934	chromosome16	55875954
ENSG00000170293	chromosome3	32255469
ENSG00000176887	chromosome2	5750305
ENSG00000124766	chromosome6	21702745
ENSG00000177732	chromosome20	254569
ENSG00000181449	chromosome3	182912843
ENSG00000134595	chromosomeX	139414892
ENSG00000182968	chromosome13	111769974
ENSG00000164736	chromosome8	55533252
ENSG00000203883	chromosome20	62151314
ENSG00000171056	chromosome8	10625354
ENSG00000134595	chromosomeX	139414892
ENSG00000182968	chromosome13	111769974
ENSG00000181449	chromosome3	182912843
ENSG00000125398	chromosome17	67629128
ENSG00000100146	chromosome22	36709738
ENSG00000005513	chromosome16	971924
ENSG00000125398	chromosome17	67629128
ENSG00000182968	chromosome13	111769974
ENSG00000100146	chromosome22	36709738
ENSG00000125398	chromosome17	67629128
ENSG00000005513	chromosome16	971924
ENSG00000125398	chromosome17	67629128
ENSG00000100146	chromosome22	36709738
ENSG00000124766	chromosome6	21702745
ENSG00000176887	chromosome2	5750305
ENSG00000100146	chromosome22	36709738
ENSG00000171056	chromosome8	10625354
ENSG00000203883	chromosome20	62151314
ENSG00000203883	chromosome20	62151314
ENSG00000171056	chromosome8	10625354
ENSG00000164736	chromosome8	55533252
ENSG00000182968	chromosome13	111769974
ENSG00000134595	chromosomeX	139414892
ENSG00000100146	chromosome22	36709738
ENSG00000181449	chromosome3	182912843
ENSG00000134595	chromosomeX	139414892

ENSG00000182968	chromosome13	111769974
ENSG00000129194	chromosome17	7433719
ENSG00000005513	chromosome16	971924
ENSG00000167535	chromosome12	47498980
ENSG00000182389	chromosome2	152663772
ENSG00000067191	chromosome17	34607275
ENSG00000165995	chromosome10	18469672
ENSG00000182389	chromosome2	152663772
ENSG00000167535	chromosome12	47498980
ENSG00000165995	chromosome10	18469672
ENSG00000067191	chromosome17	34607275
ENSG00000175832	chromosome17	38978512
ENSG00000006468	chromosome7	13995236
ENSG00000171656	chromosome3	187306352
ENSG00000134954	chromosome11	127948236
ENSG00000157557	chromosome21	39103829
ENSG00000006468	chromosome7	13995236
ENSG00000175832	chromosome17	38978512
ENSG00000006468	chromosome7	13995236
ENSG00000157557	chromosome21	39103829
ENSG00000134954	chromosome11	127948236
ENSG00000198060	chromosome10	94041232
ENSG00000148204	chromosome9	125158361
ENSG00000148204	chromosome9	125158361
ENSG00000148204	chromosome9	125158361
ENSG00000060709	chromosome12	129529512
ENSG00000183246	chromosome22	20234984
ENSG00000196934	chromosome22	20068430
ENSG00000196622	chromosome22	18841020
ENSG00000005379	chromosome17	53760281
ENSG00000108551	chromosome17	17340221
ENSG00000100302	chromosome22	34272803
ENSG00000131914	chromosome1	26609970
ENSG00000187772	chromosome6	105511741
ENSG00000131914	chromosome1	26609970
ENSG00000127084	chromosome9	94778360
ENSG00000139132	chromosome12	32620559
ENSG00000127084	chromosome9	94778360
ENSG00000146192	chromosome6	37081572
ENSG00000127084	chromosome9	94778360
ENSG00000139132	chromosome12	32620559
ENSG00000134775	chromosome18	32131797
ENSG00000135723	chromosome16	65838815
ENSG00000133110	chromosome13	37070864
ENSG00000120708	chromosome5	135392644
ENSG00000148396	chromosome9	138491889
ENSG00000120341	chromosome1	176203740
ENSG00000119231	chromosome3	198096450
ENSG00000161956	chromosome17	7407118
ENSG00000079387	chromosome12	46785504
ENSG00000163904	chromosome3	186786896
ENSG00000057704	chromosome12	93568338
ENSG00000133069	chromosome1	203464316
ENSG00000172765	chromosome3	131029912
ENSG00000133069	chromosome1	203464316
ENSG00000172765	chromosome3	131029912
ENSG00000057704	chromosome12	93568338
ENSG00000185254	chromosomeX	153173665
ENSG00000182242	chromosomeX	153135862

ENSG00000102080	chromosomeX	153098741
ENSG00000172765	chromosome3	131029912
ENSG00000133069	chromosome1	203464316
ENSG00000112033	chromosome6	35486843
ENSG00000186951	chromosome22	44972945
ENSG00000132170	chromosome3	12368092
ENSG00000186951	chromosome22	44972945
ENSG00000112033	chromosome6	35486843
ENSG00000111424	chromosome12	46559164
ENSG00000144852	chromosome3	120984295
ENSG00000143257	chromosome1	159474475
ENSG00000077782	chromosome8	38434122
ENSG00000066468	chromosome10	123343379
ENSG00000066468	chromosome10	123343379
ENSG00000077782	chromosome8	38434122
ENSG00000160867	chromosome5	176449210
ENSG00000068078	chromosome4	1764832
ENSG00000068078	chromosome4	1764832
ENSG00000160867	chromosome5	176449210
ENSG00000106004	chromosome7	27149752
ENSG00000120075	chromosome17	44026044
ENSG00000106006	chromosome7	27153894
ENSG00000108511	chromosome17	44030512
ENSG00000120075	chromosome17	44026044
ENSG00000172789	chromosome12	52713174
ENSG00000106004	chromosome7	27149752
ENSG00000120075	chromosome17	44026044
ENSG00000182742	chromosome17	44010681
ENSG00000170166	chromosome2	176724608
ENSG00000198353	chromosome12	52733974
ENSG00000108511	chromosome17	44030512
ENSG00000106006	chromosome7	27153894
ENSG00000197757	chromosome12	52708573
ENSG00000120087	chromosome17	44043280
ENSG00000122592	chromosome7	27162690
ENSG00000106004	chromosome7	27149752
ENSG00000120075	chromosome17	44026044
ENSG00000172789	chromosome12	52713174
ENSG00000106004	chromosome7	27149752
ENSG00000120075	chromosome17	44026044
ENSG00000170166	chromosome2	176724608
ENSG00000198353	chromosome12	52733974
ENSG00000182742	chromosome17	44010681
ENSG00000197576	chromosome7	27136878
ENSG00000197576	chromosome7	27136878
ENSG00000170166	chromosome2	176724608
ENSG00000198353	chromosome12	52733974
ENSG00000182742	chromosome17	44010681
ENSG00000037965	chromosome12	52689336
ENSG00000175879	chromosome2	176703341
ENSG00000120068	chromosome17	44047066
ENSG00000120075	chromosome17	44026044
ENSG00000106004	chromosome7	27149752
ENSG00000120087	chromosome17	44043280
ENSG00000122592	chromosome7	27162690
ENSG00000120075	chromosome17	44026044
ENSG00000198353	chromosome12	52733974
ENSG00000170166	chromosome2	176724608
ENSG00000120068	chromosome17	44047066

ENSG00000175879	chromosome2	176703341
ENSG00000037965	chromosome12	52689336
ENSG00000161835	chromosome12	50687071
ENSG00000115165	chromosome2	158008779
ENSG00000087495	chromosome20	57585959
ENSG00000112137	chromosome6	12826963
ENSG00000204138	chromosome1	28606546
ENSG00000112419	chromosome6	143971140
ENSG00000112419	chromosome6	143971140
ENSG00000204138	chromosome1	28606546
ENSG00000112137	chromosome6	12826963
ENSG00000087495	chromosome20	57585959
ENSG00000165046	chromosome8	38369311
ENSG00000214906	chromosome8	100777550
ENSG00000168924	chromosome4	1827476
ENSG00000078081	chromosome3	184363138
ENSG00000129226	chromosome17	7423720
ENSG00000005893	chromosomeX	119487053
ENSG00000185896	chromosome13	112999751
ENSG00000122180	chromosome1	201321713
ENSG00000111046	chromosome12	79625630
ENSG00000129152	chromosome11	17697906
ENSG00000111049	chromosome12	79634974
ENSG00000156298	chromosomeX	38305744
ENSG00000156298	chromosomeX	38305744
ENSG00000140391	chromosome15	75150352
ENSG00000140391	chromosome15	75150352
ENSG00000156298	chromosomeX	38305744
ENSG00000180549	chromosome9	139046265
ENSG00000130383	chromosome19	5818737
ENSG00000156413	chromosome19	5783579
ENSG00000171124	chromosome19	5795851
ENSG00000196371	chromosome11	93916948
ENSG00000172461	chromosome6	96757753
ENSG00000172461	chromosome6	96757753
ENSG00000196371	chromosome11	93916948
ENSG00000196371	chromosome11	93916948
ENSG00000172461	chromosome6	96757753
ENSG00000130383	chromosome19	5818737
ENSG00000156413	chromosome19	5783579
ENSG00000171124	chromosome19	5795851
ENSG00000150594	chromosome10	112827790
ENSG00000181210	chromosome2	96145616
ENSG00000181210	chromosome2	96145616
ENSG00000150594	chromosome10	112827790
ENSG00000184845	chromosome5	174802709
ENSG00000184303	chromosome2	91237637
ENSG00000164270	chromosome5	147996771
ENSG00000181210	chromosome2	96145616
ENSG00000178394	chromosome5	63293303
ENSG00000168830	chromosome6	87781772
ENSG00000179097	chromosome3	88122590
ENSG00000179546	chromosome1	23393300
ENSG00000135312	chromosome6	78229840
ENSG00000135914	chromosome2	231696723
ENSG00000102468	chromosome13	46368043
ENSG00000147246	chromosomeX	113867602
ENSG00000169252	chromosome5	148186588
ENSG00000043591	chromosome10	115793882

ENSG00000184845	chromosome5	174802709
ENSG00000184845	chromosome5	174802709
ENSG00000147246	chromosomeX	113867602
ENSG00000102468	chromosome13	46368043
ENSG00000164270	chromosome5	147996771
ENSG00000113749	chromosome5	175042843
ENSG00000184303	chromosome2	91237637
ENSG00000184845	chromosome5	174802709
ENSG00000135312	chromosome6	78229840
ENSG00000179546	chromosome1	23393300
ENSG00000043591	chromosome10	115793882
ENSG00000169252	chromosome5	148186588
ENSG00000188778	chromosome8	37943145
ENSG00000179097	chromosome3	88122590
ENSG00000168830	chromosome6	87781772
ENSG00000102468	chromosome13	46368043
ENSG00000147246	chromosomeX	113867602
ENSG00000135914	chromosome2	231696723
ENSG00000147246	chromosomeX	113867602
ENSG00000179546	chromosome1	23393300
ENSG00000135312	chromosome6	78229840
ENSG00000168830	chromosome6	87781772
ENSG00000179097	chromosome3	88122590
ENSG00000113749	chromosome5	175042843
ENSG00000184303	chromosome2	91237637
ENSG00000184845	chromosome5	174802709
ENSG00000164270	chromosome5	147996771
ENSG00000184303	chromosome2	91237637
ENSG00000184845	chromosome5	174802709
ENSG00000113749	chromosome5	175042843
ENSG00000184845	chromosome5	174802709
ENSG00000142632	chromosome1	16408137
ENSG00000066248	chromosome2	233547845
ENSG00000142632	chromosome1	16408137
ENSG00000142632	chromosome1	16408137
ENSG00000198844	chromosome17	8156083
ENSG00000066248	chromosome2	233547845
ENSG00000142632	chromosome1	16408137
ENSG00000114790	chromosome3	155322472
ENSG00000130762	chromosome1	3369509
ENSG00000066248	chromosome2	233547845
ENSG00000142632	chromosome1	16408137
ENSG00000198844	chromosome17	8156083
ENSG00000130762	chromosome1	3369509
ENSG00000114790	chromosome3	155322472
ENSG00000078018	chromosome2	210226140
ENSG00000186868	chromosome17	41395540
ENSG00000186868	chromosome17	41395540
ENSG00000078018	chromosome2	210226140
ENSG00000047849	chromosome3	48105454
ENSG00000165782	chromosome14	19999325
ENSG00000155099	chromosome8	92122154
ENSG00000123146	chromosome19	14353336
ENSG00000131355	chromosome19	14646583
ENSG00000127507	chromosome19	14750111
ENSG00000186629	chromosome19	6941810
ENSG00000174837	chromosome19	6838620
ENSG00000153292	chromosome6	47104757
ENSG00000164393	chromosome6	47732182

ENSG00000153294	chromosome6	47761559
ENSG00000069122	chromosome6	46982459
ENSG00000204818	chromosome9	44115538
ENSG00000173567	chromosome2	26422607
ENSG00000131355	chromosome19	14646583
ENSG00000127507	chromosome19	14750111
ENSG00000186629	chromosome19	6941810
ENSG00000174837	chromosome19	6838620
ENSG00000123146	chromosome19	14353336
ENSG00000162618	chromosome1	79247246
ENSG00000131355	chromosome19	14646583
ENSG00000127507	chromosome19	14750111
ENSG00000186629	chromosome19	6941810
ENSG00000174837	chromosome19	6838620
ENSG00000123146	chromosome19	14353336
ENSG00000153292	chromosome6	47104757
ENSG00000164393	chromosome6	47732182
ENSG00000153294	chromosome6	47761559
ENSG00000069122	chromosome6	46982459
ENSG00000204818	chromosome9	44115538
ENSG00000173567	chromosome2	26422607
ENSG00000173567	chromosome2	26422607
ENSG00000153292	chromosome6	47104757
ENSG00000164393	chromosome6	47732182
ENSG00000153294	chromosome6	47761559
ENSG00000069122	chromosome6	46982459
ENSG00000204818	chromosome9	44115538
ENSG00000162618	chromosome1	79247246
ENSG00000186648	chromosome14	23591203
ENSG00000159753	chromosome16	66236531
ENSG00000079691	chromosome6	25387287
ENSG00000159753	chromosome16	66236531
ENSG00000186648	chromosome14	23591203
ENSG00000102531	chromosome13	48478328
ENSG00000075420	chromosome3	173312964
ENSG00000102531	chromosome13	48478328
ENSG00000166341	chromosome11	6619421
ENSG00000083857	chromosome4	187867976
ENSG00000083857	chromosome4	187867976
ENSG00000086570	chromosome5	150928686
ENSG00000028277	chromosome19	47328404
ENSG00000143190	chromosome1	165565140
ENSG00000185668	chromosome1	38285003
ENSG00000196767	chromosomeX	82649989
ENSG00000198914	chromosome2	104838401
ENSG00000184486	chromosome6	99389471
ENSG00000152192	chromosome13	78075463
ENSG00000196767	chromosomeX	82649989
ENSG00000198914	chromosome2	104838401
ENSG00000185668	chromosome1	38285003
ENSG00000152192	chromosome13	78075463
ENSG00000091010	chromosome5	145698869
ENSG00000151615	chromosome4	147779743
ENSG00000184486	chromosome6	99389471
ENSG00000185668	chromosome1	38285003
ENSG00000196767	chromosomeX	82649989
ENSG00000198914	chromosome2	104838401
ENSG00000151615	chromosome4	147779743
ENSG00000091010	chromosome5	145698869

ENSG00000198914	chromosome2	104838401
ENSG00000196767	chromosomeX	82649989
ENSG00000124772	chromosome6	36915132
ENSG00000139117	chromosome12	37587450
ENSG00000178773	chromosome16	88169807
ENSG00000100884	chromosome14	23611986
ENSG00000139117	chromosome12	37587450
ENSG00000124772	chromosome6	36915132
ENSG00000144550	chromosome3	9720681
ENSG00000214078	chromosome20	33684260
ENSG00000085719	chromosome8	87609890
ENSG00000140848	chromosome16	55702156
ENSG00000085719	chromosome8	87609890
ENSG00000214078	chromosome20	33684260
ENSG00000196353	chromosome3	133106978
ENSG00000100884	chromosome14	23611986
ENSG00000178773	chromosome16	88169807
ENSG00000167434	chromosome17	55582178
ENSG00000215487	chromosome22	20040557
ENSG00000215501	chromosome22	18871531
ENSG00000215543	chromosome22	17402085
ENSG00000131686	chromosome1	8928533
ENSG00000107159	chromosome9	35663957
ENSG00000118298	chromosome1	148497148
ENSG00000074410	chromosome15	61460973
ENSG00000168748	chromosome16	65435892
ENSG00000174990	chromosome16	86527558
ENSG00000214729	chromosome16	29555244
ENSG00000214873	chromosome16	21469334
ENSG00000169239	chromosomeX	15678068
ENSG00000118298	chromosome1	148497148
ENSG00000074410	chromosome15	61460973
ENSG00000107159	chromosome9	35663957
ENSG00000074410	chromosome15	61460973
ENSG00000118298	chromosome1	148497148
ENSG00000167434	chromosome17	55582178
ENSG00000215487	chromosome22	20040557
ENSG00000215501	chromosome22	18871531
ENSG00000215543	chromosome22	17402085
ENSG00000167434	chromosome17	55582178
ENSG00000185015	chromosome8	86345310
ENSG00000133742	chromosome8	86441117
ENSG00000104267	chromosome8	86563563
ENSG00000164879	chromosome8	86538391
ENSG00000174990	chromosome16	86527558
ENSG00000214729	chromosome16	29555244
ENSG00000214873	chromosome16	21469334
ENSG00000169239	chromosomeX	15678068
ENSG00000168748	chromosome16	65435892
ENSG00000111605	chromosome12	67919694
ENSG00000149532	chromosome11	60953284
ENSG00000158156	chromosome1	28159168
ENSG00000198669	chromosome8	71755848
ENSG00000171044	chromosome8	11096259
ENSG00000101321	chromosome20	30019640
ENSG00000101321	chromosome20	30019640
ENSG00000171044	chromosome8	11096259
ENSG00000206579	chromosome8	56177603
ENSG00000198669	chromosome8	71755848

ENSG00000101474	chromosome20	24921325
ENSG00000188599	chromosome16	15132797
ENSG00000183458	chromosome16	16328243
ENSG00000183793	chromosome16	14930771
ENSG00000008710	chromosome16	2125692
ENSG00000130943	chromosome22	45037884
ENSG00000166473	chromosome16	79811477
ENSG00000187008	chromosome16	70591379
ENSG00000187008	chromosome16	70591379
ENSG00000166473	chromosome16	79811477
ENSG00000158683	chromosome7	47954563
ENSG00000130943	chromosome22	45037884
ENSG00000188599	chromosome16	15132797
ENSG00000183458	chromosome16	16328243
ENSG00000183793	chromosome16	14930771
ENSG00000008710	chromosome16	2125692
ENSG00000008710	chromosome16	2125692
ENSG00000188599	chromosome16	15132797
ENSG00000183458	chromosome16	16328243
ENSG00000183793	chromosome16	14930771
ENSG00000008710	chromosome16	2125692
ENSG00000008710	chromosome16	2125692
ENSG00000130943	chromosome22	45037884
ENSG00000188599	chromosome16	15132797
ENSG00000183458	chromosome16	16328243
ENSG00000183793	chromosome16	14930771
ENSG00000008710	chromosome16	2125692
ENSG00000158683	chromosome7	47954563
ENSG00000169933	chromosomeX	12067012
ENSG00000147234	chromosomeX	106652336
ENSG00000070601	chromosome9	37682639
ENSG00000070601	chromosome9	37682639
ENSG00000147234	chromosomeX	106652336
ENSG00000169933	chromosomeX	12067012
ENSG00000185513	chromosome20	41576599
ENSG00000198945	chromosome6	130405538
ENSG00000154655	chromosome18	6301625
ENSG00000011258	chromosome17	46657522
ENSG00000100395	chromosome22	39931313
ENSG00000198879	chromosome10	7463867
ENSG00000163935	chromosome3	52978185
ENSG00000198945	chromosome6	130405538
ENSG00000198945	chromosome6	130405538
ENSG00000185513	chromosome20	41576599
ENSG00000095464	chromosome10	95362473
ENSG00000132915	chromosome5	149304430
ENSG00000133256	chromosome4	609416
ENSG00000138735	chromosome4	120769275
ENSG00000133256	chromosome4	609416
ENSG00000132915	chromosome5	149304430
ENSG00000128655	chromosome2	178677437
ENSG00000184164	chromosome22	48698422
ENSG00000163703	chromosome3	9951123
ENSG00000006740	chromosome17	12633881
ENSG00000006740	chromosome17	12633881
ENSG00000100092	chromosome22	36365741
ENSG00000141384	chromosome18	22060896
ENSG00000130699	chromosome20	60074262
ENSG00000130699	chromosome20	60074262

ENSG00000141384	chromosome18	22060896
ENSG00000163950	chromosome4	1683714
ENSG00000057657	chromosome6	106642835
ENSG00000176083	chromosome1	26567605
ENSG00000135597	chromosome6	139310741
ENSG00000169891	chromosomeX	16874906
ENSG00000125814	chromosome20	23350040
ENSG00000105402	chromosome19	52710010
ENSG00000135048	chromosome9	73555110
ENSG00000103888	chromosome15	78953276
ENSG00000135048	chromosome9	73555110
ENSG00000117707	chromosome1	212236502
ENSG00000119608	chromosome14	74400291
ENSG00000169398	chromosome8	141988408
ENSG00000120899	chromosome8	27311019
ENSG00000177463	chromosome3	15020426
ENSG00000120798	chromosome12	93985292
ENSG00000054219	chromosome2	160469440
ENSG00000153246	chromosome2	160627161
ENSG00000011028	chromosome17	58058896
ENSG00000054219	chromosome2	160469440
ENSG00000153246	chromosome2	160627161
ENSG00000120586	chromosome10	18138461
ENSG00000183748	chromosome10	17891471
ENSG00000011028	chromosome17	58058896
ENSG00000054219	chromosome2	160469440
ENSG00000153246	chromosome2	160627161
ENSG00000153246	chromosome2	160627161
ENSG00000054219	chromosome2	160469440
ENSG00000165659	chromosome13	71338909
ENSG00000126733	chromosomeX	85290281
ENSG00000126733	chromosomeX	85290281
ENSG00000165659	chromosome13	71338909
ENSG00000126733	chromosomeX	85290281
ENSG00000165659	chromosome13	71338909
ENSG00000047648	chromosomeX	11592870
ENSG00000147256	chromosomeX	130020042
ENSG00000128487	chromosome17	19940557
ENSG00000128487	chromosome17	19940557
ENSG00000100014	chromosome22	23028164
ENSG00000183963	chromosome22	29809245
ENSG00000188176	chromosome17	4434650
ENSG00000214872	chromosome11	57066692
ENSG00000188176	chromosome17	4434650
ENSG00000183963	chromosome22	29809245
ENSG00000173406	chromosome1	57529291
ENSG00000153071	chromosome5	39430180
ENSG00000015285	chromosomeX	48427187
ENSG00000106299	chromosome7	123176025
ENSG00000104442	chromosome8	66702188
ENSG00000116260	chromosome1	178390659
ENSG00000165661	chromosome9	138277471
ENSG00000171680	chromosome1	6502159
ENSG00000008323	chromosome12	6291654
ENSG00000156687	chromosome8	35521508
ENSG00000113763	chromosome5	176170358
ENSG00000107731	chromosome10	72642749
ENSG00000182168	chromosome4	96689032
ENSG00000182168	chromosome4	96689032

ENSG00000107731	chromosome10	72642749
ENSG00000113763	chromosome5	176170358
ENSG00000107731	chromosome10	72642749
ENSG00000182168	chromosome4	96689032
ENSG00000156687	chromosome8	35521508
ENSG00000124602	chromosome6	41110792
ENSG00000107731	chromosome10	72642749
ENSG00000182168	chromosome4	96689032
ENSG00000113763	chromosome5	176170358
ENSG00000128610	chromosome7	121731728
ENSG00000153266	chromosome3	62333584
ENSG00000101445	chromosome20	36897983
ENSG00000160972	chromosome8	145693386
ENSG00000186205	chromosome1	219026910
ENSG00000117791	chromosome1	218988497
ENSG00000170820	chromosome2	49235061
ENSG00000138039	chromosome2	48836315
ENSG00000165409	chromosome14	80491778
ENSG00000138039	chromosome2	48836315
ENSG00000170820	chromosome2	49235061
ENSG00000205213	chromosome11	27450426
ENSG00000139292	chromosome12	70120128
ENSG00000133067	chromosome1	200429741
ENSG00000133067	chromosome1	200429741
ENSG00000139292	chromosome12	70120128
ENSG00000151276	chromosome3	65999024
ENSG00000081026	chromosome1	113735179
ENSG00000187391	chromosome7	78920573
ENSG00000187391	chromosome7	78920573
ENSG00000081026	chromosome1	113735179
ENSG00000081026	chromosome1	113735179
ENSG00000187391	chromosome7	78920573
ENSG00000151276	chromosome3	65999024
ENSG00000124215	chromosome20	57967177
ENSG00000124215	chromosome20	57967177
ENSG00000134755	chromosome18	26935933
ENSG00000134762	chromosome18	26876625
ENSG00000134765	chromosome18	26996556
ENSG00000134755	chromosome18	26935933
ENSG00000134762	chromosome18	26876625
ENSG00000134765	chromosome18	26996556
ENSG00000124215	chromosome20	57967177
ENSG00000129910	chromosome16	87765741
ENSG00000166589	chromosome16	65509470
ENSG00000079112	chromosome8	95276090
ENSG00000131089	chromosomeX	62921724
ENSG00000182957	chromosome13	23721837
ENSG00000136002	chromosome2	131405001
ENSG00000182957	chromosome13	23721837
ENSG00000131089	chromosomeX	62921724
ENSG00000145934	chromosome5	167235568
ENSG00000145934	chromosome5	167235568
ENSG00000149256	chromosome11	78458638
ENSG00000149256	chromosome11	78458638
ENSG00000145934	chromosome5	167235568
ENSG00000009694	chromosomeX	123925284
ENSG00000066933	chromosome15	70125959
ENSG00000099331	chromosome19	17073528
ENSG00000091536	chromosome17	17962840

ENSG00000145555	chromosome5	16936723
ENSG00000105877	chromosome7	21549389
ENSG00000171634	chromosome17	63252303
ENSG00000069188	chromosome17	69015333
ENSG00000146555	chromosome7	3307745
ENSG00000177103	chromosome11	117173185
ENSG00000171587	chromosome21	41140458
ENSG00000142449	chromosome19	8118365
ENSG00000138829	chromosome5	127901196
ENSG00000166147	chromosome15	46724259
ENSG00000138829	chromosome5	127901196
ENSG00000142449	chromosome19	8118365
ENSG00000111052	chromosome12	79855633
ENSG00000104863	chromosome19	54309474
ENSG00000148943	chromosome11	27484876
ENSG00000148943	chromosome11	27484876
ENSG00000104863	chromosome19	54309474
ENSG00000079482	chromosomeX	67569588
ENSG00000165895	chromosome11	100289393
ENSG00000145819	chromosome5	142130511
ENSG00000071205	chromosome4	148872903
ENSG00000165895	chromosome11	100289393
ENSG00000079482	chromosomeX	67569588
ENSG00000071205	chromosome4	148872903
ENSG00000145819	chromosome5	142130511
ENSG00000142197	chromosome21	36458902
ENSG00000083097	chromosome6	83863416
ENSG00000130787	chromosome12	121886051
ENSG00000127946	chromosome7	75206175
ENSG00000197565	chromosomeX	107569258
ENSG00000134871	chromosome13	109758252
ENSG00000081052	chromosome2	227720444
ENSG00000187498	chromosome13	109757376
ENSG00000188153	chromosomeX	107570012
ENSG00000134871	chromosome13	109758252
ENSG00000197565	chromosomeX	107569258
ENSG00000169031	chromosome2	227737687
ENSG00000188153	chromosomeX	107570012
ENSG00000187498	chromosome13	109757376
ENSG00000108510	chromosome17	57497349
ENSG00000123066	chromosome12	115199320
ENSG00000135636	chromosome2	71534637
ENSG00000138119	chromosome10	95231942
ENSG00000214272	chromosome2	96724997
ENSG00000214814	chromosome8	125037420
ENSG00000088340	chromosome20	33658815
ENSG00000115155	chromosome2	26634944
ENSG00000088340	chromosome20	33658815
ENSG00000214814	chromosome8	125037420
ENSG00000138119	chromosome10	95231942
ENSG00000135636	chromosome2	71534637
ENSG00000164796	chromosome8	114518260
ENSG00000183117	chromosome8	3258997
ENSG00000121904	chromosome1	34404002
ENSG00000183117	chromosome8	3258997
ENSG00000121904	chromosome1	34404002
ENSG00000164796	chromosome8	114518260
ENSG00000183117	chromosome8	3258997
ENSG00000121904	chromosome1	34404002

ENSG00000164796	chromosome8	114518260
ENSG00000121904	chromosome1	34404002
ENSG00000183117	chromosome8	3258997
ENSG00000183117	chromosome8	3258997
ENSG00000121904	chromosome1	34404002
ENSG00000164796	chromosome8	114518260
ENSG00000197859	chromosome9	135389684
ENSG00000215616	supercontigNT_113911	14306
ENSG00000143382	chromosome1	148791389
ENSG00000187720	chromosome15	69220921
ENSG00000197859	chromosome9	135389684
ENSG00000215616	supercontigNT_113911	14306
ENSG00000187720	chromosome15	69220921
ENSG00000143382	chromosome1	148791389
ENSG00000143382	chromosome1	148791389
ENSG00000100767	chromosome14	72776240
ENSG00000197859	chromosome9	135389684
ENSG00000215616	supercontigNT_113911	14306
ENSG00000183090	chromosome4	144841279
ENSG00000150893	chromosome13	38159482
ENSG00000150893	chromosome13	38159482
ENSG00000183090	chromosome4	144841279
ENSG00000173546	chromosome15	73792152
ENSG00000164946	chromosome9	14858976
ENSG00000164434	chromosome6	123142639
ENSG00000121769	chromosome1	31618449
ENSG00000143320	chromosome1	154941863
ENSG00000166426	chromosome15	76419826
ENSG00000114113	chromosome3	140677992
ENSG00000162444	chromosome1	9979849
ENSG00000114115	chromosome3	140741065
ENSG00000114113	chromosome3	140677992
ENSG00000121769	chromosome1	31618449
ENSG00000164434	chromosome6	123142639
ENSG00000197416	chromosome8	82606106
ENSG00000205186	chromosome8	82536314
ENSG00000170323	chromosome8	82557958
ENSG00000147588	chromosome8	82522183
ENSG00000164687	chromosome8	82355386
ENSG00000214004	chromosome7	151770663
ENSG00000164434	chromosome6	123142639
ENSG00000139194	chromosome12	7172639
ENSG00000114115	chromosome3	140741065
ENSG00000162444	chromosome1	9979849
ENSG00000114113	chromosome3	140677992
ENSG00000114115	chromosome3	140741065
ENSG00000139194	chromosome12	7172639
ENSG00000114113	chromosome3	140677992
ENSG00000108175	chromosome10	80631396
ENSG00000122515	chromosome7	44762374
ENSG00000214192	chromosome3	176919151
ENSG00000169139	chromosome8	49083568
ENSG00000169139	chromosome8	49083568
ENSG00000214192	chromosome3	176919151
ENSG00000104967	chromosome19	51168450
ENSG00000139910	chromosome14	26136483
ENSG00000167110	chromosome9	130078041
ENSG00000174450	chromosome15	21243422
ENSG00000197414	chromosome15	20287701

ENSG00000215405	chromosome15	19007038
ENSG00000140478	chromosome15	70734133
ENSG00000159289	chromosome15	72161904
ENSG00000167195	chromosome15	73337993
ENSG00000156363	chromosome15	80985698
ENSG00000184206	chromosome15	82695591
ENSG00000186322	chromosome15	82861021
ENSG00000188388	chromosome15	83584707
ENSG00000197092	supercontigNT_113927	55481
ENSG00000188532	supercontigNT_113924	71788
ENSG00000181984	chromosome15	19027812
ENSG00000215252	chromosome15	32615406
ENSG00000175265	chromosome15	32469185
ENSG00000153666	chromosome15	20806781
ENSG00000188626	chromosome15	26756511
ENSG00000214348	chromosome15	100139731
ENSG00000151514	chromosome18	74841263
ENSG00000101115	chromosome20	49852355
ENSG00000103449	chromosome16	49742654
ENSG00000101115	chromosome20	49852355
ENSG00000151514	chromosome18	74841263
ENSG00000101115	chromosome20	49852355
ENSG00000151514	chromosome18	74841263
ENSG00000103449	chromosome16	49742654
ENSG00000165821	chromosome14	21074896
ENSG00000078061	chromosomeX	47307311
ENSG00000132155	chromosome3	12635221
ENSG00000157764	chromosome7	140270973
ENSG00000132155	chromosome3	12635221
ENSG00000078061	chromosomeX	47307311
ENSG00000141068	chromosome17	22807797
ENSG00000171435	chromosome12	116777725
ENSG00000135638	chromosome2	72997321
ENSG00000135638	chromosome2	72997321
ENSG00000170370	chromosome10	119292769
ENSG00000124164	chromosome20	56397922
ENSG00000101558	chromosome18	9904254
ENSG00000132376	chromosome17	1366544
ENSG00000185133	chromosome22	29849001
ENSG00000204084	chromosome1	38184568
ENSG00000122126	chromosomeX	128502098
ENSG00000164953	chromosome8	94836319
ENSG00000178385	chromosome2	208574609
ENSG00000159266	chromosome17	60231414
ENSG00000141342	chromosome17	40915634
ENSG00000145016	chromosome3	198947988
ENSG00000102445	chromosome13	45850090
ENSG00000183579	chromosome22	27713064
ENSG00000108375	chromosome17	53847938
ENSG00000119669	chromosome14	76563889
ENSG00000168264	chromosome1	232811864
ENSG00000170604	chromosome19	51080873
ENSG00000170604	chromosome19	51080873
ENSG00000119669	chromosome14	76563889
ENSG00000168264	chromosome1	232811864
ENSG00000168264	chromosome1	232811864
ENSG00000119669	chromosome14	76563889
ENSG00000162419	chromosome1	28882712
ENSG00000101216	chromosome20	61721195

ENSG00000165794	chromosome14	20537446
ENSG00000143570	chromosome1	152201816
ENSG00000165794	chromosome14	20537446
ENSG00000135334	chromosome6	88468122
ENSG00000174574	chromosome1	39229640
ENSG00000122591	chromosome7	22997256
ENSG00000155744	chromosome2	201596978
ENSG00000137507	chromosome11	76054647
ENSG00000174004	chromosome3	197865808
ENSG00000137507	chromosome11	76054647
ENSG00000068323	chromosomeX	48787697
ENSG00000187098	chromosome3	69871439
ENSG00000187098	chromosome3	69871439
ENSG00000068323	chromosomeX	48787697
ENSG00000112561	chromosome6	41767131
ENSG00000112561	chromosome6	41767131
ENSG00000187098	chromosome3	69871439
ENSG00000068323	chromosomeX	48787697
ENSG00000105967	chromosome7	115411732
ENSG00000067208	chromosome1	93030540
ENSG00000142459	chromosome19	7817429
ENSG00000136111	chromosome13	74953905
ENSG00000065882	chromosome4	37580112
ENSG00000011454	chromosome9	124786651
ENSG00000152061	chromosome1	172454919
ENSG00000003147	chromosome7	8244576
ENSG00000163596	chromosome2	203401978
ENSG00000153002	chromosome3	150028301
ENSG00000163751	chromosome3	150065785
ENSG00000091704	chromosome7	129807598
ENSG00000158525	chromosome7	129773563
ENSG00000158525	chromosome7	129773563
ENSG00000091704	chromosome7	129807598
ENSG00000158516	chromosome7	129693964
ENSG00000128510	chromosome7	129720257
ENSG00000158516	chromosome7	129693964
ENSG00000128510	chromosome7	129720257
ENSG00000158525	chromosome7	129773563
ENSG00000091704	chromosome7	129807598
ENSG00000080618	chromosome13	45577146
ENSG00000163751	chromosome3	150065785
ENSG00000153002	chromosome3	150028301
ENSG00000165078	chromosome8	68820919
ENSG00000165078	chromosome8	68820919
ENSG00000163751	chromosome3	150065785
ENSG00000153002	chromosome3	150028301
ENSG00000144410	chromosome2	207512569
ENSG00000163751	chromosome3	150065785
ENSG00000153002	chromosome3	150028301
ENSG00000165078	chromosome8	68820919
ENSG00000080618	chromosome13	45577146
ENSG00000143401	chromosome1	148474759
ENSG00000139223	chromosome12	47152715
ENSG00000140350	chromosome15	66900139
ENSG00000184701	chromosome4	165338314
ENSG00000215332	chromosome22	43426379
ENSG00000136938	chromosome9	99785659
ENSG00000139223	chromosome12	47152715
ENSG00000140350	chromosome15	66900139

ENSG00000184701	chromosome4	165338314
ENSG00000215332	chromosome22	43426379
ENSG00000136938	chromosome9	99785659
ENSG00000215332	chromosome22	43426379
ENSG00000136938	chromosome9	99785659
ENSG00000139223	chromosome12	47152715
ENSG00000140350	chromosome15	66900139
ENSG00000184701	chromosome4	165338314
ENSG00000154118	chromosome16	86194254
ENSG00000092051	chromosome14	23116507
ENSG00000149596	chromosome20	42248760
ENSG00000104369	chromosome8	75396078
ENSG00000149596	chromosome20	42248760
ENSG00000149596	chromosome20	42248760
ENSG00000104369	chromosome8	75396078
ENSG00000092051	chromosome14	23116507
ENSG00000154118	chromosome16	86194254
ENSG00000180251	chromosome2	102456651
ENSG00000115616	chromosome2	102602740
ENSG00000066230	chromosome5	577438
ENSG00000135740	chromosome16	65840419
ENSG00000090020	chromosome1	27353413
ENSG00000180251	chromosome2	102456651
ENSG00000115616	chromosome2	102602740
ENSG00000198929	chromosome1	160306592
ENSG00000011007	chromosome1	23942581
ENSG00000183791	chromosome18	42810212
ENSG00000206182	chromosome18	42804297
ENSG00000206183	chromosome18	42798370
ENSG00000206181	chromosome18	42815634
ENSG00000084234	chromosome11	129445083
ENSG00000105290	chromosome19	41051379
ENSG00000142192	chromosome21	26464810
ENSG00000105290	chromosome19	41051379
ENSG00000084234	chromosome11	129445083
ENSG00000106392	chromosome7	7240476
ENSG00000113319	chromosome5	80292314
ENSG00000058335	chromosome15	77169896
ENSG00000107263	chromosome9	133605039
ENSG00000115904	chromosome2	39201068
ENSG00000100485	chromosome14	49767752
ENSG00000196132	chromosome20	62266497
ENSG00000186487	chromosome2	1962557
ENSG00000186487	chromosome2	1962557
ENSG00000196132	chromosome20	62266497
ENSG00000147488	chromosome8	53289371
ENSG00000138756	chromosome4	79916722
ENSG00000115977	chromosome2	69723677
ENSG00000105323	chromosome19	46462249
ENSG00000153187	chromosome1	243094233
ENSG00000214753	chromosome11	62251205
ENSG00000153187	chromosome1	243094233
ENSG00000105323	chromosome19	46462249
ENSG00000006047	chromosome17	7138544
ENSG00000214299	chromosome10	125741254
ENSG00000173616	chromosome7	105010940
ENSG00000065978	chromosome1	42920824
ENSG00000060138	chromosome12	10766978
ENSG00000060138	chromosome12	10766978

ENSG00000173616	chromosome7	105010940
ENSG00000065978	chromosome1	42920824
ENSG00000006047	chromosome17	7138544
ENSG00000214299	chromosome10	125741254
ENSG00000138942	chromosome22	29913081
ENSG00000205408	chromosome8	38577837
ENSG00000204308	chromosome6	32254267
ENSG00000167202	chromosome15	76133567
ENSG00000095383	chromosome9	100057645
ENSG00000177054	chromosome11	19095373
ENSG00000186908	chromosome12	75682148
ENSG00000169925	chromosome9	135908421
ENSG00000204256	chromosome6	33048654
ENSG00000141867	chromosome19	15244911
ENSG00000137948	chromosome1	92200864
ENSG00000137948	chromosome1	92200864
ENSG00000141867	chromosome19	15244911
ENSG00000204256	chromosome6	33048654
ENSG00000169925	chromosome9	135908421
ENSG00000132386	chromosome17	1616955
ENSG00000167711	chromosome17	1593113
ENSG00000149131	chromosome11	57122320
ENSG00000149131	chromosome11	57122320
ENSG00000132386	chromosome17	1616955
ENSG00000167711	chromosome17	1593113
ENSG00000167711	chromosome17	1593113
ENSG00000132386	chromosome17	1616955
ENSG00000102007	chromosomeX	48915292
ENSG00000166091	chromosome14	22916301
ENSG00000140931	chromosome16	65196210
ENSG00000091317	chromosome3	32519242
ENSG00000183723	chromosome16	65287930
ENSG00000140931	chromosome16	65196210
ENSG00000166091	chromosome14	22916301
ENSG00000164078	chromosome3	49916047
ENSG00000105976	chromosome7	116126375
ENSG00000167601	chromosome19	46417138
ENSG00000153208	chromosome2	112372784
ENSG00000214672	chromosome15	74340960
ENSG00000092445	chromosome15	39638748
ENSG00000214672	chromosome15	74340960
ENSG00000092445	chromosome15	39638748
ENSG00000153208	chromosome2	112372784
ENSG00000132510	chromosome17	7689598
ENSG00000183878	chromosomeY	11391420
ENSG00000147050	chromosomeX	44617742
ENSG00000100234	chromosome22	31527988
ENSG00000157150	chromosome3	12175341
ENSG00000035862	chromosome17	74432766
ENSG00000102265	chromosomeX	47326906
ENSG00000035862	chromosome17	74432766
ENSG00000157150	chromosome3	12175341
ENSG00000157150	chromosome3	12175341
ENSG00000035862	chromosome17	74432766
ENSG00000100234	chromosome22	31527988
ENSG00000110987	chromosome12	120944381
ENSG00000106635	chromosome7	72609845
ENSG00000099385	chromosome16	30812767
ENSG00000139433	chromosome12	108802563

ENSG00000113269	chromosome5	179431309
ENSG00000170153	chromosome4	142273413
ENSG00000163162	chromosome2	101291483
ENSG00000108523	chromosome17	4781829
ENSG00000105428	chromosome19	5406503
ENSG00000082996	chromosome3	151046504
ENSG00000105428	chromosome19	5406503
ENSG00000108523	chromosome17	4781829
ENSG00000163162	chromosome2	101291483
ENSG00000113269	chromosome5	179431309
ENSG00000170153	chromosome4	142273413
ENSG00000188050	chromosome7	122126209
ENSG00000133135	chromosomeX	105823889
ENSG00000170153	chromosome4	142273413
ENSG00000113269	chromosome5	179431309
ENSG00000159714	chromosome16	65999180
ENSG00000188818	chromosome5	903718
ENSG00000206077	chromosome5	809254
ENSG00000106070	chromosome7	50767514
ENSG00000115290	chromosome2	165186066
ENSG00000173166	chromosome2	204068322
ENSG00000077420	chromosome10	26821262
ENSG00000115290	chromosome2	165186066
ENSG00000106070	chromosome7	50767514
ENSG00000141738	chromosome17	35152081
ENSG00000165694	chromosomeX	131089554
ENSG00000163520	chromosome3	13586856
ENSG00000115380	chromosome2	56003080
ENSG00000172638	chromosome11	65396402
ENSG00000163520	chromosome3	13586856
ENSG00000077942	chromosome22	44277530
ENSG00000140092	chromosome14	91490500
ENSG00000172638	chromosome11	65396402
ENSG00000115380	chromosome2	56003080
ENSG00000077942	chromosome22	44277530
ENSG00000163520	chromosome3	13586856
ENSG00000119681	chromosome14	74148401
ENSG00000090006	chromosome19	45790912
ENSG00000049323	chromosome2	33025896
ENSG00000168056	chromosome11	65082007
ENSG00000090006	chromosome19	45790912
ENSG00000049323	chromosome2	33025896
ENSG00000119681	chromosome14	74148401
ENSG00000172638	chromosome11	65396402
ENSG00000115380	chromosome2	56003080
ENSG00000140092	chromosome14	91490500
ENSG00000049323	chromosome2	33025896
ENSG00000090006	chromosome19	45790912
ENSG00000090006	chromosome19	45790912
ENSG00000049323	chromosome2	33025896
ENSG00000119681	chromosome14	74148401
ENSG00000168056	chromosome11	65082007
ENSG00000128250	chromosome22	28164781
ENSG00000128276	chromosome22	31084059
ENSG00000128253	chromosome22	30928439
ENSG00000187574	chromosome19	60974983
ENSG00000188683	chromosome19	60964979
ENSG00000182805	chromosome6	112777604
ENSG00000179046	chromosome4	189263367

ENSG00000026950	chromosome6	26513771
ENSG00000111801	chromosome6	26551782
ENSG00000124549	chromosome6	26530326
ENSG00000112763	chromosome6	26566844
ENSG00000124508	chromosome6	26492029
ENSG00000124557	chromosome6	26609494
ENSG00000113303	chromosome5	180258917
ENSG00000168903	chromosome5	180348635
ENSG00000165810	chromosome5	180405096
ENSG00000214693	chromosome4	166804948
ENSG00000164010	chromosome1	43068707
ENSG00000204618	chromosome6	30151546
ENSG00000121236	chromosome11	5574704
ENSG00000132274	chromosome11	5674039
ENSG00000132256	chromosome11	5657984
ENSG00000204614	chromosome6	30212793
ENSG00000204616	chromosome6	30188562
ENSG00000121236	chromosome11	5574704
ENSG00000132274	chromosome11	5674039
ENSG00000132256	chromosome11	5657984
ENSG00000026950	chromosome6	26513771
ENSG00000111801	chromosome6	26551782
ENSG00000124549	chromosome6	26530326
ENSG00000112763	chromosome6	26566844
ENSG00000124508	chromosome6	26492029
ENSG00000124557	chromosome6	26609494
ENSG00000113303	chromosome5	180258917
ENSG00000168903	chromosome5	180348635
ENSG00000165810	chromosome5	180405096
ENSG00000214693	chromosome4	166804948
ENSG00000164010	chromosome1	43068707
ENSG00000204618	chromosome6	30151546
ENSG00000204618	chromosome6	30151546
ENSG00000026950	chromosome6	26513771
ENSG00000111801	chromosome6	26551782
ENSG00000124549	chromosome6	26530326
ENSG00000112763	chromosome6	26566844
ENSG00000124508	chromosome6	26492029
ENSG00000124557	chromosome6	26609494
ENSG00000113303	chromosome5	180258917
ENSG00000168903	chromosome5	180348635
ENSG00000165810	chromosome5	180405096
ENSG00000214693	chromosome4	166804948
ENSG00000164010	chromosome1	43068707
ENSG00000176422	chromosome12	55148643
ENSG00000121236	chromosome11	5574704
ENSG00000132274	chromosome11	5674039
ENSG00000132256	chromosome11	5657984
ENSG00000026950	chromosome6	26513771
ENSG00000111801	chromosome6	26551782
ENSG00000124549	chromosome6	26530326
ENSG00000112763	chromosome6	26566844
ENSG00000124508	chromosome6	26492029
ENSG00000124557	chromosome6	26609494
ENSG00000113303	chromosome5	180258917
ENSG00000168903	chromosome5	180348635
ENSG00000165810	chromosome5	180405096
ENSG00000214693	chromosome4	166804948
ENSG00000164010	chromosome1	43068707

ENSG00000204618	chromosome6	30151546
ENSG00000121236	chromosome11	5574704
ENSG00000132274	chromosome11	5674039
ENSG00000132256	chromosome11	5657984
ENSG00000128250	chromosome22	28164781
ENSG00000128276	chromosome22	31084059
ENSG00000128253	chromosome22	30928439
ENSG00000187574	chromosome19	60974983
ENSG00000188683	chromosome19	60964979
ENSG00000182805	chromosome6	112777604
ENSG00000179046	chromosome4	189263367
ENSG00000204618	chromosome6	30151546
ENSG00000204614	chromosome6	30212793
ENSG00000204616	chromosome6	30188562
ENSG00000128250	chromosome22	28164781
ENSG00000128276	chromosome22	31084059
ENSG00000128253	chromosome22	30928439
ENSG00000187574	chromosome19	60974983
ENSG00000188683	chromosome19	60964979
ENSG00000182805	chromosome6	112777604
ENSG00000179046	chromosome4	189263367
ENSG00000026950	chromosome6	26513771
ENSG00000111801	chromosome6	26551782
ENSG00000124549	chromosome6	26530326
ENSG00000112763	chromosome6	26566844
ENSG00000124508	chromosome6	26492029
ENSG00000124557	chromosome6	26609494
ENSG00000113303	chromosome5	180258917
ENSG00000168903	chromosome5	180348635
ENSG00000165810	chromosome5	180405096
ENSG00000214693	chromosome4	166804948
ENSG00000164010	chromosome1	43068707
ENSG00000204618	chromosome6	30151546
ENSG00000121236	chromosome11	5574704
ENSG00000132274	chromosome11	5674039
ENSG00000132256	chromosome11	5657984
ENSG00000205198	chromosome4	166088681
ENSG00000183439	chromosome4	166110605
ENSG00000139155	chromosome12	20743778
ENSG00000176463	chromosome15	90198143
ENSG00000176463	chromosome15	90198143
ENSG00000139155	chromosome12	20743778
ENSG00000134538	chromosome12	21185776
ENSG00000111700	chromosome12	20859940
ENSG00000205754	chromosome12	21059834
ENSG00000084453	chromosome12	21378849
ENSG00000174640	chromosome3	135231337
ENSG00000137491	chromosome11	74540075
ENSG00000134538	chromosome12	21185776
ENSG00000111700	chromosome12	20859940
ENSG00000205754	chromosome12	21059834
ENSG00000084453	chromosome12	21378849
ENSG00000139155	chromosome12	20743778
ENSG00000145741	chromosome5	72830249
ENSG00000118903	chromosome13	76400201
ENSG00000197407	chromosome1	225688367
ENSG00000134717	chromosome1	52294604
ENSG00000214680	chromosome4	169756559
ENSG00000134717	chromosome1	52294604

ENSG00000214680	chromosome4	169756559
ENSG00000089693	chromosome12	6731762
ENSG00000178053	chromosome3	159771784
ENSG00000111907	chromosome6	125516797
ENSG00000170777	chromosome9	6318596
ENSG00000101150	chromosome20	61959531
ENSG00000076554	chromosome8	81155244
ENSG00000076554	chromosome8	81155244
ENSG00000170777	chromosome9	6318596
ENSG00000101150	chromosome20	61959531
ENSG00000066056	chromosome1	43539330
ENSG00000120156	chromosome9	27099589
ENSG00000146963	chromosome7	138695548
ENSG00000007392	chromosome16	219340
ENSG00000175175	chromosome17	54188358
ENSG00000100034	chromosome22	20630421
ENSG00000178700	chromosome3	95263046
ENSG00000188985	chromosome18	22005249
ENSG00000214575	chromosome15	81103804
ENSG00000107864	chromosome10	93990088
ENSG00000113742	chromosome5	173249343
ENSG00000137449	chromosome4	14614707
ENSG00000113742	chromosome5	173249343
ENSG00000107864	chromosome10	93990088
ENSG00000141622	chromosome18	42168236
ENSG00000186187	chromosome16	73591071
ENSG00000180233	chromosome7	30291229
ENSG00000186187	chromosome16	73591071
ENSG00000135870	chromosome1	172228747
ENSG00000056586	chromosome9	124699610
ENSG00000176915	chromosome12	131848458
ENSG00000105991	chromosome7	27102057
ENSG00000120094	chromosome17	43963266
ENSG00000128645	chromosome2	176761650
ENSG00000120094	chromosome17	43963266
ENSG00000105991	chromosome7	27102057
ENSG00000105996	chromosome7	27108645
ENSG00000173917	chromosome17	43977273
ENSG00000158711	chromosome1	203859634
ENSG00000111145	chromosome12	95141476
ENSG00000126767	chromosomeX	47394903
ENSG00000111145	chromosome12	95141476
ENSG00000158711	chromosome1	203859634
ENSG00000133612	chromosome7	150414762
ENSG00000174194	chromosome10	50916416
ENSG00000188234	chromosome10	45662802
ENSG00000204169	chromosome10	51156208
ENSG00000204149	chromosome10	51418482
ENSG00000172650	chromosome10	75127520
ENSG00000198035	chromosome10	47828502
ENSG00000204172	chromosome10	46640862
ENSG00000151303	chromosome10	88751319
ENSG00000157985	chromosome2	236068070
ENSG00000139436	chromosome12	108918413
ENSG00000135439	chromosome12	56422122
ENSG00000174194	chromosome10	50916416
ENSG00000188234	chromosome10	45662802
ENSG00000204169	chromosome10	51156208
ENSG00000204149	chromosome10	51418482

ENSG00000172650	chromosome10	75127520
ENSG00000198035	chromosome10	47828502
ENSG00000204172	chromosome10	46640862
ENSG00000151303	chromosome10	88751319
ENSG00000157985	chromosome2	236068070
ENSG00000133612	chromosome7	150414762
ENSG00000139436	chromosome12	108918413
ENSG00000108262	chromosome17	24940535
ENSG00000135916	chromosome2	231437985
ENSG00000078596	chromosomeX	78509369
ENSG00000136156	chromosome13	47705498
ENSG00000136156	chromosome13	47705498
ENSG00000078596	chromosomeX	78509369
ENSG00000130751	chromosome19	52216145
ENSG00000151322	chromosome14	32478274
ENSG00000112246	chromosome6	101018066
ENSG00000159263	chromosome21	36993917
ENSG00000100644	chromosome14	61232276
ENSG00000124440	chromosome19	51492174
ENSG00000116016	chromosome2	46378555
ENSG00000124440	chromosome19	51492174
ENSG00000100644	chromosome14	61232276
ENSG00000159263	chromosome21	36993917
ENSG00000112246	chromosome6	101018066
ENSG00000116016	chromosome2	46378555
ENSG00000196104	chromosome4	168391900
ENSG00000152377	chromosome5	136862147
ENSG00000107742	chromosome10	73518093
ENSG00000152377	chromosome5	136862147
ENSG00000196104	chromosome4	168391900
ENSG00000019582	chromosome5	149772506
ENSG00000090674	chromosome19	7493637
ENSG00000055732	chromosome1	85283632
ENSG00000153898	chromosome1	85235144
ENSG00000055732	chromosome1	85283632
ENSG00000090674	chromosome19	7493637
ENSG00000161958	chromosome17	7283664
ENSG00000114279	chromosome3	193608707
ENSG00000102466	chromosome13	101852030
ENSG00000113578	chromosome5	141973877
ENSG00000138685	chromosome4	123967381
ENSG00000102466	chromosome13	101852030
ENSG00000114279	chromosome3	193608707
ENSG00000111241	chromosome12	4424998
ENSG00000075388	chromosome11	69299034
ENSG00000186895	chromosome11	69342639
ENSG00000185390	chromosome21	13643459
ENSG00000204837	chromosome9	41953938
ENSG00000215053	chromosome9	66284184
ENSG00000204801	chromosome9	46627626
ENSG00000204816	chromosome9	44285681
ENSG00000204817	chromosome9	44242234
ENSG00000140285	chromosome15	47503787
ENSG00000070193	chromosome5	44424542
ENSG00000070388	chromosome19	590926
ENSG00000114279	chromosome3	193608707
ENSG00000102466	chromosome13	101852030
ENSG00000161958	chromosome17	7283664
ENSG00000129682	chromosomeX	137894913

ENSG00000102678	chromosome13	21144052
ENSG00000078579	chromosome8	16903913
ENSG00000196468	chromosomeX	76596303
ENSG00000070193	chromosome5	44424542
ENSG00000185390	chromosome21	13643459
ENSG00000204837	chromosome9	41953938
ENSG00000215053	chromosome9	66284184
ENSG00000204801	chromosome9	46627626
ENSG00000204816	chromosome9	44285681
ENSG00000204817	chromosome9	44242234
ENSG00000140285	chromosome15	47503787
ENSG00000075388	chromosome11	69299034
ENSG00000111241	chromosome12	4424998
ENSG00000070388	chromosome19	590926
ENSG00000185390	chromosome21	13643459
ENSG00000204837	chromosome9	41953938
ENSG00000215053	chromosome9	66284184
ENSG00000204801	chromosome9	46627626
ENSG00000204816	chromosome9	44285681
ENSG00000204817	chromosome9	44242234
ENSG00000140285	chromosome15	47503787
ENSG00000070193	chromosome5	44424542
ENSG00000078579	chromosome8	16903913
ENSG00000102678	chromosome13	21144052
ENSG00000128709	chromosome2	176695773
ENSG00000180806	chromosome12	52680240
ENSG00000170689	chromosome17	44058631
ENSG00000078399	chromosome7	27175795
ENSG00000078399	chromosome7	27175795
ENSG00000170689	chromosome17	44058631
ENSG00000180818	chromosome12	52665311
ENSG00000128710	chromosome2	176689808
ENSG00000128709	chromosome2	176695773
ENSG00000180806	chromosome12	52680240
ENSG00000170689	chromosome17	44058631
ENSG00000078399	chromosome7	27175795
ENSG00000180818	chromosome12	52665311
ENSG00000128710	chromosome2	176689808
ENSG00000153807	chromosome7	27185800
ENSG00000128709	chromosome2	176695773
ENSG00000180806	chromosome12	52680240
ENSG00000170689	chromosome17	44058631
ENSG00000078399	chromosome7	27175795
ENSG00000180806	chromosome12	52680240
ENSG00000170689	chromosome17	44058631
ENSG00000078399	chromosome7	27175795
ENSG00000128709	chromosome2	176695773
ENSG00000128710	chromosome2	176689808
ENSG00000180818	chromosome12	52665311
ENSG00000153807	chromosome7	27185800
ENSG00000180818	chromosome12	52665311
ENSG00000128710	chromosome2	176689808
ENSG00000170689	chromosome17	44058631
ENSG00000078399	chromosome7	27175795
ENSG00000180806	chromosome12	52680240
ENSG00000143940	chromosome2	55034677
ENSG00000165521	chromosome14	88328601
ENSG00000143924	chromosome2	42250256
ENSG00000149499	chromosome11	62136506

ENSG00000125746	chromosome19	50834475
ENSG00000066629	chromosome14	99329567
ENSG00000149499	chromosome11	62136506
ENSG00000143924	chromosome2	42250256
ENSG00000066629	chromosome14	99329567
ENSG00000125746	chromosome19	50834475
ENSG00000173253	chromosome9	1041614
ENSG00000142700	chromosome1	50659571
ENSG00000176399	chromosome9	22437065
ENSG00000137090	chromosome9	831839
ENSG00000173253	chromosome9	1041614
ENSG00000162733	chromosome1	160955478
ENSG00000162733	chromosome1	160955478
ENSG00000204580	chromosome6	30964486
ENSG00000170421	chromosome12	51585033
ENSG00000215847	chromosome1	155309850
ENSG00000214752	chromosome5	122765898
ENSG00000214233	chromosome11	118979972
ENSG00000215287	chromosomeX	45377947
ENSG00000214477	chromosome3	87455273
ENSG00000214974	chromosome3	35233646
ENSG00000214323	chromosome16	76641059
ENSG00000167768	chromosome12	51360400
ENSG00000189182	chromosome12	51383486
ENSG00000172867	chromosome12	51332194
ENSG00000139648	chromosome12	51233129
ENSG00000186049	chromosome12	51298576
ENSG00000170486	chromosome12	51281504
ENSG00000170484	chromosome12	51253829
ENSG00000186442	chromosome12	51476094
ENSG00000185069	chromosome12	51457343
ENSG00000170423	chromosome12	51528982
ENSG00000170477	chromosome12	51494332
ENSG00000185640	chromosome12	51514312
ENSG00000170465	chromosome12	51153789
ENSG00000185479	chromosome12	51132130
ENSG00000205420	chromosome12	51173240
ENSG00000186081	chromosome12	51200348
ENSG00000170454	chromosome12	51114356
ENSG00000161849	chromosome12	51065637
ENSG00000161850	chromosome12	51086329
ENSG00000135443	chromosome12	51047457
ENSG00000135477	chromosome12	50938350
ENSG00000170442	chromosome12	50981968
ENSG00000205426	chromosome12	50971517
ENSG00000170523	chromosome12	51001387
ENSG00000135480	chromosome12	50913348
ENSG00000167767	chromosome12	50871954
ENSG00000170421	chromosome12	51585033
ENSG00000215847	chromosome1	155309850
ENSG00000214752	chromosome5	122765898
ENSG00000214233	chromosome11	118979972
ENSG00000215287	chromosomeX	45377947
ENSG00000214477	chromosome3	87455273
ENSG00000214974	chromosome3	35233646
ENSG00000214323	chromosome16	76641059
ENSG00000167768	chromosome12	51360400
ENSG00000189182	chromosome12	51383486
ENSG00000172867	chromosome12	51332194

ENSG00000139648	chromosome12	51233129
ENSG00000186049	chromosome12	51298576
ENSG00000170486	chromosome12	51281504
ENSG00000170484	chromosome12	51253829
ENSG00000186442	chromosome12	51476094
ENSG00000185069	chromosome12	51457343
ENSG00000170423	chromosome12	51528982
ENSG00000170477	chromosome12	51494332
ENSG00000185640	chromosome12	51514312
ENSG00000170465	chromosome12	51153789
ENSG00000185479	chromosome12	51132130
ENSG00000205420	chromosome12	51173240
ENSG00000186081	chromosome12	51200348
ENSG00000170454	chromosome12	51114356
ENSG00000161849	chromosome12	51065637
ENSG00000161850	chromosome12	51086329
ENSG00000135443	chromosome12	51047457
ENSG00000135477	chromosome12	50938350
ENSG00000170442	chromosome12	50981968
ENSG00000205426	chromosome12	50971517
ENSG00000170523	chromosome12	51001387
ENSG00000135480	chromosome12	50913348
ENSG00000167767	chromosome12	50871954
ENSG00000170421	chromosome12	51585033
ENSG00000215847	chromosome1	155309850
ENSG00000214752	chromosome5	122765898
ENSG00000214233	chromosome11	118979972
ENSG00000215287	chromosomeX	45377947
ENSG00000214477	chromosome3	87455273
ENSG00000214974	chromosome3	35233646
ENSG00000214323	chromosome16	76641059
ENSG00000167768	chromosome12	51360400
ENSG00000189182	chromosome12	51383486
ENSG00000172867	chromosome12	51332194
ENSG00000139648	chromosome12	51233129
ENSG00000186049	chromosome12	51298576
ENSG00000170486	chromosome12	51281504
ENSG00000170484	chromosome12	51253829
ENSG00000186442	chromosome12	51476094
ENSG00000185069	chromosome12	51457343
ENSG00000170423	chromosome12	51528982
ENSG00000170477	chromosome12	51494332
ENSG00000185640	chromosome12	51514312
ENSG00000170465	chromosome12	51153789
ENSG00000185479	chromosome12	51132130
ENSG00000205420	chromosome12	51173240
ENSG00000186081	chromosome12	51200348
ENSG00000170454	chromosome12	51114356
ENSG00000161849	chromosome12	51065637
ENSG00000161850	chromosome12	51086329
ENSG00000135443	chromosome12	51047457
ENSG00000135477	chromosome12	50938350
ENSG00000170442	chromosome12	50981968
ENSG00000205426	chromosome12	50971517
ENSG00000170523	chromosome12	51001387
ENSG00000135480	chromosome12	50913348
ENSG00000170421	chromosome12	51585033
ENSG00000215847	chromosome1	155309850
ENSG00000214752	chromosome5	122765898

ENSG00000214233	chromosome11	118979972
ENSG00000215287	chromosomeX	45377947
ENSG00000214477	chromosome3	87455273
ENSG00000214974	chromosome3	35233646
ENSG00000214323	chromosome16	76641059
ENSG00000167768	chromosome12	51360400
ENSG00000189182	chromosome12	51383486
ENSG00000172867	chromosome12	51332194
ENSG00000139648	chromosome12	51233129
ENSG00000186049	chromosome12	51298576
ENSG00000170486	chromosome12	51281504
ENSG00000170484	chromosome12	51253829
ENSG00000186442	chromosome12	51476094
ENSG00000185069	chromosome12	51457343
ENSG00000170423	chromosome12	51528982
ENSG00000170477	chromosome12	51494332
ENSG00000185640	chromosome12	51514312
ENSG00000170465	chromosome12	51153789
ENSG00000185479	chromosome12	51132130
ENSG00000205420	chromosome12	51173240
ENSG00000186081	chromosome12	51200348
ENSG00000170454	chromosome12	51114356
ENSG00000161849	chromosome12	51065637
ENSG00000161850	chromosome12	51086329
ENSG00000135443	chromosome12	51047457
ENSG00000135477	chromosome12	50938350
ENSG00000170442	chromosome12	50981968
ENSG00000205426	chromosome12	50971517
ENSG00000170523	chromosome12	51001387
ENSG00000135480	chromosome12	50913348
ENSG00000167767	chromosome12	50871954
ENSG00000155760	chromosome2	202607616
ENSG00000157240	chromosome7	90732132
ENSG00000180340	chromosome17	39990583
ENSG00000106483	chromosome7	37922665
ENSG00000162998	chromosome2	183439526
ENSG00000111432	chromosome12	129213441
ENSG00000188763	chromosome7	72486274
ENSG00000163251	chromosome2	208341709
ENSG00000177283	chromosome10	35970364
ENSG00000177283	chromosome10	35970364
ENSG00000162998	chromosome2	183439526
ENSG00000106483	chromosome7	37922665
ENSG00000177283	chromosome10	35970364
ENSG00000163251	chromosome2	208341709
ENSG00000104290	chromosome8	28416450
ENSG00000164930	chromosome8	104381512
ENSG00000180340	chromosome17	39990583
ENSG00000157240	chromosome7	90732132
ENSG00000138760	chromosome4	77353721
ENSG00000172164	chromosome8	121893265
ENSG00000168807	chromosome16	67778571
ENSG00000168807	chromosome16	67778571
ENSG00000172164	chromosome8	121893265
ENSG00000101400	chromosome20	31495088
ENSG00000147481	chromosome8	51469352
ENSG00000172554	chromosome2	936683
ENSG00000070423	chromosome19	614122
ENSG00000121848	chromosome1	144322597

ENSG00000168036	chromosome3	41227538
ENSG00000168036	chromosome3	41227538
ENSG00000173801	chromosome17	37181633
ENSG00000107140	chromosome9	35595617
ENSG00000070759	chromosome1	45696045
ENSG00000106683	chromosome7	73136257
ENSG00000182541	chromosome22	29938395
ENSG00000123091	chromosome1	51475017
ENSG00000206048	chromosome15	38403549
ENSG00000111404	chromosome12	18133485
ENSG00000122035	chromosome13	26743082
ENSG00000128045	chromosome4	53423432
ENSG00000122035	chromosome13	26743082
ENSG00000099204	chromosome10	116434103
ENSG00000158856	chromosome8	21980333
ENSG00000163995	chromosome4	8211443
ENSG00000173210	chromosome5	148501852
ENSG00000163995	chromosome4	8211443
ENSG00000173210	chromosome5	148501852
ENSG00000158856	chromosome8	21980333
ENSG00000173210	chromosome5	148501852
ENSG00000163995	chromosome4	8211443
ENSG00000158856	chromosome8	21980333
ENSG00000163072	chromosome2	169367403
ENSG00000138869	chromosome22	22440062
ENSG00000182699	chromosome8	55267168
ENSG00000106153	chromosome7	56141685
ENSG00000186940	chromosome9	81196039
ENSG00000160145	chromosome3	125296375
ENSG00000038382	chromosome5	14196835
ENSG00000038382	chromosome5	14196835
ENSG00000126217	chromosome13	112604518
ENSG00000053524	chromosome3	184628460
ENSG00000101977	chromosomeX	138601951
ENSG00000053524	chromosome3	184628460
ENSG00000126217	chromosome13	112604518
ENSG00000160145	chromosome3	125296375
ENSG00000179277	chromosome17	15630910
ENSG00000188013	chromosome17	20433198
ENSG00000105419	chromosome19	52614151
ENSG00000134138	chromosome15	35189741
ENSG00000134138	chromosome15	35189741
ENSG00000179277	chromosome17	15630910
ENSG00000188013	chromosome17	20433198
ENSG00000105419	chromosome19	52614151
ENSG00000177426	chromosome18	3437738
ENSG00000153779	chromosomeX	89063741
ENSG00000176679	chromosomeY	797766
ENSG00000118707	chromosome20	34640592
ENSG00000165495	chromosome11	124726412
ENSG00000160199	chromosome21	43297561
ENSG00000134138	chromosome15	35189741
ENSG00000148429	chromosome10	11679640
ENSG00000174115	chromosome17	33550663
ENSG00000197681	chromosome17	33421452
ENSG00000185128	chromosome17	33359927
ENSG00000213474	chromosome17	33600889
ENSG00000161583	chromosome17	31880632
ENSG00000189309	chromosome17	31526524

ENSG00000215625	supercontigNT_113936	142401
ENSG00000205019	chromosome17	31614568
ENSG00000188849	chromosome17	31829579
ENSG00000215748	supercontigNT_113935	25189
ENSG00000204471	chromosome17	55449642
ENSG00000188755	chromosome17	57706259
ENSG00000215730	supercontigNT_113935	44125
ENSG00000128438	chromosome17	16770162
ENSG00000161800	chromosome12	48696766
ENSG00000127152	chromosome14	98807309
ENSG00000119866	chromosome2	60634043
ENSG00000119866	chromosome2	60634043
ENSG00000127152	chromosome14	98807309
ENSG00000170684	chromosome19	50271472
ENSG00000134644	chromosome1	31311220
ENSG00000055917	chromosome2	20390603
ENSG00000146416	chromosome6	143423756
ENSG00000146416	chromosome6	143423756
ENSG00000111863	chromosome6	11886979
ENSG00000114854	chromosome3	52463072
ENSG00000114854	chromosome3	52463072
ENSG00000101470	chromosome20	43889295
ENSG00000004975	chromosome17	7078306
ENSG00000107404	chromosome1	1274309
ENSG00000215737	supercontigNT_113871	80092
ENSG00000161202	chromosome3	185356118
ENSG00000107404	chromosome1	1274309
ENSG00000215737	supercontigNT_113871	80092
ENSG00000161202	chromosome3	185356118
ENSG00000004975	chromosome17	7078306
ENSG00000161202	chromosome3	185356118
ENSG00000107404	chromosome1	1274309
ENSG00000215737	supercontigNT_113871	80092
ENSG00000205922	chromosome19	1704869
ENSG00000119547	chromosome18	53253947
ENSG00000119547	chromosome18	53253947
ENSG00000205922	chromosome19	1704869
ENSG00000169856	chromosome15	50869374
ENSG00000111249	chromosome12	109956365
ENSG00000160967	chromosome7	101246031
ENSG00000169856	chromosome15	50869374
ENSG00000119547	chromosome18	53253947
ENSG00000205922	chromosome19	1704869
ENSG00000167037	chromosome22	23559391
ENSG00000141258	chromosome17	2187733
ENSG00000198450	chromosome3	131761902
ENSG00000205221	chromosome2	36797018
ENSG00000100473	chromosome14	30413896
ENSG00000163359	chromosome2	237970200
ENSG00000198450	chromosome3	131761902
ENSG00000198450	chromosome3	131761902
ENSG00000163359	chromosome2	237970200
ENSG00000165816	chromosome10	115998463
ENSG00000099822	chromosome19	540946
ENSG00000164588	chromosome5	45731953
ENSG00000170289	chromosome8	87824972
ENSG00000070729	chromosome16	56558692
ENSG00000143630	chromosome1	153514006
ENSG00000138622	chromosome15	71447665

ENSG00000132259	chromosome11	6212700
ENSG00000183862	chromosomeX	150657612
ENSG00000144191	chromosome2	98352871
ENSG00000183862	chromosomeX	150657612
ENSG00000144191	chromosome2	98352871
ENSG00000132259	chromosome11	6212700
ENSG00000164588	chromosome5	45731953
ENSG00000099822	chromosome19	540946
ENSG00000138622	chromosome15	71447665
ENSG00000143630	chromosome1	153514006
ENSG00000144191	chromosome2	98352871
ENSG00000183862	chromosomeX	150657612
ENSG00000183570	chromosome21	46140636
ENSG00000169564	chromosome2	70168380
ENSG00000197111	chromosome12	52134852
ENSG00000090097	chromosome3	51970297
ENSG00000169564	chromosome2	70168380
ENSG00000197111	chromosome12	52134852
ENSG00000090097	chromosome3	51970297
ENSG00000183570	chromosome21	46140636
ENSG00000090097	chromosome3	51970297
ENSG00000169564	chromosome2	70168380
ENSG00000197111	chromosome12	52134852
ENSG00000131873	chromosome15	99609185
ENSG00000198108	chromosome5	129268422
ENSG00000147408	chromosome8	19407626
ENSG00000169826	chromosome10	42970604
ENSG00000123989	chromosome2	220116505
ENSG00000033100	chromosome7	150562031
ENSG00000139044	chromosome12	439804
ENSG00000182272	chromosome11	359804
ENSG00000123989	chromosome2	220116505
ENSG00000033100	chromosome7	150562031
ENSG00000114331	chromosome3	196645004
ENSG00000131584	chromosome1	1233059
ENSG00000072818	chromosome17	7180778
ENSG00000072818	chromosome17	7180778
ENSG00000114331	chromosome3	196645004
ENSG00000131584	chromosome1	1233059
ENSG00000131584	chromosome1	1233059
ENSG00000114331	chromosome3	196645004
ENSG00000148832	chromosome10	135042811
ENSG00000088826	chromosome20	4103703
ENSG00000088826	chromosome20	4103703
ENSG00000148832	chromosome10	135042811
ENSG00000148832	chromosome10	135042811
ENSG00000088826	chromosome20	4103703
ENSG00000142864	chromosome1	67668572
ENSG00000130956	chromosome9	98252383
ENSG00000099849	chromosome11	551769
ENSG00000099849	chromosome11	551769
ENSG00000123094	chromosome12	26099544
ENSG00000099849	chromosome11	551769
ENSG00000172062	chromosome5	70256687
ENSG00000205571	chromosome5	69381269
ENSG00000086544	chromosome19	45914881
ENSG00000143772	chromosome1	224991783
ENSG00000137825	chromosome15	39573418
ENSG00000143772	chromosome1	224991783

ENSG00000086544	chromosome19	45914881
ENSG00000137825	chromosome15	39573418
ENSG00000115234	chromosome2	27447115
ENSG00000174226	chromosome8	101730919
ENSG00000186001	chromosome3	199002547
ENSG00000077454	chromosome7	100021660
ENSG00000136141	chromosome13	46025533
ENSG00000130224	chromosomeX	114374861
ENSG00000130224	chromosomeX	114374861
ENSG00000136141	chromosome13	46025533
ENSG00000077454	chromosome7	100021660
ENSG00000186001	chromosome3	199002547
ENSG00000140280	chromosome15	49817112
ENSG00000163155	chromosome1	149404359
ENSG00000179094	chromosome17	7994750
ENSG00000132326	chromosome2	238851317
ENSG00000049246	chromosome1	7767525
ENSG00000132326	chromosome2	238851317
ENSG00000179094	chromosome17	7994750
ENSG00000081059	chromosome5	133478497
ENSG00000138795	chromosome4	109308373
ENSG00000138795	chromosome4	109308373
ENSG00000081059	chromosome5	133478497
ENSG00000148737	chromosome10	114700506
ENSG00000152284	chromosome2	85214319
ENSG00000148737	chromosome10	114700506
ENSG00000138795	chromosome4	109308373
ENSG00000081059	chromosome5	133478497
ENSG00000141424	chromosome18	31960969
ENSG00000196950	chromosome2	196253012
ENSG00000104635	chromosome8	22280765
ENSG00000147804	chromosome8	145612982
ENSG00000148482	chromosome10	18282212
ENSG00000196950	chromosome2	196253012
ENSG00000141424	chromosome18	31960969
ENSG00000139540	chromosome12	54911329
ENSG00000138821	chromosome4	103484843
ENSG00000104635	chromosome8	22280765
ENSG00000172209	chromosome7	106901742
ENSG00000172209	chromosome7	106901742
ENSG00000138379	chromosome2	190635568
ENSG00000135414	chromosome12	54423368
ENSG00000092969	chromosome1	216586667
ENSG00000105329	chromosome19	46550790
ENSG00000092969	chromosome1	216586667
ENSG00000171596	chromosome2	232103317
ENSG00000171596	chromosome2	232103317
ENSG00000171596	chromosome2	232103317
ENSG00000182132	chromosome5	169713459
ENSG00000185774	chromosome4	21559353
ENSG00000115041	chromosome2	95326914
ENSG00000185774	chromosome4	21559353
ENSG00000182132	chromosome5	169713459
ENSG00000120049	chromosome10	103593316
ENSG00000185774	chromosome4	21559353
ENSG00000182132	chromosome5	169713459
ENSG00000115041	chromosome2	95326914
ENSG00000114857	chromosome3	42617503
ENSG00000152591	chromosome4	88751085

ENSG00000138398	chromosome2	170168798
ENSG00000138398	chromosome2	170168798
ENSG00000152591	chromosome4	88751085
ENSG00000100083	chromosome22	36334814
ENSG00000103365	chromosome16	23429234
ENSG00000125447	chromosome17	70769264
ENSG00000103365	chromosome16	23429234
ENSG00000100083	chromosome22	36334814
ENSG00000146910	chromosome7	154994494
ENSG00000144785	chromosome12	54995269
ENSG00000133812	chromosome11	10272193
ENSG00000100241	chromosome22	49260308
ENSG00000164440	chromosome6	139651730
ENSG00000086712	chromosomeX	16714532
ENSG00000084652	chromosome1	32418534
ENSG00000084652	chromosome1	32418534
ENSG00000086712	chromosomeX	16714532
ENSG00000076864	chromosome1	21868390
ENSG00000132359	chromosome17	2646479
ENSG00000132359	chromosome17	2646479
ENSG00000076864	chromosome1	21868390
ENSG00000076864	chromosome1	21868390
ENSG00000105738	chromosome19	43264046
ENSG00000116991	chromosome1	230717709
ENSG00000197555	chromosome14	71124343
ENSG00000213445	chromosome11	65164969
ENSG00000197555	chromosome14	71124343
ENSG00000116991	chromosome1	230717709
ENSG00000116991	chromosome1	230717709
ENSG00000197555	chromosome14	71124343
ENSG00000105738	chromosome19	43264046
ENSG00000184505	chromosome15	19093971
ENSG00000187779	chromosome15	21052802
ENSG00000145860	chromosome5	158567386
ENSG00000162630	chromosome1	191417316
ENSG00000156966	chromosome2	231953837
ENSG00000176383	chromosome12	121255119
ENSG00000180561	chromosome16	65741890
ENSG00000162630	chromosome1	191417316
ENSG00000172318	chromosome2	168433796
ENSG00000170340	chromosome2	62302467
ENSG00000177191	chromosome19	46624524
ENSG00000179913	chromosome19	17779617
ENSG00000198488	chromosome11	76428244
ENSG00000176383	chromosome12	121255119
ENSG00000180561	chromosome16	65741890
ENSG00000156966	chromosome2	231953837
ENSG00000156966	chromosome2	231953837
ENSG00000162630	chromosome1	191417316
ENSG00000172318	chromosome2	168433796
ENSG00000162630	chromosome1	191417316
ENSG00000169255	chromosome3	162287237
ENSG00000183778	chromosome21	39954357
ENSG00000162630	chromosome1	191417316
ENSG00000169255	chromosome3	162287237
ENSG00000183778	chromosome21	39954357
ENSG00000172318	chromosome2	168433796
ENSG00000162630	chromosome1	191417316
ENSG00000180561	chromosome16	65741890

ENSG00000176383	chromosome12	121255119
ENSG00000179913	chromosome19	17779617
ENSG00000198488	chromosome11	76428244
ENSG00000170340	chromosome2	62302467
ENSG00000177191	chromosome19	46624524
ENSG00000177191	chromosome19	46624524
ENSG00000170340	chromosome2	62302467
ENSG00000177191	chromosome19	46624524
ENSG00000214654	chromosome9	122600367
ENSG00000204222	chromosome6	33353175
ENSG00000176383	chromosome12	121255119
ENSG00000180561	chromosome16	65741890
ENSG00000156966	chromosome2	231953837
ENSG00000170340	chromosome2	62302467
ENSG00000177191	chromosome19	46624524
ENSG00000179913	chromosome19	17779617
ENSG00000198488	chromosome11	76428244
ENSG00000170581	chromosome12	55036623
ENSG00000168610	chromosome17	37754061
ENSG00000115415	chromosome2	191583343
ENSG00000138378	chromosome2	191721175
ENSG00000138378	chromosome2	191721175
ENSG00000115415	chromosome2	191583343
ENSG00000115415	chromosome2	191583343
ENSG00000138378	chromosome2	191721175
ENSG00000170581	chromosome12	55036623
ENSG00000168610	chromosome17	37754061
ENSG00000168610	chromosome17	37754061
ENSG00000170581	chromosome12	55036623
ENSG00000135373	chromosome11	34620754
ENSG00000163435	chromosome1	200246888
ENSG00000166211	chromosome12	100394814
ENSG00000163435	chromosome1	200246888
ENSG00000135373	chromosome11	34620754
ENSG00000135374	chromosome11	34489693
ENSG00000010030	chromosome6	36476393
ENSG00000139083	chromosome12	11694329
ENSG00000102034	chromosomeX	129042986
ENSG00000120690	chromosome13	40454191
ENSG00000109381	chromosome4	140278306
ENSG00000109381	chromosome4	140278306
ENSG00000102034	chromosomeX	129042986
ENSG00000120690	chromosome13	40454191
ENSG00000163714	chromosome3	144203161
ENSG00000196504	chromosome2	153282155
ENSG00000110844	chromosome12	48310658
ENSG00000083799	chromosome16	49341111
ENSG00000083799	chromosome16	49341111
ENSG00000011201	chromosomeX	8660078
ENSG00000065923	chromosomeX	46503409
ENSG00000198689	chromosomeX	134895328
ENSG00000181804	chromosome3	145049855
ENSG00000198689	chromosomeX	134895328
ENSG00000065923	chromosomeX	46503409
ENSG00000140090	chromosome14	91858675
ENSG00000185052	chromosome20	19141487
ENSG00000155886	chromosome9	19776865
ENSG00000074621	chromosome15	63703472
ENSG00000074621	chromosome15	63703472

ENSG00000185052	chromosome20	19141487
ENSG00000140090	chromosome14	91858675
ENSG00000104237	chromosome8	55696080
ENSG00000183638	chromosome8	10518122
ENSG00000133083	chromosome13	35598275
ENSG00000077279	chromosomeX	110540859
ENSG00000146038	chromosome6	24465958
ENSG00000170390	chromosome4	151219630
ENSG00000077279	chromosomeX	110540859
ENSG00000133083	chromosome13	35598275
ENSG00000168738	chromosome18	10861359
ENSG00000196428	chromosome3	151609828
ENSG00000166925	chromosome7	99913598
ENSG00000102804	chromosome13	44048211
ENSG00000166925	chromosome7	99913598
ENSG00000196428	chromosome3	151609828
ENSG00000136810	chromosome9	112058537
ENSG00000113758	chromosome5	176833129
ENSG00000136279	chromosome7	44050862
ENSG00000113758	chromosome5	176833129
ENSG00000145687	chromosome5	81082618
ENSG00000214079	chromosome2	131827283
ENSG00000214099	chromosome2	130466932
ENSG00000215390	chromosome14	19206828
ENSG00000157216	chromosome1	54644270
ENSG00000215535	chromosome18	12058418
ENSG00000130511	chromosome19	18391459
ENSG00000214079	chromosome2	131827283
ENSG00000214099	chromosome2	130466932
ENSG00000215390	chromosome14	19206828
ENSG00000157216	chromosome1	54644270
ENSG00000145687	chromosome5	81082618
ENSG00000133884	chromosome11	64857934
ENSG00000011332	chromosome19	43406623
ENSG00000205683	chromosome14	72430550
ENSG00000205683	chromosome14	72430550
ENSG00000011332	chromosome19	43406623
ENSG00000120738	chromosome5	137829350
ENSG00000120738	chromosome5	137829350
ENSG00000179388	chromosome8	22606403
ENSG00000122877	chromosome10	64245796
ENSG00000135625	chromosome2	73373954
ENSG00000179388	chromosome8	22606403
ENSG00000122877	chromosome10	64245796
ENSG00000120738	chromosome5	137829350
ENSG00000122877	chromosome10	64245796
ENSG00000179388	chromosome8	22606403
ENSG00000141540	chromosome17	69721322
ENSG00000167614	chromosome19	59618539
ENSG00000136295	chromosome7	2638316
ENSG00000167614	chromosome19	59618539
ENSG00000141540	chromosome17	69721322
ENSG00000183701	chromosome17	57947861
ENSG00000101278	chromosome20	768621
ENSG00000215464	chromosome22	22996104
ENSG00000124614	chromosome6	34500977
ENSG00000198810	chromosome1	199755807
ENSG00000198810	chromosome1	199755807
ENSG00000183701	chromosome17	57947861

ENSG00000101278	chromosome20	768621
ENSG00000215464	chromosome22	22996104
ENSG00000124614	chromosome6	34500977
ENSG00000183091	chromosome2	152297917
ENSG00000078114	chromosome10	21502769
ENSG00000078114	chromosome10	21502769
ENSG00000183091	chromosome2	152297917
ENSG00000002834	chromosome17	34279969
ENSG00000078114	chromosome10	21502769
ENSG00000183091	chromosome2	152297917
ENSG00000151025	chromosome10	25504356
ENSG00000188888	chromosome17	33753199
ENSG00000077984	chromosome20	24878076
ENSG00000175315	chromosome11	65536092
ENSG00000101441	chromosome20	23617607
ENSG00000170369	chromosome20	23755298
ENSG00000170373	chromosome20	23679504
ENSG00000170367	chromosome20	23808314
ENSG00000101439	chromosome20	23566500
ENSG00000125823	chromosome20	23368905
ENSG00000125815	chromosome20	23420305
ENSG00000204663	chromosome20	23449513
ENSG00000125831	chromosome20	23381449
ENSG00000173335	chromosome20	23534502
ENSG00000204658	chromosome20	23512279
ENSG00000101435	chromosome20	23497088
ENSG00000204662	chromosome20	23479290
ENSG00000175315	chromosome11	65536092
ENSG00000077984	chromosome20	24878076
ENSG00000133424	chromosome22	32487464
ENSG00000165905	chromosome11	45901010
ENSG00000138600	chromosome15	48845023
ENSG00000185294	chromosome17	41278053
ENSG00000005206	chromosome19	2279709
ENSG00000005206	chromosome19	2279709
ENSG00000185294	chromosome17	41278053
ENSG00000214979	chromosomeX	126352002
ENSG00000135241	chromosome7	107943172
ENSG00000107551	chromosome10	44785657
ENSG00000101265	chromosome20	4729677
ENSG00000169435	chromosome4	74705082
ENSG00000101265	chromosome20	4729677
ENSG00000107551	chromosome10	44785657
ENSG00000136653	chromosome1	204747559
ENSG00000153179	chromosome12	63290680
ENSG00000139746	chromosome13	78877911
ENSG00000091009	chromosome5	145563522
ENSG00000069764	chromosome16	14695588
ENSG00000187980	chromosome1	20374248
ENSG00000158786	chromosome1	20338508
ENSG00000188784	chromosome1	20122640
ENSG00000117215	chromosome1	20318579
ENSG00000127472	chromosome1	20283911
ENSG00000188257	chromosome1	20177854
ENSG00000187980	chromosome1	20374248
ENSG00000158786	chromosome1	20338508
ENSG00000188784	chromosome1	20122640
ENSG00000117215	chromosome1	20318579
ENSG00000127472	chromosome1	20283911

ENSG00000188257	chromosome1	20177854
ENSG00000069764	chromosome16	14695588
ENSG00000122176	chromosome1	201584022
ENSG00000122176	chromosome1	201584022
ENSG00000122176	chromosome1	201584022
ENSG00000127083	chromosome9	94219662
ENSG00000139330	chromosome12	89974190
ENSG00000188783	chromosome1	201718936
ENSG00000188783	chromosome1	201718936
ENSG00000139330	chromosome12	89974190
ENSG00000139330	chromosome12	89974190
ENSG00000188783	chromosome1	201718936
ENSG00000127083	chromosome9	94219662
ENSG00000112319	chromosome6	133637612
ENSG00000064655	chromosome20	45040678
ENSG00000104313	chromosome8	72431276
ENSG00000158161	chromosome1	28257125
ENSG00000064655	chromosome20	45040678
ENSG00000104313	chromosome8	72431276
ENSG00000112319	chromosome6	133637612
ENSG00000104313	chromosome8	72431276
ENSG00000064655	chromosome20	45040678
ENSG00000113140	chromosome5	151035943
ENSG00000152583	chromosome4	88639751
ENSG00000113430	chromosome5	1935762
ENSG00000159387	chromosome16	53916505
ENSG00000113430	chromosome5	1935762
ENSG00000177508	chromosome16	52877464
ENSG00000176842	chromosome16	53522612
ENSG00000150051	chromosome10	28072351
ENSG00000170561	chromosome5	2804528
ENSG00000176842	chromosome16	53522612
ENSG00000170549	chromosome5	3649220
ENSG00000177508	chromosome16	52877464
ENSG00000170549	chromosome5	3649220
ENSG00000177508	chromosome16	52877464
ENSG00000170549	chromosome5	3649220
ENSG00000176842	chromosome16	53522612
ENSG00000170561	chromosome5	2804528
ENSG00000170549	chromosome5	3649220
ENSG00000177508	chromosome16	52877464
ENSG00000176842	chromosome16	53522612
ENSG00000170561	chromosome5	2804528
ENSG00000177508	chromosome16	52877464
ENSG00000170549	chromosome5	3649220
ENSG00000159387	chromosome16	53916505
ENSG00000113430	chromosome5	1935762
ENSG00000214545	chromosome15	82531761
ENSG00000188659	chromosome15	80342276
ENSG00000155875	chromosome9	19022907
ENSG00000084112	chromosome12	107775402
ENSG00000141298	chromosome17	25281145
ENSG00000141298	chromosome17	25281145
ENSG00000084112	chromosome12	107775402
ENSG00000172830	chromosome11	66827673
ENSG00000107897	chromosome10	27569771
ENSG00000181513	chromosome17	40569038
ENSG00000148704	chromosome10	118887558
ENSG00000116035	chromosome2	70981260

ENSG00000119630	chromosome14	74491699
ENSG00000112715	chromosome6	43846422
ENSG00000173511	chromosome11	63758882
ENSG00000150630	chromosome4	177950460
ENSG00000165197	chromosomeX	15311990
ENSG00000119630	chromosome14	74491699
ENSG00000112715	chromosome6	43846422
ENSG00000165197	chromosomeX	15311990
ENSG00000150630	chromosome4	177950460
ENSG00000112715	chromosome6	43846422
ENSG00000119630	chromosome14	74491699
ENSG00000100311	chromosome22	37969915
ENSG00000197461	chromosome7	525165
ENSG00000215682	supercontigNT_113902	12146
ENSG00000165406	chromosome10	45348666
ENSG00000145416	chromosome4	164994734
ENSG00000139266	chromosome12	56435579
ENSG00000144583	chromosome2	216943229
ENSG00000173926	chromosome5	126281763
ENSG00000099785	chromosome19	8392725
ENSG00000144583	chromosome2	216943229
ENSG00000139266	chromosome12	56435579
ENSG00000183654	chromosome5	16232655
ENSG00000145416	chromosome4	164994734
ENSG00000165406	chromosome10	45348666
ENSG00000099785	chromosome19	8392725
ENSG00000173926	chromosome5	126281763
ENSG00000104427	chromosome8	79740939
ENSG00000196233	chromosome10	98698805
ENSG00000178177	chromosome4	17632473
ENSG00000196233	chromosome10	98698805
ENSG00000186111	chromosome19	3651389
ENSG00000173780	chromosome6	7931769
ENSG00000143398	chromosome1	149438097
ENSG00000107242	chromosome9	70627351
ENSG00000173780	chromosome6	7931769
ENSG00000143398	chromosome1	149438097
ENSG00000186111	chromosome19	3651389
ENSG00000106069	chromosome7	29201083
ENSG00000174576	chromosome11	65945227
ENSG00000156299	chromosome21	31561160
ENSG00000146426	chromosome6	155492050
ENSG00000162704	chromosome1	181871418
ENSG00000136950	chromosome9	126671391
ENSG00000130066	chromosomeX	23711318
ENSG00000184788	chromosomeX	84250070
ENSG00000141504	chromosome17	7471899
ENSG00000184788	chromosomeX	84250070
ENSG00000130066	chromosomeX	23711318
ENSG00000137819	chromosome15	67439474
ENSG00000160781	chromosome1	154484472
ENSG00000182749	chromosome1	26062918
ENSG00000170915	chromosome6	52375956
ENSG00000172175	chromosome18	54489856
ENSG00000172175	chromosome18	54489856
ENSG00000146005	chromosome5	139169210
ENSG00000156011	chromosome8	18915374
ENSG00000059915	chromosome10	104166786
ENSG00000156011	chromosome8	18915374

ENSG00000146005	chromosome5	139169210
ENSG00000156011	chromosome8	18915374
ENSG00000146005	chromosome5	139169210
ENSG00000059915	chromosome10	104166786
ENSG00000125637	chromosome2	113656505
ENSG00000125637	chromosome2	113656505
ENSG00000156011	chromosome8	18915374
ENSG00000146005	chromosome5	139169210
ENSG00000059915	chromosome10	104166786
ENSG00000059915	chromosome10	104166786
ENSG00000101928	chromosomeX	133861130
ENSG00000106330	chromosome7	100048328
ENSG00000115109	chromosome2	120493131
ENSG00000095203	chromosome9	111122548
ENSG00000153303	chromosome6	168222624
ENSG00000163812	chromosome3	44975933
ENSG00000153786	chromosome16	83581726
ENSG00000163812	chromosome3	44975933
ENSG00000125695	chromosome17	59159462
ENSG00000082146	chromosome2	202027809
ENSG00000105663	chromosome19	40900761
ENSG00000118058	chromosome11	117812438
ENSG00000011304	chromosome19	748498
ENSG00000117569	chromosome1	96960009
ENSG00000119314	chromosome9	114135721
ENSG00000119314	chromosome9	114135721
ENSG00000011304	chromosome19	748498
ENSG00000117569	chromosome1	96960009
ENSG00000117569	chromosome1	96960009
ENSG00000011304	chromosome19	748498
ENSG00000100219	chromosome22	27526513
ENSG00000137714	chromosome11	109806055
ENSG00000111405	chromosome12	46405464
ENSG00000133136	chromosomeX	109476794
ENSG00000174021	chromosome1	84744363
ENSG00000133136	chromosomeX	109476794
ENSG00000174021	chromosome1	84744363
ENSG00000182625	chromosome15	56765902
ENSG00000168243	chromosome1	233813762
ENSG00000162188	chromosome11	62232344
ENSG00000167414	chromosome19	51829780
ENSG00000186469	chromosome14	51487147
ENSG00000213611	chromosome19	2471687
ENSG00000172380	chromosome1	67945958
ENSG00000162188	chromosome11	62232344
ENSG00000168243	chromosome1	233813762
ENSG00000167414	chromosome19	51829780
ENSG00000186469	chromosome14	51487147
ENSG00000168243	chromosome1	233813762
ENSG00000162188	chromosome11	62232344
ENSG00000186469	chromosome14	51487147
ENSG00000167414	chromosome19	51829780
ENSG00000127928	chromosome7	93373995
ENSG00000127920	chromosome7	93389386
ENSG00000167083	chromosome17	44639784
ENSG00000213611	chromosome19	2471687
ENSG00000172380	chromosome1	67945958
ENSG00000168243	chromosome1	233813762
ENSG00000162188	chromosome11	62232344

ENSG00000167414	chromosome19	51829780
ENSG00000186469	chromosome14	51487147
ENSG00000182625	chromosome15	56765902
ENSG00000133136	chromosomeX	109476794
ENSG00000174021	chromosome1	84744363
ENSG00000172380	chromosome1	67945958
ENSG00000213611	chromosome19	2471687
ENSG00000172380	chromosome1	67945958
ENSG00000143365	chromosome1	150070865
ENSG00000198963	chromosome9	76302533
ENSG00000198963	chromosome9	76302533
ENSG00000143365	chromosome1	150070865
ENSG00000198963	chromosome9	76302533
ENSG00000008056	chromosomeX	47364072
ENSG00000157152	chromosome3	12021029
ENSG00000185666	chromosome22	31732648
ENSG00000157152	chromosome3	12021029
ENSG00000008056	chromosomeX	47364072
ENSG00000198728	chromosome10	103861201
ENSG00000169744	chromosome4	16509207
ENSG00000029153	chromosome12	27377273
ENSG00000133794	chromosome11	13332432
ENSG00000143437	chromosome1	149115815
ENSG00000172379	chromosome15	78483742
ENSG00000178719	chromosome8	145137380
ENSG00000178719	chromosome8	145137380
ENSG00000135472	chromosome12	48583843
ENSG00000135472	chromosome12	48583843
ENSG00000135926	chromosome2	218855109
ENSG00000007372	chromosome11	31784536
ENSG00000007372	chromosome11	31784536
ENSG00000106331	chromosome7	127043219
ENSG00000007372	chromosome11	31784536
ENSG00000100906	chromosome14	34943602
ENSG00000146232	chromosome6	44341479
ENSG00000146232	chromosome6	44341479
ENSG00000100906	chromosome14	34943602
ENSG00000104825	chromosome19	44082513
ENSG00000198947	chromosomeX	33267304
ENSG00000152818	chromosome6	144654658
ENSG00000129351	chromosome19	10642282
ENSG00000165209	chromosome9	124986235
ENSG00000129351	chromosome19	10642282
ENSG00000165209	chromosome9	124986235
ENSG00000129351	chromosome19	10642282
ENSG00000105278	chromosome19	3785924
ENSG00000056097	chromosome5	32480522
ENSG00000056097	chromosome5	32480522
ENSG00000105278	chromosome19	3785924
ENSG00000205593	chromosome22	49100011
ENSG00000174839	chromosome3	57653786
ENSG00000136231	chromosome7	23476255
ENSG00000159217	chromosome17	44430107
ENSG00000073792	chromosome3	187025443
ENSG00000159217	chromosome17	44430107
ENSG00000136231	chromosome7	23476255
ENSG00000134709	chromosome1	60053378
ENSG00000095066	chromosome19	12747264
ENSG00000168172	chromosome8	42871432

ENSG00000115355	chromosome2	55499720
ENSG00000015133	chromosome14	90953788
ENSG00000168172	chromosome8	42871432
ENSG00000095066	chromosome19	12747264
ENSG00000055813	chromosome2	56265156
ENSG00000205476	chromosome14	99140050
ENSG00000205476	chromosome14	99140050
ENSG00000055813	chromosome2	56265156
ENSG00000175602	chromosome11	65414831
ENSG00000185176	chromosome2	241270996
ENSG00000184945	chromosome2	241279936
ENSG00000178301	chromosome11	76978686
ENSG00000130244	chromosome19	43585634
ENSG00000171262	chromosome15	36533655
ENSG00000119812	chromosome2	33677793
ENSG00000171262	chromosome15	36533655
ENSG00000130244	chromosome19	43585634
ENSG00000142279	chromosome19	39665173
ENSG00000129474	chromosome14	22521316
ENSG00000159840	chromosome7	142788460
ENSG00000162458	chromosome1	15964066
ENSG00000144791	chromosome3	45611376
ENSG00000129474	chromosome14	22521316
ENSG00000142279	chromosome19	39665173
ENSG00000087077	chromosome7	100303056
ENSG00000145012	chromosome3	189606603
ENSG00000147421	chromosome8	28877304
ENSG00000135100	chromosome12	119900955
ENSG00000108753	chromosome17	33178989
ENSG00000108753	chromosome17	33178989
ENSG00000135100	chromosome12	119900955
ENSG00000146966	chromosome7	139948667
ENSG00000166444	chromosome11	8728824
ENSG00000166444	chromosome11	8728824
ENSG00000146966	chromosome7	139948667
ENSG00000175984	chromosome1	114970129
ENSG00000162701	chromosome1	196010909
ENSG00000119522	chromosome9	125732006
ENSG00000119522	chromosome9	125732006
ENSG00000162701	chromosome1	196010909
ENSG00000205744	chromosome19	6432785
ENSG00000175984	chromosome1	114970129
ENSG00000166444	chromosome11	8728824
ENSG00000146966	chromosome7	139948667
ENSG00000162777	chromosome1	111548499
ENSG00000164776	chromosome7	56124129
ENSG00000156873	chromosome16	30667643
ENSG00000116017	chromosome19	880529
ENSG00000116017	chromosome19	880529
ENSG00000179361	chromosome15	72623331
ENSG00000117713	chromosome1	26895482
ENSG00000049618	chromosome6	157140756
ENSG00000049618	chromosome6	157140756
ENSG00000117713	chromosome1	26895482
ENSG00000049618	chromosome6	157140756
ENSG00000117713	chromosome1	26895482
ENSG00000165487	chromosome13	21076288
ENSG00000155970	chromosome8	16929160
ENSG00000197976	chromosomeX	1672356

ENSG00000214992	chromosomeX	118275662
ENSG00000131051	chromosome20	33792211
ENSG00000100461	chromosome14	22450443
ENSG00000162512	chromosome1	31154021
ENSG00000115884	chromosome2	20288110
ENSG00000169439	chromosome8	97575676
ENSG00000124145	chromosome20	43410439
ENSG00000169439	chromosome8	97575676
ENSG00000124145	chromosome20	43410439
ENSG00000169439	chromosome8	97575676
ENSG00000115884	chromosome2	20288110
ENSG00000162512	chromosome1	31154021
ENSG00000164089	chromosome4	109903504
ENSG00000175309	chromosome5	177592158
ENSG00000110693	chromosome11	16319591
ENSG00000134532	chromosome12	23993803
ENSG00000134532	chromosome12	23993803
ENSG00000110693	chromosome11	16319591
ENSG00000143842	chromosome1	202348721
ENSG00000181072	chromosome7	136350153
ENSG00000184984	chromosome15	32142211
ENSG00000168539	chromosome11	62435149
ENSG00000181072	chromosome7	136350153
ENSG00000168539	chromosome11	62435149
ENSG00000184984	chromosome15	32142211
ENSG00000133019	chromosome1	238137375
ENSG00000133019	chromosome1	238137375
ENSG00000168539	chromosome11	62435149
ENSG00000184984	chromosome15	32142211
ENSG00000181072	chromosome7	136350153
ENSG00000162290	chromosome3	53356585
ENSG00000151065	chromosome12	1983859
ENSG00000132879	chromosome1	11638480
ENSG00000116663	chromosome1	11651303
ENSG00000116661	chromosome1	11636985
ENSG00000188505	chromosome19	44379463
ENSG00000161241	chromosome19	44135279
ENSG00000161243	chromosome19	44214708
ENSG00000116661	chromosome1	11636985
ENSG00000132879	chromosome1	11638480
ENSG00000116663	chromosome1	11651303
ENSG00000161241	chromosome19	44135279
ENSG00000161243	chromosome19	44214708
ENSG00000188505	chromosome19	44379463
ENSG00000173404	chromosome20	20296912
ENSG00000168348	chromosome14	35073210
ENSG00000123352	chromosome12	48141063
ENSG00000196141	chromosome2	200985153
ENSG00000188816	chromosome10	124897885
ENSG00000188620	chromosome10	124885557
ENSG00000188620	chromosome10	124885557
ENSG00000188620	chromosome10	124885557
ENSG00000188816	chromosome10	124897885
ENSG00000215612	supercontigNT_113886	43973
ENSG00000188816	chromosome10	124897885
ENSG00000188620	chromosome10	124885557
ENSG00000064999	chromosome6	34965158
ENSG00000185046	chromosome12	98902147
ENSG00000167971	chromosome16	2186435

ENSG00000167971	chromosome16	2186435
ENSG00000177303	chromosome17	71021481
ENSG00000205981	chromosome3	182190085
ENSG00000120675	chromosome13	42495763
ENSG00000170873	chromosome8	125809378
ENSG00000132613	chromosome16	69277196
ENSG00000132613	chromosome16	69277196
ENSG00000170873	chromosome8	125809378
ENSG00000184226	chromosome13	66700574
ENSG00000099715	chromosomeY	2251218
ENSG00000102290	chromosomeX	90952908
ENSG00000156453	chromosome5	141238012
ENSG00000169851	chromosome4	30332143
ENSG00000197991	chromosome13	60887293
ENSG00000099715	chromosomeY	2251218
ENSG00000102290	chromosomeX	90952908
ENSG00000184226	chromosome13	66700574
ENSG00000169851	chromosome4	30332143
ENSG00000156453	chromosome5	141238012
ENSG00000099715	chromosomeY	2251218
ENSG00000102290	chromosomeX	90952908
ENSG00000184226	chromosome13	66700574
ENSG00000197991	chromosome13	60887293
ENSG00000197991	chromosome13	60887293
ENSG00000169851	chromosome4	30332143
ENSG00000177600	chromosome11	800235
ENSG00000173914	chromosome11	66201127
ENSG00000173933	chromosome11	66163759
ENSG00000173959	chromosome11	66140768
ENSG00000173914	chromosome11	66201127
ENSG00000173933	chromosome11	66163759
ENSG00000116962	chromosome1	234295003
ENSG00000087303	chromosome14	51606296
ENSG00000112562	chromosome6	168584900
ENSG00000198732	chromosome14	69416149
ENSG00000154556	chromosome4	186848720
ENSG00000120896	chromosome8	22467965
ENSG00000095637	chromosome10	97240839
ENSG00000095637	chromosome10	97240839
ENSG00000120896	chromosome8	22467965
ENSG00000115468	chromosome2	233206659
ENSG00000142634	chromosome1	15609055
ENSG00000142634	chromosome1	15609055
ENSG00000115468	chromosome2	233206659
ENSG00000126878	chromosome9	132961761
ENSG00000204472	chromosome6	31691086
ENSG00000159733	chromosome4	2390167
ENSG00000198355	chromosome22	48740600
ENSG00000137193	chromosome6	37246057
ENSG00000198355	chromosome22	48740600
ENSG00000137193	chromosome6	37246057
ENSG00000198355	chromosome22	48740600
ENSG00000090530	chromosome3	191321215
ENSG00000110811	chromosome12	6803326
ENSG00000117385	chromosome1	43005230
ENSG00000141696	chromosome17	37221694
ENSG00000170275	chromosome3	33130574
ENSG00000117385	chromosome1	43005230
ENSG00000110811	chromosome12	6803326

ENSG00000100068	chromosome22	24086060
ENSG00000144597	chromosome3	15444024
ENSG00000145088	chromosome3	123036823
ENSG00000118515	chromosome6	134680292
ENSG00000101049	chromosome20	41628370
ENSG00000118515	chromosome6	134680292
ENSG00000070882	chromosome7	24898617
ENSG00000079156	chromosome2	178879137
ENSG00000006025	chromosome17	43252644
ENSG00000079156	chromosome2	178879137
ENSG00000070882	chromosome7	24898617
ENSG00000101413	chromosome20	36095764
ENSG00000141425	chromosome18	31901366
ENSG00000167106	chromosome9	129782238
ENSG00000162636	chromosome1	108904667
ENSG00000215315	chromosome15	28123950
ENSG00000108639	chromosome17	73676293
ENSG00000127561	chromosome16	1980105
ENSG00000105467	chromosome19	53560912
ENSG00000100321	chromosome22	38075962
ENSG00000100321	chromosome22	38075962
ENSG00000127561	chromosome16	1980105
ENSG00000105467	chromosome19	53560912
ENSG00000124783	chromosome6	7258353
ENSG00000121775	chromosome1	32311236
ENSG00000176142	chromosome3	120663612
ENSG00000183722	chromosome13	39073354
ENSG00000182508	chromosomeX	111801275
ENSG00000197753	chromosome6	35881348
ENSG00000187416	chromosome7	103756464
ENSG00000187416	chromosome7	103756464
ENSG00000197753	chromosome6	35881348
ENSG00000156959	chromosome3	9569364
ENSG00000145685	chromosome5	77841793
ENSG00000156959	chromosome3	9569364
ENSG00000187416	chromosome7	103756464
ENSG00000197753	chromosome6	35881348
ENSG00000127191	chromosome9	138913014
ENSG00000127191	chromosome9	138913014
ENSG00000056558	chromosome9	122728175
ENSG00000131323	chromosome14	102406292
ENSG00000082512	chromosome1	209593205
ENSG00000170264	chromosome2	61922856
ENSG00000156050	chromosome14	73486483
ENSG00000156050	chromosome14	73486483
ENSG00000170264	chromosome2	61922856
ENSG00000175513	chromosome11	65469813
ENSG00000198843	chromosome3	151803840
ENSG00000021645	chromosome14	78187321
ENSG00000179915	chromosome2	51108916
ENSG00000110076	chromosome11	64237748
ENSG00000179915	chromosome2	51108916
ENSG00000021645	chromosome14	78187321
ENSG00000117481	chromosome1	46579086
ENSG00000178694	chromosome3	95264661
ENSG00000154710	chromosome7	65731487
ENSG00000111783	chromosome12	105501229
ENSG00000185002	chromosome6	117305132
ENSG00000181827	chromosome15	54222378

ENSG00000132005	chromosome19	13965656
ENSG00000080298	chromosome9	3385589
ENSG00000087903	chromosome19	5998508
ENSG00000185002	chromosome6	117305132
ENSG00000111783	chromosome12	105501229
ENSG00000087903	chromosome19	5998508
ENSG00000080298	chromosome9	3385589
ENSG00000181827	chromosome15	54222378
ENSG00000143390	chromosome1	149585421
ENSG00000188580	chromosome6	124167045
ENSG00000084628	chromosome1	31484982
ENSG00000185942	chromosome8	63324187
ENSG00000101198	chromosome20	61368919
ENSG00000101198	chromosome20	61368919
ENSG00000185942	chromosome8	63324187
ENSG00000185942	chromosome8	63324187
ENSG00000101198	chromosome20	61368919
ENSG00000188580	chromosome6	124167045
ENSG00000084628	chromosome1	31484982
ENSG00000084628	chromosome1	31484982
ENSG00000188580	chromosome6	124167045
ENSG00000184557	chromosome17	73866772
ENSG00000171150	chromosome2	46839174
ENSG00000180008	chromosome14	54579513
ENSG00000114737	chromosome3	50624086
ENSG00000120833	chromosome12	92490805
ENSG00000184557	chromosome17	73866772
ENSG00000185338	chromosome16	11256837
ENSG00000120833	chromosome12	92490805
ENSG00000114737	chromosome3	50624086
ENSG00000185338	chromosome16	11256837
ENSG00000184557	chromosome17	73866772
ENSG00000180008	chromosome14	54579513
ENSG00000171150	chromosome2	46839174
ENSG00000167491	chromosome19	19437155
ENSG00000143614	chromosome1	152067448
ENSG00000170579	chromosome18	3870069
ENSG00000080845	chromosome20	34493535
ENSG00000116544	chromosome1	35143572
ENSG00000198010	chromosome8	1436941
ENSG00000080845	chromosome20	34493535
ENSG00000170579	chromosome18	3870069
ENSG00000198010	chromosome8	1436941
ENSG00000116544	chromosome1	35143572
ENSG00000116544	chromosome1	35143572
ENSG00000198010	chromosome8	1436941
ENSG00000170579	chromosome18	3870069
ENSG00000080845	chromosome20	34493535
ENSG00000126787	chromosome14	54728014
ENSG00000170412	chromosome17	69939773
ENSG00000167191	chromosome16	19791669
ENSG00000167191	chromosome16	19791669
ENSG00000170412	chromosome17	69939773
ENSG00000013588	chromosome12	12952451
ENSG00000111291	chromosome12	12994586
ENSG00000013588	chromosome12	12952451
ENSG00000111291	chromosome12	12994586
ENSG00000167191	chromosome16	19791669
ENSG00000170412	chromosome17	69939773

ENSG00000166068	chromosome15	36332679
ENSG00000198369	chromosome2	65512626
ENSG00000188766	chromosome19	43572783
ENSG00000196405	chromosome14	99601501
ENSG00000125753	chromosome19	50702870
ENSG00000198369	chromosome2	65512626
ENSG00000166068	chromosome15	36332679
ENSG00000125753	chromosome19	50702870
ENSG00000196405	chromosome14	99601501
ENSG00000154380	chromosome1	223907157
ENSG00000125753	chromosome19	50702870
ENSG00000075568	chromosome2	97870967
ENSG00000121210	chromosome4	154678627
ENSG00000167193	chromosome17	1306162
ENSG00000099942	chromosome22	19602223
ENSG00000173467	chromosome7	16884768
ENSG00000106541	chromosome7	16807946
ENSG00000106541	chromosome7	16807946
ENSG00000173467	chromosome7	16884768
ENSG00000173467	chromosome7	16884768
ENSG00000106541	chromosome7	16807946
ENSG00000117862	chromosome1	52293357
ENSG00000173467	chromosome7	16884768
ENSG00000106541	chromosome7	16807946
ENSG00000020633	chromosome1	25164198
ENSG00000124813	chromosome6	45404038
ENSG00000159216	chromosome21	35343067
ENSG00000159216	chromosome21	35343067
ENSG00000020633	chromosome1	25164198
ENSG00000124813	chromosome6	45404038
ENSG00000124813	chromosome6	45404038
ENSG00000020633	chromosome1	25164198
ENSG00000187323	chromosome18	48121156
ENSG00000215851	chromosome1	152034311
ENSG00000174498	chromosome15	63457180
ENSG00000166450	chromosome15	53822422
ENSG00000174498	chromosome15	63457180
ENSG00000215851	chromosome1	152034311
ENSG00000187323	chromosome18	48121156
ENSG00000067141	chromosome15	71132070
ENSG00000174498	chromosome15	63457180
ENSG00000215851	chromosome1	152034311
ENSG00000166450	chromosome15	53822422
ENSG00000103742	chromosome15	63502254
ENSG00000140577	chromosome15	88874308
ENSG00000105662	chromosome19	18655513
ENSG00000160741	chromosome1	152197598
ENSG00000105662	chromosome19	18655513
ENSG00000140577	chromosome15	88874308
ENSG00000186792	chromosome3	50308038
ENSG00000114378	chromosome3	50315392
ENSG00000106304	chromosome7	123380861
ENSG00000106302	chromosome7	123295564
ENSG00000068001	chromosome3	50332925
ENSG00000106304	chromosome7	123380861
ENSG00000106302	chromosome7	123295564
ENSG00000106302	chromosome7	123295564
ENSG00000106304	chromosome7	123380861
ENSG00000106304	chromosome7	123380861

ENSG00000106302	chromosome7	123295564
ENSG00000068001	chromosome3	50332925
ENSG00000114378	chromosome3	50315392
ENSG00000068001	chromosome3	50332925
ENSG00000106304	chromosome7	123380861
ENSG00000106302	chromosome7	123295564
ENSG00000068001	chromosome3	50332925
ENSG00000188177	chromosome2	112750043
ENSG00000130749	chromosome19	52307682
ENSG00000182827	chromosome1	224441000
ENSG00000182827	chromosome1	224441000
ENSG00000100580	chromosome14	76913150
ENSG00000140262	chromosome15	54999404
ENSG00000196628	chromosome18	51454127
ENSG00000215291	chromosome9	5100914
ENSG00000071564	chromosome19	1601248
ENSG00000215291	chromosome9	5100914
ENSG00000071564	chromosome19	1601248
ENSG00000196628	chromosome18	51454127
ENSG00000117016	chromosome1	40880185
ENSG00000079841	chromosome6	72653448
ENSG00000117016	chromosome1	40880185
ENSG00000176406	chromosome8	104582291
ENSG00000176406	chromosome8	104582291
ENSG00000117016	chromosome1	40880185
ENSG00000138068	chromosome2	37269174
ENSG00000105398	chromosome19	53081327
ENSG00000088002	chromosome19	53747322
ENSG00000105398	chromosome19	53081327
ENSG00000088002	chromosome19	53747322
ENSG00000198203	chromosome2	108276556
ENSG00000198075	chromosome2	108361226
ENSG00000196228	chromosome2	108230083
ENSG00000105398	chromosome19	53081327
ENSG00000088002	chromosome19	53747322
ENSG00000198203	chromosome2	108276556
ENSG00000198075	chromosome2	108361226
ENSG00000196228	chromosome2	108230083
ENSG00000213599	chromosome16	30119551
ENSG00000213648	chromosome16	29380208
ENSG00000196502	chromosome16	28542020
ENSG00000197165	chromosome16	28514753
ENSG00000109193	chromosome4	70757952
ENSG00000173597	chromosome4	70655525
ENSG00000130540	chromosome22	42589596
ENSG00000105398	chromosome19	53081327
ENSG00000088002	chromosome19	53747322
ENSG00000156920	chromosomeX	135218603
ENSG00000112414	chromosome6	142672375
ENSG00000112414	chromosome6	142672375
ENSG00000156920	chromosomeX	135218603
ENSG00000173698	chromosomeX	18996874
ENSG00000149972	chromosome11	99195478
ENSG00000163531	chromosome1	203180067
ENSG00000198493	chromosome6	32088298
ENSG00000091129	chromosome7	107667745
ENSG00000149972	chromosome11	99195478
ENSG00000198493	chromosome6	32088298
ENSG00000091129	chromosome7	107667745

ENSG00000163531	chromosome1	203180067
ENSG00000184144	chromosome1	203288937
ENSG00000149972	chromosome11	99195478
ENSG00000184144	chromosome1	203288937
ENSG00000134121	chromosome3	336460
ENSG00000198493	chromosome6	32088298
ENSG00000091129	chromosome7	107667745
ENSG00000163531	chromosome1	203180067
ENSG00000149972	chromosome11	99195478
ENSG00000091129	chromosome7	107667745
ENSG00000198493	chromosome6	32088298
ENSG00000149972	chromosome11	99195478
ENSG00000184144	chromosome1	203288937
ENSG00000106714	chromosome9	39278062
ENSG00000185020	chromosome9	40297346
ENSG00000154529	chromosome9	43625147
ENSG00000152910	chromosome16	74901295
ENSG00000155052	chromosome2	124499566
ENSG00000174469	chromosome7	145444902
ENSG00000108797	chromosome17	38088374
ENSG00000174469	chromosome7	145444902
ENSG00000106714	chromosome9	39278062
ENSG00000185020	chromosome9	40297346
ENSG00000154529	chromosome9	43625147
ENSG00000152910	chromosome16	74901295
ENSG00000155052	chromosome2	124499566
ENSG00000116574	chromosome1	226938113
ENSG00000104140	chromosome15	38953630
ENSG00000116574	chromosome1	226938113
ENSG00000139722	chromosome12	121946563
ENSG00000176428	chromosome7	72720225
ENSG00000167987	chromosome11	60662878
ENSG00000167987	chromosome11	60662878
ENSG00000176428	chromosome7	72720225
ENSG00000107518	chromosome10	116843500
ENSG00000088812	chromosome20	3399755
ENSG00000184363	chromosome11	384293
ENSG00000057294	chromosome12	32940933
ENSG00000081277	chromosome1	199519454
ENSG00000198561	chromosome11	57315527
ENSG00000099889	chromosome22	18358318
ENSG00000081277	chromosome1	199519454
ENSG00000057294	chromosome12	32940933
ENSG00000169862	chromosome5	11956966
ENSG00000144283	chromosome2	159097943
ENSG00000152601	chromosome3	153500673
ENSG00000076770	chromosomeX	131401321
ENSG00000139793	chromosome13	96726491
ENSG00000139793	chromosome13	96726491
ENSG00000076770	chromosomeX	131401321
ENSG00000161813	chromosome12	49081061
ENSG00000107929	chromosome10	921662
ENSG00000107929	chromosome10	921662
ENSG00000103326	chromosome16	536840
ENSG00000215835	chromosome1	164513453
ENSG00000182944	chromosome22	27994326
ENSG00000089280	chromosome16	31099037
ENSG00000172660	chromosome17	31160597
ENSG00000172660	chromosome17	31160597

ENSG00000089280	chromosome16	31099037
ENSG00000177468	chromosome6	137857001
ENSG00000205927	chromosome21	33321041
ENSG00000177468	chromosome6	137857001
ENSG00000205927	chromosome21	33321041
ENSG00000184221	chromosome21	33364423
ENSG00000125533	chromosome20	61108524
ENSG00000180828	chromosome8	65655902
ENSG00000205927	chromosome21	33321041
ENSG00000177468	chromosome6	137857001
ENSG00000177468	chromosome6	137857001
ENSG00000205927	chromosome21	33321041
ENSG00000180828	chromosome8	65655902
ENSG00000125533	chromosome20	61108524
ENSG00000214717	chromosomeX	2418761
ENSG00000100426	chromosome22	48663315
ENSG00000214717	chromosomeX	2418761
ENSG00000100426	chromosome22	48663315
ENSG00000139352	chromosome12	101876153
ENSG00000183734	chromosome11	2248139
ENSG00000188108	chromosome1	199351268
ENSG00000176009	chromosome11	8916285
ENSG00000187855	chromosome12	106693126
ENSG00000176009	chromosome11	8916285
ENSG00000188108	chromosome1	199351268
ENSG00000135913	chromosome2	219131621
ENSG00000131864	chromosome19	62331856
ENSG00000134588	chromosomeX	131989915
ENSG00000115183	chromosome2	159630669
ENSG00000151458	chromosome4	125851117
ENSG00000115183	chromosome2	159630669
ENSG00000170921	chromosome17	58745720
ENSG00000170921	chromosome17	58745720
ENSG00000115183	chromosome2	159630669
ENSG00000144426	chromosome2	203708789
ENSG00000160796	chromosome3	47004542
ENSG00000186625	chromosome6	150001377
ENSG00000102781	chromosome13	29755915
ENSG00000132535	chromosome17	7062893
ENSG00000075711	chromosome3	198508473
ENSG00000082458	chromosomeX	69581777
ENSG00000150672	chromosome11	84987389
ENSG00000075711	chromosome3	198508473
ENSG00000132535	chromosome17	7062893
ENSG00000150672	chromosome11	84987389
ENSG00000082458	chromosomeX	69581777
ENSG00000113300	chromosome5	179888883
ENSG00000138767	chromosome4	78959574
ENSG00000155827	chromosome9	103337527
ENSG00000103549	chromosome16	30681368
ENSG00000160999	chromosome7	101730555
ENSG00000178188	chromosome16	28784917
ENSG00000111252	chromosome12	110340333
ENSG00000178188	chromosome16	28784917
ENSG00000160999	chromosome7	101730555
ENSG00000177565	chromosome3	178265460
ENSG00000092377	chromosomeY	4243606
ENSG00000101849	chromosomeX	9581627
ENSG00000008405	chromosome12	106010870

ENSG00000121671	chromosome11	45825618
ENSG00000008405	chromosome12	106010870
ENSG00000145390	chromosome4	120380363
ENSG00000150593	chromosome10	112625776
ENSG00000151233	chromosome12	40824716
ENSG00000172986	chromosome3	73020075
ENSG00000174032	chromosome13	44883561
ENSG00000102078	chromosomeX	129301934
ENSG00000175567	chromosome11	73367072
ENSG00000175564	chromosome11	73395736
ENSG00000109424	chromosome4	141709334
ENSG00000175564	chromosome11	73395736
ENSG00000175567	chromosome11	73367072
ENSG00000124356	chromosome2	73911492
ENSG00000138134	chromosome10	90651446
ENSG00000124356	chromosome2	73911492
ENSG00000124356	chromosome2	73911492
ENSG00000138134	chromosome10	90651446
ENSG00000070081	chromosome11	17273447
ENSG00000104805	chromosome19	54095866
ENSG00000197562	chromosome16	580293
ENSG00000102128	chromosomeX	102078903
ENSG00000172476	chromosomeX	102642341
ENSG00000141542	chromosome17	78249762
ENSG00000123600	chromosome2	171956942
ENSG00000165055	chromosome7	127904056
ENSG00000087995	chromosome17	57854996
ENSG00000165861	chromosome14	72560970
ENSG00000145990	chromosome6	13595102
ENSG00000141098	chromosome16	66277120
ENSG00000137802	chromosome15	39854766
ENSG00000075702	chromosome19	41237714
ENSG00000187257	chromosome7	77163723
ENSG00000081019	chromosome1	114156558
ENSG00000160539	chromosome9	133155206
ENSG00000205808	chromosome9	4652376
ENSG00000178202	chromosome11	107874304
ENSG00000134901	chromosome13	102249022
ENSG00000140836	chromosome16	71551546
ENSG00000091656	chromosome8	77778879
ENSG00000136367	chromosome14	23065826
ENSG00000091656	chromosome8	77778879
ENSG00000140836	chromosome16	71551546
ENSG00000104731	chromosome16	86356998
ENSG00000138308	chromosome10	74384450
ENSG00000138308	chromosome10	74384450
ENSG00000123739	chromosome4	110870415
ENSG00000169918	chromosome15	29734742
ENSG00000163113	chromosome1	148216070
ENSG00000163113	chromosome1	148216070
ENSG00000169918	chromosome15	29734742
ENSG00000214577	chromosome9	130246724
ENSG00000166889	chromosome11	59182996
ENSG00000188760	chromosome2	220117694
ENSG00000184613	chromosome12	43555920
ENSG00000165973	chromosome11	20647846
ENSG00000151967	chromosome3	160474304
ENSG00000142453	chromosome19	10843379
ENSG00000105698	chromosome19	40451845

ENSG00000158773	chromosome1	159279690
ENSG00000111843	chromosome6	10832833
ENSG00000214881	chromosome10	69974597
ENSG00000137210	chromosome6	10857465
ENSG00000189156	chromosome3	153541384
ENSG00000096092	chromosome6	52649860
ENSG00000008853	chromosome8	22917893
ENSG00000072422	chromosome10	62341307
ENSG00000008853	chromosome8	22917893
ENSG00000142687	chromosome1	35792680
ENSG00000137261	chromosome6	24709311
ENSG00000106537	chromosome7	16760118
ENSG00000135452	chromosome12	56425205
ENSG00000172057	chromosome17	35333983
ENSG00000128699	chromosome2	190355567
ENSG00000123353	chromosome12	54499051
ENSG00000128699	chromosome2	190355567
ENSG00000172057	chromosome17	35333983
ENSG00000175471	chromosome5	94646036
ENSG00000140563	chromosome15	92642499
ENSG00000102786	chromosome13	50924663
ENSG00000215130	chromosome5	39757402
ENSG00000165359	chromosomeX	134482584
ENSG00000181433	chromosomeX	134806068
ENSG00000165359	chromosomeX	134482584
ENSG00000102786	chromosome13	50924663
ENSG00000215130	chromosome5	39757402
ENSG00000185787	chromosome15	76952415
ENSG00000123562	chromosomeX	102818612
ENSG00000137817	chromosome15	70346873
ENSG00000151883	chromosome5	49998687
ENSG00000185361	chromosome19	4602882
ENSG00000183578	chromosome15	49184666
ENSG00000163154	chromosome1	149397798
ENSG00000145779	chromosome5	118752897
ENSG00000163154	chromosome1	149397798
ENSG00000183578	chromosome15	49184666
ENSG00000183578	chromosome15	49184666
ENSG00000163154	chromosome1	149397798
ENSG00000185361	chromosome19	4602882
ENSG00000167964	chromosome16	2138786
ENSG00000172794	chromosome17	70179321
ENSG00000172794	chromosome17	70179321
ENSG00000124120	chromosome20	42542054
ENSG00000137561	chromosome8	64161135
ENSG00000146352	chromosome6	123360622
ENSG00000177182	chromosome8	62374941
ENSG00000137563	chromosome8	64113882
ENSG00000171735	chromosome1	6768178
ENSG00000171735	chromosome1	6768178
ENSG00000108509	chromosome17	4831649
ENSG00000066322	chromosome1	43603868
ENSG00000164181	chromosome5	60118982
ENSG00000012660	chromosome6	53321683
ENSG00000197977	chromosome6	11152450
ENSG00000097096	chromosome1	85439034
ENSG00000105137	chromosome19	15079245
ENSG00000179387	chromosome4	141666033
ENSG00000110675	chromosome11	106992719

ENSG00000165458	chromosome11	71613677
ENSG00000165458	chromosome11	71613677
ENSG00000168918	chromosome2	233633433
ENSG00000148384	chromosome9	138453693
ENSG00000165458	chromosome11	71613677
ENSG00000170801	chromosome4	8322596
ENSG00000115317	chromosome2	74610642
ENSG00000169495	chromosome8	38950940
ENSG00000166033	chromosome10	124211159
ENSG00000170801	chromosome4	8322596
ENSG00000170801	chromosome4	8322596
ENSG00000169495	chromosome8	38950940
ENSG00000166033	chromosome10	124211159
ENSG00000166033	chromosome10	124211159
ENSG00000169495	chromosome8	38950940
ENSG00000182606	chromosome3	42107966
ENSG00000115993	chromosome2	201993476
ENSG00000115993	chromosome2	201993476
ENSG00000182606	chromosome3	42107966
ENSG00000173805	chromosome17	37144413
ENSG00000158106	chromosome8	144522533
ENSG00000215407	chromosome15	18742143
ENSG00000131941	chromosome19	38247599
ENSG00000076201	chromosome3	47397591
ENSG00000101222	chromosome20	3709935
ENSG00000163283	chromosome2	232951712
ENSG00000163286	chromosome2	232979849
ENSG00000163295	chromosome2	233029154
ENSG00000162551	chromosome1	21753162
ENSG00000163283	chromosome2	232951712
ENSG00000163286	chromosome2	232979849
ENSG00000163295	chromosome2	233029154
ENSG00000162551	chromosome1	21753162
ENSG00000163283	chromosome2	232951712
ENSG00000163286	chromosome2	232979849
ENSG00000163295	chromosome2	233029154
ENSG00000204155	chromosome10	50758083
ENSG00000171840	chromosome12	642926
ENSG00000131669	chromosome9	94936321
ENSG00000131669	chromosome9	94936321
ENSG00000171840	chromosome12	642926
ENSG00000160050	chromosome1	32440124
ENSG00000024862	chromosome6	139136505
ENSG00000185295	chromosomeX	55532098
ENSG00000180991	chromosomeX	49531315
ENSG00000124422	chromosome17	20886741
ENSG00000116793	chromosome1	114102849
ENSG00000006576	chromosome7	77307509
ENSG00000138639	chromosome4	86615553
ENSG00000128805	chromosome10	49482848
ENSG00000163219	chromosome2	68815836
ENSG00000128805	chromosome10	49482848
ENSG00000138639	chromosome4	86615553
ENSG00000147799	chromosome8	145801808
ENSG00000163219	chromosome2	68815836
ENSG00000138639	chromosome4	86615553
ENSG00000128805	chromosome10	49482848
ENSG00000147799	chromosome8	145801808
ENSG00000114395	chromosome3	50363869

ENSG00000174151	chromosome1	109838285
ENSG00000076053	chromosome11	113776604
ENSG00000185272	chromosome21	14510379
ENSG00000124201	chromosome20	47325784
ENSG00000135547	chromosome6	126112616
ENSG00000164683	chromosome8	80842454
ENSG00000163909	chromosome1	39877885
ENSG00000164683	chromosome8	80842454
ENSG00000135547	chromosome6	126112616
ENSG00000169814	chromosome3	15618254
ENSG00000093134	chromosome6	133097525
ENSG00000112299	chromosome6	133076868
ENSG00000112303	chromosome6	133120716
ENSG00000198373	chromosome16	68378415
ENSG00000123124	chromosome8	87455396
ENSG00000078747	chromosome20	32445279
ENSG00000049759	chromosome18	53862891
ENSG00000069869	chromosome15	54073346
ENSG00000078747	chromosome20	32445279
ENSG00000123124	chromosome8	87455396
ENSG00000108854	chromosome17	60088854
ENSG00000198742	chromosome7	98579664
ENSG00000138698	chromosome4	99401740
ENSG00000203805	chromosome10	122206808
ENSG00000147535	chromosome8	38245617
ENSG00000148498	chromosome10	35143930
ENSG00000116117	chromosome2	205118968
ENSG00000198876	chromosome9	34124184
ENSG00000198354	chromosomeX	125127589
ENSG00000198889	chromosomeX	125514273
ENSG00000149658	chromosome20	61317690
ENSG00000185728	chromosome8	64243991
ENSG00000185728	chromosome8	64243991
ENSG00000149658	chromosome20	61317690
ENSG00000198492	chromosome1	28936220
ENSG00000100916	chromosome14	35365474
ENSG00000174744	chromosome11	65866282
ENSG00000196482	chromosome1	214963265
ENSG00000119715	chromosome14	75975450
ENSG00000140009	chromosome14	63819457
ENSG00000091831	chromosome6	152170741
ENSG00000119715	chromosome14	75975450
ENSG00000196482	chromosome1	214963265
ENSG00000196482	chromosome1	214963265
ENSG00000119715	chromosome14	75975450
ENSG00000173153	chromosome11	63829629
ENSG00000196482	chromosome1	214963265
ENSG00000119715	chromosome14	75975450
ENSG00000091831	chromosome6	152170741
ENSG00000140009	chromosome14	63819457
ENSG00000148935	chromosome11	22652992
ENSG00000139354	chromosome12	99512814
ENSG00000185340	chromosome22	28034096
ENSG00000132139	chromosome17	31103983
ENSG00000095370	chromosome9	129580728
ENSG00000137936	chromosome1	93913075
ENSG00000125731	chromosome19	6714760
ENSG00000125731	chromosome19	6714760
ENSG00000137936	chromosome1	93913075

ENSG00000115677	chromosome2	241854958
ENSG00000122870	chromosome10	59942910
ENSG00000064601	chromosome20	43953372
ENSG00000183807	chromosome6	117193433
ENSG00000114023	chromosome3	123585803
ENSG00000078043	chromosome18	42751307
ENSG00000131788	chromosome1	144287436
ENSG00000033800	chromosome15	66133623
ENSG00000105229	chromosome19	3958759
ENSG00000033800	chromosome15	66133623
ENSG00000131788	chromosome1	144287436
ENSG00000131788	chromosome1	144287436
ENSG00000033800	chromosome15	66133623
ENSG00000078043	chromosome18	42751307
ENSG00000162972	chromosome2	200528767
ENSG00000165675	chromosomeX	129670947
ENSG00000120658	chromosome13	42885051
ENSG00000144711	chromosome3	12983952
ENSG00000124313	chromosomeX	53367047
ENSG00000120645	chromosome12	46310
ENSG00000124313	chromosomeX	53367047
ENSG00000144711	chromosome3	12983952
ENSG00000197622	chromosome1	149294831
ENSG00000158985	chromosome5	130723086
ENSG00000197622	chromosome1	149294831
ENSG00000186889	chromosome2	62586769
ENSG00000187049	chromosome11	60917979
ENSG00000177042	chromosome11	685609
ENSG00000185963	chromosome9	94566848
ENSG00000151746	chromosome12	32151449
ENSG00000151746	chromosome12	32151449
ENSG00000185963	chromosome9	94566848
ENSG00000024048	chromosome6	42640036
ENSG00000159459	chromosome15	41185513
ENSG00000144306	chromosome2	174971258
ENSG00000136193	chromosome7	29975209
ENSG00000141295	chromosome17	43273209
ENSG00000141295	chromosome17	43273209
ENSG00000136193	chromosome7	29975209
ENSG00000176783	chromosome5	178910177
ENSG00000018189	chromosome4	71807155
ENSG00000214795	chromosome5	105780842
ENSG00000204130	chromosome10	69837061
ENSG00000108309	chromosome17	39741622
ENSG00000105784	chromosome7	87096076
ENSG00000204130	chromosome10	69837061
ENSG00000018189	chromosome4	71807155
ENSG00000214795	chromosome5	105780842
ENSG00000105784	chromosome7	87096076
ENSG00000108309	chromosome17	39741622
ENSG00000139146	chromosome12	31342406
ENSG00000143363	chromosome1	149247733
ENSG00000156035	chromosome9	78710700
ENSG00000164828	chromosome7	838688
ENSG00000100242	chromosome22	37478580
ENSG00000061656	chromosome20	33667340
ENSG00000167098	chromosome20	31055808
ENSG00000164744	chromosome7	48035061
ENSG00000146926	chromosome7	150515743

ENSG00000182177	chromosome2	236814903
ENSG00000128791	chromosome18	9327228
ENSG00000105519	chromosome19	5864944
ENSG00000152611	chromosome5	35956980
ENSG00000165886	chromosome10	99248951
ENSG00000168246	chromosome5	171643275
ENSG00000168452	chromosome6	32229382
ENSG00000149679	chromosome20	60415728
ENSG00000134508	chromosome18	18969725
ENSG00000146592	chromosome7	28419059
ENSG00000115966	chromosome2	175709418
ENSG00000170653	chromosome12	52281052
ENSG00000177606	chromosome1	59021331
ENSG00000130522	chromosome19	18253295
ENSG00000115966	chromosome2	175709418
ENSG00000170653	chromosome12	52281052
ENSG00000146592	chromosome7	28419059
ENSG00000130522	chromosome19	18253295
ENSG00000177606	chromosome1	59021331
ENSG00000171223	chromosome19	12763586
ENSG00000177606	chromosome1	59021331
ENSG00000130522	chromosome19	18253295
ENSG00000170653	chromosome12	52281052
ENSG00000115966	chromosome2	175709418
ENSG00000146592	chromosome7	28419059
ENSG00000177606	chromosome1	59021331
ENSG00000168092	chromosome11	116528374
ENSG00000079462	chromosome19	47498302
ENSG00000162174	chromosome11	61862026
ENSG00000117069	chromosome1	77105949
ENSG00000160408	chromosome9	129700082
ENSG00000111728	chromosome12	22378434
ENSG00000101638	chromosome18	42590470
ENSG00000148488	chromosome10	17536261
ENSG00000177511	chromosome18	53171076
ENSG00000160408	chromosome9	129700082
ENSG00000117069	chromosome1	77105949
ENSG00000073849	chromosome3	188243186
ENSG00000144057	chromosome2	106826881
ENSG00000148488	chromosome10	17536261
ENSG00000111728	chromosome12	22378434
ENSG00000101638	chromosome18	42590470
ENSG00000177511	chromosome18	53171076
ENSG00000111728	chromosome12	22378434
ENSG00000101638	chromosome18	42590470
ENSG00000148488	chromosome10	17536261
ENSG00000101638	chromosome18	42590470
ENSG00000111728	chromosome12	22378434
ENSG00000144057	chromosome2	106826881
ENSG00000073849	chromosome3	188243186
ENSG00000160408	chromosome9	129700082
ENSG00000117069	chromosome1	77105949
ENSG00000111728	chromosome12	22378434
ENSG00000101638	chromosome18	42590470
ENSG00000148488	chromosome10	17536261
ENSG00000177511	chromosome18	53171076
ENSG00000111728	chromosome12	22378434
ENSG00000101638	chromosome18	42590470
ENSG00000148488	chromosome10	17536261

ENSG00000198208	chromosome14	74457998
ENSG00000110025	chromosome11	64551586
ENSG00000136643	chromosome1	211291370
ENSG00000136643	chromosome1	211291370
ENSG00000110025	chromosome11	64551586
ENSG00000178662	chromosome2	166159822
ENSG00000178662	chromosome2	166159822
ENSG00000110925	chromosome12	49756612
ENSG00000144655	chromosome3	39170074
ENSG00000110925	chromosome12	49756612
ENSG00000178662	chromosome2	166159822
ENSG00000198901	chromosome15	89338814
ENSG00000143126	chromosome1	109594225
ENSG00000143126	chromosome1	109594225
ENSG00000075275	chromosome22	45311732
ENSG00000008300	chromosome3	48684986
ENSG00000075275	chromosome22	45311732
ENSG00000143126	chromosome1	109594225
ENSG00000196876	chromosome12	50364210
ENSG00000136546	chromosome2	167042453
ENSG00000169432	chromosome2	166876513
ENSG00000153253	chromosome2	165741151
ENSG00000144285	chromosome2	166638378
ENSG00000136531	chromosome2	165860580
ENSG00000007314	chromosome17	59403934
ENSG00000185313	chromosome3	38810506
ENSG00000168356	chromosome3	38966858
ENSG00000183873	chromosome3	38649803
ENSG00000136546	chromosome2	167042453
ENSG00000169432	chromosome2	166876513
ENSG00000153253	chromosome2	165741151
ENSG00000144285	chromosome2	166638378
ENSG00000136531	chromosome2	165860580
ENSG00000196876	chromosome12	50364210
ENSG00000136546	chromosome2	167042453
ENSG00000169432	chromosome2	166876513
ENSG00000153253	chromosome2	165741151
ENSG00000144285	chromosome2	166638378
ENSG00000136531	chromosome2	165860580
ENSG00000196876	chromosome12	50364210
ENSG00000185313	chromosome3	38810506
ENSG00000168356	chromosome3	38966858
ENSG00000183873	chromosome3	38649803
ENSG00000139219	chromosome12	46684372
ENSG00000164692	chromosome7	93862280
ENSG00000108821	chromosome17	45633874
ENSG00000204262	chromosome2	189752576
ENSG00000168542	chromosome2	189547461
ENSG00000060718	chromosome1	103346323
ENSG00000130635	chromosome9	136673855
ENSG00000204248	chromosome6	33267996
ENSG00000060718	chromosome1	103346323
ENSG00000080573	chromosome19	9982062
ENSG00000130635	chromosome9	136673855
ENSG00000204248	chromosome6	33267996
ENSG00000060718	chromosome1	103346323
ENSG00000204248	chromosome6	33267996
ENSG00000130635	chromosome9	136673855
ENSG00000108821	chromosome17	45633874

ENSG00000164692	chromosome7	93862280
ENSG00000164692	chromosome7	93862280
ENSG00000108821	chromosome17	45633874
ENSG00000139219	chromosome12	46684372
ENSG00000204262	chromosome2	189752576
ENSG00000139219	chromosome12	46684372
ENSG00000164692	chromosome7	93862280
ENSG00000108821	chromosome17	45633874
ENSG00000168268	chromosome3	52543710
ENSG00000111696	chromosome12	102759065
ENSG00000168268	chromosome3	52543710
ENSG00000186765	chromosome17	77110153
ENSG00000075618	chromosome7	5599076
ENSG00000106328	chromosome7	127021144
ENSG00000075618	chromosome7	5599076
ENSG00000186765	chromosome17	77110153
ENSG00000163738	chromosome4	75259118
ENSG00000065911	chromosome2	74279277
ENSG00000214698	chromosome4	163463664
ENSG00000138336	chromosome10	70002102
ENSG00000012223	chromosome3	46481362
ENSG00000091513	chromosome3	134947975
ENSG00000012223	chromosome3	46481362
ENSG00000091513	chromosome3	134947975
ENSG00000137460	chromosome4	154083660
ENSG00000204178	chromosome1	25630177
ENSG00000155792	chromosome8	120955268
ENSG00000163328	chromosome2	175054931
ENSG00000155792	chromosome8	120955268
ENSG00000155792	chromosome8	120955268
ENSG00000152022	chromosome1	144188516
ENSG00000145721	chromosome5	96504037
ENSG00000047644	chromosomeX	9991579
ENSG00000151718	chromosome4	184366153
ENSG00000113645	chromosome5	167651736
ENSG00000113645	chromosome5	167651736
ENSG00000151718	chromosome4	184366153
ENSG00000185090	chromosome1	38032442
ENSG00000172469	chromosome6	96141037
ENSG00000075035	chromosome12	107113740
ENSG00000179314	chromosome17	5924703
ENSG00000186666	chromosome12	48523138
ENSG00000008130	chromosome1	1686706
ENSG00000158987	chromosome5	131160514
ENSG00000164120	chromosome4	175680178
ENSG00000158423	chromosomeX	53469950
ENSG00000128408	chromosome22	44192154
ENSG00000093100	chromosome22	16769579
ENSG00000133816	chromosome11	12140279
ENSG00000093100	chromosome22	16769579
ENSG00000093100	chromosome22	16769579
ENSG00000133816	chromosome11	12140279
ENSG00000135596	chromosome6	109882093
ENSG00000188779	chromosome15	65899096
ENSG00000188779	chromosome15	65899096
ENSG00000215474	chromosome18	43029553
ENSG00000157933	chromosome1	2150066
ENSG00000136603	chromosome3	171560814
ENSG00000121680	chromosome11	45895939

ENSG00000154309	chromosome1	221182789
ENSG00000140323	chromosome15	38437815
ENSG00000124827	chromosome6	10990013
ENSG00000137270	chromosome6	53118390
ENSG00000163141	chromosome1	149275826
ENSG00000140299	chromosome15	57761949
ENSG00000175220	chromosome11	46674234
ENSG00000106772	chromosome9	78518317
ENSG00000167654	chromosome19	3836766
ENSG00000140299	chromosome15	57761949
ENSG00000163141	chromosome1	149275826
ENSG00000167654	chromosome19	3836766
ENSG00000106772	chromosome9	78518317
ENSG00000020577	chromosome14	54104388
ENSG00000179134	chromosome19	44539374
ENSG00000104679	chromosome8	23201577
ENSG00000166024	chromosome10	99913142
ENSG00000108272	chromosome17	32022553
ENSG00000184634	chromosomeX	70255330
ENSG00000144893	chromosome3	152287404
ENSG00000146247	chromosome6	79844505
ENSG00000165288	chromosomeX	79951627
ENSG00000165288	chromosomeX	79951627
ENSG00000146247	chromosome6	79844505
ENSG00000185658	chromosome21	39607288
ENSG00000168283	chromosome10	22655385
ENSG00000056661	chromosome17	34150182
ENSG00000180628	chromosome10	92972609
ENSG00000185619	chromosome4	717461
ENSG00000100060	chromosome22	36212162
ENSG00000106003	chromosome7	2524714
ENSG00000106003	chromosome7	2524714
ENSG00000100060	chromosome22	36212162
ENSG00000169733	chromosome17	77602932
ENSG00000169733	chromosome17	77602932
ENSG00000106003	chromosome7	2524714
ENSG00000100060	chromosome22	36212162
ENSG00000100060	chromosome22	36212162
ENSG00000139641	chromosome12	54808371
ENSG00000158220	chromosome3	139636331
ENSG00000158220	chromosome3	139636331
ENSG00000139641	chromosome12	54808371
ENSG00000117868	chromosome7	158315015
ENSG00000139641	chromosome12	54808371
ENSG00000213341	chromosome10	101979280
ENSG00000104365	chromosome8	42248776
ENSG00000143466	chromosome1	204713194
ENSG00000183735	chromosome12	63135918
ENSG00000198959	chromosome20	36227111
ENSG00000166947	chromosome15	41300316
ENSG00000104055	chromosome15	41346340
ENSG00000159495	chromosome15	41381741
ENSG00000125780	chromosome20	2224710
ENSG00000166948	chromosome20	2309615
ENSG00000198959	chromosome20	36227111
ENSG00000124491	chromosome6	6263897
ENSG00000092295	chromosome14	23801399
ENSG00000166947	chromosome15	41300316
ENSG00000104055	chromosome15	41346340

ENSG00000159495	chromosome15	41381741
ENSG00000125780	chromosome20	2224710
ENSG00000166948	chromosome20	2309615
ENSG00000198959	chromosome20	36227111
ENSG00000092295	chromosome14	23801399
ENSG00000124491	chromosome6	6263897
ENSG00000166947	chromosome15	41300316
ENSG00000104055	chromosome15	41346340
ENSG00000159495	chromosome15	41381741
ENSG00000125780	chromosome20	2224710
ENSG00000166948	chromosome20	2309615
ENSG00000198959	chromosome20	36227111
ENSG00000124491	chromosome6	6263897
ENSG00000092295	chromosome14	23801399
ENSG00000163810	chromosome3	44888721
ENSG00000127527	chromosome19	16443757
ENSG00000198399	chromosome2	24404456
ENSG00000205726	chromosome21	34013004
ENSG00000127527	chromosome19	16443757
ENSG00000085832	chromosome1	51757492
ENSG00000196187	chromosome1	224131904
ENSG00000137216	chromosome6	44210300
ENSG00000165548	chromosome14	76754898
ENSG00000137216	chromosome6	44210300
ENSG00000196187	chromosome1	224131904
ENSG00000131409	chromosome19	55743908
ENSG00000148948	chromosome11	40094419
ENSG00000082153	chromosome2	201386186
ENSG00000136261	chromosome7	16671594
ENSG00000157021	chromosome15	39242613
ENSG00000188343	chromosome8	94781911
ENSG00000153789	chromosome16	83703571
ENSG00000122557	chromosome7	35700466
ENSG00000051108	chromosome16	55523658
ENSG00000111790	chromosome12	26998359
ENSG00000052723	chromosome1	115124752
ENSG00000106244	chromosome7	98844108
ENSG00000183016	chromosome7	150533474
ENSG00000132321	chromosome2	237080650
ENSG00000197712	chromosome4	38556095
ENSG00000055147	chromosome5	153394707
ENSG00000172346	chromosome22	40297916
ENSG00000153048	chromosome16	8860687
ENSG00000131482	chromosome17	38306420
ENSG00000152254	chromosome2	169466088
ENSG00000152254	chromosome2	169466088
ENSG00000131482	chromosome17	38306420
ENSG00000141349	chromosome17	39503860
ENSG00000156968	chromosome16	15397256
ENSG00000152430	chromosome2	198358973
ENSG00000092345	chromosome3	16621717
ENSG00000188120	chromosomeY	21044816
ENSG00000205916	chromosomeY	22680142
ENSG00000187191	chromosomeY	22659201
ENSG00000205944	chromosomeY	21065756
ENSG00000089250	chromosome12	116253258
ENSG00000164867	chromosome7	150321825
ENSG00000007171	chromosome17	23149963
ENSG00000164867	chromosome7	150321825

ENSG00000089250	chromosome12	116253258
ENSG00000156136	chromosome4	72078417
ENSG00000114956	chromosome2	74007546
ENSG00000156136	chromosome4	72078417
ENSG00000142494	chromosome17	19377845
ENSG00000180638	chromosome17	19560461
ENSG00000160007	chromosome19	52113773
ENSG00000100852	chromosome14	31616323
ENSG00000213533	chromosome3	52906507
ENSG00000170471	chromosome20	36550490
ENSG00000188559	chromosome20	20641271
ENSG00000174373	chromosome14	35347793
ENSG00000117280	chromosome1	204010708
ENSG00000118508	chromosome6	146906701
ENSG00000117280	chromosome1	204010708
ENSG00000107104	chromosome9	666973
ENSG00000197256	chromosome19	11166188
ENSG00000132854	chromosome1	62519825
ENSG00000132854	chromosome1	62519825
ENSG00000197256	chromosome19	11166188
ENSG00000107104	chromosome9	666973
ENSG00000186994	chromosome19	8308713
ENSG00000107104	chromosome9	666973
ENSG00000132854	chromosome1	62519825
ENSG00000197256	chromosome19	11166188
ENSG00000125434	chromosome17	8138851
ENSG00000162461	chromosome1	15935568
ENSG00000015413	chromosome16	88224320
ENSG00000141096	chromosome16	66571860
ENSG00000167261	chromosome16	66584617
ENSG00000156304	chromosome21	32025877
ENSG00000213079	chromosome6	155096727
ENSG00000170185	chromosome4	144326054
ENSG00000118369	chromosome11	77584940
ENSG00000105963	chromosome7	960640
ENSG00000105963	chromosome7	960640
ENSG00000184060	chromosome17	26273159
ENSG00000177169	chromosome12	130945500
ENSG00000083290	chromosome17	19711323
ENSG00000137478	chromosome11	72404576
ENSG00000197948	chromosome5	141011120
ENSG00000170049	chromosome17	7773479
ENSG00000169282	chromosome3	157321095
ENSG00000069424	chromosome1	6023216
ENSG00000169282	chromosome3	157321095
ENSG00000170049	chromosome17	7773479
ENSG00000170485	chromosome2	100803040
ENSG00000134852	chromosome4	56043710
ENSG00000170485	chromosome2	100803040
ENSG00000170485	chromosome2	100803040
ENSG00000134852	chromosome4	56043710
ENSG00000166049	chromosomeX	150520682
ENSG00000184774	chromosome1	201061258
ENSG00000182050	chromosome12	84907456
ENSG00000182050	chromosome12	84907456
ENSG00000184774	chromosome1	201061258
ENSG00000161013	chromosome5	179166190
ENSG00000071073	chromosome2	98709228
ENSG00000071073	chromosome2	98709228

ENSG00000161013	chromosome5	179166190
ENSG00000074590	chromosome12	105056562
ENSG00000163545	chromosome1	203557380
ENSG00000163545	chromosome1	203557380
ENSG00000074590	chromosome12	105056562
ENSG00000141378	chromosome17	55130122
ENSG00000164758	chromosome8	118602297
ENSG00000099882	chromosome22	49505930
ENSG00000161681	chromosome19	55911989
ENSG00000162105	chromosome11	70536021
ENSG00000161681	chromosome19	55911989
ENSG00000099882	chromosome22	49505930
ENSG00000197296	chromosome20	42373203
ENSG00000139914	chromosome14	23670613
ENSG00000007062	chromosome4	15686628
ENSG00000155066	chromosome2	95304061
ENSG00000007062	chromosome4	15686628
ENSG00000122873	chromosome10	59699040
ENSG00000145354	chromosome4	104009683
ENSG00000122873	chromosome10	59699040
ENSG00000180089	chromosome19	60431922
ENSG00000151117	chromosome11	18677010
ENSG00000100078	chromosome22	29866341
ENSG00000143162	chromosome1	165789684
ENSG00000175874	chromosome2	101370352
ENSG00000185818	chromosome4	2032651
ENSG00000144035	chromosome2	73722264
ENSG00000185818	chromosome4	2032651
ENSG00000144035	chromosome2	73722264
ENSG00000159167	chromosome8	23767982
ENSG00000113739	chromosome5	172687803
ENSG00000124160	chromosome20	44141471
ENSG00000009307	chromosome1	115085809
ENSG00000187147	chromosome1	44650357
ENSG00000187147	chromosome1	44650357
ENSG00000187147	chromosome1	44650357
ENSG00000120733	chromosome5	137716384
ENSG00000115548	chromosome2	86522682
ENSG00000171988	chromosome10	64895429
ENSG00000115548	chromosome2	86522682
ENSG00000120733	chromosome5	137716384
ENSG00000115548	chromosome2	86522682
ENSG00000120733	chromosome5	137716384
ENSG00000171988	chromosome10	64895429
ENSG00000168453	chromosome8	22042629
ENSG00000150347	chromosome10	63331475
ENSG00000196843	chromosome2	96566186
ENSG00000175727	chromosome12	121001143
ENSG00000009950	chromosome7	72676759
ENSG00000185716	chromosome16	21983224
ENSG00000100372	chromosome22	39545199
ENSG00000164022	chromosome4	107457154
ENSG00000034677	chromosome8	101369579
ENSG00000116514	chromosome1	33202874
ENSG00000151692	chromosome2	7054509
ENSG00000137393	chromosome6	18495724
ENSG00000116514	chromosome1	33202874
ENSG00000034677	chromosome8	101369579
ENSG00000048342	chromosome4	15090015

ENSG00000165102	chromosome8	43114803
ENSG00000137106	chromosome9	37412748
ENSG00000008382	chromosome19	4294591
ENSG00000162601	chromosome1	58938313
ENSG00000122012	chromosome5	75463332
ENSG00000159164	chromosome1	148152017
ENSG00000185518	chromosome15	89570498
ENSG00000159164	chromosome1	148152017
ENSG00000122012	chromosome5	75463332
ENSG00000185269	chromosome17	77511878
ENSG00000189203	chromosome14	104142124
ENSG00000189203	chromosome14	104142124
ENSG00000152078	chromosome1	95382046
ENSG00000167695	chromosome17	582638
ENSG00000149926	chromosome16	29949350
ENSG00000162415	chromosome1	45444610
ENSG00000130449	chromosome5	60852930
ENSG00000132003	chromosome19	13767463
ENSG00000130449	chromosome5	60852930
ENSG00000162415	chromosome1	45444610
ENSG00000157093	chromosome3	42423748
ENSG00000151033	chromosome10	30958641
ENSG00000120563	chromosome10	29618053
ENSG00000141316	chromosome17	28343070
ENSG00000171478	chromosomeX	47875014
ENSG00000171489	chromosomeX	47752145
ENSG00000161572	chromosome17	31290474
ENSG00000167531	chromosome12	47250071
ENSG00000090382	chromosome12	68028456
ENSG00000167531	chromosome12	47250071
ENSG00000157093	chromosome3	42423748
ENSG00000151033	chromosome10	30958641
ENSG00000120563	chromosome10	29618053
ENSG00000141316	chromosome17	28343070
ENSG00000171478	chromosomeX	47875014
ENSG00000171489	chromosomeX	47752145
ENSG00000161572	chromosome17	31290474
ENSG00000157093	chromosome3	42423748
ENSG00000151033	chromosome10	30958641
ENSG00000120563	chromosome10	29618053
ENSG00000141316	chromosome17	28343070
ENSG00000171478	chromosomeX	47875014
ENSG00000171489	chromosomeX	47752145
ENSG00000161572	chromosome17	31290474
ENSG00000167531	chromosome12	47250071
ENSG00000090382	chromosome12	68028456
ENSG00000090382	chromosome12	68028456
ENSG00000157093	chromosome3	42423748
ENSG00000151033	chromosome10	30958641
ENSG00000120563	chromosome10	29618053
ENSG00000141316	chromosome17	28343070
ENSG00000171478	chromosomeX	47875014
ENSG00000171489	chromosomeX	47752145
ENSG00000161572	chromosome17	31290474
ENSG00000167531	chromosome12	47250071
ENSG00000157077	chromosome1	52471467
ENSG00000039319	chromosome5	79765723
ENSG00000102471	chromosome13	78953340
ENSG00000131507	chromosome5	141468724

ENSG00000197627	chromosome15	28278803
ENSG00000131507	chromosome5	141468724
ENSG00000102471	chromosome13	78953340
ENSG00000113296	chromosome5	79367117
ENSG00000105664	chromosome19	18763079
ENSG00000137801	chromosome15	37661351
ENSG00000186340	chromosome6	169392805
ENSG00000105664	chromosome19	18763079
ENSG00000113296	chromosome5	79367117
ENSG00000169231	chromosome1	153444292
ENSG00000156395	chromosome10	106391076
ENSG00000108018	chromosome10	108914275
ENSG00000134243	chromosome1	109742038
ENSG00000108018	chromosome10	108914275
ENSG00000156395	chromosome10	106391076
ENSG00000184985	chromosome4	7245275
ENSG00000182040	chromosome17	70430932
ENSG00000175311	chromosome16	21152560
ENSG00000125686	chromosome17	34860842
ENSG00000165655	chromosome10	76831184
ENSG00000183779	chromosome8	37672656
ENSG00000082805	chromosome12	1007331
ENSG00000187672	chromosome3	56444076
ENSG00000171522	chromosome5	40716853
ENSG00000160951	chromosome19	14446133
ENSG00000122420	chromosome1	78731017
ENSG00000125384	chromosome14	51851017
ENSG00000168229	chromosome14	51804283
ENSG00000171522	chromosome5	40716853
ENSG00000122420	chromosome1	78731017
ENSG00000160951	chromosome19	14446133
ENSG00000006638	chromosome19	3551633
ENSG00000006638	chromosome19	3551633
ENSG00000122420	chromosome1	78731017
ENSG00000160951	chromosome19	14446133
ENSG00000050628	chromosome1	71285990
ENSG00000160951	chromosome19	14446133
ENSG00000160013	chromosome19	51819323
ENSG00000168229	chromosome14	51804283
ENSG00000125384	chromosome14	51851017
ENSG00000171522	chromosome5	40716853
ENSG00000168229	chromosome14	51804283
ENSG00000125384	chromosome14	51851017
ENSG00000160013	chromosome19	51819323
ENSG00000147443	chromosome8	21827059
ENSG00000146094	chromosome5	176869460
ENSG00000115325	chromosome2	74635390
ENSG00000146094	chromosome5	176869460
ENSG00000115325	chromosome2	74635390
ENSG00000147443	chromosome8	21827059
ENSG00000206052	chromosome18	65219461
ENSG00000101134	chromosome20	52525893
ENSG00000115325	chromosome2	74635390
ENSG00000146094	chromosome5	176869460
ENSG00000125170	chromosome16	56070921
ENSG00000101134	chromosome20	52525893
ENSG00000206052	chromosome18	65219461
ENSG00000166225	chromosome12	68249078
ENSG00000137218	chromosome6	41852704

ENSG00000175445	chromosome8	19841232
ENSG00000101670	chromosome18	45342677
ENSG00000166035	chromosome15	56511524
ENSG00000101670	chromosome18	45342677
ENSG00000175445	chromosome8	19841232
ENSG00000144837	chromosome3	120799451
ENSG00000188992	chromosome21	14501116
ENSG00000163898	chromosome3	186752954
ENSG00000188992	chromosome21	14501116
ENSG00000163898	chromosome3	186752954
ENSG00000144837	chromosome3	120799451
ENSG00000101670	chromosome18	45342677
ENSG00000175445	chromosome8	19841232
ENSG00000166035	chromosome15	56511524
ENSG00000175445	chromosome8	19841232
ENSG00000054277	chromosome1	239870180
ENSG00000124818	chromosome6	47857762
ENSG00000124818	chromosome6	47857762
ENSG00000180245	chromosome4	110968633
ENSG00000148604	chromosome10	85994827
ENSG00000128617	chromosome7	128203081
ENSG00000163914	chromosome3	130730267
ENSG00000166160	chromosomeX	153138479
ENSG00000147380	chromosomeX	153101361
ENSG00000102076	chromosomeX	153062952
ENSG00000124818	chromosome6	47857762
ENSG00000054277	chromosome1	239870180
ENSG00000122375	chromosome10	88404521
ENSG00000124818	chromosome6	47857762
ENSG00000166160	chromosomeX	153138479
ENSG00000147380	chromosomeX	153101361
ENSG00000102076	chromosomeX	153062952
ENSG00000163914	chromosome3	130730267
ENSG00000180245	chromosome4	110968633
ENSG00000148604	chromosome10	85994827
ENSG00000124818	chromosome6	47857762
ENSG00000163914	chromosome3	130730267
ENSG00000128617	chromosome7	128203081
ENSG00000148604	chromosome10	85994827
ENSG00000148604	chromosome10	85994827
ENSG00000180245	chromosome4	110968633
ENSG00000163914	chromosome3	130730267
ENSG00000124818	chromosome6	47857762
ENSG00000071655	chromosome19	1543631
ENSG00000134046	chromosome18	50005096
ENSG00000134046	chromosome18	50005096
ENSG00000071655	chromosome19	1543631
ENSG00000184301	chromosome19	6981623
ENSG00000205718	chromosome19	6991156
ENSG00000182315	chromosome19	7009617
ENSG00000196589	chromosome19	6972409
ENSG00000205717	chromosome19	7000385
ENSG00000170948	chromosome19	8814355
ENSG00000186862	chromosome10	102779967
ENSG00000095397	chromosome9	116306903
ENSG00000095397	chromosome9	116306903
ENSG00000186862	chromosome10	102779967
ENSG00000006611	chromosome11	17522431
ENSG00000138136	chromosome10	102978563

ENSG00000179528	chromosome2	74583495
ENSG00000138136	chromosome10	102978563
ENSG00000164056	chromosome4	124542197
ENSG00000187678	chromosome5	141679515
ENSG00000136158	chromosome13	79809842
ENSG00000136158	chromosome13	79809842
ENSG00000164056	chromosome4	124542197
ENSG00000187678	chromosome5	141679515
ENSG00000168939	chromosomeX	154656728
ENSG00000187678	chromosome5	141679515
ENSG00000164056	chromosome4	124542197
ENSG00000121653	chromosome11	45863948
ENSG00000008735	chromosome22	49386114
ENSG00000173230	chromosome3	122932509
ENSG00000173230	chromosome3	122932509
ENSG00000173230	chromosome3	122932509
ENSG00000144674	chromosome3	37260046
ENSG00000172137	chromosome16	69950197
ENSG00000104327	chromosome8	91164102
ENSG00000104327	chromosome8	91164102
ENSG00000172137	chromosome16	69950197
ENSG00000143079	chromosome1	112760311
ENSG00000077063	chromosome7	117300706
ENSG00000100503	chromosome14	50359773
ENSG00000101004	chromosome20	25455224
ENSG00000157827	chromosome2	152900364
ENSG00000161791	chromosome12	48387246
ENSG00000184922	chromosome17	40655275
ENSG00000161791	chromosome12	48387246
ENSG00000157827	chromosome2	152900364
ENSG00000157827	chromosome2	152900364
ENSG00000151693	chromosome2	9264685
ENSG00000153317	chromosome8	131483372
ENSG00000153317	chromosome8	131483372
ENSG00000151693	chromosome2	9264685
ENSG00000153317	chromosome8	131483372
ENSG00000151693	chromosome2	9264685
ENSG00000088280	chromosome1	23683214
ENSG00000090432	chromosome1	20707105
ENSG00000170633	chromosome12	120322399
ENSG00000092871	chromosome17	30377686
ENSG00000105499	chromosome19	53301599
ENSG00000168907	chromosome15	40236101
ENSG00000168970	chromosome15	39907615
ENSG00000159337	chromosome15	40173950
ENSG00000188089	chromosome15	40130194
ENSG00000168970	chromosome15	39907615
ENSG00000159337	chromosome15	40173950
ENSG00000188089	chromosome15	40130194
ENSG00000168907	chromosome15	40236101
ENSG00000137776	chromosome15	57013057
ENSG00000130254	chromosome19	5573727
ENSG00000160633	chromosome19	5574217
ENSG00000130254	chromosome19	5573727
ENSG00000160633	chromosome19	5574217
ENSG00000137776	chromosome15	57013057
ENSG00000163485	chromosome1	201364593
ENSG00000121933	chromosome1	111908098
ENSG00000170425	chromosome17	15789288

ENSG00000128271	chromosome22	23159315
ENSG00000170425	chromosome17	15789288
ENSG00000128271	chromosome22	23159315
ENSG00000163485	chromosome1	201364593
ENSG00000121933	chromosome1	111908098
ENSG00000128271	chromosome22	23159315
ENSG00000170425	chromosome17	15789288
ENSG00000121933	chromosome1	111908098
ENSG00000163485	chromosome1	201364593
ENSG00000121933	chromosome1	111908098
ENSG00000154920	chromosome17	45807569
ENSG00000197774	chromosome16	1763230
ENSG00000145919	chromosome5	172976046
ENSG00000136856	chromosome9	129199331
ENSG00000160326	chromosome9	135334003
ENSG00000107807	chromosome10	102881289
ENSG00000115297	chromosome2	74595442
ENSG00000164438	chromosome5	170668975
ENSG00000115297	chromosome2	74595442
ENSG00000107807	chromosome10	102881289
ENSG00000173744	chromosome2	228045382
ENSG00000106351	chromosome7	99974906
ENSG00000139263	chromosome12	57600284
ENSG00000198799	chromosome1	113417552
ENSG00000198799	chromosome1	113417552
ENSG00000139263	chromosome12	57600284
ENSG00000144749	chromosome3	66633522
ENSG00000152492	chromosome3	192529762
ENSG00000147262	chromosomeX	129347103
ENSG00000147262	chromosomeX	129347103
ENSG00000158715	chromosome1	203900408
ENSG00000115226	chromosome2	27571051
ENSG00000160097	chromosome1	33108938
ENSG00000160097	chromosome1	33108938
ENSG00000115226	chromosome2	27571051
ENSG00000135097	chromosome12	119291278
ENSG00000153944	chromosome17	52689103
ENSG00000156531	chromosomeX	133339314
ENSG00000136147	chromosome13	48968139
ENSG00000010318	chromosome3	52421936
ENSG00000092140	chromosome14	30120037
ENSG00000092140	chromosome14	30120037
ENSG00000010318	chromosome3	52421936
ENSG00000136147	chromosome13	48968139
ENSG00000156531	chromosomeX	133339314
ENSG00000138757	chromosome4	76806234
ENSG00000145907	chromosome5	151146375
ENSG00000047346	chromosome15	50709157
ENSG00000005238	chromosome9	35098272
ENSG00000204120	chromosome2	233308125
ENSG00000146830	chromosome7	100123798
ENSG00000115602	chromosome2	102321157
ENSG00000115594	chromosome2	102140617
ENSG00000115598	chromosome2	102170760
ENSG00000115590	chromosome2	101991470
ENSG00000115604	chromosome2	102345553
ENSG00000115607	chromosome2	102406170
ENSG00000196083	chromosome3	191764773
ENSG00000185187	chromosome11	399995

ENSG00000196083	chromosome3	191764773
ENSG00000115604	chromosome2	102345553
ENSG00000115607	chromosome2	102406170
ENSG00000115604	chromosome2	102345553
ENSG00000115607	chromosome2	102406170
ENSG00000196083	chromosome3	191764773
ENSG00000115602	chromosome2	102321157
ENSG00000115594	chromosome2	102140617
ENSG00000115598	chromosome2	102170760
ENSG00000115590	chromosome2	101991470
ENSG00000185187	chromosome11	399995
ENSG00000115602	chromosome2	102321157
ENSG00000115594	chromosome2	102140617
ENSG00000115598	chromosome2	102170760
ENSG00000115590	chromosome2	101991470
ENSG00000115604	chromosome2	102345553
ENSG00000115607	chromosome2	102406170
ENSG00000196083	chromosome3	191764773
ENSG00000196083	chromosome3	191764773
ENSG00000135144	chromosome12	111980381
ENSG00000110042	chromosome11	58705895
ENSG00000110042	chromosome11	58705895
ENSG00000135144	chromosome12	111980381
ENSG00000091073	chromosome7	75947763
ENSG00000148143	chromosome9	108725486
ENSG00000172260	chromosome1	72520766
ENSG00000185565	chromosome3	117646569
ENSG00000183715	chromosome11	132907430
ENSG00000182667	chromosome11	130745912
ENSG00000183715	chromosome11	132907430
ENSG00000182667	chromosome11	130745912
ENSG00000172260	chromosome1	72520766
ENSG00000185565	chromosome3	117646569
ENSG00000142549	chromosome19	56516573
ENSG00000185565	chromosome3	117646569
ENSG00000172260	chromosome1	72520766
ENSG00000142549	chromosome19	56516573
ENSG00000183715	chromosome11	132907430
ENSG00000182667	chromosome11	130745912
ENSG00000172260	chromosome1	72520766
ENSG00000185565	chromosome3	117646569
ENSG00000124302	chromosome19	38872008
ENSG00000154080	chromosome18	22976772
ENSG00000180767	chromosome3	127725866
ENSG00000171310	chromosome12	103375320
ENSG00000154080	chromosome18	22976772
ENSG00000124302	chromosome19	38872008
ENSG00000136213	chromosome7	2438801
ENSG00000171310	chromosome12	103375320
ENSG00000180767	chromosome3	127725866
ENSG00000136213	chromosome7	2438801
ENSG00000197183	chromosome20	30636537
ENSG00000101746	chromosome18	30057216
ENSG00000164342	chromosome4	187234768
ENSG00000196664	chromosomeX	12795619
ENSG00000173366	chromosome3	52248073
ENSG00000196664	chromosomeX	12795619
ENSG00000173366	chromosome3	52248073
ENSG00000164342	chromosome4	187234768

ENSG00000173366	chromosome3	52248073
ENSG00000196664	chromosomeX	12795619
ENSG00000135437	chromosome12	54401236
ENSG00000073737	chromosome2	169646338
ENSG00000135437	chromosome12	54401236
ENSG00000073737	chromosome2	169646338
ENSG00000135437	chromosome12	54401236
ENSG00000139547	chromosome12	55637514
ENSG00000025423	chromosome12	55453904
ENSG00000170426	chromosome12	55614313
ENSG00000176387	chromosome16	66022653
ENSG00000086696	chromosome16	80626531
ENSG00000051128	chromosome19	18910792
ENSG00000152413	chromosome5	78844390
ENSG00000103942	chromosome15	81412481
ENSG00000214396	chromosome14	105988026
ENSG00000152413	chromosome5	78844390
ENSG00000051128	chromosome19	18910792
ENSG00000162924	chromosome2	60962480
ENSG00000173039	chromosome11	65186880
ENSG00000104856	chromosome19	50196678
ENSG00000077150	chromosome10	104145707
ENSG00000109320	chromosome4	103665706
ENSG00000173039	chromosome11	65186880
ENSG00000162924	chromosome2	60962480
ENSG00000040933	chromosome2	98502944
ENSG00000109452	chromosome4	143571863
ENSG00000175866	chromosome17	76623650
ENSG00000006453	chromosome7	97868101
ENSG00000175866	chromosome17	76623650
ENSG00000006453	chromosome7	97868101
ENSG00000175866	chromosome17	76623650
ENSG00000128298	chromosome22	36836479
ENSG00000178718	chromosome15	73035978
ENSG00000164967	chromosome9	34601294
ENSG00000129535	chromosome14	23621898
ENSG00000204103	chromosome20	38750905
ENSG00000178573	chromosome16	78191301
ENSG00000182759	chromosome8	144583720
ENSG00000178573	chromosome16	78191301
ENSG00000204103	chromosome20	38750905
ENSG00000197063	chromosome17	77474354
ENSG00000215622	supercontigNT_113944	177887
ENSG00000198517	chromosome7	1545355
ENSG00000185022	chromosome22	36939807
ENSG00000197063	chromosome17	77474354
ENSG00000215622	supercontigNT_113944	177887
ENSG00000197063	chromosome17	77474354
ENSG00000215622	supercontigNT_113944	177887
ENSG00000185022	chromosome22	36939807
ENSG00000204103	chromosome20	38750905
ENSG00000178573	chromosome16	78191301
ENSG00000129535	chromosome14	23621898
ENSG00000072864	chromosome16	15651600
ENSG00000166579	chromosome17	8288315
ENSG00000131779	chromosome1	144227758
ENSG00000166821	chromosome15	88034868
ENSG00000151379	chromosome2	17861267
ENSG00000188095	chromosome15	88120593

ENSG00000166823	chromosome15	88095467
ENSG00000151379	chromosome2	17861267
ENSG00000141568	chromosome17	78071054
ENSG00000164916	chromosome7	4688466
ENSG00000131263	chromosomeX	73732538
ENSG00000127870	chromosome13	25691787
ENSG00000137075	chromosome9	36390106
ENSG00000146083	chromosome5	175892080
ENSG00000099984	chromosome22	22652389
ENSG00000133433	chromosome22	22633319
ENSG00000184490	chromosome22	22696752
ENSG00000184674	chromosome22	22714232
ENSG00000185831	chromosome22	22672401
ENSG00000206090	chromosome22	22613313
ENSG00000157404	chromosome4	55218939
ENSG00000182578	chromosome5	149446184
ENSG00000122025	chromosome13	27572648
ENSG00000037280	chromosome5	180009152
ENSG00000128052	chromosome4	55686218
ENSG00000102755	chromosome13	27966981
ENSG00000128052	chromosome4	55686218
ENSG00000037280	chromosome5	180009152
ENSG00000037280	chromosome5	180009152
ENSG00000128052	chromosome4	55686218
ENSG00000182578	chromosome5	149446184
ENSG00000157404	chromosome4	55218939
ENSG00000134853	chromosome4	54819693
ENSG00000113721	chromosome5	149496804
ENSG00000122025	chromosome13	27572648
ENSG00000157404	chromosome4	55218939
ENSG00000182578	chromosome5	149446184
ENSG00000102755	chromosome13	27966981
ENSG00000037280	chromosome5	180009152
ENSG00000128052	chromosome4	55686218
ENSG00000104213	chromosome8	17479047
ENSG00000113721	chromosome5	149496804
ENSG00000134853	chromosome4	54819693
ENSG00000169291	chromosome1	152741127
ENSG00000138606	chromosome15	43278565
ENSG00000107338	chromosome9	38058643
ENSG00000105251	chromosome19	4231061
ENSG00000107338	chromosome9	38058643
ENSG00000105251	chromosome19	4231061
ENSG00000138606	chromosome15	43278565
ENSG00000105251	chromosome19	4231061
ENSG00000107338	chromosome9	38058643
ENSG00000181781	chromosome19	425748
ENSG00000182950	chromosome15	73803596
ENSG00000205464	chromosome5	81636977
ENSG00000071553	chromosomeX	153310233
ENSG00000071553	chromosomeX	153310233
ENSG00000166145	chromosome15	38924045
ENSG00000120256	chromosome6	150226850
ENSG00000104880	chromosome19	7410827
ENSG00000038102	chromosome5	73217484
ENSG00000170776	chromosome15	83829956
ENSG00000170776	chromosome15	83829956
ENSG00000104880	chromosome19	7410827
ENSG00000038102	chromosome5	73217484

ENSG00000116584	chromosome1	154214843
ENSG00000038102	chromosome5	73217484
ENSG00000104880	chromosome19	7410827
ENSG00000166819	chromosome15	88021725
ENSG00000047634	chromosomeX	17672228
ENSG00000130590	chromosome20	62081265
ENSG00000177570	chromosome8	119703237
ENSG00000177570	chromosome8	119703237
ENSG00000130590	chromosome20	62081265
ENSG00000047634	chromosomeX	17672228
ENSG00000203943	chromosome1	84536634
ENSG00000168309	chromosome3	58530628
ENSG00000065809	chromosome10	14856669
ENSG00000166813	chromosome15	87992583
ENSG00000165115	chromosome9	85720327
ENSG00000169047	chromosome2	227371699
ENSG00000133124	chromosomeX	107866231
ENSG00000185950	chromosome13	109236402
ENSG00000169047	chromosome2	227371699
ENSG00000133124	chromosomeX	107866231
ENSG00000185950	chromosome13	109236402
ENSG00000133124	chromosomeX	107866231
ENSG00000185950	chromosome13	109236402
ENSG00000169047	chromosome2	227371699
ENSG00000185950	chromosome13	109236402
ENSG00000133124	chromosomeX	107866231
ENSG00000141052	chromosome17	12510231
ENSG00000186260	chromosome16	14141965
ENSG00000196588	chromosome22	39189178
ENSG00000196588	chromosome22	39189178
ENSG00000186260	chromosome16	14141965
ENSG00000141052	chromosome17	12510231
ENSG00000141052	chromosome17	12510231
ENSG00000196588	chromosome22	39189178
ENSG00000186260	chromosome16	14141965
ENSG00000176909	chromosome19	53914101
ENSG00000143458	chromosome1	149327290
ENSG00000104064	chromosome15	48389288
ENSG00000173273	chromosome8	9450860
ENSG00000107854	chromosome10	93548428
ENSG00000134013	chromosome8	23281810
ENSG00000138131	chromosome10	100012767
ENSG00000113083	chromosome5	121441580
ENSG00000113083	chromosome5	121441580
ENSG00000129038	chromosome15	72006178
ENSG00000138131	chromosome10	100012767
ENSG00000134013	chromosome8	23281810
ENSG00000115318	chromosome2	74633270
ENSG00000142871	chromosome1	85819256
ENSG00000112761	chromosome6	112482200
ENSG00000104415	chromosome8	134272545
ENSG00000064205	chromosome20	42777446
ENSG00000136999	chromosome8	120497954
ENSG00000118523	chromosome6	132314006
ENSG00000104415	chromosome8	134272545
ENSG00000112761	chromosome6	112482200
ENSG00000136999	chromosome8	120497954
ENSG00000118523	chromosome6	132314006
ENSG00000118523	chromosome6	132314006

ENSG00000136999	chromosome8	120497954
ENSG00000142871	chromosome1	85819256
ENSG00000136999	chromosome8	120497954
ENSG00000118523	chromosome6	132314006
ENSG00000064205	chromosome20	42777446
ENSG00000142871	chromosome1	85819256
ENSG00000186031	chromosome15	31146322
ENSG00000155816	chromosome1	238321604
ENSG00000156256	chromosome21	29322106
ENSG00000123552	chromosome6	100064818
ENSG00000186472	chromosome7	82629845
ENSG00000164061	chromosome3	49567040
ENSG00000157741	chromosome7	138567020
ENSG00000118900	chromosome16	4842920
ENSG00000115295	chromosome2	29197756
ENSG00000105270	chromosome19	41215100
ENSG00000130779	chromosome12	121430953
ENSG00000106665	chromosome7	73369813
ENSG00000105270	chromosome19	41215100
ENSG00000115295	chromosome2	29197756
ENSG00000085552	chromosome1	158179899
ENSG00000116785	chromosome1	195010640
ENSG00000134365	chromosome1	195123904
ENSG00000080910	chromosome1	195055598
ENSG00000134391	chromosome1	195179634
ENSG00000134389	chromosome1	195213346
ENSG00000000971	chromosome1	194887871
ENSG00000143278	chromosome1	195302977
ENSG00000105656	chromosome19	18493866
ENSG00000187213	chromosome1	156414163
ENSG00000118985	chromosome5	95323182
ENSG00000197822	chromosome5	68835828
ENSG00000152939	chromosome5	68750969
ENSG00000197822	chromosome5	68835828
ENSG00000197822	chromosome5	68835828
ENSG00000152939	chromosome5	68750969
ENSG00000187213	chromosome1	156414163
ENSG00000118985	chromosome5	95323182
ENSG00000105656	chromosome19	18493866
ENSG00000152939	chromosome5	68750969
ENSG00000187213	chromosome1	156414163
ENSG00000118985	chromosome5	95323182
ENSG00000105656	chromosome19	18493866
ENSG00000101188	chromosome20	60811005
ENSG00000132911	chromosome5	151764868
ENSG00000174417	chromosome8	110168918
ENSG00000174417	chromosome8	110168918
ENSG00000156675	chromosome8	37876118
ENSG00000135631	chromosome2	73193420
ENSG00000135631	chromosome2	73193420
ENSG00000156675	chromosome8	37876118
ENSG00000107560	chromosome10	119795665
ENSG00000109089	chromosome17	70495526
ENSG00000140743	chromosome16	22293132
ENSG00000171509	chromosome4	159662579
ENSG00000133105	chromosome13	31211750
ENSG00000171509	chromosome4	159662579
ENSG00000165246	chromosomeY	12533874
ENSG00000146938	chromosomeX	6079508

ENSG00000169760	chromosome3	174805083
ENSG00000169992	chromosome17	7252299
ENSG00000196338	chromosomeX	70284325
ENSG00000196338	chromosomeX	70284325
ENSG00000169760	chromosome3	174805083
ENSG00000169992	chromosome17	7252299
ENSG00000169992	chromosome17	7252299
ENSG00000169760	chromosome3	174805083
ENSG00000169760	chromosome3	174805083
ENSG00000169992	chromosome17	7252299
ENSG00000196338	chromosomeX	70284325
ENSG00000165246	chromosomeY	12533874
ENSG00000146938	chromosomeX	6079508
ENSG00000154654	chromosome21	21292753
ENSG00000149294	chromosome11	112573125
ENSG00000149294	chromosome11	112573125
ENSG00000154654	chromosome21	21292753
ENSG00000156876	chromosome1	100370959
ENSG00000072364	chromosome5	132300781
ENSG00000172493	chromosome4	88147584
ENSG00000144218	chromosome2	100087349
ENSG00000155966	chromosomeX	147390310
ENSG00000172493	chromosome4	88147584
ENSG00000072364	chromosome5	132300781
ENSG00000155966	chromosomeX	147390310
ENSG00000144218	chromosome2	100087349
ENSG00000172201	chromosome6	19945965
ENSG00000125968	chromosome20	29656852
ENSG00000115738	chromosome2	8739747
ENSG00000117318	chromosome1	23758505
ENSG00000125968	chromosome20	29656852
ENSG00000172201	chromosome6	19945965
ENSG00000115738	chromosome2	8739747
ENSG00000172201	chromosome6	19945965
ENSG00000125968	chromosome20	29656852
ENSG00000186288	chromosomeX	72215951
ENSG00000184388	chromosomeX	72140207
ENSG00000134278	chromosome18	12536799
ENSG00000204991	chromosome16	88422430
ENSG00000134278	chromosome18	12536799
ENSG00000187164	chromosome10	118754758
ENSG00000101057	chromosome20	41729338
ENSG00000118513	chromosome6	135544345
ENSG00000185697	chromosome8	67693681
ENSG00000101057	chromosome20	41729338
ENSG00000185697	chromosome8	67693681
ENSG00000118513	chromosome6	135544345
ENSG00000183060	chromosome15	98089728
ENSG00000176018	chromosome5	89856863
ENSG00000106554	chromosome7	132417238
ENSG00000159685	chromosome3	127905846
ENSG00000159685	chromosome3	127905846
ENSG00000106554	chromosome7	132417238
ENSG00000130235	chromosome14	104238748
ENSG00000075426	chromosome2	28469181
ENSG00000175592	chromosome11	65424387
ENSG00000140044	chromosome14	74974377
ENSG00000162772	chromosome1	210854987
ENSG00000125740	chromosome19	50663685

ENSG00000170345	chromosome14	74815439
ENSG00000175592	chromosome11	65424387
ENSG00000075426	chromosome2	28469181
ENSG00000170345	chromosome14	74815439
ENSG00000125740	chromosome19	50663685
ENSG00000140044	chromosome14	74974377
ENSG00000133401	chromosome5	31835113
ENSG00000172349	chromosome15	79286597
ENSG00000143341	chromosome1	183970535
ENSG00000148357	chromosome9	132250950
ENSG00000152580	chromosome3	152659188
ENSG00000168462	chromosomeY	9906767
ENSG00000101825	chromosomeX	3271875
ENSG00000155657	chromosome2	179377615
ENSG00000168462	chromosomeY	9906767
ENSG00000101825	chromosomeX	3271875
ENSG00000101825	chromosomeX	3271875
ENSG00000168462	chromosomeY	9906767
ENSG00000168462	chromosomeY	9906767
ENSG00000101825	chromosomeX	3271875
ENSG00000143341	chromosome1	183970535
ENSG00000148357	chromosome9	132250950
ENSG00000152580	chromosome3	152659188
ENSG00000168462	chromosomeY	9906767
ENSG00000101825	chromosomeX	3271875
ENSG00000143341	chromosome1	183970535
ENSG00000148357	chromosome9	132250950
ENSG00000152580	chromosome3	152659188
ENSG00000143341	chromosome1	183970535
ENSG00000148357	chromosome9	132250950
ENSG00000152580	chromosome3	152659188
ENSG00000168462	chromosomeY	9906767
ENSG00000101825	chromosomeX	3271875
ENSG00000170162	chromosome6	117693620
ENSG00000206538	chromosome3	87122584
ENSG00000206538	chromosome3	87122584
ENSG00000170162	chromosome6	117693620
ENSG00000170162	chromosome6	117693620
ENSG00000206538	chromosome3	87122584
ENSG00000102243	chromosomeX	135445846
ENSG00000165300	chromosome13	87125645
ENSG00000184564	chromosome13	85268645
ENSG00000179542	chromosomeX	142546591
ENSG00000184564	chromosome13	85268645
ENSG00000179542	chromosomeX	142546591
ENSG00000165300	chromosome13	87125645
ENSG00000179542	chromosomeX	142546591
ENSG00000184564	chromosome13	85268645
ENSG00000144488	chromosome2	238673800
ENSG00000144488	chromosome2	238673800
ENSG00000116219	chromosome1	16919240
ENSG00000187017	chromosome1	6407603
ENSG00000167522	chromosome16	87910971
ENSG00000101745	chromosome18	9172431
ENSG00000138376	chromosome2	215382539
ENSG00000101745	chromosome18	9172431
ENSG00000167522	chromosome16	87910971
ENSG00000123595	chromosomeX	13636787
ENSG00000123570	chromosomeX	102967371

ENSG00000123570	chromosomeX	102967371
ENSG00000123595	chromosomeX	13636787
ENSG00000131196	chromosome18	75257213
ENSG00000101096	chromosome20	49592446
ENSG00000100968	chromosome14	23907387
ENSG00000072736	chromosome16	66677086
ENSG00000072736	chromosome16	66677086
ENSG00000100968	chromosome14	23907387
ENSG00000102908	chromosome16	68156886
ENSG00000100968	chromosome14	23907387
ENSG00000072736	chromosome16	66677086
ENSG00000131196	chromosome18	75257213
ENSG00000101096	chromosome20	49592446
ENSG00000101096	chromosome20	49592446
ENSG00000131196	chromosome18	75257213
ENSG00000149970	chromosomeX	21302937
ENSG00000153721	chromosome6	154872941
ENSG00000074706	chromosome6	154628774
ENSG00000149970	chromosomeX	21302937
ENSG00000153721	chromosome6	154872941
ENSG00000153721	chromosome6	154872941
ENSG00000074706	chromosome6	154628774
ENSG00000149970	chromosomeX	21302937
ENSG00000142675	chromosome1	26376626
ENSG00000149970	chromosomeX	21302937
ENSG00000074706	chromosome6	154628774
ENSG00000071909	chromosome2	170743044
ENSG00000095777	chromosome10	26281046
ENSG00000174799	chromosome4	56513054
ENSG00000135951	chromosome2	99092335
ENSG00000142798	chromosome1	22136298
ENSG00000095585	chromosome10	98021146
ENSG00000152292	chromosome2	85514705
ENSG00000043462	chromosome5	169657194
ENSG00000109684	chromosome4	10278592
ENSG00000109684	chromosome4	10278592
ENSG00000043462	chromosome5	169657194
ENSG00000152292	chromosome2	85514705
ENSG00000095585	chromosome10	98021146
ENSG00000204842	chromosome12	110521702
ENSG00000168488	chromosome16	28742082
ENSG00000166173	chromosome15	68933482
ENSG00000073009	chromosomeX	153423673
ENSG00000123240	chromosome10	13191129
ENSG00000186918	chromosome8	28274561
ENSG00000125520	chromosome20	61841710
ENSG00000164684	chromosome8	81948835
ENSG00000164684	chromosome8	81948835
ENSG00000125520	chromosome20	61841710
ENSG00000125520	chromosome20	61841710
ENSG00000164684	chromosome8	81948835
ENSG00000186918	chromosome8	28274561
ENSG00000168772	chromosome4	105632409
ENSG00000168772	chromosome4	105632409
ENSG00000171604	chromosome5	139040293
ENSG00000172366	chromosome16	631993
ENSG00000144331	chromosome2	180342728
ENSG00000151789	chromosome3	21767413
ENSG00000161642	chromosome12	53064566

ENSG00000187595	chromosome17	37459394
ENSG00000161642	chromosome12	53064566
ENSG00000144331	chromosome2	180342728
ENSG00000151789	chromosome3	21767413
ENSG00000151789	chromosome3	21767413
ENSG00000144331	chromosome2	180342728
ENSG00000109113	chromosome17	24068412
ENSG00000100228	chromosome22	21817553
ENSG00000109113	chromosome17	24068412
ENSG00000124243	chromosome20	48844938
ENSG00000186222	chromosome4	6768838
ENSG00000121577	chromosome3	120861961
ENSG00000132429	chromosome6	105716478
ENSG00000135127	chromosome12	118912056
ENSG00000162069	chromosome16	3025499
ENSG00000116044	chromosome2	177837551
ENSG00000082641	chromosome17	43483423
ENSG00000123405	chromosome12	52975300
ENSG00000112182	chromosome6	90775285
ENSG00000082641	chromosome17	43483423
ENSG00000116044	chromosome2	177837551
ENSG00000082641	chromosome17	43483423
ENSG00000123405	chromosome12	52975300
ENSG00000112182	chromosome6	90775285
ENSG00000112182	chromosome6	90775285
ENSG00000156273	chromosome21	29615473
ENSG00000198324	chromosome12	110285615
ENSG00000177096	chromosome22	40803244
ENSG00000198324	chromosome12	110285615
ENSG00000177096	chromosome22	40803244
ENSG00000187550	chromosome19	60739474
ENSG00000188322	chromosome16	28236214
ENSG00000188322	chromosome16	28236214
ENSG00000187550	chromosome19	60739474
ENSG00000187550	chromosome19	60739474
ENSG00000188322	chromosome16	28236214
ENSG00000172270	chromosome19	522548
ENSG00000156642	chromosome15	71712610
ENSG00000170571	chromosome5	49772743
ENSG00000156642	chromosome15	71712610
ENSG00000172270	chromosome19	522548
ENSG00000162706	chromosome1	157408180
ENSG00000175161	chromosome3	85858322
ENSG00000109943	chromosome11	122214465
ENSG00000175161	chromosome3	85858322
ENSG00000162706	chromosome1	157408180
ENSG00000124104	chromosome20	43895966
ENSG00000167208	chromosome16	49268939
ENSG00000118976	chromosome12	3818729
ENSG00000164164	chromosome4	146320576
ENSG00000165175	chromosomeX	38549144
ENSG00000165175	chromosomeX	38549144
ENSG00000151365	chromosome11	77452576
ENSG00000181754	chromosome1	109853058
ENSG00000176020	chromosome3	49731903
ENSG00000176020	chromosome3	49731903
ENSG00000181754	chromosome1	109853058
ENSG00000139211	chromosome12	45759053
ENSG00000120727	chromosome5	138727373

ENSG00000124374	chromosome2	71283240
ENSG00000187556	chromosome19	13849063
ENSG00000188425	chromosome19	51109792
ENSG00000095627	chromosome10	115937581
ENSG00000180113	chromosome6	46763825
ENSG00000180113	chromosome6	46763825
ENSG00000095627	chromosome10	115937581
ENSG00000095627	chromosome10	115937581
ENSG00000180113	chromosome6	46763825
ENSG00000132972	chromosome13	24236342
ENSG00000180113	chromosome6	46763825
ENSG00000068650	chromosome13	112392732
ENSG00000101974	chromosomeX	138841868
ENSG00000101974	chromosomeX	138841868
ENSG00000068650	chromosome13	112392732
ENSG00000058063	chromosome3	183994242
ENSG00000144596	chromosome3	14556820
ENSG00000155974	chromosome12	65358952
ENSG00000007908	chromosome1	167969378
ENSG00000188404	chromosome1	167947303
ENSG00000159674	chromosome4	1155860
ENSG00000151327	chromosome14	34585421
ENSG00000214886	chromosome4	100096174
ENSG00000197520	chromosome1	220986511
ENSG00000189431	chromosome11	12987700
ENSG00000198774	chromosome12	84723874
ENSG00000015592	chromosome8	27157158
ENSG00000117632	chromosome1	26103755
ENSG00000015592	chromosome8	27157158
ENSG00000117632	chromosome1	26103755
ENSG00000015592	chromosome8	27157158
ENSG00000213603	chromosome20	61755143
ENSG00000104435	chromosome8	80685986
ENSG00000214241	chromosome6	155069370
ENSG00000104435	chromosome8	80685986
ENSG00000214241	chromosome6	155069370
ENSG00000213603	chromosome20	61755143
ENSG00000137693	chromosome11	101486790
ENSG00000214251	chromosome6	147769718
ENSG00000018408	chromosome3	150857784
ENSG00000128573	chromosome7	113853803
ENSG00000137166	chromosome6	41641477
ENSG00000114861	chromosome3	71330223
ENSG00000137166	chromosome6	41641477
ENSG00000128573	chromosome7	113853803
ENSG00000162434	chromosome1	65124536
ENSG00000105397	chromosome19	10350083
ENSG00000096968	chromosome9	5011988
ENSG00000105639	chromosome19	17816227
ENSG00000096968	chromosome9	5011988
ENSG00000105397	chromosome19	10350083
ENSG00000162434	chromosome1	65124536
ENSG00000096968	chromosome9	5011988
ENSG00000096968	chromosome9	5011988
ENSG00000105639	chromosome19	17816227
ENSG00000154359	chromosome8	12657301
ENSG00000154359	chromosome8	12657301
ENSG00000175556	chromosomeX	117992772
ENSG00000170500	chromosome2	100304988

ENSG00000170500	chromosome2	100304988
ENSG00000175556	chromosomeX	117992772
ENSG00000163623	chromosome4	85638406
ENSG00000148826	chromosome10	134449443
ENSG00000148826	chromosome10	134449443
ENSG00000163623	chromosome4	85638406
ENSG00000163623	chromosome4	85638406
ENSG00000148826	chromosome10	134449443
ENSG00000165066	chromosome8	41627169
ENSG00000179796	chromosome3	26726168
ENSG00000160233	chromosome21	44700956
ENSG00000160233	chromosome21	44700956
ENSG00000179796	chromosome3	26726168
ENSG00000124194	chromosome20	42309389
ENSG00000104381	chromosome8	75425252
ENSG00000152784	chromosome4	81340259
ENSG00000165388	chromosome10	47990539
ENSG00000152784	chromosome4	81340259
ENSG00000109180	chromosome4	48529399
ENSG00000145247	chromosome4	48601324
ENSG00000198952	chromosome1	154519096
ENSG00000070366	chromosome17	2153765
ENSG00000125775	chromosome20	1249061
ENSG00000137575	chromosome8	59640147
ENSG00000149571	chromosome11	125938019
ENSG00000126259	chromosome19	41039862
ENSG00000183853	chromosome1	156230091
ENSG00000126259	chromosome19	41039862
ENSG00000149571	chromosome11	125938019
ENSG00000189159	chromosome17	70662088
ENSG00000206053	chromosome16	1668315
ENSG00000167930	chromosome16	244414
ENSG00000084444	chromosome12	13088605
ENSG00000131242	chromosome17	26742997
ENSG00000090565	chromosome16	416008
ENSG00000101446	chromosome20	43577663
ENSG00000180210	chromosome11	46697362
ENSG00000115718	chromosome2	127893989
ENSG00000180210	chromosome11	46697362
ENSG00000115718	chromosome2	127893989
ENSG00000101981	chromosomeX	138440590
ENSG00000057593	chromosome13	112808157
ENSG00000126218	chromosome13	112825171
ENSG00000057593	chromosome13	112808157
ENSG00000180210	chromosome11	46697362
ENSG00000115718	chromosome2	127893989
ENSG00000101981	chromosomeX	138440590
ENSG00000115718	chromosome2	127893989
ENSG00000180210	chromosome11	46697362
ENSG00000126218	chromosome13	112825171
ENSG00000180210	chromosome11	46697362
ENSG00000115718	chromosome2	127893989
ENSG00000101981	chromosomeX	138440590
ENSG00000057593	chromosome13	112808157
ENSG00000126231	chromosome13	112860976
ENSG00000101981	chromosomeX	138440590
ENSG00000180210	chromosome11	46697362
ENSG00000115718	chromosome2	127893989
ENSG00000178075	chromosome3	115040447

ENSG00000089351	chromosome19	40183223
ENSG00000023171	chromosome11	122902067
ENSG00000023171	chromosome11	122902067
ENSG00000089351	chromosome19	40183223
ENSG00000158806	chromosome8	21938706
ENSG00000107833	chromosome10	103533138
ENSG00000182965	chromosome7	111947758
ENSG00000215280	chromosomeX	47184116
ENSG00000214246	chromosome3	151140275
ENSG00000214032	chromosome7	148662048
ENSG00000214884	chromosome8	104098148
ENSG00000181163	chromosome5	170747558
ENSG00000214774	chromosome12	9739438
ENSG00000107833	chromosome10	103533138
ENSG00000158806	chromosome8	21938706
ENSG00000068137	chromosome17	38082108
ENSG00000180423	chromosome11	46594364
ENSG00000180423	chromosome11	46594364
ENSG00000180423	chromosome11	46594364
ENSG00000075461	chromosome17	62391490
ENSG00000166862	chromosome22	35428568
ENSG00000006116	chromosome16	24175577
ENSG00000142408	chromosome19	59158209
ENSG00000204278	chromosome17	73742271
ENSG00000142408	chromosome19	59158209
ENSG00000166862	chromosome22	35428568
ENSG00000006116	chromosome16	24175577
ENSG00000204278	chromosome17	73742271
ENSG00000075461	chromosome17	62391490
ENSG00000166862	chromosome22	35428568
ENSG00000006116	chromosome16	24175577
ENSG00000142408	chromosome19	59158209
ENSG00000075429	chromosome17	62303913
ENSG00000105605	chromosome19	59107898
ENSG00000204278	chromosome17	73742271
ENSG00000166862	chromosome22	35428568
ENSG00000006116	chromosome16	24175577
ENSG00000142408	chromosome19	59158209
ENSG00000075461	chromosome17	62391490
ENSG00000105605	chromosome19	59107898
ENSG00000075429	chromosome17	62303913
ENSG00000006116	chromosome16	24175577
ENSG00000166862	chromosome22	35428568
ENSG00000166979	chromosome21	32707033
ENSG00000166979	chromosome21	32707033
ENSG00000164877	chromosome7	1465522
ENSG00000100139	chromosome22	36632376
ENSG00000120925	chromosome8	42870781
ENSG00000121957	chromosome1	109229668
ENSG00000160360	chromosome9	138341973
ENSG00000167703	chromosome17	1477919
ENSG00000149150	chromosome11	57038161
ENSG00000166153	chromosome12	99184986
ENSG00000121690	chromosome11	32994078
ENSG00000024526	chromosome1	68735271
ENSG00000035499	chromosome5	60031678
ENSG00000162390	chromosome1	54786571
ENSG00000172497	chromosome5	80725690
ENSG00000165030	chromosome9	93212838

ENSG00000042445	chromosome2	85435142
ENSG00000113811	chromosome3	53900856
ENSG00000151090	chromosome3	24245455
ENSG00000126351	chromosome17	35484268
ENSG00000077092	chromosome3	25445227
ENSG00000131759	chromosome17	35740997
ENSG00000172819	chromosome12	51907597
ENSG00000174738	chromosome3	23961957
ENSG00000126368	chromosome17	35509874
ENSG00000174738	chromosome3	23961957
ENSG00000172819	chromosome12	51907597
ENSG00000131759	chromosome17	35740997
ENSG00000174738	chromosome3	23961957
ENSG00000126368	chromosome17	35509874
ENSG00000126351	chromosome17	35484268
ENSG00000151090	chromosome3	24245455
ENSG00000131759	chromosome17	35740997
ENSG00000172819	chromosome12	51907597
ENSG00000077092	chromosome3	25445227
ENSG00000063978	chromosome4	2461917
ENSG00000147872	chromosome9	19116425
ENSG00000167676	chromosome19	4464705
ENSG00000105355	chromosome19	4812407
ENSG00000214456	chromosome19	4485087
ENSG00000196542	chromosome3	162546806
ENSG00000165389	chromosome14	34001165
ENSG00000120802	chromosome12	97433678
ENSG00000188554	chromosome17	38581343
ENSG00000215691	supercontigNT_113932	2
ENSG00000121297	chromosome19	36531966
ENSG00000179981	chromosome18	71126486
ENSG00000179981	chromosome18	71126486
ENSG00000121297	chromosome19	36531966
ENSG00000182463	chromosome20	51023240
ENSG00000143970	chromosome2	25954596
ENSG00000171456	chromosome20	30410240
ENSG00000215491	chromosome18	29565930
ENSG00000171456	chromosome20	30410240
ENSG00000143970	chromosome2	25954596
ENSG00000164081	chromosome3	51683361
ENSG00000137073	chromosome9	34007147
ENSG00000143569	chromosome1	152464224
ENSG00000143702	chromosome1	241455206
ENSG00000099814	chromosome14	104415282
ENSG00000124743	chromosome6	53628030
ENSG00000197705	chromosome18	28604553
ENSG00000185214	chromosome22	19173499
ENSG00000174010	chromosomeX	23934732
ENSG00000166853	chromosome15	63156324
ENSG00000087448	chromosome12	27824531
ENSG00000186231	chromosome6	97521658
ENSG00000135686	chromosome16	83241975
ENSG00000124743	chromosome6	53628030
ENSG00000179023	chromosome1	18680063
ENSG00000130487	chromosome22	49333462
ENSG00000176595	chromosome8	1936766
ENSG00000185915	chromosomeX	21585828
ENSG00000174010	chromosomeX	23934732
ENSG00000185214	chromosome22	19173499

ENSG00000198642	chromosome9	21324859
ENSG00000003096	chromosomeX	116990820
ENSG00000176595	chromosome8	1936766
ENSG00000130487	chromosome22	49333462
ENSG00000087448	chromosome12	27824531
ENSG00000166853	chromosome15	63156324
ENSG00000130487	chromosome22	49333462
ENSG00000176595	chromosome8	1936766
ENSG00000179023	chromosome1	18680063
ENSG00000185915	chromosomeX	21585828
ENSG00000100418	chromosome22	40346790
ENSG00000125347	chromosome5	131853070
ENSG00000168310	chromosome4	185587213
ENSG00000128604	chromosome7	128369372
ENSG00000117595	chromosome1	208041382
ENSG00000126456	chromosome19	54859908
ENSG00000185507	chromosome11	605365
ENSG00000117595	chromosome1	208041382
ENSG00000213928	chromosome14	23701194
ENSG00000140968	chromosome16	84494123
ENSG00000137265	chromosome6	338153
ENSG00000126456	chromosome19	54859908
ENSG00000185507	chromosome11	605365
ENSG00000128604	chromosome7	128369372
ENSG00000117595	chromosome1	208041382
ENSG00000140968	chromosome16	84494123
ENSG00000137265	chromosome6	338153
ENSG00000213928	chromosome14	23701194
ENSG00000137265	chromosome6	338153
ENSG00000185507	chromosome11	605365
ENSG00000126456	chromosome19	54859908
ENSG00000168310	chromosome4	185587213
ENSG00000125347	chromosome5	131853070
ENSG00000117595	chromosome1	208041382
ENSG00000128604	chromosome7	128369372
ENSG00000137265	chromosome6	338153
ENSG00000140968	chromosome16	84494123
ENSG00000101331	chromosome20	30061919
ENSG00000136280	chromosome7	45006458
ENSG00000148158	chromosome9	114553103
ENSG00000162627	chromosome1	98923041
ENSG00000153982	chromosome17	54652753
ENSG00000102886	chromosome16	30032301
ENSG00000186815	chromosome12	112148918
ENSG00000131503	chromosome5	139761737
ENSG00000132466	chromosome4	74343250
ENSG00000142599	chromosome1	8638944
ENSG00000111676	chromosome12	6913426
ENSG00000120278	chromosome6	151096511
ENSG00000126822	chromosome14	64264103
ENSG00000126822	chromosome14	64264103
ENSG00000120278	chromosome6	151096511
ENSG00000090924	chromosome19	44597489
ENSG00000155629	chromosome10	98470142
ENSG00000153064	chromosome4	102931061
ENSG00000086289	chromosome7	37926707
ENSG00000171121	chromosome3	180467193
ENSG00000215575	chromosome22	15443595
ENSG00000145936	chromosome5	169745030

ENSG00000197584	chromosome3	180007892
ENSG00000145936	chromosome5	169745030
ENSG00000197584	chromosome3	180007892
ENSG00000171121	chromosome3	180467193
ENSG00000215575	chromosome22	15443595
ENSG00000135643	chromosome12	69046782
ENSG00000125868	chromosome20	17498854
ENSG00000172757	chromosome11	65382147
ENSG00000165410	chromosome14	34253498
ENSG00000165410	chromosome14	34253498
ENSG00000172757	chromosome11	65382147
ENSG00000172757	chromosome11	65382147
ENSG00000165410	chromosome14	34253498
ENSG00000125868	chromosome20	17498854
ENSG00000143196	chromosome1	166965037
ENSG00000174945	chromosome7	2706612
ENSG00000196704	chromosome17	63757924
ENSG00000136811	chromosome9	130258192
ENSG00000122417	chromosome1	86625299
ENSG00000148572	chromosome10	64563237
ENSG00000187848	chromosome12	131705476
ENSG00000109991	chromosome11	56862601
ENSG00000099957	chromosome22	19699494
ENSG00000083454	chromosome17	3546161
ENSG00000135124	chromosome12	120132351
ENSG00000089041	chromosome12	120055157
ENSG00000083454	chromosome17	3546161
ENSG00000099957	chromosome22	19699494
ENSG00000108405	chromosome17	3766269
ENSG00000099957	chromosome22	19699494
ENSG00000083454	chromosome17	3546161
ENSG00000187848	chromosome12	131705476
ENSG00000109991	chromosome11	56862601
ENSG00000089041	chromosome12	120055157
ENSG00000135124	chromosome12	120132351
ENSG00000099957	chromosome22	19699494
ENSG00000083454	chromosome17	3546161
ENSG00000187848	chromosome12	131705476
ENSG00000109991	chromosome11	56862601
ENSG00000108405	chromosome17	3766269
ENSG00000109991	chromosome11	56862601
ENSG00000187848	chromosome12	131705476
ENSG00000095787	chromosome10	28862515
ENSG00000124126	chromosome20	46877805
ENSG00000046889	chromosome8	69027184
ENSG00000184083	chromosomeX	54226357
ENSG00000048828	chromosome9	95254019
ENSG00000135966	chromosome2	105291191
ENSG00000157500	chromosome3	57236952
ENSG00000136044	chromosome12	104153921
ENSG00000151715	chromosome11	129227588
ENSG00000181458	chromosome3	101756746
ENSG00000103056	chromosome16	66963586
ENSG00000150510	chromosome13	50694639
ENSG00000124019	chromosome2	224974730
ENSG00000132744	chromosome11	67171091
ENSG00000108381	chromosome17	3326204
ENSG00000184986	chromosome14	105066217
ENSG00000184986	chromosome14	105066217

ENSG00000130723	chromosome9	133295353
ENSG00000204469	chromosome6	31698546
ENSG00000117523	chromosome1	169747852
ENSG00000204469	chromosome6	31698546
ENSG00000130723	chromosome9	133295353
ENSG00000204469	chromosome6	31698546
ENSG00000130723	chromosome9	133295353
ENSG00000117523	chromosome1	169747852
ENSG00000138823	chromosome4	100715090
ENSG00000111837	chromosome6	10938868
ENSG00000112144	chromosome6	53013994
ENSG00000167306	chromosome18	45975162
ENSG00000197535	chromosome15	50608296
ENSG00000167306	chromosome18	45975162
ENSG00000197535	chromosome15	50608296
ENSG00000167306	chromosome18	45975162
ENSG00000128833	chromosome15	50375126
ENSG00000111859	chromosome6	11341678
ENSG00000087589	chromosome20	54420922
ENSG00000050820	chromosome16	73859380
ENSG00000100842	chromosome14	22904075
ENSG00000087589	chromosome20	54420922
ENSG00000111859	chromosome6	11341678
ENSG00000087589	chromosome20	54420922
ENSG00000111859	chromosome6	11341678
ENSG00000100842	chromosome14	22904075
ENSG00000140807	chromosome16	49139966
ENSG00000145506	chromosome5	1062173
ENSG00000188338	chromosome3	50226637
ENSG00000017483	chromosomeX	48211256
ENSG00000111371	chromosome12	44919851
ENSG00000139209	chromosome12	45473122
ENSG00000134294	chromosome12	45060678
ENSG00000139209	chromosome12	45473122
ENSG00000134294	chromosome12	45060678
ENSG00000111371	chromosome12	44919851
ENSG00000134294	chromosome12	45060678
ENSG00000139209	chromosome12	45473122
ENSG00000166558	chromosome16	82633264
ENSG00000103042	chromosome16	57271532
ENSG00000017483	chromosomeX	48211256
ENSG00000188338	chromosome3	50226637
ENSG00000164039	chromosome4	104236861
ENSG00000159788	chromosome4	3287696
ENSG00000169220	chromosome5	176717632
ENSG00000101126	chromosome20	48953941
ENSG00000101544	chromosome18	75976417
ENSG00000138593	chromosome15	47125789
ENSG00000187742	chromosome9	91123252
ENSG00000102802	chromosome13	30378653
ENSG00000158480	chromosome20	47958435
ENSG00000158480	chromosome20	47958435
ENSG00000158792	chromosome16	88295142
ENSG00000125457	chromosome17	70777872
ENSG00000139351	chromosome12	100655845
ENSG00000183304	chromosomeX	8728213
ENSG00000177138	chromosomeX	8961163
ENSG00000187268	chromosomeX	12971830
ENSG00000183304	chromosomeX	8728213

ENSG00000177138	chromosomeX	8961163
ENSG00000187268	chromosomeX	12971830
ENSG00000139351	chromosome12	100655845
ENSG00000174429	chromosome8	107851595
ENSG00000174429	chromosome8	107851595
ENSG00000103494	chromosome16	52292137
ENSG00000092200	chromosome14	20825976
ENSG00000013583	chromosome12	13044316
ENSG00000031003	chromosome5	137382700
ENSG00000138640	chromosome4	90197139
ENSG00000148541	chromosome10	60792293
ENSG00000138640	chromosome4	90197139
ENSG00000031003	chromosome5	137382700
ENSG00000106460	chromosome7	12220962
ENSG00000134291	chromosome12	46644287
ENSG00000134291	chromosome12	46644287
ENSG00000106460	chromosome7	12220962
ENSG00000184988	chromosome17	38720587
ENSG00000215684	supercontigNT_113932	21541
ENSG00000090376	chromosome12	64869347
ENSG00000134070	chromosome3	10181639
ENSG00000184216	chromosomeX	152938458
ENSG00000134070	chromosome3	10181639
ENSG00000090376	chromosome12	64869347
ENSG00000169604	chromosome2	69094136
ENSG00000163297	chromosome4	81212739
ENSG00000169604	chromosome2	69094136
ENSG00000198250	chromosome10	47128505
ENSG00000169604	chromosome2	69094136
ENSG00000163297	chromosome4	81212739
ENSG00000163659	chromosome3	157878181
ENSG00000178685	chromosome8	145132516
ENSG00000173193	chromosome3	123882421
ENSG00000138496	chromosome3	123761158
ENSG00000138496	chromosome3	123761158
ENSG00000173193	chromosome3	123882421
ENSG00000178685	chromosome8	145132516
ENSG00000163659	chromosome3	157878181
ENSG00000163947	chromosome3	57044218
ENSG00000163947	chromosome3	57044218
ENSG00000173848	chromosome10	5444656
ENSG00000102313	chromosomeX	54841369
ENSG00000123243	chromosome10	7748862
ENSG00000151655	chromosome10	7785404
ENSG00000055957	chromosome3	52786672
ENSG00000162267	chromosome3	52803860
ENSG00000055955	chromosome3	52839699
ENSG00000055955	chromosome3	52839699
ENSG00000055957	chromosome3	52786672
ENSG00000162267	chromosome3	52803860
ENSG00000151474	chromosome10	14412108
ENSG00000147144	chromosomeX	48806752
ENSG00000163362	chromosome1	199127292
ENSG00000114541	chromosome3	69445359
ENSG00000147144	chromosomeX	48806752
ENSG00000163362	chromosome1	199127292
ENSG00000151474	chromosome10	14412108
ENSG00000163362	chromosome1	199127292
ENSG00000147144	chromosomeX	48806752

ENSG00000118473	chromosome1	66772558
ENSG00000157107	chromosome5	72287680
ENSG00000157107	chromosome5	72287680
ENSG00000118473	chromosome1	66772558
ENSG00000130475	chromosome19	17726926
ENSG00000108312	chromosome17	39651124
ENSG00000204664	chromosome2	97620662
ENSG00000204699	chromosome2	95654725
ENSG00000204705	chromosome2	95480962
ENSG00000183417	chromosome11	89126783
ENSG00000204448	chromosome11	89458766
ENSG00000205042	chromosome11	49060044
ENSG00000196151	chromosome2	159847827
ENSG00000173083	chromosome4	84474960
ENSG00000172987	chromosome10	100985550
ENSG00000116005	chromosome2	70338801
ENSG00000145882	chromosome5	148717825
ENSG00000077585	chromosome1	234372546
ENSG00000173264	chromosome11	63810573
ENSG00000173264	chromosome11	63810573
ENSG00000077585	chromosome1	234372546
ENSG00000180998	chromosome14	52089757
ENSG00000166716	chromosome15	83126911
ENSG00000074657	chromosome18	54683725
ENSG00000214168	chromosome2	110555814
ENSG00000214171	chromosome2	110122559
ENSG00000143373	chromosome1	149525392
ENSG00000074657	chromosome18	54683725
ENSG00000214168	chromosome2	110555814
ENSG00000214171	chromosome2	110122559
ENSG00000166716	chromosome15	83126911
ENSG00000146250	chromosome6	84289880
ENSG00000150687	chromosome11	86196334
ENSG00000150687	chromosome11	86196334
ENSG00000135094	chromosome12	112321897
ENSG00000139410	chromosome12	112350171
ENSG00000100403	chromosome22	40046611
ENSG00000122299	chromosome16	11783712
ENSG00000155313	chromosome21	16024584
ENSG00000048028	chromosome11	113251434
ENSG00000119772	chromosome2	25390358
ENSG00000088305	chromosome20	30813915
ENSG00000142182	chromosome21	44505571
ENSG00000088305	chromosome20	30813915
ENSG00000119772	chromosome2	25390358
ENSG00000088305	chromosome20	30813915
ENSG00000182218	chromosome14	99181298
ENSG00000143512	chromosome1	220788010
ENSG00000182218	chromosome14	99181298
ENSG00000119242	chromosome12	122994806
ENSG00000123636	chromosome2	160043477
ENSG00000076108	chromosome12	55316345
ENSG00000185933	chromosome10	105208499
ENSG00000183128	chromosome10	105228780
ENSG00000164451	chromosome6	116981650
ENSG00000138172	chromosome10	105199689
ENSG00000188820	chromosome6	116889277
ENSG00000178033	chromosome6	116939553
ENSG00000164451	chromosome6	116981650

ENSG00000138172	chromosome10	105199689
ENSG00000185933	chromosome10	105208499
ENSG00000183128	chromosome10	105228780
ENSG00000183128	chromosome10	105228780
ENSG00000185933	chromosome10	105208499
ENSG00000138172	chromosome10	105199689
ENSG00000164451	chromosome6	116981650
ENSG00000178033	chromosome6	116939553
ENSG00000188820	chromosome6	116889277
ENSG00000116701	chromosome1	181826172
ENSG00000188747	chromosome9	139437803
ENSG00000109685	chromosome4	1872180
ENSG00000165671	chromosome5	176494711
ENSG00000147548	chromosome8	38324847
ENSG00000165671	chromosome5	176494711
ENSG00000109685	chromosome4	1872180
ENSG00000181284	chromosome17	7279915
ENSG00000180611	chromosome3	194118324
ENSG00000052126	chromosome12	19174001
ENSG00000143850	chromosome1	202509479
ENSG00000166689	chromosome11	16992525
ENSG00000143850	chromosome1	202509479
ENSG00000052126	chromosome12	19174001
ENSG00000143850	chromosome1	202509479
ENSG00000052126	chromosome12	19174001
ENSG00000166689	chromosome11	16992525
ENSG00000105559	chromosome19	54062690
ENSG00000154153	chromosome5	16670081
ENSG00000141699	chromosome17	38014869
ENSG00000141699	chromosome17	38014869
ENSG00000154153	chromosome5	16670081
ENSG00000144567	chromosome2	219751319
ENSG00000176208	chromosome17	26183492
ENSG00000167733	chromosome19	5635844
ENSG00000117594	chromosome1	207944911
ENSG00000159256	chromosome21	36614433
ENSG00000133131	chromosomeX	106129856
ENSG00000133422	chromosome22	29693876
ENSG00000114487	chromosome3	110319597
ENSG00000093010	chromosome22	18330050
ENSG00000093010	chromosome22	18330050
ENSG00000136933	chromosome9	127002705
ENSG00000019144	chromosome11	117989762
ENSG00000144824	chromosome3	112876783
ENSG00000204852	chromosome12	109536371
ENSG00000119977	chromosome10	97443701
ENSG00000183137	chromosome6	109573118
ENSG00000166037	chromosome11	95163511
ENSG00000176871	chromosome12	116983237
ENSG00000109046	chromosome17	22645549
ENSG00000183655	chromosome15	84114046
ENSG00000171617	chromosome5	73968067
ENSG00000213160	chromosome2	170299771
ENSG00000213160	chromosome2	170299771
ENSG00000171617	chromosome5	73968067
ENSG00000183655	chromosome15	84114046
ENSG00000175946	chromosome8	124734348
ENSG00000182359	chromosome11	105435035
ENSG00000168427	chromosome2	238714135

ENSG00000171617	chromosome5	73968067
ENSG00000157514	chromosomeX	106905306
ENSG00000157514	chromosomeX	106905306
ENSG00000178104	chromosome1	143787220
ENSG00000136861	chromosome9	122382078
ENSG00000136861	chromosome9	122382078
ENSG00000178104	chromosome1	143787220
ENSG00000089063	chromosome20	5041675
ENSG00000165006	chromosome9	34210913
ENSG00000162878	chromosome2	42129441
ENSG00000133030	chromosome17	16886888
ENSG00000214820	chromosome3	44597511
ENSG00000133030	chromosome17	16886888
ENSG00000214820	chromosome3	44597511
ENSG00000168591	chromosome17	39621941
ENSG00000164897	chromosome7	150410584
ENSG00000100605	chromosome14	92651377
ENSG00000179954	chromosome19	60691769
ENSG00000164099	chromosome4	119493324
ENSG00000187908	chromosome10	124310277
ENSG00000146700	chromosome7	75871693
ENSG00000073754	chromosome1	156078116
ENSG00000177575	chromosome12	7547554
ENSG00000177675	chromosome12	7487991
ENSG00000214279	chromosome10	135121772
ENSG00000179954	chromosome19	60691769
ENSG00000164099	chromosome4	119493324
ENSG00000013725	chromosome11	60495914
ENSG00000110448	chromosome11	60626609
ENSG00000179954	chromosome19	60691769
ENSG00000164099	chromosome4	119493324
ENSG00000187908	chromosome10	124310277
ENSG00000146700	chromosome7	75871693
ENSG00000073754	chromosome1	156078116
ENSG00000177575	chromosome12	7547554
ENSG00000177675	chromosome12	7487991
ENSG00000214279	chromosome10	135121772
ENSG00000179954	chromosome19	60691769
ENSG00000164099	chromosome4	119493324
ENSG00000179954	chromosome19	60691769
ENSG00000164099	chromosome4	119493324
ENSG00000187908	chromosome10	124310277
ENSG00000146700	chromosome7	75871693
ENSG00000073754	chromosome1	156078116
ENSG00000177575	chromosome12	7547554
ENSG00000177675	chromosome12	7487991
ENSG00000214279	chromosome10	135121772
ENSG00000164099	chromosome4	119493324
ENSG00000146700	chromosome7	75871693
ENSG00000164099	chromosome4	119493324
ENSG00000164099	chromosome4	119493324
ENSG00000179954	chromosome19	60691769
ENSG00000073754	chromosome1	156078116
ENSG00000177575	chromosome12	7547554
ENSG00000177675	chromosome12	7487991
ENSG00000214279	chromosome10	135121772
ENSG00000187908	chromosome10	124310277
ENSG00000146700	chromosome7	75871693
ENSG00000146700	chromosome7	75871693

ENSG00000187908	chromosome10	124310277
ENSG00000183255	chromosome21	45117960
ENSG00000176473	chromosome14	99917015
ENSG00000168300	chromosome8	52936265
ENSG00000203880	chromosome20	62361763
ENSG00000111913	chromosome6	24983999
ENSG00000039523	chromosome16	66129851
ENSG00000042062	chromosome20	48680792
ENSG00000039523	chromosome16	66129851
ENSG00000111913	chromosome6	24983999
ENSG00000153832	chromosome2	230495474
ENSG00000165322	chromosome10	32237790
ENSG00000185602	chromosome17	40839083
ENSG00000123329	chromosome12	56168672
ENSG00000075884	chromosome2	143629530
ENSG00000107863	chromosome10	25050832
ENSG00000167433	chromosome17	33876678
ENSG00000214627	chromosome16	31894624
ENSG00000185602	chromosome17	40839083
ENSG00000165322	chromosome10	32237790
ENSG00000075884	chromosome2	143629530
ENSG00000123329	chromosome12	56168672
ENSG00000107957	chromosome10	105605015
ENSG00000174705	chromosome5	171813962
ENSG00000196408	chromosome16	1971182
ENSG00000158517	chromosome7	73826315
ENSG00000182487	chromosome7	72272617
ENSG00000165178	chromosome7	74225684
ENSG00000159231	chromosome21	36429361
ENSG00000159228	chromosome21	36364284
ENSG00000119596	chromosome14	74299946
ENSG00000029725	chromosome17	5126422
ENSG00000177548	chromosome16	28843986
ENSG00000071246	chromosome14	76298918
ENSG00000143494	chromosome1	211191508
ENSG00000100565	chromosome14	76362592
ENSG00000160221	chromosome21	44378008
ENSG00000160221	chromosome21	44378008
ENSG00000144843	chromosome3	120783707
ENSG00000153531	chromosome13	113155754
ENSG00000144843	chromosome3	120783707
ENSG00000171681	chromosome12	14468117
ENSG00000166669	chromosome16	10431979
ENSG00000185262	chromosome17	71773182
ENSG00000153443	chromosome16	4604800
ENSG00000163013	chromosome2	73350267
ENSG00000138311	chromosome10	63805947
ENSG00000135387	chromosome11	34030544
ENSG00000110888	chromosome12	30797965
ENSG00000136144	chromosome13	49039417
ENSG00000136161	chromosome13	47994077
ENSG00000100711	chromosome14	103251932
ENSG00000033327	chromosome11	77806415
ENSG00000215568	chromosome22	15869005
ENSG00000109458	chromosome4	144477792
ENSG00000160219	chromosomeX	153632495
ENSG00000109458	chromosome4	144477792
ENSG00000160219	chromosomeX	153632495
ENSG00000033327	chromosome11	77806415

ENSG00000215568	chromosome22	15869005
ENSG00000109458	chromosome4	144477792
ENSG00000109458	chromosome4	144477792
ENSG00000033327	chromosome11	77806415
ENSG00000215568	chromosome22	15869005
ENSG00000154856	chromosome18	10444979
ENSG00000198768	chromosome20	56523329
ENSG00000132622	chromosome20	3661358
ENSG00000165868	chromosome10	118492079
ENSG00000165868	chromosome10	118492079
ENSG00000132622	chromosome20	3661358
ENSG00000106052	chromosome7	27754669
ENSG00000012822	chromosome12	52405294
ENSG00000012822	chromosome12	52405294
ENSG00000106052	chromosome7	27754669
ENSG00000136436	chromosome17	44274069
ENSG00000012822	chromosome12	52405294
ENSG00000106052	chromosome7	27754669
ENSG00000107679	chromosome10	124142707
ENSG00000169499	chromosome8	38894605
ENSG00000196208	chromosome2	11600269
ENSG00000141449	chromosome18	17217478
ENSG00000188859	chromosome1	164402110
ENSG00000126882	chromosome9	133141388
ENSG00000013563	chromosomeX	153287104
ENSG00000163687	chromosome3	58171674
ENSG00000013563	chromosomeX	153287104
ENSG00000163687	chromosome3	58171674
ENSG00000167968	chromosome16	2226819
ENSG00000213918	chromosome16	3645376
ENSG00000167968	chromosome16	2226819
ENSG00000213918	chromosome16	3645376
ENSG00000013563	chromosomeX	153287104
ENSG00000163687	chromosome3	58171674
ENSG00000144815	chromosome3	102987131
ENSG00000137634	chromosome11	113970692
ENSG00000095110	chromosome11	113906514
ENSG00000204361	chromosome11	114054508
ENSG00000144815	chromosome3	102987131
ENSG00000130038	chromosome12	3676427
ENSG00000130038	chromosome12	3676427
ENSG00000130038	chromosome12	3676427
ENSG00000177685	chromosome11	818608
ENSG00000173821	chromosome17	75928544
ENSG00000205038	chromosome8	110443986
ENSG00000170927	chromosome6	52057691
ENSG00000102924	chromosome16	47872878
ENSG00000054803	chromosome20	54012635
ENSG00000141668	chromosome18	68360376
ENSG00000163145	chromosome4	15046466
ENSG00000145861	chromosome5	159730223
ENSG00000181092	chromosome3	188053542
ENSG00000102924	chromosome16	47872878
ENSG00000165985	chromosome10	16603071
ENSG00000144119	chromosome2	119632316
ENSG00000131094	chromosome17	40400943
ENSG00000186897	chromosome12	48016528
ENSG00000186897	chromosome12	48016528
ENSG00000165985	chromosome10	16603071

ENSG00000144119	chromosome2	119632316
ENSG00000131094	chromosome17	40400943
ENSG00000173918	chromosome17	74551001
ENSG00000173372	chromosome1	22836697
ENSG00000173372	chromosome1	22836697
ENSG00000173369	chromosome1	22858537
ENSG00000159189	chromosome1	22843104
ENSG00000144119	chromosome2	119632316
ENSG00000165985	chromosome10	16603071
ENSG00000163145	chromosome4	15046466
ENSG00000145861	chromosome5	159730223
ENSG00000181092	chromosome3	188053542
ENSG00000173369	chromosome1	22858537
ENSG00000159189	chromosome1	22843104
ENSG00000173372	chromosome1	22836697
ENSG00000213192	chromosome11	118716318
ENSG00000213192	chromosome11	118716318
ENSG00000173369	chromosome1	22858537
ENSG00000159189	chromosome1	22843104
ENSG00000173372	chromosome1	22836697
ENSG00000145861	chromosome5	159730223
ENSG00000163145	chromosome4	15046466
ENSG00000054803	chromosome20	54012635
ENSG00000141668	chromosome18	68360376
ENSG00000102924	chromosome16	47872878
ENSG00000139899	chromosome14	23968101
ENSG00000181092	chromosome3	188053542
ENSG00000173369	chromosome1	22858537
ENSG00000159189	chromosome1	22843104
ENSG00000173372	chromosome1	22836697
ENSG00000213192	chromosome11	118716318
ENSG00000163145	chromosome4	15046466
ENSG00000145861	chromosome5	159730223
ENSG00000131094	chromosome17	40400943
ENSG00000165985	chromosome10	16603071
ENSG00000144119	chromosome2	119632316
ENSG00000165985	chromosome10	16603071
ENSG00000144119	chromosome2	119632316
ENSG00000131094	chromosome17	40400943
ENSG00000186897	chromosome12	48016528
ENSG00000163145	chromosome4	15046466
ENSG00000145861	chromosome5	159730223
ENSG00000181092	chromosome3	188053542
ENSG00000173369	chromosome1	22858537
ENSG00000159189	chromosome1	22843104
ENSG00000173372	chromosome1	22836697
ENSG00000213192	chromosome11	118716318
ENSG00000181092	chromosome3	188053542
ENSG00000141668	chromosome18	68360376
ENSG00000054803	chromosome20	54012635
ENSG00000173918	chromosome17	74551001
ENSG00000184471	chromosome16	1084924
ENSG00000133466	chromosome22	35914200
ENSG00000173918	chromosome17	74551001
ENSG00000173369	chromosome1	22858537
ENSG00000159189	chromosome1	22843104
ENSG00000173372	chromosome1	22836697
ENSG00000213192	chromosome11	118716318
ENSG00000181092	chromosome3	188053542

ENSG00000182240	chromosome21	41462061
ENSG00000186318	chromosome11	116691814
ENSG00000159248	chromosome15	32833982
ENSG00000152661	chromosome6	121809693
ENSG00000187513	chromosome1	35032402
ENSG00000135355	chromosome6	90660909
ENSG00000131233	chromosome1	39114358
ENSG00000143140	chromosome1	145697971
ENSG00000121743	chromosome13	19615428
ENSG00000152661	chromosome6	121809693
ENSG00000187513	chromosome1	35032402
ENSG00000121743	chromosome13	19615428
ENSG00000121634	chromosome1	145846707
ENSG00000169562	chromosomeX	70360283
ENSG00000121742	chromosome13	19695620
ENSG00000165474	chromosome13	19661721
ENSG00000152661	chromosome6	121809693
ENSG00000177291	chromosome10	35934502
ENSG00000176402	chromosome7	99365180
ENSG00000198835	chromosome1	226412083
ENSG00000182963	chromosome17	40238712
ENSG00000121742	chromosome13	19695620
ENSG00000165474	chromosome13	19661721
ENSG00000169562	chromosomeX	70360283
ENSG00000164411	chromosome6	88051350
ENSG00000189433	chromosome1	34999443
ENSG00000189280	chromosome1	34995519
ENSG00000188910	chromosome1	35022951
ENSG00000143140	chromosome1	145697971
ENSG00000135355	chromosome6	90660909
ENSG00000131233	chromosome1	39114358
ENSG00000121634	chromosome1	145846707
ENSG00000152661	chromosome6	121809693
ENSG00000187513	chromosome1	35032402
ENSG00000121743	chromosome13	19615428
ENSG00000183153	chromosome17	35773594
ENSG00000159248	chromosome15	32833982
ENSG00000143140	chromosome1	145697971
ENSG00000135355	chromosome6	90660909
ENSG00000131233	chromosome1	39114358
ENSG00000121634	chromosome1	145846707
ENSG00000152661	chromosome6	121809693
ENSG00000187513	chromosome1	35032402
ENSG00000121743	chromosome13	19615428
ENSG00000121742	chromosome13	19695620
ENSG00000165474	chromosome13	19661721
ENSG00000169562	chromosomeX	70360283
ENSG00000164411	chromosome6	88051350
ENSG00000189433	chromosome1	34999443
ENSG00000189280	chromosome1	34995519
ENSG00000188910	chromosome1	35022951
ENSG00000187513	chromosome1	35032402
ENSG00000152661	chromosome6	121809693
ENSG00000159248	chromosome15	32833982
ENSG00000189433	chromosome1	34999443
ENSG00000189280	chromosome1	34995519
ENSG00000188910	chromosome1	35022951
ENSG00000121743	chromosome13	19615428
ENSG00000152661	chromosome6	121809693

ENSG00000187513	chromosome1	35032402
ENSG00000188910	chromosome1	35022951
ENSG00000189433	chromosome1	34999443
ENSG00000189280	chromosome1	34995519
ENSG00000121742	chromosome13	19695620
ENSG00000165474	chromosome13	19661721
ENSG00000169562	chromosomeX	70360283
ENSG00000164411	chromosome6	88051350
ENSG00000189433	chromosome1	34999443
ENSG00000189280	chromosome1	34995519
ENSG00000188910	chromosome1	35022951
ENSG00000143140	chromosome1	145697971
ENSG00000135355	chromosome6	90660909
ENSG00000131233	chromosome1	39114358
ENSG00000121634	chromosome1	145846707
ENSG00000152661	chromosome6	121809693
ENSG00000187513	chromosome1	35032402
ENSG00000121743	chromosome13	19615428
ENSG00000183153	chromosome17	35773594
ENSG00000159248	chromosome15	32833982
ENSG00000176402	chromosome7	99365180
ENSG00000198835	chromosome1	226412083
ENSG00000182963	chromosome17	40238712
ENSG00000131233	chromosome1	39114358
ENSG00000135355	chromosome6	90660909
ENSG00000159248	chromosome15	32833982
ENSG00000183153	chromosome17	35773594
ENSG00000189433	chromosome1	34999443
ENSG00000189280	chromosome1	34995519
ENSG00000188910	chromosome1	35022951
ENSG00000164411	chromosome6	88051350
ENSG00000121634	chromosome1	145846707
ENSG00000152661	chromosome6	121809693
ENSG00000187513	chromosome1	35032402
ENSG00000121743	chromosome13	19615428
ENSG00000143140	chromosome1	145697971
ENSG00000135355	chromosome6	90660909
ENSG00000131233	chromosome1	39114358
ENSG00000164411	chromosome6	88051350
ENSG00000189433	chromosome1	34999443
ENSG00000189280	chromosome1	34995519
ENSG00000188910	chromosome1	35022951
ENSG00000121742	chromosome13	19695620
ENSG00000165474	chromosome13	19661721
ENSG00000169562	chromosomeX	70360283
ENSG00000182963	chromosome17	40238712
ENSG00000183153	chromosome17	35773594
ENSG00000159248	chromosome15	32833982
ENSG00000121742	chromosome13	19695620
ENSG00000165474	chromosome13	19661721
ENSG00000169562	chromosomeX	70360283
ENSG00000164411	chromosome6	88051350
ENSG00000189433	chromosome1	34999443
ENSG00000189280	chromosome1	34995519
ENSG00000188910	chromosome1	35022951
ENSG00000143140	chromosome1	145697971
ENSG00000135355	chromosome6	90660909
ENSG00000131233	chromosome1	39114358
ENSG00000121634	chromosome1	145846707

ENSG00000152661	chromosome6	121809693
ENSG00000187513	chromosome1	35032402
ENSG00000121743	chromosome13	19615428
ENSG00000198835	chromosome1	226412083
ENSG00000182963	chromosome17	40238712
ENSG00000176402	chromosome7	99365180
ENSG00000182963	chromosome17	40238712
ENSG00000198835	chromosome1	226412083
ENSG00000177291	chromosome10	35934502
ENSG00000164542	chromosome7	36396209
ENSG00000196123	chromosome16	65772015
ENSG00000172572	chromosome12	20413486
ENSG00000152270	chromosome11	14622198
ENSG00000144730	chromosome3	57174355
ENSG00000056736	chromosome3	53855656
ENSG00000177663	chromosome22	15945982
ENSG00000180447	chromosome9	88751515
ENSG00000215714	supercontigNT_113899	8974
ENSG00000095303	chromosome9	124173185
ENSG00000073756	chromosome1	184916046
ENSG00000078687	chromosome17	73556589
ENSG00000090905	chromosome16	24648613
ENSG00000090905	chromosome16	24648613
ENSG00000078687	chromosome17	73556589
ENSG00000100354	chromosome22	38851768
ENSG00000000005	chromosomeX	99726672
ENSG00000110002	chromosome11	123493429
ENSG00000145198	chromosome3	185435016
ENSG00000158816	chromosome1	20509682
ENSG00000158816	chromosome1	20509682
ENSG00000145198	chromosome3	185435016
ENSG00000211448	chromosome14	79747569
ENSG00000197406	chromosome14	101097665
ENSG00000211452	chromosome1	54132472
ENSG00000197406	chromosome14	101097665
ENSG00000211448	chromosome14	79747569
ENSG00000141441	chromosome18	28304390
ENSG00000157833	chromosome2	26249465
ENSG00000167470	chromosome19	1201296
ENSG00000180357	chromosome15	62578672
ENSG00000168916	chromosome5	124108582
ENSG00000173334	chromosome8	126512327
ENSG00000071575	chromosome2	12775885
ENSG00000101255	chromosome20	316655
ENSG00000071575	chromosome2	12775885
ENSG00000173334	chromosome8	126512327
ENSG00000005108	chromosome7	11838098
ENSG00000144229	chromosome2	137356219
ENSG00000198734	chromosome1	167822249
ENSG00000185010	chromosomeX	153904022
ENSG00000047457	chromosome3	150422270
ENSG00000089472	chromosomeX	65307138
ENSG00000181333	chromosome11	93394192
ENSG00000144214	chromosome2	99278566
ENSG00000185674	chromosome2	99237156
ENSG00000163806	chromosome2	28892385
ENSG00000136206	chromosome7	44007150
ENSG00000166667	chromosome7	101780499
ENSG00000173678	chromosome7	102082720

ENSG00000205238	chromosome7	101983496
ENSG00000170092	chromosome7	74962371
ENSG00000179994	chromosome7	71977449
ENSG00000154732	chromosome7	66383849
ENSG00000205482	chromosome7	76525131
ENSG00000185040	chromosome7	76006080
ENSG00000184616	chromosome7	73963632
ENSG00000205509	chromosome7	74334002
ENSG00000205508	chromosome7	74751929
ENSG00000186645	chromosome7	76497824
ENSG00000205505	chromosome7	74804292
ENSG00000205580	chromosome7	72164512
ENSG00000205506	chromosome7	74776265
ENSG00000198305	chromosome7	72136450
ENSG00000214300	chromosome7	99743445
ENSG00000215092	chromosome7	5044013
ENSG00000183318	chromosome17	8602444
ENSG00000204710	chromosome11	64694283
ENSG00000116981	chromosome1	39910298
ENSG00000185013	chromosome2	18634216
ENSG00000185013	chromosome2	18634216
ENSG00000116981	chromosome1	39910298
ENSG00000108423	chromosome17	55323146
ENSG00000174136	chromosome5	98132959
ENSG00000174136	chromosome5	98132959
ENSG00000168509	chromosome1	144126139
ENSG00000182175	chromosome15	91433442
ENSG00000182175	chromosome15	91433442
ENSG00000168509	chromosome1	144126139
ENSG00000109101	chromosome17	23875115
ENSG00000139445	chromosome12	108230510
ENSG00000176302	chromosome11	118347852
ENSG00000189299	chromosomeX	55666870
ENSG00000139445	chromosome12	108230510
ENSG00000109101	chromosome17	23875115
ENSG00000134369	chromosome1	199865255
ENSG00000166833	chromosome11	19691818
ENSG00000067798	chromosome12	76749373
ENSG00000067798	chromosome12	76749373
ENSG00000166833	chromosome11	19691818
ENSG00000170044	chromosome3	103636649
ENSG00000177398	chromosome21	42364495
ENSG00000170044	chromosome3	103636649
ENSG00000139880	chromosome14	22594783
ENSG00000140937	chromosome16	63596274
ENSG00000150394	chromosome16	60612809
ENSG00000140937	chromosome16	63596274
ENSG00000139880	chromosome14	22594783
ENSG00000140937	chromosome16	63596274
ENSG00000139880	chromosome14	22594783
ENSG00000150394	chromosome16	60612809
ENSG00000113100	chromosome5	27024198
ENSG00000113361	chromosome5	31303338
ENSG00000040731	chromosome5	24629357
ENSG00000154162	chromosome5	22114543
ENSG00000145526	chromosome5	19874853
ENSG00000101542	chromosome18	57308767
ENSG00000071991	chromosome18	62390422
ENSG00000149654	chromosome20	44313341

ENSG00000081138	chromosome18	61581059
ENSG00000081138	chromosome18	61581059
ENSG00000149654	chromosome20	44313341
ENSG00000179776	chromosome16	64970742
ENSG00000113100	chromosome5	27024198
ENSG00000113361	chromosome5	31303338
ENSG00000040731	chromosome5	24629357
ENSG00000154162	chromosome5	22114543
ENSG00000145526	chromosome5	19874853
ENSG00000101542	chromosome18	57308767
ENSG00000071991	chromosome18	62390422
ENSG00000149654	chromosome20	44313341
ENSG00000081138	chromosome18	61581059
ENSG00000140937	chromosome16	63596274
ENSG00000139880	chromosome14	22594783
ENSG00000150394	chromosome16	60612809
ENSG00000145526	chromosome5	19874853
ENSG00000113100	chromosome5	27024198
ENSG00000113361	chromosome5	31303338
ENSG00000040731	chromosome5	24629357
ENSG00000154162	chromosome5	22114543
ENSG00000149654	chromosome20	44313341
ENSG00000081138	chromosome18	61581059
ENSG00000071991	chromosome18	62390422
ENSG00000101542	chromosome18	57308767
ENSG00000071991	chromosome18	62390422
ENSG00000149654	chromosome20	44313341
ENSG00000081138	chromosome18	61581059
ENSG00000113100	chromosome5	27024198
ENSG00000113361	chromosome5	31303338
ENSG00000040731	chromosome5	24629357
ENSG00000154162	chromosome5	22114543
ENSG00000145526	chromosome5	19874853
ENSG00000154162	chromosome5	22114543
ENSG00000113100	chromosome5	27024198
ENSG00000113361	chromosome5	31303338
ENSG00000040731	chromosome5	24629357
ENSG00000071991	chromosome18	62390422
ENSG00000149654	chromosome20	44313341
ENSG00000081138	chromosome18	61581059
ENSG00000101542	chromosome18	57308767
ENSG00000122861	chromosome10	75341320
ENSG00000104368	chromosome8	42169861
ENSG00000173531	chromosome3	49701087
ENSG00000186715	chromosome1	16963563
ENSG00000019991	chromosome7	81237224
ENSG00000186301	chromosome1	16846045
ENSG00000104368	chromosome8	42169861
ENSG00000122861	chromosome10	75341320
ENSG00000131187	chromosome5	176769135
ENSG00000109758	chromosome4	3413527
ENSG00000148702	chromosome10	115302871
ENSG00000131187	chromosome5	176769135
ENSG00000109758	chromosome4	3413527
ENSG00000104368	chromosome8	42169861
ENSG00000122861	chromosome10	75341320
ENSG00000019991	chromosome7	81237224
ENSG00000173531	chromosome3	49701087
ENSG00000186715	chromosome1	16963563

ENSG00000186301	chromosome1	16846045
ENSG00000173531	chromosome3	49701087
ENSG00000186715	chromosome1	16963563
ENSG00000019991	chromosome7	81237224
ENSG00000122194	chromosome6	161043327
ENSG00000198670	chromosome6	161005237
ENSG00000109758	chromosome4	3413527
ENSG00000131187	chromosome5	176769135
ENSG00000204396	chromosome6	31852536
ENSG00000174453	chromosome2	214987163
ENSG00000188730	chromosome7	49785578
ENSG00000174453	chromosome2	214987163
ENSG00000188730	chromosome7	49785578
ENSG00000188730	chromosome7	49785578
ENSG00000174453	chromosome2	214987163
ENSG00000111799	chromosome6	75969229
ENSG00000187955	chromosome8	121229263
ENSG00000187955	chromosome8	121229263
ENSG00000111799	chromosome6	75969229
ENSG00000101203	chromosome20	61396905
ENSG00000101203	chromosome20	61396905
ENSG00000187955	chromosome8	121229263
ENSG00000111799	chromosome6	75969229
ENSG00000168477	chromosome6	32173954
ENSG00000116147	chromosome1	173642474
ENSG00000041982	chromosome9	116893119
ENSG00000115414	chromosome2	216008771
ENSG00000120332	chromosome1	173303613
ENSG00000116147	chromosome1	173642474
ENSG00000041982	chromosome9	116893119
ENSG00000168477	chromosome6	32173954
ENSG00000115414	chromosome2	216008771
ENSG00000041982	chromosome9	116893119
ENSG00000116147	chromosome1	173642474
ENSG00000116147	chromosome1	173642474
ENSG00000041982	chromosome9	116893119
ENSG00000168477	chromosome6	32173954
ENSG00000120332	chromosome1	173303613
ENSG00000112902	chromosome5	9433059
ENSG00000082684	chromosome3	124162801
ENSG00000167680	chromosome19	4509470
ENSG00000082684	chromosome3	124162801
ENSG00000112902	chromosome5	9433059
ENSG00000023191	chromosome11	492163
ENSG00000167984	chromosome16	3567216
ENSG00000167207	chromosome16	49288656
ENSG00000167207	chromosome16	49288656
ENSG00000167984	chromosome16	3567216
ENSG00000179873	chromosome19	61021353
ENSG00000173572	chromosome19	61135490
ENSG00000167634	chromosome19	60144892
ENSG00000022556	chromosome19	60173196
ENSG00000171487	chromosome19	61202904
ENSG00000179709	chromosome19	61151081
ENSG00000158077	chromosome11	7016394
ENSG00000185792	chromosome19	60941553
ENSG00000182261	chromosome11	7941619
ENSG00000160505	chromosome19	61055259
ENSG00000162711	chromosome1	245648720

ENSG00000142405	chromosome19	59019241
ENSG00000174885	chromosome11	268570
ENSG00000140853	chromosome16	55612126
ENSG00000023191	chromosome11	492163
ENSG00000179873	chromosome19	61021353
ENSG00000173572	chromosome19	61135490
ENSG00000167634	chromosome19	60144892
ENSG00000022556	chromosome19	60173196
ENSG00000171487	chromosome19	61202904
ENSG00000179709	chromosome19	61151081
ENSG00000158077	chromosome11	7016394
ENSG00000185792	chromosome19	60941553
ENSG00000182261	chromosome11	7941619
ENSG00000160505	chromosome19	61055259
ENSG00000162711	chromosome1	245648720
ENSG00000142405	chromosome19	59019241
ENSG00000174885	chromosome11	268570
ENSG00000140853	chromosome16	55612126
ENSG00000023191	chromosome11	492163
ENSG00000167207	chromosome16	49288656
ENSG00000167984	chromosome16	3567216
ENSG00000023191	chromosome11	492163
ENSG00000179873	chromosome19	61021353
ENSG00000173572	chromosome19	61135490
ENSG00000167634	chromosome19	60144892
ENSG00000022556	chromosome19	60173196
ENSG00000171487	chromosome19	61202904
ENSG00000179709	chromosome19	61151081
ENSG00000158077	chromosome11	7016394
ENSG00000185792	chromosome19	60941553
ENSG00000182261	chromosome11	7941619
ENSG00000160505	chromosome19	61055259
ENSG00000162711	chromosome1	245648720
ENSG00000142405	chromosome19	59019241
ENSG00000174885	chromosome11	268570
ENSG00000140853	chromosome16	55612126
ENSG00000107554	chromosome10	101721872
ENSG00000107554	chromosome10	101721872
ENSG00000183111	chromosome5	148957526
ENSG00000173080	chromosome1	154178125
ENSG00000182631	chromosome5	33972603
ENSG00000144891	chromosome3	149941513
ENSG00000180772	chromosomeX	115217562
ENSG00000154165	chromosome3	99733568
ENSG00000170128	chromosome1	199108789
ENSG00000134817	chromosome11	56761055
ENSG00000100739	chromosome14	95799773
ENSG00000168398	chromosome14	95773198
ENSG00000170128	chromosome1	199108789
ENSG00000154165	chromosome3	99733568
ENSG00000182631	chromosome5	33972603
ENSG00000173080	chromosome1	154178125
ENSG00000182631	chromosome5	33972603
ENSG00000182631	chromosome5	33972603
ENSG00000100739	chromosome14	95799773
ENSG00000168398	chromosome14	95773198
ENSG00000144891	chromosome3	149941513
ENSG00000180772	chromosomeX	115217562
ENSG00000154165	chromosome3	99733568

ENSG00000170128	chromosome1	199108789
ENSG00000134817	chromosome11	56761055
ENSG00000134817	chromosome11	56761055
ENSG00000154165	chromosome3	99733568
ENSG00000170128	chromosome1	199108789
ENSG00000134817	chromosome11	56761055
ENSG00000154165	chromosome3	99733568
ENSG00000170128	chromosome1	199108789
ENSG00000134817	chromosome11	56761055
ENSG00000144891	chromosome3	149941513
ENSG00000180772	chromosomeX	115217562
ENSG00000180772	chromosomeX	115217562
ENSG00000144891	chromosome3	149941513
ENSG00000168398	chromosome14	95773198
ENSG00000100739	chromosome14	95799773
ENSG00000112796	chromosome6	46243959
ENSG00000001561	chromosome6	46215280
ENSG00000182156	chromosome17	75319497
ENSG00000136960	chromosome8	120720202
ENSG00000154269	chromosome6	132000218
ENSG00000197594	chromosome6	132170869
ENSG00000197594	chromosome6	132170869
ENSG00000154269	chromosome6	132000218
ENSG00000174938	chromosome16	29817842
ENSG00000100095	chromosome22	24895636
ENSG00000063015	chromosome17	24357134
ENSG00000100095	chromosome22	24895636
ENSG00000174938	chromosome16	29817842
ENSG00000134874	chromosome13	95092285
ENSG00000158163	chromosome3	139305504
ENSG00000197320	chromosome7	9732395
ENSG00000197320	chromosome7	9732395
ENSG00000175898	chromosome19	10196582
ENSG00000180739	chromosome19	10486688
ENSG00000170989	chromosome1	101477129
ENSG00000213694	chromosome9	90805936
ENSG00000170989	chromosome1	101477129
ENSG00000213694	chromosome9	90805936
ENSG00000175898	chromosome19	10196582
ENSG00000180739	chromosome19	10486688
ENSG00000125910	chromosome19	3129791
ENSG00000125910	chromosome19	3129791
ENSG00000170989	chromosome1	101477129
ENSG00000213694	chromosome9	90805936
ENSG00000175898	chromosome19	10196582
ENSG00000180739	chromosome19	10486688
ENSG00000198121	chromosome9	112774219
ENSG00000064547	chromosome19	19599094
ENSG00000171517	chromosome1	85104392
ENSG00000213694	chromosome9	90805936
ENSG00000132975	chromosome13	26231965
ENSG00000198121	chromosome9	112774219
ENSG00000064547	chromosome19	19599094
ENSG00000171517	chromosome1	85104392
ENSG00000125910	chromosome19	3129791
ENSG00000170989	chromosome1	101477129
ENSG00000213694	chromosome9	90805936
ENSG00000175898	chromosome19	10196582
ENSG00000180739	chromosome19	10486688

ENSG00000118432	chromosome6	88911713
ENSG00000188822	chromosome1	24074695
ENSG00000146360	chromosome6	110407009
ENSG00000132975	chromosome13	26231965
ENSG00000181773	chromosome1	27592890
ENSG00000132975	chromosome13	26231965
ENSG00000146360	chromosome6	110407009
ENSG00000181773	chromosome1	27592890
ENSG00000146360	chromosome6	110407009
ENSG00000132975	chromosome13	26231965
ENSG00000125910	chromosome19	3129791
ENSG00000170989	chromosome1	101477129
ENSG00000213694	chromosome9	90805936
ENSG00000175898	chromosome19	10196582
ENSG00000180739	chromosome19	10486688
ENSG00000198121	chromosome9	112774219
ENSG00000064547	chromosome19	19599094
ENSG00000171517	chromosome1	85104392
ENSG00000213694	chromosome9	90805936
ENSG00000170989	chromosome1	101477129
ENSG00000180739	chromosome19	10486688
ENSG00000175898	chromosome19	10196582
ENSG00000064547	chromosome19	19599094
ENSG00000198121	chromosome9	112774219
ENSG00000171517	chromosome1	85104392
ENSG00000198121	chromosome9	112774219
ENSG00000064547	chromosome19	19599094
ENSG00000144152	chromosome2	112612704
ENSG00000132031	chromosome2	20075874
ENSG00000162510	chromosome1	30968986
ENSG00000162510	chromosome1	30968986
ENSG00000132031	chromosome2	20075874
ENSG00000124159	chromosome20	43367637
ENSG00000124159	chromosome20	43367637
ENSG00000162510	chromosome1	30968986
ENSG00000132031	chromosome2	20075874
ENSG00000132561	chromosome8	98969505
ENSG00000185924	chromosome17	1787866
ENSG00000186907	chromosome11	56984915
ENSG00000040608	chromosome22	18635616
ENSG00000186907	chromosome11	56984915
ENSG00000185924	chromosome17	1787866
ENSG00000137492	chromosome11	75769529
ENSG00000215028	chromosome8	79837127
ENSG00000137492	chromosome11	75769529
ENSG00000215028	chromosome8	79837127
ENSG00000100285	chromosome22	28206252
ENSG00000215479	chromosome20	40061187
ENSG00000104722	chromosome8	24827212
ENSG00000148798	chromosome10	105026959
ENSG00000104722	chromosome8	24827212
ENSG00000100285	chromosome22	28206252
ENSG00000215479	chromosome20	40061187
ENSG00000131095	chromosome17	40348381
ENSG00000135406	chromosome12	47975251
ENSG00000175084	chromosome2	219991429
ENSG00000026025	chromosome10	17311428
ENSG00000104725	chromosome8	24869947
ENSG00000100285	chromosome22	28206252

ENSG00000215479	chromosome20	40061187
ENSG00000104722	chromosome8	24827212
ENSG00000148798	chromosome10	105026959
ENSG00000148798	chromosome10	105026959
ENSG00000100285	chromosome22	28206252
ENSG00000215479	chromosome20	40061187
ENSG00000104722	chromosome8	24827212
ENSG00000135406	chromosome12	47975251
ENSG00000175084	chromosome2	219991429
ENSG00000131095	chromosome17	40348381
ENSG00000182253	chromosome15	97462929
ENSG00000026025	chromosome10	17311428
ENSG00000131095	chromosome17	40348381
ENSG00000135406	chromosome12	47975251
ENSG00000175084	chromosome2	219991429
ENSG00000100285	chromosome22	28206252
ENSG00000215479	chromosome20	40061187
ENSG00000104722	chromosome8	24827212
ENSG00000148798	chromosome10	105026959
ENSG00000104725	chromosome8	24869947
ENSG00000182253	chromosome15	97462929
ENSG00000175084	chromosome2	219991429
ENSG00000135406	chromosome12	47975251
ENSG00000182253	chromosome15	97462929
ENSG00000026025	chromosome10	17311428
ENSG00000131095	chromosome17	40348381
ENSG00000135406	chromosome12	47975251
ENSG00000175084	chromosome2	219991429
ENSG00000100285	chromosome22	28206252
ENSG00000215479	chromosome20	40061187
ENSG00000104722	chromosome8	24827212
ENSG00000148798	chromosome10	105026959
ENSG00000104725	chromosome8	24869947
ENSG00000100285	chromosome22	28206252
ENSG00000215479	chromosome20	40061187
ENSG00000104722	chromosome8	24827212
ENSG00000148798	chromosome10	105026959
ENSG00000104725	chromosome8	24869947
ENSG00000026025	chromosome10	17311428
ENSG00000131095	chromosome17	40348381
ENSG00000135406	chromosome12	47975251
ENSG00000175084	chromosome2	219991429
ENSG00000182253	chromosome15	97462929
ENSG00000104725	chromosome8	24869947
ENSG00000161277	chromosome19	41236960
ENSG00000131931	chromosome8	42817395
ENSG00000173451	chromosome12	70344557
ENSG00000161277	chromosome19	41236960
ENSG00000041988	chromosome1	6607866
ENSG00000041988	chromosome1	6607866
ENSG00000161277	chromosome19	41236960
ENSG00000173451	chromosome12	70344557
ENSG00000131931	chromosome8	42817395
ENSG00000138615	chromosome15	63289147
ENSG00000160161	chromosome19	19510159
ENSG00000057019	chromosome3	100102861
ENSG00000164465	chromosome6	117910509
ENSG00000140545	chromosome15	87257555
ENSG00000164176	chromosome5	83715949

ENSG00000099250	chromosome10	33663317
ENSG00000118257	chromosome2	206256260
ENSG00000102104	chromosomeX	18600110
ENSG00000164465	chromosome6	117910509
ENSG00000057019	chromosome3	100102861
ENSG00000204359	chromosome6	32021978
ENSG00000204364	chromosome6	32003509
ENSG00000215846	chromosome1	157512932
ENSG00000132703	chromosome1	157824336
ENSG00000132693	chromosome1	157950900
ENSG00000171246	chromosome17	76064842
ENSG00000106236	chromosome7	98084710
ENSG00000171246	chromosome17	76064842
ENSG00000167957	chromosome16	1478470
ENSG00000163661	chromosome3	158637417
ENSG00000215846	chromosome1	157512932
ENSG00000132703	chromosome1	157824336
ENSG00000132693	chromosome1	157950900
ENSG00000106236	chromosome7	98084710
ENSG00000171246	chromosome17	76064842
ENSG00000215846	chromosome1	157512932
ENSG00000132703	chromosome1	157824336
ENSG00000132693	chromosome1	157950900
ENSG00000106236	chromosome7	98084710
ENSG00000171246	chromosome17	76064842
ENSG00000106236	chromosome7	98084710
ENSG00000171246	chromosome17	76064842
ENSG00000163661	chromosome3	158637417
ENSG00000215846	chromosome1	157512932
ENSG00000132703	chromosome1	157824336
ENSG00000132693	chromosome1	157950900
ENSG00000215846	chromosome1	157512932
ENSG00000132703	chromosome1	157824336
ENSG00000132693	chromosome1	157950900
ENSG00000163661	chromosome3	158637417
ENSG00000163661	chromosome3	158637417
ENSG00000215846	chromosome1	157512932
ENSG00000132703	chromosome1	157824336
ENSG00000132693	chromosome1	157950900
ENSG00000106236	chromosome7	98084710
ENSG00000171246	chromosome17	76064842
ENSG00000167957	chromosome16	1478470
ENSG00000146067	chromosome5	176896078
ENSG00000125386	chromosome4	2602530
ENSG00000167604	chromosome19	41085026
ENSG00000144802	chromosome3	103051163
ENSG00000115267	chromosome2	162883064
ENSG00000108771	chromosome17	37517437
ENSG00000108771	chromosome17	37517437
ENSG00000115267	chromosome2	162883064
ENSG00000107201	chromosome9	32516165
ENSG00000119913	chromosome10	114033483
ENSG00000169347	chromosome16	20245255
ENSG00000169344	chromosome16	20269561
ENSG00000138315	chromosome10	74323563
ENSG00000119913	chromosome10	114033483
ENSG00000169347	chromosome16	20245255
ENSG00000169344	chromosome16	20269561
ENSG00000145287	chromosome4	84248099

ENSG00000105427	chromosome19	47585030
ENSG00000105427	chromosome19	47585030
ENSG00000145287	chromosome4	84248099
ENSG00000173261	chromosome5	145464068
ENSG00000173261	chromosome5	145464068
ENSG00000105427	chromosome19	47585030
ENSG00000145287	chromosome4	84248099
ENSG00000139915	chromosome14	47213543
ENSG00000112139	chromosome6	37772566
ENSG00000170231	chromosome5	159558692
ENSG00000163586	chromosome2	88208652
ENSG00000182326	chromosome12	7039425
ENSG00000159403	chromosome12	7135417
ENSG00000139178	chromosome12	7153053
ENSG00000127241	chromosome3	188492445
ENSG00000009724	chromosome1	11029876
ENSG00000159403	chromosome12	7135417
ENSG00000139178	chromosome12	7153053
ENSG00000182326	chromosome12	7039425
ENSG00000159403	chromosome12	7135417
ENSG00000139178	chromosome12	7153053
ENSG00000182326	chromosome12	7039425
ENSG00000009724	chromosome1	11029876
ENSG00000102878	chromosome16	65756216
ENSG00000185122	chromosome8	145486248
ENSG00000215701	supercontigNT_113906	30899
ENSG00000025156	chromosome6	122762582
ENSG00000214373	chromosome12	53804171
ENSG00000185122	chromosome8	145486248
ENSG00000215701	supercontigNT_113906	30899
ENSG00000102878	chromosome16	65756216
ENSG00000025156	chromosome6	122762582
ENSG00000214373	chromosome12	53804171
ENSG00000102878	chromosome16	65756216
ENSG00000134531	chromosome12	13255712
ENSG00000213853	chromosome16	10548976
ENSG00000142227	chromosome19	53521914
ENSG00000109099	chromosome17	15104770
ENSG00000213853	chromosome16	10548976
ENSG00000134531	chromosome12	13255712
ENSG00000109099	chromosome17	15104770
ENSG00000142227	chromosome19	53521914
ENSG00000168928	chromosome16	73798541
ENSG00000168925	chromosome16	73810407
ENSG00000141086	chromosome16	66523257
ENSG00000139610	chromosome12	50026690
ENSG00000162438	chromosome1	15637548
ENSG00000215704	chromosome1	15675208
ENSG00000142615	chromosome1	15655836
ENSG00000168928	chromosome16	73798541
ENSG00000168925	chromosome16	73810407
ENSG00000142789	chromosome1	22176000
ENSG00000139610	chromosome12	50026690
ENSG00000215704	chromosome1	15675208
ENSG00000142615	chromosome1	15655836
ENSG00000162438	chromosome1	15637548
ENSG00000139610	chromosome12	50026690
ENSG00000142789	chromosome1	22176000
ENSG00000215704	chromosome1	15675208

ENSG00000142615	chromosome1	15655836
ENSG00000162438	chromosome1	15637548
ENSG00000142789	chromosome1	22176000
ENSG00000139610	chromosome12	50026690
ENSG00000215704	chromosome1	15675208
ENSG00000142615	chromosome1	15655836
ENSG00000162438	chromosome1	15637548
ENSG00000142789	chromosome1	22176000
ENSG00000139610	chromosome12	50026690
ENSG00000183615	chromosome1	32485610
ENSG00000154319	chromosome8	11339331
ENSG00000118004	chromosome2	3629806
ENSG00000184374	chromosome8	120148702
ENSG00000163815	chromosome3	45042859
ENSG00000166509	chromosome16	76614025
ENSG00000166509	chromosome16	76614025
ENSG00000163815	chromosome3	45042859
ENSG00000105472	chromosome19	55918595
ENSG00000113600	chromosome5	39400324
ENSG00000180644	chromosome10	72030665
ENSG00000021852	chromosome1	57204210
ENSG00000157131	chromosome1	57093163
ENSG00000112936	chromosome5	40945353
ENSG00000180644	chromosome10	72030665
ENSG00000113600	chromosome5	39400324
ENSG00000112936	chromosome5	40945353
ENSG00000021852	chromosome1	57204210
ENSG00000157131	chromosome1	57093163
ENSG00000180644	chromosome10	72030665
ENSG00000157131	chromosome1	57093163
ENSG00000021852	chromosome1	57204210
ENSG00000021852	chromosome1	57204210
ENSG00000157131	chromosome1	57093163
ENSG00000112936	chromosome5	40945353
ENSG00000113600	chromosome5	39400324
ENSG00000180644	chromosome10	72030665
ENSG00000039537	chromosome5	41239090
ENSG00000076706	chromosome11	118693022
ENSG00000187244	chromosome19	50004222
ENSG00000160318	chromosome19	56563644
ENSG00000105370	chromosome19	56582510
ENSG00000105374	chromosome19	56567602
ENSG00000105370	chromosome19	56582510
ENSG00000105370	chromosome19	56582510
ENSG00000160318	chromosome19	56563644
ENSG00000105370	chromosome19	56582510
ENSG00000182923	chromosome3	135696853
ENSG00000165325	chromosome11	92705098
ENSG00000053328	chromosome6	110786169
ENSG00000149418	chromosome11	129535085
ENSG00000176040	chromosome3	113249359
ENSG00000149418	chromosome11	129535085
ENSG00000187045	chromosome22	35829526
ENSG00000149418	chromosome11	129535085
ENSG00000176040	chromosome3	113249359
ENSG00000149418	chromosome11	129535085
ENSG00000187045	chromosome22	35829526
ENSG00000176040	chromosome3	113249359
ENSG00000149418	chromosome11	129535085

ENSG00000205403	chromosome4	110942688
ENSG00000213199	chromosome7	150376906
ENSG00000108684	chromosome17	29507665
ENSG00000110881	chromosome12	48738817
ENSG00000108684	chromosome17	29507665
ENSG00000139517	chromosome13	27053841
ENSG00000072201	chromosome4	54134927
ENSG00000107186	chromosome9	13240315
ENSG00000132849	chromosome1	62001153
ENSG00000139517	chromosome13	27053841
ENSG00000178726	chromosome20	22978142
ENSG00000174807	chromosome11	65841075
ENSG00000125810	chromosome20	23014830
ENSG00000174807	chromosome11	65841075
ENSG00000178726	chromosome20	22978142
ENSG00000125810	chromosome20	23014830
ENSG00000178726	chromosome20	22978142
ENSG00000174807	chromosome11	65841075
ENSG00000176435	chromosome14	37794979
ENSG00000204381	chromosome11	110916779
ENSG00000154645	chromosome21	18539412
ENSG00000214424	chromosome15	95101533
ENSG00000138286	chromosome10	74598104
ENSG00000109794	chromosome4	187303091
ENSG00000159723	chromosome16	66074803
ENSG00000101440	chromosome20	32311842
ENSG00000214316	chromosome3	130203195
ENSG00000168334	chromosome3	39205941
ENSG00000163092	chromosome2	167468239
ENSG00000181104	chromosome5	76047889
ENSG00000164251	chromosome5	76150764
ENSG00000147138	chromosomeX	78313161
ENSG00000147138	chromosomeX	78313161
ENSG00000126251	chromosome19	40554102
ENSG00000185897	chromosome19	40541633
ENSG00000126262	chromosome19	40632457
ENSG00000182162	chromosomeX	1545452
ENSG00000164251	chromosome5	76150764
ENSG00000181104	chromosome5	76047889
ENSG00000164220	chromosome5	75954812
ENSG00000125245	chromosome13	98706128
ENSG00000144230	chromosome2	128124057
ENSG00000181104	chromosome5	76047889
ENSG00000144230	chromosome2	128124057
ENSG00000125245	chromosome13	98706128
ENSG00000164220	chromosome5	75954812
ENSG00000182559	chromosome3	160042568
ENSG00000171631	chromosome11	72685212
ENSG00000171631	chromosome11	72685212
ENSG00000182559	chromosome3	160042568
ENSG00000182782	chromosome12	121753784
ENSG00000196917	chromosome12	121780840
ENSG00000162881	chromosome2	42844824
ENSG00000126251	chromosome19	40554102
ENSG00000185897	chromosome19	40541633
ENSG00000126262	chromosome19	40632457
ENSG00000182162	chromosomeX	1545452
ENSG00000164251	chromosome5	76150764
ENSG00000181104	chromosome5	76047889

ENSG00000164220	chromosome5	75954812
ENSG00000125245	chromosome13	98706128
ENSG00000144230	chromosome2	128124057
ENSG00000147138	chromosomeX	78313161
ENSG00000144230	chromosome2	128124057
ENSG00000147138	chromosomeX	78313161
ENSG00000182162	chromosomeX	1545452
ENSG00000164251	chromosome5	76150764
ENSG00000181104	chromosome5	76047889
ENSG00000164220	chromosome5	75954812
ENSG00000126251	chromosome19	40554102
ENSG00000185897	chromosome19	40541633
ENSG00000126262	chromosome19	40632457
ENSG00000182782	chromosome12	121753784
ENSG00000196917	chromosome12	121780840
ENSG00000162881	chromosome2	42844824
ENSG00000126251	chromosome19	40554102
ENSG00000185897	chromosome19	40541633
ENSG00000126262	chromosome19	40632457
ENSG00000182162	chromosomeX	1545452
ENSG00000164251	chromosome5	76150764
ENSG00000181104	chromosome5	76047889
ENSG00000164220	chromosome5	75954812
ENSG00000125245	chromosome13	98706128
ENSG00000144230	chromosome2	128124057
ENSG00000147138	chromosomeX	78313161
ENSG00000171631	chromosome11	72685212
ENSG00000182559	chromosome3	160042568
ENSG00000165621	chromosome13	96438015
ENSG00000126251	chromosome19	40554102
ENSG00000185897	chromosome19	40541633
ENSG00000126262	chromosome19	40632457
ENSG00000126251	chromosome19	40554102
ENSG00000185897	chromosome19	40541633
ENSG00000126262	chromosome19	40632457
ENSG00000181104	chromosome5	76047889
ENSG00000162881	chromosome2	42844824
ENSG00000182782	chromosome12	121753784
ENSG00000196917	chromosome12	121780840
ENSG00000126251	chromosome19	40554102
ENSG00000185897	chromosome19	40541633
ENSG00000126262	chromosome19	40632457
ENSG00000182162	chromosomeX	1545452
ENSG00000164251	chromosome5	76150764
ENSG00000181104	chromosome5	76047889
ENSG00000164220	chromosome5	75954812
ENSG00000125245	chromosome13	98706128
ENSG00000144230	chromosome2	128124057
ENSG00000147138	chromosomeX	78313161
ENSG00000182782	chromosome12	121753784
ENSG00000196917	chromosome12	121780840
ENSG00000162881	chromosome2	42844824
ENSG00000164251	chromosome5	76150764
ENSG00000181104	chromosome5	76047889
ENSG00000164220	chromosome5	75954812
ENSG00000182162	chromosomeX	1545452
ENSG00000182782	chromosome12	121753784
ENSG00000196917	chromosome12	121780840
ENSG00000162881	chromosome2	42844824

ENSG00000126251	chromosome19	40554102
ENSG00000185897	chromosome19	40541633
ENSG00000126262	chromosome19	40632457
ENSG00000182162	chromosomeX	1545452
ENSG00000164251	chromosome5	76150764
ENSG00000181104	chromosome5	76047889
ENSG00000164220	chromosome5	75954812
ENSG00000125245	chromosome13	98706128
ENSG00000144230	chromosome2	128124057
ENSG00000147138	chromosomeX	78313161
ENSG00000126251	chromosome19	40554102
ENSG00000185897	chromosome19	40541633
ENSG00000126262	chromosome19	40632457
ENSG00000182162	chromosomeX	1545452
ENSG00000164251	chromosome5	76150764
ENSG00000181104	chromosome5	76047889
ENSG00000164220	chromosome5	75954812
ENSG00000125245	chromosome13	98706128
ENSG00000144230	chromosome2	128124057
ENSG00000126251	chromosome19	40554102
ENSG00000185897	chromosome19	40541633
ENSG00000126262	chromosome19	40632457
ENSG00000182162	chromosomeX	1545452
ENSG00000164251	chromosome5	76150764
ENSG00000181104	chromosome5	76047889
ENSG00000164220	chromosome5	75954812
ENSG00000125245	chromosome13	98706128
ENSG00000144230	chromosome2	128124057
ENSG00000182559	chromosome3	160042568
ENSG00000125245	chromosome13	98706128
ENSG00000144230	chromosome2	128124057
ENSG00000126251	chromosome19	40554102
ENSG00000185897	chromosome19	40541633
ENSG00000126262	chromosome19	40632457
ENSG00000182162	chromosomeX	1545452
ENSG00000164251	chromosome5	76150764
ENSG00000181104	chromosome5	76047889
ENSG00000164220	chromosome5	75954812
ENSG00000164220	chromosome5	75954812
ENSG00000164251	chromosome5	76150764
ENSG00000181104	chromosome5	76047889
ENSG00000182782	chromosome12	121753784
ENSG00000196917	chromosome12	121780840
ENSG00000162881	chromosome2	42844824
ENSG00000126251	chromosome19	40554102
ENSG00000185897	chromosome19	40541633
ENSG00000126262	chromosome19	40632457
ENSG00000182162	chromosomeX	1545452
ENSG00000164251	chromosome5	76150764
ENSG00000181104	chromosome5	76047889
ENSG00000164220	chromosome5	75954812
ENSG00000125245	chromosome13	98706128
ENSG00000144230	chromosome2	128124057
ENSG00000147138	chromosomeX	78313161
ENSG00000168143	chromosome6	54843004
ENSG00000180921	chromosome8	144884741
ENSG00000147689	chromosome8	124264278
ENSG00000125998	chromosome20	33343522
ENSG00000101447	chromosome20	36988410

ENSG00000105523	chromosome19	53808442
ENSG00000133477	chromosome22	38720993
ENSG00000188522	chromosome17	18848080
ENSG00000147689	chromosome8	124264278
ENSG00000125998	chromosome20	33343522
ENSG00000168143	chromosome6	54843004
ENSG00000180921	chromosome8	144884741
ENSG00000180921	chromosome8	144884741
ENSG00000168143	chromosome6	54843004
ENSG00000105523	chromosome19	53808442
ENSG00000133477	chromosome22	38720993
ENSG00000188522	chromosome17	18848080
ENSG00000101447	chromosome20	36988410
ENSG00000188522	chromosome17	18848080
ENSG00000133477	chromosome22	38720993
ENSG00000133477	chromosome22	38720993
ENSG00000188522	chromosome17	18848080
ENSG00000105523	chromosome19	53808442
ENSG00000125998	chromosome20	33343522
ENSG00000147689	chromosome8	124264278
ENSG00000186106	chromosome8	101611238
ENSG00000100124	chromosome22	36570192
ENSG00000166737	chromosome16	12903300
ENSG00000187902	chromosome19	60645584
ENSG00000187902	chromosome19	60645584
ENSG00000166737	chromosome16	12903300
ENSG00000180730	chromosome13	25523114
ENSG00000178343	chromosome4	42094831
ENSG00000166737	chromosome16	12903300
ENSG00000187902	chromosome19	60645584
ENSG00000188803	chromosome17	11107397
ENSG00000166737	chromosome16	12903300
ENSG00000187902	chromosome19	60645584
ENSG00000180730	chromosome13	25523114
ENSG00000178343	chromosome4	42094831
ENSG00000178343	chromosome4	42094831
ENSG00000180730	chromosome13	25523114
ENSG00000180730	chromosome13	25523114
ENSG00000178343	chromosome4	42094831
ENSG00000188803	chromosome17	11107397
ENSG00000180730	chromosome13	25523114
ENSG00000178343	chromosome4	42094831
ENSG00000166737	chromosome16	12903300
ENSG00000187902	chromosome19	60645584
ENSG00000171033	chromosome8	79673175
ENSG00000168734	chromosome20	42676612
ENSG00000135549	chromosome6	123064332
ENSG00000168734	chromosome20	42676612
ENSG00000171033	chromosome8	79673175
ENSG00000159184	chromosome17	44160955
ENSG00000106031	chromosome7	27206222
ENSG00000123364	chromosome12	52618958
ENSG00000123364	chromosome12	52618958
ENSG00000159184	chromosome17	44160955
ENSG00000106031	chromosome7	27206222
ENSG00000128714	chromosome2	176665865
ENSG00000106031	chromosome7	27206222
ENSG00000159184	chromosome17	44160955
ENSG00000198759	chromosomeX	13497902

ENSG00000168743	chromosome4	107036259
ENSG00000167992	chromosome11	60819086
ENSG00000120832	chromosome12	105896623
ENSG00000127989	chromosome7	91347334
ENSG00000168672	chromosome8	127638817
ENSG00000162981	chromosome2	14691555
ENSG00000162981	chromosome2	14691555
ENSG00000168672	chromosome8	127638817
ENSG00000133321	chromosome11	63060917
ENSG00000168004	chromosome11	63015083
ENSG00000133328	chromosome11	63087373
ENSG00000176485	chromosome11	63138063
ENSG00000127252	chromosome3	194456134
ENSG00000121207	chromosome4	155884929
ENSG00000133321	chromosome11	63060917
ENSG00000168004	chromosome11	63015083
ENSG00000133328	chromosome11	63087373
ENSG00000176485	chromosome11	63138063
ENSG00000127252	chromosome3	194456134
ENSG00000162981	chromosome2	14691555
ENSG00000168672	chromosome8	127638817
ENSG00000168672	chromosome8	127638817
ENSG00000133321	chromosome11	63060917
ENSG00000168004	chromosome11	63015083
ENSG00000133328	chromosome11	63087373
ENSG00000176485	chromosome11	63138063
ENSG00000127252	chromosome3	194456134
ENSG00000162981	chromosome2	14691555
ENSG00000168672	chromosome8	127638817
ENSG00000127252	chromosome3	194456134
ENSG00000133321	chromosome11	63060917
ENSG00000168004	chromosome11	63015083
ENSG00000133328	chromosome11	63087373
ENSG00000176485	chromosome11	63138063
ENSG00000104341	chromosome8	98857001
ENSG00000068697	chromosome2	20114763
ENSG00000068697	chromosome2	20114763
ENSG00000104341	chromosome8	98857001
ENSG00000162511	chromosome1	31003180
ENSG00000171115	chromosome7	149794720
ENSG00000133561	chromosome7	149958164
ENSG00000106560	chromosome7	150015081
ENSG00000213203	chromosome7	150047069
ENSG00000196329	chromosome7	150068906
ENSG00000133574	chromosome7	149897923
ENSG00000179144	chromosome7	149847996
ENSG00000171115	chromosome7	149794720
ENSG00000133561	chromosome7	149958164
ENSG00000106560	chromosome7	150015081
ENSG00000213203	chromosome7	150047069
ENSG00000196329	chromosome7	150068906
ENSG00000133574	chromosome7	149897923
ENSG00000179144	chromosome7	149847996
ENSG00000171115	chromosome7	149794720
ENSG00000133561	chromosome7	149958164
ENSG00000106560	chromosome7	150015081
ENSG00000213203	chromosome7	150047069
ENSG00000196329	chromosome7	150068906
ENSG00000133574	chromosome7	149897923

ENSG00000179144	chromosome7	149847996
ENSG00000144560	chromosome3	11719509
ENSG00000109686	chromosome4	152315957
ENSG00000109686	chromosome4	152315957
ENSG00000100365	chromosome22	35587160
ENSG00000112964	chromosome5	42601734
ENSG00000113494	chromosome5	35125480
ENSG00000113494	chromosome5	35125480
ENSG00000112964	chromosome5	42601734
ENSG00000038945	chromosome8	16079869
ENSG00000168079	chromosome8	27901052
ENSG00000019169	chromosome2	119416347
ENSG00000168079	chromosome8	27901052
ENSG00000038945	chromosome8	16079869
ENSG00000134107	chromosome3	4996404
ENSG00000123095	chromosome12	26168980
ENSG00000173621	chromosome11	66381792
ENSG00000204033	chromosome10	85975257
ENSG00000183423	chromosome4	110992374
ENSG00000148602	chromosome10	85991176
ENSG00000148602	chromosome10	85991176
ENSG00000183423	chromosome4	110992374
ENSG00000173621	chromosome11	66381792
ENSG00000173621	chromosome11	66381792
ENSG00000183423	chromosome4	110992374
ENSG00000148602	chromosome10	85991176
ENSG00000204033	chromosome10	85975257
ENSG00000172403	chromosome4	120029640
ENSG00000166317	chromosome10	75085687
ENSG00000171992	chromosome5	149978123
ENSG00000166317	chromosome10	75085687
ENSG00000172403	chromosome4	120029640
ENSG00000066697	chromosome9	102244042
ENSG00000144339	chromosome2	192767496
ENSG00000019102	chromosome11	124127244
ENSG00000149564	chromosome11	124137261
ENSG00000144847	chromosome3	120306730
ENSG00000143167	chromosome1	165326149
ENSG00000154639	chromosome21	17807319
ENSG00000188004	chromosome1	158098936
ENSG00000166250	chromosome11	122570859
ENSG00000144847	chromosome3	120306730
ENSG00000149564	chromosome11	124137261
ENSG00000188004	chromosome1	158098936
ENSG00000154639	chromosome21	17807319
ENSG00000154639	chromosome21	17807319
ENSG00000188004	chromosome1	158098936
ENSG00000166250	chromosome11	122570859
ENSG00000019102	chromosome11	124127244
ENSG00000149564	chromosome11	124137261
ENSG00000144847	chromosome3	120306730
ENSG00000143167	chromosome1	165326149
ENSG00000143167	chromosome1	165326149
ENSG00000019102	chromosome11	124127244
ENSG00000149564	chromosome11	124137261
ENSG00000144847	chromosome3	120306730
ENSG00000166250	chromosome11	122570859
ENSG00000154639	chromosome21	17807319
ENSG00000188004	chromosome1	158098936

ENSG00000144847	chromosome3	120306730
ENSG00000149564	chromosome11	124137261
ENSG00000144847	chromosome3	120306730
ENSG00000019102	chromosome11	124127244
ENSG00000145103	chromosome3	123223615
ENSG00000143195	chromosome1	165211130
ENSG00000105699	chromosome19	40431622
ENSG00000145103	chromosome3	123223615
ENSG00000105699	chromosome19	40431622
ENSG00000143195	chromosome1	165211130
ENSG00000172602	chromosome12	47545818
ENSG00000115963	chromosome2	151052178
ENSG00000108830	chromosome17	38430891
ENSG00000115963	chromosome2	151052178
ENSG00000172602	chromosome12	47545818
ENSG00000088386	chromosome13	98202854
ENSG00000163406	chromosome3	123096014
ENSG00000139370	chromosome12	127874442
ENSG00000110446	chromosome11	60475600
ENSG00000113916	chromosome3	188934176
ENSG00000161940	chromosome17	6867715
ENSG00000155011	chromosome4	108176198
ENSG00000107984	chromosome10	53744201
ENSG00000104371	chromosome8	42353721
ENSG00000107984	chromosome10	53744201
ENSG00000155011	chromosome4	108176198
ENSG00000103260	chromosome16	705291
ENSG00000176845	chromosome17	78630981
ENSG00000103260	chromosome16	705291
ENSG00000111341	chromosome12	14929993
ENSG00000111341	chromosome12	14929993
ENSG00000198821	chromosome1	165754456
ENSG00000158869	chromosome1	159451736
ENSG00000112130	chromosome6	37429919
ENSG00000072609	chromosome12	131973988
ENSG00000125898	chromosome20	773448
ENSG00000169122	chromosome8	59221344
ENSG00000197245	chromosome1	26360370
ENSG00000184731	chromosome2	36386
ENSG00000169122	chromosome8	59221344
ENSG00000125898	chromosome20	773448
ENSG00000184731	chromosome2	36386
ENSG00000197245	chromosome1	26360370
ENSG00000170903	chromosome11	105386855
ENSG00000173578	chromosome3	46038444
ENSG00000121807	chromosome3	46374023
ENSG00000215782	supercontigNT_113884	46106
ENSG00000160791	chromosome3	46389398
ENSG00000215778	supercontigNT_113884	61483
ENSG00000121797	chromosome3	46424166
ENSG00000215775	supercontigNT_113884	96233
ENSG00000183625	chromosome3	46281654
ENSG00000163823	chromosome3	46220809
ENSG00000183813	chromosome3	32969919
ENSG00000179934	chromosome3	39348827
ENSG00000168329	chromosome3	39298191
ENSG00000112486	chromosome6	167469613
ENSG00000126353	chromosome17	35975188
ENSG00000121966	chromosome2	136589980

ENSG00000160683	chromosome11	118259861
ENSG00000186810	chromosomeX	70755025
ENSG00000163464	chromosome2	218738180
ENSG00000180871	chromosome2	218707770
ENSG00000129048	chromosome3	133801932
ENSG00000184451	chromosome17	38087368
ENSG00000173585	chromosome3	45911430
ENSG00000172215	chromosome3	45962978
ENSG00000184451	chromosome17	38087368
ENSG00000129048	chromosome3	133801932
ENSG00000163464	chromosome2	218738180
ENSG00000180871	chromosome2	218707770
ENSG00000186810	chromosomeX	70755025
ENSG00000160683	chromosome11	118259861
ENSG00000126353	chromosome17	35975188
ENSG00000112486	chromosome6	167469613
ENSG00000184451	chromosome17	38087368
ENSG00000129048	chromosome3	133801932
ENSG00000173585	chromosome3	45911430
ENSG00000172215	chromosome3	45962978
ENSG00000160683	chromosome11	118259861
ENSG00000186810	chromosomeX	70755025
ENSG00000163464	chromosome2	218738180
ENSG00000180871	chromosome2	218707770
ENSG00000121966	chromosome2	136589980
ENSG00000144648	chromosome3	42880999
ENSG00000186810	chromosomeX	70755025
ENSG00000160683	chromosome11	118259861
ENSG00000186810	chromosomeX	70755025
ENSG00000163464	chromosome2	218738180
ENSG00000180871	chromosome2	218707770
ENSG00000121966	chromosome2	136589980
ENSG00000126353	chromosome17	35975188
ENSG00000112486	chromosome6	167469613
ENSG00000184451	chromosome17	38087368
ENSG00000129048	chromosome3	133801932
ENSG00000173585	chromosome3	45911430
ENSG00000172215	chromosome3	45962978
ENSG00000168329	chromosome3	39298191
ENSG00000121807	chromosome3	46374023
ENSG00000215782	supercontigNT_113884	46106
ENSG00000160791	chromosome3	46389398
ENSG00000215778	supercontigNT_113884	61483
ENSG00000121797	chromosome3	46424166
ENSG00000215775	supercontigNT_113884	96233
ENSG00000183625	chromosome3	46281654
ENSG00000163823	chromosome3	46220809
ENSG00000183813	chromosome3	32969919
ENSG00000179934	chromosome3	39348827
ENSG00000184451	chromosome17	38087368
ENSG00000129048	chromosome3	133801932
ENSG00000173585	chromosome3	45911430
ENSG00000172215	chromosome3	45962978
ENSG00000126353	chromosome17	35975188
ENSG00000112486	chromosome6	167469613
ENSG00000173578	chromosome3	46038444
ENSG00000144648	chromosome3	42880999
ENSG00000126353	chromosome17	35975188
ENSG00000112486	chromosome6	167469613

ENSG00000184451	chromosome17	38087368
ENSG00000129048	chromosome3	133801932
ENSG00000173585	chromosome3	45911430
ENSG00000172215	chromosome3	45962978
ENSG00000160683	chromosome11	118259861
ENSG00000186810	chromosomeX	70755025
ENSG00000163464	chromosome2	218738180
ENSG00000180871	chromosome2	218707770
ENSG00000121966	chromosome2	136589980
ENSG00000121807	chromosome3	46374023
ENSG00000215782	supercontigNT_113884	46106
ENSG00000160791	chromosome3	46389398
ENSG00000215778	supercontigNT_113884	61483
ENSG00000121797	chromosome3	46424166
ENSG00000215775	supercontigNT_113884	96233
ENSG00000183625	chromosome3	46281654
ENSG00000163823	chromosome3	46220809
ENSG00000183813	chromosome3	32969919
ENSG00000179934	chromosome3	39348827
ENSG00000168329	chromosome3	39298191
ENSG00000173578	chromosome3	46038444
ENSG00000163464	chromosome2	218738180
ENSG00000180871	chromosome2	218707770
ENSG00000160683	chromosome11	118259861
ENSG00000186810	chromosomeX	70755025
ENSG00000171659	chromosomeX	41439831
ENSG00000171659	chromosomeX	41439831
ENSG00000171659	chromosomeX	41439831
ENSG00000174946	chromosome3	152399864
ENSG00000138271	chromosome3	152500579
ENSG00000174944	chromosome3	152414795
ENSG00000181631	chromosome3	152529471
ENSG00000169313	chromosome3	152539324
ENSG00000174946	chromosome3	152399864
ENSG00000171659	chromosomeX	41439831
ENSG00000174944	chromosome3	152414795
ENSG00000138271	chromosome3	152500579
ENSG00000169313	chromosome3	152539324
ENSG00000181631	chromosome3	152529471
ENSG00000169313	chromosome3	152539324
ENSG00000181631	chromosome3	152529471
ENSG00000174944	chromosome3	152414795
ENSG00000138271	chromosome3	152500579
ENSG00000125970	chromosome20	32123542
ENSG00000184672	chromosome8	85604112
ENSG00000214634	chromosome7	63598608
ENSG00000215880	chromosome1	67899290
ENSG00000179172	chromosome1	12830730
ENSG00000092199	chromosome14	20772193
ENSG00000184672	chromosome8	85604112
ENSG00000125970	chromosome20	32123542
ENSG00000171051	chromosome19	56942060
ENSG00000187474	chromosome19	57018814
ENSG00000171049	chromosome19	56963724
ENSG00000183671	chromosome2	206750217
ENSG00000174600	chromosome12	107211499
ENSG00000171860	chromosome12	8104049
ENSG00000134830	chromosome19	52535897
ENSG00000197405	chromosome19	52504993

ENSG00000213903	chromosome14	23854698
ENSG00000213906	chromosome14	23849618
ENSG00000213903	chromosome14	23854698
ENSG00000213906	chromosome14	23849618
ENSG00000183134	chromosome11	60377772
ENSG00000171860	chromosome12	8104049
ENSG00000134830	chromosome19	52535897
ENSG00000197405	chromosome19	52504993
ENSG00000171051	chromosome19	56942060
ENSG00000187474	chromosome19	57018814
ENSG00000171049	chromosome19	56963724
ENSG00000183671	chromosome2	206750217
ENSG00000174600	chromosome12	107211499
ENSG00000183671	chromosome2	206750217
ENSG00000174600	chromosome12	107211499
ENSG00000171051	chromosome19	56942060
ENSG00000187474	chromosome19	57018814
ENSG00000171049	chromosome19	56963724
ENSG00000213903	chromosome14	23854698
ENSG00000213906	chromosome14	23849618
ENSG00000171860	chromosome12	8104049
ENSG00000134830	chromosome19	52535897
ENSG00000197405	chromosome19	52504993
ENSG00000171051	chromosome19	56942060
ENSG00000187474	chromosome19	57018814
ENSG00000171049	chromosome19	56963724
ENSG00000183671	chromosome2	206750217
ENSG00000174600	chromosome12	107211499
ENSG00000183134	chromosome11	60377772
ENSG00000183671	chromosome2	206750217
ENSG00000174600	chromosome12	107211499
ENSG00000174600	chromosome12	107211499
ENSG00000183671	chromosome2	206750217
ENSG00000134830	chromosome19	52535897
ENSG00000197405	chromosome19	52504993
ENSG00000171860	chromosome12	8104049
ENSG00000188676	chromosome8	39911873
ENSG00000131203	chromosome8	39890599
ENSG00000168795	chromosome9	37432549
ENSG00000185670	chromosome11	62278111
ENSG00000160062	chromosome1	32831120
ENSG00000215897	chromosome1	32708813
ENSG00000205189	chromosome8	81560574
ENSG00000215897	chromosome1	32708813
ENSG00000160062	chromosome1	32831120
ENSG00000168795	chromosome9	37432549
ENSG00000183186	chromosome19	360146
ENSG00000183186	chromosome19	360146
ENSG00000198535	chromosome15	60147105
ENSG00000205502	chromosome15	60244476
ENSG00000175164	chromosome9	135140427
ENSG00000204007	chromosome9	137670855
ENSG00000184389	chromosome1	33551078
ENSG00000204007	chromosome9	137670855
ENSG00000184389	chromosome1	33551078
ENSG00000175164	chromosome9	135140427
ENSG00000148288	chromosome9	135027621
ENSG00000184389	chromosome1	33551078
ENSG00000204007	chromosome9	137670855

ENSG00000164125	chromosome4	159311978
ENSG00000144649	chromosome3	43048982
ENSG00000165259	chromosomeX	83617062
ENSG00000141380	chromosome18	21924541
ENSG00000184402	chromosome20	60152272
ENSG00000184402	chromosome20	60152272
ENSG00000141380	chromosome18	21924541
ENSG00000137103	chromosome9	35819225
ENSG00000129925	chromosome16	371823
ENSG00000124226	chromosome20	47986357
ENSG00000158717	chromosome16	87300235
ENSG00000101695	chromosome18	27852825
ENSG00000134758	chromosome18	27926738
ENSG00000158717	chromosome16	87300235
ENSG00000124226	chromosome20	47986357
ENSG00000125629	chromosome2	118570603
ENSG00000186480	chromosome7	154720929
ENSG00000153233	chromosome12	69600438
ENSG00000110786	chromosome11	18750082
ENSG00000110786	chromosome11	18750082
ENSG00000153233	chromosome12	69600438
ENSG00000143851	chromosome1	200396559
ENSG00000110171	chromosome11	6443502
ENSG00000109654	chromosome4	154410988
ENSG00000187566	chromosome6	18230817
ENSG00000187566	chromosome6	18230817
ENSG00000119401	chromosome9	118499843
ENSG00000184305	chromosome4	91448459
ENSG00000107771	chromosome10	86120789
ENSG00000152409	chromosome5	78568230
ENSG00000156232	chromosome15	81275470
ENSG00000183166	chromosome7	71381725
ENSG00000100314	chromosome22	28446414
ENSG00000125967	chromosome20	31725820
ENSG00000123119	chromosome8	91873291
ENSG00000123119	chromosome8	91873291
ENSG00000125967	chromosome20	31725820
ENSG00000103154	chromosome16	82559755
ENSG00000177469	chromosome17	37828642
ENSG00000170681	chromosome9	102380247
ENSG00000170681	chromosome9	102380247
ENSG00000177469	chromosome17	37828642
ENSG00000170955	chromosome11	6298295
ENSG00000168497	chromosome2	192419897
ENSG00000177427	chromosome17	18105175
ENSG00000100335	chromosome22	38237246
ENSG00000138080	chromosome2	27155438
ENSG00000183798	chromosome20	39428689
ENSG00000132205	chromosome18	2837042
ENSG00000132205	chromosome18	2837042
ENSG00000183798	chromosome20	39428689
ENSG00000184497	chromosome13	113651847
ENSG00000125355	chromosomeX	119329193
ENSG00000154175	chromosome3	102194940
ENSG00000130695	chromosome1	26438862
ENSG00000111860	chromosome6	119080638
ENSG00000073008	chromosome19	49839237
ENSG00000130202	chromosome19	50041623
ENSG00000110400	chromosome11	119104474

ENSG00000110400	chromosome11	119104474
ENSG00000073008	chromosome19	49839237
ENSG00000130202	chromosome19	50041623
ENSG00000177707	chromosome3	112273555
ENSG00000073008	chromosome19	49839237
ENSG00000130202	chromosome19	50041623
ENSG00000110400	chromosome11	119104474
ENSG00000073008	chromosome19	49839237
ENSG00000130202	chromosome19	50041623
ENSG00000110400	chromosome11	119104474
ENSG00000177707	chromosome3	112273555
ENSG00000141293	chromosome17	43862599
ENSG00000005020	chromosome7	26870574
ENSG00000164691	chromosome6	159385167
ENSG00000137727	chromosome11	110088165
ENSG00000115488	chromosome2	233605626
ENSG00000204099	chromosome2	242401197
ENSG00000162139	chromosome11	74383207
ENSG00000204386	chromosome6	31938533
ENSG00000204099	chromosome2	242401197
ENSG00000162139	chromosome11	74383207
ENSG00000115488	chromosome2	233605626
ENSG00000162139	chromosome11	74383207
ENSG00000204099	chromosome2	242401197
ENSG00000173636	chromosome7	142136910
ENSG00000204983	chromosome7	142158416
ENSG00000204981	chromosome7	142170799
ENSG00000204982	chromosome7	142167698
ENSG00000010438	chromosome9	33740515
ENSG00000214083	chromosome7	141636134
ENSG00000173636	chromosome7	142136910
ENSG00000204983	chromosome7	142158416
ENSG00000204981	chromosome7	142170799
ENSG00000204982	chromosome7	142167698
ENSG00000010438	chromosome9	33740515
ENSG00000214083	chromosome7	141636134
ENSG00000173636	chromosome7	142136910
ENSG00000204983	chromosome7	142158416
ENSG00000204981	chromosome7	142170799
ENSG00000204982	chromosome7	142167698
ENSG00000010438	chromosome9	33740515
ENSG00000214083	chromosome7	141636134
ENSG00000173636	chromosome7	142136910
ENSG00000204983	chromosome7	142158416
ENSG00000204981	chromosome7	142170799
ENSG00000204982	chromosome7	142167698
ENSG00000010438	chromosome9	33740515
ENSG00000214083	chromosome7	141636134
ENSG00000165076	chromosome7	141187319
ENSG00000171147	chromosome7	141604005
ENSG00000173636	chromosome7	142136910
ENSG00000204983	chromosome7	142158416
ENSG00000204981	chromosome7	142170799
ENSG00000204982	chromosome7	142167698
ENSG00000010438	chromosome9	33740515
ENSG00000214083	chromosome7	141636134
ENSG00000173636	chromosome7	142136910
ENSG00000204983	chromosome7	142158416
ENSG00000204981	chromosome7	142170799

ENSG00000204982	chromosome7	142167698
ENSG00000010438	chromosome9	33740515
ENSG00000214083	chromosome7	141636134
ENSG00000132669	chromosome20	19815314
ENSG00000174791	chromosome11	65860450
ENSG00000174791	chromosome11	65860450
ENSG00000132669	chromosome20	19815314
ENSG00000100599	chromosome14	92050030
ENSG00000100599	chromosome14	92050030
ENSG00000174791	chromosome11	65860450
ENSG00000132669	chromosome20	19815314
ENSG00000187994	chromosome19	44054322
ENSG00000112297	chromosome6	107066910
ENSG00000176092	chromosome1	26541901
ENSG00000176092	chromosome1	26541901
ENSG00000112297	chromosome6	107066910
ENSG00000080200	chromosome3	99078573
ENSG00000215811	chromosome1	226766628
ENSG00000186470	chromosome6	26476390
ENSG00000215631	supercontigNT_113890	64341
ENSG00000204290	chromosome6	32482879
ENSG00000215889	chromosome1	48405667
ENSG00000204655	chromosome6	29732966
ENSG00000114455	chromosome3	109553351
ENSG00000134258	chromosome1	117555076
ENSG00000215811	chromosome1	226766628
ENSG00000186470	chromosome6	26476390
ENSG00000215631	supercontigNT_113890	64341
ENSG00000204290	chromosome6	32482879
ENSG00000215889	chromosome1	48405667
ENSG00000204655	chromosome6	29732966
ENSG00000188211	chromosome11	17330093
ENSG00000215811	chromosome1	226766628
ENSG00000186470	chromosome6	26476390
ENSG00000215631	supercontigNT_113890	64341
ENSG00000204290	chromosome6	32482879
ENSG00000215889	chromosome1	48405667
ENSG00000204655	chromosome6	29732966
ENSG00000197646	chromosome9	5500725
ENSG00000120217	chromosome9	5446114
ENSG00000197646	chromosome9	5500725
ENSG00000120217	chromosome9	5446114
ENSG00000160223	chromosome21	44485267
ENSG00000114013	chromosome3	123257027
ENSG00000134258	chromosome1	117555076
ENSG00000114455	chromosome3	109553351
ENSG00000103855	chromosome15	71779034
ENSG00000215811	chromosome1	226766628
ENSG00000186470	chromosome6	26476390
ENSG00000215631	supercontigNT_113890	64341
ENSG00000204290	chromosome6	32482879
ENSG00000215889	chromosome1	48405667
ENSG00000204655	chromosome6	29732966
ENSG00000188211	chromosome11	17330093
ENSG00000103855	chromosome15	71779034
ENSG00000134258	chromosome1	117555076
ENSG00000114455	chromosome3	109553351
ENSG00000160223	chromosome21	44485267
ENSG00000114013	chromosome3	123257027

ENSG00000197646	chromosome9	5500725
ENSG00000120217	chromosome9	5446114
ENSG00000103855	chromosome15	71779034
ENSG00000134258	chromosome1	117555076
ENSG00000114455	chromosome3	109553351
ENSG00000215811	chromosome1	226766628
ENSG00000186470	chromosome6	26476390
ENSG00000215631	supercontigNT_113890	64341
ENSG00000204290	chromosome6	32482879
ENSG00000215889	chromosome1	48405667
ENSG00000204655	chromosome6	29732966
ENSG00000188211	chromosome11	17330093
ENSG00000188211	chromosome11	17330093
ENSG00000215811	chromosome1	226766628
ENSG00000186470	chromosome6	26476390
ENSG00000215631	supercontigNT_113890	64341
ENSG00000204290	chromosome6	32482879
ENSG00000215889	chromosome1	48405667
ENSG00000204655	chromosome6	29732966
ENSG00000103855	chromosome15	71779034
ENSG00000188211	chromosome11	17330093
ENSG00000215811	chromosome1	226766628
ENSG00000186470	chromosome6	26476390
ENSG00000215631	supercontigNT_113890	64341
ENSG00000204290	chromosome6	32482879
ENSG00000215889	chromosome1	48405667
ENSG00000204655	chromosome6	29732966
ENSG00000188211	chromosome11	17330093
ENSG00000166263	chromosome17	50418580
ENSG00000179820	chromosome19	59068596
ENSG00000185105	chromosome17	77492897
ENSG00000179820	chromosome19	59068596
ENSG00000185105	chromosome17	77492897
ENSG00000185105	chromosome17	77492897
ENSG00000179820	chromosome19	59068596
ENSG00000179820	chromosome19	59068596
ENSG00000185105	chromosome17	77492897
ENSG00000096696	chromosome6	7487148
ENSG00000167880	chromosome17	71534875
ENSG00000118898	chromosome16	4927048
ENSG00000151914	chromosome6	56927364
ENSG00000127603	chromosome1	39322426
ENSG00000096696	chromosome6	7487148
ENSG00000096696	chromosome6	7487148
ENSG00000118898	chromosome16	4927048
ENSG00000167880	chromosome17	71534875
ENSG00000127603	chromosome1	39322426
ENSG00000151914	chromosome6	56927364
ENSG00000118564	chromosome4	15266009
ENSG00000151876	chromosome5	41961169
ENSG00000135052	chromosome9	87884056
ENSG00000166734	chromosome15	42368520
ENSG00000215599	chromosome13	19037041
ENSG00000152102	chromosome2	131556690
ENSG00000054965	chromosome11	72857168
ENSG00000099625	chromosome19	1187082
ENSG00000186174	chromosome11	118285859
ENSG00000116128	chromosome1	145550260
ENSG00000151835	chromosome13	22847426

ENSG00000189127	chromosome5	79891595
ENSG00000188134	chromosome15	77372685
ENSG00000181039	chromosome1	144184686
ENSG00000181039	chromosome1	144184686
ENSG00000188134	chromosome15	77372685
ENSG00000164761	chromosome8	120033242
ENSG00000164761	chromosome8	120033242
ENSG00000146072	chromosome6	47385207
ENSG00000028137	chromosome1	12149648
ENSG00000120949	chromosome1	12046243
ENSG00000146072	chromosome6	47385207
ENSG00000164761	chromosome8	120033242
ENSG00000120949	chromosome1	12046243
ENSG00000028137	chromosome1	12149648
ENSG00000157873	chromosome1	2486315
ENSG00000111321	chromosome12	6363786
ENSG00000120949	chromosome1	12046243
ENSG00000028137	chromosome1	12149648
ENSG00000146072	chromosome6	47385207
ENSG00000164761	chromosome8	120033242
ENSG00000120949	chromosome1	12046243
ENSG00000028137	chromosome1	12149648
ENSG00000146072	chromosome6	47385207
ENSG00000164761	chromosome8	120033242
ENSG00000111321	chromosome12	6363786
ENSG00000164604	chromosome7	112512013
ENSG00000184194	chromosomeX	53122529
ENSG00000170837	chromosome3	71885891
ENSG00000184194	chromosomeX	53122529
ENSG00000164604	chromosome7	112512013
ENSG00000160877	chromosome19	13107022
ENSG00000148411	chromosome9	138082189
ENSG00000177125	chromosome9	128681500
ENSG00000177125	chromosome9	128681500
ENSG00000160877	chromosome19	13107022
ENSG00000148411	chromosome9	138082189
ENSG00000173276	chromosome21	42287274
ENSG00000148411	chromosome9	138082189
ENSG00000160877	chromosome19	13107022
ENSG00000160877	chromosome19	13107022
ENSG00000148411	chromosome9	138082189
ENSG00000198522	chromosome2	27659484
ENSG00000196700	chromosome20	62069748
ENSG00000185811	chromosome7	50329203
ENSG00000161405	chromosome17	35273906
ENSG00000161405	chromosome17	35273906
ENSG00000185811	chromosome7	50329203
ENSG00000123411	chromosome12	54706852
ENSG00000030419	chromosome2	213723153
ENSG00000123411	chromosome12	54706852
ENSG00000161405	chromosome17	35273906
ENSG00000185811	chromosome7	50329203
ENSG00000182319	chromosome8	8276668
ENSG00000205639	chromosome2	24086471
ENSG00000168389	chromosome1	40193552
ENSG00000168389	chromosome1	40193552
ENSG00000205639	chromosome2	24086471
ENSG00000165478	chromosome11	124311113
ENSG00000188175	chromosome7	92693696

ENSG00000188175	chromosome7	92693696
ENSG00000165478	chromosome11	124311113
ENSG00000176395	chromosome19	49725373
ENSG00000197177	chromosome10	134734423
ENSG00000152990	chromosome4	22126506
ENSG00000152990	chromosome4	22126506
ENSG00000197177	chromosome10	134734423
ENSG00000020181	chromosome8	37773966
ENSG00000215018	chromosome7	7539032
ENSG00000142156	chromosome21	46226193
ENSG00000142173	chromosome21	46355819
ENSG00000215018	chromosome7	7539032
ENSG00000142173	chromosome21	46355819
ENSG00000142156	chromosome21	46226193
ENSG00000176771	chromosome2	133777233
ENSG00000167566	chromosome12	48477052
ENSG00000176771	chromosome2	133777233
ENSG00000122756	chromosome9	34558980
ENSG00000137070	chromosome9	34645215
ENSG00000105246	chromosome19	4180548
ENSG00000137070	chromosome9	34645215
ENSG00000105246	chromosome19	4180548
ENSG00000122756	chromosome9	34558980
ENSG00000160712	chromosome1	152644730
ENSG00000105246	chromosome19	4180548
ENSG00000137070	chromosome9	34645215
ENSG00000146674	chromosome7	45927395
ENSG00000115461	chromosome2	217267744
ENSG00000167779	chromosome12	51777769
ENSG00000167779	chromosome12	51777769
ENSG00000146674	chromosome7	45927395
ENSG00000115461	chromosome2	217267744
ENSG00000115457	chromosome2	217206492
ENSG00000141753	chromosome17	35853514
ENSG00000146678	chromosome7	45894777
ENSG00000115457	chromosome2	217206492
ENSG00000167779	chromosome12	51777769
ENSG00000146674	chromosome7	45927395
ENSG00000115461	chromosome2	217267744
ENSG00000115461	chromosome2	217267744
ENSG00000146674	chromosome7	45927395
ENSG00000146678	chromosome7	45894777
ENSG00000141753	chromosome17	35853514
ENSG00000156381	chromosome14	102043980
ENSG00000010295	chromosome12	6535457
ENSG00000169991	chromosome1	19154931
ENSG00000105696	chromosome19	18584769
ENSG00000116209	chromosome1	54291450
ENSG00000156427	chromosome5	170779809
ENSG00000156427	chromosome5	170779809
ENSG00000158815	chromosome8	21956386
ENSG00000107831	chromosome10	103525648
ENSG00000107831	chromosome10	103525648
ENSG00000158815	chromosome8	21956386
ENSG00000124209	chromosome20	56318439
ENSG00000214576	chromosome16	45162916
ENSG00000168461	chromosome18	9764874
ENSG00000174930	chromosome3	157219241
ENSG00000173612	chromosome6	117256870

ENSG00000179002	chromosome1	19058742
ENSG00000173662	chromosome1	6538021
ENSG00000174930	chromosome3	157219241
ENSG00000173612	chromosome6	117256870
ENSG00000169962	chromosome1	1256589
ENSG00000215740	supercontigNT_113871	71768
ENSG00000173662	chromosome1	6538021
ENSG00000179002	chromosome1	19058742
ENSG00000173612	chromosome6	117256870
ENSG00000169962	chromosome1	1256589
ENSG00000215740	supercontigNT_113871	71768
ENSG00000173662	chromosome1	6538021
ENSG00000179002	chromosome1	19058742
ENSG00000174930	chromosome3	157219241
ENSG00000036828	chromosome3	123455727
ENSG00000169962	chromosome1	1256589
ENSG00000215740	supercontigNT_113871	71768
ENSG00000173662	chromosome1	6538021
ENSG00000179002	chromosome1	19058742
ENSG00000173612	chromosome6	117256870
ENSG00000173662	chromosome1	6538021
ENSG00000179002	chromosome1	19058742
ENSG00000169962	chromosome1	1256589
ENSG00000215740	supercontigNT_113871	71768
ENSG00000171813	chromosome10	134060721
ENSG00000170234	chromosome5	159478974
ENSG00000137831	chromosome15	68842801
ENSG00000198483	chromosome1	144260674
ENSG00000089847	chromosome19	4134323
ENSG00000039560	chromosome5	34722782
ENSG00000198483	chromosome1	144260674
ENSG00000137831	chromosome15	68842801
ENSG00000137831	chromosome15	68842801
ENSG00000039560	chromosome5	34722782
ENSG00000089847	chromosome19	4134323
ENSG00000184163	chromosome1	1171934
ENSG00000178752	chromosome2	238737425
ENSG00000198933	chromosome17	43128406
ENSG00000163512	chromosome3	28357113
ENSG00000170775	chromosome7	124192267
ENSG00000170075	chromosome1	200358715
ENSG00000126010	chromosomeX	16051998
ENSG00000102239	chromosomeX	135397940
ENSG00000136160	chromosome13	77391752
ENSG00000151617	chromosome4	148626284
ENSG00000170075	chromosome1	200358715
ENSG00000170775	chromosome7	124192267
ENSG00000151617	chromosome4	148626284
ENSG00000136160	chromosome13	77391752
ENSG00000102239	chromosomeX	135397940
ENSG00000126010	chromosomeX	16051998
ENSG00000135577	chromosome6	142451489
ENSG00000136160	chromosome13	77391752
ENSG00000151617	chromosome4	148626284
ENSG00000136160	chromosome13	77391752
ENSG00000170075	chromosome1	200358715
ENSG00000170775	chromosome7	124192267
ENSG00000135577	chromosome6	142451489
ENSG00000102239	chromosomeX	135397940

ENSG00000126010	chromosomeX	16051998
ENSG00000134686	chromosome1	33613728
ENSG00000111752	chromosome12	8961541
ENSG00000179899	chromosome12	54094839
ENSG00000187634	chromosome1	850393
ENSG00000187033	chromosome3	171119981
ENSG00000111752	chromosome12	8961541
ENSG00000179899	chromosome12	54094839
ENSG00000134686	chromosome1	33613728
ENSG00000173889	chromosome3	171379399
ENSG00000187033	chromosome3	171119981
ENSG00000187634	chromosome1	850393
ENSG00000173889	chromosome3	171379399
ENSG00000111752	chromosome12	8961541
ENSG00000179899	chromosome12	54094839
ENSG00000134686	chromosome1	33613728
ENSG00000165617	chromosome14	58174674
ENSG00000164488	chromosome6	168463163
ENSG00000197380	chromosome19	51856162
ENSG00000164488	chromosome6	168463163
ENSG00000165617	chromosome14	58174674
ENSG00000152154	chromosome2	39746619
ENSG00000152154	chromosome2	39746619
ENSG00000152154	chromosome2	39746619
ENSG00000108557	chromosome17	17636988
ENSG00000100207	chromosome22	40941256
ENSG00000100100	chromosome22	30018331
ENSG00000131650	chromosome16	2954523
ENSG00000183762	chromosome22	27799119
ENSG00000183762	chromosome22	27799119
ENSG00000131650	chromosome16	2954523
ENSG00000100593	chromosome14	77034896
ENSG00000101230	chromosome20	13199151
ENSG00000162650	chromosome1	109828099
ENSG00000146776	chromosome7	105310264
ENSG00000163635	chromosome3	63873315
ENSG00000163635	chromosome3	63873315
ENSG00000146776	chromosome7	105310264
ENSG00000148468	chromosome10	15453065
ENSG00000161682	chromosome17	39796609
ENSG00000144369	chromosome2	187267146
ENSG00000161682	chromosome17	39796609
ENSG00000148468	chromosome10	15453065
ENSG00000081148	chromosome3	102521907
ENSG00000112706	chromosome6	76838926
ENSG00000179588	chromosome16	87047548
ENSG00000169946	chromosome8	106499975
ENSG00000170417	chromosome2	102745109
ENSG00000124701	chromosome6	41129065
ENSG00000198904	chromosome22	37740248
ENSG00000128394	chromosome22	37766837
ENSG00000100298	chromosome22	37826230
ENSG00000111701	chromosome12	7709736
ENSG00000111732	chromosome12	8656631
ENSG00000128383	chromosome22	37683643
ENSG00000179750	chromosome22	37708405
ENSG00000128383	chromosome22	37683643
ENSG00000179750	chromosome22	37708405
ENSG00000111732	chromosome12	8656631

ENSG00000198904	chromosome22	37740248
ENSG00000128394	chromosome22	37766837
ENSG00000100298	chromosome22	37826230
ENSG00000111701	chromosome12	7709736
ENSG00000124701	chromosome6	41129065
ENSG00000101463	chromosome20	24471734
ENSG00000183379	chromosome14	73946201
ENSG00000101463	chromosome20	24471734
ENSG00000183379	chromosome14	73946201
ENSG00000101463	chromosome20	24471734
ENSG00000183379	chromosome14	73946201
ENSG00000183379	chromosome14	73946201
ENSG00000101463	chromosome20	24471734
ENSG00000167371	chromosome16	29731877
ENSG00000101463	chromosome20	24471734
ENSG00000183379	chromosome14	73946201
ENSG00000167371	chromosome16	29731877
ENSG00000101463	chromosome20	24471734
ENSG00000183379	chromosome14	73946201
ENSG00000101463	chromosome20	24471734
ENSG00000183379	chromosome14	73946201
ENSG00000167371	chromosome16	29731877
ENSG00000167371	chromosome16	29731877
ENSG00000172889	chromosome9	138682556
ENSG00000172889	chromosome9	138682556
ENSG00000186212	chromosome4	78038027
ENSG00000187808	chromosomeX	118776659
ENSG00000198142	chromosome2	109729356
ENSG00000198944	chromosome5	132177213
ENSG00000187808	chromosomeX	118776659
ENSG00000198142	chromosome2	109729356
ENSG00000186212	chromosome4	78038027
ENSG00000198142	chromosome2	109729356
ENSG00000187808	chromosomeX	118776659
ENSG00000198944	chromosome5	132177213
ENSG00000186212	chromosome4	78038027
ENSG00000187808	chromosomeX	118776659
ENSG00000198142	chromosome2	109729356
ENSG00000204347	chromosome17	69869554
ENSG00000108679	chromosome17	74484869
ENSG00000146707	chromosome7	76093318
ENSG00000188372	chromosome7	75864805
ENSG00000142484	chromosome17	4621967
ENSG00000142484	chromosome17	4621967
ENSG00000145107	chromosome3	197538859
ENSG00000163762	chromosome3	150533860
ENSG00000169908	chromosome3	150578025
ENSG00000145107	chromosome3	197538859
ENSG00000142484	chromosome17	4621967
ENSG00000163762	chromosome3	150533860
ENSG00000169908	chromosome3	150578025
ENSG00000169903	chromosome3	150675355
ENSG00000169903	chromosome3	150675355
ENSG00000163762	chromosome3	150533860
ENSG00000169908	chromosome3	150578025
ENSG00000163762	chromosome3	150533860
ENSG00000169908	chromosome3	150578025
ENSG00000169903	chromosome3	150675355
ENSG00000145107	chromosome3	197538859

ENSG00000142484	chromosome17	4621967
ENSG00000168955	chromosome2	227952229
ENSG00000040633	chromosome17	7083323
ENSG00000116273	chromosome1	6596714
ENSG00000160888	chromosome19	13125001
ENSG00000162783	chromosome1	179324662
ENSG00000188483	chromosome9	130980153
ENSG00000188483	chromosome9	130980153
ENSG00000162783	chromosome1	179324662
ENSG00000060491	chromosome20	60906657
ENSG00000119900	chromosome6	72055361
ENSG00000141579	chromosome17	78383620
ENSG00000161992	chromosome16	553296
ENSG00000182223	chromosome4	48187066
ENSG00000189167	chromosome13	31784063
ENSG00000189167	chromosome13	31784063
ENSG00000182223	chromosome4	48187066
ENSG00000127863	chromosome13	23062323
ENSG00000131080	chromosomeX	65752588
ENSG00000135960	chromosome2	108913903
ENSG00000131080	chromosomeX	65752588
ENSG00000127863	chromosome13	23062323
ENSG00000214978	chromosome17	9666129
ENSG00000169181	chromosome16	27982247
ENSG00000214978	chromosome17	9666129
ENSG00000111305	chromosome12	13147714
ENSG00000214978	chromosome17	9666129
ENSG00000169181	chromosome16	27982247
ENSG00000169704	chromosome3	130263273
ENSG00000203618	chromosome22	18091093
ENSG00000204545	chromosome11	74630211
ENSG00000203618	chromosome22	18091093
ENSG00000169704	chromosome3	130263273
ENSG00000168386	chromosome3	101132555
ENSG00000118407	chromosome6	76181409
ENSG00000169641	chromosome1	23293342
ENSG00000118407	chromosome6	76181409
ENSG00000168386	chromosome3	101132555
ENSG00000163254	chromosome2	208702762
ENSG00000168582	chromosome2	208736526
ENSG00000118231	chromosome2	208697443
ENSG00000182187	chromosome2	208719090
ENSG00000163254	chromosome2	208702762
ENSG00000168582	chromosome2	208736526
ENSG00000118231	chromosome2	208697443
ENSG00000182187	chromosome2	208719090
ENSG00000213139	chromosome3	187744810
ENSG00000196431	chromosome22	25348561
ENSG00000108255	chromosome17	24598008
ENSG00000163499	chromosome2	219566143
ENSG00000163254	chromosome2	208702762
ENSG00000168582	chromosome2	208736526
ENSG00000118231	chromosome2	208697443
ENSG00000182187	chromosome2	208719090
ENSG00000196431	chromosome22	25348561
ENSG00000108255	chromosome17	24598008
ENSG00000100053	chromosome22	23927364
ENSG00000100122	chromosome22	25343838
ENSG00000100058	chromosome22	23947397

ENSG00000100122	chromosome22	25343838
ENSG00000100053	chromosome22	23927364
ENSG00000127377	chromosome7	150767937
ENSG00000213139	chromosome3	187744810
ENSG00000163254	chromosome2	208702762
ENSG00000168582	chromosome2	208736526
ENSG00000118231	chromosome2	208697443
ENSG00000182187	chromosome2	208719090
ENSG00000163499	chromosome2	219566143
ENSG00000196431	chromosome22	25348561
ENSG00000108255	chromosome17	24598008
ENSG00000100058	chromosome22	23947397
ENSG00000100053	chromosome22	23927364
ENSG00000100122	chromosome22	25343838
ENSG00000163254	chromosome2	208702762
ENSG00000168582	chromosome2	208736526
ENSG00000118231	chromosome2	208697443
ENSG00000182187	chromosome2	208719090
ENSG00000100058	chromosome22	23947397
ENSG00000100053	chromosome22	23927364
ENSG00000100122	chromosome22	25343838
ENSG00000163499	chromosome2	219566143
ENSG00000196431	chromosome22	25348561
ENSG00000108255	chromosome17	24598008
ENSG00000163499	chromosome2	219566143
ENSG00000196431	chromosome22	25348561
ENSG00000108255	chromosome17	24598008
ENSG00000100058	chromosome22	23947397
ENSG00000100053	chromosome22	23927364
ENSG00000100122	chromosome22	25343838
ENSG00000127377	chromosome7	150767937
ENSG00000213139	chromosome3	187744810
ENSG00000163254	chromosome2	208702762
ENSG00000168582	chromosome2	208736526
ENSG00000118231	chromosome2	208697443
ENSG00000182187	chromosome2	208719090
ENSG00000100122	chromosome22	25343838
ENSG00000100122	chromosome22	25343838
ENSG00000213139	chromosome3	187744810
ENSG00000163254	chromosome2	208702762
ENSG00000168582	chromosome2	208736526
ENSG00000118231	chromosome2	208697443
ENSG00000182187	chromosome2	208719090
ENSG00000127377	chromosome7	150767937
ENSG00000163254	chromosome2	208702762
ENSG00000168582	chromosome2	208736526
ENSG00000118231	chromosome2	208697443
ENSG00000182187	chromosome2	208719090
ENSG00000108255	chromosome17	24598008
ENSG00000196431	chromosome22	25348561
ENSG00000146410	chromosome6	136611931
ENSG00000066855	chromosome8	66744742
ENSG00000066855	chromosome8	66744742
ENSG00000146410	chromosome6	136611931
ENSG00000117640	chromosome1	26022183
ENSG00000146410	chromosome6	136611931
ENSG00000066855	chromosome8	66744742
ENSG00000151468	chromosome10	13083577
ENSG00000135148	chromosome12	111052710

ENSG00000132530	chromosome17	6600122
ENSG00000088899	chromosome20	3095810
ENSG00000145911	chromosome5	177479191
ENSG00000061337	chromosome8	20156973
ENSG00000107816	chromosome10	102752286
ENSG00000145911	chromosome5	177479191
ENSG00000088899	chromosome20	3095810
ENSG00000107816	chromosome10	102752286
ENSG00000061337	chromosome8	20156973
ENSG00000074964	chromosome1	17779678
ENSG00000104728	chromosome8	1761684
ENSG00000050393	chromosome6	13922641
ENSG00000137500	chromosome11	82674664
ENSG00000137500	chromosome11	82674664
ENSG00000050393	chromosome6	13922641
ENSG00000171017	chromosome19	7866489
ENSG00000171488	chromosome1	89924621
ENSG00000197147	chromosome1	89820798
ENSG00000136802	chromosome9	130709265
ENSG00000171492	chromosome1	90171216
ENSG00000136802	chromosome9	130709265
ENSG00000197147	chromosome1	89820798
ENSG00000171492	chromosome1	90171216
ENSG00000171017	chromosome19	7866489
ENSG00000171488	chromosome1	89924621
ENSG00000197147	chromosome1	89820798
ENSG00000136802	chromosome9	130709265
ENSG00000197147	chromosome1	89820798
ENSG00000136802	chromosome9	130709265
ENSG00000171017	chromosome19	7866489
ENSG00000171488	chromosome1	89924621
ENSG00000171488	chromosome1	89924621
ENSG00000171017	chromosome19	7866489
ENSG00000138207	chromosome10	95351464
ENSG00000160679	chromosome1	151875689
ENSG00000154721	chromosome21	25934005
ENSG00000154721	chromosome21	25934005
ENSG00000166086	chromosome11	133444054
ENSG00000047597	chromosomeX	37430134
ENSG00000172967	chromosome22	15668964
ENSG00000182489	chromosomeX	100069989
ENSG00000114993	chromosome2	74522452
ENSG00000182010	chromosome10	63698376
ENSG00000157601	chromosome21	41725868
ENSG00000183486	chromosome21	41670704
ENSG00000136011	chromosome12	102505385
ENSG00000010327	chromosome3	52504470
ENSG00000102935	chromosome16	48414098
ENSG00000198795	chromosome18	21186109
ENSG00000139438	chromosome12	108666335
ENSG00000173065	chromosome17	24117812
ENSG00000140548	chromosome15	88411374
ENSG00000178175	chromosome5	71793080
ENSG00000135365	chromosome11	46062347
ENSG00000056487	chromosome22	43784095
ENSG00000175182	chromosome3	185542230
ENSG00000159784	chromosome7	142767309
ENSG00000175182	chromosome3	185542230
ENSG00000175182	chromosome3	185542230

ENSG00000159784	chromosome7	142767309
ENSG00000185519	chromosome1	16272525
ENSG00000166780	chromosome16	15436012
ENSG00000166780	chromosome16	15436012
ENSG00000166780	chromosome16	15436012
ENSG00000123496	chromosomeX	114158089
ENSG00000091181	chromosome3	3121669
ENSG00000185291	chromosomeX	1420659
ENSG00000198223	chromosomeX	1361597
ENSG00000131724	chromosomeX	117745630
ENSG00000186326	chromosome19	37859010
ENSG00000186326	chromosome19	37859010
ENSG00000135426	chromosome12	53646136
ENSG00000138434	chromosome2	182465039
ENSG00000180347	chromosome7	31559164
ENSG00000198570	chromosome1	209721381
ENSG00000125848	chromosome20	14256153
ENSG00000126500	chromosome11	63640316
ENSG00000186105	chromosome5	61911023
ENSG00000126500	chromosome11	63640316
ENSG00000125848	chromosome20	14256153
ENSG00000186105	chromosome5	61911023
ENSG00000125848	chromosome20	14256153
ENSG00000126500	chromosome11	63640316
ENSG00000170011	chromosome3	39917312
ENSG00000115648	chromosome2	238066809
ENSG00000162670	chromosome1	188690644
ENSG00000078725	chromosome9	121115455
ENSG00000078725	chromosome9	121115455
ENSG00000162670	chromosome1	188690644
ENSG00000198797	chromosome1	175465636
ENSG00000213512	chromosome1	89410207
ENSG00000162654	chromosome1	89437106
ENSG00000183347	chromosome1	89606699
ENSG00000162645	chromosome1	89360238
ENSG00000117228	chromosome1	89301506
ENSG00000117226	chromosome1	89258993
ENSG00000154451	chromosome1	89507827
ENSG00000213512	chromosome1	89410207
ENSG00000162654	chromosome1	89437106
ENSG00000183347	chromosome1	89606699
ENSG00000162645	chromosome1	89360238
ENSG00000117228	chromosome1	89301506
ENSG00000117226	chromosome1	89258993
ENSG00000154451	chromosome1	89507827
ENSG00000157617	chromosome21	42246827
ENSG00000172375	chromosome11	118483662
ENSG00000183092	chromosome14	100104015
ENSG00000137221	chromosome6	43574718
ENSG00000109171	chromosome4	48038421
ENSG00000109171	chromosome4	48038421
ENSG00000139737	chromosome13	77170050
ENSG00000139737	chromosome13	77170050
ENSG00000109171	chromosome4	48038421
ENSG00000143367	chromosome1	149779467
ENSG00000143367	chromosome1	149779467
ENSG00000166510	chromosome18	50761021
ENSG00000166510	chromosome18	50761021
ENSG00000143367	chromosome1	149779467

ENSG00000135298	chromosome6	69405289
ENSG00000181790	chromosome8	143542562
ENSG00000181790	chromosome8	143542562
ENSG00000135298	chromosome6	69405289
ENSG00000121753	chromosome1	31997539
ENSG00000182752	chromosome9	117956273
ENSG00000116183	chromosome1	174792082
ENSG00000187398	chromosome11	24475366
ENSG00000137142	chromosome9	38414422
ENSG00000163453	chromosome4	57671275
ENSG00000107821	chromosome10	102812340
ENSG00000163453	chromosome4	57671275
ENSG00000137142	chromosome9	38414422
ENSG00000107821	chromosome10	102812340
ENSG00000137142	chromosome9	38414422
ENSG00000163453	chromosome4	57671275
ENSG00000151892	chromosome10	118021532
ENSG00000151892	chromosome10	118021532
ENSG00000168546	chromosome8	21701618
ENSG00000151892	chromosome10	118021532
ENSG00000125861	chromosome20	3592047
ENSG00000168546	chromosome8	21701618
ENSG00000151892	chromosome10	118021532
ENSG00000146013	chromosome5	137638013
ENSG00000187871	chromosome6	55300312
ENSG00000168546	chromosome8	21701618
ENSG00000151892	chromosome10	118021532
ENSG00000125861	chromosome20	3592047
ENSG00000182482	chromosome22	29015487
ENSG00000183086	chromosome7	74017019
ENSG00000198750	chromosome7	74705278
ENSG00000160844	chromosome7	99707543
ENSG00000163126	chromosome2	96873457
ENSG00000148677	chromosome10	92670765
ENSG00000165887	chromosome10	99322455
ENSG00000148677	chromosome10	92670765
ENSG00000163126	chromosome2	96873457
ENSG00000108878	chromosome17	62471239
ENSG00000130433	chromosome19	59187944
ENSG00000108878	chromosome17	62471239
ENSG00000141084	chromosome16	66397941
ENSG00000010017	chromosome6	13819717
ENSG00000162437	chromosome1	64983444
ENSG00000161847	chromosome19	10305184
ENSG00000138100	chromosome2	27359104
ENSG00000147573	chromosome8	67202058
ENSG00000158022	chromosome1	26266573
ENSG00000147573	chromosome8	67202058
ENSG00000204977	chromosome13	49473243
ENSG00000213186	chromosome3	161639666
ENSG00000147573	chromosome8	67202058
ENSG00000138100	chromosome2	27359104
ENSG00000178974	chromosome14	54886862
ENSG00000168675	chromosome18	13377638
ENSG00000124225	chromosome20	55719037
ENSG00000112619	chromosome6	42798051
ENSG00000149489	chromosome11	62137330
ENSG00000112619	chromosome6	42798051
ENSG00000103495	chromosome16	29725524

ENSG00000136451	chromosome17	53420472
ENSG00000100105	chromosome22	30071589
ENSG00000136451	chromosome17	53420472
ENSG00000103495	chromosome16	29725524
ENSG00000176641	chromosome18	57634677
ENSG00000178828	chromosome1	20014182
ENSG00000180537	chromosome6	14085330
ENSG00000180537	chromosome6	14085330
ENSG00000180537	chromosome6	14085330
ENSG00000178828	chromosome1	20014182
ENSG00000176641	chromosome18	57634677
ENSG00000168412	chromosome4	187713514
ENSG00000134640	chromosome11	92342540
ENSG00000168412	chromosome4	187713514
ENSG00000134640	chromosome11	92342540
ENSG00000168412	chromosome4	187713514
ENSG00000134640	chromosome11	92342540
ENSG00000168412	chromosome4	187713514
ENSG00000102195	chromosomeX	150095852
ENSG00000143442	chromosome1	149681305
ENSG00000056277	chromosomeX	129222105
ENSG00000137871	chromosome15	54813424
ENSG00000169548	chromosome22	21199955
ENSG00000198477	chromosome22	21173724
ENSG00000196169	chromosome17	69833546
ENSG00000149798	chromosome11	64844946
ENSG00000163171	chromosome2	37727235
ENSG00000167617	chromosome19	59668544
ENSG00000128283	chromosome22	36292303
ENSG00000179604	chromosome17	68794235
ENSG00000149798	chromosome11	64844946
ENSG00000179604	chromosome17	68794235
ENSG00000128283	chromosome22	36292303
ENSG00000128283	chromosome22	36292303
ENSG00000179604	chromosome17	68794235
ENSG00000167617	chromosome19	59668544
ENSG00000149798	chromosome11	64844946
ENSG00000163171	chromosome2	37727235
ENSG00000163171	chromosome2	37727235
ENSG00000149798	chromosome11	64844946
ENSG00000167617	chromosome19	59668544
ENSG00000128283	chromosome22	36292303
ENSG00000179604	chromosome17	68794235
ENSG00000149798	chromosome11	64844946
ENSG00000163171	chromosome2	37727235
ENSG00000160588	chromosome11	117628220
ENSG00000160593	chromosome11	117590792
ENSG00000158887	chromosome1	159546350
ENSG00000197965	chromosome1	165957827
ENSG00000149573	chromosome11	117640079
ENSG00000160593	chromosome11	117590792
ENSG00000160588	chromosome11	117628220
ENSG00000160593	chromosome11	117590792
ENSG00000160588	chromosome11	117628220
ENSG00000149573	chromosome11	117640079
ENSG00000197965	chromosome1	165957827
ENSG00000158887	chromosome1	159546350
ENSG00000197965	chromosome1	165957827
ENSG00000158887	chromosome1	159546350

ENSG00000160593	chromosome11	117590792
ENSG00000160588	chromosome11	117628220
ENSG00000149573	chromosome11	117640079
ENSG00000149575	chromosome11	117552357
ENSG00000177098	chromosome11	117528599
ENSG00000160593	chromosome11	117590792
ENSG00000160588	chromosome11	117628220
ENSG00000149573	chromosome11	117640079
ENSG00000197965	chromosome1	165957827
ENSG00000158887	chromosome1	159546350
ENSG00000177098	chromosome11	117528599
ENSG00000149575	chromosome11	117552357
ENSG00000187135	chromosome19	34709331
ENSG00000132821	chromosome20	35965167
ENSG00000170419	chromosome7	54577921
ENSG00000132821	chromosome20	35965167
ENSG00000187135	chromosome19	34709331
ENSG00000070087	chromosome3	151171334
ENSG00000070087	chromosome3	151171334
ENSG00000070087	chromosome3	151171334
ENSG00000108518	chromosome17	4792435
ENSG00000196570	chromosome5	176760184
ENSG00000172399	chromosome4	120277129
ENSG00000164591	chromosome5	150022697
ENSG00000177791	chromosome10	75069782
ENSG00000164591	chromosome5	150022697
ENSG00000172399	chromosome4	120277129
ENSG00000183662	chromosome3	68138460
ENSG00000198673	chromosome12	60547474
ENSG00000163377	chromosome3	69017030
ENSG00000184599	chromosome1	113066379
ENSG00000163377	chromosome3	69017030
ENSG00000184599	chromosome1	113066379
ENSG00000198673	chromosome12	60547474
ENSG00000198673	chromosome12	60547474
ENSG00000163377	chromosome3	69017030
ENSG00000184599	chromosome1	113066379
ENSG00000183662	chromosome3	68138460
ENSG00000184599	chromosome1	113066379
ENSG00000163377	chromosome3	69017030
ENSG00000183662	chromosome3	68138460
ENSG00000163377	chromosome3	69017030
ENSG00000165660	chromosome10	126480389
ENSG00000163322	chromosome4	84625250
ENSG00000204442	chromosome13	107316946
ENSG00000130054	chromosomeX	68641851
ENSG00000169554	chromosome2	144991388
ENSG00000148516	chromosome10	31648170
ENSG00000148516	chromosome10	31648170
ENSG00000169554	chromosome2	144991388
ENSG00000162702	chromosome1	198645457
ENSG00000163848	chromosome3	126515175
ENSG00000115762	chromosome2	131594985
ENSG00000021300	chromosome11	73023592
ENSG00000072952	chromosome11	10630361
ENSG00000118308	chromosome12	25110604
ENSG00000003393	chromosome2	202341854
ENSG00000178038	chromosome3	46705935
ENSG00000164896	chromosome7	150408787

ENSG00000169218	chromosome1	37868161
ENSG00000101282	chromosome20	930808
ENSG00000147655	chromosome8	109164043
ENSG00000147655	chromosome8	109164043
ENSG00000101282	chromosome20	930808
ENSG00000183484	chromosome14	104592803
ENSG00000119714	chromosome14	90771178
ENSG00000177464	chromosome19	50786965
ENSG00000183484	chromosome14	104592803
ENSG00000140030	chromosome14	87546945
ENSG00000177464	chromosome19	50786965
ENSG00000119714	chromosome14	90771178
ENSG00000183484	chromosome14	104592803
ENSG00000177464	chromosome19	50786965
ENSG00000119714	chromosome14	90771178
ENSG00000140030	chromosome14	87546945
ENSG00000165186	chromosomeX	23262914
ENSG00000178729	chromosome6	48144351
ENSG00000124814	chromosome6	47955531
ENSG00000160570	chromosome19	47413000
ENSG00000158796	chromosome1	159360877
ENSG00000184828	chromosome18	43821477
ENSG00000178951	chromosome19	4006231
ENSG00000178951	chromosome19	4006231
ENSG00000184828	chromosome18	43821477
ENSG00000160685	chromosome1	153253761
ENSG00000101605	chromosome18	3205222
ENSG00000036448	chromosome8	1986288
ENSG00000036448	chromosome8	1986288
ENSG00000101605	chromosome18	3205222
ENSG00000142661	chromosome1	24307714
ENSG00000206011	chromosomeY	20251463
ENSG00000169800	chromosomeY	20026985
ENSG00000169811	chromosomeY	19355273
ENSG00000206046	chromosomeY	19777562
ENSG00000174622	chromosomeY	19375105
ENSG00000213516	chromosome1	89222098
ENSG00000215210	chromosome9	30679103
ENSG00000214442	chromosome11	82194044
ENSG00000147274	chromosomeX	135789253
ENSG00000170748	chromosome11	7066928
ENSG00000010818	chromosome6	143137569
ENSG00000127124	chromosome1	41823056
ENSG00000127124	chromosome1	41823056
ENSG00000010818	chromosome6	143137569
ENSG00000095951	chromosome6	12123848
ENSG00000168917	chromosome3	138055993
ENSG00000164729	chromosome17	30545440
ENSG00000177710	chromosome8	11226026
ENSG00000177164	chromosome18	11599595
ENSG00000168917	chromosome3	138055993
ENSG00000177409	chromosome7	92603221
ENSG00000205413	chromosome7	92573347
ENSG00000137868	chromosome15	72281662
ENSG00000168502	chromosome18	8696676
ENSG00000214338	chromosome6	127879453
ENSG00000149639	chromosome20	34925162
ENSG00000214338	chromosome6	127879453
ENSG00000149639	chromosome20	34925162

ENSG00000168502	chromosome18	8696676
ENSG00000214338	chromosome6	127879453
ENSG00000214338	chromosome6	127879453
ENSG00000168502	chromosome18	8696676
ENSG00000095574	chromosome10	124748132
ENSG00000173320	chromosome4	185064866
ENSG00000213578	chromosome15	72906098
ENSG00000166569	chromosome18	55136675
ENSG00000166569	chromosome18	55136675
ENSG00000166569	chromosome18	55136675
ENSG00000213578	chromosome15	72906098
ENSG00000212864	supercontigNT_113917	155
ENSG00000196435	chromosome9	139235486
ENSG00000167100	chromosome17	45557323
ENSG00000205162	chromosome8	86909379
ENSG00000205168	chromosome8	86873085
ENSG00000205164	chromosome8	86897189
ENSG00000205170	chromosome8	86860894
ENSG00000205178	chromosome8	86743163
ENSG00000205166	chromosome8	86885275
ENSG00000205173	chromosome8	86761908
ENSG00000212867	supercontigNT_113908	8828
ENSG00000205175	chromosome8	86755071
ENSG00000171773	chromosome19	17432679
ENSG00000130045	chromosome9	90340170
ENSG00000130045	chromosome9	90340170
ENSG00000171773	chromosome19	17432679
ENSG00000188022	chromosome4	17125988
ENSG00000163646	chromosome3	152173186
ENSG00000180745	chromosome10	129581039
ENSG00000163646	chromosome3	152173186
ENSG00000188022	chromosome4	17125988
ENSG00000206004	chromosome12	3613158
ENSG00000112090	chromosome6	36798350
ENSG00000112090	chromosome6	36798350
ENSG00000206004	chromosome12	3613158
ENSG00000165370	chromosomeX	135941500
ENSG00000143147	chromosome1	166340713
ENSG00000183935	chromosome12	13044079
ENSG00000143147	chromosome1	166340713
ENSG00000165370	chromosomeX	135941500
ENSG00000143147	chromosome1	166340713
ENSG00000171873	chromosome20	4177605
ENSG00000170214	chromosome5	159276491
ENSG00000120907	chromosome8	26778404
ENSG00000183935	chromosome12	13044079
ENSG00000165370	chromosomeX	135941500
ENSG00000143147	chromosome1	166340713
ENSG00000170214	chromosome5	159276491
ENSG00000171873	chromosome20	4177605
ENSG00000124449	chromosome19	48914551
ENSG00000167378	chromosome19	48791331
ENSG00000124449	chromosome19	48914551
ENSG00000183808	chromosome8	94817815
ENSG00000125976	chromosome20	33706659
ENSG00000188385	chromosome10	133768303
ENSG00000176049	chromosome5	147031563
ENSG00000152969	chromosome4	6165479
ENSG00000152969	chromosome4	6165479

ENSG00000176049	chromosome5	147031563
ENSG00000125931	chromosomeX	71439445
ENSG00000164442	chromosome6	139736775
ENSG00000164442	chromosome6	139736775
ENSG00000125931	chromosomeX	71439445
ENSG00000164442	chromosome6	139736775
ENSG00000125931	chromosomeX	71439445
ENSG00000179862	chromosome1	41100387
ENSG00000174521	chromosome19	45416129
ENSG00000133985	chromosome14	70178258
ENSG00000133985	chromosome14	70178258
ENSG00000174521	chromosome19	45416129
ENSG00000162222	chromosome11	62252412
ENSG00000162222	chromosome11	62252412
ENSG00000133985	chromosome14	70178258
ENSG00000174521	chromosome19	45416129
ENSG00000164970	chromosome9	34448262
ENSG00000178761	chromosome15	72986195
ENSG00000158321	chromosome7	68702576
ENSG00000112787	chromosome12	131657121
ENSG00000156860	chromosome16	30578341
ENSG00000112787	chromosome12	131657121
ENSG00000158321	chromosome7	68702576
ENSG00000135119	chromosome12	115672216
ENSG00000187870	chromosome17	20599004
ENSG00000189050	chromosome17	55396807
ENSG00000184697	chromosome16	3006024
ENSG00000213937	chromosome16	3003365
ENSG00000184113	chromosome22	17891779
ENSG00000165215	chromosome7	72822316
ENSG00000189143	chromosome7	72883468
ENSG00000156282	chromosome21	30460807
ENSG00000156284	chromosome21	30510115
ENSG00000134873	chromosome13	94884089
ENSG00000106404	chromosome7	100667583
ENSG00000165215	chromosome7	72822316
ENSG00000189143	chromosome7	72883468
ENSG00000184697	chromosome16	3006024
ENSG00000213937	chromosome16	3003365
ENSG00000184113	chromosome22	17891779
ENSG00000165376	chromosomeX	106058115
ENSG00000159261	chromosome21	36755864
ENSG00000134873	chromosome13	94884089
ENSG00000106404	chromosome7	100667583
ENSG00000134873	chromosome13	94884089
ENSG00000171217	chromosome6	155638546
ENSG00000159261	chromosome21	36755864
ENSG00000165376	chromosomeX	106058115
ENSG00000106404	chromosome7	100667583
ENSG00000134873	chromosome13	94884089
ENSG00000156282	chromosome21	30460807
ENSG00000156284	chromosome21	30510115
ENSG00000184697	chromosome16	3006024
ENSG00000213937	chromosome16	3003365
ENSG00000184113	chromosome22	17891779
ENSG00000165215	chromosome7	72822316
ENSG00000189143	chromosome7	72883468
ENSG00000177300	chromosome4	184478366
ENSG00000185758	chromosome4	184480574

ENSG00000156282	chromosome21	30460807
ENSG00000156284	chromosome21	30510115
ENSG00000184697	chromosome16	3006024
ENSG00000213937	chromosome16	3003365
ENSG00000184113	chromosome22	17891779
ENSG00000165215	chromosome7	72822316
ENSG00000189143	chromosome7	72883468
ENSG00000159261	chromosome21	36755864
ENSG00000165376	chromosomeX	106058115
ENSG00000171217	chromosome6	155638546
ENSG00000134873	chromosome13	94884089
ENSG00000181885	chromosome17	7106087
ENSG00000163347	chromosome3	191522690
ENSG00000159261	chromosome21	36755864
ENSG00000165376	chromosomeX	106058115
ENSG00000171217	chromosome6	155638546
ENSG00000177300	chromosome4	184478366
ENSG00000185758	chromosome4	184480574
ENSG00000156282	chromosome21	30460807
ENSG00000156284	chromosome21	30510115
ENSG00000184697	chromosome16	3006024
ENSG00000213937	chromosome16	3003365
ENSG00000184113	chromosome22	17891779
ENSG00000165215	chromosome7	72822316
ENSG00000189143	chromosome7	72883468
ENSG00000163347	chromosome3	191522690
ENSG00000181885	chromosome17	7106087
ENSG00000134873	chromosome13	94884089
ENSG00000184697	chromosome16	3006024
ENSG00000213937	chromosome16	3003365
ENSG00000184113	chromosome22	17891779
ENSG00000164007	chromosome1	42978322
ENSG00000106404	chromosome7	100667583
ENSG00000134873	chromosome13	94884089
ENSG00000163347	chromosome3	191522690
ENSG00000181885	chromosome17	7106087
ENSG00000159261	chromosome21	36755864
ENSG00000165376	chromosomeX	106058115
ENSG00000171217	chromosome6	155638546
ENSG00000177300	chromosome4	184478366
ENSG00000185758	chromosome4	184480574
ENSG00000156282	chromosome21	30460807
ENSG00000156284	chromosome21	30510115
ENSG00000184697	chromosome16	3006024
ENSG00000213937	chromosome16	3003365
ENSG00000184113	chromosome22	17891779
ENSG00000165215	chromosome7	72822316
ENSG00000189143	chromosome7	72883468
ENSG00000184113	chromosome22	17891779
ENSG00000184697	chromosome16	3006024
ENSG00000213937	chromosome16	3003365
ENSG00000106404	chromosome7	100667583
ENSG00000134873	chromosome13	94884089
ENSG00000164007	chromosome1	42978322
ENSG00000165215	chromosome7	72822316
ENSG00000189143	chromosome7	72883468
ENSG00000156282	chromosome21	30460807
ENSG00000156284	chromosome21	30510115
ENSG00000156282	chromosome21	30460807

ENSG00000156284	chromosome21	30510115
ENSG00000184697	chromosome16	3006024
ENSG00000213937	chromosome16	3003365
ENSG00000184113	chromosome22	17891779
ENSG00000165215	chromosome7	72822316
ENSG00000189143	chromosome7	72883468
ENSG00000177300	chromosome4	184478366
ENSG00000185758	chromosome4	184480574
ENSG00000164007	chromosome1	42978322
ENSG00000106404	chromosome7	100667583
ENSG00000134873	chromosome13	94884089
ENSG00000177300	chromosome4	184478366
ENSG00000185758	chromosome4	184480574
ENSG00000116157	chromosome1	52840670
ENSG00000164294	chromosome5	54491778
ENSG00000198881	chromosomeX	63362256
ENSG00000065802	chromosome2	239000451
ENSG00000164122	chromosome4	177427254
ENSG00000164122	chromosome4	177427254
ENSG00000102048	chromosomeX	15197918
ENSG00000214426	chromosome15	91139881
ENSG00000165192	chromosomeX	15243649
ENSG00000196372	chromosome10	5748521
ENSG00000102048	chromosomeX	15197918
ENSG00000214426	chromosome15	91139881
ENSG00000165192	chromosomeX	15243649
ENSG00000164122	chromosome4	177427254
ENSG00000165192	chromosomeX	15243649
ENSG00000102048	chromosomeX	15197918
ENSG00000214426	chromosome15	91139881
ENSG00000166106	chromosome11	129824079
ENSG00000158859	chromosome1	159435042
ENSG00000154734	chromosome21	27139145
ENSG00000134917	chromosome11	129803392
ENSG00000134917	chromosome11	129803392
ENSG00000158859	chromosome1	159435042
ENSG00000154734	chromosome21	27139145
ENSG00000158859	chromosome1	159435042
ENSG00000154734	chromosome21	27139145
ENSG00000134917	chromosome11	129803392
ENSG00000166106	chromosome11	129824079
ENSG00000154736	chromosome21	27260582
ENSG00000154734	chromosome21	27139145
ENSG00000158859	chromosome1	159435042
ENSG00000092978	chromosome1	215870937
ENSG00000089916	chromosome14	75690460
ENSG00000154358	chromosome1	226466108
ENSG00000072195	chromosome2	220007944
ENSG00000178502	chromosome17	37275150
ENSG00000185972	chromosome9	36159500
ENSG00000148120	chromosome9	96561887
ENSG00000055208	chromosome6	149732827
ENSG00000157625	chromosomeX	30787627
ENSG00000164236	chromosome5	10617580
ENSG00000167612	chromosome12	50568238
ENSG00000126003	chromosome20	30253643
ENSG00000118495	chromosome6	144310967
ENSG00000181690	chromosome8	57243383
ENSG00000181690	chromosome8	57243383

ENSG00000118495	chromosome6	144310967
ENSG00000157214	chromosome7	89692333
ENSG00000115107	chromosome2	119704768
ENSG00000105889	chromosome7	22501000
ENSG00000164647	chromosome7	89627039
ENSG00000127954	chromosome7	87751521
ENSG00000105889	chromosome7	22501000
ENSG00000164647	chromosome7	89627039
ENSG00000157214	chromosome7	89692333
ENSG00000115107	chromosome2	119704768
ENSG00000115107	chromosome2	119704768
ENSG00000157214	chromosome7	89692333
ENSG00000152092	chromosome1	175400436
ENSG00000148219	chromosome9	119217038
ENSG00000155324	chromosome5	125787198
ENSG00000166292	chromosome17	51153431
ENSG00000178078	chromosome19	4289751
ENSG00000035720	chromosome4	68107123
ENSG00000171357	chromosome1	46441686
ENSG00000171357	chromosome1	46441686
ENSG00000153714	chromosome9	12765715
ENSG00000152954	chromosome6	24242535
ENSG00000125841	chromosome20	278288
ENSG00000174137	chromosome4	1640267
ENSG00000189319	chromosome10	126385273
ENSG00000120709	chromosome5	137704924
ENSG00000189319	chromosome10	126385273
ENSG00000174137	chromosome4	1640267
ENSG00000105711	chromosome19	40213565
ENSG00000166257	chromosome11	123029720
ENSG00000203833	chromosome1	146559962
ENSG00000135272	chromosome7	114349708
ENSG00000112559	chromosome6	41714466
ENSG00000112559	chromosome6	41714466
ENSG00000135272	chromosome7	114349708
ENSG00000135272	chromosome7	114349708
ENSG00000112559	chromosome6	41714466
ENSG00000135272	chromosome7	114349708
ENSG00000112559	chromosome6	41714466
ENSG00000086300	chromosome7	26352588
ENSG00000002919	chromosome17	43544405
ENSG00000135378	chromosome11	32808652
ENSG00000126460	chromosome19	54778289
ENSG00000135378	chromosome11	32808652
ENSG00000130962	chromosomeX	37150424
ENSG00000130032	chromosomeX	150617943
ENSG00000130032	chromosomeX	150617943
ENSG00000130962	chromosomeX	37150424
ENSG00000168894	chromosome2	85676398
ENSG00000214209	chromosome7	104957892
ENSG00000185036	chromosome17	52478497
ENSG00000170962	chromosome11	103539866
ENSG00000145431	chromosome4	158111506
ENSG00000132702	chromosome1	154859907
ENSG00000140511	chromosome15	87231534
ENSG00000187664	chromosome19	19234542
ENSG00000145681	chromosome5	83005099
ENSG00000038427	chromosome5	82815094
ENSG00000130287	chromosome19	19188763

ENSG00000157766	chromosome15	87180442
ENSG00000130287	chromosome19	19188763
ENSG00000132702	chromosome1	154859907
ENSG00000140511	chromosome15	87231534
ENSG00000187664	chromosome19	19234542
ENSG00000145681	chromosome5	83005099
ENSG00000038427	chromosome5	82815094
ENSG00000038427	chromosome5	82815094
ENSG00000132702	chromosome1	154859907
ENSG00000140511	chromosome15	87231534
ENSG00000187664	chromosome19	19234542
ENSG00000145681	chromosome5	83005099
ENSG00000145681	chromosome5	83005099
ENSG00000187664	chromosome19	19234542
ENSG00000145681	chromosome5	83005099
ENSG00000140511	chromosome15	87231534
ENSG00000140511	chromosome15	87231534
ENSG00000187664	chromosome19	19234542
ENSG00000145681	chromosome5	83005099
ENSG00000132702	chromosome1	154859907
ENSG00000157766	chromosome15	87180442
ENSG00000132702	chromosome1	154859907
ENSG00000140511	chromosome15	87231534
ENSG00000187664	chromosome19	19234542
ENSG00000145681	chromosome5	83005099
ENSG00000038427	chromosome5	82815094
ENSG00000130287	chromosome19	19188763
ENSG00000132702	chromosome1	154859907
ENSG00000140511	chromosome15	87231534
ENSG00000187664	chromosome19	19234542
ENSG00000145681	chromosome5	83005099
ENSG00000038427	chromosome5	82815094
ENSG00000130287	chromosome19	19188763
ENSG00000157766	chromosome15	87180442
ENSG00000132692	chromosome1	154882471
ENSG00000145681	chromosome5	83005099
ENSG00000187664	chromosome19	19234542
ENSG00000171475	chromosome17	35666238
ENSG00000115935	chromosome2	175158548
ENSG00000115935	chromosome2	175158548
ENSG00000171475	chromosome17	35666238
ENSG00000122574	chromosome7	29840866
ENSG00000171475	chromosome17	35666238
ENSG00000147650	chromosome8	105670302
ENSG00000130881	chromosome19	38377532
ENSG00000197324	chromosome14	22411353
ENSG00000130881	chromosome19	38377532
ENSG00000147650	chromosome8	105670302
ENSG00000064042	chromosome4	41057615
ENSG00000136153	chromosome13	75093831
ENSG00000125735	chromosome19	6621081
ENSG00000117560	chromosome1	170894965
ENSG00000121858	chromosome3	173723869
ENSG00000120659	chromosome13	42046440
ENSG00000121858	chromosome3	173723869
ENSG00000102245	chromosomeX	135558074
ENSG00000102245	chromosomeX	135558074
ENSG00000120659	chromosome13	42046440
ENSG00000121858	chromosome3	173723869

ENSG00000181634	chromosome9	116608114
ENSG00000204490	chromosome6	31651498
ENSG00000204496	chromosome6	31648499
ENSG00000117560	chromosome1	170894965
ENSG00000125735	chromosome19	6621081
ENSG00000117560	chromosome1	170894965
ENSG00000125735	chromosome19	6621081
ENSG00000181634	chromosome9	116608114
ENSG00000204490	chromosome6	31651498
ENSG00000204496	chromosome6	31648499
ENSG00000120659	chromosome13	42046440
ENSG00000121858	chromosome3	173723869
ENSG00000204490	chromosome6	31651498
ENSG00000204496	chromosome6	31648499
ENSG00000204490	chromosome6	31651498
ENSG00000204496	chromosome6	31648499
ENSG00000181634	chromosome9	116608114
ENSG00000121858	chromosome3	173723869
ENSG00000120659	chromosome13	42046440
ENSG00000214447	chromosome17	40336724
ENSG00000177558	chromosome19	40411424
ENSG00000070047	chromosome11	571498
ENSG00000139218	chromosome12	44644218
ENSG00000126461	chromosome19	54840096
ENSG00000126461	chromosome19	54840096
ENSG00000139218	chromosome12	44644218
ENSG00000121853	chromosome3	173648898
ENSG00000121853	chromosome3	173648898
ENSG00000069275	chromosome1	203985920
ENSG00000186060	chromosome17	18571596
ENSG00000089775	chromosome14	64027005
ENSG00000181472	chromosome6	151736466
ENSG00000126804	chromosome14	64057976
ENSG00000181472	chromosome6	151736466
ENSG00000089775	chromosome14	64027005
ENSG00000181472	chromosome6	151736466
ENSG00000168824	chromosome4	4440258
ENSG00000170091	chromosome5	173406365
ENSG00000170091	chromosome5	173406365
ENSG00000168824	chromosome4	4440258
ENSG00000168824	chromosome4	4440258
ENSG00000170091	chromosome5	173406365
ENSG00000130643	chromosome10	134992484
ENSG00000212121	chromosome6	11213530
ENSG00000197604	chromosome7	91937632
ENSG00000184992	chromosome12	124044290
ENSG00000110108	chromosome11	60443742
ENSG00000165804	chromosome14	20631591
ENSG00000198597	chromosome19	35626310
ENSG00000101493	chromosome18	72283999
ENSG00000101493	chromosome18	72283999
ENSG00000165804	chromosome14	20631591
ENSG00000198597	chromosome19	35626310
ENSG00000101493	chromosome18	72283999
ENSG00000165804	chromosome14	20631591
ENSG00000198597	chromosome19	35626310
ENSG00000171940	chromosome20	51632773
ENSG00000198597	chromosome19	35626310
ENSG00000165804	chromosome14	20631591

ENSG00000115956	chromosome2	68445988
ENSG00000100558	chromosome14	66948530
ENSG00000128849	chromosome15	55517490
ENSG00000143375	chromosome1	149757620
ENSG00000017373	chromosome17	33988593
ENSG00000120549	chromosome10	24538129
ENSG00000064309	chromosome11	125398582
ENSG00000144857	chromosome3	114451340
ENSG00000135525	chromosome6	136913240
ENSG00000116871	chromosome1	36394576
ENSG00000184368	chromosomeX	20044919
ENSG00000116871	chromosome1	36394576
ENSG00000135525	chromosome6	136913240
ENSG00000184368	chromosomeX	20044919
ENSG00000135525	chromosome6	136913240
ENSG00000116871	chromosome1	36394576
ENSG00000129680	chromosomeX	135161184
ENSG00000099284	chromosome10	71505421
ENSG00000113648	chromosome5	134752683
ENSG00000148841	chromosome10	106088122
ENSG00000198885	chromosome2	96356073
ENSG00000198885	chromosome2	96356073
ENSG00000148841	chromosome10	106088122
ENSG00000205730	chromosome16	19033285
ENSG00000164715	chromosome7	97574426
ENSG00000181409	chromosome17	76754388
ENSG00000142235	chromosome19	53706646
ENSG00000181409	chromosome17	76754388
ENSG00000164715	chromosome7	97574426
ENSG00000143643	chromosome1	229181200
ENSG00000139865	chromosome14	37326153
ENSG00000102794	chromosome13	76420695
ENSG00000135454	chromosome12	56312183
ENSG00000167080	chromosome17	44565154
ENSG00000204969	chromosome5	140154734
ENSG00000204970	chromosome5	140146060
ENSG00000204961	chromosome5	140208265
ENSG00000204963	chromosome5	140194153
ENSG00000204967	chromosome5	140166957
ENSG00000204968	chromosome5	140160967
ENSG00000204964	chromosome5	140187861
ENSG00000204962	chromosome5	140201091
ENSG00000081842	chromosome5	140215818
ENSG00000214600	chromosome5	140235242
ENSG00000204965	chromosome5	140181545
ENSG00000214608	chromosome5	140228873
ENSG00000204969	chromosome5	140154734
ENSG00000204970	chromosome5	140146060
ENSG00000204961	chromosome5	140208265
ENSG00000204963	chromosome5	140194153
ENSG00000204967	chromosome5	140166957
ENSG00000204968	chromosome5	140160967
ENSG00000204964	chromosome5	140187861
ENSG00000204962	chromosome5	140201091
ENSG00000081842	chromosome5	140215818
ENSG00000214600	chromosome5	140235242
ENSG00000204965	chromosome5	140181545
ENSG00000214608	chromosome5	140228873
ENSG00000204969	chromosome5	140154734

ENSG00000204970	chromosome5	140146060
ENSG00000204961	chromosome5	140208265
ENSG00000204963	chromosome5	140194153
ENSG00000204967	chromosome5	140166957
ENSG00000204968	chromosome5	140160967
ENSG00000204964	chromosome5	140187861
ENSG00000204962	chromosome5	140201091
ENSG00000081842	chromosome5	140215818
ENSG00000214600	chromosome5	140235242
ENSG00000204965	chromosome5	140181545
ENSG00000214608	chromosome5	140228873
ENSG00000081853	chromosome5	140790511
ENSG00000214567	chromosome5	140780979
ENSG00000214574	chromosome5	140752565
ENSG00000214580	chromosome5	140733835
ENSG00000204955	chromosome5	140698723
ENSG00000214594	chromosome5	140703698
ENSG00000204956	chromosome5	140690436
ENSG00000214583	chromosome5	140730146
ENSG00000214570	chromosome5	140777611
ENSG00000204969	chromosome5	140154734
ENSG00000204970	chromosome5	140146060
ENSG00000204961	chromosome5	140208265
ENSG00000204963	chromosome5	140194153
ENSG00000204967	chromosome5	140166957
ENSG00000204968	chromosome5	140160967
ENSG00000204964	chromosome5	140187861
ENSG00000204962	chromosome5	140201091
ENSG00000081842	chromosome5	140215818
ENSG00000214600	chromosome5	140235242
ENSG00000204965	chromosome5	140181545
ENSG00000214608	chromosome5	140228873
ENSG00000120322	chromosome5	140537800
ENSG00000187372	chromosome5	140573880
ENSG00000196963	chromosome5	140542319
ENSG00000113205	chromosome5	140460418
ENSG00000113248	chromosome5	140605331
ENSG00000113209	chromosome5	140495201
ENSG00000113211	chromosome5	140510023
ENSG00000081818	chromosome5	140481765
ENSG00000120328	chromosome5	140568664
ENSG00000197479	chromosome5	140559532
ENSG00000120324	chromosome5	140552310
ENSG00000113212	chromosome5	140532601
ENSG00000112852	chromosome5	140454559
ENSG00000146001	chromosome5	140594470
ENSG00000120327	chromosome5	140583262
ENSG00000171815	chromosome5	140411240
ENSG00000204969	chromosome5	140154734
ENSG00000204970	chromosome5	140146060
ENSG00000204961	chromosome5	140208265
ENSG00000204963	chromosome5	140194153
ENSG00000204967	chromosome5	140166957
ENSG00000204968	chromosome5	140160967
ENSG00000204964	chromosome5	140187861
ENSG00000204962	chromosome5	140201091
ENSG00000081842	chromosome5	140215818
ENSG00000214600	chromosome5	140235242
ENSG00000204965	chromosome5	140181545

ENSG00000214608	chromosome5	140228873
ENSG00000081853	chromosome5	140790511
ENSG00000214567	chromosome5	140780979
ENSG00000214574	chromosome5	140752565
ENSG00000214580	chromosome5	140733835
ENSG00000204955	chromosome5	140698723
ENSG00000214594	chromosome5	140703698
ENSG00000204956	chromosome5	140690436
ENSG00000214583	chromosome5	140730146
ENSG00000214570	chromosome5	140777611
ENSG00000204969	chromosome5	140154734
ENSG00000204970	chromosome5	140146060
ENSG00000204961	chromosome5	140208265
ENSG00000204963	chromosome5	140194153
ENSG00000204967	chromosome5	140166957
ENSG00000204968	chromosome5	140160967
ENSG00000204964	chromosome5	140187861
ENSG00000204962	chromosome5	140201091
ENSG00000081842	chromosome5	140215818
ENSG00000214600	chromosome5	140235242
ENSG00000204965	chromosome5	140181545
ENSG00000214608	chromosome5	140228873
ENSG00000060971	chromosome3	38153531
ENSG00000174307	chromosome1	199704538
ENSG00000139289	chromosome12	74711789
ENSG00000181649	chromosome11	2907171
ENSG00000139289	chromosome12	74711789
ENSG00000174307	chromosome1	199704538
ENSG00000206177	chromosome16	155997
ENSG00000188536	chromosome16	162912
ENSG00000206172	chromosome16	166716
ENSG00000086506	chromosome16	170486
ENSG00000130656	chromosome16	142909
ENSG00000206178	chromosome16	153145
ENSG00000102524	chromosome13	107720245
ENSG00000161955	chromosome17	7393195
ENSG00000115263	chromosome2	162713935
ENSG00000159224	chromosome17	44399594
ENSG00000181826	chromosome4	37364305
ENSG00000164620	chromosome5	140997977
ENSG00000054967	chromosome11	72777830
ENSG00000164620	chromosome5	140997977
ENSG00000181826	chromosome4	37364305
ENSG00000123685	chromosome1	210939728
ENSG00000156127	chromosome14	75058779
ENSG00000168062	chromosome11	64520963
ENSG00000156127	chromosome14	75058779
ENSG00000123685	chromosome1	210939728
ENSG00000122543	chromosome7	5887047
ENSG00000135175	chromosome7	97457353
ENSG00000122543	chromosome7	5887047
ENSG00000135175	chromosome7	97457353
ENSG00000100362	chromosome22	35542996
ENSG00000117407	chromosome1	44173916
ENSG00000125650	chromosome19	6326861
ENSG00000171119	chromosome19	5775177
ENSG00000168621	chromosome5	37871518
ENSG00000125650	chromosome19	6326861
ENSG00000117407	chromosome1	44173916

ENSG00000168621	chromosome5	37871518
ENSG00000171119	chromosome19	5775177
ENSG00000188394	chromosome9	124836667
ENSG00000203737	chromosome1	172683873
ENSG00000110492	chromosome11	46360184
ENSG00000105894	chromosome7	136678979
ENSG00000131910	chromosome1	27113019
ENSG00000169297	chromosomeX	30237402
ENSG00000131910	chromosome1	27113019
ENSG00000178882	chromosome12	123345929
ENSG00000183688	chromosome17	295959
ENSG00000101298	chromosome20	1166741
ENSG00000147642	chromosome8	110733799
ENSG00000146411	chromosome6	134415312
ENSG00000197496	chromosome20	44771783
ENSG00000064652	chromosome5	122209228
ENSG00000157734	chromosome15	62231028
ENSG00000136444	chromosome17	45911245
ENSG00000158270	chromosome18	490689
ENSG00000168077	chromosome8	27547499
ENSG00000120820	chromosome12	102937557
ENSG00000016864	chromosome3	52709517
ENSG00000155269	chromosome4	8633610
ENSG00000154478	chromosome10	125415914
ENSG00000105695	chromosome19	40478152
ENSG00000129450	chromosome19	56320044
ENSG00000168995	chromosome19	56337439
ENSG00000160296	chromosome19	56696800
ENSG00000105366	chromosome19	56653454
ENSG00000105383	chromosome19	56420187
ENSG00000171101	chromosome19	56362398
ENSG00000167764	chromosome19	56703639
ENSG00000105492	chromosome19	56726715
ENSG00000105501	chromosome19	56825401
ENSG00000197865	chromosome19	56841825
ENSG00000161640	chromosome19	55156223
ENSG00000161643	chromosome19	55164771
ENSG00000142512	chromosome19	56612648
ENSG00000187123	chromosome2	150002469
ENSG00000150556	chromosome2	149725236
ENSG00000143502	chromosome1	221603391
ENSG00000100647	chromosome14	69195063
ENSG00000100647	chromosome14	69195063
ENSG00000143502	chromosome1	221603391
ENSG00000158458	chromosome5	139402852
ENSG00000157168	chromosome8	32525787
ENSG00000119865	chromosome2	68400037
ENSG00000103044	chromosome16	67700800
ENSG00000170961	chromosome8	122710762
ENSG00000170961	chromosome8	122710762
ENSG00000103044	chromosome16	67700800
ENSG00000105509	chromosome19	56918999
ENSG00000105509	chromosome19	56918999
ENSG00000170961	chromosome8	122710762
ENSG00000103044	chromosome16	67700800
ENSG00000140832	chromosome16	70217634
ENSG00000004468	chromosome4	15389136
ENSG00000109743	chromosome4	15313866
ENSG00000142166	chromosome21	33619231

ENSG00000159113	chromosome21	33560641
ENSG00000174564	chromosome3	138159646
ENSG00000142166	chromosome21	33619231
ENSG00000159113	chromosome21	33560641
ENSG00000174564	chromosome3	138159646
ENSG00000159113	chromosome21	33560641
ENSG00000142166	chromosome21	33619231
ENSG00000174564	chromosome3	138159646
ENSG00000142166	chromosome21	33619231
ENSG00000159113	chromosome21	33560641
ENSG00000142166	chromosome21	33619231
ENSG00000159113	chromosome21	33560641
ENSG00000174564	chromosome3	138159646
ENSG00000198892	chromosome1	200124746
ENSG00000198892	chromosome1	200124746
ENSG00000164054	chromosome3	48516532
ENSG00000135447	chromosome12	53268714
ENSG00000150722	chromosome2	182559083
ENSG00000131771	chromosome17	35037172
ENSG00000150722	chromosome2	182559083
ENSG00000135447	chromosome12	53268714
ENSG00000198780	chromosome5	74173929
ENSG00000185087	chromosome15	96841533
ENSG00000188038	chromosome16	66476308
ENSG00000188038	chromosome16	66476308
ENSG00000124785	chromosome6	5951982
ENSG00000173250	chromosome5	145875870
ENSG00000204414	chromosome17	59342289
ENSG00000136488	chromosome17	59327604
ENSG00000213218	chromosome17	59304707
ENSG00000189162	chromosome17	59349869
ENSG00000136487	chromosome17	59312894
ENSG00000204414	chromosome17	59342289
ENSG00000136488	chromosome17	59327604
ENSG00000213218	chromosome17	59304707
ENSG00000189162	chromosome17	59349869
ENSG00000136487	chromosome17	59312894
ENSG00000204414	chromosome17	59342289
ENSG00000136488	chromosome17	59327604
ENSG00000213218	chromosome17	59304707
ENSG00000189162	chromosome17	59349869
ENSG00000136487	chromosome17	59312894
ENSG00000172179	chromosome6	22405191
ENSG00000162591	chromosome1	3517693
ENSG00000187800	chromosome1	155140343
ENSG00000145794	chromosome5	126694900
ENSG00000157890	chromosome15	64203367
ENSG00000074660	chromosome17	1495742
ENSG00000099910	chromosome22	19122042
ENSG00000099910	chromosome22	19122042
ENSG00000074660	chromosome17	1495742
ENSG00000145794	chromosome5	126694900
ENSG00000157890	chromosome15	64203367
ENSG00000187800	chromosome1	155140343
ENSG00000157890	chromosome15	64203367
ENSG00000145794	chromosome5	126694900
ENSG00000162591	chromosome1	3517693
ENSG00000074660	chromosome17	1495742
ENSG00000099910	chromosome22	19122042

ENSG00000187024	chromosome9	129517740
ENSG00000103852	chromosome15	97586441
ENSG00000205838	chromosome5	34875031
ENSG00000181195	chromosome8	57521067
ENSG00000101327	chromosome20	1911731
ENSG00000168081	chromosome8	28242594
ENSG00000101327	chromosome20	1911731
ENSG00000181195	chromosome8	57521067
ENSG00000196526	chromosome4	7924706
ENSG00000157510	chromosome5	148631725
ENSG00000169129	chromosome10	116154205
ENSG00000157510	chromosome5	148631725
ENSG00000196526	chromosome4	7924706
ENSG00000164484	chromosome6	130803261
ENSG00000206432	chromosome18	5882063
ENSG00000187975	chromosome1	29320928
ENSG00000206432	chromosome18	5882063
ENSG00000164484	chromosome6	130803261
ENSG00000185231	chromosome18	13875518
ENSG00000124089	chromosome20	54257196
ENSG00000176136	chromosome18	13815765
ENSG00000166603	chromosome18	56190563
ENSG00000124089	chromosome20	54257196
ENSG00000176136	chromosome18	13815765
ENSG00000166603	chromosome18	56190563
ENSG00000176136	chromosome18	13815765
ENSG00000124089	chromosome20	54257196
ENSG00000166603	chromosome18	56190563
ENSG00000124089	chromosome20	54257196
ENSG00000176136	chromosome18	13815765
ENSG00000174145	chromosome4	37121740
ENSG00000124151	chromosome20	45684399
ENSG00000140396	chromosome8	71291535
ENSG00000084676	chromosome2	24735051
ENSG00000140396	chromosome8	71291535
ENSG00000124151	chromosome20	45684399
ENSG00000198740	chromosome17	44750087
ENSG00000114853	chromosome3	42674852
ENSG00000136457	chromosome17	45901174
ENSG00000136457	chromosome17	45901174
ENSG00000146006	chromosome5	138238146
ENSG00000162951	chromosome2	80384456
ENSG00000176204	chromosome2	77600503
ENSG00000198739	chromosome10	68356336
ENSG00000136457	chromosome17	45901174
ENSG00000176204	chromosome2	77600503
ENSG00000198739	chromosome10	68356336
ENSG00000146006	chromosome5	138238146
ENSG00000162951	chromosome2	80384456
ENSG00000162951	chromosome2	80384456
ENSG00000146006	chromosome5	138238146
ENSG00000186660	chromosome11	58103331
ENSG00000171163	chromosome1	247119132
ENSG00000161914	chromosome19	11477602
ENSG00000158805	chromosome16	88316460
ENSG00000158805	chromosome16	88316460
ENSG00000161914	chromosome19	11477602
ENSG00000171163	chromosome1	247119132
ENSG00000186660	chromosome11	58103331

ENSG00000186787	chromosomeX	57163788
ENSG00000147059	chromosomeX	57179756
ENSG00000186767	chromosomeX	62487424
ENSG00000204271	chromosomeX	57038106
ENSG00000106723	chromosome9	90231275
ENSG00000204271	chromosomeX	57038106
ENSG00000106723	chromosome9	90231275
ENSG00000186787	chromosomeX	57163788
ENSG00000147059	chromosomeX	57179756
ENSG00000186767	chromosomeX	62487424
ENSG00000185278	chromosome1	172105987
ENSG00000180610	chromosome4	39449396
ENSG00000204366	chromosome6	31977062
ENSG00000169155	chromosome9	128634610
ENSG00000213588	chromosome6	33530856
ENSG00000204210	chromosome6	33393489
ENSG00000180610	chromosome4	39449396
ENSG00000204366	chromosome6	31977062
ENSG00000185278	chromosome1	172105987
ENSG00000213588	chromosome6	33530856
ENSG00000204210	chromosome6	33393489
ENSG00000169155	chromosome9	128634610
ENSG00000180929	chromosome3	51964709
ENSG00000156097	chromosome1	109887168
ENSG00000163376	chromosome3	67132157
ENSG00000170852	chromosome7	32885742
ENSG00000120696	chromosome13	40666394
ENSG00000165572	chromosome13	40604648
ENSG00000170852	chromosome7	32885742
ENSG00000163376	chromosome3	67132157
ENSG00000168038	chromosome3	41971256
ENSG00000168038	chromosome3	41971256
ENSG00000122778	chromosome7	138254705
ENSG00000110427	chromosome11	33520577
ENSG00000162729	chromosome1	158334887
ENSG00000134256	chromosome1	117345963
ENSG00000143061	chromosome1	117010532
ENSG00000134247	chromosome1	117254208
ENSG00000134256	chromosome1	117345963
ENSG00000143061	chromosome1	117010532
ENSG00000134256	chromosome1	117345963
ENSG00000143061	chromosome1	117010532
ENSG00000162729	chromosome1	158334887
ENSG00000143061	chromosome1	117010532
ENSG00000134256	chromosome1	117345963
ENSG00000185028	chromosome5	244618
ENSG00000116708	chromosome1	12865803
ENSG00000157358	chromosome1	13517387
ENSG00000204501	chromosome1	13296590
ENSG00000204502	chromosome1	13237478
ENSG00000204507	chromosome1	13035268
ENSG00000204509	chromosome1	12924936
ENSG00000204513	chromosome1	12811111
ENSG00000204478	chromosome1	13615399
ENSG00000204486	chromosome1	13394560
ENSG00000204510	chromosome1	12900100
ENSG00000182330	chromosome1	13262290
ENSG00000204485	chromosome1	13483075
ENSG00000116726	chromosome1	12757598

ENSG00000204481	chromosome1	13544279
ENSG00000204495	chromosome1	13323424
ENSG00000116721	chromosome1	12775964
ENSG00000120952	chromosome1	12841452
ENSG00000204480	chromosome1	13570946
ENSG00000204491	chromosome1	13350110
ENSG00000204503	chromosome1	13204253
ENSG00000204508	chromosome1	12958130
ENSG00000204505	chromosome1	13063043
ENSG00000179704	chromosome1	145779720
ENSG00000204479	chromosome1	13588705
ENSG00000204488	chromosome1	13367871
ENSG00000187545	chromosome1	12878266
ENSG00000185686	chromosome22	21229253
ENSG00000197549	chromosome22	20678843
ENSG00000160959	chromosome8	145715918
ENSG00000182568	chromosome3	18437470
ENSG00000182568	chromosome3	18437470
ENSG00000119042	chromosome2	200029006
ENSG00000179673	chromosome17	42411373
ENSG00000177519	chromosome2	154043326
ENSG00000177519	chromosome2	154043326
ENSG00000179673	chromosome17	42411373
ENSG00000125879	chromosome20	16677047
ENSG00000213054	chromosome19	45973288
ENSG00000150526	chromosome14	38773070
ENSG00000213054	chromosome19	45973288
ENSG00000125879	chromosome20	16677047
ENSG00000150526	chromosome14	38773070
ENSG00000160678	chromosome1	151867173
ENSG00000171643	chromosome5	76206941
ENSG00000163191	chromosome1	150276016
ENSG00000188643	chromosome1	151847252
ENSG00000189171	chromosome1	151865573
ENSG00000189334	chromosome1	151854959
ENSG00000197747	chromosome1	150225331
ENSG00000160678	chromosome1	151867173
ENSG00000171643	chromosome5	76206941
ENSG00000163221	chromosome1	151613702
ENSG00000143546	chromosome1	151629636
ENSG00000163220	chromosome1	151597384
ENSG00000160307	chromosome21	46846757
ENSG00000163993	chromosome4	6746561
ENSG00000160678	chromosome1	151867173
ENSG00000171643	chromosome5	76206941
ENSG00000163191	chromosome1	150276016
ENSG00000188643	chromosome1	151847252
ENSG00000189171	chromosome1	151865573
ENSG00000189334	chromosome1	151854959
ENSG00000197747	chromosome1	150225331
ENSG00000160307	chromosome21	46846757
ENSG00000189334	chromosome1	151854959
ENSG00000189171	chromosome1	151865573
ENSG00000189171	chromosome1	151865573
ENSG00000189334	chromosome1	151854959
ENSG00000188643	chromosome1	151847252
ENSG00000196754	chromosome1	151802978
ENSG00000196154	chromosome1	151783895
ENSG00000188015	chromosome1	151787586

ENSG00000197956	chromosome1	151774440
ENSG00000196420	chromosome1	151780525
ENSG00000163221	chromosome1	151613702
ENSG00000143546	chromosome1	151629636
ENSG00000163220	chromosome1	151597384
ENSG00000160307	chromosome21	46846757
ENSG00000163993	chromosome4	6746561
ENSG00000163191	chromosome1	150276016
ENSG00000188643	chromosome1	151847252
ENSG00000189171	chromosome1	151865573
ENSG00000189334	chromosome1	151854959
ENSG00000197747	chromosome1	150225331
ENSG00000160678	chromosome1	151867173
ENSG00000171643	chromosome5	76206941
ENSG00000169906	chromosomeX	16579051
ENSG00000197747	chromosome1	150225331
ENSG00000163191	chromosome1	150276016
ENSG00000188643	chromosome1	151847252
ENSG00000189171	chromosome1	151865573
ENSG00000189334	chromosome1	151854959
ENSG00000171643	chromosome5	76206941
ENSG00000160678	chromosome1	151867173
ENSG00000163221	chromosome1	151613702
ENSG00000143546	chromosome1	151629636
ENSG00000163220	chromosome1	151597384
ENSG00000160307	chromosome21	46846757
ENSG00000163993	chromosome4	6746561
ENSG00000163191	chromosome1	150276016
ENSG00000188643	chromosome1	151847252
ENSG00000189171	chromosome1	151865573
ENSG00000189334	chromosome1	151854959
ENSG00000197747	chromosome1	150225331
ENSG00000160678	chromosome1	151867173
ENSG00000171643	chromosome5	76206941
ENSG00000196754	chromosome1	151802978
ENSG00000196154	chromosome1	151783895
ENSG00000188015	chromosome1	151787586
ENSG00000197956	chromosome1	151774440
ENSG00000196420	chromosome1	151780525
ENSG00000160307	chromosome21	46846757
ENSG00000163993	chromosome4	6746561
ENSG00000163221	chromosome1	151613702
ENSG00000143546	chromosome1	151629636
ENSG00000163220	chromosome1	151597384
ENSG00000188643	chromosome1	151847252
ENSG00000189171	chromosome1	151865573
ENSG00000189334	chromosome1	151854959
ENSG00000163191	chromosome1	150276016
ENSG00000163221	chromosome1	151613702
ENSG00000143546	chromosome1	151629636
ENSG00000163220	chromosome1	151597384
ENSG00000163993	chromosome4	6746561
ENSG00000160307	chromosome21	46846757
ENSG00000163421	chromosome3	71916894
ENSG00000143125	chromosome1	110795362
ENSG00000160767	chromosome1	153491293
ENSG00000104059	chromosome15	27276046
ENSG00000135063	chromosome9	71140994
ENSG00000104059	chromosome15	27276046

ENSG00000160767	chromosome1	153491293
ENSG00000170819	chromosome3	134601534
ENSG00000111057	chromosome12	51629225
ENSG00000214220	chromosome3	158364416
ENSG00000214959	chromosome9	80842201
ENSG00000213951	chromosome2	189885644
ENSG00000214454	chromosome2	74388644
ENSG00000187686	chromosome11	124489066
ENSG00000111057	chromosome12	51629225
ENSG00000214220	chromosome3	158364416
ENSG00000214959	chromosome9	80842201
ENSG00000213951	chromosome2	189885644
ENSG00000214454	chromosome2	74388644
ENSG00000187686	chromosome11	124489066
ENSG00000170819	chromosome3	134601534
ENSG00000108244	chromosome17	36346382
ENSG00000197079	chromosome17	36890876
ENSG00000108759	chromosome17	36877104
ENSG00000006059	chromosome17	36760546
ENSG00000131737	chromosome17	36792151
ENSG00000131738	chromosome17	36779532
ENSG00000094796	chromosome17	36807324
ENSG00000204889	chromosome17	36394052
ENSG00000215773	supercontigNT_113931	144464
ENSG00000215772	supercontigNT_113931	161881
ENSG00000196859	chromosome17	36376635
ENSG00000108417	chromosome17	36834302
ENSG00000171360	chromosome17	36850700
ENSG00000126337	chromosome17	36902112
ENSG00000171431	chromosome17	36294964
ENSG00000197079	chromosome17	36890876
ENSG00000108759	chromosome17	36877104
ENSG00000006059	chromosome17	36760546
ENSG00000131737	chromosome17	36792151
ENSG00000131738	chromosome17	36779532
ENSG00000094796	chromosome17	36807324
ENSG00000204889	chromosome17	36394052
ENSG00000215773	supercontigNT_113931	144464
ENSG00000215772	supercontigNT_113931	161881
ENSG00000196859	chromosome17	36376635
ENSG00000108417	chromosome17	36834302
ENSG00000171360	chromosome17	36850700
ENSG00000126337	chromosome17	36902112
ENSG00000171431	chromosome17	36294964
ENSG00000111057	chromosome12	51629225
ENSG00000214220	chromosome3	158364416
ENSG00000214959	chromosome9	80842201
ENSG00000213951	chromosome2	189885644
ENSG00000214454	chromosome2	74388644
ENSG00000187686	chromosome11	124489066
ENSG00000170819	chromosome3	134601534
ENSG00000108244	chromosome17	36346382
ENSG00000171345	chromosome17	36938026
ENSG00000186832	chromosome17	37022467
ENSG00000131885	chromosome17	18270966
ENSG00000128422	chromosome17	16689855
ENSG00000186831	chromosome17	37034288
ENSG00000186847	chromosome17	36996613
ENSG00000171401	chromosome17	36915329

ENSG00000171346	chromosome17	36928606
ENSG00000167916	chromosome17	36113472
ENSG00000173908	chromosome17	36209672
ENSG00000171446	chromosome17	36192272
ENSG00000204897	chromosome17	36165050
ENSG00000186393	chromosome17	36181892
ENSG00000186395	chromosome17	36232364
ENSG00000187242	chromosome17	36276965
ENSG00000171403	chromosome17	36981771
ENSG00000111057	chromosome12	51629225
ENSG00000214220	chromosome3	158364416
ENSG00000214959	chromosome9	80842201
ENSG00000213951	chromosome2	189885644
ENSG00000214454	chromosome2	74388644
ENSG00000187686	chromosome11	124489066
ENSG00000170819	chromosome3	134601534
ENSG00000108244	chromosome17	36346382
ENSG00000111057	chromosome12	51629225
ENSG00000214220	chromosome3	158364416
ENSG00000214959	chromosome9	80842201
ENSG00000213951	chromosome2	189885644
ENSG00000214454	chromosome2	74388644
ENSG00000187686	chromosome11	124489066
ENSG00000170819	chromosome3	134601534
ENSG00000170819	chromosome3	134601534
ENSG00000111057	chromosome12	51629225
ENSG00000214220	chromosome3	158364416
ENSG00000214959	chromosome9	80842201
ENSG00000213951	chromosome2	189885644
ENSG00000214454	chromosome2	74388644
ENSG00000187686	chromosome11	124489066
ENSG00000170819	chromosome3	134601534
ENSG00000197079	chromosome17	36890876
ENSG00000108759	chromosome17	36877104
ENSG00000006059	chromosome17	36760546
ENSG00000131737	chromosome17	36792151
ENSG00000131738	chromosome17	36779532
ENSG00000094796	chromosome17	36807324
ENSG00000204889	chromosome17	36394052
ENSG00000215773	supercontigNT_113931	144464
ENSG00000215772	supercontigNT_113931	161881
ENSG00000196859	chromosome17	36376635
ENSG00000108417	chromosome17	36834302
ENSG00000171360	chromosome17	36850700
ENSG00000126337	chromosome17	36902112
ENSG00000171431	chromosome17	36294964
ENSG00000111057	chromosome12	51629225
ENSG00000214220	chromosome3	158364416
ENSG00000214959	chromosome9	80842201
ENSG00000213951	chromosome2	189885644
ENSG00000214454	chromosome2	74388644
ENSG00000187686	chromosome11	124489066
ENSG00000170819	chromosome3	134601534
ENSG00000108244	chromosome17	36346382
ENSG00000167874	chromosome17	7699118
ENSG00000205116	chromosome1	1351371
ENSG00000184838	chromosome5	119844798
ENSG00000123609	chromosome2	151847709
ENSG00000068079	chromosome17	38412491

ENSG00000088543	chromosome3	50578135
ENSG00000162194	chromosome11	62195321
ENSG00000213626	chromosome2	30308109
ENSG00000213626	chromosome2	30308109
ENSG00000121900	chromosome1	33149273
ENSG00000157992	chromosome2	27518784
ENSG00000113070	chromosome5	139706098
ENSG00000205595	chromosome4	75699863
ENSG00000109321	chromosome4	75529927
ENSG00000173267	chromosome10	88708435
ENSG00000145335	chromosome4	90975842
ENSG00000074317	chromosome5	175989262
ENSG00000122585	chromosome7	24291385
ENSG00000131096	chromosome17	39399197
ENSG00000204474	chromosomeX	49807547
ENSG00000131096	chromosome17	39399197
ENSG00000204474	chromosomeX	49807547
ENSG00000122585	chromosome7	24291385
ENSG00000108849	chromosome17	39374549
ENSG00000109072	chromosome17	23721352
ENSG00000116690	chromosome1	184532631
ENSG00000116690	chromosome1	184532631
ENSG00000109072	chromosome17	23721352
ENSG00000103310	chromosome16	21130370
ENSG00000149506	chromosome11	60391611
ENSG00000116996	chromosome1	236120559
ENSG00000149506	chromosome11	60391611
ENSG00000116996	chromosome1	236120559
ENSG00000149506	chromosome11	60391611
ENSG00000116996	chromosome1	236120559
ENSG00000103310	chromosome16	21130370
ENSG00000163961	chromosome3	197714442
ENSG00000166439	chromosome11	74137574
ENSG00000141433	chromosome18	895388
ENSG00000146469	chromosome6	153115006
ENSG00000146469	chromosome6	153115006
ENSG00000175130	chromosome1	32574222
ENSG00000155130	chromosome6	114285615
ENSG00000155130	chromosome6	114285615
ENSG00000175130	chromosome1	32574222
ENSG00000155130	chromosome6	114285615
ENSG00000175130	chromosome1	32574222
ENSG00000155130	chromosome6	114285615
ENSG00000155130	chromosome6	114285615
ENSG00000155130	chromosome6	114285615
ENSG00000155130	chromosome6	114285615
ENSG00000175130	chromosome1	32574222
ENSG00000167244	chromosome11	2113330
ENSG00000017427	chromosome12	101398290
ENSG00000017427	chromosome12	101398290
ENSG00000167244	chromosome11	2113330
ENSG00000129965	chromosome11	2138778
ENSG00000178026	chromosome22	23319007
ENSG00000181350	chromosome17	16335889
ENSG00000181350	chromosome17	16335889
ENSG00000178026	chromosome22	23319007
ENSG00000108256	chromosome17	24645204
ENSG00000185614	chromosome3	49815824
ENSG00000197852	chromosome1	112083388

ENSG00000177853	chromosome10	97906070
ENSG00000178163	chromosome4	10057051
ENSG00000204950	chromosome11	61033113
ENSG00000198812	chromosome12	68290886
ENSG00000188833	chromosome9	139452559
ENSG00000054179	chromosome9	139068271
ENSG00000138185	chromosome10	97461704
ENSG00000054179	chromosome9	139068271
ENSG00000188833	chromosome9	139452559
ENSG00000138185	chromosome10	97461704
ENSG00000188833	chromosome9	139452559
ENSG00000054179	chromosome9	139068271
ENSG00000168032	chromosome3	40404553
ENSG00000185482	chromosome12	55929687
ENSG00000144681	chromosome3	36397140
ENSG00000141750	chromosome17	34635282
ENSG00000141750	chromosome17	34635282
ENSG00000144681	chromosome3	36397140
ENSG00000182743	chromosome7	1750780
ENSG00000166897	chromosome22	36101521
ENSG00000128606	chromosome7	102361510
ENSG00000187122	chromosome10	98935422
ENSG00000145147	chromosome4	19864537
ENSG00000184347	chromosome5	168660292
ENSG00000184347	chromosome5	168660292
ENSG00000145147	chromosome4	19864537
ENSG00000128606	chromosome7	102361510
ENSG00000182743	chromosome7	1750780
ENSG00000166897	chromosome22	36101521
ENSG00000187122	chromosome10	98935422
ENSG00000182743	chromosome7	1750780
ENSG00000166897	chromosome22	36101521
ENSG00000128606	chromosome7	102361510
ENSG00000166897	chromosome22	36101521
ENSG00000182743	chromosome7	1750780
ENSG00000084636	chromosome1	31940382
ENSG00000049089	chromosome1	40555457
ENSG00000112280	chromosome6	71069349
ENSG00000084636	chromosome1	31940382
ENSG00000092758	chromosome20	60918862
ENSG00000084636	chromosome1	31940382
ENSG00000092758	chromosome20	60918862
ENSG00000049089	chromosome1	40555457
ENSG00000112280	chromosome6	71069349
ENSG00000092758	chromosome20	60918862
ENSG00000084636	chromosome1	31940382
ENSG00000112280	chromosome6	71069349
ENSG00000049089	chromosome1	40555457
ENSG00000144810	chromosome3	100992217
ENSG00000171812	chromosome1	36338431
ENSG00000171812	chromosome1	36338431
ENSG00000144810	chromosome3	100992217
ENSG00000123500	chromosome6	116553349
ENSG00000148600	chromosome10	85944497
ENSG00000074276	chromosome5	175924960
ENSG00000196782	chromosome4	141031581
ENSG00000161021	chromosome5	179092472
ENSG00000184384	chromosome11	95466330
ENSG00000161021	chromosome5	179092472

ENSG00000196782	chromosome4	141031581
ENSG00000184384	chromosome11	95466330
ENSG00000196782	chromosome4	141031581
ENSG00000161021	chromosome5	179092472
ENSG00000013619	chromosomeX	149364441
ENSG00000124749	chromosome6	56155376
ENSG00000169436	chromosome8	139964598
ENSG00000124749	chromosome6	56155376
ENSG00000163235	chromosome2	70634408
ENSG00000134200	chromosome1	115377507
ENSG00000131808	chromosome11	30210026
ENSG00000131808	chromosome11	30210026
ENSG00000134200	chromosome1	115377507
ENSG00000204748	chromosome19	54231830
ENSG00000104818	chromosome19	54227116
ENSG00000189052	chromosome19	54239279
ENSG00000213030	chromosome19	54243809
ENSG00000104827	chromosome19	54219041
ENSG00000196337	chromosome19	54250445
ENSG00000104826	chromosome19	54212151
ENSG00000179600	chromosome14	62854317
ENSG00000204748	chromosome19	54231830
ENSG00000104818	chromosome19	54227116
ENSG00000189052	chromosome19	54239279
ENSG00000213030	chromosome19	54243809
ENSG00000104827	chromosome19	54219041
ENSG00000196337	chromosome19	54250445
ENSG00000104826	chromosome19	54212151
ENSG00000131808	chromosome11	30210026
ENSG00000134200	chromosome1	115377507
ENSG00000179600	chromosome14	62854317
ENSG00000164326	chromosome5	71050877
ENSG00000143157	chromosome1	165076818
ENSG00000147571	chromosome8	67252267
ENSG00000163794	chromosome2	27384268
ENSG00000064199	chromosome11	124050371
ENSG00000154040	chromosome18	19977077
ENSG00000161544	chromosome17	72045220
ENSG00000161544	chromosome17	72045220
ENSG00000198125	chromosome22	34343251
ENSG00000047579	chromosome6	15771080
ENSG00000204070	chromosome20	43468506
ENSG00000003249	chromosome16	88613251
ENSG00000047579	chromosome6	15771080
ENSG00000204070	chromosome20	43468506
ENSG00000047579	chromosome6	15771080
ENSG00000169607	chromosome2	113238647
ENSG00000136108	chromosome13	51927693
ENSG00000165566	chromosome13	24643758
ENSG00000184675	chromosomeX	63329892
ENSG00000178171	chromosome2	131236116
ENSG00000184675	chromosomeX	63329892
ENSG00000165566	chromosome13	24643758
ENSG00000122034	chromosome13	26896700
ENSG00000078804	chromosome20	32760205
ENSG00000164938	chromosome8	96022346
ENSG00000163348	chromosome1	153200677
ENSG00000171016	chromosome15	53668343
ENSG00000079101	chromosome18	587047

ENSG00000120885	chromosome8	27528216
ENSG00000151612	chromosome4	147079010
ENSG00000020256	chromosome20	50241583
ENSG00000182575	chromosome17	45008581
ENSG00000144227	chromosome2	139145718
ENSG00000182379	chromosome12	55897020
ENSG00000122584	chromosome7	8757163
ENSG00000122584	chromosome7	8757163
ENSG00000144227	chromosome2	139145718
ENSG00000182379	chromosome12	55897020
ENSG00000182379	chromosome12	55897020
ENSG00000144227	chromosome2	139145718
ENSG00000169372	chromosome12	92596682
ENSG00000143340	chromosome1	178048856
ENSG00000196990	chromosome9	135435167
ENSG00000215611	supercontigNT_113911	27615
ENSG00000213996	chromosome19	19245025
ENSG00000136404	chromosome15	81567437
ENSG00000177311	chromosome3	142643921
ENSG00000174282	chromosome17	7310845
ENSG00000198081	chromosome18	5283246
ENSG00000198081	chromosome18	5283246
ENSG00000174282	chromosome17	7310845
ENSG00000177311	chromosome3	142643921
ENSG00000177485	chromosomeX	119271299
ENSG00000198081	chromosome18	5283246
ENSG00000198081	chromosome18	5283246
ENSG00000174282	chromosome17	7310845
ENSG00000177311	chromosome3	142643921
ENSG00000177485	chromosomeX	119271299
ENSG00000177485	chromosomeX	119271299
ENSG00000174282	chromosome17	7310845
ENSG00000177311	chromosome3	142643921
ENSG00000064490	chromosome19	19165756
ENSG00000164331	chromosome5	72894463
ENSG00000023902	chromosome1	148388391
ENSG00000169094	chromosome15	62921269
ENSG00000023902	chromosome1	148388391
ENSG00000154305	chromosome1	220858076
ENSG00000150527	chromosome14	38804350
ENSG00000183287	chromosome18	55515555
ENSG00000183287	chromosome18	55515555
ENSG00000126464	chromosome19	54790591
ENSG00000060749	chromosome11	32906149
ENSG00000154143	chromosome11	123986663
ENSG00000110218	chromosome11	93502127
ENSG00000073150	chromosome22	48951317
ENSG00000110218	chromosome11	93502127
ENSG00000154143	chromosome11	123986663
ENSG00000147526	chromosome8	38734008
ENSG00000138162	chromosome10	123771487
ENSG00000138162	chromosome10	123771487
ENSG00000147526	chromosome8	38734008
ENSG00000013810	chromosome4	1694947
ENSG00000108231	chromosome10	95507892
ENSG00000168481	chromosome8	22070001
ENSG00000153012	chromosome4	24641414
ENSG00000153012	chromosome4	24641414
ENSG00000168481	chromosome8	22070001

ENSG00000168481	chromosome8	22070001
ENSG00000153012	chromosome4	24641414
ENSG00000108231	chromosome10	95507892
ENSG00000153902	chromosome19	40317425
ENSG00000121104	chromosome17	45196449
ENSG00000138439	chromosome2	203208454
ENSG00000106415	chromosome7	7975507
ENSG00000106415	chromosome7	7975507
ENSG00000138439	chromosome2	203208454
ENSG00000133789	chromosome11	9642303
ENSG00000023892	chromosome6	35373612
ENSG00000023892	chromosome6	35373612
ENSG00000171448	chromosome9	124722035
ENSG00000186130	chromosome9	124714173
ENSG00000186130	chromosome9	124714173
ENSG00000171448	chromosome9	124722035
ENSG00000169247	chromosome5	148422779
ENSG00000125089	chromosome4	8257822
ENSG00000162545	chromosome1	20684460
ENSG00000163888	chromosome3	185461768
ENSG00000127129	chromosome1	41722860
ENSG00000124205	chromosome20	57309263
ENSG00000078401	chromosome6	12398849
ENSG00000124205	chromosome20	57309263
ENSG00000127129	chromosome1	41722860
ENSG00000157005	chromosome3	188870774
ENSG00000167183	chromosome17	43385600
ENSG00000176532	chromosome7	29572471
ENSG00000214882	chromosome15	53468915
ENSG00000163346	chromosome1	153192822
ENSG00000198948	chromosome4	171163604
ENSG00000037749	chromosome5	153409476
ENSG00000132604	chromosome16	67977250
ENSG00000187589	chromosome18	14369048
ENSG00000147601	chromosome8	74083676
ENSG00000184862	chromosome13	18152635
ENSG00000146278	chromosome6	89847333
ENSG00000189266	chromosome1	24160535
ENSG00000215700	supercontigNT_113878	31290
ENSG00000172020	chromosome3	116825227
ENSG00000179071	chromosome11	85074822
ENSG00000129654	chromosome17	71648072
ENSG00000198815	chromosome1	42549352
ENSG00000065970	chromosome12	8083696
ENSG00000136634	chromosome1	205012404
ENSG00000162892	chromosome1	205137801
ENSG00000162891	chromosome1	205105821
ENSG00000142224	chromosome1	205038863
ENSG00000162892	chromosome1	205137801
ENSG00000162891	chromosome1	205105821
ENSG00000142224	chromosome1	205038863
ENSG00000136634	chromosome1	205012404
ENSG00000111536	chromosome12	66905804
ENSG00000127318	chromosome12	66933496
ENSG00000182103	chromosome11	82122420
ENSG00000140067	chromosome14	93461371
ENSG00000131378	chromosome3	16510381
ENSG00000162944	chromosome2	198248428
ENSG00000131477	chromosome17	38166806

ENSG00000122679	chromosome7	45163953
ENSG00000122679	chromosome7	45163953
ENSG00000131477	chromosome17	38166806
ENSG00000132329	chromosome2	238433058
ENSG00000132329	chromosome2	238433058
ENSG00000122679	chromosome7	45163953
ENSG00000131477	chromosome17	38166806
ENSG00000185527	chromosome17	77234019
ENSG00000139053	chromosome12	15022214
ENSG00000185527	chromosome17	77234019
ENSG00000155085	chromosome6	110103642
ENSG00000197453	chromosome6	109978187
ENSG00000129355	chromosome19	10540330
ENSG00000123080	chromosome1	51208629
ENSG00000198435	chromosome9	139316202
ENSG00000129355	chromosome19	10540330
ENSG00000123080	chromosome1	51208629
ENSG00000129355	chromosome19	10540330
ENSG00000123080	chromosome1	51208629
ENSG00000123080	chromosome1	51208629
ENSG00000129355	chromosome19	10540330
ENSG00000183337	chromosomeX	39822127
ENSG00000085185	chromosomeX	128966889
ENSG00000175449	chromosome5	95013732
ENSG00000149218	chromosome11	94462740
ENSG00000135540	chromosome6	138934742
ENSG00000188158	chromosomeX	17303802
ENSG00000135540	chromosome6	138934742
ENSG00000135540	chromosome6	138934742
ENSG00000188158	chromosomeX	17303802
ENSG00000204131	chromosomeX	71275222
ENSG00000204131	chromosomeX	71275222
ENSG00000135540	chromosome6	138934742
ENSG00000188158	chromosomeX	17303802
ENSG00000162522	chromosome1	32980143
ENSG00000131067	chromosome20	32924280
ENSG00000135842	chromosome1	183210112
ENSG00000136830	chromosome9	129381038
ENSG00000167483	chromosome19	17495248
ENSG00000136830	chromosome9	129381038
ENSG00000135842	chromosome1	183210112
ENSG00000174306	chromosome20	39266971
ENSG00000165156	chromosome8	124337368
ENSG00000178764	chromosome8	124032932
ENSG00000215271	chromosome14	22816205
ENSG00000165156	chromosome8	124337368
ENSG00000178764	chromosome8	124032932
ENSG00000174306	chromosome20	39266971
ENSG00000178764	chromosome8	124032932
ENSG00000165156	chromosome8	124337368
ENSG00000114019	chromosome3	135572966
ENSG00000126016	chromosomeX	111953011
ENSG00000126016	chromosomeX	111953011
ENSG00000114019	chromosome3	135572966
ENSG00000166025	chromosome11	94141308
ENSG00000158352	chromosomeX	50573759
ENSG00000146950	chromosomeX	9714586
ENSG00000138771	chromosome4	77576230
ENSG00000146950	chromosomeX	9714586

ENSG00000158352	chromosomeX	50573759
ENSG00000174950	chromosome1	27582317
ENSG00000135535	chromosome6	109810275
ENSG00000178804	chromosome3	130744752
ENSG00000189320	chromosome7	135083869
ENSG00000196666	chromosome11	47564822
ENSG00000189292	chromosome2	277836
ENSG00000196711	chromosome8	53640370
ENSG00000178531	chromosome19	7896424
ENSG00000205279	chromosome5	127021113
ENSG00000205279	chromosome5	127021113
ENSG00000172410	chromosome1	67039493
ENSG00000171136	chromosome19	14000017
ENSG00000171136	chromosome19	14000017
ENSG00000172410	chromosome1	67039493
ENSG00000172410	chromosome1	67039493
ENSG00000171136	chromosome19	14000017
ENSG00000172410	chromosome1	67039493
ENSG00000171136	chromosome19	14000017
ENSG00000107018	chromosome9	5329747
ENSG00000107014	chromosome9	5294581
ENSG00000120211	chromosome9	5221524
ENSG00000006128	chromosome7	97199861
ENSG00000129422	chromosome8	17657597
ENSG00000132938	chromosome13	28496800
ENSG00000132938	chromosome13	28496800
ENSG00000129422	chromosome8	17657597
ENSG00000198624	chromosome5	150583725
ENSG00000117122	chromosome1	17177356
ENSG00000197614	chromosome12	8705968
ENSG00000187094	chromosome3	42280127
ENSG00000053702	chromosome12	2814411
ENSG00000175352	chromosome11	8982059
ENSG00000211446	chromosome5	42844213
ENSG00000169248	chromosome4	77176165
ENSG00000169429	chromosome4	74825147
ENSG00000124875	chromosome4	74921332
ENSG00000163735	chromosome4	75083286
ENSG00000109272	chromosome4	74937944
ENSG00000163737	chromosome4	75066535
ENSG00000163736	chromosome4	75072685
ENSG00000081041	chromosome4	75183702
ENSG00000163739	chromosome4	74954052
ENSG00000163734	chromosome4	75123193
ENSG00000169429	chromosome4	74825147
ENSG00000124875	chromosome4	74921332
ENSG00000163735	chromosome4	75083286
ENSG00000109272	chromosome4	74937944
ENSG00000163737	chromosome4	75066535
ENSG00000163736	chromosome4	75072685
ENSG00000081041	chromosome4	75183702
ENSG00000163739	chromosome4	74954052
ENSG00000163734	chromosome4	75123193
ENSG00000124875	chromosome4	74921332
ENSG00000163735	chromosome4	75083286
ENSG00000109272	chromosome4	74937944
ENSG00000163737	chromosome4	75066535
ENSG00000163736	chromosome4	75072685
ENSG00000081041	chromosome4	75183702

ENSG00000163739	chromosome4	74954052
ENSG00000163734	chromosome4	75123193
ENSG00000169429	chromosome4	74825147
ENSG00000138755	chromosome4	77147627
ENSG00000169245	chromosome4	77163609
ENSG00000169248	chromosome4	77176165
ENSG00000138755	chromosome4	77147627
ENSG00000169245	chromosome4	77163609
ENSG00000124875	chromosome4	74921332
ENSG00000163735	chromosome4	75083286
ENSG00000109272	chromosome4	74937944
ENSG00000163737	chromosome4	75066535
ENSG00000163736	chromosome4	75072685
ENSG00000081041	chromosome4	75183702
ENSG00000163739	chromosome4	74954052
ENSG00000163734	chromosome4	75123193
ENSG00000169429	chromosome4	74825147
ENSG00000137726	chromosome11	117218702
ENSG00000182707	chromosomeX	73011628
ENSG00000150201	chromosome10	43189201
ENSG00000089356	chromosome19	40302121
ENSG00000137731	chromosome11	117203939
ENSG00000126258	chromosome19	40322824
ENSG00000150201	chromosome10	43189201
ENSG00000089356	chromosome19	40302121
ENSG00000137726	chromosome11	117218702
ENSG00000182707	chromosomeX	73011628
ENSG00000174844	chromosome3	57503638
ENSG00000188916	chromosome10	128864650
ENSG00000204767	chromosome5	169243481
ENSG00000188916	chromosome10	128864650
ENSG00000158109	chromosome1	3531497
ENSG00000158109	chromosome1	3531497
ENSG00000188001	chromosome3	190407868
ENSG00000172673	chromosome6	128263771
ENSG00000130775	chromosome1	28071670
ENSG00000130775	chromosome1	28071670
ENSG00000172673	chromosome6	128263771
ENSG00000169635	chromosome22	20127121
ENSG00000169635	chromosome22	20127121
ENSG00000177374	chromosome17	1906354
ENSG00000144476	chromosome2	237153848
ENSG00000166856	chromosome12	55675261
ENSG00000082438	chromosome2	165406777
ENSG00000106078	chromosome7	51351825
ENSG00000171206	chromosome10	104394365
ENSG00000171206	chromosome10	104394365
ENSG00000119725	chromosome14	73428632
ENSG00000065029	chromosome6	35356907
ENSG00000166478	chromosome11	9449432
ENSG00000166478	chromosome11	9449432
ENSG00000065029	chromosome6	35356907
ENSG00000164850	chromosome7	1097891
ENSG00000164849	chromosome7	1063678
ENSG00000105835	chromosome7	105712567
ENSG00000149962	chromosome10	36853169
ENSG00000135164	chromosome7	86632194
ENSG00000125482	chromosome9	134268030
ENSG00000114631	chromosome3	128830753

ENSG00000128567	chromosome7	130891659
ENSG00000125844	chromosome20	17589153
ENSG00000126777	chromosome14	55148520
ENSG00000133392	chromosome16	15839611
ENSG00000100345	chromosome22	35075228
ENSG00000133026	chromosome17	8467290
ENSG00000105357	chromosome19	55405435
ENSG00000100345	chromosome22	35075228
ENSG00000133392	chromosome16	15839611
ENSG00000105357	chromosome19	55405435
ENSG00000133026	chromosome17	8467290
ENSG00000189045	chromosome5	74951884
ENSG00000166839	chromosome15	62991183
ENSG00000173302	chromosome2	131203195
ENSG00000122477	chromosome1	100406661
ENSG00000163827	chromosome3	46568086
ENSG00000197766	chromosome19	810690
ENSG00000185198	chromosome19	646431
ENSG00000113088	chromosome5	54355908
ENSG00000145649	chromosome5	54434268
ENSG00000100448	chromosome14	24115270
ENSG00000100450	chromosome14	24148660
ENSG00000100453	chromosome14	24173309
ENSG00000092009	chromosome14	24047282
ENSG00000197766	chromosome19	810690
ENSG00000185198	chromosome19	646431
ENSG00000113088	chromosome5	54355908
ENSG00000145649	chromosome5	54434268
ENSG00000100448	chromosome14	24115270
ENSG00000100450	chromosome14	24148660
ENSG00000100453	chromosome14	24173309
ENSG00000092009	chromosome14	24047282
ENSG00000113088	chromosome5	54355908
ENSG00000145649	chromosome5	54434268
ENSG00000197766	chromosome19	810690
ENSG00000185198	chromosome19	646431
ENSG00000185198	chromosome19	646431
ENSG00000197766	chromosome19	810690
ENSG00000165323	chromosome11	92213484
ENSG00000106701	chromosome9	107250323
ENSG00000105255	chromosome19	4255744
ENSG00000105255	chromosome19	4255744
ENSG00000106701	chromosome9	107250323
ENSG00000101871	chromosomeX	10495588
ENSG00000080561	chromosomeX	106970612
ENSG00000122482	chromosome1	91220499
ENSG00000011451	chromosome19	15420119
ENSG00000145192	chromosome3	187813625
ENSG00000090512	chromosome3	187840944
ENSG00000113905	chromosome3	187866515
ENSG00000145192	chromosome3	187813625
ENSG00000145192	chromosome3	187813625
ENSG00000090512	chromosome3	187840944
ENSG00000113905	chromosome3	187866515
ENSG00000177770	chromosome1	224749746
ENSG00000214740	chromosome2	26903893
ENSG00000145835	chromosome5	133775445
ENSG00000177770	chromosome1	224749746
ENSG00000214740	chromosome2	26903893

ENSG00000145835	chromosome5	133775445
ENSG00000168564	chromosome4	184602945
ENSG00000160282	chromosome21	46399866
ENSG00000169306	chromosomeX	28717382
ENSG00000189108	chromosomeX	103790251
ENSG00000104689	chromosome8	23138520
ENSG00000173530	chromosome8	23077394
ENSG00000173535	chromosome8	23016460
ENSG00000120889	chromosome8	22982353
ENSG00000215788	chromosome1	6448755
ENSG00000067182	chromosome12	6321242
ENSG00000026103	chromosome10	90739206
ENSG00000104689	chromosome8	23138520
ENSG00000173530	chromosome8	23077394
ENSG00000173535	chromosome8	23016460
ENSG00000120889	chromosome8	22982353
ENSG00000104689	chromosome8	23138520
ENSG00000173530	chromosome8	23077394
ENSG00000173535	chromosome8	23016460
ENSG00000120889	chromosome8	22982353
ENSG00000026103	chromosome10	90739206
ENSG00000015479	chromosome5	138671004
ENSG00000203867	chromosome10	112547256
ENSG00000075292	chromosome2	71429593
ENSG00000203867	chromosome10	112547256
ENSG00000015479	chromosome5	138671004
ENSG00000196358	chromosome9	134032040
ENSG00000162631	chromosome1	107492739
ENSG00000048545	chromosome6	42249330
ENSG00000138472	chromosome3	110155300
ENSG00000138472	chromosome3	110155300
ENSG00000048545	chromosome6	42249330
ENSG00000112599	chromosome6	42270537
ENSG00000112599	chromosome6	42270537
ENSG00000112599	chromosome6	42270537
ENSG00000048545	chromosome6	42249330
ENSG00000138472	chromosome3	110155300
ENSG00000205694	chromosome4	56971439
ENSG00000205850	chromosome13	23788142
ENSG00000205863	chromosome13	23369126
ENSG00000182447	chromosome3	162697290
ENSG00000182447	chromosome3	162697290
ENSG00000205850	chromosome13	23788142
ENSG00000205863	chromosome13	23369126
ENSG00000177764	chromosome20	226228
ENSG00000177764	chromosome20	226228
ENSG00000171451	chromosome18	63332856
ENSG00000111817	chromosome6	116827107
ENSG00000091583	chromosome17	61655960
ENSG00000117335	chromosome1	205992181
ENSG00000123838	chromosome1	205352994
ENSG00000197721	chromosome1	205909413
ENSG00000196352	chromosome1	205561734
ENSG00000091583	chromosome17	61655960
ENSG00000166710	chromosome15	42791037
ENSG00000133800	chromosome11	10546656
ENSG00000026508	chromosome11	35117427
ENSG00000123610	chromosome2	151922427
ENSG00000026508	chromosome11	35117427

ENSG00000133800	chromosome11	10546656
ENSG00000181408	chromosome17	77925490
ENSG00000181408	chromosome17	77925490
ENSG00000181408	chromosome17	77925490
ENSG00000178102	chromosome17	74078649
ENSG00000178102	chromosome17	74078649
ENSG00000112493	chromosome6	33389797
ENSG00000139192	chromosome12	6431676
ENSG00000139192	chromosome12	6431676
ENSG00000112493	chromosome6	33389797
ENSG00000139192	chromosome12	6431676
ENSG00000180269	chromosome16	19992440
ENSG00000180269	chromosome16	19992440
ENSG00000136305	chromosome14	23846905
ENSG00000176194	chromosome18	12244383
ENSG00000187288	chromosome3	9895171
ENSG00000176194	chromosome18	12244383
ENSG00000136305	chromosome14	23846905
ENSG00000153404	chromosome5	193375
ENSG00000149633	chromosome20	36322317
ENSG00000153404	chromosome5	193375
ENSG00000196155	chromosome16	65871449
ENSG00000165801	chromosome14	20608396
ENSG00000153404	chromosome5	193375
ENSG00000149633	chromosome20	36322317
ENSG00000196155	chromosome16	65871449
ENSG00000165801	chromosome14	20608396
ENSG00000165801	chromosome14	20608396
ENSG00000196155	chromosome16	65871449
ENSG00000170382	chromosome1	202855744
ENSG00000173114	chromosome7	110550065
ENSG00000175928	chromosome3	3861326
ENSG00000183014	chromosome19	2242824
ENSG00000174482	chromosome9	27940670
ENSG00000169783	chromosome15	75695286
ENSG00000174482	chromosome9	27940670
ENSG00000183014	chromosome19	2242824
ENSG00000128594	chromosome7	127457930
ENSG00000213171	chromosome1	150041805
ENSG00000183014	chromosome19	2242824
ENSG00000174482	chromosome9	27940670
ENSG00000169783	chromosome15	75695286
ENSG00000170382	chromosome1	202855744
ENSG00000173114	chromosome7	110550065
ENSG00000175928	chromosome3	3861326
ENSG00000173114	chromosome7	110550065
ENSG00000170382	chromosome1	202855744
ENSG00000128594	chromosome7	127457930
ENSG00000213171	chromosome1	150041805
ENSG00000183014	chromosome19	2242824
ENSG00000174482	chromosome9	27940670
ENSG00000169783	chromosome15	75695286
ENSG00000128594	chromosome7	127457930
ENSG00000169783	chromosome15	75695286
ENSG00000183014	chromosome19	2242824
ENSG00000174482	chromosome9	27940670
ENSG00000183014	chromosome19	2242824
ENSG00000174482	chromosome9	27940670
ENSG00000169783	chromosome15	75695286

ENSG00000213171	chromosome1	150041805
ENSG00000175928	chromosome3	3861326
ENSG00000170382	chromosome1	202855744
ENSG00000173114	chromosome7	110550065
ENSG00000069696	chromosome11	627305
ENSG00000149295	chromosome11	112800584
ENSG00000069696	chromosome11	627305
ENSG00000151577	chromosome3	115373530
ENSG00000149295	chromosome11	112800584
ENSG00000149295	chromosome11	112800584
ENSG00000151577	chromosome3	115373530
ENSG00000096264	chromosome6	41411593
ENSG00000095970	chromosome6	41238822
ENSG00000161649	chromosome17	39280111
ENSG00000186407	chromosome17	70131437
ENSG00000167850	chromosome17	70053517
ENSG00000167851	chromosome17	69974438
ENSG00000186074	chromosome17	70220601
ENSG00000178789	chromosome17	70039196
ENSG00000204345	chromosome17	70099937
ENSG00000162897	chromosome1	205210094
ENSG00000162896	chromosome1	205180500
ENSG00000161649	chromosome17	39280111
ENSG00000186407	chromosome17	70131437
ENSG00000167850	chromosome17	70053517
ENSG00000167851	chromosome17	69974438
ENSG00000186074	chromosome17	70220601
ENSG00000178789	chromosome17	70039196
ENSG00000204345	chromosome17	70099937
ENSG00000096264	chromosome6	41411593
ENSG00000095970	chromosome6	41238822
ENSG00000118137	chromosome11	116213405
ENSG00000215756	supercontigNT_113960	13733
ENSG00000110243	chromosome11	116167787
ENSG00000215754	supercontigNT_113960	27917
ENSG00000110244	chromosome11	116199118
ENSG00000130203	chromosome19	50101722
ENSG00000130203	chromosome19	50101722
ENSG00000118137	chromosome11	116213405
ENSG00000215756	supercontigNT_113960	13733
ENSG00000110243	chromosome11	116167787
ENSG00000215754	supercontigNT_113960	27917
ENSG00000110244	chromosome11	116199118
ENSG00000110243	chromosome11	116167787
ENSG00000215754	supercontigNT_113960	27917
ENSG00000110244	chromosome11	116199118
ENSG00000118137	chromosome11	116213405
ENSG00000215756	supercontigNT_113960	13733
ENSG00000091972	chromosome3	113534672
ENSG00000011465	chromosome12	90096461
ENSG00000182492	chromosomeX	152423284
ENSG00000106819	chromosome9	94277001
ENSG00000182492	chromosomeX	152423284
ENSG00000106819	chromosome9	94277001
ENSG00000011465	chromosome12	90096461
ENSG00000106819	chromosome9	94277001
ENSG00000182492	chromosomeX	152423284
ENSG00000011465	chromosome12	90096461
ENSG00000182492	chromosomeX	152423284

ENSG00000106819	chromosome9	94277001
ENSG00000139329	chromosome12	90026888
ENSG00000105737	chromosome19	47261763
ENSG00000178878	chromosome12	12770202
ENSG00000178878	chromosome12	12770202
ENSG00000128335	chromosome22	34959155
ENSG00000100342	chromosome22	34979955
ENSG00000100336	chromosome22	34928029
ENSG00000128284	chromosome22	34886886
ENSG00000128313	chromosome22	34443865
ENSG00000196785	chromosome22	34382419
ENSG00000178878	chromosome12	12770202
ENSG00000162763	chromosome1	163780158
ENSG00000183908	chromosome11	56705944
ENSG00000162494	chromosome1	13712746
ENSG00000183908	chromosome11	56705944
ENSG00000162494	chromosome1	13712746
ENSG00000162763	chromosome1	163780158
ENSG00000184709	chromosome9	139184217
ENSG00000162494	chromosome1	13712746
ENSG00000183908	chromosome11	56705944
ENSG00000118242	chromosome2	216586296
ENSG00000127418	chromosome4	996274
ENSG00000124006	chromosome2	220144199
ENSG00000124006	chromosome2	220144199
ENSG00000167791	chromosome11	67047374
ENSG00000167791	chromosome11	67047374
ENSG00000157782	chromosome12	119562872
ENSG00000105507	chromosome19	53238992
ENSG00000175544	chromosome11	66979471
ENSG00000157782	chromosome12	119562872
ENSG00000105507	chromosome19	53238992
ENSG00000167791	chromosome11	67047374
ENSG00000105507	chromosome19	53238992
ENSG00000157782	chromosome12	119562872
ENSG00000157782	chromosome12	119562872
ENSG00000105507	chromosome19	53238992
ENSG00000167791	chromosome11	67047374
ENSG00000175544	chromosome11	66979471
ENSG00000088827	chromosome20	3635776
ENSG00000012124	chromosome19	40514784
ENSG00000185664	chromosome12	54646063
ENSG00000136235	chromosome7	23253002
ENSG00000108702	chromosome17	29714294
ENSG00000172156	chromosome17	29636941
ENSG00000108688	chromosome17	29621393
ENSG00000108691	chromosome17	29606482
ENSG00000181374	chromosome17	29707659
ENSG00000108700	chromosome17	29670604
ENSG00000006074	chromosome17	31415816
ENSG00000215629	supercontigNT_113936	14179
ENSG00000205017	chromosome17	31649760
ENSG00000205021	chromosome17	31548186
ENSG00000006075	chromosome17	31441518
ENSG00000167236	chromosome17	31369048
ENSG00000161574	chromosome17	31352645
ENSG00000213494	chromosome17	31337799
ENSG00000205020	chromosome17	31562657
ENSG00000215627	supercontigNT_113936	28631

ENSG00000197262	chromosome17	31664223
ENSG00000129277	chromosome17	31455412
ENSG00000161570	chromosome17	31231423
ENSG00000161573	chromosome17	31332570
ENSG00000106178	chromosome7	75280970
ENSG00000006606	chromosome7	75239431
ENSG00000115009	chromosome2	228386872
ENSG00000102970	chromosome16	56005346
ENSG00000102962	chromosome16	55950230
ENSG00000006074	chromosome17	31415816
ENSG00000215629	supercontigNT_113936	14179
ENSG00000205017	chromosome17	31649760
ENSG00000205021	chromosome17	31548186
ENSG00000006075	chromosome17	31441518
ENSG00000167236	chromosome17	31369048
ENSG00000161574	chromosome17	31352645
ENSG00000213494	chromosome17	31337799
ENSG00000205020	chromosome17	31562657
ENSG00000215627	supercontigNT_113936	28631
ENSG00000197262	chromosome17	31664223
ENSG00000129277	chromosome17	31455412
ENSG00000161570	chromosome17	31231423
ENSG00000161573	chromosome17	31332570
ENSG00000106178	chromosome7	75280970
ENSG00000006606	chromosome7	75239431
ENSG00000172156	chromosome17	29636941
ENSG00000108688	chromosome17	29621393
ENSG00000108691	chromosome17	29606482
ENSG00000181374	chromosome17	29707659
ENSG00000108700	chromosome17	29670604
ENSG00000172724	chromosome9	34681137
ENSG00000161570	chromosome17	31231423
ENSG00000006074	chromosome17	31415816
ENSG00000215629	supercontigNT_113936	14179
ENSG00000205017	chromosome17	31649760
ENSG00000205021	chromosome17	31548186
ENSG00000006075	chromosome17	31441518
ENSG00000167236	chromosome17	31369048
ENSG00000161574	chromosome17	31352645
ENSG00000213494	chromosome17	31337799
ENSG00000205020	chromosome17	31562657
ENSG00000215627	supercontigNT_113936	28631
ENSG00000197262	chromosome17	31664223
ENSG00000129277	chromosome17	31455412
ENSG00000172724	chromosome9	34681137
ENSG00000143184	chromosome1	166812500
ENSG00000143185	chromosome1	166779827
ENSG00000102970	chromosome16	56005346
ENSG00000102962	chromosome16	55950230
ENSG00000115009	chromosome2	228386872
ENSG00000172156	chromosome17	29636941
ENSG00000108688	chromosome17	29621393
ENSG00000108691	chromosome17	29606482
ENSG00000181374	chromosome17	29707659
ENSG00000108700	chromosome17	29670604
ENSG00000006074	chromosome17	31415816
ENSG00000215629	supercontigNT_113936	14179
ENSG00000205017	chromosome17	31649760
ENSG00000205021	chromosome17	31548186

ENSG00000006075	chromosome17	31441518
ENSG00000167236	chromosome17	31369048
ENSG00000161574	chromosome17	31352645
ENSG00000213494	chromosome17	31337799
ENSG00000205020	chromosome17	31562657
ENSG00000215627	supercontigNT_113936	28631
ENSG00000197262	chromosome17	31664223
ENSG00000129277	chromosome17	31455412
ENSG00000161570	chromosome17	31231423
ENSG00000161573	chromosome17	31332570
ENSG00000106178	chromosome7	75280970
ENSG00000006606	chromosome7	75239431
ENSG00000108702	chromosome17	29714294
ENSG00000143184	chromosome1	166812500
ENSG00000143185	chromosome1	166779827
ENSG00000102970	chromosome16	56005346
ENSG00000102962	chromosome16	55950230
ENSG00000115009	chromosome2	228386872
ENSG00000172724	chromosome9	34681137
ENSG00000108702	chromosome17	29714294
ENSG00000161573	chromosome17	31332570
ENSG00000106178	chromosome7	75280970
ENSG00000006606	chromosome7	75239431
ENSG00000006074	chromosome17	31415816
ENSG00000215629	supercontigNT_113936	14179
ENSG00000205017	chromosome17	31649760
ENSG00000205021	chromosome17	31548186
ENSG00000006075	chromosome17	31441518
ENSG00000167236	chromosome17	31369048
ENSG00000161574	chromosome17	31352645
ENSG00000213494	chromosome17	31337799
ENSG00000205020	chromosome17	31562657
ENSG00000215627	supercontigNT_113936	28631
ENSG00000197262	chromosome17	31664223
ENSG00000129277	chromosome17	31455412
ENSG00000161570	chromosome17	31231423
ENSG00000102970	chromosome16	56005346
ENSG00000102962	chromosome16	55950230
ENSG00000115009	chromosome2	228386872
ENSG00000143184	chromosome1	166812500
ENSG00000143185	chromosome1	166779827
ENSG00000206013	chromosome11	289491
ENSG00000185885	chromosome11	301317
ENSG00000215096	chromosome8	64484177
ENSG00000142089	chromosome11	310814
ENSG00000185201	chromosome11	298193
ENSG00000188082	chromosome11	1728249
ENSG00000185885	chromosome11	301317
ENSG00000215096	chromosome8	64484177
ENSG00000142089	chromosome11	310814
ENSG00000185201	chromosome11	298193
ENSG00000188082	chromosome11	1728249
ENSG00000206013	chromosome11	289491
ENSG00000185885	chromosome11	301317
ENSG00000215096	chromosome8	64484177
ENSG00000142089	chromosome11	310814
ENSG00000185201	chromosome11	298193
ENSG00000188082	chromosome11	1728249
ENSG00000206013	chromosome11	289491

ENSG00000188082	chromosome11	1728249
ENSG00000185885	chromosome11	301317
ENSG00000215096	chromosome8	64484177
ENSG00000142089	chromosome11	310814
ENSG00000185201	chromosome11	298193
ENSG00000072694	chromosome1	159899656
ENSG00000143226	chromosome1	159741882
ENSG00000162747	chromosome1	159867617
ENSG00000203747	chromosome1	159786259
ENSG00000179639	chromosome1	157538770
ENSG00000150337	chromosome1	148020924
ENSG00000203820	chromosome1	147635968
ENSG00000198019	chromosome1	120737418
ENSG00000132185	chromosome1	159943628
ENSG00000072694	chromosome1	159899656
ENSG00000143226	chromosome1	159741882
ENSG00000162747	chromosome1	159867617
ENSG00000203747	chromosome1	159786259
ENSG00000179639	chromosome1	157538770
ENSG00000150337	chromosome1	148020924
ENSG00000203820	chromosome1	147635968
ENSG00000198019	chromosome1	120737418
ENSG00000132185	chromosome1	159943628
ENSG00000181036	chromosome1	158037012
ENSG00000072694	chromosome1	159899656
ENSG00000143226	chromosome1	159741882
ENSG00000162747	chromosome1	159867617
ENSG00000203747	chromosome1	159786259
ENSG00000179639	chromosome1	157538770
ENSG00000150337	chromosome1	148020924
ENSG00000203820	chromosome1	147635968
ENSG00000198019	chromosome1	120737418
ENSG00000132185	chromosome1	159943628
ENSG00000072694	chromosome1	159899656
ENSG00000143226	chromosome1	159741882
ENSG00000162747	chromosome1	159867617
ENSG00000203747	chromosome1	159786259
ENSG00000179639	chromosome1	157538770
ENSG00000150337	chromosome1	148020924
ENSG00000203820	chromosome1	147635968
ENSG00000198019	chromosome1	120737418
ENSG00000132185	chromosome1	159943628
ENSG00000181036	chromosome1	158037012
ENSG00000072694	chromosome1	159899656
ENSG00000143226	chromosome1	159741882
ENSG00000162747	chromosome1	159867617
ENSG00000203747	chromosome1	159786259
ENSG00000179639	chromosome1	157538770
ENSG00000150337	chromosome1	148020924
ENSG00000203820	chromosome1	147635968
ENSG00000198019	chromosome1	120737418
ENSG00000132185	chromosome1	159943628
ENSG00000136155	chromosome13	77028012
ENSG00000147394	chromosomeX	151837329
ENSG00000179300	chromosomeX	77800574
ENSG00000187823	chromosomeX	111584613
ENSG00000179300	chromosomeX	77800574
ENSG00000187823	chromosomeX	111584613
ENSG00000179300	chromosomeX	77800574

ENSG00000156738	chromosome11	59986424
ENSG00000166926	chromosome11	59858945
ENSG00000110077	chromosome11	59705777
ENSG00000166927	chromosome11	59907191
ENSG00000149516	chromosome11	59585210
ENSG00000110079	chromosome11	59804725
ENSG00000214787	chromosome11	59754099
ENSG00000166930	chromosome11	59953724
ENSG00000149534	chromosome11	59612815
ENSG00000071203	chromosome11	60021368
ENSG00000166959	chromosome11	60224910
ENSG00000166961	chromosome11	60291636
ENSG00000166926	chromosome11	59858945
ENSG00000110077	chromosome11	59705777
ENSG00000166927	chromosome11	59907191
ENSG00000149516	chromosome11	59585210
ENSG00000110079	chromosome11	59804725
ENSG00000214787	chromosome11	59754099
ENSG00000166930	chromosome11	59953724
ENSG00000149534	chromosome11	59612815
ENSG00000071203	chromosome11	60021368
ENSG00000166959	chromosome11	60224910
ENSG00000166961	chromosome11	60291636
ENSG00000156738	chromosome11	59986424
ENSG00000178209	chromosome8	145121526
ENSG00000165959	chromosome14	94855883
ENSG00000178209	chromosome8	145121526
ENSG00000162804	chromosome2	241622721
ENSG00000162804	chromosome2	241622721
ENSG00000205365	chromosome11	6693807
ENSG00000187878	chromosome11	6697158
ENSG00000106608	chromosome7	43894155
ENSG00000132357	chromosome5	40877242
ENSG00000106608	chromosome7	43894155
ENSG00000132357	chromosome5	40877242
ENSG00000205365	chromosome11	6693807
ENSG00000106608	chromosome7	43894155
ENSG00000132357	chromosome5	40877242
ENSG00000187878	chromosome11	6697158
ENSG00000169994	chromosome2	128038198
ENSG00000137474	chromosome11	76531241
ENSG00000130702	chromosome20	60375697
ENSG00000053747	chromosome18	19523646
ENSG00000053747	chromosome18	19523646
ENSG00000130702	chromosome20	60375697
ENSG00000053747	chromosome18	19523646
ENSG00000130702	chromosome20	60375697
ENSG00000112769	chromosome6	112682046
ENSG00000196569	chromosome6	129246084
ENSG00000101680	chromosome18	7107720
ENSG00000063169	chromosome19	52874394
ENSG00000112624	chromosome6	42897739
ENSG00000211898	chromosome14	105383056
ENSG00000211675	chromosome22	21567555
ENSG00000211677	chromosome22	21573156
ENSG00000211679	chromosome22	21578512
ENSG00000211685	chromosome22	21594766
ENSG00000211683	chromosome22	21591707
ENSG00000128322	chromosome22	22252378

ENSG00000206066	chromosome22	24043988
ENSG00000211592	chromosome2	88938312
ENSG00000211592	chromosome2	88938312
ENSG00000211675	chromosome22	21567555
ENSG00000211677	chromosome22	21573156
ENSG00000211679	chromosome22	21578512
ENSG00000211685	chromosome22	21594766
ENSG00000211683	chromosome22	21591707
ENSG00000128322	chromosome22	22252378
ENSG00000206066	chromosome22	24043988
ENSG00000211675	chromosome22	21567555
ENSG00000211677	chromosome22	21573156
ENSG00000211679	chromosome22	21578512
ENSG00000211685	chromosome22	21594766
ENSG00000211683	chromosome22	21591707
ENSG00000128322	chromosome22	22252378
ENSG00000206066	chromosome22	24043988
ENSG00000211592	chromosome2	88938312
ENSG00000211675	chromosome22	21567555
ENSG00000211677	chromosome22	21573156
ENSG00000211679	chromosome22	21578512
ENSG00000211685	chromosome22	21594766
ENSG00000211683	chromosome22	21591707
ENSG00000128322	chromosome22	22252378
ENSG00000206066	chromosome22	24043988
ENSG00000147180	chromosomeX	84389235
ENSG00000005889	chromosomeX	24100781
ENSG00000067646	chromosomeY	172458
ENSG00000121864	chromosome3	180528776
ENSG00000187775	chromosome17	73970725
ENSG00000140948	chromosome16	86082935
ENSG00000141664	chromosome18	58342475
ENSG00000153347	chromosome5	94752850
ENSG00000157470	chromosome15	57538127
ENSG00000181722	chromosome3	115582325
ENSG00000119574	chromosome19	63720853
ENSG00000204924	chromosome19	48808093
ENSG00000167978	chromosome16	2746367
ENSG00000139767	chromosome12	117903780
ENSG00000177679	chromosome7	75702321
ENSG00000139767	chromosome12	117903780
ENSG00000177679	chromosome7	75702321
ENSG00000204924	chromosome19	48808093
ENSG00000167978	chromosome16	2746367
ENSG00000177679	chromosome7	75702321
ENSG00000139767	chromosome12	117903780
ENSG00000164841	chromosome8	109866504
ENSG00000125895	chromosome20	1112446
ENSG00000174749	chromosome4	113286186
ENSG00000171224	chromosome10	71060399
ENSG00000176244	chromosome10	15170734
ENSG00000155368	chromosome2	119841098
ENSG00000177683	chromosome7	107992573
ENSG00000174796	chromosome4	76659687
ENSG00000184436	chromosome22	19686201
ENSG00000184436	chromosome22	19686201
ENSG00000174796	chromosome4	76659687
ENSG00000141371	chromosome17	55854652
ENSG00000118972	chromosome12	4359010

ENSG00000105550	chromosome19	53951306
ENSG00000162344	chromosome11	69227826
ENSG00000162344	chromosome11	69227826
ENSG00000105550	chromosome19	53951306
ENSG00000137440	chromosome4	15547354
ENSG00000137441	chromosome4	15573851
ENSG00000174721	chromosome10	93658707
ENSG00000137441	chromosome4	15573851
ENSG00000137440	chromosome4	15547354
ENSG00000198851	chromosome11	117680878
ENSG00000160654	chromosome11	117720349
ENSG00000167286	chromosome11	117718533
ENSG00000173681	chromosomeX	19898304
ENSG00000029363	chromosome6	136642698
ENSG00000054118	chromosome1	36520752
ENSG00000054118	chromosome1	36520752
ENSG00000029363	chromosome6	136642698
ENSG00000172568	chromosome5	156703123
ENSG00000171282	chromosome17	77025447
ENSG00000182095	chromosome7	5377385
ENSG00000182095	chromosome7	5377385
ENSG00000171282	chromosome17	77025447
ENSG00000007312	chromosome17	59363354
ENSG00000105369	chromosome19	47073215
ENSG00000183145	chromosome21	37300943
ENSG00000147223	chromosomeX	106033222
ENSG00000203877	chromosome6	84619855
ENSG00000203877	chromosome6	84619855
ENSG00000147223	chromosomeX	106033222
ENSG00000102891	chromosome16	55156542
ENSG00000205362	chromosome16	55230152
ENSG00000125144	chromosome16	55259407
ENSG00000125148	chromosome16	55200069
ENSG00000205358	chromosome16	55261298
ENSG00000169688	chromosome16	55243367
ENSG00000198417	chromosome16	55249472
ENSG00000205364	chromosome16	55224145
ENSG00000169715	chromosome16	55217265
ENSG00000187193	chromosome16	55267603
ENSG00000205360	chromosome16	55239661
ENSG00000087250	chromosome16	55181004
ENSG00000163946	chromosome3	56670058
ENSG00000183036	chromosome21	40161298
ENSG00000173126	chromosome1	159520073
ENSG00000176919	chromosome9	138959594
ENSG00000176919	chromosome9	138959594
ENSG00000184925	chromosome9	138966601
ENSG00000148346	chromosome9	129951626
ENSG00000107317	chromosome9	138991852
ENSG00000187922	chromosome9	138757177
ENSG00000214402	chromosome9	138997339
ENSG00000177984	chromosome9	138778762
ENSG00000187922	chromosome9	138757177
ENSG00000214402	chromosome9	138997339
ENSG00000177984	chromosome9	138778762
ENSG00000184925	chromosome9	138966601
ENSG00000148346	chromosome9	129951626
ENSG00000107317	chromosome9	138991852
ENSG00000113249	chromosome5	156417533

ENSG00000145850	chromosome5	156322788
ENSG00000113249	chromosome5	156417533
ENSG00000145850	chromosome5	156322788
ENSG00000135077	chromosome5	156468573
ENSG00000164136	chromosome4	142845215
ENSG00000105321	chromosome19	52453498
ENSG00000188549	chromosome15	38420445
ENSG00000102760	chromosome13	40929844
ENSG00000132000	chromosome19	13910016
ENSG00000174348	chromosome1	53300480
ENSG00000174348	chromosome1	53300480
ENSG00000132000	chromosome19	13910016
ENSG00000006042	chromosome17	28282660
ENSG00000166946	chromosome15	41264989
ENSG00000198133	chromosome14	67010394
ENSG00000198133	chromosome14	67010394
ENSG00000120071	chromosome17	41605287
ENSG00000144445	chromosome2	210727552
ENSG00000101955	chromosomeX	37965641
ENSG00000102359	chromosomeX	99787976
ENSG00000215182	chromosome11	1152663
ENSG00000117400	chromosome1	43576107
ENSG00000187266	chromosome19	11355884
ENSG00000168685	chromosome5	35892837
ENSG00000187266	chromosome19	11355884
ENSG00000117400	chromosome1	43576107
ENSG00000186998	chromosome22	27932028
ENSG00000160963	chromosome7	100793034
ENSG00000211774	chromosome14	21159897
ENSG00000211775	chromosome14	21181033
ENSG00000211799	chromosome14	21545708
ENSG00000211792	chromosome14	21462154
ENSG00000211816	chromosome14	21809694
ENSG00000211817	chromosome14	21818874
ENSG00000211804	chromosome14	21634167
ENSG00000211774	chromosome14	21159897
ENSG00000211775	chromosome14	21181033
ENSG00000211799	chromosome14	21545708
ENSG00000211792	chromosome14	21462154
ENSG00000211816	chromosome14	21809694
ENSG00000211817	chromosome14	21818874
ENSG00000211804	chromosome14	21634167
ENSG00000211776	chromosome14	21250394
ENSG00000211782	chromosome14	21335431
ENSG00000211787	chromosome14	21390575
ENSG00000211808	chromosome14	21670355
ENSG00000211786	chromosome14	21384784
ENSG00000211790	chromosome14	21432581
ENSG00000211795	chromosome14	21516759
ENSG00000211777	chromosome14	21261976
ENSG00000211796	chromosome14	21528575
ENSG00000211798	chromosome14	21541290
ENSG00000211819	chromosome14	21852762
ENSG00000211783	chromosome14	21349514
ENSG00000211793	chromosome14	21479209
ENSG00000211830	chromosome14	22008447
ENSG00000211695	chromosome7	38323619
ENSG00000211774	chromosome14	21159897
ENSG00000211775	chromosome14	21181033

ENSG00000211799	chromosome14	21545708
ENSG00000211792	chromosome14	21462154
ENSG00000211816	chromosome14	21809694
ENSG00000211817	chromosome14	21818874
ENSG00000211804	chromosome14	21634167
ENSG00000211776	chromosome14	21250394
ENSG00000211782	chromosome14	21335431
ENSG00000211787	chromosome14	21390575
ENSG00000211808	chromosome14	21670355
ENSG00000211786	chromosome14	21384784
ENSG00000211790	chromosome14	21432581
ENSG00000211795	chromosome14	21516759
ENSG00000211777	chromosome14	21261976
ENSG00000211796	chromosome14	21528575
ENSG00000211798	chromosome14	21541290
ENSG00000211819	chromosome14	21852762
ENSG00000211783	chromosome14	21349514
ENSG00000211793	chromosome14	21479209
ENSG00000211830	chromosome14	22008447
ENSG00000211776	chromosome14	21250394
ENSG00000211782	chromosome14	21335431
ENSG00000211787	chromosome14	21390575
ENSG00000211808	chromosome14	21670355
ENSG00000211786	chromosome14	21384784
ENSG00000211790	chromosome14	21432581
ENSG00000211795	chromosome14	21516759
ENSG00000211777	chromosome14	21261976
ENSG00000211796	chromosome14	21528575
ENSG00000211798	chromosome14	21541290
ENSG00000211819	chromosome14	21852762
ENSG00000211783	chromosome14	21349514
ENSG00000211793	chromosome14	21479209
ENSG00000211774	chromosome14	21159897
ENSG00000211775	chromosome14	21181033
ENSG00000211799	chromosome14	21545708
ENSG00000211792	chromosome14	21462154
ENSG00000211816	chromosome14	21809694
ENSG00000211817	chromosome14	21818874
ENSG00000211804	chromosome14	21634167
ENSG00000211830	chromosome14	22008447
ENSG00000211774	chromosome14	21159897
ENSG00000211775	chromosome14	21181033
ENSG00000211799	chromosome14	21545708
ENSG00000211792	chromosome14	21462154
ENSG00000211816	chromosome14	21809694
ENSG00000211817	chromosome14	21818874
ENSG00000211804	chromosome14	21634167
ENSG00000211776	chromosome14	21250394
ENSG00000211782	chromosome14	21335431
ENSG00000211787	chromosome14	21390575
ENSG00000211808	chromosome14	21670355
ENSG00000211786	chromosome14	21384784
ENSG00000211790	chromosome14	21432581
ENSG00000211795	chromosome14	21516759
ENSG00000211777	chromosome14	21261976
ENSG00000211796	chromosome14	21528575
ENSG00000211798	chromosome14	21541290
ENSG00000211819	chromosome14	21852762
ENSG00000211783	chromosome14	21349514

ENSG00000211793	chromosome14	21479209
ENSG00000186834	chromosome17	40582341
ENSG00000168517	chromosome17	40595944
ENSG00000196684	chromosome19	16115543
ENSG00000027869	chromosome1	155053125
ENSG00000178217	chromosome10	82288068
ENSG00000196684	chromosome19	16115543
ENSG00000027869	chromosome1	155053125
ENSG00000104611	chromosome8	19221339
ENSG00000178217	chromosome10	82288068
ENSG00000178217	chromosome10	82288068
ENSG00000104611	chromosome8	19221339
ENSG00000027869	chromosome1	155053125
ENSG00000196684	chromosome19	16115543
ENSG00000184454	chromosome1	24794509
ENSG00000163867	chromosome1	35268828
ENSG00000146463	chromosome1	35507235
ENSG00000147130	chromosomeX	70389831
ENSG00000121741	chromosome13	19465213
ENSG00000132950	chromosome13	19324321
ENSG00000198218	chromosome3	49089455
ENSG00000147130	chromosomeX	70389831
ENSG00000121741	chromosome13	19465213
ENSG00000132950	chromosome13	19324321
ENSG00000163867	chromosome1	35268828
ENSG00000146463	chromosome1	35507235
ENSG00000121741	chromosome13	19465213
ENSG00000132950	chromosome13	19324321
ENSG00000147130	chromosomeX	70389831
ENSG00000198218	chromosome3	49089455
ENSG00000082074	chromosome5	39238820
ENSG00000133246	chromosome19	8473476
ENSG00000187889	chromosome1	57057552
ENSG00000186198	chromosome15	63129396
ENSG00000136732	chromosome2	127130310
ENSG00000136732	chromosome2	127130310
ENSG00000169258	chromosome5	175959442
ENSG00000185477	chromosome4	90390285
ENSG00000204175	chromosome10	46418887
ENSG00000185477	chromosome4	90390285
ENSG00000169258	chromosome5	175959442
ENSG00000159314	chromosome17	40863429
ENSG00000069702	chromosome1	92099677
ENSG00000106991	chromosome9	129656456
ENSG00000179456	chromosome1	242281357
ENSG00000214438	chromosome14	104338553
ENSG00000179456	chromosome1	242281357
ENSG00000100399	chromosome22	39966832
ENSG00000188937	chromosomeX	41192087
ENSG00000214475	chromosome3	98016440
ENSG00000214475	chromosome3	98016440
ENSG00000214475	chromosome3	98016440
ENSG00000214475	chromosome3	98016440
ENSG00000214475	chromosome3	98016440
ENSG00000214475	chromosome3	98016440
ENSG00000100385	chromosome22	35870159
ENSG00000103522	chromosome16	27348894
ENSG00000124334	chromosomeX	154880603
ENSG00000215303	chromosome10	122225

ENSG00000103522	chromosome16	27348894
ENSG00000100385	chromosome22	35870159
ENSG00000165526	chromosome11	125586744
ENSG00000156990	chromosome3	9860699
ENSG00000214686	chromosome3	51788541
ENSG00000173389	chromosome3	51912329
ENSG00000214681	chromosome3	51886182
ENSG00000184345	chromosome3	51870723
ENSG00000206115	chromosome3	170582017
ENSG00000206115	chromosome3	170582017
ENSG00000126856	chromosome16	88656094
ENSG00000184935	chromosome15	40655206
ENSG00000184935	chromosome15	40655206
ENSG00000145623	chromosome5	38904904
ENSG00000113594	chromosome5	38566507
ENSG00000104998	chromosome19	14003685
ENSG00000119535	chromosome1	36717685
ENSG00000164509	chromosome5	55183156
ENSG00000134352	chromosome5	55307864
ENSG00000081985	chromosome1	67558640
ENSG00000164509	chromosome5	55183156
ENSG00000134352	chromosome5	55307864
ENSG00000119535	chromosome1	36717685
ENSG00000081985	chromosome1	67558640
ENSG00000119535	chromosome1	36717685
ENSG00000164509	chromosome5	55183156
ENSG00000134352	chromosome5	55307864
ENSG00000134352	chromosome5	55307864
ENSG00000164509	chromosome5	55183156
ENSG00000119535	chromosome1	36717685
ENSG00000164509	chromosome5	55183156
ENSG00000134352	chromosome5	55307864
ENSG00000081985	chromosome1	67558640
ENSG00000104998	chromosome19	14003685
ENSG00000184516	chromosome1	243596894
ENSG00000188517	chromosome4	110442625
ENSG00000050767	chromosome5	177949805
ENSG00000050767	chromosome5	177949805
ENSG00000188517	chromosome4	110442625
ENSG00000197467	chromosome10	71232186
ENSG00000176907	chromosome8	40130209
ENSG00000109255	chromosome4	56197117
ENSG00000204640	chromosome2	100453383
ENSG00000171533	chromosome11	75057063
ENSG00000180834	chromosome3	185026030
ENSG00000105939	chromosome7	138444618
ENSG00000146858	chromosome7	138371304
ENSG00000105939	chromosome7	138444618
ENSG00000146858	chromosome7	138371304
ENSG00000059378	chromosome7	139409117
ENSG00000111224	chromosome12	3809443
ENSG00000105939	chromosome7	138444618
ENSG00000146858	chromosome7	138371304
ENSG00000111224	chromosome12	3809443
ENSG00000111224	chromosome12	3809443
ENSG00000059378	chromosome7	139409117
ENSG00000186369	chromosome14	61664288
ENSG00000141979	chromosome19	16467941
ENSG00000179218	chromosome19	12910494

ENSG00000179218	chromosome19	12910494
ENSG00000141979	chromosome19	16467941
ENSG00000099364	chromosome16	30844577
ENSG00000186352	chromosome4	186555072
ENSG00000179766	chromosome9	35434401
ENSG00000205351	chromosome17	10647979
ENSG00000205351	chromosome17	10647979
ENSG00000205795	chromosome2	10137802
ENSG00000205795	chromosome2	10137802
ENSG00000196535	chromosome17	24518085
ENSG00000133454	chromosome22	24487060
ENSG00000197025	chromosome19	61580428
ENSG00000184294	chromosome19	63522443
ENSG00000169740	chromosome10	43461588
ENSG00000197008	chromosome7	63913430
ENSG00000213976	chromosome19	21630059
ENSG00000213980	chromosome19	21358699
ENSG00000215180	chromosome8	47902840
ENSG00000215510	chromosomeY	22832468
ENSG00000215537	chromosomeY	22347167
ENSG00000215598	chromosomeY	5638422
ENSG00000215600	chromosomeY	5569473
ENSG00000142396	chromosome19	58165447
ENSG00000186777	chromosome4	255717
ENSG00000198155	chromosome4	237906
ENSG00000204756	chromosome8	145903923
ENSG00000186446	chromosome3	44750918
ENSG00000174255	chromosome3	115438612
ENSG00000197062	chromosome6	28348468
ENSG00000197332	chromosome19	11657042
ENSG00000179195	chromosome12	123062645
ENSG00000179965	chromosome16	30326876
ENSG00000179965	chromosome16	30326876
ENSG00000095932	chromosome19	3434402
ENSG00000198697	chromosome2	97907279
ENSG00000137501	chromosome11	85146417
ENSG00000142765	chromosome1	27544413
ENSG00000142765	chromosome1	27544413
ENSG00000102362	chromosomeX	99843690
ENSG00000147041	chromosomeX	37778087
ENSG00000142765	chromosome1	27544413
ENSG00000137501	chromosome11	85146417
ENSG00000147041	chromosomeX	37778087
ENSG00000102362	chromosomeX	99843690
ENSG00000183812	chromosome9	45332710
ENSG00000197833	chromosome9	46280273
ENSG00000204806	chromosome9	46004060
ENSG00000205613	chromosome7	57886449
ENSG00000204808	chromosome7	57714170
ENSG00000204805	chromosome9	46276999
ENSG00000204807	chromosome9	45623584
ENSG00000215061	chromosome9	45617849
ENSG00000172264	chromosome20	13924410
ENSG00000133315	chromosome11	63690043
ENSG00000133315	chromosome11	63690043
ENSG00000172264	chromosome20	13924410
ENSG00000184968	chromosome16	65141086
ENSG00000205066	chromosome8	104327947
ENSG00000182257	chromosome22	44828638

ENSG00000198230	chromosome11	117405789
ENSG00000197791	chromosome2	201742765
ENSG00000197382	chromosome21	24783141
ENSG00000178769	chromosome20	5404722
ENSG00000163820	chromosome3	46001407
ENSG00000188282	chromosome2	218639930
ENSG00000184635	chromosome19	19806955
ENSG00000205245	chromosome19	23806924
ENSG00000213799	chromosome19	58399078
ENSG00000176584	chromosome10	124529587
ENSG00000176584	chromosome10	124529587
ENSG00000176584	chromosome10	124529587
ENSG00000128656	chromosome2	175493978
ENSG00000197412	chromosome7	39615672
ENSG00000187605	chromosome2	74127906
ENSG00000174792	chromosome4	76700317
ENSG00000187605	chromosome2	74127906
ENSG00000187605	chromosome2	74127906
ENSG00000174792	chromosome4	76700317
ENSG00000205699	chromosome10	1372283
ENSG00000179349	chromosome4	75242950
ENSG00000116525	chromosome1	33419621
ENSG00000185880	chromosome15	42816195
ENSG00000155428	chromosome7	72074625
ENSG00000178809	chromosome7	74866154
ENSG00000146755	chromosome7	72376722
ENSG00000177238	chromosome16	31133561
ENSG00000116525	chromosome1	33419621
ENSG00000185880	chromosome15	42816195
ENSG00000116525	chromosome1	33419621
ENSG00000119917	chromosome10	91077811
ENSG00000119922	chromosome10	91051861
ENSG00000204010	chromosome10	91127873
ENSG00000185745	chromosome10	91142450
ENSG00000152778	chromosome10	91164536
ENSG00000100300	chromosome22	41885189
ENSG00000112212	chromosome6	41118703
ENSG00000160916	chromosome7	98901701
ENSG00000186390	chromosome9	78845152
ENSG00000122863	chromosome10	73435607
ENSG00000175264	chromosome11	45629050
ENSG00000183196	chromosome16	74071228
ENSG00000135702	chromosome16	74121784
ENSG00000140835	chromosome16	70128082
ENSG00000175040	chromosome3	144322349
ENSG00000147119	chromosomeX	46318311
ENSG00000147119	chromosomeX	46318311
ENSG00000175040	chromosome3	144322349
ENSG00000175264	chromosome11	45629050
ENSG00000175264	chromosome11	45629050
ENSG00000122863	chromosome10	73435607
ENSG00000175040	chromosome3	144322349
ENSG00000147119	chromosomeX	46318311
ENSG00000183196	chromosome16	74071228
ENSG00000135702	chromosome16	74121784
ENSG00000140835	chromosome16	70128082
ENSG00000183196	chromosome16	74071228
ENSG00000135702	chromosome16	74121784
ENSG00000140835	chromosome16	70128082

ENSG00000181031	chromosome17	177335
ENSG00000148840	chromosome10	103882816
ENSG00000155846	chromosome5	149090099
ENSG00000109819	chromosome4	23500679
ENSG00000148840	chromosome10	103882816
ENSG00000109819	chromosome4	23500679
ENSG00000155846	chromosome5	149090099
ENSG00000180263	chromosome12	94135148
ENSG00000154783	chromosome3	14835583
ENSG00000154783	chromosome3	14835583
ENSG00000180263	chromosome12	94135148
ENSG00000102302	chromosomeX	54538591
ENSG00000170099	chromosome14	93850739
ENSG00000140093	chromosome14	93826684
ENSG00000099937	chromosome22	19463601
ENSG00000140093	chromosome14	93826684
ENSG00000170099	chromosome14	93850739
ENSG00000183918	chromosomeX	123308174
ENSG00000198574	chromosome1	160648431
ENSG00000146399	chromosome6	133008836
ENSG00000146378	chromosome6	132987108
ENSG00000146399	chromosome6	133008836
ENSG00000146378	chromosome6	132987108
ENSG00000146399	chromosome6	133008836
ENSG00000146383	chromosome6	132933154
ENSG00000146385	chromosome6	132915525
ENSG00000135569	chromosome6	132952916
ENSG00000153468	chromosome6	167504209
ENSG00000151849	chromosome13	24385164
ENSG00000187876	chromosome2	174910398
ENSG00000204235	chromosome11	134360603
ENSG00000204707	chromosome19	54695593
ENSG00000205461	chromosome14	100381654
ENSG00000187876	chromosome2	174910398
ENSG00000204235	chromosome11	134360603
ENSG00000204707	chromosome19	54695593
ENSG00000205461	chromosome14	100381654
ENSG00000138347	chromosome10	69536488
ENSG00000129116	chromosome4	169669231
ENSG00000138347	chromosome10	69536488
ENSG00000120729	chromosome5	137234240
ENSG00000138347	chromosome10	69536488
ENSG00000129116	chromosome4	169669231
ENSG00000165951	chromosome14	93902789
ENSG00000197249	chromosome14	93919328
ENSG00000187483	chromosome14	94177408
ENSG00000186910	chromosome14	93984865
ENSG00000170054	chromosome14	94012263
ENSG00000123561	chromosomeX	105167706
ENSG00000100665	chromosome14	94099573
ENSG00000196136	chromosome14	94148471
ENSG00000188488	chromosome14	94123453
ENSG00000165953	chromosome14	94034488
ENSG00000135744	chromosome1	228913220
ENSG00000165951	chromosome14	93902789
ENSG00000197249	chromosome14	93919328
ENSG00000187483	chromosome14	94177408
ENSG00000186910	chromosome14	93984865
ENSG00000170054	chromosome14	94012263

ENSG00000123561	chromosomeX	105167706
ENSG00000100665	chromosome14	94099573
ENSG00000196136	chromosome14	94148471
ENSG00000188488	chromosome14	94123453
ENSG00000165953	chromosome14	94034488
ENSG00000165951	chromosome14	93902789
ENSG00000197249	chromosome14	93919328
ENSG00000187483	chromosome14	94177408
ENSG00000186910	chromosome14	93984865
ENSG00000170054	chromosome14	94012263
ENSG00000123561	chromosomeX	105167706
ENSG00000100665	chromosome14	94099573
ENSG00000196136	chromosome14	94148471
ENSG00000188488	chromosome14	94123453
ENSG00000165953	chromosome14	94034488
ENSG00000118496	chromosome6	146169235
ENSG00000163833	chromosome3	122822113
ENSG00000118496	chromosome6	146169235
ENSG00000184405	chromosome15	19264747
ENSG00000204239	chromosome11	133652526
ENSG00000181996	chromosome1	176961974
ENSG00000206061	chromosome16	1602811
ENSG00000205663	chromosomeX	3810323
ENSG00000205664	chromosomeX	3771899
ENSG00000206061	chromosome16	1602811
ENSG00000205663	chromosomeX	3810323
ENSG00000205664	chromosomeX	3771899
ENSG00000206061	chromosome16	1602811
ENSG00000205663	chromosomeX	3810323
ENSG00000205664	chromosomeX	3771899
ENSG00000184405	chromosome15	19264747
ENSG00000204239	chromosome11	133652526
ENSG00000181996	chromosome1	176961974
ENSG00000171863	chromosome2	3601075
ENSG00000215342	chromosome4	4559708
ENSG00000174121	chromosome13	21101155
ENSG00000183405	chromosome17	23818967
ENSG00000162596	chromosome1	68481327
ENSG00000215804	chromosome1	238242577
ENSG00000213240	chromosome1	143920466
ENSG00000164619	chromosome7	33911751
ENSG00000214028	chromosome7	149110240
ENSG00000214028	chromosome7	149110240
ENSG00000164619	chromosome7	33911751
ENSG00000171877	chromosome15	42274545
ENSG00000172159	chromosome9	85342967
ENSG00000106105	chromosome7	30601063
ENSG00000204202	chromosomeY	9643140
ENSG00000204204	chromosome10	41676853
ENSG00000205737	chromosome4	48789608
ENSG00000205731	chromosome4	49332236
ENSG00000198361	chromosome20	29293221
ENSG00000206163	chromosomeY	9209829
ENSG00000204205	chromosome10	39127100
ENSG00000204207	chromosome10	38814442
ENSG00000214559	chromosome6	64632210
ENSG00000118479	chromosome6	65679357
ENSG00000214559	chromosome6	64632210
ENSG00000197475	chromosome8	39451343

ENSG00000204336	chromosome2	171279200
ENSG00000185251	chromosome7	97395928
ENSG00000168993	chromosome4	776360
ENSG00000185251	chromosome7	97395928
ENSG00000204336	chromosome2	171279200
ENSG00000214435	chromosome10	104619322
ENSG00000011083	chromosome5	149550084
ENSG00000174358	chromosome5	1254766
ENSG00000157103	chromosome3	11033898
ENSG00000214617	chromosome16	32798537
ENSG00000132164	chromosome3	10832951
ENSG00000111181	chromosome12	189414
ENSG00000164363	chromosome5	1278593
ENSG00000174358	chromosome5	1254766
ENSG00000163817	chromosome3	45812916
ENSG00000174358	chromosome5	1254766
ENSG00000164363	chromosome5	1278593
ENSG00000108576	chromosome17	25573966
ENSG00000111181	chromosome12	189414
ENSG00000132164	chromosome3	10832951
ENSG00000010379	chromosome12	239480
ENSG00000174358	chromosome5	1254766
ENSG00000111181	chromosome12	189414
ENSG00000132164	chromosome3	10832951
ENSG00000010379	chromosome12	239480
ENSG00000010379	chromosome12	239480
ENSG00000111181	chromosome12	189414
ENSG00000132164	chromosome3	10832951
ENSG00000142319	chromosome5	1496313
ENSG00000103546	chromosome16	54248108
ENSG00000130821	chromosomeX	152607224
ENSG00000198555	chromosome16	33686170
ENSG00000188782	chromosome1	26389706
ENSG00000152705	chromosome5	134331581
ENSG00000134769	chromosome18	30589939
ENSG00000134769	chromosome18	30589939
ENSG00000205012	chromosome5	138758658
ENSG00000196739	chromosome9	115958052
ENSG00000171502	chromosome1	86394668
ENSG00000171502	chromosome1	86394668
ENSG00000196739	chromosome9	115958052
ENSG00000205399	chromosome14	105240922
ENSG00000214421	chromosome14	104515620
ENSG00000214421	chromosome14	104515620
ENSG00000203774	chromosome10	134638085
ENSG00000198829	chromosome3	153080371
ENSG00000169860	chromosome3	154036262
ENSG00000175591	chromosome11	72622853
ENSG00000186912	chromosomeX	69396200
ENSG00000198829	chromosome3	153080371
ENSG00000173198	chromosomeX	77415900
ENSG00000152207	chromosome13	48178955
ENSG00000152207	chromosome13	48178955
ENSG00000173198	chromosomeX	77415900
ENSG00000078589	chromosomeX	78102674
ENSG00000078589	chromosomeX	78102674
ENSG00000184574	chromosome12	6600676
ENSG00000147145	chromosomeX	77897023
ENSG00000139679	chromosome13	47884561

ENSG00000169508	chromosome13	98746401
ENSG00000169508	chromosome13	98746401
ENSG00000184574	chromosome12	6600676
ENSG00000147145	chromosomeX	77897023
ENSG00000139679	chromosome13	47884561
ENSG00000078589	chromosomeX	78102674
ENSG00000169403	chromosome1	28350120
ENSG00000175591	chromosome11	72622853
ENSG00000186912	chromosomeX	69396200
ENSG00000169860	chromosome3	154036262
ENSG00000169403	chromosome1	28350120
ENSG00000147145	chromosomeX	77897023
ENSG00000139679	chromosome13	47884561
ENSG00000184574	chromosome12	6600676
ENSG00000186912	chromosomeX	69396200
ENSG00000175591	chromosome11	72622853
ENSG00000169403	chromosome1	28350120
ENSG00000184574	chromosome12	6600676
ENSG00000147145	chromosomeX	77897023
ENSG00000139679	chromosome13	47884561
ENSG00000078589	chromosomeX	78102674
ENSG00000152207	chromosome13	48178955
ENSG00000173198	chromosomeX	77415900
ENSG00000184574	chromosome12	6600676
ENSG00000147145	chromosomeX	77897023
ENSG00000139679	chromosome13	47884561
ENSG00000078589	chromosomeX	78102674
ENSG00000169403	chromosome1	28350120
ENSG00000169508	chromosome13	98746401
ENSG00000139679	chromosome13	47884561
ENSG00000147145	chromosomeX	77897023
ENSG00000169860	chromosome3	154036262
ENSG00000175591	chromosome11	72622853
ENSG00000186912	chromosomeX	69396200
ENSG00000198829	chromosome3	153080371
ENSG00000184574	chromosome12	6600676
ENSG00000147145	chromosomeX	77897023
ENSG00000139679	chromosome13	47884561
ENSG00000078589	chromosomeX	78102674
ENSG00000169403	chromosome1	28350120
ENSG00000169508	chromosome13	98746401
ENSG00000152207	chromosome13	48178955
ENSG00000173198	chromosomeX	77415900
ENSG00000163795	chromosome2	27456898
ENSG00000113761	chromosome5	176382346
ENSG00000165061	chromosome8	40802353
ENSG00000165061	chromosome8	40802353
ENSG00000165061	chromosome8	40802353
ENSG00000113761	chromosome5	176382346
ENSG00000113761	chromosome5	176382346
ENSG00000165061	chromosome8	40802353
ENSG00000172667	chromosome3	180268235
ENSG00000214766	chromosome3	49011886
ENSG00000120057	chromosome10	99521581
ENSG00000104332	chromosome8	41285836
ENSG00000145423	chromosome4	154929438
ENSG00000145423	chromosome4	154929438
ENSG00000104332	chromosome8	41285836
ENSG00000120057	chromosome10	99521581

ENSG00000112701	chromosome6	76368961
ENSG00000138468	chromosome3	102714665
ENSG00000214572	chromosome19	2288035
ENSG00000215393	chromosome15	19501231
ENSG00000205063	chromosome1	2248441
ENSG00000215176	chromosome7	1014883
ENSG00000206202	chromosome15	19281770
ENSG00000205134	chromosome4	186146224
ENSG00000205134	chromosome4	186146224
ENSG00000205063	chromosome1	2248441
ENSG00000215176	chromosome7	1014883
ENSG00000206202	chromosome15	19281770
ENSG00000215242	chromosome5	1598109
ENSG00000205063	chromosome1	2248441
ENSG00000215176	chromosome7	1014883
ENSG00000206202	chromosome15	19281770
ENSG00000205134	chromosome4	186146224
ENSG00000214572	chromosome19	2288035
ENSG00000215393	chromosome15	19501231
ENSG00000172845	chromosome2	174538146
ENSG00000185591	chromosome12	52060343
ENSG00000105866	chromosome7	21434395
ENSG00000105866	chromosome7	21434395
ENSG00000185591	chromosome12	52060343
ENSG00000185591	chromosome12	52060343
ENSG00000105866	chromosome7	21434395
ENSG00000172845	chromosome2	174538146
ENSG00000156564	chromosome6	40508831
ENSG00000165379	chromosome14	41425579
ENSG00000126243	chromosome19	41122168
ENSG00000128011	chromosome19	44497604
ENSG00000165379	chromosome14	41425579
ENSG00000156564	chromosome6	40508831
ENSG00000128011	chromosome19	44497604
ENSG00000126243	chromosome19	41122168
ENSG00000126243	chromosome19	41122168
ENSG00000198087	chromosome6	47553817
ENSG00000147010	chromosomeX	19815488
ENSG00000106571	chromosome7	42229378
ENSG00000074047	chromosome2	121271367
ENSG00000111087	chromosome12	56143742
ENSG00000074047	chromosome2	121271367
ENSG00000106571	chromosome7	42229378
ENSG00000107249	chromosome9	4276426
ENSG00000174332	chromosome1	53833164
ENSG00000130479	chromosome19	17691312
ENSG00000131711	chromosome5	71439115
ENSG00000166963	chromosome15	41590479
ENSG00000166963	chromosome15	41590479
ENSG00000131711	chromosome5	71439115
ENSG00000040341	chromosome8	74813091
ENSG00000124214	chromosome20	47216146
ENSG00000173068	chromosome9	16860647
ENSG00000169594	chromosome15	81744385
ENSG00000165417	chromosome14	80756616
ENSG00000172367	chromosome11	118561414
ENSG00000174827	chromosome1	144458401
ENSG00000215859	chromosome1	145948449
ENSG00000215860	chromosome1	144651471

ENSG00000109062	chromosome17	70256581
ENSG00000065054	chromosome16	2017008
ENSG00000015153	chromosome12	40918250
ENSG00000163602	chromosome3	72578465
ENSG00000108468	chromosome17	43509366
ENSG00000122565	chromosome7	26209144
ENSG00000122565	chromosome7	26209144
ENSG00000108468	chromosome17	43509366
ENSG00000094916	chromosome12	52937702
ENSG00000122565	chromosome7	26209144
ENSG00000151702	chromosome11	128069286
ENSG00000157554	chromosome21	38869495
ENSG00000122257	chromosome16	24459449
ENSG00000102349	chromosomeX	56276232
ENSG00000118922	chromosome13	73467161
ENSG00000109787	chromosome4	38358642
ENSG00000118922	chromosome13	73467161
ENSG00000102349	chromosomeX	56276232
ENSG00000163884	chromosome3	127554456
ENSG00000134668	chromosome1	32053522
ENSG00000118482	chromosome6	64414416
ENSG00000101191	chromosome20	61013410
ENSG00000101191	chromosome20	61013410
ENSG00000134668	chromosome1	32053522
ENSG00000118482	chromosome6	64414416
ENSG00000105227	chromosome19	45605680
ENSG00000214427	chromosome14	104490817
ENSG00000124942	chromosome11	62060147
ENSG00000124942	chromosome11	62060147
ENSG00000105227	chromosome19	45605680
ENSG00000214427	chromosome14	104490817
ENSG00000124942	chromosome11	62060147
ENSG00000105880	chromosome7	96491872
ENSG00000064195	chromosome17	45427362
ENSG00000115844	chromosome2	172675513
ENSG00000115844	chromosome2	172675513
ENSG00000064195	chromosome17	45427362
ENSG00000105538	chromosome19	53935352
ENSG00000157927	chromosome7	4884297
ENSG00000124788	chromosome6	16436521
ENSG00000124788	chromosome6	16436521
ENSG00000123388	chromosome12	52653293
ENSG00000128713	chromosome2	176680330
ENSG00000005073	chromosome7	27191289
ENSG00000128713	chromosome2	176680330
ENSG00000005073	chromosome7	27191289
ENSG00000123388	chromosome12	52653293
ENSG00000005073	chromosome7	27191289
ENSG00000128713	chromosome2	176680330
ENSG00000120149	chromosome5	174084269
ENSG00000163132	chromosome4	4912528
ENSG00000163132	chromosome4	4912528
ENSG00000120149	chromosome5	174084269
ENSG00000106536	chromosome7	39091991
ENSG00000184271	chromosome12	49886888
ENSG00000111961	chromosome6	148635186
ENSG00000155307	chromosome21	14877969
ENSG00000122122	chromosomeX	128741755
ENSG00000122122	chromosomeX	128741755

ENSG00000155307	chromosome21	14877969
ENSG00000130396	chromosome6	167970662
ENSG00000043039	chromosome11	128751141
ENSG00000131668	chromosome9	95757250
ENSG00000136931	chromosome9	126305496
ENSG00000116833	chromosome1	198263599
ENSG00000148200	chromosome9	126573313
ENSG00000116833	chromosome1	198263599
ENSG00000136931	chromosome9	126305496
ENSG00000118526	chromosome6	134252229
ENSG00000178860	chromosome8	72918968
ENSG00000178860	chromosome8	72918968
ENSG00000118526	chromosome6	134252229
ENSG00000163792	chromosome2	27225506
ENSG00000187728	chromosome8	68036516
ENSG00000119614	chromosome14	73776018
ENSG00000100987	chromosome20	25010733
ENSG00000052850	chromosome11	44288189
ENSG00000180318	chromosome12	84198171
ENSG00000180318	chromosome12	84198171
ENSG00000052850	chromosome11	44288189
ENSG00000156150	chromosome1	110414758
ENSG00000156150	chromosome1	110414758
ENSG00000180318	chromosome12	84198171
ENSG00000052850	chromosome11	44288189
ENSG00000004848	chromosomeX	24943776
ENSG00000002587	chromosome4	11010728
ENSG00000002587	chromosome4	11010728
ENSG00000175818	chromosome6	114490703
ENSG00000153976	chromosome17	13445172
ENSG00000125430	chromosome17	14145561
ENSG00000182601	chromosome16	25611240
ENSG00000122254	chromosome16	22733409
ENSG00000162040	chromosome16	1908326
ENSG00000162040	chromosome16	1908326
ENSG00000182601	chromosome16	25611240
ENSG00000122254	chromosome16	22733409
ENSG00000002587	chromosome4	11010728
ENSG00000122254	chromosome16	22733409
ENSG00000182601	chromosome16	25611240
ENSG00000185960	chromosomeX	511633
ENSG00000168779	chromosome3	159306508
ENSG00000162676	chromosome1	92721633
ENSG00000165702	chromosome9	134851887
ENSG00000188994	chromosome6	87922029
ENSG00000117000	chromosome1	40399659
ENSG00000117000	chromosome1	40399659
ENSG00000188994	chromosome6	87922029
ENSG00000166860	chromosome12	55684969
ENSG00000011590	chromosome19	40897369
ENSG00000109906	chromosome11	113439233
ENSG00000109906	chromosome11	113439233
ENSG00000109906	chromosome11	113439233
ENSG00000011590	chromosome19	40897369
ENSG00000143867	chromosome2	19417048
ENSG00000164920	chromosome8	100030357
ENSG00000172273	chromosome11	118502865
ENSG00000169926	chromosome15	29406708
ENSG00000119138	chromosome9	72218100

ENSG00000185008	chromosome3	77172627
ENSG00000154134	chromosome11	124240684
ENSG00000169855	chromosome3	79721752
ENSG00000154133	chromosome11	124272902
ENSG00000169855	chromosome3	79721752
ENSG00000185008	chromosome3	77172627
ENSG00000154134	chromosome11	124240684
ENSG00000154134	chromosome11	124240684
ENSG00000185008	chromosome3	77172627
ENSG00000105722	chromosome19	47450992
ENSG00000105722	chromosome19	47450992
ENSG00000172018	chromosome1	155335940
ENSG00000166831	chromosome15	62854572
ENSG00000157110	chromosome8	30362151
ENSG00000048540	chromosome12	16649091
ENSG00000166407	chromosome11	8241594
ENSG00000135363	chromosome11	33847509
ENSG00000166407	chromosome11	8241594
ENSG00000048540	chromosome12	16649091
ENSG00000121060	chromosome17	52346349
ENSG00000121060	chromosome17	52346349
ENSG00000132481	chromosome17	71386225
ENSG00000137699	chromosome11	119513950
ENSG00000121060	chromosome17	52346349
ENSG00000121060	chromosome17	52346349
ENSG00000137699	chromosome11	119513950
ENSG00000137699	chromosome11	119513950
ENSG00000134115	chromosome3	1164693
ENSG00000144619	chromosome3	2588188
ENSG00000113805	chromosome3	74653029
ENSG00000018236	chromosome12	39588502
ENSG00000144619	chromosome3	2588188
ENSG00000113805	chromosome3	74653029
ENSG00000134115	chromosome3	1164693
ENSG00000134115	chromosome3	1164693
ENSG00000144619	chromosome3	2588188
ENSG00000113805	chromosome3	74653029
ENSG00000113805	chromosome3	74653029
ENSG00000144619	chromosome3	2588188
ENSG00000101916	chromosomeX	12834747
ENSG00000174123	chromosome4	38453607
ENSG00000174125	chromosome4	38476848
ENSG00000174130	chromosome4	38507490
ENSG00000137462	chromosome4	154843510
ENSG00000101916	chromosomeX	12834747
ENSG00000187554	chromosome1	221352997
ENSG00000134061	chromosome5	66528226
ENSG00000136869	chromosome9	119506572
ENSG00000136869	chromosome9	119506572
ENSG00000134061	chromosome5	66528226
ENSG00000137462	chromosome4	154843510
ENSG00000174123	chromosome4	38453607
ENSG00000174125	chromosome4	38476848
ENSG00000174130	chromosome4	38507490
ENSG00000101916	chromosomeX	12834747
ENSG00000174123	chromosome4	38453607
ENSG00000174125	chromosome4	38476848
ENSG00000174130	chromosome4	38507490
ENSG00000137462	chromosome4	154843510

ENSG00000174123	chromosome4	38453607
ENSG00000174125	chromosome4	38476848
ENSG00000174130	chromosome4	38507490
ENSG00000174123	chromosome4	38453607
ENSG00000174125	chromosome4	38476848
ENSG00000174130	chromosome4	38507490
ENSG00000137462	chromosome4	154843510
ENSG00000101916	chromosomeX	12834747
ENSG00000187554	chromosome1	221352997
ENSG00000187554	chromosome1	221352997
ENSG00000101916	chromosomeX	12834747
ENSG00000101916	chromosomeX	12834747
ENSG00000187554	chromosome1	221352997
ENSG00000174123	chromosome4	38453607
ENSG00000174125	chromosome4	38476848
ENSG00000174130	chromosome4	38507490
ENSG00000137462	chromosome4	154843510
ENSG00000144771	chromosome3	54936979
ENSG00000166159	chromosome12	1807576
ENSG00000185245	chromosome17	4776680
ENSG00000166159	chromosome12	1807576
ENSG00000144771	chromosome3	54936979
ENSG00000146242	chromosome6	83131398
ENSG00000167657	chromosome19	3920734
ENSG00000196730	chromosome9	89303813
ENSG00000035664	chromosome15	62119514
ENSG00000167657	chromosome19	3920734
ENSG00000145949	chromosome6	2694528
ENSG00000140795	chromosome16	45339607
ENSG00000140795	chromosome16	45339607
ENSG00000145949	chromosome6	2694528
ENSG00000101306	chromosome20	29871045
ENSG00000167657	chromosome19	3920734
ENSG00000035664	chromosome15	62119514
ENSG00000035664	chromosome15	62119514
ENSG00000167657	chromosome19	3920734
ENSG00000196730	chromosome9	89303813
ENSG00000065534	chromosome3	124995379
ENSG00000178732	chromosome3	195600301
ENSG00000172061	chromosome3	195563068
ENSG00000178772	chromosome3	195545127
ENSG00000172061	chromosome3	195563068
ENSG00000178732	chromosome3	195600301
ENSG00000156218	chromosome15	82115518
ENSG00000178031	chromosome9	18464231
ENSG00000143469	chromosome1	208178233
ENSG00000139973	chromosome14	61532491
ENSG00000171132	chromosome2	45732744
ENSG00000145901	chromosome5	150424850
ENSG00000050730	chromosome4	122304731
ENSG00000198286	chromosome7	2964718
ENSG00000187796	chromosome9	138386352
ENSG00000187796	chromosome9	138386352
ENSG00000198286	chromosome7	2964718
ENSG00000100065	chromosome22	36245154
ENSG00000100065	chromosome22	36245154
ENSG00000187796	chromosome9	138386352
ENSG00000198286	chromosome7	2964718
ENSG00000125733	chromosome19	6690773

ENSG00000137942	chromosome1	93686427
ENSG00000187239	chromosome9	131845076
ENSG00000137942	chromosome1	93686427
ENSG00000125733	chromosome19	6690773
ENSG00000165588	chromosome14	56341928
ENSG00000186103	chromosome3	122772251
ENSG00000105392	chromosome19	53029513
ENSG00000165588	chromosome14	56341928
ENSG00000115507	chromosome2	63133630
ENSG00000186103	chromosome3	122772251
ENSG00000105392	chromosome19	53029513
ENSG00000165588	chromosome14	56341928
ENSG00000115507	chromosome2	63133630
ENSG00000180053	chromosome8	23620057
ENSG00000119919	chromosome10	101282879
ENSG00000136352	chromosome14	36059086
ENSG00000125816	chromosome20	21326038
ENSG00000125816	chromosome20	21326038
ENSG00000136352	chromosome14	36059086
ENSG00000125820	chromosome20	21442308
ENSG00000136327	chromosome14	36121346
ENSG00000136327	chromosome14	36121346
ENSG00000119919	chromosome10	101282879
ENSG00000180053	chromosome8	23620057
ENSG00000183072	chromosome5	172594693
ENSG00000136327	chromosome14	36121346
ENSG00000125820	chromosome20	21442308
ENSG00000125816	chromosome20	21326038
ENSG00000136352	chromosome14	36059086
ENSG00000109705	chromosome4	13155137
ENSG00000185585	chromosome9	126579371
ENSG00000162745	chromosome1	160259845
ENSG00000185585	chromosome9	126579371
ENSG00000152495	chromosome5	110588081
ENSG00000145362	chromosome4	114190334
ENSG00000151150	chromosome10	61819303
ENSG00000029534	chromosome8	41873156
ENSG00000029534	chromosome8	41873156
ENSG00000151150	chromosome10	61819303
ENSG00000152767	chromosome13	97663498
ENSG00000006607	chromosome2	241961196
ENSG00000187957	chromosome2	230287384
ENSG00000188107	chromosome6	66262025
ENSG00000124193	chromosome20	41520088
ENSG00000116350	chromosome1	29380852
ENSG00000100650	chromosome14	69304627
ENSG00000122566	chromosome7	26206897
ENSG00000188174	chromosomeX	138942254
ENSG00000214653	chromosome6	48225621
ENSG00000215885	chromosome1	54214296
ENSG00000170144	chromosome2	177785774
ENSG00000215876	chromosome1	81665075
ENSG00000176825	chromosome10	43605776
ENSG00000177219	chromosome3	75346391
ENSG00000215222	chromosome15	33480231
ENSG00000215870	chromosome1	101180056
ENSG00000212961	chromosome16	50237230
ENSG00000135486	chromosome12	52960859
ENSG00000214592	chromosome9	127397812

ENSG00000139675	chromosome13	52114629
ENSG00000215492	chromosome18	28247138
ENSG00000206228	chromosome8	83367173
ENSG00000214284	chromosome12	66714616
ENSG00000214361	chromosome3	123519206
ENSG00000215222	chromosome15	33480231
ENSG00000215870	chromosome1	101180056
ENSG00000212961	chromosome16	50237230
ENSG00000135486	chromosome12	52960859
ENSG00000214592	chromosome9	127397812
ENSG00000139675	chromosome13	52114629
ENSG00000215492	chromosome18	28247138
ENSG00000206228	chromosome8	83367173
ENSG00000214284	chromosome12	66714616
ENSG00000214361	chromosome3	123519206
ENSG00000188174	chromosomeX	138942254
ENSG00000214653	chromosome6	48225621
ENSG00000215885	chromosome1	54214296
ENSG00000170144	chromosome2	177785774
ENSG00000215876	chromosome1	81665075
ENSG00000176825	chromosome10	43605776
ENSG00000177219	chromosome3	75346391
ENSG00000144485	chromosome2	238813286
ENSG00000144485	chromosome2	238813286
ENSG00000197921	chromosome1	2451464
ENSG00000197921	chromosome1	2451464
ENSG00000179111	chromosome17	7968128
ENSG00000114315	chromosome3	195336864
ENSG00000188290	chromosome1	925217
ENSG00000197921	chromosome1	2451464
ENSG00000144485	chromosome2	238813286
ENSG00000179111	chromosome17	7968128
ENSG00000197921	chromosome1	2451464
ENSG00000173673	chromosome1	6227013
ENSG00000179111	chromosome17	7968128
ENSG00000144485	chromosome2	238813286
ENSG00000179111	chromosome17	7968128
ENSG00000197921	chromosome1	2451464
ENSG00000144485	chromosome2	238813286
ENSG00000114315	chromosome3	195336864
ENSG00000188290	chromosome1	925217
ENSG00000154065	chromosome18	19496693
ENSG00000145780	chromosome5	114907090
ENSG00000215524	chromosome18	14383909
ENSG00000215602	chromosome13	18137371
ENSG00000141965	chromosome19	4742867
ENSG00000152402	chromosome11	106393992
ENSG00000043355	chromosome13	99432320
ENSG00000156925	chromosomeX	136476517
ENSG00000152977	chromosome3	148610590
ENSG00000156925	chromosomeX	136476517
ENSG00000043355	chromosome13	99432320
ENSG00000139800	chromosome13	99421931
ENSG00000174963	chromosome3	148603275
ENSG00000152977	chromosome3	148610590
ENSG00000043355	chromosome13	99432320
ENSG00000156925	chromosomeX	136476517
ENSG00000174963	chromosome3	148603275
ENSG00000139800	chromosome13	99421931

ENSG00000174963	chromosome3	148603275
ENSG00000139800	chromosome13	99421931
ENSG00000152977	chromosome3	148610590
ENSG00000043355	chromosome13	99432320
ENSG00000156925	chromosomeX	136476517
ENSG00000180900	chromosome8	144969531
ENSG00000137269	chromosome6	53768014
ENSG00000112851	chromosome5	65324303
ENSG00000033122	chromosome1	69806911
ENSG00000147324	chromosome8	8787979
ENSG00000188306	chromosome3	171022395
ENSG00000149930	chromosome16	29896595
ENSG00000160551	chromosome17	24802693
ENSG00000160551	chromosome17	24802693
ENSG00000149930	chromosome16	29896595
ENSG00000135090	chromosome12	117177756
ENSG00000072786	chromosome5	171547652
ENSG00000065613	chromosome10	105717494
ENSG00000065613	chromosome10	105717494
ENSG00000072786	chromosome5	171547652
ENSG00000072786	chromosome5	171547652
ENSG00000065613	chromosome10	105717494
ENSG00000137962	chromosome1	94469756
ENSG00000180448	chromosome19	1018405
ENSG00000180448	chromosome19	1018405
ENSG00000137962	chromosome1	94469756
ENSG00000089639	chromosome19	19615339
ENSG00000031081	chromosome3	120496442
ENSG00000186517	chromosome1	159306039
ENSG00000134909	chromosome11	128539427
ENSG00000004777	chromosome19	40958401
ENSG00000004777	chromosome19	40958401
ENSG00000134909	chromosome11	128539427
ENSG00000186517	chromosome1	159306039
ENSG00000031081	chromosome3	120496442
ENSG00000134909	chromosome11	128539427
ENSG00000167460	chromosome19	16039435
ENSG00000198467	chromosome9	35679830
ENSG00000183022	chromosome19	58637394
ENSG00000214869	chromosome7	27029882
ENSG00000177152	chromosome7	116400202
ENSG00000187536	chromosome2	231146871
ENSG00000183022	chromosome19	58637394
ENSG00000214869	chromosome7	27029882
ENSG00000177152	chromosome7	116400202
ENSG00000187536	chromosome2	231146871
ENSG00000167460	chromosome19	16039435
ENSG00000183022	chromosome19	58637394
ENSG00000214869	chromosome7	27029882
ENSG00000177152	chromosome7	116400202
ENSG00000187536	chromosome2	231146871
ENSG00000198467	chromosome9	35679830
ENSG00000140416	chromosome15	61122082
ENSG00000143549	chromosome1	152431119
ENSG00000183022	chromosome19	58637394
ENSG00000214869	chromosome7	27029882
ENSG00000177152	chromosome7	116400202
ENSG00000187536	chromosome2	231146871
ENSG00000183022	chromosome19	58637394

ENSG00000214869	chromosome7	27029882
ENSG00000177152	chromosome7	116400202
ENSG00000187536	chromosome2	231146871
ENSG00000183022	chromosome19	58637394
ENSG00000214869	chromosome7	27029882
ENSG00000177152	chromosome7	116400202
ENSG00000187536	chromosome2	231146871
ENSG00000183022	chromosome19	58637394
ENSG00000214869	chromosome7	27029882
ENSG00000177152	chromosome7	116400202
ENSG00000187536	chromosome2	231146871
ENSG00000198467	chromosome9	35679830
ENSG00000167460	chromosome19	16039435
ENSG00000140416	chromosome15	61122082
ENSG00000167460	chromosome19	16039435
ENSG00000183022	chromosome19	58637394
ENSG00000214869	chromosome7	27029882
ENSG00000177152	chromosome7	116400202
ENSG00000187536	chromosome2	231146871
ENSG00000198467	chromosome9	35679830
ENSG00000168505	chromosome2	236741354
ENSG00000164900	chromosome7	150495569
ENSG00000164900	chromosome7	150495569
ENSG00000168505	chromosome2	236741354
ENSG00000130675	chromosome7	156495806
ENSG00000131023	chromosome6	150064956
ENSG00000150457	chromosome13	20518166
ENSG00000160808	chromosome3	46879885
ENSG00000198336	chromosome17	42641788
ENSG00000160808	chromosome3	46879885
ENSG00000198336	chromosome17	42641788
ENSG00000196465	chromosome12	54833970
ENSG00000092841	chromosome12	54838453
ENSG00000198336	chromosome17	42641788
ENSG00000160808	chromosome3	46879885
ENSG00000137273	chromosome6	1335182
ENSG00000137273	chromosome6	1335182
ENSG00000103241	chromosome16	85101677
ENSG00000005102	chromosome17	39094429
ENSG00000123407	chromosome12	52634981
ENSG00000170178	chromosome2	176672776
ENSG00000113722	chromosome5	149526556
ENSG00000165556	chromosome13	27441144
ENSG00000131264	chromosomeX	72583815
ENSG00000131264	chromosomeX	72583815
ENSG00000165556	chromosome13	27441144
ENSG00000151650	chromosome10	134901409
ENSG00000197587	chromosome1	46745270
ENSG00000135903	chromosome2	222871579
ENSG00000009709	chromosome1	18830685
ENSG00000116132	chromosome1	168899984
ENSG00000167157	chromosome9	131467968
ENSG00000109132	chromosome4	41445385
ENSG00000165462	chromosome11	71632697
ENSG00000133937	chromosome14	94306106
ENSG00000063515	chromosome22	17517797
ENSG00000133937	chromosome14	94306106
ENSG00000133937	chromosome14	94306106
ENSG00000063515	chromosome22	17517797

ENSG00000171540	chromosome5	76970131
ENSG00000149948	chromosome12	64505318
ENSG00000137309	chromosome6	34316536
ENSG00000159023	chromosome1	29186537
ENSG00000079819	chromosome6	131319328
ENSG00000079819	chromosome6	131319328
ENSG00000159023	chromosome1	29186537
ENSG00000082397	chromosome18	5620641
ENSG00000088367	chromosome20	34176780
ENSG00000082397	chromosome18	5620641
ENSG00000079819	chromosome6	131319328
ENSG00000159023	chromosome1	29186537
ENSG00000115085	chromosome2	97706932
ENSG00000165025	chromosome9	92646002
ENSG00000071051	chromosome2	105837952
ENSG00000158092	chromosome3	138129534
ENSG00000215917	chromosome1	989531
ENSG00000111679	chromosome12	6926150
ENSG00000111679	chromosome12	6926150
ENSG00000215917	chromosome1	989531
ENSG00000179295	chromosome12	111341299
ENSG00000010810	chromosome6	112147948
ENSG00000182866	chromosome1	32512518
ENSG00000136573	chromosome8	11438143
ENSG00000101336	chromosome20	30103889
ENSG00000147507	chromosome8	57016973
ENSG00000101336	chromosome20	30103889
ENSG00000147507	chromosome8	57016973
ENSG00000136573	chromosome8	11438143
ENSG00000010810	chromosome6	112147948
ENSG00000000938	chromosome1	27823015
ENSG00000147507	chromosome8	57016973
ENSG00000101336	chromosome20	30103889
ENSG00000197122	chromosome20	35445971
ENSG00000176105	chromosome18	746828
ENSG00000000938	chromosome1	27823015
ENSG00000010810	chromosome6	112147948
ENSG00000176105	chromosome18	746828
ENSG00000197122	chromosome20	35445971
ENSG00000062524	chromosome15	39593200
ENSG00000171094	chromosome2	29997030
ENSG00000171105	chromosome19	7244903
ENSG00000027644	chromosome1	155095038
ENSG00000140443	chromosome15	97010334
ENSG00000140443	chromosome15	97010334
ENSG00000027644	chromosome1	155095038
ENSG00000074181	chromosome19	15172717
ENSG00000134250	chromosome1	120413544
ENSG00000148400	chromosome9	138560060
ENSG00000134250	chromosome1	120413544
ENSG00000074181	chromosome19	15172717
ENSG00000154474	chromosome2	170074535
ENSG00000157119	chromosome3	42702115
ENSG00000122550	chromosome7	23112171
ENSG00000157119	chromosome3	42702115
ENSG00000154474	chromosome2	170074535
ENSG00000119771	chromosome2	23718883
ENSG00000172578	chromosome3	184756136
ENSG00000149243	chromosome11	74818885

ENSG00000172578	chromosome3	184756136
ENSG00000119771	chromosome2	23718883
ENSG00000114796	chromosome3	184850839
ENSG00000149243	chromosome11	74818885
ENSG00000119771	chromosome2	23718883
ENSG00000172578	chromosome3	184756136
ENSG00000159307	chromosome22	42069212
ENSG00000146197	chromosome6	35290174
ENSG00000146197	chromosome6	35290174
ENSG00000159307	chromosome22	42069212
ENSG00000175356	chromosome11	9069652
ENSG00000172460	chromosome16	2831330
ENSG00000116176	chromosome16	1215254
ENSG00000103355	chromosome16	2777452
ENSG00000095917	chromosome16	1246283
ENSG00000172236	chromosome16	1230941
ENSG00000197253	chromosome16	1219954
ENSG00000196364	chromosome16	1266750
ENSG00000189099	chromosome4	152417775
ENSG00000215148	chromosome16	2788487
ENSG00000007038	chromosome16	2807271
ENSG00000178055	chromosome3	46850590
ENSG00000188086	chromosome3	46761250
ENSG00000185888	chromosome1	226070041
ENSG00000214877	chromosome9	93991642
ENSG00000206549	chromosome3	46734319
ENSG00000206549	chromosome3	46734319
ENSG00000189099	chromosome4	152417775
ENSG00000215148	chromosome16	2788487
ENSG00000007038	chromosome16	2807271
ENSG00000178055	chromosome3	46850590
ENSG00000188086	chromosome3	46761250
ENSG00000185888	chromosome1	226070041
ENSG00000214877	chromosome9	93991642
ENSG00000172382	chromosome16	2710163
ENSG00000005001	chromosome16	2848107
ENSG00000052344	chromosome16	31054321
ENSG00000189099	chromosome4	152417775
ENSG00000215148	chromosome16	2788487
ENSG00000007038	chromosome16	2807271
ENSG00000178055	chromosome3	46850590
ENSG00000188086	chromosome3	46761250
ENSG00000185888	chromosome1	226070041
ENSG00000214877	chromosome9	93991642
ENSG00000206549	chromosome3	46734319
ENSG00000172460	chromosome16	2831330
ENSG00000116176	chromosome16	1215254
ENSG00000103355	chromosome16	2777452
ENSG00000095917	chromosome16	1246283
ENSG00000172236	chromosome16	1230941
ENSG00000197253	chromosome16	1219954
ENSG00000196364	chromosome16	1266750
ENSG00000189099	chromosome4	152417775
ENSG00000215148	chromosome16	2788487
ENSG00000007038	chromosome16	2807271
ENSG00000178055	chromosome3	46850590
ENSG00000188086	chromosome3	46761250
ENSG00000185888	chromosome1	226070041
ENSG00000214877	chromosome9	93991642

ENSG00000206549	chromosome3	46734319
ENSG00000172460	chromosome16	2831330
ENSG00000116176	chromosome16	1215254
ENSG00000103355	chromosome16	2777452
ENSG00000095917	chromosome16	1246283
ENSG00000172236	chromosome16	1230941
ENSG00000197253	chromosome16	1219954
ENSG00000196364	chromosome16	1266750
ENSG00000172382	chromosome16	2710163
ENSG00000005001	chromosome16	2848107
ENSG00000052344	chromosome16	31054321
ENSG00000160183	chromosome21	42689321
ENSG00000184012	chromosome21	41791931
ENSG00000166682	chromosome11	113082157
ENSG00000137648	chromosome11	117453111
ENSG00000137747	chromosome11	117305233
ENSG00000105707	chromosome19	40224566
ENSG00000184012	chromosome21	41791931
ENSG00000160183	chromosome21	42689321
ENSG00000105707	chromosome19	40224566
ENSG00000137747	chromosome11	117305233
ENSG00000166682	chromosome11	113082157
ENSG00000137648	chromosome11	117453111
ENSG00000160183	chromosome21	42689321
ENSG00000184012	chromosome21	41791931
ENSG00000137747	chromosome11	117305233
ENSG00000105707	chromosome19	40224566
ENSG00000160183	chromosome21	42689321
ENSG00000184012	chromosome21	41791931
ENSG00000166682	chromosome11	113082157
ENSG00000137648	chromosome11	117453111
ENSG00000137648	chromosome11	117453111
ENSG00000166682	chromosome11	113082157
ENSG00000130164	chromosome19	11061225
ENSG00000157193	chromosome1	53566173
ENSG00000147852	chromosome9	2611856
ENSG00000157193	chromosome1	53566173
ENSG00000130164	chromosome19	11061225
ENSG00000147852	chromosome9	2611856
ENSG00000130164	chromosome19	11061225
ENSG00000157193	chromosome1	53566173
ENSG00000146530	chromosome7	12376234
ENSG00000125285	chromosome13	94162305
ENSG00000168875	chromosome3	138966317
ENSG00000168875	chromosome3	138966317
ENSG00000125285	chromosome13	94162305
ENSG00000140107	chromosome14	99859445
ENSG00000162241	chromosome11	64905999
ENSG00000101180	chromosome20	60228422
ENSG00000134489	chromosome18	20294691
ENSG00000101180	chromosome20	60228422
ENSG00000125845	chromosome20	6698774
ENSG00000125378	chromosome14	53488691
ENSG00000125378	chromosome14	53488691
ENSG00000125845	chromosome20	6698774
ENSG00000122641	chromosome7	41706498
ENSG00000163083	chromosome2	120820235
ENSG00000163083	chromosome2	120820235
ENSG00000122641	chromosome7	41706498

ENSG00000175189	chromosome12	56114937
ENSG00000121871	chromosome3	166391313
ENSG00000185985	chromosomeX	144711636
ENSG00000178235	chromosome13	83353644
ENSG00000178235	chromosome13	83353644
ENSG00000185985	chromosomeX	144711636
ENSG00000132334	chromosome10	129729136
ENSG00000132670	chromosome20	2892924
ENSG00000184916	chromosome14	104705803
ENSG00000101384	chromosome20	10602179
ENSG00000128917	chromosome15	39008937
ENSG00000128917	chromosome15	39008937
ENSG00000198719	chromosome6	170441153
ENSG00000171462	chromosome6	43530542
ENSG00000185559	chromosome14	100263159
ENSG00000089169	chromosome12	111750507
ENSG00000215770	supercontigNT_113933	17990
ENSG00000083782	chromosome12	89920474
ENSG00000188770	chromosome1	201731757
ENSG00000106809	chromosome9	94205511
ENSG00000188770	chromosome1	201731757
ENSG00000083782	chromosome12	89920474
ENSG00000149177	chromosome11	47959041
ENSG00000080031	chromosome19	60412614
ENSG00000080031	chromosome19	60412614
ENSG00000149177	chromosome11	47959041
ENSG00000127329	chromosome12	69317443
ENSG00000151490	chromosome12	15366928
ENSG00000080031	chromosome19	60412614
ENSG00000149177	chromosome11	47959041
ENSG00000080031	chromosome19	60412614
ENSG00000149177	chromosome11	47959041
ENSG00000127329	chromosome12	69317443
ENSG00000080031	chromosome19	60412614
ENSG00000149177	chromosome11	47959041
ENSG00000069011	chromosome5	134397471
ENSG00000164093	chromosome4	111773604
ENSG00000107859	chromosome10	103981828
ENSG00000164093	chromosome4	111773604
ENSG00000069011	chromosome5	134397471
ENSG00000179580	chromosome16	1957276
ENSG00000179580	chromosome16	1957276
ENSG00000197789	chromosome17	21835139
ENSG00000205557	chromosome14	87718404
ENSG00000197789	chromosome17	21835139
ENSG00000108370	chromosome17	60564121
ENSG00000076344	chromosome16	265913
ENSG00000182732	chromosome14	71501262
ENSG00000182901	chromosome1	239585700
ENSG00000054356	chromosome2	219882299
ENSG00000155093	chromosome7	158073123
ENSG00000134242	chromosome1	114215769
ENSG00000127947	chromosome7	77004800
ENSG00000127947	chromosome7	77004800
ENSG00000134242	chromosome1	114215769
ENSG00000072135	chromosome2	130830151
ENSG00000106278	chromosome7	121300520
ENSG00000144724	chromosome3	61523002
ENSG00000163788	chromosome3	43319700

ENSG00000111452	chromosome12	130004964
ENSG00000180264	chromosome9	126253244
ENSG00000117114	chromosome1	82075258
ENSG00000072071	chromosome19	14155415
ENSG00000072071	chromosome19	14155415
ENSG00000117114	chromosome1	82075258
ENSG00000150471	chromosome4	62045607
ENSG00000136099	chromosome13	52320573
ENSG00000189184	chromosome4	138672693
ENSG00000136099	chromosome13	52320573
ENSG00000165194	chromosomeX	99550252
ENSG00000118946	chromosome13	57104682
ENSG00000138650	chromosome4	134290746
ENSG00000189184	chromosome4	138672693
ENSG00000138650	chromosome4	134290746
ENSG00000165194	chromosomeX	99550252
ENSG00000118946	chromosome13	57104682
ENSG00000136099	chromosome13	52320573
ENSG00000118946	chromosome13	57104682
ENSG00000165194	chromosomeX	99550252
ENSG00000189184	chromosome4	138672693
ENSG00000165194	chromosomeX	99550252
ENSG00000118946	chromosome13	57104682
ENSG00000138650	chromosome4	134290746
ENSG00000153487	chromosome13	110163322
ENSG00000168556	chromosome4	184663343
ENSG00000168395	chromosome2	242290154
ENSG00000215693	supercontigNT_113880	118604
ENSG00000111653	chromosome12	6642529
ENSG00000137845	chromosome15	56829026
ENSG00000087470	chromosome12	32723526
ENSG00000211599	chromosome2	88977926
ENSG00000211605	chromosome2	89108297
ENSG00000211632	chromosome2	89849045
ENSG00000211610	chromosome2	89223707
ENSG00000211625	chromosome2	89715082
ENSG00000211602	chromosome2	89059619
ENSG00000211636	chromosome2	89911009
ENSG00000211607	chromosome2	89166302
ENSG00000211629	chromosome2	89791050
ENSG00000204776	chromosome9	69634917
ENSG00000204780	chromosome9	69434439
ENSG00000156755	chromosome9	69067579
ENSG00000183719	chromosome2	91586562
ENSG00000204670	chromosome2	97075615
ENSG00000136700	chromosome2	113880633
ENSG00000211617	chromosome2	89378611
ENSG00000211613	chromosome2	89294498
ENSG00000211603	chromosome2	89073513
ENSG00000211635	chromosome2	89897079
ENSG00000211608	chromosome2	89180943
ENSG00000211628	chromosome2	89776410
ENSG00000211606	chromosome2	89121311
ENSG00000211631	chromosome2	89836021
ENSG00000211627	chromosome2	89758963
ENSG00000211601	chromosome2	89047373
ENSG00000211609	chromosome2	89198424
ENSG00000211630	chromosome2	89830253
ENSG00000211618	chromosome2	89400973

ENSG00000211604	chromosome2	89091070
ENSG00000211633	chromosome2	89866370
ENSG00000211634	chromosome2	89886225
ENSG00000211600	chromosome2	89028410
ENSG00000211616	chromosome2	89349348
ENSG00000211620	chromosome2	89589921
ENSG00000211626	chromosome2	89745835
ENSG00000211611	chromosome2	89240902
ENSG00000197794	chromosome2	88996310
ENSG00000106546	chromosome7	17305414
ENSG00000106546	chromosome7	17305414
ENSG00000063438	chromosome5	324836
ENSG00000108669	chromosome17	74289973
ENSG00000008256	chromosome7	6278664
ENSG00000008256	chromosome7	6278664
ENSG00000108669	chromosome17	74289973
ENSG00000105443	chromosome19	53664577
ENSG00000101076	chromosome20	42417859
ENSG00000164749	chromosome8	76614783
ENSG00000175745	chromosome5	92946486
ENSG00000185551	chromosome15	94670543
ENSG00000204231	chromosome6	33276232
ENSG00000143171	chromosome1	163680755
ENSG00000186350	chromosome9	136358299
ENSG00000185551	chromosome15	94670543
ENSG00000175745	chromosome5	92946486
ENSG00000186350	chromosome9	136358299
ENSG00000143171	chromosome1	163680755
ENSG00000204231	chromosome6	33276232
ENSG00000164749	chromosome8	76614783
ENSG00000101076	chromosome20	42417859
ENSG00000138784	chromosome4	106693372
ENSG00000106100	chromosome7	30463063
ENSG00000179583	chromosome16	10867648
ENSG00000116191	chromosome1	177012523
ENSG00000136828	chromosome9	128764390
ENSG00000184500	chromosome3	95175416
ENSG00000183087	chromosome13	113547050
ENSG00000025434	chromosome11	47237344
ENSG00000131408	chromosome19	55572675
ENSG00000136878	chromosome9	131654656
ENSG00000077254	chromosome1	77983736
ENSG00000214661	chromosome11	65140359
ENSG00000163597	chromosome2	203614730
ENSG00000145723	chromosome5	102472311
ENSG00000182771	chromosome10	88116130
ENSG00000152208	chromosome4	93444831
ENSG00000065320	chromosome17	8866416
ENSG00000162068	chromosome16	2461704
ENSG00000162068	chromosome16	2461704
ENSG00000065320	chromosome17	8866416
ENSG00000142233	chromosome19	53866056
ENSG00000066336	chromosome11	47356481
ENSG00000142539	chromosome19	55614032
ENSG00000129159	chromosome11	17714126
ENSG00000131398	chromosome19	55524152
ENSG00000116396	chromosome1	110555645
ENSG00000116396	chromosome1	110555645
ENSG00000129159	chromosome11	17714126

ENSG00000131398	chromosome19	55524152
ENSG00000166006	chromosome12	73888031
ENSG00000131398	chromosome19	55524152
ENSG00000129159	chromosome11	17714126
ENSG00000139088	chromosome12	9616894
ENSG00000186611	chromosome12	9444162
ENSG00000005812	chromosome13	76493997
ENSG00000164616	chromosome5	135300351
ENSG00000151239	chromosome12	42486418
ENSG00000021355	chromosome6	2785820
ENSG00000170542	chromosome6	2845845
ENSG00000166401	chromosome18	59788239
ENSG00000124570	chromosome6	2911722
ENSG00000057149	chromosome18	59461792
ENSG00000206073	chromosome18	59479431
ENSG00000197641	chromosome18	59406882
ENSG00000166634	chromosome18	59374373
ENSG00000166396	chromosome18	59600587
ENSG00000206072	chromosome18	59528408
ENSG00000166404	chromosome18	59733725
ENSG00000197632	chromosome18	59709659
ENSG00000206075	chromosome18	59302642
ENSG00000215461	chromosome13	50813217
ENSG00000106366	chromosome7	100558395
ENSG00000135919	chromosome2	224574862
ENSG00000163536	chromosome3	168989611
ENSG00000163536	chromosome3	168989611
ENSG00000106366	chromosome7	100558395
ENSG00000135919	chromosome2	224574862
ENSG00000163536	chromosome3	168989611
ENSG00000135919	chromosome2	224574862
ENSG00000106366	chromosome7	100558395
ENSG00000001617	chromosome3	50167528
ENSG00000001617	chromosome3	50167528
ENSG00000075223	chromosome7	80384034
ENSG00000075223	chromosome7	80384034
ENSG00000001617	chromosome3	50167528
ENSG00000119514	chromosome9	100609802
ENSG00000072110	chromosome14	68515527
ENSG00000130402	chromosome19	43830226
ENSG00000204633	chromosome11	66070988
ENSG00000077522	chromosome1	234916597
ENSG00000077522	chromosome1	234916597
ENSG00000204633	chromosome11	66070988
ENSG00000130402	chromosome19	43830226
ENSG00000072110	chromosome14	68515527
ENSG00000077522	chromosome1	234916597
ENSG00000204633	chromosome11	66070988
ENSG00000072041	chromosome12	83810031
ENSG00000197106	chromosome1	110511075
ENSG00000063127	chromosome19	54506417
ENSG00000072041	chromosome12	83810031
ENSG00000197106	chromosome1	110511075
ENSG00000197106	chromosome1	110511075
ENSG00000072041	chromosome12	83810031
ENSG00000136840	chromosome9	129718520
ENSG00000184005	chromosome1	76313140
ENSG00000140557	chromosome15	90738299
ENSG00000113532	chromosome5	100266559

ENSG00000105928	chromosome7	24755919
ENSG00000204311	chromosome2	179026383
ENSG00000117834	chromosome1	48461017
ENSG00000154025	chromosome17	18796244
ENSG00000100191	chromosome22	30981317
ENSG00000100170	chromosome22	30769269
ENSG00000140675	chromosome16	31401959
ENSG00000154025	chromosome17	18796244
ENSG00000117834	chromosome1	48461017
ENSG00000140675	chromosome16	31401959
ENSG00000100191	chromosome22	30981317
ENSG00000100170	chromosome22	30769269
ENSG00000114739	chromosome3	38470818
ENSG00000121989	chromosome2	148319192
ENSG00000214063	chromosome11	840305
ENSG00000011105	chromosome12	3180621
ENSG00000143119	chromosome1	111235554
ENSG00000117472	chromosome1	46419367
ENSG00000177697	chromosome11	826070
ENSG00000110900	chromosome12	30998193
ENSG00000177697	chromosome11	826070
ENSG00000085117	chromosome11	44572789
ENSG00000104894	chromosome19	54530610
ENSG00000011105	chromosome12	3180621
ENSG00000214063	chromosome11	840305
ENSG00000117472	chromosome1	46419367
ENSG00000157570	chromosome11	44884544
ENSG00000157570	chromosome11	44884544
ENSG00000214063	chromosome11	840305
ENSG00000110651	chromosome11	2355356
ENSG00000134198	chromosome1	115433577
ENSG00000134198	chromosome1	115433577
ENSG00000110651	chromosome11	2355356
ENSG00000010278	chromosome12	6179927
ENSG00000010278	chromosome12	6179927
ENSG00000134198	chromosome1	115433577
ENSG00000110651	chromosome11	2355356
ENSG00000135622	chromosome2	74735012
ENSG00000154975	chromosome17	47590146
ENSG00000063180	chromosome19	53840702
ENSG00000115363	chromosome2	75598775
ENSG00000142694	chromosome1	36561226
ENSG00000142694	chromosome1	36561226
ENSG00000115363	chromosome2	75598775
ENSG00000142694	chromosome1	36561226
ENSG00000171243	chromosome7	16471819
ENSG00000167941	chromosome17	39191636
ENSG00000102796	chromosome13	51271797
ENSG00000158125	chromosome2	31491037
ENSG00000138356	chromosome2	201159077
ENSG00000138356	chromosome2	201159077
ENSG00000111911	chromosome6	126319817
ENSG00000205142	chromosome4	184955839
ENSG00000071889	chromosomeX	153397291
ENSG00000198643	chromosome3	58616331
ENSG00000198643	chromosome3	58616331
ENSG00000071889	chromosomeX	153397291
ENSG00000174028	chromosomeX	23003796
ENSG00000196937	chromosome7	120810260

ENSG00000183844	chromosome21	41610527
ENSG00000198643	chromosome3	58616331
ENSG00000071889	chromosomeX	153397291
ENSG00000174028	chromosomeX	23003796
ENSG00000196937	chromosome7	120810260
ENSG00000174028	chromosomeX	23003796
ENSG00000196937	chromosome7	120810260
ENSG00000198643	chromosome3	58616331
ENSG00000071889	chromosomeX	153397291
ENSG00000183691	chromosome17	52026584
ENSG00000183691	chromosome17	52026584
ENSG00000175806	chromosome8	9949437
ENSG00000159202	chromosome17	44340884
ENSG00000196911	chromosome6	117109056
ENSG00000025800	chromosome1	32346319
ENSG00000114030	chromosome3	123698103
ENSG00000025800	chromosome1	32346319
ENSG00000196911	chromosome6	117109056
ENSG00000184571	chromosome22	23488467
ENSG00000125207	chromosome12	129393090
ENSG00000134627	chromosome11	93940333
ENSG00000101109	chromosome20	43028624
ENSG00000104375	chromosome8	99906945
ENSG00000181991	chromosome15	86811953
ENSG00000164032	chromosome4	101090351
ENSG00000105968	chromosome7	44854240
ENSG00000105968	chromosome7	44854240
ENSG00000164032	chromosome4	101090351
ENSG00000154930	chromosome20	24986739
ENSG00000144908	chromosome3	127382502
ENSG00000136010	chromosome12	104002345
ENSG00000180138	chromosome13	36577394
ENSG00000113712	chromosome5	148910793
ENSG00000113712	chromosome5	148910793
ENSG00000180138	chromosome13	36577394
ENSG00000017260	chromosome3	132096304
ENSG00000064270	chromosome16	82959723
ENSG00000206190	chromosome15	23659337
ENSG00000118322	chromosome5	160047660
ENSG00000145246	chromosome4	47209315
ENSG00000145246	chromosome4	47209315
ENSG00000118322	chromosome5	160047660
ENSG00000156599	chromosome11	57197139
ENSG00000099904	chromosome22	18499471
ENSG00000099904	chromosome22	18499471
ENSG00000156599	chromosome11	57197139
ENSG00000099904	chromosome22	18499471
ENSG00000156599	chromosome11	57197139
ENSG00000163958	chromosome3	197422584
ENSG00000139780	chromosome13	102144850
ENSG00000165366	chromosome13	102330521
ENSG00000109184	chromosome4	52404166
ENSG00000137692	chromosome11	102467812
ENSG00000187446	chromosome15	39310873
ENSG00000166869	chromosome16	23673872
ENSG00000214530	chromosome11	72169875
ENSG00000214530	chromosome11	72169875
ENSG00000130377	chromosome19	6092168
ENSG00000103740	chromosome15	76313899

ENSG00000103740	chromosome15	76313899
ENSG00000130377	chromosome19	6092168
ENSG00000101082	chromosome20	34703153
ENSG00000155926	chromosome8	134141588
ENSG00000100351	chromosome22	38673057
ENSG00000101082	chromosome20	34703153
ENSG00000155926	chromosome8	134141588
ENSG00000100351	chromosome22	38673057
ENSG00000154016	chromosome17	18890965
ENSG00000155926	chromosome8	134141588
ENSG00000101082	chromosome20	34703153
ENSG00000176170	chromosome17	71892782
ENSG00000063176	chromosome19	53815584
ENSG00000159082	chromosome21	33021078
ENSG00000078269	chromosome6	158322982
ENSG00000166908	chromosome12	56271340
ENSG00000141720	chromosome17	34209204
ENSG00000150867	chromosome10	23043262
ENSG00000150867	chromosome10	23043262
ENSG00000141720	chromosome17	34209204
ENSG00000055609	chromosome7	151763805
ENSG00000167548	chromosome12	47735375
ENSG00000143889	chromosome2	38683514
ENSG00000104824	chromosome19	44032447
ENSG00000215255	chromosome15	32570039
ENSG00000184792	chromosome22	29420897
ENSG00000110048	chromosome11	59139750
ENSG00000110080	chromosome11	125781261
ENSG00000064225	chromosome3	99969975
ENSG00000157350	chromosome16	68989935
ENSG00000126091	chromosome1	43974521
ENSG00000064225	chromosome3	99969975
ENSG00000110080	chromosome11	125781261
ENSG00000064225	chromosome3	99969975
ENSG00000110080	chromosome11	125781261
ENSG00000126091	chromosome1	43974521
ENSG00000008513	chromosome8	134557450
ENSG00000157350	chromosome16	68989935
ENSG00000126091	chromosome1	43974521
ENSG00000181026	chromosome15	86970445
ENSG00000143319	chromosome1	154964069
ENSG00000143319	chromosome1	154964069
ENSG00000181026	chromosome15	86970445
ENSG00000181026	chromosome15	86970445
ENSG00000143319	chromosome1	154964069
ENSG00000172183	chromosome15	86983602
ENSG00000197217	chromosome8	23363359
ENSG00000198018	chromosome10	101409704
