# Supplementary material for: 2R and remodeling of vertebrate signal transduction engine
Source: BMC Biol. 2010 Dec 13;8:146. doi: 10.1186/1741-7007-8-146 (PMC3238295; doi:10.1186/1741-7007-8-146)
Supplement: Additional file 25 — TableS12. Fractions contained in 2RO-defined multiplicons by chromosome breakdown, paralog regions cover 83% of the human genome proving a WGD. [file 1741-7007-8-146-S25.pdf]

Chromosome	Gene_count	Genes_in_multiplicons	Fraction_in_multiplicons
chromosome1	2092	2077	99.282982791587%
chromosome10	681	623	91.4831130690162%
chromosome11	1051	699	66.5080875356803%
chromosome12	1022	928	90.8023483365949%
chromosome13	364	306	84.0659340659341%
chromosome14	616	594	96.4285714285714%
chromosome15	510	447	87.6470588235294%
chromosome16	660	615	93.1818181818182%
chromosome17	1110	770	69.3693693693694%
chromosome18	265	0	0%
chromosome19	1115	984	88.2511210762332%
chromosome2	1248	1002	80.2884615384615%
chromosome20	537	362	67.4115456238361%
chromosome21	177	0	0%
chromosome22	437	382	87.4141876430206%
chromosome3	1072	673	62.7798507462687%
chromosome4	735	685	93.1972789115646%
chromosome5	949	841	88.6195995785037%
chromosome6	973	932	95.7862281603289%
chromosome7	901	828	91.8978912319645%
chromosome8	653	565	86.5237366003063%
chromosome9	663	661	99.6983408748115%
chromosomeX	797	607	76.1606022584693%
chromosomeY	38	0	0%

83.4726240222865% of the genome of homo_sapiens is duplicated.
