# Supplementary material for: 2R and remodeling of vertebrate signal transduction engine
Source: BMC Biol. 2010 Dec 13;8:146. doi: 10.1186/1741-7007-8-146 (PMC3238295; doi:10.1186/1741-7007-8-146)
Supplement: Additional file 26 — TableS13. Chromosomal clusters for gene duplications mapped to Chordata. [file 1741-7007-8-146-S26.pdf]

	ChrMapID	Pvalue	OddsRatio	ExpCount	Count	Size
11q22.3	11q22.3	2.735059e-06	50.97784	0.971263	7	8
