# Supplementary material for: 2R and remodeling of vertebrate signal transduction engine
Source: BMC Biol. 2010 Dec 13;8:146. doi: 10.1186/1741-7007-8-146 (PMC3238295; doi:10.1186/1741-7007-8-146)
Supplement: Additional file 27 — TableS14. Chromosomal clusters for gene duplications mapped to Amniota/Tetrapoda. [file 1741-7007-8-146-S27.pdf]

	ChrMapID	Pvalue	OddsRatio	ExpCount	Count	Size
9p22	9p22	7.423929e-10	20.746689	1.3722433	12	20
6p22.1	6p22.1	2.208531e-07	48.045977	0.6175095	7	9
6p2	6p2	1.210757e-06	2.425920	20.6522611	44	301
19q13.4	19q13.4	5.527083e-06	4.828580	3.9795055	15	58
