# Supplementary material for: 2R and remodeling of vertebrate signal transduction engine
Source: BMC Biol. 2010 Dec 13;8:146. doi: 10.1186/1741-7007-8-146 (PMC3238295; doi:10.1186/1741-7007-8-146)
Supplement: Additional file 28 — TableS15. Chromosomal clusters for duplications mapped to Mammalia/Eutheria/Theria. [file 1741-7007-8-146-S28.pdf]

	ChrMapID	Pvalue	OddsRatio	ExpCount	Count	Size
6p2	6p2	5.873906e-21	3.609446	39.0917799	101	301
6p21.3	6p21.3	7.633477e-18	4.552952	21.4290488	65	165
19q13.4	19q13.4	9.737743e-12	6.845207	7.5326353	29	58
19q1	19q1	1.423177e-11	2.622732	39.0917799	82	301
6	6	3.632226e-10	2.044829	69.4820673	120	535
19	19	4.290806e-10	2.013207	72.7288928	124	560
6p22.1	6p22.1	1.023225e-08	Inf	1.1688572	9	9
9p22	9p22	8.992901e-08	12.571552	2.5974605	13	20
Xp21.3	Xp21.3	4.745081e-06	Inf	0.7792381	6	6
Xq	Xq	7.802268e-06	2.136875	28.4421920	52	219
