# Supplementary material for: 2R and remodeling of vertebrate signal transduction engine
Source: BMC Biol. 2010 Dec 13;8:146. doi: 10.1186/1741-7007-8-146 (PMC3238295; doi:10.1186/1741-7007-8-146)
Supplement: Additional file 29 — TableS16. Chromosomal clusters for human-specific duplications. [file 1741-7007-8-146-S29.pdf]

	ChrMapID	Pvalue	OddsRatio	ExpCount	Count	Size
X	X	5.569701e-07	3.332946	9.3155491	27	389
6p2	6p2	7.876626e-07	3.656231	7.2081755	23	301
Xp11	Xp11	8.414195e-07	6.941020	2.0594787	12	86
Y	Y	1.612955e-06	12.056591	0.8621074	8	36
Yq1	Yq1	2.567277e-06	20.935407	0.4310537	6	18
Yq11.2	Yq11.2	3.088997e-06	34.750000	0.2634217	5	11
Xq28	Xq28	9.480134e-06	6.630335	1.7721096	10	74
