# Supplementary material for: 2R and remodeling of vertebrate signal transduction engine
Source: BMC Biol. 2010 Dec 13;8:146. doi: 10.1186/1741-7007-8-146 (PMC3238295; doi:10.1186/1741-7007-8-146)
Supplement: Additional file 24 — TableS11. Paralogy relationship between human 2ROs. [file 1741-7007-8-146-S24.pdf]

paralog1	paralog2
ENSG00000000005	ENSG00000136110
ENSG00000000938	ENSG00000010810
ENSG00000000938	ENSG00000101336
ENSG00000000938	ENSG00000111816
ENSG00000000938	ENSG00000136573
ENSG00000000938	ENSG00000147507
ENSG00000000938	ENSG00000176105
ENSG00000000938	ENSG00000182866
ENSG00000000938	ENSG00000197122
ENSG00000000971	ENSG00000080910
ENSG00000000971	ENSG00000116785
ENSG00000000971	ENSG00000134365
ENSG00000000971	ENSG00000134389
ENSG00000000971	ENSG00000134391
ENSG00000000971	ENSG00000143278
ENSG00000001461	ENSG00000104361
ENSG00000001461	ENSG00000140157
ENSG00000001461	ENSG00000163293
ENSG00000001461	ENSG00000170113
ENSG00000001461	ENSG00000172548
ENSG00000001561	ENSG00000112796
ENSG00000001561	ENSG00000136960
ENSG00000001561	ENSG00000154269
ENSG00000001561	ENSG00000164303
ENSG00000001561	ENSG00000182156
ENSG00000001561	ENSG00000197594
ENSG00000001617	ENSG00000075223
ENSG00000002587	ENSG00000122254
ENSG00000002587	ENSG00000125430
ENSG00000002587	ENSG00000153976
ENSG00000002587	ENSG00000162040
ENSG00000002587	ENSG00000175818
ENSG00000002587	ENSG00000182601
ENSG00000002726	ENSG00000131471
ENSG00000002726	ENSG00000131480
ENSG00000002746	ENSG00000138411
ENSG00000002834	ENSG00000078114
ENSG00000002834	ENSG00000183091
ENSG00000002919	ENSG00000086300
ENSG00000003096	ENSG00000087448
ENSG00000003096	ENSG00000124743
ENSG00000003096	ENSG00000130487
ENSG00000003096	ENSG00000135686
ENSG00000003096	ENSG00000166853
ENSG00000003096	ENSG00000167487
ENSG00000003096	ENSG00000174010
ENSG00000003096	ENSG00000176595
ENSG00000003096	ENSG00000179023
ENSG00000003096	ENSG00000185214
ENSG00000003096	ENSG00000185915
ENSG00000003096	ENSG00000186231
ENSG00000003096	ENSG00000197705
ENSG00000003096	ENSG00000198642
ENSG00000003137	ENSG00000095596
ENSG00000003137	ENSG00000187553
ENSG00000003147	ENSG00000163596
ENSG00000003249	ENSG00000047579
ENSG00000003249	ENSG00000204070

ENSG00000003393	ENSG00000178038
ENSG00000003400	ENSG00000064012
ENSG00000003400	ENSG00000105141
ENSG00000003400	ENSG00000106144
ENSG00000003400	ENSG00000132906
ENSG00000003400	ENSG00000137752
ENSG00000003400	ENSG00000137757
ENSG00000003400	ENSG00000138794
ENSG00000003400	ENSG00000164305
ENSG00000003400	ENSG00000165806
ENSG00000003400	ENSG00000196954
ENSG00000003400	ENSG00000204403
ENSG00000003436	ENSG00000105825
ENSG00000003436	ENSG00000127578
ENSG00000003436	ENSG00000173714
ENSG00000003756	ENSG00000004534
ENSG00000003756	ENSG00000164252
ENSG00000003756	ENSG00000182872
ENSG00000003756	ENSG00000214603
ENSG00000003987	ENSG00000014914
ENSG00000003987	ENSG00000063601
ENSG00000003987	ENSG00000087053
ENSG00000003987	ENSG00000100330
ENSG00000003987	ENSG00000102043
ENSG00000003987	ENSG00000104643
ENSG00000003987	ENSG00000108389
ENSG00000003987	ENSG00000139505
ENSG00000003987	ENSG00000150712
ENSG00000003987	ENSG00000166912
ENSG00000003987	ENSG00000171100
ENSG00000003987	ENSG00000184416
ENSG00000003989	ENSG00000013293
ENSG00000003989	ENSG00000099960
ENSG00000003989	ENSG00000139514
ENSG00000003989	ENSG00000165349
ENSG00000004059	ENSG00000134287
ENSG00000004059	ENSG00000143761
ENSG00000004059	ENSG00000165527
ENSG00000004059	ENSG00000168374
ENSG00000004399	ENSG00000076356
ENSG00000004399	ENSG00000114554
ENSG00000004399	ENSG00000130827
ENSG00000004399	ENSG00000136040
ENSG00000004399	ENSG00000164050
ENSG00000004399	ENSG00000189437
ENSG00000004399	ENSG00000196576
ENSG00000004399	ENSG00000198753
ENSG00000004468	ENSG00000109743
ENSG00000004478	ENSG00000096060
ENSG00000004534	ENSG00000003756
ENSG00000004534	ENSG00000164252
ENSG00000004534	ENSG00000182872
ENSG00000004534	ENSG00000214603
ENSG00000004660	ENSG00000110931
ENSG00000004776	ENSG00000106211
ENSG00000004776	ENSG00000109846
ENSG00000004776	ENSG00000152137
ENSG00000004776	ENSG00000160202
ENSG00000004776	ENSG00000169271

ENSG00000004776	ENSG00000170276
ENSG00000004777	ENSG00000031081
ENSG00000004777	ENSG00000134909
ENSG00000004777	ENSG00000186517
ENSG00000004799	ENSG00000005882
ENSG00000004799	ENSG00000067992
ENSG00000004799	ENSG00000152256
ENSG00000004846	ENSG00000005471
ENSG00000004846	ENSG00000073734
ENSG00000004846	ENSG00000085563
ENSG00000004848	ENSG00000052850
ENSG00000004848	ENSG00000100987
ENSG00000004848	ENSG00000119614
ENSG00000004848	ENSG00000156150
ENSG00000004848	ENSG00000180318
ENSG00000004864	ENSG00000115840
ENSG00000004864	ENSG00000177542
ENSG00000004864	ENSG00000182902
ENSG00000004866	ENSG00000007341
ENSG00000004939	ENSG00000033867
ENSG00000004939	ENSG00000050438
ENSG00000004939	ENSG00000080493
ENSG00000004939	ENSG00000088836
ENSG00000004939	ENSG00000113073
ENSG00000004939	ENSG00000114923
ENSG00000004939	ENSG00000144290
ENSG00000004939	ENSG00000164889
ENSG00000004939	ENSG00000188687
ENSG00000004948	ENSG00000010310
ENSG00000004948	ENSG00000064989
ENSG00000004948	ENSG00000065325
ENSG00000004948	ENSG00000078549
ENSG00000004948	ENSG00000080293
ENSG00000004948	ENSG00000106018
ENSG00000004948	ENSG00000106113
ENSG00000004948	ENSG00000106128
ENSG00000004948	ENSG00000112164
ENSG00000004948	ENSG00000114812
ENSG00000004948	ENSG00000120088
ENSG00000004948	ENSG00000144407
ENSG00000004948	ENSG00000160801
ENSG00000004948	ENSG00000215644
ENSG00000004975	ENSG00000107404
ENSG00000004975	ENSG00000150764
ENSG00000004975	ENSG00000161202
ENSG00000004975	ENSG00000215737
ENSG00000005001	ENSG00000007038
ENSG00000005001	ENSG00000052344
ENSG00000005001	ENSG00000095917
ENSG00000005001	ENSG00000103355
ENSG00000005001	ENSG00000116176
ENSG00000005001	ENSG00000172236
ENSG00000005001	ENSG00000172382
ENSG00000005001	ENSG00000172460
ENSG00000005001	ENSG00000178055
ENSG00000005001	ENSG00000178226
ENSG00000005001	ENSG00000185888
ENSG00000005001	ENSG00000188086
ENSG00000005001	ENSG00000189099

ENSG00000005001	ENSG00000196364
ENSG00000005001	ENSG00000197253
ENSG00000005001	ENSG00000206549
ENSG00000005001	ENSG00000214877
ENSG00000005001	ENSG00000215148
ENSG00000005020	ENSG00000141293
ENSG00000005022	ENSG00000151475
ENSG00000005022	ENSG00000151729
ENSG00000005022	ENSG00000169100
ENSG00000005073	ENSG00000123388
ENSG00000005073	ENSG00000128713
ENSG00000005108	ENSG00000144229
ENSG00000005206	ENSG00000138600
ENSG00000005206	ENSG00000185294
ENSG00000005238	ENSG00000047346
ENSG00000005243	ENSG00000111481
ENSG00000005249	ENSG00000108946
ENSG00000005249	ENSG00000114302
ENSG00000005249	ENSG00000188191
ENSG00000005339	ENSG00000100393
ENSG00000005379	ENSG00000060709
ENSG00000005379	ENSG00000183246
ENSG00000005379	ENSG00000196622
ENSG00000005379	ENSG00000196934
ENSG00000005381	ENSG00000115705
ENSG00000005381	ENSG00000121053
ENSG00000005381	ENSG00000130508
ENSG00000005381	ENSG00000147485
ENSG00000005381	ENSG00000167419
ENSG00000005436	ENSG00000159086
ENSG00000005471	ENSG00000004846
ENSG00000005471	ENSG00000073734
ENSG00000005471	ENSG00000085563
ENSG00000005483	ENSG00000168137
ENSG00000005513	ENSG00000100146
ENSG00000005513	ENSG00000124766
ENSG00000005513	ENSG00000125398
ENSG00000005513	ENSG00000129194
ENSG00000005513	ENSG00000134595
ENSG00000005513	ENSG00000164736
ENSG00000005513	ENSG00000171056
ENSG00000005513	ENSG00000176887
ENSG00000005513	ENSG00000177732
ENSG00000005513	ENSG00000181449
ENSG00000005513	ENSG00000182968
ENSG00000005513	ENSG00000184895
ENSG00000005513	ENSG00000203883
ENSG00000005812	ENSG00000164616
ENSG00000005844	ENSG00000005884
ENSG00000005844	ENSG00000005961
ENSG00000005844	ENSG00000077943
ENSG00000005844	ENSG00000083457
ENSG00000005844	ENSG00000091409
ENSG00000005844	ENSG00000115232
ENSG00000005844	ENSG00000135424
ENSG00000005844	ENSG00000137809
ENSG00000005844	ENSG00000138448
ENSG00000005844	ENSG00000140678
ENSG00000005844	ENSG00000143127

ENSG00000005844	ENSG00000144668
ENSG00000005844	ENSG00000156886
ENSG00000005844	ENSG00000161638
ENSG00000005844	ENSG00000164171
ENSG00000005844	ENSG00000169896
ENSG00000005844	ENSG00000213949
ENSG00000005882	ENSG00000004799
ENSG00000005882	ENSG00000067992
ENSG00000005882	ENSG00000152256
ENSG00000005884	ENSG00000005844
ENSG00000005884	ENSG00000005961
ENSG00000005884	ENSG00000077943
ENSG00000005884	ENSG00000083457
ENSG00000005884	ENSG00000091409
ENSG00000005884	ENSG00000115232
ENSG00000005884	ENSG00000135424
ENSG00000005884	ENSG00000137809
ENSG00000005884	ENSG00000138448
ENSG00000005884	ENSG00000140678
ENSG00000005884	ENSG00000143127
ENSG00000005884	ENSG00000144668
ENSG00000005884	ENSG00000156886
ENSG00000005884	ENSG00000161638
ENSG00000005884	ENSG00000164171
ENSG00000005884	ENSG00000169896
ENSG00000005884	ENSG00000213949
ENSG00000005889	ENSG00000067646
ENSG00000005889	ENSG00000121864
ENSG00000005889	ENSG00000147180
ENSG00000005893	ENSG00000078081
ENSG00000005893	ENSG00000129226
ENSG00000005893	ENSG00000185896
ENSG00000005961	ENSG00000005844
ENSG00000005961	ENSG00000005884
ENSG00000005961	ENSG00000077943
ENSG00000005961	ENSG00000083457
ENSG00000005961	ENSG00000091409
ENSG00000005961	ENSG00000115232
ENSG00000005961	ENSG00000135424
ENSG00000005961	ENSG00000137809
ENSG00000005961	ENSG00000138448
ENSG00000005961	ENSG00000140678
ENSG00000005961	ENSG00000143127
ENSG00000005961	ENSG00000144668
ENSG00000005961	ENSG00000156886
ENSG00000005961	ENSG00000161638
ENSG00000005961	ENSG00000164171
ENSG00000005961	ENSG00000169896
ENSG00000005961	ENSG00000213949
ENSG00000006016	ENSG00000116678
ENSG00000006025	ENSG00000070882
ENSG00000006025	ENSG00000079156
ENSG00000006042	ENSG00000166946
ENSG00000006047	ENSG00000060138
ENSG00000006047	ENSG00000065978
ENSG00000006047	ENSG00000173616
ENSG00000006047	ENSG00000183443
ENSG00000006047	ENSG00000214299
ENSG00000006059	ENSG00000094796

ENSG00000006059	ENSG00000108244
ENSG00000006059	ENSG00000108417
ENSG00000006059	ENSG00000108759
ENSG00000006059	ENSG00000111057
ENSG00000006059	ENSG00000126337
ENSG00000006059	ENSG00000128422
ENSG00000006059	ENSG00000131737
ENSG00000006059	ENSG00000131738
ENSG00000006059	ENSG00000131885
ENSG00000006059	ENSG00000167916
ENSG00000006059	ENSG00000170819
ENSG00000006059	ENSG00000171345
ENSG00000006059	ENSG00000171346
ENSG00000006059	ENSG00000171360
ENSG00000006059	ENSG00000171401
ENSG00000006059	ENSG00000171403
ENSG00000006059	ENSG00000171431
ENSG00000006059	ENSG00000171446
ENSG00000006059	ENSG00000173908
ENSG00000006059	ENSG00000186393
ENSG00000006059	ENSG00000186395
ENSG00000006059	ENSG00000186831
ENSG00000006059	ENSG00000186832
ENSG00000006059	ENSG00000186847
ENSG00000006059	ENSG00000187242
ENSG00000006059	ENSG00000187686
ENSG00000006059	ENSG00000196859
ENSG00000006059	ENSG00000197079
ENSG00000006059	ENSG00000204889
ENSG00000006059	ENSG00000204897
ENSG00000006059	ENSG00000213951
ENSG00000006059	ENSG00000214220
ENSG00000006059	ENSG00000214454
ENSG00000006059	ENSG00000214959
ENSG00000006059	ENSG00000215772
ENSG00000006059	ENSG00000215773
ENSG00000006071	ENSG00000069431
ENSG00000006074	ENSG00000006075
ENSG00000006074	ENSG00000006606
ENSG00000006074	ENSG00000102962
ENSG00000006074	ENSG00000102970
ENSG00000006074	ENSG00000106178
ENSG00000006074	ENSG00000108688
ENSG00000006074	ENSG00000108691
ENSG00000006074	ENSG00000108700
ENSG00000006074	ENSG00000108702
ENSG00000006074	ENSG00000115009
ENSG00000006074	ENSG00000129277
ENSG00000006074	ENSG00000143184
ENSG00000006074	ENSG00000143185
ENSG00000006074	ENSG00000161570
ENSG00000006074	ENSG00000161573
ENSG00000006074	ENSG00000161574
ENSG00000006074	ENSG00000167236
ENSG00000006074	ENSG00000172156
ENSG00000006074	ENSG00000172724
ENSG00000006074	ENSG00000181374
ENSG00000006074	ENSG00000197262
ENSG00000006074	ENSG00000205017

ENSG00000006074	ENSG00000205020
ENSG00000006074	ENSG00000205021
ENSG00000006074	ENSG00000213494
ENSG00000006074	ENSG00000215627
ENSG00000006074	ENSG00000215629
ENSG00000006075	ENSG00000006074
ENSG00000006075	ENSG00000006606
ENSG00000006075	ENSG00000102962
ENSG00000006075	ENSG00000102970
ENSG00000006075	ENSG00000106178
ENSG00000006075	ENSG00000108688
ENSG00000006075	ENSG00000108691
ENSG00000006075	ENSG00000108700
ENSG00000006075	ENSG00000108702
ENSG00000006075	ENSG00000115009
ENSG00000006075	ENSG00000129277
ENSG00000006075	ENSG00000143184
ENSG00000006075	ENSG00000143185
ENSG00000006075	ENSG00000161570
ENSG00000006075	ENSG00000161573
ENSG00000006075	ENSG00000161574
ENSG00000006075	ENSG00000167236
ENSG00000006075	ENSG00000172156
ENSG00000006075	ENSG00000172724
ENSG00000006075	ENSG00000181374
ENSG00000006075	ENSG00000197262
ENSG00000006075	ENSG00000205017
ENSG00000006075	ENSG00000205020
ENSG00000006075	ENSG00000205021
ENSG00000006075	ENSG00000213494
ENSG00000006075	ENSG00000215627
ENSG00000006075	ENSG00000215629
ENSG00000006116	ENSG00000075429
ENSG00000006116	ENSG00000075461
ENSG00000006116	ENSG00000105605
ENSG00000006116	ENSG00000142408
ENSG00000006116	ENSG00000166862
ENSG00000006116	ENSG00000204278
ENSG00000006118	ENSG00000139364
ENSG00000006118	ENSG00000151952
ENSG00000006118	ENSG00000181234
ENSG00000006118	ENSG00000181291
ENSG00000006125	ENSG00000100280
ENSG00000006283	ENSG00000100346
ENSG00000006283	ENSG00000196557
ENSG00000006377	ENSG00000108813
ENSG00000006377	ENSG00000144355
ENSG00000006377	ENSG00000167034
ENSG00000006432	ENSG00000130758
ENSG00000006432	ENSG00000143674
ENSG00000006432	ENSG00000173327
ENSG00000006451	ENSG00000126458
ENSG00000006451	ENSG00000133703
ENSG00000006451	ENSG00000133818
ENSG00000006451	ENSG00000144118
ENSG00000006451	ENSG00000158186
ENSG00000006451	ENSG00000174775
ENSG00000006451	ENSG00000187682
ENSG00000006451	ENSG00000213281

ENSG00000006453	ENSG00000128298
ENSG00000006453	ENSG00000175866
ENSG00000006459	ENSG00000089094
ENSG00000006459	ENSG00000172943
ENSG00000006459	ENSG00000173120
ENSG00000006459	ENSG00000197724
ENSG00000006468	ENSG00000105672
ENSG00000006468	ENSG00000134954
ENSG00000006468	ENSG00000157557
ENSG00000006468	ENSG00000163497
ENSG00000006468	ENSG00000171656
ENSG00000006468	ENSG00000175832
ENSG00000006534	ENSG00000072210
ENSG00000006534	ENSG00000108602
ENSG00000006534	ENSG00000132746
ENSG00000006576	ENSG00000116793
ENSG00000006606	ENSG00000006074
ENSG00000006606	ENSG00000006075
ENSG00000006606	ENSG00000102962
ENSG00000006606	ENSG00000102970
ENSG00000006606	ENSG00000106178
ENSG00000006606	ENSG00000108688
ENSG00000006606	ENSG00000108691
ENSG00000006606	ENSG00000108700
ENSG00000006606	ENSG00000108702
ENSG00000006606	ENSG00000115009
ENSG00000006606	ENSG00000129277
ENSG00000006606	ENSG00000143184
ENSG00000006606	ENSG00000143185
ENSG00000006606	ENSG00000161570
ENSG00000006606	ENSG00000161573
ENSG00000006606	ENSG00000161574
ENSG00000006606	ENSG00000167236
ENSG00000006606	ENSG00000172156
ENSG00000006606	ENSG00000172724
ENSG00000006606	ENSG00000181374
ENSG00000006606	ENSG00000197262
ENSG00000006606	ENSG00000205017
ENSG00000006606	ENSG00000205020
ENSG00000006606	ENSG00000205021
ENSG00000006606	ENSG00000213494
ENSG00000006606	ENSG00000215627
ENSG00000006606	ENSG00000215629
ENSG00000006607	ENSG00000152767
ENSG00000006611	ENSG00000095397
ENSG00000006611	ENSG00000186862
ENSG00000006638	ENSG00000050628
ENSG00000006638	ENSG00000122420
ENSG00000006638	ENSG00000125384
ENSG00000006638	ENSG00000160013
ENSG00000006638	ENSG00000160951
ENSG00000006638	ENSG00000168229
ENSG00000006638	ENSG00000171522
ENSG00000006652	ENSG00000214706
ENSG00000006659	ENSG00000100079
ENSG00000006659	ENSG00000100097
ENSG00000006659	ENSG00000105198
ENSG00000006659	ENSG00000105205
ENSG00000006659	ENSG00000116977

ENSG00000006659	ENSG00000119862
ENSG00000006659	ENSG00000131981
ENSG00000006659	ENSG00000133317
ENSG00000006659	ENSG00000168961
ENSG00000006659	ENSG00000170298
ENSG00000006659	ENSG00000171747
ENSG00000006659	ENSG00000171916
ENSG00000006659	ENSG00000178934
ENSG00000006659	ENSG00000197778
ENSG00000006659	ENSG00000205076
ENSG00000006659	ENSG00000215132
ENSG00000006740	ENSG00000100092
ENSG00000006747	ENSG00000042493
ENSG00000006747	ENSG00000127831
ENSG00000006747	ENSG00000135407
ENSG00000006747	ENSG00000136059
ENSG00000006747	ENSG00000148180
ENSG00000006747	ENSG00000177731
ENSG00000006756	ENSG00000062096
ENSG00000006756	ENSG00000100299
ENSG00000006756	ENSG00000101846
ENSG00000006756	ENSG00000113273
ENSG00000006756	ENSG00000141012
ENSG00000006756	ENSG00000141337
ENSG00000006756	ENSG00000157399
ENSG00000006756	ENSG00000180801
ENSG00000006756	ENSG00000183876
ENSG00000006756	ENSG00000205667
ENSG00000006788	ENSG00000002079
ENSG00000006788	ENSG00000078814
ENSG00000006788	ENSG00000092054
ENSG00000006788	ENSG00000109061
ENSG00000006788	ENSG00000109063
ENSG00000006788	ENSG00000125414
ENSG00000006788	ENSG00000133020
ENSG00000006788	ENSG00000141048
ENSG00000006788	ENSG00000144821
ENSG00000006788	ENSG00000197616
ENSG00000006831	ENSG00000159346
ENSG00000006831	ENSG00000163291
ENSG00000006837	ENSG00000100490
ENSG00000006837	ENSG00000138769
ENSG00000006837	ENSG00000205111
ENSG00000007001	ENSG00000183696
ENSG00000007038	ENSG00000005001
ENSG00000007038	ENSG00000052344
ENSG00000007038	ENSG00000095917
ENSG00000007038	ENSG00000103355
ENSG00000007038	ENSG00000116176
ENSG00000007038	ENSG00000172236
ENSG00000007038	ENSG00000172382
ENSG00000007038	ENSG00000172460
ENSG00000007038	ENSG00000178055
ENSG00000007038	ENSG00000178226
ENSG00000007038	ENSG00000185888
ENSG00000007038	ENSG00000188086
ENSG00000007038	ENSG00000189099
ENSG00000007038	ENSG00000196364
ENSG00000007038	ENSG00000197253

ENSG00000007038	ENSG00000206549
ENSG00000007038	ENSG00000214877
ENSG00000007038	ENSG00000215148
ENSG00000007047	ENSG00000072518
ENSG00000007047	ENSG00000075413
ENSG00000007047	ENSG00000116141
ENSG00000007047	ENSG00000142178
ENSG00000007047	ENSG00000160584
ENSG00000007047	ENSG00000170145
ENSG00000007062	ENSG00000155066
ENSG00000007171	ENSG00000089250
ENSG00000007171	ENSG00000164867
ENSG00000007216	ENSG00000081800
ENSG00000007216	ENSG00000141485
ENSG00000007216	ENSG00000158296
ENSG00000007216	ENSG00000164707
ENSG00000007255	ENSG00000182400
ENSG00000007264	ENSG00000101213
ENSG00000007264	ENSG00000103653
ENSG00000007264	ENSG00000125508
ENSG00000007312	ENSG00000105369
ENSG00000007314	ENSG00000136531
ENSG00000007314	ENSG00000136546
ENSG00000007314	ENSG00000144285
ENSG00000007314	ENSG00000153253
ENSG00000007314	ENSG00000168356
ENSG00000007314	ENSG00000169432
ENSG00000007314	ENSG00000183873
ENSG00000007314	ENSG00000185313
ENSG00000007314	ENSG00000196876
ENSG00000007341	ENSG00000004866
ENSG00000007350	ENSG00000151005
ENSG00000007350	ENSG00000163931
ENSG00000007372	ENSG00000106331
ENSG00000007384	ENSG00000129667
ENSG00000007384	ENSG00000163902
ENSG00000007392	ENSG00000146963
ENSG00000007402	ENSG00000151062
ENSG00000007402	ENSG00000153956
ENSG00000007402	ENSG00000157445
ENSG00000007402	ENSG00000158966
ENSG00000007516	ENSG00000092929
ENSG00000007866	ENSG00000074219
ENSG00000007866	ENSG00000187079
ENSG00000007866	ENSG00000197905
ENSG00000007908	ENSG00000188404
ENSG00000007952	ENSG00000074771
ENSG00000007952	ENSG00000086991
ENSG00000007952	ENSG00000165168
ENSG00000007968	ENSG00000101412
ENSG00000007968	ENSG00000112242
ENSG00000007968	ENSG00000133740
ENSG00000007968	ENSG00000169016
ENSG00000007968	ENSG00000188841
ENSG00000007968	ENSG00000205250
ENSG00000008056	ENSG00000157152
ENSG00000008056	ENSG00000185666
ENSG00000008118	ENSG00000081320
ENSG00000008118	ENSG00000130822

ENSG00000008118	ENSG00000134072
ENSG00000008118	ENSG00000147613
ENSG00000008118	ENSG00000159792
ENSG00000008118	ENSG00000164076
ENSG00000008118	ENSG00000164543
ENSG00000008118	ENSG00000183049
ENSG00000008196	ENSG00000008197
ENSG00000008196	ENSG00000087510
ENSG00000008196	ENSG00000116819
ENSG00000008196	ENSG00000137203
ENSG00000008197	ENSG00000008196
ENSG00000008197	ENSG00000087510
ENSG00000008197	ENSG00000116819
ENSG00000008197	ENSG00000137203
ENSG00000008256	ENSG00000105443
ENSG00000008256	ENSG00000108669
ENSG00000008277	ENSG00000042980
ENSG00000008277	ENSG00000069206
ENSG00000008277	ENSG00000073670
ENSG00000008277	ENSG00000104755
ENSG00000008277	ENSG00000114948
ENSG00000008277	ENSG00000134007
ENSG00000008277	ENSG00000134028
ENSG00000008277	ENSG00000134249
ENSG00000008277	ENSG00000135074
ENSG00000008277	ENSG00000139985
ENSG00000008277	ENSG00000143537
ENSG00000008277	ENSG00000148848
ENSG00000008277	ENSG00000149451
ENSG00000008277	ENSG00000151651
ENSG00000008277	ENSG00000151694
ENSG00000008277	ENSG00000168594
ENSG00000008277	ENSG00000168615
ENSG00000008277	ENSG00000168619
ENSG00000008277	ENSG00000196115
ENSG00000008277	ENSG00000197140
ENSG00000008277	ENSG00000214137
ENSG00000008282	ENSG00000102003
ENSG00000008282	ENSG00000143028
ENSG00000008282	ENSG00000163630
ENSG00000008294	ENSG00000138834
ENSG00000008300	ENSG00000075275
ENSG00000008300	ENSG00000143126
ENSG00000008323	ENSG00000171680
ENSG00000008323	ENSG00000187510
ENSG00000008382	ENSG00000162601
ENSG00000008405	ENSG00000121671
ENSG00000008441	ENSG00000141905
ENSG00000008441	ENSG00000147862
ENSG00000008441	ENSG00000162599
ENSG00000008513	ENSG00000064225
ENSG00000008513	ENSG00000110080
ENSG00000008513	ENSG00000126091
ENSG00000008513	ENSG00000157350
ENSG00000008516	ENSG00000087245
ENSG00000008516	ENSG00000099953
ENSG00000008516	ENSG00000100985
ENSG00000008516	ENSG00000102996
ENSG00000008516	ENSG00000110347

ENSG00000008516	ENSG00000118113
ENSG00000008516	ENSG00000123342
ENSG00000008516	ENSG00000125966
ENSG00000008516	ENSG00000129270
ENSG00000008516	ENSG00000137673
ENSG00000008516	ENSG00000137674
ENSG00000008516	ENSG00000137675
ENSG00000008516	ENSG00000137745
ENSG00000008516	ENSG00000149968
ENSG00000008516	ENSG00000154485
ENSG00000008516	ENSG00000156103
ENSG00000008516	ENSG00000157227
ENSG00000008516	ENSG00000166670
ENSG00000008516	ENSG00000167346
ENSG00000008516	ENSG00000189409
ENSG00000008516	ENSG00000196611
ENSG00000008516	ENSG00000198598
ENSG00000008516	ENSG00000215914
ENSG00000008710	ENSG00000078795
ENSG00000008710	ENSG00000107593
ENSG00000008710	ENSG00000118762
ENSG00000008710	ENSG00000130943
ENSG00000008710	ENSG00000158683
ENSG00000008710	ENSG00000166473
ENSG00000008710	ENSG00000183458
ENSG00000008710	ENSG00000183793
ENSG00000008710	ENSG00000187008
ENSG00000008710	ENSG00000188599
ENSG00000008735	ENSG00000121653
ENSG00000008853	ENSG00000072422
ENSG00000008853	ENSG00000164292
ENSG00000008869	ENSG00000129493
ENSG00000009694	ENSG00000145934
ENSG00000009694	ENSG00000149256
ENSG00000009709	ENSG00000135903
ENSG00000009724	ENSG00000127241
ENSG00000009724	ENSG00000139178
ENSG00000009724	ENSG00000159403
ENSG00000009724	ENSG00000182326
ENSG00000009780	ENSG00000077458
ENSG00000009950	ENSG00000175727
ENSG00000010017	ENSG00000141084
ENSG00000010017	ENSG00000167778
ENSG00000010030	ENSG00000102034
ENSG00000010030	ENSG00000109381
ENSG00000010030	ENSG00000120690
ENSG00000010030	ENSG00000124664
ENSG00000010030	ENSG00000135373
ENSG00000010030	ENSG00000135374
ENSG00000010030	ENSG00000139083
ENSG00000010030	ENSG00000163435
ENSG00000010030	ENSG00000166211
ENSG00000010256	ENSG00000105819
ENSG00000010278	ENSG00000110651
ENSG00000010278	ENSG00000127324
ENSG00000010278	ENSG00000134198
ENSG00000010295	ENSG00000169991
ENSG00000010310	ENSG00000004948
ENSG00000010310	ENSG00000064989

ENSG00000010310	ENSG00000065325
ENSG00000010310	ENSG00000078549
ENSG00000010310	ENSG00000080293
ENSG00000010310	ENSG00000106018
ENSG00000010310	ENSG00000106113
ENSG00000010310	ENSG00000106128
ENSG00000010310	ENSG00000112164
ENSG00000010310	ENSG00000114812
ENSG00000010310	ENSG00000120088
ENSG00000010310	ENSG00000144407
ENSG00000010310	ENSG00000160801
ENSG00000010310	ENSG00000215644
ENSG00000010318	ENSG00000092140
ENSG00000010318	ENSG00000136147
ENSG00000010318	ENSG00000156531
ENSG00000010319	ENSG00000012171
ENSG00000010319	ENSG00000075213
ENSG00000010319	ENSG00000092421
ENSG00000010319	ENSG00000095539
ENSG00000010319	ENSG00000137872
ENSG00000010319	ENSG00000143434
ENSG00000010319	ENSG00000153993
ENSG00000010319	ENSG00000168758
ENSG00000010319	ENSG00000170381
ENSG00000010319	ENSG00000185033
ENSG00000010319	ENSG00000187764
ENSG00000010319	ENSG00000196189
ENSG00000010327	ENSG00000136011
ENSG00000010379	ENSG00000011083
ENSG00000010379	ENSG00000087916
ENSG00000010379	ENSG00000103546
ENSG00000010379	ENSG00000108576
ENSG00000010379	ENSG00000111181
ENSG00000010379	ENSG00000130821
ENSG00000010379	ENSG00000131389
ENSG00000010379	ENSG00000132164
ENSG00000010379	ENSG00000142319
ENSG00000010379	ENSG00000157103
ENSG00000010379	ENSG00000163817
ENSG00000010379	ENSG00000164363
ENSG00000010379	ENSG00000165970
ENSG00000010379	ENSG00000174358
ENSG00000010379	ENSG00000196517
ENSG00000010379	ENSG00000198555
ENSG00000010379	ENSG00000214617
ENSG00000010438	ENSG00000129437
ENSG00000010438	ENSG00000129451
ENSG00000010438	ENSG00000129455
ENSG00000010438	ENSG00000142515
ENSG00000010438	ENSG00000165076
ENSG00000010438	ENSG00000167748
ENSG00000010438	ENSG00000167749
ENSG00000010438	ENSG00000167751
ENSG00000010438	ENSG00000167754
ENSG00000010438	ENSG00000167755
ENSG00000010438	ENSG00000167757
ENSG00000010438	ENSG00000167759
ENSG00000010438	ENSG00000169035
ENSG00000010438	ENSG00000171147

ENSG00000010438	ENSG00000173636
ENSG00000010438	ENSG00000174562
ENSG00000010438	ENSG00000186474
ENSG00000010438	ENSG00000204981
ENSG00000010438	ENSG00000204982
ENSG00000010438	ENSG00000204983
ENSG00000010438	ENSG00000213022
ENSG00000010438	ENSG00000214083
ENSG00000010671	ENSG00000074966
ENSG00000010671	ENSG00000102010
ENSG00000010671	ENSG00000113263
ENSG00000010671	ENSG00000135605
ENSG00000010671	ENSG00000151422
ENSG00000010671	ENSG00000182511
ENSG00000010803	ENSG00000102098
ENSG00000010803	ENSG00000146285
ENSG00000010810	ENSG00000000938
ENSG00000010810	ENSG00000101336
ENSG00000010810	ENSG00000111816
ENSG00000010810	ENSG00000136573
ENSG00000010810	ENSG00000147507
ENSG00000010810	ENSG00000176105
ENSG00000010810	ENSG00000182866
ENSG00000010810	ENSG00000197122
ENSG00000010818	ENSG00000095951
ENSG00000010818	ENSG00000127124
ENSG00000011007	ENSG00000183791
ENSG00000011007	ENSG00000206181
ENSG00000011007	ENSG00000206182
ENSG00000011007	ENSG00000206183
ENSG00000011009	ENSG00000120992
ENSG00000011009	ENSG00000143353
ENSG00000011028	ENSG00000054219
ENSG00000011028	ENSG00000120586
ENSG00000011028	ENSG00000153246
ENSG00000011028	ENSG00000183748
ENSG00000011052	ENSG00000103024
ENSG00000011052	ENSG00000103200
ENSG00000011052	ENSG00000123009
ENSG00000011083	ENSG00000010379
ENSG00000011083	ENSG00000087916
ENSG00000011083	ENSG00000103546
ENSG00000011083	ENSG00000108576
ENSG00000011083	ENSG00000111181
ENSG00000011083	ENSG00000130821
ENSG00000011083	ENSG00000131389
ENSG00000011083	ENSG00000132164
ENSG00000011083	ENSG00000142319
ENSG00000011083	ENSG00000157103
ENSG00000011083	ENSG00000163817
ENSG00000011083	ENSG00000164363
ENSG00000011083	ENSG00000165970
ENSG00000011083	ENSG00000174358
ENSG00000011083	ENSG00000196517
ENSG00000011083	ENSG00000198555
ENSG00000011083	ENSG00000214617
ENSG00000011105	ENSG00000085117
ENSG00000011105	ENSG00000104894
ENSG00000011105	ENSG00000110900

ENSG00000011105	ENSG00000117472
ENSG00000011105	ENSG00000143119
ENSG00000011105	ENSG00000157570
ENSG00000011105	ENSG00000177697
ENSG00000011105	ENSG00000214063
ENSG00000011132	ENSG00000034053
ENSG00000011132	ENSG00000107282
ENSG00000011177	ENSG00000141744
ENSG00000011177	ENSG00000166741
ENSG00000011198	ENSG00000100439
ENSG00000011243	ENSG00000105127
ENSG00000011243	ENSG00000162664
ENSG00000011258	ENSG00000100395
ENSG00000011258	ENSG00000154655
ENSG00000011258	ENSG00000163935
ENSG00000011258	ENSG00000185513
ENSG00000011258	ENSG00000198879
ENSG00000011258	ENSG00000198945
ENSG00000011304	ENSG00000117569
ENSG00000011304	ENSG00000119314
ENSG00000011332	ENSG00000130024
ENSG00000011332	ENSG00000133884
ENSG00000011332	ENSG00000205683
ENSG00000011405	ENSG00000051382
ENSG00000011405	ENSG00000105851
ENSG00000011405	ENSG00000121879
ENSG00000011405	ENSG00000133056
ENSG00000011405	ENSG00000139144
ENSG00000011405	ENSG00000171608
ENSG00000011451	ENSG00000122482
ENSG00000011454	ENSG00000065882
ENSG00000011454	ENSG00000067208
ENSG00000011454	ENSG00000136111
ENSG00000011454	ENSG00000142459
ENSG00000011454	ENSG00000152061
ENSG00000011465	ENSG00000106819
ENSG00000011465	ENSG00000139329
ENSG00000011465	ENSG00000182492
ENSG00000011478	ENSG00000115828
ENSG00000011566	ENSG00000012983
ENSG00000011566	ENSG00000104814
ENSG00000011566	ENSG00000168067
ENSG00000011590	ENSG00000109906
ENSG00000011590	ENSG00000166860
ENSG00000011677	ENSG00000022355
ENSG00000011677	ENSG00000094755
ENSG00000011677	ENSG00000101958
ENSG00000011677	ENSG00000102287
ENSG00000011677	ENSG00000109158
ENSG00000011677	ENSG00000109738
ENSG00000011677	ENSG00000111886
ENSG00000011677	ENSG00000113327
ENSG00000011677	ENSG00000145451
ENSG00000011677	ENSG00000145863
ENSG00000011677	ENSG00000145864
ENSG00000011677	ENSG00000145888
ENSG00000011677	ENSG00000146276
ENSG00000011677	ENSG00000147402
ENSG00000011677	ENSG00000151834

ENSG00000011677	ENSG00000163285
ENSG00000011677	ENSG00000163288
ENSG00000011677	ENSG00000166206
ENSG00000011677	ENSG00000182256
ENSG00000011677	ENSG00000183185
ENSG00000011677	ENSG00000186297
ENSG00000011677	ENSG00000187730
ENSG00000011677	ENSG00000188828
ENSG00000012124	ENSG00000088827
ENSG00000012171	ENSG00000010319
ENSG00000012171	ENSG00000075213
ENSG00000012171	ENSG00000092421
ENSG00000012171	ENSG00000095539
ENSG00000012171	ENSG00000137872
ENSG00000012171	ENSG00000143434
ENSG00000012171	ENSG00000153993
ENSG00000012171	ENSG00000168758
ENSG00000012171	ENSG00000170381
ENSG00000012171	ENSG00000185033
ENSG00000012171	ENSG00000187764
ENSG00000012171	ENSG00000196189
ENSG00000012211	ENSG00000071282
ENSG00000012211	ENSG00000124593
ENSG00000012211	ENSG00000135269
ENSG00000012211	ENSG00000139174
ENSG00000012211	ENSG00000163637
ENSG00000012223	ENSG00000091513
ENSG00000012223	ENSG00000163975
ENSG00000012660	ENSG00000066322
ENSG00000012660	ENSG00000118402
ENSG00000012660	ENSG00000164181
ENSG00000012660	ENSG00000197977
ENSG00000012779	ENSG00000108839
ENSG00000012779	ENSG00000161905
ENSG00000012779	ENSG00000179148
ENSG00000012779	ENSG00000179477
ENSG00000012779	ENSG00000179593
ENSG00000012779	ENSG00000188101
ENSG00000012779	ENSG00000214859
ENSG00000012817	ENSG00000073614
ENSG00000012817	ENSG00000117139
ENSG00000012817	ENSG00000126012
ENSG00000012822	ENSG00000106052
ENSG00000012822	ENSG00000136436
ENSG00000012983	ENSG00000011566
ENSG00000012983	ENSG00000104814
ENSG00000012983	ENSG00000168067
ENSG00000013016	ENSG00000024422
ENSG00000013016	ENSG00000103966
ENSG00000013016	ENSG00000110047
ENSG00000013306	ENSG00000075303
ENSG00000013441	ENSG00000113240
ENSG00000013441	ENSG00000176444
ENSG00000013441	ENSG00000178340
ENSG00000013441	ENSG00000179335
ENSG00000013441	ENSG00000215793
ENSG00000013455	ENSG00000130429
ENSG00000013523	ENSG00000174606
ENSG00000013563	ENSG00000163687

ENSG00000013563	ENSG00000167968
ENSG00000013563	ENSG00000213918
ENSG00000013583	ENSG00000051620
ENSG00000013588	ENSG00000111291
ENSG00000013588	ENSG00000167191
ENSG00000013588	ENSG00000170412
ENSG00000013619	ENSG00000161021
ENSG00000013619	ENSG00000184384
ENSG00000013619	ENSG00000196782
ENSG00000013725	ENSG00000073754
ENSG00000013725	ENSG00000110448
ENSG00000013725	ENSG00000146700
ENSG00000013725	ENSG00000164099
ENSG00000013725	ENSG00000177575
ENSG00000013725	ENSG00000177675
ENSG00000013725	ENSG00000179954
ENSG00000013725	ENSG00000187908
ENSG00000013725	ENSG00000214279
ENSG00000013810	ENSG00000138162
ENSG00000013810	ENSG00000147526
ENSG00000014216	ENSG00000077274
ENSG00000014216	ENSG00000092529
ENSG00000014216	ENSG00000135773
ENSG00000014216	ENSG00000137225
ENSG00000014216	ENSG00000142330
ENSG00000014216	ENSG00000149260
ENSG00000014216	ENSG00000162909
ENSG00000014216	ENSG00000162949
ENSG00000014216	ENSG00000182472
ENSG00000014216	ENSG00000203697
ENSG00000014216	ENSG00000214711
ENSG00000014257	ENSG00000134575
ENSG00000014257	ENSG00000142513
ENSG00000014914	ENSG00000003987
ENSG00000014914	ENSG00000063601
ENSG00000014914	ENSG00000087053
ENSG00000014914	ENSG00000100330
ENSG00000014914	ENSG00000102043
ENSG00000014914	ENSG00000104643
ENSG00000014914	ENSG00000108389
ENSG00000014914	ENSG00000139505
ENSG00000014914	ENSG00000150712
ENSG00000014914	ENSG00000166912
ENSG00000014914	ENSG00000171100
ENSG00000014914	ENSG00000184416
ENSG00000015133	ENSG00000095066
ENSG00000015133	ENSG00000115355
ENSG00000015133	ENSG00000134709
ENSG00000015133	ENSG00000168071
ENSG00000015133	ENSG00000168172
ENSG00000015153	ENSG00000163602
ENSG00000015285	ENSG00000106299
ENSG00000015413	ENSG00000141096
ENSG00000015413	ENSG00000167261
ENSG00000015479	ENSG00000075292
ENSG00000015479	ENSG00000203867
ENSG00000015532	ENSG00000103489
ENSG00000015532	ENSG00000111846
ENSG00000015532	ENSG00000124091

ENSG00000015532	ENSG00000140297
ENSG00000015532	ENSG00000176928
ENSG00000015532	ENSG00000187210
ENSG00000015532	ENSG00000205318
ENSG00000015592	ENSG00000104435
ENSG00000015592	ENSG00000117632
ENSG00000015592	ENSG00000213603
ENSG00000015592	ENSG00000214241
ENSG00000016082	ENSG00000089116
ENSG00000016082	ENSG00000106689
ENSG00000016082	ENSG00000106852
ENSG00000016082	ENSG00000107187
ENSG00000016082	ENSG00000121454
ENSG00000016082	ENSG00000132130
ENSG00000016082	ENSG00000136944
ENSG00000016082	ENSG00000143013
ENSG00000016082	ENSG00000143355
ENSG00000016082	ENSG00000159556
ENSG00000016082	ENSG00000162624
ENSG00000016082	ENSG00000162761
ENSG00000016864	ENSG00000120820
ENSG00000017260	ENSG00000064270
ENSG00000017373	ENSG00000120549
ENSG00000017427	ENSG00000129965
ENSG00000017427	ENSG00000167244
ENSG00000017483	ENSG00000103042
ENSG00000017483	ENSG00000111371
ENSG00000017483	ENSG00000134294
ENSG00000017483	ENSG00000139209
ENSG00000017483	ENSG00000139974
ENSG00000017483	ENSG00000166558
ENSG00000017483	ENSG00000169507
ENSG00000017483	ENSG00000188338
ENSG00000017621	ENSG00000150175
ENSG00000017621	ENSG00000197704
ENSG00000017621	ENSG00000213463
ENSG00000018189	ENSG00000105784
ENSG00000018189	ENSG00000108309
ENSG00000018189	ENSG00000176783
ENSG00000018189	ENSG00000204130
ENSG00000018189	ENSG00000214795
ENSG00000018236	ENSG00000113805
ENSG00000018236	ENSG00000134115
ENSG00000018236	ENSG00000144619
ENSG00000018280	ENSG00000110911
ENSG00000018408	ENSG00000137693
ENSG00000018408	ENSG00000214251
ENSG00000018625	ENSG00000075673
ENSG00000018625	ENSG00000105409
ENSG00000018625	ENSG00000105675
ENSG00000018625	ENSG00000132681
ENSG00000018625	ENSG00000163399
ENSG00000019102	ENSG00000143167
ENSG00000019102	ENSG00000144847
ENSG00000019102	ENSG00000149564
ENSG00000019102	ENSG00000154639
ENSG00000019102	ENSG00000166250
ENSG00000019102	ENSG00000188004
ENSG00000019144	ENSG00000144824

ENSG00000019169	ENSG00000038945
ENSG00000019169	ENSG00000168079
ENSG00000019186	ENSG00000111012
ENSG00000019186	ENSG00000135929
ENSG00000019186	ENSG00000140459
ENSG00000019186	ENSG00000160882
ENSG00000019186	ENSG00000179142
ENSG00000019186	ENSG00000186684
ENSG00000019549	ENSG00000124216
ENSG00000019549	ENSG00000170616
ENSG00000019549	ENSG00000177835
ENSG00000019549	ENSG00000185669
ENSG00000019549	ENSG00000215397
ENSG00000019549	ENSG00000215713
ENSG00000019582	ENSG00000107742
ENSG00000019582	ENSG00000152377
ENSG00000019582	ENSG00000196104
ENSG00000019991	ENSG00000104368
ENSG00000019991	ENSG00000109758
ENSG00000019991	ENSG00000122194
ENSG00000019991	ENSG00000122861
ENSG00000019991	ENSG00000131187
ENSG00000019991	ENSG00000148702
ENSG00000019991	ENSG00000173531
ENSG00000019991	ENSG00000186301
ENSG00000019991	ENSG00000186715
ENSG00000019991	ENSG00000198670
ENSG00000020181	ENSG00000152990
ENSG00000020181	ENSG00000197177
ENSG00000020256	ENSG00000151612
ENSG00000020577	ENSG00000179134
ENSG00000020633	ENSG00000124813
ENSG00000020633	ENSG00000159216
ENSG00000021300	ENSG00000115762
ENSG00000021355	ENSG00000057149
ENSG00000021355	ENSG00000124570
ENSG00000021355	ENSG00000166396
ENSG00000021355	ENSG00000166401
ENSG00000021355	ENSG00000166404
ENSG00000021355	ENSG00000166634
ENSG00000021355	ENSG00000170542
ENSG00000021355	ENSG00000197632
ENSG00000021355	ENSG00000197641
ENSG00000021355	ENSG00000206072
ENSG00000021355	ENSG00000206073
ENSG00000021355	ENSG00000206075
ENSG00000021461	ENSG00000059377
ENSG00000021461	ENSG00000106258
ENSG00000021461	ENSG00000160868
ENSG00000021461	ENSG00000160870
ENSG00000021645	ENSG00000110076
ENSG00000021645	ENSG00000179915
ENSG00000021762	ENSG00000091039
ENSG00000021762	ENSG00000117859
ENSG00000021762	ENSG00000144645
ENSG00000021762	ENSG00000144909
ENSG00000021852	ENSG00000039537
ENSG00000021852	ENSG00000112936
ENSG00000021852	ENSG00000113600

ENSG00000021852	ENSG00000157131
ENSG00000021852	ENSG00000180644
ENSG00000022355	ENSG00000011677
ENSG00000022355	ENSG00000094755
ENSG00000022355	ENSG00000101958
ENSG00000022355	ENSG00000102287
ENSG00000022355	ENSG00000109158
ENSG00000022355	ENSG00000109738
ENSG00000022355	ENSG00000111886
ENSG00000022355	ENSG00000113327
ENSG00000022355	ENSG00000145451
ENSG00000022355	ENSG00000145863
ENSG00000022355	ENSG00000145864
ENSG00000022355	ENSG00000145888
ENSG00000022355	ENSG00000146276
ENSG00000022355	ENSG00000147402
ENSG00000022355	ENSG00000151834
ENSG00000022355	ENSG00000163285
ENSG00000022355	ENSG00000163288
ENSG00000022355	ENSG00000166206
ENSG00000022355	ENSG00000182256
ENSG00000022355	ENSG00000183185
ENSG00000022355	ENSG00000186297
ENSG00000022355	ENSG00000187730
ENSG00000022355	ENSG00000188828
ENSG00000022556	ENSG00000023191
ENSG00000022556	ENSG00000140853
ENSG00000022556	ENSG00000142405
ENSG00000022556	ENSG00000158077
ENSG00000022556	ENSG00000160505
ENSG00000022556	ENSG00000162711
ENSG00000022556	ENSG00000167207
ENSG00000022556	ENSG00000167634
ENSG00000022556	ENSG00000167984
ENSG00000022556	ENSG00000171487
ENSG00000022556	ENSG00000173572
ENSG00000022556	ENSG00000174885
ENSG00000022556	ENSG00000179709
ENSG00000022556	ENSG00000179873
ENSG00000022556	ENSG00000182261
ENSG00000022556	ENSG00000185792
ENSG00000023171	ENSG00000089351
ENSG00000023171	ENSG00000175318
ENSG00000023171	ENSG00000178075
ENSG00000023191	ENSG00000022556
ENSG00000023191	ENSG00000140853
ENSG00000023191	ENSG00000142405
ENSG00000023191	ENSG00000158077
ENSG00000023191	ENSG00000160505
ENSG00000023191	ENSG00000162711
ENSG00000023191	ENSG00000167207
ENSG00000023191	ENSG00000167634
ENSG00000023191	ENSG00000167984
ENSG00000023191	ENSG00000171487
ENSG00000023191	ENSG00000173572
ENSG00000023191	ENSG00000174885
ENSG00000023191	ENSG00000179709
ENSG00000023191	ENSG00000179873
ENSG00000023191	ENSG00000182261

ENSG00000023191	ENSG00000185792
ENSG00000023330	ENSG00000158578
ENSG00000023445	ENSG00000081770
ENSG00000023445	ENSG00000101197
ENSG00000023445	ENSG00000101966
ENSG00000023445	ENSG00000110330
ENSG00000023445	ENSG00000179978
ENSG00000023445	ENSG00000180152
ENSG00000023839	ENSG00000091262
ENSG00000023839	ENSG00000103222
ENSG00000023839	ENSG00000108846
ENSG00000023892	ENSG00000133789
ENSG00000023902	ENSG00000169094
ENSG00000024048	ENSG00000144357
ENSG00000024048	ENSG00000159459
ENSG00000024422	ENSG00000013016
ENSG00000024422	ENSG00000103966
ENSG00000024422	ENSG00000110047
ENSG00000024526	ENSG00000035499
ENSG00000024526	ENSG00000121690
ENSG00000024526	ENSG00000166153
ENSG00000024862	ENSG00000160050
ENSG00000025039	ENSG00000116954
ENSG00000025156	ENSG00000102878
ENSG00000025156	ENSG00000169953
ENSG00000025156	ENSG00000171116
ENSG00000025156	ENSG00000171129
ENSG00000025156	ENSG00000172468
ENSG00000025156	ENSG00000176160
ENSG00000025156	ENSG00000185122
ENSG00000025156	ENSG00000214373
ENSG00000025156	ENSG00000215574
ENSG00000025156	ENSG00000215701
ENSG00000025293	ENSG00000129292
ENSG00000025423	ENSG00000073737
ENSG00000025423	ENSG00000086696
ENSG00000025423	ENSG00000135437
ENSG00000025423	ENSG00000139547
ENSG00000025423	ENSG00000161267
ENSG00000025423	ENSG00000170426
ENSG00000025423	ENSG00000176387
ENSG00000025434	ENSG00000131408
ENSG00000025800	ENSG00000114030
ENSG00000025800	ENSG00000196911
ENSG00000026025	ENSG00000100285
ENSG00000026025	ENSG00000104722
ENSG00000026025	ENSG00000104725
ENSG00000026025	ENSG00000131095
ENSG00000026025	ENSG00000135406
ENSG00000026025	ENSG00000148798
ENSG00000026025	ENSG00000175084
ENSG00000026025	ENSG00000182253
ENSG00000026025	ENSG00000215479
ENSG00000026103	ENSG00000067182
ENSG00000026103	ENSG00000104689
ENSG00000026103	ENSG00000120889
ENSG00000026103	ENSG00000173530
ENSG00000026103	ENSG00000173535
ENSG00000026103	ENSG00000215788

ENSG00000026508	ENSG00000123610
ENSG00000026508	ENSG00000133800
ENSG00000026559	ENSG00000102057
ENSG00000026559	ENSG00000104848
ENSG00000026559	ENSG00000111262
ENSG00000026559	ENSG00000124134
ENSG00000026559	ENSG00000130037
ENSG00000026559	ENSG00000143105
ENSG00000026559	ENSG00000151079
ENSG00000026559	ENSG00000156486
ENSG00000026559	ENSG00000158445
ENSG00000026559	ENSG00000162975
ENSG00000026559	ENSG00000164794
ENSG00000026559	ENSG00000168263
ENSG00000026559	ENSG00000168418
ENSG00000026559	ENSG00000170745
ENSG00000026559	ENSG00000171126
ENSG00000026559	ENSG00000171385
ENSG00000026559	ENSG00000177272
ENSG00000026559	ENSG00000177301
ENSG00000026559	ENSG00000178342
ENSG00000026559	ENSG00000182255
ENSG00000026559	ENSG00000182674
ENSG00000026559	ENSG00000184408
ENSG00000026652	ENSG00000160216
ENSG00000026950	ENSG00000111801
ENSG00000026950	ENSG00000112763
ENSG00000026950	ENSG00000113303
ENSG00000026950	ENSG00000121236
ENSG00000026950	ENSG00000124508
ENSG00000026950	ENSG00000124549
ENSG00000026950	ENSG00000124557
ENSG00000026950	ENSG00000128250
ENSG00000026950	ENSG00000128253
ENSG00000026950	ENSG00000128276
ENSG00000026950	ENSG00000128482
ENSG00000026950	ENSG00000132256
ENSG00000026950	ENSG00000132274
ENSG00000026950	ENSG00000164010
ENSG00000026950	ENSG00000165810
ENSG00000026950	ENSG00000168903
ENSG00000026950	ENSG00000176422
ENSG00000026950	ENSG00000179046
ENSG00000026950	ENSG00000182805
ENSG00000026950	ENSG00000183439
ENSG00000026950	ENSG00000183718
ENSG00000026950	ENSG00000187574
ENSG00000026950	ENSG00000188683
ENSG00000026950	ENSG00000204614
ENSG00000026950	ENSG00000204616
ENSG00000026950	ENSG00000204618
ENSG00000026950	ENSG00000205198
ENSG00000026950	ENSG00000214693
ENSG00000027644	ENSG00000047936
ENSG00000027644	ENSG00000062524
ENSG00000027644	ENSG00000140443
ENSG00000027644	ENSG00000171094
ENSG00000027644	ENSG00000171105
ENSG00000027869	ENSG00000104611

ENSG00000027869	ENSG00000178217
ENSG00000027869	ENSG00000196684
ENSG00000028116	ENSG00000100749
ENSG00000028116	ENSG00000105053
ENSG00000028137	ENSG00000101017
ENSG00000028137	ENSG00000111321
ENSG00000028137	ENSG00000120949
ENSG00000028137	ENSG00000141655
ENSG00000028137	ENSG00000146072
ENSG00000028137	ENSG00000157873
ENSG00000028137	ENSG00000164761
ENSG00000028277	ENSG00000064835
ENSG00000028277	ENSG00000091010
ENSG00000028277	ENSG00000137709
ENSG00000028277	ENSG00000143190
ENSG00000028277	ENSG00000151615
ENSG00000028277	ENSG00000152192
ENSG00000028277	ENSG00000184486
ENSG00000028277	ENSG00000185668
ENSG00000028277	ENSG00000196767
ENSG00000028277	ENSG00000198914
ENSG00000028277	ENSG00000204531
ENSG00000028277	ENSG00000212993
ENSG00000028310	ENSG00000166164
ENSG00000028310	ENSG00000214423
ENSG00000028310	ENSG00000215415
ENSG00000028528	ENSG00000089006
ENSG00000028528	ENSG00000129515
ENSG00000028528	ENSG00000172803
ENSG00000028528	ENSG00000205302
ENSG00000029153	ENSG00000133794
ENSG00000029153	ENSG00000143437
ENSG00000029153	ENSG00000172379
ENSG00000029363	ENSG00000054118
ENSG00000029363	ENSG00000173681
ENSG00000029534	ENSG00000145362
ENSG00000029534	ENSG00000151150
ENSG00000029534	ENSG00000185722
ENSG00000029725	ENSG00000177548
ENSG00000029993	ENSG00000100118
ENSG00000029993	ENSG00000124097
ENSG00000029993	ENSG00000132967
ENSG00000029993	ENSG00000164104
ENSG00000029993	ENSG00000173961
ENSG00000029993	ENSG00000176256
ENSG00000029993	ENSG00000180212
ENSG00000029993	ENSG00000182181
ENSG00000029993	ENSG00000182711
ENSG00000029993	ENSG00000189403
ENSG00000029993	ENSG00000214364
ENSG00000029993	ENSG00000214852
ENSG00000029993	ENSG00000215308
ENSG00000030419	ENSG00000123411
ENSG00000030419	ENSG00000161405
ENSG00000030419	ENSG00000185811
ENSG00000031003	ENSG00000138640
ENSG00000031003	ENSG00000148541
ENSG00000031081	ENSG00000004777
ENSG00000031081	ENSG00000134909

ENSG00000031081	ENSG00000186517
ENSG00000031823	ENSG00000164188
ENSG00000032219	ENSG00000054267
ENSG00000032444	ENSG00000130653
ENSG00000033100	ENSG00000123989
ENSG00000033100	ENSG00000131873
ENSG00000033100	ENSG00000139044
ENSG00000033100	ENSG00000147408
ENSG00000033100	ENSG00000169826
ENSG00000033100	ENSG00000182272
ENSG00000033100	ENSG00000198108
ENSG00000033122	ENSG00000112851
ENSG00000033122	ENSG00000137269
ENSG00000033122	ENSG00000147324
ENSG00000033122	ENSG00000180900
ENSG00000033122	ENSG00000188306
ENSG00000033327	ENSG00000109458
ENSG00000033327	ENSG00000160219
ENSG00000033327	ENSG00000215568
ENSG00000033627	ENSG00000105929
ENSG00000033627	ENSG00000110719
ENSG00000033627	ENSG00000185344
ENSG00000033800	ENSG00000078043
ENSG00000033800	ENSG00000105229
ENSG00000033800	ENSG00000131788
ENSG00000033867	ENSG00000004939
ENSG00000033867	ENSG00000050438
ENSG00000033867	ENSG00000080493
ENSG00000033867	ENSG00000088836
ENSG00000033867	ENSG00000113073
ENSG00000033867	ENSG00000114923
ENSG00000033867	ENSG00000144290
ENSG00000033867	ENSG00000164889
ENSG00000033867	ENSG00000188687
ENSG00000034053	ENSG00000011132
ENSG00000034053	ENSG00000107282
ENSG00000034063	ENSG00000147854
ENSG00000034677	ENSG00000116514
ENSG00000034677	ENSG00000137393
ENSG00000034677	ENSG00000151692
ENSG00000035403	ENSG00000044115
ENSG00000035403	ENSG00000066032
ENSG00000035403	ENSG00000119326
ENSG00000035403	ENSG00000183230
ENSG00000035499	ENSG00000024526
ENSG00000035499	ENSG00000121690
ENSG00000035499	ENSG00000166153
ENSG00000035664	ENSG00000065534
ENSG00000035664	ENSG00000101306
ENSG00000035664	ENSG00000140795
ENSG00000035664	ENSG00000145949
ENSG00000035664	ENSG00000167657
ENSG00000035664	ENSG00000196730
ENSG00000035687	ENSG00000185100
ENSG00000035720	ENSG00000178078
ENSG00000035862	ENSG00000100234
ENSG00000035862	ENSG00000102265
ENSG00000035862	ENSG00000157150
ENSG00000036448	ENSG00000101605

ENSG00000036448	ENSG00000142661
ENSG00000036565	ENSG00000146409
ENSG00000036565	ENSG00000165646
ENSG00000036565	ENSG00000187714
ENSG00000036672	ENSG00000138592
ENSG00000036672	ENSG00000143258
ENSG00000036672	ENSG00000170236
ENSG00000036828	ENSG00000169962
ENSG00000036828	ENSG00000173612
ENSG00000036828	ENSG00000173662
ENSG00000036828	ENSG00000174930
ENSG00000036828	ENSG00000179002
ENSG00000036828	ENSG00000215740
ENSG00000037042	ENSG00000131462
ENSG00000037280	ENSG00000102755
ENSG00000037280	ENSG00000104213
ENSG00000037280	ENSG00000113721
ENSG00000037280	ENSG00000122025
ENSG00000037280	ENSG00000128052
ENSG00000037280	ENSG00000134853
ENSG00000037280	ENSG00000157404
ENSG00000037280	ENSG00000182578
ENSG00000037749	ENSG00000198948
ENSG00000037965	ENSG00000106004
ENSG00000037965	ENSG00000106006
ENSG00000037965	ENSG00000108511
ENSG00000037965	ENSG00000120068
ENSG00000037965	ENSG00000120075
ENSG00000037965	ENSG00000120087
ENSG00000037965	ENSG00000122592
ENSG00000037965	ENSG00000170166
ENSG00000037965	ENSG00000172789
ENSG00000037965	ENSG00000175879
ENSG00000037965	ENSG00000182742
ENSG00000037965	ENSG00000197576
ENSG00000037965	ENSG00000197757
ENSG00000037965	ENSG00000198353
ENSG00000038102	ENSG00000104880
ENSG00000038102	ENSG00000116584
ENSG00000038102	ENSG00000170776
ENSG00000038210	ENSG00000043822
ENSG00000038295	ENSG00000095587
ENSG00000038295	ENSG00000106333
ENSG00000038295	ENSG00000107611
ENSG00000038295	ENSG00000157211
ENSG00000038295	ENSG00000163710
ENSG00000038295	ENSG00000166342
ENSG00000038295	ENSG00000168487
ENSG00000038295	ENSG00000171208
ENSG00000038295	ENSG00000184824
ENSG00000038382	ENSG00000053524
ENSG00000038382	ENSG00000101977
ENSG00000038382	ENSG00000126217
ENSG00000038382	ENSG00000160145
ENSG00000038427	ENSG00000130287
ENSG00000038427	ENSG00000132692
ENSG00000038427	ENSG00000132702
ENSG00000038427	ENSG00000140511
ENSG00000038427	ENSG00000145681

ENSG00000038427	ENSG00000157766
ENSG00000038427	ENSG00000187664
ENSG00000038945	ENSG00000019169
ENSG00000038945	ENSG00000168079
ENSG00000039319	ENSG00000157077
ENSG00000039523	ENSG00000042062
ENSG00000039523	ENSG00000111913
ENSG00000039537	ENSG00000021852
ENSG00000039537	ENSG00000112936
ENSG00000039537	ENSG00000113600
ENSG00000039537	ENSG00000157131
ENSG00000039537	ENSG00000180644
ENSG00000039560	ENSG00000089847
ENSG00000039560	ENSG00000137831
ENSG00000039560	ENSG00000198483
ENSG00000039987	ENSG00000127325
ENSG00000039987	ENSG00000142959
ENSG00000039987	ENSG00000167995
ENSG00000040199	ENSG00000081913
ENSG00000040341	ENSG00000124214
ENSG00000040608	ENSG00000185924
ENSG00000040608	ENSG00000186907
ENSG00000040633	ENSG00000116273
ENSG00000040731	ENSG00000071991
ENSG00000040731	ENSG00000081138
ENSG00000040731	ENSG00000101542
ENSG00000040731	ENSG00000113100
ENSG00000040731	ENSG00000113361
ENSG00000040731	ENSG00000139880
ENSG00000040731	ENSG00000140937
ENSG00000040731	ENSG00000145526
ENSG00000040731	ENSG00000149654
ENSG00000040731	ENSG00000150394
ENSG00000040731	ENSG00000154162
ENSG00000040731	ENSG00000179776
ENSG00000040933	ENSG00000109452
ENSG00000041353	ENSG00000069974
ENSG00000041982	ENSG00000115414
ENSG00000041982	ENSG00000116147
ENSG00000041982	ENSG00000120332
ENSG00000041982	ENSG00000168477
ENSG00000041988	ENSG00000129028
ENSG00000041988	ENSG00000131931
ENSG00000041988	ENSG00000161277
ENSG00000041988	ENSG00000173451
ENSG00000042062	ENSG00000039523
ENSG00000042062	ENSG00000111913
ENSG00000042493	ENSG00000006747
ENSG00000042493	ENSG00000127831
ENSG00000042493	ENSG00000135407
ENSG00000042493	ENSG00000136059
ENSG00000042493	ENSG00000148180
ENSG00000042493	ENSG00000177731
ENSG00000042980	ENSG00000008277
ENSG00000042980	ENSG00000069206
ENSG00000042980	ENSG00000073670
ENSG00000042980	ENSG00000104755
ENSG00000042980	ENSG00000114948
ENSG00000042980	ENSG00000134007

ENSG00000042980	ENSG00000134028
ENSG00000042980	ENSG00000134249
ENSG00000042980	ENSG00000135074
ENSG00000042980	ENSG00000139985
ENSG00000042980	ENSG00000143537
ENSG00000042980	ENSG00000148848
ENSG00000042980	ENSG00000149451
ENSG00000042980	ENSG00000151651
ENSG00000042980	ENSG00000151694
ENSG00000042980	ENSG00000168594
ENSG00000042980	ENSG00000168615
ENSG00000042980	ENSG00000168619
ENSG00000042980	ENSG00000196115
ENSG00000042980	ENSG00000197140
ENSG00000042980	ENSG00000214137
ENSG00000043039	ENSG00000109851
ENSG00000043039	ENSG00000131668
ENSG00000043039	ENSG00000136630
ENSG00000043039	ENSG00000185610
ENSG00000043039	ENSG00000188909
ENSG00000043093	ENSG00000150401
ENSG00000043093	ENSG00000188215
ENSG00000043143	ENSG00000077684
ENSG00000043143	ENSG00000078403
ENSG00000043143	ENSG00000096070
ENSG00000043143	ENSG00000100425
ENSG00000043143	ENSG00000102221
ENSG00000043143	ENSG00000106443
ENSG00000043143	ENSG00000108292
ENSG00000043143	ENSG00000156983
ENSG00000043355	ENSG00000139800
ENSG00000043355	ENSG00000152977
ENSG00000043355	ENSG00000156925
ENSG00000043355	ENSG00000174963
ENSG00000043462	ENSG00000095585
ENSG00000043462	ENSG00000109684
ENSG00000043462	ENSG00000152292
ENSG00000043591	ENSG00000102468
ENSG00000043591	ENSG00000113749
ENSG00000043591	ENSG00000125631
ENSG00000043591	ENSG00000135312
ENSG00000043591	ENSG00000135914
ENSG00000043591	ENSG00000147246
ENSG00000043591	ENSG00000150594
ENSG00000043591	ENSG00000157219
ENSG00000043591	ENSG00000164270
ENSG00000043591	ENSG00000168830
ENSG00000043591	ENSG00000169252
ENSG00000043591	ENSG00000175658
ENSG00000043591	ENSG00000178394
ENSG00000043591	ENSG00000179097
ENSG00000043591	ENSG00000179546
ENSG00000043591	ENSG00000181210
ENSG00000043591	ENSG00000184303
ENSG00000043591	ENSG00000184845
ENSG00000043591	ENSG00000185321
ENSG00000043591	ENSG00000188778
ENSG00000043822	ENSG00000038210
ENSG00000044115	ENSG00000035403

ENSG00000044115	ENSG00000066032
ENSG00000044115	ENSG00000119326
ENSG00000044115	ENSG00000183230
ENSG00000044446	ENSG00000067177
ENSG00000044446	ENSG00000102893
ENSG00000044524	ENSG00000070886
ENSG00000044524	ENSG00000080224
ENSG00000044524	ENSG00000106123
ENSG00000044524	ENSG00000116106
ENSG00000044524	ENSG00000133216
ENSG00000044524	ENSG00000135333
ENSG00000044524	ENSG00000142627
ENSG00000044524	ENSG00000145242
ENSG00000044524	ENSG00000146904
ENSG00000044524	ENSG00000154928
ENSG00000044524	ENSG00000182580
ENSG00000044524	ENSG00000183317
ENSG00000044524	ENSG00000196411
ENSG00000046653	ENSG00000123560
ENSG00000046653	ENSG00000150625
ENSG00000046889	ENSG00000124126
ENSG00000047230	ENSG00000171793
ENSG00000047346	ENSG00000005238
ENSG00000047365	ENSG00000120318
ENSG00000047365	ENSG00000186635
ENSG00000047457	ENSG00000089472
ENSG00000047457	ENSG00000181333
ENSG00000047457	ENSG00000185010
ENSG00000047457	ENSG00000198734
ENSG00000047579	ENSG00000003249
ENSG00000047579	ENSG00000204070
ENSG00000047597	ENSG00000172967
ENSG00000047597	ENSG00000182489
ENSG00000047617	ENSG00000074855
ENSG00000047617	ENSG00000131620
ENSG00000047617	ENSG00000134343
ENSG00000047617	ENSG00000146205
ENSG00000047617	ENSG00000151572
ENSG00000047617	ENSG00000160746
ENSG00000047617	ENSG00000171714
ENSG00000047617	ENSG00000177119
ENSG00000047617	ENSG00000185101
ENSG00000047634	ENSG00000130590
ENSG00000047634	ENSG00000177570
ENSG00000047634	ENSG00000203943
ENSG00000047644	ENSG00000113645
ENSG00000047644	ENSG00000151718
ENSG00000047648	ENSG00000147256
ENSG00000047662	ENSG00000111879
ENSG00000047849	ENSG00000078018
ENSG00000047849	ENSG00000186868
ENSG00000048028	ENSG00000155313
ENSG00000048052	ENSG00000061273
ENSG00000048052	ENSG00000068024
ENSG00000048052	ENSG00000108840
ENSG00000048140	ENSG00000099282
ENSG00000048140	ENSG00000108219
ENSG00000048140	ENSG00000158457
ENSG00000048140	ENSG00000168785

ENSG00000048140	ENSG00000182612
ENSG00000048392	ENSG00000171848
ENSG00000048540	ENSG00000135363
ENSG00000048540	ENSG00000166407
ENSG00000048545	ENSG00000112599
ENSG00000048545	ENSG00000138472
ENSG00000048740	ENSG00000101489
ENSG00000048740	ENSG00000140488
ENSG00000048740	ENSG00000149187
ENSG00000048740	ENSG00000159409
ENSG00000048740	ENSG00000161082
ENSG00000048828	ENSG00000184083
ENSG00000048991	ENSG00000172995
ENSG00000048991	ENSG00000179912
ENSG00000049089	ENSG00000084636
ENSG00000049089	ENSG00000092758
ENSG00000049089	ENSG00000112280
ENSG00000049192	ENSG00000087116
ENSG00000049192	ENSG00000136378
ENSG00000049192	ENSG00000138316
ENSG00000049192	ENSG00000140470
ENSG00000049192	ENSG00000140873
ENSG00000049192	ENSG00000142303
ENSG00000049192	ENSG00000145536
ENSG00000049192	ENSG00000145808
ENSG00000049192	ENSG00000151388
ENSG00000049192	ENSG00000156140
ENSG00000049192	ENSG00000160323
ENSG00000049192	ENSG00000163638
ENSG00000049192	ENSG00000173157
ENSG00000049192	ENSG00000214644
ENSG00000049192	ENSG00000215743
ENSG00000049246	ENSG00000132326
ENSG00000049246	ENSG00000179094
ENSG00000049283	ENSG00000063245
ENSG00000049283	ENSG00000072134
ENSG00000049283	ENSG00000113282
ENSG00000049283	ENSG00000176177
ENSG00000049323	ENSG00000077942
ENSG00000049323	ENSG00000090006
ENSG00000049323	ENSG00000115380
ENSG00000049323	ENSG00000119681
ENSG00000049323	ENSG00000140092
ENSG00000049323	ENSG00000163520
ENSG00000049323	ENSG00000168056
ENSG00000049323	ENSG00000172638
ENSG00000049323	ENSG00000214536
ENSG00000049449	ENSG00000078808
ENSG00000049449	ENSG00000117906
ENSG00000049449	ENSG00000128595
ENSG00000049449	ENSG00000142552
ENSG00000049618	ENSG00000116017
ENSG00000049618	ENSG00000117713
ENSG00000049618	ENSG00000179361
ENSG00000049618	ENSG00000205143
ENSG00000049759	ENSG00000069869
ENSG00000049759	ENSG00000078747
ENSG00000049759	ENSG00000108854
ENSG00000049759	ENSG00000123124

ENSG00000049759	ENSG00000198373
ENSG00000049759	ENSG00000198742
ENSG00000049860	ENSG00000213614
ENSG00000050393	ENSG00000137500
ENSG00000050438	ENSG00000004939
ENSG00000050438	ENSG00000033867
ENSG00000050438	ENSG00000080493
ENSG00000050438	ENSG00000088836
ENSG00000050438	ENSG00000113073
ENSG00000050438	ENSG00000114923
ENSG00000050438	ENSG00000144290
ENSG00000050438	ENSG00000164889
ENSG00000050438	ENSG00000188687
ENSG00000050555	ENSG00000058085
ENSG00000050555	ENSG00000074527
ENSG00000050555	ENSG00000091128
ENSG00000050555	ENSG00000091136
ENSG00000050555	ENSG00000106780
ENSG00000050555	ENSG00000135862
ENSG00000050555	ENSG00000172037
ENSG00000050555	ENSG00000196878
ENSG00000050628	ENSG00000006638
ENSG00000050628	ENSG00000122420
ENSG00000050628	ENSG00000125384
ENSG00000050628	ENSG00000160013
ENSG00000050628	ENSG00000160951
ENSG00000050628	ENSG00000168229
ENSG00000050628	ENSG00000171522
ENSG00000050730	ENSG00000145901
ENSG00000050748	ENSG00000107643
ENSG00000050748	ENSG00000109339
ENSG00000050748	ENSG00000112062
ENSG00000050748	ENSG00000156711
ENSG00000050748	ENSG00000185386
ENSG00000050748	ENSG00000188130
ENSG00000050767	ENSG00000188517
ENSG00000050767	ENSG00000197467
ENSG00000050820	ENSG00000087589
ENSG00000050820	ENSG00000100842
ENSG00000050820	ENSG00000111859
ENSG00000051009	ENSG00000151553
ENSG00000051009	ENSG00000158863
ENSG00000051009	ENSG00000164142
ENSG00000051108	ENSG00000122557
ENSG00000051128	ENSG00000103942
ENSG00000051128	ENSG00000152413
ENSG00000051128	ENSG00000214396
ENSG00000051382	ENSG00000011405
ENSG00000051382	ENSG00000105851
ENSG00000051382	ENSG00000121879
ENSG00000051382	ENSG00000133056
ENSG00000051382	ENSG00000139144
ENSG00000051382	ENSG00000171608
ENSG00000052126	ENSG00000105559
ENSG00000052126	ENSG00000143850
ENSG00000052126	ENSG00000166689
ENSG00000052344	ENSG00000005001
ENSG00000052344	ENSG00000007038
ENSG00000052344	ENSG00000095917

ENSG00000052344	ENSG00000103355
ENSG00000052344	ENSG00000116176
ENSG00000052344	ENSG00000172236
ENSG00000052344	ENSG00000172382
ENSG00000052344	ENSG00000172460
ENSG00000052344	ENSG00000178055
ENSG00000052344	ENSG00000178226
ENSG00000052344	ENSG00000185888
ENSG00000052344	ENSG00000188086
ENSG00000052344	ENSG00000189099
ENSG00000052344	ENSG00000196364
ENSG00000052344	ENSG00000197253
ENSG00000052344	ENSG00000206549
ENSG00000052344	ENSG00000214877
ENSG00000052344	ENSG00000215148
ENSG00000052723	ENSG00000111790
ENSG00000052850	ENSG00000004848
ENSG00000052850	ENSG00000100987
ENSG00000052850	ENSG00000119614
ENSG00000052850	ENSG00000156150
ENSG00000052850	ENSG00000180318
ENSG00000053108	ENSG00000070404
ENSG00000053108	ENSG00000134363
ENSG00000053108	ENSG00000163430
ENSG00000053254	ENSG00000170802
ENSG00000053524	ENSG00000038382
ENSG00000053524	ENSG00000101977
ENSG00000053524	ENSG00000126217
ENSG00000053524	ENSG00000160145
ENSG00000053702	ENSG00000170967
ENSG00000053702	ENSG00000175352
ENSG00000053702	ENSG00000197312
ENSG00000053747	ENSG00000101680
ENSG00000053747	ENSG00000112769
ENSG00000053747	ENSG00000130702
ENSG00000053747	ENSG00000196569
ENSG00000053918	ENSG00000075043
ENSG00000053918	ENSG00000117013
ENSG00000053918	ENSG00000184156
ENSG00000053918	ENSG00000185760
ENSG00000054116	ENSG00000173626
ENSG00000054118	ENSG00000029363
ENSG00000054118	ENSG00000173681
ENSG00000054179	ENSG00000138185
ENSG00000054179	ENSG00000168032
ENSG00000054179	ENSG00000188833
ENSG00000054219	ENSG00000011028
ENSG00000054219	ENSG00000120586
ENSG00000054219	ENSG00000153246
ENSG00000054219	ENSG00000183748
ENSG00000054267	ENSG00000032219
ENSG00000054277	ENSG00000102076
ENSG00000054277	ENSG00000122375
ENSG00000054277	ENSG00000124818
ENSG00000054277	ENSG00000128617
ENSG00000054277	ENSG00000147380
ENSG00000054277	ENSG00000148604
ENSG00000054277	ENSG00000163914
ENSG00000054277	ENSG00000166160

ENSG00000054277	ENSG00000180245
ENSG00000054356	ENSG00000155093
ENSG00000054523	ENSG00000089177
ENSG00000054523	ENSG00000118193
ENSG00000054523	ENSG00000129250
ENSG00000054523	ENSG00000130294
ENSG00000054523	ENSG00000137177
ENSG00000054523	ENSG00000197892
ENSG00000054598	ENSG00000125798
ENSG00000054598	ENSG00000129514
ENSG00000054598	ENSG00000164379
ENSG00000054598	ENSG00000168269
ENSG00000054598	ENSG00000170122
ENSG00000054598	ENSG00000170608
ENSG00000054598	ENSG00000176165
ENSG00000054598	ENSG00000176678
ENSG00000054598	ENSG00000176692
ENSG00000054598	ENSG00000178919
ENSG00000054598	ENSG00000179772
ENSG00000054598	ENSG00000183770
ENSG00000054598	ENSG00000184492
ENSG00000054598	ENSG00000184659
ENSG00000054598	ENSG00000186564
ENSG00000054598	ENSG00000186766
ENSG00000054598	ENSG00000186790
ENSG00000054598	ENSG00000187140
ENSG00000054598	ENSG00000187559
ENSG00000054598	ENSG00000204612
ENSG00000054598	ENSG00000204779
ENSG00000054598	ENSG00000204793
ENSG00000054598	ENSG00000204828
ENSG00000054598	ENSG00000214336
ENSG00000054611	ENSG00000065491
ENSG00000054690	ENSG00000152527
ENSG00000054793	ENSG00000166377
ENSG00000054803	ENSG00000102924
ENSG00000054803	ENSG00000131094
ENSG00000054803	ENSG00000133466
ENSG00000054803	ENSG00000139899
ENSG00000054803	ENSG00000141668
ENSG00000054803	ENSG00000144119
ENSG00000054803	ENSG00000145861
ENSG00000054803	ENSG00000159189
ENSG00000054803	ENSG00000163145
ENSG00000054803	ENSG00000165985
ENSG00000054803	ENSG00000172247
ENSG00000054803	ENSG00000173369
ENSG00000054803	ENSG00000173372
ENSG00000054803	ENSG00000173918
ENSG00000054803	ENSG00000181092
ENSG00000054803	ENSG00000184471
ENSG00000054803	ENSG00000186897
ENSG00000054803	ENSG00000213192
ENSG00000054938	ENSG00000090539
ENSG00000054938	ENSG00000101938
ENSG00000054938	ENSG00000135253
ENSG00000054938	ENSG00000150938
ENSG00000054965	ENSG00000152102
ENSG00000054967	ENSG00000164620

ENSG00000054967	ENSG00000181826
ENSG00000055118	ENSG00000089558
ENSG00000055118	ENSG00000135519
ENSG00000055118	ENSG00000140015
ENSG00000055118	ENSG00000143473
ENSG00000055118	ENSG00000173826
ENSG00000055118	ENSG00000183960
ENSG00000055118	ENSG00000184611
ENSG00000055147	ENSG00000197712
ENSG00000055163	ENSG00000068793
ENSG00000055208	ENSG00000157625
ENSG00000055609	ENSG00000167548
ENSG00000055732	ENSG00000090674
ENSG00000055732	ENSG00000153898
ENSG00000055813	ENSG00000175602
ENSG00000055813	ENSG00000205476
ENSG00000055917	ENSG00000134644
ENSG00000055955	ENSG00000055957
ENSG00000055955	ENSG00000102313
ENSG00000055955	ENSG00000123243
ENSG00000055955	ENSG00000151655
ENSG00000055955	ENSG00000162267
ENSG00000055957	ENSG00000055955
ENSG00000055957	ENSG00000102313
ENSG00000055957	ENSG00000123243
ENSG00000055957	ENSG00000151655
ENSG00000055957	ENSG00000162267
ENSG00000056097	ENSG00000105278
ENSG00000056097	ENSG00000129351
ENSG00000056097	ENSG00000143621
ENSG00000056097	ENSG00000165209
ENSG00000056277	ENSG00000137871
ENSG00000056277	ENSG00000143442
ENSG00000056277	ENSG00000169548
ENSG00000056277	ENSG00000198477
ENSG00000056291	ENSG00000075073
ENSG00000056291	ENSG00000101292
ENSG00000056291	ENSG00000110148
ENSG00000056291	ENSG00000115353
ENSG00000056291	ENSG00000119973
ENSG00000056291	ENSG00000121764
ENSG00000056291	ENSG00000123901
ENSG00000056291	ENSG00000137252
ENSG00000056291	ENSG00000148734
ENSG00000056291	ENSG00000159279
ENSG00000056291	ENSG00000163394
ENSG00000056291	ENSG00000164128
ENSG00000056291	ENSG00000164129
ENSG00000056291	ENSG00000169618
ENSG00000056291	ENSG00000169836
ENSG00000056291	ENSG00000185149
ENSG00000056291	ENSG00000186867
ENSG00000056291	ENSG00000204174
ENSG00000056291	ENSG00000215167
ENSG00000056345	ENSG00000082781
ENSG00000056345	ENSG00000105855
ENSG00000056345	ENSG00000115221
ENSG00000056345	ENSG00000132470
ENSG00000056345	ENSG00000139626

ENSG00000056345	ENSG00000150093
ENSG00000056345	ENSG00000160255
ENSG00000056487	ENSG00000135365
ENSG00000056558	ENSG00000076604
ENSG00000056558	ENSG00000082512
ENSG00000056558	ENSG00000127191
ENSG00000056558	ENSG00000131323
ENSG00000056558	ENSG00000175104
ENSG00000056586	ENSG00000135870
ENSG00000056661	ENSG00000115289
ENSG00000056661	ENSG00000156374
ENSG00000056661	ENSG00000168283
ENSG00000056661	ENSG00000180628
ENSG00000056661	ENSG00000185619
ENSG00000056736	ENSG00000144730
ENSG00000056736	ENSG00000177663
ENSG00000056998	ENSG00000163754
ENSG00000057019	ENSG00000099250
ENSG00000057019	ENSG00000102104
ENSG00000057019	ENSG00000118257
ENSG00000057019	ENSG00000140545
ENSG00000057019	ENSG00000164176
ENSG00000057019	ENSG00000164465
ENSG00000057149	ENSG00000021355
ENSG00000057149	ENSG00000124570
ENSG00000057149	ENSG00000166396
ENSG00000057149	ENSG00000166401
ENSG00000057149	ENSG00000166404
ENSG00000057149	ENSG00000166634
ENSG00000057149	ENSG00000170542
ENSG00000057149	ENSG00000197632
ENSG00000057149	ENSG00000197641
ENSG00000057149	ENSG00000206072
ENSG00000057149	ENSG00000206073
ENSG00000057149	ENSG00000206075
ENSG00000057294	ENSG00000081277
ENSG00000057294	ENSG00000099889
ENSG00000057294	ENSG00000144283
ENSG00000057294	ENSG00000169862
ENSG00000057294	ENSG00000184363
ENSG00000057294	ENSG00000198561
ENSG00000057593	ENSG00000101981
ENSG00000057593	ENSG00000115718
ENSG00000057593	ENSG00000126218
ENSG00000057593	ENSG00000126231
ENSG00000057593	ENSG00000180210
ENSG00000057608	ENSG00000203879
ENSG00000057657	ENSG00000176083
ENSG00000057704	ENSG00000102080
ENSG00000057704	ENSG00000133069
ENSG00000057704	ENSG00000172765
ENSG00000057704	ENSG00000182242
ENSG00000057704	ENSG00000185254
ENSG00000057935	ENSG00000149480
ENSG00000057935	ENSG00000182979
ENSG00000058056	ENSG00000111667
ENSG00000058063	ENSG00000068650
ENSG00000058063	ENSG00000101974
ENSG00000058085	ENSG00000050555

ENSG00000058085	ENSG00000074527
ENSG00000058085	ENSG00000091128
ENSG00000058085	ENSG00000091136
ENSG00000058085	ENSG00000106780
ENSG00000058085	ENSG00000135862
ENSG00000058085	ENSG00000172037
ENSG00000058085	ENSG00000196878
ENSG00000058091	ENSG00000059758
ENSG00000058091	ENSG00000102225
ENSG00000058091	ENSG00000117266
ENSG00000058091	ENSG00000138395
ENSG00000058262	ENSG00000065665
ENSG00000058272	ENSG00000077157
ENSG00000058272	ENSG00000125503
ENSG00000058335	ENSG00000100485
ENSG00000058335	ENSG00000107263
ENSG00000058335	ENSG00000113319
ENSG00000058335	ENSG00000115904
ENSG00000058404	ENSG00000070808
ENSG00000058404	ENSG00000145349
ENSG00000058404	ENSG00000148660
ENSG00000058453	ENSG00000126001
ENSG00000058668	ENSG00000067842
ENSG00000058668	ENSG00000070961
ENSG00000058668	ENSG00000157087
ENSG00000058799	ENSG00000130733
ENSG00000058866	ENSG00000065357
ENSG00000058866	ENSG00000077044
ENSG00000058866	ENSG00000102780
ENSG00000058866	ENSG00000136267
ENSG00000058866	ENSG00000153933
ENSG00000058866	ENSG00000204466
ENSG00000059145	ENSG00000132478
ENSG00000059377	ENSG00000021461
ENSG00000059377	ENSG00000106258
ENSG00000059377	ENSG00000160868
ENSG00000059377	ENSG00000160870
ENSG00000059378	ENSG00000105939
ENSG00000059378	ENSG00000111224
ENSG00000059378	ENSG00000146858
ENSG00000059728	ENSG00000070444
ENSG00000059728	ENSG00000119950
ENSG00000059728	ENSG00000123933
ENSG00000059728	ENSG00000213347
ENSG00000059758	ENSG00000058091
ENSG00000059758	ENSG00000102225
ENSG00000059758	ENSG00000117266
ENSG00000059758	ENSG00000138395
ENSG00000059804	ENSG00000109667
ENSG00000059804	ENSG00000117394
ENSG00000059804	ENSG00000133460
ENSG00000059804	ENSG00000142583
ENSG00000059804	ENSG00000163581
ENSG00000059804	ENSG00000173262
ENSG00000059804	ENSG00000181856
ENSG00000059804	ENSG00000197241
ENSG00000059915	ENSG00000125637
ENSG00000059915	ENSG00000146005
ENSG00000059915	ENSG00000156011

ENSG00000060138	ENSG00000006047
ENSG00000060138	ENSG00000065978
ENSG00000060138	ENSG00000173616
ENSG00000060138	ENSG00000183443
ENSG00000060138	ENSG00000214299
ENSG00000060237	ENSG00000115216
ENSG00000060237	ENSG00000126562
ENSG00000060237	ENSG00000165238
ENSG00000060237	ENSG00000196632
ENSG00000060491	ENSG00000119900
ENSG00000060566	ENSG00000107175
ENSG00000060566	ENSG00000118217
ENSG00000060566	ENSG00000143578
ENSG00000060566	ENSG00000157613
ENSG00000060566	ENSG00000182158
ENSG00000060566	ENSG00000213676
ENSG00000060656	ENSG00000105426
ENSG00000060656	ENSG00000142949
ENSG00000060656	ENSG00000152894
ENSG00000060656	ENSG00000153707
ENSG00000060656	ENSG00000173482
ENSG00000060656	ENSG00000196090
ENSG00000060709	ENSG00000005379
ENSG00000060709	ENSG00000183246
ENSG00000060709	ENSG00000196622
ENSG00000060709	ENSG00000196934
ENSG00000060718	ENSG00000080573
ENSG00000060718	ENSG00000108821
ENSG00000060718	ENSG00000130635
ENSG00000060718	ENSG00000139219
ENSG00000060718	ENSG00000164692
ENSG00000060718	ENSG00000168542
ENSG00000060718	ENSG00000204248
ENSG00000060718	ENSG00000204262
ENSG00000060749	ENSG00000126464
ENSG00000060982	ENSG00000105552
ENSG00000061273	ENSG00000048052
ENSG00000061273	ENSG00000068024
ENSG00000061273	ENSG00000108840
ENSG00000061337	ENSG00000088899
ENSG00000061337	ENSG00000107816
ENSG00000061337	ENSG00000145911
ENSG00000061492	ENSG00000002745
ENSG00000061492	ENSG00000075290
ENSG00000061492	ENSG00000085741
ENSG00000061492	ENSG00000105989
ENSG00000061492	ENSG00000108379
ENSG00000061492	ENSG00000111186
ENSG00000061492	ENSG00000114251
ENSG00000061492	ENSG00000115596
ENSG00000061492	ENSG00000125084
ENSG00000061492	ENSG00000134245
ENSG00000061492	ENSG00000135925
ENSG00000061492	ENSG00000143816
ENSG00000061492	ENSG00000154342
ENSG00000061492	ENSG00000154764
ENSG00000061492	ENSG00000158955
ENSG00000061492	ENSG00000162552
ENSG00000061492	ENSG00000169884

ENSG00000061492	ENSG00000188064
ENSG00000061656	ENSG00000100242
ENSG00000061656	ENSG00000164744
ENSG00000061656	ENSG00000164828
ENSG00000061656	ENSG00000167098
ENSG00000061676	ENSG00000123338
ENSG00000062096	ENSG00000006756
ENSG00000062096	ENSG00000100299
ENSG00000062096	ENSG00000101846
ENSG00000062096	ENSG00000113273
ENSG00000062096	ENSG00000141012
ENSG00000062096	ENSG00000141337
ENSG00000062096	ENSG00000157399
ENSG00000062096	ENSG00000180801
ENSG00000062096	ENSG00000183876
ENSG00000062096	ENSG00000205667
ENSG00000062282	ENSG00000106384
ENSG00000062282	ENSG00000124003
ENSG00000062282	ENSG00000147160
ENSG00000062282	ENSG00000166391
ENSG00000062282	ENSG00000184210
ENSG00000062282	ENSG00000204195
ENSG00000062282	ENSG00000205267
ENSG00000062524	ENSG00000027644
ENSG00000062524	ENSG00000047936
ENSG00000062524	ENSG00000140443
ENSG00000062524	ENSG00000171094
ENSG00000062524	ENSG00000171105
ENSG00000062598	ENSG00000102890
ENSG00000062598	ENSG00000155849
ENSG00000063015	ENSG00000100095
ENSG00000063015	ENSG00000174938
ENSG00000063127	ENSG00000072041
ENSG00000063127	ENSG00000197106
ENSG00000063169	ENSG00000112624
ENSG00000063176	ENSG00000176170
ENSG00000063180	ENSG00000154975
ENSG00000063245	ENSG00000049283
ENSG00000063245	ENSG00000072134
ENSG00000063245	ENSG00000113282
ENSG00000063245	ENSG00000176177
ENSG00000063438	ENSG00000106546
ENSG00000063515	ENSG00000133937
ENSG00000063601	ENSG00000003987
ENSG00000063601	ENSG00000014914
ENSG00000063601	ENSG00000087053
ENSG00000063601	ENSG00000100330
ENSG00000063601	ENSG00000102043
ENSG00000063601	ENSG00000104643
ENSG00000063601	ENSG00000108389
ENSG00000063601	ENSG00000139505
ENSG00000063601	ENSG00000150712
ENSG00000063601	ENSG00000166912
ENSG00000063601	ENSG00000171100
ENSG00000063601	ENSG00000184416
ENSG00000063660	ENSG00000076716
ENSG00000063660	ENSG00000147257
ENSG00000063660	ENSG00000179399
ENSG00000063660	ENSG00000183098

ENSG00000063660	ENSG00000213420
ENSG00000063854	ENSG00000103253
ENSG00000063854	ENSG00000127838
ENSG00000064012	ENSG00000003400
ENSG00000064012	ENSG00000105141
ENSG00000064012	ENSG00000106144
ENSG00000064012	ENSG00000132906
ENSG00000064012	ENSG00000137752
ENSG00000064012	ENSG00000137757
ENSG00000064012	ENSG00000138794
ENSG00000064012	ENSG00000164305
ENSG00000064012	ENSG00000165806
ENSG00000064012	ENSG00000196954
ENSG00000064012	ENSG00000204403
ENSG00000064042	ENSG00000136153
ENSG00000064195	ENSG00000105880
ENSG00000064195	ENSG00000115844
ENSG00000064199	ENSG00000154040
ENSG00000064205	ENSG00000104415
ENSG00000064205	ENSG00000112761
ENSG00000064205	ENSG00000118523
ENSG00000064205	ENSG00000136999
ENSG00000064205	ENSG00000142871
ENSG00000064225	ENSG00000008513
ENSG00000064225	ENSG00000110080
ENSG00000064225	ENSG00000126091
ENSG00000064225	ENSG00000157350
ENSG00000064270	ENSG00000017260
ENSG00000064300	ENSG00000214778
ENSG00000064309	ENSG00000144857
ENSG00000064393	ENSG00000110422
ENSG00000064393	ENSG00000160396
ENSG00000064393	ENSG00000163349
ENSG00000064489	ENSG00000068305
ENSG00000064489	ENSG00000081189
ENSG00000064489	ENSG00000116604
ENSG00000064490	ENSG00000164331
ENSG00000064545	ENSG00000164180
ENSG00000064547	ENSG00000118432
ENSG00000064547	ENSG00000125910
ENSG00000064547	ENSG00000132975
ENSG00000064547	ENSG00000146360
ENSG00000064547	ENSG00000170989
ENSG00000064547	ENSG00000171517
ENSG00000064547	ENSG00000175898
ENSG00000064547	ENSG00000180739
ENSG00000064547	ENSG00000181773
ENSG00000064547	ENSG00000188822
ENSG00000064547	ENSG00000198121
ENSG00000064547	ENSG00000213694
ENSG00000064651	ENSG00000070915
ENSG00000064651	ENSG00000074803
ENSG00000064651	ENSG00000146828
ENSG00000064652	ENSG00000157734
ENSG00000064655	ENSG00000104313
ENSG00000064655	ENSG00000112319
ENSG00000064655	ENSG00000158161
ENSG00000064666	ENSG00000117519
ENSG00000064666	ENSG00000130176

ENSG00000064666	ENSG00000144834
ENSG00000064666	ENSG00000149591
ENSG00000064666	ENSG00000158710
ENSG00000064666	ENSG00000204782
ENSG00000064666	ENSG00000204826
ENSG00000064666	ENSG00000215433
ENSG00000064687	ENSG00000107331
ENSG00000064687	ENSG00000144452
ENSG00000064687	ENSG00000165029
ENSG00000064687	ENSG00000167972
ENSG00000064687	ENSG00000179869
ENSG00000064687	ENSG00000198691
ENSG00000064726	ENSG00000132640
ENSG00000064726	ENSG00000133243
ENSG00000064726	ENSG00000184887
ENSG00000064763	ENSG00000197601
ENSG00000064886	ENSG00000085465
ENSG00000064886	ENSG00000133048
ENSG00000064886	ENSG00000133063
ENSG00000064886	ENSG00000134216
ENSG00000064886	ENSG00000203878
ENSG00000064932	ENSG00000139697
ENSG00000064961	ENSG00000140382
ENSG00000064989	ENSG00000004948
ENSG00000064989	ENSG00000010310
ENSG00000064989	ENSG00000065325
ENSG00000064989	ENSG00000078549
ENSG00000064989	ENSG00000080293
ENSG00000064989	ENSG00000106018
ENSG00000064989	ENSG00000106113
ENSG00000064989	ENSG00000106128
ENSG00000064989	ENSG00000112164
ENSG00000064989	ENSG00000114812
ENSG00000064989	ENSG00000120088
ENSG00000064989	ENSG00000144407
ENSG00000064989	ENSG00000160801
ENSG00000064989	ENSG00000215644
ENSG00000064999	ENSG00000167971
ENSG00000064999	ENSG00000177303
ENSG00000064999	ENSG00000185046
ENSG00000065029	ENSG00000119725
ENSG00000065029	ENSG00000166478
ENSG00000065054	ENSG00000109062
ENSG00000065054	ENSG00000172367
ENSG00000065054	ENSG00000174827
ENSG00000065054	ENSG00000215859
ENSG00000065054	ENSG00000215860
ENSG00000065060	ENSG00000111647
ENSG00000065135	ENSG00000060558
ENSG00000065135	ENSG00000087258
ENSG00000065135	ENSG00000087460
ENSG00000065135	ENSG00000088256
ENSG00000065135	ENSG00000114349
ENSG00000065135	ENSG00000114353
ENSG00000065135	ENSG00000120063
ENSG00000065135	ENSG00000127955
ENSG00000065135	ENSG00000128266
ENSG00000065135	ENSG00000134183
ENSG00000065135	ENSG00000141404

ENSG00000065135	ENSG00000146535
ENSG00000065135	ENSG00000156049
ENSG00000065135	ENSG00000156052
ENSG00000065135	ENSG00000214415
ENSG00000065135	ENSG00000214622
ENSG00000065243	ENSG00000123143
ENSG00000065243	ENSG00000160447
ENSG00000065308	ENSG00000067167
ENSG00000065308	ENSG00000090661
ENSG00000065308	ENSG00000139624
ENSG00000065308	ENSG00000143418
ENSG00000065308	ENSG00000154227
ENSG00000065308	ENSG00000172292
ENSG00000065308	ENSG00000174599
ENSG00000065320	ENSG00000142233
ENSG00000065320	ENSG00000162068
ENSG00000065325	ENSG00000004948
ENSG00000065325	ENSG00000010310
ENSG00000065325	ENSG00000064989
ENSG00000065325	ENSG00000078549
ENSG00000065325	ENSG00000080293
ENSG00000065325	ENSG00000106018
ENSG00000065325	ENSG00000106113
ENSG00000065325	ENSG00000106128
ENSG00000065325	ENSG00000112164
ENSG00000065325	ENSG00000114812
ENSG00000065325	ENSG00000120088
ENSG00000065325	ENSG00000144407
ENSG00000065325	ENSG00000160801
ENSG00000065325	ENSG00000215644
ENSG00000065357	ENSG00000058866
ENSG00000065357	ENSG00000077044
ENSG00000065357	ENSG00000102780
ENSG00000065357	ENSG00000136267
ENSG00000065357	ENSG00000153933
ENSG00000065357	ENSG00000204466
ENSG00000065361	ENSG00000141736
ENSG00000065361	ENSG00000146648
ENSG00000065361	ENSG00000178568
ENSG00000065371	ENSG00000114547
ENSG00000065371	ENSG00000145491
ENSG00000065413	ENSG00000119509
ENSG00000065413	ENSG00000139645
ENSG00000065413	ENSG00000206560
ENSG00000065491	ENSG00000054611
ENSG00000065534	ENSG00000035664
ENSG00000065534	ENSG00000101306
ENSG00000065534	ENSG00000140795
ENSG00000065534	ENSG00000145949
ENSG00000065534	ENSG00000167657
ENSG00000065534	ENSG00000196730
ENSG00000065609	ENSG00000073921
ENSG00000065613	ENSG00000072786
ENSG00000065665	ENSG00000058262
ENSG00000065675	ENSG00000027075
ENSG00000065675	ENSG00000067606
ENSG00000065675	ENSG00000105221
ENSG00000065675	ENSG00000117020
ENSG00000065675	ENSG00000126583

ENSG00000065675	ENSG00000142208
ENSG00000065675	ENSG00000154229
ENSG00000065675	ENSG00000163558
ENSG00000065675	ENSG00000163932
ENSG00000065675	ENSG00000166501
ENSG00000065717	ENSG00000104953
ENSG00000065717	ENSG00000104964
ENSG00000065717	ENSG00000106829
ENSG00000065717	ENSG00000140332
ENSG00000065717	ENSG00000196781
ENSG00000065802	ENSG00000102048
ENSG00000065802	ENSG00000164122
ENSG00000065802	ENSG00000165192
ENSG00000065802	ENSG00000196372
ENSG00000065802	ENSG00000198881
ENSG00000065802	ENSG00000214426
ENSG00000065809	ENSG00000168309
ENSG00000065833	ENSG00000082212
ENSG00000065833	ENSG00000151376
ENSG00000065882	ENSG00000011454
ENSG00000065882	ENSG00000067208
ENSG00000065882	ENSG00000136111
ENSG00000065882	ENSG00000142459
ENSG00000065882	ENSG00000152061
ENSG00000065883	ENSG00000167258
ENSG00000065911	ENSG00000163738
ENSG00000065911	ENSG00000214698
ENSG00000065923	ENSG00000181804
ENSG00000065923	ENSG00000198689
ENSG00000065970	ENSG00000111206
ENSG00000065970	ENSG00000129654
ENSG00000065970	ENSG00000198815
ENSG00000065978	ENSG00000006047
ENSG00000065978	ENSG00000060138
ENSG00000065978	ENSG00000173616
ENSG00000065978	ENSG00000183443
ENSG00000065978	ENSG00000214299
ENSG00000065989	ENSG00000073417
ENSG00000065989	ENSG00000105650
ENSG00000065989	ENSG00000113231
ENSG00000065989	ENSG00000113448
ENSG00000065989	ENSG00000115252
ENSG00000065989	ENSG00000123360
ENSG00000065989	ENSG00000154678
ENSG00000065989	ENSG00000160191
ENSG00000065989	ENSG00000171408
ENSG00000065989	ENSG00000184588
ENSG00000065989	ENSG00000205268
ENSG00000066027	ENSG00000068971
ENSG00000066027	ENSG00000078304
ENSG00000066027	ENSG00000112640
ENSG00000066027	ENSG00000154001
ENSG00000066032	ENSG00000035403
ENSG00000066032	ENSG00000044115
ENSG00000066032	ENSG00000119326
ENSG00000066032	ENSG00000183230
ENSG00000066044	ENSG00000107105
ENSG00000066044	ENSG00000162374
ENSG00000066044	ENSG00000196361

ENSG00000066056	ENSG00000120156
ENSG00000066084	ENSG00000151240
ENSG00000066084	ENSG00000160305
ENSG00000066117	ENSG00000082014
ENSG00000066117	ENSG00000108604
ENSG00000066135	ENSG00000107077
ENSG00000066135	ENSG00000127663
ENSG00000066135	ENSG00000186280
ENSG00000066230	ENSG00000090020
ENSG00000066230	ENSG00000115616
ENSG00000066230	ENSG00000135740
ENSG00000066230	ENSG00000180251
ENSG00000066248	ENSG00000114790
ENSG00000066248	ENSG00000130762
ENSG00000066248	ENSG00000142632
ENSG00000066248	ENSG00000198844
ENSG00000066322	ENSG00000012660
ENSG00000066322	ENSG00000118402
ENSG00000066322	ENSG00000164181
ENSG00000066322	ENSG00000197977
ENSG00000066336	ENSG00000142539
ENSG00000066382	ENSG00000186732
ENSG00000066468	ENSG00000068078
ENSG00000066468	ENSG00000077782
ENSG00000066468	ENSG00000160867
ENSG00000066629	ENSG00000125746
ENSG00000066629	ENSG00000143924
ENSG00000066629	ENSG00000143940
ENSG00000066629	ENSG00000149499
ENSG00000066629	ENSG00000165521
ENSG00000066629	ENSG00000214595
ENSG00000066697	ENSG00000144339
ENSG00000066735	ENSG00000162849
ENSG00000066739	ENSG00000110046
ENSG00000066777	ENSG00000112379
ENSG00000066777	ENSG00000124198
ENSG00000066855	ENSG00000117640
ENSG00000066855	ENSG00000146410
ENSG00000066923	ENSG00000101972
ENSG00000066923	ENSG00000118007
ENSG00000066933	ENSG00000091536
ENSG00000066933	ENSG00000099331
ENSG00000066933	ENSG00000145555
ENSG00000067057	ENSG00000141959
ENSG00000067057	ENSG00000152556
ENSG00000067082	ENSG00000102554
ENSG00000067082	ENSG00000105610
ENSG00000067082	ENSG00000127528
ENSG00000067082	ENSG00000129911
ENSG00000067082	ENSG00000136826
ENSG00000067082	ENSG00000139651
ENSG00000067082	ENSG00000155090
ENSG00000067082	ENSG00000164651
ENSG00000067082	ENSG00000167182
ENSG00000067082	ENSG00000170374
ENSG00000067082	ENSG00000171872
ENSG00000067082	ENSG00000172059
ENSG00000067082	ENSG00000174595
ENSG00000067082	ENSG00000189120

ENSG00000067082	ENSG00000197294
ENSG00000067082	ENSG00000197550
ENSG00000067082	ENSG00000204335
ENSG00000067113	ENSG00000105520
ENSG00000067113	ENSG00000117598
ENSG00000067113	ENSG00000117600
ENSG00000067113	ENSG00000129951
ENSG00000067113	ENSG00000141934
ENSG00000067113	ENSG00000148123
ENSG00000067113	ENSG00000162407
ENSG00000067141	ENSG00000103742
ENSG00000067141	ENSG00000166450
ENSG00000067141	ENSG00000174498
ENSG00000067141	ENSG00000187323
ENSG00000067141	ENSG00000215851
ENSG00000067167	ENSG00000065308
ENSG00000067167	ENSG00000090661
ENSG00000067167	ENSG00000139624
ENSG00000067167	ENSG00000143418
ENSG00000067167	ENSG00000154227
ENSG00000067167	ENSG00000172292
ENSG00000067167	ENSG00000174599
ENSG00000067177	ENSG00000044446
ENSG00000067177	ENSG00000102893
ENSG00000067182	ENSG00000026103
ENSG00000067182	ENSG00000104689
ENSG00000067182	ENSG00000120889
ENSG00000067182	ENSG00000173530
ENSG00000067182	ENSG00000173535
ENSG00000067182	ENSG00000215788
ENSG00000067191	ENSG00000165995
ENSG00000067191	ENSG00000167535
ENSG00000067191	ENSG00000182389
ENSG00000067208	ENSG00000011454
ENSG00000067208	ENSG00000065882
ENSG00000067208	ENSG00000136111
ENSG00000067208	ENSG00000142459
ENSG00000067208	ENSG00000152061
ENSG00000067225	ENSG00000143627
ENSG00000067560	ENSG00000143878
ENSG00000067560	ENSG00000155366
ENSG00000067606	ENSG00000027075
ENSG00000067606	ENSG00000065675
ENSG00000067606	ENSG00000105221
ENSG00000067606	ENSG00000117020
ENSG00000067606	ENSG00000126583
ENSG00000067606	ENSG00000142208
ENSG00000067606	ENSG00000154229
ENSG00000067606	ENSG00000163558
ENSG00000067606	ENSG00000163932
ENSG00000067606	ENSG00000166501
ENSG00000067646	ENSG00000005889
ENSG00000067646	ENSG00000121864
ENSG00000067646	ENSG00000147180
ENSG00000067715	ENSG00000011347
ENSG00000067715	ENSG00000019505
ENSG00000067715	ENSG00000101098
ENSG00000067715	ENSG00000103528
ENSG00000067715	ENSG00000110975

ENSG00000067715	ENSG00000129990
ENSG00000067715	ENSG00000132718
ENSG00000067715	ENSG00000132872
ENSG00000067715	ENSG00000134207
ENSG00000067715	ENSG00000143858
ENSG00000067715	ENSG00000149043
ENSG00000067715	ENSG00000149927
ENSG00000067715	ENSG00000170743
ENSG00000067715	ENSG00000173227
ENSG00000067715	ENSG00000204176
ENSG00000067715	ENSG00000213023
ENSG00000067715	ENSG00000214408
ENSG00000067715	ENSG00000214409
ENSG00000067715	ENSG00000214569
ENSG00000067715	ENSG00000214783
ENSG00000067798	ENSG00000134369
ENSG00000067798	ENSG00000166833
ENSG00000067840	ENSG00000121440
ENSG00000067840	ENSG00000165966
ENSG00000067842	ENSG00000058668
ENSG00000067842	ENSG00000070961
ENSG00000067842	ENSG00000157087
ENSG00000067900	ENSG00000134318
ENSG00000067900	ENSG00000143776
ENSG00000067900	ENSG00000171219
ENSG00000067900	ENSG00000198752
ENSG00000067992	ENSG00000004799
ENSG00000067992	ENSG00000005882
ENSG00000067992	ENSG00000152256
ENSG00000068001	ENSG00000106302
ENSG00000068001	ENSG00000106304
ENSG00000068001	ENSG00000114378
ENSG00000068001	ENSG00000186792
ENSG00000068024	ENSG00000048052
ENSG00000068024	ENSG00000061273
ENSG00000068024	ENSG00000108840
ENSG00000068078	ENSG00000066468
ENSG00000068078	ENSG00000077782
ENSG00000068078	ENSG00000160867
ENSG00000068079	ENSG00000123609
ENSG00000068305	ENSG00000064489
ENSG00000068305	ENSG00000081189
ENSG00000068305	ENSG00000116604
ENSG00000068323	ENSG00000105967
ENSG00000068323	ENSG00000112561
ENSG00000068323	ENSG00000187098
ENSG00000068366	ENSG00000123983
ENSG00000068615	ENSG00000132563
ENSG00000068615	ENSG00000165476
ENSG00000068615	ENSG00000168476
ENSG00000068650	ENSG00000058063
ENSG00000068650	ENSG00000101974
ENSG00000068697	ENSG00000104341
ENSG00000068697	ENSG00000162511
ENSG00000068724	ENSG00000165914
ENSG00000068793	ENSG00000055163
ENSG00000068796	ENSG00000141200
ENSG00000068796	ENSG00000142945
ENSG00000068831	ENSG00000152689

ENSG00000068831	ENSG00000171777
ENSG00000068831	ENSG00000172575
ENSG00000068971	ENSG00000066027
ENSG00000068971	ENSG00000078304
ENSG00000068971	ENSG00000112640
ENSG00000068971	ENSG00000154001
ENSG00000068976	ENSG00000100504
ENSG00000068976	ENSG00000100994
ENSG00000069011	ENSG00000107859
ENSG00000069011	ENSG00000164093
ENSG00000069018	ENSG00000072315
ENSG00000069018	ENSG00000133107
ENSG00000069018	ENSG00000137672
ENSG00000069018	ENSG00000138741
ENSG00000069018	ENSG00000144935
ENSG00000069020	ENSG00000086015
ENSG00000069020	ENSG00000099308
ENSG00000069020	ENSG00000105613
ENSG00000069020	ENSG00000120539
ENSG00000069122	ENSG00000123146
ENSG00000069122	ENSG00000127507
ENSG00000069122	ENSG00000131355
ENSG00000069122	ENSG00000153292
ENSG00000069122	ENSG00000153294
ENSG00000069122	ENSG00000162618
ENSG00000069122	ENSG00000164393
ENSG00000069122	ENSG00000173567
ENSG00000069122	ENSG00000174837
ENSG00000069122	ENSG00000186629
ENSG00000069122	ENSG00000204818
ENSG00000069188	ENSG00000146555
ENSG00000069188	ENSG00000171587
ENSG00000069188	ENSG00000177103
ENSG00000069206	ENSG00000008277
ENSG00000069206	ENSG00000042980
ENSG00000069206	ENSG00000073670
ENSG00000069206	ENSG00000104755
ENSG00000069206	ENSG00000114948
ENSG00000069206	ENSG00000134007
ENSG00000069206	ENSG00000134028
ENSG00000069206	ENSG00000134249
ENSG00000069206	ENSG00000135074
ENSG00000069206	ENSG00000139985
ENSG00000069206	ENSG00000143537
ENSG00000069206	ENSG00000148848
ENSG00000069206	ENSG00000149451
ENSG00000069206	ENSG00000151651
ENSG00000069206	ENSG00000151694
ENSG00000069206	ENSG00000168594
ENSG00000069206	ENSG00000168615
ENSG00000069206	ENSG00000168619
ENSG00000069206	ENSG00000196115
ENSG00000069206	ENSG00000197140
ENSG00000069206	ENSG00000214137
ENSG00000069424	ENSG00000169282
ENSG00000069424	ENSG00000170049
ENSG00000069431	ENSG00000006071
ENSG00000069509	ENSG00000165775
ENSG00000069509	ENSG00000182814

ENSG00000069535	ENSG00000104951
ENSG00000069535	ENSG00000189221
ENSG00000069696	ENSG00000149295
ENSG00000069696	ENSG00000151577
ENSG00000069702	ENSG00000106991
ENSG00000069764	ENSG00000117215
ENSG00000069764	ENSG00000127472
ENSG00000069764	ENSG00000158786
ENSG00000069764	ENSG00000170890
ENSG00000069764	ENSG00000187980
ENSG00000069764	ENSG00000188257
ENSG00000069764	ENSG00000188784
ENSG00000069849	ENSG00000101892
ENSG00000069849	ENSG00000129244
ENSG00000069849	ENSG00000143153
ENSG00000069849	ENSG00000186009
ENSG00000069869	ENSG00000049759
ENSG00000069869	ENSG00000078747
ENSG00000069869	ENSG00000108854
ENSG00000069869	ENSG00000123124
ENSG00000069869	ENSG00000198373
ENSG00000069869	ENSG00000198742
ENSG00000069956	ENSG00000141639
ENSG00000069956	ENSG00000215542
ENSG00000069974	ENSG00000041353
ENSG00000070018	ENSG00000081479
ENSG00000070018	ENSG00000123384
ENSG00000070018	ENSG00000134569
ENSG00000070018	ENSG00000138798
ENSG00000070018	ENSG00000162337
ENSG00000070018	ENSG00000168702
ENSG00000070047	ENSG00000126461
ENSG00000070047	ENSG00000139218
ENSG00000070081	ENSG00000104805
ENSG00000070087	ENSG00000108518
ENSG00000070087	ENSG00000196570
ENSG00000070159	ENSG00000070778
ENSG00000070159	ENSG00000088179
ENSG00000070159	ENSG00000152104
ENSG00000070182	ENSG00000115306
ENSG00000070182	ENSG00000137877
ENSG00000070182	ENSG00000160460
ENSG00000070182	ENSG00000173898
ENSG00000070193	ENSG00000070388
ENSG00000070193	ENSG00000075388
ENSG00000070193	ENSG00000078579
ENSG00000070193	ENSG00000102466
ENSG00000070193	ENSG00000102678
ENSG00000070193	ENSG00000111241
ENSG00000070193	ENSG00000113578
ENSG00000070193	ENSG00000114279
ENSG00000070193	ENSG00000129682
ENSG00000070193	ENSG00000138675
ENSG00000070193	ENSG00000138685
ENSG00000070193	ENSG00000140285
ENSG00000070193	ENSG00000161958
ENSG00000070193	ENSG00000185390
ENSG00000070193	ENSG00000186895
ENSG00000070193	ENSG00000196468

ENSG00000070193	ENSG00000204801
ENSG00000070193	ENSG00000204816
ENSG00000070193	ENSG00000204817
ENSG00000070193	ENSG00000204837
ENSG00000070193	ENSG00000215053
ENSG00000070214	ENSG00000129353
ENSG00000070214	ENSG00000137968
ENSG00000070214	ENSG00000143036
ENSG00000070214	ENSG00000204385
ENSG00000070366	ENSG00000116698
ENSG00000070366	ENSG00000198952
ENSG00000070371	ENSG00000141367
ENSG00000070388	ENSG00000070193
ENSG00000070388	ENSG00000075388
ENSG00000070388	ENSG00000078579
ENSG00000070388	ENSG00000102466
ENSG00000070388	ENSG00000102678
ENSG00000070388	ENSG00000111241
ENSG00000070388	ENSG00000113578
ENSG00000070388	ENSG00000114279
ENSG00000070388	ENSG00000129682
ENSG00000070388	ENSG00000138675
ENSG00000070388	ENSG00000138685
ENSG00000070388	ENSG00000140285
ENSG00000070388	ENSG00000161958
ENSG00000070388	ENSG00000185390
ENSG00000070388	ENSG00000186895
ENSG00000070388	ENSG00000196468
ENSG00000070388	ENSG00000204801
ENSG00000070388	ENSG00000204816
ENSG00000070388	ENSG00000204817
ENSG00000070388	ENSG00000204837
ENSG00000070388	ENSG00000215053
ENSG00000070404	ENSG00000053108
ENSG00000070404	ENSG00000134363
ENSG00000070404	ENSG00000163430
ENSG00000070423	ENSG00000121848
ENSG00000070540	ENSG00000157954
ENSG00000070601	ENSG00000147234
ENSG00000070601	ENSG00000169933
ENSG00000070614	ENSG00000138653
ENSG00000070614	ENSG00000164100
ENSG00000070614	ENSG00000166507
ENSG00000070718	ENSG00000185009
ENSG00000070729	ENSG00000099822
ENSG00000070729	ENSG00000132259
ENSG00000070729	ENSG00000138622
ENSG00000070729	ENSG00000143630
ENSG00000070729	ENSG00000144191
ENSG00000070729	ENSG00000164588
ENSG00000070729	ENSG00000170289
ENSG00000070729	ENSG00000183862
ENSG00000070756	ENSG00000090621
ENSG00000070756	ENSG00000101104
ENSG00000070756	ENSG00000151846
ENSG00000070756	ENSG00000174740
ENSG00000070756	ENSG00000215587
ENSG00000070759	ENSG00000106683
ENSG00000070759	ENSG00000107140

ENSG00000070759	ENSG00000182541
ENSG00000070770	ENSG00000101266
ENSG00000070778	ENSG00000070159
ENSG00000070778	ENSG00000088179
ENSG00000070778	ENSG00000152104
ENSG00000070808	ENSG00000058404
ENSG00000070808	ENSG00000145349
ENSG00000070808	ENSG00000148660
ENSG00000070831	ENSG00000119729
ENSG00000070831	ENSG00000126785
ENSG00000070831	ENSG00000128340
ENSG00000070831	ENSG00000136238
ENSG00000070831	ENSG00000169750
ENSG00000070831	ENSG00000177105
ENSG00000070882	ENSG00000006025
ENSG00000070882	ENSG00000079156
ENSG00000070886	ENSG00000044524
ENSG00000070886	ENSG00000080224
ENSG00000070886	ENSG00000106123
ENSG00000070886	ENSG00000116106
ENSG00000070886	ENSG00000133216
ENSG00000070886	ENSG00000135333
ENSG00000070886	ENSG00000142627
ENSG00000070886	ENSG00000145242
ENSG00000070886	ENSG00000146904
ENSG00000070886	ENSG00000154928
ENSG00000070886	ENSG00000182580
ENSG00000070886	ENSG00000183317
ENSG00000070886	ENSG00000196411
ENSG00000070915	ENSG00000064651
ENSG00000070915	ENSG00000074803
ENSG00000070915	ENSG00000146828
ENSG00000070961	ENSG00000058668
ENSG00000070961	ENSG00000067842
ENSG00000070961	ENSG00000157087
ENSG00000070985	ENSG00000083067
ENSG00000070985	ENSG00000092439
ENSG00000070985	ENSG00000119121
ENSG00000070985	ENSG00000130529
ENSG00000070985	ENSG00000134160
ENSG00000070985	ENSG00000142185
ENSG00000070985	ENSG00000144481
ENSG00000071051	ENSG00000158092
ENSG00000071054	ENSG00000123572
ENSG00000071054	ENSG00000141503
ENSG00000071054	ENSG00000154310
ENSG00000071073	ENSG00000161013
ENSG00000071073	ENSG00000182050
ENSG00000071073	ENSG00000184774
ENSG00000071203	ENSG00000110077
ENSG00000071203	ENSG00000110079
ENSG00000071203	ENSG00000149516
ENSG00000071203	ENSG00000149534
ENSG00000071203	ENSG00000156738
ENSG00000071203	ENSG00000166926
ENSG00000071203	ENSG00000166927
ENSG00000071203	ENSG00000166930
ENSG00000071203	ENSG00000166959
ENSG00000071203	ENSG00000166961

ENSG00000071203	ENSG00000214787
ENSG00000071205	ENSG00000079482
ENSG00000071205	ENSG00000145819
ENSG00000071205	ENSG00000165895
ENSG00000071242	ENSG00000072133
ENSG00000071242	ENSG00000100784
ENSG00000071242	ENSG00000108443
ENSG00000071242	ENSG00000117676
ENSG00000071242	ENSG00000162302
ENSG00000071242	ENSG00000175634
ENSG00000071242	ENSG00000177189
ENSG00000071242	ENSG00000214817
ENSG00000071246	ENSG00000143494
ENSG00000071282	ENSG00000012211
ENSG00000071282	ENSG00000124593
ENSG00000071282	ENSG00000135269
ENSG00000071282	ENSG00000139174
ENSG00000071282	ENSG00000163637
ENSG00000071537	ENSG00000091490
ENSG00000071537	ENSG00000101251
ENSG00000071537	ENSG00000109771
ENSG00000071553	ENSG00000205464
ENSG00000071564	ENSG00000140262
ENSG00000071564	ENSG00000196628
ENSG00000071564	ENSG00000215291
ENSG00000071575	ENSG00000101255
ENSG00000071575	ENSG00000173334
ENSG00000071575	ENSG00000196182
ENSG00000071655	ENSG00000134046
ENSG00000071655	ENSG00000170948
ENSG00000071655	ENSG00000182315
ENSG00000071655	ENSG00000184301
ENSG00000071655	ENSG00000196589
ENSG00000071655	ENSG00000205717
ENSG00000071655	ENSG00000205718
ENSG00000071889	ENSG00000174028
ENSG00000071889	ENSG00000183844
ENSG00000071889	ENSG00000196937
ENSG00000071889	ENSG00000198643
ENSG00000071909	ENSG00000095777
ENSG00000071991	ENSG00000040731
ENSG00000071991	ENSG00000081138
ENSG00000071991	ENSG00000101542
ENSG00000071991	ENSG00000113100
ENSG00000071991	ENSG00000113361
ENSG00000071991	ENSG00000139880
ENSG00000071991	ENSG00000140937
ENSG00000071991	ENSG00000145526
ENSG00000071991	ENSG00000149654
ENSG00000071991	ENSG00000150394
ENSG00000071991	ENSG00000154162
ENSG00000071991	ENSG00000179776
ENSG00000072041	ENSG00000063127
ENSG00000072041	ENSG00000197106
ENSG00000072042	ENSG00000139988
ENSG00000072042	ENSG00000160439
ENSG00000072042	ENSG00000167536
ENSG00000072042	ENSG00000169084
ENSG00000072062	ENSG00000099725

ENSG00000072062	ENSG00000142875
ENSG00000072062	ENSG00000165059
ENSG00000072062	ENSG00000183943
ENSG00000072071	ENSG00000111452
ENSG00000072071	ENSG00000117114
ENSG00000072071	ENSG00000150471
ENSG00000072071	ENSG00000180264
ENSG00000072110	ENSG00000077522
ENSG00000072110	ENSG00000130402
ENSG00000072110	ENSG00000204633
ENSG00000072133	ENSG00000071242
ENSG00000072133	ENSG00000100784
ENSG00000072133	ENSG00000108443
ENSG00000072133	ENSG00000117676
ENSG00000072133	ENSG00000162302
ENSG00000072133	ENSG00000175634
ENSG00000072133	ENSG00000177189
ENSG00000072133	ENSG00000214817
ENSG00000072134	ENSG00000049283
ENSG00000072134	ENSG00000063245
ENSG00000072134	ENSG00000113282
ENSG00000072134	ENSG00000176177
ENSG00000072135	ENSG00000127947
ENSG00000072135	ENSG00000134242
ENSG00000072163	ENSG00000089159
ENSG00000072163	ENSG00000110031
ENSG00000072163	ENSG00000112214
ENSG00000072163	ENSG00000140682
ENSG00000072163	ENSG00000169756
ENSG00000072163	ENSG00000183386
ENSG00000072195	ENSG00000154358
ENSG00000072201	ENSG00000107186
ENSG00000072201	ENSG00000132849
ENSG00000072201	ENSG00000139517
ENSG00000072210	ENSG00000006534
ENSG00000072210	ENSG00000108602
ENSG00000072210	ENSG00000132746
ENSG00000072274	ENSG00000077616
ENSG00000072274	ENSG00000086205
ENSG00000072274	ENSG00000106327
ENSG00000072274	ENSG00000134612
ENSG00000072274	ENSG00000168060
ENSG00000072274	ENSG00000177694
ENSG00000072310	ENSG00000198911
ENSG00000072315	ENSG00000069018
ENSG00000072315	ENSG00000133107
ENSG00000072315	ENSG00000137672
ENSG00000072315	ENSG00000138741
ENSG00000072315	ENSG00000144935
ENSG00000072364	ENSG00000144218
ENSG00000072364	ENSG00000155966
ENSG00000072364	ENSG00000172493
ENSG00000072401	ENSG00000078967
ENSG00000072401	ENSG00000109332
ENSG00000072401	ENSG00000131508
ENSG00000072401	ENSG00000184888
ENSG00000072422	ENSG00000008853
ENSG00000072422	ENSG00000164292
ENSG00000072501	ENSG00000077935

ENSG00000072518	ENSG00000007047
ENSG00000072518	ENSG00000075413
ENSG00000072518	ENSG00000116141
ENSG00000072518	ENSG00000142178
ENSG00000072518	ENSG00000160584
ENSG00000072518	ENSG00000170145
ENSG00000072609	ENSG00000112130
ENSG00000072682	ENSG00000122884
ENSG00000072682	ENSG00000149380
ENSG00000072694	ENSG00000132185
ENSG00000072694	ENSG00000143226
ENSG00000072694	ENSG00000150337
ENSG00000072694	ENSG00000162747
ENSG00000072694	ENSG00000179639
ENSG00000072694	ENSG00000181036
ENSG00000072694	ENSG00000198019
ENSG00000072694	ENSG00000203747
ENSG00000072694	ENSG00000203820
ENSG00000072736	ENSG00000100968
ENSG00000072736	ENSG00000101096
ENSG00000072736	ENSG00000102908
ENSG00000072736	ENSG00000131196
ENSG00000072786	ENSG00000065613
ENSG00000072803	ENSG00000166167
ENSG00000072818	ENSG00000114331
ENSG00000072818	ENSG00000131584
ENSG00000072832	ENSG00000092964
ENSG00000072832	ENSG00000113657
ENSG00000072832	ENSG00000147647
ENSG00000072832	ENSG00000151640
ENSG00000072832	ENSG00000157851
ENSG00000072849	ENSG00000099958
ENSG00000072858	ENSG00000149577
ENSG00000072864	ENSG00000166579
ENSG00000072952	ENSG00000118308
ENSG00000072954	ENSG00000095209
ENSG00000072958	ENSG00000129354
ENSG00000072958	ENSG00000140022
ENSG00000073008	ENSG00000110400
ENSG00000073008	ENSG00000130202
ENSG00000073008	ENSG00000177707
ENSG00000073009	ENSG00000123240
ENSG00000073150	ENSG00000110218
ENSG00000073150	ENSG00000154143
ENSG00000073282	ENSG00000078900
ENSG00000073282	ENSG00000141510
ENSG00000073331	ENSG00000103319
ENSG00000073350	ENSG00000131899
ENSG00000073350	ENSG00000145087
ENSG00000073350	ENSG00000164506
ENSG00000073417	ENSG00000065989
ENSG00000073417	ENSG00000105650
ENSG00000073417	ENSG00000113231
ENSG00000073417	ENSG00000113448
ENSG00000073417	ENSG00000115252
ENSG00000073417	ENSG00000123360
ENSG00000073417	ENSG00000154678
ENSG00000073417	ENSG00000160191
ENSG00000073417	ENSG00000171408

ENSG00000073417	ENSG00000184588
ENSG00000073417	ENSG00000205268
ENSG00000073464	ENSG00000011021
ENSG00000073464	ENSG00000103249
ENSG00000073464	ENSG00000109572
ENSG00000073464	ENSG00000171365
ENSG00000073614	ENSG00000012817
ENSG00000073614	ENSG00000117139
ENSG00000073614	ENSG00000126012
ENSG00000073670	ENSG00000008277
ENSG00000073670	ENSG00000042980
ENSG00000073670	ENSG00000069206
ENSG00000073670	ENSG00000104755
ENSG00000073670	ENSG00000114948
ENSG00000073670	ENSG00000134007
ENSG00000073670	ENSG00000134028
ENSG00000073670	ENSG00000134249
ENSG00000073670	ENSG00000135074
ENSG00000073670	ENSG00000139985
ENSG00000073670	ENSG00000143537
ENSG00000073670	ENSG00000148848
ENSG00000073670	ENSG00000149451
ENSG00000073670	ENSG00000151651
ENSG00000073670	ENSG00000151694
ENSG00000073670	ENSG00000168594
ENSG00000073670	ENSG00000168615
ENSG00000073670	ENSG00000168619
ENSG00000073670	ENSG00000196115
ENSG00000073670	ENSG00000197140
ENSG00000073670	ENSG00000214137
ENSG00000073711	ENSG00000167393
ENSG00000073712	ENSG00000101311
ENSG00000073712	ENSG00000137076
ENSG00000073712	ENSG00000149781
ENSG00000073712	ENSG00000171914
ENSG00000073734	ENSG00000004846
ENSG00000073734	ENSG00000005471
ENSG00000073734	ENSG00000085563
ENSG00000073737	ENSG00000025423
ENSG00000073737	ENSG00000086696
ENSG00000073737	ENSG00000135437
ENSG00000073737	ENSG00000139547
ENSG00000073737	ENSG00000161267
ENSG00000073737	ENSG00000170426
ENSG00000073737	ENSG00000176387
ENSG00000073754	ENSG00000013725
ENSG00000073754	ENSG00000110448
ENSG00000073754	ENSG00000146700
ENSG00000073754	ENSG00000164099
ENSG00000073754	ENSG00000177575
ENSG00000073754	ENSG00000177675
ENSG00000073754	ENSG00000179954
ENSG00000073754	ENSG00000187908
ENSG00000073754	ENSG00000214279
ENSG00000073756	ENSG00000095303
ENSG00000073792	ENSG00000136231
ENSG00000073792	ENSG00000159217
ENSG00000073803	ENSG00000139625
ENSG00000073849	ENSG00000101638

ENSG00000073849	ENSG00000111728
ENSG00000073849	ENSG00000117069
ENSG00000073849	ENSG00000144057
ENSG00000073849	ENSG00000148488
ENSG00000073849	ENSG00000160408
ENSG00000073849	ENSG00000177511
ENSG00000073861	ENSG00000089225
ENSG00000073861	ENSG00000092607
ENSG00000073861	ENSG00000112837
ENSG00000073861	ENSG00000121068
ENSG00000073861	ENSG00000121075
ENSG00000073861	ENSG00000122145
ENSG00000073861	ENSG00000135111
ENSG00000073861	ENSG00000136535
ENSG00000073861	ENSG00000143178
ENSG00000073861	ENSG00000149922
ENSG00000073861	ENSG00000163508
ENSG00000073861	ENSG00000164458
ENSG00000073861	ENSG00000164532
ENSG00000073861	ENSG00000167800
ENSG00000073861	ENSG00000174197
ENSG00000073861	ENSG00000184058
ENSG00000073910	ENSG00000075539
ENSG00000073921	ENSG00000065609
ENSG00000074047	ENSG00000106571
ENSG00000074047	ENSG00000107249
ENSG00000074047	ENSG00000111087
ENSG00000074047	ENSG00000126603
ENSG00000074047	ENSG00000174332
ENSG00000074054	ENSG00000163539
ENSG00000074181	ENSG00000134250
ENSG00000074181	ENSG00000134376
ENSG00000074181	ENSG00000148400
ENSG00000074181	ENSG00000204301
ENSG00000074211	ENSG00000156475
ENSG00000074211	ENSG00000175470
ENSG00000074211	ENSG00000214122
ENSG00000074219	ENSG00000007866
ENSG00000074219	ENSG00000187079
ENSG00000074219	ENSG00000197905
ENSG00000074276	ENSG00000148600
ENSG00000074317	ENSG00000145335
ENSG00000074317	ENSG00000173267
ENSG00000074370	ENSG00000174437
ENSG00000074370	ENSG00000196296
ENSG00000074410	ENSG00000104267
ENSG00000074410	ENSG00000107159
ENSG00000074410	ENSG00000118298
ENSG00000074410	ENSG00000131686
ENSG00000074410	ENSG00000133742
ENSG00000074410	ENSG00000164879
ENSG00000074410	ENSG00000167434
ENSG00000074410	ENSG00000168748
ENSG00000074410	ENSG00000169239
ENSG00000074410	ENSG00000174990
ENSG00000074410	ENSG00000178538
ENSG00000074410	ENSG00000185015
ENSG00000074410	ENSG00000214729
ENSG00000074410	ENSG00000214873

ENSG00000074410	ENSG00000215487
ENSG00000074410	ENSG00000215501
ENSG00000074410	ENSG00000215543
ENSG00000074527	ENSG00000050555
ENSG00000074527	ENSG00000058085
ENSG00000074527	ENSG00000091128
ENSG00000074527	ENSG00000091136
ENSG00000074527	ENSG00000106780
ENSG00000074527	ENSG00000135862
ENSG00000074527	ENSG00000172037
ENSG00000074527	ENSG00000196878
ENSG00000074590	ENSG00000163545
ENSG00000074603	ENSG00000078098
ENSG00000074603	ENSG00000130226
ENSG00000074603	ENSG00000142002
ENSG00000074603	ENSG00000175497
ENSG00000074603	ENSG00000197635
ENSG00000074621	ENSG00000140090
ENSG00000074621	ENSG00000155886
ENSG00000074621	ENSG00000185052
ENSG00000074621	ENSG00000188467
ENSG00000074657	ENSG00000143373
ENSG00000074657	ENSG00000166716
ENSG00000074657	ENSG00000214168
ENSG00000074657	ENSG00000214171
ENSG00000074660	ENSG00000099910
ENSG00000074660	ENSG00000145794
ENSG00000074660	ENSG00000157890
ENSG00000074660	ENSG00000162591
ENSG00000074660	ENSG00000187800
ENSG00000074695	ENSG00000114988
ENSG00000074695	ENSG00000140506
ENSG00000074695	ENSG00000169223
ENSG00000074696	ENSG00000165996
ENSG00000074696	ENSG00000188921
ENSG00000074696	ENSG00000206527
ENSG00000074706	ENSG00000142675
ENSG00000074706	ENSG00000149970
ENSG00000074706	ENSG00000153721
ENSG00000074771	ENSG00000007952
ENSG00000074771	ENSG00000086991
ENSG00000074771	ENSG00000165168
ENSG00000074800	ENSG00000108515
ENSG00000074800	ENSG00000111674
ENSG00000074800	ENSG00000214431
ENSG00000074800	ENSG00000215809
ENSG00000074803	ENSG00000064651
ENSG00000074803	ENSG00000070915
ENSG00000074803	ENSG00000146828
ENSG00000074964	ENSG00000104728
ENSG00000074966	ENSG00000010671
ENSG00000074966	ENSG00000102010
ENSG00000074966	ENSG00000113263
ENSG00000074966	ENSG00000135605
ENSG00000074966	ENSG00000151422
ENSG00000074966	ENSG00000182511
ENSG00000075035	ENSG00000179314
ENSG00000075043	ENSG00000053918
ENSG00000075043	ENSG00000117013

ENSG00000075043	ENSG00000184156
ENSG00000075043	ENSG00000185760
ENSG00000075073	ENSG00000056291
ENSG00000075073	ENSG00000101292
ENSG00000075073	ENSG00000110148
ENSG00000075073	ENSG00000115353
ENSG00000075073	ENSG00000119973
ENSG00000075073	ENSG00000121764
ENSG00000075073	ENSG00000123901
ENSG00000075073	ENSG00000137252
ENSG00000075073	ENSG00000148734
ENSG00000075073	ENSG00000159279
ENSG00000075073	ENSG00000163394
ENSG00000075073	ENSG00000164128
ENSG00000075073	ENSG00000164129
ENSG00000075073	ENSG00000169618
ENSG00000075073	ENSG00000169836
ENSG00000075073	ENSG00000185149
ENSG00000075073	ENSG00000186867
ENSG00000075073	ENSG00000204174
ENSG00000075073	ENSG00000215167
ENSG00000075142	ENSG00000115271
ENSG00000075142	ENSG00000126247
ENSG00000075142	ENSG00000162517
ENSG00000075151	ENSG00000114867
ENSG00000075213	ENSG00000010319
ENSG00000075213	ENSG00000012171
ENSG00000075213	ENSG00000092421
ENSG00000075213	ENSG00000095539
ENSG00000075213	ENSG00000137872
ENSG00000075213	ENSG00000143434
ENSG00000075213	ENSG00000153993
ENSG00000075213	ENSG00000168758
ENSG00000075213	ENSG00000170381
ENSG00000075213	ENSG00000185033
ENSG00000075213	ENSG00000187764
ENSG00000075213	ENSG00000196189
ENSG00000075223	ENSG00000001617
ENSG00000075275	ENSG00000008300
ENSG00000075275	ENSG00000143126
ENSG00000075290	ENSG00000002745
ENSG00000075290	ENSG00000061492
ENSG00000075290	ENSG00000085741
ENSG00000075290	ENSG00000105989
ENSG00000075290	ENSG00000108379
ENSG00000075290	ENSG00000111186
ENSG00000075290	ENSG00000114251
ENSG00000075290	ENSG00000115596
ENSG00000075290	ENSG00000125084
ENSG00000075290	ENSG00000134245
ENSG00000075290	ENSG00000135925
ENSG00000075290	ENSG00000143816
ENSG00000075290	ENSG00000154342
ENSG00000075290	ENSG00000154764
ENSG00000075290	ENSG00000158955
ENSG00000075290	ENSG00000162552
ENSG00000075290	ENSG00000169884
ENSG00000075290	ENSG00000188064
ENSG00000075292	ENSG00000015479

ENSG00000075292	ENSG00000203867
ENSG00000075303	ENSG00000013306
ENSG00000075340	ENSG00000087274
ENSG00000075340	ENSG00000148700
ENSG00000075388	ENSG00000070193
ENSG00000075388	ENSG00000070388
ENSG00000075388	ENSG00000078579
ENSG00000075388	ENSG00000102466
ENSG00000075388	ENSG00000102678
ENSG00000075388	ENSG00000111241
ENSG00000075388	ENSG00000113578
ENSG00000075388	ENSG00000114279
ENSG00000075388	ENSG00000129682
ENSG00000075388	ENSG00000138675
ENSG00000075388	ENSG00000138685
ENSG00000075388	ENSG00000140285
ENSG00000075388	ENSG00000161958
ENSG00000075388	ENSG00000185390
ENSG00000075388	ENSG00000186895
ENSG00000075388	ENSG00000196468
ENSG00000075388	ENSG00000204801
ENSG00000075388	ENSG00000204816
ENSG00000075388	ENSG00000204817
ENSG00000075388	ENSG00000204837
ENSG00000075388	ENSG00000215053
ENSG00000075391	ENSG00000105122
ENSG00000075391	ENSG00000136848
ENSG00000075391	ENSG00000197283
ENSG00000075413	ENSG00000007047
ENSG00000075413	ENSG00000072518
ENSG00000075413	ENSG00000116141
ENSG00000075413	ENSG00000142178
ENSG00000075413	ENSG00000160584
ENSG00000075413	ENSG00000170145
ENSG00000075420	ENSG00000102531
ENSG00000075426	ENSG00000125740
ENSG00000075426	ENSG00000140044
ENSG00000075426	ENSG00000162772
ENSG00000075426	ENSG00000170345
ENSG00000075426	ENSG00000175592
ENSG00000075429	ENSG00000006116
ENSG00000075429	ENSG00000075461
ENSG00000075429	ENSG00000105605
ENSG00000075429	ENSG00000142408
ENSG00000075429	ENSG00000166862
ENSG00000075429	ENSG00000204278
ENSG00000075461	ENSG00000006116
ENSG00000075461	ENSG00000075429
ENSG00000075461	ENSG00000105605
ENSG00000075461	ENSG00000142408
ENSG00000075461	ENSG00000166862
ENSG00000075461	ENSG00000204278
ENSG00000075539	ENSG00000073910
ENSG00000075568	ENSG00000121210
ENSG00000075618	ENSG00000106328
ENSG00000075618	ENSG00000186765
ENSG00000075624	ENSG00000107796
ENSG00000075624	ENSG00000143632
ENSG00000075624	ENSG00000159251

ENSG00000075624	ENSG00000163017
ENSG00000075624	ENSG00000169067
ENSG00000075624	ENSG00000184009
ENSG00000075624	ENSG00000204434
ENSG00000075624	ENSG00000214555
ENSG00000075624	ENSG00000215141
ENSG00000075651	ENSG00000129219
ENSG00000075673	ENSG00000018625
ENSG00000075673	ENSG00000105409
ENSG00000075673	ENSG00000105675
ENSG00000075673	ENSG00000132681
ENSG00000075673	ENSG00000163399
ENSG00000075702	ENSG00000137802
ENSG00000075702	ENSG00000166596
ENSG00000075711	ENSG00000082458
ENSG00000075711	ENSG00000132535
ENSG00000075711	ENSG00000150672
ENSG00000075711	ENSG00000151208
ENSG00000075790	ENSG00000185825
ENSG00000075826	ENSG00000138674
ENSG00000075884	ENSG00000107863
ENSG00000075884	ENSG00000123329
ENSG00000075884	ENSG00000165322
ENSG00000075884	ENSG00000167433
ENSG00000075884	ENSG00000185602
ENSG00000075884	ENSG00000214627
ENSG00000075891	ENSG00000125618
ENSG00000075891	ENSG00000125813
ENSG00000075891	ENSG00000196092
ENSG00000075891	ENSG00000198807
ENSG00000075975	ENSG00000133606
ENSG00000075975	ENSG00000179455
ENSG00000076053	ENSG00000185272
ENSG00000076067	ENSG00000144642
ENSG00000076067	ENSG00000153250
ENSG00000076108	ENSG00000123636
ENSG00000076201	ENSG00000131941
ENSG00000076201	ENSG00000158106
ENSG00000076201	ENSG00000170248
ENSG00000076201	ENSG00000215407
ENSG00000076344	ENSG00000108370
ENSG00000076344	ENSG00000182732
ENSG00000076344	ENSG00000182901
ENSG00000076351	ENSG00000119457
ENSG00000076351	ENSG00000139508
ENSG00000076356	ENSG00000004399
ENSG00000076356	ENSG00000114554
ENSG00000076356	ENSG00000130827
ENSG00000076356	ENSG00000136040
ENSG00000076356	ENSG00000164050
ENSG00000076356	ENSG00000189437
ENSG00000076356	ENSG00000196576
ENSG00000076356	ENSG00000198753
ENSG00000076513	ENSG00000118454
ENSG00000076513	ENSG00000172932
ENSG00000076513	ENSG00000198720
ENSG00000076554	ENSG00000101150
ENSG00000076554	ENSG00000111907
ENSG00000076554	ENSG00000170777

ENSG00000076555	ENSG00000132142
ENSG00000076685	ENSG00000144130
ENSG00000076706	ENSG00000187244
ENSG00000076716	ENSG00000063660
ENSG00000076716	ENSG00000147257
ENSG00000076716	ENSG00000179399
ENSG00000076716	ENSG00000183098
ENSG00000076716	ENSG00000213420
ENSG00000076770	ENSG00000135482
ENSG00000076770	ENSG00000139793
ENSG00000076770	ENSG00000152601
ENSG00000076826	ENSG00000118200
ENSG00000076826	ENSG00000130559
ENSG00000076864	ENSG00000105738
ENSG00000076864	ENSG00000116991
ENSG00000076864	ENSG00000132359
ENSG00000076864	ENSG00000136895
ENSG00000076864	ENSG00000197555
ENSG00000076864	ENSG00000213445
ENSG00000076928	ENSG00000132694
ENSG00000076928	ENSG00000196914
ENSG00000076944	ENSG00000116266
ENSG00000076944	ENSG00000136854
ENSG00000077009	ENSG00000106733
ENSG00000077009	ENSG00000139597
ENSG00000077044	ENSG00000058866
ENSG00000077044	ENSG00000065357
ENSG00000077044	ENSG00000102780
ENSG00000077044	ENSG00000136267
ENSG00000077044	ENSG00000153933
ENSG00000077044	ENSG00000204466
ENSG00000077063	ENSG00000143079
ENSG00000077080	ENSG00000136518
ENSG00000077092	ENSG00000126351
ENSG00000077092	ENSG00000126368
ENSG00000077092	ENSG00000131759
ENSG00000077092	ENSG00000151090
ENSG00000077092	ENSG00000172819
ENSG00000077092	ENSG00000174738
ENSG00000077097	ENSG00000131747
ENSG00000077150	ENSG00000104856
ENSG00000077150	ENSG00000109320
ENSG00000077150	ENSG00000162924
ENSG00000077150	ENSG00000173039
ENSG00000077157	ENSG00000058272
ENSG00000077157	ENSG00000125503
ENSG00000077254	ENSG00000136878
ENSG00000077264	ENSG00000149269
ENSG00000077264	ENSG00000180370
ENSG00000077274	ENSG00000014216
ENSG00000077274	ENSG00000092529
ENSG00000077274	ENSG00000135773
ENSG00000077274	ENSG00000137225
ENSG00000077274	ENSG00000142330
ENSG00000077274	ENSG00000149260
ENSG00000077274	ENSG00000162909
ENSG00000077274	ENSG00000162949
ENSG00000077274	ENSG00000182472
ENSG00000077274	ENSG00000203697

ENSG00000077274	ENSG00000214711
ENSG00000077279	ENSG00000104237
ENSG00000077279	ENSG00000130413
ENSG00000077279	ENSG00000133083
ENSG00000077279	ENSG00000146038
ENSG00000077279	ENSG00000163673
ENSG00000077279	ENSG00000170390
ENSG00000077279	ENSG00000183638
ENSG00000077380	ENSG00000119333
ENSG00000077380	ENSG00000122735
ENSG00000077380	ENSG00000152763
ENSG00000077380	ENSG00000158560
ENSG00000077380	ENSG00000171595
ENSG00000077420	ENSG00000106070
ENSG00000077420	ENSG00000115290
ENSG00000077420	ENSG00000141738
ENSG00000077420	ENSG00000173166
ENSG00000077454	ENSG00000130224
ENSG00000077454	ENSG00000136141
ENSG00000077454	ENSG00000186001
ENSG00000077458	ENSG00000009780
ENSG00000077522	ENSG00000072110
ENSG00000077522	ENSG00000130402
ENSG00000077522	ENSG00000204633
ENSG00000077585	ENSG00000173264
ENSG00000077585	ENSG00000180998
ENSG00000077616	ENSG00000072274
ENSG00000077616	ENSG00000086205
ENSG00000077616	ENSG00000106327
ENSG00000077616	ENSG00000134612
ENSG00000077616	ENSG00000168060
ENSG00000077616	ENSG00000177694
ENSG00000077684	ENSG00000043143
ENSG00000077684	ENSG00000078403
ENSG00000077684	ENSG00000096070
ENSG00000077684	ENSG00000100425
ENSG00000077684	ENSG00000102221
ENSG00000077684	ENSG00000106443
ENSG00000077684	ENSG00000108292
ENSG00000077684	ENSG00000156983
ENSG00000077721	ENSG00000119048
ENSG00000077782	ENSG00000066468
ENSG00000077782	ENSG00000068078
ENSG00000077782	ENSG00000160867
ENSG00000077935	ENSG00000072501
ENSG00000077942	ENSG00000049323
ENSG00000077942	ENSG00000090006
ENSG00000077942	ENSG00000115380
ENSG00000077942	ENSG00000119681
ENSG00000077942	ENSG00000140092
ENSG00000077942	ENSG00000163520
ENSG00000077942	ENSG00000168056
ENSG00000077942	ENSG00000172638
ENSG00000077942	ENSG00000214536
ENSG00000077943	ENSG00000005844
ENSG00000077943	ENSG00000005884
ENSG00000077943	ENSG00000005961
ENSG00000077943	ENSG00000083457
ENSG00000077943	ENSG00000091409

ENSG00000077943	ENSG00000115232
ENSG00000077943	ENSG00000135424
ENSG00000077943	ENSG00000137809
ENSG00000077943	ENSG00000138448
ENSG00000077943	ENSG00000140678
ENSG00000077943	ENSG00000143127
ENSG00000077943	ENSG00000144668
ENSG00000077943	ENSG00000156886
ENSG00000077943	ENSG00000161638
ENSG00000077943	ENSG00000164171
ENSG00000077943	ENSG00000169896
ENSG00000077943	ENSG00000213949
ENSG00000077984	ENSG00000101435
ENSG00000077984	ENSG00000101439
ENSG00000077984	ENSG00000101441
ENSG00000077984	ENSG00000125815
ENSG00000077984	ENSG00000125823
ENSG00000077984	ENSG00000125831
ENSG00000077984	ENSG00000170367
ENSG00000077984	ENSG00000170369
ENSG00000077984	ENSG00000170373
ENSG00000077984	ENSG00000173335
ENSG00000077984	ENSG00000175315
ENSG00000077984	ENSG00000204658
ENSG00000077984	ENSG00000204662
ENSG00000077984	ENSG00000204663
ENSG00000078018	ENSG00000047849
ENSG00000078018	ENSG00000186868
ENSG00000078043	ENSG00000033800
ENSG00000078043	ENSG00000105229
ENSG00000078043	ENSG00000131788
ENSG00000078053	ENSG00000110934
ENSG00000078053	ENSG00000136717
ENSG00000078061	ENSG00000132155
ENSG00000078061	ENSG00000141068
ENSG00000078061	ENSG00000157764
ENSG00000078061	ENSG00000171435
ENSG00000078081	ENSG00000005893
ENSG00000078081	ENSG00000129226
ENSG00000078081	ENSG00000185896
ENSG00000078114	ENSG00000002834
ENSG00000078114	ENSG00000183091
ENSG00000078246	ENSG00000104804
ENSG00000078246	ENSG00000112041
ENSG00000078246	ENSG00000118965
ENSG00000078246	ENSG00000130338
ENSG00000078246	ENSG00000166402
ENSG00000078269	ENSG00000159082
ENSG00000078295	ENSG00000121281
ENSG00000078295	ENSG00000129467
ENSG00000078295	ENSG00000138031
ENSG00000078295	ENSG00000155897
ENSG00000078295	ENSG00000162104
ENSG00000078295	ENSG00000164742
ENSG00000078295	ENSG00000173175
ENSG00000078295	ENSG00000174233
ENSG00000078304	ENSG00000066027
ENSG00000078304	ENSG00000068971
ENSG00000078304	ENSG00000112640

ENSG00000078304	ENSG00000154001
ENSG00000078328	ENSG00000100320
ENSG00000078328	ENSG00000167281
ENSG00000078369	ENSG00000069966
ENSG00000078369	ENSG00000111664
ENSG00000078369	ENSG00000114450
ENSG00000078369	ENSG00000172354
ENSG00000078399	ENSG00000128709
ENSG00000078399	ENSG00000128710
ENSG00000078399	ENSG00000153807
ENSG00000078399	ENSG00000170689
ENSG00000078399	ENSG00000180806
ENSG00000078399	ENSG00000180818
ENSG00000078401	ENSG00000124205
ENSG00000078401	ENSG00000127129
ENSG00000078403	ENSG00000043143
ENSG00000078403	ENSG00000077684
ENSG00000078403	ENSG00000096070
ENSG00000078403	ENSG00000100425
ENSG00000078403	ENSG00000102221
ENSG00000078403	ENSG00000106443
ENSG00000078403	ENSG00000108292
ENSG00000078403	ENSG00000156983
ENSG00000078549	ENSG00000004948
ENSG00000078549	ENSG00000010310
ENSG00000078549	ENSG00000064989
ENSG00000078549	ENSG00000065325
ENSG00000078549	ENSG00000080293
ENSG00000078549	ENSG00000106018
ENSG00000078549	ENSG00000106113
ENSG00000078549	ENSG00000106128
ENSG00000078549	ENSG00000112164
ENSG00000078549	ENSG00000114812
ENSG00000078549	ENSG00000120088
ENSG00000078549	ENSG00000144407
ENSG00000078549	ENSG00000160801
ENSG00000078549	ENSG00000215644
ENSG00000078579	ENSG00000070193
ENSG00000078579	ENSG00000070388
ENSG00000078579	ENSG00000075388
ENSG00000078579	ENSG00000102466
ENSG00000078579	ENSG00000102678
ENSG00000078579	ENSG00000111241
ENSG00000078579	ENSG00000113578
ENSG00000078579	ENSG00000114279
ENSG00000078579	ENSG00000129682
ENSG00000078579	ENSG00000138675
ENSG00000078579	ENSG00000138685
ENSG00000078579	ENSG00000140285
ENSG00000078579	ENSG00000161958
ENSG00000078579	ENSG00000185390
ENSG00000078579	ENSG00000186895
ENSG00000078579	ENSG00000196468
ENSG00000078579	ENSG00000204801
ENSG00000078579	ENSG00000204816
ENSG00000078579	ENSG00000204817
ENSG00000078579	ENSG00000204837
ENSG00000078579	ENSG00000215053
ENSG00000078589	ENSG00000139679

ENSG00000078589	ENSG00000147145
ENSG00000078589	ENSG00000152207
ENSG00000078589	ENSG00000169403
ENSG00000078589	ENSG00000169508
ENSG00000078589	ENSG00000169860
ENSG00000078589	ENSG00000173198
ENSG00000078589	ENSG00000175591
ENSG00000078589	ENSG00000184574
ENSG00000078589	ENSG00000186912
ENSG00000078589	ENSG00000198829
ENSG00000078596	ENSG00000135916
ENSG00000078596	ENSG00000136156
ENSG00000078668	ENSG00000165637
ENSG00000078668	ENSG00000172974
ENSG00000078668	ENSG00000213585
ENSG00000078668	ENSG00000215818
ENSG00000078687	ENSG00000090905
ENSG00000078687	ENSG00000100354
ENSG00000078699	ENSG00000079102
ENSG00000078699	ENSG00000129993
ENSG00000078725	ENSG00000162670
ENSG00000078725	ENSG00000198797
ENSG00000078747	ENSG00000049759
ENSG00000078747	ENSG00000069869
ENSG00000078747	ENSG00000108854
ENSG00000078747	ENSG00000123124
ENSG00000078747	ENSG00000198373
ENSG00000078747	ENSG00000198742
ENSG00000078804	ENSG00000164938
ENSG00000078814	ENSG00000002079
ENSG00000078814	ENSG00000006788
ENSG00000078814	ENSG00000092054
ENSG00000078814	ENSG00000109061
ENSG00000078814	ENSG00000109063
ENSG00000078814	ENSG00000125414
ENSG00000078814	ENSG00000133020
ENSG00000078814	ENSG00000141048
ENSG00000078814	ENSG00000144821
ENSG00000078814	ENSG00000197616
ENSG00000078898	ENSG00000100979
ENSG00000078898	ENSG00000101425
ENSG00000078898	ENSG00000129988
ENSG00000078898	ENSG00000167104
ENSG00000078898	ENSG00000184459
ENSG00000078898	ENSG00000186190
ENSG00000078898	ENSG00000186191
ENSG00000078900	ENSG00000073282
ENSG00000078900	ENSG00000141510
ENSG00000078967	ENSG00000072401
ENSG00000078967	ENSG00000109332
ENSG00000078967	ENSG00000131508
ENSG00000078967	ENSG00000184888
ENSG00000079101	ENSG00000120885
ENSG00000079102	ENSG00000078699
ENSG00000079102	ENSG00000129993
ENSG00000079112	ENSG00000039068
ENSG00000079112	ENSG00000062038
ENSG00000079112	ENSG00000124215
ENSG00000079112	ENSG00000129910

ENSG00000079112	ENSG00000134755
ENSG00000079112	ENSG00000134762
ENSG00000079112	ENSG00000134765
ENSG00000079112	ENSG00000140945
ENSG00000079112	ENSG00000166589
ENSG00000079112	ENSG00000170558
ENSG00000079112	ENSG00000179242
ENSG00000079150	ENSG00000106080
ENSG00000079150	ENSG00000122642
ENSG00000079150	ENSG00000134285
ENSG00000079150	ENSG00000141756
ENSG00000079150	ENSG00000176826
ENSG00000079156	ENSG00000006025
ENSG00000079156	ENSG00000070882
ENSG00000079215	ENSG00000105143
ENSG00000079215	ENSG00000105281
ENSG00000079215	ENSG00000106688
ENSG00000079215	ENSG00000110436
ENSG00000079215	ENSG00000115902
ENSG00000079215	ENSG00000162383
ENSG00000079277	ENSG00000099875
ENSG00000079308	ENSG00000111077
ENSG00000079308	ENSG00000131746
ENSG00000079308	ENSG00000136205
ENSG00000079332	ENSG00000152700
ENSG00000079332	ENSG00000173386
ENSG00000079335	ENSG00000081377
ENSG00000079337	ENSG00000091428
ENSG00000079337	ENSG00000108352
ENSG00000079337	ENSG00000109756
ENSG00000079337	ENSG00000136237
ENSG00000079387	ENSG00000119231
ENSG00000079387	ENSG00000161956
ENSG00000079387	ENSG00000163904
ENSG00000079393	ENSG00000108861
ENSG00000079393	ENSG00000133878
ENSG00000079393	ENSG00000188542
ENSG00000079393	ENSG00000188716
ENSG00000079462	ENSG00000168092
ENSG00000079482	ENSG00000071205
ENSG00000079482	ENSG00000145819
ENSG00000079482	ENSG00000165895
ENSG00000079691	ENSG00000159753
ENSG00000079691	ENSG00000186648
ENSG00000079739	ENSG00000154330
ENSG00000079739	ENSG00000204794
ENSG00000079805	ENSG00000106976
ENSG00000079805	ENSG00000197959
ENSG00000079819	ENSG00000082397
ENSG00000079819	ENSG00000088367
ENSG00000079819	ENSG00000159023
ENSG00000079841	ENSG00000117016
ENSG00000079841	ENSG00000176406
ENSG00000079950	ENSG00000117758
ENSG00000080031	ENSG00000127329
ENSG00000080031	ENSG00000149177
ENSG00000080031	ENSG00000151490
ENSG00000080166	ENSG00000077498
ENSG00000080166	ENSG00000107165

ENSG00000080200	ENSG00000112297
ENSG00000080200	ENSG00000176092
ENSG00000080224	ENSG00000044524
ENSG00000080224	ENSG00000070886
ENSG00000080224	ENSG00000106123
ENSG00000080224	ENSG00000116106
ENSG00000080224	ENSG00000133216
ENSG00000080224	ENSG00000135333
ENSG00000080224	ENSG00000142627
ENSG00000080224	ENSG00000145242
ENSG00000080224	ENSG00000146904
ENSG00000080224	ENSG00000154928
ENSG00000080224	ENSG00000182580
ENSG00000080224	ENSG00000183317
ENSG00000080224	ENSG00000196411
ENSG00000080293	ENSG00000004948
ENSG00000080293	ENSG00000010310
ENSG00000080293	ENSG00000064989
ENSG00000080293	ENSG00000065325
ENSG00000080293	ENSG00000078549
ENSG00000080293	ENSG00000106018
ENSG00000080293	ENSG00000106113
ENSG00000080293	ENSG00000106128
ENSG00000080293	ENSG00000112164
ENSG00000080293	ENSG00000114812
ENSG00000080293	ENSG00000120088
ENSG00000080293	ENSG00000144407
ENSG00000080293	ENSG00000160801
ENSG00000080293	ENSG00000215644
ENSG00000080298	ENSG00000087903
ENSG00000080298	ENSG00000111783
ENSG00000080298	ENSG00000132005
ENSG00000080298	ENSG00000143390
ENSG00000080298	ENSG00000181827
ENSG00000080298	ENSG00000185002
ENSG00000080493	ENSG00000004939
ENSG00000080493	ENSG00000033867
ENSG00000080493	ENSG00000050438
ENSG00000080493	ENSG00000088836
ENSG00000080493	ENSG00000113073
ENSG00000080493	ENSG00000114923
ENSG00000080493	ENSG00000144290
ENSG00000080493	ENSG00000164889
ENSG00000080493	ENSG00000188687
ENSG00000080503	ENSG00000127616
ENSG00000080511	ENSG00000108785
ENSG00000080511	ENSG00000108786
ENSG00000080546	ENSG00000130766
ENSG00000080546	ENSG00000149212
ENSG00000080561	ENSG00000101871
ENSG00000080561	ENSG00000105255
ENSG00000080561	ENSG00000106701
ENSG00000080573	ENSG00000060718
ENSG00000080573	ENSG00000108821
ENSG00000080573	ENSG00000130635
ENSG00000080573	ENSG00000139219
ENSG00000080573	ENSG00000164692
ENSG00000080573	ENSG00000168542
ENSG00000080573	ENSG00000204248

ENSG00000080573	ENSG00000204262
ENSG00000080603	ENSG00000183495
ENSG00000080618	ENSG00000091704
ENSG00000080618	ENSG00000128510
ENSG00000080618	ENSG00000144410
ENSG00000080618	ENSG00000153002
ENSG00000080618	ENSG00000158516
ENSG00000080618	ENSG00000158525
ENSG00000080618	ENSG00000163751
ENSG00000080618	ENSG00000165078
ENSG00000080644	ENSG00000101204
ENSG00000080644	ENSG00000108556
ENSG00000080644	ENSG00000117971
ENSG00000080644	ENSG00000120903
ENSG00000080644	ENSG00000129749
ENSG00000080644	ENSG00000135902
ENSG00000080644	ENSG00000138435
ENSG00000080644	ENSG00000147432
ENSG00000080644	ENSG00000147434
ENSG00000080644	ENSG00000149305
ENSG00000080644	ENSG00000160716
ENSG00000080644	ENSG00000166664
ENSG00000080644	ENSG00000166736
ENSG00000080644	ENSG00000169684
ENSG00000080644	ENSG00000170175
ENSG00000080644	ENSG00000174343
ENSG00000080644	ENSG00000175344
ENSG00000080644	ENSG00000178084
ENSG00000080644	ENSG00000186038
ENSG00000080644	ENSG00000186090
ENSG00000080644	ENSG00000186919
ENSG00000080644	ENSG00000196811
ENSG00000080709	ENSG00000104783
ENSG00000080709	ENSG00000105642
ENSG00000080709	ENSG00000143603
ENSG00000080815	ENSG00000143801
ENSG00000080824	ENSG00000096384
ENSG00000080824	ENSG00000183199
ENSG00000080824	ENSG00000205100
ENSG00000080824	ENSG00000205527
ENSG00000080824	ENSG00000205940
ENSG00000080839	ENSG00000103479
ENSG00000080839	ENSG00000139687
ENSG00000080845	ENSG00000116544
ENSG00000080845	ENSG00000126787
ENSG00000080845	ENSG00000170579
ENSG00000080845	ENSG00000198010
ENSG00000080910	ENSG00000000971
ENSG00000080910	ENSG00000116785
ENSG00000080910	ENSG00000134365
ENSG00000080910	ENSG00000134389
ENSG00000080910	ENSG00000134391
ENSG00000080910	ENSG00000143278
ENSG00000081019	ENSG00000187257
ENSG00000081026	ENSG00000151276
ENSG00000081026	ENSG00000187391
ENSG00000081041	ENSG00000109272
ENSG00000081041	ENSG00000124875
ENSG00000081041	ENSG00000138755

ENSG00000081041	ENSG00000163734
ENSG00000081041	ENSG00000163735
ENSG00000081041	ENSG00000163736
ENSG00000081041	ENSG00000163737
ENSG00000081041	ENSG00000163739
ENSG00000081041	ENSG00000169245
ENSG00000081041	ENSG00000169248
ENSG00000081041	ENSG00000169429
ENSG00000081052	ENSG00000134871
ENSG00000081052	ENSG00000169031
ENSG00000081052	ENSG00000187498
ENSG00000081052	ENSG00000188153
ENSG00000081052	ENSG00000197565
ENSG00000081059	ENSG00000138795
ENSG00000081059	ENSG00000148737
ENSG00000081059	ENSG00000152284
ENSG00000081138	ENSG00000040731
ENSG00000081138	ENSG00000071991
ENSG00000081138	ENSG00000101542
ENSG00000081138	ENSG00000113100
ENSG00000081138	ENSG00000113361
ENSG00000081138	ENSG00000139880
ENSG00000081138	ENSG00000140937
ENSG00000081138	ENSG00000145526
ENSG00000081138	ENSG00000149654
ENSG00000081138	ENSG00000150394
ENSG00000081138	ENSG00000154162
ENSG00000081138	ENSG00000179776
ENSG00000081148	ENSG00000112706
ENSG00000081181	ENSG00000118520
ENSG00000081189	ENSG00000064489
ENSG00000081189	ENSG00000068305
ENSG00000081189	ENSG00000116604
ENSG00000081248	ENSG00000102001
ENSG00000081248	ENSG00000141837
ENSG00000081248	ENSG00000148408
ENSG00000081248	ENSG00000151067
ENSG00000081248	ENSG00000157388
ENSG00000081248	ENSG00000198216
ENSG00000081277	ENSG00000057294
ENSG00000081277	ENSG00000099889
ENSG00000081277	ENSG00000144283
ENSG00000081277	ENSG00000169862
ENSG00000081277	ENSG00000184363
ENSG00000081277	ENSG00000198561
ENSG00000081320	ENSG00000008118
ENSG00000081320	ENSG00000130822
ENSG00000081320	ENSG00000134072
ENSG00000081320	ENSG00000147613
ENSG00000081320	ENSG00000159792
ENSG00000081320	ENSG00000164076
ENSG00000081320	ENSG00000164543
ENSG00000081320	ENSG00000183049
ENSG00000081377	ENSG00000079335
ENSG00000081479	ENSG00000070018
ENSG00000081479	ENSG00000123384
ENSG00000081479	ENSG00000134569
ENSG00000081479	ENSG00000138798
ENSG00000081479	ENSG00000162337

ENSG00000081479	ENSG00000168702
ENSG00000081770	ENSG00000023445
ENSG00000081770	ENSG00000101197
ENSG00000081770	ENSG00000101966
ENSG00000081770	ENSG00000110330
ENSG00000081770	ENSG00000179978
ENSG00000081770	ENSG00000180152
ENSG00000081800	ENSG00000007216
ENSG00000081800	ENSG00000141485
ENSG00000081800	ENSG00000158296
ENSG00000081800	ENSG00000164707
ENSG00000081803	ENSG00000163618
ENSG00000081818	ENSG00000081842
ENSG00000081818	ENSG00000081853
ENSG00000081818	ENSG00000112852
ENSG00000081818	ENSG00000113205
ENSG00000081818	ENSG00000113209
ENSG00000081818	ENSG00000113211
ENSG00000081818	ENSG00000113212
ENSG00000081818	ENSG00000113248
ENSG00000081818	ENSG00000120322
ENSG00000081818	ENSG00000120324
ENSG00000081818	ENSG00000120327
ENSG00000081818	ENSG00000120328
ENSG00000081818	ENSG00000146001
ENSG00000081818	ENSG00000171815
ENSG00000081818	ENSG00000187372
ENSG00000081818	ENSG00000196963
ENSG00000081818	ENSG00000197479
ENSG00000081818	ENSG00000204955
ENSG00000081818	ENSG00000204956
ENSG00000081818	ENSG00000204961
ENSG00000081818	ENSG00000204962
ENSG00000081818	ENSG00000204963
ENSG00000081818	ENSG00000204964
ENSG00000081818	ENSG00000204965
ENSG00000081818	ENSG00000204967
ENSG00000081818	ENSG00000204968
ENSG00000081818	ENSG00000204969
ENSG00000081818	ENSG00000204970
ENSG00000081818	ENSG00000214567
ENSG00000081818	ENSG00000214570
ENSG00000081818	ENSG00000214574
ENSG00000081818	ENSG00000214580
ENSG00000081818	ENSG00000214583
ENSG00000081818	ENSG00000214594
ENSG00000081818	ENSG00000214600
ENSG00000081818	ENSG00000214608
ENSG00000081842	ENSG00000081818
ENSG00000081842	ENSG00000081853
ENSG00000081842	ENSG00000112852
ENSG00000081842	ENSG00000113205
ENSG00000081842	ENSG00000113209
ENSG00000081842	ENSG00000113211
ENSG00000081842	ENSG00000113212
ENSG00000081842	ENSG00000113248
ENSG00000081842	ENSG00000120322
ENSG00000081842	ENSG00000120324
ENSG00000081842	ENSG00000120327

ENSG00000081842	ENSG00000120328
ENSG00000081842	ENSG00000146001
ENSG00000081842	ENSG00000171815
ENSG00000081842	ENSG00000187372
ENSG00000081842	ENSG00000196963
ENSG00000081842	ENSG00000197479
ENSG00000081842	ENSG00000204955
ENSG00000081842	ENSG00000204956
ENSG00000081842	ENSG00000204961
ENSG00000081842	ENSG00000204962
ENSG00000081842	ENSG00000204963
ENSG00000081842	ENSG00000204964
ENSG00000081842	ENSG00000204965
ENSG00000081842	ENSG00000204967
ENSG00000081842	ENSG00000204968
ENSG00000081842	ENSG00000204969
ENSG00000081842	ENSG00000204970
ENSG00000081842	ENSG00000214567
ENSG00000081842	ENSG00000214570
ENSG00000081842	ENSG00000214574
ENSG00000081842	ENSG00000214580
ENSG00000081842	ENSG00000214583
ENSG00000081842	ENSG00000214594
ENSG00000081842	ENSG00000214600
ENSG00000081842	ENSG00000214608
ENSG00000081853	ENSG00000081818
ENSG00000081853	ENSG00000081842
ENSG00000081853	ENSG00000112852
ENSG00000081853	ENSG00000113205
ENSG00000081853	ENSG00000113209
ENSG00000081853	ENSG00000113211
ENSG00000081853	ENSG00000113212
ENSG00000081853	ENSG00000113248
ENSG00000081853	ENSG00000120322
ENSG00000081853	ENSG00000120324
ENSG00000081853	ENSG00000120327
ENSG00000081853	ENSG00000120328
ENSG00000081853	ENSG00000146001
ENSG00000081853	ENSG00000171815
ENSG00000081853	ENSG00000187372
ENSG00000081853	ENSG00000196963
ENSG00000081853	ENSG00000197479
ENSG00000081853	ENSG00000204955
ENSG00000081853	ENSG00000204956
ENSG00000081853	ENSG00000204961
ENSG00000081853	ENSG00000204962
ENSG00000081853	ENSG00000204963
ENSG00000081853	ENSG00000204964
ENSG00000081853	ENSG00000204965
ENSG00000081853	ENSG00000204967
ENSG00000081853	ENSG00000204968
ENSG00000081853	ENSG00000204969
ENSG00000081853	ENSG00000204970
ENSG00000081853	ENSG00000214567
ENSG00000081853	ENSG00000214570
ENSG00000081853	ENSG00000214574
ENSG00000081853	ENSG00000214580
ENSG00000081853	ENSG00000214583
ENSG00000081853	ENSG00000214594

ENSG00000081853	ENSG00000214600
ENSG00000081853	ENSG00000214608
ENSG00000081913	ENSG00000040199
ENSG00000081985	ENSG00000104998
ENSG00000081985	ENSG00000113594
ENSG00000081985	ENSG00000119535
ENSG00000081985	ENSG00000134352
ENSG00000081985	ENSG00000145623
ENSG00000081985	ENSG00000164509
ENSG00000082014	ENSG00000066117
ENSG00000082014	ENSG00000108604
ENSG00000082074	ENSG00000133246
ENSG00000082074	ENSG00000187889
ENSG00000082126	ENSG00000072415
ENSG00000082126	ENSG00000105926
ENSG00000082126	ENSG00000108852
ENSG00000082126	ENSG00000130830
ENSG00000082126	ENSG00000147044
ENSG00000082126	ENSG00000150054
ENSG00000082126	ENSG00000161647
ENSG00000082146	ENSG00000125695
ENSG00000082153	ENSG00000136261
ENSG00000082175	ENSG00000113580
ENSG00000082175	ENSG00000151623
ENSG00000082212	ENSG00000065833
ENSG00000082212	ENSG00000151376
ENSG00000082258	ENSG00000129315
ENSG00000082269	ENSG00000147724
ENSG00000082397	ENSG00000079819
ENSG00000082397	ENSG00000088367
ENSG00000082397	ENSG00000159023
ENSG00000082438	ENSG00000106078
ENSG00000082458	ENSG00000075711
ENSG00000082458	ENSG00000132535
ENSG00000082458	ENSG00000150672
ENSG00000082458	ENSG00000151208
ENSG00000082482	ENSG00000095981
ENSG00000082482	ENSG00000099337
ENSG00000082482	ENSG00000100433
ENSG00000082482	ENSG00000124249
ENSG00000082482	ENSG00000124780
ENSG00000082482	ENSG00000135750
ENSG00000082482	ENSG00000152315
ENSG00000082482	ENSG00000164626
ENSG00000082482	ENSG00000169427
ENSG00000082482	ENSG00000171303
ENSG00000082482	ENSG00000173338
ENSG00000082482	ENSG00000182450
ENSG00000082482	ENSG00000184261
ENSG00000082512	ENSG00000056558
ENSG00000082512	ENSG00000076604
ENSG00000082512	ENSG00000127191
ENSG00000082512	ENSG00000131323
ENSG00000082512	ENSG00000175104
ENSG00000082556	ENSG00000112038
ENSG00000082556	ENSG00000116014
ENSG00000082556	ENSG00000116329
ENSG00000082556	ENSG00000125510
ENSG00000082556	ENSG00000125522

ENSG00000082556	ENSG00000128285
ENSG00000082556	ENSG00000128310
ENSG00000082556	ENSG00000132671
ENSG00000082556	ENSG00000139874
ENSG00000082556	ENSG00000152034
ENSG00000082556	ENSG00000162009
ENSG00000082556	ENSG00000166573
ENSG00000082556	ENSG00000180616
ENSG00000082556	ENSG00000182687
ENSG00000082556	ENSG00000183473
ENSG00000082556	ENSG00000183729
ENSG00000082641	ENSG00000112182
ENSG00000082641	ENSG00000116044
ENSG00000082641	ENSG00000123405
ENSG00000082641	ENSG00000156273
ENSG00000082684	ENSG00000112902
ENSG00000082684	ENSG00000167680
ENSG00000082701	ENSG00000105723
ENSG00000082781	ENSG00000056345
ENSG00000082781	ENSG00000105855
ENSG00000082781	ENSG00000115221
ENSG00000082781	ENSG00000132470
ENSG00000082781	ENSG00000139626
ENSG00000082781	ENSG00000150093
ENSG00000082781	ENSG00000160255
ENSG00000082805	ENSG00000187672
ENSG00000082996	ENSG00000105428
ENSG00000082996	ENSG00000108523
ENSG00000082996	ENSG00000113269
ENSG00000082996	ENSG00000133135
ENSG00000082996	ENSG00000163162
ENSG00000082996	ENSG00000170153
ENSG00000082996	ENSG00000188050
ENSG00000083067	ENSG00000070985
ENSG00000083067	ENSG00000092439
ENSG00000083067	ENSG00000119121
ENSG00000083067	ENSG00000130529
ENSG00000083067	ENSG00000134160
ENSG00000083067	ENSG00000142185
ENSG00000083067	ENSG00000144481
ENSG00000083097	ENSG00000142197
ENSG00000083168	ENSG00000156650
ENSG00000083223	ENSG00000134744
ENSG00000083223	ENSG00000164329
ENSG00000083290	ENSG00000140474
ENSG00000083290	ENSG00000177169
ENSG00000083307	ENSG00000115112
ENSG00000083307	ENSG00000134317
ENSG00000083307	ENSG00000135457
ENSG00000083307	ENSG00000153560
ENSG00000083307	ENSG00000158055
ENSG00000083307	ENSG00000214547
ENSG00000083312	ENSG00000105576
ENSG00000083444	ENSG00000106397
ENSG00000083444	ENSG00000130309
ENSG00000083444	ENSG00000152952
ENSG00000083444	ENSG00000167123
ENSG00000083444	ENSG00000198756
ENSG00000083454	ENSG00000089041

ENSG00000083454	ENSG00000099957
ENSG00000083454	ENSG00000108405
ENSG00000083454	ENSG00000109991
ENSG00000083454	ENSG00000135124
ENSG00000083454	ENSG00000187848
ENSG00000083457	ENSG00000005844
ENSG00000083457	ENSG00000005884
ENSG00000083457	ENSG00000005961
ENSG00000083457	ENSG00000077943
ENSG00000083457	ENSG00000091409
ENSG00000083457	ENSG00000115232
ENSG00000083457	ENSG00000135424
ENSG00000083457	ENSG00000137809
ENSG00000083457	ENSG00000138448
ENSG00000083457	ENSG00000140678
ENSG00000083457	ENSG00000143127
ENSG00000083457	ENSG00000144668
ENSG00000083457	ENSG00000156886
ENSG00000083457	ENSG00000161638
ENSG00000083457	ENSG00000164171
ENSG00000083457	ENSG00000169896
ENSG00000083457	ENSG00000213949
ENSG00000083642	ENSG00000121892
ENSG00000083782	ENSG00000106809
ENSG00000083782	ENSG00000188770
ENSG00000083807	ENSG00000113396
ENSG00000083807	ENSG00000130304
ENSG00000083807	ENSG00000140284
ENSG00000083807	ENSG00000143554
ENSG00000083807	ENSG00000167114
ENSG00000083857	ENSG00000086570
ENSG00000083857	ENSG00000107736
ENSG00000083857	ENSG00000166341
ENSG00000084070	ENSG00000112305
ENSG00000084112	ENSG00000141298
ENSG00000084112	ENSG00000172830
ENSG00000084234	ENSG00000105290
ENSG00000084234	ENSG00000142192
ENSG00000084444	ENSG00000167930
ENSG00000084453	ENSG00000101187
ENSG00000084453	ENSG00000111700
ENSG00000084453	ENSG00000134538
ENSG00000084453	ENSG00000137491
ENSG00000084453	ENSG00000137571
ENSG00000084453	ENSG00000139155
ENSG00000084453	ENSG00000173930
ENSG00000084453	ENSG00000174640
ENSG00000084453	ENSG00000176463
ENSG00000084453	ENSG00000205359
ENSG00000084453	ENSG00000205754
ENSG00000084628	ENSG00000101198
ENSG00000084628	ENSG00000185942
ENSG00000084628	ENSG00000188580
ENSG00000084636	ENSG00000049089
ENSG00000084636	ENSG00000092758
ENSG00000084636	ENSG00000112280
ENSG00000084652	ENSG00000086712
ENSG00000084652	ENSG00000164440
ENSG00000084676	ENSG00000124151

ENSG00000084676	ENSG00000140396
ENSG00000084710	ENSG00000132294
ENSG00000084731	ENSG00000101350
ENSG00000084731	ENSG00000117245
ENSG00000084731	ENSG00000131437
ENSG00000084764	ENSG00000101367
ENSG00000084764	ENSG00000166974
ENSG00000085117	ENSG00000011105
ENSG00000085117	ENSG00000104894
ENSG00000085117	ENSG00000110900
ENSG00000085117	ENSG00000117472
ENSG00000085117	ENSG00000143119
ENSG00000085117	ENSG00000157570
ENSG00000085117	ENSG00000177697
ENSG00000085117	ENSG00000214063
ENSG00000085185	ENSG00000183337
ENSG00000085224	ENSG00000164080
ENSG00000085224	ENSG00000215766
ENSG00000085276	ENSG00000142611
ENSG00000085365	ENSG00000116521
ENSG00000085365	ENSG00000140497
ENSG00000085365	ENSG00000198794
ENSG00000085449	ENSG00000139668
ENSG00000085449	ENSG00000185359
ENSG00000085465	ENSG00000064886
ENSG00000085465	ENSG00000133048
ENSG00000085465	ENSG00000133063
ENSG00000085465	ENSG00000134216
ENSG00000085465	ENSG00000203878
ENSG00000085491	ENSG00000125648
ENSG00000085491	ENSG00000148339
ENSG00000085491	ENSG00000181240
ENSG00000085563	ENSG00000004846
ENSG00000085563	ENSG00000005471
ENSG00000085563	ENSG00000073734
ENSG00000085719	ENSG00000100884
ENSG00000085719	ENSG00000124772
ENSG00000085719	ENSG00000139117
ENSG00000085719	ENSG00000140848
ENSG00000085719	ENSG00000144550
ENSG00000085719	ENSG00000178773
ENSG00000085719	ENSG00000196353
ENSG00000085719	ENSG00000214078
ENSG00000085741	ENSG00000002745
ENSG00000085741	ENSG00000061492
ENSG00000085741	ENSG00000075290
ENSG00000085741	ENSG00000105989
ENSG00000085741	ENSG00000108379
ENSG00000085741	ENSG00000111186
ENSG00000085741	ENSG00000114251
ENSG00000085741	ENSG00000115596
ENSG00000085741	ENSG00000125084
ENSG00000085741	ENSG00000134245
ENSG00000085741	ENSG00000135925
ENSG00000085741	ENSG00000143816
ENSG00000085741	ENSG00000154342
ENSG00000085741	ENSG00000154764
ENSG00000085741	ENSG00000158955
ENSG00000085741	ENSG00000162552

ENSG00000085741	ENSG00000169884
ENSG00000085741	ENSG00000188064
ENSG00000085788	ENSG00000100523
ENSG00000085788	ENSG00000107651
ENSG00000085831	ENSG00000155158
ENSG00000085831	ENSG00000168234
ENSG00000085832	ENSG00000127527
ENSG00000085832	ENSG00000198399
ENSG00000085832	ENSG00000205726
ENSG00000085871	ENSG00000132965
ENSG00000085871	ENSG00000143198
ENSG00000085871	ENSG00000213316
ENSG00000085978	ENSG00000168010
ENSG00000086015	ENSG00000069020
ENSG00000086015	ENSG00000099308
ENSG00000086015	ENSG00000105613
ENSG00000086015	ENSG00000120539
ENSG00000086061	ENSG00000069345
ENSG00000086061	ENSG00000140403
ENSG00000086062	ENSG00000027847
ENSG00000086062	ENSG00000117411
ENSG00000086062	ENSG00000118276
ENSG00000086062	ENSG00000121578
ENSG00000086062	ENSG00000158470
ENSG00000086062	ENSG00000158850
ENSG00000086159	ENSG00000103375
ENSG00000086159	ENSG00000106125
ENSG00000086159	ENSG00000135517
ENSG00000086159	ENSG00000161798
ENSG00000086159	ENSG00000167580
ENSG00000086159	ENSG00000171885
ENSG00000086205	ENSG00000072274
ENSG00000086205	ENSG00000077616
ENSG00000086205	ENSG00000106327
ENSG00000086205	ENSG00000134612
ENSG00000086205	ENSG00000168060
ENSG00000086205	ENSG00000177694
ENSG00000086288	ENSG00000112981
ENSG00000086288	ENSG00000143156
ENSG00000086288	ENSG00000172113
ENSG00000086288	ENSG00000181322
ENSG00000086300	ENSG00000002919
ENSG00000086475	ENSG00000179918
ENSG00000086506	ENSG00000130656
ENSG00000086506	ENSG00000188536
ENSG00000086506	ENSG00000206172
ENSG00000086506	ENSG00000206177
ENSG00000086506	ENSG00000206178
ENSG00000086544	ENSG00000137825
ENSG00000086544	ENSG00000143772
ENSG00000086570	ENSG00000083857
ENSG00000086570	ENSG00000107736
ENSG00000086570	ENSG00000166341
ENSG00000086598	ENSG00000099203
ENSG00000086598	ENSG00000117500
ENSG00000086598	ENSG00000134970
ENSG00000086598	ENSG00000157315
ENSG00000086598	ENSG00000166557
ENSG00000086619	ENSG00000197930

ENSG00000086666	ENSG00000107372
ENSG00000086696	ENSG00000025423
ENSG00000086696	ENSG00000073737
ENSG00000086696	ENSG00000135437
ENSG00000086696	ENSG00000139547
ENSG00000086696	ENSG00000161267
ENSG00000086696	ENSG00000170426
ENSG00000086696	ENSG00000176387
ENSG00000086712	ENSG00000084652
ENSG00000086712	ENSG00000164440
ENSG00000086717	ENSG00000156194
ENSG00000087053	ENSG00000003987
ENSG00000087053	ENSG00000014914
ENSG00000087053	ENSG00000063601
ENSG00000087053	ENSG00000100330
ENSG00000087053	ENSG00000102043
ENSG00000087053	ENSG00000104643
ENSG00000087053	ENSG00000108389
ENSG00000087053	ENSG00000139505
ENSG00000087053	ENSG00000150712
ENSG00000087053	ENSG00000166912
ENSG00000087053	ENSG00000171100
ENSG00000087053	ENSG00000184416
ENSG00000087077	ENSG00000129474
ENSG00000087077	ENSG00000142279
ENSG00000087077	ENSG00000144791
ENSG00000087077	ENSG00000145012
ENSG00000087077	ENSG00000159840
ENSG00000087077	ENSG00000162458
ENSG00000087085	ENSG00000114200
ENSG00000087085	ENSG00000159398
ENSG00000087085	ENSG00000170835
ENSG00000087085	ENSG00000172824
ENSG00000087085	ENSG00000172828
ENSG00000087085	ENSG00000172831
ENSG00000087085	ENSG00000196959
ENSG00000087085	ENSG00000198848
ENSG00000087085	ENSG00000215478
ENSG00000087086	ENSG00000132446
ENSG00000087086	ENSG00000167996
ENSG00000087086	ENSG00000171878
ENSG00000087086	ENSG00000173477
ENSG00000087086	ENSG00000176778
ENSG00000087086	ENSG00000180795
ENSG00000087086	ENSG00000181867
ENSG00000087086	ENSG00000197518
ENSG00000087086	ENSG00000215234
ENSG00000087088	ENSG00000030110
ENSG00000087088	ENSG00000129473
ENSG00000087088	ENSG00000140379
ENSG00000087088	ENSG00000143384
ENSG00000087088	ENSG00000171552
ENSG00000087088	ENSG00000171791
ENSG00000087088	ENSG00000176720
ENSG00000087116	ENSG00000049192
ENSG00000087116	ENSG00000136378
ENSG00000087116	ENSG00000138316
ENSG00000087116	ENSG00000140470
ENSG00000087116	ENSG00000140873

ENSG00000087116	ENSG00000142303
ENSG00000087116	ENSG00000145536
ENSG00000087116	ENSG00000145808
ENSG00000087116	ENSG00000151388
ENSG00000087116	ENSG00000156140
ENSG00000087116	ENSG00000160323
ENSG00000087116	ENSG00000163638
ENSG00000087116	ENSG00000173157
ENSG00000087116	ENSG00000214644
ENSG00000087116	ENSG00000215743
ENSG00000087245	ENSG00000008516
ENSG00000087245	ENSG00000099953
ENSG00000087245	ENSG00000100985
ENSG00000087245	ENSG00000102996
ENSG00000087245	ENSG00000110347
ENSG00000087245	ENSG00000118113
ENSG00000087245	ENSG00000123342
ENSG00000087245	ENSG00000125966
ENSG00000087245	ENSG00000129270
ENSG00000087245	ENSG00000137673
ENSG00000087245	ENSG00000137674
ENSG00000087245	ENSG00000137675
ENSG00000087245	ENSG00000137745
ENSG00000087245	ENSG00000149968
ENSG00000087245	ENSG00000154485
ENSG00000087245	ENSG00000156103
ENSG00000087245	ENSG00000157227
ENSG00000087245	ENSG00000166670
ENSG00000087245	ENSG00000167346
ENSG00000087245	ENSG00000189409
ENSG00000087245	ENSG00000196611
ENSG00000087245	ENSG00000198598
ENSG00000087245	ENSG00000215914
ENSG00000087250	ENSG00000102891
ENSG00000087250	ENSG00000125144
ENSG00000087250	ENSG00000125148
ENSG00000087250	ENSG00000169688
ENSG00000087250	ENSG00000169715
ENSG00000087250	ENSG00000187193
ENSG00000087250	ENSG00000198417
ENSG00000087250	ENSG00000205358
ENSG00000087250	ENSG00000205360
ENSG00000087250	ENSG00000205362
ENSG00000087250	ENSG00000205364
ENSG00000087258	ENSG00000060558
ENSG00000087258	ENSG00000065135
ENSG00000087258	ENSG00000087460
ENSG00000087258	ENSG00000088256
ENSG00000087258	ENSG00000114349
ENSG00000087258	ENSG00000114353
ENSG00000087258	ENSG00000120063
ENSG00000087258	ENSG00000127955
ENSG00000087258	ENSG00000128266
ENSG00000087258	ENSG00000134183
ENSG00000087258	ENSG00000141404
ENSG00000087258	ENSG00000146535
ENSG00000087258	ENSG00000156049
ENSG00000087258	ENSG00000156052
ENSG00000087258	ENSG00000214415

ENSG00000087258	ENSG00000214622
ENSG00000087274	ENSG00000075340
ENSG00000087274	ENSG00000148700
ENSG00000087303	ENSG00000112562
ENSG00000087303	ENSG00000116962
ENSG00000087303	ENSG00000198732
ENSG00000087338	ENSG00000138152
ENSG00000087448	ENSG00000003096
ENSG00000087448	ENSG00000124743
ENSG00000087448	ENSG00000130487
ENSG00000087448	ENSG00000135686
ENSG00000087448	ENSG00000166853
ENSG00000087448	ENSG00000167487
ENSG00000087448	ENSG00000174010
ENSG00000087448	ENSG00000176595
ENSG00000087448	ENSG00000179023
ENSG00000087448	ENSG00000185214
ENSG00000087448	ENSG00000185915
ENSG00000087448	ENSG00000186231
ENSG00000087448	ENSG00000197705
ENSG00000087448	ENSG00000198642
ENSG00000087460	ENSG00000060558
ENSG00000087460	ENSG00000065135
ENSG00000087460	ENSG00000087258
ENSG00000087460	ENSG00000088256
ENSG00000087460	ENSG00000114349
ENSG00000087460	ENSG00000114353
ENSG00000087460	ENSG00000120063
ENSG00000087460	ENSG00000127955
ENSG00000087460	ENSG00000128266
ENSG00000087460	ENSG00000134183
ENSG00000087460	ENSG00000141404
ENSG00000087460	ENSG00000146535
ENSG00000087460	ENSG00000156049
ENSG00000087460	ENSG00000156052
ENSG00000087460	ENSG00000214415
ENSG00000087460	ENSG00000214622
ENSG00000087495	ENSG00000112137
ENSG00000087495	ENSG00000112419
ENSG00000087495	ENSG00000204138
ENSG00000087510	ENSG00000008196
ENSG00000087510	ENSG00000008197
ENSG00000087510	ENSG00000116819
ENSG00000087510	ENSG00000137203
ENSG00000087586	ENSG00000105146
ENSG00000087589	ENSG00000050820
ENSG00000087589	ENSG00000100842
ENSG00000087589	ENSG00000111859
ENSG00000087903	ENSG00000080298
ENSG00000087903	ENSG00000111783
ENSG00000087903	ENSG00000132005
ENSG00000087903	ENSG00000143390
ENSG00000087903	ENSG00000181827
ENSG00000087903	ENSG00000185002
ENSG00000087995	ENSG00000123600
ENSG00000087995	ENSG00000165055
ENSG00000088002	ENSG00000105398
ENSG00000088002	ENSG00000109193
ENSG00000088002	ENSG00000130540

ENSG00000088002	ENSG00000138068
ENSG00000088002	ENSG00000173597
ENSG00000088002	ENSG00000196228
ENSG00000088002	ENSG00000196502
ENSG00000088002	ENSG00000197165
ENSG00000088002	ENSG00000198075
ENSG00000088002	ENSG00000198203
ENSG00000088002	ENSG00000213599
ENSG00000088002	ENSG00000213648
ENSG00000088179	ENSG00000070159
ENSG00000088179	ENSG00000070778
ENSG00000088179	ENSG00000152104
ENSG00000088247	ENSG00000107164
ENSG00000088247	ENSG00000162613
ENSG00000088247	ENSG00000215224
ENSG00000088256	ENSG00000060558
ENSG00000088256	ENSG00000065135
ENSG00000088256	ENSG00000087258
ENSG00000088256	ENSG00000087460
ENSG00000088256	ENSG00000114349
ENSG00000088256	ENSG00000114353
ENSG00000088256	ENSG00000120063
ENSG00000088256	ENSG00000127955
ENSG00000088256	ENSG00000128266
ENSG00000088256	ENSG00000134183
ENSG00000088256	ENSG00000141404
ENSG00000088256	ENSG00000146535
ENSG00000088256	ENSG00000156049
ENSG00000088256	ENSG00000156052
ENSG00000088256	ENSG00000214415
ENSG00000088256	ENSG00000214622
ENSG00000088280	ENSG00000151693
ENSG00000088280	ENSG00000153317
ENSG00000088305	ENSG00000119772
ENSG00000088305	ENSG00000142182
ENSG00000088320	ENSG00000139890
ENSG00000088320	ENSG00000164949
ENSG00000088320	ENSG00000166592
ENSG00000088340	ENSG00000115155
ENSG00000088340	ENSG00000135636
ENSG00000088340	ENSG00000138119
ENSG00000088340	ENSG00000214272
ENSG00000088340	ENSG00000214814
ENSG00000088367	ENSG00000079819
ENSG00000088367	ENSG00000082397
ENSG00000088367	ENSG00000159023
ENSG00000088386	ENSG00000110446
ENSG00000088386	ENSG00000139370
ENSG00000088386	ENSG00000163406
ENSG00000088387	ENSG00000107099
ENSG00000088387	ENSG00000116641
ENSG00000088387	ENSG00000130158
ENSG00000088387	ENSG00000135905
ENSG00000088387	ENSG00000147251
ENSG00000088538	ENSG00000128512
ENSG00000088538	ENSG00000134516
ENSG00000088538	ENSG00000147459
ENSG00000088538	ENSG00000150760
ENSG00000088756	ENSG00000124143

ENSG00000088756	ENSG00000130052
ENSG00000088756	ENSG00000133121
ENSG00000088756	ENSG00000146376
ENSG00000088756	ENSG00000164741
ENSG00000088808	ENSG00000104881
ENSG00000088808	ENSG00000143514
ENSG00000088812	ENSG00000105429
ENSG00000088812	ENSG00000107518
ENSG00000088826	ENSG00000148832
ENSG00000088827	ENSG00000012124
ENSG00000088832	ENSG00000119782
ENSG00000088832	ENSG00000146722
ENSG00000088832	ENSG00000198225
ENSG00000088833	ENSG00000173960
ENSG00000088833	ENSG00000215114
ENSG00000088881	ENSG00000108001
ENSG00000088881	ENSG00000134025
ENSG00000088881	ENSG00000164330
ENSG00000088882	ENSG00000106624
ENSG00000088882	ENSG00000108582
ENSG00000088882	ENSG00000109472
ENSG00000088882	ENSG00000109625
ENSG00000088882	ENSG00000120054
ENSG00000088882	ENSG00000121898
ENSG00000088882	ENSG00000135678
ENSG00000088899	ENSG00000061337
ENSG00000088899	ENSG00000107816
ENSG00000088899	ENSG00000145911
ENSG00000088986	ENSG00000121083
ENSG00000089006	ENSG00000028528
ENSG00000089006	ENSG00000129515
ENSG00000089006	ENSG00000172803
ENSG00000089006	ENSG00000205302
ENSG00000089041	ENSG00000083454
ENSG00000089041	ENSG00000099957
ENSG00000089041	ENSG00000108405
ENSG00000089041	ENSG00000109991
ENSG00000089041	ENSG00000135124
ENSG00000089041	ENSG00000187848
ENSG00000089057	ENSG00000170482
ENSG00000089057	ENSG00000213901
ENSG00000089063	ENSG00000088356
ENSG00000089094	ENSG00000006459
ENSG00000089094	ENSG00000172943
ENSG00000089094	ENSG00000173120
ENSG00000089094	ENSG00000197724
ENSG00000089116	ENSG00000016082
ENSG00000089116	ENSG00000106689
ENSG00000089116	ENSG00000106852
ENSG00000089116	ENSG00000107187
ENSG00000089116	ENSG00000121454
ENSG00000089116	ENSG00000132130
ENSG00000089116	ENSG00000136944
ENSG00000089116	ENSG00000143013
ENSG00000089116	ENSG00000143355
ENSG00000089116	ENSG00000159556
ENSG00000089116	ENSG00000162624
ENSG00000089116	ENSG00000162761
ENSG00000089159	ENSG00000072163

ENSG00000089159	ENSG00000110031
ENSG00000089159	ENSG00000112214
ENSG00000089159	ENSG00000140682
ENSG00000089159	ENSG00000169756
ENSG00000089159	ENSG00000183386
ENSG00000089169	ENSG00000215770
ENSG00000089177	ENSG00000054523
ENSG00000089177	ENSG00000118193
ENSG00000089177	ENSG00000129250
ENSG00000089177	ENSG00000130294
ENSG00000089177	ENSG00000137177
ENSG00000089177	ENSG00000197892
ENSG00000089225	ENSG00000073861
ENSG00000089225	ENSG00000092607
ENSG00000089225	ENSG00000112837
ENSG00000089225	ENSG00000121068
ENSG00000089225	ENSG00000121075
ENSG00000089225	ENSG00000122145
ENSG00000089225	ENSG00000135111
ENSG00000089225	ENSG00000136535
ENSG00000089225	ENSG00000143178
ENSG00000089225	ENSG00000149922
ENSG00000089225	ENSG00000163508
ENSG00000089225	ENSG00000164458
ENSG00000089225	ENSG00000164532
ENSG00000089225	ENSG00000167800
ENSG00000089225	ENSG00000174197
ENSG00000089225	ENSG00000184058
ENSG00000089250	ENSG00000007171
ENSG00000089250	ENSG00000164867
ENSG00000089280	ENSG00000172660
ENSG00000089280	ENSG00000182944
ENSG00000089280	ENSG00000215835
ENSG00000089351	ENSG00000023171
ENSG00000089351	ENSG00000175318
ENSG00000089351	ENSG00000178075
ENSG00000089356	ENSG00000126258
ENSG00000089356	ENSG00000137726
ENSG00000089356	ENSG00000137731
ENSG00000089356	ENSG00000150201
ENSG00000089356	ENSG00000182707
ENSG00000089472	ENSG00000047457
ENSG00000089472	ENSG00000181333
ENSG00000089472	ENSG00000185010
ENSG00000089472	ENSG00000198734
ENSG00000089486	ENSG00000189067
ENSG00000089486	ENSG00000215079
ENSG00000089558	ENSG00000055118
ENSG00000089558	ENSG00000135519
ENSG00000089558	ENSG00000140015
ENSG00000089558	ENSG00000143473
ENSG00000089558	ENSG00000173826
ENSG00000089558	ENSG00000183960
ENSG00000089558	ENSG00000184611
ENSG00000089597	ENSG00000214013
ENSG00000089639	ENSG00000137962
ENSG00000089639	ENSG00000180448
ENSG00000089693	ENSG00000178053
ENSG00000089723	ENSG00000167770

ENSG00000089775	ENSG00000126804
ENSG00000089775	ENSG00000181472
ENSG00000089818	ENSG00000157191
ENSG00000089820	ENSG00000163486
ENSG00000089820	ENSG00000196220
ENSG00000089820	ENSG00000196935
ENSG00000089847	ENSG00000039560
ENSG00000089847	ENSG00000137831
ENSG00000089847	ENSG00000198483
ENSG00000089876	ENSG00000109606
ENSG00000089876	ENSG00000144045
ENSG00000089902	ENSG00000117625
ENSG00000089902	ENSG00000167771
ENSG00000089916	ENSG00000092978
ENSG00000090006	ENSG00000049323
ENSG00000090006	ENSG00000077942
ENSG00000090006	ENSG00000115380
ENSG00000090006	ENSG00000119681
ENSG00000090006	ENSG00000140092
ENSG00000090006	ENSG00000163520
ENSG00000090006	ENSG00000168056
ENSG00000090006	ENSG00000172638
ENSG00000090006	ENSG00000214536
ENSG00000090020	ENSG00000066230
ENSG00000090020	ENSG00000115616
ENSG00000090020	ENSG00000135740
ENSG00000090020	ENSG00000180251
ENSG00000090060	ENSG00000115421
ENSG00000090097	ENSG00000169564
ENSG00000090097	ENSG00000183570
ENSG00000090097	ENSG00000197111
ENSG00000090104	ENSG00000091844
ENSG00000090104	ENSG00000116741
ENSG00000090104	ENSG00000117152
ENSG00000090104	ENSG00000127074
ENSG00000090104	ENSG00000135824
ENSG00000090104	ENSG00000143248
ENSG00000090104	ENSG00000143333
ENSG00000090104	ENSG00000147509
ENSG00000090104	ENSG00000148908
ENSG00000090104	ENSG00000150681
ENSG00000090104	ENSG00000171700
ENSG00000090104	ENSG00000215825
ENSG00000090238	ENSG00000100027
ENSG00000090238	ENSG00000166793
ENSG00000090238	ENSG00000175155
ENSG00000090372	ENSG00000115808
ENSG00000090372	ENSG00000196792
ENSG00000090376	ENSG00000134070
ENSG00000090376	ENSG00000184216
ENSG00000090382	ENSG00000120563
ENSG00000090382	ENSG00000141316
ENSG00000090382	ENSG00000151033
ENSG00000090382	ENSG00000157093
ENSG00000090382	ENSG00000161572
ENSG00000090382	ENSG00000167531
ENSG00000090382	ENSG00000171478
ENSG00000090382	ENSG00000171489
ENSG00000090432	ENSG00000092871

ENSG00000090432	ENSG00000170633
ENSG00000090512	ENSG00000113905
ENSG00000090512	ENSG00000145192
ENSG00000090530	ENSG00000110811
ENSG00000090530	ENSG00000117385
ENSG00000090530	ENSG00000141696
ENSG00000090530	ENSG00000170275
ENSG00000090565	ENSG00000131242
ENSG00000090621	ENSG00000070756
ENSG00000090621	ENSG00000101104
ENSG00000090621	ENSG00000151846
ENSG00000090621	ENSG00000174740
ENSG00000090621	ENSG00000215587
ENSG00000090661	ENSG00000065308
ENSG00000090661	ENSG00000067167
ENSG00000090661	ENSG00000139624
ENSG00000090661	ENSG00000143418
ENSG00000090661	ENSG00000154227
ENSG00000090661	ENSG00000172292
ENSG00000090661	ENSG00000174599
ENSG00000090674	ENSG00000055732
ENSG00000090674	ENSG00000153898
ENSG00000090776	ENSG00000099617
ENSG00000090776	ENSG00000108947
ENSG00000090776	ENSG00000125266
ENSG00000090776	ENSG00000143590
ENSG00000090776	ENSG00000143620
ENSG00000090776	ENSG00000169242
ENSG00000090776	ENSG00000184349
ENSG00000090905	ENSG00000078687
ENSG00000090905	ENSG00000100354
ENSG00000090920	ENSG00000109927
ENSG00000090920	ENSG00000110799
ENSG00000090920	ENSG00000117983
ENSG00000090920	ENSG00000146839
ENSG00000090920	ENSG00000184956
ENSG00000090920	ENSG00000188162
ENSG00000090920	ENSG00000196292
ENSG00000090920	ENSG00000197558
ENSG00000090920	ENSG00000198788
ENSG00000090924	ENSG00000120278
ENSG00000090924	ENSG00000126822
ENSG00000090975	ENSG00000091622
ENSG00000090975	ENSG00000110697
ENSG00000090989	ENSG00000168952
ENSG00000091009	ENSG00000139746
ENSG00000091010	ENSG00000028277
ENSG00000091010	ENSG00000064835
ENSG00000091010	ENSG00000137709
ENSG00000091010	ENSG00000143190
ENSG00000091010	ENSG00000151615
ENSG00000091010	ENSG00000152192
ENSG00000091010	ENSG00000184486
ENSG00000091010	ENSG00000185668
ENSG00000091010	ENSG00000196767
ENSG00000091010	ENSG00000198914
ENSG00000091010	ENSG00000204531
ENSG00000091010	ENSG00000212993
ENSG00000091039	ENSG00000021762

ENSG00000091039	ENSG00000117859
ENSG00000091039	ENSG00000144645
ENSG00000091039	ENSG00000144909
ENSG00000091073	ENSG00000110042
ENSG00000091073	ENSG00000135144
ENSG00000091073	ENSG00000163840
ENSG00000091073	ENSG00000178498
ENSG00000091129	ENSG00000134121
ENSG00000091129	ENSG00000149972
ENSG00000091129	ENSG00000163531
ENSG00000091129	ENSG00000184144
ENSG00000091129	ENSG00000198493
ENSG00000091136	ENSG00000050555
ENSG00000091136	ENSG00000058085
ENSG00000091136	ENSG00000074527
ENSG00000091136	ENSG00000091128
ENSG00000091136	ENSG00000106780
ENSG00000091136	ENSG00000135862
ENSG00000091136	ENSG00000172037
ENSG00000091136	ENSG00000196878
ENSG00000091137	ENSG00000091138
ENSG00000091137	ENSG00000112053
ENSG00000091137	ENSG00000135502
ENSG00000091137	ENSG00000145217
ENSG00000091137	ENSG00000147606
ENSG00000091137	ENSG00000155850
ENSG00000091137	ENSG00000170615
ENSG00000091137	ENSG00000174502
ENSG00000091138	ENSG00000091137
ENSG00000091138	ENSG00000112053
ENSG00000091138	ENSG00000135502
ENSG00000091138	ENSG00000145217
ENSG00000091138	ENSG00000147606
ENSG00000091138	ENSG00000155850
ENSG00000091138	ENSG00000170615
ENSG00000091138	ENSG00000174502
ENSG00000091157	ENSG00000166415
ENSG00000091181	ENSG00000123496
ENSG00000091181	ENSG00000131724
ENSG00000091181	ENSG00000185291
ENSG00000091181	ENSG00000198223
ENSG00000091262	ENSG00000023839
ENSG00000091262	ENSG00000103222
ENSG00000091262	ENSG00000108846
ENSG00000091317	ENSG00000102007
ENSG00000091317	ENSG00000140931
ENSG00000091317	ENSG00000153551
ENSG00000091317	ENSG00000166091
ENSG00000091317	ENSG00000183723
ENSG00000091409	ENSG00000005844
ENSG00000091409	ENSG00000005884
ENSG00000091409	ENSG00000005961
ENSG00000091409	ENSG00000077943
ENSG00000091409	ENSG00000083457
ENSG00000091409	ENSG00000115232
ENSG00000091409	ENSG00000135424
ENSG00000091409	ENSG00000137809
ENSG00000091409	ENSG00000138448
ENSG00000091409	ENSG00000140678

ENSG00000091409	ENSG00000143127
ENSG00000091409	ENSG00000144668
ENSG00000091409	ENSG00000156886
ENSG00000091409	ENSG00000161638
ENSG00000091409	ENSG00000164171
ENSG00000091409	ENSG00000169896
ENSG00000091409	ENSG00000213949
ENSG00000091428	ENSG00000079337
ENSG00000091428	ENSG00000108352
ENSG00000091428	ENSG00000109756
ENSG00000091428	ENSG00000136237
ENSG00000091490	ENSG00000071537
ENSG00000091490	ENSG00000101251
ENSG00000091490	ENSG00000109771
ENSG00000091513	ENSG00000012223
ENSG00000091513	ENSG00000163975
ENSG00000091536	ENSG00000066933
ENSG00000091536	ENSG00000099331
ENSG00000091536	ENSG00000145555
ENSG00000091583	ENSG00000117335
ENSG00000091583	ENSG00000123838
ENSG00000091583	ENSG00000196352
ENSG00000091583	ENSG00000197721
ENSG00000091622	ENSG00000090975
ENSG00000091622	ENSG00000110697
ENSG00000091656	ENSG00000136367
ENSG00000091656	ENSG00000140836
ENSG00000091664	ENSG00000104888
ENSG00000091664	ENSG00000112337
ENSG00000091664	ENSG00000119899
ENSG00000091664	ENSG00000124564
ENSG00000091664	ENSG00000124568
ENSG00000091664	ENSG00000146039
ENSG00000091664	ENSG00000179520
ENSG00000091704	ENSG00000080618
ENSG00000091704	ENSG00000128510
ENSG00000091704	ENSG00000144410
ENSG00000091704	ENSG00000153002
ENSG00000091704	ENSG00000158516
ENSG00000091704	ENSG00000158525
ENSG00000091704	ENSG00000163751
ENSG00000091704	ENSG00000165078
ENSG00000091831	ENSG00000119715
ENSG00000091831	ENSG00000140009
ENSG00000091831	ENSG00000173153
ENSG00000091831	ENSG00000196482
ENSG00000091844	ENSG00000090104
ENSG00000091844	ENSG00000116741
ENSG00000091844	ENSG00000117152
ENSG00000091844	ENSG00000127074
ENSG00000091844	ENSG00000135824
ENSG00000091844	ENSG00000143248
ENSG00000091844	ENSG00000143333
ENSG00000091844	ENSG00000147509
ENSG00000091844	ENSG00000148908
ENSG00000091844	ENSG00000150681
ENSG00000091844	ENSG00000171700
ENSG00000091844	ENSG00000215825
ENSG00000092009	ENSG00000100448

ENSG00000092009	ENSG00000100450
ENSG00000092009	ENSG00000100453
ENSG00000092009	ENSG00000113088
ENSG00000092009	ENSG00000145649
ENSG00000092009	ENSG00000185198
ENSG00000092009	ENSG00000197766
ENSG00000092010	ENSG00000100911
ENSG00000092010	ENSG00000131467
ENSG00000092010	ENSG00000174144
ENSG00000092051	ENSG00000104369
ENSG00000092051	ENSG00000139714
ENSG00000092051	ENSG00000149596
ENSG00000092051	ENSG00000154118
ENSG00000092054	ENSG00000002079
ENSG00000092054	ENSG00000006788
ENSG00000092054	ENSG00000078814
ENSG00000092054	ENSG00000109061
ENSG00000092054	ENSG00000109063
ENSG00000092054	ENSG00000125414
ENSG00000092054	ENSG00000133020
ENSG00000092054	ENSG00000141048
ENSG00000092054	ENSG00000144821
ENSG00000092054	ENSG00000197616
ENSG00000092067	ENSG00000172216
ENSG00000092067	ENSG00000180733
ENSG00000092068	ENSG00000021488
ENSG00000092068	ENSG00000103064
ENSG00000092068	ENSG00000103257
ENSG00000092068	ENSG00000130876
ENSG00000092068	ENSG00000151012
ENSG00000092068	ENSG00000155465
ENSG00000092068	ENSG00000164893
ENSG00000092140	ENSG00000010318
ENSG00000092140	ENSG00000136147
ENSG00000092140	ENSG00000156531
ENSG00000092199	ENSG00000125970
ENSG00000092199	ENSG00000179172
ENSG00000092199	ENSG00000184672
ENSG00000092199	ENSG00000214634
ENSG00000092199	ENSG00000215880
ENSG00000092200	ENSG00000103494
ENSG00000092203	ENSG00000103460
ENSG00000092203	ENSG00000124191
ENSG00000092203	ENSG00000198846
ENSG00000092295	ENSG00000104055
ENSG00000092295	ENSG00000124491
ENSG00000092295	ENSG00000125780
ENSG00000092295	ENSG00000159495
ENSG00000092295	ENSG00000163810
ENSG00000092295	ENSG00000166947
ENSG00000092295	ENSG00000166948
ENSG00000092295	ENSG00000198959
ENSG00000092345	ENSG00000152430
ENSG00000092345	ENSG00000187191
ENSG00000092345	ENSG00000188120
ENSG00000092345	ENSG00000205916
ENSG00000092345	ENSG00000205944
ENSG00000092377	ENSG00000101849
ENSG00000092377	ENSG00000150627

ENSG00000092377	ENSG00000177565
ENSG00000092421	ENSG00000010319
ENSG00000092421	ENSG00000012171
ENSG00000092421	ENSG00000075213
ENSG00000092421	ENSG00000095539
ENSG00000092421	ENSG00000137872
ENSG00000092421	ENSG00000143434
ENSG00000092421	ENSG00000153993
ENSG00000092421	ENSG00000168758
ENSG00000092421	ENSG00000170381
ENSG00000092421	ENSG00000185033
ENSG00000092421	ENSG00000187764
ENSG00000092421	ENSG00000196189
ENSG00000092439	ENSG00000070985
ENSG00000092439	ENSG00000083067
ENSG00000092439	ENSG00000119121
ENSG00000092439	ENSG00000130529
ENSG00000092439	ENSG00000134160
ENSG00000092439	ENSG00000142185
ENSG00000092439	ENSG00000144481
ENSG00000092445	ENSG00000105976
ENSG00000092445	ENSG00000153208
ENSG00000092445	ENSG00000163785
ENSG00000092445	ENSG00000164078
ENSG00000092445	ENSG00000167601
ENSG00000092445	ENSG00000214672
ENSG00000092529	ENSG00000014216
ENSG00000092529	ENSG00000077274
ENSG00000092529	ENSG00000135773
ENSG00000092529	ENSG00000137225
ENSG00000092529	ENSG00000142330
ENSG00000092529	ENSG00000149260
ENSG00000092529	ENSG00000162909
ENSG00000092529	ENSG00000162949
ENSG00000092529	ENSG00000182472
ENSG00000092529	ENSG00000203697
ENSG00000092529	ENSG00000214711
ENSG00000092531	ENSG00000132639
ENSG00000092607	ENSG00000073861
ENSG00000092607	ENSG00000089225
ENSG00000092607	ENSG00000112837
ENSG00000092607	ENSG00000121068
ENSG00000092607	ENSG00000121075
ENSG00000092607	ENSG00000122145
ENSG00000092607	ENSG00000135111
ENSG00000092607	ENSG00000136535
ENSG00000092607	ENSG00000143178
ENSG00000092607	ENSG00000149922
ENSG00000092607	ENSG00000163508
ENSG00000092607	ENSG00000164458
ENSG00000092607	ENSG00000164532
ENSG00000092607	ENSG00000167800
ENSG00000092607	ENSG00000174197
ENSG00000092607	ENSG00000184058
ENSG00000092758	ENSG00000049089
ENSG00000092758	ENSG00000084636
ENSG00000092758	ENSG00000112280
ENSG00000092820	ENSG00000137710
ENSG00000092820	ENSG00000147065

ENSG00000092820	ENSG00000186575
ENSG00000092841	ENSG00000160808
ENSG00000092841	ENSG00000168530
ENSG00000092841	ENSG00000196465
ENSG00000092841	ENSG00000198336
ENSG00000092847	ENSG00000123908
ENSG00000092847	ENSG00000126070
ENSG00000092847	ENSG00000134698
ENSG00000092871	ENSG00000090432
ENSG00000092871	ENSG00000170633
ENSG00000092929	ENSG00000007516
ENSG00000092964	ENSG00000072832
ENSG00000092964	ENSG00000113657
ENSG00000092964	ENSG00000147647
ENSG00000092964	ENSG00000151640
ENSG00000092964	ENSG00000157851
ENSG00000092969	ENSG00000105329
ENSG00000092969	ENSG00000135414
ENSG00000092969	ENSG00000138379
ENSG00000092969	ENSG00000139269
ENSG00000092978	ENSG00000089916
ENSG00000093100	ENSG00000133816
ENSG00000093100	ENSG00000135596
ENSG00000093134	ENSG00000112299
ENSG00000093134	ENSG00000112303
ENSG00000093134	ENSG00000169814
ENSG00000093167	ENSG00000124831
ENSG00000093183	ENSG00000121542
ENSG00000094755	ENSG00000011677
ENSG00000094755	ENSG00000022355
ENSG00000094755	ENSG00000101958
ENSG00000094755	ENSG00000102287
ENSG00000094755	ENSG00000109158
ENSG00000094755	ENSG00000109738
ENSG00000094755	ENSG00000111886
ENSG00000094755	ENSG00000113327
ENSG00000094755	ENSG00000145451
ENSG00000094755	ENSG00000145863
ENSG00000094755	ENSG00000145864
ENSG00000094755	ENSG00000145888
ENSG00000094755	ENSG00000146276
ENSG00000094755	ENSG00000147402
ENSG00000094755	ENSG00000151834
ENSG00000094755	ENSG00000163285
ENSG00000094755	ENSG00000163288
ENSG00000094755	ENSG00000166206
ENSG00000094755	ENSG00000182256
ENSG00000094755	ENSG00000183185
ENSG00000094755	ENSG00000186297
ENSG00000094755	ENSG00000187730
ENSG00000094755	ENSG00000188828
ENSG00000094796	ENSG00000006059
ENSG00000094796	ENSG00000108244
ENSG00000094796	ENSG00000108417
ENSG00000094796	ENSG00000108759
ENSG00000094796	ENSG00000111057
ENSG00000094796	ENSG00000126337
ENSG00000094796	ENSG00000128422
ENSG00000094796	ENSG00000131737

ENSG00000094796	ENSG00000131738
ENSG00000094796	ENSG00000131885
ENSG00000094796	ENSG00000167916
ENSG00000094796	ENSG00000170819
ENSG00000094796	ENSG00000171345
ENSG00000094796	ENSG00000171346
ENSG00000094796	ENSG00000171360
ENSG00000094796	ENSG00000171401
ENSG00000094796	ENSG00000171403
ENSG00000094796	ENSG00000171431
ENSG00000094796	ENSG00000171446
ENSG00000094796	ENSG00000173908
ENSG00000094796	ENSG00000186393
ENSG00000094796	ENSG00000186395
ENSG00000094796	ENSG00000186831
ENSG00000094796	ENSG00000186832
ENSG00000094796	ENSG00000186847
ENSG00000094796	ENSG00000187242
ENSG00000094796	ENSG00000187686
ENSG00000094796	ENSG00000196859
ENSG00000094796	ENSG00000197079
ENSG00000094796	ENSG00000204889
ENSG00000094796	ENSG00000204897
ENSG00000094796	ENSG00000213951
ENSG00000094796	ENSG00000214220
ENSG00000094796	ENSG00000214454
ENSG00000094796	ENSG00000214959
ENSG00000094796	ENSG00000215772
ENSG00000094796	ENSG00000215773
ENSG00000094916	ENSG00000108468
ENSG00000094916	ENSG00000122565
ENSG00000095066	ENSG00000015133
ENSG00000095066	ENSG00000115355
ENSG00000095066	ENSG00000134709
ENSG00000095066	ENSG00000168071
ENSG00000095066	ENSG00000168172
ENSG00000095110	ENSG00000137634
ENSG00000095110	ENSG00000144815
ENSG00000095110	ENSG00000204361
ENSG00000095203	ENSG00000115109
ENSG00000095203	ENSG00000129595
ENSG00000095203	ENSG00000153303
ENSG00000095209	ENSG00000072954
ENSG00000095303	ENSG00000073756
ENSG00000095319	ENSG00000144554
ENSG00000095321	ENSG00000005469
ENSG00000095321	ENSG00000070748
ENSG00000095321	ENSG00000110090
ENSG00000095321	ENSG00000169169
ENSG00000095321	ENSG00000205560
ENSG00000095370	ENSG00000125731
ENSG00000095370	ENSG00000137936
ENSG00000095383	ENSG00000100359
ENSG00000095383	ENSG00000111490
ENSG00000095383	ENSG00000167202
ENSG00000095397	ENSG00000006611
ENSG00000095397	ENSG00000186862
ENSG00000095464	ENSG00000112541
ENSG00000095464	ENSG00000128655

ENSG00000095464	ENSG00000132915
ENSG00000095464	ENSG00000133256
ENSG00000095464	ENSG00000138735
ENSG00000095464	ENSG00000186642
ENSG00000095539	ENSG00000010319
ENSG00000095539	ENSG00000012171
ENSG00000095539	ENSG00000075213
ENSG00000095539	ENSG00000092421
ENSG00000095539	ENSG00000137872
ENSG00000095539	ENSG00000143434
ENSG00000095539	ENSG00000153993
ENSG00000095539	ENSG00000168758
ENSG00000095539	ENSG00000170381
ENSG00000095539	ENSG00000185033
ENSG00000095539	ENSG00000187764
ENSG00000095539	ENSG00000196189
ENSG00000095585	ENSG00000043462
ENSG00000095585	ENSG00000109684
ENSG00000095585	ENSG00000152292
ENSG00000095587	ENSG00000038295
ENSG00000095587	ENSG00000106333
ENSG00000095587	ENSG00000107611
ENSG00000095587	ENSG00000157211
ENSG00000095587	ENSG00000163710
ENSG00000095587	ENSG00000166342
ENSG00000095587	ENSG00000168487
ENSG00000095587	ENSG00000171208
ENSG00000095587	ENSG00000184824
ENSG00000095596	ENSG00000003137
ENSG00000095596	ENSG00000187553
ENSG00000095627	ENSG00000132972
ENSG00000095627	ENSG00000180113
ENSG00000095627	ENSG00000182134
ENSG00000095627	ENSG00000196116
ENSG00000095637	ENSG00000120896
ENSG00000095637	ENSG00000154556
ENSG00000095777	ENSG00000071909
ENSG00000095794	ENSG00000118260
ENSG00000095794	ENSG00000123268
ENSG00000095917	ENSG00000005001
ENSG00000095917	ENSG00000007038
ENSG00000095917	ENSG00000052344
ENSG00000095917	ENSG00000103355
ENSG00000095917	ENSG00000116176
ENSG00000095917	ENSG00000172236
ENSG00000095917	ENSG00000172382
ENSG00000095917	ENSG00000172460
ENSG00000095917	ENSG00000178055
ENSG00000095917	ENSG00000178226
ENSG00000095917	ENSG00000185888
ENSG00000095917	ENSG00000188086
ENSG00000095917	ENSG00000189099
ENSG00000095917	ENSG00000196364
ENSG00000095917	ENSG00000197253
ENSG00000095917	ENSG00000206549
ENSG00000095917	ENSG00000214877
ENSG00000095917	ENSG00000215148
ENSG00000095951	ENSG00000010818
ENSG00000095951	ENSG00000127124

ENSG00000095970	ENSG00000096264
ENSG00000095970	ENSG00000161649
ENSG00000095970	ENSG00000162896
ENSG00000095970	ENSG00000162897
ENSG00000095970	ENSG00000167850
ENSG00000095970	ENSG00000167851
ENSG00000095970	ENSG00000178789
ENSG00000095970	ENSG00000186074
ENSG00000095970	ENSG00000186407
ENSG00000095970	ENSG00000204345
ENSG00000095981	ENSG00000082482
ENSG00000095981	ENSG00000099337
ENSG00000095981	ENSG00000100433
ENSG00000095981	ENSG00000124249
ENSG00000095981	ENSG00000124780
ENSG00000095981	ENSG00000135750
ENSG00000095981	ENSG00000152315
ENSG00000095981	ENSG00000164626
ENSG00000095981	ENSG00000169427
ENSG00000095981	ENSG00000171303
ENSG00000095981	ENSG00000173338
ENSG00000095981	ENSG00000182450
ENSG00000095981	ENSG00000184261
ENSG00000096060	ENSG00000004478
ENSG00000096063	ENSG00000135250
ENSG00000096063	ENSG00000184343
ENSG00000096070	ENSG00000043143
ENSG00000096070	ENSG00000077684
ENSG00000096070	ENSG00000078403
ENSG00000096070	ENSG00000100425
ENSG00000096070	ENSG00000102221
ENSG00000096070	ENSG00000106443
ENSG00000096070	ENSG00000108292
ENSG00000096070	ENSG00000156983
ENSG00000096088	ENSG00000117984
ENSG00000096088	ENSG00000131400
ENSG00000096088	ENSG00000131401
ENSG00000096088	ENSG00000143839
ENSG00000096088	ENSG00000172647
ENSG00000096088	ENSG00000180860
ENSG00000096088	ENSG00000184488
ENSG00000096088	ENSG00000196188
ENSG00000096088	ENSG00000204973
ENSG00000096088	ENSG00000215868
ENSG00000096092	ENSG00000111843
ENSG00000096092	ENSG00000137210
ENSG00000096092	ENSG00000189156
ENSG00000096092	ENSG00000214881
ENSG00000096264	ENSG00000095970
ENSG00000096264	ENSG00000161649
ENSG00000096264	ENSG00000162896
ENSG00000096264	ENSG00000162897
ENSG00000096264	ENSG00000167850
ENSG00000096264	ENSG00000167851
ENSG00000096264	ENSG00000178789
ENSG00000096264	ENSG00000186074
ENSG00000096264	ENSG00000186407
ENSG00000096264	ENSG00000204345
ENSG00000096384	ENSG00000080824

ENSG00000096384	ENSG00000183199
ENSG00000096384	ENSG00000205100
ENSG00000096384	ENSG00000205527
ENSG00000096384	ENSG00000205940
ENSG00000096433	ENSG00000123104
ENSG00000096433	ENSG00000150995
ENSG00000096696	ENSG00000118898
ENSG00000096696	ENSG00000127603
ENSG00000096696	ENSG00000151914
ENSG00000096696	ENSG00000167880
ENSG00000096746	ENSG00000103067
ENSG00000096746	ENSG00000104413
ENSG00000096746	ENSG00000126945
ENSG00000096746	ENSG00000132463
ENSG00000096746	ENSG00000169045
ENSG00000096746	ENSG00000169813
ENSG00000096968	ENSG00000105397
ENSG00000096968	ENSG00000105639
ENSG00000096968	ENSG00000162434
ENSG00000097007	ENSG00000143322
ENSG00000097096	ENSG00000105137
ENSG00000099139	ENSG00000115257
ENSG00000099139	ENSG00000125851
ENSG00000099139	ENSG00000140479
ENSG00000099139	ENSG00000140564
ENSG00000099139	ENSG00000160613
ENSG00000099139	ENSG00000175426
ENSG00000099203	ENSG00000086598
ENSG00000099203	ENSG00000117500
ENSG00000099203	ENSG00000134970
ENSG00000099203	ENSG00000157315
ENSG00000099203	ENSG00000166557
ENSG00000099204	ENSG00000158856
ENSG00000099204	ENSG00000163995
ENSG00000099204	ENSG00000173210
ENSG00000099250	ENSG00000057019
ENSG00000099250	ENSG00000102104
ENSG00000099250	ENSG00000118257
ENSG00000099250	ENSG00000140545
ENSG00000099250	ENSG00000164176
ENSG00000099250	ENSG00000164465
ENSG00000099256	ENSG00000165704
ENSG00000099260	ENSG00000099864
ENSG00000099260	ENSG00000157654
ENSG00000099282	ENSG00000048140
ENSG00000099282	ENSG00000108219
ENSG00000099282	ENSG00000158457
ENSG00000099282	ENSG00000168785
ENSG00000099282	ENSG00000182612
ENSG00000099284	ENSG00000113648
ENSG00000099308	ENSG00000069020
ENSG00000099308	ENSG00000086015
ENSG00000099308	ENSG00000105613
ENSG00000099308	ENSG00000120539
ENSG00000099331	ENSG00000066933
ENSG00000099331	ENSG00000091536
ENSG00000099331	ENSG00000145555
ENSG00000099337	ENSG00000082482
ENSG00000099337	ENSG00000095981

ENSG00000099337	ENSG00000100433
ENSG00000099337	ENSG00000124249
ENSG00000099337	ENSG00000124780
ENSG00000099337	ENSG00000135750
ENSG00000099337	ENSG00000152315
ENSG00000099337	ENSG00000164626
ENSG00000099337	ENSG00000169427
ENSG00000099337	ENSG00000171303
ENSG00000099337	ENSG00000173338
ENSG00000099337	ENSG00000182450
ENSG00000099337	ENSG00000184261
ENSG00000099365	ENSG00000103496
ENSG00000099365	ENSG00000106089
ENSG00000099365	ENSG00000111450
ENSG00000099365	ENSG00000135604
ENSG00000099365	ENSG00000166900
ENSG00000099365	ENSG00000178750
ENSG00000099381	ENSG00000139718
ENSG00000099385	ENSG00000106635
ENSG00000099385	ENSG00000110987
ENSG00000099617	ENSG00000090776
ENSG00000099617	ENSG00000108947
ENSG00000099617	ENSG00000125266
ENSG00000099617	ENSG00000143590
ENSG00000099617	ENSG00000143620
ENSG00000099617	ENSG00000169242
ENSG00000099617	ENSG00000184349
ENSG00000099715	ENSG00000102290
ENSG00000099715	ENSG00000113555
ENSG00000099715	ENSG00000156453
ENSG00000099715	ENSG00000169851
ENSG00000099715	ENSG00000184226
ENSG00000099715	ENSG00000197991
ENSG00000099715	ENSG00000215442
ENSG00000099783	ENSG00000104177
ENSG00000099783	ENSG00000215013
ENSG00000099785	ENSG00000139266
ENSG00000099785	ENSG00000144583
ENSG00000099785	ENSG00000145416
ENSG00000099785	ENSG00000165406
ENSG00000099785	ENSG00000173926
ENSG00000099785	ENSG00000183654
ENSG00000099797	ENSG00000205678
ENSG00000099804	ENSG00000107341
ENSG00000099814	ENSG00000143702
ENSG00000099822	ENSG00000070729
ENSG00000099822	ENSG00000132259
ENSG00000099822	ENSG00000138622
ENSG00000099822	ENSG00000143630
ENSG00000099822	ENSG00000144191
ENSG00000099822	ENSG00000164588
ENSG00000099822	ENSG00000170289
ENSG00000099822	ENSG00000183862
ENSG00000099849	ENSG00000123094
ENSG00000099860	ENSG00000112306
ENSG00000099860	ENSG00000116717
ENSG00000099860	ENSG00000130222
ENSG00000099860	ENSG00000170250
ENSG00000099864	ENSG00000099260

ENSG00000099864	ENSG00000157654
ENSG00000099875	ENSG00000079277
ENSG00000099882	ENSG00000161681
ENSG00000099882	ENSG00000162105
ENSG00000099889	ENSG00000057294
ENSG00000099889	ENSG00000081277
ENSG00000099889	ENSG00000144283
ENSG00000099889	ENSG00000169862
ENSG00000099889	ENSG00000184363
ENSG00000099889	ENSG00000198561
ENSG00000099904	ENSG00000156599
ENSG00000099904	ENSG00000163958
ENSG00000099910	ENSG00000074660
ENSG00000099910	ENSG00000145794
ENSG00000099910	ENSG00000157890
ENSG00000099910	ENSG00000162591
ENSG00000099910	ENSG00000187800
ENSG00000099937	ENSG00000114204
ENSG00000099937	ENSG00000117601
ENSG00000099937	ENSG00000140093
ENSG00000099937	ENSG00000149257
ENSG00000099937	ENSG00000170099
ENSG00000099942	ENSG00000167193
ENSG00000099957	ENSG00000083454
ENSG00000099957	ENSG00000089041
ENSG00000099957	ENSG00000108405
ENSG00000099957	ENSG00000109991
ENSG00000099957	ENSG00000135124
ENSG00000099957	ENSG00000187848
ENSG00000099958	ENSG00000072849
ENSG00000099984	ENSG00000133433
ENSG00000099984	ENSG00000184490
ENSG00000099984	ENSG00000184674
ENSG00000099984	ENSG00000185831
ENSG00000099984	ENSG00000206090
ENSG00000099992	ENSG00000169221
ENSG00000099992	ENSG00000175463
ENSG00000099998	ENSG00000100031
ENSG00000099998	ENSG00000100121
ENSG00000099998	ENSG00000133475
ENSG00000099998	ENSG00000149435
ENSG00000099998	ENSG00000183038
ENSG00000099998	ENSG00000197421
ENSG00000099998	ENSG00000215466
ENSG00000099998	ENSG00000215500
ENSG00000099998	ENSG00000215610
ENSG00000100014	ENSG00000128487
ENSG00000100014	ENSG00000183963
ENSG00000100014	ENSG00000188176
ENSG00000100014	ENSG00000214872
ENSG00000100027	ENSG00000090238
ENSG00000100027	ENSG00000166793
ENSG00000100027	ENSG00000175155
ENSG00000100030	ENSG00000102882
ENSG00000100031	ENSG00000099998
ENSG00000100031	ENSG00000100121
ENSG00000100031	ENSG00000133475
ENSG00000100031	ENSG00000149435
ENSG00000100031	ENSG00000183038

ENSG00000100031	ENSG00000197421
ENSG00000100031	ENSG00000215466
ENSG00000100031	ENSG00000215500
ENSG00000100031	ENSG00000215610
ENSG00000100034	ENSG00000175175
ENSG00000100053	ENSG00000100058
ENSG00000100053	ENSG00000100122
ENSG00000100053	ENSG00000108255
ENSG00000100053	ENSG00000118231
ENSG00000100053	ENSG00000127377
ENSG00000100053	ENSG00000163254
ENSG00000100053	ENSG00000163499
ENSG00000100053	ENSG00000168582
ENSG00000100053	ENSG00000182187
ENSG00000100053	ENSG00000196431
ENSG00000100053	ENSG00000213139
ENSG00000100058	ENSG00000100053
ENSG00000100058	ENSG00000100122
ENSG00000100058	ENSG00000108255
ENSG00000100058	ENSG00000118231
ENSG00000100058	ENSG00000127377
ENSG00000100058	ENSG00000163254
ENSG00000100058	ENSG00000163499
ENSG00000100058	ENSG00000168582
ENSG00000100058	ENSG00000182187
ENSG00000100058	ENSG00000196431
ENSG00000100058	ENSG00000213139
ENSG00000100060	ENSG00000106003
ENSG00000100060	ENSG00000143891
ENSG00000100060	ENSG00000169733
ENSG00000100065	ENSG00000187796
ENSG00000100065	ENSG00000198286
ENSG00000100077	ENSG00000114124
ENSG00000100077	ENSG00000125388
ENSG00000100077	ENSG00000173020
ENSG00000100077	ENSG00000185974
ENSG00000100077	ENSG00000198055
ENSG00000100077	ENSG00000198873
ENSG00000100079	ENSG00000006659
ENSG00000100079	ENSG00000100097
ENSG00000100079	ENSG00000105198
ENSG00000100079	ENSG00000105205
ENSG00000100079	ENSG00000116977
ENSG00000100079	ENSG00000119862
ENSG00000100079	ENSG00000131981
ENSG00000100079	ENSG00000133317
ENSG00000100079	ENSG00000168961
ENSG00000100079	ENSG00000170298
ENSG00000100079	ENSG00000171747
ENSG00000100079	ENSG00000171916
ENSG00000100079	ENSG00000178934
ENSG00000100079	ENSG00000197778
ENSG00000100079	ENSG00000205076
ENSG00000100079	ENSG00000215132
ENSG00000100083	ENSG00000103365
ENSG00000100083	ENSG00000125447
ENSG00000100092	ENSG00000006740
ENSG00000100095	ENSG00000063015
ENSG00000100095	ENSG00000174938

ENSG00000100097	ENSG00000006659
ENSG00000100097	ENSG00000100079
ENSG00000100097	ENSG00000105198
ENSG00000100097	ENSG00000105205
ENSG00000100097	ENSG00000116977
ENSG00000100097	ENSG00000119862
ENSG00000100097	ENSG00000131981
ENSG00000100097	ENSG00000133317
ENSG00000100097	ENSG00000168961
ENSG00000100097	ENSG00000170298
ENSG00000100097	ENSG00000171747
ENSG00000100097	ENSG00000171916
ENSG00000100097	ENSG00000178934
ENSG00000100097	ENSG00000197778
ENSG00000100097	ENSG00000205076
ENSG00000100097	ENSG00000215132
ENSG00000100100	ENSG00000131650
ENSG00000100100	ENSG00000183762
ENSG00000100105	ENSG00000103495
ENSG00000100105	ENSG00000136451
ENSG00000100118	ENSG00000029993
ENSG00000100118	ENSG00000124097
ENSG00000100118	ENSG00000132967
ENSG00000100118	ENSG00000164104
ENSG00000100118	ENSG00000173961
ENSG00000100118	ENSG00000176256
ENSG00000100118	ENSG00000180212
ENSG00000100118	ENSG00000182181
ENSG00000100118	ENSG00000182711
ENSG00000100118	ENSG00000189403
ENSG00000100118	ENSG00000214364
ENSG00000100118	ENSG00000214852
ENSG00000100118	ENSG00000215308
ENSG00000100121	ENSG00000099998
ENSG00000100121	ENSG00000100031
ENSG00000100121	ENSG00000133475
ENSG00000100121	ENSG00000149435
ENSG00000100121	ENSG00000183038
ENSG00000100121	ENSG00000197421
ENSG00000100121	ENSG00000215466
ENSG00000100121	ENSG00000215500
ENSG00000100121	ENSG00000215610
ENSG00000100122	ENSG00000100053
ENSG00000100122	ENSG00000100058
ENSG00000100122	ENSG00000108255
ENSG00000100122	ENSG00000118231
ENSG00000100122	ENSG00000127377
ENSG00000100122	ENSG00000163254
ENSG00000100122	ENSG00000163499
ENSG00000100122	ENSG00000168582
ENSG00000100122	ENSG00000182187
ENSG00000100122	ENSG00000196431
ENSG00000100122	ENSG00000213139
ENSG00000100124	ENSG00000186106
ENSG00000100139	ENSG00000164877
ENSG00000100146	ENSG00000005513
ENSG00000100146	ENSG00000124766
ENSG00000100146	ENSG00000125398
ENSG00000100146	ENSG00000129194

ENSG00000100146	ENSG00000134595
ENSG00000100146	ENSG00000164736
ENSG00000100146	ENSG00000171056
ENSG00000100146	ENSG00000176887
ENSG00000100146	ENSG00000177732
ENSG00000100146	ENSG00000181449
ENSG00000100146	ENSG00000182968
ENSG00000100146	ENSG00000184895
ENSG00000100146	ENSG00000203883
ENSG00000100156	ENSG00000108932
ENSG00000100156	ENSG00000112394
ENSG00000100156	ENSG00000118596
ENSG00000100156	ENSG00000141526
ENSG00000100156	ENSG00000147100
ENSG00000100156	ENSG00000152779
ENSG00000100156	ENSG00000155380
ENSG00000100156	ENSG00000163053
ENSG00000100156	ENSG00000165449
ENSG00000100156	ENSG00000168679
ENSG00000100156	ENSG00000170190
ENSG00000100156	ENSG00000174326
ENSG00000100156	ENSG00000174327
ENSG00000100167	ENSG00000140623
ENSG00000100167	ENSG00000184640
ENSG00000100170	ENSG00000100191
ENSG00000100170	ENSG00000117834
ENSG00000100170	ENSG00000140675
ENSG00000100170	ENSG00000154025
ENSG00000100170	ENSG00000158865
ENSG00000100170	ENSG00000198743
ENSG00000100191	ENSG00000100170
ENSG00000100191	ENSG00000117834
ENSG00000100191	ENSG00000140675
ENSG00000100191	ENSG00000154025
ENSG00000100191	ENSG00000158865
ENSG00000100191	ENSG00000198743
ENSG00000100196	ENSG00000105438
ENSG00000100196	ENSG00000136240
ENSG00000100201	ENSG00000108654
ENSG00000100207	ENSG00000108557
ENSG00000100221	ENSG00000161677
ENSG00000100228	ENSG00000109113
ENSG00000100234	ENSG00000035862
ENSG00000100234	ENSG00000102265
ENSG00000100234	ENSG00000157150
ENSG00000100239	ENSG00000105063
ENSG00000100239	ENSG00000110075
ENSG00000100241	ENSG00000110514
ENSG00000100241	ENSG00000133812
ENSG00000100242	ENSG00000061656
ENSG00000100242	ENSG00000164744
ENSG00000100242	ENSG00000164828
ENSG00000100242	ENSG00000167098
ENSG00000100243	ENSG00000159348
ENSG00000100243	ENSG00000166394
ENSG00000100262	ENSG00000149182
ENSG00000100266	ENSG00000124507
ENSG00000100266	ENSG00000140368
ENSG00000100266	ENSG00000152229

ENSG00000100266	ENSG00000165912
ENSG00000100280	ENSG00000006125
ENSG00000100284	ENSG00000141198
ENSG00000100284	ENSG00000175662
ENSG00000100285	ENSG00000026025
ENSG00000100285	ENSG00000104722
ENSG00000100285	ENSG00000104725
ENSG00000100285	ENSG00000131095
ENSG00000100285	ENSG00000135406
ENSG00000100285	ENSG00000148798
ENSG00000100285	ENSG00000175084
ENSG00000100285	ENSG00000182253
ENSG00000100285	ENSG00000215479
ENSG00000100288	ENSG00000110721
ENSG00000100288	ENSG00000139163
ENSG00000100288	ENSG00000143845
ENSG00000100292	ENSG00000103415
ENSG00000100298	ENSG00000111701
ENSG00000100298	ENSG00000111732
ENSG00000100298	ENSG00000124701
ENSG00000100298	ENSG00000128383
ENSG00000100298	ENSG00000128394
ENSG00000100298	ENSG00000179750
ENSG00000100298	ENSG00000198904
ENSG00000100300	ENSG00000112212
ENSG00000100302	ENSG00000108551
ENSG00000100307	ENSG00000141570
ENSG00000100307	ENSG00000141582
ENSG00000100307	ENSG00000173894
ENSG00000100307	ENSG00000183741
ENSG00000100311	ENSG00000112715
ENSG00000100311	ENSG00000119630
ENSG00000100311	ENSG00000150630
ENSG00000100311	ENSG00000165197
ENSG00000100311	ENSG00000173511
ENSG00000100311	ENSG00000197461
ENSG00000100311	ENSG00000215682
ENSG00000100314	ENSG00000183166
ENSG00000100316	ENSG00000140986
ENSG00000100320	ENSG00000078328
ENSG00000100320	ENSG00000167281
ENSG00000100321	ENSG00000105467
ENSG00000100321	ENSG00000108639
ENSG00000100321	ENSG00000127561
ENSG00000100321	ENSG00000215315
ENSG00000100330	ENSG00000003987
ENSG00000100330	ENSG00000014914
ENSG00000100330	ENSG00000063601
ENSG00000100330	ENSG00000087053
ENSG00000100330	ENSG00000102043
ENSG00000100330	ENSG00000104643
ENSG00000100330	ENSG00000108389
ENSG00000100330	ENSG00000139505
ENSG00000100330	ENSG00000150712
ENSG00000100330	ENSG00000166912
ENSG00000100330	ENSG00000171100
ENSG00000100330	ENSG00000184416
ENSG00000100335	ENSG00000177427
ENSG00000100336	ENSG00000100342

ENSG00000100336	ENSG00000128284
ENSG00000100336	ENSG00000128313
ENSG00000100336	ENSG00000128335
ENSG00000100336	ENSG00000178878
ENSG00000100336	ENSG00000196785
ENSG00000100341	ENSG00000006757
ENSG00000100341	ENSG00000100344
ENSG00000100341	ENSG00000177666
ENSG00000100341	ENSG00000180316
ENSG00000100342	ENSG00000100336
ENSG00000100342	ENSG00000128284
ENSG00000100342	ENSG00000128313
ENSG00000100342	ENSG00000128335
ENSG00000100342	ENSG00000178878
ENSG00000100342	ENSG00000196785
ENSG00000100344	ENSG00000006757
ENSG00000100344	ENSG00000100341
ENSG00000100344	ENSG00000177666
ENSG00000100344	ENSG00000180316
ENSG00000100345	ENSG00000105357
ENSG00000100345	ENSG00000133026
ENSG00000100345	ENSG00000133392
ENSG00000100346	ENSG00000006283
ENSG00000100346	ENSG00000196557
ENSG00000100351	ENSG00000101082
ENSG00000100351	ENSG00000154016
ENSG00000100351	ENSG00000155926
ENSG00000100351	ENSG00000177885
ENSG00000100354	ENSG00000078687
ENSG00000100354	ENSG00000090905
ENSG00000100362	ENSG00000122543
ENSG00000100362	ENSG00000135175
ENSG00000100365	ENSG00000109686
ENSG00000100385	ENSG00000103522
ENSG00000100385	ENSG00000124334
ENSG00000100385	ENSG00000215303
ENSG00000100393	ENSG00000005339
ENSG00000100395	ENSG00000011258
ENSG00000100395	ENSG00000154655
ENSG00000100395	ENSG00000163935
ENSG00000100395	ENSG00000185513
ENSG00000100395	ENSG00000198879
ENSG00000100395	ENSG00000198945
ENSG00000100399	ENSG00000188937
ENSG00000100403	ENSG00000122299
ENSG00000100417	ENSG00000140650
ENSG00000100422	ENSG00000188452
ENSG00000100425	ENSG00000043143
ENSG00000100425	ENSG00000077684
ENSG00000100425	ENSG00000078403
ENSG00000100425	ENSG00000096070
ENSG00000100425	ENSG00000102221
ENSG00000100425	ENSG00000106443
ENSG00000100425	ENSG00000108292
ENSG00000100425	ENSG00000156983
ENSG00000100426	ENSG00000214717
ENSG00000100433	ENSG00000082482
ENSG00000100433	ENSG00000095981
ENSG00000100433	ENSG00000099337

ENSG00000100433	ENSG00000124249
ENSG00000100433	ENSG00000124780
ENSG00000100433	ENSG00000135750
ENSG00000100433	ENSG00000152315
ENSG00000100433	ENSG00000164626
ENSG00000100433	ENSG00000169427
ENSG00000100433	ENSG00000171303
ENSG00000100433	ENSG00000173338
ENSG00000100433	ENSG00000182450
ENSG00000100433	ENSG00000184261
ENSG00000100439	ENSG00000011198
ENSG00000100441	ENSG00000102053
ENSG00000100441	ENSG00000102921
ENSG00000100441	ENSG00000149289
ENSG00000100441	ENSG00000163874
ENSG00000100441	ENSG00000178199
ENSG00000100441	ENSG00000205978
ENSG00000100448	ENSG00000092009
ENSG00000100448	ENSG00000100450
ENSG00000100448	ENSG00000100453
ENSG00000100448	ENSG00000113088
ENSG00000100448	ENSG00000145649
ENSG00000100448	ENSG00000185198
ENSG00000100448	ENSG00000197766
ENSG00000100450	ENSG00000092009
ENSG00000100450	ENSG00000100448
ENSG00000100450	ENSG00000100453
ENSG00000100450	ENSG00000113088
ENSG00000100450	ENSG00000145649
ENSG00000100450	ENSG00000185198
ENSG00000100450	ENSG00000197766
ENSG00000100453	ENSG00000092009
ENSG00000100453	ENSG00000100448
ENSG00000100453	ENSG00000100450
ENSG00000100453	ENSG00000113088
ENSG00000100453	ENSG00000145649
ENSG00000100453	ENSG00000185198
ENSG00000100453	ENSG00000197766
ENSG00000100461	ENSG00000131051
ENSG00000100473	ENSG00000163359
ENSG00000100473	ENSG00000165816
ENSG00000100473	ENSG00000198450
ENSG00000100473	ENSG00000205221
ENSG00000100485	ENSG00000058335
ENSG00000100485	ENSG00000107263
ENSG00000100485	ENSG00000113319
ENSG00000100485	ENSG00000115904
ENSG00000100503	ENSG00000101004
ENSG00000100504	ENSG00000068976
ENSG00000100504	ENSG00000100994
ENSG00000100505	ENSG00000119283
ENSG00000100505	ENSG00000152503
ENSG00000100505	ENSG00000163462
ENSG00000100505	ENSG00000186628
ENSG00000100528	ENSG00000143771
ENSG00000100528	ENSG00000143786
ENSG00000100528	ENSG00000174871
ENSG00000100558	ENSG00000115956
ENSG00000100565	ENSG00000187905

ENSG00000100580	ENSG00000182827
ENSG00000100591	ENSG00000173209
ENSG00000100592	ENSG00000146122
ENSG00000100593	ENSG00000101230
ENSG00000100596	ENSG00000172296
ENSG00000100599	ENSG00000132669
ENSG00000100599	ENSG00000174791
ENSG00000100599	ENSG00000187994
ENSG00000100614	ENSG00000138032
ENSG00000100614	ENSG00000177886
ENSG00000100625	ENSG00000126778
ENSG00000100625	ENSG00000138083
ENSG00000100625	ENSG00000170577
ENSG00000100625	ENSG00000177045
ENSG00000100625	ENSG00000184302
ENSG00000100626	ENSG00000106648
ENSG00000100626	ENSG00000110328
ENSG00000100626	ENSG00000115339
ENSG00000100626	ENSG00000131386
ENSG00000100626	ENSG00000136542
ENSG00000100626	ENSG00000139629
ENSG00000100626	ENSG00000141429
ENSG00000100626	ENSG00000143641
ENSG00000100626	ENSG00000144278
ENSG00000100626	ENSG00000158089
ENSG00000100626	ENSG00000164574
ENSG00000100626	ENSG00000174473
ENSG00000100626	ENSG00000178234
ENSG00000100626	ENSG00000182870
ENSG00000100626	ENSG00000185274
ENSG00000100644	ENSG00000112246
ENSG00000100644	ENSG00000116016
ENSG00000100644	ENSG00000124440
ENSG00000100644	ENSG00000130751
ENSG00000100644	ENSG00000151322
ENSG00000100644	ENSG00000159263
ENSG00000100647	ENSG00000143502
ENSG00000100650	ENSG00000116350
ENSG00000100650	ENSG00000124193
ENSG00000100665	ENSG00000123561
ENSG00000100665	ENSG00000135744
ENSG00000100665	ENSG00000165951
ENSG00000100665	ENSG00000165953
ENSG00000100665	ENSG00000170054
ENSG00000100665	ENSG00000186910
ENSG00000100665	ENSG00000187483
ENSG00000100665	ENSG00000188488
ENSG00000100665	ENSG00000196136
ENSG00000100665	ENSG00000197249
ENSG00000100678	ENSG00000118160
ENSG00000100678	ENSG00000183023
ENSG00000100714	ENSG00000120254
ENSG00000100731	ENSG00000126773
ENSG00000100731	ENSG00000135749
ENSG00000100731	ENSG00000197136
ENSG00000100739	ENSG00000134817
ENSG00000100739	ENSG00000144891
ENSG00000100739	ENSG00000154165
ENSG00000100739	ENSG00000168398

ENSG00000100739	ENSG00000170128
ENSG00000100739	ENSG00000173080
ENSG00000100739	ENSG00000180772
ENSG00000100739	ENSG00000182631
ENSG00000100749	ENSG00000028116
ENSG00000100749	ENSG00000105053
ENSG00000100767	ENSG00000143382
ENSG00000100767	ENSG00000185761
ENSG00000100767	ENSG00000187720
ENSG00000100767	ENSG00000197859
ENSG00000100767	ENSG00000215616
ENSG00000100784	ENSG00000071242
ENSG00000100784	ENSG00000072133
ENSG00000100784	ENSG00000108443
ENSG00000100784	ENSG00000117676
ENSG00000100784	ENSG00000162302
ENSG00000100784	ENSG00000175634
ENSG00000100784	ENSG00000177189
ENSG00000100784	ENSG00000214817
ENSG00000100796	ENSG00000138041
ENSG00000100796	ENSG00000182112
ENSG00000100804	ENSG00000129543
ENSG00000100804	ENSG00000204264
ENSG00000100811	ENSG00000179059
ENSG00000100811	ENSG00000188786
ENSG00000100811	ENSG00000198767
ENSG00000100829	ENSG00000119938
ENSG00000100829	ENSG00000132825
ENSG00000100829	ENSG00000154415
ENSG00000100829	ENSG00000173281
ENSG00000100836	ENSG00000205022
ENSG00000100842	ENSG00000050820
ENSG00000100842	ENSG00000087589
ENSG00000100842	ENSG00000111859
ENSG00000100852	ENSG00000160007
ENSG00000100884	ENSG00000085719
ENSG00000100884	ENSG00000124772
ENSG00000100884	ENSG00000139117
ENSG00000100884	ENSG00000140848
ENSG00000100884	ENSG00000144550
ENSG00000100884	ENSG00000178773
ENSG00000100884	ENSG00000196353
ENSG00000100884	ENSG00000214078
ENSG00000100888	ENSG00000124177
ENSG00000100888	ENSG00000171316
ENSG00000100888	ENSG00000177200
ENSG00000100889	ENSG00000124253
ENSG00000100906	ENSG00000069399
ENSG00000100906	ENSG00000104825
ENSG00000100906	ENSG00000146232
ENSG00000100908	ENSG00000131148
ENSG00000100911	ENSG00000092010
ENSG00000100911	ENSG00000131467
ENSG00000100911	ENSG00000174144
ENSG00000100916	ENSG00000111707
ENSG00000100916	ENSG00000174744
ENSG00000100931	ENSG00000101421
ENSG00000100931	ENSG00000164695
ENSG00000100934	ENSG00000101310

ENSG00000100938	ENSG00000106348
ENSG00000100938	ENSG00000137198
ENSG00000100938	ENSG00000178035
ENSG00000100968	ENSG00000072736
ENSG00000100968	ENSG00000101096
ENSG00000100968	ENSG00000102908
ENSG00000100968	ENSG00000131196
ENSG00000100979	ENSG00000078898
ENSG00000100979	ENSG00000101425
ENSG00000100979	ENSG00000129988
ENSG00000100979	ENSG00000167104
ENSG00000100979	ENSG00000184459
ENSG00000100979	ENSG00000186190
ENSG00000100979	ENSG00000186191
ENSG00000100985	ENSG00000008516
ENSG00000100985	ENSG00000087245
ENSG00000100985	ENSG00000099953
ENSG00000100985	ENSG00000102996
ENSG00000100985	ENSG00000110347
ENSG00000100985	ENSG00000118113
ENSG00000100985	ENSG00000123342
ENSG00000100985	ENSG00000125966
ENSG00000100985	ENSG00000129270
ENSG00000100985	ENSG00000137673
ENSG00000100985	ENSG00000137674
ENSG00000100985	ENSG00000137675
ENSG00000100985	ENSG00000137745
ENSG00000100985	ENSG00000149968
ENSG00000100985	ENSG00000154485
ENSG00000100985	ENSG00000156103
ENSG00000100985	ENSG00000157227
ENSG00000100985	ENSG00000166670
ENSG00000100985	ENSG00000167346
ENSG00000100985	ENSG00000189409
ENSG00000100985	ENSG00000196611
ENSG00000100985	ENSG00000198598
ENSG00000100985	ENSG00000215914
ENSG00000100987	ENSG00000004848
ENSG00000100987	ENSG00000052850
ENSG00000100987	ENSG00000119614
ENSG00000100987	ENSG00000156150
ENSG00000100987	ENSG00000180318
ENSG00000100994	ENSG00000068976
ENSG00000100994	ENSG00000100504
ENSG00000100997	ENSG00000131969
ENSG00000100997	ENSG00000139826
ENSG00000101004	ENSG00000100503
ENSG00000101049	ENSG00000104205
ENSG00000101049	ENSG00000118515
ENSG00000101057	ENSG00000118513
ENSG00000101057	ENSG00000185697
ENSG00000101074	ENSG00000096006
ENSG00000101074	ENSG00000103196
ENSG00000101074	ENSG00000121005
ENSG00000101074	ENSG00000124490
ENSG00000101074	ENSG00000124812
ENSG00000101074	ENSG00000137558
ENSG00000101074	ENSG00000139278
ENSG00000101074	ENSG00000164530

ENSG00000101074	ENSG00000173401
ENSG00000101074	ENSG00000180481
ENSG00000101076	ENSG00000143171
ENSG00000101076	ENSG00000160113
ENSG00000101076	ENSG00000164749
ENSG00000101076	ENSG00000175745
ENSG00000101076	ENSG00000185551
ENSG00000101076	ENSG00000186350
ENSG00000101076	ENSG00000204231
ENSG00000101079	ENSG00000103034
ENSG00000101079	ENSG00000104419
ENSG00000101079	ENSG00000165795
ENSG00000101082	ENSG00000100351
ENSG00000101082	ENSG00000154016
ENSG00000101082	ENSG00000155926
ENSG00000101082	ENSG00000177885
ENSG00000101096	ENSG00000072736
ENSG00000101096	ENSG00000100968
ENSG00000101096	ENSG00000102908
ENSG00000101096	ENSG00000131196
ENSG00000101098	ENSG00000011347
ENSG00000101098	ENSG00000019505
ENSG00000101098	ENSG00000067715
ENSG00000101098	ENSG00000103528
ENSG00000101098	ENSG00000110975
ENSG00000101098	ENSG00000129990
ENSG00000101098	ENSG00000132718
ENSG00000101098	ENSG00000132872
ENSG00000101098	ENSG00000134207
ENSG00000101098	ENSG00000143858
ENSG00000101098	ENSG00000149043
ENSG00000101098	ENSG00000149927
ENSG00000101098	ENSG00000170743
ENSG00000101098	ENSG00000173227
ENSG00000101098	ENSG00000204176
ENSG00000101098	ENSG00000213023
ENSG00000101098	ENSG00000214408
ENSG00000101098	ENSG00000214409
ENSG00000101098	ENSG00000214569
ENSG00000101098	ENSG00000214783
ENSG00000101104	ENSG00000070756
ENSG00000101104	ENSG00000090621
ENSG00000101104	ENSG00000151846
ENSG00000101104	ENSG00000174740
ENSG00000101104	ENSG00000215587
ENSG00000101109	ENSG00000104375
ENSG00000101115	ENSG00000103449
ENSG00000101115	ENSG00000151514
ENSG00000101115	ENSG00000165821
ENSG00000101126	ENSG00000101544
ENSG00000101134	ENSG00000115325
ENSG00000101134	ENSG00000125170
ENSG00000101134	ENSG00000137218
ENSG00000101134	ENSG00000146094
ENSG00000101134	ENSG00000147443
ENSG00000101134	ENSG00000166225
ENSG00000101134	ENSG00000206052
ENSG00000101144	ENSG00000107623
ENSG00000101144	ENSG00000112175

ENSG00000101144	ENSG00000116985
ENSG00000101144	ENSG00000125965
ENSG00000101144	ENSG00000128802
ENSG00000101144	ENSG00000130385
ENSG00000101144	ENSG00000143869
ENSG00000101144	ENSG00000152785
ENSG00000101144	ENSG00000153162
ENSG00000101144	ENSG00000156466
ENSG00000101144	ENSG00000156574
ENSG00000101144	ENSG00000163217
ENSG00000101144	ENSG00000183682
ENSG00000101150	ENSG00000076554
ENSG00000101150	ENSG00000111907
ENSG00000101150	ENSG00000170777
ENSG00000101152	ENSG00000147570
ENSG00000101152	ENSG00000163793
ENSG00000101160	ENSG00000109861
ENSG00000101166	ENSG00000141391
ENSG00000101180	ENSG00000134489
ENSG00000101188	ENSG00000132911
ENSG00000101188	ENSG00000174417
ENSG00000101191	ENSG00000118482
ENSG00000101191	ENSG00000134668
ENSG00000101197	ENSG00000023445
ENSG00000101197	ENSG00000081770
ENSG00000101197	ENSG00000101966
ENSG00000101197	ENSG00000110330
ENSG00000101197	ENSG00000179978
ENSG00000101197	ENSG00000180152
ENSG00000101198	ENSG00000084628
ENSG00000101198	ENSG00000185942
ENSG00000101198	ENSG00000188580
ENSG00000101203	ENSG00000111799
ENSG00000101203	ENSG00000187955
ENSG00000101204	ENSG00000080644
ENSG00000101204	ENSG00000108556
ENSG00000101204	ENSG00000117971
ENSG00000101204	ENSG00000120903
ENSG00000101204	ENSG00000129749
ENSG00000101204	ENSG00000135902
ENSG00000101204	ENSG00000138435
ENSG00000101204	ENSG00000147432
ENSG00000101204	ENSG00000147434
ENSG00000101204	ENSG00000149305
ENSG00000101204	ENSG00000160716
ENSG00000101204	ENSG00000166664
ENSG00000101204	ENSG00000166736
ENSG00000101204	ENSG00000169684
ENSG00000101204	ENSG00000170175
ENSG00000101204	ENSG00000174343
ENSG00000101204	ENSG00000175344
ENSG00000101204	ENSG00000178084
ENSG00000101204	ENSG00000186038
ENSG00000101204	ENSG00000186090
ENSG00000101204	ENSG00000186919
ENSG00000101204	ENSG00000196811
ENSG00000101210	ENSG00000156508
ENSG00000101210	ENSG00000185637
ENSG00000101210	ENSG00000214744

ENSG00000101210	ENSG00000215093
ENSG00000101213	ENSG00000007264
ENSG00000101213	ENSG00000103653
ENSG00000101213	ENSG00000125508
ENSG00000101216	ENSG00000162419
ENSG00000101222	ENSG00000150628
ENSG00000101224	ENSG00000158402
ENSG00000101224	ENSG00000164045
ENSG00000101230	ENSG00000100593
ENSG00000101251	ENSG00000071537
ENSG00000101251	ENSG00000091490
ENSG00000101251	ENSG00000109771
ENSG00000101255	ENSG00000071575
ENSG00000101255	ENSG00000173334
ENSG00000101255	ENSG00000196182
ENSG00000101265	ENSG00000068028
ENSG00000101265	ENSG00000107551
ENSG00000101265	ENSG00000136653
ENSG00000101265	ENSG00000153179
ENSG00000101265	ENSG00000169435
ENSG00000101266	ENSG00000070770
ENSG00000101276	ENSG00000132517
ENSG00000101276	ENSG00000185803
ENSG00000101278	ENSG00000124614
ENSG00000101278	ENSG00000183701
ENSG00000101278	ENSG00000198810
ENSG00000101278	ENSG00000215464
ENSG00000101282	ENSG00000146374
ENSG00000101282	ENSG00000147655
ENSG00000101282	ENSG00000169218
ENSG00000101290	ENSG00000163624
ENSG00000101292	ENSG00000056291
ENSG00000101292	ENSG00000075073
ENSG00000101292	ENSG00000110148
ENSG00000101292	ENSG00000115353
ENSG00000101292	ENSG00000119973
ENSG00000101292	ENSG00000121764
ENSG00000101292	ENSG00000123901
ENSG00000101292	ENSG00000137252
ENSG00000101292	ENSG00000148734
ENSG00000101292	ENSG00000159279
ENSG00000101292	ENSG00000163394
ENSG00000101292	ENSG00000164128
ENSG00000101292	ENSG00000164129
ENSG00000101292	ENSG00000169618
ENSG00000101292	ENSG00000169836
ENSG00000101292	ENSG00000185149
ENSG00000101292	ENSG00000186867
ENSG00000101292	ENSG00000204174
ENSG00000101292	ENSG00000215167
ENSG00000101298	ENSG00000147642
ENSG00000101306	ENSG00000035664
ENSG00000101306	ENSG00000065534
ENSG00000101306	ENSG00000140795
ENSG00000101306	ENSG00000145949
ENSG00000101306	ENSG00000167657
ENSG00000101306	ENSG00000196730
ENSG00000101310	ENSG00000100934
ENSG00000101311	ENSG00000073712

ENSG00000101311	ENSG00000137076
ENSG00000101311	ENSG00000149781
ENSG00000101311	ENSG00000171914
ENSG00000101321	ENSG00000158156
ENSG00000101321	ENSG00000171044
ENSG00000101321	ENSG00000186530
ENSG00000101321	ENSG00000198669
ENSG00000101321	ENSG00000206579
ENSG00000101327	ENSG00000168081
ENSG00000101327	ENSG00000181195
ENSG00000101331	ENSG00000136280
ENSG00000101335	ENSG00000101608
ENSG00000101335	ENSG00000106436
ENSG00000101335	ENSG00000106631
ENSG00000101335	ENSG00000111245
ENSG00000101335	ENSG00000118680
ENSG00000101335	ENSG00000180209
ENSG00000101335	ENSG00000215375
ENSG00000101336	ENSG00000000938
ENSG00000101336	ENSG00000010810
ENSG00000101336	ENSG00000111816
ENSG00000101336	ENSG00000136573
ENSG00000101336	ENSG00000147507
ENSG00000101336	ENSG00000176105
ENSG00000101336	ENSG00000182866
ENSG00000101336	ENSG00000197122
ENSG00000101349	ENSG00000130669
ENSG00000101349	ENSG00000137843
ENSG00000101350	ENSG00000084731
ENSG00000101350	ENSG00000117245
ENSG00000101350	ENSG00000131437
ENSG00000101367	ENSG00000084764
ENSG00000101367	ENSG00000166974
ENSG00000101384	ENSG00000090932
ENSG00000101384	ENSG00000128917
ENSG00000101384	ENSG00000171462
ENSG00000101384	ENSG00000184916
ENSG00000101384	ENSG00000185559
ENSG00000101384	ENSG00000198719
ENSG00000101400	ENSG00000047932
ENSG00000101400	ENSG00000147481
ENSG00000101400	ENSG00000168807
ENSG00000101400	ENSG00000172164
ENSG00000101400	ENSG00000172554
ENSG00000101412	ENSG00000007968
ENSG00000101412	ENSG00000112242
ENSG00000101412	ENSG00000133740
ENSG00000101412	ENSG00000169016
ENSG00000101412	ENSG00000188841
ENSG00000101412	ENSG00000205250
ENSG00000101413	ENSG00000141425
ENSG00000101413	ENSG00000163125
ENSG00000101421	ENSG00000100931
ENSG00000101421	ENSG00000164695
ENSG00000101425	ENSG00000078898
ENSG00000101425	ENSG00000100979
ENSG00000101425	ENSG00000129988
ENSG00000101425	ENSG00000167104
ENSG00000101425	ENSG00000184459

ENSG00000101425	ENSG00000186190
ENSG00000101425	ENSG00000186191
ENSG00000101435	ENSG00000077984
ENSG00000101435	ENSG00000101439
ENSG00000101435	ENSG00000101441
ENSG00000101435	ENSG00000125815
ENSG00000101435	ENSG00000125823
ENSG00000101435	ENSG00000125831
ENSG00000101435	ENSG00000170367
ENSG00000101435	ENSG00000170369
ENSG00000101435	ENSG00000170373
ENSG00000101435	ENSG00000173335
ENSG00000101435	ENSG00000175315
ENSG00000101435	ENSG00000204658
ENSG00000101435	ENSG00000204662
ENSG00000101435	ENSG00000204663
ENSG00000101439	ENSG00000077984
ENSG00000101439	ENSG00000101435
ENSG00000101439	ENSG00000101441
ENSG00000101439	ENSG00000125815
ENSG00000101439	ENSG00000125823
ENSG00000101439	ENSG00000125831
ENSG00000101439	ENSG00000170367
ENSG00000101439	ENSG00000170369
ENSG00000101439	ENSG00000170373
ENSG00000101439	ENSG00000173335
ENSG00000101439	ENSG00000175315
ENSG00000101439	ENSG00000204658
ENSG00000101439	ENSG00000204662
ENSG00000101439	ENSG00000204663
ENSG00000101440	ENSG00000159723
ENSG00000101441	ENSG00000077984
ENSG00000101441	ENSG00000101435
ENSG00000101441	ENSG00000101439
ENSG00000101441	ENSG00000125815
ENSG00000101441	ENSG00000125823
ENSG00000101441	ENSG00000125831
ENSG00000101441	ENSG00000170367
ENSG00000101441	ENSG00000170369
ENSG00000101441	ENSG00000170373
ENSG00000101441	ENSG00000173335
ENSG00000101441	ENSG00000175315
ENSG00000101441	ENSG00000204658
ENSG00000101441	ENSG00000204662
ENSG00000101441	ENSG00000204663
ENSG00000101445	ENSG00000160972
ENSG00000101446	ENSG00000168630
ENSG00000101447	ENSG00000105523
ENSG00000101447	ENSG00000125998
ENSG00000101447	ENSG00000133477
ENSG00000101447	ENSG00000147689
ENSG00000101447	ENSG00000168143
ENSG00000101447	ENSG00000180921
ENSG00000101447	ENSG00000188522
ENSG00000101460	ENSG00000034713
ENSG00000101460	ENSG00000140941
ENSG00000101460	ENSG00000171471
ENSG00000101460	ENSG00000197769
ENSG00000101463	ENSG00000167371

ENSG00000101463	ENSG00000183379
ENSG00000101470	ENSG00000114854
ENSG00000101489	ENSG00000048740
ENSG00000101489	ENSG00000140488
ENSG00000101489	ENSG00000149187
ENSG00000101489	ENSG00000159409
ENSG00000101489	ENSG00000161082
ENSG00000101493	ENSG00000165804
ENSG00000101493	ENSG00000171940
ENSG00000101493	ENSG00000198597
ENSG00000101542	ENSG00000040731
ENSG00000101542	ENSG00000071991
ENSG00000101542	ENSG00000081138
ENSG00000101542	ENSG00000113100
ENSG00000101542	ENSG00000113361
ENSG00000101542	ENSG00000139880
ENSG00000101542	ENSG00000140937
ENSG00000101542	ENSG00000145526
ENSG00000101542	ENSG00000149654
ENSG00000101542	ENSG00000150394
ENSG00000101542	ENSG00000154162
ENSG00000101542	ENSG00000179776
ENSG00000101544	ENSG00000101126
ENSG00000101558	ENSG00000124164
ENSG00000101577	ENSG00000132793
ENSG00000101577	ENSG00000134324
ENSG00000101605	ENSG00000036448
ENSG00000101605	ENSG00000142661
ENSG00000101608	ENSG00000101335
ENSG00000101608	ENSG00000106436
ENSG00000101608	ENSG00000106631
ENSG00000101608	ENSG00000111245
ENSG00000101608	ENSG00000118680
ENSG00000101608	ENSG00000180209
ENSG00000101608	ENSG00000215375
ENSG00000101638	ENSG00000073849
ENSG00000101638	ENSG00000111728
ENSG00000101638	ENSG00000117069
ENSG00000101638	ENSG00000144057
ENSG00000101638	ENSG00000148488
ENSG00000101638	ENSG00000160408
ENSG00000101638	ENSG00000177511
ENSG00000101665	ENSG00000113658
ENSG00000101665	ENSG00000120693
ENSG00000101665	ENSG00000137834
ENSG00000101665	ENSG00000141646
ENSG00000101665	ENSG00000166949
ENSG00000101665	ENSG00000170365
ENSG00000101665	ENSG00000175387
ENSG00000101670	ENSG00000144837
ENSG00000101670	ENSG00000163898
ENSG00000101670	ENSG00000165862
ENSG00000101670	ENSG00000166035
ENSG00000101670	ENSG00000175445
ENSG00000101670	ENSG00000175535
ENSG00000101670	ENSG00000187021
ENSG00000101670	ENSG00000188992
ENSG00000101670	ENSG00000203837
ENSG00000101680	ENSG00000053747

ENSG00000101680	ENSG00000112769
ENSG00000101680	ENSG00000130702
ENSG00000101680	ENSG00000196569
ENSG00000101695	ENSG00000124226
ENSG00000101695	ENSG00000134758
ENSG00000101695	ENSG00000158717
ENSG00000101745	ENSG00000138376
ENSG00000101745	ENSG00000145700
ENSG00000101745	ENSG00000160949
ENSG00000101745	ENSG00000167522
ENSG00000101746	ENSG00000197183
ENSG00000101812	ENSG00000123569
ENSG00000101812	ENSG00000124635
ENSG00000101812	ENSG00000146047
ENSG00000101812	ENSG00000158373
ENSG00000101812	ENSG00000168242
ENSG00000101812	ENSG00000172450
ENSG00000101812	ENSG00000174258
ENSG00000101812	ENSG00000180596
ENSG00000101812	ENSG00000184678
ENSG00000101812	ENSG00000185130
ENSG00000101812	ENSG00000187990
ENSG00000101812	ENSG00000196226
ENSG00000101812	ENSG00000196331
ENSG00000101812	ENSG00000196374
ENSG00000101812	ENSG00000196501
ENSG00000101812	ENSG00000196890
ENSG00000101812	ENSG00000197034
ENSG00000101812	ENSG00000197459
ENSG00000101812	ENSG00000197597
ENSG00000101812	ENSG00000197697
ENSG00000101812	ENSG00000197846
ENSG00000101812	ENSG00000197903
ENSG00000101812	ENSG00000203814
ENSG00000101825	ENSG00000143341
ENSG00000101825	ENSG00000148357
ENSG00000101825	ENSG00000152580
ENSG00000101825	ENSG00000155657
ENSG00000101825	ENSG00000168462
ENSG00000101844	ENSG00000125703
ENSG00000101844	ENSG00000130734
ENSG00000101844	ENSG00000168397
ENSG00000101844	ENSG00000215697
ENSG00000101846	ENSG00000006756
ENSG00000101846	ENSG00000062096
ENSG00000101846	ENSG00000100299
ENSG00000101846	ENSG00000113273
ENSG00000101846	ENSG00000141012
ENSG00000101846	ENSG00000141337
ENSG00000101846	ENSG00000157399
ENSG00000101846	ENSG00000180801
ENSG00000101846	ENSG00000183876
ENSG00000101846	ENSG00000205667
ENSG00000101849	ENSG00000092377
ENSG00000101849	ENSG00000150627
ENSG00000101849	ENSG00000177565
ENSG00000101856	ENSG00000164040
ENSG00000101871	ENSG00000080561
ENSG00000101871	ENSG00000105255

ENSG00000101871	ENSG00000106701
ENSG00000101890	ENSG00000070019
ENSG00000101890	ENSG00000123201
ENSG00000101890	ENSG00000132518
ENSG00000101890	ENSG00000159899
ENSG00000101890	ENSG00000164116
ENSG00000101890	ENSG00000169418
ENSG00000101892	ENSG00000069849
ENSG00000101892	ENSG00000129244
ENSG00000101892	ENSG00000143153
ENSG00000101892	ENSG00000186009
ENSG00000101911	ENSG00000147224
ENSG00000101916	ENSG00000134061
ENSG00000101916	ENSG00000136869
ENSG00000101916	ENSG00000137462
ENSG00000101916	ENSG00000174123
ENSG00000101916	ENSG00000174125
ENSG00000101916	ENSG00000174130
ENSG00000101916	ENSG00000187554
ENSG00000101928	ENSG00000106330
ENSG00000101938	ENSG00000054938
ENSG00000101938	ENSG00000090539
ENSG00000101938	ENSG00000135253
ENSG00000101938	ENSG00000150938
ENSG00000101945	ENSG00000152455
ENSG00000101955	ENSG00000102359
ENSG00000101958	ENSG00000011677
ENSG00000101958	ENSG00000022355
ENSG00000101958	ENSG00000094755
ENSG00000101958	ENSG00000102287
ENSG00000101958	ENSG00000109158
ENSG00000101958	ENSG00000109738
ENSG00000101958	ENSG00000111886
ENSG00000101958	ENSG00000113327
ENSG00000101958	ENSG00000145451
ENSG00000101958	ENSG00000145863
ENSG00000101958	ENSG00000145864
ENSG00000101958	ENSG00000145888
ENSG00000101958	ENSG00000146276
ENSG00000101958	ENSG00000147402
ENSG00000101958	ENSG00000151834
ENSG00000101958	ENSG00000163285
ENSG00000101958	ENSG00000163288
ENSG00000101958	ENSG00000166206
ENSG00000101958	ENSG00000182256
ENSG00000101958	ENSG00000183185
ENSG00000101958	ENSG00000186297
ENSG00000101958	ENSG00000187730
ENSG00000101958	ENSG00000188828
ENSG00000101966	ENSG00000023445
ENSG00000101966	ENSG00000081770
ENSG00000101966	ENSG00000101197
ENSG00000101966	ENSG00000110330
ENSG00000101966	ENSG00000179978
ENSG00000101966	ENSG00000180152
ENSG00000101972	ENSG00000066923
ENSG00000101972	ENSG00000118007
ENSG00000101974	ENSG00000058063
ENSG00000101974	ENSG00000068650

ENSG00000101977	ENSG00000038382
ENSG00000101977	ENSG00000053524
ENSG00000101977	ENSG00000126217
ENSG00000101977	ENSG00000160145
ENSG00000101981	ENSG00000057593
ENSG00000101981	ENSG00000115718
ENSG00000101981	ENSG00000126218
ENSG00000101981	ENSG00000126231
ENSG00000101981	ENSG00000180210
ENSG00000101986	ENSG00000117528
ENSG00000101986	ENSG00000173208
ENSG00000102001	ENSG00000081248
ENSG00000102001	ENSG00000141837
ENSG00000102001	ENSG00000148408
ENSG00000102001	ENSG00000151067
ENSG00000102001	ENSG00000157388
ENSG00000102001	ENSG00000198216
ENSG00000102003	ENSG00000008282
ENSG00000102003	ENSG00000143028
ENSG00000102003	ENSG00000163630
ENSG00000102007	ENSG00000091317
ENSG00000102007	ENSG00000140931
ENSG00000102007	ENSG00000153551
ENSG00000102007	ENSG00000166091
ENSG00000102007	ENSG00000183723
ENSG00000102010	ENSG00000010671
ENSG00000102010	ENSG00000074966
ENSG00000102010	ENSG00000113263
ENSG00000102010	ENSG00000135605
ENSG00000102010	ENSG00000151422
ENSG00000102010	ENSG00000182511
ENSG00000102024	ENSG00000120756
ENSG00000102024	ENSG00000136167
ENSG00000102034	ENSG00000010030
ENSG00000102034	ENSG00000109381
ENSG00000102034	ENSG00000120690
ENSG00000102034	ENSG00000124664
ENSG00000102034	ENSG00000135373
ENSG00000102034	ENSG00000135374
ENSG00000102034	ENSG00000139083
ENSG00000102034	ENSG00000163435
ENSG00000102034	ENSG00000166211
ENSG00000102038	ENSG00000153147
ENSG00000102043	ENSG00000003987
ENSG00000102043	ENSG00000014914
ENSG00000102043	ENSG00000063601
ENSG00000102043	ENSG00000087053
ENSG00000102043	ENSG00000100330
ENSG00000102043	ENSG00000104643
ENSG00000102043	ENSG00000108389
ENSG00000102043	ENSG00000139505
ENSG00000102043	ENSG00000150712
ENSG00000102043	ENSG00000166912
ENSG00000102043	ENSG00000171100
ENSG00000102043	ENSG00000184416
ENSG00000102048	ENSG00000065802
ENSG00000102048	ENSG00000164122
ENSG00000102048	ENSG00000165192
ENSG00000102048	ENSG00000196372

ENSG00000102048	ENSG00000198881
ENSG00000102048	ENSG00000214426
ENSG00000102050	ENSG00000144746
ENSG00000102053	ENSG00000100441
ENSG00000102053	ENSG00000102921
ENSG00000102053	ENSG00000149289
ENSG00000102053	ENSG00000163874
ENSG00000102053	ENSG00000178199
ENSG00000102053	ENSG00000205978
ENSG00000102054	ENSG00000162521
ENSG00000102055	ENSG00000184203
ENSG00000102055	ENSG00000215031
ENSG00000102055	ENSG00000215471
ENSG00000102057	ENSG00000026559
ENSG00000102057	ENSG00000104848
ENSG00000102057	ENSG00000111262
ENSG00000102057	ENSG00000124134
ENSG00000102057	ENSG00000130037
ENSG00000102057	ENSG00000143105
ENSG00000102057	ENSG00000151079
ENSG00000102057	ENSG00000156486
ENSG00000102057	ENSG00000158445
ENSG00000102057	ENSG00000162975
ENSG00000102057	ENSG00000164794
ENSG00000102057	ENSG00000168263
ENSG00000102057	ENSG00000168418
ENSG00000102057	ENSG00000170745
ENSG00000102057	ENSG00000171126
ENSG00000102057	ENSG00000171385
ENSG00000102057	ENSG00000177272
ENSG00000102057	ENSG00000177301
ENSG00000102057	ENSG00000178342
ENSG00000102057	ENSG00000182255
ENSG00000102057	ENSG00000182674
ENSG00000102057	ENSG00000184408
ENSG00000102076	ENSG00000054277
ENSG00000102076	ENSG00000122375
ENSG00000102076	ENSG00000124818
ENSG00000102076	ENSG00000128617
ENSG00000102076	ENSG00000147380
ENSG00000102076	ENSG00000148604
ENSG00000102076	ENSG00000163914
ENSG00000102076	ENSG00000166160
ENSG00000102076	ENSG00000180245
ENSG00000102078	ENSG00000109424
ENSG00000102078	ENSG00000174032
ENSG00000102078	ENSG00000175564
ENSG00000102078	ENSG00000175567
ENSG00000102080	ENSG00000057704
ENSG00000102080	ENSG00000133069
ENSG00000102080	ENSG00000172765
ENSG00000102080	ENSG00000182242
ENSG00000102080	ENSG00000185254
ENSG00000102081	ENSG00000114416
ENSG00000102081	ENSG00000129245
ENSG00000102098	ENSG00000010803
ENSG00000102098	ENSG00000146285
ENSG00000102104	ENSG00000057019
ENSG00000102104	ENSG00000099250

ENSG00000102104	ENSG00000118257
ENSG00000102104	ENSG00000140545
ENSG00000102104	ENSG00000164176
ENSG00000102104	ENSG00000164465
ENSG00000102128	ENSG00000141542
ENSG00000102128	ENSG00000172476
ENSG00000102128	ENSG00000197562
ENSG00000102145	ENSG00000107485
ENSG00000102145	ENSG00000130700
ENSG00000102145	ENSG00000136574
ENSG00000102145	ENSG00000141448
ENSG00000102145	ENSG00000179348
ENSG00000102158	ENSG00000104723
ENSG00000102158	ENSG00000215762
ENSG00000102195	ENSG00000134640
ENSG00000102195	ENSG00000168412
ENSG00000102221	ENSG00000043143
ENSG00000102221	ENSG00000077684
ENSG00000102221	ENSG00000078403
ENSG00000102221	ENSG00000096070
ENSG00000102221	ENSG00000100425
ENSG00000102221	ENSG00000106443
ENSG00000102221	ENSG00000108292
ENSG00000102221	ENSG00000156983
ENSG00000102225	ENSG00000058091
ENSG00000102225	ENSG00000059758
ENSG00000102225	ENSG00000117266
ENSG00000102225	ENSG00000138395
ENSG00000102226	ENSG00000114316
ENSG00000102226	ENSG00000135655
ENSG00000102226	ENSG00000172046
ENSG00000102230	ENSG00000161217
ENSG00000102239	ENSG00000126010
ENSG00000102239	ENSG00000135577
ENSG00000102239	ENSG00000136160
ENSG00000102239	ENSG00000151617
ENSG00000102239	ENSG00000170075
ENSG00000102239	ENSG00000170775
ENSG00000102243	ENSG00000170162
ENSG00000102243	ENSG00000206538
ENSG00000102245	ENSG00000117560
ENSG00000102245	ENSG00000120659
ENSG00000102245	ENSG00000121858
ENSG00000102245	ENSG00000125735
ENSG00000102245	ENSG00000181634
ENSG00000102245	ENSG00000204490
ENSG00000102245	ENSG00000204496
ENSG00000102265	ENSG00000035862
ENSG00000102265	ENSG00000100234
ENSG00000102265	ENSG00000157150
ENSG00000102287	ENSG00000011677
ENSG00000102287	ENSG00000022355
ENSG00000102287	ENSG00000094755
ENSG00000102287	ENSG00000101958
ENSG00000102287	ENSG00000109158
ENSG00000102287	ENSG00000109738
ENSG00000102287	ENSG00000111886
ENSG00000102287	ENSG00000113327
ENSG00000102287	ENSG00000145451

ENSG00000102287	ENSG00000145863
ENSG00000102287	ENSG00000145864
ENSG00000102287	ENSG00000145888
ENSG00000102287	ENSG00000146276
ENSG00000102287	ENSG00000147402
ENSG00000102287	ENSG00000151834
ENSG00000102287	ENSG00000163285
ENSG00000102287	ENSG00000163288
ENSG00000102287	ENSG00000166206
ENSG00000102287	ENSG00000182256
ENSG00000102287	ENSG00000183185
ENSG00000102287	ENSG00000186297
ENSG00000102287	ENSG00000187730
ENSG00000102287	ENSG00000188828
ENSG00000102290	ENSG00000099715
ENSG00000102290	ENSG00000113555
ENSG00000102290	ENSG00000156453
ENSG00000102290	ENSG00000169851
ENSG00000102290	ENSG00000184226
ENSG00000102290	ENSG00000197991
ENSG00000102290	ENSG00000215442
ENSG00000102302	ENSG00000154783
ENSG00000102302	ENSG00000180263
ENSG00000102313	ENSG00000055955
ENSG00000102313	ENSG00000055957
ENSG00000102313	ENSG00000123243
ENSG00000102313	ENSG00000151655
ENSG00000102313	ENSG00000162267
ENSG00000102349	ENSG00000109787
ENSG00000102349	ENSG00000118263
ENSG00000102349	ENSG00000118922
ENSG00000102349	ENSG00000163884
ENSG00000102359	ENSG00000101955
ENSG00000102362	ENSG00000137501
ENSG00000102362	ENSG00000142765
ENSG00000102362	ENSG00000147041
ENSG00000102362	ENSG00000164674
ENSG00000102383	ENSG00000104219
ENSG00000102383	ENSG00000180776
ENSG00000102387	ENSG00000178913
ENSG00000102393	ENSG00000198951
ENSG00000102445	ENSG00000140995
ENSG00000102445	ENSG00000141342
ENSG00000102445	ENSG00000145016
ENSG00000102445	ENSG00000159266
ENSG00000102445	ENSG00000178385
ENSG00000102466	ENSG00000070193
ENSG00000102466	ENSG00000070388
ENSG00000102466	ENSG00000075388
ENSG00000102466	ENSG00000078579
ENSG00000102466	ENSG00000102678
ENSG00000102466	ENSG00000111241
ENSG00000102466	ENSG00000113578
ENSG00000102466	ENSG00000114279
ENSG00000102466	ENSG00000129682
ENSG00000102466	ENSG00000138675
ENSG00000102466	ENSG00000138685
ENSG00000102466	ENSG00000140285
ENSG00000102466	ENSG00000161958

ENSG00000102466	ENSG00000185390
ENSG00000102466	ENSG00000186895
ENSG00000102466	ENSG00000196468
ENSG00000102466	ENSG00000204801
ENSG00000102466	ENSG00000204816
ENSG00000102466	ENSG00000204817
ENSG00000102466	ENSG00000204837
ENSG00000102466	ENSG00000215053
ENSG00000102468	ENSG00000043591
ENSG00000102468	ENSG00000113749
ENSG00000102468	ENSG00000125631
ENSG00000102468	ENSG00000135312
ENSG00000102468	ENSG00000135914
ENSG00000102468	ENSG00000147246
ENSG00000102468	ENSG00000150594
ENSG00000102468	ENSG00000157219
ENSG00000102468	ENSG00000164270
ENSG00000102468	ENSG00000168830
ENSG00000102468	ENSG00000169252
ENSG00000102468	ENSG00000175658
ENSG00000102468	ENSG00000178394
ENSG00000102468	ENSG00000179097
ENSG00000102468	ENSG00000179546
ENSG00000102468	ENSG00000181210
ENSG00000102468	ENSG00000184303
ENSG00000102468	ENSG00000184845
ENSG00000102468	ENSG00000185321
ENSG00000102468	ENSG00000188778
ENSG00000102471	ENSG00000131507
ENSG00000102471	ENSG00000197627
ENSG00000102524	ENSG00000161955
ENSG00000102531	ENSG00000075420
ENSG00000102547	ENSG00000135932
ENSG00000102554	ENSG00000067082
ENSG00000102554	ENSG00000105610
ENSG00000102554	ENSG00000127528
ENSG00000102554	ENSG00000129911
ENSG00000102554	ENSG00000136826
ENSG00000102554	ENSG00000139651
ENSG00000102554	ENSG00000155090
ENSG00000102554	ENSG00000164651
ENSG00000102554	ENSG00000167182
ENSG00000102554	ENSG00000170374
ENSG00000102554	ENSG00000171872
ENSG00000102554	ENSG00000172059
ENSG00000102554	ENSG00000174595
ENSG00000102554	ENSG00000189120
ENSG00000102554	ENSG00000197294
ENSG00000102554	ENSG00000197550
ENSG00000102554	ENSG00000204335
ENSG00000102572	ENSG00000115694
ENSG00000102572	ENSG00000134602
ENSG00000102595	ENSG00000136731
ENSG00000102606	ENSG00000129675
ENSG00000102606	ENSG00000187583
ENSG00000102678	ENSG00000070193
ENSG00000102678	ENSG00000070388
ENSG00000102678	ENSG00000075388
ENSG00000102678	ENSG00000078579

ENSG00000102678	ENSG00000102466
ENSG00000102678	ENSG00000111241
ENSG00000102678	ENSG00000113578
ENSG00000102678	ENSG00000114279
ENSG00000102678	ENSG00000129682
ENSG00000102678	ENSG00000138675
ENSG00000102678	ENSG00000138685
ENSG00000102678	ENSG00000140285
ENSG00000102678	ENSG00000161958
ENSG00000102678	ENSG00000185390
ENSG00000102678	ENSG00000186895
ENSG00000102678	ENSG00000196468
ENSG00000102678	ENSG00000204801
ENSG00000102678	ENSG00000204816
ENSG00000102678	ENSG00000204817
ENSG00000102678	ENSG00000204837
ENSG00000102678	ENSG00000215053
ENSG00000102683	ENSG00000163069
ENSG00000102683	ENSG00000170624
ENSG00000102683	ENSG00000185053
ENSG00000102743	ENSG00000120329
ENSG00000102753	ENSG00000182481
ENSG00000102753	ENSG00000185467
ENSG00000102753	ENSG00000186432
ENSG00000102753	ENSG00000214035
ENSG00000102753	ENSG00000214180
ENSG00000102753	ENSG00000215769
ENSG00000102755	ENSG00000037280
ENSG00000102755	ENSG00000104213
ENSG00000102755	ENSG00000113721
ENSG00000102755	ENSG00000122025
ENSG00000102755	ENSG00000128052
ENSG00000102755	ENSG00000134853
ENSG00000102755	ENSG00000157404
ENSG00000102755	ENSG00000182578
ENSG00000102780	ENSG00000058866
ENSG00000102780	ENSG00000065357
ENSG00000102780	ENSG00000077044
ENSG00000102780	ENSG00000136267
ENSG00000102780	ENSG00000153933
ENSG00000102780	ENSG00000204466
ENSG00000102781	ENSG00000186625
ENSG00000102786	ENSG00000165359
ENSG00000102786	ENSG00000181433
ENSG00000102786	ENSG00000215130
ENSG00000102804	ENSG00000166925
ENSG00000102804	ENSG00000196428
ENSG00000102837	ENSG00000034971
ENSG00000102837	ENSG00000105088
ENSG00000102837	ENSG00000118733
ENSG00000102837	ENSG00000130558
ENSG00000102837	ENSG00000183801
ENSG00000102837	ENSG00000186417
ENSG00000102837	ENSG00000215081
ENSG00000102858	ENSG00000141576
ENSG00000102878	ENSG00000025156
ENSG00000102878	ENSG00000169953
ENSG00000102878	ENSG00000171116
ENSG00000102878	ENSG00000171129

ENSG00000102878	ENSG00000172468
ENSG00000102878	ENSG00000176160
ENSG00000102878	ENSG00000185122
ENSG00000102878	ENSG00000214373
ENSG00000102878	ENSG00000215574
ENSG00000102878	ENSG00000215701
ENSG00000102879	ENSG00000103426
ENSG00000102879	ENSG00000103647
ENSG00000102879	ENSG00000106789
ENSG00000102879	ENSG00000110880
ENSG00000102879	ENSG00000167549
ENSG00000102879	ENSG00000172725
ENSG00000102882	ENSG00000100030
ENSG00000102886	ENSG00000153982
ENSG00000102890	ENSG00000062598
ENSG00000102890	ENSG00000155849
ENSG00000102891	ENSG00000087250
ENSG00000102891	ENSG00000125144
ENSG00000102891	ENSG00000125148
ENSG00000102891	ENSG00000169688
ENSG00000102891	ENSG00000169715
ENSG00000102891	ENSG00000187193
ENSG00000102891	ENSG00000198417
ENSG00000102891	ENSG00000205358
ENSG00000102891	ENSG00000205360
ENSG00000102891	ENSG00000205362
ENSG00000102891	ENSG00000205364
ENSG00000102908	ENSG00000072736
ENSG00000102908	ENSG00000100968
ENSG00000102908	ENSG00000101096
ENSG00000102908	ENSG00000131196
ENSG00000102921	ENSG00000100441
ENSG00000102921	ENSG00000102053
ENSG00000102921	ENSG00000149289
ENSG00000102921	ENSG00000163874
ENSG00000102921	ENSG00000178199
ENSG00000102921	ENSG00000205978
ENSG00000102924	ENSG00000054803
ENSG00000102924	ENSG00000131094
ENSG00000102924	ENSG00000133466
ENSG00000102924	ENSG00000139899
ENSG00000102924	ENSG00000141668
ENSG00000102924	ENSG00000144119
ENSG00000102924	ENSG00000145861
ENSG00000102924	ENSG00000159189
ENSG00000102924	ENSG00000163145
ENSG00000102924	ENSG00000165985
ENSG00000102924	ENSG00000172247
ENSG00000102924	ENSG00000173369
ENSG00000102924	ENSG00000173372
ENSG00000102924	ENSG00000173918
ENSG00000102924	ENSG00000181092
ENSG00000102924	ENSG00000184471
ENSG00000102924	ENSG00000186897
ENSG00000102924	ENSG00000213192
ENSG00000102934	ENSG00000144063
ENSG00000102934	ENSG00000147676
ENSG00000102934	ENSG00000155254
ENSG00000102934	ENSG00000170293

ENSG00000102934	ENSG00000172005
ENSG00000102935	ENSG00000198795
ENSG00000102962	ENSG00000006074
ENSG00000102962	ENSG00000006075
ENSG00000102962	ENSG00000006606
ENSG00000102962	ENSG00000102970
ENSG00000102962	ENSG00000106178
ENSG00000102962	ENSG00000108688
ENSG00000102962	ENSG00000108691
ENSG00000102962	ENSG00000108700
ENSG00000102962	ENSG00000108702
ENSG00000102962	ENSG00000115009
ENSG00000102962	ENSG00000129277
ENSG00000102962	ENSG00000143184
ENSG00000102962	ENSG00000143185
ENSG00000102962	ENSG00000161570
ENSG00000102962	ENSG00000161573
ENSG00000102962	ENSG00000161574
ENSG00000102962	ENSG00000167236
ENSG00000102962	ENSG00000172156
ENSG00000102962	ENSG00000172724
ENSG00000102962	ENSG00000181374
ENSG00000102962	ENSG00000197262
ENSG00000102962	ENSG00000205017
ENSG00000102962	ENSG00000205020
ENSG00000102962	ENSG00000205021
ENSG00000102962	ENSG00000213494
ENSG00000102962	ENSG00000215627
ENSG00000102962	ENSG00000215629
ENSG00000102970	ENSG00000006074
ENSG00000102970	ENSG00000006075
ENSG00000102970	ENSG00000006606
ENSG00000102970	ENSG00000102962
ENSG00000102970	ENSG00000106178
ENSG00000102970	ENSG00000108688
ENSG00000102970	ENSG00000108691
ENSG00000102970	ENSG00000108700
ENSG00000102970	ENSG00000108702
ENSG00000102970	ENSG00000115009
ENSG00000102970	ENSG00000129277
ENSG00000102970	ENSG00000143184
ENSG00000102970	ENSG00000143185
ENSG00000102970	ENSG00000161570
ENSG00000102970	ENSG00000161573
ENSG00000102970	ENSG00000161574
ENSG00000102970	ENSG00000167236
ENSG00000102970	ENSG00000172156
ENSG00000102970	ENSG00000172724
ENSG00000102970	ENSG00000181374
ENSG00000102970	ENSG00000197262
ENSG00000102970	ENSG00000205017
ENSG00000102970	ENSG00000205020
ENSG00000102970	ENSG00000205021
ENSG00000102970	ENSG00000213494
ENSG00000102970	ENSG00000215627
ENSG00000102970	ENSG00000215629
ENSG00000102974	ENSG00000124092
ENSG00000102981	ENSG00000124171
ENSG00000102981	ENSG00000178184

ENSG00000102996	ENSG00000008516
ENSG00000102996	ENSG00000087245
ENSG00000102996	ENSG00000099953
ENSG00000102996	ENSG00000100985
ENSG00000102996	ENSG00000110347
ENSG00000102996	ENSG00000118113
ENSG00000102996	ENSG00000123342
ENSG00000102996	ENSG00000125966
ENSG00000102996	ENSG00000129270
ENSG00000102996	ENSG00000137673
ENSG00000102996	ENSG00000137674
ENSG00000102996	ENSG00000137675
ENSG00000102996	ENSG00000137745
ENSG00000102996	ENSG00000149968
ENSG00000102996	ENSG00000154485
ENSG00000102996	ENSG00000156103
ENSG00000102996	ENSG00000157227
ENSG00000102996	ENSG00000166670
ENSG00000102996	ENSG00000167346
ENSG00000102996	ENSG00000189409
ENSG00000102996	ENSG00000196611
ENSG00000102996	ENSG00000198598
ENSG00000102996	ENSG00000215914
ENSG00000103018	ENSG00000166347
ENSG00000103024	ENSG00000011052
ENSG00000103024	ENSG00000103200
ENSG00000103024	ENSG00000123009
ENSG00000103034	ENSG00000101079
ENSG00000103034	ENSG00000104419
ENSG00000103034	ENSG00000165795
ENSG00000103042	ENSG00000017483
ENSG00000103042	ENSG00000111371
ENSG00000103042	ENSG00000134294
ENSG00000103042	ENSG00000139209
ENSG00000103042	ENSG00000139974
ENSG00000103042	ENSG00000166558
ENSG00000103042	ENSG00000169507
ENSG00000103042	ENSG00000188338
ENSG00000103044	ENSG00000105509
ENSG00000103044	ENSG00000170961
ENSG00000103064	ENSG00000021488
ENSG00000103064	ENSG00000092068
ENSG00000103064	ENSG00000103257
ENSG00000103064	ENSG00000130876
ENSG00000103064	ENSG00000151012
ENSG00000103064	ENSG00000155465
ENSG00000103064	ENSG00000164893
ENSG00000103066	ENSG00000213398
ENSG00000103067	ENSG00000096746
ENSG00000103067	ENSG00000104413
ENSG00000103067	ENSG00000126945
ENSG00000103067	ENSG00000132463
ENSG00000103067	ENSG00000169045
ENSG00000103067	ENSG00000169813
ENSG00000103111	ENSG00000164077
ENSG00000103126	ENSG00000168646
ENSG00000103154	ENSG00000123119
ENSG00000103154	ENSG00000125967
ENSG00000103184	ENSG00000100003

ENSG00000103184	ENSG00000100012
ENSG00000103184	ENSG00000129657
ENSG00000103184	ENSG00000133488
ENSG00000103196	ENSG00000096006
ENSG00000103196	ENSG00000101074
ENSG00000103196	ENSG00000121005
ENSG00000103196	ENSG00000124490
ENSG00000103196	ENSG00000124812
ENSG00000103196	ENSG00000137558
ENSG00000103196	ENSG00000139278
ENSG00000103196	ENSG00000164530
ENSG00000103196	ENSG00000173401
ENSG00000103196	ENSG00000180481
ENSG00000103200	ENSG00000011052
ENSG00000103200	ENSG00000103024
ENSG00000103200	ENSG00000123009
ENSG00000103222	ENSG00000023839
ENSG00000103222	ENSG00000091262
ENSG00000103222	ENSG00000108846
ENSG00000103241	ENSG00000137273
ENSG00000103245	ENSG00000141562
ENSG00000103253	ENSG00000063854
ENSG00000103253	ENSG00000127838
ENSG00000103254	ENSG00000150756
ENSG00000103257	ENSG00000021488
ENSG00000103257	ENSG00000092068
ENSG00000103257	ENSG00000103064
ENSG00000103257	ENSG00000130876
ENSG00000103257	ENSG00000151012
ENSG00000103257	ENSG00000155465
ENSG00000103257	ENSG00000164893
ENSG00000103260	ENSG00000176845
ENSG00000103269	ENSG00000141314
ENSG00000103269	ENSG00000158315
ENSG00000103310	ENSG00000116996
ENSG00000103310	ENSG00000149506
ENSG00000103319	ENSG00000073331
ENSG00000103326	ENSG00000131375
ENSG00000103335	ENSG00000154864
ENSG00000103335	ENSG00000175388
ENSG00000103355	ENSG00000005001
ENSG00000103355	ENSG00000007038
ENSG00000103355	ENSG00000052344
ENSG00000103355	ENSG00000095917
ENSG00000103355	ENSG00000116176
ENSG00000103355	ENSG00000172236
ENSG00000103355	ENSG00000172382
ENSG00000103355	ENSG00000172460
ENSG00000103355	ENSG00000178055
ENSG00000103355	ENSG00000178226
ENSG00000103355	ENSG00000185888
ENSG00000103355	ENSG00000188086
ENSG00000103355	ENSG00000189099
ENSG00000103355	ENSG00000196364
ENSG00000103355	ENSG00000197253
ENSG00000103355	ENSG00000206549
ENSG00000103355	ENSG00000214877
ENSG00000103355	ENSG00000215148
ENSG00000103365	ENSG00000100083

ENSG00000103365	ENSG00000125447
ENSG00000103375	ENSG00000086159
ENSG00000103375	ENSG00000106125
ENSG00000103375	ENSG00000135517
ENSG00000103375	ENSG00000161798
ENSG00000103375	ENSG00000167580
ENSG00000103375	ENSG00000171885
ENSG00000103404	ENSG00000154914
ENSG00000103415	ENSG00000100292
ENSG00000103449	ENSG00000101115
ENSG00000103449	ENSG00000151514
ENSG00000103449	ENSG00000165821
ENSG00000103460	ENSG00000092203
ENSG00000103460	ENSG00000124191
ENSG00000103460	ENSG00000198846
ENSG00000103479	ENSG00000080839
ENSG00000103479	ENSG00000139687
ENSG00000103489	ENSG00000015532
ENSG00000103489	ENSG00000111846
ENSG00000103489	ENSG00000124091
ENSG00000103489	ENSG00000140297
ENSG00000103489	ENSG00000176928
ENSG00000103489	ENSG00000187210
ENSG00000103489	ENSG00000205318
ENSG00000103494	ENSG00000092200
ENSG00000103495	ENSG00000100105
ENSG00000103495	ENSG00000136451
ENSG00000103522	ENSG00000100385
ENSG00000103522	ENSG00000124334
ENSG00000103522	ENSG00000215303
ENSG00000103534	ENSG00000141524
ENSG00000103534	ENSG00000149488
ENSG00000103534	ENSG00000165091
ENSG00000103534	ENSG00000167608
ENSG00000103534	ENSG00000167895
ENSG00000103534	ENSG00000170537
ENSG00000103534	ENSG00000188869
ENSG00000103546	ENSG00000010379
ENSG00000103546	ENSG00000011083
ENSG00000103546	ENSG00000087916
ENSG00000103546	ENSG00000108576
ENSG00000103546	ENSG00000111181
ENSG00000103546	ENSG00000130821
ENSG00000103546	ENSG00000131389
ENSG00000103546	ENSG00000132164
ENSG00000103546	ENSG00000142319
ENSG00000103546	ENSG00000157103
ENSG00000103546	ENSG00000163817
ENSG00000103546	ENSG00000164363
ENSG00000103546	ENSG00000165970
ENSG00000103546	ENSG00000174358
ENSG00000103546	ENSG00000196517
ENSG00000103546	ENSG00000198555
ENSG00000103546	ENSG00000214617
ENSG00000103549	ENSG00000155827
ENSG00000103569	ENSG00000143595
ENSG00000103569	ENSG00000165269
ENSG00000103569	ENSG00000165272
ENSG00000103569	ENSG00000176115

ENSG00000103569	ENSG00000181997
ENSG00000103569	ENSG00000186466
ENSG00000103647	ENSG00000102879
ENSG00000103647	ENSG00000103426
ENSG00000103647	ENSG00000106789
ENSG00000103647	ENSG00000110880
ENSG00000103647	ENSG00000167549
ENSG00000103647	ENSG00000172725
ENSG00000103653	ENSG00000007264
ENSG00000103653	ENSG00000101213
ENSG00000103653	ENSG00000125508
ENSG00000103723	ENSG00000132842
ENSG00000103740	ENSG00000130377
ENSG00000103742	ENSG00000067141
ENSG00000103742	ENSG00000166450
ENSG00000103742	ENSG00000174498
ENSG00000103742	ENSG00000187323
ENSG00000103742	ENSG00000215851
ENSG00000103769	ENSG00000132698
ENSG00000103769	ENSG00000185236
ENSG00000103852	ENSG00000205838
ENSG00000103855	ENSG00000114013
ENSG00000103855	ENSG00000114455
ENSG00000103855	ENSG00000120217
ENSG00000103855	ENSG00000134258
ENSG00000103855	ENSG00000160223
ENSG00000103855	ENSG00000186470
ENSG00000103855	ENSG00000188211
ENSG00000103855	ENSG00000197646
ENSG00000103855	ENSG00000204290
ENSG00000103855	ENSG00000204655
ENSG00000103855	ENSG00000215631
ENSG00000103855	ENSG00000215811
ENSG00000103855	ENSG00000215889
ENSG00000103888	ENSG00000135048
ENSG00000103942	ENSG00000051128
ENSG00000103942	ENSG00000152413
ENSG00000103942	ENSG00000214396
ENSG00000103966	ENSG00000013016
ENSG00000103966	ENSG00000024422
ENSG00000103966	ENSG00000110047
ENSG00000103978	ENSG00000153214
ENSG00000104043	ENSG00000081923
ENSG00000104043	ENSG00000124406
ENSG00000104043	ENSG00000130270
ENSG00000104043	ENSG00000132932
ENSG00000104043	ENSG00000143515
ENSG00000104055	ENSG00000092295
ENSG00000104055	ENSG00000124491
ENSG00000104055	ENSG00000125780
ENSG00000104055	ENSG00000159495
ENSG00000104055	ENSG00000163810
ENSG00000104055	ENSG00000166947
ENSG00000104055	ENSG00000166948
ENSG00000104055	ENSG00000198959
ENSG00000104059	ENSG00000135063
ENSG00000104059	ENSG00000160767
ENSG00000104064	ENSG00000107854
ENSG00000104064	ENSG00000143458

ENSG00000104064	ENSG00000173273
ENSG00000104067	ENSG00000119139
ENSG00000104093	ENSG00000172869
ENSG00000104140	ENSG00000116574
ENSG00000104177	ENSG00000099783
ENSG00000104177	ENSG00000215013
ENSG00000104213	ENSG00000037280
ENSG00000104213	ENSG00000102755
ENSG00000104213	ENSG00000113721
ENSG00000104213	ENSG00000122025
ENSG00000104213	ENSG00000128052
ENSG00000104213	ENSG00000134853
ENSG00000104213	ENSG00000157404
ENSG00000104213	ENSG00000182578
ENSG00000104219	ENSG00000102383
ENSG00000104219	ENSG00000180776
ENSG00000104237	ENSG00000077279
ENSG00000104237	ENSG00000130413
ENSG00000104237	ENSG00000133083
ENSG00000104237	ENSG00000146038
ENSG00000104237	ENSG00000163673
ENSG00000104237	ENSG00000170390
ENSG00000104237	ENSG00000183638
ENSG00000104267	ENSG00000074410
ENSG00000104267	ENSG00000107159
ENSG00000104267	ENSG00000118298
ENSG00000104267	ENSG00000131686
ENSG00000104267	ENSG00000133742
ENSG00000104267	ENSG00000164879
ENSG00000104267	ENSG00000167434
ENSG00000104267	ENSG00000168748
ENSG00000104267	ENSG00000169239
ENSG00000104267	ENSG00000174990
ENSG00000104267	ENSG00000178538
ENSG00000104267	ENSG00000185015
ENSG00000104267	ENSG00000214729
ENSG00000104267	ENSG00000214873
ENSG00000104267	ENSG00000215487
ENSG00000104267	ENSG00000215501
ENSG00000104267	ENSG00000215543
ENSG00000104290	ENSG00000106483
ENSG00000104290	ENSG00000111432
ENSG00000104290	ENSG00000155760
ENSG00000104290	ENSG00000157240
ENSG00000104290	ENSG00000162998
ENSG00000104290	ENSG00000163251
ENSG00000104290	ENSG00000164930
ENSG00000104290	ENSG00000174804
ENSG00000104290	ENSG00000177283
ENSG00000104290	ENSG00000180340
ENSG00000104290	ENSG00000188763
ENSG00000104312	ENSG00000129465
ENSG00000104312	ENSG00000137275
ENSG00000104312	ENSG00000170209
ENSG00000104312	ENSG00000183421
ENSG00000104313	ENSG00000064655
ENSG00000104313	ENSG00000112319
ENSG00000104313	ENSG00000158161
ENSG00000104327	ENSG00000079689

ENSG00000104327	ENSG00000172137
ENSG00000104331	ENSG00000151689
ENSG00000104331	ENSG00000162813
ENSG00000104332	ENSG00000120057
ENSG00000104332	ENSG00000145423
ENSG00000104332	ENSG00000214766
ENSG00000104341	ENSG00000068697
ENSG00000104341	ENSG00000162511
ENSG00000104361	ENSG00000001461
ENSG00000104361	ENSG00000140157
ENSG00000104361	ENSG00000163293
ENSG00000104361	ENSG00000170113
ENSG00000104361	ENSG00000172548
ENSG00000104365	ENSG00000143466
ENSG00000104365	ENSG00000183735
ENSG00000104365	ENSG00000213341
ENSG00000104368	ENSG00000019991
ENSG00000104368	ENSG00000109758
ENSG00000104368	ENSG00000122194
ENSG00000104368	ENSG00000122861
ENSG00000104368	ENSG00000131187
ENSG00000104368	ENSG00000148702
ENSG00000104368	ENSG00000173531
ENSG00000104368	ENSG00000186301
ENSG00000104368	ENSG00000186715
ENSG00000104368	ENSG00000198670
ENSG00000104369	ENSG00000092051
ENSG00000104369	ENSG00000139714
ENSG00000104369	ENSG00000149596
ENSG00000104369	ENSG00000154118
ENSG00000104371	ENSG00000107984
ENSG00000104371	ENSG00000155011
ENSG00000104375	ENSG00000101109
ENSG00000104381	ENSG00000124194
ENSG00000104413	ENSG00000096746
ENSG00000104413	ENSG00000103067
ENSG00000104413	ENSG00000126945
ENSG00000104413	ENSG00000132463
ENSG00000104413	ENSG00000169045
ENSG00000104413	ENSG00000169813
ENSG00000104415	ENSG00000064205
ENSG00000104415	ENSG00000112761
ENSG00000104415	ENSG00000118523
ENSG00000104415	ENSG00000136999
ENSG00000104415	ENSG00000142871
ENSG00000104419	ENSG00000101079
ENSG00000104419	ENSG00000103034
ENSG00000104419	ENSG00000165795
ENSG00000104427	ENSG00000119703
ENSG00000104435	ENSG00000015592
ENSG00000104435	ENSG00000117632
ENSG00000104435	ENSG00000213603
ENSG00000104435	ENSG00000214241
ENSG00000104490	ENSG00000107130
ENSG00000104490	ENSG00000109047
ENSG00000104490	ENSG00000115756
ENSG00000104490	ENSG00000116983
ENSG00000104490	ENSG00000121905
ENSG00000104490	ENSG00000163032

ENSG00000104611	ENSG00000027869
ENSG00000104611	ENSG00000178217
ENSG00000104611	ENSG00000196684
ENSG00000104635	ENSG00000112473
ENSG00000104635	ENSG00000138821
ENSG00000104635	ENSG00000139540
ENSG00000104635	ENSG00000141424
ENSG00000104635	ENSG00000147804
ENSG00000104635	ENSG00000148482
ENSG00000104635	ENSG00000165915
ENSG00000104635	ENSG00000196950
ENSG00000104643	ENSG00000003987
ENSG00000104643	ENSG00000014914
ENSG00000104643	ENSG00000063601
ENSG00000104643	ENSG00000087053
ENSG00000104643	ENSG00000100330
ENSG00000104643	ENSG00000102043
ENSG00000104643	ENSG00000108389
ENSG00000104643	ENSG00000139505
ENSG00000104643	ENSG00000150712
ENSG00000104643	ENSG00000166912
ENSG00000104643	ENSG00000171100
ENSG00000104643	ENSG00000184416
ENSG00000104660	ENSG00000213625
ENSG00000104679	ENSG00000166024
ENSG00000104689	ENSG00000026103
ENSG00000104689	ENSG00000067182
ENSG00000104689	ENSG00000120889
ENSG00000104689	ENSG00000173530
ENSG00000104689	ENSG00000173535
ENSG00000104689	ENSG00000215788
ENSG00000104695	ENSG00000113575
ENSG00000104722	ENSG00000026025
ENSG00000104722	ENSG00000100285
ENSG00000104722	ENSG00000104725
ENSG00000104722	ENSG00000131095
ENSG00000104722	ENSG00000135406
ENSG00000104722	ENSG00000148798
ENSG00000104722	ENSG00000175084
ENSG00000104722	ENSG00000182253
ENSG00000104722	ENSG00000215479
ENSG00000104723	ENSG00000102158
ENSG00000104723	ENSG00000215762
ENSG00000104725	ENSG00000026025
ENSG00000104725	ENSG00000100285
ENSG00000104725	ENSG00000104722
ENSG00000104725	ENSG00000131095
ENSG00000104725	ENSG00000135406
ENSG00000104725	ENSG00000148798
ENSG00000104725	ENSG00000175084
ENSG00000104725	ENSG00000182253
ENSG00000104725	ENSG00000215479
ENSG00000104728	ENSG00000074964
ENSG00000104755	ENSG00000008277
ENSG00000104755	ENSG00000042980
ENSG00000104755	ENSG00000069206
ENSG00000104755	ENSG00000073670
ENSG00000104755	ENSG00000114948
ENSG00000104755	ENSG00000134007

ENSG00000104755	ENSG00000134028
ENSG00000104755	ENSG00000134249
ENSG00000104755	ENSG00000135074
ENSG00000104755	ENSG00000139985
ENSG00000104755	ENSG00000143537
ENSG00000104755	ENSG00000148848
ENSG00000104755	ENSG00000149451
ENSG00000104755	ENSG00000151651
ENSG00000104755	ENSG00000151694
ENSG00000104755	ENSG00000168594
ENSG00000104755	ENSG00000168615
ENSG00000104755	ENSG00000168619
ENSG00000104755	ENSG00000196115
ENSG00000104755	ENSG00000197140
ENSG00000104755	ENSG00000214137
ENSG00000104765	ENSG00000176171
ENSG00000104804	ENSG00000078246
ENSG00000104804	ENSG00000112041
ENSG00000104804	ENSG00000118965
ENSG00000104804	ENSG00000130338
ENSG00000104804	ENSG00000166402
ENSG00000104805	ENSG00000070081
ENSG00000104812	ENSG00000111713
ENSG00000104814	ENSG00000011566
ENSG00000104814	ENSG00000012983
ENSG00000104814	ENSG00000168067
ENSG00000104818	ENSG00000104826
ENSG00000104818	ENSG00000104827
ENSG00000104818	ENSG00000131808
ENSG00000104818	ENSG00000134200
ENSG00000104818	ENSG00000179600
ENSG00000104818	ENSG00000189052
ENSG00000104818	ENSG00000196337
ENSG00000104818	ENSG00000204748
ENSG00000104818	ENSG00000213030
ENSG00000104824	ENSG00000143889
ENSG00000104824	ENSG00000215255
ENSG00000104825	ENSG00000069399
ENSG00000104825	ENSG00000100906
ENSG00000104825	ENSG00000146232
ENSG00000104826	ENSG00000104818
ENSG00000104826	ENSG00000104827
ENSG00000104826	ENSG00000131808
ENSG00000104826	ENSG00000134200
ENSG00000104826	ENSG00000179600
ENSG00000104826	ENSG00000189052
ENSG00000104826	ENSG00000196337
ENSG00000104826	ENSG00000204748
ENSG00000104826	ENSG00000213030
ENSG00000104827	ENSG00000104818
ENSG00000104827	ENSG00000104826
ENSG00000104827	ENSG00000131808
ENSG00000104827	ENSG00000134200
ENSG00000104827	ENSG00000179600
ENSG00000104827	ENSG00000189052
ENSG00000104827	ENSG00000196337
ENSG00000104827	ENSG00000204748
ENSG00000104827	ENSG00000213030
ENSG00000104848	ENSG00000026559

ENSG00000104848	ENSG00000102057
ENSG00000104848	ENSG00000111262
ENSG00000104848	ENSG00000124134
ENSG00000104848	ENSG00000130037
ENSG00000104848	ENSG00000143105
ENSG00000104848	ENSG00000151079
ENSG00000104848	ENSG00000156486
ENSG00000104848	ENSG00000158445
ENSG00000104848	ENSG00000162975
ENSG00000104848	ENSG00000164794
ENSG00000104848	ENSG00000168263
ENSG00000104848	ENSG00000168418
ENSG00000104848	ENSG00000170745
ENSG00000104848	ENSG00000171126
ENSG00000104848	ENSG00000171385
ENSG00000104848	ENSG00000177272
ENSG00000104848	ENSG00000177301
ENSG00000104848	ENSG00000178342
ENSG00000104848	ENSG00000182255
ENSG00000104848	ENSG00000182674
ENSG00000104848	ENSG00000184408
ENSG00000104856	ENSG00000077150
ENSG00000104856	ENSG00000109320
ENSG00000104856	ENSG00000162924
ENSG00000104856	ENSG00000173039
ENSG00000104863	ENSG00000111052
ENSG00000104863	ENSG00000148943
ENSG00000104879	ENSG00000131730
ENSG00000104879	ENSG00000166165
ENSG00000104879	ENSG00000166998
ENSG00000104879	ENSG00000168775
ENSG00000104880	ENSG00000038102
ENSG00000104880	ENSG00000116584
ENSG00000104880	ENSG00000170776
ENSG00000104888	ENSG00000091664
ENSG00000104888	ENSG00000112337
ENSG00000104888	ENSG00000119899
ENSG00000104888	ENSG00000124564
ENSG00000104888	ENSG00000124568
ENSG00000104888	ENSG00000146039
ENSG00000104888	ENSG00000179520
ENSG00000104892	ENSG00000011295
ENSG00000104892	ENSG00000062725
ENSG00000104892	ENSG00000126214
ENSG00000104892	ENSG00000137171
ENSG00000104892	ENSG00000174996
ENSG00000104894	ENSG00000011105
ENSG00000104894	ENSG00000085117
ENSG00000104894	ENSG00000110900
ENSG00000104894	ENSG00000117472
ENSG00000104894	ENSG00000143119
ENSG00000104894	ENSG00000157570
ENSG00000104894	ENSG00000177697
ENSG00000104894	ENSG00000214063
ENSG00000104903	ENSG00000113196
ENSG00000104903	ENSG00000122691
ENSG00000104903	ENSG00000122859
ENSG00000104903	ENSG00000123307
ENSG00000104903	ENSG00000125878

ENSG00000104903	ENSG00000146618
ENSG00000104903	ENSG00000162367
ENSG00000104903	ENSG00000162992
ENSG00000104903	ENSG00000164107
ENSG00000104903	ENSG00000164600
ENSG00000104903	ENSG00000168267
ENSG00000104903	ENSG00000171532
ENSG00000104903	ENSG00000171786
ENSG00000104903	ENSG00000172238
ENSG00000104903	ENSG00000177551
ENSG00000104903	ENSG00000178403
ENSG00000104903	ENSG00000179774
ENSG00000104903	ENSG00000180535
ENSG00000104903	ENSG00000181965
ENSG00000104903	ENSG00000186051
ENSG00000104903	ENSG00000187786
ENSG00000104903	ENSG00000188686
ENSG00000104904	ENSG00000143450
ENSG00000104904	ENSG00000180304
ENSG00000104915	ENSG00000135823
ENSG00000104936	ENSG00000112079
ENSG00000104936	ENSG00000211455
ENSG00000104946	ENSG00000121749
ENSG00000104951	ENSG00000069535
ENSG00000104951	ENSG00000189221
ENSG00000104964	ENSG00000065717
ENSG00000104964	ENSG00000104953
ENSG00000104964	ENSG00000106829
ENSG00000104964	ENSG00000140332
ENSG00000104964	ENSG00000196781
ENSG00000104967	ENSG00000139910
ENSG00000104969	ENSG00000197860
ENSG00000104998	ENSG00000081985
ENSG00000104998	ENSG00000113594
ENSG00000104998	ENSG00000119535
ENSG00000104998	ENSG00000134352
ENSG00000104998	ENSG00000145623
ENSG00000104998	ENSG00000164509
ENSG00000105011	ENSG00000111875
ENSG00000105048	ENSG00000118194
ENSG00000105048	ENSG00000130595
ENSG00000105053	ENSG00000028116
ENSG00000105053	ENSG00000100749
ENSG00000105063	ENSG00000100239
ENSG00000105063	ENSG00000110075
ENSG00000105088	ENSG00000034971
ENSG00000105088	ENSG00000102837
ENSG00000105088	ENSG00000118733
ENSG00000105088	ENSG00000130558
ENSG00000105088	ENSG00000183801
ENSG00000105088	ENSG00000186417
ENSG00000105088	ENSG00000215081
ENSG00000105122	ENSG00000075391
ENSG00000105122	ENSG00000136848
ENSG00000105122	ENSG00000197283
ENSG00000105127	ENSG00000011243
ENSG00000105127	ENSG00000162664
ENSG00000105131	ENSG00000172031
ENSG00000105137	ENSG00000097096

ENSG00000105141	ENSG00000003400
ENSG00000105141	ENSG00000064012
ENSG00000105141	ENSG00000106144
ENSG00000105141	ENSG00000132906
ENSG00000105141	ENSG00000137752
ENSG00000105141	ENSG00000137757
ENSG00000105141	ENSG00000138794
ENSG00000105141	ENSG00000164305
ENSG00000105141	ENSG00000165806
ENSG00000105141	ENSG00000196954
ENSG00000105141	ENSG00000204403
ENSG00000105143	ENSG00000079215
ENSG00000105143	ENSG00000105281
ENSG00000105143	ENSG00000106688
ENSG00000105143	ENSG00000110436
ENSG00000105143	ENSG00000115902
ENSG00000105143	ENSG00000162383
ENSG00000105146	ENSG00000087586
ENSG00000105173	ENSG00000175305
ENSG00000105198	ENSG00000006659
ENSG00000105198	ENSG00000100079
ENSG00000105198	ENSG00000100097
ENSG00000105198	ENSG00000105205
ENSG00000105198	ENSG00000116977
ENSG00000105198	ENSG00000119862
ENSG00000105198	ENSG00000131981
ENSG00000105198	ENSG00000133317
ENSG00000105198	ENSG00000168961
ENSG00000105198	ENSG00000170298
ENSG00000105198	ENSG00000171747
ENSG00000105198	ENSG00000171916
ENSG00000105198	ENSG00000178934
ENSG00000105198	ENSG00000197778
ENSG00000105198	ENSG00000205076
ENSG00000105198	ENSG00000215132
ENSG00000105204	ENSG00000010219
ENSG00000105204	ENSG00000127334
ENSG00000105204	ENSG00000143479
ENSG00000105204	ENSG00000157540
ENSG00000105205	ENSG00000006659
ENSG00000105205	ENSG00000100079
ENSG00000105205	ENSG00000100097
ENSG00000105205	ENSG00000105198
ENSG00000105205	ENSG00000116977
ENSG00000105205	ENSG00000119862
ENSG00000105205	ENSG00000131981
ENSG00000105205	ENSG00000133317
ENSG00000105205	ENSG00000168961
ENSG00000105205	ENSG00000170298
ENSG00000105205	ENSG00000171747
ENSG00000105205	ENSG00000171916
ENSG00000105205	ENSG00000178934
ENSG00000105205	ENSG00000197778
ENSG00000105205	ENSG00000205076
ENSG00000105205	ENSG00000215132
ENSG00000105216	ENSG00000149657
ENSG00000105221	ENSG00000027075
ENSG00000105221	ENSG00000065675
ENSG00000105221	ENSG00000067606

ENSG00000105221	ENSG00000117020
ENSG00000105221	ENSG00000126583
ENSG00000105221	ENSG00000142208
ENSG00000105221	ENSG00000154229
ENSG00000105221	ENSG00000163558
ENSG00000105221	ENSG00000163932
ENSG00000105221	ENSG00000166501
ENSG00000105223	ENSG00000166428
ENSG00000105223	ENSG00000180287
ENSG00000105227	ENSG00000124942
ENSG00000105227	ENSG00000214427
ENSG00000105229	ENSG00000033800
ENSG00000105229	ENSG00000078043
ENSG00000105229	ENSG00000131788
ENSG00000105245	ENSG00000133961
ENSG00000105245	ENSG00000144366
ENSG00000105245	ENSG00000157978
ENSG00000105245	ENSG00000183114
ENSG00000105245	ENSG00000185112
ENSG00000105246	ENSG00000122756
ENSG00000105246	ENSG00000137070
ENSG00000105246	ENSG00000160712
ENSG00000105251	ENSG00000107338
ENSG00000105251	ENSG00000138606
ENSG00000105251	ENSG00000169291
ENSG00000105255	ENSG00000080561
ENSG00000105255	ENSG00000101871
ENSG00000105255	ENSG00000106701
ENSG00000105270	ENSG00000106665
ENSG00000105270	ENSG00000115295
ENSG00000105270	ENSG00000130779
ENSG00000105278	ENSG00000056097
ENSG00000105278	ENSG00000129351
ENSG00000105278	ENSG00000143621
ENSG00000105278	ENSG00000165209
ENSG00000105281	ENSG00000079215
ENSG00000105281	ENSG00000105143
ENSG00000105281	ENSG00000106688
ENSG00000105281	ENSG00000110436
ENSG00000105281	ENSG00000115902
ENSG00000105281	ENSG00000162383
ENSG00000105287	ENSG00000115825
ENSG00000105287	ENSG00000184304
ENSG00000105289	ENSG00000141527
ENSG00000105290	ENSG00000084234
ENSG00000105290	ENSG00000142192
ENSG00000105321	ENSG00000188549
ENSG00000105323	ENSG00000153187
ENSG00000105323	ENSG00000214753
ENSG00000105329	ENSG00000092969
ENSG00000105329	ENSG00000135414
ENSG00000105329	ENSG00000138379
ENSG00000105329	ENSG00000139269
ENSG00000105355	ENSG00000147872
ENSG00000105355	ENSG00000167676
ENSG00000105355	ENSG00000214456
ENSG00000105357	ENSG00000100345
ENSG00000105357	ENSG00000133026
ENSG00000105357	ENSG00000133392

ENSG00000105366	ENSG00000105383
ENSG00000105366	ENSG00000105492
ENSG00000105366	ENSG00000105501
ENSG00000105366	ENSG00000105695
ENSG00000105366	ENSG00000129450
ENSG00000105366	ENSG00000142512
ENSG00000105366	ENSG00000160296
ENSG00000105366	ENSG00000161640
ENSG00000105366	ENSG00000161643
ENSG00000105366	ENSG00000167764
ENSG00000105366	ENSG00000168995
ENSG00000105366	ENSG00000171101
ENSG00000105366	ENSG00000197865
ENSG00000105369	ENSG00000007312
ENSG00000105370	ENSG00000105374
ENSG00000105370	ENSG00000160318
ENSG00000105374	ENSG00000105370
ENSG00000105374	ENSG00000160318
ENSG00000105383	ENSG00000105366
ENSG00000105383	ENSG00000105492
ENSG00000105383	ENSG00000105501
ENSG00000105383	ENSG00000105695
ENSG00000105383	ENSG00000129450
ENSG00000105383	ENSG00000142512
ENSG00000105383	ENSG00000160296
ENSG00000105383	ENSG00000161640
ENSG00000105383	ENSG00000161643
ENSG00000105383	ENSG00000167764
ENSG00000105383	ENSG00000168995
ENSG00000105383	ENSG00000171101
ENSG00000105383	ENSG00000197865
ENSG00000105392	ENSG00000115507
ENSG00000105392	ENSG00000165588
ENSG00000105392	ENSG00000186103
ENSG00000105397	ENSG00000096968
ENSG00000105397	ENSG00000105639
ENSG00000105397	ENSG00000162434
ENSG00000105398	ENSG00000088002
ENSG00000105398	ENSG00000109193
ENSG00000105398	ENSG00000130540
ENSG00000105398	ENSG00000138068
ENSG00000105398	ENSG00000173597
ENSG00000105398	ENSG00000196228
ENSG00000105398	ENSG00000196502
ENSG00000105398	ENSG00000197165
ENSG00000105398	ENSG00000198075
ENSG00000105398	ENSG00000198203
ENSG00000105398	ENSG00000213599
ENSG00000105398	ENSG00000213648
ENSG00000105401	ENSG00000106993
ENSG00000105401	ENSG00000196347
ENSG00000105401	ENSG00000196585
ENSG00000105402	ENSG00000125814
ENSG00000105409	ENSG00000018625
ENSG00000105409	ENSG00000075673
ENSG00000105409	ENSG00000105675
ENSG00000105409	ENSG00000132681
ENSG00000105409	ENSG00000163399
ENSG00000105419	ENSG00000118707

ENSG00000105419	ENSG00000134138
ENSG00000105419	ENSG00000143995
ENSG00000105419	ENSG00000153779
ENSG00000105419	ENSG00000160199
ENSG00000105419	ENSG00000165495
ENSG00000105419	ENSG00000176679
ENSG00000105419	ENSG00000177426
ENSG00000105419	ENSG00000179277
ENSG00000105419	ENSG00000188013
ENSG00000105426	ENSG00000060656
ENSG00000105426	ENSG00000142949
ENSG00000105426	ENSG00000152894
ENSG00000105426	ENSG00000153707
ENSG00000105426	ENSG00000173482
ENSG00000105426	ENSG00000196090
ENSG00000105427	ENSG00000145287
ENSG00000105427	ENSG00000173261
ENSG00000105428	ENSG00000082996
ENSG00000105428	ENSG00000108523
ENSG00000105428	ENSG00000113269
ENSG00000105428	ENSG00000133135
ENSG00000105428	ENSG00000163162
ENSG00000105428	ENSG00000170153
ENSG00000105428	ENSG00000188050
ENSG00000105438	ENSG00000100196
ENSG00000105438	ENSG00000136240
ENSG00000105443	ENSG00000008256
ENSG00000105443	ENSG00000108669
ENSG00000105464	ENSG00000116032
ENSG00000105464	ENSG00000150086
ENSG00000105464	ENSG00000161509
ENSG00000105464	ENSG00000176884
ENSG00000105464	ENSG00000183454
ENSG00000105464	ENSG00000198785
ENSG00000105467	ENSG00000100321
ENSG00000105467	ENSG00000108639
ENSG00000105467	ENSG00000127561
ENSG00000105467	ENSG00000215315
ENSG00000105472	ENSG00000118004
ENSG00000105472	ENSG00000122852
ENSG00000105472	ENSG00000122854
ENSG00000105472	ENSG00000133661
ENSG00000105472	ENSG00000163815
ENSG00000105472	ENSG00000165471
ENSG00000105472	ENSG00000166509
ENSG00000105472	ENSG00000182314
ENSG00000105472	ENSG00000184374
ENSG00000105472	ENSG00000185303
ENSG00000105492	ENSG00000105366
ENSG00000105492	ENSG00000105383
ENSG00000105492	ENSG00000105501
ENSG00000105492	ENSG00000105695
ENSG00000105492	ENSG00000129450
ENSG00000105492	ENSG00000142512
ENSG00000105492	ENSG00000160296
ENSG00000105492	ENSG00000161640
ENSG00000105492	ENSG00000161643
ENSG00000105492	ENSG00000167764
ENSG00000105492	ENSG00000168995

ENSG00000105492	ENSG00000171101
ENSG00000105492	ENSG00000197865
ENSG00000105499	ENSG00000116711
ENSG00000105499	ENSG00000159337
ENSG00000105499	ENSG00000168907
ENSG00000105499	ENSG00000168970
ENSG00000105499	ENSG00000188089
ENSG00000105501	ENSG00000105366
ENSG00000105501	ENSG00000105383
ENSG00000105501	ENSG00000105492
ENSG00000105501	ENSG00000105695
ENSG00000105501	ENSG00000129450
ENSG00000105501	ENSG00000142512
ENSG00000105501	ENSG00000160296
ENSG00000105501	ENSG00000161640
ENSG00000105501	ENSG00000161643
ENSG00000105501	ENSG00000167764
ENSG00000105501	ENSG00000168995
ENSG00000105501	ENSG00000171101
ENSG00000105501	ENSG00000197865
ENSG00000105507	ENSG00000157782
ENSG00000105507	ENSG00000167791
ENSG00000105507	ENSG00000175544
ENSG00000105509	ENSG00000103044
ENSG00000105509	ENSG00000170961
ENSG00000105514	ENSG00000105649
ENSG00000105514	ENSG00000152932
ENSG00000105514	ENSG00000169213
ENSG00000105516	ENSG00000108924
ENSG00000105516	ENSG00000167074
ENSG00000105519	ENSG00000152611
ENSG00000105519	ENSG00000180881
ENSG00000105520	ENSG00000067113
ENSG00000105520	ENSG00000117598
ENSG00000105520	ENSG00000117600
ENSG00000105520	ENSG00000129951
ENSG00000105520	ENSG00000141934
ENSG00000105520	ENSG00000148123
ENSG00000105520	ENSG00000162407
ENSG00000105523	ENSG00000101447
ENSG00000105523	ENSG00000125998
ENSG00000105523	ENSG00000133477
ENSG00000105523	ENSG00000147689
ENSG00000105523	ENSG00000168143
ENSG00000105523	ENSG00000180921
ENSG00000105523	ENSG00000188522
ENSG00000105538	ENSG00000157927
ENSG00000105550	ENSG00000118972
ENSG00000105550	ENSG00000162344
ENSG00000105552	ENSG00000060982
ENSG00000105556	ENSG00000155545
ENSG00000105556	ENSG00000198160
ENSG00000105559	ENSG00000052126
ENSG00000105559	ENSG00000143850
ENSG00000105559	ENSG00000166689
ENSG00000105568	ENSG00000137713
ENSG00000105576	ENSG00000083312
ENSG00000105605	ENSG00000006116
ENSG00000105605	ENSG00000075429

ENSG00000105605	ENSG00000075461
ENSG00000105605	ENSG00000142408
ENSG00000105605	ENSG00000166862
ENSG00000105605	ENSG00000204278
ENSG00000105610	ENSG00000067082
ENSG00000105610	ENSG00000102554
ENSG00000105610	ENSG00000127528
ENSG00000105610	ENSG00000129911
ENSG00000105610	ENSG00000136826
ENSG00000105610	ENSG00000139651
ENSG00000105610	ENSG00000155090
ENSG00000105610	ENSG00000164651
ENSG00000105610	ENSG00000167182
ENSG00000105610	ENSG00000170374
ENSG00000105610	ENSG00000171872
ENSG00000105610	ENSG00000172059
ENSG00000105610	ENSG00000174595
ENSG00000105610	ENSG00000189120
ENSG00000105610	ENSG00000197294
ENSG00000105610	ENSG00000197550
ENSG00000105610	ENSG00000204335
ENSG00000105612	ENSG00000137976
ENSG00000105613	ENSG00000069020
ENSG00000105613	ENSG00000086015
ENSG00000105613	ENSG00000099308
ENSG00000105613	ENSG00000120539
ENSG00000105639	ENSG00000096968
ENSG00000105639	ENSG00000105397
ENSG00000105639	ENSG00000162434
ENSG00000105642	ENSG00000080709
ENSG00000105642	ENSG00000104783
ENSG00000105642	ENSG00000143603
ENSG00000105643	ENSG00000113369
ENSG00000105643	ENSG00000117289
ENSG00000105643	ENSG00000140450
ENSG00000105643	ENSG00000197070
ENSG00000105643	ENSG00000205784
ENSG00000105647	ENSG00000117461
ENSG00000105647	ENSG00000145675
ENSG00000105649	ENSG00000105514
ENSG00000105649	ENSG00000152932
ENSG00000105649	ENSG00000169213
ENSG00000105650	ENSG00000065989
ENSG00000105650	ENSG00000073417
ENSG00000105650	ENSG00000113231
ENSG00000105650	ENSG00000113448
ENSG00000105650	ENSG00000115252
ENSG00000105650	ENSG00000123360
ENSG00000105650	ENSG00000154678
ENSG00000105650	ENSG00000160191
ENSG00000105650	ENSG00000171408
ENSG00000105650	ENSG00000184588
ENSG00000105650	ENSG00000205268
ENSG00000105656	ENSG00000118985
ENSG00000105656	ENSG00000152939
ENSG00000105656	ENSG00000187213
ENSG00000105656	ENSG00000197822
ENSG00000105662	ENSG00000140577
ENSG00000105662	ENSG00000160741

ENSG00000105663	ENSG00000118058
ENSG00000105664	ENSG00000113296
ENSG00000105664	ENSG00000137801
ENSG00000105664	ENSG00000169231
ENSG00000105664	ENSG00000186340
ENSG00000105675	ENSG00000018625
ENSG00000105675	ENSG00000075673
ENSG00000105675	ENSG00000105409
ENSG00000105675	ENSG00000132681
ENSG00000105675	ENSG00000163399
ENSG00000105694	ENSG00000154582
ENSG00000105695	ENSG00000105366
ENSG00000105695	ENSG00000105383
ENSG00000105695	ENSG00000105492
ENSG00000105695	ENSG00000105501
ENSG00000105695	ENSG00000129450
ENSG00000105695	ENSG00000142512
ENSG00000105695	ENSG00000160296
ENSG00000105695	ENSG00000161640
ENSG00000105695	ENSG00000161643
ENSG00000105695	ENSG00000167764
ENSG00000105695	ENSG00000168995
ENSG00000105695	ENSG00000171101
ENSG00000105695	ENSG00000197865
ENSG00000105696	ENSG00000116209
ENSG00000105698	ENSG00000158773
ENSG00000105699	ENSG00000143195
ENSG00000105699	ENSG00000145103
ENSG00000105707	ENSG00000137648
ENSG00000105707	ENSG00000137747
ENSG00000105707	ENSG00000145244
ENSG00000105707	ENSG00000154646
ENSG00000105707	ENSG00000160183
ENSG00000105707	ENSG00000166682
ENSG00000105707	ENSG00000184012
ENSG00000105711	ENSG00000166257
ENSG00000105717	ENSG00000167081
ENSG00000105717	ENSG00000185630
ENSG00000105717	ENSG00000204304
ENSG00000105722	ENSG00000172018
ENSG00000105723	ENSG00000082701
ENSG00000105738	ENSG00000076864
ENSG00000105738	ENSG00000116991
ENSG00000105738	ENSG00000132359
ENSG00000105738	ENSG00000136895
ENSG00000105738	ENSG00000197555
ENSG00000105738	ENSG00000213445
ENSG00000105784	ENSG00000018189
ENSG00000105784	ENSG00000108309
ENSG00000105784	ENSG00000176783
ENSG00000105784	ENSG00000204130
ENSG00000105784	ENSG00000214795
ENSG00000105808	ENSG00000111344
ENSG00000105808	ENSG00000155903
ENSG00000105808	ENSG00000170667
ENSG00000105808	ENSG00000185989
ENSG00000105810	ENSG00000135446
ENSG00000105819	ENSG00000010256
ENSG00000105825	ENSG00000003436

ENSG00000105825	ENSG00000127578
ENSG00000105825	ENSG00000173714
ENSG00000105835	ENSG00000149962
ENSG00000105851	ENSG00000011405
ENSG00000105851	ENSG00000051382
ENSG00000105851	ENSG00000121879
ENSG00000105851	ENSG00000133056
ENSG00000105851	ENSG00000139144
ENSG00000105851	ENSG00000171608
ENSG00000105855	ENSG00000056345
ENSG00000105855	ENSG00000082781
ENSG00000105855	ENSG00000115221
ENSG00000105855	ENSG00000132470
ENSG00000105855	ENSG00000139626
ENSG00000105855	ENSG00000150093
ENSG00000105855	ENSG00000160255
ENSG00000105866	ENSG00000172845
ENSG00000105866	ENSG00000185591
ENSG00000105877	ENSG00000007174
ENSG00000105877	ENSG00000039139
ENSG00000105877	ENSG00000114841
ENSG00000105877	ENSG00000118997
ENSG00000105877	ENSG00000124721
ENSG00000105877	ENSG00000158486
ENSG00000105877	ENSG00000183914
ENSG00000105877	ENSG00000197653
ENSG00000105880	ENSG00000064195
ENSG00000105880	ENSG00000115844
ENSG00000105889	ENSG00000115107
ENSG00000105889	ENSG00000127954
ENSG00000105889	ENSG00000157214
ENSG00000105889	ENSG00000164647
ENSG00000105894	ENSG00000110492
ENSG00000105926	ENSG00000072415
ENSG00000105926	ENSG00000082126
ENSG00000105926	ENSG00000108852
ENSG00000105926	ENSG00000130830
ENSG00000105926	ENSG00000147044
ENSG00000105926	ENSG00000150054
ENSG00000105926	ENSG00000161647
ENSG00000105928	ENSG00000204311
ENSG00000105929	ENSG00000033627
ENSG00000105929	ENSG00000110719
ENSG00000105929	ENSG00000185344
ENSG00000105939	ENSG00000059378
ENSG00000105939	ENSG00000111224
ENSG00000105939	ENSG00000146858
ENSG00000105953	ENSG00000197444
ENSG00000105963	ENSG00000184060
ENSG00000105967	ENSG00000068323
ENSG00000105967	ENSG00000112561
ENSG00000105967	ENSG00000187098
ENSG00000105968	ENSG00000164032
ENSG00000105971	ENSG00000105974
ENSG00000105971	ENSG00000182533
ENSG00000105974	ENSG00000105971
ENSG00000105974	ENSG00000182533
ENSG00000105976	ENSG00000092445
ENSG00000105976	ENSG00000153208

ENSG00000105976	ENSG00000163785
ENSG00000105976	ENSG00000164078
ENSG00000105976	ENSG00000167601
ENSG00000105976	ENSG00000214672
ENSG00000105983	ENSG00000139636
ENSG00000105989	ENSG00000002745
ENSG00000105989	ENSG00000061492
ENSG00000105989	ENSG00000075290
ENSG00000105989	ENSG00000085741
ENSG00000105989	ENSG00000108379
ENSG00000105989	ENSG00000111186
ENSG00000105989	ENSG00000114251
ENSG00000105989	ENSG00000115596
ENSG00000105989	ENSG00000125084
ENSG00000105989	ENSG00000134245
ENSG00000105989	ENSG00000135925
ENSG00000105989	ENSG00000143816
ENSG00000105989	ENSG00000154342
ENSG00000105989	ENSG00000154764
ENSG00000105989	ENSG00000158955
ENSG00000105989	ENSG00000162552
ENSG00000105989	ENSG00000169884
ENSG00000105989	ENSG00000188064
ENSG00000105991	ENSG00000105996
ENSG00000105991	ENSG00000120094
ENSG00000105991	ENSG00000128645
ENSG00000105991	ENSG00000173917
ENSG00000105993	ENSG00000135924
ENSG00000105993	ENSG00000172404
ENSG00000105993	ENSG00000179407
ENSG00000105996	ENSG00000105991
ENSG00000105996	ENSG00000120094
ENSG00000105996	ENSG00000128645
ENSG00000105996	ENSG00000173917
ENSG00000105997	ENSG00000106038
ENSG00000105997	ENSG00000120093
ENSG00000105997	ENSG00000128652
ENSG00000105997	ENSG00000169840
ENSG00000105997	ENSG00000174279
ENSG00000105997	ENSG00000180613
ENSG00000106003	ENSG00000100060
ENSG00000106003	ENSG00000143891
ENSG00000106003	ENSG00000169733
ENSG00000106004	ENSG00000037965
ENSG00000106004	ENSG00000106006
ENSG00000106004	ENSG00000108511
ENSG00000106004	ENSG00000120068
ENSG00000106004	ENSG00000120075
ENSG00000106004	ENSG00000120087
ENSG00000106004	ENSG00000122592
ENSG00000106004	ENSG00000170166
ENSG00000106004	ENSG00000172789
ENSG00000106004	ENSG00000175879
ENSG00000106004	ENSG00000182742
ENSG00000106004	ENSG00000197576
ENSG00000106004	ENSG00000197757
ENSG00000106004	ENSG00000198353
ENSG00000106006	ENSG00000037965
ENSG00000106006	ENSG00000106004

ENSG00000106006	ENSG00000108511
ENSG00000106006	ENSG00000120068
ENSG00000106006	ENSG00000120075
ENSG00000106006	ENSG00000120087
ENSG00000106006	ENSG00000122592
ENSG00000106006	ENSG00000170166
ENSG00000106006	ENSG00000172789
ENSG00000106006	ENSG00000175879
ENSG00000106006	ENSG00000182742
ENSG00000106006	ENSG00000197576
ENSG00000106006	ENSG00000197757
ENSG00000106006	ENSG00000198353
ENSG00000106018	ENSG00000004948
ENSG00000106018	ENSG00000010310
ENSG00000106018	ENSG00000064989
ENSG00000106018	ENSG00000065325
ENSG00000106018	ENSG00000078549
ENSG00000106018	ENSG00000080293
ENSG00000106018	ENSG00000106113
ENSG00000106018	ENSG00000106128
ENSG00000106018	ENSG00000112164
ENSG00000106018	ENSG00000114812
ENSG00000106018	ENSG00000120088
ENSG00000106018	ENSG00000144407
ENSG00000106018	ENSG00000160801
ENSG00000106018	ENSG00000215644
ENSG00000106031	ENSG00000123364
ENSG00000106031	ENSG00000128714
ENSG00000106031	ENSG00000159184
ENSG00000106038	ENSG00000105997
ENSG00000106038	ENSG00000120093
ENSG00000106038	ENSG00000128652
ENSG00000106038	ENSG00000169840
ENSG00000106038	ENSG00000174279
ENSG00000106038	ENSG00000180613
ENSG00000106052	ENSG00000012822
ENSG00000106052	ENSG00000136436
ENSG00000106070	ENSG00000077420
ENSG00000106070	ENSG00000115290
ENSG00000106070	ENSG00000141738
ENSG00000106070	ENSG00000173166
ENSG00000106078	ENSG00000082438
ENSG00000106080	ENSG00000079150
ENSG00000106080	ENSG00000122642
ENSG00000106080	ENSG00000134285
ENSG00000106080	ENSG00000141756
ENSG00000106080	ENSG00000176826
ENSG00000106089	ENSG00000099365
ENSG00000106089	ENSG00000103496
ENSG00000106089	ENSG00000111450
ENSG00000106089	ENSG00000135604
ENSG00000106089	ENSG00000166900
ENSG00000106089	ENSG00000178750
ENSG00000106100	ENSG00000179583
ENSG00000106113	ENSG00000004948
ENSG00000106113	ENSG00000010310
ENSG00000106113	ENSG00000064989
ENSG00000106113	ENSG00000065325
ENSG00000106113	ENSG00000078549

ENSG00000106113	ENSG00000080293
ENSG00000106113	ENSG00000106018
ENSG00000106113	ENSG00000106128
ENSG00000106113	ENSG00000112164
ENSG00000106113	ENSG00000114812
ENSG00000106113	ENSG00000120088
ENSG00000106113	ENSG00000144407
ENSG00000106113	ENSG00000160801
ENSG00000106113	ENSG00000215644
ENSG00000106123	ENSG00000044524
ENSG00000106123	ENSG00000070886
ENSG00000106123	ENSG00000080224
ENSG00000106123	ENSG00000116106
ENSG00000106123	ENSG00000133216
ENSG00000106123	ENSG00000135333
ENSG00000106123	ENSG00000142627
ENSG00000106123	ENSG00000145242
ENSG00000106123	ENSG00000146904
ENSG00000106123	ENSG00000154928
ENSG00000106123	ENSG00000182580
ENSG00000106123	ENSG00000183317
ENSG00000106123	ENSG00000196411
ENSG00000106125	ENSG00000086159
ENSG00000106125	ENSG00000103375
ENSG00000106125	ENSG00000135517
ENSG00000106125	ENSG00000161798
ENSG00000106125	ENSG00000167580
ENSG00000106125	ENSG00000171885
ENSG00000106128	ENSG00000004948
ENSG00000106128	ENSG00000010310
ENSG00000106128	ENSG00000064989
ENSG00000106128	ENSG00000065325
ENSG00000106128	ENSG00000078549
ENSG00000106128	ENSG00000080293
ENSG00000106128	ENSG00000106018
ENSG00000106128	ENSG00000106113
ENSG00000106128	ENSG00000112164
ENSG00000106128	ENSG00000114812
ENSG00000106128	ENSG00000120088
ENSG00000106128	ENSG00000144407
ENSG00000106128	ENSG00000160801
ENSG00000106128	ENSG00000215644
ENSG00000106153	ENSG00000138869
ENSG00000106153	ENSG00000182699
ENSG00000106153	ENSG00000186940
ENSG00000106153	ENSG00000203682
ENSG00000106178	ENSG00000006074
ENSG00000106178	ENSG00000006075
ENSG00000106178	ENSG00000006606
ENSG00000106178	ENSG00000102962
ENSG00000106178	ENSG00000102970
ENSG00000106178	ENSG00000108688
ENSG00000106178	ENSG00000108691
ENSG00000106178	ENSG00000108700
ENSG00000106178	ENSG00000108702
ENSG00000106178	ENSG00000115009
ENSG00000106178	ENSG00000129277
ENSG00000106178	ENSG00000143184
ENSG00000106178	ENSG00000143185

ENSG00000106178	ENSG00000161570
ENSG00000106178	ENSG00000161573
ENSG00000106178	ENSG00000161574
ENSG00000106178	ENSG00000167236
ENSG00000106178	ENSG00000172156
ENSG00000106178	ENSG00000172724
ENSG00000106178	ENSG00000181374
ENSG00000106178	ENSG00000197262
ENSG00000106178	ENSG00000205017
ENSG00000106178	ENSG00000205020
ENSG00000106178	ENSG00000205021
ENSG00000106178	ENSG00000213494
ENSG00000106178	ENSG00000215627
ENSG00000106178	ENSG00000215629
ENSG00000106211	ENSG00000004776
ENSG00000106211	ENSG00000109846
ENSG00000106211	ENSG00000152137
ENSG00000106211	ENSG00000160202
ENSG00000106211	ENSG00000169271
ENSG00000106211	ENSG00000170276
ENSG00000106236	ENSG00000132693
ENSG00000106236	ENSG00000132703
ENSG00000106236	ENSG00000163661
ENSG00000106236	ENSG00000167957
ENSG00000106236	ENSG00000171246
ENSG00000106236	ENSG00000215846
ENSG00000106258	ENSG00000021461
ENSG00000106258	ENSG00000059377
ENSG00000106258	ENSG00000160868
ENSG00000106258	ENSG00000160870
ENSG00000106278	ENSG00000144724
ENSG00000106299	ENSG00000015285
ENSG00000106302	ENSG00000068001
ENSG00000106302	ENSG00000106304
ENSG00000106302	ENSG00000114378
ENSG00000106302	ENSG00000186792
ENSG00000106304	ENSG00000068001
ENSG00000106304	ENSG00000106302
ENSG00000106304	ENSG00000114378
ENSG00000106304	ENSG00000186792
ENSG00000106327	ENSG00000072274
ENSG00000106327	ENSG00000077616
ENSG00000106327	ENSG00000086205
ENSG00000106327	ENSG00000134612
ENSG00000106327	ENSG00000168060
ENSG00000106327	ENSG00000177694
ENSG00000106328	ENSG00000075618
ENSG00000106328	ENSG00000186765
ENSG00000106330	ENSG00000101928
ENSG00000106331	ENSG00000007372
ENSG00000106333	ENSG00000038295
ENSG00000106333	ENSG00000095587
ENSG00000106333	ENSG00000107611
ENSG00000106333	ENSG00000157211
ENSG00000106333	ENSG00000163710
ENSG00000106333	ENSG00000166342
ENSG00000106333	ENSG00000168487
ENSG00000106333	ENSG00000171208
ENSG00000106333	ENSG00000184824

ENSG00000106348	ENSG00000100938
ENSG00000106348	ENSG00000137198
ENSG00000106348	ENSG00000178035
ENSG00000106351	ENSG00000173744
ENSG00000106366	ENSG00000135919
ENSG00000106366	ENSG00000163536
ENSG00000106366	ENSG00000215461
ENSG00000106367	ENSG00000152056
ENSG00000106367	ENSG00000182287
ENSG00000106384	ENSG00000062282
ENSG00000106384	ENSG00000124003
ENSG00000106384	ENSG00000147160
ENSG00000106384	ENSG00000166391
ENSG00000106384	ENSG00000184210
ENSG00000106384	ENSG00000204195
ENSG00000106384	ENSG00000205267
ENSG00000106392	ENSG00000162887
ENSG00000106392	ENSG00000171155
ENSG00000106397	ENSG00000083444
ENSG00000106397	ENSG00000130309
ENSG00000106397	ENSG00000152952
ENSG00000106397	ENSG00000167123
ENSG00000106397	ENSG00000198756
ENSG00000106404	ENSG00000013297
ENSG00000106404	ENSG00000066405
ENSG00000106404	ENSG00000113946
ENSG00000106404	ENSG00000134873
ENSG00000106404	ENSG00000156282
ENSG00000106404	ENSG00000156284
ENSG00000106404	ENSG00000159261
ENSG00000106404	ENSG00000163347
ENSG00000106404	ENSG00000164007
ENSG00000106404	ENSG00000165215
ENSG00000106404	ENSG00000165376
ENSG00000106404	ENSG00000171217
ENSG00000106404	ENSG00000177300
ENSG00000106404	ENSG00000181885
ENSG00000106404	ENSG00000184113
ENSG00000106404	ENSG00000184697
ENSG00000106404	ENSG00000185758
ENSG00000106404	ENSG00000189118
ENSG00000106404	ENSG00000189143
ENSG00000106404	ENSG00000213937
ENSG00000106415	ENSG00000121104
ENSG00000106415	ENSG00000138439
ENSG00000106436	ENSG00000101335
ENSG00000106436	ENSG00000101608
ENSG00000106436	ENSG00000106631
ENSG00000106436	ENSG00000111245
ENSG00000106436	ENSG00000118680
ENSG00000106436	ENSG00000180209
ENSG00000106436	ENSG00000215375
ENSG00000106460	ENSG00000134291
ENSG00000106460	ENSG00000184988
ENSG00000106460	ENSG00000215684
ENSG00000106462	ENSG00000108799
ENSG00000106483	ENSG00000104290
ENSG00000106483	ENSG00000111432
ENSG00000106483	ENSG00000155760

ENSG00000106483	ENSG00000157240
ENSG00000106483	ENSG00000162998
ENSG00000106483	ENSG00000163251
ENSG00000106483	ENSG00000164930
ENSG00000106483	ENSG00000174804
ENSG00000106483	ENSG00000177283
ENSG00000106483	ENSG00000180340
ENSG00000106483	ENSG00000188763
ENSG00000106526	ENSG00000115091
ENSG00000106526	ENSG00000133627
ENSG00000106536	ENSG00000184271
ENSG00000106537	ENSG00000135452
ENSG00000106541	ENSG00000117862
ENSG00000106541	ENSG00000173467
ENSG00000106546	ENSG00000063438
ENSG00000106554	ENSG00000159685
ENSG00000106560	ENSG00000133561
ENSG00000106560	ENSG00000133574
ENSG00000106560	ENSG00000171115
ENSG00000106560	ENSG00000179144
ENSG00000106560	ENSG00000196329
ENSG00000106560	ENSG00000213203
ENSG00000106571	ENSG00000074047
ENSG00000106571	ENSG00000107249
ENSG00000106571	ENSG00000111087
ENSG00000106571	ENSG00000126603
ENSG00000106571	ENSG00000174332
ENSG00000106608	ENSG00000132357
ENSG00000106608	ENSG00000187878
ENSG00000106608	ENSG00000205365
ENSG00000106615	ENSG00000167550
ENSG00000106617	ENSG00000115592
ENSG00000106617	ENSG00000181929
ENSG00000106624	ENSG00000088882
ENSG00000106624	ENSG00000108582
ENSG00000106624	ENSG00000109472
ENSG00000106624	ENSG00000109625
ENSG00000106624	ENSG00000120054
ENSG00000106624	ENSG00000121898
ENSG00000106624	ENSG00000135678
ENSG00000106631	ENSG00000101335
ENSG00000106631	ENSG00000101608
ENSG00000106631	ENSG00000106436
ENSG00000106631	ENSG00000111245
ENSG00000106631	ENSG00000118680
ENSG00000106631	ENSG00000180209
ENSG00000106631	ENSG00000215375
ENSG00000106633	ENSG00000156510
ENSG00000106633	ENSG00000156515
ENSG00000106633	ENSG00000159399
ENSG00000106633	ENSG00000160883
ENSG00000106635	ENSG00000099385
ENSG00000106635	ENSG00000110987
ENSG00000106648	ENSG00000100626
ENSG00000106648	ENSG00000110328
ENSG00000106648	ENSG00000115339
ENSG00000106648	ENSG00000131386
ENSG00000106648	ENSG00000136542
ENSG00000106648	ENSG00000139629

ENSG00000106648	ENSG00000141429
ENSG00000106648	ENSG00000143641
ENSG00000106648	ENSG00000144278
ENSG00000106648	ENSG00000158089
ENSG00000106648	ENSG00000164574
ENSG00000106648	ENSG00000174473
ENSG00000106648	ENSG00000178234
ENSG00000106648	ENSG00000182870
ENSG00000106648	ENSG00000185274
ENSG00000106665	ENSG00000105270
ENSG00000106665	ENSG00000115295
ENSG00000106665	ENSG00000130779
ENSG00000106683	ENSG00000070759
ENSG00000106683	ENSG00000107140
ENSG00000106683	ENSG00000182541
ENSG00000106688	ENSG00000079215
ENSG00000106688	ENSG00000105143
ENSG00000106688	ENSG00000105281
ENSG00000106688	ENSG00000110436
ENSG00000106688	ENSG00000115902
ENSG00000106688	ENSG00000162383
ENSG00000106689	ENSG00000016082
ENSG00000106689	ENSG00000089116
ENSG00000106689	ENSG00000106852
ENSG00000106689	ENSG00000107187
ENSG00000106689	ENSG00000121454
ENSG00000106689	ENSG00000132130
ENSG00000106689	ENSG00000136944
ENSG00000106689	ENSG00000143013
ENSG00000106689	ENSG00000143355
ENSG00000106689	ENSG00000159556
ENSG00000106689	ENSG00000162624
ENSG00000106689	ENSG00000162761
ENSG00000106701	ENSG00000080561
ENSG00000106701	ENSG00000101871
ENSG00000106701	ENSG00000105255
ENSG00000106714	ENSG00000108797
ENSG00000106714	ENSG00000152910
ENSG00000106714	ENSG00000154529
ENSG00000106714	ENSG00000155052
ENSG00000106714	ENSG00000174469
ENSG00000106714	ENSG00000185020
ENSG00000106723	ENSG00000147059
ENSG00000106723	ENSG00000186767
ENSG00000106723	ENSG00000186787
ENSG00000106723	ENSG00000204271
ENSG00000106733	ENSG00000077009
ENSG00000106733	ENSG00000139597
ENSG00000106772	ENSG00000140299
ENSG00000106772	ENSG00000163141
ENSG00000106772	ENSG00000167654
ENSG00000106772	ENSG00000175220
ENSG00000106772	ENSG00000196505
ENSG00000106780	ENSG00000050555
ENSG00000106780	ENSG00000058085
ENSG00000106780	ENSG00000074527
ENSG00000106780	ENSG00000091128
ENSG00000106780	ENSG00000091136
ENSG00000106780	ENSG00000135862

ENSG00000106780	ENSG00000172037
ENSG00000106780	ENSG00000196878
ENSG00000106789	ENSG00000102879
ENSG00000106789	ENSG00000103426
ENSG00000106789	ENSG00000103647
ENSG00000106789	ENSG00000110880
ENSG00000106789	ENSG00000167549
ENSG00000106789	ENSG00000172725
ENSG00000106799	ENSG00000107779
ENSG00000106799	ENSG00000115170
ENSG00000106799	ENSG00000123612
ENSG00000106799	ENSG00000135409
ENSG00000106799	ENSG00000135503
ENSG00000106799	ENSG00000138696
ENSG00000106799	ENSG00000139567
ENSG00000106799	ENSG00000163513
ENSG00000106799	ENSG00000204217
ENSG00000106804	ENSG00000125730
ENSG00000106804	ENSG00000126838
ENSG00000106804	ENSG00000156535
ENSG00000106804	ENSG00000160111
ENSG00000106804	ENSG00000166535
ENSG00000106804	ENSG00000175899
ENSG00000106804	ENSG00000177359
ENSG00000106804	ENSG00000204319
ENSG00000106804	ENSG00000204342
ENSG00000106804	ENSG00000214776
ENSG00000106809	ENSG00000083782
ENSG00000106809	ENSG00000188770
ENSG00000106819	ENSG00000011465
ENSG00000106819	ENSG00000139329
ENSG00000106819	ENSG00000182492
ENSG00000106829	ENSG00000065717
ENSG00000106829	ENSG00000104953
ENSG00000106829	ENSG00000104964
ENSG00000106829	ENSG00000140332
ENSG00000106829	ENSG00000196781
ENSG00000106852	ENSG00000016082
ENSG00000106852	ENSG00000089116
ENSG00000106852	ENSG00000106689
ENSG00000106852	ENSG00000107187
ENSG00000106852	ENSG00000121454
ENSG00000106852	ENSG00000132130
ENSG00000106852	ENSG00000136944
ENSG00000106852	ENSG00000143013
ENSG00000106852	ENSG00000143355
ENSG00000106852	ENSG00000159556
ENSG00000106852	ENSG00000162624
ENSG00000106852	ENSG00000162761
ENSG00000106976	ENSG00000079805
ENSG00000106976	ENSG00000197959
ENSG00000106991	ENSG00000069702
ENSG00000106992	ENSG00000154027
ENSG00000106992	ENSG00000214347
ENSG00000106993	ENSG00000105401
ENSG00000106993	ENSG00000196347
ENSG00000106993	ENSG00000196585
ENSG00000107014	ENSG00000107018
ENSG00000107014	ENSG00000120211

ENSG00000107014	ENSG00000171136
ENSG00000107014	ENSG00000172410
ENSG00000107018	ENSG00000107014
ENSG00000107018	ENSG00000120211
ENSG00000107018	ENSG00000171136
ENSG00000107018	ENSG00000172410
ENSG00000107077	ENSG00000066135
ENSG00000107077	ENSG00000127663
ENSG00000107077	ENSG00000186280
ENSG00000107099	ENSG00000088387
ENSG00000107099	ENSG00000116641
ENSG00000107099	ENSG00000130158
ENSG00000107099	ENSG00000135905
ENSG00000107099	ENSG00000147251
ENSG00000107104	ENSG00000132854
ENSG00000107104	ENSG00000186994
ENSG00000107104	ENSG00000197256
ENSG00000107105	ENSG00000066044
ENSG00000107105	ENSG00000162374
ENSG00000107105	ENSG00000196361
ENSG00000107140	ENSG00000070759
ENSG00000107140	ENSG00000106683
ENSG00000107140	ENSG00000182541
ENSG00000107147	ENSG00000156113
ENSG00000107147	ENSG00000162687
ENSG00000107147	ENSG00000215262
ENSG00000107159	ENSG00000074410
ENSG00000107159	ENSG00000104267
ENSG00000107159	ENSG00000118298
ENSG00000107159	ENSG00000131686
ENSG00000107159	ENSG00000133742
ENSG00000107159	ENSG00000164879
ENSG00000107159	ENSG00000167434
ENSG00000107159	ENSG00000168748
ENSG00000107159	ENSG00000169239
ENSG00000107159	ENSG00000174990
ENSG00000107159	ENSG00000178538
ENSG00000107159	ENSG00000185015
ENSG00000107159	ENSG00000214729
ENSG00000107159	ENSG00000214873
ENSG00000107159	ENSG00000215487
ENSG00000107159	ENSG00000215501
ENSG00000107159	ENSG00000215543
ENSG00000107164	ENSG00000088247
ENSG00000107164	ENSG00000162613
ENSG00000107164	ENSG00000215224
ENSG00000107165	ENSG00000077498
ENSG00000107165	ENSG00000080166
ENSG00000107175	ENSG00000060566
ENSG00000107175	ENSG00000118217
ENSG00000107175	ENSG00000143578
ENSG00000107175	ENSG00000157613
ENSG00000107175	ENSG00000182158
ENSG00000107175	ENSG00000213676
ENSG00000107186	ENSG00000072201
ENSG00000107186	ENSG00000132849
ENSG00000107186	ENSG00000139517
ENSG00000107187	ENSG00000016082
ENSG00000107187	ENSG00000089116

ENSG00000107187	ENSG00000106689
ENSG00000107187	ENSG00000106852
ENSG00000107187	ENSG00000121454
ENSG00000107187	ENSG00000132130
ENSG00000107187	ENSG00000136944
ENSG00000107187	ENSG00000143013
ENSG00000107187	ENSG00000143355
ENSG00000107187	ENSG00000159556
ENSG00000107187	ENSG00000162624
ENSG00000107187	ENSG00000162761
ENSG00000107201	ENSG00000100697
ENSG00000107201	ENSG00000108771
ENSG00000107201	ENSG00000115267
ENSG00000107242	ENSG00000143398
ENSG00000107242	ENSG00000173780
ENSG00000107242	ENSG00000186111
ENSG00000107249	ENSG00000074047
ENSG00000107249	ENSG00000106571
ENSG00000107249	ENSG00000111087
ENSG00000107249	ENSG00000126603
ENSG00000107249	ENSG00000174332
ENSG00000107263	ENSG00000058335
ENSG00000107263	ENSG00000100485
ENSG00000107263	ENSG00000113319
ENSG00000107263	ENSG00000115904
ENSG00000107282	ENSG00000011132
ENSG00000107282	ENSG00000034053
ENSG00000107295	ENSG00000097033
ENSG00000107295	ENSG00000140600
ENSG00000107295	ENSG00000141985
ENSG00000107295	ENSG00000148341
ENSG00000107317	ENSG00000148346
ENSG00000107317	ENSG00000176919
ENSG00000107317	ENSG00000177984
ENSG00000107317	ENSG00000184925
ENSG00000107317	ENSG00000187922
ENSG00000107317	ENSG00000214402
ENSG00000107331	ENSG00000064687
ENSG00000107331	ENSG00000144452
ENSG00000107331	ENSG00000165029
ENSG00000107331	ENSG00000167972
ENSG00000107331	ENSG00000179869
ENSG00000107331	ENSG00000198691
ENSG00000107338	ENSG00000105251
ENSG00000107338	ENSG00000138606
ENSG00000107338	ENSG00000169291
ENSG00000107341	ENSG00000099804
ENSG00000107362	ENSG00000129968
ENSG00000107362	ENSG00000136379
ENSG00000107362	ENSG00000182502
ENSG00000107362	ENSG00000182556
ENSG00000107362	ENSG00000198658
ENSG00000107362	ENSG00000203835
ENSG00000107372	ENSG00000086666
ENSG00000107404	ENSG00000004975
ENSG00000107404	ENSG00000150764
ENSG00000107404	ENSG00000161202
ENSG00000107404	ENSG00000215737
ENSG00000107438	ENSG00000120913

ENSG00000107438	ENSG00000122367
ENSG00000107438	ENSG00000131435
ENSG00000107438	ENSG00000154553
ENSG00000107438	ENSG00000163110
ENSG00000107438	ENSG00000196923
ENSG00000107438	ENSG00000214895
ENSG00000107443	ENSG00000135083
ENSG00000107447	ENSG00000122678
ENSG00000107485	ENSG00000102145
ENSG00000107485	ENSG00000130700
ENSG00000107485	ENSG00000136574
ENSG00000107485	ENSG00000141448
ENSG00000107485	ENSG00000179348
ENSG00000107518	ENSG00000088812
ENSG00000107518	ENSG00000105429
ENSG00000107551	ENSG00000068028
ENSG00000107551	ENSG00000101265
ENSG00000107551	ENSG00000136653
ENSG00000107551	ENSG00000153179
ENSG00000107551	ENSG00000169435
ENSG00000107554	ENSG00000183111
ENSG00000107560	ENSG00000135631
ENSG00000107560	ENSG00000156675
ENSG00000107566	ENSG00000147475
ENSG00000107611	ENSG00000038295
ENSG00000107611	ENSG00000095587
ENSG00000107611	ENSG00000106333
ENSG00000107611	ENSG00000157211
ENSG00000107611	ENSG00000163710
ENSG00000107611	ENSG00000166342
ENSG00000107611	ENSG00000168487
ENSG00000107611	ENSG00000171208
ENSG00000107611	ENSG00000184824
ENSG00000107623	ENSG00000101144
ENSG00000107623	ENSG00000112175
ENSG00000107623	ENSG00000116985
ENSG00000107623	ENSG00000125965
ENSG00000107623	ENSG00000128802
ENSG00000107623	ENSG00000130385
ENSG00000107623	ENSG00000143869
ENSG00000107623	ENSG00000152785
ENSG00000107623	ENSG00000153162
ENSG00000107623	ENSG00000156466
ENSG00000107623	ENSG00000156574
ENSG00000107623	ENSG00000163217
ENSG00000107623	ENSG00000183682
ENSG00000107643	ENSG00000050748
ENSG00000107643	ENSG00000109339
ENSG00000107643	ENSG00000112062
ENSG00000107643	ENSG00000156711
ENSG00000107643	ENSG00000185386
ENSG00000107643	ENSG00000188130
ENSG00000107651	ENSG00000085788
ENSG00000107651	ENSG00000100523
ENSG00000107679	ENSG00000169499
ENSG00000107731	ENSG00000113763
ENSG00000107731	ENSG00000124602
ENSG00000107731	ENSG00000156687
ENSG00000107731	ENSG00000182168

ENSG00000107742	ENSG00000019582
ENSG00000107742	ENSG00000152377
ENSG00000107742	ENSG00000196104
ENSG00000107758	ENSG00000120910
ENSG00000107758	ENSG00000138814
ENSG00000107771	ENSG00000184305
ENSG00000107779	ENSG00000106799
ENSG00000107779	ENSG00000115170
ENSG00000107779	ENSG00000123612
ENSG00000107779	ENSG00000135409
ENSG00000107779	ENSG00000135503
ENSG00000107779	ENSG00000138696
ENSG00000107779	ENSG00000139567
ENSG00000107779	ENSG00000163513
ENSG00000107779	ENSG00000204217
ENSG00000107796	ENSG00000075624
ENSG00000107796	ENSG00000143632
ENSG00000107796	ENSG00000159251
ENSG00000107796	ENSG00000163017
ENSG00000107796	ENSG00000169067
ENSG00000107796	ENSG00000184009
ENSG00000107796	ENSG00000204434
ENSG00000107796	ENSG00000214555
ENSG00000107796	ENSG00000215141
ENSG00000107807	ENSG00000115297
ENSG00000107807	ENSG00000164438
ENSG00000107816	ENSG00000061337
ENSG00000107816	ENSG00000088899
ENSG00000107816	ENSG00000145911
ENSG00000107819	ENSG00000144040
ENSG00000107819	ENSG00000156398
ENSG00000107819	ENSG00000164466
ENSG00000107819	ENSG00000183605
ENSG00000107821	ENSG00000137142
ENSG00000107821	ENSG00000163453
ENSG00000107831	ENSG00000156427
ENSG00000107831	ENSG00000158815
ENSG00000107833	ENSG00000158806
ENSG00000107833	ENSG00000181163
ENSG00000107833	ENSG00000182965
ENSG00000107833	ENSG00000214032
ENSG00000107833	ENSG00000214246
ENSG00000107833	ENSG00000214774
ENSG00000107833	ENSG00000214884
ENSG00000107833	ENSG00000215280
ENSG00000107854	ENSG00000104064
ENSG00000107854	ENSG00000143458
ENSG00000107854	ENSG00000173273
ENSG00000107859	ENSG00000069011
ENSG00000107859	ENSG00000164093
ENSG00000107863	ENSG00000075884
ENSG00000107863	ENSG00000123329
ENSG00000107863	ENSG00000165322
ENSG00000107863	ENSG00000167433
ENSG00000107863	ENSG00000185602
ENSG00000107863	ENSG00000214627
ENSG00000107864	ENSG00000113742
ENSG00000107864	ENSG00000137449
ENSG00000107864	ENSG00000214575

ENSG00000107897	ENSG00000181513
ENSG00000107929	ENSG00000161813
ENSG00000107954	ENSG00000124257
ENSG00000107954	ENSG00000214357
ENSG00000107954	ENSG00000215041
ENSG00000107957	ENSG00000158517
ENSG00000107957	ENSG00000165178
ENSG00000107957	ENSG00000174705
ENSG00000107957	ENSG00000182487
ENSG00000107957	ENSG00000196408
ENSG00000107984	ENSG00000104371
ENSG00000107984	ENSG00000155011
ENSG00000108001	ENSG00000088881
ENSG00000108001	ENSG00000134025
ENSG00000108001	ENSG00000164330
ENSG00000108018	ENSG00000134243
ENSG00000108018	ENSG00000137642
ENSG00000108018	ENSG00000156395
ENSG00000108018	ENSG00000184985
ENSG00000108100	ENSG00000163249
ENSG00000108100	ENSG00000179296
ENSG00000108100	ENSG00000182632
ENSG00000108100	ENSG00000184766
ENSG00000108100	ENSG00000204150
ENSG00000108100	ENSG00000215169
ENSG00000108175	ENSG00000122515
ENSG00000108179	ENSG00000150276
ENSG00000108179	ENSG00000196262
ENSG00000108179	ENSG00000198161
ENSG00000108179	ENSG00000198360
ENSG00000108179	ENSG00000198618
ENSG00000108179	ENSG00000198936
ENSG00000108179	ENSG00000203847
ENSG00000108179	ENSG00000214975
ENSG00000108179	ENSG00000215551
ENSG00000108219	ENSG00000048140
ENSG00000108219	ENSG00000099282
ENSG00000108219	ENSG00000158457
ENSG00000108219	ENSG00000168785
ENSG00000108219	ENSG00000182612
ENSG00000108231	ENSG00000153012
ENSG00000108231	ENSG00000153902
ENSG00000108231	ENSG00000168481
ENSG00000108239	ENSG00000132405
ENSG00000108244	ENSG00000006059
ENSG00000108244	ENSG00000094796
ENSG00000108244	ENSG00000108417
ENSG00000108244	ENSG00000108759
ENSG00000108244	ENSG00000111057
ENSG00000108244	ENSG00000126337
ENSG00000108244	ENSG00000128422
ENSG00000108244	ENSG00000131737
ENSG00000108244	ENSG00000131738
ENSG00000108244	ENSG00000131885
ENSG00000108244	ENSG00000167916
ENSG00000108244	ENSG00000170819
ENSG00000108244	ENSG00000171345
ENSG00000108244	ENSG00000171346
ENSG00000108244	ENSG00000171360

ENSG00000108244	ENSG00000171401
ENSG00000108244	ENSG00000171403
ENSG00000108244	ENSG00000171431
ENSG00000108244	ENSG00000171446
ENSG00000108244	ENSG00000173908
ENSG00000108244	ENSG00000186393
ENSG00000108244	ENSG00000186395
ENSG00000108244	ENSG00000186831
ENSG00000108244	ENSG00000186832
ENSG00000108244	ENSG00000186847
ENSG00000108244	ENSG00000187242
ENSG00000108244	ENSG00000187686
ENSG00000108244	ENSG00000196859
ENSG00000108244	ENSG00000197079
ENSG00000108244	ENSG00000204889
ENSG00000108244	ENSG00000204897
ENSG00000108244	ENSG00000213951
ENSG00000108244	ENSG00000214220
ENSG00000108244	ENSG00000214454
ENSG00000108244	ENSG00000214959
ENSG00000108244	ENSG00000215772
ENSG00000108244	ENSG00000215773
ENSG00000108255	ENSG00000100053
ENSG00000108255	ENSG00000100058
ENSG00000108255	ENSG00000100122
ENSG00000108255	ENSG00000118231
ENSG00000108255	ENSG00000127377
ENSG00000108255	ENSG00000163254
ENSG00000108255	ENSG00000163499
ENSG00000108255	ENSG00000168582
ENSG00000108255	ENSG00000182187
ENSG00000108255	ENSG00000196431
ENSG00000108255	ENSG00000213139
ENSG00000108262	ENSG00000133612
ENSG00000108262	ENSG00000135439
ENSG00000108262	ENSG00000139436
ENSG00000108262	ENSG00000151303
ENSG00000108262	ENSG00000157985
ENSG00000108262	ENSG00000172650
ENSG00000108262	ENSG00000174194
ENSG00000108262	ENSG00000188234
ENSG00000108262	ENSG00000198035
ENSG00000108262	ENSG00000204149
ENSG00000108262	ENSG00000204169
ENSG00000108262	ENSG00000204172
ENSG00000108292	ENSG00000043143
ENSG00000108292	ENSG00000077684
ENSG00000108292	ENSG00000078403
ENSG00000108292	ENSG00000096070
ENSG00000108292	ENSG00000100425
ENSG00000108292	ENSG00000102221
ENSG00000108292	ENSG00000106443
ENSG00000108292	ENSG00000156983
ENSG00000108309	ENSG00000018189
ENSG00000108309	ENSG00000105784
ENSG00000108309	ENSG00000176783
ENSG00000108309	ENSG00000204130
ENSG00000108309	ENSG00000214795
ENSG00000108312	ENSG00000183417

ENSG00000108312	ENSG00000204448
ENSG00000108312	ENSG00000204664
ENSG00000108312	ENSG00000204699
ENSG00000108312	ENSG00000204705
ENSG00000108312	ENSG00000205042
ENSG00000108352	ENSG00000079337
ENSG00000108352	ENSG00000091428
ENSG00000108352	ENSG00000109756
ENSG00000108352	ENSG00000136237
ENSG00000108370	ENSG00000076344
ENSG00000108370	ENSG00000182732
ENSG00000108370	ENSG00000182901
ENSG00000108375	ENSG00000099999
ENSG00000108375	ENSG00000183579
ENSG00000108379	ENSG00000002745
ENSG00000108379	ENSG00000061492
ENSG00000108379	ENSG00000075290
ENSG00000108379	ENSG00000085741
ENSG00000108379	ENSG00000105989
ENSG00000108379	ENSG00000111186
ENSG00000108379	ENSG00000114251
ENSG00000108379	ENSG00000115596
ENSG00000108379	ENSG00000125084
ENSG00000108379	ENSG00000134245
ENSG00000108379	ENSG00000135925
ENSG00000108379	ENSG00000143816
ENSG00000108379	ENSG00000154342
ENSG00000108379	ENSG00000154764
ENSG00000108379	ENSG00000158955
ENSG00000108379	ENSG00000162552
ENSG00000108379	ENSG00000169884
ENSG00000108379	ENSG00000188064
ENSG00000108381	ENSG00000132744
ENSG00000108387	ENSG00000122545
ENSG00000108387	ENSG00000168385
ENSG00000108387	ENSG00000180096
ENSG00000108387	ENSG00000184702
ENSG00000108387	ENSG00000214005
ENSG00000108387	ENSG00000214765
ENSG00000108387	ENSG00000214834
ENSG00000108389	ENSG00000003987
ENSG00000108389	ENSG00000014914
ENSG00000108389	ENSG00000063601
ENSG00000108389	ENSG00000087053
ENSG00000108389	ENSG00000100330
ENSG00000108389	ENSG00000102043
ENSG00000108389	ENSG00000104643
ENSG00000108389	ENSG00000139505
ENSG00000108389	ENSG00000150712
ENSG00000108389	ENSG00000166912
ENSG00000108389	ENSG00000171100
ENSG00000108389	ENSG00000184416
ENSG00000108405	ENSG00000083454
ENSG00000108405	ENSG00000089041
ENSG00000108405	ENSG00000099957
ENSG00000108405	ENSG00000109991
ENSG00000108405	ENSG00000135124
ENSG00000108405	ENSG00000187848
ENSG00000108417	ENSG00000006059

ENSG00000108417	ENSG00000094796
ENSG00000108417	ENSG00000108244
ENSG00000108417	ENSG00000108759
ENSG00000108417	ENSG00000111057
ENSG00000108417	ENSG00000126337
ENSG00000108417	ENSG00000128422
ENSG00000108417	ENSG00000131737
ENSG00000108417	ENSG00000131738
ENSG00000108417	ENSG00000131885
ENSG00000108417	ENSG00000167916
ENSG00000108417	ENSG00000170819
ENSG00000108417	ENSG00000171345
ENSG00000108417	ENSG00000171346
ENSG00000108417	ENSG00000171360
ENSG00000108417	ENSG00000171401
ENSG00000108417	ENSG00000171403
ENSG00000108417	ENSG00000171431
ENSG00000108417	ENSG00000171446
ENSG00000108417	ENSG00000173908
ENSG00000108417	ENSG00000186393
ENSG00000108417	ENSG00000186395
ENSG00000108417	ENSG00000186831
ENSG00000108417	ENSG00000186832
ENSG00000108417	ENSG00000186847
ENSG00000108417	ENSG00000187242
ENSG00000108417	ENSG00000187686
ENSG00000108417	ENSG00000196859
ENSG00000108417	ENSG00000197079
ENSG00000108417	ENSG00000204889
ENSG00000108417	ENSG00000204897
ENSG00000108417	ENSG00000213951
ENSG00000108417	ENSG00000214220
ENSG00000108417	ENSG00000214454
ENSG00000108417	ENSG00000214959
ENSG00000108417	ENSG00000215772
ENSG00000108417	ENSG00000215773
ENSG00000108442	ENSG00000166676
ENSG00000108442	ENSG00000171928
ENSG00000108443	ENSG00000071242
ENSG00000108443	ENSG00000072133
ENSG00000108443	ENSG00000100784
ENSG00000108443	ENSG00000117676
ENSG00000108443	ENSG00000162302
ENSG00000108443	ENSG00000175634
ENSG00000108443	ENSG00000177189
ENSG00000108443	ENSG00000214817
ENSG00000108468	ENSG00000094916
ENSG00000108468	ENSG00000122565
ENSG00000108504	ENSG00000123374
ENSG00000108504	ENSG00000170312
ENSG00000108509	ENSG00000171735
ENSG00000108510	ENSG00000123066
ENSG00000108511	ENSG00000037965
ENSG00000108511	ENSG00000106004
ENSG00000108511	ENSG00000106006
ENSG00000108511	ENSG00000120068
ENSG00000108511	ENSG00000120075
ENSG00000108511	ENSG00000120087
ENSG00000108511	ENSG00000122592

ENSG00000108511	ENSG00000170166
ENSG00000108511	ENSG00000172789
ENSG00000108511	ENSG00000175879
ENSG00000108511	ENSG00000182742
ENSG00000108511	ENSG00000197576
ENSG00000108511	ENSG00000197757
ENSG00000108511	ENSG00000198353
ENSG00000108515	ENSG00000074800
ENSG00000108515	ENSG00000111674
ENSG00000108515	ENSG00000214431
ENSG00000108515	ENSG00000215809
ENSG00000108518	ENSG00000070087
ENSG00000108518	ENSG00000196570
ENSG00000108523	ENSG00000082996
ENSG00000108523	ENSG00000105428
ENSG00000108523	ENSG00000113269
ENSG00000108523	ENSG00000133135
ENSG00000108523	ENSG00000163162
ENSG00000108523	ENSG00000170153
ENSG00000108523	ENSG00000188050
ENSG00000108551	ENSG00000100302
ENSG00000108556	ENSG00000080644
ENSG00000108556	ENSG00000101204
ENSG00000108556	ENSG00000117971
ENSG00000108556	ENSG00000120903
ENSG00000108556	ENSG00000129749
ENSG00000108556	ENSG00000135902
ENSG00000108556	ENSG00000138435
ENSG00000108556	ENSG00000147432
ENSG00000108556	ENSG00000147434
ENSG00000108556	ENSG00000149305
ENSG00000108556	ENSG00000160716
ENSG00000108556	ENSG00000166664
ENSG00000108556	ENSG00000166736
ENSG00000108556	ENSG00000169684
ENSG00000108556	ENSG00000170175
ENSG00000108556	ENSG00000174343
ENSG00000108556	ENSG00000175344
ENSG00000108556	ENSG00000178084
ENSG00000108556	ENSG00000186038
ENSG00000108556	ENSG00000186090
ENSG00000108556	ENSG00000186919
ENSG00000108556	ENSG00000196811
ENSG00000108557	ENSG00000100207
ENSG00000108576	ENSG00000010379
ENSG00000108576	ENSG00000011083
ENSG00000108576	ENSG00000087916
ENSG00000108576	ENSG00000103546
ENSG00000108576	ENSG00000111181
ENSG00000108576	ENSG00000130821
ENSG00000108576	ENSG00000131389
ENSG00000108576	ENSG00000132164
ENSG00000108576	ENSG00000142319
ENSG00000108576	ENSG00000157103
ENSG00000108576	ENSG00000163817
ENSG00000108576	ENSG00000164363
ENSG00000108576	ENSG00000165970
ENSG00000108576	ENSG00000174358
ENSG00000108576	ENSG00000196517

ENSG00000108576	ENSG00000198555
ENSG00000108576	ENSG00000214617
ENSG00000108602	ENSG00000006534
ENSG00000108602	ENSG00000072210
ENSG00000108602	ENSG00000132746
ENSG00000108604	ENSG00000066117
ENSG00000108604	ENSG00000082014
ENSG00000108639	ENSG00000100321
ENSG00000108639	ENSG00000105467
ENSG00000108639	ENSG00000127561
ENSG00000108639	ENSG00000215315
ENSG00000108654	ENSG00000100201
ENSG00000108669	ENSG00000008256
ENSG00000108669	ENSG00000105443
ENSG00000108679	ENSG00000204347
ENSG00000108684	ENSG00000072182
ENSG00000108684	ENSG00000110881
ENSG00000108684	ENSG00000213199
ENSG00000108688	ENSG00000006074
ENSG00000108688	ENSG00000006075
ENSG00000108688	ENSG00000006606
ENSG00000108688	ENSG00000102962
ENSG00000108688	ENSG00000102970
ENSG00000108688	ENSG00000106178
ENSG00000108688	ENSG00000108691
ENSG00000108688	ENSG00000108700
ENSG00000108688	ENSG00000108702
ENSG00000108688	ENSG00000115009
ENSG00000108688	ENSG00000129277
ENSG00000108688	ENSG00000143184
ENSG00000108688	ENSG00000143185
ENSG00000108688	ENSG00000161570
ENSG00000108688	ENSG00000161573
ENSG00000108688	ENSG00000161574
ENSG00000108688	ENSG00000167236
ENSG00000108688	ENSG00000172156
ENSG00000108688	ENSG00000172724
ENSG00000108688	ENSG00000181374
ENSG00000108688	ENSG00000197262
ENSG00000108688	ENSG00000205017
ENSG00000108688	ENSG00000205020
ENSG00000108688	ENSG00000205021
ENSG00000108688	ENSG00000213494
ENSG00000108688	ENSG00000215627
ENSG00000108688	ENSG00000215629
ENSG00000108691	ENSG00000006074
ENSG00000108691	ENSG00000006075
ENSG00000108691	ENSG00000006606
ENSG00000108691	ENSG00000102962
ENSG00000108691	ENSG00000102970
ENSG00000108691	ENSG00000106178
ENSG00000108691	ENSG00000108688
ENSG00000108691	ENSG00000108700
ENSG00000108691	ENSG00000108702
ENSG00000108691	ENSG00000115009
ENSG00000108691	ENSG00000129277
ENSG00000108691	ENSG00000143184
ENSG00000108691	ENSG00000143185
ENSG00000108691	ENSG00000161570

ENSG00000108691	ENSG00000161573
ENSG00000108691	ENSG00000161574
ENSG00000108691	ENSG00000167236
ENSG00000108691	ENSG00000172156
ENSG00000108691	ENSG00000172724
ENSG00000108691	ENSG00000181374
ENSG00000108691	ENSG00000197262
ENSG00000108691	ENSG00000205017
ENSG00000108691	ENSG00000205020
ENSG00000108691	ENSG00000205021
ENSG00000108691	ENSG00000213494
ENSG00000108691	ENSG00000215627
ENSG00000108691	ENSG00000215629
ENSG00000108700	ENSG00000006074
ENSG00000108700	ENSG00000006075
ENSG00000108700	ENSG00000006606
ENSG00000108700	ENSG00000102962
ENSG00000108700	ENSG00000102970
ENSG00000108700	ENSG00000106178
ENSG00000108700	ENSG00000108688
ENSG00000108700	ENSG00000108691
ENSG00000108700	ENSG00000108702
ENSG00000108700	ENSG00000115009
ENSG00000108700	ENSG00000129277
ENSG00000108700	ENSG00000143184
ENSG00000108700	ENSG00000143185
ENSG00000108700	ENSG00000161570
ENSG00000108700	ENSG00000161573
ENSG00000108700	ENSG00000161574
ENSG00000108700	ENSG00000167236
ENSG00000108700	ENSG00000172156
ENSG00000108700	ENSG00000172724
ENSG00000108700	ENSG00000181374
ENSG00000108700	ENSG00000197262
ENSG00000108700	ENSG00000205017
ENSG00000108700	ENSG00000205020
ENSG00000108700	ENSG00000205021
ENSG00000108700	ENSG00000213494
ENSG00000108700	ENSG00000215627
ENSG00000108700	ENSG00000215629
ENSG00000108702	ENSG00000006074
ENSG00000108702	ENSG00000006075
ENSG00000108702	ENSG00000006606
ENSG00000108702	ENSG00000102962
ENSG00000108702	ENSG00000102970
ENSG00000108702	ENSG00000106178
ENSG00000108702	ENSG00000108688
ENSG00000108702	ENSG00000108691
ENSG00000108702	ENSG00000108700
ENSG00000108702	ENSG00000115009
ENSG00000108702	ENSG00000129277
ENSG00000108702	ENSG00000143184
ENSG00000108702	ENSG00000143185
ENSG00000108702	ENSG00000161570
ENSG00000108702	ENSG00000161573
ENSG00000108702	ENSG00000161574
ENSG00000108702	ENSG00000167236
ENSG00000108702	ENSG00000172156
ENSG00000108702	ENSG00000172724

ENSG00000108702	ENSG00000181374
ENSG00000108702	ENSG00000197262
ENSG00000108702	ENSG00000205017
ENSG00000108702	ENSG00000205020
ENSG00000108702	ENSG00000205021
ENSG00000108702	ENSG00000213494
ENSG00000108702	ENSG00000215627
ENSG00000108702	ENSG00000215629
ENSG00000108753	ENSG00000135100
ENSG00000108753	ENSG00000147421
ENSG00000108759	ENSG00000006059
ENSG00000108759	ENSG00000094796
ENSG00000108759	ENSG00000108244
ENSG00000108759	ENSG00000108417
ENSG00000108759	ENSG00000111057
ENSG00000108759	ENSG00000126337
ENSG00000108759	ENSG00000128422
ENSG00000108759	ENSG00000131737
ENSG00000108759	ENSG00000131738
ENSG00000108759	ENSG00000131885
ENSG00000108759	ENSG00000167916
ENSG00000108759	ENSG00000170819
ENSG00000108759	ENSG00000171345
ENSG00000108759	ENSG00000171346
ENSG00000108759	ENSG00000171360
ENSG00000108759	ENSG00000171401
ENSG00000108759	ENSG00000171403
ENSG00000108759	ENSG00000171431
ENSG00000108759	ENSG00000171446
ENSG00000108759	ENSG00000173908
ENSG00000108759	ENSG00000186393
ENSG00000108759	ENSG00000186395
ENSG00000108759	ENSG00000186831
ENSG00000108759	ENSG00000186832
ENSG00000108759	ENSG00000186847
ENSG00000108759	ENSG00000187242
ENSG00000108759	ENSG00000187686
ENSG00000108759	ENSG00000196859
ENSG00000108759	ENSG00000197079
ENSG00000108759	ENSG00000204889
ENSG00000108759	ENSG00000204897
ENSG00000108759	ENSG00000213951
ENSG00000108759	ENSG00000214220
ENSG00000108759	ENSG00000214454
ENSG00000108759	ENSG00000214959
ENSG00000108759	ENSG00000215772
ENSG00000108759	ENSG00000215773
ENSG00000108771	ENSG00000100697
ENSG00000108771	ENSG00000107201
ENSG00000108771	ENSG00000115267
ENSG00000108773	ENSG00000114166
ENSG00000108774	ENSG00000080371
ENSG00000108774	ENSG00000111540
ENSG00000108774	ENSG00000124839
ENSG00000108774	ENSG00000144566
ENSG00000108774	ENSG00000169228
ENSG00000108785	ENSG00000080511
ENSG00000108785	ENSG00000108786
ENSG00000108786	ENSG00000080511

ENSG00000108786	ENSG00000108785
ENSG00000108797	ENSG00000106714
ENSG00000108797	ENSG00000152910
ENSG00000108797	ENSG00000154529
ENSG00000108797	ENSG00000155052
ENSG00000108797	ENSG00000174469
ENSG00000108797	ENSG00000185020
ENSG00000108798	ENSG00000136754
ENSG00000108798	ENSG00000138443
ENSG00000108799	ENSG00000106462
ENSG00000108813	ENSG00000006377
ENSG00000108813	ENSG00000144355
ENSG00000108813	ENSG00000167034
ENSG00000108819	ENSG00000158528
ENSG00000108821	ENSG00000060718
ENSG00000108821	ENSG00000080573
ENSG00000108821	ENSG00000130635
ENSG00000108821	ENSG00000139219
ENSG00000108821	ENSG00000164692
ENSG00000108821	ENSG00000168542
ENSG00000108821	ENSG00000204248
ENSG00000108821	ENSG00000204262
ENSG00000108823	ENSG00000127990
ENSG00000108830	ENSG00000115963
ENSG00000108830	ENSG00000172602
ENSG00000108839	ENSG00000012779
ENSG00000108839	ENSG00000161905
ENSG00000108839	ENSG00000179148
ENSG00000108839	ENSG00000179477
ENSG00000108839	ENSG00000179593
ENSG00000108839	ENSG00000188101
ENSG00000108839	ENSG00000214859
ENSG00000108840	ENSG00000048052
ENSG00000108840	ENSG00000061273
ENSG00000108840	ENSG00000068024
ENSG00000108846	ENSG00000023839
ENSG00000108846	ENSG00000091262
ENSG00000108846	ENSG00000103222
ENSG00000108849	ENSG00000122585
ENSG00000108849	ENSG00000131096
ENSG00000108849	ENSG00000204474
ENSG00000108852	ENSG00000072415
ENSG00000108852	ENSG00000082126
ENSG00000108852	ENSG00000105926
ENSG00000108852	ENSG00000130830
ENSG00000108852	ENSG00000147044
ENSG00000108852	ENSG00000150054
ENSG00000108852	ENSG00000161647
ENSG00000108854	ENSG00000049759
ENSG00000108854	ENSG00000069869
ENSG00000108854	ENSG00000078747
ENSG00000108854	ENSG00000123124
ENSG00000108854	ENSG00000198373
ENSG00000108854	ENSG00000198742
ENSG00000108861	ENSG00000079393
ENSG00000108861	ENSG00000133878
ENSG00000108861	ENSG00000188542
ENSG00000108861	ENSG00000188716
ENSG00000108878	ENSG00000130433

ENSG00000108924	ENSG00000105516
ENSG00000108924	ENSG00000167074
ENSG00000108932	ENSG00000100156
ENSG00000108932	ENSG00000112394
ENSG00000108932	ENSG00000118596
ENSG00000108932	ENSG00000141526
ENSG00000108932	ENSG00000147100
ENSG00000108932	ENSG00000152779
ENSG00000108932	ENSG00000155380
ENSG00000108932	ENSG00000163053
ENSG00000108932	ENSG00000165449
ENSG00000108932	ENSG00000168679
ENSG00000108932	ENSG00000170190
ENSG00000108932	ENSG00000174326
ENSG00000108932	ENSG00000174327
ENSG00000108946	ENSG00000005249
ENSG00000108946	ENSG00000114302
ENSG00000108946	ENSG00000188191
ENSG00000108947	ENSG00000090776
ENSG00000108947	ENSG00000099617
ENSG00000108947	ENSG00000125266
ENSG00000108947	ENSG00000143590
ENSG00000108947	ENSG00000143620
ENSG00000108947	ENSG00000169242
ENSG00000108947	ENSG00000184349
ENSG00000108950	ENSG00000116199
ENSG00000108960	ENSG00000136297
ENSG00000109016	ENSG00000184544
ENSG00000109046	ENSG00000176871
ENSG00000109047	ENSG00000104490
ENSG00000109047	ENSG00000107130
ENSG00000109047	ENSG00000115756
ENSG00000109047	ENSG00000116983
ENSG00000109047	ENSG00000121905
ENSG00000109047	ENSG00000163032
ENSG00000109061	ENSG00000002079
ENSG00000109061	ENSG00000006788
ENSG00000109061	ENSG00000078814
ENSG00000109061	ENSG00000092054
ENSG00000109061	ENSG00000109063
ENSG00000109061	ENSG00000125414
ENSG00000109061	ENSG00000133020
ENSG00000109061	ENSG00000141048
ENSG00000109061	ENSG00000144821
ENSG00000109061	ENSG00000197616
ENSG00000109062	ENSG00000065054
ENSG00000109062	ENSG00000172367
ENSG00000109062	ENSG00000174827
ENSG00000109062	ENSG00000215859
ENSG00000109062	ENSG00000215860
ENSG00000109063	ENSG00000002079
ENSG00000109063	ENSG00000006788
ENSG00000109063	ENSG00000078814
ENSG00000109063	ENSG00000092054
ENSG00000109063	ENSG00000109061
ENSG00000109063	ENSG00000125414
ENSG00000109063	ENSG00000133020
ENSG00000109063	ENSG00000141048
ENSG00000109063	ENSG00000144821

ENSG00000109063	ENSG00000197616
ENSG00000109072	ENSG00000116690
ENSG00000109079	ENSG00000110906
ENSG00000109079	ENSG00000174943
ENSG00000109079	ENSG00000181819
ENSG00000109089	ENSG00000140743
ENSG00000109099	ENSG00000134531
ENSG00000109099	ENSG00000142227
ENSG00000109099	ENSG00000213853
ENSG00000109101	ENSG00000139445
ENSG00000109101	ENSG00000176302
ENSG00000109101	ENSG00000189299
ENSG00000109103	ENSG00000156973
ENSG00000109103	ENSG00000175970
ENSG00000109107	ENSG00000136872
ENSG00000109107	ENSG00000149925
ENSG00000109113	ENSG00000100228
ENSG00000109132	ENSG00000116132
ENSG00000109132	ENSG00000165462
ENSG00000109132	ENSG00000165606
ENSG00000109132	ENSG00000167157
ENSG00000109158	ENSG00000011677
ENSG00000109158	ENSG00000022355
ENSG00000109158	ENSG00000094755
ENSG00000109158	ENSG00000101958
ENSG00000109158	ENSG00000102287
ENSG00000109158	ENSG00000109738
ENSG00000109158	ENSG00000111886
ENSG00000109158	ENSG00000113327
ENSG00000109158	ENSG00000145451
ENSG00000109158	ENSG00000145863
ENSG00000109158	ENSG00000145864
ENSG00000109158	ENSG00000145888
ENSG00000109158	ENSG00000146276
ENSG00000109158	ENSG00000147402
ENSG00000109158	ENSG00000151834
ENSG00000109158	ENSG00000163285
ENSG00000109158	ENSG00000163288
ENSG00000109158	ENSG00000166206
ENSG00000109158	ENSG00000182256
ENSG00000109158	ENSG00000183185
ENSG00000109158	ENSG00000186297
ENSG00000109158	ENSG00000187730
ENSG00000109158	ENSG00000188828
ENSG00000109163	ENSG00000126895
ENSG00000109163	ENSG00000166148
ENSG00000109163	ENSG00000178015
ENSG00000109163	ENSG00000180914
ENSG00000109163	ENSG00000187258
ENSG00000109163	ENSG00000198049
ENSG00000109163	ENSG00000211451
ENSG00000109171	ENSG00000139737
ENSG00000109180	ENSG00000145247
ENSG00000109181	ENSG00000135220
ENSG00000109181	ENSG00000135226
ENSG00000109181	ENSG00000145626
ENSG00000109181	ENSG00000156096
ENSG00000109181	ENSG00000167165
ENSG00000109181	ENSG00000168671

ENSG00000109181	ENSG00000171234
ENSG00000109181	ENSG00000173610
ENSG00000109181	ENSG00000174607
ENSG00000109181	ENSG00000196620
ENSG00000109181	ENSG00000197592
ENSG00000109181	ENSG00000197888
ENSG00000109181	ENSG00000198277
ENSG00000109181	ENSG00000213759
ENSG00000109184	ENSG00000137692
ENSG00000109189	ENSG00000152484
ENSG00000109193	ENSG00000088002
ENSG00000109193	ENSG00000105398
ENSG00000109193	ENSG00000130540
ENSG00000109193	ENSG00000138068
ENSG00000109193	ENSG00000173597
ENSG00000109193	ENSG00000196228
ENSG00000109193	ENSG00000196502
ENSG00000109193	ENSG00000197165
ENSG00000109193	ENSG00000198075
ENSG00000109193	ENSG00000198203
ENSG00000109193	ENSG00000213599
ENSG00000109193	ENSG00000213648
ENSG00000109220	ENSG00000204116
ENSG00000109255	ENSG00000204640
ENSG00000109272	ENSG00000081041
ENSG00000109272	ENSG00000124875
ENSG00000109272	ENSG00000138755
ENSG00000109272	ENSG00000163734
ENSG00000109272	ENSG00000163735
ENSG00000109272	ENSG00000163736
ENSG00000109272	ENSG00000163737
ENSG00000109272	ENSG00000163739
ENSG00000109272	ENSG00000169245
ENSG00000109272	ENSG00000169248
ENSG00000109272	ENSG00000169429
ENSG00000109320	ENSG00000077150
ENSG00000109320	ENSG00000104856
ENSG00000109320	ENSG00000162924
ENSG00000109320	ENSG00000173039
ENSG00000109321	ENSG00000113070
ENSG00000109321	ENSG00000205595
ENSG00000109332	ENSG00000072401
ENSG00000109332	ENSG00000078967
ENSG00000109332	ENSG00000131508
ENSG00000109332	ENSG00000184888
ENSG00000109339	ENSG00000050748
ENSG00000109339	ENSG00000107643
ENSG00000109339	ENSG00000112062
ENSG00000109339	ENSG00000156711
ENSG00000109339	ENSG00000185386
ENSG00000109339	ENSG00000188130
ENSG00000109381	ENSG00000010030
ENSG00000109381	ENSG00000102034
ENSG00000109381	ENSG00000120690
ENSG00000109381	ENSG00000124664
ENSG00000109381	ENSG00000135373
ENSG00000109381	ENSG00000135374
ENSG00000109381	ENSG00000139083
ENSG00000109381	ENSG00000163435

ENSG00000109381	ENSG00000166211
ENSG00000109424	ENSG00000102078
ENSG00000109424	ENSG00000174032
ENSG00000109424	ENSG00000175564
ENSG00000109424	ENSG00000175567
ENSG00000109436	ENSG00000133138
ENSG00000109436	ENSG00000197226
ENSG00000109436	ENSG00000204634
ENSG00000109452	ENSG00000040933
ENSG00000109458	ENSG00000033327
ENSG00000109458	ENSG00000160219
ENSG00000109458	ENSG00000215568
ENSG00000109511	ENSG00000104537
ENSG00000109511	ENSG00000122359
ENSG00000109511	ENSG00000135046
ENSG00000109511	ENSG00000138279
ENSG00000109511	ENSG00000138772
ENSG00000109511	ENSG00000143412
ENSG00000109511	ENSG00000150165
ENSG00000109511	ENSG00000164111
ENSG00000109511	ENSG00000165390
ENSG00000109511	ENSG00000182718
ENSG00000109511	ENSG00000183059
ENSG00000109511	ENSG00000186807
ENSG00000109511	ENSG00000196975
ENSG00000109511	ENSG00000197043
ENSG00000109519	ENSG00000164284
ENSG00000109519	ENSG00000214621
ENSG00000109572	ENSG00000011021
ENSG00000109572	ENSG00000073464
ENSG00000109572	ENSG00000103249
ENSG00000109572	ENSG00000171365
ENSG00000109654	ENSG00000110171
ENSG00000109654	ENSG00000119401
ENSG00000109654	ENSG00000187566
ENSG00000109654	ENSG00000188811
ENSG00000109654	ENSG00000206557
ENSG00000109667	ENSG00000059804
ENSG00000109667	ENSG00000117394
ENSG00000109667	ENSG00000133460
ENSG00000109667	ENSG00000142583
ENSG00000109667	ENSG00000163581
ENSG00000109667	ENSG00000173262
ENSG00000109667	ENSG00000181856
ENSG00000109667	ENSG00000197241
ENSG00000109684	ENSG00000043462
ENSG00000109684	ENSG00000095585
ENSG00000109684	ENSG00000152292
ENSG00000109685	ENSG00000147548
ENSG00000109685	ENSG00000165671
ENSG00000109686	ENSG00000100365
ENSG00000109689	ENSG00000167323
ENSG00000109705	ENSG00000119919
ENSG00000109705	ENSG00000125816
ENSG00000109705	ENSG00000125820
ENSG00000109705	ENSG00000136327
ENSG00000109705	ENSG00000136352
ENSG00000109705	ENSG00000180053
ENSG00000109705	ENSG00000183072

ENSG00000109738	ENSG00000011677
ENSG00000109738	ENSG00000022355
ENSG00000109738	ENSG00000094755
ENSG00000109738	ENSG00000101958
ENSG00000109738	ENSG00000102287
ENSG00000109738	ENSG00000109158
ENSG00000109738	ENSG00000111886
ENSG00000109738	ENSG00000113327
ENSG00000109738	ENSG00000145451
ENSG00000109738	ENSG00000145863
ENSG00000109738	ENSG00000145864
ENSG00000109738	ENSG00000145888
ENSG00000109738	ENSG00000146276
ENSG00000109738	ENSG00000147402
ENSG00000109738	ENSG00000151834
ENSG00000109738	ENSG00000163285
ENSG00000109738	ENSG00000163288
ENSG00000109738	ENSG00000166206
ENSG00000109738	ENSG00000182256
ENSG00000109738	ENSG00000183185
ENSG00000109738	ENSG00000186297
ENSG00000109738	ENSG00000187730
ENSG00000109738	ENSG00000188828
ENSG00000109743	ENSG00000004468
ENSG00000109756	ENSG00000079337
ENSG00000109756	ENSG00000091428
ENSG00000109756	ENSG00000108352
ENSG00000109756	ENSG00000136237
ENSG00000109758	ENSG00000019991
ENSG00000109758	ENSG00000104368
ENSG00000109758	ENSG00000122194
ENSG00000109758	ENSG00000122861
ENSG00000109758	ENSG00000131187
ENSG00000109758	ENSG00000148702
ENSG00000109758	ENSG00000173531
ENSG00000109758	ENSG00000186301
ENSG00000109758	ENSG00000186715
ENSG00000109758	ENSG00000198670
ENSG00000109787	ENSG00000102349
ENSG00000109787	ENSG00000118263
ENSG00000109787	ENSG00000118922
ENSG00000109787	ENSG00000163884
ENSG00000109794	ENSG00000138286
ENSG00000109794	ENSG00000214424
ENSG00000109819	ENSG00000148840
ENSG00000109819	ENSG00000155846
ENSG00000109832	ENSG00000157349
ENSG00000109832	ENSG00000168872
ENSG00000109846	ENSG00000004776
ENSG00000109846	ENSG00000106211
ENSG00000109846	ENSG00000152137
ENSG00000109846	ENSG00000160202
ENSG00000109846	ENSG00000169271
ENSG00000109846	ENSG00000170276
ENSG00000109906	ENSG00000011590
ENSG00000109906	ENSG00000166860
ENSG00000109919	ENSG00000137409
ENSG00000109919	ENSG00000214315
ENSG00000109927	ENSG00000090920

ENSG00000109927	ENSG00000110799
ENSG00000109927	ENSG00000117983
ENSG00000109927	ENSG00000146839
ENSG00000109927	ENSG00000184956
ENSG00000109927	ENSG00000188162
ENSG00000109927	ENSG00000196292
ENSG00000109927	ENSG00000197558
ENSG00000109927	ENSG00000198788
ENSG00000109943	ENSG00000162706
ENSG00000109943	ENSG00000175161
ENSG00000109956	ENSG00000112309
ENSG00000109956	ENSG00000149541
ENSG00000109971	ENSG00000126803
ENSG00000109971	ENSG00000173110
ENSG00000109971	ENSG00000204388
ENSG00000109971	ENSG00000204389
ENSG00000109971	ENSG00000204390
ENSG00000109991	ENSG00000083454
ENSG00000109991	ENSG00000089041
ENSG00000109991	ENSG00000099957
ENSG00000109991	ENSG00000108405
ENSG00000109991	ENSG00000135124
ENSG00000109991	ENSG00000187848
ENSG00000110002	ENSG00000102699
ENSG00000110002	ENSG00000145198
ENSG00000110002	ENSG00000158816
ENSG00000110025	ENSG00000136643
ENSG00000110025	ENSG00000198208
ENSG00000110031	ENSG00000072163
ENSG00000110031	ENSG00000089159
ENSG00000110031	ENSG00000112214
ENSG00000110031	ENSG00000140682
ENSG00000110031	ENSG00000169756
ENSG00000110031	ENSG00000183386
ENSG00000110042	ENSG00000091073
ENSG00000110042	ENSG00000135144
ENSG00000110042	ENSG00000163840
ENSG00000110042	ENSG00000178498
ENSG00000110046	ENSG00000066739
ENSG00000110047	ENSG00000013016
ENSG00000110047	ENSG00000024422
ENSG00000110047	ENSG00000103966
ENSG00000110048	ENSG00000184792
ENSG00000110057	ENSG00000112494
ENSG00000110057	ENSG00000155070
ENSG00000110057	ENSG00000172971
ENSG00000110057	ENSG00000184795
ENSG00000110066	ENSG00000133247
ENSG00000110075	ENSG00000100239
ENSG00000110075	ENSG00000105063
ENSG00000110076	ENSG00000021645
ENSG00000110076	ENSG00000179915
ENSG00000110077	ENSG00000071203
ENSG00000110077	ENSG00000110079
ENSG00000110077	ENSG00000149516
ENSG00000110077	ENSG00000149534
ENSG00000110077	ENSG00000156738
ENSG00000110077	ENSG00000166926
ENSG00000110077	ENSG00000166927

ENSG00000110077	ENSG00000166930
ENSG00000110077	ENSG00000166959
ENSG00000110077	ENSG00000166961
ENSG00000110077	ENSG00000214787
ENSG00000110079	ENSG00000071203
ENSG00000110079	ENSG00000110077
ENSG00000110079	ENSG00000149516
ENSG00000110079	ENSG00000149534
ENSG00000110079	ENSG00000156738
ENSG00000110079	ENSG00000166926
ENSG00000110079	ENSG00000166927
ENSG00000110079	ENSG00000166930
ENSG00000110079	ENSG00000166959
ENSG00000110079	ENSG00000166961
ENSG00000110079	ENSG00000214787
ENSG00000110080	ENSG00000008513
ENSG00000110080	ENSG00000064225
ENSG00000110080	ENSG00000126091
ENSG00000110080	ENSG00000157350
ENSG00000110090	ENSG00000005469
ENSG00000110090	ENSG00000070748
ENSG00000110090	ENSG00000095321
ENSG00000110090	ENSG00000169169
ENSG00000110090	ENSG00000205560
ENSG00000110092	ENSG00000112576
ENSG00000110092	ENSG00000118971
ENSG00000110108	ENSG00000184992
ENSG00000110148	ENSG00000056291
ENSG00000110148	ENSG00000075073
ENSG00000110148	ENSG00000101292
ENSG00000110148	ENSG00000115353
ENSG00000110148	ENSG00000119973
ENSG00000110148	ENSG00000121764
ENSG00000110148	ENSG00000123901
ENSG00000110148	ENSG00000137252
ENSG00000110148	ENSG00000148734
ENSG00000110148	ENSG00000159279
ENSG00000110148	ENSG00000163394
ENSG00000110148	ENSG00000164128
ENSG00000110148	ENSG00000164129
ENSG00000110148	ENSG00000169618
ENSG00000110148	ENSG00000169836
ENSG00000110148	ENSG00000185149
ENSG00000110148	ENSG00000186867
ENSG00000110148	ENSG00000204174
ENSG00000110148	ENSG00000215167
ENSG00000110171	ENSG00000109654
ENSG00000110171	ENSG00000119401
ENSG00000110171	ENSG00000187566
ENSG00000110171	ENSG00000188811
ENSG00000110171	ENSG00000206557
ENSG00000110172	ENSG00000147166
ENSG00000110218	ENSG00000073150
ENSG00000110218	ENSG00000154143
ENSG00000110243	ENSG00000110244
ENSG00000110243	ENSG00000118137
ENSG00000110243	ENSG00000130203
ENSG00000110243	ENSG00000215754
ENSG00000110243	ENSG00000215756

ENSG00000110244	ENSG00000110243
ENSG00000110244	ENSG00000118137
ENSG00000110244	ENSG00000130203
ENSG00000110244	ENSG00000215754
ENSG00000110244	ENSG00000215756
ENSG00000110328	ENSG00000100626
ENSG00000110328	ENSG00000106648
ENSG00000110328	ENSG00000115339
ENSG00000110328	ENSG00000131386
ENSG00000110328	ENSG00000136542
ENSG00000110328	ENSG00000139629
ENSG00000110328	ENSG00000141429
ENSG00000110328	ENSG00000143641
ENSG00000110328	ENSG00000144278
ENSG00000110328	ENSG00000158089
ENSG00000110328	ENSG00000164574
ENSG00000110328	ENSG00000174473
ENSG00000110328	ENSG00000178234
ENSG00000110328	ENSG00000182870
ENSG00000110328	ENSG00000185274
ENSG00000110330	ENSG00000023445
ENSG00000110330	ENSG00000081770
ENSG00000110330	ENSG00000101197
ENSG00000110330	ENSG00000101966
ENSG00000110330	ENSG00000179978
ENSG00000110330	ENSG00000180152
ENSG00000110347	ENSG00000008516
ENSG00000110347	ENSG00000087245
ENSG00000110347	ENSG00000099953
ENSG00000110347	ENSG00000100985
ENSG00000110347	ENSG00000102996
ENSG00000110347	ENSG00000118113
ENSG00000110347	ENSG00000123342
ENSG00000110347	ENSG00000125966
ENSG00000110347	ENSG00000129270
ENSG00000110347	ENSG00000137673
ENSG00000110347	ENSG00000137674
ENSG00000110347	ENSG00000137675
ENSG00000110347	ENSG00000137745
ENSG00000110347	ENSG00000149968
ENSG00000110347	ENSG00000154485
ENSG00000110347	ENSG00000156103
ENSG00000110347	ENSG00000157227
ENSG00000110347	ENSG00000166670
ENSG00000110347	ENSG00000167346
ENSG00000110347	ENSG00000189409
ENSG00000110347	ENSG00000196611
ENSG00000110347	ENSG00000198598
ENSG00000110347	ENSG00000215914
ENSG00000110395	ENSG00000114423
ENSG00000110395	ENSG00000142273
ENSG00000110400	ENSG00000073008
ENSG00000110400	ENSG00000130202
ENSG00000110400	ENSG00000177707
ENSG00000110422	ENSG00000064393
ENSG00000110422	ENSG00000160396
ENSG00000110422	ENSG00000163349
ENSG00000110427	ENSG00000122778
ENSG00000110436	ENSG00000079215

ENSG00000110436	ENSG00000105143
ENSG00000110436	ENSG00000105281
ENSG00000110436	ENSG00000106688
ENSG00000110436	ENSG00000115902
ENSG00000110436	ENSG00000162383
ENSG00000110446	ENSG00000088386
ENSG00000110446	ENSG00000139370
ENSG00000110446	ENSG00000163406
ENSG00000110448	ENSG00000013725
ENSG00000110448	ENSG00000073754
ENSG00000110448	ENSG00000146700
ENSG00000110448	ENSG00000164099
ENSG00000110448	ENSG00000177575
ENSG00000110448	ENSG00000177675
ENSG00000110448	ENSG00000179954
ENSG00000110448	ENSG00000187908
ENSG00000110448	ENSG00000214279
ENSG00000110492	ENSG00000105894
ENSG00000110651	ENSG00000010278
ENSG00000110651	ENSG00000127324
ENSG00000110651	ENSG00000134198
ENSG00000110660	ENSG00000196376
ENSG00000110675	ENSG00000115459
ENSG00000110675	ENSG00000179387
ENSG00000110693	ENSG00000134532
ENSG00000110693	ENSG00000143842
ENSG00000110697	ENSG00000090975
ENSG00000110697	ENSG00000091622
ENSG00000110711	ENSG00000129221
ENSG00000110719	ENSG00000033627
ENSG00000110719	ENSG00000105929
ENSG00000110719	ENSG00000185344
ENSG00000110721	ENSG00000100288
ENSG00000110721	ENSG00000139163
ENSG00000110721	ENSG00000143845
ENSG00000110786	ENSG00000143851
ENSG00000110786	ENSG00000153233
ENSG00000110811	ENSG00000090530
ENSG00000110811	ENSG00000117385
ENSG00000110811	ENSG00000141696
ENSG00000110811	ENSG00000170275
ENSG00000110841	ENSG00000131626
ENSG00000110841	ENSG00000139220
ENSG00000110841	ENSG00000143847
ENSG00000110841	ENSG00000166387
ENSG00000110841	ENSG00000177380
ENSG00000110844	ENSG00000196504
ENSG00000110880	ENSG00000102879
ENSG00000110880	ENSG00000103426
ENSG00000110880	ENSG00000103647
ENSG00000110880	ENSG00000106789
ENSG00000110880	ENSG00000167549
ENSG00000110880	ENSG00000172725
ENSG00000110881	ENSG00000072182
ENSG00000110881	ENSG00000108684
ENSG00000110881	ENSG00000213199
ENSG00000110887	ENSG00000203797
ENSG00000110888	ENSG00000135387
ENSG00000110900	ENSG00000011105

ENSG00000110900	ENSG00000085117
ENSG00000110900	ENSG00000104894
ENSG00000110900	ENSG00000117472
ENSG00000110900	ENSG00000143119
ENSG00000110900	ENSG00000157570
ENSG00000110900	ENSG00000177697
ENSG00000110900	ENSG00000214063
ENSG00000110906	ENSG00000109079
ENSG00000110906	ENSG00000174943
ENSG00000110906	ENSG00000181819
ENSG00000110911	ENSG00000018280
ENSG00000110925	ENSG00000144655
ENSG00000110925	ENSG00000178662
ENSG00000110931	ENSG00000004660
ENSG00000110934	ENSG00000078053
ENSG00000110934	ENSG00000136717
ENSG00000110975	ENSG00000011347
ENSG00000110975	ENSG00000019505
ENSG00000110975	ENSG00000067715
ENSG00000110975	ENSG00000101098
ENSG00000110975	ENSG00000103528
ENSG00000110975	ENSG00000129990
ENSG00000110975	ENSG00000132718
ENSG00000110975	ENSG00000132872
ENSG00000110975	ENSG00000134207
ENSG00000110975	ENSG00000143858
ENSG00000110975	ENSG00000149043
ENSG00000110975	ENSG00000149927
ENSG00000110975	ENSG00000170743
ENSG00000110975	ENSG00000173227
ENSG00000110975	ENSG00000204176
ENSG00000110975	ENSG00000213023
ENSG00000110975	ENSG00000214408
ENSG00000110975	ENSG00000214409
ENSG00000110975	ENSG00000214569
ENSG00000110975	ENSG00000214783
ENSG00000110987	ENSG00000099385
ENSG00000110987	ENSG00000106635
ENSG00000111012	ENSG00000019186
ENSG00000111012	ENSG00000135929
ENSG00000111012	ENSG00000140459
ENSG00000111012	ENSG00000160882
ENSG00000111012	ENSG00000179142
ENSG00000111012	ENSG00000186684
ENSG00000111046	ENSG00000111049
ENSG00000111046	ENSG00000122180
ENSG00000111046	ENSG00000129152
ENSG00000111049	ENSG00000111046
ENSG00000111049	ENSG00000122180
ENSG00000111049	ENSG00000129152
ENSG00000111052	ENSG00000104863
ENSG00000111052	ENSG00000148943
ENSG00000111057	ENSG00000006059
ENSG00000111057	ENSG00000094796
ENSG00000111057	ENSG00000108244
ENSG00000111057	ENSG00000108417
ENSG00000111057	ENSG00000108759
ENSG00000111057	ENSG00000126337
ENSG00000111057	ENSG00000128422

ENSG00000111057	ENSG00000131737
ENSG00000111057	ENSG00000131738
ENSG00000111057	ENSG00000131885
ENSG00000111057	ENSG00000167916
ENSG00000111057	ENSG00000170819
ENSG00000111057	ENSG00000171345
ENSG00000111057	ENSG00000171346
ENSG00000111057	ENSG00000171360
ENSG00000111057	ENSG00000171401
ENSG00000111057	ENSG00000171403
ENSG00000111057	ENSG00000171431
ENSG00000111057	ENSG00000171446
ENSG00000111057	ENSG00000173908
ENSG00000111057	ENSG00000186393
ENSG00000111057	ENSG00000186395
ENSG00000111057	ENSG00000186831
ENSG00000111057	ENSG00000186832
ENSG00000111057	ENSG00000186847
ENSG00000111057	ENSG00000187242
ENSG00000111057	ENSG00000187686
ENSG00000111057	ENSG00000196859
ENSG00000111057	ENSG00000197079
ENSG00000111057	ENSG00000204889
ENSG00000111057	ENSG00000204897
ENSG00000111057	ENSG00000213951
ENSG00000111057	ENSG00000214220
ENSG00000111057	ENSG00000214454
ENSG00000111057	ENSG00000214959
ENSG00000111057	ENSG00000215772
ENSG00000111057	ENSG00000215773
ENSG00000111077	ENSG00000079308
ENSG00000111077	ENSG00000131746
ENSG00000111077	ENSG00000136205
ENSG00000111087	ENSG00000074047
ENSG00000111087	ENSG00000106571
ENSG00000111087	ENSG00000107249
ENSG00000111087	ENSG00000126603
ENSG00000111087	ENSG00000174332
ENSG00000111110	ENSG00000155367
ENSG00000111110	ENSG00000164088
ENSG00000111145	ENSG00000117036
ENSG00000111145	ENSG00000126767
ENSG00000111145	ENSG00000158711
ENSG00000111181	ENSG00000010379
ENSG00000111181	ENSG00000011083
ENSG00000111181	ENSG00000087916
ENSG00000111181	ENSG00000103546
ENSG00000111181	ENSG00000108576
ENSG00000111181	ENSG00000130821
ENSG00000111181	ENSG00000131389
ENSG00000111181	ENSG00000132164
ENSG00000111181	ENSG00000142319
ENSG00000111181	ENSG00000157103
ENSG00000111181	ENSG00000163817
ENSG00000111181	ENSG00000164363
ENSG00000111181	ENSG00000165970
ENSG00000111181	ENSG00000174358
ENSG00000111181	ENSG00000196517
ENSG00000111181	ENSG00000198555

ENSG00000111181	ENSG00000214617
ENSG00000111186	ENSG00000002745
ENSG00000111186	ENSG00000061492
ENSG00000111186	ENSG00000075290
ENSG00000111186	ENSG00000085741
ENSG00000111186	ENSG00000105989
ENSG00000111186	ENSG00000108379
ENSG00000111186	ENSG00000114251
ENSG00000111186	ENSG00000115596
ENSG00000111186	ENSG00000125084
ENSG00000111186	ENSG00000134245
ENSG00000111186	ENSG00000135925
ENSG00000111186	ENSG00000143816
ENSG00000111186	ENSG00000154342
ENSG00000111186	ENSG00000154764
ENSG00000111186	ENSG00000158955
ENSG00000111186	ENSG00000162552
ENSG00000111186	ENSG00000169884
ENSG00000111186	ENSG00000188064
ENSG00000111199	ENSG00000127412
ENSG00000111199	ENSG00000165125
ENSG00000111199	ENSG00000167723
ENSG00000111199	ENSG00000187688
ENSG00000111199	ENSG00000196689
ENSG00000111218	ENSG00000126457
ENSG00000111224	ENSG00000059378
ENSG00000111224	ENSG00000105939
ENSG00000111224	ENSG00000146858
ENSG00000111241	ENSG00000070193
ENSG00000111241	ENSG00000070388
ENSG00000111241	ENSG00000075388
ENSG00000111241	ENSG00000078579
ENSG00000111241	ENSG00000102466
ENSG00000111241	ENSG00000102678
ENSG00000111241	ENSG00000113578
ENSG00000111241	ENSG00000114279
ENSG00000111241	ENSG00000129682
ENSG00000111241	ENSG00000138675
ENSG00000111241	ENSG00000138685
ENSG00000111241	ENSG00000140285
ENSG00000111241	ENSG00000161958
ENSG00000111241	ENSG00000185390
ENSG00000111241	ENSG00000186895
ENSG00000111241	ENSG00000196468
ENSG00000111241	ENSG00000204801
ENSG00000111241	ENSG00000204816
ENSG00000111241	ENSG00000204817
ENSG00000111241	ENSG00000204837
ENSG00000111241	ENSG00000215053
ENSG00000111245	ENSG00000101335
ENSG00000111245	ENSG00000101608
ENSG00000111245	ENSG00000106436
ENSG00000111245	ENSG00000106631
ENSG00000111245	ENSG00000118680
ENSG00000111245	ENSG00000180209
ENSG00000111245	ENSG00000215375
ENSG00000111249	ENSG00000119547
ENSG00000111249	ENSG00000160967
ENSG00000111249	ENSG00000169856

ENSG00000111249	ENSG00000205922
ENSG00000111252	ENSG00000160999
ENSG00000111252	ENSG00000178188
ENSG00000111262	ENSG00000026559
ENSG00000111262	ENSG00000102057
ENSG00000111262	ENSG00000104848
ENSG00000111262	ENSG00000124134
ENSG00000111262	ENSG00000130037
ENSG00000111262	ENSG00000143105
ENSG00000111262	ENSG00000151079
ENSG00000111262	ENSG00000156486
ENSG00000111262	ENSG00000158445
ENSG00000111262	ENSG00000162975
ENSG00000111262	ENSG00000164794
ENSG00000111262	ENSG00000168263
ENSG00000111262	ENSG00000168418
ENSG00000111262	ENSG00000170745
ENSG00000111262	ENSG00000171126
ENSG00000111262	ENSG00000171385
ENSG00000111262	ENSG00000177272
ENSG00000111262	ENSG00000177301
ENSG00000111262	ENSG00000178342
ENSG00000111262	ENSG00000182255
ENSG00000111262	ENSG00000182674
ENSG00000111262	ENSG00000184408
ENSG00000111266	ENSG00000120129
ENSG00000111266	ENSG00000120875
ENSG00000111266	ENSG00000130829
ENSG00000111266	ENSG00000138166
ENSG00000111266	ENSG00000139318
ENSG00000111266	ENSG00000143507
ENSG00000111266	ENSG00000158050
ENSG00000111266	ENSG00000164086
ENSG00000111266	ENSG00000184545
ENSG00000111275	ENSG00000128918
ENSG00000111275	ENSG00000137124
ENSG00000111275	ENSG00000165092
ENSG00000111275	ENSG00000184254
ENSG00000111276	ENSG00000124762
ENSG00000111291	ENSG00000013588
ENSG00000111291	ENSG00000167191
ENSG00000111291	ENSG00000170412
ENSG00000111305	ENSG00000169181
ENSG00000111305	ENSG00000214978
ENSG00000111321	ENSG00000028137
ENSG00000111321	ENSG00000101017
ENSG00000111321	ENSG00000120949
ENSG00000111321	ENSG00000141655
ENSG00000111321	ENSG00000146072
ENSG00000111321	ENSG00000157873
ENSG00000111321	ENSG00000164761
ENSG00000111328	ENSG00000167797
ENSG00000111332	ENSG00000124642
ENSG00000111332	ENSG00000137259
ENSG00000111332	ENSG00000158403
ENSG00000111332	ENSG00000164508
ENSG00000111332	ENSG00000168274
ENSG00000111332	ENSG00000180573
ENSG00000111332	ENSG00000181218

ENSG00000111332	ENSG00000182611
ENSG00000111332	ENSG00000183558
ENSG00000111332	ENSG00000184260
ENSG00000111332	ENSG00000184270
ENSG00000111332	ENSG00000184348
ENSG00000111332	ENSG00000184825
ENSG00000111332	ENSG00000185978
ENSG00000111332	ENSG00000188486
ENSG00000111332	ENSG00000196747
ENSG00000111332	ENSG00000196787
ENSG00000111332	ENSG00000196866
ENSG00000111332	ENSG00000198082
ENSG00000111332	ENSG00000198307
ENSG00000111332	ENSG00000198374
ENSG00000111332	ENSG00000203812
ENSG00000111332	ENSG00000205070
ENSG00000111332	ENSG00000205079
ENSG00000111344	ENSG00000105808
ENSG00000111344	ENSG00000155903
ENSG00000111344	ENSG00000170667
ENSG00000111344	ENSG00000185989
ENSG00000111348	ENSG00000141522
ENSG00000111348	ENSG00000206156
ENSG00000111348	ENSG00000215642
ENSG00000111371	ENSG00000017483
ENSG00000111371	ENSG00000103042
ENSG00000111371	ENSG00000134294
ENSG00000111371	ENSG00000139209
ENSG00000111371	ENSG00000139974
ENSG00000111371	ENSG00000166558
ENSG00000111371	ENSG00000169507
ENSG00000111371	ENSG00000188338
ENSG00000111404	ENSG00000103710
ENSG00000111404	ENSG00000122035
ENSG00000111404	ENSG00000128045
ENSG00000111404	ENSG00000134533
ENSG00000111424	ENSG00000012504
ENSG00000111424	ENSG00000112033
ENSG00000111424	ENSG00000132170
ENSG00000111424	ENSG00000143257
ENSG00000111424	ENSG00000144852
ENSG00000111424	ENSG00000186951
ENSG00000111432	ENSG00000104290
ENSG00000111432	ENSG00000106483
ENSG00000111432	ENSG00000155760
ENSG00000111432	ENSG00000157240
ENSG00000111432	ENSG00000162998
ENSG00000111432	ENSG00000163251
ENSG00000111432	ENSG00000164930
ENSG00000111432	ENSG00000174804
ENSG00000111432	ENSG00000177283
ENSG00000111432	ENSG00000180340
ENSG00000111432	ENSG00000188763
ENSG00000111450	ENSG00000099365
ENSG00000111450	ENSG00000103496
ENSG00000111450	ENSG00000106089
ENSG00000111450	ENSG00000135604
ENSG00000111450	ENSG00000166900
ENSG00000111450	ENSG00000178750

ENSG00000111452	ENSG00000072071
ENSG00000111452	ENSG00000117114
ENSG00000111452	ENSG00000150471
ENSG00000111452	ENSG00000180264
ENSG00000111481	ENSG00000005243
ENSG00000111530	ENSG00000144712
ENSG00000111536	ENSG00000127318
ENSG00000111536	ENSG00000136634
ENSG00000111536	ENSG00000142224
ENSG00000111536	ENSG00000162891
ENSG00000111536	ENSG00000162892
ENSG00000111540	ENSG00000080371
ENSG00000111540	ENSG00000108774
ENSG00000111540	ENSG00000124839
ENSG00000111540	ENSG00000144566
ENSG00000111540	ENSG00000169228
ENSG00000111605	ENSG00000149532
ENSG00000111642	ENSG00000116254
ENSG00000111642	ENSG00000170004
ENSG00000111647	ENSG00000065060
ENSG00000111653	ENSG00000153487
ENSG00000111653	ENSG00000168395
ENSG00000111653	ENSG00000168556
ENSG00000111653	ENSG00000215693
ENSG00000111666	ENSG00000134255
ENSG00000111666	ENSG00000138018
ENSG00000111667	ENSG00000058056
ENSG00000111671	ENSG00000162032
ENSG00000111671	ENSG00000171621
ENSG00000111671	ENSG00000174013
ENSG00000111671	ENSG00000175093
ENSG00000111674	ENSG00000074800
ENSG00000111674	ENSG00000108515
ENSG00000111674	ENSG00000214431
ENSG00000111674	ENSG00000215809
ENSG00000111676	ENSG00000142599
ENSG00000111679	ENSG00000179295
ENSG00000111679	ENSG00000215917
ENSG00000111696	ENSG00000168268
ENSG00000111700	ENSG00000084453
ENSG00000111700	ENSG00000101187
ENSG00000111700	ENSG00000134538
ENSG00000111700	ENSG00000137491
ENSG00000111700	ENSG00000137571
ENSG00000111700	ENSG00000139155
ENSG00000111700	ENSG00000173930
ENSG00000111700	ENSG00000174640
ENSG00000111700	ENSG00000176463
ENSG00000111700	ENSG00000205359
ENSG00000111700	ENSG00000205754
ENSG00000111701	ENSG00000100298
ENSG00000111701	ENSG00000111732
ENSG00000111701	ENSG00000124701
ENSG00000111701	ENSG00000128383
ENSG00000111701	ENSG00000128394
ENSG00000111701	ENSG00000179750
ENSG00000111701	ENSG00000198904
ENSG00000111711	ENSG00000174567
ENSG00000111713	ENSG00000104812

ENSG00000111716	ENSG00000134333
ENSG00000111716	ENSG00000151116
ENSG00000111716	ENSG00000166796
ENSG00000111716	ENSG00000166800
ENSG00000111716	ENSG00000171989
ENSG00000111725	ENSG00000131791
ENSG00000111727	ENSG00000172534
ENSG00000111728	ENSG00000073849
ENSG00000111728	ENSG00000101638
ENSG00000111728	ENSG00000117069
ENSG00000111728	ENSG00000144057
ENSG00000111728	ENSG00000148488
ENSG00000111728	ENSG00000160408
ENSG00000111728	ENSG00000177511
ENSG00000111732	ENSG00000100298
ENSG00000111732	ENSG00000111701
ENSG00000111732	ENSG00000124701
ENSG00000111732	ENSG00000128383
ENSG00000111732	ENSG00000128394
ENSG00000111732	ENSG00000179750
ENSG00000111732	ENSG00000198904
ENSG00000111752	ENSG00000134686
ENSG00000111752	ENSG00000173889
ENSG00000111752	ENSG00000179899
ENSG00000111752	ENSG00000187033
ENSG00000111752	ENSG00000187634
ENSG00000111775	ENSG00000156885
ENSG00000111775	ENSG00000164605
ENSG00000111783	ENSG00000080298
ENSG00000111783	ENSG00000087903
ENSG00000111783	ENSG00000132005
ENSG00000111783	ENSG00000143390
ENSG00000111783	ENSG00000181827
ENSG00000111783	ENSG00000185002
ENSG00000111785	ENSG00000177963
ENSG00000111786	ENSG00000136450
ENSG00000111790	ENSG00000052723
ENSG00000111799	ENSG00000101203
ENSG00000111799	ENSG00000187955
ENSG00000111801	ENSG00000026950
ENSG00000111801	ENSG00000112763
ENSG00000111801	ENSG00000113303
ENSG00000111801	ENSG00000121236
ENSG00000111801	ENSG00000124508
ENSG00000111801	ENSG00000124549
ENSG00000111801	ENSG00000124557
ENSG00000111801	ENSG00000128250
ENSG00000111801	ENSG00000128253
ENSG00000111801	ENSG00000128276
ENSG00000111801	ENSG00000128482
ENSG00000111801	ENSG00000132256
ENSG00000111801	ENSG00000132274
ENSG00000111801	ENSG00000164010
ENSG00000111801	ENSG00000165810
ENSG00000111801	ENSG00000168903
ENSG00000111801	ENSG00000176422
ENSG00000111801	ENSG00000179046
ENSG00000111801	ENSG00000182805
ENSG00000111801	ENSG00000183439

ENSG00000111801	ENSG00000183718
ENSG00000111801	ENSG00000187574
ENSG00000111801	ENSG00000188683
ENSG00000111801	ENSG00000204614
ENSG00000111801	ENSG00000204616
ENSG00000111801	ENSG00000204618
ENSG00000111801	ENSG00000205198
ENSG00000111801	ENSG00000214693
ENSG00000111817	ENSG00000171451
ENSG00000111837	ENSG00000080823
ENSG00000111837	ENSG00000112144
ENSG00000111843	ENSG00000096092
ENSG00000111843	ENSG00000137210
ENSG00000111843	ENSG00000189156
ENSG00000111843	ENSG00000214881
ENSG00000111846	ENSG00000015532
ENSG00000111846	ENSG00000103489
ENSG00000111846	ENSG00000124091
ENSG00000111846	ENSG00000140297
ENSG00000111846	ENSG00000176928
ENSG00000111846	ENSG00000187210
ENSG00000111846	ENSG00000205318
ENSG00000111859	ENSG00000050820
ENSG00000111859	ENSG00000087589
ENSG00000111859	ENSG00000100842
ENSG00000111860	ENSG00000130695
ENSG00000111863	ENSG00000146416
ENSG00000111875	ENSG00000105011
ENSG00000111879	ENSG00000047662
ENSG00000111885	ENSG00000117643
ENSG00000111885	ENSG00000198162
ENSG00000111886	ENSG00000011677
ENSG00000111886	ENSG00000022355
ENSG00000111886	ENSG00000094755
ENSG00000111886	ENSG00000101958
ENSG00000111886	ENSG00000102287
ENSG00000111886	ENSG00000109158
ENSG00000111886	ENSG00000109738
ENSG00000111886	ENSG00000113327
ENSG00000111886	ENSG00000145451
ENSG00000111886	ENSG00000145863
ENSG00000111886	ENSG00000145864
ENSG00000111886	ENSG00000145888
ENSG00000111886	ENSG00000146276
ENSG00000111886	ENSG00000147402
ENSG00000111886	ENSG00000151834
ENSG00000111886	ENSG00000163285
ENSG00000111886	ENSG00000163288
ENSG00000111886	ENSG00000166206
ENSG00000111886	ENSG00000182256
ENSG00000111886	ENSG00000183185
ENSG00000111886	ENSG00000186297
ENSG00000111886	ENSG00000187730
ENSG00000111886	ENSG00000188828
ENSG00000111897	ENSG00000132824
ENSG00000111897	ENSG00000164300
ENSG00000111897	ENSG00000168528
ENSG00000111897	ENSG00000184716
ENSG00000111907	ENSG00000076554

ENSG00000111907	ENSG00000101150
ENSG00000111907	ENSG00000170777
ENSG00000111912	ENSG00000164830
ENSG00000111913	ENSG00000039523
ENSG00000111913	ENSG00000042062
ENSG00000111961	ENSG00000122122
ENSG00000111961	ENSG00000155307
ENSG00000112029	ENSG00000156509
ENSG00000112031	ENSG00000120662
ENSG00000112031	ENSG00000180015
ENSG00000112033	ENSG00000012504
ENSG00000112033	ENSG00000111424
ENSG00000112033	ENSG00000132170
ENSG00000112033	ENSG00000143257
ENSG00000112033	ENSG00000144852
ENSG00000112033	ENSG00000186951
ENSG00000112038	ENSG00000082556
ENSG00000112038	ENSG00000116014
ENSG00000112038	ENSG00000116329
ENSG00000112038	ENSG00000125510
ENSG00000112038	ENSG00000125522
ENSG00000112038	ENSG00000128285
ENSG00000112038	ENSG00000128310
ENSG00000112038	ENSG00000132671
ENSG00000112038	ENSG00000139874
ENSG00000112038	ENSG00000152034
ENSG00000112038	ENSG00000162009
ENSG00000112038	ENSG00000166573
ENSG00000112038	ENSG00000180616
ENSG00000112038	ENSG00000182687
ENSG00000112038	ENSG00000183473
ENSG00000112038	ENSG00000183729
ENSG00000112041	ENSG00000078246
ENSG00000112041	ENSG00000104804
ENSG00000112041	ENSG00000118965
ENSG00000112041	ENSG00000130338
ENSG00000112041	ENSG00000166402
ENSG00000112062	ENSG00000050748
ENSG00000112062	ENSG00000107643
ENSG00000112062	ENSG00000109339
ENSG00000112062	ENSG00000156711
ENSG00000112062	ENSG00000185386
ENSG00000112062	ENSG00000188130
ENSG00000112077	ENSG00000132677
ENSG00000112077	ENSG00000140519
ENSG00000112077	ENSG00000187010
ENSG00000112077	ENSG00000188672
ENSG00000112078	ENSG00000148925
ENSG00000112079	ENSG00000104936
ENSG00000112079	ENSG00000211455
ENSG00000112081	ENSG00000115875
ENSG00000112081	ENSG00000139168
ENSG00000112081	ENSG00000204183
ENSG00000112090	ENSG00000206004
ENSG00000112115	ENSG00000112116
ENSG00000112115	ENSG00000124391
ENSG00000112115	ENSG00000127743
ENSG00000112115	ENSG00000166090
ENSG00000112115	ENSG00000172458

ENSG00000112116	ENSG00000112115
ENSG00000112116	ENSG00000124391
ENSG00000112116	ENSG00000127743
ENSG00000112116	ENSG00000166090
ENSG00000112116	ENSG00000172458
ENSG00000112130	ENSG00000072609
ENSG00000112137	ENSG00000087495
ENSG00000112137	ENSG00000112419
ENSG00000112137	ENSG00000204138
ENSG00000112139	ENSG00000139915
ENSG00000112139	ENSG00000165072
ENSG00000112139	ENSG00000177943
ENSG00000112139	ENSG00000204741
ENSG00000112144	ENSG00000080823
ENSG00000112144	ENSG00000111837
ENSG00000112164	ENSG00000004948
ENSG00000112164	ENSG00000010310
ENSG00000112164	ENSG00000064989
ENSG00000112164	ENSG00000065325
ENSG00000112164	ENSG00000078549
ENSG00000112164	ENSG00000080293
ENSG00000112164	ENSG00000106018
ENSG00000112164	ENSG00000106113
ENSG00000112164	ENSG00000106128
ENSG00000112164	ENSG00000114812
ENSG00000112164	ENSG00000120088
ENSG00000112164	ENSG00000144407
ENSG00000112164	ENSG00000160801
ENSG00000112164	ENSG00000215644
ENSG00000112175	ENSG00000101144
ENSG00000112175	ENSG00000107623
ENSG00000112175	ENSG00000116985
ENSG00000112175	ENSG00000125965
ENSG00000112175	ENSG00000128802
ENSG00000112175	ENSG00000130385
ENSG00000112175	ENSG00000143869
ENSG00000112175	ENSG00000152785
ENSG00000112175	ENSG00000153162
ENSG00000112175	ENSG00000156466
ENSG00000112175	ENSG00000156574
ENSG00000112175	ENSG00000163217
ENSG00000112175	ENSG00000183682
ENSG00000112182	ENSG00000082641
ENSG00000112182	ENSG00000116044
ENSG00000112182	ENSG00000123405
ENSG00000112182	ENSG00000156273
ENSG00000112183	ENSG00000132819
ENSG00000112186	ENSG00000131236
ENSG00000112212	ENSG00000100300
ENSG00000112214	ENSG00000072163
ENSG00000112214	ENSG00000089159
ENSG00000112214	ENSG00000110031
ENSG00000112214	ENSG00000140682
ENSG00000112214	ENSG00000169756
ENSG00000112214	ENSG00000183386
ENSG00000112232	ENSG00000112531
ENSG00000112232	ENSG00000121774
ENSG00000112232	ENSG00000131773
ENSG00000112242	ENSG00000007968

ENSG00000112242	ENSG00000101412
ENSG00000112242	ENSG00000133740
ENSG00000112242	ENSG00000169016
ENSG00000112242	ENSG00000188841
ENSG00000112242	ENSG00000205250
ENSG00000112245	ENSG00000184007
ENSG00000112245	ENSG00000184489
ENSG00000112246	ENSG00000100644
ENSG00000112246	ENSG00000116016
ENSG00000112246	ENSG00000124440
ENSG00000112246	ENSG00000130751
ENSG00000112246	ENSG00000151322
ENSG00000112246	ENSG00000159263
ENSG00000112273	ENSG00000143321
ENSG00000112273	ENSG00000164985
ENSG00000112273	ENSG00000166503
ENSG00000112273	ENSG00000167674
ENSG00000112280	ENSG00000049089
ENSG00000112280	ENSG00000084636
ENSG00000112280	ENSG00000092758
ENSG00000112290	ENSG00000132970
ENSG00000112290	ENSG00000158195
ENSG00000112290	ENSG00000188459
ENSG00000112297	ENSG00000080200
ENSG00000112297	ENSG00000176092
ENSG00000112299	ENSG00000093134
ENSG00000112299	ENSG00000112303
ENSG00000112299	ENSG00000169814
ENSG00000112303	ENSG00000093134
ENSG00000112303	ENSG00000112299
ENSG00000112303	ENSG00000169814
ENSG00000112305	ENSG00000084070
ENSG00000112309	ENSG00000109956
ENSG00000112309	ENSG00000149541
ENSG00000112319	ENSG00000064655
ENSG00000112319	ENSG00000104313
ENSG00000112319	ENSG00000158161
ENSG00000112335	ENSG00000147164
ENSG00000112337	ENSG00000091664
ENSG00000112337	ENSG00000104888
ENSG00000112337	ENSG00000119899
ENSG00000112337	ENSG00000124564
ENSG00000112337	ENSG00000124568
ENSG00000112337	ENSG00000146039
ENSG00000112337	ENSG00000179520
ENSG00000112378	ENSG00000147027
ENSG00000112378	ENSG00000176711
ENSG00000112394	ENSG00000100156
ENSG00000112394	ENSG00000108932
ENSG00000112394	ENSG00000118596
ENSG00000112394	ENSG00000141526
ENSG00000112394	ENSG00000147100
ENSG00000112394	ENSG00000152779
ENSG00000112394	ENSG00000155380
ENSG00000112394	ENSG00000163053
ENSG00000112394	ENSG00000165449
ENSG00000112394	ENSG00000168679
ENSG00000112394	ENSG00000170190
ENSG00000112394	ENSG00000174326

ENSG00000112394	ENSG00000174327
ENSG00000112414	ENSG00000156920
ENSG00000112414	ENSG00000159618
ENSG00000112414	ENSG00000173698
ENSG00000112414	ENSG00000182885
ENSG00000112414	ENSG00000205336
ENSG00000112419	ENSG00000087495
ENSG00000112419	ENSG00000112137
ENSG00000112419	ENSG00000204138
ENSG00000112486	ENSG00000121797
ENSG00000112486	ENSG00000121807
ENSG00000112486	ENSG00000121966
ENSG00000112486	ENSG00000126353
ENSG00000112486	ENSG00000129048
ENSG00000112486	ENSG00000144648
ENSG00000112486	ENSG00000160683
ENSG00000112486	ENSG00000160791
ENSG00000112486	ENSG00000163464
ENSG00000112486	ENSG00000163823
ENSG00000112486	ENSG00000168329
ENSG00000112486	ENSG00000172215
ENSG00000112486	ENSG00000173578
ENSG00000112486	ENSG00000173585
ENSG00000112486	ENSG00000179934
ENSG00000112486	ENSG00000180871
ENSG00000112486	ENSG00000183625
ENSG00000112486	ENSG00000183813
ENSG00000112486	ENSG00000184451
ENSG00000112486	ENSG00000186810
ENSG00000112486	ENSG00000215775
ENSG00000112486	ENSG00000215778
ENSG00000112486	ENSG00000215782
ENSG00000112493	ENSG00000139192
ENSG00000112511	ENSG00000119403
ENSG00000112511	ENSG00000143033
ENSG00000112531	ENSG00000112232
ENSG00000112531	ENSG00000121774
ENSG00000112531	ENSG00000131773
ENSG00000112559	ENSG00000135272
ENSG00000112561	ENSG00000068323
ENSG00000112561	ENSG00000105967
ENSG00000112561	ENSG00000187098
ENSG00000112562	ENSG00000087303
ENSG00000112562	ENSG00000116962
ENSG00000112562	ENSG00000198732
ENSG00000112576	ENSG00000110092
ENSG00000112576	ENSG00000118971
ENSG00000112592	ENSG00000028839
ENSG00000112592	ENSG00000182521
ENSG00000112599	ENSG00000048545
ENSG00000112599	ENSG00000138472
ENSG00000112619	ENSG00000149489
ENSG00000112624	ENSG00000063169
ENSG00000112640	ENSG00000066027
ENSG00000112640	ENSG00000068971
ENSG00000112640	ENSG00000078304
ENSG00000112640	ENSG00000154001
ENSG00000112664	ENSG00000122824
ENSG00000112664	ENSG00000173598

ENSG00000112664	ENSG00000196368
ENSG00000112679	ENSG00000149599
ENSG00000112695	ENSG00000115944
ENSG00000112695	ENSG00000161281
ENSG00000112697	ENSG00000182107
ENSG00000112701	ENSG00000138468
ENSG00000112706	ENSG00000081148
ENSG00000112715	ENSG00000100311
ENSG00000112715	ENSG00000119630
ENSG00000112715	ENSG00000150630
ENSG00000112715	ENSG00000165197
ENSG00000112715	ENSG00000173511
ENSG00000112715	ENSG00000197461
ENSG00000112715	ENSG00000215682
ENSG00000112759	ENSG00000164638
ENSG00000112759	ENSG00000174669
ENSG00000112759	ENSG00000198246
ENSG00000112759	ENSG00000214660
ENSG00000112759	ENSG00000214668
ENSG00000112761	ENSG00000064205
ENSG00000112761	ENSG00000104415
ENSG00000112761	ENSG00000118523
ENSG00000112761	ENSG00000136999
ENSG00000112761	ENSG00000142871
ENSG00000112763	ENSG00000026950
ENSG00000112763	ENSG00000111801
ENSG00000112763	ENSG00000113303
ENSG00000112763	ENSG00000121236
ENSG00000112763	ENSG00000124508
ENSG00000112763	ENSG00000124549
ENSG00000112763	ENSG00000124557
ENSG00000112763	ENSG00000128250
ENSG00000112763	ENSG00000128253
ENSG00000112763	ENSG00000128276
ENSG00000112763	ENSG00000128482
ENSG00000112763	ENSG00000132256
ENSG00000112763	ENSG00000132274
ENSG00000112763	ENSG00000164010
ENSG00000112763	ENSG00000165810
ENSG00000112763	ENSG00000168903
ENSG00000112763	ENSG00000176422
ENSG00000112763	ENSG00000179046
ENSG00000112763	ENSG00000182805
ENSG00000112763	ENSG00000183439
ENSG00000112763	ENSG00000183718
ENSG00000112763	ENSG00000187574
ENSG00000112763	ENSG00000188683
ENSG00000112763	ENSG00000204614
ENSG00000112763	ENSG00000204616
ENSG00000112763	ENSG00000204618
ENSG00000112763	ENSG00000205198
ENSG00000112763	ENSG00000214693
ENSG00000112769	ENSG00000053747
ENSG00000112769	ENSG00000101680
ENSG00000112769	ENSG00000130702
ENSG00000112769	ENSG00000196569
ENSG00000112773	ENSG00000158246
ENSG00000112773	ENSG00000174016
ENSG00000112773	ENSG00000183508

ENSG00000112782	ENSG00000155962
ENSG00000112782	ENSG00000159212
ENSG00000112782	ENSG00000169504
ENSG00000112782	ENSG00000169583
ENSG00000112782	ENSG00000213719
ENSG00000112782	ENSG00000214258
ENSG00000112787	ENSG00000156860
ENSG00000112787	ENSG00000158321
ENSG00000112796	ENSG00000001561
ENSG00000112796	ENSG00000136960
ENSG00000112796	ENSG00000154269
ENSG00000112796	ENSG00000164303
ENSG00000112796	ENSG00000182156
ENSG00000112796	ENSG00000197594
ENSG00000112818	ENSG00000141434
ENSG00000112818	ENSG00000188886
ENSG00000112837	ENSG00000073861
ENSG00000112837	ENSG00000089225
ENSG00000112837	ENSG00000092607
ENSG00000112837	ENSG00000121068
ENSG00000112837	ENSG00000121075
ENSG00000112837	ENSG00000122145
ENSG00000112837	ENSG00000135111
ENSG00000112837	ENSG00000136535
ENSG00000112837	ENSG00000143178
ENSG00000112837	ENSG00000149922
ENSG00000112837	ENSG00000163508
ENSG00000112837	ENSG00000164458
ENSG00000112837	ENSG00000164532
ENSG00000112837	ENSG00000167800
ENSG00000112837	ENSG00000174197
ENSG00000112837	ENSG00000184058
ENSG00000112851	ENSG00000033122
ENSG00000112851	ENSG00000137269
ENSG00000112851	ENSG00000147324
ENSG00000112851	ENSG00000180900
ENSG00000112851	ENSG00000188306
ENSG00000112852	ENSG00000081818
ENSG00000112852	ENSG00000081842
ENSG00000112852	ENSG00000081853
ENSG00000112852	ENSG00000113205
ENSG00000112852	ENSG00000113209
ENSG00000112852	ENSG00000113211
ENSG00000112852	ENSG00000113212
ENSG00000112852	ENSG00000113248
ENSG00000112852	ENSG00000120322
ENSG00000112852	ENSG00000120324
ENSG00000112852	ENSG00000120327
ENSG00000112852	ENSG00000120328
ENSG00000112852	ENSG00000146001
ENSG00000112852	ENSG00000171815
ENSG00000112852	ENSG00000187372
ENSG00000112852	ENSG00000196963
ENSG00000112852	ENSG00000197479
ENSG00000112852	ENSG00000204955
ENSG00000112852	ENSG00000204956
ENSG00000112852	ENSG00000204961
ENSG00000112852	ENSG00000204962
ENSG00000112852	ENSG00000204963

ENSG00000112852	ENSG00000204964
ENSG00000112852	ENSG00000204965
ENSG00000112852	ENSG00000204967
ENSG00000112852	ENSG00000204968
ENSG00000112852	ENSG00000204969
ENSG00000112852	ENSG00000204970
ENSG00000112852	ENSG00000214567
ENSG00000112852	ENSG00000214570
ENSG00000112852	ENSG00000214574
ENSG00000112852	ENSG00000214580
ENSG00000112852	ENSG00000214583
ENSG00000112852	ENSG00000214594
ENSG00000112852	ENSG00000214600
ENSG00000112852	ENSG00000214608
ENSG00000112855	ENSG00000170445
ENSG00000112893	ENSG00000196547
ENSG00000112902	ENSG00000082684
ENSG00000112902	ENSG00000167680
ENSG00000112936	ENSG00000021852
ENSG00000112936	ENSG00000039537
ENSG00000112936	ENSG00000113600
ENSG00000112936	ENSG00000157131
ENSG00000112936	ENSG00000180644
ENSG00000112941	ENSG00000121274
ENSG00000112964	ENSG00000113494
ENSG00000112972	ENSG00000134240
ENSG00000112984	ENSG00000137807
ENSG00000112984	ENSG00000138182
ENSG00000113070	ENSG00000109321
ENSG00000113070	ENSG00000205595
ENSG00000113073	ENSG00000004939
ENSG00000113073	ENSG00000033867
ENSG00000113073	ENSG00000050438
ENSG00000113073	ENSG00000080493
ENSG00000113073	ENSG00000088836
ENSG00000113073	ENSG00000114923
ENSG00000113073	ENSG00000144290
ENSG00000113073	ENSG00000164889
ENSG00000113073	ENSG00000188687
ENSG00000113083	ENSG00000115318
ENSG00000113083	ENSG00000129038
ENSG00000113083	ENSG00000134013
ENSG00000113083	ENSG00000138131
ENSG00000113088	ENSG00000092009
ENSG00000113088	ENSG00000100448
ENSG00000113088	ENSG00000100450
ENSG00000113088	ENSG00000100453
ENSG00000113088	ENSG00000145649
ENSG00000113088	ENSG00000185198
ENSG00000113088	ENSG00000197766
ENSG00000113100	ENSG00000040731
ENSG00000113100	ENSG00000071991
ENSG00000113100	ENSG00000081138
ENSG00000113100	ENSG00000101542
ENSG00000113100	ENSG00000113361
ENSG00000113100	ENSG00000139880
ENSG00000113100	ENSG00000140937
ENSG00000113100	ENSG00000145526
ENSG00000113100	ENSG00000149654

ENSG00000113100	ENSG00000150394
ENSG00000113100	ENSG00000154162
ENSG00000113100	ENSG00000179776
ENSG00000113108	ENSG00000163697
ENSG00000113108	ENSG00000166313
ENSG00000113140	ENSG00000152583
ENSG00000113196	ENSG00000104903
ENSG00000113196	ENSG00000122691
ENSG00000113196	ENSG00000122859
ENSG00000113196	ENSG00000123307
ENSG00000113196	ENSG00000125878
ENSG00000113196	ENSG00000146618
ENSG00000113196	ENSG00000162367
ENSG00000113196	ENSG00000162992
ENSG00000113196	ENSG00000164107
ENSG00000113196	ENSG00000164600
ENSG00000113196	ENSG00000168267
ENSG00000113196	ENSG00000171532
ENSG00000113196	ENSG00000171786
ENSG00000113196	ENSG00000172238
ENSG00000113196	ENSG00000177551
ENSG00000113196	ENSG00000178403
ENSG00000113196	ENSG00000179774
ENSG00000113196	ENSG00000180535
ENSG00000113196	ENSG00000181965
ENSG00000113196	ENSG00000186051
ENSG00000113196	ENSG00000187786
ENSG00000113196	ENSG00000188686
ENSG00000113205	ENSG00000081818
ENSG00000113205	ENSG00000081842
ENSG00000113205	ENSG00000081853
ENSG00000113205	ENSG00000112852
ENSG00000113205	ENSG00000113209
ENSG00000113205	ENSG00000113211
ENSG00000113205	ENSG00000113212
ENSG00000113205	ENSG00000113248
ENSG00000113205	ENSG00000120322
ENSG00000113205	ENSG00000120324
ENSG00000113205	ENSG00000120327
ENSG00000113205	ENSG00000120328
ENSG00000113205	ENSG00000146001
ENSG00000113205	ENSG00000171815
ENSG00000113205	ENSG00000187372
ENSG00000113205	ENSG00000196963
ENSG00000113205	ENSG00000197479
ENSG00000113205	ENSG00000204955
ENSG00000113205	ENSG00000204956
ENSG00000113205	ENSG00000204961
ENSG00000113205	ENSG00000204962
ENSG00000113205	ENSG00000204963
ENSG00000113205	ENSG00000204964
ENSG00000113205	ENSG00000204965
ENSG00000113205	ENSG00000204967
ENSG00000113205	ENSG00000204968
ENSG00000113205	ENSG00000204969
ENSG00000113205	ENSG00000204970
ENSG00000113205	ENSG00000214567
ENSG00000113205	ENSG00000214570
ENSG00000113205	ENSG00000214574

ENSG00000113205	ENSG00000214580
ENSG00000113205	ENSG00000214583
ENSG00000113205	ENSG00000214594
ENSG00000113205	ENSG00000214600
ENSG00000113205	ENSG00000214608
ENSG00000113209	ENSG00000081818
ENSG00000113209	ENSG00000081842
ENSG00000113209	ENSG00000081853
ENSG00000113209	ENSG00000112852
ENSG00000113209	ENSG00000113205
ENSG00000113209	ENSG00000113211
ENSG00000113209	ENSG00000113212
ENSG00000113209	ENSG00000113248
ENSG00000113209	ENSG00000120322
ENSG00000113209	ENSG00000120324
ENSG00000113209	ENSG00000120327
ENSG00000113209	ENSG00000120328
ENSG00000113209	ENSG00000146001
ENSG00000113209	ENSG00000171815
ENSG00000113209	ENSG00000187372
ENSG00000113209	ENSG00000196963
ENSG00000113209	ENSG00000197479
ENSG00000113209	ENSG00000204955
ENSG00000113209	ENSG00000204956
ENSG00000113209	ENSG00000204961
ENSG00000113209	ENSG00000204962
ENSG00000113209	ENSG00000204963
ENSG00000113209	ENSG00000204964
ENSG00000113209	ENSG00000204965
ENSG00000113209	ENSG00000204967
ENSG00000113209	ENSG00000204968
ENSG00000113209	ENSG00000204969
ENSG00000113209	ENSG00000204970
ENSG00000113209	ENSG00000214567
ENSG00000113209	ENSG00000214570
ENSG00000113209	ENSG00000214574
ENSG00000113209	ENSG00000214580
ENSG00000113209	ENSG00000214583
ENSG00000113209	ENSG00000214594
ENSG00000113209	ENSG00000214600
ENSG00000113209	ENSG00000214608
ENSG00000113211	ENSG00000081818
ENSG00000113211	ENSG00000081842
ENSG00000113211	ENSG00000081853
ENSG00000113211	ENSG00000112852
ENSG00000113211	ENSG00000113205
ENSG00000113211	ENSG00000113209
ENSG00000113211	ENSG00000113212
ENSG00000113211	ENSG00000113248
ENSG00000113211	ENSG00000120322
ENSG00000113211	ENSG00000120324
ENSG00000113211	ENSG00000120327
ENSG00000113211	ENSG00000120328
ENSG00000113211	ENSG00000146001
ENSG00000113211	ENSG00000171815
ENSG00000113211	ENSG00000187372
ENSG00000113211	ENSG00000196963
ENSG00000113211	ENSG00000197479
ENSG00000113211	ENSG00000204955

ENSG00000113211	ENSG00000204956
ENSG00000113211	ENSG00000204961
ENSG00000113211	ENSG00000204962
ENSG00000113211	ENSG00000204963
ENSG00000113211	ENSG00000204964
ENSG00000113211	ENSG00000204965
ENSG00000113211	ENSG00000204967
ENSG00000113211	ENSG00000204968
ENSG00000113211	ENSG00000204969
ENSG00000113211	ENSG00000204970
ENSG00000113211	ENSG00000214567
ENSG00000113211	ENSG00000214570
ENSG00000113211	ENSG00000214574
ENSG00000113211	ENSG00000214580
ENSG00000113211	ENSG00000214583
ENSG00000113211	ENSG00000214594
ENSG00000113211	ENSG00000214600
ENSG00000113211	ENSG00000214608
ENSG00000113212	ENSG00000081818
ENSG00000113212	ENSG00000081842
ENSG00000113212	ENSG00000081853
ENSG00000113212	ENSG00000112852
ENSG00000113212	ENSG00000113205
ENSG00000113212	ENSG00000113209
ENSG00000113212	ENSG00000113211
ENSG00000113212	ENSG00000113248
ENSG00000113212	ENSG00000120322
ENSG00000113212	ENSG00000120324
ENSG00000113212	ENSG00000120327
ENSG00000113212	ENSG00000120328
ENSG00000113212	ENSG00000146001
ENSG00000113212	ENSG00000171815
ENSG00000113212	ENSG00000187372
ENSG00000113212	ENSG00000196963
ENSG00000113212	ENSG00000197479
ENSG00000113212	ENSG00000204955
ENSG00000113212	ENSG00000204956
ENSG00000113212	ENSG00000204961
ENSG00000113212	ENSG00000204962
ENSG00000113212	ENSG00000204963
ENSG00000113212	ENSG00000204964
ENSG00000113212	ENSG00000204965
ENSG00000113212	ENSG00000204967
ENSG00000113212	ENSG00000204968
ENSG00000113212	ENSG00000204969
ENSG00000113212	ENSG00000204970
ENSG00000113212	ENSG00000214567
ENSG00000113212	ENSG00000214570
ENSG00000113212	ENSG00000214574
ENSG00000113212	ENSG00000214580
ENSG00000113212	ENSG00000214583
ENSG00000113212	ENSG00000214594
ENSG00000113212	ENSG00000214600
ENSG00000113212	ENSG00000214608
ENSG00000113231	ENSG00000065989
ENSG00000113231	ENSG00000073417
ENSG00000113231	ENSG00000105650
ENSG00000113231	ENSG00000113448
ENSG00000113231	ENSG00000115252

ENSG00000113231	ENSG00000123360
ENSG00000113231	ENSG00000154678
ENSG00000113231	ENSG00000160191
ENSG00000113231	ENSG00000171408
ENSG00000113231	ENSG00000184588
ENSG00000113231	ENSG00000205268
ENSG00000113240	ENSG00000013441
ENSG00000113240	ENSG00000176444
ENSG00000113240	ENSG00000178340
ENSG00000113240	ENSG00000179335
ENSG00000113240	ENSG00000215793
ENSG00000113248	ENSG00000081818
ENSG00000113248	ENSG00000081842
ENSG00000113248	ENSG00000081853
ENSG00000113248	ENSG00000112852
ENSG00000113248	ENSG00000113205
ENSG00000113248	ENSG00000113209
ENSG00000113248	ENSG00000113211
ENSG00000113248	ENSG00000113212
ENSG00000113248	ENSG00000120322
ENSG00000113248	ENSG00000120324
ENSG00000113248	ENSG00000120327
ENSG00000113248	ENSG00000120328
ENSG00000113248	ENSG00000146001
ENSG00000113248	ENSG00000171815
ENSG00000113248	ENSG00000187372
ENSG00000113248	ENSG00000196963
ENSG00000113248	ENSG00000197479
ENSG00000113248	ENSG00000204955
ENSG00000113248	ENSG00000204956
ENSG00000113248	ENSG00000204961
ENSG00000113248	ENSG00000204962
ENSG00000113248	ENSG00000204963
ENSG00000113248	ENSG00000204964
ENSG00000113248	ENSG00000204965
ENSG00000113248	ENSG00000204967
ENSG00000113248	ENSG00000204968
ENSG00000113248	ENSG00000204969
ENSG00000113248	ENSG00000204970
ENSG00000113248	ENSG00000214567
ENSG00000113248	ENSG00000214570
ENSG00000113248	ENSG00000214574
ENSG00000113248	ENSG00000214580
ENSG00000113248	ENSG00000214583
ENSG00000113248	ENSG00000214594
ENSG00000113248	ENSG00000214600
ENSG00000113248	ENSG00000214608
ENSG00000113249	ENSG00000135077
ENSG00000113249	ENSG00000145850
ENSG00000113262	ENSG00000124493
ENSG00000113262	ENSG00000152822
ENSG00000113262	ENSG00000164082
ENSG00000113262	ENSG00000168959
ENSG00000113262	ENSG00000179603
ENSG00000113262	ENSG00000196277
ENSG00000113262	ENSG00000198822
ENSG00000113263	ENSG00000010671
ENSG00000113263	ENSG00000074966
ENSG00000113263	ENSG00000102010

ENSG00000113263	ENSG00000135605
ENSG00000113263	ENSG00000151422
ENSG00000113263	ENSG00000182511
ENSG00000113269	ENSG00000082996
ENSG00000113269	ENSG00000105428
ENSG00000113269	ENSG00000108523
ENSG00000113269	ENSG00000133135
ENSG00000113269	ENSG00000163162
ENSG00000113269	ENSG00000170153
ENSG00000113269	ENSG00000188050
ENSG00000113273	ENSG00000006756
ENSG00000113273	ENSG00000062096
ENSG00000113273	ENSG00000100299
ENSG00000113273	ENSG00000101846
ENSG00000113273	ENSG00000141012
ENSG00000113273	ENSG00000141337
ENSG00000113273	ENSG00000157399
ENSG00000113273	ENSG00000180801
ENSG00000113273	ENSG00000183876
ENSG00000113273	ENSG00000205667
ENSG00000113282	ENSG00000049283
ENSG00000113282	ENSG00000063245
ENSG00000113282	ENSG00000072134
ENSG00000113282	ENSG00000176177
ENSG00000113296	ENSG00000105664
ENSG00000113296	ENSG00000137801
ENSG00000113296	ENSG00000169231
ENSG00000113296	ENSG00000186340
ENSG00000113300	ENSG00000138767
ENSG00000113300	ENSG00000151014
ENSG00000113300	ENSG00000174840
ENSG00000113303	ENSG00000026950
ENSG00000113303	ENSG00000111801
ENSG00000113303	ENSG00000112763
ENSG00000113303	ENSG00000121236
ENSG00000113303	ENSG00000124508
ENSG00000113303	ENSG00000124549
ENSG00000113303	ENSG00000124557
ENSG00000113303	ENSG00000128250
ENSG00000113303	ENSG00000128253
ENSG00000113303	ENSG00000128276
ENSG00000113303	ENSG00000128482
ENSG00000113303	ENSG00000132256
ENSG00000113303	ENSG00000132274
ENSG00000113303	ENSG00000164010
ENSG00000113303	ENSG00000165810
ENSG00000113303	ENSG00000168903
ENSG00000113303	ENSG00000176422
ENSG00000113303	ENSG00000179046
ENSG00000113303	ENSG00000182805
ENSG00000113303	ENSG00000183439
ENSG00000113303	ENSG00000183718
ENSG00000113303	ENSG00000187574
ENSG00000113303	ENSG00000188683
ENSG00000113303	ENSG00000204614
ENSG00000113303	ENSG00000204616
ENSG00000113303	ENSG00000204618
ENSG00000113303	ENSG00000205198
ENSG00000113303	ENSG00000214693

ENSG00000113319	ENSG00000058335
ENSG00000113319	ENSG00000100485
ENSG00000113319	ENSG00000107263
ENSG00000113319	ENSG00000115904
ENSG00000113327	ENSG00000011677
ENSG00000113327	ENSG00000022355
ENSG00000113327	ENSG00000094755
ENSG00000113327	ENSG00000101958
ENSG00000113327	ENSG00000102287
ENSG00000113327	ENSG00000109158
ENSG00000113327	ENSG00000109738
ENSG00000113327	ENSG00000111886
ENSG00000113327	ENSG00000145451
ENSG00000113327	ENSG00000145863
ENSG00000113327	ENSG00000145864
ENSG00000113327	ENSG00000145888
ENSG00000113327	ENSG00000146276
ENSG00000113327	ENSG00000147402
ENSG00000113327	ENSG00000151834
ENSG00000113327	ENSG00000163285
ENSG00000113327	ENSG00000163288
ENSG00000113327	ENSG00000166206
ENSG00000113327	ENSG00000182256
ENSG00000113327	ENSG00000183185
ENSG00000113327	ENSG00000186297
ENSG00000113327	ENSG00000187730
ENSG00000113327	ENSG00000188828
ENSG00000113328	ENSG00000118816
ENSG00000113328	ENSG00000138764
ENSG00000113328	ENSG00000205089
ENSG00000113356	ENSG00000121851
ENSG00000113361	ENSG00000040731
ENSG00000113361	ENSG00000071991
ENSG00000113361	ENSG00000081138
ENSG00000113361	ENSG00000101542
ENSG00000113361	ENSG00000113100
ENSG00000113361	ENSG00000139880
ENSG00000113361	ENSG00000140937
ENSG00000113361	ENSG00000145526
ENSG00000113361	ENSG00000149654
ENSG00000113361	ENSG00000150394
ENSG00000113361	ENSG00000154162
ENSG00000113361	ENSG00000179776
ENSG00000113368	ENSG00000160789
ENSG00000113368	ENSG00000176619
ENSG00000113369	ENSG00000105643
ENSG00000113369	ENSG00000117289
ENSG00000113369	ENSG00000140450
ENSG00000113369	ENSG00000197070
ENSG00000113369	ENSG00000205784
ENSG00000113384	ENSG00000143457
ENSG00000113391	ENSG00000175841
ENSG00000113396	ENSG00000083807
ENSG00000113396	ENSG00000130304
ENSG00000113396	ENSG00000140284
ENSG00000113396	ENSG00000143554
ENSG00000113396	ENSG00000167114
ENSG00000113407	ENSG00000143374
ENSG00000113407	ENSG00000154719

ENSG00000113407	ENSG00000185418
ENSG00000113430	ENSG00000150051
ENSG00000113430	ENSG00000159387
ENSG00000113430	ENSG00000170549
ENSG00000113430	ENSG00000170561
ENSG00000113430	ENSG00000176842
ENSG00000113430	ENSG00000177508
ENSG00000113441	ENSG00000072657
ENSG00000113441	ENSG00000138792
ENSG00000113441	ENSG00000141279
ENSG00000113441	ENSG00000164307
ENSG00000113441	ENSG00000164308
ENSG00000113441	ENSG00000166825
ENSG00000113441	ENSG00000172901
ENSG00000113441	ENSG00000174093
ENSG00000113441	ENSG00000215728
ENSG00000113448	ENSG00000065989
ENSG00000113448	ENSG00000073417
ENSG00000113448	ENSG00000105650
ENSG00000113448	ENSG00000113231
ENSG00000113448	ENSG00000115252
ENSG00000113448	ENSG00000123360
ENSG00000113448	ENSG00000154678
ENSG00000113448	ENSG00000160191
ENSG00000113448	ENSG00000171408
ENSG00000113448	ENSG00000184588
ENSG00000113448	ENSG00000205268
ENSG00000113494	ENSG00000112964
ENSG00000113504	ENSG00000124067
ENSG00000113504	ENSG00000124140
ENSG00000113504	ENSG00000140199
ENSG00000113532	ENSG00000140557
ENSG00000113552	ENSG00000163281
ENSG00000113575	ENSG00000104695
ENSG00000113578	ENSG00000070193
ENSG00000113578	ENSG00000070388
ENSG00000113578	ENSG00000075388
ENSG00000113578	ENSG00000078579
ENSG00000113578	ENSG00000102466
ENSG00000113578	ENSG00000102678
ENSG00000113578	ENSG00000111241
ENSG00000113578	ENSG00000114279
ENSG00000113578	ENSG00000129682
ENSG00000113578	ENSG00000138675
ENSG00000113578	ENSG00000138685
ENSG00000113578	ENSG00000140285
ENSG00000113578	ENSG00000161958
ENSG00000113578	ENSG00000185390
ENSG00000113578	ENSG00000186895
ENSG00000113578	ENSG00000196468
ENSG00000113578	ENSG00000204801
ENSG00000113578	ENSG00000204816
ENSG00000113578	ENSG00000204817
ENSG00000113578	ENSG00000204837
ENSG00000113578	ENSG00000215053
ENSG00000113580	ENSG00000082175
ENSG00000113580	ENSG00000151623
ENSG00000113594	ENSG00000081985
ENSG00000113594	ENSG00000104998

ENSG00000113594	ENSG00000119535
ENSG00000113594	ENSG00000134352
ENSG00000113594	ENSG00000145623
ENSG00000113594	ENSG00000164509
ENSG00000113600	ENSG00000021852
ENSG00000113600	ENSG00000039537
ENSG00000113600	ENSG00000112936
ENSG00000113600	ENSG00000157131
ENSG00000113600	ENSG00000180644
ENSG00000113645	ENSG00000047644
ENSG00000113645	ENSG00000151718
ENSG00000113648	ENSG00000099284
ENSG00000113657	ENSG00000072832
ENSG00000113657	ENSG00000092964
ENSG00000113657	ENSG00000147647
ENSG00000113657	ENSG00000151640
ENSG00000113657	ENSG00000157851
ENSG00000113658	ENSG00000101665
ENSG00000113658	ENSG00000120693
ENSG00000113658	ENSG00000137834
ENSG00000113658	ENSG00000141646
ENSG00000113658	ENSG00000166949
ENSG00000113658	ENSG00000170365
ENSG00000113658	ENSG00000175387
ENSG00000113712	ENSG00000180138
ENSG00000113721	ENSG00000037280
ENSG00000113721	ENSG00000102755
ENSG00000113721	ENSG00000104213
ENSG00000113721	ENSG00000122025
ENSG00000113721	ENSG00000128052
ENSG00000113721	ENSG00000134853
ENSG00000113721	ENSG00000157404
ENSG00000113721	ENSG00000182578
ENSG00000113722	ENSG00000131264
ENSG00000113722	ENSG00000165556
ENSG00000113739	ENSG00000159167
ENSG00000113742	ENSG00000107864
ENSG00000113742	ENSG00000137449
ENSG00000113742	ENSG00000214575
ENSG00000113749	ENSG00000043591
ENSG00000113749	ENSG00000102468
ENSG00000113749	ENSG00000125631
ENSG00000113749	ENSG00000135312
ENSG00000113749	ENSG00000135914
ENSG00000113749	ENSG00000147246
ENSG00000113749	ENSG00000150594
ENSG00000113749	ENSG00000157219
ENSG00000113749	ENSG00000164270
ENSG00000113749	ENSG00000168830
ENSG00000113749	ENSG00000169252
ENSG00000113749	ENSG00000175658
ENSG00000113749	ENSG00000178394
ENSG00000113749	ENSG00000179097
ENSG00000113749	ENSG00000179546
ENSG00000113749	ENSG00000181210
ENSG00000113749	ENSG00000184303
ENSG00000113749	ENSG00000184845
ENSG00000113749	ENSG00000185321
ENSG00000113749	ENSG00000188778

ENSG00000113758	ENSG00000085733
ENSG00000113758	ENSG00000136279
ENSG00000113758	ENSG00000180353
ENSG00000113761	ENSG00000165061
ENSG00000113761	ENSG00000172667
ENSG00000113763	ENSG00000107731
ENSG00000113763	ENSG00000124602
ENSG00000113763	ENSG00000156687
ENSG00000113763	ENSG00000182168
ENSG00000113805	ENSG00000018236
ENSG00000113805	ENSG00000134115
ENSG00000113805	ENSG00000144619
ENSG00000113905	ENSG00000090512
ENSG00000113905	ENSG00000145192
ENSG00000113916	ENSG00000161940
ENSG00000114013	ENSG00000103855
ENSG00000114013	ENSG00000114455
ENSG00000114013	ENSG00000120217
ENSG00000114013	ENSG00000134258
ENSG00000114013	ENSG00000160223
ENSG00000114013	ENSG00000186470
ENSG00000114013	ENSG00000188211
ENSG00000114013	ENSG00000197646
ENSG00000114013	ENSG00000204290
ENSG00000114013	ENSG00000204655
ENSG00000114013	ENSG00000215631
ENSG00000114013	ENSG00000215811
ENSG00000114013	ENSG00000215889
ENSG00000114019	ENSG00000126016
ENSG00000114019	ENSG00000166025
ENSG00000114023	ENSG00000183807
ENSG00000114030	ENSG00000025800
ENSG00000114030	ENSG00000196911
ENSG00000114062	ENSG00000138641
ENSG00000114062	ENSG00000138642
ENSG00000114062	ENSG00000138646
ENSG00000114062	ENSG00000148634
ENSG00000114062	ENSG00000165338
ENSG00000114113	ENSG00000114115
ENSG00000114113	ENSG00000121769
ENSG00000114113	ENSG00000139194
ENSG00000114113	ENSG00000143320
ENSG00000114113	ENSG00000145384
ENSG00000114113	ENSG00000147588
ENSG00000114113	ENSG00000162444
ENSG00000114113	ENSG00000164434
ENSG00000114113	ENSG00000164687
ENSG00000114113	ENSG00000166426
ENSG00000114113	ENSG00000170323
ENSG00000114113	ENSG00000197416
ENSG00000114113	ENSG00000205186
ENSG00000114113	ENSG00000214004
ENSG00000114115	ENSG00000114113
ENSG00000114115	ENSG00000121769
ENSG00000114115	ENSG00000139194
ENSG00000114115	ENSG00000143320
ENSG00000114115	ENSG00000145384
ENSG00000114115	ENSG00000147588
ENSG00000114115	ENSG00000162444

ENSG00000114115	ENSG00000164434
ENSG00000114115	ENSG00000164687
ENSG00000114115	ENSG00000166426
ENSG00000114115	ENSG00000170323
ENSG00000114115	ENSG00000197416
ENSG00000114115	ENSG00000205186
ENSG00000114115	ENSG00000214004
ENSG00000114120	ENSG00000171612
ENSG00000114124	ENSG00000100077
ENSG00000114124	ENSG00000125388
ENSG00000114124	ENSG00000173020
ENSG00000114124	ENSG00000185974
ENSG00000114124	ENSG00000198055
ENSG00000114124	ENSG00000198873
ENSG00000114126	ENSG00000198176
ENSG00000114166	ENSG00000108773
ENSG00000114200	ENSG00000087085
ENSG00000114200	ENSG00000159398
ENSG00000114200	ENSG00000170835
ENSG00000114200	ENSG00000172824
ENSG00000114200	ENSG00000172828
ENSG00000114200	ENSG00000172831
ENSG00000114200	ENSG00000196959
ENSG00000114200	ENSG00000198848
ENSG00000114200	ENSG00000215478
ENSG00000114251	ENSG00000002745
ENSG00000114251	ENSG00000061492
ENSG00000114251	ENSG00000075290
ENSG00000114251	ENSG00000085741
ENSG00000114251	ENSG00000105989
ENSG00000114251	ENSG00000108379
ENSG00000114251	ENSG00000111186
ENSG00000114251	ENSG00000115596
ENSG00000114251	ENSG00000125084
ENSG00000114251	ENSG00000134245
ENSG00000114251	ENSG00000135925
ENSG00000114251	ENSG00000143816
ENSG00000114251	ENSG00000154342
ENSG00000114251	ENSG00000154764
ENSG00000114251	ENSG00000158955
ENSG00000114251	ENSG00000162552
ENSG00000114251	ENSG00000169884
ENSG00000114251	ENSG00000188064
ENSG00000114268	ENSG00000123836
ENSG00000114268	ENSG00000158571
ENSG00000114268	ENSG00000170525
ENSG00000114279	ENSG00000070193
ENSG00000114279	ENSG00000070388
ENSG00000114279	ENSG00000075388
ENSG00000114279	ENSG00000078579
ENSG00000114279	ENSG00000102466
ENSG00000114279	ENSG00000102678
ENSG00000114279	ENSG00000111241
ENSG00000114279	ENSG00000113578
ENSG00000114279	ENSG00000129682
ENSG00000114279	ENSG00000138675
ENSG00000114279	ENSG00000138685
ENSG00000114279	ENSG00000140285
ENSG00000114279	ENSG00000161958

ENSG00000114279	ENSG00000185390
ENSG00000114279	ENSG00000186895
ENSG00000114279	ENSG00000196468
ENSG00000114279	ENSG00000204801
ENSG00000114279	ENSG00000204816
ENSG00000114279	ENSG00000204817
ENSG00000114279	ENSG00000204837
ENSG00000114279	ENSG00000215053
ENSG00000114302	ENSG00000005249
ENSG00000114302	ENSG00000108946
ENSG00000114302	ENSG00000188191
ENSG00000114315	ENSG00000069812
ENSG00000114315	ENSG00000144485
ENSG00000114315	ENSG00000173673
ENSG00000114315	ENSG00000179111
ENSG00000114315	ENSG00000188290
ENSG00000114315	ENSG00000197921
ENSG00000114316	ENSG00000102226
ENSG00000114316	ENSG00000135655
ENSG00000114316	ENSG00000172046
ENSG00000114331	ENSG00000072818
ENSG00000114331	ENSG00000131584
ENSG00000114349	ENSG00000060558
ENSG00000114349	ENSG00000065135
ENSG00000114349	ENSG00000087258
ENSG00000114349	ENSG00000087460
ENSG00000114349	ENSG00000088256
ENSG00000114349	ENSG00000114353
ENSG00000114349	ENSG00000120063
ENSG00000114349	ENSG00000127955
ENSG00000114349	ENSG00000128266
ENSG00000114349	ENSG00000134183
ENSG00000114349	ENSG00000141404
ENSG00000114349	ENSG00000146535
ENSG00000114349	ENSG00000156049
ENSG00000114349	ENSG00000156052
ENSG00000114349	ENSG00000214415
ENSG00000114349	ENSG00000214622
ENSG00000114353	ENSG00000060558
ENSG00000114353	ENSG00000065135
ENSG00000114353	ENSG00000087258
ENSG00000114353	ENSG00000087460
ENSG00000114353	ENSG00000088256
ENSG00000114353	ENSG00000114349
ENSG00000114353	ENSG00000120063
ENSG00000114353	ENSG00000127955
ENSG00000114353	ENSG00000128266
ENSG00000114353	ENSG00000134183
ENSG00000114353	ENSG00000141404
ENSG00000114353	ENSG00000146535
ENSG00000114353	ENSG00000156049
ENSG00000114353	ENSG00000156052
ENSG00000114353	ENSG00000214415
ENSG00000114353	ENSG00000214622
ENSG00000114378	ENSG00000068001
ENSG00000114378	ENSG00000106302
ENSG00000114378	ENSG00000106304
ENSG00000114378	ENSG00000186792
ENSG00000114395	ENSG00000174151

ENSG00000114416	ENSG00000102081
ENSG00000114416	ENSG00000129245
ENSG00000114423	ENSG00000110395
ENSG00000114423	ENSG00000142273
ENSG00000114450	ENSG00000069966
ENSG00000114450	ENSG00000078369
ENSG00000114450	ENSG00000111664
ENSG00000114450	ENSG00000172354
ENSG00000114455	ENSG00000103855
ENSG00000114455	ENSG00000114013
ENSG00000114455	ENSG00000120217
ENSG00000114455	ENSG00000134258
ENSG00000114455	ENSG00000160223
ENSG00000114455	ENSG00000186470
ENSG00000114455	ENSG00000188211
ENSG00000114455	ENSG00000197646
ENSG00000114455	ENSG00000204290
ENSG00000114455	ENSG00000204655
ENSG00000114455	ENSG00000215631
ENSG00000114455	ENSG00000215811
ENSG00000114455	ENSG00000215889
ENSG00000114487	ENSG00000133131
ENSG00000114487	ENSG00000133422
ENSG00000114487	ENSG00000159256
ENSG00000114541	ENSG00000147144
ENSG00000114541	ENSG00000151474
ENSG00000114541	ENSG00000163362
ENSG00000114544	ENSG00000133065
ENSG00000114544	ENSG00000136052
ENSG00000114547	ENSG00000065371
ENSG00000114547	ENSG00000145491
ENSG00000114554	ENSG00000004399
ENSG00000114554	ENSG00000076356
ENSG00000114554	ENSG00000130827
ENSG00000114554	ENSG00000136040
ENSG00000114554	ENSG00000164050
ENSG00000114554	ENSG00000189437
ENSG00000114554	ENSG00000196576
ENSG00000114554	ENSG00000198753
ENSG00000114631	ENSG00000128567
ENSG00000114698	ENSG00000163746
ENSG00000114698	ENSG00000187838
ENSG00000114698	ENSG00000188313
ENSG00000114737	ENSG00000120833
ENSG00000114737	ENSG00000170677
ENSG00000114737	ENSG00000171150
ENSG00000114737	ENSG00000174111
ENSG00000114737	ENSG00000180008
ENSG00000114737	ENSG00000184557
ENSG00000114737	ENSG00000185338
ENSG00000114738	ENSG00000089022
ENSG00000114738	ENSG00000162889
ENSG00000114739	ENSG00000121989
ENSG00000114757	ENSG00000139197
ENSG00000114771	ENSG00000144959
ENSG00000114771	ENSG00000188984
ENSG00000114771	ENSG00000197953
ENSG00000114771	ENSG00000204518
ENSG00000114784	ENSG00000173812

ENSG00000114790	ENSG00000066248
ENSG00000114790	ENSG00000130762
ENSG00000114790	ENSG00000142632
ENSG00000114790	ENSG00000198844
ENSG00000114796	ENSG00000119771
ENSG00000114796	ENSG00000149243
ENSG00000114796	ENSG00000172578
ENSG00000114805	ENSG00000101333
ENSG00000114805	ENSG00000115556
ENSG00000114805	ENSG00000115896
ENSG00000114805	ENSG00000124181
ENSG00000114805	ENSG00000137841
ENSG00000114805	ENSG00000139151
ENSG00000114805	ENSG00000149527
ENSG00000114805	ENSG00000149782
ENSG00000114805	ENSG00000154822
ENSG00000114805	ENSG00000161714
ENSG00000114805	ENSG00000182621
ENSG00000114805	ENSG00000187091
ENSG00000114805	ENSG00000197943
ENSG00000114812	ENSG00000004948
ENSG00000114812	ENSG00000010310
ENSG00000114812	ENSG00000064989
ENSG00000114812	ENSG00000065325
ENSG00000114812	ENSG00000078549
ENSG00000114812	ENSG00000080293
ENSG00000114812	ENSG00000106018
ENSG00000114812	ENSG00000106113
ENSG00000114812	ENSG00000106128
ENSG00000114812	ENSG00000112164
ENSG00000114812	ENSG00000120088
ENSG00000114812	ENSG00000144407
ENSG00000114812	ENSG00000160801
ENSG00000114812	ENSG00000215644
ENSG00000114853	ENSG00000198740
ENSG00000114854	ENSG00000101470
ENSG00000114857	ENSG00000138398
ENSG00000114857	ENSG00000152591
ENSG00000114859	ENSG00000184908
ENSG00000114859	ENSG00000186510
ENSG00000114861	ENSG00000049768
ENSG00000114861	ENSG00000128573
ENSG00000114861	ENSG00000137166
ENSG00000114867	ENSG00000075151
ENSG00000114923	ENSG00000004939
ENSG00000114923	ENSG00000033867
ENSG00000114923	ENSG00000050438
ENSG00000114923	ENSG00000080493
ENSG00000114923	ENSG00000088836
ENSG00000114923	ENSG00000113073
ENSG00000114923	ENSG00000144290
ENSG00000114923	ENSG00000164889
ENSG00000114923	ENSG00000188687
ENSG00000114948	ENSG00000008277
ENSG00000114948	ENSG00000042980
ENSG00000114948	ENSG00000069206
ENSG00000114948	ENSG00000073670
ENSG00000114948	ENSG00000104755
ENSG00000114948	ENSG00000134007

ENSG00000114948	ENSG00000134028
ENSG00000114948	ENSG00000134249
ENSG00000114948	ENSG00000135074
ENSG00000114948	ENSG00000139985
ENSG00000114948	ENSG00000143537
ENSG00000114948	ENSG00000148848
ENSG00000114948	ENSG00000149451
ENSG00000114948	ENSG00000151651
ENSG00000114948	ENSG00000151694
ENSG00000114948	ENSG00000168594
ENSG00000114948	ENSG00000168615
ENSG00000114948	ENSG00000168619
ENSG00000114948	ENSG00000196115
ENSG00000114948	ENSG00000197140
ENSG00000114948	ENSG00000214137
ENSG00000114956	ENSG00000156136
ENSG00000114956	ENSG00000166548
ENSG00000114978	ENSG00000120162
ENSG00000114978	ENSG00000142961
ENSG00000114978	ENSG00000172081
ENSG00000114978	ENSG00000173542
ENSG00000114978	ENSG00000182208
ENSG00000114988	ENSG00000074695
ENSG00000114988	ENSG00000140506
ENSG00000114988	ENSG00000169223
ENSG00000114993	ENSG00000182010
ENSG00000115009	ENSG00000006074
ENSG00000115009	ENSG00000006075
ENSG00000115009	ENSG00000006606
ENSG00000115009	ENSG00000102962
ENSG00000115009	ENSG00000102970
ENSG00000115009	ENSG00000106178
ENSG00000115009	ENSG00000108688
ENSG00000115009	ENSG00000108691
ENSG00000115009	ENSG00000108700
ENSG00000115009	ENSG00000108702
ENSG00000115009	ENSG00000129277
ENSG00000115009	ENSG00000143184
ENSG00000115009	ENSG00000143185
ENSG00000115009	ENSG00000161570
ENSG00000115009	ENSG00000161573
ENSG00000115009	ENSG00000161574
ENSG00000115009	ENSG00000167236
ENSG00000115009	ENSG00000172156
ENSG00000115009	ENSG00000172724
ENSG00000115009	ENSG00000181374
ENSG00000115009	ENSG00000197262
ENSG00000115009	ENSG00000205017
ENSG00000115009	ENSG00000205020
ENSG00000115009	ENSG00000205021
ENSG00000115009	ENSG00000213494
ENSG00000115009	ENSG00000215627
ENSG00000115009	ENSG00000215629
ENSG00000115041	ENSG00000120049
ENSG00000115041	ENSG00000182132
ENSG00000115041	ENSG00000185774
ENSG00000115073	ENSG00000138107
ENSG00000115085	ENSG00000165025
ENSG00000115091	ENSG00000106526

ENSG00000115091	ENSG00000133627
ENSG00000115107	ENSG00000105889
ENSG00000115107	ENSG00000127954
ENSG00000115107	ENSG00000157214
ENSG00000115107	ENSG00000164647
ENSG00000115109	ENSG00000095203
ENSG00000115109	ENSG00000129595
ENSG00000115109	ENSG00000153303
ENSG00000115112	ENSG00000083307
ENSG00000115112	ENSG00000134317
ENSG00000115112	ENSG00000135457
ENSG00000115112	ENSG00000153560
ENSG00000115112	ENSG00000158055
ENSG00000115112	ENSG00000214547
ENSG00000115145	ENSG00000136738
ENSG00000115155	ENSG00000088340
ENSG00000115155	ENSG00000135636
ENSG00000115155	ENSG00000138119
ENSG00000115155	ENSG00000214272
ENSG00000115155	ENSG00000214814
ENSG00000115165	ENSG00000161835
ENSG00000115170	ENSG00000106799
ENSG00000115170	ENSG00000107779
ENSG00000115170	ENSG00000123612
ENSG00000115170	ENSG00000135409
ENSG00000115170	ENSG00000135503
ENSG00000115170	ENSG00000138696
ENSG00000115170	ENSG00000139567
ENSG00000115170	ENSG00000163513
ENSG00000115170	ENSG00000204217
ENSG00000115183	ENSG00000151458
ENSG00000115183	ENSG00000170921
ENSG00000115194	ENSG00000104154
ENSG00000115194	ENSG00000158014
ENSG00000115194	ENSG00000164756
ENSG00000115216	ENSG00000060237
ENSG00000115216	ENSG00000126562
ENSG00000115216	ENSG00000165238
ENSG00000115216	ENSG00000196632
ENSG00000115221	ENSG00000056345
ENSG00000115221	ENSG00000082781
ENSG00000115221	ENSG00000105855
ENSG00000115221	ENSG00000132470
ENSG00000115221	ENSG00000139626
ENSG00000115221	ENSG00000150093
ENSG00000115221	ENSG00000160255
ENSG00000115226	ENSG00000160097
ENSG00000115232	ENSG00000005844
ENSG00000115232	ENSG00000005884
ENSG00000115232	ENSG00000005961
ENSG00000115232	ENSG00000077943
ENSG00000115232	ENSG00000083457
ENSG00000115232	ENSG00000091409
ENSG00000115232	ENSG00000135424
ENSG00000115232	ENSG00000137809
ENSG00000115232	ENSG00000138448
ENSG00000115232	ENSG00000140678
ENSG00000115232	ENSG00000143127
ENSG00000115232	ENSG00000144668

ENSG00000115232	ENSG00000156886
ENSG00000115232	ENSG00000161638
ENSG00000115232	ENSG00000164171
ENSG00000115232	ENSG00000169896
ENSG00000115232	ENSG00000213949
ENSG00000115234	ENSG00000143376
ENSG00000115234	ENSG00000174226
ENSG00000115252	ENSG00000065989
ENSG00000115252	ENSG00000073417
ENSG00000115252	ENSG00000105650
ENSG00000115252	ENSG00000113231
ENSG00000115252	ENSG00000113448
ENSG00000115252	ENSG00000123360
ENSG00000115252	ENSG00000154678
ENSG00000115252	ENSG00000160191
ENSG00000115252	ENSG00000171408
ENSG00000115252	ENSG00000184588
ENSG00000115252	ENSG00000205268
ENSG00000115255	ENSG00000129625
ENSG00000115257	ENSG00000099139
ENSG00000115257	ENSG00000125851
ENSG00000115257	ENSG00000140479
ENSG00000115257	ENSG00000140564
ENSG00000115257	ENSG00000160613
ENSG00000115257	ENSG00000175426
ENSG00000115263	ENSG00000159224
ENSG00000115266	ENSG00000134982
ENSG00000115267	ENSG00000100697
ENSG00000115267	ENSG00000107201
ENSG00000115267	ENSG00000108771
ENSG00000115271	ENSG00000075142
ENSG00000115271	ENSG00000126247
ENSG00000115271	ENSG00000162517
ENSG00000115290	ENSG00000077420
ENSG00000115290	ENSG00000106070
ENSG00000115290	ENSG00000141738
ENSG00000115290	ENSG00000173166
ENSG00000115295	ENSG00000105270
ENSG00000115295	ENSG00000106665
ENSG00000115295	ENSG00000130779
ENSG00000115297	ENSG00000107807
ENSG00000115297	ENSG00000164438
ENSG00000115306	ENSG00000070182
ENSG00000115306	ENSG00000137877
ENSG00000115306	ENSG00000160460
ENSG00000115306	ENSG00000173898
ENSG00000115310	ENSG00000125744
ENSG00000115310	ENSG00000133318
ENSG00000115310	ENSG00000139970
ENSG00000115317	ENSG00000166033
ENSG00000115317	ENSG00000169495
ENSG00000115317	ENSG00000170801
ENSG00000115318	ENSG00000113083
ENSG00000115318	ENSG00000129038
ENSG00000115318	ENSG00000134013
ENSG00000115318	ENSG00000138131
ENSG00000115325	ENSG00000101134
ENSG00000115325	ENSG00000125170
ENSG00000115325	ENSG00000137218

ENSG00000115325	ENSG00000146094
ENSG00000115325	ENSG00000147443
ENSG00000115325	ENSG00000166225
ENSG00000115325	ENSG00000206052
ENSG00000115339	ENSG00000100626
ENSG00000115339	ENSG00000106648
ENSG00000115339	ENSG00000110328
ENSG00000115339	ENSG00000131386
ENSG00000115339	ENSG00000136542
ENSG00000115339	ENSG00000139629
ENSG00000115339	ENSG00000141429
ENSG00000115339	ENSG00000143641
ENSG00000115339	ENSG00000144278
ENSG00000115339	ENSG00000158089
ENSG00000115339	ENSG00000164574
ENSG00000115339	ENSG00000174473
ENSG00000115339	ENSG00000178234
ENSG00000115339	ENSG00000182870
ENSG00000115339	ENSG00000185274
ENSG00000115353	ENSG00000056291
ENSG00000115353	ENSG00000075073
ENSG00000115353	ENSG00000101292
ENSG00000115353	ENSG00000110148
ENSG00000115353	ENSG00000119973
ENSG00000115353	ENSG00000121764
ENSG00000115353	ENSG00000123901
ENSG00000115353	ENSG00000137252
ENSG00000115353	ENSG00000148734
ENSG00000115353	ENSG00000159279
ENSG00000115353	ENSG00000163394
ENSG00000115353	ENSG00000164128
ENSG00000115353	ENSG00000164129
ENSG00000115353	ENSG00000169618
ENSG00000115353	ENSG00000169836
ENSG00000115353	ENSG00000185149
ENSG00000115353	ENSG00000186867
ENSG00000115353	ENSG00000204174
ENSG00000115353	ENSG00000215167
ENSG00000115355	ENSG00000015133
ENSG00000115355	ENSG00000095066
ENSG00000115355	ENSG00000134709
ENSG00000115355	ENSG00000168071
ENSG00000115355	ENSG00000168172
ENSG00000115363	ENSG00000142694
ENSG00000115365	ENSG00000132434
ENSG00000115365	ENSG00000147036
ENSG00000115380	ENSG00000049323
ENSG00000115380	ENSG00000077942
ENSG00000115380	ENSG00000090006
ENSG00000115380	ENSG00000119681
ENSG00000115380	ENSG00000140092
ENSG00000115380	ENSG00000163520
ENSG00000115380	ENSG00000168056
ENSG00000115380	ENSG00000172638
ENSG00000115380	ENSG00000214536
ENSG00000115414	ENSG00000041982
ENSG00000115414	ENSG00000116147
ENSG00000115414	ENSG00000120332
ENSG00000115414	ENSG00000168477

ENSG00000115415	ENSG00000126561
ENSG00000115415	ENSG00000138378
ENSG00000115415	ENSG00000166888
ENSG00000115415	ENSG00000168610
ENSG00000115415	ENSG00000170581
ENSG00000115415	ENSG00000173757
ENSG00000115419	ENSG00000135423
ENSG00000115421	ENSG00000090060
ENSG00000115457	ENSG00000115461
ENSG00000115457	ENSG00000141753
ENSG00000115457	ENSG00000146674
ENSG00000115457	ENSG00000146678
ENSG00000115457	ENSG00000167779
ENSG00000115461	ENSG00000115457
ENSG00000115461	ENSG00000141753
ENSG00000115461	ENSG00000146674
ENSG00000115461	ENSG00000146678
ENSG00000115461	ENSG00000167779
ENSG00000115468	ENSG00000126878
ENSG00000115468	ENSG00000142634
ENSG00000115468	ENSG00000204472
ENSG00000115474	ENSG00000120457
ENSG00000115474	ENSG00000121361
ENSG00000115474	ENSG00000123700
ENSG00000115474	ENSG00000151704
ENSG00000115474	ENSG00000153822
ENSG00000115474	ENSG00000157542
ENSG00000115474	ENSG00000157551
ENSG00000115474	ENSG00000162728
ENSG00000115474	ENSG00000162989
ENSG00000115474	ENSG00000168135
ENSG00000115474	ENSG00000177807
ENSG00000115474	ENSG00000182324
ENSG00000115474	ENSG00000184185
ENSG00000115474	ENSG00000187486
ENSG00000115488	ENSG00000162139
ENSG00000115488	ENSG00000204099
ENSG00000115488	ENSG00000204386
ENSG00000115504	ENSG00000173442
ENSG00000115507	ENSG00000105392
ENSG00000115507	ENSG00000165588
ENSG00000115507	ENSG00000186103
ENSG00000115520	ENSG00000135469
ENSG00000115539	ENSG00000116703
ENSG00000115539	ENSG00000136940
ENSG00000115539	ENSG00000163440
ENSG00000115539	ENSG00000215109
ENSG00000115548	ENSG00000120733
ENSG00000115548	ENSG00000168453
ENSG00000115548	ENSG00000171988
ENSG00000115556	ENSG00000101333
ENSG00000115556	ENSG00000114805
ENSG00000115556	ENSG00000115896
ENSG00000115556	ENSG00000124181
ENSG00000115556	ENSG00000137841
ENSG00000115556	ENSG00000139151
ENSG00000115556	ENSG00000149527
ENSG00000115556	ENSG00000149782
ENSG00000115556	ENSG00000154822

ENSG00000115556	ENSG00000161714
ENSG00000115556	ENSG00000182621
ENSG00000115556	ENSG00000187091
ENSG00000115556	ENSG00000197943
ENSG00000115590	ENSG00000115594
ENSG00000115590	ENSG00000115598
ENSG00000115590	ENSG00000115602
ENSG00000115590	ENSG00000115604
ENSG00000115590	ENSG00000115607
ENSG00000115590	ENSG00000185187
ENSG00000115590	ENSG00000196083
ENSG00000115592	ENSG00000106617
ENSG00000115592	ENSG00000181929
ENSG00000115593	ENSG00000143499
ENSG00000115593	ENSG00000185420
ENSG00000115594	ENSG00000115590
ENSG00000115594	ENSG00000115598
ENSG00000115594	ENSG00000115602
ENSG00000115594	ENSG00000115604
ENSG00000115594	ENSG00000115607
ENSG00000115594	ENSG00000185187
ENSG00000115594	ENSG00000196083
ENSG00000115598	ENSG00000115590
ENSG00000115598	ENSG00000115594
ENSG00000115598	ENSG00000115602
ENSG00000115598	ENSG00000115604
ENSG00000115598	ENSG00000115607
ENSG00000115598	ENSG00000185187
ENSG00000115598	ENSG00000196083
ENSG00000115602	ENSG00000115590
ENSG00000115602	ENSG00000115594
ENSG00000115602	ENSG00000115598
ENSG00000115602	ENSG00000115604
ENSG00000115602	ENSG00000115607
ENSG00000115602	ENSG00000185187
ENSG00000115602	ENSG00000196083
ENSG00000115604	ENSG00000115590
ENSG00000115604	ENSG00000115594
ENSG00000115604	ENSG00000115598
ENSG00000115604	ENSG00000115602
ENSG00000115604	ENSG00000115607
ENSG00000115604	ENSG00000185187
ENSG00000115604	ENSG00000196083
ENSG00000115607	ENSG00000115590
ENSG00000115607	ENSG00000115594
ENSG00000115607	ENSG00000115598
ENSG00000115607	ENSG00000115602
ENSG00000115607	ENSG00000115604
ENSG00000115607	ENSG00000185187
ENSG00000115607	ENSG00000196083
ENSG00000115616	ENSG00000066230
ENSG00000115616	ENSG00000090020
ENSG00000115616	ENSG00000135740
ENSG00000115616	ENSG00000180251
ENSG00000115648	ENSG00000170011
ENSG00000115677	ENSG00000122870
ENSG00000115694	ENSG00000102572
ENSG00000115694	ENSG00000134602
ENSG00000115718	ENSG00000057593

ENSG00000115718	ENSG00000101981
ENSG00000115718	ENSG00000126218
ENSG00000115718	ENSG00000126231
ENSG00000115718	ENSG00000180210
ENSG00000115738	ENSG00000117318
ENSG00000115738	ENSG00000125968
ENSG00000115738	ENSG00000172201
ENSG00000115756	ENSG00000104490
ENSG00000115756	ENSG00000107130
ENSG00000115756	ENSG00000109047
ENSG00000115756	ENSG00000116983
ENSG00000115756	ENSG00000121905
ENSG00000115756	ENSG00000163032
ENSG00000115758	ENSG00000142920
ENSG00000115758	ENSG00000155096
ENSG00000115762	ENSG00000021300
ENSG00000115808	ENSG00000090372
ENSG00000115808	ENSG00000196792
ENSG00000115825	ENSG00000105287
ENSG00000115825	ENSG00000184304
ENSG00000115828	ENSG00000011478
ENSG00000115840	ENSG00000004864
ENSG00000115840	ENSG00000177542
ENSG00000115840	ENSG00000182902
ENSG00000115841	ENSG00000137824
ENSG00000115841	ENSG00000176623
ENSG00000115844	ENSG00000064195
ENSG00000115844	ENSG00000105880
ENSG00000115884	ENSG00000124145
ENSG00000115884	ENSG00000162512
ENSG00000115884	ENSG00000169439
ENSG00000115896	ENSG00000101333
ENSG00000115896	ENSG00000114805
ENSG00000115896	ENSG00000115556
ENSG00000115896	ENSG00000124181
ENSG00000115896	ENSG00000137841
ENSG00000115896	ENSG00000139151
ENSG00000115896	ENSG00000149527
ENSG00000115896	ENSG00000149782
ENSG00000115896	ENSG00000154822
ENSG00000115896	ENSG00000161714
ENSG00000115896	ENSG00000182621
ENSG00000115896	ENSG00000187091
ENSG00000115896	ENSG00000197943
ENSG00000115902	ENSG00000079215
ENSG00000115902	ENSG00000105143
ENSG00000115902	ENSG00000105281
ENSG00000115902	ENSG00000106688
ENSG00000115902	ENSG00000110436
ENSG00000115902	ENSG00000162383
ENSG00000115904	ENSG00000058335
ENSG00000115904	ENSG00000100485
ENSG00000115904	ENSG00000107263
ENSG00000115904	ENSG00000113319
ENSG00000115935	ENSG00000122574
ENSG00000115935	ENSG00000171475
ENSG00000115935	ENSG00000205090
ENSG00000115944	ENSG00000112695
ENSG00000115944	ENSG00000161281

ENSG00000115956	ENSG00000100558
ENSG00000115963	ENSG00000108830
ENSG00000115963	ENSG00000172602
ENSG00000115966	ENSG00000130522
ENSG00000115966	ENSG00000146592
ENSG00000115966	ENSG00000170653
ENSG00000115966	ENSG00000171223
ENSG00000115966	ENSG00000177606
ENSG00000115977	ENSG00000138756
ENSG00000115993	ENSG00000173805
ENSG00000115993	ENSG00000182606
ENSG00000116001	ENSG00000151923
ENSG00000116005	ENSG00000145882
ENSG00000116014	ENSG00000082556
ENSG00000116014	ENSG00000112038
ENSG00000116014	ENSG00000116329
ENSG00000116014	ENSG00000125510
ENSG00000116014	ENSG00000125522
ENSG00000116014	ENSG00000128285
ENSG00000116014	ENSG00000128310
ENSG00000116014	ENSG00000132671
ENSG00000116014	ENSG00000139874
ENSG00000116014	ENSG00000152034
ENSG00000116014	ENSG00000162009
ENSG00000116014	ENSG00000166573
ENSG00000116014	ENSG00000180616
ENSG00000116014	ENSG00000182687
ENSG00000116014	ENSG00000183473
ENSG00000116014	ENSG00000183729
ENSG00000116016	ENSG00000100644
ENSG00000116016	ENSG00000112246
ENSG00000116016	ENSG00000124440
ENSG00000116016	ENSG00000130751
ENSG00000116016	ENSG00000151322
ENSG00000116016	ENSG00000159263
ENSG00000116017	ENSG00000049618
ENSG00000116017	ENSG00000117713
ENSG00000116017	ENSG00000179361
ENSG00000116017	ENSG00000205143
ENSG00000116032	ENSG00000105464
ENSG00000116032	ENSG00000150086
ENSG00000116032	ENSG00000161509
ENSG00000116032	ENSG00000176884
ENSG00000116032	ENSG00000183454
ENSG00000116032	ENSG00000198785
ENSG00000116035	ENSG00000148704
ENSG00000116039	ENSG00000147416
ENSG00000116044	ENSG00000082641
ENSG00000116044	ENSG00000112182
ENSG00000116044	ENSG00000123405
ENSG00000116044	ENSG00000156273
ENSG00000116106	ENSG00000044524
ENSG00000116106	ENSG00000070886
ENSG00000116106	ENSG00000080224
ENSG00000116106	ENSG00000106123
ENSG00000116106	ENSG00000133216
ENSG00000116106	ENSG00000135333
ENSG00000116106	ENSG00000142627
ENSG00000116106	ENSG00000145242

ENSG00000116106	ENSG00000146904
ENSG00000116106	ENSG00000154928
ENSG00000116106	ENSG00000182580
ENSG00000116106	ENSG00000183317
ENSG00000116106	ENSG00000196411
ENSG00000116117	ENSG00000148498
ENSG00000116128	ENSG00000186174
ENSG00000116132	ENSG00000109132
ENSG00000116132	ENSG00000165462
ENSG00000116132	ENSG00000165606
ENSG00000116132	ENSG00000167157
ENSG00000116141	ENSG00000007047
ENSG00000116141	ENSG00000072518
ENSG00000116141	ENSG00000075413
ENSG00000116141	ENSG00000142178
ENSG00000116141	ENSG00000160584
ENSG00000116141	ENSG00000170145
ENSG00000116147	ENSG00000041982
ENSG00000116147	ENSG00000115414
ENSG00000116147	ENSG00000120332
ENSG00000116147	ENSG00000168477
ENSG00000116148	ENSG00000163660
ENSG00000116148	ENSG00000215726
ENSG00000116157	ENSG00000164294
ENSG00000116176	ENSG00000005001
ENSG00000116176	ENSG00000007038
ENSG00000116176	ENSG00000052344
ENSG00000116176	ENSG00000095917
ENSG00000116176	ENSG00000103355
ENSG00000116176	ENSG00000172236
ENSG00000116176	ENSG00000172382
ENSG00000116176	ENSG00000172460
ENSG00000116176	ENSG00000178055
ENSG00000116176	ENSG00000178226
ENSG00000116176	ENSG00000185888
ENSG00000116176	ENSG00000188086
ENSG00000116176	ENSG00000189099
ENSG00000116176	ENSG00000196364
ENSG00000116176	ENSG00000197253
ENSG00000116176	ENSG00000206549
ENSG00000116176	ENSG00000214877
ENSG00000116176	ENSG00000215148
ENSG00000116183	ENSG00000182752
ENSG00000116191	ENSG00000136828
ENSG00000116209	ENSG00000105696
ENSG00000116219	ENSG00000144488
ENSG00000116219	ENSG00000187017
ENSG00000116254	ENSG00000111642
ENSG00000116254	ENSG00000170004
ENSG00000116260	ENSG00000165661
ENSG00000116266	ENSG00000076944
ENSG00000116266	ENSG00000136854
ENSG00000116273	ENSG00000040633
ENSG00000116299	ENSG00000164659
ENSG00000116329	ENSG00000082556
ENSG00000116329	ENSG00000112038
ENSG00000116329	ENSG00000116014
ENSG00000116329	ENSG00000125510
ENSG00000116329	ENSG00000125522

ENSG00000116329	ENSG00000128285
ENSG00000116329	ENSG00000128310
ENSG00000116329	ENSG00000132671
ENSG00000116329	ENSG00000139874
ENSG00000116329	ENSG00000152034
ENSG00000116329	ENSG00000162009
ENSG00000116329	ENSG00000166573
ENSG00000116329	ENSG00000180616
ENSG00000116329	ENSG00000182687
ENSG00000116329	ENSG00000183473
ENSG00000116329	ENSG00000183729
ENSG00000116350	ENSG00000100650
ENSG00000116350	ENSG00000124193
ENSG00000116396	ENSG00000129159
ENSG00000116396	ENSG00000131398
ENSG00000116396	ENSG00000166006
ENSG00000116473	ENSG00000123728
ENSG00000116473	ENSG00000125249
ENSG00000116473	ENSG00000127314
ENSG00000116473	ENSG00000162595
ENSG00000116473	ENSG00000165023
ENSG00000116473	ENSG00000176276
ENSG00000116473	ENSG00000176490
ENSG00000116473	ENSG00000181467
ENSG00000116473	ENSG00000214405
ENSG00000116473	ENSG00000214492
ENSG00000116478	ENSG00000196591
ENSG00000116489	ENSG00000177938
ENSG00000116489	ENSG00000198898
ENSG00000116514	ENSG00000034677
ENSG00000116514	ENSG00000137393
ENSG00000116514	ENSG00000151692
ENSG00000116521	ENSG00000085365
ENSG00000116521	ENSG00000140497
ENSG00000116521	ENSG00000198794
ENSG00000116525	ENSG00000146755
ENSG00000116525	ENSG00000155428
ENSG00000116525	ENSG00000177238
ENSG00000116525	ENSG00000178809
ENSG00000116525	ENSG00000185880
ENSG00000116539	ENSG00000152217
ENSG00000116544	ENSG00000080845
ENSG00000116544	ENSG00000126787
ENSG00000116544	ENSG00000170579
ENSG00000116544	ENSG00000198010
ENSG00000116560	ENSG00000121390
ENSG00000116560	ENSG00000147140
ENSG00000116574	ENSG00000104140
ENSG00000116584	ENSG00000038102
ENSG00000116584	ENSG00000104880
ENSG00000116584	ENSG00000170776
ENSG00000116604	ENSG00000064489
ENSG00000116604	ENSG00000068305
ENSG00000116604	ENSG00000081189
ENSG00000116641	ENSG00000088387
ENSG00000116641	ENSG00000107099
ENSG00000116641	ENSG00000130158
ENSG00000116641	ENSG00000135905
ENSG00000116641	ENSG00000147251

ENSG00000116661	ENSG00000116663
ENSG00000116661	ENSG00000132879
ENSG00000116661	ENSG00000161241
ENSG00000116661	ENSG00000161243
ENSG00000116661	ENSG00000188505
ENSG00000116663	ENSG00000116661
ENSG00000116663	ENSG00000132879
ENSG00000116663	ENSG00000161241
ENSG00000116663	ENSG00000161243
ENSG00000116663	ENSG00000188505
ENSG00000116675	ENSG00000178950
ENSG00000116678	ENSG00000006016
ENSG00000116688	ENSG00000171109
ENSG00000116690	ENSG00000109072
ENSG00000116701	ENSG00000188747
ENSG00000116703	ENSG00000115539
ENSG00000116703	ENSG00000136940
ENSG00000116703	ENSG00000163440
ENSG00000116703	ENSG00000215109
ENSG00000116704	ENSG00000130958
ENSG00000116704	ENSG00000182747
ENSG00000116708	ENSG00000116721
ENSG00000116708	ENSG00000116726
ENSG00000116708	ENSG00000120952
ENSG00000116708	ENSG00000157358
ENSG00000116708	ENSG00000160959
ENSG00000116708	ENSG00000179704
ENSG00000116708	ENSG00000182330
ENSG00000116708	ENSG00000185028
ENSG00000116708	ENSG00000185686
ENSG00000116708	ENSG00000187545
ENSG00000116708	ENSG00000197549
ENSG00000116708	ENSG00000204478
ENSG00000116708	ENSG00000204479
ENSG00000116708	ENSG00000204480
ENSG00000116708	ENSG00000204481
ENSG00000116708	ENSG00000204485
ENSG00000116708	ENSG00000204486
ENSG00000116708	ENSG00000204488
ENSG00000116708	ENSG00000204491
ENSG00000116708	ENSG00000204495
ENSG00000116708	ENSG00000204501
ENSG00000116708	ENSG00000204502
ENSG00000116708	ENSG00000204503
ENSG00000116708	ENSG00000204505
ENSG00000116708	ENSG00000204507
ENSG00000116708	ENSG00000204508
ENSG00000116708	ENSG00000204509
ENSG00000116708	ENSG00000204510
ENSG00000116708	ENSG00000204513
ENSG00000116717	ENSG00000099860
ENSG00000116717	ENSG00000112306
ENSG00000116717	ENSG00000130222
ENSG00000116717	ENSG00000170250
ENSG00000116721	ENSG00000116708
ENSG00000116721	ENSG00000116726
ENSG00000116721	ENSG00000120952
ENSG00000116721	ENSG00000157358
ENSG00000116721	ENSG00000160959

ENSG00000116721	ENSG00000179704
ENSG00000116721	ENSG00000182330
ENSG00000116721	ENSG00000185028
ENSG00000116721	ENSG00000185686
ENSG00000116721	ENSG00000187545
ENSG00000116721	ENSG00000197549
ENSG00000116721	ENSG00000204478
ENSG00000116721	ENSG00000204479
ENSG00000116721	ENSG00000204480
ENSG00000116721	ENSG00000204481
ENSG00000116721	ENSG00000204485
ENSG00000116721	ENSG00000204486
ENSG00000116721	ENSG00000204488
ENSG00000116721	ENSG00000204491
ENSG00000116721	ENSG00000204495
ENSG00000116721	ENSG00000204501
ENSG00000116721	ENSG00000204502
ENSG00000116721	ENSG00000204503
ENSG00000116721	ENSG00000204505
ENSG00000116721	ENSG00000204507
ENSG00000116721	ENSG00000204508
ENSG00000116721	ENSG00000204509
ENSG00000116721	ENSG00000204510
ENSG00000116721	ENSG00000204513
ENSG00000116726	ENSG00000116708
ENSG00000116726	ENSG00000116721
ENSG00000116726	ENSG00000120952
ENSG00000116726	ENSG00000157358
ENSG00000116726	ENSG00000160959
ENSG00000116726	ENSG00000179704
ENSG00000116726	ENSG00000182330
ENSG00000116726	ENSG00000185028
ENSG00000116726	ENSG00000185686
ENSG00000116726	ENSG00000187545
ENSG00000116726	ENSG00000197549
ENSG00000116726	ENSG00000204478
ENSG00000116726	ENSG00000204479
ENSG00000116726	ENSG00000204480
ENSG00000116726	ENSG00000204481
ENSG00000116726	ENSG00000204485
ENSG00000116726	ENSG00000204486
ENSG00000116726	ENSG00000204488
ENSG00000116726	ENSG00000204491
ENSG00000116726	ENSG00000204495
ENSG00000116726	ENSG00000204501
ENSG00000116726	ENSG00000204502
ENSG00000116726	ENSG00000204503
ENSG00000116726	ENSG00000204505
ENSG00000116726	ENSG00000204507
ENSG00000116726	ENSG00000204508
ENSG00000116726	ENSG00000204509
ENSG00000116726	ENSG00000204510
ENSG00000116726	ENSG00000204513
ENSG00000116741	ENSG00000090104
ENSG00000116741	ENSG00000091844
ENSG00000116741	ENSG00000117152
ENSG00000116741	ENSG00000127074
ENSG00000116741	ENSG00000135824
ENSG00000116741	ENSG00000143248

ENSG00000116741	ENSG00000143333
ENSG00000116741	ENSG00000147509
ENSG00000116741	ENSG00000148908
ENSG00000116741	ENSG00000150681
ENSG00000116741	ENSG00000171700
ENSG00000116741	ENSG00000215825
ENSG00000116745	ENSG00000135697
ENSG00000116745	ENSG00000197580
ENSG00000116748	ENSG00000116337
ENSG00000116748	ENSG00000133805
ENSG00000116754	ENSG00000153914
ENSG00000116785	ENSG00000000971
ENSG00000116785	ENSG00000080910
ENSG00000116785	ENSG00000134365
ENSG00000116785	ENSG00000134389
ENSG00000116785	ENSG00000134391
ENSG00000116785	ENSG00000143278
ENSG00000116793	ENSG00000006576
ENSG00000116819	ENSG00000008196
ENSG00000116819	ENSG00000008197
ENSG00000116819	ENSG00000087510
ENSG00000116819	ENSG00000137203
ENSG00000116833	ENSG00000136931
ENSG00000116833	ENSG00000148200
ENSG00000116852	ENSG00000090889
ENSG00000116852	ENSG00000139116
ENSG00000116857	ENSG00000175348
ENSG00000116871	ENSG00000129680
ENSG00000116871	ENSG00000135525
ENSG00000116871	ENSG00000184368
ENSG00000116906	ENSG00000119927
ENSG00000116906	ENSG00000186281
ENSG00000116954	ENSG00000025039
ENSG00000116962	ENSG00000087303
ENSG00000116962	ENSG00000112562
ENSG00000116962	ENSG00000198732
ENSG00000116977	ENSG00000006659
ENSG00000116977	ENSG00000100079
ENSG00000116977	ENSG00000100097
ENSG00000116977	ENSG00000105198
ENSG00000116977	ENSG00000105205
ENSG00000116977	ENSG00000119862
ENSG00000116977	ENSG00000131981
ENSG00000116977	ENSG00000133317
ENSG00000116977	ENSG00000168961
ENSG00000116977	ENSG00000170298
ENSG00000116977	ENSG00000171747
ENSG00000116977	ENSG00000171916
ENSG00000116977	ENSG00000178934
ENSG00000116977	ENSG00000197778
ENSG00000116977	ENSG00000205076
ENSG00000116977	ENSG00000215132
ENSG00000116981	ENSG00000185013
ENSG00000116983	ENSG00000104490
ENSG00000116983	ENSG00000107130
ENSG00000116983	ENSG00000109047
ENSG00000116983	ENSG00000115756
ENSG00000116983	ENSG00000121905
ENSG00000116983	ENSG00000163032

ENSG00000116985	ENSG00000101144
ENSG00000116985	ENSG00000107623
ENSG00000116985	ENSG00000112175
ENSG00000116985	ENSG00000125965
ENSG00000116985	ENSG00000128802
ENSG00000116985	ENSG00000130385
ENSG00000116985	ENSG00000143869
ENSG00000116985	ENSG00000152785
ENSG00000116985	ENSG00000153162
ENSG00000116985	ENSG00000156466
ENSG00000116985	ENSG00000156574
ENSG00000116985	ENSG00000163217
ENSG00000116985	ENSG00000183682
ENSG00000116990	ENSG00000134323
ENSG00000116990	ENSG00000136997
ENSG00000116991	ENSG00000076864
ENSG00000116991	ENSG00000105738
ENSG00000116991	ENSG00000132359
ENSG00000116991	ENSG00000136895
ENSG00000116991	ENSG00000197555
ENSG00000116991	ENSG00000213445
ENSG00000116996	ENSG00000103310
ENSG00000116996	ENSG00000149506
ENSG00000117000	ENSG00000188994
ENSG00000117013	ENSG00000053918
ENSG00000117013	ENSG00000075043
ENSG00000117013	ENSG00000184156
ENSG00000117013	ENSG00000185760
ENSG00000117016	ENSG00000079841
ENSG00000117016	ENSG00000176406
ENSG00000117020	ENSG00000027075
ENSG00000117020	ENSG00000065675
ENSG00000117020	ENSG00000067606
ENSG00000117020	ENSG00000105221
ENSG00000117020	ENSG00000126583
ENSG00000117020	ENSG00000142208
ENSG00000117020	ENSG00000154229
ENSG00000117020	ENSG00000163558
ENSG00000117020	ENSG00000163932
ENSG00000117020	ENSG00000166501
ENSG00000117069	ENSG00000073849
ENSG00000117069	ENSG00000101638
ENSG00000117069	ENSG00000111728
ENSG00000117069	ENSG00000144057
ENSG00000117069	ENSG00000148488
ENSG00000117069	ENSG00000160408
ENSG00000117069	ENSG00000177511
ENSG00000117114	ENSG00000072071
ENSG00000117114	ENSG00000111452
ENSG00000117114	ENSG00000150471
ENSG00000117114	ENSG00000180264
ENSG00000117122	ENSG00000197614
ENSG00000117139	ENSG00000012817
ENSG00000117139	ENSG00000073614
ENSG00000117139	ENSG00000126012
ENSG00000117143	ENSG00000197355
ENSG00000117152	ENSG00000090104
ENSG00000117152	ENSG00000091844
ENSG00000117152	ENSG00000116741

ENSG00000117152	ENSG00000127074
ENSG00000117152	ENSG00000135824
ENSG00000117152	ENSG00000143248
ENSG00000117152	ENSG00000143333
ENSG00000117152	ENSG00000147509
ENSG00000117152	ENSG00000148908
ENSG00000117152	ENSG00000150681
ENSG00000117152	ENSG00000171700
ENSG00000117152	ENSG00000215825
ENSG00000117215	ENSG00000069764
ENSG00000117215	ENSG00000127472
ENSG00000117215	ENSG00000158786
ENSG00000117215	ENSG00000170890
ENSG00000117215	ENSG00000187980
ENSG00000117215	ENSG00000188257
ENSG00000117215	ENSG00000188784
ENSG00000117226	ENSG00000117228
ENSG00000117226	ENSG00000154451
ENSG00000117226	ENSG00000162645
ENSG00000117226	ENSG00000162654
ENSG00000117226	ENSG00000183347
ENSG00000117226	ENSG00000213512
ENSG00000117228	ENSG00000117226
ENSG00000117228	ENSG00000154451
ENSG00000117228	ENSG00000162645
ENSG00000117228	ENSG00000162654
ENSG00000117228	ENSG00000183347
ENSG00000117228	ENSG00000213512
ENSG00000117266	ENSG00000058091
ENSG00000117266	ENSG00000059758
ENSG00000117266	ENSG00000102225
ENSG00000117266	ENSG00000138395
ENSG00000117280	ENSG00000118508
ENSG00000117280	ENSG00000123892
ENSG00000117289	ENSG00000105643
ENSG00000117289	ENSG00000113369
ENSG00000117289	ENSG00000140450
ENSG00000117289	ENSG00000197070
ENSG00000117289	ENSG00000205784
ENSG00000117298	ENSG00000102174
ENSG00000117298	ENSG00000142606
ENSG00000117298	ENSG00000145194
ENSG00000117298	ENSG00000171551
ENSG00000117298	ENSG00000196549
ENSG00000117298	ENSG00000197993
ENSG00000117305	ENSG00000146151
ENSG00000117318	ENSG00000115738
ENSG00000117318	ENSG00000125968
ENSG00000117318	ENSG00000172201
ENSG00000117335	ENSG00000091583
ENSG00000117335	ENSG00000123838
ENSG00000117335	ENSG00000196352
ENSG00000117335	ENSG00000197721
ENSG00000117362	ENSG00000138613
ENSG00000117385	ENSG00000090530
ENSG00000117385	ENSG00000110811
ENSG00000117385	ENSG00000141696
ENSG00000117385	ENSG00000170275
ENSG00000117394	ENSG00000059804

ENSG00000117394	ENSG00000109667
ENSG00000117394	ENSG00000133460
ENSG00000117394	ENSG00000142583
ENSG00000117394	ENSG00000163581
ENSG00000117394	ENSG00000173262
ENSG00000117394	ENSG00000181856
ENSG00000117394	ENSG00000197241
ENSG00000117400	ENSG00000168685
ENSG00000117400	ENSG00000187266
ENSG00000117407	ENSG00000125650
ENSG00000117407	ENSG00000168621
ENSG00000117407	ENSG00000171119
ENSG00000117408	ENSG00000064419
ENSG00000117411	ENSG00000027847
ENSG00000117411	ENSG00000086062
ENSG00000117411	ENSG00000118276
ENSG00000117411	ENSG00000121578
ENSG00000117411	ENSG00000158470
ENSG00000117411	ENSG00000158850
ENSG00000117425	ENSG00000185920
ENSG00000117448	ENSG00000085662
ENSG00000117448	ENSG00000122787
ENSG00000117448	ENSG00000151631
ENSG00000117448	ENSG00000151632
ENSG00000117448	ENSG00000165568
ENSG00000117448	ENSG00000187134
ENSG00000117448	ENSG00000196139
ENSG00000117448	ENSG00000196326
ENSG00000117448	ENSG00000198074
ENSG00000117448	ENSG00000198610
ENSG00000117448	ENSG00000215267
ENSG00000117450	ENSG00000123131
ENSG00000117450	ENSG00000165672
ENSG00000117450	ENSG00000167815
ENSG00000117461	ENSG00000105647
ENSG00000117461	ENSG00000145675
ENSG00000117472	ENSG00000011105
ENSG00000117472	ENSG00000085117
ENSG00000117472	ENSG00000104894
ENSG00000117472	ENSG00000110900
ENSG00000117472	ENSG00000143119
ENSG00000117472	ENSG00000157570
ENSG00000117472	ENSG00000177697
ENSG00000117472	ENSG00000214063
ENSG00000117479	ENSG00000135917
ENSG00000117479	ENSG00000173638
ENSG00000117481	ENSG00000178694
ENSG00000117500	ENSG00000086598
ENSG00000117500	ENSG00000099203
ENSG00000117500	ENSG00000134970
ENSG00000117500	ENSG00000157315
ENSG00000117500	ENSG00000166557
ENSG00000117519	ENSG00000064666
ENSG00000117519	ENSG00000130176
ENSG00000117519	ENSG00000144834
ENSG00000117519	ENSG00000149591
ENSG00000117519	ENSG00000158710
ENSG00000117519	ENSG00000204782
ENSG00000117519	ENSG00000204826

ENSG00000117519	ENSG00000215433
ENSG00000117523	ENSG00000130723
ENSG00000117523	ENSG00000204469
ENSG00000117560	ENSG00000102245
ENSG00000117560	ENSG00000120659
ENSG00000117560	ENSG00000121858
ENSG00000117560	ENSG00000125735
ENSG00000117560	ENSG00000181634
ENSG00000117560	ENSG00000204490
ENSG00000117560	ENSG00000204496
ENSG00000117569	ENSG00000011304
ENSG00000117569	ENSG00000119314
ENSG00000117594	ENSG00000167733
ENSG00000117595	ENSG00000125347
ENSG00000117595	ENSG00000126456
ENSG00000117595	ENSG00000128604
ENSG00000117595	ENSG00000137265
ENSG00000117595	ENSG00000140968
ENSG00000117595	ENSG00000168310
ENSG00000117595	ENSG00000185507
ENSG00000117595	ENSG00000213928
ENSG00000117598	ENSG00000067113
ENSG00000117598	ENSG00000105520
ENSG00000117598	ENSG00000117600
ENSG00000117598	ENSG00000129951
ENSG00000117598	ENSG00000141934
ENSG00000117598	ENSG00000148123
ENSG00000117598	ENSG00000162407
ENSG00000117600	ENSG00000067113
ENSG00000117600	ENSG00000105520
ENSG00000117600	ENSG00000117598
ENSG00000117600	ENSG00000129951
ENSG00000117600	ENSG00000141934
ENSG00000117600	ENSG00000148123
ENSG00000117600	ENSG00000162407
ENSG00000117602	ENSG00000159200
ENSG00000117602	ENSG00000172348
ENSG00000117625	ENSG00000089902
ENSG00000117625	ENSG00000167771
ENSG00000117632	ENSG00000015592
ENSG00000117632	ENSG00000104435
ENSG00000117632	ENSG00000213603
ENSG00000117632	ENSG00000214241
ENSG00000117640	ENSG00000066855
ENSG00000117640	ENSG00000146410
ENSG00000117643	ENSG00000111885
ENSG00000117643	ENSG00000198162
ENSG00000117676	ENSG00000071242
ENSG00000117676	ENSG00000072133
ENSG00000117676	ENSG00000100784
ENSG00000117676	ENSG00000108443
ENSG00000117676	ENSG00000162302
ENSG00000117676	ENSG00000175634
ENSG00000117676	ENSG00000177189
ENSG00000117676	ENSG00000214817
ENSG00000117707	ENSG00000119608
ENSG00000117713	ENSG00000049618
ENSG00000117713	ENSG00000116017
ENSG00000117713	ENSG00000179361

ENSG00000117713	ENSG00000205143
ENSG00000117748	ENSG00000204086
ENSG00000117758	ENSG00000079950
ENSG00000117791	ENSG00000186205
ENSG00000117834	ENSG00000100170
ENSG00000117834	ENSG00000100191
ENSG00000117834	ENSG00000140675
ENSG00000117834	ENSG00000154025
ENSG00000117834	ENSG00000158865
ENSG00000117834	ENSG00000198743
ENSG00000117862	ENSG00000106541
ENSG00000117862	ENSG00000173467
ENSG00000117868	ENSG00000139641
ENSG00000117868	ENSG00000158220
ENSG00000117971	ENSG00000080644
ENSG00000117971	ENSG00000101204
ENSG00000117971	ENSG00000108556
ENSG00000117971	ENSG00000120903
ENSG00000117971	ENSG00000129749
ENSG00000117971	ENSG00000135902
ENSG00000117971	ENSG00000138435
ENSG00000117971	ENSG00000147432
ENSG00000117971	ENSG00000147434
ENSG00000117971	ENSG00000149305
ENSG00000117971	ENSG00000160716
ENSG00000117971	ENSG00000166664
ENSG00000117971	ENSG00000166736
ENSG00000117971	ENSG00000169684
ENSG00000117971	ENSG00000170175
ENSG00000117971	ENSG00000174343
ENSG00000117971	ENSG00000175344
ENSG00000117971	ENSG00000178084
ENSG00000117971	ENSG00000186038
ENSG00000117971	ENSG00000186090
ENSG00000117971	ENSG00000186919
ENSG00000117971	ENSG00000196811
ENSG00000117983	ENSG00000090920
ENSG00000117983	ENSG00000109927
ENSG00000117983	ENSG00000110799
ENSG00000117983	ENSG00000146839
ENSG00000117983	ENSG00000184956
ENSG00000117983	ENSG00000188162
ENSG00000117983	ENSG00000196292
ENSG00000117983	ENSG00000197558
ENSG00000117983	ENSG00000198788
ENSG00000117984	ENSG00000096088
ENSG00000117984	ENSG00000131400
ENSG00000117984	ENSG00000131401
ENSG00000117984	ENSG00000143839
ENSG00000117984	ENSG00000172647
ENSG00000117984	ENSG00000180860
ENSG00000117984	ENSG00000184488
ENSG00000117984	ENSG00000196188
ENSG00000117984	ENSG00000204973
ENSG00000117984	ENSG00000215868
ENSG00000118004	ENSG00000105472
ENSG00000118004	ENSG00000122852
ENSG00000118004	ENSG00000122854
ENSG00000118004	ENSG00000133661

ENSG00000118004	ENSG00000163815
ENSG00000118004	ENSG00000165471
ENSG00000118004	ENSG00000166509
ENSG00000118004	ENSG00000182314
ENSG00000118004	ENSG00000184374
ENSG00000118004	ENSG00000185303
ENSG00000118007	ENSG00000066923
ENSG00000118007	ENSG00000101972
ENSG00000118058	ENSG00000105663
ENSG00000118113	ENSG00000008516
ENSG00000118113	ENSG00000087245
ENSG00000118113	ENSG00000099953
ENSG00000118113	ENSG00000100985
ENSG00000118113	ENSG00000102996
ENSG00000118113	ENSG00000110347
ENSG00000118113	ENSG00000123342
ENSG00000118113	ENSG00000125966
ENSG00000118113	ENSG00000129270
ENSG00000118113	ENSG00000137673
ENSG00000118113	ENSG00000137674
ENSG00000118113	ENSG00000137675
ENSG00000118113	ENSG00000137745
ENSG00000118113	ENSG00000149968
ENSG00000118113	ENSG00000154485
ENSG00000118113	ENSG00000156103
ENSG00000118113	ENSG00000157227
ENSG00000118113	ENSG00000166670
ENSG00000118113	ENSG00000167346
ENSG00000118113	ENSG00000189409
ENSG00000118113	ENSG00000196611
ENSG00000118113	ENSG00000198598
ENSG00000118113	ENSG00000215914
ENSG00000118137	ENSG00000110243
ENSG00000118137	ENSG00000110244
ENSG00000118137	ENSG00000130203
ENSG00000118137	ENSG00000215754
ENSG00000118137	ENSG00000215756
ENSG00000118156	ENSG00000124496
ENSG00000118156	ENSG00000156030
ENSG00000118160	ENSG00000100678
ENSG00000118160	ENSG00000183023
ENSG00000118181	ENSG00000188094
ENSG00000118181	ENSG00000198940
ENSG00000118194	ENSG00000105048
ENSG00000118194	ENSG00000130595
ENSG00000118200	ENSG00000076826
ENSG00000118200	ENSG00000130559
ENSG00000118217	ENSG00000060566
ENSG00000118217	ENSG00000107175
ENSG00000118217	ENSG00000143578
ENSG00000118217	ENSG00000157613
ENSG00000118217	ENSG00000182158
ENSG00000118217	ENSG00000213676
ENSG00000118231	ENSG00000100053
ENSG00000118231	ENSG00000100058
ENSG00000118231	ENSG00000100122
ENSG00000118231	ENSG00000108255
ENSG00000118231	ENSG00000127377
ENSG00000118231	ENSG00000163254

ENSG00000118231	ENSG00000163499
ENSG00000118231	ENSG00000168582
ENSG00000118231	ENSG00000182187
ENSG00000118231	ENSG00000196431
ENSG00000118231	ENSG00000213139
ENSG00000118257	ENSG00000057019
ENSG00000118257	ENSG00000099250
ENSG00000118257	ENSG00000102104
ENSG00000118257	ENSG00000140545
ENSG00000118257	ENSG00000164176
ENSG00000118257	ENSG00000164465
ENSG00000118260	ENSG00000095794
ENSG00000118260	ENSG00000123268
ENSG00000118276	ENSG00000027847
ENSG00000118276	ENSG00000086062
ENSG00000118276	ENSG00000117411
ENSG00000118276	ENSG00000121578
ENSG00000118276	ENSG00000158470
ENSG00000118276	ENSG00000158850
ENSG00000118298	ENSG00000074410
ENSG00000118298	ENSG00000104267
ENSG00000118298	ENSG00000107159
ENSG00000118298	ENSG00000131686
ENSG00000118298	ENSG00000133742
ENSG00000118298	ENSG00000164879
ENSG00000118298	ENSG00000167434
ENSG00000118298	ENSG00000168748
ENSG00000118298	ENSG00000169239
ENSG00000118298	ENSG00000174990
ENSG00000118298	ENSG00000178538
ENSG00000118298	ENSG00000185015
ENSG00000118298	ENSG00000214729
ENSG00000118298	ENSG00000214873
ENSG00000118298	ENSG00000215487
ENSG00000118298	ENSG00000215501
ENSG00000118298	ENSG00000215543
ENSG00000118308	ENSG00000072952
ENSG00000118322	ENSG00000145246
ENSG00000118322	ENSG00000206190
ENSG00000118369	ENSG00000170185
ENSG00000118407	ENSG00000168386
ENSG00000118407	ENSG00000169641
ENSG00000118418	ENSG00000176982
ENSG00000118418	ENSG00000180150
ENSG00000118418	ENSG00000182952
ENSG00000118418	ENSG00000198830
ENSG00000118418	ENSG00000212769
ENSG00000118418	ENSG00000214578
ENSG00000118432	ENSG00000064547
ENSG00000118432	ENSG00000125910
ENSG00000118432	ENSG00000132975
ENSG00000118432	ENSG00000146360
ENSG00000118432	ENSG00000170989
ENSG00000118432	ENSG00000171517
ENSG00000118432	ENSG00000175898
ENSG00000118432	ENSG00000180739
ENSG00000118432	ENSG00000181773
ENSG00000118432	ENSG00000188822
ENSG00000118432	ENSG00000198121

ENSG00000118432	ENSG00000213694
ENSG00000118473	ENSG00000007237
ENSG00000118473	ENSG00000130475
ENSG00000118473	ENSG00000157107
ENSG00000118479	ENSG00000214559
ENSG00000118482	ENSG00000101191
ENSG00000118482	ENSG00000134668
ENSG00000118495	ENSG00000126003
ENSG00000118495	ENSG00000181690
ENSG00000118496	ENSG00000163833
ENSG00000118508	ENSG00000117280
ENSG00000118508	ENSG00000123892
ENSG00000118513	ENSG00000101057
ENSG00000118513	ENSG00000185697
ENSG00000118515	ENSG00000101049
ENSG00000118515	ENSG00000104205
ENSG00000118520	ENSG00000081181
ENSG00000118523	ENSG00000064205
ENSG00000118523	ENSG00000104415
ENSG00000118523	ENSG00000112761
ENSG00000118523	ENSG00000136999
ENSG00000118523	ENSG00000142871
ENSG00000118526	ENSG00000163792
ENSG00000118526	ENSG00000178860
ENSG00000118526	ENSG00000187728
ENSG00000118564	ENSG00000151876
ENSG00000118596	ENSG00000100156
ENSG00000118596	ENSG00000108932
ENSG00000118596	ENSG00000112394
ENSG00000118596	ENSG00000141526
ENSG00000118596	ENSG00000147100
ENSG00000118596	ENSG00000152779
ENSG00000118596	ENSG00000155380
ENSG00000118596	ENSG00000163053
ENSG00000118596	ENSG00000165449
ENSG00000118596	ENSG00000168679
ENSG00000118596	ENSG00000170190
ENSG00000118596	ENSG00000174326
ENSG00000118596	ENSG00000174327
ENSG00000118680	ENSG00000101335
ENSG00000118680	ENSG00000101608
ENSG00000118680	ENSG00000106436
ENSG00000118680	ENSG00000106631
ENSG00000118680	ENSG00000111245
ENSG00000118680	ENSG00000180209
ENSG00000118680	ENSG00000215375
ENSG00000118689	ENSG00000150907
ENSG00000118689	ENSG00000184481
ENSG00000118689	ENSG00000204060
ENSG00000118707	ENSG00000105419
ENSG00000118707	ENSG00000134138
ENSG00000118707	ENSG00000143995
ENSG00000118707	ENSG00000153779
ENSG00000118707	ENSG00000160199
ENSG00000118707	ENSG00000165495
ENSG00000118707	ENSG00000176679
ENSG00000118707	ENSG00000177426
ENSG00000118707	ENSG00000179277
ENSG00000118707	ENSG00000188013

ENSG00000118729	ENSG00000143318
ENSG00000118733	ENSG00000034971
ENSG00000118733	ENSG00000102837
ENSG00000118733	ENSG00000105088
ENSG00000118733	ENSG00000130558
ENSG00000118733	ENSG00000183801
ENSG00000118733	ENSG00000186417
ENSG00000118733	ENSG00000215081
ENSG00000118816	ENSG00000113328
ENSG00000118816	ENSG00000138764
ENSG00000118816	ENSG00000205089
ENSG00000118898	ENSG00000096696
ENSG00000118898	ENSG00000127603
ENSG00000118898	ENSG00000151914
ENSG00000118898	ENSG00000167880
ENSG00000118900	ENSG00000157741
ENSG00000118903	ENSG00000134717
ENSG00000118903	ENSG00000145741
ENSG00000118903	ENSG00000197407
ENSG00000118903	ENSG00000214680
ENSG00000118922	ENSG00000102349
ENSG00000118922	ENSG00000109787
ENSG00000118922	ENSG00000118263
ENSG00000118922	ENSG00000163884
ENSG00000118939	ENSG00000154277
ENSG00000118946	ENSG00000136099
ENSG00000118946	ENSG00000138650
ENSG00000118946	ENSG00000165194
ENSG00000118946	ENSG00000189184
ENSG00000118971	ENSG00000110092
ENSG00000118971	ENSG00000112576
ENSG00000118972	ENSG00000105550
ENSG00000118972	ENSG00000162344
ENSG00000118975	ENSG00000184319
ENSG00000118975	ENSG00000185822
ENSG00000118975	ENSG00000186239
ENSG00000118975	ENSG00000186698
ENSG00000118975	ENSG00000198242
ENSG00000118975	ENSG00000206147
ENSG00000118975	ENSG00000212932
ENSG00000118975	ENSG00000213753
ENSG00000118975	ENSG00000214230
ENSG00000118975	ENSG00000215001
ENSG00000118975	ENSG00000215140
ENSG00000118975	ENSG00000215387
ENSG00000118976	ENSG00000068308
ENSG00000118976	ENSG00000164164
ENSG00000118985	ENSG00000105656
ENSG00000118985	ENSG00000152939
ENSG00000118985	ENSG00000187213
ENSG00000118985	ENSG00000197822
ENSG00000119042	ENSG00000182568
ENSG00000119048	ENSG00000077721
ENSG00000119121	ENSG00000070985
ENSG00000119121	ENSG00000083067
ENSG00000119121	ENSG00000092439
ENSG00000119121	ENSG00000130529
ENSG00000119121	ENSG00000134160
ENSG00000119121	ENSG00000142185

ENSG00000119121	ENSG00000144481
ENSG00000119138	ENSG00000169926
ENSG00000119139	ENSG00000104067
ENSG00000119231	ENSG00000079387
ENSG00000119231	ENSG00000161956
ENSG00000119231	ENSG00000163904
ENSG00000119283	ENSG00000100505
ENSG00000119283	ENSG00000152503
ENSG00000119283	ENSG00000163462
ENSG00000119283	ENSG00000186628
ENSG00000119314	ENSG00000011304
ENSG00000119314	ENSG00000117569
ENSG00000119318	ENSG00000179262
ENSG00000119335	ENSG00000125979
ENSG00000119335	ENSG00000168692
ENSG00000119335	ENSG00000168757
ENSG00000119335	ENSG00000169849
ENSG00000119335	ENSG00000178021
ENSG00000119335	ENSG00000180543
ENSG00000119335	ENSG00000184205
ENSG00000119335	ENSG00000187189
ENSG00000119335	ENSG00000187657
ENSG00000119335	ENSG00000188656
ENSG00000119335	ENSG00000189241
ENSG00000119335	ENSG00000203911
ENSG00000119335	ENSG00000205405
ENSG00000119335	ENSG00000206167
ENSG00000119335	ENSG00000206184
ENSG00000119335	ENSG00000214236
ENSG00000119335	ENSG00000215569
ENSG00000119335	ENSG00000215591
ENSG00000119335	ENSG00000215592
ENSG00000119335	ENSG00000215593
ENSG00000119335	ENSG00000215594
ENSG00000119335	ENSG00000215601
ENSG00000119335	ENSG00000215605
ENSG00000119335	ENSG00000215829
ENSG00000119396	ENSG00000104388
ENSG00000119396	ENSG00000129472
ENSG00000119396	ENSG00000155961
ENSG00000119396	ENSG00000167578
ENSG00000119396	ENSG00000168118
ENSG00000119396	ENSG00000179331
ENSG00000119396	ENSG00000183940
ENSG00000119396	ENSG00000188060
ENSG00000119401	ENSG00000109654
ENSG00000119401	ENSG00000110171
ENSG00000119401	ENSG00000187566
ENSG00000119401	ENSG00000188811
ENSG00000119401	ENSG00000206557
ENSG00000119403	ENSG00000112511
ENSG00000119403	ENSG00000143033
ENSG00000119457	ENSG00000076351
ENSG00000119457	ENSG00000139508
ENSG00000119508	ENSG00000123358
ENSG00000119508	ENSG00000153234
ENSG00000119509	ENSG00000065413
ENSG00000119509	ENSG00000139645
ENSG00000119509	ENSG00000206560

ENSG00000119522	ENSG00000146966
ENSG00000119522	ENSG00000162701
ENSG00000119522	ENSG00000162777
ENSG00000119522	ENSG00000166444
ENSG00000119522	ENSG00000175984
ENSG00000119522	ENSG00000205744
ENSG00000119535	ENSG00000081985
ENSG00000119535	ENSG00000104998
ENSG00000119535	ENSG00000113594
ENSG00000119535	ENSG00000134352
ENSG00000119535	ENSG00000145623
ENSG00000119535	ENSG00000164509
ENSG00000119541	ENSG00000132612
ENSG00000119547	ENSG00000111249
ENSG00000119547	ENSG00000160967
ENSG00000119547	ENSG00000169856
ENSG00000119547	ENSG00000205922
ENSG00000119574	ENSG00000181722
ENSG00000119608	ENSG00000117707
ENSG00000119614	ENSG00000004848
ENSG00000119614	ENSG00000052850
ENSG00000119614	ENSG00000100987
ENSG00000119614	ENSG00000156150
ENSG00000119614	ENSG00000180318
ENSG00000119630	ENSG00000100311
ENSG00000119630	ENSG00000112715
ENSG00000119630	ENSG00000150630
ENSG00000119630	ENSG00000165197
ENSG00000119630	ENSG00000173511
ENSG00000119630	ENSG00000197461
ENSG00000119630	ENSG00000215682
ENSG00000119640	ENSG00000170634
ENSG00000119669	ENSG00000168264
ENSG00000119669	ENSG00000170604
ENSG00000119681	ENSG00000049323
ENSG00000119681	ENSG00000077942
ENSG00000119681	ENSG00000090006
ENSG00000119681	ENSG00000115380
ENSG00000119681	ENSG00000140092
ENSG00000119681	ENSG00000163520
ENSG00000119681	ENSG00000168056
ENSG00000119681	ENSG00000172638
ENSG00000119681	ENSG00000214536
ENSG00000119686	ENSG00000138463
ENSG00000119686	ENSG00000162769
ENSG00000119686	ENSG00000169026
ENSG00000119714	ENSG00000140030
ENSG00000119714	ENSG00000177464
ENSG00000119714	ENSG00000183484
ENSG00000119715	ENSG00000091831
ENSG00000119715	ENSG00000140009
ENSG00000119715	ENSG00000173153
ENSG00000119715	ENSG00000196482
ENSG00000119725	ENSG00000065029
ENSG00000119725	ENSG00000166478
ENSG00000119729	ENSG00000070831
ENSG00000119729	ENSG00000126785
ENSG00000119729	ENSG00000128340
ENSG00000119729	ENSG00000136238

ENSG00000119729	ENSG00000169750
ENSG00000119729	ENSG00000177105
ENSG00000119771	ENSG00000114796
ENSG00000119771	ENSG00000149243
ENSG00000119771	ENSG00000172578
ENSG00000119772	ENSG00000088305
ENSG00000119772	ENSG00000142182
ENSG00000119782	ENSG00000088832
ENSG00000119782	ENSG00000146722
ENSG00000119782	ENSG00000198225
ENSG00000119787	ENSG00000184743
ENSG00000119787	ENSG00000198513
ENSG00000119812	ENSG00000130244
ENSG00000119812	ENSG00000171262
ENSG00000119862	ENSG00000006659
ENSG00000119862	ENSG00000100079
ENSG00000119862	ENSG00000100097
ENSG00000119862	ENSG00000105198
ENSG00000119862	ENSG00000105205
ENSG00000119862	ENSG00000116977
ENSG00000119862	ENSG00000131981
ENSG00000119862	ENSG00000133317
ENSG00000119862	ENSG00000168961
ENSG00000119862	ENSG00000170298
ENSG00000119862	ENSG00000171747
ENSG00000119862	ENSG00000171916
ENSG00000119862	ENSG00000178934
ENSG00000119862	ENSG00000197778
ENSG00000119862	ENSG00000205076
ENSG00000119862	ENSG00000215132
ENSG00000119866	ENSG00000127152
ENSG00000119866	ENSG00000170684
ENSG00000119899	ENSG00000091664
ENSG00000119899	ENSG00000104888
ENSG00000119899	ENSG00000112337
ENSG00000119899	ENSG00000124564
ENSG00000119899	ENSG00000124568
ENSG00000119899	ENSG00000146039
ENSG00000119899	ENSG00000179520
ENSG00000119900	ENSG00000060491
ENSG00000119913	ENSG00000138315
ENSG00000119913	ENSG00000169344
ENSG00000119913	ENSG00000169347
ENSG00000119917	ENSG00000119922
ENSG00000119917	ENSG00000152778
ENSG00000119917	ENSG00000185745
ENSG00000119917	ENSG00000204010
ENSG00000119919	ENSG00000109705
ENSG00000119919	ENSG00000125816
ENSG00000119919	ENSG00000125820
ENSG00000119919	ENSG00000136327
ENSG00000119919	ENSG00000136352
ENSG00000119919	ENSG00000180053
ENSG00000119919	ENSG00000183072
ENSG00000119922	ENSG00000119917
ENSG00000119922	ENSG00000152778
ENSG00000119922	ENSG00000185745
ENSG00000119922	ENSG00000204010
ENSG00000119927	ENSG00000116906

ENSG00000119927	ENSG00000186281
ENSG00000119938	ENSG00000100829
ENSG00000119938	ENSG00000132825
ENSG00000119938	ENSG00000154415
ENSG00000119938	ENSG00000173281
ENSG00000119946	ENSG00000148842
ENSG00000119946	ENSG00000158158
ENSG00000119946	ENSG00000168763
ENSG00000119950	ENSG00000059728
ENSG00000119950	ENSG00000070444
ENSG00000119950	ENSG00000123933
ENSG00000119950	ENSG00000213347
ENSG00000119973	ENSG00000056291
ENSG00000119973	ENSG00000075073
ENSG00000119973	ENSG00000101292
ENSG00000119973	ENSG00000110148
ENSG00000119973	ENSG00000115353
ENSG00000119973	ENSG00000121764
ENSG00000119973	ENSG00000123901
ENSG00000119973	ENSG00000137252
ENSG00000119973	ENSG00000148734
ENSG00000119973	ENSG00000159279
ENSG00000119973	ENSG00000163394
ENSG00000119973	ENSG00000164128
ENSG00000119973	ENSG00000164129
ENSG00000119973	ENSG00000169618
ENSG00000119973	ENSG00000169836
ENSG00000119973	ENSG00000185149
ENSG00000119973	ENSG00000186867
ENSG00000119973	ENSG00000204174
ENSG00000119973	ENSG00000215167
ENSG00000119977	ENSG00000168778
ENSG00000119977	ENSG00000204852
ENSG00000120049	ENSG00000115041
ENSG00000120049	ENSG00000182132
ENSG00000120049	ENSG00000185774
ENSG00000120053	ENSG00000169154
ENSG00000120053	ENSG00000215618
ENSG00000120057	ENSG00000104332
ENSG00000120057	ENSG00000145423
ENSG00000120057	ENSG00000214766
ENSG00000120063	ENSG00000060558
ENSG00000120063	ENSG00000065135
ENSG00000120063	ENSG00000087258
ENSG00000120063	ENSG00000087460
ENSG00000120063	ENSG00000088256
ENSG00000120063	ENSG00000114349
ENSG00000120063	ENSG00000114353
ENSG00000120063	ENSG00000127955
ENSG00000120063	ENSG00000128266
ENSG00000120063	ENSG00000134183
ENSG00000120063	ENSG00000141404
ENSG00000120063	ENSG00000146535
ENSG00000120063	ENSG00000156049
ENSG00000120063	ENSG00000156052
ENSG00000120063	ENSG00000214415
ENSG00000120063	ENSG00000214622
ENSG00000120068	ENSG00000037965
ENSG00000120068	ENSG00000106004

ENSG00000120068	ENSG00000106006
ENSG00000120068	ENSG00000108511
ENSG00000120068	ENSG00000120075
ENSG00000120068	ENSG00000120087
ENSG00000120068	ENSG00000122592
ENSG00000120068	ENSG00000170166
ENSG00000120068	ENSG00000172789
ENSG00000120068	ENSG00000175879
ENSG00000120068	ENSG00000182742
ENSG00000120068	ENSG00000197576
ENSG00000120068	ENSG00000197757
ENSG00000120068	ENSG00000198353
ENSG00000120071	ENSG00000144445
ENSG00000120075	ENSG00000037965
ENSG00000120075	ENSG00000106004
ENSG00000120075	ENSG00000106006
ENSG00000120075	ENSG00000108511
ENSG00000120075	ENSG00000120068
ENSG00000120075	ENSG00000120087
ENSG00000120075	ENSG00000122592
ENSG00000120075	ENSG00000170166
ENSG00000120075	ENSG00000172789
ENSG00000120075	ENSG00000175879
ENSG00000120075	ENSG00000182742
ENSG00000120075	ENSG00000197576
ENSG00000120075	ENSG00000197757
ENSG00000120075	ENSG00000198353
ENSG00000120087	ENSG00000037965
ENSG00000120087	ENSG00000106004
ENSG00000120087	ENSG00000106006
ENSG00000120087	ENSG00000108511
ENSG00000120087	ENSG00000120068
ENSG00000120087	ENSG00000120075
ENSG00000120087	ENSG00000122592
ENSG00000120087	ENSG00000170166
ENSG00000120087	ENSG00000172789
ENSG00000120087	ENSG00000175879
ENSG00000120087	ENSG00000182742
ENSG00000120087	ENSG00000197576
ENSG00000120087	ENSG00000197757
ENSG00000120087	ENSG00000198353
ENSG00000120088	ENSG00000004948
ENSG00000120088	ENSG00000010310
ENSG00000120088	ENSG00000064989
ENSG00000120088	ENSG00000065325
ENSG00000120088	ENSG00000078549
ENSG00000120088	ENSG00000080293
ENSG00000120088	ENSG00000106018
ENSG00000120088	ENSG00000106113
ENSG00000120088	ENSG00000106128
ENSG00000120088	ENSG00000112164
ENSG00000120088	ENSG00000114812
ENSG00000120088	ENSG00000144407
ENSG00000120088	ENSG00000160801
ENSG00000120088	ENSG00000215644
ENSG00000120093	ENSG00000105997
ENSG00000120093	ENSG00000106038
ENSG00000120093	ENSG00000128652
ENSG00000120093	ENSG00000169840

ENSG00000120093	ENSG00000174279
ENSG00000120093	ENSG00000180613
ENSG00000120094	ENSG00000105991
ENSG00000120094	ENSG00000105996
ENSG00000120094	ENSG00000128645
ENSG00000120094	ENSG00000173917
ENSG00000120129	ENSG00000111266
ENSG00000120129	ENSG00000120875
ENSG00000120129	ENSG00000130829
ENSG00000120129	ENSG00000138166
ENSG00000120129	ENSG00000139318
ENSG00000120129	ENSG00000143507
ENSG00000120129	ENSG00000158050
ENSG00000120129	ENSG00000164086
ENSG00000120129	ENSG00000184545
ENSG00000120137	ENSG00000125779
ENSG00000120137	ENSG00000152782
ENSG00000120149	ENSG00000163132
ENSG00000120156	ENSG00000066056
ENSG00000120162	ENSG00000114978
ENSG00000120162	ENSG00000142961
ENSG00000120162	ENSG00000172081
ENSG00000120162	ENSG00000173542
ENSG00000120162	ENSG00000182208
ENSG00000120211	ENSG00000107014
ENSG00000120211	ENSG00000107018
ENSG00000120211	ENSG00000171136
ENSG00000120211	ENSG00000172410
ENSG00000120217	ENSG00000103855
ENSG00000120217	ENSG00000114013
ENSG00000120217	ENSG00000114455
ENSG00000120217	ENSG00000134258
ENSG00000120217	ENSG00000160223
ENSG00000120217	ENSG00000186470
ENSG00000120217	ENSG00000188211
ENSG00000120217	ENSG00000197646
ENSG00000120217	ENSG00000204290
ENSG00000120217	ENSG00000204655
ENSG00000120217	ENSG00000215631
ENSG00000120217	ENSG00000215811
ENSG00000120217	ENSG00000215889
ENSG00000120251	ENSG00000125675
ENSG00000120251	ENSG00000149403
ENSG00000120251	ENSG00000152578
ENSG00000120251	ENSG00000155511
ENSG00000120251	ENSG00000163873
ENSG00000120251	ENSG00000164418
ENSG00000120251	ENSG00000171189
ENSG00000120254	ENSG00000100714
ENSG00000120256	ENSG00000166145
ENSG00000120278	ENSG00000090924
ENSG00000120278	ENSG00000126822
ENSG00000120318	ENSG00000047365
ENSG00000120318	ENSG00000186635
ENSG00000120322	ENSG00000081818
ENSG00000120322	ENSG00000081842
ENSG00000120322	ENSG00000081853
ENSG00000120322	ENSG00000112852
ENSG00000120322	ENSG00000113205

ENSG00000120322	ENSG00000113209
ENSG00000120322	ENSG00000113211
ENSG00000120322	ENSG00000113212
ENSG00000120322	ENSG00000113248
ENSG00000120322	ENSG00000120324
ENSG00000120322	ENSG00000120327
ENSG00000120322	ENSG00000120328
ENSG00000120322	ENSG00000146001
ENSG00000120322	ENSG00000171815
ENSG00000120322	ENSG00000187372
ENSG00000120322	ENSG00000196963
ENSG00000120322	ENSG00000197479
ENSG00000120322	ENSG00000204955
ENSG00000120322	ENSG00000204956
ENSG00000120322	ENSG00000204961
ENSG00000120322	ENSG00000204962
ENSG00000120322	ENSG00000204963
ENSG00000120322	ENSG00000204964
ENSG00000120322	ENSG00000204965
ENSG00000120322	ENSG00000204967
ENSG00000120322	ENSG00000204968
ENSG00000120322	ENSG00000204969
ENSG00000120322	ENSG00000204970
ENSG00000120322	ENSG00000214567
ENSG00000120322	ENSG00000214570
ENSG00000120322	ENSG00000214574
ENSG00000120322	ENSG00000214580
ENSG00000120322	ENSG00000214583
ENSG00000120322	ENSG00000214594
ENSG00000120322	ENSG00000214600
ENSG00000120322	ENSG00000214608
ENSG00000120324	ENSG00000081818
ENSG00000120324	ENSG00000081842
ENSG00000120324	ENSG00000081853
ENSG00000120324	ENSG00000112852
ENSG00000120324	ENSG00000113205
ENSG00000120324	ENSG00000113209
ENSG00000120324	ENSG00000113211
ENSG00000120324	ENSG00000113212
ENSG00000120324	ENSG00000113248
ENSG00000120324	ENSG00000120322
ENSG00000120324	ENSG00000120327
ENSG00000120324	ENSG00000120328
ENSG00000120324	ENSG00000146001
ENSG00000120324	ENSG00000171815
ENSG00000120324	ENSG00000187372
ENSG00000120324	ENSG00000196963
ENSG00000120324	ENSG00000197479
ENSG00000120324	ENSG00000204955
ENSG00000120324	ENSG00000204956
ENSG00000120324	ENSG00000204961
ENSG00000120324	ENSG00000204962
ENSG00000120324	ENSG00000204963
ENSG00000120324	ENSG00000204964
ENSG00000120324	ENSG00000204965
ENSG00000120324	ENSG00000204967
ENSG00000120324	ENSG00000204968
ENSG00000120324	ENSG00000204969
ENSG00000120324	ENSG00000204970

ENSG00000120324	ENSG00000214567
ENSG00000120324	ENSG00000214570
ENSG00000120324	ENSG00000214574
ENSG00000120324	ENSG00000214580
ENSG00000120324	ENSG00000214583
ENSG00000120324	ENSG00000214594
ENSG00000120324	ENSG00000214600
ENSG00000120324	ENSG00000214608
ENSG00000120327	ENSG00000081818
ENSG00000120327	ENSG00000081842
ENSG00000120327	ENSG00000081853
ENSG00000120327	ENSG00000112852
ENSG00000120327	ENSG00000113205
ENSG00000120327	ENSG00000113209
ENSG00000120327	ENSG00000113211
ENSG00000120327	ENSG00000113212
ENSG00000120327	ENSG00000113248
ENSG00000120327	ENSG00000120322
ENSG00000120327	ENSG00000120324
ENSG00000120327	ENSG00000120328
ENSG00000120327	ENSG00000146001
ENSG00000120327	ENSG00000171815
ENSG00000120327	ENSG00000187372
ENSG00000120327	ENSG00000196963
ENSG00000120327	ENSG00000197479
ENSG00000120327	ENSG00000204955
ENSG00000120327	ENSG00000204956
ENSG00000120327	ENSG00000204961
ENSG00000120327	ENSG00000204962
ENSG00000120327	ENSG00000204963
ENSG00000120327	ENSG00000204964
ENSG00000120327	ENSG00000204965
ENSG00000120327	ENSG00000204967
ENSG00000120327	ENSG00000204968
ENSG00000120327	ENSG00000204969
ENSG00000120327	ENSG00000204970
ENSG00000120327	ENSG00000214567
ENSG00000120327	ENSG00000214570
ENSG00000120327	ENSG00000214574
ENSG00000120327	ENSG00000214580
ENSG00000120327	ENSG00000214583
ENSG00000120327	ENSG00000214594
ENSG00000120327	ENSG00000214600
ENSG00000120327	ENSG00000214608
ENSG00000120328	ENSG00000081818
ENSG00000120328	ENSG00000081842
ENSG00000120328	ENSG00000081853
ENSG00000120328	ENSG00000112852
ENSG00000120328	ENSG00000113205
ENSG00000120328	ENSG00000113209
ENSG00000120328	ENSG00000113211
ENSG00000120328	ENSG00000113212
ENSG00000120328	ENSG00000113248
ENSG00000120328	ENSG00000120322
ENSG00000120328	ENSG00000120324
ENSG00000120328	ENSG00000120327
ENSG00000120328	ENSG00000146001
ENSG00000120328	ENSG00000171815
ENSG00000120328	ENSG00000187372

ENSG00000120328	ENSG00000196963
ENSG00000120328	ENSG00000197479
ENSG00000120328	ENSG00000204955
ENSG00000120328	ENSG00000204956
ENSG00000120328	ENSG00000204961
ENSG00000120328	ENSG00000204962
ENSG00000120328	ENSG00000204963
ENSG00000120328	ENSG00000204964
ENSG00000120328	ENSG00000204965
ENSG00000120328	ENSG00000204967
ENSG00000120328	ENSG00000204968
ENSG00000120328	ENSG00000204969
ENSG00000120328	ENSG00000204970
ENSG00000120328	ENSG00000214567
ENSG00000120328	ENSG00000214570
ENSG00000120328	ENSG00000214574
ENSG00000120328	ENSG00000214580
ENSG00000120328	ENSG00000214583
ENSG00000120328	ENSG00000214594
ENSG00000120328	ENSG00000214600
ENSG00000120328	ENSG00000214608
ENSG00000120329	ENSG00000102743
ENSG00000120332	ENSG00000041982
ENSG00000120332	ENSG00000115414
ENSG00000120332	ENSG00000116147
ENSG00000120332	ENSG00000168477
ENSG00000120341	ENSG00000148396
ENSG00000120457	ENSG00000115474
ENSG00000120457	ENSG00000121361
ENSG00000120457	ENSG00000123700
ENSG00000120457	ENSG00000151704
ENSG00000120457	ENSG00000153822
ENSG00000120457	ENSG00000157542
ENSG00000120457	ENSG00000157551
ENSG00000120457	ENSG00000162728
ENSG00000120457	ENSG00000162989
ENSG00000120457	ENSG00000168135
ENSG00000120457	ENSG00000177807
ENSG00000120457	ENSG00000182324
ENSG00000120457	ENSG00000184185
ENSG00000120457	ENSG00000187486
ENSG00000120500	ENSG00000130561
ENSG00000120500	ENSG00000137486
ENSG00000120500	ENSG00000141480
ENSG00000120549	ENSG00000017373
ENSG00000120563	ENSG00000090382
ENSG00000120563	ENSG00000141316
ENSG00000120563	ENSG00000151033
ENSG00000120563	ENSG00000157093
ENSG00000120563	ENSG00000161572
ENSG00000120563	ENSG00000167531
ENSG00000120563	ENSG00000171478
ENSG00000120563	ENSG00000171489
ENSG00000120586	ENSG00000011028
ENSG00000120586	ENSG00000054219
ENSG00000120586	ENSG00000153246
ENSG00000120586	ENSG00000183748
ENSG00000120594	ENSG00000161381
ENSG00000120616	ENSG00000135999

ENSG00000120645	ENSG00000124313
ENSG00000120645	ENSG00000144711
ENSG00000120645	ENSG00000164117
ENSG00000120658	ENSG00000165675
ENSG00000120659	ENSG00000102245
ENSG00000120659	ENSG00000117560
ENSG00000120659	ENSG00000121858
ENSG00000120659	ENSG00000125735
ENSG00000120659	ENSG00000181634
ENSG00000120659	ENSG00000204490
ENSG00000120659	ENSG00000204496
ENSG00000120662	ENSG00000112031
ENSG00000120662	ENSG00000180015
ENSG00000120675	ENSG00000205981
ENSG00000120690	ENSG00000010030
ENSG00000120690	ENSG00000102034
ENSG00000120690	ENSG00000109381
ENSG00000120690	ENSG00000124664
ENSG00000120690	ENSG00000135373
ENSG00000120690	ENSG00000135374
ENSG00000120690	ENSG00000139083
ENSG00000120690	ENSG00000163435
ENSG00000120690	ENSG00000166211
ENSG00000120693	ENSG00000101665
ENSG00000120693	ENSG00000113658
ENSG00000120693	ENSG00000137834
ENSG00000120693	ENSG00000141646
ENSG00000120693	ENSG00000166949
ENSG00000120693	ENSG00000170365
ENSG00000120693	ENSG00000175387
ENSG00000120694	ENSG00000164070
ENSG00000120694	ENSG00000170606
ENSG00000120696	ENSG00000163376
ENSG00000120696	ENSG00000165572
ENSG00000120696	ENSG00000170852
ENSG00000120708	ENSG00000133110
ENSG00000120709	ENSG00000174137
ENSG00000120709	ENSG00000189319
ENSG00000120727	ENSG00000124374
ENSG00000120729	ENSG00000129116
ENSG00000120729	ENSG00000138347
ENSG00000120733	ENSG00000115548
ENSG00000120733	ENSG00000168453
ENSG00000120733	ENSG00000171988
ENSG00000120738	ENSG00000122877
ENSG00000120738	ENSG00000135625
ENSG00000120738	ENSG00000179388
ENSG00000120738	ENSG00000184937
ENSG00000120742	ENSG00000151778
ENSG00000120756	ENSG00000102024
ENSG00000120756	ENSG00000136167
ENSG00000120798	ENSG00000177463
ENSG00000120820	ENSG00000016864
ENSG00000120832	ENSG00000127989
ENSG00000120833	ENSG00000114737
ENSG00000120833	ENSG00000170677
ENSG00000120833	ENSG00000171150
ENSG00000120833	ENSG00000174111
ENSG00000120833	ENSG00000180008

ENSG00000120833	ENSG00000184557
ENSG00000120833	ENSG00000185338
ENSG00000120875	ENSG00000111266
ENSG00000120875	ENSG00000120129
ENSG00000120875	ENSG00000130829
ENSG00000120875	ENSG00000138166
ENSG00000120875	ENSG00000139318
ENSG00000120875	ENSG00000143507
ENSG00000120875	ENSG00000158050
ENSG00000120875	ENSG00000164086
ENSG00000120875	ENSG00000184545
ENSG00000120885	ENSG00000079101
ENSG00000120889	ENSG00000026103
ENSG00000120889	ENSG00000067182
ENSG00000120889	ENSG00000104689
ENSG00000120889	ENSG00000173530
ENSG00000120889	ENSG00000173535
ENSG00000120889	ENSG00000215788
ENSG00000120896	ENSG00000095637
ENSG00000120896	ENSG00000154556
ENSG00000120899	ENSG00000061938
ENSG00000120899	ENSG00000169398
ENSG00000120899	ENSG00000174292
ENSG00000120903	ENSG00000080644
ENSG00000120903	ENSG00000101204
ENSG00000120903	ENSG00000108556
ENSG00000120903	ENSG00000117971
ENSG00000120903	ENSG00000129749
ENSG00000120903	ENSG00000135902
ENSG00000120903	ENSG00000138435
ENSG00000120903	ENSG00000147432
ENSG00000120903	ENSG00000147434
ENSG00000120903	ENSG00000149305
ENSG00000120903	ENSG00000160716
ENSG00000120903	ENSG00000166664
ENSG00000120903	ENSG00000166736
ENSG00000120903	ENSG00000169684
ENSG00000120903	ENSG00000170175
ENSG00000120903	ENSG00000174343
ENSG00000120903	ENSG00000175344
ENSG00000120903	ENSG00000178084
ENSG00000120903	ENSG00000186038
ENSG00000120903	ENSG00000186090
ENSG00000120903	ENSG00000186919
ENSG00000120903	ENSG00000196811
ENSG00000120907	ENSG00000143147
ENSG00000120907	ENSG00000148680
ENSG00000120907	ENSG00000165370
ENSG00000120907	ENSG00000170214
ENSG00000120907	ENSG00000171873
ENSG00000120907	ENSG00000183935
ENSG00000120910	ENSG00000107758
ENSG00000120910	ENSG00000138814
ENSG00000120913	ENSG00000107438
ENSG00000120913	ENSG00000122367
ENSG00000120913	ENSG00000131435
ENSG00000120913	ENSG00000154553
ENSG00000120913	ENSG00000163110
ENSG00000120913	ENSG00000196923

ENSG00000120913	ENSG00000214895
ENSG00000120937	ENSG00000175206
ENSG00000120949	ENSG00000028137
ENSG00000120949	ENSG00000101017
ENSG00000120949	ENSG00000111321
ENSG00000120949	ENSG00000141655
ENSG00000120949	ENSG00000146072
ENSG00000120949	ENSG00000157873
ENSG00000120949	ENSG00000164761
ENSG00000120952	ENSG00000116708
ENSG00000120952	ENSG00000116721
ENSG00000120952	ENSG00000116726
ENSG00000120952	ENSG00000157358
ENSG00000120952	ENSG00000160959
ENSG00000120952	ENSG00000179704
ENSG00000120952	ENSG00000182330
ENSG00000120952	ENSG00000185028
ENSG00000120952	ENSG00000185686
ENSG00000120952	ENSG00000187545
ENSG00000120952	ENSG00000197549
ENSG00000120952	ENSG00000204478
ENSG00000120952	ENSG00000204479
ENSG00000120952	ENSG00000204480
ENSG00000120952	ENSG00000204481
ENSG00000120952	ENSG00000204485
ENSG00000120952	ENSG00000204486
ENSG00000120952	ENSG00000204488
ENSG00000120952	ENSG00000204491
ENSG00000120952	ENSG00000204495
ENSG00000120952	ENSG00000204501
ENSG00000120952	ENSG00000204502
ENSG00000120952	ENSG00000204503
ENSG00000120952	ENSG00000204505
ENSG00000120952	ENSG00000204507
ENSG00000120952	ENSG00000204508
ENSG00000120952	ENSG00000204509
ENSG00000120952	ENSG00000204510
ENSG00000120952	ENSG00000204513
ENSG00000120992	ENSG00000011009
ENSG00000120992	ENSG00000143353
ENSG00000121005	ENSG00000096006
ENSG00000121005	ENSG00000101074
ENSG00000121005	ENSG00000103196
ENSG00000121005	ENSG00000124490
ENSG00000121005	ENSG00000124812
ENSG00000121005	ENSG00000137558
ENSG00000121005	ENSG00000139278
ENSG00000121005	ENSG00000164530
ENSG00000121005	ENSG00000173401
ENSG00000121005	ENSG00000180481
ENSG00000121039	ENSG00000162496
ENSG00000121039	ENSG00000170509
ENSG00000121039	ENSG00000170786
ENSG00000121039	ENSG00000198189
ENSG00000121053	ENSG00000005381
ENSG00000121053	ENSG00000115705
ENSG00000121053	ENSG00000130508
ENSG00000121053	ENSG00000147485
ENSG00000121053	ENSG00000167419

ENSG00000121060	ENSG00000132481
ENSG00000121060	ENSG00000137699
ENSG00000121067	ENSG00000144228
ENSG00000121068	ENSG00000073861
ENSG00000121068	ENSG00000089225
ENSG00000121068	ENSG00000092607
ENSG00000121068	ENSG00000112837
ENSG00000121068	ENSG00000121075
ENSG00000121068	ENSG00000122145
ENSG00000121068	ENSG00000135111
ENSG00000121068	ENSG00000136535
ENSG00000121068	ENSG00000143178
ENSG00000121068	ENSG00000149922
ENSG00000121068	ENSG00000163508
ENSG00000121068	ENSG00000164458
ENSG00000121068	ENSG00000164532
ENSG00000121068	ENSG00000167800
ENSG00000121068	ENSG00000174197
ENSG00000121068	ENSG00000184058
ENSG00000121075	ENSG00000073861
ENSG00000121075	ENSG00000089225
ENSG00000121075	ENSG00000092607
ENSG00000121075	ENSG00000112837
ENSG00000121075	ENSG00000121068
ENSG00000121075	ENSG00000122145
ENSG00000121075	ENSG00000135111
ENSG00000121075	ENSG00000136535
ENSG00000121075	ENSG00000143178
ENSG00000121075	ENSG00000149922
ENSG00000121075	ENSG00000163508
ENSG00000121075	ENSG00000164458
ENSG00000121075	ENSG00000164532
ENSG00000121075	ENSG00000167800
ENSG00000121075	ENSG00000174197
ENSG00000121075	ENSG00000184058
ENSG00000121083	ENSG00000088986
ENSG00000121089	ENSG00000136274
ENSG00000121089	ENSG00000196531
ENSG00000121089	ENSG00000196861
ENSG00000121104	ENSG00000106415
ENSG00000121104	ENSG00000138439
ENSG00000121207	ENSG00000127252
ENSG00000121207	ENSG00000133321
ENSG00000121207	ENSG00000133328
ENSG00000121207	ENSG00000162981
ENSG00000121207	ENSG00000168004
ENSG00000121207	ENSG00000168672
ENSG00000121207	ENSG00000176485
ENSG00000121210	ENSG00000075568
ENSG00000121236	ENSG00000026950
ENSG00000121236	ENSG00000111801
ENSG00000121236	ENSG00000112763
ENSG00000121236	ENSG00000113303
ENSG00000121236	ENSG00000124508
ENSG00000121236	ENSG00000124549
ENSG00000121236	ENSG00000124557
ENSG00000121236	ENSG00000128250
ENSG00000121236	ENSG00000128253
ENSG00000121236	ENSG00000128276

ENSG00000121236	ENSG00000128482
ENSG00000121236	ENSG00000132256
ENSG00000121236	ENSG00000132274
ENSG00000121236	ENSG00000164010
ENSG00000121236	ENSG00000165810
ENSG00000121236	ENSG00000168903
ENSG00000121236	ENSG00000176422
ENSG00000121236	ENSG00000179046
ENSG00000121236	ENSG00000182805
ENSG00000121236	ENSG00000183439
ENSG00000121236	ENSG00000183718
ENSG00000121236	ENSG00000187574
ENSG00000121236	ENSG00000188683
ENSG00000121236	ENSG00000204614
ENSG00000121236	ENSG00000204616
ENSG00000121236	ENSG00000204618
ENSG00000121236	ENSG00000205198
ENSG00000121236	ENSG00000214693
ENSG00000121274	ENSG00000112941
ENSG00000121281	ENSG00000078295
ENSG00000121281	ENSG00000129467
ENSG00000121281	ENSG00000138031
ENSG00000121281	ENSG00000155897
ENSG00000121281	ENSG00000162104
ENSG00000121281	ENSG00000164742
ENSG00000121281	ENSG00000173175
ENSG00000121281	ENSG00000174233
ENSG00000121297	ENSG00000179981
ENSG00000121297	ENSG00000182463
ENSG00000121361	ENSG00000115474
ENSG00000121361	ENSG00000120457
ENSG00000121361	ENSG00000123700
ENSG00000121361	ENSG00000151704
ENSG00000121361	ENSG00000153822
ENSG00000121361	ENSG00000157542
ENSG00000121361	ENSG00000157551
ENSG00000121361	ENSG00000162728
ENSG00000121361	ENSG00000162989
ENSG00000121361	ENSG00000168135
ENSG00000121361	ENSG00000177807
ENSG00000121361	ENSG00000182324
ENSG00000121361	ENSG00000184185
ENSG00000121361	ENSG00000187486
ENSG00000121390	ENSG00000116560
ENSG00000121390	ENSG00000147140
ENSG00000121440	ENSG00000067840
ENSG00000121440	ENSG00000165966
ENSG00000121454	ENSG00000016082
ENSG00000121454	ENSG00000089116
ENSG00000121454	ENSG00000106689
ENSG00000121454	ENSG00000106852
ENSG00000121454	ENSG00000107187
ENSG00000121454	ENSG00000132130
ENSG00000121454	ENSG00000136944
ENSG00000121454	ENSG00000143013
ENSG00000121454	ENSG00000143355
ENSG00000121454	ENSG00000159556
ENSG00000121454	ENSG00000162624
ENSG00000121454	ENSG00000162761

ENSG00000121481	ENSG00000204227
ENSG00000121542	ENSG00000093183
ENSG00000121577	ENSG00000112276
ENSG00000121577	ENSG00000132429
ENSG00000121578	ENSG00000027847
ENSG00000121578	ENSG00000086062
ENSG00000121578	ENSG00000117411
ENSG00000121578	ENSG00000118276
ENSG00000121578	ENSG00000158470
ENSG00000121578	ENSG00000158850
ENSG00000121621	ENSG00000189285
ENSG00000121634	ENSG00000121742
ENSG00000121634	ENSG00000121743
ENSG00000121634	ENSG00000131233
ENSG00000121634	ENSG00000135355
ENSG00000121634	ENSG00000143140
ENSG00000121634	ENSG00000152661
ENSG00000121634	ENSG00000159248
ENSG00000121634	ENSG00000164411
ENSG00000121634	ENSG00000165474
ENSG00000121634	ENSG00000169562
ENSG00000121634	ENSG00000176402
ENSG00000121634	ENSG00000177291
ENSG00000121634	ENSG00000182963
ENSG00000121634	ENSG00000183153
ENSG00000121634	ENSG00000187513
ENSG00000121634	ENSG00000188910
ENSG00000121634	ENSG00000189280
ENSG00000121634	ENSG00000189433
ENSG00000121634	ENSG00000198835
ENSG00000121634	ENSG00000203733
ENSG00000121653	ENSG00000008735
ENSG00000121671	ENSG00000008405
ENSG00000121690	ENSG00000024526
ENSG00000121690	ENSG00000035499
ENSG00000121690	ENSG00000166153
ENSG00000121741	ENSG00000132950
ENSG00000121741	ENSG00000146463
ENSG00000121741	ENSG00000147130
ENSG00000121741	ENSG00000163867
ENSG00000121741	ENSG00000198218
ENSG00000121742	ENSG00000121634
ENSG00000121742	ENSG00000121743
ENSG00000121742	ENSG00000131233
ENSG00000121742	ENSG00000135355
ENSG00000121742	ENSG00000143140
ENSG00000121742	ENSG00000152661
ENSG00000121742	ENSG00000159248
ENSG00000121742	ENSG00000164411
ENSG00000121742	ENSG00000165474
ENSG00000121742	ENSG00000169562
ENSG00000121742	ENSG00000176402
ENSG00000121742	ENSG00000177291
ENSG00000121742	ENSG00000182963
ENSG00000121742	ENSG00000183153
ENSG00000121742	ENSG00000187513
ENSG00000121742	ENSG00000188910
ENSG00000121742	ENSG00000189280
ENSG00000121742	ENSG00000189433

ENSG00000121742	ENSG00000198835
ENSG00000121742	ENSG00000203733
ENSG00000121743	ENSG00000121634
ENSG00000121743	ENSG00000121742
ENSG00000121743	ENSG00000131233
ENSG00000121743	ENSG00000135355
ENSG00000121743	ENSG00000143140
ENSG00000121743	ENSG00000152661
ENSG00000121743	ENSG00000159248
ENSG00000121743	ENSG00000164411
ENSG00000121743	ENSG00000165474
ENSG00000121743	ENSG00000169562
ENSG00000121743	ENSG00000176402
ENSG00000121743	ENSG00000177291
ENSG00000121743	ENSG00000182963
ENSG00000121743	ENSG00000183153
ENSG00000121743	ENSG00000187513
ENSG00000121743	ENSG00000188910
ENSG00000121743	ENSG00000189280
ENSG00000121743	ENSG00000189433
ENSG00000121743	ENSG00000198835
ENSG00000121743	ENSG00000203733
ENSG00000121749	ENSG00000104946
ENSG00000121753	ENSG00000135298
ENSG00000121753	ENSG00000181790
ENSG00000121764	ENSG00000056291
ENSG00000121764	ENSG00000075073
ENSG00000121764	ENSG00000101292
ENSG00000121764	ENSG00000110148
ENSG00000121764	ENSG00000115353
ENSG00000121764	ENSG00000119973
ENSG00000121764	ENSG00000123901
ENSG00000121764	ENSG00000137252
ENSG00000121764	ENSG00000148734
ENSG00000121764	ENSG00000159279
ENSG00000121764	ENSG00000163394
ENSG00000121764	ENSG00000164128
ENSG00000121764	ENSG00000164129
ENSG00000121764	ENSG00000169618
ENSG00000121764	ENSG00000169836
ENSG00000121764	ENSG00000185149
ENSG00000121764	ENSG00000186867
ENSG00000121764	ENSG00000204174
ENSG00000121764	ENSG00000215167
ENSG00000121769	ENSG00000114113
ENSG00000121769	ENSG00000114115
ENSG00000121769	ENSG00000139194
ENSG00000121769	ENSG00000143320
ENSG00000121769	ENSG00000145384
ENSG00000121769	ENSG00000147588
ENSG00000121769	ENSG00000162444
ENSG00000121769	ENSG00000164434
ENSG00000121769	ENSG00000164687
ENSG00000121769	ENSG00000166426
ENSG00000121769	ENSG00000170323
ENSG00000121769	ENSG00000197416
ENSG00000121769	ENSG00000205186
ENSG00000121769	ENSG00000214004
ENSG00000121774	ENSG00000112232

ENSG00000121774	ENSG00000112531
ENSG00000121774	ENSG00000131773
ENSG00000121775	ENSG00000176142
ENSG00000121797	ENSG00000112486
ENSG00000121797	ENSG00000121807
ENSG00000121797	ENSG00000121966
ENSG00000121797	ENSG00000126353
ENSG00000121797	ENSG00000129048
ENSG00000121797	ENSG00000144648
ENSG00000121797	ENSG00000160683
ENSG00000121797	ENSG00000160791
ENSG00000121797	ENSG00000163464
ENSG00000121797	ENSG00000163823
ENSG00000121797	ENSG00000168329
ENSG00000121797	ENSG00000172215
ENSG00000121797	ENSG00000173578
ENSG00000121797	ENSG00000173585
ENSG00000121797	ENSG00000179934
ENSG00000121797	ENSG00000180871
ENSG00000121797	ENSG00000183625
ENSG00000121797	ENSG00000183813
ENSG00000121797	ENSG00000184451
ENSG00000121797	ENSG00000186810
ENSG00000121797	ENSG00000215775
ENSG00000121797	ENSG00000215778
ENSG00000121797	ENSG00000215782
ENSG00000121807	ENSG00000112486
ENSG00000121807	ENSG00000121797
ENSG00000121807	ENSG00000121966
ENSG00000121807	ENSG00000126353
ENSG00000121807	ENSG00000129048
ENSG00000121807	ENSG00000144648
ENSG00000121807	ENSG00000160683
ENSG00000121807	ENSG00000160791
ENSG00000121807	ENSG00000163464
ENSG00000121807	ENSG00000163823
ENSG00000121807	ENSG00000168329
ENSG00000121807	ENSG00000172215
ENSG00000121807	ENSG00000173578
ENSG00000121807	ENSG00000173585
ENSG00000121807	ENSG00000179934
ENSG00000121807	ENSG00000180871
ENSG00000121807	ENSG00000183625
ENSG00000121807	ENSG00000183813
ENSG00000121807	ENSG00000184451
ENSG00000121807	ENSG00000186810
ENSG00000121807	ENSG00000215775
ENSG00000121807	ENSG00000215778
ENSG00000121807	ENSG00000215782
ENSG00000121848	ENSG00000070423
ENSG00000121851	ENSG00000113356
ENSG00000121858	ENSG00000102245
ENSG00000121858	ENSG00000117560
ENSG00000121858	ENSG00000120659
ENSG00000121858	ENSG00000125735
ENSG00000121858	ENSG00000181634
ENSG00000121858	ENSG00000204490
ENSG00000121858	ENSG00000204496
ENSG00000121864	ENSG00000005889

ENSG00000121864	ENSG00000067646
ENSG00000121864	ENSG00000147180
ENSG00000121871	ENSG00000178235
ENSG00000121871	ENSG00000185985
ENSG00000121879	ENSG00000011405
ENSG00000121879	ENSG00000051382
ENSG00000121879	ENSG00000105851
ENSG00000121879	ENSG00000133056
ENSG00000121879	ENSG00000139144
ENSG00000121879	ENSG00000171608
ENSG00000121892	ENSG00000083642
ENSG00000121898	ENSG00000088882
ENSG00000121898	ENSG00000106624
ENSG00000121898	ENSG00000108582
ENSG00000121898	ENSG00000109472
ENSG00000121898	ENSG00000109625
ENSG00000121898	ENSG00000120054
ENSG00000121898	ENSG00000135678
ENSG00000121900	ENSG00000157992
ENSG00000121904	ENSG00000117322
ENSG00000121904	ENSG00000164796
ENSG00000121904	ENSG00000174175
ENSG00000121904	ENSG00000183117
ENSG00000121904	ENSG00000203710
ENSG00000121905	ENSG00000104490
ENSG00000121905	ENSG00000107130
ENSG00000121905	ENSG00000109047
ENSG00000121905	ENSG00000115756
ENSG00000121905	ENSG00000116983
ENSG00000121905	ENSG00000163032
ENSG00000121933	ENSG00000128271
ENSG00000121933	ENSG00000163485
ENSG00000121933	ENSG00000170425
ENSG00000121957	ENSG00000100154
ENSG00000121957	ENSG00000137473
ENSG00000121957	ENSG00000160360
ENSG00000121957	ENSG00000165917
ENSG00000121957	ENSG00000187862
ENSG00000121966	ENSG00000112486
ENSG00000121966	ENSG00000121797
ENSG00000121966	ENSG00000121807
ENSG00000121966	ENSG00000126353
ENSG00000121966	ENSG00000129048
ENSG00000121966	ENSG00000144648
ENSG00000121966	ENSG00000160683
ENSG00000121966	ENSG00000160791
ENSG00000121966	ENSG00000163464
ENSG00000121966	ENSG00000163823
ENSG00000121966	ENSG00000168329
ENSG00000121966	ENSG00000172215
ENSG00000121966	ENSG00000173578
ENSG00000121966	ENSG00000173585
ENSG00000121966	ENSG00000179934
ENSG00000121966	ENSG00000180871
ENSG00000121966	ENSG00000183625
ENSG00000121966	ENSG00000183813
ENSG00000121966	ENSG00000184451
ENSG00000121966	ENSG00000186810
ENSG00000121966	ENSG00000215775

ENSG00000121966	ENSG00000215778
ENSG00000121966	ENSG00000215782
ENSG00000121989	ENSG00000114739
ENSG00000122012	ENSG00000159164
ENSG00000122012	ENSG00000185518
ENSG00000122025	ENSG00000037280
ENSG00000122025	ENSG00000102755
ENSG00000122025	ENSG00000104213
ENSG00000122025	ENSG00000113721
ENSG00000122025	ENSG00000128052
ENSG00000122025	ENSG00000134853
ENSG00000122025	ENSG00000157404
ENSG00000122025	ENSG00000182578
ENSG00000122035	ENSG00000103710
ENSG00000122035	ENSG00000111404
ENSG00000122035	ENSG00000128045
ENSG00000122035	ENSG00000134533
ENSG00000122122	ENSG00000111961
ENSG00000122122	ENSG00000155307
ENSG00000122126	ENSG00000132376
ENSG00000122126	ENSG00000185133
ENSG00000122126	ENSG00000204084
ENSG00000122145	ENSG00000073861
ENSG00000122145	ENSG00000089225
ENSG00000122145	ENSG00000092607
ENSG00000122145	ENSG00000112837
ENSG00000122145	ENSG00000121068
ENSG00000122145	ENSG00000121075
ENSG00000122145	ENSG00000135111
ENSG00000122145	ENSG00000136535
ENSG00000122145	ENSG00000143178
ENSG00000122145	ENSG00000149922
ENSG00000122145	ENSG00000163508
ENSG00000122145	ENSG00000164458
ENSG00000122145	ENSG00000164532
ENSG00000122145	ENSG00000167800
ENSG00000122145	ENSG00000174197
ENSG00000122145	ENSG00000184058
ENSG00000122176	ENSG00000127083
ENSG00000122176	ENSG00000139330
ENSG00000122176	ENSG00000188783
ENSG00000122180	ENSG00000111046
ENSG00000122180	ENSG00000111049
ENSG00000122180	ENSG00000129152
ENSG00000122194	ENSG00000019991
ENSG00000122194	ENSG00000104368
ENSG00000122194	ENSG00000109758
ENSG00000122194	ENSG00000122861
ENSG00000122194	ENSG00000131187
ENSG00000122194	ENSG00000148702
ENSG00000122194	ENSG00000173531
ENSG00000122194	ENSG00000186301
ENSG00000122194	ENSG00000186715
ENSG00000122194	ENSG00000198670
ENSG00000122254	ENSG00000002587
ENSG00000122254	ENSG00000125430
ENSG00000122254	ENSG00000153976
ENSG00000122254	ENSG00000162040
ENSG00000122254	ENSG00000175818

ENSG00000122254	ENSG00000182601
ENSG00000122299	ENSG00000100403
ENSG00000122359	ENSG00000104537
ENSG00000122359	ENSG00000109511
ENSG00000122359	ENSG00000135046
ENSG00000122359	ENSG00000138279
ENSG00000122359	ENSG00000138772
ENSG00000122359	ENSG00000143412
ENSG00000122359	ENSG00000150165
ENSG00000122359	ENSG00000164111
ENSG00000122359	ENSG00000165390
ENSG00000122359	ENSG00000182718
ENSG00000122359	ENSG00000183059
ENSG00000122359	ENSG00000186807
ENSG00000122359	ENSG00000196975
ENSG00000122359	ENSG00000197043
ENSG00000122367	ENSG00000107438
ENSG00000122367	ENSG00000120913
ENSG00000122367	ENSG00000131435
ENSG00000122367	ENSG00000154553
ENSG00000122367	ENSG00000163110
ENSG00000122367	ENSG00000196923
ENSG00000122367	ENSG00000214895
ENSG00000122375	ENSG00000054277
ENSG00000122375	ENSG00000102076
ENSG00000122375	ENSG00000124818
ENSG00000122375	ENSG00000128617
ENSG00000122375	ENSG00000147380
ENSG00000122375	ENSG00000148604
ENSG00000122375	ENSG00000163914
ENSG00000122375	ENSG00000166160
ENSG00000122375	ENSG00000180245
ENSG00000122417	ENSG00000136811
ENSG00000122420	ENSG00000006638
ENSG00000122420	ENSG00000050628
ENSG00000122420	ENSG00000125384
ENSG00000122420	ENSG00000160013
ENSG00000122420	ENSG00000160951
ENSG00000122420	ENSG00000168229
ENSG00000122420	ENSG00000171522
ENSG00000122477	ENSG00000163827
ENSG00000122477	ENSG00000206422
ENSG00000122482	ENSG00000011451
ENSG00000122515	ENSG00000108175
ENSG00000122543	ENSG00000100362
ENSG00000122543	ENSG00000135175
ENSG00000122545	ENSG00000108387
ENSG00000122545	ENSG00000168385
ENSG00000122545	ENSG00000180096
ENSG00000122545	ENSG00000184702
ENSG00000122545	ENSG00000214005
ENSG00000122545	ENSG00000214765
ENSG00000122545	ENSG00000214834
ENSG00000122550	ENSG00000154474
ENSG00000122550	ENSG00000157119
ENSG00000122557	ENSG00000051108
ENSG00000122565	ENSG00000094916
ENSG00000122565	ENSG00000108468
ENSG00000122566	ENSG00000135486

ENSG00000122566	ENSG00000139675
ENSG00000122566	ENSG00000170144
ENSG00000122566	ENSG00000176825
ENSG00000122566	ENSG00000177219
ENSG00000122566	ENSG00000177733
ENSG00000122566	ENSG00000188174
ENSG00000122566	ENSG00000206228
ENSG00000122566	ENSG00000212961
ENSG00000122566	ENSG00000214284
ENSG00000122566	ENSG00000214361
ENSG00000122566	ENSG00000214592
ENSG00000122566	ENSG00000214653
ENSG00000122566	ENSG00000215222
ENSG00000122566	ENSG00000215492
ENSG00000122566	ENSG00000215870
ENSG00000122566	ENSG00000215876
ENSG00000122566	ENSG00000215885
ENSG00000122574	ENSG00000115935
ENSG00000122574	ENSG00000171475
ENSG00000122574	ENSG00000205090
ENSG00000122584	ENSG00000144227
ENSG00000122584	ENSG00000182379
ENSG00000122584	ENSG00000182575
ENSG00000122585	ENSG00000108849
ENSG00000122585	ENSG00000131096
ENSG00000122585	ENSG00000204474
ENSG00000122591	ENSG00000155744
ENSG00000122592	ENSG00000037965
ENSG00000122592	ENSG00000106004
ENSG00000122592	ENSG00000106006
ENSG00000122592	ENSG00000108511
ENSG00000122592	ENSG00000120068
ENSG00000122592	ENSG00000120075
ENSG00000122592	ENSG00000120087
ENSG00000122592	ENSG00000170166
ENSG00000122592	ENSG00000172789
ENSG00000122592	ENSG00000175879
ENSG00000122592	ENSG00000182742
ENSG00000122592	ENSG00000197576
ENSG00000122592	ENSG00000197757
ENSG00000122592	ENSG00000198353
ENSG00000122641	ENSG00000163083
ENSG00000122641	ENSG00000175189
ENSG00000122642	ENSG00000079150
ENSG00000122642	ENSG00000106080
ENSG00000122642	ENSG00000134285
ENSG00000122642	ENSG00000141756
ENSG00000122642	ENSG00000176826
ENSG00000122643	ENSG00000141698
ENSG00000122644	ENSG00000122872
ENSG00000122644	ENSG00000175906
ENSG00000122644	ENSG00000188042
ENSG00000122678	ENSG00000107447
ENSG00000122679	ENSG00000131477
ENSG00000122679	ENSG00000132329
ENSG00000122691	ENSG00000104903
ENSG00000122691	ENSG00000113196
ENSG00000122691	ENSG00000122859
ENSG00000122691	ENSG00000123307

ENSG00000122691	ENSG00000125878
ENSG00000122691	ENSG00000146618
ENSG00000122691	ENSG00000162367
ENSG00000122691	ENSG00000162992
ENSG00000122691	ENSG00000164107
ENSG00000122691	ENSG00000164600
ENSG00000122691	ENSG00000168267
ENSG00000122691	ENSG00000171532
ENSG00000122691	ENSG00000171786
ENSG00000122691	ENSG00000172238
ENSG00000122691	ENSG00000177551
ENSG00000122691	ENSG00000178403
ENSG00000122691	ENSG00000179774
ENSG00000122691	ENSG00000180535
ENSG00000122691	ENSG00000181965
ENSG00000122691	ENSG00000186051
ENSG00000122691	ENSG00000187786
ENSG00000122691	ENSG00000188686
ENSG00000122696	ENSG00000141437
ENSG00000122696	ENSG00000176274
ENSG00000122696	ENSG00000189332
ENSG00000122705	ENSG00000175416
ENSG00000122728	ENSG00000147133
ENSG00000122729	ENSG00000136381
ENSG00000122756	ENSG00000105246
ENSG00000122756	ENSG00000137070
ENSG00000122756	ENSG00000160712
ENSG00000122778	ENSG00000110427
ENSG00000122779	ENSG00000130726
ENSG00000122779	ENSG00000166436
ENSG00000122779	ENSG00000197323
ENSG00000122787	ENSG00000085662
ENSG00000122787	ENSG00000117448
ENSG00000122787	ENSG00000151631
ENSG00000122787	ENSG00000151632
ENSG00000122787	ENSG00000165568
ENSG00000122787	ENSG00000187134
ENSG00000122787	ENSG00000196139
ENSG00000122787	ENSG00000196326
ENSG00000122787	ENSG00000198074
ENSG00000122787	ENSG00000198610
ENSG00000122787	ENSG00000215267
ENSG00000122824	ENSG00000112664
ENSG00000122824	ENSG00000173598
ENSG00000122824	ENSG00000196368
ENSG00000122859	ENSG00000104903
ENSG00000122859	ENSG00000113196
ENSG00000122859	ENSG00000122691
ENSG00000122859	ENSG00000123307
ENSG00000122859	ENSG00000125878
ENSG00000122859	ENSG00000146618
ENSG00000122859	ENSG00000162367
ENSG00000122859	ENSG00000162992
ENSG00000122859	ENSG00000164107
ENSG00000122859	ENSG00000164600
ENSG00000122859	ENSG00000168267
ENSG00000122859	ENSG00000171532
ENSG00000122859	ENSG00000171786
ENSG00000122859	ENSG00000172238

ENSG00000122859	ENSG00000177551
ENSG00000122859	ENSG00000178403
ENSG00000122859	ENSG00000179774
ENSG00000122859	ENSG00000180535
ENSG00000122859	ENSG00000181965
ENSG00000122859	ENSG00000186051
ENSG00000122859	ENSG00000187786
ENSG00000122859	ENSG00000188686
ENSG00000122861	ENSG00000019991
ENSG00000122861	ENSG00000104368
ENSG00000122861	ENSG00000109758
ENSG00000122861	ENSG00000122194
ENSG00000122861	ENSG00000131187
ENSG00000122861	ENSG00000148702
ENSG00000122861	ENSG00000173531
ENSG00000122861	ENSG00000186301
ENSG00000122861	ENSG00000186715
ENSG00000122861	ENSG00000198670
ENSG00000122863	ENSG00000135702
ENSG00000122863	ENSG00000140835
ENSG00000122863	ENSG00000147119
ENSG00000122863	ENSG00000175040
ENSG00000122863	ENSG00000175264
ENSG00000122863	ENSG00000183196
ENSG00000122870	ENSG00000115677
ENSG00000122872	ENSG00000122644
ENSG00000122872	ENSG00000175906
ENSG00000122872	ENSG00000188042
ENSG00000122873	ENSG00000145354
ENSG00000122877	ENSG00000120738
ENSG00000122877	ENSG00000135625
ENSG00000122877	ENSG00000179388
ENSG00000122877	ENSG00000184937
ENSG00000122884	ENSG00000072682
ENSG00000122884	ENSG00000149380
ENSG00000123009	ENSG00000011052
ENSG00000123009	ENSG00000103024
ENSG00000123009	ENSG00000103200
ENSG00000123066	ENSG00000108510
ENSG00000123080	ENSG00000129355
ENSG00000123080	ENSG00000198435
ENSG00000123094	ENSG00000099849
ENSG00000123095	ENSG00000134107
ENSG00000123104	ENSG00000096433
ENSG00000123104	ENSG00000150995
ENSG00000123119	ENSG00000103154
ENSG00000123119	ENSG00000125967
ENSG00000123124	ENSG00000049759
ENSG00000123124	ENSG00000069869
ENSG00000123124	ENSG00000078747
ENSG00000123124	ENSG00000108854
ENSG00000123124	ENSG00000198373
ENSG00000123124	ENSG00000198742
ENSG00000123136	ENSG00000198563
ENSG00000123143	ENSG00000065243
ENSG00000123143	ENSG00000160447
ENSG00000123146	ENSG00000069122
ENSG00000123146	ENSG00000127507
ENSG00000123146	ENSG00000131355

ENSG00000123146	ENSG00000153292
ENSG00000123146	ENSG00000153294
ENSG00000123146	ENSG00000162618
ENSG00000123146	ENSG00000164393
ENSG00000123146	ENSG00000173567
ENSG00000123146	ENSG00000174837
ENSG00000123146	ENSG00000186629
ENSG00000123146	ENSG00000204818
ENSG00000123159	ENSG00000137960
ENSG00000123159	ENSG00000179855
ENSG00000123179	ENSG00000147155
ENSG00000123179	ENSG00000214469
ENSG00000123191	ENSG00000165240
ENSG00000123191	ENSG00000215763
ENSG00000123213	ENSG00000172009
ENSG00000123240	ENSG00000073009
ENSG00000123243	ENSG00000055955
ENSG00000123243	ENSG00000055957
ENSG00000123243	ENSG00000102313
ENSG00000123243	ENSG00000151655
ENSG00000123243	ENSG00000162267
ENSG00000123268	ENSG00000095794
ENSG00000123268	ENSG00000118260
ENSG00000123307	ENSG00000104903
ENSG00000123307	ENSG00000113196
ENSG00000123307	ENSG00000122691
ENSG00000123307	ENSG00000122859
ENSG00000123307	ENSG00000125878
ENSG00000123307	ENSG00000146618
ENSG00000123307	ENSG00000162367
ENSG00000123307	ENSG00000162992
ENSG00000123307	ENSG00000164107
ENSG00000123307	ENSG00000164600
ENSG00000123307	ENSG00000168267
ENSG00000123307	ENSG00000171532
ENSG00000123307	ENSG00000171786
ENSG00000123307	ENSG00000172238
ENSG00000123307	ENSG00000177551
ENSG00000123307	ENSG00000178403
ENSG00000123307	ENSG00000179774
ENSG00000123307	ENSG00000180535
ENSG00000123307	ENSG00000181965
ENSG00000123307	ENSG00000186051
ENSG00000123307	ENSG00000187786
ENSG00000123307	ENSG00000188686
ENSG00000123329	ENSG00000075884
ENSG00000123329	ENSG00000107863
ENSG00000123329	ENSG00000165322
ENSG00000123329	ENSG00000167433
ENSG00000123329	ENSG00000185602
ENSG00000123329	ENSG00000214627
ENSG00000123338	ENSG00000061676
ENSG00000123342	ENSG00000008516
ENSG00000123342	ENSG00000087245
ENSG00000123342	ENSG00000099953
ENSG00000123342	ENSG00000100985
ENSG00000123342	ENSG00000102996
ENSG00000123342	ENSG00000110347
ENSG00000123342	ENSG00000118113

ENSG00000123342	ENSG00000125966
ENSG00000123342	ENSG00000129270
ENSG00000123342	ENSG00000137673
ENSG00000123342	ENSG00000137674
ENSG00000123342	ENSG00000137675
ENSG00000123342	ENSG00000137745
ENSG00000123342	ENSG00000149968
ENSG00000123342	ENSG00000154485
ENSG00000123342	ENSG00000156103
ENSG00000123342	ENSG00000157227
ENSG00000123342	ENSG00000166670
ENSG00000123342	ENSG00000167346
ENSG00000123342	ENSG00000189409
ENSG00000123342	ENSG00000196611
ENSG00000123342	ENSG00000198598
ENSG00000123342	ENSG00000215914
ENSG00000123352	ENSG00000196141
ENSG00000123353	ENSG00000128699
ENSG00000123353	ENSG00000172057
ENSG00000123358	ENSG00000119508
ENSG00000123358	ENSG00000153234
ENSG00000123360	ENSG00000065989
ENSG00000123360	ENSG00000073417
ENSG00000123360	ENSG00000105650
ENSG00000123360	ENSG00000113231
ENSG00000123360	ENSG00000113448
ENSG00000123360	ENSG00000115252
ENSG00000123360	ENSG00000154678
ENSG00000123360	ENSG00000160191
ENSG00000123360	ENSG00000171408
ENSG00000123360	ENSG00000184588
ENSG00000123360	ENSG00000205268
ENSG00000123364	ENSG00000106031
ENSG00000123364	ENSG00000128714
ENSG00000123364	ENSG00000159184
ENSG00000123374	ENSG00000108504
ENSG00000123374	ENSG00000170312
ENSG00000123384	ENSG00000070018
ENSG00000123384	ENSG00000081479
ENSG00000123384	ENSG00000134569
ENSG00000123384	ENSG00000138798
ENSG00000123384	ENSG00000162337
ENSG00000123384	ENSG00000168702
ENSG00000123388	ENSG00000005073
ENSG00000123388	ENSG00000128713
ENSG00000123405	ENSG00000082641
ENSG00000123405	ENSG00000112182
ENSG00000123405	ENSG00000116044
ENSG00000123405	ENSG00000156273
ENSG00000123407	ENSG00000170178
ENSG00000123411	ENSG00000030419
ENSG00000123411	ENSG00000161405
ENSG00000123411	ENSG00000185811
ENSG00000123416	ENSG00000075886
ENSG00000123416	ENSG00000127824
ENSG00000123416	ENSG00000152086
ENSG00000123416	ENSG00000163526
ENSG00000123416	ENSG00000167552
ENSG00000123416	ENSG00000167553

ENSG00000123416	ENSG00000178462
ENSG00000123416	ENSG00000183785
ENSG00000123416	ENSG00000198033
ENSG00000123416	ENSG00000214391
ENSG00000123416	ENSG00000215823
ENSG00000123427	ENSG00000144401
ENSG00000123496	ENSG00000091181
ENSG00000123496	ENSG00000131724
ENSG00000123496	ENSG00000185291
ENSG00000123496	ENSG00000198223
ENSG00000123500	ENSG00000144810
ENSG00000123500	ENSG00000171812
ENSG00000123552	ENSG00000156256
ENSG00000123560	ENSG00000046653
ENSG00000123560	ENSG00000150625
ENSG00000123561	ENSG00000100665
ENSG00000123561	ENSG00000135744
ENSG00000123561	ENSG00000165951
ENSG00000123561	ENSG00000165953
ENSG00000123561	ENSG00000170054
ENSG00000123561	ENSG00000186910
ENSG00000123561	ENSG00000187483
ENSG00000123561	ENSG00000188488
ENSG00000123561	ENSG00000196136
ENSG00000123561	ENSG00000197249
ENSG00000123562	ENSG00000005302
ENSG00000123562	ENSG00000185787
ENSG00000123569	ENSG00000101812
ENSG00000123569	ENSG00000124635
ENSG00000123569	ENSG00000146047
ENSG00000123569	ENSG00000158373
ENSG00000123569	ENSG00000168242
ENSG00000123569	ENSG00000172450
ENSG00000123569	ENSG00000174258
ENSG00000123569	ENSG00000180596
ENSG00000123569	ENSG00000184678
ENSG00000123569	ENSG00000185130
ENSG00000123569	ENSG00000187990
ENSG00000123569	ENSG00000196226
ENSG00000123569	ENSG00000196331
ENSG00000123569	ENSG00000196374
ENSG00000123569	ENSG00000196501
ENSG00000123569	ENSG00000196890
ENSG00000123569	ENSG00000197034
ENSG00000123569	ENSG00000197459
ENSG00000123569	ENSG00000197597
ENSG00000123569	ENSG00000197697
ENSG00000123569	ENSG00000197846
ENSG00000123569	ENSG00000197903
ENSG00000123569	ENSG00000203814
ENSG00000123570	ENSG00000123595
ENSG00000123572	ENSG00000071054
ENSG00000123572	ENSG00000141503
ENSG00000123572	ENSG00000154310
ENSG00000123595	ENSG00000123570
ENSG00000123600	ENSG00000087995
ENSG00000123600	ENSG00000165055
ENSG00000123607	ENSG00000168026
ENSG00000123609	ENSG00000068079

ENSG00000123610	ENSG00000026508
ENSG00000123610	ENSG00000133800
ENSG00000123612	ENSG00000106799
ENSG00000123612	ENSG00000107779
ENSG00000123612	ENSG00000115170
ENSG00000123612	ENSG00000135409
ENSG00000123612	ENSG00000135503
ENSG00000123612	ENSG00000138696
ENSG00000123612	ENSG00000139567
ENSG00000123612	ENSG00000163513
ENSG00000123612	ENSG00000204217
ENSG00000123636	ENSG00000076108
ENSG00000123643	ENSG00000180773
ENSG00000123643	ENSG00000186334
ENSG00000123643	ENSG00000186335
ENSG00000123685	ENSG00000156127
ENSG00000123685	ENSG00000168062
ENSG00000123700	ENSG00000115474
ENSG00000123700	ENSG00000120457
ENSG00000123700	ENSG00000121361
ENSG00000123700	ENSG00000151704
ENSG00000123700	ENSG00000153822
ENSG00000123700	ENSG00000157542
ENSG00000123700	ENSG00000157551
ENSG00000123700	ENSG00000162728
ENSG00000123700	ENSG00000162989
ENSG00000123700	ENSG00000168135
ENSG00000123700	ENSG00000177807
ENSG00000123700	ENSG00000182324
ENSG00000123700	ENSG00000184185
ENSG00000123700	ENSG00000187486
ENSG00000123728	ENSG00000116473
ENSG00000123728	ENSG00000125249
ENSG00000123728	ENSG00000127314
ENSG00000123728	ENSG00000162595
ENSG00000123728	ENSG00000165023
ENSG00000123728	ENSG00000176276
ENSG00000123728	ENSG00000176490
ENSG00000123728	ENSG00000181467
ENSG00000123728	ENSG00000214405
ENSG00000123728	ENSG00000214492
ENSG00000123739	ENSG00000138308
ENSG00000123815	ENSG00000163050
ENSG00000123836	ENSG00000114268
ENSG00000123836	ENSG00000158571
ENSG00000123836	ENSG00000170525
ENSG00000123838	ENSG00000091583
ENSG00000123838	ENSG00000117335
ENSG00000123838	ENSG00000196352
ENSG00000123838	ENSG00000197721
ENSG00000123908	ENSG00000092847
ENSG00000123908	ENSG00000126070
ENSG00000123908	ENSG00000134698
ENSG00000123933	ENSG00000059728
ENSG00000123933	ENSG00000070444
ENSG00000123933	ENSG00000119950
ENSG00000123933	ENSG00000213347
ENSG00000123975	ENSG00000173207
ENSG00000123975	ENSG00000178556

ENSG00000123983	ENSG00000068366
ENSG00000123989	ENSG00000033100
ENSG00000123989	ENSG00000131873
ENSG00000123989	ENSG00000139044
ENSG00000123989	ENSG00000147408
ENSG00000123989	ENSG00000169826
ENSG00000123989	ENSG00000182272
ENSG00000123989	ENSG00000198108
ENSG00000124003	ENSG00000062282
ENSG00000124003	ENSG00000106384
ENSG00000124003	ENSG00000147160
ENSG00000124003	ENSG00000166391
ENSG00000124003	ENSG00000184210
ENSG00000124003	ENSG00000204195
ENSG00000124003	ENSG00000205267
ENSG00000124006	ENSG00000127418
ENSG00000124019	ENSG00000150510
ENSG00000124067	ENSG00000113504
ENSG00000124067	ENSG00000124140
ENSG00000124067	ENSG00000140199
ENSG00000124089	ENSG00000166603
ENSG00000124089	ENSG00000176136
ENSG00000124089	ENSG00000185231
ENSG00000124091	ENSG00000015532
ENSG00000124091	ENSG00000103489
ENSG00000124091	ENSG00000111846
ENSG00000124091	ENSG00000140297
ENSG00000124091	ENSG00000176928
ENSG00000124091	ENSG00000187210
ENSG00000124091	ENSG00000205318
ENSG00000124092	ENSG00000102974
ENSG00000124097	ENSG00000029993
ENSG00000124097	ENSG00000100118
ENSG00000124097	ENSG00000132967
ENSG00000124097	ENSG00000164104
ENSG00000124097	ENSG00000173961
ENSG00000124097	ENSG00000176256
ENSG00000124097	ENSG00000180212
ENSG00000124097	ENSG00000182181
ENSG00000124097	ENSG00000182711
ENSG00000124097	ENSG00000189403
ENSG00000124097	ENSG00000214364
ENSG00000124097	ENSG00000214852
ENSG00000124097	ENSG00000215308
ENSG00000124104	ENSG00000167208
ENSG00000124120	ENSG00000137561
ENSG00000124120	ENSG00000140522
ENSG00000124120	ENSG00000146352
ENSG00000124120	ENSG00000177182
ENSG00000124126	ENSG00000046889
ENSG00000124134	ENSG00000026559
ENSG00000124134	ENSG00000102057
ENSG00000124134	ENSG00000104848
ENSG00000124134	ENSG00000111262
ENSG00000124134	ENSG00000130037
ENSG00000124134	ENSG00000143105
ENSG00000124134	ENSG00000151079
ENSG00000124134	ENSG00000156486
ENSG00000124134	ENSG00000158445

ENSG00000124134	ENSG00000162975
ENSG00000124134	ENSG00000164794
ENSG00000124134	ENSG00000168263
ENSG00000124134	ENSG00000168418
ENSG00000124134	ENSG00000170745
ENSG00000124134	ENSG00000171126
ENSG00000124134	ENSG00000171385
ENSG00000124134	ENSG00000177272
ENSG00000124134	ENSG00000177301
ENSG00000124134	ENSG00000178342
ENSG00000124134	ENSG00000182255
ENSG00000124134	ENSG00000182674
ENSG00000124134	ENSG00000184408
ENSG00000124140	ENSG00000113504
ENSG00000124140	ENSG00000124067
ENSG00000124140	ENSG00000140199
ENSG00000124143	ENSG00000088756
ENSG00000124143	ENSG00000130052
ENSG00000124143	ENSG00000133121
ENSG00000124143	ENSG00000146376
ENSG00000124143	ENSG00000164741
ENSG00000124145	ENSG00000115884
ENSG00000124145	ENSG00000162512
ENSG00000124145	ENSG00000169439
ENSG00000124151	ENSG00000084676
ENSG00000124151	ENSG00000140396
ENSG00000124159	ENSG00000132031
ENSG00000124159	ENSG00000132561
ENSG00000124159	ENSG00000162510
ENSG00000124159	ENSG00000172752
ENSG00000124164	ENSG00000101558
ENSG00000124171	ENSG00000102981
ENSG00000124171	ENSG00000178184
ENSG00000124177	ENSG00000100888
ENSG00000124177	ENSG00000171316
ENSG00000124177	ENSG00000177200
ENSG00000124181	ENSG00000101333
ENSG00000124181	ENSG00000114805
ENSG00000124181	ENSG00000115556
ENSG00000124181	ENSG00000115896
ENSG00000124181	ENSG00000137841
ENSG00000124181	ENSG00000139151
ENSG00000124181	ENSG00000149527
ENSG00000124181	ENSG00000149782
ENSG00000124181	ENSG00000154822
ENSG00000124181	ENSG00000161714
ENSG00000124181	ENSG00000182621
ENSG00000124181	ENSG00000187091
ENSG00000124181	ENSG00000197943
ENSG00000124191	ENSG00000092203
ENSG00000124191	ENSG00000103460
ENSG00000124191	ENSG00000198846
ENSG00000124193	ENSG00000100650
ENSG00000124193	ENSG00000116350
ENSG00000124194	ENSG00000104381
ENSG00000124198	ENSG00000066777
ENSG00000124198	ENSG00000112379
ENSG00000124201	ENSG00000170448
ENSG00000124205	ENSG00000078401

ENSG00000124205	ENSG00000127129
ENSG00000124209	ENSG00000168461
ENSG00000124209	ENSG00000214576
ENSG00000124212	ENSG00000146233
ENSG00000124212	ENSG00000167910
ENSG00000124212	ENSG00000172817
ENSG00000124212	ENSG00000180432
ENSG00000124214	ENSG00000040341
ENSG00000124215	ENSG00000039068
ENSG00000124215	ENSG00000062038
ENSG00000124215	ENSG00000079112
ENSG00000124215	ENSG00000129910
ENSG00000124215	ENSG00000134755
ENSG00000124215	ENSG00000134762
ENSG00000124215	ENSG00000134765
ENSG00000124215	ENSG00000140945
ENSG00000124215	ENSG00000166589
ENSG00000124215	ENSG00000170558
ENSG00000124215	ENSG00000179242
ENSG00000124216	ENSG00000019549
ENSG00000124216	ENSG00000170616
ENSG00000124216	ENSG00000177835
ENSG00000124216	ENSG00000185669
ENSG00000124216	ENSG00000215397
ENSG00000124216	ENSG00000215713
ENSG00000124224	ENSG00000154845
ENSG00000124224	ENSG00000215443
ENSG00000124225	ENSG00000168675
ENSG00000124226	ENSG00000101695
ENSG00000124226	ENSG00000134758
ENSG00000124226	ENSG00000158717
ENSG00000124243	ENSG00000186222
ENSG00000124249	ENSG00000082482
ENSG00000124249	ENSG00000095981
ENSG00000124249	ENSG00000099337
ENSG00000124249	ENSG00000100433
ENSG00000124249	ENSG00000124780
ENSG00000124249	ENSG00000135750
ENSG00000124249	ENSG00000152315
ENSG00000124249	ENSG00000164626
ENSG00000124249	ENSG00000169427
ENSG00000124249	ENSG00000171303
ENSG00000124249	ENSG00000173338
ENSG00000124249	ENSG00000182450
ENSG00000124249	ENSG00000184261
ENSG00000124253	ENSG00000100889
ENSG00000124302	ENSG00000115526
ENSG00000124302	ENSG00000136213
ENSG00000124302	ENSG00000154080
ENSG00000124302	ENSG00000169105
ENSG00000124302	ENSG00000171310
ENSG00000124302	ENSG00000180767
ENSG00000124313	ENSG00000120645
ENSG00000124313	ENSG00000144711
ENSG00000124313	ENSG00000164117
ENSG00000124334	ENSG00000100385
ENSG00000124334	ENSG00000103522
ENSG00000124334	ENSG00000215303
ENSG00000124356	ENSG00000138134

ENSG00000124374	ENSG00000120727
ENSG00000124391	ENSG00000112115
ENSG00000124391	ENSG00000112116
ENSG00000124391	ENSG00000127743
ENSG00000124391	ENSG00000166090
ENSG00000124391	ENSG00000172458
ENSG00000124406	ENSG00000081923
ENSG00000124406	ENSG00000104043
ENSG00000124406	ENSG00000130270
ENSG00000124406	ENSG00000132932
ENSG00000124406	ENSG00000143515
ENSG00000124422	ENSG00000180991
ENSG00000124422	ENSG00000185295
ENSG00000124440	ENSG00000100644
ENSG00000124440	ENSG00000112246
ENSG00000124440	ENSG00000116016
ENSG00000124440	ENSG00000130751
ENSG00000124440	ENSG00000151322
ENSG00000124440	ENSG00000159263
ENSG00000124449	ENSG00000167378
ENSG00000124491	ENSG00000092295
ENSG00000124491	ENSG00000104055
ENSG00000124491	ENSG00000125780
ENSG00000124491	ENSG00000159495
ENSG00000124491	ENSG00000163810
ENSG00000124491	ENSG00000166947
ENSG00000124491	ENSG00000166948
ENSG00000124491	ENSG00000198959
ENSG00000124493	ENSG00000113262
ENSG00000124493	ENSG00000152822
ENSG00000124493	ENSG00000164082
ENSG00000124493	ENSG00000168959
ENSG00000124493	ENSG00000179603
ENSG00000124493	ENSG00000196277
ENSG00000124493	ENSG00000198822
ENSG00000124496	ENSG00000118156
ENSG00000124496	ENSG00000156030
ENSG00000124507	ENSG00000100266
ENSG00000124507	ENSG00000140368
ENSG00000124507	ENSG00000152229
ENSG00000124507	ENSG00000165912
ENSG00000124508	ENSG00000026950
ENSG00000124508	ENSG00000111801
ENSG00000124508	ENSG00000112763
ENSG00000124508	ENSG00000113303
ENSG00000124508	ENSG00000121236
ENSG00000124508	ENSG00000124549
ENSG00000124508	ENSG00000124557
ENSG00000124508	ENSG00000128250
ENSG00000124508	ENSG00000128253
ENSG00000124508	ENSG00000128276
ENSG00000124508	ENSG00000128482
ENSG00000124508	ENSG00000132256
ENSG00000124508	ENSG00000132274
ENSG00000124508	ENSG00000164010
ENSG00000124508	ENSG00000165810
ENSG00000124508	ENSG00000168903
ENSG00000124508	ENSG00000176422
ENSG00000124508	ENSG00000179046

ENSG00000124508	ENSG00000182805
ENSG00000124508	ENSG00000183439
ENSG00000124508	ENSG00000183718
ENSG00000124508	ENSG00000187574
ENSG00000124508	ENSG00000188683
ENSG00000124508	ENSG00000204614
ENSG00000124508	ENSG00000204616
ENSG00000124508	ENSG00000204618
ENSG00000124508	ENSG00000205198
ENSG00000124508	ENSG00000214693
ENSG00000124529	ENSG00000124578
ENSG00000124529	ENSG00000158406
ENSG00000124529	ENSG00000182217
ENSG00000124529	ENSG00000183941
ENSG00000124529	ENSG00000188987
ENSG00000124529	ENSG00000196176
ENSG00000124529	ENSG00000197061
ENSG00000124529	ENSG00000197238
ENSG00000124529	ENSG00000197837
ENSG00000124529	ENSG00000197914
ENSG00000124529	ENSG00000198327
ENSG00000124529	ENSG00000198339
ENSG00000124529	ENSG00000198518
ENSG00000124529	ENSG00000198558
ENSG00000124549	ENSG00000026950
ENSG00000124549	ENSG00000111801
ENSG00000124549	ENSG00000112763
ENSG00000124549	ENSG00000113303
ENSG00000124549	ENSG00000121236
ENSG00000124549	ENSG00000124508
ENSG00000124549	ENSG00000124557
ENSG00000124549	ENSG00000128250
ENSG00000124549	ENSG00000128253
ENSG00000124549	ENSG00000128276
ENSG00000124549	ENSG00000128482
ENSG00000124549	ENSG00000132256
ENSG00000124549	ENSG00000132274
ENSG00000124549	ENSG00000164010
ENSG00000124549	ENSG00000165810
ENSG00000124549	ENSG00000168903
ENSG00000124549	ENSG00000176422
ENSG00000124549	ENSG00000179046
ENSG00000124549	ENSG00000182805
ENSG00000124549	ENSG00000183439
ENSG00000124549	ENSG00000183718
ENSG00000124549	ENSG00000187574
ENSG00000124549	ENSG00000188683
ENSG00000124549	ENSG00000204614
ENSG00000124549	ENSG00000204616
ENSG00000124549	ENSG00000204618
ENSG00000124549	ENSG00000205198
ENSG00000124549	ENSG00000214693
ENSG00000124557	ENSG00000026950
ENSG00000124557	ENSG00000111801
ENSG00000124557	ENSG00000112763
ENSG00000124557	ENSG00000113303
ENSG00000124557	ENSG00000121236
ENSG00000124557	ENSG00000124508
ENSG00000124557	ENSG00000124549

ENSG00000124557	ENSG00000128250
ENSG00000124557	ENSG00000128253
ENSG00000124557	ENSG00000128276
ENSG00000124557	ENSG00000128482
ENSG00000124557	ENSG00000132256
ENSG00000124557	ENSG00000132274
ENSG00000124557	ENSG00000164010
ENSG00000124557	ENSG00000165810
ENSG00000124557	ENSG00000168903
ENSG00000124557	ENSG00000176422
ENSG00000124557	ENSG00000179046
ENSG00000124557	ENSG00000182805
ENSG00000124557	ENSG00000183439
ENSG00000124557	ENSG00000183718
ENSG00000124557	ENSG00000187574
ENSG00000124557	ENSG00000188683
ENSG00000124557	ENSG00000204614
ENSG00000124557	ENSG00000204616
ENSG00000124557	ENSG00000204618
ENSG00000124557	ENSG00000205198
ENSG00000124557	ENSG00000214693
ENSG00000124564	ENSG00000091664
ENSG00000124564	ENSG00000104888
ENSG00000124564	ENSG00000112337
ENSG00000124564	ENSG00000119899
ENSG00000124564	ENSG00000124568
ENSG00000124564	ENSG00000146039
ENSG00000124564	ENSG00000179520
ENSG00000124568	ENSG00000091664
ENSG00000124568	ENSG00000104888
ENSG00000124568	ENSG00000112337
ENSG00000124568	ENSG00000119899
ENSG00000124568	ENSG00000124564
ENSG00000124568	ENSG00000146039
ENSG00000124568	ENSG00000179520
ENSG00000124570	ENSG00000021355
ENSG00000124570	ENSG00000057149
ENSG00000124570	ENSG00000166396
ENSG00000124570	ENSG00000166401
ENSG00000124570	ENSG00000166404
ENSG00000124570	ENSG00000166634
ENSG00000124570	ENSG00000170542
ENSG00000124570	ENSG00000197632
ENSG00000124570	ENSG00000197641
ENSG00000124570	ENSG00000206072
ENSG00000124570	ENSG00000206073
ENSG00000124570	ENSG00000206075
ENSG00000124575	ENSG00000124610
ENSG00000124575	ENSG00000168298
ENSG00000124575	ENSG00000184357
ENSG00000124575	ENSG00000184897
ENSG00000124575	ENSG00000187475
ENSG00000124575	ENSG00000187837
ENSG00000124575	ENSG00000189060
ENSG00000124575	ENSG00000196440
ENSG00000124578	ENSG00000124529
ENSG00000124578	ENSG00000158406
ENSG00000124578	ENSG00000182217
ENSG00000124578	ENSG00000183941

ENSG00000124578	ENSG00000188987
ENSG00000124578	ENSG00000196176
ENSG00000124578	ENSG00000197061
ENSG00000124578	ENSG00000197238
ENSG00000124578	ENSG00000197837
ENSG00000124578	ENSG00000197914
ENSG00000124578	ENSG00000198327
ENSG00000124578	ENSG00000198339
ENSG00000124578	ENSG00000198518
ENSG00000124578	ENSG00000198558
ENSG00000124588	ENSG00000181019
ENSG00000124593	ENSG00000012211
ENSG00000124593	ENSG00000071282
ENSG00000124593	ENSG00000135269
ENSG00000124593	ENSG00000139174
ENSG00000124593	ENSG00000163637
ENSG00000124602	ENSG00000107731
ENSG00000124602	ENSG00000113763
ENSG00000124602	ENSG00000156687
ENSG00000124602	ENSG00000182168
ENSG00000124610	ENSG00000124575
ENSG00000124610	ENSG00000168298
ENSG00000124610	ENSG00000184357
ENSG00000124610	ENSG00000184897
ENSG00000124610	ENSG00000187475
ENSG00000124610	ENSG00000187837
ENSG00000124610	ENSG00000189060
ENSG00000124610	ENSG00000196440
ENSG00000124614	ENSG00000101278
ENSG00000124614	ENSG00000183701
ENSG00000124614	ENSG00000198810
ENSG00000124614	ENSG00000215464
ENSG00000124642	ENSG00000111332
ENSG00000124642	ENSG00000137259
ENSG00000124642	ENSG00000158403
ENSG00000124642	ENSG00000164508
ENSG00000124642	ENSG00000168274
ENSG00000124642	ENSG00000180573
ENSG00000124642	ENSG00000181218
ENSG00000124642	ENSG00000182611
ENSG00000124642	ENSG00000183558
ENSG00000124642	ENSG00000184260
ENSG00000124642	ENSG00000184270
ENSG00000124642	ENSG00000184348
ENSG00000124642	ENSG00000184825
ENSG00000124642	ENSG00000185978
ENSG00000124642	ENSG00000188486
ENSG00000124642	ENSG00000196747
ENSG00000124642	ENSG00000196787
ENSG00000124642	ENSG00000196866
ENSG00000124642	ENSG00000198082
ENSG00000124642	ENSG00000198307
ENSG00000124642	ENSG00000198374
ENSG00000124642	ENSG00000203812
ENSG00000124642	ENSG00000205070
ENSG00000124642	ENSG00000205079
ENSG00000124678	ENSG00000166046
ENSG00000124678	ENSG00000176148
ENSG00000124678	ENSG00000215029

ENSG00000124678	ENSG00000215046
ENSG00000124701	ENSG00000100298
ENSG00000124701	ENSG00000111701
ENSG00000124701	ENSG00000111732
ENSG00000124701	ENSG00000128383
ENSG00000124701	ENSG00000128394
ENSG00000124701	ENSG00000179750
ENSG00000124701	ENSG00000198904
ENSG00000124743	ENSG00000003096
ENSG00000124743	ENSG00000087448
ENSG00000124743	ENSG00000130487
ENSG00000124743	ENSG00000135686
ENSG00000124743	ENSG00000166853
ENSG00000124743	ENSG00000167487
ENSG00000124743	ENSG00000174010
ENSG00000124743	ENSG00000176595
ENSG00000124743	ENSG00000179023
ENSG00000124743	ENSG00000185214
ENSG00000124743	ENSG00000185915
ENSG00000124743	ENSG00000186231
ENSG00000124743	ENSG00000197705
ENSG00000124743	ENSG00000198642
ENSG00000124749	ENSG00000169436
ENSG00000124762	ENSG00000111276
ENSG00000124766	ENSG00000005513
ENSG00000124766	ENSG00000100146
ENSG00000124766	ENSG00000125398
ENSG00000124766	ENSG00000129194
ENSG00000124766	ENSG00000134595
ENSG00000124766	ENSG00000164736
ENSG00000124766	ENSG00000171056
ENSG00000124766	ENSG00000176887
ENSG00000124766	ENSG00000177732
ENSG00000124766	ENSG00000181449
ENSG00000124766	ENSG00000182968
ENSG00000124766	ENSG00000184895
ENSG00000124766	ENSG00000203883
ENSG00000124772	ENSG00000085719
ENSG00000124772	ENSG00000100884
ENSG00000124772	ENSG00000139117
ENSG00000124772	ENSG00000140848
ENSG00000124772	ENSG00000144550
ENSG00000124772	ENSG00000178773
ENSG00000124772	ENSG00000196353
ENSG00000124772	ENSG00000214078
ENSG00000124780	ENSG00000082482
ENSG00000124780	ENSG00000095981
ENSG00000124780	ENSG00000099337
ENSG00000124780	ENSG00000100433
ENSG00000124780	ENSG00000124249
ENSG00000124780	ENSG00000135750
ENSG00000124780	ENSG00000152315
ENSG00000124780	ENSG00000164626
ENSG00000124780	ENSG00000169427
ENSG00000124780	ENSG00000171303
ENSG00000124780	ENSG00000173338
ENSG00000124780	ENSG00000182450
ENSG00000124780	ENSG00000184261
ENSG00000124785	ENSG00000188038

ENSG00000124813	ENSG00000020633
ENSG00000124813	ENSG00000159216
ENSG00000124814	ENSG00000165186
ENSG00000124814	ENSG00000178729
ENSG00000124814	ENSG00000182077
ENSG00000124818	ENSG00000054277
ENSG00000124818	ENSG00000102076
ENSG00000124818	ENSG00000122375
ENSG00000124818	ENSG00000128617
ENSG00000124818	ENSG00000147380
ENSG00000124818	ENSG00000148604
ENSG00000124818	ENSG00000163914
ENSG00000124818	ENSG00000166160
ENSG00000124818	ENSG00000180245
ENSG00000124827	ENSG00000137270
ENSG00000124831	ENSG00000093167
ENSG00000124875	ENSG00000081041
ENSG00000124875	ENSG00000109272
ENSG00000124875	ENSG00000138755
ENSG00000124875	ENSG00000163734
ENSG00000124875	ENSG00000163735
ENSG00000124875	ENSG00000163736
ENSG00000124875	ENSG00000163737
ENSG00000124875	ENSG00000163739
ENSG00000124875	ENSG00000169245
ENSG00000124875	ENSG00000169248
ENSG00000124875	ENSG00000169429
ENSG00000124942	ENSG00000105227
ENSG00000124942	ENSG00000214427
ENSG00000125089	ENSG00000169247
ENSG00000125144	ENSG00000087250
ENSG00000125144	ENSG00000102891
ENSG00000125144	ENSG00000125148
ENSG00000125144	ENSG00000169688
ENSG00000125144	ENSG00000169715
ENSG00000125144	ENSG00000187193
ENSG00000125144	ENSG00000198417
ENSG00000125144	ENSG00000205358
ENSG00000125144	ENSG00000205360
ENSG00000125144	ENSG00000205362
ENSG00000125144	ENSG00000205364
ENSG00000125148	ENSG00000087250
ENSG00000125148	ENSG00000102891
ENSG00000125148	ENSG00000125144
ENSG00000125148	ENSG00000169688
ENSG00000125148	ENSG00000169715
ENSG00000125148	ENSG00000187193
ENSG00000125148	ENSG00000198417
ENSG00000125148	ENSG00000205358
ENSG00000125148	ENSG00000205360
ENSG00000125148	ENSG00000205362
ENSG00000125148	ENSG00000205364
ENSG00000125170	ENSG00000101134
ENSG00000125170	ENSG00000115325
ENSG00000125170	ENSG00000137218
ENSG00000125170	ENSG00000146094
ENSG00000125170	ENSG00000147443
ENSG00000125170	ENSG00000166225
ENSG00000125170	ENSG00000206052

ENSG00000125207	ENSG00000134627
ENSG00000125207	ENSG00000184571
ENSG00000125207	ENSG00000197181
ENSG00000125245	ENSG00000126251
ENSG00000125245	ENSG00000126262
ENSG00000125245	ENSG00000144230
ENSG00000125245	ENSG00000147138
ENSG00000125245	ENSG00000162881
ENSG00000125245	ENSG00000164220
ENSG00000125245	ENSG00000164251
ENSG00000125245	ENSG00000165621
ENSG00000125245	ENSG00000171631
ENSG00000125245	ENSG00000181104
ENSG00000125245	ENSG00000182162
ENSG00000125245	ENSG00000182559
ENSG00000125245	ENSG00000182782
ENSG00000125245	ENSG00000185897
ENSG00000125245	ENSG00000196917
ENSG00000125249	ENSG00000116473
ENSG00000125249	ENSG00000123728
ENSG00000125249	ENSG00000127314
ENSG00000125249	ENSG00000162595
ENSG00000125249	ENSG00000165023
ENSG00000125249	ENSG00000176276
ENSG00000125249	ENSG00000176490
ENSG00000125249	ENSG00000181467
ENSG00000125249	ENSG00000214405
ENSG00000125249	ENSG00000214492
ENSG00000125255	ENSG00000100652
ENSG00000125255	ENSG00000126903
ENSG00000125255	ENSG00000145248
ENSG00000125255	ENSG00000145283
ENSG00000125255	ENSG00000205184
ENSG00000125266	ENSG00000090776
ENSG00000125266	ENSG00000099617
ENSG00000125266	ENSG00000108947
ENSG00000125266	ENSG00000143590
ENSG00000125266	ENSG00000143620
ENSG00000125266	ENSG00000169242
ENSG00000125266	ENSG00000184349
ENSG00000125285	ENSG00000168875
ENSG00000125347	ENSG00000117595
ENSG00000125347	ENSG00000126456
ENSG00000125347	ENSG00000128604
ENSG00000125347	ENSG00000137265
ENSG00000125347	ENSG00000140968
ENSG00000125347	ENSG00000168310
ENSG00000125347	ENSG00000185507
ENSG00000125347	ENSG00000213928
ENSG00000125351	ENSG00000169062
ENSG00000125354	ENSG00000138758
ENSG00000125354	ENSG00000154997
ENSG00000125354	ENSG00000164402
ENSG00000125354	ENSG00000186522
ENSG00000125355	ENSG00000184497
ENSG00000125378	ENSG00000125845
ENSG00000125378	ENSG00000130283
ENSG00000125378	ENSG00000184344
ENSG00000125384	ENSG00000006638

ENSG00000125384	ENSG00000050628
ENSG00000125384	ENSG00000122420
ENSG00000125384	ENSG00000160013
ENSG00000125384	ENSG00000160951
ENSG00000125384	ENSG00000168229
ENSG00000125384	ENSG00000171522
ENSG00000125386	ENSG00000146067
ENSG00000125388	ENSG00000100077
ENSG00000125388	ENSG00000114124
ENSG00000125388	ENSG00000173020
ENSG00000125388	ENSG00000185974
ENSG00000125388	ENSG00000198055
ENSG00000125388	ENSG00000198873
ENSG00000125398	ENSG00000005513
ENSG00000125398	ENSG00000100146
ENSG00000125398	ENSG00000124766
ENSG00000125398	ENSG00000129194
ENSG00000125398	ENSG00000134595
ENSG00000125398	ENSG00000164736
ENSG00000125398	ENSG00000171056
ENSG00000125398	ENSG00000176887
ENSG00000125398	ENSG00000177732
ENSG00000125398	ENSG00000181449
ENSG00000125398	ENSG00000182968
ENSG00000125398	ENSG00000184895
ENSG00000125398	ENSG00000203883
ENSG00000125414	ENSG00000002079
ENSG00000125414	ENSG00000006788
ENSG00000125414	ENSG00000078814
ENSG00000125414	ENSG00000092054
ENSG00000125414	ENSG00000109061
ENSG00000125414	ENSG00000109063
ENSG00000125414	ENSG00000133020
ENSG00000125414	ENSG00000141048
ENSG00000125414	ENSG00000144821
ENSG00000125414	ENSG00000197616
ENSG00000125430	ENSG00000002587
ENSG00000125430	ENSG00000122254
ENSG00000125430	ENSG00000153976
ENSG00000125430	ENSG00000162040
ENSG00000125430	ENSG00000175818
ENSG00000125430	ENSG00000182601
ENSG00000125434	ENSG00000162461
ENSG00000125447	ENSG00000100083
ENSG00000125447	ENSG00000103365
ENSG00000125482	ENSG00000135164
ENSG00000125492	ENSG00000143032
ENSG00000125492	ENSG00000206148
ENSG00000125503	ENSG00000058272
ENSG00000125503	ENSG00000077157
ENSG00000125508	ENSG00000007264
ENSG00000125508	ENSG00000101213
ENSG00000125508	ENSG00000103653
ENSG00000125510	ENSG00000082556
ENSG00000125510	ENSG00000112038
ENSG00000125510	ENSG00000116014
ENSG00000125510	ENSG00000116329
ENSG00000125510	ENSG00000125522
ENSG00000125510	ENSG00000128285

ENSG00000125510	ENSG00000128310
ENSG00000125510	ENSG00000132671
ENSG00000125510	ENSG00000139874
ENSG00000125510	ENSG00000152034
ENSG00000125510	ENSG00000162009
ENSG00000125510	ENSG00000166573
ENSG00000125510	ENSG00000180616
ENSG00000125510	ENSG00000182687
ENSG00000125510	ENSG00000183473
ENSG00000125510	ENSG00000183729
ENSG00000125520	ENSG00000164684
ENSG00000125520	ENSG00000186918
ENSG00000125522	ENSG00000082556
ENSG00000125522	ENSG00000112038
ENSG00000125522	ENSG00000116014
ENSG00000125522	ENSG00000116329
ENSG00000125522	ENSG00000125510
ENSG00000125522	ENSG00000128285
ENSG00000125522	ENSG00000128310
ENSG00000125522	ENSG00000132671
ENSG00000125522	ENSG00000139874
ENSG00000125522	ENSG00000152034
ENSG00000125522	ENSG00000162009
ENSG00000125522	ENSG00000166573
ENSG00000125522	ENSG00000180616
ENSG00000125522	ENSG00000182687
ENSG00000125522	ENSG00000183473
ENSG00000125522	ENSG00000183729
ENSG00000125533	ENSG00000177468
ENSG00000125533	ENSG00000180828
ENSG00000125533	ENSG00000184221
ENSG00000125533	ENSG00000205927
ENSG00000125618	ENSG00000075891
ENSG00000125618	ENSG00000125813
ENSG00000125618	ENSG00000196092
ENSG00000125618	ENSG00000198807
ENSG00000125629	ENSG00000186480
ENSG00000125637	ENSG00000059915
ENSG00000125637	ENSG00000146005
ENSG00000125637	ENSG00000156011
ENSG00000125648	ENSG00000085491
ENSG00000125648	ENSG00000148339
ENSG00000125648	ENSG00000181240
ENSG00000125650	ENSG00000117407
ENSG00000125650	ENSG00000168621
ENSG00000125650	ENSG00000171119
ENSG00000125675	ENSG00000120251
ENSG00000125675	ENSG00000149403
ENSG00000125675	ENSG00000152578
ENSG00000125675	ENSG00000155511
ENSG00000125675	ENSG00000163873
ENSG00000125675	ENSG00000164418
ENSG00000125675	ENSG00000171189
ENSG00000125695	ENSG00000082146
ENSG00000125703	ENSG00000101844
ENSG00000125703	ENSG00000130734
ENSG00000125703	ENSG00000168397
ENSG00000125703	ENSG00000215697
ENSG00000125730	ENSG00000106804

ENSG00000125730	ENSG00000126838
ENSG00000125730	ENSG00000156535
ENSG00000125730	ENSG00000160111
ENSG00000125730	ENSG00000166535
ENSG00000125730	ENSG00000175899
ENSG00000125730	ENSG00000177359
ENSG00000125730	ENSG00000204319
ENSG00000125730	ENSG00000204342
ENSG00000125730	ENSG00000214776
ENSG00000125731	ENSG00000095370
ENSG00000125731	ENSG00000137936
ENSG00000125733	ENSG00000137942
ENSG00000125733	ENSG00000187239
ENSG00000125734	ENSG00000148358
ENSG00000125735	ENSG00000102245
ENSG00000125735	ENSG00000117560
ENSG00000125735	ENSG00000120659
ENSG00000125735	ENSG00000121858
ENSG00000125735	ENSG00000181634
ENSG00000125735	ENSG00000204490
ENSG00000125735	ENSG00000204496
ENSG00000125740	ENSG00000075426
ENSG00000125740	ENSG00000140044
ENSG00000125740	ENSG00000162772
ENSG00000125740	ENSG00000170345
ENSG00000125740	ENSG00000175592
ENSG00000125746	ENSG00000066629
ENSG00000125746	ENSG00000143924
ENSG00000125746	ENSG00000143940
ENSG00000125746	ENSG00000149499
ENSG00000125746	ENSG00000165521
ENSG00000125746	ENSG00000214595
ENSG00000125753	ENSG00000154380
ENSG00000125753	ENSG00000166068
ENSG00000125753	ENSG00000188766
ENSG00000125753	ENSG00000196405
ENSG00000125753	ENSG00000198369
ENSG00000125775	ENSG00000137575
ENSG00000125779	ENSG00000120137
ENSG00000125779	ENSG00000152782
ENSG00000125780	ENSG00000092295
ENSG00000125780	ENSG00000104055
ENSG00000125780	ENSG00000124491
ENSG00000125780	ENSG00000159495
ENSG00000125780	ENSG00000163810
ENSG00000125780	ENSG00000166947
ENSG00000125780	ENSG00000166948
ENSG00000125780	ENSG00000198959
ENSG00000125798	ENSG00000054598
ENSG00000125798	ENSG00000129514
ENSG00000125798	ENSG00000164379
ENSG00000125798	ENSG00000168269
ENSG00000125798	ENSG00000170122
ENSG00000125798	ENSG00000170608
ENSG00000125798	ENSG00000176165
ENSG00000125798	ENSG00000176678
ENSG00000125798	ENSG00000176692
ENSG00000125798	ENSG00000178919
ENSG00000125798	ENSG00000179772

ENSG00000125798	ENSG00000183770
ENSG00000125798	ENSG00000184492
ENSG00000125798	ENSG00000184659
ENSG00000125798	ENSG00000186564
ENSG00000125798	ENSG00000186766
ENSG00000125798	ENSG00000186790
ENSG00000125798	ENSG00000187140
ENSG00000125798	ENSG00000187559
ENSG00000125798	ENSG00000204612
ENSG00000125798	ENSG00000204779
ENSG00000125798	ENSG00000204793
ENSG00000125798	ENSG00000204828
ENSG00000125798	ENSG00000214336
ENSG00000125810	ENSG00000174807
ENSG00000125810	ENSG00000176435
ENSG00000125810	ENSG00000178726
ENSG00000125813	ENSG00000075891
ENSG00000125813	ENSG00000125618
ENSG00000125813	ENSG00000196092
ENSG00000125813	ENSG00000198807
ENSG00000125814	ENSG00000105402
ENSG00000125815	ENSG00000077984
ENSG00000125815	ENSG00000101435
ENSG00000125815	ENSG00000101439
ENSG00000125815	ENSG00000101441
ENSG00000125815	ENSG00000125823
ENSG00000125815	ENSG00000125831
ENSG00000125815	ENSG00000170367
ENSG00000125815	ENSG00000170369
ENSG00000125815	ENSG00000170373
ENSG00000125815	ENSG00000173335
ENSG00000125815	ENSG00000175315
ENSG00000125815	ENSG00000204658
ENSG00000125815	ENSG00000204662
ENSG00000125815	ENSG00000204663
ENSG00000125816	ENSG00000109705
ENSG00000125816	ENSG00000119919
ENSG00000125816	ENSG00000125820
ENSG00000125816	ENSG00000136327
ENSG00000125816	ENSG00000136352
ENSG00000125816	ENSG00000180053
ENSG00000125816	ENSG00000183072
ENSG00000125820	ENSG00000109705
ENSG00000125820	ENSG00000119919
ENSG00000125820	ENSG00000125816
ENSG00000125820	ENSG00000136327
ENSG00000125820	ENSG00000136352
ENSG00000125820	ENSG00000180053
ENSG00000125820	ENSG00000183072
ENSG00000125823	ENSG00000077984
ENSG00000125823	ENSG00000101435
ENSG00000125823	ENSG00000101439
ENSG00000125823	ENSG00000101441
ENSG00000125823	ENSG00000125815
ENSG00000125823	ENSG00000125831
ENSG00000125823	ENSG00000170367
ENSG00000125823	ENSG00000170369
ENSG00000125823	ENSG00000170373
ENSG00000125823	ENSG00000173335

ENSG00000125823	ENSG00000175315
ENSG00000125823	ENSG00000204658
ENSG00000125823	ENSG00000204662
ENSG00000125823	ENSG00000204663
ENSG00000125827	ENSG00000139921
ENSG00000125831	ENSG00000077984
ENSG00000125831	ENSG00000101435
ENSG00000125831	ENSG00000101439
ENSG00000125831	ENSG00000101441
ENSG00000125831	ENSG00000125815
ENSG00000125831	ENSG00000125823
ENSG00000125831	ENSG00000170367
ENSG00000125831	ENSG00000170369
ENSG00000125831	ENSG00000170373
ENSG00000125831	ENSG00000173335
ENSG00000125831	ENSG00000175315
ENSG00000125831	ENSG00000204658
ENSG00000125831	ENSG00000204662
ENSG00000125831	ENSG00000204663
ENSG00000125834	ENSG00000175087
ENSG00000125835	ENSG00000128739
ENSG00000125835	ENSG00000138101
ENSG00000125841	ENSG00000152954
ENSG00000125844	ENSG00000126777
ENSG00000125845	ENSG00000125378
ENSG00000125845	ENSG00000130283
ENSG00000125845	ENSG00000184344
ENSG00000125848	ENSG00000126500
ENSG00000125848	ENSG00000186105
ENSG00000125850	ENSG00000172818
ENSG00000125861	ENSG00000146013
ENSG00000125861	ENSG00000151892
ENSG00000125861	ENSG00000168546
ENSG00000125861	ENSG00000187871
ENSG00000125868	ENSG00000165410
ENSG00000125868	ENSG00000172757
ENSG00000125878	ENSG00000104903
ENSG00000125878	ENSG00000113196
ENSG00000125878	ENSG00000122691
ENSG00000125878	ENSG00000122859
ENSG00000125878	ENSG00000123307
ENSG00000125878	ENSG00000146618
ENSG00000125878	ENSG00000162367
ENSG00000125878	ENSG00000162992
ENSG00000125878	ENSG00000164107
ENSG00000125878	ENSG00000164600
ENSG00000125878	ENSG00000168267
ENSG00000125878	ENSG00000171532
ENSG00000125878	ENSG00000171786
ENSG00000125878	ENSG00000172238
ENSG00000125878	ENSG00000177551
ENSG00000125878	ENSG00000178403
ENSG00000125878	ENSG00000179774
ENSG00000125878	ENSG00000180535
ENSG00000125878	ENSG00000181965
ENSG00000125878	ENSG00000186051
ENSG00000125878	ENSG00000187786
ENSG00000125878	ENSG00000188686
ENSG00000125879	ENSG00000150526

ENSG00000125879	ENSG00000213054
ENSG00000125888	ENSG00000175334
ENSG00000125895	ENSG00000164841
ENSG00000125898	ENSG00000169122
ENSG00000125898	ENSG00000184731
ENSG00000125898	ENSG00000197245
ENSG00000125910	ENSG00000064547
ENSG00000125910	ENSG00000118432
ENSG00000125910	ENSG00000132975
ENSG00000125910	ENSG00000146360
ENSG00000125910	ENSG00000170989
ENSG00000125910	ENSG00000171517
ENSG00000125910	ENSG00000175898
ENSG00000125910	ENSG00000180739
ENSG00000125910	ENSG00000181773
ENSG00000125910	ENSG00000188822
ENSG00000125910	ENSG00000198121
ENSG00000125910	ENSG00000213694
ENSG00000125931	ENSG00000164442
ENSG00000125931	ENSG00000179862
ENSG00000125944	ENSG00000135316
ENSG00000125944	ENSG00000148584
ENSG00000125944	ENSG00000151962
ENSG00000125944	ENSG00000163694
ENSG00000125944	ENSG00000183403
ENSG00000125965	ENSG00000101144
ENSG00000125965	ENSG00000107623
ENSG00000125965	ENSG00000112175
ENSG00000125965	ENSG00000116985
ENSG00000125965	ENSG00000128802
ENSG00000125965	ENSG00000130385
ENSG00000125965	ENSG00000143869
ENSG00000125965	ENSG00000152785
ENSG00000125965	ENSG00000153162
ENSG00000125965	ENSG00000156466
ENSG00000125965	ENSG00000156574
ENSG00000125965	ENSG00000163217
ENSG00000125965	ENSG00000183682
ENSG00000125966	ENSG00000008516
ENSG00000125966	ENSG00000087245
ENSG00000125966	ENSG00000099953
ENSG00000125966	ENSG00000100985
ENSG00000125966	ENSG00000102996
ENSG00000125966	ENSG00000110347
ENSG00000125966	ENSG00000118113
ENSG00000125966	ENSG00000123342
ENSG00000125966	ENSG00000129270
ENSG00000125966	ENSG00000137673
ENSG00000125966	ENSG00000137674
ENSG00000125966	ENSG00000137675
ENSG00000125966	ENSG00000137745
ENSG00000125966	ENSG00000149968
ENSG00000125966	ENSG00000154485
ENSG00000125966	ENSG00000156103
ENSG00000125966	ENSG00000157227
ENSG00000125966	ENSG00000166670
ENSG00000125966	ENSG00000167346
ENSG00000125966	ENSG00000189409
ENSG00000125966	ENSG00000196611

ENSG00000125966	ENSG00000198598
ENSG00000125966	ENSG00000215914
ENSG00000125967	ENSG00000103154
ENSG00000125967	ENSG00000123119
ENSG00000125968	ENSG00000115738
ENSG00000125968	ENSG00000117318
ENSG00000125968	ENSG00000172201
ENSG00000125970	ENSG00000092199
ENSG00000125970	ENSG00000179172
ENSG00000125970	ENSG00000184672
ENSG00000125970	ENSG00000214634
ENSG00000125970	ENSG00000215880
ENSG00000125976	ENSG00000183808
ENSG00000125998	ENSG00000101447
ENSG00000125998	ENSG00000105523
ENSG00000125998	ENSG00000133477
ENSG00000125998	ENSG00000147689
ENSG00000125998	ENSG00000168143
ENSG00000125998	ENSG00000180921
ENSG00000125998	ENSG00000188522
ENSG00000126003	ENSG00000118495
ENSG00000126003	ENSG00000181690
ENSG00000126010	ENSG00000102239
ENSG00000126010	ENSG00000135577
ENSG00000126010	ENSG00000136160
ENSG00000126010	ENSG00000151617
ENSG00000126010	ENSG00000170075
ENSG00000126010	ENSG00000170775
ENSG00000126012	ENSG00000012817
ENSG00000126012	ENSG00000073614
ENSG00000126012	ENSG00000117139
ENSG00000126016	ENSG00000114019
ENSG00000126016	ENSG00000166025
ENSG00000126070	ENSG00000092847
ENSG00000126070	ENSG00000123908
ENSG00000126070	ENSG00000134698
ENSG00000126091	ENSG00000008513
ENSG00000126091	ENSG00000064225
ENSG00000126091	ENSG00000110080
ENSG00000126091	ENSG00000157350
ENSG00000126214	ENSG00000011295
ENSG00000126214	ENSG00000062725
ENSG00000126214	ENSG00000104892
ENSG00000126214	ENSG00000137171
ENSG00000126214	ENSG00000174996
ENSG00000126217	ENSG00000038382
ENSG00000126217	ENSG00000053524
ENSG00000126217	ENSG00000101977
ENSG00000126217	ENSG00000160145
ENSG00000126218	ENSG00000057593
ENSG00000126218	ENSG00000101981
ENSG00000126218	ENSG00000115718
ENSG00000126218	ENSG00000126231
ENSG00000126218	ENSG00000180210
ENSG00000126231	ENSG00000057593
ENSG00000126231	ENSG00000101981
ENSG00000126231	ENSG00000115718
ENSG00000126231	ENSG00000126218
ENSG00000126231	ENSG00000180210

ENSG00000126243	ENSG00000128011
ENSG00000126243	ENSG00000156564
ENSG00000126243	ENSG00000165379
ENSG00000126247	ENSG00000075142
ENSG00000126247	ENSG00000115271
ENSG00000126247	ENSG00000162517
ENSG00000126251	ENSG00000125245
ENSG00000126251	ENSG00000126262
ENSG00000126251	ENSG00000144230
ENSG00000126251	ENSG00000147138
ENSG00000126251	ENSG00000162881
ENSG00000126251	ENSG00000164220
ENSG00000126251	ENSG00000164251
ENSG00000126251	ENSG00000165621
ENSG00000126251	ENSG00000171631
ENSG00000126251	ENSG00000181104
ENSG00000126251	ENSG00000182162
ENSG00000126251	ENSG00000182559
ENSG00000126251	ENSG00000182782
ENSG00000126251	ENSG00000185897
ENSG00000126251	ENSG00000196917
ENSG00000126258	ENSG00000089356
ENSG00000126258	ENSG00000137726
ENSG00000126258	ENSG00000137731
ENSG00000126258	ENSG00000150201
ENSG00000126258	ENSG00000182707
ENSG00000126259	ENSG00000149571
ENSG00000126259	ENSG00000161270
ENSG00000126259	ENSG00000183853
ENSG00000126262	ENSG00000125245
ENSG00000126262	ENSG00000126251
ENSG00000126262	ENSG00000144230
ENSG00000126262	ENSG00000147138
ENSG00000126262	ENSG00000162881
ENSG00000126262	ENSG00000164220
ENSG00000126262	ENSG00000164251
ENSG00000126262	ENSG00000165621
ENSG00000126262	ENSG00000171631
ENSG00000126262	ENSG00000181104
ENSG00000126262	ENSG00000182162
ENSG00000126262	ENSG00000182559
ENSG00000126262	ENSG00000182782
ENSG00000126262	ENSG00000185897
ENSG00000126262	ENSG00000196917
ENSG00000126267	ENSG00000160471
ENSG00000126267	ENSG00000172912
ENSG00000126337	ENSG00000006059
ENSG00000126337	ENSG00000094796
ENSG00000126337	ENSG00000108244
ENSG00000126337	ENSG00000108417
ENSG00000126337	ENSG00000108759
ENSG00000126337	ENSG00000111057
ENSG00000126337	ENSG00000128422
ENSG00000126337	ENSG00000131737
ENSG00000126337	ENSG00000131738
ENSG00000126337	ENSG00000131885
ENSG00000126337	ENSG00000167916
ENSG00000126337	ENSG00000170819
ENSG00000126337	ENSG00000171345

ENSG00000126337	ENSG00000171346
ENSG00000126337	ENSG00000171360
ENSG00000126337	ENSG00000171401
ENSG00000126337	ENSG00000171403
ENSG00000126337	ENSG00000171431
ENSG00000126337	ENSG00000171446
ENSG00000126337	ENSG00000173908
ENSG00000126337	ENSG00000186393
ENSG00000126337	ENSG00000186395
ENSG00000126337	ENSG00000186831
ENSG00000126337	ENSG00000186832
ENSG00000126337	ENSG00000186847
ENSG00000126337	ENSG00000187242
ENSG00000126337	ENSG00000187686
ENSG00000126337	ENSG00000196859
ENSG00000126337	ENSG00000197079
ENSG00000126337	ENSG00000204889
ENSG00000126337	ENSG00000204897
ENSG00000126337	ENSG00000213951
ENSG00000126337	ENSG00000214220
ENSG00000126337	ENSG00000214454
ENSG00000126337	ENSG00000214959
ENSG00000126337	ENSG00000215772
ENSG00000126337	ENSG00000215773
ENSG00000126351	ENSG00000077092
ENSG00000126351	ENSG00000126368
ENSG00000126351	ENSG00000131759
ENSG00000126351	ENSG00000151090
ENSG00000126351	ENSG00000172819
ENSG00000126351	ENSG00000174738
ENSG00000126353	ENSG00000112486
ENSG00000126353	ENSG00000121797
ENSG00000126353	ENSG00000121807
ENSG00000126353	ENSG00000121966
ENSG00000126353	ENSG00000129048
ENSG00000126353	ENSG00000144648
ENSG00000126353	ENSG00000160683
ENSG00000126353	ENSG00000160791
ENSG00000126353	ENSG00000163464
ENSG00000126353	ENSG00000163823
ENSG00000126353	ENSG00000168329
ENSG00000126353	ENSG00000172215
ENSG00000126353	ENSG00000173578
ENSG00000126353	ENSG00000173585
ENSG00000126353	ENSG00000179934
ENSG00000126353	ENSG00000180871
ENSG00000126353	ENSG00000183625
ENSG00000126353	ENSG00000183813
ENSG00000126353	ENSG00000184451
ENSG00000126353	ENSG00000186810
ENSG00000126353	ENSG00000215775
ENSG00000126353	ENSG00000215778
ENSG00000126353	ENSG00000215782
ENSG00000126368	ENSG00000077092
ENSG00000126368	ENSG00000126351
ENSG00000126368	ENSG00000131759
ENSG00000126368	ENSG00000151090
ENSG00000126368	ENSG00000172819
ENSG00000126368	ENSG00000174738

ENSG00000126456	ENSG00000117595
ENSG00000126456	ENSG00000125347
ENSG00000126456	ENSG00000128604
ENSG00000126456	ENSG00000137265
ENSG00000126456	ENSG00000140968
ENSG00000126456	ENSG00000168310
ENSG00000126456	ENSG00000185507
ENSG00000126456	ENSG00000213928
ENSG00000126457	ENSG00000111218
ENSG00000126458	ENSG00000006451
ENSG00000126458	ENSG00000133703
ENSG00000126458	ENSG00000133818
ENSG00000126458	ENSG00000144118
ENSG00000126458	ENSG00000158186
ENSG00000126458	ENSG00000174775
ENSG00000126458	ENSG00000187682
ENSG00000126458	ENSG00000213281
ENSG00000126460	ENSG00000130032
ENSG00000126460	ENSG00000130962
ENSG00000126460	ENSG00000135378
ENSG00000126461	ENSG00000070047
ENSG00000126461	ENSG00000139218
ENSG00000126464	ENSG00000060749
ENSG00000126500	ENSG00000125848
ENSG00000126500	ENSG00000186105
ENSG00000126562	ENSG00000060237
ENSG00000126562	ENSG00000115216
ENSG00000126562	ENSG00000165238
ENSG00000126562	ENSG00000196632
ENSG00000126583	ENSG00000027075
ENSG00000126583	ENSG00000065675
ENSG00000126583	ENSG00000067606
ENSG00000126583	ENSG00000105221
ENSG00000126583	ENSG00000117020
ENSG00000126583	ENSG00000142208
ENSG00000126583	ENSG00000154229
ENSG00000126583	ENSG00000163558
ENSG00000126583	ENSG00000163932
ENSG00000126583	ENSG00000166501
ENSG00000126656	ENSG00000101883
ENSG00000126656	ENSG00000123576
ENSG00000126656	ENSG00000134438
ENSG00000126656	ENSG00000173976
ENSG00000126656	ENSG00000175325
ENSG00000126656	ENSG00000175329
ENSG00000126656	ENSG00000204595
ENSG00000126733	ENSG00000165659
ENSG00000126767	ENSG00000111145
ENSG00000126767	ENSG00000117036
ENSG00000126767	ENSG00000158711
ENSG00000126768	ENSG00000134375
ENSG00000126777	ENSG00000125844
ENSG00000126778	ENSG00000100625
ENSG00000126778	ENSG00000138083
ENSG00000126778	ENSG00000170577
ENSG00000126778	ENSG00000177045
ENSG00000126778	ENSG00000184302
ENSG00000126785	ENSG00000070831
ENSG00000126785	ENSG00000119729

ENSG00000126785	ENSG00000128340
ENSG00000126785	ENSG00000136238
ENSG00000126785	ENSG00000169750
ENSG00000126785	ENSG00000177105
ENSG00000126787	ENSG00000080845
ENSG00000126787	ENSG00000116544
ENSG00000126787	ENSG00000170579
ENSG00000126787	ENSG00000198010
ENSG00000126803	ENSG00000109971
ENSG00000126803	ENSG00000173110
ENSG00000126803	ENSG00000204388
ENSG00000126803	ENSG00000204389
ENSG00000126803	ENSG00000204390
ENSG00000126804	ENSG00000089775
ENSG00000126804	ENSG00000181472
ENSG00000126822	ENSG00000090924
ENSG00000126822	ENSG00000120278
ENSG00000126856	ENSG00000206115
ENSG00000126858	ENSG00000140983
ENSG00000126878	ENSG00000115468
ENSG00000126878	ENSG00000142634
ENSG00000126878	ENSG00000204472
ENSG00000126882	ENSG00000188859
ENSG00000126895	ENSG00000109163
ENSG00000126895	ENSG00000166148
ENSG00000126895	ENSG00000178015
ENSG00000126895	ENSG00000180914
ENSG00000126895	ENSG00000187258
ENSG00000126895	ENSG00000198049
ENSG00000126895	ENSG00000211451
ENSG00000126903	ENSG00000100652
ENSG00000126903	ENSG00000125255
ENSG00000126903	ENSG00000145248
ENSG00000126903	ENSG00000145283
ENSG00000126903	ENSG00000205184
ENSG00000126934	ENSG00000169032
ENSG00000126934	ENSG00000212825
ENSG00000126945	ENSG00000096746
ENSG00000126945	ENSG00000103067
ENSG00000126945	ENSG00000104413
ENSG00000126945	ENSG00000132463
ENSG00000126945	ENSG00000169045
ENSG00000126945	ENSG00000169813
ENSG00000126952	ENSG00000147206
ENSG00000126952	ENSG00000162231
ENSG00000126952	ENSG00000185554
ENSG00000126952	ENSG00000185945
ENSG00000126952	ENSG00000196970
ENSG00000126953	ENSG00000150779
ENSG00000127022	ENSG00000153132
ENSG00000127074	ENSG00000090104
ENSG00000127074	ENSG00000091844
ENSG00000127074	ENSG00000116741
ENSG00000127074	ENSG00000117152
ENSG00000127074	ENSG00000135824
ENSG00000127074	ENSG00000143248
ENSG00000127074	ENSG00000143333
ENSG00000127074	ENSG00000147509
ENSG00000127074	ENSG00000148908

ENSG00000127074	ENSG00000150681
ENSG00000127074	ENSG00000171700
ENSG00000127074	ENSG00000215825
ENSG00000127083	ENSG00000122176
ENSG00000127083	ENSG00000139330
ENSG00000127083	ENSG00000188783
ENSG00000127084	ENSG00000139132
ENSG00000127084	ENSG00000146192
ENSG00000127124	ENSG00000010818
ENSG00000127124	ENSG00000095951
ENSG00000127129	ENSG00000078401
ENSG00000127129	ENSG00000124205
ENSG00000127152	ENSG00000119866
ENSG00000127152	ENSG00000170684
ENSG00000127191	ENSG00000056558
ENSG00000127191	ENSG00000076604
ENSG00000127191	ENSG00000082512
ENSG00000127191	ENSG00000131323
ENSG00000127191	ENSG00000175104
ENSG00000127241	ENSG00000009724
ENSG00000127241	ENSG00000139178
ENSG00000127241	ENSG00000159403
ENSG00000127241	ENSG00000182326
ENSG00000127249	ENSG00000133657
ENSG00000127249	ENSG00000159363
ENSG00000127249	ENSG00000187527
ENSG00000127252	ENSG00000121207
ENSG00000127252	ENSG00000133321
ENSG00000127252	ENSG00000133328
ENSG00000127252	ENSG00000162981
ENSG00000127252	ENSG00000168004
ENSG00000127252	ENSG00000168672
ENSG00000127252	ENSG00000176485
ENSG00000127314	ENSG00000116473
ENSG00000127314	ENSG00000123728
ENSG00000127314	ENSG00000125249
ENSG00000127314	ENSG00000162595
ENSG00000127314	ENSG00000165023
ENSG00000127314	ENSG00000176276
ENSG00000127314	ENSG00000176490
ENSG00000127314	ENSG00000181467
ENSG00000127314	ENSG00000214405
ENSG00000127314	ENSG00000214492
ENSG00000127318	ENSG00000111536
ENSG00000127318	ENSG00000136634
ENSG00000127318	ENSG00000142224
ENSG00000127318	ENSG00000162891
ENSG00000127318	ENSG00000162892
ENSG00000127325	ENSG00000039987
ENSG00000127325	ENSG00000142959
ENSG00000127325	ENSG00000167995
ENSG00000127328	ENSG00000167994
ENSG00000127329	ENSG00000080031
ENSG00000127329	ENSG00000149177
ENSG00000127329	ENSG00000151490
ENSG00000127334	ENSG00000010219
ENSG00000127334	ENSG00000105204
ENSG00000127334	ENSG00000143479
ENSG00000127334	ENSG00000157540

ENSG00000127377	ENSG00000100053
ENSG00000127377	ENSG00000100058
ENSG00000127377	ENSG00000100122
ENSG00000127377	ENSG00000108255
ENSG00000127377	ENSG00000118231
ENSG00000127377	ENSG00000163254
ENSG00000127377	ENSG00000163499
ENSG00000127377	ENSG00000168582
ENSG00000127377	ENSG00000182187
ENSG00000127377	ENSG00000196431
ENSG00000127377	ENSG00000213139
ENSG00000127412	ENSG00000111199
ENSG00000127412	ENSG00000165125
ENSG00000127412	ENSG00000167723
ENSG00000127412	ENSG00000187688
ENSG00000127412	ENSG00000196689
ENSG00000127418	ENSG00000124006
ENSG00000127472	ENSG00000069764
ENSG00000127472	ENSG00000117215
ENSG00000127472	ENSG00000158786
ENSG00000127472	ENSG00000170890
ENSG00000127472	ENSG00000187980
ENSG00000127472	ENSG00000188257
ENSG00000127472	ENSG00000188784
ENSG00000127507	ENSG00000069122
ENSG00000127507	ENSG00000123146
ENSG00000127507	ENSG00000131355
ENSG00000127507	ENSG00000153292
ENSG00000127507	ENSG00000153294
ENSG00000127507	ENSG00000162618
ENSG00000127507	ENSG00000164393
ENSG00000127507	ENSG00000173567
ENSG00000127507	ENSG00000174837
ENSG00000127507	ENSG00000186629
ENSG00000127507	ENSG00000204818
ENSG00000127511	ENSG00000169375
ENSG00000127527	ENSG00000085832
ENSG00000127527	ENSG00000198399
ENSG00000127527	ENSG00000205726
ENSG00000127528	ENSG00000067082
ENSG00000127528	ENSG00000102554
ENSG00000127528	ENSG00000105610
ENSG00000127528	ENSG00000129911
ENSG00000127528	ENSG00000136826
ENSG00000127528	ENSG00000139651
ENSG00000127528	ENSG00000155090
ENSG00000127528	ENSG00000164651
ENSG00000127528	ENSG00000167182
ENSG00000127528	ENSG00000170374
ENSG00000127528	ENSG00000171872
ENSG00000127528	ENSG00000172059
ENSG00000127528	ENSG00000174595
ENSG00000127528	ENSG00000189120
ENSG00000127528	ENSG00000197294
ENSG00000127528	ENSG00000197550
ENSG00000127528	ENSG00000204335
ENSG00000127561	ENSG00000100321
ENSG00000127561	ENSG00000105467
ENSG00000127561	ENSG00000108639

ENSG00000127561	ENSG00000215315
ENSG00000127578	ENSG00000003436
ENSG00000127578	ENSG00000105825
ENSG00000127578	ENSG00000173714
ENSG00000127589	ENSG00000101162
ENSG00000127589	ENSG00000104833
ENSG00000127589	ENSG00000137267
ENSG00000127589	ENSG00000137285
ENSG00000127589	ENSG00000159247
ENSG00000127589	ENSG00000173213
ENSG00000127589	ENSG00000173876
ENSG00000127589	ENSG00000176014
ENSG00000127589	ENSG00000188229
ENSG00000127589	ENSG00000196230
ENSG00000127589	ENSG00000198211
ENSG00000127589	ENSG00000215801
ENSG00000127589	ENSG00000215813
ENSG00000127589	ENSG00000215814
ENSG00000127603	ENSG00000096696
ENSG00000127603	ENSG00000118898
ENSG00000127603	ENSG00000151914
ENSG00000127603	ENSG00000167880
ENSG00000127616	ENSG00000080503
ENSG00000127663	ENSG00000066135
ENSG00000127663	ENSG00000107077
ENSG00000127663	ENSG00000186280
ENSG00000127824	ENSG00000075886
ENSG00000127824	ENSG00000123416
ENSG00000127824	ENSG00000152086
ENSG00000127824	ENSG00000163526
ENSG00000127824	ENSG00000167552
ENSG00000127824	ENSG00000167553
ENSG00000127824	ENSG00000178462
ENSG00000127824	ENSG00000183785
ENSG00000127824	ENSG00000198033
ENSG00000127824	ENSG00000214391
ENSG00000127824	ENSG00000215823
ENSG00000127831	ENSG00000006747
ENSG00000127831	ENSG00000042493
ENSG00000127831	ENSG00000135407
ENSG00000127831	ENSG00000136059
ENSG00000127831	ENSG00000148180
ENSG00000127831	ENSG00000177731
ENSG00000127863	ENSG00000131080
ENSG00000127863	ENSG00000135960
ENSG00000127870	ENSG00000131263
ENSG00000127870	ENSG00000137075
ENSG00000127870	ENSG00000146083
ENSG00000127920	ENSG00000127588
ENSG00000127920	ENSG00000127928
ENSG00000127920	ENSG00000133136
ENSG00000127920	ENSG00000162188
ENSG00000127920	ENSG00000167083
ENSG00000127920	ENSG00000167414
ENSG00000127920	ENSG00000168243
ENSG00000127920	ENSG00000172380
ENSG00000127920	ENSG00000174021
ENSG00000127920	ENSG00000182625
ENSG00000127920	ENSG00000186469

ENSG00000127920	ENSG00000213611
ENSG00000127928	ENSG00000127588
ENSG00000127928	ENSG00000127920
ENSG00000127928	ENSG00000133136
ENSG00000127928	ENSG00000162188
ENSG00000127928	ENSG00000167083
ENSG00000127928	ENSG00000167414
ENSG00000127928	ENSG00000168243
ENSG00000127928	ENSG00000172380
ENSG00000127928	ENSG00000174021
ENSG00000127928	ENSG00000182625
ENSG00000127928	ENSG00000186469
ENSG00000127928	ENSG00000213611
ENSG00000127946	ENSG00000130787
ENSG00000127947	ENSG00000072135
ENSG00000127947	ENSG00000134242
ENSG00000127954	ENSG00000105889
ENSG00000127954	ENSG00000115107
ENSG00000127954	ENSG00000157214
ENSG00000127954	ENSG00000164647
ENSG00000127955	ENSG00000060558
ENSG00000127955	ENSG00000065135
ENSG00000127955	ENSG00000087258
ENSG00000127955	ENSG00000087460
ENSG00000127955	ENSG00000088256
ENSG00000127955	ENSG00000114349
ENSG00000127955	ENSG00000114353
ENSG00000127955	ENSG00000120063
ENSG00000127955	ENSG00000128266
ENSG00000127955	ENSG00000134183
ENSG00000127955	ENSG00000141404
ENSG00000127955	ENSG00000146535
ENSG00000127955	ENSG00000156049
ENSG00000127955	ENSG00000156052
ENSG00000127955	ENSG00000214415
ENSG00000127955	ENSG00000214622
ENSG00000127989	ENSG00000120832
ENSG00000127990	ENSG00000108823
ENSG00000128011	ENSG00000126243
ENSG00000128011	ENSG00000156564
ENSG00000128011	ENSG00000165379
ENSG00000128016	ENSG00000152518
ENSG00000128016	ENSG00000185650
ENSG00000128040	ENSG00000122711
ENSG00000128040	ENSG00000145879
ENSG00000128040	ENSG00000164266
ENSG00000128040	ENSG00000178172
ENSG00000128040	ENSG00000196800
ENSG00000128040	ENSG00000204909
ENSG00000128040	ENSG00000214510
ENSG00000128045	ENSG00000103710
ENSG00000128045	ENSG00000111404
ENSG00000128045	ENSG00000122035
ENSG00000128045	ENSG00000134533
ENSG00000128052	ENSG00000037280
ENSG00000128052	ENSG00000102755
ENSG00000128052	ENSG00000104213
ENSG00000128052	ENSG00000113721
ENSG00000128052	ENSG00000122025

ENSG00000128052	ENSG00000134853
ENSG00000128052	ENSG00000157404
ENSG00000128052	ENSG00000182578
ENSG00000128203	ENSG00000174939
ENSG00000128203	ENSG00000198363
ENSG00000128228	ENSG00000132581
ENSG00000128242	ENSG00000154252
ENSG00000128242	ENSG00000175229
ENSG00000128242	ENSG00000197093
ENSG00000128245	ENSG00000108953
ENSG00000128245	ENSG00000134308
ENSG00000128245	ENSG00000164924
ENSG00000128245	ENSG00000166913
ENSG00000128245	ENSG00000170027
ENSG00000128245	ENSG00000175793
ENSG00000128245	ENSG00000214637
ENSG00000128245	ENSG00000215108
ENSG00000128250	ENSG00000026950
ENSG00000128250	ENSG00000111801
ENSG00000128250	ENSG00000112763
ENSG00000128250	ENSG00000113303
ENSG00000128250	ENSG00000121236
ENSG00000128250	ENSG00000124508
ENSG00000128250	ENSG00000124549
ENSG00000128250	ENSG00000124557
ENSG00000128250	ENSG00000128253
ENSG00000128250	ENSG00000128276
ENSG00000128250	ENSG00000128482
ENSG00000128250	ENSG00000132256
ENSG00000128250	ENSG00000132274
ENSG00000128250	ENSG00000164010
ENSG00000128250	ENSG00000165810
ENSG00000128250	ENSG00000168903
ENSG00000128250	ENSG00000176422
ENSG00000128250	ENSG00000179046
ENSG00000128250	ENSG00000182805
ENSG00000128250	ENSG00000183439
ENSG00000128250	ENSG00000183718
ENSG00000128250	ENSG00000187574
ENSG00000128250	ENSG00000188683
ENSG00000128250	ENSG00000204614
ENSG00000128250	ENSG00000204616
ENSG00000128250	ENSG00000204618
ENSG00000128250	ENSG00000205198
ENSG00000128250	ENSG00000214693
ENSG00000128253	ENSG00000026950
ENSG00000128253	ENSG00000111801
ENSG00000128253	ENSG00000112763
ENSG00000128253	ENSG00000113303
ENSG00000128253	ENSG00000121236
ENSG00000128253	ENSG00000124508
ENSG00000128253	ENSG00000124549
ENSG00000128253	ENSG00000124557
ENSG00000128253	ENSG00000128250
ENSG00000128253	ENSG00000128276
ENSG00000128253	ENSG00000128482
ENSG00000128253	ENSG00000132256
ENSG00000128253	ENSG00000132274
ENSG00000128253	ENSG00000164010

ENSG00000128253	ENSG00000165810
ENSG00000128253	ENSG00000168903
ENSG00000128253	ENSG00000176422
ENSG00000128253	ENSG00000179046
ENSG00000128253	ENSG00000182805
ENSG00000128253	ENSG00000183439
ENSG00000128253	ENSG00000183718
ENSG00000128253	ENSG00000187574
ENSG00000128253	ENSG00000188683
ENSG00000128253	ENSG00000204614
ENSG00000128253	ENSG00000204616
ENSG00000128253	ENSG00000204618
ENSG00000128253	ENSG00000205198
ENSG00000128253	ENSG00000214693
ENSG00000128271	ENSG00000121933
ENSG00000128271	ENSG00000163485
ENSG00000128271	ENSG00000170425
ENSG00000128272	ENSG00000169136
ENSG00000128276	ENSG00000026950
ENSG00000128276	ENSG00000111801
ENSG00000128276	ENSG00000112763
ENSG00000128276	ENSG00000113303
ENSG00000128276	ENSG00000121236
ENSG00000128276	ENSG00000124508
ENSG00000128276	ENSG00000124549
ENSG00000128276	ENSG00000124557
ENSG00000128276	ENSG00000128250
ENSG00000128276	ENSG00000128253
ENSG00000128276	ENSG00000128482
ENSG00000128276	ENSG00000132256
ENSG00000128276	ENSG00000132274
ENSG00000128276	ENSG00000164010
ENSG00000128276	ENSG00000165810
ENSG00000128276	ENSG00000168903
ENSG00000128276	ENSG00000176422
ENSG00000128276	ENSG00000179046
ENSG00000128276	ENSG00000182805
ENSG00000128276	ENSG00000183439
ENSG00000128276	ENSG00000183718
ENSG00000128276	ENSG00000187574
ENSG00000128276	ENSG00000188683
ENSG00000128276	ENSG00000204614
ENSG00000128276	ENSG00000204616
ENSG00000128276	ENSG00000204618
ENSG00000128276	ENSG00000205198
ENSG00000128276	ENSG00000214693
ENSG00000128283	ENSG00000149798
ENSG00000128283	ENSG00000163171
ENSG00000128283	ENSG00000167617
ENSG00000128283	ENSG00000179604
ENSG00000128284	ENSG00000100336
ENSG00000128284	ENSG00000100342
ENSG00000128284	ENSG00000128313
ENSG00000128284	ENSG00000128335
ENSG00000128284	ENSG00000178878
ENSG00000128284	ENSG00000196785
ENSG00000128285	ENSG00000082556
ENSG00000128285	ENSG00000112038
ENSG00000128285	ENSG00000116014

ENSG00000128285	ENSG00000116329
ENSG00000128285	ENSG00000125510
ENSG00000128285	ENSG00000125522
ENSG00000128285	ENSG00000128310
ENSG00000128285	ENSG00000132671
ENSG00000128285	ENSG00000139874
ENSG00000128285	ENSG00000152034
ENSG00000128285	ENSG00000162009
ENSG00000128285	ENSG00000166573
ENSG00000128285	ENSG00000180616
ENSG00000128285	ENSG00000182687
ENSG00000128285	ENSG00000183473
ENSG00000128285	ENSG00000183729
ENSG00000128294	ENSG00000169902
ENSG00000128298	ENSG00000006453
ENSG00000128298	ENSG00000175866
ENSG00000128310	ENSG00000082556
ENSG00000128310	ENSG00000112038
ENSG00000128310	ENSG00000116014
ENSG00000128310	ENSG00000116329
ENSG00000128310	ENSG00000125510
ENSG00000128310	ENSG00000125522
ENSG00000128310	ENSG00000128285
ENSG00000128310	ENSG00000132671
ENSG00000128310	ENSG00000139874
ENSG00000128310	ENSG00000152034
ENSG00000128310	ENSG00000162009
ENSG00000128310	ENSG00000166573
ENSG00000128310	ENSG00000180616
ENSG00000128310	ENSG00000182687
ENSG00000128310	ENSG00000183473
ENSG00000128310	ENSG00000183729
ENSG00000128313	ENSG00000100336
ENSG00000128313	ENSG00000100342
ENSG00000128313	ENSG00000128284
ENSG00000128313	ENSG00000128335
ENSG00000128313	ENSG00000178878
ENSG00000128313	ENSG00000196785
ENSG00000128322	ENSG00000206066
ENSG00000128322	ENSG00000211592
ENSG00000128322	ENSG00000211675
ENSG00000128322	ENSG00000211677
ENSG00000128322	ENSG00000211679
ENSG00000128322	ENSG00000211683
ENSG00000128322	ENSG00000211685
ENSG00000128322	ENSG00000211898
ENSG00000128335	ENSG00000100336
ENSG00000128335	ENSG00000100342
ENSG00000128335	ENSG00000128284
ENSG00000128335	ENSG00000128313
ENSG00000128335	ENSG00000178878
ENSG00000128335	ENSG00000196785
ENSG00000128340	ENSG00000070831
ENSG00000128340	ENSG00000119729
ENSG00000128340	ENSG00000126785
ENSG00000128340	ENSG00000136238
ENSG00000128340	ENSG00000169750
ENSG00000128340	ENSG00000177105
ENSG00000128383	ENSG00000100298

ENSG00000128383	ENSG00000111701
ENSG00000128383	ENSG00000111732
ENSG00000128383	ENSG00000124701
ENSG00000128383	ENSG00000128394
ENSG00000128383	ENSG00000179750
ENSG00000128383	ENSG00000198904
ENSG00000128394	ENSG00000100298
ENSG00000128394	ENSG00000111701
ENSG00000128394	ENSG00000111732
ENSG00000128394	ENSG00000124701
ENSG00000128394	ENSG00000128383
ENSG00000128394	ENSG00000179750
ENSG00000128394	ENSG00000198904
ENSG00000128408	ENSG00000158423
ENSG00000128422	ENSG00000006059
ENSG00000128422	ENSG00000094796
ENSG00000128422	ENSG00000108244
ENSG00000128422	ENSG00000108417
ENSG00000128422	ENSG00000108759
ENSG00000128422	ENSG00000111057
ENSG00000128422	ENSG00000126337
ENSG00000128422	ENSG00000131737
ENSG00000128422	ENSG00000131738
ENSG00000128422	ENSG00000131885
ENSG00000128422	ENSG00000167916
ENSG00000128422	ENSG00000170819
ENSG00000128422	ENSG00000171345
ENSG00000128422	ENSG00000171346
ENSG00000128422	ENSG00000171360
ENSG00000128422	ENSG00000171401
ENSG00000128422	ENSG00000171403
ENSG00000128422	ENSG00000171431
ENSG00000128422	ENSG00000171446
ENSG00000128422	ENSG00000173908
ENSG00000128422	ENSG00000186393
ENSG00000128422	ENSG00000186395
ENSG00000128422	ENSG00000186831
ENSG00000128422	ENSG00000186832
ENSG00000128422	ENSG00000186847
ENSG00000128422	ENSG00000187242
ENSG00000128422	ENSG00000187686
ENSG00000128422	ENSG00000196859
ENSG00000128422	ENSG00000197079
ENSG00000128422	ENSG00000204889
ENSG00000128422	ENSG00000204897
ENSG00000128422	ENSG00000213951
ENSG00000128422	ENSG00000214220
ENSG00000128422	ENSG00000214454
ENSG00000128422	ENSG00000214959
ENSG00000128422	ENSG00000215772
ENSG00000128422	ENSG00000215773
ENSG00000128438	ENSG00000148429
ENSG00000128438	ENSG00000161583
ENSG00000128438	ENSG00000174115
ENSG00000128438	ENSG00000185128
ENSG00000128438	ENSG00000188755
ENSG00000128438	ENSG00000188849
ENSG00000128438	ENSG00000189309
ENSG00000128438	ENSG00000197681

ENSG00000128438	ENSG00000204471
ENSG00000128438	ENSG00000205019
ENSG00000128438	ENSG00000213474
ENSG00000128438	ENSG00000215625
ENSG00000128438	ENSG00000215730
ENSG00000128438	ENSG00000215748
ENSG00000128487	ENSG00000100014
ENSG00000128487	ENSG00000183963
ENSG00000128487	ENSG00000188176
ENSG00000128487	ENSG00000214872
ENSG00000128510	ENSG00000080618
ENSG00000128510	ENSG00000091704
ENSG00000128510	ENSG00000144410
ENSG00000128510	ENSG00000153002
ENSG00000128510	ENSG00000158516
ENSG00000128510	ENSG00000158525
ENSG00000128510	ENSG00000163751
ENSG00000128510	ENSG00000165078
ENSG00000128512	ENSG00000088538
ENSG00000128512	ENSG00000134516
ENSG00000128512	ENSG00000147459
ENSG00000128512	ENSG00000150760
ENSG00000128567	ENSG00000114631
ENSG00000128573	ENSG00000049768
ENSG00000128573	ENSG00000114861
ENSG00000128573	ENSG00000137166
ENSG00000128578	ENSG00000143093
ENSG00000128591	ENSG00000136068
ENSG00000128591	ENSG00000196924
ENSG00000128594	ENSG00000169783
ENSG00000128594	ENSG00000170382
ENSG00000128594	ENSG00000173114
ENSG00000128594	ENSG00000174482
ENSG00000128594	ENSG00000175928
ENSG00000128594	ENSG00000183014
ENSG00000128594	ENSG00000213171
ENSG00000128595	ENSG00000049449
ENSG00000128595	ENSG00000078808
ENSG00000128595	ENSG00000117906
ENSG00000128595	ENSG00000142552
ENSG00000128604	ENSG00000117595
ENSG00000128604	ENSG00000125347
ENSG00000128604	ENSG00000126456
ENSG00000128604	ENSG00000137265
ENSG00000128604	ENSG00000140968
ENSG00000128604	ENSG00000168310
ENSG00000128604	ENSG00000185507
ENSG00000128604	ENSG00000213928
ENSG00000128606	ENSG00000145147
ENSG00000128606	ENSG00000166897
ENSG00000128606	ENSG00000182743
ENSG00000128606	ENSG00000184347
ENSG00000128606	ENSG00000187122
ENSG00000128610	ENSG00000153266
ENSG00000128617	ENSG00000054277
ENSG00000128617	ENSG00000102076
ENSG00000128617	ENSG00000122375
ENSG00000128617	ENSG00000124818
ENSG00000128617	ENSG00000147380

ENSG00000128617	ENSG00000148604
ENSG00000128617	ENSG00000163914
ENSG00000128617	ENSG00000166160
ENSG00000128617	ENSG00000180245
ENSG00000128641	ENSG00000136286
ENSG00000128641	ENSG00000142347
ENSG00000128641	ENSG00000157483
ENSG00000128641	ENSG00000166866
ENSG00000128641	ENSG00000176658
ENSG00000128641	ENSG00000197879
ENSG00000128645	ENSG00000105991
ENSG00000128645	ENSG00000105996
ENSG00000128645	ENSG00000120094
ENSG00000128645	ENSG00000173917
ENSG00000128652	ENSG00000105997
ENSG00000128652	ENSG00000106038
ENSG00000128652	ENSG00000120093
ENSG00000128652	ENSG00000169840
ENSG00000128652	ENSG00000174279
ENSG00000128652	ENSG00000180613
ENSG00000128655	ENSG00000095464
ENSG00000128655	ENSG00000112541
ENSG00000128655	ENSG00000132915
ENSG00000128655	ENSG00000133256
ENSG00000128655	ENSG00000138735
ENSG00000128655	ENSG00000186642
ENSG00000128683	ENSG00000136750
ENSG00000128683	ENSG00000139631
ENSG00000128683	ENSG00000144644
ENSG00000128683	ENSG00000215438
ENSG00000128699	ENSG00000123353
ENSG00000128699	ENSG00000172057
ENSG00000128709	ENSG00000078399
ENSG00000128709	ENSG00000128710
ENSG00000128709	ENSG00000153807
ENSG00000128709	ENSG00000170689
ENSG00000128709	ENSG00000180806
ENSG00000128709	ENSG00000180818
ENSG00000128710	ENSG00000078399
ENSG00000128710	ENSG00000128709
ENSG00000128710	ENSG00000153807
ENSG00000128710	ENSG00000170689
ENSG00000128710	ENSG00000180806
ENSG00000128710	ENSG00000180818
ENSG00000128713	ENSG00000005073
ENSG00000128713	ENSG00000123388
ENSG00000128714	ENSG00000106031
ENSG00000128714	ENSG00000123364
ENSG00000128714	ENSG00000159184
ENSG00000128739	ENSG00000125835
ENSG00000128739	ENSG00000138101
ENSG00000128802	ENSG00000101144
ENSG00000128802	ENSG00000107623
ENSG00000128802	ENSG00000112175
ENSG00000128802	ENSG00000116985
ENSG00000128802	ENSG00000125965
ENSG00000128802	ENSG00000130385
ENSG00000128802	ENSG00000143869
ENSG00000128802	ENSG00000152785

ENSG00000128802	ENSG00000153162
ENSG00000128802	ENSG00000156466
ENSG00000128802	ENSG00000156574
ENSG00000128802	ENSG00000163217
ENSG00000128802	ENSG00000183682
ENSG00000128805	ENSG00000138639
ENSG00000128805	ENSG00000147799
ENSG00000128805	ENSG00000163219
ENSG00000128815	ENSG00000143669
ENSG00000128815	ENSG00000163625
ENSG00000128833	ENSG00000141140
ENSG00000128833	ENSG00000167306
ENSG00000128833	ENSG00000197535
ENSG00000128849	ENSG00000143375
ENSG00000128872	ENSG00000136842
ENSG00000128872	ENSG00000138594
ENSG00000128872	ENSG00000163157
ENSG00000128872	ENSG00000163380
ENSG00000128872	ENSG00000163431
ENSG00000128872	ENSG00000170807
ENSG00000128917	ENSG00000090932
ENSG00000128917	ENSG00000101384
ENSG00000128917	ENSG00000171462
ENSG00000128917	ENSG00000184916
ENSG00000128917	ENSG00000185559
ENSG00000128917	ENSG00000198719
ENSG00000128918	ENSG00000111275
ENSG00000128918	ENSG00000137124
ENSG00000128918	ENSG00000165092
ENSG00000128918	ENSG00000184254
ENSG00000128923	ENSG00000143409
ENSG00000128989	ENSG00000143420
ENSG00000129003	ENSG00000048707
ENSG00000129003	ENSG00000197969
ENSG00000129038	ENSG00000113083
ENSG00000129038	ENSG00000115318
ENSG00000129038	ENSG00000134013
ENSG00000129038	ENSG00000138131
ENSG00000129048	ENSG00000112486
ENSG00000129048	ENSG00000121797
ENSG00000129048	ENSG00000121807
ENSG00000129048	ENSG00000121966
ENSG00000129048	ENSG00000126353
ENSG00000129048	ENSG00000144648
ENSG00000129048	ENSG00000160683
ENSG00000129048	ENSG00000160791
ENSG00000129048	ENSG00000163464
ENSG00000129048	ENSG00000163823
ENSG00000129048	ENSG00000168329
ENSG00000129048	ENSG00000172215
ENSG00000129048	ENSG00000173578
ENSG00000129048	ENSG00000173585
ENSG00000129048	ENSG00000179934
ENSG00000129048	ENSG00000180871
ENSG00000129048	ENSG00000183625
ENSG00000129048	ENSG00000183813
ENSG00000129048	ENSG00000184451
ENSG00000129048	ENSG00000186810
ENSG00000129048	ENSG00000215775

ENSG00000129048	ENSG00000215778
ENSG00000129048	ENSG00000215782
ENSG00000129116	ENSG00000120729
ENSG00000129116	ENSG00000138347
ENSG00000129152	ENSG00000111046
ENSG00000129152	ENSG00000111049
ENSG00000129152	ENSG00000122180
ENSG00000129159	ENSG00000116396
ENSG00000129159	ENSG00000131398
ENSG00000129159	ENSG00000166006
ENSG00000129167	ENSG00000139287
ENSG00000129167	ENSG00000171759
ENSG00000129167	ENSG00000180176
ENSG00000129170	ENSG00000136490
ENSG00000129170	ENSG00000146215
ENSG00000129170	ENSG00000159176
ENSG00000129170	ENSG00000175183
ENSG00000129170	ENSG00000182809
ENSG00000129170	ENSG00000197893
ENSG00000129170	ENSG00000213145
ENSG00000129173	ENSG00000165891
ENSG00000129194	ENSG00000005513
ENSG00000129194	ENSG00000100146
ENSG00000129194	ENSG00000124766
ENSG00000129194	ENSG00000125398
ENSG00000129194	ENSG00000134595
ENSG00000129194	ENSG00000164736
ENSG00000129194	ENSG00000171056
ENSG00000129194	ENSG00000176887
ENSG00000129194	ENSG00000177732
ENSG00000129194	ENSG00000181449
ENSG00000129194	ENSG00000182968
ENSG00000129194	ENSG00000184895
ENSG00000129194	ENSG00000203883
ENSG00000129219	ENSG00000075651
ENSG00000129221	ENSG00000110711
ENSG00000129226	ENSG00000005893
ENSG00000129226	ENSG00000078081
ENSG00000129226	ENSG00000185896
ENSG00000129244	ENSG00000069849
ENSG00000129244	ENSG00000101892
ENSG00000129244	ENSG00000143153
ENSG00000129244	ENSG00000186009
ENSG00000129245	ENSG00000102081
ENSG00000129245	ENSG00000114416
ENSG00000129270	ENSG00000008516
ENSG00000129270	ENSG00000087245
ENSG00000129270	ENSG00000099953
ENSG00000129270	ENSG00000100985
ENSG00000129270	ENSG00000102996
ENSG00000129270	ENSG00000110347
ENSG00000129270	ENSG00000118113
ENSG00000129270	ENSG00000123342
ENSG00000129270	ENSG00000125966
ENSG00000129270	ENSG00000137673
ENSG00000129270	ENSG00000137674
ENSG00000129270	ENSG00000137675
ENSG00000129270	ENSG00000137745
ENSG00000129270	ENSG00000149968

ENSG00000129270	ENSG00000154485
ENSG00000129270	ENSG00000156103
ENSG00000129270	ENSG00000157227
ENSG00000129270	ENSG00000166670
ENSG00000129270	ENSG00000167346
ENSG00000129270	ENSG00000189409
ENSG00000129270	ENSG00000196611
ENSG00000129270	ENSG00000198598
ENSG00000129270	ENSG00000215914
ENSG00000129277	ENSG00000006074
ENSG00000129277	ENSG00000006075
ENSG00000129277	ENSG00000006606
ENSG00000129277	ENSG00000102962
ENSG00000129277	ENSG00000102970
ENSG00000129277	ENSG00000106178
ENSG00000129277	ENSG00000108688
ENSG00000129277	ENSG00000108691
ENSG00000129277	ENSG00000108700
ENSG00000129277	ENSG00000108702
ENSG00000129277	ENSG00000115009
ENSG00000129277	ENSG00000143184
ENSG00000129277	ENSG00000143185
ENSG00000129277	ENSG00000161570
ENSG00000129277	ENSG00000161573
ENSG00000129277	ENSG00000161574
ENSG00000129277	ENSG00000167236
ENSG00000129277	ENSG00000172156
ENSG00000129277	ENSG00000172724
ENSG00000129277	ENSG00000181374
ENSG00000129277	ENSG00000197262
ENSG00000129277	ENSG00000205017
ENSG00000129277	ENSG00000205020
ENSG00000129277	ENSG00000205021
ENSG00000129277	ENSG00000213494
ENSG00000129277	ENSG00000215627
ENSG00000129277	ENSG00000215629
ENSG00000129292	ENSG00000025293
ENSG00000129315	ENSG00000082258
ENSG00000129351	ENSG00000056097
ENSG00000129351	ENSG00000105278
ENSG00000129351	ENSG00000143621
ENSG00000129351	ENSG00000165209
ENSG00000129353	ENSG00000070214
ENSG00000129353	ENSG00000137968
ENSG00000129353	ENSG00000143036
ENSG00000129353	ENSG00000204385
ENSG00000129354	ENSG00000072958
ENSG00000129354	ENSG00000140022
ENSG00000129355	ENSG00000123080
ENSG00000129355	ENSG00000198435
ENSG00000129422	ENSG00000132938
ENSG00000129422	ENSG00000198624
ENSG00000129450	ENSG00000105366
ENSG00000129450	ENSG00000105383
ENSG00000129450	ENSG00000105492
ENSG00000129450	ENSG00000105501
ENSG00000129450	ENSG00000105695
ENSG00000129450	ENSG00000142512
ENSG00000129450	ENSG00000160296

ENSG00000129450	ENSG00000161640
ENSG00000129450	ENSG00000161643
ENSG00000129450	ENSG00000167764
ENSG00000129450	ENSG00000168995
ENSG00000129450	ENSG00000171101
ENSG00000129450	ENSG00000197865
ENSG00000129465	ENSG00000104312
ENSG00000129465	ENSG00000137275
ENSG00000129465	ENSG00000170209
ENSG00000129465	ENSG00000183421
ENSG00000129467	ENSG00000078295
ENSG00000129467	ENSG00000121281
ENSG00000129467	ENSG00000138031
ENSG00000129467	ENSG00000155897
ENSG00000129467	ENSG00000162104
ENSG00000129467	ENSG00000164742
ENSG00000129467	ENSG00000173175
ENSG00000129467	ENSG00000174233
ENSG00000129473	ENSG00000030110
ENSG00000129473	ENSG00000087088
ENSG00000129473	ENSG00000140379
ENSG00000129473	ENSG00000143384
ENSG00000129473	ENSG00000171552
ENSG00000129473	ENSG00000171791
ENSG00000129473	ENSG00000176720
ENSG00000129474	ENSG00000087077
ENSG00000129474	ENSG00000142279
ENSG00000129474	ENSG00000144791
ENSG00000129474	ENSG00000145012
ENSG00000129474	ENSG00000159840
ENSG00000129474	ENSG00000162458
ENSG00000129493	ENSG00000008869
ENSG00000129514	ENSG00000054598
ENSG00000129514	ENSG00000125798
ENSG00000129514	ENSG00000164379
ENSG00000129514	ENSG00000168269
ENSG00000129514	ENSG00000170122
ENSG00000129514	ENSG00000170608
ENSG00000129514	ENSG00000176165
ENSG00000129514	ENSG00000176678
ENSG00000129514	ENSG00000176692
ENSG00000129514	ENSG00000178919
ENSG00000129514	ENSG00000179772
ENSG00000129514	ENSG00000183770
ENSG00000129514	ENSG00000184492
ENSG00000129514	ENSG00000184659
ENSG00000129514	ENSG00000186564
ENSG00000129514	ENSG00000186766
ENSG00000129514	ENSG00000186790
ENSG00000129514	ENSG00000187140
ENSG00000129514	ENSG00000187559
ENSG00000129514	ENSG00000204612
ENSG00000129514	ENSG00000204779
ENSG00000129514	ENSG00000204793
ENSG00000129514	ENSG00000204828
ENSG00000129514	ENSG00000214336
ENSG00000129515	ENSG00000028528
ENSG00000129515	ENSG00000089006
ENSG00000129515	ENSG00000172803

ENSG00000129515	ENSG00000205302
ENSG00000129521	ENSG00000135766
ENSG00000129521	ENSG00000171570
ENSG00000129535	ENSG00000178573
ENSG00000129535	ENSG00000182759
ENSG00000129535	ENSG00000185022
ENSG00000129535	ENSG00000197063
ENSG00000129535	ENSG00000198517
ENSG00000129535	ENSG00000204103
ENSG00000129535	ENSG00000215622
ENSG00000129543	ENSG00000100804
ENSG00000129543	ENSG00000204264
ENSG00000129625	ENSG00000115255
ENSG00000129654	ENSG00000065970
ENSG00000129654	ENSG00000111206
ENSG00000129654	ENSG00000198815
ENSG00000129657	ENSG00000100003
ENSG00000129657	ENSG00000100012
ENSG00000129657	ENSG00000103184
ENSG00000129657	ENSG00000133488
ENSG00000129667	ENSG00000007384
ENSG00000129667	ENSG00000163902
ENSG00000129675	ENSG00000102606
ENSG00000129675	ENSG00000187583
ENSG00000129680	ENSG00000116871
ENSG00000129680	ENSG00000135525
ENSG00000129680	ENSG00000184368
ENSG00000129682	ENSG00000070193
ENSG00000129682	ENSG00000070388
ENSG00000129682	ENSG00000075388
ENSG00000129682	ENSG00000078579
ENSG00000129682	ENSG00000102466
ENSG00000129682	ENSG00000102678
ENSG00000129682	ENSG00000111241
ENSG00000129682	ENSG00000113578
ENSG00000129682	ENSG00000114279
ENSG00000129682	ENSG00000138675
ENSG00000129682	ENSG00000138685
ENSG00000129682	ENSG00000140285
ENSG00000129682	ENSG00000161958
ENSG00000129682	ENSG00000185390
ENSG00000129682	ENSG00000186895
ENSG00000129682	ENSG00000196468
ENSG00000129682	ENSG00000204801
ENSG00000129682	ENSG00000204816
ENSG00000129682	ENSG00000204817
ENSG00000129682	ENSG00000204837
ENSG00000129682	ENSG00000215053
ENSG00000129749	ENSG00000080644
ENSG00000129749	ENSG00000101204
ENSG00000129749	ENSG00000108556
ENSG00000129749	ENSG00000117971
ENSG00000129749	ENSG00000120903
ENSG00000129749	ENSG00000135902
ENSG00000129749	ENSG00000138435
ENSG00000129749	ENSG00000147432
ENSG00000129749	ENSG00000147434
ENSG00000129749	ENSG00000149305
ENSG00000129749	ENSG00000160716

ENSG00000129749	ENSG00000166664
ENSG00000129749	ENSG00000166736
ENSG00000129749	ENSG00000169684
ENSG00000129749	ENSG00000170175
ENSG00000129749	ENSG00000174343
ENSG00000129749	ENSG00000175344
ENSG00000129749	ENSG00000178084
ENSG00000129749	ENSG00000186038
ENSG00000129749	ENSG00000186090
ENSG00000129749	ENSG00000186919
ENSG00000129749	ENSG00000196811
ENSG00000129873	ENSG00000153046
ENSG00000129873	ENSG00000166446
ENSG00000129873	ENSG00000172288
ENSG00000129873	ENSG00000172352
ENSG00000129873	ENSG00000182415
ENSG00000129873	ENSG00000198721
ENSG00000129873	ENSG00000215563
ENSG00000129910	ENSG00000039068
ENSG00000129910	ENSG00000062038
ENSG00000129910	ENSG00000079112
ENSG00000129910	ENSG00000124215
ENSG00000129910	ENSG00000134755
ENSG00000129910	ENSG00000134762
ENSG00000129910	ENSG00000134765
ENSG00000129910	ENSG00000140945
ENSG00000129910	ENSG00000166589
ENSG00000129910	ENSG00000170558
ENSG00000129910	ENSG00000179242
ENSG00000129925	ENSG00000137103
ENSG00000129946	ENSG00000148082
ENSG00000129946	ENSG00000160691
ENSG00000129946	ENSG00000185634
ENSG00000129951	ENSG00000067113
ENSG00000129951	ENSG00000105520
ENSG00000129951	ENSG00000117598
ENSG00000129951	ENSG00000117600
ENSG00000129951	ENSG00000141934
ENSG00000129951	ENSG00000148123
ENSG00000129951	ENSG00000162407
ENSG00000129965	ENSG00000017427
ENSG00000129965	ENSG00000167244
ENSG00000129968	ENSG00000107362
ENSG00000129968	ENSG00000136379
ENSG00000129968	ENSG00000182502
ENSG00000129968	ENSG00000182556
ENSG00000129968	ENSG00000198658
ENSG00000129968	ENSG00000203835
ENSG00000129988	ENSG00000078898
ENSG00000129988	ENSG00000100979
ENSG00000129988	ENSG00000101425
ENSG00000129988	ENSG00000167104
ENSG00000129988	ENSG00000184459
ENSG00000129988	ENSG00000186190
ENSG00000129988	ENSG00000186191
ENSG00000129990	ENSG00000011347
ENSG00000129990	ENSG00000019505
ENSG00000129990	ENSG00000067715
ENSG00000129990	ENSG00000101098

ENSG00000129990	ENSG00000103528
ENSG00000129990	ENSG00000110975
ENSG00000129990	ENSG00000132718
ENSG00000129990	ENSG00000132872
ENSG00000129990	ENSG00000134207
ENSG00000129990	ENSG00000143858
ENSG00000129990	ENSG00000149043
ENSG00000129990	ENSG00000149927
ENSG00000129990	ENSG00000170743
ENSG00000129990	ENSG00000173227
ENSG00000129990	ENSG00000204176
ENSG00000129990	ENSG00000213023
ENSG00000129990	ENSG00000214408
ENSG00000129990	ENSG00000214409
ENSG00000129990	ENSG00000214569
ENSG00000129990	ENSG00000214783
ENSG00000129991	ENSG00000130598
ENSG00000129991	ENSG00000159173
ENSG00000129993	ENSG00000078699
ENSG00000129993	ENSG00000079102
ENSG00000130032	ENSG00000126460
ENSG00000130032	ENSG00000130962
ENSG00000130032	ENSG00000135378
ENSG00000130037	ENSG00000026559
ENSG00000130037	ENSG00000102057
ENSG00000130037	ENSG00000104848
ENSG00000130037	ENSG00000111262
ENSG00000130037	ENSG00000124134
ENSG00000130037	ENSG00000143105
ENSG00000130037	ENSG00000151079
ENSG00000130037	ENSG00000156486
ENSG00000130037	ENSG00000158445
ENSG00000130037	ENSG00000162975
ENSG00000130037	ENSG00000164794
ENSG00000130037	ENSG00000168263
ENSG00000130037	ENSG00000168418
ENSG00000130037	ENSG00000170745
ENSG00000130037	ENSG00000171126
ENSG00000130037	ENSG00000171385
ENSG00000130037	ENSG00000177272
ENSG00000130037	ENSG00000177301
ENSG00000130037	ENSG00000178342
ENSG00000130037	ENSG00000182255
ENSG00000130037	ENSG00000182674
ENSG00000130037	ENSG00000184408
ENSG00000130038	ENSG00000177685
ENSG00000130041	ENSG00000163144
ENSG00000130041	ENSG00000177954
ENSG00000130041	ENSG00000185088
ENSG00000130041	ENSG00000198731
ENSG00000130045	ENSG00000167693
ENSG00000130045	ENSG00000171773
ENSG00000130052	ENSG00000088756
ENSG00000130052	ENSG00000124143
ENSG00000130052	ENSG00000133121
ENSG00000130052	ENSG00000146376
ENSG00000130052	ENSG00000164741
ENSG00000130054	ENSG00000204442
ENSG00000130055	ENSG00000006007

ENSG00000130055	ENSG00000158555
ENSG00000130055	ENSG00000178795
ENSG00000130066	ENSG00000141504
ENSG00000130066	ENSG00000184788
ENSG00000130119	ENSG00000163938
ENSG00000130147	ENSG00000183742
ENSG00000130158	ENSG00000088387
ENSG00000130158	ENSG00000107099
ENSG00000130158	ENSG00000116641
ENSG00000130158	ENSG00000135905
ENSG00000130158	ENSG00000147251
ENSG00000130164	ENSG00000147852
ENSG00000130164	ENSG00000157193
ENSG00000130176	ENSG00000064666
ENSG00000130176	ENSG00000117519
ENSG00000130176	ENSG00000144834
ENSG00000130176	ENSG00000149591
ENSG00000130176	ENSG00000158710
ENSG00000130176	ENSG00000204782
ENSG00000130176	ENSG00000204826
ENSG00000130176	ENSG00000215433
ENSG00000130201	ENSG00000179044
ENSG00000130201	ENSG00000180104
ENSG00000130201	ENSG00000185215
ENSG00000130201	ENSG00000205436
ENSG00000130202	ENSG00000073008
ENSG00000130202	ENSG00000110400
ENSG00000130202	ENSG00000177707
ENSG00000130203	ENSG00000110243
ENSG00000130203	ENSG00000110244
ENSG00000130203	ENSG00000118137
ENSG00000130203	ENSG00000215754
ENSG00000130203	ENSG00000215756
ENSG00000130204	ENSG00000158882
ENSG00000130222	ENSG00000099860
ENSG00000130222	ENSG00000112306
ENSG00000130222	ENSG00000116717
ENSG00000130222	ENSG00000170250
ENSG00000130224	ENSG00000077454
ENSG00000130224	ENSG00000136141
ENSG00000130224	ENSG00000186001
ENSG00000130226	ENSG00000074603
ENSG00000130226	ENSG00000078098
ENSG00000130226	ENSG00000142002
ENSG00000130226	ENSG00000175497
ENSG00000130226	ENSG00000197635
ENSG00000130227	ENSG00000204764
ENSG00000130244	ENSG00000119812
ENSG00000130244	ENSG00000171262
ENSG00000130254	ENSG00000137776
ENSG00000130254	ENSG00000160633
ENSG00000130287	ENSG00000038427
ENSG00000130287	ENSG00000132692
ENSG00000130287	ENSG00000132702
ENSG00000130287	ENSG00000140511
ENSG00000130287	ENSG00000145681
ENSG00000130287	ENSG00000157766
ENSG00000130287	ENSG00000187664
ENSG00000130294	ENSG00000054523

ENSG00000130294	ENSG00000089177
ENSG00000130294	ENSG00000118193
ENSG00000130294	ENSG00000129250
ENSG00000130294	ENSG00000137177
ENSG00000130294	ENSG00000197892
ENSG00000130304	ENSG00000083807
ENSG00000130304	ENSG00000113396
ENSG00000130304	ENSG00000140284
ENSG00000130304	ENSG00000143554
ENSG00000130304	ENSG00000167114
ENSG00000130309	ENSG00000083444
ENSG00000130309	ENSG00000106397
ENSG00000130309	ENSG00000152952
ENSG00000130309	ENSG00000167123
ENSG00000130309	ENSG00000198756
ENSG00000130377	ENSG00000103740
ENSG00000130382	ENSG00000127337
ENSG00000130382	ENSG00000163872
ENSG00000130382	ENSG00000171843
ENSG00000130383	ENSG00000156413
ENSG00000130383	ENSG00000171124
ENSG00000130383	ENSG00000172461
ENSG00000130383	ENSG00000172728
ENSG00000130383	ENSG00000180549
ENSG00000130383	ENSG00000196371
ENSG00000130383	ENSG00000196968
ENSG00000130385	ENSG00000101144
ENSG00000130385	ENSG00000107623
ENSG00000130385	ENSG00000112175
ENSG00000130385	ENSG00000116985
ENSG00000130385	ENSG00000125965
ENSG00000130385	ENSG00000128802
ENSG00000130385	ENSG00000143869
ENSG00000130385	ENSG00000152785
ENSG00000130385	ENSG00000153162
ENSG00000130385	ENSG00000156466
ENSG00000130385	ENSG00000156574
ENSG00000130385	ENSG00000163217
ENSG00000130385	ENSG00000183682
ENSG00000130402	ENSG00000072110
ENSG00000130402	ENSG00000077522
ENSG00000130402	ENSG00000204633
ENSG00000130429	ENSG00000013455
ENSG00000130433	ENSG00000108878
ENSG00000130449	ENSG00000132003
ENSG00000130449	ENSG00000162415
ENSG00000130449	ENSG00000214655
ENSG00000130475	ENSG00000007237
ENSG00000130475	ENSG00000118473
ENSG00000130475	ENSG00000157107
ENSG00000130477	ENSG00000137766
ENSG00000130477	ENSG00000198722
ENSG00000130479	ENSG00000131711
ENSG00000130479	ENSG00000166963
ENSG00000130487	ENSG00000003096
ENSG00000130487	ENSG00000087448
ENSG00000130487	ENSG00000124743
ENSG00000130487	ENSG00000135686
ENSG00000130487	ENSG00000166853

ENSG00000130487	ENSG00000167487
ENSG00000130487	ENSG00000174010
ENSG00000130487	ENSG00000176595
ENSG00000130487	ENSG00000179023
ENSG00000130487	ENSG00000185214
ENSG00000130487	ENSG00000185915
ENSG00000130487	ENSG00000186231
ENSG00000130487	ENSG00000197705
ENSG00000130487	ENSG00000198642
ENSG00000130489	ENSG00000133028
ENSG00000130489	ENSG00000214586
ENSG00000130508	ENSG00000005381
ENSG00000130508	ENSG00000115705
ENSG00000130508	ENSG00000121053
ENSG00000130508	ENSG00000147485
ENSG00000130508	ENSG00000167419
ENSG00000130511	ENSG00000145687
ENSG00000130511	ENSG00000157216
ENSG00000130511	ENSG00000214079
ENSG00000130511	ENSG00000214099
ENSG00000130511	ENSG00000215390
ENSG00000130511	ENSG00000215535
ENSG00000130517	ENSG00000183571
ENSG00000130522	ENSG00000115966
ENSG00000130522	ENSG00000146592
ENSG00000130522	ENSG00000170653
ENSG00000130522	ENSG00000171223
ENSG00000130522	ENSG00000177606
ENSG00000130529	ENSG00000070985
ENSG00000130529	ENSG00000083067
ENSG00000130529	ENSG00000092439
ENSG00000130529	ENSG00000119121
ENSG00000130529	ENSG00000134160
ENSG00000130529	ENSG00000142185
ENSG00000130529	ENSG00000144481
ENSG00000130540	ENSG00000088002
ENSG00000130540	ENSG00000105398
ENSG00000130540	ENSG00000109193
ENSG00000130540	ENSG00000138068
ENSG00000130540	ENSG00000173597
ENSG00000130540	ENSG00000196228
ENSG00000130540	ENSG00000196502
ENSG00000130540	ENSG00000197165
ENSG00000130540	ENSG00000198075
ENSG00000130540	ENSG00000198203
ENSG00000130540	ENSG00000213599
ENSG00000130540	ENSG00000213648
ENSG00000130558	ENSG00000034971
ENSG00000130558	ENSG00000102837
ENSG00000130558	ENSG00000105088
ENSG00000130558	ENSG00000118733
ENSG00000130558	ENSG00000183801
ENSG00000130558	ENSG00000186417
ENSG00000130558	ENSG00000215081
ENSG00000130559	ENSG00000076826
ENSG00000130559	ENSG00000118200
ENSG00000130561	ENSG00000120500
ENSG00000130561	ENSG00000137486
ENSG00000130561	ENSG00000141480

ENSG00000130590	ENSG00000047634
ENSG00000130590	ENSG00000177570
ENSG00000130590	ENSG00000203943
ENSG00000130595	ENSG00000105048
ENSG00000130595	ENSG00000118194
ENSG00000130598	ENSG00000129991
ENSG00000130598	ENSG00000159173
ENSG00000130635	ENSG00000060718
ENSG00000130635	ENSG00000080573
ENSG00000130635	ENSG00000108821
ENSG00000130635	ENSG00000139219
ENSG00000130635	ENSG00000164692
ENSG00000130635	ENSG00000168542
ENSG00000130635	ENSG00000204248
ENSG00000130635	ENSG00000204262
ENSG00000130643	ENSG00000168824
ENSG00000130643	ENSG00000170091
ENSG00000130653	ENSG00000032444
ENSG00000130656	ENSG00000086506
ENSG00000130656	ENSG00000188536
ENSG00000130656	ENSG00000206172
ENSG00000130656	ENSG00000206177
ENSG00000130656	ENSG00000206178
ENSG00000130669	ENSG00000101349
ENSG00000130669	ENSG00000137843
ENSG00000130675	ENSG00000164900
ENSG00000130675	ENSG00000168505
ENSG00000130695	ENSG00000111860
ENSG00000130699	ENSG00000141384
ENSG00000130700	ENSG00000102145
ENSG00000130700	ENSG00000107485
ENSG00000130700	ENSG00000136574
ENSG00000130700	ENSG00000141448
ENSG00000130700	ENSG00000179348
ENSG00000130702	ENSG00000053747
ENSG00000130702	ENSG00000101680
ENSG00000130702	ENSG00000112769
ENSG00000130702	ENSG00000196569
ENSG00000130723	ENSG00000117523
ENSG00000130723	ENSG00000204469
ENSG00000130726	ENSG00000122779
ENSG00000130726	ENSG00000166436
ENSG00000130726	ENSG00000197323
ENSG00000130733	ENSG00000058799
ENSG00000130734	ENSG00000101844
ENSG00000130734	ENSG00000125703
ENSG00000130734	ENSG00000168397
ENSG00000130734	ENSG00000215697
ENSG00000130749	ENSG00000188177
ENSG00000130751	ENSG00000100644
ENSG00000130751	ENSG00000112246
ENSG00000130751	ENSG00000116016
ENSG00000130751	ENSG00000124440
ENSG00000130751	ENSG00000151322
ENSG00000130751	ENSG00000159263
ENSG00000130755	ENSG00000197045
ENSG00000130758	ENSG00000006432
ENSG00000130758	ENSG00000143674
ENSG00000130758	ENSG00000173327

ENSG00000130762	ENSG00000066248
ENSG00000130762	ENSG00000114790
ENSG00000130762	ENSG00000142632
ENSG00000130762	ENSG00000198844
ENSG00000130766	ENSG00000080546
ENSG00000130766	ENSG00000149212
ENSG00000130775	ENSG00000172673
ENSG00000130779	ENSG00000105270
ENSG00000130779	ENSG00000106665
ENSG00000130779	ENSG00000115295
ENSG00000130787	ENSG00000127946
ENSG00000130821	ENSG00000010379
ENSG00000130821	ENSG00000011083
ENSG00000130821	ENSG00000087916
ENSG00000130821	ENSG00000103546
ENSG00000130821	ENSG00000108576
ENSG00000130821	ENSG00000111181
ENSG00000130821	ENSG00000131389
ENSG00000130821	ENSG00000132164
ENSG00000130821	ENSG00000142319
ENSG00000130821	ENSG00000157103
ENSG00000130821	ENSG00000163817
ENSG00000130821	ENSG00000164363
ENSG00000130821	ENSG00000165970
ENSG00000130821	ENSG00000174358
ENSG00000130821	ENSG00000196517
ENSG00000130821	ENSG00000198555
ENSG00000130821	ENSG00000214617
ENSG00000130822	ENSG00000008118
ENSG00000130822	ENSG00000081320
ENSG00000130822	ENSG00000134072
ENSG00000130822	ENSG00000147613
ENSG00000130822	ENSG00000159792
ENSG00000130822	ENSG00000164076
ENSG00000130822	ENSG00000164543
ENSG00000130822	ENSG00000183049
ENSG00000130827	ENSG00000004399
ENSG00000130827	ENSG00000076356
ENSG00000130827	ENSG00000114554
ENSG00000130827	ENSG00000136040
ENSG00000130827	ENSG00000164050
ENSG00000130827	ENSG00000189437
ENSG00000130827	ENSG00000196576
ENSG00000130827	ENSG00000198753
ENSG00000130829	ENSG00000111266
ENSG00000130829	ENSG00000120129
ENSG00000130829	ENSG00000120875
ENSG00000130829	ENSG00000138166
ENSG00000130829	ENSG00000139318
ENSG00000130829	ENSG00000143507
ENSG00000130829	ENSG00000158050
ENSG00000130829	ENSG00000164086
ENSG00000130829	ENSG00000184545
ENSG00000130876	ENSG00000021488
ENSG00000130876	ENSG00000092068
ENSG00000130876	ENSG00000103064
ENSG00000130876	ENSG00000103257
ENSG00000130876	ENSG00000151012
ENSG00000130876	ENSG00000155465

ENSG00000130876	ENSG00000164893
ENSG00000130881	ENSG00000147650
ENSG00000130881	ENSG00000197324
ENSG00000130943	ENSG00000008710
ENSG00000130943	ENSG00000078795
ENSG00000130943	ENSG00000107593
ENSG00000130943	ENSG00000118762
ENSG00000130943	ENSG00000158683
ENSG00000130943	ENSG00000166473
ENSG00000130943	ENSG00000183458
ENSG00000130943	ENSG00000183793
ENSG00000130943	ENSG00000187008
ENSG00000130943	ENSG00000188599
ENSG00000130948	ENSG00000103160
ENSG00000130948	ENSG00000149084
ENSG00000130956	ENSG00000142864
ENSG00000130957	ENSG00000165140
ENSG00000130958	ENSG00000116704
ENSG00000130958	ENSG00000182747
ENSG00000130962	ENSG00000126460
ENSG00000130962	ENSG00000130032
ENSG00000130962	ENSG00000135378
ENSG00000130985	ENSG00000033178
ENSG00000130985	ENSG00000182179
ENSG00000131023	ENSG00000150457
ENSG00000131037	ENSG00000151491
ENSG00000131037	ENSG00000177106
ENSG00000131037	ENSG00000198758
ENSG00000131051	ENSG00000100461
ENSG00000131055	ENSG00000131143
ENSG00000131080	ENSG00000127863
ENSG00000131080	ENSG00000135960
ENSG00000131089	ENSG00000136002
ENSG00000131089	ENSG00000182957
ENSG00000131094	ENSG00000054803
ENSG00000131094	ENSG00000102924
ENSG00000131094	ENSG00000133466
ENSG00000131094	ENSG00000139899
ENSG00000131094	ENSG00000141668
ENSG00000131094	ENSG00000144119
ENSG00000131094	ENSG00000145861
ENSG00000131094	ENSG00000159189
ENSG00000131094	ENSG00000163145
ENSG00000131094	ENSG00000165985
ENSG00000131094	ENSG00000172247
ENSG00000131094	ENSG00000173369
ENSG00000131094	ENSG00000173372
ENSG00000131094	ENSG00000173918
ENSG00000131094	ENSG00000181092
ENSG00000131094	ENSG00000184471
ENSG00000131094	ENSG00000186897
ENSG00000131094	ENSG00000213192
ENSG00000131095	ENSG00000026025
ENSG00000131095	ENSG00000100285
ENSG00000131095	ENSG00000104722
ENSG00000131095	ENSG00000104725
ENSG00000131095	ENSG00000135406
ENSG00000131095	ENSG00000148798
ENSG00000131095	ENSG00000175084

ENSG00000131095	ENSG00000182253
ENSG00000131095	ENSG00000215479
ENSG00000131096	ENSG00000108849
ENSG00000131096	ENSG00000122585
ENSG00000131096	ENSG00000204474
ENSG00000131097	ENSG00000146066
ENSG00000131097	ENSG00000175202
ENSG00000131097	ENSG00000181061
ENSG00000131097	ENSG00000212646
ENSG00000131097	ENSG00000214511
ENSG00000131143	ENSG00000131055
ENSG00000131148	ENSG00000100908
ENSG00000131171	ENSG00000142669
ENSG00000131171	ENSG00000185437
ENSG00000131171	ENSG00000198478
ENSG00000131171	ENSG00000214636
ENSG00000131183	ENSG00000157765
ENSG00000131183	ENSG00000198569
ENSG00000131187	ENSG00000019991
ENSG00000131187	ENSG00000104368
ENSG00000131187	ENSG00000109758
ENSG00000131187	ENSG00000122194
ENSG00000131187	ENSG00000122861
ENSG00000131187	ENSG00000148702
ENSG00000131187	ENSG00000173531
ENSG00000131187	ENSG00000186301
ENSG00000131187	ENSG00000186715
ENSG00000131187	ENSG00000198670
ENSG00000131196	ENSG00000072736
ENSG00000131196	ENSG00000100968
ENSG00000131196	ENSG00000101096
ENSG00000131196	ENSG00000102908
ENSG00000131203	ENSG00000188676
ENSG00000131233	ENSG00000121634
ENSG00000131233	ENSG00000121742
ENSG00000131233	ENSG00000121743
ENSG00000131233	ENSG00000135355
ENSG00000131233	ENSG00000143140
ENSG00000131233	ENSG00000152661
ENSG00000131233	ENSG00000159248
ENSG00000131233	ENSG00000164411
ENSG00000131233	ENSG00000165474
ENSG00000131233	ENSG00000169562
ENSG00000131233	ENSG00000176402
ENSG00000131233	ENSG00000177291
ENSG00000131233	ENSG00000182963
ENSG00000131233	ENSG00000183153
ENSG00000131233	ENSG00000187513
ENSG00000131233	ENSG00000188910
ENSG00000131233	ENSG00000189280
ENSG00000131233	ENSG00000189433
ENSG00000131233	ENSG00000198835
ENSG00000131233	ENSG00000203733
ENSG00000131236	ENSG00000112186
ENSG00000131242	ENSG00000090565
ENSG00000131263	ENSG00000127870
ENSG00000131263	ENSG00000137075
ENSG00000131263	ENSG00000146083
ENSG00000131264	ENSG00000113722

ENSG00000131264	ENSG00000165556
ENSG00000131323	ENSG00000056558
ENSG00000131323	ENSG00000076604
ENSG00000131323	ENSG00000082512
ENSG00000131323	ENSG00000127191
ENSG00000131323	ENSG00000175104
ENSG00000131355	ENSG00000069122
ENSG00000131355	ENSG00000123146
ENSG00000131355	ENSG00000127507
ENSG00000131355	ENSG00000153292
ENSG00000131355	ENSG00000153294
ENSG00000131355	ENSG00000162618
ENSG00000131355	ENSG00000164393
ENSG00000131355	ENSG00000173567
ENSG00000131355	ENSG00000174837
ENSG00000131355	ENSG00000186629
ENSG00000131355	ENSG00000204818
ENSG00000131370	ENSG00000175137
ENSG00000131378	ENSG00000162944
ENSG00000131398	ENSG00000116396
ENSG00000131398	ENSG00000129159
ENSG00000131398	ENSG00000166006
ENSG00000131400	ENSG00000096088
ENSG00000131400	ENSG00000117984
ENSG00000131400	ENSG00000131401
ENSG00000131400	ENSG00000143839
ENSG00000131400	ENSG00000172647
ENSG00000131400	ENSG00000180860
ENSG00000131400	ENSG00000184488
ENSG00000131400	ENSG00000196188
ENSG00000131400	ENSG00000204973
ENSG00000131400	ENSG00000215868
ENSG00000131401	ENSG00000096088
ENSG00000131401	ENSG00000117984
ENSG00000131401	ENSG00000131400
ENSG00000131401	ENSG00000143839
ENSG00000131401	ENSG00000172647
ENSG00000131401	ENSG00000180860
ENSG00000131401	ENSG00000184488
ENSG00000131401	ENSG00000196188
ENSG00000131401	ENSG00000204973
ENSG00000131401	ENSG00000215868
ENSG00000131408	ENSG00000025434
ENSG00000131409	ENSG00000148948
ENSG00000131435	ENSG00000107438
ENSG00000131435	ENSG00000120913
ENSG00000131435	ENSG00000122367
ENSG00000131435	ENSG00000154553
ENSG00000131435	ENSG00000163110
ENSG00000131435	ENSG00000196923
ENSG00000131435	ENSG00000214895
ENSG00000131459	ENSG00000198380
ENSG00000131462	ENSG00000037042
ENSG00000131467	ENSG00000092010
ENSG00000131467	ENSG00000100911
ENSG00000131467	ENSG00000174144
ENSG00000131471	ENSG00000002726
ENSG00000131471	ENSG00000131480
ENSG00000131477	ENSG00000122679

ENSG00000131477	ENSG00000132329
ENSG00000131480	ENSG00000002726
ENSG00000131480	ENSG00000131471
ENSG00000131482	ENSG00000141349
ENSG00000131482	ENSG00000152254
ENSG00000131503	ENSG00000132466
ENSG00000131503	ENSG00000165138
ENSG00000131504	ENSG00000139734
ENSG00000131504	ENSG00000147202
ENSG00000131507	ENSG00000102471
ENSG00000131507	ENSG00000197627
ENSG00000131508	ENSG00000072401
ENSG00000131508	ENSG00000078967
ENSG00000131508	ENSG00000109332
ENSG00000131508	ENSG00000184888
ENSG00000131584	ENSG00000072818
ENSG00000131584	ENSG00000114331
ENSG00000131620	ENSG00000047617
ENSG00000131620	ENSG00000074855
ENSG00000131620	ENSG00000134343
ENSG00000131620	ENSG00000146205
ENSG00000131620	ENSG00000151572
ENSG00000131620	ENSG00000160746
ENSG00000131620	ENSG00000171714
ENSG00000131620	ENSG00000177119
ENSG00000131620	ENSG00000185101
ENSG00000131626	ENSG00000110841
ENSG00000131626	ENSG00000139220
ENSG00000131626	ENSG00000143847
ENSG00000131626	ENSG00000166387
ENSG00000131626	ENSG00000177380
ENSG00000131650	ENSG00000100100
ENSG00000131650	ENSG00000183762
ENSG00000131668	ENSG00000043039
ENSG00000131668	ENSG00000109851
ENSG00000131668	ENSG00000136630
ENSG00000131668	ENSG00000185610
ENSG00000131668	ENSG00000188909
ENSG00000131669	ENSG00000171840
ENSG00000131686	ENSG00000074410
ENSG00000131686	ENSG00000104267
ENSG00000131686	ENSG00000107159
ENSG00000131686	ENSG00000118298
ENSG00000131686	ENSG00000133742
ENSG00000131686	ENSG00000164879
ENSG00000131686	ENSG00000167434
ENSG00000131686	ENSG00000168748
ENSG00000131686	ENSG00000169239
ENSG00000131686	ENSG00000174990
ENSG00000131686	ENSG00000178538
ENSG00000131686	ENSG00000185015
ENSG00000131686	ENSG00000214729
ENSG00000131686	ENSG00000214873
ENSG00000131686	ENSG00000215487
ENSG00000131686	ENSG00000215501
ENSG00000131686	ENSG00000215543
ENSG00000131711	ENSG00000130479
ENSG00000131711	ENSG00000166963
ENSG00000131724	ENSG00000091181

ENSG00000131724	ENSG00000123496
ENSG00000131724	ENSG00000185291
ENSG00000131724	ENSG00000198223
ENSG00000131730	ENSG00000104879
ENSG00000131730	ENSG00000166165
ENSG00000131730	ENSG00000166998
ENSG00000131730	ENSG00000168775
ENSG00000131737	ENSG00000006059
ENSG00000131737	ENSG00000094796
ENSG00000131737	ENSG00000108244
ENSG00000131737	ENSG00000108417
ENSG00000131737	ENSG00000108759
ENSG00000131737	ENSG00000111057
ENSG00000131737	ENSG00000126337
ENSG00000131737	ENSG00000128422
ENSG00000131737	ENSG00000131738
ENSG00000131737	ENSG00000131885
ENSG00000131737	ENSG00000167916
ENSG00000131737	ENSG00000170819
ENSG00000131737	ENSG00000171345
ENSG00000131737	ENSG00000171346
ENSG00000131737	ENSG00000171360
ENSG00000131737	ENSG00000171401
ENSG00000131737	ENSG00000171403
ENSG00000131737	ENSG00000171431
ENSG00000131737	ENSG00000171446
ENSG00000131737	ENSG00000173908
ENSG00000131737	ENSG00000186393
ENSG00000131737	ENSG00000186395
ENSG00000131737	ENSG00000186831
ENSG00000131737	ENSG00000186832
ENSG00000131737	ENSG00000186847
ENSG00000131737	ENSG00000187242
ENSG00000131737	ENSG00000187686
ENSG00000131737	ENSG00000196859
ENSG00000131737	ENSG00000197079
ENSG00000131737	ENSG00000204889
ENSG00000131737	ENSG00000204897
ENSG00000131737	ENSG00000213951
ENSG00000131737	ENSG00000214220
ENSG00000131737	ENSG00000214454
ENSG00000131737	ENSG00000214959
ENSG00000131737	ENSG00000215772
ENSG00000131737	ENSG00000215773
ENSG00000131738	ENSG00000006059
ENSG00000131738	ENSG00000094796
ENSG00000131738	ENSG00000108244
ENSG00000131738	ENSG00000108417
ENSG00000131738	ENSG00000108759
ENSG00000131738	ENSG00000111057
ENSG00000131738	ENSG00000126337
ENSG00000131738	ENSG00000128422
ENSG00000131738	ENSG00000131737
ENSG00000131738	ENSG00000131885
ENSG00000131738	ENSG00000167916
ENSG00000131738	ENSG00000170819
ENSG00000131738	ENSG00000171345
ENSG00000131738	ENSG00000171346
ENSG00000131738	ENSG00000171360

ENSG00000131738	ENSG00000171401
ENSG00000131738	ENSG00000171403
ENSG00000131738	ENSG00000171431
ENSG00000131738	ENSG00000171446
ENSG00000131738	ENSG00000173908
ENSG00000131738	ENSG00000186393
ENSG00000131738	ENSG00000186395
ENSG00000131738	ENSG00000186831
ENSG00000131738	ENSG00000186832
ENSG00000131738	ENSG00000186847
ENSG00000131738	ENSG00000187242
ENSG00000131738	ENSG00000187686
ENSG00000131738	ENSG00000196859
ENSG00000131738	ENSG00000197079
ENSG00000131738	ENSG00000204889
ENSG00000131738	ENSG00000204897
ENSG00000131738	ENSG00000213951
ENSG00000131738	ENSG00000214220
ENSG00000131738	ENSG00000214454
ENSG00000131738	ENSG00000214959
ENSG00000131738	ENSG00000215772
ENSG00000131738	ENSG00000215773
ENSG00000131746	ENSG00000079308
ENSG00000131746	ENSG00000111077
ENSG00000131746	ENSG00000136205
ENSG00000131747	ENSG00000077097
ENSG00000131748	ENSG00000147465
ENSG00000131759	ENSG00000077092
ENSG00000131759	ENSG00000126351
ENSG00000131759	ENSG00000126368
ENSG00000131759	ENSG00000151090
ENSG00000131759	ENSG00000172819
ENSG00000131759	ENSG00000174738
ENSG00000131771	ENSG00000135447
ENSG00000131771	ENSG00000150722
ENSG00000131773	ENSG00000112232
ENSG00000131773	ENSG00000112531
ENSG00000131773	ENSG00000121774
ENSG00000131779	ENSG00000166821
ENSG00000131788	ENSG00000033800
ENSG00000131788	ENSG00000078043
ENSG00000131788	ENSG00000105229
ENSG00000131791	ENSG00000111725
ENSG00000131808	ENSG00000104818
ENSG00000131808	ENSG00000104826
ENSG00000131808	ENSG00000104827
ENSG00000131808	ENSG00000134200
ENSG00000131808	ENSG00000179600
ENSG00000131808	ENSG00000189052
ENSG00000131808	ENSG00000196337
ENSG00000131808	ENSG00000204748
ENSG00000131808	ENSG00000213030
ENSG00000131864	ENSG00000134588
ENSG00000131864	ENSG00000135913
ENSG00000131873	ENSG00000033100
ENSG00000131873	ENSG00000123989
ENSG00000131873	ENSG00000139044
ENSG00000131873	ENSG00000147408
ENSG00000131873	ENSG00000169826

ENSG00000131873	ENSG00000182272
ENSG00000131873	ENSG00000198108
ENSG00000131885	ENSG00000006059
ENSG00000131885	ENSG00000094796
ENSG00000131885	ENSG00000108244
ENSG00000131885	ENSG00000108417
ENSG00000131885	ENSG00000108759
ENSG00000131885	ENSG00000111057
ENSG00000131885	ENSG00000126337
ENSG00000131885	ENSG00000128422
ENSG00000131885	ENSG00000131737
ENSG00000131885	ENSG00000131738
ENSG00000131885	ENSG00000167916
ENSG00000131885	ENSG00000170819
ENSG00000131885	ENSG00000171345
ENSG00000131885	ENSG00000171346
ENSG00000131885	ENSG00000171360
ENSG00000131885	ENSG00000171401
ENSG00000131885	ENSG00000171403
ENSG00000131885	ENSG00000171431
ENSG00000131885	ENSG00000171446
ENSG00000131885	ENSG00000173908
ENSG00000131885	ENSG00000186393
ENSG00000131885	ENSG00000186395
ENSG00000131885	ENSG00000186831
ENSG00000131885	ENSG00000186832
ENSG00000131885	ENSG00000186847
ENSG00000131885	ENSG00000187242
ENSG00000131885	ENSG00000187686
ENSG00000131885	ENSG00000196859
ENSG00000131885	ENSG00000197079
ENSG00000131885	ENSG00000204889
ENSG00000131885	ENSG00000204897
ENSG00000131885	ENSG00000213951
ENSG00000131885	ENSG00000214220
ENSG00000131885	ENSG00000214454
ENSG00000131885	ENSG00000214959
ENSG00000131885	ENSG00000215772
ENSG00000131885	ENSG00000215773
ENSG00000131899	ENSG00000073350
ENSG00000131899	ENSG00000145087
ENSG00000131899	ENSG00000164506
ENSG00000131910	ENSG00000169297
ENSG00000131914	ENSG00000187772
ENSG00000131931	ENSG00000041988
ENSG00000131931	ENSG00000129028
ENSG00000131931	ENSG00000161277
ENSG00000131931	ENSG00000173451
ENSG00000131941	ENSG00000076201
ENSG00000131941	ENSG00000158106
ENSG00000131941	ENSG00000170248
ENSG00000131941	ENSG00000215407
ENSG00000131969	ENSG00000100997
ENSG00000131969	ENSG00000139826
ENSG00000131981	ENSG00000006659
ENSG00000131981	ENSG00000100079
ENSG00000131981	ENSG00000100097
ENSG00000131981	ENSG00000105198
ENSG00000131981	ENSG00000105205

ENSG00000131981	ENSG00000116977
ENSG00000131981	ENSG00000119862
ENSG00000131981	ENSG00000133317
ENSG00000131981	ENSG00000168961
ENSG00000131981	ENSG00000170298
ENSG00000131981	ENSG00000171747
ENSG00000131981	ENSG00000171916
ENSG00000131981	ENSG00000178934
ENSG00000131981	ENSG00000197778
ENSG00000131981	ENSG00000205076
ENSG00000131981	ENSG00000215132
ENSG00000131982	ENSG00000156587
ENSG00000131982	ENSG00000185651
ENSG00000132000	ENSG00000174348
ENSG00000132002	ENSG00000137094
ENSG00000132002	ENSG00000162616
ENSG00000132002	ENSG00000187726
ENSG00000132003	ENSG00000130449
ENSG00000132003	ENSG00000162415
ENSG00000132003	ENSG00000214655
ENSG00000132005	ENSG00000080298
ENSG00000132005	ENSG00000087903
ENSG00000132005	ENSG00000111783
ENSG00000132005	ENSG00000143390
ENSG00000132005	ENSG00000181827
ENSG00000132005	ENSG00000185002
ENSG00000132024	ENSG00000154222
ENSG00000132031	ENSG00000124159
ENSG00000132031	ENSG00000132561
ENSG00000132031	ENSG00000162510
ENSG00000132031	ENSG00000172752
ENSG00000132130	ENSG00000016082
ENSG00000132130	ENSG00000089116
ENSG00000132130	ENSG00000106689
ENSG00000132130	ENSG00000106852
ENSG00000132130	ENSG00000107187
ENSG00000132130	ENSG00000121454
ENSG00000132130	ENSG00000136944
ENSG00000132130	ENSG00000143013
ENSG00000132130	ENSG00000143355
ENSG00000132130	ENSG00000159556
ENSG00000132130	ENSG00000162624
ENSG00000132130	ENSG00000162761
ENSG00000132139	ENSG00000139354
ENSG00000132139	ENSG00000148935
ENSG00000132139	ENSG00000185340
ENSG00000132142	ENSG00000076555
ENSG00000132155	ENSG00000078061
ENSG00000132155	ENSG00000141068
ENSG00000132155	ENSG00000157764
ENSG00000132155	ENSG00000171435
ENSG00000132164	ENSG00000010379
ENSG00000132164	ENSG00000011083
ENSG00000132164	ENSG00000087916
ENSG00000132164	ENSG00000103546
ENSG00000132164	ENSG00000108576
ENSG00000132164	ENSG00000111181
ENSG00000132164	ENSG00000130821
ENSG00000132164	ENSG00000131389

ENSG00000132164	ENSG00000142319
ENSG00000132164	ENSG00000157103
ENSG00000132164	ENSG00000163817
ENSG00000132164	ENSG00000164363
ENSG00000132164	ENSG00000165970
ENSG00000132164	ENSG00000174358
ENSG00000132164	ENSG00000196517
ENSG00000132164	ENSG00000198555
ENSG00000132164	ENSG00000214617
ENSG00000132170	ENSG00000012504
ENSG00000132170	ENSG00000111424
ENSG00000132170	ENSG00000112033
ENSG00000132170	ENSG00000143257
ENSG00000132170	ENSG00000144852
ENSG00000132170	ENSG00000186951
ENSG00000132185	ENSG00000072694
ENSG00000132185	ENSG00000143226
ENSG00000132185	ENSG00000150337
ENSG00000132185	ENSG00000162747
ENSG00000132185	ENSG00000179639
ENSG00000132185	ENSG00000181036
ENSG00000132185	ENSG00000198019
ENSG00000132185	ENSG00000203747
ENSG00000132185	ENSG00000203820
ENSG00000132205	ENSG00000138080
ENSG00000132205	ENSG00000183798
ENSG00000132254	ENSG00000100151
ENSG00000132254	ENSG00000164144
ENSG00000132256	ENSG00000026950
ENSG00000132256	ENSG00000111801
ENSG00000132256	ENSG00000112763
ENSG00000132256	ENSG00000113303
ENSG00000132256	ENSG00000121236
ENSG00000132256	ENSG00000124508
ENSG00000132256	ENSG00000124549
ENSG00000132256	ENSG00000124557
ENSG00000132256	ENSG00000128250
ENSG00000132256	ENSG00000128253
ENSG00000132256	ENSG00000128276
ENSG00000132256	ENSG00000128482
ENSG00000132256	ENSG00000132274
ENSG00000132256	ENSG00000164010
ENSG00000132256	ENSG00000165810
ENSG00000132256	ENSG00000168903
ENSG00000132256	ENSG00000176422
ENSG00000132256	ENSG00000179046
ENSG00000132256	ENSG00000182805
ENSG00000132256	ENSG00000183439
ENSG00000132256	ENSG00000183718
ENSG00000132256	ENSG00000187574
ENSG00000132256	ENSG00000188683
ENSG00000132256	ENSG00000204614
ENSG00000132256	ENSG00000204616
ENSG00000132256	ENSG00000204618
ENSG00000132256	ENSG00000205198
ENSG00000132256	ENSG00000214693
ENSG00000132259	ENSG00000070729
ENSG00000132259	ENSG00000099822
ENSG00000132259	ENSG00000138622

ENSG00000132259	ENSG00000143630
ENSG00000132259	ENSG00000144191
ENSG00000132259	ENSG00000164588
ENSG00000132259	ENSG00000170289
ENSG00000132259	ENSG00000183862
ENSG00000132274	ENSG00000026950
ENSG00000132274	ENSG00000111801
ENSG00000132274	ENSG00000112763
ENSG00000132274	ENSG00000113303
ENSG00000132274	ENSG00000121236
ENSG00000132274	ENSG00000124508
ENSG00000132274	ENSG00000124549
ENSG00000132274	ENSG00000124557
ENSG00000132274	ENSG00000128250
ENSG00000132274	ENSG00000128253
ENSG00000132274	ENSG00000128276
ENSG00000132274	ENSG00000128482
ENSG00000132274	ENSG00000132256
ENSG00000132274	ENSG00000164010
ENSG00000132274	ENSG00000165810
ENSG00000132274	ENSG00000168903
ENSG00000132274	ENSG00000176422
ENSG00000132274	ENSG00000179046
ENSG00000132274	ENSG00000182805
ENSG00000132274	ENSG00000183439
ENSG00000132274	ENSG00000183718
ENSG00000132274	ENSG00000187574
ENSG00000132274	ENSG00000188683
ENSG00000132274	ENSG00000204614
ENSG00000132274	ENSG00000204616
ENSG00000132274	ENSG00000204618
ENSG00000132274	ENSG00000205198
ENSG00000132274	ENSG00000214693
ENSG00000132294	ENSG00000084710
ENSG00000132321	ENSG00000183016
ENSG00000132326	ENSG00000049246
ENSG00000132326	ENSG00000179094
ENSG00000132329	ENSG00000122679
ENSG00000132329	ENSG00000131477
ENSG00000132334	ENSG00000081237
ENSG00000132334	ENSG00000132670
ENSG00000132356	ENSG00000162409
ENSG00000132356	ENSG00000165304
ENSG00000132357	ENSG00000106608
ENSG00000132357	ENSG00000187878
ENSG00000132357	ENSG00000205365
ENSG00000132359	ENSG00000076864
ENSG00000132359	ENSG00000105738
ENSG00000132359	ENSG00000116991
ENSG00000132359	ENSG00000136895
ENSG00000132359	ENSG00000197555
ENSG00000132359	ENSG00000213445
ENSG00000132376	ENSG00000122126
ENSG00000132376	ENSG00000185133
ENSG00000132376	ENSG00000204084
ENSG00000132386	ENSG00000149131
ENSG00000132386	ENSG00000167711
ENSG00000132388	ENSG00000184787
ENSG00000132405	ENSG00000108239

ENSG00000132429	ENSG00000112276
ENSG00000132429	ENSG00000121577
ENSG00000132434	ENSG00000115365
ENSG00000132434	ENSG00000147036
ENSG00000132446	ENSG00000087086
ENSG00000132446	ENSG00000167996
ENSG00000132446	ENSG00000171878
ENSG00000132446	ENSG00000173477
ENSG00000132446	ENSG00000176778
ENSG00000132446	ENSG00000180795
ENSG00000132446	ENSG00000181867
ENSG00000132446	ENSG00000197518
ENSG00000132446	ENSG00000215234
ENSG00000132463	ENSG00000096746
ENSG00000132463	ENSG00000103067
ENSG00000132463	ENSG00000104413
ENSG00000132463	ENSG00000126945
ENSG00000132463	ENSG00000169045
ENSG00000132463	ENSG00000169813
ENSG00000132466	ENSG00000131503
ENSG00000132466	ENSG00000165138
ENSG00000132471	ENSG00000183066
ENSG00000132471	ENSG00000186433
ENSG00000132478	ENSG00000059145
ENSG00000132481	ENSG00000121060
ENSG00000132481	ENSG00000137699
ENSG00000132507	ENSG00000163577
ENSG00000132507	ENSG00000196513
ENSG00000132510	ENSG00000147050
ENSG00000132510	ENSG00000183878
ENSG00000132517	ENSG00000101276
ENSG00000132517	ENSG00000185803
ENSG00000132518	ENSG00000070019
ENSG00000132518	ENSG00000101890
ENSG00000132518	ENSG00000123201
ENSG00000132518	ENSG00000159899
ENSG00000132518	ENSG00000164116
ENSG00000132518	ENSG00000169418
ENSG00000132530	ENSG00000135148
ENSG00000132535	ENSG00000075711
ENSG00000132535	ENSG00000082458
ENSG00000132535	ENSG00000150672
ENSG00000132535	ENSG00000151208
ENSG00000132561	ENSG00000124159
ENSG00000132561	ENSG00000132031
ENSG00000132561	ENSG00000162510
ENSG00000132561	ENSG00000172752
ENSG00000132563	ENSG00000068615
ENSG00000132563	ENSG00000165476
ENSG00000132563	ENSG00000168476
ENSG00000132570	ENSG00000166228
ENSG00000132581	ENSG00000128228
ENSG00000132604	ENSG00000147601
ENSG00000132604	ENSG00000184862
ENSG00000132604	ENSG00000187589
ENSG00000132612	ENSG00000119541
ENSG00000132613	ENSG00000170873
ENSG00000132622	ENSG00000165868
ENSG00000132639	ENSG00000092531

ENSG00000132640	ENSG00000064726
ENSG00000132640	ENSG00000133243
ENSG00000132640	ENSG00000184887
ENSG00000132669	ENSG00000100599
ENSG00000132669	ENSG00000174791
ENSG00000132669	ENSG00000187994
ENSG00000132670	ENSG00000081237
ENSG00000132670	ENSG00000132334
ENSG00000132671	ENSG00000082556
ENSG00000132671	ENSG00000112038
ENSG00000132671	ENSG00000116014
ENSG00000132671	ENSG00000116329
ENSG00000132671	ENSG00000125510
ENSG00000132671	ENSG00000125522
ENSG00000132671	ENSG00000128285
ENSG00000132671	ENSG00000128310
ENSG00000132671	ENSG00000139874
ENSG00000132671	ENSG00000152034
ENSG00000132671	ENSG00000162009
ENSG00000132671	ENSG00000166573
ENSG00000132671	ENSG00000180616
ENSG00000132671	ENSG00000182687
ENSG00000132671	ENSG00000183473
ENSG00000132671	ENSG00000183729
ENSG00000132677	ENSG00000112077
ENSG00000132677	ENSG00000140519
ENSG00000132677	ENSG00000187010
ENSG00000132677	ENSG00000188672
ENSG00000132681	ENSG00000018625
ENSG00000132681	ENSG00000075673
ENSG00000132681	ENSG00000105409
ENSG00000132681	ENSG00000105675
ENSG00000132681	ENSG00000163399
ENSG00000132692	ENSG00000038427
ENSG00000132692	ENSG00000130287
ENSG00000132692	ENSG00000132702
ENSG00000132692	ENSG00000140511
ENSG00000132692	ENSG00000145681
ENSG00000132692	ENSG00000157766
ENSG00000132692	ENSG00000187664
ENSG00000132693	ENSG00000106236
ENSG00000132693	ENSG00000132703
ENSG00000132693	ENSG00000163661
ENSG00000132693	ENSG00000167957
ENSG00000132693	ENSG00000171246
ENSG00000132693	ENSG00000215846
ENSG00000132694	ENSG00000076928
ENSG00000132694	ENSG00000196914
ENSG00000132702	ENSG00000038427
ENSG00000132702	ENSG00000130287
ENSG00000132702	ENSG00000132692
ENSG00000132702	ENSG00000140511
ENSG00000132702	ENSG00000145681
ENSG00000132702	ENSG00000157766
ENSG00000132702	ENSG00000187664
ENSG00000132703	ENSG00000106236
ENSG00000132703	ENSG00000132693
ENSG00000132703	ENSG00000163661
ENSG00000132703	ENSG00000167957

ENSG00000132703	ENSG00000171246
ENSG00000132703	ENSG00000215846
ENSG00000132718	ENSG00000011347
ENSG00000132718	ENSG00000019505
ENSG00000132718	ENSG00000067715
ENSG00000132718	ENSG00000101098
ENSG00000132718	ENSG00000103528
ENSG00000132718	ENSG00000110975
ENSG00000132718	ENSG00000129990
ENSG00000132718	ENSG00000132872
ENSG00000132718	ENSG00000134207
ENSG00000132718	ENSG00000143858
ENSG00000132718	ENSG00000149043
ENSG00000132718	ENSG00000149927
ENSG00000132718	ENSG00000170743
ENSG00000132718	ENSG00000173227
ENSG00000132718	ENSG00000204176
ENSG00000132718	ENSG00000213023
ENSG00000132718	ENSG00000214408
ENSG00000132718	ENSG00000214409
ENSG00000132718	ENSG00000214569
ENSG00000132718	ENSG00000214783
ENSG00000132744	ENSG00000108381
ENSG00000132746	ENSG00000006534
ENSG00000132746	ENSG00000072210
ENSG00000132746	ENSG00000108602
ENSG00000132793	ENSG00000101577
ENSG00000132793	ENSG00000134324
ENSG00000132819	ENSG00000112183
ENSG00000132821	ENSG00000170419
ENSG00000132821	ENSG00000187135
ENSG00000132824	ENSG00000111897
ENSG00000132824	ENSG00000164300
ENSG00000132824	ENSG00000168528
ENSG00000132824	ENSG00000184716
ENSG00000132825	ENSG00000100829
ENSG00000132825	ENSG00000119938
ENSG00000132825	ENSG00000154415
ENSG00000132825	ENSG00000173281
ENSG00000132842	ENSG00000103723
ENSG00000132849	ENSG00000072201
ENSG00000132849	ENSG00000107186
ENSG00000132849	ENSG00000139517
ENSG00000132854	ENSG00000107104
ENSG00000132854	ENSG00000186994
ENSG00000132854	ENSG00000197256
ENSG00000132872	ENSG00000011347
ENSG00000132872	ENSG00000019505
ENSG00000132872	ENSG00000067715
ENSG00000132872	ENSG00000101098
ENSG00000132872	ENSG00000103528
ENSG00000132872	ENSG00000110975
ENSG00000132872	ENSG00000129990
ENSG00000132872	ENSG00000132718
ENSG00000132872	ENSG00000134207
ENSG00000132872	ENSG00000143858
ENSG00000132872	ENSG00000149043
ENSG00000132872	ENSG00000149927
ENSG00000132872	ENSG00000170743

ENSG00000132872	ENSG00000173227
ENSG00000132872	ENSG00000204176
ENSG00000132872	ENSG00000213023
ENSG00000132872	ENSG00000214408
ENSG00000132872	ENSG00000214409
ENSG00000132872	ENSG00000214569
ENSG00000132872	ENSG00000214783
ENSG00000132879	ENSG00000116661
ENSG00000132879	ENSG00000116663
ENSG00000132879	ENSG00000161241
ENSG00000132879	ENSG00000161243
ENSG00000132879	ENSG00000188505
ENSG00000132911	ENSG00000101188
ENSG00000132911	ENSG00000174417
ENSG00000132915	ENSG00000095464
ENSG00000132915	ENSG00000112541
ENSG00000132915	ENSG00000128655
ENSG00000132915	ENSG00000133256
ENSG00000132915	ENSG00000138735
ENSG00000132915	ENSG00000186642
ENSG00000132932	ENSG00000081923
ENSG00000132932	ENSG00000104043
ENSG00000132932	ENSG00000124406
ENSG00000132932	ENSG00000130270
ENSG00000132932	ENSG00000143515
ENSG00000132938	ENSG00000129422
ENSG00000132938	ENSG00000198624
ENSG00000132950	ENSG00000121741
ENSG00000132950	ENSG00000146463
ENSG00000132950	ENSG00000147130
ENSG00000132950	ENSG00000163867
ENSG00000132950	ENSG00000198218
ENSG00000132964	ENSG00000155111
ENSG00000132965	ENSG00000085871
ENSG00000132965	ENSG00000143198
ENSG00000132965	ENSG00000213316
ENSG00000132967	ENSG00000029993
ENSG00000132967	ENSG00000100118
ENSG00000132967	ENSG00000124097
ENSG00000132967	ENSG00000164104
ENSG00000132967	ENSG00000173961
ENSG00000132967	ENSG00000176256
ENSG00000132967	ENSG00000180212
ENSG00000132967	ENSG00000182181
ENSG00000132967	ENSG00000182711
ENSG00000132967	ENSG00000189403
ENSG00000132967	ENSG00000214364
ENSG00000132967	ENSG00000214852
ENSG00000132967	ENSG00000215308
ENSG00000132970	ENSG00000112290
ENSG00000132970	ENSG00000158195
ENSG00000132970	ENSG00000188459
ENSG00000132972	ENSG00000095627
ENSG00000132972	ENSG00000180113
ENSG00000132972	ENSG00000182134
ENSG00000132972	ENSG00000196116
ENSG00000132975	ENSG00000064547
ENSG00000132975	ENSG00000118432
ENSG00000132975	ENSG00000125910

ENSG00000132975	ENSG00000146360
ENSG00000132975	ENSG00000170989
ENSG00000132975	ENSG00000171517
ENSG00000132975	ENSG00000175898
ENSG00000132975	ENSG00000180739
ENSG00000132975	ENSG00000181773
ENSG00000132975	ENSG00000188822
ENSG00000132975	ENSG00000198121
ENSG00000132975	ENSG00000213694
ENSG00000133019	ENSG00000168539
ENSG00000133019	ENSG00000181072
ENSG00000133019	ENSG00000184984
ENSG00000133020	ENSG00000002079
ENSG00000133020	ENSG00000006788
ENSG00000133020	ENSG00000078814
ENSG00000133020	ENSG00000092054
ENSG00000133020	ENSG00000109061
ENSG00000133020	ENSG00000109063
ENSG00000133020	ENSG00000125414
ENSG00000133020	ENSG00000141048
ENSG00000133020	ENSG00000144821
ENSG00000133020	ENSG00000197616
ENSG00000133026	ENSG00000100345
ENSG00000133026	ENSG00000105357
ENSG00000133026	ENSG00000133392
ENSG00000133028	ENSG00000130489
ENSG00000133028	ENSG00000214586
ENSG00000133030	ENSG00000214820
ENSG00000133048	ENSG00000064886
ENSG00000133048	ENSG00000085465
ENSG00000133048	ENSG00000133063
ENSG00000133048	ENSG00000134216
ENSG00000133048	ENSG00000203878
ENSG00000133056	ENSG00000011405
ENSG00000133056	ENSG00000051382
ENSG00000133056	ENSG00000105851
ENSG00000133056	ENSG00000121879
ENSG00000133056	ENSG00000139144
ENSG00000133056	ENSG00000171608
ENSG00000133063	ENSG00000064886
ENSG00000133063	ENSG00000085465
ENSG00000133063	ENSG00000133048
ENSG00000133063	ENSG00000134216
ENSG00000133063	ENSG00000203878
ENSG00000133065	ENSG00000114544
ENSG00000133065	ENSG00000136052
ENSG00000133067	ENSG00000138039
ENSG00000133067	ENSG00000139292
ENSG00000133067	ENSG00000165409
ENSG00000133067	ENSG00000170820
ENSG00000133067	ENSG00000205213
ENSG00000133069	ENSG00000057704
ENSG00000133069	ENSG00000102080
ENSG00000133069	ENSG00000172765
ENSG00000133069	ENSG00000182242
ENSG00000133069	ENSG00000185254
ENSG00000133083	ENSG00000077279
ENSG00000133083	ENSG00000104237
ENSG00000133083	ENSG00000130413

ENSG00000133083	ENSG00000146038
ENSG00000133083	ENSG00000163673
ENSG00000133083	ENSG00000170390
ENSG00000133083	ENSG00000183638
ENSG00000133101	ENSG00000145386
ENSG00000133105	ENSG00000171509
ENSG00000133107	ENSG00000069018
ENSG00000133107	ENSG00000072315
ENSG00000133107	ENSG00000137672
ENSG00000133107	ENSG00000138741
ENSG00000133107	ENSG00000144935
ENSG00000133110	ENSG00000120708
ENSG00000133115	ENSG00000067221
ENSG00000133115	ENSG00000116218
ENSG00000133115	ENSG00000148175
ENSG00000133115	ENSG00000165283
ENSG00000133116	ENSG00000115850
ENSG00000133116	ENSG00000134962
ENSG00000133116	ENSG00000176201
ENSG00000133116	ENSG00000188501
ENSG00000133121	ENSG00000088756
ENSG00000133121	ENSG00000124143
ENSG00000133121	ENSG00000130052
ENSG00000133121	ENSG00000146376
ENSG00000133121	ENSG00000164741
ENSG00000133124	ENSG00000169047
ENSG00000133124	ENSG00000185950
ENSG00000133131	ENSG00000114487
ENSG00000133131	ENSG00000133422
ENSG00000133131	ENSG00000159256
ENSG00000133135	ENSG00000082996
ENSG00000133135	ENSG00000105428
ENSG00000133135	ENSG00000108523
ENSG00000133135	ENSG00000113269
ENSG00000133135	ENSG00000163162
ENSG00000133135	ENSG00000170153
ENSG00000133135	ENSG00000188050
ENSG00000133136	ENSG00000127588
ENSG00000133136	ENSG00000127920
ENSG00000133136	ENSG00000127928
ENSG00000133136	ENSG00000162188
ENSG00000133136	ENSG00000167083
ENSG00000133136	ENSG00000167414
ENSG00000133136	ENSG00000168243
ENSG00000133136	ENSG00000172380
ENSG00000133136	ENSG00000174021
ENSG00000133136	ENSG00000182625
ENSG00000133136	ENSG00000186469
ENSG00000133136	ENSG00000213611
ENSG00000133138	ENSG00000109436
ENSG00000133138	ENSG00000197226
ENSG00000133138	ENSG00000204634
ENSG00000133216	ENSG00000044524
ENSG00000133216	ENSG00000070886
ENSG00000133216	ENSG00000080224
ENSG00000133216	ENSG00000106123
ENSG00000133216	ENSG00000116106
ENSG00000133216	ENSG00000135333
ENSG00000133216	ENSG00000142627

ENSG00000133216	ENSG00000145242
ENSG00000133216	ENSG00000146904
ENSG00000133216	ENSG00000154928
ENSG00000133216	ENSG00000182580
ENSG00000133216	ENSG00000183317
ENSG00000133216	ENSG00000196411
ENSG00000133243	ENSG00000064726
ENSG00000133243	ENSG00000132640
ENSG00000133243	ENSG00000184887
ENSG00000133246	ENSG00000082074
ENSG00000133246	ENSG00000187889
ENSG00000133247	ENSG00000110066
ENSG00000133256	ENSG00000095464
ENSG00000133256	ENSG00000112541
ENSG00000133256	ENSG00000128655
ENSG00000133256	ENSG00000132915
ENSG00000133256	ENSG00000138735
ENSG00000133256	ENSG00000186642
ENSG00000133275	ENSG00000151292
ENSG00000133275	ENSG00000169118
ENSG00000133315	ENSG00000172264
ENSG00000133317	ENSG00000006659
ENSG00000133317	ENSG00000100079
ENSG00000133317	ENSG00000100097
ENSG00000133317	ENSG00000105198
ENSG00000133317	ENSG00000105205
ENSG00000133317	ENSG00000116977
ENSG00000133317	ENSG00000119862
ENSG00000133317	ENSG00000131981
ENSG00000133317	ENSG00000168961
ENSG00000133317	ENSG00000170298
ENSG00000133317	ENSG00000171747
ENSG00000133317	ENSG00000171916
ENSG00000133317	ENSG00000178934
ENSG00000133317	ENSG00000197778
ENSG00000133317	ENSG00000205076
ENSG00000133317	ENSG00000215132
ENSG00000133318	ENSG00000115310
ENSG00000133318	ENSG00000125744
ENSG00000133318	ENSG00000139970
ENSG00000133321	ENSG00000121207
ENSG00000133321	ENSG00000127252
ENSG00000133321	ENSG00000133328
ENSG00000133321	ENSG00000162981
ENSG00000133321	ENSG00000168004
ENSG00000133321	ENSG00000168672
ENSG00000133321	ENSG00000176485
ENSG00000133328	ENSG00000121207
ENSG00000133328	ENSG00000127252
ENSG00000133328	ENSG00000133321
ENSG00000133328	ENSG00000162981
ENSG00000133328	ENSG00000168004
ENSG00000133328	ENSG00000168672
ENSG00000133328	ENSG00000176485
ENSG00000133392	ENSG00000100345
ENSG00000133392	ENSG00000105357
ENSG00000133392	ENSG00000133026
ENSG00000133401	ENSG00000172349
ENSG00000133422	ENSG00000114487

ENSG00000133422	ENSG00000133131
ENSG00000133422	ENSG00000159256
ENSG00000133424	ENSG00000165905
ENSG00000133424	ENSG00000174684
ENSG00000133433	ENSG00000099984
ENSG00000133433	ENSG00000184490
ENSG00000133433	ENSG00000184674
ENSG00000133433	ENSG00000185831
ENSG00000133433	ENSG00000206090
ENSG00000133454	ENSG00000196535
ENSG00000133460	ENSG00000059804
ENSG00000133460	ENSG00000109667
ENSG00000133460	ENSG00000117394
ENSG00000133460	ENSG00000142583
ENSG00000133460	ENSG00000163581
ENSG00000133460	ENSG00000173262
ENSG00000133460	ENSG00000181856
ENSG00000133460	ENSG00000197241
ENSG00000133466	ENSG00000054803
ENSG00000133466	ENSG00000102924
ENSG00000133466	ENSG00000131094
ENSG00000133466	ENSG00000139899
ENSG00000133466	ENSG00000141668
ENSG00000133466	ENSG00000144119
ENSG00000133466	ENSG00000145861
ENSG00000133466	ENSG00000159189
ENSG00000133466	ENSG00000163145
ENSG00000133466	ENSG00000165985
ENSG00000133466	ENSG00000172247
ENSG00000133466	ENSG00000173369
ENSG00000133466	ENSG00000173372
ENSG00000133466	ENSG00000173918
ENSG00000133466	ENSG00000181092
ENSG00000133466	ENSG00000184471
ENSG00000133466	ENSG00000186897
ENSG00000133466	ENSG00000213192
ENSG00000133475	ENSG00000099998
ENSG00000133475	ENSG00000100031
ENSG00000133475	ENSG00000100121
ENSG00000133475	ENSG00000149435
ENSG00000133475	ENSG00000183038
ENSG00000133475	ENSG00000197421
ENSG00000133475	ENSG00000215466
ENSG00000133475	ENSG00000215500
ENSG00000133475	ENSG00000215610
ENSG00000133477	ENSG00000101447
ENSG00000133477	ENSG00000105523
ENSG00000133477	ENSG00000125998
ENSG00000133477	ENSG00000147689
ENSG00000133477	ENSG00000168143
ENSG00000133477	ENSG00000180921
ENSG00000133477	ENSG00000188522
ENSG00000133561	ENSG00000106560
ENSG00000133561	ENSG00000133574
ENSG00000133561	ENSG00000171115
ENSG00000133561	ENSG00000179144
ENSG00000133561	ENSG00000196329
ENSG00000133561	ENSG00000213203
ENSG00000133574	ENSG00000106560

ENSG00000133574	ENSG00000133561
ENSG00000133574	ENSG00000171115
ENSG00000133574	ENSG00000179144
ENSG00000133574	ENSG00000196329
ENSG00000133574	ENSG00000213203
ENSG00000133606	ENSG00000075975
ENSG00000133606	ENSG00000179455
ENSG00000133612	ENSG00000108262
ENSG00000133612	ENSG00000135439
ENSG00000133612	ENSG00000139436
ENSG00000133612	ENSG00000151303
ENSG00000133612	ENSG00000157985
ENSG00000133612	ENSG00000172650
ENSG00000133612	ENSG00000174194
ENSG00000133612	ENSG00000188234
ENSG00000133612	ENSG00000198035
ENSG00000133612	ENSG00000204149
ENSG00000133612	ENSG00000204169
ENSG00000133612	ENSG00000204172
ENSG00000133627	ENSG00000106526
ENSG00000133627	ENSG00000115091
ENSG00000133639	ENSG00000137707
ENSG00000133639	ENSG00000154640
ENSG00000133639	ENSG00000159388
ENSG00000133657	ENSG00000127249
ENSG00000133657	ENSG00000159363
ENSG00000133657	ENSG00000187527
ENSG00000133703	ENSG00000006451
ENSG00000133703	ENSG00000126458
ENSG00000133703	ENSG00000133818
ENSG00000133703	ENSG00000144118
ENSG00000133703	ENSG00000158186
ENSG00000133703	ENSG00000174775
ENSG00000133703	ENSG00000187682
ENSG00000133703	ENSG00000213281
ENSG00000133704	ENSG00000205339
ENSG00000133731	ENSG00000141401
ENSG00000133742	ENSG00000074410
ENSG00000133742	ENSG00000104267
ENSG00000133742	ENSG00000107159
ENSG00000133742	ENSG00000118298
ENSG00000133742	ENSG00000131686
ENSG00000133742	ENSG00000164879
ENSG00000133742	ENSG00000167434
ENSG00000133742	ENSG00000168748
ENSG00000133742	ENSG00000169239
ENSG00000133742	ENSG00000174990
ENSG00000133742	ENSG00000178538
ENSG00000133742	ENSG00000185015
ENSG00000133742	ENSG00000214729
ENSG00000133742	ENSG00000214873
ENSG00000133742	ENSG00000215487
ENSG00000133742	ENSG00000215501
ENSG00000133742	ENSG00000215543
ENSG00000133789	ENSG00000023892
ENSG00000133794	ENSG00000029153
ENSG00000133794	ENSG00000143437
ENSG00000133794	ENSG00000172379
ENSG00000133800	ENSG00000026508

ENSG00000133800	ENSG00000123610
ENSG00000133805	ENSG00000116337
ENSG00000133805	ENSG00000116748
ENSG00000133812	ENSG00000100241
ENSG00000133812	ENSG00000110514
ENSG00000133816	ENSG00000093100
ENSG00000133816	ENSG00000135596
ENSG00000133818	ENSG00000006451
ENSG00000133818	ENSG00000126458
ENSG00000133818	ENSG00000133703
ENSG00000133818	ENSG00000144118
ENSG00000133818	ENSG00000158186
ENSG00000133818	ENSG00000174775
ENSG00000133818	ENSG00000187682
ENSG00000133818	ENSG00000213281
ENSG00000133878	ENSG00000079393
ENSG00000133878	ENSG00000108861
ENSG00000133878	ENSG00000188542
ENSG00000133878	ENSG00000188716
ENSG00000133884	ENSG00000011332
ENSG00000133884	ENSG00000130024
ENSG00000133884	ENSG00000205683
ENSG00000133937	ENSG00000063515
ENSG00000133961	ENSG00000105245
ENSG00000133961	ENSG00000144366
ENSG00000133961	ENSG00000157978
ENSG00000133961	ENSG00000183114
ENSG00000133961	ENSG00000185112
ENSG00000133985	ENSG00000162222
ENSG00000133985	ENSG00000174521
ENSG00000134007	ENSG00000008277
ENSG00000134007	ENSG00000042980
ENSG00000134007	ENSG00000069206
ENSG00000134007	ENSG00000073670
ENSG00000134007	ENSG00000104755
ENSG00000134007	ENSG00000114948
ENSG00000134007	ENSG00000134028
ENSG00000134007	ENSG00000134249
ENSG00000134007	ENSG00000135074
ENSG00000134007	ENSG00000139985
ENSG00000134007	ENSG00000143537
ENSG00000134007	ENSG00000148848
ENSG00000134007	ENSG00000149451
ENSG00000134007	ENSG00000151651
ENSG00000134007	ENSG00000151694
ENSG00000134007	ENSG00000168594
ENSG00000134007	ENSG00000168615
ENSG00000134007	ENSG00000168619
ENSG00000134007	ENSG00000196115
ENSG00000134007	ENSG00000197140
ENSG00000134007	ENSG00000214137
ENSG00000134013	ENSG00000113083
ENSG00000134013	ENSG00000115318
ENSG00000134013	ENSG00000129038
ENSG00000134013	ENSG00000138131
ENSG00000134025	ENSG00000088881
ENSG00000134025	ENSG00000108001
ENSG00000134025	ENSG00000164330
ENSG00000134028	ENSG00000008277

ENSG00000134028	ENSG00000042980
ENSG00000134028	ENSG00000069206
ENSG00000134028	ENSG00000073670
ENSG00000134028	ENSG00000104755
ENSG00000134028	ENSG00000114948
ENSG00000134028	ENSG00000134007
ENSG00000134028	ENSG00000134249
ENSG00000134028	ENSG00000135074
ENSG00000134028	ENSG00000139985
ENSG00000134028	ENSG00000143537
ENSG00000134028	ENSG00000148848
ENSG00000134028	ENSG00000149451
ENSG00000134028	ENSG00000151651
ENSG00000134028	ENSG00000151694
ENSG00000134028	ENSG00000168594
ENSG00000134028	ENSG00000168615
ENSG00000134028	ENSG00000168619
ENSG00000134028	ENSG00000196115
ENSG00000134028	ENSG00000197140
ENSG00000134028	ENSG00000214137
ENSG00000134046	ENSG00000071655
ENSG00000134046	ENSG00000170948
ENSG00000134046	ENSG00000182315
ENSG00000134046	ENSG00000184301
ENSG00000134046	ENSG00000196589
ENSG00000134046	ENSG00000205717
ENSG00000134046	ENSG00000205718
ENSG00000134057	ENSG00000147082
ENSG00000134057	ENSG00000157456
ENSG00000134061	ENSG00000101916
ENSG00000134061	ENSG00000136869
ENSG00000134061	ENSG00000137462
ENSG00000134061	ENSG00000174123
ENSG00000134061	ENSG00000174125
ENSG00000134061	ENSG00000174130
ENSG00000134061	ENSG00000187554
ENSG00000134070	ENSG00000090376
ENSG00000134070	ENSG00000184216
ENSG00000134072	ENSG00000008118
ENSG00000134072	ENSG00000081320
ENSG00000134072	ENSG00000130822
ENSG00000134072	ENSG00000147613
ENSG00000134072	ENSG00000159792
ENSG00000134072	ENSG00000164076
ENSG00000134072	ENSG00000164543
ENSG00000134072	ENSG00000183049
ENSG00000134077	ENSG00000138050
ENSG00000134107	ENSG00000123095
ENSG00000134108	ENSG00000143862
ENSG00000134115	ENSG00000018236
ENSG00000134115	ENSG00000113805
ENSG00000134115	ENSG00000144619
ENSG00000134121	ENSG00000091129
ENSG00000134121	ENSG00000149972
ENSG00000134121	ENSG00000163531
ENSG00000134121	ENSG00000184144
ENSG00000134121	ENSG00000198493
ENSG00000134138	ENSG00000105419
ENSG00000134138	ENSG00000118707

ENSG00000134138	ENSG00000143995
ENSG00000134138	ENSG00000153779
ENSG00000134138	ENSG00000160199
ENSG00000134138	ENSG00000165495
ENSG00000134138	ENSG00000176679
ENSG00000134138	ENSG00000177426
ENSG00000134138	ENSG00000179277
ENSG00000134138	ENSG00000188013
ENSG00000134160	ENSG00000070985
ENSG00000134160	ENSG00000083067
ENSG00000134160	ENSG00000092439
ENSG00000134160	ENSG00000119121
ENSG00000134160	ENSG00000130529
ENSG00000134160	ENSG00000142185
ENSG00000134160	ENSG00000144481
ENSG00000134183	ENSG00000060558
ENSG00000134183	ENSG00000065135
ENSG00000134183	ENSG00000087258
ENSG00000134183	ENSG00000087460
ENSG00000134183	ENSG00000088256
ENSG00000134183	ENSG00000114349
ENSG00000134183	ENSG00000114353
ENSG00000134183	ENSG00000120063
ENSG00000134183	ENSG00000127955
ENSG00000134183	ENSG00000128266
ENSG00000134183	ENSG00000141404
ENSG00000134183	ENSG00000146535
ENSG00000134183	ENSG00000156049
ENSG00000134183	ENSG00000156052
ENSG00000134183	ENSG00000214415
ENSG00000134183	ENSG00000214622
ENSG00000134198	ENSG00000010278
ENSG00000134198	ENSG00000110651
ENSG00000134198	ENSG00000127324
ENSG00000134200	ENSG00000104818
ENSG00000134200	ENSG00000104826
ENSG00000134200	ENSG00000104827
ENSG00000134200	ENSG00000131808
ENSG00000134200	ENSG00000179600
ENSG00000134200	ENSG00000189052
ENSG00000134200	ENSG00000196337
ENSG00000134200	ENSG00000204748
ENSG00000134200	ENSG00000213030
ENSG00000134207	ENSG00000011347
ENSG00000134207	ENSG00000019505
ENSG00000134207	ENSG00000067715
ENSG00000134207	ENSG00000101098
ENSG00000134207	ENSG00000103528
ENSG00000134207	ENSG00000110975
ENSG00000134207	ENSG00000129990
ENSG00000134207	ENSG00000132718
ENSG00000134207	ENSG00000132872
ENSG00000134207	ENSG00000143858
ENSG00000134207	ENSG00000149043
ENSG00000134207	ENSG00000149927
ENSG00000134207	ENSG00000170743
ENSG00000134207	ENSG00000173227
ENSG00000134207	ENSG00000204176
ENSG00000134207	ENSG00000213023

ENSG00000134207	ENSG00000214408
ENSG00000134207	ENSG00000214409
ENSG00000134207	ENSG00000214569
ENSG00000134207	ENSG00000214783
ENSG00000134215	ENSG00000141968
ENSG00000134215	ENSG00000160293
ENSG00000134216	ENSG00000064886
ENSG00000134216	ENSG00000085465
ENSG00000134216	ENSG00000133048
ENSG00000134216	ENSG00000133063
ENSG00000134216	ENSG00000203878
ENSG00000134240	ENSG00000112972
ENSG00000134242	ENSG00000072135
ENSG00000134242	ENSG00000127947
ENSG00000134243	ENSG00000108018
ENSG00000134243	ENSG00000137642
ENSG00000134243	ENSG00000156395
ENSG00000134243	ENSG00000184985
ENSG00000134245	ENSG00000002745
ENSG00000134245	ENSG00000061492
ENSG00000134245	ENSG00000075290
ENSG00000134245	ENSG00000085741
ENSG00000134245	ENSG00000105989
ENSG00000134245	ENSG00000108379
ENSG00000134245	ENSG00000111186
ENSG00000134245	ENSG00000114251
ENSG00000134245	ENSG00000115596
ENSG00000134245	ENSG00000125084
ENSG00000134245	ENSG00000135925
ENSG00000134245	ENSG00000143816
ENSG00000134245	ENSG00000154342
ENSG00000134245	ENSG00000154764
ENSG00000134245	ENSG00000158955
ENSG00000134245	ENSG00000162552
ENSG00000134245	ENSG00000169884
ENSG00000134245	ENSG00000188064
ENSG00000134247	ENSG00000134256
ENSG00000134247	ENSG00000143061
ENSG00000134247	ENSG00000162729
ENSG00000134249	ENSG00000008277
ENSG00000134249	ENSG00000042980
ENSG00000134249	ENSG00000069206
ENSG00000134249	ENSG00000073670
ENSG00000134249	ENSG00000104755
ENSG00000134249	ENSG00000114948
ENSG00000134249	ENSG00000134007
ENSG00000134249	ENSG00000134028
ENSG00000134249	ENSG00000135074
ENSG00000134249	ENSG00000139985
ENSG00000134249	ENSG00000143537
ENSG00000134249	ENSG00000148848
ENSG00000134249	ENSG00000149451
ENSG00000134249	ENSG00000151651
ENSG00000134249	ENSG00000151694
ENSG00000134249	ENSG00000168594
ENSG00000134249	ENSG00000168615
ENSG00000134249	ENSG00000168619
ENSG00000134249	ENSG00000196115
ENSG00000134249	ENSG00000197140

ENSG00000134249	ENSG00000214137
ENSG00000134250	ENSG00000074181
ENSG00000134250	ENSG00000134376
ENSG00000134250	ENSG00000148400
ENSG00000134250	ENSG00000204301
ENSG00000134255	ENSG00000111666
ENSG00000134255	ENSG00000138018
ENSG00000134256	ENSG00000134247
ENSG00000134256	ENSG00000143061
ENSG00000134256	ENSG00000162729
ENSG00000134258	ENSG00000103855
ENSG00000134258	ENSG00000114013
ENSG00000134258	ENSG00000114455
ENSG00000134258	ENSG00000120217
ENSG00000134258	ENSG00000160223
ENSG00000134258	ENSG00000186470
ENSG00000134258	ENSG00000188211
ENSG00000134258	ENSG00000197646
ENSG00000134258	ENSG00000204290
ENSG00000134258	ENSG00000204655
ENSG00000134258	ENSG00000215631
ENSG00000134258	ENSG00000215811
ENSG00000134258	ENSG00000215889
ENSG00000134259	ENSG00000167744
ENSG00000134259	ENSG00000176697
ENSG00000134259	ENSG00000185652
ENSG00000134259	ENSG00000212844
ENSG00000134278	ENSG00000204991
ENSG00000134287	ENSG00000004059
ENSG00000134287	ENSG00000143761
ENSG00000134287	ENSG00000165527
ENSG00000134287	ENSG00000168374
ENSG00000134291	ENSG00000106460
ENSG00000134291	ENSG00000184988
ENSG00000134291	ENSG00000215684
ENSG00000134294	ENSG00000017483
ENSG00000134294	ENSG00000103042
ENSG00000134294	ENSG00000111371
ENSG00000134294	ENSG00000139209
ENSG00000134294	ENSG00000139974
ENSG00000134294	ENSG00000166558
ENSG00000134294	ENSG00000169507
ENSG00000134294	ENSG00000188338
ENSG00000134308	ENSG00000108953
ENSG00000134308	ENSG00000128245
ENSG00000134308	ENSG00000164924
ENSG00000134308	ENSG00000166913
ENSG00000134308	ENSG00000170027
ENSG00000134308	ENSG00000175793
ENSG00000134308	ENSG00000214637
ENSG00000134308	ENSG00000215108
ENSG00000134317	ENSG00000083307
ENSG00000134317	ENSG00000115112
ENSG00000134317	ENSG00000135457
ENSG00000134317	ENSG00000153560
ENSG00000134317	ENSG00000158055
ENSG00000134317	ENSG00000214547
ENSG00000134318	ENSG00000067900
ENSG00000134318	ENSG00000143776

ENSG00000134318	ENSG00000171219
ENSG00000134318	ENSG00000198752
ENSG00000134323	ENSG00000116990
ENSG00000134323	ENSG00000136997
ENSG00000134324	ENSG00000101577
ENSG00000134324	ENSG00000132793
ENSG00000134333	ENSG00000111716
ENSG00000134333	ENSG00000151116
ENSG00000134333	ENSG00000166796
ENSG00000134333	ENSG00000166800
ENSG00000134333	ENSG00000171989
ENSG00000134343	ENSG00000047617
ENSG00000134343	ENSG00000074855
ENSG00000134343	ENSG00000131620
ENSG00000134343	ENSG00000146205
ENSG00000134343	ENSG00000151572
ENSG00000134343	ENSG00000160746
ENSG00000134343	ENSG00000171714
ENSG00000134343	ENSG00000177119
ENSG00000134343	ENSG00000185101
ENSG00000134352	ENSG00000081985
ENSG00000134352	ENSG00000104998
ENSG00000134352	ENSG00000113594
ENSG00000134352	ENSG00000119535
ENSG00000134352	ENSG00000145623
ENSG00000134352	ENSG00000164509
ENSG00000134363	ENSG00000053108
ENSG00000134363	ENSG00000070404
ENSG00000134363	ENSG00000163430
ENSG00000134365	ENSG00000000971
ENSG00000134365	ENSG00000080910
ENSG00000134365	ENSG00000116785
ENSG00000134365	ENSG00000134389
ENSG00000134365	ENSG00000134391
ENSG00000134365	ENSG00000143278
ENSG00000134369	ENSG00000067798
ENSG00000134369	ENSG00000166833
ENSG00000134375	ENSG00000126768
ENSG00000134389	ENSG00000000971
ENSG00000134389	ENSG00000080910
ENSG00000134389	ENSG00000116785
ENSG00000134389	ENSG00000134365
ENSG00000134389	ENSG00000134391
ENSG00000134389	ENSG00000143278
ENSG00000134391	ENSG00000000971
ENSG00000134391	ENSG00000080910
ENSG00000134391	ENSG00000116785
ENSG00000134391	ENSG00000134365
ENSG00000134391	ENSG00000134389
ENSG00000134391	ENSG00000143278
ENSG00000134398	ENSG00000178607
ENSG00000134438	ENSG00000101883
ENSG00000134438	ENSG00000123576
ENSG00000134438	ENSG00000126656
ENSG00000134438	ENSG00000173976
ENSG00000134438	ENSG00000175325
ENSG00000134438	ENSG00000175329
ENSG00000134438	ENSG00000204595
ENSG00000134489	ENSG00000101180

ENSG00000134504	ENSG00000151364
ENSG00000134504	ENSG00000153885
ENSG00000134504	ENSG00000155729
ENSG00000134504	ENSG00000168301
ENSG00000134504	ENSG00000168676
ENSG00000134504	ENSG00000178695
ENSG00000134504	ENSG00000180332
ENSG00000134504	ENSG00000183775
ENSG00000134504	ENSG00000183783
ENSG00000134504	ENSG00000188997
ENSG00000134504	ENSG00000198553
ENSG00000134504	ENSG00000213859
ENSG00000134508	ENSG00000149679
ENSG00000134516	ENSG00000088538
ENSG00000134516	ENSG00000128512
ENSG00000134516	ENSG00000147459
ENSG00000134516	ENSG00000150760
ENSG00000134531	ENSG00000109099
ENSG00000134531	ENSG00000142227
ENSG00000134531	ENSG00000213853
ENSG00000134532	ENSG00000110693
ENSG00000134532	ENSG00000143842
ENSG00000134538	ENSG00000084453
ENSG00000134538	ENSG00000101187
ENSG00000134538	ENSG00000111700
ENSG00000134538	ENSG00000137491
ENSG00000134538	ENSG00000137571
ENSG00000134538	ENSG00000139155
ENSG00000134538	ENSG00000173930
ENSG00000134538	ENSG00000174640
ENSG00000134538	ENSG00000176463
ENSG00000134538	ENSG00000205359
ENSG00000134538	ENSG00000205754
ENSG00000134575	ENSG00000014257
ENSG00000134575	ENSG00000142513
ENSG00000134588	ENSG00000131864
ENSG00000134588	ENSG00000135913
ENSG00000134594	ENSG00000137502
ENSG00000134594	ENSG00000138069
ENSG00000134594	ENSG00000146955
ENSG00000134594	ENSG00000172007
ENSG00000134594	ENSG00000174903
ENSG00000134594	ENSG00000185051
ENSG00000134595	ENSG00000005513
ENSG00000134595	ENSG00000100146
ENSG00000134595	ENSG00000124766
ENSG00000134595	ENSG00000125398
ENSG00000134595	ENSG00000129194
ENSG00000134595	ENSG00000164736
ENSG00000134595	ENSG00000171056
ENSG00000134595	ENSG00000176887
ENSG00000134595	ENSG00000177732
ENSG00000134595	ENSG00000181449
ENSG00000134595	ENSG00000182968
ENSG00000134595	ENSG00000184895
ENSG00000134595	ENSG00000203883
ENSG00000134602	ENSG00000102572
ENSG00000134602	ENSG00000115694
ENSG00000134612	ENSG00000072274

ENSG00000134612	ENSG00000077616
ENSG00000134612	ENSG00000086205
ENSG00000134612	ENSG00000106327
ENSG00000134612	ENSG00000168060
ENSG00000134612	ENSG00000177694
ENSG00000134627	ENSG00000125207
ENSG00000134627	ENSG00000184571
ENSG00000134627	ENSG00000197181
ENSG00000134640	ENSG00000102195
ENSG00000134640	ENSG00000168412
ENSG00000134644	ENSG00000055917
ENSG00000134668	ENSG00000101191
ENSG00000134668	ENSG00000118482
ENSG00000134686	ENSG00000111752
ENSG00000134686	ENSG00000173889
ENSG00000134686	ENSG00000179899
ENSG00000134686	ENSG00000187033
ENSG00000134686	ENSG00000187634
ENSG00000134698	ENSG00000092847
ENSG00000134698	ENSG00000123908
ENSG00000134698	ENSG00000126070
ENSG00000134709	ENSG00000015133
ENSG00000134709	ENSG00000095066
ENSG00000134709	ENSG00000115355
ENSG00000134709	ENSG00000168071
ENSG00000134709	ENSG00000168172
ENSG00000134717	ENSG00000118903
ENSG00000134717	ENSG00000145741
ENSG00000134717	ENSG00000197407
ENSG00000134717	ENSG00000214680
ENSG00000134744	ENSG00000083223
ENSG00000134744	ENSG00000164329
ENSG00000134755	ENSG00000039068
ENSG00000134755	ENSG00000062038
ENSG00000134755	ENSG00000079112
ENSG00000134755	ENSG00000124215
ENSG00000134755	ENSG00000129910
ENSG00000134755	ENSG00000134762
ENSG00000134755	ENSG00000134765
ENSG00000134755	ENSG00000140945
ENSG00000134755	ENSG00000166589
ENSG00000134755	ENSG00000170558
ENSG00000134755	ENSG00000179242
ENSG00000134758	ENSG00000101695
ENSG00000134758	ENSG00000124226
ENSG00000134758	ENSG00000158717
ENSG00000134762	ENSG00000039068
ENSG00000134762	ENSG00000062038
ENSG00000134762	ENSG00000079112
ENSG00000134762	ENSG00000124215
ENSG00000134762	ENSG00000129910
ENSG00000134762	ENSG00000134755
ENSG00000134762	ENSG00000134765
ENSG00000134762	ENSG00000140945
ENSG00000134762	ENSG00000166589
ENSG00000134762	ENSG00000170558
ENSG00000134762	ENSG00000179242
ENSG00000134765	ENSG00000039068
ENSG00000134765	ENSG00000062038

ENSG00000134765	ENSG00000079112
ENSG00000134765	ENSG00000124215
ENSG00000134765	ENSG00000129910
ENSG00000134765	ENSG00000134755
ENSG00000134765	ENSG00000134762
ENSG00000134765	ENSG00000140945
ENSG00000134765	ENSG00000166589
ENSG00000134765	ENSG00000170558
ENSG00000134765	ENSG00000179242
ENSG00000134775	ENSG00000135723
ENSG00000134817	ENSG00000100739
ENSG00000134817	ENSG00000144891
ENSG00000134817	ENSG00000154165
ENSG00000134817	ENSG00000168398
ENSG00000134817	ENSG00000170128
ENSG00000134817	ENSG00000173080
ENSG00000134817	ENSG00000180772
ENSG00000134817	ENSG00000182631
ENSG00000134824	ENSG00000149485
ENSG00000134824	ENSG00000214876
ENSG00000134830	ENSG00000171049
ENSG00000134830	ENSG00000171051
ENSG00000134830	ENSG00000171860
ENSG00000134830	ENSG00000174600
ENSG00000134830	ENSG00000183134
ENSG00000134830	ENSG00000183671
ENSG00000134830	ENSG00000187474
ENSG00000134830	ENSG00000197405
ENSG00000134830	ENSG00000213903
ENSG00000134830	ENSG00000213906
ENSG00000134852	ENSG00000166049
ENSG00000134852	ENSG00000170485
ENSG00000134853	ENSG00000037280
ENSG00000134853	ENSG00000102755
ENSG00000134853	ENSG00000104213
ENSG00000134853	ENSG00000113721
ENSG00000134853	ENSG00000122025
ENSG00000134853	ENSG00000128052
ENSG00000134853	ENSG00000157404
ENSG00000134853	ENSG00000182578
ENSG00000134871	ENSG00000081052
ENSG00000134871	ENSG00000169031
ENSG00000134871	ENSG00000187498
ENSG00000134871	ENSG00000188153
ENSG00000134871	ENSG00000197565
ENSG00000134873	ENSG00000013297
ENSG00000134873	ENSG00000066405
ENSG00000134873	ENSG00000106404
ENSG00000134873	ENSG00000113946
ENSG00000134873	ENSG00000156282
ENSG00000134873	ENSG00000156284
ENSG00000134873	ENSG00000159261
ENSG00000134873	ENSG00000163347
ENSG00000134873	ENSG00000164007
ENSG00000134873	ENSG00000165215
ENSG00000134873	ENSG00000165376
ENSG00000134873	ENSG00000171217
ENSG00000134873	ENSG00000177300
ENSG00000134873	ENSG00000181885

ENSG00000134873	ENSG00000184113
ENSG00000134873	ENSG00000184697
ENSG00000134873	ENSG00000185758
ENSG00000134873	ENSG00000189118
ENSG00000134873	ENSG00000189143
ENSG00000134873	ENSG00000213937
ENSG00000134874	ENSG00000158163
ENSG00000134901	ENSG00000163389
ENSG00000134901	ENSG00000178202
ENSG00000134909	ENSG00000004777
ENSG00000134909	ENSG00000031081
ENSG00000134909	ENSG00000186517
ENSG00000134917	ENSG00000154734
ENSG00000134917	ENSG00000154736
ENSG00000134917	ENSG00000158859
ENSG00000134917	ENSG00000166106
ENSG00000134954	ENSG00000006468
ENSG00000134954	ENSG00000105672
ENSG00000134954	ENSG00000157557
ENSG00000134954	ENSG00000163497
ENSG00000134954	ENSG00000171656
ENSG00000134954	ENSG00000175832
ENSG00000134955	ENSG00000137700
ENSG00000134955	ENSG00000157800
ENSG00000134955	ENSG00000160190
ENSG00000134962	ENSG00000115850
ENSG00000134962	ENSG00000133116
ENSG00000134962	ENSG00000176201
ENSG00000134962	ENSG00000188501
ENSG00000134970	ENSG00000086598
ENSG00000134970	ENSG00000099203
ENSG00000134970	ENSG00000117500
ENSG00000134970	ENSG00000157315
ENSG00000134970	ENSG00000166557
ENSG00000134982	ENSG00000115266
ENSG00000135018	ENSG00000160803
ENSG00000135018	ENSG00000175518
ENSG00000135018	ENSG00000175520
ENSG00000135018	ENSG00000188021
ENSG00000135046	ENSG00000104537
ENSG00000135046	ENSG00000109511
ENSG00000135046	ENSG00000122359
ENSG00000135046	ENSG00000138279
ENSG00000135046	ENSG00000138772
ENSG00000135046	ENSG00000143412
ENSG00000135046	ENSG00000150165
ENSG00000135046	ENSG00000164111
ENSG00000135046	ENSG00000165390
ENSG00000135046	ENSG00000182718
ENSG00000135046	ENSG00000183059
ENSG00000135046	ENSG00000186807
ENSG00000135046	ENSG00000196975
ENSG00000135046	ENSG00000197043
ENSG00000135047	ENSG00000136943
ENSG00000135047	ENSG00000143387
ENSG00000135047	ENSG00000163131
ENSG00000135047	ENSG00000188029
ENSG00000135047	ENSG00000204437
ENSG00000135048	ENSG00000103888

ENSG00000135049	ENSG00000146856
ENSG00000135049	ENSG00000165923
ENSG00000135049	ENSG00000166748
ENSG00000135052	ENSG00000166734
ENSG00000135052	ENSG00000215599
ENSG00000135063	ENSG00000104059
ENSG00000135063	ENSG00000160767
ENSG00000135074	ENSG00000008277
ENSG00000135074	ENSG00000042980
ENSG00000135074	ENSG00000069206
ENSG00000135074	ENSG00000073670
ENSG00000135074	ENSG00000104755
ENSG00000135074	ENSG00000114948
ENSG00000135074	ENSG00000134007
ENSG00000135074	ENSG00000134028
ENSG00000135074	ENSG00000134249
ENSG00000135074	ENSG00000139985
ENSG00000135074	ENSG00000143537
ENSG00000135074	ENSG00000148848
ENSG00000135074	ENSG00000149451
ENSG00000135074	ENSG00000151651
ENSG00000135074	ENSG00000151694
ENSG00000135074	ENSG00000168594
ENSG00000135074	ENSG00000168615
ENSG00000135074	ENSG00000168619
ENSG00000135074	ENSG00000196115
ENSG00000135074	ENSG00000197140
ENSG00000135074	ENSG00000214137
ENSG00000135077	ENSG00000113249
ENSG00000135077	ENSG00000145850
ENSG00000135083	ENSG00000107443
ENSG00000135090	ENSG00000149930
ENSG00000135090	ENSG00000160551
ENSG00000135094	ENSG00000139410
ENSG00000135097	ENSG00000153944
ENSG00000135100	ENSG00000108753
ENSG00000135100	ENSG00000147421
ENSG00000135111	ENSG00000073861
ENSG00000135111	ENSG00000089225
ENSG00000135111	ENSG00000092607
ENSG00000135111	ENSG00000112837
ENSG00000135111	ENSG00000121068
ENSG00000135111	ENSG00000121075
ENSG00000135111	ENSG00000122145
ENSG00000135111	ENSG00000136535
ENSG00000135111	ENSG00000143178
ENSG00000135111	ENSG00000149922
ENSG00000135111	ENSG00000163508
ENSG00000135111	ENSG00000164458
ENSG00000135111	ENSG00000164532
ENSG00000135111	ENSG00000167800
ENSG00000135111	ENSG00000174197
ENSG00000135111	ENSG00000184058
ENSG00000135119	ENSG00000187870
ENSG00000135119	ENSG00000189050
ENSG00000135124	ENSG00000083454
ENSG00000135124	ENSG00000089041
ENSG00000135124	ENSG00000099957
ENSG00000135124	ENSG00000108405

ENSG00000135124	ENSG00000109991
ENSG00000135124	ENSG00000187848
ENSG00000135127	ENSG00000162069
ENSG00000135144	ENSG00000091073
ENSG00000135144	ENSG00000110042
ENSG00000135144	ENSG00000163840
ENSG00000135144	ENSG00000178498
ENSG00000135148	ENSG00000132530
ENSG00000135164	ENSG00000125482
ENSG00000135175	ENSG00000100362
ENSG00000135175	ENSG00000122543
ENSG00000135220	ENSG00000109181
ENSG00000135220	ENSG00000135226
ENSG00000135220	ENSG00000145626
ENSG00000135220	ENSG00000156096
ENSG00000135220	ENSG00000167165
ENSG00000135220	ENSG00000168671
ENSG00000135220	ENSG00000171234
ENSG00000135220	ENSG00000173610
ENSG00000135220	ENSG00000174607
ENSG00000135220	ENSG00000196620
ENSG00000135220	ENSG00000197592
ENSG00000135220	ENSG00000197888
ENSG00000135220	ENSG00000198277
ENSG00000135220	ENSG00000213759
ENSG00000135226	ENSG00000109181
ENSG00000135226	ENSG00000135220
ENSG00000135226	ENSG00000145626
ENSG00000135226	ENSG00000156096
ENSG00000135226	ENSG00000167165
ENSG00000135226	ENSG00000168671
ENSG00000135226	ENSG00000171234
ENSG00000135226	ENSG00000173610
ENSG00000135226	ENSG00000174607
ENSG00000135226	ENSG00000196620
ENSG00000135226	ENSG00000197592
ENSG00000135226	ENSG00000197888
ENSG00000135226	ENSG00000198277
ENSG00000135226	ENSG00000213759
ENSG00000135241	ENSG00000184381
ENSG00000135241	ENSG00000214979
ENSG00000135250	ENSG00000096063
ENSG00000135250	ENSG00000184343
ENSG00000135269	ENSG00000012211
ENSG00000135269	ENSG00000071282
ENSG00000135269	ENSG00000124593
ENSG00000135269	ENSG00000139174
ENSG00000135269	ENSG00000163637
ENSG00000135272	ENSG00000112559
ENSG00000135298	ENSG00000121753
ENSG00000135298	ENSG00000181790
ENSG00000135312	ENSG00000043591
ENSG00000135312	ENSG00000102468
ENSG00000135312	ENSG00000113749
ENSG00000135312	ENSG00000125631
ENSG00000135312	ENSG00000135914
ENSG00000135312	ENSG00000147246
ENSG00000135312	ENSG00000150594
ENSG00000135312	ENSG00000157219

ENSG00000135312	ENSG00000164270
ENSG00000135312	ENSG00000168830
ENSG00000135312	ENSG00000169252
ENSG00000135312	ENSG00000175658
ENSG00000135312	ENSG00000178394
ENSG00000135312	ENSG00000179097
ENSG00000135312	ENSG00000179546
ENSG00000135312	ENSG00000181210
ENSG00000135312	ENSG00000184303
ENSG00000135312	ENSG00000184845
ENSG00000135312	ENSG00000185321
ENSG00000135312	ENSG00000188778
ENSG00000135316	ENSG00000125944
ENSG00000135316	ENSG00000148584
ENSG00000135316	ENSG00000151962
ENSG00000135316	ENSG00000163694
ENSG00000135316	ENSG00000183403
ENSG00000135333	ENSG00000044524
ENSG00000135333	ENSG00000070886
ENSG00000135333	ENSG00000080224
ENSG00000135333	ENSG00000106123
ENSG00000135333	ENSG00000116106
ENSG00000135333	ENSG00000133216
ENSG00000135333	ENSG00000142627
ENSG00000135333	ENSG00000145242
ENSG00000135333	ENSG00000146904
ENSG00000135333	ENSG00000154928
ENSG00000135333	ENSG00000182580
ENSG00000135333	ENSG00000183317
ENSG00000135333	ENSG00000196411
ENSG00000135334	ENSG00000174574
ENSG00000135355	ENSG00000121634
ENSG00000135355	ENSG00000121742
ENSG00000135355	ENSG00000121743
ENSG00000135355	ENSG00000131233
ENSG00000135355	ENSG00000143140
ENSG00000135355	ENSG00000152661
ENSG00000135355	ENSG00000159248
ENSG00000135355	ENSG00000164411
ENSG00000135355	ENSG00000165474
ENSG00000135355	ENSG00000169562
ENSG00000135355	ENSG00000176402
ENSG00000135355	ENSG00000177291
ENSG00000135355	ENSG00000182963
ENSG00000135355	ENSG00000183153
ENSG00000135355	ENSG00000187513
ENSG00000135355	ENSG00000188910
ENSG00000135355	ENSG00000189280
ENSG00000135355	ENSG00000189433
ENSG00000135355	ENSG00000198835
ENSG00000135355	ENSG00000203733
ENSG00000135362	ENSG00000186654
ENSG00000135363	ENSG00000048540
ENSG00000135363	ENSG00000166407
ENSG00000135365	ENSG00000056487
ENSG00000135373	ENSG00000010030
ENSG00000135373	ENSG00000102034
ENSG00000135373	ENSG00000109381
ENSG00000135373	ENSG00000120690

ENSG00000135373	ENSG00000124664
ENSG00000135373	ENSG00000135374
ENSG00000135373	ENSG00000139083
ENSG00000135373	ENSG00000163435
ENSG00000135373	ENSG00000166211
ENSG00000135374	ENSG00000010030
ENSG00000135374	ENSG00000102034
ENSG00000135374	ENSG00000109381
ENSG00000135374	ENSG00000120690
ENSG00000135374	ENSG00000124664
ENSG00000135374	ENSG00000135373
ENSG00000135374	ENSG00000139083
ENSG00000135374	ENSG00000163435
ENSG00000135374	ENSG00000166211
ENSG00000135378	ENSG00000126460
ENSG00000135378	ENSG00000130032
ENSG00000135378	ENSG00000130962
ENSG00000135387	ENSG00000110888
ENSG00000135390	ENSG00000154518
ENSG00000135390	ENSG00000159199
ENSG00000135406	ENSG00000026025
ENSG00000135406	ENSG00000100285
ENSG00000135406	ENSG00000104722
ENSG00000135406	ENSG00000104725
ENSG00000135406	ENSG00000131095
ENSG00000135406	ENSG00000148798
ENSG00000135406	ENSG00000175084
ENSG00000135406	ENSG00000182253
ENSG00000135406	ENSG00000215479
ENSG00000135407	ENSG00000006747
ENSG00000135407	ENSG00000042493
ENSG00000135407	ENSG00000127831
ENSG00000135407	ENSG00000136059
ENSG00000135407	ENSG00000148180
ENSG00000135407	ENSG00000177731
ENSG00000135414	ENSG00000092969
ENSG00000135414	ENSG00000105329
ENSG00000135414	ENSG00000138379
ENSG00000135414	ENSG00000139269
ENSG00000135423	ENSG00000115419
ENSG00000135424	ENSG00000005844
ENSG00000135424	ENSG00000005884
ENSG00000135424	ENSG00000005961
ENSG00000135424	ENSG00000077943
ENSG00000135424	ENSG00000083457
ENSG00000135424	ENSG00000091409
ENSG00000135424	ENSG00000115232
ENSG00000135424	ENSG00000137809
ENSG00000135424	ENSG00000138448
ENSG00000135424	ENSG00000140678
ENSG00000135424	ENSG00000143127
ENSG00000135424	ENSG00000144668
ENSG00000135424	ENSG00000156886
ENSG00000135424	ENSG00000161638
ENSG00000135424	ENSG00000164171
ENSG00000135424	ENSG00000169896
ENSG00000135424	ENSG00000213949
ENSG00000135426	ENSG00000138434
ENSG00000135426	ENSG00000180347

ENSG00000135437	ENSG00000025423
ENSG00000135437	ENSG00000073737
ENSG00000135437	ENSG00000086696
ENSG00000135437	ENSG00000139547
ENSG00000135437	ENSG00000161267
ENSG00000135437	ENSG00000170426
ENSG00000135437	ENSG00000176387
ENSG00000135439	ENSG00000108262
ENSG00000135439	ENSG00000133612
ENSG00000135439	ENSG00000139436
ENSG00000135439	ENSG00000151303
ENSG00000135439	ENSG00000157985
ENSG00000135439	ENSG00000172650
ENSG00000135439	ENSG00000174194
ENSG00000135439	ENSG00000188234
ENSG00000135439	ENSG00000198035
ENSG00000135439	ENSG00000204149
ENSG00000135439	ENSG00000204169
ENSG00000135439	ENSG00000204172
ENSG00000135443	ENSG00000135477
ENSG00000135443	ENSG00000135480
ENSG00000135443	ENSG00000139648
ENSG00000135443	ENSG00000161849
ENSG00000135443	ENSG00000161850
ENSG00000135443	ENSG00000167767
ENSG00000135443	ENSG00000167768
ENSG00000135443	ENSG00000170421
ENSG00000135443	ENSG00000170423
ENSG00000135443	ENSG00000170442
ENSG00000135443	ENSG00000170454
ENSG00000135443	ENSG00000170465
ENSG00000135443	ENSG00000170477
ENSG00000135443	ENSG00000170484
ENSG00000135443	ENSG00000170486
ENSG00000135443	ENSG00000170523
ENSG00000135443	ENSG00000172867
ENSG00000135443	ENSG00000185069
ENSG00000135443	ENSG00000185479
ENSG00000135443	ENSG00000185640
ENSG00000135443	ENSG00000186049
ENSG00000135443	ENSG00000186081
ENSG00000135443	ENSG00000186442
ENSG00000135443	ENSG00000189182
ENSG00000135443	ENSG00000205420
ENSG00000135443	ENSG00000205426
ENSG00000135443	ENSG00000214233
ENSG00000135443	ENSG00000214323
ENSG00000135443	ENSG00000214477
ENSG00000135443	ENSG00000214752
ENSG00000135443	ENSG00000214974
ENSG00000135443	ENSG00000215287
ENSG00000135443	ENSG00000215847
ENSG00000135447	ENSG00000131771
ENSG00000135447	ENSG00000150722
ENSG00000135452	ENSG00000106537
ENSG00000135454	ENSG00000167080
ENSG00000135457	ENSG00000083307
ENSG00000135457	ENSG00000115112
ENSG00000135457	ENSG00000134317

ENSG00000135457	ENSG00000153560
ENSG00000135457	ENSG00000158055
ENSG00000135457	ENSG00000214547
ENSG00000135469	ENSG00000115520
ENSG00000135472	ENSG00000135926
ENSG00000135472	ENSG00000178719
ENSG00000135477	ENSG00000135443
ENSG00000135477	ENSG00000135480
ENSG00000135477	ENSG00000139648
ENSG00000135477	ENSG00000161849
ENSG00000135477	ENSG00000161850
ENSG00000135477	ENSG00000167767
ENSG00000135477	ENSG00000167768
ENSG00000135477	ENSG00000170421
ENSG00000135477	ENSG00000170423
ENSG00000135477	ENSG00000170442
ENSG00000135477	ENSG00000170454
ENSG00000135477	ENSG00000170465
ENSG00000135477	ENSG00000170477
ENSG00000135477	ENSG00000170484
ENSG00000135477	ENSG00000170486
ENSG00000135477	ENSG00000170523
ENSG00000135477	ENSG00000172867
ENSG00000135477	ENSG00000185069
ENSG00000135477	ENSG00000185479
ENSG00000135477	ENSG00000185640
ENSG00000135477	ENSG00000186049
ENSG00000135477	ENSG00000186081
ENSG00000135477	ENSG00000186442
ENSG00000135477	ENSG00000189182
ENSG00000135477	ENSG00000205420
ENSG00000135477	ENSG00000205426
ENSG00000135477	ENSG00000214233
ENSG00000135477	ENSG00000214323
ENSG00000135477	ENSG00000214477
ENSG00000135477	ENSG00000214752
ENSG00000135477	ENSG00000214974
ENSG00000135477	ENSG00000215287
ENSG00000135477	ENSG00000215847
ENSG00000135480	ENSG00000135443
ENSG00000135480	ENSG00000135477
ENSG00000135480	ENSG00000139648
ENSG00000135480	ENSG00000161849
ENSG00000135480	ENSG00000161850
ENSG00000135480	ENSG00000167767
ENSG00000135480	ENSG00000167768
ENSG00000135480	ENSG00000170421
ENSG00000135480	ENSG00000170423
ENSG00000135480	ENSG00000170442
ENSG00000135480	ENSG00000170454
ENSG00000135480	ENSG00000170465
ENSG00000135480	ENSG00000170477
ENSG00000135480	ENSG00000170484
ENSG00000135480	ENSG00000170486
ENSG00000135480	ENSG00000170523
ENSG00000135480	ENSG00000172867
ENSG00000135480	ENSG00000185069
ENSG00000135480	ENSG00000185479
ENSG00000135480	ENSG00000185640

ENSG00000135480	ENSG00000186049
ENSG00000135480	ENSG00000186081
ENSG00000135480	ENSG00000186442
ENSG00000135480	ENSG00000189182
ENSG00000135480	ENSG00000205420
ENSG00000135480	ENSG00000205426
ENSG00000135480	ENSG00000214233
ENSG00000135480	ENSG00000214323
ENSG00000135480	ENSG00000214477
ENSG00000135480	ENSG00000214752
ENSG00000135480	ENSG00000214974
ENSG00000135480	ENSG00000215287
ENSG00000135480	ENSG00000215847
ENSG00000135486	ENSG00000122566
ENSG00000135486	ENSG00000139675
ENSG00000135486	ENSG00000170144
ENSG00000135486	ENSG00000176825
ENSG00000135486	ENSG00000177219
ENSG00000135486	ENSG00000177733
ENSG00000135486	ENSG00000188174
ENSG00000135486	ENSG00000206228
ENSG00000135486	ENSG00000212961
ENSG00000135486	ENSG00000214284
ENSG00000135486	ENSG00000214361
ENSG00000135486	ENSG00000214592
ENSG00000135486	ENSG00000214653
ENSG00000135486	ENSG00000215222
ENSG00000135486	ENSG00000215492
ENSG00000135486	ENSG00000215870
ENSG00000135486	ENSG00000215876
ENSG00000135486	ENSG00000215885
ENSG00000135502	ENSG00000091137
ENSG00000135502	ENSG00000091138
ENSG00000135502	ENSG00000112053
ENSG00000135502	ENSG00000145217
ENSG00000135502	ENSG00000147606
ENSG00000135502	ENSG00000155850
ENSG00000135502	ENSG00000170615
ENSG00000135502	ENSG00000174502
ENSG00000135503	ENSG00000106799
ENSG00000135503	ENSG00000107779
ENSG00000135503	ENSG00000115170
ENSG00000135503	ENSG00000123612
ENSG00000135503	ENSG00000135409
ENSG00000135503	ENSG00000138696
ENSG00000135503	ENSG00000139567
ENSG00000135503	ENSG00000163513
ENSG00000135503	ENSG00000204217
ENSG00000135517	ENSG00000086159
ENSG00000135517	ENSG00000103375
ENSG00000135517	ENSG00000106125
ENSG00000135517	ENSG00000161798
ENSG00000135517	ENSG00000167580
ENSG00000135517	ENSG00000171885
ENSG00000135519	ENSG00000055118
ENSG00000135519	ENSG00000089558
ENSG00000135519	ENSG00000140015
ENSG00000135519	ENSG00000143473
ENSG00000135519	ENSG00000173826

ENSG00000135519	ENSG00000183960
ENSG00000135519	ENSG00000184611
ENSG00000135525	ENSG00000116871
ENSG00000135525	ENSG00000129680
ENSG00000135525	ENSG00000184368
ENSG00000135535	ENSG00000174950
ENSG00000135540	ENSG00000162522
ENSG00000135540	ENSG00000188158
ENSG00000135540	ENSG00000204131
ENSG00000135547	ENSG00000163909
ENSG00000135547	ENSG00000164683
ENSG00000135547	ENSG00000187821
ENSG00000135549	ENSG00000168734
ENSG00000135549	ENSG00000171033
ENSG00000135569	ENSG00000146378
ENSG00000135569	ENSG00000146383
ENSG00000135569	ENSG00000146385
ENSG00000135569	ENSG00000146399
ENSG00000135577	ENSG00000102239
ENSG00000135577	ENSG00000126010
ENSG00000135577	ENSG00000136160
ENSG00000135577	ENSG00000151617
ENSG00000135577	ENSG00000170075
ENSG00000135577	ENSG00000170775
ENSG00000135596	ENSG00000093100
ENSG00000135596	ENSG00000133816
ENSG00000135597	ENSG00000169891
ENSG00000135604	ENSG00000099365
ENSG00000135604	ENSG00000103496
ENSG00000135604	ENSG00000106089
ENSG00000135604	ENSG00000111450
ENSG00000135604	ENSG00000166900
ENSG00000135604	ENSG00000178750
ENSG00000135605	ENSG00000010671
ENSG00000135605	ENSG00000074966
ENSG00000135605	ENSG00000102010
ENSG00000135605	ENSG00000113263
ENSG00000135605	ENSG00000151422
ENSG00000135605	ENSG00000182511
ENSG00000135625	ENSG00000120738
ENSG00000135625	ENSG00000122877
ENSG00000135625	ENSG00000179388
ENSG00000135625	ENSG00000184937
ENSG00000135631	ENSG00000107560
ENSG00000135631	ENSG00000156675
ENSG00000135636	ENSG00000088340
ENSG00000135636	ENSG00000115155
ENSG00000135636	ENSG00000138119
ENSG00000135636	ENSG00000214272
ENSG00000135636	ENSG00000214814
ENSG00000135638	ENSG00000170370
ENSG00000135643	ENSG00000145936
ENSG00000135643	ENSG00000171121
ENSG00000135643	ENSG00000197584
ENSG00000135643	ENSG00000215575
ENSG00000135655	ENSG00000102226
ENSG00000135655	ENSG00000114316
ENSG00000135655	ENSG00000172046
ENSG00000135677	ENSG00000137573

ENSG00000135677	ENSG00000164291
ENSG00000135677	ENSG00000196562
ENSG00000135679	ENSG00000198625
ENSG00000135686	ENSG00000003096
ENSG00000135686	ENSG00000087448
ENSG00000135686	ENSG00000124743
ENSG00000135686	ENSG00000130487
ENSG00000135686	ENSG00000166853
ENSG00000135686	ENSG00000167487
ENSG00000135686	ENSG00000174010
ENSG00000135686	ENSG00000176595
ENSG00000135686	ENSG00000179023
ENSG00000135686	ENSG00000185214
ENSG00000135686	ENSG00000185915
ENSG00000135686	ENSG00000186231
ENSG00000135686	ENSG00000197705
ENSG00000135686	ENSG00000198642
ENSG00000135697	ENSG00000116745
ENSG00000135697	ENSG00000197580
ENSG00000135702	ENSG00000122863
ENSG00000135702	ENSG00000140835
ENSG00000135702	ENSG00000147119
ENSG00000135702	ENSG00000175040
ENSG00000135702	ENSG00000175264
ENSG00000135702	ENSG00000183196
ENSG00000135723	ENSG00000134775
ENSG00000135740	ENSG00000066230
ENSG00000135740	ENSG00000090020
ENSG00000135740	ENSG00000115616
ENSG00000135740	ENSG00000180251
ENSG00000135744	ENSG00000100665
ENSG00000135744	ENSG00000123561
ENSG00000135744	ENSG00000165951
ENSG00000135744	ENSG00000165953
ENSG00000135744	ENSG00000170054
ENSG00000135744	ENSG00000186910
ENSG00000135744	ENSG00000187483
ENSG00000135744	ENSG00000188488
ENSG00000135744	ENSG00000196136
ENSG00000135744	ENSG00000197249
ENSG00000135749	ENSG00000100731
ENSG00000135749	ENSG00000126773
ENSG00000135749	ENSG00000197136
ENSG00000135750	ENSG00000082482
ENSG00000135750	ENSG00000095981
ENSG00000135750	ENSG00000099337
ENSG00000135750	ENSG00000100433
ENSG00000135750	ENSG00000124249
ENSG00000135750	ENSG00000124780
ENSG00000135750	ENSG00000152315
ENSG00000135750	ENSG00000164626
ENSG00000135750	ENSG00000169427
ENSG00000135750	ENSG00000171303
ENSG00000135750	ENSG00000173338
ENSG00000135750	ENSG00000182450
ENSG00000135750	ENSG00000184261
ENSG00000135766	ENSG00000129521
ENSG00000135766	ENSG00000171570
ENSG00000135773	ENSG00000014216

ENSG00000135773	ENSG00000077274
ENSG00000135773	ENSG00000092529
ENSG00000135773	ENSG00000137225
ENSG00000135773	ENSG00000142330
ENSG00000135773	ENSG00000149260
ENSG00000135773	ENSG00000162909
ENSG00000135773	ENSG00000162949
ENSG00000135773	ENSG00000182472
ENSG00000135773	ENSG00000203697
ENSG00000135773	ENSG00000214711
ENSG00000135823	ENSG00000104915
ENSG00000135824	ENSG00000090104
ENSG00000135824	ENSG00000091844
ENSG00000135824	ENSG00000116741
ENSG00000135824	ENSG00000117152
ENSG00000135824	ENSG00000127074
ENSG00000135824	ENSG00000143248
ENSG00000135824	ENSG00000143333
ENSG00000135824	ENSG00000147509
ENSG00000135824	ENSG00000148908
ENSG00000135824	ENSG00000150681
ENSG00000135824	ENSG00000171700
ENSG00000135824	ENSG00000215825
ENSG00000135842	ENSG00000136830
ENSG00000135842	ENSG00000167483
ENSG00000135862	ENSG00000050555
ENSG00000135862	ENSG00000058085
ENSG00000135862	ENSG00000074527
ENSG00000135862	ENSG00000091128
ENSG00000135862	ENSG00000091136
ENSG00000135862	ENSG00000106780
ENSG00000135862	ENSG00000172037
ENSG00000135862	ENSG00000196878
ENSG00000135870	ENSG00000056586
ENSG00000135902	ENSG00000080644
ENSG00000135902	ENSG00000101204
ENSG00000135902	ENSG00000108556
ENSG00000135902	ENSG00000117971
ENSG00000135902	ENSG00000120903
ENSG00000135902	ENSG00000129749
ENSG00000135902	ENSG00000138435
ENSG00000135902	ENSG00000147432
ENSG00000135902	ENSG00000147434
ENSG00000135902	ENSG00000149305
ENSG00000135902	ENSG00000160716
ENSG00000135902	ENSG00000166664
ENSG00000135902	ENSG00000166736
ENSG00000135902	ENSG00000169684
ENSG00000135902	ENSG00000170175
ENSG00000135902	ENSG00000174343
ENSG00000135902	ENSG00000175344
ENSG00000135902	ENSG00000178084
ENSG00000135902	ENSG00000186038
ENSG00000135902	ENSG00000186090
ENSG00000135902	ENSG00000186919
ENSG00000135902	ENSG00000196811
ENSG00000135903	ENSG00000009709
ENSG00000135905	ENSG00000088387
ENSG00000135905	ENSG00000107099

ENSG00000135905	ENSG00000116641
ENSG00000135905	ENSG00000130158
ENSG00000135905	ENSG00000147251
ENSG00000135913	ENSG00000131864
ENSG00000135913	ENSG00000134588
ENSG00000135914	ENSG00000043591
ENSG00000135914	ENSG00000102468
ENSG00000135914	ENSG00000113749
ENSG00000135914	ENSG00000125631
ENSG00000135914	ENSG00000135312
ENSG00000135914	ENSG00000147246
ENSG00000135914	ENSG00000150594
ENSG00000135914	ENSG00000157219
ENSG00000135914	ENSG00000164270
ENSG00000135914	ENSG00000168830
ENSG00000135914	ENSG00000169252
ENSG00000135914	ENSG00000175658
ENSG00000135914	ENSG00000178394
ENSG00000135914	ENSG00000179097
ENSG00000135914	ENSG00000179546
ENSG00000135914	ENSG00000181210
ENSG00000135914	ENSG00000184303
ENSG00000135914	ENSG00000184845
ENSG00000135914	ENSG00000185321
ENSG00000135914	ENSG00000188778
ENSG00000135916	ENSG00000078596
ENSG00000135916	ENSG00000136156
ENSG00000135917	ENSG00000117479
ENSG00000135917	ENSG00000173638
ENSG00000135919	ENSG00000106366
ENSG00000135919	ENSG00000163536
ENSG00000135919	ENSG00000215461
ENSG00000135925	ENSG00000002745
ENSG00000135925	ENSG00000061492
ENSG00000135925	ENSG00000075290
ENSG00000135925	ENSG00000085741
ENSG00000135925	ENSG00000105989
ENSG00000135925	ENSG00000108379
ENSG00000135925	ENSG00000111186
ENSG00000135925	ENSG00000114251
ENSG00000135925	ENSG00000115596
ENSG00000135925	ENSG00000125084
ENSG00000135925	ENSG00000134245
ENSG00000135925	ENSG00000143816
ENSG00000135925	ENSG00000154342
ENSG00000135925	ENSG00000154764
ENSG00000135925	ENSG00000158955
ENSG00000135925	ENSG00000162552
ENSG00000135925	ENSG00000169884
ENSG00000135925	ENSG00000188064
ENSG00000135926	ENSG00000135472
ENSG00000135926	ENSG00000178719
ENSG00000135929	ENSG00000019186
ENSG00000135929	ENSG00000111012
ENSG00000135929	ENSG00000140459
ENSG00000135929	ENSG00000160882
ENSG00000135929	ENSG00000179142
ENSG00000135929	ENSG00000186684
ENSG00000135932	ENSG00000102547

ENSG00000135951	ENSG00000174799
ENSG00000135960	ENSG00000127863
ENSG00000135960	ENSG00000131080
ENSG00000135999	ENSG00000120616
ENSG00000136002	ENSG00000131089
ENSG00000136002	ENSG00000182957
ENSG00000136010	ENSG00000144908
ENSG00000136011	ENSG00000010327
ENSG00000136014	ENSG00000140455
ENSG00000136014	ENSG00000164663
ENSG00000136040	ENSG00000004399
ENSG00000136040	ENSG00000076356
ENSG00000136040	ENSG00000114554
ENSG00000136040	ENSG00000130827
ENSG00000136040	ENSG00000164050
ENSG00000136040	ENSG00000189437
ENSG00000136040	ENSG00000196576
ENSG00000136040	ENSG00000198753
ENSG00000136044	ENSG00000157500
ENSG00000136052	ENSG00000114544
ENSG00000136052	ENSG00000133065
ENSG00000136059	ENSG00000006747
ENSG00000136059	ENSG00000042493
ENSG00000136059	ENSG00000127831
ENSG00000136059	ENSG00000135407
ENSG00000136059	ENSG00000148180
ENSG00000136059	ENSG00000177731
ENSG00000136068	ENSG00000128591
ENSG00000136068	ENSG00000196924
ENSG00000136098	ENSG00000114670
ENSG00000136098	ENSG00000114904
ENSG00000136098	ENSG00000119638
ENSG00000136098	ENSG00000160602
ENSG00000136098	ENSG00000197168
ENSG00000136099	ENSG00000118946
ENSG00000136099	ENSG00000138650
ENSG00000136099	ENSG00000165194
ENSG00000136099	ENSG00000189184
ENSG00000136108	ENSG00000169607
ENSG00000136111	ENSG00000011454
ENSG00000136111	ENSG00000065882
ENSG00000136111	ENSG00000067208
ENSG00000136111	ENSG00000142459
ENSG00000136111	ENSG00000152061
ENSG00000136141	ENSG00000077454
ENSG00000136141	ENSG00000130224
ENSG00000136141	ENSG00000186001
ENSG00000136144	ENSG00000136161
ENSG00000136147	ENSG00000010318
ENSG00000136147	ENSG00000092140
ENSG00000136147	ENSG00000156531
ENSG00000136153	ENSG00000064042
ENSG00000136155	ENSG00000147394
ENSG00000136156	ENSG00000078596
ENSG00000136156	ENSG00000135916
ENSG00000136158	ENSG00000164056
ENSG00000136158	ENSG00000168939
ENSG00000136158	ENSG00000187678
ENSG00000136160	ENSG00000102239

ENSG00000136160	ENSG00000126010
ENSG00000136160	ENSG00000135577
ENSG00000136160	ENSG00000151617
ENSG00000136160	ENSG00000170075
ENSG00000136160	ENSG00000170775
ENSG00000136161	ENSG00000136144
ENSG00000136167	ENSG00000102024
ENSG00000136167	ENSG00000120756
ENSG00000136169	ENSG00000143379
ENSG00000136193	ENSG00000141295
ENSG00000136193	ENSG00000144306
ENSG00000136205	ENSG00000079308
ENSG00000136205	ENSG00000111077
ENSG00000136205	ENSG00000131746
ENSG00000136206	ENSG00000154732
ENSG00000136206	ENSG00000163806
ENSG00000136206	ENSG00000166667
ENSG00000136206	ENSG00000170092
ENSG00000136206	ENSG00000173678
ENSG00000136206	ENSG00000179994
ENSG00000136206	ENSG00000183318
ENSG00000136206	ENSG00000184616
ENSG00000136206	ENSG00000185040
ENSG00000136206	ENSG00000186645
ENSG00000136206	ENSG00000198305
ENSG00000136206	ENSG00000204710
ENSG00000136206	ENSG00000205238
ENSG00000136206	ENSG00000205482
ENSG00000136206	ENSG00000205505
ENSG00000136206	ENSG00000205506
ENSG00000136206	ENSG00000205508
ENSG00000136206	ENSG00000205509
ENSG00000136206	ENSG00000205580
ENSG00000136206	ENSG00000214300
ENSG00000136206	ENSG00000215092
ENSG00000136213	ENSG00000115526
ENSG00000136213	ENSG00000124302
ENSG00000136213	ENSG00000154080
ENSG00000136213	ENSG00000169105
ENSG00000136213	ENSG00000171310
ENSG00000136213	ENSG00000180767
ENSG00000136231	ENSG00000073792
ENSG00000136231	ENSG00000159217
ENSG00000136235	ENSG00000185664
ENSG00000136237	ENSG00000079337
ENSG00000136237	ENSG00000091428
ENSG00000136237	ENSG00000108352
ENSG00000136237	ENSG00000109756
ENSG00000136238	ENSG00000070831
ENSG00000136238	ENSG00000119729
ENSG00000136238	ENSG00000126785
ENSG00000136238	ENSG00000128340
ENSG00000136238	ENSG00000169750
ENSG00000136238	ENSG00000177105
ENSG00000136240	ENSG00000100196
ENSG00000136240	ENSG00000105438
ENSG00000136261	ENSG00000082153
ENSG00000136267	ENSG00000058866
ENSG00000136267	ENSG00000065357

ENSG00000136267	ENSG00000077044
ENSG00000136267	ENSG00000102780
ENSG00000136267	ENSG00000153933
ENSG00000136267	ENSG00000204466
ENSG00000136274	ENSG00000121089
ENSG00000136274	ENSG00000196531
ENSG00000136274	ENSG00000196861
ENSG00000136279	ENSG00000085733
ENSG00000136279	ENSG00000113758
ENSG00000136279	ENSG00000180353
ENSG00000136280	ENSG00000101331
ENSG00000136286	ENSG00000128641
ENSG00000136286	ENSG00000142347
ENSG00000136286	ENSG00000157483
ENSG00000136286	ENSG00000166866
ENSG00000136286	ENSG00000176658
ENSG00000136286	ENSG00000197879
ENSG00000136295	ENSG00000141540
ENSG00000136295	ENSG00000167614
ENSG00000136297	ENSG00000108960
ENSG00000136305	ENSG00000176194
ENSG00000136305	ENSG00000187288
ENSG00000136327	ENSG00000109705
ENSG00000136327	ENSG00000119919
ENSG00000136327	ENSG00000125816
ENSG00000136327	ENSG00000125820
ENSG00000136327	ENSG00000136352
ENSG00000136327	ENSG00000180053
ENSG00000136327	ENSG00000183072
ENSG00000136352	ENSG00000109705
ENSG00000136352	ENSG00000119919
ENSG00000136352	ENSG00000125816
ENSG00000136352	ENSG00000125820
ENSG00000136352	ENSG00000136327
ENSG00000136352	ENSG00000180053
ENSG00000136352	ENSG00000183072
ENSG00000136367	ENSG00000091656
ENSG00000136367	ENSG00000140836
ENSG00000136378	ENSG00000049192
ENSG00000136378	ENSG00000087116
ENSG00000136378	ENSG00000138316
ENSG00000136378	ENSG00000140470
ENSG00000136378	ENSG00000140873
ENSG00000136378	ENSG00000142303
ENSG00000136378	ENSG00000145536
ENSG00000136378	ENSG00000145808
ENSG00000136378	ENSG00000151388
ENSG00000136378	ENSG00000156140
ENSG00000136378	ENSG00000160323
ENSG00000136378	ENSG00000163638
ENSG00000136378	ENSG00000173157
ENSG00000136378	ENSG00000214644
ENSG00000136378	ENSG00000215743
ENSG00000136379	ENSG00000107362
ENSG00000136379	ENSG00000129968
ENSG00000136379	ENSG00000182502
ENSG00000136379	ENSG00000182556
ENSG00000136379	ENSG00000198658
ENSG00000136379	ENSG00000203835

ENSG00000136381	ENSG00000122729
ENSG00000136404	ENSG00000213996
ENSG00000136425	ENSG00000141977
ENSG00000136425	ENSG00000157884
ENSG00000136425	ENSG00000185043
ENSG00000136436	ENSG00000012822
ENSG00000136436	ENSG00000106052
ENSG00000136448	ENSG00000152465
ENSG00000136450	ENSG00000111786
ENSG00000136451	ENSG00000100105
ENSG00000136451	ENSG00000103495
ENSG00000136457	ENSG00000146006
ENSG00000136457	ENSG00000162951
ENSG00000136457	ENSG00000176204
ENSG00000136457	ENSG00000198739
ENSG00000136487	ENSG00000136488
ENSG00000136487	ENSG00000172179
ENSG00000136487	ENSG00000189162
ENSG00000136487	ENSG00000204414
ENSG00000136487	ENSG00000213218
ENSG00000136488	ENSG00000136487
ENSG00000136488	ENSG00000172179
ENSG00000136488	ENSG00000189162
ENSG00000136488	ENSG00000204414
ENSG00000136488	ENSG00000213218
ENSG00000136490	ENSG00000129170
ENSG00000136490	ENSG00000146215
ENSG00000136490	ENSG00000159176
ENSG00000136490	ENSG00000175183
ENSG00000136490	ENSG00000182809
ENSG00000136490	ENSG00000197893
ENSG00000136490	ENSG00000213145
ENSG00000136518	ENSG00000077080
ENSG00000136527	ENSG00000164548
ENSG00000136531	ENSG00000007314
ENSG00000136531	ENSG00000136546
ENSG00000136531	ENSG00000144285
ENSG00000136531	ENSG00000153253
ENSG00000136531	ENSG00000168356
ENSG00000136531	ENSG00000169432
ENSG00000136531	ENSG00000183873
ENSG00000136531	ENSG00000185313
ENSG00000136531	ENSG00000196876
ENSG00000136535	ENSG00000073861
ENSG00000136535	ENSG00000089225
ENSG00000136535	ENSG00000092607
ENSG00000136535	ENSG00000112837
ENSG00000136535	ENSG00000121068
ENSG00000136535	ENSG00000121075
ENSG00000136535	ENSG00000122145
ENSG00000136535	ENSG00000135111
ENSG00000136535	ENSG00000143178
ENSG00000136535	ENSG00000149922
ENSG00000136535	ENSG00000163508
ENSG00000136535	ENSG00000164458
ENSG00000136535	ENSG00000164532
ENSG00000136535	ENSG00000167800
ENSG00000136535	ENSG00000174197
ENSG00000136535	ENSG00000184058

ENSG00000136546	ENSG00000007314
ENSG00000136546	ENSG00000136531
ENSG00000136546	ENSG00000144285
ENSG00000136546	ENSG00000153253
ENSG00000136546	ENSG00000168356
ENSG00000136546	ENSG00000169432
ENSG00000136546	ENSG00000183873
ENSG00000136546	ENSG00000185313
ENSG00000136546	ENSG00000196876
ENSG00000136573	ENSG00000000938
ENSG00000136573	ENSG00000010810
ENSG00000136573	ENSG00000101336
ENSG00000136573	ENSG00000111816
ENSG00000136573	ENSG00000147507
ENSG00000136573	ENSG00000176105
ENSG00000136573	ENSG00000182866
ENSG00000136573	ENSG00000197122
ENSG00000136574	ENSG00000102145
ENSG00000136574	ENSG00000107485
ENSG00000136574	ENSG00000130700
ENSG00000136574	ENSG00000141448
ENSG00000136574	ENSG00000179348
ENSG00000136603	ENSG00000157933
ENSG00000136603	ENSG00000188779
ENSG00000136603	ENSG00000215474
ENSG00000136634	ENSG00000111536
ENSG00000136634	ENSG00000127318
ENSG00000136634	ENSG00000142224
ENSG00000136634	ENSG00000162891
ENSG00000136634	ENSG00000162892
ENSG00000136643	ENSG00000110025
ENSG00000136643	ENSG00000198208
ENSG00000136653	ENSG00000068028
ENSG00000136653	ENSG00000101265
ENSG00000136653	ENSG00000107551
ENSG00000136653	ENSG00000153179
ENSG00000136653	ENSG00000169435
ENSG00000136700	ENSG00000156755
ENSG00000136700	ENSG00000183719
ENSG00000136700	ENSG00000197794
ENSG00000136700	ENSG00000204670
ENSG00000136700	ENSG00000204776
ENSG00000136700	ENSG00000204780
ENSG00000136700	ENSG00000211599
ENSG00000136700	ENSG00000211600
ENSG00000136700	ENSG00000211601
ENSG00000136700	ENSG00000211602
ENSG00000136700	ENSG00000211603
ENSG00000136700	ENSG00000211604
ENSG00000136700	ENSG00000211605
ENSG00000136700	ENSG00000211606
ENSG00000136700	ENSG00000211607
ENSG00000136700	ENSG00000211608
ENSG00000136700	ENSG00000211609
ENSG00000136700	ENSG00000211610
ENSG00000136700	ENSG00000211611
ENSG00000136700	ENSG00000211613
ENSG00000136700	ENSG00000211616
ENSG00000136700	ENSG00000211617

ENSG00000136700	ENSG00000211618
ENSG00000136700	ENSG00000211620
ENSG00000136700	ENSG00000211625
ENSG00000136700	ENSG00000211626
ENSG00000136700	ENSG00000211627
ENSG00000136700	ENSG00000211628
ENSG00000136700	ENSG00000211629
ENSG00000136700	ENSG00000211630
ENSG00000136700	ENSG00000211631
ENSG00000136700	ENSG00000211632
ENSG00000136700	ENSG00000211633
ENSG00000136700	ENSG00000211634
ENSG00000136700	ENSG00000211635
ENSG00000136700	ENSG00000211636
ENSG00000136717	ENSG00000078053
ENSG00000136717	ENSG00000110934
ENSG00000136720	ENSG00000171004
ENSG00000136720	ENSG00000185352
ENSG00000136731	ENSG00000102595
ENSG00000136738	ENSG00000115145
ENSG00000136750	ENSG00000128683
ENSG00000136750	ENSG00000139631
ENSG00000136750	ENSG00000144644
ENSG00000136750	ENSG00000215438
ENSG00000136754	ENSG00000108798
ENSG00000136754	ENSG00000138443
ENSG00000136802	ENSG00000171017
ENSG00000136802	ENSG00000171488
ENSG00000136802	ENSG00000171492
ENSG00000136802	ENSG00000197147
ENSG00000136811	ENSG00000122417
ENSG00000136816	ENSG00000136827
ENSG00000136816	ENSG00000160404
ENSG00000136816	ENSG00000186283
ENSG00000136816	ENSG00000198113
ENSG00000136826	ENSG00000067082
ENSG00000136826	ENSG00000102554
ENSG00000136826	ENSG00000105610
ENSG00000136826	ENSG00000127528
ENSG00000136826	ENSG00000129911
ENSG00000136826	ENSG00000139651
ENSG00000136826	ENSG00000155090
ENSG00000136826	ENSG00000164651
ENSG00000136826	ENSG00000167182
ENSG00000136826	ENSG00000170374
ENSG00000136826	ENSG00000171872
ENSG00000136826	ENSG00000172059
ENSG00000136826	ENSG00000174595
ENSG00000136826	ENSG00000189120
ENSG00000136826	ENSG00000197294
ENSG00000136826	ENSG00000197550
ENSG00000136826	ENSG00000204335
ENSG00000136827	ENSG00000136816
ENSG00000136827	ENSG00000160404
ENSG00000136827	ENSG00000186283
ENSG00000136827	ENSG00000198113
ENSG00000136828	ENSG00000116191
ENSG00000136830	ENSG00000135842
ENSG00000136830	ENSG00000167483

ENSG00000136840	ENSG00000184005
ENSG00000136842	ENSG00000128872
ENSG00000136842	ENSG00000138594
ENSG00000136842	ENSG00000163157
ENSG00000136842	ENSG00000163380
ENSG00000136842	ENSG00000163431
ENSG00000136842	ENSG00000170807
ENSG00000136848	ENSG00000075391
ENSG00000136848	ENSG00000105122
ENSG00000136848	ENSG00000197283
ENSG00000136854	ENSG00000076944
ENSG00000136854	ENSG00000116266
ENSG00000136856	ENSG00000160326
ENSG00000136861	ENSG00000178104
ENSG00000136869	ENSG00000101916
ENSG00000136869	ENSG00000134061
ENSG00000136869	ENSG00000137462
ENSG00000136869	ENSG00000174123
ENSG00000136869	ENSG00000174125
ENSG00000136869	ENSG00000174130
ENSG00000136869	ENSG00000187554
ENSG00000136872	ENSG00000109107
ENSG00000136872	ENSG00000149925
ENSG00000136878	ENSG00000077254
ENSG00000136928	ENSG00000175697
ENSG00000136928	ENSG00000204681
ENSG00000136930	ENSG00000205220
ENSG00000136931	ENSG00000116833
ENSG00000136931	ENSG00000148200
ENSG00000136938	ENSG00000139223
ENSG00000136938	ENSG00000140350
ENSG00000136938	ENSG00000143401
ENSG00000136938	ENSG00000184701
ENSG00000136938	ENSG00000215332
ENSG00000136940	ENSG00000115539
ENSG00000136940	ENSG00000116703
ENSG00000136940	ENSG00000163440
ENSG00000136940	ENSG00000215109
ENSG00000136943	ENSG00000135047
ENSG00000136943	ENSG00000143387
ENSG00000136943	ENSG00000163131
ENSG00000136943	ENSG00000188029
ENSG00000136943	ENSG00000204437
ENSG00000136944	ENSG00000016082
ENSG00000136944	ENSG00000089116
ENSG00000136944	ENSG00000106689
ENSG00000136944	ENSG00000106852
ENSG00000136944	ENSG00000107187
ENSG00000136944	ENSG00000121454
ENSG00000136944	ENSG00000132130
ENSG00000136944	ENSG00000143013
ENSG00000136944	ENSG00000143355
ENSG00000136944	ENSG00000159556
ENSG00000136944	ENSG00000162624
ENSG00000136944	ENSG00000162761
ENSG00000136950	ENSG00000162704
ENSG00000136960	ENSG00000001561
ENSG00000136960	ENSG00000112796
ENSG00000136960	ENSG00000154269

ENSG00000136960	ENSG00000164303
ENSG00000136960	ENSG00000182156
ENSG00000136960	ENSG00000197594
ENSG00000136997	ENSG00000116990
ENSG00000136997	ENSG00000134323
ENSG00000136999	ENSG00000064205
ENSG00000136999	ENSG00000104415
ENSG00000136999	ENSG00000112761
ENSG00000136999	ENSG00000118523
ENSG00000136999	ENSG00000142871
ENSG00000137070	ENSG00000105246
ENSG00000137070	ENSG00000122756
ENSG00000137070	ENSG00000160712
ENSG00000137073	ENSG00000143569
ENSG00000137075	ENSG00000127870
ENSG00000137075	ENSG00000131263
ENSG00000137075	ENSG00000146083
ENSG00000137076	ENSG00000073712
ENSG00000137076	ENSG00000101311
ENSG00000137076	ENSG00000149781
ENSG00000137076	ENSG00000171914
ENSG00000137090	ENSG00000064218
ENSG00000137090	ENSG00000142025
ENSG00000137090	ENSG00000142700
ENSG00000137090	ENSG00000143006
ENSG00000137090	ENSG00000173253
ENSG00000137090	ENSG00000176399
ENSG00000137094	ENSG00000132002
ENSG00000137094	ENSG00000162616
ENSG00000137094	ENSG00000187726
ENSG00000137103	ENSG00000129925
ENSG00000137124	ENSG00000111275
ENSG00000137124	ENSG00000128918
ENSG00000137124	ENSG00000165092
ENSG00000137124	ENSG00000184254
ENSG00000137142	ENSG00000107821
ENSG00000137142	ENSG00000163453
ENSG00000137145	ENSG00000170456
ENSG00000137145	ENSG00000174485
ENSG00000137145	ENSG00000184014
ENSG00000137145	ENSG00000198837
ENSG00000137154	ENSG00000173322
ENSG00000137154	ENSG00000214908
ENSG00000137166	ENSG00000049768
ENSG00000137166	ENSG00000114861
ENSG00000137166	ENSG00000128573
ENSG00000137171	ENSG00000011295
ENSG00000137171	ENSG00000062725
ENSG00000137171	ENSG00000104892
ENSG00000137171	ENSG00000126214
ENSG00000137171	ENSG00000174996
ENSG00000137177	ENSG00000054523
ENSG00000137177	ENSG00000089177
ENSG00000137177	ENSG00000118193
ENSG00000137177	ENSG00000129250
ENSG00000137177	ENSG00000130294
ENSG00000137177	ENSG00000197892
ENSG00000137193	ENSG00000102096
ENSG00000137193	ENSG00000198355

ENSG00000137198	ENSG00000100938
ENSG00000137198	ENSG00000106348
ENSG00000137198	ENSG00000178035
ENSG00000137203	ENSG00000008196
ENSG00000137203	ENSG00000008197
ENSG00000137203	ENSG00000087510
ENSG00000137203	ENSG00000116819
ENSG00000137210	ENSG00000096092
ENSG00000137210	ENSG00000111843
ENSG00000137210	ENSG00000189156
ENSG00000137210	ENSG00000214881
ENSG00000137216	ENSG00000165548
ENSG00000137216	ENSG00000196187
ENSG00000137218	ENSG00000101134
ENSG00000137218	ENSG00000115325
ENSG00000137218	ENSG00000125170
ENSG00000137218	ENSG00000146094
ENSG00000137218	ENSG00000147443
ENSG00000137218	ENSG00000166225
ENSG00000137218	ENSG00000206052
ENSG00000137221	ENSG00000183092
ENSG00000137225	ENSG00000014216
ENSG00000137225	ENSG00000077274
ENSG00000137225	ENSG00000092529
ENSG00000137225	ENSG00000135773
ENSG00000137225	ENSG00000142330
ENSG00000137225	ENSG00000149260
ENSG00000137225	ENSG00000162909
ENSG00000137225	ENSG00000162949
ENSG00000137225	ENSG00000182472
ENSG00000137225	ENSG00000203697
ENSG00000137225	ENSG00000214711
ENSG00000137251	ENSG00000142910
ENSG00000137252	ENSG00000056291
ENSG00000137252	ENSG00000075073
ENSG00000137252	ENSG00000101292
ENSG00000137252	ENSG00000110148
ENSG00000137252	ENSG00000115353
ENSG00000137252	ENSG00000119973
ENSG00000137252	ENSG00000121764
ENSG00000137252	ENSG00000123901
ENSG00000137252	ENSG00000148734
ENSG00000137252	ENSG00000159279
ENSG00000137252	ENSG00000163394
ENSG00000137252	ENSG00000164128
ENSG00000137252	ENSG00000164129
ENSG00000137252	ENSG00000169618
ENSG00000137252	ENSG00000169836
ENSG00000137252	ENSG00000185149
ENSG00000137252	ENSG00000186867
ENSG00000137252	ENSG00000204174
ENSG00000137252	ENSG00000215167
ENSG00000137259	ENSG00000111332
ENSG00000137259	ENSG00000124642
ENSG00000137259	ENSG00000158403
ENSG00000137259	ENSG00000164508
ENSG00000137259	ENSG00000168274
ENSG00000137259	ENSG00000180573
ENSG00000137259	ENSG00000181218

ENSG00000137259	ENSG00000182611
ENSG00000137259	ENSG00000183558
ENSG00000137259	ENSG00000184260
ENSG00000137259	ENSG00000184270
ENSG00000137259	ENSG00000184348
ENSG00000137259	ENSG00000184825
ENSG00000137259	ENSG00000185978
ENSG00000137259	ENSG00000188486
ENSG00000137259	ENSG00000196747
ENSG00000137259	ENSG00000196787
ENSG00000137259	ENSG00000196866
ENSG00000137259	ENSG00000198082
ENSG00000137259	ENSG00000198307
ENSG00000137259	ENSG00000198374
ENSG00000137259	ENSG00000203812
ENSG00000137259	ENSG00000205070
ENSG00000137259	ENSG00000205079
ENSG00000137261	ENSG00000142687
ENSG00000137265	ENSG00000117595
ENSG00000137265	ENSG00000125347
ENSG00000137265	ENSG00000126456
ENSG00000137265	ENSG00000128604
ENSG00000137265	ENSG00000140968
ENSG00000137265	ENSG00000168310
ENSG00000137265	ENSG00000185507
ENSG00000137265	ENSG00000213928
ENSG00000137267	ENSG00000101162
ENSG00000137267	ENSG00000104833
ENSG00000137267	ENSG00000127589
ENSG00000137267	ENSG00000137285
ENSG00000137267	ENSG00000159247
ENSG00000137267	ENSG00000173213
ENSG00000137267	ENSG00000173876
ENSG00000137267	ENSG00000176014
ENSG00000137267	ENSG00000188229
ENSG00000137267	ENSG00000196230
ENSG00000137267	ENSG00000198211
ENSG00000137267	ENSG00000215801
ENSG00000137267	ENSG00000215813
ENSG00000137267	ENSG00000215814
ENSG00000137269	ENSG00000033122
ENSG00000137269	ENSG00000112851
ENSG00000137269	ENSG00000147324
ENSG00000137269	ENSG00000180900
ENSG00000137269	ENSG00000188306
ENSG00000137270	ENSG00000124827
ENSG00000137273	ENSG00000103241
ENSG00000137275	ENSG00000104312
ENSG00000137275	ENSG00000129465
ENSG00000137275	ENSG00000170209
ENSG00000137275	ENSG00000183421
ENSG00000137285	ENSG00000101162
ENSG00000137285	ENSG00000104833
ENSG00000137285	ENSG00000127589
ENSG00000137285	ENSG00000137267
ENSG00000137285	ENSG00000159247
ENSG00000137285	ENSG00000173213
ENSG00000137285	ENSG00000173876
ENSG00000137285	ENSG00000176014

ENSG00000137285	ENSG00000188229
ENSG00000137285	ENSG00000196230
ENSG00000137285	ENSG00000198211
ENSG00000137285	ENSG00000215801
ENSG00000137285	ENSG00000215813
ENSG00000137285	ENSG00000215814
ENSG00000137309	ENSG00000149948
ENSG00000137393	ENSG00000034677
ENSG00000137393	ENSG00000116514
ENSG00000137393	ENSG00000151692
ENSG00000137409	ENSG00000109919
ENSG00000137409	ENSG00000214315
ENSG00000137440	ENSG00000137441
ENSG00000137440	ENSG00000174721
ENSG00000137441	ENSG00000137440
ENSG00000137441	ENSG00000174721
ENSG00000137449	ENSG00000107864
ENSG00000137449	ENSG00000113742
ENSG00000137449	ENSG00000214575
ENSG00000137462	ENSG00000101916
ENSG00000137462	ENSG00000134061
ENSG00000137462	ENSG00000136869
ENSG00000137462	ENSG00000174123
ENSG00000137462	ENSG00000174125
ENSG00000137462	ENSG00000174130
ENSG00000137462	ENSG00000187554
ENSG00000137474	ENSG00000169994
ENSG00000137478	ENSG00000197948
ENSG00000137486	ENSG00000120500
ENSG00000137486	ENSG00000130561
ENSG00000137486	ENSG00000141480
ENSG00000137491	ENSG00000084453
ENSG00000137491	ENSG00000101187
ENSG00000137491	ENSG00000111700
ENSG00000137491	ENSG00000134538
ENSG00000137491	ENSG00000137571
ENSG00000137491	ENSG00000139155
ENSG00000137491	ENSG00000173930
ENSG00000137491	ENSG00000174640
ENSG00000137491	ENSG00000176463
ENSG00000137491	ENSG00000205359
ENSG00000137491	ENSG00000205754
ENSG00000137492	ENSG00000197056
ENSG00000137492	ENSG00000215028
ENSG00000137500	ENSG00000050393
ENSG00000137501	ENSG00000102362
ENSG00000137501	ENSG00000142765
ENSG00000137501	ENSG00000147041
ENSG00000137501	ENSG00000164674
ENSG00000137507	ENSG00000174004
ENSG00000137522	ENSG00000145428
ENSG00000137558	ENSG00000096006
ENSG00000137558	ENSG00000101074
ENSG00000137558	ENSG00000103196
ENSG00000137558	ENSG00000121005
ENSG00000137558	ENSG00000124490
ENSG00000137558	ENSG00000124812
ENSG00000137558	ENSG00000139278
ENSG00000137558	ENSG00000164530

ENSG00000137558	ENSG00000173401
ENSG00000137558	ENSG00000180481
ENSG00000137561	ENSG00000124120
ENSG00000137561	ENSG00000140522
ENSG00000137561	ENSG00000146352
ENSG00000137561	ENSG00000177182
ENSG00000137573	ENSG00000135677
ENSG00000137573	ENSG00000164291
ENSG00000137573	ENSG00000196562
ENSG00000137575	ENSG00000125775
ENSG00000137634	ENSG00000095110
ENSG00000137634	ENSG00000144815
ENSG00000137634	ENSG00000204361
ENSG00000137648	ENSG00000105707
ENSG00000137648	ENSG00000137747
ENSG00000137648	ENSG00000145244
ENSG00000137648	ENSG00000154646
ENSG00000137648	ENSG00000160183
ENSG00000137648	ENSG00000166682
ENSG00000137648	ENSG00000184012
ENSG00000137672	ENSG00000069018
ENSG00000137672	ENSG00000072315
ENSG00000137672	ENSG00000133107
ENSG00000137672	ENSG00000138741
ENSG00000137672	ENSG00000144935
ENSG00000137673	ENSG00000008516
ENSG00000137673	ENSG00000087245
ENSG00000137673	ENSG00000099953
ENSG00000137673	ENSG00000100985
ENSG00000137673	ENSG00000102996
ENSG00000137673	ENSG00000110347
ENSG00000137673	ENSG00000118113
ENSG00000137673	ENSG00000123342
ENSG00000137673	ENSG00000125966
ENSG00000137673	ENSG00000129270
ENSG00000137673	ENSG00000137674
ENSG00000137673	ENSG00000137675
ENSG00000137673	ENSG00000137745
ENSG00000137673	ENSG00000149968
ENSG00000137673	ENSG00000154485
ENSG00000137673	ENSG00000156103
ENSG00000137673	ENSG00000157227
ENSG00000137673	ENSG00000166670
ENSG00000137673	ENSG00000167346
ENSG00000137673	ENSG00000189409
ENSG00000137673	ENSG00000196611
ENSG00000137673	ENSG00000198598
ENSG00000137673	ENSG00000215914
ENSG00000137674	ENSG00000008516
ENSG00000137674	ENSG00000087245
ENSG00000137674	ENSG00000099953
ENSG00000137674	ENSG00000100985
ENSG00000137674	ENSG00000102996
ENSG00000137674	ENSG00000110347
ENSG00000137674	ENSG00000118113
ENSG00000137674	ENSG00000123342
ENSG00000137674	ENSG00000125966
ENSG00000137674	ENSG00000129270
ENSG00000137674	ENSG00000137673

ENSG00000137674	ENSG00000137675
ENSG00000137674	ENSG00000137745
ENSG00000137674	ENSG00000149968
ENSG00000137674	ENSG00000154485
ENSG00000137674	ENSG00000156103
ENSG00000137674	ENSG00000157227
ENSG00000137674	ENSG00000166670
ENSG00000137674	ENSG00000167346
ENSG00000137674	ENSG00000189409
ENSG00000137674	ENSG00000196611
ENSG00000137674	ENSG00000198598
ENSG00000137674	ENSG00000215914
ENSG00000137675	ENSG00000008516
ENSG00000137675	ENSG00000087245
ENSG00000137675	ENSG00000099953
ENSG00000137675	ENSG00000100985
ENSG00000137675	ENSG00000102996
ENSG00000137675	ENSG00000110347
ENSG00000137675	ENSG00000118113
ENSG00000137675	ENSG00000123342
ENSG00000137675	ENSG00000125966
ENSG00000137675	ENSG00000129270
ENSG00000137675	ENSG00000137673
ENSG00000137675	ENSG00000137674
ENSG00000137675	ENSG00000137745
ENSG00000137675	ENSG00000149968
ENSG00000137675	ENSG00000154485
ENSG00000137675	ENSG00000156103
ENSG00000137675	ENSG00000157227
ENSG00000137675	ENSG00000166670
ENSG00000137675	ENSG00000167346
ENSG00000137675	ENSG00000189409
ENSG00000137675	ENSG00000196611
ENSG00000137675	ENSG00000198598
ENSG00000137675	ENSG00000215914
ENSG00000137692	ENSG00000109184
ENSG00000137693	ENSG00000018408
ENSG00000137693	ENSG00000214251
ENSG00000137699	ENSG00000121060
ENSG00000137699	ENSG00000132481
ENSG00000137707	ENSG00000133639
ENSG00000137707	ENSG00000154640
ENSG00000137707	ENSG00000159388
ENSG00000137710	ENSG00000092820
ENSG00000137710	ENSG00000147065
ENSG00000137710	ENSG00000186575
ENSG00000137713	ENSG00000105568
ENSG00000137726	ENSG00000089356
ENSG00000137726	ENSG00000126258
ENSG00000137726	ENSG00000137731
ENSG00000137726	ENSG00000150201
ENSG00000137726	ENSG00000182707
ENSG00000137727	ENSG00000164691
ENSG00000137731	ENSG00000089356
ENSG00000137731	ENSG00000126258
ENSG00000137731	ENSG00000137726
ENSG00000137731	ENSG00000150201
ENSG00000137731	ENSG00000182707
ENSG00000137745	ENSG00000008516

ENSG00000137745	ENSG00000087245
ENSG00000137745	ENSG00000099953
ENSG00000137745	ENSG00000100985
ENSG00000137745	ENSG00000102996
ENSG00000137745	ENSG00000110347
ENSG00000137745	ENSG00000118113
ENSG00000137745	ENSG00000123342
ENSG00000137745	ENSG00000125966
ENSG00000137745	ENSG00000129270
ENSG00000137745	ENSG00000137673
ENSG00000137745	ENSG00000137674
ENSG00000137745	ENSG00000137675
ENSG00000137745	ENSG00000149968
ENSG00000137745	ENSG00000154485
ENSG00000137745	ENSG00000156103
ENSG00000137745	ENSG00000157227
ENSG00000137745	ENSG00000166670
ENSG00000137745	ENSG00000167346
ENSG00000137745	ENSG00000189409
ENSG00000137745	ENSG00000196611
ENSG00000137745	ENSG00000198598
ENSG00000137745	ENSG00000215914
ENSG00000137747	ENSG00000105707
ENSG00000137747	ENSG00000137648
ENSG00000137747	ENSG00000145244
ENSG00000137747	ENSG00000154646
ENSG00000137747	ENSG00000160183
ENSG00000137747	ENSG00000166682
ENSG00000137747	ENSG00000184012
ENSG00000137752	ENSG00000003400
ENSG00000137752	ENSG00000064012
ENSG00000137752	ENSG00000105141
ENSG00000137752	ENSG00000106144
ENSG00000137752	ENSG00000132906
ENSG00000137752	ENSG00000137757
ENSG00000137752	ENSG00000138794
ENSG00000137752	ENSG00000164305
ENSG00000137752	ENSG00000165806
ENSG00000137752	ENSG00000196954
ENSG00000137752	ENSG00000204403
ENSG00000137757	ENSG00000003400
ENSG00000137757	ENSG00000064012
ENSG00000137757	ENSG00000105141
ENSG00000137757	ENSG00000106144
ENSG00000137757	ENSG00000132906
ENSG00000137757	ENSG00000137752
ENSG00000137757	ENSG00000138794
ENSG00000137757	ENSG00000164305
ENSG00000137757	ENSG00000165806
ENSG00000137757	ENSG00000196954
ENSG00000137757	ENSG00000204403
ENSG00000137766	ENSG00000130477
ENSG00000137766	ENSG00000198722
ENSG00000137776	ENSG00000130254
ENSG00000137776	ENSG00000160633
ENSG00000137801	ENSG00000105664
ENSG00000137801	ENSG00000113296
ENSG00000137801	ENSG00000169231
ENSG00000137801	ENSG00000186340

ENSG00000137802	ENSG00000075702
ENSG00000137802	ENSG00000166596
ENSG00000137809	ENSG00000005844
ENSG00000137809	ENSG00000005884
ENSG00000137809	ENSG00000005961
ENSG00000137809	ENSG00000077943
ENSG00000137809	ENSG00000083457
ENSG00000137809	ENSG00000091409
ENSG00000137809	ENSG00000115232
ENSG00000137809	ENSG00000135424
ENSG00000137809	ENSG00000138448
ENSG00000137809	ENSG00000140678
ENSG00000137809	ENSG00000143127
ENSG00000137809	ENSG00000144668
ENSG00000137809	ENSG00000156886
ENSG00000137809	ENSG00000161638
ENSG00000137809	ENSG00000164171
ENSG00000137809	ENSG00000169896
ENSG00000137809	ENSG00000213949
ENSG00000137817	ENSG00000138617
ENSG00000137817	ENSG00000151883
ENSG00000137819	ENSG00000160781
ENSG00000137819	ENSG00000170915
ENSG00000137819	ENSG00000182749
ENSG00000137819	ENSG00000188582
ENSG00000137824	ENSG00000115841
ENSG00000137824	ENSG00000176623
ENSG00000137825	ENSG00000086544
ENSG00000137825	ENSG00000143772
ENSG00000137831	ENSG00000039560
ENSG00000137831	ENSG00000089847
ENSG00000137831	ENSG00000198483
ENSG00000137834	ENSG00000101665
ENSG00000137834	ENSG00000113658
ENSG00000137834	ENSG00000120693
ENSG00000137834	ENSG00000141646
ENSG00000137834	ENSG00000166949
ENSG00000137834	ENSG00000170365
ENSG00000137834	ENSG00000175387
ENSG00000137841	ENSG00000101333
ENSG00000137841	ENSG00000114805
ENSG00000137841	ENSG00000115556
ENSG00000137841	ENSG00000115896
ENSG00000137841	ENSG00000124181
ENSG00000137841	ENSG00000139151
ENSG00000137841	ENSG00000149527
ENSG00000137841	ENSG00000149782
ENSG00000137841	ENSG00000154822
ENSG00000137841	ENSG00000161714
ENSG00000137841	ENSG00000182621
ENSG00000137841	ENSG00000187091
ENSG00000137841	ENSG00000197943
ENSG00000137843	ENSG00000101349
ENSG00000137843	ENSG00000130669
ENSG00000137860	ENSG00000156222
ENSG00000137860	ENSG00000197506
ENSG00000137871	ENSG00000056277
ENSG00000137871	ENSG00000143442
ENSG00000137871	ENSG00000169548

ENSG00000137871	ENSG00000198477
ENSG00000137872	ENSG00000010319
ENSG00000137872	ENSG00000012171
ENSG00000137872	ENSG00000075213
ENSG00000137872	ENSG00000092421
ENSG00000137872	ENSG00000095539
ENSG00000137872	ENSG00000143434
ENSG00000137872	ENSG00000153993
ENSG00000137872	ENSG00000168758
ENSG00000137872	ENSG00000170381
ENSG00000137872	ENSG00000185033
ENSG00000137872	ENSG00000187764
ENSG00000137872	ENSG00000196189
ENSG00000137936	ENSG00000095370
ENSG00000137936	ENSG00000125731
ENSG00000137942	ENSG00000125733
ENSG00000137942	ENSG00000187239
ENSG00000137948	ENSG00000141867
ENSG00000137948	ENSG00000169925
ENSG00000137948	ENSG00000204256
ENSG00000137960	ENSG00000123159
ENSG00000137960	ENSG00000179855
ENSG00000137962	ENSG00000089639
ENSG00000137962	ENSG00000180448
ENSG00000137968	ENSG00000070214
ENSG00000137968	ENSG00000129353
ENSG00000137968	ENSG00000143036
ENSG00000137968	ENSG00000204385
ENSG00000137970	ENSG00000146223
ENSG00000137970	ENSG00000147604
ENSG00000137970	ENSG00000169216
ENSG00000137970	ENSG00000171549
ENSG00000137970	ENSG00000181416
ENSG00000137970	ENSG00000182520
ENSG00000137970	ENSG00000214485
ENSG00000137970	ENSG00000214557
ENSG00000137970	ENSG00000214932
ENSG00000137970	ENSG00000215297
ENSG00000137970	ENSG00000215402
ENSG00000137976	ENSG00000105612
ENSG00000138018	ENSG00000111666
ENSG00000138018	ENSG00000134255
ENSG00000138031	ENSG00000078295
ENSG00000138031	ENSG00000121281
ENSG00000138031	ENSG00000129467
ENSG00000138031	ENSG00000155897
ENSG00000138031	ENSG00000162104
ENSG00000138031	ENSG00000164742
ENSG00000138031	ENSG00000173175
ENSG00000138031	ENSG00000174233
ENSG00000138032	ENSG00000100614
ENSG00000138032	ENSG00000177886
ENSG00000138039	ENSG00000133067
ENSG00000138039	ENSG00000139292
ENSG00000138039	ENSG00000165409
ENSG00000138039	ENSG00000170820
ENSG00000138039	ENSG00000205213
ENSG00000138041	ENSG00000100796
ENSG00000138041	ENSG00000182112

ENSG00000138050	ENSG00000134077
ENSG00000138068	ENSG00000088002
ENSG00000138068	ENSG00000105398
ENSG00000138068	ENSG00000109193
ENSG00000138068	ENSG00000130540
ENSG00000138068	ENSG00000173597
ENSG00000138068	ENSG00000196228
ENSG00000138068	ENSG00000196502
ENSG00000138068	ENSG00000197165
ENSG00000138068	ENSG00000198075
ENSG00000138068	ENSG00000198203
ENSG00000138068	ENSG00000213599
ENSG00000138068	ENSG00000213648
ENSG00000138069	ENSG00000134594
ENSG00000138069	ENSG00000137502
ENSG00000138069	ENSG00000146955
ENSG00000138069	ENSG00000172007
ENSG00000138069	ENSG00000174903
ENSG00000138069	ENSG00000185051
ENSG00000138071	ENSG00000213985
ENSG00000138075	ENSG00000143921
ENSG00000138080	ENSG00000132205
ENSG00000138080	ENSG00000183798
ENSG00000138081	ENSG00000147912
ENSG00000138083	ENSG00000100625
ENSG00000138083	ENSG00000126778
ENSG00000138083	ENSG00000170577
ENSG00000138083	ENSG00000177045
ENSG00000138083	ENSG00000184302
ENSG00000138100	ENSG00000147573
ENSG00000138100	ENSG00000158022
ENSG00000138100	ENSG00000204977
ENSG00000138100	ENSG00000213186
ENSG00000138101	ENSG00000125835
ENSG00000138101	ENSG00000128739
ENSG00000138107	ENSG00000115073
ENSG00000138119	ENSG00000088340
ENSG00000138119	ENSG00000115155
ENSG00000138119	ENSG00000135636
ENSG00000138119	ENSG00000214272
ENSG00000138119	ENSG00000214814
ENSG00000138131	ENSG00000113083
ENSG00000138131	ENSG00000115318
ENSG00000138131	ENSG00000129038
ENSG00000138131	ENSG00000134013
ENSG00000138134	ENSG00000124356
ENSG00000138136	ENSG00000152804
ENSG00000138136	ENSG00000179528
ENSG00000138162	ENSG00000013810
ENSG00000138162	ENSG00000147526
ENSG00000138166	ENSG00000111266
ENSG00000138166	ENSG00000120129
ENSG00000138166	ENSG00000120875
ENSG00000138166	ENSG00000130829
ENSG00000138166	ENSG00000139318
ENSG00000138166	ENSG00000143507
ENSG00000138166	ENSG00000158050
ENSG00000138166	ENSG00000164086
ENSG00000138166	ENSG00000184545

ENSG00000138172	ENSG00000164451
ENSG00000138172	ENSG00000178033
ENSG00000138172	ENSG00000183128
ENSG00000138172	ENSG00000185933
ENSG00000138172	ENSG00000188820
ENSG00000138182	ENSG00000112984
ENSG00000138182	ENSG00000137807
ENSG00000138185	ENSG00000054179
ENSG00000138185	ENSG00000168032
ENSG00000138185	ENSG00000188833
ENSG00000138190	ENSG00000144036
ENSG00000138271	ENSG00000169313
ENSG00000138271	ENSG00000171659
ENSG00000138271	ENSG00000174944
ENSG00000138271	ENSG00000174946
ENSG00000138271	ENSG00000181631
ENSG00000138286	ENSG00000109794
ENSG00000138286	ENSG00000214424
ENSG00000138308	ENSG00000123739
ENSG00000138311	ENSG00000163013
ENSG00000138315	ENSG00000119913
ENSG00000138315	ENSG00000169344
ENSG00000138315	ENSG00000169347
ENSG00000138316	ENSG00000049192
ENSG00000138316	ENSG00000087116
ENSG00000138316	ENSG00000136378
ENSG00000138316	ENSG00000140470
ENSG00000138316	ENSG00000140873
ENSG00000138316	ENSG00000142303
ENSG00000138316	ENSG00000145536
ENSG00000138316	ENSG00000145808
ENSG00000138316	ENSG00000151388
ENSG00000138316	ENSG00000156140
ENSG00000138316	ENSG00000160323
ENSG00000138316	ENSG00000163638
ENSG00000138316	ENSG00000173157
ENSG00000138316	ENSG00000214644
ENSG00000138316	ENSG00000215743
ENSG00000138347	ENSG00000120729
ENSG00000138347	ENSG00000129116
ENSG00000138356	ENSG00000158125
ENSG00000138376	ENSG00000101745
ENSG00000138376	ENSG00000145700
ENSG00000138376	ENSG00000160949
ENSG00000138376	ENSG00000167522
ENSG00000138378	ENSG00000115415
ENSG00000138378	ENSG00000126561
ENSG00000138378	ENSG00000166888
ENSG00000138378	ENSG00000168610
ENSG00000138378	ENSG00000170581
ENSG00000138378	ENSG00000173757
ENSG00000138379	ENSG00000092969
ENSG00000138379	ENSG00000105329
ENSG00000138379	ENSG00000135414
ENSG00000138379	ENSG00000139269
ENSG00000138386	ENSG00000166886
ENSG00000138386	ENSG00000214916
ENSG00000138395	ENSG00000058091
ENSG00000138395	ENSG00000059758

ENSG00000138395	ENSG00000102225
ENSG00000138395	ENSG00000117266
ENSG00000138398	ENSG00000114857
ENSG00000138398	ENSG00000152591
ENSG00000138411	ENSG00000002746
ENSG00000138434	ENSG00000135426
ENSG00000138434	ENSG00000180347
ENSG00000138439	ENSG00000106415
ENSG00000138439	ENSG00000121104
ENSG00000138443	ENSG00000108798
ENSG00000138443	ENSG00000136754
ENSG00000138448	ENSG00000005844
ENSG00000138448	ENSG00000005884
ENSG00000138448	ENSG00000005961
ENSG00000138448	ENSG00000077943
ENSG00000138448	ENSG00000083457
ENSG00000138448	ENSG00000091409
ENSG00000138448	ENSG00000115232
ENSG00000138448	ENSG00000135424
ENSG00000138448	ENSG00000137809
ENSG00000138448	ENSG00000140678
ENSG00000138448	ENSG00000143127
ENSG00000138448	ENSG00000144668
ENSG00000138448	ENSG00000156886
ENSG00000138448	ENSG00000161638
ENSG00000138448	ENSG00000164171
ENSG00000138448	ENSG00000169896
ENSG00000138448	ENSG00000213949
ENSG00000138463	ENSG00000119686
ENSG00000138463	ENSG00000162769
ENSG00000138463	ENSG00000169026
ENSG00000138468	ENSG00000112701
ENSG00000138472	ENSG00000048545
ENSG00000138472	ENSG00000112599
ENSG00000138496	ENSG00000163659
ENSG00000138496	ENSG00000173193
ENSG00000138496	ENSG00000178685
ENSG00000138593	ENSG00000187742
ENSG00000138594	ENSG00000128872
ENSG00000138594	ENSG00000136842
ENSG00000138594	ENSG00000163157
ENSG00000138594	ENSG00000163380
ENSG00000138594	ENSG00000163431
ENSG00000138594	ENSG00000170807
ENSG00000138600	ENSG00000005206
ENSG00000138600	ENSG00000185294
ENSG00000138606	ENSG00000105251
ENSG00000138606	ENSG00000107338
ENSG00000138606	ENSG00000169291
ENSG00000138613	ENSG00000117362
ENSG00000138615	ENSG00000160161
ENSG00000138622	ENSG00000070729
ENSG00000138622	ENSG00000099822
ENSG00000138622	ENSG00000132259
ENSG00000138622	ENSG00000143630
ENSG00000138622	ENSG00000144191
ENSG00000138622	ENSG00000164588
ENSG00000138622	ENSG00000170289
ENSG00000138622	ENSG00000183862

ENSG00000138639	ENSG00000128805
ENSG00000138639	ENSG00000147799
ENSG00000138639	ENSG00000163219
ENSG00000138640	ENSG00000031003
ENSG00000138640	ENSG00000148541
ENSG00000138641	ENSG00000114062
ENSG00000138641	ENSG00000138642
ENSG00000138641	ENSG00000138646
ENSG00000138641	ENSG00000148634
ENSG00000138641	ENSG00000165338
ENSG00000138642	ENSG00000114062
ENSG00000138642	ENSG00000138641
ENSG00000138642	ENSG00000138646
ENSG00000138642	ENSG00000148634
ENSG00000138642	ENSG00000165338
ENSG00000138646	ENSG00000114062
ENSG00000138646	ENSG00000138641
ENSG00000138646	ENSG00000138642
ENSG00000138646	ENSG00000148634
ENSG00000138646	ENSG00000165338
ENSG00000138650	ENSG00000118946
ENSG00000138650	ENSG00000136099
ENSG00000138650	ENSG00000165194
ENSG00000138650	ENSG00000189184
ENSG00000138653	ENSG00000070614
ENSG00000138653	ENSG00000164100
ENSG00000138653	ENSG00000166507
ENSG00000138668	ENSG00000071626
ENSG00000138668	ENSG00000152795
ENSG00000138668	ENSG00000197451
ENSG00000138668	ENSG00000215173
ENSG00000138669	ENSG00000185532
ENSG00000138670	ENSG00000146090
ENSG00000138670	ENSG00000198915
ENSG00000138674	ENSG00000075826
ENSG00000138678	ENSG00000158669
ENSG00000138685	ENSG00000070193
ENSG00000138685	ENSG00000070388
ENSG00000138685	ENSG00000075388
ENSG00000138685	ENSG00000078579
ENSG00000138685	ENSG00000102466
ENSG00000138685	ENSG00000102678
ENSG00000138685	ENSG00000111241
ENSG00000138685	ENSG00000113578
ENSG00000138685	ENSG00000114279
ENSG00000138685	ENSG00000129682
ENSG00000138685	ENSG00000138675
ENSG00000138685	ENSG00000140285
ENSG00000138685	ENSG00000161958
ENSG00000138685	ENSG00000185390
ENSG00000138685	ENSG00000186895
ENSG00000138685	ENSG00000196468
ENSG00000138685	ENSG00000204801
ENSG00000138685	ENSG00000204816
ENSG00000138685	ENSG00000204817
ENSG00000138685	ENSG00000204837
ENSG00000138685	ENSG00000215053
ENSG00000138696	ENSG00000106799
ENSG00000138696	ENSG00000107779

ENSG00000138696	ENSG00000115170
ENSG00000138696	ENSG00000123612
ENSG00000138696	ENSG00000135409
ENSG00000138696	ENSG00000135503
ENSG00000138696	ENSG00000139567
ENSG00000138696	ENSG00000163513
ENSG00000138696	ENSG00000204217
ENSG00000138709	ENSG00000155506
ENSG00000138735	ENSG00000095464
ENSG00000138735	ENSG00000112541
ENSG00000138735	ENSG00000128655
ENSG00000138735	ENSG00000132915
ENSG00000138735	ENSG00000133256
ENSG00000138735	ENSG00000186642
ENSG00000138741	ENSG00000069018
ENSG00000138741	ENSG00000072315
ENSG00000138741	ENSG00000133107
ENSG00000138741	ENSG00000137672
ENSG00000138741	ENSG00000144935
ENSG00000138755	ENSG00000081041
ENSG00000138755	ENSG00000109272
ENSG00000138755	ENSG00000124875
ENSG00000138755	ENSG00000163734
ENSG00000138755	ENSG00000163735
ENSG00000138755	ENSG00000163736
ENSG00000138755	ENSG00000163737
ENSG00000138755	ENSG00000163739
ENSG00000138755	ENSG00000169245
ENSG00000138755	ENSG00000169248
ENSG00000138755	ENSG00000169429
ENSG00000138756	ENSG00000115977
ENSG00000138757	ENSG00000145907
ENSG00000138758	ENSG00000125354
ENSG00000138758	ENSG00000154997
ENSG00000138758	ENSG00000164402
ENSG00000138758	ENSG00000186522
ENSG00000138760	ENSG00000073060
ENSG00000138760	ENSG00000135218
ENSG00000138764	ENSG00000113328
ENSG00000138764	ENSG00000118816
ENSG00000138764	ENSG00000205089
ENSG00000138767	ENSG00000113300
ENSG00000138767	ENSG00000151014
ENSG00000138767	ENSG00000174840
ENSG00000138769	ENSG00000006837
ENSG00000138769	ENSG00000100490
ENSG00000138769	ENSG00000205111
ENSG00000138771	ENSG00000146950
ENSG00000138771	ENSG00000158352
ENSG00000138772	ENSG00000104537
ENSG00000138772	ENSG00000109511
ENSG00000138772	ENSG00000122359
ENSG00000138772	ENSG00000135046
ENSG00000138772	ENSG00000138279
ENSG00000138772	ENSG00000143412
ENSG00000138772	ENSG00000150165
ENSG00000138772	ENSG00000164111
ENSG00000138772	ENSG00000165390
ENSG00000138772	ENSG00000182718

ENSG00000138772	ENSG00000183059
ENSG00000138772	ENSG00000186807
ENSG00000138772	ENSG00000196975
ENSG00000138772	ENSG00000197043
ENSG00000138777	ENSG00000180817
ENSG00000138795	ENSG00000081059
ENSG00000138795	ENSG00000148737
ENSG00000138795	ENSG00000152284
ENSG00000138801	ENSG00000198682
ENSG00000138814	ENSG00000107758
ENSG00000138814	ENSG00000120910
ENSG00000138821	ENSG00000104635
ENSG00000138821	ENSG00000112473
ENSG00000138821	ENSG00000139540
ENSG00000138821	ENSG00000141424
ENSG00000138821	ENSG00000147804
ENSG00000138821	ENSG00000148482
ENSG00000138821	ENSG00000165915
ENSG00000138821	ENSG00000196950
ENSG00000138829	ENSG00000142449
ENSG00000138829	ENSG00000166147
ENSG00000138834	ENSG00000008294
ENSG00000138869	ENSG00000106153
ENSG00000138869	ENSG00000182699
ENSG00000138869	ENSG00000186940
ENSG00000138869	ENSG00000203682
ENSG00000138892	ENSG00000100271
ENSG00000138892	ENSG00000119685
ENSG00000138892	ENSG00000120440
ENSG00000138892	ENSG00000131044
ENSG00000138892	ENSG00000135912
ENSG00000138892	ENSG00000137941
ENSG00000138892	ENSG00000170703
ENSG00000138892	ENSG00000175764
ENSG00000138892	ENSG00000213471
ENSG00000138892	ENSG00000214021
ENSG00000138942	ENSG00000204308
ENSG00000138942	ENSG00000205408
ENSG00000139044	ENSG00000033100
ENSG00000139044	ENSG00000123989
ENSG00000139044	ENSG00000131873
ENSG00000139044	ENSG00000147408
ENSG00000139044	ENSG00000169826
ENSG00000139044	ENSG00000182272
ENSG00000139044	ENSG00000198108
ENSG00000139053	ENSG00000185527
ENSG00000139055	ENSG00000169340
ENSG00000139055	ENSG00000185615
ENSG00000139055	ENSG00000185624
ENSG00000139083	ENSG00000010030
ENSG00000139083	ENSG00000102034
ENSG00000139083	ENSG00000109381
ENSG00000139083	ENSG00000120690
ENSG00000139083	ENSG00000124664
ENSG00000139083	ENSG00000135373
ENSG00000139083	ENSG00000135374
ENSG00000139083	ENSG00000163435
ENSG00000139083	ENSG00000166211
ENSG00000139088	ENSG00000186611

ENSG00000139116	ENSG00000090889
ENSG00000139116	ENSG00000116852
ENSG00000139117	ENSG00000085719
ENSG00000139117	ENSG00000100884
ENSG00000139117	ENSG00000124772
ENSG00000139117	ENSG00000140848
ENSG00000139117	ENSG00000144550
ENSG00000139117	ENSG00000178773
ENSG00000139117	ENSG00000196353
ENSG00000139117	ENSG00000214078
ENSG00000139132	ENSG00000127084
ENSG00000139132	ENSG00000146192
ENSG00000139144	ENSG00000011405
ENSG00000139144	ENSG00000051382
ENSG00000139144	ENSG00000105851
ENSG00000139144	ENSG00000121879
ENSG00000139144	ENSG00000133056
ENSG00000139144	ENSG00000171608
ENSG00000139151	ENSG00000101333
ENSG00000139151	ENSG00000114805
ENSG00000139151	ENSG00000115556
ENSG00000139151	ENSG00000115896
ENSG00000139151	ENSG00000124181
ENSG00000139151	ENSG00000137841
ENSG00000139151	ENSG00000149527
ENSG00000139151	ENSG00000149782
ENSG00000139151	ENSG00000154822
ENSG00000139151	ENSG00000161714
ENSG00000139151	ENSG00000182621
ENSG00000139151	ENSG00000187091
ENSG00000139151	ENSG00000197943
ENSG00000139155	ENSG00000084453
ENSG00000139155	ENSG00000101187
ENSG00000139155	ENSG00000111700
ENSG00000139155	ENSG00000134538
ENSG00000139155	ENSG00000137491
ENSG00000139155	ENSG00000137571
ENSG00000139155	ENSG00000173930
ENSG00000139155	ENSG00000174640
ENSG00000139155	ENSG00000176463
ENSG00000139155	ENSG00000205359
ENSG00000139155	ENSG00000205754
ENSG00000139163	ENSG00000100288
ENSG00000139163	ENSG00000110721
ENSG00000139163	ENSG00000143845
ENSG00000139174	ENSG00000012211
ENSG00000139174	ENSG00000071282
ENSG00000139174	ENSG00000124593
ENSG00000139174	ENSG00000135269
ENSG00000139174	ENSG00000163637
ENSG00000139178	ENSG00000009724
ENSG00000139178	ENSG00000127241
ENSG00000139178	ENSG00000159403
ENSG00000139178	ENSG00000182326
ENSG00000139182	ENSG00000158258
ENSG00000139182	ENSG00000171603
ENSG00000139190	ENSG00000049245
ENSG00000139190	ENSG00000117533
ENSG00000139190	ENSG00000168899

ENSG00000139190	ENSG00000179036
ENSG00000139190	ENSG00000214719
ENSG00000139192	ENSG00000112493
ENSG00000139194	ENSG00000114113
ENSG00000139194	ENSG00000114115
ENSG00000139194	ENSG00000121769
ENSG00000139194	ENSG00000143320
ENSG00000139194	ENSG00000145384
ENSG00000139194	ENSG00000147588
ENSG00000139194	ENSG00000162444
ENSG00000139194	ENSG00000164434
ENSG00000139194	ENSG00000164687
ENSG00000139194	ENSG00000166426
ENSG00000139194	ENSG00000170323
ENSG00000139194	ENSG00000197416
ENSG00000139194	ENSG00000205186
ENSG00000139194	ENSG00000214004
ENSG00000139197	ENSG00000114757
ENSG00000139209	ENSG00000017483
ENSG00000139209	ENSG00000103042
ENSG00000139209	ENSG00000111371
ENSG00000139209	ENSG00000134294
ENSG00000139209	ENSG00000139974
ENSG00000139209	ENSG00000166558
ENSG00000139209	ENSG00000169507
ENSG00000139209	ENSG00000188338
ENSG00000139211	ENSG00000176020
ENSG00000139211	ENSG00000181754
ENSG00000139218	ENSG00000070047
ENSG00000139218	ENSG00000126461
ENSG00000139219	ENSG00000060718
ENSG00000139219	ENSG00000080573
ENSG00000139219	ENSG00000108821
ENSG00000139219	ENSG00000130635
ENSG00000139219	ENSG00000164692
ENSG00000139219	ENSG00000168542
ENSG00000139219	ENSG00000204248
ENSG00000139219	ENSG00000204262
ENSG00000139220	ENSG00000110841
ENSG00000139220	ENSG00000131626
ENSG00000139220	ENSG00000143847
ENSG00000139220	ENSG00000166387
ENSG00000139220	ENSG00000177380
ENSG00000139223	ENSG00000136938
ENSG00000139223	ENSG00000140350
ENSG00000139223	ENSG00000143401
ENSG00000139223	ENSG00000184701
ENSG00000139223	ENSG00000215332
ENSG00000139263	ENSG00000144749
ENSG00000139263	ENSG00000198799
ENSG00000139266	ENSG00000099785
ENSG00000139266	ENSG00000144583
ENSG00000139266	ENSG00000145416
ENSG00000139266	ENSG00000165406
ENSG00000139266	ENSG00000173926
ENSG00000139266	ENSG00000183654
ENSG00000139278	ENSG00000096006
ENSG00000139278	ENSG00000101074
ENSG00000139278	ENSG00000103196

ENSG00000139278	ENSG00000121005
ENSG00000139278	ENSG00000124490
ENSG00000139278	ENSG00000124812
ENSG00000139278	ENSG00000137558
ENSG00000139278	ENSG00000164530
ENSG00000139278	ENSG00000173401
ENSG00000139278	ENSG00000180481
ENSG00000139287	ENSG00000129167
ENSG00000139287	ENSG00000171759
ENSG00000139287	ENSG00000180176
ENSG00000139289	ENSG00000174307
ENSG00000139289	ENSG00000181649
ENSG00000139292	ENSG00000133067
ENSG00000139292	ENSG00000138039
ENSG00000139292	ENSG00000165409
ENSG00000139292	ENSG00000170820
ENSG00000139292	ENSG00000205213
ENSG00000139318	ENSG00000111266
ENSG00000139318	ENSG00000120129
ENSG00000139318	ENSG00000120875
ENSG00000139318	ENSG00000130829
ENSG00000139318	ENSG00000138166
ENSG00000139318	ENSG00000143507
ENSG00000139318	ENSG00000158050
ENSG00000139318	ENSG00000164086
ENSG00000139318	ENSG00000184545
ENSG00000139329	ENSG00000011465
ENSG00000139329	ENSG00000106819
ENSG00000139329	ENSG00000182492
ENSG00000139330	ENSG00000122176
ENSG00000139330	ENSG00000127083
ENSG00000139330	ENSG00000188783
ENSG00000139351	ENSG00000177138
ENSG00000139351	ENSG00000183304
ENSG00000139351	ENSG00000187268
ENSG00000139352	ENSG00000176009
ENSG00000139352	ENSG00000183734
ENSG00000139352	ENSG00000187855
ENSG00000139352	ENSG00000188108
ENSG00000139354	ENSG00000132139
ENSG00000139354	ENSG00000148935
ENSG00000139354	ENSG00000185340
ENSG00000139364	ENSG00000006118
ENSG00000139364	ENSG00000151952
ENSG00000139364	ENSG00000181234
ENSG00000139364	ENSG00000181291
ENSG00000139370	ENSG00000088386
ENSG00000139370	ENSG00000110446
ENSG00000139370	ENSG00000163406
ENSG00000139410	ENSG00000135094
ENSG00000139433	ENSG00000106086
ENSG00000139433	ENSG00000116095
ENSG00000139433	ENSG00000134297
ENSG00000139433	ENSG00000213907
ENSG00000139436	ENSG00000108262
ENSG00000139436	ENSG00000133612
ENSG00000139436	ENSG00000135439
ENSG00000139436	ENSG00000151303
ENSG00000139436	ENSG00000157985

ENSG00000139436	ENSG00000172650
ENSG00000139436	ENSG00000174194
ENSG00000139436	ENSG00000188234
ENSG00000139436	ENSG00000198035
ENSG00000139436	ENSG00000204149
ENSG00000139436	ENSG00000204169
ENSG00000139436	ENSG00000204172
ENSG00000139438	ENSG00000173065
ENSG00000139445	ENSG00000109101
ENSG00000139445	ENSG00000176302
ENSG00000139445	ENSG00000189299
ENSG00000139505	ENSG00000003987
ENSG00000139505	ENSG00000014914
ENSG00000139505	ENSG00000063601
ENSG00000139505	ENSG00000087053
ENSG00000139505	ENSG00000100330
ENSG00000139505	ENSG00000102043
ENSG00000139505	ENSG00000104643
ENSG00000139505	ENSG00000108389
ENSG00000139505	ENSG00000150712
ENSG00000139505	ENSG00000166912
ENSG00000139505	ENSG00000171100
ENSG00000139505	ENSG00000184416
ENSG00000139508	ENSG00000076351
ENSG00000139508	ENSG00000119457
ENSG00000139514	ENSG00000003989
ENSG00000139514	ENSG00000013293
ENSG00000139514	ENSG00000099960
ENSG00000139514	ENSG00000165349
ENSG00000139517	ENSG00000072201
ENSG00000139517	ENSG00000107186
ENSG00000139517	ENSG00000132849
ENSG00000139540	ENSG00000104635
ENSG00000139540	ENSG00000112473
ENSG00000139540	ENSG00000138821
ENSG00000139540	ENSG00000141424
ENSG00000139540	ENSG00000147804
ENSG00000139540	ENSG00000148482
ENSG00000139540	ENSG00000165915
ENSG00000139540	ENSG00000196950
ENSG00000139546	ENSG00000180228
ENSG00000139547	ENSG00000025423
ENSG00000139547	ENSG00000073737
ENSG00000139547	ENSG00000086696
ENSG00000139547	ENSG00000135437
ENSG00000139547	ENSG00000161267
ENSG00000139547	ENSG00000170426
ENSG00000139547	ENSG00000176387
ENSG00000139549	ENSG00000163501
ENSG00000139549	ENSG00000164690
ENSG00000139567	ENSG00000106799
ENSG00000139567	ENSG00000107779
ENSG00000139567	ENSG00000115170
ENSG00000139567	ENSG00000123612
ENSG00000139567	ENSG00000135409
ENSG00000139567	ENSG00000135503
ENSG00000139567	ENSG00000138696
ENSG00000139567	ENSG00000163513
ENSG00000139567	ENSG00000204217

ENSG00000139579	ENSG00000173559
ENSG00000139597	ENSG00000077009
ENSG00000139597	ENSG00000106733
ENSG00000139610	ENSG00000141086
ENSG00000139610	ENSG00000142615
ENSG00000139610	ENSG00000142789
ENSG00000139610	ENSG00000162438
ENSG00000139610	ENSG00000168925
ENSG00000139610	ENSG00000168928
ENSG00000139610	ENSG00000215704
ENSG00000139613	ENSG00000173473
ENSG00000139624	ENSG00000065308
ENSG00000139624	ENSG00000067167
ENSG00000139624	ENSG00000090661
ENSG00000139624	ENSG00000143418
ENSG00000139624	ENSG00000154227
ENSG00000139624	ENSG00000172292
ENSG00000139624	ENSG00000174599
ENSG00000139625	ENSG00000073803
ENSG00000139626	ENSG00000056345
ENSG00000139626	ENSG00000082781
ENSG00000139626	ENSG00000105855
ENSG00000139626	ENSG00000115221
ENSG00000139626	ENSG00000132470
ENSG00000139626	ENSG00000150093
ENSG00000139626	ENSG00000160255
ENSG00000139629	ENSG00000100626
ENSG00000139629	ENSG00000106648
ENSG00000139629	ENSG00000110328
ENSG00000139629	ENSG00000115339
ENSG00000139629	ENSG00000131386
ENSG00000139629	ENSG00000136542
ENSG00000139629	ENSG00000141429
ENSG00000139629	ENSG00000143641
ENSG00000139629	ENSG00000144278
ENSG00000139629	ENSG00000158089
ENSG00000139629	ENSG00000164574
ENSG00000139629	ENSG00000174473
ENSG00000139629	ENSG00000178234
ENSG00000139629	ENSG00000182870
ENSG00000139629	ENSG00000185274
ENSG00000139631	ENSG00000128683
ENSG00000139631	ENSG00000136750
ENSG00000139631	ENSG00000144644
ENSG00000139631	ENSG00000215438
ENSG00000139636	ENSG00000105983
ENSG00000139641	ENSG00000117868
ENSG00000139641	ENSG00000158220
ENSG00000139645	ENSG00000065413
ENSG00000139645	ENSG00000119509
ENSG00000139645	ENSG00000206560
ENSG00000139648	ENSG00000135443
ENSG00000139648	ENSG00000135477
ENSG00000139648	ENSG00000135480
ENSG00000139648	ENSG00000161849
ENSG00000139648	ENSG00000161850
ENSG00000139648	ENSG00000167767
ENSG00000139648	ENSG00000167768
ENSG00000139648	ENSG00000170421

ENSG00000139648	ENSG00000170423
ENSG00000139648	ENSG00000170442
ENSG00000139648	ENSG00000170454
ENSG00000139648	ENSG00000170465
ENSG00000139648	ENSG00000170477
ENSG00000139648	ENSG00000170484
ENSG00000139648	ENSG00000170486
ENSG00000139648	ENSG00000170523
ENSG00000139648	ENSG00000172867
ENSG00000139648	ENSG00000185069
ENSG00000139648	ENSG00000185479
ENSG00000139648	ENSG00000185640
ENSG00000139648	ENSG00000186049
ENSG00000139648	ENSG00000186081
ENSG00000139648	ENSG00000186442
ENSG00000139648	ENSG00000189182
ENSG00000139648	ENSG00000205420
ENSG00000139648	ENSG00000205426
ENSG00000139648	ENSG00000214233
ENSG00000139648	ENSG00000214323
ENSG00000139648	ENSG00000214477
ENSG00000139648	ENSG00000214752
ENSG00000139648	ENSG00000214974
ENSG00000139648	ENSG00000215287
ENSG00000139648	ENSG00000215847
ENSG00000139651	ENSG00000067082
ENSG00000139651	ENSG00000102554
ENSG00000139651	ENSG00000105610
ENSG00000139651	ENSG00000127528
ENSG00000139651	ENSG00000129911
ENSG00000139651	ENSG00000136826
ENSG00000139651	ENSG00000155090
ENSG00000139651	ENSG00000164651
ENSG00000139651	ENSG00000167182
ENSG00000139651	ENSG00000170374
ENSG00000139651	ENSG00000171872
ENSG00000139651	ENSG00000172059
ENSG00000139651	ENSG00000174595
ENSG00000139651	ENSG00000189120
ENSG00000139651	ENSG00000197294
ENSG00000139651	ENSG00000197550
ENSG00000139651	ENSG00000204335
ENSG00000139668	ENSG00000085449
ENSG00000139668	ENSG00000185359
ENSG00000139675	ENSG00000122566
ENSG00000139675	ENSG00000135486
ENSG00000139675	ENSG00000170144
ENSG00000139675	ENSG00000176825
ENSG00000139675	ENSG00000177219
ENSG00000139675	ENSG00000177733
ENSG00000139675	ENSG00000188174
ENSG00000139675	ENSG00000206228
ENSG00000139675	ENSG00000212961
ENSG00000139675	ENSG00000214284
ENSG00000139675	ENSG00000214361
ENSG00000139675	ENSG00000214592
ENSG00000139675	ENSG00000214653
ENSG00000139675	ENSG00000215222
ENSG00000139675	ENSG00000215492

ENSG00000139675	ENSG00000215870
ENSG00000139675	ENSG00000215876
ENSG00000139675	ENSG00000215885
ENSG00000139679	ENSG00000078589
ENSG00000139679	ENSG00000147145
ENSG00000139679	ENSG00000152207
ENSG00000139679	ENSG00000169403
ENSG00000139679	ENSG00000169508
ENSG00000139679	ENSG00000169860
ENSG00000139679	ENSG00000173198
ENSG00000139679	ENSG00000175591
ENSG00000139679	ENSG00000184574
ENSG00000139679	ENSG00000186912
ENSG00000139679	ENSG00000198829
ENSG00000139697	ENSG00000064932
ENSG00000139718	ENSG00000099381
ENSG00000139722	ENSG00000167987
ENSG00000139722	ENSG00000176428
ENSG00000139734	ENSG00000131504
ENSG00000139734	ENSG00000147202
ENSG00000139737	ENSG00000109171
ENSG00000139746	ENSG00000091009
ENSG00000139767	ENSG00000167978
ENSG00000139767	ENSG00000177679
ENSG00000139767	ENSG00000204924
ENSG00000139780	ENSG00000165366
ENSG00000139793	ENSG00000076770
ENSG00000139793	ENSG00000135482
ENSG00000139793	ENSG00000152601
ENSG00000139800	ENSG00000043355
ENSG00000139800	ENSG00000152977
ENSG00000139800	ENSG00000156925
ENSG00000139800	ENSG00000174963
ENSG00000139842	ENSG00000158290
ENSG00000139865	ENSG00000143643
ENSG00000139874	ENSG00000082556
ENSG00000139874	ENSG00000112038
ENSG00000139874	ENSG00000116014
ENSG00000139874	ENSG00000116329
ENSG00000139874	ENSG00000125510
ENSG00000139874	ENSG00000125522
ENSG00000139874	ENSG00000128285
ENSG00000139874	ENSG00000128310
ENSG00000139874	ENSG00000132671
ENSG00000139874	ENSG00000152034
ENSG00000139874	ENSG00000162009
ENSG00000139874	ENSG00000166573
ENSG00000139874	ENSG00000180616
ENSG00000139874	ENSG00000182687
ENSG00000139874	ENSG00000183473
ENSG00000139874	ENSG00000183729
ENSG00000139880	ENSG00000040731
ENSG00000139880	ENSG00000071991
ENSG00000139880	ENSG00000081138
ENSG00000139880	ENSG00000101542
ENSG00000139880	ENSG00000113100
ENSG00000139880	ENSG00000113361
ENSG00000139880	ENSG00000140937
ENSG00000139880	ENSG00000145526

ENSG00000139880	ENSG00000149654
ENSG00000139880	ENSG00000150394
ENSG00000139880	ENSG00000154162
ENSG00000139880	ENSG00000179776
ENSG00000139890	ENSG00000088320
ENSG00000139890	ENSG00000164949
ENSG00000139890	ENSG00000166592
ENSG00000139899	ENSG00000054803
ENSG00000139899	ENSG00000102924
ENSG00000139899	ENSG00000131094
ENSG00000139899	ENSG00000133466
ENSG00000139899	ENSG00000141668
ENSG00000139899	ENSG00000144119
ENSG00000139899	ENSG00000145861
ENSG00000139899	ENSG00000159189
ENSG00000139899	ENSG00000163145
ENSG00000139899	ENSG00000165985
ENSG00000139899	ENSG00000172247
ENSG00000139899	ENSG00000173369
ENSG00000139899	ENSG00000173372
ENSG00000139899	ENSG00000173918
ENSG00000139899	ENSG00000181092
ENSG00000139899	ENSG00000184471
ENSG00000139899	ENSG00000186897
ENSG00000139899	ENSG00000213192
ENSG00000139910	ENSG00000104967
ENSG00000139914	ENSG00000197296
ENSG00000139915	ENSG00000112139
ENSG00000139915	ENSG00000165072
ENSG00000139915	ENSG00000177943
ENSG00000139915	ENSG00000204741
ENSG00000139921	ENSG00000125827
ENSG00000139926	ENSG00000163629
ENSG00000139926	ENSG00000170324
ENSG00000139946	ENSG00000174516
ENSG00000139946	ENSG00000197329
ENSG00000139970	ENSG00000115310
ENSG00000139970	ENSG00000125744
ENSG00000139970	ENSG00000133318
ENSG00000139973	ENSG00000143469
ENSG00000139985	ENSG00000008277
ENSG00000139985	ENSG00000042980
ENSG00000139985	ENSG00000069206
ENSG00000139985	ENSG00000073670
ENSG00000139985	ENSG00000104755
ENSG00000139985	ENSG00000114948
ENSG00000139985	ENSG00000134007
ENSG00000139985	ENSG00000134028
ENSG00000139985	ENSG00000134249
ENSG00000139985	ENSG00000135074
ENSG00000139985	ENSG00000143537
ENSG00000139985	ENSG00000148848
ENSG00000139985	ENSG00000149451
ENSG00000139985	ENSG00000151651
ENSG00000139985	ENSG00000151694
ENSG00000139985	ENSG00000168594
ENSG00000139985	ENSG00000168615
ENSG00000139985	ENSG00000168619
ENSG00000139985	ENSG00000196115

ENSG00000139985	ENSG00000197140
ENSG00000139985	ENSG00000214137
ENSG00000139988	ENSG00000072042
ENSG00000139988	ENSG00000160439
ENSG00000139988	ENSG00000167536
ENSG00000139988	ENSG00000169084
ENSG00000139998	ENSG00000084733
ENSG00000139998	ENSG00000143545
ENSG00000139998	ENSG00000166128
ENSG00000139998	ENSG00000167461
ENSG00000140009	ENSG00000091831
ENSG00000140009	ENSG00000119715
ENSG00000140009	ENSG00000173153
ENSG00000140009	ENSG00000196482
ENSG00000140015	ENSG00000055118
ENSG00000140015	ENSG00000089558
ENSG00000140015	ENSG00000135519
ENSG00000140015	ENSG00000143473
ENSG00000140015	ENSG00000173826
ENSG00000140015	ENSG00000183960
ENSG00000140015	ENSG00000184611
ENSG00000140022	ENSG00000072958
ENSG00000140022	ENSG00000129354
ENSG00000140030	ENSG00000119714
ENSG00000140030	ENSG00000177464
ENSG00000140030	ENSG00000183484
ENSG00000140044	ENSG00000075426
ENSG00000140044	ENSG00000125740
ENSG00000140044	ENSG00000162772
ENSG00000140044	ENSG00000170345
ENSG00000140044	ENSG00000175592
ENSG00000140067	ENSG00000182103
ENSG00000140090	ENSG00000074621
ENSG00000140090	ENSG00000155886
ENSG00000140090	ENSG00000185052
ENSG00000140090	ENSG00000188467
ENSG00000140092	ENSG00000049323
ENSG00000140092	ENSG00000077942
ENSG00000140092	ENSG00000090006
ENSG00000140092	ENSG00000115380
ENSG00000140092	ENSG00000119681
ENSG00000140092	ENSG00000163520
ENSG00000140092	ENSG00000168056
ENSG00000140092	ENSG00000172638
ENSG00000140092	ENSG00000214536
ENSG00000140093	ENSG00000099937
ENSG00000140093	ENSG00000114204
ENSG00000140093	ENSG00000117601
ENSG00000140093	ENSG00000149257
ENSG00000140093	ENSG00000170099
ENSG00000140107	ENSG00000145832
ENSG00000140107	ENSG00000162241
ENSG00000140107	ENSG00000197119
ENSG00000140153	ENSG00000185800
ENSG00000140157	ENSG00000001461
ENSG00000140157	ENSG00000104361
ENSG00000140157	ENSG00000163293
ENSG00000140157	ENSG00000170113
ENSG00000140157	ENSG00000172548

ENSG00000140199	ENSG00000113504
ENSG00000140199	ENSG00000124067
ENSG00000140199	ENSG00000124140
ENSG00000140262	ENSG00000071564
ENSG00000140262	ENSG00000196628
ENSG00000140262	ENSG00000215291
ENSG00000140280	ENSG00000163155
ENSG00000140284	ENSG00000083807
ENSG00000140284	ENSG00000113396
ENSG00000140284	ENSG00000130304
ENSG00000140284	ENSG00000143554
ENSG00000140284	ENSG00000167114
ENSG00000140285	ENSG00000070193
ENSG00000140285	ENSG00000070388
ENSG00000140285	ENSG00000075388
ENSG00000140285	ENSG00000078579
ENSG00000140285	ENSG00000102466
ENSG00000140285	ENSG00000102678
ENSG00000140285	ENSG00000111241
ENSG00000140285	ENSG00000113578
ENSG00000140285	ENSG00000114279
ENSG00000140285	ENSG00000129682
ENSG00000140285	ENSG00000138675
ENSG00000140285	ENSG00000138685
ENSG00000140285	ENSG00000161958
ENSG00000140285	ENSG00000185390
ENSG00000140285	ENSG00000186895
ENSG00000140285	ENSG00000196468
ENSG00000140285	ENSG00000204801
ENSG00000140285	ENSG00000204816
ENSG00000140285	ENSG00000204817
ENSG00000140285	ENSG00000204837
ENSG00000140285	ENSG00000215053
ENSG00000140297	ENSG00000015532
ENSG00000140297	ENSG00000103489
ENSG00000140297	ENSG00000111846
ENSG00000140297	ENSG00000124091
ENSG00000140297	ENSG00000176928
ENSG00000140297	ENSG00000187210
ENSG00000140297	ENSG00000205318
ENSG00000140299	ENSG00000106772
ENSG00000140299	ENSG00000163141
ENSG00000140299	ENSG00000167654
ENSG00000140299	ENSG00000175220
ENSG00000140299	ENSG00000196505
ENSG00000140323	ENSG00000154309
ENSG00000140332	ENSG00000065717
ENSG00000140332	ENSG00000104953
ENSG00000140332	ENSG00000104964
ENSG00000140332	ENSG00000106829
ENSG00000140332	ENSG00000196781
ENSG00000140350	ENSG00000136938
ENSG00000140350	ENSG00000139223
ENSG00000140350	ENSG00000143401
ENSG00000140350	ENSG00000184701
ENSG00000140350	ENSG00000215332
ENSG00000140367	ENSG00000160714
ENSG00000140367	ENSG00000189136
ENSG00000140367	ENSG00000215218

ENSG00000140368	ENSG00000100266
ENSG00000140368	ENSG00000124507
ENSG00000140368	ENSG00000152229
ENSG00000140368	ENSG00000165912
ENSG00000140379	ENSG00000030110
ENSG00000140379	ENSG00000087088
ENSG00000140379	ENSG00000129473
ENSG00000140379	ENSG00000143384
ENSG00000140379	ENSG00000171552
ENSG00000140379	ENSG00000171791
ENSG00000140379	ENSG00000176720
ENSG00000140382	ENSG00000064961
ENSG00000140391	ENSG00000106025
ENSG00000140391	ENSG00000130167
ENSG00000140391	ENSG00000135404
ENSG00000140391	ENSG00000156298
ENSG00000140396	ENSG00000084676
ENSG00000140396	ENSG00000124151
ENSG00000140403	ENSG00000069345
ENSG00000140403	ENSG00000086061
ENSG00000140416	ENSG00000143549
ENSG00000140416	ENSG00000167460
ENSG00000140416	ENSG00000177152
ENSG00000140416	ENSG00000183022
ENSG00000140416	ENSG00000187536
ENSG00000140416	ENSG00000198467
ENSG00000140416	ENSG00000214869
ENSG00000140443	ENSG00000027644
ENSG00000140443	ENSG00000047936
ENSG00000140443	ENSG00000062524
ENSG00000140443	ENSG00000171094
ENSG00000140443	ENSG00000171105
ENSG00000140450	ENSG00000105643
ENSG00000140450	ENSG00000113369
ENSG00000140450	ENSG00000117289
ENSG00000140450	ENSG00000197070
ENSG00000140450	ENSG00000205784
ENSG00000140459	ENSG00000019186
ENSG00000140459	ENSG00000111012
ENSG00000140459	ENSG00000135929
ENSG00000140459	ENSG00000160882
ENSG00000140459	ENSG00000179142
ENSG00000140459	ENSG00000186684
ENSG00000140470	ENSG00000049192
ENSG00000140470	ENSG00000087116
ENSG00000140470	ENSG00000136378
ENSG00000140470	ENSG00000138316
ENSG00000140470	ENSG00000140873
ENSG00000140470	ENSG00000142303
ENSG00000140470	ENSG00000145536
ENSG00000140470	ENSG00000145808
ENSG00000140470	ENSG00000151388
ENSG00000140470	ENSG00000156140
ENSG00000140470	ENSG00000160323
ENSG00000140470	ENSG00000163638
ENSG00000140470	ENSG00000173157
ENSG00000140470	ENSG00000214644
ENSG00000140470	ENSG00000215743
ENSG00000140478	ENSG00000153666

ENSG00000140478	ENSG00000156363
ENSG00000140478	ENSG00000159289
ENSG00000140478	ENSG00000167110
ENSG00000140478	ENSG00000167195
ENSG00000140478	ENSG00000174450
ENSG00000140478	ENSG00000175265
ENSG00000140478	ENSG00000181984
ENSG00000140478	ENSG00000184206
ENSG00000140478	ENSG00000186322
ENSG00000140478	ENSG00000188388
ENSG00000140478	ENSG00000188532
ENSG00000140478	ENSG00000188626
ENSG00000140478	ENSG00000197092
ENSG00000140478	ENSG00000197414
ENSG00000140478	ENSG00000214348
ENSG00000140478	ENSG00000215252
ENSG00000140478	ENSG00000215405
ENSG00000140479	ENSG00000099139
ENSG00000140479	ENSG00000115257
ENSG00000140479	ENSG00000125851
ENSG00000140479	ENSG00000140564
ENSG00000140479	ENSG00000160613
ENSG00000140479	ENSG00000175426
ENSG00000140488	ENSG00000048740
ENSG00000140488	ENSG00000101489
ENSG00000140488	ENSG00000149187
ENSG00000140488	ENSG00000159409
ENSG00000140488	ENSG00000161082
ENSG00000140497	ENSG00000085365
ENSG00000140497	ENSG00000116521
ENSG00000140497	ENSG00000198794
ENSG00000140506	ENSG00000074695
ENSG00000140506	ENSG00000114988
ENSG00000140506	ENSG00000169223
ENSG00000140511	ENSG00000038427
ENSG00000140511	ENSG00000130287
ENSG00000140511	ENSG00000132692
ENSG00000140511	ENSG00000132702
ENSG00000140511	ENSG00000145681
ENSG00000140511	ENSG00000157766
ENSG00000140511	ENSG00000187664
ENSG00000140519	ENSG00000112077
ENSG00000140519	ENSG00000132677
ENSG00000140519	ENSG00000187010
ENSG00000140519	ENSG00000188672
ENSG00000140526	ENSG00000143994
ENSG00000140526	ENSG00000158201
ENSG00000140538	ENSG00000030304
ENSG00000140538	ENSG00000148053
ENSG00000140538	ENSG00000169071
ENSG00000140538	ENSG00000185483
ENSG00000140538	ENSG00000198400
ENSG00000140545	ENSG00000057019
ENSG00000140545	ENSG00000099250
ENSG00000140545	ENSG00000102104
ENSG00000140545	ENSG00000118257
ENSG00000140545	ENSG00000164176
ENSG00000140545	ENSG00000164465
ENSG00000140548	ENSG00000178175

ENSG00000140553	ENSG00000141161
ENSG00000140557	ENSG00000113532
ENSG00000140563	ENSG00000175471
ENSG00000140564	ENSG00000099139
ENSG00000140564	ENSG00000115257
ENSG00000140564	ENSG00000125851
ENSG00000140564	ENSG00000140479
ENSG00000140564	ENSG00000160613
ENSG00000140564	ENSG00000175426
ENSG00000140575	ENSG00000145703
ENSG00000140575	ENSG00000183856
ENSG00000140577	ENSG00000105662
ENSG00000140577	ENSG00000160741
ENSG00000140600	ENSG00000097033
ENSG00000140600	ENSG00000107295
ENSG00000140600	ENSG00000141985
ENSG00000140600	ENSG00000148341
ENSG00000140612	ENSG00000166562
ENSG00000140623	ENSG00000100167
ENSG00000140623	ENSG00000184640
ENSG00000140650	ENSG00000100417
ENSG00000140675	ENSG00000100170
ENSG00000140675	ENSG00000100191
ENSG00000140675	ENSG00000117834
ENSG00000140675	ENSG00000154025
ENSG00000140675	ENSG00000158865
ENSG00000140675	ENSG00000198743
ENSG00000140678	ENSG00000005844
ENSG00000140678	ENSG00000005884
ENSG00000140678	ENSG00000005961
ENSG00000140678	ENSG00000077943
ENSG00000140678	ENSG00000083457
ENSG00000140678	ENSG00000091409
ENSG00000140678	ENSG00000115232
ENSG00000140678	ENSG00000135424
ENSG00000140678	ENSG00000137809
ENSG00000140678	ENSG00000138448
ENSG00000140678	ENSG00000143127
ENSG00000140678	ENSG00000144668
ENSG00000140678	ENSG00000156886
ENSG00000140678	ENSG00000161638
ENSG00000140678	ENSG00000164171
ENSG00000140678	ENSG00000169896
ENSG00000140678	ENSG00000213949
ENSG00000140682	ENSG00000072163
ENSG00000140682	ENSG00000089159
ENSG00000140682	ENSG00000110031
ENSG00000140682	ENSG00000112214
ENSG00000140682	ENSG00000169756
ENSG00000140682	ENSG00000183386
ENSG00000140743	ENSG00000109089
ENSG00000140795	ENSG00000035664
ENSG00000140795	ENSG00000065534
ENSG00000140795	ENSG00000101306
ENSG00000140795	ENSG00000145949
ENSG00000140795	ENSG00000167657
ENSG00000140795	ENSG00000196730
ENSG00000140807	ENSG00000145506
ENSG00000140835	ENSG00000122863

ENSG00000140835	ENSG00000135702
ENSG00000140835	ENSG00000147119
ENSG00000140835	ENSG00000175040
ENSG00000140835	ENSG00000175264
ENSG00000140835	ENSG00000183196
ENSG00000140836	ENSG00000091656
ENSG00000140836	ENSG00000136367
ENSG00000140848	ENSG00000085719
ENSG00000140848	ENSG00000100884
ENSG00000140848	ENSG00000124772
ENSG00000140848	ENSG00000139117
ENSG00000140848	ENSG00000144550
ENSG00000140848	ENSG00000178773
ENSG00000140848	ENSG00000196353
ENSG00000140848	ENSG00000214078
ENSG00000140853	ENSG00000022556
ENSG00000140853	ENSG00000023191
ENSG00000140853	ENSG00000142405
ENSG00000140853	ENSG00000158077
ENSG00000140853	ENSG00000160505
ENSG00000140853	ENSG00000162711
ENSG00000140853	ENSG00000167207
ENSG00000140853	ENSG00000167634
ENSG00000140853	ENSG00000167984
ENSG00000140853	ENSG00000171487
ENSG00000140853	ENSG00000173572
ENSG00000140853	ENSG00000174885
ENSG00000140853	ENSG00000179709
ENSG00000140853	ENSG00000179873
ENSG00000140853	ENSG00000182261
ENSG00000140853	ENSG00000185792
ENSG00000140859	ENSG00000167702
ENSG00000140873	ENSG00000049192
ENSG00000140873	ENSG00000087116
ENSG00000140873	ENSG00000136378
ENSG00000140873	ENSG00000138316
ENSG00000140873	ENSG00000140470
ENSG00000140873	ENSG00000142303
ENSG00000140873	ENSG00000145536
ENSG00000140873	ENSG00000145808
ENSG00000140873	ENSG00000151388
ENSG00000140873	ENSG00000156140
ENSG00000140873	ENSG00000160323
ENSG00000140873	ENSG00000163638
ENSG00000140873	ENSG00000173157
ENSG00000140873	ENSG00000214644
ENSG00000140873	ENSG00000215743
ENSG00000140931	ENSG00000091317
ENSG00000140931	ENSG00000102007
ENSG00000140931	ENSG00000153551
ENSG00000140931	ENSG00000166091
ENSG00000140931	ENSG00000183723
ENSG00000140937	ENSG00000040731
ENSG00000140937	ENSG00000071991
ENSG00000140937	ENSG00000081138
ENSG00000140937	ENSG00000101542
ENSG00000140937	ENSG00000113100
ENSG00000140937	ENSG00000113361
ENSG00000140937	ENSG00000139880

ENSG00000140937	ENSG00000145526
ENSG00000140937	ENSG00000149654
ENSG00000140937	ENSG00000150394
ENSG00000140937	ENSG00000154162
ENSG00000140937	ENSG00000179776
ENSG00000140941	ENSG00000034713
ENSG00000140941	ENSG00000101460
ENSG00000140941	ENSG00000171471
ENSG00000140941	ENSG00000197769
ENSG00000140948	ENSG00000141664
ENSG00000140955	ENSG00000065457
ENSG00000140955	ENSG00000160710
ENSG00000140955	ENSG00000164113
ENSG00000140955	ENSG00000185736
ENSG00000140955	ENSG00000197381
ENSG00000140961	ENSG00000164823
ENSG00000140968	ENSG00000117595
ENSG00000140968	ENSG00000125347
ENSG00000140968	ENSG00000126456
ENSG00000140968	ENSG00000128604
ENSG00000140968	ENSG00000137265
ENSG00000140968	ENSG00000168310
ENSG00000140968	ENSG00000185507
ENSG00000140968	ENSG00000213928
ENSG00000140983	ENSG00000126858
ENSG00000140986	ENSG00000100316
ENSG00000141027	ENSG00000196498
ENSG00000141048	ENSG00000002079
ENSG00000141048	ENSG00000006788
ENSG00000141048	ENSG00000078814
ENSG00000141048	ENSG00000092054
ENSG00000141048	ENSG00000109061
ENSG00000141048	ENSG00000109063
ENSG00000141048	ENSG00000125414
ENSG00000141048	ENSG00000133020
ENSG00000141048	ENSG00000144821
ENSG00000141048	ENSG00000197616
ENSG00000141052	ENSG00000176909
ENSG00000141052	ENSG00000186260
ENSG00000141052	ENSG00000196588
ENSG00000141068	ENSG00000078061
ENSG00000141068	ENSG00000132155
ENSG00000141068	ENSG00000157764
ENSG00000141068	ENSG00000171435
ENSG00000141084	ENSG00000010017
ENSG00000141084	ENSG00000167778
ENSG00000141086	ENSG00000139610
ENSG00000141086	ENSG00000142615
ENSG00000141086	ENSG00000142789
ENSG00000141086	ENSG00000162438
ENSG00000141086	ENSG00000168925
ENSG00000141086	ENSG00000168928
ENSG00000141086	ENSG00000215704
ENSG00000141096	ENSG00000015413
ENSG00000141096	ENSG00000167261
ENSG00000141098	ENSG00000145990
ENSG00000141127	ENSG00000161542
ENSG00000141161	ENSG00000140553
ENSG00000141200	ENSG00000068796

ENSG00000141200	ENSG00000142945
ENSG00000141232	ENSG00000183864
ENSG00000141258	ENSG00000167037
ENSG00000141293	ENSG00000005020
ENSG00000141295	ENSG00000136193
ENSG00000141295	ENSG00000144306
ENSG00000141298	ENSG00000084112
ENSG00000141298	ENSG00000172830
ENSG00000141314	ENSG00000103269
ENSG00000141314	ENSG00000158315
ENSG00000141316	ENSG00000090382
ENSG00000141316	ENSG00000120563
ENSG00000141316	ENSG00000151033
ENSG00000141316	ENSG00000157093
ENSG00000141316	ENSG00000161572
ENSG00000141316	ENSG00000167531
ENSG00000141316	ENSG00000171478
ENSG00000141316	ENSG00000171489
ENSG00000141342	ENSG00000102445
ENSG00000141342	ENSG00000140995
ENSG00000141342	ENSG00000145016
ENSG00000141342	ENSG00000159266
ENSG00000141342	ENSG00000178385
ENSG00000141349	ENSG00000131482
ENSG00000141349	ENSG00000152254
ENSG00000141367	ENSG00000070371
ENSG00000141380	ENSG00000184402
ENSG00000141384	ENSG00000130699
ENSG00000141391	ENSG00000101166
ENSG00000141401	ENSG00000133731
ENSG00000141404	ENSG00000060558
ENSG00000141404	ENSG00000065135
ENSG00000141404	ENSG00000087258
ENSG00000141404	ENSG00000087460
ENSG00000141404	ENSG00000088256
ENSG00000141404	ENSG00000114349
ENSG00000141404	ENSG00000114353
ENSG00000141404	ENSG00000120063
ENSG00000141404	ENSG00000127955
ENSG00000141404	ENSG00000128266
ENSG00000141404	ENSG00000134183
ENSG00000141404	ENSG00000146535
ENSG00000141404	ENSG00000156049
ENSG00000141404	ENSG00000156052
ENSG00000141404	ENSG00000214415
ENSG00000141404	ENSG00000214622
ENSG00000141424	ENSG00000104635
ENSG00000141424	ENSG00000112473
ENSG00000141424	ENSG00000138821
ENSG00000141424	ENSG00000139540
ENSG00000141424	ENSG00000147804
ENSG00000141424	ENSG00000148482
ENSG00000141424	ENSG00000165915
ENSG00000141424	ENSG00000196950
ENSG00000141425	ENSG00000101413
ENSG00000141425	ENSG00000163125
ENSG00000141429	ENSG00000100626
ENSG00000141429	ENSG00000106648
ENSG00000141429	ENSG00000110328

ENSG00000141429	ENSG00000115339
ENSG00000141429	ENSG00000131386
ENSG00000141429	ENSG00000136542
ENSG00000141429	ENSG00000139629
ENSG00000141429	ENSG00000143641
ENSG00000141429	ENSG00000144278
ENSG00000141429	ENSG00000158089
ENSG00000141429	ENSG00000164574
ENSG00000141429	ENSG00000174473
ENSG00000141429	ENSG00000178234
ENSG00000141429	ENSG00000182870
ENSG00000141429	ENSG00000185274
ENSG00000141433	ENSG00000146469
ENSG00000141434	ENSG00000112818
ENSG00000141434	ENSG00000188886
ENSG00000141437	ENSG00000122696
ENSG00000141437	ENSG00000176274
ENSG00000141437	ENSG00000189332
ENSG00000141441	ENSG00000157833
ENSG00000141446	ENSG00000171320
ENSG00000141448	ENSG00000102145
ENSG00000141448	ENSG00000107485
ENSG00000141448	ENSG00000130700
ENSG00000141448	ENSG00000136574
ENSG00000141448	ENSG00000179348
ENSG00000141449	ENSG00000196208
ENSG00000141480	ENSG00000120500
ENSG00000141480	ENSG00000130561
ENSG00000141480	ENSG00000137486
ENSG00000141485	ENSG00000007216
ENSG00000141485	ENSG00000081800
ENSG00000141485	ENSG00000158296
ENSG00000141485	ENSG00000164707
ENSG00000141503	ENSG00000071054
ENSG00000141503	ENSG00000123572
ENSG00000141503	ENSG00000154310
ENSG00000141504	ENSG00000130066
ENSG00000141504	ENSG00000184788
ENSG00000141506	ENSG00000174083
ENSG00000141510	ENSG00000073282
ENSG00000141510	ENSG00000078900
ENSG00000141522	ENSG00000111348
ENSG00000141522	ENSG00000206156
ENSG00000141522	ENSG00000215642
ENSG00000141524	ENSG00000103534
ENSG00000141524	ENSG00000149488
ENSG00000141524	ENSG00000165091
ENSG00000141524	ENSG00000167608
ENSG00000141524	ENSG00000167895
ENSG00000141524	ENSG00000170537
ENSG00000141524	ENSG00000188869
ENSG00000141526	ENSG00000100156
ENSG00000141526	ENSG00000108932
ENSG00000141526	ENSG00000112394
ENSG00000141526	ENSG00000118596
ENSG00000141526	ENSG00000147100
ENSG00000141526	ENSG00000152779
ENSG00000141526	ENSG00000155380
ENSG00000141526	ENSG00000163053

ENSG00000141526	ENSG00000165449
ENSG00000141526	ENSG00000168679
ENSG00000141526	ENSG00000170190
ENSG00000141526	ENSG00000174326
ENSG00000141526	ENSG00000174327
ENSG00000141527	ENSG00000105289
ENSG00000141540	ENSG00000136295
ENSG00000141540	ENSG00000167614
ENSG00000141542	ENSG00000102128
ENSG00000141542	ENSG00000172476
ENSG00000141542	ENSG00000197562
ENSG00000141551	ENSG00000213923
ENSG00000141562	ENSG00000103245
ENSG00000141568	ENSG00000164916
ENSG00000141570	ENSG00000100307
ENSG00000141570	ENSG00000141582
ENSG00000141570	ENSG00000173894
ENSG00000141570	ENSG00000183741
ENSG00000141576	ENSG00000102858
ENSG00000141579	ENSG00000161992
ENSG00000141582	ENSG00000100307
ENSG00000141582	ENSG00000141570
ENSG00000141582	ENSG00000173894
ENSG00000141582	ENSG00000183741
ENSG00000141622	ENSG00000180233
ENSG00000141622	ENSG00000186187
ENSG00000141639	ENSG00000069956
ENSG00000141639	ENSG00000215542
ENSG00000141646	ENSG00000101665
ENSG00000141646	ENSG00000113658
ENSG00000141646	ENSG00000120693
ENSG00000141646	ENSG00000137834
ENSG00000141646	ENSG00000166949
ENSG00000141646	ENSG00000170365
ENSG00000141646	ENSG00000175387
ENSG00000141664	ENSG00000140948
ENSG00000141668	ENSG00000054803
ENSG00000141668	ENSG00000102924
ENSG00000141668	ENSG00000131094
ENSG00000141668	ENSG00000133466
ENSG00000141668	ENSG00000139899
ENSG00000141668	ENSG00000144119
ENSG00000141668	ENSG00000145861
ENSG00000141668	ENSG00000159189
ENSG00000141668	ENSG00000163145
ENSG00000141668	ENSG00000165985
ENSG00000141668	ENSG00000172247
ENSG00000141668	ENSG00000173369
ENSG00000141668	ENSG00000173372
ENSG00000141668	ENSG00000173918
ENSG00000141668	ENSG00000181092
ENSG00000141668	ENSG00000184471
ENSG00000141668	ENSG00000186897
ENSG00000141668	ENSG00000213192
ENSG00000141696	ENSG00000090530
ENSG00000141696	ENSG00000110811
ENSG00000141696	ENSG00000117385
ENSG00000141696	ENSG00000170275
ENSG00000141698	ENSG00000122643

ENSG00000141699	ENSG00000144567
ENSG00000141699	ENSG00000154153
ENSG00000141720	ENSG00000150867
ENSG00000141720	ENSG00000166908
ENSG00000141720	ENSG00000167103
ENSG00000141736	ENSG00000065361
ENSG00000141736	ENSG00000146648
ENSG00000141736	ENSG00000178568
ENSG00000141738	ENSG00000077420
ENSG00000141738	ENSG00000106070
ENSG00000141738	ENSG00000115290
ENSG00000141738	ENSG00000173166
ENSG00000141744	ENSG00000011177
ENSG00000141744	ENSG00000166741
ENSG00000141748	ENSG00000162980
ENSG00000141748	ENSG00000165997
ENSG00000141748	ENSG00000215451
ENSG00000141750	ENSG00000144681
ENSG00000141750	ENSG00000185482
ENSG00000141753	ENSG00000115457
ENSG00000141753	ENSG00000115461
ENSG00000141753	ENSG00000146674
ENSG00000141753	ENSG00000146678
ENSG00000141753	ENSG00000167779
ENSG00000141756	ENSG00000079150
ENSG00000141756	ENSG00000106080
ENSG00000141756	ENSG00000122642
ENSG00000141756	ENSG00000134285
ENSG00000141756	ENSG00000176826
ENSG00000141837	ENSG00000081248
ENSG00000141837	ENSG00000102001
ENSG00000141837	ENSG00000148408
ENSG00000141837	ENSG00000151067
ENSG00000141837	ENSG00000157388
ENSG00000141837	ENSG00000198216
ENSG00000141867	ENSG00000137948
ENSG00000141867	ENSG00000169925
ENSG00000141867	ENSG00000204256
ENSG00000141905	ENSG00000008441
ENSG00000141905	ENSG00000147862
ENSG00000141905	ENSG00000162599
ENSG00000141934	ENSG00000067113
ENSG00000141934	ENSG00000105520
ENSG00000141934	ENSG00000117598
ENSG00000141934	ENSG00000117600
ENSG00000141934	ENSG00000129951
ENSG00000141934	ENSG00000148123
ENSG00000141934	ENSG00000162407
ENSG00000141959	ENSG00000067057
ENSG00000141959	ENSG00000152556
ENSG00000141965	ENSG00000145780
ENSG00000141965	ENSG00000169018
ENSG00000141965	ENSG00000215524
ENSG00000141965	ENSG00000215602
ENSG00000141968	ENSG00000134215
ENSG00000141968	ENSG00000160293
ENSG00000141971	ENSG00000196814
ENSG00000141977	ENSG00000136425
ENSG00000141977	ENSG00000157884

ENSG00000141977	ENSG00000185043
ENSG00000141979	ENSG00000179218
ENSG00000141985	ENSG00000097033
ENSG00000141985	ENSG00000107295
ENSG00000141985	ENSG00000140600
ENSG00000141985	ENSG00000148341
ENSG00000142002	ENSG00000074603
ENSG00000142002	ENSG00000078098
ENSG00000142002	ENSG00000130226
ENSG00000142002	ENSG00000175497
ENSG00000142002	ENSG00000197635
ENSG00000142082	ENSG00000068903
ENSG00000142089	ENSG00000185201
ENSG00000142089	ENSG00000185885
ENSG00000142089	ENSG00000188082
ENSG00000142089	ENSG00000206013
ENSG00000142089	ENSG00000215096
ENSG00000142156	ENSG00000142173
ENSG00000142156	ENSG00000215018
ENSG00000142166	ENSG00000159113
ENSG00000142166	ENSG00000174564
ENSG00000142173	ENSG00000142156
ENSG00000142173	ENSG00000215018
ENSG00000142178	ENSG00000007047
ENSG00000142178	ENSG00000072518
ENSG00000142178	ENSG00000075413
ENSG00000142178	ENSG00000116141
ENSG00000142178	ENSG00000160584
ENSG00000142178	ENSG00000170145
ENSG00000142182	ENSG00000088305
ENSG00000142182	ENSG00000119772
ENSG00000142185	ENSG00000070985
ENSG00000142185	ENSG00000083067
ENSG00000142185	ENSG00000092439
ENSG00000142185	ENSG00000119121
ENSG00000142185	ENSG00000130529
ENSG00000142185	ENSG00000134160
ENSG00000142185	ENSG00000144481
ENSG00000142188	ENSG00000183726
ENSG00000142192	ENSG00000084234
ENSG00000142192	ENSG00000105290
ENSG00000142197	ENSG00000083097
ENSG00000142208	ENSG00000027075
ENSG00000142208	ENSG00000065675
ENSG00000142208	ENSG00000067606
ENSG00000142208	ENSG00000105221
ENSG00000142208	ENSG00000117020
ENSG00000142208	ENSG00000126583
ENSG00000142208	ENSG00000154229
ENSG00000142208	ENSG00000163558
ENSG00000142208	ENSG00000163932
ENSG00000142208	ENSG00000166501
ENSG00000142224	ENSG00000111536
ENSG00000142224	ENSG00000127318
ENSG00000142224	ENSG00000136634
ENSG00000142224	ENSG00000162891
ENSG00000142224	ENSG00000162892
ENSG00000142227	ENSG00000109099
ENSG00000142227	ENSG00000134531

ENSG00000142227	ENSG00000213853
ENSG00000142233	ENSG00000065320
ENSG00000142233	ENSG00000162068
ENSG00000142235	ENSG00000164715
ENSG00000142235	ENSG00000181409
ENSG00000142273	ENSG00000110395
ENSG00000142273	ENSG00000114423
ENSG00000142279	ENSG00000087077
ENSG00000142279	ENSG00000129474
ENSG00000142279	ENSG00000144791
ENSG00000142279	ENSG00000145012
ENSG00000142279	ENSG00000159840
ENSG00000142279	ENSG00000162458
ENSG00000142303	ENSG00000049192
ENSG00000142303	ENSG00000087116
ENSG00000142303	ENSG00000136378
ENSG00000142303	ENSG00000138316
ENSG00000142303	ENSG00000140470
ENSG00000142303	ENSG00000140873
ENSG00000142303	ENSG00000145536
ENSG00000142303	ENSG00000145808
ENSG00000142303	ENSG00000151388
ENSG00000142303	ENSG00000156140
ENSG00000142303	ENSG00000160323
ENSG00000142303	ENSG00000163638
ENSG00000142303	ENSG00000173157
ENSG00000142303	ENSG00000214644
ENSG00000142303	ENSG00000215743
ENSG00000142319	ENSG00000010379
ENSG00000142319	ENSG00000011083
ENSG00000142319	ENSG00000087916
ENSG00000142319	ENSG00000103546
ENSG00000142319	ENSG00000108576
ENSG00000142319	ENSG00000111181
ENSG00000142319	ENSG00000130821
ENSG00000142319	ENSG00000131389
ENSG00000142319	ENSG00000132164
ENSG00000142319	ENSG00000157103
ENSG00000142319	ENSG00000163817
ENSG00000142319	ENSG00000164363
ENSG00000142319	ENSG00000165970
ENSG00000142319	ENSG00000174358
ENSG00000142319	ENSG00000196517
ENSG00000142319	ENSG00000198555
ENSG00000142319	ENSG00000214617
ENSG00000142327	ENSG00000111144
ENSG00000142327	ENSG00000176393
ENSG00000142347	ENSG00000128641
ENSG00000142347	ENSG00000136286
ENSG00000142347	ENSG00000157483
ENSG00000142347	ENSG00000166866
ENSG00000142347	ENSG00000176658
ENSG00000142347	ENSG00000197879
ENSG00000142396	ENSG00000169740
ENSG00000142396	ENSG00000174255
ENSG00000142396	ENSG00000179195
ENSG00000142396	ENSG00000179965
ENSG00000142396	ENSG00000184294
ENSG00000142396	ENSG00000186446

ENSG00000142396	ENSG00000186777
ENSG00000142396	ENSG00000197008
ENSG00000142396	ENSG00000197025
ENSG00000142396	ENSG00000197062
ENSG00000142396	ENSG00000197332
ENSG00000142396	ENSG00000198155
ENSG00000142396	ENSG00000204756
ENSG00000142396	ENSG00000212805
ENSG00000142396	ENSG00000213976
ENSG00000142396	ENSG00000213980
ENSG00000142396	ENSG00000215180
ENSG00000142396	ENSG00000215510
ENSG00000142396	ENSG00000215537
ENSG00000142396	ENSG00000215598
ENSG00000142396	ENSG00000215600
ENSG00000142405	ENSG00000022556
ENSG00000142405	ENSG00000023191
ENSG00000142405	ENSG00000140853
ENSG00000142405	ENSG00000158077
ENSG00000142405	ENSG00000160505
ENSG00000142405	ENSG00000162711
ENSG00000142405	ENSG00000167207
ENSG00000142405	ENSG00000167634
ENSG00000142405	ENSG00000167984
ENSG00000142405	ENSG00000171487
ENSG00000142405	ENSG00000173572
ENSG00000142405	ENSG00000174885
ENSG00000142405	ENSG00000179709
ENSG00000142405	ENSG00000179873
ENSG00000142405	ENSG00000182261
ENSG00000142405	ENSG00000185792
ENSG00000142408	ENSG00000006116
ENSG00000142408	ENSG00000075429
ENSG00000142408	ENSG00000075461
ENSG00000142408	ENSG00000105605
ENSG00000142408	ENSG00000166862
ENSG00000142408	ENSG00000204278
ENSG00000142449	ENSG00000138829
ENSG00000142449	ENSG00000166147
ENSG00000142459	ENSG00000011454
ENSG00000142459	ENSG00000065882
ENSG00000142459	ENSG00000067208
ENSG00000142459	ENSG00000136111
ENSG00000142459	ENSG00000152061
ENSG00000142484	ENSG00000145107
ENSG00000142484	ENSG00000163762
ENSG00000142484	ENSG00000168955
ENSG00000142484	ENSG00000169903
ENSG00000142484	ENSG00000169908
ENSG00000142494	ENSG00000180638
ENSG00000142507	ENSG00000204261
ENSG00000142512	ENSG00000105366
ENSG00000142512	ENSG00000105383
ENSG00000142512	ENSG00000105492
ENSG00000142512	ENSG00000105501
ENSG00000142512	ENSG00000105695
ENSG00000142512	ENSG00000129450
ENSG00000142512	ENSG00000160296
ENSG00000142512	ENSG00000161640

ENSG00000142512	ENSG00000161643
ENSG00000142512	ENSG00000167764
ENSG00000142512	ENSG00000168995
ENSG00000142512	ENSG00000171101
ENSG00000142512	ENSG00000197865
ENSG00000142513	ENSG00000014257
ENSG00000142513	ENSG00000134575
ENSG00000142539	ENSG00000066336
ENSG00000142549	ENSG00000172260
ENSG00000142549	ENSG00000182667
ENSG00000142549	ENSG00000183715
ENSG00000142549	ENSG00000185565
ENSG00000142552	ENSG00000049449
ENSG00000142552	ENSG00000078808
ENSG00000142552	ENSG00000117906
ENSG00000142552	ENSG00000128595
ENSG00000142599	ENSG00000111676
ENSG00000142606	ENSG00000102174
ENSG00000142606	ENSG00000117298
ENSG00000142606	ENSG00000145194
ENSG00000142606	ENSG00000171551
ENSG00000142606	ENSG00000196549
ENSG00000142606	ENSG00000197993
ENSG00000142611	ENSG00000085276
ENSG00000142615	ENSG00000139610
ENSG00000142615	ENSG00000141086
ENSG00000142615	ENSG00000142789
ENSG00000142615	ENSG00000162438
ENSG00000142615	ENSG00000168925
ENSG00000142615	ENSG00000168928
ENSG00000142615	ENSG00000215704
ENSG00000142627	ENSG00000044524
ENSG00000142627	ENSG00000070886
ENSG00000142627	ENSG00000080224
ENSG00000142627	ENSG00000106123
ENSG00000142627	ENSG00000116106
ENSG00000142627	ENSG00000133216
ENSG00000142627	ENSG00000135333
ENSG00000142627	ENSG00000145242
ENSG00000142627	ENSG00000146904
ENSG00000142627	ENSG00000154928
ENSG00000142627	ENSG00000182580
ENSG00000142627	ENSG00000183317
ENSG00000142627	ENSG00000196411
ENSG00000142632	ENSG00000066248
ENSG00000142632	ENSG00000114790
ENSG00000142632	ENSG00000130762
ENSG00000142632	ENSG00000198844
ENSG00000142634	ENSG00000115468
ENSG00000142634	ENSG00000126878
ENSG00000142634	ENSG00000204472
ENSG00000142661	ENSG00000036448
ENSG00000142661	ENSG00000101605
ENSG00000142669	ENSG00000131171
ENSG00000142669	ENSG00000185437
ENSG00000142669	ENSG00000198478
ENSG00000142669	ENSG00000214636
ENSG00000142675	ENSG00000074706
ENSG00000142675	ENSG00000149970

ENSG00000142675	ENSG00000153721
ENSG00000142687	ENSG00000137261
ENSG00000142694	ENSG00000115363
ENSG00000142700	ENSG00000064218
ENSG00000142700	ENSG00000137090
ENSG00000142700	ENSG00000142025
ENSG00000142700	ENSG00000143006
ENSG00000142700	ENSG00000173253
ENSG00000142700	ENSG00000176399
ENSG00000142733	ENSG00000180815
ENSG00000142733	ENSG00000197442
ENSG00000142765	ENSG00000102362
ENSG00000142765	ENSG00000137501
ENSG00000142765	ENSG00000147041
ENSG00000142765	ENSG00000164674
ENSG00000142789	ENSG00000139610
ENSG00000142789	ENSG00000141086
ENSG00000142789	ENSG00000142615
ENSG00000142789	ENSG00000162438
ENSG00000142789	ENSG00000168925
ENSG00000142789	ENSG00000168928
ENSG00000142789	ENSG00000215704
ENSG00000142798	ENSG00000164318
ENSG00000142798	ENSG00000188157
ENSG00000142864	ENSG00000130956
ENSG00000142871	ENSG00000064205
ENSG00000142871	ENSG00000104415
ENSG00000142871	ENSG00000112761
ENSG00000142871	ENSG00000118523
ENSG00000142871	ENSG00000136999
ENSG00000142875	ENSG00000072062
ENSG00000142875	ENSG00000099725
ENSG00000142875	ENSG00000165059
ENSG00000142875	ENSG00000183943
ENSG00000142910	ENSG00000137251
ENSG00000142920	ENSG00000115758
ENSG00000142920	ENSG00000155096
ENSG00000142945	ENSG00000068796
ENSG00000142945	ENSG00000141200
ENSG00000142949	ENSG00000060656
ENSG00000142949	ENSG00000105426
ENSG00000142949	ENSG00000152894
ENSG00000142949	ENSG00000153707
ENSG00000142949	ENSG00000173482
ENSG00000142949	ENSG00000196090
ENSG00000142959	ENSG00000039987
ENSG00000142959	ENSG00000127325
ENSG00000142959	ENSG00000167995
ENSG00000142961	ENSG00000114978
ENSG00000142961	ENSG00000120162
ENSG00000142961	ENSG00000172081
ENSG00000142961	ENSG00000173542
ENSG00000142961	ENSG00000182208
ENSG00000142973	ENSG00000145476
ENSG00000142973	ENSG00000154198
ENSG00000142973	ENSG00000162365
ENSG00000142973	ENSG00000171903
ENSG00000142973	ENSG00000171954
ENSG00000142973	ENSG00000186115

ENSG00000142973	ENSG00000186160
ENSG00000142973	ENSG00000186204
ENSG00000142973	ENSG00000186377
ENSG00000142973	ENSG00000186526
ENSG00000142973	ENSG00000186529
ENSG00000142973	ENSG00000187048
ENSG00000143013	ENSG00000016082
ENSG00000143013	ENSG00000089116
ENSG00000143013	ENSG00000106689
ENSG00000143013	ENSG00000106852
ENSG00000143013	ENSG00000107187
ENSG00000143013	ENSG00000121454
ENSG00000143013	ENSG00000132130
ENSG00000143013	ENSG00000136944
ENSG00000143013	ENSG00000143355
ENSG00000143013	ENSG00000159556
ENSG00000143013	ENSG00000162624
ENSG00000143013	ENSG00000162761
ENSG00000143028	ENSG00000008282
ENSG00000143028	ENSG00000102003
ENSG00000143028	ENSG00000163630
ENSG00000143032	ENSG00000125492
ENSG00000143032	ENSG00000206148
ENSG00000143033	ENSG00000112511
ENSG00000143033	ENSG00000119403
ENSG00000143036	ENSG00000070214
ENSG00000143036	ENSG00000129353
ENSG00000143036	ENSG00000137968
ENSG00000143036	ENSG00000204385
ENSG00000143061	ENSG00000134247
ENSG00000143061	ENSG00000134256
ENSG00000143061	ENSG00000162729
ENSG00000143079	ENSG00000077063
ENSG00000143093	ENSG00000128578
ENSG00000143105	ENSG00000026559
ENSG00000143105	ENSG00000102057
ENSG00000143105	ENSG00000104848
ENSG00000143105	ENSG00000111262
ENSG00000143105	ENSG00000124134
ENSG00000143105	ENSG00000130037
ENSG00000143105	ENSG00000151079
ENSG00000143105	ENSG00000156486
ENSG00000143105	ENSG00000158445
ENSG00000143105	ENSG00000162975
ENSG00000143105	ENSG00000164794
ENSG00000143105	ENSG00000168263
ENSG00000143105	ENSG00000168418
ENSG00000143105	ENSG00000170745
ENSG00000143105	ENSG00000171126
ENSG00000143105	ENSG00000171385
ENSG00000143105	ENSG00000177272
ENSG00000143105	ENSG00000177301
ENSG00000143105	ENSG00000178342
ENSG00000143105	ENSG00000182255
ENSG00000143105	ENSG00000182674
ENSG00000143105	ENSG00000184408
ENSG00000143119	ENSG00000011105
ENSG00000143119	ENSG00000085117
ENSG00000143119	ENSG00000104894

ENSG00000143119	ENSG00000110900
ENSG00000143119	ENSG00000117472
ENSG00000143119	ENSG00000157570
ENSG00000143119	ENSG00000177697
ENSG00000143119	ENSG00000214063
ENSG00000143125	ENSG00000163421
ENSG00000143126	ENSG00000008300
ENSG00000143126	ENSG00000075275
ENSG00000143127	ENSG00000005844
ENSG00000143127	ENSG00000005884
ENSG00000143127	ENSG00000005961
ENSG00000143127	ENSG00000077943
ENSG00000143127	ENSG00000083457
ENSG00000143127	ENSG00000091409
ENSG00000143127	ENSG00000115232
ENSG00000143127	ENSG00000135424
ENSG00000143127	ENSG00000137809
ENSG00000143127	ENSG00000138448
ENSG00000143127	ENSG00000140678
ENSG00000143127	ENSG00000144668
ENSG00000143127	ENSG00000156886
ENSG00000143127	ENSG00000161638
ENSG00000143127	ENSG00000164171
ENSG00000143127	ENSG00000169896
ENSG00000143127	ENSG00000213949
ENSG00000143140	ENSG00000121634
ENSG00000143140	ENSG00000121742
ENSG00000143140	ENSG00000121743
ENSG00000143140	ENSG00000131233
ENSG00000143140	ENSG00000135355
ENSG00000143140	ENSG00000152661
ENSG00000143140	ENSG00000159248
ENSG00000143140	ENSG00000164411
ENSG00000143140	ENSG00000165474
ENSG00000143140	ENSG00000169562
ENSG00000143140	ENSG00000176402
ENSG00000143140	ENSG00000177291
ENSG00000143140	ENSG00000182963
ENSG00000143140	ENSG00000183153
ENSG00000143140	ENSG00000187513
ENSG00000143140	ENSG00000188910
ENSG00000143140	ENSG00000189280
ENSG00000143140	ENSG00000189433
ENSG00000143140	ENSG00000198835
ENSG00000143140	ENSG00000203733
ENSG00000143147	ENSG00000120907
ENSG00000143147	ENSG00000148680
ENSG00000143147	ENSG00000165370
ENSG00000143147	ENSG00000170214
ENSG00000143147	ENSG00000171873
ENSG00000143147	ENSG00000183935
ENSG00000143153	ENSG00000069849
ENSG00000143153	ENSG00000101892
ENSG00000143153	ENSG00000129244
ENSG00000143153	ENSG00000186009
ENSG00000143162	ENSG00000175874
ENSG00000143167	ENSG00000019102
ENSG00000143167	ENSG00000144847
ENSG00000143167	ENSG00000149564

ENSG00000143167	ENSG00000154639
ENSG00000143167	ENSG00000166250
ENSG00000143167	ENSG00000188004
ENSG00000143171	ENSG00000101076
ENSG00000143171	ENSG00000160113
ENSG00000143171	ENSG00000164749
ENSG00000143171	ENSG00000175745
ENSG00000143171	ENSG00000185551
ENSG00000143171	ENSG00000186350
ENSG00000143171	ENSG00000204231
ENSG00000143178	ENSG00000073861
ENSG00000143178	ENSG00000089225
ENSG00000143178	ENSG00000092607
ENSG00000143178	ENSG00000112837
ENSG00000143178	ENSG00000121068
ENSG00000143178	ENSG00000121075
ENSG00000143178	ENSG00000122145
ENSG00000143178	ENSG00000135111
ENSG00000143178	ENSG00000136535
ENSG00000143178	ENSG00000149922
ENSG00000143178	ENSG00000163508
ENSG00000143178	ENSG00000164458
ENSG00000143178	ENSG00000164532
ENSG00000143178	ENSG00000167800
ENSG00000143178	ENSG00000174197
ENSG00000143178	ENSG00000184058
ENSG00000143184	ENSG00000006074
ENSG00000143184	ENSG00000006075
ENSG00000143184	ENSG00000006606
ENSG00000143184	ENSG00000102962
ENSG00000143184	ENSG00000102970
ENSG00000143184	ENSG00000106178
ENSG00000143184	ENSG00000108688
ENSG00000143184	ENSG00000108691
ENSG00000143184	ENSG00000108700
ENSG00000143184	ENSG00000108702
ENSG00000143184	ENSG00000115009
ENSG00000143184	ENSG00000129277
ENSG00000143184	ENSG00000143185
ENSG00000143184	ENSG00000161570
ENSG00000143184	ENSG00000161573
ENSG00000143184	ENSG00000161574
ENSG00000143184	ENSG00000167236
ENSG00000143184	ENSG00000172156
ENSG00000143184	ENSG00000172724
ENSG00000143184	ENSG00000181374
ENSG00000143184	ENSG00000197262
ENSG00000143184	ENSG00000205017
ENSG00000143184	ENSG00000205020
ENSG00000143184	ENSG00000205021
ENSG00000143184	ENSG00000213494
ENSG00000143184	ENSG00000215627
ENSG00000143184	ENSG00000215629
ENSG00000143185	ENSG00000006074
ENSG00000143185	ENSG00000006075
ENSG00000143185	ENSG00000006606
ENSG00000143185	ENSG00000102962
ENSG00000143185	ENSG00000102970
ENSG00000143185	ENSG00000106178

ENSG00000143185	ENSG00000108688
ENSG00000143185	ENSG00000108691
ENSG00000143185	ENSG00000108700
ENSG00000143185	ENSG00000108702
ENSG00000143185	ENSG00000115009
ENSG00000143185	ENSG00000129277
ENSG00000143185	ENSG00000143184
ENSG00000143185	ENSG00000161570
ENSG00000143185	ENSG00000161573
ENSG00000143185	ENSG00000161574
ENSG00000143185	ENSG00000167236
ENSG00000143185	ENSG00000172156
ENSG00000143185	ENSG00000172724
ENSG00000143185	ENSG00000181374
ENSG00000143185	ENSG00000197262
ENSG00000143185	ENSG00000205017
ENSG00000143185	ENSG00000205020
ENSG00000143185	ENSG00000205021
ENSG00000143185	ENSG00000213494
ENSG00000143185	ENSG00000215627
ENSG00000143185	ENSG00000215629
ENSG00000143190	ENSG00000028277
ENSG00000143190	ENSG00000064835
ENSG00000143190	ENSG00000091010
ENSG00000143190	ENSG00000137709
ENSG00000143190	ENSG00000151615
ENSG00000143190	ENSG00000152192
ENSG00000143190	ENSG00000184486
ENSG00000143190	ENSG00000185668
ENSG00000143190	ENSG00000196767
ENSG00000143190	ENSG00000198914
ENSG00000143190	ENSG00000204531
ENSG00000143190	ENSG00000212993
ENSG00000143195	ENSG00000105699
ENSG00000143195	ENSG00000145103
ENSG00000143226	ENSG00000072694
ENSG00000143226	ENSG00000132185
ENSG00000143226	ENSG00000150337
ENSG00000143226	ENSG00000162747
ENSG00000143226	ENSG00000179639
ENSG00000143226	ENSG00000181036
ENSG00000143226	ENSG00000198019
ENSG00000143226	ENSG00000203747
ENSG00000143226	ENSG00000203820
ENSG00000143248	ENSG00000090104
ENSG00000143248	ENSG00000091844
ENSG00000143248	ENSG00000116741
ENSG00000143248	ENSG00000117152
ENSG00000143248	ENSG00000127074
ENSG00000143248	ENSG00000135824
ENSG00000143248	ENSG00000143333
ENSG00000143248	ENSG00000147509
ENSG00000143248	ENSG00000148908
ENSG00000143248	ENSG00000150681
ENSG00000143248	ENSG00000171700
ENSG00000143248	ENSG00000215825
ENSG00000143257	ENSG00000012504
ENSG00000143257	ENSG00000111424
ENSG00000143257	ENSG00000112033

ENSG00000143257	ENSG00000132170
ENSG00000143257	ENSG00000144852
ENSG00000143257	ENSG00000186951
ENSG00000143258	ENSG00000036672
ENSG00000143258	ENSG00000138592
ENSG00000143258	ENSG00000170236
ENSG00000143278	ENSG00000000971
ENSG00000143278	ENSG00000080910
ENSG00000143278	ENSG00000116785
ENSG00000143278	ENSG00000134365
ENSG00000143278	ENSG00000134389
ENSG00000143278	ENSG00000134391
ENSG00000143318	ENSG00000118729
ENSG00000143319	ENSG00000172183
ENSG00000143319	ENSG00000181026
ENSG00000143320	ENSG00000114113
ENSG00000143320	ENSG00000114115
ENSG00000143320	ENSG00000121769
ENSG00000143320	ENSG00000139194
ENSG00000143320	ENSG00000145384
ENSG00000143320	ENSG00000147588
ENSG00000143320	ENSG00000162444
ENSG00000143320	ENSG00000164434
ENSG00000143320	ENSG00000164687
ENSG00000143320	ENSG00000166426
ENSG00000143320	ENSG00000170323
ENSG00000143320	ENSG00000197416
ENSG00000143320	ENSG00000205186
ENSG00000143320	ENSG00000214004
ENSG00000143321	ENSG00000112273
ENSG00000143321	ENSG00000164985
ENSG00000143321	ENSG00000166503
ENSG00000143321	ENSG00000167674
ENSG00000143322	ENSG00000097007
ENSG00000143333	ENSG00000090104
ENSG00000143333	ENSG00000091844
ENSG00000143333	ENSG00000116741
ENSG00000143333	ENSG00000117152
ENSG00000143333	ENSG00000127074
ENSG00000143333	ENSG00000135824
ENSG00000143333	ENSG00000143248
ENSG00000143333	ENSG00000147509
ENSG00000143333	ENSG00000148908
ENSG00000143333	ENSG00000150681
ENSG00000143333	ENSG00000171700
ENSG00000143333	ENSG00000215825
ENSG00000143340	ENSG00000196990
ENSG00000143340	ENSG00000215611
ENSG00000143341	ENSG00000101825
ENSG00000143341	ENSG00000148357
ENSG00000143341	ENSG00000152580
ENSG00000143341	ENSG00000155657
ENSG00000143341	ENSG00000168462
ENSG00000143344	ENSG00000160271
ENSG00000143344	ENSG00000204218
ENSG00000143344	ENSG00000205517
ENSG00000143355	ENSG00000016082
ENSG00000143355	ENSG00000089116
ENSG00000143355	ENSG00000106689

ENSG00000143355	ENSG00000106852
ENSG00000143355	ENSG00000107187
ENSG00000143355	ENSG00000121454
ENSG00000143355	ENSG00000132130
ENSG00000143355	ENSG00000136944
ENSG00000143355	ENSG00000143013
ENSG00000143355	ENSG00000159556
ENSG00000143355	ENSG00000162624
ENSG00000143355	ENSG00000162761
ENSG00000143363	ENSG00000156035
ENSG00000143365	ENSG00000198963
ENSG00000143367	ENSG00000166510
ENSG00000143373	ENSG00000074657
ENSG00000143373	ENSG00000166716
ENSG00000143373	ENSG00000214168
ENSG00000143373	ENSG00000214171
ENSG00000143374	ENSG00000113407
ENSG00000143374	ENSG00000154719
ENSG00000143374	ENSG00000185418
ENSG00000143375	ENSG00000128849
ENSG00000143379	ENSG00000136169
ENSG00000143382	ENSG00000100767
ENSG00000143382	ENSG00000185761
ENSG00000143382	ENSG00000187720
ENSG00000143382	ENSG00000197859
ENSG00000143382	ENSG00000215616
ENSG00000143384	ENSG00000030110
ENSG00000143384	ENSG00000087088
ENSG00000143384	ENSG00000129473
ENSG00000143384	ENSG00000140379
ENSG00000143384	ENSG00000171552
ENSG00000143384	ENSG00000171791
ENSG00000143384	ENSG00000176720
ENSG00000143387	ENSG00000135047
ENSG00000143387	ENSG00000136943
ENSG00000143387	ENSG00000163131
ENSG00000143387	ENSG00000188029
ENSG00000143387	ENSG00000204437
ENSG00000143390	ENSG00000080298
ENSG00000143390	ENSG00000087903
ENSG00000143390	ENSG00000111783
ENSG00000143390	ENSG00000132005
ENSG00000143390	ENSG00000181827
ENSG00000143390	ENSG00000185002
ENSG00000143398	ENSG00000107242
ENSG00000143398	ENSG00000173780
ENSG00000143398	ENSG00000186111
ENSG00000143401	ENSG00000136938
ENSG00000143401	ENSG00000139223
ENSG00000143401	ENSG00000140350
ENSG00000143401	ENSG00000184701
ENSG00000143401	ENSG00000215332
ENSG00000143409	ENSG00000128923
ENSG00000143412	ENSG00000104537
ENSG00000143412	ENSG00000109511
ENSG00000143412	ENSG00000122359
ENSG00000143412	ENSG00000135046
ENSG00000143412	ENSG00000138279
ENSG00000143412	ENSG00000138772

ENSG00000143412	ENSG00000150165
ENSG00000143412	ENSG00000164111
ENSG00000143412	ENSG00000165390
ENSG00000143412	ENSG00000182718
ENSG00000143412	ENSG00000183059
ENSG00000143412	ENSG00000186807
ENSG00000143412	ENSG00000196975
ENSG00000143412	ENSG00000197043
ENSG00000143418	ENSG00000065308
ENSG00000143418	ENSG00000067167
ENSG00000143418	ENSG00000090661
ENSG00000143418	ENSG00000139624
ENSG00000143418	ENSG00000154227
ENSG00000143418	ENSG00000172292
ENSG00000143418	ENSG00000174599
ENSG00000143420	ENSG00000128989
ENSG00000143434	ENSG00000010319
ENSG00000143434	ENSG00000012171
ENSG00000143434	ENSG00000075213
ENSG00000143434	ENSG00000092421
ENSG00000143434	ENSG00000095539
ENSG00000143434	ENSG00000137872
ENSG00000143434	ENSG00000153993
ENSG00000143434	ENSG00000168758
ENSG00000143434	ENSG00000170381
ENSG00000143434	ENSG00000185033
ENSG00000143434	ENSG00000187764
ENSG00000143434	ENSG00000196189
ENSG00000143437	ENSG00000029153
ENSG00000143437	ENSG00000133794
ENSG00000143437	ENSG00000172379
ENSG00000143442	ENSG00000056277
ENSG00000143442	ENSG00000137871
ENSG00000143442	ENSG00000169548
ENSG00000143442	ENSG00000198477
ENSG00000143450	ENSG00000104904
ENSG00000143450	ENSG00000180304
ENSG00000143452	ENSG00000176635
ENSG00000143457	ENSG00000113384
ENSG00000143458	ENSG00000104064
ENSG00000143458	ENSG00000107854
ENSG00000143458	ENSG00000173273
ENSG00000143466	ENSG00000104365
ENSG00000143466	ENSG00000183735
ENSG00000143466	ENSG00000213341
ENSG00000143469	ENSG00000139973
ENSG00000143473	ENSG00000055118
ENSG00000143473	ENSG00000089558
ENSG00000143473	ENSG00000135519
ENSG00000143473	ENSG00000140015
ENSG00000143473	ENSG00000173826
ENSG00000143473	ENSG00000183960
ENSG00000143473	ENSG00000184611
ENSG00000143479	ENSG00000010219
ENSG00000143479	ENSG00000105204
ENSG00000143479	ENSG00000127334
ENSG00000143479	ENSG00000157540
ENSG00000143494	ENSG00000071246
ENSG00000143499	ENSG00000115593

ENSG00000143499	ENSG00000185420
ENSG00000143502	ENSG00000100647
ENSG00000143507	ENSG00000111266
ENSG00000143507	ENSG00000120129
ENSG00000143507	ENSG00000120875
ENSG00000143507	ENSG00000130829
ENSG00000143507	ENSG00000138166
ENSG00000143507	ENSG00000139318
ENSG00000143507	ENSG00000158050
ENSG00000143507	ENSG00000164086
ENSG00000143507	ENSG00000184545
ENSG00000143512	ENSG00000164161
ENSG00000143512	ENSG00000182218
ENSG00000143514	ENSG00000088808
ENSG00000143514	ENSG00000104881
ENSG00000143515	ENSG00000081923
ENSG00000143515	ENSG00000104043
ENSG00000143515	ENSG00000124406
ENSG00000143515	ENSG00000130270
ENSG00000143515	ENSG00000132932
ENSG00000143537	ENSG00000008277
ENSG00000143537	ENSG00000042980
ENSG00000143537	ENSG00000069206
ENSG00000143537	ENSG00000073670
ENSG00000143537	ENSG00000104755
ENSG00000143537	ENSG00000114948
ENSG00000143537	ENSG00000134007
ENSG00000143537	ENSG00000134028
ENSG00000143537	ENSG00000134249
ENSG00000143537	ENSG00000135074
ENSG00000143537	ENSG00000139985
ENSG00000143537	ENSG00000148848
ENSG00000143537	ENSG00000149451
ENSG00000143537	ENSG00000151651
ENSG00000143537	ENSG00000151694
ENSG00000143537	ENSG00000168594
ENSG00000143537	ENSG00000168615
ENSG00000143537	ENSG00000168619
ENSG00000143537	ENSG00000196115
ENSG00000143537	ENSG00000197140
ENSG00000143537	ENSG00000214137
ENSG00000143545	ENSG00000084733
ENSG00000143545	ENSG00000139998
ENSG00000143545	ENSG00000166128
ENSG00000143545	ENSG00000167461
ENSG00000143546	ENSG00000160307
ENSG00000143546	ENSG00000160678
ENSG00000143546	ENSG00000163191
ENSG00000143546	ENSG00000163220
ENSG00000143546	ENSG00000163221
ENSG00000143546	ENSG00000163993
ENSG00000143546	ENSG00000169906
ENSG00000143546	ENSG00000171643
ENSG00000143546	ENSG00000188015
ENSG00000143546	ENSG00000188643
ENSG00000143546	ENSG00000189171
ENSG00000143546	ENSG00000189334
ENSG00000143546	ENSG00000196154
ENSG00000143546	ENSG00000196420

ENSG00000143546	ENSG00000196754
ENSG00000143546	ENSG00000197747
ENSG00000143546	ENSG00000197956
ENSG00000143549	ENSG00000140416
ENSG00000143549	ENSG00000167460
ENSG00000143549	ENSG00000177152
ENSG00000143549	ENSG00000183022
ENSG00000143549	ENSG00000187536
ENSG00000143549	ENSG00000198467
ENSG00000143549	ENSG00000214869
ENSG00000143554	ENSG00000083807
ENSG00000143554	ENSG00000113396
ENSG00000143554	ENSG00000130304
ENSG00000143554	ENSG00000140284
ENSG00000143554	ENSG00000167114
ENSG00000143569	ENSG00000137073
ENSG00000143570	ENSG00000141873
ENSG00000143570	ENSG00000165794
ENSG00000143578	ENSG00000060566
ENSG00000143578	ENSG00000107175
ENSG00000143578	ENSG00000118217
ENSG00000143578	ENSG00000157613
ENSG00000143578	ENSG00000182158
ENSG00000143578	ENSG00000213676
ENSG00000143590	ENSG00000090776
ENSG00000143590	ENSG00000099617
ENSG00000143590	ENSG00000108947
ENSG00000143590	ENSG00000125266
ENSG00000143590	ENSG00000143620
ENSG00000143590	ENSG00000169242
ENSG00000143590	ENSG00000184349
ENSG00000143595	ENSG00000103569
ENSG00000143595	ENSG00000165269
ENSG00000143595	ENSG00000165272
ENSG00000143595	ENSG00000176115
ENSG00000143595	ENSG00000181997
ENSG00000143595	ENSG00000186466
ENSG00000143603	ENSG00000080709
ENSG00000143603	ENSG00000104783
ENSG00000143603	ENSG00000105642
ENSG00000143614	ENSG00000167491
ENSG00000143620	ENSG00000090776
ENSG00000143620	ENSG00000099617
ENSG00000143620	ENSG00000108947
ENSG00000143620	ENSG00000125266
ENSG00000143620	ENSG00000143590
ENSG00000143620	ENSG00000169242
ENSG00000143620	ENSG00000184349
ENSG00000143622	ENSG00000152214
ENSG00000143627	ENSG00000067225
ENSG00000143630	ENSG00000070729
ENSG00000143630	ENSG00000099822
ENSG00000143630	ENSG00000132259
ENSG00000143630	ENSG00000138622
ENSG00000143630	ENSG00000144191
ENSG00000143630	ENSG00000164588
ENSG00000143630	ENSG00000170289
ENSG00000143630	ENSG00000183862
ENSG00000143632	ENSG00000075624

ENSG00000143632	ENSG00000107796
ENSG00000143632	ENSG00000159251
ENSG00000143632	ENSG00000163017
ENSG00000143632	ENSG00000169067
ENSG00000143632	ENSG00000184009
ENSG00000143632	ENSG00000204434
ENSG00000143632	ENSG00000214555
ENSG00000143632	ENSG00000215141
ENSG00000143643	ENSG00000139865
ENSG00000143674	ENSG00000006432
ENSG00000143674	ENSG00000130758
ENSG00000143674	ENSG00000173327
ENSG00000143702	ENSG00000099814
ENSG00000143753	ENSG00000168350
ENSG00000143761	ENSG00000004059
ENSG00000143761	ENSG00000134287
ENSG00000143761	ENSG00000165527
ENSG00000143761	ENSG00000168374
ENSG00000143772	ENSG00000086544
ENSG00000143772	ENSG00000137825
ENSG00000143776	ENSG00000067900
ENSG00000143776	ENSG00000134318
ENSG00000143776	ENSG00000171219
ENSG00000143776	ENSG00000198752
ENSG00000143786	ENSG00000100528
ENSG00000143786	ENSG00000143771
ENSG00000143786	ENSG00000174871
ENSG00000143797	ENSG00000172197
ENSG00000143797	ENSG00000177669
ENSG00000143801	ENSG00000080815
ENSG00000143815	ENSG00000149809
ENSG00000143816	ENSG00000002745
ENSG00000143816	ENSG00000061492
ENSG00000143816	ENSG00000075290
ENSG00000143816	ENSG00000085741
ENSG00000143816	ENSG00000105989
ENSG00000143816	ENSG00000108379
ENSG00000143816	ENSG00000111186
ENSG00000143816	ENSG00000114251
ENSG00000143816	ENSG00000115596
ENSG00000143816	ENSG00000125084
ENSG00000143816	ENSG00000134245
ENSG00000143816	ENSG00000135925
ENSG00000143816	ENSG00000154342
ENSG00000143816	ENSG00000154764
ENSG00000143816	ENSG00000158955
ENSG00000143816	ENSG00000162552
ENSG00000143816	ENSG00000169884
ENSG00000143816	ENSG00000188064
ENSG00000143839	ENSG00000096088
ENSG00000143839	ENSG00000117984
ENSG00000143839	ENSG00000131400
ENSG00000143839	ENSG00000131401
ENSG00000143839	ENSG00000172647
ENSG00000143839	ENSG00000180860
ENSG00000143839	ENSG00000184488
ENSG00000143839	ENSG00000196188
ENSG00000143839	ENSG00000204973
ENSG00000143839	ENSG00000215868

ENSG00000143842	ENSG00000110693
ENSG00000143842	ENSG00000134532
ENSG00000143845	ENSG00000100288
ENSG00000143845	ENSG00000110721
ENSG00000143845	ENSG00000139163
ENSG00000143847	ENSG00000110841
ENSG00000143847	ENSG00000131626
ENSG00000143847	ENSG00000139220
ENSG00000143847	ENSG00000166387
ENSG00000143847	ENSG00000177380
ENSG00000143850	ENSG00000052126
ENSG00000143850	ENSG00000105559
ENSG00000143850	ENSG00000166689
ENSG00000143851	ENSG00000110786
ENSG00000143851	ENSG00000153233
ENSG00000143858	ENSG00000011347
ENSG00000143858	ENSG00000019505
ENSG00000143858	ENSG00000067715
ENSG00000143858	ENSG00000101098
ENSG00000143858	ENSG00000103528
ENSG00000143858	ENSG00000110975
ENSG00000143858	ENSG00000129990
ENSG00000143858	ENSG00000132718
ENSG00000143858	ENSG00000132872
ENSG00000143858	ENSG00000134207
ENSG00000143858	ENSG00000149043
ENSG00000143858	ENSG00000149927
ENSG00000143858	ENSG00000170743
ENSG00000143858	ENSG00000173227
ENSG00000143858	ENSG00000204176
ENSG00000143858	ENSG00000213023
ENSG00000143858	ENSG00000214408
ENSG00000143858	ENSG00000214409
ENSG00000143858	ENSG00000214569
ENSG00000143858	ENSG00000214783
ENSG00000143867	ENSG00000164920
ENSG00000143869	ENSG00000101144
ENSG00000143869	ENSG00000107623
ENSG00000143869	ENSG00000112175
ENSG00000143869	ENSG00000116985
ENSG00000143869	ENSG00000125965
ENSG00000143869	ENSG00000128802
ENSG00000143869	ENSG00000130385
ENSG00000143869	ENSG00000152785
ENSG00000143869	ENSG00000153162
ENSG00000143869	ENSG00000156466
ENSG00000143869	ENSG00000156574
ENSG00000143869	ENSG00000163217
ENSG00000143869	ENSG00000183682
ENSG00000143878	ENSG00000067560
ENSG00000143878	ENSG00000155366
ENSG00000143882	ENSG00000155097
ENSG00000143889	ENSG00000104824
ENSG00000143889	ENSG00000215255
ENSG00000143921	ENSG00000138075
ENSG00000143924	ENSG00000066629
ENSG00000143924	ENSG00000125746
ENSG00000143924	ENSG00000143940
ENSG00000143924	ENSG00000149499

ENSG00000143924	ENSG00000165521
ENSG00000143924	ENSG00000214595
ENSG00000143933	ENSG00000129007
ENSG00000143933	ENSG00000160014
ENSG00000143933	ENSG00000178363
ENSG00000143933	ENSG00000178372
ENSG00000143933	ENSG00000198668
ENSG00000143940	ENSG00000066629
ENSG00000143940	ENSG00000125746
ENSG00000143940	ENSG00000143924
ENSG00000143940	ENSG00000149499
ENSG00000143940	ENSG00000165521
ENSG00000143940	ENSG00000214595
ENSG00000143970	ENSG00000171456
ENSG00000143970	ENSG00000215491
ENSG00000143994	ENSG00000140526
ENSG00000143994	ENSG00000158201
ENSG00000144035	ENSG00000185818
ENSG00000144036	ENSG00000138190
ENSG00000144040	ENSG00000107819
ENSG00000144040	ENSG00000156398
ENSG00000144040	ENSG00000164466
ENSG00000144040	ENSG00000183605
ENSG00000144045	ENSG00000089876
ENSG00000144045	ENSG00000109606
ENSG00000144057	ENSG00000073849
ENSG00000144057	ENSG00000101638
ENSG00000144057	ENSG00000111728
ENSG00000144057	ENSG00000117069
ENSG00000144057	ENSG00000148488
ENSG00000144057	ENSG00000160408
ENSG00000144057	ENSG00000177511
ENSG00000144063	ENSG00000102934
ENSG00000144063	ENSG00000147676
ENSG00000144063	ENSG00000155254
ENSG00000144063	ENSG00000170293
ENSG00000144063	ENSG00000172005
ENSG00000144118	ENSG00000006451
ENSG00000144118	ENSG00000126458
ENSG00000144118	ENSG00000133703
ENSG00000144118	ENSG00000133818
ENSG00000144118	ENSG00000158186
ENSG00000144118	ENSG00000174775
ENSG00000144118	ENSG00000187682
ENSG00000144118	ENSG00000213281
ENSG00000144119	ENSG00000054803
ENSG00000144119	ENSG00000102924
ENSG00000144119	ENSG00000131094
ENSG00000144119	ENSG00000133466
ENSG00000144119	ENSG00000139899
ENSG00000144119	ENSG00000141668
ENSG00000144119	ENSG00000145861
ENSG00000144119	ENSG00000159189
ENSG00000144119	ENSG00000163145
ENSG00000144119	ENSG00000165985
ENSG00000144119	ENSG00000172247
ENSG00000144119	ENSG00000173369
ENSG00000144119	ENSG00000173372
ENSG00000144119	ENSG00000173918

ENSG00000144119	ENSG00000181092
ENSG00000144119	ENSG00000184471
ENSG00000144119	ENSG00000186897
ENSG00000144119	ENSG00000213192
ENSG00000144130	ENSG00000076685
ENSG00000144136	ENSG00000168575
ENSG00000144191	ENSG00000070729
ENSG00000144191	ENSG00000099822
ENSG00000144191	ENSG00000132259
ENSG00000144191	ENSG00000138622
ENSG00000144191	ENSG00000143630
ENSG00000144191	ENSG00000164588
ENSG00000144191	ENSG00000170289
ENSG00000144191	ENSG00000183862
ENSG00000144214	ENSG00000185674
ENSG00000144218	ENSG00000072364
ENSG00000144218	ENSG00000155966
ENSG00000144218	ENSG00000172493
ENSG00000144227	ENSG00000122584
ENSG00000144227	ENSG00000182379
ENSG00000144227	ENSG00000182575
ENSG00000144228	ENSG00000121067
ENSG00000144229	ENSG00000005108
ENSG00000144230	ENSG00000125245
ENSG00000144230	ENSG00000126251
ENSG00000144230	ENSG00000126262
ENSG00000144230	ENSG00000147138
ENSG00000144230	ENSG00000162881
ENSG00000144230	ENSG00000164220
ENSG00000144230	ENSG00000164251
ENSG00000144230	ENSG00000165621
ENSG00000144230	ENSG00000171631
ENSG00000144230	ENSG00000181104
ENSG00000144230	ENSG00000182162
ENSG00000144230	ENSG00000182559
ENSG00000144230	ENSG00000182782
ENSG00000144230	ENSG00000185897
ENSG00000144230	ENSG00000196917
ENSG00000144278	ENSG00000100626
ENSG00000144278	ENSG00000106648
ENSG00000144278	ENSG00000110328
ENSG00000144278	ENSG00000115339
ENSG00000144278	ENSG00000131386
ENSG00000144278	ENSG00000136542
ENSG00000144278	ENSG00000139629
ENSG00000144278	ENSG00000141429
ENSG00000144278	ENSG00000143641
ENSG00000144278	ENSG00000158089
ENSG00000144278	ENSG00000164574
ENSG00000144278	ENSG00000174473
ENSG00000144278	ENSG00000178234
ENSG00000144278	ENSG00000182870
ENSG00000144278	ENSG00000185274
ENSG00000144283	ENSG00000057294
ENSG00000144283	ENSG00000081277
ENSG00000144283	ENSG00000099889
ENSG00000144283	ENSG00000169862
ENSG00000144283	ENSG00000184363
ENSG00000144283	ENSG00000198561

ENSG00000144285	ENSG00000007314
ENSG00000144285	ENSG00000136531
ENSG00000144285	ENSG00000136546
ENSG00000144285	ENSG00000153253
ENSG00000144285	ENSG00000168356
ENSG00000144285	ENSG00000169432
ENSG00000144285	ENSG00000183873
ENSG00000144285	ENSG00000185313
ENSG00000144285	ENSG00000196876
ENSG00000144290	ENSG00000004939
ENSG00000144290	ENSG00000033867
ENSG00000144290	ENSG00000050438
ENSG00000144290	ENSG00000080493
ENSG00000144290	ENSG00000088836
ENSG00000144290	ENSG00000113073
ENSG00000144290	ENSG00000114923
ENSG00000144290	ENSG00000164889
ENSG00000144290	ENSG00000188687
ENSG00000144306	ENSG00000136193
ENSG00000144306	ENSG00000141295
ENSG00000144331	ENSG00000151789
ENSG00000144331	ENSG00000161642
ENSG00000144331	ENSG00000187595
ENSG00000144339	ENSG00000066697
ENSG00000144354	ENSG00000164649
ENSG00000144355	ENSG00000006377
ENSG00000144355	ENSG00000108813
ENSG00000144355	ENSG00000167034
ENSG00000144362	ENSG00000173868
ENSG00000144366	ENSG00000105245
ENSG00000144366	ENSG00000133961
ENSG00000144366	ENSG00000157978
ENSG00000144366	ENSG00000183114
ENSG00000144366	ENSG00000185112
ENSG00000144369	ENSG00000148468
ENSG00000144369	ENSG00000161682
ENSG00000144401	ENSG00000123427
ENSG00000144407	ENSG00000004948
ENSG00000144407	ENSG00000010310
ENSG00000144407	ENSG00000064989
ENSG00000144407	ENSG00000065325
ENSG00000144407	ENSG00000078549
ENSG00000144407	ENSG00000080293
ENSG00000144407	ENSG00000106018
ENSG00000144407	ENSG00000106113
ENSG00000144407	ENSG00000106128
ENSG00000144407	ENSG00000112164
ENSG00000144407	ENSG00000114812
ENSG00000144407	ENSG00000120088
ENSG00000144407	ENSG00000160801
ENSG00000144407	ENSG00000215644
ENSG00000144410	ENSG00000080618
ENSG00000144410	ENSG00000091704
ENSG00000144410	ENSG00000128510
ENSG00000144410	ENSG00000153002
ENSG00000144410	ENSG00000158516
ENSG00000144410	ENSG00000158525
ENSG00000144410	ENSG00000163751
ENSG00000144410	ENSG00000165078

ENSG00000144426	ENSG00000160796
ENSG00000144445	ENSG00000120071
ENSG00000144452	ENSG00000064687
ENSG00000144452	ENSG00000107331
ENSG00000144452	ENSG00000165029
ENSG00000144452	ENSG00000167972
ENSG00000144452	ENSG00000179869
ENSG00000144452	ENSG00000198691
ENSG00000144476	ENSG00000166856
ENSG00000144481	ENSG00000070985
ENSG00000144481	ENSG00000083067
ENSG00000144481	ENSG00000092439
ENSG00000144481	ENSG00000119121
ENSG00000144481	ENSG00000130529
ENSG00000144481	ENSG00000134160
ENSG00000144481	ENSG00000142185
ENSG00000144485	ENSG00000069812
ENSG00000144485	ENSG00000114315
ENSG00000144485	ENSG00000173673
ENSG00000144485	ENSG00000179111
ENSG00000144485	ENSG00000188290
ENSG00000144485	ENSG00000197921
ENSG00000144488	ENSG00000116219
ENSG00000144488	ENSG00000187017
ENSG00000144550	ENSG00000085719
ENSG00000144550	ENSG00000100884
ENSG00000144550	ENSG00000124772
ENSG00000144550	ENSG00000139117
ENSG00000144550	ENSG00000140848
ENSG00000144550	ENSG00000178773
ENSG00000144550	ENSG00000196353
ENSG00000144550	ENSG00000214078
ENSG00000144554	ENSG00000095319
ENSG00000144566	ENSG00000080371
ENSG00000144566	ENSG00000108774
ENSG00000144566	ENSG00000111540
ENSG00000144566	ENSG00000124839
ENSG00000144566	ENSG00000169228
ENSG00000144567	ENSG00000141699
ENSG00000144567	ENSG00000154153
ENSG00000144579	ENSG00000144677
ENSG00000144579	ENSG00000175215
ENSG00000144583	ENSG00000099785
ENSG00000144583	ENSG00000139266
ENSG00000144583	ENSG00000145416
ENSG00000144583	ENSG00000165406
ENSG00000144583	ENSG00000173926
ENSG00000144583	ENSG00000183654
ENSG00000144596	ENSG00000155974
ENSG00000144597	ENSG00000145088
ENSG00000144619	ENSG00000018236
ENSG00000144619	ENSG00000113805
ENSG00000144619	ENSG00000134115
ENSG00000144635	ENSG00000138036
ENSG00000144642	ENSG00000076067
ENSG00000144642	ENSG00000153250
ENSG00000144644	ENSG00000128683
ENSG00000144644	ENSG00000136750
ENSG00000144644	ENSG00000139631

ENSG00000144644	ENSG00000215438
ENSG00000144645	ENSG00000021762
ENSG00000144645	ENSG00000091039
ENSG00000144645	ENSG00000117859
ENSG00000144645	ENSG00000144909
ENSG00000144648	ENSG00000112486
ENSG00000144648	ENSG00000121797
ENSG00000144648	ENSG00000121807
ENSG00000144648	ENSG00000121966
ENSG00000144648	ENSG00000126353
ENSG00000144648	ENSG00000129048
ENSG00000144648	ENSG00000160683
ENSG00000144648	ENSG00000160791
ENSG00000144648	ENSG00000163464
ENSG00000144648	ENSG00000163823
ENSG00000144648	ENSG00000168329
ENSG00000144648	ENSG00000172215
ENSG00000144648	ENSG00000173578
ENSG00000144648	ENSG00000173585
ENSG00000144648	ENSG00000179934
ENSG00000144648	ENSG00000180871
ENSG00000144648	ENSG00000183625
ENSG00000144648	ENSG00000183813
ENSG00000144648	ENSG00000184451
ENSG00000144648	ENSG00000186810
ENSG00000144648	ENSG00000215775
ENSG00000144648	ENSG00000215778
ENSG00000144648	ENSG00000215782
ENSG00000144649	ENSG00000164125
ENSG00000144655	ENSG00000110925
ENSG00000144655	ENSG00000178662
ENSG00000144668	ENSG00000005844
ENSG00000144668	ENSG00000005884
ENSG00000144668	ENSG00000005961
ENSG00000144668	ENSG00000077943
ENSG00000144668	ENSG00000083457
ENSG00000144668	ENSG00000091409
ENSG00000144668	ENSG00000115232
ENSG00000144668	ENSG00000135424
ENSG00000144668	ENSG00000137809
ENSG00000144668	ENSG00000138448
ENSG00000144668	ENSG00000140678
ENSG00000144668	ENSG00000143127
ENSG00000144668	ENSG00000156886
ENSG00000144668	ENSG00000161638
ENSG00000144668	ENSG00000164171
ENSG00000144668	ENSG00000169896
ENSG00000144668	ENSG00000213949
ENSG00000144674	ENSG00000173230
ENSG00000144677	ENSG00000144579
ENSG00000144677	ENSG00000175215
ENSG00000144681	ENSG00000141750
ENSG00000144681	ENSG00000185482
ENSG00000144711	ENSG00000120645
ENSG00000144711	ENSG00000124313
ENSG00000144711	ENSG00000164117
ENSG00000144712	ENSG00000111530
ENSG00000144724	ENSG00000106278
ENSG00000144730	ENSG00000056736

ENSG00000144730	ENSG00000177663
ENSG00000144746	ENSG00000102050
ENSG00000144749	ENSG00000139263
ENSG00000144749	ENSG00000198799
ENSG00000144771	ENSG00000166159
ENSG00000144771	ENSG00000185245
ENSG00000144785	ENSG00000146910
ENSG00000144791	ENSG00000087077
ENSG00000144791	ENSG00000129474
ENSG00000144791	ENSG00000142279
ENSG00000144791	ENSG00000145012
ENSG00000144791	ENSG00000159840
ENSG00000144791	ENSG00000162458
ENSG00000144802	ENSG00000167604
ENSG00000144810	ENSG00000123500
ENSG00000144810	ENSG00000171812
ENSG00000144815	ENSG00000095110
ENSG00000144815	ENSG00000137634
ENSG00000144815	ENSG00000204361
ENSG00000144821	ENSG00000002079
ENSG00000144821	ENSG00000006788
ENSG00000144821	ENSG00000078814
ENSG00000144821	ENSG00000092054
ENSG00000144821	ENSG00000109061
ENSG00000144821	ENSG00000109063
ENSG00000144821	ENSG00000125414
ENSG00000144821	ENSG00000133020
ENSG00000144821	ENSG00000141048
ENSG00000144821	ENSG00000197616
ENSG00000144824	ENSG00000019144
ENSG00000144834	ENSG00000064666
ENSG00000144834	ENSG00000117519
ENSG00000144834	ENSG00000130176
ENSG00000144834	ENSG00000149591
ENSG00000144834	ENSG00000158710
ENSG00000144834	ENSG00000204782
ENSG00000144834	ENSG00000204826
ENSG00000144834	ENSG00000215433
ENSG00000144837	ENSG00000101670
ENSG00000144837	ENSG00000163898
ENSG00000144837	ENSG00000165862
ENSG00000144837	ENSG00000166035
ENSG00000144837	ENSG00000175445
ENSG00000144837	ENSG00000175535
ENSG00000144837	ENSG00000187021
ENSG00000144837	ENSG00000188992
ENSG00000144837	ENSG00000203837
ENSG00000144843	ENSG00000153531
ENSG00000144847	ENSG00000019102
ENSG00000144847	ENSG00000143167
ENSG00000144847	ENSG00000149564
ENSG00000144847	ENSG00000154639
ENSG00000144847	ENSG00000166250
ENSG00000144847	ENSG00000188004
ENSG00000144852	ENSG00000012504
ENSG00000144852	ENSG00000111424
ENSG00000144852	ENSG00000112033
ENSG00000144852	ENSG00000132170
ENSG00000144852	ENSG00000143257

ENSG00000144852	ENSG00000186951
ENSG00000144857	ENSG00000064309
ENSG00000144891	ENSG00000100739
ENSG00000144891	ENSG00000134817
ENSG00000144891	ENSG00000154165
ENSG00000144891	ENSG00000168398
ENSG00000144891	ENSG00000170128
ENSG00000144891	ENSG00000173080
ENSG00000144891	ENSG00000180772
ENSG00000144891	ENSG00000182631
ENSG00000144893	ENSG00000184634
ENSG00000144908	ENSG00000136010
ENSG00000144909	ENSG00000021762
ENSG00000144909	ENSG00000091039
ENSG00000144909	ENSG00000117859
ENSG00000144909	ENSG00000144645
ENSG00000144935	ENSG00000069018
ENSG00000144935	ENSG00000072315
ENSG00000144935	ENSG00000133107
ENSG00000144935	ENSG00000137672
ENSG00000144935	ENSG00000138741
ENSG00000144959	ENSG00000114771
ENSG00000144959	ENSG00000188984
ENSG00000144959	ENSG00000197953
ENSG00000144959	ENSG00000204518
ENSG00000145012	ENSG00000087077
ENSG00000145012	ENSG00000129474
ENSG00000145012	ENSG00000142279
ENSG00000145012	ENSG00000144791
ENSG00000145012	ENSG00000159840
ENSG00000145012	ENSG00000162458
ENSG00000145016	ENSG00000102445
ENSG00000145016	ENSG00000140995
ENSG00000145016	ENSG00000141342
ENSG00000145016	ENSG00000159266
ENSG00000145016	ENSG00000178385
ENSG00000145050	ENSG00000185267
ENSG00000145087	ENSG00000073350
ENSG00000145087	ENSG00000131899
ENSG00000145087	ENSG00000164506
ENSG00000145088	ENSG00000144597
ENSG00000145103	ENSG00000105699
ENSG00000145103	ENSG00000143195
ENSG00000145107	ENSG00000142484
ENSG00000145107	ENSG00000163762
ENSG00000145107	ENSG00000168955
ENSG00000145107	ENSG00000169903
ENSG00000145107	ENSG00000169908
ENSG00000145147	ENSG00000128606
ENSG00000145147	ENSG00000166897
ENSG00000145147	ENSG00000182743
ENSG00000145147	ENSG00000184347
ENSG00000145147	ENSG00000187122
ENSG00000145192	ENSG00000090512
ENSG00000145192	ENSG00000113905
ENSG00000145194	ENSG00000102174
ENSG00000145194	ENSG00000117298
ENSG00000145194	ENSG00000142606
ENSG00000145194	ENSG00000171551

ENSG00000145194	ENSG00000196549
ENSG00000145194	ENSG00000197993
ENSG00000145198	ENSG00000102699
ENSG00000145198	ENSG00000110002
ENSG00000145198	ENSG00000158816
ENSG00000145217	ENSG00000091137
ENSG00000145217	ENSG00000091138
ENSG00000145217	ENSG00000112053
ENSG00000145217	ENSG00000135502
ENSG00000145217	ENSG00000147606
ENSG00000145217	ENSG00000155850
ENSG00000145217	ENSG00000170615
ENSG00000145217	ENSG00000174502
ENSG00000145242	ENSG00000044524
ENSG00000145242	ENSG00000070886
ENSG00000145242	ENSG00000080224
ENSG00000145242	ENSG00000106123
ENSG00000145242	ENSG00000116106
ENSG00000145242	ENSG00000133216
ENSG00000145242	ENSG00000135333
ENSG00000145242	ENSG00000142627
ENSG00000145242	ENSG00000146904
ENSG00000145242	ENSG00000154928
ENSG00000145242	ENSG00000182580
ENSG00000145242	ENSG00000183317
ENSG00000145242	ENSG00000196411
ENSG00000145246	ENSG00000118322
ENSG00000145246	ENSG00000206190
ENSG00000145247	ENSG00000109180
ENSG00000145283	ENSG00000100652
ENSG00000145283	ENSG00000125255
ENSG00000145283	ENSG00000126903
ENSG00000145283	ENSG00000145248
ENSG00000145283	ENSG00000205184
ENSG00000145287	ENSG00000105427
ENSG00000145287	ENSG00000173261
ENSG00000145335	ENSG00000074317
ENSG00000145335	ENSG00000173267
ENSG00000145349	ENSG00000058404
ENSG00000145349	ENSG00000070808
ENSG00000145349	ENSG00000148660
ENSG00000145354	ENSG00000122873
ENSG00000145358	ENSG00000168209
ENSG00000145362	ENSG00000029534
ENSG00000145362	ENSG00000151150
ENSG00000145362	ENSG00000185722
ENSG00000145386	ENSG00000133101
ENSG00000145416	ENSG00000099785
ENSG00000145416	ENSG00000139266
ENSG00000145416	ENSG00000144583
ENSG00000145416	ENSG00000165406
ENSG00000145416	ENSG00000173926
ENSG00000145416	ENSG00000183654
ENSG00000145423	ENSG00000104332
ENSG00000145423	ENSG00000120057
ENSG00000145423	ENSG00000214766
ENSG00000145425	ENSG00000178429
ENSG00000145425	ENSG00000187899
ENSG00000145425	ENSG00000215836

ENSG00000145428	ENSG00000137522
ENSG00000145431	ENSG00000170962
ENSG00000145451	ENSG00000011677
ENSG00000145451	ENSG00000022355
ENSG00000145451	ENSG00000094755
ENSG00000145451	ENSG00000101958
ENSG00000145451	ENSG00000102287
ENSG00000145451	ENSG00000109158
ENSG00000145451	ENSG00000109738
ENSG00000145451	ENSG00000111886
ENSG00000145451	ENSG00000113327
ENSG00000145451	ENSG00000145863
ENSG00000145451	ENSG00000145864
ENSG00000145451	ENSG00000145888
ENSG00000145451	ENSG00000146276
ENSG00000145451	ENSG00000147402
ENSG00000145451	ENSG00000151834
ENSG00000145451	ENSG00000163285
ENSG00000145451	ENSG00000163288
ENSG00000145451	ENSG00000166206
ENSG00000145451	ENSG00000182256
ENSG00000145451	ENSG00000183185
ENSG00000145451	ENSG00000186297
ENSG00000145451	ENSG00000187730
ENSG00000145451	ENSG00000188828
ENSG00000145491	ENSG00000065371
ENSG00000145491	ENSG00000114547
ENSG00000145506	ENSG00000140807
ENSG00000145526	ENSG00000040731
ENSG00000145526	ENSG00000071991
ENSG00000145526	ENSG00000081138
ENSG00000145526	ENSG00000101542
ENSG00000145526	ENSG00000113100
ENSG00000145526	ENSG00000113361
ENSG00000145526	ENSG00000139880
ENSG00000145526	ENSG00000140937
ENSG00000145526	ENSG00000149654
ENSG00000145526	ENSG00000150394
ENSG00000145526	ENSG00000154162
ENSG00000145526	ENSG00000179776
ENSG00000145536	ENSG00000049192
ENSG00000145536	ENSG00000087116
ENSG00000145536	ENSG00000136378
ENSG00000145536	ENSG00000138316
ENSG00000145536	ENSG00000140470
ENSG00000145536	ENSG00000140873
ENSG00000145536	ENSG00000142303
ENSG00000145536	ENSG00000145808
ENSG00000145536	ENSG00000151388
ENSG00000145536	ENSG00000156140
ENSG00000145536	ENSG00000160323
ENSG00000145536	ENSG00000163638
ENSG00000145536	ENSG00000173157
ENSG00000145536	ENSG00000214644
ENSG00000145536	ENSG00000215743
ENSG00000145555	ENSG00000066933
ENSG00000145555	ENSG00000091536
ENSG00000145555	ENSG00000099331
ENSG00000145623	ENSG00000081985

ENSG00000145623	ENSG00000104998
ENSG00000145623	ENSG00000113594
ENSG00000145623	ENSG00000119535
ENSG00000145623	ENSG00000134352
ENSG00000145623	ENSG00000164509
ENSG00000145626	ENSG00000109181
ENSG00000145626	ENSG00000135220
ENSG00000145626	ENSG00000135226
ENSG00000145626	ENSG00000156096
ENSG00000145626	ENSG00000167165
ENSG00000145626	ENSG00000168671
ENSG00000145626	ENSG00000171234
ENSG00000145626	ENSG00000173610
ENSG00000145626	ENSG00000174607
ENSG00000145626	ENSG00000196620
ENSG00000145626	ENSG00000197592
ENSG00000145626	ENSG00000197888
ENSG00000145626	ENSG00000198277
ENSG00000145626	ENSG00000213759
ENSG00000145632	ENSG00000166851
ENSG00000145632	ENSG00000173846
ENSG00000145632	ENSG00000185988
ENSG00000145632	ENSG00000214270
ENSG00000145649	ENSG00000092009
ENSG00000145649	ENSG00000100448
ENSG00000145649	ENSG00000100450
ENSG00000145649	ENSG00000100453
ENSG00000145649	ENSG00000113088
ENSG00000145649	ENSG00000185198
ENSG00000145649	ENSG00000197766
ENSG00000145675	ENSG00000105647
ENSG00000145675	ENSG00000117461
ENSG00000145681	ENSG00000038427
ENSG00000145681	ENSG00000130287
ENSG00000145681	ENSG00000132692
ENSG00000145681	ENSG00000132702
ENSG00000145681	ENSG00000140511
ENSG00000145681	ENSG00000157766
ENSG00000145681	ENSG00000187664
ENSG00000145685	ENSG00000156959
ENSG00000145685	ENSG00000182508
ENSG00000145685	ENSG00000183722
ENSG00000145685	ENSG00000187416
ENSG00000145685	ENSG00000197753
ENSG00000145687	ENSG00000130511
ENSG00000145687	ENSG00000157216
ENSG00000145687	ENSG00000214079
ENSG00000145687	ENSG00000214099
ENSG00000145687	ENSG00000215390
ENSG00000145687	ENSG00000215535
ENSG00000145703	ENSG00000140575
ENSG00000145703	ENSG00000183856
ENSG00000145721	ENSG00000152022
ENSG00000145725	ENSG00000168781
ENSG00000145741	ENSG00000118903
ENSG00000145741	ENSG00000134717
ENSG00000145741	ENSG00000197407
ENSG00000145741	ENSG00000214680
ENSG00000145779	ENSG00000163154

ENSG00000145779	ENSG00000183578
ENSG00000145779	ENSG00000185361
ENSG00000145780	ENSG00000141965
ENSG00000145780	ENSG00000169018
ENSG00000145780	ENSG00000215524
ENSG00000145780	ENSG00000215602
ENSG00000145794	ENSG00000074660
ENSG00000145794	ENSG00000099910
ENSG00000145794	ENSG00000157890
ENSG00000145794	ENSG00000162591
ENSG00000145794	ENSG00000187800
ENSG00000145808	ENSG00000049192
ENSG00000145808	ENSG00000087116
ENSG00000145808	ENSG00000136378
ENSG00000145808	ENSG00000138316
ENSG00000145808	ENSG00000140470
ENSG00000145808	ENSG00000140873
ENSG00000145808	ENSG00000142303
ENSG00000145808	ENSG00000145536
ENSG00000145808	ENSG00000151388
ENSG00000145808	ENSG00000156140
ENSG00000145808	ENSG00000160323
ENSG00000145808	ENSG00000163638
ENSG00000145808	ENSG00000173157
ENSG00000145808	ENSG00000214644
ENSG00000145808	ENSG00000215743
ENSG00000145817	ENSG00000177752
ENSG00000145819	ENSG00000071205
ENSG00000145819	ENSG00000079482
ENSG00000145819	ENSG00000165895
ENSG00000145835	ENSG00000168564
ENSG00000145835	ENSG00000177770
ENSG00000145835	ENSG00000214740
ENSG00000145850	ENSG00000113249
ENSG00000145850	ENSG00000135077
ENSG00000145860	ENSG00000162298
ENSG00000145860	ENSG00000170881
ENSG00000145860	ENSG00000184505
ENSG00000145860	ENSG00000187779
ENSG00000145861	ENSG00000054803
ENSG00000145861	ENSG00000102924
ENSG00000145861	ENSG00000131094
ENSG00000145861	ENSG00000133466
ENSG00000145861	ENSG00000139899
ENSG00000145861	ENSG00000141668
ENSG00000145861	ENSG00000144119
ENSG00000145861	ENSG00000159189
ENSG00000145861	ENSG00000163145
ENSG00000145861	ENSG00000165985
ENSG00000145861	ENSG00000172247
ENSG00000145861	ENSG00000173369
ENSG00000145861	ENSG00000173372
ENSG00000145861	ENSG00000173918
ENSG00000145861	ENSG00000181092
ENSG00000145861	ENSG00000184471
ENSG00000145861	ENSG00000186897
ENSG00000145861	ENSG00000213192
ENSG00000145863	ENSG00000011677
ENSG00000145863	ENSG00000022355

ENSG00000145863	ENSG00000094755
ENSG00000145863	ENSG00000101958
ENSG00000145863	ENSG00000102287
ENSG00000145863	ENSG00000109158
ENSG00000145863	ENSG00000109738
ENSG00000145863	ENSG00000111886
ENSG00000145863	ENSG00000113327
ENSG00000145863	ENSG00000145451
ENSG00000145863	ENSG00000145864
ENSG00000145863	ENSG00000145888
ENSG00000145863	ENSG00000146276
ENSG00000145863	ENSG00000147402
ENSG00000145863	ENSG00000151834
ENSG00000145863	ENSG00000163285
ENSG00000145863	ENSG00000163288
ENSG00000145863	ENSG00000166206
ENSG00000145863	ENSG00000182256
ENSG00000145863	ENSG00000183185
ENSG00000145863	ENSG00000186297
ENSG00000145863	ENSG00000187730
ENSG00000145863	ENSG00000188828
ENSG00000145864	ENSG00000011677
ENSG00000145864	ENSG00000022355
ENSG00000145864	ENSG00000094755
ENSG00000145864	ENSG00000101958
ENSG00000145864	ENSG00000102287
ENSG00000145864	ENSG00000109158
ENSG00000145864	ENSG00000109738
ENSG00000145864	ENSG00000111886
ENSG00000145864	ENSG00000113327
ENSG00000145864	ENSG00000145451
ENSG00000145864	ENSG00000145863
ENSG00000145864	ENSG00000145888
ENSG00000145864	ENSG00000146276
ENSG00000145864	ENSG00000147402
ENSG00000145864	ENSG00000151834
ENSG00000145864	ENSG00000163285
ENSG00000145864	ENSG00000163288
ENSG00000145864	ENSG00000166206
ENSG00000145864	ENSG00000182256
ENSG00000145864	ENSG00000183185
ENSG00000145864	ENSG00000186297
ENSG00000145864	ENSG00000187730
ENSG00000145864	ENSG00000188828
ENSG00000145882	ENSG00000116005
ENSG00000145888	ENSG00000011677
ENSG00000145888	ENSG00000022355
ENSG00000145888	ENSG00000094755
ENSG00000145888	ENSG00000101958
ENSG00000145888	ENSG00000102287
ENSG00000145888	ENSG00000109158
ENSG00000145888	ENSG00000109738
ENSG00000145888	ENSG00000111886
ENSG00000145888	ENSG00000113327
ENSG00000145888	ENSG00000145451
ENSG00000145888	ENSG00000145863
ENSG00000145888	ENSG00000145864
ENSG00000145888	ENSG00000146276
ENSG00000145888	ENSG00000147402

ENSG00000145888	ENSG00000151834
ENSG00000145888	ENSG00000163285
ENSG00000145888	ENSG00000163288
ENSG00000145888	ENSG00000166206
ENSG00000145888	ENSG00000182256
ENSG00000145888	ENSG00000183185
ENSG00000145888	ENSG00000186297
ENSG00000145888	ENSG00000187730
ENSG00000145888	ENSG00000188828
ENSG00000145901	ENSG00000050730
ENSG00000145907	ENSG00000138757
ENSG00000145911	ENSG00000061337
ENSG00000145911	ENSG00000088899
ENSG00000145911	ENSG00000107816
ENSG00000145916	ENSG00000153561
ENSG00000145934	ENSG00000009694
ENSG00000145934	ENSG00000149256
ENSG00000145936	ENSG00000135643
ENSG00000145936	ENSG00000171121
ENSG00000145936	ENSG00000197584
ENSG00000145936	ENSG00000215575
ENSG00000145949	ENSG00000035664
ENSG00000145949	ENSG00000065534
ENSG00000145949	ENSG00000101306
ENSG00000145949	ENSG00000140795
ENSG00000145949	ENSG00000167657
ENSG00000145949	ENSG00000196730
ENSG00000145990	ENSG00000141098
ENSG00000146001	ENSG00000081818
ENSG00000146001	ENSG00000081842
ENSG00000146001	ENSG00000081853
ENSG00000146001	ENSG00000112852
ENSG00000146001	ENSG00000113205
ENSG00000146001	ENSG00000113209
ENSG00000146001	ENSG00000113211
ENSG00000146001	ENSG00000113212
ENSG00000146001	ENSG00000113248
ENSG00000146001	ENSG00000120322
ENSG00000146001	ENSG00000120324
ENSG00000146001	ENSG00000120327
ENSG00000146001	ENSG00000120328
ENSG00000146001	ENSG00000171815
ENSG00000146001	ENSG00000187372
ENSG00000146001	ENSG00000196963
ENSG00000146001	ENSG00000197479
ENSG00000146001	ENSG00000204955
ENSG00000146001	ENSG00000204956
ENSG00000146001	ENSG00000204961
ENSG00000146001	ENSG00000204962
ENSG00000146001	ENSG00000204963
ENSG00000146001	ENSG00000204964
ENSG00000146001	ENSG00000204965
ENSG00000146001	ENSG00000204967
ENSG00000146001	ENSG00000204968
ENSG00000146001	ENSG00000204969
ENSG00000146001	ENSG00000204970
ENSG00000146001	ENSG00000214567
ENSG00000146001	ENSG00000214570
ENSG00000146001	ENSG00000214574

ENSG00000146001	ENSG00000214580
ENSG00000146001	ENSG00000214583
ENSG00000146001	ENSG00000214594
ENSG00000146001	ENSG00000214600
ENSG00000146001	ENSG00000214608
ENSG00000146005	ENSG00000059915
ENSG00000146005	ENSG00000125637
ENSG00000146005	ENSG00000156011
ENSG00000146006	ENSG00000136457
ENSG00000146006	ENSG00000162951
ENSG00000146006	ENSG00000176204
ENSG00000146006	ENSG00000198739
ENSG00000146013	ENSG00000125861
ENSG00000146013	ENSG00000151892
ENSG00000146013	ENSG00000168546
ENSG00000146013	ENSG00000187871
ENSG00000146038	ENSG00000077279
ENSG00000146038	ENSG00000104237
ENSG00000146038	ENSG00000130413
ENSG00000146038	ENSG00000133083
ENSG00000146038	ENSG00000163673
ENSG00000146038	ENSG00000170390
ENSG00000146038	ENSG00000183638
ENSG00000146039	ENSG00000091664
ENSG00000146039	ENSG00000104888
ENSG00000146039	ENSG00000112337
ENSG00000146039	ENSG00000119899
ENSG00000146039	ENSG00000124564
ENSG00000146039	ENSG00000124568
ENSG00000146039	ENSG00000179520
ENSG00000146067	ENSG00000125386
ENSG00000146070	ENSG00000158006
ENSG00000146072	ENSG00000028137
ENSG00000146072	ENSG00000101017
ENSG00000146072	ENSG00000111321
ENSG00000146072	ENSG00000120949
ENSG00000146072	ENSG00000141655
ENSG00000146072	ENSG00000157873
ENSG00000146072	ENSG00000164761
ENSG00000146083	ENSG00000127870
ENSG00000146083	ENSG00000131263
ENSG00000146083	ENSG00000137075
ENSG00000146090	ENSG00000138670
ENSG00000146090	ENSG00000198915
ENSG00000146094	ENSG00000101134
ENSG00000146094	ENSG00000115325
ENSG00000146094	ENSG00000125170
ENSG00000146094	ENSG00000137218
ENSG00000146094	ENSG00000147443
ENSG00000146094	ENSG00000166225
ENSG00000146094	ENSG00000206052
ENSG00000146122	ENSG00000100592
ENSG00000146151	ENSG00000117305
ENSG00000146192	ENSG00000127084
ENSG00000146192	ENSG00000139132
ENSG00000146197	ENSG00000159307
ENSG00000146197	ENSG00000175356
ENSG00000146205	ENSG00000047617
ENSG00000146205	ENSG00000074855

ENSG00000146205	ENSG00000131620
ENSG00000146205	ENSG00000134343
ENSG00000146205	ENSG00000151572
ENSG00000146205	ENSG00000160746
ENSG00000146205	ENSG00000171714
ENSG00000146205	ENSG00000177119
ENSG00000146205	ENSG00000185101
ENSG00000146215	ENSG00000129170
ENSG00000146215	ENSG00000136490
ENSG00000146215	ENSG00000159176
ENSG00000146215	ENSG00000175183
ENSG00000146215	ENSG00000182809
ENSG00000146215	ENSG00000197893
ENSG00000146215	ENSG00000213145
ENSG00000146223	ENSG00000137970
ENSG00000146223	ENSG00000147604
ENSG00000146223	ENSG00000169216
ENSG00000146223	ENSG00000171549
ENSG00000146223	ENSG00000181416
ENSG00000146223	ENSG00000182520
ENSG00000146223	ENSG00000214485
ENSG00000146223	ENSG00000214557
ENSG00000146223	ENSG00000214932
ENSG00000146223	ENSG00000215297
ENSG00000146223	ENSG00000215402
ENSG00000146232	ENSG00000069399
ENSG00000146232	ENSG00000100906
ENSG00000146232	ENSG00000104825
ENSG00000146233	ENSG00000124212
ENSG00000146233	ENSG00000167910
ENSG00000146233	ENSG00000172817
ENSG00000146233	ENSG00000180432
ENSG00000146247	ENSG00000165288
ENSG00000146247	ENSG00000185658
ENSG00000146250	ENSG00000150687
ENSG00000146276	ENSG00000011677
ENSG00000146276	ENSG00000022355
ENSG00000146276	ENSG00000094755
ENSG00000146276	ENSG00000101958
ENSG00000146276	ENSG00000102287
ENSG00000146276	ENSG00000109158
ENSG00000146276	ENSG00000109738
ENSG00000146276	ENSG00000111886
ENSG00000146276	ENSG00000113327
ENSG00000146276	ENSG00000145451
ENSG00000146276	ENSG00000145863
ENSG00000146276	ENSG00000145864
ENSG00000146276	ENSG00000145888
ENSG00000146276	ENSG00000147402
ENSG00000146276	ENSG00000151834
ENSG00000146276	ENSG00000163285
ENSG00000146276	ENSG00000163288
ENSG00000146276	ENSG00000166206
ENSG00000146276	ENSG00000182256
ENSG00000146276	ENSG00000183185
ENSG00000146276	ENSG00000186297
ENSG00000146276	ENSG00000187730
ENSG00000146276	ENSG00000188828
ENSG00000146278	ENSG00000189266

ENSG00000146278	ENSG00000215700
ENSG00000146285	ENSG00000010803
ENSG00000146285	ENSG00000102098
ENSG00000146352	ENSG00000124120
ENSG00000146352	ENSG00000137561
ENSG00000146352	ENSG00000140522
ENSG00000146352	ENSG00000177182
ENSG00000146360	ENSG00000064547
ENSG00000146360	ENSG00000118432
ENSG00000146360	ENSG00000125910
ENSG00000146360	ENSG00000132975
ENSG00000146360	ENSG00000170989
ENSG00000146360	ENSG00000171517
ENSG00000146360	ENSG00000175898
ENSG00000146360	ENSG00000180739
ENSG00000146360	ENSG00000181773
ENSG00000146360	ENSG00000188822
ENSG00000146360	ENSG00000198121
ENSG00000146360	ENSG00000213694
ENSG00000146376	ENSG00000088756
ENSG00000146376	ENSG00000124143
ENSG00000146376	ENSG00000130052
ENSG00000146376	ENSG00000133121
ENSG00000146376	ENSG00000164741
ENSG00000146378	ENSG00000135569
ENSG00000146378	ENSG00000146383
ENSG00000146378	ENSG00000146385
ENSG00000146378	ENSG00000146399
ENSG00000146383	ENSG00000135569
ENSG00000146383	ENSG00000146378
ENSG00000146383	ENSG00000146385
ENSG00000146383	ENSG00000146399
ENSG00000146385	ENSG00000135569
ENSG00000146385	ENSG00000146378
ENSG00000146385	ENSG00000146383
ENSG00000146385	ENSG00000146399
ENSG00000146399	ENSG00000135569
ENSG00000146399	ENSG00000146378
ENSG00000146399	ENSG00000146383
ENSG00000146399	ENSG00000146385
ENSG00000146410	ENSG00000066855
ENSG00000146410	ENSG00000117640
ENSG00000146411	ENSG00000197496
ENSG00000146416	ENSG00000111863
ENSG00000146426	ENSG00000156299
ENSG00000146463	ENSG00000121741
ENSG00000146463	ENSG00000132950
ENSG00000146463	ENSG00000147130
ENSG00000146463	ENSG00000163867
ENSG00000146463	ENSG00000198218
ENSG00000146469	ENSG00000141433
ENSG00000146535	ENSG00000060558
ENSG00000146535	ENSG00000065135
ENSG00000146535	ENSG00000087258
ENSG00000146535	ENSG00000087460
ENSG00000146535	ENSG00000088256
ENSG00000146535	ENSG00000114349
ENSG00000146535	ENSG00000114353
ENSG00000146535	ENSG00000120063

ENSG00000146535	ENSG00000127955
ENSG00000146535	ENSG00000128266
ENSG00000146535	ENSG00000134183
ENSG00000146535	ENSG00000141404
ENSG00000146535	ENSG00000156049
ENSG00000146535	ENSG00000156052
ENSG00000146535	ENSG00000214415
ENSG00000146535	ENSG00000214622
ENSG00000146555	ENSG00000069188
ENSG00000146555	ENSG00000171587
ENSG00000146555	ENSG00000177103
ENSG00000146592	ENSG00000115966
ENSG00000146592	ENSG00000130522
ENSG00000146592	ENSG00000170653
ENSG00000146592	ENSG00000171223
ENSG00000146592	ENSG00000177606
ENSG00000146648	ENSG00000065361
ENSG00000146648	ENSG00000141736
ENSG00000146648	ENSG00000178568
ENSG00000146674	ENSG00000115457
ENSG00000146674	ENSG00000115461
ENSG00000146674	ENSG00000141753
ENSG00000146674	ENSG00000146678
ENSG00000146674	ENSG00000167779
ENSG00000146676	ENSG00000172733
ENSG00000146676	ENSG00000185129
ENSG00000146678	ENSG00000115457
ENSG00000146678	ENSG00000115461
ENSG00000146678	ENSG00000141753
ENSG00000146678	ENSG00000146674
ENSG00000146678	ENSG00000167779
ENSG00000146700	ENSG00000013725
ENSG00000146700	ENSG00000073754
ENSG00000146700	ENSG00000110448
ENSG00000146700	ENSG00000164099
ENSG00000146700	ENSG00000177575
ENSG00000146700	ENSG00000177675
ENSG00000146700	ENSG00000179954
ENSG00000146700	ENSG00000187908
ENSG00000146700	ENSG00000214279
ENSG00000146707	ENSG00000188372
ENSG00000146729	ENSG00000136783
ENSG00000146729	ENSG00000165028
ENSG00000146729	ENSG00000184117
ENSG00000146755	ENSG00000116525
ENSG00000146755	ENSG00000155428
ENSG00000146755	ENSG00000177238
ENSG00000146755	ENSG00000178809
ENSG00000146755	ENSG00000185880
ENSG00000146776	ENSG00000162650
ENSG00000146776	ENSG00000163635
ENSG00000146809	ENSG00000100628
ENSG00000146809	ENSG00000115239
ENSG00000146809	ENSG00000168387
ENSG00000146828	ENSG00000064651
ENSG00000146828	ENSG00000070915
ENSG00000146828	ENSG00000074803
ENSG00000146830	ENSG00000204120
ENSG00000146839	ENSG00000090920

ENSG00000146839	ENSG00000109927
ENSG00000146839	ENSG00000110799
ENSG00000146839	ENSG00000117983
ENSG00000146839	ENSG00000184956
ENSG00000146839	ENSG00000188162
ENSG00000146839	ENSG00000196292
ENSG00000146839	ENSG00000197558
ENSG00000146839	ENSG00000198788
ENSG00000146856	ENSG00000135049
ENSG00000146856	ENSG00000165923
ENSG00000146856	ENSG00000166748
ENSG00000146858	ENSG00000059378
ENSG00000146858	ENSG00000105939
ENSG00000146858	ENSG00000111224
ENSG00000146872	ENSG00000198586
ENSG00000146872	ENSG00000214705
ENSG00000146872	ENSG00000215170
ENSG00000146904	ENSG00000044524
ENSG00000146904	ENSG00000070886
ENSG00000146904	ENSG00000080224
ENSG00000146904	ENSG00000106123
ENSG00000146904	ENSG00000116106
ENSG00000146904	ENSG00000133216
ENSG00000146904	ENSG00000135333
ENSG00000146904	ENSG00000142627
ENSG00000146904	ENSG00000145242
ENSG00000146904	ENSG00000154928
ENSG00000146904	ENSG00000182580
ENSG00000146904	ENSG00000183317
ENSG00000146904	ENSG00000196411
ENSG00000146910	ENSG00000144785
ENSG00000146926	ENSG00000182177
ENSG00000146938	ENSG00000165246
ENSG00000146938	ENSG00000169760
ENSG00000146938	ENSG00000169992
ENSG00000146938	ENSG00000196338
ENSG00000146950	ENSG00000138771
ENSG00000146950	ENSG00000158352
ENSG00000146955	ENSG00000134594
ENSG00000146955	ENSG00000137502
ENSG00000146955	ENSG00000138069
ENSG00000146955	ENSG00000172007
ENSG00000146955	ENSG00000174903
ENSG00000146955	ENSG00000185051
ENSG00000146963	ENSG00000007392
ENSG00000146966	ENSG00000119522
ENSG00000146966	ENSG00000162701
ENSG00000146966	ENSG00000162777
ENSG00000146966	ENSG00000166444
ENSG00000146966	ENSG00000175984
ENSG00000146966	ENSG00000205744
ENSG00000147010	ENSG00000198087
ENSG00000147027	ENSG00000112378
ENSG00000147027	ENSG00000176711
ENSG00000147041	ENSG00000102362
ENSG00000147041	ENSG00000137501
ENSG00000147041	ENSG00000142765
ENSG00000147041	ENSG00000164674
ENSG00000147050	ENSG00000132510

ENSG00000147050	ENSG00000183878
ENSG00000147059	ENSG00000106723
ENSG00000147059	ENSG00000186767
ENSG00000147059	ENSG00000186787
ENSG00000147059	ENSG00000204271
ENSG00000147065	ENSG00000092820
ENSG00000147065	ENSG00000137710
ENSG00000147065	ENSG00000186575
ENSG00000147100	ENSG00000100156
ENSG00000147100	ENSG00000108932
ENSG00000147100	ENSG00000112394
ENSG00000147100	ENSG00000118596
ENSG00000147100	ENSG00000141526
ENSG00000147100	ENSG00000152779
ENSG00000147100	ENSG00000155380
ENSG00000147100	ENSG00000163053
ENSG00000147100	ENSG00000165449
ENSG00000147100	ENSG00000168679
ENSG00000147100	ENSG00000170190
ENSG00000147100	ENSG00000174326
ENSG00000147100	ENSG00000174327
ENSG00000147119	ENSG00000122863
ENSG00000147119	ENSG00000135702
ENSG00000147119	ENSG00000140835
ENSG00000147119	ENSG00000175040
ENSG00000147119	ENSG00000175264
ENSG00000147119	ENSG00000183196
ENSG00000147127	ENSG00000154917
ENSG00000147127	ENSG00000175582
ENSG00000147130	ENSG00000121741
ENSG00000147130	ENSG00000132950
ENSG00000147130	ENSG00000146463
ENSG00000147130	ENSG00000163867
ENSG00000147130	ENSG00000198218
ENSG00000147133	ENSG00000122728
ENSG00000147138	ENSG00000125245
ENSG00000147138	ENSG00000126251
ENSG00000147138	ENSG00000126262
ENSG00000147138	ENSG00000144230
ENSG00000147138	ENSG00000162881
ENSG00000147138	ENSG00000164220
ENSG00000147138	ENSG00000164251
ENSG00000147138	ENSG00000165621
ENSG00000147138	ENSG00000171631
ENSG00000147138	ENSG00000181104
ENSG00000147138	ENSG00000182162
ENSG00000147138	ENSG00000182559
ENSG00000147138	ENSG00000182782
ENSG00000147138	ENSG00000185897
ENSG00000147138	ENSG00000196917
ENSG00000147140	ENSG00000116560
ENSG00000147140	ENSG00000121390
ENSG00000147144	ENSG00000114541
ENSG00000147144	ENSG00000151474
ENSG00000147144	ENSG00000163362
ENSG00000147145	ENSG00000078589
ENSG00000147145	ENSG00000139679
ENSG00000147145	ENSG00000152207
ENSG00000147145	ENSG00000169403

ENSG00000147145	ENSG00000169508
ENSG00000147145	ENSG00000169860
ENSG00000147145	ENSG00000173198
ENSG00000147145	ENSG00000175591
ENSG00000147145	ENSG00000184574
ENSG00000147145	ENSG00000186912
ENSG00000147145	ENSG00000198829
ENSG00000147155	ENSG00000123179
ENSG00000147155	ENSG00000214469
ENSG00000147160	ENSG00000062282
ENSG00000147160	ENSG00000106384
ENSG00000147160	ENSG00000124003
ENSG00000147160	ENSG00000166391
ENSG00000147160	ENSG00000184210
ENSG00000147160	ENSG00000204195
ENSG00000147160	ENSG00000205267
ENSG00000147164	ENSG00000112335
ENSG00000147166	ENSG00000110172
ENSG00000147180	ENSG00000005889
ENSG00000147180	ENSG00000067646
ENSG00000147180	ENSG00000121864
ENSG00000147202	ENSG00000131504
ENSG00000147202	ENSG00000139734
ENSG00000147206	ENSG00000126952
ENSG00000147206	ENSG00000162231
ENSG00000147206	ENSG00000185554
ENSG00000147206	ENSG00000185945
ENSG00000147206	ENSG00000196970
ENSG00000147223	ENSG00000183145
ENSG00000147223	ENSG00000203877
ENSG00000147224	ENSG00000101911
ENSG00000147234	ENSG00000070601
ENSG00000147234	ENSG00000169933
ENSG00000147246	ENSG00000043591
ENSG00000147246	ENSG00000102468
ENSG00000147246	ENSG00000113749
ENSG00000147246	ENSG00000125631
ENSG00000147246	ENSG00000135312
ENSG00000147246	ENSG00000135914
ENSG00000147246	ENSG00000150594
ENSG00000147246	ENSG00000157219
ENSG00000147246	ENSG00000164270
ENSG00000147246	ENSG00000168830
ENSG00000147246	ENSG00000169252
ENSG00000147246	ENSG00000175658
ENSG00000147246	ENSG00000178394
ENSG00000147246	ENSG00000179097
ENSG00000147246	ENSG00000179546
ENSG00000147246	ENSG00000181210
ENSG00000147246	ENSG00000184303
ENSG00000147246	ENSG00000184845
ENSG00000147246	ENSG00000185321
ENSG00000147246	ENSG00000188778
ENSG00000147251	ENSG00000088387
ENSG00000147251	ENSG00000107099
ENSG00000147251	ENSG00000116641
ENSG00000147251	ENSG00000130158
ENSG00000147251	ENSG00000135905
ENSG00000147256	ENSG00000047648

ENSG00000147257	ENSG00000063660
ENSG00000147257	ENSG00000076716
ENSG00000147257	ENSG00000179399
ENSG00000147257	ENSG00000183098
ENSG00000147257	ENSG00000213420
ENSG00000147274	ENSG00000169800
ENSG00000147274	ENSG00000169811
ENSG00000147274	ENSG00000170748
ENSG00000147274	ENSG00000174622
ENSG00000147274	ENSG00000206011
ENSG00000147274	ENSG00000206046
ENSG00000147274	ENSG00000213516
ENSG00000147274	ENSG00000214442
ENSG00000147274	ENSG00000215210
ENSG00000147324	ENSG00000033122
ENSG00000147324	ENSG00000112851
ENSG00000147324	ENSG00000137269
ENSG00000147324	ENSG00000180900
ENSG00000147324	ENSG00000188306
ENSG00000147364	ENSG00000156804
ENSG00000147380	ENSG00000054277
ENSG00000147380	ENSG00000102076
ENSG00000147380	ENSG00000122375
ENSG00000147380	ENSG00000124818
ENSG00000147380	ENSG00000128617
ENSG00000147380	ENSG00000148604
ENSG00000147380	ENSG00000163914
ENSG00000147380	ENSG00000166160
ENSG00000147380	ENSG00000180245
ENSG00000147394	ENSG00000136155
ENSG00000147402	ENSG00000011677
ENSG00000147402	ENSG00000022355
ENSG00000147402	ENSG00000094755
ENSG00000147402	ENSG00000101958
ENSG00000147402	ENSG00000102287
ENSG00000147402	ENSG00000109158
ENSG00000147402	ENSG00000109738
ENSG00000147402	ENSG00000111886
ENSG00000147402	ENSG00000113327
ENSG00000147402	ENSG00000145451
ENSG00000147402	ENSG00000145863
ENSG00000147402	ENSG00000145864
ENSG00000147402	ENSG00000145888
ENSG00000147402	ENSG00000146276
ENSG00000147402	ENSG00000151834
ENSG00000147402	ENSG00000163285
ENSG00000147402	ENSG00000163288
ENSG00000147402	ENSG00000166206
ENSG00000147402	ENSG00000182256
ENSG00000147402	ENSG00000183185
ENSG00000147402	ENSG00000186297
ENSG00000147402	ENSG00000187730
ENSG00000147402	ENSG00000188828
ENSG00000147408	ENSG00000033100
ENSG00000147408	ENSG00000123989
ENSG00000147408	ENSG00000131873
ENSG00000147408	ENSG00000139044
ENSG00000147408	ENSG00000169826
ENSG00000147408	ENSG00000182272

ENSG00000147408	ENSG00000198108
ENSG00000147416	ENSG00000116039
ENSG00000147421	ENSG00000108753
ENSG00000147421	ENSG00000135100
ENSG00000147432	ENSG00000080644
ENSG00000147432	ENSG00000101204
ENSG00000147432	ENSG00000108556
ENSG00000147432	ENSG00000117971
ENSG00000147432	ENSG00000120903
ENSG00000147432	ENSG00000129749
ENSG00000147432	ENSG00000135902
ENSG00000147432	ENSG00000138435
ENSG00000147432	ENSG00000147434
ENSG00000147432	ENSG00000149305
ENSG00000147432	ENSG00000160716
ENSG00000147432	ENSG00000166664
ENSG00000147432	ENSG00000166736
ENSG00000147432	ENSG00000169684
ENSG00000147432	ENSG00000170175
ENSG00000147432	ENSG00000174343
ENSG00000147432	ENSG00000175344
ENSG00000147432	ENSG00000178084
ENSG00000147432	ENSG00000186038
ENSG00000147432	ENSG00000186090
ENSG00000147432	ENSG00000186919
ENSG00000147432	ENSG00000196811
ENSG00000147434	ENSG00000080644
ENSG00000147434	ENSG00000101204
ENSG00000147434	ENSG00000108556
ENSG00000147434	ENSG00000117971
ENSG00000147434	ENSG00000120903
ENSG00000147434	ENSG00000129749
ENSG00000147434	ENSG00000135902
ENSG00000147434	ENSG00000138435
ENSG00000147434	ENSG00000147432
ENSG00000147434	ENSG00000149305
ENSG00000147434	ENSG00000160716
ENSG00000147434	ENSG00000166664
ENSG00000147434	ENSG00000166736
ENSG00000147434	ENSG00000169684
ENSG00000147434	ENSG00000170175
ENSG00000147434	ENSG00000174343
ENSG00000147434	ENSG00000175344
ENSG00000147434	ENSG00000178084
ENSG00000147434	ENSG00000186038
ENSG00000147434	ENSG00000186090
ENSG00000147434	ENSG00000186919
ENSG00000147434	ENSG00000196811
ENSG00000147443	ENSG00000101134
ENSG00000147443	ENSG00000115325
ENSG00000147443	ENSG00000125170
ENSG00000147443	ENSG00000137218
ENSG00000147443	ENSG00000146094
ENSG00000147443	ENSG00000166225
ENSG00000147443	ENSG00000206052
ENSG00000147454	ENSG00000155287
ENSG00000147459	ENSG00000088538
ENSG00000147459	ENSG00000128512
ENSG00000147459	ENSG00000134516

ENSG00000147459	ENSG00000150760
ENSG00000147465	ENSG00000131748
ENSG00000147475	ENSG00000107566
ENSG00000147481	ENSG00000047932
ENSG00000147481	ENSG00000101400
ENSG00000147481	ENSG00000168807
ENSG00000147481	ENSG00000172164
ENSG00000147481	ENSG00000172554
ENSG00000147485	ENSG00000005381
ENSG00000147485	ENSG00000115705
ENSG00000147485	ENSG00000121053
ENSG00000147485	ENSG00000130508
ENSG00000147485	ENSG00000167419
ENSG00000147488	ENSG00000186487
ENSG00000147488	ENSG00000196132
ENSG00000147507	ENSG00000000938
ENSG00000147507	ENSG00000010810
ENSG00000147507	ENSG00000101336
ENSG00000147507	ENSG00000111816
ENSG00000147507	ENSG00000136573
ENSG00000147507	ENSG00000176105
ENSG00000147507	ENSG00000182866
ENSG00000147507	ENSG00000197122
ENSG00000147509	ENSG00000090104
ENSG00000147509	ENSG00000091844
ENSG00000147509	ENSG00000116741
ENSG00000147509	ENSG00000117152
ENSG00000147509	ENSG00000127074
ENSG00000147509	ENSG00000135824
ENSG00000147509	ENSG00000143248
ENSG00000147509	ENSG00000143333
ENSG00000147509	ENSG00000148908
ENSG00000147509	ENSG00000150681
ENSG00000147509	ENSG00000171700
ENSG00000147509	ENSG00000215825
ENSG00000147526	ENSG00000013810
ENSG00000147526	ENSG00000138162
ENSG00000147533	ENSG00000155265
ENSG00000147533	ENSG00000205596
ENSG00000147535	ENSG00000203805
ENSG00000147548	ENSG00000109685
ENSG00000147548	ENSG00000165671
ENSG00000147570	ENSG00000101152
ENSG00000147570	ENSG00000163793
ENSG00000147571	ENSG00000163794
ENSG00000147573	ENSG00000138100
ENSG00000147573	ENSG00000158022
ENSG00000147573	ENSG00000204977
ENSG00000147573	ENSG00000213186
ENSG00000147588	ENSG00000114113
ENSG00000147588	ENSG00000114115
ENSG00000147588	ENSG00000121769
ENSG00000147588	ENSG00000139194
ENSG00000147588	ENSG00000143320
ENSG00000147588	ENSG00000145384
ENSG00000147588	ENSG00000162444
ENSG00000147588	ENSG00000164434
ENSG00000147588	ENSG00000164687
ENSG00000147588	ENSG00000166426

ENSG00000147588	ENSG00000170323
ENSG00000147588	ENSG00000197416
ENSG00000147588	ENSG00000205186
ENSG00000147588	ENSG00000214004
ENSG00000147601	ENSG00000132604
ENSG00000147601	ENSG00000184862
ENSG00000147601	ENSG00000187589
ENSG00000147604	ENSG00000137970
ENSG00000147604	ENSG00000146223
ENSG00000147604	ENSG00000169216
ENSG00000147604	ENSG00000171549
ENSG00000147604	ENSG00000181416
ENSG00000147604	ENSG00000182520
ENSG00000147604	ENSG00000214485
ENSG00000147604	ENSG00000214557
ENSG00000147604	ENSG00000214932
ENSG00000147604	ENSG00000215297
ENSG00000147604	ENSG00000215402
ENSG00000147606	ENSG00000091137
ENSG00000147606	ENSG00000091138
ENSG00000147606	ENSG00000112053
ENSG00000147606	ENSG00000135502
ENSG00000147606	ENSG00000145217
ENSG00000147606	ENSG00000155850
ENSG00000147606	ENSG00000170615
ENSG00000147606	ENSG00000174502
ENSG00000147613	ENSG00000008118
ENSG00000147613	ENSG00000081320
ENSG00000147613	ENSG00000130822
ENSG00000147613	ENSG00000134072
ENSG00000147613	ENSG00000159792
ENSG00000147613	ENSG00000164076
ENSG00000147613	ENSG00000164543
ENSG00000147613	ENSG00000183049
ENSG00000147614	ENSG00000159720
ENSG00000147642	ENSG00000101298
ENSG00000147650	ENSG00000130881
ENSG00000147650	ENSG00000197324
ENSG00000147655	ENSG00000101282
ENSG00000147655	ENSG00000146374
ENSG00000147655	ENSG00000169218
ENSG00000147676	ENSG00000102934
ENSG00000147676	ENSG00000144063
ENSG00000147676	ENSG00000155254
ENSG00000147676	ENSG00000170293
ENSG00000147676	ENSG00000172005
ENSG00000147689	ENSG00000101447
ENSG00000147689	ENSG00000105523
ENSG00000147689	ENSG00000125998
ENSG00000147689	ENSG00000133477
ENSG00000147689	ENSG00000168143
ENSG00000147689	ENSG00000180921
ENSG00000147689	ENSG00000188522
ENSG00000147724	ENSG00000082269
ENSG00000147799	ENSG00000128805
ENSG00000147799	ENSG00000138639
ENSG00000147799	ENSG00000163219
ENSG00000147804	ENSG00000104635
ENSG00000147804	ENSG00000112473

ENSG00000147804	ENSG00000138821
ENSG00000147804	ENSG00000139540
ENSG00000147804	ENSG00000141424
ENSG00000147804	ENSG00000148482
ENSG00000147804	ENSG00000165915
ENSG00000147804	ENSG00000196950
ENSG00000147852	ENSG00000130164
ENSG00000147852	ENSG00000157193
ENSG00000147853	ENSG00000162433
ENSG00000147854	ENSG00000034063
ENSG00000147862	ENSG00000008441
ENSG00000147862	ENSG00000141905
ENSG00000147862	ENSG00000162599
ENSG00000147869	ENSG00000158747
ENSG00000147869	ENSG00000166923
ENSG00000147869	ENSG00000179284
ENSG00000147869	ENSG00000180875
ENSG00000147872	ENSG00000105355
ENSG00000147872	ENSG00000167676
ENSG00000147872	ENSG00000214456
ENSG00000148053	ENSG00000030304
ENSG00000148053	ENSG00000140538
ENSG00000148053	ENSG00000169071
ENSG00000148053	ENSG00000185483
ENSG00000148053	ENSG00000198400
ENSG00000148082	ENSG00000129946
ENSG00000148082	ENSG00000160691
ENSG00000148082	ENSG00000185634
ENSG00000148110	ENSG00000156875
ENSG00000148123	ENSG00000067113
ENSG00000148123	ENSG00000105520
ENSG00000148123	ENSG00000117598
ENSG00000148123	ENSG00000117600
ENSG00000148123	ENSG00000129951
ENSG00000148123	ENSG00000141934
ENSG00000148123	ENSG00000162407
ENSG00000148158	ENSG00000114520
ENSG00000148158	ENSG00000162627
ENSG00000148175	ENSG00000067221
ENSG00000148175	ENSG00000116218
ENSG00000148175	ENSG00000133115
ENSG00000148175	ENSG00000165283
ENSG00000148180	ENSG00000006747
ENSG00000148180	ENSG00000042493
ENSG00000148180	ENSG00000127831
ENSG00000148180	ENSG00000135407
ENSG00000148180	ENSG00000136059
ENSG00000148180	ENSG00000177731
ENSG00000148200	ENSG00000116833
ENSG00000148200	ENSG00000136931
ENSG00000148204	ENSG00000156076
ENSG00000148219	ENSG00000152092
ENSG00000148288	ENSG00000175164
ENSG00000148288	ENSG00000184389
ENSG00000148288	ENSG00000204007
ENSG00000148335	ENSG00000203740
ENSG00000148339	ENSG00000085491
ENSG00000148339	ENSG00000125648
ENSG00000148339	ENSG00000181240

ENSG00000148343	ENSG00000180488
ENSG00000148346	ENSG00000107317
ENSG00000148346	ENSG00000176919
ENSG00000148346	ENSG00000177984
ENSG00000148346	ENSG00000184925
ENSG00000148346	ENSG00000187922
ENSG00000148346	ENSG00000214402
ENSG00000148357	ENSG00000101825
ENSG00000148357	ENSG00000143341
ENSG00000148357	ENSG00000152580
ENSG00000148357	ENSG00000155657
ENSG00000148357	ENSG00000168462
ENSG00000148358	ENSG00000125734
ENSG00000148384	ENSG00000165458
ENSG00000148384	ENSG00000168918
ENSG00000148396	ENSG00000120341
ENSG00000148400	ENSG00000074181
ENSG00000148400	ENSG00000134250
ENSG00000148400	ENSG00000134376
ENSG00000148400	ENSG00000204301
ENSG00000148408	ENSG00000081248
ENSG00000148408	ENSG00000102001
ENSG00000148408	ENSG00000141837
ENSG00000148408	ENSG00000151067
ENSG00000148408	ENSG00000157388
ENSG00000148408	ENSG00000198216
ENSG00000148411	ENSG00000160877
ENSG00000148411	ENSG00000173276
ENSG00000148411	ENSG00000177125
ENSG00000148429	ENSG00000128438
ENSG00000148429	ENSG00000161583
ENSG00000148429	ENSG00000174115
ENSG00000148429	ENSG00000185128
ENSG00000148429	ENSG00000188755
ENSG00000148429	ENSG00000188849
ENSG00000148429	ENSG00000189309
ENSG00000148429	ENSG00000197681
ENSG00000148429	ENSG00000204471
ENSG00000148429	ENSG00000205019
ENSG00000148429	ENSG00000213474
ENSG00000148429	ENSG00000215625
ENSG00000148429	ENSG00000215730
ENSG00000148429	ENSG00000215748
ENSG00000148468	ENSG00000144369
ENSG00000148468	ENSG00000161682
ENSG00000148482	ENSG00000104635
ENSG00000148482	ENSG00000112473
ENSG00000148482	ENSG00000138821
ENSG00000148482	ENSG00000139540
ENSG00000148482	ENSG00000141424
ENSG00000148482	ENSG00000147804
ENSG00000148482	ENSG00000165915
ENSG00000148482	ENSG00000196950
ENSG00000148488	ENSG00000073849
ENSG00000148488	ENSG00000101638
ENSG00000148488	ENSG00000111728
ENSG00000148488	ENSG00000117069
ENSG00000148488	ENSG00000144057
ENSG00000148488	ENSG00000160408

ENSG00000148488	ENSG00000177511
ENSG00000148498	ENSG00000116117
ENSG00000148516	ENSG00000169554
ENSG00000148541	ENSG00000031003
ENSG00000148541	ENSG00000138640
ENSG00000148600	ENSG00000074276
ENSG00000148602	ENSG00000173621
ENSG00000148602	ENSG00000183423
ENSG00000148602	ENSG00000204033
ENSG00000148604	ENSG00000054277
ENSG00000148604	ENSG00000102076
ENSG00000148604	ENSG00000122375
ENSG00000148604	ENSG00000124818
ENSG00000148604	ENSG00000128617
ENSG00000148604	ENSG00000147380
ENSG00000148604	ENSG00000163914
ENSG00000148604	ENSG00000166160
ENSG00000148604	ENSG00000180245
ENSG00000148634	ENSG00000114062
ENSG00000148634	ENSG00000138641
ENSG00000148634	ENSG00000138642
ENSG00000148634	ENSG00000138646
ENSG00000148634	ENSG00000165338
ENSG00000148660	ENSG00000058404
ENSG00000148660	ENSG00000070808
ENSG00000148660	ENSG00000145349
ENSG00000148677	ENSG00000163126
ENSG00000148677	ENSG00000165887
ENSG00000148700	ENSG00000075340
ENSG00000148700	ENSG00000087274
ENSG00000148702	ENSG00000019991
ENSG00000148702	ENSG00000104368
ENSG00000148702	ENSG00000109758
ENSG00000148702	ENSG00000122194
ENSG00000148702	ENSG00000122861
ENSG00000148702	ENSG00000131187
ENSG00000148702	ENSG00000173531
ENSG00000148702	ENSG00000186301
ENSG00000148702	ENSG00000186715
ENSG00000148702	ENSG00000198670
ENSG00000148704	ENSG00000116035
ENSG00000148719	ENSG00000164031
ENSG00000148719	ENSG00000170464
ENSG00000148730	ENSG00000187840
ENSG00000148734	ENSG00000056291
ENSG00000148734	ENSG00000075073
ENSG00000148734	ENSG00000101292
ENSG00000148734	ENSG00000110148
ENSG00000148734	ENSG00000115353
ENSG00000148734	ENSG00000119973
ENSG00000148734	ENSG00000121764
ENSG00000148734	ENSG00000123901
ENSG00000148734	ENSG00000137252
ENSG00000148734	ENSG00000159279
ENSG00000148734	ENSG00000163394
ENSG00000148734	ENSG00000164128
ENSG00000148734	ENSG00000164129
ENSG00000148734	ENSG00000169618
ENSG00000148734	ENSG00000169836

ENSG00000148734	ENSG00000185149
ENSG00000148734	ENSG00000186867
ENSG00000148734	ENSG00000204174
ENSG00000148734	ENSG00000215167
ENSG00000148737	ENSG00000081059
ENSG00000148737	ENSG00000138795
ENSG00000148737	ENSG00000152284
ENSG00000148795	ENSG00000198457
ENSG00000148798	ENSG00000026025
ENSG00000148798	ENSG00000100285
ENSG00000148798	ENSG00000104722
ENSG00000148798	ENSG00000104725
ENSG00000148798	ENSG00000131095
ENSG00000148798	ENSG00000135406
ENSG00000148798	ENSG00000175084
ENSG00000148798	ENSG00000182253
ENSG00000148798	ENSG00000215479
ENSG00000148826	ENSG00000163623
ENSG00000148826	ENSG00000165066
ENSG00000148832	ENSG00000088826
ENSG00000148840	ENSG00000109819
ENSG00000148840	ENSG00000155846
ENSG00000148841	ENSG00000198885
ENSG00000148841	ENSG00000205730
ENSG00000148842	ENSG00000119946
ENSG00000148842	ENSG00000158158
ENSG00000148842	ENSG00000168763
ENSG00000148848	ENSG00000008277
ENSG00000148848	ENSG00000042980
ENSG00000148848	ENSG00000069206
ENSG00000148848	ENSG00000073670
ENSG00000148848	ENSG00000104755
ENSG00000148848	ENSG00000114948
ENSG00000148848	ENSG00000134007
ENSG00000148848	ENSG00000134028
ENSG00000148848	ENSG00000134249
ENSG00000148848	ENSG00000135074
ENSG00000148848	ENSG00000139985
ENSG00000148848	ENSG00000143537
ENSG00000148848	ENSG00000149451
ENSG00000148848	ENSG00000151651
ENSG00000148848	ENSG00000151694
ENSG00000148848	ENSG00000168594
ENSG00000148848	ENSG00000168615
ENSG00000148848	ENSG00000168619
ENSG00000148848	ENSG00000196115
ENSG00000148848	ENSG00000197140
ENSG00000148848	ENSG00000214137
ENSG00000148908	ENSG00000090104
ENSG00000148908	ENSG00000091844
ENSG00000148908	ENSG00000116741
ENSG00000148908	ENSG00000117152
ENSG00000148908	ENSG00000127074
ENSG00000148908	ENSG00000135824
ENSG00000148908	ENSG00000143248
ENSG00000148908	ENSG00000143333
ENSG00000148908	ENSG00000147509
ENSG00000148908	ENSG00000150681
ENSG00000148908	ENSG00000171700

ENSG00000148908	ENSG00000215825
ENSG00000148925	ENSG00000112078
ENSG00000148935	ENSG00000132139
ENSG00000148935	ENSG00000139354
ENSG00000148935	ENSG00000185340
ENSG00000148943	ENSG00000104863
ENSG00000148943	ENSG00000111052
ENSG00000148948	ENSG00000131409
ENSG00000149091	ENSG00000145214
ENSG00000149091	ENSG00000157680
ENSG00000149131	ENSG00000132386
ENSG00000149131	ENSG00000167711
ENSG00000149150	ENSG00000134802
ENSG00000149150	ENSG00000167703
ENSG00000149177	ENSG00000080031
ENSG00000149177	ENSG00000127329
ENSG00000149177	ENSG00000151490
ENSG00000149182	ENSG00000100262
ENSG00000149187	ENSG00000048740
ENSG00000149187	ENSG00000101489
ENSG00000149187	ENSG00000140488
ENSG00000149187	ENSG00000159409
ENSG00000149187	ENSG00000161082
ENSG00000149212	ENSG00000080546
ENSG00000149212	ENSG00000130766
ENSG00000149243	ENSG00000114796
ENSG00000149243	ENSG00000119771
ENSG00000149243	ENSG00000172578
ENSG00000149256	ENSG00000009694
ENSG00000149256	ENSG00000145934
ENSG00000149260	ENSG00000014216
ENSG00000149260	ENSG00000077274
ENSG00000149260	ENSG00000092529
ENSG00000149260	ENSG00000135773
ENSG00000149260	ENSG00000137225
ENSG00000149260	ENSG00000142330
ENSG00000149260	ENSG00000162909
ENSG00000149260	ENSG00000162949
ENSG00000149260	ENSG00000182472
ENSG00000149260	ENSG00000203697
ENSG00000149260	ENSG00000214711
ENSG00000149269	ENSG00000077264
ENSG00000149269	ENSG00000180370
ENSG00000149289	ENSG00000100441
ENSG00000149289	ENSG00000102053
ENSG00000149289	ENSG00000102921
ENSG00000149289	ENSG00000163874
ENSG00000149289	ENSG00000178199
ENSG00000149289	ENSG00000205978
ENSG00000149294	ENSG00000154654
ENSG00000149295	ENSG00000069696
ENSG00000149295	ENSG00000151577
ENSG00000149305	ENSG00000080644
ENSG00000149305	ENSG00000101204
ENSG00000149305	ENSG00000108556
ENSG00000149305	ENSG00000117971
ENSG00000149305	ENSG00000120903
ENSG00000149305	ENSG00000129749
ENSG00000149305	ENSG00000135902

ENSG00000149305	ENSG00000138435
ENSG00000149305	ENSG00000147432
ENSG00000149305	ENSG00000147434
ENSG00000149305	ENSG00000160716
ENSG00000149305	ENSG00000166664
ENSG00000149305	ENSG00000166736
ENSG00000149305	ENSG00000169684
ENSG00000149305	ENSG00000170175
ENSG00000149305	ENSG00000174343
ENSG00000149305	ENSG00000175344
ENSG00000149305	ENSG00000178084
ENSG00000149305	ENSG00000186038
ENSG00000149305	ENSG00000186090
ENSG00000149305	ENSG00000186919
ENSG00000149305	ENSG00000196811
ENSG00000149403	ENSG00000120251
ENSG00000149403	ENSG00000125675
ENSG00000149403	ENSG00000152578
ENSG00000149403	ENSG00000155511
ENSG00000149403	ENSG00000163873
ENSG00000149403	ENSG00000164418
ENSG00000149403	ENSG00000171189
ENSG00000149418	ENSG00000176040
ENSG00000149418	ENSG00000178297
ENSG00000149418	ENSG00000187045
ENSG00000149418	ENSG00000205403
ENSG00000149435	ENSG00000099998
ENSG00000149435	ENSG00000100031
ENSG00000149435	ENSG00000100121
ENSG00000149435	ENSG00000133475
ENSG00000149435	ENSG00000183038
ENSG00000149435	ENSG00000197421
ENSG00000149435	ENSG00000215466
ENSG00000149435	ENSG00000215500
ENSG00000149435	ENSG00000215610
ENSG00000149451	ENSG00000008277
ENSG00000149451	ENSG00000042980
ENSG00000149451	ENSG00000069206
ENSG00000149451	ENSG00000073670
ENSG00000149451	ENSG00000104755
ENSG00000149451	ENSG00000114948
ENSG00000149451	ENSG00000134007
ENSG00000149451	ENSG00000134028
ENSG00000149451	ENSG00000134249
ENSG00000149451	ENSG00000135074
ENSG00000149451	ENSG00000139985
ENSG00000149451	ENSG00000143537
ENSG00000149451	ENSG00000148848
ENSG00000149451	ENSG00000151651
ENSG00000149451	ENSG00000151694
ENSG00000149451	ENSG00000168594
ENSG00000149451	ENSG00000168615
ENSG00000149451	ENSG00000168619
ENSG00000149451	ENSG00000196115
ENSG00000149451	ENSG00000197140
ENSG00000149451	ENSG00000214137
ENSG00000149480	ENSG00000057935
ENSG00000149480	ENSG00000182979
ENSG00000149485	ENSG00000134824

ENSG00000149485	ENSG00000214876
ENSG00000149488	ENSG00000103534
ENSG00000149488	ENSG00000141524
ENSG00000149488	ENSG00000165091
ENSG00000149488	ENSG00000167608
ENSG00000149488	ENSG00000167895
ENSG00000149488	ENSG00000170537
ENSG00000149488	ENSG00000188869
ENSG00000149489	ENSG00000112619
ENSG00000149499	ENSG00000066629
ENSG00000149499	ENSG00000125746
ENSG00000149499	ENSG00000143924
ENSG00000149499	ENSG00000143940
ENSG00000149499	ENSG00000165521
ENSG00000149499	ENSG00000214595
ENSG00000149506	ENSG00000103310
ENSG00000149506	ENSG00000116996
ENSG00000149516	ENSG00000071203
ENSG00000149516	ENSG00000110077
ENSG00000149516	ENSG00000110079
ENSG00000149516	ENSG00000149534
ENSG00000149516	ENSG00000156738
ENSG00000149516	ENSG00000166926
ENSG00000149516	ENSG00000166927
ENSG00000149516	ENSG00000166930
ENSG00000149516	ENSG00000166959
ENSG00000149516	ENSG00000166961
ENSG00000149516	ENSG00000214787
ENSG00000149527	ENSG00000101333
ENSG00000149527	ENSG00000114805
ENSG00000149527	ENSG00000115556
ENSG00000149527	ENSG00000115896
ENSG00000149527	ENSG00000124181
ENSG00000149527	ENSG00000137841
ENSG00000149527	ENSG00000139151
ENSG00000149527	ENSG00000149782
ENSG00000149527	ENSG00000154822
ENSG00000149527	ENSG00000161714
ENSG00000149527	ENSG00000182621
ENSG00000149527	ENSG00000187091
ENSG00000149527	ENSG00000197943
ENSG00000149532	ENSG00000111605
ENSG00000149534	ENSG00000071203
ENSG00000149534	ENSG00000110077
ENSG00000149534	ENSG00000110079
ENSG00000149534	ENSG00000149516
ENSG00000149534	ENSG00000156738
ENSG00000149534	ENSG00000166926
ENSG00000149534	ENSG00000166927
ENSG00000149534	ENSG00000166930
ENSG00000149534	ENSG00000166959
ENSG00000149534	ENSG00000166961
ENSG00000149534	ENSG00000214787
ENSG00000149541	ENSG00000109956
ENSG00000149541	ENSG00000112309
ENSG00000149557	ENSG00000171055
ENSG00000149564	ENSG00000019102
ENSG00000149564	ENSG00000143167
ENSG00000149564	ENSG00000144847

ENSG00000149564	ENSG00000154639
ENSG00000149564	ENSG00000166250
ENSG00000149564	ENSG00000188004
ENSG00000149571	ENSG00000126259
ENSG00000149571	ENSG00000161270
ENSG00000149571	ENSG00000183853
ENSG00000149573	ENSG00000149575
ENSG00000149573	ENSG00000158887
ENSG00000149573	ENSG00000160588
ENSG00000149573	ENSG00000160593
ENSG00000149573	ENSG00000177098
ENSG00000149573	ENSG00000197965
ENSG00000149575	ENSG00000149573
ENSG00000149575	ENSG00000158887
ENSG00000149575	ENSG00000160588
ENSG00000149575	ENSG00000160593
ENSG00000149575	ENSG00000177098
ENSG00000149575	ENSG00000197965
ENSG00000149577	ENSG00000072858
ENSG00000149591	ENSG00000064666
ENSG00000149591	ENSG00000117519
ENSG00000149591	ENSG00000130176
ENSG00000149591	ENSG00000144834
ENSG00000149591	ENSG00000158710
ENSG00000149591	ENSG00000204782
ENSG00000149591	ENSG00000204826
ENSG00000149591	ENSG00000215433
ENSG00000149596	ENSG00000092051
ENSG00000149596	ENSG00000104369
ENSG00000149596	ENSG00000139714
ENSG00000149596	ENSG00000154118
ENSG00000149599	ENSG00000112679
ENSG00000149633	ENSG00000153404
ENSG00000149633	ENSG00000165801
ENSG00000149633	ENSG00000196155
ENSG00000149639	ENSG00000168502
ENSG00000149639	ENSG00000214338
ENSG00000149654	ENSG00000040731
ENSG00000149654	ENSG00000071991
ENSG00000149654	ENSG00000081138
ENSG00000149654	ENSG00000101542
ENSG00000149654	ENSG00000113100
ENSG00000149654	ENSG00000113361
ENSG00000149654	ENSG00000139880
ENSG00000149654	ENSG00000140937
ENSG00000149654	ENSG00000145526
ENSG00000149654	ENSG00000150394
ENSG00000149654	ENSG00000154162
ENSG00000149654	ENSG00000179776
ENSG00000149657	ENSG00000105216
ENSG00000149658	ENSG00000185728
ENSG00000149658	ENSG00000198492
ENSG00000149679	ENSG00000134508
ENSG00000149781	ENSG00000073712
ENSG00000149781	ENSG00000101311
ENSG00000149781	ENSG00000137076
ENSG00000149781	ENSG00000171914
ENSG00000149782	ENSG00000101333
ENSG00000149782	ENSG00000114805

ENSG00000149782	ENSG00000115556
ENSG00000149782	ENSG00000115896
ENSG00000149782	ENSG00000124181
ENSG00000149782	ENSG00000137841
ENSG00000149782	ENSG00000139151
ENSG00000149782	ENSG00000149527
ENSG00000149782	ENSG00000154822
ENSG00000149782	ENSG00000161714
ENSG00000149782	ENSG00000182621
ENSG00000149782	ENSG00000187091
ENSG00000149782	ENSG00000197943
ENSG00000149798	ENSG00000128283
ENSG00000149798	ENSG00000163171
ENSG00000149798	ENSG00000167617
ENSG00000149798	ENSG00000179604
ENSG00000149809	ENSG00000143815
ENSG00000149922	ENSG00000073861
ENSG00000149922	ENSG00000089225
ENSG00000149922	ENSG00000092607
ENSG00000149922	ENSG00000112837
ENSG00000149922	ENSG00000121068
ENSG00000149922	ENSG00000121075
ENSG00000149922	ENSG00000122145
ENSG00000149922	ENSG00000135111
ENSG00000149922	ENSG00000136535
ENSG00000149922	ENSG00000143178
ENSG00000149922	ENSG00000163508
ENSG00000149922	ENSG00000164458
ENSG00000149922	ENSG00000164532
ENSG00000149922	ENSG00000167800
ENSG00000149922	ENSG00000174197
ENSG00000149922	ENSG00000184058
ENSG00000149925	ENSG00000109107
ENSG00000149925	ENSG00000136872
ENSG00000149926	ENSG00000152078
ENSG00000149926	ENSG00000167695
ENSG00000149927	ENSG00000011347
ENSG00000149927	ENSG00000019505
ENSG00000149927	ENSG00000067715
ENSG00000149927	ENSG00000101098
ENSG00000149927	ENSG00000103528
ENSG00000149927	ENSG00000110975
ENSG00000149927	ENSG00000129990
ENSG00000149927	ENSG00000132718
ENSG00000149927	ENSG00000132872
ENSG00000149927	ENSG00000134207
ENSG00000149927	ENSG00000143858
ENSG00000149927	ENSG00000149043
ENSG00000149927	ENSG00000170743
ENSG00000149927	ENSG00000173227
ENSG00000149927	ENSG00000204176
ENSG00000149927	ENSG00000213023
ENSG00000149927	ENSG00000214408
ENSG00000149927	ENSG00000214409
ENSG00000149927	ENSG00000214569
ENSG00000149927	ENSG00000214783
ENSG00000149930	ENSG00000135090
ENSG00000149930	ENSG00000160551
ENSG00000149948	ENSG00000137309

ENSG00000149962	ENSG00000105835
ENSG00000149968	ENSG00000008516
ENSG00000149968	ENSG00000087245
ENSG00000149968	ENSG00000099953
ENSG00000149968	ENSG00000100985
ENSG00000149968	ENSG00000102996
ENSG00000149968	ENSG00000110347
ENSG00000149968	ENSG00000118113
ENSG00000149968	ENSG00000123342
ENSG00000149968	ENSG00000125966
ENSG00000149968	ENSG00000129270
ENSG00000149968	ENSG00000137673
ENSG00000149968	ENSG00000137674
ENSG00000149968	ENSG00000137675
ENSG00000149968	ENSG00000137745
ENSG00000149968	ENSG00000154485
ENSG00000149968	ENSG00000156103
ENSG00000149968	ENSG00000157227
ENSG00000149968	ENSG00000166670
ENSG00000149968	ENSG00000167346
ENSG00000149968	ENSG00000189409
ENSG00000149968	ENSG00000196611
ENSG00000149968	ENSG00000198598
ENSG00000149968	ENSG00000215914
ENSG00000149970	ENSG00000074706
ENSG00000149970	ENSG00000142675
ENSG00000149970	ENSG00000153721
ENSG00000149972	ENSG00000091129
ENSG00000149972	ENSG00000134121
ENSG00000149972	ENSG00000163531
ENSG00000149972	ENSG00000184144
ENSG00000149972	ENSG00000198493
ENSG00000150051	ENSG00000113430
ENSG00000150051	ENSG00000159387
ENSG00000150051	ENSG00000170549
ENSG00000150051	ENSG00000170561
ENSG00000150051	ENSG00000176842
ENSG00000150051	ENSG00000177508
ENSG00000150054	ENSG00000072415
ENSG00000150054	ENSG00000082126
ENSG00000150054	ENSG00000105926
ENSG00000150054	ENSG00000108852
ENSG00000150054	ENSG00000130830
ENSG00000150054	ENSG00000147044
ENSG00000150054	ENSG00000161647
ENSG00000150086	ENSG00000105464
ENSG00000150086	ENSG00000116032
ENSG00000150086	ENSG00000161509
ENSG00000150086	ENSG00000176884
ENSG00000150086	ENSG00000183454
ENSG00000150086	ENSG00000198785
ENSG00000150093	ENSG00000056345
ENSG00000150093	ENSG00000082781
ENSG00000150093	ENSG00000105855
ENSG00000150093	ENSG00000115221
ENSG00000150093	ENSG00000132470
ENSG00000150093	ENSG00000139626
ENSG00000150093	ENSG00000160255
ENSG00000150165	ENSG00000104537

ENSG00000150165	ENSG00000109511
ENSG00000150165	ENSG00000122359
ENSG00000150165	ENSG00000135046
ENSG00000150165	ENSG00000138279
ENSG00000150165	ENSG00000138772
ENSG00000150165	ENSG00000143412
ENSG00000150165	ENSG00000164111
ENSG00000150165	ENSG00000165390
ENSG00000150165	ENSG00000182718
ENSG00000150165	ENSG00000183059
ENSG00000150165	ENSG00000186807
ENSG00000150165	ENSG00000196975
ENSG00000150165	ENSG00000197043
ENSG00000150201	ENSG00000089356
ENSG00000150201	ENSG00000126258
ENSG00000150201	ENSG00000137726
ENSG00000150201	ENSG00000137731
ENSG00000150201	ENSG00000182707
ENSG00000150276	ENSG00000108179
ENSG00000150276	ENSG00000196262
ENSG00000150276	ENSG00000198161
ENSG00000150276	ENSG00000198360
ENSG00000150276	ENSG00000198618
ENSG00000150276	ENSG00000198936
ENSG00000150276	ENSG00000203847
ENSG00000150276	ENSG00000214975
ENSG00000150276	ENSG00000215551
ENSG00000150337	ENSG00000072694
ENSG00000150337	ENSG00000132185
ENSG00000150337	ENSG00000143226
ENSG00000150337	ENSG00000162747
ENSG00000150337	ENSG00000179639
ENSG00000150337	ENSG00000181036
ENSG00000150337	ENSG00000198019
ENSG00000150337	ENSG00000203747
ENSG00000150337	ENSG00000203820
ENSG00000150347	ENSG00000196843
ENSG00000150394	ENSG00000040731
ENSG00000150394	ENSG00000071991
ENSG00000150394	ENSG00000081138
ENSG00000150394	ENSG00000101542
ENSG00000150394	ENSG00000113100
ENSG00000150394	ENSG00000113361
ENSG00000150394	ENSG00000139880
ENSG00000150394	ENSG00000140937
ENSG00000150394	ENSG00000145526
ENSG00000150394	ENSG00000149654
ENSG00000150394	ENSG00000154162
ENSG00000150394	ENSG00000179776
ENSG00000150401	ENSG00000043093
ENSG00000150401	ENSG00000188215
ENSG00000150457	ENSG00000131023
ENSG00000150471	ENSG00000072071
ENSG00000150471	ENSG00000111452
ENSG00000150471	ENSG00000117114
ENSG00000150471	ENSG00000180264
ENSG00000150510	ENSG00000124019
ENSG00000150526	ENSG00000125879
ENSG00000150526	ENSG00000213054

ENSG00000150527	ENSG00000154305
ENSG00000150556	ENSG00000187123
ENSG00000150594	ENSG00000043591
ENSG00000150594	ENSG00000102468
ENSG00000150594	ENSG00000113749
ENSG00000150594	ENSG00000125631
ENSG00000150594	ENSG00000135312
ENSG00000150594	ENSG00000135914
ENSG00000150594	ENSG00000147246
ENSG00000150594	ENSG00000157219
ENSG00000150594	ENSG00000164270
ENSG00000150594	ENSG00000168830
ENSG00000150594	ENSG00000169252
ENSG00000150594	ENSG00000175658
ENSG00000150594	ENSG00000178394
ENSG00000150594	ENSG00000179097
ENSG00000150594	ENSG00000179546
ENSG00000150594	ENSG00000181210
ENSG00000150594	ENSG00000184303
ENSG00000150594	ENSG00000184845
ENSG00000150594	ENSG00000185321
ENSG00000150594	ENSG00000188778
ENSG00000150625	ENSG00000046653
ENSG00000150625	ENSG00000123560
ENSG00000150630	ENSG00000100311
ENSG00000150630	ENSG00000112715
ENSG00000150630	ENSG00000119630
ENSG00000150630	ENSG00000165197
ENSG00000150630	ENSG00000173511
ENSG00000150630	ENSG00000197461
ENSG00000150630	ENSG00000215682
ENSG00000150672	ENSG00000075711
ENSG00000150672	ENSG00000082458
ENSG00000150672	ENSG00000132535
ENSG00000150672	ENSG00000151208
ENSG00000150681	ENSG00000090104
ENSG00000150681	ENSG00000091844
ENSG00000150681	ENSG00000116741
ENSG00000150681	ENSG00000117152
ENSG00000150681	ENSG00000127074
ENSG00000150681	ENSG00000135824
ENSG00000150681	ENSG00000143248
ENSG00000150681	ENSG00000143333
ENSG00000150681	ENSG00000147509
ENSG00000150681	ENSG00000148908
ENSG00000150681	ENSG00000171700
ENSG00000150681	ENSG00000215825
ENSG00000150687	ENSG00000146250
ENSG00000150712	ENSG00000003987
ENSG00000150712	ENSG00000014914
ENSG00000150712	ENSG00000063601
ENSG00000150712	ENSG00000087053
ENSG00000150712	ENSG00000100330
ENSG00000150712	ENSG00000102043
ENSG00000150712	ENSG00000104643
ENSG00000150712	ENSG00000108389
ENSG00000150712	ENSG00000139505
ENSG00000150712	ENSG00000166912
ENSG00000150712	ENSG00000171100

ENSG00000150712	ENSG00000184416
ENSG00000150722	ENSG00000131771
ENSG00000150722	ENSG00000135447
ENSG00000150756	ENSG00000103254
ENSG00000150760	ENSG00000088538
ENSG00000150760	ENSG00000128512
ENSG00000150760	ENSG00000134516
ENSG00000150760	ENSG00000147459
ENSG00000150779	ENSG00000126953
ENSG00000150867	ENSG00000141720
ENSG00000150867	ENSG00000166908
ENSG00000150867	ENSG00000167103
ENSG00000150893	ENSG00000138759
ENSG00000150893	ENSG00000164946
ENSG00000150893	ENSG00000173546
ENSG00000150893	ENSG00000183090
ENSG00000150893	ENSG00000215060
ENSG00000150907	ENSG00000118689
ENSG00000150907	ENSG00000184481
ENSG00000150907	ENSG00000204060
ENSG00000150961	ENSG00000176986
ENSG00000150967	ENSG00000168394
ENSG00000150967	ENSG00000204267
ENSG00000150977	ENSG00000167705
ENSG00000150977	ENSG00000188026
ENSG00000150995	ENSG00000096433
ENSG00000150995	ENSG00000123104
ENSG00000151005	ENSG00000007350
ENSG00000151005	ENSG00000163931
ENSG00000151012	ENSG00000021488
ENSG00000151012	ENSG00000092068
ENSG00000151012	ENSG00000103064
ENSG00000151012	ENSG00000103257
ENSG00000151012	ENSG00000130876
ENSG00000151012	ENSG00000155465
ENSG00000151012	ENSG00000164893
ENSG00000151025	ENSG00000188888
ENSG00000151033	ENSG00000090382
ENSG00000151033	ENSG00000120563
ENSG00000151033	ENSG00000141316
ENSG00000151033	ENSG00000157093
ENSG00000151033	ENSG00000161572
ENSG00000151033	ENSG00000167531
ENSG00000151033	ENSG00000171478
ENSG00000151033	ENSG00000171489
ENSG00000151062	ENSG00000007402
ENSG00000151062	ENSG00000153956
ENSG00000151062	ENSG00000157445
ENSG00000151062	ENSG00000158966
ENSG00000151065	ENSG00000162290
ENSG00000151067	ENSG00000081248
ENSG00000151067	ENSG00000102001
ENSG00000151067	ENSG00000141837
ENSG00000151067	ENSG00000148408
ENSG00000151067	ENSG00000157388
ENSG00000151067	ENSG00000198216
ENSG00000151079	ENSG00000026559
ENSG00000151079	ENSG00000102057
ENSG00000151079	ENSG00000104848

ENSG00000151079	ENSG00000111262
ENSG00000151079	ENSG00000124134
ENSG00000151079	ENSG00000130037
ENSG00000151079	ENSG00000143105
ENSG00000151079	ENSG00000156486
ENSG00000151079	ENSG00000158445
ENSG00000151079	ENSG00000162975
ENSG00000151079	ENSG00000164794
ENSG00000151079	ENSG00000168263
ENSG00000151079	ENSG00000168418
ENSG00000151079	ENSG00000170745
ENSG00000151079	ENSG00000171126
ENSG00000151079	ENSG00000171385
ENSG00000151079	ENSG00000177272
ENSG00000151079	ENSG00000177301
ENSG00000151079	ENSG00000178342
ENSG00000151079	ENSG00000182255
ENSG00000151079	ENSG00000182674
ENSG00000151079	ENSG00000184408
ENSG00000151090	ENSG00000077092
ENSG00000151090	ENSG00000126351
ENSG00000151090	ENSG00000126368
ENSG00000151090	ENSG00000131759
ENSG00000151090	ENSG00000172819
ENSG00000151090	ENSG00000174738
ENSG00000151093	ENSG00000169710
ENSG00000151117	ENSG00000180089
ENSG00000151136	ENSG00000166016
ENSG00000151150	ENSG00000029534
ENSG00000151150	ENSG00000145362
ENSG00000151150	ENSG00000185722
ENSG00000151164	ENSG00000172613
ENSG00000151224	ENSG00000168906
ENSG00000151233	ENSG00000172986
ENSG00000151233	ENSG00000173950
ENSG00000151240	ENSG00000066084
ENSG00000151240	ENSG00000160305
ENSG00000151247	ENSG00000175766
ENSG00000151276	ENSG00000081026
ENSG00000151276	ENSG00000187391
ENSG00000151292	ENSG00000133275
ENSG00000151292	ENSG00000169118
ENSG00000151303	ENSG00000108262
ENSG00000151303	ENSG00000133612
ENSG00000151303	ENSG00000135439
ENSG00000151303	ENSG00000139436
ENSG00000151303	ENSG00000157985
ENSG00000151303	ENSG00000172650
ENSG00000151303	ENSG00000174194
ENSG00000151303	ENSG00000188234
ENSG00000151303	ENSG00000198035
ENSG00000151303	ENSG00000204149
ENSG00000151303	ENSG00000204169
ENSG00000151303	ENSG00000204172
ENSG00000151322	ENSG00000100644
ENSG00000151322	ENSG00000112246
ENSG00000151322	ENSG00000116016
ENSG00000151322	ENSG00000124440
ENSG00000151322	ENSG00000130751

ENSG00000151322	ENSG00000159263
ENSG00000151327	ENSG00000197520
ENSG00000151327	ENSG00000214886
ENSG00000151365	ENSG00000165175
ENSG00000151376	ENSG00000065833
ENSG00000151376	ENSG00000082212
ENSG00000151379	ENSG00000166823
ENSG00000151379	ENSG00000188095
ENSG00000151388	ENSG00000049192
ENSG00000151388	ENSG00000087116
ENSG00000151388	ENSG00000136378
ENSG00000151388	ENSG00000138316
ENSG00000151388	ENSG00000140470
ENSG00000151388	ENSG00000140873
ENSG00000151388	ENSG00000142303
ENSG00000151388	ENSG00000145536
ENSG00000151388	ENSG00000145808
ENSG00000151388	ENSG00000156140
ENSG00000151388	ENSG00000160323
ENSG00000151388	ENSG00000163638
ENSG00000151388	ENSG00000173157
ENSG00000151388	ENSG00000214644
ENSG00000151388	ENSG00000215743
ENSG00000151422	ENSG00000010671
ENSG00000151422	ENSG00000074966
ENSG00000151422	ENSG00000102010
ENSG00000151422	ENSG00000113263
ENSG00000151422	ENSG00000135605
ENSG00000151422	ENSG00000182511
ENSG00000151458	ENSG00000115183
ENSG00000151458	ENSG00000170921
ENSG00000151474	ENSG00000114541
ENSG00000151474	ENSG00000147144
ENSG00000151474	ENSG00000163362
ENSG00000151490	ENSG00000080031
ENSG00000151490	ENSG00000127329
ENSG00000151490	ENSG00000149177
ENSG00000151491	ENSG00000131037
ENSG00000151491	ENSG00000177106
ENSG00000151491	ENSG00000198758
ENSG00000151514	ENSG00000101115
ENSG00000151514	ENSG00000103449
ENSG00000151514	ENSG00000165821
ENSG00000151553	ENSG00000051009
ENSG00000151553	ENSG00000158863
ENSG00000151553	ENSG00000164142
ENSG00000151572	ENSG00000047617
ENSG00000151572	ENSG00000074855
ENSG00000151572	ENSG00000131620
ENSG00000151572	ENSG00000134343
ENSG00000151572	ENSG00000146205
ENSG00000151572	ENSG00000160746
ENSG00000151572	ENSG00000171714
ENSG00000151572	ENSG00000177119
ENSG00000151572	ENSG00000185101
ENSG00000151577	ENSG00000069696
ENSG00000151577	ENSG00000149295
ENSG00000151612	ENSG00000020256
ENSG00000151615	ENSG00000028277

ENSG00000151615	ENSG00000064835
ENSG00000151615	ENSG00000091010
ENSG00000151615	ENSG00000137709
ENSG00000151615	ENSG00000143190
ENSG00000151615	ENSG00000152192
ENSG00000151615	ENSG00000184486
ENSG00000151615	ENSG00000185668
ENSG00000151615	ENSG00000196767
ENSG00000151615	ENSG00000198914
ENSG00000151615	ENSG00000204531
ENSG00000151615	ENSG00000212993
ENSG00000151617	ENSG00000102239
ENSG00000151617	ENSG00000126010
ENSG00000151617	ENSG00000135577
ENSG00000151617	ENSG00000136160
ENSG00000151617	ENSG00000170075
ENSG00000151617	ENSG00000170775
ENSG00000151623	ENSG00000082175
ENSG00000151623	ENSG00000113580
ENSG00000151631	ENSG00000085662
ENSG00000151631	ENSG00000117448
ENSG00000151631	ENSG00000122787
ENSG00000151631	ENSG00000151632
ENSG00000151631	ENSG00000165568
ENSG00000151631	ENSG00000187134
ENSG00000151631	ENSG00000196139
ENSG00000151631	ENSG00000196326
ENSG00000151631	ENSG00000198074
ENSG00000151631	ENSG00000198610
ENSG00000151631	ENSG00000215267
ENSG00000151632	ENSG00000085662
ENSG00000151632	ENSG00000117448
ENSG00000151632	ENSG00000122787
ENSG00000151632	ENSG00000151631
ENSG00000151632	ENSG00000165568
ENSG00000151632	ENSG00000187134
ENSG00000151632	ENSG00000196139
ENSG00000151632	ENSG00000196326
ENSG00000151632	ENSG00000198074
ENSG00000151632	ENSG00000198610
ENSG00000151632	ENSG00000215267
ENSG00000151640	ENSG00000072832
ENSG00000151640	ENSG00000092964
ENSG00000151640	ENSG00000113657
ENSG00000151640	ENSG00000147647
ENSG00000151640	ENSG00000157851
ENSG00000151651	ENSG00000008277
ENSG00000151651	ENSG00000042980
ENSG00000151651	ENSG00000069206
ENSG00000151651	ENSG00000073670
ENSG00000151651	ENSG00000104755
ENSG00000151651	ENSG00000114948
ENSG00000151651	ENSG00000134007
ENSG00000151651	ENSG00000134028
ENSG00000151651	ENSG00000134249
ENSG00000151651	ENSG00000135074
ENSG00000151651	ENSG00000139985
ENSG00000151651	ENSG00000143537
ENSG00000151651	ENSG00000148848

ENSG00000151651	ENSG00000149451
ENSG00000151651	ENSG00000151694
ENSG00000151651	ENSG00000168594
ENSG00000151651	ENSG00000168615
ENSG00000151651	ENSG00000168619
ENSG00000151651	ENSG00000196115
ENSG00000151651	ENSG00000197140
ENSG00000151651	ENSG00000214137
ENSG00000151655	ENSG00000055955
ENSG00000151655	ENSG00000055957
ENSG00000151655	ENSG00000102313
ENSG00000151655	ENSG00000123243
ENSG00000151655	ENSG00000162267
ENSG00000151692	ENSG00000034677
ENSG00000151692	ENSG00000116514
ENSG00000151692	ENSG00000137393
ENSG00000151693	ENSG00000088280
ENSG00000151693	ENSG00000153317
ENSG00000151702	ENSG00000154727
ENSG00000151702	ENSG00000157554
ENSG00000151704	ENSG00000115474
ENSG00000151704	ENSG00000120457
ENSG00000151704	ENSG00000121361
ENSG00000151704	ENSG00000123700
ENSG00000151704	ENSG00000153822
ENSG00000151704	ENSG00000157542
ENSG00000151704	ENSG00000157551
ENSG00000151704	ENSG00000162728
ENSG00000151704	ENSG00000162989
ENSG00000151704	ENSG00000168135
ENSG00000151704	ENSG00000177807
ENSG00000151704	ENSG00000182324
ENSG00000151704	ENSG00000184185
ENSG00000151704	ENSG00000187486
ENSG00000151715	ENSG00000181458
ENSG00000151715	ENSG00000203730
ENSG00000151715	ENSG00000214154
ENSG00000151718	ENSG00000047644
ENSG00000151718	ENSG00000113645
ENSG00000151726	ENSG00000164398
ENSG00000151726	ENSG00000197142
ENSG00000151729	ENSG00000005022
ENSG00000151729	ENSG00000151475
ENSG00000151729	ENSG00000169100
ENSG00000151746	ENSG00000185963
ENSG00000151778	ENSG00000120742
ENSG00000151789	ENSG00000144331
ENSG00000151789	ENSG00000161642
ENSG00000151789	ENSG00000187595
ENSG00000151812	ENSG00000183780
ENSG00000151834	ENSG00000011677
ENSG00000151834	ENSG00000022355
ENSG00000151834	ENSG00000094755
ENSG00000151834	ENSG00000101958
ENSG00000151834	ENSG00000102287
ENSG00000151834	ENSG00000109158
ENSG00000151834	ENSG00000109738
ENSG00000151834	ENSG00000111886
ENSG00000151834	ENSG00000113327

ENSG00000151834	ENSG00000145451
ENSG00000151834	ENSG00000145863
ENSG00000151834	ENSG00000145864
ENSG00000151834	ENSG00000145888
ENSG00000151834	ENSG00000146276
ENSG00000151834	ENSG00000147402
ENSG00000151834	ENSG00000163285
ENSG00000151834	ENSG00000163288
ENSG00000151834	ENSG00000166206
ENSG00000151834	ENSG00000182256
ENSG00000151834	ENSG00000183185
ENSG00000151834	ENSG00000186297
ENSG00000151834	ENSG00000187730
ENSG00000151834	ENSG00000188828
ENSG00000151846	ENSG00000070756
ENSG00000151846	ENSG00000090621
ENSG00000151846	ENSG00000101104
ENSG00000151846	ENSG00000174740
ENSG00000151846	ENSG00000215587
ENSG00000151849	ENSG00000153468
ENSG00000151876	ENSG00000118564
ENSG00000151883	ENSG00000137817
ENSG00000151883	ENSG00000138617
ENSG00000151892	ENSG00000125861
ENSG00000151892	ENSG00000146013
ENSG00000151892	ENSG00000168546
ENSG00000151892	ENSG00000187871
ENSG00000151914	ENSG00000096696
ENSG00000151914	ENSG00000118898
ENSG00000151914	ENSG00000127603
ENSG00000151914	ENSG00000167880
ENSG00000151923	ENSG00000116001
ENSG00000151929	ENSG00000156735
ENSG00000151952	ENSG00000006118
ENSG00000151952	ENSG00000139364
ENSG00000151952	ENSG00000181234
ENSG00000151952	ENSG00000181291
ENSG00000152022	ENSG00000145721
ENSG00000152034	ENSG00000082556
ENSG00000152034	ENSG00000112038
ENSG00000152034	ENSG00000116014
ENSG00000152034	ENSG00000116329
ENSG00000152034	ENSG00000125510
ENSG00000152034	ENSG00000125522
ENSG00000152034	ENSG00000128285
ENSG00000152034	ENSG00000128310
ENSG00000152034	ENSG00000132671
ENSG00000152034	ENSG00000139874
ENSG00000152034	ENSG00000162009
ENSG00000152034	ENSG00000166573
ENSG00000152034	ENSG00000180616
ENSG00000152034	ENSG00000182687
ENSG00000152034	ENSG00000183473
ENSG00000152034	ENSG00000183729
ENSG00000152056	ENSG00000106367
ENSG00000152056	ENSG00000182287
ENSG00000152061	ENSG00000011454
ENSG00000152061	ENSG00000065882
ENSG00000152061	ENSG00000067208

ENSG00000152061	ENSG00000136111
ENSG00000152061	ENSG00000142459
ENSG00000152078	ENSG00000149926
ENSG00000152078	ENSG00000167695
ENSG00000152092	ENSG00000148219
ENSG00000152102	ENSG00000054965
ENSG00000152104	ENSG00000070159
ENSG00000152104	ENSG00000070778
ENSG00000152104	ENSG00000088179
ENSG00000152127	ENSG00000167889
ENSG00000152137	ENSG00000004776
ENSG00000152137	ENSG00000106211
ENSG00000152137	ENSG00000109846
ENSG00000152137	ENSG00000160202
ENSG00000152137	ENSG00000169271
ENSG00000152137	ENSG00000170276
ENSG00000152192	ENSG00000028277
ENSG00000152192	ENSG00000064835
ENSG00000152192	ENSG00000091010
ENSG00000152192	ENSG00000137709
ENSG00000152192	ENSG00000143190
ENSG00000152192	ENSG00000151615
ENSG00000152192	ENSG00000184486
ENSG00000152192	ENSG00000185668
ENSG00000152192	ENSG00000196767
ENSG00000152192	ENSG00000198914
ENSG00000152192	ENSG00000204531
ENSG00000152192	ENSG00000212993
ENSG00000152207	ENSG00000078589
ENSG00000152207	ENSG00000139679
ENSG00000152207	ENSG00000147145
ENSG00000152207	ENSG00000169403
ENSG00000152207	ENSG00000169508
ENSG00000152207	ENSG00000169860
ENSG00000152207	ENSG00000173198
ENSG00000152207	ENSG00000175591
ENSG00000152207	ENSG00000184574
ENSG00000152207	ENSG00000186912
ENSG00000152207	ENSG00000198829
ENSG00000152208	ENSG00000182771
ENSG00000152214	ENSG00000143622
ENSG00000152217	ENSG00000116539
ENSG00000152229	ENSG00000100266
ENSG00000152229	ENSG00000124507
ENSG00000152229	ENSG00000140368
ENSG00000152229	ENSG00000165912
ENSG00000152254	ENSG00000131482
ENSG00000152254	ENSG00000141349
ENSG00000152256	ENSG00000004799
ENSG00000152256	ENSG00000005882
ENSG00000152256	ENSG00000067992
ENSG00000152270	ENSG00000172572
ENSG00000152284	ENSG00000081059
ENSG00000152284	ENSG00000138795
ENSG00000152284	ENSG00000148737
ENSG00000152292	ENSG00000043462
ENSG00000152292	ENSG00000095585
ENSG00000152292	ENSG00000109684
ENSG00000152315	ENSG00000082482

ENSG00000152315	ENSG00000095981
ENSG00000152315	ENSG00000099337
ENSG00000152315	ENSG00000100433
ENSG00000152315	ENSG00000124249
ENSG00000152315	ENSG00000124780
ENSG00000152315	ENSG00000135750
ENSG00000152315	ENSG00000164626
ENSG00000152315	ENSG00000169427
ENSG00000152315	ENSG00000171303
ENSG00000152315	ENSG00000173338
ENSG00000152315	ENSG00000182450
ENSG00000152315	ENSG00000184261
ENSG00000152377	ENSG00000019582
ENSG00000152377	ENSG00000107742
ENSG00000152377	ENSG00000196104
ENSG00000152380	ENSG00000162391
ENSG00000152402	ENSG00000061918
ENSG00000152409	ENSG00000156232
ENSG00000152413	ENSG00000051128
ENSG00000152413	ENSG00000103942
ENSG00000152413	ENSG00000214396
ENSG00000152430	ENSG00000092345
ENSG00000152430	ENSG00000187191
ENSG00000152430	ENSG00000188120
ENSG00000152430	ENSG00000205916
ENSG00000152430	ENSG00000205944
ENSG00000152455	ENSG00000101945
ENSG00000152465	ENSG00000136448
ENSG00000152484	ENSG00000109189
ENSG00000152503	ENSG00000100505
ENSG00000152503	ENSG00000119283
ENSG00000152503	ENSG00000163462
ENSG00000152503	ENSG00000186628
ENSG00000152518	ENSG00000128016
ENSG00000152518	ENSG00000185650
ENSG00000152527	ENSG00000054690
ENSG00000152556	ENSG00000067057
ENSG00000152556	ENSG00000141959
ENSG00000152578	ENSG00000120251
ENSG00000152578	ENSG00000125675
ENSG00000152578	ENSG00000149403
ENSG00000152578	ENSG00000155511
ENSG00000152578	ENSG00000163873
ENSG00000152578	ENSG00000164418
ENSG00000152578	ENSG00000171189
ENSG00000152580	ENSG00000101825
ENSG00000152580	ENSG00000143341
ENSG00000152580	ENSG00000148357
ENSG00000152580	ENSG00000155657
ENSG00000152580	ENSG00000168462
ENSG00000152583	ENSG00000113140
ENSG00000152591	ENSG00000114857
ENSG00000152591	ENSG00000138398
ENSG00000152601	ENSG00000076770
ENSG00000152601	ENSG00000135482
ENSG00000152601	ENSG00000139793
ENSG00000152611	ENSG00000105519
ENSG00000152611	ENSG00000180881
ENSG00000152642	ENSG00000167588

ENSG00000152661	ENSG00000121634
ENSG00000152661	ENSG00000121742
ENSG00000152661	ENSG00000121743
ENSG00000152661	ENSG00000131233
ENSG00000152661	ENSG00000135355
ENSG00000152661	ENSG00000143140
ENSG00000152661	ENSG00000159248
ENSG00000152661	ENSG00000164411
ENSG00000152661	ENSG00000165474
ENSG00000152661	ENSG00000169562
ENSG00000152661	ENSG00000176402
ENSG00000152661	ENSG00000177291
ENSG00000152661	ENSG00000182963
ENSG00000152661	ENSG00000183153
ENSG00000152661	ENSG00000187513
ENSG00000152661	ENSG00000188910
ENSG00000152661	ENSG00000189280
ENSG00000152661	ENSG00000189433
ENSG00000152661	ENSG00000198835
ENSG00000152661	ENSG00000203733
ENSG00000152689	ENSG00000068831
ENSG00000152689	ENSG00000171777
ENSG00000152689	ENSG00000172575
ENSG00000152700	ENSG00000079332
ENSG00000152700	ENSG00000173386
ENSG00000152705	ENSG00000188782
ENSG00000152767	ENSG00000006607
ENSG00000152778	ENSG00000119917
ENSG00000152778	ENSG00000119922
ENSG00000152778	ENSG00000185745
ENSG00000152778	ENSG00000204010
ENSG00000152782	ENSG00000120137
ENSG00000152782	ENSG00000125779
ENSG00000152784	ENSG00000165388
ENSG00000152785	ENSG00000101144
ENSG00000152785	ENSG00000107623
ENSG00000152785	ENSG00000112175
ENSG00000152785	ENSG00000116985
ENSG00000152785	ENSG00000125965
ENSG00000152785	ENSG00000128802
ENSG00000152785	ENSG00000130385
ENSG00000152785	ENSG00000143869
ENSG00000152785	ENSG00000153162
ENSG00000152785	ENSG00000156466
ENSG00000152785	ENSG00000156574
ENSG00000152785	ENSG00000163217
ENSG00000152785	ENSG00000183682
ENSG00000152795	ENSG00000071626
ENSG00000152795	ENSG00000138668
ENSG00000152795	ENSG00000197451
ENSG00000152795	ENSG00000215173
ENSG00000152818	ENSG00000198947
ENSG00000152822	ENSG00000113262
ENSG00000152822	ENSG00000124493
ENSG00000152822	ENSG00000164082
ENSG00000152822	ENSG00000168959
ENSG00000152822	ENSG00000179603
ENSG00000152822	ENSG00000196277
ENSG00000152822	ENSG00000198822

ENSG00000152894	ENSG00000060656
ENSG00000152894	ENSG00000105426
ENSG00000152894	ENSG00000142949
ENSG00000152894	ENSG00000153707
ENSG00000152894	ENSG00000173482
ENSG00000152894	ENSG00000196090
ENSG00000152910	ENSG00000106714
ENSG00000152910	ENSG00000108797
ENSG00000152910	ENSG00000154529
ENSG00000152910	ENSG00000155052
ENSG00000152910	ENSG00000174469
ENSG00000152910	ENSG00000185020
ENSG00000152932	ENSG00000105514
ENSG00000152932	ENSG00000105649
ENSG00000152932	ENSG00000169213
ENSG00000152939	ENSG00000105656
ENSG00000152939	ENSG00000118985
ENSG00000152939	ENSG00000187213
ENSG00000152939	ENSG00000197822
ENSG00000152952	ENSG00000083444
ENSG00000152952	ENSG00000106397
ENSG00000152952	ENSG00000130309
ENSG00000152952	ENSG00000167123
ENSG00000152952	ENSG00000198756
ENSG00000152953	ENSG00000165752
ENSG00000152953	ENSG00000169302
ENSG00000152954	ENSG00000125841
ENSG00000152969	ENSG00000176049
ENSG00000152969	ENSG00000188385
ENSG00000152977	ENSG00000043355
ENSG00000152977	ENSG00000139800
ENSG00000152977	ENSG00000156925
ENSG00000152977	ENSG00000174963
ENSG00000152990	ENSG00000020181
ENSG00000152990	ENSG00000197177
ENSG00000153002	ENSG00000080618
ENSG00000153002	ENSG00000091704
ENSG00000153002	ENSG00000128510
ENSG00000153002	ENSG00000144410
ENSG00000153002	ENSG00000158516
ENSG00000153002	ENSG00000158525
ENSG00000153002	ENSG00000163751
ENSG00000153002	ENSG00000165078
ENSG00000153012	ENSG00000108231
ENSG00000153012	ENSG00000153902
ENSG00000153012	ENSG00000168481
ENSG00000153046	ENSG00000129873
ENSG00000153046	ENSG00000166446
ENSG00000153046	ENSG00000172288
ENSG00000153046	ENSG00000172352
ENSG00000153046	ENSG00000182415
ENSG00000153046	ENSG00000198721
ENSG00000153046	ENSG00000215563
ENSG00000153048	ENSG00000172346
ENSG00000153064	ENSG00000155629
ENSG00000153071	ENSG00000173406
ENSG00000153132	ENSG00000127022
ENSG00000153147	ENSG00000102038
ENSG00000153162	ENSG00000101144

ENSG00000153162	ENSG00000107623
ENSG00000153162	ENSG00000112175
ENSG00000153162	ENSG00000116985
ENSG00000153162	ENSG00000125965
ENSG00000153162	ENSG00000128802
ENSG00000153162	ENSG00000130385
ENSG00000153162	ENSG00000143869
ENSG00000153162	ENSG00000152785
ENSG00000153162	ENSG00000156466
ENSG00000153162	ENSG00000156574
ENSG00000153162	ENSG00000163217
ENSG00000153162	ENSG00000183682
ENSG00000153179	ENSG00000068028
ENSG00000153179	ENSG00000101265
ENSG00000153179	ENSG00000107551
ENSG00000153179	ENSG00000136653
ENSG00000153179	ENSG00000169435
ENSG00000153187	ENSG00000105323
ENSG00000153187	ENSG00000214753
ENSG00000153208	ENSG00000092445
ENSG00000153208	ENSG00000105976
ENSG00000153208	ENSG00000163785
ENSG00000153208	ENSG00000164078
ENSG00000153208	ENSG00000167601
ENSG00000153208	ENSG00000214672
ENSG00000153214	ENSG00000103978
ENSG00000153233	ENSG00000110786
ENSG00000153233	ENSG00000143851
ENSG00000153234	ENSG00000119508
ENSG00000153234	ENSG00000123358
ENSG00000153246	ENSG00000011028
ENSG00000153246	ENSG00000054219
ENSG00000153246	ENSG00000120586
ENSG00000153246	ENSG00000183748
ENSG00000153250	ENSG00000076067
ENSG00000153250	ENSG00000144642
ENSG00000153253	ENSG00000007314
ENSG00000153253	ENSG00000136531
ENSG00000153253	ENSG00000136546
ENSG00000153253	ENSG00000144285
ENSG00000153253	ENSG00000168356
ENSG00000153253	ENSG00000169432
ENSG00000153253	ENSG00000183873
ENSG00000153253	ENSG00000185313
ENSG00000153253	ENSG00000196876
ENSG00000153266	ENSG00000128610
ENSG00000153292	ENSG00000069122
ENSG00000153292	ENSG00000123146
ENSG00000153292	ENSG00000127507
ENSG00000153292	ENSG00000131355
ENSG00000153292	ENSG00000153294
ENSG00000153292	ENSG00000162618
ENSG00000153292	ENSG00000164393
ENSG00000153292	ENSG00000173567
ENSG00000153292	ENSG00000174837
ENSG00000153292	ENSG00000186629
ENSG00000153292	ENSG00000204818
ENSG00000153294	ENSG00000069122
ENSG00000153294	ENSG00000123146

ENSG00000153294	ENSG00000127507
ENSG00000153294	ENSG00000131355
ENSG00000153294	ENSG00000153292
ENSG00000153294	ENSG00000162618
ENSG00000153294	ENSG00000164393
ENSG00000153294	ENSG00000173567
ENSG00000153294	ENSG00000174837
ENSG00000153294	ENSG00000186629
ENSG00000153294	ENSG00000204818
ENSG00000153303	ENSG00000095203
ENSG00000153303	ENSG00000115109
ENSG00000153303	ENSG00000129595
ENSG00000153310	ENSG00000197872
ENSG00000153317	ENSG00000088280
ENSG00000153317	ENSG00000151693
ENSG00000153347	ENSG00000157470
ENSG00000153404	ENSG00000149633
ENSG00000153404	ENSG00000165801
ENSG00000153404	ENSG00000196155
ENSG00000153443	ENSG00000185262
ENSG00000153468	ENSG00000151849
ENSG00000153487	ENSG00000111653
ENSG00000153487	ENSG00000168395
ENSG00000153487	ENSG00000168556
ENSG00000153487	ENSG00000215693
ENSG00000153531	ENSG00000144843
ENSG00000153560	ENSG00000083307
ENSG00000153560	ENSG00000115112
ENSG00000153560	ENSG00000134317
ENSG00000153560	ENSG00000135457
ENSG00000153560	ENSG00000158055
ENSG00000153560	ENSG00000214547
ENSG00000153561	ENSG00000145916
ENSG00000153666	ENSG00000140478
ENSG00000153666	ENSG00000156363
ENSG00000153666	ENSG00000159289
ENSG00000153666	ENSG00000167110
ENSG00000153666	ENSG00000167195
ENSG00000153666	ENSG00000174450
ENSG00000153666	ENSG00000175265
ENSG00000153666	ENSG00000181984
ENSG00000153666	ENSG00000184206
ENSG00000153666	ENSG00000186322
ENSG00000153666	ENSG00000188388
ENSG00000153666	ENSG00000188532
ENSG00000153666	ENSG00000188626
ENSG00000153666	ENSG00000197092
ENSG00000153666	ENSG00000197414
ENSG00000153666	ENSG00000214348
ENSG00000153666	ENSG00000215252
ENSG00000153666	ENSG00000215405
ENSG00000153707	ENSG00000060656
ENSG00000153707	ENSG00000105426
ENSG00000153707	ENSG00000142949
ENSG00000153707	ENSG00000152894
ENSG00000153707	ENSG00000173482
ENSG00000153707	ENSG00000196090
ENSG00000153714	ENSG00000171357
ENSG00000153721	ENSG00000074706

ENSG00000153721	ENSG00000142675
ENSG00000153721	ENSG00000149970
ENSG00000153779	ENSG00000105419
ENSG00000153779	ENSG00000118707
ENSG00000153779	ENSG00000134138
ENSG00000153779	ENSG00000143995
ENSG00000153779	ENSG00000160199
ENSG00000153779	ENSG00000165495
ENSG00000153779	ENSG00000176679
ENSG00000153779	ENSG00000177426
ENSG00000153779	ENSG00000179277
ENSG00000153779	ENSG00000188013
ENSG00000153786	ENSG00000163812
ENSG00000153786	ENSG00000175893
ENSG00000153789	ENSG00000157021
ENSG00000153789	ENSG00000188343
ENSG00000153807	ENSG00000078399
ENSG00000153807	ENSG00000128709
ENSG00000153807	ENSG00000128710
ENSG00000153807	ENSG00000170689
ENSG00000153807	ENSG00000180806
ENSG00000153807	ENSG00000180818
ENSG00000153822	ENSG00000115474
ENSG00000153822	ENSG00000120457
ENSG00000153822	ENSG00000121361
ENSG00000153822	ENSG00000123700
ENSG00000153822	ENSG00000151704
ENSG00000153822	ENSG00000157542
ENSG00000153822	ENSG00000157551
ENSG00000153822	ENSG00000162728
ENSG00000153822	ENSG00000162989
ENSG00000153822	ENSG00000168135
ENSG00000153822	ENSG00000177807
ENSG00000153822	ENSG00000182324
ENSG00000153822	ENSG00000184185
ENSG00000153822	ENSG00000187486
ENSG00000153885	ENSG00000134504
ENSG00000153885	ENSG00000151364
ENSG00000153885	ENSG00000155729
ENSG00000153885	ENSG00000168301
ENSG00000153885	ENSG00000168676
ENSG00000153885	ENSG00000178695
ENSG00000153885	ENSG00000180332
ENSG00000153885	ENSG00000183775
ENSG00000153885	ENSG00000183783
ENSG00000153885	ENSG00000188997
ENSG00000153885	ENSG00000198553
ENSG00000153885	ENSG00000213859
ENSG00000153898	ENSG00000055732
ENSG00000153898	ENSG00000090674
ENSG00000153902	ENSG00000108231
ENSG00000153902	ENSG00000153012
ENSG00000153902	ENSG00000168481
ENSG00000153904	ENSG00000213722
ENSG00000153914	ENSG00000116754
ENSG00000153922	ENSG00000173575
ENSG00000153944	ENSG00000135097
ENSG00000153956	ENSG00000007402
ENSG00000153956	ENSG00000151062

ENSG00000153956	ENSG00000157445
ENSG00000153956	ENSG00000158966
ENSG00000153976	ENSG00000002587
ENSG00000153976	ENSG00000122254
ENSG00000153976	ENSG00000125430
ENSG00000153976	ENSG00000162040
ENSG00000153976	ENSG00000175818
ENSG00000153976	ENSG00000182601
ENSG00000153982	ENSG00000102886
ENSG00000153993	ENSG00000010319
ENSG00000153993	ENSG00000012171
ENSG00000153993	ENSG00000075213
ENSG00000153993	ENSG00000092421
ENSG00000153993	ENSG00000095539
ENSG00000153993	ENSG00000137872
ENSG00000153993	ENSG00000143434
ENSG00000153993	ENSG00000168758
ENSG00000153993	ENSG00000170381
ENSG00000153993	ENSG00000185033
ENSG00000153993	ENSG00000187764
ENSG00000153993	ENSG00000196189
ENSG00000154001	ENSG00000066027
ENSG00000154001	ENSG00000068971
ENSG00000154001	ENSG00000078304
ENSG00000154001	ENSG00000112640
ENSG00000154016	ENSG00000100351
ENSG00000154016	ENSG00000101082
ENSG00000154016	ENSG00000155926
ENSG00000154016	ENSG00000177885
ENSG00000154025	ENSG00000100170
ENSG00000154025	ENSG00000100191
ENSG00000154025	ENSG00000117834
ENSG00000154025	ENSG00000140675
ENSG00000154025	ENSG00000158865
ENSG00000154025	ENSG00000198743
ENSG00000154027	ENSG00000106992
ENSG00000154027	ENSG00000214347
ENSG00000154040	ENSG00000064199
ENSG00000154080	ENSG00000115526
ENSG00000154080	ENSG00000124302
ENSG00000154080	ENSG00000136213
ENSG00000154080	ENSG00000169105
ENSG00000154080	ENSG00000171310
ENSG00000154080	ENSG00000180767
ENSG00000154118	ENSG00000092051
ENSG00000154118	ENSG00000104369
ENSG00000154118	ENSG00000139714
ENSG00000154118	ENSG00000149596
ENSG00000154127	ENSG00000160185
ENSG00000154133	ENSG00000154134
ENSG00000154133	ENSG00000169855
ENSG00000154133	ENSG00000185008
ENSG00000154134	ENSG00000154133
ENSG00000154134	ENSG00000169855
ENSG00000154134	ENSG00000185008
ENSG00000154143	ENSG00000073150
ENSG00000154143	ENSG00000110218
ENSG00000154153	ENSG00000141699
ENSG00000154153	ENSG00000144567

ENSG00000154162	ENSG00000040731
ENSG00000154162	ENSG00000071991
ENSG00000154162	ENSG00000081138
ENSG00000154162	ENSG00000101542
ENSG00000154162	ENSG00000113100
ENSG00000154162	ENSG00000113361
ENSG00000154162	ENSG00000139880
ENSG00000154162	ENSG00000140937
ENSG00000154162	ENSG00000145526
ENSG00000154162	ENSG00000149654
ENSG00000154162	ENSG00000150394
ENSG00000154162	ENSG00000179776
ENSG00000154165	ENSG00000100739
ENSG00000154165	ENSG00000134817
ENSG00000154165	ENSG00000144891
ENSG00000154165	ENSG00000168398
ENSG00000154165	ENSG00000170128
ENSG00000154165	ENSG00000173080
ENSG00000154165	ENSG00000180772
ENSG00000154165	ENSG00000182631
ENSG00000154198	ENSG00000142973
ENSG00000154198	ENSG00000145476
ENSG00000154198	ENSG00000162365
ENSG00000154198	ENSG00000171903
ENSG00000154198	ENSG00000171954
ENSG00000154198	ENSG00000186115
ENSG00000154198	ENSG00000186160
ENSG00000154198	ENSG00000186204
ENSG00000154198	ENSG00000186377
ENSG00000154198	ENSG00000186526
ENSG00000154198	ENSG00000186529
ENSG00000154198	ENSG00000187048
ENSG00000154217	ENSG00000174238
ENSG00000154217	ENSG00000180957
ENSG00000154222	ENSG00000132024
ENSG00000154227	ENSG00000065308
ENSG00000154227	ENSG00000067167
ENSG00000154227	ENSG00000090661
ENSG00000154227	ENSG00000139624
ENSG00000154227	ENSG00000143418
ENSG00000154227	ENSG00000172292
ENSG00000154227	ENSG00000174599
ENSG00000154229	ENSG00000027075
ENSG00000154229	ENSG00000065675
ENSG00000154229	ENSG00000067606
ENSG00000154229	ENSG00000105221
ENSG00000154229	ENSG00000117020
ENSG00000154229	ENSG00000126583
ENSG00000154229	ENSG00000142208
ENSG00000154229	ENSG00000163558
ENSG00000154229	ENSG00000163932
ENSG00000154229	ENSG00000166501
ENSG00000154237	ENSG00000188906
ENSG00000154252	ENSG00000128242
ENSG00000154252	ENSG00000175229
ENSG00000154252	ENSG00000197093
ENSG00000154269	ENSG00000001561
ENSG00000154269	ENSG00000112796
ENSG00000154269	ENSG00000136960

ENSG00000154269	ENSG00000164303
ENSG00000154269	ENSG00000182156
ENSG00000154269	ENSG00000197594
ENSG00000154277	ENSG00000118939
ENSG00000154305	ENSG00000150527
ENSG00000154309	ENSG00000140323
ENSG00000154310	ENSG00000071054
ENSG00000154310	ENSG00000123572
ENSG00000154310	ENSG00000141503
ENSG00000154319	ENSG00000183615
ENSG00000154330	ENSG00000079739
ENSG00000154330	ENSG00000204794
ENSG00000154342	ENSG00000002745
ENSG00000154342	ENSG00000061492
ENSG00000154342	ENSG00000075290
ENSG00000154342	ENSG00000085741
ENSG00000154342	ENSG00000105989
ENSG00000154342	ENSG00000108379
ENSG00000154342	ENSG00000111186
ENSG00000154342	ENSG00000114251
ENSG00000154342	ENSG00000115596
ENSG00000154342	ENSG00000125084
ENSG00000154342	ENSG00000134245
ENSG00000154342	ENSG00000135925
ENSG00000154342	ENSG00000143816
ENSG00000154342	ENSG00000154764
ENSG00000154342	ENSG00000158955
ENSG00000154342	ENSG00000162552
ENSG00000154342	ENSG00000169884
ENSG00000154342	ENSG00000188064
ENSG00000154358	ENSG00000072195
ENSG00000154359	ENSG00000170500
ENSG00000154359	ENSG00000175556
ENSG00000154380	ENSG00000125753
ENSG00000154380	ENSG00000166068
ENSG00000154380	ENSG00000188766
ENSG00000154380	ENSG00000196405
ENSG00000154380	ENSG00000198369
ENSG00000154415	ENSG00000100829
ENSG00000154415	ENSG00000119938
ENSG00000154415	ENSG00000132825
ENSG00000154415	ENSG00000173281
ENSG00000154447	ENSG00000156463
ENSG00000154447	ENSG00000172985
ENSG00000154451	ENSG00000117226
ENSG00000154451	ENSG00000117228
ENSG00000154451	ENSG00000162645
ENSG00000154451	ENSG00000162654
ENSG00000154451	ENSG00000183347
ENSG00000154451	ENSG00000213512
ENSG00000154474	ENSG00000122550
ENSG00000154474	ENSG00000157119
ENSG00000154478	ENSG00000155269
ENSG00000154511	ENSG00000165716
ENSG00000154511	ENSG00000181744
ENSG00000154511	ENSG00000187773
ENSG00000154518	ENSG00000135390
ENSG00000154518	ENSG00000159199
ENSG00000154529	ENSG00000106714

ENSG00000154529	ENSG00000108797
ENSG00000154529	ENSG00000152910
ENSG00000154529	ENSG00000155052
ENSG00000154529	ENSG00000174469
ENSG00000154529	ENSG00000185020
ENSG00000154553	ENSG00000107438
ENSG00000154553	ENSG00000120913
ENSG00000154553	ENSG00000122367
ENSG00000154553	ENSG00000131435
ENSG00000154553	ENSG00000163110
ENSG00000154553	ENSG00000196923
ENSG00000154553	ENSG00000214895
ENSG00000154556	ENSG00000095637
ENSG00000154556	ENSG00000120896
ENSG00000154582	ENSG00000105694
ENSG00000154639	ENSG00000019102
ENSG00000154639	ENSG00000143167
ENSG00000154639	ENSG00000144847
ENSG00000154639	ENSG00000149564
ENSG00000154639	ENSG00000166250
ENSG00000154639	ENSG00000188004
ENSG00000154640	ENSG00000133639
ENSG00000154640	ENSG00000137707
ENSG00000154640	ENSG00000159388
ENSG00000154645	ENSG00000204381
ENSG00000154654	ENSG00000149294
ENSG00000154655	ENSG00000011258
ENSG00000154655	ENSG00000100395
ENSG00000154655	ENSG00000163935
ENSG00000154655	ENSG00000185513
ENSG00000154655	ENSG00000198879
ENSG00000154655	ENSG00000198945
ENSG00000154678	ENSG00000065989
ENSG00000154678	ENSG00000073417
ENSG00000154678	ENSG00000105650
ENSG00000154678	ENSG00000113231
ENSG00000154678	ENSG00000113448
ENSG00000154678	ENSG00000115252
ENSG00000154678	ENSG00000123360
ENSG00000154678	ENSG00000160191
ENSG00000154678	ENSG00000171408
ENSG00000154678	ENSG00000184588
ENSG00000154678	ENSG00000205268
ENSG00000154721	ENSG00000166086
ENSG00000154732	ENSG00000136206
ENSG00000154732	ENSG00000163806
ENSG00000154732	ENSG00000166667
ENSG00000154732	ENSG00000170092
ENSG00000154732	ENSG00000173678
ENSG00000154732	ENSG00000179994
ENSG00000154732	ENSG00000183318
ENSG00000154732	ENSG00000184616
ENSG00000154732	ENSG00000185040
ENSG00000154732	ENSG00000186645
ENSG00000154732	ENSG00000198305
ENSG00000154732	ENSG00000204710
ENSG00000154732	ENSG00000205238
ENSG00000154732	ENSG00000205482
ENSG00000154732	ENSG00000205505

ENSG00000154732	ENSG00000205506
ENSG00000154732	ENSG00000205508
ENSG00000154732	ENSG00000205509
ENSG00000154732	ENSG00000205580
ENSG00000154732	ENSG00000214300
ENSG00000154732	ENSG00000215092
ENSG00000154734	ENSG00000134917
ENSG00000154734	ENSG00000154736
ENSG00000154734	ENSG00000158859
ENSG00000154734	ENSG00000166106
ENSG00000154736	ENSG00000134917
ENSG00000154736	ENSG00000154734
ENSG00000154736	ENSG00000158859
ENSG00000154736	ENSG00000166106
ENSG00000154764	ENSG00000002745
ENSG00000154764	ENSG00000061492
ENSG00000154764	ENSG00000075290
ENSG00000154764	ENSG00000085741
ENSG00000154764	ENSG00000105989
ENSG00000154764	ENSG00000108379
ENSG00000154764	ENSG00000111186
ENSG00000154764	ENSG00000114251
ENSG00000154764	ENSG00000115596
ENSG00000154764	ENSG00000125084
ENSG00000154764	ENSG00000134245
ENSG00000154764	ENSG00000135925
ENSG00000154764	ENSG00000143816
ENSG00000154764	ENSG00000154342
ENSG00000154764	ENSG00000158955
ENSG00000154764	ENSG00000162552
ENSG00000154764	ENSG00000169884
ENSG00000154764	ENSG00000188064
ENSG00000154783	ENSG00000102302
ENSG00000154783	ENSG00000180263
ENSG00000154822	ENSG00000101333
ENSG00000154822	ENSG00000114805
ENSG00000154822	ENSG00000115556
ENSG00000154822	ENSG00000115896
ENSG00000154822	ENSG00000124181
ENSG00000154822	ENSG00000137841
ENSG00000154822	ENSG00000139151
ENSG00000154822	ENSG00000149527
ENSG00000154822	ENSG00000149782
ENSG00000154822	ENSG00000161714
ENSG00000154822	ENSG00000182621
ENSG00000154822	ENSG00000187091
ENSG00000154822	ENSG00000197943
ENSG00000154845	ENSG00000124224
ENSG00000154845	ENSG00000215443
ENSG00000154856	ENSG00000198768
ENSG00000154864	ENSG00000103335
ENSG00000154864	ENSG00000175388
ENSG00000154914	ENSG00000103404
ENSG00000154917	ENSG00000147127
ENSG00000154917	ENSG00000175582
ENSG00000154920	ENSG00000197774
ENSG00000154928	ENSG00000044524
ENSG00000154928	ENSG00000070886
ENSG00000154928	ENSG00000080224

ENSG00000154928	ENSG00000106123
ENSG00000154928	ENSG00000116106
ENSG00000154928	ENSG00000133216
ENSG00000154928	ENSG00000135333
ENSG00000154928	ENSG00000142627
ENSG00000154928	ENSG00000145242
ENSG00000154928	ENSG00000146904
ENSG00000154928	ENSG00000182580
ENSG00000154928	ENSG00000183317
ENSG00000154928	ENSG00000196411
ENSG00000154975	ENSG00000063180
ENSG00000154997	ENSG00000125354
ENSG00000154997	ENSG00000138758
ENSG00000154997	ENSG00000164402
ENSG00000154997	ENSG00000186522
ENSG00000155008	ENSG00000184831
ENSG00000155011	ENSG00000104371
ENSG00000155011	ENSG00000107984
ENSG00000155052	ENSG00000106714
ENSG00000155052	ENSG00000108797
ENSG00000155052	ENSG00000152910
ENSG00000155052	ENSG00000154529
ENSG00000155052	ENSG00000174469
ENSG00000155052	ENSG00000185020
ENSG00000155066	ENSG00000007062
ENSG00000155070	ENSG00000110057
ENSG00000155070	ENSG00000112494
ENSG00000155070	ENSG00000172971
ENSG00000155070	ENSG00000184795
ENSG00000155085	ENSG00000197453
ENSG00000155090	ENSG00000067082
ENSG00000155090	ENSG00000102554
ENSG00000155090	ENSG00000105610
ENSG00000155090	ENSG00000127528
ENSG00000155090	ENSG00000129911
ENSG00000155090	ENSG00000136826
ENSG00000155090	ENSG00000139651
ENSG00000155090	ENSG00000164651
ENSG00000155090	ENSG00000167182
ENSG00000155090	ENSG00000170374
ENSG00000155090	ENSG00000171872
ENSG00000155090	ENSG00000172059
ENSG00000155090	ENSG00000174595
ENSG00000155090	ENSG00000189120
ENSG00000155090	ENSG00000197294
ENSG00000155090	ENSG00000197550
ENSG00000155090	ENSG00000204335
ENSG00000155093	ENSG00000054356
ENSG00000155096	ENSG00000115758
ENSG00000155096	ENSG00000142920
ENSG00000155097	ENSG00000143882
ENSG00000155099	ENSG00000165782
ENSG00000155111	ENSG00000132964
ENSG00000155130	ENSG00000175130
ENSG00000155158	ENSG00000085831
ENSG00000155158	ENSG00000168234
ENSG00000155254	ENSG00000102934
ENSG00000155254	ENSG00000144063
ENSG00000155254	ENSG00000147676

ENSG00000155254	ENSG00000170293
ENSG00000155254	ENSG00000172005
ENSG00000155265	ENSG00000147533
ENSG00000155265	ENSG00000205596
ENSG00000155269	ENSG00000154478
ENSG00000155287	ENSG00000147454
ENSG00000155307	ENSG00000111961
ENSG00000155307	ENSG00000122122
ENSG00000155313	ENSG00000048028
ENSG00000155366	ENSG00000067560
ENSG00000155366	ENSG00000143878
ENSG00000155367	ENSG00000111110
ENSG00000155367	ENSG00000164088
ENSG00000155368	ENSG00000176244
ENSG00000155380	ENSG00000100156
ENSG00000155380	ENSG00000108932
ENSG00000155380	ENSG00000112394
ENSG00000155380	ENSG00000118596
ENSG00000155380	ENSG00000141526
ENSG00000155380	ENSG00000147100
ENSG00000155380	ENSG00000152779
ENSG00000155380	ENSG00000163053
ENSG00000155380	ENSG00000165449
ENSG00000155380	ENSG00000168679
ENSG00000155380	ENSG00000170190
ENSG00000155380	ENSG00000174326
ENSG00000155380	ENSG00000174327
ENSG00000155428	ENSG00000116525
ENSG00000155428	ENSG00000146755
ENSG00000155428	ENSG00000177238
ENSG00000155428	ENSG00000178809
ENSG00000155428	ENSG00000185880
ENSG00000155465	ENSG00000021488
ENSG00000155465	ENSG00000092068
ENSG00000155465	ENSG00000103064
ENSG00000155465	ENSG00000103257
ENSG00000155465	ENSG00000130876
ENSG00000155465	ENSG00000151012
ENSG00000155465	ENSG00000164893
ENSG00000155506	ENSG00000138709
ENSG00000155508	ENSG00000198791
ENSG00000155511	ENSG00000120251
ENSG00000155511	ENSG00000125675
ENSG00000155511	ENSG00000149403
ENSG00000155511	ENSG00000152578
ENSG00000155511	ENSG00000163873
ENSG00000155511	ENSG00000164418
ENSG00000155511	ENSG00000171189
ENSG00000155545	ENSG00000105556
ENSG00000155545	ENSG00000198160
ENSG00000155629	ENSG00000153064
ENSG00000155657	ENSG00000101825
ENSG00000155657	ENSG00000143341
ENSG00000155657	ENSG00000148357
ENSG00000155657	ENSG00000152580
ENSG00000155657	ENSG00000168462
ENSG00000155744	ENSG00000122591
ENSG00000155760	ENSG00000104290
ENSG00000155760	ENSG00000106483

ENSG00000155760	ENSG00000111432
ENSG00000155760	ENSG00000157240
ENSG00000155760	ENSG00000162998
ENSG00000155760	ENSG00000163251
ENSG00000155760	ENSG00000164930
ENSG00000155760	ENSG00000174804
ENSG00000155760	ENSG00000177283
ENSG00000155760	ENSG00000180340
ENSG00000155760	ENSG00000188763
ENSG00000155792	ENSG00000163328
ENSG00000155816	ENSG00000186031
ENSG00000155827	ENSG00000103549
ENSG00000155846	ENSG00000109819
ENSG00000155846	ENSG00000148840
ENSG00000155849	ENSG00000062598
ENSG00000155849	ENSG00000102890
ENSG00000155850	ENSG00000091137
ENSG00000155850	ENSG00000091138
ENSG00000155850	ENSG00000112053
ENSG00000155850	ENSG00000135502
ENSG00000155850	ENSG00000145217
ENSG00000155850	ENSG00000147606
ENSG00000155850	ENSG00000170615
ENSG00000155850	ENSG00000174502
ENSG00000155875	ENSG00000188659
ENSG00000155875	ENSG00000214545
ENSG00000155886	ENSG00000074621
ENSG00000155886	ENSG00000140090
ENSG00000155886	ENSG00000185052
ENSG00000155886	ENSG00000188467
ENSG00000155897	ENSG00000078295
ENSG00000155897	ENSG00000121281
ENSG00000155897	ENSG00000129467
ENSG00000155897	ENSG00000138031
ENSG00000155897	ENSG00000162104
ENSG00000155897	ENSG00000164742
ENSG00000155897	ENSG00000173175
ENSG00000155897	ENSG00000174233
ENSG00000155903	ENSG00000105808
ENSG00000155903	ENSG00000111344
ENSG00000155903	ENSG00000170667
ENSG00000155903	ENSG00000185989
ENSG00000155926	ENSG00000100351
ENSG00000155926	ENSG00000101082
ENSG00000155926	ENSG00000154016
ENSG00000155926	ENSG00000177885
ENSG00000155961	ENSG00000104388
ENSG00000155961	ENSG00000119396
ENSG00000155961	ENSG00000129472
ENSG00000155961	ENSG00000167578
ENSG00000155961	ENSG00000168118
ENSG00000155961	ENSG00000179331
ENSG00000155961	ENSG00000183940
ENSG00000155961	ENSG00000188060
ENSG00000155962	ENSG00000112782
ENSG00000155962	ENSG00000159212
ENSG00000155962	ENSG00000169504
ENSG00000155962	ENSG00000169583
ENSG00000155962	ENSG00000213719

ENSG00000155962	ENSG00000214258
ENSG00000155966	ENSG00000072364
ENSG00000155966	ENSG00000144218
ENSG00000155966	ENSG00000172493
ENSG00000155970	ENSG00000165487
ENSG00000155974	ENSG00000144596
ENSG00000155980	ENSG00000168280
ENSG00000155980	ENSG00000170759
ENSG00000156006	ENSG00000171428
ENSG00000156011	ENSG00000059915
ENSG00000156011	ENSG00000125637
ENSG00000156011	ENSG00000146005
ENSG00000156030	ENSG00000118156
ENSG00000156030	ENSG00000124496
ENSG00000156035	ENSG00000143363
ENSG00000156049	ENSG00000060558
ENSG00000156049	ENSG00000065135
ENSG00000156049	ENSG00000087258
ENSG00000156049	ENSG00000087460
ENSG00000156049	ENSG00000088256
ENSG00000156049	ENSG00000114349
ENSG00000156049	ENSG00000114353
ENSG00000156049	ENSG00000120063
ENSG00000156049	ENSG00000127955
ENSG00000156049	ENSG00000128266
ENSG00000156049	ENSG00000134183
ENSG00000156049	ENSG00000141404
ENSG00000156049	ENSG00000146535
ENSG00000156049	ENSG00000156052
ENSG00000156049	ENSG00000214415
ENSG00000156049	ENSG00000214622
ENSG00000156050	ENSG00000170264
ENSG00000156050	ENSG00000175513
ENSG00000156052	ENSG00000060558
ENSG00000156052	ENSG00000065135
ENSG00000156052	ENSG00000087258
ENSG00000156052	ENSG00000087460
ENSG00000156052	ENSG00000088256
ENSG00000156052	ENSG00000114349
ENSG00000156052	ENSG00000114353
ENSG00000156052	ENSG00000120063
ENSG00000156052	ENSG00000127955
ENSG00000156052	ENSG00000128266
ENSG00000156052	ENSG00000134183
ENSG00000156052	ENSG00000141404
ENSG00000156052	ENSG00000146535
ENSG00000156052	ENSG00000156049
ENSG00000156052	ENSG00000214415
ENSG00000156052	ENSG00000214622
ENSG00000156096	ENSG00000109181
ENSG00000156096	ENSG00000135220
ENSG00000156096	ENSG00000135226
ENSG00000156096	ENSG00000145626
ENSG00000156096	ENSG00000167165
ENSG00000156096	ENSG00000168671
ENSG00000156096	ENSG00000171234
ENSG00000156096	ENSG00000173610
ENSG00000156096	ENSG00000174607
ENSG00000156096	ENSG00000196620

ENSG00000156096	ENSG00000197592
ENSG00000156096	ENSG00000197888
ENSG00000156096	ENSG00000198277
ENSG00000156096	ENSG00000213759
ENSG00000156097	ENSG00000180929
ENSG00000156103	ENSG00000008516
ENSG00000156103	ENSG00000087245
ENSG00000156103	ENSG00000099953
ENSG00000156103	ENSG00000100985
ENSG00000156103	ENSG00000102996
ENSG00000156103	ENSG00000110347
ENSG00000156103	ENSG00000118113
ENSG00000156103	ENSG00000123342
ENSG00000156103	ENSG00000125966
ENSG00000156103	ENSG00000129270
ENSG00000156103	ENSG00000137673
ENSG00000156103	ENSG00000137674
ENSG00000156103	ENSG00000137675
ENSG00000156103	ENSG00000137745
ENSG00000156103	ENSG00000149968
ENSG00000156103	ENSG00000154485
ENSG00000156103	ENSG00000157227
ENSG00000156103	ENSG00000166670
ENSG00000156103	ENSG00000167346
ENSG00000156103	ENSG00000189409
ENSG00000156103	ENSG00000196611
ENSG00000156103	ENSG00000198598
ENSG00000156103	ENSG00000215914
ENSG00000156113	ENSG00000107147
ENSG00000156113	ENSG00000162687
ENSG00000156113	ENSG00000215262
ENSG00000156127	ENSG00000123685
ENSG00000156127	ENSG00000168062
ENSG00000156136	ENSG00000114956
ENSG00000156136	ENSG00000166548
ENSG00000156140	ENSG00000049192
ENSG00000156140	ENSG00000087116
ENSG00000156140	ENSG00000136378
ENSG00000156140	ENSG00000138316
ENSG00000156140	ENSG00000140470
ENSG00000156140	ENSG00000140873
ENSG00000156140	ENSG00000142303
ENSG00000156140	ENSG00000145536
ENSG00000156140	ENSG00000145808
ENSG00000156140	ENSG00000151388
ENSG00000156140	ENSG00000160323
ENSG00000156140	ENSG00000163638
ENSG00000156140	ENSG00000173157
ENSG00000156140	ENSG00000214644
ENSG00000156140	ENSG00000215743
ENSG00000156150	ENSG00000004848
ENSG00000156150	ENSG00000052850
ENSG00000156150	ENSG00000100987
ENSG00000156150	ENSG00000119614
ENSG00000156150	ENSG00000180318
ENSG00000156162	ENSG00000170629
ENSG00000156162	ENSG00000173852
ENSG00000156162	ENSG00000177990
ENSG00000156162	ENSG00000178904

ENSG00000156194	ENSG00000086717
ENSG00000156218	ENSG00000178031
ENSG00000156222	ENSG00000137860
ENSG00000156222	ENSG00000197506
ENSG00000156232	ENSG00000152409
ENSG00000156256	ENSG00000123552
ENSG00000156273	ENSG00000082641
ENSG00000156273	ENSG00000112182
ENSG00000156273	ENSG00000116044
ENSG00000156273	ENSG00000123405
ENSG00000156282	ENSG00000013297
ENSG00000156282	ENSG00000066405
ENSG00000156282	ENSG00000106404
ENSG00000156282	ENSG00000113946
ENSG00000156282	ENSG00000134873
ENSG00000156282	ENSG00000156284
ENSG00000156282	ENSG00000159261
ENSG00000156282	ENSG00000163347
ENSG00000156282	ENSG00000164007
ENSG00000156282	ENSG00000165215
ENSG00000156282	ENSG00000165376
ENSG00000156282	ENSG00000171217
ENSG00000156282	ENSG00000177300
ENSG00000156282	ENSG00000181885
ENSG00000156282	ENSG00000184113
ENSG00000156282	ENSG00000184697
ENSG00000156282	ENSG00000185758
ENSG00000156282	ENSG00000189118
ENSG00000156282	ENSG00000189143
ENSG00000156282	ENSG00000213937
ENSG00000156284	ENSG00000013297
ENSG00000156284	ENSG00000066405
ENSG00000156284	ENSG00000106404
ENSG00000156284	ENSG00000113946
ENSG00000156284	ENSG00000134873
ENSG00000156284	ENSG00000156282
ENSG00000156284	ENSG00000159261
ENSG00000156284	ENSG00000163347
ENSG00000156284	ENSG00000164007
ENSG00000156284	ENSG00000165215
ENSG00000156284	ENSG00000165376
ENSG00000156284	ENSG00000171217
ENSG00000156284	ENSG00000177300
ENSG00000156284	ENSG00000181885
ENSG00000156284	ENSG00000184113
ENSG00000156284	ENSG00000184697
ENSG00000156284	ENSG00000185758
ENSG00000156284	ENSG00000189118
ENSG00000156284	ENSG00000189143
ENSG00000156284	ENSG00000213937
ENSG00000156298	ENSG00000106025
ENSG00000156298	ENSG00000130167
ENSG00000156298	ENSG00000135404
ENSG00000156298	ENSG00000140391
ENSG00000156299	ENSG00000146426
ENSG00000156304	ENSG00000213079
ENSG00000156363	ENSG00000140478
ENSG00000156363	ENSG00000153666
ENSG00000156363	ENSG00000159289

ENSG00000156363	ENSG00000167110
ENSG00000156363	ENSG00000167195
ENSG00000156363	ENSG00000174450
ENSG00000156363	ENSG00000175265
ENSG00000156363	ENSG00000181984
ENSG00000156363	ENSG00000184206
ENSG00000156363	ENSG00000186322
ENSG00000156363	ENSG00000188388
ENSG00000156363	ENSG00000188532
ENSG00000156363	ENSG00000188626
ENSG00000156363	ENSG00000197092
ENSG00000156363	ENSG00000197414
ENSG00000156363	ENSG00000214348
ENSG00000156363	ENSG00000215252
ENSG00000156363	ENSG00000215405
ENSG00000156395	ENSG00000108018
ENSG00000156395	ENSG00000134243
ENSG00000156395	ENSG00000137642
ENSG00000156395	ENSG00000184985
ENSG00000156413	ENSG00000130383
ENSG00000156413	ENSG00000171124
ENSG00000156413	ENSG00000172461
ENSG00000156413	ENSG00000172728
ENSG00000156413	ENSG00000180549
ENSG00000156413	ENSG00000196371
ENSG00000156413	ENSG00000196968
ENSG00000156427	ENSG00000107831
ENSG00000156427	ENSG00000158815
ENSG00000156453	ENSG00000099715
ENSG00000156453	ENSG00000102290
ENSG00000156453	ENSG00000113555
ENSG00000156453	ENSG00000169851
ENSG00000156453	ENSG00000184226
ENSG00000156453	ENSG00000197991
ENSG00000156453	ENSG00000215442
ENSG00000156463	ENSG00000154447
ENSG00000156463	ENSG00000172985
ENSG00000156466	ENSG00000101144
ENSG00000156466	ENSG00000107623
ENSG00000156466	ENSG00000112175
ENSG00000156466	ENSG00000116985
ENSG00000156466	ENSG00000125965
ENSG00000156466	ENSG00000128802
ENSG00000156466	ENSG00000130385
ENSG00000156466	ENSG00000143869
ENSG00000156466	ENSG00000152785
ENSG00000156466	ENSG00000153162
ENSG00000156466	ENSG00000156574
ENSG00000156466	ENSG00000163217
ENSG00000156466	ENSG00000183682
ENSG00000156475	ENSG00000074211
ENSG00000156475	ENSG00000175470
ENSG00000156475	ENSG00000214122
ENSG00000156482	ENSG00000172786
ENSG00000156482	ENSG00000173180
ENSG00000156486	ENSG00000026559
ENSG00000156486	ENSG00000102057
ENSG00000156486	ENSG00000104848
ENSG00000156486	ENSG00000111262

ENSG00000156486	ENSG00000124134
ENSG00000156486	ENSG00000130037
ENSG00000156486	ENSG00000143105
ENSG00000156486	ENSG00000151079
ENSG00000156486	ENSG00000158445
ENSG00000156486	ENSG00000162975
ENSG00000156486	ENSG00000164794
ENSG00000156486	ENSG00000168263
ENSG00000156486	ENSG00000168418
ENSG00000156486	ENSG00000170745
ENSG00000156486	ENSG00000171126
ENSG00000156486	ENSG00000171385
ENSG00000156486	ENSG00000177272
ENSG00000156486	ENSG00000177301
ENSG00000156486	ENSG00000178342
ENSG00000156486	ENSG00000182255
ENSG00000156486	ENSG00000182674
ENSG00000156486	ENSG00000184408
ENSG00000156508	ENSG00000101210
ENSG00000156508	ENSG00000185637
ENSG00000156508	ENSG00000214744
ENSG00000156508	ENSG00000215093
ENSG00000156509	ENSG00000112029
ENSG00000156510	ENSG00000106633
ENSG00000156510	ENSG00000156515
ENSG00000156510	ENSG00000159399
ENSG00000156510	ENSG00000160883
ENSG00000156515	ENSG00000106633
ENSG00000156515	ENSG00000156510
ENSG00000156515	ENSG00000159399
ENSG00000156515	ENSG00000160883
ENSG00000156531	ENSG00000010318
ENSG00000156531	ENSG00000092140
ENSG00000156531	ENSG00000136147
ENSG00000156564	ENSG00000126243
ENSG00000156564	ENSG00000128011
ENSG00000156564	ENSG00000165379
ENSG00000156599	ENSG00000099904
ENSG00000156599	ENSG00000163958
ENSG00000156642	ENSG00000170571
ENSG00000156642	ENSG00000172270
ENSG00000156650	ENSG00000083168
ENSG00000156675	ENSG00000107560
ENSG00000156675	ENSG00000135631
ENSG00000156687	ENSG00000107731
ENSG00000156687	ENSG00000113763
ENSG00000156687	ENSG00000124602
ENSG00000156687	ENSG00000182168
ENSG00000156711	ENSG00000050748
ENSG00000156711	ENSG00000107643
ENSG00000156711	ENSG00000109339
ENSG00000156711	ENSG00000112062
ENSG00000156711	ENSG00000185386
ENSG00000156711	ENSG00000188130
ENSG00000156735	ENSG00000151929
ENSG00000156738	ENSG00000071203
ENSG00000156738	ENSG00000110077
ENSG00000156738	ENSG00000110079
ENSG00000156738	ENSG00000149516

ENSG00000156738	ENSG00000149534
ENSG00000156738	ENSG00000166926
ENSG00000156738	ENSG00000166927
ENSG00000156738	ENSG00000166930
ENSG00000156738	ENSG00000166959
ENSG00000156738	ENSG00000166961
ENSG00000156738	ENSG00000214787
ENSG00000156755	ENSG00000136700
ENSG00000156755	ENSG00000183719
ENSG00000156755	ENSG00000197794
ENSG00000156755	ENSG00000204670
ENSG00000156755	ENSG00000204776
ENSG00000156755	ENSG00000204780
ENSG00000156755	ENSG00000211599
ENSG00000156755	ENSG00000211600
ENSG00000156755	ENSG00000211601
ENSG00000156755	ENSG00000211602
ENSG00000156755	ENSG00000211603
ENSG00000156755	ENSG00000211604
ENSG00000156755	ENSG00000211605
ENSG00000156755	ENSG00000211606
ENSG00000156755	ENSG00000211607
ENSG00000156755	ENSG00000211608
ENSG00000156755	ENSG00000211609
ENSG00000156755	ENSG00000211610
ENSG00000156755	ENSG00000211611
ENSG00000156755	ENSG00000211613
ENSG00000156755	ENSG00000211616
ENSG00000156755	ENSG00000211617
ENSG00000156755	ENSG00000211618
ENSG00000156755	ENSG00000211620
ENSG00000156755	ENSG00000211625
ENSG00000156755	ENSG00000211626
ENSG00000156755	ENSG00000211627
ENSG00000156755	ENSG00000211628
ENSG00000156755	ENSG00000211629
ENSG00000156755	ENSG00000211630
ENSG00000156755	ENSG00000211631
ENSG00000156755	ENSG00000211632
ENSG00000156755	ENSG00000211633
ENSG00000156755	ENSG00000211634
ENSG00000156755	ENSG00000211635
ENSG00000156755	ENSG00000211636
ENSG00000156804	ENSG00000147364
ENSG00000156860	ENSG00000112787
ENSG00000156860	ENSG00000158321
ENSG00000156869	ENSG00000136805
ENSG00000156873	ENSG00000164776
ENSG00000156875	ENSG00000148110
ENSG00000156885	ENSG00000111775
ENSG00000156885	ENSG00000164605
ENSG00000156886	ENSG00000005844
ENSG00000156886	ENSG00000005884
ENSG00000156886	ENSG00000005961
ENSG00000156886	ENSG00000077943
ENSG00000156886	ENSG00000083457
ENSG00000156886	ENSG00000091409
ENSG00000156886	ENSG00000115232
ENSG00000156886	ENSG00000135424

ENSG00000156886	ENSG00000137809
ENSG00000156886	ENSG00000138448
ENSG00000156886	ENSG00000140678
ENSG00000156886	ENSG00000143127
ENSG00000156886	ENSG00000144668
ENSG00000156886	ENSG00000161638
ENSG00000156886	ENSG00000164171
ENSG00000156886	ENSG00000169896
ENSG00000156886	ENSG00000213949
ENSG00000156920	ENSG00000112414
ENSG00000156920	ENSG00000159618
ENSG00000156920	ENSG00000173698
ENSG00000156920	ENSG00000182885
ENSG00000156920	ENSG00000205336
ENSG00000156925	ENSG00000043355
ENSG00000156925	ENSG00000139800
ENSG00000156925	ENSG00000152977
ENSG00000156925	ENSG00000174963
ENSG00000156959	ENSG00000145685
ENSG00000156959	ENSG00000182508
ENSG00000156959	ENSG00000183722
ENSG00000156959	ENSG00000187416
ENSG00000156959	ENSG00000197753
ENSG00000156966	ENSG00000162630
ENSG00000156966	ENSG00000169255
ENSG00000156966	ENSG00000170340
ENSG00000156966	ENSG00000172318
ENSG00000156966	ENSG00000176383
ENSG00000156966	ENSG00000176597
ENSG00000156966	ENSG00000177191
ENSG00000156966	ENSG00000179913
ENSG00000156966	ENSG00000180561
ENSG00000156966	ENSG00000183778
ENSG00000156966	ENSG00000198488
ENSG00000156966	ENSG00000204222
ENSG00000156966	ENSG00000214654
ENSG00000156968	ENSG00000127226
ENSG00000156976	ENSG00000161960
ENSG00000156983	ENSG00000043143
ENSG00000156983	ENSG00000077684
ENSG00000156983	ENSG00000078403
ENSG00000156983	ENSG00000096070
ENSG00000156983	ENSG00000100425
ENSG00000156983	ENSG00000102221
ENSG00000156983	ENSG00000106443
ENSG00000156983	ENSG00000108292
ENSG00000156990	ENSG00000165526
ENSG00000157021	ENSG00000153789
ENSG00000157021	ENSG00000188343
ENSG00000157077	ENSG00000039319
ENSG00000157087	ENSG00000058668
ENSG00000157087	ENSG00000067842
ENSG00000157087	ENSG00000070961
ENSG00000157093	ENSG00000090382
ENSG00000157093	ENSG00000120563
ENSG00000157093	ENSG00000141316
ENSG00000157093	ENSG00000151033
ENSG00000157093	ENSG00000161572
ENSG00000157093	ENSG00000167531

ENSG00000157093	ENSG00000171478
ENSG00000157093	ENSG00000171489
ENSG00000157103	ENSG00000010379
ENSG00000157103	ENSG00000011083
ENSG00000157103	ENSG00000087916
ENSG00000157103	ENSG00000103546
ENSG00000157103	ENSG00000108576
ENSG00000157103	ENSG00000111181
ENSG00000157103	ENSG00000130821
ENSG00000157103	ENSG00000131389
ENSG00000157103	ENSG00000132164
ENSG00000157103	ENSG00000142319
ENSG00000157103	ENSG00000163817
ENSG00000157103	ENSG00000164363
ENSG00000157103	ENSG00000165970
ENSG00000157103	ENSG00000174358
ENSG00000157103	ENSG00000196517
ENSG00000157103	ENSG00000198555
ENSG00000157103	ENSG00000214617
ENSG00000157107	ENSG00000007237
ENSG00000157107	ENSG00000118473
ENSG00000157107	ENSG00000130475
ENSG00000157110	ENSG00000166831
ENSG00000157119	ENSG00000122550
ENSG00000157119	ENSG00000154474
ENSG00000157131	ENSG00000021852
ENSG00000157131	ENSG00000039537
ENSG00000157131	ENSG00000112936
ENSG00000157131	ENSG00000113600
ENSG00000157131	ENSG00000180644
ENSG00000157150	ENSG00000035862
ENSG00000157150	ENSG00000100234
ENSG00000157150	ENSG00000102265
ENSG00000157152	ENSG00000008056
ENSG00000157152	ENSG00000185666
ENSG00000157168	ENSG00000158458
ENSG00000157191	ENSG00000089818
ENSG00000157193	ENSG00000130164
ENSG00000157193	ENSG00000147852
ENSG00000157214	ENSG00000105889
ENSG00000157214	ENSG00000115107
ENSG00000157214	ENSG00000127954
ENSG00000157214	ENSG00000164647
ENSG00000157216	ENSG00000130511
ENSG00000157216	ENSG00000145687
ENSG00000157216	ENSG00000214079
ENSG00000157216	ENSG00000214099
ENSG00000157216	ENSG00000215390
ENSG00000157216	ENSG00000215535
ENSG00000157227	ENSG00000008516
ENSG00000157227	ENSG00000087245
ENSG00000157227	ENSG00000099953
ENSG00000157227	ENSG00000100985
ENSG00000157227	ENSG00000102996
ENSG00000157227	ENSG00000110347
ENSG00000157227	ENSG00000118113
ENSG00000157227	ENSG00000123342
ENSG00000157227	ENSG00000125966
ENSG00000157227	ENSG00000129270

ENSG00000157227	ENSG00000137673
ENSG00000157227	ENSG00000137674
ENSG00000157227	ENSG00000137675
ENSG00000157227	ENSG00000137745
ENSG00000157227	ENSG00000149968
ENSG00000157227	ENSG00000154485
ENSG00000157227	ENSG00000156103
ENSG00000157227	ENSG00000166670
ENSG00000157227	ENSG00000167346
ENSG00000157227	ENSG00000189409
ENSG00000157227	ENSG00000196611
ENSG00000157227	ENSG00000198598
ENSG00000157227	ENSG00000215914
ENSG00000157240	ENSG00000104290
ENSG00000157240	ENSG00000106483
ENSG00000157240	ENSG00000111432
ENSG00000157240	ENSG00000155760
ENSG00000157240	ENSG00000162998
ENSG00000157240	ENSG00000163251
ENSG00000157240	ENSG00000164930
ENSG00000157240	ENSG00000174804
ENSG00000157240	ENSG00000177283
ENSG00000157240	ENSG00000180340
ENSG00000157240	ENSG00000188763
ENSG00000157349	ENSG00000109832
ENSG00000157349	ENSG00000168872
ENSG00000157350	ENSG00000008513
ENSG00000157350	ENSG00000064225
ENSG00000157350	ENSG00000110080
ENSG00000157350	ENSG00000126091
ENSG00000157358	ENSG00000116708
ENSG00000157358	ENSG00000116721
ENSG00000157358	ENSG00000116726
ENSG00000157358	ENSG00000120952
ENSG00000157358	ENSG00000160959
ENSG00000157358	ENSG00000179704
ENSG00000157358	ENSG00000182330
ENSG00000157358	ENSG00000185028
ENSG00000157358	ENSG00000185686
ENSG00000157358	ENSG00000187545
ENSG00000157358	ENSG00000197549
ENSG00000157358	ENSG00000204478
ENSG00000157358	ENSG00000204479
ENSG00000157358	ENSG00000204480
ENSG00000157358	ENSG00000204481
ENSG00000157358	ENSG00000204485
ENSG00000157358	ENSG00000204486
ENSG00000157358	ENSG00000204488
ENSG00000157358	ENSG00000204491
ENSG00000157358	ENSG00000204495
ENSG00000157358	ENSG00000204501
ENSG00000157358	ENSG00000204502
ENSG00000157358	ENSG00000204503
ENSG00000157358	ENSG00000204505
ENSG00000157358	ENSG00000204507
ENSG00000157358	ENSG00000204508
ENSG00000157358	ENSG00000204509
ENSG00000157358	ENSG00000204510
ENSG00000157358	ENSG00000204513

ENSG00000157388	ENSG00000081248
ENSG00000157388	ENSG00000102001
ENSG00000157388	ENSG00000141837
ENSG00000157388	ENSG00000148408
ENSG00000157388	ENSG00000151067
ENSG00000157388	ENSG00000198216
ENSG00000157399	ENSG00000006756
ENSG00000157399	ENSG00000062096
ENSG00000157399	ENSG00000100299
ENSG00000157399	ENSG00000101846
ENSG00000157399	ENSG00000113273
ENSG00000157399	ENSG00000141012
ENSG00000157399	ENSG00000141337
ENSG00000157399	ENSG00000180801
ENSG00000157399	ENSG00000183876
ENSG00000157399	ENSG00000205667
ENSG00000157404	ENSG00000037280
ENSG00000157404	ENSG00000102755
ENSG00000157404	ENSG00000104213
ENSG00000157404	ENSG00000113721
ENSG00000157404	ENSG00000122025
ENSG00000157404	ENSG00000128052
ENSG00000157404	ENSG00000134853
ENSG00000157404	ENSG00000182578
ENSG00000157445	ENSG00000007402
ENSG00000157445	ENSG00000151062
ENSG00000157445	ENSG00000153956
ENSG00000157445	ENSG00000158966
ENSG00000157456	ENSG00000134057
ENSG00000157456	ENSG00000147082
ENSG00000157470	ENSG00000153347
ENSG00000157483	ENSG00000128641
ENSG00000157483	ENSG00000136286
ENSG00000157483	ENSG00000142347
ENSG00000157483	ENSG00000166866
ENSG00000157483	ENSG00000176658
ENSG00000157483	ENSG00000197879
ENSG00000157500	ENSG00000136044
ENSG00000157510	ENSG00000169129
ENSG00000157510	ENSG00000196526
ENSG00000157514	ENSG00000214114
ENSG00000157540	ENSG00000010219
ENSG00000157540	ENSG00000105204
ENSG00000157540	ENSG00000127334
ENSG00000157540	ENSG00000143479
ENSG00000157542	ENSG00000115474
ENSG00000157542	ENSG00000120457
ENSG00000157542	ENSG00000121361
ENSG00000157542	ENSG00000123700
ENSG00000157542	ENSG00000151704
ENSG00000157542	ENSG00000153822
ENSG00000157542	ENSG00000157551
ENSG00000157542	ENSG00000162728
ENSG00000157542	ENSG00000162989
ENSG00000157542	ENSG00000168135
ENSG00000157542	ENSG00000177807
ENSG00000157542	ENSG00000182324
ENSG00000157542	ENSG00000184185
ENSG00000157542	ENSG00000187486

ENSG00000157551	ENSG00000115474
ENSG00000157551	ENSG00000120457
ENSG00000157551	ENSG00000121361
ENSG00000157551	ENSG00000123700
ENSG00000157551	ENSG00000151704
ENSG00000157551	ENSG00000153822
ENSG00000157551	ENSG00000157542
ENSG00000157551	ENSG00000162728
ENSG00000157551	ENSG00000162989
ENSG00000157551	ENSG00000168135
ENSG00000157551	ENSG00000177807
ENSG00000157551	ENSG00000182324
ENSG00000157551	ENSG00000184185
ENSG00000157551	ENSG00000187486
ENSG00000157554	ENSG00000151702
ENSG00000157554	ENSG00000154727
ENSG00000157557	ENSG00000006468
ENSG00000157557	ENSG00000105672
ENSG00000157557	ENSG00000134954
ENSG00000157557	ENSG00000163497
ENSG00000157557	ENSG00000171656
ENSG00000157557	ENSG00000175832
ENSG00000157570	ENSG00000011105
ENSG00000157570	ENSG00000085117
ENSG00000157570	ENSG00000104894
ENSG00000157570	ENSG00000110900
ENSG00000157570	ENSG00000117472
ENSG00000157570	ENSG00000143119
ENSG00000157570	ENSG00000177697
ENSG00000157570	ENSG00000214063
ENSG00000157576	ENSG00000172186
ENSG00000157576	ENSG00000182765
ENSG00000157576	ENSG00000184409
ENSG00000157576	ENSG00000197366
ENSG00000157576	ENSG00000198157
ENSG00000157576	ENSG00000198875
ENSG00000157601	ENSG00000183486
ENSG00000157613	ENSG00000060566
ENSG00000157613	ENSG00000107175
ENSG00000157613	ENSG00000118217
ENSG00000157613	ENSG00000143578
ENSG00000157613	ENSG00000182158
ENSG00000157613	ENSG00000213676
ENSG00000157617	ENSG00000172375
ENSG00000157625	ENSG00000055208
ENSG00000157654	ENSG00000099260
ENSG00000157654	ENSG00000099864
ENSG00000157680	ENSG00000145214
ENSG00000157680	ENSG00000149091
ENSG00000157734	ENSG00000064652
ENSG00000157741	ENSG00000118900
ENSG00000157764	ENSG00000078061
ENSG00000157764	ENSG00000132155
ENSG00000157764	ENSG00000141068
ENSG00000157764	ENSG00000171435
ENSG00000157765	ENSG00000131183
ENSG00000157765	ENSG00000198569
ENSG00000157766	ENSG00000038427
ENSG00000157766	ENSG00000130287

ENSG00000157766	ENSG00000132692
ENSG00000157766	ENSG00000132702
ENSG00000157766	ENSG00000140511
ENSG00000157766	ENSG00000145681
ENSG00000157766	ENSG00000187664
ENSG00000157782	ENSG00000105507
ENSG00000157782	ENSG00000167791
ENSG00000157782	ENSG00000175544
ENSG00000157823	ENSG00000177879
ENSG00000157823	ENSG00000214446
ENSG00000157827	ENSG00000161791
ENSG00000157827	ENSG00000184922
ENSG00000157833	ENSG00000141441
ENSG00000157873	ENSG00000028137
ENSG00000157873	ENSG00000101017
ENSG00000157873	ENSG00000111321
ENSG00000157873	ENSG00000120949
ENSG00000157873	ENSG00000141655
ENSG00000157873	ENSG00000146072
ENSG00000157873	ENSG00000164761
ENSG00000157884	ENSG00000136425
ENSG00000157884	ENSG00000141977
ENSG00000157884	ENSG00000185043
ENSG00000157890	ENSG00000074660
ENSG00000157890	ENSG00000099910
ENSG00000157890	ENSG00000145794
ENSG00000157890	ENSG00000162591
ENSG00000157890	ENSG00000187800
ENSG00000157927	ENSG00000105538
ENSG00000157933	ENSG00000136603
ENSG00000157933	ENSG00000188779
ENSG00000157933	ENSG00000215474
ENSG00000157954	ENSG00000070540
ENSG00000157978	ENSG00000105245
ENSG00000157978	ENSG00000133961
ENSG00000157978	ENSG00000144366
ENSG00000157978	ENSG00000183114
ENSG00000157978	ENSG00000185112
ENSG00000157985	ENSG00000108262
ENSG00000157985	ENSG00000133612
ENSG00000157985	ENSG00000135439
ENSG00000157985	ENSG00000139436
ENSG00000157985	ENSG00000151303
ENSG00000157985	ENSG00000172650
ENSG00000157985	ENSG00000174194
ENSG00000157985	ENSG00000188234
ENSG00000157985	ENSG00000198035
ENSG00000157985	ENSG00000204149
ENSG00000157985	ENSG00000204169
ENSG00000157985	ENSG00000204172
ENSG00000157992	ENSG00000121900
ENSG00000158006	ENSG00000146070
ENSG00000158008	ENSG00000012232
ENSG00000158008	ENSG00000151348
ENSG00000158008	ENSG00000162694
ENSG00000158008	ENSG00000182197
ENSG00000158014	ENSG00000104154
ENSG00000158014	ENSG00000115194
ENSG00000158014	ENSG00000164756

ENSG00000158022	ENSG00000138100
ENSG00000158022	ENSG00000147573
ENSG00000158022	ENSG00000204977
ENSG00000158022	ENSG00000213186
ENSG00000158050	ENSG00000111266
ENSG00000158050	ENSG00000120129
ENSG00000158050	ENSG00000120875
ENSG00000158050	ENSG00000130829
ENSG00000158050	ENSG00000138166
ENSG00000158050	ENSG00000139318
ENSG00000158050	ENSG00000143507
ENSG00000158050	ENSG00000164086
ENSG00000158050	ENSG00000184545
ENSG00000158055	ENSG00000083307
ENSG00000158055	ENSG00000115112
ENSG00000158055	ENSG00000134317
ENSG00000158055	ENSG00000135457
ENSG00000158055	ENSG00000153560
ENSG00000158055	ENSG00000214547
ENSG00000158077	ENSG00000022556
ENSG00000158077	ENSG00000023191
ENSG00000158077	ENSG00000140853
ENSG00000158077	ENSG00000142405
ENSG00000158077	ENSG00000160505
ENSG00000158077	ENSG00000162711
ENSG00000158077	ENSG00000167207
ENSG00000158077	ENSG00000167634
ENSG00000158077	ENSG00000167984
ENSG00000158077	ENSG00000171487
ENSG00000158077	ENSG00000173572
ENSG00000158077	ENSG00000174885
ENSG00000158077	ENSG00000179709
ENSG00000158077	ENSG00000179873
ENSG00000158077	ENSG00000182261
ENSG00000158077	ENSG00000185792
ENSG00000158089	ENSG00000100626
ENSG00000158089	ENSG00000106648
ENSG00000158089	ENSG00000110328
ENSG00000158089	ENSG00000115339
ENSG00000158089	ENSG00000131386
ENSG00000158089	ENSG00000136542
ENSG00000158089	ENSG00000139629
ENSG00000158089	ENSG00000141429
ENSG00000158089	ENSG00000143641
ENSG00000158089	ENSG00000144278
ENSG00000158089	ENSG00000164574
ENSG00000158089	ENSG00000174473
ENSG00000158089	ENSG00000178234
ENSG00000158089	ENSG00000182870
ENSG00000158089	ENSG00000185274
ENSG00000158092	ENSG00000071051
ENSG00000158104	ENSG00000186603
ENSG00000158106	ENSG00000076201
ENSG00000158106	ENSG00000131941
ENSG00000158106	ENSG00000170248
ENSG00000158106	ENSG00000215407
ENSG00000158109	ENSG00000188001
ENSG00000158125	ENSG00000138356
ENSG00000158156	ENSG00000101321

ENSG00000158156	ENSG00000171044
ENSG00000158156	ENSG00000186530
ENSG00000158156	ENSG00000198669
ENSG00000158156	ENSG00000206579
ENSG00000158158	ENSG00000119946
ENSG00000158158	ENSG00000148842
ENSG00000158158	ENSG00000168763
ENSG00000158161	ENSG00000064655
ENSG00000158161	ENSG00000104313
ENSG00000158161	ENSG00000112319
ENSG00000158163	ENSG00000134874
ENSG00000158195	ENSG00000112290
ENSG00000158195	ENSG00000132970
ENSG00000158195	ENSG00000188459
ENSG00000158201	ENSG00000140526
ENSG00000158201	ENSG00000143994
ENSG00000158220	ENSG00000117868
ENSG00000158220	ENSG00000139641
ENSG00000158246	ENSG00000112773
ENSG00000158246	ENSG00000174016
ENSG00000158246	ENSG00000183508
ENSG00000158258	ENSG00000139182
ENSG00000158258	ENSG00000171603
ENSG00000158270	ENSG00000168077
ENSG00000158290	ENSG00000139842
ENSG00000158321	ENSG00000112787
ENSG00000158321	ENSG00000156860
ENSG00000158352	ENSG00000138771
ENSG00000158352	ENSG00000146950
ENSG00000158402	ENSG00000101224
ENSG00000158402	ENSG00000164045
ENSG00000158403	ENSG00000111332
ENSG00000158403	ENSG00000124642
ENSG00000158403	ENSG00000137259
ENSG00000158403	ENSG00000164508
ENSG00000158403	ENSG00000168274
ENSG00000158403	ENSG00000180573
ENSG00000158403	ENSG00000181218
ENSG00000158403	ENSG00000182611
ENSG00000158403	ENSG00000183558
ENSG00000158403	ENSG00000184260
ENSG00000158403	ENSG00000184270
ENSG00000158403	ENSG00000184348
ENSG00000158403	ENSG00000184825
ENSG00000158403	ENSG00000185978
ENSG00000158403	ENSG00000188486
ENSG00000158403	ENSG00000196747
ENSG00000158403	ENSG00000196787
ENSG00000158403	ENSG00000196866
ENSG00000158403	ENSG00000198082
ENSG00000158403	ENSG00000198307
ENSG00000158403	ENSG00000198374
ENSG00000158403	ENSG00000203812
ENSG00000158403	ENSG00000205070
ENSG00000158403	ENSG00000205079
ENSG00000158406	ENSG00000124529
ENSG00000158406	ENSG00000124578
ENSG00000158406	ENSG00000182217
ENSG00000158406	ENSG00000183941

ENSG00000158406	ENSG00000188987
ENSG00000158406	ENSG00000196176
ENSG00000158406	ENSG00000197061
ENSG00000158406	ENSG00000197238
ENSG00000158406	ENSG00000197837
ENSG00000158406	ENSG00000197914
ENSG00000158406	ENSG00000198327
ENSG00000158406	ENSG00000198339
ENSG00000158406	ENSG00000198518
ENSG00000158406	ENSG00000198558
ENSG00000158423	ENSG00000128408
ENSG00000158457	ENSG00000048140
ENSG00000158457	ENSG00000099282
ENSG00000158457	ENSG00000108219
ENSG00000158457	ENSG00000168785
ENSG00000158457	ENSG00000182612
ENSG00000158458	ENSG00000157168
ENSG00000158467	ENSG00000101444
ENSG00000158467	ENSG00000168710
ENSG00000158467	ENSG00000214066
ENSG00000158470	ENSG00000027847
ENSG00000158470	ENSG00000086062
ENSG00000158470	ENSG00000117411
ENSG00000158470	ENSG00000118276
ENSG00000158470	ENSG00000121578
ENSG00000158470	ENSG00000158850
ENSG00000158480	ENSG00000158792
ENSG00000158516	ENSG00000080618
ENSG00000158516	ENSG00000091704
ENSG00000158516	ENSG00000128510
ENSG00000158516	ENSG00000144410
ENSG00000158516	ENSG00000153002
ENSG00000158516	ENSG00000158525
ENSG00000158516	ENSG00000163751
ENSG00000158516	ENSG00000165078
ENSG00000158517	ENSG00000107957
ENSG00000158517	ENSG00000165178
ENSG00000158517	ENSG00000174705
ENSG00000158517	ENSG00000182487
ENSG00000158517	ENSG00000196408
ENSG00000158525	ENSG00000080618
ENSG00000158525	ENSG00000091704
ENSG00000158525	ENSG00000128510
ENSG00000158525	ENSG00000144410
ENSG00000158525	ENSG00000153002
ENSG00000158525	ENSG00000158516
ENSG00000158525	ENSG00000163751
ENSG00000158525	ENSG00000165078
ENSG00000158528	ENSG00000108819
ENSG00000158555	ENSG00000006007
ENSG00000158555	ENSG00000130055
ENSG00000158555	ENSG00000178795
ENSG00000158560	ENSG00000077380
ENSG00000158560	ENSG00000119333
ENSG00000158560	ENSG00000122735
ENSG00000158560	ENSG00000152763
ENSG00000158560	ENSG00000171595
ENSG00000158571	ENSG00000114268
ENSG00000158571	ENSG00000123836

ENSG00000158571	ENSG00000170525
ENSG00000158578	ENSG00000023330
ENSG00000158604	ENSG00000184840
ENSG00000158604	ENSG00000215367
ENSG00000158669	ENSG00000138678
ENSG00000158683	ENSG00000008710
ENSG00000158683	ENSG00000078795
ENSG00000158683	ENSG00000107593
ENSG00000158683	ENSG00000118762
ENSG00000158683	ENSG00000130943
ENSG00000158683	ENSG00000166473
ENSG00000158683	ENSG00000183458
ENSG00000158683	ENSG00000183793
ENSG00000158683	ENSG00000187008
ENSG00000158683	ENSG00000188599
ENSG00000158710	ENSG00000064666
ENSG00000158710	ENSG00000117519
ENSG00000158710	ENSG00000130176
ENSG00000158710	ENSG00000144834
ENSG00000158710	ENSG00000149591
ENSG00000158710	ENSG00000204782
ENSG00000158710	ENSG00000204826
ENSG00000158710	ENSG00000215433
ENSG00000158711	ENSG00000111145
ENSG00000158711	ENSG00000117036
ENSG00000158711	ENSG00000126767
ENSG00000158715	ENSG00000022567
ENSG00000158715	ENSG00000162426
ENSG00000158715	ENSG00000164175
ENSG00000158717	ENSG00000101695
ENSG00000158717	ENSG00000124226
ENSG00000158717	ENSG00000134758
ENSG00000158773	ENSG00000105698
ENSG00000158786	ENSG00000069764
ENSG00000158786	ENSG00000117215
ENSG00000158786	ENSG00000127472
ENSG00000158786	ENSG00000170890
ENSG00000158786	ENSG00000187980
ENSG00000158786	ENSG00000188257
ENSG00000158786	ENSG00000188784
ENSG00000158792	ENSG00000158480
ENSG00000158796	ENSG00000160570
ENSG00000158805	ENSG00000161914
ENSG00000158805	ENSG00000171163
ENSG00000158805	ENSG00000186660
ENSG00000158806	ENSG00000107833
ENSG00000158806	ENSG00000181163
ENSG00000158806	ENSG00000182965
ENSG00000158806	ENSG00000214032
ENSG00000158806	ENSG00000214246
ENSG00000158806	ENSG00000214774
ENSG00000158806	ENSG00000214884
ENSG00000158806	ENSG00000215280
ENSG00000158815	ENSG00000107831
ENSG00000158815	ENSG00000156427
ENSG00000158816	ENSG00000102699
ENSG00000158816	ENSG00000110002
ENSG00000158816	ENSG00000145198
ENSG00000158850	ENSG00000027847

ENSG00000158850	ENSG00000086062
ENSG00000158850	ENSG00000117411
ENSG00000158850	ENSG00000118276
ENSG00000158850	ENSG00000121578
ENSG00000158850	ENSG00000158470
ENSG00000158856	ENSG00000099204
ENSG00000158856	ENSG00000163995
ENSG00000158856	ENSG00000173210
ENSG00000158859	ENSG00000134917
ENSG00000158859	ENSG00000154734
ENSG00000158859	ENSG00000154736
ENSG00000158859	ENSG00000166106
ENSG00000158863	ENSG00000051009
ENSG00000158863	ENSG00000151553
ENSG00000158863	ENSG00000164142
ENSG00000158869	ENSG00000198821
ENSG00000158882	ENSG00000130204
ENSG00000158887	ENSG00000149573
ENSG00000158887	ENSG00000149575
ENSG00000158887	ENSG00000160588
ENSG00000158887	ENSG00000160593
ENSG00000158887	ENSG00000177098
ENSG00000158887	ENSG00000197965
ENSG00000158955	ENSG00000002745
ENSG00000158955	ENSG00000061492
ENSG00000158955	ENSG00000075290
ENSG00000158955	ENSG00000085741
ENSG00000158955	ENSG00000105989
ENSG00000158955	ENSG00000108379
ENSG00000158955	ENSG00000111186
ENSG00000158955	ENSG00000114251
ENSG00000158955	ENSG00000115596
ENSG00000158955	ENSG00000125084
ENSG00000158955	ENSG00000134245
ENSG00000158955	ENSG00000135925
ENSG00000158955	ENSG00000143816
ENSG00000158955	ENSG00000154342
ENSG00000158955	ENSG00000154764
ENSG00000158955	ENSG00000162552
ENSG00000158955	ENSG00000169884
ENSG00000158955	ENSG00000188064
ENSG00000158985	ENSG00000197622
ENSG00000159023	ENSG00000079819
ENSG00000159023	ENSG00000082397
ENSG00000159023	ENSG00000088367
ENSG00000159082	ENSG00000078269
ENSG00000159086	ENSG00000005436
ENSG00000159113	ENSG00000142166
ENSG00000159113	ENSG00000174564
ENSG00000159164	ENSG00000122012
ENSG00000159164	ENSG00000185518
ENSG00000159167	ENSG00000113739
ENSG00000159173	ENSG00000129991
ENSG00000159173	ENSG00000130598
ENSG00000159176	ENSG00000129170
ENSG00000159176	ENSG00000136490
ENSG00000159176	ENSG00000146215
ENSG00000159176	ENSG00000175183
ENSG00000159176	ENSG00000182809

ENSG00000159176	ENSG00000197893
ENSG00000159176	ENSG00000213145
ENSG00000159184	ENSG00000106031
ENSG00000159184	ENSG00000123364
ENSG00000159184	ENSG00000128714
ENSG00000159189	ENSG00000054803
ENSG00000159189	ENSG00000102924
ENSG00000159189	ENSG00000131094
ENSG00000159189	ENSG00000133466
ENSG00000159189	ENSG00000139899
ENSG00000159189	ENSG00000141668
ENSG00000159189	ENSG00000144119
ENSG00000159189	ENSG00000145861
ENSG00000159189	ENSG00000163145
ENSG00000159189	ENSG00000165985
ENSG00000159189	ENSG00000172247
ENSG00000159189	ENSG00000173369
ENSG00000159189	ENSG00000173372
ENSG00000159189	ENSG00000173918
ENSG00000159189	ENSG00000181092
ENSG00000159189	ENSG00000184471
ENSG00000159189	ENSG00000186897
ENSG00000159189	ENSG00000213192
ENSG00000159199	ENSG00000135390
ENSG00000159199	ENSG00000154518
ENSG00000159200	ENSG00000117602
ENSG00000159200	ENSG00000172348
ENSG00000159212	ENSG00000112782
ENSG00000159212	ENSG00000155962
ENSG00000159212	ENSG00000169504
ENSG00000159212	ENSG00000169583
ENSG00000159212	ENSG00000213719
ENSG00000159212	ENSG00000214258
ENSG00000159216	ENSG00000020633
ENSG00000159216	ENSG00000124813
ENSG00000159217	ENSG00000073792
ENSG00000159217	ENSG00000136231
ENSG00000159224	ENSG00000115263
ENSG00000159228	ENSG00000159231
ENSG00000159231	ENSG00000159228
ENSG00000159247	ENSG00000101162
ENSG00000159247	ENSG00000104833
ENSG00000159247	ENSG00000127589
ENSG00000159247	ENSG00000137267
ENSG00000159247	ENSG00000137285
ENSG00000159247	ENSG00000173213
ENSG00000159247	ENSG00000173876
ENSG00000159247	ENSG00000176014
ENSG00000159247	ENSG00000188229
ENSG00000159247	ENSG00000196230
ENSG00000159247	ENSG00000198211
ENSG00000159247	ENSG00000215801
ENSG00000159247	ENSG00000215813
ENSG00000159247	ENSG00000215814
ENSG00000159248	ENSG00000121634
ENSG00000159248	ENSG00000121742
ENSG00000159248	ENSG00000121743
ENSG00000159248	ENSG00000131233
ENSG00000159248	ENSG00000135355

ENSG00000159248	ENSG00000143140
ENSG00000159248	ENSG00000152661
ENSG00000159248	ENSG00000164411
ENSG00000159248	ENSG00000165474
ENSG00000159248	ENSG00000169562
ENSG00000159248	ENSG00000176402
ENSG00000159248	ENSG00000177291
ENSG00000159248	ENSG00000182963
ENSG00000159248	ENSG00000183153
ENSG00000159248	ENSG00000187513
ENSG00000159248	ENSG00000188910
ENSG00000159248	ENSG00000189280
ENSG00000159248	ENSG00000189433
ENSG00000159248	ENSG00000198835
ENSG00000159248	ENSG00000203733
ENSG00000159251	ENSG00000075624
ENSG00000159251	ENSG00000107796
ENSG00000159251	ENSG00000143632
ENSG00000159251	ENSG00000163017
ENSG00000159251	ENSG00000169067
ENSG00000159251	ENSG00000184009
ENSG00000159251	ENSG00000204434
ENSG00000159251	ENSG00000214555
ENSG00000159251	ENSG00000215141
ENSG00000159256	ENSG00000114487
ENSG00000159256	ENSG00000133131
ENSG00000159256	ENSG00000133422
ENSG00000159261	ENSG00000013297
ENSG00000159261	ENSG00000066405
ENSG00000159261	ENSG00000106404
ENSG00000159261	ENSG00000113946
ENSG00000159261	ENSG00000134873
ENSG00000159261	ENSG00000156282
ENSG00000159261	ENSG00000156284
ENSG00000159261	ENSG00000163347
ENSG00000159261	ENSG00000164007
ENSG00000159261	ENSG00000165215
ENSG00000159261	ENSG00000165376
ENSG00000159261	ENSG00000171217
ENSG00000159261	ENSG00000177300
ENSG00000159261	ENSG00000181885
ENSG00000159261	ENSG00000184113
ENSG00000159261	ENSG00000184697
ENSG00000159261	ENSG00000185758
ENSG00000159261	ENSG00000189118
ENSG00000159261	ENSG00000189143
ENSG00000159261	ENSG00000213937
ENSG00000159263	ENSG00000100644
ENSG00000159263	ENSG00000112246
ENSG00000159263	ENSG00000116016
ENSG00000159263	ENSG00000124440
ENSG00000159263	ENSG00000130751
ENSG00000159263	ENSG00000151322
ENSG00000159266	ENSG00000102445
ENSG00000159266	ENSG00000140995
ENSG00000159266	ENSG00000141342
ENSG00000159266	ENSG00000145016
ENSG00000159266	ENSG00000178385
ENSG00000159279	ENSG00000056291

ENSG00000159279	ENSG00000075073
ENSG00000159279	ENSG00000101292
ENSG00000159279	ENSG00000110148
ENSG00000159279	ENSG00000115353
ENSG00000159279	ENSG00000119973
ENSG00000159279	ENSG00000121764
ENSG00000159279	ENSG00000123901
ENSG00000159279	ENSG00000137252
ENSG00000159279	ENSG00000148734
ENSG00000159279	ENSG00000163394
ENSG00000159279	ENSG00000164128
ENSG00000159279	ENSG00000164129
ENSG00000159279	ENSG00000169618
ENSG00000159279	ENSG00000169836
ENSG00000159279	ENSG00000185149
ENSG00000159279	ENSG00000186867
ENSG00000159279	ENSG00000204174
ENSG00000159279	ENSG00000215167
ENSG00000159289	ENSG00000140478
ENSG00000159289	ENSG00000153666
ENSG00000159289	ENSG00000156363
ENSG00000159289	ENSG00000167110
ENSG00000159289	ENSG00000167195
ENSG00000159289	ENSG00000174450
ENSG00000159289	ENSG00000175265
ENSG00000159289	ENSG00000181984
ENSG00000159289	ENSG00000184206
ENSG00000159289	ENSG00000186322
ENSG00000159289	ENSG00000188388
ENSG00000159289	ENSG00000188532
ENSG00000159289	ENSG00000188626
ENSG00000159289	ENSG00000197092
ENSG00000159289	ENSG00000197414
ENSG00000159289	ENSG00000214348
ENSG00000159289	ENSG00000215252
ENSG00000159289	ENSG00000215405
ENSG00000159307	ENSG00000146197
ENSG00000159307	ENSG00000175356
ENSG00000159337	ENSG00000105499
ENSG00000159337	ENSG00000116711
ENSG00000159337	ENSG00000168907
ENSG00000159337	ENSG00000168970
ENSG00000159337	ENSG00000188089
ENSG00000159346	ENSG00000006831
ENSG00000159346	ENSG00000163291
ENSG00000159348	ENSG00000100243
ENSG00000159348	ENSG00000166394
ENSG00000159363	ENSG00000127249
ENSG00000159363	ENSG00000133657
ENSG00000159363	ENSG00000187527
ENSG00000159387	ENSG00000113430
ENSG00000159387	ENSG00000150051
ENSG00000159387	ENSG00000170549
ENSG00000159387	ENSG00000170561
ENSG00000159387	ENSG00000176842
ENSG00000159387	ENSG00000177508
ENSG00000159388	ENSG00000133639
ENSG00000159388	ENSG00000137707
ENSG00000159388	ENSG00000154640

ENSG00000159399	ENSG00000106633
ENSG00000159399	ENSG00000156510
ENSG00000159399	ENSG00000156515
ENSG00000159399	ENSG00000160883
ENSG00000159403	ENSG00000009724
ENSG00000159403	ENSG00000127241
ENSG00000159403	ENSG00000139178
ENSG00000159403	ENSG00000182326
ENSG00000159409	ENSG00000048740
ENSG00000159409	ENSG00000101489
ENSG00000159409	ENSG00000140488
ENSG00000159409	ENSG00000149187
ENSG00000159409	ENSG00000161082
ENSG00000159459	ENSG00000024048
ENSG00000159459	ENSG00000144357
ENSG00000159495	ENSG00000092295
ENSG00000159495	ENSG00000104055
ENSG00000159495	ENSG00000124491
ENSG00000159495	ENSG00000125780
ENSG00000159495	ENSG00000163810
ENSG00000159495	ENSG00000166947
ENSG00000159495	ENSG00000166948
ENSG00000159495	ENSG00000198959
ENSG00000159556	ENSG00000016082
ENSG00000159556	ENSG00000089116
ENSG00000159556	ENSG00000106689
ENSG00000159556	ENSG00000106852
ENSG00000159556	ENSG00000107187
ENSG00000159556	ENSG00000121454
ENSG00000159556	ENSG00000132130
ENSG00000159556	ENSG00000136944
ENSG00000159556	ENSG00000143013
ENSG00000159556	ENSG00000143355
ENSG00000159556	ENSG00000162624
ENSG00000159556	ENSG00000162761
ENSG00000159640	ENSG00000130234
ENSG00000159685	ENSG00000106554
ENSG00000159692	ENSG00000175029
ENSG00000159713	ENSG00000171368
ENSG00000159713	ENSG00000179636
ENSG00000159714	ENSG00000188818
ENSG00000159714	ENSG00000206077
ENSG00000159720	ENSG00000147614
ENSG00000159723	ENSG00000101440
ENSG00000159753	ENSG00000079691
ENSG00000159753	ENSG00000186648
ENSG00000159784	ENSG00000175182
ENSG00000159784	ENSG00000185519
ENSG00000159788	ENSG00000169220
ENSG00000159792	ENSG00000008118
ENSG00000159792	ENSG00000081320
ENSG00000159792	ENSG00000130822
ENSG00000159792	ENSG00000134072
ENSG00000159792	ENSG00000147613
ENSG00000159792	ENSG00000164076
ENSG00000159792	ENSG00000164543
ENSG00000159792	ENSG00000183049
ENSG00000159840	ENSG00000087077
ENSG00000159840	ENSG00000129474

ENSG00000159840	ENSG00000142279
ENSG00000159840	ENSG00000144791
ENSG00000159840	ENSG00000145012
ENSG00000159840	ENSG00000162458
ENSG00000159842	ENSG00000186716
ENSG00000159899	ENSG00000070019
ENSG00000159899	ENSG00000101890
ENSG00000159899	ENSG00000123201
ENSG00000159899	ENSG00000132518
ENSG00000159899	ENSG00000164116
ENSG00000159899	ENSG00000169418
ENSG00000160007	ENSG00000100852
ENSG00000160013	ENSG00000006638
ENSG00000160013	ENSG00000050628
ENSG00000160013	ENSG00000122420
ENSG00000160013	ENSG00000125384
ENSG00000160013	ENSG00000160951
ENSG00000160013	ENSG00000168229
ENSG00000160013	ENSG00000171522
ENSG00000160014	ENSG00000129007
ENSG00000160014	ENSG00000143933
ENSG00000160014	ENSG00000178363
ENSG00000160014	ENSG00000178372
ENSG00000160014	ENSG00000198668
ENSG00000160050	ENSG00000024862
ENSG00000160062	ENSG00000168795
ENSG00000160062	ENSG00000185670
ENSG00000160062	ENSG00000205189
ENSG00000160062	ENSG00000215897
ENSG00000160075	ENSG00000176960
ENSG00000160075	ENSG00000186217
ENSG00000160075	ENSG00000186223
ENSG00000160075	ENSG00000186232
ENSG00000160097	ENSG00000115226
ENSG00000160145	ENSG00000038382
ENSG00000160145	ENSG00000053524
ENSG00000160145	ENSG00000101977
ENSG00000160145	ENSG00000126217
ENSG00000160161	ENSG00000138615
ENSG00000160179	ENSG00000172350
ENSG00000160183	ENSG00000105707
ENSG00000160183	ENSG00000137648
ENSG00000160183	ENSG00000137747
ENSG00000160183	ENSG00000145244
ENSG00000160183	ENSG00000154646
ENSG00000160183	ENSG00000166682
ENSG00000160183	ENSG00000184012
ENSG00000160185	ENSG00000154127
ENSG00000160190	ENSG00000134955
ENSG00000160190	ENSG00000137700
ENSG00000160190	ENSG00000157800
ENSG00000160199	ENSG00000105419
ENSG00000160199	ENSG00000118707
ENSG00000160199	ENSG00000134138
ENSG00000160199	ENSG00000143995
ENSG00000160199	ENSG00000153779
ENSG00000160199	ENSG00000165495
ENSG00000160199	ENSG00000176679
ENSG00000160199	ENSG00000177426

ENSG00000160199	ENSG00000179277
ENSG00000160199	ENSG00000188013
ENSG00000160201	ENSG00000161265
ENSG00000160201	ENSG00000179315
ENSG00000160202	ENSG00000004776
ENSG00000160202	ENSG00000106211
ENSG00000160202	ENSG00000109846
ENSG00000160202	ENSG00000152137
ENSG00000160202	ENSG00000169271
ENSG00000160202	ENSG00000170276
ENSG00000160216	ENSG00000026652
ENSG00000160219	ENSG00000033327
ENSG00000160219	ENSG00000109458
ENSG00000160219	ENSG00000215568
ENSG00000160223	ENSG00000103855
ENSG00000160223	ENSG00000114013
ENSG00000160223	ENSG00000114455
ENSG00000160223	ENSG00000120217
ENSG00000160223	ENSG00000134258
ENSG00000160223	ENSG00000186470
ENSG00000160223	ENSG00000188211
ENSG00000160223	ENSG00000197646
ENSG00000160223	ENSG00000204290
ENSG00000160223	ENSG00000204655
ENSG00000160223	ENSG00000215631
ENSG00000160223	ENSG00000215811
ENSG00000160223	ENSG00000215889
ENSG00000160233	ENSG00000179796
ENSG00000160255	ENSG00000056345
ENSG00000160255	ENSG00000082781
ENSG00000160255	ENSG00000105855
ENSG00000160255	ENSG00000115221
ENSG00000160255	ENSG00000132470
ENSG00000160255	ENSG00000139626
ENSG00000160255	ENSG00000150093
ENSG00000160271	ENSG00000143344
ENSG00000160271	ENSG00000204218
ENSG00000160271	ENSG00000205517
ENSG00000160293	ENSG00000134215
ENSG00000160293	ENSG00000141968
ENSG00000160296	ENSG00000105366
ENSG00000160296	ENSG00000105383
ENSG00000160296	ENSG00000105492
ENSG00000160296	ENSG00000105501
ENSG00000160296	ENSG00000105695
ENSG00000160296	ENSG00000129450
ENSG00000160296	ENSG00000142512
ENSG00000160296	ENSG00000161640
ENSG00000160296	ENSG00000161643
ENSG00000160296	ENSG00000167764
ENSG00000160296	ENSG00000168995
ENSG00000160296	ENSG00000171101
ENSG00000160296	ENSG00000197865
ENSG00000160305	ENSG00000066084
ENSG00000160305	ENSG00000151240
ENSG00000160307	ENSG00000143546
ENSG00000160307	ENSG00000160678
ENSG00000160307	ENSG00000163191
ENSG00000160307	ENSG00000163220

ENSG00000160307	ENSG00000163221
ENSG00000160307	ENSG00000163993
ENSG00000160307	ENSG00000169906
ENSG00000160307	ENSG00000171643
ENSG00000160307	ENSG00000188015
ENSG00000160307	ENSG00000188643
ENSG00000160307	ENSG00000189171
ENSG00000160307	ENSG00000189334
ENSG00000160307	ENSG00000196154
ENSG00000160307	ENSG00000196420
ENSG00000160307	ENSG00000196754
ENSG00000160307	ENSG00000197747
ENSG00000160307	ENSG00000197956
ENSG00000160318	ENSG00000105370
ENSG00000160318	ENSG00000105374
ENSG00000160323	ENSG00000049192
ENSG00000160323	ENSG00000087116
ENSG00000160323	ENSG00000136378
ENSG00000160323	ENSG00000138316
ENSG00000160323	ENSG00000140470
ENSG00000160323	ENSG00000140873
ENSG00000160323	ENSG00000142303
ENSG00000160323	ENSG00000145536
ENSG00000160323	ENSG00000145808
ENSG00000160323	ENSG00000151388
ENSG00000160323	ENSG00000156140
ENSG00000160323	ENSG00000163638
ENSG00000160323	ENSG00000173157
ENSG00000160323	ENSG00000214644
ENSG00000160323	ENSG00000215743
ENSG00000160326	ENSG00000136856
ENSG00000160360	ENSG00000100154
ENSG00000160360	ENSG00000121957
ENSG00000160360	ENSG00000137473
ENSG00000160360	ENSG00000165917
ENSG00000160360	ENSG00000187862
ENSG00000160396	ENSG00000064393
ENSG00000160396	ENSG00000110422
ENSG00000160396	ENSG00000163349
ENSG00000160404	ENSG00000136816
ENSG00000160404	ENSG00000136827
ENSG00000160404	ENSG00000186283
ENSG00000160404	ENSG00000198113
ENSG00000160408	ENSG00000073849
ENSG00000160408	ENSG00000101638
ENSG00000160408	ENSG00000111728
ENSG00000160408	ENSG00000117069
ENSG00000160408	ENSG00000144057
ENSG00000160408	ENSG00000148488
ENSG00000160408	ENSG00000177511
ENSG00000160439	ENSG00000072042
ENSG00000160439	ENSG00000139988
ENSG00000160439	ENSG00000167536
ENSG00000160439	ENSG00000169084
ENSG00000160447	ENSG00000065243
ENSG00000160447	ENSG00000123143
ENSG00000160460	ENSG00000070182
ENSG00000160460	ENSG00000115306
ENSG00000160460	ENSG00000137877

ENSG00000160460	ENSG00000173898
ENSG00000160469	ENSG00000174672
ENSG00000160471	ENSG00000126267
ENSG00000160471	ENSG00000172912
ENSG00000160505	ENSG00000022556
ENSG00000160505	ENSG00000023191
ENSG00000160505	ENSG00000140853
ENSG00000160505	ENSG00000142405
ENSG00000160505	ENSG00000158077
ENSG00000160505	ENSG00000162711
ENSG00000160505	ENSG00000167207
ENSG00000160505	ENSG00000167634
ENSG00000160505	ENSG00000167984
ENSG00000160505	ENSG00000171487
ENSG00000160505	ENSG00000173572
ENSG00000160505	ENSG00000174885
ENSG00000160505	ENSG00000179709
ENSG00000160505	ENSG00000179873
ENSG00000160505	ENSG00000182261
ENSG00000160505	ENSG00000185792
ENSG00000160516	ENSG00000182953
ENSG00000160516	ENSG00000197303
ENSG00000160539	ENSG00000205808
ENSG00000160551	ENSG00000135090
ENSG00000160551	ENSG00000149930
ENSG00000160570	ENSG00000158796
ENSG00000160588	ENSG00000149573
ENSG00000160588	ENSG00000149575
ENSG00000160588	ENSG00000158887
ENSG00000160588	ENSG00000160593
ENSG00000160588	ENSG00000177098
ENSG00000160588	ENSG00000197965
ENSG00000160593	ENSG00000149573
ENSG00000160593	ENSG00000149575
ENSG00000160593	ENSG00000158887
ENSG00000160593	ENSG00000160588
ENSG00000160593	ENSG00000177098
ENSG00000160593	ENSG00000197965
ENSG00000160633	ENSG00000130254
ENSG00000160633	ENSG00000137776
ENSG00000160654	ENSG00000167286
ENSG00000160654	ENSG00000198851
ENSG00000160678	ENSG00000143546
ENSG00000160678	ENSG00000160307
ENSG00000160678	ENSG00000163191
ENSG00000160678	ENSG00000163220
ENSG00000160678	ENSG00000163221
ENSG00000160678	ENSG00000163993
ENSG00000160678	ENSG00000169906
ENSG00000160678	ENSG00000171643
ENSG00000160678	ENSG00000188015
ENSG00000160678	ENSG00000188643
ENSG00000160678	ENSG00000189171
ENSG00000160678	ENSG00000189334
ENSG00000160678	ENSG00000196154
ENSG00000160678	ENSG00000196420
ENSG00000160678	ENSG00000196754
ENSG00000160678	ENSG00000197747
ENSG00000160678	ENSG00000197956

ENSG00000160683	ENSG00000112486
ENSG00000160683	ENSG00000121797
ENSG00000160683	ENSG00000121807
ENSG00000160683	ENSG00000121966
ENSG00000160683	ENSG00000126353
ENSG00000160683	ENSG00000129048
ENSG00000160683	ENSG00000144648
ENSG00000160683	ENSG00000160791
ENSG00000160683	ENSG00000163464
ENSG00000160683	ENSG00000163823
ENSG00000160683	ENSG00000168329
ENSG00000160683	ENSG00000172215
ENSG00000160683	ENSG00000173578
ENSG00000160683	ENSG00000173585
ENSG00000160683	ENSG00000179934
ENSG00000160683	ENSG00000180871
ENSG00000160683	ENSG00000183625
ENSG00000160683	ENSG00000183813
ENSG00000160683	ENSG00000184451
ENSG00000160683	ENSG00000186810
ENSG00000160683	ENSG00000215775
ENSG00000160683	ENSG00000215778
ENSG00000160683	ENSG00000215782
ENSG00000160685	ENSG00000178951
ENSG00000160685	ENSG00000184828
ENSG00000160691	ENSG00000129946
ENSG00000160691	ENSG00000148082
ENSG00000160691	ENSG00000185634
ENSG00000160710	ENSG00000065457
ENSG00000160710	ENSG00000140955
ENSG00000160710	ENSG00000164113
ENSG00000160710	ENSG00000185736
ENSG00000160710	ENSG00000197381
ENSG00000160712	ENSG00000105246
ENSG00000160712	ENSG00000122756
ENSG00000160712	ENSG00000137070
ENSG00000160714	ENSG00000140367
ENSG00000160714	ENSG00000189136
ENSG00000160714	ENSG00000215218
ENSG00000160716	ENSG00000080644
ENSG00000160716	ENSG00000101204
ENSG00000160716	ENSG00000108556
ENSG00000160716	ENSG00000117971
ENSG00000160716	ENSG00000120903
ENSG00000160716	ENSG00000129749
ENSG00000160716	ENSG00000135902
ENSG00000160716	ENSG00000138435
ENSG00000160716	ENSG00000147432
ENSG00000160716	ENSG00000147434
ENSG00000160716	ENSG00000149305
ENSG00000160716	ENSG00000166664
ENSG00000160716	ENSG00000166736
ENSG00000160716	ENSG00000169684
ENSG00000160716	ENSG00000170175
ENSG00000160716	ENSG00000174343
ENSG00000160716	ENSG00000175344
ENSG00000160716	ENSG00000178084
ENSG00000160716	ENSG00000186038
ENSG00000160716	ENSG00000186090

ENSG00000160716	ENSG00000186919
ENSG00000160716	ENSG00000196811
ENSG00000160741	ENSG00000105662
ENSG00000160741	ENSG00000140577
ENSG00000160746	ENSG00000047617
ENSG00000160746	ENSG00000074855
ENSG00000160746	ENSG00000131620
ENSG00000160746	ENSG00000134343
ENSG00000160746	ENSG00000146205
ENSG00000160746	ENSG00000151572
ENSG00000160746	ENSG00000171714
ENSG00000160746	ENSG00000177119
ENSG00000160746	ENSG00000185101
ENSG00000160767	ENSG00000104059
ENSG00000160767	ENSG00000135063
ENSG00000160781	ENSG00000137819
ENSG00000160781	ENSG00000170915
ENSG00000160781	ENSG00000182749
ENSG00000160781	ENSG00000188582
ENSG00000160789	ENSG00000113368
ENSG00000160789	ENSG00000176619
ENSG00000160791	ENSG00000112486
ENSG00000160791	ENSG00000121797
ENSG00000160791	ENSG00000121807
ENSG00000160791	ENSG00000121966
ENSG00000160791	ENSG00000126353
ENSG00000160791	ENSG00000129048
ENSG00000160791	ENSG00000144648
ENSG00000160791	ENSG00000160683
ENSG00000160791	ENSG00000163464
ENSG00000160791	ENSG00000163823
ENSG00000160791	ENSG00000168329
ENSG00000160791	ENSG00000172215
ENSG00000160791	ENSG00000173578
ENSG00000160791	ENSG00000173585
ENSG00000160791	ENSG00000179934
ENSG00000160791	ENSG00000180871
ENSG00000160791	ENSG00000183625
ENSG00000160791	ENSG00000183813
ENSG00000160791	ENSG00000184451
ENSG00000160791	ENSG00000186810
ENSG00000160791	ENSG00000215775
ENSG00000160791	ENSG00000215778
ENSG00000160791	ENSG00000215782
ENSG00000160796	ENSG00000144426
ENSG00000160801	ENSG00000004948
ENSG00000160801	ENSG00000010310
ENSG00000160801	ENSG00000064989
ENSG00000160801	ENSG00000065325
ENSG00000160801	ENSG00000078549
ENSG00000160801	ENSG00000080293
ENSG00000160801	ENSG00000106018
ENSG00000160801	ENSG00000106113
ENSG00000160801	ENSG00000106128
ENSG00000160801	ENSG00000112164
ENSG00000160801	ENSG00000114812
ENSG00000160801	ENSG00000120088
ENSG00000160801	ENSG00000144407
ENSG00000160801	ENSG00000215644

ENSG00000160803	ENSG00000135018
ENSG00000160803	ENSG00000175518
ENSG00000160803	ENSG00000175520
ENSG00000160803	ENSG00000188021
ENSG00000160808	ENSG00000092841
ENSG00000160808	ENSG00000168530
ENSG00000160808	ENSG00000196465
ENSG00000160808	ENSG00000198336
ENSG00000160844	ENSG00000182482
ENSG00000160844	ENSG00000183086
ENSG00000160844	ENSG00000198750
ENSG00000160867	ENSG00000066468
ENSG00000160867	ENSG00000068078
ENSG00000160867	ENSG00000077782
ENSG00000160868	ENSG00000021461
ENSG00000160868	ENSG00000059377
ENSG00000160868	ENSG00000106258
ENSG00000160868	ENSG00000160870
ENSG00000160870	ENSG00000021461
ENSG00000160870	ENSG00000059377
ENSG00000160870	ENSG00000106258
ENSG00000160870	ENSG00000160868
ENSG00000160877	ENSG00000148411
ENSG00000160877	ENSG00000173276
ENSG00000160877	ENSG00000177125
ENSG00000160882	ENSG00000019186
ENSG00000160882	ENSG00000111012
ENSG00000160882	ENSG00000135929
ENSG00000160882	ENSG00000140459
ENSG00000160882	ENSG00000179142
ENSG00000160882	ENSG00000186684
ENSG00000160883	ENSG00000106633
ENSG00000160883	ENSG00000156510
ENSG00000160883	ENSG00000156515
ENSG00000160883	ENSG00000159399
ENSG00000160888	ENSG00000162783
ENSG00000160888	ENSG00000188483
ENSG00000160916	ENSG00000186390
ENSG00000160951	ENSG00000006638
ENSG00000160951	ENSG00000050628
ENSG00000160951	ENSG00000122420
ENSG00000160951	ENSG00000125384
ENSG00000160951	ENSG00000160013
ENSG00000160951	ENSG00000168229
ENSG00000160951	ENSG00000171522
ENSG00000160959	ENSG00000116708
ENSG00000160959	ENSG00000116721
ENSG00000160959	ENSG00000116726
ENSG00000160959	ENSG00000120952
ENSG00000160959	ENSG00000157358
ENSG00000160959	ENSG00000179704
ENSG00000160959	ENSG00000182330
ENSG00000160959	ENSG00000185028
ENSG00000160959	ENSG00000185686
ENSG00000160959	ENSG00000187545
ENSG00000160959	ENSG00000197549
ENSG00000160959	ENSG00000204478
ENSG00000160959	ENSG00000204479
ENSG00000160959	ENSG00000204480

ENSG00000160959	ENSG00000204481
ENSG00000160959	ENSG00000204485
ENSG00000160959	ENSG00000204486
ENSG00000160959	ENSG00000204488
ENSG00000160959	ENSG00000204491
ENSG00000160959	ENSG00000204495
ENSG00000160959	ENSG00000204501
ENSG00000160959	ENSG00000204502
ENSG00000160959	ENSG00000204503
ENSG00000160959	ENSG00000204505
ENSG00000160959	ENSG00000204507
ENSG00000160959	ENSG00000204508
ENSG00000160959	ENSG00000204509
ENSG00000160959	ENSG00000204510
ENSG00000160959	ENSG00000204513
ENSG00000160963	ENSG00000186998
ENSG00000160967	ENSG00000111249
ENSG00000160967	ENSG00000119547
ENSG00000160967	ENSG00000169856
ENSG00000160967	ENSG00000205922
ENSG00000160972	ENSG00000101445
ENSG00000160991	ENSG00000175938
ENSG00000160991	ENSG00000182500
ENSG00000160999	ENSG00000111252
ENSG00000160999	ENSG00000178188
ENSG00000161013	ENSG00000071073
ENSG00000161013	ENSG00000182050
ENSG00000161013	ENSG00000184774
ENSG00000161021	ENSG00000013619
ENSG00000161021	ENSG00000184384
ENSG00000161021	ENSG00000196782
ENSG00000161082	ENSG00000048740
ENSG00000161082	ENSG00000101489
ENSG00000161082	ENSG00000140488
ENSG00000161082	ENSG00000149187
ENSG00000161082	ENSG00000159409
ENSG00000161202	ENSG00000004975
ENSG00000161202	ENSG00000107404
ENSG00000161202	ENSG00000150764
ENSG00000161202	ENSG00000215737
ENSG00000161217	ENSG00000102230
ENSG00000161241	ENSG00000116661
ENSG00000161241	ENSG00000116663
ENSG00000161241	ENSG00000132879
ENSG00000161241	ENSG00000161243
ENSG00000161241	ENSG00000188505
ENSG00000161243	ENSG00000116661
ENSG00000161243	ENSG00000116663
ENSG00000161243	ENSG00000132879
ENSG00000161243	ENSG00000161241
ENSG00000161243	ENSG00000188505
ENSG00000161265	ENSG00000160201
ENSG00000161265	ENSG00000179315
ENSG00000161277	ENSG00000041988
ENSG00000161277	ENSG00000129028
ENSG00000161277	ENSG00000131931
ENSG00000161277	ENSG00000173451
ENSG00000161281	ENSG00000112695
ENSG00000161281	ENSG00000115944

ENSG00000161326	ENSG00000167065
ENSG00000161326	ENSG00000189037
ENSG00000161381	ENSG00000120594
ENSG00000161405	ENSG00000030419
ENSG00000161405	ENSG00000123411
ENSG00000161405	ENSG00000185811
ENSG00000161509	ENSG00000105464
ENSG00000161509	ENSG00000116032
ENSG00000161509	ENSG00000150086
ENSG00000161509	ENSG00000176884
ENSG00000161509	ENSG00000183454
ENSG00000161509	ENSG00000198785
ENSG00000161542	ENSG00000141127
ENSG00000161544	ENSG00000198125
ENSG00000161547	ENSG00000164493
ENSG00000161547	ENSG00000206197
ENSG00000161547	ENSG00000214754
ENSG00000161570	ENSG00000006074
ENSG00000161570	ENSG00000006075
ENSG00000161570	ENSG00000006606
ENSG00000161570	ENSG00000102962
ENSG00000161570	ENSG00000102970
ENSG00000161570	ENSG00000106178
ENSG00000161570	ENSG00000108688
ENSG00000161570	ENSG00000108691
ENSG00000161570	ENSG00000108700
ENSG00000161570	ENSG00000108702
ENSG00000161570	ENSG00000115009
ENSG00000161570	ENSG00000129277
ENSG00000161570	ENSG00000143184
ENSG00000161570	ENSG00000143185
ENSG00000161570	ENSG00000161573
ENSG00000161570	ENSG00000161574
ENSG00000161570	ENSG00000167236
ENSG00000161570	ENSG00000172156
ENSG00000161570	ENSG00000172724
ENSG00000161570	ENSG00000181374
ENSG00000161570	ENSG00000197262
ENSG00000161570	ENSG00000205017
ENSG00000161570	ENSG00000205020
ENSG00000161570	ENSG00000205021
ENSG00000161570	ENSG00000213494
ENSG00000161570	ENSG00000215627
ENSG00000161570	ENSG00000215629
ENSG00000161572	ENSG00000090382
ENSG00000161572	ENSG00000120563
ENSG00000161572	ENSG00000141316
ENSG00000161572	ENSG00000151033
ENSG00000161572	ENSG00000157093
ENSG00000161572	ENSG00000167531
ENSG00000161572	ENSG00000171478
ENSG00000161572	ENSG00000171489
ENSG00000161573	ENSG00000006074
ENSG00000161573	ENSG00000006075
ENSG00000161573	ENSG00000006606
ENSG00000161573	ENSG00000102962
ENSG00000161573	ENSG00000102970
ENSG00000161573	ENSG00000106178
ENSG00000161573	ENSG00000108688

ENSG00000161573	ENSG00000108691
ENSG00000161573	ENSG00000108700
ENSG00000161573	ENSG00000108702
ENSG00000161573	ENSG00000115009
ENSG00000161573	ENSG00000129277
ENSG00000161573	ENSG00000143184
ENSG00000161573	ENSG00000143185
ENSG00000161573	ENSG00000161570
ENSG00000161573	ENSG00000161574
ENSG00000161573	ENSG00000167236
ENSG00000161573	ENSG00000172156
ENSG00000161573	ENSG00000172724
ENSG00000161573	ENSG00000181374
ENSG00000161573	ENSG00000197262
ENSG00000161573	ENSG00000205017
ENSG00000161573	ENSG00000205020
ENSG00000161573	ENSG00000205021
ENSG00000161573	ENSG00000213494
ENSG00000161573	ENSG00000215627
ENSG00000161573	ENSG00000215629
ENSG00000161574	ENSG00000006074
ENSG00000161574	ENSG00000006075
ENSG00000161574	ENSG00000006606
ENSG00000161574	ENSG00000102962
ENSG00000161574	ENSG00000102970
ENSG00000161574	ENSG00000106178
ENSG00000161574	ENSG00000108688
ENSG00000161574	ENSG00000108691
ENSG00000161574	ENSG00000108700
ENSG00000161574	ENSG00000108702
ENSG00000161574	ENSG00000115009
ENSG00000161574	ENSG00000129277
ENSG00000161574	ENSG00000143184
ENSG00000161574	ENSG00000143185
ENSG00000161574	ENSG00000161570
ENSG00000161574	ENSG00000161573
ENSG00000161574	ENSG00000167236
ENSG00000161574	ENSG00000172156
ENSG00000161574	ENSG00000172724
ENSG00000161574	ENSG00000181374
ENSG00000161574	ENSG00000197262
ENSG00000161574	ENSG00000205017
ENSG00000161574	ENSG00000205020
ENSG00000161574	ENSG00000205021
ENSG00000161574	ENSG00000213494
ENSG00000161574	ENSG00000215627
ENSG00000161574	ENSG00000215629
ENSG00000161583	ENSG00000128438
ENSG00000161583	ENSG00000148429
ENSG00000161583	ENSG00000174115
ENSG00000161583	ENSG00000185128
ENSG00000161583	ENSG00000188755
ENSG00000161583	ENSG00000188849
ENSG00000161583	ENSG00000189309
ENSG00000161583	ENSG00000197681
ENSG00000161583	ENSG00000204471
ENSG00000161583	ENSG00000205019
ENSG00000161583	ENSG00000213474
ENSG00000161583	ENSG00000215625

ENSG00000161583	ENSG00000215730
ENSG00000161583	ENSG00000215748
ENSG00000161638	ENSG00000005844
ENSG00000161638	ENSG00000005884
ENSG00000161638	ENSG00000005961
ENSG00000161638	ENSG00000077943
ENSG00000161638	ENSG00000083457
ENSG00000161638	ENSG00000091409
ENSG00000161638	ENSG00000115232
ENSG00000161638	ENSG00000135424
ENSG00000161638	ENSG00000137809
ENSG00000161638	ENSG00000138448
ENSG00000161638	ENSG00000140678
ENSG00000161638	ENSG00000143127
ENSG00000161638	ENSG00000144668
ENSG00000161638	ENSG00000156886
ENSG00000161638	ENSG00000164171
ENSG00000161638	ENSG00000169896
ENSG00000161638	ENSG00000213949
ENSG00000161640	ENSG00000105366
ENSG00000161640	ENSG00000105383
ENSG00000161640	ENSG00000105492
ENSG00000161640	ENSG00000105501
ENSG00000161640	ENSG00000105695
ENSG00000161640	ENSG00000129450
ENSG00000161640	ENSG00000142512
ENSG00000161640	ENSG00000160296
ENSG00000161640	ENSG00000161643
ENSG00000161640	ENSG00000167764
ENSG00000161640	ENSG00000168995
ENSG00000161640	ENSG00000171101
ENSG00000161640	ENSG00000197865
ENSG00000161642	ENSG00000144331
ENSG00000161642	ENSG00000151789
ENSG00000161642	ENSG00000187595
ENSG00000161643	ENSG00000105366
ENSG00000161643	ENSG00000105383
ENSG00000161643	ENSG00000105492
ENSG00000161643	ENSG00000105501
ENSG00000161643	ENSG00000105695
ENSG00000161643	ENSG00000129450
ENSG00000161643	ENSG00000142512
ENSG00000161643	ENSG00000160296
ENSG00000161643	ENSG00000161640
ENSG00000161643	ENSG00000167764
ENSG00000161643	ENSG00000168995
ENSG00000161643	ENSG00000171101
ENSG00000161643	ENSG00000197865
ENSG00000161647	ENSG00000072415
ENSG00000161647	ENSG00000082126
ENSG00000161647	ENSG00000105926
ENSG00000161647	ENSG00000108852
ENSG00000161647	ENSG00000130830
ENSG00000161647	ENSG00000147044
ENSG00000161647	ENSG00000150054
ENSG00000161649	ENSG00000095970
ENSG00000161649	ENSG00000096264
ENSG00000161649	ENSG00000162896
ENSG00000161649	ENSG00000162897

ENSG00000161649	ENSG00000167850
ENSG00000161649	ENSG00000167851
ENSG00000161649	ENSG00000178789
ENSG00000161649	ENSG00000186074
ENSG00000161649	ENSG00000186407
ENSG00000161649	ENSG00000204345
ENSG00000161677	ENSG00000100221
ENSG00000161681	ENSG00000099882
ENSG00000161681	ENSG00000162105
ENSG00000161682	ENSG00000144369
ENSG00000161682	ENSG00000148468
ENSG00000161714	ENSG00000101333
ENSG00000161714	ENSG00000114805
ENSG00000161714	ENSG00000115556
ENSG00000161714	ENSG00000115896
ENSG00000161714	ENSG00000124181
ENSG00000161714	ENSG00000137841
ENSG00000161714	ENSG00000139151
ENSG00000161714	ENSG00000149527
ENSG00000161714	ENSG00000149782
ENSG00000161714	ENSG00000154822
ENSG00000161714	ENSG00000182621
ENSG00000161714	ENSG00000187091
ENSG00000161714	ENSG00000197943
ENSG00000161791	ENSG00000157827
ENSG00000161791	ENSG00000184922
ENSG00000161798	ENSG00000086159
ENSG00000161798	ENSG00000103375
ENSG00000161798	ENSG00000106125
ENSG00000161798	ENSG00000135517
ENSG00000161798	ENSG00000167580
ENSG00000161798	ENSG00000171885
ENSG00000161813	ENSG00000107929
ENSG00000161835	ENSG00000115165
ENSG00000161847	ENSG00000162437
ENSG00000161849	ENSG00000135443
ENSG00000161849	ENSG00000135477
ENSG00000161849	ENSG00000135480
ENSG00000161849	ENSG00000139648
ENSG00000161849	ENSG00000161850
ENSG00000161849	ENSG00000167767
ENSG00000161849	ENSG00000167768
ENSG00000161849	ENSG00000170421
ENSG00000161849	ENSG00000170423
ENSG00000161849	ENSG00000170442
ENSG00000161849	ENSG00000170454
ENSG00000161849	ENSG00000170465
ENSG00000161849	ENSG00000170477
ENSG00000161849	ENSG00000170484
ENSG00000161849	ENSG00000170486
ENSG00000161849	ENSG00000170523
ENSG00000161849	ENSG00000172867
ENSG00000161849	ENSG00000185069
ENSG00000161849	ENSG00000185479
ENSG00000161849	ENSG00000185640
ENSG00000161849	ENSG00000186049
ENSG00000161849	ENSG00000186081
ENSG00000161849	ENSG00000186442
ENSG00000161849	ENSG00000189182

ENSG00000161849	ENSG00000205420
ENSG00000161849	ENSG00000205426
ENSG00000161849	ENSG00000214233
ENSG00000161849	ENSG00000214323
ENSG00000161849	ENSG00000214477
ENSG00000161849	ENSG00000214752
ENSG00000161849	ENSG00000214974
ENSG00000161849	ENSG00000215287
ENSG00000161849	ENSG00000215847
ENSG00000161850	ENSG00000135443
ENSG00000161850	ENSG00000135477
ENSG00000161850	ENSG00000135480
ENSG00000161850	ENSG00000139648
ENSG00000161850	ENSG00000161849
ENSG00000161850	ENSG00000167767
ENSG00000161850	ENSG00000167768
ENSG00000161850	ENSG00000170421
ENSG00000161850	ENSG00000170423
ENSG00000161850	ENSG00000170442
ENSG00000161850	ENSG00000170454
ENSG00000161850	ENSG00000170465
ENSG00000161850	ENSG00000170477
ENSG00000161850	ENSG00000170484
ENSG00000161850	ENSG00000170486
ENSG00000161850	ENSG00000170523
ENSG00000161850	ENSG00000172867
ENSG00000161850	ENSG00000185069
ENSG00000161850	ENSG00000185479
ENSG00000161850	ENSG00000185640
ENSG00000161850	ENSG00000186049
ENSG00000161850	ENSG00000186081
ENSG00000161850	ENSG00000186442
ENSG00000161850	ENSG00000189182
ENSG00000161850	ENSG00000205420
ENSG00000161850	ENSG00000205426
ENSG00000161850	ENSG00000214233
ENSG00000161850	ENSG00000214323
ENSG00000161850	ENSG00000214477
ENSG00000161850	ENSG00000214752
ENSG00000161850	ENSG00000214974
ENSG00000161850	ENSG00000215287
ENSG00000161850	ENSG00000215847
ENSG00000161896	ENSG00000068745
ENSG00000161896	ENSG00000176095
ENSG00000161905	ENSG00000012779
ENSG00000161905	ENSG00000108839
ENSG00000161905	ENSG00000179148
ENSG00000161905	ENSG00000179477
ENSG00000161905	ENSG00000179593
ENSG00000161905	ENSG00000188101
ENSG00000161905	ENSG00000214859
ENSG00000161914	ENSG00000158805
ENSG00000161914	ENSG00000171163
ENSG00000161914	ENSG00000186660
ENSG00000161940	ENSG00000113916
ENSG00000161955	ENSG00000102524
ENSG00000161956	ENSG00000079387
ENSG00000161956	ENSG00000119231
ENSG00000161956	ENSG00000163904

ENSG00000161958	ENSG00000070193
ENSG00000161958	ENSG00000070388
ENSG00000161958	ENSG00000075388
ENSG00000161958	ENSG00000078579
ENSG00000161958	ENSG00000102466
ENSG00000161958	ENSG00000102678
ENSG00000161958	ENSG00000111241
ENSG00000161958	ENSG00000113578
ENSG00000161958	ENSG00000114279
ENSG00000161958	ENSG00000129682
ENSG00000161958	ENSG00000138675
ENSG00000161958	ENSG00000138685
ENSG00000161958	ENSG00000140285
ENSG00000161958	ENSG00000185390
ENSG00000161958	ENSG00000186895
ENSG00000161958	ENSG00000196468
ENSG00000161958	ENSG00000204801
ENSG00000161958	ENSG00000204816
ENSG00000161958	ENSG00000204817
ENSG00000161958	ENSG00000204837
ENSG00000161958	ENSG00000215053
ENSG00000161960	ENSG00000156976
ENSG00000161992	ENSG00000141579
ENSG00000162009	ENSG00000082556
ENSG00000162009	ENSG00000112038
ENSG00000162009	ENSG00000116014
ENSG00000162009	ENSG00000116329
ENSG00000162009	ENSG00000125510
ENSG00000162009	ENSG00000125522
ENSG00000162009	ENSG00000128285
ENSG00000162009	ENSG00000128310
ENSG00000162009	ENSG00000132671
ENSG00000162009	ENSG00000139874
ENSG00000162009	ENSG00000152034
ENSG00000162009	ENSG00000166573
ENSG00000162009	ENSG00000180616
ENSG00000162009	ENSG00000182687
ENSG00000162009	ENSG00000183473
ENSG00000162009	ENSG00000183729
ENSG00000162032	ENSG00000111671
ENSG00000162032	ENSG00000171621
ENSG00000162032	ENSG00000174013
ENSG00000162032	ENSG00000175093
ENSG00000162040	ENSG00000002587
ENSG00000162040	ENSG00000122254
ENSG00000162040	ENSG00000125430
ENSG00000162040	ENSG00000153976
ENSG00000162040	ENSG00000175818
ENSG00000162040	ENSG00000182601
ENSG00000162068	ENSG00000065320
ENSG00000162068	ENSG00000142233
ENSG00000162069	ENSG00000135127
ENSG00000162104	ENSG00000078295
ENSG00000162104	ENSG00000121281
ENSG00000162104	ENSG00000129467
ENSG00000162104	ENSG00000138031
ENSG00000162104	ENSG00000155897
ENSG00000162104	ENSG00000164742
ENSG00000162104	ENSG00000173175

ENSG00000162104	ENSG00000174233
ENSG00000162105	ENSG00000099882
ENSG00000162105	ENSG00000161681
ENSG00000162139	ENSG00000115488
ENSG00000162139	ENSG00000204099
ENSG00000162139	ENSG00000204386
ENSG00000162188	ENSG00000127588
ENSG00000162188	ENSG00000127920
ENSG00000162188	ENSG00000127928
ENSG00000162188	ENSG00000133136
ENSG00000162188	ENSG00000167083
ENSG00000162188	ENSG00000167414
ENSG00000162188	ENSG00000168243
ENSG00000162188	ENSG00000172380
ENSG00000162188	ENSG00000174021
ENSG00000162188	ENSG00000182625
ENSG00000162188	ENSG00000186469
ENSG00000162188	ENSG00000213611
ENSG00000162194	ENSG00000213626
ENSG00000162222	ENSG00000133985
ENSG00000162222	ENSG00000174521
ENSG00000162231	ENSG00000126952
ENSG00000162231	ENSG00000147206
ENSG00000162231	ENSG00000185554
ENSG00000162231	ENSG00000185945
ENSG00000162231	ENSG00000196970
ENSG00000162241	ENSG00000140107
ENSG00000162241	ENSG00000145832
ENSG00000162241	ENSG00000197119
ENSG00000162267	ENSG00000055955
ENSG00000162267	ENSG00000055957
ENSG00000162267	ENSG00000102313
ENSG00000162267	ENSG00000123243
ENSG00000162267	ENSG00000151655
ENSG00000162290	ENSG00000151065
ENSG00000162302	ENSG00000071242
ENSG00000162302	ENSG00000072133
ENSG00000162302	ENSG00000100784
ENSG00000162302	ENSG00000108443
ENSG00000162302	ENSG00000117676
ENSG00000162302	ENSG00000175634
ENSG00000162302	ENSG00000177189
ENSG00000162302	ENSG00000214817
ENSG00000162337	ENSG00000070018
ENSG00000162337	ENSG00000081479
ENSG00000162337	ENSG00000123384
ENSG00000162337	ENSG00000134569
ENSG00000162337	ENSG00000138798
ENSG00000162337	ENSG00000168702
ENSG00000162344	ENSG00000105550
ENSG00000162344	ENSG00000118972
ENSG00000162365	ENSG00000142973
ENSG00000162365	ENSG00000145476
ENSG00000162365	ENSG00000154198
ENSG00000162365	ENSG00000171903
ENSG00000162365	ENSG00000171954
ENSG00000162365	ENSG00000186115
ENSG00000162365	ENSG00000186160
ENSG00000162365	ENSG00000186204

ENSG00000162365	ENSG00000186377
ENSG00000162365	ENSG00000186526
ENSG00000162365	ENSG00000186529
ENSG00000162365	ENSG00000187048
ENSG00000162367	ENSG00000104903
ENSG00000162367	ENSG00000113196
ENSG00000162367	ENSG00000122691
ENSG00000162367	ENSG00000122859
ENSG00000162367	ENSG00000123307
ENSG00000162367	ENSG00000125878
ENSG00000162367	ENSG00000146618
ENSG00000162367	ENSG00000162992
ENSG00000162367	ENSG00000164107
ENSG00000162367	ENSG00000164600
ENSG00000162367	ENSG00000168267
ENSG00000162367	ENSG00000171532
ENSG00000162367	ENSG00000171786
ENSG00000162367	ENSG00000172238
ENSG00000162367	ENSG00000177551
ENSG00000162367	ENSG00000178403
ENSG00000162367	ENSG00000179774
ENSG00000162367	ENSG00000180535
ENSG00000162367	ENSG00000181965
ENSG00000162367	ENSG00000186051
ENSG00000162367	ENSG00000187786
ENSG00000162367	ENSG00000188686
ENSG00000162374	ENSG00000066044
ENSG00000162374	ENSG00000107105
ENSG00000162374	ENSG00000196361
ENSG00000162383	ENSG00000079215
ENSG00000162383	ENSG00000105143
ENSG00000162383	ENSG00000105281
ENSG00000162383	ENSG00000106688
ENSG00000162383	ENSG00000110436
ENSG00000162383	ENSG00000115902
ENSG00000162390	ENSG00000172497
ENSG00000162391	ENSG00000152380
ENSG00000162407	ENSG00000067113
ENSG00000162407	ENSG00000105520
ENSG00000162407	ENSG00000117598
ENSG00000162407	ENSG00000117600
ENSG00000162407	ENSG00000129951
ENSG00000162407	ENSG00000141934
ENSG00000162407	ENSG00000148123
ENSG00000162409	ENSG00000132356
ENSG00000162409	ENSG00000165304
ENSG00000162415	ENSG00000130449
ENSG00000162415	ENSG00000132003
ENSG00000162415	ENSG00000214655
ENSG00000162419	ENSG00000101216
ENSG00000162433	ENSG00000147853
ENSG00000162434	ENSG00000096968
ENSG00000162434	ENSG00000105397
ENSG00000162434	ENSG00000105639
ENSG00000162437	ENSG00000161847
ENSG00000162438	ENSG00000139610
ENSG00000162438	ENSG00000141086
ENSG00000162438	ENSG00000142615
ENSG00000162438	ENSG00000142789

ENSG00000162438	ENSG00000168925
ENSG00000162438	ENSG00000168928
ENSG00000162438	ENSG00000215704
ENSG00000162441	ENSG00000178585
ENSG00000162444	ENSG00000114113
ENSG00000162444	ENSG00000114115
ENSG00000162444	ENSG00000121769
ENSG00000162444	ENSG00000139194
ENSG00000162444	ENSG00000143320
ENSG00000162444	ENSG00000145384
ENSG00000162444	ENSG00000147588
ENSG00000162444	ENSG00000164434
ENSG00000162444	ENSG00000164687
ENSG00000162444	ENSG00000166426
ENSG00000162444	ENSG00000170323
ENSG00000162444	ENSG00000197416
ENSG00000162444	ENSG00000205186
ENSG00000162444	ENSG00000214004
ENSG00000162458	ENSG00000087077
ENSG00000162458	ENSG00000129474
ENSG00000162458	ENSG00000142279
ENSG00000162458	ENSG00000144791
ENSG00000162458	ENSG00000145012
ENSG00000162458	ENSG00000159840
ENSG00000162461	ENSG00000125434
ENSG00000162494	ENSG00000162763
ENSG00000162494	ENSG00000183908
ENSG00000162494	ENSG00000184709
ENSG00000162510	ENSG00000124159
ENSG00000162510	ENSG00000132031
ENSG00000162510	ENSG00000132561
ENSG00000162510	ENSG00000172752
ENSG00000162511	ENSG00000068697
ENSG00000162511	ENSG00000104341
ENSG00000162512	ENSG00000115884
ENSG00000162512	ENSG00000124145
ENSG00000162512	ENSG00000169439
ENSG00000162521	ENSG00000102054
ENSG00000162522	ENSG00000135540
ENSG00000162522	ENSG00000188158
ENSG00000162522	ENSG00000204131
ENSG00000162545	ENSG00000163888
ENSG00000162551	ENSG00000163283
ENSG00000162551	ENSG00000163286
ENSG00000162551	ENSG00000163295
ENSG00000162552	ENSG00000002745
ENSG00000162552	ENSG00000061492
ENSG00000162552	ENSG00000075290
ENSG00000162552	ENSG00000085741
ENSG00000162552	ENSG00000105989
ENSG00000162552	ENSG00000108379
ENSG00000162552	ENSG00000111186
ENSG00000162552	ENSG00000114251
ENSG00000162552	ENSG00000115596
ENSG00000162552	ENSG00000125084
ENSG00000162552	ENSG00000134245
ENSG00000162552	ENSG00000135925
ENSG00000162552	ENSG00000143816
ENSG00000162552	ENSG00000154342

ENSG00000162552	ENSG00000154764
ENSG00000162552	ENSG00000158955
ENSG00000162552	ENSG00000169884
ENSG00000162552	ENSG00000188064
ENSG00000162591	ENSG00000074660
ENSG00000162591	ENSG00000099910
ENSG00000162591	ENSG00000145794
ENSG00000162591	ENSG00000157890
ENSG00000162591	ENSG00000187800
ENSG00000162595	ENSG00000116473
ENSG00000162595	ENSG00000123728
ENSG00000162595	ENSG00000125249
ENSG00000162595	ENSG00000127314
ENSG00000162595	ENSG00000165023
ENSG00000162595	ENSG00000176276
ENSG00000162595	ENSG00000176490
ENSG00000162595	ENSG00000181467
ENSG00000162595	ENSG00000214405
ENSG00000162595	ENSG00000214492
ENSG00000162596	ENSG00000171863
ENSG00000162596	ENSG00000174121
ENSG00000162596	ENSG00000183405
ENSG00000162596	ENSG00000215342
ENSG00000162596	ENSG00000215804
ENSG00000162599	ENSG00000008441
ENSG00000162599	ENSG00000141905
ENSG00000162599	ENSG00000147862
ENSG00000162601	ENSG00000008382
ENSG00000162613	ENSG00000088247
ENSG00000162613	ENSG00000107164
ENSG00000162613	ENSG00000215224
ENSG00000162616	ENSG00000132002
ENSG00000162616	ENSG00000137094
ENSG00000162616	ENSG00000187726
ENSG00000162618	ENSG00000069122
ENSG00000162618	ENSG00000123146
ENSG00000162618	ENSG00000127507
ENSG00000162618	ENSG00000131355
ENSG00000162618	ENSG00000153292
ENSG00000162618	ENSG00000153294
ENSG00000162618	ENSG00000164393
ENSG00000162618	ENSG00000173567
ENSG00000162618	ENSG00000174837
ENSG00000162618	ENSG00000186629
ENSG00000162618	ENSG00000204818
ENSG00000162624	ENSG00000016082
ENSG00000162624	ENSG00000089116
ENSG00000162624	ENSG00000106689
ENSG00000162624	ENSG00000106852
ENSG00000162624	ENSG00000107187
ENSG00000162624	ENSG00000121454
ENSG00000162624	ENSG00000132130
ENSG00000162624	ENSG00000136944
ENSG00000162624	ENSG00000143013
ENSG00000162624	ENSG00000143355
ENSG00000162624	ENSG00000159556
ENSG00000162624	ENSG00000162761
ENSG00000162627	ENSG00000114520
ENSG00000162627	ENSG00000148158

ENSG00000162630	ENSG00000156966
ENSG00000162630	ENSG00000169255
ENSG00000162630	ENSG00000170340
ENSG00000162630	ENSG00000172318
ENSG00000162630	ENSG00000176383
ENSG00000162630	ENSG00000176597
ENSG00000162630	ENSG00000177191
ENSG00000162630	ENSG00000179913
ENSG00000162630	ENSG00000180561
ENSG00000162630	ENSG00000183778
ENSG00000162630	ENSG00000198488
ENSG00000162630	ENSG00000204222
ENSG00000162630	ENSG00000214654
ENSG00000162631	ENSG00000196358
ENSG00000162636	ENSG00000167106
ENSG00000162645	ENSG00000117226
ENSG00000162645	ENSG00000117228
ENSG00000162645	ENSG00000154451
ENSG00000162645	ENSG00000162654
ENSG00000162645	ENSG00000183347
ENSG00000162645	ENSG00000213512
ENSG00000162647	ENSG00000174695
ENSG00000162647	ENSG00000215717
ENSG00000162650	ENSG00000146776
ENSG00000162650	ENSG00000163635
ENSG00000162654	ENSG00000117226
ENSG00000162654	ENSG00000117228
ENSG00000162654	ENSG00000154451
ENSG00000162654	ENSG00000162645
ENSG00000162654	ENSG00000183347
ENSG00000162654	ENSG00000213512
ENSG00000162664	ENSG00000011243
ENSG00000162664	ENSG00000105127
ENSG00000162670	ENSG00000078725
ENSG00000162670	ENSG00000198797
ENSG00000162676	ENSG00000165702
ENSG00000162687	ENSG00000107147
ENSG00000162687	ENSG00000156113
ENSG00000162687	ENSG00000215262
ENSG00000162701	ENSG00000119522
ENSG00000162701	ENSG00000146966
ENSG00000162701	ENSG00000162777
ENSG00000162701	ENSG00000166444
ENSG00000162701	ENSG00000175984
ENSG00000162701	ENSG00000205744
ENSG00000162702	ENSG00000163848
ENSG00000162704	ENSG00000136950
ENSG00000162706	ENSG00000109943
ENSG00000162706	ENSG00000175161
ENSG00000162711	ENSG00000022556
ENSG00000162711	ENSG00000023191
ENSG00000162711	ENSG00000140853
ENSG00000162711	ENSG00000142405
ENSG00000162711	ENSG00000158077
ENSG00000162711	ENSG00000160505
ENSG00000162711	ENSG00000167207
ENSG00000162711	ENSG00000167634
ENSG00000162711	ENSG00000167984
ENSG00000162711	ENSG00000171487

ENSG00000162711	ENSG00000173572
ENSG00000162711	ENSG00000174885
ENSG00000162711	ENSG00000179709
ENSG00000162711	ENSG00000179873
ENSG00000162711	ENSG00000182261
ENSG00000162711	ENSG00000185792
ENSG00000162728	ENSG00000115474
ENSG00000162728	ENSG00000120457
ENSG00000162728	ENSG00000121361
ENSG00000162728	ENSG00000123700
ENSG00000162728	ENSG00000151704
ENSG00000162728	ENSG00000153822
ENSG00000162728	ENSG00000157542
ENSG00000162728	ENSG00000157551
ENSG00000162728	ENSG00000162989
ENSG00000162728	ENSG00000168135
ENSG00000162728	ENSG00000177807
ENSG00000162728	ENSG00000182324
ENSG00000162728	ENSG00000184185
ENSG00000162728	ENSG00000187486
ENSG00000162729	ENSG00000134247
ENSG00000162729	ENSG00000134256
ENSG00000162729	ENSG00000143061
ENSG00000162733	ENSG00000204580
ENSG00000162738	ENSG00000173218
ENSG00000162745	ENSG00000185585
ENSG00000162747	ENSG00000072694
ENSG00000162747	ENSG00000132185
ENSG00000162747	ENSG00000143226
ENSG00000162747	ENSG00000150337
ENSG00000162747	ENSG00000179639
ENSG00000162747	ENSG00000181036
ENSG00000162747	ENSG00000198019
ENSG00000162747	ENSG00000203747
ENSG00000162747	ENSG00000203820
ENSG00000162761	ENSG00000016082
ENSG00000162761	ENSG00000089116
ENSG00000162761	ENSG00000106689
ENSG00000162761	ENSG00000106852
ENSG00000162761	ENSG00000107187
ENSG00000162761	ENSG00000121454
ENSG00000162761	ENSG00000132130
ENSG00000162761	ENSG00000136944
ENSG00000162761	ENSG00000143013
ENSG00000162761	ENSG00000143355
ENSG00000162761	ENSG00000159556
ENSG00000162761	ENSG00000162624
ENSG00000162763	ENSG00000162494
ENSG00000162763	ENSG00000183908
ENSG00000162763	ENSG00000184709
ENSG00000162769	ENSG00000119686
ENSG00000162769	ENSG00000138463
ENSG00000162769	ENSG00000169026
ENSG00000162772	ENSG00000075426
ENSG00000162772	ENSG00000125740
ENSG00000162772	ENSG00000140044
ENSG00000162772	ENSG00000170345
ENSG00000162772	ENSG00000175592
ENSG00000162775	ENSG00000065526

ENSG00000162775	ENSG00000179837
ENSG00000162777	ENSG00000119522
ENSG00000162777	ENSG00000146966
ENSG00000162777	ENSG00000162701
ENSG00000162777	ENSG00000166444
ENSG00000162777	ENSG00000175984
ENSG00000162777	ENSG00000205744
ENSG00000162783	ENSG00000160888
ENSG00000162783	ENSG00000188483
ENSG00000162849	ENSG00000066735
ENSG00000162881	ENSG00000125245
ENSG00000162881	ENSG00000126251
ENSG00000162881	ENSG00000126262
ENSG00000162881	ENSG00000144230
ENSG00000162881	ENSG00000147138
ENSG00000162881	ENSG00000164220
ENSG00000162881	ENSG00000164251
ENSG00000162881	ENSG00000165621
ENSG00000162881	ENSG00000171631
ENSG00000162881	ENSG00000181104
ENSG00000162881	ENSG00000182162
ENSG00000162881	ENSG00000182559
ENSG00000162881	ENSG00000182782
ENSG00000162881	ENSG00000185897
ENSG00000162881	ENSG00000196917
ENSG00000162889	ENSG00000089022
ENSG00000162889	ENSG00000114738
ENSG00000162891	ENSG00000111536
ENSG00000162891	ENSG00000127318
ENSG00000162891	ENSG00000136634
ENSG00000162891	ENSG00000142224
ENSG00000162891	ENSG00000162892
ENSG00000162892	ENSG00000111536
ENSG00000162892	ENSG00000127318
ENSG00000162892	ENSG00000136634
ENSG00000162892	ENSG00000142224
ENSG00000162892	ENSG00000162891
ENSG00000162896	ENSG00000095970
ENSG00000162896	ENSG00000096264
ENSG00000162896	ENSG00000161649
ENSG00000162896	ENSG00000162897
ENSG00000162896	ENSG00000167850
ENSG00000162896	ENSG00000167851
ENSG00000162896	ENSG00000178789
ENSG00000162896	ENSG00000186074
ENSG00000162896	ENSG00000186407
ENSG00000162896	ENSG00000204345
ENSG00000162897	ENSG00000095970
ENSG00000162897	ENSG00000096264
ENSG00000162897	ENSG00000161649
ENSG00000162897	ENSG00000162896
ENSG00000162897	ENSG00000167850
ENSG00000162897	ENSG00000167851
ENSG00000162897	ENSG00000178789
ENSG00000162897	ENSG00000186074
ENSG00000162897	ENSG00000186407
ENSG00000162897	ENSG00000204345
ENSG00000162909	ENSG00000014216
ENSG00000162909	ENSG00000077274

ENSG00000162909	ENSG00000092529
ENSG00000162909	ENSG00000135773
ENSG00000162909	ENSG00000137225
ENSG00000162909	ENSG00000142330
ENSG00000162909	ENSG00000149260
ENSG00000162909	ENSG00000162949
ENSG00000162909	ENSG00000182472
ENSG00000162909	ENSG00000203697
ENSG00000162909	ENSG00000214711
ENSG00000162924	ENSG00000077150
ENSG00000162924	ENSG00000104856
ENSG00000162924	ENSG00000109320
ENSG00000162924	ENSG00000173039
ENSG00000162944	ENSG00000131378
ENSG00000162949	ENSG00000014216
ENSG00000162949	ENSG00000077274
ENSG00000162949	ENSG00000092529
ENSG00000162949	ENSG00000135773
ENSG00000162949	ENSG00000137225
ENSG00000162949	ENSG00000142330
ENSG00000162949	ENSG00000149260
ENSG00000162949	ENSG00000162909
ENSG00000162949	ENSG00000182472
ENSG00000162949	ENSG00000203697
ENSG00000162949	ENSG00000214711
ENSG00000162951	ENSG00000136457
ENSG00000162951	ENSG00000146006
ENSG00000162951	ENSG00000176204
ENSG00000162951	ENSG00000198739
ENSG00000162980	ENSG00000141748
ENSG00000162980	ENSG00000165997
ENSG00000162980	ENSG00000215451
ENSG00000162981	ENSG00000121207
ENSG00000162981	ENSG00000127252
ENSG00000162981	ENSG00000133321
ENSG00000162981	ENSG00000133328
ENSG00000162981	ENSG00000168004
ENSG00000162981	ENSG00000168672
ENSG00000162981	ENSG00000176485
ENSG00000162989	ENSG00000115474
ENSG00000162989	ENSG00000120457
ENSG00000162989	ENSG00000121361
ENSG00000162989	ENSG00000123700
ENSG00000162989	ENSG00000151704
ENSG00000162989	ENSG00000153822
ENSG00000162989	ENSG00000157542
ENSG00000162989	ENSG00000157551
ENSG00000162989	ENSG00000162728
ENSG00000162989	ENSG00000168135
ENSG00000162989	ENSG00000177807
ENSG00000162989	ENSG00000182324
ENSG00000162989	ENSG00000184185
ENSG00000162989	ENSG00000187486
ENSG00000162992	ENSG00000104903
ENSG00000162992	ENSG00000113196
ENSG00000162992	ENSG00000122691
ENSG00000162992	ENSG00000122859
ENSG00000162992	ENSG00000123307
ENSG00000162992	ENSG00000125878

ENSG00000162992	ENSG00000146618
ENSG00000162992	ENSG00000162367
ENSG00000162992	ENSG00000164107
ENSG00000162992	ENSG00000164600
ENSG00000162992	ENSG00000168267
ENSG00000162992	ENSG00000171532
ENSG00000162992	ENSG00000171786
ENSG00000162992	ENSG00000172238
ENSG00000162992	ENSG00000177551
ENSG00000162992	ENSG00000178403
ENSG00000162992	ENSG00000179774
ENSG00000162992	ENSG00000180535
ENSG00000162992	ENSG00000181965
ENSG00000162992	ENSG00000186051
ENSG00000162992	ENSG00000187786
ENSG00000162992	ENSG00000188686
ENSG00000162998	ENSG00000104290
ENSG00000162998	ENSG00000106483
ENSG00000162998	ENSG00000111432
ENSG00000162998	ENSG00000155760
ENSG00000162998	ENSG00000157240
ENSG00000162998	ENSG00000163251
ENSG00000162998	ENSG00000164930
ENSG00000162998	ENSG00000174804
ENSG00000162998	ENSG00000177283
ENSG00000162998	ENSG00000180340
ENSG00000162998	ENSG00000188763
ENSG00000163013	ENSG00000138311
ENSG00000163017	ENSG00000075624
ENSG00000163017	ENSG00000107796
ENSG00000163017	ENSG00000143632
ENSG00000163017	ENSG00000159251
ENSG00000163017	ENSG00000169067
ENSG00000163017	ENSG00000184009
ENSG00000163017	ENSG00000204434
ENSG00000163017	ENSG00000214555
ENSG00000163017	ENSG00000215141
ENSG00000163032	ENSG00000104490
ENSG00000163032	ENSG00000107130
ENSG00000163032	ENSG00000109047
ENSG00000163032	ENSG00000115756
ENSG00000163032	ENSG00000116983
ENSG00000163032	ENSG00000121905
ENSG00000163041	ENSG00000112727
ENSG00000163041	ENSG00000124693
ENSG00000163041	ENSG00000132475
ENSG00000163041	ENSG00000168148
ENSG00000163041	ENSG00000178458
ENSG00000163041	ENSG00000182431
ENSG00000163041	ENSG00000182572
ENSG00000163041	ENSG00000182915
ENSG00000163041	ENSG00000183598
ENSG00000163041	ENSG00000188375
ENSG00000163041	ENSG00000196285
ENSG00000163041	ENSG00000196532
ENSG00000163041	ENSG00000196966
ENSG00000163041	ENSG00000197153
ENSG00000163041	ENSG00000197409
ENSG00000163041	ENSG00000198366

ENSG00000163041	ENSG00000203811
ENSG00000163041	ENSG00000203813
ENSG00000163041	ENSG00000203818
ENSG00000163041	ENSG00000203852
ENSG00000163050	ENSG00000123815
ENSG00000163053	ENSG00000100156
ENSG00000163053	ENSG00000108932
ENSG00000163053	ENSG00000112394
ENSG00000163053	ENSG00000118596
ENSG00000163053	ENSG00000141526
ENSG00000163053	ENSG00000147100
ENSG00000163053	ENSG00000152779
ENSG00000163053	ENSG00000155380
ENSG00000163053	ENSG00000165449
ENSG00000163053	ENSG00000168679
ENSG00000163053	ENSG00000170190
ENSG00000163053	ENSG00000174326
ENSG00000163053	ENSG00000174327
ENSG00000163064	ENSG00000164778
ENSG00000163083	ENSG00000122641
ENSG00000163083	ENSG00000175189
ENSG00000163092	ENSG00000168334
ENSG00000163110	ENSG00000107438
ENSG00000163110	ENSG00000120913
ENSG00000163110	ENSG00000122367
ENSG00000163110	ENSG00000131435
ENSG00000163110	ENSG00000154553
ENSG00000163110	ENSG00000196923
ENSG00000163110	ENSG00000214895
ENSG00000163113	ENSG00000019995
ENSG00000163113	ENSG00000118503
ENSG00000163113	ENSG00000169918
ENSG00000163126	ENSG00000148677
ENSG00000163126	ENSG00000165887
ENSG00000163131	ENSG00000135047
ENSG00000163131	ENSG00000136943
ENSG00000163131	ENSG00000143387
ENSG00000163131	ENSG00000188029
ENSG00000163131	ENSG00000204437
ENSG00000163132	ENSG00000120149
ENSG00000163141	ENSG00000106772
ENSG00000163141	ENSG00000140299
ENSG00000163141	ENSG00000167654
ENSG00000163141	ENSG00000175220
ENSG00000163141	ENSG00000196505
ENSG00000163144	ENSG00000130041
ENSG00000163144	ENSG00000177954
ENSG00000163144	ENSG00000185088
ENSG00000163144	ENSG00000198731
ENSG00000163145	ENSG00000054803
ENSG00000163145	ENSG00000102924
ENSG00000163145	ENSG00000131094
ENSG00000163145	ENSG00000133466
ENSG00000163145	ENSG00000139899
ENSG00000163145	ENSG00000141668
ENSG00000163145	ENSG00000144119
ENSG00000163145	ENSG00000145861
ENSG00000163145	ENSG00000159189
ENSG00000163145	ENSG00000165985

ENSG00000163145	ENSG00000172247
ENSG00000163145	ENSG00000173369
ENSG00000163145	ENSG00000173372
ENSG00000163145	ENSG00000173918
ENSG00000163145	ENSG00000181092
ENSG00000163145	ENSG00000184471
ENSG00000163145	ENSG00000186897
ENSG00000163145	ENSG00000213192
ENSG00000163154	ENSG00000145779
ENSG00000163154	ENSG00000183578
ENSG00000163154	ENSG00000185361
ENSG00000163155	ENSG00000140280
ENSG00000163157	ENSG00000128872
ENSG00000163157	ENSG00000136842
ENSG00000163157	ENSG00000138594
ENSG00000163157	ENSG00000163380
ENSG00000163157	ENSG00000163431
ENSG00000163157	ENSG00000170807
ENSG00000163162	ENSG00000082996
ENSG00000163162	ENSG00000105428
ENSG00000163162	ENSG00000108523
ENSG00000163162	ENSG00000113269
ENSG00000163162	ENSG00000133135
ENSG00000163162	ENSG00000170153
ENSG00000163162	ENSG00000188050
ENSG00000163171	ENSG00000128283
ENSG00000163171	ENSG00000149798
ENSG00000163171	ENSG00000167617
ENSG00000163171	ENSG00000179604
ENSG00000163191	ENSG00000143546
ENSG00000163191	ENSG00000160307
ENSG00000163191	ENSG00000160678
ENSG00000163191	ENSG00000163220
ENSG00000163191	ENSG00000163221
ENSG00000163191	ENSG00000163993
ENSG00000163191	ENSG00000169906
ENSG00000163191	ENSG00000171643
ENSG00000163191	ENSG00000188015
ENSG00000163191	ENSG00000188643
ENSG00000163191	ENSG00000189171
ENSG00000163191	ENSG00000189334
ENSG00000163191	ENSG00000196154
ENSG00000163191	ENSG00000196420
ENSG00000163191	ENSG00000196754
ENSG00000163191	ENSG00000197747
ENSG00000163191	ENSG00000197956
ENSG00000163217	ENSG00000101144
ENSG00000163217	ENSG00000107623
ENSG00000163217	ENSG00000112175
ENSG00000163217	ENSG00000116985
ENSG00000163217	ENSG00000125965
ENSG00000163217	ENSG00000128802
ENSG00000163217	ENSG00000130385
ENSG00000163217	ENSG00000143869
ENSG00000163217	ENSG00000152785
ENSG00000163217	ENSG00000153162
ENSG00000163217	ENSG00000156466
ENSG00000163217	ENSG00000156574
ENSG00000163217	ENSG00000183682

ENSG00000163219	ENSG00000128805
ENSG00000163219	ENSG00000138639
ENSG00000163219	ENSG00000147799
ENSG00000163220	ENSG00000143546
ENSG00000163220	ENSG00000160307
ENSG00000163220	ENSG00000160678
ENSG00000163220	ENSG00000163191
ENSG00000163220	ENSG00000163221
ENSG00000163220	ENSG00000163993
ENSG00000163220	ENSG00000169906
ENSG00000163220	ENSG00000171643
ENSG00000163220	ENSG00000188015
ENSG00000163220	ENSG00000188643
ENSG00000163220	ENSG00000189171
ENSG00000163220	ENSG00000189334
ENSG00000163220	ENSG00000196154
ENSG00000163220	ENSG00000196420
ENSG00000163220	ENSG00000196754
ENSG00000163220	ENSG00000197747
ENSG00000163220	ENSG00000197956
ENSG00000163221	ENSG00000143546
ENSG00000163221	ENSG00000160307
ENSG00000163221	ENSG00000160678
ENSG00000163221	ENSG00000163191
ENSG00000163221	ENSG00000163220
ENSG00000163221	ENSG00000163993
ENSG00000163221	ENSG00000169906
ENSG00000163221	ENSG00000171643
ENSG00000163221	ENSG00000188015
ENSG00000163221	ENSG00000188643
ENSG00000163221	ENSG00000189171
ENSG00000163221	ENSG00000189334
ENSG00000163221	ENSG00000196154
ENSG00000163221	ENSG00000196420
ENSG00000163221	ENSG00000196754
ENSG00000163221	ENSG00000197747
ENSG00000163221	ENSG00000197956
ENSG00000163235	ENSG00000174808
ENSG00000163249	ENSG00000108100
ENSG00000163249	ENSG00000179296
ENSG00000163249	ENSG00000182632
ENSG00000163249	ENSG00000184766
ENSG00000163249	ENSG00000204150
ENSG00000163249	ENSG00000215169
ENSG00000163251	ENSG00000104290
ENSG00000163251	ENSG00000106483
ENSG00000163251	ENSG00000111432
ENSG00000163251	ENSG00000155760
ENSG00000163251	ENSG00000157240
ENSG00000163251	ENSG00000162998
ENSG00000163251	ENSG00000164930
ENSG00000163251	ENSG00000174804
ENSG00000163251	ENSG00000177283
ENSG00000163251	ENSG00000180340
ENSG00000163251	ENSG00000188763
ENSG00000163254	ENSG00000100053
ENSG00000163254	ENSG00000100058
ENSG00000163254	ENSG00000100122
ENSG00000163254	ENSG00000108255

ENSG00000163254	ENSG00000118231
ENSG00000163254	ENSG00000127377
ENSG00000163254	ENSG00000163499
ENSG00000163254	ENSG00000168582
ENSG00000163254	ENSG00000182187
ENSG00000163254	ENSG00000196431
ENSG00000163254	ENSG00000213139
ENSG00000163281	ENSG00000113552
ENSG00000163283	ENSG00000162551
ENSG00000163283	ENSG00000163286
ENSG00000163283	ENSG00000163295
ENSG00000163285	ENSG00000011677
ENSG00000163285	ENSG00000022355
ENSG00000163285	ENSG00000094755
ENSG00000163285	ENSG00000101958
ENSG00000163285	ENSG00000102287
ENSG00000163285	ENSG00000109158
ENSG00000163285	ENSG00000109738
ENSG00000163285	ENSG00000111886
ENSG00000163285	ENSG00000113327
ENSG00000163285	ENSG00000145451
ENSG00000163285	ENSG00000145863
ENSG00000163285	ENSG00000145864
ENSG00000163285	ENSG00000145888
ENSG00000163285	ENSG00000146276
ENSG00000163285	ENSG00000147402
ENSG00000163285	ENSG00000151834
ENSG00000163285	ENSG00000163288
ENSG00000163285	ENSG00000166206
ENSG00000163285	ENSG00000182256
ENSG00000163285	ENSG00000183185
ENSG00000163285	ENSG00000186297
ENSG00000163285	ENSG00000187730
ENSG00000163285	ENSG00000188828
ENSG00000163286	ENSG00000162551
ENSG00000163286	ENSG00000163283
ENSG00000163286	ENSG00000163295
ENSG00000163288	ENSG00000011677
ENSG00000163288	ENSG00000022355
ENSG00000163288	ENSG00000094755
ENSG00000163288	ENSG00000101958
ENSG00000163288	ENSG00000102287
ENSG00000163288	ENSG00000109158
ENSG00000163288	ENSG00000109738
ENSG00000163288	ENSG00000111886
ENSG00000163288	ENSG00000113327
ENSG00000163288	ENSG00000145451
ENSG00000163288	ENSG00000145863
ENSG00000163288	ENSG00000145864
ENSG00000163288	ENSG00000145888
ENSG00000163288	ENSG00000146276
ENSG00000163288	ENSG00000147402
ENSG00000163288	ENSG00000151834
ENSG00000163288	ENSG00000163285
ENSG00000163288	ENSG00000166206
ENSG00000163288	ENSG00000182256
ENSG00000163288	ENSG00000183185
ENSG00000163288	ENSG00000186297
ENSG00000163288	ENSG00000187730

ENSG00000163288	ENSG00000188828
ENSG00000163293	ENSG00000001461
ENSG00000163293	ENSG00000104361
ENSG00000163293	ENSG00000140157
ENSG00000163293	ENSG00000170113
ENSG00000163293	ENSG00000172548
ENSG00000163295	ENSG00000162551
ENSG00000163295	ENSG00000163283
ENSG00000163295	ENSG00000163286
ENSG00000163297	ENSG00000169604
ENSG00000163297	ENSG00000198250
ENSG00000163322	ENSG00000165660
ENSG00000163328	ENSG00000155792
ENSG00000163346	ENSG00000214882
ENSG00000163347	ENSG00000013297
ENSG00000163347	ENSG00000066405
ENSG00000163347	ENSG00000106404
ENSG00000163347	ENSG00000113946
ENSG00000163347	ENSG00000134873
ENSG00000163347	ENSG00000156282
ENSG00000163347	ENSG00000156284
ENSG00000163347	ENSG00000159261
ENSG00000163347	ENSG00000164007
ENSG00000163347	ENSG00000165215
ENSG00000163347	ENSG00000165376
ENSG00000163347	ENSG00000171217
ENSG00000163347	ENSG00000177300
ENSG00000163347	ENSG00000181885
ENSG00000163347	ENSG00000184113
ENSG00000163347	ENSG00000184697
ENSG00000163347	ENSG00000185758
ENSG00000163347	ENSG00000189118
ENSG00000163347	ENSG00000189143
ENSG00000163347	ENSG00000213937
ENSG00000163348	ENSG00000171016
ENSG00000163349	ENSG00000064393
ENSG00000163349	ENSG00000110422
ENSG00000163349	ENSG00000160396
ENSG00000163359	ENSG00000100473
ENSG00000163359	ENSG00000165816
ENSG00000163359	ENSG00000198450
ENSG00000163359	ENSG00000205221
ENSG00000163362	ENSG00000114541
ENSG00000163362	ENSG00000147144
ENSG00000163362	ENSG00000151474
ENSG00000163376	ENSG00000120696
ENSG00000163376	ENSG00000165572
ENSG00000163376	ENSG00000170852
ENSG00000163377	ENSG00000183662
ENSG00000163377	ENSG00000184599
ENSG00000163377	ENSG00000198673
ENSG00000163380	ENSG00000128872
ENSG00000163380	ENSG00000136842
ENSG00000163380	ENSG00000138594
ENSG00000163380	ENSG00000163157
ENSG00000163380	ENSG00000163431
ENSG00000163380	ENSG00000170807
ENSG00000163382	ENSG00000186010
ENSG00000163394	ENSG00000056291

ENSG00000163394	ENSG00000075073
ENSG00000163394	ENSG00000101292
ENSG00000163394	ENSG00000110148
ENSG00000163394	ENSG00000115353
ENSG00000163394	ENSG00000119973
ENSG00000163394	ENSG00000121764
ENSG00000163394	ENSG00000123901
ENSG00000163394	ENSG00000137252
ENSG00000163394	ENSG00000148734
ENSG00000163394	ENSG00000159279
ENSG00000163394	ENSG00000164128
ENSG00000163394	ENSG00000164129
ENSG00000163394	ENSG00000169618
ENSG00000163394	ENSG00000169836
ENSG00000163394	ENSG00000185149
ENSG00000163394	ENSG00000186867
ENSG00000163394	ENSG00000204174
ENSG00000163394	ENSG00000215167
ENSG00000163399	ENSG00000018625
ENSG00000163399	ENSG00000075673
ENSG00000163399	ENSG00000105409
ENSG00000163399	ENSG00000105675
ENSG00000163399	ENSG00000132681
ENSG00000163406	ENSG00000088386
ENSG00000163406	ENSG00000110446
ENSG00000163406	ENSG00000139370
ENSG00000163421	ENSG00000143125
ENSG00000163430	ENSG00000053108
ENSG00000163430	ENSG00000070404
ENSG00000163430	ENSG00000134363
ENSG00000163431	ENSG00000128872
ENSG00000163431	ENSG00000136842
ENSG00000163431	ENSG00000138594
ENSG00000163431	ENSG00000163157
ENSG00000163431	ENSG00000163380
ENSG00000163431	ENSG00000170807
ENSG00000163435	ENSG00000010030
ENSG00000163435	ENSG00000102034
ENSG00000163435	ENSG00000109381
ENSG00000163435	ENSG00000120690
ENSG00000163435	ENSG00000124664
ENSG00000163435	ENSG00000135373
ENSG00000163435	ENSG00000135374
ENSG00000163435	ENSG00000139083
ENSG00000163435	ENSG00000166211
ENSG00000163440	ENSG00000115539
ENSG00000163440	ENSG00000116703
ENSG00000163440	ENSG00000136940
ENSG00000163440	ENSG00000215109
ENSG00000163453	ENSG00000107821
ENSG00000163453	ENSG00000137142
ENSG00000163462	ENSG00000100505
ENSG00000163462	ENSG00000119283
ENSG00000163462	ENSG00000152503
ENSG00000163462	ENSG00000186628
ENSG00000163464	ENSG00000112486
ENSG00000163464	ENSG00000121797
ENSG00000163464	ENSG00000121807
ENSG00000163464	ENSG00000121966

ENSG00000163464	ENSG00000126353
ENSG00000163464	ENSG00000129048
ENSG00000163464	ENSG00000144648
ENSG00000163464	ENSG00000160683
ENSG00000163464	ENSG00000160791
ENSG00000163464	ENSG00000163823
ENSG00000163464	ENSG00000168329
ENSG00000163464	ENSG00000172215
ENSG00000163464	ENSG00000173578
ENSG00000163464	ENSG00000173585
ENSG00000163464	ENSG00000179934
ENSG00000163464	ENSG00000180871
ENSG00000163464	ENSG00000183625
ENSG00000163464	ENSG00000183813
ENSG00000163464	ENSG00000184451
ENSG00000163464	ENSG00000186810
ENSG00000163464	ENSG00000215775
ENSG00000163464	ENSG00000215778
ENSG00000163464	ENSG00000215782
ENSG00000163485	ENSG00000121933
ENSG00000163485	ENSG00000128271
ENSG00000163485	ENSG00000170425
ENSG00000163486	ENSG00000089820
ENSG00000163486	ENSG00000196220
ENSG00000163486	ENSG00000196935
ENSG00000163499	ENSG00000100053
ENSG00000163499	ENSG00000100058
ENSG00000163499	ENSG00000100122
ENSG00000163499	ENSG00000108255
ENSG00000163499	ENSG00000118231
ENSG00000163499	ENSG00000127377
ENSG00000163499	ENSG00000163254
ENSG00000163499	ENSG00000168582
ENSG00000163499	ENSG00000182187
ENSG00000163499	ENSG00000196431
ENSG00000163499	ENSG00000213139
ENSG00000163501	ENSG00000139549
ENSG00000163501	ENSG00000164690
ENSG00000163508	ENSG00000073861
ENSG00000163508	ENSG00000089225
ENSG00000163508	ENSG00000092607
ENSG00000163508	ENSG00000112837
ENSG00000163508	ENSG00000121068
ENSG00000163508	ENSG00000121075
ENSG00000163508	ENSG00000122145
ENSG00000163508	ENSG00000135111
ENSG00000163508	ENSG00000136535
ENSG00000163508	ENSG00000143178
ENSG00000163508	ENSG00000149922
ENSG00000163508	ENSG00000164458
ENSG00000163508	ENSG00000164532
ENSG00000163508	ENSG00000167800
ENSG00000163508	ENSG00000174197
ENSG00000163508	ENSG00000184058
ENSG00000163512	ENSG00000198933
ENSG00000163513	ENSG00000106799
ENSG00000163513	ENSG00000107779
ENSG00000163513	ENSG00000115170
ENSG00000163513	ENSG00000123612

ENSG00000163513	ENSG00000135409
ENSG00000163513	ENSG00000135503
ENSG00000163513	ENSG00000138696
ENSG00000163513	ENSG00000139567
ENSG00000163513	ENSG00000204217
ENSG00000163520	ENSG00000049323
ENSG00000163520	ENSG00000077942
ENSG00000163520	ENSG00000090006
ENSG00000163520	ENSG00000115380
ENSG00000163520	ENSG00000119681
ENSG00000163520	ENSG00000140092
ENSG00000163520	ENSG00000168056
ENSG00000163520	ENSG00000172638
ENSG00000163520	ENSG00000214536
ENSG00000163521	ENSG00000170266
ENSG00000163526	ENSG00000075886
ENSG00000163526	ENSG00000123416
ENSG00000163526	ENSG00000127824
ENSG00000163526	ENSG00000152086
ENSG00000163526	ENSG00000167552
ENSG00000163526	ENSG00000167553
ENSG00000163526	ENSG00000178462
ENSG00000163526	ENSG00000183785
ENSG00000163526	ENSG00000198033
ENSG00000163526	ENSG00000214391
ENSG00000163526	ENSG00000215823
ENSG00000163531	ENSG00000091129
ENSG00000163531	ENSG00000134121
ENSG00000163531	ENSG00000149972
ENSG00000163531	ENSG00000184144
ENSG00000163531	ENSG00000198493
ENSG00000163536	ENSG00000106366
ENSG00000163536	ENSG00000135919
ENSG00000163536	ENSG00000215461
ENSG00000163539	ENSG00000074054
ENSG00000163545	ENSG00000074590
ENSG00000163558	ENSG00000027075
ENSG00000163558	ENSG00000065675
ENSG00000163558	ENSG00000067606
ENSG00000163558	ENSG00000105221
ENSG00000163558	ENSG00000117020
ENSG00000163558	ENSG00000126583
ENSG00000163558	ENSG00000142208
ENSG00000163558	ENSG00000154229
ENSG00000163558	ENSG00000163932
ENSG00000163558	ENSG00000166501
ENSG00000163577	ENSG00000132507
ENSG00000163577	ENSG00000196513
ENSG00000163581	ENSG00000059804
ENSG00000163581	ENSG00000109667
ENSG00000163581	ENSG00000117394
ENSG00000163581	ENSG00000133460
ENSG00000163581	ENSG00000142583
ENSG00000163581	ENSG00000173262
ENSG00000163581	ENSG00000181856
ENSG00000163581	ENSG00000197241
ENSG00000163586	ENSG00000170231
ENSG00000163596	ENSG00000003147
ENSG00000163602	ENSG00000015153

ENSG00000163618	ENSG00000081803
ENSG00000163623	ENSG00000148826
ENSG00000163623	ENSG00000165066
ENSG00000163624	ENSG00000101290
ENSG00000163625	ENSG00000128815
ENSG00000163625	ENSG00000143669
ENSG00000163629	ENSG00000139926
ENSG00000163629	ENSG00000170324
ENSG00000163630	ENSG00000008282
ENSG00000163630	ENSG00000102003
ENSG00000163630	ENSG00000143028
ENSG00000163635	ENSG00000146776
ENSG00000163635	ENSG00000162650
ENSG00000163637	ENSG00000012211
ENSG00000163637	ENSG00000071282
ENSG00000163637	ENSG00000124593
ENSG00000163637	ENSG00000135269
ENSG00000163637	ENSG00000139174
ENSG00000163638	ENSG00000049192
ENSG00000163638	ENSG00000087116
ENSG00000163638	ENSG00000136378
ENSG00000163638	ENSG00000138316
ENSG00000163638	ENSG00000140470
ENSG00000163638	ENSG00000140873
ENSG00000163638	ENSG00000142303
ENSG00000163638	ENSG00000145536
ENSG00000163638	ENSG00000145808
ENSG00000163638	ENSG00000151388
ENSG00000163638	ENSG00000156140
ENSG00000163638	ENSG00000160323
ENSG00000163638	ENSG00000173157
ENSG00000163638	ENSG00000214644
ENSG00000163638	ENSG00000215743
ENSG00000163646	ENSG00000180745
ENSG00000163646	ENSG00000188022
ENSG00000163659	ENSG00000138496
ENSG00000163659	ENSG00000173193
ENSG00000163659	ENSG00000178685
ENSG00000163660	ENSG00000116148
ENSG00000163660	ENSG00000215726
ENSG00000163661	ENSG00000106236
ENSG00000163661	ENSG00000132693
ENSG00000163661	ENSG00000132703
ENSG00000163661	ENSG00000167957
ENSG00000163661	ENSG00000171246
ENSG00000163661	ENSG00000215846
ENSG00000163687	ENSG00000013563
ENSG00000163687	ENSG00000167968
ENSG00000163687	ENSG00000213918
ENSG00000163697	ENSG00000113108
ENSG00000163697	ENSG00000166313
ENSG00000163703	ENSG00000184164
ENSG00000163710	ENSG00000038295
ENSG00000163710	ENSG00000095587
ENSG00000163710	ENSG00000106333
ENSG00000163710	ENSG00000107611
ENSG00000163710	ENSG00000157211
ENSG00000163710	ENSG00000166342
ENSG00000163710	ENSG00000168487

ENSG00000163710	ENSG00000171208
ENSG00000163710	ENSG00000184824
ENSG00000163734	ENSG00000081041
ENSG00000163734	ENSG00000109272
ENSG00000163734	ENSG00000124875
ENSG00000163734	ENSG00000138755
ENSG00000163734	ENSG00000163735
ENSG00000163734	ENSG00000163736
ENSG00000163734	ENSG00000163737
ENSG00000163734	ENSG00000163739
ENSG00000163734	ENSG00000169245
ENSG00000163734	ENSG00000169248
ENSG00000163734	ENSG00000169429
ENSG00000163735	ENSG00000081041
ENSG00000163735	ENSG00000109272
ENSG00000163735	ENSG00000124875
ENSG00000163735	ENSG00000138755
ENSG00000163735	ENSG00000163734
ENSG00000163735	ENSG00000163736
ENSG00000163735	ENSG00000163737
ENSG00000163735	ENSG00000163739
ENSG00000163735	ENSG00000169245
ENSG00000163735	ENSG00000169248
ENSG00000163735	ENSG00000169429
ENSG00000163736	ENSG00000081041
ENSG00000163736	ENSG00000109272
ENSG00000163736	ENSG00000124875
ENSG00000163736	ENSG00000138755
ENSG00000163736	ENSG00000163734
ENSG00000163736	ENSG00000163735
ENSG00000163736	ENSG00000163737
ENSG00000163736	ENSG00000163739
ENSG00000163736	ENSG00000169245
ENSG00000163736	ENSG00000169248
ENSG00000163736	ENSG00000169429
ENSG00000163737	ENSG00000081041
ENSG00000163737	ENSG00000109272
ENSG00000163737	ENSG00000124875
ENSG00000163737	ENSG00000138755
ENSG00000163737	ENSG00000163734
ENSG00000163737	ENSG00000163735
ENSG00000163737	ENSG00000163736
ENSG00000163737	ENSG00000163739
ENSG00000163737	ENSG00000169245
ENSG00000163737	ENSG00000169248
ENSG00000163737	ENSG00000169429
ENSG00000163738	ENSG00000065911
ENSG00000163738	ENSG00000214698
ENSG00000163739	ENSG00000081041
ENSG00000163739	ENSG00000109272
ENSG00000163739	ENSG00000124875
ENSG00000163739	ENSG00000138755
ENSG00000163739	ENSG00000163734
ENSG00000163739	ENSG00000163735
ENSG00000163739	ENSG00000163736
ENSG00000163739	ENSG00000163737
ENSG00000163739	ENSG00000169245
ENSG00000163739	ENSG00000169248
ENSG00000163739	ENSG00000169429

ENSG00000163746	ENSG00000114698
ENSG00000163746	ENSG00000187838
ENSG00000163746	ENSG00000188313
ENSG00000163751	ENSG00000080618
ENSG00000163751	ENSG00000091704
ENSG00000163751	ENSG00000128510
ENSG00000163751	ENSG00000144410
ENSG00000163751	ENSG00000153002
ENSG00000163751	ENSG00000158516
ENSG00000163751	ENSG00000158525
ENSG00000163751	ENSG00000165078
ENSG00000163754	ENSG00000056998
ENSG00000163762	ENSG00000142484
ENSG00000163762	ENSG00000145107
ENSG00000163762	ENSG00000168955
ENSG00000163762	ENSG00000169903
ENSG00000163762	ENSG00000169908
ENSG00000163792	ENSG00000118526
ENSG00000163792	ENSG00000178860
ENSG00000163792	ENSG00000187728
ENSG00000163794	ENSG00000147571
ENSG00000163806	ENSG00000136206
ENSG00000163806	ENSG00000154732
ENSG00000163806	ENSG00000166667
ENSG00000163806	ENSG00000170092
ENSG00000163806	ENSG00000173678
ENSG00000163806	ENSG00000179994
ENSG00000163806	ENSG00000183318
ENSG00000163806	ENSG00000184616
ENSG00000163806	ENSG00000185040
ENSG00000163806	ENSG00000186645
ENSG00000163806	ENSG00000198305
ENSG00000163806	ENSG00000204710
ENSG00000163806	ENSG00000205238
ENSG00000163806	ENSG00000205482
ENSG00000163806	ENSG00000205505
ENSG00000163806	ENSG00000205506
ENSG00000163806	ENSG00000205508
ENSG00000163806	ENSG00000205509
ENSG00000163806	ENSG00000205580
ENSG00000163806	ENSG00000214300
ENSG00000163806	ENSG00000215092
ENSG00000163810	ENSG00000092295
ENSG00000163810	ENSG00000104055
ENSG00000163810	ENSG00000124491
ENSG00000163810	ENSG00000125780
ENSG00000163810	ENSG00000159495
ENSG00000163810	ENSG00000166947
ENSG00000163810	ENSG00000166948
ENSG00000163810	ENSG00000198959
ENSG00000163812	ENSG00000153786
ENSG00000163812	ENSG00000175893
ENSG00000163815	ENSG00000105472
ENSG00000163815	ENSG00000118004
ENSG00000163815	ENSG00000122852
ENSG00000163815	ENSG00000122854
ENSG00000163815	ENSG00000133661
ENSG00000163815	ENSG00000165471
ENSG00000163815	ENSG00000166509

ENSG00000163815	ENSG00000182314
ENSG00000163815	ENSG00000184374
ENSG00000163815	ENSG00000185303
ENSG00000163817	ENSG00000010379
ENSG00000163817	ENSG00000011083
ENSG00000163817	ENSG00000087916
ENSG00000163817	ENSG00000103546
ENSG00000163817	ENSG00000108576
ENSG00000163817	ENSG00000111181
ENSG00000163817	ENSG00000130821
ENSG00000163817	ENSG00000131389
ENSG00000163817	ENSG00000132164
ENSG00000163817	ENSG00000142319
ENSG00000163817	ENSG00000157103
ENSG00000163817	ENSG00000164363
ENSG00000163817	ENSG00000165970
ENSG00000163817	ENSG00000174358
ENSG00000163817	ENSG00000196517
ENSG00000163817	ENSG00000198555
ENSG00000163817	ENSG00000214617
ENSG00000163820	ENSG00000188282
ENSG00000163823	ENSG00000112486
ENSG00000163823	ENSG00000121797
ENSG00000163823	ENSG00000121807
ENSG00000163823	ENSG00000121966
ENSG00000163823	ENSG00000126353
ENSG00000163823	ENSG00000129048
ENSG00000163823	ENSG00000144648
ENSG00000163823	ENSG00000160683
ENSG00000163823	ENSG00000160791
ENSG00000163823	ENSG00000163464
ENSG00000163823	ENSG00000168329
ENSG00000163823	ENSG00000172215
ENSG00000163823	ENSG00000173578
ENSG00000163823	ENSG00000173585
ENSG00000163823	ENSG00000179934
ENSG00000163823	ENSG00000180871
ENSG00000163823	ENSG00000183625
ENSG00000163823	ENSG00000183813
ENSG00000163823	ENSG00000184451
ENSG00000163823	ENSG00000186810
ENSG00000163823	ENSG00000215775
ENSG00000163823	ENSG00000215778
ENSG00000163823	ENSG00000215782
ENSG00000163827	ENSG00000122477
ENSG00000163827	ENSG00000206422
ENSG00000163833	ENSG00000118496
ENSG00000163848	ENSG00000162702
ENSG00000163864	ENSG00000157064
ENSG00000163864	ENSG00000173614
ENSG00000163867	ENSG00000121741
ENSG00000163867	ENSG00000132950
ENSG00000163867	ENSG00000146463
ENSG00000163867	ENSG00000147130
ENSG00000163867	ENSG00000198218
ENSG00000163873	ENSG00000120251
ENSG00000163873	ENSG00000125675
ENSG00000163873	ENSG00000149403
ENSG00000163873	ENSG00000152578

ENSG00000163873	ENSG00000155511
ENSG00000163873	ENSG00000164418
ENSG00000163873	ENSG00000171189
ENSG00000163874	ENSG00000100441
ENSG00000163874	ENSG00000102053
ENSG00000163874	ENSG00000102921
ENSG00000163874	ENSG00000149289
ENSG00000163874	ENSG00000178199
ENSG00000163874	ENSG00000205978
ENSG00000163884	ENSG00000102349
ENSG00000163884	ENSG00000109787
ENSG00000163884	ENSG00000118263
ENSG00000163884	ENSG00000118922
ENSG00000163888	ENSG00000162545
ENSG00000163898	ENSG00000101670
ENSG00000163898	ENSG00000144837
ENSG00000163898	ENSG00000165862
ENSG00000163898	ENSG00000166035
ENSG00000163898	ENSG00000175445
ENSG00000163898	ENSG00000175535
ENSG00000163898	ENSG00000187021
ENSG00000163898	ENSG00000188992
ENSG00000163898	ENSG00000203837
ENSG00000163904	ENSG00000079387
ENSG00000163904	ENSG00000119231
ENSG00000163904	ENSG00000161956
ENSG00000163909	ENSG00000135547
ENSG00000163909	ENSG00000164683
ENSG00000163909	ENSG00000187821
ENSG00000163914	ENSG00000054277
ENSG00000163914	ENSG00000102076
ENSG00000163914	ENSG00000122375
ENSG00000163914	ENSG00000124818
ENSG00000163914	ENSG00000128617
ENSG00000163914	ENSG00000147380
ENSG00000163914	ENSG00000148604
ENSG00000163914	ENSG00000166160
ENSG00000163914	ENSG00000180245
ENSG00000163923	ENSG00000198918
ENSG00000163923	ENSG00000214289
ENSG00000163931	ENSG00000007350
ENSG00000163931	ENSG00000151005
ENSG00000163932	ENSG00000027075
ENSG00000163932	ENSG00000065675
ENSG00000163932	ENSG00000067606
ENSG00000163932	ENSG00000105221
ENSG00000163932	ENSG00000117020
ENSG00000163932	ENSG00000126583
ENSG00000163932	ENSG00000142208
ENSG00000163932	ENSG00000154229
ENSG00000163932	ENSG00000163558
ENSG00000163932	ENSG00000166501
ENSG00000163935	ENSG00000011258
ENSG00000163935	ENSG00000100395
ENSG00000163935	ENSG00000154655
ENSG00000163935	ENSG00000185513
ENSG00000163935	ENSG00000198879
ENSG00000163935	ENSG00000198945
ENSG00000163938	ENSG00000130119

ENSG00000163947	ENSG00000173848
ENSG00000163958	ENSG00000099904
ENSG00000163958	ENSG00000156599
ENSG00000163961	ENSG00000166439
ENSG00000163982	ENSG00000182938
ENSG00000163982	ENSG00000183034
ENSG00000163982	ENSG00000214333
ENSG00000163993	ENSG00000143546
ENSG00000163993	ENSG00000160307
ENSG00000163993	ENSG00000160678
ENSG00000163993	ENSG00000163191
ENSG00000163993	ENSG00000163220
ENSG00000163993	ENSG00000163221
ENSG00000163993	ENSG00000169906
ENSG00000163993	ENSG00000171643
ENSG00000163993	ENSG00000188015
ENSG00000163993	ENSG00000188643
ENSG00000163993	ENSG00000189171
ENSG00000163993	ENSG00000189334
ENSG00000163993	ENSG00000196154
ENSG00000163993	ENSG00000196420
ENSG00000163993	ENSG00000196754
ENSG00000163993	ENSG00000197747
ENSG00000163993	ENSG00000197956
ENSG00000163995	ENSG00000099204
ENSG00000163995	ENSG00000158856
ENSG00000163995	ENSG00000173210
ENSG00000164007	ENSG00000013297
ENSG00000164007	ENSG00000066405
ENSG00000164007	ENSG00000106404
ENSG00000164007	ENSG00000113946
ENSG00000164007	ENSG00000134873
ENSG00000164007	ENSG00000156282
ENSG00000164007	ENSG00000156284
ENSG00000164007	ENSG00000159261
ENSG00000164007	ENSG00000163347
ENSG00000164007	ENSG00000165215
ENSG00000164007	ENSG00000165376
ENSG00000164007	ENSG00000171217
ENSG00000164007	ENSG00000177300
ENSG00000164007	ENSG00000181885
ENSG00000164007	ENSG00000184113
ENSG00000164007	ENSG00000184697
ENSG00000164007	ENSG00000185758
ENSG00000164007	ENSG00000189118
ENSG00000164007	ENSG00000189143
ENSG00000164007	ENSG00000213937
ENSG00000164010	ENSG00000026950
ENSG00000164010	ENSG00000111801
ENSG00000164010	ENSG00000112763
ENSG00000164010	ENSG00000113303
ENSG00000164010	ENSG00000121236
ENSG00000164010	ENSG00000124508
ENSG00000164010	ENSG00000124549
ENSG00000164010	ENSG00000124557
ENSG00000164010	ENSG00000128250
ENSG00000164010	ENSG00000128253
ENSG00000164010	ENSG00000128276
ENSG00000164010	ENSG00000128482

ENSG00000164010	ENSG00000132256
ENSG00000164010	ENSG00000132274
ENSG00000164010	ENSG00000165810
ENSG00000164010	ENSG00000168903
ENSG00000164010	ENSG00000176422
ENSG00000164010	ENSG00000179046
ENSG00000164010	ENSG00000182805
ENSG00000164010	ENSG00000183439
ENSG00000164010	ENSG00000183718
ENSG00000164010	ENSG00000187574
ENSG00000164010	ENSG00000188683
ENSG00000164010	ENSG00000204614
ENSG00000164010	ENSG00000204616
ENSG00000164010	ENSG00000204618
ENSG00000164010	ENSG00000205198
ENSG00000164010	ENSG00000214693
ENSG00000164023	ENSG00000156671
ENSG00000164023	ENSG00000198964
ENSG00000164031	ENSG00000148719
ENSG00000164031	ENSG00000170464
ENSG00000164032	ENSG00000105968
ENSG00000164040	ENSG00000101856
ENSG00000164045	ENSG00000101224
ENSG00000164045	ENSG00000158402
ENSG00000164050	ENSG00000004399
ENSG00000164050	ENSG00000076356
ENSG00000164050	ENSG00000114554
ENSG00000164050	ENSG00000130827
ENSG00000164050	ENSG00000136040
ENSG00000164050	ENSG00000189437
ENSG00000164050	ENSG00000196576
ENSG00000164050	ENSG00000198753
ENSG00000164054	ENSG00000198892
ENSG00000164056	ENSG00000136158
ENSG00000164056	ENSG00000168939
ENSG00000164056	ENSG00000187678
ENSG00000164061	ENSG00000186472
ENSG00000164070	ENSG00000120694
ENSG00000164070	ENSG00000170606
ENSG00000164076	ENSG00000008118
ENSG00000164076	ENSG00000081320
ENSG00000164076	ENSG00000130822
ENSG00000164076	ENSG00000134072
ENSG00000164076	ENSG00000147613
ENSG00000164076	ENSG00000159792
ENSG00000164076	ENSG00000164543
ENSG00000164076	ENSG00000183049
ENSG00000164077	ENSG00000103111
ENSG00000164078	ENSG00000092445
ENSG00000164078	ENSG00000105976
ENSG00000164078	ENSG00000153208
ENSG00000164078	ENSG00000163785
ENSG00000164078	ENSG00000167601
ENSG00000164078	ENSG00000214672
ENSG00000164082	ENSG00000113262
ENSG00000164082	ENSG00000124493
ENSG00000164082	ENSG00000152822
ENSG00000164082	ENSG00000168959
ENSG00000164082	ENSG00000179603

ENSG00000164082	ENSG00000196277
ENSG00000164082	ENSG00000198822
ENSG00000164086	ENSG00000111266
ENSG00000164086	ENSG00000120129
ENSG00000164086	ENSG00000120875
ENSG00000164086	ENSG00000130829
ENSG00000164086	ENSG00000138166
ENSG00000164086	ENSG00000139318
ENSG00000164086	ENSG00000143507
ENSG00000164086	ENSG00000158050
ENSG00000164086	ENSG00000184545
ENSG00000164088	ENSG00000111110
ENSG00000164088	ENSG00000155367
ENSG00000164089	ENSG00000175309
ENSG00000164093	ENSG00000069011
ENSG00000164093	ENSG00000107859
ENSG00000164099	ENSG00000013725
ENSG00000164099	ENSG00000073754
ENSG00000164099	ENSG00000110448
ENSG00000164099	ENSG00000146700
ENSG00000164099	ENSG00000177575
ENSG00000164099	ENSG00000177675
ENSG00000164099	ENSG00000179954
ENSG00000164099	ENSG00000187908
ENSG00000164099	ENSG00000214279
ENSG00000164100	ENSG00000070614
ENSG00000164100	ENSG00000138653
ENSG00000164100	ENSG00000166507
ENSG00000164104	ENSG00000029993
ENSG00000164104	ENSG00000100118
ENSG00000164104	ENSG00000124097
ENSG00000164104	ENSG00000132967
ENSG00000164104	ENSG00000173961
ENSG00000164104	ENSG00000176256
ENSG00000164104	ENSG00000180212
ENSG00000164104	ENSG00000182181
ENSG00000164104	ENSG00000182711
ENSG00000164104	ENSG00000189403
ENSG00000164104	ENSG00000214364
ENSG00000164104	ENSG00000214852
ENSG00000164104	ENSG00000215308
ENSG00000164107	ENSG00000104903
ENSG00000164107	ENSG00000113196
ENSG00000164107	ENSG00000122691
ENSG00000164107	ENSG00000122859
ENSG00000164107	ENSG00000123307
ENSG00000164107	ENSG00000125878
ENSG00000164107	ENSG00000146618
ENSG00000164107	ENSG00000162367
ENSG00000164107	ENSG00000162992
ENSG00000164107	ENSG00000164600
ENSG00000164107	ENSG00000168267
ENSG00000164107	ENSG00000171532
ENSG00000164107	ENSG00000171786
ENSG00000164107	ENSG00000172238
ENSG00000164107	ENSG00000177551
ENSG00000164107	ENSG00000178403
ENSG00000164107	ENSG00000179774
ENSG00000164107	ENSG00000180535

ENSG00000164107	ENSG00000181965
ENSG00000164107	ENSG00000186051
ENSG00000164107	ENSG00000187786
ENSG00000164107	ENSG00000188686
ENSG00000164111	ENSG00000104537
ENSG00000164111	ENSG00000109511
ENSG00000164111	ENSG00000122359
ENSG00000164111	ENSG00000135046
ENSG00000164111	ENSG00000138279
ENSG00000164111	ENSG00000138772
ENSG00000164111	ENSG00000143412
ENSG00000164111	ENSG00000150165
ENSG00000164111	ENSG00000165390
ENSG00000164111	ENSG00000182718
ENSG00000164111	ENSG00000183059
ENSG00000164111	ENSG00000186807
ENSG00000164111	ENSG00000196975
ENSG00000164111	ENSG00000197043
ENSG00000164113	ENSG00000065457
ENSG00000164113	ENSG00000140955
ENSG00000164113	ENSG00000160710
ENSG00000164113	ENSG00000185736
ENSG00000164113	ENSG00000197381
ENSG00000164116	ENSG00000070019
ENSG00000164116	ENSG00000101890
ENSG00000164116	ENSG00000123201
ENSG00000164116	ENSG00000132518
ENSG00000164116	ENSG00000159899
ENSG00000164116	ENSG00000169418
ENSG00000164122	ENSG00000065802
ENSG00000164122	ENSG00000102048
ENSG00000164122	ENSG00000165192
ENSG00000164122	ENSG00000196372
ENSG00000164122	ENSG00000198881
ENSG00000164122	ENSG00000214426
ENSG00000164125	ENSG00000144649
ENSG00000164128	ENSG00000056291
ENSG00000164128	ENSG00000075073
ENSG00000164128	ENSG00000101292
ENSG00000164128	ENSG00000110148
ENSG00000164128	ENSG00000115353
ENSG00000164128	ENSG00000119973
ENSG00000164128	ENSG00000121764
ENSG00000164128	ENSG00000123901
ENSG00000164128	ENSG00000137252
ENSG00000164128	ENSG00000148734
ENSG00000164128	ENSG00000159279
ENSG00000164128	ENSG00000163394
ENSG00000164128	ENSG00000164129
ENSG00000164128	ENSG00000169618
ENSG00000164128	ENSG00000169836
ENSG00000164128	ENSG00000185149
ENSG00000164128	ENSG00000186867
ENSG00000164128	ENSG00000204174
ENSG00000164128	ENSG00000215167
ENSG00000164129	ENSG00000056291
ENSG00000164129	ENSG00000075073
ENSG00000164129	ENSG00000101292
ENSG00000164129	ENSG00000110148

ENSG00000164129	ENSG00000115353
ENSG00000164129	ENSG00000119973
ENSG00000164129	ENSG00000121764
ENSG00000164129	ENSG00000123901
ENSG00000164129	ENSG00000137252
ENSG00000164129	ENSG00000148734
ENSG00000164129	ENSG00000159279
ENSG00000164129	ENSG00000163394
ENSG00000164129	ENSG00000164128
ENSG00000164129	ENSG00000169618
ENSG00000164129	ENSG00000169836
ENSG00000164129	ENSG00000185149
ENSG00000164129	ENSG00000186867
ENSG00000164129	ENSG00000204174
ENSG00000164129	ENSG00000215167
ENSG00000164134	ENSG00000172766
ENSG00000164142	ENSG00000051009
ENSG00000164142	ENSG00000151553
ENSG00000164142	ENSG00000158863
ENSG00000164144	ENSG00000100151
ENSG00000164144	ENSG00000132254
ENSG00000164164	ENSG00000068308
ENSG00000164164	ENSG00000118976
ENSG00000164171	ENSG00000005844
ENSG00000164171	ENSG00000005884
ENSG00000164171	ENSG00000005961
ENSG00000164171	ENSG00000077943
ENSG00000164171	ENSG00000083457
ENSG00000164171	ENSG00000091409
ENSG00000164171	ENSG00000115232
ENSG00000164171	ENSG00000135424
ENSG00000164171	ENSG00000137809
ENSG00000164171	ENSG00000138448
ENSG00000164171	ENSG00000140678
ENSG00000164171	ENSG00000143127
ENSG00000164171	ENSG00000144668
ENSG00000164171	ENSG00000156886
ENSG00000164171	ENSG00000161638
ENSG00000164171	ENSG00000169896
ENSG00000164171	ENSG00000213949
ENSG00000164176	ENSG00000057019
ENSG00000164176	ENSG00000099250
ENSG00000164176	ENSG00000102104
ENSG00000164176	ENSG00000118257
ENSG00000164176	ENSG00000140545
ENSG00000164176	ENSG00000164465
ENSG00000164180	ENSG00000064545
ENSG00000164181	ENSG00000012660
ENSG00000164181	ENSG00000066322
ENSG00000164181	ENSG00000118402
ENSG00000164181	ENSG00000197977
ENSG00000164188	ENSG00000031823
ENSG00000164220	ENSG00000125245
ENSG00000164220	ENSG00000126251
ENSG00000164220	ENSG00000126262
ENSG00000164220	ENSG00000144230
ENSG00000164220	ENSG00000147138
ENSG00000164220	ENSG00000162881
ENSG00000164220	ENSG00000164251

ENSG00000164220	ENSG00000165621
ENSG00000164220	ENSG00000171631
ENSG00000164220	ENSG00000181104
ENSG00000164220	ENSG00000182162
ENSG00000164220	ENSG00000182559
ENSG00000164220	ENSG00000182782
ENSG00000164220	ENSG00000185897
ENSG00000164220	ENSG00000196917
ENSG00000164236	ENSG00000167612
ENSG00000164251	ENSG00000125245
ENSG00000164251	ENSG00000126251
ENSG00000164251	ENSG00000126262
ENSG00000164251	ENSG00000144230
ENSG00000164251	ENSG00000147138
ENSG00000164251	ENSG00000162881
ENSG00000164251	ENSG00000164220
ENSG00000164251	ENSG00000165621
ENSG00000164251	ENSG00000171631
ENSG00000164251	ENSG00000181104
ENSG00000164251	ENSG00000182162
ENSG00000164251	ENSG00000182559
ENSG00000164251	ENSG00000182782
ENSG00000164251	ENSG00000185897
ENSG00000164251	ENSG00000196917
ENSG00000164266	ENSG00000122711
ENSG00000164266	ENSG00000128040
ENSG00000164266	ENSG00000145879
ENSG00000164266	ENSG00000178172
ENSG00000164266	ENSG00000196800
ENSG00000164266	ENSG00000204909
ENSG00000164266	ENSG00000214510
ENSG00000164270	ENSG00000043591
ENSG00000164270	ENSG00000102468
ENSG00000164270	ENSG00000113749
ENSG00000164270	ENSG00000125631
ENSG00000164270	ENSG00000135312
ENSG00000164270	ENSG00000135914
ENSG00000164270	ENSG00000147246
ENSG00000164270	ENSG00000150594
ENSG00000164270	ENSG00000157219
ENSG00000164270	ENSG00000168830
ENSG00000164270	ENSG00000169252
ENSG00000164270	ENSG00000175658
ENSG00000164270	ENSG00000178394
ENSG00000164270	ENSG00000179097
ENSG00000164270	ENSG00000179546
ENSG00000164270	ENSG00000181210
ENSG00000164270	ENSG00000184303
ENSG00000164270	ENSG00000184845
ENSG00000164270	ENSG00000185321
ENSG00000164270	ENSG00000188778
ENSG00000164284	ENSG00000109519
ENSG00000164284	ENSG00000214621
ENSG00000164294	ENSG00000116157
ENSG00000164300	ENSG00000111897
ENSG00000164300	ENSG00000132824
ENSG00000164300	ENSG00000168528
ENSG00000164300	ENSG00000184716
ENSG00000164305	ENSG00000003400

ENSG00000164305	ENSG00000064012
ENSG00000164305	ENSG00000105141
ENSG00000164305	ENSG00000106144
ENSG00000164305	ENSG00000132906
ENSG00000164305	ENSG00000137752
ENSG00000164305	ENSG00000137757
ENSG00000164305	ENSG00000138794
ENSG00000164305	ENSG00000165806
ENSG00000164305	ENSG00000196954
ENSG00000164305	ENSG00000204403
ENSG00000164307	ENSG00000072657
ENSG00000164307	ENSG00000113441
ENSG00000164307	ENSG00000138792
ENSG00000164307	ENSG00000141279
ENSG00000164307	ENSG00000164308
ENSG00000164307	ENSG00000166825
ENSG00000164307	ENSG00000172901
ENSG00000164307	ENSG00000174093
ENSG00000164307	ENSG00000215728
ENSG00000164308	ENSG00000072657
ENSG00000164308	ENSG00000113441
ENSG00000164308	ENSG00000138792
ENSG00000164308	ENSG00000141279
ENSG00000164308	ENSG00000164307
ENSG00000164308	ENSG00000166825
ENSG00000164308	ENSG00000172901
ENSG00000164308	ENSG00000174093
ENSG00000164308	ENSG00000215728
ENSG00000164330	ENSG00000088881
ENSG00000164330	ENSG00000108001
ENSG00000164330	ENSG00000134025
ENSG00000164331	ENSG00000064490
ENSG00000164342	ENSG00000173366
ENSG00000164342	ENSG00000196664
ENSG00000164363	ENSG00000010379
ENSG00000164363	ENSG00000011083
ENSG00000164363	ENSG00000087916
ENSG00000164363	ENSG00000103546
ENSG00000164363	ENSG00000108576
ENSG00000164363	ENSG00000111181
ENSG00000164363	ENSG00000130821
ENSG00000164363	ENSG00000131389
ENSG00000164363	ENSG00000132164
ENSG00000164363	ENSG00000142319
ENSG00000164363	ENSG00000157103
ENSG00000164363	ENSG00000163817
ENSG00000164363	ENSG00000165970
ENSG00000164363	ENSG00000174358
ENSG00000164363	ENSG00000196517
ENSG00000164363	ENSG00000198555
ENSG00000164363	ENSG00000214617
ENSG00000164393	ENSG00000069122
ENSG00000164393	ENSG00000123146
ENSG00000164393	ENSG00000127507
ENSG00000164393	ENSG00000131355
ENSG00000164393	ENSG00000153292
ENSG00000164393	ENSG00000153294
ENSG00000164393	ENSG00000162618
ENSG00000164393	ENSG00000173567

ENSG00000164393	ENSG00000174837
ENSG00000164393	ENSG00000186629
ENSG00000164393	ENSG00000204818
ENSG00000164398	ENSG00000151726
ENSG00000164398	ENSG00000197142
ENSG00000164402	ENSG00000125354
ENSG00000164402	ENSG00000138758
ENSG00000164402	ENSG00000154997
ENSG00000164402	ENSG00000186522
ENSG00000164411	ENSG00000121634
ENSG00000164411	ENSG00000121742
ENSG00000164411	ENSG00000121743
ENSG00000164411	ENSG00000131233
ENSG00000164411	ENSG00000135355
ENSG00000164411	ENSG00000143140
ENSG00000164411	ENSG00000152661
ENSG00000164411	ENSG00000159248
ENSG00000164411	ENSG00000165474
ENSG00000164411	ENSG00000169562
ENSG00000164411	ENSG00000176402
ENSG00000164411	ENSG00000177291
ENSG00000164411	ENSG00000182963
ENSG00000164411	ENSG00000183153
ENSG00000164411	ENSG00000187513
ENSG00000164411	ENSG00000188910
ENSG00000164411	ENSG00000189280
ENSG00000164411	ENSG00000189433
ENSG00000164411	ENSG00000198835
ENSG00000164411	ENSG00000203733
ENSG00000164418	ENSG00000120251
ENSG00000164418	ENSG00000125675
ENSG00000164418	ENSG00000149403
ENSG00000164418	ENSG00000152578
ENSG00000164418	ENSG00000155511
ENSG00000164418	ENSG00000163873
ENSG00000164418	ENSG00000171189
ENSG00000164434	ENSG00000114113
ENSG00000164434	ENSG00000114115
ENSG00000164434	ENSG00000121769
ENSG00000164434	ENSG00000139194
ENSG00000164434	ENSG00000143320
ENSG00000164434	ENSG00000145384
ENSG00000164434	ENSG00000147588
ENSG00000164434	ENSG00000162444
ENSG00000164434	ENSG00000164687
ENSG00000164434	ENSG00000166426
ENSG00000164434	ENSG00000170323
ENSG00000164434	ENSG00000197416
ENSG00000164434	ENSG00000205186
ENSG00000164434	ENSG00000214004
ENSG00000164438	ENSG00000107807
ENSG00000164438	ENSG00000115297
ENSG00000164440	ENSG00000084652
ENSG00000164440	ENSG00000086712
ENSG00000164442	ENSG00000125931
ENSG00000164442	ENSG00000179862
ENSG00000164451	ENSG00000138172
ENSG00000164451	ENSG00000178033
ENSG00000164451	ENSG00000183128

ENSG00000164451	ENSG00000185933
ENSG00000164451	ENSG00000188820
ENSG00000164458	ENSG00000073861
ENSG00000164458	ENSG00000089225
ENSG00000164458	ENSG00000092607
ENSG00000164458	ENSG00000112837
ENSG00000164458	ENSG00000121068
ENSG00000164458	ENSG00000121075
ENSG00000164458	ENSG00000122145
ENSG00000164458	ENSG00000135111
ENSG00000164458	ENSG00000136535
ENSG00000164458	ENSG00000143178
ENSG00000164458	ENSG00000149922
ENSG00000164458	ENSG00000163508
ENSG00000164458	ENSG00000164532
ENSG00000164458	ENSG00000167800
ENSG00000164458	ENSG00000174197
ENSG00000164458	ENSG00000184058
ENSG00000164465	ENSG00000057019
ENSG00000164465	ENSG00000099250
ENSG00000164465	ENSG00000102104
ENSG00000164465	ENSG00000118257
ENSG00000164465	ENSG00000140545
ENSG00000164465	ENSG00000164176
ENSG00000164466	ENSG00000107819
ENSG00000164466	ENSG00000144040
ENSG00000164466	ENSG00000156398
ENSG00000164466	ENSG00000183605
ENSG00000164484	ENSG00000187975
ENSG00000164484	ENSG00000206432
ENSG00000164488	ENSG00000165617
ENSG00000164488	ENSG00000197380
ENSG00000164506	ENSG00000073350
ENSG00000164506	ENSG00000131899
ENSG00000164506	ENSG00000145087
ENSG00000164508	ENSG00000111332
ENSG00000164508	ENSG00000124642
ENSG00000164508	ENSG00000137259
ENSG00000164508	ENSG00000158403
ENSG00000164508	ENSG00000168274
ENSG00000164508	ENSG00000180573
ENSG00000164508	ENSG00000181218
ENSG00000164508	ENSG00000182611
ENSG00000164508	ENSG00000183558
ENSG00000164508	ENSG00000184260
ENSG00000164508	ENSG00000184270
ENSG00000164508	ENSG00000184348
ENSG00000164508	ENSG00000184825
ENSG00000164508	ENSG00000185978
ENSG00000164508	ENSG00000188486
ENSG00000164508	ENSG00000196747
ENSG00000164508	ENSG00000196787
ENSG00000164508	ENSG00000196866
ENSG00000164508	ENSG00000198082
ENSG00000164508	ENSG00000198307
ENSG00000164508	ENSG00000198374
ENSG00000164508	ENSG00000203812
ENSG00000164508	ENSG00000205070
ENSG00000164508	ENSG00000205079

ENSG00000164509	ENSG00000081985
ENSG00000164509	ENSG00000104998
ENSG00000164509	ENSG00000113594
ENSG00000164509	ENSG00000119535
ENSG00000164509	ENSG00000134352
ENSG00000164509	ENSG00000145623
ENSG00000164542	ENSG00000196123
ENSG00000164543	ENSG00000008118
ENSG00000164543	ENSG00000081320
ENSG00000164543	ENSG00000130822
ENSG00000164543	ENSG00000134072
ENSG00000164543	ENSG00000147613
ENSG00000164543	ENSG00000159792
ENSG00000164543	ENSG00000164076
ENSG00000164543	ENSG00000183049
ENSG00000164548	ENSG00000136527
ENSG00000164588	ENSG00000070729
ENSG00000164588	ENSG00000099822
ENSG00000164588	ENSG00000132259
ENSG00000164588	ENSG00000138622
ENSG00000164588	ENSG00000143630
ENSG00000164588	ENSG00000144191
ENSG00000164588	ENSG00000170289
ENSG00000164588	ENSG00000183862
ENSG00000164591	ENSG00000172399
ENSG00000164591	ENSG00000177791
ENSG00000164600	ENSG00000104903
ENSG00000164600	ENSG00000113196
ENSG00000164600	ENSG00000122691
ENSG00000164600	ENSG00000122859
ENSG00000164600	ENSG00000123307
ENSG00000164600	ENSG00000125878
ENSG00000164600	ENSG00000146618
ENSG00000164600	ENSG00000162367
ENSG00000164600	ENSG00000162992
ENSG00000164600	ENSG00000164107
ENSG00000164600	ENSG00000168267
ENSG00000164600	ENSG00000171532
ENSG00000164600	ENSG00000171786
ENSG00000164600	ENSG00000172238
ENSG00000164600	ENSG00000177551
ENSG00000164600	ENSG00000178403
ENSG00000164600	ENSG00000179774
ENSG00000164600	ENSG00000180535
ENSG00000164600	ENSG00000181965
ENSG00000164600	ENSG00000186051
ENSG00000164600	ENSG00000187786
ENSG00000164600	ENSG00000188686
ENSG00000164604	ENSG00000170837
ENSG00000164604	ENSG00000184194
ENSG00000164605	ENSG00000111775
ENSG00000164605	ENSG00000156885
ENSG00000164616	ENSG00000005812
ENSG00000164619	ENSG00000179066
ENSG00000164619	ENSG00000214028
ENSG00000164620	ENSG00000054967
ENSG00000164620	ENSG00000181826
ENSG00000164626	ENSG00000082482
ENSG00000164626	ENSG00000095981

ENSG00000164626	ENSG00000099337
ENSG00000164626	ENSG00000100433
ENSG00000164626	ENSG00000124249
ENSG00000164626	ENSG00000124780
ENSG00000164626	ENSG00000135750
ENSG00000164626	ENSG00000152315
ENSG00000164626	ENSG00000169427
ENSG00000164626	ENSG00000171303
ENSG00000164626	ENSG00000173338
ENSG00000164626	ENSG00000182450
ENSG00000164626	ENSG00000184261
ENSG00000164647	ENSG00000105889
ENSG00000164647	ENSG00000115107
ENSG00000164647	ENSG00000127954
ENSG00000164647	ENSG00000157214
ENSG00000164649	ENSG00000144354
ENSG00000164651	ENSG00000067082
ENSG00000164651	ENSG00000102554
ENSG00000164651	ENSG00000105610
ENSG00000164651	ENSG00000127528
ENSG00000164651	ENSG00000129911
ENSG00000164651	ENSG00000136826
ENSG00000164651	ENSG00000139651
ENSG00000164651	ENSG00000155090
ENSG00000164651	ENSG00000167182
ENSG00000164651	ENSG00000170374
ENSG00000164651	ENSG00000171872
ENSG00000164651	ENSG00000172059
ENSG00000164651	ENSG00000174595
ENSG00000164651	ENSG00000189120
ENSG00000164651	ENSG00000197294
ENSG00000164651	ENSG00000197550
ENSG00000164651	ENSG00000204335
ENSG00000164659	ENSG00000116299
ENSG00000164663	ENSG00000136014
ENSG00000164663	ENSG00000140455
ENSG00000164683	ENSG00000135547
ENSG00000164683	ENSG00000163909
ENSG00000164683	ENSG00000187821
ENSG00000164684	ENSG00000125520
ENSG00000164684	ENSG00000186918
ENSG00000164687	ENSG00000114113
ENSG00000164687	ENSG00000114115
ENSG00000164687	ENSG00000121769
ENSG00000164687	ENSG00000139194
ENSG00000164687	ENSG00000143320
ENSG00000164687	ENSG00000145384
ENSG00000164687	ENSG00000147588
ENSG00000164687	ENSG00000162444
ENSG00000164687	ENSG00000164434
ENSG00000164687	ENSG00000166426
ENSG00000164687	ENSG00000170323
ENSG00000164687	ENSG00000197416
ENSG00000164687	ENSG00000205186
ENSG00000164687	ENSG00000214004
ENSG00000164690	ENSG00000139549
ENSG00000164690	ENSG00000163501
ENSG00000164691	ENSG00000137727
ENSG00000164692	ENSG00000060718

ENSG00000164692	ENSG00000080573
ENSG00000164692	ENSG00000108821
ENSG00000164692	ENSG00000130635
ENSG00000164692	ENSG00000139219
ENSG00000164692	ENSG00000168542
ENSG00000164692	ENSG00000204248
ENSG00000164692	ENSG00000204262
ENSG00000164695	ENSG00000100931
ENSG00000164695	ENSG00000101421
ENSG00000164707	ENSG00000007216
ENSG00000164707	ENSG00000081800
ENSG00000164707	ENSG00000141485
ENSG00000164707	ENSG00000158296
ENSG00000164708	ENSG00000171314
ENSG00000164708	ENSG00000172331
ENSG00000164708	ENSG00000186076
ENSG00000164708	ENSG00000197041
ENSG00000164708	ENSG00000214206
ENSG00000164708	ENSG00000214824
ENSG00000164708	ENSG00000215178
ENSG00000164715	ENSG00000142235
ENSG00000164715	ENSG00000181409
ENSG00000164729	ENSG00000168917
ENSG00000164729	ENSG00000177164
ENSG00000164729	ENSG00000177710
ENSG00000164736	ENSG00000005513
ENSG00000164736	ENSG00000100146
ENSG00000164736	ENSG00000124766
ENSG00000164736	ENSG00000125398
ENSG00000164736	ENSG00000129194
ENSG00000164736	ENSG00000134595
ENSG00000164736	ENSG00000171056
ENSG00000164736	ENSG00000176887
ENSG00000164736	ENSG00000177732
ENSG00000164736	ENSG00000181449
ENSG00000164736	ENSG00000182968
ENSG00000164736	ENSG00000184895
ENSG00000164736	ENSG00000203883
ENSG00000164741	ENSG00000088756
ENSG00000164741	ENSG00000124143
ENSG00000164741	ENSG00000130052
ENSG00000164741	ENSG00000133121
ENSG00000164741	ENSG00000146376
ENSG00000164742	ENSG00000078295
ENSG00000164742	ENSG00000121281
ENSG00000164742	ENSG00000129467
ENSG00000164742	ENSG00000138031
ENSG00000164742	ENSG00000155897
ENSG00000164742	ENSG00000162104
ENSG00000164742	ENSG00000173175
ENSG00000164742	ENSG00000174233
ENSG00000164744	ENSG00000061656
ENSG00000164744	ENSG00000100242
ENSG00000164744	ENSG00000164828
ENSG00000164744	ENSG00000167098
ENSG00000164749	ENSG00000101076
ENSG00000164749	ENSG00000143171
ENSG00000164749	ENSG00000160113
ENSG00000164749	ENSG00000175745

ENSG00000164749	ENSG00000185551
ENSG00000164749	ENSG00000186350
ENSG00000164749	ENSG00000204231
ENSG00000164756	ENSG00000104154
ENSG00000164756	ENSG00000115194
ENSG00000164756	ENSG00000158014
ENSG00000164761	ENSG00000028137
ENSG00000164761	ENSG00000101017
ENSG00000164761	ENSG00000111321
ENSG00000164761	ENSG00000120949
ENSG00000164761	ENSG00000141655
ENSG00000164761	ENSG00000146072
ENSG00000164761	ENSG00000157873
ENSG00000164776	ENSG00000156873
ENSG00000164778	ENSG00000163064
ENSG00000164794	ENSG00000026559
ENSG00000164794	ENSG00000102057
ENSG00000164794	ENSG00000104848
ENSG00000164794	ENSG00000111262
ENSG00000164794	ENSG00000124134
ENSG00000164794	ENSG00000130037
ENSG00000164794	ENSG00000143105
ENSG00000164794	ENSG00000151079
ENSG00000164794	ENSG00000156486
ENSG00000164794	ENSG00000158445
ENSG00000164794	ENSG00000162975
ENSG00000164794	ENSG00000168263
ENSG00000164794	ENSG00000168418
ENSG00000164794	ENSG00000170745
ENSG00000164794	ENSG00000171126
ENSG00000164794	ENSG00000171385
ENSG00000164794	ENSG00000177272
ENSG00000164794	ENSG00000177301
ENSG00000164794	ENSG00000178342
ENSG00000164794	ENSG00000182255
ENSG00000164794	ENSG00000182674
ENSG00000164794	ENSG00000184408
ENSG00000164796	ENSG00000117322
ENSG00000164796	ENSG00000121904
ENSG00000164796	ENSG00000174175
ENSG00000164796	ENSG00000183117
ENSG00000164796	ENSG00000203710
ENSG00000164823	ENSG00000140961
ENSG00000164828	ENSG00000061656
ENSG00000164828	ENSG00000100242
ENSG00000164828	ENSG00000164744
ENSG00000164828	ENSG00000167098
ENSG00000164830	ENSG00000111912
ENSG00000164841	ENSG00000125895
ENSG00000164849	ENSG00000164850
ENSG00000164850	ENSG00000164849
ENSG00000164867	ENSG00000007171
ENSG00000164867	ENSG00000089250
ENSG00000164877	ENSG00000100139
ENSG00000164879	ENSG00000074410
ENSG00000164879	ENSG00000104267
ENSG00000164879	ENSG00000107159
ENSG00000164879	ENSG00000118298
ENSG00000164879	ENSG00000131686

ENSG00000164879	ENSG00000133742
ENSG00000164879	ENSG00000167434
ENSG00000164879	ENSG00000168748
ENSG00000164879	ENSG00000169239
ENSG00000164879	ENSG00000174990
ENSG00000164879	ENSG00000178538
ENSG00000164879	ENSG00000185015
ENSG00000164879	ENSG00000214729
ENSG00000164879	ENSG00000214873
ENSG00000164879	ENSG00000215487
ENSG00000164879	ENSG00000215501
ENSG00000164879	ENSG00000215543
ENSG00000164889	ENSG00000004939
ENSG00000164889	ENSG00000033867
ENSG00000164889	ENSG00000050438
ENSG00000164889	ENSG00000080493
ENSG00000164889	ENSG00000088836
ENSG00000164889	ENSG00000113073
ENSG00000164889	ENSG00000114923
ENSG00000164889	ENSG00000144290
ENSG00000164889	ENSG00000188687
ENSG00000164897	ENSG00000168591
ENSG00000164900	ENSG00000130675
ENSG00000164900	ENSG00000168505
ENSG00000164916	ENSG00000141568
ENSG00000164920	ENSG00000143867
ENSG00000164924	ENSG00000108953
ENSG00000164924	ENSG00000128245
ENSG00000164924	ENSG00000134308
ENSG00000164924	ENSG00000166913
ENSG00000164924	ENSG00000170027
ENSG00000164924	ENSG00000175793
ENSG00000164924	ENSG00000214637
ENSG00000164924	ENSG00000215108
ENSG00000164930	ENSG00000104290
ENSG00000164930	ENSG00000106483
ENSG00000164930	ENSG00000111432
ENSG00000164930	ENSG00000155760
ENSG00000164930	ENSG00000157240
ENSG00000164930	ENSG00000162998
ENSG00000164930	ENSG00000163251
ENSG00000164930	ENSG00000174804
ENSG00000164930	ENSG00000177283
ENSG00000164930	ENSG00000180340
ENSG00000164930	ENSG00000188763
ENSG00000164938	ENSG00000078804
ENSG00000164946	ENSG00000138759
ENSG00000164946	ENSG00000150893
ENSG00000164946	ENSG00000173546
ENSG00000164946	ENSG00000183090
ENSG00000164946	ENSG00000215060
ENSG00000164949	ENSG00000088320
ENSG00000164949	ENSG00000139890
ENSG00000164949	ENSG00000166592
ENSG00000164951	ENSG00000172840
ENSG00000164967	ENSG00000178718
ENSG00000164970	ENSG00000178761
ENSG00000164985	ENSG00000112273
ENSG00000164985	ENSG00000143321

ENSG00000164985	ENSG00000166503
ENSG00000164985	ENSG00000167674
ENSG00000165023	ENSG00000116473
ENSG00000165023	ENSG00000123728
ENSG00000165023	ENSG00000125249
ENSG00000165023	ENSG00000127314
ENSG00000165023	ENSG00000162595
ENSG00000165023	ENSG00000176276
ENSG00000165023	ENSG00000176490
ENSG00000165023	ENSG00000181467
ENSG00000165023	ENSG00000214405
ENSG00000165023	ENSG00000214492
ENSG00000165025	ENSG00000115085
ENSG00000165029	ENSG00000064687
ENSG00000165029	ENSG00000107331
ENSG00000165029	ENSG00000144452
ENSG00000165029	ENSG00000167972
ENSG00000165029	ENSG00000179869
ENSG00000165029	ENSG00000198691
ENSG00000165046	ENSG00000168924
ENSG00000165046	ENSG00000214906
ENSG00000165055	ENSG00000087995
ENSG00000165055	ENSG00000123600
ENSG00000165059	ENSG00000072062
ENSG00000165059	ENSG00000099725
ENSG00000165059	ENSG00000142875
ENSG00000165059	ENSG00000183943
ENSG00000165061	ENSG00000113761
ENSG00000165061	ENSG00000172667
ENSG00000165066	ENSG00000148826
ENSG00000165066	ENSG00000163623
ENSG00000165076	ENSG00000010438
ENSG00000165076	ENSG00000129437
ENSG00000165076	ENSG00000129451
ENSG00000165076	ENSG00000129455
ENSG00000165076	ENSG00000142515
ENSG00000165076	ENSG00000167748
ENSG00000165076	ENSG00000167749
ENSG00000165076	ENSG00000167751
ENSG00000165076	ENSG00000167754
ENSG00000165076	ENSG00000167755
ENSG00000165076	ENSG00000167757
ENSG00000165076	ENSG00000167759
ENSG00000165076	ENSG00000169035
ENSG00000165076	ENSG00000171147
ENSG00000165076	ENSG00000173636
ENSG00000165076	ENSG00000174562
ENSG00000165076	ENSG00000186474
ENSG00000165076	ENSG00000204981
ENSG00000165076	ENSG00000204982
ENSG00000165076	ENSG00000204983
ENSG00000165076	ENSG00000213022
ENSG00000165076	ENSG00000214083
ENSG00000165078	ENSG00000080618
ENSG00000165078	ENSG00000091704
ENSG00000165078	ENSG00000128510
ENSG00000165078	ENSG00000144410
ENSG00000165078	ENSG00000153002
ENSG00000165078	ENSG00000158516

ENSG00000165078	ENSG00000158525
ENSG00000165078	ENSG00000163751
ENSG00000165091	ENSG00000103534
ENSG00000165091	ENSG00000141524
ENSG00000165091	ENSG00000149488
ENSG00000165091	ENSG00000167608
ENSG00000165091	ENSG00000167895
ENSG00000165091	ENSG00000170537
ENSG00000165091	ENSG00000188869
ENSG00000165092	ENSG00000111275
ENSG00000165092	ENSG00000128918
ENSG00000165092	ENSG00000137124
ENSG00000165092	ENSG00000184254
ENSG00000165115	ENSG00000166813
ENSG00000165125	ENSG00000111199
ENSG00000165125	ENSG00000127412
ENSG00000165125	ENSG00000167723
ENSG00000165125	ENSG00000187688
ENSG00000165125	ENSG00000196689
ENSG00000165140	ENSG00000130957
ENSG00000165156	ENSG00000174306
ENSG00000165156	ENSG00000178764
ENSG00000165156	ENSG00000215271
ENSG00000165168	ENSG00000007952
ENSG00000165168	ENSG00000074771
ENSG00000165168	ENSG00000086991
ENSG00000165175	ENSG00000151365
ENSG00000165178	ENSG00000107957
ENSG00000165178	ENSG00000158517
ENSG00000165178	ENSG00000174705
ENSG00000165178	ENSG00000182487
ENSG00000165178	ENSG00000196408
ENSG00000165186	ENSG00000124814
ENSG00000165186	ENSG00000178729
ENSG00000165186	ENSG00000182077
ENSG00000165192	ENSG00000065802
ENSG00000165192	ENSG00000102048
ENSG00000165192	ENSG00000164122
ENSG00000165192	ENSG00000196372
ENSG00000165192	ENSG00000198881
ENSG00000165192	ENSG00000214426
ENSG00000165194	ENSG00000118946
ENSG00000165194	ENSG00000136099
ENSG00000165194	ENSG00000138650
ENSG00000165194	ENSG00000189184
ENSG00000165197	ENSG00000100311
ENSG00000165197	ENSG00000112715
ENSG00000165197	ENSG00000119630
ENSG00000165197	ENSG00000150630
ENSG00000165197	ENSG00000173511
ENSG00000165197	ENSG00000197461
ENSG00000165197	ENSG00000215682
ENSG00000165209	ENSG00000056097
ENSG00000165209	ENSG00000105278
ENSG00000165209	ENSG00000129351
ENSG00000165209	ENSG00000143621
ENSG00000165215	ENSG00000013297
ENSG00000165215	ENSG00000066405
ENSG00000165215	ENSG00000106404

ENSG00000165215	ENSG00000113946
ENSG00000165215	ENSG00000134873
ENSG00000165215	ENSG00000156282
ENSG00000165215	ENSG00000156284
ENSG00000165215	ENSG00000159261
ENSG00000165215	ENSG00000163347
ENSG00000165215	ENSG00000164007
ENSG00000165215	ENSG00000165376
ENSG00000165215	ENSG00000171217
ENSG00000165215	ENSG00000177300
ENSG00000165215	ENSG00000181885
ENSG00000165215	ENSG00000184113
ENSG00000165215	ENSG00000184697
ENSG00000165215	ENSG00000185758
ENSG00000165215	ENSG00000189118
ENSG00000165215	ENSG00000189143
ENSG00000165215	ENSG00000213937
ENSG00000165238	ENSG00000060237
ENSG00000165238	ENSG00000115216
ENSG00000165238	ENSG00000126562
ENSG00000165238	ENSG00000196632
ENSG00000165240	ENSG00000123191
ENSG00000165240	ENSG00000215763
ENSG00000165246	ENSG00000146938
ENSG00000165246	ENSG00000169760
ENSG00000165246	ENSG00000169992
ENSG00000165246	ENSG00000196338
ENSG00000165269	ENSG00000103569
ENSG00000165269	ENSG00000143595
ENSG00000165269	ENSG00000165272
ENSG00000165269	ENSG00000176115
ENSG00000165269	ENSG00000181997
ENSG00000165269	ENSG00000186466
ENSG00000165272	ENSG00000103569
ENSG00000165272	ENSG00000143595
ENSG00000165272	ENSG00000165269
ENSG00000165272	ENSG00000176115
ENSG00000165272	ENSG00000181997
ENSG00000165272	ENSG00000186466
ENSG00000165288	ENSG00000146247
ENSG00000165288	ENSG00000185658
ENSG00000165300	ENSG00000179542
ENSG00000165300	ENSG00000184564
ENSG00000165322	ENSG00000075884
ENSG00000165322	ENSG00000107863
ENSG00000165322	ENSG00000123329
ENSG00000165322	ENSG00000167433
ENSG00000165322	ENSG00000185602
ENSG00000165322	ENSG00000214627
ENSG00000165325	ENSG00000182923
ENSG00000165349	ENSG00000003989
ENSG00000165349	ENSG00000013293
ENSG00000165349	ENSG00000099960
ENSG00000165349	ENSG00000139514
ENSG00000165359	ENSG00000102786
ENSG00000165359	ENSG00000181433
ENSG00000165359	ENSG00000215130
ENSG00000165366	ENSG00000139780
ENSG00000165370	ENSG00000120907

ENSG00000165370	ENSG00000143147
ENSG00000165370	ENSG00000148680
ENSG00000165370	ENSG00000170214
ENSG00000165370	ENSG00000171873
ENSG00000165370	ENSG00000183935
ENSG00000165376	ENSG00000013297
ENSG00000165376	ENSG00000066405
ENSG00000165376	ENSG00000106404
ENSG00000165376	ENSG00000113946
ENSG00000165376	ENSG00000134873
ENSG00000165376	ENSG00000156282
ENSG00000165376	ENSG00000156284
ENSG00000165376	ENSG00000159261
ENSG00000165376	ENSG00000163347
ENSG00000165376	ENSG00000164007
ENSG00000165376	ENSG00000165215
ENSG00000165376	ENSG00000171217
ENSG00000165376	ENSG00000177300
ENSG00000165376	ENSG00000181885
ENSG00000165376	ENSG00000184113
ENSG00000165376	ENSG00000184697
ENSG00000165376	ENSG00000185758
ENSG00000165376	ENSG00000189118
ENSG00000165376	ENSG00000189143
ENSG00000165376	ENSG00000213937
ENSG00000165379	ENSG00000126243
ENSG00000165379	ENSG00000128011
ENSG00000165379	ENSG00000156564
ENSG00000165388	ENSG00000152784
ENSG00000165389	ENSG00000196542
ENSG00000165390	ENSG00000104537
ENSG00000165390	ENSG00000109511
ENSG00000165390	ENSG00000122359
ENSG00000165390	ENSG00000135046
ENSG00000165390	ENSG00000138279
ENSG00000165390	ENSG00000138772
ENSG00000165390	ENSG00000143412
ENSG00000165390	ENSG00000150165
ENSG00000165390	ENSG00000164111
ENSG00000165390	ENSG00000182718
ENSG00000165390	ENSG00000183059
ENSG00000165390	ENSG00000186807
ENSG00000165390	ENSG00000196975
ENSG00000165390	ENSG00000197043
ENSG00000165406	ENSG00000099785
ENSG00000165406	ENSG00000139266
ENSG00000165406	ENSG00000144583
ENSG00000165406	ENSG00000145416
ENSG00000165406	ENSG00000173926
ENSG00000165406	ENSG00000183654
ENSG00000165409	ENSG00000133067
ENSG00000165409	ENSG00000138039
ENSG00000165409	ENSG00000139292
ENSG00000165409	ENSG00000170820
ENSG00000165409	ENSG00000205213
ENSG00000165410	ENSG00000125868
ENSG00000165410	ENSG00000172757
ENSG00000165434	ENSG00000169299
ENSG00000165443	ENSG00000168490

ENSG00000165449	ENSG00000100156
ENSG00000165449	ENSG00000108932
ENSG00000165449	ENSG00000112394
ENSG00000165449	ENSG00000118596
ENSG00000165449	ENSG00000141526
ENSG00000165449	ENSG00000147100
ENSG00000165449	ENSG00000152779
ENSG00000165449	ENSG00000155380
ENSG00000165449	ENSG00000163053
ENSG00000165449	ENSG00000168679
ENSG00000165449	ENSG00000170190
ENSG00000165449	ENSG00000174326
ENSG00000165449	ENSG00000174327
ENSG00000165458	ENSG00000148384
ENSG00000165458	ENSG00000168918
ENSG00000165462	ENSG00000109132
ENSG00000165462	ENSG00000116132
ENSG00000165462	ENSG00000165606
ENSG00000165462	ENSG00000167157
ENSG00000165474	ENSG00000121634
ENSG00000165474	ENSG00000121742
ENSG00000165474	ENSG00000121743
ENSG00000165474	ENSG00000131233
ENSG00000165474	ENSG00000135355
ENSG00000165474	ENSG00000143140
ENSG00000165474	ENSG00000152661
ENSG00000165474	ENSG00000159248
ENSG00000165474	ENSG00000164411
ENSG00000165474	ENSG00000169562
ENSG00000165474	ENSG00000176402
ENSG00000165474	ENSG00000177291
ENSG00000165474	ENSG00000182963
ENSG00000165474	ENSG00000183153
ENSG00000165474	ENSG00000187513
ENSG00000165474	ENSG00000188910
ENSG00000165474	ENSG00000189280
ENSG00000165474	ENSG00000189433
ENSG00000165474	ENSG00000198835
ENSG00000165474	ENSG00000203733
ENSG00000165476	ENSG00000068615
ENSG00000165476	ENSG00000132563
ENSG00000165476	ENSG00000168476
ENSG00000165478	ENSG00000176395
ENSG00000165478	ENSG00000188175
ENSG00000165487	ENSG00000155970
ENSG00000165495	ENSG00000105419
ENSG00000165495	ENSG00000118707
ENSG00000165495	ENSG00000134138
ENSG00000165495	ENSG00000143995
ENSG00000165495	ENSG00000153779
ENSG00000165495	ENSG00000160199
ENSG00000165495	ENSG00000176679
ENSG00000165495	ENSG00000177426
ENSG00000165495	ENSG00000179277
ENSG00000165495	ENSG00000188013
ENSG00000165516	ENSG00000099949
ENSG00000165516	ENSG00000128607
ENSG00000165516	ENSG00000197776
ENSG00000165521	ENSG00000066629

ENSG00000165521	ENSG00000125746
ENSG00000165521	ENSG00000143924
ENSG00000165521	ENSG00000143940
ENSG00000165521	ENSG00000149499
ENSG00000165521	ENSG00000214595
ENSG00000165526	ENSG00000156990
ENSG00000165527	ENSG00000004059
ENSG00000165527	ENSG00000134287
ENSG00000165527	ENSG00000143761
ENSG00000165527	ENSG00000168374
ENSG00000165548	ENSG00000137216
ENSG00000165548	ENSG00000196187
ENSG00000165556	ENSG00000113722
ENSG00000165556	ENSG00000131264
ENSG00000165566	ENSG00000178171
ENSG00000165566	ENSG00000184675
ENSG00000165572	ENSG00000120696
ENSG00000165572	ENSG00000163376
ENSG00000165572	ENSG00000170852
ENSG00000165588	ENSG00000105392
ENSG00000165588	ENSG00000115507
ENSG00000165588	ENSG00000186103
ENSG00000165617	ENSG00000164488
ENSG00000165617	ENSG00000197380
ENSG00000165621	ENSG00000125245
ENSG00000165621	ENSG00000126251
ENSG00000165621	ENSG00000126262
ENSG00000165621	ENSG00000144230
ENSG00000165621	ENSG00000147138
ENSG00000165621	ENSG00000162881
ENSG00000165621	ENSG00000164220
ENSG00000165621	ENSG00000164251
ENSG00000165621	ENSG00000171631
ENSG00000165621	ENSG00000181104
ENSG00000165621	ENSG00000182162
ENSG00000165621	ENSG00000182559
ENSG00000165621	ENSG00000182782
ENSG00000165621	ENSG00000185897
ENSG00000165621	ENSG00000196917
ENSG00000165637	ENSG00000078668
ENSG00000165637	ENSG00000172974
ENSG00000165637	ENSG00000213585
ENSG00000165637	ENSG00000215818
ENSG00000165646	ENSG00000036565
ENSG00000165646	ENSG00000146409
ENSG00000165646	ENSG00000187714
ENSG00000165655	ENSG00000183779
ENSG00000165659	ENSG00000126733
ENSG00000165660	ENSG00000163322
ENSG00000165661	ENSG00000116260
ENSG00000165671	ENSG00000109685
ENSG00000165671	ENSG00000147548
ENSG00000165675	ENSG00000120658
ENSG00000165702	ENSG00000162676
ENSG00000165704	ENSG00000099256
ENSG00000165716	ENSG00000154511
ENSG00000165716	ENSG00000181744
ENSG00000165716	ENSG00000187773
ENSG00000165752	ENSG00000152953

ENSG00000165752	ENSG00000169302
ENSG00000165775	ENSG00000069509
ENSG00000165775	ENSG00000182814
ENSG00000165782	ENSG00000155099
ENSG00000165794	ENSG00000141873
ENSG00000165794	ENSG00000143570
ENSG00000165795	ENSG00000101079
ENSG00000165795	ENSG00000103034
ENSG00000165795	ENSG00000104419
ENSG00000165801	ENSG00000149633
ENSG00000165801	ENSG00000153404
ENSG00000165801	ENSG00000196155
ENSG00000165804	ENSG00000101493
ENSG00000165804	ENSG00000171940
ENSG00000165804	ENSG00000198597
ENSG00000165806	ENSG00000003400
ENSG00000165806	ENSG00000064012
ENSG00000165806	ENSG00000105141
ENSG00000165806	ENSG00000106144
ENSG00000165806	ENSG00000132906
ENSG00000165806	ENSG00000137752
ENSG00000165806	ENSG00000137757
ENSG00000165806	ENSG00000138794
ENSG00000165806	ENSG00000164305
ENSG00000165806	ENSG00000196954
ENSG00000165806	ENSG00000204403
ENSG00000165810	ENSG00000026950
ENSG00000165810	ENSG00000111801
ENSG00000165810	ENSG00000112763
ENSG00000165810	ENSG00000113303
ENSG00000165810	ENSG00000121236
ENSG00000165810	ENSG00000124508
ENSG00000165810	ENSG00000124549
ENSG00000165810	ENSG00000124557
ENSG00000165810	ENSG00000128250
ENSG00000165810	ENSG00000128253
ENSG00000165810	ENSG00000128276
ENSG00000165810	ENSG00000128482
ENSG00000165810	ENSG00000132256
ENSG00000165810	ENSG00000132274
ENSG00000165810	ENSG00000164010
ENSG00000165810	ENSG00000168903
ENSG00000165810	ENSG00000176422
ENSG00000165810	ENSG00000179046
ENSG00000165810	ENSG00000182805
ENSG00000165810	ENSG00000183439
ENSG00000165810	ENSG00000183718
ENSG00000165810	ENSG00000187574
ENSG00000165810	ENSG00000188683
ENSG00000165810	ENSG00000204614
ENSG00000165810	ENSG00000204616
ENSG00000165810	ENSG00000204618
ENSG00000165810	ENSG00000205198
ENSG00000165810	ENSG00000214693
ENSG00000165816	ENSG00000100473
ENSG00000165816	ENSG00000163359
ENSG00000165816	ENSG00000198450
ENSG00000165816	ENSG00000205221
ENSG00000165821	ENSG00000101115

ENSG00000165821	ENSG00000103449
ENSG00000165821	ENSG00000151514
ENSG00000165868	ENSG00000132622
ENSG00000165886	ENSG00000168246
ENSG00000165887	ENSG00000148677
ENSG00000165887	ENSG00000163126
ENSG00000165891	ENSG00000129173
ENSG00000165895	ENSG00000071205
ENSG00000165895	ENSG00000079482
ENSG00000165895	ENSG00000145819
ENSG00000165905	ENSG00000133424
ENSG00000165905	ENSG00000174684
ENSG00000165912	ENSG00000100266
ENSG00000165912	ENSG00000124507
ENSG00000165912	ENSG00000140368
ENSG00000165912	ENSG00000152229
ENSG00000165914	ENSG00000068724
ENSG00000165923	ENSG00000135049
ENSG00000165923	ENSG00000146856
ENSG00000165923	ENSG00000166748
ENSG00000165951	ENSG00000100665
ENSG00000165951	ENSG00000123561
ENSG00000165951	ENSG00000135744
ENSG00000165951	ENSG00000165953
ENSG00000165951	ENSG00000170054
ENSG00000165951	ENSG00000186910
ENSG00000165951	ENSG00000187483
ENSG00000165951	ENSG00000188488
ENSG00000165951	ENSG00000196136
ENSG00000165951	ENSG00000197249
ENSG00000165953	ENSG00000100665
ENSG00000165953	ENSG00000123561
ENSG00000165953	ENSG00000135744
ENSG00000165953	ENSG00000165951
ENSG00000165953	ENSG00000170054
ENSG00000165953	ENSG00000186910
ENSG00000165953	ENSG00000187483
ENSG00000165953	ENSG00000188488
ENSG00000165953	ENSG00000196136
ENSG00000165953	ENSG00000197249
ENSG00000165959	ENSG00000178209
ENSG00000165966	ENSG00000067840
ENSG00000165966	ENSG00000121440
ENSG00000165973	ENSG00000184613
ENSG00000165985	ENSG00000054803
ENSG00000165985	ENSG00000102924
ENSG00000165985	ENSG00000131094
ENSG00000165985	ENSG00000133466
ENSG00000165985	ENSG00000139899
ENSG00000165985	ENSG00000141668
ENSG00000165985	ENSG00000144119
ENSG00000165985	ENSG00000145861
ENSG00000165985	ENSG00000159189
ENSG00000165985	ENSG00000163145
ENSG00000165985	ENSG00000172247
ENSG00000165985	ENSG00000173369
ENSG00000165985	ENSG00000173372
ENSG00000165985	ENSG00000173918
ENSG00000165985	ENSG00000181092

ENSG00000165985	ENSG00000184471
ENSG00000165985	ENSG00000186897
ENSG00000165985	ENSG00000213192
ENSG00000165995	ENSG00000067191
ENSG00000165995	ENSG00000167535
ENSG00000165995	ENSG00000182389
ENSG00000165996	ENSG00000074696
ENSG00000165996	ENSG00000188921
ENSG00000165996	ENSG00000206527
ENSG00000165997	ENSG00000141748
ENSG00000165997	ENSG00000162980
ENSG00000165997	ENSG00000215451
ENSG00000166006	ENSG00000116396
ENSG00000166006	ENSG00000129159
ENSG00000166006	ENSG00000131398
ENSG00000166016	ENSG00000151136
ENSG00000166024	ENSG00000104679
ENSG00000166025	ENSG00000114019
ENSG00000166025	ENSG00000126016
ENSG00000166033	ENSG00000115317
ENSG00000166033	ENSG00000169495
ENSG00000166033	ENSG00000170801
ENSG00000166035	ENSG00000101670
ENSG00000166035	ENSG00000144837
ENSG00000166035	ENSG00000163898
ENSG00000166035	ENSG00000165862
ENSG00000166035	ENSG00000175445
ENSG00000166035	ENSG00000175535
ENSG00000166035	ENSG00000187021
ENSG00000166035	ENSG00000188992
ENSG00000166035	ENSG00000203837
ENSG00000166037	ENSG00000183137
ENSG00000166046	ENSG00000124678
ENSG00000166046	ENSG00000176148
ENSG00000166046	ENSG00000215029
ENSG00000166046	ENSG00000215046
ENSG00000166049	ENSG00000134852
ENSG00000166049	ENSG00000170485
ENSG00000166068	ENSG00000125753
ENSG00000166068	ENSG00000154380
ENSG00000166068	ENSG00000188766
ENSG00000166068	ENSG00000196405
ENSG00000166068	ENSG00000198369
ENSG00000166086	ENSG00000154721
ENSG00000166090	ENSG00000112115
ENSG00000166090	ENSG00000112116
ENSG00000166090	ENSG00000124391
ENSG00000166090	ENSG00000127743
ENSG00000166090	ENSG00000172458
ENSG00000166091	ENSG00000091317
ENSG00000166091	ENSG00000102007
ENSG00000166091	ENSG00000140931
ENSG00000166091	ENSG00000153551
ENSG00000166091	ENSG00000183723
ENSG00000166106	ENSG00000134917
ENSG00000166106	ENSG00000154734
ENSG00000166106	ENSG00000154736
ENSG00000166106	ENSG00000158859
ENSG00000166111	ENSG00000157703

ENSG00000166123	ENSG00000167701
ENSG00000166128	ENSG00000084733
ENSG00000166128	ENSG00000139998
ENSG00000166128	ENSG00000143545
ENSG00000166128	ENSG00000167461
ENSG00000166143	ENSG00000167641
ENSG00000166143	ENSG00000173457
ENSG00000166143	ENSG00000198729
ENSG00000166145	ENSG00000120256
ENSG00000166147	ENSG00000138829
ENSG00000166147	ENSG00000142449
ENSG00000166148	ENSG00000109163
ENSG00000166148	ENSG00000126895
ENSG00000166148	ENSG00000178015
ENSG00000166148	ENSG00000180914
ENSG00000166148	ENSG00000187258
ENSG00000166148	ENSG00000198049
ENSG00000166148	ENSG00000211451
ENSG00000166153	ENSG00000024526
ENSG00000166153	ENSG00000035499
ENSG00000166153	ENSG00000121690
ENSG00000166159	ENSG00000144771
ENSG00000166159	ENSG00000185245
ENSG00000166160	ENSG00000054277
ENSG00000166160	ENSG00000102076
ENSG00000166160	ENSG00000122375
ENSG00000166160	ENSG00000124818
ENSG00000166160	ENSG00000128617
ENSG00000166160	ENSG00000147380
ENSG00000166160	ENSG00000148604
ENSG00000166160	ENSG00000163914
ENSG00000166160	ENSG00000180245
ENSG00000166164	ENSG00000028310
ENSG00000166164	ENSG00000214423
ENSG00000166164	ENSG00000215415
ENSG00000166165	ENSG00000104879
ENSG00000166165	ENSG00000131730
ENSG00000166165	ENSG00000166998
ENSG00000166165	ENSG00000168775
ENSG00000166167	ENSG00000072803
ENSG00000166206	ENSG00000011677
ENSG00000166206	ENSG00000022355
ENSG00000166206	ENSG00000094755
ENSG00000166206	ENSG00000101958
ENSG00000166206	ENSG00000102287
ENSG00000166206	ENSG00000109158
ENSG00000166206	ENSG00000109738
ENSG00000166206	ENSG00000111886
ENSG00000166206	ENSG00000113327
ENSG00000166206	ENSG00000145451
ENSG00000166206	ENSG00000145863
ENSG00000166206	ENSG00000145864
ENSG00000166206	ENSG00000145888
ENSG00000166206	ENSG00000146276
ENSG00000166206	ENSG00000147402
ENSG00000166206	ENSG00000151834
ENSG00000166206	ENSG00000163285
ENSG00000166206	ENSG00000163288
ENSG00000166206	ENSG00000182256

ENSG00000166206	ENSG00000183185
ENSG00000166206	ENSG00000186297
ENSG00000166206	ENSG00000187730
ENSG00000166206	ENSG00000188828
ENSG00000166211	ENSG00000010030
ENSG00000166211	ENSG00000102034
ENSG00000166211	ENSG00000109381
ENSG00000166211	ENSG00000120690
ENSG00000166211	ENSG00000124664
ENSG00000166211	ENSG00000135373
ENSG00000166211	ENSG00000135374
ENSG00000166211	ENSG00000139083
ENSG00000166211	ENSG00000163435
ENSG00000166225	ENSG00000101134
ENSG00000166225	ENSG00000115325
ENSG00000166225	ENSG00000125170
ENSG00000166225	ENSG00000137218
ENSG00000166225	ENSG00000146094
ENSG00000166225	ENSG00000147443
ENSG00000166225	ENSG00000206052
ENSG00000166228	ENSG00000132570
ENSG00000166250	ENSG00000019102
ENSG00000166250	ENSG00000143167
ENSG00000166250	ENSG00000144847
ENSG00000166250	ENSG00000149564
ENSG00000166250	ENSG00000154639
ENSG00000166250	ENSG00000188004
ENSG00000166257	ENSG00000105711
ENSG00000166289	ENSG00000175895
ENSG00000166313	ENSG00000113108
ENSG00000166313	ENSG00000163697
ENSG00000166317	ENSG00000171992
ENSG00000166317	ENSG00000172403
ENSG00000166341	ENSG00000083857
ENSG00000166341	ENSG00000086570
ENSG00000166341	ENSG00000107736
ENSG00000166342	ENSG00000038295
ENSG00000166342	ENSG00000095587
ENSG00000166342	ENSG00000106333
ENSG00000166342	ENSG00000107611
ENSG00000166342	ENSG00000157211
ENSG00000166342	ENSG00000163710
ENSG00000166342	ENSG00000168487
ENSG00000166342	ENSG00000171208
ENSG00000166342	ENSG00000184824
ENSG00000166347	ENSG00000103018
ENSG00000166377	ENSG00000054793
ENSG00000166387	ENSG00000110841
ENSG00000166387	ENSG00000131626
ENSG00000166387	ENSG00000139220
ENSG00000166387	ENSG00000143847
ENSG00000166387	ENSG00000177380
ENSG00000166391	ENSG00000062282
ENSG00000166391	ENSG00000106384
ENSG00000166391	ENSG00000124003
ENSG00000166391	ENSG00000147160
ENSG00000166391	ENSG00000184210
ENSG00000166391	ENSG00000204195
ENSG00000166391	ENSG00000205267

ENSG00000166394	ENSG00000100243
ENSG00000166394	ENSG00000159348
ENSG00000166396	ENSG00000021355
ENSG00000166396	ENSG00000057149
ENSG00000166396	ENSG00000124570
ENSG00000166396	ENSG00000166401
ENSG00000166396	ENSG00000166404
ENSG00000166396	ENSG00000166634
ENSG00000166396	ENSG00000170542
ENSG00000166396	ENSG00000197632
ENSG00000166396	ENSG00000197641
ENSG00000166396	ENSG00000206072
ENSG00000166396	ENSG00000206073
ENSG00000166396	ENSG00000206075
ENSG00000166401	ENSG00000021355
ENSG00000166401	ENSG00000057149
ENSG00000166401	ENSG00000124570
ENSG00000166401	ENSG00000166396
ENSG00000166401	ENSG00000166404
ENSG00000166401	ENSG00000166634
ENSG00000166401	ENSG00000170542
ENSG00000166401	ENSG00000197632
ENSG00000166401	ENSG00000197641
ENSG00000166401	ENSG00000206072
ENSG00000166401	ENSG00000206073
ENSG00000166401	ENSG00000206075
ENSG00000166402	ENSG00000078246
ENSG00000166402	ENSG00000104804
ENSG00000166402	ENSG00000112041
ENSG00000166402	ENSG00000118965
ENSG00000166402	ENSG00000130338
ENSG00000166404	ENSG00000021355
ENSG00000166404	ENSG00000057149
ENSG00000166404	ENSG00000124570
ENSG00000166404	ENSG00000166396
ENSG00000166404	ENSG00000166401
ENSG00000166404	ENSG00000166634
ENSG00000166404	ENSG00000170542
ENSG00000166404	ENSG00000197632
ENSG00000166404	ENSG00000197641
ENSG00000166404	ENSG00000206072
ENSG00000166404	ENSG00000206073
ENSG00000166404	ENSG00000206075
ENSG00000166407	ENSG00000048540
ENSG00000166407	ENSG00000135363
ENSG00000166415	ENSG00000091157
ENSG00000166426	ENSG00000114113
ENSG00000166426	ENSG00000114115
ENSG00000166426	ENSG00000121769
ENSG00000166426	ENSG00000139194
ENSG00000166426	ENSG00000143320
ENSG00000166426	ENSG00000145384
ENSG00000166426	ENSG00000147588
ENSG00000166426	ENSG00000162444
ENSG00000166426	ENSG00000164434
ENSG00000166426	ENSG00000164687
ENSG00000166426	ENSG00000170323
ENSG00000166426	ENSG00000197416
ENSG00000166426	ENSG00000205186

ENSG00000166426	ENSG00000214004
ENSG00000166428	ENSG00000105223
ENSG00000166428	ENSG00000180287
ENSG00000166436	ENSG00000122779
ENSG00000166436	ENSG00000130726
ENSG00000166436	ENSG00000197323
ENSG00000166439	ENSG00000163961
ENSG00000166444	ENSG00000119522
ENSG00000166444	ENSG00000146966
ENSG00000166444	ENSG00000162701
ENSG00000166444	ENSG00000162777
ENSG00000166444	ENSG00000175984
ENSG00000166444	ENSG00000205744
ENSG00000166446	ENSG00000129873
ENSG00000166446	ENSG00000153046
ENSG00000166446	ENSG00000172288
ENSG00000166446	ENSG00000172352
ENSG00000166446	ENSG00000182415
ENSG00000166446	ENSG00000198721
ENSG00000166446	ENSG00000215563
ENSG00000166450	ENSG00000067141
ENSG00000166450	ENSG00000103742
ENSG00000166450	ENSG00000174498
ENSG00000166450	ENSG00000187323
ENSG00000166450	ENSG00000215851
ENSG00000166473	ENSG00000008710
ENSG00000166473	ENSG00000078795
ENSG00000166473	ENSG00000107593
ENSG00000166473	ENSG00000118762
ENSG00000166473	ENSG00000130943
ENSG00000166473	ENSG00000158683
ENSG00000166473	ENSG00000183458
ENSG00000166473	ENSG00000183793
ENSG00000166473	ENSG00000187008
ENSG00000166473	ENSG00000188599
ENSG00000166478	ENSG00000065029
ENSG00000166478	ENSG00000119725
ENSG00000166483	ENSG00000214102
ENSG00000166501	ENSG00000027075
ENSG00000166501	ENSG00000065675
ENSG00000166501	ENSG00000067606
ENSG00000166501	ENSG00000105221
ENSG00000166501	ENSG00000117020
ENSG00000166501	ENSG00000126583
ENSG00000166501	ENSG00000142208
ENSG00000166501	ENSG00000154229
ENSG00000166501	ENSG00000163558
ENSG00000166501	ENSG00000163932
ENSG00000166503	ENSG00000112273
ENSG00000166503	ENSG00000143321
ENSG00000166503	ENSG00000164985
ENSG00000166503	ENSG00000167674
ENSG00000166507	ENSG00000070614
ENSG00000166507	ENSG00000138653
ENSG00000166507	ENSG00000164100
ENSG00000166509	ENSG00000105472
ENSG00000166509	ENSG00000118004
ENSG00000166509	ENSG00000122852
ENSG00000166509	ENSG00000122854

ENSG00000166509	ENSG00000133661
ENSG00000166509	ENSG00000163815
ENSG00000166509	ENSG00000165471
ENSG00000166509	ENSG00000182314
ENSG00000166509	ENSG00000184374
ENSG00000166509	ENSG00000185303
ENSG00000166510	ENSG00000143367
ENSG00000166530	ENSG00000168999
ENSG00000166557	ENSG00000086598
ENSG00000166557	ENSG00000099203
ENSG00000166557	ENSG00000117500
ENSG00000166557	ENSG00000134970
ENSG00000166557	ENSG00000157315
ENSG00000166558	ENSG00000017483
ENSG00000166558	ENSG00000103042
ENSG00000166558	ENSG00000111371
ENSG00000166558	ENSG00000134294
ENSG00000166558	ENSG00000139209
ENSG00000166558	ENSG00000139974
ENSG00000166558	ENSG00000169507
ENSG00000166558	ENSG00000188338
ENSG00000166562	ENSG00000140612
ENSG00000166569	ENSG00000213578
ENSG00000166573	ENSG00000082556
ENSG00000166573	ENSG00000112038
ENSG00000166573	ENSG00000116014
ENSG00000166573	ENSG00000116329
ENSG00000166573	ENSG00000125510
ENSG00000166573	ENSG00000125522
ENSG00000166573	ENSG00000128285
ENSG00000166573	ENSG00000128310
ENSG00000166573	ENSG00000132671
ENSG00000166573	ENSG00000139874
ENSG00000166573	ENSG00000152034
ENSG00000166573	ENSG00000162009
ENSG00000166573	ENSG00000180616
ENSG00000166573	ENSG00000182687
ENSG00000166573	ENSG00000183473
ENSG00000166573	ENSG00000183729
ENSG00000166579	ENSG00000072864
ENSG00000166589	ENSG00000039068
ENSG00000166589	ENSG00000062038
ENSG00000166589	ENSG00000079112
ENSG00000166589	ENSG00000124215
ENSG00000166589	ENSG00000129910
ENSG00000166589	ENSG00000134755
ENSG00000166589	ENSG00000134762
ENSG00000166589	ENSG00000134765
ENSG00000166589	ENSG00000140945
ENSG00000166589	ENSG00000170558
ENSG00000166589	ENSG00000179242
ENSG00000166592	ENSG00000088320
ENSG00000166592	ENSG00000139890
ENSG00000166592	ENSG00000164949
ENSG00000166603	ENSG00000124089
ENSG00000166603	ENSG00000176136
ENSG00000166603	ENSG00000185231
ENSG00000166634	ENSG00000021355
ENSG00000166634	ENSG00000057149

ENSG00000166634	ENSG00000124570
ENSG00000166634	ENSG00000166396
ENSG00000166634	ENSG00000166401
ENSG00000166634	ENSG00000166404
ENSG00000166634	ENSG00000170542
ENSG00000166634	ENSG00000197632
ENSG00000166634	ENSG00000197641
ENSG00000166634	ENSG00000206072
ENSG00000166634	ENSG00000206073
ENSG00000166634	ENSG00000206075
ENSG00000166664	ENSG00000080644
ENSG00000166664	ENSG00000101204
ENSG00000166664	ENSG00000108556
ENSG00000166664	ENSG00000117971
ENSG00000166664	ENSG00000120903
ENSG00000166664	ENSG00000129749
ENSG00000166664	ENSG00000135902
ENSG00000166664	ENSG00000138435
ENSG00000166664	ENSG00000147432
ENSG00000166664	ENSG00000147434
ENSG00000166664	ENSG00000149305
ENSG00000166664	ENSG00000160716
ENSG00000166664	ENSG00000166736
ENSG00000166664	ENSG00000169684
ENSG00000166664	ENSG00000170175
ENSG00000166664	ENSG00000174343
ENSG00000166664	ENSG00000175344
ENSG00000166664	ENSG00000178084
ENSG00000166664	ENSG00000186038
ENSG00000166664	ENSG00000186090
ENSG00000166664	ENSG00000186919
ENSG00000166664	ENSG00000196811
ENSG00000166667	ENSG00000136206
ENSG00000166667	ENSG00000154732
ENSG00000166667	ENSG00000163806
ENSG00000166667	ENSG00000170092
ENSG00000166667	ENSG00000173678
ENSG00000166667	ENSG00000179994
ENSG00000166667	ENSG00000183318
ENSG00000166667	ENSG00000184616
ENSG00000166667	ENSG00000185040
ENSG00000166667	ENSG00000186645
ENSG00000166667	ENSG00000198305
ENSG00000166667	ENSG00000204710
ENSG00000166667	ENSG00000205238
ENSG00000166667	ENSG00000205482
ENSG00000166667	ENSG00000205505
ENSG00000166667	ENSG00000205506
ENSG00000166667	ENSG00000205508
ENSG00000166667	ENSG00000205509
ENSG00000166667	ENSG00000205580
ENSG00000166667	ENSG00000214300
ENSG00000166667	ENSG00000215092
ENSG00000166669	ENSG00000171681
ENSG00000166670	ENSG00000008516
ENSG00000166670	ENSG00000087245
ENSG00000166670	ENSG00000099953
ENSG00000166670	ENSG00000100985
ENSG00000166670	ENSG00000102996

ENSG00000166670	ENSG00000110347
ENSG00000166670	ENSG00000118113
ENSG00000166670	ENSG00000123342
ENSG00000166670	ENSG00000125966
ENSG00000166670	ENSG00000129270
ENSG00000166670	ENSG00000137673
ENSG00000166670	ENSG00000137674
ENSG00000166670	ENSG00000137675
ENSG00000166670	ENSG00000137745
ENSG00000166670	ENSG00000149968
ENSG00000166670	ENSG00000154485
ENSG00000166670	ENSG00000156103
ENSG00000166670	ENSG00000157227
ENSG00000166670	ENSG00000167346
ENSG00000166670	ENSG00000189409
ENSG00000166670	ENSG00000196611
ENSG00000166670	ENSG00000198598
ENSG00000166670	ENSG00000215914
ENSG00000166676	ENSG00000108442
ENSG00000166676	ENSG00000171928
ENSG00000166682	ENSG00000105707
ENSG00000166682	ENSG00000137648
ENSG00000166682	ENSG00000137747
ENSG00000166682	ENSG00000145244
ENSG00000166682	ENSG00000154646
ENSG00000166682	ENSG00000160183
ENSG00000166682	ENSG00000184012
ENSG00000166689	ENSG00000052126
ENSG00000166689	ENSG00000105559
ENSG00000166689	ENSG00000143850
ENSG00000166716	ENSG00000074657
ENSG00000166716	ENSG00000143373
ENSG00000166716	ENSG00000214168
ENSG00000166716	ENSG00000214171
ENSG00000166734	ENSG00000135052
ENSG00000166734	ENSG00000215599
ENSG00000166736	ENSG00000080644
ENSG00000166736	ENSG00000101204
ENSG00000166736	ENSG00000108556
ENSG00000166736	ENSG00000117971
ENSG00000166736	ENSG00000120903
ENSG00000166736	ENSG00000129749
ENSG00000166736	ENSG00000135902
ENSG00000166736	ENSG00000138435
ENSG00000166736	ENSG00000147432
ENSG00000166736	ENSG00000147434
ENSG00000166736	ENSG00000149305
ENSG00000166736	ENSG00000160716
ENSG00000166736	ENSG00000166664
ENSG00000166736	ENSG00000169684
ENSG00000166736	ENSG00000170175
ENSG00000166736	ENSG00000174343
ENSG00000166736	ENSG00000175344
ENSG00000166736	ENSG00000178084
ENSG00000166736	ENSG00000186038
ENSG00000166736	ENSG00000186090
ENSG00000166736	ENSG00000186919
ENSG00000166736	ENSG00000196811
ENSG00000166737	ENSG00000178343

ENSG00000166737	ENSG00000180730
ENSG00000166737	ENSG00000187902
ENSG00000166737	ENSG00000188803
ENSG00000166741	ENSG00000011177
ENSG00000166741	ENSG00000141744
ENSG00000166747	ENSG00000213983
ENSG00000166748	ENSG00000135049
ENSG00000166748	ENSG00000146856
ENSG00000166748	ENSG00000165923
ENSG00000166793	ENSG00000090238
ENSG00000166793	ENSG00000100027
ENSG00000166793	ENSG00000175155
ENSG00000166796	ENSG00000111716
ENSG00000166796	ENSG00000134333
ENSG00000166796	ENSG00000151116
ENSG00000166796	ENSG00000166800
ENSG00000166796	ENSG00000171989
ENSG00000166800	ENSG00000111716
ENSG00000166800	ENSG00000134333
ENSG00000166800	ENSG00000151116
ENSG00000166800	ENSG00000166796
ENSG00000166800	ENSG00000171989
ENSG00000166813	ENSG00000165115
ENSG00000166821	ENSG00000131779
ENSG00000166822	ENSG00000205269
ENSG00000166823	ENSG00000151379
ENSG00000166823	ENSG00000188095
ENSG00000166825	ENSG00000072657
ENSG00000166825	ENSG00000113441
ENSG00000166825	ENSG00000138792
ENSG00000166825	ENSG00000141279
ENSG00000166825	ENSG00000164307
ENSG00000166825	ENSG00000164308
ENSG00000166825	ENSG00000172901
ENSG00000166825	ENSG00000174093
ENSG00000166825	ENSG00000215728
ENSG00000166831	ENSG00000157110
ENSG00000166833	ENSG00000067798
ENSG00000166833	ENSG00000134369
ENSG00000166839	ENSG00000189045
ENSG00000166853	ENSG00000003096
ENSG00000166853	ENSG00000087448
ENSG00000166853	ENSG00000124743
ENSG00000166853	ENSG00000130487
ENSG00000166853	ENSG00000135686
ENSG00000166853	ENSG00000167487
ENSG00000166853	ENSG00000174010
ENSG00000166853	ENSG00000176595
ENSG00000166853	ENSG00000179023
ENSG00000166853	ENSG00000185214
ENSG00000166853	ENSG00000185915
ENSG00000166853	ENSG00000186231
ENSG00000166853	ENSG00000197705
ENSG00000166853	ENSG00000198642
ENSG00000166856	ENSG00000144476
ENSG00000166860	ENSG00000011590
ENSG00000166860	ENSG00000109906
ENSG00000166862	ENSG00000006116
ENSG00000166862	ENSG00000075429

ENSG00000166862	ENSG00000075461
ENSG00000166862	ENSG00000105605
ENSG00000166862	ENSG00000142408
ENSG00000166862	ENSG00000204278
ENSG00000166866	ENSG00000128641
ENSG00000166866	ENSG00000136286
ENSG00000166866	ENSG00000142347
ENSG00000166866	ENSG00000157483
ENSG00000166866	ENSG00000176658
ENSG00000166866	ENSG00000197879
ENSG00000166869	ENSG00000088992
ENSG00000166869	ENSG00000187446
ENSG00000166881	ENSG00000189362
ENSG00000166886	ENSG00000138386
ENSG00000166886	ENSG00000214916
ENSG00000166889	ENSG00000214577
ENSG00000166897	ENSG00000128606
ENSG00000166897	ENSG00000145147
ENSG00000166897	ENSG00000182743
ENSG00000166897	ENSG00000184347
ENSG00000166897	ENSG00000187122
ENSG00000166900	ENSG00000099365
ENSG00000166900	ENSG00000103496
ENSG00000166900	ENSG00000106089
ENSG00000166900	ENSG00000111450
ENSG00000166900	ENSG00000135604
ENSG00000166900	ENSG00000178750
ENSG00000166908	ENSG00000141720
ENSG00000166908	ENSG00000150867
ENSG00000166908	ENSG00000167103
ENSG00000166912	ENSG00000003987
ENSG00000166912	ENSG00000014914
ENSG00000166912	ENSG00000063601
ENSG00000166912	ENSG00000087053
ENSG00000166912	ENSG00000100330
ENSG00000166912	ENSG00000102043
ENSG00000166912	ENSG00000104643
ENSG00000166912	ENSG00000108389
ENSG00000166912	ENSG00000139505
ENSG00000166912	ENSG00000150712
ENSG00000166912	ENSG00000171100
ENSG00000166912	ENSG00000184416
ENSG00000166913	ENSG00000108953
ENSG00000166913	ENSG00000128245
ENSG00000166913	ENSG00000134308
ENSG00000166913	ENSG00000164924
ENSG00000166913	ENSG00000170027
ENSG00000166913	ENSG00000175793
ENSG00000166913	ENSG00000214637
ENSG00000166913	ENSG00000215108
ENSG00000166923	ENSG00000147869
ENSG00000166923	ENSG00000158747
ENSG00000166923	ENSG00000179284
ENSG00000166923	ENSG00000180875
ENSG00000166925	ENSG00000102804
ENSG00000166925	ENSG00000196428
ENSG00000166926	ENSG00000071203
ENSG00000166926	ENSG00000110077
ENSG00000166926	ENSG00000110079

ENSG00000166926	ENSG00000149516
ENSG00000166926	ENSG00000149534
ENSG00000166926	ENSG00000156738
ENSG00000166926	ENSG00000166927
ENSG00000166926	ENSG00000166930
ENSG00000166926	ENSG00000166959
ENSG00000166926	ENSG00000166961
ENSG00000166926	ENSG00000214787
ENSG00000166927	ENSG00000071203
ENSG00000166927	ENSG00000110077
ENSG00000166927	ENSG00000110079
ENSG00000166927	ENSG00000149516
ENSG00000166927	ENSG00000149534
ENSG00000166927	ENSG00000156738
ENSG00000166927	ENSG00000166926
ENSG00000166927	ENSG00000166930
ENSG00000166927	ENSG00000166959
ENSG00000166927	ENSG00000166961
ENSG00000166927	ENSG00000214787
ENSG00000166930	ENSG00000071203
ENSG00000166930	ENSG00000110077
ENSG00000166930	ENSG00000110079
ENSG00000166930	ENSG00000149516
ENSG00000166930	ENSG00000149534
ENSG00000166930	ENSG00000156738
ENSG00000166930	ENSG00000166926
ENSG00000166930	ENSG00000166927
ENSG00000166930	ENSG00000166959
ENSG00000166930	ENSG00000166961
ENSG00000166930	ENSG00000214787
ENSG00000166946	ENSG00000006042
ENSG00000166947	ENSG00000092295
ENSG00000166947	ENSG00000104055
ENSG00000166947	ENSG00000124491
ENSG00000166947	ENSG00000125780
ENSG00000166947	ENSG00000159495
ENSG00000166947	ENSG00000163810
ENSG00000166947	ENSG00000166948
ENSG00000166947	ENSG00000198959
ENSG00000166948	ENSG00000092295
ENSG00000166948	ENSG00000104055
ENSG00000166948	ENSG00000124491
ENSG00000166948	ENSG00000125780
ENSG00000166948	ENSG00000159495
ENSG00000166948	ENSG00000163810
ENSG00000166948	ENSG00000166947
ENSG00000166948	ENSG00000198959
ENSG00000166949	ENSG00000101665
ENSG00000166949	ENSG00000113658
ENSG00000166949	ENSG00000120693
ENSG00000166949	ENSG00000137834
ENSG00000166949	ENSG00000141646
ENSG00000166949	ENSG00000170365
ENSG00000166949	ENSG00000175387
ENSG00000166959	ENSG00000071203
ENSG00000166959	ENSG00000110077
ENSG00000166959	ENSG00000110079
ENSG00000166959	ENSG00000149516
ENSG00000166959	ENSG00000149534

ENSG00000166959	ENSG00000156738
ENSG00000166959	ENSG00000166926
ENSG00000166959	ENSG00000166927
ENSG00000166959	ENSG00000166930
ENSG00000166959	ENSG00000166961
ENSG00000166959	ENSG00000214787
ENSG00000166961	ENSG00000071203
ENSG00000166961	ENSG00000110077
ENSG00000166961	ENSG00000110079
ENSG00000166961	ENSG00000149516
ENSG00000166961	ENSG00000149534
ENSG00000166961	ENSG00000156738
ENSG00000166961	ENSG00000166926
ENSG00000166961	ENSG00000166927
ENSG00000166961	ENSG00000166930
ENSG00000166961	ENSG00000166959
ENSG00000166961	ENSG00000214787
ENSG00000166963	ENSG00000130479
ENSG00000166963	ENSG00000131711
ENSG00000166974	ENSG00000084764
ENSG00000166974	ENSG00000101367
ENSG00000166998	ENSG00000104879
ENSG00000166998	ENSG00000131730
ENSG00000166998	ENSG00000166165
ENSG00000166998	ENSG00000168775
ENSG00000167004	ENSG00000155660
ENSG00000167034	ENSG00000006377
ENSG00000167034	ENSG00000108813
ENSG00000167034	ENSG00000144355
ENSG00000167037	ENSG00000141258
ENSG00000167065	ENSG00000161326
ENSG00000167065	ENSG00000189037
ENSG00000167074	ENSG00000105516
ENSG00000167074	ENSG00000108924
ENSG00000167080	ENSG00000135454
ENSG00000167081	ENSG00000105717
ENSG00000167081	ENSG00000185630
ENSG00000167081	ENSG00000204304
ENSG00000167083	ENSG00000127588
ENSG00000167083	ENSG00000127920
ENSG00000167083	ENSG00000127928
ENSG00000167083	ENSG00000133136
ENSG00000167083	ENSG00000162188
ENSG00000167083	ENSG00000167414
ENSG00000167083	ENSG00000168243
ENSG00000167083	ENSG00000172380
ENSG00000167083	ENSG00000174021
ENSG00000167083	ENSG00000182625
ENSG00000167083	ENSG00000186469
ENSG00000167083	ENSG00000213611
ENSG00000167098	ENSG00000061656
ENSG00000167098	ENSG00000100242
ENSG00000167098	ENSG00000164744
ENSG00000167098	ENSG00000164828
ENSG00000167104	ENSG00000078898
ENSG00000167104	ENSG00000100979
ENSG00000167104	ENSG00000101425
ENSG00000167104	ENSG00000129988
ENSG00000167104	ENSG00000184459

ENSG00000167104	ENSG00000186190
ENSG00000167104	ENSG00000186191
ENSG00000167106	ENSG00000162636
ENSG00000167110	ENSG00000140478
ENSG00000167110	ENSG00000153666
ENSG00000167110	ENSG00000156363
ENSG00000167110	ENSG00000159289
ENSG00000167110	ENSG00000167195
ENSG00000167110	ENSG00000174450
ENSG00000167110	ENSG00000175265
ENSG00000167110	ENSG00000181984
ENSG00000167110	ENSG00000184206
ENSG00000167110	ENSG00000186322
ENSG00000167110	ENSG00000188388
ENSG00000167110	ENSG00000188532
ENSG00000167110	ENSG00000188626
ENSG00000167110	ENSG00000197092
ENSG00000167110	ENSG00000197414
ENSG00000167110	ENSG00000214348
ENSG00000167110	ENSG00000215252
ENSG00000167110	ENSG00000215405
ENSG00000167114	ENSG00000083807
ENSG00000167114	ENSG00000113396
ENSG00000167114	ENSG00000130304
ENSG00000167114	ENSG00000140284
ENSG00000167114	ENSG00000143554
ENSG00000167123	ENSG00000083444
ENSG00000167123	ENSG00000106397
ENSG00000167123	ENSG00000130309
ENSG00000167123	ENSG00000152952
ENSG00000167123	ENSG00000198756
ENSG00000167157	ENSG00000109132
ENSG00000167157	ENSG00000116132
ENSG00000167157	ENSG00000165462
ENSG00000167157	ENSG00000165606
ENSG00000167165	ENSG00000109181
ENSG00000167165	ENSG00000135220
ENSG00000167165	ENSG00000135226
ENSG00000167165	ENSG00000145626
ENSG00000167165	ENSG00000156096
ENSG00000167165	ENSG00000168671
ENSG00000167165	ENSG00000171234
ENSG00000167165	ENSG00000173610
ENSG00000167165	ENSG00000174607
ENSG00000167165	ENSG00000196620
ENSG00000167165	ENSG00000197592
ENSG00000167165	ENSG00000197888
ENSG00000167165	ENSG00000198277
ENSG00000167165	ENSG00000213759
ENSG00000167183	ENSG00000176532
ENSG00000167191	ENSG00000013588
ENSG00000167191	ENSG00000111291
ENSG00000167191	ENSG00000170412
ENSG00000167193	ENSG00000099942
ENSG00000167195	ENSG00000140478
ENSG00000167195	ENSG00000153666
ENSG00000167195	ENSG00000156363
ENSG00000167195	ENSG00000159289
ENSG00000167195	ENSG00000167110

ENSG00000167195	ENSG00000174450
ENSG00000167195	ENSG00000175265
ENSG00000167195	ENSG00000181984
ENSG00000167195	ENSG00000184206
ENSG00000167195	ENSG00000186322
ENSG00000167195	ENSG00000188388
ENSG00000167195	ENSG00000188532
ENSG00000167195	ENSG00000188626
ENSG00000167195	ENSG00000197092
ENSG00000167195	ENSG00000197414
ENSG00000167195	ENSG00000214348
ENSG00000167195	ENSG00000215252
ENSG00000167195	ENSG00000215405
ENSG00000167202	ENSG00000095383
ENSG00000167202	ENSG00000100359
ENSG00000167202	ENSG00000111490
ENSG00000167207	ENSG00000022556
ENSG00000167207	ENSG00000023191
ENSG00000167207	ENSG00000140853
ENSG00000167207	ENSG00000142405
ENSG00000167207	ENSG00000158077
ENSG00000167207	ENSG00000160505
ENSG00000167207	ENSG00000162711
ENSG00000167207	ENSG00000167634
ENSG00000167207	ENSG00000167984
ENSG00000167207	ENSG00000171487
ENSG00000167207	ENSG00000173572
ENSG00000167207	ENSG00000174885
ENSG00000167207	ENSG00000179709
ENSG00000167207	ENSG00000179873
ENSG00000167207	ENSG00000182261
ENSG00000167207	ENSG00000185792
ENSG00000167208	ENSG00000124104
ENSG00000167236	ENSG00000006074
ENSG00000167236	ENSG00000006075
ENSG00000167236	ENSG00000006606
ENSG00000167236	ENSG00000102962
ENSG00000167236	ENSG00000102970
ENSG00000167236	ENSG00000106178
ENSG00000167236	ENSG00000108688
ENSG00000167236	ENSG00000108691
ENSG00000167236	ENSG00000108700
ENSG00000167236	ENSG00000108702
ENSG00000167236	ENSG00000115009
ENSG00000167236	ENSG00000129277
ENSG00000167236	ENSG00000143184
ENSG00000167236	ENSG00000143185
ENSG00000167236	ENSG00000161570
ENSG00000167236	ENSG00000161573
ENSG00000167236	ENSG00000161574
ENSG00000167236	ENSG00000172156
ENSG00000167236	ENSG00000172724
ENSG00000167236	ENSG00000181374
ENSG00000167236	ENSG00000197262
ENSG00000167236	ENSG00000205017
ENSG00000167236	ENSG00000205020
ENSG00000167236	ENSG00000205021
ENSG00000167236	ENSG00000213494
ENSG00000167236	ENSG00000215627

ENSG00000167236	ENSG00000215629
ENSG00000167244	ENSG00000017427
ENSG00000167244	ENSG00000129965
ENSG00000167258	ENSG00000065883
ENSG00000167261	ENSG00000015413
ENSG00000167261	ENSG00000141096
ENSG00000167281	ENSG00000078328
ENSG00000167281	ENSG00000100320
ENSG00000167286	ENSG00000160654
ENSG00000167286	ENSG00000198851
ENSG00000167306	ENSG00000128833
ENSG00000167306	ENSG00000141140
ENSG00000167306	ENSG00000197535
ENSG00000167323	ENSG00000109689
ENSG00000167371	ENSG00000101463
ENSG00000167371	ENSG00000183379
ENSG00000167378	ENSG00000124449
ENSG00000167393	ENSG00000073711
ENSG00000167414	ENSG00000127588
ENSG00000167414	ENSG00000127920
ENSG00000167414	ENSG00000127928
ENSG00000167414	ENSG00000133136
ENSG00000167414	ENSG00000162188
ENSG00000167414	ENSG00000167083
ENSG00000167414	ENSG00000168243
ENSG00000167414	ENSG00000172380
ENSG00000167414	ENSG00000174021
ENSG00000167414	ENSG00000182625
ENSG00000167414	ENSG00000186469
ENSG00000167414	ENSG00000213611
ENSG00000167419	ENSG00000005381
ENSG00000167419	ENSG00000115705
ENSG00000167419	ENSG00000121053
ENSG00000167419	ENSG00000130508
ENSG00000167419	ENSG00000147485
ENSG00000167433	ENSG00000075884
ENSG00000167433	ENSG00000107863
ENSG00000167433	ENSG00000123329
ENSG00000167433	ENSG00000165322
ENSG00000167433	ENSG00000185602
ENSG00000167433	ENSG00000214627
ENSG00000167434	ENSG00000074410
ENSG00000167434	ENSG00000104267
ENSG00000167434	ENSG00000107159
ENSG00000167434	ENSG00000118298
ENSG00000167434	ENSG00000131686
ENSG00000167434	ENSG00000133742
ENSG00000167434	ENSG00000164879
ENSG00000167434	ENSG00000168748
ENSG00000167434	ENSG00000169239
ENSG00000167434	ENSG00000174990
ENSG00000167434	ENSG00000178538
ENSG00000167434	ENSG00000185015
ENSG00000167434	ENSG00000214729
ENSG00000167434	ENSG00000214873
ENSG00000167434	ENSG00000215487
ENSG00000167434	ENSG00000215501
ENSG00000167434	ENSG00000215543
ENSG00000167460	ENSG00000140416

ENSG00000167460	ENSG00000143549
ENSG00000167460	ENSG00000177152
ENSG00000167460	ENSG00000183022
ENSG00000167460	ENSG00000187536
ENSG00000167460	ENSG00000198467
ENSG00000167460	ENSG00000214869
ENSG00000167461	ENSG00000084733
ENSG00000167461	ENSG00000139998
ENSG00000167461	ENSG00000143545
ENSG00000167461	ENSG00000166128
ENSG00000167483	ENSG00000135842
ENSG00000167483	ENSG00000136830
ENSG00000167491	ENSG00000143614
ENSG00000167522	ENSG00000101745
ENSG00000167522	ENSG00000138376
ENSG00000167522	ENSG00000145700
ENSG00000167522	ENSG00000160949
ENSG00000167531	ENSG00000090382
ENSG00000167531	ENSG00000120563
ENSG00000167531	ENSG00000141316
ENSG00000167531	ENSG00000151033
ENSG00000167531	ENSG00000157093
ENSG00000167531	ENSG00000161572
ENSG00000167531	ENSG00000171478
ENSG00000167531	ENSG00000171489
ENSG00000167535	ENSG00000067191
ENSG00000167535	ENSG00000165995
ENSG00000167535	ENSG00000182389
ENSG00000167536	ENSG00000072042
ENSG00000167536	ENSG00000139988
ENSG00000167536	ENSG00000160439
ENSG00000167536	ENSG00000169084
ENSG00000167548	ENSG00000055609
ENSG00000167549	ENSG00000102879
ENSG00000167549	ENSG00000103426
ENSG00000167549	ENSG00000103647
ENSG00000167549	ENSG00000106789
ENSG00000167549	ENSG00000110880
ENSG00000167549	ENSG00000172725
ENSG00000167550	ENSG00000106615
ENSG00000167565	ENSG00000170779
ENSG00000167565	ENSG00000197019
ENSG00000167566	ENSG00000176771
ENSG00000167578	ENSG00000104388
ENSG00000167578	ENSG00000119396
ENSG00000167578	ENSG00000129472
ENSG00000167578	ENSG00000155961
ENSG00000167578	ENSG00000168118
ENSG00000167578	ENSG00000179331
ENSG00000167578	ENSG00000183940
ENSG00000167578	ENSG00000188060
ENSG00000167580	ENSG00000086159
ENSG00000167580	ENSG00000103375
ENSG00000167580	ENSG00000106125
ENSG00000167580	ENSG00000135517
ENSG00000167580	ENSG00000161798
ENSG00000167580	ENSG00000171885
ENSG00000167588	ENSG00000152642
ENSG00000167601	ENSG00000092445

ENSG00000167601	ENSG00000105976
ENSG00000167601	ENSG00000153208
ENSG00000167601	ENSG00000163785
ENSG00000167601	ENSG00000164078
ENSG00000167601	ENSG00000214672
ENSG00000167604	ENSG00000144802
ENSG00000167608	ENSG00000103534
ENSG00000167608	ENSG00000141524
ENSG00000167608	ENSG00000149488
ENSG00000167608	ENSG00000165091
ENSG00000167608	ENSG00000167895
ENSG00000167608	ENSG00000170537
ENSG00000167608	ENSG00000188869
ENSG00000167612	ENSG00000164236
ENSG00000167614	ENSG00000136295
ENSG00000167614	ENSG00000141540
ENSG00000167617	ENSG00000128283
ENSG00000167617	ENSG00000149798
ENSG00000167617	ENSG00000163171
ENSG00000167617	ENSG00000179604
ENSG00000167634	ENSG00000022556
ENSG00000167634	ENSG00000023191
ENSG00000167634	ENSG00000140853
ENSG00000167634	ENSG00000142405
ENSG00000167634	ENSG00000158077
ENSG00000167634	ENSG00000160505
ENSG00000167634	ENSG00000162711
ENSG00000167634	ENSG00000167207
ENSG00000167634	ENSG00000167984
ENSG00000167634	ENSG00000171487
ENSG00000167634	ENSG00000173572
ENSG00000167634	ENSG00000174885
ENSG00000167634	ENSG00000179709
ENSG00000167634	ENSG00000179873
ENSG00000167634	ENSG00000182261
ENSG00000167634	ENSG00000185792
ENSG00000167641	ENSG00000166143
ENSG00000167641	ENSG00000173457
ENSG00000167641	ENSG00000198729
ENSG00000167645	ENSG00000174851
ENSG00000167654	ENSG00000106772
ENSG00000167654	ENSG00000140299
ENSG00000167654	ENSG00000163141
ENSG00000167654	ENSG00000175220
ENSG00000167654	ENSG00000196505
ENSG00000167657	ENSG00000035664
ENSG00000167657	ENSG00000065534
ENSG00000167657	ENSG00000101306
ENSG00000167657	ENSG00000140795
ENSG00000167657	ENSG00000145949
ENSG00000167657	ENSG00000196730
ENSG00000167674	ENSG00000112273
ENSG00000167674	ENSG00000143321
ENSG00000167674	ENSG00000164985
ENSG00000167674	ENSG00000166503
ENSG00000167676	ENSG00000105355
ENSG00000167676	ENSG00000147872
ENSG00000167676	ENSG00000214456
ENSG00000167680	ENSG00000082684

ENSG00000167680	ENSG00000112902
ENSG00000167695	ENSG00000149926
ENSG00000167695	ENSG00000152078
ENSG00000167701	ENSG00000166123
ENSG00000167702	ENSG00000140859
ENSG00000167703	ENSG00000134802
ENSG00000167703	ENSG00000149150
ENSG00000167705	ENSG00000150977
ENSG00000167705	ENSG00000188026
ENSG00000167711	ENSG00000132386
ENSG00000167711	ENSG00000149131
ENSG00000167723	ENSG00000111199
ENSG00000167723	ENSG00000127412
ENSG00000167723	ENSG00000165125
ENSG00000167723	ENSG00000187688
ENSG00000167723	ENSG00000196689
ENSG00000167733	ENSG00000117594
ENSG00000167744	ENSG00000134259
ENSG00000167744	ENSG00000176697
ENSG00000167744	ENSG00000185652
ENSG00000167744	ENSG00000212844
ENSG00000167764	ENSG00000105366
ENSG00000167764	ENSG00000105383
ENSG00000167764	ENSG00000105492
ENSG00000167764	ENSG00000105501
ENSG00000167764	ENSG00000105695
ENSG00000167764	ENSG00000129450
ENSG00000167764	ENSG00000142512
ENSG00000167764	ENSG00000160296
ENSG00000167764	ENSG00000161640
ENSG00000167764	ENSG00000161643
ENSG00000167764	ENSG00000168995
ENSG00000167764	ENSG00000171101
ENSG00000167764	ENSG00000197865
ENSG00000167767	ENSG00000135443
ENSG00000167767	ENSG00000135477
ENSG00000167767	ENSG00000135480
ENSG00000167767	ENSG00000139648
ENSG00000167767	ENSG00000161849
ENSG00000167767	ENSG00000161850
ENSG00000167767	ENSG00000167768
ENSG00000167767	ENSG00000170421
ENSG00000167767	ENSG00000170423
ENSG00000167767	ENSG00000170442
ENSG00000167767	ENSG00000170454
ENSG00000167767	ENSG00000170465
ENSG00000167767	ENSG00000170477
ENSG00000167767	ENSG00000170484
ENSG00000167767	ENSG00000170486
ENSG00000167767	ENSG00000170523
ENSG00000167767	ENSG00000172867
ENSG00000167767	ENSG00000185069
ENSG00000167767	ENSG00000185479
ENSG00000167767	ENSG00000185640
ENSG00000167767	ENSG00000186049
ENSG00000167767	ENSG00000186081
ENSG00000167767	ENSG00000186442
ENSG00000167767	ENSG00000189182
ENSG00000167767	ENSG00000205420

ENSG00000167767	ENSG00000205426
ENSG00000167767	ENSG00000214233
ENSG00000167767	ENSG00000214323
ENSG00000167767	ENSG00000214477
ENSG00000167767	ENSG00000214752
ENSG00000167767	ENSG00000214974
ENSG00000167767	ENSG00000215287
ENSG00000167767	ENSG00000215847
ENSG00000167768	ENSG00000135443
ENSG00000167768	ENSG00000135477
ENSG00000167768	ENSG00000135480
ENSG00000167768	ENSG00000139648
ENSG00000167768	ENSG00000161849
ENSG00000167768	ENSG00000161850
ENSG00000167768	ENSG00000167767
ENSG00000167768	ENSG00000170421
ENSG00000167768	ENSG00000170423
ENSG00000167768	ENSG00000170442
ENSG00000167768	ENSG00000170454
ENSG00000167768	ENSG00000170465
ENSG00000167768	ENSG00000170477
ENSG00000167768	ENSG00000170484
ENSG00000167768	ENSG00000170486
ENSG00000167768	ENSG00000170523
ENSG00000167768	ENSG00000172867
ENSG00000167768	ENSG00000185069
ENSG00000167768	ENSG00000185479
ENSG00000167768	ENSG00000185640
ENSG00000167768	ENSG00000186049
ENSG00000167768	ENSG00000186081
ENSG00000167768	ENSG00000186442
ENSG00000167768	ENSG00000189182
ENSG00000167768	ENSG00000205420
ENSG00000167768	ENSG00000205426
ENSG00000167768	ENSG00000214233
ENSG00000167768	ENSG00000214323
ENSG00000167768	ENSG00000214477
ENSG00000167768	ENSG00000214752
ENSG00000167768	ENSG00000214974
ENSG00000167768	ENSG00000215287
ENSG00000167768	ENSG00000215847
ENSG00000167770	ENSG00000089723
ENSG00000167771	ENSG00000089902
ENSG00000167771	ENSG00000117625
ENSG00000167779	ENSG00000115457
ENSG00000167779	ENSG00000115461
ENSG00000167779	ENSG00000141753
ENSG00000167779	ENSG00000146674
ENSG00000167779	ENSG00000146678
ENSG00000167791	ENSG00000105507
ENSG00000167791	ENSG00000157782
ENSG00000167791	ENSG00000175544
ENSG00000167797	ENSG00000111328
ENSG00000167800	ENSG00000073861
ENSG00000167800	ENSG00000089225
ENSG00000167800	ENSG00000092607
ENSG00000167800	ENSG00000112837
ENSG00000167800	ENSG00000121068
ENSG00000167800	ENSG00000121075

ENSG00000167800	ENSG00000122145
ENSG00000167800	ENSG00000135111
ENSG00000167800	ENSG00000136535
ENSG00000167800	ENSG00000143178
ENSG00000167800	ENSG00000149922
ENSG00000167800	ENSG00000163508
ENSG00000167800	ENSG00000164458
ENSG00000167800	ENSG00000164532
ENSG00000167800	ENSG00000174197
ENSG00000167800	ENSG00000184058
ENSG00000167815	ENSG00000117450
ENSG00000167815	ENSG00000123131
ENSG00000167815	ENSG00000165672
ENSG00000167850	ENSG00000095970
ENSG00000167850	ENSG00000096264
ENSG00000167850	ENSG00000161649
ENSG00000167850	ENSG00000162896
ENSG00000167850	ENSG00000162897
ENSG00000167850	ENSG00000167851
ENSG00000167850	ENSG00000178789
ENSG00000167850	ENSG00000186074
ENSG00000167850	ENSG00000186407
ENSG00000167850	ENSG00000204345
ENSG00000167851	ENSG00000095970
ENSG00000167851	ENSG00000096264
ENSG00000167851	ENSG00000161649
ENSG00000167851	ENSG00000162896
ENSG00000167851	ENSG00000162897
ENSG00000167851	ENSG00000167850
ENSG00000167851	ENSG00000178789
ENSG00000167851	ENSG00000186074
ENSG00000167851	ENSG00000186407
ENSG00000167851	ENSG00000204345
ENSG00000167874	ENSG00000205116
ENSG00000167880	ENSG00000096696
ENSG00000167880	ENSG00000118898
ENSG00000167880	ENSG00000127603
ENSG00000167880	ENSG00000151914
ENSG00000167889	ENSG00000152127
ENSG00000167895	ENSG00000103534
ENSG00000167895	ENSG00000141524
ENSG00000167895	ENSG00000149488
ENSG00000167895	ENSG00000165091
ENSG00000167895	ENSG00000167608
ENSG00000167895	ENSG00000170537
ENSG00000167895	ENSG00000188869
ENSG00000167910	ENSG00000124212
ENSG00000167910	ENSG00000146233
ENSG00000167910	ENSG00000172817
ENSG00000167910	ENSG00000180432
ENSG00000167916	ENSG00000006059
ENSG00000167916	ENSG00000094796
ENSG00000167916	ENSG00000108244
ENSG00000167916	ENSG00000108417
ENSG00000167916	ENSG00000108759
ENSG00000167916	ENSG00000111057
ENSG00000167916	ENSG00000126337
ENSG00000167916	ENSG00000128422
ENSG00000167916	ENSG00000131737

ENSG00000167916	ENSG00000131738
ENSG00000167916	ENSG00000131885
ENSG00000167916	ENSG00000170819
ENSG00000167916	ENSG00000171345
ENSG00000167916	ENSG00000171346
ENSG00000167916	ENSG00000171360
ENSG00000167916	ENSG00000171401
ENSG00000167916	ENSG00000171403
ENSG00000167916	ENSG00000171431
ENSG00000167916	ENSG00000171446
ENSG00000167916	ENSG00000173908
ENSG00000167916	ENSG00000186393
ENSG00000167916	ENSG00000186395
ENSG00000167916	ENSG00000186831
ENSG00000167916	ENSG00000186832
ENSG00000167916	ENSG00000186847
ENSG00000167916	ENSG00000187242
ENSG00000167916	ENSG00000187686
ENSG00000167916	ENSG00000196859
ENSG00000167916	ENSG00000197079
ENSG00000167916	ENSG00000204889
ENSG00000167916	ENSG00000204897
ENSG00000167916	ENSG00000213951
ENSG00000167916	ENSG00000214220
ENSG00000167916	ENSG00000214454
ENSG00000167916	ENSG00000214959
ENSG00000167916	ENSG00000215772
ENSG00000167916	ENSG00000215773
ENSG00000167930	ENSG00000084444
ENSG00000167941	ENSG00000171243
ENSG00000167957	ENSG00000106236
ENSG00000167957	ENSG00000132693
ENSG00000167957	ENSG00000132703
ENSG00000167957	ENSG00000163661
ENSG00000167957	ENSG00000171246
ENSG00000167957	ENSG00000215846
ENSG00000167964	ENSG00000172794
ENSG00000167968	ENSG00000013563
ENSG00000167968	ENSG00000163687
ENSG00000167968	ENSG00000213918
ENSG00000167971	ENSG00000064999
ENSG00000167971	ENSG00000177303
ENSG00000167971	ENSG00000185046
ENSG00000167972	ENSG00000064687
ENSG00000167972	ENSG00000107331
ENSG00000167972	ENSG00000144452
ENSG00000167972	ENSG00000165029
ENSG00000167972	ENSG00000179869
ENSG00000167972	ENSG00000198691
ENSG00000167977	ENSG00000100379
ENSG00000167977	ENSG00000104756
ENSG00000167977	ENSG00000136636
ENSG00000167977	ENSG00000160410
ENSG00000167977	ENSG00000180901
ENSG00000167977	ENSG00000187074
ENSG00000167977	ENSG00000187988
ENSG00000167978	ENSG00000139767
ENSG00000167978	ENSG00000177679
ENSG00000167978	ENSG00000204924

ENSG00000167984	ENSG00000022556
ENSG00000167984	ENSG00000023191
ENSG00000167984	ENSG00000140853
ENSG00000167984	ENSG00000142405
ENSG00000167984	ENSG00000158077
ENSG00000167984	ENSG00000160505
ENSG00000167984	ENSG00000162711
ENSG00000167984	ENSG00000167207
ENSG00000167984	ENSG00000167634
ENSG00000167984	ENSG00000171487
ENSG00000167984	ENSG00000173572
ENSG00000167984	ENSG00000174885
ENSG00000167984	ENSG00000179709
ENSG00000167984	ENSG00000179873
ENSG00000167984	ENSG00000182261
ENSG00000167984	ENSG00000185792
ENSG00000167987	ENSG00000139722
ENSG00000167987	ENSG00000176428
ENSG00000167992	ENSG00000168743
ENSG00000167992	ENSG00000198759
ENSG00000167994	ENSG00000127328
ENSG00000167995	ENSG00000039987
ENSG00000167995	ENSG00000127325
ENSG00000167995	ENSG00000142959
ENSG00000167996	ENSG00000087086
ENSG00000167996	ENSG00000132446
ENSG00000167996	ENSG00000171878
ENSG00000167996	ENSG00000173477
ENSG00000167996	ENSG00000176778
ENSG00000167996	ENSG00000180795
ENSG00000167996	ENSG00000181867
ENSG00000167996	ENSG00000197518
ENSG00000167996	ENSG00000215234
ENSG00000168003	ENSG00000138079
ENSG00000168004	ENSG00000121207
ENSG00000168004	ENSG00000127252
ENSG00000168004	ENSG00000133321
ENSG00000168004	ENSG00000133328
ENSG00000168004	ENSG00000162981
ENSG00000168004	ENSG00000168672
ENSG00000168004	ENSG00000176485
ENSG00000168010	ENSG00000085978
ENSG00000168026	ENSG00000123607
ENSG00000168032	ENSG00000054179
ENSG00000168032	ENSG00000138185
ENSG00000168032	ENSG00000188833
ENSG00000168036	ENSG00000173801
ENSG00000168056	ENSG00000049323
ENSG00000168056	ENSG00000077942
ENSG00000168056	ENSG00000090006
ENSG00000168056	ENSG00000115380
ENSG00000168056	ENSG00000119681
ENSG00000168056	ENSG00000140092
ENSG00000168056	ENSG00000163520
ENSG00000168056	ENSG00000172638
ENSG00000168056	ENSG00000214536
ENSG00000168060	ENSG00000072274
ENSG00000168060	ENSG00000077616
ENSG00000168060	ENSG00000086205

ENSG00000168060	ENSG00000106327
ENSG00000168060	ENSG00000134612
ENSG00000168060	ENSG00000177694
ENSG00000168062	ENSG00000123685
ENSG00000168062	ENSG00000156127
ENSG00000168067	ENSG00000011566
ENSG00000168067	ENSG00000012983
ENSG00000168067	ENSG00000104814
ENSG00000168077	ENSG00000158270
ENSG00000168079	ENSG00000019169
ENSG00000168079	ENSG00000038945
ENSG00000168081	ENSG00000101327
ENSG00000168081	ENSG00000181195
ENSG00000168092	ENSG00000079462
ENSG00000168118	ENSG00000104388
ENSG00000168118	ENSG00000119396
ENSG00000168118	ENSG00000129472
ENSG00000168118	ENSG00000155961
ENSG00000168118	ENSG00000167578
ENSG00000168118	ENSG00000179331
ENSG00000168118	ENSG00000183940
ENSG00000168118	ENSG00000188060
ENSG00000168135	ENSG00000115474
ENSG00000168135	ENSG00000120457
ENSG00000168135	ENSG00000121361
ENSG00000168135	ENSG00000123700
ENSG00000168135	ENSG00000151704
ENSG00000168135	ENSG00000153822
ENSG00000168135	ENSG00000157542
ENSG00000168135	ENSG00000157551
ENSG00000168135	ENSG00000162728
ENSG00000168135	ENSG00000162989
ENSG00000168135	ENSG00000177807
ENSG00000168135	ENSG00000182324
ENSG00000168135	ENSG00000184185
ENSG00000168135	ENSG00000187486
ENSG00000168137	ENSG00000005483
ENSG00000168143	ENSG00000101447
ENSG00000168143	ENSG00000105523
ENSG00000168143	ENSG00000125998
ENSG00000168143	ENSG00000133477
ENSG00000168143	ENSG00000147689
ENSG00000168143	ENSG00000180921
ENSG00000168143	ENSG00000188522
ENSG00000168172	ENSG00000015133
ENSG00000168172	ENSG00000095066
ENSG00000168172	ENSG00000115355
ENSG00000168172	ENSG00000134709
ENSG00000168172	ENSG00000168071
ENSG00000168209	ENSG00000145358
ENSG00000168214	ENSG00000124232
ENSG00000168214	ENSG00000215133
ENSG00000168229	ENSG00000006638
ENSG00000168229	ENSG00000050628
ENSG00000168229	ENSG00000122420
ENSG00000168229	ENSG00000125384
ENSG00000168229	ENSG00000160013
ENSG00000168229	ENSG00000160951
ENSG00000168229	ENSG00000171522

ENSG00000168243	ENSG00000127588
ENSG00000168243	ENSG00000127920
ENSG00000168243	ENSG00000127928
ENSG00000168243	ENSG00000133136
ENSG00000168243	ENSG00000162188
ENSG00000168243	ENSG00000167083
ENSG00000168243	ENSG00000167414
ENSG00000168243	ENSG00000172380
ENSG00000168243	ENSG00000174021
ENSG00000168243	ENSG00000182625
ENSG00000168243	ENSG00000186469
ENSG00000168243	ENSG00000213611
ENSG00000168246	ENSG00000165886
ENSG00000168256	ENSG00000197885
ENSG00000168263	ENSG00000026559
ENSG00000168263	ENSG00000102057
ENSG00000168263	ENSG00000104848
ENSG00000168263	ENSG00000111262
ENSG00000168263	ENSG00000124134
ENSG00000168263	ENSG00000130037
ENSG00000168263	ENSG00000143105
ENSG00000168263	ENSG00000151079
ENSG00000168263	ENSG00000156486
ENSG00000168263	ENSG00000158445
ENSG00000168263	ENSG00000162975
ENSG00000168263	ENSG00000164794
ENSG00000168263	ENSG00000168418
ENSG00000168263	ENSG00000170745
ENSG00000168263	ENSG00000171126
ENSG00000168263	ENSG00000171385
ENSG00000168263	ENSG00000177272
ENSG00000168263	ENSG00000177301
ENSG00000168263	ENSG00000178342
ENSG00000168263	ENSG00000182255
ENSG00000168263	ENSG00000182674
ENSG00000168263	ENSG00000184408
ENSG00000168264	ENSG00000119669
ENSG00000168264	ENSG00000170604
ENSG00000168268	ENSG00000111696
ENSG00000168269	ENSG00000054598
ENSG00000168269	ENSG00000125798
ENSG00000168269	ENSG00000129514
ENSG00000168269	ENSG00000164379
ENSG00000168269	ENSG00000170122
ENSG00000168269	ENSG00000170608
ENSG00000168269	ENSG00000176165
ENSG00000168269	ENSG00000176678
ENSG00000168269	ENSG00000176692
ENSG00000168269	ENSG00000178919
ENSG00000168269	ENSG00000179772
ENSG00000168269	ENSG00000183770
ENSG00000168269	ENSG00000184492
ENSG00000168269	ENSG00000184659
ENSG00000168269	ENSG00000186564
ENSG00000168269	ENSG00000186766
ENSG00000168269	ENSG00000186790
ENSG00000168269	ENSG00000187140
ENSG00000168269	ENSG00000187559
ENSG00000168269	ENSG00000204612

ENSG00000168269	ENSG00000204779
ENSG00000168269	ENSG00000204793
ENSG00000168269	ENSG00000204828
ENSG00000168269	ENSG00000214336
ENSG00000168274	ENSG00000111332
ENSG00000168274	ENSG00000124642
ENSG00000168274	ENSG00000137259
ENSG00000168274	ENSG00000158403
ENSG00000168274	ENSG00000164508
ENSG00000168274	ENSG00000180573
ENSG00000168274	ENSG00000181218
ENSG00000168274	ENSG00000182611
ENSG00000168274	ENSG00000183558
ENSG00000168274	ENSG00000184260
ENSG00000168274	ENSG00000184270
ENSG00000168274	ENSG00000184348
ENSG00000168274	ENSG00000184825
ENSG00000168274	ENSG00000185978
ENSG00000168274	ENSG00000188486
ENSG00000168274	ENSG00000196747
ENSG00000168274	ENSG00000196787
ENSG00000168274	ENSG00000196866
ENSG00000168274	ENSG00000198082
ENSG00000168274	ENSG00000198307
ENSG00000168274	ENSG00000198374
ENSG00000168274	ENSG00000203812
ENSG00000168274	ENSG00000205070
ENSG00000168274	ENSG00000205079
ENSG00000168280	ENSG00000155980
ENSG00000168280	ENSG00000170759
ENSG00000168283	ENSG00000056661
ENSG00000168283	ENSG00000115289
ENSG00000168283	ENSG00000156374
ENSG00000168283	ENSG00000180628
ENSG00000168283	ENSG00000185619
ENSG00000168298	ENSG00000124575
ENSG00000168298	ENSG00000124610
ENSG00000168298	ENSG00000184357
ENSG00000168298	ENSG00000184897
ENSG00000168298	ENSG00000187475
ENSG00000168298	ENSG00000187837
ENSG00000168298	ENSG00000189060
ENSG00000168298	ENSG00000196440
ENSG00000168300	ENSG00000203880
ENSG00000168301	ENSG00000134504
ENSG00000168301	ENSG00000151364
ENSG00000168301	ENSG00000153885
ENSG00000168301	ENSG00000155729
ENSG00000168301	ENSG00000168676
ENSG00000168301	ENSG00000178695
ENSG00000168301	ENSG00000180332
ENSG00000168301	ENSG00000183775
ENSG00000168301	ENSG00000183783
ENSG00000168301	ENSG00000188997
ENSG00000168301	ENSG00000198553
ENSG00000168301	ENSG00000213859
ENSG00000168309	ENSG00000065809
ENSG00000168310	ENSG00000117595
ENSG00000168310	ENSG00000125347

ENSG00000168310	ENSG00000126456
ENSG00000168310	ENSG00000128604
ENSG00000168310	ENSG00000137265
ENSG00000168310	ENSG00000140968
ENSG00000168310	ENSG00000185507
ENSG00000168310	ENSG00000213928
ENSG00000168329	ENSG00000112486
ENSG00000168329	ENSG00000121797
ENSG00000168329	ENSG00000121807
ENSG00000168329	ENSG00000121966
ENSG00000168329	ENSG00000126353
ENSG00000168329	ENSG00000129048
ENSG00000168329	ENSG00000144648
ENSG00000168329	ENSG00000160683
ENSG00000168329	ENSG00000160791
ENSG00000168329	ENSG00000163464
ENSG00000168329	ENSG00000163823
ENSG00000168329	ENSG00000172215
ENSG00000168329	ENSG00000173578
ENSG00000168329	ENSG00000173585
ENSG00000168329	ENSG00000179934
ENSG00000168329	ENSG00000180871
ENSG00000168329	ENSG00000183625
ENSG00000168329	ENSG00000183813
ENSG00000168329	ENSG00000184451
ENSG00000168329	ENSG00000186810
ENSG00000168329	ENSG00000215775
ENSG00000168329	ENSG00000215778
ENSG00000168329	ENSG00000215782
ENSG00000168334	ENSG00000163092
ENSG00000168348	ENSG00000173404
ENSG00000168350	ENSG00000143753
ENSG00000168356	ENSG00000007314
ENSG00000168356	ENSG00000136531
ENSG00000168356	ENSG00000136546
ENSG00000168356	ENSG00000144285
ENSG00000168356	ENSG00000153253
ENSG00000168356	ENSG00000169432
ENSG00000168356	ENSG00000183873
ENSG00000168356	ENSG00000185313
ENSG00000168356	ENSG00000196876
ENSG00000168374	ENSG00000004059
ENSG00000168374	ENSG00000134287
ENSG00000168374	ENSG00000143761
ENSG00000168374	ENSG00000165527
ENSG00000168385	ENSG00000108387
ENSG00000168385	ENSG00000122545
ENSG00000168385	ENSG00000180096
ENSG00000168385	ENSG00000184702
ENSG00000168385	ENSG00000214005
ENSG00000168385	ENSG00000214765
ENSG00000168385	ENSG00000214834
ENSG00000168386	ENSG00000118407
ENSG00000168386	ENSG00000169641
ENSG00000168387	ENSG00000100628
ENSG00000168387	ENSG00000115239
ENSG00000168387	ENSG00000146809
ENSG00000168389	ENSG00000205639
ENSG00000168394	ENSG00000150967

ENSG00000168394	ENSG00000204267
ENSG00000168395	ENSG00000111653
ENSG00000168395	ENSG00000153487
ENSG00000168395	ENSG00000168556
ENSG00000168395	ENSG00000215693
ENSG00000168397	ENSG00000101844
ENSG00000168397	ENSG00000125703
ENSG00000168397	ENSG00000130734
ENSG00000168397	ENSG00000215697
ENSG00000168398	ENSG00000100739
ENSG00000168398	ENSG00000134817
ENSG00000168398	ENSG00000144891
ENSG00000168398	ENSG00000154165
ENSG00000168398	ENSG00000170128
ENSG00000168398	ENSG00000173080
ENSG00000168398	ENSG00000180772
ENSG00000168398	ENSG00000182631
ENSG00000168412	ENSG00000102195
ENSG00000168412	ENSG00000134640
ENSG00000168418	ENSG00000026559
ENSG00000168418	ENSG00000102057
ENSG00000168418	ENSG00000104848
ENSG00000168418	ENSG00000111262
ENSG00000168418	ENSG00000124134
ENSG00000168418	ENSG00000130037
ENSG00000168418	ENSG00000143105
ENSG00000168418	ENSG00000151079
ENSG00000168418	ENSG00000156486
ENSG00000168418	ENSG00000158445
ENSG00000168418	ENSG00000162975
ENSG00000168418	ENSG00000164794
ENSG00000168418	ENSG00000168263
ENSG00000168418	ENSG00000170745
ENSG00000168418	ENSG00000171126
ENSG00000168418	ENSG00000171385
ENSG00000168418	ENSG00000177272
ENSG00000168418	ENSG00000177301
ENSG00000168418	ENSG00000178342
ENSG00000168418	ENSG00000182255
ENSG00000168418	ENSG00000182674
ENSG00000168418	ENSG00000184408
ENSG00000168427	ENSG00000162413
ENSG00000168427	ENSG00000171617
ENSG00000168427	ENSG00000175946
ENSG00000168427	ENSG00000182359
ENSG00000168427	ENSG00000183655
ENSG00000168427	ENSG00000213160
ENSG00000168452	ENSG00000131238
ENSG00000168453	ENSG00000115548
ENSG00000168453	ENSG00000120733
ENSG00000168453	ENSG00000171988
ENSG00000168461	ENSG00000124209
ENSG00000168461	ENSG00000214576
ENSG00000168462	ENSG00000101825
ENSG00000168462	ENSG00000143341
ENSG00000168462	ENSG00000148357
ENSG00000168462	ENSG00000152580
ENSG00000168462	ENSG00000155657
ENSG00000168476	ENSG00000068615

ENSG00000168476	ENSG00000132563
ENSG00000168476	ENSG00000165476
ENSG00000168477	ENSG00000041982
ENSG00000168477	ENSG00000115414
ENSG00000168477	ENSG00000116147
ENSG00000168477	ENSG00000120332
ENSG00000168481	ENSG00000108231
ENSG00000168481	ENSG00000153012
ENSG00000168481	ENSG00000153902
ENSG00000168487	ENSG00000038295
ENSG00000168487	ENSG00000095587
ENSG00000168487	ENSG00000106333
ENSG00000168487	ENSG00000107611
ENSG00000168487	ENSG00000157211
ENSG00000168487	ENSG00000163710
ENSG00000168487	ENSG00000166342
ENSG00000168487	ENSG00000171208
ENSG00000168487	ENSG00000184824
ENSG00000168488	ENSG00000204842
ENSG00000168490	ENSG00000165443
ENSG00000168497	ENSG00000170681
ENSG00000168497	ENSG00000170955
ENSG00000168497	ENSG00000177469
ENSG00000168502	ENSG00000149639
ENSG00000168502	ENSG00000214338
ENSG00000168505	ENSG00000130675
ENSG00000168505	ENSG00000164900
ENSG00000168509	ENSG00000174136
ENSG00000168509	ENSG00000182175
ENSG00000168517	ENSG00000186834
ENSG00000168528	ENSG00000111897
ENSG00000168528	ENSG00000132824
ENSG00000168528	ENSG00000164300
ENSG00000168528	ENSG00000184716
ENSG00000168539	ENSG00000133019
ENSG00000168539	ENSG00000181072
ENSG00000168539	ENSG00000184984
ENSG00000168542	ENSG00000060718
ENSG00000168542	ENSG00000080573
ENSG00000168542	ENSG00000108821
ENSG00000168542	ENSG00000130635
ENSG00000168542	ENSG00000139219
ENSG00000168542	ENSG00000164692
ENSG00000168542	ENSG00000204248
ENSG00000168542	ENSG00000204262
ENSG00000168546	ENSG00000125861
ENSG00000168546	ENSG00000146013
ENSG00000168546	ENSG00000151892
ENSG00000168546	ENSG00000187871
ENSG00000168556	ENSG00000111653
ENSG00000168556	ENSG00000153487
ENSG00000168556	ENSG00000168395
ENSG00000168556	ENSG00000215693
ENSG00000168564	ENSG00000145835
ENSG00000168564	ENSG00000177770
ENSG00000168564	ENSG00000214740
ENSG00000168575	ENSG00000144136
ENSG00000168582	ENSG00000100053
ENSG00000168582	ENSG00000100058

ENSG00000168582	ENSG00000100122
ENSG00000168582	ENSG00000108255
ENSG00000168582	ENSG00000118231
ENSG00000168582	ENSG00000127377
ENSG00000168582	ENSG00000163254
ENSG00000168582	ENSG00000163499
ENSG00000168582	ENSG00000182187
ENSG00000168582	ENSG00000196431
ENSG00000168582	ENSG00000213139
ENSG00000168591	ENSG00000164897
ENSG00000168594	ENSG00000008277
ENSG00000168594	ENSG00000042980
ENSG00000168594	ENSG00000069206
ENSG00000168594	ENSG00000073670
ENSG00000168594	ENSG00000104755
ENSG00000168594	ENSG00000114948
ENSG00000168594	ENSG00000134007
ENSG00000168594	ENSG00000134028
ENSG00000168594	ENSG00000134249
ENSG00000168594	ENSG00000135074
ENSG00000168594	ENSG00000139985
ENSG00000168594	ENSG00000143537
ENSG00000168594	ENSG00000148848
ENSG00000168594	ENSG00000149451
ENSG00000168594	ENSG00000151651
ENSG00000168594	ENSG00000151694
ENSG00000168594	ENSG00000168615
ENSG00000168594	ENSG00000168619
ENSG00000168594	ENSG00000196115
ENSG00000168594	ENSG00000197140
ENSG00000168594	ENSG00000214137
ENSG00000168610	ENSG00000115415
ENSG00000168610	ENSG00000126561
ENSG00000168610	ENSG00000138378
ENSG00000168610	ENSG00000166888
ENSG00000168610	ENSG00000170581
ENSG00000168610	ENSG00000173757
ENSG00000168615	ENSG00000008277
ENSG00000168615	ENSG00000042980
ENSG00000168615	ENSG00000069206
ENSG00000168615	ENSG00000073670
ENSG00000168615	ENSG00000104755
ENSG00000168615	ENSG00000114948
ENSG00000168615	ENSG00000134007
ENSG00000168615	ENSG00000134028
ENSG00000168615	ENSG00000134249
ENSG00000168615	ENSG00000135074
ENSG00000168615	ENSG00000139985
ENSG00000168615	ENSG00000143537
ENSG00000168615	ENSG00000148848
ENSG00000168615	ENSG00000149451
ENSG00000168615	ENSG00000151651
ENSG00000168615	ENSG00000151694
ENSG00000168615	ENSG00000168594
ENSG00000168615	ENSG00000168619
ENSG00000168615	ENSG00000196115
ENSG00000168615	ENSG00000197140
ENSG00000168615	ENSG00000214137
ENSG00000168619	ENSG00000008277

ENSG00000168619	ENSG00000042980
ENSG00000168619	ENSG00000069206
ENSG00000168619	ENSG00000073670
ENSG00000168619	ENSG00000104755
ENSG00000168619	ENSG00000114948
ENSG00000168619	ENSG00000134007
ENSG00000168619	ENSG00000134028
ENSG00000168619	ENSG00000134249
ENSG00000168619	ENSG00000135074
ENSG00000168619	ENSG00000139985
ENSG00000168619	ENSG00000143537
ENSG00000168619	ENSG00000148848
ENSG00000168619	ENSG00000149451
ENSG00000168619	ENSG00000151651
ENSG00000168619	ENSG00000151694
ENSG00000168619	ENSG00000168594
ENSG00000168619	ENSG00000168615
ENSG00000168619	ENSG00000196115
ENSG00000168619	ENSG00000197140
ENSG00000168619	ENSG00000214137
ENSG00000168621	ENSG00000117407
ENSG00000168621	ENSG00000125650
ENSG00000168621	ENSG00000171119
ENSG00000168646	ENSG00000103126
ENSG00000168671	ENSG00000109181
ENSG00000168671	ENSG00000135220
ENSG00000168671	ENSG00000135226
ENSG00000168671	ENSG00000145626
ENSG00000168671	ENSG00000156096
ENSG00000168671	ENSG00000167165
ENSG00000168671	ENSG00000171234
ENSG00000168671	ENSG00000173610
ENSG00000168671	ENSG00000174607
ENSG00000168671	ENSG00000196620
ENSG00000168671	ENSG00000197592
ENSG00000168671	ENSG00000197888
ENSG00000168671	ENSG00000198277
ENSG00000168671	ENSG00000213759
ENSG00000168672	ENSG00000121207
ENSG00000168672	ENSG00000127252
ENSG00000168672	ENSG00000133321
ENSG00000168672	ENSG00000133328
ENSG00000168672	ENSG00000162981
ENSG00000168672	ENSG00000168004
ENSG00000168672	ENSG00000176485
ENSG00000168675	ENSG00000124225
ENSG00000168685	ENSG00000117400
ENSG00000168685	ENSG00000187266
ENSG00000168702	ENSG00000070018
ENSG00000168702	ENSG00000081479
ENSG00000168702	ENSG00000123384
ENSG00000168702	ENSG00000134569
ENSG00000168702	ENSG00000138798
ENSG00000168702	ENSG00000162337
ENSG00000168710	ENSG00000101444
ENSG00000168710	ENSG00000158467
ENSG00000168710	ENSG00000214066
ENSG00000168734	ENSG00000135549
ENSG00000168734	ENSG00000171033

ENSG00000168743	ENSG00000167992
ENSG00000168743	ENSG00000198759
ENSG00000168748	ENSG00000074410
ENSG00000168748	ENSG00000104267
ENSG00000168748	ENSG00000107159
ENSG00000168748	ENSG00000118298
ENSG00000168748	ENSG00000131686
ENSG00000168748	ENSG00000133742
ENSG00000168748	ENSG00000164879
ENSG00000168748	ENSG00000167434
ENSG00000168748	ENSG00000169239
ENSG00000168748	ENSG00000174990
ENSG00000168748	ENSG00000178538
ENSG00000168748	ENSG00000185015
ENSG00000168748	ENSG00000214729
ENSG00000168748	ENSG00000214873
ENSG00000168748	ENSG00000215487
ENSG00000168748	ENSG00000215501
ENSG00000168748	ENSG00000215543
ENSG00000168758	ENSG00000010319
ENSG00000168758	ENSG00000012171
ENSG00000168758	ENSG00000075213
ENSG00000168758	ENSG00000092421
ENSG00000168758	ENSG00000095539
ENSG00000168758	ENSG00000137872
ENSG00000168758	ENSG00000143434
ENSG00000168758	ENSG00000153993
ENSG00000168758	ENSG00000170381
ENSG00000168758	ENSG00000185033
ENSG00000168758	ENSG00000187764
ENSG00000168758	ENSG00000196189
ENSG00000168763	ENSG00000119946
ENSG00000168763	ENSG00000148842
ENSG00000168763	ENSG00000158158
ENSG00000168772	ENSG00000171604
ENSG00000168775	ENSG00000104879
ENSG00000168775	ENSG00000131730
ENSG00000168775	ENSG00000166165
ENSG00000168775	ENSG00000166998
ENSG00000168779	ENSG00000185960
ENSG00000168781	ENSG00000145725
ENSG00000168785	ENSG00000048140
ENSG00000168785	ENSG00000099282
ENSG00000168785	ENSG00000108219
ENSG00000168785	ENSG00000158457
ENSG00000168785	ENSG00000182612
ENSG00000168795	ENSG00000160062
ENSG00000168795	ENSG00000185670
ENSG00000168795	ENSG00000205189
ENSG00000168795	ENSG00000215897
ENSG00000168807	ENSG00000047932
ENSG00000168807	ENSG00000101400
ENSG00000168807	ENSG00000147481
ENSG00000168807	ENSG00000172164
ENSG00000168807	ENSG00000172554
ENSG00000168824	ENSG00000130643
ENSG00000168824	ENSG00000170091
ENSG00000168830	ENSG00000043591
ENSG00000168830	ENSG00000102468

ENSG00000168830	ENSG00000113749
ENSG00000168830	ENSG00000125631
ENSG00000168830	ENSG00000135312
ENSG00000168830	ENSG00000135914
ENSG00000168830	ENSG00000147246
ENSG00000168830	ENSG00000150594
ENSG00000168830	ENSG00000157219
ENSG00000168830	ENSG00000164270
ENSG00000168830	ENSG00000169252
ENSG00000168830	ENSG00000175658
ENSG00000168830	ENSG00000178394
ENSG00000168830	ENSG00000179097
ENSG00000168830	ENSG00000179546
ENSG00000168830	ENSG00000181210
ENSG00000168830	ENSG00000184303
ENSG00000168830	ENSG00000184845
ENSG00000168830	ENSG00000185321
ENSG00000168830	ENSG00000188778
ENSG00000168872	ENSG00000109832
ENSG00000168872	ENSG00000157349
ENSG00000168875	ENSG00000125285
ENSG00000168890	ENSG00000136048
ENSG00000168890	ENSG00000156171
ENSG00000168890	ENSG00000180061
ENSG00000168894	ENSG00000185036
ENSG00000168894	ENSG00000214209
ENSG00000168903	ENSG00000026950
ENSG00000168903	ENSG00000111801
ENSG00000168903	ENSG00000112763
ENSG00000168903	ENSG00000113303
ENSG00000168903	ENSG00000121236
ENSG00000168903	ENSG00000124508
ENSG00000168903	ENSG00000124549
ENSG00000168903	ENSG00000124557
ENSG00000168903	ENSG00000128250
ENSG00000168903	ENSG00000128253
ENSG00000168903	ENSG00000128276
ENSG00000168903	ENSG00000128482
ENSG00000168903	ENSG00000132256
ENSG00000168903	ENSG00000132274
ENSG00000168903	ENSG00000164010
ENSG00000168903	ENSG00000165810
ENSG00000168903	ENSG00000176422
ENSG00000168903	ENSG00000179046
ENSG00000168903	ENSG00000182805
ENSG00000168903	ENSG00000183439
ENSG00000168903	ENSG00000183718
ENSG00000168903	ENSG00000187574
ENSG00000168903	ENSG00000188683
ENSG00000168903	ENSG00000204614
ENSG00000168903	ENSG00000204616
ENSG00000168903	ENSG00000204618
ENSG00000168903	ENSG00000205198
ENSG00000168903	ENSG00000214693
ENSG00000168906	ENSG00000151224
ENSG00000168907	ENSG00000105499
ENSG00000168907	ENSG00000116711
ENSG00000168907	ENSG00000159337
ENSG00000168907	ENSG00000168970

ENSG00000168907	ENSG00000188089
ENSG00000168916	ENSG00000180357
ENSG00000168917	ENSG00000164729
ENSG00000168917	ENSG00000177164
ENSG00000168917	ENSG00000177710
ENSG00000168918	ENSG00000148384
ENSG00000168918	ENSG00000165458
ENSG00000168924	ENSG00000165046
ENSG00000168924	ENSG00000214906
ENSG00000168925	ENSG00000139610
ENSG00000168925	ENSG00000141086
ENSG00000168925	ENSG00000142615
ENSG00000168925	ENSG00000142789
ENSG00000168925	ENSG00000162438
ENSG00000168925	ENSG00000168928
ENSG00000168925	ENSG00000215704
ENSG00000168928	ENSG00000139610
ENSG00000168928	ENSG00000141086
ENSG00000168928	ENSG00000142615
ENSG00000168928	ENSG00000142789
ENSG00000168928	ENSG00000162438
ENSG00000168928	ENSG00000168925
ENSG00000168928	ENSG00000215704
ENSG00000168939	ENSG00000136158
ENSG00000168939	ENSG00000164056
ENSG00000168939	ENSG00000187678
ENSG00000168952	ENSG00000090989
ENSG00000168955	ENSG00000142484
ENSG00000168955	ENSG00000145107
ENSG00000168955	ENSG00000163762
ENSG00000168955	ENSG00000169903
ENSG00000168955	ENSG00000169908
ENSG00000168959	ENSG00000113262
ENSG00000168959	ENSG00000124493
ENSG00000168959	ENSG00000152822
ENSG00000168959	ENSG00000164082
ENSG00000168959	ENSG00000179603
ENSG00000168959	ENSG00000196277
ENSG00000168959	ENSG00000198822
ENSG00000168961	ENSG00000006659
ENSG00000168961	ENSG00000100079
ENSG00000168961	ENSG00000100097
ENSG00000168961	ENSG00000105198
ENSG00000168961	ENSG00000105205
ENSG00000168961	ENSG00000116977
ENSG00000168961	ENSG00000119862
ENSG00000168961	ENSG00000131981
ENSG00000168961	ENSG00000133317
ENSG00000168961	ENSG00000170298
ENSG00000168961	ENSG00000171747
ENSG00000168961	ENSG00000171916
ENSG00000168961	ENSG00000178934
ENSG00000168961	ENSG00000197778
ENSG00000168961	ENSG00000205076
ENSG00000168961	ENSG00000215132
ENSG00000168970	ENSG00000105499
ENSG00000168970	ENSG00000116711
ENSG00000168970	ENSG00000159337
ENSG00000168970	ENSG00000168907

ENSG00000168970	ENSG00000188089
ENSG00000168993	ENSG00000185251
ENSG00000168993	ENSG00000204336
ENSG00000168993	ENSG00000205861
ENSG00000168995	ENSG00000105366
ENSG00000168995	ENSG00000105383
ENSG00000168995	ENSG00000105492
ENSG00000168995	ENSG00000105501
ENSG00000168995	ENSG00000105695
ENSG00000168995	ENSG00000129450
ENSG00000168995	ENSG00000142512
ENSG00000168995	ENSG00000160296
ENSG00000168995	ENSG00000161640
ENSG00000168995	ENSG00000161643
ENSG00000168995	ENSG00000167764
ENSG00000168995	ENSG00000171101
ENSG00000168995	ENSG00000197865
ENSG00000168999	ENSG00000166530
ENSG00000169016	ENSG00000007968
ENSG00000169016	ENSG00000101412
ENSG00000169016	ENSG00000112242
ENSG00000169016	ENSG00000133740
ENSG00000169016	ENSG00000188841
ENSG00000169016	ENSG00000205250
ENSG00000169031	ENSG00000081052
ENSG00000169031	ENSG00000134871
ENSG00000169031	ENSG00000187498
ENSG00000169031	ENSG00000188153
ENSG00000169031	ENSG00000197565
ENSG00000169032	ENSG00000126934
ENSG00000169032	ENSG00000212825
ENSG00000169045	ENSG00000096746
ENSG00000169045	ENSG00000103067
ENSG00000169045	ENSG00000104413
ENSG00000169045	ENSG00000126945
ENSG00000169045	ENSG00000132463
ENSG00000169045	ENSG00000169813
ENSG00000169047	ENSG00000133124
ENSG00000169047	ENSG00000185950
ENSG00000169062	ENSG00000125351
ENSG00000169071	ENSG00000030304
ENSG00000169071	ENSG00000140538
ENSG00000169071	ENSG00000148053
ENSG00000169071	ENSG00000185483
ENSG00000169071	ENSG00000198400
ENSG00000169093	ENSG00000196433
ENSG00000169094	ENSG00000023902
ENSG00000169100	ENSG00000005022
ENSG00000169100	ENSG00000151475
ENSG00000169100	ENSG00000151729
ENSG00000169118	ENSG00000133275
ENSG00000169118	ENSG00000151292
ENSG00000169122	ENSG00000125898
ENSG00000169122	ENSG00000184731
ENSG00000169122	ENSG00000197245
ENSG00000169129	ENSG00000157510
ENSG00000169129	ENSG00000196526
ENSG00000169136	ENSG00000128272
ENSG00000169139	ENSG00000214192

ENSG00000169154	ENSG00000120053
ENSG00000169154	ENSG00000215618
ENSG00000169155	ENSG00000180610
ENSG00000169155	ENSG00000185278
ENSG00000169155	ENSG00000204210
ENSG00000169155	ENSG00000204366
ENSG00000169155	ENSG00000213588
ENSG00000169169	ENSG00000005469
ENSG00000169169	ENSG00000070748
ENSG00000169169	ENSG00000095321
ENSG00000169169	ENSG00000110090
ENSG00000169169	ENSG00000205560
ENSG00000169181	ENSG00000111305
ENSG00000169181	ENSG00000214978
ENSG00000169213	ENSG00000105514
ENSG00000169213	ENSG00000105649
ENSG00000169213	ENSG00000152932
ENSG00000169216	ENSG00000137970
ENSG00000169216	ENSG00000146223
ENSG00000169216	ENSG00000147604
ENSG00000169216	ENSG00000171549
ENSG00000169216	ENSG00000181416
ENSG00000169216	ENSG00000182520
ENSG00000169216	ENSG00000214485
ENSG00000169216	ENSG00000214557
ENSG00000169216	ENSG00000214932
ENSG00000169216	ENSG00000215297
ENSG00000169216	ENSG00000215402
ENSG00000169218	ENSG00000101282
ENSG00000169218	ENSG00000146374
ENSG00000169218	ENSG00000147655
ENSG00000169220	ENSG00000159788
ENSG00000169221	ENSG00000099992
ENSG00000169221	ENSG00000175463
ENSG00000169223	ENSG00000074695
ENSG00000169223	ENSG00000114988
ENSG00000169223	ENSG00000140506
ENSG00000169231	ENSG00000105664
ENSG00000169231	ENSG00000113296
ENSG00000169231	ENSG00000137801
ENSG00000169231	ENSG00000186340
ENSG00000169239	ENSG00000074410
ENSG00000169239	ENSG00000104267
ENSG00000169239	ENSG00000107159
ENSG00000169239	ENSG00000118298
ENSG00000169239	ENSG00000131686
ENSG00000169239	ENSG00000133742
ENSG00000169239	ENSG00000164879
ENSG00000169239	ENSG00000167434
ENSG00000169239	ENSG00000168748
ENSG00000169239	ENSG00000174990
ENSG00000169239	ENSG00000178538
ENSG00000169239	ENSG00000185015
ENSG00000169239	ENSG00000214729
ENSG00000169239	ENSG00000214873
ENSG00000169239	ENSG00000215487
ENSG00000169239	ENSG00000215501
ENSG00000169239	ENSG00000215543
ENSG00000169242	ENSG00000090776

ENSG00000169242	ENSG00000099617
ENSG00000169242	ENSG00000108947
ENSG00000169242	ENSG00000125266
ENSG00000169242	ENSG00000143590
ENSG00000169242	ENSG00000143620
ENSG00000169242	ENSG00000184349
ENSG00000169245	ENSG00000081041
ENSG00000169245	ENSG00000109272
ENSG00000169245	ENSG00000124875
ENSG00000169245	ENSG00000138755
ENSG00000169245	ENSG00000163734
ENSG00000169245	ENSG00000163735
ENSG00000169245	ENSG00000163736
ENSG00000169245	ENSG00000163737
ENSG00000169245	ENSG00000163739
ENSG00000169245	ENSG00000169248
ENSG00000169245	ENSG00000169429
ENSG00000169247	ENSG00000125089
ENSG00000169248	ENSG00000081041
ENSG00000169248	ENSG00000109272
ENSG00000169248	ENSG00000124875
ENSG00000169248	ENSG00000138755
ENSG00000169248	ENSG00000163734
ENSG00000169248	ENSG00000163735
ENSG00000169248	ENSG00000163736
ENSG00000169248	ENSG00000163737
ENSG00000169248	ENSG00000163739
ENSG00000169248	ENSG00000169245
ENSG00000169248	ENSG00000169429
ENSG00000169252	ENSG00000043591
ENSG00000169252	ENSG00000102468
ENSG00000169252	ENSG00000113749
ENSG00000169252	ENSG00000125631
ENSG00000169252	ENSG00000135312
ENSG00000169252	ENSG00000135914
ENSG00000169252	ENSG00000147246
ENSG00000169252	ENSG00000150594
ENSG00000169252	ENSG00000157219
ENSG00000169252	ENSG00000164270
ENSG00000169252	ENSG00000168830
ENSG00000169252	ENSG00000175658
ENSG00000169252	ENSG00000178394
ENSG00000169252	ENSG00000179097
ENSG00000169252	ENSG00000179546
ENSG00000169252	ENSG00000181210
ENSG00000169252	ENSG00000184303
ENSG00000169252	ENSG00000184845
ENSG00000169252	ENSG00000185321
ENSG00000169252	ENSG00000188778
ENSG00000169255	ENSG00000156966
ENSG00000169255	ENSG00000162630
ENSG00000169255	ENSG00000170340
ENSG00000169255	ENSG00000172318
ENSG00000169255	ENSG00000176383
ENSG00000169255	ENSG00000176597
ENSG00000169255	ENSG00000177191
ENSG00000169255	ENSG00000179913
ENSG00000169255	ENSG00000180561
ENSG00000169255	ENSG00000183778

ENSG00000169255	ENSG00000198488
ENSG00000169255	ENSG00000204222
ENSG00000169255	ENSG00000214654
ENSG00000169258	ENSG00000185477
ENSG00000169258	ENSG00000204175
ENSG00000169271	ENSG00000004776
ENSG00000169271	ENSG00000106211
ENSG00000169271	ENSG00000109846
ENSG00000169271	ENSG00000152137
ENSG00000169271	ENSG00000160202
ENSG00000169271	ENSG00000170276
ENSG00000169282	ENSG00000069424
ENSG00000169282	ENSG00000170049
ENSG00000169291	ENSG00000105251
ENSG00000169291	ENSG00000107338
ENSG00000169291	ENSG00000138606
ENSG00000169297	ENSG00000131910
ENSG00000169299	ENSG00000165434
ENSG00000169302	ENSG00000152953
ENSG00000169302	ENSG00000165752
ENSG00000169306	ENSG00000189108
ENSG00000169313	ENSG00000138271
ENSG00000169313	ENSG00000171659
ENSG00000169313	ENSG00000174944
ENSG00000169313	ENSG00000174946
ENSG00000169313	ENSG00000181631
ENSG00000169340	ENSG00000139055
ENSG00000169340	ENSG00000185615
ENSG00000169340	ENSG00000185624
ENSG00000169344	ENSG00000119913
ENSG00000169344	ENSG00000138315
ENSG00000169344	ENSG00000169347
ENSG00000169347	ENSG00000119913
ENSG00000169347	ENSG00000138315
ENSG00000169347	ENSG00000169344
ENSG00000169375	ENSG00000127511
ENSG00000169379	ENSG00000174225
ENSG00000169398	ENSG00000061938
ENSG00000169398	ENSG00000120899
ENSG00000169398	ENSG00000174292
ENSG00000169403	ENSG00000078589
ENSG00000169403	ENSG00000139679
ENSG00000169403	ENSG00000147145
ENSG00000169403	ENSG00000152207
ENSG00000169403	ENSG00000169508
ENSG00000169403	ENSG00000169860
ENSG00000169403	ENSG00000173198
ENSG00000169403	ENSG00000175591
ENSG00000169403	ENSG00000184574
ENSG00000169403	ENSG00000186912
ENSG00000169403	ENSG00000198829
ENSG00000169410	ENSG00000139304
ENSG00000169410	ENSG00000175354
ENSG00000169410	ENSG00000196396
ENSG00000169418	ENSG00000070019
ENSG00000169418	ENSG00000101890
ENSG00000169418	ENSG00000123201
ENSG00000169418	ENSG00000132518
ENSG00000169418	ENSG00000159899

ENSG00000169418	ENSG00000164116
ENSG00000169427	ENSG00000082482
ENSG00000169427	ENSG00000095981
ENSG00000169427	ENSG00000099337
ENSG00000169427	ENSG00000100433
ENSG00000169427	ENSG00000124249
ENSG00000169427	ENSG00000124780
ENSG00000169427	ENSG00000135750
ENSG00000169427	ENSG00000152315
ENSG00000169427	ENSG00000164626
ENSG00000169427	ENSG00000171303
ENSG00000169427	ENSG00000173338
ENSG00000169427	ENSG00000182450
ENSG00000169427	ENSG00000184261
ENSG00000169429	ENSG00000081041
ENSG00000169429	ENSG00000109272
ENSG00000169429	ENSG00000124875
ENSG00000169429	ENSG00000138755
ENSG00000169429	ENSG00000163734
ENSG00000169429	ENSG00000163735
ENSG00000169429	ENSG00000163736
ENSG00000169429	ENSG00000163737
ENSG00000169429	ENSG00000163739
ENSG00000169429	ENSG00000169245
ENSG00000169429	ENSG00000169248
ENSG00000169432	ENSG00000007314
ENSG00000169432	ENSG00000136531
ENSG00000169432	ENSG00000136546
ENSG00000169432	ENSG00000144285
ENSG00000169432	ENSG00000153253
ENSG00000169432	ENSG00000168356
ENSG00000169432	ENSG00000183873
ENSG00000169432	ENSG00000185313
ENSG00000169432	ENSG00000196876
ENSG00000169435	ENSG00000068028
ENSG00000169435	ENSG00000101265
ENSG00000169435	ENSG00000107551
ENSG00000169435	ENSG00000136653
ENSG00000169435	ENSG00000153179
ENSG00000169436	ENSG00000124749
ENSG00000169439	ENSG00000115884
ENSG00000169439	ENSG00000124145
ENSG00000169439	ENSG00000162512
ENSG00000169495	ENSG00000115317
ENSG00000169495	ENSG00000166033
ENSG00000169495	ENSG00000170801
ENSG00000169499	ENSG00000107679
ENSG00000169504	ENSG00000112782
ENSG00000169504	ENSG00000155962
ENSG00000169504	ENSG00000159212
ENSG00000169504	ENSG00000169583
ENSG00000169504	ENSG00000213719
ENSG00000169504	ENSG00000214258
ENSG00000169508	ENSG00000078589
ENSG00000169508	ENSG00000139679
ENSG00000169508	ENSG00000147145
ENSG00000169508	ENSG00000152207
ENSG00000169508	ENSG00000169403
ENSG00000169508	ENSG00000169860

ENSG00000169508	ENSG00000173198
ENSG00000169508	ENSG00000175591
ENSG00000169508	ENSG00000184574
ENSG00000169508	ENSG00000186912
ENSG00000169508	ENSG00000198829
ENSG00000169548	ENSG00000056277
ENSG00000169548	ENSG00000137871
ENSG00000169548	ENSG00000143442
ENSG00000169548	ENSG00000198477
ENSG00000169554	ENSG00000148516
ENSG00000169562	ENSG00000121634
ENSG00000169562	ENSG00000121742
ENSG00000169562	ENSG00000121743
ENSG00000169562	ENSG00000131233
ENSG00000169562	ENSG00000135355
ENSG00000169562	ENSG00000143140
ENSG00000169562	ENSG00000152661
ENSG00000169562	ENSG00000159248
ENSG00000169562	ENSG00000164411
ENSG00000169562	ENSG00000165474
ENSG00000169562	ENSG00000176402
ENSG00000169562	ENSG00000177291
ENSG00000169562	ENSG00000182963
ENSG00000169562	ENSG00000183153
ENSG00000169562	ENSG00000187513
ENSG00000169562	ENSG00000188910
ENSG00000169562	ENSG00000189280
ENSG00000169562	ENSG00000189433
ENSG00000169562	ENSG00000198835
ENSG00000169562	ENSG00000203733
ENSG00000169564	ENSG00000090097
ENSG00000169564	ENSG00000183570
ENSG00000169564	ENSG00000197111
ENSG00000169583	ENSG00000112782
ENSG00000169583	ENSG00000155962
ENSG00000169583	ENSG00000159212
ENSG00000169583	ENSG00000169504
ENSG00000169583	ENSG00000213719
ENSG00000169583	ENSG00000214258
ENSG00000169594	ENSG00000173068
ENSG00000169604	ENSG00000163297
ENSG00000169604	ENSG00000198250
ENSG00000169607	ENSG00000136108
ENSG00000169618	ENSG00000056291
ENSG00000169618	ENSG00000075073
ENSG00000169618	ENSG00000101292
ENSG00000169618	ENSG00000110148
ENSG00000169618	ENSG00000115353
ENSG00000169618	ENSG00000119973
ENSG00000169618	ENSG00000121764
ENSG00000169618	ENSG00000123901
ENSG00000169618	ENSG00000137252
ENSG00000169618	ENSG00000148734
ENSG00000169618	ENSG00000159279
ENSG00000169618	ENSG00000163394
ENSG00000169618	ENSG00000164128
ENSG00000169618	ENSG00000164129
ENSG00000169618	ENSG00000169836
ENSG00000169618	ENSG00000185149

ENSG00000169618	ENSG00000186867
ENSG00000169618	ENSG00000204174
ENSG00000169618	ENSG00000215167
ENSG00000169635	ENSG00000177374
ENSG00000169641	ENSG00000118407
ENSG00000169641	ENSG00000168386
ENSG00000169682	ENSG00000182557
ENSG00000169682	ENSG00000183018
ENSG00000169684	ENSG00000080644
ENSG00000169684	ENSG00000101204
ENSG00000169684	ENSG00000108556
ENSG00000169684	ENSG00000117971
ENSG00000169684	ENSG00000120903
ENSG00000169684	ENSG00000129749
ENSG00000169684	ENSG00000135902
ENSG00000169684	ENSG00000138435
ENSG00000169684	ENSG00000147432
ENSG00000169684	ENSG00000147434
ENSG00000169684	ENSG00000149305
ENSG00000169684	ENSG00000160716
ENSG00000169684	ENSG00000166664
ENSG00000169684	ENSG00000166736
ENSG00000169684	ENSG00000170175
ENSG00000169684	ENSG00000174343
ENSG00000169684	ENSG00000175344
ENSG00000169684	ENSG00000178084
ENSG00000169684	ENSG00000186038
ENSG00000169684	ENSG00000186090
ENSG00000169684	ENSG00000186919
ENSG00000169684	ENSG00000196811
ENSG00000169688	ENSG00000087250
ENSG00000169688	ENSG00000102891
ENSG00000169688	ENSG00000125144
ENSG00000169688	ENSG00000125148
ENSG00000169688	ENSG00000169715
ENSG00000169688	ENSG00000187193
ENSG00000169688	ENSG00000198417
ENSG00000169688	ENSG00000205358
ENSG00000169688	ENSG00000205360
ENSG00000169688	ENSG00000205362
ENSG00000169688	ENSG00000205364
ENSG00000169692	ENSG00000204310
ENSG00000169704	ENSG00000203618
ENSG00000169704	ENSG00000204545
ENSG00000169715	ENSG00000087250
ENSG00000169715	ENSG00000102891
ENSG00000169715	ENSG00000125144
ENSG00000169715	ENSG00000125148
ENSG00000169715	ENSG00000169688
ENSG00000169715	ENSG00000187193
ENSG00000169715	ENSG00000198417
ENSG00000169715	ENSG00000205358
ENSG00000169715	ENSG00000205360
ENSG00000169715	ENSG00000205362
ENSG00000169715	ENSG00000205364
ENSG00000169733	ENSG00000100060
ENSG00000169733	ENSG00000106003
ENSG00000169733	ENSG00000143891
ENSG00000169740	ENSG00000142396

ENSG00000169740	ENSG00000174255
ENSG00000169740	ENSG00000179195
ENSG00000169740	ENSG00000179965
ENSG00000169740	ENSG00000184294
ENSG00000169740	ENSG00000186446
ENSG00000169740	ENSG00000186777
ENSG00000169740	ENSG00000197008
ENSG00000169740	ENSG00000197025
ENSG00000169740	ENSG00000197062
ENSG00000169740	ENSG00000197332
ENSG00000169740	ENSG00000198155
ENSG00000169740	ENSG00000204756
ENSG00000169740	ENSG00000212805
ENSG00000169740	ENSG00000213976
ENSG00000169740	ENSG00000213980
ENSG00000169740	ENSG00000215180
ENSG00000169740	ENSG00000215510
ENSG00000169740	ENSG00000215537
ENSG00000169740	ENSG00000215598
ENSG00000169740	ENSG00000215600
ENSG00000169744	ENSG00000198728
ENSG00000169750	ENSG00000070831
ENSG00000169750	ENSG00000119729
ENSG00000169750	ENSG00000126785
ENSG00000169750	ENSG00000128340
ENSG00000169750	ENSG00000136238
ENSG00000169750	ENSG00000177105
ENSG00000169756	ENSG00000072163
ENSG00000169756	ENSG00000089159
ENSG00000169756	ENSG00000110031
ENSG00000169756	ENSG00000112214
ENSG00000169756	ENSG00000140682
ENSG00000169756	ENSG00000183386
ENSG00000169760	ENSG00000146938
ENSG00000169760	ENSG00000165246
ENSG00000169760	ENSG00000169992
ENSG00000169760	ENSG00000196338
ENSG00000169783	ENSG00000128594
ENSG00000169783	ENSG00000170382
ENSG00000169783	ENSG00000173114
ENSG00000169783	ENSG00000174482
ENSG00000169783	ENSG00000175928
ENSG00000169783	ENSG00000183014
ENSG00000169783	ENSG00000213171
ENSG00000169800	ENSG00000147274
ENSG00000169800	ENSG00000169811
ENSG00000169800	ENSG00000170748
ENSG00000169800	ENSG00000174622
ENSG00000169800	ENSG00000206011
ENSG00000169800	ENSG00000206046
ENSG00000169800	ENSG00000213516
ENSG00000169800	ENSG00000214442
ENSG00000169800	ENSG00000215210
ENSG00000169811	ENSG00000147274
ENSG00000169811	ENSG00000169800
ENSG00000169811	ENSG00000170748
ENSG00000169811	ENSG00000174622
ENSG00000169811	ENSG00000206011
ENSG00000169811	ENSG00000206046

ENSG00000169811	ENSG00000213516
ENSG00000169811	ENSG00000214442
ENSG00000169811	ENSG00000215210
ENSG00000169813	ENSG00000096746
ENSG00000169813	ENSG00000103067
ENSG00000169813	ENSG00000104413
ENSG00000169813	ENSG00000126945
ENSG00000169813	ENSG00000132463
ENSG00000169813	ENSG00000169045
ENSG00000169814	ENSG00000093134
ENSG00000169814	ENSG00000112299
ENSG00000169814	ENSG00000112303
ENSG00000169826	ENSG00000033100
ENSG00000169826	ENSG00000123989
ENSG00000169826	ENSG00000131873
ENSG00000169826	ENSG00000139044
ENSG00000169826	ENSG00000147408
ENSG00000169826	ENSG00000182272
ENSG00000169826	ENSG00000198108
ENSG00000169836	ENSG00000056291
ENSG00000169836	ENSG00000075073
ENSG00000169836	ENSG00000101292
ENSG00000169836	ENSG00000110148
ENSG00000169836	ENSG00000115353
ENSG00000169836	ENSG00000119973
ENSG00000169836	ENSG00000121764
ENSG00000169836	ENSG00000123901
ENSG00000169836	ENSG00000137252
ENSG00000169836	ENSG00000148734
ENSG00000169836	ENSG00000159279
ENSG00000169836	ENSG00000163394
ENSG00000169836	ENSG00000164128
ENSG00000169836	ENSG00000164129
ENSG00000169836	ENSG00000169618
ENSG00000169836	ENSG00000185149
ENSG00000169836	ENSG00000186867
ENSG00000169836	ENSG00000204174
ENSG00000169836	ENSG00000215167
ENSG00000169840	ENSG00000105997
ENSG00000169840	ENSG00000106038
ENSG00000169840	ENSG00000120093
ENSG00000169840	ENSG00000128652
ENSG00000169840	ENSG00000174279
ENSG00000169840	ENSG00000180613
ENSG00000169851	ENSG00000099715
ENSG00000169851	ENSG00000102290
ENSG00000169851	ENSG00000113555
ENSG00000169851	ENSG00000156453
ENSG00000169851	ENSG00000184226
ENSG00000169851	ENSG00000197991
ENSG00000169851	ENSG00000215442
ENSG00000169855	ENSG00000154133
ENSG00000169855	ENSG00000154134
ENSG00000169855	ENSG00000185008
ENSG00000169856	ENSG00000111249
ENSG00000169856	ENSG00000119547
ENSG00000169856	ENSG00000160967
ENSG00000169856	ENSG00000205922
ENSG00000169860	ENSG00000078589

ENSG00000169860	ENSG00000139679
ENSG00000169860	ENSG00000147145
ENSG00000169860	ENSG00000152207
ENSG00000169860	ENSG00000169403
ENSG00000169860	ENSG00000169508
ENSG00000169860	ENSG00000173198
ENSG00000169860	ENSG00000175591
ENSG00000169860	ENSG00000184574
ENSG00000169860	ENSG00000186912
ENSG00000169860	ENSG00000198829
ENSG00000169862	ENSG00000057294
ENSG00000169862	ENSG00000081277
ENSG00000169862	ENSG00000099889
ENSG00000169862	ENSG00000144283
ENSG00000169862	ENSG00000184363
ENSG00000169862	ENSG00000198561
ENSG00000169884	ENSG00000002745
ENSG00000169884	ENSG00000061492
ENSG00000169884	ENSG00000075290
ENSG00000169884	ENSG00000085741
ENSG00000169884	ENSG00000105989
ENSG00000169884	ENSG00000108379
ENSG00000169884	ENSG00000111186
ENSG00000169884	ENSG00000114251
ENSG00000169884	ENSG00000115596
ENSG00000169884	ENSG00000125084
ENSG00000169884	ENSG00000134245
ENSG00000169884	ENSG00000135925
ENSG00000169884	ENSG00000143816
ENSG00000169884	ENSG00000154342
ENSG00000169884	ENSG00000154764
ENSG00000169884	ENSG00000158955
ENSG00000169884	ENSG00000162552
ENSG00000169884	ENSG00000188064
ENSG00000169891	ENSG00000135597
ENSG00000169896	ENSG00000005844
ENSG00000169896	ENSG00000005884
ENSG00000169896	ENSG00000005961
ENSG00000169896	ENSG00000077943
ENSG00000169896	ENSG00000083457
ENSG00000169896	ENSG00000091409
ENSG00000169896	ENSG00000115232
ENSG00000169896	ENSG00000135424
ENSG00000169896	ENSG00000137809
ENSG00000169896	ENSG00000138448
ENSG00000169896	ENSG00000140678
ENSG00000169896	ENSG00000143127
ENSG00000169896	ENSG00000144668
ENSG00000169896	ENSG00000156886
ENSG00000169896	ENSG00000161638
ENSG00000169896	ENSG00000164171
ENSG00000169896	ENSG00000213949
ENSG00000169902	ENSG00000128294
ENSG00000169903	ENSG00000142484
ENSG00000169903	ENSG00000145107
ENSG00000169903	ENSG00000163762
ENSG00000169903	ENSG00000168955
ENSG00000169903	ENSG00000169908
ENSG00000169906	ENSG00000143546

ENSG00000169906	ENSG00000160307
ENSG00000169906	ENSG00000160678
ENSG00000169906	ENSG00000163191
ENSG00000169906	ENSG00000163220
ENSG00000169906	ENSG00000163221
ENSG00000169906	ENSG00000163993
ENSG00000169906	ENSG00000171643
ENSG00000169906	ENSG00000188015
ENSG00000169906	ENSG00000188643
ENSG00000169906	ENSG00000189171
ENSG00000169906	ENSG00000189334
ENSG00000169906	ENSG00000196154
ENSG00000169906	ENSG00000196420
ENSG00000169906	ENSG00000196754
ENSG00000169906	ENSG00000197747
ENSG00000169906	ENSG00000197956
ENSG00000169908	ENSG00000142484
ENSG00000169908	ENSG00000145107
ENSG00000169908	ENSG00000163762
ENSG00000169908	ENSG00000168955
ENSG00000169908	ENSG00000169903
ENSG00000169918	ENSG00000019995
ENSG00000169918	ENSG00000118503
ENSG00000169918	ENSG00000163113
ENSG00000169925	ENSG00000137948
ENSG00000169925	ENSG00000141867
ENSG00000169925	ENSG00000204256
ENSG00000169926	ENSG00000119138
ENSG00000169933	ENSG00000070601
ENSG00000169933	ENSG00000147234
ENSG00000169946	ENSG00000179588
ENSG00000169962	ENSG00000036828
ENSG00000169962	ENSG00000173612
ENSG00000169962	ENSG00000173662
ENSG00000169962	ENSG00000174930
ENSG00000169962	ENSG00000179002
ENSG00000169962	ENSG00000215740
ENSG00000169967	ENSG00000198909
ENSG00000169991	ENSG00000010295
ENSG00000169992	ENSG00000146938
ENSG00000169992	ENSG00000165246
ENSG00000169992	ENSG00000169760
ENSG00000169992	ENSG00000196338
ENSG00000169994	ENSG00000137474
ENSG00000170004	ENSG00000111642
ENSG00000170004	ENSG00000116254
ENSG00000170011	ENSG00000115648
ENSG00000170027	ENSG00000108953
ENSG00000170027	ENSG00000128245
ENSG00000170027	ENSG00000134308
ENSG00000170027	ENSG00000164924
ENSG00000170027	ENSG00000166913
ENSG00000170027	ENSG00000175793
ENSG00000170027	ENSG00000214637
ENSG00000170027	ENSG00000215108
ENSG00000170035	ENSG00000170142
ENSG00000170035	ENSG00000182247
ENSG00000170044	ENSG00000177398
ENSG00000170049	ENSG00000069424

ENSG00000170049	ENSG00000169282
ENSG00000170054	ENSG00000100665
ENSG00000170054	ENSG00000123561
ENSG00000170054	ENSG00000135744
ENSG00000170054	ENSG00000165951
ENSG00000170054	ENSG00000165953
ENSG00000170054	ENSG00000186910
ENSG00000170054	ENSG00000187483
ENSG00000170054	ENSG00000188488
ENSG00000170054	ENSG00000196136
ENSG00000170054	ENSG00000197249
ENSG00000170075	ENSG00000102239
ENSG00000170075	ENSG00000126010
ENSG00000170075	ENSG00000135577
ENSG00000170075	ENSG00000136160
ENSG00000170075	ENSG00000151617
ENSG00000170075	ENSG00000170775
ENSG00000170091	ENSG00000130643
ENSG00000170091	ENSG00000168824
ENSG00000170092	ENSG00000136206
ENSG00000170092	ENSG00000154732
ENSG00000170092	ENSG00000163806
ENSG00000170092	ENSG00000166667
ENSG00000170092	ENSG00000173678
ENSG00000170092	ENSG00000179994
ENSG00000170092	ENSG00000183318
ENSG00000170092	ENSG00000184616
ENSG00000170092	ENSG00000185040
ENSG00000170092	ENSG00000186645
ENSG00000170092	ENSG00000198305
ENSG00000170092	ENSG00000204710
ENSG00000170092	ENSG00000205238
ENSG00000170092	ENSG00000205482
ENSG00000170092	ENSG00000205505
ENSG00000170092	ENSG00000205506
ENSG00000170092	ENSG00000205508
ENSG00000170092	ENSG00000205509
ENSG00000170092	ENSG00000205580
ENSG00000170092	ENSG00000214300
ENSG00000170092	ENSG00000215092
ENSG00000170099	ENSG00000099937
ENSG00000170099	ENSG00000114204
ENSG00000170099	ENSG00000117601
ENSG00000170099	ENSG00000140093
ENSG00000170099	ENSG00000149257
ENSG00000170122	ENSG00000054598
ENSG00000170122	ENSG00000125798
ENSG00000170122	ENSG00000129514
ENSG00000170122	ENSG00000164379
ENSG00000170122	ENSG00000168269
ENSG00000170122	ENSG00000170608
ENSG00000170122	ENSG00000176165
ENSG00000170122	ENSG00000176678
ENSG00000170122	ENSG00000176692
ENSG00000170122	ENSG00000178919
ENSG00000170122	ENSG00000179772
ENSG00000170122	ENSG00000183770
ENSG00000170122	ENSG00000184492
ENSG00000170122	ENSG00000184659

ENSG00000170122	ENSG00000186564
ENSG00000170122	ENSG00000186766
ENSG00000170122	ENSG00000186790
ENSG00000170122	ENSG00000187140
ENSG00000170122	ENSG00000187559
ENSG00000170122	ENSG00000204612
ENSG00000170122	ENSG00000204779
ENSG00000170122	ENSG00000204793
ENSG00000170122	ENSG00000204828
ENSG00000170122	ENSG00000214336
ENSG00000170128	ENSG00000100739
ENSG00000170128	ENSG00000134817
ENSG00000170128	ENSG00000144891
ENSG00000170128	ENSG00000154165
ENSG00000170128	ENSG00000168398
ENSG00000170128	ENSG00000173080
ENSG00000170128	ENSG00000180772
ENSG00000170128	ENSG00000182631
ENSG00000170142	ENSG00000170035
ENSG00000170142	ENSG00000182247
ENSG00000170144	ENSG00000122566
ENSG00000170144	ENSG00000135486
ENSG00000170144	ENSG00000139675
ENSG00000170144	ENSG00000176825
ENSG00000170144	ENSG00000177219
ENSG00000170144	ENSG00000177733
ENSG00000170144	ENSG00000188174
ENSG00000170144	ENSG00000206228
ENSG00000170144	ENSG00000212961
ENSG00000170144	ENSG00000214284
ENSG00000170144	ENSG00000214361
ENSG00000170144	ENSG00000214592
ENSG00000170144	ENSG00000214653
ENSG00000170144	ENSG00000215222
ENSG00000170144	ENSG00000215492
ENSG00000170144	ENSG00000215870
ENSG00000170144	ENSG00000215876
ENSG00000170144	ENSG00000215885
ENSG00000170145	ENSG00000007047
ENSG00000170145	ENSG00000072518
ENSG00000170145	ENSG00000075413
ENSG00000170145	ENSG00000116141
ENSG00000170145	ENSG00000142178
ENSG00000170145	ENSG00000160584
ENSG00000170153	ENSG00000082996
ENSG00000170153	ENSG00000105428
ENSG00000170153	ENSG00000108523
ENSG00000170153	ENSG00000113269
ENSG00000170153	ENSG00000133135
ENSG00000170153	ENSG00000163162
ENSG00000170153	ENSG00000188050
ENSG00000170162	ENSG00000102243
ENSG00000170162	ENSG00000206538
ENSG00000170166	ENSG00000037965
ENSG00000170166	ENSG00000106004
ENSG00000170166	ENSG00000106006
ENSG00000170166	ENSG00000108511
ENSG00000170166	ENSG00000120068
ENSG00000170166	ENSG00000120075

ENSG00000170166	ENSG00000120087
ENSG00000170166	ENSG00000122592
ENSG00000170166	ENSG00000172789
ENSG00000170166	ENSG00000175879
ENSG00000170166	ENSG00000182742
ENSG00000170166	ENSG00000197576
ENSG00000170166	ENSG00000197757
ENSG00000170166	ENSG00000198353
ENSG00000170175	ENSG00000080644
ENSG00000170175	ENSG00000101204
ENSG00000170175	ENSG00000108556
ENSG00000170175	ENSG00000117971
ENSG00000170175	ENSG00000120903
ENSG00000170175	ENSG00000129749
ENSG00000170175	ENSG00000135902
ENSG00000170175	ENSG00000138435
ENSG00000170175	ENSG00000147432
ENSG00000170175	ENSG00000147434
ENSG00000170175	ENSG00000149305
ENSG00000170175	ENSG00000160716
ENSG00000170175	ENSG00000166664
ENSG00000170175	ENSG00000166736
ENSG00000170175	ENSG00000169684
ENSG00000170175	ENSG00000174343
ENSG00000170175	ENSG00000175344
ENSG00000170175	ENSG00000178084
ENSG00000170175	ENSG00000186038
ENSG00000170175	ENSG00000186090
ENSG00000170175	ENSG00000186919
ENSG00000170175	ENSG00000196811
ENSG00000170178	ENSG00000123407
ENSG00000170185	ENSG00000118369
ENSG00000170190	ENSG00000100156
ENSG00000170190	ENSG00000108932
ENSG00000170190	ENSG00000112394
ENSG00000170190	ENSG00000118596
ENSG00000170190	ENSG00000141526
ENSG00000170190	ENSG00000147100
ENSG00000170190	ENSG00000152779
ENSG00000170190	ENSG00000155380
ENSG00000170190	ENSG00000163053
ENSG00000170190	ENSG00000165449
ENSG00000170190	ENSG00000168679
ENSG00000170190	ENSG00000174326
ENSG00000170190	ENSG00000174327
ENSG00000170209	ENSG00000104312
ENSG00000170209	ENSG00000129465
ENSG00000170209	ENSG00000137275
ENSG00000170209	ENSG00000183421
ENSG00000170214	ENSG00000120907
ENSG00000170214	ENSG00000143147
ENSG00000170214	ENSG00000148680
ENSG00000170214	ENSG00000165370
ENSG00000170214	ENSG00000171873
ENSG00000170214	ENSG00000183935
ENSG00000170231	ENSG00000163586
ENSG00000170234	ENSG00000171813
ENSG00000170264	ENSG00000156050
ENSG00000170264	ENSG00000175513

ENSG00000170266	ENSG00000163521
ENSG00000170275	ENSG00000090530
ENSG00000170275	ENSG00000110811
ENSG00000170275	ENSG00000117385
ENSG00000170275	ENSG00000141696
ENSG00000170276	ENSG00000004776
ENSG00000170276	ENSG00000106211
ENSG00000170276	ENSG00000109846
ENSG00000170276	ENSG00000152137
ENSG00000170276	ENSG00000160202
ENSG00000170276	ENSG00000169271
ENSG00000170289	ENSG00000070729
ENSG00000170289	ENSG00000099822
ENSG00000170289	ENSG00000132259
ENSG00000170289	ENSG00000138622
ENSG00000170289	ENSG00000143630
ENSG00000170289	ENSG00000144191
ENSG00000170289	ENSG00000164588
ENSG00000170289	ENSG00000183862
ENSG00000170293	ENSG00000102934
ENSG00000170293	ENSG00000144063
ENSG00000170293	ENSG00000147676
ENSG00000170293	ENSG00000155254
ENSG00000170293	ENSG00000172005
ENSG00000170298	ENSG00000006659
ENSG00000170298	ENSG00000100079
ENSG00000170298	ENSG00000100097
ENSG00000170298	ENSG00000105198
ENSG00000170298	ENSG00000105205
ENSG00000170298	ENSG00000116977
ENSG00000170298	ENSG00000119862
ENSG00000170298	ENSG00000131981
ENSG00000170298	ENSG00000133317
ENSG00000170298	ENSG00000168961
ENSG00000170298	ENSG00000171747
ENSG00000170298	ENSG00000171916
ENSG00000170298	ENSG00000178934
ENSG00000170298	ENSG00000197778
ENSG00000170298	ENSG00000205076
ENSG00000170298	ENSG00000215132
ENSG00000170323	ENSG00000114113
ENSG00000170323	ENSG00000114115
ENSG00000170323	ENSG00000121769
ENSG00000170323	ENSG00000139194
ENSG00000170323	ENSG00000143320
ENSG00000170323	ENSG00000145384
ENSG00000170323	ENSG00000147588
ENSG00000170323	ENSG00000162444
ENSG00000170323	ENSG00000164434
ENSG00000170323	ENSG00000164687
ENSG00000170323	ENSG00000166426
ENSG00000170323	ENSG00000197416
ENSG00000170323	ENSG00000205186
ENSG00000170323	ENSG00000214004
ENSG00000170324	ENSG00000139926
ENSG00000170324	ENSG00000163629
ENSG00000170340	ENSG00000156966
ENSG00000170340	ENSG00000162630
ENSG00000170340	ENSG00000169255

ENSG00000170340	ENSG00000172318
ENSG00000170340	ENSG00000176383
ENSG00000170340	ENSG00000176597
ENSG00000170340	ENSG00000177191
ENSG00000170340	ENSG00000179913
ENSG00000170340	ENSG00000180561
ENSG00000170340	ENSG00000183778
ENSG00000170340	ENSG00000198488
ENSG00000170340	ENSG00000204222
ENSG00000170340	ENSG00000214654
ENSG00000170345	ENSG00000075426
ENSG00000170345	ENSG00000125740
ENSG00000170345	ENSG00000140044
ENSG00000170345	ENSG00000162772
ENSG00000170345	ENSG00000175592
ENSG00000170365	ENSG00000101665
ENSG00000170365	ENSG00000113658
ENSG00000170365	ENSG00000120693
ENSG00000170365	ENSG00000137834
ENSG00000170365	ENSG00000141646
ENSG00000170365	ENSG00000166949
ENSG00000170365	ENSG00000175387
ENSG00000170367	ENSG00000077984
ENSG00000170367	ENSG00000101435
ENSG00000170367	ENSG00000101439
ENSG00000170367	ENSG00000101441
ENSG00000170367	ENSG00000125815
ENSG00000170367	ENSG00000125823
ENSG00000170367	ENSG00000125831
ENSG00000170367	ENSG00000170369
ENSG00000170367	ENSG00000170373
ENSG00000170367	ENSG00000173335
ENSG00000170367	ENSG00000175315
ENSG00000170367	ENSG00000204658
ENSG00000170367	ENSG00000204662
ENSG00000170367	ENSG00000204663
ENSG00000170369	ENSG00000077984
ENSG00000170369	ENSG00000101435
ENSG00000170369	ENSG00000101439
ENSG00000170369	ENSG00000101441
ENSG00000170369	ENSG00000125815
ENSG00000170369	ENSG00000125823
ENSG00000170369	ENSG00000125831
ENSG00000170369	ENSG00000170367
ENSG00000170369	ENSG00000170373
ENSG00000170369	ENSG00000173335
ENSG00000170369	ENSG00000175315
ENSG00000170369	ENSG00000204658
ENSG00000170369	ENSG00000204662
ENSG00000170369	ENSG00000204663
ENSG00000170370	ENSG00000135638
ENSG00000170373	ENSG00000077984
ENSG00000170373	ENSG00000101435
ENSG00000170373	ENSG00000101439
ENSG00000170373	ENSG00000101441
ENSG00000170373	ENSG00000125815
ENSG00000170373	ENSG00000125823
ENSG00000170373	ENSG00000125831
ENSG00000170373	ENSG00000170367

ENSG00000170373	ENSG00000170369
ENSG00000170373	ENSG00000173335
ENSG00000170373	ENSG00000175315
ENSG00000170373	ENSG00000204658
ENSG00000170373	ENSG00000204662
ENSG00000170373	ENSG00000204663
ENSG00000170374	ENSG00000067082
ENSG00000170374	ENSG00000102554
ENSG00000170374	ENSG00000105610
ENSG00000170374	ENSG00000127528
ENSG00000170374	ENSG00000129911
ENSG00000170374	ENSG00000136826
ENSG00000170374	ENSG00000139651
ENSG00000170374	ENSG00000155090
ENSG00000170374	ENSG00000164651
ENSG00000170374	ENSG00000167182
ENSG00000170374	ENSG00000171872
ENSG00000170374	ENSG00000172059
ENSG00000170374	ENSG00000174595
ENSG00000170374	ENSG00000189120
ENSG00000170374	ENSG00000197294
ENSG00000170374	ENSG00000197550
ENSG00000170374	ENSG00000204335
ENSG00000170381	ENSG00000010319
ENSG00000170381	ENSG00000012171
ENSG00000170381	ENSG00000075213
ENSG00000170381	ENSG00000092421
ENSG00000170381	ENSG00000095539
ENSG00000170381	ENSG00000137872
ENSG00000170381	ENSG00000143434
ENSG00000170381	ENSG00000153993
ENSG00000170381	ENSG00000168758
ENSG00000170381	ENSG00000185033
ENSG00000170381	ENSG00000187764
ENSG00000170381	ENSG00000196189
ENSG00000170382	ENSG00000128594
ENSG00000170382	ENSG00000169783
ENSG00000170382	ENSG00000173114
ENSG00000170382	ENSG00000174482
ENSG00000170382	ENSG00000175928
ENSG00000170382	ENSG00000183014
ENSG00000170382	ENSG00000213171
ENSG00000170385	ENSG00000196660
ENSG00000170390	ENSG00000077279
ENSG00000170390	ENSG00000104237
ENSG00000170390	ENSG00000130413
ENSG00000170390	ENSG00000133083
ENSG00000170390	ENSG00000146038
ENSG00000170390	ENSG00000163673
ENSG00000170390	ENSG00000183638
ENSG00000170412	ENSG00000013588
ENSG00000170412	ENSG00000111291
ENSG00000170412	ENSG00000167191
ENSG00000170419	ENSG00000132821
ENSG00000170419	ENSG00000187135
ENSG00000170421	ENSG00000135443
ENSG00000170421	ENSG00000135477
ENSG00000170421	ENSG00000135480
ENSG00000170421	ENSG00000139648

ENSG00000170421	ENSG00000161849
ENSG00000170421	ENSG00000161850
ENSG00000170421	ENSG00000167767
ENSG00000170421	ENSG00000167768
ENSG00000170421	ENSG00000170423
ENSG00000170421	ENSG00000170442
ENSG00000170421	ENSG00000170454
ENSG00000170421	ENSG00000170465
ENSG00000170421	ENSG00000170477
ENSG00000170421	ENSG00000170484
ENSG00000170421	ENSG00000170486
ENSG00000170421	ENSG00000170523
ENSG00000170421	ENSG00000172867
ENSG00000170421	ENSG00000185069
ENSG00000170421	ENSG00000185479
ENSG00000170421	ENSG00000185640
ENSG00000170421	ENSG00000186049
ENSG00000170421	ENSG00000186081
ENSG00000170421	ENSG00000186442
ENSG00000170421	ENSG00000189182
ENSG00000170421	ENSG00000205420
ENSG00000170421	ENSG00000205426
ENSG00000170421	ENSG00000214233
ENSG00000170421	ENSG00000214323
ENSG00000170421	ENSG00000214477
ENSG00000170421	ENSG00000214752
ENSG00000170421	ENSG00000214974
ENSG00000170421	ENSG00000215287
ENSG00000170421	ENSG00000215847
ENSG00000170423	ENSG00000135443
ENSG00000170423	ENSG00000135477
ENSG00000170423	ENSG00000135480
ENSG00000170423	ENSG00000139648
ENSG00000170423	ENSG00000161849
ENSG00000170423	ENSG00000161850
ENSG00000170423	ENSG00000167767
ENSG00000170423	ENSG00000167768
ENSG00000170423	ENSG00000170421
ENSG00000170423	ENSG00000170442
ENSG00000170423	ENSG00000170454
ENSG00000170423	ENSG00000170465
ENSG00000170423	ENSG00000170477
ENSG00000170423	ENSG00000170484
ENSG00000170423	ENSG00000170486
ENSG00000170423	ENSG00000170523
ENSG00000170423	ENSG00000172867
ENSG00000170423	ENSG00000185069
ENSG00000170423	ENSG00000185479
ENSG00000170423	ENSG00000185640
ENSG00000170423	ENSG00000186049
ENSG00000170423	ENSG00000186081
ENSG00000170423	ENSG00000186442
ENSG00000170423	ENSG00000189182
ENSG00000170423	ENSG00000205420
ENSG00000170423	ENSG00000205426
ENSG00000170423	ENSG00000214233
ENSG00000170423	ENSG00000214323
ENSG00000170423	ENSG00000214477
ENSG00000170423	ENSG00000214752

ENSG00000170423	ENSG00000214974
ENSG00000170423	ENSG00000215287
ENSG00000170423	ENSG00000215847
ENSG00000170425	ENSG00000121933
ENSG00000170425	ENSG00000128271
ENSG00000170425	ENSG00000163485
ENSG00000170426	ENSG00000025423
ENSG00000170426	ENSG00000073737
ENSG00000170426	ENSG00000086696
ENSG00000170426	ENSG00000135437
ENSG00000170426	ENSG00000139547
ENSG00000170426	ENSG00000161267
ENSG00000170426	ENSG00000176387
ENSG00000170442	ENSG00000135443
ENSG00000170442	ENSG00000135477
ENSG00000170442	ENSG00000135480
ENSG00000170442	ENSG00000139648
ENSG00000170442	ENSG00000161849
ENSG00000170442	ENSG00000161850
ENSG00000170442	ENSG00000167767
ENSG00000170442	ENSG00000167768
ENSG00000170442	ENSG00000170421
ENSG00000170442	ENSG00000170423
ENSG00000170442	ENSG00000170454
ENSG00000170442	ENSG00000170465
ENSG00000170442	ENSG00000170477
ENSG00000170442	ENSG00000170484
ENSG00000170442	ENSG00000170486
ENSG00000170442	ENSG00000170523
ENSG00000170442	ENSG00000172867
ENSG00000170442	ENSG00000185069
ENSG00000170442	ENSG00000185479
ENSG00000170442	ENSG00000185640
ENSG00000170442	ENSG00000186049
ENSG00000170442	ENSG00000186081
ENSG00000170442	ENSG00000186442
ENSG00000170442	ENSG00000189182
ENSG00000170442	ENSG00000205420
ENSG00000170442	ENSG00000205426
ENSG00000170442	ENSG00000214233
ENSG00000170442	ENSG00000214323
ENSG00000170442	ENSG00000214477
ENSG00000170442	ENSG00000214752
ENSG00000170442	ENSG00000214974
ENSG00000170442	ENSG00000215287
ENSG00000170442	ENSG00000215847
ENSG00000170445	ENSG00000112855
ENSG00000170454	ENSG00000135443
ENSG00000170454	ENSG00000135477
ENSG00000170454	ENSG00000135480
ENSG00000170454	ENSG00000139648
ENSG00000170454	ENSG00000161849
ENSG00000170454	ENSG00000161850
ENSG00000170454	ENSG00000167767
ENSG00000170454	ENSG00000167768
ENSG00000170454	ENSG00000170421
ENSG00000170454	ENSG00000170423
ENSG00000170454	ENSG00000170442
ENSG00000170454	ENSG00000170465

ENSG00000170454	ENSG00000170477
ENSG00000170454	ENSG00000170484
ENSG00000170454	ENSG00000170486
ENSG00000170454	ENSG00000170523
ENSG00000170454	ENSG00000172867
ENSG00000170454	ENSG00000185069
ENSG00000170454	ENSG00000185479
ENSG00000170454	ENSG00000185640
ENSG00000170454	ENSG00000186049
ENSG00000170454	ENSG00000186081
ENSG00000170454	ENSG00000186442
ENSG00000170454	ENSG00000189182
ENSG00000170454	ENSG00000205420
ENSG00000170454	ENSG00000205426
ENSG00000170454	ENSG00000214233
ENSG00000170454	ENSG00000214323
ENSG00000170454	ENSG00000214477
ENSG00000170454	ENSG00000214752
ENSG00000170454	ENSG00000214974
ENSG00000170454	ENSG00000215287
ENSG00000170454	ENSG00000215847
ENSG00000170456	ENSG00000137145
ENSG00000170456	ENSG00000174485
ENSG00000170456	ENSG00000184014
ENSG00000170456	ENSG00000198837
ENSG00000170464	ENSG00000148719
ENSG00000170464	ENSG00000164031
ENSG00000170465	ENSG00000135443
ENSG00000170465	ENSG00000135477
ENSG00000170465	ENSG00000135480
ENSG00000170465	ENSG00000139648
ENSG00000170465	ENSG00000161849
ENSG00000170465	ENSG00000161850
ENSG00000170465	ENSG00000167767
ENSG00000170465	ENSG00000167768
ENSG00000170465	ENSG00000170421
ENSG00000170465	ENSG00000170423
ENSG00000170465	ENSG00000170442
ENSG00000170465	ENSG00000170454
ENSG00000170465	ENSG00000170477
ENSG00000170465	ENSG00000170484
ENSG00000170465	ENSG00000170486
ENSG00000170465	ENSG00000170523
ENSG00000170465	ENSG00000172867
ENSG00000170465	ENSG00000185069
ENSG00000170465	ENSG00000185479
ENSG00000170465	ENSG00000185640
ENSG00000170465	ENSG00000186049
ENSG00000170465	ENSG00000186081
ENSG00000170465	ENSG00000186442
ENSG00000170465	ENSG00000189182
ENSG00000170465	ENSG00000205420
ENSG00000170465	ENSG00000205426
ENSG00000170465	ENSG00000214233
ENSG00000170465	ENSG00000214323
ENSG00000170465	ENSG00000214477
ENSG00000170465	ENSG00000214752
ENSG00000170465	ENSG00000214974
ENSG00000170465	ENSG00000215287

ENSG00000170465	ENSG00000215847
ENSG00000170477	ENSG00000135443
ENSG00000170477	ENSG00000135477
ENSG00000170477	ENSG00000135480
ENSG00000170477	ENSG00000139648
ENSG00000170477	ENSG00000161849
ENSG00000170477	ENSG00000161850
ENSG00000170477	ENSG00000167767
ENSG00000170477	ENSG00000167768
ENSG00000170477	ENSG00000170421
ENSG00000170477	ENSG00000170423
ENSG00000170477	ENSG00000170442
ENSG00000170477	ENSG00000170454
ENSG00000170477	ENSG00000170465
ENSG00000170477	ENSG00000170484
ENSG00000170477	ENSG00000170486
ENSG00000170477	ENSG00000170523
ENSG00000170477	ENSG00000172867
ENSG00000170477	ENSG00000185069
ENSG00000170477	ENSG00000185479
ENSG00000170477	ENSG00000185640
ENSG00000170477	ENSG00000186049
ENSG00000170477	ENSG00000186081
ENSG00000170477	ENSG00000186442
ENSG00000170477	ENSG00000189182
ENSG00000170477	ENSG00000205420
ENSG00000170477	ENSG00000205426
ENSG00000170477	ENSG00000214233
ENSG00000170477	ENSG00000214323
ENSG00000170477	ENSG00000214477
ENSG00000170477	ENSG00000214752
ENSG00000170477	ENSG00000214974
ENSG00000170477	ENSG00000215287
ENSG00000170477	ENSG00000215847
ENSG00000170482	ENSG00000089057
ENSG00000170482	ENSG00000213901
ENSG00000170484	ENSG00000135443
ENSG00000170484	ENSG00000135477
ENSG00000170484	ENSG00000135480
ENSG00000170484	ENSG00000139648
ENSG00000170484	ENSG00000161849
ENSG00000170484	ENSG00000161850
ENSG00000170484	ENSG00000167767
ENSG00000170484	ENSG00000167768
ENSG00000170484	ENSG00000170421
ENSG00000170484	ENSG00000170423
ENSG00000170484	ENSG00000170442
ENSG00000170484	ENSG00000170454
ENSG00000170484	ENSG00000170465
ENSG00000170484	ENSG00000170477
ENSG00000170484	ENSG00000170486
ENSG00000170484	ENSG00000170523
ENSG00000170484	ENSG00000172867
ENSG00000170484	ENSG00000185069
ENSG00000170484	ENSG00000185479
ENSG00000170484	ENSG00000185640
ENSG00000170484	ENSG00000186049
ENSG00000170484	ENSG00000186081
ENSG00000170484	ENSG00000186442

ENSG00000170484	ENSG00000189182
ENSG00000170484	ENSG00000205420
ENSG00000170484	ENSG00000205426
ENSG00000170484	ENSG00000214233
ENSG00000170484	ENSG00000214323
ENSG00000170484	ENSG00000214477
ENSG00000170484	ENSG00000214752
ENSG00000170484	ENSG00000214974
ENSG00000170484	ENSG00000215287
ENSG00000170484	ENSG00000215847
ENSG00000170485	ENSG00000134852
ENSG00000170485	ENSG00000166049
ENSG00000170486	ENSG00000135443
ENSG00000170486	ENSG00000135477
ENSG00000170486	ENSG00000135480
ENSG00000170486	ENSG00000139648
ENSG00000170486	ENSG00000161849
ENSG00000170486	ENSG00000161850
ENSG00000170486	ENSG00000167767
ENSG00000170486	ENSG00000167768
ENSG00000170486	ENSG00000170421
ENSG00000170486	ENSG00000170423
ENSG00000170486	ENSG00000170442
ENSG00000170486	ENSG00000170454
ENSG00000170486	ENSG00000170465
ENSG00000170486	ENSG00000170477
ENSG00000170486	ENSG00000170484
ENSG00000170486	ENSG00000170523
ENSG00000170486	ENSG00000172867
ENSG00000170486	ENSG00000185069
ENSG00000170486	ENSG00000185479
ENSG00000170486	ENSG00000185640
ENSG00000170486	ENSG00000186049
ENSG00000170486	ENSG00000186081
ENSG00000170486	ENSG00000186442
ENSG00000170486	ENSG00000189182
ENSG00000170486	ENSG00000205420
ENSG00000170486	ENSG00000205426
ENSG00000170486	ENSG00000214233
ENSG00000170486	ENSG00000214323
ENSG00000170486	ENSG00000214477
ENSG00000170486	ENSG00000214752
ENSG00000170486	ENSG00000214974
ENSG00000170486	ENSG00000215287
ENSG00000170486	ENSG00000215847
ENSG00000170500	ENSG00000154359
ENSG00000170500	ENSG00000175556
ENSG00000170522	ENSG00000119915
ENSG00000170523	ENSG00000135443
ENSG00000170523	ENSG00000135477
ENSG00000170523	ENSG00000135480
ENSG00000170523	ENSG00000139648
ENSG00000170523	ENSG00000161849
ENSG00000170523	ENSG00000161850
ENSG00000170523	ENSG00000167767
ENSG00000170523	ENSG00000167768
ENSG00000170523	ENSG00000170421
ENSG00000170523	ENSG00000170423
ENSG00000170523	ENSG00000170442

ENSG00000170523	ENSG00000170454
ENSG00000170523	ENSG00000170465
ENSG00000170523	ENSG00000170477
ENSG00000170523	ENSG00000170484
ENSG00000170523	ENSG00000170486
ENSG00000170523	ENSG00000172867
ENSG00000170523	ENSG00000185069
ENSG00000170523	ENSG00000185479
ENSG00000170523	ENSG00000185640
ENSG00000170523	ENSG00000186049
ENSG00000170523	ENSG00000186081
ENSG00000170523	ENSG00000186442
ENSG00000170523	ENSG00000189182
ENSG00000170523	ENSG00000205420
ENSG00000170523	ENSG00000205426
ENSG00000170523	ENSG00000214233
ENSG00000170523	ENSG00000214323
ENSG00000170523	ENSG00000214477
ENSG00000170523	ENSG00000214752
ENSG00000170523	ENSG00000214974
ENSG00000170523	ENSG00000215287
ENSG00000170523	ENSG00000215847
ENSG00000170525	ENSG00000114268
ENSG00000170525	ENSG00000123836
ENSG00000170525	ENSG00000158571
ENSG00000170537	ENSG00000103534
ENSG00000170537	ENSG00000141524
ENSG00000170537	ENSG00000149488
ENSG00000170537	ENSG00000165091
ENSG00000170537	ENSG00000167608
ENSG00000170537	ENSG00000167895
ENSG00000170537	ENSG00000188869
ENSG00000170542	ENSG00000021355
ENSG00000170542	ENSG00000057149
ENSG00000170542	ENSG00000124570
ENSG00000170542	ENSG00000166396
ENSG00000170542	ENSG00000166401
ENSG00000170542	ENSG00000166404
ENSG00000170542	ENSG00000166634
ENSG00000170542	ENSG00000197632
ENSG00000170542	ENSG00000197641
ENSG00000170542	ENSG00000206072
ENSG00000170542	ENSG00000206073
ENSG00000170542	ENSG00000206075
ENSG00000170549	ENSG00000113430
ENSG00000170549	ENSG00000150051
ENSG00000170549	ENSG00000159387
ENSG00000170549	ENSG00000170561
ENSG00000170549	ENSG00000176842
ENSG00000170549	ENSG00000177508
ENSG00000170561	ENSG00000113430
ENSG00000170561	ENSG00000150051
ENSG00000170561	ENSG00000159387
ENSG00000170561	ENSG00000170549
ENSG00000170561	ENSG00000176842
ENSG00000170561	ENSG00000177508
ENSG00000170571	ENSG00000156642
ENSG00000170571	ENSG00000172270
ENSG00000170577	ENSG00000100625

ENSG00000170577	ENSG00000126778
ENSG00000170577	ENSG00000138083
ENSG00000170577	ENSG00000177045
ENSG00000170577	ENSG00000184302
ENSG00000170579	ENSG00000080845
ENSG00000170579	ENSG00000116544
ENSG00000170579	ENSG00000126787
ENSG00000170579	ENSG00000198010
ENSG00000170581	ENSG00000115415
ENSG00000170581	ENSG00000126561
ENSG00000170581	ENSG00000138378
ENSG00000170581	ENSG00000166888
ENSG00000170581	ENSG00000168610
ENSG00000170581	ENSG00000173757
ENSG00000170604	ENSG00000119669
ENSG00000170604	ENSG00000168264
ENSG00000170606	ENSG00000120694
ENSG00000170606	ENSG00000164070
ENSG00000170608	ENSG00000054598
ENSG00000170608	ENSG00000125798
ENSG00000170608	ENSG00000129514
ENSG00000170608	ENSG00000164379
ENSG00000170608	ENSG00000168269
ENSG00000170608	ENSG00000170122
ENSG00000170608	ENSG00000176165
ENSG00000170608	ENSG00000176678
ENSG00000170608	ENSG00000176692
ENSG00000170608	ENSG00000178919
ENSG00000170608	ENSG00000179772
ENSG00000170608	ENSG00000183770
ENSG00000170608	ENSG00000184492
ENSG00000170608	ENSG00000184659
ENSG00000170608	ENSG00000186564
ENSG00000170608	ENSG00000186766
ENSG00000170608	ENSG00000186790
ENSG00000170608	ENSG00000187140
ENSG00000170608	ENSG00000187559
ENSG00000170608	ENSG00000204612
ENSG00000170608	ENSG00000204779
ENSG00000170608	ENSG00000204793
ENSG00000170608	ENSG00000204828
ENSG00000170608	ENSG00000214336
ENSG00000170615	ENSG00000091137
ENSG00000170615	ENSG00000091138
ENSG00000170615	ENSG00000112053
ENSG00000170615	ENSG00000135502
ENSG00000170615	ENSG00000145217
ENSG00000170615	ENSG00000147606
ENSG00000170615	ENSG00000155850
ENSG00000170615	ENSG00000174502
ENSG00000170624	ENSG00000102683
ENSG00000170624	ENSG00000163069
ENSG00000170624	ENSG00000185053
ENSG00000170633	ENSG00000090432
ENSG00000170633	ENSG00000092871
ENSG00000170634	ENSG00000119640
ENSG00000170653	ENSG00000115966
ENSG00000170653	ENSG00000130522
ENSG00000170653	ENSG00000146592

ENSG00000170653	ENSG00000171223
ENSG00000170653	ENSG00000177606
ENSG00000170667	ENSG00000105808
ENSG00000170667	ENSG00000111344
ENSG00000170667	ENSG00000155903
ENSG00000170667	ENSG00000185989
ENSG00000170681	ENSG00000168497
ENSG00000170681	ENSG00000170955
ENSG00000170681	ENSG00000177469
ENSG00000170684	ENSG00000119866
ENSG00000170684	ENSG00000127152
ENSG00000170689	ENSG00000078399
ENSG00000170689	ENSG00000128709
ENSG00000170689	ENSG00000128710
ENSG00000170689	ENSG00000153807
ENSG00000170689	ENSG00000180806
ENSG00000170689	ENSG00000180818
ENSG00000170703	ENSG00000100271
ENSG00000170703	ENSG00000119685
ENSG00000170703	ENSG00000120440
ENSG00000170703	ENSG00000131044
ENSG00000170703	ENSG00000135912
ENSG00000170703	ENSG00000137941
ENSG00000170703	ENSG00000138892
ENSG00000170703	ENSG00000175764
ENSG00000170703	ENSG00000213471
ENSG00000170703	ENSG00000214021
ENSG00000170743	ENSG00000011347
ENSG00000170743	ENSG00000019505
ENSG00000170743	ENSG00000067715
ENSG00000170743	ENSG00000101098
ENSG00000170743	ENSG00000103528
ENSG00000170743	ENSG00000110975
ENSG00000170743	ENSG00000129990
ENSG00000170743	ENSG00000132718
ENSG00000170743	ENSG00000132872
ENSG00000170743	ENSG00000134207
ENSG00000170743	ENSG00000143858
ENSG00000170743	ENSG00000149043
ENSG00000170743	ENSG00000149927
ENSG00000170743	ENSG00000173227
ENSG00000170743	ENSG00000204176
ENSG00000170743	ENSG00000213023
ENSG00000170743	ENSG00000214408
ENSG00000170743	ENSG00000214409
ENSG00000170743	ENSG00000214569
ENSG00000170743	ENSG00000214783
ENSG00000170745	ENSG00000026559
ENSG00000170745	ENSG00000102057
ENSG00000170745	ENSG00000104848
ENSG00000170745	ENSG00000111262
ENSG00000170745	ENSG00000124134
ENSG00000170745	ENSG00000130037
ENSG00000170745	ENSG00000143105
ENSG00000170745	ENSG00000151079
ENSG00000170745	ENSG00000156486
ENSG00000170745	ENSG00000158445
ENSG00000170745	ENSG00000162975
ENSG00000170745	ENSG00000164794

ENSG00000170745	ENSG00000168263
ENSG00000170745	ENSG00000168418
ENSG00000170745	ENSG00000171126
ENSG00000170745	ENSG00000171385
ENSG00000170745	ENSG00000177272
ENSG00000170745	ENSG00000177301
ENSG00000170745	ENSG00000178342
ENSG00000170745	ENSG00000182255
ENSG00000170745	ENSG00000182674
ENSG00000170745	ENSG00000184408
ENSG00000170748	ENSG00000147274
ENSG00000170748	ENSG00000169800
ENSG00000170748	ENSG00000169811
ENSG00000170748	ENSG00000174622
ENSG00000170748	ENSG00000206011
ENSG00000170748	ENSG00000206046
ENSG00000170748	ENSG00000213516
ENSG00000170748	ENSG00000214442
ENSG00000170748	ENSG00000215210
ENSG00000170759	ENSG00000155980
ENSG00000170759	ENSG00000168280
ENSG00000170775	ENSG00000102239
ENSG00000170775	ENSG00000126010
ENSG00000170775	ENSG00000135577
ENSG00000170775	ENSG00000136160
ENSG00000170775	ENSG00000151617
ENSG00000170775	ENSG00000170075
ENSG00000170776	ENSG00000038102
ENSG00000170776	ENSG00000104880
ENSG00000170776	ENSG00000116584
ENSG00000170777	ENSG00000076554
ENSG00000170777	ENSG00000101150
ENSG00000170777	ENSG00000111907
ENSG00000170779	ENSG00000167565
ENSG00000170779	ENSG00000197019
ENSG00000170786	ENSG00000121039
ENSG00000170786	ENSG00000162496
ENSG00000170786	ENSG00000170509
ENSG00000170786	ENSG00000198189
ENSG00000170801	ENSG00000115317
ENSG00000170801	ENSG00000166033
ENSG00000170801	ENSG00000169495
ENSG00000170802	ENSG00000053254
ENSG00000170807	ENSG00000128872
ENSG00000170807	ENSG00000136842
ENSG00000170807	ENSG00000138594
ENSG00000170807	ENSG00000163157
ENSG00000170807	ENSG00000163380
ENSG00000170807	ENSG00000163431
ENSG00000170819	ENSG00000006059
ENSG00000170819	ENSG00000094796
ENSG00000170819	ENSG00000108244
ENSG00000170819	ENSG00000108417
ENSG00000170819	ENSG00000108759
ENSG00000170819	ENSG00000111057
ENSG00000170819	ENSG00000126337
ENSG00000170819	ENSG00000128422
ENSG00000170819	ENSG00000131737
ENSG00000170819	ENSG00000131738

ENSG00000170819	ENSG00000131885
ENSG00000170819	ENSG00000167916
ENSG00000170819	ENSG00000171345
ENSG00000170819	ENSG00000171346
ENSG00000170819	ENSG00000171360
ENSG00000170819	ENSG00000171401
ENSG00000170819	ENSG00000171403
ENSG00000170819	ENSG00000171431
ENSG00000170819	ENSG00000171446
ENSG00000170819	ENSG00000173908
ENSG00000170819	ENSG00000186393
ENSG00000170819	ENSG00000186395
ENSG00000170819	ENSG00000186831
ENSG00000170819	ENSG00000186832
ENSG00000170819	ENSG00000186847
ENSG00000170819	ENSG00000187242
ENSG00000170819	ENSG00000187686
ENSG00000170819	ENSG00000196859
ENSG00000170819	ENSG00000197079
ENSG00000170819	ENSG00000204889
ENSG00000170819	ENSG00000204897
ENSG00000170819	ENSG00000213951
ENSG00000170819	ENSG00000214220
ENSG00000170819	ENSG00000214454
ENSG00000170819	ENSG00000214959
ENSG00000170819	ENSG00000215772
ENSG00000170819	ENSG00000215773
ENSG00000170820	ENSG00000133067
ENSG00000170820	ENSG00000138039
ENSG00000170820	ENSG00000139292
ENSG00000170820	ENSG00000165409
ENSG00000170820	ENSG00000205213
ENSG00000170837	ENSG00000164604
ENSG00000170837	ENSG00000184194
ENSG00000170852	ENSG00000120696
ENSG00000170852	ENSG00000163376
ENSG00000170852	ENSG00000165572
ENSG00000170873	ENSG00000132613
ENSG00000170915	ENSG00000137819
ENSG00000170915	ENSG00000160781
ENSG00000170915	ENSG00000182749
ENSG00000170915	ENSG00000188582
ENSG00000170921	ENSG00000115183
ENSG00000170921	ENSG00000151458
ENSG00000170927	ENSG00000205038
ENSG00000170948	ENSG00000071655
ENSG00000170948	ENSG00000134046
ENSG00000170948	ENSG00000182315
ENSG00000170948	ENSG00000184301
ENSG00000170948	ENSG00000196589
ENSG00000170948	ENSG00000205717
ENSG00000170948	ENSG00000205718
ENSG00000170955	ENSG00000168497
ENSG00000170955	ENSG00000170681
ENSG00000170955	ENSG00000177469
ENSG00000170961	ENSG00000103044
ENSG00000170961	ENSG00000105509
ENSG00000170962	ENSG00000145431
ENSG00000170989	ENSG00000064547

ENSG00000170989	ENSG00000118432
ENSG00000170989	ENSG00000125910
ENSG00000170989	ENSG00000132975
ENSG00000170989	ENSG00000146360
ENSG00000170989	ENSG00000171517
ENSG00000170989	ENSG00000175898
ENSG00000170989	ENSG00000180739
ENSG00000170989	ENSG00000181773
ENSG00000170989	ENSG00000188822
ENSG00000170989	ENSG00000198121
ENSG00000170989	ENSG00000213694
ENSG00000171004	ENSG00000136720
ENSG00000171004	ENSG00000185352
ENSG00000171016	ENSG00000163348
ENSG00000171017	ENSG00000136802
ENSG00000171017	ENSG00000171488
ENSG00000171017	ENSG00000171492
ENSG00000171017	ENSG00000197147
ENSG00000171033	ENSG00000135549
ENSG00000171033	ENSG00000168734
ENSG00000171044	ENSG00000101321
ENSG00000171044	ENSG00000158156
ENSG00000171044	ENSG00000186530
ENSG00000171044	ENSG00000198669
ENSG00000171044	ENSG00000206579
ENSG00000171049	ENSG00000134830
ENSG00000171049	ENSG00000171051
ENSG00000171049	ENSG00000171860
ENSG00000171049	ENSG00000174600
ENSG00000171049	ENSG00000183134
ENSG00000171049	ENSG00000183671
ENSG00000171049	ENSG00000187474
ENSG00000171049	ENSG00000197405
ENSG00000171049	ENSG00000213903
ENSG00000171049	ENSG00000213906
ENSG00000171051	ENSG00000134830
ENSG00000171051	ENSG00000171049
ENSG00000171051	ENSG00000171860
ENSG00000171051	ENSG00000174600
ENSG00000171051	ENSG00000183134
ENSG00000171051	ENSG00000183671
ENSG00000171051	ENSG00000187474
ENSG00000171051	ENSG00000197405
ENSG00000171051	ENSG00000213903
ENSG00000171051	ENSG00000213906
ENSG00000171055	ENSG00000149557
ENSG00000171056	ENSG00000005513
ENSG00000171056	ENSG00000100146
ENSG00000171056	ENSG00000124766
ENSG00000171056	ENSG00000125398
ENSG00000171056	ENSG00000129194
ENSG00000171056	ENSG00000134595
ENSG00000171056	ENSG00000164736
ENSG00000171056	ENSG00000176887
ENSG00000171056	ENSG00000177732
ENSG00000171056	ENSG00000181449
ENSG00000171056	ENSG00000182968
ENSG00000171056	ENSG00000184895
ENSG00000171056	ENSG00000203883

ENSG00000171094	ENSG00000027644
ENSG00000171094	ENSG00000047936
ENSG00000171094	ENSG00000062524
ENSG00000171094	ENSG00000140443
ENSG00000171094	ENSG00000171105
ENSG00000171100	ENSG00000003987
ENSG00000171100	ENSG00000014914
ENSG00000171100	ENSG00000063601
ENSG00000171100	ENSG00000087053
ENSG00000171100	ENSG00000100330
ENSG00000171100	ENSG00000102043
ENSG00000171100	ENSG00000104643
ENSG00000171100	ENSG00000108389
ENSG00000171100	ENSG00000139505
ENSG00000171100	ENSG00000150712
ENSG00000171100	ENSG00000166912
ENSG00000171100	ENSG00000184416
ENSG00000171101	ENSG00000105366
ENSG00000171101	ENSG00000105383
ENSG00000171101	ENSG00000105492
ENSG00000171101	ENSG00000105501
ENSG00000171101	ENSG00000105695
ENSG00000171101	ENSG00000129450
ENSG00000171101	ENSG00000142512
ENSG00000171101	ENSG00000160296
ENSG00000171101	ENSG00000161640
ENSG00000171101	ENSG00000161643
ENSG00000171101	ENSG00000167764
ENSG00000171101	ENSG00000168995
ENSG00000171101	ENSG00000197865
ENSG00000171105	ENSG00000027644
ENSG00000171105	ENSG00000047936
ENSG00000171105	ENSG00000062524
ENSG00000171105	ENSG00000140443
ENSG00000171105	ENSG00000171094
ENSG00000171109	ENSG00000116688
ENSG00000171115	ENSG00000106560
ENSG00000171115	ENSG00000133561
ENSG00000171115	ENSG00000133574
ENSG00000171115	ENSG00000179144
ENSG00000171115	ENSG00000196329
ENSG00000171115	ENSG00000213203
ENSG00000171119	ENSG00000117407
ENSG00000171119	ENSG00000125650
ENSG00000171119	ENSG00000168621
ENSG00000171121	ENSG00000135643
ENSG00000171121	ENSG00000145936
ENSG00000171121	ENSG00000197584
ENSG00000171121	ENSG00000215575
ENSG00000171124	ENSG00000130383
ENSG00000171124	ENSG00000156413
ENSG00000171124	ENSG00000172461
ENSG00000171124	ENSG00000172728
ENSG00000171124	ENSG00000180549
ENSG00000171124	ENSG00000196371
ENSG00000171124	ENSG00000196968
ENSG00000171126	ENSG00000026559
ENSG00000171126	ENSG00000102057
ENSG00000171126	ENSG00000104848

ENSG00000171126	ENSG00000111262
ENSG00000171126	ENSG00000124134
ENSG00000171126	ENSG00000130037
ENSG00000171126	ENSG00000143105
ENSG00000171126	ENSG00000151079
ENSG00000171126	ENSG00000156486
ENSG00000171126	ENSG00000158445
ENSG00000171126	ENSG00000162975
ENSG00000171126	ENSG00000164794
ENSG00000171126	ENSG00000168263
ENSG00000171126	ENSG00000168418
ENSG00000171126	ENSG00000170745
ENSG00000171126	ENSG00000171385
ENSG00000171126	ENSG00000177272
ENSG00000171126	ENSG00000177301
ENSG00000171126	ENSG00000178342
ENSG00000171126	ENSG00000182255
ENSG00000171126	ENSG00000182674
ENSG00000171126	ENSG00000184408
ENSG00000171136	ENSG00000107014
ENSG00000171136	ENSG00000107018
ENSG00000171136	ENSG00000120211
ENSG00000171136	ENSG00000172410
ENSG00000171147	ENSG00000010438
ENSG00000171147	ENSG00000129437
ENSG00000171147	ENSG00000129451
ENSG00000171147	ENSG00000129455
ENSG00000171147	ENSG00000142515
ENSG00000171147	ENSG00000165076
ENSG00000171147	ENSG00000167748
ENSG00000171147	ENSG00000167749
ENSG00000171147	ENSG00000167751
ENSG00000171147	ENSG00000167754
ENSG00000171147	ENSG00000167755
ENSG00000171147	ENSG00000167757
ENSG00000171147	ENSG00000167759
ENSG00000171147	ENSG00000169035
ENSG00000171147	ENSG00000173636
ENSG00000171147	ENSG00000174562
ENSG00000171147	ENSG00000186474
ENSG00000171147	ENSG00000204981
ENSG00000171147	ENSG00000204982
ENSG00000171147	ENSG00000204983
ENSG00000171147	ENSG00000213022
ENSG00000171147	ENSG00000214083
ENSG00000171150	ENSG00000114737
ENSG00000171150	ENSG00000120833
ENSG00000171150	ENSG00000170677
ENSG00000171150	ENSG00000174111
ENSG00000171150	ENSG00000180008
ENSG00000171150	ENSG00000184557
ENSG00000171150	ENSG00000185338
ENSG00000171163	ENSG00000158805
ENSG00000171163	ENSG00000161914
ENSG00000171163	ENSG00000186660
ENSG00000171189	ENSG00000120251
ENSG00000171189	ENSG00000125675
ENSG00000171189	ENSG00000149403
ENSG00000171189	ENSG00000152578

ENSG00000171189	ENSG00000155511
ENSG00000171189	ENSG00000163873
ENSG00000171189	ENSG00000164418
ENSG00000171208	ENSG00000038295
ENSG00000171208	ENSG00000095587
ENSG00000171208	ENSG00000106333
ENSG00000171208	ENSG00000107611
ENSG00000171208	ENSG00000157211
ENSG00000171208	ENSG00000163710
ENSG00000171208	ENSG00000166342
ENSG00000171208	ENSG00000168487
ENSG00000171208	ENSG00000184824
ENSG00000171217	ENSG00000013297
ENSG00000171217	ENSG00000066405
ENSG00000171217	ENSG00000106404
ENSG00000171217	ENSG00000113946
ENSG00000171217	ENSG00000134873
ENSG00000171217	ENSG00000156282
ENSG00000171217	ENSG00000156284
ENSG00000171217	ENSG00000159261
ENSG00000171217	ENSG00000163347
ENSG00000171217	ENSG00000164007
ENSG00000171217	ENSG00000165215
ENSG00000171217	ENSG00000165376
ENSG00000171217	ENSG00000177300
ENSG00000171217	ENSG00000181885
ENSG00000171217	ENSG00000184113
ENSG00000171217	ENSG00000184697
ENSG00000171217	ENSG00000185758
ENSG00000171217	ENSG00000189118
ENSG00000171217	ENSG00000189143
ENSG00000171217	ENSG00000213937
ENSG00000171219	ENSG00000067900
ENSG00000171219	ENSG00000134318
ENSG00000171219	ENSG00000143776
ENSG00000171219	ENSG00000198752
ENSG00000171223	ENSG00000115966
ENSG00000171223	ENSG00000130522
ENSG00000171223	ENSG00000146592
ENSG00000171223	ENSG00000170653
ENSG00000171223	ENSG00000177606
ENSG00000171224	ENSG00000174749
ENSG00000171234	ENSG00000109181
ENSG00000171234	ENSG00000135220
ENSG00000171234	ENSG00000135226
ENSG00000171234	ENSG00000145626
ENSG00000171234	ENSG00000156096
ENSG00000171234	ENSG00000167165
ENSG00000171234	ENSG00000168671
ENSG00000171234	ENSG00000173610
ENSG00000171234	ENSG00000174607
ENSG00000171234	ENSG00000196620
ENSG00000171234	ENSG00000197592
ENSG00000171234	ENSG00000197888
ENSG00000171234	ENSG00000198277
ENSG00000171234	ENSG00000213759
ENSG00000171243	ENSG00000167941
ENSG00000171246	ENSG00000106236
ENSG00000171246	ENSG00000132693

ENSG00000171246	ENSG00000132703
ENSG00000171246	ENSG00000163661
ENSG00000171246	ENSG00000167957
ENSG00000171246	ENSG00000215846
ENSG00000171262	ENSG00000119812
ENSG00000171262	ENSG00000130244
ENSG00000171282	ENSG00000182095
ENSG00000171298	ENSG00000090402
ENSG00000171298	ENSG00000179087
ENSG00000171298	ENSG00000204987
ENSG00000171298	ENSG00000214088
ENSG00000171303	ENSG00000082482
ENSG00000171303	ENSG00000095981
ENSG00000171303	ENSG00000099337
ENSG00000171303	ENSG00000100433
ENSG00000171303	ENSG00000124249
ENSG00000171303	ENSG00000124780
ENSG00000171303	ENSG00000135750
ENSG00000171303	ENSG00000152315
ENSG00000171303	ENSG00000164626
ENSG00000171303	ENSG00000169427
ENSG00000171303	ENSG00000173338
ENSG00000171303	ENSG00000182450
ENSG00000171303	ENSG00000184261
ENSG00000171310	ENSG00000115526
ENSG00000171310	ENSG00000124302
ENSG00000171310	ENSG00000136213
ENSG00000171310	ENSG00000154080
ENSG00000171310	ENSG00000169105
ENSG00000171310	ENSG00000180767
ENSG00000171314	ENSG00000164708
ENSG00000171314	ENSG00000172331
ENSG00000171314	ENSG00000186076
ENSG00000171314	ENSG00000197041
ENSG00000171314	ENSG00000214206
ENSG00000171314	ENSG00000214824
ENSG00000171314	ENSG00000215178
ENSG00000171316	ENSG00000100888
ENSG00000171316	ENSG00000124177
ENSG00000171316	ENSG00000177200
ENSG00000171320	ENSG00000141446
ENSG00000171345	ENSG00000006059
ENSG00000171345	ENSG00000094796
ENSG00000171345	ENSG00000108244
ENSG00000171345	ENSG00000108417
ENSG00000171345	ENSG00000108759
ENSG00000171345	ENSG00000111057
ENSG00000171345	ENSG00000126337
ENSG00000171345	ENSG00000128422
ENSG00000171345	ENSG00000131737
ENSG00000171345	ENSG00000131738
ENSG00000171345	ENSG00000131885
ENSG00000171345	ENSG00000167916
ENSG00000171345	ENSG00000170819
ENSG00000171345	ENSG00000171346
ENSG00000171345	ENSG00000171360
ENSG00000171345	ENSG00000171401
ENSG00000171345	ENSG00000171403
ENSG00000171345	ENSG00000171431

ENSG00000171345	ENSG00000171446
ENSG00000171345	ENSG00000173908
ENSG00000171345	ENSG00000186393
ENSG00000171345	ENSG00000186395
ENSG00000171345	ENSG00000186831
ENSG00000171345	ENSG00000186832
ENSG00000171345	ENSG00000186847
ENSG00000171345	ENSG00000187242
ENSG00000171345	ENSG00000187686
ENSG00000171345	ENSG00000196859
ENSG00000171345	ENSG00000197079
ENSG00000171345	ENSG00000204889
ENSG00000171345	ENSG00000204897
ENSG00000171345	ENSG00000213951
ENSG00000171345	ENSG00000214220
ENSG00000171345	ENSG00000214454
ENSG00000171345	ENSG00000214959
ENSG00000171345	ENSG00000215772
ENSG00000171345	ENSG00000215773
ENSG00000171346	ENSG00000006059
ENSG00000171346	ENSG00000094796
ENSG00000171346	ENSG00000108244
ENSG00000171346	ENSG00000108417
ENSG00000171346	ENSG00000108759
ENSG00000171346	ENSG00000111057
ENSG00000171346	ENSG00000126337
ENSG00000171346	ENSG00000128422
ENSG00000171346	ENSG00000131737
ENSG00000171346	ENSG00000131738
ENSG00000171346	ENSG00000131885
ENSG00000171346	ENSG00000167916
ENSG00000171346	ENSG00000170819
ENSG00000171346	ENSG00000171345
ENSG00000171346	ENSG00000171360
ENSG00000171346	ENSG00000171401
ENSG00000171346	ENSG00000171403
ENSG00000171346	ENSG00000171431
ENSG00000171346	ENSG00000171446
ENSG00000171346	ENSG00000173908
ENSG00000171346	ENSG00000186393
ENSG00000171346	ENSG00000186395
ENSG00000171346	ENSG00000186831
ENSG00000171346	ENSG00000186832
ENSG00000171346	ENSG00000186847
ENSG00000171346	ENSG00000187242
ENSG00000171346	ENSG00000187686
ENSG00000171346	ENSG00000196859
ENSG00000171346	ENSG00000197079
ENSG00000171346	ENSG00000204889
ENSG00000171346	ENSG00000204897
ENSG00000171346	ENSG00000213951
ENSG00000171346	ENSG00000214220
ENSG00000171346	ENSG00000214454
ENSG00000171346	ENSG00000214959
ENSG00000171346	ENSG00000215772
ENSG00000171346	ENSG00000215773
ENSG00000171357	ENSG00000153714
ENSG00000171360	ENSG00000006059
ENSG00000171360	ENSG00000094796

ENSG00000171360	ENSG00000108244
ENSG00000171360	ENSG00000108417
ENSG00000171360	ENSG00000108759
ENSG00000171360	ENSG00000111057
ENSG00000171360	ENSG00000126337
ENSG00000171360	ENSG00000128422
ENSG00000171360	ENSG00000131737
ENSG00000171360	ENSG00000131738
ENSG00000171360	ENSG00000131885
ENSG00000171360	ENSG00000167916
ENSG00000171360	ENSG00000170819
ENSG00000171360	ENSG00000171345
ENSG00000171360	ENSG00000171346
ENSG00000171360	ENSG00000171401
ENSG00000171360	ENSG00000171403
ENSG00000171360	ENSG00000171431
ENSG00000171360	ENSG00000171446
ENSG00000171360	ENSG00000173908
ENSG00000171360	ENSG00000186393
ENSG00000171360	ENSG00000186395
ENSG00000171360	ENSG00000186831
ENSG00000171360	ENSG00000186832
ENSG00000171360	ENSG00000186847
ENSG00000171360	ENSG00000187242
ENSG00000171360	ENSG00000187686
ENSG00000171360	ENSG00000196859
ENSG00000171360	ENSG00000197079
ENSG00000171360	ENSG00000204889
ENSG00000171360	ENSG00000204897
ENSG00000171360	ENSG00000213951
ENSG00000171360	ENSG00000214220
ENSG00000171360	ENSG00000214454
ENSG00000171360	ENSG00000214959
ENSG00000171360	ENSG00000215772
ENSG00000171360	ENSG00000215773
ENSG00000171365	ENSG00000011021
ENSG00000171365	ENSG00000073464
ENSG00000171365	ENSG00000103249
ENSG00000171365	ENSG00000109572
ENSG00000171368	ENSG00000159713
ENSG00000171368	ENSG00000179636
ENSG00000171385	ENSG00000026559
ENSG00000171385	ENSG00000102057
ENSG00000171385	ENSG00000104848
ENSG00000171385	ENSG00000111262
ENSG00000171385	ENSG00000124134
ENSG00000171385	ENSG00000130037
ENSG00000171385	ENSG00000143105
ENSG00000171385	ENSG00000151079
ENSG00000171385	ENSG00000156486
ENSG00000171385	ENSG00000158445
ENSG00000171385	ENSG00000162975
ENSG00000171385	ENSG00000164794
ENSG00000171385	ENSG00000168263
ENSG00000171385	ENSG00000168418
ENSG00000171385	ENSG00000170745
ENSG00000171385	ENSG00000171126
ENSG00000171385	ENSG00000177272
ENSG00000171385	ENSG00000177301

ENSG00000171385	ENSG00000178342
ENSG00000171385	ENSG00000182255
ENSG00000171385	ENSG00000182674
ENSG00000171385	ENSG00000184408
ENSG00000171401	ENSG00000006059
ENSG00000171401	ENSG00000094796
ENSG00000171401	ENSG00000108244
ENSG00000171401	ENSG00000108417
ENSG00000171401	ENSG00000108759
ENSG00000171401	ENSG00000111057
ENSG00000171401	ENSG00000126337
ENSG00000171401	ENSG00000128422
ENSG00000171401	ENSG00000131737
ENSG00000171401	ENSG00000131738
ENSG00000171401	ENSG00000131885
ENSG00000171401	ENSG00000167916
ENSG00000171401	ENSG00000170819
ENSG00000171401	ENSG00000171345
ENSG00000171401	ENSG00000171346
ENSG00000171401	ENSG00000171360
ENSG00000171401	ENSG00000171403
ENSG00000171401	ENSG00000171431
ENSG00000171401	ENSG00000171446
ENSG00000171401	ENSG00000173908
ENSG00000171401	ENSG00000186393
ENSG00000171401	ENSG00000186395
ENSG00000171401	ENSG00000186831
ENSG00000171401	ENSG00000186832
ENSG00000171401	ENSG00000186847
ENSG00000171401	ENSG00000187242
ENSG00000171401	ENSG00000187686
ENSG00000171401	ENSG00000196859
ENSG00000171401	ENSG00000197079
ENSG00000171401	ENSG00000204889
ENSG00000171401	ENSG00000204897
ENSG00000171401	ENSG00000213951
ENSG00000171401	ENSG00000214220
ENSG00000171401	ENSG00000214454
ENSG00000171401	ENSG00000214959
ENSG00000171401	ENSG00000215772
ENSG00000171401	ENSG00000215773
ENSG00000171403	ENSG00000006059
ENSG00000171403	ENSG00000094796
ENSG00000171403	ENSG00000108244
ENSG00000171403	ENSG00000108417
ENSG00000171403	ENSG00000108759
ENSG00000171403	ENSG00000111057
ENSG00000171403	ENSG00000126337
ENSG00000171403	ENSG00000128422
ENSG00000171403	ENSG00000131737
ENSG00000171403	ENSG00000131738
ENSG00000171403	ENSG00000131885
ENSG00000171403	ENSG00000167916
ENSG00000171403	ENSG00000170819
ENSG00000171403	ENSG00000171345
ENSG00000171403	ENSG00000171346
ENSG00000171403	ENSG00000171360
ENSG00000171403	ENSG00000171401
ENSG00000171403	ENSG00000171431

ENSG00000171403	ENSG00000171446
ENSG00000171403	ENSG00000173908
ENSG00000171403	ENSG00000186393
ENSG00000171403	ENSG00000186395
ENSG00000171403	ENSG00000186831
ENSG00000171403	ENSG00000186832
ENSG00000171403	ENSG00000186847
ENSG00000171403	ENSG00000187242
ENSG00000171403	ENSG00000187686
ENSG00000171403	ENSG00000196859
ENSG00000171403	ENSG00000197079
ENSG00000171403	ENSG00000204889
ENSG00000171403	ENSG00000204897
ENSG00000171403	ENSG00000213951
ENSG00000171403	ENSG00000214220
ENSG00000171403	ENSG00000214454
ENSG00000171403	ENSG00000214959
ENSG00000171403	ENSG00000215772
ENSG00000171403	ENSG00000215773
ENSG00000171408	ENSG00000065989
ENSG00000171408	ENSG00000073417
ENSG00000171408	ENSG00000105650
ENSG00000171408	ENSG00000113231
ENSG00000171408	ENSG00000113448
ENSG00000171408	ENSG00000115252
ENSG00000171408	ENSG00000123360
ENSG00000171408	ENSG00000154678
ENSG00000171408	ENSG00000160191
ENSG00000171408	ENSG00000184588
ENSG00000171408	ENSG00000205268
ENSG00000171428	ENSG00000156006
ENSG00000171431	ENSG00000006059
ENSG00000171431	ENSG00000094796
ENSG00000171431	ENSG00000108244
ENSG00000171431	ENSG00000108417
ENSG00000171431	ENSG00000108759
ENSG00000171431	ENSG00000111057
ENSG00000171431	ENSG00000126337
ENSG00000171431	ENSG00000128422
ENSG00000171431	ENSG00000131737
ENSG00000171431	ENSG00000131738
ENSG00000171431	ENSG00000131885
ENSG00000171431	ENSG00000167916
ENSG00000171431	ENSG00000170819
ENSG00000171431	ENSG00000171345
ENSG00000171431	ENSG00000171346
ENSG00000171431	ENSG00000171360
ENSG00000171431	ENSG00000171401
ENSG00000171431	ENSG00000171403
ENSG00000171431	ENSG00000171446
ENSG00000171431	ENSG00000173908
ENSG00000171431	ENSG00000186393
ENSG00000171431	ENSG00000186395
ENSG00000171431	ENSG00000186831
ENSG00000171431	ENSG00000186832
ENSG00000171431	ENSG00000186847
ENSG00000171431	ENSG00000187242
ENSG00000171431	ENSG00000187686
ENSG00000171431	ENSG00000196859

ENSG00000171431	ENSG00000197079
ENSG00000171431	ENSG00000204889
ENSG00000171431	ENSG00000204897
ENSG00000171431	ENSG00000213951
ENSG00000171431	ENSG00000214220
ENSG00000171431	ENSG00000214454
ENSG00000171431	ENSG00000214959
ENSG00000171431	ENSG00000215772
ENSG00000171431	ENSG00000215773
ENSG00000171435	ENSG00000078061
ENSG00000171435	ENSG00000132155
ENSG00000171435	ENSG00000141068
ENSG00000171435	ENSG00000157764
ENSG00000171446	ENSG00000006059
ENSG00000171446	ENSG00000094796
ENSG00000171446	ENSG00000108244
ENSG00000171446	ENSG00000108417
ENSG00000171446	ENSG00000108759
ENSG00000171446	ENSG00000111057
ENSG00000171446	ENSG00000126337
ENSG00000171446	ENSG00000128422
ENSG00000171446	ENSG00000131737
ENSG00000171446	ENSG00000131738
ENSG00000171446	ENSG00000131885
ENSG00000171446	ENSG00000167916
ENSG00000171446	ENSG00000170819
ENSG00000171446	ENSG00000171345
ENSG00000171446	ENSG00000171346
ENSG00000171446	ENSG00000171360
ENSG00000171446	ENSG00000171401
ENSG00000171446	ENSG00000171403
ENSG00000171446	ENSG00000171431
ENSG00000171446	ENSG00000173908
ENSG00000171446	ENSG00000186393
ENSG00000171446	ENSG00000186395
ENSG00000171446	ENSG00000186831
ENSG00000171446	ENSG00000186832
ENSG00000171446	ENSG00000186847
ENSG00000171446	ENSG00000187242
ENSG00000171446	ENSG00000187686
ENSG00000171446	ENSG00000196859
ENSG00000171446	ENSG00000197079
ENSG00000171446	ENSG00000204889
ENSG00000171446	ENSG00000204897
ENSG00000171446	ENSG00000213951
ENSG00000171446	ENSG00000214220
ENSG00000171446	ENSG00000214454
ENSG00000171446	ENSG00000214959
ENSG00000171446	ENSG00000215772
ENSG00000171446	ENSG00000215773
ENSG00000171448	ENSG00000186130
ENSG00000171450	ENSG00000176749
ENSG00000171451	ENSG00000111817
ENSG00000171456	ENSG00000143970
ENSG00000171456	ENSG00000215491
ENSG00000171462	ENSG00000090932
ENSG00000171462	ENSG00000101384
ENSG00000171462	ENSG00000128917
ENSG00000171462	ENSG00000184916

ENSG00000171462	ENSG00000185559
ENSG00000171462	ENSG00000198719
ENSG00000171471	ENSG00000034713
ENSG00000171471	ENSG00000101460
ENSG00000171471	ENSG00000140941
ENSG00000171471	ENSG00000197769
ENSG00000171475	ENSG00000115935
ENSG00000171475	ENSG00000122574
ENSG00000171475	ENSG00000205090
ENSG00000171478	ENSG00000090382
ENSG00000171478	ENSG00000120563
ENSG00000171478	ENSG00000141316
ENSG00000171478	ENSG00000151033
ENSG00000171478	ENSG00000157093
ENSG00000171478	ENSG00000161572
ENSG00000171478	ENSG00000167531
ENSG00000171478	ENSG00000171489
ENSG00000171487	ENSG00000022556
ENSG00000171487	ENSG00000023191
ENSG00000171487	ENSG00000140853
ENSG00000171487	ENSG00000142405
ENSG00000171487	ENSG00000158077
ENSG00000171487	ENSG00000160505
ENSG00000171487	ENSG00000162711
ENSG00000171487	ENSG00000167207
ENSG00000171487	ENSG00000167634
ENSG00000171487	ENSG00000167984
ENSG00000171487	ENSG00000173572
ENSG00000171487	ENSG00000174885
ENSG00000171487	ENSG00000179709
ENSG00000171487	ENSG00000179873
ENSG00000171487	ENSG00000182261
ENSG00000171487	ENSG00000185792
ENSG00000171488	ENSG00000136802
ENSG00000171488	ENSG00000171017
ENSG00000171488	ENSG00000171492
ENSG00000171488	ENSG00000197147
ENSG00000171489	ENSG00000090382
ENSG00000171489	ENSG00000120563
ENSG00000171489	ENSG00000141316
ENSG00000171489	ENSG00000151033
ENSG00000171489	ENSG00000157093
ENSG00000171489	ENSG00000161572
ENSG00000171489	ENSG00000167531
ENSG00000171489	ENSG00000171478
ENSG00000171492	ENSG00000136802
ENSG00000171492	ENSG00000171017
ENSG00000171492	ENSG00000171488
ENSG00000171492	ENSG00000197147
ENSG00000171495	ENSG00000179832
ENSG00000171495	ENSG00000185038
ENSG00000171502	ENSG00000196739
ENSG00000171509	ENSG00000133105
ENSG00000171517	ENSG00000064547
ENSG00000171517	ENSG00000118432
ENSG00000171517	ENSG00000125910
ENSG00000171517	ENSG00000132975
ENSG00000171517	ENSG00000146360
ENSG00000171517	ENSG00000170989

ENSG00000171517	ENSG00000175898
ENSG00000171517	ENSG00000180739
ENSG00000171517	ENSG00000181773
ENSG00000171517	ENSG00000188822
ENSG00000171517	ENSG00000198121
ENSG00000171517	ENSG00000213694
ENSG00000171522	ENSG00000006638
ENSG00000171522	ENSG00000050628
ENSG00000171522	ENSG00000122420
ENSG00000171522	ENSG00000125384
ENSG00000171522	ENSG00000160013
ENSG00000171522	ENSG00000160951
ENSG00000171522	ENSG00000168229
ENSG00000171532	ENSG00000104903
ENSG00000171532	ENSG00000113196
ENSG00000171532	ENSG00000122691
ENSG00000171532	ENSG00000122859
ENSG00000171532	ENSG00000123307
ENSG00000171532	ENSG00000125878
ENSG00000171532	ENSG00000146618
ENSG00000171532	ENSG00000162367
ENSG00000171532	ENSG00000162992
ENSG00000171532	ENSG00000164107
ENSG00000171532	ENSG00000164600
ENSG00000171532	ENSG00000168267
ENSG00000171532	ENSG00000171786
ENSG00000171532	ENSG00000172238
ENSG00000171532	ENSG00000177551
ENSG00000171532	ENSG00000178403
ENSG00000171532	ENSG00000179774
ENSG00000171532	ENSG00000180535
ENSG00000171532	ENSG00000181965
ENSG00000171532	ENSG00000186051
ENSG00000171532	ENSG00000187786
ENSG00000171532	ENSG00000188686
ENSG00000171533	ENSG00000180834
ENSG00000171549	ENSG00000137970
ENSG00000171549	ENSG00000146223
ENSG00000171549	ENSG00000147604
ENSG00000171549	ENSG00000169216
ENSG00000171549	ENSG00000181416
ENSG00000171549	ENSG00000182520
ENSG00000171549	ENSG00000214485
ENSG00000171549	ENSG00000214557
ENSG00000171549	ENSG00000214932
ENSG00000171549	ENSG00000215297
ENSG00000171549	ENSG00000215402
ENSG00000171552	ENSG00000030110
ENSG00000171552	ENSG00000087088
ENSG00000171552	ENSG00000129473
ENSG00000171552	ENSG00000140379
ENSG00000171552	ENSG00000143384
ENSG00000171552	ENSG00000171791
ENSG00000171552	ENSG00000176720
ENSG00000171570	ENSG00000129521
ENSG00000171570	ENSG00000135766
ENSG00000171587	ENSG00000069188
ENSG00000171587	ENSG00000146555
ENSG00000171587	ENSG00000177103

ENSG00000171603	ENSG00000139182
ENSG00000171603	ENSG00000158258
ENSG00000171604	ENSG00000168772
ENSG00000171608	ENSG00000011405
ENSG00000171608	ENSG00000051382
ENSG00000171608	ENSG00000105851
ENSG00000171608	ENSG00000121879
ENSG00000171608	ENSG00000133056
ENSG00000171608	ENSG00000139144
ENSG00000171612	ENSG00000114120
ENSG00000171617	ENSG00000162413
ENSG00000171617	ENSG00000168427
ENSG00000171617	ENSG00000175946
ENSG00000171617	ENSG00000182359
ENSG00000171617	ENSG00000183655
ENSG00000171617	ENSG00000213160
ENSG00000171621	ENSG00000111671
ENSG00000171621	ENSG00000162032
ENSG00000171621	ENSG00000174013
ENSG00000171621	ENSG00000175093
ENSG00000171631	ENSG00000125245
ENSG00000171631	ENSG00000126251
ENSG00000171631	ENSG00000126262
ENSG00000171631	ENSG00000144230
ENSG00000171631	ENSG00000147138
ENSG00000171631	ENSG00000162881
ENSG00000171631	ENSG00000164220
ENSG00000171631	ENSG00000164251
ENSG00000171631	ENSG00000165621
ENSG00000171631	ENSG00000181104
ENSG00000171631	ENSG00000182162
ENSG00000171631	ENSG00000182559
ENSG00000171631	ENSG00000182782
ENSG00000171631	ENSG00000185897
ENSG00000171631	ENSG00000196917
ENSG00000171643	ENSG00000143546
ENSG00000171643	ENSG00000160307
ENSG00000171643	ENSG00000160678
ENSG00000171643	ENSG00000163191
ENSG00000171643	ENSG00000163220
ENSG00000171643	ENSG00000163221
ENSG00000171643	ENSG00000163993
ENSG00000171643	ENSG00000169906
ENSG00000171643	ENSG00000188015
ENSG00000171643	ENSG00000188643
ENSG00000171643	ENSG00000189171
ENSG00000171643	ENSG00000189334
ENSG00000171643	ENSG00000196154
ENSG00000171643	ENSG00000196420
ENSG00000171643	ENSG00000196754
ENSG00000171643	ENSG00000197747
ENSG00000171643	ENSG00000197956
ENSG00000171656	ENSG00000006468
ENSG00000171656	ENSG00000105672
ENSG00000171656	ENSG00000134954
ENSG00000171656	ENSG00000157557
ENSG00000171656	ENSG00000163497
ENSG00000171656	ENSG00000175832
ENSG00000171659	ENSG00000138271

ENSG00000171659	ENSG00000169313
ENSG00000171659	ENSG00000174944
ENSG00000171659	ENSG00000174946
ENSG00000171659	ENSG00000181631
ENSG00000171680	ENSG00000008323
ENSG00000171680	ENSG00000187510
ENSG00000171681	ENSG00000166669
ENSG00000171700	ENSG00000090104
ENSG00000171700	ENSG00000091844
ENSG00000171700	ENSG00000116741
ENSG00000171700	ENSG00000117152
ENSG00000171700	ENSG00000127074
ENSG00000171700	ENSG00000135824
ENSG00000171700	ENSG00000143248
ENSG00000171700	ENSG00000143333
ENSG00000171700	ENSG00000147509
ENSG00000171700	ENSG00000148908
ENSG00000171700	ENSG00000150681
ENSG00000171700	ENSG00000215825
ENSG00000171703	ENSG00000176896
ENSG00000171703	ENSG00000187735
ENSG00000171703	ENSG00000204219
ENSG00000171714	ENSG00000047617
ENSG00000171714	ENSG00000074855
ENSG00000171714	ENSG00000131620
ENSG00000171714	ENSG00000134343
ENSG00000171714	ENSG00000146205
ENSG00000171714	ENSG00000151572
ENSG00000171714	ENSG00000160746
ENSG00000171714	ENSG00000177119
ENSG00000171714	ENSG00000185101
ENSG00000171735	ENSG00000108509
ENSG00000171747	ENSG00000006659
ENSG00000171747	ENSG00000100079
ENSG00000171747	ENSG00000100097
ENSG00000171747	ENSG00000105198
ENSG00000171747	ENSG00000105205
ENSG00000171747	ENSG00000116977
ENSG00000171747	ENSG00000119862
ENSG00000171747	ENSG00000131981
ENSG00000171747	ENSG00000133317
ENSG00000171747	ENSG00000168961
ENSG00000171747	ENSG00000170298
ENSG00000171747	ENSG00000171916
ENSG00000171747	ENSG00000178934
ENSG00000171747	ENSG00000197778
ENSG00000171747	ENSG00000205076
ENSG00000171747	ENSG00000215132
ENSG00000171773	ENSG00000130045
ENSG00000171773	ENSG00000167693
ENSG00000171777	ENSG00000068831
ENSG00000171777	ENSG00000152689
ENSG00000171777	ENSG00000172575
ENSG00000171786	ENSG00000104903
ENSG00000171786	ENSG00000113196
ENSG00000171786	ENSG00000122691
ENSG00000171786	ENSG00000122859
ENSG00000171786	ENSG00000123307
ENSG00000171786	ENSG00000125878

ENSG00000171786	ENSG00000146618
ENSG00000171786	ENSG00000162367
ENSG00000171786	ENSG00000162992
ENSG00000171786	ENSG00000164107
ENSG00000171786	ENSG00000164600
ENSG00000171786	ENSG00000168267
ENSG00000171786	ENSG00000171532
ENSG00000171786	ENSG00000172238
ENSG00000171786	ENSG00000177551
ENSG00000171786	ENSG00000178403
ENSG00000171786	ENSG00000179774
ENSG00000171786	ENSG00000180535
ENSG00000171786	ENSG00000181965
ENSG00000171786	ENSG00000186051
ENSG00000171786	ENSG00000187786
ENSG00000171786	ENSG00000188686
ENSG00000171791	ENSG00000030110
ENSG00000171791	ENSG00000087088
ENSG00000171791	ENSG00000129473
ENSG00000171791	ENSG00000140379
ENSG00000171791	ENSG00000143384
ENSG00000171791	ENSG00000171552
ENSG00000171791	ENSG00000176720
ENSG00000171793	ENSG00000047230
ENSG00000171812	ENSG00000123500
ENSG00000171812	ENSG00000144810
ENSG00000171813	ENSG00000170234
ENSG00000171815	ENSG00000081818
ENSG00000171815	ENSG00000081842
ENSG00000171815	ENSG00000081853
ENSG00000171815	ENSG00000112852
ENSG00000171815	ENSG00000113205
ENSG00000171815	ENSG00000113209
ENSG00000171815	ENSG00000113211
ENSG00000171815	ENSG00000113212
ENSG00000171815	ENSG00000113248
ENSG00000171815	ENSG00000120322
ENSG00000171815	ENSG00000120324
ENSG00000171815	ENSG00000120327
ENSG00000171815	ENSG00000120328
ENSG00000171815	ENSG00000146001
ENSG00000171815	ENSG00000187372
ENSG00000171815	ENSG00000196963
ENSG00000171815	ENSG00000197479
ENSG00000171815	ENSG00000204955
ENSG00000171815	ENSG00000204956
ENSG00000171815	ENSG00000204961
ENSG00000171815	ENSG00000204962
ENSG00000171815	ENSG00000204963
ENSG00000171815	ENSG00000204964
ENSG00000171815	ENSG00000204965
ENSG00000171815	ENSG00000204967
ENSG00000171815	ENSG00000204968
ENSG00000171815	ENSG00000204969
ENSG00000171815	ENSG00000204970
ENSG00000171815	ENSG00000214567
ENSG00000171815	ENSG00000214570
ENSG00000171815	ENSG00000214574
ENSG00000171815	ENSG00000214580

ENSG00000171815	ENSG00000214583
ENSG00000171815	ENSG00000214594
ENSG00000171815	ENSG00000214600
ENSG00000171815	ENSG00000214608
ENSG00000171840	ENSG00000131669
ENSG00000171843	ENSG00000127337
ENSG00000171843	ENSG00000130382
ENSG00000171843	ENSG00000163872
ENSG00000171848	ENSG00000048392
ENSG00000171860	ENSG00000134830
ENSG00000171860	ENSG00000171049
ENSG00000171860	ENSG00000171051
ENSG00000171860	ENSG00000174600
ENSG00000171860	ENSG00000183134
ENSG00000171860	ENSG00000183671
ENSG00000171860	ENSG00000187474
ENSG00000171860	ENSG00000197405
ENSG00000171860	ENSG00000213903
ENSG00000171860	ENSG00000213906
ENSG00000171863	ENSG00000162596
ENSG00000171863	ENSG00000174121
ENSG00000171863	ENSG00000183405
ENSG00000171863	ENSG00000215342
ENSG00000171863	ENSG00000215804
ENSG00000171873	ENSG00000120907
ENSG00000171873	ENSG00000143147
ENSG00000171873	ENSG00000148680
ENSG00000171873	ENSG00000165370
ENSG00000171873	ENSG00000170214
ENSG00000171873	ENSG00000183935
ENSG00000171877	ENSG00000172159
ENSG00000171878	ENSG00000087086
ENSG00000171878	ENSG00000132446
ENSG00000171878	ENSG00000167996
ENSG00000171878	ENSG00000173477
ENSG00000171878	ENSG00000176778
ENSG00000171878	ENSG00000180795
ENSG00000171878	ENSG00000181867
ENSG00000171878	ENSG00000197518
ENSG00000171878	ENSG00000215234
ENSG00000171885	ENSG00000086159
ENSG00000171885	ENSG00000103375
ENSG00000171885	ENSG00000106125
ENSG00000171885	ENSG00000135517
ENSG00000171885	ENSG00000161798
ENSG00000171885	ENSG00000167580
ENSG00000171903	ENSG00000142973
ENSG00000171903	ENSG00000145476
ENSG00000171903	ENSG00000154198
ENSG00000171903	ENSG00000162365
ENSG00000171903	ENSG00000171954
ENSG00000171903	ENSG00000186115
ENSG00000171903	ENSG00000186160
ENSG00000171903	ENSG00000186204
ENSG00000171903	ENSG00000186377
ENSG00000171903	ENSG00000186526
ENSG00000171903	ENSG00000186529
ENSG00000171903	ENSG00000187048
ENSG00000171914	ENSG00000073712

ENSG00000171914	ENSG00000101311
ENSG00000171914	ENSG00000137076
ENSG00000171914	ENSG00000149781
ENSG00000171916	ENSG00000006659
ENSG00000171916	ENSG00000100079
ENSG00000171916	ENSG00000100097
ENSG00000171916	ENSG00000105198
ENSG00000171916	ENSG00000105205
ENSG00000171916	ENSG00000116977
ENSG00000171916	ENSG00000119862
ENSG00000171916	ENSG00000131981
ENSG00000171916	ENSG00000133317
ENSG00000171916	ENSG00000168961
ENSG00000171916	ENSG00000170298
ENSG00000171916	ENSG00000171747
ENSG00000171916	ENSG00000178934
ENSG00000171916	ENSG00000197778
ENSG00000171916	ENSG00000205076
ENSG00000171916	ENSG00000215132
ENSG00000171928	ENSG00000108442
ENSG00000171928	ENSG00000166676
ENSG00000171940	ENSG00000101493
ENSG00000171940	ENSG00000165804
ENSG00000171940	ENSG00000198597
ENSG00000171954	ENSG00000142973
ENSG00000171954	ENSG00000145476
ENSG00000171954	ENSG00000154198
ENSG00000171954	ENSG00000162365
ENSG00000171954	ENSG00000171903
ENSG00000171954	ENSG00000186115
ENSG00000171954	ENSG00000186160
ENSG00000171954	ENSG00000186204
ENSG00000171954	ENSG00000186377
ENSG00000171954	ENSG00000186526
ENSG00000171954	ENSG00000186529
ENSG00000171954	ENSG00000187048
ENSG00000171988	ENSG00000115548
ENSG00000171988	ENSG00000120733
ENSG00000171988	ENSG00000168453
ENSG00000171989	ENSG00000111716
ENSG00000171989	ENSG00000134333
ENSG00000171989	ENSG00000151116
ENSG00000171989	ENSG00000166796
ENSG00000171989	ENSG00000166800
ENSG00000171992	ENSG00000166317
ENSG00000171992	ENSG00000172403
ENSG00000172005	ENSG00000102934
ENSG00000172005	ENSG00000144063
ENSG00000172005	ENSG00000147676
ENSG00000172005	ENSG00000155254
ENSG00000172005	ENSG00000170293
ENSG00000172007	ENSG00000134594
ENSG00000172007	ENSG00000137502
ENSG00000172007	ENSG00000138069
ENSG00000172007	ENSG00000146955
ENSG00000172007	ENSG00000174903
ENSG00000172007	ENSG00000185051
ENSG00000172009	ENSG00000123213
ENSG00000172018	ENSG00000105722

ENSG00000172031	ENSG00000105131
ENSG00000172037	ENSG00000050555
ENSG00000172037	ENSG00000058085
ENSG00000172037	ENSG00000074527
ENSG00000172037	ENSG00000091128
ENSG00000172037	ENSG00000091136
ENSG00000172037	ENSG00000106780
ENSG00000172037	ENSG00000135862
ENSG00000172037	ENSG00000196878
ENSG00000172057	ENSG00000123353
ENSG00000172057	ENSG00000128699
ENSG00000172059	ENSG00000067082
ENSG00000172059	ENSG00000102554
ENSG00000172059	ENSG00000105610
ENSG00000172059	ENSG00000127528
ENSG00000172059	ENSG00000129911
ENSG00000172059	ENSG00000136826
ENSG00000172059	ENSG00000139651
ENSG00000172059	ENSG00000155090
ENSG00000172059	ENSG00000164651
ENSG00000172059	ENSG00000167182
ENSG00000172059	ENSG00000170374
ENSG00000172059	ENSG00000171872
ENSG00000172059	ENSG00000174595
ENSG00000172059	ENSG00000189120
ENSG00000172059	ENSG00000197294
ENSG00000172059	ENSG00000197550
ENSG00000172059	ENSG00000204335
ENSG00000172061	ENSG00000099769
ENSG00000172061	ENSG00000178732
ENSG00000172061	ENSG00000178772
ENSG00000172062	ENSG00000205571
ENSG00000172081	ENSG00000114978
ENSG00000172081	ENSG00000120162
ENSG00000172081	ENSG00000142961
ENSG00000172081	ENSG00000173542
ENSG00000172081	ENSG00000182208
ENSG00000172115	ENSG00000174440
ENSG00000172115	ENSG00000188512
ENSG00000172115	ENSG00000214810
ENSG00000172137	ENSG00000079689
ENSG00000172137	ENSG00000104327
ENSG00000172156	ENSG00000006074
ENSG00000172156	ENSG00000006075
ENSG00000172156	ENSG00000006606
ENSG00000172156	ENSG00000102962
ENSG00000172156	ENSG00000102970
ENSG00000172156	ENSG00000106178
ENSG00000172156	ENSG00000108688
ENSG00000172156	ENSG00000108691
ENSG00000172156	ENSG00000108700
ENSG00000172156	ENSG00000108702
ENSG00000172156	ENSG00000115009
ENSG00000172156	ENSG00000129277
ENSG00000172156	ENSG00000143184
ENSG00000172156	ENSG00000143185
ENSG00000172156	ENSG00000161570
ENSG00000172156	ENSG00000161573
ENSG00000172156	ENSG00000161574

ENSG00000172156	ENSG00000167236
ENSG00000172156	ENSG00000172724
ENSG00000172156	ENSG00000181374
ENSG00000172156	ENSG00000197262
ENSG00000172156	ENSG00000205017
ENSG00000172156	ENSG00000205020
ENSG00000172156	ENSG00000205021
ENSG00000172156	ENSG00000213494
ENSG00000172156	ENSG00000215627
ENSG00000172156	ENSG00000215629
ENSG00000172159	ENSG00000171877
ENSG00000172164	ENSG00000047932
ENSG00000172164	ENSG00000101400
ENSG00000172164	ENSG00000147481
ENSG00000172164	ENSG00000168807
ENSG00000172164	ENSG00000172554
ENSG00000172179	ENSG00000136487
ENSG00000172179	ENSG00000136488
ENSG00000172179	ENSG00000189162
ENSG00000172179	ENSG00000204414
ENSG00000172179	ENSG00000213218
ENSG00000172183	ENSG00000143319
ENSG00000172183	ENSG00000181026
ENSG00000172186	ENSG00000157576
ENSG00000172186	ENSG00000182765
ENSG00000172186	ENSG00000184409
ENSG00000172186	ENSG00000197366
ENSG00000172186	ENSG00000198157
ENSG00000172186	ENSG00000198875
ENSG00000172197	ENSG00000143797
ENSG00000172197	ENSG00000177669
ENSG00000172201	ENSG00000115738
ENSG00000172201	ENSG00000117318
ENSG00000172201	ENSG00000125968
ENSG00000172215	ENSG00000112486
ENSG00000172215	ENSG00000121797
ENSG00000172215	ENSG00000121807
ENSG00000172215	ENSG00000121966
ENSG00000172215	ENSG00000126353
ENSG00000172215	ENSG00000129048
ENSG00000172215	ENSG00000144648
ENSG00000172215	ENSG00000160683
ENSG00000172215	ENSG00000160791
ENSG00000172215	ENSG00000163464
ENSG00000172215	ENSG00000163823
ENSG00000172215	ENSG00000168329
ENSG00000172215	ENSG00000173578
ENSG00000172215	ENSG00000173585
ENSG00000172215	ENSG00000179934
ENSG00000172215	ENSG00000180871
ENSG00000172215	ENSG00000183625
ENSG00000172215	ENSG00000183813
ENSG00000172215	ENSG00000184451
ENSG00000172215	ENSG00000186810
ENSG00000172215	ENSG00000215775
ENSG00000172215	ENSG00000215778
ENSG00000172215	ENSG00000215782
ENSG00000172216	ENSG00000092067
ENSG00000172216	ENSG00000180733

ENSG00000172236	ENSG00000005001
ENSG00000172236	ENSG00000007038
ENSG00000172236	ENSG00000052344
ENSG00000172236	ENSG00000095917
ENSG00000172236	ENSG00000103355
ENSG00000172236	ENSG00000116176
ENSG00000172236	ENSG00000172382
ENSG00000172236	ENSG00000172460
ENSG00000172236	ENSG00000178055
ENSG00000172236	ENSG00000178226
ENSG00000172236	ENSG00000185888
ENSG00000172236	ENSG00000188086
ENSG00000172236	ENSG00000189099
ENSG00000172236	ENSG00000196364
ENSG00000172236	ENSG00000197253
ENSG00000172236	ENSG00000206549
ENSG00000172236	ENSG00000214877
ENSG00000172236	ENSG00000215148
ENSG00000172260	ENSG00000142549
ENSG00000172260	ENSG00000182667
ENSG00000172260	ENSG00000183715
ENSG00000172260	ENSG00000185565
ENSG00000172264	ENSG00000133315
ENSG00000172270	ENSG00000156642
ENSG00000172270	ENSG00000170571
ENSG00000172288	ENSG00000129873
ENSG00000172288	ENSG00000153046
ENSG00000172288	ENSG00000166446
ENSG00000172288	ENSG00000172352
ENSG00000172288	ENSG00000182415
ENSG00000172288	ENSG00000198721
ENSG00000172288	ENSG00000215563
ENSG00000172292	ENSG00000065308
ENSG00000172292	ENSG00000067167
ENSG00000172292	ENSG00000090661
ENSG00000172292	ENSG00000139624
ENSG00000172292	ENSG00000143418
ENSG00000172292	ENSG00000154227
ENSG00000172292	ENSG00000174599
ENSG00000172296	ENSG00000100596
ENSG00000172318	ENSG00000156966
ENSG00000172318	ENSG00000162630
ENSG00000172318	ENSG00000169255
ENSG00000172318	ENSG00000170340
ENSG00000172318	ENSG00000176383
ENSG00000172318	ENSG00000176597
ENSG00000172318	ENSG00000177191
ENSG00000172318	ENSG00000179913
ENSG00000172318	ENSG00000180561
ENSG00000172318	ENSG00000183778
ENSG00000172318	ENSG00000198488
ENSG00000172318	ENSG00000204222
ENSG00000172318	ENSG00000214654
ENSG00000172346	ENSG00000153048
ENSG00000172348	ENSG00000117602
ENSG00000172348	ENSG00000159200
ENSG00000172349	ENSG00000133401
ENSG00000172350	ENSG00000160179
ENSG00000172352	ENSG00000129873

ENSG00000172352	ENSG00000153046
ENSG00000172352	ENSG00000166446
ENSG00000172352	ENSG00000172288
ENSG00000172352	ENSG00000182415
ENSG00000172352	ENSG00000198721
ENSG00000172352	ENSG00000215563
ENSG00000172354	ENSG00000069966
ENSG00000172354	ENSG00000078369
ENSG00000172354	ENSG00000111664
ENSG00000172354	ENSG00000114450
ENSG00000172367	ENSG00000065054
ENSG00000172367	ENSG00000109062
ENSG00000172367	ENSG00000174827
ENSG00000172367	ENSG00000215859
ENSG00000172367	ENSG00000215860
ENSG00000172375	ENSG00000157617
ENSG00000172379	ENSG00000029153
ENSG00000172379	ENSG00000133794
ENSG00000172379	ENSG00000143437
ENSG00000172380	ENSG00000127588
ENSG00000172380	ENSG00000127920
ENSG00000172380	ENSG00000127928
ENSG00000172380	ENSG00000133136
ENSG00000172380	ENSG00000162188
ENSG00000172380	ENSG00000167083
ENSG00000172380	ENSG00000167414
ENSG00000172380	ENSG00000168243
ENSG00000172380	ENSG00000174021
ENSG00000172380	ENSG00000182625
ENSG00000172380	ENSG00000186469
ENSG00000172380	ENSG00000213611
ENSG00000172382	ENSG00000005001
ENSG00000172382	ENSG00000007038
ENSG00000172382	ENSG00000052344
ENSG00000172382	ENSG00000095917
ENSG00000172382	ENSG00000103355
ENSG00000172382	ENSG00000116176
ENSG00000172382	ENSG00000172236
ENSG00000172382	ENSG00000172460
ENSG00000172382	ENSG00000178055
ENSG00000172382	ENSG00000178226
ENSG00000172382	ENSG00000185888
ENSG00000172382	ENSG00000188086
ENSG00000172382	ENSG00000189099
ENSG00000172382	ENSG00000196364
ENSG00000172382	ENSG00000197253
ENSG00000172382	ENSG00000206549
ENSG00000172382	ENSG00000214877
ENSG00000172382	ENSG00000215148
ENSG00000172399	ENSG00000164591
ENSG00000172399	ENSG00000177791
ENSG00000172403	ENSG00000166317
ENSG00000172403	ENSG00000171992
ENSG00000172404	ENSG00000105993
ENSG00000172404	ENSG00000135924
ENSG00000172404	ENSG00000179407
ENSG00000172410	ENSG00000107014
ENSG00000172410	ENSG00000107018
ENSG00000172410	ENSG00000120211

ENSG00000172410	ENSG00000171136
ENSG00000172450	ENSG00000101812
ENSG00000172450	ENSG00000123569
ENSG00000172450	ENSG00000124635
ENSG00000172450	ENSG00000146047
ENSG00000172450	ENSG00000158373
ENSG00000172450	ENSG00000168242
ENSG00000172450	ENSG00000174258
ENSG00000172450	ENSG00000180596
ENSG00000172450	ENSG00000184678
ENSG00000172450	ENSG00000185130
ENSG00000172450	ENSG00000187990
ENSG00000172450	ENSG00000196226
ENSG00000172450	ENSG00000196331
ENSG00000172450	ENSG00000196374
ENSG00000172450	ENSG00000196501
ENSG00000172450	ENSG00000196890
ENSG00000172450	ENSG00000197034
ENSG00000172450	ENSG00000197459
ENSG00000172450	ENSG00000197597
ENSG00000172450	ENSG00000197697
ENSG00000172450	ENSG00000197846
ENSG00000172450	ENSG00000197903
ENSG00000172450	ENSG00000203814
ENSG00000172460	ENSG00000005001
ENSG00000172460	ENSG00000007038
ENSG00000172460	ENSG00000052344
ENSG00000172460	ENSG00000095917
ENSG00000172460	ENSG00000103355
ENSG00000172460	ENSG00000116176
ENSG00000172460	ENSG00000172236
ENSG00000172460	ENSG00000172382
ENSG00000172460	ENSG00000178055
ENSG00000172460	ENSG00000178226
ENSG00000172460	ENSG00000185888
ENSG00000172460	ENSG00000188086
ENSG00000172460	ENSG00000189099
ENSG00000172460	ENSG00000196364
ENSG00000172460	ENSG00000197253
ENSG00000172460	ENSG00000206549
ENSG00000172460	ENSG00000214877
ENSG00000172460	ENSG00000215148
ENSG00000172461	ENSG00000130383
ENSG00000172461	ENSG00000156413
ENSG00000172461	ENSG00000171124
ENSG00000172461	ENSG00000172728
ENSG00000172461	ENSG00000180549
ENSG00000172461	ENSG00000196371
ENSG00000172461	ENSG00000196968
ENSG00000172469	ENSG00000185090
ENSG00000172476	ENSG00000102128
ENSG00000172476	ENSG00000141542
ENSG00000172476	ENSG00000197562
ENSG00000172493	ENSG00000072364
ENSG00000172493	ENSG00000144218
ENSG00000172493	ENSG00000155966
ENSG00000172497	ENSG00000162390
ENSG00000172534	ENSG00000111727
ENSG00000172548	ENSG00000001461

ENSG00000172548	ENSG00000104361
ENSG00000172548	ENSG00000140157
ENSG00000172548	ENSG00000163293
ENSG00000172548	ENSG00000170113
ENSG00000172554	ENSG00000047932
ENSG00000172554	ENSG00000101400
ENSG00000172554	ENSG00000147481
ENSG00000172554	ENSG00000168807
ENSG00000172554	ENSG00000172164
ENSG00000172572	ENSG00000152270
ENSG00000172575	ENSG00000068831
ENSG00000172575	ENSG00000152689
ENSG00000172575	ENSG00000171777
ENSG00000172578	ENSG00000114796
ENSG00000172578	ENSG00000119771
ENSG00000172578	ENSG00000149243
ENSG00000172602	ENSG00000108830
ENSG00000172602	ENSG00000115963
ENSG00000172613	ENSG00000151164
ENSG00000172638	ENSG00000049323
ENSG00000172638	ENSG00000077942
ENSG00000172638	ENSG00000090006
ENSG00000172638	ENSG00000115380
ENSG00000172638	ENSG00000119681
ENSG00000172638	ENSG00000140092
ENSG00000172638	ENSG00000163520
ENSG00000172638	ENSG00000168056
ENSG00000172638	ENSG00000214536
ENSG00000172647	ENSG00000096088
ENSG00000172647	ENSG00000117984
ENSG00000172647	ENSG00000131400
ENSG00000172647	ENSG00000131401
ENSG00000172647	ENSG00000143839
ENSG00000172647	ENSG00000180860
ENSG00000172647	ENSG00000184488
ENSG00000172647	ENSG00000196188
ENSG00000172647	ENSG00000204973
ENSG00000172647	ENSG00000215868
ENSG00000172650	ENSG00000108262
ENSG00000172650	ENSG00000133612
ENSG00000172650	ENSG00000135439
ENSG00000172650	ENSG00000139436
ENSG00000172650	ENSG00000151303
ENSG00000172650	ENSG00000157985
ENSG00000172650	ENSG00000174194
ENSG00000172650	ENSG00000188234
ENSG00000172650	ENSG00000198035
ENSG00000172650	ENSG00000204149
ENSG00000172650	ENSG00000204169
ENSG00000172650	ENSG00000204172
ENSG00000172660	ENSG00000089280
ENSG00000172660	ENSG00000182944
ENSG00000172660	ENSG00000215835
ENSG00000172667	ENSG00000113761
ENSG00000172667	ENSG00000165061
ENSG00000172673	ENSG00000130775
ENSG00000172724	ENSG00000006074
ENSG00000172724	ENSG00000006075
ENSG00000172724	ENSG00000006606

ENSG00000172724	ENSG00000102962
ENSG00000172724	ENSG00000102970
ENSG00000172724	ENSG00000106178
ENSG00000172724	ENSG00000108688
ENSG00000172724	ENSG00000108691
ENSG00000172724	ENSG00000108700
ENSG00000172724	ENSG00000108702
ENSG00000172724	ENSG00000115009
ENSG00000172724	ENSG00000129277
ENSG00000172724	ENSG00000143184
ENSG00000172724	ENSG00000143185
ENSG00000172724	ENSG00000161570
ENSG00000172724	ENSG00000161573
ENSG00000172724	ENSG00000161574
ENSG00000172724	ENSG00000167236
ENSG00000172724	ENSG00000172156
ENSG00000172724	ENSG00000181374
ENSG00000172724	ENSG00000197262
ENSG00000172724	ENSG00000205017
ENSG00000172724	ENSG00000205020
ENSG00000172724	ENSG00000205021
ENSG00000172724	ENSG00000213494
ENSG00000172724	ENSG00000215627
ENSG00000172724	ENSG00000215629
ENSG00000172725	ENSG00000102879
ENSG00000172725	ENSG00000103426
ENSG00000172725	ENSG00000103647
ENSG00000172725	ENSG00000106789
ENSG00000172725	ENSG00000110880
ENSG00000172725	ENSG00000167549
ENSG00000172733	ENSG00000146676
ENSG00000172733	ENSG00000185129
ENSG00000172757	ENSG00000125868
ENSG00000172757	ENSG00000165410
ENSG00000172765	ENSG00000057704
ENSG00000172765	ENSG00000102080
ENSG00000172765	ENSG00000133069
ENSG00000172765	ENSG00000182242
ENSG00000172765	ENSG00000185254
ENSG00000172766	ENSG00000164134
ENSG00000172786	ENSG00000156482
ENSG00000172786	ENSG00000173180
ENSG00000172789	ENSG00000037965
ENSG00000172789	ENSG00000106004
ENSG00000172789	ENSG00000106006
ENSG00000172789	ENSG00000108511
ENSG00000172789	ENSG00000120068
ENSG00000172789	ENSG00000120075
ENSG00000172789	ENSG00000120087
ENSG00000172789	ENSG00000122592
ENSG00000172789	ENSG00000170166
ENSG00000172789	ENSG00000175879
ENSG00000172789	ENSG00000182742
ENSG00000172789	ENSG00000197576
ENSG00000172789	ENSG00000197757
ENSG00000172789	ENSG00000198353
ENSG00000172794	ENSG00000167964
ENSG00000172803	ENSG00000028528
ENSG00000172803	ENSG00000089006

ENSG00000172803	ENSG00000129515
ENSG00000172803	ENSG00000205302
ENSG00000172817	ENSG00000124212
ENSG00000172817	ENSG00000146233
ENSG00000172817	ENSG00000167910
ENSG00000172817	ENSG00000180432
ENSG00000172818	ENSG00000125850
ENSG00000172819	ENSG00000077092
ENSG00000172819	ENSG00000126351
ENSG00000172819	ENSG00000126368
ENSG00000172819	ENSG00000131759
ENSG00000172819	ENSG00000151090
ENSG00000172819	ENSG00000174738
ENSG00000172830	ENSG00000084112
ENSG00000172830	ENSG00000141298
ENSG00000172840	ENSG00000164951
ENSG00000172845	ENSG00000105866
ENSG00000172845	ENSG00000185591
ENSG00000172867	ENSG00000135443
ENSG00000172867	ENSG00000135477
ENSG00000172867	ENSG00000135480
ENSG00000172867	ENSG00000139648
ENSG00000172867	ENSG00000161849
ENSG00000172867	ENSG00000161850
ENSG00000172867	ENSG00000167767
ENSG00000172867	ENSG00000167768
ENSG00000172867	ENSG00000170421
ENSG00000172867	ENSG00000170423
ENSG00000172867	ENSG00000170442
ENSG00000172867	ENSG00000170454
ENSG00000172867	ENSG00000170465
ENSG00000172867	ENSG00000170477
ENSG00000172867	ENSG00000170484
ENSG00000172867	ENSG00000170486
ENSG00000172867	ENSG00000170523
ENSG00000172867	ENSG00000185069
ENSG00000172867	ENSG00000185479
ENSG00000172867	ENSG00000185640
ENSG00000172867	ENSG00000186049
ENSG00000172867	ENSG00000186081
ENSG00000172867	ENSG00000186442
ENSG00000172867	ENSG00000189182
ENSG00000172867	ENSG00000205420
ENSG00000172867	ENSG00000205426
ENSG00000172867	ENSG00000214233
ENSG00000172867	ENSG00000214323
ENSG00000172867	ENSG00000214477
ENSG00000172867	ENSG00000214752
ENSG00000172867	ENSG00000214974
ENSG00000172867	ENSG00000215287
ENSG00000172867	ENSG00000215847
ENSG00000172869	ENSG00000104093
ENSG00000172912	ENSG00000126267
ENSG00000172912	ENSG00000160471
ENSG00000172915	ENSG00000198589
ENSG00000172932	ENSG00000076513
ENSG00000172932	ENSG00000118454
ENSG00000172932	ENSG00000198720
ENSG00000172939	ENSG00000198648

ENSG00000172943	ENSG00000006459
ENSG00000172943	ENSG00000089094
ENSG00000172943	ENSG00000173120
ENSG00000172943	ENSG00000197724
ENSG00000172955	ENSG00000187758
ENSG00000172955	ENSG00000196344
ENSG00000172955	ENSG00000196616
ENSG00000172955	ENSG00000197894
ENSG00000172955	ENSG00000198099
ENSG00000172967	ENSG00000047597
ENSG00000172967	ENSG00000182489
ENSG00000172971	ENSG00000110057
ENSG00000172971	ENSG00000112494
ENSG00000172971	ENSG00000155070
ENSG00000172971	ENSG00000184795
ENSG00000172974	ENSG00000078668
ENSG00000172974	ENSG00000165637
ENSG00000172974	ENSG00000213585
ENSG00000172974	ENSG00000215818
ENSG00000172985	ENSG00000154447
ENSG00000172985	ENSG00000156463
ENSG00000172986	ENSG00000151233
ENSG00000172986	ENSG00000173950
ENSG00000172987	ENSG00000173083
ENSG00000172995	ENSG00000048991
ENSG00000172995	ENSG00000179912
ENSG00000173020	ENSG00000100077
ENSG00000173020	ENSG00000114124
ENSG00000173020	ENSG00000125388
ENSG00000173020	ENSG00000185974
ENSG00000173020	ENSG00000198055
ENSG00000173020	ENSG00000198873
ENSG00000173039	ENSG00000077150
ENSG00000173039	ENSG00000104856
ENSG00000173039	ENSG00000109320
ENSG00000173039	ENSG00000162924
ENSG00000173065	ENSG00000139438
ENSG00000173068	ENSG00000169594
ENSG00000173080	ENSG00000100739
ENSG00000173080	ENSG00000134817
ENSG00000173080	ENSG00000144891
ENSG00000173080	ENSG00000154165
ENSG00000173080	ENSG00000168398
ENSG00000173080	ENSG00000170128
ENSG00000173080	ENSG00000180772
ENSG00000173080	ENSG00000182631
ENSG00000173083	ENSG00000172987
ENSG00000173110	ENSG00000109971
ENSG00000173110	ENSG00000126803
ENSG00000173110	ENSG00000204388
ENSG00000173110	ENSG00000204389
ENSG00000173110	ENSG00000204390
ENSG00000173114	ENSG00000128594
ENSG00000173114	ENSG00000169783
ENSG00000173114	ENSG00000170382
ENSG00000173114	ENSG00000174482
ENSG00000173114	ENSG00000175928
ENSG00000173114	ENSG00000183014
ENSG00000173114	ENSG00000213171

ENSG00000173120	ENSG00000006459
ENSG00000173120	ENSG00000089094
ENSG00000173120	ENSG00000172943
ENSG00000173120	ENSG00000197724
ENSG00000173126	ENSG00000183036
ENSG00000173153	ENSG00000091831
ENSG00000173153	ENSG00000119715
ENSG00000173153	ENSG00000140009
ENSG00000173153	ENSG00000196482
ENSG00000173157	ENSG00000049192
ENSG00000173157	ENSG00000087116
ENSG00000173157	ENSG00000136378
ENSG00000173157	ENSG00000138316
ENSG00000173157	ENSG00000140470
ENSG00000173157	ENSG00000140873
ENSG00000173157	ENSG00000142303
ENSG00000173157	ENSG00000145536
ENSG00000173157	ENSG00000145808
ENSG00000173157	ENSG00000151388
ENSG00000173157	ENSG00000156140
ENSG00000173157	ENSG00000160323
ENSG00000173157	ENSG00000163638
ENSG00000173157	ENSG00000214644
ENSG00000173157	ENSG00000215743
ENSG00000173166	ENSG00000077420
ENSG00000173166	ENSG00000106070
ENSG00000173166	ENSG00000115290
ENSG00000173166	ENSG00000141738
ENSG00000173171	ENSG00000128654
ENSG00000173171	ENSG00000177034
ENSG00000173175	ENSG00000078295
ENSG00000173175	ENSG00000121281
ENSG00000173175	ENSG00000129467
ENSG00000173175	ENSG00000138031
ENSG00000173175	ENSG00000155897
ENSG00000173175	ENSG00000162104
ENSG00000173175	ENSG00000164742
ENSG00000173175	ENSG00000174233
ENSG00000173180	ENSG00000156482
ENSG00000173180	ENSG00000172786
ENSG00000173193	ENSG00000138496
ENSG00000173193	ENSG00000163659
ENSG00000173193	ENSG00000178685
ENSG00000173198	ENSG00000078589
ENSG00000173198	ENSG00000139679
ENSG00000173198	ENSG00000147145
ENSG00000173198	ENSG00000152207
ENSG00000173198	ENSG00000169403
ENSG00000173198	ENSG00000169508
ENSG00000173198	ENSG00000169860
ENSG00000173198	ENSG00000175591
ENSG00000173198	ENSG00000184574
ENSG00000173198	ENSG00000186912
ENSG00000173198	ENSG00000198829
ENSG00000173207	ENSG00000123975
ENSG00000173207	ENSG00000178556
ENSG00000173208	ENSG00000101986
ENSG00000173208	ENSG00000117528
ENSG00000173209	ENSG00000100591

ENSG00000173210	ENSG00000099204
ENSG00000173210	ENSG00000158856
ENSG00000173210	ENSG00000163995
ENSG00000173213	ENSG00000101162
ENSG00000173213	ENSG00000104833
ENSG00000173213	ENSG00000127589
ENSG00000173213	ENSG00000137267
ENSG00000173213	ENSG00000137285
ENSG00000173213	ENSG00000159247
ENSG00000173213	ENSG00000173876
ENSG00000173213	ENSG00000176014
ENSG00000173213	ENSG00000188229
ENSG00000173213	ENSG00000196230
ENSG00000173213	ENSG00000198211
ENSG00000173213	ENSG00000215801
ENSG00000173213	ENSG00000215813
ENSG00000173213	ENSG00000215814
ENSG00000173218	ENSG00000162738
ENSG00000173230	ENSG00000144674
ENSG00000173253	ENSG00000064218
ENSG00000173253	ENSG00000137090
ENSG00000173253	ENSG00000142025
ENSG00000173253	ENSG00000142700
ENSG00000173253	ENSG00000143006
ENSG00000173253	ENSG00000176399
ENSG00000173261	ENSG00000105427
ENSG00000173261	ENSG00000145287
ENSG00000173262	ENSG00000059804
ENSG00000173262	ENSG00000109667
ENSG00000173262	ENSG00000117394
ENSG00000173262	ENSG00000133460
ENSG00000173262	ENSG00000142583
ENSG00000173262	ENSG00000163581
ENSG00000173262	ENSG00000181856
ENSG00000173262	ENSG00000197241
ENSG00000173264	ENSG00000077585
ENSG00000173264	ENSG00000180998
ENSG00000173267	ENSG00000074317
ENSG00000173267	ENSG00000145335
ENSG00000173273	ENSG00000104064
ENSG00000173273	ENSG00000107854
ENSG00000173273	ENSG00000143458
ENSG00000173276	ENSG00000148411
ENSG00000173276	ENSG00000160877
ENSG00000173276	ENSG00000177125
ENSG00000173322	ENSG00000137154
ENSG00000173322	ENSG00000214908
ENSG00000173327	ENSG00000006432
ENSG00000173327	ENSG00000130758
ENSG00000173327	ENSG00000143674
ENSG00000173334	ENSG00000071575
ENSG00000173334	ENSG00000101255
ENSG00000173334	ENSG00000196182
ENSG00000173335	ENSG00000077984
ENSG00000173335	ENSG00000101435
ENSG00000173335	ENSG00000101439
ENSG00000173335	ENSG00000101441
ENSG00000173335	ENSG00000125815
ENSG00000173335	ENSG00000125823

ENSG00000173335	ENSG00000125831
ENSG00000173335	ENSG00000170367
ENSG00000173335	ENSG00000170369
ENSG00000173335	ENSG00000170373
ENSG00000173335	ENSG00000175315
ENSG00000173335	ENSG00000204658
ENSG00000173335	ENSG00000204662
ENSG00000173335	ENSG00000204663
ENSG00000173338	ENSG00000082482
ENSG00000173338	ENSG00000095981
ENSG00000173338	ENSG00000099337
ENSG00000173338	ENSG00000100433
ENSG00000173338	ENSG00000124249
ENSG00000173338	ENSG00000124780
ENSG00000173338	ENSG00000135750
ENSG00000173338	ENSG00000152315
ENSG00000173338	ENSG00000164626
ENSG00000173338	ENSG00000169427
ENSG00000173338	ENSG00000171303
ENSG00000173338	ENSG00000182450
ENSG00000173338	ENSG00000184261
ENSG00000173366	ENSG00000164342
ENSG00000173366	ENSG00000196664
ENSG00000173369	ENSG00000054803
ENSG00000173369	ENSG00000102924
ENSG00000173369	ENSG00000131094
ENSG00000173369	ENSG00000133466
ENSG00000173369	ENSG00000139899
ENSG00000173369	ENSG00000141668
ENSG00000173369	ENSG00000144119
ENSG00000173369	ENSG00000145861
ENSG00000173369	ENSG00000159189
ENSG00000173369	ENSG00000163145
ENSG00000173369	ENSG00000165985
ENSG00000173369	ENSG00000172247
ENSG00000173369	ENSG00000173372
ENSG00000173369	ENSG00000173918
ENSG00000173369	ENSG00000181092
ENSG00000173369	ENSG00000184471
ENSG00000173369	ENSG00000186897
ENSG00000173369	ENSG00000213192
ENSG00000173372	ENSG00000054803
ENSG00000173372	ENSG00000102924
ENSG00000173372	ENSG00000131094
ENSG00000173372	ENSG00000133466
ENSG00000173372	ENSG00000139899
ENSG00000173372	ENSG00000141668
ENSG00000173372	ENSG00000144119
ENSG00000173372	ENSG00000145861
ENSG00000173372	ENSG00000159189
ENSG00000173372	ENSG00000163145
ENSG00000173372	ENSG00000165985
ENSG00000173372	ENSG00000172247
ENSG00000173372	ENSG00000173369
ENSG00000173372	ENSG00000173918
ENSG00000173372	ENSG00000181092
ENSG00000173372	ENSG00000184471
ENSG00000173372	ENSG00000186897
ENSG00000173372	ENSG00000213192

ENSG00000173386	ENSG00000079332
ENSG00000173386	ENSG00000152700
ENSG00000173389	ENSG00000184345
ENSG00000173389	ENSG00000214681
ENSG00000173389	ENSG00000214686
ENSG00000173401	ENSG00000096006
ENSG00000173401	ENSG00000101074
ENSG00000173401	ENSG00000103196
ENSG00000173401	ENSG00000121005
ENSG00000173401	ENSG00000124490
ENSG00000173401	ENSG00000124812
ENSG00000173401	ENSG00000137558
ENSG00000173401	ENSG00000139278
ENSG00000173401	ENSG00000164530
ENSG00000173401	ENSG00000180481
ENSG00000173404	ENSG00000168348
ENSG00000173406	ENSG00000153071
ENSG00000173442	ENSG00000115504
ENSG00000173451	ENSG00000041988
ENSG00000173451	ENSG00000129028
ENSG00000173451	ENSG00000131931
ENSG00000173451	ENSG00000161277
ENSG00000173457	ENSG00000166143
ENSG00000173457	ENSG00000167641
ENSG00000173457	ENSG00000198729
ENSG00000173467	ENSG00000106541
ENSG00000173467	ENSG00000117862
ENSG00000173473	ENSG00000139613
ENSG00000173477	ENSG00000087086
ENSG00000173477	ENSG00000132446
ENSG00000173477	ENSG00000167996
ENSG00000173477	ENSG00000171878
ENSG00000173477	ENSG00000176778
ENSG00000173477	ENSG00000180795
ENSG00000173477	ENSG00000181867
ENSG00000173477	ENSG00000197518
ENSG00000173477	ENSG00000215234
ENSG00000173482	ENSG00000060656
ENSG00000173482	ENSG00000105426
ENSG00000173482	ENSG00000142949
ENSG00000173482	ENSG00000152894
ENSG00000173482	ENSG00000153707
ENSG00000173482	ENSG00000196090
ENSG00000173511	ENSG00000100311
ENSG00000173511	ENSG00000112715
ENSG00000173511	ENSG00000119630
ENSG00000173511	ENSG00000150630
ENSG00000173511	ENSG00000165197
ENSG00000173511	ENSG00000197461
ENSG00000173511	ENSG00000215682
ENSG00000173530	ENSG00000026103
ENSG00000173530	ENSG00000067182
ENSG00000173530	ENSG00000104689
ENSG00000173530	ENSG00000120889
ENSG00000173530	ENSG00000173535
ENSG00000173530	ENSG00000215788
ENSG00000173531	ENSG00000019991
ENSG00000173531	ENSG00000104368
ENSG00000173531	ENSG00000109758

ENSG00000173531	ENSG00000122194
ENSG00000173531	ENSG00000122861
ENSG00000173531	ENSG00000131187
ENSG00000173531	ENSG00000148702
ENSG00000173531	ENSG00000186301
ENSG00000173531	ENSG00000186715
ENSG00000173531	ENSG00000198670
ENSG00000173535	ENSG00000026103
ENSG00000173535	ENSG00000067182
ENSG00000173535	ENSG00000104689
ENSG00000173535	ENSG00000120889
ENSG00000173535	ENSG00000173530
ENSG00000173535	ENSG00000215788
ENSG00000173542	ENSG00000114978
ENSG00000173542	ENSG00000120162
ENSG00000173542	ENSG00000142961
ENSG00000173542	ENSG00000172081
ENSG00000173542	ENSG00000182208
ENSG00000173546	ENSG00000138759
ENSG00000173546	ENSG00000150893
ENSG00000173546	ENSG00000164946
ENSG00000173546	ENSG00000183090
ENSG00000173546	ENSG00000215060
ENSG00000173548	ENSG00000106266
ENSG00000173548	ENSG00000130340
ENSG00000173548	ENSG00000178996
ENSG00000173559	ENSG00000139579
ENSG00000173567	ENSG00000069122
ENSG00000173567	ENSG00000123146
ENSG00000173567	ENSG00000127507
ENSG00000173567	ENSG00000131355
ENSG00000173567	ENSG00000153292
ENSG00000173567	ENSG00000153294
ENSG00000173567	ENSG00000162618
ENSG00000173567	ENSG00000164393
ENSG00000173567	ENSG00000174837
ENSG00000173567	ENSG00000186629
ENSG00000173567	ENSG00000204818
ENSG00000173572	ENSG00000022556
ENSG00000173572	ENSG00000023191
ENSG00000173572	ENSG00000140853
ENSG00000173572	ENSG00000142405
ENSG00000173572	ENSG00000158077
ENSG00000173572	ENSG00000160505
ENSG00000173572	ENSG00000162711
ENSG00000173572	ENSG00000167207
ENSG00000173572	ENSG00000167634
ENSG00000173572	ENSG00000167984
ENSG00000173572	ENSG00000171487
ENSG00000173572	ENSG00000174885
ENSG00000173572	ENSG00000179709
ENSG00000173572	ENSG00000179873
ENSG00000173572	ENSG00000182261
ENSG00000173572	ENSG00000185792
ENSG00000173575	ENSG00000153922
ENSG00000173578	ENSG00000112486
ENSG00000173578	ENSG00000121797
ENSG00000173578	ENSG00000121807
ENSG00000173578	ENSG00000121966

ENSG00000173578	ENSG00000126353
ENSG00000173578	ENSG00000129048
ENSG00000173578	ENSG00000144648
ENSG00000173578	ENSG00000160683
ENSG00000173578	ENSG00000160791
ENSG00000173578	ENSG00000163464
ENSG00000173578	ENSG00000163823
ENSG00000173578	ENSG00000168329
ENSG00000173578	ENSG00000172215
ENSG00000173578	ENSG00000173585
ENSG00000173578	ENSG00000179934
ENSG00000173578	ENSG00000180871
ENSG00000173578	ENSG00000183625
ENSG00000173578	ENSG00000183813
ENSG00000173578	ENSG00000184451
ENSG00000173578	ENSG00000186810
ENSG00000173578	ENSG00000215775
ENSG00000173578	ENSG00000215778
ENSG00000173578	ENSG00000215782
ENSG00000173585	ENSG00000112486
ENSG00000173585	ENSG00000121797
ENSG00000173585	ENSG00000121807
ENSG00000173585	ENSG00000121966
ENSG00000173585	ENSG00000126353
ENSG00000173585	ENSG00000129048
ENSG00000173585	ENSG00000144648
ENSG00000173585	ENSG00000160683
ENSG00000173585	ENSG00000160791
ENSG00000173585	ENSG00000163464
ENSG00000173585	ENSG00000163823
ENSG00000173585	ENSG00000168329
ENSG00000173585	ENSG00000172215
ENSG00000173585	ENSG00000173578
ENSG00000173585	ENSG00000179934
ENSG00000173585	ENSG00000180871
ENSG00000173585	ENSG00000183625
ENSG00000173585	ENSG00000183813
ENSG00000173585	ENSG00000184451
ENSG00000173585	ENSG00000186810
ENSG00000173585	ENSG00000215775
ENSG00000173585	ENSG00000215778
ENSG00000173585	ENSG00000215782
ENSG00000173597	ENSG00000088002
ENSG00000173597	ENSG00000105398
ENSG00000173597	ENSG00000109193
ENSG00000173597	ENSG00000130540
ENSG00000173597	ENSG00000138068
ENSG00000173597	ENSG00000196228
ENSG00000173597	ENSG00000196502
ENSG00000173597	ENSG00000197165
ENSG00000173597	ENSG00000198075
ENSG00000173597	ENSG00000198203
ENSG00000173597	ENSG00000213599
ENSG00000173597	ENSG00000213648
ENSG00000173598	ENSG00000112664
ENSG00000173598	ENSG00000122824
ENSG00000173598	ENSG00000196368
ENSG00000173610	ENSG00000109181
ENSG00000173610	ENSG00000135220

ENSG00000173610	ENSG00000135226
ENSG00000173610	ENSG00000145626
ENSG00000173610	ENSG00000156096
ENSG00000173610	ENSG00000167165
ENSG00000173610	ENSG00000168671
ENSG00000173610	ENSG00000171234
ENSG00000173610	ENSG00000174607
ENSG00000173610	ENSG00000196620
ENSG00000173610	ENSG00000197592
ENSG00000173610	ENSG00000197888
ENSG00000173610	ENSG00000198277
ENSG00000173610	ENSG00000213759
ENSG00000173612	ENSG00000036828
ENSG00000173612	ENSG00000169962
ENSG00000173612	ENSG00000173662
ENSG00000173612	ENSG00000174930
ENSG00000173612	ENSG00000179002
ENSG00000173612	ENSG00000215740
ENSG00000173614	ENSG00000157064
ENSG00000173614	ENSG00000163864
ENSG00000173616	ENSG00000006047
ENSG00000173616	ENSG00000060138
ENSG00000173616	ENSG00000065978
ENSG00000173616	ENSG00000183443
ENSG00000173616	ENSG00000214299
ENSG00000173621	ENSG00000148602
ENSG00000173621	ENSG00000183423
ENSG00000173621	ENSG00000204033
ENSG00000173626	ENSG00000054116
ENSG00000173636	ENSG00000010438
ENSG00000173636	ENSG00000129437
ENSG00000173636	ENSG00000129451
ENSG00000173636	ENSG00000129455
ENSG00000173636	ENSG00000142515
ENSG00000173636	ENSG00000165076
ENSG00000173636	ENSG00000167748
ENSG00000173636	ENSG00000167749
ENSG00000173636	ENSG00000167751
ENSG00000173636	ENSG00000167754
ENSG00000173636	ENSG00000167755
ENSG00000173636	ENSG00000167757
ENSG00000173636	ENSG00000167759
ENSG00000173636	ENSG00000169035
ENSG00000173636	ENSG00000171147
ENSG00000173636	ENSG00000174562
ENSG00000173636	ENSG00000186474
ENSG00000173636	ENSG00000204981
ENSG00000173636	ENSG00000204982
ENSG00000173636	ENSG00000204983
ENSG00000173636	ENSG00000213022
ENSG00000173636	ENSG00000214083
ENSG00000173638	ENSG00000117479
ENSG00000173638	ENSG00000135917
ENSG00000173662	ENSG00000036828
ENSG00000173662	ENSG00000169962
ENSG00000173662	ENSG00000173612
ENSG00000173662	ENSG00000174930
ENSG00000173662	ENSG00000179002
ENSG00000173662	ENSG00000215740

ENSG00000173673	ENSG00000069812
ENSG00000173673	ENSG00000114315
ENSG00000173673	ENSG00000144485
ENSG00000173673	ENSG00000179111
ENSG00000173673	ENSG00000188290
ENSG00000173673	ENSG00000197921
ENSG00000173678	ENSG00000136206
ENSG00000173678	ENSG00000154732
ENSG00000173678	ENSG00000163806
ENSG00000173678	ENSG00000166667
ENSG00000173678	ENSG00000170092
ENSG00000173678	ENSG00000179994
ENSG00000173678	ENSG00000183318
ENSG00000173678	ENSG00000184616
ENSG00000173678	ENSG00000185040
ENSG00000173678	ENSG00000186645
ENSG00000173678	ENSG00000198305
ENSG00000173678	ENSG00000204710
ENSG00000173678	ENSG00000205238
ENSG00000173678	ENSG00000205482
ENSG00000173678	ENSG00000205505
ENSG00000173678	ENSG00000205506
ENSG00000173678	ENSG00000205508
ENSG00000173678	ENSG00000205509
ENSG00000173678	ENSG00000205580
ENSG00000173678	ENSG00000214300
ENSG00000173678	ENSG00000215092
ENSG00000173681	ENSG00000029363
ENSG00000173681	ENSG00000054118
ENSG00000173698	ENSG00000112414
ENSG00000173698	ENSG00000156920
ENSG00000173698	ENSG00000159618
ENSG00000173698	ENSG00000182885
ENSG00000173698	ENSG00000205336
ENSG00000173714	ENSG00000003436
ENSG00000173714	ENSG00000105825
ENSG00000173714	ENSG00000127578
ENSG00000173726	ENSG00000196860
ENSG00000173744	ENSG00000106351
ENSG00000173780	ENSG00000107242
ENSG00000173780	ENSG00000143398
ENSG00000173780	ENSG00000186111
ENSG00000173801	ENSG00000168036
ENSG00000173805	ENSG00000115993
ENSG00000173805	ENSG00000182606
ENSG00000173812	ENSG00000114784
ENSG00000173826	ENSG00000055118
ENSG00000173826	ENSG00000089558
ENSG00000173826	ENSG00000135519
ENSG00000173826	ENSG00000140015
ENSG00000173826	ENSG00000143473
ENSG00000173826	ENSG00000183960
ENSG00000173826	ENSG00000184611
ENSG00000173846	ENSG00000145632
ENSG00000173846	ENSG00000166851
ENSG00000173846	ENSG00000185988
ENSG00000173846	ENSG00000214270
ENSG00000173848	ENSG00000163947
ENSG00000173868	ENSG00000144362

ENSG00000173876	ENSG00000101162
ENSG00000173876	ENSG00000104833
ENSG00000173876	ENSG00000127589
ENSG00000173876	ENSG00000137267
ENSG00000173876	ENSG00000137285
ENSG00000173876	ENSG00000159247
ENSG00000173876	ENSG00000173213
ENSG00000173876	ENSG00000176014
ENSG00000173876	ENSG00000188229
ENSG00000173876	ENSG00000196230
ENSG00000173876	ENSG00000198211
ENSG00000173876	ENSG00000215801
ENSG00000173876	ENSG00000215813
ENSG00000173876	ENSG00000215814
ENSG00000173889	ENSG00000111752
ENSG00000173889	ENSG00000134686
ENSG00000173889	ENSG00000179899
ENSG00000173889	ENSG00000187033
ENSG00000173889	ENSG00000187634
ENSG00000173894	ENSG00000100307
ENSG00000173894	ENSG00000141570
ENSG00000173894	ENSG00000141582
ENSG00000173894	ENSG00000183741
ENSG00000173898	ENSG00000070182
ENSG00000173898	ENSG00000115306
ENSG00000173898	ENSG00000137877
ENSG00000173898	ENSG00000160460
ENSG00000173908	ENSG00000006059
ENSG00000173908	ENSG00000094796
ENSG00000173908	ENSG00000108244
ENSG00000173908	ENSG00000108417
ENSG00000173908	ENSG00000108759
ENSG00000173908	ENSG00000111057
ENSG00000173908	ENSG00000126337
ENSG00000173908	ENSG00000128422
ENSG00000173908	ENSG00000131737
ENSG00000173908	ENSG00000131738
ENSG00000173908	ENSG00000131885
ENSG00000173908	ENSG00000167916
ENSG00000173908	ENSG00000170819
ENSG00000173908	ENSG00000171345
ENSG00000173908	ENSG00000171346
ENSG00000173908	ENSG00000171360
ENSG00000173908	ENSG00000171401
ENSG00000173908	ENSG00000171403
ENSG00000173908	ENSG00000171431
ENSG00000173908	ENSG00000171446
ENSG00000173908	ENSG00000186393
ENSG00000173908	ENSG00000186395
ENSG00000173908	ENSG00000186831
ENSG00000173908	ENSG00000186832
ENSG00000173908	ENSG00000186847
ENSG00000173908	ENSG00000187242
ENSG00000173908	ENSG00000187686
ENSG00000173908	ENSG00000196859
ENSG00000173908	ENSG00000197079
ENSG00000173908	ENSG00000204889
ENSG00000173908	ENSG00000204897
ENSG00000173908	ENSG00000213951

ENSG00000173908	ENSG00000214220
ENSG00000173908	ENSG00000214454
ENSG00000173908	ENSG00000214959
ENSG00000173908	ENSG00000215772
ENSG00000173908	ENSG00000215773
ENSG00000173914	ENSG00000173933
ENSG00000173914	ENSG00000173959
ENSG00000173917	ENSG00000105991
ENSG00000173917	ENSG00000105996
ENSG00000173917	ENSG00000120094
ENSG00000173917	ENSG00000128645
ENSG00000173918	ENSG00000054803
ENSG00000173918	ENSG00000102924
ENSG00000173918	ENSG00000131094
ENSG00000173918	ENSG00000133466
ENSG00000173918	ENSG00000139899
ENSG00000173918	ENSG00000141668
ENSG00000173918	ENSG00000144119
ENSG00000173918	ENSG00000145861
ENSG00000173918	ENSG00000159189
ENSG00000173918	ENSG00000163145
ENSG00000173918	ENSG00000165985
ENSG00000173918	ENSG00000172247
ENSG00000173918	ENSG00000173369
ENSG00000173918	ENSG00000173372
ENSG00000173918	ENSG00000181092
ENSG00000173918	ENSG00000184471
ENSG00000173918	ENSG00000186897
ENSG00000173918	ENSG00000213192
ENSG00000173926	ENSG00000099785
ENSG00000173926	ENSG00000139266
ENSG00000173926	ENSG00000144583
ENSG00000173926	ENSG00000145416
ENSG00000173926	ENSG00000165406
ENSG00000173926	ENSG00000183654
ENSG00000173933	ENSG00000173914
ENSG00000173933	ENSG00000173959
ENSG00000173959	ENSG00000173914
ENSG00000173959	ENSG00000173933
ENSG00000173960	ENSG00000088833
ENSG00000173960	ENSG00000215114
ENSG00000173961	ENSG00000029993
ENSG00000173961	ENSG00000100118
ENSG00000173961	ENSG00000124097
ENSG00000173961	ENSG00000132967
ENSG00000173961	ENSG00000164104
ENSG00000173961	ENSG00000176256
ENSG00000173961	ENSG00000180212
ENSG00000173961	ENSG00000182181
ENSG00000173961	ENSG00000182711
ENSG00000173961	ENSG00000189403
ENSG00000173961	ENSG00000214364
ENSG00000173961	ENSG00000214852
ENSG00000173961	ENSG00000215308
ENSG00000173976	ENSG00000101883
ENSG00000173976	ENSG00000123576
ENSG00000173976	ENSG00000126656
ENSG00000173976	ENSG00000134438
ENSG00000173976	ENSG00000175325

ENSG00000173976	ENSG00000175329
ENSG00000173976	ENSG00000204595
ENSG00000174004	ENSG00000137507
ENSG00000174010	ENSG00000003096
ENSG00000174010	ENSG00000087448
ENSG00000174010	ENSG00000124743
ENSG00000174010	ENSG00000130487
ENSG00000174010	ENSG00000135686
ENSG00000174010	ENSG00000166853
ENSG00000174010	ENSG00000167487
ENSG00000174010	ENSG00000176595
ENSG00000174010	ENSG00000179023
ENSG00000174010	ENSG00000185214
ENSG00000174010	ENSG00000185915
ENSG00000174010	ENSG00000186231
ENSG00000174010	ENSG00000197705
ENSG00000174010	ENSG00000198642
ENSG00000174016	ENSG00000112773
ENSG00000174016	ENSG00000158246
ENSG00000174016	ENSG00000183508
ENSG00000174021	ENSG00000127588
ENSG00000174021	ENSG00000127920
ENSG00000174021	ENSG00000127928
ENSG00000174021	ENSG00000133136
ENSG00000174021	ENSG00000162188
ENSG00000174021	ENSG00000167083
ENSG00000174021	ENSG00000167414
ENSG00000174021	ENSG00000168243
ENSG00000174021	ENSG00000172380
ENSG00000174021	ENSG00000182625
ENSG00000174021	ENSG00000186469
ENSG00000174021	ENSG00000213611
ENSG00000174028	ENSG00000071889
ENSG00000174028	ENSG00000183844
ENSG00000174028	ENSG00000196937
ENSG00000174028	ENSG00000198643
ENSG00000174032	ENSG00000102078
ENSG00000174032	ENSG00000109424
ENSG00000174032	ENSG00000175564
ENSG00000174032	ENSG00000175567
ENSG00000174083	ENSG00000141506
ENSG00000174115	ENSG00000128438
ENSG00000174115	ENSG00000148429
ENSG00000174115	ENSG00000161583
ENSG00000174115	ENSG00000185128
ENSG00000174115	ENSG00000188755
ENSG00000174115	ENSG00000188849
ENSG00000174115	ENSG00000189309
ENSG00000174115	ENSG00000197681
ENSG00000174115	ENSG00000204471
ENSG00000174115	ENSG00000205019
ENSG00000174115	ENSG00000213474
ENSG00000174115	ENSG00000215625
ENSG00000174115	ENSG00000215730
ENSG00000174115	ENSG00000215748
ENSG00000174121	ENSG00000162596
ENSG00000174121	ENSG00000171863
ENSG00000174121	ENSG00000183405
ENSG00000174121	ENSG00000215342

ENSG00000174121	ENSG00000215804
ENSG00000174123	ENSG00000101916
ENSG00000174123	ENSG00000134061
ENSG00000174123	ENSG00000136869
ENSG00000174123	ENSG00000137462
ENSG00000174123	ENSG00000174125
ENSG00000174123	ENSG00000174130
ENSG00000174123	ENSG00000187554
ENSG00000174125	ENSG00000101916
ENSG00000174125	ENSG00000134061
ENSG00000174125	ENSG00000136869
ENSG00000174125	ENSG00000137462
ENSG00000174125	ENSG00000174123
ENSG00000174125	ENSG00000174130
ENSG00000174125	ENSG00000187554
ENSG00000174130	ENSG00000101916
ENSG00000174130	ENSG00000134061
ENSG00000174130	ENSG00000136869
ENSG00000174130	ENSG00000137462
ENSG00000174130	ENSG00000174123
ENSG00000174130	ENSG00000174125
ENSG00000174130	ENSG00000187554
ENSG00000174136	ENSG00000168509
ENSG00000174136	ENSG00000182175
ENSG00000174137	ENSG00000120709
ENSG00000174137	ENSG00000189319
ENSG00000174144	ENSG00000092010
ENSG00000174144	ENSG00000100911
ENSG00000174144	ENSG00000131467
ENSG00000174145	ENSG00000188039
ENSG00000174151	ENSG00000114395
ENSG00000174194	ENSG00000108262
ENSG00000174194	ENSG00000133612
ENSG00000174194	ENSG00000135439
ENSG00000174194	ENSG00000139436
ENSG00000174194	ENSG00000151303
ENSG00000174194	ENSG00000157985
ENSG00000174194	ENSG00000172650
ENSG00000174194	ENSG00000188234
ENSG00000174194	ENSG00000198035
ENSG00000174194	ENSG00000204149
ENSG00000174194	ENSG00000204169
ENSG00000174194	ENSG00000204172
ENSG00000174197	ENSG00000073861
ENSG00000174197	ENSG00000089225
ENSG00000174197	ENSG00000092607
ENSG00000174197	ENSG00000112837
ENSG00000174197	ENSG00000121068
ENSG00000174197	ENSG00000121075
ENSG00000174197	ENSG00000122145
ENSG00000174197	ENSG00000135111
ENSG00000174197	ENSG00000136535
ENSG00000174197	ENSG00000143178
ENSG00000174197	ENSG00000149922
ENSG00000174197	ENSG00000163508
ENSG00000174197	ENSG00000164458
ENSG00000174197	ENSG00000164532
ENSG00000174197	ENSG00000167800
ENSG00000174197	ENSG00000184058

ENSG00000174225	ENSG00000169379
ENSG00000174226	ENSG00000115234
ENSG00000174226	ENSG00000143376
ENSG00000174233	ENSG00000078295
ENSG00000174233	ENSG00000121281
ENSG00000174233	ENSG00000129467
ENSG00000174233	ENSG00000138031
ENSG00000174233	ENSG00000155897
ENSG00000174233	ENSG00000162104
ENSG00000174233	ENSG00000164742
ENSG00000174233	ENSG00000173175
ENSG00000174238	ENSG00000154217
ENSG00000174238	ENSG00000180957
ENSG00000174255	ENSG00000142396
ENSG00000174255	ENSG00000169740
ENSG00000174255	ENSG00000179195
ENSG00000174255	ENSG00000179965
ENSG00000174255	ENSG00000184294
ENSG00000174255	ENSG00000186446
ENSG00000174255	ENSG00000186777
ENSG00000174255	ENSG00000197008
ENSG00000174255	ENSG00000197025
ENSG00000174255	ENSG00000197062
ENSG00000174255	ENSG00000197332
ENSG00000174255	ENSG00000198155
ENSG00000174255	ENSG00000204756
ENSG00000174255	ENSG00000212805
ENSG00000174255	ENSG00000213976
ENSG00000174255	ENSG00000213980
ENSG00000174255	ENSG00000215180
ENSG00000174255	ENSG00000215510
ENSG00000174255	ENSG00000215537
ENSG00000174255	ENSG00000215598
ENSG00000174255	ENSG00000215600
ENSG00000174279	ENSG00000105997
ENSG00000174279	ENSG00000106038
ENSG00000174279	ENSG00000120093
ENSG00000174279	ENSG00000128652
ENSG00000174279	ENSG00000169840
ENSG00000174279	ENSG00000180613
ENSG00000174282	ENSG00000177311
ENSG00000174282	ENSG00000177485
ENSG00000174282	ENSG00000198081
ENSG00000174306	ENSG00000165156
ENSG00000174306	ENSG00000178764
ENSG00000174306	ENSG00000215271
ENSG00000174307	ENSG00000139289
ENSG00000174307	ENSG00000181649
ENSG00000174326	ENSG00000100156
ENSG00000174326	ENSG00000108932
ENSG00000174326	ENSG00000112394
ENSG00000174326	ENSG00000118596
ENSG00000174326	ENSG00000141526
ENSG00000174326	ENSG00000147100
ENSG00000174326	ENSG00000152779
ENSG00000174326	ENSG00000155380
ENSG00000174326	ENSG00000163053
ENSG00000174326	ENSG00000165449
ENSG00000174326	ENSG00000168679

ENSG00000174326	ENSG00000170190
ENSG00000174326	ENSG00000174327
ENSG00000174327	ENSG00000100156
ENSG00000174327	ENSG00000108932
ENSG00000174327	ENSG00000112394
ENSG00000174327	ENSG00000118596
ENSG00000174327	ENSG00000141526
ENSG00000174327	ENSG00000147100
ENSG00000174327	ENSG00000152779
ENSG00000174327	ENSG00000155380
ENSG00000174327	ENSG00000163053
ENSG00000174327	ENSG00000165449
ENSG00000174327	ENSG00000168679
ENSG00000174327	ENSG00000170190
ENSG00000174327	ENSG00000174326
ENSG00000174332	ENSG00000074047
ENSG00000174332	ENSG00000106571
ENSG00000174332	ENSG00000107249
ENSG00000174332	ENSG00000111087
ENSG00000174332	ENSG00000126603
ENSG00000174343	ENSG00000080644
ENSG00000174343	ENSG00000101204
ENSG00000174343	ENSG00000108556
ENSG00000174343	ENSG00000117971
ENSG00000174343	ENSG00000120903
ENSG00000174343	ENSG00000129749
ENSG00000174343	ENSG00000135902
ENSG00000174343	ENSG00000138435
ENSG00000174343	ENSG00000147432
ENSG00000174343	ENSG00000147434
ENSG00000174343	ENSG00000149305
ENSG00000174343	ENSG00000160716
ENSG00000174343	ENSG00000166664
ENSG00000174343	ENSG00000166736
ENSG00000174343	ENSG00000169684
ENSG00000174343	ENSG00000170175
ENSG00000174343	ENSG00000175344
ENSG00000174343	ENSG00000178084
ENSG00000174343	ENSG00000186038
ENSG00000174343	ENSG00000186090
ENSG00000174343	ENSG00000186919
ENSG00000174343	ENSG00000196811
ENSG00000174348	ENSG00000132000
ENSG00000174358	ENSG00000010379
ENSG00000174358	ENSG00000011083
ENSG00000174358	ENSG00000087916
ENSG00000174358	ENSG00000103546
ENSG00000174358	ENSG00000108576
ENSG00000174358	ENSG00000111181
ENSG00000174358	ENSG00000130821
ENSG00000174358	ENSG00000131389
ENSG00000174358	ENSG00000132164
ENSG00000174358	ENSG00000142319
ENSG00000174358	ENSG00000157103
ENSG00000174358	ENSG00000163817
ENSG00000174358	ENSG00000164363
ENSG00000174358	ENSG00000165970
ENSG00000174358	ENSG00000196517
ENSG00000174358	ENSG00000198555

ENSG00000174358	ENSG00000214617
ENSG00000174373	ENSG00000103197
ENSG00000174373	ENSG00000188559
ENSG00000174417	ENSG00000101188
ENSG00000174417	ENSG00000132911
ENSG00000174437	ENSG00000074370
ENSG00000174437	ENSG00000196296
ENSG00000174440	ENSG00000172115
ENSG00000174440	ENSG00000188512
ENSG00000174440	ENSG00000214810
ENSG00000174450	ENSG00000140478
ENSG00000174450	ENSG00000153666
ENSG00000174450	ENSG00000156363
ENSG00000174450	ENSG00000159289
ENSG00000174450	ENSG00000167110
ENSG00000174450	ENSG00000167195
ENSG00000174450	ENSG00000175265
ENSG00000174450	ENSG00000181984
ENSG00000174450	ENSG00000184206
ENSG00000174450	ENSG00000186322
ENSG00000174450	ENSG00000188388
ENSG00000174450	ENSG00000188532
ENSG00000174450	ENSG00000188626
ENSG00000174450	ENSG00000197092
ENSG00000174450	ENSG00000197414
ENSG00000174450	ENSG00000214348
ENSG00000174450	ENSG00000215252
ENSG00000174450	ENSG00000215405
ENSG00000174453	ENSG00000188730
ENSG00000174469	ENSG00000106714
ENSG00000174469	ENSG00000108797
ENSG00000174469	ENSG00000152910
ENSG00000174469	ENSG00000154529
ENSG00000174469	ENSG00000155052
ENSG00000174469	ENSG00000185020
ENSG00000174482	ENSG00000128594
ENSG00000174482	ENSG00000169783
ENSG00000174482	ENSG00000170382
ENSG00000174482	ENSG00000173114
ENSG00000174482	ENSG00000175928
ENSG00000174482	ENSG00000183014
ENSG00000174482	ENSG00000213171
ENSG00000174485	ENSG00000137145
ENSG00000174485	ENSG00000170456
ENSG00000174485	ENSG00000184014
ENSG00000174485	ENSG00000198837
ENSG00000174498	ENSG00000067141
ENSG00000174498	ENSG00000103742
ENSG00000174498	ENSG00000166450
ENSG00000174498	ENSG00000187323
ENSG00000174498	ENSG00000215851
ENSG00000174502	ENSG00000091137
ENSG00000174502	ENSG00000091138
ENSG00000174502	ENSG00000112053
ENSG00000174502	ENSG00000135502
ENSG00000174502	ENSG00000145217
ENSG00000174502	ENSG00000147606
ENSG00000174502	ENSG00000155850
ENSG00000174502	ENSG00000170615

ENSG00000174516	ENSG00000139946
ENSG00000174516	ENSG00000197329
ENSG00000174521	ENSG00000133985
ENSG00000174521	ENSG00000162222
ENSG00000174564	ENSG00000142166
ENSG00000174564	ENSG00000159113
ENSG00000174567	ENSG00000111711
ENSG00000174574	ENSG00000135334
ENSG00000174599	ENSG00000065308
ENSG00000174599	ENSG00000067167
ENSG00000174599	ENSG00000090661
ENSG00000174599	ENSG00000139624
ENSG00000174599	ENSG00000143418
ENSG00000174599	ENSG00000154227
ENSG00000174599	ENSG00000172292
ENSG00000174600	ENSG00000134830
ENSG00000174600	ENSG00000171049
ENSG00000174600	ENSG00000171051
ENSG00000174600	ENSG00000171860
ENSG00000174600	ENSG00000183134
ENSG00000174600	ENSG00000183671
ENSG00000174600	ENSG00000187474
ENSG00000174600	ENSG00000197405
ENSG00000174600	ENSG00000213903
ENSG00000174600	ENSG00000213906
ENSG00000174606	ENSG00000013523
ENSG00000174607	ENSG00000109181
ENSG00000174607	ENSG00000135220
ENSG00000174607	ENSG00000135226
ENSG00000174607	ENSG00000145626
ENSG00000174607	ENSG00000156096
ENSG00000174607	ENSG00000167165
ENSG00000174607	ENSG00000168671
ENSG00000174607	ENSG00000171234
ENSG00000174607	ENSG00000173610
ENSG00000174607	ENSG00000196620
ENSG00000174607	ENSG00000197592
ENSG00000174607	ENSG00000197888
ENSG00000174607	ENSG00000198277
ENSG00000174607	ENSG00000213759
ENSG00000174622	ENSG00000147274
ENSG00000174622	ENSG00000169800
ENSG00000174622	ENSG00000169811
ENSG00000174622	ENSG00000170748
ENSG00000174622	ENSG00000206011
ENSG00000174622	ENSG00000206046
ENSG00000174622	ENSG00000213516
ENSG00000174622	ENSG00000214442
ENSG00000174622	ENSG00000215210
ENSG00000174640	ENSG00000084453
ENSG00000174640	ENSG00000101187
ENSG00000174640	ENSG00000111700
ENSG00000174640	ENSG00000134538
ENSG00000174640	ENSG00000137491
ENSG00000174640	ENSG00000137571
ENSG00000174640	ENSG00000139155
ENSG00000174640	ENSG00000173930
ENSG00000174640	ENSG00000176463
ENSG00000174640	ENSG00000205359

ENSG00000174640	ENSG00000205754
ENSG00000174669	ENSG00000112759
ENSG00000174669	ENSG00000164638
ENSG00000174669	ENSG00000198246
ENSG00000174669	ENSG00000214660
ENSG00000174669	ENSG00000214668
ENSG00000174672	ENSG00000160469
ENSG00000174705	ENSG00000107957
ENSG00000174705	ENSG00000158517
ENSG00000174705	ENSG00000165178
ENSG00000174705	ENSG00000182487
ENSG00000174705	ENSG00000196408
ENSG00000174721	ENSG00000137440
ENSG00000174721	ENSG00000137441
ENSG00000174738	ENSG00000077092
ENSG00000174738	ENSG00000126351
ENSG00000174738	ENSG00000126368
ENSG00000174738	ENSG00000131759
ENSG00000174738	ENSG00000151090
ENSG00000174738	ENSG00000172819
ENSG00000174740	ENSG00000070756
ENSG00000174740	ENSG00000090621
ENSG00000174740	ENSG00000101104
ENSG00000174740	ENSG00000151846
ENSG00000174740	ENSG00000215587
ENSG00000174744	ENSG00000100916
ENSG00000174744	ENSG00000111707
ENSG00000174749	ENSG00000171224
ENSG00000174775	ENSG00000006451
ENSG00000174775	ENSG00000126458
ENSG00000174775	ENSG00000133703
ENSG00000174775	ENSG00000133818
ENSG00000174775	ENSG00000144118
ENSG00000174775	ENSG00000158186
ENSG00000174775	ENSG00000187682
ENSG00000174775	ENSG00000213281
ENSG00000174791	ENSG00000100599
ENSG00000174791	ENSG00000132669
ENSG00000174791	ENSG00000187994
ENSG00000174792	ENSG00000187605
ENSG00000174796	ENSG00000177683
ENSG00000174796	ENSG00000184436
ENSG00000174799	ENSG00000135951
ENSG00000174807	ENSG00000125810
ENSG00000174807	ENSG00000176435
ENSG00000174807	ENSG00000178726
ENSG00000174827	ENSG00000065054
ENSG00000174827	ENSG00000109062
ENSG00000174827	ENSG00000172367
ENSG00000174827	ENSG00000215859
ENSG00000174827	ENSG00000215860
ENSG00000174837	ENSG00000069122
ENSG00000174837	ENSG00000123146
ENSG00000174837	ENSG00000127507
ENSG00000174837	ENSG00000131355
ENSG00000174837	ENSG00000153292
ENSG00000174837	ENSG00000153294
ENSG00000174837	ENSG00000162618
ENSG00000174837	ENSG00000164393

ENSG00000174837	ENSG00000173567
ENSG00000174837	ENSG00000186629
ENSG00000174837	ENSG00000204818
ENSG00000174839	ENSG00000205593
ENSG00000174851	ENSG00000167645
ENSG00000174871	ENSG00000100528
ENSG00000174871	ENSG00000143771
ENSG00000174871	ENSG00000143786
ENSG00000174885	ENSG00000022556
ENSG00000174885	ENSG00000023191
ENSG00000174885	ENSG00000140853
ENSG00000174885	ENSG00000142405
ENSG00000174885	ENSG00000158077
ENSG00000174885	ENSG00000160505
ENSG00000174885	ENSG00000162711
ENSG00000174885	ENSG00000167207
ENSG00000174885	ENSG00000167634
ENSG00000174885	ENSG00000167984
ENSG00000174885	ENSG00000171487
ENSG00000174885	ENSG00000173572
ENSG00000174885	ENSG00000179709
ENSG00000174885	ENSG00000179873
ENSG00000174885	ENSG00000182261
ENSG00000174885	ENSG00000185792
ENSG00000174903	ENSG00000134594
ENSG00000174903	ENSG00000137502
ENSG00000174903	ENSG00000138069
ENSG00000174903	ENSG00000146955
ENSG00000174903	ENSG00000172007
ENSG00000174903	ENSG00000185051
ENSG00000174930	ENSG00000036828
ENSG00000174930	ENSG00000169962
ENSG00000174930	ENSG00000173612
ENSG00000174930	ENSG00000173662
ENSG00000174930	ENSG00000179002
ENSG00000174930	ENSG00000215740
ENSG00000174938	ENSG00000063015
ENSG00000174938	ENSG00000100095
ENSG00000174939	ENSG00000128203
ENSG00000174939	ENSG00000198363
ENSG00000174943	ENSG00000109079
ENSG00000174943	ENSG00000110906
ENSG00000174943	ENSG00000181819
ENSG00000174944	ENSG00000138271
ENSG00000174944	ENSG00000169313
ENSG00000174944	ENSG00000171659
ENSG00000174944	ENSG00000174946
ENSG00000174944	ENSG00000181631
ENSG00000174945	ENSG00000196704
ENSG00000174946	ENSG00000138271
ENSG00000174946	ENSG00000169313
ENSG00000174946	ENSG00000171659
ENSG00000174946	ENSG00000174944
ENSG00000174946	ENSG00000181631
ENSG00000174950	ENSG00000135535
ENSG00000174963	ENSG00000043355
ENSG00000174963	ENSG00000139800
ENSG00000174963	ENSG00000152977
ENSG00000174963	ENSG00000156925

ENSG00000174990	ENSG00000074410
ENSG00000174990	ENSG00000104267
ENSG00000174990	ENSG00000107159
ENSG00000174990	ENSG00000118298
ENSG00000174990	ENSG00000131686
ENSG00000174990	ENSG00000133742
ENSG00000174990	ENSG00000164879
ENSG00000174990	ENSG00000167434
ENSG00000174990	ENSG00000168748
ENSG00000174990	ENSG00000169239
ENSG00000174990	ENSG00000178538
ENSG00000174990	ENSG00000185015
ENSG00000174990	ENSG00000214729
ENSG00000174990	ENSG00000214873
ENSG00000174990	ENSG00000215487
ENSG00000174990	ENSG00000215501
ENSG00000174990	ENSG00000215543
ENSG00000174996	ENSG00000011295
ENSG00000174996	ENSG00000062725
ENSG00000174996	ENSG00000104892
ENSG00000174996	ENSG00000126214
ENSG00000174996	ENSG00000137171
ENSG00000175029	ENSG00000159692
ENSG00000175040	ENSG00000122863
ENSG00000175040	ENSG00000135702
ENSG00000175040	ENSG00000140835
ENSG00000175040	ENSG00000147119
ENSG00000175040	ENSG00000175264
ENSG00000175040	ENSG00000183196
ENSG00000175048	ENSG00000188706
ENSG00000175048	ENSG00000204160
ENSG00000175084	ENSG00000026025
ENSG00000175084	ENSG00000100285
ENSG00000175084	ENSG00000104722
ENSG00000175084	ENSG00000104725
ENSG00000175084	ENSG00000131095
ENSG00000175084	ENSG00000135406
ENSG00000175084	ENSG00000148798
ENSG00000175084	ENSG00000182253
ENSG00000175084	ENSG00000215479
ENSG00000175087	ENSG00000125834
ENSG00000175093	ENSG00000111671
ENSG00000175093	ENSG00000162032
ENSG00000175093	ENSG00000171621
ENSG00000175093	ENSG00000174013
ENSG00000175115	ENSG00000179364
ENSG00000175130	ENSG00000155130
ENSG00000175155	ENSG00000090238
ENSG00000175155	ENSG00000100027
ENSG00000175155	ENSG00000166793
ENSG00000175161	ENSG00000109943
ENSG00000175161	ENSG00000162706
ENSG00000175164	ENSG00000148288
ENSG00000175164	ENSG00000184389
ENSG00000175164	ENSG00000204007
ENSG00000175175	ENSG00000100034
ENSG00000175182	ENSG00000159784
ENSG00000175182	ENSG00000185519
ENSG00000175183	ENSG00000129170

ENSG00000175183	ENSG00000136490
ENSG00000175183	ENSG00000146215
ENSG00000175183	ENSG00000159176
ENSG00000175183	ENSG00000182809
ENSG00000175183	ENSG00000197893
ENSG00000175183	ENSG00000213145
ENSG00000175189	ENSG00000122641
ENSG00000175189	ENSG00000163083
ENSG00000175206	ENSG00000120937
ENSG00000175215	ENSG00000144579
ENSG00000175215	ENSG00000144677
ENSG00000175220	ENSG00000106772
ENSG00000175220	ENSG00000140299
ENSG00000175220	ENSG00000163141
ENSG00000175220	ENSG00000167654
ENSG00000175220	ENSG00000196505
ENSG00000175229	ENSG00000128242
ENSG00000175229	ENSG00000154252
ENSG00000175229	ENSG00000197093
ENSG00000175264	ENSG00000122863
ENSG00000175264	ENSG00000135702
ENSG00000175264	ENSG00000140835
ENSG00000175264	ENSG00000147119
ENSG00000175264	ENSG00000175040
ENSG00000175264	ENSG00000183196
ENSG00000175265	ENSG00000140478
ENSG00000175265	ENSG00000153666
ENSG00000175265	ENSG00000156363
ENSG00000175265	ENSG00000159289
ENSG00000175265	ENSG00000167110
ENSG00000175265	ENSG00000167195
ENSG00000175265	ENSG00000174450
ENSG00000175265	ENSG00000181984
ENSG00000175265	ENSG00000184206
ENSG00000175265	ENSG00000186322
ENSG00000175265	ENSG00000188388
ENSG00000175265	ENSG00000188532
ENSG00000175265	ENSG00000188626
ENSG00000175265	ENSG00000197092
ENSG00000175265	ENSG00000197414
ENSG00000175265	ENSG00000214348
ENSG00000175265	ENSG00000215252
ENSG00000175265	ENSG00000215405
ENSG00000175305	ENSG00000105173
ENSG00000175309	ENSG00000164089
ENSG00000175311	ENSG00000182040
ENSG00000175315	ENSG00000077984
ENSG00000175315	ENSG00000101435
ENSG00000175315	ENSG00000101439
ENSG00000175315	ENSG00000101441
ENSG00000175315	ENSG00000125815
ENSG00000175315	ENSG00000125823
ENSG00000175315	ENSG00000125831
ENSG00000175315	ENSG00000170367
ENSG00000175315	ENSG00000170369
ENSG00000175315	ENSG00000170373
ENSG00000175315	ENSG00000173335
ENSG00000175315	ENSG00000204658
ENSG00000175315	ENSG00000204662

ENSG00000175315	ENSG00000204663
ENSG00000175334	ENSG00000125888
ENSG00000175344	ENSG00000080644
ENSG00000175344	ENSG00000101204
ENSG00000175344	ENSG00000108556
ENSG00000175344	ENSG00000117971
ENSG00000175344	ENSG00000120903
ENSG00000175344	ENSG00000129749
ENSG00000175344	ENSG00000135902
ENSG00000175344	ENSG00000138435
ENSG00000175344	ENSG00000147432
ENSG00000175344	ENSG00000147434
ENSG00000175344	ENSG00000149305
ENSG00000175344	ENSG00000160716
ENSG00000175344	ENSG00000166664
ENSG00000175344	ENSG00000166736
ENSG00000175344	ENSG00000169684
ENSG00000175344	ENSG00000170175
ENSG00000175344	ENSG00000174343
ENSG00000175344	ENSG00000178084
ENSG00000175344	ENSG00000186038
ENSG00000175344	ENSG00000186090
ENSG00000175344	ENSG00000186919
ENSG00000175344	ENSG00000196811
ENSG00000175348	ENSG00000116857
ENSG00000175352	ENSG00000053702
ENSG00000175352	ENSG00000170967
ENSG00000175352	ENSG00000197312
ENSG00000175354	ENSG00000139304
ENSG00000175354	ENSG00000169410
ENSG00000175354	ENSG00000196396
ENSG00000175356	ENSG00000146197
ENSG00000175356	ENSG00000159307
ENSG00000175387	ENSG00000101665
ENSG00000175387	ENSG00000113658
ENSG00000175387	ENSG00000120693
ENSG00000175387	ENSG00000137834
ENSG00000175387	ENSG00000141646
ENSG00000175387	ENSG00000166949
ENSG00000175387	ENSG00000170365
ENSG00000175388	ENSG00000103335
ENSG00000175388	ENSG00000154864
ENSG00000175414	ENSG00000196503
ENSG00000175416	ENSG00000122705
ENSG00000175445	ENSG00000101670
ENSG00000175445	ENSG00000144837
ENSG00000175445	ENSG00000163898
ENSG00000175445	ENSG00000165862
ENSG00000175445	ENSG00000166035
ENSG00000175445	ENSG00000175535
ENSG00000175445	ENSG00000187021
ENSG00000175445	ENSG00000188992
ENSG00000175445	ENSG00000203837
ENSG00000175463	ENSG00000099992
ENSG00000175463	ENSG00000169221
ENSG00000175470	ENSG00000074211
ENSG00000175470	ENSG00000156475
ENSG00000175470	ENSG00000214122
ENSG00000175471	ENSG00000140563

ENSG00000175497	ENSG00000074603
ENSG00000175497	ENSG00000078098
ENSG00000175497	ENSG00000130226
ENSG00000175497	ENSG00000142002
ENSG00000175497	ENSG00000197635
ENSG00000175513	ENSG00000156050
ENSG00000175513	ENSG00000170264
ENSG00000175518	ENSG00000135018
ENSG00000175518	ENSG00000160803
ENSG00000175518	ENSG00000175520
ENSG00000175518	ENSG00000188021
ENSG00000175520	ENSG00000135018
ENSG00000175520	ENSG00000160803
ENSG00000175520	ENSG00000175518
ENSG00000175520	ENSG00000188021
ENSG00000175544	ENSG00000105507
ENSG00000175544	ENSG00000157782
ENSG00000175544	ENSG00000167791
ENSG00000175556	ENSG00000154359
ENSG00000175556	ENSG00000170500
ENSG00000175564	ENSG00000102078
ENSG00000175564	ENSG00000109424
ENSG00000175564	ENSG00000174032
ENSG00000175564	ENSG00000175567
ENSG00000175567	ENSG00000102078
ENSG00000175567	ENSG00000109424
ENSG00000175567	ENSG00000174032
ENSG00000175567	ENSG00000175564
ENSG00000175582	ENSG00000147127
ENSG00000175582	ENSG00000154917
ENSG00000175591	ENSG00000078589
ENSG00000175591	ENSG00000139679
ENSG00000175591	ENSG00000147145
ENSG00000175591	ENSG00000152207
ENSG00000175591	ENSG00000169403
ENSG00000175591	ENSG00000169508
ENSG00000175591	ENSG00000169860
ENSG00000175591	ENSG00000173198
ENSG00000175591	ENSG00000184574
ENSG00000175591	ENSG00000186912
ENSG00000175591	ENSG00000198829
ENSG00000175592	ENSG00000075426
ENSG00000175592	ENSG00000125740
ENSG00000175592	ENSG00000140044
ENSG00000175592	ENSG00000162772
ENSG00000175592	ENSG00000170345
ENSG00000175602	ENSG00000055813
ENSG00000175602	ENSG00000205476
ENSG00000175634	ENSG00000071242
ENSG00000175634	ENSG00000072133
ENSG00000175634	ENSG00000100784
ENSG00000175634	ENSG00000108443
ENSG00000175634	ENSG00000117676
ENSG00000175634	ENSG00000162302
ENSG00000175634	ENSG00000177189
ENSG00000175634	ENSG00000214817
ENSG00000175662	ENSG00000100284
ENSG00000175662	ENSG00000141198
ENSG00000175697	ENSG00000136928

ENSG00000175697	ENSG00000204681
ENSG00000175727	ENSG00000009950
ENSG00000175745	ENSG00000101076
ENSG00000175745	ENSG00000143171
ENSG00000175745	ENSG00000160113
ENSG00000175745	ENSG00000164749
ENSG00000175745	ENSG00000185551
ENSG00000175745	ENSG00000186350
ENSG00000175745	ENSG00000204231
ENSG00000175766	ENSG00000151247
ENSG00000175793	ENSG00000108953
ENSG00000175793	ENSG00000128245
ENSG00000175793	ENSG00000134308
ENSG00000175793	ENSG00000164924
ENSG00000175793	ENSG00000166913
ENSG00000175793	ENSG00000170027
ENSG00000175793	ENSG00000214637
ENSG00000175793	ENSG00000215108
ENSG00000175818	ENSG00000002587
ENSG00000175818	ENSG00000122254
ENSG00000175818	ENSG00000125430
ENSG00000175818	ENSG00000153976
ENSG00000175818	ENSG00000162040
ENSG00000175818	ENSG00000182601
ENSG00000175832	ENSG00000006468
ENSG00000175832	ENSG00000105672
ENSG00000175832	ENSG00000134954
ENSG00000175832	ENSG00000157557
ENSG00000175832	ENSG00000163497
ENSG00000175832	ENSG00000171656
ENSG00000175841	ENSG00000113391
ENSG00000175866	ENSG00000006453
ENSG00000175866	ENSG00000128298
ENSG00000175874	ENSG00000143162
ENSG00000175879	ENSG00000037965
ENSG00000175879	ENSG00000106004
ENSG00000175879	ENSG00000106006
ENSG00000175879	ENSG00000108511
ENSG00000175879	ENSG00000120068
ENSG00000175879	ENSG00000120075
ENSG00000175879	ENSG00000120087
ENSG00000175879	ENSG00000122592
ENSG00000175879	ENSG00000170166
ENSG00000175879	ENSG00000172789
ENSG00000175879	ENSG00000182742
ENSG00000175879	ENSG00000197576
ENSG00000175879	ENSG00000197757
ENSG00000175879	ENSG00000198353
ENSG00000175895	ENSG00000166289
ENSG00000175898	ENSG00000064547
ENSG00000175898	ENSG00000118432
ENSG00000175898	ENSG00000125910
ENSG00000175898	ENSG00000132975
ENSG00000175898	ENSG00000146360
ENSG00000175898	ENSG00000170989
ENSG00000175898	ENSG00000171517
ENSG00000175898	ENSG00000180739
ENSG00000175898	ENSG00000181773
ENSG00000175898	ENSG00000188822

ENSG00000175898	ENSG00000198121
ENSG00000175898	ENSG00000213694
ENSG00000175906	ENSG00000122644
ENSG00000175906	ENSG00000122872
ENSG00000175906	ENSG00000188042
ENSG00000175928	ENSG00000128594
ENSG00000175928	ENSG00000169783
ENSG00000175928	ENSG00000170382
ENSG00000175928	ENSG00000173114
ENSG00000175928	ENSG00000174482
ENSG00000175928	ENSG00000183014
ENSG00000175928	ENSG00000213171
ENSG00000175938	ENSG00000160991
ENSG00000175938	ENSG00000182500
ENSG00000175946	ENSG00000162413
ENSG00000175946	ENSG00000168427
ENSG00000175946	ENSG00000171617
ENSG00000175946	ENSG00000182359
ENSG00000175946	ENSG00000183655
ENSG00000175946	ENSG00000213160
ENSG00000175970	ENSG00000109103
ENSG00000175970	ENSG00000156973
ENSG00000175984	ENSG00000119522
ENSG00000175984	ENSG00000146966
ENSG00000175984	ENSG00000162701
ENSG00000175984	ENSG00000162777
ENSG00000175984	ENSG00000166444
ENSG00000175984	ENSG00000205744
ENSG00000176009	ENSG00000139352
ENSG00000176009	ENSG00000183734
ENSG00000176009	ENSG00000187855
ENSG00000176009	ENSG00000188108
ENSG00000176014	ENSG00000101162
ENSG00000176014	ENSG00000104833
ENSG00000176014	ENSG00000127589
ENSG00000176014	ENSG00000137267
ENSG00000176014	ENSG00000137285
ENSG00000176014	ENSG00000159247
ENSG00000176014	ENSG00000173213
ENSG00000176014	ENSG00000173876
ENSG00000176014	ENSG00000188229
ENSG00000176014	ENSG00000196230
ENSG00000176014	ENSG00000198211
ENSG00000176014	ENSG00000215801
ENSG00000176014	ENSG00000215813
ENSG00000176014	ENSG00000215814
ENSG00000176018	ENSG00000183060
ENSG00000176020	ENSG00000139211
ENSG00000176020	ENSG00000181754
ENSG00000176040	ENSG00000149418
ENSG00000176040	ENSG00000178297
ENSG00000176040	ENSG00000187045
ENSG00000176040	ENSG00000205403
ENSG00000176049	ENSG00000152969
ENSG00000176049	ENSG00000188385
ENSG00000176083	ENSG00000057657
ENSG00000176092	ENSG00000080200
ENSG00000176092	ENSG00000112297
ENSG00000176095	ENSG00000068745

ENSG00000176095	ENSG00000161896
ENSG00000176105	ENSG00000000938
ENSG00000176105	ENSG00000010810
ENSG00000176105	ENSG00000101336
ENSG00000176105	ENSG00000111816
ENSG00000176105	ENSG00000136573
ENSG00000176105	ENSG00000147507
ENSG00000176105	ENSG00000182866
ENSG00000176105	ENSG00000197122
ENSG00000176115	ENSG00000103569
ENSG00000176115	ENSG00000143595
ENSG00000176115	ENSG00000165269
ENSG00000176115	ENSG00000165272
ENSG00000176115	ENSG00000181997
ENSG00000176115	ENSG00000186466
ENSG00000176136	ENSG00000124089
ENSG00000176136	ENSG00000166603
ENSG00000176136	ENSG00000185231
ENSG00000176142	ENSG00000121775
ENSG00000176148	ENSG00000124678
ENSG00000176148	ENSG00000166046
ENSG00000176148	ENSG00000215029
ENSG00000176148	ENSG00000215046
ENSG00000176153	ENSG00000079782
ENSG00000176153	ENSG00000197582
ENSG00000176153	ENSG00000198704
ENSG00000176153	ENSG00000211445
ENSG00000176165	ENSG00000054598
ENSG00000176165	ENSG00000125798
ENSG00000176165	ENSG00000129514
ENSG00000176165	ENSG00000164379
ENSG00000176165	ENSG00000168269
ENSG00000176165	ENSG00000170122
ENSG00000176165	ENSG00000170608
ENSG00000176165	ENSG00000176678
ENSG00000176165	ENSG00000176692
ENSG00000176165	ENSG00000178919
ENSG00000176165	ENSG00000179772
ENSG00000176165	ENSG00000183770
ENSG00000176165	ENSG00000184492
ENSG00000176165	ENSG00000184659
ENSG00000176165	ENSG00000186564
ENSG00000176165	ENSG00000186766
ENSG00000176165	ENSG00000186790
ENSG00000176165	ENSG00000187140
ENSG00000176165	ENSG00000187559
ENSG00000176165	ENSG00000204612
ENSG00000176165	ENSG00000204779
ENSG00000176165	ENSG00000204793
ENSG00000176165	ENSG00000204828
ENSG00000176165	ENSG00000214336
ENSG00000176170	ENSG00000063176
ENSG00000176171	ENSG00000104765
ENSG00000176194	ENSG00000136305
ENSG00000176194	ENSG00000187288
ENSG00000176204	ENSG00000136457
ENSG00000176204	ENSG00000146006
ENSG00000176204	ENSG00000162951
ENSG00000176204	ENSG00000198739

ENSG00000176244	ENSG00000155368
ENSG00000176274	ENSG00000122696
ENSG00000176274	ENSG00000141437
ENSG00000176274	ENSG00000189332
ENSG00000176276	ENSG00000116473
ENSG00000176276	ENSG00000123728
ENSG00000176276	ENSG00000125249
ENSG00000176276	ENSG00000127314
ENSG00000176276	ENSG00000162595
ENSG00000176276	ENSG00000165023
ENSG00000176276	ENSG00000176490
ENSG00000176276	ENSG00000181467
ENSG00000176276	ENSG00000214405
ENSG00000176276	ENSG00000214492
ENSG00000176302	ENSG00000109101
ENSG00000176302	ENSG00000139445
ENSG00000176302	ENSG00000189299
ENSG00000176340	ENSG00000187581
ENSG00000176343	ENSG00000197756
ENSG00000176383	ENSG00000156966
ENSG00000176383	ENSG00000162630
ENSG00000176383	ENSG00000169255
ENSG00000176383	ENSG00000170340
ENSG00000176383	ENSG00000172318
ENSG00000176383	ENSG00000176597
ENSG00000176383	ENSG00000177191
ENSG00000176383	ENSG00000179913
ENSG00000176383	ENSG00000180561
ENSG00000176383	ENSG00000183778
ENSG00000176383	ENSG00000198488
ENSG00000176383	ENSG00000204222
ENSG00000176383	ENSG00000214654
ENSG00000176387	ENSG00000025423
ENSG00000176387	ENSG00000073737
ENSG00000176387	ENSG00000086696
ENSG00000176387	ENSG00000135437
ENSG00000176387	ENSG00000139547
ENSG00000176387	ENSG00000161267
ENSG00000176387	ENSG00000170426
ENSG00000176393	ENSG00000111144
ENSG00000176393	ENSG00000142327
ENSG00000176395	ENSG00000165478
ENSG00000176395	ENSG00000188175
ENSG00000176399	ENSG00000064218
ENSG00000176399	ENSG00000137090
ENSG00000176399	ENSG00000142025
ENSG00000176399	ENSG00000142700
ENSG00000176399	ENSG00000143006
ENSG00000176399	ENSG00000173253
ENSG00000176402	ENSG00000121634
ENSG00000176402	ENSG00000121742
ENSG00000176402	ENSG00000121743
ENSG00000176402	ENSG00000131233
ENSG00000176402	ENSG00000135355
ENSG00000176402	ENSG00000143140
ENSG00000176402	ENSG00000152661
ENSG00000176402	ENSG00000159248
ENSG00000176402	ENSG00000164411
ENSG00000176402	ENSG00000165474

ENSG00000176402	ENSG00000169562
ENSG00000176402	ENSG00000177291
ENSG00000176402	ENSG00000182963
ENSG00000176402	ENSG00000183153
ENSG00000176402	ENSG00000187513
ENSG00000176402	ENSG00000188910
ENSG00000176402	ENSG00000189280
ENSG00000176402	ENSG00000189433
ENSG00000176402	ENSG00000198835
ENSG00000176402	ENSG00000203733
ENSG00000176406	ENSG00000079841
ENSG00000176406	ENSG00000117016
ENSG00000176422	ENSG00000026950
ENSG00000176422	ENSG00000111801
ENSG00000176422	ENSG00000112763
ENSG00000176422	ENSG00000113303
ENSG00000176422	ENSG00000121236
ENSG00000176422	ENSG00000124508
ENSG00000176422	ENSG00000124549
ENSG00000176422	ENSG00000124557
ENSG00000176422	ENSG00000128250
ENSG00000176422	ENSG00000128253
ENSG00000176422	ENSG00000128276
ENSG00000176422	ENSG00000128482
ENSG00000176422	ENSG00000132256
ENSG00000176422	ENSG00000132274
ENSG00000176422	ENSG00000164010
ENSG00000176422	ENSG00000165810
ENSG00000176422	ENSG00000168903
ENSG00000176422	ENSG00000179046
ENSG00000176422	ENSG00000182805
ENSG00000176422	ENSG00000183439
ENSG00000176422	ENSG00000183718
ENSG00000176422	ENSG00000187574
ENSG00000176422	ENSG00000188683
ENSG00000176422	ENSG00000204614
ENSG00000176422	ENSG00000204616
ENSG00000176422	ENSG00000204618
ENSG00000176422	ENSG00000205198
ENSG00000176422	ENSG00000214693
ENSG00000176428	ENSG00000139722
ENSG00000176428	ENSG00000167987
ENSG00000176435	ENSG00000125810
ENSG00000176435	ENSG00000174807
ENSG00000176435	ENSG00000178726
ENSG00000176444	ENSG00000013441
ENSG00000176444	ENSG00000113240
ENSG00000176444	ENSG00000178340
ENSG00000176444	ENSG00000179335
ENSG00000176444	ENSG00000215793
ENSG00000176463	ENSG00000084453
ENSG00000176463	ENSG00000101187
ENSG00000176463	ENSG00000111700
ENSG00000176463	ENSG00000134538
ENSG00000176463	ENSG00000137491
ENSG00000176463	ENSG00000137571
ENSG00000176463	ENSG00000139155
ENSG00000176463	ENSG00000173930
ENSG00000176463	ENSG00000174640

ENSG00000176463	ENSG00000205359
ENSG00000176463	ENSG00000205754
ENSG00000176485	ENSG00000121207
ENSG00000176485	ENSG00000127252
ENSG00000176485	ENSG00000133321
ENSG00000176485	ENSG00000133328
ENSG00000176485	ENSG00000162981
ENSG00000176485	ENSG00000168004
ENSG00000176485	ENSG00000168672
ENSG00000176490	ENSG00000116473
ENSG00000176490	ENSG00000123728
ENSG00000176490	ENSG00000125249
ENSG00000176490	ENSG00000127314
ENSG00000176490	ENSG00000162595
ENSG00000176490	ENSG00000165023
ENSG00000176490	ENSG00000176276
ENSG00000176490	ENSG00000181467
ENSG00000176490	ENSG00000214405
ENSG00000176490	ENSG00000214492
ENSG00000176532	ENSG00000167183
ENSG00000176584	ENSG00000148805
ENSG00000176595	ENSG00000003096
ENSG00000176595	ENSG00000087448
ENSG00000176595	ENSG00000124743
ENSG00000176595	ENSG00000130487
ENSG00000176595	ENSG00000135686
ENSG00000176595	ENSG00000166853
ENSG00000176595	ENSG00000167487
ENSG00000176595	ENSG00000174010
ENSG00000176595	ENSG00000179023
ENSG00000176595	ENSG00000185214
ENSG00000176595	ENSG00000185915
ENSG00000176595	ENSG00000186231
ENSG00000176595	ENSG00000197705
ENSG00000176595	ENSG00000198642
ENSG00000176619	ENSG00000113368
ENSG00000176619	ENSG00000160789
ENSG00000176624	ENSG00000181588
ENSG00000176624	ENSG00000183496
ENSG00000176624	ENSG00000203759
ENSG00000176635	ENSG00000143452
ENSG00000176641	ENSG00000178828
ENSG00000176641	ENSG00000180537
ENSG00000176658	ENSG00000128641
ENSG00000176658	ENSG00000136286
ENSG00000176658	ENSG00000142347
ENSG00000176658	ENSG00000157483
ENSG00000176658	ENSG00000166866
ENSG00000176658	ENSG00000197879
ENSG00000176679	ENSG00000105419
ENSG00000176679	ENSG00000118707
ENSG00000176679	ENSG00000134138
ENSG00000176679	ENSG00000143995
ENSG00000176679	ENSG00000153779
ENSG00000176679	ENSG00000160199
ENSG00000176679	ENSG00000165495
ENSG00000176679	ENSG00000177426
ENSG00000176679	ENSG00000179277
ENSG00000176679	ENSG00000188013

ENSG00000176692	ENSG00000054598
ENSG00000176692	ENSG00000125798
ENSG00000176692	ENSG00000129514
ENSG00000176692	ENSG00000164379
ENSG00000176692	ENSG00000168269
ENSG00000176692	ENSG00000170122
ENSG00000176692	ENSG00000170608
ENSG00000176692	ENSG00000176165
ENSG00000176692	ENSG00000176678
ENSG00000176692	ENSG00000178919
ENSG00000176692	ENSG00000179772
ENSG00000176692	ENSG00000183770
ENSG00000176692	ENSG00000184492
ENSG00000176692	ENSG00000184659
ENSG00000176692	ENSG00000186564
ENSG00000176692	ENSG00000186766
ENSG00000176692	ENSG00000186790
ENSG00000176692	ENSG00000187140
ENSG00000176692	ENSG00000187559
ENSG00000176692	ENSG00000204612
ENSG00000176692	ENSG00000204779
ENSG00000176692	ENSG00000204793
ENSG00000176692	ENSG00000204828
ENSG00000176692	ENSG00000214336
ENSG00000176697	ENSG00000134259
ENSG00000176697	ENSG00000167744
ENSG00000176697	ENSG00000185652
ENSG00000176697	ENSG00000212844
ENSG00000176711	ENSG00000112378
ENSG00000176711	ENSG00000147027
ENSG00000176749	ENSG00000171450
ENSG00000176771	ENSG00000167566
ENSG00000176778	ENSG00000087086
ENSG00000176778	ENSG00000132446
ENSG00000176778	ENSG00000167996
ENSG00000176778	ENSG00000171878
ENSG00000176778	ENSG00000173477
ENSG00000176778	ENSG00000180795
ENSG00000176778	ENSG00000181867
ENSG00000176778	ENSG00000197518
ENSG00000176778	ENSG00000215234
ENSG00000176783	ENSG00000018189
ENSG00000176783	ENSG00000105784
ENSG00000176783	ENSG00000108309
ENSG00000176783	ENSG00000204130
ENSG00000176783	ENSG00000214795
ENSG00000176825	ENSG00000122566
ENSG00000176825	ENSG00000135486
ENSG00000176825	ENSG00000139675
ENSG00000176825	ENSG00000170144
ENSG00000176825	ENSG00000177219
ENSG00000176825	ENSG00000177733
ENSG00000176825	ENSG00000188174
ENSG00000176825	ENSG00000206228
ENSG00000176825	ENSG00000212961
ENSG00000176825	ENSG00000214284
ENSG00000176825	ENSG00000214361
ENSG00000176825	ENSG00000214592
ENSG00000176825	ENSG00000214653

ENSG00000176825	ENSG00000215222
ENSG00000176825	ENSG00000215492
ENSG00000176825	ENSG00000215870
ENSG00000176825	ENSG00000215876
ENSG00000176825	ENSG00000215885
ENSG00000176826	ENSG00000079150
ENSG00000176826	ENSG00000106080
ENSG00000176826	ENSG00000122642
ENSG00000176826	ENSG00000134285
ENSG00000176826	ENSG00000141756
ENSG00000176842	ENSG00000113430
ENSG00000176842	ENSG00000150051
ENSG00000176842	ENSG00000159387
ENSG00000176842	ENSG00000170549
ENSG00000176842	ENSG00000170561
ENSG00000176842	ENSG00000177508
ENSG00000176845	ENSG00000103260
ENSG00000176871	ENSG00000109046
ENSG00000176887	ENSG00000005513
ENSG00000176887	ENSG00000100146
ENSG00000176887	ENSG00000124766
ENSG00000176887	ENSG00000125398
ENSG00000176887	ENSG00000129194
ENSG00000176887	ENSG00000134595
ENSG00000176887	ENSG00000164736
ENSG00000176887	ENSG00000171056
ENSG00000176887	ENSG00000177732
ENSG00000176887	ENSG00000181449
ENSG00000176887	ENSG00000182968
ENSG00000176887	ENSG00000184895
ENSG00000176887	ENSG00000203883
ENSG00000176909	ENSG00000141052
ENSG00000176909	ENSG00000186260
ENSG00000176909	ENSG00000196588
ENSG00000176919	ENSG00000107317
ENSG00000176919	ENSG00000148346
ENSG00000176919	ENSG00000177984
ENSG00000176919	ENSG00000184925
ENSG00000176919	ENSG00000187922
ENSG00000176919	ENSG00000214402
ENSG00000176960	ENSG00000160075
ENSG00000176960	ENSG00000186217
ENSG00000176960	ENSG00000186223
ENSG00000176960	ENSG00000186232
ENSG00000176982	ENSG00000118418
ENSG00000176982	ENSG00000180150
ENSG00000176982	ENSG00000182952
ENSG00000176982	ENSG00000198830
ENSG00000176982	ENSG00000212769
ENSG00000176982	ENSG00000214578
ENSG00000176986	ENSG00000150961
ENSG00000177034	ENSG00000128654
ENSG00000177034	ENSG00000173171
ENSG00000177042	ENSG00000186889
ENSG00000177042	ENSG00000187049
ENSG00000177045	ENSG00000100625
ENSG00000177045	ENSG00000126778
ENSG00000177045	ENSG00000138083
ENSG00000177045	ENSG00000170577

ENSG00000177045	ENSG00000184302
ENSG00000177054	ENSG00000186908
ENSG00000177096	ENSG00000198324
ENSG00000177098	ENSG00000149573
ENSG00000177098	ENSG00000149575
ENSG00000177098	ENSG00000158887
ENSG00000177098	ENSG00000160588
ENSG00000177098	ENSG00000160593
ENSG00000177098	ENSG00000197965
ENSG00000177103	ENSG00000069188
ENSG00000177103	ENSG00000146555
ENSG00000177103	ENSG00000171587
ENSG00000177105	ENSG00000070831
ENSG00000177105	ENSG00000119729
ENSG00000177105	ENSG00000126785
ENSG00000177105	ENSG00000128340
ENSG00000177105	ENSG00000136238
ENSG00000177105	ENSG00000169750
ENSG00000177106	ENSG00000131037
ENSG00000177106	ENSG00000151491
ENSG00000177106	ENSG00000198758
ENSG00000177119	ENSG00000047617
ENSG00000177119	ENSG00000074855
ENSG00000177119	ENSG00000131620
ENSG00000177119	ENSG00000134343
ENSG00000177119	ENSG00000146205
ENSG00000177119	ENSG00000151572
ENSG00000177119	ENSG00000160746
ENSG00000177119	ENSG00000171714
ENSG00000177119	ENSG00000185101
ENSG00000177125	ENSG00000148411
ENSG00000177125	ENSG00000160877
ENSG00000177125	ENSG00000173276
ENSG00000177138	ENSG00000139351
ENSG00000177138	ENSG00000183304
ENSG00000177138	ENSG00000187268
ENSG00000177152	ENSG00000140416
ENSG00000177152	ENSG00000143549
ENSG00000177152	ENSG00000167460
ENSG00000177152	ENSG00000183022
ENSG00000177152	ENSG00000187536
ENSG00000177152	ENSG00000198467
ENSG00000177152	ENSG00000214869
ENSG00000177164	ENSG00000164729
ENSG00000177164	ENSG00000168917
ENSG00000177164	ENSG00000177710
ENSG00000177169	ENSG00000083290
ENSG00000177169	ENSG00000140474
ENSG00000177182	ENSG00000124120
ENSG00000177182	ENSG00000137561
ENSG00000177182	ENSG00000140522
ENSG00000177182	ENSG00000146352
ENSG00000177189	ENSG00000071242
ENSG00000177189	ENSG00000072133
ENSG00000177189	ENSG00000100784
ENSG00000177189	ENSG00000108443
ENSG00000177189	ENSG00000117676
ENSG00000177189	ENSG00000162302
ENSG00000177189	ENSG00000175634

ENSG00000177189	ENSG00000214817
ENSG00000177191	ENSG00000156966
ENSG00000177191	ENSG00000162630
ENSG00000177191	ENSG00000169255
ENSG00000177191	ENSG00000170340
ENSG00000177191	ENSG00000172318
ENSG00000177191	ENSG00000176383
ENSG00000177191	ENSG00000176597
ENSG00000177191	ENSG00000179913
ENSG00000177191	ENSG00000180561
ENSG00000177191	ENSG00000183778
ENSG00000177191	ENSG00000198488
ENSG00000177191	ENSG00000204222
ENSG00000177191	ENSG00000214654
ENSG00000177200	ENSG00000100888
ENSG00000177200	ENSG00000124177
ENSG00000177200	ENSG00000171316
ENSG00000177219	ENSG00000122566
ENSG00000177219	ENSG00000135486
ENSG00000177219	ENSG00000139675
ENSG00000177219	ENSG00000170144
ENSG00000177219	ENSG00000176825
ENSG00000177219	ENSG00000177733
ENSG00000177219	ENSG00000188174
ENSG00000177219	ENSG00000206228
ENSG00000177219	ENSG00000212961
ENSG00000177219	ENSG00000214284
ENSG00000177219	ENSG00000214361
ENSG00000177219	ENSG00000214592
ENSG00000177219	ENSG00000214653
ENSG00000177219	ENSG00000215222
ENSG00000177219	ENSG00000215492
ENSG00000177219	ENSG00000215870
ENSG00000177219	ENSG00000215876
ENSG00000177219	ENSG00000215885
ENSG00000177238	ENSG00000116525
ENSG00000177238	ENSG00000146755
ENSG00000177238	ENSG00000155428
ENSG00000177238	ENSG00000178809
ENSG00000177238	ENSG00000185880
ENSG00000177272	ENSG00000026559
ENSG00000177272	ENSG00000102057
ENSG00000177272	ENSG00000104848
ENSG00000177272	ENSG00000111262
ENSG00000177272	ENSG00000124134
ENSG00000177272	ENSG00000130037
ENSG00000177272	ENSG00000143105
ENSG00000177272	ENSG00000151079
ENSG00000177272	ENSG00000156486
ENSG00000177272	ENSG00000158445
ENSG00000177272	ENSG00000162975
ENSG00000177272	ENSG00000164794
ENSG00000177272	ENSG00000168263
ENSG00000177272	ENSG00000168418
ENSG00000177272	ENSG00000170745
ENSG00000177272	ENSG00000171126
ENSG00000177272	ENSG00000171385
ENSG00000177272	ENSG00000177301
ENSG00000177272	ENSG00000178342

ENSG00000177272	ENSG00000182255
ENSG00000177272	ENSG00000182674
ENSG00000177272	ENSG00000184408
ENSG00000177283	ENSG00000104290
ENSG00000177283	ENSG00000106483
ENSG00000177283	ENSG00000111432
ENSG00000177283	ENSG00000155760
ENSG00000177283	ENSG00000157240
ENSG00000177283	ENSG00000162998
ENSG00000177283	ENSG00000163251
ENSG00000177283	ENSG00000164930
ENSG00000177283	ENSG00000174804
ENSG00000177283	ENSG00000180340
ENSG00000177283	ENSG00000188763
ENSG00000177291	ENSG00000121634
ENSG00000177291	ENSG00000121742
ENSG00000177291	ENSG00000121743
ENSG00000177291	ENSG00000131233
ENSG00000177291	ENSG00000135355
ENSG00000177291	ENSG00000143140
ENSG00000177291	ENSG00000152661
ENSG00000177291	ENSG00000159248
ENSG00000177291	ENSG00000164411
ENSG00000177291	ENSG00000165474
ENSG00000177291	ENSG00000169562
ENSG00000177291	ENSG00000176402
ENSG00000177291	ENSG00000182963
ENSG00000177291	ENSG00000183153
ENSG00000177291	ENSG00000187513
ENSG00000177291	ENSG00000188910
ENSG00000177291	ENSG00000189280
ENSG00000177291	ENSG00000189433
ENSG00000177291	ENSG00000198835
ENSG00000177291	ENSG00000203733
ENSG00000177300	ENSG00000013297
ENSG00000177300	ENSG00000066405
ENSG00000177300	ENSG00000106404
ENSG00000177300	ENSG00000113946
ENSG00000177300	ENSG00000134873
ENSG00000177300	ENSG00000156282
ENSG00000177300	ENSG00000156284
ENSG00000177300	ENSG00000159261
ENSG00000177300	ENSG00000163347
ENSG00000177300	ENSG00000164007
ENSG00000177300	ENSG00000165215
ENSG00000177300	ENSG00000165376
ENSG00000177300	ENSG00000171217
ENSG00000177300	ENSG00000181885
ENSG00000177300	ENSG00000184113
ENSG00000177300	ENSG00000184697
ENSG00000177300	ENSG00000185758
ENSG00000177300	ENSG00000189118
ENSG00000177300	ENSG00000189143
ENSG00000177300	ENSG00000213937
ENSG00000177301	ENSG00000026559
ENSG00000177301	ENSG00000102057
ENSG00000177301	ENSG00000104848
ENSG00000177301	ENSG00000111262
ENSG00000177301	ENSG00000124134

ENSG00000177301	ENSG00000130037
ENSG00000177301	ENSG00000143105
ENSG00000177301	ENSG00000151079
ENSG00000177301	ENSG00000156486
ENSG00000177301	ENSG00000158445
ENSG00000177301	ENSG00000162975
ENSG00000177301	ENSG00000164794
ENSG00000177301	ENSG00000168263
ENSG00000177301	ENSG00000168418
ENSG00000177301	ENSG00000170745
ENSG00000177301	ENSG00000171126
ENSG00000177301	ENSG00000171385
ENSG00000177301	ENSG00000177272
ENSG00000177301	ENSG00000178342
ENSG00000177301	ENSG00000182255
ENSG00000177301	ENSG00000182674
ENSG00000177301	ENSG00000184408
ENSG00000177303	ENSG00000064999
ENSG00000177303	ENSG00000167971
ENSG00000177303	ENSG00000185046
ENSG00000177311	ENSG00000174282
ENSG00000177311	ENSG00000177485
ENSG00000177311	ENSG00000198081
ENSG00000177374	ENSG00000169635
ENSG00000177380	ENSG00000110841
ENSG00000177380	ENSG00000131626
ENSG00000177380	ENSG00000139220
ENSG00000177380	ENSG00000143847
ENSG00000177380	ENSG00000166387
ENSG00000177398	ENSG00000170044
ENSG00000177409	ENSG00000205413
ENSG00000177426	ENSG00000105419
ENSG00000177426	ENSG00000118707
ENSG00000177426	ENSG00000134138
ENSG00000177426	ENSG00000143995
ENSG00000177426	ENSG00000153779
ENSG00000177426	ENSG00000160199
ENSG00000177426	ENSG00000165495
ENSG00000177426	ENSG00000176679
ENSG00000177426	ENSG00000179277
ENSG00000177426	ENSG00000188013
ENSG00000177427	ENSG00000100335
ENSG00000177432	ENSG00000186310
ENSG00000177432	ENSG00000186462
ENSG00000177432	ENSG00000187109
ENSG00000177432	ENSG00000205531
ENSG00000177463	ENSG00000120798
ENSG00000177464	ENSG00000119714
ENSG00000177464	ENSG00000140030
ENSG00000177464	ENSG00000183484
ENSG00000177468	ENSG00000125533
ENSG00000177468	ENSG00000180828
ENSG00000177468	ENSG00000184221
ENSG00000177468	ENSG00000205927
ENSG00000177469	ENSG00000168497
ENSG00000177469	ENSG00000170681
ENSG00000177469	ENSG00000170955
ENSG00000177485	ENSG00000174282
ENSG00000177485	ENSG00000177311

ENSG00000177485	ENSG00000198081
ENSG00000177508	ENSG00000113430
ENSG00000177508	ENSG00000150051
ENSG00000177508	ENSG00000159387
ENSG00000177508	ENSG00000170549
ENSG00000177508	ENSG00000170561
ENSG00000177508	ENSG00000176842
ENSG00000177511	ENSG00000073849
ENSG00000177511	ENSG00000101638
ENSG00000177511	ENSG00000111728
ENSG00000177511	ENSG00000117069
ENSG00000177511	ENSG00000144057
ENSG00000177511	ENSG00000148488
ENSG00000177511	ENSG00000160408
ENSG00000177519	ENSG00000179673
ENSG00000177542	ENSG00000004864
ENSG00000177542	ENSG00000115840
ENSG00000177542	ENSG00000182902
ENSG00000177548	ENSG00000029725
ENSG00000177551	ENSG00000104903
ENSG00000177551	ENSG00000113196
ENSG00000177551	ENSG00000122691
ENSG00000177551	ENSG00000122859
ENSG00000177551	ENSG00000123307
ENSG00000177551	ENSG00000125878
ENSG00000177551	ENSG00000146618
ENSG00000177551	ENSG00000162367
ENSG00000177551	ENSG00000162992
ENSG00000177551	ENSG00000164107
ENSG00000177551	ENSG00000164600
ENSG00000177551	ENSG00000168267
ENSG00000177551	ENSG00000171532
ENSG00000177551	ENSG00000171786
ENSG00000177551	ENSG00000172238
ENSG00000177551	ENSG00000178403
ENSG00000177551	ENSG00000179774
ENSG00000177551	ENSG00000180535
ENSG00000177551	ENSG00000181965
ENSG00000177551	ENSG00000186051
ENSG00000177551	ENSG00000187786
ENSG00000177551	ENSG00000188686
ENSG00000177558	ENSG00000214447
ENSG00000177565	ENSG00000092377
ENSG00000177565	ENSG00000101849
ENSG00000177565	ENSG00000150627
ENSG00000177570	ENSG00000047634
ENSG00000177570	ENSG00000130590
ENSG00000177570	ENSG00000203943
ENSG00000177575	ENSG00000013725
ENSG00000177575	ENSG00000073754
ENSG00000177575	ENSG00000110448
ENSG00000177575	ENSG00000146700
ENSG00000177575	ENSG00000164099
ENSG00000177575	ENSG00000177675
ENSG00000177575	ENSG00000179954
ENSG00000177575	ENSG00000187908
ENSG00000177575	ENSG00000214279
ENSG00000177600	ENSG00000179220
ENSG00000177606	ENSG00000115966

ENSG00000177606	ENSG00000130522
ENSG00000177606	ENSG00000146592
ENSG00000177606	ENSG00000170653
ENSG00000177606	ENSG00000171223
ENSG00000177663	ENSG00000056736
ENSG00000177663	ENSG00000144730
ENSG00000177666	ENSG00000006757
ENSG00000177666	ENSG00000100341
ENSG00000177666	ENSG00000100344
ENSG00000177666	ENSG00000180316
ENSG00000177669	ENSG00000143797
ENSG00000177669	ENSG00000172197
ENSG00000177675	ENSG00000013725
ENSG00000177675	ENSG00000073754
ENSG00000177675	ENSG00000110448
ENSG00000177675	ENSG00000146700
ENSG00000177675	ENSG00000164099
ENSG00000177675	ENSG00000177575
ENSG00000177675	ENSG00000179954
ENSG00000177675	ENSG00000187908
ENSG00000177675	ENSG00000214279
ENSG00000177679	ENSG00000139767
ENSG00000177679	ENSG00000167978
ENSG00000177679	ENSG00000204924
ENSG00000177683	ENSG00000174796
ENSG00000177683	ENSG00000184436
ENSG00000177685	ENSG00000130038
ENSG00000177688	ENSG00000116030
ENSG00000177688	ENSG00000184763
ENSG00000177688	ENSG00000184900
ENSG00000177688	ENSG00000188612
ENSG00000177694	ENSG00000072274
ENSG00000177694	ENSG00000077616
ENSG00000177694	ENSG00000086205
ENSG00000177694	ENSG00000106327
ENSG00000177694	ENSG00000134612
ENSG00000177694	ENSG00000168060
ENSG00000177697	ENSG00000011105
ENSG00000177697	ENSG00000085117
ENSG00000177697	ENSG00000104894
ENSG00000177697	ENSG00000110900
ENSG00000177697	ENSG00000117472
ENSG00000177697	ENSG00000143119
ENSG00000177697	ENSG00000157570
ENSG00000177697	ENSG00000214063
ENSG00000177707	ENSG00000073008
ENSG00000177707	ENSG00000110400
ENSG00000177707	ENSG00000130202
ENSG00000177710	ENSG00000164729
ENSG00000177710	ENSG00000168917
ENSG00000177710	ENSG00000177164
ENSG00000177732	ENSG00000005513
ENSG00000177732	ENSG00000100146
ENSG00000177732	ENSG00000124766
ENSG00000177732	ENSG00000125398
ENSG00000177732	ENSG00000129194
ENSG00000177732	ENSG00000134595
ENSG00000177732	ENSG00000164736
ENSG00000177732	ENSG00000171056

ENSG00000177732	ENSG00000176887
ENSG00000177732	ENSG00000181449
ENSG00000177732	ENSG00000182968
ENSG00000177732	ENSG00000184895
ENSG00000177732	ENSG00000203883
ENSG00000177752	ENSG00000145817
ENSG00000177770	ENSG00000145835
ENSG00000177770	ENSG00000168564
ENSG00000177770	ENSG00000214740
ENSG00000177791	ENSG00000164591
ENSG00000177791	ENSG00000172399
ENSG00000177807	ENSG00000115474
ENSG00000177807	ENSG00000120457
ENSG00000177807	ENSG00000121361
ENSG00000177807	ENSG00000123700
ENSG00000177807	ENSG00000151704
ENSG00000177807	ENSG00000153822
ENSG00000177807	ENSG00000157542
ENSG00000177807	ENSG00000157551
ENSG00000177807	ENSG00000162728
ENSG00000177807	ENSG00000162989
ENSG00000177807	ENSG00000168135
ENSG00000177807	ENSG00000182324
ENSG00000177807	ENSG00000184185
ENSG00000177807	ENSG00000187486
ENSG00000177835	ENSG00000019549
ENSG00000177835	ENSG00000124216
ENSG00000177835	ENSG00000170616
ENSG00000177835	ENSG00000185669
ENSG00000177835	ENSG00000215397
ENSG00000177835	ENSG00000215713
ENSG00000177853	ENSG00000178163
ENSG00000177879	ENSG00000157823
ENSG00000177879	ENSG00000214446
ENSG00000177886	ENSG00000100614
ENSG00000177886	ENSG00000138032
ENSG00000177938	ENSG00000116489
ENSG00000177938	ENSG00000198898
ENSG00000177954	ENSG00000130041
ENSG00000177954	ENSG00000163144
ENSG00000177954	ENSG00000185088
ENSG00000177954	ENSG00000198731
ENSG00000177963	ENSG00000111785
ENSG00000177984	ENSG00000107317
ENSG00000177984	ENSG00000148346
ENSG00000177984	ENSG00000176919
ENSG00000177984	ENSG00000184925
ENSG00000177984	ENSG00000187922
ENSG00000177984	ENSG00000214402
ENSG00000178015	ENSG00000109163
ENSG00000178015	ENSG00000126895
ENSG00000178015	ENSG00000166148
ENSG00000178015	ENSG00000180914
ENSG00000178015	ENSG00000187258
ENSG00000178015	ENSG00000198049
ENSG00000178015	ENSG00000211451
ENSG00000178026	ENSG00000181350
ENSG00000178031	ENSG00000156218
ENSG00000178033	ENSG00000138172

ENSG00000178033	ENSG00000164451
ENSG00000178033	ENSG00000183128
ENSG00000178033	ENSG00000185933
ENSG00000178033	ENSG00000188820
ENSG00000178035	ENSG00000100938
ENSG00000178035	ENSG00000106348
ENSG00000178035	ENSG00000137198
ENSG00000178038	ENSG00000003393
ENSG00000178053	ENSG00000089693
ENSG00000178055	ENSG00000005001
ENSG00000178055	ENSG00000007038
ENSG00000178055	ENSG00000052344
ENSG00000178055	ENSG00000095917
ENSG00000178055	ENSG00000103355
ENSG00000178055	ENSG00000116176
ENSG00000178055	ENSG00000172236
ENSG00000178055	ENSG00000172382
ENSG00000178055	ENSG00000172460
ENSG00000178055	ENSG00000178226
ENSG00000178055	ENSG00000185888
ENSG00000178055	ENSG00000188086
ENSG00000178055	ENSG00000189099
ENSG00000178055	ENSG00000196364
ENSG00000178055	ENSG00000197253
ENSG00000178055	ENSG00000206549
ENSG00000178055	ENSG00000214877
ENSG00000178055	ENSG00000215148
ENSG00000178075	ENSG00000023171
ENSG00000178075	ENSG00000089351
ENSG00000178075	ENSG00000175318
ENSG00000178078	ENSG00000035720
ENSG00000178084	ENSG00000080644
ENSG00000178084	ENSG00000101204
ENSG00000178084	ENSG00000108556
ENSG00000178084	ENSG00000117971
ENSG00000178084	ENSG00000120903
ENSG00000178084	ENSG00000129749
ENSG00000178084	ENSG00000135902
ENSG00000178084	ENSG00000138435
ENSG00000178084	ENSG00000147432
ENSG00000178084	ENSG00000147434
ENSG00000178084	ENSG00000149305
ENSG00000178084	ENSG00000160716
ENSG00000178084	ENSG00000166664
ENSG00000178084	ENSG00000166736
ENSG00000178084	ENSG00000169684
ENSG00000178084	ENSG00000170175
ENSG00000178084	ENSG00000174343
ENSG00000178084	ENSG00000175344
ENSG00000178084	ENSG00000186038
ENSG00000178084	ENSG00000186090
ENSG00000178084	ENSG00000186919
ENSG00000178084	ENSG00000196811
ENSG00000178093	ENSG00000139908
ENSG00000178093	ENSG00000212122
ENSG00000178093	ENSG00000214601
ENSG00000178093	ENSG00000214624
ENSG00000178104	ENSG00000136861
ENSG00000178163	ENSG00000177853

ENSG00000178171	ENSG00000165566
ENSG00000178171	ENSG00000184675
ENSG00000178175	ENSG00000140548
ENSG00000178177	ENSG00000196233
ENSG00000178184	ENSG00000102981
ENSG00000178184	ENSG00000124171
ENSG00000178188	ENSG00000111252
ENSG00000178188	ENSG00000160999
ENSG00000178199	ENSG00000100441
ENSG00000178199	ENSG00000102053
ENSG00000178199	ENSG00000102921
ENSG00000178199	ENSG00000149289
ENSG00000178199	ENSG00000163874
ENSG00000178199	ENSG00000205978
ENSG00000178202	ENSG00000134901
ENSG00000178202	ENSG00000163389
ENSG00000178209	ENSG00000165959
ENSG00000178217	ENSG00000027869
ENSG00000178217	ENSG00000104611
ENSG00000178217	ENSG00000196684
ENSG00000178233	ENSG00000179292
ENSG00000178234	ENSG00000100626
ENSG00000178234	ENSG00000106648
ENSG00000178234	ENSG00000110328
ENSG00000178234	ENSG00000115339
ENSG00000178234	ENSG00000131386
ENSG00000178234	ENSG00000136542
ENSG00000178234	ENSG00000139629
ENSG00000178234	ENSG00000141429
ENSG00000178234	ENSG00000143641
ENSG00000178234	ENSG00000144278
ENSG00000178234	ENSG00000158089
ENSG00000178234	ENSG00000164574
ENSG00000178234	ENSG00000174473
ENSG00000178234	ENSG00000182870
ENSG00000178234	ENSG00000185274
ENSG00000178235	ENSG00000121871
ENSG00000178235	ENSG00000185985
ENSG00000178301	ENSG00000184945
ENSG00000178301	ENSG00000185176
ENSG00000178340	ENSG00000013441
ENSG00000178340	ENSG00000113240
ENSG00000178340	ENSG00000176444
ENSG00000178340	ENSG00000179335
ENSG00000178340	ENSG00000215793
ENSG00000178342	ENSG00000026559
ENSG00000178342	ENSG00000102057
ENSG00000178342	ENSG00000104848
ENSG00000178342	ENSG00000111262
ENSG00000178342	ENSG00000124134
ENSG00000178342	ENSG00000130037
ENSG00000178342	ENSG00000143105
ENSG00000178342	ENSG00000151079
ENSG00000178342	ENSG00000156486
ENSG00000178342	ENSG00000158445
ENSG00000178342	ENSG00000162975
ENSG00000178342	ENSG00000164794
ENSG00000178342	ENSG00000168263
ENSG00000178342	ENSG00000168418

ENSG00000178342	ENSG00000170745
ENSG00000178342	ENSG00000171126
ENSG00000178342	ENSG00000171385
ENSG00000178342	ENSG00000177272
ENSG00000178342	ENSG00000177301
ENSG00000178342	ENSG00000182255
ENSG00000178342	ENSG00000182674
ENSG00000178342	ENSG00000184408
ENSG00000178343	ENSG00000166737
ENSG00000178343	ENSG00000180730
ENSG00000178343	ENSG00000187902
ENSG00000178343	ENSG00000188803
ENSG00000178363	ENSG00000129007
ENSG00000178363	ENSG00000143933
ENSG00000178363	ENSG00000160014
ENSG00000178363	ENSG00000178372
ENSG00000178363	ENSG00000198668
ENSG00000178372	ENSG00000129007
ENSG00000178372	ENSG00000143933
ENSG00000178372	ENSG00000160014
ENSG00000178372	ENSG00000178363
ENSG00000178372	ENSG00000198668
ENSG00000178385	ENSG00000102445
ENSG00000178385	ENSG00000140995
ENSG00000178385	ENSG00000141342
ENSG00000178385	ENSG00000145016
ENSG00000178385	ENSG00000159266
ENSG00000178394	ENSG00000043591
ENSG00000178394	ENSG00000102468
ENSG00000178394	ENSG00000113749
ENSG00000178394	ENSG00000125631
ENSG00000178394	ENSG00000135312
ENSG00000178394	ENSG00000135914
ENSG00000178394	ENSG00000147246
ENSG00000178394	ENSG00000150594
ENSG00000178394	ENSG00000157219
ENSG00000178394	ENSG00000164270
ENSG00000178394	ENSG00000168830
ENSG00000178394	ENSG00000169252
ENSG00000178394	ENSG00000175658
ENSG00000178394	ENSG00000179097
ENSG00000178394	ENSG00000179546
ENSG00000178394	ENSG00000181210
ENSG00000178394	ENSG00000184303
ENSG00000178394	ENSG00000184845
ENSG00000178394	ENSG00000185321
ENSG00000178394	ENSG00000188778
ENSG00000178403	ENSG00000104903
ENSG00000178403	ENSG00000113196
ENSG00000178403	ENSG00000122691
ENSG00000178403	ENSG00000122859
ENSG00000178403	ENSG00000123307
ENSG00000178403	ENSG00000125878
ENSG00000178403	ENSG00000146618
ENSG00000178403	ENSG00000162367
ENSG00000178403	ENSG00000162992
ENSG00000178403	ENSG00000164107
ENSG00000178403	ENSG00000164600
ENSG00000178403	ENSG00000168267

ENSG00000178403	ENSG00000171532
ENSG00000178403	ENSG00000171786
ENSG00000178403	ENSG00000172238
ENSG00000178403	ENSG00000177551
ENSG00000178403	ENSG00000179774
ENSG00000178403	ENSG00000180535
ENSG00000178403	ENSG00000181965
ENSG00000178403	ENSG00000186051
ENSG00000178403	ENSG00000187786
ENSG00000178403	ENSG00000188686
ENSG00000178429	ENSG00000145425
ENSG00000178429	ENSG00000187899
ENSG00000178429	ENSG00000215836
ENSG00000178502	ENSG00000185972
ENSG00000178531	ENSG00000205279
ENSG00000178556	ENSG00000123975
ENSG00000178556	ENSG00000173207
ENSG00000178568	ENSG00000065361
ENSG00000178568	ENSG00000141736
ENSG00000178568	ENSG00000146648
ENSG00000178573	ENSG00000129535
ENSG00000178573	ENSG00000182759
ENSG00000178573	ENSG00000185022
ENSG00000178573	ENSG00000197063
ENSG00000178573	ENSG00000198517
ENSG00000178573	ENSG00000204103
ENSG00000178573	ENSG00000215622
ENSG00000178585	ENSG00000162441
ENSG00000178607	ENSG00000134398
ENSG00000178662	ENSG00000110925
ENSG00000178662	ENSG00000144655
ENSG00000178685	ENSG00000138496
ENSG00000178685	ENSG00000163659
ENSG00000178685	ENSG00000173193
ENSG00000178694	ENSG00000117481
ENSG00000178695	ENSG00000134504
ENSG00000178695	ENSG00000151364
ENSG00000178695	ENSG00000153885
ENSG00000178695	ENSG00000155729
ENSG00000178695	ENSG00000168301
ENSG00000178695	ENSG00000168676
ENSG00000178695	ENSG00000180332
ENSG00000178695	ENSG00000183775
ENSG00000178695	ENSG00000183783
ENSG00000178695	ENSG00000188997
ENSG00000178695	ENSG00000198553
ENSG00000178695	ENSG00000213859
ENSG00000178700	ENSG00000188985
ENSG00000178718	ENSG00000164967
ENSG00000178719	ENSG00000135472
ENSG00000178719	ENSG00000135926
ENSG00000178726	ENSG00000125810
ENSG00000178726	ENSG00000174807
ENSG00000178726	ENSG00000176435
ENSG00000178729	ENSG00000124814
ENSG00000178729	ENSG00000165186
ENSG00000178729	ENSG00000182077
ENSG00000178732	ENSG00000099769
ENSG00000178732	ENSG00000172061

ENSG00000178732	ENSG00000178772
ENSG00000178750	ENSG00000099365
ENSG00000178750	ENSG00000103496
ENSG00000178750	ENSG00000106089
ENSG00000178750	ENSG00000111450
ENSG00000178750	ENSG00000135604
ENSG00000178750	ENSG00000166900
ENSG00000178752	ENSG00000184163
ENSG00000178761	ENSG00000164970
ENSG00000178764	ENSG00000165156
ENSG00000178764	ENSG00000174306
ENSG00000178764	ENSG00000215271
ENSG00000178769	ENSG00000197382
ENSG00000178769	ENSG00000197791
ENSG00000178769	ENSG00000198230
ENSG00000178772	ENSG00000099769
ENSG00000178772	ENSG00000172061
ENSG00000178772	ENSG00000178732
ENSG00000178773	ENSG00000085719
ENSG00000178773	ENSG00000100884
ENSG00000178773	ENSG00000124772
ENSG00000178773	ENSG00000139117
ENSG00000178773	ENSG00000140848
ENSG00000178773	ENSG00000144550
ENSG00000178773	ENSG00000196353
ENSG00000178773	ENSG00000214078
ENSG00000178789	ENSG00000095970
ENSG00000178789	ENSG00000096264
ENSG00000178789	ENSG00000161649
ENSG00000178789	ENSG00000162896
ENSG00000178789	ENSG00000162897
ENSG00000178789	ENSG00000167850
ENSG00000178789	ENSG00000167851
ENSG00000178789	ENSG00000186074
ENSG00000178789	ENSG00000186407
ENSG00000178789	ENSG00000204345
ENSG00000178795	ENSG00000006007
ENSG00000178795	ENSG00000130055
ENSG00000178795	ENSG00000158555
ENSG00000178809	ENSG00000116525
ENSG00000178809	ENSG00000146755
ENSG00000178809	ENSG00000155428
ENSG00000178809	ENSG00000177238
ENSG00000178809	ENSG00000185880
ENSG00000178828	ENSG00000176641
ENSG00000178828	ENSG00000180537
ENSG00000178860	ENSG00000118526
ENSG00000178860	ENSG00000163792
ENSG00000178860	ENSG00000187728
ENSG00000178878	ENSG00000100336
ENSG00000178878	ENSG00000100342
ENSG00000178878	ENSG00000128284
ENSG00000178878	ENSG00000128313
ENSG00000178878	ENSG00000128335
ENSG00000178878	ENSG00000196785
ENSG00000178882	ENSG00000183688
ENSG00000178904	ENSG00000156162
ENSG00000178904	ENSG00000170629
ENSG00000178904	ENSG00000173852

ENSG00000178904	ENSG00000177990
ENSG00000178913	ENSG00000102387
ENSG00000178934	ENSG00000006659
ENSG00000178934	ENSG00000100079
ENSG00000178934	ENSG00000100097
ENSG00000178934	ENSG00000105198
ENSG00000178934	ENSG00000105205
ENSG00000178934	ENSG00000116977
ENSG00000178934	ENSG00000119862
ENSG00000178934	ENSG00000131981
ENSG00000178934	ENSG00000133317
ENSG00000178934	ENSG00000168961
ENSG00000178934	ENSG00000170298
ENSG00000178934	ENSG00000171747
ENSG00000178934	ENSG00000171916
ENSG00000178934	ENSG00000197778
ENSG00000178934	ENSG00000205076
ENSG00000178934	ENSG00000215132
ENSG00000178950	ENSG00000116675
ENSG00000178951	ENSG00000160685
ENSG00000178951	ENSG00000184828
ENSG00000178952	ENSG00000132394
ENSG00000178996	ENSG00000106266
ENSG00000178996	ENSG00000130340
ENSG00000178996	ENSG00000173548
ENSG00000179002	ENSG00000036828
ENSG00000179002	ENSG00000169962
ENSG00000179002	ENSG00000173612
ENSG00000179002	ENSG00000173662
ENSG00000179002	ENSG00000174930
ENSG00000179002	ENSG00000215740
ENSG00000179023	ENSG00000003096
ENSG00000179023	ENSG00000087448
ENSG00000179023	ENSG00000124743
ENSG00000179023	ENSG00000130487
ENSG00000179023	ENSG00000135686
ENSG00000179023	ENSG00000166853
ENSG00000179023	ENSG00000167487
ENSG00000179023	ENSG00000174010
ENSG00000179023	ENSG00000176595
ENSG00000179023	ENSG00000185214
ENSG00000179023	ENSG00000185915
ENSG00000179023	ENSG00000186231
ENSG00000179023	ENSG00000197705
ENSG00000179023	ENSG00000198642
ENSG00000179036	ENSG00000049245
ENSG00000179036	ENSG00000117533
ENSG00000179036	ENSG00000139190
ENSG00000179036	ENSG00000168899
ENSG00000179036	ENSG00000214719
ENSG00000179044	ENSG00000130201
ENSG00000179044	ENSG00000180104
ENSG00000179044	ENSG00000185215
ENSG00000179044	ENSG00000205436
ENSG00000179046	ENSG00000026950
ENSG00000179046	ENSG00000111801
ENSG00000179046	ENSG00000112763
ENSG00000179046	ENSG00000113303
ENSG00000179046	ENSG00000121236

ENSG00000179046	ENSG00000124508
ENSG00000179046	ENSG00000124549
ENSG00000179046	ENSG00000124557
ENSG00000179046	ENSG00000128250
ENSG00000179046	ENSG00000128253
ENSG00000179046	ENSG00000128276
ENSG00000179046	ENSG00000128482
ENSG00000179046	ENSG00000132256
ENSG00000179046	ENSG00000132274
ENSG00000179046	ENSG00000164010
ENSG00000179046	ENSG00000165810
ENSG00000179046	ENSG00000168903
ENSG00000179046	ENSG00000176422
ENSG00000179046	ENSG00000182805
ENSG00000179046	ENSG00000183439
ENSG00000179046	ENSG00000183718
ENSG00000179046	ENSG00000187574
ENSG00000179046	ENSG00000188683
ENSG00000179046	ENSG00000204614
ENSG00000179046	ENSG00000204616
ENSG00000179046	ENSG00000204618
ENSG00000179046	ENSG00000205198
ENSG00000179046	ENSG00000214693
ENSG00000179059	ENSG00000100811
ENSG00000179059	ENSG00000188786
ENSG00000179059	ENSG00000198767
ENSG00000179094	ENSG00000049246
ENSG00000179094	ENSG00000132326
ENSG00000179097	ENSG00000043591
ENSG00000179097	ENSG00000102468
ENSG00000179097	ENSG00000113749
ENSG00000179097	ENSG00000125631
ENSG00000179097	ENSG00000135312
ENSG00000179097	ENSG00000135914
ENSG00000179097	ENSG00000147246
ENSG00000179097	ENSG00000150594
ENSG00000179097	ENSG00000157219
ENSG00000179097	ENSG00000164270
ENSG00000179097	ENSG00000168830
ENSG00000179097	ENSG00000169252
ENSG00000179097	ENSG00000175658
ENSG00000179097	ENSG00000178394
ENSG00000179097	ENSG00000179546
ENSG00000179097	ENSG00000181210
ENSG00000179097	ENSG00000184303
ENSG00000179097	ENSG00000184845
ENSG00000179097	ENSG00000185321
ENSG00000179097	ENSG00000188778
ENSG00000179111	ENSG00000069812
ENSG00000179111	ENSG00000114315
ENSG00000179111	ENSG00000144485
ENSG00000179111	ENSG00000173673
ENSG00000179111	ENSG00000188290
ENSG00000179111	ENSG00000197921
ENSG00000179134	ENSG00000020577
ENSG00000179142	ENSG00000019186
ENSG00000179142	ENSG00000111012
ENSG00000179142	ENSG00000135929
ENSG00000179142	ENSG00000140459

ENSG00000179142	ENSG00000160882
ENSG00000179142	ENSG00000186684
ENSG00000179144	ENSG00000106560
ENSG00000179144	ENSG00000133561
ENSG00000179144	ENSG00000133574
ENSG00000179144	ENSG00000171115
ENSG00000179144	ENSG00000196329
ENSG00000179144	ENSG00000213203
ENSG00000179148	ENSG00000012779
ENSG00000179148	ENSG00000108839
ENSG00000179148	ENSG00000161905
ENSG00000179148	ENSG00000179477
ENSG00000179148	ENSG00000179593
ENSG00000179148	ENSG00000188101
ENSG00000179148	ENSG00000214859
ENSG00000179172	ENSG00000092199
ENSG00000179172	ENSG00000125970
ENSG00000179172	ENSG00000184672
ENSG00000179172	ENSG00000214634
ENSG00000179172	ENSG00000215880
ENSG00000179195	ENSG00000142396
ENSG00000179195	ENSG00000169740
ENSG00000179195	ENSG00000174255
ENSG00000179195	ENSG00000179965
ENSG00000179195	ENSG00000184294
ENSG00000179195	ENSG00000186446
ENSG00000179195	ENSG00000186777
ENSG00000179195	ENSG00000197008
ENSG00000179195	ENSG00000197025
ENSG00000179195	ENSG00000197062
ENSG00000179195	ENSG00000197332
ENSG00000179195	ENSG00000198155
ENSG00000179195	ENSG00000204756
ENSG00000179195	ENSG00000212805
ENSG00000179195	ENSG00000213976
ENSG00000179195	ENSG00000213980
ENSG00000179195	ENSG00000215180
ENSG00000179195	ENSG00000215510
ENSG00000179195	ENSG00000215537
ENSG00000179195	ENSG00000215598
ENSG00000179195	ENSG00000215600
ENSG00000179218	ENSG00000141979
ENSG00000179262	ENSG00000119318
ENSG00000179277	ENSG00000105419
ENSG00000179277	ENSG00000118707
ENSG00000179277	ENSG00000134138
ENSG00000179277	ENSG00000143995
ENSG00000179277	ENSG00000153779
ENSG00000179277	ENSG00000160199
ENSG00000179277	ENSG00000165495
ENSG00000179277	ENSG00000176679
ENSG00000179277	ENSG00000177426
ENSG00000179277	ENSG00000188013
ENSG00000179284	ENSG00000147869
ENSG00000179284	ENSG00000158747
ENSG00000179284	ENSG00000166923
ENSG00000179284	ENSG00000180875
ENSG00000179292	ENSG00000178233
ENSG00000179295	ENSG00000111679

ENSG00000179295	ENSG00000215917
ENSG00000179296	ENSG00000108100
ENSG00000179296	ENSG00000163249
ENSG00000179296	ENSG00000182632
ENSG00000179296	ENSG00000184766
ENSG00000179296	ENSG00000204150
ENSG00000179296	ENSG00000215169
ENSG00000179300	ENSG00000187823
ENSG00000179314	ENSG00000075035
ENSG00000179315	ENSG00000160201
ENSG00000179315	ENSG00000161265
ENSG00000179331	ENSG00000104388
ENSG00000179331	ENSG00000119396
ENSG00000179331	ENSG00000129472
ENSG00000179331	ENSG00000155961
ENSG00000179331	ENSG00000167578
ENSG00000179331	ENSG00000168118
ENSG00000179331	ENSG00000183940
ENSG00000179331	ENSG00000188060
ENSG00000179335	ENSG00000013441
ENSG00000179335	ENSG00000113240
ENSG00000179335	ENSG00000176444
ENSG00000179335	ENSG00000178340
ENSG00000179335	ENSG00000215793
ENSG00000179348	ENSG00000102145
ENSG00000179348	ENSG00000107485
ENSG00000179348	ENSG00000130700
ENSG00000179348	ENSG00000136574
ENSG00000179348	ENSG00000141448
ENSG00000179349	ENSG00000205699
ENSG00000179361	ENSG00000049618
ENSG00000179361	ENSG00000116017
ENSG00000179361	ENSG00000117713
ENSG00000179361	ENSG00000205143
ENSG00000179364	ENSG00000175115
ENSG00000179387	ENSG00000110675
ENSG00000179387	ENSG00000115459
ENSG00000179388	ENSG00000120738
ENSG00000179388	ENSG00000122877
ENSG00000179388	ENSG00000135625
ENSG00000179388	ENSG00000184937
ENSG00000179399	ENSG00000063660
ENSG00000179399	ENSG00000076716
ENSG00000179399	ENSG00000147257
ENSG00000179399	ENSG00000183098
ENSG00000179399	ENSG00000213420
ENSG00000179407	ENSG00000105993
ENSG00000179407	ENSG00000135924
ENSG00000179407	ENSG00000172404
ENSG00000179455	ENSG00000075975
ENSG00000179455	ENSG00000133606
ENSG00000179456	ENSG00000214438
ENSG00000179477	ENSG00000012779
ENSG00000179477	ENSG00000108839
ENSG00000179477	ENSG00000161905
ENSG00000179477	ENSG00000179148
ENSG00000179477	ENSG00000179593
ENSG00000179477	ENSG00000188101
ENSG00000179477	ENSG00000214859

ENSG00000179520	ENSG00000091664
ENSG00000179520	ENSG00000104888
ENSG00000179520	ENSG00000112337
ENSG00000179520	ENSG00000119899
ENSG00000179520	ENSG00000124564
ENSG00000179520	ENSG00000124568
ENSG00000179520	ENSG00000146039
ENSG00000179528	ENSG00000138136
ENSG00000179528	ENSG00000152804
ENSG00000179542	ENSG00000165300
ENSG00000179542	ENSG00000184564
ENSG00000179546	ENSG00000043591
ENSG00000179546	ENSG00000102468
ENSG00000179546	ENSG00000113749
ENSG00000179546	ENSG00000125631
ENSG00000179546	ENSG00000135312
ENSG00000179546	ENSG00000135914
ENSG00000179546	ENSG00000147246
ENSG00000179546	ENSG00000150594
ENSG00000179546	ENSG00000157219
ENSG00000179546	ENSG00000164270
ENSG00000179546	ENSG00000168830
ENSG00000179546	ENSG00000169252
ENSG00000179546	ENSG00000175658
ENSG00000179546	ENSG00000178394
ENSG00000179546	ENSG00000179097
ENSG00000179546	ENSG00000181210
ENSG00000179546	ENSG00000184303
ENSG00000179546	ENSG00000184845
ENSG00000179546	ENSG00000185321
ENSG00000179546	ENSG00000188778
ENSG00000179580	ENSG00000181852
ENSG00000179583	ENSG00000106100
ENSG00000179588	ENSG00000169946
ENSG00000179593	ENSG00000012779
ENSG00000179593	ENSG00000108839
ENSG00000179593	ENSG00000161905
ENSG00000179593	ENSG00000179148
ENSG00000179593	ENSG00000179477
ENSG00000179593	ENSG00000188101
ENSG00000179593	ENSG00000214859
ENSG00000179600	ENSG00000104818
ENSG00000179600	ENSG00000104826
ENSG00000179600	ENSG00000104827
ENSG00000179600	ENSG00000131808
ENSG00000179600	ENSG00000134200
ENSG00000179600	ENSG00000189052
ENSG00000179600	ENSG00000196337
ENSG00000179600	ENSG00000204748
ENSG00000179600	ENSG00000213030
ENSG00000179603	ENSG00000113262
ENSG00000179603	ENSG00000124493
ENSG00000179603	ENSG00000152822
ENSG00000179603	ENSG00000164082
ENSG00000179603	ENSG00000168959
ENSG00000179603	ENSG00000196277
ENSG00000179603	ENSG00000198822
ENSG00000179604	ENSG00000128283
ENSG00000179604	ENSG00000149798

ENSG00000179604	ENSG00000163171
ENSG00000179604	ENSG00000167617
ENSG00000179636	ENSG00000159713
ENSG00000179636	ENSG00000171368
ENSG00000179639	ENSG00000072694
ENSG00000179639	ENSG00000132185
ENSG00000179639	ENSG00000143226
ENSG00000179639	ENSG00000150337
ENSG00000179639	ENSG00000162747
ENSG00000179639	ENSG00000181036
ENSG00000179639	ENSG00000198019
ENSG00000179639	ENSG00000203747
ENSG00000179639	ENSG00000203820
ENSG00000179673	ENSG00000177519
ENSG00000179704	ENSG00000116708
ENSG00000179704	ENSG00000116721
ENSG00000179704	ENSG00000116726
ENSG00000179704	ENSG00000120952
ENSG00000179704	ENSG00000157358
ENSG00000179704	ENSG00000160959
ENSG00000179704	ENSG00000182330
ENSG00000179704	ENSG00000185028
ENSG00000179704	ENSG00000185686
ENSG00000179704	ENSG00000187545
ENSG00000179704	ENSG00000197549
ENSG00000179704	ENSG00000204478
ENSG00000179704	ENSG00000204479
ENSG00000179704	ENSG00000204480
ENSG00000179704	ENSG00000204481
ENSG00000179704	ENSG00000204485
ENSG00000179704	ENSG00000204486
ENSG00000179704	ENSG00000204488
ENSG00000179704	ENSG00000204491
ENSG00000179704	ENSG00000204495
ENSG00000179704	ENSG00000204501
ENSG00000179704	ENSG00000204502
ENSG00000179704	ENSG00000204503
ENSG00000179704	ENSG00000204505
ENSG00000179704	ENSG00000204507
ENSG00000179704	ENSG00000204508
ENSG00000179704	ENSG00000204509
ENSG00000179704	ENSG00000204510
ENSG00000179704	ENSG00000204513
ENSG00000179709	ENSG00000022556
ENSG00000179709	ENSG00000023191
ENSG00000179709	ENSG00000140853
ENSG00000179709	ENSG00000142405
ENSG00000179709	ENSG00000158077
ENSG00000179709	ENSG00000160505
ENSG00000179709	ENSG00000162711
ENSG00000179709	ENSG00000167207
ENSG00000179709	ENSG00000167634
ENSG00000179709	ENSG00000167984
ENSG00000179709	ENSG00000171487
ENSG00000179709	ENSG00000173572
ENSG00000179709	ENSG00000174885
ENSG00000179709	ENSG00000179873
ENSG00000179709	ENSG00000182261
ENSG00000179709	ENSG00000185792

ENSG00000179750	ENSG00000100298
ENSG00000179750	ENSG00000111701
ENSG00000179750	ENSG00000111732
ENSG00000179750	ENSG00000124701
ENSG00000179750	ENSG00000128383
ENSG00000179750	ENSG00000128394
ENSG00000179750	ENSG00000198904
ENSG00000179772	ENSG00000054598
ENSG00000179772	ENSG00000125798
ENSG00000179772	ENSG00000129514
ENSG00000179772	ENSG00000164379
ENSG00000179772	ENSG00000168269
ENSG00000179772	ENSG00000170122
ENSG00000179772	ENSG00000170608
ENSG00000179772	ENSG00000176165
ENSG00000179772	ENSG00000176678
ENSG00000179772	ENSG00000176692
ENSG00000179772	ENSG00000178919
ENSG00000179772	ENSG00000183770
ENSG00000179772	ENSG00000184492
ENSG00000179772	ENSG00000184659
ENSG00000179772	ENSG00000186564
ENSG00000179772	ENSG00000186766
ENSG00000179772	ENSG00000186790
ENSG00000179772	ENSG00000187140
ENSG00000179772	ENSG00000187559
ENSG00000179772	ENSG00000204612
ENSG00000179772	ENSG00000204779
ENSG00000179772	ENSG00000204793
ENSG00000179772	ENSG00000204828
ENSG00000179772	ENSG00000214336
ENSG00000179776	ENSG00000040731
ENSG00000179776	ENSG00000071991
ENSG00000179776	ENSG00000081138
ENSG00000179776	ENSG00000101542
ENSG00000179776	ENSG00000113100
ENSG00000179776	ENSG00000113361
ENSG00000179776	ENSG00000139880
ENSG00000179776	ENSG00000140937
ENSG00000179776	ENSG00000145526
ENSG00000179776	ENSG00000149654
ENSG00000179776	ENSG00000150394
ENSG00000179776	ENSG00000154162
ENSG00000179796	ENSG00000160233
ENSG00000179820	ENSG00000185105
ENSG00000179832	ENSG00000171495
ENSG00000179832	ENSG00000185038
ENSG00000179837	ENSG00000065526
ENSG00000179837	ENSG00000162775
ENSG00000179855	ENSG00000123159
ENSG00000179855	ENSG00000137960
ENSG00000179862	ENSG00000125931
ENSG00000179862	ENSG00000164442
ENSG00000179869	ENSG00000064687
ENSG00000179869	ENSG00000107331
ENSG00000179869	ENSG00000144452
ENSG00000179869	ENSG00000165029
ENSG00000179869	ENSG00000167972
ENSG00000179869	ENSG00000198691

ENSG00000179873	ENSG00000022556
ENSG00000179873	ENSG00000023191
ENSG00000179873	ENSG00000140853
ENSG00000179873	ENSG00000142405
ENSG00000179873	ENSG00000158077
ENSG00000179873	ENSG00000160505
ENSG00000179873	ENSG00000162711
ENSG00000179873	ENSG00000167207
ENSG00000179873	ENSG00000167634
ENSG00000179873	ENSG00000167984
ENSG00000179873	ENSG00000171487
ENSG00000179873	ENSG00000173572
ENSG00000179873	ENSG00000174885
ENSG00000179873	ENSG00000179709
ENSG00000179873	ENSG00000182261
ENSG00000179873	ENSG00000185792
ENSG00000179899	ENSG00000111752
ENSG00000179899	ENSG00000134686
ENSG00000179899	ENSG00000173889
ENSG00000179899	ENSG00000187033
ENSG00000179899	ENSG00000187634
ENSG00000179912	ENSG00000048991
ENSG00000179912	ENSG00000172995
ENSG00000179913	ENSG00000156966
ENSG00000179913	ENSG00000162630
ENSG00000179913	ENSG00000169255
ENSG00000179913	ENSG00000170340
ENSG00000179913	ENSG00000172318
ENSG00000179913	ENSG00000176383
ENSG00000179913	ENSG00000176597
ENSG00000179913	ENSG00000177191
ENSG00000179913	ENSG00000180561
ENSG00000179913	ENSG00000183778
ENSG00000179913	ENSG00000198488
ENSG00000179913	ENSG00000204222
ENSG00000179913	ENSG00000214654
ENSG00000179915	ENSG00000021645
ENSG00000179915	ENSG00000110076
ENSG00000179918	ENSG00000086475
ENSG00000179934	ENSG00000112486
ENSG00000179934	ENSG00000121797
ENSG00000179934	ENSG00000121807
ENSG00000179934	ENSG00000121966
ENSG00000179934	ENSG00000126353
ENSG00000179934	ENSG00000129048
ENSG00000179934	ENSG00000144648
ENSG00000179934	ENSG00000160683
ENSG00000179934	ENSG00000160791
ENSG00000179934	ENSG00000163464
ENSG00000179934	ENSG00000163823
ENSG00000179934	ENSG00000168329
ENSG00000179934	ENSG00000172215
ENSG00000179934	ENSG00000173578
ENSG00000179934	ENSG00000173585
ENSG00000179934	ENSG00000180871
ENSG00000179934	ENSG00000183625
ENSG00000179934	ENSG00000183813
ENSG00000179934	ENSG00000184451
ENSG00000179934	ENSG00000186810

ENSG00000179934	ENSG00000215775
ENSG00000179934	ENSG00000215778
ENSG00000179934	ENSG00000215782
ENSG00000179954	ENSG00000013725
ENSG00000179954	ENSG00000073754
ENSG00000179954	ENSG00000110448
ENSG00000179954	ENSG00000146700
ENSG00000179954	ENSG00000164099
ENSG00000179954	ENSG00000177575
ENSG00000179954	ENSG00000177675
ENSG00000179954	ENSG00000187908
ENSG00000179954	ENSG00000214279
ENSG00000179965	ENSG00000142396
ENSG00000179965	ENSG00000169740
ENSG00000179965	ENSG00000174255
ENSG00000179965	ENSG00000179195
ENSG00000179965	ENSG00000184294
ENSG00000179965	ENSG00000186446
ENSG00000179965	ENSG00000186777
ENSG00000179965	ENSG00000197008
ENSG00000179965	ENSG00000197025
ENSG00000179965	ENSG00000197062
ENSG00000179965	ENSG00000197332
ENSG00000179965	ENSG00000198155
ENSG00000179965	ENSG00000204756
ENSG00000179965	ENSG00000212805
ENSG00000179965	ENSG00000213976
ENSG00000179965	ENSG00000213980
ENSG00000179965	ENSG00000215180
ENSG00000179965	ENSG00000215510
ENSG00000179965	ENSG00000215537
ENSG00000179965	ENSG00000215598
ENSG00000179965	ENSG00000215600
ENSG00000179978	ENSG00000023445
ENSG00000179978	ENSG00000081770
ENSG00000179978	ENSG00000101197
ENSG00000179978	ENSG00000101966
ENSG00000179978	ENSG00000110330
ENSG00000179978	ENSG00000180152
ENSG00000179981	ENSG00000121297
ENSG00000179981	ENSG00000182463
ENSG00000179994	ENSG00000136206
ENSG00000179994	ENSG00000154732
ENSG00000179994	ENSG00000163806
ENSG00000179994	ENSG00000166667
ENSG00000179994	ENSG00000170092
ENSG00000179994	ENSG00000173678
ENSG00000179994	ENSG00000183318
ENSG00000179994	ENSG00000184616
ENSG00000179994	ENSG00000185040
ENSG00000179994	ENSG00000186645
ENSG00000179994	ENSG00000198305
ENSG00000179994	ENSG00000204710
ENSG00000179994	ENSG00000205238
ENSG00000179994	ENSG00000205482
ENSG00000179994	ENSG00000205505
ENSG00000179994	ENSG00000205506
ENSG00000179994	ENSG00000205508
ENSG00000179994	ENSG00000205509

ENSG00000179994	ENSG00000205580
ENSG00000179994	ENSG00000214300
ENSG00000179994	ENSG00000215092
ENSG00000180008	ENSG00000114737
ENSG00000180008	ENSG00000120833
ENSG00000180008	ENSG00000170677
ENSG00000180008	ENSG00000171150
ENSG00000180008	ENSG00000174111
ENSG00000180008	ENSG00000184557
ENSG00000180008	ENSG00000185338
ENSG00000180015	ENSG00000112031
ENSG00000180015	ENSG00000120662
ENSG00000180053	ENSG00000109705
ENSG00000180053	ENSG00000119919
ENSG00000180053	ENSG00000125816
ENSG00000180053	ENSG00000125820
ENSG00000180053	ENSG00000136327
ENSG00000180053	ENSG00000136352
ENSG00000180053	ENSG00000183072
ENSG00000180061	ENSG00000136048
ENSG00000180061	ENSG00000156171
ENSG00000180061	ENSG00000168890
ENSG00000180089	ENSG00000151117
ENSG00000180096	ENSG00000108387
ENSG00000180096	ENSG00000122545
ENSG00000180096	ENSG00000168385
ENSG00000180096	ENSG00000184702
ENSG00000180096	ENSG00000214005
ENSG00000180096	ENSG00000214765
ENSG00000180096	ENSG00000214834
ENSG00000180104	ENSG00000130201
ENSG00000180104	ENSG00000179044
ENSG00000180104	ENSG00000185215
ENSG00000180104	ENSG00000205436
ENSG00000180113	ENSG00000095627
ENSG00000180113	ENSG00000132972
ENSG00000180113	ENSG00000182134
ENSG00000180113	ENSG00000196116
ENSG00000180138	ENSG00000113712
ENSG00000180150	ENSG00000118418
ENSG00000180150	ENSG00000176982
ENSG00000180150	ENSG00000182952
ENSG00000180150	ENSG00000198830
ENSG00000180150	ENSG00000212769
ENSG00000180150	ENSG00000214578
ENSG00000180152	ENSG00000023445
ENSG00000180152	ENSG00000081770
ENSG00000180152	ENSG00000101197
ENSG00000180152	ENSG00000101966
ENSG00000180152	ENSG00000110330
ENSG00000180152	ENSG00000179978
ENSG00000180176	ENSG00000129167
ENSG00000180176	ENSG00000139287
ENSG00000180176	ENSG00000171759
ENSG00000180209	ENSG00000101335
ENSG00000180209	ENSG00000101608
ENSG00000180209	ENSG00000106436
ENSG00000180209	ENSG00000106631
ENSG00000180209	ENSG00000111245

ENSG00000180209	ENSG00000118680
ENSG00000180209	ENSG00000215375
ENSG00000180210	ENSG00000057593
ENSG00000180210	ENSG00000101981
ENSG00000180210	ENSG00000115718
ENSG00000180210	ENSG00000126218
ENSG00000180210	ENSG00000126231
ENSG00000180212	ENSG00000029993
ENSG00000180212	ENSG00000100118
ENSG00000180212	ENSG00000124097
ENSG00000180212	ENSG00000132967
ENSG00000180212	ENSG00000164104
ENSG00000180212	ENSG00000173961
ENSG00000180212	ENSG00000176256
ENSG00000180212	ENSG00000182181
ENSG00000180212	ENSG00000182711
ENSG00000180212	ENSG00000189403
ENSG00000180212	ENSG00000214364
ENSG00000180212	ENSG00000214852
ENSG00000180212	ENSG00000215308
ENSG00000180228	ENSG00000139546
ENSG00000180233	ENSG00000141622
ENSG00000180233	ENSG00000186187
ENSG00000180245	ENSG00000054277
ENSG00000180245	ENSG00000102076
ENSG00000180245	ENSG00000122375
ENSG00000180245	ENSG00000124818
ENSG00000180245	ENSG00000128617
ENSG00000180245	ENSG00000147380
ENSG00000180245	ENSG00000148604
ENSG00000180245	ENSG00000163914
ENSG00000180245	ENSG00000166160
ENSG00000180251	ENSG00000066230
ENSG00000180251	ENSG00000090020
ENSG00000180251	ENSG00000115616
ENSG00000180251	ENSG00000135740
ENSG00000180263	ENSG00000102302
ENSG00000180263	ENSG00000154783
ENSG00000180264	ENSG00000072071
ENSG00000180264	ENSG00000111452
ENSG00000180264	ENSG00000117114
ENSG00000180264	ENSG00000150471
ENSG00000180287	ENSG00000105223
ENSG00000180287	ENSG00000166428
ENSG00000180304	ENSG00000104904
ENSG00000180304	ENSG00000143450
ENSG00000180316	ENSG00000006757
ENSG00000180316	ENSG00000100341
ENSG00000180316	ENSG00000100344
ENSG00000180316	ENSG00000177666
ENSG00000180318	ENSG00000004848
ENSG00000180318	ENSG00000052850
ENSG00000180318	ENSG00000100987
ENSG00000180318	ENSG00000119614
ENSG00000180318	ENSG00000156150
ENSG00000180332	ENSG00000134504
ENSG00000180332	ENSG00000151364
ENSG00000180332	ENSG00000153885
ENSG00000180332	ENSG00000155729

ENSG00000180332	ENSG00000168301
ENSG00000180332	ENSG00000168676
ENSG00000180332	ENSG00000178695
ENSG00000180332	ENSG00000183775
ENSG00000180332	ENSG00000183783
ENSG00000180332	ENSG00000188997
ENSG00000180332	ENSG00000198553
ENSG00000180332	ENSG00000213859
ENSG00000180340	ENSG00000104290
ENSG00000180340	ENSG00000106483
ENSG00000180340	ENSG00000111432
ENSG00000180340	ENSG00000155760
ENSG00000180340	ENSG00000157240
ENSG00000180340	ENSG00000162998
ENSG00000180340	ENSG00000163251
ENSG00000180340	ENSG00000164930
ENSG00000180340	ENSG00000174804
ENSG00000180340	ENSG00000177283
ENSG00000180340	ENSG00000188763
ENSG00000180347	ENSG00000135426
ENSG00000180347	ENSG00000138434
ENSG00000180357	ENSG00000168916
ENSG00000180370	ENSG00000077264
ENSG00000180370	ENSG00000149269
ENSG00000180432	ENSG00000124212
ENSG00000180432	ENSG00000146233
ENSG00000180432	ENSG00000167910
ENSG00000180432	ENSG00000172817
ENSG00000180448	ENSG00000089639
ENSG00000180448	ENSG00000137962
ENSG00000180481	ENSG00000096006
ENSG00000180481	ENSG00000101074
ENSG00000180481	ENSG00000103196
ENSG00000180481	ENSG00000121005
ENSG00000180481	ENSG00000124490
ENSG00000180481	ENSG00000124812
ENSG00000180481	ENSG00000137558
ENSG00000180481	ENSG00000139278
ENSG00000180481	ENSG00000164530
ENSG00000180481	ENSG00000173401
ENSG00000180488	ENSG00000148343
ENSG00000180537	ENSG00000176641
ENSG00000180537	ENSG00000178828
ENSG00000180549	ENSG00000130383
ENSG00000180549	ENSG00000156413
ENSG00000180549	ENSG00000171124
ENSG00000180549	ENSG00000172461
ENSG00000180549	ENSG00000172728
ENSG00000180549	ENSG00000196371
ENSG00000180549	ENSG00000196968
ENSG00000180561	ENSG00000156966
ENSG00000180561	ENSG00000162630
ENSG00000180561	ENSG00000169255
ENSG00000180561	ENSG00000170340
ENSG00000180561	ENSG00000172318
ENSG00000180561	ENSG00000176383
ENSG00000180561	ENSG00000176597
ENSG00000180561	ENSG00000177191
ENSG00000180561	ENSG00000179913

ENSG00000180561	ENSG00000183778
ENSG00000180561	ENSG00000198488
ENSG00000180561	ENSG00000204222
ENSG00000180561	ENSG00000214654
ENSG00000180573	ENSG00000111332
ENSG00000180573	ENSG00000124642
ENSG00000180573	ENSG00000137259
ENSG00000180573	ENSG00000158403
ENSG00000180573	ENSG00000164508
ENSG00000180573	ENSG00000168274
ENSG00000180573	ENSG00000181218
ENSG00000180573	ENSG00000182611
ENSG00000180573	ENSG00000183558
ENSG00000180573	ENSG00000184260
ENSG00000180573	ENSG00000184270
ENSG00000180573	ENSG00000184348
ENSG00000180573	ENSG00000184825
ENSG00000180573	ENSG00000185978
ENSG00000180573	ENSG00000188486
ENSG00000180573	ENSG00000196747
ENSG00000180573	ENSG00000196787
ENSG00000180573	ENSG00000196866
ENSG00000180573	ENSG00000198082
ENSG00000180573	ENSG00000198307
ENSG00000180573	ENSG00000198374
ENSG00000180573	ENSG00000203812
ENSG00000180573	ENSG00000205070
ENSG00000180573	ENSG00000205079
ENSG00000180610	ENSG00000169155
ENSG00000180610	ENSG00000185278
ENSG00000180610	ENSG00000204210
ENSG00000180610	ENSG00000204366
ENSG00000180610	ENSG00000213588
ENSG00000180611	ENSG00000181284
ENSG00000180613	ENSG00000105997
ENSG00000180613	ENSG00000106038
ENSG00000180613	ENSG00000120093
ENSG00000180613	ENSG00000128652
ENSG00000180613	ENSG00000169840
ENSG00000180613	ENSG00000174279
ENSG00000180616	ENSG00000082556
ENSG00000180616	ENSG00000112038
ENSG00000180616	ENSG00000116014
ENSG00000180616	ENSG00000116329
ENSG00000180616	ENSG00000125510
ENSG00000180616	ENSG00000125522
ENSG00000180616	ENSG00000128285
ENSG00000180616	ENSG00000128310
ENSG00000180616	ENSG00000132671
ENSG00000180616	ENSG00000139874
ENSG00000180616	ENSG00000152034
ENSG00000180616	ENSG00000162009
ENSG00000180616	ENSG00000166573
ENSG00000180616	ENSG00000182687
ENSG00000180616	ENSG00000183473
ENSG00000180616	ENSG00000183729
ENSG00000180628	ENSG00000056661
ENSG00000180628	ENSG00000115289
ENSG00000180628	ENSG00000156374

ENSG00000180628	ENSG00000168283
ENSG00000180628	ENSG00000185619
ENSG00000180638	ENSG00000142494
ENSG00000180644	ENSG00000021852
ENSG00000180644	ENSG00000039537
ENSG00000180644	ENSG00000112936
ENSG00000180644	ENSG00000113600
ENSG00000180644	ENSG00000157131
ENSG00000180660	ENSG00000172478
ENSG00000180660	ENSG00000173212
ENSG00000180660	ENSG00000181541
ENSG00000180730	ENSG00000166737
ENSG00000180730	ENSG00000178343
ENSG00000180730	ENSG00000187902
ENSG00000180730	ENSG00000188803
ENSG00000180733	ENSG00000092067
ENSG00000180733	ENSG00000172216
ENSG00000180739	ENSG00000064547
ENSG00000180739	ENSG00000118432
ENSG00000180739	ENSG00000125910
ENSG00000180739	ENSG00000132975
ENSG00000180739	ENSG00000146360
ENSG00000180739	ENSG00000170989
ENSG00000180739	ENSG00000171517
ENSG00000180739	ENSG00000175898
ENSG00000180739	ENSG00000181773
ENSG00000180739	ENSG00000188822
ENSG00000180739	ENSG00000198121
ENSG00000180739	ENSG00000213694
ENSG00000180745	ENSG00000163646
ENSG00000180745	ENSG00000188022
ENSG00000180767	ENSG00000115526
ENSG00000180767	ENSG00000124302
ENSG00000180767	ENSG00000136213
ENSG00000180767	ENSG00000154080
ENSG00000180767	ENSG00000169105
ENSG00000180767	ENSG00000171310
ENSG00000180772	ENSG00000100739
ENSG00000180772	ENSG00000134817
ENSG00000180772	ENSG00000144891
ENSG00000180772	ENSG00000154165
ENSG00000180772	ENSG00000168398
ENSG00000180772	ENSG00000170128
ENSG00000180772	ENSG00000173080
ENSG00000180772	ENSG00000182631
ENSG00000180773	ENSG00000123643
ENSG00000180773	ENSG00000186334
ENSG00000180773	ENSG00000186335
ENSG00000180776	ENSG00000102383
ENSG00000180776	ENSG00000104219
ENSG00000180795	ENSG00000087086
ENSG00000180795	ENSG00000132446
ENSG00000180795	ENSG00000167996
ENSG00000180795	ENSG00000171878
ENSG00000180795	ENSG00000173477
ENSG00000180795	ENSG00000176778
ENSG00000180795	ENSG00000181867
ENSG00000180795	ENSG00000197518
ENSG00000180795	ENSG00000215234

ENSG00000180801	ENSG00000006756
ENSG00000180801	ENSG00000062096
ENSG00000180801	ENSG00000100299
ENSG00000180801	ENSG00000101846
ENSG00000180801	ENSG00000113273
ENSG00000180801	ENSG00000141012
ENSG00000180801	ENSG00000141337
ENSG00000180801	ENSG00000157399
ENSG00000180801	ENSG00000183876
ENSG00000180801	ENSG00000205667
ENSG00000180806	ENSG00000078399
ENSG00000180806	ENSG00000128709
ENSG00000180806	ENSG00000128710
ENSG00000180806	ENSG00000153807
ENSG00000180806	ENSG00000170689
ENSG00000180806	ENSG00000180818
ENSG00000180815	ENSG00000142733
ENSG00000180815	ENSG00000197442
ENSG00000180817	ENSG00000138777
ENSG00000180818	ENSG00000078399
ENSG00000180818	ENSG00000128709
ENSG00000180818	ENSG00000128710
ENSG00000180818	ENSG00000153807
ENSG00000180818	ENSG00000170689
ENSG00000180818	ENSG00000180806
ENSG00000180828	ENSG00000125533
ENSG00000180828	ENSG00000177468
ENSG00000180828	ENSG00000184221
ENSG00000180828	ENSG00000205927
ENSG00000180834	ENSG00000171533
ENSG00000180860	ENSG00000096088
ENSG00000180860	ENSG00000117984
ENSG00000180860	ENSG00000131400
ENSG00000180860	ENSG00000131401
ENSG00000180860	ENSG00000143839
ENSG00000180860	ENSG00000172647
ENSG00000180860	ENSG00000184488
ENSG00000180860	ENSG00000196188
ENSG00000180860	ENSG00000204973
ENSG00000180860	ENSG00000215868
ENSG00000180871	ENSG00000112486
ENSG00000180871	ENSG00000121797
ENSG00000180871	ENSG00000121807
ENSG00000180871	ENSG00000121966
ENSG00000180871	ENSG00000126353
ENSG00000180871	ENSG00000129048
ENSG00000180871	ENSG00000144648
ENSG00000180871	ENSG00000160683
ENSG00000180871	ENSG00000160791
ENSG00000180871	ENSG00000163464
ENSG00000180871	ENSG00000163823
ENSG00000180871	ENSG00000168329
ENSG00000180871	ENSG00000172215
ENSG00000180871	ENSG00000173578
ENSG00000180871	ENSG00000173585
ENSG00000180871	ENSG00000179934
ENSG00000180871	ENSG00000183625
ENSG00000180871	ENSG00000183813
ENSG00000180871	ENSG00000184451

ENSG00000180871	ENSG00000186810
ENSG00000180871	ENSG00000215775
ENSG00000180871	ENSG00000215778
ENSG00000180871	ENSG00000215782
ENSG00000180875	ENSG00000147869
ENSG00000180875	ENSG00000158747
ENSG00000180875	ENSG00000166923
ENSG00000180875	ENSG00000179284
ENSG00000180900	ENSG00000033122
ENSG00000180900	ENSG00000112851
ENSG00000180900	ENSG00000137269
ENSG00000180900	ENSG00000147324
ENSG00000180900	ENSG00000188306
ENSG00000180901	ENSG00000100379
ENSG00000180901	ENSG00000104756
ENSG00000180901	ENSG00000136636
ENSG00000180901	ENSG00000160410
ENSG00000180901	ENSG00000167977
ENSG00000180901	ENSG00000187074
ENSG00000180901	ENSG00000187988
ENSG00000180914	ENSG00000109163
ENSG00000180914	ENSG00000126895
ENSG00000180914	ENSG00000166148
ENSG00000180914	ENSG00000178015
ENSG00000180914	ENSG00000187258
ENSG00000180914	ENSG00000198049
ENSG00000180914	ENSG00000211451
ENSG00000180921	ENSG00000101447
ENSG00000180921	ENSG00000105523
ENSG00000180921	ENSG00000125998
ENSG00000180921	ENSG00000133477
ENSG00000180921	ENSG00000147689
ENSG00000180921	ENSG00000168143
ENSG00000180921	ENSG00000188522
ENSG00000180929	ENSG00000156097
ENSG00000180957	ENSG00000154217
ENSG00000180957	ENSG00000174238
ENSG00000180991	ENSG00000124422
ENSG00000180991	ENSG00000185295
ENSG00000180998	ENSG00000077585
ENSG00000180998	ENSG00000173264
ENSG00000181019	ENSG00000124588
ENSG00000181026	ENSG00000143319
ENSG00000181026	ENSG00000172183
ENSG00000181036	ENSG00000072694
ENSG00000181036	ENSG00000132185
ENSG00000181036	ENSG00000143226
ENSG00000181036	ENSG00000150337
ENSG00000181036	ENSG00000162747
ENSG00000181036	ENSG00000179639
ENSG00000181036	ENSG00000198019
ENSG00000181036	ENSG00000203747
ENSG00000181036	ENSG00000203820
ENSG00000181039	ENSG00000188134
ENSG00000181039	ENSG00000189127
ENSG00000181061	ENSG00000131097
ENSG00000181061	ENSG00000146066
ENSG00000181061	ENSG00000175202
ENSG00000181061	ENSG00000212646

ENSG00000181061	ENSG00000214511
ENSG00000181072	ENSG00000133019
ENSG00000181072	ENSG00000168539
ENSG00000181072	ENSG00000184984
ENSG00000181090	ENSG00000204371
ENSG00000181092	ENSG00000054803
ENSG00000181092	ENSG00000102924
ENSG00000181092	ENSG00000131094
ENSG00000181092	ENSG00000133466
ENSG00000181092	ENSG00000139899
ENSG00000181092	ENSG00000141668
ENSG00000181092	ENSG00000144119
ENSG00000181092	ENSG00000145861
ENSG00000181092	ENSG00000159189
ENSG00000181092	ENSG00000163145
ENSG00000181092	ENSG00000165985
ENSG00000181092	ENSG00000172247
ENSG00000181092	ENSG00000173369
ENSG00000181092	ENSG00000173372
ENSG00000181092	ENSG00000173918
ENSG00000181092	ENSG00000184471
ENSG00000181092	ENSG00000186897
ENSG00000181092	ENSG00000213192
ENSG00000181104	ENSG00000125245
ENSG00000181104	ENSG00000126251
ENSG00000181104	ENSG00000126262
ENSG00000181104	ENSG00000144230
ENSG00000181104	ENSG00000147138
ENSG00000181104	ENSG00000162881
ENSG00000181104	ENSG00000164220
ENSG00000181104	ENSG00000164251
ENSG00000181104	ENSG00000165621
ENSG00000181104	ENSG00000171631
ENSG00000181104	ENSG00000182162
ENSG00000181104	ENSG00000182559
ENSG00000181104	ENSG00000182782
ENSG00000181104	ENSG00000185897
ENSG00000181104	ENSG00000196917
ENSG00000181163	ENSG00000107833
ENSG00000181163	ENSG00000158806
ENSG00000181163	ENSG00000182965
ENSG00000181163	ENSG00000214032
ENSG00000181163	ENSG00000214246
ENSG00000181163	ENSG00000214774
ENSG00000181163	ENSG00000214884
ENSG00000181163	ENSG00000215280
ENSG00000181195	ENSG00000101327
ENSG00000181195	ENSG00000168081
ENSG00000181210	ENSG00000043591
ENSG00000181210	ENSG00000102468
ENSG00000181210	ENSG00000113749
ENSG00000181210	ENSG00000125631
ENSG00000181210	ENSG00000135312
ENSG00000181210	ENSG00000135914
ENSG00000181210	ENSG00000147246
ENSG00000181210	ENSG00000150594
ENSG00000181210	ENSG00000157219
ENSG00000181210	ENSG00000164270
ENSG00000181210	ENSG00000168830

ENSG00000181210	ENSG00000169252
ENSG00000181210	ENSG00000175658
ENSG00000181210	ENSG00000178394
ENSG00000181210	ENSG00000179097
ENSG00000181210	ENSG00000179546
ENSG00000181210	ENSG00000184303
ENSG00000181210	ENSG00000184845
ENSG00000181210	ENSG00000185321
ENSG00000181210	ENSG00000188778
ENSG00000181218	ENSG00000111332
ENSG00000181218	ENSG00000124642
ENSG00000181218	ENSG00000137259
ENSG00000181218	ENSG00000158403
ENSG00000181218	ENSG00000164508
ENSG00000181218	ENSG00000168274
ENSG00000181218	ENSG00000180573
ENSG00000181218	ENSG00000182611
ENSG00000181218	ENSG00000183558
ENSG00000181218	ENSG00000184260
ENSG00000181218	ENSG00000184270
ENSG00000181218	ENSG00000184348
ENSG00000181218	ENSG00000184825
ENSG00000181218	ENSG00000185978
ENSG00000181218	ENSG00000188486
ENSG00000181218	ENSG00000196747
ENSG00000181218	ENSG00000196787
ENSG00000181218	ENSG00000196866
ENSG00000181218	ENSG00000198082
ENSG00000181218	ENSG00000198307
ENSG00000181218	ENSG00000198374
ENSG00000181218	ENSG00000203812
ENSG00000181218	ENSG00000205070
ENSG00000181218	ENSG00000205079
ENSG00000181234	ENSG00000006118
ENSG00000181234	ENSG00000139364
ENSG00000181234	ENSG00000151952
ENSG00000181234	ENSG00000181291
ENSG00000181240	ENSG00000085491
ENSG00000181240	ENSG00000125648
ENSG00000181240	ENSG00000148339
ENSG00000181284	ENSG00000180611
ENSG00000181291	ENSG00000006118
ENSG00000181291	ENSG00000139364
ENSG00000181291	ENSG00000151952
ENSG00000181291	ENSG00000181234
ENSG00000181322	ENSG00000086288
ENSG00000181322	ENSG00000112981
ENSG00000181322	ENSG00000143156
ENSG00000181322	ENSG00000172113
ENSG00000181333	ENSG00000047457
ENSG00000181333	ENSG00000089472
ENSG00000181333	ENSG00000185010
ENSG00000181333	ENSG00000198734
ENSG00000181350	ENSG00000178026
ENSG00000181374	ENSG00000006074
ENSG00000181374	ENSG00000006075
ENSG00000181374	ENSG00000006606
ENSG00000181374	ENSG00000102962
ENSG00000181374	ENSG00000102970

ENSG00000181374	ENSG00000106178
ENSG00000181374	ENSG00000108688
ENSG00000181374	ENSG00000108691
ENSG00000181374	ENSG00000108700
ENSG00000181374	ENSG00000108702
ENSG00000181374	ENSG00000115009
ENSG00000181374	ENSG00000129277
ENSG00000181374	ENSG00000143184
ENSG00000181374	ENSG00000143185
ENSG00000181374	ENSG00000161570
ENSG00000181374	ENSG00000161573
ENSG00000181374	ENSG00000161574
ENSG00000181374	ENSG00000167236
ENSG00000181374	ENSG00000172156
ENSG00000181374	ENSG00000172724
ENSG00000181374	ENSG00000197262
ENSG00000181374	ENSG00000205017
ENSG00000181374	ENSG00000205020
ENSG00000181374	ENSG00000205021
ENSG00000181374	ENSG00000213494
ENSG00000181374	ENSG00000215627
ENSG00000181374	ENSG00000215629
ENSG00000181409	ENSG00000142235
ENSG00000181409	ENSG00000164715
ENSG00000181416	ENSG00000137970
ENSG00000181416	ENSG00000146223
ENSG00000181416	ENSG00000147604
ENSG00000181416	ENSG00000169216
ENSG00000181416	ENSG00000171549
ENSG00000181416	ENSG00000182520
ENSG00000181416	ENSG00000214485
ENSG00000181416	ENSG00000214557
ENSG00000181416	ENSG00000214932
ENSG00000181416	ENSG00000215297
ENSG00000181416	ENSG00000215402
ENSG00000181433	ENSG00000102786
ENSG00000181433	ENSG00000165359
ENSG00000181433	ENSG00000215130
ENSG00000181449	ENSG00000005513
ENSG00000181449	ENSG00000100146
ENSG00000181449	ENSG00000124766
ENSG00000181449	ENSG00000125398
ENSG00000181449	ENSG00000129194
ENSG00000181449	ENSG00000134595
ENSG00000181449	ENSG00000164736
ENSG00000181449	ENSG00000171056
ENSG00000181449	ENSG00000176887
ENSG00000181449	ENSG00000177732
ENSG00000181449	ENSG00000182968
ENSG00000181449	ENSG00000184895
ENSG00000181449	ENSG00000203883
ENSG00000181458	ENSG00000151715
ENSG00000181458	ENSG00000203730
ENSG00000181458	ENSG00000214154
ENSG00000181467	ENSG00000116473
ENSG00000181467	ENSG00000123728
ENSG00000181467	ENSG00000125249
ENSG00000181467	ENSG00000127314
ENSG00000181467	ENSG00000162595

ENSG00000181467	ENSG00000165023
ENSG00000181467	ENSG00000176276
ENSG00000181467	ENSG00000176490
ENSG00000181467	ENSG00000214405
ENSG00000181467	ENSG00000214492
ENSG00000181472	ENSG00000089775
ENSG00000181472	ENSG00000126804
ENSG00000181513	ENSG00000107897
ENSG00000181541	ENSG00000172478
ENSG00000181541	ENSG00000173212
ENSG00000181541	ENSG00000180660
ENSG00000181588	ENSG00000176624
ENSG00000181588	ENSG00000183496
ENSG00000181588	ENSG00000203759
ENSG00000181631	ENSG00000138271
ENSG00000181631	ENSG00000169313
ENSG00000181631	ENSG00000171659
ENSG00000181631	ENSG00000174944
ENSG00000181631	ENSG00000174946
ENSG00000181634	ENSG00000102245
ENSG00000181634	ENSG00000117560
ENSG00000181634	ENSG00000120659
ENSG00000181634	ENSG00000121858
ENSG00000181634	ENSG00000125735
ENSG00000181634	ENSG00000204490
ENSG00000181634	ENSG00000204496
ENSG00000181649	ENSG00000139289
ENSG00000181649	ENSG00000174307
ENSG00000181652	ENSG00000198925
ENSG00000181690	ENSG00000118495
ENSG00000181690	ENSG00000126003
ENSG00000181722	ENSG00000119574
ENSG00000181754	ENSG00000139211
ENSG00000181754	ENSG00000176020
ENSG00000181773	ENSG00000064547
ENSG00000181773	ENSG00000118432
ENSG00000181773	ENSG00000125910
ENSG00000181773	ENSG00000132975
ENSG00000181773	ENSG00000146360
ENSG00000181773	ENSG00000170989
ENSG00000181773	ENSG00000171517
ENSG00000181773	ENSG00000175898
ENSG00000181773	ENSG00000180739
ENSG00000181773	ENSG00000188822
ENSG00000181773	ENSG00000198121
ENSG00000181773	ENSG00000213694
ENSG00000181781	ENSG00000177947
ENSG00000181781	ENSG00000177989
ENSG00000181781	ENSG00000182950
ENSG00000181788	ENSG00000196470
ENSG00000181788	ENSG00000215475
ENSG00000181790	ENSG00000121753
ENSG00000181790	ENSG00000135298
ENSG00000181804	ENSG00000065923
ENSG00000181804	ENSG00000198689
ENSG00000181826	ENSG00000054967
ENSG00000181826	ENSG00000164620
ENSG00000181827	ENSG00000080298
ENSG00000181827	ENSG00000087903

ENSG00000181827	ENSG00000111783
ENSG00000181827	ENSG00000132005
ENSG00000181827	ENSG00000143390
ENSG00000181827	ENSG00000185002
ENSG00000181856	ENSG00000059804
ENSG00000181856	ENSG00000109667
ENSG00000181856	ENSG00000117394
ENSG00000181856	ENSG00000133460
ENSG00000181856	ENSG00000142583
ENSG00000181856	ENSG00000163581
ENSG00000181856	ENSG00000173262
ENSG00000181856	ENSG00000197241
ENSG00000181867	ENSG00000087086
ENSG00000181867	ENSG00000132446
ENSG00000181867	ENSG00000167996
ENSG00000181867	ENSG00000171878
ENSG00000181867	ENSG00000173477
ENSG00000181867	ENSG00000176778
ENSG00000181867	ENSG00000180795
ENSG00000181867	ENSG00000197518
ENSG00000181867	ENSG00000215234
ENSG00000181885	ENSG00000013297
ENSG00000181885	ENSG00000066405
ENSG00000181885	ENSG00000106404
ENSG00000181885	ENSG00000113946
ENSG00000181885	ENSG00000134873
ENSG00000181885	ENSG00000156282
ENSG00000181885	ENSG00000156284
ENSG00000181885	ENSG00000159261
ENSG00000181885	ENSG00000163347
ENSG00000181885	ENSG00000164007
ENSG00000181885	ENSG00000165215
ENSG00000181885	ENSG00000165376
ENSG00000181885	ENSG00000171217
ENSG00000181885	ENSG00000177300
ENSG00000181885	ENSG00000184113
ENSG00000181885	ENSG00000184697
ENSG00000181885	ENSG00000185758
ENSG00000181885	ENSG00000189118
ENSG00000181885	ENSG00000189143
ENSG00000181885	ENSG00000213937
ENSG00000181929	ENSG00000106617
ENSG00000181929	ENSG00000115592
ENSG00000181965	ENSG00000104903
ENSG00000181965	ENSG00000113196
ENSG00000181965	ENSG00000122691
ENSG00000181965	ENSG00000122859
ENSG00000181965	ENSG00000123307
ENSG00000181965	ENSG00000125878
ENSG00000181965	ENSG00000146618
ENSG00000181965	ENSG00000162367
ENSG00000181965	ENSG00000162992
ENSG00000181965	ENSG00000164107
ENSG00000181965	ENSG00000164600
ENSG00000181965	ENSG00000168267
ENSG00000181965	ENSG00000171532
ENSG00000181965	ENSG00000171786
ENSG00000181965	ENSG00000172238
ENSG00000181965	ENSG00000177551

ENSG00000181965	ENSG00000178403
ENSG00000181965	ENSG00000179774
ENSG00000181965	ENSG00000180535
ENSG00000181965	ENSG00000186051
ENSG00000181965	ENSG00000187786
ENSG00000181965	ENSG00000188686
ENSG00000181984	ENSG00000140478
ENSG00000181984	ENSG00000153666
ENSG00000181984	ENSG00000156363
ENSG00000181984	ENSG00000159289
ENSG00000181984	ENSG00000167110
ENSG00000181984	ENSG00000167195
ENSG00000181984	ENSG00000174450
ENSG00000181984	ENSG00000175265
ENSG00000181984	ENSG00000184206
ENSG00000181984	ENSG00000186322
ENSG00000181984	ENSG00000188388
ENSG00000181984	ENSG00000188532
ENSG00000181984	ENSG00000188626
ENSG00000181984	ENSG00000197092
ENSG00000181984	ENSG00000197414
ENSG00000181984	ENSG00000214348
ENSG00000181984	ENSG00000215252
ENSG00000181984	ENSG00000215405
ENSG00000181996	ENSG00000184405
ENSG00000181996	ENSG00000204239
ENSG00000181996	ENSG00000205663
ENSG00000181996	ENSG00000205664
ENSG00000181996	ENSG00000206061
ENSG00000181997	ENSG00000103569
ENSG00000181997	ENSG00000143595
ENSG00000181997	ENSG00000165269
ENSG00000181997	ENSG00000165272
ENSG00000181997	ENSG00000176115
ENSG00000181997	ENSG00000186466
ENSG00000182010	ENSG00000114993
ENSG00000182040	ENSG00000175311
ENSG00000182050	ENSG00000071073
ENSG00000182050	ENSG00000161013
ENSG00000182050	ENSG00000184774
ENSG00000182095	ENSG00000171282
ENSG00000182103	ENSG00000140067
ENSG00000182107	ENSG00000112697
ENSG00000182112	ENSG00000100796
ENSG00000182112	ENSG00000138041
ENSG00000182132	ENSG00000115041
ENSG00000182132	ENSG00000120049
ENSG00000182132	ENSG00000185774
ENSG00000182156	ENSG00000001561
ENSG00000182156	ENSG00000112796
ENSG00000182156	ENSG00000136960
ENSG00000182156	ENSG00000154269
ENSG00000182156	ENSG00000164303
ENSG00000182156	ENSG00000197594
ENSG00000182158	ENSG00000060566
ENSG00000182158	ENSG00000107175
ENSG00000182158	ENSG00000118217
ENSG00000182158	ENSG00000143578
ENSG00000182158	ENSG00000157613

ENSG00000182158	ENSG00000213676
ENSG00000182162	ENSG00000125245
ENSG00000182162	ENSG00000126251
ENSG00000182162	ENSG00000126262
ENSG00000182162	ENSG00000144230
ENSG00000182162	ENSG00000147138
ENSG00000182162	ENSG00000162881
ENSG00000182162	ENSG00000164220
ENSG00000182162	ENSG00000164251
ENSG00000182162	ENSG00000165621
ENSG00000182162	ENSG00000171631
ENSG00000182162	ENSG00000181104
ENSG00000182162	ENSG00000182559
ENSG00000182162	ENSG00000182782
ENSG00000182162	ENSG00000185897
ENSG00000182162	ENSG00000196917
ENSG00000182168	ENSG00000107731
ENSG00000182168	ENSG00000113763
ENSG00000182168	ENSG00000124602
ENSG00000182168	ENSG00000156687
ENSG00000182175	ENSG00000168509
ENSG00000182175	ENSG00000174136
ENSG00000182177	ENSG00000146926
ENSG00000182179	ENSG00000033178
ENSG00000182179	ENSG00000130985
ENSG00000182181	ENSG00000029993
ENSG00000182181	ENSG00000100118
ENSG00000182181	ENSG00000124097
ENSG00000182181	ENSG00000132967
ENSG00000182181	ENSG00000164104
ENSG00000182181	ENSG00000173961
ENSG00000182181	ENSG00000176256
ENSG00000182181	ENSG00000180212
ENSG00000182181	ENSG00000182711
ENSG00000182181	ENSG00000189403
ENSG00000182181	ENSG00000214364
ENSG00000182181	ENSG00000214852
ENSG00000182181	ENSG00000215308
ENSG00000182187	ENSG00000100053
ENSG00000182187	ENSG00000100058
ENSG00000182187	ENSG00000100122
ENSG00000182187	ENSG00000108255
ENSG00000182187	ENSG00000118231
ENSG00000182187	ENSG00000127377
ENSG00000182187	ENSG00000163254
ENSG00000182187	ENSG00000163499
ENSG00000182187	ENSG00000168582
ENSG00000182187	ENSG00000196431
ENSG00000182187	ENSG00000213139
ENSG00000182197	ENSG00000012232
ENSG00000182197	ENSG00000151348
ENSG00000182197	ENSG00000158008
ENSG00000182197	ENSG00000162694
ENSG00000182208	ENSG00000114978
ENSG00000182208	ENSG00000120162
ENSG00000182208	ENSG00000142961
ENSG00000182208	ENSG00000172081
ENSG00000182208	ENSG00000173542
ENSG00000182217	ENSG00000124529

ENSG00000182217	ENSG00000124578
ENSG00000182217	ENSG00000158406
ENSG00000182217	ENSG00000183941
ENSG00000182217	ENSG00000188987
ENSG00000182217	ENSG00000196176
ENSG00000182217	ENSG00000197061
ENSG00000182217	ENSG00000197238
ENSG00000182217	ENSG00000197837
ENSG00000182217	ENSG00000197914
ENSG00000182217	ENSG00000198327
ENSG00000182217	ENSG00000198339
ENSG00000182217	ENSG00000198518
ENSG00000182217	ENSG00000198558
ENSG00000182218	ENSG00000143512
ENSG00000182218	ENSG00000164161
ENSG00000182223	ENSG00000189167
ENSG00000182240	ENSG00000186318
ENSG00000182242	ENSG00000057704
ENSG00000182242	ENSG00000102080
ENSG00000182242	ENSG00000133069
ENSG00000182242	ENSG00000172765
ENSG00000182242	ENSG00000185254
ENSG00000182247	ENSG00000170035
ENSG00000182247	ENSG00000170142
ENSG00000182253	ENSG00000026025
ENSG00000182253	ENSG00000100285
ENSG00000182253	ENSG00000104722
ENSG00000182253	ENSG00000104725
ENSG00000182253	ENSG00000131095
ENSG00000182253	ENSG00000135406
ENSG00000182253	ENSG00000148798
ENSG00000182253	ENSG00000175084
ENSG00000182253	ENSG00000215479
ENSG00000182255	ENSG00000026559
ENSG00000182255	ENSG00000102057
ENSG00000182255	ENSG00000104848
ENSG00000182255	ENSG00000111262
ENSG00000182255	ENSG00000124134
ENSG00000182255	ENSG00000130037
ENSG00000182255	ENSG00000143105
ENSG00000182255	ENSG00000151079
ENSG00000182255	ENSG00000156486
ENSG00000182255	ENSG00000158445
ENSG00000182255	ENSG00000162975
ENSG00000182255	ENSG00000164794
ENSG00000182255	ENSG00000168263
ENSG00000182255	ENSG00000168418
ENSG00000182255	ENSG00000170745
ENSG00000182255	ENSG00000171126
ENSG00000182255	ENSG00000171385
ENSG00000182255	ENSG00000177272
ENSG00000182255	ENSG00000177301
ENSG00000182255	ENSG00000178342
ENSG00000182255	ENSG00000182674
ENSG00000182255	ENSG00000184408
ENSG00000182256	ENSG00000011677
ENSG00000182256	ENSG00000022355
ENSG00000182256	ENSG00000094755
ENSG00000182256	ENSG00000101958

ENSG00000182256	ENSG00000102287
ENSG00000182256	ENSG00000109158
ENSG00000182256	ENSG00000109738
ENSG00000182256	ENSG00000111886
ENSG00000182256	ENSG00000113327
ENSG00000182256	ENSG00000145451
ENSG00000182256	ENSG00000145863
ENSG00000182256	ENSG00000145864
ENSG00000182256	ENSG00000145888
ENSG00000182256	ENSG00000146276
ENSG00000182256	ENSG00000147402
ENSG00000182256	ENSG00000151834
ENSG00000182256	ENSG00000163285
ENSG00000182256	ENSG00000163288
ENSG00000182256	ENSG00000166206
ENSG00000182256	ENSG00000183185
ENSG00000182256	ENSG00000186297
ENSG00000182256	ENSG00000187730
ENSG00000182256	ENSG00000188828
ENSG00000182257	ENSG00000184968
ENSG00000182257	ENSG00000205066
ENSG00000182261	ENSG00000022556
ENSG00000182261	ENSG00000023191
ENSG00000182261	ENSG00000140853
ENSG00000182261	ENSG00000142405
ENSG00000182261	ENSG00000158077
ENSG00000182261	ENSG00000160505
ENSG00000182261	ENSG00000162711
ENSG00000182261	ENSG00000167207
ENSG00000182261	ENSG00000167634
ENSG00000182261	ENSG00000167984
ENSG00000182261	ENSG00000171487
ENSG00000182261	ENSG00000173572
ENSG00000182261	ENSG00000174885
ENSG00000182261	ENSG00000179709
ENSG00000182261	ENSG00000179873
ENSG00000182261	ENSG00000185792
ENSG00000182263	ENSG00000205432
ENSG00000182272	ENSG00000033100
ENSG00000182272	ENSG00000123989
ENSG00000182272	ENSG00000131873
ENSG00000182272	ENSG00000139044
ENSG00000182272	ENSG00000147408
ENSG00000182272	ENSG00000169826
ENSG00000182272	ENSG00000198108
ENSG00000182287	ENSG00000106367
ENSG00000182287	ENSG00000152056
ENSG00000182315	ENSG00000071655
ENSG00000182315	ENSG00000134046
ENSG00000182315	ENSG00000170948
ENSG00000182315	ENSG00000184301
ENSG00000182315	ENSG00000196589
ENSG00000182315	ENSG00000205717
ENSG00000182315	ENSG00000205718
ENSG00000182324	ENSG00000115474
ENSG00000182324	ENSG00000120457
ENSG00000182324	ENSG00000121361
ENSG00000182324	ENSG00000123700
ENSG00000182324	ENSG00000151704

ENSG00000182324	ENSG00000153822
ENSG00000182324	ENSG00000157542
ENSG00000182324	ENSG00000157551
ENSG00000182324	ENSG00000162728
ENSG00000182324	ENSG00000162989
ENSG00000182324	ENSG00000168135
ENSG00000182324	ENSG00000177807
ENSG00000182324	ENSG00000184185
ENSG00000182324	ENSG00000187486
ENSG00000182326	ENSG00000009724
ENSG00000182326	ENSG00000127241
ENSG00000182326	ENSG00000139178
ENSG00000182326	ENSG00000159403
ENSG00000182327	ENSG00000215747
ENSG00000182327	ENSG00000215792
ENSG00000182330	ENSG00000116708
ENSG00000182330	ENSG00000116721
ENSG00000182330	ENSG00000116726
ENSG00000182330	ENSG00000120952
ENSG00000182330	ENSG00000157358
ENSG00000182330	ENSG00000160959
ENSG00000182330	ENSG00000179704
ENSG00000182330	ENSG00000185028
ENSG00000182330	ENSG00000185686
ENSG00000182330	ENSG00000187545
ENSG00000182330	ENSG00000197549
ENSG00000182330	ENSG00000204478
ENSG00000182330	ENSG00000204479
ENSG00000182330	ENSG00000204480
ENSG00000182330	ENSG00000204481
ENSG00000182330	ENSG00000204485
ENSG00000182330	ENSG00000204486
ENSG00000182330	ENSG00000204488
ENSG00000182330	ENSG00000204491
ENSG00000182330	ENSG00000204495
ENSG00000182330	ENSG00000204501
ENSG00000182330	ENSG00000204502
ENSG00000182330	ENSG00000204503
ENSG00000182330	ENSG00000204505
ENSG00000182330	ENSG00000204507
ENSG00000182330	ENSG00000204508
ENSG00000182330	ENSG00000204509
ENSG00000182330	ENSG00000204510
ENSG00000182330	ENSG00000204513
ENSG00000182359	ENSG00000162413
ENSG00000182359	ENSG00000168427
ENSG00000182359	ENSG00000171617
ENSG00000182359	ENSG00000175946
ENSG00000182359	ENSG00000183655
ENSG00000182359	ENSG00000213160
ENSG00000182378	ENSG00000182836
ENSG00000182379	ENSG00000122584
ENSG00000182379	ENSG00000144227
ENSG00000182379	ENSG00000182575
ENSG00000182389	ENSG00000067191
ENSG00000182389	ENSG00000165995
ENSG00000182389	ENSG00000167535
ENSG00000182400	ENSG00000007255
ENSG00000182415	ENSG00000129873

ENSG00000182415	ENSG00000153046
ENSG00000182415	ENSG00000166446
ENSG00000182415	ENSG00000172288
ENSG00000182415	ENSG00000172352
ENSG00000182415	ENSG00000198721
ENSG00000182415	ENSG00000215563
ENSG00000182447	ENSG00000205850
ENSG00000182447	ENSG00000205863
ENSG00000182450	ENSG00000082482
ENSG00000182450	ENSG00000095981
ENSG00000182450	ENSG00000099337
ENSG00000182450	ENSG00000100433
ENSG00000182450	ENSG00000124249
ENSG00000182450	ENSG00000124780
ENSG00000182450	ENSG00000135750
ENSG00000182450	ENSG00000152315
ENSG00000182450	ENSG00000164626
ENSG00000182450	ENSG00000169427
ENSG00000182450	ENSG00000171303
ENSG00000182450	ENSG00000173338
ENSG00000182450	ENSG00000184261
ENSG00000182463	ENSG00000121297
ENSG00000182463	ENSG00000179981
ENSG00000182472	ENSG00000014216
ENSG00000182472	ENSG00000077274
ENSG00000182472	ENSG00000092529
ENSG00000182472	ENSG00000135773
ENSG00000182472	ENSG00000137225
ENSG00000182472	ENSG00000142330
ENSG00000182472	ENSG00000149260
ENSG00000182472	ENSG00000162909
ENSG00000182472	ENSG00000162949
ENSG00000182472	ENSG00000203697
ENSG00000182472	ENSG00000214711
ENSG00000182481	ENSG00000102753
ENSG00000182481	ENSG00000185467
ENSG00000182481	ENSG00000186432
ENSG00000182481	ENSG00000214035
ENSG00000182481	ENSG00000214180
ENSG00000182481	ENSG00000215769
ENSG00000182482	ENSG00000160844
ENSG00000182482	ENSG00000183086
ENSG00000182482	ENSG00000198750
ENSG00000182487	ENSG00000107957
ENSG00000182487	ENSG00000158517
ENSG00000182487	ENSG00000165178
ENSG00000182487	ENSG00000174705
ENSG00000182487	ENSG00000196408
ENSG00000182489	ENSG00000047597
ENSG00000182489	ENSG00000172967
ENSG00000182492	ENSG00000011465
ENSG00000182492	ENSG00000106819
ENSG00000182492	ENSG00000139329
ENSG00000182500	ENSG00000160991
ENSG00000182500	ENSG00000175938
ENSG00000182502	ENSG00000107362
ENSG00000182502	ENSG00000129968
ENSG00000182502	ENSG00000136379
ENSG00000182502	ENSG00000182556

ENSG00000182502	ENSG00000198658
ENSG00000182502	ENSG00000203835
ENSG00000182508	ENSG00000145685
ENSG00000182508	ENSG00000156959
ENSG00000182508	ENSG00000183722
ENSG00000182508	ENSG00000187416
ENSG00000182508	ENSG00000197753
ENSG00000182511	ENSG00000010671
ENSG00000182511	ENSG00000074966
ENSG00000182511	ENSG00000102010
ENSG00000182511	ENSG00000113263
ENSG00000182511	ENSG00000135605
ENSG00000182511	ENSG00000151422
ENSG00000182520	ENSG00000137970
ENSG00000182520	ENSG00000146223
ENSG00000182520	ENSG00000147604
ENSG00000182520	ENSG00000169216
ENSG00000182520	ENSG00000171549
ENSG00000182520	ENSG00000181416
ENSG00000182520	ENSG00000214485
ENSG00000182520	ENSG00000214557
ENSG00000182520	ENSG00000214932
ENSG00000182520	ENSG00000215297
ENSG00000182520	ENSG00000215402
ENSG00000182521	ENSG00000028839
ENSG00000182521	ENSG00000112592
ENSG00000182533	ENSG00000105971
ENSG00000182533	ENSG00000105974
ENSG00000182541	ENSG00000070759
ENSG00000182541	ENSG00000106683
ENSG00000182541	ENSG00000107140
ENSG00000182556	ENSG00000107362
ENSG00000182556	ENSG00000129968
ENSG00000182556	ENSG00000136379
ENSG00000182556	ENSG00000182502
ENSG00000182556	ENSG00000198658
ENSG00000182556	ENSG00000203835
ENSG00000182557	ENSG00000169682
ENSG00000182557	ENSG00000183018
ENSG00000182559	ENSG00000125245
ENSG00000182559	ENSG00000126251
ENSG00000182559	ENSG00000126262
ENSG00000182559	ENSG00000144230
ENSG00000182559	ENSG00000147138
ENSG00000182559	ENSG00000162881
ENSG00000182559	ENSG00000164220
ENSG00000182559	ENSG00000164251
ENSG00000182559	ENSG00000165621
ENSG00000182559	ENSG00000171631
ENSG00000182559	ENSG00000181104
ENSG00000182559	ENSG00000182162
ENSG00000182559	ENSG00000182782
ENSG00000182559	ENSG00000185897
ENSG00000182559	ENSG00000196917
ENSG00000182568	ENSG00000119042
ENSG00000182575	ENSG00000122584
ENSG00000182575	ENSG00000144227
ENSG00000182575	ENSG00000182379
ENSG00000182578	ENSG00000037280

ENSG00000182578	ENSG00000102755
ENSG00000182578	ENSG00000104213
ENSG00000182578	ENSG00000113721
ENSG00000182578	ENSG00000122025
ENSG00000182578	ENSG00000128052
ENSG00000182578	ENSG00000134853
ENSG00000182578	ENSG00000157404
ENSG00000182580	ENSG00000044524
ENSG00000182580	ENSG00000070886
ENSG00000182580	ENSG00000080224
ENSG00000182580	ENSG00000106123
ENSG00000182580	ENSG00000116106
ENSG00000182580	ENSG00000133216
ENSG00000182580	ENSG00000135333
ENSG00000182580	ENSG00000142627
ENSG00000182580	ENSG00000145242
ENSG00000182580	ENSG00000146904
ENSG00000182580	ENSG00000154928
ENSG00000182580	ENSG00000183317
ENSG00000182580	ENSG00000196411
ENSG00000182601	ENSG00000002587
ENSG00000182601	ENSG00000122254
ENSG00000182601	ENSG00000125430
ENSG00000182601	ENSG00000153976
ENSG00000182601	ENSG00000162040
ENSG00000182601	ENSG00000175818
ENSG00000182606	ENSG00000115993
ENSG00000182606	ENSG00000173805
ENSG00000182611	ENSG00000111332
ENSG00000182611	ENSG00000124642
ENSG00000182611	ENSG00000137259
ENSG00000182611	ENSG00000158403
ENSG00000182611	ENSG00000164508
ENSG00000182611	ENSG00000168274
ENSG00000182611	ENSG00000180573
ENSG00000182611	ENSG00000181218
ENSG00000182611	ENSG00000183558
ENSG00000182611	ENSG00000184260
ENSG00000182611	ENSG00000184270
ENSG00000182611	ENSG00000184348
ENSG00000182611	ENSG00000184825
ENSG00000182611	ENSG00000185978
ENSG00000182611	ENSG00000188486
ENSG00000182611	ENSG00000196747
ENSG00000182611	ENSG00000196787
ENSG00000182611	ENSG00000196866
ENSG00000182611	ENSG00000198082
ENSG00000182611	ENSG00000198307
ENSG00000182611	ENSG00000198374
ENSG00000182611	ENSG00000203812
ENSG00000182611	ENSG00000205070
ENSG00000182611	ENSG00000205079
ENSG00000182612	ENSG00000048140
ENSG00000182612	ENSG00000099282
ENSG00000182612	ENSG00000108219
ENSG00000182612	ENSG00000158457
ENSG00000182612	ENSG00000168785
ENSG00000182621	ENSG00000101333
ENSG00000182621	ENSG00000114805

ENSG00000182621	ENSG00000115556
ENSG00000182621	ENSG00000115896
ENSG00000182621	ENSG00000124181
ENSG00000182621	ENSG00000137841
ENSG00000182621	ENSG00000139151
ENSG00000182621	ENSG00000149527
ENSG00000182621	ENSG00000149782
ENSG00000182621	ENSG00000154822
ENSG00000182621	ENSG00000161714
ENSG00000182621	ENSG00000187091
ENSG00000182621	ENSG00000197943
ENSG00000182625	ENSG00000127588
ENSG00000182625	ENSG00000127920
ENSG00000182625	ENSG00000127928
ENSG00000182625	ENSG00000133136
ENSG00000182625	ENSG00000162188
ENSG00000182625	ENSG00000167083
ENSG00000182625	ENSG00000167414
ENSG00000182625	ENSG00000168243
ENSG00000182625	ENSG00000172380
ENSG00000182625	ENSG00000174021
ENSG00000182625	ENSG00000186469
ENSG00000182625	ENSG00000213611
ENSG00000182631	ENSG00000100739
ENSG00000182631	ENSG00000134817
ENSG00000182631	ENSG00000144891
ENSG00000182631	ENSG00000154165
ENSG00000182631	ENSG00000168398
ENSG00000182631	ENSG00000170128
ENSG00000182631	ENSG00000173080
ENSG00000182631	ENSG00000180772
ENSG00000182632	ENSG00000108100
ENSG00000182632	ENSG00000163249
ENSG00000182632	ENSG00000179296
ENSG00000182632	ENSG00000184766
ENSG00000182632	ENSG00000204150
ENSG00000182632	ENSG00000215169
ENSG00000182667	ENSG00000142549
ENSG00000182667	ENSG00000172260
ENSG00000182667	ENSG00000183715
ENSG00000182667	ENSG00000185565
ENSG00000182687	ENSG00000082556
ENSG00000182687	ENSG00000112038
ENSG00000182687	ENSG00000116014
ENSG00000182687	ENSG00000116329
ENSG00000182687	ENSG00000125510
ENSG00000182687	ENSG00000125522
ENSG00000182687	ENSG00000128285
ENSG00000182687	ENSG00000128310
ENSG00000182687	ENSG00000132671
ENSG00000182687	ENSG00000139874
ENSG00000182687	ENSG00000152034
ENSG00000182687	ENSG00000162009
ENSG00000182687	ENSG00000166573
ENSG00000182687	ENSG00000180616
ENSG00000182687	ENSG00000183473
ENSG00000182687	ENSG00000183729
ENSG00000182699	ENSG00000106153
ENSG00000182699	ENSG00000138869

ENSG00000182699	ENSG00000186940
ENSG00000182699	ENSG00000203682
ENSG00000182707	ENSG00000089356
ENSG00000182707	ENSG00000126258
ENSG00000182707	ENSG00000137726
ENSG00000182707	ENSG00000137731
ENSG00000182707	ENSG00000150201
ENSG00000182711	ENSG00000029993
ENSG00000182711	ENSG00000100118
ENSG00000182711	ENSG00000124097
ENSG00000182711	ENSG00000132967
ENSG00000182711	ENSG00000164104
ENSG00000182711	ENSG00000173961
ENSG00000182711	ENSG00000176256
ENSG00000182711	ENSG00000180212
ENSG00000182711	ENSG00000182181
ENSG00000182711	ENSG00000189403
ENSG00000182711	ENSG00000214364
ENSG00000182711	ENSG00000214852
ENSG00000182711	ENSG00000215308
ENSG00000182718	ENSG00000104537
ENSG00000182718	ENSG00000109511
ENSG00000182718	ENSG00000122359
ENSG00000182718	ENSG00000135046
ENSG00000182718	ENSG00000138279
ENSG00000182718	ENSG00000138772
ENSG00000182718	ENSG00000143412
ENSG00000182718	ENSG00000150165
ENSG00000182718	ENSG00000164111
ENSG00000182718	ENSG00000165390
ENSG00000182718	ENSG00000183059
ENSG00000182718	ENSG00000186807
ENSG00000182718	ENSG00000196975
ENSG00000182718	ENSG00000197043
ENSG00000182732	ENSG00000076344
ENSG00000182732	ENSG00000108370
ENSG00000182732	ENSG00000182901
ENSG00000182742	ENSG00000037965
ENSG00000182742	ENSG00000106004
ENSG00000182742	ENSG00000106006
ENSG00000182742	ENSG00000108511
ENSG00000182742	ENSG00000120068
ENSG00000182742	ENSG00000120075
ENSG00000182742	ENSG00000120087
ENSG00000182742	ENSG00000122592
ENSG00000182742	ENSG00000170166
ENSG00000182742	ENSG00000172789
ENSG00000182742	ENSG00000175879
ENSG00000182742	ENSG00000197576
ENSG00000182742	ENSG00000197757
ENSG00000182742	ENSG00000198353
ENSG00000182743	ENSG00000128606
ENSG00000182743	ENSG00000145147
ENSG00000182743	ENSG00000166897
ENSG00000182743	ENSG00000184347
ENSG00000182743	ENSG00000187122
ENSG00000182749	ENSG00000137819
ENSG00000182749	ENSG00000160781
ENSG00000182749	ENSG00000170915

ENSG00000182749	ENSG00000188582
ENSG00000182752	ENSG00000116183
ENSG00000182759	ENSG00000129535
ENSG00000182759	ENSG00000178573
ENSG00000182759	ENSG00000185022
ENSG00000182759	ENSG00000197063
ENSG00000182759	ENSG00000198517
ENSG00000182759	ENSG00000204103
ENSG00000182759	ENSG00000215622
ENSG00000182765	ENSG00000157576
ENSG00000182765	ENSG00000172186
ENSG00000182765	ENSG00000184409
ENSG00000182765	ENSG00000197366
ENSG00000182765	ENSG00000198157
ENSG00000182765	ENSG00000198875
ENSG00000182771	ENSG00000152208
ENSG00000182782	ENSG00000125245
ENSG00000182782	ENSG00000126251
ENSG00000182782	ENSG00000126262
ENSG00000182782	ENSG00000144230
ENSG00000182782	ENSG00000147138
ENSG00000182782	ENSG00000162881
ENSG00000182782	ENSG00000164220
ENSG00000182782	ENSG00000164251
ENSG00000182782	ENSG00000165621
ENSG00000182782	ENSG00000171631
ENSG00000182782	ENSG00000181104
ENSG00000182782	ENSG00000182162
ENSG00000182782	ENSG00000182559
ENSG00000182782	ENSG00000185897
ENSG00000182782	ENSG00000196917
ENSG00000182805	ENSG00000026950
ENSG00000182805	ENSG00000111801
ENSG00000182805	ENSG00000112763
ENSG00000182805	ENSG00000113303
ENSG00000182805	ENSG00000121236
ENSG00000182805	ENSG00000124508
ENSG00000182805	ENSG00000124549
ENSG00000182805	ENSG00000124557
ENSG00000182805	ENSG00000128250
ENSG00000182805	ENSG00000128253
ENSG00000182805	ENSG00000128276
ENSG00000182805	ENSG00000128482
ENSG00000182805	ENSG00000132256
ENSG00000182805	ENSG00000132274
ENSG00000182805	ENSG00000164010
ENSG00000182805	ENSG00000165810
ENSG00000182805	ENSG00000168903
ENSG00000182805	ENSG00000176422
ENSG00000182805	ENSG00000179046
ENSG00000182805	ENSG00000183439
ENSG00000182805	ENSG00000183718
ENSG00000182805	ENSG00000187574
ENSG00000182805	ENSG00000188683
ENSG00000182805	ENSG00000204614
ENSG00000182805	ENSG00000204616
ENSG00000182805	ENSG00000204618
ENSG00000182805	ENSG00000205198
ENSG00000182805	ENSG00000214693

ENSG00000182809	ENSG00000129170
ENSG00000182809	ENSG00000136490
ENSG00000182809	ENSG00000146215
ENSG00000182809	ENSG00000159176
ENSG00000182809	ENSG00000175183
ENSG00000182809	ENSG00000197893
ENSG00000182809	ENSG00000213145
ENSG00000182814	ENSG00000069509
ENSG00000182814	ENSG00000165775
ENSG00000182827	ENSG00000100580
ENSG00000182836	ENSG00000182378
ENSG00000182866	ENSG00000000938
ENSG00000182866	ENSG00000010810
ENSG00000182866	ENSG00000101336
ENSG00000182866	ENSG00000111816
ENSG00000182866	ENSG00000136573
ENSG00000182866	ENSG00000147507
ENSG00000182866	ENSG00000176105
ENSG00000182866	ENSG00000197122
ENSG00000182870	ENSG00000100626
ENSG00000182870	ENSG00000106648
ENSG00000182870	ENSG00000110328
ENSG00000182870	ENSG00000115339
ENSG00000182870	ENSG00000131386
ENSG00000182870	ENSG00000136542
ENSG00000182870	ENSG00000139629
ENSG00000182870	ENSG00000141429
ENSG00000182870	ENSG00000143641
ENSG00000182870	ENSG00000144278
ENSG00000182870	ENSG00000158089
ENSG00000182870	ENSG00000164574
ENSG00000182870	ENSG00000174473
ENSG00000182870	ENSG00000178234
ENSG00000182870	ENSG00000185274
ENSG00000182871	ENSG00000204291
ENSG00000182872	ENSG00000003756
ENSG00000182872	ENSG00000004534
ENSG00000182872	ENSG00000164252
ENSG00000182872	ENSG00000214603
ENSG00000182901	ENSG00000076344
ENSG00000182901	ENSG00000108370
ENSG00000182901	ENSG00000182732
ENSG00000182902	ENSG00000004864
ENSG00000182902	ENSG00000115840
ENSG00000182902	ENSG00000177542
ENSG00000182923	ENSG00000165325
ENSG00000182938	ENSG00000163982
ENSG00000182938	ENSG00000183034
ENSG00000182938	ENSG00000214333
ENSG00000182944	ENSG00000089280
ENSG00000182944	ENSG00000172660
ENSG00000182944	ENSG00000215835
ENSG00000182950	ENSG00000177947
ENSG00000182950	ENSG00000177989
ENSG00000182950	ENSG00000181781
ENSG00000182952	ENSG00000118418
ENSG00000182952	ENSG00000176982
ENSG00000182952	ENSG00000180150
ENSG00000182952	ENSG00000198830

ENSG00000182952	ENSG00000212769
ENSG00000182952	ENSG00000214578
ENSG00000182953	ENSG00000160516
ENSG00000182953	ENSG00000197303
ENSG00000182957	ENSG00000131089
ENSG00000182957	ENSG00000136002
ENSG00000182963	ENSG00000121634
ENSG00000182963	ENSG00000121742
ENSG00000182963	ENSG00000121743
ENSG00000182963	ENSG00000131233
ENSG00000182963	ENSG00000135355
ENSG00000182963	ENSG00000143140
ENSG00000182963	ENSG00000152661
ENSG00000182963	ENSG00000159248
ENSG00000182963	ENSG00000164411
ENSG00000182963	ENSG00000165474
ENSG00000182963	ENSG00000169562
ENSG00000182963	ENSG00000176402
ENSG00000182963	ENSG00000177291
ENSG00000182963	ENSG00000183153
ENSG00000182963	ENSG00000187513
ENSG00000182963	ENSG00000188910
ENSG00000182963	ENSG00000189280
ENSG00000182963	ENSG00000189433
ENSG00000182963	ENSG00000198835
ENSG00000182963	ENSG00000203733
ENSG00000182965	ENSG00000107833
ENSG00000182965	ENSG00000158806
ENSG00000182965	ENSG00000181163
ENSG00000182965	ENSG00000214032
ENSG00000182965	ENSG00000214246
ENSG00000182965	ENSG00000214774
ENSG00000182965	ENSG00000214884
ENSG00000182965	ENSG00000215280
ENSG00000182968	ENSG00000005513
ENSG00000182968	ENSG00000100146
ENSG00000182968	ENSG00000124766
ENSG00000182968	ENSG00000125398
ENSG00000182968	ENSG00000129194
ENSG00000182968	ENSG00000134595
ENSG00000182968	ENSG00000164736
ENSG00000182968	ENSG00000171056
ENSG00000182968	ENSG00000176887
ENSG00000182968	ENSG00000177732
ENSG00000182968	ENSG00000181449
ENSG00000182968	ENSG00000184895
ENSG00000182968	ENSG00000203883
ENSG00000182979	ENSG00000057935
ENSG00000182979	ENSG00000149480
ENSG00000183014	ENSG00000128594
ENSG00000183014	ENSG00000169783
ENSG00000183014	ENSG00000170382
ENSG00000183014	ENSG00000173114
ENSG00000183014	ENSG00000174482
ENSG00000183014	ENSG00000175928
ENSG00000183014	ENSG00000213171
ENSG00000183016	ENSG00000132321
ENSG00000183018	ENSG00000169682
ENSG00000183018	ENSG00000182557

ENSG00000183020	ENSG00000196961
ENSG00000183022	ENSG00000140416
ENSG00000183022	ENSG00000143549
ENSG00000183022	ENSG00000167460
ENSG00000183022	ENSG00000177152
ENSG00000183022	ENSG00000187536
ENSG00000183022	ENSG00000198467
ENSG00000183022	ENSG00000214869
ENSG00000183023	ENSG00000100678
ENSG00000183023	ENSG00000118160
ENSG00000183034	ENSG00000163982
ENSG00000183034	ENSG00000182938
ENSG00000183034	ENSG00000214333
ENSG00000183036	ENSG00000173126
ENSG00000183038	ENSG00000099998
ENSG00000183038	ENSG00000100031
ENSG00000183038	ENSG00000100121
ENSG00000183038	ENSG00000133475
ENSG00000183038	ENSG00000149435
ENSG00000183038	ENSG00000197421
ENSG00000183038	ENSG00000215466
ENSG00000183038	ENSG00000215500
ENSG00000183038	ENSG00000215610
ENSG00000183049	ENSG00000008118
ENSG00000183049	ENSG00000081320
ENSG00000183049	ENSG00000130822
ENSG00000183049	ENSG00000134072
ENSG00000183049	ENSG00000147613
ENSG00000183049	ENSG00000159792
ENSG00000183049	ENSG00000164076
ENSG00000183049	ENSG00000164543
ENSG00000183059	ENSG00000104537
ENSG00000183059	ENSG00000109511
ENSG00000183059	ENSG00000122359
ENSG00000183059	ENSG00000135046
ENSG00000183059	ENSG00000138279
ENSG00000183059	ENSG00000138772
ENSG00000183059	ENSG00000143412
ENSG00000183059	ENSG00000150165
ENSG00000183059	ENSG00000164111
ENSG00000183059	ENSG00000165390
ENSG00000183059	ENSG00000182718
ENSG00000183059	ENSG00000186807
ENSG00000183059	ENSG00000196975
ENSG00000183059	ENSG00000197043
ENSG00000183060	ENSG00000176018
ENSG00000183066	ENSG00000132471
ENSG00000183066	ENSG00000186433
ENSG00000183072	ENSG00000109705
ENSG00000183072	ENSG00000119919
ENSG00000183072	ENSG00000125816
ENSG00000183072	ENSG00000125820
ENSG00000183072	ENSG00000136327
ENSG00000183072	ENSG00000136352
ENSG00000183072	ENSG00000180053
ENSG00000183086	ENSG00000160844
ENSG00000183086	ENSG00000182482
ENSG00000183086	ENSG00000198750
ENSG00000183087	ENSG00000184500

ENSG00000183090	ENSG00000138759
ENSG00000183090	ENSG00000150893
ENSG00000183090	ENSG00000164946
ENSG00000183090	ENSG00000173546
ENSG00000183090	ENSG00000215060
ENSG00000183091	ENSG00000002834
ENSG00000183091	ENSG00000078114
ENSG00000183092	ENSG00000137221
ENSG00000183098	ENSG00000063660
ENSG00000183098	ENSG00000076716
ENSG00000183098	ENSG00000147257
ENSG00000183098	ENSG00000179399
ENSG00000183098	ENSG00000213420
ENSG00000183111	ENSG00000107554
ENSG00000183114	ENSG00000105245
ENSG00000183114	ENSG00000133961
ENSG00000183114	ENSG00000144366
ENSG00000183114	ENSG00000157978
ENSG00000183114	ENSG00000185112
ENSG00000183117	ENSG00000117322
ENSG00000183117	ENSG00000121904
ENSG00000183117	ENSG00000164796
ENSG00000183117	ENSG00000174175
ENSG00000183117	ENSG00000203710
ENSG00000183128	ENSG00000138172
ENSG00000183128	ENSG00000164451
ENSG00000183128	ENSG00000178033
ENSG00000183128	ENSG00000185933
ENSG00000183128	ENSG00000188820
ENSG00000183134	ENSG00000134830
ENSG00000183134	ENSG00000171049
ENSG00000183134	ENSG00000171051
ENSG00000183134	ENSG00000171860
ENSG00000183134	ENSG00000174600
ENSG00000183134	ENSG00000183671
ENSG00000183134	ENSG00000187474
ENSG00000183134	ENSG00000197405
ENSG00000183134	ENSG00000213903
ENSG00000183134	ENSG00000213906
ENSG00000183137	ENSG00000166037
ENSG00000183145	ENSG00000147223
ENSG00000183145	ENSG00000203877
ENSG00000183153	ENSG00000121634
ENSG00000183153	ENSG00000121742
ENSG00000183153	ENSG00000121743
ENSG00000183153	ENSG00000131233
ENSG00000183153	ENSG00000135355
ENSG00000183153	ENSG00000143140
ENSG00000183153	ENSG00000152661
ENSG00000183153	ENSG00000159248
ENSG00000183153	ENSG00000164411
ENSG00000183153	ENSG00000165474
ENSG00000183153	ENSG00000169562
ENSG00000183153	ENSG00000176402
ENSG00000183153	ENSG00000177291
ENSG00000183153	ENSG00000182963
ENSG00000183153	ENSG00000187513
ENSG00000183153	ENSG00000188910
ENSG00000183153	ENSG00000189280

ENSG00000183153	ENSG00000189433
ENSG00000183153	ENSG00000198835
ENSG00000183153	ENSG00000203733
ENSG00000183166	ENSG00000100314
ENSG00000183185	ENSG00000011677
ENSG00000183185	ENSG00000022355
ENSG00000183185	ENSG00000094755
ENSG00000183185	ENSG00000101958
ENSG00000183185	ENSG00000102287
ENSG00000183185	ENSG00000109158
ENSG00000183185	ENSG00000109738
ENSG00000183185	ENSG00000111886
ENSG00000183185	ENSG00000113327
ENSG00000183185	ENSG00000145451
ENSG00000183185	ENSG00000145863
ENSG00000183185	ENSG00000145864
ENSG00000183185	ENSG00000145888
ENSG00000183185	ENSG00000146276
ENSG00000183185	ENSG00000147402
ENSG00000183185	ENSG00000151834
ENSG00000183185	ENSG00000163285
ENSG00000183185	ENSG00000163288
ENSG00000183185	ENSG00000166206
ENSG00000183185	ENSG00000182256
ENSG00000183185	ENSG00000186297
ENSG00000183185	ENSG00000187730
ENSG00000183185	ENSG00000188828
ENSG00000183186	ENSG00000198535
ENSG00000183186	ENSG00000205502
ENSG00000183196	ENSG00000122863
ENSG00000183196	ENSG00000135702
ENSG00000183196	ENSG00000140835
ENSG00000183196	ENSG00000147119
ENSG00000183196	ENSG00000175040
ENSG00000183196	ENSG00000175264
ENSG00000183199	ENSG00000080824
ENSG00000183199	ENSG00000096384
ENSG00000183199	ENSG00000205100
ENSG00000183199	ENSG00000205527
ENSG00000183199	ENSG00000205940
ENSG00000183230	ENSG00000035403
ENSG00000183230	ENSG00000044115
ENSG00000183230	ENSG00000066032
ENSG00000183230	ENSG00000119326
ENSG00000183246	ENSG00000005379
ENSG00000183246	ENSG00000060709
ENSG00000183246	ENSG00000196622
ENSG00000183246	ENSG00000196934
ENSG00000183304	ENSG00000139351
ENSG00000183304	ENSG00000177138
ENSG00000183304	ENSG00000187268
ENSG00000183317	ENSG00000044524
ENSG00000183317	ENSG00000070886
ENSG00000183317	ENSG00000080224
ENSG00000183317	ENSG00000106123
ENSG00000183317	ENSG00000116106
ENSG00000183317	ENSG00000133216
ENSG00000183317	ENSG00000135333
ENSG00000183317	ENSG00000142627

ENSG00000183317	ENSG00000145242
ENSG00000183317	ENSG00000146904
ENSG00000183317	ENSG00000154928
ENSG00000183317	ENSG00000182580
ENSG00000183317	ENSG00000196411
ENSG00000183318	ENSG00000136206
ENSG00000183318	ENSG00000154732
ENSG00000183318	ENSG00000163806
ENSG00000183318	ENSG00000166667
ENSG00000183318	ENSG00000170092
ENSG00000183318	ENSG00000173678
ENSG00000183318	ENSG00000179994
ENSG00000183318	ENSG00000184616
ENSG00000183318	ENSG00000185040
ENSG00000183318	ENSG00000186645
ENSG00000183318	ENSG00000198305
ENSG00000183318	ENSG00000204710
ENSG00000183318	ENSG00000205238
ENSG00000183318	ENSG00000205482
ENSG00000183318	ENSG00000205505
ENSG00000183318	ENSG00000205506
ENSG00000183318	ENSG00000205508
ENSG00000183318	ENSG00000205509
ENSG00000183318	ENSG00000205580
ENSG00000183318	ENSG00000214300
ENSG00000183318	ENSG00000215092
ENSG00000183337	ENSG00000085185
ENSG00000183347	ENSG00000117226
ENSG00000183347	ENSG00000117228
ENSG00000183347	ENSG00000154451
ENSG00000183347	ENSG00000162645
ENSG00000183347	ENSG00000162654
ENSG00000183347	ENSG00000213512
ENSG00000183379	ENSG00000101463
ENSG00000183379	ENSG00000167371
ENSG00000183386	ENSG00000072163
ENSG00000183386	ENSG00000089159
ENSG00000183386	ENSG00000110031
ENSG00000183386	ENSG00000112214
ENSG00000183386	ENSG00000140682
ENSG00000183386	ENSG00000169756
ENSG00000183405	ENSG00000162596
ENSG00000183405	ENSG00000171863
ENSG00000183405	ENSG00000174121
ENSG00000183405	ENSG00000215342
ENSG00000183405	ENSG00000215804
ENSG00000183417	ENSG00000108312
ENSG00000183417	ENSG00000204448
ENSG00000183417	ENSG00000204664
ENSG00000183417	ENSG00000204699
ENSG00000183417	ENSG00000204705
ENSG00000183417	ENSG00000205042
ENSG00000183421	ENSG00000104312
ENSG00000183421	ENSG00000129465
ENSG00000183421	ENSG00000137275
ENSG00000183421	ENSG00000170209
ENSG00000183423	ENSG00000148602
ENSG00000183423	ENSG00000173621
ENSG00000183423	ENSG00000204033

ENSG00000183439	ENSG00000026950
ENSG00000183439	ENSG00000111801
ENSG00000183439	ENSG00000112763
ENSG00000183439	ENSG00000113303
ENSG00000183439	ENSG00000121236
ENSG00000183439	ENSG00000124508
ENSG00000183439	ENSG00000124549
ENSG00000183439	ENSG00000124557
ENSG00000183439	ENSG00000128250
ENSG00000183439	ENSG00000128253
ENSG00000183439	ENSG00000128276
ENSG00000183439	ENSG00000128482
ENSG00000183439	ENSG00000132256
ENSG00000183439	ENSG00000132274
ENSG00000183439	ENSG00000164010
ENSG00000183439	ENSG00000165810
ENSG00000183439	ENSG00000168903
ENSG00000183439	ENSG00000176422
ENSG00000183439	ENSG00000179046
ENSG00000183439	ENSG00000182805
ENSG00000183439	ENSG00000183718
ENSG00000183439	ENSG00000187574
ENSG00000183439	ENSG00000188683
ENSG00000183439	ENSG00000204614
ENSG00000183439	ENSG00000204616
ENSG00000183439	ENSG00000204618
ENSG00000183439	ENSG00000205198
ENSG00000183439	ENSG00000214693
ENSG00000183454	ENSG00000105464
ENSG00000183454	ENSG00000116032
ENSG00000183454	ENSG00000150086
ENSG00000183454	ENSG00000161509
ENSG00000183454	ENSG00000176884
ENSG00000183454	ENSG00000198785
ENSG00000183458	ENSG00000008710
ENSG00000183458	ENSG00000078795
ENSG00000183458	ENSG00000107593
ENSG00000183458	ENSG00000118762
ENSG00000183458	ENSG00000130943
ENSG00000183458	ENSG00000158683
ENSG00000183458	ENSG00000166473
ENSG00000183458	ENSG00000183793
ENSG00000183458	ENSG00000187008
ENSG00000183458	ENSG00000188599
ENSG00000183473	ENSG00000082556
ENSG00000183473	ENSG00000112038
ENSG00000183473	ENSG00000116014
ENSG00000183473	ENSG00000116329
ENSG00000183473	ENSG00000125510
ENSG00000183473	ENSG00000125522
ENSG00000183473	ENSG00000128285
ENSG00000183473	ENSG00000128310
ENSG00000183473	ENSG00000132671
ENSG00000183473	ENSG00000139874
ENSG00000183473	ENSG00000152034
ENSG00000183473	ENSG00000162009
ENSG00000183473	ENSG00000166573
ENSG00000183473	ENSG00000180616
ENSG00000183473	ENSG00000182687

ENSG00000183473	ENSG00000183729
ENSG00000183484	ENSG00000119714
ENSG00000183484	ENSG00000140030
ENSG00000183484	ENSG00000177464
ENSG00000183486	ENSG00000157601
ENSG00000183495	ENSG00000080603
ENSG00000183496	ENSG00000176624
ENSG00000183496	ENSG00000181588
ENSG00000183496	ENSG00000203759
ENSG00000183508	ENSG00000112773
ENSG00000183508	ENSG00000158246
ENSG00000183508	ENSG00000174016
ENSG00000183558	ENSG00000111332
ENSG00000183558	ENSG00000124642
ENSG00000183558	ENSG00000137259
ENSG00000183558	ENSG00000158403
ENSG00000183558	ENSG00000164508
ENSG00000183558	ENSG00000168274
ENSG00000183558	ENSG00000180573
ENSG00000183558	ENSG00000181218
ENSG00000183558	ENSG00000182611
ENSG00000183558	ENSG00000184260
ENSG00000183558	ENSG00000184270
ENSG00000183558	ENSG00000184348
ENSG00000183558	ENSG00000184825
ENSG00000183558	ENSG00000185978
ENSG00000183558	ENSG00000188486
ENSG00000183558	ENSG00000196747
ENSG00000183558	ENSG00000196787
ENSG00000183558	ENSG00000196866
ENSG00000183558	ENSG00000198082
ENSG00000183558	ENSG00000198307
ENSG00000183558	ENSG00000198374
ENSG00000183558	ENSG00000203812
ENSG00000183558	ENSG00000205070
ENSG00000183558	ENSG00000205079
ENSG00000183570	ENSG00000090097
ENSG00000183570	ENSG00000169564
ENSG00000183570	ENSG00000197111
ENSG00000183571	ENSG00000130517
ENSG00000183578	ENSG00000145779
ENSG00000183578	ENSG00000163154
ENSG00000183578	ENSG00000185361
ENSG00000183579	ENSG00000099999
ENSG00000183579	ENSG00000108375
ENSG00000183598	ENSG00000112727
ENSG00000183598	ENSG00000124693
ENSG00000183598	ENSG00000132475
ENSG00000183598	ENSG00000163041
ENSG00000183598	ENSG00000168148
ENSG00000183598	ENSG00000178458
ENSG00000183598	ENSG00000182431
ENSG00000183598	ENSG00000182572
ENSG00000183598	ENSG00000182915
ENSG00000183598	ENSG00000188375
ENSG00000183598	ENSG00000196285
ENSG00000183598	ENSG00000196532
ENSG00000183598	ENSG00000196966
ENSG00000183598	ENSG00000197153

ENSG00000183598	ENSG00000197409
ENSG00000183598	ENSG00000198366
ENSG00000183598	ENSG00000203811
ENSG00000183598	ENSG00000203813
ENSG00000183598	ENSG00000203818
ENSG00000183598	ENSG00000203852
ENSG00000183615	ENSG00000154319
ENSG00000183625	ENSG00000112486
ENSG00000183625	ENSG00000121797
ENSG00000183625	ENSG00000121807
ENSG00000183625	ENSG00000121966
ENSG00000183625	ENSG00000126353
ENSG00000183625	ENSG00000129048
ENSG00000183625	ENSG00000144648
ENSG00000183625	ENSG00000160683
ENSG00000183625	ENSG00000160791
ENSG00000183625	ENSG00000163464
ENSG00000183625	ENSG00000163823
ENSG00000183625	ENSG00000168329
ENSG00000183625	ENSG00000172215
ENSG00000183625	ENSG00000173578
ENSG00000183625	ENSG00000173585
ENSG00000183625	ENSG00000179934
ENSG00000183625	ENSG00000180871
ENSG00000183625	ENSG00000183813
ENSG00000183625	ENSG00000184451
ENSG00000183625	ENSG00000186810
ENSG00000183625	ENSG00000215775
ENSG00000183625	ENSG00000215778
ENSG00000183625	ENSG00000215782
ENSG00000183638	ENSG00000077279
ENSG00000183638	ENSG00000104237
ENSG00000183638	ENSG00000130413
ENSG00000183638	ENSG00000133083
ENSG00000183638	ENSG00000146038
ENSG00000183638	ENSG00000163673
ENSG00000183638	ENSG00000170390
ENSG00000183654	ENSG00000099785
ENSG00000183654	ENSG00000139266
ENSG00000183654	ENSG00000144583
ENSG00000183654	ENSG00000145416
ENSG00000183654	ENSG00000165406
ENSG00000183654	ENSG00000173926
ENSG00000183655	ENSG00000162413
ENSG00000183655	ENSG00000168427
ENSG00000183655	ENSG00000171617
ENSG00000183655	ENSG00000175946
ENSG00000183655	ENSG00000182359
ENSG00000183655	ENSG00000213160
ENSG00000183662	ENSG00000163377
ENSG00000183662	ENSG00000184599
ENSG00000183662	ENSG00000198673
ENSG00000183671	ENSG00000134830
ENSG00000183671	ENSG00000171049
ENSG00000183671	ENSG00000171051
ENSG00000183671	ENSG00000171860
ENSG00000183671	ENSG00000174600
ENSG00000183671	ENSG00000183134
ENSG00000183671	ENSG00000187474

ENSG00000183671	ENSG00000197405
ENSG00000183671	ENSG00000213903
ENSG00000183671	ENSG00000213906
ENSG00000183682	ENSG00000101144
ENSG00000183682	ENSG00000107623
ENSG00000183682	ENSG00000112175
ENSG00000183682	ENSG00000116985
ENSG00000183682	ENSG00000125965
ENSG00000183682	ENSG00000128802
ENSG00000183682	ENSG00000130385
ENSG00000183682	ENSG00000143869
ENSG00000183682	ENSG00000152785
ENSG00000183682	ENSG00000153162
ENSG00000183682	ENSG00000156466
ENSG00000183682	ENSG00000156574
ENSG00000183682	ENSG00000163217
ENSG00000183688	ENSG00000178882
ENSG00000183696	ENSG00000007001
ENSG00000183701	ENSG00000101278
ENSG00000183701	ENSG00000124614
ENSG00000183701	ENSG00000198810
ENSG00000183701	ENSG00000215464
ENSG00000183715	ENSG00000142549
ENSG00000183715	ENSG00000172260
ENSG00000183715	ENSG00000182667
ENSG00000183715	ENSG00000185565
ENSG00000183719	ENSG00000136700
ENSG00000183719	ENSG00000156755
ENSG00000183719	ENSG00000197794
ENSG00000183719	ENSG00000204670
ENSG00000183719	ENSG00000204776
ENSG00000183719	ENSG00000204780
ENSG00000183719	ENSG00000211599
ENSG00000183719	ENSG00000211600
ENSG00000183719	ENSG00000211601
ENSG00000183719	ENSG00000211602
ENSG00000183719	ENSG00000211603
ENSG00000183719	ENSG00000211604
ENSG00000183719	ENSG00000211605
ENSG00000183719	ENSG00000211606
ENSG00000183719	ENSG00000211607
ENSG00000183719	ENSG00000211608
ENSG00000183719	ENSG00000211609
ENSG00000183719	ENSG00000211610
ENSG00000183719	ENSG00000211611
ENSG00000183719	ENSG00000211613
ENSG00000183719	ENSG00000211616
ENSG00000183719	ENSG00000211617
ENSG00000183719	ENSG00000211618
ENSG00000183719	ENSG00000211620
ENSG00000183719	ENSG00000211625
ENSG00000183719	ENSG00000211626
ENSG00000183719	ENSG00000211627
ENSG00000183719	ENSG00000211628
ENSG00000183719	ENSG00000211629
ENSG00000183719	ENSG00000211630
ENSG00000183719	ENSG00000211631
ENSG00000183719	ENSG00000211632
ENSG00000183719	ENSG00000211633

ENSG00000183719	ENSG00000211634
ENSG00000183719	ENSG00000211635
ENSG00000183719	ENSG00000211636
ENSG00000183722	ENSG00000145685
ENSG00000183722	ENSG00000156959
ENSG00000183722	ENSG00000182508
ENSG00000183722	ENSG00000187416
ENSG00000183722	ENSG00000197753
ENSG00000183723	ENSG00000091317
ENSG00000183723	ENSG00000102007
ENSG00000183723	ENSG00000140931
ENSG00000183723	ENSG00000153551
ENSG00000183723	ENSG00000166091
ENSG00000183726	ENSG00000142188
ENSG00000183729	ENSG00000082556
ENSG00000183729	ENSG00000112038
ENSG00000183729	ENSG00000116014
ENSG00000183729	ENSG00000116329
ENSG00000183729	ENSG00000125510
ENSG00000183729	ENSG00000125522
ENSG00000183729	ENSG00000128285
ENSG00000183729	ENSG00000128310
ENSG00000183729	ENSG00000132671
ENSG00000183729	ENSG00000139874
ENSG00000183729	ENSG00000152034
ENSG00000183729	ENSG00000162009
ENSG00000183729	ENSG00000166573
ENSG00000183729	ENSG00000180616
ENSG00000183729	ENSG00000182687
ENSG00000183729	ENSG00000183473
ENSG00000183734	ENSG00000139352
ENSG00000183734	ENSG00000176009
ENSG00000183734	ENSG00000187855
ENSG00000183734	ENSG00000188108
ENSG00000183735	ENSG00000104365
ENSG00000183735	ENSG00000143466
ENSG00000183735	ENSG00000213341
ENSG00000183741	ENSG00000100307
ENSG00000183741	ENSG00000141570
ENSG00000183741	ENSG00000141582
ENSG00000183741	ENSG00000173894
ENSG00000183742	ENSG00000130147
ENSG00000183748	ENSG00000011028
ENSG00000183748	ENSG00000054219
ENSG00000183748	ENSG00000120586
ENSG00000183748	ENSG00000153246
ENSG00000183762	ENSG00000100100
ENSG00000183762	ENSG00000131650
ENSG00000183770	ENSG00000054598
ENSG00000183770	ENSG00000125798
ENSG00000183770	ENSG00000129514
ENSG00000183770	ENSG00000164379
ENSG00000183770	ENSG00000168269
ENSG00000183770	ENSG00000170122
ENSG00000183770	ENSG00000170608
ENSG00000183770	ENSG00000176165
ENSG00000183770	ENSG00000176678
ENSG00000183770	ENSG00000176692
ENSG00000183770	ENSG00000178919

ENSG00000183770	ENSG00000179772
ENSG00000183770	ENSG00000184492
ENSG00000183770	ENSG00000184659
ENSG00000183770	ENSG00000186564
ENSG00000183770	ENSG00000186766
ENSG00000183770	ENSG00000186790
ENSG00000183770	ENSG00000187140
ENSG00000183770	ENSG00000187559
ENSG00000183770	ENSG00000204612
ENSG00000183770	ENSG00000204779
ENSG00000183770	ENSG00000204793
ENSG00000183770	ENSG00000204828
ENSG00000183770	ENSG00000214336
ENSG00000183773	ENSG00000156709
ENSG00000183775	ENSG00000134504
ENSG00000183775	ENSG00000151364
ENSG00000183775	ENSG00000153885
ENSG00000183775	ENSG00000155729
ENSG00000183775	ENSG00000168301
ENSG00000183775	ENSG00000168676
ENSG00000183775	ENSG00000178695
ENSG00000183775	ENSG00000180332
ENSG00000183775	ENSG00000183783
ENSG00000183775	ENSG00000188997
ENSG00000183775	ENSG00000198553
ENSG00000183775	ENSG00000213859
ENSG00000183778	ENSG00000156966
ENSG00000183778	ENSG00000162630
ENSG00000183778	ENSG00000169255
ENSG00000183778	ENSG00000170340
ENSG00000183778	ENSG00000172318
ENSG00000183778	ENSG00000176383
ENSG00000183778	ENSG00000176597
ENSG00000183778	ENSG00000177191
ENSG00000183778	ENSG00000179913
ENSG00000183778	ENSG00000180561
ENSG00000183778	ENSG00000198488
ENSG00000183778	ENSG00000204222
ENSG00000183778	ENSG00000214654
ENSG00000183779	ENSG00000165655
ENSG00000183780	ENSG00000151812
ENSG00000183783	ENSG00000134504
ENSG00000183783	ENSG00000151364
ENSG00000183783	ENSG00000153885
ENSG00000183783	ENSG00000155729
ENSG00000183783	ENSG00000168301
ENSG00000183783	ENSG00000168676
ENSG00000183783	ENSG00000178695
ENSG00000183783	ENSG00000180332
ENSG00000183783	ENSG00000183775
ENSG00000183783	ENSG00000188997
ENSG00000183783	ENSG00000198553
ENSG00000183783	ENSG00000213859
ENSG00000183791	ENSG00000011007
ENSG00000183791	ENSG00000206181
ENSG00000183791	ENSG00000206182
ENSG00000183791	ENSG00000206183
ENSG00000183793	ENSG00000008710
ENSG00000183793	ENSG00000078795

ENSG00000183793	ENSG00000107593
ENSG00000183793	ENSG00000118762
ENSG00000183793	ENSG00000130943
ENSG00000183793	ENSG00000158683
ENSG00000183793	ENSG00000166473
ENSG00000183793	ENSG00000183458
ENSG00000183793	ENSG00000187008
ENSG00000183793	ENSG00000188599
ENSG00000183798	ENSG00000132205
ENSG00000183798	ENSG00000138080
ENSG00000183807	ENSG00000114023
ENSG00000183808	ENSG00000125976
ENSG00000183812	ENSG00000197833
ENSG00000183812	ENSG00000204805
ENSG00000183812	ENSG00000204806
ENSG00000183812	ENSG00000204807
ENSG00000183812	ENSG00000204808
ENSG00000183812	ENSG00000205613
ENSG00000183812	ENSG00000215061
ENSG00000183813	ENSG00000112486
ENSG00000183813	ENSG00000121797
ENSG00000183813	ENSG00000121807
ENSG00000183813	ENSG00000121966
ENSG00000183813	ENSG00000126353
ENSG00000183813	ENSG00000129048
ENSG00000183813	ENSG00000144648
ENSG00000183813	ENSG00000160683
ENSG00000183813	ENSG00000160791
ENSG00000183813	ENSG00000163464
ENSG00000183813	ENSG00000163823
ENSG00000183813	ENSG00000168329
ENSG00000183813	ENSG00000172215
ENSG00000183813	ENSG00000173578
ENSG00000183813	ENSG00000173585
ENSG00000183813	ENSG00000179934
ENSG00000183813	ENSG00000180871
ENSG00000183813	ENSG00000183625
ENSG00000183813	ENSG00000184451
ENSG00000183813	ENSG00000186810
ENSG00000183813	ENSG00000215775
ENSG00000183813	ENSG00000215778
ENSG00000183813	ENSG00000215782
ENSG00000183844	ENSG00000071889
ENSG00000183844	ENSG00000174028
ENSG00000183844	ENSG00000196937
ENSG00000183844	ENSG00000198643
ENSG00000183853	ENSG00000126259
ENSG00000183853	ENSG00000149571
ENSG00000183853	ENSG00000161270
ENSG00000183856	ENSG00000140575
ENSG00000183856	ENSG00000145703
ENSG00000183862	ENSG00000070729
ENSG00000183862	ENSG00000099822
ENSG00000183862	ENSG00000132259
ENSG00000183862	ENSG00000138622
ENSG00000183862	ENSG00000143630
ENSG00000183862	ENSG00000144191
ENSG00000183862	ENSG00000164588
ENSG00000183862	ENSG00000170289

ENSG00000183864	ENSG00000141232
ENSG00000183873	ENSG00000007314
ENSG00000183873	ENSG00000136531
ENSG00000183873	ENSG00000136546
ENSG00000183873	ENSG00000144285
ENSG00000183873	ENSG00000153253
ENSG00000183873	ENSG00000168356
ENSG00000183873	ENSG00000169432
ENSG00000183873	ENSG00000185313
ENSG00000183873	ENSG00000196876
ENSG00000183876	ENSG00000006756
ENSG00000183876	ENSG00000062096
ENSG00000183876	ENSG00000100299
ENSG00000183876	ENSG00000101846
ENSG00000183876	ENSG00000113273
ENSG00000183876	ENSG00000141012
ENSG00000183876	ENSG00000141337
ENSG00000183876	ENSG00000157399
ENSG00000183876	ENSG00000180801
ENSG00000183876	ENSG00000205667
ENSG00000183878	ENSG00000132510
ENSG00000183878	ENSG00000147050
ENSG00000183908	ENSG00000162494
ENSG00000183908	ENSG00000162763
ENSG00000183908	ENSG00000184709
ENSG00000183918	ENSG00000198574
ENSG00000183921	ENSG00000184860
ENSG00000183935	ENSG00000120907
ENSG00000183935	ENSG00000143147
ENSG00000183935	ENSG00000148680
ENSG00000183935	ENSG00000165370
ENSG00000183935	ENSG00000170214
ENSG00000183935	ENSG00000171873
ENSG00000183940	ENSG00000104388
ENSG00000183940	ENSG00000119396
ENSG00000183940	ENSG00000129472
ENSG00000183940	ENSG00000155961
ENSG00000183940	ENSG00000167578
ENSG00000183940	ENSG00000168118
ENSG00000183940	ENSG00000179331
ENSG00000183940	ENSG00000188060
ENSG00000183941	ENSG00000124529
ENSG00000183941	ENSG00000124578
ENSG00000183941	ENSG00000158406
ENSG00000183941	ENSG00000182217
ENSG00000183941	ENSG00000188987
ENSG00000183941	ENSG00000196176
ENSG00000183941	ENSG00000197061
ENSG00000183941	ENSG00000197238
ENSG00000183941	ENSG00000197837
ENSG00000183941	ENSG00000197914
ENSG00000183941	ENSG00000198327
ENSG00000183941	ENSG00000198339
ENSG00000183941	ENSG00000198518
ENSG00000183941	ENSG00000198558
ENSG00000183960	ENSG00000055118
ENSG00000183960	ENSG00000089558
ENSG00000183960	ENSG00000135519
ENSG00000183960	ENSG00000140015

ENSG00000183960	ENSG00000143473
ENSG00000183960	ENSG00000173826
ENSG00000183960	ENSG00000184611
ENSG00000183963	ENSG00000100014
ENSG00000183963	ENSG00000128487
ENSG00000183963	ENSG00000188176
ENSG00000183963	ENSG00000214872
ENSG00000184005	ENSG00000136840
ENSG00000184007	ENSG00000112245
ENSG00000184007	ENSG00000184489
ENSG00000184009	ENSG00000075624
ENSG00000184009	ENSG00000107796
ENSG00000184009	ENSG00000143632
ENSG00000184009	ENSG00000159251
ENSG00000184009	ENSG00000163017
ENSG00000184009	ENSG00000169067
ENSG00000184009	ENSG00000204434
ENSG00000184009	ENSG00000214555
ENSG00000184009	ENSG00000215141
ENSG00000184012	ENSG00000105707
ENSG00000184012	ENSG00000137648
ENSG00000184012	ENSG00000137747
ENSG00000184012	ENSG00000145244
ENSG00000184012	ENSG00000154646
ENSG00000184012	ENSG00000160183
ENSG00000184012	ENSG00000166682
ENSG00000184014	ENSG00000137145
ENSG00000184014	ENSG00000170456
ENSG00000184014	ENSG00000174485
ENSG00000184014	ENSG00000198837
ENSG00000184058	ENSG00000073861
ENSG00000184058	ENSG00000089225
ENSG00000184058	ENSG00000092607
ENSG00000184058	ENSG00000112837
ENSG00000184058	ENSG00000121068
ENSG00000184058	ENSG00000121075
ENSG00000184058	ENSG00000122145
ENSG00000184058	ENSG00000135111
ENSG00000184058	ENSG00000136535
ENSG00000184058	ENSG00000143178
ENSG00000184058	ENSG00000149922
ENSG00000184058	ENSG00000163508
ENSG00000184058	ENSG00000164458
ENSG00000184058	ENSG00000164532
ENSG00000184058	ENSG00000167800
ENSG00000184058	ENSG00000174197
ENSG00000184060	ENSG00000105963
ENSG00000184083	ENSG00000048828
ENSG00000184113	ENSG00000013297
ENSG00000184113	ENSG00000066405
ENSG00000184113	ENSG00000106404
ENSG00000184113	ENSG00000113946
ENSG00000184113	ENSG00000134873
ENSG00000184113	ENSG00000156282
ENSG00000184113	ENSG00000156284
ENSG00000184113	ENSG00000159261
ENSG00000184113	ENSG00000163347
ENSG00000184113	ENSG00000164007
ENSG00000184113	ENSG00000165215

ENSG00000184113	ENSG00000165376
ENSG00000184113	ENSG00000171217
ENSG00000184113	ENSG00000177300
ENSG00000184113	ENSG00000181885
ENSG00000184113	ENSG00000184697
ENSG00000184113	ENSG00000185758
ENSG00000184113	ENSG00000189118
ENSG00000184113	ENSG00000189143
ENSG00000184113	ENSG00000213937
ENSG00000184117	ENSG00000136783
ENSG00000184117	ENSG00000146729
ENSG00000184117	ENSG00000165028
ENSG00000184144	ENSG00000091129
ENSG00000184144	ENSG00000134121
ENSG00000184144	ENSG00000149972
ENSG00000184144	ENSG00000163531
ENSG00000184144	ENSG00000198493
ENSG00000184156	ENSG00000053918
ENSG00000184156	ENSG00000075043
ENSG00000184156	ENSG00000117013
ENSG00000184156	ENSG00000185760
ENSG00000184163	ENSG00000178752
ENSG00000184164	ENSG00000163703
ENSG00000184185	ENSG00000115474
ENSG00000184185	ENSG00000120457
ENSG00000184185	ENSG00000121361
ENSG00000184185	ENSG00000123700
ENSG00000184185	ENSG00000151704
ENSG00000184185	ENSG00000153822
ENSG00000184185	ENSG00000157542
ENSG00000184185	ENSG00000157551
ENSG00000184185	ENSG00000162728
ENSG00000184185	ENSG00000162989
ENSG00000184185	ENSG00000168135
ENSG00000184185	ENSG00000177807
ENSG00000184185	ENSG00000182324
ENSG00000184185	ENSG00000187486
ENSG00000184194	ENSG00000164604
ENSG00000184194	ENSG00000170837
ENSG00000184203	ENSG00000102055
ENSG00000184203	ENSG00000215031
ENSG00000184203	ENSG00000215471
ENSG00000184206	ENSG00000140478
ENSG00000184206	ENSG00000153666
ENSG00000184206	ENSG00000156363
ENSG00000184206	ENSG00000159289
ENSG00000184206	ENSG00000167110
ENSG00000184206	ENSG00000167195
ENSG00000184206	ENSG00000174450
ENSG00000184206	ENSG00000175265
ENSG00000184206	ENSG00000181984
ENSG00000184206	ENSG00000186322
ENSG00000184206	ENSG00000188388
ENSG00000184206	ENSG00000188532
ENSG00000184206	ENSG00000188626
ENSG00000184206	ENSG00000197092
ENSG00000184206	ENSG00000197414
ENSG00000184206	ENSG00000214348
ENSG00000184206	ENSG00000215252

ENSG00000184206	ENSG00000215405
ENSG00000184207	ENSG00000107902
ENSG00000184207	ENSG00000167220
ENSG00000184210	ENSG00000062282
ENSG00000184210	ENSG00000106384
ENSG00000184210	ENSG00000124003
ENSG00000184210	ENSG00000147160
ENSG00000184210	ENSG00000166391
ENSG00000184210	ENSG00000204195
ENSG00000184210	ENSG00000205267
ENSG00000184216	ENSG00000090376
ENSG00000184216	ENSG00000134070
ENSG00000184221	ENSG00000125533
ENSG00000184221	ENSG00000177468
ENSG00000184221	ENSG00000180828
ENSG00000184221	ENSG00000205927
ENSG00000184226	ENSG00000099715
ENSG00000184226	ENSG00000102290
ENSG00000184226	ENSG00000113555
ENSG00000184226	ENSG00000156453
ENSG00000184226	ENSG00000169851
ENSG00000184226	ENSG00000197991
ENSG00000184226	ENSG00000215442
ENSG00000184254	ENSG00000111275
ENSG00000184254	ENSG00000128918
ENSG00000184254	ENSG00000137124
ENSG00000184254	ENSG00000165092
ENSG00000184260	ENSG00000111332
ENSG00000184260	ENSG00000124642
ENSG00000184260	ENSG00000137259
ENSG00000184260	ENSG00000158403
ENSG00000184260	ENSG00000164508
ENSG00000184260	ENSG00000168274
ENSG00000184260	ENSG00000180573
ENSG00000184260	ENSG00000181218
ENSG00000184260	ENSG00000182611
ENSG00000184260	ENSG00000183558
ENSG00000184260	ENSG00000184270
ENSG00000184260	ENSG00000184348
ENSG00000184260	ENSG00000184825
ENSG00000184260	ENSG00000185978
ENSG00000184260	ENSG00000188486
ENSG00000184260	ENSG00000196747
ENSG00000184260	ENSG00000196787
ENSG00000184260	ENSG00000196866
ENSG00000184260	ENSG00000198082
ENSG00000184260	ENSG00000198307
ENSG00000184260	ENSG00000198374
ENSG00000184260	ENSG00000203812
ENSG00000184260	ENSG00000205070
ENSG00000184260	ENSG00000205079
ENSG00000184261	ENSG00000082482
ENSG00000184261	ENSG00000095981
ENSG00000184261	ENSG00000099337
ENSG00000184261	ENSG00000100433
ENSG00000184261	ENSG00000124249
ENSG00000184261	ENSG00000124780
ENSG00000184261	ENSG00000135750
ENSG00000184261	ENSG00000152315

ENSG00000184261	ENSG00000164626
ENSG00000184261	ENSG00000169427
ENSG00000184261	ENSG00000171303
ENSG00000184261	ENSG00000173338
ENSG00000184261	ENSG00000182450
ENSG00000184270	ENSG00000111332
ENSG00000184270	ENSG00000124642
ENSG00000184270	ENSG00000137259
ENSG00000184270	ENSG00000158403
ENSG00000184270	ENSG00000164508
ENSG00000184270	ENSG00000168274
ENSG00000184270	ENSG00000180573
ENSG00000184270	ENSG00000181218
ENSG00000184270	ENSG00000182611
ENSG00000184270	ENSG00000183558
ENSG00000184270	ENSG00000184260
ENSG00000184270	ENSG00000184348
ENSG00000184270	ENSG00000184825
ENSG00000184270	ENSG00000185978
ENSG00000184270	ENSG00000188486
ENSG00000184270	ENSG00000196747
ENSG00000184270	ENSG00000196787
ENSG00000184270	ENSG00000196866
ENSG00000184270	ENSG00000198082
ENSG00000184270	ENSG00000198307
ENSG00000184270	ENSG00000198374
ENSG00000184270	ENSG00000203812
ENSG00000184270	ENSG00000205070
ENSG00000184270	ENSG00000205079
ENSG00000184271	ENSG00000106536
ENSG00000184294	ENSG00000142396
ENSG00000184294	ENSG00000169740
ENSG00000184294	ENSG00000174255
ENSG00000184294	ENSG00000179195
ENSG00000184294	ENSG00000179965
ENSG00000184294	ENSG00000186446
ENSG00000184294	ENSG00000186777
ENSG00000184294	ENSG00000197008
ENSG00000184294	ENSG00000197025
ENSG00000184294	ENSG00000197062
ENSG00000184294	ENSG00000197332
ENSG00000184294	ENSG00000198155
ENSG00000184294	ENSG00000204756
ENSG00000184294	ENSG00000212805
ENSG00000184294	ENSG00000213976
ENSG00000184294	ENSG00000213980
ENSG00000184294	ENSG00000215180
ENSG00000184294	ENSG00000215510
ENSG00000184294	ENSG00000215537
ENSG00000184294	ENSG00000215598
ENSG00000184294	ENSG00000215600
ENSG00000184301	ENSG00000071655
ENSG00000184301	ENSG00000134046
ENSG00000184301	ENSG00000170948
ENSG00000184301	ENSG00000182315
ENSG00000184301	ENSG00000196589
ENSG00000184301	ENSG00000205717
ENSG00000184301	ENSG00000205718
ENSG00000184302	ENSG00000100625

ENSG00000184302	ENSG00000126778
ENSG00000184302	ENSG00000138083
ENSG00000184302	ENSG00000170577
ENSG00000184302	ENSG00000177045
ENSG00000184303	ENSG00000043591
ENSG00000184303	ENSG00000102468
ENSG00000184303	ENSG00000113749
ENSG00000184303	ENSG00000125631
ENSG00000184303	ENSG00000135312
ENSG00000184303	ENSG00000135914
ENSG00000184303	ENSG00000147246
ENSG00000184303	ENSG00000150594
ENSG00000184303	ENSG00000157219
ENSG00000184303	ENSG00000164270
ENSG00000184303	ENSG00000168830
ENSG00000184303	ENSG00000169252
ENSG00000184303	ENSG00000175658
ENSG00000184303	ENSG00000178394
ENSG00000184303	ENSG00000179097
ENSG00000184303	ENSG00000179546
ENSG00000184303	ENSG00000181210
ENSG00000184303	ENSG00000184845
ENSG00000184303	ENSG00000185321
ENSG00000184303	ENSG00000188778
ENSG00000184304	ENSG00000105287
ENSG00000184304	ENSG00000115825
ENSG00000184305	ENSG00000107771
ENSG00000184319	ENSG00000118975
ENSG00000184319	ENSG00000185822
ENSG00000184319	ENSG00000186239
ENSG00000184319	ENSG00000186698
ENSG00000184319	ENSG00000198242
ENSG00000184319	ENSG00000206147
ENSG00000184319	ENSG00000212932
ENSG00000184319	ENSG00000213753
ENSG00000184319	ENSG00000214230
ENSG00000184319	ENSG00000215001
ENSG00000184319	ENSG00000215140
ENSG00000184319	ENSG00000215387
ENSG00000184343	ENSG00000096063
ENSG00000184343	ENSG00000135250
ENSG00000184345	ENSG00000173389
ENSG00000184345	ENSG00000214681
ENSG00000184345	ENSG00000214686
ENSG00000184347	ENSG00000128606
ENSG00000184347	ENSG00000145147
ENSG00000184347	ENSG00000166897
ENSG00000184347	ENSG00000182743
ENSG00000184347	ENSG00000187122
ENSG00000184348	ENSG00000111332
ENSG00000184348	ENSG00000124642
ENSG00000184348	ENSG00000137259
ENSG00000184348	ENSG00000158403
ENSG00000184348	ENSG00000164508
ENSG00000184348	ENSG00000168274
ENSG00000184348	ENSG00000180573
ENSG00000184348	ENSG00000181218
ENSG00000184348	ENSG00000182611
ENSG00000184348	ENSG00000183558

ENSG00000184348	ENSG00000184260
ENSG00000184348	ENSG00000184270
ENSG00000184348	ENSG00000184825
ENSG00000184348	ENSG00000185978
ENSG00000184348	ENSG00000188486
ENSG00000184348	ENSG00000196747
ENSG00000184348	ENSG00000196787
ENSG00000184348	ENSG00000196866
ENSG00000184348	ENSG00000198082
ENSG00000184348	ENSG00000198307
ENSG00000184348	ENSG00000198374
ENSG00000184348	ENSG00000203812
ENSG00000184348	ENSG00000205070
ENSG00000184348	ENSG00000205079
ENSG00000184349	ENSG00000090776
ENSG00000184349	ENSG00000099617
ENSG00000184349	ENSG00000108947
ENSG00000184349	ENSG00000125266
ENSG00000184349	ENSG00000143590
ENSG00000184349	ENSG00000143620
ENSG00000184349	ENSG00000169242
ENSG00000184357	ENSG00000124575
ENSG00000184357	ENSG00000124610
ENSG00000184357	ENSG00000168298
ENSG00000184357	ENSG00000184897
ENSG00000184357	ENSG00000187475
ENSG00000184357	ENSG00000187837
ENSG00000184357	ENSG00000189060
ENSG00000184357	ENSG00000196440
ENSG00000184363	ENSG00000057294
ENSG00000184363	ENSG00000081277
ENSG00000184363	ENSG00000099889
ENSG00000184363	ENSG00000144283
ENSG00000184363	ENSG00000169862
ENSG00000184363	ENSG00000198561
ENSG00000184368	ENSG00000116871
ENSG00000184368	ENSG00000129680
ENSG00000184368	ENSG00000135525
ENSG00000184374	ENSG00000105472
ENSG00000184374	ENSG00000118004
ENSG00000184374	ENSG00000122852
ENSG00000184374	ENSG00000122854
ENSG00000184374	ENSG00000133661
ENSG00000184374	ENSG00000163815
ENSG00000184374	ENSG00000165471
ENSG00000184374	ENSG00000166509
ENSG00000184374	ENSG00000182314
ENSG00000184374	ENSG00000185303
ENSG00000184384	ENSG00000013619
ENSG00000184384	ENSG00000161021
ENSG00000184384	ENSG00000196782
ENSG00000184388	ENSG00000186288
ENSG00000184389	ENSG00000148288
ENSG00000184389	ENSG00000175164
ENSG00000184389	ENSG00000204007
ENSG00000184402	ENSG00000141380
ENSG00000184405	ENSG00000181996
ENSG00000184405	ENSG00000204239
ENSG00000184405	ENSG00000205663

ENSG00000184405	ENSG00000205664
ENSG00000184405	ENSG00000206061
ENSG00000184408	ENSG00000026559
ENSG00000184408	ENSG00000102057
ENSG00000184408	ENSG00000104848
ENSG00000184408	ENSG00000111262
ENSG00000184408	ENSG00000124134
ENSG00000184408	ENSG00000130037
ENSG00000184408	ENSG00000143105
ENSG00000184408	ENSG00000151079
ENSG00000184408	ENSG00000156486
ENSG00000184408	ENSG00000158445
ENSG00000184408	ENSG00000162975
ENSG00000184408	ENSG00000164794
ENSG00000184408	ENSG00000168263
ENSG00000184408	ENSG00000168418
ENSG00000184408	ENSG00000170745
ENSG00000184408	ENSG00000171126
ENSG00000184408	ENSG00000171385
ENSG00000184408	ENSG00000177272
ENSG00000184408	ENSG00000177301
ENSG00000184408	ENSG00000178342
ENSG00000184408	ENSG00000182255
ENSG00000184408	ENSG00000182674
ENSG00000184409	ENSG00000157576
ENSG00000184409	ENSG00000172186
ENSG00000184409	ENSG00000182765
ENSG00000184409	ENSG00000197366
ENSG00000184409	ENSG00000198157
ENSG00000184409	ENSG00000198875
ENSG00000184416	ENSG00000003987
ENSG00000184416	ENSG00000014914
ENSG00000184416	ENSG00000063601
ENSG00000184416	ENSG00000087053
ENSG00000184416	ENSG00000100330
ENSG00000184416	ENSG00000102043
ENSG00000184416	ENSG00000104643
ENSG00000184416	ENSG00000108389
ENSG00000184416	ENSG00000139505
ENSG00000184416	ENSG00000150712
ENSG00000184416	ENSG00000166912
ENSG00000184416	ENSG00000171100
ENSG00000184428	ENSG00000198900
ENSG00000184436	ENSG00000174796
ENSG00000184436	ENSG00000177683
ENSG00000184451	ENSG00000112486
ENSG00000184451	ENSG00000121797
ENSG00000184451	ENSG00000121807
ENSG00000184451	ENSG00000121966
ENSG00000184451	ENSG00000126353
ENSG00000184451	ENSG00000129048
ENSG00000184451	ENSG00000144648
ENSG00000184451	ENSG00000160683
ENSG00000184451	ENSG00000160791
ENSG00000184451	ENSG00000163464
ENSG00000184451	ENSG00000163823
ENSG00000184451	ENSG00000168329
ENSG00000184451	ENSG00000172215
ENSG00000184451	ENSG00000173578

ENSG00000184451	ENSG00000173585
ENSG00000184451	ENSG00000179934
ENSG00000184451	ENSG00000180871
ENSG00000184451	ENSG00000183625
ENSG00000184451	ENSG00000183813
ENSG00000184451	ENSG00000186810
ENSG00000184451	ENSG00000215775
ENSG00000184451	ENSG00000215778
ENSG00000184451	ENSG00000215782
ENSG00000184459	ENSG00000078898
ENSG00000184459	ENSG00000100979
ENSG00000184459	ENSG00000101425
ENSG00000184459	ENSG00000129988
ENSG00000184459	ENSG00000167104
ENSG00000184459	ENSG00000186190
ENSG00000184459	ENSG00000186191
ENSG00000184471	ENSG00000054803
ENSG00000184471	ENSG00000102924
ENSG00000184471	ENSG00000131094
ENSG00000184471	ENSG00000133466
ENSG00000184471	ENSG00000139899
ENSG00000184471	ENSG00000141668
ENSG00000184471	ENSG00000144119
ENSG00000184471	ENSG00000145861
ENSG00000184471	ENSG00000159189
ENSG00000184471	ENSG00000163145
ENSG00000184471	ENSG00000165985
ENSG00000184471	ENSG00000172247
ENSG00000184471	ENSG00000173369
ENSG00000184471	ENSG00000173372
ENSG00000184471	ENSG00000173918
ENSG00000184471	ENSG00000181092
ENSG00000184471	ENSG00000186897
ENSG00000184471	ENSG00000213192
ENSG00000184481	ENSG00000118689
ENSG00000184481	ENSG00000150907
ENSG00000184481	ENSG00000204060
ENSG00000184486	ENSG00000028277
ENSG00000184486	ENSG00000064835
ENSG00000184486	ENSG00000091010
ENSG00000184486	ENSG00000137709
ENSG00000184486	ENSG00000143190
ENSG00000184486	ENSG00000151615
ENSG00000184486	ENSG00000152192
ENSG00000184486	ENSG00000185668
ENSG00000184486	ENSG00000196767
ENSG00000184486	ENSG00000198914
ENSG00000184486	ENSG00000204531
ENSG00000184486	ENSG00000212993
ENSG00000184488	ENSG00000096088
ENSG00000184488	ENSG00000117984
ENSG00000184488	ENSG00000131400
ENSG00000184488	ENSG00000131401
ENSG00000184488	ENSG00000143839
ENSG00000184488	ENSG00000172647
ENSG00000184488	ENSG00000180860
ENSG00000184488	ENSG00000196188
ENSG00000184488	ENSG00000204973
ENSG00000184488	ENSG00000215868

ENSG00000184489	ENSG00000112245
ENSG00000184489	ENSG00000184007
ENSG00000184490	ENSG00000099984
ENSG00000184490	ENSG00000133433
ENSG00000184490	ENSG00000184674
ENSG00000184490	ENSG00000185831
ENSG00000184490	ENSG00000206090
ENSG00000184492	ENSG00000054598
ENSG00000184492	ENSG00000125798
ENSG00000184492	ENSG00000129514
ENSG00000184492	ENSG00000164379
ENSG00000184492	ENSG00000168269
ENSG00000184492	ENSG00000170122
ENSG00000184492	ENSG00000170608
ENSG00000184492	ENSG00000176165
ENSG00000184492	ENSG00000176678
ENSG00000184492	ENSG00000176692
ENSG00000184492	ENSG00000178919
ENSG00000184492	ENSG00000179772
ENSG00000184492	ENSG00000183770
ENSG00000184492	ENSG00000184659
ENSG00000184492	ENSG00000186564
ENSG00000184492	ENSG00000186766
ENSG00000184492	ENSG00000186790
ENSG00000184492	ENSG00000187140
ENSG00000184492	ENSG00000187559
ENSG00000184492	ENSG00000204612
ENSG00000184492	ENSG00000204779
ENSG00000184492	ENSG00000204793
ENSG00000184492	ENSG00000204828
ENSG00000184492	ENSG00000214336
ENSG00000184497	ENSG00000125355
ENSG00000184500	ENSG00000183087
ENSG00000184505	ENSG00000145860
ENSG00000184505	ENSG00000162298
ENSG00000184505	ENSG00000170881
ENSG00000184505	ENSG00000187779
ENSG00000184544	ENSG00000109016
ENSG00000184545	ENSG00000111266
ENSG00000184545	ENSG00000120129
ENSG00000184545	ENSG00000120875
ENSG00000184545	ENSG00000130829
ENSG00000184545	ENSG00000138166
ENSG00000184545	ENSG00000139318
ENSG00000184545	ENSG00000143507
ENSG00000184545	ENSG00000158050
ENSG00000184545	ENSG00000164086
ENSG00000184557	ENSG00000114737
ENSG00000184557	ENSG00000120833
ENSG00000184557	ENSG00000170677
ENSG00000184557	ENSG00000171150
ENSG00000184557	ENSG00000174111
ENSG00000184557	ENSG00000180008
ENSG00000184557	ENSG00000185338
ENSG00000184564	ENSG00000165300
ENSG00000184564	ENSG00000179542
ENSG00000184571	ENSG00000125207
ENSG00000184571	ENSG00000134627
ENSG00000184571	ENSG00000197181

ENSG00000184574	ENSG00000078589
ENSG00000184574	ENSG00000139679
ENSG00000184574	ENSG00000147145
ENSG00000184574	ENSG00000152207
ENSG00000184574	ENSG00000169403
ENSG00000184574	ENSG00000169508
ENSG00000184574	ENSG00000169860
ENSG00000184574	ENSG00000173198
ENSG00000184574	ENSG00000175591
ENSG00000184574	ENSG00000186912
ENSG00000184574	ENSG00000198829
ENSG00000184588	ENSG00000065989
ENSG00000184588	ENSG00000073417
ENSG00000184588	ENSG00000105650
ENSG00000184588	ENSG00000113231
ENSG00000184588	ENSG00000113448
ENSG00000184588	ENSG00000115252
ENSG00000184588	ENSG00000123360
ENSG00000184588	ENSG00000154678
ENSG00000184588	ENSG00000160191
ENSG00000184588	ENSG00000171408
ENSG00000184588	ENSG00000205268
ENSG00000184599	ENSG00000163377
ENSG00000184599	ENSG00000183662
ENSG00000184599	ENSG00000198673
ENSG00000184611	ENSG00000055118
ENSG00000184611	ENSG00000089558
ENSG00000184611	ENSG00000135519
ENSG00000184611	ENSG00000140015
ENSG00000184611	ENSG00000143473
ENSG00000184611	ENSG00000173826
ENSG00000184611	ENSG00000183960
ENSG00000184613	ENSG00000165973
ENSG00000184616	ENSG00000136206
ENSG00000184616	ENSG00000154732
ENSG00000184616	ENSG00000163806
ENSG00000184616	ENSG00000166667
ENSG00000184616	ENSG00000170092
ENSG00000184616	ENSG00000173678
ENSG00000184616	ENSG00000179994
ENSG00000184616	ENSG00000183318
ENSG00000184616	ENSG00000185040
ENSG00000184616	ENSG00000186645
ENSG00000184616	ENSG00000198305
ENSG00000184616	ENSG00000204710
ENSG00000184616	ENSG00000205238
ENSG00000184616	ENSG00000205482
ENSG00000184616	ENSG00000205505
ENSG00000184616	ENSG00000205506
ENSG00000184616	ENSG00000205508
ENSG00000184616	ENSG00000205509
ENSG00000184616	ENSG00000205580
ENSG00000184616	ENSG00000214300
ENSG00000184616	ENSG00000215092
ENSG00000184634	ENSG00000144893
ENSG00000184635	ENSG00000205245
ENSG00000184635	ENSG00000213799
ENSG00000184640	ENSG00000100167
ENSG00000184640	ENSG00000140623

ENSG00000184659	ENSG00000054598
ENSG00000184659	ENSG00000125798
ENSG00000184659	ENSG00000129514
ENSG00000184659	ENSG00000164379
ENSG00000184659	ENSG00000168269
ENSG00000184659	ENSG00000170122
ENSG00000184659	ENSG00000170608
ENSG00000184659	ENSG00000176165
ENSG00000184659	ENSG00000176678
ENSG00000184659	ENSG00000176692
ENSG00000184659	ENSG00000178919
ENSG00000184659	ENSG00000179772
ENSG00000184659	ENSG00000183770
ENSG00000184659	ENSG00000184492
ENSG00000184659	ENSG00000186564
ENSG00000184659	ENSG00000186766
ENSG00000184659	ENSG00000186790
ENSG00000184659	ENSG00000187140
ENSG00000184659	ENSG00000187559
ENSG00000184659	ENSG00000204612
ENSG00000184659	ENSG00000204779
ENSG00000184659	ENSG00000204793
ENSG00000184659	ENSG00000204828
ENSG00000184659	ENSG00000214336
ENSG00000184672	ENSG00000092199
ENSG00000184672	ENSG00000125970
ENSG00000184672	ENSG00000179172
ENSG00000184672	ENSG00000214634
ENSG00000184672	ENSG00000215880
ENSG00000184674	ENSG00000099984
ENSG00000184674	ENSG00000133433
ENSG00000184674	ENSG00000184490
ENSG00000184674	ENSG00000185831
ENSG00000184674	ENSG00000206090
ENSG00000184675	ENSG00000165566
ENSG00000184675	ENSG00000178171
ENSG00000184697	ENSG00000013297
ENSG00000184697	ENSG00000066405
ENSG00000184697	ENSG00000106404
ENSG00000184697	ENSG00000113946
ENSG00000184697	ENSG00000134873
ENSG00000184697	ENSG00000156282
ENSG00000184697	ENSG00000156284
ENSG00000184697	ENSG00000159261
ENSG00000184697	ENSG00000163347
ENSG00000184697	ENSG00000164007
ENSG00000184697	ENSG00000165215
ENSG00000184697	ENSG00000165376
ENSG00000184697	ENSG00000171217
ENSG00000184697	ENSG00000177300
ENSG00000184697	ENSG00000181885
ENSG00000184697	ENSG00000184113
ENSG00000184697	ENSG00000185758
ENSG00000184697	ENSG00000189118
ENSG00000184697	ENSG00000189143
ENSG00000184697	ENSG00000213937
ENSG00000184701	ENSG00000136938
ENSG00000184701	ENSG00000139223
ENSG00000184701	ENSG00000140350

ENSG00000184701	ENSG00000143401
ENSG00000184701	ENSG00000215332
ENSG00000184702	ENSG00000108387
ENSG00000184702	ENSG00000122545
ENSG00000184702	ENSG00000168385
ENSG00000184702	ENSG00000180096
ENSG00000184702	ENSG00000214005
ENSG00000184702	ENSG00000214765
ENSG00000184702	ENSG00000214834
ENSG00000184709	ENSG00000162494
ENSG00000184709	ENSG00000162763
ENSG00000184709	ENSG00000183908
ENSG00000184716	ENSG00000111897
ENSG00000184716	ENSG00000132824
ENSG00000184716	ENSG00000164300
ENSG00000184716	ENSG00000168528
ENSG00000184731	ENSG00000125898
ENSG00000184731	ENSG00000169122
ENSG00000184731	ENSG00000197245
ENSG00000184743	ENSG00000119787
ENSG00000184743	ENSG00000198513
ENSG00000184763	ENSG00000116030
ENSG00000184763	ENSG00000177688
ENSG00000184763	ENSG00000184900
ENSG00000184763	ENSG00000188612
ENSG00000184766	ENSG00000108100
ENSG00000184766	ENSG00000163249
ENSG00000184766	ENSG00000179296
ENSG00000184766	ENSG00000182632
ENSG00000184766	ENSG00000204150
ENSG00000184766	ENSG00000215169
ENSG00000184774	ENSG00000071073
ENSG00000184774	ENSG00000161013
ENSG00000184774	ENSG00000182050
ENSG00000184788	ENSG00000130066
ENSG00000184788	ENSG00000141504
ENSG00000184792	ENSG00000110048
ENSG00000184795	ENSG00000110057
ENSG00000184795	ENSG00000112494
ENSG00000184795	ENSG00000155070
ENSG00000184795	ENSG00000172971
ENSG00000184825	ENSG00000111332
ENSG00000184825	ENSG00000124642
ENSG00000184825	ENSG00000137259
ENSG00000184825	ENSG00000158403
ENSG00000184825	ENSG00000164508
ENSG00000184825	ENSG00000168274
ENSG00000184825	ENSG00000180573
ENSG00000184825	ENSG00000181218
ENSG00000184825	ENSG00000182611
ENSG00000184825	ENSG00000183558
ENSG00000184825	ENSG00000184260
ENSG00000184825	ENSG00000184270
ENSG00000184825	ENSG00000184348
ENSG00000184825	ENSG00000185978
ENSG00000184825	ENSG00000188486
ENSG00000184825	ENSG00000196747
ENSG00000184825	ENSG00000196787
ENSG00000184825	ENSG00000196866

ENSG00000184825	ENSG00000198082
ENSG00000184825	ENSG00000198307
ENSG00000184825	ENSG00000198374
ENSG00000184825	ENSG00000203812
ENSG00000184825	ENSG00000205070
ENSG00000184825	ENSG00000205079
ENSG00000184828	ENSG00000160685
ENSG00000184828	ENSG00000178951
ENSG00000184831	ENSG00000155008
ENSG00000184840	ENSG00000158604
ENSG00000184840	ENSG00000215367
ENSG00000184845	ENSG00000043591
ENSG00000184845	ENSG00000102468
ENSG00000184845	ENSG00000113749
ENSG00000184845	ENSG00000125631
ENSG00000184845	ENSG00000135312
ENSG00000184845	ENSG00000135914
ENSG00000184845	ENSG00000147246
ENSG00000184845	ENSG00000150594
ENSG00000184845	ENSG00000157219
ENSG00000184845	ENSG00000164270
ENSG00000184845	ENSG00000168830
ENSG00000184845	ENSG00000169252
ENSG00000184845	ENSG00000175658
ENSG00000184845	ENSG00000178394
ENSG00000184845	ENSG00000179097
ENSG00000184845	ENSG00000179546
ENSG00000184845	ENSG00000181210
ENSG00000184845	ENSG00000184303
ENSG00000184845	ENSG00000185321
ENSG00000184845	ENSG00000188778
ENSG00000184860	ENSG00000183921
ENSG00000184862	ENSG00000132604
ENSG00000184862	ENSG00000147601
ENSG00000184862	ENSG00000187589
ENSG00000184887	ENSG00000064726
ENSG00000184887	ENSG00000132640
ENSG00000184887	ENSG00000133243
ENSG00000184888	ENSG00000072401
ENSG00000184888	ENSG00000078967
ENSG00000184888	ENSG00000109332
ENSG00000184888	ENSG00000131508
ENSG00000184897	ENSG00000124575
ENSG00000184897	ENSG00000124610
ENSG00000184897	ENSG00000168298
ENSG00000184897	ENSG00000184357
ENSG00000184897	ENSG00000187475
ENSG00000184897	ENSG00000187837
ENSG00000184897	ENSG00000189060
ENSG00000184897	ENSG00000196440
ENSG00000184900	ENSG00000116030
ENSG00000184900	ENSG00000177688
ENSG00000184900	ENSG00000184763
ENSG00000184900	ENSG00000188612
ENSG00000184908	ENSG00000114859
ENSG00000184908	ENSG00000186510
ENSG00000184916	ENSG00000090932
ENSG00000184916	ENSG00000101384
ENSG00000184916	ENSG00000128917

ENSG00000184916	ENSG00000171462
ENSG00000184916	ENSG00000185559
ENSG00000184916	ENSG00000198719
ENSG00000184922	ENSG00000157827
ENSG00000184922	ENSG00000161791
ENSG00000184925	ENSG00000107317
ENSG00000184925	ENSG00000148346
ENSG00000184925	ENSG00000176919
ENSG00000184925	ENSG00000177984
ENSG00000184925	ENSG00000187922
ENSG00000184925	ENSG00000214402
ENSG00000184945	ENSG00000178301
ENSG00000184945	ENSG00000185176
ENSG00000184956	ENSG00000090920
ENSG00000184956	ENSG00000109927
ENSG00000184956	ENSG00000110799
ENSG00000184956	ENSG00000117983
ENSG00000184956	ENSG00000146839
ENSG00000184956	ENSG00000188162
ENSG00000184956	ENSG00000196292
ENSG00000184956	ENSG00000197558
ENSG00000184956	ENSG00000198788
ENSG00000184968	ENSG00000182257
ENSG00000184968	ENSG00000205066
ENSG00000184984	ENSG00000133019
ENSG00000184984	ENSG00000168539
ENSG00000184984	ENSG00000181072
ENSG00000184985	ENSG00000108018
ENSG00000184985	ENSG00000134243
ENSG00000184985	ENSG00000137642
ENSG00000184985	ENSG00000156395
ENSG00000184988	ENSG00000106460
ENSG00000184988	ENSG00000134291
ENSG00000184988	ENSG00000215684
ENSG00000184992	ENSG00000110108
ENSG00000185002	ENSG00000080298
ENSG00000185002	ENSG00000087903
ENSG00000185002	ENSG00000111783
ENSG00000185002	ENSG00000132005
ENSG00000185002	ENSG00000143390
ENSG00000185002	ENSG00000181827
ENSG00000185008	ENSG00000154133
ENSG00000185008	ENSG00000154134
ENSG00000185008	ENSG00000169855
ENSG00000185009	ENSG00000070718
ENSG00000185010	ENSG00000047457
ENSG00000185010	ENSG00000089472
ENSG00000185010	ENSG00000181333
ENSG00000185010	ENSG00000198734
ENSG00000185013	ENSG00000116981
ENSG00000185015	ENSG00000074410
ENSG00000185015	ENSG00000104267
ENSG00000185015	ENSG00000107159
ENSG00000185015	ENSG00000118298
ENSG00000185015	ENSG00000131686
ENSG00000185015	ENSG00000133742
ENSG00000185015	ENSG00000164879
ENSG00000185015	ENSG00000167434
ENSG00000185015	ENSG00000168748

ENSG00000185015	ENSG00000169239
ENSG00000185015	ENSG00000174990
ENSG00000185015	ENSG00000178538
ENSG00000185015	ENSG00000214729
ENSG00000185015	ENSG00000214873
ENSG00000185015	ENSG00000215487
ENSG00000185015	ENSG00000215501
ENSG00000185015	ENSG00000215543
ENSG00000185020	ENSG00000106714
ENSG00000185020	ENSG00000108797
ENSG00000185020	ENSG00000152910
ENSG00000185020	ENSG00000154529
ENSG00000185020	ENSG00000155052
ENSG00000185020	ENSG00000174469
ENSG00000185022	ENSG00000129535
ENSG00000185022	ENSG00000178573
ENSG00000185022	ENSG00000182759
ENSG00000185022	ENSG00000197063
ENSG00000185022	ENSG00000198517
ENSG00000185022	ENSG00000204103
ENSG00000185022	ENSG00000215622
ENSG00000185028	ENSG00000116708
ENSG00000185028	ENSG00000116721
ENSG00000185028	ENSG00000116726
ENSG00000185028	ENSG00000120952
ENSG00000185028	ENSG00000157358
ENSG00000185028	ENSG00000160959
ENSG00000185028	ENSG00000179704
ENSG00000185028	ENSG00000182330
ENSG00000185028	ENSG00000185686
ENSG00000185028	ENSG00000187545
ENSG00000185028	ENSG00000197549
ENSG00000185028	ENSG00000204478
ENSG00000185028	ENSG00000204479
ENSG00000185028	ENSG00000204480
ENSG00000185028	ENSG00000204481
ENSG00000185028	ENSG00000204485
ENSG00000185028	ENSG00000204486
ENSG00000185028	ENSG00000204488
ENSG00000185028	ENSG00000204491
ENSG00000185028	ENSG00000204495
ENSG00000185028	ENSG00000204501
ENSG00000185028	ENSG00000204502
ENSG00000185028	ENSG00000204503
ENSG00000185028	ENSG00000204505
ENSG00000185028	ENSG00000204507
ENSG00000185028	ENSG00000204508
ENSG00000185028	ENSG00000204509
ENSG00000185028	ENSG00000204510
ENSG00000185028	ENSG00000204513
ENSG00000185033	ENSG00000010319
ENSG00000185033	ENSG00000012171
ENSG00000185033	ENSG00000075213
ENSG00000185033	ENSG00000092421
ENSG00000185033	ENSG00000095539
ENSG00000185033	ENSG00000137872
ENSG00000185033	ENSG00000143434
ENSG00000185033	ENSG00000153993
ENSG00000185033	ENSG00000168758

ENSG00000185033	ENSG00000170381
ENSG00000185033	ENSG00000187764
ENSG00000185033	ENSG00000196189
ENSG00000185036	ENSG00000168894
ENSG00000185036	ENSG00000214209
ENSG00000185038	ENSG00000171495
ENSG00000185038	ENSG00000179832
ENSG00000185040	ENSG00000136206
ENSG00000185040	ENSG00000154732
ENSG00000185040	ENSG00000163806
ENSG00000185040	ENSG00000166667
ENSG00000185040	ENSG00000170092
ENSG00000185040	ENSG00000173678
ENSG00000185040	ENSG00000179994
ENSG00000185040	ENSG00000183318
ENSG00000185040	ENSG00000184616
ENSG00000185040	ENSG00000186645
ENSG00000185040	ENSG00000198305
ENSG00000185040	ENSG00000204710
ENSG00000185040	ENSG00000205238
ENSG00000185040	ENSG00000205482
ENSG00000185040	ENSG00000205505
ENSG00000185040	ENSG00000205506
ENSG00000185040	ENSG00000205508
ENSG00000185040	ENSG00000205509
ENSG00000185040	ENSG00000205580
ENSG00000185040	ENSG00000214300
ENSG00000185040	ENSG00000215092
ENSG00000185043	ENSG00000136425
ENSG00000185043	ENSG00000141977
ENSG00000185043	ENSG00000157884
ENSG00000185046	ENSG00000064999
ENSG00000185046	ENSG00000167971
ENSG00000185046	ENSG00000177303
ENSG00000185051	ENSG00000134594
ENSG00000185051	ENSG00000137502
ENSG00000185051	ENSG00000138069
ENSG00000185051	ENSG00000146955
ENSG00000185051	ENSG00000172007
ENSG00000185051	ENSG00000174903
ENSG00000185052	ENSG00000074621
ENSG00000185052	ENSG00000140090
ENSG00000185052	ENSG00000155886
ENSG00000185052	ENSG00000188467
ENSG00000185053	ENSG00000102683
ENSG00000185053	ENSG00000163069
ENSG00000185053	ENSG00000170624
ENSG00000185069	ENSG00000135443
ENSG00000185069	ENSG00000135477
ENSG00000185069	ENSG00000135480
ENSG00000185069	ENSG00000139648
ENSG00000185069	ENSG00000161849
ENSG00000185069	ENSG00000161850
ENSG00000185069	ENSG00000167767
ENSG00000185069	ENSG00000167768
ENSG00000185069	ENSG00000170421
ENSG00000185069	ENSG00000170423
ENSG00000185069	ENSG00000170442
ENSG00000185069	ENSG00000170454

ENSG00000185069	ENSG00000170465
ENSG00000185069	ENSG00000170477
ENSG00000185069	ENSG00000170484
ENSG00000185069	ENSG00000170486
ENSG00000185069	ENSG00000170523
ENSG00000185069	ENSG00000172867
ENSG00000185069	ENSG00000185479
ENSG00000185069	ENSG00000185640
ENSG00000185069	ENSG00000186049
ENSG00000185069	ENSG00000186081
ENSG00000185069	ENSG00000186442
ENSG00000185069	ENSG00000189182
ENSG00000185069	ENSG00000205420
ENSG00000185069	ENSG00000205426
ENSG00000185069	ENSG00000214233
ENSG00000185069	ENSG00000214323
ENSG00000185069	ENSG00000214477
ENSG00000185069	ENSG00000214752
ENSG00000185069	ENSG00000214974
ENSG00000185069	ENSG00000215287
ENSG00000185069	ENSG00000215847
ENSG00000185087	ENSG00000198780
ENSG00000185088	ENSG00000130041
ENSG00000185088	ENSG00000163144
ENSG00000185088	ENSG00000177954
ENSG00000185088	ENSG00000198731
ENSG00000185090	ENSG00000172469
ENSG00000185100	ENSG00000035687
ENSG00000185101	ENSG00000047617
ENSG00000185101	ENSG00000074855
ENSG00000185101	ENSG00000131620
ENSG00000185101	ENSG00000134343
ENSG00000185101	ENSG00000146205
ENSG00000185101	ENSG00000151572
ENSG00000185101	ENSG00000160746
ENSG00000185101	ENSG00000171714
ENSG00000185101	ENSG00000177119
ENSG00000185105	ENSG00000179820
ENSG00000185112	ENSG00000105245
ENSG00000185112	ENSG00000133961
ENSG00000185112	ENSG00000144366
ENSG00000185112	ENSG00000157978
ENSG00000185112	ENSG00000183114
ENSG00000185122	ENSG00000025156
ENSG00000185122	ENSG00000102878
ENSG00000185122	ENSG00000169953
ENSG00000185122	ENSG00000171116
ENSG00000185122	ENSG00000171129
ENSG00000185122	ENSG00000172468
ENSG00000185122	ENSG00000176160
ENSG00000185122	ENSG00000214373
ENSG00000185122	ENSG00000215574
ENSG00000185122	ENSG00000215701
ENSG00000185128	ENSG00000128438
ENSG00000185128	ENSG00000148429
ENSG00000185128	ENSG00000161583
ENSG00000185128	ENSG00000174115
ENSG00000185128	ENSG00000188755
ENSG00000185128	ENSG00000188849

ENSG00000185128	ENSG00000189309
ENSG00000185128	ENSG00000197681
ENSG00000185128	ENSG00000204471
ENSG00000185128	ENSG00000205019
ENSG00000185128	ENSG00000213474
ENSG00000185128	ENSG00000215625
ENSG00000185128	ENSG00000215730
ENSG00000185128	ENSG00000215748
ENSG00000185129	ENSG00000146676
ENSG00000185129	ENSG00000172733
ENSG00000185133	ENSG00000122126
ENSG00000185133	ENSG00000132376
ENSG00000185133	ENSG00000204084
ENSG00000185149	ENSG00000056291
ENSG00000185149	ENSG00000075073
ENSG00000185149	ENSG00000101292
ENSG00000185149	ENSG00000110148
ENSG00000185149	ENSG00000115353
ENSG00000185149	ENSG00000119973
ENSG00000185149	ENSG00000121764
ENSG00000185149	ENSG00000123901
ENSG00000185149	ENSG00000137252
ENSG00000185149	ENSG00000148734
ENSG00000185149	ENSG00000159279
ENSG00000185149	ENSG00000163394
ENSG00000185149	ENSG00000164128
ENSG00000185149	ENSG00000164129
ENSG00000185149	ENSG00000169618
ENSG00000185149	ENSG00000169836
ENSG00000185149	ENSG00000186867
ENSG00000185149	ENSG00000204174
ENSG00000185149	ENSG00000215167
ENSG00000185176	ENSG00000178301
ENSG00000185176	ENSG00000184945
ENSG00000185187	ENSG00000115590
ENSG00000185187	ENSG00000115594
ENSG00000185187	ENSG00000115598
ENSG00000185187	ENSG00000115602
ENSG00000185187	ENSG00000115604
ENSG00000185187	ENSG00000115607
ENSG00000185187	ENSG00000196083
ENSG00000185198	ENSG00000092009
ENSG00000185198	ENSG00000100448
ENSG00000185198	ENSG00000100450
ENSG00000185198	ENSG00000100453
ENSG00000185198	ENSG00000113088
ENSG00000185198	ENSG00000145649
ENSG00000185198	ENSG00000197766
ENSG00000185201	ENSG00000142089
ENSG00000185201	ENSG00000185885
ENSG00000185201	ENSG00000188082
ENSG00000185201	ENSG00000206013
ENSG00000185201	ENSG00000215096
ENSG00000185214	ENSG00000003096
ENSG00000185214	ENSG00000087448
ENSG00000185214	ENSG00000124743
ENSG00000185214	ENSG00000130487
ENSG00000185214	ENSG00000135686
ENSG00000185214	ENSG00000166853

ENSG00000185214	ENSG00000167487
ENSG00000185214	ENSG00000174010
ENSG00000185214	ENSG00000176595
ENSG00000185214	ENSG00000179023
ENSG00000185214	ENSG00000185915
ENSG00000185214	ENSG00000186231
ENSG00000185214	ENSG00000197705
ENSG00000185214	ENSG00000198642
ENSG00000185215	ENSG00000130201
ENSG00000185215	ENSG00000179044
ENSG00000185215	ENSG00000180104
ENSG00000185215	ENSG00000205436
ENSG00000185231	ENSG00000124089
ENSG00000185231	ENSG00000166603
ENSG00000185231	ENSG00000176136
ENSG00000185236	ENSG00000103769
ENSG00000185236	ENSG00000132698
ENSG00000185245	ENSG00000144771
ENSG00000185245	ENSG00000166159
ENSG00000185251	ENSG00000168993
ENSG00000185251	ENSG00000204336
ENSG00000185251	ENSG00000205861
ENSG00000185254	ENSG00000057704
ENSG00000185254	ENSG00000102080
ENSG00000185254	ENSG00000133069
ENSG00000185254	ENSG00000172765
ENSG00000185254	ENSG00000182242
ENSG00000185262	ENSG00000153443
ENSG00000185267	ENSG00000145050
ENSG00000185272	ENSG00000076053
ENSG00000185274	ENSG00000100626
ENSG00000185274	ENSG00000106648
ENSG00000185274	ENSG00000110328
ENSG00000185274	ENSG00000115339
ENSG00000185274	ENSG00000131386
ENSG00000185274	ENSG00000136542
ENSG00000185274	ENSG00000139629
ENSG00000185274	ENSG00000141429
ENSG00000185274	ENSG00000143641
ENSG00000185274	ENSG00000144278
ENSG00000185274	ENSG00000158089
ENSG00000185274	ENSG00000164574
ENSG00000185274	ENSG00000174473
ENSG00000185274	ENSG00000178234
ENSG00000185274	ENSG00000182870
ENSG00000185278	ENSG00000169155
ENSG00000185278	ENSG00000180610
ENSG00000185278	ENSG00000204210
ENSG00000185278	ENSG00000204366
ENSG00000185278	ENSG00000213588
ENSG00000185291	ENSG00000091181
ENSG00000185291	ENSG00000123496
ENSG00000185291	ENSG00000131724
ENSG00000185291	ENSG00000198223
ENSG00000185294	ENSG00000005206
ENSG00000185294	ENSG00000138600
ENSG00000185295	ENSG00000124422
ENSG00000185295	ENSG00000180991
ENSG00000185313	ENSG00000007314

ENSG00000185313	ENSG00000136531
ENSG00000185313	ENSG00000136546
ENSG00000185313	ENSG00000144285
ENSG00000185313	ENSG00000153253
ENSG00000185313	ENSG00000168356
ENSG00000185313	ENSG00000169432
ENSG00000185313	ENSG00000183873
ENSG00000185313	ENSG00000196876
ENSG00000185338	ENSG00000114737
ENSG00000185338	ENSG00000120833
ENSG00000185338	ENSG00000170677
ENSG00000185338	ENSG00000171150
ENSG00000185338	ENSG00000174111
ENSG00000185338	ENSG00000180008
ENSG00000185338	ENSG00000184557
ENSG00000185340	ENSG00000132139
ENSG00000185340	ENSG00000139354
ENSG00000185340	ENSG00000148935
ENSG00000185344	ENSG00000033627
ENSG00000185344	ENSG00000105929
ENSG00000185344	ENSG00000110719
ENSG00000185352	ENSG00000136720
ENSG00000185352	ENSG00000171004
ENSG00000185361	ENSG00000145779
ENSG00000185361	ENSG00000163154
ENSG00000185361	ENSG00000183578
ENSG00000185386	ENSG00000050748
ENSG00000185386	ENSG00000107643
ENSG00000185386	ENSG00000109339
ENSG00000185386	ENSG00000112062
ENSG00000185386	ENSG00000156711
ENSG00000185386	ENSG00000188130
ENSG00000185390	ENSG00000070193
ENSG00000185390	ENSG00000070388
ENSG00000185390	ENSG00000075388
ENSG00000185390	ENSG00000078579
ENSG00000185390	ENSG00000102466
ENSG00000185390	ENSG00000102678
ENSG00000185390	ENSG00000111241
ENSG00000185390	ENSG00000113578
ENSG00000185390	ENSG00000114279
ENSG00000185390	ENSG00000129682
ENSG00000185390	ENSG00000138675
ENSG00000185390	ENSG00000138685
ENSG00000185390	ENSG00000140285
ENSG00000185390	ENSG00000161958
ENSG00000185390	ENSG00000186895
ENSG00000185390	ENSG00000196468
ENSG00000185390	ENSG00000204801
ENSG00000185390	ENSG00000204816
ENSG00000185390	ENSG00000204817
ENSG00000185390	ENSG00000204837
ENSG00000185390	ENSG00000215053
ENSG00000185418	ENSG00000113407
ENSG00000185418	ENSG00000143374
ENSG00000185418	ENSG00000154719
ENSG00000185437	ENSG00000131171
ENSG00000185437	ENSG00000142669
ENSG00000185437	ENSG00000198478

ENSG00000185437	ENSG00000214636
ENSG00000185467	ENSG00000102753
ENSG00000185467	ENSG00000182481
ENSG00000185467	ENSG00000186432
ENSG00000185467	ENSG00000214035
ENSG00000185467	ENSG00000214180
ENSG00000185467	ENSG00000215769
ENSG00000185477	ENSG00000169258
ENSG00000185477	ENSG00000204175
ENSG00000185479	ENSG00000135443
ENSG00000185479	ENSG00000135477
ENSG00000185479	ENSG00000135480
ENSG00000185479	ENSG00000139648
ENSG00000185479	ENSG00000161849
ENSG00000185479	ENSG00000161850
ENSG00000185479	ENSG00000167767
ENSG00000185479	ENSG00000167768
ENSG00000185479	ENSG00000170421
ENSG00000185479	ENSG00000170423
ENSG00000185479	ENSG00000170442
ENSG00000185479	ENSG00000170454
ENSG00000185479	ENSG00000170465
ENSG00000185479	ENSG00000170477
ENSG00000185479	ENSG00000170484
ENSG00000185479	ENSG00000170486
ENSG00000185479	ENSG00000170523
ENSG00000185479	ENSG00000172867
ENSG00000185479	ENSG00000185069
ENSG00000185479	ENSG00000185640
ENSG00000185479	ENSG00000186049
ENSG00000185479	ENSG00000186081
ENSG00000185479	ENSG00000186442
ENSG00000185479	ENSG00000189182
ENSG00000185479	ENSG00000205420
ENSG00000185479	ENSG00000205426
ENSG00000185479	ENSG00000214233
ENSG00000185479	ENSG00000214323
ENSG00000185479	ENSG00000214477
ENSG00000185479	ENSG00000214752
ENSG00000185479	ENSG00000214974
ENSG00000185479	ENSG00000215287
ENSG00000185479	ENSG00000215847
ENSG00000185482	ENSG00000141750
ENSG00000185482	ENSG00000144681
ENSG00000185483	ENSG00000030304
ENSG00000185483	ENSG00000140538
ENSG00000185483	ENSG00000148053
ENSG00000185483	ENSG00000169071
ENSG00000185483	ENSG00000198400
ENSG00000185507	ENSG00000117595
ENSG00000185507	ENSG00000125347
ENSG00000185507	ENSG00000126456
ENSG00000185507	ENSG00000128604
ENSG00000185507	ENSG00000137265
ENSG00000185507	ENSG00000140968
ENSG00000185507	ENSG00000168310
ENSG00000185507	ENSG00000213928
ENSG00000185513	ENSG00000011258
ENSG00000185513	ENSG00000100395

ENSG00000185513	ENSG00000154655
ENSG00000185513	ENSG00000163935
ENSG00000185513	ENSG00000198879
ENSG00000185513	ENSG00000198945
ENSG00000185518	ENSG00000122012
ENSG00000185518	ENSG00000159164
ENSG00000185519	ENSG00000159784
ENSG00000185519	ENSG00000175182
ENSG00000185527	ENSG00000139053
ENSG00000185532	ENSG00000138669
ENSG00000185551	ENSG00000101076
ENSG00000185551	ENSG00000143171
ENSG00000185551	ENSG00000160113
ENSG00000185551	ENSG00000164749
ENSG00000185551	ENSG00000175745
ENSG00000185551	ENSG00000186350
ENSG00000185551	ENSG00000204231
ENSG00000185554	ENSG00000126952
ENSG00000185554	ENSG00000147206
ENSG00000185554	ENSG00000162231
ENSG00000185554	ENSG00000185945
ENSG00000185554	ENSG00000196970
ENSG00000185559	ENSG00000090932
ENSG00000185559	ENSG00000101384
ENSG00000185559	ENSG00000128917
ENSG00000185559	ENSG00000171462
ENSG00000185559	ENSG00000184916
ENSG00000185559	ENSG00000198719
ENSG00000185565	ENSG00000142549
ENSG00000185565	ENSG00000172260
ENSG00000185565	ENSG00000182667
ENSG00000185565	ENSG00000183715
ENSG00000185585	ENSG00000162745
ENSG00000185591	ENSG00000105866
ENSG00000185591	ENSG00000172845
ENSG00000185602	ENSG00000075884
ENSG00000185602	ENSG00000107863
ENSG00000185602	ENSG00000123329
ENSG00000185602	ENSG00000165322
ENSG00000185602	ENSG00000167433
ENSG00000185602	ENSG00000214627
ENSG00000185614	ENSG00000197852
ENSG00000185615	ENSG00000139055
ENSG00000185615	ENSG00000169340
ENSG00000185615	ENSG00000185624
ENSG00000185619	ENSG00000056661
ENSG00000185619	ENSG00000115289
ENSG00000185619	ENSG00000156374
ENSG00000185619	ENSG00000168283
ENSG00000185619	ENSG00000180628
ENSG00000185624	ENSG00000139055
ENSG00000185624	ENSG00000169340
ENSG00000185624	ENSG00000185615
ENSG00000185630	ENSG00000105717
ENSG00000185630	ENSG00000167081
ENSG00000185630	ENSG00000204304
ENSG00000185633	ENSG00000166920
ENSG00000185633	ENSG00000189043
ENSG00000185633	ENSG00000212665

ENSG00000185634	ENSG00000129946
ENSG00000185634	ENSG00000148082
ENSG00000185634	ENSG00000160691
ENSG00000185637	ENSG00000101210
ENSG00000185637	ENSG00000156508
ENSG00000185637	ENSG00000214744
ENSG00000185637	ENSG00000215093
ENSG00000185640	ENSG00000135443
ENSG00000185640	ENSG00000135477
ENSG00000185640	ENSG00000135480
ENSG00000185640	ENSG00000139648
ENSG00000185640	ENSG00000161849
ENSG00000185640	ENSG00000161850
ENSG00000185640	ENSG00000167767
ENSG00000185640	ENSG00000167768
ENSG00000185640	ENSG00000170421
ENSG00000185640	ENSG00000170423
ENSG00000185640	ENSG00000170442
ENSG00000185640	ENSG00000170454
ENSG00000185640	ENSG00000170465
ENSG00000185640	ENSG00000170477
ENSG00000185640	ENSG00000170484
ENSG00000185640	ENSG00000170486
ENSG00000185640	ENSG00000170523
ENSG00000185640	ENSG00000172867
ENSG00000185640	ENSG00000185069
ENSG00000185640	ENSG00000185479
ENSG00000185640	ENSG00000186049
ENSG00000185640	ENSG00000186081
ENSG00000185640	ENSG00000186442
ENSG00000185640	ENSG00000189182
ENSG00000185640	ENSG00000205420
ENSG00000185640	ENSG00000205426
ENSG00000185640	ENSG00000214233
ENSG00000185640	ENSG00000214323
ENSG00000185640	ENSG00000214477
ENSG00000185640	ENSG00000214752
ENSG00000185640	ENSG00000214974
ENSG00000185640	ENSG00000215287
ENSG00000185640	ENSG00000215847
ENSG00000185650	ENSG00000128016
ENSG00000185650	ENSG00000152518
ENSG00000185651	ENSG00000131982
ENSG00000185651	ENSG00000156587
ENSG00000185652	ENSG00000134259
ENSG00000185652	ENSG00000167744
ENSG00000185652	ENSG00000176697
ENSG00000185652	ENSG00000212844
ENSG00000185658	ENSG00000146247
ENSG00000185658	ENSG00000165288
ENSG00000185664	ENSG00000136235
ENSG00000185666	ENSG00000008056
ENSG00000185666	ENSG00000157152
ENSG00000185668	ENSG00000028277
ENSG00000185668	ENSG00000064835
ENSG00000185668	ENSG00000091010
ENSG00000185668	ENSG00000137709
ENSG00000185668	ENSG00000143190
ENSG00000185668	ENSG00000151615

ENSG00000185668	ENSG00000152192
ENSG00000185668	ENSG00000184486
ENSG00000185668	ENSG00000196767
ENSG00000185668	ENSG00000198914
ENSG00000185668	ENSG00000204531
ENSG00000185668	ENSG00000212993
ENSG00000185669	ENSG00000019549
ENSG00000185669	ENSG00000124216
ENSG00000185669	ENSG00000170616
ENSG00000185669	ENSG00000177835
ENSG00000185669	ENSG00000215397
ENSG00000185669	ENSG00000215713
ENSG00000185670	ENSG00000160062
ENSG00000185670	ENSG00000168795
ENSG00000185670	ENSG00000205189
ENSG00000185670	ENSG00000215897
ENSG00000185674	ENSG00000144214
ENSG00000185686	ENSG00000116708
ENSG00000185686	ENSG00000116721
ENSG00000185686	ENSG00000116726
ENSG00000185686	ENSG00000120952
ENSG00000185686	ENSG00000157358
ENSG00000185686	ENSG00000160959
ENSG00000185686	ENSG00000179704
ENSG00000185686	ENSG00000182330
ENSG00000185686	ENSG00000185028
ENSG00000185686	ENSG00000187545
ENSG00000185686	ENSG00000197549
ENSG00000185686	ENSG00000204478
ENSG00000185686	ENSG00000204479
ENSG00000185686	ENSG00000204480
ENSG00000185686	ENSG00000204481
ENSG00000185686	ENSG00000204485
ENSG00000185686	ENSG00000204486
ENSG00000185686	ENSG00000204488
ENSG00000185686	ENSG00000204491
ENSG00000185686	ENSG00000204495
ENSG00000185686	ENSG00000204501
ENSG00000185686	ENSG00000204502
ENSG00000185686	ENSG00000204503
ENSG00000185686	ENSG00000204505
ENSG00000185686	ENSG00000204507
ENSG00000185686	ENSG00000204508
ENSG00000185686	ENSG00000204509
ENSG00000185686	ENSG00000204510
ENSG00000185686	ENSG00000204513
ENSG00000185697	ENSG00000101057
ENSG00000185697	ENSG00000118513
ENSG00000185728	ENSG00000149658
ENSG00000185728	ENSG00000198492
ENSG00000185736	ENSG00000065457
ENSG00000185736	ENSG00000140955
ENSG00000185736	ENSG00000160710
ENSG00000185736	ENSG00000164113
ENSG00000185736	ENSG00000197381
ENSG00000185745	ENSG00000119917
ENSG00000185745	ENSG00000119922
ENSG00000185745	ENSG00000152778
ENSG00000185745	ENSG00000204010

ENSG00000185758	ENSG00000013297
ENSG00000185758	ENSG00000066405
ENSG00000185758	ENSG00000106404
ENSG00000185758	ENSG00000113946
ENSG00000185758	ENSG00000134873
ENSG00000185758	ENSG00000156282
ENSG00000185758	ENSG00000156284
ENSG00000185758	ENSG00000159261
ENSG00000185758	ENSG00000163347
ENSG00000185758	ENSG00000164007
ENSG00000185758	ENSG00000165215
ENSG00000185758	ENSG00000165376
ENSG00000185758	ENSG00000171217
ENSG00000185758	ENSG00000177300
ENSG00000185758	ENSG00000181885
ENSG00000185758	ENSG00000184113
ENSG00000185758	ENSG00000184697
ENSG00000185758	ENSG00000189118
ENSG00000185758	ENSG00000189143
ENSG00000185758	ENSG00000213937
ENSG00000185760	ENSG00000053918
ENSG00000185760	ENSG00000075043
ENSG00000185760	ENSG00000117013
ENSG00000185760	ENSG00000184156
ENSG00000185774	ENSG00000115041
ENSG00000185774	ENSG00000120049
ENSG00000185774	ENSG00000182132
ENSG00000185787	ENSG00000005302
ENSG00000185787	ENSG00000123562
ENSG00000185792	ENSG00000022556
ENSG00000185792	ENSG00000023191
ENSG00000185792	ENSG00000140853
ENSG00000185792	ENSG00000142405
ENSG00000185792	ENSG00000158077
ENSG00000185792	ENSG00000160505
ENSG00000185792	ENSG00000162711
ENSG00000185792	ENSG00000167207
ENSG00000185792	ENSG00000167634
ENSG00000185792	ENSG00000167984
ENSG00000185792	ENSG00000171487
ENSG00000185792	ENSG00000173572
ENSG00000185792	ENSG00000174885
ENSG00000185792	ENSG00000179709
ENSG00000185792	ENSG00000179873
ENSG00000185792	ENSG00000182261
ENSG00000185800	ENSG00000140153
ENSG00000185803	ENSG00000101276
ENSG00000185803	ENSG00000132517
ENSG00000185811	ENSG00000030419
ENSG00000185811	ENSG00000123411
ENSG00000185811	ENSG00000161405
ENSG00000185818	ENSG00000144035
ENSG00000185822	ENSG00000118975
ENSG00000185822	ENSG00000184319
ENSG00000185822	ENSG00000186239
ENSG00000185822	ENSG00000186698
ENSG00000185822	ENSG00000198242
ENSG00000185822	ENSG00000206147
ENSG00000185822	ENSG00000212932

ENSG00000185822	ENSG00000213753
ENSG00000185822	ENSG00000214230
ENSG00000185822	ENSG00000215001
ENSG00000185822	ENSG00000215140
ENSG00000185822	ENSG00000215387
ENSG00000185825	ENSG00000075790
ENSG00000185831	ENSG00000099984
ENSG00000185831	ENSG00000133433
ENSG00000185831	ENSG00000184490
ENSG00000185831	ENSG00000184674
ENSG00000185831	ENSG00000206090
ENSG00000185880	ENSG00000116525
ENSG00000185880	ENSG00000146755
ENSG00000185880	ENSG00000155428
ENSG00000185880	ENSG00000177238
ENSG00000185880	ENSG00000178809
ENSG00000185885	ENSG00000142089
ENSG00000185885	ENSG00000185201
ENSG00000185885	ENSG00000188082
ENSG00000185885	ENSG00000206013
ENSG00000185885	ENSG00000215096
ENSG00000185888	ENSG00000005001
ENSG00000185888	ENSG00000007038
ENSG00000185888	ENSG00000052344
ENSG00000185888	ENSG00000095917
ENSG00000185888	ENSG00000103355
ENSG00000185888	ENSG00000116176
ENSG00000185888	ENSG00000172236
ENSG00000185888	ENSG00000172382
ENSG00000185888	ENSG00000172460
ENSG00000185888	ENSG00000178055
ENSG00000185888	ENSG00000178226
ENSG00000185888	ENSG00000188086
ENSG00000185888	ENSG00000189099
ENSG00000185888	ENSG00000196364
ENSG00000185888	ENSG00000197253
ENSG00000185888	ENSG00000206549
ENSG00000185888	ENSG00000214877
ENSG00000185888	ENSG00000215148
ENSG00000185896	ENSG00000005893
ENSG00000185896	ENSG00000078081
ENSG00000185896	ENSG00000129226
ENSG00000185897	ENSG00000125245
ENSG00000185897	ENSG00000126251
ENSG00000185897	ENSG00000126262
ENSG00000185897	ENSG00000144230
ENSG00000185897	ENSG00000147138
ENSG00000185897	ENSG00000162881
ENSG00000185897	ENSG00000164220
ENSG00000185897	ENSG00000164251
ENSG00000185897	ENSG00000165621
ENSG00000185897	ENSG00000171631
ENSG00000185897	ENSG00000181104
ENSG00000185897	ENSG00000182162
ENSG00000185897	ENSG00000182559
ENSG00000185897	ENSG00000182782
ENSG00000185897	ENSG00000196917
ENSG00000185915	ENSG00000003096
ENSG00000185915	ENSG00000087448

ENSG00000185915	ENSG00000124743
ENSG00000185915	ENSG00000130487
ENSG00000185915	ENSG00000135686
ENSG00000185915	ENSG00000166853
ENSG00000185915	ENSG00000167487
ENSG00000185915	ENSG00000174010
ENSG00000185915	ENSG00000176595
ENSG00000185915	ENSG00000179023
ENSG00000185915	ENSG00000185214
ENSG00000185915	ENSG00000186231
ENSG00000185915	ENSG00000197705
ENSG00000185915	ENSG00000198642
ENSG00000185920	ENSG00000117425
ENSG00000185924	ENSG00000040608
ENSG00000185924	ENSG00000186907
ENSG00000185933	ENSG00000138172
ENSG00000185933	ENSG00000164451
ENSG00000185933	ENSG00000178033
ENSG00000185933	ENSG00000183128
ENSG00000185933	ENSG00000188820
ENSG00000185942	ENSG00000084628
ENSG00000185942	ENSG00000101198
ENSG00000185942	ENSG00000188580
ENSG00000185945	ENSG00000126952
ENSG00000185945	ENSG00000147206
ENSG00000185945	ENSG00000162231
ENSG00000185945	ENSG00000185554
ENSG00000185945	ENSG00000196970
ENSG00000185950	ENSG00000133124
ENSG00000185950	ENSG00000169047
ENSG00000185960	ENSG00000168779
ENSG00000185963	ENSG00000151746
ENSG00000185972	ENSG00000178502
ENSG00000185974	ENSG00000100077
ENSG00000185974	ENSG00000114124
ENSG00000185974	ENSG00000125388
ENSG00000185974	ENSG00000173020
ENSG00000185974	ENSG00000198055
ENSG00000185974	ENSG00000198873
ENSG00000185978	ENSG00000111332
ENSG00000185978	ENSG00000124642
ENSG00000185978	ENSG00000137259
ENSG00000185978	ENSG00000158403
ENSG00000185978	ENSG00000164508
ENSG00000185978	ENSG00000168274
ENSG00000185978	ENSG00000180573
ENSG00000185978	ENSG00000181218
ENSG00000185978	ENSG00000182611
ENSG00000185978	ENSG00000183558
ENSG00000185978	ENSG00000184260
ENSG00000185978	ENSG00000184270
ENSG00000185978	ENSG00000184348
ENSG00000185978	ENSG00000184825
ENSG00000185978	ENSG00000188486
ENSG00000185978	ENSG00000196747
ENSG00000185978	ENSG00000196787
ENSG00000185978	ENSG00000196866
ENSG00000185978	ENSG00000198082
ENSG00000185978	ENSG00000198307

ENSG00000185978	ENSG00000198374
ENSG00000185978	ENSG00000203812
ENSG00000185978	ENSG00000205070
ENSG00000185978	ENSG00000205079
ENSG00000185985	ENSG00000121871
ENSG00000185985	ENSG00000178235
ENSG00000185988	ENSG00000145632
ENSG00000185988	ENSG00000166851
ENSG00000185988	ENSG00000173846
ENSG00000185988	ENSG00000214270
ENSG00000185989	ENSG00000105808
ENSG00000185989	ENSG00000111344
ENSG00000185989	ENSG00000155903
ENSG00000185989	ENSG00000170667
ENSG00000186001	ENSG00000077454
ENSG00000186001	ENSG00000130224
ENSG00000186001	ENSG00000136141
ENSG00000186009	ENSG00000069849
ENSG00000186009	ENSG00000101892
ENSG00000186009	ENSG00000129244
ENSG00000186009	ENSG00000143153
ENSG00000186010	ENSG00000163382
ENSG00000186031	ENSG00000155816
ENSG00000186038	ENSG00000080644
ENSG00000186038	ENSG00000101204
ENSG00000186038	ENSG00000108556
ENSG00000186038	ENSG00000117971
ENSG00000186038	ENSG00000120903
ENSG00000186038	ENSG00000129749
ENSG00000186038	ENSG00000135902
ENSG00000186038	ENSG00000138435
ENSG00000186038	ENSG00000147432
ENSG00000186038	ENSG00000147434
ENSG00000186038	ENSG00000149305
ENSG00000186038	ENSG00000160716
ENSG00000186038	ENSG00000166664
ENSG00000186038	ENSG00000166736
ENSG00000186038	ENSG00000169684
ENSG00000186038	ENSG00000170175
ENSG00000186038	ENSG00000174343
ENSG00000186038	ENSG00000175344
ENSG00000186038	ENSG00000178084
ENSG00000186038	ENSG00000186090
ENSG00000186038	ENSG00000186919
ENSG00000186038	ENSG00000196811
ENSG00000186049	ENSG00000135443
ENSG00000186049	ENSG00000135477
ENSG00000186049	ENSG00000135480
ENSG00000186049	ENSG00000139648
ENSG00000186049	ENSG00000161849
ENSG00000186049	ENSG00000161850
ENSG00000186049	ENSG00000167767
ENSG00000186049	ENSG00000167768
ENSG00000186049	ENSG00000170421
ENSG00000186049	ENSG00000170423
ENSG00000186049	ENSG00000170442
ENSG00000186049	ENSG00000170454
ENSG00000186049	ENSG00000170465
ENSG00000186049	ENSG00000170477

ENSG00000186049	ENSG00000170484
ENSG00000186049	ENSG00000170486
ENSG00000186049	ENSG00000170523
ENSG00000186049	ENSG00000172867
ENSG00000186049	ENSG00000185069
ENSG00000186049	ENSG00000185479
ENSG00000186049	ENSG00000185640
ENSG00000186049	ENSG00000186081
ENSG00000186049	ENSG00000186442
ENSG00000186049	ENSG00000189182
ENSG00000186049	ENSG00000205420
ENSG00000186049	ENSG00000205426
ENSG00000186049	ENSG00000214233
ENSG00000186049	ENSG00000214323
ENSG00000186049	ENSG00000214477
ENSG00000186049	ENSG00000214752
ENSG00000186049	ENSG00000214974
ENSG00000186049	ENSG00000215287
ENSG00000186049	ENSG00000215847
ENSG00000186051	ENSG00000104903
ENSG00000186051	ENSG00000113196
ENSG00000186051	ENSG00000122691
ENSG00000186051	ENSG00000122859
ENSG00000186051	ENSG00000123307
ENSG00000186051	ENSG00000125878
ENSG00000186051	ENSG00000146618
ENSG00000186051	ENSG00000162367
ENSG00000186051	ENSG00000162992
ENSG00000186051	ENSG00000164107
ENSG00000186051	ENSG00000164600
ENSG00000186051	ENSG00000168267
ENSG00000186051	ENSG00000171532
ENSG00000186051	ENSG00000171786
ENSG00000186051	ENSG00000172238
ENSG00000186051	ENSG00000177551
ENSG00000186051	ENSG00000178403
ENSG00000186051	ENSG00000179774
ENSG00000186051	ENSG00000180535
ENSG00000186051	ENSG00000181965
ENSG00000186051	ENSG00000187786
ENSG00000186051	ENSG00000188686
ENSG00000186074	ENSG00000095970
ENSG00000186074	ENSG00000096264
ENSG00000186074	ENSG00000161649
ENSG00000186074	ENSG00000162896
ENSG00000186074	ENSG00000162897
ENSG00000186074	ENSG00000167850
ENSG00000186074	ENSG00000167851
ENSG00000186074	ENSG00000178789
ENSG00000186074	ENSG00000186407
ENSG00000186074	ENSG00000204345
ENSG00000186076	ENSG00000164708
ENSG00000186076	ENSG00000171314
ENSG00000186076	ENSG00000172331
ENSG00000186076	ENSG00000197041
ENSG00000186076	ENSG00000214206
ENSG00000186076	ENSG00000214824
ENSG00000186076	ENSG00000215178
ENSG00000186081	ENSG00000135443

ENSG00000186081	ENSG00000135477
ENSG00000186081	ENSG00000135480
ENSG00000186081	ENSG00000139648
ENSG00000186081	ENSG00000161849
ENSG00000186081	ENSG00000161850
ENSG00000186081	ENSG00000167767
ENSG00000186081	ENSG00000167768
ENSG00000186081	ENSG00000170421
ENSG00000186081	ENSG00000170423
ENSG00000186081	ENSG00000170442
ENSG00000186081	ENSG00000170454
ENSG00000186081	ENSG00000170465
ENSG00000186081	ENSG00000170477
ENSG00000186081	ENSG00000170484
ENSG00000186081	ENSG00000170486
ENSG00000186081	ENSG00000170523
ENSG00000186081	ENSG00000172867
ENSG00000186081	ENSG00000185069
ENSG00000186081	ENSG00000185479
ENSG00000186081	ENSG00000185640
ENSG00000186081	ENSG00000186049
ENSG00000186081	ENSG00000186442
ENSG00000186081	ENSG00000189182
ENSG00000186081	ENSG00000205420
ENSG00000186081	ENSG00000205426
ENSG00000186081	ENSG00000214233
ENSG00000186081	ENSG00000214323
ENSG00000186081	ENSG00000214477
ENSG00000186081	ENSG00000214752
ENSG00000186081	ENSG00000214974
ENSG00000186081	ENSG00000215287
ENSG00000186081	ENSG00000215847
ENSG00000186090	ENSG00000080644
ENSG00000186090	ENSG00000101204
ENSG00000186090	ENSG00000108556
ENSG00000186090	ENSG00000117971
ENSG00000186090	ENSG00000120903
ENSG00000186090	ENSG00000129749
ENSG00000186090	ENSG00000135902
ENSG00000186090	ENSG00000138435
ENSG00000186090	ENSG00000147432
ENSG00000186090	ENSG00000147434
ENSG00000186090	ENSG00000149305
ENSG00000186090	ENSG00000160716
ENSG00000186090	ENSG00000166664
ENSG00000186090	ENSG00000166736
ENSG00000186090	ENSG00000169684
ENSG00000186090	ENSG00000170175
ENSG00000186090	ENSG00000174343
ENSG00000186090	ENSG00000175344
ENSG00000186090	ENSG00000178084
ENSG00000186090	ENSG00000186038
ENSG00000186090	ENSG00000186919
ENSG00000186090	ENSG00000196811
ENSG00000186103	ENSG00000105392
ENSG00000186103	ENSG00000115507
ENSG00000186103	ENSG00000165588
ENSG00000186105	ENSG00000125848
ENSG00000186105	ENSG00000126500

ENSG00000186106	ENSG00000100124
ENSG00000186111	ENSG00000107242
ENSG00000186111	ENSG00000143398
ENSG00000186111	ENSG00000173780
ENSG00000186115	ENSG00000142973
ENSG00000186115	ENSG00000145476
ENSG00000186115	ENSG00000154198
ENSG00000186115	ENSG00000162365
ENSG00000186115	ENSG00000171903
ENSG00000186115	ENSG00000171954
ENSG00000186115	ENSG00000186160
ENSG00000186115	ENSG00000186204
ENSG00000186115	ENSG00000186377
ENSG00000186115	ENSG00000186526
ENSG00000186115	ENSG00000186529
ENSG00000186115	ENSG00000187048
ENSG00000186130	ENSG00000171448
ENSG00000186160	ENSG00000142973
ENSG00000186160	ENSG00000145476
ENSG00000186160	ENSG00000154198
ENSG00000186160	ENSG00000162365
ENSG00000186160	ENSG00000171903
ENSG00000186160	ENSG00000171954
ENSG00000186160	ENSG00000186115
ENSG00000186160	ENSG00000186204
ENSG00000186160	ENSG00000186377
ENSG00000186160	ENSG00000186526
ENSG00000186160	ENSG00000186529
ENSG00000186160	ENSG00000187048
ENSG00000186174	ENSG00000116128
ENSG00000186187	ENSG00000141622
ENSG00000186187	ENSG00000180233
ENSG00000186190	ENSG00000078898
ENSG00000186190	ENSG00000100979
ENSG00000186190	ENSG00000101425
ENSG00000186190	ENSG00000129988
ENSG00000186190	ENSG00000167104
ENSG00000186190	ENSG00000184459
ENSG00000186190	ENSG00000186191
ENSG00000186191	ENSG00000078898
ENSG00000186191	ENSG00000100979
ENSG00000186191	ENSG00000101425
ENSG00000186191	ENSG00000129988
ENSG00000186191	ENSG00000167104
ENSG00000186191	ENSG00000184459
ENSG00000186191	ENSG00000186190
ENSG00000186204	ENSG00000142973
ENSG00000186204	ENSG00000145476
ENSG00000186204	ENSG00000154198
ENSG00000186204	ENSG00000162365
ENSG00000186204	ENSG00000171903
ENSG00000186204	ENSG00000171954
ENSG00000186204	ENSG00000186115
ENSG00000186204	ENSG00000186160
ENSG00000186204	ENSG00000186377
ENSG00000186204	ENSG00000186526
ENSG00000186204	ENSG00000186529
ENSG00000186204	ENSG00000187048
ENSG00000186205	ENSG00000117791

ENSG00000186212	ENSG00000187808
ENSG00000186212	ENSG00000198142
ENSG00000186212	ENSG00000198944
ENSG00000186217	ENSG00000160075
ENSG00000186217	ENSG00000176960
ENSG00000186217	ENSG00000186223
ENSG00000186217	ENSG00000186232
ENSG00000186222	ENSG00000124243
ENSG00000186223	ENSG00000160075
ENSG00000186223	ENSG00000176960
ENSG00000186223	ENSG00000186217
ENSG00000186223	ENSG00000186232
ENSG00000186231	ENSG00000003096
ENSG00000186231	ENSG00000087448
ENSG00000186231	ENSG00000124743
ENSG00000186231	ENSG00000130487
ENSG00000186231	ENSG00000135686
ENSG00000186231	ENSG00000166853
ENSG00000186231	ENSG00000167487
ENSG00000186231	ENSG00000174010
ENSG00000186231	ENSG00000176595
ENSG00000186231	ENSG00000179023
ENSG00000186231	ENSG00000185214
ENSG00000186231	ENSG00000185915
ENSG00000186231	ENSG00000197705
ENSG00000186231	ENSG00000198642
ENSG00000186232	ENSG00000160075
ENSG00000186232	ENSG00000176960
ENSG00000186232	ENSG00000186217
ENSG00000186232	ENSG00000186223
ENSG00000186239	ENSG00000118975
ENSG00000186239	ENSG00000184319
ENSG00000186239	ENSG00000185822
ENSG00000186239	ENSG00000186698
ENSG00000186239	ENSG00000198242
ENSG00000186239	ENSG00000206147
ENSG00000186239	ENSG00000212932
ENSG00000186239	ENSG00000213753
ENSG00000186239	ENSG00000214230
ENSG00000186239	ENSG00000215001
ENSG00000186239	ENSG00000215140
ENSG00000186239	ENSG00000215387
ENSG00000186260	ENSG00000141052
ENSG00000186260	ENSG00000176909
ENSG00000186260	ENSG00000196588
ENSG00000186280	ENSG00000066135
ENSG00000186280	ENSG00000107077
ENSG00000186280	ENSG00000127663
ENSG00000186281	ENSG00000116906
ENSG00000186281	ENSG00000119927
ENSG00000186283	ENSG00000136816
ENSG00000186283	ENSG00000136827
ENSG00000186283	ENSG00000160404
ENSG00000186283	ENSG00000198113
ENSG00000186288	ENSG00000184388
ENSG00000186297	ENSG00000011677
ENSG00000186297	ENSG00000022355
ENSG00000186297	ENSG00000094755
ENSG00000186297	ENSG00000101958

ENSG00000186297	ENSG00000102287
ENSG00000186297	ENSG00000109158
ENSG00000186297	ENSG00000109738
ENSG00000186297	ENSG00000111886
ENSG00000186297	ENSG00000113327
ENSG00000186297	ENSG00000145451
ENSG00000186297	ENSG00000145863
ENSG00000186297	ENSG00000145864
ENSG00000186297	ENSG00000145888
ENSG00000186297	ENSG00000146276
ENSG00000186297	ENSG00000147402
ENSG00000186297	ENSG00000151834
ENSG00000186297	ENSG00000163285
ENSG00000186297	ENSG00000163288
ENSG00000186297	ENSG00000166206
ENSG00000186297	ENSG00000182256
ENSG00000186297	ENSG00000183185
ENSG00000186297	ENSG00000187730
ENSG00000186297	ENSG00000188828
ENSG00000186301	ENSG00000019991
ENSG00000186301	ENSG00000104368
ENSG00000186301	ENSG00000109758
ENSG00000186301	ENSG00000122194
ENSG00000186301	ENSG00000122861
ENSG00000186301	ENSG00000131187
ENSG00000186301	ENSG00000148702
ENSG00000186301	ENSG00000173531
ENSG00000186301	ENSG00000186715
ENSG00000186301	ENSG00000198670
ENSG00000186310	ENSG00000177432
ENSG00000186310	ENSG00000186462
ENSG00000186310	ENSG00000187109
ENSG00000186310	ENSG00000205531
ENSG00000186318	ENSG00000182240
ENSG00000186322	ENSG00000140478
ENSG00000186322	ENSG00000153666
ENSG00000186322	ENSG00000156363
ENSG00000186322	ENSG00000159289
ENSG00000186322	ENSG00000167110
ENSG00000186322	ENSG00000167195
ENSG00000186322	ENSG00000174450
ENSG00000186322	ENSG00000175265
ENSG00000186322	ENSG00000181984
ENSG00000186322	ENSG00000184206
ENSG00000186322	ENSG00000188388
ENSG00000186322	ENSG00000188532
ENSG00000186322	ENSG00000188626
ENSG00000186322	ENSG00000197092
ENSG00000186322	ENSG00000197414
ENSG00000186322	ENSG00000214348
ENSG00000186322	ENSG00000215252
ENSG00000186322	ENSG00000215405
ENSG00000186334	ENSG00000123643
ENSG00000186334	ENSG00000180773
ENSG00000186334	ENSG00000186335
ENSG00000186335	ENSG00000123643
ENSG00000186335	ENSG00000180773
ENSG00000186335	ENSG00000186334
ENSG00000186340	ENSG00000105664

ENSG00000186340	ENSG00000113296
ENSG00000186340	ENSG00000137801
ENSG00000186340	ENSG00000169231
ENSG00000186350	ENSG00000101076
ENSG00000186350	ENSG00000143171
ENSG00000186350	ENSG00000160113
ENSG00000186350	ENSG00000164749
ENSG00000186350	ENSG00000175745
ENSG00000186350	ENSG00000185551
ENSG00000186350	ENSG00000204231
ENSG00000186377	ENSG00000142973
ENSG00000186377	ENSG00000145476
ENSG00000186377	ENSG00000154198
ENSG00000186377	ENSG00000162365
ENSG00000186377	ENSG00000171903
ENSG00000186377	ENSG00000171954
ENSG00000186377	ENSG00000186115
ENSG00000186377	ENSG00000186160
ENSG00000186377	ENSG00000186204
ENSG00000186377	ENSG00000186526
ENSG00000186377	ENSG00000186529
ENSG00000186377	ENSG00000187048
ENSG00000186390	ENSG00000160916
ENSG00000186393	ENSG00000006059
ENSG00000186393	ENSG00000094796
ENSG00000186393	ENSG00000108244
ENSG00000186393	ENSG00000108417
ENSG00000186393	ENSG00000108759
ENSG00000186393	ENSG00000111057
ENSG00000186393	ENSG00000126337
ENSG00000186393	ENSG00000128422
ENSG00000186393	ENSG00000131737
ENSG00000186393	ENSG00000131738
ENSG00000186393	ENSG00000131885
ENSG00000186393	ENSG00000167916
ENSG00000186393	ENSG00000170819
ENSG00000186393	ENSG00000171345
ENSG00000186393	ENSG00000171346
ENSG00000186393	ENSG00000171360
ENSG00000186393	ENSG00000171401
ENSG00000186393	ENSG00000171403
ENSG00000186393	ENSG00000171431
ENSG00000186393	ENSG00000171446
ENSG00000186393	ENSG00000173908
ENSG00000186393	ENSG00000186395
ENSG00000186393	ENSG00000186831
ENSG00000186393	ENSG00000186832
ENSG00000186393	ENSG00000186847
ENSG00000186393	ENSG00000187242
ENSG00000186393	ENSG00000187686
ENSG00000186393	ENSG00000196859
ENSG00000186393	ENSG00000197079
ENSG00000186393	ENSG00000204889
ENSG00000186393	ENSG00000204897
ENSG00000186393	ENSG00000213951
ENSG00000186393	ENSG00000214220
ENSG00000186393	ENSG00000214454
ENSG00000186393	ENSG00000214959
ENSG00000186393	ENSG00000215772

ENSG00000186393	ENSG00000215773
ENSG00000186395	ENSG00000006059
ENSG00000186395	ENSG00000094796
ENSG00000186395	ENSG00000108244
ENSG00000186395	ENSG00000108417
ENSG00000186395	ENSG00000108759
ENSG00000186395	ENSG00000111057
ENSG00000186395	ENSG00000126337
ENSG00000186395	ENSG00000128422
ENSG00000186395	ENSG00000131737
ENSG00000186395	ENSG00000131738
ENSG00000186395	ENSG00000131885
ENSG00000186395	ENSG00000167916
ENSG00000186395	ENSG00000170819
ENSG00000186395	ENSG00000171345
ENSG00000186395	ENSG00000171346
ENSG00000186395	ENSG00000171360
ENSG00000186395	ENSG00000171401
ENSG00000186395	ENSG00000171403
ENSG00000186395	ENSG00000171431
ENSG00000186395	ENSG00000171446
ENSG00000186395	ENSG00000173908
ENSG00000186395	ENSG00000186393
ENSG00000186395	ENSG00000186831
ENSG00000186395	ENSG00000186832
ENSG00000186395	ENSG00000186847
ENSG00000186395	ENSG00000187242
ENSG00000186395	ENSG00000187686
ENSG00000186395	ENSG00000196859
ENSG00000186395	ENSG00000197079
ENSG00000186395	ENSG00000204889
ENSG00000186395	ENSG00000204897
ENSG00000186395	ENSG00000213951
ENSG00000186395	ENSG00000214220
ENSG00000186395	ENSG00000214454
ENSG00000186395	ENSG00000214959
ENSG00000186395	ENSG00000215772
ENSG00000186395	ENSG00000215773
ENSG00000186407	ENSG00000095970
ENSG00000186407	ENSG00000096264
ENSG00000186407	ENSG00000161649
ENSG00000186407	ENSG00000162896
ENSG00000186407	ENSG00000162897
ENSG00000186407	ENSG00000167850
ENSG00000186407	ENSG00000167851
ENSG00000186407	ENSG00000178789
ENSG00000186407	ENSG00000186074
ENSG00000186407	ENSG00000204345
ENSG00000186417	ENSG00000034971
ENSG00000186417	ENSG00000102837
ENSG00000186417	ENSG00000105088
ENSG00000186417	ENSG00000118733
ENSG00000186417	ENSG00000130558
ENSG00000186417	ENSG00000183801
ENSG00000186417	ENSG00000215081
ENSG00000186432	ENSG00000102753
ENSG00000186432	ENSG00000182481
ENSG00000186432	ENSG00000185467
ENSG00000186432	ENSG00000214035

ENSG00000186432	ENSG00000214180
ENSG00000186432	ENSG00000215769
ENSG00000186433	ENSG00000132471
ENSG00000186433	ENSG00000183066
ENSG00000186442	ENSG00000135443
ENSG00000186442	ENSG00000135477
ENSG00000186442	ENSG00000135480
ENSG00000186442	ENSG00000139648
ENSG00000186442	ENSG00000161849
ENSG00000186442	ENSG00000161850
ENSG00000186442	ENSG00000167767
ENSG00000186442	ENSG00000167768
ENSG00000186442	ENSG00000170421
ENSG00000186442	ENSG00000170423
ENSG00000186442	ENSG00000170442
ENSG00000186442	ENSG00000170454
ENSG00000186442	ENSG00000170465
ENSG00000186442	ENSG00000170477
ENSG00000186442	ENSG00000170484
ENSG00000186442	ENSG00000170486
ENSG00000186442	ENSG00000170523
ENSG00000186442	ENSG00000172867
ENSG00000186442	ENSG00000185069
ENSG00000186442	ENSG00000185479
ENSG00000186442	ENSG00000185640
ENSG00000186442	ENSG00000186049
ENSG00000186442	ENSG00000186081
ENSG00000186442	ENSG00000189182
ENSG00000186442	ENSG00000205420
ENSG00000186442	ENSG00000205426
ENSG00000186442	ENSG00000214233
ENSG00000186442	ENSG00000214323
ENSG00000186442	ENSG00000214477
ENSG00000186442	ENSG00000214752
ENSG00000186442	ENSG00000214974
ENSG00000186442	ENSG00000215287
ENSG00000186442	ENSG00000215847
ENSG00000186446	ENSG00000142396
ENSG00000186446	ENSG00000169740
ENSG00000186446	ENSG00000174255
ENSG00000186446	ENSG00000179195
ENSG00000186446	ENSG00000179965
ENSG00000186446	ENSG00000184294
ENSG00000186446	ENSG00000186777
ENSG00000186446	ENSG00000197008
ENSG00000186446	ENSG00000197025
ENSG00000186446	ENSG00000197062
ENSG00000186446	ENSG00000197332
ENSG00000186446	ENSG00000198155
ENSG00000186446	ENSG00000204756
ENSG00000186446	ENSG00000212805
ENSG00000186446	ENSG00000213976
ENSG00000186446	ENSG00000213980
ENSG00000186446	ENSG00000215180
ENSG00000186446	ENSG00000215510
ENSG00000186446	ENSG00000215537
ENSG00000186446	ENSG00000215598
ENSG00000186446	ENSG00000215600
ENSG00000186462	ENSG00000177432

ENSG00000186462	ENSG00000186310
ENSG00000186462	ENSG00000187109
ENSG00000186462	ENSG00000205531
ENSG00000186466	ENSG00000103569
ENSG00000186466	ENSG00000143595
ENSG00000186466	ENSG00000165269
ENSG00000186466	ENSG00000165272
ENSG00000186466	ENSG00000176115
ENSG00000186466	ENSG00000181997
ENSG00000186469	ENSG00000127588
ENSG00000186469	ENSG00000127920
ENSG00000186469	ENSG00000127928
ENSG00000186469	ENSG00000133136
ENSG00000186469	ENSG00000162188
ENSG00000186469	ENSG00000167083
ENSG00000186469	ENSG00000167414
ENSG00000186469	ENSG00000168243
ENSG00000186469	ENSG00000172380
ENSG00000186469	ENSG00000174021
ENSG00000186469	ENSG00000182625
ENSG00000186469	ENSG00000213611
ENSG00000186470	ENSG00000103855
ENSG00000186470	ENSG00000114013
ENSG00000186470	ENSG00000114455
ENSG00000186470	ENSG00000120217
ENSG00000186470	ENSG00000134258
ENSG00000186470	ENSG00000160223
ENSG00000186470	ENSG00000188211
ENSG00000186470	ENSG00000197646
ENSG00000186470	ENSG00000204290
ENSG00000186470	ENSG00000204655
ENSG00000186470	ENSG00000215631
ENSG00000186470	ENSG00000215811
ENSG00000186470	ENSG00000215889
ENSG00000186472	ENSG00000164061
ENSG00000186480	ENSG00000125629
ENSG00000186487	ENSG00000147488
ENSG00000186487	ENSG00000196132
ENSG00000186510	ENSG00000114859
ENSG00000186510	ENSG00000184908
ENSG00000186517	ENSG00000004777
ENSG00000186517	ENSG00000031081
ENSG00000186517	ENSG00000134909
ENSG00000186522	ENSG00000125354
ENSG00000186522	ENSG00000138758
ENSG00000186522	ENSG00000154997
ENSG00000186522	ENSG00000164402
ENSG00000186526	ENSG00000142973
ENSG00000186526	ENSG00000145476
ENSG00000186526	ENSG00000154198
ENSG00000186526	ENSG00000162365
ENSG00000186526	ENSG00000171903
ENSG00000186526	ENSG00000171954
ENSG00000186526	ENSG00000186115
ENSG00000186526	ENSG00000186160
ENSG00000186526	ENSG00000186204
ENSG00000186526	ENSG00000186377
ENSG00000186526	ENSG00000186529
ENSG00000186526	ENSG00000187048

ENSG00000186529	ENSG00000142973
ENSG00000186529	ENSG00000145476
ENSG00000186529	ENSG00000154198
ENSG00000186529	ENSG00000162365
ENSG00000186529	ENSG00000171903
ENSG00000186529	ENSG00000171954
ENSG00000186529	ENSG00000186115
ENSG00000186529	ENSG00000186160
ENSG00000186529	ENSG00000186204
ENSG00000186529	ENSG00000186377
ENSG00000186529	ENSG00000186526
ENSG00000186529	ENSG00000187048
ENSG00000186564	ENSG00000054598
ENSG00000186564	ENSG00000125798
ENSG00000186564	ENSG00000129514
ENSG00000186564	ENSG00000164379
ENSG00000186564	ENSG00000168269
ENSG00000186564	ENSG00000170122
ENSG00000186564	ENSG00000170608
ENSG00000186564	ENSG00000176165
ENSG00000186564	ENSG00000176678
ENSG00000186564	ENSG00000176692
ENSG00000186564	ENSG00000178919
ENSG00000186564	ENSG00000179772
ENSG00000186564	ENSG00000183770
ENSG00000186564	ENSG00000184492
ENSG00000186564	ENSG00000184659
ENSG00000186564	ENSG00000186766
ENSG00000186564	ENSG00000186790
ENSG00000186564	ENSG00000187140
ENSG00000186564	ENSG00000187559
ENSG00000186564	ENSG00000204612
ENSG00000186564	ENSG00000204779
ENSG00000186564	ENSG00000204793
ENSG00000186564	ENSG00000204828
ENSG00000186564	ENSG00000214336
ENSG00000186575	ENSG00000092820
ENSG00000186575	ENSG00000137710
ENSG00000186575	ENSG00000147065
ENSG00000186611	ENSG00000139088
ENSG00000186625	ENSG00000102781
ENSG00000186628	ENSG00000100505
ENSG00000186628	ENSG00000119283
ENSG00000186628	ENSG00000152503
ENSG00000186628	ENSG00000163462
ENSG00000186629	ENSG00000069122
ENSG00000186629	ENSG00000123146
ENSG00000186629	ENSG00000127507
ENSG00000186629	ENSG00000131355
ENSG00000186629	ENSG00000153292
ENSG00000186629	ENSG00000153294
ENSG00000186629	ENSG00000162618
ENSG00000186629	ENSG00000164393
ENSG00000186629	ENSG00000173567
ENSG00000186629	ENSG00000174837
ENSG00000186629	ENSG00000204818
ENSG00000186635	ENSG00000047365
ENSG00000186635	ENSG00000120318
ENSG00000186645	ENSG00000136206

ENSG00000186645	ENSG00000154732
ENSG00000186645	ENSG00000163806
ENSG00000186645	ENSG00000166667
ENSG00000186645	ENSG00000170092
ENSG00000186645	ENSG00000173678
ENSG00000186645	ENSG00000179994
ENSG00000186645	ENSG00000183318
ENSG00000186645	ENSG00000184616
ENSG00000186645	ENSG00000185040
ENSG00000186645	ENSG00000198305
ENSG00000186645	ENSG00000204710
ENSG00000186645	ENSG00000205238
ENSG00000186645	ENSG00000205482
ENSG00000186645	ENSG00000205505
ENSG00000186645	ENSG00000205506
ENSG00000186645	ENSG00000205508
ENSG00000186645	ENSG00000205509
ENSG00000186645	ENSG00000205580
ENSG00000186645	ENSG00000214300
ENSG00000186645	ENSG00000215092
ENSG00000186648	ENSG00000079691
ENSG00000186648	ENSG00000159753
ENSG00000186654	ENSG00000135362
ENSG00000186660	ENSG00000158805
ENSG00000186660	ENSG00000161914
ENSG00000186660	ENSG00000171163
ENSG00000186666	ENSG00000146834
ENSG00000186684	ENSG00000019186
ENSG00000186684	ENSG00000111012
ENSG00000186684	ENSG00000135929
ENSG00000186684	ENSG00000140459
ENSG00000186684	ENSG00000160882
ENSG00000186684	ENSG00000179142
ENSG00000186698	ENSG00000118975
ENSG00000186698	ENSG00000184319
ENSG00000186698	ENSG00000185822
ENSG00000186698	ENSG00000186239
ENSG00000186698	ENSG00000198242
ENSG00000186698	ENSG00000206147
ENSG00000186698	ENSG00000212932
ENSG00000186698	ENSG00000213753
ENSG00000186698	ENSG00000214230
ENSG00000186698	ENSG00000215001
ENSG00000186698	ENSG00000215140
ENSG00000186698	ENSG00000215387
ENSG00000186715	ENSG00000019991
ENSG00000186715	ENSG00000104368
ENSG00000186715	ENSG00000109758
ENSG00000186715	ENSG00000122194
ENSG00000186715	ENSG00000122861
ENSG00000186715	ENSG00000131187
ENSG00000186715	ENSG00000148702
ENSG00000186715	ENSG00000173531
ENSG00000186715	ENSG00000186301
ENSG00000186715	ENSG00000198670
ENSG00000186716	ENSG00000159842
ENSG00000186732	ENSG00000066382
ENSG00000186765	ENSG00000075618
ENSG00000186765	ENSG00000106328

ENSG00000186766	ENSG00000054598
ENSG00000186766	ENSG00000125798
ENSG00000186766	ENSG00000129514
ENSG00000186766	ENSG00000164379
ENSG00000186766	ENSG00000168269
ENSG00000186766	ENSG00000170122
ENSG00000186766	ENSG00000170608
ENSG00000186766	ENSG00000176165
ENSG00000186766	ENSG00000176678
ENSG00000186766	ENSG00000176692
ENSG00000186766	ENSG00000178919
ENSG00000186766	ENSG00000179772
ENSG00000186766	ENSG00000183770
ENSG00000186766	ENSG00000184492
ENSG00000186766	ENSG00000184659
ENSG00000186766	ENSG00000186564
ENSG00000186766	ENSG00000186790
ENSG00000186766	ENSG00000187140
ENSG00000186766	ENSG00000187559
ENSG00000186766	ENSG00000204612
ENSG00000186766	ENSG00000204779
ENSG00000186766	ENSG00000204793
ENSG00000186766	ENSG00000204828
ENSG00000186766	ENSG00000214336
ENSG00000186767	ENSG00000106723
ENSG00000186767	ENSG00000147059
ENSG00000186767	ENSG00000186787
ENSG00000186767	ENSG00000204271
ENSG00000186777	ENSG00000142396
ENSG00000186777	ENSG00000169740
ENSG00000186777	ENSG00000174255
ENSG00000186777	ENSG00000179195
ENSG00000186777	ENSG00000179965
ENSG00000186777	ENSG00000184294
ENSG00000186777	ENSG00000186446
ENSG00000186777	ENSG00000197008
ENSG00000186777	ENSG00000197025
ENSG00000186777	ENSG00000197062
ENSG00000186777	ENSG00000197332
ENSG00000186777	ENSG00000198155
ENSG00000186777	ENSG00000204756
ENSG00000186777	ENSG00000212805
ENSG00000186777	ENSG00000213976
ENSG00000186777	ENSG00000213980
ENSG00000186777	ENSG00000215180
ENSG00000186777	ENSG00000215510
ENSG00000186777	ENSG00000215537
ENSG00000186777	ENSG00000215598
ENSG00000186777	ENSG00000215600
ENSG00000186787	ENSG00000106723
ENSG00000186787	ENSG00000147059
ENSG00000186787	ENSG00000186767
ENSG00000186787	ENSG00000204271
ENSG00000186792	ENSG00000068001
ENSG00000186792	ENSG00000106302
ENSG00000186792	ENSG00000106304
ENSG00000186792	ENSG00000114378
ENSG00000186807	ENSG00000104537
ENSG00000186807	ENSG00000109511

ENSG00000186807	ENSG00000122359
ENSG00000186807	ENSG00000135046
ENSG00000186807	ENSG00000138279
ENSG00000186807	ENSG00000138772
ENSG00000186807	ENSG00000143412
ENSG00000186807	ENSG00000150165
ENSG00000186807	ENSG00000164111
ENSG00000186807	ENSG00000165390
ENSG00000186807	ENSG00000182718
ENSG00000186807	ENSG00000183059
ENSG00000186807	ENSG00000196975
ENSG00000186807	ENSG00000197043
ENSG00000186810	ENSG00000112486
ENSG00000186810	ENSG00000121797
ENSG00000186810	ENSG00000121807
ENSG00000186810	ENSG00000121966
ENSG00000186810	ENSG00000126353
ENSG00000186810	ENSG00000129048
ENSG00000186810	ENSG00000144648
ENSG00000186810	ENSG00000160683
ENSG00000186810	ENSG00000160791
ENSG00000186810	ENSG00000163464
ENSG00000186810	ENSG00000163823
ENSG00000186810	ENSG00000168329
ENSG00000186810	ENSG00000172215
ENSG00000186810	ENSG00000173578
ENSG00000186810	ENSG00000173585
ENSG00000186810	ENSG00000179934
ENSG00000186810	ENSG00000180871
ENSG00000186810	ENSG00000183625
ENSG00000186810	ENSG00000183813
ENSG00000186810	ENSG00000184451
ENSG00000186810	ENSG00000215775
ENSG00000186810	ENSG00000215778
ENSG00000186810	ENSG00000215782
ENSG00000186815	ENSG00000162341
ENSG00000186831	ENSG00000006059
ENSG00000186831	ENSG00000094796
ENSG00000186831	ENSG00000108244
ENSG00000186831	ENSG00000108417
ENSG00000186831	ENSG00000108759
ENSG00000186831	ENSG00000111057
ENSG00000186831	ENSG00000126337
ENSG00000186831	ENSG00000128422
ENSG00000186831	ENSG00000131737
ENSG00000186831	ENSG00000131738
ENSG00000186831	ENSG00000131885
ENSG00000186831	ENSG00000167916
ENSG00000186831	ENSG00000170819
ENSG00000186831	ENSG00000171345
ENSG00000186831	ENSG00000171346
ENSG00000186831	ENSG00000171360
ENSG00000186831	ENSG00000171401
ENSG00000186831	ENSG00000171403
ENSG00000186831	ENSG00000171431
ENSG00000186831	ENSG00000171446
ENSG00000186831	ENSG00000173908
ENSG00000186831	ENSG00000186393
ENSG00000186831	ENSG00000186395

ENSG00000186831	ENSG00000186832
ENSG00000186831	ENSG00000186847
ENSG00000186831	ENSG00000187242
ENSG00000186831	ENSG00000187686
ENSG00000186831	ENSG00000196859
ENSG00000186831	ENSG00000197079
ENSG00000186831	ENSG00000204889
ENSG00000186831	ENSG00000204897
ENSG00000186831	ENSG00000213951
ENSG00000186831	ENSG00000214220
ENSG00000186831	ENSG00000214454
ENSG00000186831	ENSG00000214959
ENSG00000186831	ENSG00000215772
ENSG00000186831	ENSG00000215773
ENSG00000186832	ENSG00000006059
ENSG00000186832	ENSG00000094796
ENSG00000186832	ENSG00000108244
ENSG00000186832	ENSG00000108417
ENSG00000186832	ENSG00000108759
ENSG00000186832	ENSG00000111057
ENSG00000186832	ENSG00000126337
ENSG00000186832	ENSG00000128422
ENSG00000186832	ENSG00000131737
ENSG00000186832	ENSG00000131738
ENSG00000186832	ENSG00000131885
ENSG00000186832	ENSG00000167916
ENSG00000186832	ENSG00000170819
ENSG00000186832	ENSG00000171345
ENSG00000186832	ENSG00000171346
ENSG00000186832	ENSG00000171360
ENSG00000186832	ENSG00000171401
ENSG00000186832	ENSG00000171403
ENSG00000186832	ENSG00000171431
ENSG00000186832	ENSG00000171446
ENSG00000186832	ENSG00000173908
ENSG00000186832	ENSG00000186393
ENSG00000186832	ENSG00000186395
ENSG00000186832	ENSG00000186831
ENSG00000186832	ENSG00000186847
ENSG00000186832	ENSG00000187242
ENSG00000186832	ENSG00000187686
ENSG00000186832	ENSG00000196859
ENSG00000186832	ENSG00000197079
ENSG00000186832	ENSG00000204889
ENSG00000186832	ENSG00000204897
ENSG00000186832	ENSG00000213951
ENSG00000186832	ENSG00000214220
ENSG00000186832	ENSG00000214454
ENSG00000186832	ENSG00000214959
ENSG00000186832	ENSG00000215772
ENSG00000186832	ENSG00000215773
ENSG00000186834	ENSG00000168517
ENSG00000186847	ENSG00000006059
ENSG00000186847	ENSG00000094796
ENSG00000186847	ENSG00000108244
ENSG00000186847	ENSG00000108417
ENSG00000186847	ENSG00000108759
ENSG00000186847	ENSG00000111057
ENSG00000186847	ENSG00000126337

ENSG00000186847	ENSG00000128422
ENSG00000186847	ENSG00000131737
ENSG00000186847	ENSG00000131738
ENSG00000186847	ENSG00000131885
ENSG00000186847	ENSG00000167916
ENSG00000186847	ENSG00000170819
ENSG00000186847	ENSG00000171345
ENSG00000186847	ENSG00000171346
ENSG00000186847	ENSG00000171360
ENSG00000186847	ENSG00000171401
ENSG00000186847	ENSG00000171403
ENSG00000186847	ENSG00000171431
ENSG00000186847	ENSG00000171446
ENSG00000186847	ENSG00000173908
ENSG00000186847	ENSG00000186393
ENSG00000186847	ENSG00000186395
ENSG00000186847	ENSG00000186831
ENSG00000186847	ENSG00000186832
ENSG00000186847	ENSG00000187242
ENSG00000186847	ENSG00000187686
ENSG00000186847	ENSG00000196859
ENSG00000186847	ENSG00000197079
ENSG00000186847	ENSG00000204889
ENSG00000186847	ENSG00000204897
ENSG00000186847	ENSG00000213951
ENSG00000186847	ENSG00000214220
ENSG00000186847	ENSG00000214454
ENSG00000186847	ENSG00000214959
ENSG00000186847	ENSG00000215772
ENSG00000186847	ENSG00000215773
ENSG00000186854	ENSG00000204018
ENSG00000186862	ENSG00000006611
ENSG00000186862	ENSG00000095397
ENSG00000186867	ENSG00000056291
ENSG00000186867	ENSG00000075073
ENSG00000186867	ENSG00000101292
ENSG00000186867	ENSG00000110148
ENSG00000186867	ENSG00000115353
ENSG00000186867	ENSG00000119973
ENSG00000186867	ENSG00000121764
ENSG00000186867	ENSG00000123901
ENSG00000186867	ENSG00000137252
ENSG00000186867	ENSG00000148734
ENSG00000186867	ENSG00000159279
ENSG00000186867	ENSG00000163394
ENSG00000186867	ENSG00000164128
ENSG00000186867	ENSG00000164129
ENSG00000186867	ENSG00000169618
ENSG00000186867	ENSG00000169836
ENSG00000186867	ENSG00000185149
ENSG00000186867	ENSG00000204174
ENSG00000186867	ENSG00000215167
ENSG00000186868	ENSG00000047849
ENSG00000186868	ENSG00000078018
ENSG00000186889	ENSG00000177042
ENSG00000186889	ENSG00000187049
ENSG00000186895	ENSG00000070193
ENSG00000186895	ENSG00000070388
ENSG00000186895	ENSG00000075388

ENSG00000186895	ENSG00000078579
ENSG00000186895	ENSG00000102466
ENSG00000186895	ENSG00000102678
ENSG00000186895	ENSG00000111241
ENSG00000186895	ENSG00000113578
ENSG00000186895	ENSG00000114279
ENSG00000186895	ENSG00000129682
ENSG00000186895	ENSG00000138675
ENSG00000186895	ENSG00000138685
ENSG00000186895	ENSG00000140285
ENSG00000186895	ENSG00000161958
ENSG00000186895	ENSG00000185390
ENSG00000186895	ENSG00000196468
ENSG00000186895	ENSG00000204801
ENSG00000186895	ENSG00000204816
ENSG00000186895	ENSG00000204817
ENSG00000186895	ENSG00000204837
ENSG00000186895	ENSG00000215053
ENSG00000186897	ENSG00000054803
ENSG00000186897	ENSG00000102924
ENSG00000186897	ENSG00000131094
ENSG00000186897	ENSG00000133466
ENSG00000186897	ENSG00000139899
ENSG00000186897	ENSG00000141668
ENSG00000186897	ENSG00000144119
ENSG00000186897	ENSG00000145861
ENSG00000186897	ENSG00000159189
ENSG00000186897	ENSG00000163145
ENSG00000186897	ENSG00000165985
ENSG00000186897	ENSG00000172247
ENSG00000186897	ENSG00000173369
ENSG00000186897	ENSG00000173372
ENSG00000186897	ENSG00000173918
ENSG00000186897	ENSG00000181092
ENSG00000186897	ENSG00000184471
ENSG00000186897	ENSG00000213192
ENSG00000186907	ENSG00000040608
ENSG00000186907	ENSG00000185924
ENSG00000186908	ENSG00000177054
ENSG00000186910	ENSG00000100665
ENSG00000186910	ENSG00000123561
ENSG00000186910	ENSG00000135744
ENSG00000186910	ENSG00000165951
ENSG00000186910	ENSG00000165953
ENSG00000186910	ENSG00000170054
ENSG00000186910	ENSG00000187483
ENSG00000186910	ENSG00000188488
ENSG00000186910	ENSG00000196136
ENSG00000186910	ENSG00000197249
ENSG00000186912	ENSG00000078589
ENSG00000186912	ENSG00000139679
ENSG00000186912	ENSG00000147145
ENSG00000186912	ENSG00000152207
ENSG00000186912	ENSG00000169403
ENSG00000186912	ENSG00000169508
ENSG00000186912	ENSG00000169860
ENSG00000186912	ENSG00000173198
ENSG00000186912	ENSG00000175591
ENSG00000186912	ENSG00000184574

ENSG00000186912	ENSG00000198829
ENSG00000186918	ENSG00000125520
ENSG00000186918	ENSG00000164684
ENSG00000186940	ENSG00000106153
ENSG00000186940	ENSG00000138869
ENSG00000186940	ENSG00000182699
ENSG00000186940	ENSG00000203682
ENSG00000186951	ENSG00000012504
ENSG00000186951	ENSG00000111424
ENSG00000186951	ENSG00000112033
ENSG00000186951	ENSG00000132170
ENSG00000186951	ENSG00000143257
ENSG00000186951	ENSG00000144852
ENSG00000186994	ENSG00000107104
ENSG00000186994	ENSG00000132854
ENSG00000186994	ENSG00000197256
ENSG00000186998	ENSG00000160963
ENSG00000187008	ENSG00000008710
ENSG00000187008	ENSG00000078795
ENSG00000187008	ENSG00000107593
ENSG00000187008	ENSG00000118762
ENSG00000187008	ENSG00000130943
ENSG00000187008	ENSG00000158683
ENSG00000187008	ENSG00000166473
ENSG00000187008	ENSG00000183458
ENSG00000187008	ENSG00000183793
ENSG00000187008	ENSG00000188599
ENSG00000187010	ENSG00000112077
ENSG00000187010	ENSG00000132677
ENSG00000187010	ENSG00000140519
ENSG00000187010	ENSG00000188672
ENSG00000187017	ENSG00000116219
ENSG00000187017	ENSG00000144488
ENSG00000187033	ENSG00000111752
ENSG00000187033	ENSG00000134686
ENSG00000187033	ENSG00000173889
ENSG00000187033	ENSG00000179899
ENSG00000187033	ENSG00000187634
ENSG00000187045	ENSG00000149418
ENSG00000187045	ENSG00000176040
ENSG00000187045	ENSG00000178297
ENSG00000187045	ENSG00000205403
ENSG00000187048	ENSG00000142973
ENSG00000187048	ENSG00000145476
ENSG00000187048	ENSG00000154198
ENSG00000187048	ENSG00000162365
ENSG00000187048	ENSG00000171903
ENSG00000187048	ENSG00000171954
ENSG00000187048	ENSG00000186115
ENSG00000187048	ENSG00000186160
ENSG00000187048	ENSG00000186204
ENSG00000187048	ENSG00000186377
ENSG00000187048	ENSG00000186526
ENSG00000187048	ENSG00000186529
ENSG00000187049	ENSG00000177042
ENSG00000187049	ENSG00000186889
ENSG00000187079	ENSG00000007866
ENSG00000187079	ENSG00000074219
ENSG00000187079	ENSG00000197905

ENSG00000187091	ENSG00000101333
ENSG00000187091	ENSG00000114805
ENSG00000187091	ENSG00000115556
ENSG00000187091	ENSG00000115896
ENSG00000187091	ENSG00000124181
ENSG00000187091	ENSG00000137841
ENSG00000187091	ENSG00000139151
ENSG00000187091	ENSG00000149527
ENSG00000187091	ENSG00000149782
ENSG00000187091	ENSG00000154822
ENSG00000187091	ENSG00000161714
ENSG00000187091	ENSG00000182621
ENSG00000187091	ENSG00000197943
ENSG00000187097	ENSG00000197586
ENSG00000187098	ENSG00000068323
ENSG00000187098	ENSG00000105967
ENSG00000187098	ENSG00000112561
ENSG00000187109	ENSG00000177432
ENSG00000187109	ENSG00000186310
ENSG00000187109	ENSG00000186462
ENSG00000187109	ENSG00000205531
ENSG00000187122	ENSG00000128606
ENSG00000187122	ENSG00000145147
ENSG00000187122	ENSG00000166897
ENSG00000187122	ENSG00000182743
ENSG00000187122	ENSG00000184347
ENSG00000187123	ENSG00000150556
ENSG00000187134	ENSG00000085662
ENSG00000187134	ENSG00000117448
ENSG00000187134	ENSG00000122787
ENSG00000187134	ENSG00000151631
ENSG00000187134	ENSG00000151632
ENSG00000187134	ENSG00000165568
ENSG00000187134	ENSG00000196139
ENSG00000187134	ENSG00000196326
ENSG00000187134	ENSG00000198074
ENSG00000187134	ENSG00000198610
ENSG00000187134	ENSG00000215267
ENSG00000187135	ENSG00000132821
ENSG00000187135	ENSG00000170419
ENSG00000187140	ENSG00000054598
ENSG00000187140	ENSG00000125798
ENSG00000187140	ENSG00000129514
ENSG00000187140	ENSG00000164379
ENSG00000187140	ENSG00000168269
ENSG00000187140	ENSG00000170122
ENSG00000187140	ENSG00000170608
ENSG00000187140	ENSG00000176165
ENSG00000187140	ENSG00000176678
ENSG00000187140	ENSG00000176692
ENSG00000187140	ENSG00000178919
ENSG00000187140	ENSG00000179772
ENSG00000187140	ENSG00000183770
ENSG00000187140	ENSG00000184492
ENSG00000187140	ENSG00000184659
ENSG00000187140	ENSG00000186564
ENSG00000187140	ENSG00000186766
ENSG00000187140	ENSG00000186790
ENSG00000187140	ENSG00000187559

ENSG00000187140	ENSG00000204612
ENSG00000187140	ENSG00000204779
ENSG00000187140	ENSG00000204793
ENSG00000187140	ENSG00000204828
ENSG00000187140	ENSG00000214336
ENSG00000187191	ENSG00000092345
ENSG00000187191	ENSG00000152430
ENSG00000187191	ENSG00000188120
ENSG00000187191	ENSG00000205916
ENSG00000187191	ENSG00000205944
ENSG00000187193	ENSG00000087250
ENSG00000187193	ENSG00000102891
ENSG00000187193	ENSG00000125144
ENSG00000187193	ENSG00000125148
ENSG00000187193	ENSG00000169688
ENSG00000187193	ENSG00000169715
ENSG00000187193	ENSG00000198417
ENSG00000187193	ENSG00000205358
ENSG00000187193	ENSG00000205360
ENSG00000187193	ENSG00000205362
ENSG00000187193	ENSG00000205364
ENSG00000187210	ENSG00000015532
ENSG00000187210	ENSG00000103489
ENSG00000187210	ENSG00000111846
ENSG00000187210	ENSG00000124091
ENSG00000187210	ENSG00000140297
ENSG00000187210	ENSG00000176928
ENSG00000187210	ENSG00000205318
ENSG00000187213	ENSG00000105656
ENSG00000187213	ENSG00000118985
ENSG00000187213	ENSG00000152939
ENSG00000187213	ENSG00000197822
ENSG00000187239	ENSG00000125733
ENSG00000187239	ENSG00000137942
ENSG00000187242	ENSG00000006059
ENSG00000187242	ENSG00000094796
ENSG00000187242	ENSG00000108244
ENSG00000187242	ENSG00000108417
ENSG00000187242	ENSG00000108759
ENSG00000187242	ENSG00000111057
ENSG00000187242	ENSG00000126337
ENSG00000187242	ENSG00000128422
ENSG00000187242	ENSG00000131737
ENSG00000187242	ENSG00000131738
ENSG00000187242	ENSG00000131885
ENSG00000187242	ENSG00000167916
ENSG00000187242	ENSG00000170819
ENSG00000187242	ENSG00000171345
ENSG00000187242	ENSG00000171346
ENSG00000187242	ENSG00000171360
ENSG00000187242	ENSG00000171401
ENSG00000187242	ENSG00000171403
ENSG00000187242	ENSG00000171431
ENSG00000187242	ENSG00000171446
ENSG00000187242	ENSG00000173908
ENSG00000187242	ENSG00000186393
ENSG00000187242	ENSG00000186395
ENSG00000187242	ENSG00000186831
ENSG00000187242	ENSG00000186832

ENSG00000187242	ENSG00000186847
ENSG00000187242	ENSG00000187686
ENSG00000187242	ENSG00000196859
ENSG00000187242	ENSG00000197079
ENSG00000187242	ENSG00000204889
ENSG00000187242	ENSG00000204897
ENSG00000187242	ENSG00000213951
ENSG00000187242	ENSG00000214220
ENSG00000187242	ENSG00000214454
ENSG00000187242	ENSG00000214959
ENSG00000187242	ENSG00000215772
ENSG00000187242	ENSG00000215773
ENSG00000187244	ENSG00000076706
ENSG00000187257	ENSG00000081019
ENSG00000187266	ENSG00000117400
ENSG00000187266	ENSG00000168685
ENSG00000187268	ENSG00000139351
ENSG00000187268	ENSG00000177138
ENSG00000187268	ENSG00000183304
ENSG00000187288	ENSG00000136305
ENSG00000187288	ENSG00000176194
ENSG00000187323	ENSG00000067141
ENSG00000187323	ENSG00000103742
ENSG00000187323	ENSG00000166450
ENSG00000187323	ENSG00000174498
ENSG00000187323	ENSG00000215851
ENSG00000187372	ENSG00000081818
ENSG00000187372	ENSG00000081842
ENSG00000187372	ENSG00000081853
ENSG00000187372	ENSG00000112852
ENSG00000187372	ENSG00000113205
ENSG00000187372	ENSG00000113209
ENSG00000187372	ENSG00000113211
ENSG00000187372	ENSG00000113212
ENSG00000187372	ENSG00000113248
ENSG00000187372	ENSG00000120322
ENSG00000187372	ENSG00000120324
ENSG00000187372	ENSG00000120327
ENSG00000187372	ENSG00000120328
ENSG00000187372	ENSG00000146001
ENSG00000187372	ENSG00000171815
ENSG00000187372	ENSG00000196963
ENSG00000187372	ENSG00000197479
ENSG00000187372	ENSG00000204955
ENSG00000187372	ENSG00000204956
ENSG00000187372	ENSG00000204961
ENSG00000187372	ENSG00000204962
ENSG00000187372	ENSG00000204963
ENSG00000187372	ENSG00000204964
ENSG00000187372	ENSG00000204965
ENSG00000187372	ENSG00000204967
ENSG00000187372	ENSG00000204968
ENSG00000187372	ENSG00000204969
ENSG00000187372	ENSG00000204970
ENSG00000187372	ENSG00000214567
ENSG00000187372	ENSG00000214570
ENSG00000187372	ENSG00000214574
ENSG00000187372	ENSG00000214580
ENSG00000187372	ENSG00000214583

ENSG00000187372	ENSG00000214594
ENSG00000187372	ENSG00000214600
ENSG00000187372	ENSG00000214608
ENSG00000187391	ENSG00000081026
ENSG00000187391	ENSG00000151276
ENSG00000187416	ENSG00000145685
ENSG00000187416	ENSG00000156959
ENSG00000187416	ENSG00000182508
ENSG00000187416	ENSG00000183722
ENSG00000187416	ENSG00000197753
ENSG00000187446	ENSG00000088992
ENSG00000187446	ENSG00000166869
ENSG00000187474	ENSG00000134830
ENSG00000187474	ENSG00000171049
ENSG00000187474	ENSG00000171051
ENSG00000187474	ENSG00000171860
ENSG00000187474	ENSG00000174600
ENSG00000187474	ENSG00000183134
ENSG00000187474	ENSG00000183671
ENSG00000187474	ENSG00000197405
ENSG00000187474	ENSG00000213903
ENSG00000187474	ENSG00000213906
ENSG00000187475	ENSG00000124575
ENSG00000187475	ENSG00000124610
ENSG00000187475	ENSG00000168298
ENSG00000187475	ENSG00000184357
ENSG00000187475	ENSG00000184897
ENSG00000187475	ENSG00000187837
ENSG00000187475	ENSG00000189060
ENSG00000187475	ENSG00000196440
ENSG00000187483	ENSG00000100665
ENSG00000187483	ENSG00000123561
ENSG00000187483	ENSG00000135744
ENSG00000187483	ENSG00000165951
ENSG00000187483	ENSG00000165953
ENSG00000187483	ENSG00000170054
ENSG00000187483	ENSG00000186910
ENSG00000187483	ENSG00000188488
ENSG00000187483	ENSG00000196136
ENSG00000187483	ENSG00000197249
ENSG00000187486	ENSG00000115474
ENSG00000187486	ENSG00000120457
ENSG00000187486	ENSG00000121361
ENSG00000187486	ENSG00000123700
ENSG00000187486	ENSG00000151704
ENSG00000187486	ENSG00000153822
ENSG00000187486	ENSG00000157542
ENSG00000187486	ENSG00000157551
ENSG00000187486	ENSG00000162728
ENSG00000187486	ENSG00000162989
ENSG00000187486	ENSG00000168135
ENSG00000187486	ENSG00000177807
ENSG00000187486	ENSG00000182324
ENSG00000187486	ENSG00000184185
ENSG00000187498	ENSG00000081052
ENSG00000187498	ENSG00000134871
ENSG00000187498	ENSG00000169031
ENSG00000187498	ENSG00000188153
ENSG00000187498	ENSG00000197565

ENSG00000187513	ENSG00000121634
ENSG00000187513	ENSG00000121742
ENSG00000187513	ENSG00000121743
ENSG00000187513	ENSG00000131233
ENSG00000187513	ENSG00000135355
ENSG00000187513	ENSG00000143140
ENSG00000187513	ENSG00000152661
ENSG00000187513	ENSG00000159248
ENSG00000187513	ENSG00000164411
ENSG00000187513	ENSG00000165474
ENSG00000187513	ENSG00000169562
ENSG00000187513	ENSG00000176402
ENSG00000187513	ENSG00000177291
ENSG00000187513	ENSG00000182963
ENSG00000187513	ENSG00000183153
ENSG00000187513	ENSG00000188910
ENSG00000187513	ENSG00000189280
ENSG00000187513	ENSG00000189433
ENSG00000187513	ENSG00000198835
ENSG00000187513	ENSG00000203733
ENSG00000187527	ENSG00000127249
ENSG00000187527	ENSG00000133657
ENSG00000187527	ENSG00000159363
ENSG00000187536	ENSG00000140416
ENSG00000187536	ENSG00000143549
ENSG00000187536	ENSG00000167460
ENSG00000187536	ENSG00000177152
ENSG00000187536	ENSG00000183022
ENSG00000187536	ENSG00000198467
ENSG00000187536	ENSG00000214869
ENSG00000187545	ENSG00000116708
ENSG00000187545	ENSG00000116721
ENSG00000187545	ENSG00000116726
ENSG00000187545	ENSG00000120952
ENSG00000187545	ENSG00000157358
ENSG00000187545	ENSG00000160959
ENSG00000187545	ENSG00000179704
ENSG00000187545	ENSG00000182330
ENSG00000187545	ENSG00000185028
ENSG00000187545	ENSG00000185686
ENSG00000187545	ENSG00000197549
ENSG00000187545	ENSG00000204478
ENSG00000187545	ENSG00000204479
ENSG00000187545	ENSG00000204480
ENSG00000187545	ENSG00000204481
ENSG00000187545	ENSG00000204485
ENSG00000187545	ENSG00000204486
ENSG00000187545	ENSG00000204488
ENSG00000187545	ENSG00000204491
ENSG00000187545	ENSG00000204495
ENSG00000187545	ENSG00000204501
ENSG00000187545	ENSG00000204502
ENSG00000187545	ENSG00000204503
ENSG00000187545	ENSG00000204505
ENSG00000187545	ENSG00000204507
ENSG00000187545	ENSG00000204508
ENSG00000187545	ENSG00000204509
ENSG00000187545	ENSG00000204510
ENSG00000187545	ENSG00000204513

ENSG00000187550	ENSG00000188322
ENSG00000187553	ENSG00000003137
ENSG00000187553	ENSG00000095596
ENSG00000187554	ENSG00000101916
ENSG00000187554	ENSG00000134061
ENSG00000187554	ENSG00000136869
ENSG00000187554	ENSG00000137462
ENSG00000187554	ENSG00000174123
ENSG00000187554	ENSG00000174125
ENSG00000187554	ENSG00000174130
ENSG00000187556	ENSG00000188425
ENSG00000187556	ENSG00000188613
ENSG00000187559	ENSG00000054598
ENSG00000187559	ENSG00000125798
ENSG00000187559	ENSG00000129514
ENSG00000187559	ENSG00000164379
ENSG00000187559	ENSG00000168269
ENSG00000187559	ENSG00000170122
ENSG00000187559	ENSG00000170608
ENSG00000187559	ENSG00000176165
ENSG00000187559	ENSG00000176678
ENSG00000187559	ENSG00000176692
ENSG00000187559	ENSG00000178919
ENSG00000187559	ENSG00000179772
ENSG00000187559	ENSG00000183770
ENSG00000187559	ENSG00000184492
ENSG00000187559	ENSG00000184659
ENSG00000187559	ENSG00000186564
ENSG00000187559	ENSG00000186766
ENSG00000187559	ENSG00000186790
ENSG00000187559	ENSG00000187140
ENSG00000187559	ENSG00000204612
ENSG00000187559	ENSG00000204779
ENSG00000187559	ENSG00000204793
ENSG00000187559	ENSG00000204828
ENSG00000187559	ENSG00000214336
ENSG00000187566	ENSG00000109654
ENSG00000187566	ENSG00000110171
ENSG00000187566	ENSG00000119401
ENSG00000187566	ENSG00000188811
ENSG00000187566	ENSG00000206557
ENSG00000187574	ENSG00000026950
ENSG00000187574	ENSG00000111801
ENSG00000187574	ENSG00000112763
ENSG00000187574	ENSG00000113303
ENSG00000187574	ENSG00000121236
ENSG00000187574	ENSG00000124508
ENSG00000187574	ENSG00000124549
ENSG00000187574	ENSG00000124557
ENSG00000187574	ENSG00000128250
ENSG00000187574	ENSG00000128253
ENSG00000187574	ENSG00000128276
ENSG00000187574	ENSG00000128482
ENSG00000187574	ENSG00000132256
ENSG00000187574	ENSG00000132274
ENSG00000187574	ENSG00000164010
ENSG00000187574	ENSG00000165810
ENSG00000187574	ENSG00000168903
ENSG00000187574	ENSG00000176422

ENSG00000187574	ENSG00000179046
ENSG00000187574	ENSG00000182805
ENSG00000187574	ENSG00000183439
ENSG00000187574	ENSG00000183718
ENSG00000187574	ENSG00000188683
ENSG00000187574	ENSG00000204614
ENSG00000187574	ENSG00000204616
ENSG00000187574	ENSG00000204618
ENSG00000187574	ENSG00000205198
ENSG00000187574	ENSG00000214693
ENSG00000187581	ENSG00000176340
ENSG00000187583	ENSG00000102606
ENSG00000187583	ENSG00000129675
ENSG00000187589	ENSG00000132604
ENSG00000187589	ENSG00000147601
ENSG00000187589	ENSG00000184862
ENSG00000187595	ENSG00000144331
ENSG00000187595	ENSG00000151789
ENSG00000187595	ENSG00000161642
ENSG00000187605	ENSG00000174792
ENSG00000187634	ENSG00000111752
ENSG00000187634	ENSG00000134686
ENSG00000187634	ENSG00000173889
ENSG00000187634	ENSG00000179899
ENSG00000187634	ENSG00000187033
ENSG00000187664	ENSG00000038427
ENSG00000187664	ENSG00000130287
ENSG00000187664	ENSG00000132692
ENSG00000187664	ENSG00000132702
ENSG00000187664	ENSG00000140511
ENSG00000187664	ENSG00000145681
ENSG00000187664	ENSG00000157766
ENSG00000187672	ENSG00000082805
ENSG00000187678	ENSG00000136158
ENSG00000187678	ENSG00000164056
ENSG00000187678	ENSG00000168939
ENSG00000187682	ENSG00000006451
ENSG00000187682	ENSG00000126458
ENSG00000187682	ENSG00000133703
ENSG00000187682	ENSG00000133818
ENSG00000187682	ENSG00000144118
ENSG00000187682	ENSG00000158186
ENSG00000187682	ENSG00000174775
ENSG00000187682	ENSG00000213281
ENSG00000187686	ENSG00000006059
ENSG00000187686	ENSG00000094796
ENSG00000187686	ENSG00000108244
ENSG00000187686	ENSG00000108417
ENSG00000187686	ENSG00000108759
ENSG00000187686	ENSG00000111057
ENSG00000187686	ENSG00000126337
ENSG00000187686	ENSG00000128422
ENSG00000187686	ENSG00000131737
ENSG00000187686	ENSG00000131738
ENSG00000187686	ENSG00000131885
ENSG00000187686	ENSG00000167916
ENSG00000187686	ENSG00000170819
ENSG00000187686	ENSG00000171345
ENSG00000187686	ENSG00000171346

ENSG00000187686	ENSG00000171360
ENSG00000187686	ENSG00000171401
ENSG00000187686	ENSG00000171403
ENSG00000187686	ENSG00000171431
ENSG00000187686	ENSG00000171446
ENSG00000187686	ENSG00000173908
ENSG00000187686	ENSG00000186393
ENSG00000187686	ENSG00000186395
ENSG00000187686	ENSG00000186831
ENSG00000187686	ENSG00000186832
ENSG00000187686	ENSG00000186847
ENSG00000187686	ENSG00000187242
ENSG00000187686	ENSG00000196859
ENSG00000187686	ENSG00000197079
ENSG00000187686	ENSG00000204889
ENSG00000187686	ENSG00000204897
ENSG00000187686	ENSG00000213951
ENSG00000187686	ENSG00000214220
ENSG00000187686	ENSG00000214454
ENSG00000187686	ENSG00000214959
ENSG00000187686	ENSG00000215772
ENSG00000187686	ENSG00000215773
ENSG00000187688	ENSG00000111199
ENSG00000187688	ENSG00000127412
ENSG00000187688	ENSG00000165125
ENSG00000187688	ENSG00000167723
ENSG00000187688	ENSG00000196689
ENSG00000187720	ENSG00000100767
ENSG00000187720	ENSG00000143382
ENSG00000187720	ENSG00000185761
ENSG00000187720	ENSG00000197859
ENSG00000187720	ENSG00000215616
ENSG00000187728	ENSG00000118526
ENSG00000187728	ENSG00000163792
ENSG00000187728	ENSG00000178860
ENSG00000187730	ENSG00000011677
ENSG00000187730	ENSG00000022355
ENSG00000187730	ENSG00000094755
ENSG00000187730	ENSG00000101958
ENSG00000187730	ENSG00000102287
ENSG00000187730	ENSG00000109158
ENSG00000187730	ENSG00000109738
ENSG00000187730	ENSG00000111886
ENSG00000187730	ENSG00000113327
ENSG00000187730	ENSG00000145451
ENSG00000187730	ENSG00000145863
ENSG00000187730	ENSG00000145864
ENSG00000187730	ENSG00000145888
ENSG00000187730	ENSG00000146276
ENSG00000187730	ENSG00000147402
ENSG00000187730	ENSG00000151834
ENSG00000187730	ENSG00000163285
ENSG00000187730	ENSG00000163288
ENSG00000187730	ENSG00000166206
ENSG00000187730	ENSG00000182256
ENSG00000187730	ENSG00000183185
ENSG00000187730	ENSG00000186297
ENSG00000187730	ENSG00000188828
ENSG00000187735	ENSG00000171703

ENSG00000187735	ENSG00000176896
ENSG00000187735	ENSG00000204219
ENSG00000187742	ENSG00000138593
ENSG00000187758	ENSG00000172955
ENSG00000187758	ENSG00000196344
ENSG00000187758	ENSG00000196616
ENSG00000187758	ENSG00000197894
ENSG00000187758	ENSG00000198099
ENSG00000187764	ENSG00000010319
ENSG00000187764	ENSG00000012171
ENSG00000187764	ENSG00000075213
ENSG00000187764	ENSG00000092421
ENSG00000187764	ENSG00000095539
ENSG00000187764	ENSG00000137872
ENSG00000187764	ENSG00000143434
ENSG00000187764	ENSG00000153993
ENSG00000187764	ENSG00000168758
ENSG00000187764	ENSG00000170381
ENSG00000187764	ENSG00000185033
ENSG00000187764	ENSG00000196189
ENSG00000187772	ENSG00000131914
ENSG00000187775	ENSG00000187240
ENSG00000187779	ENSG00000145860
ENSG00000187779	ENSG00000162298
ENSG00000187779	ENSG00000170881
ENSG00000187779	ENSG00000184505
ENSG00000187786	ENSG00000104903
ENSG00000187786	ENSG00000113196
ENSG00000187786	ENSG00000122691
ENSG00000187786	ENSG00000122859
ENSG00000187786	ENSG00000123307
ENSG00000187786	ENSG00000125878
ENSG00000187786	ENSG00000146618
ENSG00000187786	ENSG00000162367
ENSG00000187786	ENSG00000162992
ENSG00000187786	ENSG00000164107
ENSG00000187786	ENSG00000164600
ENSG00000187786	ENSG00000168267
ENSG00000187786	ENSG00000171532
ENSG00000187786	ENSG00000171786
ENSG00000187786	ENSG00000172238
ENSG00000187786	ENSG00000177551
ENSG00000187786	ENSG00000178403
ENSG00000187786	ENSG00000179774
ENSG00000187786	ENSG00000180535
ENSG00000187786	ENSG00000181965
ENSG00000187786	ENSG00000186051
ENSG00000187786	ENSG00000188686
ENSG00000187796	ENSG00000100065
ENSG00000187796	ENSG00000198286
ENSG00000187800	ENSG00000074660
ENSG00000187800	ENSG00000099910
ENSG00000187800	ENSG00000145794
ENSG00000187800	ENSG00000157890
ENSG00000187800	ENSG00000162591
ENSG00000187808	ENSG00000186212
ENSG00000187808	ENSG00000198142
ENSG00000187808	ENSG00000198944
ENSG00000187823	ENSG00000179300

ENSG00000187837	ENSG00000124575
ENSG00000187837	ENSG00000124610
ENSG00000187837	ENSG00000168298
ENSG00000187837	ENSG00000184357
ENSG00000187837	ENSG00000184897
ENSG00000187837	ENSG00000187475
ENSG00000187837	ENSG00000189060
ENSG00000187837	ENSG00000196440
ENSG00000187838	ENSG00000114698
ENSG00000187838	ENSG00000163746
ENSG00000187838	ENSG00000188313
ENSG00000187840	ENSG00000148730
ENSG00000187848	ENSG00000083454
ENSG00000187848	ENSG00000089041
ENSG00000187848	ENSG00000099957
ENSG00000187848	ENSG00000108405
ENSG00000187848	ENSG00000109991
ENSG00000187848	ENSG00000135124
ENSG00000187855	ENSG00000139352
ENSG00000187855	ENSG00000176009
ENSG00000187855	ENSG00000183734
ENSG00000187855	ENSG00000188108
ENSG00000187870	ENSG00000135119
ENSG00000187870	ENSG00000189050
ENSG00000187871	ENSG00000125861
ENSG00000187871	ENSG00000146013
ENSG00000187871	ENSG00000151892
ENSG00000187871	ENSG00000168546
ENSG00000187876	ENSG00000204235
ENSG00000187876	ENSG00000204544
ENSG00000187876	ENSG00000204707
ENSG00000187876	ENSG00000205461
ENSG00000187878	ENSG00000106608
ENSG00000187878	ENSG00000132357
ENSG00000187878	ENSG00000205365
ENSG00000187889	ENSG00000082074
ENSG00000187889	ENSG00000133246
ENSG00000187899	ENSG00000145425
ENSG00000187899	ENSG00000178429
ENSG00000187899	ENSG00000215836
ENSG00000187902	ENSG00000166737
ENSG00000187902	ENSG00000178343
ENSG00000187902	ENSG00000180730
ENSG00000187902	ENSG00000188803
ENSG00000187908	ENSG00000013725
ENSG00000187908	ENSG00000073754
ENSG00000187908	ENSG00000110448
ENSG00000187908	ENSG00000146700
ENSG00000187908	ENSG00000164099
ENSG00000187908	ENSG00000177575
ENSG00000187908	ENSG00000177675
ENSG00000187908	ENSG00000179954
ENSG00000187908	ENSG00000214279
ENSG00000187922	ENSG00000107317
ENSG00000187922	ENSG00000148346
ENSG00000187922	ENSG00000176919
ENSG00000187922	ENSG00000177984
ENSG00000187922	ENSG00000184925
ENSG00000187922	ENSG00000214402

ENSG00000187955	ENSG00000101203
ENSG00000187955	ENSG00000111799
ENSG00000187957	ENSG00000188107
ENSG00000187975	ENSG00000164484
ENSG00000187975	ENSG00000206432
ENSG00000187980	ENSG00000069764
ENSG00000187980	ENSG00000117215
ENSG00000187980	ENSG00000127472
ENSG00000187980	ENSG00000158786
ENSG00000187980	ENSG00000170890
ENSG00000187980	ENSG00000188257
ENSG00000187980	ENSG00000188784
ENSG00000187994	ENSG00000100599
ENSG00000187994	ENSG00000132669
ENSG00000187994	ENSG00000174791
ENSG00000188001	ENSG00000158109
ENSG00000188004	ENSG00000019102
ENSG00000188004	ENSG00000143167
ENSG00000188004	ENSG00000144847
ENSG00000188004	ENSG00000149564
ENSG00000188004	ENSG00000154639
ENSG00000188004	ENSG00000166250
ENSG00000188013	ENSG00000105419
ENSG00000188013	ENSG00000118707
ENSG00000188013	ENSG00000134138
ENSG00000188013	ENSG00000143995
ENSG00000188013	ENSG00000153779
ENSG00000188013	ENSG00000160199
ENSG00000188013	ENSG00000165495
ENSG00000188013	ENSG00000176679
ENSG00000188013	ENSG00000177426
ENSG00000188013	ENSG00000179277
ENSG00000188015	ENSG00000143546
ENSG00000188015	ENSG00000160307
ENSG00000188015	ENSG00000160678
ENSG00000188015	ENSG00000163191
ENSG00000188015	ENSG00000163220
ENSG00000188015	ENSG00000163221
ENSG00000188015	ENSG00000163993
ENSG00000188015	ENSG00000169906
ENSG00000188015	ENSG00000171643
ENSG00000188015	ENSG00000188643
ENSG00000188015	ENSG00000189171
ENSG00000188015	ENSG00000189334
ENSG00000188015	ENSG00000196154
ENSG00000188015	ENSG00000196420
ENSG00000188015	ENSG00000196754
ENSG00000188015	ENSG00000197747
ENSG00000188015	ENSG00000197956
ENSG00000188021	ENSG00000135018
ENSG00000188021	ENSG00000160803
ENSG00000188021	ENSG00000175518
ENSG00000188021	ENSG00000175520
ENSG00000188022	ENSG00000163646
ENSG00000188022	ENSG00000180745
ENSG00000188026	ENSG00000150977
ENSG00000188026	ENSG00000167705
ENSG00000188029	ENSG00000135047
ENSG00000188029	ENSG00000136943

ENSG00000188029	ENSG00000143387
ENSG00000188029	ENSG00000163131
ENSG00000188029	ENSG00000204437
ENSG00000188038	ENSG00000124785
ENSG00000188042	ENSG00000122644
ENSG00000188042	ENSG00000122872
ENSG00000188042	ENSG00000175906
ENSG00000188050	ENSG00000082996
ENSG00000188050	ENSG00000105428
ENSG00000188050	ENSG00000108523
ENSG00000188050	ENSG00000113269
ENSG00000188050	ENSG00000133135
ENSG00000188050	ENSG00000163162
ENSG00000188050	ENSG00000170153
ENSG00000188060	ENSG00000104388
ENSG00000188060	ENSG00000119396
ENSG00000188060	ENSG00000129472
ENSG00000188060	ENSG00000155961
ENSG00000188060	ENSG00000167578
ENSG00000188060	ENSG00000168118
ENSG00000188060	ENSG00000179331
ENSG00000188060	ENSG00000183940
ENSG00000188064	ENSG00000002745
ENSG00000188064	ENSG00000061492
ENSG00000188064	ENSG00000075290
ENSG00000188064	ENSG00000085741
ENSG00000188064	ENSG00000105989
ENSG00000188064	ENSG00000108379
ENSG00000188064	ENSG00000111186
ENSG00000188064	ENSG00000114251
ENSG00000188064	ENSG00000115596
ENSG00000188064	ENSG00000125084
ENSG00000188064	ENSG00000134245
ENSG00000188064	ENSG00000135925
ENSG00000188064	ENSG00000143816
ENSG00000188064	ENSG00000154342
ENSG00000188064	ENSG00000154764
ENSG00000188064	ENSG00000158955
ENSG00000188064	ENSG00000162552
ENSG00000188064	ENSG00000169884
ENSG00000188082	ENSG00000142089
ENSG00000188082	ENSG00000185201
ENSG00000188082	ENSG00000185885
ENSG00000188082	ENSG00000206013
ENSG00000188082	ENSG00000215096
ENSG00000188086	ENSG00000005001
ENSG00000188086	ENSG00000007038
ENSG00000188086	ENSG00000052344
ENSG00000188086	ENSG00000095917
ENSG00000188086	ENSG00000103355
ENSG00000188086	ENSG00000116176
ENSG00000188086	ENSG00000172236
ENSG00000188086	ENSG00000172382
ENSG00000188086	ENSG00000172460
ENSG00000188086	ENSG00000178055
ENSG00000188086	ENSG00000178226
ENSG00000188086	ENSG00000185888
ENSG00000188086	ENSG00000189099
ENSG00000188086	ENSG00000196364

ENSG00000188086	ENSG00000197253
ENSG00000188086	ENSG00000206549
ENSG00000188086	ENSG00000214877
ENSG00000188086	ENSG00000215148
ENSG00000188089	ENSG00000105499
ENSG00000188089	ENSG00000116711
ENSG00000188089	ENSG00000159337
ENSG00000188089	ENSG00000168907
ENSG00000188089	ENSG00000168970
ENSG00000188094	ENSG00000118181
ENSG00000188094	ENSG00000198940
ENSG00000188095	ENSG00000151379
ENSG00000188095	ENSG00000166823
ENSG00000188101	ENSG00000012779
ENSG00000188101	ENSG00000108839
ENSG00000188101	ENSG00000161905
ENSG00000188101	ENSG00000179148
ENSG00000188101	ENSG00000179477
ENSG00000188101	ENSG00000179593
ENSG00000188101	ENSG00000214859
ENSG00000188107	ENSG00000187957
ENSG00000188108	ENSG00000139352
ENSG00000188108	ENSG00000176009
ENSG00000188108	ENSG00000183734
ENSG00000188108	ENSG00000187855
ENSG00000188120	ENSG00000092345
ENSG00000188120	ENSG00000152430
ENSG00000188120	ENSG00000187191
ENSG00000188120	ENSG00000205916
ENSG00000188120	ENSG00000205944
ENSG00000188130	ENSG00000050748
ENSG00000188130	ENSG00000107643
ENSG00000188130	ENSG00000109339
ENSG00000188130	ENSG00000112062
ENSG00000188130	ENSG00000156711
ENSG00000188130	ENSG00000185386
ENSG00000188134	ENSG00000181039
ENSG00000188134	ENSG00000189127
ENSG00000188153	ENSG00000081052
ENSG00000188153	ENSG00000134871
ENSG00000188153	ENSG00000169031
ENSG00000188153	ENSG00000187498
ENSG00000188153	ENSG00000197565
ENSG00000188158	ENSG00000135540
ENSG00000188158	ENSG00000162522
ENSG00000188158	ENSG00000204131
ENSG00000188162	ENSG00000090920
ENSG00000188162	ENSG00000109927
ENSG00000188162	ENSG00000110799
ENSG00000188162	ENSG00000117983
ENSG00000188162	ENSG00000146839
ENSG00000188162	ENSG00000184956
ENSG00000188162	ENSG00000196292
ENSG00000188162	ENSG00000197558
ENSG00000188162	ENSG00000198788
ENSG00000188174	ENSG00000122566
ENSG00000188174	ENSG00000135486
ENSG00000188174	ENSG00000139675
ENSG00000188174	ENSG00000170144

ENSG00000188174	ENSG00000176825
ENSG00000188174	ENSG00000177219
ENSG00000188174	ENSG00000177733
ENSG00000188174	ENSG00000206228
ENSG00000188174	ENSG00000212961
ENSG00000188174	ENSG00000214284
ENSG00000188174	ENSG00000214361
ENSG00000188174	ENSG00000214592
ENSG00000188174	ENSG00000214653
ENSG00000188174	ENSG00000215222
ENSG00000188174	ENSG00000215492
ENSG00000188174	ENSG00000215870
ENSG00000188174	ENSG00000215876
ENSG00000188174	ENSG00000215885
ENSG00000188175	ENSG00000165478
ENSG00000188175	ENSG00000176395
ENSG00000188176	ENSG00000100014
ENSG00000188176	ENSG00000128487
ENSG00000188176	ENSG00000183963
ENSG00000188176	ENSG00000214872
ENSG00000188177	ENSG00000130749
ENSG00000188191	ENSG00000005249
ENSG00000188191	ENSG00000108946
ENSG00000188191	ENSG00000114302
ENSG00000188211	ENSG00000103855
ENSG00000188211	ENSG00000114013
ENSG00000188211	ENSG00000114455
ENSG00000188211	ENSG00000120217
ENSG00000188211	ENSG00000134258
ENSG00000188211	ENSG00000160223
ENSG00000188211	ENSG00000186470
ENSG00000188211	ENSG00000197646
ENSG00000188211	ENSG00000204290
ENSG00000188211	ENSG00000204655
ENSG00000188211	ENSG00000215631
ENSG00000188211	ENSG00000215811
ENSG00000188211	ENSG00000215889
ENSG00000188234	ENSG00000108262
ENSG00000188234	ENSG00000133612
ENSG00000188234	ENSG00000135439
ENSG00000188234	ENSG00000139436
ENSG00000188234	ENSG00000151303
ENSG00000188234	ENSG00000157985
ENSG00000188234	ENSG00000172650
ENSG00000188234	ENSG00000174194
ENSG00000188234	ENSG00000198035
ENSG00000188234	ENSG00000204149
ENSG00000188234	ENSG00000204169
ENSG00000188234	ENSG00000204172
ENSG00000188257	ENSG00000069764
ENSG00000188257	ENSG00000117215
ENSG00000188257	ENSG00000127472
ENSG00000188257	ENSG00000158786
ENSG00000188257	ENSG00000170890
ENSG00000188257	ENSG00000187980
ENSG00000188257	ENSG00000188784
ENSG00000188282	ENSG00000163820
ENSG00000188290	ENSG00000069812
ENSG00000188290	ENSG00000114315

ENSG00000188290	ENSG00000144485
ENSG00000188290	ENSG00000173673
ENSG00000188290	ENSG00000179111
ENSG00000188290	ENSG00000197921
ENSG00000188306	ENSG00000033122
ENSG00000188306	ENSG00000112851
ENSG00000188306	ENSG00000137269
ENSG00000188306	ENSG00000147324
ENSG00000188306	ENSG00000180900
ENSG00000188313	ENSG00000114698
ENSG00000188313	ENSG00000163746
ENSG00000188313	ENSG00000187838
ENSG00000188322	ENSG00000187550
ENSG00000188338	ENSG00000017483
ENSG00000188338	ENSG00000103042
ENSG00000188338	ENSG00000111371
ENSG00000188338	ENSG00000134294
ENSG00000188338	ENSG00000139209
ENSG00000188338	ENSG00000139974
ENSG00000188338	ENSG00000166558
ENSG00000188338	ENSG00000169507
ENSG00000188343	ENSG00000153789
ENSG00000188343	ENSG00000157021
ENSG00000188372	ENSG00000146707
ENSG00000188385	ENSG00000152969
ENSG00000188385	ENSG00000176049
ENSG00000188388	ENSG00000140478
ENSG00000188388	ENSG00000153666
ENSG00000188388	ENSG00000156363
ENSG00000188388	ENSG00000159289
ENSG00000188388	ENSG00000167110
ENSG00000188388	ENSG00000167195
ENSG00000188388	ENSG00000174450
ENSG00000188388	ENSG00000175265
ENSG00000188388	ENSG00000181984
ENSG00000188388	ENSG00000184206
ENSG00000188388	ENSG00000186322
ENSG00000188388	ENSG00000188532
ENSG00000188388	ENSG00000188626
ENSG00000188388	ENSG00000197092
ENSG00000188388	ENSG00000197414
ENSG00000188388	ENSG00000214348
ENSG00000188388	ENSG00000215252
ENSG00000188388	ENSG00000215405
ENSG00000188394	ENSG00000203737
ENSG00000188404	ENSG00000007908
ENSG00000188425	ENSG00000187556
ENSG00000188425	ENSG00000188613
ENSG00000188459	ENSG00000112290
ENSG00000188459	ENSG00000132970
ENSG00000188459	ENSG00000158195
ENSG00000188483	ENSG00000160888
ENSG00000188483	ENSG00000162783
ENSG00000188486	ENSG00000111332
ENSG00000188486	ENSG00000124642
ENSG00000188486	ENSG00000137259
ENSG00000188486	ENSG00000158403
ENSG00000188486	ENSG00000164508
ENSG00000188486	ENSG00000168274

ENSG00000188486	ENSG00000180573
ENSG00000188486	ENSG00000181218
ENSG00000188486	ENSG00000182611
ENSG00000188486	ENSG00000183558
ENSG00000188486	ENSG00000184260
ENSG00000188486	ENSG00000184270
ENSG00000188486	ENSG00000184348
ENSG00000188486	ENSG00000184825
ENSG00000188486	ENSG00000185978
ENSG00000188486	ENSG00000196747
ENSG00000188486	ENSG00000196787
ENSG00000188486	ENSG00000196866
ENSG00000188486	ENSG00000198082
ENSG00000188486	ENSG00000198307
ENSG00000188486	ENSG00000198374
ENSG00000188486	ENSG00000203812
ENSG00000188486	ENSG00000205070
ENSG00000188486	ENSG00000205079
ENSG00000188488	ENSG00000100665
ENSG00000188488	ENSG00000123561
ENSG00000188488	ENSG00000135744
ENSG00000188488	ENSG00000165951
ENSG00000188488	ENSG00000165953
ENSG00000188488	ENSG00000170054
ENSG00000188488	ENSG00000186910
ENSG00000188488	ENSG00000187483
ENSG00000188488	ENSG00000196136
ENSG00000188488	ENSG00000197249
ENSG00000188505	ENSG00000116661
ENSG00000188505	ENSG00000116663
ENSG00000188505	ENSG00000132879
ENSG00000188505	ENSG00000161241
ENSG00000188505	ENSG00000161243
ENSG00000188512	ENSG00000172115
ENSG00000188512	ENSG00000174440
ENSG00000188512	ENSG00000214810
ENSG00000188517	ENSG00000050767
ENSG00000188517	ENSG00000197467
ENSG00000188522	ENSG00000101447
ENSG00000188522	ENSG00000105523
ENSG00000188522	ENSG00000125998
ENSG00000188522	ENSG00000133477
ENSG00000188522	ENSG00000147689
ENSG00000188522	ENSG00000168143
ENSG00000188522	ENSG00000180921
ENSG00000188532	ENSG00000140478
ENSG00000188532	ENSG00000153666
ENSG00000188532	ENSG00000156363
ENSG00000188532	ENSG00000159289
ENSG00000188532	ENSG00000167110
ENSG00000188532	ENSG00000167195
ENSG00000188532	ENSG00000174450
ENSG00000188532	ENSG00000175265
ENSG00000188532	ENSG00000181984
ENSG00000188532	ENSG00000184206
ENSG00000188532	ENSG00000186322
ENSG00000188532	ENSG00000188388
ENSG00000188532	ENSG00000188626
ENSG00000188532	ENSG00000197092

ENSG00000188532	ENSG00000197414
ENSG00000188532	ENSG00000214348
ENSG00000188532	ENSG00000215252
ENSG00000188532	ENSG00000215405
ENSG00000188536	ENSG00000086506
ENSG00000188536	ENSG00000130656
ENSG00000188536	ENSG00000206172
ENSG00000188536	ENSG00000206177
ENSG00000188536	ENSG00000206178
ENSG00000188549	ENSG00000105321
ENSG00000188554	ENSG00000215691
ENSG00000188559	ENSG00000103197
ENSG00000188559	ENSG00000174373
ENSG00000188580	ENSG00000084628
ENSG00000188580	ENSG00000101198
ENSG00000188580	ENSG00000185942
ENSG00000188599	ENSG00000008710
ENSG00000188599	ENSG00000078795
ENSG00000188599	ENSG00000107593
ENSG00000188599	ENSG00000118762
ENSG00000188599	ENSG00000130943
ENSG00000188599	ENSG00000158683
ENSG00000188599	ENSG00000166473
ENSG00000188599	ENSG00000183458
ENSG00000188599	ENSG00000183793
ENSG00000188599	ENSG00000187008
ENSG00000188612	ENSG00000116030
ENSG00000188612	ENSG00000177688
ENSG00000188612	ENSG00000184763
ENSG00000188612	ENSG00000184900
ENSG00000188620	ENSG00000188816
ENSG00000188620	ENSG00000215612
ENSG00000188626	ENSG00000140478
ENSG00000188626	ENSG00000153666
ENSG00000188626	ENSG00000156363
ENSG00000188626	ENSG00000159289
ENSG00000188626	ENSG00000167110
ENSG00000188626	ENSG00000167195
ENSG00000188626	ENSG00000174450
ENSG00000188626	ENSG00000175265
ENSG00000188626	ENSG00000181984
ENSG00000188626	ENSG00000184206
ENSG00000188626	ENSG00000186322
ENSG00000188626	ENSG00000188388
ENSG00000188626	ENSG00000188532
ENSG00000188626	ENSG00000197092
ENSG00000188626	ENSG00000197414
ENSG00000188626	ENSG00000214348
ENSG00000188626	ENSG00000215252
ENSG00000188626	ENSG00000215405
ENSG00000188643	ENSG00000143546
ENSG00000188643	ENSG00000160307
ENSG00000188643	ENSG00000160678
ENSG00000188643	ENSG00000163191
ENSG00000188643	ENSG00000163220
ENSG00000188643	ENSG00000163221
ENSG00000188643	ENSG00000163993
ENSG00000188643	ENSG00000169906
ENSG00000188643	ENSG00000171643

ENSG00000188643	ENSG00000188015
ENSG00000188643	ENSG00000189171
ENSG00000188643	ENSG00000189334
ENSG00000188643	ENSG00000196154
ENSG00000188643	ENSG00000196420
ENSG00000188643	ENSG00000196754
ENSG00000188643	ENSG00000197747
ENSG00000188643	ENSG00000197956
ENSG00000188659	ENSG00000155875
ENSG00000188659	ENSG00000214545
ENSG00000188672	ENSG00000112077
ENSG00000188672	ENSG00000132677
ENSG00000188672	ENSG00000140519
ENSG00000188672	ENSG00000187010
ENSG00000188676	ENSG00000131203
ENSG00000188677	ENSG00000138964
ENSG00000188677	ENSG00000197702
ENSG00000188683	ENSG00000026950
ENSG00000188683	ENSG00000111801
ENSG00000188683	ENSG00000112763
ENSG00000188683	ENSG00000113303
ENSG00000188683	ENSG00000121236
ENSG00000188683	ENSG00000124508
ENSG00000188683	ENSG00000124549
ENSG00000188683	ENSG00000124557
ENSG00000188683	ENSG00000128250
ENSG00000188683	ENSG00000128253
ENSG00000188683	ENSG00000128276
ENSG00000188683	ENSG00000128482
ENSG00000188683	ENSG00000132256
ENSG00000188683	ENSG00000132274
ENSG00000188683	ENSG00000164010
ENSG00000188683	ENSG00000165810
ENSG00000188683	ENSG00000168903
ENSG00000188683	ENSG00000176422
ENSG00000188683	ENSG00000179046
ENSG00000188683	ENSG00000182805
ENSG00000188683	ENSG00000183439
ENSG00000188683	ENSG00000183718
ENSG00000188683	ENSG00000187574
ENSG00000188683	ENSG00000204614
ENSG00000188683	ENSG00000204616
ENSG00000188683	ENSG00000204618
ENSG00000188683	ENSG00000205198
ENSG00000188683	ENSG00000214693
ENSG00000188686	ENSG00000104903
ENSG00000188686	ENSG00000113196
ENSG00000188686	ENSG00000122691
ENSG00000188686	ENSG00000122859
ENSG00000188686	ENSG00000123307
ENSG00000188686	ENSG00000125878
ENSG00000188686	ENSG00000146618
ENSG00000188686	ENSG00000162367
ENSG00000188686	ENSG00000162992
ENSG00000188686	ENSG00000164107
ENSG00000188686	ENSG00000164600
ENSG00000188686	ENSG00000168267
ENSG00000188686	ENSG00000171532
ENSG00000188686	ENSG00000171786

ENSG00000188686	ENSG00000172238
ENSG00000188686	ENSG00000177551
ENSG00000188686	ENSG00000178403
ENSG00000188686	ENSG00000179774
ENSG00000188686	ENSG00000180535
ENSG00000188686	ENSG00000181965
ENSG00000188686	ENSG00000186051
ENSG00000188686	ENSG00000187786
ENSG00000188687	ENSG00000004939
ENSG00000188687	ENSG00000033867
ENSG00000188687	ENSG00000050438
ENSG00000188687	ENSG00000080493
ENSG00000188687	ENSG00000088836
ENSG00000188687	ENSG00000113073
ENSG00000188687	ENSG00000114923
ENSG00000188687	ENSG00000144290
ENSG00000188687	ENSG00000164889
ENSG00000188706	ENSG00000175048
ENSG00000188706	ENSG00000204160
ENSG00000188716	ENSG00000079393
ENSG00000188716	ENSG00000108861
ENSG00000188716	ENSG00000133878
ENSG00000188716	ENSG00000188542
ENSG00000188730	ENSG00000174453
ENSG00000188735	ENSG00000189077
ENSG00000188747	ENSG00000116701
ENSG00000188755	ENSG00000128438
ENSG00000188755	ENSG00000148429
ENSG00000188755	ENSG00000161583
ENSG00000188755	ENSG00000174115
ENSG00000188755	ENSG00000185128
ENSG00000188755	ENSG00000188849
ENSG00000188755	ENSG00000189309
ENSG00000188755	ENSG00000197681
ENSG00000188755	ENSG00000204471
ENSG00000188755	ENSG00000205019
ENSG00000188755	ENSG00000213474
ENSG00000188755	ENSG00000215625
ENSG00000188755	ENSG00000215730
ENSG00000188755	ENSG00000215748
ENSG00000188763	ENSG00000104290
ENSG00000188763	ENSG00000106483
ENSG00000188763	ENSG00000111432
ENSG00000188763	ENSG00000155760
ENSG00000188763	ENSG00000157240
ENSG00000188763	ENSG00000162998
ENSG00000188763	ENSG00000163251
ENSG00000188763	ENSG00000164930
ENSG00000188763	ENSG00000174804
ENSG00000188763	ENSG00000177283
ENSG00000188763	ENSG00000180340
ENSG00000188766	ENSG00000125753
ENSG00000188766	ENSG00000154380
ENSG00000188766	ENSG00000166068
ENSG00000188766	ENSG00000196405
ENSG00000188766	ENSG00000198369
ENSG00000188770	ENSG00000083782
ENSG00000188770	ENSG00000106809
ENSG00000188778	ENSG00000043591

ENSG00000188778	ENSG00000102468
ENSG00000188778	ENSG00000113749
ENSG00000188778	ENSG00000125631
ENSG00000188778	ENSG00000135312
ENSG00000188778	ENSG00000135914
ENSG00000188778	ENSG00000147246
ENSG00000188778	ENSG00000150594
ENSG00000188778	ENSG00000157219
ENSG00000188778	ENSG00000164270
ENSG00000188778	ENSG00000168830
ENSG00000188778	ENSG00000169252
ENSG00000188778	ENSG00000175658
ENSG00000188778	ENSG00000178394
ENSG00000188778	ENSG00000179097
ENSG00000188778	ENSG00000179546
ENSG00000188778	ENSG00000181210
ENSG00000188778	ENSG00000184303
ENSG00000188778	ENSG00000184845
ENSG00000188778	ENSG00000185321
ENSG00000188779	ENSG00000136603
ENSG00000188779	ENSG00000157933
ENSG00000188779	ENSG00000215474
ENSG00000188782	ENSG00000152705
ENSG00000188783	ENSG00000122176
ENSG00000188783	ENSG00000127083
ENSG00000188783	ENSG00000139330
ENSG00000188784	ENSG00000069764
ENSG00000188784	ENSG00000117215
ENSG00000188784	ENSG00000127472
ENSG00000188784	ENSG00000158786
ENSG00000188784	ENSG00000170890
ENSG00000188784	ENSG00000187980
ENSG00000188784	ENSG00000188257
ENSG00000188803	ENSG00000166737
ENSG00000188803	ENSG00000178343
ENSG00000188803	ENSG00000180730
ENSG00000188803	ENSG00000187902
ENSG00000188816	ENSG00000188620
ENSG00000188816	ENSG00000215612
ENSG00000188818	ENSG00000159714
ENSG00000188818	ENSG00000206077
ENSG00000188820	ENSG00000138172
ENSG00000188820	ENSG00000164451
ENSG00000188820	ENSG00000178033
ENSG00000188820	ENSG00000183128
ENSG00000188820	ENSG00000185933
ENSG00000188822	ENSG00000064547
ENSG00000188822	ENSG00000118432
ENSG00000188822	ENSG00000125910
ENSG00000188822	ENSG00000132975
ENSG00000188822	ENSG00000146360
ENSG00000188822	ENSG00000170989
ENSG00000188822	ENSG00000171517
ENSG00000188822	ENSG00000175898
ENSG00000188822	ENSG00000180739
ENSG00000188822	ENSG00000181773
ENSG00000188822	ENSG00000198121
ENSG00000188822	ENSG00000213694
ENSG00000188828	ENSG00000011677

ENSG00000188828	ENSG00000022355
ENSG00000188828	ENSG00000094755
ENSG00000188828	ENSG00000101958
ENSG00000188828	ENSG00000102287
ENSG00000188828	ENSG00000109158
ENSG00000188828	ENSG00000109738
ENSG00000188828	ENSG00000111886
ENSG00000188828	ENSG00000113327
ENSG00000188828	ENSG00000145451
ENSG00000188828	ENSG00000145863
ENSG00000188828	ENSG00000145864
ENSG00000188828	ENSG00000145888
ENSG00000188828	ENSG00000146276
ENSG00000188828	ENSG00000147402
ENSG00000188828	ENSG00000151834
ENSG00000188828	ENSG00000163285
ENSG00000188828	ENSG00000163288
ENSG00000188828	ENSG00000166206
ENSG00000188828	ENSG00000182256
ENSG00000188828	ENSG00000183185
ENSG00000188828	ENSG00000186297
ENSG00000188828	ENSG00000187730
ENSG00000188833	ENSG00000054179
ENSG00000188833	ENSG00000138185
ENSG00000188833	ENSG00000168032
ENSG00000188841	ENSG00000007968
ENSG00000188841	ENSG00000101412
ENSG00000188841	ENSG00000112242
ENSG00000188841	ENSG00000133740
ENSG00000188841	ENSG00000169016
ENSG00000188841	ENSG00000205250
ENSG00000188849	ENSG00000128438
ENSG00000188849	ENSG00000148429
ENSG00000188849	ENSG00000161583
ENSG00000188849	ENSG00000174115
ENSG00000188849	ENSG00000185128
ENSG00000188849	ENSG00000188755
ENSG00000188849	ENSG00000189309
ENSG00000188849	ENSG00000197681
ENSG00000188849	ENSG00000204471
ENSG00000188849	ENSG00000205019
ENSG00000188849	ENSG00000213474
ENSG00000188849	ENSG00000215625
ENSG00000188849	ENSG00000215730
ENSG00000188849	ENSG00000215748
ENSG00000188859	ENSG00000126882
ENSG00000188888	ENSG00000151025
ENSG00000188906	ENSG00000154237
ENSG00000188910	ENSG00000121634
ENSG00000188910	ENSG00000121742
ENSG00000188910	ENSG00000121743
ENSG00000188910	ENSG00000131233
ENSG00000188910	ENSG00000135355
ENSG00000188910	ENSG00000143140
ENSG00000188910	ENSG00000152661
ENSG00000188910	ENSG00000159248
ENSG00000188910	ENSG00000164411
ENSG00000188910	ENSG00000165474
ENSG00000188910	ENSG00000169562

ENSG00000188910	ENSG00000176402
ENSG00000188910	ENSG00000177291
ENSG00000188910	ENSG00000182963
ENSG00000188910	ENSG00000183153
ENSG00000188910	ENSG00000187513
ENSG00000188910	ENSG00000189280
ENSG00000188910	ENSG00000189433
ENSG00000188910	ENSG00000198835
ENSG00000188910	ENSG00000203733
ENSG00000188916	ENSG00000204767
ENSG00000188921	ENSG00000074696
ENSG00000188921	ENSG00000165996
ENSG00000188921	ENSG00000206527
ENSG00000188937	ENSG00000100399
ENSG00000188984	ENSG00000114771
ENSG00000188984	ENSG00000144959
ENSG00000188984	ENSG00000197953
ENSG00000188984	ENSG00000204518
ENSG00000188985	ENSG00000178700
ENSG00000188987	ENSG00000124529
ENSG00000188987	ENSG00000124578
ENSG00000188987	ENSG00000158406
ENSG00000188987	ENSG00000182217
ENSG00000188987	ENSG00000183941
ENSG00000188987	ENSG00000196176
ENSG00000188987	ENSG00000197061
ENSG00000188987	ENSG00000197238
ENSG00000188987	ENSG00000197837
ENSG00000188987	ENSG00000197914
ENSG00000188987	ENSG00000198327
ENSG00000188987	ENSG00000198339
ENSG00000188987	ENSG00000198518
ENSG00000188987	ENSG00000198558
ENSG00000188992	ENSG00000101670
ENSG00000188992	ENSG00000144837
ENSG00000188992	ENSG00000163898
ENSG00000188992	ENSG00000165862
ENSG00000188992	ENSG00000166035
ENSG00000188992	ENSG00000175445
ENSG00000188992	ENSG00000175535
ENSG00000188992	ENSG00000187021
ENSG00000188992	ENSG00000203837
ENSG00000188994	ENSG00000117000
ENSG00000188997	ENSG00000134504
ENSG00000188997	ENSG00000151364
ENSG00000188997	ENSG00000153885
ENSG00000188997	ENSG00000155729
ENSG00000188997	ENSG00000168301
ENSG00000188997	ENSG00000168676
ENSG00000188997	ENSG00000178695
ENSG00000188997	ENSG00000180332
ENSG00000188997	ENSG00000183775
ENSG00000188997	ENSG00000183783
ENSG00000188997	ENSG00000198553
ENSG00000188997	ENSG00000213859
ENSG00000189037	ENSG00000161326
ENSG00000189037	ENSG00000167065
ENSG00000189043	ENSG00000166920
ENSG00000189043	ENSG00000185633

ENSG00000189043	ENSG00000212665
ENSG00000189045	ENSG00000166839
ENSG00000189050	ENSG00000135119
ENSG00000189050	ENSG00000187870
ENSG00000189052	ENSG00000104818
ENSG00000189052	ENSG00000104826
ENSG00000189052	ENSG00000104827
ENSG00000189052	ENSG00000131808
ENSG00000189052	ENSG00000134200
ENSG00000189052	ENSG00000179600
ENSG00000189052	ENSG00000196337
ENSG00000189052	ENSG00000204748
ENSG00000189052	ENSG00000213030
ENSG00000189067	ENSG00000089486
ENSG00000189067	ENSG00000215079
ENSG00000189077	ENSG00000188735
ENSG00000189099	ENSG00000005001
ENSG00000189099	ENSG00000007038
ENSG00000189099	ENSG00000052344
ENSG00000189099	ENSG00000095917
ENSG00000189099	ENSG00000103355
ENSG00000189099	ENSG00000116176
ENSG00000189099	ENSG00000172236
ENSG00000189099	ENSG00000172382
ENSG00000189099	ENSG00000172460
ENSG00000189099	ENSG00000178055
ENSG00000189099	ENSG00000178226
ENSG00000189099	ENSG00000185888
ENSG00000189099	ENSG00000188086
ENSG00000189099	ENSG00000196364
ENSG00000189099	ENSG00000197253
ENSG00000189099	ENSG00000206549
ENSG00000189099	ENSG00000214877
ENSG00000189099	ENSG00000215148
ENSG00000189108	ENSG00000169306
ENSG00000189120	ENSG00000067082
ENSG00000189120	ENSG00000102554
ENSG00000189120	ENSG00000105610
ENSG00000189120	ENSG00000127528
ENSG00000189120	ENSG00000129911
ENSG00000189120	ENSG00000136826
ENSG00000189120	ENSG00000139651
ENSG00000189120	ENSG00000155090
ENSG00000189120	ENSG00000164651
ENSG00000189120	ENSG00000167182
ENSG00000189120	ENSG00000170374
ENSG00000189120	ENSG00000171872
ENSG00000189120	ENSG00000172059
ENSG00000189120	ENSG00000174595
ENSG00000189120	ENSG00000197294
ENSG00000189120	ENSG00000197550
ENSG00000189120	ENSG00000204335
ENSG00000189127	ENSG00000181039
ENSG00000189127	ENSG00000188134
ENSG00000189136	ENSG00000140367
ENSG00000189136	ENSG00000160714
ENSG00000189136	ENSG00000215218
ENSG00000189143	ENSG00000013297
ENSG00000189143	ENSG00000066405

ENSG00000189143	ENSG00000106404
ENSG00000189143	ENSG00000113946
ENSG00000189143	ENSG00000134873
ENSG00000189143	ENSG00000156282
ENSG00000189143	ENSG00000156284
ENSG00000189143	ENSG00000159261
ENSG00000189143	ENSG00000163347
ENSG00000189143	ENSG00000164007
ENSG00000189143	ENSG00000165215
ENSG00000189143	ENSG00000165376
ENSG00000189143	ENSG00000171217
ENSG00000189143	ENSG00000177300
ENSG00000189143	ENSG00000181885
ENSG00000189143	ENSG00000184113
ENSG00000189143	ENSG00000184697
ENSG00000189143	ENSG00000185758
ENSG00000189143	ENSG00000189118
ENSG00000189143	ENSG00000213937
ENSG00000189156	ENSG00000096092
ENSG00000189156	ENSG00000111843
ENSG00000189156	ENSG00000137210
ENSG00000189156	ENSG00000214881
ENSG00000189159	ENSG00000206053
ENSG00000189162	ENSG00000136487
ENSG00000189162	ENSG00000136488
ENSG00000189162	ENSG00000172179
ENSG00000189162	ENSG00000204414
ENSG00000189162	ENSG00000213218
ENSG00000189167	ENSG00000182223
ENSG00000189171	ENSG00000143546
ENSG00000189171	ENSG00000160307
ENSG00000189171	ENSG00000160678
ENSG00000189171	ENSG00000163191
ENSG00000189171	ENSG00000163220
ENSG00000189171	ENSG00000163221
ENSG00000189171	ENSG00000163993
ENSG00000189171	ENSG00000169906
ENSG00000189171	ENSG00000171643
ENSG00000189171	ENSG00000188015
ENSG00000189171	ENSG00000188643
ENSG00000189171	ENSG00000189334
ENSG00000189171	ENSG00000196154
ENSG00000189171	ENSG00000196420
ENSG00000189171	ENSG00000196754
ENSG00000189171	ENSG00000197747
ENSG00000189171	ENSG00000197956
ENSG00000189182	ENSG00000135443
ENSG00000189182	ENSG00000135477
ENSG00000189182	ENSG00000135480
ENSG00000189182	ENSG00000139648
ENSG00000189182	ENSG00000161849
ENSG00000189182	ENSG00000161850
ENSG00000189182	ENSG00000167767
ENSG00000189182	ENSG00000167768
ENSG00000189182	ENSG00000170421
ENSG00000189182	ENSG00000170423
ENSG00000189182	ENSG00000170442
ENSG00000189182	ENSG00000170454
ENSG00000189182	ENSG00000170465

ENSG00000189182	ENSG00000170477
ENSG00000189182	ENSG00000170484
ENSG00000189182	ENSG00000170486
ENSG00000189182	ENSG00000170523
ENSG00000189182	ENSG00000172867
ENSG00000189182	ENSG00000185069
ENSG00000189182	ENSG00000185479
ENSG00000189182	ENSG00000185640
ENSG00000189182	ENSG00000186049
ENSG00000189182	ENSG00000186081
ENSG00000189182	ENSG00000186442
ENSG00000189182	ENSG00000205420
ENSG00000189182	ENSG00000205426
ENSG00000189182	ENSG00000214233
ENSG00000189182	ENSG00000214323
ENSG00000189182	ENSG00000214477
ENSG00000189182	ENSG00000214752
ENSG00000189182	ENSG00000214974
ENSG00000189182	ENSG00000215287
ENSG00000189182	ENSG00000215847
ENSG00000189184	ENSG00000118946
ENSG00000189184	ENSG00000136099
ENSG00000189184	ENSG00000138650
ENSG00000189184	ENSG00000165194
ENSG00000189203	ENSG00000185475
ENSG00000189221	ENSG00000069535
ENSG00000189221	ENSG00000104951
ENSG00000189266	ENSG00000146278
ENSG00000189266	ENSG00000215700
ENSG00000189280	ENSG00000121634
ENSG00000189280	ENSG00000121742
ENSG00000189280	ENSG00000121743
ENSG00000189280	ENSG00000131233
ENSG00000189280	ENSG00000135355
ENSG00000189280	ENSG00000143140
ENSG00000189280	ENSG00000152661
ENSG00000189280	ENSG00000159248
ENSG00000189280	ENSG00000164411
ENSG00000189280	ENSG00000165474
ENSG00000189280	ENSG00000169562
ENSG00000189280	ENSG00000176402
ENSG00000189280	ENSG00000177291
ENSG00000189280	ENSG00000182963
ENSG00000189280	ENSG00000183153
ENSG00000189280	ENSG00000187513
ENSG00000189280	ENSG00000188910
ENSG00000189280	ENSG00000189433
ENSG00000189280	ENSG00000198835
ENSG00000189280	ENSG00000203733
ENSG00000189285	ENSG00000121621
ENSG00000189292	ENSG00000196711
ENSG00000189299	ENSG00000109101
ENSG00000189299	ENSG00000139445
ENSG00000189299	ENSG00000176302
ENSG00000189309	ENSG00000128438
ENSG00000189309	ENSG00000148429
ENSG00000189309	ENSG00000161583
ENSG00000189309	ENSG00000174115
ENSG00000189309	ENSG00000185128

ENSG00000189309	ENSG00000188755
ENSG00000189309	ENSG00000188849
ENSG00000189309	ENSG00000197681
ENSG00000189309	ENSG00000204471
ENSG00000189309	ENSG00000205019
ENSG00000189309	ENSG00000213474
ENSG00000189309	ENSG00000215625
ENSG00000189309	ENSG00000215730
ENSG00000189309	ENSG00000215748
ENSG00000189319	ENSG00000120709
ENSG00000189319	ENSG00000174137
ENSG00000189320	ENSG00000196666
ENSG00000189332	ENSG00000122696
ENSG00000189332	ENSG00000141437
ENSG00000189332	ENSG00000176274
ENSG00000189334	ENSG00000143546
ENSG00000189334	ENSG00000160307
ENSG00000189334	ENSG00000160678
ENSG00000189334	ENSG00000163191
ENSG00000189334	ENSG00000163220
ENSG00000189334	ENSG00000163221
ENSG00000189334	ENSG00000163993
ENSG00000189334	ENSG00000169906
ENSG00000189334	ENSG00000171643
ENSG00000189334	ENSG00000188015
ENSG00000189334	ENSG00000188643
ENSG00000189334	ENSG00000189171
ENSG00000189334	ENSG00000196154
ENSG00000189334	ENSG00000196420
ENSG00000189334	ENSG00000196754
ENSG00000189334	ENSG00000197747
ENSG00000189334	ENSG00000197956
ENSG00000189350	ENSG00000198718
ENSG00000189362	ENSG00000166881
ENSG00000189403	ENSG00000029993
ENSG00000189403	ENSG00000100118
ENSG00000189403	ENSG00000124097
ENSG00000189403	ENSG00000132967
ENSG00000189403	ENSG00000164104
ENSG00000189403	ENSG00000173961
ENSG00000189403	ENSG00000176256
ENSG00000189403	ENSG00000180212
ENSG00000189403	ENSG00000182181
ENSG00000189403	ENSG00000182711
ENSG00000189403	ENSG00000214364
ENSG00000189403	ENSG00000214852
ENSG00000189403	ENSG00000215308
ENSG00000189409	ENSG00000008516
ENSG00000189409	ENSG00000087245
ENSG00000189409	ENSG00000099953
ENSG00000189409	ENSG00000100985
ENSG00000189409	ENSG00000102996
ENSG00000189409	ENSG00000110347
ENSG00000189409	ENSG00000118113
ENSG00000189409	ENSG00000123342
ENSG00000189409	ENSG00000125966
ENSG00000189409	ENSG00000129270
ENSG00000189409	ENSG00000137673
ENSG00000189409	ENSG00000137674

ENSG00000189409	ENSG00000137675
ENSG00000189409	ENSG00000137745
ENSG00000189409	ENSG00000149968
ENSG00000189409	ENSG00000154485
ENSG00000189409	ENSG00000156103
ENSG00000189409	ENSG00000157227
ENSG00000189409	ENSG00000166670
ENSG00000189409	ENSG00000167346
ENSG00000189409	ENSG00000196611
ENSG00000189409	ENSG00000198598
ENSG00000189409	ENSG00000215914
ENSG00000189431	ENSG00000198774
ENSG00000189433	ENSG00000121634
ENSG00000189433	ENSG00000121742
ENSG00000189433	ENSG00000121743
ENSG00000189433	ENSG00000131233
ENSG00000189433	ENSG00000135355
ENSG00000189433	ENSG00000143140
ENSG00000189433	ENSG00000152661
ENSG00000189433	ENSG00000159248
ENSG00000189433	ENSG00000164411
ENSG00000189433	ENSG00000165474
ENSG00000189433	ENSG00000169562
ENSG00000189433	ENSG00000176402
ENSG00000189433	ENSG00000177291
ENSG00000189433	ENSG00000182963
ENSG00000189433	ENSG00000183153
ENSG00000189433	ENSG00000187513
ENSG00000189433	ENSG00000188910
ENSG00000189433	ENSG00000189280
ENSG00000189433	ENSG00000198835
ENSG00000189433	ENSG00000203733
ENSG00000189437	ENSG00000004399
ENSG00000189437	ENSG00000076356
ENSG00000189437	ENSG00000114554
ENSG00000189437	ENSG00000130827
ENSG00000189437	ENSG00000136040
ENSG00000189437	ENSG00000164050
ENSG00000189437	ENSG00000196576
ENSG00000189437	ENSG00000198753
ENSG00000196083	ENSG00000115590
ENSG00000196083	ENSG00000115594
ENSG00000196083	ENSG00000115598
ENSG00000196083	ENSG00000115602
ENSG00000196083	ENSG00000115604
ENSG00000196083	ENSG00000115607
ENSG00000196083	ENSG00000185187
ENSG00000196090	ENSG00000060656
ENSG00000196090	ENSG00000105426
ENSG00000196090	ENSG00000142949
ENSG00000196090	ENSG00000152894
ENSG00000196090	ENSG00000153707
ENSG00000196090	ENSG00000173482
ENSG00000196092	ENSG00000075891
ENSG00000196092	ENSG00000125618
ENSG00000196092	ENSG00000125813
ENSG00000196092	ENSG00000198807
ENSG00000196104	ENSG00000019582
ENSG00000196104	ENSG00000107742

ENSG00000196104	ENSG00000152377
ENSG00000196115	ENSG00000008277
ENSG00000196115	ENSG00000042980
ENSG00000196115	ENSG00000069206
ENSG00000196115	ENSG00000073670
ENSG00000196115	ENSG00000104755
ENSG00000196115	ENSG00000114948
ENSG00000196115	ENSG00000134007
ENSG00000196115	ENSG00000134028
ENSG00000196115	ENSG00000134249
ENSG00000196115	ENSG00000135074
ENSG00000196115	ENSG00000139985
ENSG00000196115	ENSG00000143537
ENSG00000196115	ENSG00000148848
ENSG00000196115	ENSG00000149451
ENSG00000196115	ENSG00000151651
ENSG00000196115	ENSG00000151694
ENSG00000196115	ENSG00000168594
ENSG00000196115	ENSG00000168615
ENSG00000196115	ENSG00000168619
ENSG00000196115	ENSG00000197140
ENSG00000196115	ENSG00000214137
ENSG00000196123	ENSG00000164542
ENSG00000196132	ENSG00000147488
ENSG00000196132	ENSG00000186487
ENSG00000196136	ENSG00000100665
ENSG00000196136	ENSG00000123561
ENSG00000196136	ENSG00000135744
ENSG00000196136	ENSG00000165951
ENSG00000196136	ENSG00000165953
ENSG00000196136	ENSG00000170054
ENSG00000196136	ENSG00000186910
ENSG00000196136	ENSG00000187483
ENSG00000196136	ENSG00000188488
ENSG00000196136	ENSG00000197249
ENSG00000196139	ENSG00000085662
ENSG00000196139	ENSG00000117448
ENSG00000196139	ENSG00000122787
ENSG00000196139	ENSG00000151631
ENSG00000196139	ENSG00000151632
ENSG00000196139	ENSG00000165568
ENSG00000196139	ENSG00000187134
ENSG00000196139	ENSG00000196326
ENSG00000196139	ENSG00000198074
ENSG00000196139	ENSG00000198610
ENSG00000196139	ENSG00000215267
ENSG00000196141	ENSG00000123352
ENSG00000196154	ENSG00000143546
ENSG00000196154	ENSG00000160307
ENSG00000196154	ENSG00000160678
ENSG00000196154	ENSG00000163191
ENSG00000196154	ENSG00000163220
ENSG00000196154	ENSG00000163221
ENSG00000196154	ENSG00000163993
ENSG00000196154	ENSG00000169906
ENSG00000196154	ENSG00000171643
ENSG00000196154	ENSG00000188015
ENSG00000196154	ENSG00000188643
ENSG00000196154	ENSG00000189171

ENSG00000196154	ENSG00000189334
ENSG00000196154	ENSG00000196420
ENSG00000196154	ENSG00000196754
ENSG00000196154	ENSG00000197747
ENSG00000196154	ENSG00000197956
ENSG00000196155	ENSG00000149633
ENSG00000196155	ENSG00000153404
ENSG00000196155	ENSG00000165801
ENSG00000196176	ENSG00000124529
ENSG00000196176	ENSG00000124578
ENSG00000196176	ENSG00000158406
ENSG00000196176	ENSG00000182217
ENSG00000196176	ENSG00000183941
ENSG00000196176	ENSG00000188987
ENSG00000196176	ENSG00000197061
ENSG00000196176	ENSG00000197238
ENSG00000196176	ENSG00000197837
ENSG00000196176	ENSG00000197914
ENSG00000196176	ENSG00000198327
ENSG00000196176	ENSG00000198339
ENSG00000196176	ENSG00000198518
ENSG00000196176	ENSG00000198558
ENSG00000196187	ENSG00000137216
ENSG00000196187	ENSG00000165548
ENSG00000196188	ENSG00000096088
ENSG00000196188	ENSG00000117984
ENSG00000196188	ENSG00000131400
ENSG00000196188	ENSG00000131401
ENSG00000196188	ENSG00000143839
ENSG00000196188	ENSG00000172647
ENSG00000196188	ENSG00000180860
ENSG00000196188	ENSG00000184488
ENSG00000196188	ENSG00000204973
ENSG00000196188	ENSG00000215868
ENSG00000196189	ENSG00000010319
ENSG00000196189	ENSG00000012171
ENSG00000196189	ENSG00000075213
ENSG00000196189	ENSG00000092421
ENSG00000196189	ENSG00000095539
ENSG00000196189	ENSG00000137872
ENSG00000196189	ENSG00000143434
ENSG00000196189	ENSG00000153993
ENSG00000196189	ENSG00000168758
ENSG00000196189	ENSG00000170381
ENSG00000196189	ENSG00000185033
ENSG00000196189	ENSG00000187764
ENSG00000196208	ENSG00000141449
ENSG00000196218	ENSG00000198626
ENSG00000196218	ENSG00000198838
ENSG00000196220	ENSG00000089820
ENSG00000196220	ENSG00000163486
ENSG00000196220	ENSG00000196935
ENSG00000196228	ENSG00000088002
ENSG00000196228	ENSG00000105398
ENSG00000196228	ENSG00000109193
ENSG00000196228	ENSG00000130540
ENSG00000196228	ENSG00000138068
ENSG00000196228	ENSG00000173597
ENSG00000196228	ENSG00000196502

ENSG00000196228	ENSG00000197165
ENSG00000196228	ENSG00000198075
ENSG00000196228	ENSG00000198203
ENSG00000196228	ENSG00000213599
ENSG00000196228	ENSG00000213648
ENSG00000196233	ENSG00000178177
ENSG00000196262	ENSG00000108179
ENSG00000196262	ENSG00000150276
ENSG00000196262	ENSG00000198161
ENSG00000196262	ENSG00000198360
ENSG00000196262	ENSG00000198618
ENSG00000196262	ENSG00000198936
ENSG00000196262	ENSG00000203847
ENSG00000196262	ENSG00000214975
ENSG00000196262	ENSG00000215551
ENSG00000196277	ENSG00000113262
ENSG00000196277	ENSG00000124493
ENSG00000196277	ENSG00000152822
ENSG00000196277	ENSG00000164082
ENSG00000196277	ENSG00000168959
ENSG00000196277	ENSG00000179603
ENSG00000196277	ENSG00000198822
ENSG00000196285	ENSG00000112727
ENSG00000196285	ENSG00000124693
ENSG00000196285	ENSG00000132475
ENSG00000196285	ENSG00000163041
ENSG00000196285	ENSG00000168148
ENSG00000196285	ENSG00000178458
ENSG00000196285	ENSG00000182431
ENSG00000196285	ENSG00000182572
ENSG00000196285	ENSG00000182915
ENSG00000196285	ENSG00000183598
ENSG00000196285	ENSG00000188375
ENSG00000196285	ENSG00000196532
ENSG00000196285	ENSG00000196966
ENSG00000196285	ENSG00000197153
ENSG00000196285	ENSG00000197409
ENSG00000196285	ENSG00000198366
ENSG00000196285	ENSG00000203811
ENSG00000196285	ENSG00000203813
ENSG00000196285	ENSG00000203818
ENSG00000196285	ENSG00000203852
ENSG00000196292	ENSG00000090920
ENSG00000196292	ENSG00000109927
ENSG00000196292	ENSG00000110799
ENSG00000196292	ENSG00000117983
ENSG00000196292	ENSG00000146839
ENSG00000196292	ENSG00000184956
ENSG00000196292	ENSG00000188162
ENSG00000196292	ENSG00000197558
ENSG00000196292	ENSG00000198788
ENSG00000196296	ENSG00000074370
ENSG00000196296	ENSG00000174437
ENSG00000196326	ENSG00000085662
ENSG00000196326	ENSG00000117448
ENSG00000196326	ENSG00000122787
ENSG00000196326	ENSG00000151631
ENSG00000196326	ENSG00000151632
ENSG00000196326	ENSG00000165568

ENSG00000196326	ENSG00000187134
ENSG00000196326	ENSG00000196139
ENSG00000196326	ENSG00000198074
ENSG00000196326	ENSG00000198610
ENSG00000196326	ENSG00000215267
ENSG00000196329	ENSG00000106560
ENSG00000196329	ENSG00000133561
ENSG00000196329	ENSG00000133574
ENSG00000196329	ENSG00000171115
ENSG00000196329	ENSG00000179144
ENSG00000196329	ENSG00000213203
ENSG00000196337	ENSG00000104818
ENSG00000196337	ENSG00000104826
ENSG00000196337	ENSG00000104827
ENSG00000196337	ENSG00000131808
ENSG00000196337	ENSG00000134200
ENSG00000196337	ENSG00000179600
ENSG00000196337	ENSG00000189052
ENSG00000196337	ENSG00000204748
ENSG00000196337	ENSG00000213030
ENSG00000196338	ENSG00000146938
ENSG00000196338	ENSG00000165246
ENSG00000196338	ENSG00000169760
ENSG00000196338	ENSG00000169992
ENSG00000196344	ENSG00000172955
ENSG00000196344	ENSG00000187758
ENSG00000196344	ENSG00000196616
ENSG00000196344	ENSG00000197894
ENSG00000196344	ENSG00000198099
ENSG00000196347	ENSG00000105401
ENSG00000196347	ENSG00000106993
ENSG00000196347	ENSG00000196585
ENSG00000196352	ENSG00000091583
ENSG00000196352	ENSG00000117335
ENSG00000196352	ENSG00000123838
ENSG00000196352	ENSG00000197721
ENSG00000196353	ENSG00000085719
ENSG00000196353	ENSG00000100884
ENSG00000196353	ENSG00000124772
ENSG00000196353	ENSG00000139117
ENSG00000196353	ENSG00000140848
ENSG00000196353	ENSG00000144550
ENSG00000196353	ENSG00000178773
ENSG00000196353	ENSG00000214078
ENSG00000196358	ENSG00000162631
ENSG00000196361	ENSG00000066044
ENSG00000196361	ENSG00000107105
ENSG00000196361	ENSG00000162374
ENSG00000196364	ENSG00000005001
ENSG00000196364	ENSG00000007038
ENSG00000196364	ENSG00000052344
ENSG00000196364	ENSG00000095917
ENSG00000196364	ENSG00000103355
ENSG00000196364	ENSG00000116176
ENSG00000196364	ENSG00000172236
ENSG00000196364	ENSG00000172382
ENSG00000196364	ENSG00000172460
ENSG00000196364	ENSG00000178055
ENSG00000196364	ENSG00000178226

ENSG00000196364	ENSG00000185888
ENSG00000196364	ENSG00000188086
ENSG00000196364	ENSG00000189099
ENSG00000196364	ENSG00000197253
ENSG00000196364	ENSG00000206549
ENSG00000196364	ENSG00000214877
ENSG00000196364	ENSG00000215148
ENSG00000196368	ENSG00000112664
ENSG00000196368	ENSG00000122824
ENSG00000196368	ENSG00000173598
ENSG00000196371	ENSG00000130383
ENSG00000196371	ENSG00000156413
ENSG00000196371	ENSG00000171124
ENSG00000196371	ENSG00000172461
ENSG00000196371	ENSG00000172728
ENSG00000196371	ENSG00000180549
ENSG00000196371	ENSG00000196968
ENSG00000196372	ENSG00000065802
ENSG00000196372	ENSG00000102048
ENSG00000196372	ENSG00000164122
ENSG00000196372	ENSG00000165192
ENSG00000196372	ENSG00000198881
ENSG00000196372	ENSG00000214426
ENSG00000196376	ENSG00000110660
ENSG00000196396	ENSG00000139304
ENSG00000196396	ENSG00000169410
ENSG00000196396	ENSG00000175354
ENSG00000196405	ENSG00000125753
ENSG00000196405	ENSG00000154380
ENSG00000196405	ENSG00000166068
ENSG00000196405	ENSG00000188766
ENSG00000196405	ENSG00000198369
ENSG00000196408	ENSG00000107957
ENSG00000196408	ENSG00000158517
ENSG00000196408	ENSG00000165178
ENSG00000196408	ENSG00000174705
ENSG00000196408	ENSG00000182487
ENSG00000196411	ENSG00000044524
ENSG00000196411	ENSG00000070886
ENSG00000196411	ENSG00000080224
ENSG00000196411	ENSG00000106123
ENSG00000196411	ENSG00000116106
ENSG00000196411	ENSG00000133216
ENSG00000196411	ENSG00000135333
ENSG00000196411	ENSG00000142627
ENSG00000196411	ENSG00000145242
ENSG00000196411	ENSG00000146904
ENSG00000196411	ENSG00000154928
ENSG00000196411	ENSG00000182580
ENSG00000196411	ENSG00000183317
ENSG00000196420	ENSG00000143546
ENSG00000196420	ENSG00000160307
ENSG00000196420	ENSG00000160678
ENSG00000196420	ENSG00000163191
ENSG00000196420	ENSG00000163220
ENSG00000196420	ENSG00000163221
ENSG00000196420	ENSG00000163993
ENSG00000196420	ENSG00000169906
ENSG00000196420	ENSG00000171643

ENSG00000196420	ENSG00000188015
ENSG00000196420	ENSG00000188643
ENSG00000196420	ENSG00000189171
ENSG00000196420	ENSG00000189334
ENSG00000196420	ENSG00000196154
ENSG00000196420	ENSG00000196754
ENSG00000196420	ENSG00000197747
ENSG00000196420	ENSG00000197956
ENSG00000196428	ENSG00000102804
ENSG00000196428	ENSG00000166925
ENSG00000196431	ENSG00000100053
ENSG00000196431	ENSG00000100058
ENSG00000196431	ENSG00000100122
ENSG00000196431	ENSG00000108255
ENSG00000196431	ENSG00000118231
ENSG00000196431	ENSG00000127377
ENSG00000196431	ENSG00000163254
ENSG00000196431	ENSG00000163499
ENSG00000196431	ENSG00000168582
ENSG00000196431	ENSG00000182187
ENSG00000196431	ENSG00000213139
ENSG00000196433	ENSG00000169093
ENSG00000196435	ENSG00000212864
ENSG00000196440	ENSG00000124575
ENSG00000196440	ENSG00000124610
ENSG00000196440	ENSG00000168298
ENSG00000196440	ENSG00000184357
ENSG00000196440	ENSG00000184897
ENSG00000196440	ENSG00000187475
ENSG00000196440	ENSG00000187837
ENSG00000196440	ENSG00000189060
ENSG00000196465	ENSG00000092841
ENSG00000196465	ENSG00000160808
ENSG00000196465	ENSG00000168530
ENSG00000196465	ENSG00000198336
ENSG00000196468	ENSG00000070193
ENSG00000196468	ENSG00000070388
ENSG00000196468	ENSG00000075388
ENSG00000196468	ENSG00000078579
ENSG00000196468	ENSG00000102466
ENSG00000196468	ENSG00000102678
ENSG00000196468	ENSG00000111241
ENSG00000196468	ENSG00000113578
ENSG00000196468	ENSG00000114279
ENSG00000196468	ENSG00000129682
ENSG00000196468	ENSG00000138675
ENSG00000196468	ENSG00000138685
ENSG00000196468	ENSG00000140285
ENSG00000196468	ENSG00000161958
ENSG00000196468	ENSG00000185390
ENSG00000196468	ENSG00000186895
ENSG00000196468	ENSG00000204801
ENSG00000196468	ENSG00000204816
ENSG00000196468	ENSG00000204817
ENSG00000196468	ENSG00000204837
ENSG00000196468	ENSG00000215053
ENSG00000196470	ENSG00000181788
ENSG00000196470	ENSG00000215475
ENSG00000196482	ENSG00000091831

ENSG00000196482	ENSG00000119715
ENSG00000196482	ENSG00000140009
ENSG00000196482	ENSG00000173153
ENSG00000196498	ENSG00000141027
ENSG00000196502	ENSG00000088002
ENSG00000196502	ENSG00000105398
ENSG00000196502	ENSG00000109193
ENSG00000196502	ENSG00000130540
ENSG00000196502	ENSG00000138068
ENSG00000196502	ENSG00000173597
ENSG00000196502	ENSG00000196228
ENSG00000196502	ENSG00000197165
ENSG00000196502	ENSG00000198075
ENSG00000196502	ENSG00000198203
ENSG00000196502	ENSG00000213599
ENSG00000196502	ENSG00000213648
ENSG00000196503	ENSG00000175414
ENSG00000196504	ENSG00000110844
ENSG00000196513	ENSG00000132507
ENSG00000196513	ENSG00000163577
ENSG00000196526	ENSG00000157510
ENSG00000196526	ENSG00000169129
ENSG00000196531	ENSG00000121089
ENSG00000196531	ENSG00000136274
ENSG00000196531	ENSG00000196861
ENSG00000196535	ENSG00000133454
ENSG00000196542	ENSG00000165389
ENSG00000196547	ENSG00000112893
ENSG00000196549	ENSG00000102174
ENSG00000196549	ENSG00000117298
ENSG00000196549	ENSG00000142606
ENSG00000196549	ENSG00000145194
ENSG00000196549	ENSG00000171551
ENSG00000196549	ENSG00000197993
ENSG00000196557	ENSG00000006283
ENSG00000196557	ENSG00000100346
ENSG00000196562	ENSG00000135677
ENSG00000196562	ENSG00000137573
ENSG00000196562	ENSG00000164291
ENSG00000196569	ENSG00000053747
ENSG00000196569	ENSG00000101680
ENSG00000196569	ENSG00000112769
ENSG00000196569	ENSG00000130702
ENSG00000196570	ENSG00000070087
ENSG00000196570	ENSG00000108518
ENSG00000196576	ENSG00000004399
ENSG00000196576	ENSG00000076356
ENSG00000196576	ENSG00000114554
ENSG00000196576	ENSG00000130827
ENSG00000196576	ENSG00000136040
ENSG00000196576	ENSG00000164050
ENSG00000196576	ENSG00000189437
ENSG00000196576	ENSG00000198753
ENSG00000196585	ENSG00000105401
ENSG00000196585	ENSG00000106993
ENSG00000196585	ENSG00000196347
ENSG00000196588	ENSG00000141052
ENSG00000196588	ENSG00000176909
ENSG00000196588	ENSG00000186260

ENSG00000196589	ENSG00000071655
ENSG00000196589	ENSG00000134046
ENSG00000196589	ENSG00000170948
ENSG00000196589	ENSG00000182315
ENSG00000196589	ENSG00000184301
ENSG00000196589	ENSG00000205717
ENSG00000196589	ENSG00000205718
ENSG00000196591	ENSG00000116478
ENSG00000196611	ENSG00000008516
ENSG00000196611	ENSG00000087245
ENSG00000196611	ENSG00000099953
ENSG00000196611	ENSG00000100985
ENSG00000196611	ENSG00000102996
ENSG00000196611	ENSG00000110347
ENSG00000196611	ENSG00000118113
ENSG00000196611	ENSG00000123342
ENSG00000196611	ENSG00000125966
ENSG00000196611	ENSG00000129270
ENSG00000196611	ENSG00000137673
ENSG00000196611	ENSG00000137674
ENSG00000196611	ENSG00000137675
ENSG00000196611	ENSG00000137745
ENSG00000196611	ENSG00000149968
ENSG00000196611	ENSG00000154485
ENSG00000196611	ENSG00000156103
ENSG00000196611	ENSG00000157227
ENSG00000196611	ENSG00000166670
ENSG00000196611	ENSG00000167346
ENSG00000196611	ENSG00000189409
ENSG00000196611	ENSG00000198598
ENSG00000196611	ENSG00000215914
ENSG00000196616	ENSG00000172955
ENSG00000196616	ENSG00000187758
ENSG00000196616	ENSG00000196344
ENSG00000196616	ENSG00000197894
ENSG00000196616	ENSG00000198099
ENSG00000196620	ENSG00000109181
ENSG00000196620	ENSG00000135220
ENSG00000196620	ENSG00000135226
ENSG00000196620	ENSG00000145626
ENSG00000196620	ENSG00000156096
ENSG00000196620	ENSG00000167165
ENSG00000196620	ENSG00000168671
ENSG00000196620	ENSG00000171234
ENSG00000196620	ENSG00000173610
ENSG00000196620	ENSG00000174607
ENSG00000196620	ENSG00000197592
ENSG00000196620	ENSG00000197888
ENSG00000196620	ENSG00000198277
ENSG00000196620	ENSG00000213759
ENSG00000196622	ENSG00000005379
ENSG00000196622	ENSG00000060709
ENSG00000196622	ENSG00000183246
ENSG00000196622	ENSG00000196934
ENSG00000196628	ENSG00000071564
ENSG00000196628	ENSG00000140262
ENSG00000196628	ENSG00000215291
ENSG00000196632	ENSG00000060237
ENSG00000196632	ENSG00000115216

ENSG00000196632	ENSG00000126562
ENSG00000196632	ENSG00000165238
ENSG00000196664	ENSG00000164342
ENSG00000196664	ENSG00000173366
ENSG00000196666	ENSG00000189320
ENSG00000196684	ENSG00000027869
ENSG00000196684	ENSG00000104611
ENSG00000196684	ENSG00000178217
ENSG00000196689	ENSG00000111199
ENSG00000196689	ENSG00000127412
ENSG00000196689	ENSG00000165125
ENSG00000196689	ENSG00000167723
ENSG00000196689	ENSG00000187688
ENSG00000196700	ENSG00000198522
ENSG00000196704	ENSG00000174945
ENSG00000196711	ENSG00000189292
ENSG00000196730	ENSG00000035664
ENSG00000196730	ENSG00000065534
ENSG00000196730	ENSG00000101306
ENSG00000196730	ENSG00000140795
ENSG00000196730	ENSG00000145949
ENSG00000196730	ENSG00000167657
ENSG00000196739	ENSG00000171502
ENSG00000196747	ENSG00000111332
ENSG00000196747	ENSG00000124642
ENSG00000196747	ENSG00000137259
ENSG00000196747	ENSG00000158403
ENSG00000196747	ENSG00000164508
ENSG00000196747	ENSG00000168274
ENSG00000196747	ENSG00000180573
ENSG00000196747	ENSG00000181218
ENSG00000196747	ENSG00000182611
ENSG00000196747	ENSG00000183558
ENSG00000196747	ENSG00000184260
ENSG00000196747	ENSG00000184270
ENSG00000196747	ENSG00000184348
ENSG00000196747	ENSG00000184825
ENSG00000196747	ENSG00000185978
ENSG00000196747	ENSG00000188486
ENSG00000196747	ENSG00000196787
ENSG00000196747	ENSG00000196866
ENSG00000196747	ENSG00000198082
ENSG00000196747	ENSG00000198307
ENSG00000196747	ENSG00000198374
ENSG00000196747	ENSG00000203812
ENSG00000196747	ENSG00000205070
ENSG00000196747	ENSG00000205079
ENSG00000196754	ENSG00000143546
ENSG00000196754	ENSG00000160307
ENSG00000196754	ENSG00000160678
ENSG00000196754	ENSG00000163191
ENSG00000196754	ENSG00000163220
ENSG00000196754	ENSG00000163221
ENSG00000196754	ENSG00000163993
ENSG00000196754	ENSG00000169906
ENSG00000196754	ENSG00000171643
ENSG00000196754	ENSG00000188015
ENSG00000196754	ENSG00000188643
ENSG00000196754	ENSG00000189171

ENSG00000196754	ENSG00000189334
ENSG00000196754	ENSG00000196154
ENSG00000196754	ENSG00000196420
ENSG00000196754	ENSG00000197747
ENSG00000196754	ENSG00000197956
ENSG00000196767	ENSG00000028277
ENSG00000196767	ENSG00000064835
ENSG00000196767	ENSG00000091010
ENSG00000196767	ENSG00000137709
ENSG00000196767	ENSG00000143190
ENSG00000196767	ENSG00000151615
ENSG00000196767	ENSG00000152192
ENSG00000196767	ENSG00000184486
ENSG00000196767	ENSG00000185668
ENSG00000196767	ENSG00000198914
ENSG00000196767	ENSG00000204531
ENSG00000196767	ENSG00000212993
ENSG00000196781	ENSG00000065717
ENSG00000196781	ENSG00000104953
ENSG00000196781	ENSG00000104964
ENSG00000196781	ENSG00000106829
ENSG00000196781	ENSG00000140332
ENSG00000196782	ENSG00000013619
ENSG00000196782	ENSG00000161021
ENSG00000196782	ENSG00000184384
ENSG00000196785	ENSG00000100336
ENSG00000196785	ENSG00000100342
ENSG00000196785	ENSG00000128284
ENSG00000196785	ENSG00000128313
ENSG00000196785	ENSG00000128335
ENSG00000196785	ENSG00000178878
ENSG00000196787	ENSG00000111332
ENSG00000196787	ENSG00000124642
ENSG00000196787	ENSG00000137259
ENSG00000196787	ENSG00000158403
ENSG00000196787	ENSG00000164508
ENSG00000196787	ENSG00000168274
ENSG00000196787	ENSG00000180573
ENSG00000196787	ENSG00000181218
ENSG00000196787	ENSG00000182611
ENSG00000196787	ENSG00000183558
ENSG00000196787	ENSG00000184260
ENSG00000196787	ENSG00000184270
ENSG00000196787	ENSG00000184348
ENSG00000196787	ENSG00000184825
ENSG00000196787	ENSG00000185978
ENSG00000196787	ENSG00000188486
ENSG00000196787	ENSG00000196747
ENSG00000196787	ENSG00000196866
ENSG00000196787	ENSG00000198082
ENSG00000196787	ENSG00000198307
ENSG00000196787	ENSG00000198374
ENSG00000196787	ENSG00000203812
ENSG00000196787	ENSG00000205070
ENSG00000196787	ENSG00000205079
ENSG00000196792	ENSG00000090372
ENSG00000196792	ENSG00000115808
ENSG00000196811	ENSG00000080644
ENSG00000196811	ENSG00000101204

ENSG00000196811	ENSG00000108556
ENSG00000196811	ENSG00000117971
ENSG00000196811	ENSG00000120903
ENSG00000196811	ENSG00000129749
ENSG00000196811	ENSG00000135902
ENSG00000196811	ENSG00000138435
ENSG00000196811	ENSG00000147432
ENSG00000196811	ENSG00000147434
ENSG00000196811	ENSG00000149305
ENSG00000196811	ENSG00000160716
ENSG00000196811	ENSG00000166664
ENSG00000196811	ENSG00000166736
ENSG00000196811	ENSG00000169684
ENSG00000196811	ENSG00000170175
ENSG00000196811	ENSG00000174343
ENSG00000196811	ENSG00000175344
ENSG00000196811	ENSG00000178084
ENSG00000196811	ENSG00000186038
ENSG00000196811	ENSG00000186090
ENSG00000196811	ENSG00000186919
ENSG00000196814	ENSG00000141971
ENSG00000196843	ENSG00000150347
ENSG00000196859	ENSG00000006059
ENSG00000196859	ENSG00000094796
ENSG00000196859	ENSG00000108244
ENSG00000196859	ENSG00000108417
ENSG00000196859	ENSG00000108759
ENSG00000196859	ENSG00000111057
ENSG00000196859	ENSG00000126337
ENSG00000196859	ENSG00000128422
ENSG00000196859	ENSG00000131737
ENSG00000196859	ENSG00000131738
ENSG00000196859	ENSG00000131885
ENSG00000196859	ENSG00000167916
ENSG00000196859	ENSG00000170819
ENSG00000196859	ENSG00000171345
ENSG00000196859	ENSG00000171346
ENSG00000196859	ENSG00000171360
ENSG00000196859	ENSG00000171401
ENSG00000196859	ENSG00000171403
ENSG00000196859	ENSG00000171431
ENSG00000196859	ENSG00000171446
ENSG00000196859	ENSG00000173908
ENSG00000196859	ENSG00000186393
ENSG00000196859	ENSG00000186395
ENSG00000196859	ENSG00000186831
ENSG00000196859	ENSG00000186832
ENSG00000196859	ENSG00000186847
ENSG00000196859	ENSG00000187242
ENSG00000196859	ENSG00000187686
ENSG00000196859	ENSG00000197079
ENSG00000196859	ENSG00000204889
ENSG00000196859	ENSG00000204897
ENSG00000196859	ENSG00000213951
ENSG00000196859	ENSG00000214220
ENSG00000196859	ENSG00000214454
ENSG00000196859	ENSG00000214959
ENSG00000196859	ENSG00000215772
ENSG00000196859	ENSG00000215773

ENSG00000196860	ENSG00000173726
ENSG00000196861	ENSG00000121089
ENSG00000196861	ENSG00000136274
ENSG00000196861	ENSG00000196531
ENSG00000196866	ENSG00000111332
ENSG00000196866	ENSG00000124642
ENSG00000196866	ENSG00000137259
ENSG00000196866	ENSG00000158403
ENSG00000196866	ENSG00000164508
ENSG00000196866	ENSG00000168274
ENSG00000196866	ENSG00000180573
ENSG00000196866	ENSG00000181218
ENSG00000196866	ENSG00000182611
ENSG00000196866	ENSG00000183558
ENSG00000196866	ENSG00000184260
ENSG00000196866	ENSG00000184270
ENSG00000196866	ENSG00000184348
ENSG00000196866	ENSG00000184825
ENSG00000196866	ENSG00000185978
ENSG00000196866	ENSG00000188486
ENSG00000196866	ENSG00000196747
ENSG00000196866	ENSG00000196787
ENSG00000196866	ENSG00000198082
ENSG00000196866	ENSG00000198307
ENSG00000196866	ENSG00000198374
ENSG00000196866	ENSG00000203812
ENSG00000196866	ENSG00000205070
ENSG00000196866	ENSG00000205079
ENSG00000196876	ENSG00000007314
ENSG00000196876	ENSG00000136531
ENSG00000196876	ENSG00000136546
ENSG00000196876	ENSG00000144285
ENSG00000196876	ENSG00000153253
ENSG00000196876	ENSG00000168356
ENSG00000196876	ENSG00000169432
ENSG00000196876	ENSG00000183873
ENSG00000196876	ENSG00000185313
ENSG00000196911	ENSG00000025800
ENSG00000196911	ENSG00000114030
ENSG00000196914	ENSG00000076928
ENSG00000196914	ENSG00000132694
ENSG00000196917	ENSG00000125245
ENSG00000196917	ENSG00000126251
ENSG00000196917	ENSG00000126262
ENSG00000196917	ENSG00000144230
ENSG00000196917	ENSG00000147138
ENSG00000196917	ENSG00000162881
ENSG00000196917	ENSG00000164220
ENSG00000196917	ENSG00000164251
ENSG00000196917	ENSG00000165621
ENSG00000196917	ENSG00000171631
ENSG00000196917	ENSG00000181104
ENSG00000196917	ENSG00000182162
ENSG00000196917	ENSG00000182559
ENSG00000196917	ENSG00000182782
ENSG00000196917	ENSG00000185897
ENSG00000196923	ENSG00000107438
ENSG00000196923	ENSG00000120913
ENSG00000196923	ENSG00000122367

ENSG00000196923	ENSG00000131435
ENSG00000196923	ENSG00000154553
ENSG00000196923	ENSG00000163110
ENSG00000196923	ENSG00000214895
ENSG00000196924	ENSG00000128591
ENSG00000196924	ENSG00000136068
ENSG00000196934	ENSG00000005379
ENSG00000196934	ENSG00000060709
ENSG00000196934	ENSG00000183246
ENSG00000196934	ENSG00000196622
ENSG00000196935	ENSG00000089820
ENSG00000196935	ENSG00000163486
ENSG00000196935	ENSG00000196220
ENSG00000196937	ENSG00000071889
ENSG00000196937	ENSG00000174028
ENSG00000196937	ENSG00000183844
ENSG00000196937	ENSG00000198643
ENSG00000196950	ENSG00000104635
ENSG00000196950	ENSG00000112473
ENSG00000196950	ENSG00000138821
ENSG00000196950	ENSG00000139540
ENSG00000196950	ENSG00000141424
ENSG00000196950	ENSG00000147804
ENSG00000196950	ENSG00000148482
ENSG00000196950	ENSG00000165915
ENSG00000196954	ENSG00000003400
ENSG00000196954	ENSG00000064012
ENSG00000196954	ENSG00000105141
ENSG00000196954	ENSG00000106144
ENSG00000196954	ENSG00000132906
ENSG00000196954	ENSG00000137752
ENSG00000196954	ENSG00000137757
ENSG00000196954	ENSG00000138794
ENSG00000196954	ENSG00000164305
ENSG00000196954	ENSG00000165806
ENSG00000196954	ENSG00000204403
ENSG00000196961	ENSG00000183020
ENSG00000196963	ENSG00000081818
ENSG00000196963	ENSG00000081842
ENSG00000196963	ENSG00000081853
ENSG00000196963	ENSG00000112852
ENSG00000196963	ENSG00000113205
ENSG00000196963	ENSG00000113209
ENSG00000196963	ENSG00000113211
ENSG00000196963	ENSG00000113212
ENSG00000196963	ENSG00000113248
ENSG00000196963	ENSG00000120322
ENSG00000196963	ENSG00000120324
ENSG00000196963	ENSG00000120327
ENSG00000196963	ENSG00000120328
ENSG00000196963	ENSG00000146001
ENSG00000196963	ENSG00000171815
ENSG00000196963	ENSG00000187372
ENSG00000196963	ENSG00000197479
ENSG00000196963	ENSG00000204955
ENSG00000196963	ENSG00000204956
ENSG00000196963	ENSG00000204961
ENSG00000196963	ENSG00000204962
ENSG00000196963	ENSG00000204963

ENSG00000196963	ENSG00000204964
ENSG00000196963	ENSG00000204965
ENSG00000196963	ENSG00000204967
ENSG00000196963	ENSG00000204968
ENSG00000196963	ENSG00000204969
ENSG00000196963	ENSG00000204970
ENSG00000196963	ENSG00000214567
ENSG00000196963	ENSG00000214570
ENSG00000196963	ENSG00000214574
ENSG00000196963	ENSG00000214580
ENSG00000196963	ENSG00000214583
ENSG00000196963	ENSG00000214594
ENSG00000196963	ENSG00000214600
ENSG00000196963	ENSG00000214608
ENSG00000196970	ENSG00000126952
ENSG00000196970	ENSG00000147206
ENSG00000196970	ENSG00000162231
ENSG00000196970	ENSG00000185554
ENSG00000196970	ENSG00000185945
ENSG00000196975	ENSG00000104537
ENSG00000196975	ENSG00000109511
ENSG00000196975	ENSG00000122359
ENSG00000196975	ENSG00000135046
ENSG00000196975	ENSG00000138279
ENSG00000196975	ENSG00000138772
ENSG00000196975	ENSG00000143412
ENSG00000196975	ENSG00000150165
ENSG00000196975	ENSG00000164111
ENSG00000196975	ENSG00000165390
ENSG00000196975	ENSG00000182718
ENSG00000196975	ENSG00000183059
ENSG00000196975	ENSG00000186807
ENSG00000196975	ENSG00000197043
ENSG00000196990	ENSG00000143340
ENSG00000196990	ENSG00000215611
ENSG00000197008	ENSG00000142396
ENSG00000197008	ENSG00000169740
ENSG00000197008	ENSG00000174255
ENSG00000197008	ENSG00000179195
ENSG00000197008	ENSG00000179965
ENSG00000197008	ENSG00000184294
ENSG00000197008	ENSG00000186446
ENSG00000197008	ENSG00000186777
ENSG00000197008	ENSG00000197025
ENSG00000197008	ENSG00000197062
ENSG00000197008	ENSG00000197332
ENSG00000197008	ENSG00000198155
ENSG00000197008	ENSG00000204756
ENSG00000197008	ENSG00000212805
ENSG00000197008	ENSG00000213976
ENSG00000197008	ENSG00000213980
ENSG00000197008	ENSG00000215180
ENSG00000197008	ENSG00000215510
ENSG00000197008	ENSG00000215537
ENSG00000197008	ENSG00000215598
ENSG00000197008	ENSG00000215600
ENSG00000197019	ENSG00000167565
ENSG00000197019	ENSG00000170779
ENSG00000197025	ENSG00000142396

ENSG00000197025	ENSG00000169740
ENSG00000197025	ENSG00000174255
ENSG00000197025	ENSG00000179195
ENSG00000197025	ENSG00000179965
ENSG00000197025	ENSG00000184294
ENSG00000197025	ENSG00000186446
ENSG00000197025	ENSG00000186777
ENSG00000197025	ENSG00000197008
ENSG00000197025	ENSG00000197062
ENSG00000197025	ENSG00000197332
ENSG00000197025	ENSG00000198155
ENSG00000197025	ENSG00000204756
ENSG00000197025	ENSG00000212805
ENSG00000197025	ENSG00000213976
ENSG00000197025	ENSG00000213980
ENSG00000197025	ENSG00000215180
ENSG00000197025	ENSG00000215510
ENSG00000197025	ENSG00000215537
ENSG00000197025	ENSG00000215598
ENSG00000197025	ENSG00000215600
ENSG00000197041	ENSG00000164708
ENSG00000197041	ENSG00000171314
ENSG00000197041	ENSG00000172331
ENSG00000197041	ENSG00000186076
ENSG00000197041	ENSG00000214206
ENSG00000197041	ENSG00000214824
ENSG00000197041	ENSG00000215178
ENSG00000197043	ENSG00000104537
ENSG00000197043	ENSG00000109511
ENSG00000197043	ENSG00000122359
ENSG00000197043	ENSG00000135046
ENSG00000197043	ENSG00000138279
ENSG00000197043	ENSG00000138772
ENSG00000197043	ENSG00000143412
ENSG00000197043	ENSG00000150165
ENSG00000197043	ENSG00000164111
ENSG00000197043	ENSG00000165390
ENSG00000197043	ENSG00000182718
ENSG00000197043	ENSG00000183059
ENSG00000197043	ENSG00000186807
ENSG00000197043	ENSG00000196975
ENSG00000197045	ENSG00000130755
ENSG00000197061	ENSG00000124529
ENSG00000197061	ENSG00000124578
ENSG00000197061	ENSG00000158406
ENSG00000197061	ENSG00000182217
ENSG00000197061	ENSG00000183941
ENSG00000197061	ENSG00000188987
ENSG00000197061	ENSG00000196176
ENSG00000197061	ENSG00000197238
ENSG00000197061	ENSG00000197837
ENSG00000197061	ENSG00000197914
ENSG00000197061	ENSG00000198327
ENSG00000197061	ENSG00000198339
ENSG00000197061	ENSG00000198518
ENSG00000197061	ENSG00000198558
ENSG00000197062	ENSG00000142396
ENSG00000197062	ENSG00000169740
ENSG00000197062	ENSG00000174255

ENSG00000197062	ENSG00000179195
ENSG00000197062	ENSG00000179965
ENSG00000197062	ENSG00000184294
ENSG00000197062	ENSG00000186446
ENSG00000197062	ENSG00000186777
ENSG00000197062	ENSG00000197008
ENSG00000197062	ENSG00000197025
ENSG00000197062	ENSG00000197332
ENSG00000197062	ENSG00000198155
ENSG00000197062	ENSG00000204756
ENSG00000197062	ENSG00000212805
ENSG00000197062	ENSG00000213976
ENSG00000197062	ENSG00000213980
ENSG00000197062	ENSG00000215180
ENSG00000197062	ENSG00000215510
ENSG00000197062	ENSG00000215537
ENSG00000197062	ENSG00000215598
ENSG00000197062	ENSG00000215600
ENSG00000197063	ENSG00000129535
ENSG00000197063	ENSG00000178573
ENSG00000197063	ENSG00000182759
ENSG00000197063	ENSG00000185022
ENSG00000197063	ENSG00000198517
ENSG00000197063	ENSG00000204103
ENSG00000197063	ENSG00000215622
ENSG00000197070	ENSG00000105643
ENSG00000197070	ENSG00000113369
ENSG00000197070	ENSG00000117289
ENSG00000197070	ENSG00000140450
ENSG00000197070	ENSG00000205784
ENSG00000197079	ENSG00000006059
ENSG00000197079	ENSG00000094796
ENSG00000197079	ENSG00000108244
ENSG00000197079	ENSG00000108417
ENSG00000197079	ENSG00000108759
ENSG00000197079	ENSG00000111057
ENSG00000197079	ENSG00000126337
ENSG00000197079	ENSG00000128422
ENSG00000197079	ENSG00000131737
ENSG00000197079	ENSG00000131738
ENSG00000197079	ENSG00000131885
ENSG00000197079	ENSG00000167916
ENSG00000197079	ENSG00000170819
ENSG00000197079	ENSG00000171345
ENSG00000197079	ENSG00000171346
ENSG00000197079	ENSG00000171360
ENSG00000197079	ENSG00000171401
ENSG00000197079	ENSG00000171403
ENSG00000197079	ENSG00000171431
ENSG00000197079	ENSG00000171446
ENSG00000197079	ENSG00000173908
ENSG00000197079	ENSG00000186393
ENSG00000197079	ENSG00000186395
ENSG00000197079	ENSG00000186831
ENSG00000197079	ENSG00000186832
ENSG00000197079	ENSG00000186847
ENSG00000197079	ENSG00000187242
ENSG00000197079	ENSG00000187686
ENSG00000197079	ENSG00000196859

ENSG00000197079	ENSG00000204889
ENSG00000197079	ENSG00000204897
ENSG00000197079	ENSG00000213951
ENSG00000197079	ENSG00000214220
ENSG00000197079	ENSG00000214454
ENSG00000197079	ENSG00000214959
ENSG00000197079	ENSG00000215772
ENSG00000197079	ENSG00000215773
ENSG00000197092	ENSG00000140478
ENSG00000197092	ENSG00000153666
ENSG00000197092	ENSG00000156363
ENSG00000197092	ENSG00000159289
ENSG00000197092	ENSG00000167110
ENSG00000197092	ENSG00000167195
ENSG00000197092	ENSG00000174450
ENSG00000197092	ENSG00000175265
ENSG00000197092	ENSG00000181984
ENSG00000197092	ENSG00000184206
ENSG00000197092	ENSG00000186322
ENSG00000197092	ENSG00000188388
ENSG00000197092	ENSG00000188532
ENSG00000197092	ENSG00000188626
ENSG00000197092	ENSG00000197414
ENSG00000197092	ENSG00000214348
ENSG00000197092	ENSG00000215252
ENSG00000197092	ENSG00000215405
ENSG00000197093	ENSG00000128242
ENSG00000197093	ENSG00000154252
ENSG00000197093	ENSG00000175229
ENSG00000197106	ENSG00000063127
ENSG00000197106	ENSG00000072041
ENSG00000197111	ENSG00000090097
ENSG00000197111	ENSG00000169564
ENSG00000197111	ENSG00000183570
ENSG00000197122	ENSG00000000938
ENSG00000197122	ENSG00000010810
ENSG00000197122	ENSG00000101336
ENSG00000197122	ENSG00000111816
ENSG00000197122	ENSG00000136573
ENSG00000197122	ENSG00000147507
ENSG00000197122	ENSG00000176105
ENSG00000197122	ENSG00000182866
ENSG00000197136	ENSG00000100731
ENSG00000197136	ENSG00000126773
ENSG00000197136	ENSG00000135749
ENSG00000197140	ENSG00000008277
ENSG00000197140	ENSG00000042980
ENSG00000197140	ENSG00000069206
ENSG00000197140	ENSG00000073670
ENSG00000197140	ENSG00000104755
ENSG00000197140	ENSG00000114948
ENSG00000197140	ENSG00000134007
ENSG00000197140	ENSG00000134028
ENSG00000197140	ENSG00000134249
ENSG00000197140	ENSG00000135074
ENSG00000197140	ENSG00000139985
ENSG00000197140	ENSG00000143537
ENSG00000197140	ENSG00000148848
ENSG00000197140	ENSG00000149451

ENSG00000197140	ENSG00000151651
ENSG00000197140	ENSG00000151694
ENSG00000197140	ENSG00000168594
ENSG00000197140	ENSG00000168615
ENSG00000197140	ENSG00000168619
ENSG00000197140	ENSG00000196115
ENSG00000197140	ENSG00000214137
ENSG00000197142	ENSG00000151726
ENSG00000197142	ENSG00000164398
ENSG00000197147	ENSG00000136802
ENSG00000197147	ENSG00000171017
ENSG00000197147	ENSG00000171488
ENSG00000197147	ENSG00000171492
ENSG00000197165	ENSG00000088002
ENSG00000197165	ENSG00000105398
ENSG00000197165	ENSG00000109193
ENSG00000197165	ENSG00000130540
ENSG00000197165	ENSG00000138068
ENSG00000197165	ENSG00000173597
ENSG00000197165	ENSG00000196228
ENSG00000197165	ENSG00000196502
ENSG00000197165	ENSG00000198075
ENSG00000197165	ENSG00000198203
ENSG00000197165	ENSG00000213599
ENSG00000197165	ENSG00000213648
ENSG00000197168	ENSG00000114670
ENSG00000197168	ENSG00000114904
ENSG00000197168	ENSG00000119638
ENSG00000197168	ENSG00000136098
ENSG00000197168	ENSG00000160602
ENSG00000197177	ENSG00000020181
ENSG00000197177	ENSG00000152990
ENSG00000197183	ENSG00000101746
ENSG00000197217	ENSG00000198018
ENSG00000197226	ENSG00000109436
ENSG00000197226	ENSG00000133138
ENSG00000197226	ENSG00000204634
ENSG00000197238	ENSG00000124529
ENSG00000197238	ENSG00000124578
ENSG00000197238	ENSG00000158406
ENSG00000197238	ENSG00000182217
ENSG00000197238	ENSG00000183941
ENSG00000197238	ENSG00000188987
ENSG00000197238	ENSG00000196176
ENSG00000197238	ENSG00000197061
ENSG00000197238	ENSG00000197837
ENSG00000197238	ENSG00000197914
ENSG00000197238	ENSG00000198327
ENSG00000197238	ENSG00000198339
ENSG00000197238	ENSG00000198518
ENSG00000197238	ENSG00000198558
ENSG00000197245	ENSG00000125898
ENSG00000197245	ENSG00000169122
ENSG00000197245	ENSG00000184731
ENSG00000197249	ENSG00000100665
ENSG00000197249	ENSG00000123561
ENSG00000197249	ENSG00000135744
ENSG00000197249	ENSG00000165951
ENSG00000197249	ENSG00000165953

ENSG00000197249	ENSG00000170054
ENSG00000197249	ENSG00000186910
ENSG00000197249	ENSG00000187483
ENSG00000197249	ENSG00000188488
ENSG00000197249	ENSG00000196136
ENSG00000197253	ENSG00000005001
ENSG00000197253	ENSG00000007038
ENSG00000197253	ENSG00000052344
ENSG00000197253	ENSG00000095917
ENSG00000197253	ENSG00000103355
ENSG00000197253	ENSG00000116176
ENSG00000197253	ENSG00000172236
ENSG00000197253	ENSG00000172382
ENSG00000197253	ENSG00000172460
ENSG00000197253	ENSG00000178055
ENSG00000197253	ENSG00000178226
ENSG00000197253	ENSG00000185888
ENSG00000197253	ENSG00000188086
ENSG00000197253	ENSG00000189099
ENSG00000197253	ENSG00000196364
ENSG00000197253	ENSG00000206549
ENSG00000197253	ENSG00000214877
ENSG00000197253	ENSG00000215148
ENSG00000197256	ENSG00000107104
ENSG00000197256	ENSG00000132854
ENSG00000197256	ENSG00000186994
ENSG00000197262	ENSG00000006074
ENSG00000197262	ENSG00000006075
ENSG00000197262	ENSG00000006606
ENSG00000197262	ENSG00000102962
ENSG00000197262	ENSG00000102970
ENSG00000197262	ENSG00000106178
ENSG00000197262	ENSG00000108688
ENSG00000197262	ENSG00000108691
ENSG00000197262	ENSG00000108700
ENSG00000197262	ENSG00000108702
ENSG00000197262	ENSG00000115009
ENSG00000197262	ENSG00000129277
ENSG00000197262	ENSG00000143184
ENSG00000197262	ENSG00000143185
ENSG00000197262	ENSG00000161570
ENSG00000197262	ENSG00000161573
ENSG00000197262	ENSG00000161574
ENSG00000197262	ENSG00000167236
ENSG00000197262	ENSG00000172156
ENSG00000197262	ENSG00000172724
ENSG00000197262	ENSG00000181374
ENSG00000197262	ENSG00000205017
ENSG00000197262	ENSG00000205020
ENSG00000197262	ENSG00000205021
ENSG00000197262	ENSG00000213494
ENSG00000197262	ENSG00000215627
ENSG00000197262	ENSG00000215629
ENSG00000197283	ENSG00000075391
ENSG00000197283	ENSG00000105122
ENSG00000197283	ENSG00000136848
ENSG00000197294	ENSG00000067082
ENSG00000197294	ENSG00000102554
ENSG00000197294	ENSG00000105610

ENSG00000197294	ENSG00000127528
ENSG00000197294	ENSG00000129911
ENSG00000197294	ENSG00000136826
ENSG00000197294	ENSG00000139651
ENSG00000197294	ENSG00000155090
ENSG00000197294	ENSG00000164651
ENSG00000197294	ENSG00000167182
ENSG00000197294	ENSG00000170374
ENSG00000197294	ENSG00000171872
ENSG00000197294	ENSG00000172059
ENSG00000197294	ENSG00000174595
ENSG00000197294	ENSG00000189120
ENSG00000197294	ENSG00000197550
ENSG00000197294	ENSG00000204335
ENSG00000197296	ENSG00000139914
ENSG00000197303	ENSG00000160516
ENSG00000197303	ENSG00000182953
ENSG00000197323	ENSG00000122779
ENSG00000197323	ENSG00000130726
ENSG00000197323	ENSG00000166436
ENSG00000197324	ENSG00000130881
ENSG00000197324	ENSG00000147650
ENSG00000197329	ENSG00000139946
ENSG00000197329	ENSG00000174516
ENSG00000197332	ENSG00000142396
ENSG00000197332	ENSG00000169740
ENSG00000197332	ENSG00000174255
ENSG00000197332	ENSG00000179195
ENSG00000197332	ENSG00000179965
ENSG00000197332	ENSG00000184294
ENSG00000197332	ENSG00000186446
ENSG00000197332	ENSG00000186777
ENSG00000197332	ENSG00000197008
ENSG00000197332	ENSG00000197025
ENSG00000197332	ENSG00000197062
ENSG00000197332	ENSG00000198155
ENSG00000197332	ENSG00000204756
ENSG00000197332	ENSG00000212805
ENSG00000197332	ENSG00000213976
ENSG00000197332	ENSG00000213980
ENSG00000197332	ENSG00000215180
ENSG00000197332	ENSG00000215510
ENSG00000197332	ENSG00000215537
ENSG00000197332	ENSG00000215598
ENSG00000197332	ENSG00000215600
ENSG00000197355	ENSG00000117143
ENSG00000197366	ENSG00000157576
ENSG00000197366	ENSG00000172186
ENSG00000197366	ENSG00000182765
ENSG00000197366	ENSG00000184409
ENSG00000197366	ENSG00000198157
ENSG00000197366	ENSG00000198875
ENSG00000197380	ENSG00000164488
ENSG00000197380	ENSG00000165617
ENSG00000197381	ENSG00000065457
ENSG00000197381	ENSG00000140955
ENSG00000197381	ENSG00000160710
ENSG00000197381	ENSG00000164113
ENSG00000197381	ENSG00000185736

ENSG00000197382	ENSG00000178769
ENSG00000197382	ENSG00000197791
ENSG00000197382	ENSG00000198230
ENSG00000197405	ENSG00000134830
ENSG00000197405	ENSG00000171049
ENSG00000197405	ENSG00000171051
ENSG00000197405	ENSG00000171860
ENSG00000197405	ENSG00000174600
ENSG00000197405	ENSG00000183134
ENSG00000197405	ENSG00000183671
ENSG00000197405	ENSG00000187474
ENSG00000197405	ENSG00000213903
ENSG00000197405	ENSG00000213906
ENSG00000197406	ENSG00000211448
ENSG00000197406	ENSG00000211452
ENSG00000197407	ENSG00000118903
ENSG00000197407	ENSG00000134717
ENSG00000197407	ENSG00000145741
ENSG00000197407	ENSG00000214680
ENSG00000197414	ENSG00000140478
ENSG00000197414	ENSG00000153666
ENSG00000197414	ENSG00000156363
ENSG00000197414	ENSG00000159289
ENSG00000197414	ENSG00000167110
ENSG00000197414	ENSG00000167195
ENSG00000197414	ENSG00000174450
ENSG00000197414	ENSG00000175265
ENSG00000197414	ENSG00000181984
ENSG00000197414	ENSG00000184206
ENSG00000197414	ENSG00000186322
ENSG00000197414	ENSG00000188388
ENSG00000197414	ENSG00000188532
ENSG00000197414	ENSG00000188626
ENSG00000197414	ENSG00000197092
ENSG00000197414	ENSG00000214348
ENSG00000197414	ENSG00000215252
ENSG00000197414	ENSG00000215405
ENSG00000197416	ENSG00000114113
ENSG00000197416	ENSG00000114115
ENSG00000197416	ENSG00000121769
ENSG00000197416	ENSG00000139194
ENSG00000197416	ENSG00000143320
ENSG00000197416	ENSG00000145384
ENSG00000197416	ENSG00000147588
ENSG00000197416	ENSG00000162444
ENSG00000197416	ENSG00000164434
ENSG00000197416	ENSG00000164687
ENSG00000197416	ENSG00000166426
ENSG00000197416	ENSG00000170323
ENSG00000197416	ENSG00000205186
ENSG00000197416	ENSG00000214004
ENSG00000197421	ENSG00000099998
ENSG00000197421	ENSG00000100031
ENSG00000197421	ENSG00000100121
ENSG00000197421	ENSG00000133475
ENSG00000197421	ENSG00000149435
ENSG00000197421	ENSG00000183038
ENSG00000197421	ENSG00000215466
ENSG00000197421	ENSG00000215500

ENSG00000197421	ENSG00000215610
ENSG00000197442	ENSG00000142733
ENSG00000197442	ENSG00000180815
ENSG00000197444	ENSG00000105953
ENSG00000197451	ENSG00000071626
ENSG00000197451	ENSG00000138668
ENSG00000197451	ENSG00000152795
ENSG00000197451	ENSG00000215173
ENSG00000197453	ENSG00000155085
ENSG00000197461	ENSG00000100311
ENSG00000197461	ENSG00000112715
ENSG00000197461	ENSG00000119630
ENSG00000197461	ENSG00000150630
ENSG00000197461	ENSG00000165197
ENSG00000197461	ENSG00000173511
ENSG00000197461	ENSG00000215682
ENSG00000197467	ENSG00000050767
ENSG00000197467	ENSG00000188517
ENSG00000197479	ENSG00000081818
ENSG00000197479	ENSG00000081842
ENSG00000197479	ENSG00000081853
ENSG00000197479	ENSG00000112852
ENSG00000197479	ENSG00000113205
ENSG00000197479	ENSG00000113209
ENSG00000197479	ENSG00000113211
ENSG00000197479	ENSG00000113212
ENSG00000197479	ENSG00000113248
ENSG00000197479	ENSG00000120322
ENSG00000197479	ENSG00000120324
ENSG00000197479	ENSG00000120327
ENSG00000197479	ENSG00000120328
ENSG00000197479	ENSG00000146001
ENSG00000197479	ENSG00000171815
ENSG00000197479	ENSG00000187372
ENSG00000197479	ENSG00000196963
ENSG00000197479	ENSG00000204955
ENSG00000197479	ENSG00000204956
ENSG00000197479	ENSG00000204961
ENSG00000197479	ENSG00000204962
ENSG00000197479	ENSG00000204963
ENSG00000197479	ENSG00000204964
ENSG00000197479	ENSG00000204965
ENSG00000197479	ENSG00000204967
ENSG00000197479	ENSG00000204968
ENSG00000197479	ENSG00000204969
ENSG00000197479	ENSG00000204970
ENSG00000197479	ENSG00000214567
ENSG00000197479	ENSG00000214570
ENSG00000197479	ENSG00000214574
ENSG00000197479	ENSG00000214580
ENSG00000197479	ENSG00000214583
ENSG00000197479	ENSG00000214594
ENSG00000197479	ENSG00000214600
ENSG00000197479	ENSG00000214608
ENSG00000197496	ENSG00000146411
ENSG00000197506	ENSG00000137860
ENSG00000197506	ENSG00000156222
ENSG00000197518	ENSG00000087086
ENSG00000197518	ENSG00000132446

ENSG00000197518	ENSG00000167996
ENSG00000197518	ENSG00000171878
ENSG00000197518	ENSG00000173477
ENSG00000197518	ENSG00000176778
ENSG00000197518	ENSG00000180795
ENSG00000197518	ENSG00000181867
ENSG00000197518	ENSG00000215234
ENSG00000197520	ENSG00000151327
ENSG00000197520	ENSG00000214886
ENSG00000197535	ENSG00000128833
ENSG00000197535	ENSG00000141140
ENSG00000197535	ENSG00000167306
ENSG00000197549	ENSG00000116708
ENSG00000197549	ENSG00000116721
ENSG00000197549	ENSG00000116726
ENSG00000197549	ENSG00000120952
ENSG00000197549	ENSG00000157358
ENSG00000197549	ENSG00000160959
ENSG00000197549	ENSG00000179704
ENSG00000197549	ENSG00000182330
ENSG00000197549	ENSG00000185028
ENSG00000197549	ENSG00000185686
ENSG00000197549	ENSG00000187545
ENSG00000197549	ENSG00000204478
ENSG00000197549	ENSG00000204479
ENSG00000197549	ENSG00000204480
ENSG00000197549	ENSG00000204481
ENSG00000197549	ENSG00000204485
ENSG00000197549	ENSG00000204486
ENSG00000197549	ENSG00000204488
ENSG00000197549	ENSG00000204491
ENSG00000197549	ENSG00000204495
ENSG00000197549	ENSG00000204501
ENSG00000197549	ENSG00000204502
ENSG00000197549	ENSG00000204503
ENSG00000197549	ENSG00000204505
ENSG00000197549	ENSG00000204507
ENSG00000197549	ENSG00000204508
ENSG00000197549	ENSG00000204509
ENSG00000197549	ENSG00000204510
ENSG00000197549	ENSG00000204513
ENSG00000197550	ENSG00000067082
ENSG00000197550	ENSG00000102554
ENSG00000197550	ENSG00000105610
ENSG00000197550	ENSG00000127528
ENSG00000197550	ENSG00000129911
ENSG00000197550	ENSG00000136826
ENSG00000197550	ENSG00000139651
ENSG00000197550	ENSG00000155090
ENSG00000197550	ENSG00000164651
ENSG00000197550	ENSG00000167182
ENSG00000197550	ENSG00000170374
ENSG00000197550	ENSG00000171872
ENSG00000197550	ENSG00000172059
ENSG00000197550	ENSG00000174595
ENSG00000197550	ENSG00000189120
ENSG00000197550	ENSG00000197294
ENSG00000197550	ENSG00000204335
ENSG00000197555	ENSG00000076864

ENSG00000197555	ENSG00000105738
ENSG00000197555	ENSG00000116991
ENSG00000197555	ENSG00000132359
ENSG00000197555	ENSG00000136895
ENSG00000197555	ENSG00000213445
ENSG00000197558	ENSG00000090920
ENSG00000197558	ENSG00000109927
ENSG00000197558	ENSG00000110799
ENSG00000197558	ENSG00000117983
ENSG00000197558	ENSG00000146839
ENSG00000197558	ENSG00000184956
ENSG00000197558	ENSG00000188162
ENSG00000197558	ENSG00000196292
ENSG00000197558	ENSG00000198788
ENSG00000197562	ENSG00000102128
ENSG00000197562	ENSG00000141542
ENSG00000197562	ENSG00000172476
ENSG00000197565	ENSG00000081052
ENSG00000197565	ENSG00000134871
ENSG00000197565	ENSG00000169031
ENSG00000197565	ENSG00000187498
ENSG00000197565	ENSG00000188153
ENSG00000197576	ENSG00000037965
ENSG00000197576	ENSG00000106004
ENSG00000197576	ENSG00000106006
ENSG00000197576	ENSG00000108511
ENSG00000197576	ENSG00000120068
ENSG00000197576	ENSG00000120075
ENSG00000197576	ENSG00000120087
ENSG00000197576	ENSG00000122592
ENSG00000197576	ENSG00000170166
ENSG00000197576	ENSG00000172789
ENSG00000197576	ENSG00000175879
ENSG00000197576	ENSG00000182742
ENSG00000197576	ENSG00000197757
ENSG00000197576	ENSG00000198353
ENSG00000197580	ENSG00000116745
ENSG00000197580	ENSG00000135697
ENSG00000197582	ENSG00000079782
ENSG00000197582	ENSG00000176153
ENSG00000197582	ENSG00000198704
ENSG00000197582	ENSG00000211445
ENSG00000197584	ENSG00000135643
ENSG00000197584	ENSG00000145936
ENSG00000197584	ENSG00000171121
ENSG00000197584	ENSG00000215575
ENSG00000197586	ENSG00000187097
ENSG00000197592	ENSG00000109181
ENSG00000197592	ENSG00000135220
ENSG00000197592	ENSG00000135226
ENSG00000197592	ENSG00000145626
ENSG00000197592	ENSG00000156096
ENSG00000197592	ENSG00000167165
ENSG00000197592	ENSG00000168671
ENSG00000197592	ENSG00000171234
ENSG00000197592	ENSG00000173610
ENSG00000197592	ENSG00000174607
ENSG00000197592	ENSG00000196620
ENSG00000197592	ENSG00000197888

ENSG00000197592	ENSG00000198277
ENSG00000197592	ENSG00000213759
ENSG00000197594	ENSG00000001561
ENSG00000197594	ENSG00000112796
ENSG00000197594	ENSG00000136960
ENSG00000197594	ENSG00000154269
ENSG00000197594	ENSG00000164303
ENSG00000197594	ENSG00000182156
ENSG00000197601	ENSG00000064763
ENSG00000197604	ENSG00000212121
ENSG00000197614	ENSG00000117122
ENSG00000197616	ENSG00000002079
ENSG00000197616	ENSG00000006788
ENSG00000197616	ENSG00000078814
ENSG00000197616	ENSG00000092054
ENSG00000197616	ENSG00000109061
ENSG00000197616	ENSG00000109063
ENSG00000197616	ENSG00000125414
ENSG00000197616	ENSG00000133020
ENSG00000197616	ENSG00000141048
ENSG00000197616	ENSG00000144821
ENSG00000197622	ENSG00000158985
ENSG00000197627	ENSG00000102471
ENSG00000197627	ENSG00000131507
ENSG00000197632	ENSG00000021355
ENSG00000197632	ENSG00000057149
ENSG00000197632	ENSG00000124570
ENSG00000197632	ENSG00000166396
ENSG00000197632	ENSG00000166401
ENSG00000197632	ENSG00000166404
ENSG00000197632	ENSG00000166634
ENSG00000197632	ENSG00000170542
ENSG00000197632	ENSG00000197641
ENSG00000197632	ENSG00000206072
ENSG00000197632	ENSG00000206073
ENSG00000197632	ENSG00000206075
ENSG00000197641	ENSG00000021355
ENSG00000197641	ENSG00000057149
ENSG00000197641	ENSG00000124570
ENSG00000197641	ENSG00000166396
ENSG00000197641	ENSG00000166401
ENSG00000197641	ENSG00000166404
ENSG00000197641	ENSG00000166634
ENSG00000197641	ENSG00000170542
ENSG00000197641	ENSG00000197632
ENSG00000197641	ENSG00000206072
ENSG00000197641	ENSG00000206073
ENSG00000197641	ENSG00000206075
ENSG00000197646	ENSG00000103855
ENSG00000197646	ENSG00000114013
ENSG00000197646	ENSG00000114455
ENSG00000197646	ENSG00000120217
ENSG00000197646	ENSG00000134258
ENSG00000197646	ENSG00000160223
ENSG00000197646	ENSG00000186470
ENSG00000197646	ENSG00000188211
ENSG00000197646	ENSG00000204290
ENSG00000197646	ENSG00000204655
ENSG00000197646	ENSG00000215631

ENSG00000197646	ENSG00000215811
ENSG00000197646	ENSG00000215889
ENSG00000197681	ENSG00000128438
ENSG00000197681	ENSG00000148429
ENSG00000197681	ENSG00000161583
ENSG00000197681	ENSG00000174115
ENSG00000197681	ENSG00000185128
ENSG00000197681	ENSG00000188755
ENSG00000197681	ENSG00000188849
ENSG00000197681	ENSG00000189309
ENSG00000197681	ENSG00000204471
ENSG00000197681	ENSG00000205019
ENSG00000197681	ENSG00000213474
ENSG00000197681	ENSG00000215625
ENSG00000197681	ENSG00000215730
ENSG00000197681	ENSG00000215748
ENSG00000197702	ENSG00000138964
ENSG00000197702	ENSG00000188677
ENSG00000197705	ENSG00000003096
ENSG00000197705	ENSG00000087448
ENSG00000197705	ENSG00000124743
ENSG00000197705	ENSG00000130487
ENSG00000197705	ENSG00000135686
ENSG00000197705	ENSG00000166853
ENSG00000197705	ENSG00000167487
ENSG00000197705	ENSG00000174010
ENSG00000197705	ENSG00000176595
ENSG00000197705	ENSG00000179023
ENSG00000197705	ENSG00000185214
ENSG00000197705	ENSG00000185915
ENSG00000197705	ENSG00000186231
ENSG00000197705	ENSG00000198642
ENSG00000197712	ENSG00000055147
ENSG00000197721	ENSG00000091583
ENSG00000197721	ENSG00000117335
ENSG00000197721	ENSG00000123838
ENSG00000197721	ENSG00000196352
ENSG00000197724	ENSG00000006459
ENSG00000197724	ENSG00000089094
ENSG00000197724	ENSG00000172943
ENSG00000197724	ENSG00000173120
ENSG00000197747	ENSG00000143546
ENSG00000197747	ENSG00000160307
ENSG00000197747	ENSG00000160678
ENSG00000197747	ENSG00000163191
ENSG00000197747	ENSG00000163220
ENSG00000197747	ENSG00000163221
ENSG00000197747	ENSG00000163993
ENSG00000197747	ENSG00000169906
ENSG00000197747	ENSG00000171643
ENSG00000197747	ENSG00000188015
ENSG00000197747	ENSG00000188643
ENSG00000197747	ENSG00000189171
ENSG00000197747	ENSG00000189334
ENSG00000197747	ENSG00000196154
ENSG00000197747	ENSG00000196420
ENSG00000197747	ENSG00000196754
ENSG00000197747	ENSG00000197956
ENSG00000197753	ENSG00000145685

ENSG00000197753	ENSG00000156959
ENSG00000197753	ENSG00000182508
ENSG00000197753	ENSG00000183722
ENSG00000197753	ENSG00000187416
ENSG00000197756	ENSG00000176343
ENSG00000197757	ENSG00000037965
ENSG00000197757	ENSG00000106004
ENSG00000197757	ENSG00000106006
ENSG00000197757	ENSG00000108511
ENSG00000197757	ENSG00000120068
ENSG00000197757	ENSG00000120075
ENSG00000197757	ENSG00000120087
ENSG00000197757	ENSG00000122592
ENSG00000197757	ENSG00000170166
ENSG00000197757	ENSG00000172789
ENSG00000197757	ENSG00000175879
ENSG00000197757	ENSG00000182742
ENSG00000197757	ENSG00000197576
ENSG00000197757	ENSG00000198353
ENSG00000197763	ENSG00000184470
ENSG00000197763	ENSG00000198431
ENSG00000197766	ENSG00000092009
ENSG00000197766	ENSG00000100448
ENSG00000197766	ENSG00000100450
ENSG00000197766	ENSG00000100453
ENSG00000197766	ENSG00000113088
ENSG00000197766	ENSG00000145649
ENSG00000197766	ENSG00000185198
ENSG00000197774	ENSG00000154920
ENSG00000197776	ENSG00000099949
ENSG00000197776	ENSG00000128607
ENSG00000197776	ENSG00000165516
ENSG00000197778	ENSG00000006659
ENSG00000197778	ENSG00000100079
ENSG00000197778	ENSG00000100097
ENSG00000197778	ENSG00000105198
ENSG00000197778	ENSG00000105205
ENSG00000197778	ENSG00000116977
ENSG00000197778	ENSG00000119862
ENSG00000197778	ENSG00000131981
ENSG00000197778	ENSG00000133317
ENSG00000197778	ENSG00000168961
ENSG00000197778	ENSG00000170298
ENSG00000197778	ENSG00000171747
ENSG00000197778	ENSG00000171916
ENSG00000197778	ENSG00000178934
ENSG00000197778	ENSG00000205076
ENSG00000197778	ENSG00000215132
ENSG00000197789	ENSG00000205557
ENSG00000197791	ENSG00000178769
ENSG00000197791	ENSG00000197382
ENSG00000197791	ENSG00000198230
ENSG00000197794	ENSG00000136700
ENSG00000197794	ENSG00000156755
ENSG00000197794	ENSG00000183719
ENSG00000197794	ENSG00000204670
ENSG00000197794	ENSG00000204776
ENSG00000197794	ENSG00000204780
ENSG00000197794	ENSG00000211599

ENSG00000197794	ENSG00000211600
ENSG00000197794	ENSG00000211601
ENSG00000197794	ENSG00000211602
ENSG00000197794	ENSG00000211603
ENSG00000197794	ENSG00000211604
ENSG00000197794	ENSG00000211605
ENSG00000197794	ENSG00000211606
ENSG00000197794	ENSG00000211607
ENSG00000197794	ENSG00000211608
ENSG00000197794	ENSG00000211609
ENSG00000197794	ENSG00000211610
ENSG00000197794	ENSG00000211611
ENSG00000197794	ENSG00000211613
ENSG00000197794	ENSG00000211616
ENSG00000197794	ENSG00000211617
ENSG00000197794	ENSG00000211618
ENSG00000197794	ENSG00000211620
ENSG00000197794	ENSG00000211625
ENSG00000197794	ENSG00000211626
ENSG00000197794	ENSG00000211627
ENSG00000197794	ENSG00000211628
ENSG00000197794	ENSG00000211629
ENSG00000197794	ENSG00000211630
ENSG00000197794	ENSG00000211631
ENSG00000197794	ENSG00000211632
ENSG00000197794	ENSG00000211633
ENSG00000197794	ENSG00000211634
ENSG00000197794	ENSG00000211635
ENSG00000197794	ENSG00000211636
ENSG00000197822	ENSG00000105656
ENSG00000197822	ENSG00000118985
ENSG00000197822	ENSG00000152939
ENSG00000197822	ENSG00000187213
ENSG00000197833	ENSG00000183812
ENSG00000197833	ENSG00000204805
ENSG00000197833	ENSG00000204806
ENSG00000197833	ENSG00000204807
ENSG00000197833	ENSG00000204808
ENSG00000197833	ENSG00000205613
ENSG00000197833	ENSG00000215061
ENSG00000197837	ENSG00000124529
ENSG00000197837	ENSG00000124578
ENSG00000197837	ENSG00000158406
ENSG00000197837	ENSG00000182217
ENSG00000197837	ENSG00000183941
ENSG00000197837	ENSG00000188987
ENSG00000197837	ENSG00000196176
ENSG00000197837	ENSG00000197061
ENSG00000197837	ENSG00000197238
ENSG00000197837	ENSG00000197914
ENSG00000197837	ENSG00000198327
ENSG00000197837	ENSG00000198339
ENSG00000197837	ENSG00000198518
ENSG00000197837	ENSG00000198558
ENSG00000197852	ENSG00000185614
ENSG00000197859	ENSG00000100767
ENSG00000197859	ENSG00000143382
ENSG00000197859	ENSG00000185761
ENSG00000197859	ENSG00000187720

ENSG00000197859	ENSG00000215616
ENSG00000197860	ENSG00000104969
ENSG00000197865	ENSG00000105366
ENSG00000197865	ENSG00000105383
ENSG00000197865	ENSG00000105492
ENSG00000197865	ENSG00000105501
ENSG00000197865	ENSG00000105695
ENSG00000197865	ENSG00000129450
ENSG00000197865	ENSG00000142512
ENSG00000197865	ENSG00000160296
ENSG00000197865	ENSG00000161640
ENSG00000197865	ENSG00000161643
ENSG00000197865	ENSG00000167764
ENSG00000197865	ENSG00000168995
ENSG00000197865	ENSG00000171101
ENSG00000197872	ENSG00000153310
ENSG00000197879	ENSG00000128641
ENSG00000197879	ENSG00000136286
ENSG00000197879	ENSG00000142347
ENSG00000197879	ENSG00000157483
ENSG00000197879	ENSG00000166866
ENSG00000197879	ENSG00000176658
ENSG00000197885	ENSG00000168256
ENSG00000197888	ENSG00000109181
ENSG00000197888	ENSG00000135220
ENSG00000197888	ENSG00000135226
ENSG00000197888	ENSG00000145626
ENSG00000197888	ENSG00000156096
ENSG00000197888	ENSG00000167165
ENSG00000197888	ENSG00000168671
ENSG00000197888	ENSG00000171234
ENSG00000197888	ENSG00000173610
ENSG00000197888	ENSG00000174607
ENSG00000197888	ENSG00000196620
ENSG00000197888	ENSG00000197592
ENSG00000197888	ENSG00000198277
ENSG00000197888	ENSG00000213759
ENSG00000197892	ENSG00000054523
ENSG00000197892	ENSG00000089177
ENSG00000197892	ENSG00000118193
ENSG00000197892	ENSG00000129250
ENSG00000197892	ENSG00000130294
ENSG00000197892	ENSG00000137177
ENSG00000197893	ENSG00000129170
ENSG00000197893	ENSG00000136490
ENSG00000197893	ENSG00000146215
ENSG00000197893	ENSG00000159176
ENSG00000197893	ENSG00000175183
ENSG00000197893	ENSG00000182809
ENSG00000197893	ENSG00000213145
ENSG00000197894	ENSG00000172955
ENSG00000197894	ENSG00000187758
ENSG00000197894	ENSG00000196344
ENSG00000197894	ENSG00000196616
ENSG00000197894	ENSG00000198099
ENSG00000197914	ENSG00000124529
ENSG00000197914	ENSG00000124578
ENSG00000197914	ENSG00000158406
ENSG00000197914	ENSG00000182217

ENSG00000197914	ENSG00000183941
ENSG00000197914	ENSG00000188987
ENSG00000197914	ENSG00000196176
ENSG00000197914	ENSG00000197061
ENSG00000197914	ENSG00000197238
ENSG00000197914	ENSG00000197837
ENSG00000197914	ENSG00000198327
ENSG00000197914	ENSG00000198339
ENSG00000197914	ENSG00000198518
ENSG00000197914	ENSG00000198558
ENSG00000197921	ENSG00000069812
ENSG00000197921	ENSG00000114315
ENSG00000197921	ENSG00000144485
ENSG00000197921	ENSG00000173673
ENSG00000197921	ENSG00000179111
ENSG00000197921	ENSG00000188290
ENSG00000197930	ENSG00000086619
ENSG00000197943	ENSG00000101333
ENSG00000197943	ENSG00000114805
ENSG00000197943	ENSG00000115556
ENSG00000197943	ENSG00000115896
ENSG00000197943	ENSG00000124181
ENSG00000197943	ENSG00000137841
ENSG00000197943	ENSG00000139151
ENSG00000197943	ENSG00000149527
ENSG00000197943	ENSG00000149782
ENSG00000197943	ENSG00000154822
ENSG00000197943	ENSG00000161714
ENSG00000197943	ENSG00000182621
ENSG00000197943	ENSG00000187091
ENSG00000197948	ENSG00000137478
ENSG00000197953	ENSG00000114771
ENSG00000197953	ENSG00000144959
ENSG00000197953	ENSG00000188984
ENSG00000197953	ENSG00000204518
ENSG00000197956	ENSG00000143546
ENSG00000197956	ENSG00000160307
ENSG00000197956	ENSG00000160678
ENSG00000197956	ENSG00000163191
ENSG00000197956	ENSG00000163220
ENSG00000197956	ENSG00000163221
ENSG00000197956	ENSG00000163993
ENSG00000197956	ENSG00000169906
ENSG00000197956	ENSG00000171643
ENSG00000197956	ENSG00000188015
ENSG00000197956	ENSG00000188643
ENSG00000197956	ENSG00000189171
ENSG00000197956	ENSG00000189334
ENSG00000197956	ENSG00000196154
ENSG00000197956	ENSG00000196420
ENSG00000197956	ENSG00000196754
ENSG00000197956	ENSG00000197747
ENSG00000197959	ENSG00000079805
ENSG00000197959	ENSG00000106976
ENSG00000197965	ENSG00000149573
ENSG00000197965	ENSG00000149575
ENSG00000197965	ENSG00000158887
ENSG00000197965	ENSG00000160588
ENSG00000197965	ENSG00000160593

ENSG00000197965	ENSG00000177098
ENSG00000197969	ENSG00000048707
ENSG00000197969	ENSG00000129003
ENSG00000197976	ENSG00000214992
ENSG00000197977	ENSG00000012660
ENSG00000197977	ENSG00000066322
ENSG00000197977	ENSG00000118402
ENSG00000197977	ENSG00000164181
ENSG00000197991	ENSG00000099715
ENSG00000197991	ENSG00000102290
ENSG00000197991	ENSG00000113555
ENSG00000197991	ENSG00000156453
ENSG00000197991	ENSG00000169851
ENSG00000197991	ENSG00000184226
ENSG00000197991	ENSG00000215442
ENSG00000197993	ENSG00000102174
ENSG00000197993	ENSG00000117298
ENSG00000197993	ENSG00000142606
ENSG00000197993	ENSG00000145194
ENSG00000197993	ENSG00000171551
ENSG00000197993	ENSG00000196549
ENSG00000198010	ENSG00000080845
ENSG00000198010	ENSG00000116544
ENSG00000198010	ENSG00000126787
ENSG00000198010	ENSG00000170579
ENSG00000198018	ENSG00000197217
ENSG00000198019	ENSG00000072694
ENSG00000198019	ENSG00000132185
ENSG00000198019	ENSG00000143226
ENSG00000198019	ENSG00000150337
ENSG00000198019	ENSG00000162747
ENSG00000198019	ENSG00000179639
ENSG00000198019	ENSG00000181036
ENSG00000198019	ENSG00000203747
ENSG00000198019	ENSG00000203820
ENSG00000198035	ENSG00000108262
ENSG00000198035	ENSG00000133612
ENSG00000198035	ENSG00000135439
ENSG00000198035	ENSG00000139436
ENSG00000198035	ENSG00000151303
ENSG00000198035	ENSG00000157985
ENSG00000198035	ENSG00000172650
ENSG00000198035	ENSG00000174194
ENSG00000198035	ENSG00000188234
ENSG00000198035	ENSG00000204149
ENSG00000198035	ENSG00000204169
ENSG00000198035	ENSG00000204172
ENSG00000198049	ENSG00000109163
ENSG00000198049	ENSG00000126895
ENSG00000198049	ENSG00000166148
ENSG00000198049	ENSG00000178015
ENSG00000198049	ENSG00000180914
ENSG00000198049	ENSG00000187258
ENSG00000198049	ENSG00000211451
ENSG00000198055	ENSG00000100077
ENSG00000198055	ENSG00000114124
ENSG00000198055	ENSG00000125388
ENSG00000198055	ENSG00000173020
ENSG00000198055	ENSG00000185974

ENSG00000198055	ENSG00000198873
ENSG00000198075	ENSG00000088002
ENSG00000198075	ENSG00000105398
ENSG00000198075	ENSG00000109193
ENSG00000198075	ENSG00000130540
ENSG00000198075	ENSG00000138068
ENSG00000198075	ENSG00000173597
ENSG00000198075	ENSG00000196228
ENSG00000198075	ENSG00000196502
ENSG00000198075	ENSG00000197165
ENSG00000198075	ENSG00000198203
ENSG00000198075	ENSG00000213599
ENSG00000198075	ENSG00000213648
ENSG00000198081	ENSG00000174282
ENSG00000198081	ENSG00000177311
ENSG00000198081	ENSG00000177485
ENSG00000198082	ENSG00000111332
ENSG00000198082	ENSG00000124642
ENSG00000198082	ENSG00000137259
ENSG00000198082	ENSG00000158403
ENSG00000198082	ENSG00000164508
ENSG00000198082	ENSG00000168274
ENSG00000198082	ENSG00000180573
ENSG00000198082	ENSG00000181218
ENSG00000198082	ENSG00000182611
ENSG00000198082	ENSG00000183558
ENSG00000198082	ENSG00000184260
ENSG00000198082	ENSG00000184270
ENSG00000198082	ENSG00000184348
ENSG00000198082	ENSG00000184825
ENSG00000198082	ENSG00000185978
ENSG00000198082	ENSG00000188486
ENSG00000198082	ENSG00000196747
ENSG00000198082	ENSG00000196787
ENSG00000198082	ENSG00000196866
ENSG00000198082	ENSG00000198307
ENSG00000198082	ENSG00000198374
ENSG00000198082	ENSG00000203812
ENSG00000198082	ENSG00000205070
ENSG00000198082	ENSG00000205079
ENSG00000198087	ENSG00000147010
ENSG00000198099	ENSG00000172955
ENSG00000198099	ENSG00000187758
ENSG00000198099	ENSG00000196344
ENSG00000198099	ENSG00000196616
ENSG00000198099	ENSG00000197894
ENSG00000198108	ENSG00000033100
ENSG00000198108	ENSG00000123989
ENSG00000198108	ENSG00000131873
ENSG00000198108	ENSG00000139044
ENSG00000198108	ENSG00000147408
ENSG00000198108	ENSG00000169826
ENSG00000198108	ENSG00000182272
ENSG00000198113	ENSG00000136816
ENSG00000198113	ENSG00000136827
ENSG00000198113	ENSG00000160404
ENSG00000198113	ENSG00000186283
ENSG00000198121	ENSG00000064547
ENSG00000198121	ENSG00000118432

ENSG00000198121	ENSG00000125910
ENSG00000198121	ENSG00000132975
ENSG00000198121	ENSG00000146360
ENSG00000198121	ENSG00000170989
ENSG00000198121	ENSG00000171517
ENSG00000198121	ENSG00000175898
ENSG00000198121	ENSG00000180739
ENSG00000198121	ENSG00000181773
ENSG00000198121	ENSG00000188822
ENSG00000198121	ENSG00000213694
ENSG00000198125	ENSG00000161544
ENSG00000198142	ENSG00000186212
ENSG00000198142	ENSG00000187808
ENSG00000198142	ENSG00000198944
ENSG00000198155	ENSG00000142396
ENSG00000198155	ENSG00000169740
ENSG00000198155	ENSG00000174255
ENSG00000198155	ENSG00000179195
ENSG00000198155	ENSG00000179965
ENSG00000198155	ENSG00000184294
ENSG00000198155	ENSG00000186446
ENSG00000198155	ENSG00000186777
ENSG00000198155	ENSG00000197008
ENSG00000198155	ENSG00000197025
ENSG00000198155	ENSG00000197062
ENSG00000198155	ENSG00000197332
ENSG00000198155	ENSG00000204756
ENSG00000198155	ENSG00000212805
ENSG00000198155	ENSG00000213976
ENSG00000198155	ENSG00000213980
ENSG00000198155	ENSG00000215180
ENSG00000198155	ENSG00000215510
ENSG00000198155	ENSG00000215537
ENSG00000198155	ENSG00000215598
ENSG00000198155	ENSG00000215600
ENSG00000198157	ENSG00000157576
ENSG00000198157	ENSG00000172186
ENSG00000198157	ENSG00000182765
ENSG00000198157	ENSG00000184409
ENSG00000198157	ENSG00000197366
ENSG00000198157	ENSG00000198875
ENSG00000198160	ENSG00000105556
ENSG00000198160	ENSG00000155545
ENSG00000198161	ENSG00000108179
ENSG00000198161	ENSG00000150276
ENSG00000198161	ENSG00000196262
ENSG00000198161	ENSG00000198360
ENSG00000198161	ENSG00000198618
ENSG00000198161	ENSG00000198936
ENSG00000198161	ENSG00000203847
ENSG00000198161	ENSG00000214975
ENSG00000198161	ENSG00000215551
ENSG00000198162	ENSG00000111885
ENSG00000198162	ENSG00000117643
ENSG00000198176	ENSG00000114126
ENSG00000198203	ENSG00000088002
ENSG00000198203	ENSG00000105398
ENSG00000198203	ENSG00000109193
ENSG00000198203	ENSG00000130540

ENSG00000198203	ENSG00000138068
ENSG00000198203	ENSG00000173597
ENSG00000198203	ENSG00000196228
ENSG00000198203	ENSG00000196502
ENSG00000198203	ENSG00000197165
ENSG00000198203	ENSG00000198075
ENSG00000198203	ENSG00000213599
ENSG00000198203	ENSG00000213648
ENSG00000198208	ENSG00000110025
ENSG00000198208	ENSG00000136643
ENSG00000198211	ENSG00000101162
ENSG00000198211	ENSG00000104833
ENSG00000198211	ENSG00000127589
ENSG00000198211	ENSG00000137267
ENSG00000198211	ENSG00000137285
ENSG00000198211	ENSG00000159247
ENSG00000198211	ENSG00000173213
ENSG00000198211	ENSG00000173876
ENSG00000198211	ENSG00000176014
ENSG00000198211	ENSG00000188229
ENSG00000198211	ENSG00000196230
ENSG00000198211	ENSG00000215801
ENSG00000198211	ENSG00000215813
ENSG00000198211	ENSG00000215814
ENSG00000198216	ENSG00000081248
ENSG00000198216	ENSG00000102001
ENSG00000198216	ENSG00000141837
ENSG00000198216	ENSG00000148408
ENSG00000198216	ENSG00000151067
ENSG00000198216	ENSG00000157388
ENSG00000198218	ENSG00000121741
ENSG00000198218	ENSG00000132950
ENSG00000198218	ENSG00000146463
ENSG00000198218	ENSG00000147130
ENSG00000198218	ENSG00000163867
ENSG00000198223	ENSG00000091181
ENSG00000198223	ENSG00000123496
ENSG00000198223	ENSG00000131724
ENSG00000198223	ENSG00000185291
ENSG00000198225	ENSG00000088832
ENSG00000198225	ENSG00000119782
ENSG00000198225	ENSG00000146722
ENSG00000198230	ENSG00000178769
ENSG00000198230	ENSG00000197382
ENSG00000198230	ENSG00000197791
ENSG00000198242	ENSG00000118975
ENSG00000198242	ENSG00000184319
ENSG00000198242	ENSG00000185822
ENSG00000198242	ENSG00000186239
ENSG00000198242	ENSG00000186698
ENSG00000198242	ENSG00000206147
ENSG00000198242	ENSG00000212932
ENSG00000198242	ENSG00000213753
ENSG00000198242	ENSG00000214230
ENSG00000198242	ENSG00000215001
ENSG00000198242	ENSG00000215140
ENSG00000198242	ENSG00000215387
ENSG00000198250	ENSG00000163297
ENSG00000198250	ENSG00000169604

ENSG00000198277	ENSG00000109181
ENSG00000198277	ENSG00000135220
ENSG00000198277	ENSG00000135226
ENSG00000198277	ENSG00000145626
ENSG00000198277	ENSG00000156096
ENSG00000198277	ENSG00000167165
ENSG00000198277	ENSG00000168671
ENSG00000198277	ENSG00000171234
ENSG00000198277	ENSG00000173610
ENSG00000198277	ENSG00000174607
ENSG00000198277	ENSG00000196620
ENSG00000198277	ENSG00000197592
ENSG00000198277	ENSG00000197888
ENSG00000198277	ENSG00000213759
ENSG00000198286	ENSG00000100065
ENSG00000198286	ENSG00000187796
ENSG00000198305	ENSG00000136206
ENSG00000198305	ENSG00000154732
ENSG00000198305	ENSG00000163806
ENSG00000198305	ENSG00000166667
ENSG00000198305	ENSG00000170092
ENSG00000198305	ENSG00000173678
ENSG00000198305	ENSG00000179994
ENSG00000198305	ENSG00000183318
ENSG00000198305	ENSG00000184616
ENSG00000198305	ENSG00000185040
ENSG00000198305	ENSG00000186645
ENSG00000198305	ENSG00000204710
ENSG00000198305	ENSG00000205238
ENSG00000198305	ENSG00000205482
ENSG00000198305	ENSG00000205505
ENSG00000198305	ENSG00000205506
ENSG00000198305	ENSG00000205508
ENSG00000198305	ENSG00000205509
ENSG00000198305	ENSG00000205580
ENSG00000198305	ENSG00000214300
ENSG00000198305	ENSG00000215092
ENSG00000198307	ENSG00000111332
ENSG00000198307	ENSG00000124642
ENSG00000198307	ENSG00000137259
ENSG00000198307	ENSG00000158403
ENSG00000198307	ENSG00000164508
ENSG00000198307	ENSG00000168274
ENSG00000198307	ENSG00000180573
ENSG00000198307	ENSG00000181218
ENSG00000198307	ENSG00000182611
ENSG00000198307	ENSG00000183558
ENSG00000198307	ENSG00000184260
ENSG00000198307	ENSG00000184270
ENSG00000198307	ENSG00000184348
ENSG00000198307	ENSG00000184825
ENSG00000198307	ENSG00000185978
ENSG00000198307	ENSG00000188486
ENSG00000198307	ENSG00000196747
ENSG00000198307	ENSG00000196787
ENSG00000198307	ENSG00000196866
ENSG00000198307	ENSG00000198082
ENSG00000198307	ENSG00000198374
ENSG00000198307	ENSG00000203812

ENSG00000198307	ENSG00000205070
ENSG00000198307	ENSG00000205079
ENSG00000198324	ENSG00000177096
ENSG00000198327	ENSG00000124529
ENSG00000198327	ENSG00000124578
ENSG00000198327	ENSG00000158406
ENSG00000198327	ENSG00000182217
ENSG00000198327	ENSG00000183941
ENSG00000198327	ENSG00000188987
ENSG00000198327	ENSG00000196176
ENSG00000198327	ENSG00000197061
ENSG00000198327	ENSG00000197238
ENSG00000198327	ENSG00000197837
ENSG00000198327	ENSG00000197914
ENSG00000198327	ENSG00000198339
ENSG00000198327	ENSG00000198518
ENSG00000198327	ENSG00000198558
ENSG00000198336	ENSG00000092841
ENSG00000198336	ENSG00000160808
ENSG00000198336	ENSG00000168530
ENSG00000198336	ENSG00000196465
ENSG00000198339	ENSG00000124529
ENSG00000198339	ENSG00000124578
ENSG00000198339	ENSG00000158406
ENSG00000198339	ENSG00000182217
ENSG00000198339	ENSG00000183941
ENSG00000198339	ENSG00000188987
ENSG00000198339	ENSG00000196176
ENSG00000198339	ENSG00000197061
ENSG00000198339	ENSG00000197238
ENSG00000198339	ENSG00000197837
ENSG00000198339	ENSG00000197914
ENSG00000198339	ENSG00000198327
ENSG00000198339	ENSG00000198518
ENSG00000198339	ENSG00000198558
ENSG00000198353	ENSG00000037965
ENSG00000198353	ENSG00000106004
ENSG00000198353	ENSG00000106006
ENSG00000198353	ENSG00000108511
ENSG00000198353	ENSG00000120068
ENSG00000198353	ENSG00000120075
ENSG00000198353	ENSG00000120087
ENSG00000198353	ENSG00000122592
ENSG00000198353	ENSG00000170166
ENSG00000198353	ENSG00000172789
ENSG00000198353	ENSG00000175879
ENSG00000198353	ENSG00000182742
ENSG00000198353	ENSG00000197576
ENSG00000198353	ENSG00000197757
ENSG00000198354	ENSG00000198876
ENSG00000198354	ENSG00000198889
ENSG00000198355	ENSG00000102096
ENSG00000198355	ENSG00000137193
ENSG00000198360	ENSG00000108179
ENSG00000198360	ENSG00000150276
ENSG00000198360	ENSG00000196262
ENSG00000198360	ENSG00000198161
ENSG00000198360	ENSG00000198618
ENSG00000198360	ENSG00000198936

ENSG00000198360	ENSG00000203847
ENSG00000198360	ENSG00000214975
ENSG00000198360	ENSG00000215551
ENSG00000198361	ENSG00000198684
ENSG00000198361	ENSG00000204202
ENSG00000198361	ENSG00000204203
ENSG00000198361	ENSG00000204204
ENSG00000198361	ENSG00000204205
ENSG00000198361	ENSG00000204206
ENSG00000198361	ENSG00000204207
ENSG00000198361	ENSG00000205731
ENSG00000198361	ENSG00000205735
ENSG00000198361	ENSG00000205737
ENSG00000198361	ENSG00000206163
ENSG00000198369	ENSG00000125753
ENSG00000198369	ENSG00000154380
ENSG00000198369	ENSG00000166068
ENSG00000198369	ENSG00000188766
ENSG00000198369	ENSG00000196405
ENSG00000198373	ENSG00000049759
ENSG00000198373	ENSG00000069869
ENSG00000198373	ENSG00000078747
ENSG00000198373	ENSG00000108854
ENSG00000198373	ENSG00000123124
ENSG00000198373	ENSG00000198742
ENSG00000198374	ENSG00000111332
ENSG00000198374	ENSG00000124642
ENSG00000198374	ENSG00000137259
ENSG00000198374	ENSG00000158403
ENSG00000198374	ENSG00000164508
ENSG00000198374	ENSG00000168274
ENSG00000198374	ENSG00000180573
ENSG00000198374	ENSG00000181218
ENSG00000198374	ENSG00000182611
ENSG00000198374	ENSG00000183558
ENSG00000198374	ENSG00000184260
ENSG00000198374	ENSG00000184270
ENSG00000198374	ENSG00000184348
ENSG00000198374	ENSG00000184825
ENSG00000198374	ENSG00000185978
ENSG00000198374	ENSG00000188486
ENSG00000198374	ENSG00000196747
ENSG00000198374	ENSG00000196787
ENSG00000198374	ENSG00000196866
ENSG00000198374	ENSG00000198082
ENSG00000198374	ENSG00000198307
ENSG00000198374	ENSG00000203812
ENSG00000198374	ENSG00000205070
ENSG00000198374	ENSG00000205079
ENSG00000198380	ENSG00000131459
ENSG00000198399	ENSG00000085832
ENSG00000198399	ENSG00000127527
ENSG00000198399	ENSG00000205726
ENSG00000198400	ENSG00000030304
ENSG00000198400	ENSG00000140538
ENSG00000198400	ENSG00000148053
ENSG00000198400	ENSG00000169071
ENSG00000198400	ENSG00000185483
ENSG00000198417	ENSG00000087250

ENSG00000198417	ENSG00000102891
ENSG00000198417	ENSG00000125144
ENSG00000198417	ENSG00000125148
ENSG00000198417	ENSG00000169688
ENSG00000198417	ENSG00000169715
ENSG00000198417	ENSG00000187193
ENSG00000198417	ENSG00000205358
ENSG00000198417	ENSG00000205360
ENSG00000198417	ENSG00000205362
ENSG00000198417	ENSG00000205364
ENSG00000198431	ENSG00000184470
ENSG00000198431	ENSG00000197763
ENSG00000198435	ENSG00000123080
ENSG00000198435	ENSG00000129355
ENSG00000198450	ENSG00000100473
ENSG00000198450	ENSG00000163359
ENSG00000198450	ENSG00000165816
ENSG00000198450	ENSG00000205221
ENSG00000198457	ENSG00000148795
ENSG00000198467	ENSG00000140416
ENSG00000198467	ENSG00000143549
ENSG00000198467	ENSG00000167460
ENSG00000198467	ENSG00000177152
ENSG00000198467	ENSG00000183022
ENSG00000198467	ENSG00000187536
ENSG00000198467	ENSG00000214869
ENSG00000198477	ENSG00000056277
ENSG00000198477	ENSG00000137871
ENSG00000198477	ENSG00000143442
ENSG00000198477	ENSG00000169548
ENSG00000198478	ENSG00000131171
ENSG00000198478	ENSG00000142669
ENSG00000198478	ENSG00000185437
ENSG00000198478	ENSG00000214636
ENSG00000198483	ENSG00000039560
ENSG00000198483	ENSG00000089847
ENSG00000198483	ENSG00000137831
ENSG00000198488	ENSG00000156966
ENSG00000198488	ENSG00000162630
ENSG00000198488	ENSG00000169255
ENSG00000198488	ENSG00000170340
ENSG00000198488	ENSG00000172318
ENSG00000198488	ENSG00000176383
ENSG00000198488	ENSG00000176597
ENSG00000198488	ENSG00000177191
ENSG00000198488	ENSG00000179913
ENSG00000198488	ENSG00000180561
ENSG00000198488	ENSG00000183778
ENSG00000198488	ENSG00000204222
ENSG00000198488	ENSG00000214654
ENSG00000198492	ENSG00000149658
ENSG00000198492	ENSG00000185728
ENSG00000198493	ENSG00000091129
ENSG00000198493	ENSG00000134121
ENSG00000198493	ENSG00000149972
ENSG00000198493	ENSG00000163531
ENSG00000198493	ENSG00000184144
ENSG00000198513	ENSG00000119787
ENSG00000198513	ENSG00000184743

ENSG00000198517	ENSG00000129535
ENSG00000198517	ENSG00000178573
ENSG00000198517	ENSG00000182759
ENSG00000198517	ENSG00000185022
ENSG00000198517	ENSG00000197063
ENSG00000198517	ENSG00000204103
ENSG00000198517	ENSG00000215622
ENSG00000198518	ENSG00000124529
ENSG00000198518	ENSG00000124578
ENSG00000198518	ENSG00000158406
ENSG00000198518	ENSG00000182217
ENSG00000198518	ENSG00000183941
ENSG00000198518	ENSG00000188987
ENSG00000198518	ENSG00000196176
ENSG00000198518	ENSG00000197061
ENSG00000198518	ENSG00000197238
ENSG00000198518	ENSG00000197837
ENSG00000198518	ENSG00000197914
ENSG00000198518	ENSG00000198327
ENSG00000198518	ENSG00000198339
ENSG00000198518	ENSG00000198558
ENSG00000198522	ENSG00000196700
ENSG00000198535	ENSG00000183186
ENSG00000198535	ENSG00000205502
ENSG00000198555	ENSG00000010379
ENSG00000198555	ENSG00000011083
ENSG00000198555	ENSG00000087916
ENSG00000198555	ENSG00000103546
ENSG00000198555	ENSG00000108576
ENSG00000198555	ENSG00000111181
ENSG00000198555	ENSG00000130821
ENSG00000198555	ENSG00000131389
ENSG00000198555	ENSG00000132164
ENSG00000198555	ENSG00000142319
ENSG00000198555	ENSG00000157103
ENSG00000198555	ENSG00000163817
ENSG00000198555	ENSG00000164363
ENSG00000198555	ENSG00000165970
ENSG00000198555	ENSG00000174358
ENSG00000198555	ENSG00000196517
ENSG00000198555	ENSG00000214617
ENSG00000198558	ENSG00000124529
ENSG00000198558	ENSG00000124578
ENSG00000198558	ENSG00000158406
ENSG00000198558	ENSG00000182217
ENSG00000198558	ENSG00000183941
ENSG00000198558	ENSG00000188987
ENSG00000198558	ENSG00000196176
ENSG00000198558	ENSG00000197061
ENSG00000198558	ENSG00000197238
ENSG00000198558	ENSG00000197837
ENSG00000198558	ENSG00000197914
ENSG00000198558	ENSG00000198327
ENSG00000198558	ENSG00000198339
ENSG00000198558	ENSG00000198518
ENSG00000198561	ENSG00000057294
ENSG00000198561	ENSG00000081277
ENSG00000198561	ENSG00000099889
ENSG00000198561	ENSG00000144283

ENSG00000198561	ENSG00000169862
ENSG00000198561	ENSG00000184363
ENSG00000198563	ENSG00000123136
ENSG00000198569	ENSG00000131183
ENSG00000198569	ENSG00000157765
ENSG00000198574	ENSG00000183918
ENSG00000198586	ENSG00000146872
ENSG00000198586	ENSG00000214705
ENSG00000198586	ENSG00000215170
ENSG00000198589	ENSG00000172915
ENSG00000198597	ENSG00000101493
ENSG00000198597	ENSG00000165804
ENSG00000198597	ENSG00000171940
ENSG00000198598	ENSG00000008516
ENSG00000198598	ENSG00000087245
ENSG00000198598	ENSG00000099953
ENSG00000198598	ENSG00000100985
ENSG00000198598	ENSG00000102996
ENSG00000198598	ENSG00000110347
ENSG00000198598	ENSG00000118113
ENSG00000198598	ENSG00000123342
ENSG00000198598	ENSG00000125966
ENSG00000198598	ENSG00000129270
ENSG00000198598	ENSG00000137673
ENSG00000198598	ENSG00000137674
ENSG00000198598	ENSG00000137675
ENSG00000198598	ENSG00000137745
ENSG00000198598	ENSG00000149968
ENSG00000198598	ENSG00000154485
ENSG00000198598	ENSG00000156103
ENSG00000198598	ENSG00000157227
ENSG00000198598	ENSG00000166670
ENSG00000198598	ENSG00000167346
ENSG00000198598	ENSG00000189409
ENSG00000198598	ENSG00000196611
ENSG00000198598	ENSG00000215914
ENSG00000198610	ENSG00000085662
ENSG00000198610	ENSG00000117448
ENSG00000198610	ENSG00000122787
ENSG00000198610	ENSG00000151631
ENSG00000198610	ENSG00000151632
ENSG00000198610	ENSG00000165568
ENSG00000198610	ENSG00000187134
ENSG00000198610	ENSG00000196139
ENSG00000198610	ENSG00000196326
ENSG00000198610	ENSG00000198074
ENSG00000198610	ENSG00000215267
ENSG00000198618	ENSG00000108179
ENSG00000198618	ENSG00000150276
ENSG00000198618	ENSG00000196262
ENSG00000198618	ENSG00000198161
ENSG00000198618	ENSG00000198360
ENSG00000198618	ENSG00000198936
ENSG00000198618	ENSG00000203847
ENSG00000198618	ENSG00000214975
ENSG00000198618	ENSG00000215551
ENSG00000198624	ENSG00000129422
ENSG00000198624	ENSG00000132938
ENSG00000198625	ENSG00000135679

ENSG00000198626	ENSG00000196218
ENSG00000198626	ENSG00000198838
ENSG00000198642	ENSG00000003096
ENSG00000198642	ENSG00000087448
ENSG00000198642	ENSG00000124743
ENSG00000198642	ENSG00000130487
ENSG00000198642	ENSG00000135686
ENSG00000198642	ENSG00000166853
ENSG00000198642	ENSG00000167487
ENSG00000198642	ENSG00000174010
ENSG00000198642	ENSG00000176595
ENSG00000198642	ENSG00000179023
ENSG00000198642	ENSG00000185214
ENSG00000198642	ENSG00000185915
ENSG00000198642	ENSG00000186231
ENSG00000198642	ENSG00000197705
ENSG00000198643	ENSG00000071889
ENSG00000198643	ENSG00000174028
ENSG00000198643	ENSG00000183844
ENSG00000198643	ENSG00000196937
ENSG00000198648	ENSG00000172939
ENSG00000198658	ENSG00000107362
ENSG00000198658	ENSG00000129968
ENSG00000198658	ENSG00000136379
ENSG00000198658	ENSG00000182502
ENSG00000198658	ENSG00000182556
ENSG00000198658	ENSG00000203835
ENSG00000198668	ENSG00000129007
ENSG00000198668	ENSG00000143933
ENSG00000198668	ENSG00000160014
ENSG00000198668	ENSG00000178363
ENSG00000198668	ENSG00000178372
ENSG00000198669	ENSG00000101321
ENSG00000198669	ENSG00000158156
ENSG00000198669	ENSG00000171044
ENSG00000198669	ENSG00000186530
ENSG00000198669	ENSG00000206579
ENSG00000198670	ENSG00000019991
ENSG00000198670	ENSG00000104368
ENSG00000198670	ENSG00000109758
ENSG00000198670	ENSG00000122194
ENSG00000198670	ENSG00000122861
ENSG00000198670	ENSG00000131187
ENSG00000198670	ENSG00000148702
ENSG00000198670	ENSG00000173531
ENSG00000198670	ENSG00000186301
ENSG00000198670	ENSG00000186715
ENSG00000198673	ENSG00000163377
ENSG00000198673	ENSG00000183662
ENSG00000198673	ENSG00000184599
ENSG00000198682	ENSG00000138801
ENSG00000198689	ENSG00000065923
ENSG00000198689	ENSG00000181804
ENSG00000198691	ENSG00000064687
ENSG00000198691	ENSG00000107331
ENSG00000198691	ENSG00000144452
ENSG00000198691	ENSG00000165029
ENSG00000198691	ENSG00000167972
ENSG00000198691	ENSG00000179869

ENSG00000198718	ENSG00000189350
ENSG00000198719	ENSG00000090932
ENSG00000198719	ENSG00000101384
ENSG00000198719	ENSG00000128917
ENSG00000198719	ENSG00000171462
ENSG00000198719	ENSG00000184916
ENSG00000198719	ENSG00000185559
ENSG00000198720	ENSG00000076513
ENSG00000198720	ENSG00000118454
ENSG00000198720	ENSG00000172932
ENSG00000198722	ENSG00000130477
ENSG00000198722	ENSG00000137766
ENSG00000198728	ENSG00000169744
ENSG00000198729	ENSG00000166143
ENSG00000198729	ENSG00000167641
ENSG00000198729	ENSG00000173457
ENSG00000198731	ENSG00000130041
ENSG00000198731	ENSG00000163144
ENSG00000198731	ENSG00000177954
ENSG00000198731	ENSG00000185088
ENSG00000198732	ENSG00000087303
ENSG00000198732	ENSG00000112562
ENSG00000198732	ENSG00000116962
ENSG00000198734	ENSG00000047457
ENSG00000198734	ENSG00000089472
ENSG00000198734	ENSG00000181333
ENSG00000198734	ENSG00000185010
ENSG00000198739	ENSG00000136457
ENSG00000198739	ENSG00000146006
ENSG00000198739	ENSG00000162951
ENSG00000198739	ENSG00000176204
ENSG00000198740	ENSG00000114853
ENSG00000198742	ENSG00000049759
ENSG00000198742	ENSG00000069869
ENSG00000198742	ENSG00000078747
ENSG00000198742	ENSG00000108854
ENSG00000198742	ENSG00000123124
ENSG00000198742	ENSG00000198373
ENSG00000198750	ENSG00000160844
ENSG00000198750	ENSG00000182482
ENSG00000198750	ENSG00000183086
ENSG00000198752	ENSG00000067900
ENSG00000198752	ENSG00000134318
ENSG00000198752	ENSG00000143776
ENSG00000198752	ENSG00000171219
ENSG00000198753	ENSG00000004399
ENSG00000198753	ENSG00000076356
ENSG00000198753	ENSG00000114554
ENSG00000198753	ENSG00000130827
ENSG00000198753	ENSG00000136040
ENSG00000198753	ENSG00000164050
ENSG00000198753	ENSG00000189437
ENSG00000198753	ENSG00000196576
ENSG00000198756	ENSG00000083444
ENSG00000198756	ENSG00000106397
ENSG00000198756	ENSG00000130309
ENSG00000198756	ENSG00000152952
ENSG00000198756	ENSG00000167123
ENSG00000198758	ENSG00000131037

ENSG00000198758	ENSG00000151491
ENSG00000198758	ENSG00000177106
ENSG00000198759	ENSG00000167992
ENSG00000198759	ENSG00000168743
ENSG00000198767	ENSG00000100811
ENSG00000198767	ENSG00000179059
ENSG00000198767	ENSG00000188786
ENSG00000198768	ENSG00000154856
ENSG00000198774	ENSG00000189431
ENSG00000198780	ENSG00000185087
ENSG00000198785	ENSG00000105464
ENSG00000198785	ENSG00000116032
ENSG00000198785	ENSG00000150086
ENSG00000198785	ENSG00000161509
ENSG00000198785	ENSG00000176884
ENSG00000198785	ENSG00000183454
ENSG00000198788	ENSG00000090920
ENSG00000198788	ENSG00000109927
ENSG00000198788	ENSG00000110799
ENSG00000198788	ENSG00000117983
ENSG00000198788	ENSG00000146839
ENSG00000198788	ENSG00000184956
ENSG00000198788	ENSG00000188162
ENSG00000198788	ENSG00000196292
ENSG00000198788	ENSG00000197558
ENSG00000198791	ENSG00000155508
ENSG00000198792	ENSG00000215155
ENSG00000198794	ENSG00000085365
ENSG00000198794	ENSG00000116521
ENSG00000198794	ENSG00000140497
ENSG00000198795	ENSG00000102935
ENSG00000198797	ENSG00000078725
ENSG00000198797	ENSG00000162670
ENSG00000198799	ENSG00000139263
ENSG00000198799	ENSG00000144749
ENSG00000198807	ENSG00000075891
ENSG00000198807	ENSG00000125618
ENSG00000198807	ENSG00000125813
ENSG00000198807	ENSG00000196092
ENSG00000198810	ENSG00000101278
ENSG00000198810	ENSG00000124614
ENSG00000198810	ENSG00000183701
ENSG00000198810	ENSG00000215464
ENSG00000198812	ENSG00000204950
ENSG00000198815	ENSG00000065970
ENSG00000198815	ENSG00000111206
ENSG00000198815	ENSG00000129654
ENSG00000198818	ENSG00000213064
ENSG00000198821	ENSG00000158869
ENSG00000198822	ENSG00000113262
ENSG00000198822	ENSG00000124493
ENSG00000198822	ENSG00000152822
ENSG00000198822	ENSG00000164082
ENSG00000198822	ENSG00000168959
ENSG00000198822	ENSG00000179603
ENSG00000198822	ENSG00000196277
ENSG00000198829	ENSG00000078589
ENSG00000198829	ENSG00000139679
ENSG00000198829	ENSG00000147145

ENSG00000198829	ENSG00000152207
ENSG00000198829	ENSG00000169403
ENSG00000198829	ENSG00000169508
ENSG00000198829	ENSG00000169860
ENSG00000198829	ENSG00000173198
ENSG00000198829	ENSG00000175591
ENSG00000198829	ENSG00000184574
ENSG00000198829	ENSG00000186912
ENSG00000198830	ENSG00000118418
ENSG00000198830	ENSG00000176982
ENSG00000198830	ENSG00000180150
ENSG00000198830	ENSG00000182952
ENSG00000198830	ENSG00000212769
ENSG00000198830	ENSG00000214578
ENSG00000198835	ENSG00000121634
ENSG00000198835	ENSG00000121742
ENSG00000198835	ENSG00000121743
ENSG00000198835	ENSG00000131233
ENSG00000198835	ENSG00000135355
ENSG00000198835	ENSG00000143140
ENSG00000198835	ENSG00000152661
ENSG00000198835	ENSG00000159248
ENSG00000198835	ENSG00000164411
ENSG00000198835	ENSG00000165474
ENSG00000198835	ENSG00000169562
ENSG00000198835	ENSG00000176402
ENSG00000198835	ENSG00000177291
ENSG00000198835	ENSG00000182963
ENSG00000198835	ENSG00000183153
ENSG00000198835	ENSG00000187513
ENSG00000198835	ENSG00000188910
ENSG00000198835	ENSG00000189280
ENSG00000198835	ENSG00000189433
ENSG00000198835	ENSG00000203733
ENSG00000198837	ENSG00000137145
ENSG00000198837	ENSG00000170456
ENSG00000198837	ENSG00000174485
ENSG00000198837	ENSG00000184014
ENSG00000198838	ENSG00000196218
ENSG00000198838	ENSG00000198626
ENSG00000198844	ENSG00000066248
ENSG00000198844	ENSG00000114790
ENSG00000198844	ENSG00000130762
ENSG00000198844	ENSG00000142632
ENSG00000198846	ENSG00000092203
ENSG00000198846	ENSG00000103460
ENSG00000198846	ENSG00000124191
ENSG00000198851	ENSG00000160654
ENSG00000198851	ENSG00000167286
ENSG00000198873	ENSG00000100077
ENSG00000198873	ENSG00000114124
ENSG00000198873	ENSG00000125388
ENSG00000198873	ENSG00000173020
ENSG00000198873	ENSG00000185974
ENSG00000198873	ENSG00000198055
ENSG00000198875	ENSG00000157576
ENSG00000198875	ENSG00000172186
ENSG00000198875	ENSG00000182765
ENSG00000198875	ENSG00000184409

ENSG00000198875	ENSG00000197366
ENSG00000198875	ENSG00000198157
ENSG00000198876	ENSG00000198354
ENSG00000198876	ENSG00000198889
ENSG00000198879	ENSG00000011258
ENSG00000198879	ENSG00000100395
ENSG00000198879	ENSG00000154655
ENSG00000198879	ENSG00000163935
ENSG00000198879	ENSG00000185513
ENSG00000198879	ENSG00000198945
ENSG00000198881	ENSG00000065802
ENSG00000198881	ENSG00000102048
ENSG00000198881	ENSG00000164122
ENSG00000198881	ENSG00000165192
ENSG00000198881	ENSG00000196372
ENSG00000198881	ENSG00000214426
ENSG00000198885	ENSG00000148841
ENSG00000198885	ENSG00000205730
ENSG00000198889	ENSG00000198354
ENSG00000198889	ENSG00000198876
ENSG00000198892	ENSG00000164054
ENSG00000198898	ENSG00000116489
ENSG00000198898	ENSG00000177938
ENSG00000198900	ENSG00000184428
ENSG00000198904	ENSG00000100298
ENSG00000198904	ENSG00000111701
ENSG00000198904	ENSG00000111732
ENSG00000198904	ENSG00000124701
ENSG00000198904	ENSG00000128383
ENSG00000198904	ENSG00000128394
ENSG00000198904	ENSG00000179750
ENSG00000198909	ENSG00000169967
ENSG00000198911	ENSG00000072310
ENSG00000198914	ENSG00000028277
ENSG00000198914	ENSG00000064835
ENSG00000198914	ENSG00000091010
ENSG00000198914	ENSG00000137709
ENSG00000198914	ENSG00000143190
ENSG00000198914	ENSG00000151615
ENSG00000198914	ENSG00000152192
ENSG00000198914	ENSG00000184486
ENSG00000198914	ENSG00000185668
ENSG00000198914	ENSG00000196767
ENSG00000198914	ENSG00000204531
ENSG00000198914	ENSG00000212993
ENSG00000198915	ENSG00000138670
ENSG00000198915	ENSG00000146090
ENSG00000198918	ENSG00000163923
ENSG00000198918	ENSG00000214289
ENSG00000198925	ENSG00000181652
ENSG00000198933	ENSG00000163512
ENSG00000198936	ENSG00000108179
ENSG00000198936	ENSG00000150276
ENSG00000198936	ENSG00000196262
ENSG00000198936	ENSG00000198161
ENSG00000198936	ENSG00000198360
ENSG00000198936	ENSG00000198618
ENSG00000198936	ENSG00000203847
ENSG00000198936	ENSG00000214975

ENSG00000198936	ENSG00000215551
ENSG00000198940	ENSG00000118181
ENSG00000198940	ENSG00000188094
ENSG00000198944	ENSG00000186212
ENSG00000198944	ENSG00000187808
ENSG00000198944	ENSG00000198142
ENSG00000198945	ENSG00000011258
ENSG00000198945	ENSG00000100395
ENSG00000198945	ENSG00000154655
ENSG00000198945	ENSG00000163935
ENSG00000198945	ENSG00000185513
ENSG00000198945	ENSG00000198879
ENSG00000198947	ENSG00000152818
ENSG00000198948	ENSG00000037749
ENSG00000198951	ENSG00000102393
ENSG00000198952	ENSG00000070366
ENSG00000198952	ENSG00000116698
ENSG00000198959	ENSG00000092295
ENSG00000198959	ENSG00000104055
ENSG00000198959	ENSG00000124491
ENSG00000198959	ENSG00000125780
ENSG00000198959	ENSG00000159495
ENSG00000198959	ENSG00000163810
ENSG00000198959	ENSG00000166947
ENSG00000198959	ENSG00000166948
ENSG00000198963	ENSG00000143365
ENSG00000198964	ENSG00000156671
ENSG00000198964	ENSG00000164023
ENSG00000203618	ENSG00000169704
ENSG00000203618	ENSG00000204545
ENSG00000203697	ENSG00000014216
ENSG00000203697	ENSG00000077274
ENSG00000203697	ENSG00000092529
ENSG00000203697	ENSG00000135773
ENSG00000203697	ENSG00000137225
ENSG00000203697	ENSG00000142330
ENSG00000203697	ENSG00000149260
ENSG00000203697	ENSG00000162909
ENSG00000203697	ENSG00000162949
ENSG00000203697	ENSG00000182472
ENSG00000203697	ENSG00000214711
ENSG00000203737	ENSG00000188394
ENSG00000203740	ENSG00000148335
ENSG00000203747	ENSG00000072694
ENSG00000203747	ENSG00000132185
ENSG00000203747	ENSG00000143226
ENSG00000203747	ENSG00000150337
ENSG00000203747	ENSG00000162747
ENSG00000203747	ENSG00000179639
ENSG00000203747	ENSG00000181036
ENSG00000203747	ENSG00000198019
ENSG00000203747	ENSG00000203820
ENSG00000203759	ENSG00000176624
ENSG00000203759	ENSG00000181588
ENSG00000203759	ENSG00000183496
ENSG00000203774	ENSG00000162012
ENSG00000203774	ENSG00000175718
ENSG00000203797	ENSG00000110887
ENSG00000203805	ENSG00000147535

ENSG00000203811	ENSG00000112727
ENSG00000203811	ENSG00000124693
ENSG00000203811	ENSG00000132475
ENSG00000203811	ENSG00000163041
ENSG00000203811	ENSG00000168148
ENSG00000203811	ENSG00000178458
ENSG00000203811	ENSG00000182431
ENSG00000203811	ENSG00000182572
ENSG00000203811	ENSG00000182915
ENSG00000203811	ENSG00000183598
ENSG00000203811	ENSG00000188375
ENSG00000203811	ENSG00000196285
ENSG00000203811	ENSG00000196532
ENSG00000203811	ENSG00000196966
ENSG00000203811	ENSG00000197153
ENSG00000203811	ENSG00000197409
ENSG00000203811	ENSG00000198366
ENSG00000203811	ENSG00000203813
ENSG00000203811	ENSG00000203818
ENSG00000203811	ENSG00000203852
ENSG00000203812	ENSG00000111332
ENSG00000203812	ENSG00000124642
ENSG00000203812	ENSG00000137259
ENSG00000203812	ENSG00000158403
ENSG00000203812	ENSG00000164508
ENSG00000203812	ENSG00000168274
ENSG00000203812	ENSG00000180573
ENSG00000203812	ENSG00000181218
ENSG00000203812	ENSG00000182611
ENSG00000203812	ENSG00000183558
ENSG00000203812	ENSG00000184260
ENSG00000203812	ENSG00000184270
ENSG00000203812	ENSG00000184348
ENSG00000203812	ENSG00000184825
ENSG00000203812	ENSG00000185978
ENSG00000203812	ENSG00000188486
ENSG00000203812	ENSG00000196747
ENSG00000203812	ENSG00000196787
ENSG00000203812	ENSG00000196866
ENSG00000203812	ENSG00000198082
ENSG00000203812	ENSG00000198307
ENSG00000203812	ENSG00000198374
ENSG00000203812	ENSG00000205070
ENSG00000203812	ENSG00000205079
ENSG00000203820	ENSG00000072694
ENSG00000203820	ENSG00000132185
ENSG00000203820	ENSG00000143226
ENSG00000203820	ENSG00000150337
ENSG00000203820	ENSG00000162747
ENSG00000203820	ENSG00000179639
ENSG00000203820	ENSG00000181036
ENSG00000203820	ENSG00000198019
ENSG00000203820	ENSG00000203747
ENSG00000203835	ENSG00000107362
ENSG00000203835	ENSG00000129968
ENSG00000203835	ENSG00000136379
ENSG00000203835	ENSG00000182502
ENSG00000203835	ENSG00000182556
ENSG00000203835	ENSG00000198658

ENSG00000203847	ENSG00000108179
ENSG00000203847	ENSG00000150276
ENSG00000203847	ENSG00000196262
ENSG00000203847	ENSG00000198161
ENSG00000203847	ENSG00000198360
ENSG00000203847	ENSG00000198618
ENSG00000203847	ENSG00000198936
ENSG00000203847	ENSG00000214975
ENSG00000203847	ENSG00000215551
ENSG00000203852	ENSG00000112727
ENSG00000203852	ENSG00000124693
ENSG00000203852	ENSG00000132475
ENSG00000203852	ENSG00000163041
ENSG00000203852	ENSG00000168148
ENSG00000203852	ENSG00000178458
ENSG00000203852	ENSG00000182431
ENSG00000203852	ENSG00000182572
ENSG00000203852	ENSG00000182915
ENSG00000203852	ENSG00000183598
ENSG00000203852	ENSG00000188375
ENSG00000203852	ENSG00000196285
ENSG00000203852	ENSG00000196532
ENSG00000203852	ENSG00000196966
ENSG00000203852	ENSG00000197153
ENSG00000203852	ENSG00000197409
ENSG00000203852	ENSG00000198366
ENSG00000203852	ENSG00000203811
ENSG00000203852	ENSG00000203813
ENSG00000203852	ENSG00000203818
ENSG00000203867	ENSG00000015479
ENSG00000203867	ENSG00000075292
ENSG00000203877	ENSG00000147223
ENSG00000203877	ENSG00000183145
ENSG00000203878	ENSG00000064886
ENSG00000203878	ENSG00000085465
ENSG00000203878	ENSG00000133048
ENSG00000203878	ENSG00000133063
ENSG00000203878	ENSG00000134216
ENSG00000203879	ENSG00000057608
ENSG00000203880	ENSG00000168300
ENSG00000203883	ENSG00000005513
ENSG00000203883	ENSG00000100146
ENSG00000203883	ENSG00000124766
ENSG00000203883	ENSG00000125398
ENSG00000203883	ENSG00000129194
ENSG00000203883	ENSG00000134595
ENSG00000203883	ENSG00000164736
ENSG00000203883	ENSG00000171056
ENSG00000203883	ENSG00000176887
ENSG00000203883	ENSG00000177732
ENSG00000203883	ENSG00000181449
ENSG00000203883	ENSG00000182968
ENSG00000203883	ENSG00000184895
ENSG00000203911	ENSG00000119335
ENSG00000203911	ENSG00000125979
ENSG00000203911	ENSG00000168692
ENSG00000203911	ENSG00000168757
ENSG00000203911	ENSG00000169849
ENSG00000203911	ENSG00000178021

ENSG00000203911	ENSG00000180543
ENSG00000203911	ENSG00000184205
ENSG00000203911	ENSG00000187189
ENSG00000203911	ENSG00000187657
ENSG00000203911	ENSG00000188656
ENSG00000203911	ENSG00000189241
ENSG00000203911	ENSG00000205405
ENSG00000203911	ENSG00000206167
ENSG00000203911	ENSG00000206184
ENSG00000203911	ENSG00000214236
ENSG00000203911	ENSG00000215569
ENSG00000203911	ENSG00000215591
ENSG00000203911	ENSG00000215592
ENSG00000203911	ENSG00000215593
ENSG00000203911	ENSG00000215594
ENSG00000203911	ENSG00000215601
ENSG00000203911	ENSG00000215605
ENSG00000203911	ENSG00000215829
ENSG00000203943	ENSG00000047634
ENSG00000203943	ENSG00000130590
ENSG00000203943	ENSG00000177570
ENSG00000204007	ENSG00000148288
ENSG00000204007	ENSG00000175164
ENSG00000204007	ENSG00000184389
ENSG00000204010	ENSG00000119917
ENSG00000204010	ENSG00000119922
ENSG00000204010	ENSG00000152778
ENSG00000204010	ENSG00000185745
ENSG00000204018	ENSG00000186854
ENSG00000204033	ENSG00000148602
ENSG00000204033	ENSG00000173621
ENSG00000204033	ENSG00000183423
ENSG00000204060	ENSG00000118689
ENSG00000204060	ENSG00000150907
ENSG00000204060	ENSG00000184481
ENSG00000204070	ENSG00000003249
ENSG00000204070	ENSG00000047579
ENSG00000204084	ENSG00000122126
ENSG00000204084	ENSG00000132376
ENSG00000204084	ENSG00000185133
ENSG00000204086	ENSG00000117748
ENSG00000204099	ENSG00000115488
ENSG00000204099	ENSG00000162139
ENSG00000204099	ENSG00000204386
ENSG00000204103	ENSG00000129535
ENSG00000204103	ENSG00000178573
ENSG00000204103	ENSG00000182759
ENSG00000204103	ENSG00000185022
ENSG00000204103	ENSG00000197063
ENSG00000204103	ENSG00000198517
ENSG00000204103	ENSG00000215622
ENSG00000204116	ENSG00000109220
ENSG00000204120	ENSG00000146830
ENSG00000204130	ENSG00000018189
ENSG00000204130	ENSG00000105784
ENSG00000204130	ENSG00000108309
ENSG00000204130	ENSG00000176783
ENSG00000204130	ENSG00000214795
ENSG00000204131	ENSG00000135540

ENSG00000204131	ENSG00000162522
ENSG00000204131	ENSG00000188158
ENSG00000204138	ENSG00000087495
ENSG00000204138	ENSG00000112137
ENSG00000204138	ENSG00000112419
ENSG00000204149	ENSG00000108262
ENSG00000204149	ENSG00000133612
ENSG00000204149	ENSG00000135439
ENSG00000204149	ENSG00000139436
ENSG00000204149	ENSG00000151303
ENSG00000204149	ENSG00000157985
ENSG00000204149	ENSG00000172650
ENSG00000204149	ENSG00000174194
ENSG00000204149	ENSG00000188234
ENSG00000204149	ENSG00000198035
ENSG00000204149	ENSG00000204169
ENSG00000204149	ENSG00000204172
ENSG00000204150	ENSG00000108100
ENSG00000204150	ENSG00000163249
ENSG00000204150	ENSG00000179296
ENSG00000204150	ENSG00000182632
ENSG00000204150	ENSG00000184766
ENSG00000204150	ENSG00000215169
ENSG00000204160	ENSG00000175048
ENSG00000204160	ENSG00000188706
ENSG00000204169	ENSG00000108262
ENSG00000204169	ENSG00000133612
ENSG00000204169	ENSG00000135439
ENSG00000204169	ENSG00000139436
ENSG00000204169	ENSG00000151303
ENSG00000204169	ENSG00000157985
ENSG00000204169	ENSG00000172650
ENSG00000204169	ENSG00000174194
ENSG00000204169	ENSG00000188234
ENSG00000204169	ENSG00000198035
ENSG00000204169	ENSG00000204149
ENSG00000204169	ENSG00000204172
ENSG00000204172	ENSG00000108262
ENSG00000204172	ENSG00000133612
ENSG00000204172	ENSG00000135439
ENSG00000204172	ENSG00000139436
ENSG00000204172	ENSG00000151303
ENSG00000204172	ENSG00000157985
ENSG00000204172	ENSG00000172650
ENSG00000204172	ENSG00000174194
ENSG00000204172	ENSG00000188234
ENSG00000204172	ENSG00000198035
ENSG00000204172	ENSG00000204149
ENSG00000204172	ENSG00000204169
ENSG00000204174	ENSG00000056291
ENSG00000204174	ENSG00000075073
ENSG00000204174	ENSG00000101292
ENSG00000204174	ENSG00000110148
ENSG00000204174	ENSG00000115353
ENSG00000204174	ENSG00000119973
ENSG00000204174	ENSG00000121764
ENSG00000204174	ENSG00000123901
ENSG00000204174	ENSG00000137252
ENSG00000204174	ENSG00000148734

ENSG00000204174	ENSG00000159279
ENSG00000204174	ENSG00000163394
ENSG00000204174	ENSG00000164128
ENSG00000204174	ENSG00000164129
ENSG00000204174	ENSG00000169618
ENSG00000204174	ENSG00000169836
ENSG00000204174	ENSG00000185149
ENSG00000204174	ENSG00000186867
ENSG00000204174	ENSG00000215167
ENSG00000204175	ENSG00000169258
ENSG00000204175	ENSG00000185477
ENSG00000204195	ENSG00000062282
ENSG00000204195	ENSG00000106384
ENSG00000204195	ENSG00000124003
ENSG00000204195	ENSG00000147160
ENSG00000204195	ENSG00000166391
ENSG00000204195	ENSG00000184210
ENSG00000204195	ENSG00000205267
ENSG00000204202	ENSG00000198361
ENSG00000204202	ENSG00000198684
ENSG00000204202	ENSG00000204203
ENSG00000204202	ENSG00000204204
ENSG00000204202	ENSG00000204205
ENSG00000204202	ENSG00000204206
ENSG00000204202	ENSG00000204207
ENSG00000204202	ENSG00000205731
ENSG00000204202	ENSG00000205735
ENSG00000204202	ENSG00000205737
ENSG00000204202	ENSG00000206163
ENSG00000204204	ENSG00000198361
ENSG00000204204	ENSG00000198684
ENSG00000204204	ENSG00000204202
ENSG00000204204	ENSG00000204203
ENSG00000204204	ENSG00000204205
ENSG00000204204	ENSG00000204206
ENSG00000204204	ENSG00000204207
ENSG00000204204	ENSG00000205731
ENSG00000204204	ENSG00000205735
ENSG00000204204	ENSG00000205737
ENSG00000204204	ENSG00000206163
ENSG00000204205	ENSG00000198361
ENSG00000204205	ENSG00000198684
ENSG00000204205	ENSG00000204202
ENSG00000204205	ENSG00000204203
ENSG00000204205	ENSG00000204204
ENSG00000204205	ENSG00000204206
ENSG00000204205	ENSG00000204207
ENSG00000204205	ENSG00000205731
ENSG00000204205	ENSG00000205735
ENSG00000204205	ENSG00000205737
ENSG00000204205	ENSG00000206163
ENSG00000204207	ENSG00000198361
ENSG00000204207	ENSG00000198684
ENSG00000204207	ENSG00000204202
ENSG00000204207	ENSG00000204203
ENSG00000204207	ENSG00000204204
ENSG00000204207	ENSG00000204205
ENSG00000204207	ENSG00000204206
ENSG00000204207	ENSG00000205731

ENSG00000204207	ENSG00000205735
ENSG00000204207	ENSG00000205737
ENSG00000204207	ENSG00000206163
ENSG00000204210	ENSG00000169155
ENSG00000204210	ENSG00000180610
ENSG00000204210	ENSG00000185278
ENSG00000204210	ENSG00000204366
ENSG00000204210	ENSG00000213588
ENSG00000204217	ENSG00000106799
ENSG00000204217	ENSG00000107779
ENSG00000204217	ENSG00000115170
ENSG00000204217	ENSG00000123612
ENSG00000204217	ENSG00000135409
ENSG00000204217	ENSG00000135503
ENSG00000204217	ENSG00000138696
ENSG00000204217	ENSG00000139567
ENSG00000204217	ENSG00000163513
ENSG00000204218	ENSG00000143344
ENSG00000204218	ENSG00000160271
ENSG00000204218	ENSG00000205517
ENSG00000204219	ENSG00000171703
ENSG00000204219	ENSG00000176896
ENSG00000204219	ENSG00000187735
ENSG00000204222	ENSG00000156966
ENSG00000204222	ENSG00000162630
ENSG00000204222	ENSG00000169255
ENSG00000204222	ENSG00000170340
ENSG00000204222	ENSG00000172318
ENSG00000204222	ENSG00000176383
ENSG00000204222	ENSG00000176597
ENSG00000204222	ENSG00000177191
ENSG00000204222	ENSG00000179913
ENSG00000204222	ENSG00000180561
ENSG00000204222	ENSG00000183778
ENSG00000204222	ENSG00000198488
ENSG00000204222	ENSG00000214654
ENSG00000204227	ENSG00000121481
ENSG00000204231	ENSG00000101076
ENSG00000204231	ENSG00000143171
ENSG00000204231	ENSG00000160113
ENSG00000204231	ENSG00000164749
ENSG00000204231	ENSG00000175745
ENSG00000204231	ENSG00000185551
ENSG00000204231	ENSG00000186350
ENSG00000204235	ENSG00000187876
ENSG00000204235	ENSG00000204544
ENSG00000204235	ENSG00000204707
ENSG00000204235	ENSG00000205461
ENSG00000204239	ENSG00000181996
ENSG00000204239	ENSG00000184405
ENSG00000204239	ENSG00000205663
ENSG00000204239	ENSG00000205664
ENSG00000204239	ENSG00000206061
ENSG00000204248	ENSG00000060718
ENSG00000204248	ENSG00000080573
ENSG00000204248	ENSG00000108821
ENSG00000204248	ENSG00000130635
ENSG00000204248	ENSG00000139219
ENSG00000204248	ENSG00000164692

ENSG00000204248	ENSG00000168542
ENSG00000204248	ENSG00000204262
ENSG00000204256	ENSG00000137948
ENSG00000204256	ENSG00000141867
ENSG00000204256	ENSG00000169925
ENSG00000204261	ENSG00000142507
ENSG00000204262	ENSG00000060718
ENSG00000204262	ENSG00000080573
ENSG00000204262	ENSG00000108821
ENSG00000204262	ENSG00000130635
ENSG00000204262	ENSG00000139219
ENSG00000204262	ENSG00000164692
ENSG00000204262	ENSG00000168542
ENSG00000204262	ENSG00000204248
ENSG00000204264	ENSG00000100804
ENSG00000204264	ENSG00000129543
ENSG00000204267	ENSG00000150967
ENSG00000204267	ENSG00000168394
ENSG00000204271	ENSG00000106723
ENSG00000204271	ENSG00000147059
ENSG00000204271	ENSG00000186767
ENSG00000204271	ENSG00000186787
ENSG00000204278	ENSG00000006116
ENSG00000204278	ENSG00000075429
ENSG00000204278	ENSG00000075461
ENSG00000204278	ENSG00000105605
ENSG00000204278	ENSG00000142408
ENSG00000204278	ENSG00000166862
ENSG00000204290	ENSG00000103855
ENSG00000204290	ENSG00000114013
ENSG00000204290	ENSG00000114455
ENSG00000204290	ENSG00000120217
ENSG00000204290	ENSG00000134258
ENSG00000204290	ENSG00000160223
ENSG00000204290	ENSG00000186470
ENSG00000204290	ENSG00000188211
ENSG00000204290	ENSG00000197646
ENSG00000204290	ENSG00000204655
ENSG00000204290	ENSG00000215631
ENSG00000204290	ENSG00000215811
ENSG00000204290	ENSG00000215889
ENSG00000204291	ENSG00000182871
ENSG00000204304	ENSG00000105717
ENSG00000204304	ENSG00000167081
ENSG00000204304	ENSG00000185630
ENSG00000204308	ENSG00000138942
ENSG00000204308	ENSG00000205408
ENSG00000204310	ENSG00000169692
ENSG00000204311	ENSG00000105928
ENSG00000204319	ENSG00000106804
ENSG00000204319	ENSG00000125730
ENSG00000204319	ENSG00000126838
ENSG00000204319	ENSG00000156535
ENSG00000204319	ENSG00000160111
ENSG00000204319	ENSG00000166535
ENSG00000204319	ENSG00000175899
ENSG00000204319	ENSG00000177359
ENSG00000204319	ENSG00000204342
ENSG00000204319	ENSG00000214776

ENSG00000204335	ENSG00000067082
ENSG00000204335	ENSG00000102554
ENSG00000204335	ENSG00000105610
ENSG00000204335	ENSG00000127528
ENSG00000204335	ENSG00000129911
ENSG00000204335	ENSG00000136826
ENSG00000204335	ENSG00000139651
ENSG00000204335	ENSG00000155090
ENSG00000204335	ENSG00000164651
ENSG00000204335	ENSG00000167182
ENSG00000204335	ENSG00000170374
ENSG00000204335	ENSG00000171872
ENSG00000204335	ENSG00000172059
ENSG00000204335	ENSG00000174595
ENSG00000204335	ENSG00000189120
ENSG00000204335	ENSG00000197294
ENSG00000204335	ENSG00000197550
ENSG00000204336	ENSG00000168993
ENSG00000204336	ENSG00000185251
ENSG00000204336	ENSG00000205861
ENSG00000204342	ENSG00000106804
ENSG00000204342	ENSG00000125730
ENSG00000204342	ENSG00000126838
ENSG00000204342	ENSG00000156535
ENSG00000204342	ENSG00000160111
ENSG00000204342	ENSG00000166535
ENSG00000204342	ENSG00000175899
ENSG00000204342	ENSG00000177359
ENSG00000204342	ENSG00000204319
ENSG00000204342	ENSG00000214776
ENSG00000204345	ENSG00000095970
ENSG00000204345	ENSG00000096264
ENSG00000204345	ENSG00000161649
ENSG00000204345	ENSG00000162896
ENSG00000204345	ENSG00000162897
ENSG00000204345	ENSG00000167850
ENSG00000204345	ENSG00000167851
ENSG00000204345	ENSG00000178789
ENSG00000204345	ENSG00000186074
ENSG00000204345	ENSG00000186407
ENSG00000204347	ENSG00000108679
ENSG00000204359	ENSG00000204364
ENSG00000204361	ENSG00000095110
ENSG00000204361	ENSG00000137634
ENSG00000204361	ENSG00000144815
ENSG00000204364	ENSG00000204359
ENSG00000204366	ENSG00000169155
ENSG00000204366	ENSG00000180610
ENSG00000204366	ENSG00000185278
ENSG00000204366	ENSG00000204210
ENSG00000204366	ENSG00000213588
ENSG00000204371	ENSG00000181090
ENSG00000204381	ENSG00000154645
ENSG00000204385	ENSG00000070214
ENSG00000204385	ENSG00000129353
ENSG00000204385	ENSG00000137968
ENSG00000204385	ENSG00000143036
ENSG00000204386	ENSG00000115488
ENSG00000204386	ENSG00000162139

ENSG00000204386	ENSG00000204099
ENSG00000204388	ENSG00000109971
ENSG00000204388	ENSG00000126803
ENSG00000204388	ENSG00000173110
ENSG00000204388	ENSG00000204389
ENSG00000204388	ENSG00000204390
ENSG00000204389	ENSG00000109971
ENSG00000204389	ENSG00000126803
ENSG00000204389	ENSG00000173110
ENSG00000204389	ENSG00000204388
ENSG00000204389	ENSG00000204390
ENSG00000204390	ENSG00000109971
ENSG00000204390	ENSG00000126803
ENSG00000204390	ENSG00000173110
ENSG00000204390	ENSG00000204388
ENSG00000204390	ENSG00000204389
ENSG00000204403	ENSG00000003400
ENSG00000204403	ENSG00000064012
ENSG00000204403	ENSG00000105141
ENSG00000204403	ENSG00000106144
ENSG00000204403	ENSG00000132906
ENSG00000204403	ENSG00000137752
ENSG00000204403	ENSG00000137757
ENSG00000204403	ENSG00000138794
ENSG00000204403	ENSG00000164305
ENSG00000204403	ENSG00000165806
ENSG00000204403	ENSG00000196954
ENSG00000204414	ENSG00000136487
ENSG00000204414	ENSG00000136488
ENSG00000204414	ENSG00000172179
ENSG00000204414	ENSG00000189162
ENSG00000204414	ENSG00000213218
ENSG00000204434	ENSG00000075624
ENSG00000204434	ENSG00000107796
ENSG00000204434	ENSG00000143632
ENSG00000204434	ENSG00000159251
ENSG00000204434	ENSG00000163017
ENSG00000204434	ENSG00000169067
ENSG00000204434	ENSG00000184009
ENSG00000204434	ENSG00000214555
ENSG00000204434	ENSG00000215141
ENSG00000204437	ENSG00000135047
ENSG00000204437	ENSG00000136943
ENSG00000204437	ENSG00000143387
ENSG00000204437	ENSG00000163131
ENSG00000204437	ENSG00000188029
ENSG00000204442	ENSG00000130054
ENSG00000204448	ENSG00000108312
ENSG00000204448	ENSG00000183417
ENSG00000204448	ENSG00000204664
ENSG00000204448	ENSG00000204699
ENSG00000204448	ENSG00000204705
ENSG00000204448	ENSG00000205042
ENSG00000204469	ENSG00000117523
ENSG00000204469	ENSG00000130723
ENSG00000204471	ENSG00000128438
ENSG00000204471	ENSG00000148429
ENSG00000204471	ENSG00000161583
ENSG00000204471	ENSG00000174115

ENSG00000204471	ENSG00000185128
ENSG00000204471	ENSG00000188755
ENSG00000204471	ENSG00000188849
ENSG00000204471	ENSG00000189309
ENSG00000204471	ENSG00000197681
ENSG00000204471	ENSG00000205019
ENSG00000204471	ENSG00000213474
ENSG00000204471	ENSG00000215625
ENSG00000204471	ENSG00000215730
ENSG00000204471	ENSG00000215748
ENSG00000204472	ENSG00000115468
ENSG00000204472	ENSG00000126878
ENSG00000204472	ENSG00000142634
ENSG00000204474	ENSG00000108849
ENSG00000204474	ENSG00000122585
ENSG00000204474	ENSG00000131096
ENSG00000204478	ENSG00000116708
ENSG00000204478	ENSG00000116721
ENSG00000204478	ENSG00000116726
ENSG00000204478	ENSG00000120952
ENSG00000204478	ENSG00000157358
ENSG00000204478	ENSG00000160959
ENSG00000204478	ENSG00000179704
ENSG00000204478	ENSG00000182330
ENSG00000204478	ENSG00000185028
ENSG00000204478	ENSG00000185686
ENSG00000204478	ENSG00000187545
ENSG00000204478	ENSG00000197549
ENSG00000204478	ENSG00000204479
ENSG00000204478	ENSG00000204480
ENSG00000204478	ENSG00000204481
ENSG00000204478	ENSG00000204485
ENSG00000204478	ENSG00000204486
ENSG00000204478	ENSG00000204488
ENSG00000204478	ENSG00000204491
ENSG00000204478	ENSG00000204495
ENSG00000204478	ENSG00000204501
ENSG00000204478	ENSG00000204502
ENSG00000204478	ENSG00000204503
ENSG00000204478	ENSG00000204505
ENSG00000204478	ENSG00000204507
ENSG00000204478	ENSG00000204508
ENSG00000204478	ENSG00000204509
ENSG00000204478	ENSG00000204510
ENSG00000204478	ENSG00000204513
ENSG00000204479	ENSG00000116708
ENSG00000204479	ENSG00000116721
ENSG00000204479	ENSG00000116726
ENSG00000204479	ENSG00000120952
ENSG00000204479	ENSG00000157358
ENSG00000204479	ENSG00000160959
ENSG00000204479	ENSG00000179704
ENSG00000204479	ENSG00000182330
ENSG00000204479	ENSG00000185028
ENSG00000204479	ENSG00000185686
ENSG00000204479	ENSG00000187545
ENSG00000204479	ENSG00000197549
ENSG00000204479	ENSG00000204478
ENSG00000204479	ENSG00000204480

ENSG00000204479	ENSG00000204481
ENSG00000204479	ENSG00000204485
ENSG00000204479	ENSG00000204486
ENSG00000204479	ENSG00000204488
ENSG00000204479	ENSG00000204491
ENSG00000204479	ENSG00000204495
ENSG00000204479	ENSG00000204501
ENSG00000204479	ENSG00000204502
ENSG00000204479	ENSG00000204503
ENSG00000204479	ENSG00000204505
ENSG00000204479	ENSG00000204507
ENSG00000204479	ENSG00000204508
ENSG00000204479	ENSG00000204509
ENSG00000204479	ENSG00000204510
ENSG00000204479	ENSG00000204513
ENSG00000204480	ENSG00000116708
ENSG00000204480	ENSG00000116721
ENSG00000204480	ENSG00000116726
ENSG00000204480	ENSG00000120952
ENSG00000204480	ENSG00000157358
ENSG00000204480	ENSG00000160959
ENSG00000204480	ENSG00000179704
ENSG00000204480	ENSG00000182330
ENSG00000204480	ENSG00000185028
ENSG00000204480	ENSG00000185686
ENSG00000204480	ENSG00000187545
ENSG00000204480	ENSG00000197549
ENSG00000204480	ENSG00000204478
ENSG00000204480	ENSG00000204479
ENSG00000204480	ENSG00000204481
ENSG00000204480	ENSG00000204485
ENSG00000204480	ENSG00000204486
ENSG00000204480	ENSG00000204488
ENSG00000204480	ENSG00000204491
ENSG00000204480	ENSG00000204495
ENSG00000204480	ENSG00000204501
ENSG00000204480	ENSG00000204502
ENSG00000204480	ENSG00000204503
ENSG00000204480	ENSG00000204505
ENSG00000204480	ENSG00000204507
ENSG00000204480	ENSG00000204508
ENSG00000204480	ENSG00000204509
ENSG00000204480	ENSG00000204510
ENSG00000204480	ENSG00000204513
ENSG00000204481	ENSG00000116708
ENSG00000204481	ENSG00000116721
ENSG00000204481	ENSG00000116726
ENSG00000204481	ENSG00000120952
ENSG00000204481	ENSG00000157358
ENSG00000204481	ENSG00000160959
ENSG00000204481	ENSG00000179704
ENSG00000204481	ENSG00000182330
ENSG00000204481	ENSG00000185028
ENSG00000204481	ENSG00000185686
ENSG00000204481	ENSG00000187545
ENSG00000204481	ENSG00000197549
ENSG00000204481	ENSG00000204478
ENSG00000204481	ENSG00000204479
ENSG00000204481	ENSG00000204480

ENSG00000204481	ENSG00000204485
ENSG00000204481	ENSG00000204486
ENSG00000204481	ENSG00000204488
ENSG00000204481	ENSG00000204491
ENSG00000204481	ENSG00000204495
ENSG00000204481	ENSG00000204501
ENSG00000204481	ENSG00000204502
ENSG00000204481	ENSG00000204503
ENSG00000204481	ENSG00000204505
ENSG00000204481	ENSG00000204507
ENSG00000204481	ENSG00000204508
ENSG00000204481	ENSG00000204509
ENSG00000204481	ENSG00000204510
ENSG00000204481	ENSG00000204513
ENSG00000204485	ENSG00000116708
ENSG00000204485	ENSG00000116721
ENSG00000204485	ENSG00000116726
ENSG00000204485	ENSG00000120952
ENSG00000204485	ENSG00000157358
ENSG00000204485	ENSG00000160959
ENSG00000204485	ENSG00000179704
ENSG00000204485	ENSG00000182330
ENSG00000204485	ENSG00000185028
ENSG00000204485	ENSG00000185686
ENSG00000204485	ENSG00000187545
ENSG00000204485	ENSG00000197549
ENSG00000204485	ENSG00000204478
ENSG00000204485	ENSG00000204479
ENSG00000204485	ENSG00000204480
ENSG00000204485	ENSG00000204481
ENSG00000204485	ENSG00000204486
ENSG00000204485	ENSG00000204488
ENSG00000204485	ENSG00000204491
ENSG00000204485	ENSG00000204495
ENSG00000204485	ENSG00000204501
ENSG00000204485	ENSG00000204502
ENSG00000204485	ENSG00000204503
ENSG00000204485	ENSG00000204505
ENSG00000204485	ENSG00000204507
ENSG00000204485	ENSG00000204508
ENSG00000204485	ENSG00000204509
ENSG00000204485	ENSG00000204510
ENSG00000204485	ENSG00000204513
ENSG00000204486	ENSG00000116708
ENSG00000204486	ENSG00000116721
ENSG00000204486	ENSG00000116726
ENSG00000204486	ENSG00000120952
ENSG00000204486	ENSG00000157358
ENSG00000204486	ENSG00000160959
ENSG00000204486	ENSG00000179704
ENSG00000204486	ENSG00000182330
ENSG00000204486	ENSG00000185028
ENSG00000204486	ENSG00000185686
ENSG00000204486	ENSG00000187545
ENSG00000204486	ENSG00000197549
ENSG00000204486	ENSG00000204478
ENSG00000204486	ENSG00000204479
ENSG00000204486	ENSG00000204480
ENSG00000204486	ENSG00000204481

ENSG00000204486	ENSG00000204485
ENSG00000204486	ENSG00000204488
ENSG00000204486	ENSG00000204491
ENSG00000204486	ENSG00000204495
ENSG00000204486	ENSG00000204501
ENSG00000204486	ENSG00000204502
ENSG00000204486	ENSG00000204503
ENSG00000204486	ENSG00000204505
ENSG00000204486	ENSG00000204507
ENSG00000204486	ENSG00000204508
ENSG00000204486	ENSG00000204509
ENSG00000204486	ENSG00000204510
ENSG00000204486	ENSG00000204513
ENSG00000204488	ENSG00000116708
ENSG00000204488	ENSG00000116721
ENSG00000204488	ENSG00000116726
ENSG00000204488	ENSG00000120952
ENSG00000204488	ENSG00000157358
ENSG00000204488	ENSG00000160959
ENSG00000204488	ENSG00000179704
ENSG00000204488	ENSG00000182330
ENSG00000204488	ENSG00000185028
ENSG00000204488	ENSG00000185686
ENSG00000204488	ENSG00000187545
ENSG00000204488	ENSG00000197549
ENSG00000204488	ENSG00000204478
ENSG00000204488	ENSG00000204479
ENSG00000204488	ENSG00000204480
ENSG00000204488	ENSG00000204481
ENSG00000204488	ENSG00000204485
ENSG00000204488	ENSG00000204486
ENSG00000204488	ENSG00000204491
ENSG00000204488	ENSG00000204495
ENSG00000204488	ENSG00000204501
ENSG00000204488	ENSG00000204502
ENSG00000204488	ENSG00000204503
ENSG00000204488	ENSG00000204505
ENSG00000204488	ENSG00000204507
ENSG00000204488	ENSG00000204508
ENSG00000204488	ENSG00000204509
ENSG00000204488	ENSG00000204510
ENSG00000204488	ENSG00000204513
ENSG00000204490	ENSG00000102245
ENSG00000204490	ENSG00000117560
ENSG00000204490	ENSG00000120659
ENSG00000204490	ENSG00000121858
ENSG00000204490	ENSG00000125735
ENSG00000204490	ENSG00000181634
ENSG00000204490	ENSG00000204496
ENSG00000204491	ENSG00000116708
ENSG00000204491	ENSG00000116721
ENSG00000204491	ENSG00000116726
ENSG00000204491	ENSG00000120952
ENSG00000204491	ENSG00000157358
ENSG00000204491	ENSG00000160959
ENSG00000204491	ENSG00000179704
ENSG00000204491	ENSG00000182330
ENSG00000204491	ENSG00000185028
ENSG00000204491	ENSG00000185686

ENSG00000204491	ENSG00000187545
ENSG00000204491	ENSG00000197549
ENSG00000204491	ENSG00000204478
ENSG00000204491	ENSG00000204479
ENSG00000204491	ENSG00000204480
ENSG00000204491	ENSG00000204481
ENSG00000204491	ENSG00000204485
ENSG00000204491	ENSG00000204486
ENSG00000204491	ENSG00000204488
ENSG00000204491	ENSG00000204495
ENSG00000204491	ENSG00000204501
ENSG00000204491	ENSG00000204502
ENSG00000204491	ENSG00000204503
ENSG00000204491	ENSG00000204505
ENSG00000204491	ENSG00000204507
ENSG00000204491	ENSG00000204508
ENSG00000204491	ENSG00000204509
ENSG00000204491	ENSG00000204510
ENSG00000204491	ENSG00000204513
ENSG00000204495	ENSG00000116708
ENSG00000204495	ENSG00000116721
ENSG00000204495	ENSG00000116726
ENSG00000204495	ENSG00000120952
ENSG00000204495	ENSG00000157358
ENSG00000204495	ENSG00000160959
ENSG00000204495	ENSG00000179704
ENSG00000204495	ENSG00000182330
ENSG00000204495	ENSG00000185028
ENSG00000204495	ENSG00000185686
ENSG00000204495	ENSG00000187545
ENSG00000204495	ENSG00000197549
ENSG00000204495	ENSG00000204478
ENSG00000204495	ENSG00000204479
ENSG00000204495	ENSG00000204480
ENSG00000204495	ENSG00000204481
ENSG00000204495	ENSG00000204485
ENSG00000204495	ENSG00000204486
ENSG00000204495	ENSG00000204488
ENSG00000204495	ENSG00000204491
ENSG00000204495	ENSG00000204501
ENSG00000204495	ENSG00000204502
ENSG00000204495	ENSG00000204503
ENSG00000204495	ENSG00000204505
ENSG00000204495	ENSG00000204507
ENSG00000204495	ENSG00000204508
ENSG00000204495	ENSG00000204509
ENSG00000204495	ENSG00000204510
ENSG00000204495	ENSG00000204513
ENSG00000204496	ENSG00000102245
ENSG00000204496	ENSG00000117560
ENSG00000204496	ENSG00000120659
ENSG00000204496	ENSG00000121858
ENSG00000204496	ENSG00000125735
ENSG00000204496	ENSG00000181634
ENSG00000204496	ENSG00000204490
ENSG00000204501	ENSG00000116708
ENSG00000204501	ENSG00000116721
ENSG00000204501	ENSG00000116726
ENSG00000204501	ENSG00000120952

ENSG00000204501	ENSG00000157358
ENSG00000204501	ENSG00000160959
ENSG00000204501	ENSG00000179704
ENSG00000204501	ENSG00000182330
ENSG00000204501	ENSG00000185028
ENSG00000204501	ENSG00000185686
ENSG00000204501	ENSG00000187545
ENSG00000204501	ENSG00000197549
ENSG00000204501	ENSG00000204478
ENSG00000204501	ENSG00000204479
ENSG00000204501	ENSG00000204480
ENSG00000204501	ENSG00000204481
ENSG00000204501	ENSG00000204485
ENSG00000204501	ENSG00000204486
ENSG00000204501	ENSG00000204488
ENSG00000204501	ENSG00000204491
ENSG00000204501	ENSG00000204495
ENSG00000204501	ENSG00000204502
ENSG00000204501	ENSG00000204503
ENSG00000204501	ENSG00000204505
ENSG00000204501	ENSG00000204507
ENSG00000204501	ENSG00000204508
ENSG00000204501	ENSG00000204509
ENSG00000204501	ENSG00000204510
ENSG00000204501	ENSG00000204513
ENSG00000204502	ENSG00000116708
ENSG00000204502	ENSG00000116721
ENSG00000204502	ENSG00000116726
ENSG00000204502	ENSG00000120952
ENSG00000204502	ENSG00000157358
ENSG00000204502	ENSG00000160959
ENSG00000204502	ENSG00000179704
ENSG00000204502	ENSG00000182330
ENSG00000204502	ENSG00000185028
ENSG00000204502	ENSG00000185686
ENSG00000204502	ENSG00000187545
ENSG00000204502	ENSG00000197549
ENSG00000204502	ENSG00000204478
ENSG00000204502	ENSG00000204479
ENSG00000204502	ENSG00000204480
ENSG00000204502	ENSG00000204481
ENSG00000204502	ENSG00000204485
ENSG00000204502	ENSG00000204486
ENSG00000204502	ENSG00000204488
ENSG00000204502	ENSG00000204491
ENSG00000204502	ENSG00000204495
ENSG00000204502	ENSG00000204501
ENSG00000204502	ENSG00000204503
ENSG00000204502	ENSG00000204505
ENSG00000204502	ENSG00000204507
ENSG00000204502	ENSG00000204508
ENSG00000204502	ENSG00000204509
ENSG00000204502	ENSG00000204510
ENSG00000204502	ENSG00000204513
ENSG00000204503	ENSG00000116708
ENSG00000204503	ENSG00000116721
ENSG00000204503	ENSG00000116726
ENSG00000204503	ENSG00000120952
ENSG00000204503	ENSG00000157358

ENSG00000204503	ENSG00000160959
ENSG00000204503	ENSG00000179704
ENSG00000204503	ENSG00000182330
ENSG00000204503	ENSG00000185028
ENSG00000204503	ENSG00000185686
ENSG00000204503	ENSG00000187545
ENSG00000204503	ENSG00000197549
ENSG00000204503	ENSG00000204478
ENSG00000204503	ENSG00000204479
ENSG00000204503	ENSG00000204480
ENSG00000204503	ENSG00000204481
ENSG00000204503	ENSG00000204485
ENSG00000204503	ENSG00000204486
ENSG00000204503	ENSG00000204488
ENSG00000204503	ENSG00000204491
ENSG00000204503	ENSG00000204495
ENSG00000204503	ENSG00000204501
ENSG00000204503	ENSG00000204502
ENSG00000204503	ENSG00000204505
ENSG00000204503	ENSG00000204507
ENSG00000204503	ENSG00000204508
ENSG00000204503	ENSG00000204509
ENSG00000204503	ENSG00000204510
ENSG00000204503	ENSG00000204513
ENSG00000204505	ENSG00000116708
ENSG00000204505	ENSG00000116721
ENSG00000204505	ENSG00000116726
ENSG00000204505	ENSG00000120952
ENSG00000204505	ENSG00000157358
ENSG00000204505	ENSG00000160959
ENSG00000204505	ENSG00000179704
ENSG00000204505	ENSG00000182330
ENSG00000204505	ENSG00000185028
ENSG00000204505	ENSG00000185686
ENSG00000204505	ENSG00000187545
ENSG00000204505	ENSG00000197549
ENSG00000204505	ENSG00000204478
ENSG00000204505	ENSG00000204479
ENSG00000204505	ENSG00000204480
ENSG00000204505	ENSG00000204481
ENSG00000204505	ENSG00000204485
ENSG00000204505	ENSG00000204486
ENSG00000204505	ENSG00000204488
ENSG00000204505	ENSG00000204491
ENSG00000204505	ENSG00000204495
ENSG00000204505	ENSG00000204501
ENSG00000204505	ENSG00000204502
ENSG00000204505	ENSG00000204503
ENSG00000204505	ENSG00000204507
ENSG00000204505	ENSG00000204508
ENSG00000204505	ENSG00000204509
ENSG00000204505	ENSG00000204510
ENSG00000204505	ENSG00000204513
ENSG00000204507	ENSG00000116708
ENSG00000204507	ENSG00000116721
ENSG00000204507	ENSG00000116726
ENSG00000204507	ENSG00000120952
ENSG00000204507	ENSG00000157358
ENSG00000204507	ENSG00000160959

ENSG00000204507	ENSG00000179704
ENSG00000204507	ENSG00000182330
ENSG00000204507	ENSG00000185028
ENSG00000204507	ENSG00000185686
ENSG00000204507	ENSG00000187545
ENSG00000204507	ENSG00000197549
ENSG00000204507	ENSG00000204478
ENSG00000204507	ENSG00000204479
ENSG00000204507	ENSG00000204480
ENSG00000204507	ENSG00000204481
ENSG00000204507	ENSG00000204485
ENSG00000204507	ENSG00000204486
ENSG00000204507	ENSG00000204488
ENSG00000204507	ENSG00000204491
ENSG00000204507	ENSG00000204495
ENSG00000204507	ENSG00000204501
ENSG00000204507	ENSG00000204502
ENSG00000204507	ENSG00000204503
ENSG00000204507	ENSG00000204505
ENSG00000204507	ENSG00000204508
ENSG00000204507	ENSG00000204509
ENSG00000204507	ENSG00000204510
ENSG00000204507	ENSG00000204513
ENSG00000204508	ENSG00000116708
ENSG00000204508	ENSG00000116721
ENSG00000204508	ENSG00000116726
ENSG00000204508	ENSG00000120952
ENSG00000204508	ENSG00000157358
ENSG00000204508	ENSG00000160959
ENSG00000204508	ENSG00000179704
ENSG00000204508	ENSG00000182330
ENSG00000204508	ENSG00000185028
ENSG00000204508	ENSG00000185686
ENSG00000204508	ENSG00000187545
ENSG00000204508	ENSG00000197549
ENSG00000204508	ENSG00000204478
ENSG00000204508	ENSG00000204479
ENSG00000204508	ENSG00000204480
ENSG00000204508	ENSG00000204481
ENSG00000204508	ENSG00000204485
ENSG00000204508	ENSG00000204486
ENSG00000204508	ENSG00000204488
ENSG00000204508	ENSG00000204491
ENSG00000204508	ENSG00000204495
ENSG00000204508	ENSG00000204501
ENSG00000204508	ENSG00000204502
ENSG00000204508	ENSG00000204503
ENSG00000204508	ENSG00000204505
ENSG00000204508	ENSG00000204507
ENSG00000204508	ENSG00000204509
ENSG00000204508	ENSG00000204510
ENSG00000204508	ENSG00000204513
ENSG00000204509	ENSG00000116708
ENSG00000204509	ENSG00000116721
ENSG00000204509	ENSG00000116726
ENSG00000204509	ENSG00000120952
ENSG00000204509	ENSG00000157358
ENSG00000204509	ENSG00000160959
ENSG00000204509	ENSG00000179704

ENSG00000204509	ENSG00000182330
ENSG00000204509	ENSG00000185028
ENSG00000204509	ENSG00000185686
ENSG00000204509	ENSG00000187545
ENSG00000204509	ENSG00000197549
ENSG00000204509	ENSG00000204478
ENSG00000204509	ENSG00000204479
ENSG00000204509	ENSG00000204480
ENSG00000204509	ENSG00000204481
ENSG00000204509	ENSG00000204485
ENSG00000204509	ENSG00000204486
ENSG00000204509	ENSG00000204488
ENSG00000204509	ENSG00000204491
ENSG00000204509	ENSG00000204495
ENSG00000204509	ENSG00000204501
ENSG00000204509	ENSG00000204502
ENSG00000204509	ENSG00000204503
ENSG00000204509	ENSG00000204505
ENSG00000204509	ENSG00000204507
ENSG00000204509	ENSG00000204508
ENSG00000204509	ENSG00000204510
ENSG00000204509	ENSG00000204513
ENSG00000204510	ENSG00000116708
ENSG00000204510	ENSG00000116721
ENSG00000204510	ENSG00000116726
ENSG00000204510	ENSG00000120952
ENSG00000204510	ENSG00000157358
ENSG00000204510	ENSG00000160959
ENSG00000204510	ENSG00000179704
ENSG00000204510	ENSG00000182330
ENSG00000204510	ENSG00000185028
ENSG00000204510	ENSG00000185686
ENSG00000204510	ENSG00000187545
ENSG00000204510	ENSG00000197549
ENSG00000204510	ENSG00000204478
ENSG00000204510	ENSG00000204479
ENSG00000204510	ENSG00000204480
ENSG00000204510	ENSG00000204481
ENSG00000204510	ENSG00000204485
ENSG00000204510	ENSG00000204486
ENSG00000204510	ENSG00000204488
ENSG00000204510	ENSG00000204491
ENSG00000204510	ENSG00000204495
ENSG00000204510	ENSG00000204501
ENSG00000204510	ENSG00000204502
ENSG00000204510	ENSG00000204503
ENSG00000204510	ENSG00000204505
ENSG00000204510	ENSG00000204507
ENSG00000204510	ENSG00000204508
ENSG00000204510	ENSG00000204509
ENSG00000204510	ENSG00000204513
ENSG00000204513	ENSG00000116708
ENSG00000204513	ENSG00000116721
ENSG00000204513	ENSG00000116726
ENSG00000204513	ENSG00000120952
ENSG00000204513	ENSG00000157358
ENSG00000204513	ENSG00000160959
ENSG00000204513	ENSG00000179704
ENSG00000204513	ENSG00000182330

ENSG00000204513	ENSG00000185028
ENSG00000204513	ENSG00000185686
ENSG00000204513	ENSG00000187545
ENSG00000204513	ENSG00000197549
ENSG00000204513	ENSG00000204478
ENSG00000204513	ENSG00000204479
ENSG00000204513	ENSG00000204480
ENSG00000204513	ENSG00000204481
ENSG00000204513	ENSG00000204485
ENSG00000204513	ENSG00000204486
ENSG00000204513	ENSG00000204488
ENSG00000204513	ENSG00000204491
ENSG00000204513	ENSG00000204495
ENSG00000204513	ENSG00000204501
ENSG00000204513	ENSG00000204502
ENSG00000204513	ENSG00000204503
ENSG00000204513	ENSG00000204505
ENSG00000204513	ENSG00000204507
ENSG00000204513	ENSG00000204508
ENSG00000204513	ENSG00000204509
ENSG00000204513	ENSG00000204510
ENSG00000204518	ENSG00000114771
ENSG00000204518	ENSG00000144959
ENSG00000204518	ENSG00000188984
ENSG00000204518	ENSG00000197953
ENSG00000204545	ENSG00000169704
ENSG00000204545	ENSG00000203618
ENSG00000204580	ENSG00000162733
ENSG00000204614	ENSG00000026950
ENSG00000204614	ENSG00000111801
ENSG00000204614	ENSG00000112763
ENSG00000204614	ENSG00000113303
ENSG00000204614	ENSG00000121236
ENSG00000204614	ENSG00000124508
ENSG00000204614	ENSG00000124549
ENSG00000204614	ENSG00000124557
ENSG00000204614	ENSG00000128250
ENSG00000204614	ENSG00000128253
ENSG00000204614	ENSG00000128276
ENSG00000204614	ENSG00000128482
ENSG00000204614	ENSG00000132256
ENSG00000204614	ENSG00000132274
ENSG00000204614	ENSG00000164010
ENSG00000204614	ENSG00000165810
ENSG00000204614	ENSG00000168903
ENSG00000204614	ENSG00000176422
ENSG00000204614	ENSG00000179046
ENSG00000204614	ENSG00000182805
ENSG00000204614	ENSG00000183439
ENSG00000204614	ENSG00000183718
ENSG00000204614	ENSG00000187574
ENSG00000204614	ENSG00000188683
ENSG00000204614	ENSG00000204616
ENSG00000204614	ENSG00000204618
ENSG00000204614	ENSG00000205198
ENSG00000204614	ENSG00000214693
ENSG00000204616	ENSG00000026950
ENSG00000204616	ENSG00000111801
ENSG00000204616	ENSG00000112763

ENSG00000204616	ENSG00000113303
ENSG00000204616	ENSG00000121236
ENSG00000204616	ENSG00000124508
ENSG00000204616	ENSG00000124549
ENSG00000204616	ENSG00000124557
ENSG00000204616	ENSG00000128250
ENSG00000204616	ENSG00000128253
ENSG00000204616	ENSG00000128276
ENSG00000204616	ENSG00000128482
ENSG00000204616	ENSG00000132256
ENSG00000204616	ENSG00000132274
ENSG00000204616	ENSG00000164010
ENSG00000204616	ENSG00000165810
ENSG00000204616	ENSG00000168903
ENSG00000204616	ENSG00000176422
ENSG00000204616	ENSG00000179046
ENSG00000204616	ENSG00000182805
ENSG00000204616	ENSG00000183439
ENSG00000204616	ENSG00000183718
ENSG00000204616	ENSG00000187574
ENSG00000204616	ENSG00000188683
ENSG00000204616	ENSG00000204614
ENSG00000204616	ENSG00000204618
ENSG00000204616	ENSG00000205198
ENSG00000204616	ENSG00000214693
ENSG00000204618	ENSG00000026950
ENSG00000204618	ENSG00000111801
ENSG00000204618	ENSG00000112763
ENSG00000204618	ENSG00000113303
ENSG00000204618	ENSG00000121236
ENSG00000204618	ENSG00000124508
ENSG00000204618	ENSG00000124549
ENSG00000204618	ENSG00000124557
ENSG00000204618	ENSG00000128250
ENSG00000204618	ENSG00000128253
ENSG00000204618	ENSG00000128276
ENSG00000204618	ENSG00000128482
ENSG00000204618	ENSG00000132256
ENSG00000204618	ENSG00000132274
ENSG00000204618	ENSG00000164010
ENSG00000204618	ENSG00000165810
ENSG00000204618	ENSG00000168903
ENSG00000204618	ENSG00000176422
ENSG00000204618	ENSG00000179046
ENSG00000204618	ENSG00000182805
ENSG00000204618	ENSG00000183439
ENSG00000204618	ENSG00000183718
ENSG00000204618	ENSG00000187574
ENSG00000204618	ENSG00000188683
ENSG00000204618	ENSG00000204614
ENSG00000204618	ENSG00000204616
ENSG00000204618	ENSG00000205198
ENSG00000204618	ENSG00000214693
ENSG00000204633	ENSG00000072110
ENSG00000204633	ENSG00000077522
ENSG00000204633	ENSG00000130402
ENSG00000204634	ENSG00000109436
ENSG00000204634	ENSG00000133138
ENSG00000204634	ENSG00000197226

ENSG00000204640	ENSG00000109255
ENSG00000204655	ENSG00000103855
ENSG00000204655	ENSG00000114013
ENSG00000204655	ENSG00000114455
ENSG00000204655	ENSG00000120217
ENSG00000204655	ENSG00000134258
ENSG00000204655	ENSG00000160223
ENSG00000204655	ENSG00000186470
ENSG00000204655	ENSG00000188211
ENSG00000204655	ENSG00000197646
ENSG00000204655	ENSG00000204290
ENSG00000204655	ENSG00000215631
ENSG00000204655	ENSG00000215811
ENSG00000204655	ENSG00000215889
ENSG00000204658	ENSG00000077984
ENSG00000204658	ENSG00000101435
ENSG00000204658	ENSG00000101439
ENSG00000204658	ENSG00000101441
ENSG00000204658	ENSG00000125815
ENSG00000204658	ENSG00000125823
ENSG00000204658	ENSG00000125831
ENSG00000204658	ENSG00000170367
ENSG00000204658	ENSG00000170369
ENSG00000204658	ENSG00000170373
ENSG00000204658	ENSG00000173335
ENSG00000204658	ENSG00000175315
ENSG00000204658	ENSG00000204662
ENSG00000204658	ENSG00000204663
ENSG00000204662	ENSG00000077984
ENSG00000204662	ENSG00000101435
ENSG00000204662	ENSG00000101439
ENSG00000204662	ENSG00000101441
ENSG00000204662	ENSG00000125815
ENSG00000204662	ENSG00000125823
ENSG00000204662	ENSG00000125831
ENSG00000204662	ENSG00000170367
ENSG00000204662	ENSG00000170369
ENSG00000204662	ENSG00000170373
ENSG00000204662	ENSG00000173335
ENSG00000204662	ENSG00000175315
ENSG00000204662	ENSG00000204658
ENSG00000204662	ENSG00000204663
ENSG00000204663	ENSG00000077984
ENSG00000204663	ENSG00000101435
ENSG00000204663	ENSG00000101439
ENSG00000204663	ENSG00000101441
ENSG00000204663	ENSG00000125815
ENSG00000204663	ENSG00000125823
ENSG00000204663	ENSG00000125831
ENSG00000204663	ENSG00000170367
ENSG00000204663	ENSG00000170369
ENSG00000204663	ENSG00000170373
ENSG00000204663	ENSG00000173335
ENSG00000204663	ENSG00000175315
ENSG00000204663	ENSG00000204658
ENSG00000204663	ENSG00000204662
ENSG00000204664	ENSG00000108312
ENSG00000204664	ENSG00000183417
ENSG00000204664	ENSG00000204448

ENSG00000204664	ENSG00000204699
ENSG00000204664	ENSG00000204705
ENSG00000204664	ENSG00000205042
ENSG00000204670	ENSG00000136700
ENSG00000204670	ENSG00000156755
ENSG00000204670	ENSG00000183719
ENSG00000204670	ENSG00000197794
ENSG00000204670	ENSG00000204776
ENSG00000204670	ENSG00000204780
ENSG00000204670	ENSG00000211599
ENSG00000204670	ENSG00000211600
ENSG00000204670	ENSG00000211601
ENSG00000204670	ENSG00000211602
ENSG00000204670	ENSG00000211603
ENSG00000204670	ENSG00000211604
ENSG00000204670	ENSG00000211605
ENSG00000204670	ENSG00000211606
ENSG00000204670	ENSG00000211607
ENSG00000204670	ENSG00000211608
ENSG00000204670	ENSG00000211609
ENSG00000204670	ENSG00000211610
ENSG00000204670	ENSG00000211611
ENSG00000204670	ENSG00000211613
ENSG00000204670	ENSG00000211616
ENSG00000204670	ENSG00000211617
ENSG00000204670	ENSG00000211618
ENSG00000204670	ENSG00000211620
ENSG00000204670	ENSG00000211625
ENSG00000204670	ENSG00000211626
ENSG00000204670	ENSG00000211627
ENSG00000204670	ENSG00000211628
ENSG00000204670	ENSG00000211629
ENSG00000204670	ENSG00000211630
ENSG00000204670	ENSG00000211631
ENSG00000204670	ENSG00000211632
ENSG00000204670	ENSG00000211633
ENSG00000204670	ENSG00000211634
ENSG00000204670	ENSG00000211635
ENSG00000204670	ENSG00000211636
ENSG00000204699	ENSG00000108312
ENSG00000204699	ENSG00000183417
ENSG00000204699	ENSG00000204448
ENSG00000204699	ENSG00000204664
ENSG00000204699	ENSG00000204705
ENSG00000204699	ENSG00000205042
ENSG00000204705	ENSG00000108312
ENSG00000204705	ENSG00000183417
ENSG00000204705	ENSG00000204448
ENSG00000204705	ENSG00000204664
ENSG00000204705	ENSG00000204699
ENSG00000204705	ENSG00000205042
ENSG00000204707	ENSG00000187876
ENSG00000204707	ENSG00000204235
ENSG00000204707	ENSG00000204544
ENSG00000204707	ENSG00000205461
ENSG00000204710	ENSG00000136206
ENSG00000204710	ENSG00000154732
ENSG00000204710	ENSG00000163806
ENSG00000204710	ENSG00000166667

ENSG00000204710	ENSG00000170092
ENSG00000204710	ENSG00000173678
ENSG00000204710	ENSG00000179994
ENSG00000204710	ENSG00000183318
ENSG00000204710	ENSG00000184616
ENSG00000204710	ENSG00000185040
ENSG00000204710	ENSG00000186645
ENSG00000204710	ENSG00000198305
ENSG00000204710	ENSG00000205238
ENSG00000204710	ENSG00000205482
ENSG00000204710	ENSG00000205505
ENSG00000204710	ENSG00000205506
ENSG00000204710	ENSG00000205508
ENSG00000204710	ENSG00000205509
ENSG00000204710	ENSG00000205580
ENSG00000204710	ENSG00000214300
ENSG00000204710	ENSG00000215092
ENSG00000204748	ENSG00000104818
ENSG00000204748	ENSG00000104826
ENSG00000204748	ENSG00000104827
ENSG00000204748	ENSG00000131808
ENSG00000204748	ENSG00000134200
ENSG00000204748	ENSG00000179600
ENSG00000204748	ENSG00000189052
ENSG00000204748	ENSG00000196337
ENSG00000204748	ENSG00000213030
ENSG00000204756	ENSG00000142396
ENSG00000204756	ENSG00000169740
ENSG00000204756	ENSG00000174255
ENSG00000204756	ENSG00000179195
ENSG00000204756	ENSG00000179965
ENSG00000204756	ENSG00000184294
ENSG00000204756	ENSG00000186446
ENSG00000204756	ENSG00000186777
ENSG00000204756	ENSG00000197008
ENSG00000204756	ENSG00000197025
ENSG00000204756	ENSG00000197062
ENSG00000204756	ENSG00000197332
ENSG00000204756	ENSG00000198155
ENSG00000204756	ENSG00000212805
ENSG00000204756	ENSG00000213976
ENSG00000204756	ENSG00000213980
ENSG00000204756	ENSG00000215180
ENSG00000204756	ENSG00000215510
ENSG00000204756	ENSG00000215537
ENSG00000204756	ENSG00000215598
ENSG00000204756	ENSG00000215600
ENSG00000204764	ENSG00000130227
ENSG00000204767	ENSG00000188916
ENSG00000204776	ENSG00000136700
ENSG00000204776	ENSG00000156755
ENSG00000204776	ENSG00000183719
ENSG00000204776	ENSG00000197794
ENSG00000204776	ENSG00000204670
ENSG00000204776	ENSG00000204780
ENSG00000204776	ENSG00000211599
ENSG00000204776	ENSG00000211600
ENSG00000204776	ENSG00000211601
ENSG00000204776	ENSG00000211602

ENSG00000204776	ENSG00000211603
ENSG00000204776	ENSG00000211604
ENSG00000204776	ENSG00000211605
ENSG00000204776	ENSG00000211606
ENSG00000204776	ENSG00000211607
ENSG00000204776	ENSG00000211608
ENSG00000204776	ENSG00000211609
ENSG00000204776	ENSG00000211610
ENSG00000204776	ENSG00000211611
ENSG00000204776	ENSG00000211613
ENSG00000204776	ENSG00000211616
ENSG00000204776	ENSG00000211617
ENSG00000204776	ENSG00000211618
ENSG00000204776	ENSG00000211620
ENSG00000204776	ENSG00000211625
ENSG00000204776	ENSG00000211626
ENSG00000204776	ENSG00000211627
ENSG00000204776	ENSG00000211628
ENSG00000204776	ENSG00000211629
ENSG00000204776	ENSG00000211630
ENSG00000204776	ENSG00000211631
ENSG00000204776	ENSG00000211632
ENSG00000204776	ENSG00000211633
ENSG00000204776	ENSG00000211634
ENSG00000204776	ENSG00000211635
ENSG00000204776	ENSG00000211636
ENSG00000204779	ENSG00000054598
ENSG00000204779	ENSG00000125798
ENSG00000204779	ENSG00000129514
ENSG00000204779	ENSG00000164379
ENSG00000204779	ENSG00000168269
ENSG00000204779	ENSG00000170122
ENSG00000204779	ENSG00000170608
ENSG00000204779	ENSG00000176165
ENSG00000204779	ENSG00000176678
ENSG00000204779	ENSG00000176692
ENSG00000204779	ENSG00000178919
ENSG00000204779	ENSG00000179772
ENSG00000204779	ENSG00000183770
ENSG00000204779	ENSG00000184492
ENSG00000204779	ENSG00000184659
ENSG00000204779	ENSG00000186564
ENSG00000204779	ENSG00000186766
ENSG00000204779	ENSG00000186790
ENSG00000204779	ENSG00000187140
ENSG00000204779	ENSG00000187559
ENSG00000204779	ENSG00000204612
ENSG00000204779	ENSG00000204793
ENSG00000204779	ENSG00000204828
ENSG00000204779	ENSG00000214336
ENSG00000204780	ENSG00000136700
ENSG00000204780	ENSG00000156755
ENSG00000204780	ENSG00000183719
ENSG00000204780	ENSG00000197794
ENSG00000204780	ENSG00000204670
ENSG00000204780	ENSG00000204776
ENSG00000204780	ENSG00000211599
ENSG00000204780	ENSG00000211600
ENSG00000204780	ENSG00000211601

ENSG00000204780	ENSG00000211602
ENSG00000204780	ENSG00000211603
ENSG00000204780	ENSG00000211604
ENSG00000204780	ENSG00000211605
ENSG00000204780	ENSG00000211606
ENSG00000204780	ENSG00000211607
ENSG00000204780	ENSG00000211608
ENSG00000204780	ENSG00000211609
ENSG00000204780	ENSG00000211610
ENSG00000204780	ENSG00000211611
ENSG00000204780	ENSG00000211613
ENSG00000204780	ENSG00000211616
ENSG00000204780	ENSG00000211617
ENSG00000204780	ENSG00000211618
ENSG00000204780	ENSG00000211620
ENSG00000204780	ENSG00000211625
ENSG00000204780	ENSG00000211626
ENSG00000204780	ENSG00000211627
ENSG00000204780	ENSG00000211628
ENSG00000204780	ENSG00000211629
ENSG00000204780	ENSG00000211630
ENSG00000204780	ENSG00000211631
ENSG00000204780	ENSG00000211632
ENSG00000204780	ENSG00000211633
ENSG00000204780	ENSG00000211634
ENSG00000204780	ENSG00000211635
ENSG00000204780	ENSG00000211636
ENSG00000204782	ENSG00000064666
ENSG00000204782	ENSG00000117519
ENSG00000204782	ENSG00000130176
ENSG00000204782	ENSG00000144834
ENSG00000204782	ENSG00000149591
ENSG00000204782	ENSG00000158710
ENSG00000204782	ENSG00000204826
ENSG00000204782	ENSG00000215433
ENSG00000204793	ENSG00000054598
ENSG00000204793	ENSG00000125798
ENSG00000204793	ENSG00000129514
ENSG00000204793	ENSG00000164379
ENSG00000204793	ENSG00000168269
ENSG00000204793	ENSG00000170122
ENSG00000204793	ENSG00000170608
ENSG00000204793	ENSG00000176165
ENSG00000204793	ENSG00000176678
ENSG00000204793	ENSG00000176692
ENSG00000204793	ENSG00000178919
ENSG00000204793	ENSG00000179772
ENSG00000204793	ENSG00000183770
ENSG00000204793	ENSG00000184492
ENSG00000204793	ENSG00000184659
ENSG00000204793	ENSG00000186564
ENSG00000204793	ENSG00000186766
ENSG00000204793	ENSG00000186790
ENSG00000204793	ENSG00000187140
ENSG00000204793	ENSG00000187559
ENSG00000204793	ENSG00000204612
ENSG00000204793	ENSG00000204779
ENSG00000204793	ENSG00000204828
ENSG00000204793	ENSG00000214336

ENSG00000204794	ENSG00000079739
ENSG00000204794	ENSG00000154330
ENSG00000204801	ENSG00000070193
ENSG00000204801	ENSG00000070388
ENSG00000204801	ENSG00000075388
ENSG00000204801	ENSG00000078579
ENSG00000204801	ENSG00000102466
ENSG00000204801	ENSG00000102678
ENSG00000204801	ENSG00000111241
ENSG00000204801	ENSG00000113578
ENSG00000204801	ENSG00000114279
ENSG00000204801	ENSG00000129682
ENSG00000204801	ENSG00000138675
ENSG00000204801	ENSG00000138685
ENSG00000204801	ENSG00000140285
ENSG00000204801	ENSG00000161958
ENSG00000204801	ENSG00000185390
ENSG00000204801	ENSG00000186895
ENSG00000204801	ENSG00000196468
ENSG00000204801	ENSG00000204816
ENSG00000204801	ENSG00000204817
ENSG00000204801	ENSG00000204837
ENSG00000204801	ENSG00000215053
ENSG00000204805	ENSG00000183812
ENSG00000204805	ENSG00000197833
ENSG00000204805	ENSG00000204806
ENSG00000204805	ENSG00000204807
ENSG00000204805	ENSG00000204808
ENSG00000204805	ENSG00000205613
ENSG00000204805	ENSG00000215061
ENSG00000204806	ENSG00000183812
ENSG00000204806	ENSG00000197833
ENSG00000204806	ENSG00000204805
ENSG00000204806	ENSG00000204807
ENSG00000204806	ENSG00000204808
ENSG00000204806	ENSG00000205613
ENSG00000204806	ENSG00000215061
ENSG00000204807	ENSG00000183812
ENSG00000204807	ENSG00000197833
ENSG00000204807	ENSG00000204805
ENSG00000204807	ENSG00000204806
ENSG00000204807	ENSG00000204808
ENSG00000204807	ENSG00000205613
ENSG00000204807	ENSG00000215061
ENSG00000204808	ENSG00000183812
ENSG00000204808	ENSG00000197833
ENSG00000204808	ENSG00000204805
ENSG00000204808	ENSG00000204806
ENSG00000204808	ENSG00000204807
ENSG00000204808	ENSG00000205613
ENSG00000204808	ENSG00000215061
ENSG00000204816	ENSG00000070193
ENSG00000204816	ENSG00000070388
ENSG00000204816	ENSG00000075388
ENSG00000204816	ENSG00000078579
ENSG00000204816	ENSG00000102466
ENSG00000204816	ENSG00000102678
ENSG00000204816	ENSG00000111241
ENSG00000204816	ENSG00000113578

ENSG00000204816	ENSG00000114279
ENSG00000204816	ENSG00000129682
ENSG00000204816	ENSG00000138675
ENSG00000204816	ENSG00000138685
ENSG00000204816	ENSG00000140285
ENSG00000204816	ENSG00000161958
ENSG00000204816	ENSG00000185390
ENSG00000204816	ENSG00000186895
ENSG00000204816	ENSG00000196468
ENSG00000204816	ENSG00000204801
ENSG00000204816	ENSG00000204817
ENSG00000204816	ENSG00000204837
ENSG00000204816	ENSG00000215053
ENSG00000204817	ENSG00000070193
ENSG00000204817	ENSG00000070388
ENSG00000204817	ENSG00000075388
ENSG00000204817	ENSG00000078579
ENSG00000204817	ENSG00000102466
ENSG00000204817	ENSG00000102678
ENSG00000204817	ENSG00000111241
ENSG00000204817	ENSG00000113578
ENSG00000204817	ENSG00000114279
ENSG00000204817	ENSG00000129682
ENSG00000204817	ENSG00000138675
ENSG00000204817	ENSG00000138685
ENSG00000204817	ENSG00000140285
ENSG00000204817	ENSG00000161958
ENSG00000204817	ENSG00000185390
ENSG00000204817	ENSG00000186895
ENSG00000204817	ENSG00000196468
ENSG00000204817	ENSG00000204801
ENSG00000204817	ENSG00000204816
ENSG00000204817	ENSG00000204837
ENSG00000204817	ENSG00000215053
ENSG00000204818	ENSG00000069122
ENSG00000204818	ENSG00000123146
ENSG00000204818	ENSG00000127507
ENSG00000204818	ENSG00000131355
ENSG00000204818	ENSG00000153292
ENSG00000204818	ENSG00000153294
ENSG00000204818	ENSG00000162618
ENSG00000204818	ENSG00000164393
ENSG00000204818	ENSG00000173567
ENSG00000204818	ENSG00000174837
ENSG00000204818	ENSG00000186629
ENSG00000204826	ENSG00000064666
ENSG00000204826	ENSG00000117519
ENSG00000204826	ENSG00000130176
ENSG00000204826	ENSG00000144834
ENSG00000204826	ENSG00000149591
ENSG00000204826	ENSG00000158710
ENSG00000204826	ENSG00000204782
ENSG00000204826	ENSG00000215433
ENSG00000204828	ENSG00000054598
ENSG00000204828	ENSG00000125798
ENSG00000204828	ENSG00000129514
ENSG00000204828	ENSG00000164379
ENSG00000204828	ENSG00000168269
ENSG00000204828	ENSG00000170122

ENSG00000204828	ENSG00000170608
ENSG00000204828	ENSG00000176165
ENSG00000204828	ENSG00000176678
ENSG00000204828	ENSG00000176692
ENSG00000204828	ENSG00000178919
ENSG00000204828	ENSG00000179772
ENSG00000204828	ENSG00000183770
ENSG00000204828	ENSG00000184492
ENSG00000204828	ENSG00000184659
ENSG00000204828	ENSG00000186564
ENSG00000204828	ENSG00000186766
ENSG00000204828	ENSG00000186790
ENSG00000204828	ENSG00000187140
ENSG00000204828	ENSG00000187559
ENSG00000204828	ENSG00000204612
ENSG00000204828	ENSG00000204779
ENSG00000204828	ENSG00000204793
ENSG00000204828	ENSG00000214336
ENSG00000204837	ENSG00000070193
ENSG00000204837	ENSG00000070388
ENSG00000204837	ENSG00000075388
ENSG00000204837	ENSG00000078579
ENSG00000204837	ENSG00000102466
ENSG00000204837	ENSG00000102678
ENSG00000204837	ENSG00000111241
ENSG00000204837	ENSG00000113578
ENSG00000204837	ENSG00000114279
ENSG00000204837	ENSG00000129682
ENSG00000204837	ENSG00000138675
ENSG00000204837	ENSG00000138685
ENSG00000204837	ENSG00000140285
ENSG00000204837	ENSG00000161958
ENSG00000204837	ENSG00000185390
ENSG00000204837	ENSG00000186895
ENSG00000204837	ENSG00000196468
ENSG00000204837	ENSG00000204801
ENSG00000204837	ENSG00000204816
ENSG00000204837	ENSG00000204817
ENSG00000204837	ENSG00000215053
ENSG00000204842	ENSG00000168488
ENSG00000204852	ENSG00000119977
ENSG00000204852	ENSG00000168778
ENSG00000204889	ENSG00000006059
ENSG00000204889	ENSG00000094796
ENSG00000204889	ENSG00000108244
ENSG00000204889	ENSG00000108417
ENSG00000204889	ENSG00000108759
ENSG00000204889	ENSG00000111057
ENSG00000204889	ENSG00000126337
ENSG00000204889	ENSG00000128422
ENSG00000204889	ENSG00000131737
ENSG00000204889	ENSG00000131738
ENSG00000204889	ENSG00000131885
ENSG00000204889	ENSG00000167916
ENSG00000204889	ENSG00000170819
ENSG00000204889	ENSG00000171345
ENSG00000204889	ENSG00000171346
ENSG00000204889	ENSG00000171360
ENSG00000204889	ENSG00000171401

ENSG00000204889	ENSG00000171403
ENSG00000204889	ENSG00000171431
ENSG00000204889	ENSG00000171446
ENSG00000204889	ENSG00000173908
ENSG00000204889	ENSG00000186393
ENSG00000204889	ENSG00000186395
ENSG00000204889	ENSG00000186831
ENSG00000204889	ENSG00000186832
ENSG00000204889	ENSG00000186847
ENSG00000204889	ENSG00000187242
ENSG00000204889	ENSG00000187686
ENSG00000204889	ENSG00000196859
ENSG00000204889	ENSG00000197079
ENSG00000204889	ENSG00000204897
ENSG00000204889	ENSG00000213951
ENSG00000204889	ENSG00000214220
ENSG00000204889	ENSG00000214454
ENSG00000204889	ENSG00000214959
ENSG00000204889	ENSG00000215772
ENSG00000204889	ENSG00000215773
ENSG00000204897	ENSG00000006059
ENSG00000204897	ENSG00000094796
ENSG00000204897	ENSG00000108244
ENSG00000204897	ENSG00000108417
ENSG00000204897	ENSG00000108759
ENSG00000204897	ENSG00000111057
ENSG00000204897	ENSG00000126337
ENSG00000204897	ENSG00000128422
ENSG00000204897	ENSG00000131737
ENSG00000204897	ENSG00000131738
ENSG00000204897	ENSG00000131885
ENSG00000204897	ENSG00000167916
ENSG00000204897	ENSG00000170819
ENSG00000204897	ENSG00000171345
ENSG00000204897	ENSG00000171346
ENSG00000204897	ENSG00000171360
ENSG00000204897	ENSG00000171401
ENSG00000204897	ENSG00000171403
ENSG00000204897	ENSG00000171431
ENSG00000204897	ENSG00000171446
ENSG00000204897	ENSG00000173908
ENSG00000204897	ENSG00000186393
ENSG00000204897	ENSG00000186395
ENSG00000204897	ENSG00000186831
ENSG00000204897	ENSG00000186832
ENSG00000204897	ENSG00000186847
ENSG00000204897	ENSG00000187242
ENSG00000204897	ENSG00000187686
ENSG00000204897	ENSG00000196859
ENSG00000204897	ENSG00000197079
ENSG00000204897	ENSG00000204889
ENSG00000204897	ENSG00000213951
ENSG00000204897	ENSG00000214220
ENSG00000204897	ENSG00000214454
ENSG00000204897	ENSG00000214959
ENSG00000204897	ENSG00000215772
ENSG00000204897	ENSG00000215773
ENSG00000204924	ENSG00000139767
ENSG00000204924	ENSG00000167978

ENSG00000204924	ENSG00000177679
ENSG00000204928	ENSG00000215203
ENSG00000204950	ENSG00000198812
ENSG00000204955	ENSG00000081818
ENSG00000204955	ENSG00000081842
ENSG00000204955	ENSG00000081853
ENSG00000204955	ENSG00000112852
ENSG00000204955	ENSG00000113205
ENSG00000204955	ENSG00000113209
ENSG00000204955	ENSG00000113211
ENSG00000204955	ENSG00000113212
ENSG00000204955	ENSG00000113248
ENSG00000204955	ENSG00000120322
ENSG00000204955	ENSG00000120324
ENSG00000204955	ENSG00000120327
ENSG00000204955	ENSG00000120328
ENSG00000204955	ENSG00000146001
ENSG00000204955	ENSG00000171815
ENSG00000204955	ENSG00000187372
ENSG00000204955	ENSG00000196963
ENSG00000204955	ENSG00000197479
ENSG00000204955	ENSG00000204956
ENSG00000204955	ENSG00000204961
ENSG00000204955	ENSG00000204962
ENSG00000204955	ENSG00000204963
ENSG00000204955	ENSG00000204964
ENSG00000204955	ENSG00000204965
ENSG00000204955	ENSG00000204967
ENSG00000204955	ENSG00000204968
ENSG00000204955	ENSG00000204969
ENSG00000204955	ENSG00000204970
ENSG00000204955	ENSG00000214567
ENSG00000204955	ENSG00000214570
ENSG00000204955	ENSG00000214574
ENSG00000204955	ENSG00000214580
ENSG00000204955	ENSG00000214583
ENSG00000204955	ENSG00000214594
ENSG00000204955	ENSG00000214600
ENSG00000204955	ENSG00000214608
ENSG00000204956	ENSG00000081818
ENSG00000204956	ENSG00000081842
ENSG00000204956	ENSG00000081853
ENSG00000204956	ENSG00000112852
ENSG00000204956	ENSG00000113205
ENSG00000204956	ENSG00000113209
ENSG00000204956	ENSG00000113211
ENSG00000204956	ENSG00000113212
ENSG00000204956	ENSG00000113248
ENSG00000204956	ENSG00000120322
ENSG00000204956	ENSG00000120324
ENSG00000204956	ENSG00000120327
ENSG00000204956	ENSG00000120328
ENSG00000204956	ENSG00000146001
ENSG00000204956	ENSG00000171815
ENSG00000204956	ENSG00000187372
ENSG00000204956	ENSG00000196963
ENSG00000204956	ENSG00000197479
ENSG00000204956	ENSG00000204955
ENSG00000204956	ENSG00000204961

ENSG00000204956	ENSG00000204962
ENSG00000204956	ENSG00000204963
ENSG00000204956	ENSG00000204964
ENSG00000204956	ENSG00000204965
ENSG00000204956	ENSG00000204967
ENSG00000204956	ENSG00000204968
ENSG00000204956	ENSG00000204969
ENSG00000204956	ENSG00000204970
ENSG00000204956	ENSG00000214567
ENSG00000204956	ENSG00000214570
ENSG00000204956	ENSG00000214574
ENSG00000204956	ENSG00000214580
ENSG00000204956	ENSG00000214583
ENSG00000204956	ENSG00000214594
ENSG00000204956	ENSG00000214600
ENSG00000204956	ENSG00000214608
ENSG00000204961	ENSG00000081818
ENSG00000204961	ENSG00000081842
ENSG00000204961	ENSG00000081853
ENSG00000204961	ENSG00000112852
ENSG00000204961	ENSG00000113205
ENSG00000204961	ENSG00000113209
ENSG00000204961	ENSG00000113211
ENSG00000204961	ENSG00000113212
ENSG00000204961	ENSG00000113248
ENSG00000204961	ENSG00000120322
ENSG00000204961	ENSG00000120324
ENSG00000204961	ENSG00000120327
ENSG00000204961	ENSG00000120328
ENSG00000204961	ENSG00000146001
ENSG00000204961	ENSG00000171815
ENSG00000204961	ENSG00000187372
ENSG00000204961	ENSG00000196963
ENSG00000204961	ENSG00000197479
ENSG00000204961	ENSG00000204955
ENSG00000204961	ENSG00000204956
ENSG00000204961	ENSG00000204962
ENSG00000204961	ENSG00000204963
ENSG00000204961	ENSG00000204964
ENSG00000204961	ENSG00000204965
ENSG00000204961	ENSG00000204967
ENSG00000204961	ENSG00000204968
ENSG00000204961	ENSG00000204969
ENSG00000204961	ENSG00000204970
ENSG00000204961	ENSG00000214567
ENSG00000204961	ENSG00000214570
ENSG00000204961	ENSG00000214574
ENSG00000204961	ENSG00000214580
ENSG00000204961	ENSG00000214583
ENSG00000204961	ENSG00000214594
ENSG00000204961	ENSG00000214600
ENSG00000204961	ENSG00000214608
ENSG00000204962	ENSG00000081818
ENSG00000204962	ENSG00000081842
ENSG00000204962	ENSG00000081853
ENSG00000204962	ENSG00000112852
ENSG00000204962	ENSG00000113205
ENSG00000204962	ENSG00000113209
ENSG00000204962	ENSG00000113211

ENSG00000204962	ENSG00000113212
ENSG00000204962	ENSG00000113248
ENSG00000204962	ENSG00000120322
ENSG00000204962	ENSG00000120324
ENSG00000204962	ENSG00000120327
ENSG00000204962	ENSG00000120328
ENSG00000204962	ENSG00000146001
ENSG00000204962	ENSG00000171815
ENSG00000204962	ENSG00000187372
ENSG00000204962	ENSG00000196963
ENSG00000204962	ENSG00000197479
ENSG00000204962	ENSG00000204955
ENSG00000204962	ENSG00000204956
ENSG00000204962	ENSG00000204961
ENSG00000204962	ENSG00000204963
ENSG00000204962	ENSG00000204964
ENSG00000204962	ENSG00000204965
ENSG00000204962	ENSG00000204967
ENSG00000204962	ENSG00000204968
ENSG00000204962	ENSG00000204969
ENSG00000204962	ENSG00000204970
ENSG00000204962	ENSG00000214567
ENSG00000204962	ENSG00000214570
ENSG00000204962	ENSG00000214574
ENSG00000204962	ENSG00000214580
ENSG00000204962	ENSG00000214583
ENSG00000204962	ENSG00000214594
ENSG00000204962	ENSG00000214600
ENSG00000204962	ENSG00000214608
ENSG00000204963	ENSG00000081818
ENSG00000204963	ENSG00000081842
ENSG00000204963	ENSG00000081853
ENSG00000204963	ENSG00000112852
ENSG00000204963	ENSG00000113205
ENSG00000204963	ENSG00000113209
ENSG00000204963	ENSG00000113211
ENSG00000204963	ENSG00000113212
ENSG00000204963	ENSG00000113248
ENSG00000204963	ENSG00000120322
ENSG00000204963	ENSG00000120324
ENSG00000204963	ENSG00000120327
ENSG00000204963	ENSG00000120328
ENSG00000204963	ENSG00000146001
ENSG00000204963	ENSG00000171815
ENSG00000204963	ENSG00000187372
ENSG00000204963	ENSG00000196963
ENSG00000204963	ENSG00000197479
ENSG00000204963	ENSG00000204955
ENSG00000204963	ENSG00000204956
ENSG00000204963	ENSG00000204961
ENSG00000204963	ENSG00000204962
ENSG00000204963	ENSG00000204964
ENSG00000204963	ENSG00000204965
ENSG00000204963	ENSG00000204967
ENSG00000204963	ENSG00000204968
ENSG00000204963	ENSG00000204969
ENSG00000204963	ENSG00000204970
ENSG00000204963	ENSG00000214567
ENSG00000204963	ENSG00000214570

ENSG00000204963	ENSG00000214574
ENSG00000204963	ENSG00000214580
ENSG00000204963	ENSG00000214583
ENSG00000204963	ENSG00000214594
ENSG00000204963	ENSG00000214600
ENSG00000204963	ENSG00000214608
ENSG00000204964	ENSG00000081818
ENSG00000204964	ENSG00000081842
ENSG00000204964	ENSG00000081853
ENSG00000204964	ENSG00000112852
ENSG00000204964	ENSG00000113205
ENSG00000204964	ENSG00000113209
ENSG00000204964	ENSG00000113211
ENSG00000204964	ENSG00000113212
ENSG00000204964	ENSG00000113248
ENSG00000204964	ENSG00000120322
ENSG00000204964	ENSG00000120324
ENSG00000204964	ENSG00000120327
ENSG00000204964	ENSG00000120328
ENSG00000204964	ENSG00000146001
ENSG00000204964	ENSG00000171815
ENSG00000204964	ENSG00000187372
ENSG00000204964	ENSG00000196963
ENSG00000204964	ENSG00000197479
ENSG00000204964	ENSG00000204955
ENSG00000204964	ENSG00000204956
ENSG00000204964	ENSG00000204961
ENSG00000204964	ENSG00000204962
ENSG00000204964	ENSG00000204963
ENSG00000204964	ENSG00000204965
ENSG00000204964	ENSG00000204967
ENSG00000204964	ENSG00000204968
ENSG00000204964	ENSG00000204969
ENSG00000204964	ENSG00000204970
ENSG00000204964	ENSG00000214567
ENSG00000204964	ENSG00000214570
ENSG00000204964	ENSG00000214574
ENSG00000204964	ENSG00000214580
ENSG00000204964	ENSG00000214583
ENSG00000204964	ENSG00000214594
ENSG00000204964	ENSG00000214600
ENSG00000204964	ENSG00000214608
ENSG00000204965	ENSG00000081818
ENSG00000204965	ENSG00000081842
ENSG00000204965	ENSG00000081853
ENSG00000204965	ENSG00000112852
ENSG00000204965	ENSG00000113205
ENSG00000204965	ENSG00000113209
ENSG00000204965	ENSG00000113211
ENSG00000204965	ENSG00000113212
ENSG00000204965	ENSG00000113248
ENSG00000204965	ENSG00000120322
ENSG00000204965	ENSG00000120324
ENSG00000204965	ENSG00000120327
ENSG00000204965	ENSG00000120328
ENSG00000204965	ENSG00000146001
ENSG00000204965	ENSG00000171815
ENSG00000204965	ENSG00000187372
ENSG00000204965	ENSG00000196963

ENSG00000204965	ENSG00000197479
ENSG00000204965	ENSG00000204955
ENSG00000204965	ENSG00000204956
ENSG00000204965	ENSG00000204961
ENSG00000204965	ENSG00000204962
ENSG00000204965	ENSG00000204963
ENSG00000204965	ENSG00000204964
ENSG00000204965	ENSG00000204967
ENSG00000204965	ENSG00000204968
ENSG00000204965	ENSG00000204969
ENSG00000204965	ENSG00000204970
ENSG00000204965	ENSG00000214567
ENSG00000204965	ENSG00000214570
ENSG00000204965	ENSG00000214574
ENSG00000204965	ENSG00000214580
ENSG00000204965	ENSG00000214583
ENSG00000204965	ENSG00000214594
ENSG00000204965	ENSG00000214600
ENSG00000204965	ENSG00000214608
ENSG00000204967	ENSG00000081818
ENSG00000204967	ENSG00000081842
ENSG00000204967	ENSG00000081853
ENSG00000204967	ENSG00000112852
ENSG00000204967	ENSG00000113205
ENSG00000204967	ENSG00000113209
ENSG00000204967	ENSG00000113211
ENSG00000204967	ENSG00000113212
ENSG00000204967	ENSG00000113248
ENSG00000204967	ENSG00000120322
ENSG00000204967	ENSG00000120324
ENSG00000204967	ENSG00000120327
ENSG00000204967	ENSG00000120328
ENSG00000204967	ENSG00000146001
ENSG00000204967	ENSG00000171815
ENSG00000204967	ENSG00000187372
ENSG00000204967	ENSG00000196963
ENSG00000204967	ENSG00000197479
ENSG00000204967	ENSG00000204955
ENSG00000204967	ENSG00000204956
ENSG00000204967	ENSG00000204961
ENSG00000204967	ENSG00000204962
ENSG00000204967	ENSG00000204963
ENSG00000204967	ENSG00000204964
ENSG00000204967	ENSG00000204965
ENSG00000204967	ENSG00000204968
ENSG00000204967	ENSG00000204969
ENSG00000204967	ENSG00000204970
ENSG00000204967	ENSG00000214567
ENSG00000204967	ENSG00000214570
ENSG00000204967	ENSG00000214574
ENSG00000204967	ENSG00000214580
ENSG00000204967	ENSG00000214583
ENSG00000204967	ENSG00000214594
ENSG00000204967	ENSG00000214600
ENSG00000204967	ENSG00000214608
ENSG00000204968	ENSG00000081818
ENSG00000204968	ENSG00000081842
ENSG00000204968	ENSG00000081853
ENSG00000204968	ENSG00000112852

ENSG00000204968	ENSG00000113205
ENSG00000204968	ENSG00000113209
ENSG00000204968	ENSG00000113211
ENSG00000204968	ENSG00000113212
ENSG00000204968	ENSG00000113248
ENSG00000204968	ENSG00000120322
ENSG00000204968	ENSG00000120324
ENSG00000204968	ENSG00000120327
ENSG00000204968	ENSG00000120328
ENSG00000204968	ENSG00000146001
ENSG00000204968	ENSG00000171815
ENSG00000204968	ENSG00000187372
ENSG00000204968	ENSG00000196963
ENSG00000204968	ENSG00000197479
ENSG00000204968	ENSG00000204955
ENSG00000204968	ENSG00000204956
ENSG00000204968	ENSG00000204961
ENSG00000204968	ENSG00000204962
ENSG00000204968	ENSG00000204963
ENSG00000204968	ENSG00000204964
ENSG00000204968	ENSG00000204965
ENSG00000204968	ENSG00000204967
ENSG00000204968	ENSG00000204969
ENSG00000204968	ENSG00000204970
ENSG00000204968	ENSG00000214567
ENSG00000204968	ENSG00000214570
ENSG00000204968	ENSG00000214574
ENSG00000204968	ENSG00000214580
ENSG00000204968	ENSG00000214583
ENSG00000204968	ENSG00000214594
ENSG00000204968	ENSG00000214600
ENSG00000204968	ENSG00000214608
ENSG00000204969	ENSG00000081818
ENSG00000204969	ENSG00000081842
ENSG00000204969	ENSG00000081853
ENSG00000204969	ENSG00000112852
ENSG00000204969	ENSG00000113205
ENSG00000204969	ENSG00000113209
ENSG00000204969	ENSG00000113211
ENSG00000204969	ENSG00000113212
ENSG00000204969	ENSG00000113248
ENSG00000204969	ENSG00000120322
ENSG00000204969	ENSG00000120324
ENSG00000204969	ENSG00000120327
ENSG00000204969	ENSG00000120328
ENSG00000204969	ENSG00000146001
ENSG00000204969	ENSG00000171815
ENSG00000204969	ENSG00000187372
ENSG00000204969	ENSG00000196963
ENSG00000204969	ENSG00000197479
ENSG00000204969	ENSG00000204955
ENSG00000204969	ENSG00000204956
ENSG00000204969	ENSG00000204961
ENSG00000204969	ENSG00000204962
ENSG00000204969	ENSG00000204963
ENSG00000204969	ENSG00000204964
ENSG00000204969	ENSG00000204965
ENSG00000204969	ENSG00000204967
ENSG00000204969	ENSG00000204968

ENSG00000204969	ENSG00000204970
ENSG00000204969	ENSG00000214567
ENSG00000204969	ENSG00000214570
ENSG00000204969	ENSG00000214574
ENSG00000204969	ENSG00000214580
ENSG00000204969	ENSG00000214583
ENSG00000204969	ENSG00000214594
ENSG00000204969	ENSG00000214600
ENSG00000204969	ENSG00000214608
ENSG00000204970	ENSG00000081818
ENSG00000204970	ENSG00000081842
ENSG00000204970	ENSG00000081853
ENSG00000204970	ENSG00000112852
ENSG00000204970	ENSG00000113205
ENSG00000204970	ENSG00000113209
ENSG00000204970	ENSG00000113211
ENSG00000204970	ENSG00000113212
ENSG00000204970	ENSG00000113248
ENSG00000204970	ENSG00000120322
ENSG00000204970	ENSG00000120324
ENSG00000204970	ENSG00000120327
ENSG00000204970	ENSG00000120328
ENSG00000204970	ENSG00000146001
ENSG00000204970	ENSG00000171815
ENSG00000204970	ENSG00000187372
ENSG00000204970	ENSG00000196963
ENSG00000204970	ENSG00000197479
ENSG00000204970	ENSG00000204955
ENSG00000204970	ENSG00000204956
ENSG00000204970	ENSG00000204961
ENSG00000204970	ENSG00000204962
ENSG00000204970	ENSG00000204963
ENSG00000204970	ENSG00000204964
ENSG00000204970	ENSG00000204965
ENSG00000204970	ENSG00000204967
ENSG00000204970	ENSG00000204968
ENSG00000204970	ENSG00000204969
ENSG00000204970	ENSG00000214567
ENSG00000204970	ENSG00000214570
ENSG00000204970	ENSG00000214574
ENSG00000204970	ENSG00000214580
ENSG00000204970	ENSG00000214583
ENSG00000204970	ENSG00000214594
ENSG00000204970	ENSG00000214600
ENSG00000204970	ENSG00000214608
ENSG00000204973	ENSG00000096088
ENSG00000204973	ENSG00000117984
ENSG00000204973	ENSG00000131400
ENSG00000204973	ENSG00000131401
ENSG00000204973	ENSG00000143839
ENSG00000204973	ENSG00000172647
ENSG00000204973	ENSG00000180860
ENSG00000204973	ENSG00000184488
ENSG00000204973	ENSG00000196188
ENSG00000204973	ENSG00000215868
ENSG00000204977	ENSG00000138100
ENSG00000204977	ENSG00000147573
ENSG00000204977	ENSG00000158022
ENSG00000204977	ENSG00000213186

ENSG00000204981	ENSG00000010438
ENSG00000204981	ENSG00000129437
ENSG00000204981	ENSG00000129451
ENSG00000204981	ENSG00000129455
ENSG00000204981	ENSG00000142515
ENSG00000204981	ENSG00000165076
ENSG00000204981	ENSG00000167748
ENSG00000204981	ENSG00000167749
ENSG00000204981	ENSG00000167751
ENSG00000204981	ENSG00000167754
ENSG00000204981	ENSG00000167755
ENSG00000204981	ENSG00000167757
ENSG00000204981	ENSG00000167759
ENSG00000204981	ENSG00000169035
ENSG00000204981	ENSG00000171147
ENSG00000204981	ENSG00000173636
ENSG00000204981	ENSG00000174562
ENSG00000204981	ENSG00000186474
ENSG00000204981	ENSG00000204982
ENSG00000204981	ENSG00000204983
ENSG00000204981	ENSG00000213022
ENSG00000204981	ENSG00000214083
ENSG00000204982	ENSG00000010438
ENSG00000204982	ENSG00000129437
ENSG00000204982	ENSG00000129451
ENSG00000204982	ENSG00000129455
ENSG00000204982	ENSG00000142515
ENSG00000204982	ENSG00000165076
ENSG00000204982	ENSG00000167748
ENSG00000204982	ENSG00000167749
ENSG00000204982	ENSG00000167751
ENSG00000204982	ENSG00000167754
ENSG00000204982	ENSG00000167755
ENSG00000204982	ENSG00000167757
ENSG00000204982	ENSG00000167759
ENSG00000204982	ENSG00000169035
ENSG00000204982	ENSG00000171147
ENSG00000204982	ENSG00000173636
ENSG00000204982	ENSG00000174562
ENSG00000204982	ENSG00000186474
ENSG00000204982	ENSG00000204981
ENSG00000204982	ENSG00000204983
ENSG00000204982	ENSG00000213022
ENSG00000204982	ENSG00000214083
ENSG00000204983	ENSG00000010438
ENSG00000204983	ENSG00000129437
ENSG00000204983	ENSG00000129451
ENSG00000204983	ENSG00000129455
ENSG00000204983	ENSG00000142515
ENSG00000204983	ENSG00000165076
ENSG00000204983	ENSG00000167748
ENSG00000204983	ENSG00000167749
ENSG00000204983	ENSG00000167751
ENSG00000204983	ENSG00000167754
ENSG00000204983	ENSG00000167755
ENSG00000204983	ENSG00000167757
ENSG00000204983	ENSG00000167759
ENSG00000204983	ENSG00000169035
ENSG00000204983	ENSG00000171147

ENSG00000204983	ENSG00000173636
ENSG00000204983	ENSG00000174562
ENSG00000204983	ENSG00000186474
ENSG00000204983	ENSG00000204981
ENSG00000204983	ENSG00000204982
ENSG00000204983	ENSG00000213022
ENSG00000204983	ENSG00000214083
ENSG00000204991	ENSG00000134278
ENSG00000205017	ENSG00000006074
ENSG00000205017	ENSG00000006075
ENSG00000205017	ENSG00000006606
ENSG00000205017	ENSG00000102962
ENSG00000205017	ENSG00000102970
ENSG00000205017	ENSG00000106178
ENSG00000205017	ENSG00000108688
ENSG00000205017	ENSG00000108691
ENSG00000205017	ENSG00000108700
ENSG00000205017	ENSG00000108702
ENSG00000205017	ENSG00000115009
ENSG00000205017	ENSG00000129277
ENSG00000205017	ENSG00000143184
ENSG00000205017	ENSG00000143185
ENSG00000205017	ENSG00000161570
ENSG00000205017	ENSG00000161573
ENSG00000205017	ENSG00000161574
ENSG00000205017	ENSG00000167236
ENSG00000205017	ENSG00000172156
ENSG00000205017	ENSG00000172724
ENSG00000205017	ENSG00000181374
ENSG00000205017	ENSG00000197262
ENSG00000205017	ENSG00000205020
ENSG00000205017	ENSG00000205021
ENSG00000205017	ENSG00000213494
ENSG00000205017	ENSG00000215627
ENSG00000205017	ENSG00000215629
ENSG00000205019	ENSG00000128438
ENSG00000205019	ENSG00000148429
ENSG00000205019	ENSG00000161583
ENSG00000205019	ENSG00000174115
ENSG00000205019	ENSG00000185128
ENSG00000205019	ENSG00000188755
ENSG00000205019	ENSG00000188849
ENSG00000205019	ENSG00000189309
ENSG00000205019	ENSG00000197681
ENSG00000205019	ENSG00000204471
ENSG00000205019	ENSG00000213474
ENSG00000205019	ENSG00000215625
ENSG00000205019	ENSG00000215730
ENSG00000205019	ENSG00000215748
ENSG00000205020	ENSG00000006074
ENSG00000205020	ENSG00000006075
ENSG00000205020	ENSG00000006606
ENSG00000205020	ENSG00000102962
ENSG00000205020	ENSG00000102970
ENSG00000205020	ENSG00000106178
ENSG00000205020	ENSG00000108688
ENSG00000205020	ENSG00000108691
ENSG00000205020	ENSG00000108700
ENSG00000205020	ENSG00000108702

ENSG00000205020	ENSG00000115009
ENSG00000205020	ENSG00000129277
ENSG00000205020	ENSG00000143184
ENSG00000205020	ENSG00000143185
ENSG00000205020	ENSG00000161570
ENSG00000205020	ENSG00000161573
ENSG00000205020	ENSG00000161574
ENSG00000205020	ENSG00000167236
ENSG00000205020	ENSG00000172156
ENSG00000205020	ENSG00000172724
ENSG00000205020	ENSG00000181374
ENSG00000205020	ENSG00000197262
ENSG00000205020	ENSG00000205017
ENSG00000205020	ENSG00000205021
ENSG00000205020	ENSG00000213494
ENSG00000205020	ENSG00000215627
ENSG00000205020	ENSG00000215629
ENSG00000205021	ENSG00000006074
ENSG00000205021	ENSG00000006075
ENSG00000205021	ENSG00000006606
ENSG00000205021	ENSG00000102962
ENSG00000205021	ENSG00000102970
ENSG00000205021	ENSG00000106178
ENSG00000205021	ENSG00000108688
ENSG00000205021	ENSG00000108691
ENSG00000205021	ENSG00000108700
ENSG00000205021	ENSG00000108702
ENSG00000205021	ENSG00000115009
ENSG00000205021	ENSG00000129277
ENSG00000205021	ENSG00000143184
ENSG00000205021	ENSG00000143185
ENSG00000205021	ENSG00000161570
ENSG00000205021	ENSG00000161573
ENSG00000205021	ENSG00000161574
ENSG00000205021	ENSG00000167236
ENSG00000205021	ENSG00000172156
ENSG00000205021	ENSG00000172724
ENSG00000205021	ENSG00000181374
ENSG00000205021	ENSG00000197262
ENSG00000205021	ENSG00000205017
ENSG00000205021	ENSG00000205020
ENSG00000205021	ENSG00000213494
ENSG00000205021	ENSG00000215627
ENSG00000205021	ENSG00000215629
ENSG00000205022	ENSG00000100836
ENSG00000205038	ENSG00000170927
ENSG00000205042	ENSG00000108312
ENSG00000205042	ENSG00000183417
ENSG00000205042	ENSG00000204448
ENSG00000205042	ENSG00000204664
ENSG00000205042	ENSG00000204699
ENSG00000205042	ENSG00000204705
ENSG00000205063	ENSG00000196225
ENSG00000205063	ENSG00000205134
ENSG00000205063	ENSG00000206202
ENSG00000205063	ENSG00000214572
ENSG00000205063	ENSG00000215176
ENSG00000205063	ENSG00000215242
ENSG00000205063	ENSG00000215393

ENSG00000205066	ENSG00000182257
ENSG00000205066	ENSG00000184968
ENSG00000205070	ENSG00000111332
ENSG00000205070	ENSG00000124642
ENSG00000205070	ENSG00000137259
ENSG00000205070	ENSG00000158403
ENSG00000205070	ENSG00000164508
ENSG00000205070	ENSG00000168274
ENSG00000205070	ENSG00000180573
ENSG00000205070	ENSG00000181218
ENSG00000205070	ENSG00000182611
ENSG00000205070	ENSG00000183558
ENSG00000205070	ENSG00000184260
ENSG00000205070	ENSG00000184270
ENSG00000205070	ENSG00000184348
ENSG00000205070	ENSG00000184825
ENSG00000205070	ENSG00000185978
ENSG00000205070	ENSG00000188486
ENSG00000205070	ENSG00000196747
ENSG00000205070	ENSG00000196787
ENSG00000205070	ENSG00000196866
ENSG00000205070	ENSG00000198082
ENSG00000205070	ENSG00000198307
ENSG00000205070	ENSG00000198374
ENSG00000205070	ENSG00000203812
ENSG00000205070	ENSG00000205079
ENSG00000205076	ENSG00000006659
ENSG00000205076	ENSG00000100079
ENSG00000205076	ENSG00000100097
ENSG00000205076	ENSG00000105198
ENSG00000205076	ENSG00000105205
ENSG00000205076	ENSG00000116977
ENSG00000205076	ENSG00000119862
ENSG00000205076	ENSG00000131981
ENSG00000205076	ENSG00000133317
ENSG00000205076	ENSG00000168961
ENSG00000205076	ENSG00000170298
ENSG00000205076	ENSG00000171747
ENSG00000205076	ENSG00000171916
ENSG00000205076	ENSG00000178934
ENSG00000205076	ENSG00000197778
ENSG00000205076	ENSG00000215132
ENSG00000205079	ENSG00000111332
ENSG00000205079	ENSG00000124642
ENSG00000205079	ENSG00000137259
ENSG00000205079	ENSG00000158403
ENSG00000205079	ENSG00000164508
ENSG00000205079	ENSG00000168274
ENSG00000205079	ENSG00000180573
ENSG00000205079	ENSG00000181218
ENSG00000205079	ENSG00000182611
ENSG00000205079	ENSG00000183558
ENSG00000205079	ENSG00000184260
ENSG00000205079	ENSG00000184270
ENSG00000205079	ENSG00000184348
ENSG00000205079	ENSG00000184825
ENSG00000205079	ENSG00000185978
ENSG00000205079	ENSG00000188486
ENSG00000205079	ENSG00000196747

ENSG00000205079	ENSG00000196787
ENSG00000205079	ENSG00000196866
ENSG00000205079	ENSG00000198082
ENSG00000205079	ENSG00000198307
ENSG00000205079	ENSG00000198374
ENSG00000205079	ENSG00000203812
ENSG00000205079	ENSG00000205070
ENSG00000205089	ENSG00000113328
ENSG00000205089	ENSG00000118816
ENSG00000205089	ENSG00000138764
ENSG00000205100	ENSG00000080824
ENSG00000205100	ENSG00000096384
ENSG00000205100	ENSG00000183199
ENSG00000205100	ENSG00000205527
ENSG00000205100	ENSG00000205940
ENSG00000205116	ENSG00000167874
ENSG00000205134	ENSG00000196225
ENSG00000205134	ENSG00000205063
ENSG00000205134	ENSG00000206202
ENSG00000205134	ENSG00000214572
ENSG00000205134	ENSG00000215176
ENSG00000205134	ENSG00000215242
ENSG00000205134	ENSG00000215393
ENSG00000205162	ENSG00000205164
ENSG00000205162	ENSG00000205166
ENSG00000205162	ENSG00000205168
ENSG00000205162	ENSG00000205170
ENSG00000205162	ENSG00000205173
ENSG00000205162	ENSG00000205175
ENSG00000205162	ENSG00000205178
ENSG00000205162	ENSG00000212867
ENSG00000205164	ENSG00000205162
ENSG00000205164	ENSG00000205166
ENSG00000205164	ENSG00000205168
ENSG00000205164	ENSG00000205170
ENSG00000205164	ENSG00000205173
ENSG00000205164	ENSG00000205175
ENSG00000205164	ENSG00000205178
ENSG00000205164	ENSG00000212867
ENSG00000205166	ENSG00000205162
ENSG00000205166	ENSG00000205164
ENSG00000205166	ENSG00000205168
ENSG00000205166	ENSG00000205170
ENSG00000205166	ENSG00000205173
ENSG00000205166	ENSG00000205175
ENSG00000205166	ENSG00000205178
ENSG00000205166	ENSG00000212867
ENSG00000205168	ENSG00000205162
ENSG00000205168	ENSG00000205164
ENSG00000205168	ENSG00000205166
ENSG00000205168	ENSG00000205170
ENSG00000205168	ENSG00000205173
ENSG00000205168	ENSG00000205175
ENSG00000205168	ENSG00000205178
ENSG00000205168	ENSG00000212867
ENSG00000205170	ENSG00000205162
ENSG00000205170	ENSG00000205164
ENSG00000205170	ENSG00000205166
ENSG00000205170	ENSG00000205168

ENSG00000205170	ENSG00000205173
ENSG00000205170	ENSG00000205175
ENSG00000205170	ENSG00000205178
ENSG00000205170	ENSG00000212867
ENSG00000205173	ENSG00000205162
ENSG00000205173	ENSG00000205164
ENSG00000205173	ENSG00000205166
ENSG00000205173	ENSG00000205168
ENSG00000205173	ENSG00000205170
ENSG00000205173	ENSG00000205175
ENSG00000205173	ENSG00000205178
ENSG00000205173	ENSG00000212867
ENSG00000205175	ENSG00000205162
ENSG00000205175	ENSG00000205164
ENSG00000205175	ENSG00000205166
ENSG00000205175	ENSG00000205168
ENSG00000205175	ENSG00000205170
ENSG00000205175	ENSG00000205173
ENSG00000205175	ENSG00000205178
ENSG00000205175	ENSG00000212867
ENSG00000205178	ENSG00000205162
ENSG00000205178	ENSG00000205164
ENSG00000205178	ENSG00000205166
ENSG00000205178	ENSG00000205168
ENSG00000205178	ENSG00000205170
ENSG00000205178	ENSG00000205173
ENSG00000205178	ENSG00000205175
ENSG00000205178	ENSG00000212867
ENSG00000205184	ENSG00000100652
ENSG00000205184	ENSG00000125255
ENSG00000205184	ENSG00000126903
ENSG00000205184	ENSG00000145248
ENSG00000205184	ENSG00000145283
ENSG00000205186	ENSG00000114113
ENSG00000205186	ENSG00000114115
ENSG00000205186	ENSG00000121769
ENSG00000205186	ENSG00000139194
ENSG00000205186	ENSG00000143320
ENSG00000205186	ENSG00000145384
ENSG00000205186	ENSG00000147588
ENSG00000205186	ENSG00000162444
ENSG00000205186	ENSG00000164434
ENSG00000205186	ENSG00000164687
ENSG00000205186	ENSG00000166426
ENSG00000205186	ENSG00000170323
ENSG00000205186	ENSG00000197416
ENSG00000205186	ENSG00000214004
ENSG00000205189	ENSG00000160062
ENSG00000205189	ENSG00000168795
ENSG00000205189	ENSG00000185670
ENSG00000205189	ENSG00000215897
ENSG00000205198	ENSG00000026950
ENSG00000205198	ENSG00000111801
ENSG00000205198	ENSG00000112763
ENSG00000205198	ENSG00000113303
ENSG00000205198	ENSG00000121236
ENSG00000205198	ENSG00000124508
ENSG00000205198	ENSG00000124549
ENSG00000205198	ENSG00000124557

ENSG00000205198	ENSG00000128250
ENSG00000205198	ENSG00000128253
ENSG00000205198	ENSG00000128276
ENSG00000205198	ENSG00000128482
ENSG00000205198	ENSG00000132256
ENSG00000205198	ENSG00000132274
ENSG00000205198	ENSG00000164010
ENSG00000205198	ENSG00000165810
ENSG00000205198	ENSG00000168903
ENSG00000205198	ENSG00000176422
ENSG00000205198	ENSG00000179046
ENSG00000205198	ENSG00000182805
ENSG00000205198	ENSG00000183439
ENSG00000205198	ENSG00000183718
ENSG00000205198	ENSG00000187574
ENSG00000205198	ENSG00000188683
ENSG00000205198	ENSG00000204614
ENSG00000205198	ENSG00000204616
ENSG00000205198	ENSG00000204618
ENSG00000205198	ENSG00000214693
ENSG00000205213	ENSG00000133067
ENSG00000205213	ENSG00000138039
ENSG00000205213	ENSG00000139292
ENSG00000205213	ENSG00000165409
ENSG00000205213	ENSG00000170820
ENSG00000205220	ENSG00000136930
ENSG00000205221	ENSG00000100473
ENSG00000205221	ENSG00000163359
ENSG00000205221	ENSG00000165816
ENSG00000205221	ENSG00000198450
ENSG00000205238	ENSG00000136206
ENSG00000205238	ENSG00000154732
ENSG00000205238	ENSG00000163806
ENSG00000205238	ENSG00000166667
ENSG00000205238	ENSG00000170092
ENSG00000205238	ENSG00000173678
ENSG00000205238	ENSG00000179994
ENSG00000205238	ENSG00000183318
ENSG00000205238	ENSG00000184616
ENSG00000205238	ENSG00000185040
ENSG00000205238	ENSG00000186645
ENSG00000205238	ENSG00000198305
ENSG00000205238	ENSG00000204710
ENSG00000205238	ENSG00000205482
ENSG00000205238	ENSG00000205505
ENSG00000205238	ENSG00000205506
ENSG00000205238	ENSG00000205508
ENSG00000205238	ENSG00000205509
ENSG00000205238	ENSG00000205580
ENSG00000205238	ENSG00000214300
ENSG00000205238	ENSG00000215092
ENSG00000205245	ENSG00000184635
ENSG00000205245	ENSG00000213799
ENSG00000205267	ENSG00000062282
ENSG00000205267	ENSG00000106384
ENSG00000205267	ENSG00000124003
ENSG00000205267	ENSG00000147160
ENSG00000205267	ENSG00000166391
ENSG00000205267	ENSG00000184210

ENSG00000205267	ENSG00000204195
ENSG00000205268	ENSG00000065989
ENSG00000205268	ENSG00000073417
ENSG00000205268	ENSG00000105650
ENSG00000205268	ENSG00000113231
ENSG00000205268	ENSG00000113448
ENSG00000205268	ENSG00000115252
ENSG00000205268	ENSG00000123360
ENSG00000205268	ENSG00000154678
ENSG00000205268	ENSG00000160191
ENSG00000205268	ENSG00000171408
ENSG00000205268	ENSG00000184588
ENSG00000205269	ENSG00000166822
ENSG00000205279	ENSG00000178531
ENSG00000205302	ENSG00000028528
ENSG00000205302	ENSG00000089006
ENSG00000205302	ENSG00000129515
ENSG00000205302	ENSG00000172803
ENSG00000205318	ENSG00000015532
ENSG00000205318	ENSG00000103489
ENSG00000205318	ENSG00000111846
ENSG00000205318	ENSG00000124091
ENSG00000205318	ENSG00000140297
ENSG00000205318	ENSG00000176928
ENSG00000205318	ENSG00000187210
ENSG00000205339	ENSG00000133704
ENSG00000205358	ENSG00000087250
ENSG00000205358	ENSG00000102891
ENSG00000205358	ENSG00000125144
ENSG00000205358	ENSG00000125148
ENSG00000205358	ENSG00000169688
ENSG00000205358	ENSG00000169715
ENSG00000205358	ENSG00000187193
ENSG00000205358	ENSG00000198417
ENSG00000205358	ENSG00000205360
ENSG00000205358	ENSG00000205362
ENSG00000205358	ENSG00000205364
ENSG00000205360	ENSG00000087250
ENSG00000205360	ENSG00000102891
ENSG00000205360	ENSG00000125144
ENSG00000205360	ENSG00000125148
ENSG00000205360	ENSG00000169688
ENSG00000205360	ENSG00000169715
ENSG00000205360	ENSG00000187193
ENSG00000205360	ENSG00000198417
ENSG00000205360	ENSG00000205358
ENSG00000205360	ENSG00000205362
ENSG00000205360	ENSG00000205364
ENSG00000205362	ENSG00000087250
ENSG00000205362	ENSG00000102891
ENSG00000205362	ENSG00000125144
ENSG00000205362	ENSG00000125148
ENSG00000205362	ENSG00000169688
ENSG00000205362	ENSG00000169715
ENSG00000205362	ENSG00000187193
ENSG00000205362	ENSG00000198417
ENSG00000205362	ENSG00000205358
ENSG00000205362	ENSG00000205360
ENSG00000205362	ENSG00000205364

ENSG00000205364	ENSG00000087250
ENSG00000205364	ENSG00000102891
ENSG00000205364	ENSG00000125144
ENSG00000205364	ENSG00000125148
ENSG00000205364	ENSG00000169688
ENSG00000205364	ENSG00000169715
ENSG00000205364	ENSG00000187193
ENSG00000205364	ENSG00000198417
ENSG00000205364	ENSG00000205358
ENSG00000205364	ENSG00000205360
ENSG00000205364	ENSG00000205362
ENSG00000205365	ENSG00000106608
ENSG00000205365	ENSG00000132357
ENSG00000205365	ENSG00000187878
ENSG00000205403	ENSG00000149418
ENSG00000205403	ENSG00000176040
ENSG00000205403	ENSG00000178297
ENSG00000205403	ENSG00000187045
ENSG00000205405	ENSG00000119335
ENSG00000205405	ENSG00000125979
ENSG00000205405	ENSG00000168692
ENSG00000205405	ENSG00000168757
ENSG00000205405	ENSG00000169849
ENSG00000205405	ENSG00000178021
ENSG00000205405	ENSG00000180543
ENSG00000205405	ENSG00000184205
ENSG00000205405	ENSG00000187189
ENSG00000205405	ENSG00000187657
ENSG00000205405	ENSG00000188656
ENSG00000205405	ENSG00000189241
ENSG00000205405	ENSG00000203911
ENSG00000205405	ENSG00000206167
ENSG00000205405	ENSG00000206184
ENSG00000205405	ENSG00000214236
ENSG00000205405	ENSG00000215569
ENSG00000205405	ENSG00000215591
ENSG00000205405	ENSG00000215592
ENSG00000205405	ENSG00000215593
ENSG00000205405	ENSG00000215594
ENSG00000205405	ENSG00000215601
ENSG00000205405	ENSG00000215605
ENSG00000205405	ENSG00000215829
ENSG00000205408	ENSG00000138942
ENSG00000205408	ENSG00000204308
ENSG00000205413	ENSG00000177409
ENSG00000205420	ENSG00000135443
ENSG00000205420	ENSG00000135477
ENSG00000205420	ENSG00000135480
ENSG00000205420	ENSG00000139648
ENSG00000205420	ENSG00000161849
ENSG00000205420	ENSG00000161850
ENSG00000205420	ENSG00000167767
ENSG00000205420	ENSG00000167768
ENSG00000205420	ENSG00000170421
ENSG00000205420	ENSG00000170423
ENSG00000205420	ENSG00000170442
ENSG00000205420	ENSG00000170454
ENSG00000205420	ENSG00000170465
ENSG00000205420	ENSG00000170477

ENSG00000205420	ENSG00000170484
ENSG00000205420	ENSG00000170486
ENSG00000205420	ENSG00000170523
ENSG00000205420	ENSG00000172867
ENSG00000205420	ENSG00000185069
ENSG00000205420	ENSG00000185479
ENSG00000205420	ENSG00000185640
ENSG00000205420	ENSG00000186049
ENSG00000205420	ENSG00000186081
ENSG00000205420	ENSG00000186442
ENSG00000205420	ENSG00000189182
ENSG00000205420	ENSG00000205426
ENSG00000205420	ENSG00000214233
ENSG00000205420	ENSG00000214323
ENSG00000205420	ENSG00000214477
ENSG00000205420	ENSG00000214752
ENSG00000205420	ENSG00000214974
ENSG00000205420	ENSG00000215287
ENSG00000205420	ENSG00000215847
ENSG00000205426	ENSG00000135443
ENSG00000205426	ENSG00000135477
ENSG00000205426	ENSG00000135480
ENSG00000205426	ENSG00000139648
ENSG00000205426	ENSG00000161849
ENSG00000205426	ENSG00000161850
ENSG00000205426	ENSG00000167767
ENSG00000205426	ENSG00000167768
ENSG00000205426	ENSG00000170421
ENSG00000205426	ENSG00000170423
ENSG00000205426	ENSG00000170442
ENSG00000205426	ENSG00000170454
ENSG00000205426	ENSG00000170465
ENSG00000205426	ENSG00000170477
ENSG00000205426	ENSG00000170484
ENSG00000205426	ENSG00000170486
ENSG00000205426	ENSG00000170523
ENSG00000205426	ENSG00000172867
ENSG00000205426	ENSG00000185069
ENSG00000205426	ENSG00000185479
ENSG00000205426	ENSG00000185640
ENSG00000205426	ENSG00000186049
ENSG00000205426	ENSG00000186081
ENSG00000205426	ENSG00000186442
ENSG00000205426	ENSG00000189182
ENSG00000205426	ENSG00000205420
ENSG00000205426	ENSG00000214233
ENSG00000205426	ENSG00000214323
ENSG00000205426	ENSG00000214477
ENSG00000205426	ENSG00000214752
ENSG00000205426	ENSG00000214974
ENSG00000205426	ENSG00000215287
ENSG00000205426	ENSG00000215847
ENSG00000205432	ENSG00000182263
ENSG00000205436	ENSG00000130201
ENSG00000205436	ENSG00000179044
ENSG00000205436	ENSG00000180104
ENSG00000205436	ENSG00000185215
ENSG00000205461	ENSG00000187876
ENSG00000205461	ENSG00000204235

ENSG00000205461	ENSG00000204544
ENSG00000205461	ENSG00000204707
ENSG00000205464	ENSG00000071553
ENSG00000205476	ENSG00000055813
ENSG00000205476	ENSG00000175602
ENSG00000205482	ENSG00000136206
ENSG00000205482	ENSG00000154732
ENSG00000205482	ENSG00000163806
ENSG00000205482	ENSG00000166667
ENSG00000205482	ENSG00000170092
ENSG00000205482	ENSG00000173678
ENSG00000205482	ENSG00000179994
ENSG00000205482	ENSG00000183318
ENSG00000205482	ENSG00000184616
ENSG00000205482	ENSG00000185040
ENSG00000205482	ENSG00000186645
ENSG00000205482	ENSG00000198305
ENSG00000205482	ENSG00000204710
ENSG00000205482	ENSG00000205238
ENSG00000205482	ENSG00000205505
ENSG00000205482	ENSG00000205506
ENSG00000205482	ENSG00000205508
ENSG00000205482	ENSG00000205509
ENSG00000205482	ENSG00000205580
ENSG00000205482	ENSG00000214300
ENSG00000205482	ENSG00000215092
ENSG00000205502	ENSG00000183186
ENSG00000205502	ENSG00000198535
ENSG00000205505	ENSG00000136206
ENSG00000205505	ENSG00000154732
ENSG00000205505	ENSG00000163806
ENSG00000205505	ENSG00000166667
ENSG00000205505	ENSG00000170092
ENSG00000205505	ENSG00000173678
ENSG00000205505	ENSG00000179994
ENSG00000205505	ENSG00000183318
ENSG00000205505	ENSG00000184616
ENSG00000205505	ENSG00000185040
ENSG00000205505	ENSG00000186645
ENSG00000205505	ENSG00000198305
ENSG00000205505	ENSG00000204710
ENSG00000205505	ENSG00000205238
ENSG00000205505	ENSG00000205482
ENSG00000205505	ENSG00000205506
ENSG00000205505	ENSG00000205508
ENSG00000205505	ENSG00000205509
ENSG00000205505	ENSG00000205580
ENSG00000205505	ENSG00000214300
ENSG00000205505	ENSG00000215092
ENSG00000205506	ENSG00000136206
ENSG00000205506	ENSG00000154732
ENSG00000205506	ENSG00000163806
ENSG00000205506	ENSG00000166667
ENSG00000205506	ENSG00000170092
ENSG00000205506	ENSG00000173678
ENSG00000205506	ENSG00000179994
ENSG00000205506	ENSG00000183318
ENSG00000205506	ENSG00000184616
ENSG00000205506	ENSG00000185040

ENSG00000205506	ENSG00000186645
ENSG00000205506	ENSG00000198305
ENSG00000205506	ENSG00000204710
ENSG00000205506	ENSG00000205238
ENSG00000205506	ENSG00000205482
ENSG00000205506	ENSG00000205505
ENSG00000205506	ENSG00000205508
ENSG00000205506	ENSG00000205509
ENSG00000205506	ENSG00000205580
ENSG00000205506	ENSG00000214300
ENSG00000205506	ENSG00000215092
ENSG00000205508	ENSG00000136206
ENSG00000205508	ENSG00000154732
ENSG00000205508	ENSG00000163806
ENSG00000205508	ENSG00000166667
ENSG00000205508	ENSG00000170092
ENSG00000205508	ENSG00000173678
ENSG00000205508	ENSG00000179994
ENSG00000205508	ENSG00000183318
ENSG00000205508	ENSG00000184616
ENSG00000205508	ENSG00000185040
ENSG00000205508	ENSG00000186645
ENSG00000205508	ENSG00000198305
ENSG00000205508	ENSG00000204710
ENSG00000205508	ENSG00000205238
ENSG00000205508	ENSG00000205482
ENSG00000205508	ENSG00000205505
ENSG00000205508	ENSG00000205506
ENSG00000205508	ENSG00000205509
ENSG00000205508	ENSG00000205580
ENSG00000205508	ENSG00000214300
ENSG00000205508	ENSG00000215092
ENSG00000205509	ENSG00000136206
ENSG00000205509	ENSG00000154732
ENSG00000205509	ENSG00000163806
ENSG00000205509	ENSG00000166667
ENSG00000205509	ENSG00000170092
ENSG00000205509	ENSG00000173678
ENSG00000205509	ENSG00000179994
ENSG00000205509	ENSG00000183318
ENSG00000205509	ENSG00000184616
ENSG00000205509	ENSG00000185040
ENSG00000205509	ENSG00000186645
ENSG00000205509	ENSG00000198305
ENSG00000205509	ENSG00000204710
ENSG00000205509	ENSG00000205238
ENSG00000205509	ENSG00000205482
ENSG00000205509	ENSG00000205505
ENSG00000205509	ENSG00000205506
ENSG00000205509	ENSG00000205508
ENSG00000205509	ENSG00000205580
ENSG00000205509	ENSG00000214300
ENSG00000205509	ENSG00000215092
ENSG00000205517	ENSG00000143344
ENSG00000205517	ENSG00000160271
ENSG00000205517	ENSG00000204218
ENSG00000205527	ENSG00000080824
ENSG00000205527	ENSG00000096384
ENSG00000205527	ENSG00000183199

ENSG00000205527	ENSG00000205100
ENSG00000205527	ENSG00000205940
ENSG00000205531	ENSG00000177432
ENSG00000205531	ENSG00000186310
ENSG00000205531	ENSG00000186462
ENSG00000205531	ENSG00000187109
ENSG00000205557	ENSG00000197789
ENSG00000205560	ENSG00000005469
ENSG00000205560	ENSG00000070748
ENSG00000205560	ENSG00000095321
ENSG00000205560	ENSG00000110090
ENSG00000205560	ENSG00000169169
ENSG00000205571	ENSG00000172062
ENSG00000205580	ENSG00000136206
ENSG00000205580	ENSG00000154732
ENSG00000205580	ENSG00000163806
ENSG00000205580	ENSG00000166667
ENSG00000205580	ENSG00000170092
ENSG00000205580	ENSG00000173678
ENSG00000205580	ENSG00000179994
ENSG00000205580	ENSG00000183318
ENSG00000205580	ENSG00000184616
ENSG00000205580	ENSG00000185040
ENSG00000205580	ENSG00000186645
ENSG00000205580	ENSG00000198305
ENSG00000205580	ENSG00000204710
ENSG00000205580	ENSG00000205238
ENSG00000205580	ENSG00000205482
ENSG00000205580	ENSG00000205505
ENSG00000205580	ENSG00000205506
ENSG00000205580	ENSG00000205508
ENSG00000205580	ENSG00000205509
ENSG00000205580	ENSG00000214300
ENSG00000205580	ENSG00000215092
ENSG00000205593	ENSG00000174839
ENSG00000205595	ENSG00000109321
ENSG00000205595	ENSG00000113070
ENSG00000205596	ENSG00000147533
ENSG00000205596	ENSG00000155265
ENSG00000205613	ENSG00000183812
ENSG00000205613	ENSG00000197833
ENSG00000205613	ENSG00000204805
ENSG00000205613	ENSG00000204806
ENSG00000205613	ENSG00000204807
ENSG00000205613	ENSG00000204808
ENSG00000205613	ENSG00000215061
ENSG00000205639	ENSG00000168389
ENSG00000205663	ENSG00000181996
ENSG00000205663	ENSG00000184405
ENSG00000205663	ENSG00000204239
ENSG00000205663	ENSG00000205664
ENSG00000205663	ENSG00000206061
ENSG00000205664	ENSG00000181996
ENSG00000205664	ENSG00000184405
ENSG00000205664	ENSG00000204239
ENSG00000205664	ENSG00000205663
ENSG00000205664	ENSG00000206061
ENSG00000205667	ENSG00000006756
ENSG00000205667	ENSG00000062096

ENSG00000205667	ENSG00000100299
ENSG00000205667	ENSG00000101846
ENSG00000205667	ENSG00000113273
ENSG00000205667	ENSG00000141012
ENSG00000205667	ENSG00000141337
ENSG00000205667	ENSG00000157399
ENSG00000205667	ENSG00000180801
ENSG00000205667	ENSG00000183876
ENSG00000205678	ENSG00000099797
ENSG00000205683	ENSG00000011332
ENSG00000205683	ENSG00000130024
ENSG00000205683	ENSG00000133884
ENSG00000205699	ENSG00000179349
ENSG00000205717	ENSG00000071655
ENSG00000205717	ENSG00000134046
ENSG00000205717	ENSG00000170948
ENSG00000205717	ENSG00000182315
ENSG00000205717	ENSG00000184301
ENSG00000205717	ENSG00000196589
ENSG00000205717	ENSG00000205718
ENSG00000205718	ENSG00000071655
ENSG00000205718	ENSG00000134046
ENSG00000205718	ENSG00000170948
ENSG00000205718	ENSG00000182315
ENSG00000205718	ENSG00000184301
ENSG00000205718	ENSG00000196589
ENSG00000205718	ENSG00000205717
ENSG00000205726	ENSG00000085832
ENSG00000205726	ENSG00000127527
ENSG00000205726	ENSG00000198399
ENSG00000205730	ENSG00000148841
ENSG00000205730	ENSG00000198885
ENSG00000205731	ENSG00000198361
ENSG00000205731	ENSG00000198684
ENSG00000205731	ENSG00000204202
ENSG00000205731	ENSG00000204203
ENSG00000205731	ENSG00000204204
ENSG00000205731	ENSG00000204205
ENSG00000205731	ENSG00000204206
ENSG00000205731	ENSG00000204207
ENSG00000205731	ENSG00000205735
ENSG00000205731	ENSG00000205737
ENSG00000205731	ENSG00000206163
ENSG00000205737	ENSG00000198361
ENSG00000205737	ENSG00000198684
ENSG00000205737	ENSG00000204202
ENSG00000205737	ENSG00000204203
ENSG00000205737	ENSG00000204204
ENSG00000205737	ENSG00000204205
ENSG00000205737	ENSG00000204206
ENSG00000205737	ENSG00000204207
ENSG00000205737	ENSG00000205731
ENSG00000205737	ENSG00000205735
ENSG00000205737	ENSG00000206163
ENSG00000205744	ENSG00000119522
ENSG00000205744	ENSG00000146966
ENSG00000205744	ENSG00000162701
ENSG00000205744	ENSG00000162777
ENSG00000205744	ENSG00000166444

ENSG00000205744	ENSG00000175984
ENSG00000205754	ENSG00000084453
ENSG00000205754	ENSG00000101187
ENSG00000205754	ENSG00000111700
ENSG00000205754	ENSG00000134538
ENSG00000205754	ENSG00000137491
ENSG00000205754	ENSG00000137571
ENSG00000205754	ENSG00000139155
ENSG00000205754	ENSG00000173930
ENSG00000205754	ENSG00000174640
ENSG00000205754	ENSG00000176463
ENSG00000205754	ENSG00000205359
ENSG00000205808	ENSG00000160539
ENSG00000205838	ENSG00000103852
ENSG00000205850	ENSG00000182447
ENSG00000205850	ENSG00000205863
ENSG00000205863	ENSG00000182447
ENSG00000205863	ENSG00000205850
ENSG00000205916	ENSG00000092345
ENSG00000205916	ENSG00000152430
ENSG00000205916	ENSG00000187191
ENSG00000205916	ENSG00000188120
ENSG00000205916	ENSG00000205944
ENSG00000205922	ENSG00000111249
ENSG00000205922	ENSG00000119547
ENSG00000205922	ENSG00000160967
ENSG00000205922	ENSG00000169856
ENSG00000205927	ENSG00000125533
ENSG00000205927	ENSG00000177468
ENSG00000205927	ENSG00000180828
ENSG00000205927	ENSG00000184221
ENSG00000205940	ENSG00000080824
ENSG00000205940	ENSG00000096384
ENSG00000205940	ENSG00000183199
ENSG00000205940	ENSG00000205100
ENSG00000205940	ENSG00000205527
ENSG00000205944	ENSG00000092345
ENSG00000205944	ENSG00000152430
ENSG00000205944	ENSG00000187191
ENSG00000205944	ENSG00000188120
ENSG00000205944	ENSG00000205916
ENSG00000205981	ENSG00000120675
ENSG00000206004	ENSG00000112090
ENSG00000206011	ENSG00000147274
ENSG00000206011	ENSG00000169800
ENSG00000206011	ENSG00000169811
ENSG00000206011	ENSG00000170748
ENSG00000206011	ENSG00000174622
ENSG00000206011	ENSG00000206046
ENSG00000206011	ENSG00000213516
ENSG00000206011	ENSG00000214442
ENSG00000206011	ENSG00000215210
ENSG00000206013	ENSG00000142089
ENSG00000206013	ENSG00000185201
ENSG00000206013	ENSG00000185885
ENSG00000206013	ENSG00000188082
ENSG00000206013	ENSG00000215096
ENSG00000206046	ENSG00000147274
ENSG00000206046	ENSG00000169800

ENSG00000206046	ENSG00000169811
ENSG00000206046	ENSG00000170748
ENSG00000206046	ENSG00000174622
ENSG00000206046	ENSG00000206011
ENSG00000206046	ENSG00000213516
ENSG00000206046	ENSG00000214442
ENSG00000206046	ENSG00000215210
ENSG00000206052	ENSG00000101134
ENSG00000206052	ENSG00000115325
ENSG00000206052	ENSG00000125170
ENSG00000206052	ENSG00000137218
ENSG00000206052	ENSG00000146094
ENSG00000206052	ENSG00000147443
ENSG00000206052	ENSG00000166225
ENSG00000206053	ENSG00000189159
ENSG00000206061	ENSG00000181996
ENSG00000206061	ENSG00000184405
ENSG00000206061	ENSG00000204239
ENSG00000206061	ENSG00000205663
ENSG00000206061	ENSG00000205664
ENSG00000206066	ENSG00000128322
ENSG00000206066	ENSG00000211592
ENSG00000206066	ENSG00000211675
ENSG00000206066	ENSG00000211677
ENSG00000206066	ENSG00000211679
ENSG00000206066	ENSG00000211683
ENSG00000206066	ENSG00000211685
ENSG00000206066	ENSG00000211898
ENSG00000206072	ENSG00000021355
ENSG00000206072	ENSG00000057149
ENSG00000206072	ENSG00000124570
ENSG00000206072	ENSG00000166396
ENSG00000206072	ENSG00000166401
ENSG00000206072	ENSG00000166404
ENSG00000206072	ENSG00000166634
ENSG00000206072	ENSG00000170542
ENSG00000206072	ENSG00000197632
ENSG00000206072	ENSG00000197641
ENSG00000206072	ENSG00000206073
ENSG00000206072	ENSG00000206075
ENSG00000206073	ENSG00000021355
ENSG00000206073	ENSG00000057149
ENSG00000206073	ENSG00000124570
ENSG00000206073	ENSG00000166396
ENSG00000206073	ENSG00000166401
ENSG00000206073	ENSG00000166404
ENSG00000206073	ENSG00000166634
ENSG00000206073	ENSG00000170542
ENSG00000206073	ENSG00000197632
ENSG00000206073	ENSG00000197641
ENSG00000206073	ENSG00000206072
ENSG00000206073	ENSG00000206075
ENSG00000206075	ENSG00000021355
ENSG00000206075	ENSG00000057149
ENSG00000206075	ENSG00000124570
ENSG00000206075	ENSG00000166396
ENSG00000206075	ENSG00000166401
ENSG00000206075	ENSG00000166404
ENSG00000206075	ENSG00000166634

ENSG00000206075	ENSG00000170542
ENSG00000206075	ENSG00000197632
ENSG00000206075	ENSG00000197641
ENSG00000206075	ENSG00000206072
ENSG00000206075	ENSG00000206073
ENSG00000206077	ENSG00000159714
ENSG00000206077	ENSG00000188818
ENSG00000206090	ENSG00000099984
ENSG00000206090	ENSG00000133433
ENSG00000206090	ENSG00000184490
ENSG00000206090	ENSG00000184674
ENSG00000206090	ENSG00000185831
ENSG00000206115	ENSG00000126856
ENSG00000206147	ENSG00000118975
ENSG00000206147	ENSG00000184319
ENSG00000206147	ENSG00000185822
ENSG00000206147	ENSG00000186239
ENSG00000206147	ENSG00000186698
ENSG00000206147	ENSG00000198242
ENSG00000206147	ENSG00000212932
ENSG00000206147	ENSG00000213753
ENSG00000206147	ENSG00000214230
ENSG00000206147	ENSG00000215001
ENSG00000206147	ENSG00000215140
ENSG00000206147	ENSG00000215387
ENSG00000206148	ENSG00000125492
ENSG00000206148	ENSG00000143032
ENSG00000206156	ENSG00000111348
ENSG00000206156	ENSG00000141522
ENSG00000206156	ENSG00000215642
ENSG00000206163	ENSG00000198361
ENSG00000206163	ENSG00000198684
ENSG00000206163	ENSG00000204202
ENSG00000206163	ENSG00000204203
ENSG00000206163	ENSG00000204204
ENSG00000206163	ENSG00000204205
ENSG00000206163	ENSG00000204206
ENSG00000206163	ENSG00000204207
ENSG00000206163	ENSG00000205731
ENSG00000206163	ENSG00000205735
ENSG00000206163	ENSG00000205737
ENSG00000206172	ENSG00000086506
ENSG00000206172	ENSG00000130656
ENSG00000206172	ENSG00000188536
ENSG00000206172	ENSG00000206177
ENSG00000206172	ENSG00000206178
ENSG00000206177	ENSG00000086506
ENSG00000206177	ENSG00000130656
ENSG00000206177	ENSG00000188536
ENSG00000206177	ENSG00000206172
ENSG00000206177	ENSG00000206178
ENSG00000206178	ENSG00000086506
ENSG00000206178	ENSG00000130656
ENSG00000206178	ENSG00000188536
ENSG00000206178	ENSG00000206172
ENSG00000206178	ENSG00000206177
ENSG00000206181	ENSG00000011007
ENSG00000206181	ENSG00000183791
ENSG00000206181	ENSG00000206182

ENSG00000206181	ENSG00000206183
ENSG00000206182	ENSG00000011007
ENSG00000206182	ENSG00000183791
ENSG00000206182	ENSG00000206181
ENSG00000206182	ENSG00000206183
ENSG00000206183	ENSG00000011007
ENSG00000206183	ENSG00000183791
ENSG00000206183	ENSG00000206181
ENSG00000206183	ENSG00000206182
ENSG00000206190	ENSG00000118322
ENSG00000206190	ENSG00000145246
ENSG00000206202	ENSG00000196225
ENSG00000206202	ENSG00000205063
ENSG00000206202	ENSG00000205134
ENSG00000206202	ENSG00000214572
ENSG00000206202	ENSG00000215176
ENSG00000206202	ENSG00000215242
ENSG00000206202	ENSG00000215393
ENSG00000206228	ENSG00000122566
ENSG00000206228	ENSG00000135486
ENSG00000206228	ENSG00000139675
ENSG00000206228	ENSG00000170144
ENSG00000206228	ENSG00000176825
ENSG00000206228	ENSG00000177219
ENSG00000206228	ENSG00000177733
ENSG00000206228	ENSG00000188174
ENSG00000206228	ENSG00000212961
ENSG00000206228	ENSG00000214284
ENSG00000206228	ENSG00000214361
ENSG00000206228	ENSG00000214592
ENSG00000206228	ENSG00000214653
ENSG00000206228	ENSG00000215222
ENSG00000206228	ENSG00000215492
ENSG00000206228	ENSG00000215870
ENSG00000206228	ENSG00000215876
ENSG00000206228	ENSG00000215885
ENSG00000206432	ENSG00000164484
ENSG00000206432	ENSG00000187975
ENSG00000206527	ENSG00000074696
ENSG00000206527	ENSG00000165996
ENSG00000206527	ENSG00000188921
ENSG00000206538	ENSG00000102243
ENSG00000206538	ENSG00000170162
ENSG00000206549	ENSG00000005001
ENSG00000206549	ENSG00000007038
ENSG00000206549	ENSG00000052344
ENSG00000206549	ENSG00000095917
ENSG00000206549	ENSG00000103355
ENSG00000206549	ENSG00000116176
ENSG00000206549	ENSG00000172236
ENSG00000206549	ENSG00000172382
ENSG00000206549	ENSG00000172460
ENSG00000206549	ENSG00000178055
ENSG00000206549	ENSG00000178226
ENSG00000206549	ENSG00000185888
ENSG00000206549	ENSG00000188086
ENSG00000206549	ENSG00000189099
ENSG00000206549	ENSG00000196364
ENSG00000206549	ENSG00000197253

ENSG00000206549	ENSG00000214877
ENSG00000206549	ENSG00000215148
ENSG00000206560	ENSG00000065413
ENSG00000206560	ENSG00000119509
ENSG00000206560	ENSG00000139645
ENSG00000206579	ENSG00000101321
ENSG00000206579	ENSG00000158156
ENSG00000206579	ENSG00000171044
ENSG00000206579	ENSG00000186530
ENSG00000206579	ENSG00000198669
ENSG00000211448	ENSG00000197406
ENSG00000211448	ENSG00000211452
ENSG00000211451	ENSG00000109163
ENSG00000211451	ENSG00000126895
ENSG00000211451	ENSG00000166148
ENSG00000211451	ENSG00000178015
ENSG00000211451	ENSG00000180914
ENSG00000211451	ENSG00000187258
ENSG00000211451	ENSG00000198049
ENSG00000211452	ENSG00000197406
ENSG00000211452	ENSG00000211448
ENSG00000211455	ENSG00000104936
ENSG00000211455	ENSG00000112079
ENSG00000211592	ENSG00000128322
ENSG00000211592	ENSG00000206066
ENSG00000211592	ENSG00000211675
ENSG00000211592	ENSG00000211677
ENSG00000211592	ENSG00000211679
ENSG00000211592	ENSG00000211683
ENSG00000211592	ENSG00000211685
ENSG00000211592	ENSG00000211898
ENSG00000211599	ENSG00000136700
ENSG00000211599	ENSG00000156755
ENSG00000211599	ENSG00000183719
ENSG00000211599	ENSG00000197794
ENSG00000211599	ENSG00000204670
ENSG00000211599	ENSG00000204776
ENSG00000211599	ENSG00000204780
ENSG00000211599	ENSG00000211600
ENSG00000211599	ENSG00000211601
ENSG00000211599	ENSG00000211602
ENSG00000211599	ENSG00000211603
ENSG00000211599	ENSG00000211604
ENSG00000211599	ENSG00000211605
ENSG00000211599	ENSG00000211606
ENSG00000211599	ENSG00000211607
ENSG00000211599	ENSG00000211608
ENSG00000211599	ENSG00000211609
ENSG00000211599	ENSG00000211610
ENSG00000211599	ENSG00000211611
ENSG00000211599	ENSG00000211613
ENSG00000211599	ENSG00000211616
ENSG00000211599	ENSG00000211617
ENSG00000211599	ENSG00000211618
ENSG00000211599	ENSG00000211620
ENSG00000211599	ENSG00000211625
ENSG00000211599	ENSG00000211626
ENSG00000211599	ENSG00000211627
ENSG00000211599	ENSG00000211628

ENSG00000211599	ENSG00000211629
ENSG00000211599	ENSG00000211630
ENSG00000211599	ENSG00000211631
ENSG00000211599	ENSG00000211632
ENSG00000211599	ENSG00000211633
ENSG00000211599	ENSG00000211634
ENSG00000211599	ENSG00000211635
ENSG00000211599	ENSG00000211636
ENSG00000211600	ENSG00000136700
ENSG00000211600	ENSG00000156755
ENSG00000211600	ENSG00000183719
ENSG00000211600	ENSG00000197794
ENSG00000211600	ENSG00000204670
ENSG00000211600	ENSG00000204776
ENSG00000211600	ENSG00000204780
ENSG00000211600	ENSG00000211599
ENSG00000211600	ENSG00000211601
ENSG00000211600	ENSG00000211602
ENSG00000211600	ENSG00000211603
ENSG00000211600	ENSG00000211604
ENSG00000211600	ENSG00000211605
ENSG00000211600	ENSG00000211606
ENSG00000211600	ENSG00000211607
ENSG00000211600	ENSG00000211608
ENSG00000211600	ENSG00000211609
ENSG00000211600	ENSG00000211610
ENSG00000211600	ENSG00000211611
ENSG00000211600	ENSG00000211613
ENSG00000211600	ENSG00000211616
ENSG00000211600	ENSG00000211617
ENSG00000211600	ENSG00000211618
ENSG00000211600	ENSG00000211620
ENSG00000211600	ENSG00000211625
ENSG00000211600	ENSG00000211626
ENSG00000211600	ENSG00000211627
ENSG00000211600	ENSG00000211628
ENSG00000211600	ENSG00000211629
ENSG00000211600	ENSG00000211630
ENSG00000211600	ENSG00000211631
ENSG00000211600	ENSG00000211632
ENSG00000211600	ENSG00000211633
ENSG00000211600	ENSG00000211634
ENSG00000211600	ENSG00000211635
ENSG00000211600	ENSG00000211636
ENSG00000211601	ENSG00000136700
ENSG00000211601	ENSG00000156755
ENSG00000211601	ENSG00000183719
ENSG00000211601	ENSG00000197794
ENSG00000211601	ENSG00000204670
ENSG00000211601	ENSG00000204776
ENSG00000211601	ENSG00000204780
ENSG00000211601	ENSG00000211599
ENSG00000211601	ENSG00000211600
ENSG00000211601	ENSG00000211602
ENSG00000211601	ENSG00000211603
ENSG00000211601	ENSG00000211604
ENSG00000211601	ENSG00000211605
ENSG00000211601	ENSG00000211606
ENSG00000211601	ENSG00000211607

ENSG00000211601	ENSG00000211608
ENSG00000211601	ENSG00000211609
ENSG00000211601	ENSG00000211610
ENSG00000211601	ENSG00000211611
ENSG00000211601	ENSG00000211613
ENSG00000211601	ENSG00000211616
ENSG00000211601	ENSG00000211617
ENSG00000211601	ENSG00000211618
ENSG00000211601	ENSG00000211620
ENSG00000211601	ENSG00000211625
ENSG00000211601	ENSG00000211626
ENSG00000211601	ENSG00000211627
ENSG00000211601	ENSG00000211628
ENSG00000211601	ENSG00000211629
ENSG00000211601	ENSG00000211630
ENSG00000211601	ENSG00000211631
ENSG00000211601	ENSG00000211632
ENSG00000211601	ENSG00000211633
ENSG00000211601	ENSG00000211634
ENSG00000211601	ENSG00000211635
ENSG00000211601	ENSG00000211636
ENSG00000211602	ENSG00000136700
ENSG00000211602	ENSG00000156755
ENSG00000211602	ENSG00000183719
ENSG00000211602	ENSG00000197794
ENSG00000211602	ENSG00000204670
ENSG00000211602	ENSG00000204776
ENSG00000211602	ENSG00000204780
ENSG00000211602	ENSG00000211599
ENSG00000211602	ENSG00000211600
ENSG00000211602	ENSG00000211601
ENSG00000211602	ENSG00000211603
ENSG00000211602	ENSG00000211604
ENSG00000211602	ENSG00000211605
ENSG00000211602	ENSG00000211606
ENSG00000211602	ENSG00000211607
ENSG00000211602	ENSG00000211608
ENSG00000211602	ENSG00000211609
ENSG00000211602	ENSG00000211610
ENSG00000211602	ENSG00000211611
ENSG00000211602	ENSG00000211613
ENSG00000211602	ENSG00000211616
ENSG00000211602	ENSG00000211617
ENSG00000211602	ENSG00000211618
ENSG00000211602	ENSG00000211620
ENSG00000211602	ENSG00000211625
ENSG00000211602	ENSG00000211626
ENSG00000211602	ENSG00000211627
ENSG00000211602	ENSG00000211628
ENSG00000211602	ENSG00000211629
ENSG00000211602	ENSG00000211630
ENSG00000211602	ENSG00000211631
ENSG00000211602	ENSG00000211632
ENSG00000211602	ENSG00000211633
ENSG00000211602	ENSG00000211634
ENSG00000211602	ENSG00000211635
ENSG00000211602	ENSG00000211636
ENSG00000211603	ENSG00000136700
ENSG00000211603	ENSG00000156755

ENSG00000211603	ENSG00000183719
ENSG00000211603	ENSG00000197794
ENSG00000211603	ENSG00000204670
ENSG00000211603	ENSG00000204776
ENSG00000211603	ENSG00000204780
ENSG00000211603	ENSG00000211599
ENSG00000211603	ENSG00000211600
ENSG00000211603	ENSG00000211601
ENSG00000211603	ENSG00000211602
ENSG00000211603	ENSG00000211604
ENSG00000211603	ENSG00000211605
ENSG00000211603	ENSG00000211606
ENSG00000211603	ENSG00000211607
ENSG00000211603	ENSG00000211608
ENSG00000211603	ENSG00000211609
ENSG00000211603	ENSG00000211610
ENSG00000211603	ENSG00000211611
ENSG00000211603	ENSG00000211613
ENSG00000211603	ENSG00000211616
ENSG00000211603	ENSG00000211617
ENSG00000211603	ENSG00000211618
ENSG00000211603	ENSG00000211620
ENSG00000211603	ENSG00000211625
ENSG00000211603	ENSG00000211626
ENSG00000211603	ENSG00000211627
ENSG00000211603	ENSG00000211628
ENSG00000211603	ENSG00000211629
ENSG00000211603	ENSG00000211630
ENSG00000211603	ENSG00000211631
ENSG00000211603	ENSG00000211632
ENSG00000211603	ENSG00000211633
ENSG00000211603	ENSG00000211634
ENSG00000211603	ENSG00000211635
ENSG00000211603	ENSG00000211636
ENSG00000211604	ENSG00000136700
ENSG00000211604	ENSG00000156755
ENSG00000211604	ENSG00000183719
ENSG00000211604	ENSG00000197794
ENSG00000211604	ENSG00000204670
ENSG00000211604	ENSG00000204776
ENSG00000211604	ENSG00000204780
ENSG00000211604	ENSG00000211599
ENSG00000211604	ENSG00000211600
ENSG00000211604	ENSG00000211601
ENSG00000211604	ENSG00000211602
ENSG00000211604	ENSG00000211603
ENSG00000211604	ENSG00000211605
ENSG00000211604	ENSG00000211606
ENSG00000211604	ENSG00000211607
ENSG00000211604	ENSG00000211608
ENSG00000211604	ENSG00000211609
ENSG00000211604	ENSG00000211610
ENSG00000211604	ENSG00000211611
ENSG00000211604	ENSG00000211613
ENSG00000211604	ENSG00000211616
ENSG00000211604	ENSG00000211617
ENSG00000211604	ENSG00000211618
ENSG00000211604	ENSG00000211620
ENSG00000211604	ENSG00000211625

ENSG00000211604	ENSG00000211626
ENSG00000211604	ENSG00000211627
ENSG00000211604	ENSG00000211628
ENSG00000211604	ENSG00000211629
ENSG00000211604	ENSG00000211630
ENSG00000211604	ENSG00000211631
ENSG00000211604	ENSG00000211632
ENSG00000211604	ENSG00000211633
ENSG00000211604	ENSG00000211634
ENSG00000211604	ENSG00000211635
ENSG00000211604	ENSG00000211636
ENSG00000211605	ENSG00000136700
ENSG00000211605	ENSG00000156755
ENSG00000211605	ENSG00000183719
ENSG00000211605	ENSG00000197794
ENSG00000211605	ENSG00000204670
ENSG00000211605	ENSG00000204776
ENSG00000211605	ENSG00000204780
ENSG00000211605	ENSG00000211599
ENSG00000211605	ENSG00000211600
ENSG00000211605	ENSG00000211601
ENSG00000211605	ENSG00000211602
ENSG00000211605	ENSG00000211603
ENSG00000211605	ENSG00000211604
ENSG00000211605	ENSG00000211606
ENSG00000211605	ENSG00000211607
ENSG00000211605	ENSG00000211608
ENSG00000211605	ENSG00000211609
ENSG00000211605	ENSG00000211610
ENSG00000211605	ENSG00000211611
ENSG00000211605	ENSG00000211613
ENSG00000211605	ENSG00000211616
ENSG00000211605	ENSG00000211617
ENSG00000211605	ENSG00000211618
ENSG00000211605	ENSG00000211620
ENSG00000211605	ENSG00000211625
ENSG00000211605	ENSG00000211626
ENSG00000211605	ENSG00000211627
ENSG00000211605	ENSG00000211628
ENSG00000211605	ENSG00000211629
ENSG00000211605	ENSG00000211630
ENSG00000211605	ENSG00000211631
ENSG00000211605	ENSG00000211632
ENSG00000211605	ENSG00000211633
ENSG00000211605	ENSG00000211634
ENSG00000211605	ENSG00000211635
ENSG00000211605	ENSG00000211636
ENSG00000211606	ENSG00000136700
ENSG00000211606	ENSG00000156755
ENSG00000211606	ENSG00000183719
ENSG00000211606	ENSG00000197794
ENSG00000211606	ENSG00000204670
ENSG00000211606	ENSG00000204776
ENSG00000211606	ENSG00000204780
ENSG00000211606	ENSG00000211599
ENSG00000211606	ENSG00000211600
ENSG00000211606	ENSG00000211601
ENSG00000211606	ENSG00000211602
ENSG00000211606	ENSG00000211603

ENSG00000211606	ENSG00000211604
ENSG00000211606	ENSG00000211605
ENSG00000211606	ENSG00000211607
ENSG00000211606	ENSG00000211608
ENSG00000211606	ENSG00000211609
ENSG00000211606	ENSG00000211610
ENSG00000211606	ENSG00000211611
ENSG00000211606	ENSG00000211613
ENSG00000211606	ENSG00000211616
ENSG00000211606	ENSG00000211617
ENSG00000211606	ENSG00000211618
ENSG00000211606	ENSG00000211620
ENSG00000211606	ENSG00000211625
ENSG00000211606	ENSG00000211626
ENSG00000211606	ENSG00000211627
ENSG00000211606	ENSG00000211628
ENSG00000211606	ENSG00000211629
ENSG00000211606	ENSG00000211630
ENSG00000211606	ENSG00000211631
ENSG00000211606	ENSG00000211632
ENSG00000211606	ENSG00000211633
ENSG00000211606	ENSG00000211634
ENSG00000211606	ENSG00000211635
ENSG00000211606	ENSG00000211636
ENSG00000211607	ENSG00000136700
ENSG00000211607	ENSG00000156755
ENSG00000211607	ENSG00000183719
ENSG00000211607	ENSG00000197794
ENSG00000211607	ENSG00000204670
ENSG00000211607	ENSG00000204776
ENSG00000211607	ENSG00000204780
ENSG00000211607	ENSG00000211599
ENSG00000211607	ENSG00000211600
ENSG00000211607	ENSG00000211601
ENSG00000211607	ENSG00000211602
ENSG00000211607	ENSG00000211603
ENSG00000211607	ENSG00000211604
ENSG00000211607	ENSG00000211605
ENSG00000211607	ENSG00000211606
ENSG00000211607	ENSG00000211608
ENSG00000211607	ENSG00000211609
ENSG00000211607	ENSG00000211610
ENSG00000211607	ENSG00000211611
ENSG00000211607	ENSG00000211613
ENSG00000211607	ENSG00000211616
ENSG00000211607	ENSG00000211617
ENSG00000211607	ENSG00000211618
ENSG00000211607	ENSG00000211620
ENSG00000211607	ENSG00000211625
ENSG00000211607	ENSG00000211626
ENSG00000211607	ENSG00000211627
ENSG00000211607	ENSG00000211628
ENSG00000211607	ENSG00000211629
ENSG00000211607	ENSG00000211630
ENSG00000211607	ENSG00000211631
ENSG00000211607	ENSG00000211632
ENSG00000211607	ENSG00000211633
ENSG00000211607	ENSG00000211634
ENSG00000211607	ENSG00000211635

ENSG00000211607	ENSG00000211636
ENSG00000211608	ENSG00000136700
ENSG00000211608	ENSG00000156755
ENSG00000211608	ENSG00000183719
ENSG00000211608	ENSG00000197794
ENSG00000211608	ENSG00000204670
ENSG00000211608	ENSG00000204776
ENSG00000211608	ENSG00000204780
ENSG00000211608	ENSG00000211599
ENSG00000211608	ENSG00000211600
ENSG00000211608	ENSG00000211601
ENSG00000211608	ENSG00000211602
ENSG00000211608	ENSG00000211603
ENSG00000211608	ENSG00000211604
ENSG00000211608	ENSG00000211605
ENSG00000211608	ENSG00000211606
ENSG00000211608	ENSG00000211607
ENSG00000211608	ENSG00000211609
ENSG00000211608	ENSG00000211610
ENSG00000211608	ENSG00000211611
ENSG00000211608	ENSG00000211613
ENSG00000211608	ENSG00000211616
ENSG00000211608	ENSG00000211617
ENSG00000211608	ENSG00000211618
ENSG00000211608	ENSG00000211620
ENSG00000211608	ENSG00000211625
ENSG00000211608	ENSG00000211626
ENSG00000211608	ENSG00000211627
ENSG00000211608	ENSG00000211628
ENSG00000211608	ENSG00000211629
ENSG00000211608	ENSG00000211630
ENSG00000211608	ENSG00000211631
ENSG00000211608	ENSG00000211632
ENSG00000211608	ENSG00000211633
ENSG00000211608	ENSG00000211634
ENSG00000211608	ENSG00000211635
ENSG00000211608	ENSG00000211636
ENSG00000211609	ENSG00000136700
ENSG00000211609	ENSG00000156755
ENSG00000211609	ENSG00000183719
ENSG00000211609	ENSG00000197794
ENSG00000211609	ENSG00000204670
ENSG00000211609	ENSG00000204776
ENSG00000211609	ENSG00000204780
ENSG00000211609	ENSG00000211599
ENSG00000211609	ENSG00000211600
ENSG00000211609	ENSG00000211601
ENSG00000211609	ENSG00000211602
ENSG00000211609	ENSG00000211603
ENSG00000211609	ENSG00000211604
ENSG00000211609	ENSG00000211605
ENSG00000211609	ENSG00000211606
ENSG00000211609	ENSG00000211607
ENSG00000211609	ENSG00000211608
ENSG00000211609	ENSG00000211610
ENSG00000211609	ENSG00000211611
ENSG00000211609	ENSG00000211613
ENSG00000211609	ENSG00000211616
ENSG00000211609	ENSG00000211617

ENSG00000211609	ENSG00000211618
ENSG00000211609	ENSG00000211620
ENSG00000211609	ENSG00000211625
ENSG00000211609	ENSG00000211626
ENSG00000211609	ENSG00000211627
ENSG00000211609	ENSG00000211628
ENSG00000211609	ENSG00000211629
ENSG00000211609	ENSG00000211630
ENSG00000211609	ENSG00000211631
ENSG00000211609	ENSG00000211632
ENSG00000211609	ENSG00000211633
ENSG00000211609	ENSG00000211634
ENSG00000211609	ENSG00000211635
ENSG00000211609	ENSG00000211636
ENSG00000211610	ENSG00000136700
ENSG00000211610	ENSG00000156755
ENSG00000211610	ENSG00000183719
ENSG00000211610	ENSG00000197794
ENSG00000211610	ENSG00000204670
ENSG00000211610	ENSG00000204776
ENSG00000211610	ENSG00000204780
ENSG00000211610	ENSG00000211599
ENSG00000211610	ENSG00000211600
ENSG00000211610	ENSG00000211601
ENSG00000211610	ENSG00000211602
ENSG00000211610	ENSG00000211603
ENSG00000211610	ENSG00000211604
ENSG00000211610	ENSG00000211605
ENSG00000211610	ENSG00000211606
ENSG00000211610	ENSG00000211607
ENSG00000211610	ENSG00000211608
ENSG00000211610	ENSG00000211609
ENSG00000211610	ENSG00000211611
ENSG00000211610	ENSG00000211613
ENSG00000211610	ENSG00000211616
ENSG00000211610	ENSG00000211617
ENSG00000211610	ENSG00000211618
ENSG00000211610	ENSG00000211620
ENSG00000211610	ENSG00000211625
ENSG00000211610	ENSG00000211626
ENSG00000211610	ENSG00000211627
ENSG00000211610	ENSG00000211628
ENSG00000211610	ENSG00000211629
ENSG00000211610	ENSG00000211630
ENSG00000211610	ENSG00000211631
ENSG00000211610	ENSG00000211632
ENSG00000211610	ENSG00000211633
ENSG00000211610	ENSG00000211634
ENSG00000211610	ENSG00000211635
ENSG00000211610	ENSG00000211636
ENSG00000211611	ENSG00000136700
ENSG00000211611	ENSG00000156755
ENSG00000211611	ENSG00000183719
ENSG00000211611	ENSG00000197794
ENSG00000211611	ENSG00000204670
ENSG00000211611	ENSG00000204776
ENSG00000211611	ENSG00000204780
ENSG00000211611	ENSG00000211599
ENSG00000211611	ENSG00000211600

ENSG00000211611	ENSG00000211601
ENSG00000211611	ENSG00000211602
ENSG00000211611	ENSG00000211603
ENSG00000211611	ENSG00000211604
ENSG00000211611	ENSG00000211605
ENSG00000211611	ENSG00000211606
ENSG00000211611	ENSG00000211607
ENSG00000211611	ENSG00000211608
ENSG00000211611	ENSG00000211609
ENSG00000211611	ENSG00000211610
ENSG00000211611	ENSG00000211613
ENSG00000211611	ENSG00000211616
ENSG00000211611	ENSG00000211617
ENSG00000211611	ENSG00000211618
ENSG00000211611	ENSG00000211620
ENSG00000211611	ENSG00000211625
ENSG00000211611	ENSG00000211626
ENSG00000211611	ENSG00000211627
ENSG00000211611	ENSG00000211628
ENSG00000211611	ENSG00000211629
ENSG00000211611	ENSG00000211630
ENSG00000211611	ENSG00000211631
ENSG00000211611	ENSG00000211632
ENSG00000211611	ENSG00000211633
ENSG00000211611	ENSG00000211634
ENSG00000211611	ENSG00000211635
ENSG00000211611	ENSG00000211636
ENSG00000211613	ENSG00000136700
ENSG00000211613	ENSG00000156755
ENSG00000211613	ENSG00000183719
ENSG00000211613	ENSG00000197794
ENSG00000211613	ENSG00000204670
ENSG00000211613	ENSG00000204776
ENSG00000211613	ENSG00000204780
ENSG00000211613	ENSG00000211599
ENSG00000211613	ENSG00000211600
ENSG00000211613	ENSG00000211601
ENSG00000211613	ENSG00000211602
ENSG00000211613	ENSG00000211603
ENSG00000211613	ENSG00000211604
ENSG00000211613	ENSG00000211605
ENSG00000211613	ENSG00000211606
ENSG00000211613	ENSG00000211607
ENSG00000211613	ENSG00000211608
ENSG00000211613	ENSG00000211609
ENSG00000211613	ENSG00000211610
ENSG00000211613	ENSG00000211611
ENSG00000211613	ENSG00000211616
ENSG00000211613	ENSG00000211617
ENSG00000211613	ENSG00000211618
ENSG00000211613	ENSG00000211620
ENSG00000211613	ENSG00000211625
ENSG00000211613	ENSG00000211626
ENSG00000211613	ENSG00000211627
ENSG00000211613	ENSG00000211628
ENSG00000211613	ENSG00000211629
ENSG00000211613	ENSG00000211630
ENSG00000211613	ENSG00000211631
ENSG00000211613	ENSG00000211632

ENSG00000211613	ENSG00000211633
ENSG00000211613	ENSG00000211634
ENSG00000211613	ENSG00000211635
ENSG00000211613	ENSG00000211636
ENSG00000211616	ENSG00000136700
ENSG00000211616	ENSG00000156755
ENSG00000211616	ENSG00000183719
ENSG00000211616	ENSG00000197794
ENSG00000211616	ENSG00000204670
ENSG00000211616	ENSG00000204776
ENSG00000211616	ENSG00000204780
ENSG00000211616	ENSG00000211599
ENSG00000211616	ENSG00000211600
ENSG00000211616	ENSG00000211601
ENSG00000211616	ENSG00000211602
ENSG00000211616	ENSG00000211603
ENSG00000211616	ENSG00000211604
ENSG00000211616	ENSG00000211605
ENSG00000211616	ENSG00000211606
ENSG00000211616	ENSG00000211607
ENSG00000211616	ENSG00000211608
ENSG00000211616	ENSG00000211609
ENSG00000211616	ENSG00000211610
ENSG00000211616	ENSG00000211611
ENSG00000211616	ENSG00000211613
ENSG00000211616	ENSG00000211617
ENSG00000211616	ENSG00000211618
ENSG00000211616	ENSG00000211620
ENSG00000211616	ENSG00000211625
ENSG00000211616	ENSG00000211626
ENSG00000211616	ENSG00000211627
ENSG00000211616	ENSG00000211628
ENSG00000211616	ENSG00000211629
ENSG00000211616	ENSG00000211630
ENSG00000211616	ENSG00000211631
ENSG00000211616	ENSG00000211632
ENSG00000211616	ENSG00000211633
ENSG00000211616	ENSG00000211634
ENSG00000211616	ENSG00000211635
ENSG00000211616	ENSG00000211636
ENSG00000211617	ENSG00000136700
ENSG00000211617	ENSG00000156755
ENSG00000211617	ENSG00000183719
ENSG00000211617	ENSG00000197794
ENSG00000211617	ENSG00000204670
ENSG00000211617	ENSG00000204776
ENSG00000211617	ENSG00000204780
ENSG00000211617	ENSG00000211599
ENSG00000211617	ENSG00000211600
ENSG00000211617	ENSG00000211601
ENSG00000211617	ENSG00000211602
ENSG00000211617	ENSG00000211603
ENSG00000211617	ENSG00000211604
ENSG00000211617	ENSG00000211605
ENSG00000211617	ENSG00000211606
ENSG00000211617	ENSG00000211607
ENSG00000211617	ENSG00000211608
ENSG00000211617	ENSG00000211609
ENSG00000211617	ENSG00000211610

ENSG00000211617	ENSG00000211611
ENSG00000211617	ENSG00000211613
ENSG00000211617	ENSG00000211616
ENSG00000211617	ENSG00000211618
ENSG00000211617	ENSG00000211620
ENSG00000211617	ENSG00000211625
ENSG00000211617	ENSG00000211626
ENSG00000211617	ENSG00000211627
ENSG00000211617	ENSG00000211628
ENSG00000211617	ENSG00000211629
ENSG00000211617	ENSG00000211630
ENSG00000211617	ENSG00000211631
ENSG00000211617	ENSG00000211632
ENSG00000211617	ENSG00000211633
ENSG00000211617	ENSG00000211634
ENSG00000211617	ENSG00000211635
ENSG00000211617	ENSG00000211636
ENSG00000211618	ENSG00000136700
ENSG00000211618	ENSG00000156755
ENSG00000211618	ENSG00000183719
ENSG00000211618	ENSG00000197794
ENSG00000211618	ENSG00000204670
ENSG00000211618	ENSG00000204776
ENSG00000211618	ENSG00000204780
ENSG00000211618	ENSG00000211599
ENSG00000211618	ENSG00000211600
ENSG00000211618	ENSG00000211601
ENSG00000211618	ENSG00000211602
ENSG00000211618	ENSG00000211603
ENSG00000211618	ENSG00000211604
ENSG00000211618	ENSG00000211605
ENSG00000211618	ENSG00000211606
ENSG00000211618	ENSG00000211607
ENSG00000211618	ENSG00000211608
ENSG00000211618	ENSG00000211609
ENSG00000211618	ENSG00000211610
ENSG00000211618	ENSG00000211611
ENSG00000211618	ENSG00000211613
ENSG00000211618	ENSG00000211616
ENSG00000211618	ENSG00000211617
ENSG00000211618	ENSG00000211620
ENSG00000211618	ENSG00000211625
ENSG00000211618	ENSG00000211626
ENSG00000211618	ENSG00000211627
ENSG00000211618	ENSG00000211628
ENSG00000211618	ENSG00000211629
ENSG00000211618	ENSG00000211630
ENSG00000211618	ENSG00000211631
ENSG00000211618	ENSG00000211632
ENSG00000211618	ENSG00000211633
ENSG00000211618	ENSG00000211634
ENSG00000211618	ENSG00000211635
ENSG00000211618	ENSG00000211636
ENSG00000211620	ENSG00000136700
ENSG00000211620	ENSG00000156755
ENSG00000211620	ENSG00000183719
ENSG00000211620	ENSG00000197794
ENSG00000211620	ENSG00000204670
ENSG00000211620	ENSG00000204776

ENSG00000211620	ENSG00000204780
ENSG00000211620	ENSG00000211599
ENSG00000211620	ENSG00000211600
ENSG00000211620	ENSG00000211601
ENSG00000211620	ENSG00000211602
ENSG00000211620	ENSG00000211603
ENSG00000211620	ENSG00000211604
ENSG00000211620	ENSG00000211605
ENSG00000211620	ENSG00000211606
ENSG00000211620	ENSG00000211607
ENSG00000211620	ENSG00000211608
ENSG00000211620	ENSG00000211609
ENSG00000211620	ENSG00000211610
ENSG00000211620	ENSG00000211611
ENSG00000211620	ENSG00000211613
ENSG00000211620	ENSG00000211616
ENSG00000211620	ENSG00000211617
ENSG00000211620	ENSG00000211618
ENSG00000211620	ENSG00000211625
ENSG00000211620	ENSG00000211626
ENSG00000211620	ENSG00000211627
ENSG00000211620	ENSG00000211628
ENSG00000211620	ENSG00000211629
ENSG00000211620	ENSG00000211630
ENSG00000211620	ENSG00000211631
ENSG00000211620	ENSG00000211632
ENSG00000211620	ENSG00000211633
ENSG00000211620	ENSG00000211634
ENSG00000211620	ENSG00000211635
ENSG00000211620	ENSG00000211636
ENSG00000211625	ENSG00000136700
ENSG00000211625	ENSG00000156755
ENSG00000211625	ENSG00000183719
ENSG00000211625	ENSG00000197794
ENSG00000211625	ENSG00000204670
ENSG00000211625	ENSG00000204776
ENSG00000211625	ENSG00000204780
ENSG00000211625	ENSG00000211599
ENSG00000211625	ENSG00000211600
ENSG00000211625	ENSG00000211601
ENSG00000211625	ENSG00000211602
ENSG00000211625	ENSG00000211603
ENSG00000211625	ENSG00000211604
ENSG00000211625	ENSG00000211605
ENSG00000211625	ENSG00000211606
ENSG00000211625	ENSG00000211607
ENSG00000211625	ENSG00000211608
ENSG00000211625	ENSG00000211609
ENSG00000211625	ENSG00000211610
ENSG00000211625	ENSG00000211611
ENSG00000211625	ENSG00000211613
ENSG00000211625	ENSG00000211616
ENSG00000211625	ENSG00000211617
ENSG00000211625	ENSG00000211618
ENSG00000211625	ENSG00000211620
ENSG00000211625	ENSG00000211626
ENSG00000211625	ENSG00000211627
ENSG00000211625	ENSG00000211628
ENSG00000211625	ENSG00000211629

ENSG00000211625	ENSG00000211630
ENSG00000211625	ENSG00000211631
ENSG00000211625	ENSG00000211632
ENSG00000211625	ENSG00000211633
ENSG00000211625	ENSG00000211634
ENSG00000211625	ENSG00000211635
ENSG00000211625	ENSG00000211636
ENSG00000211626	ENSG00000136700
ENSG00000211626	ENSG00000156755
ENSG00000211626	ENSG00000183719
ENSG00000211626	ENSG00000197794
ENSG00000211626	ENSG00000204670
ENSG00000211626	ENSG00000204776
ENSG00000211626	ENSG00000204780
ENSG00000211626	ENSG00000211599
ENSG00000211626	ENSG00000211600
ENSG00000211626	ENSG00000211601
ENSG00000211626	ENSG00000211602
ENSG00000211626	ENSG00000211603
ENSG00000211626	ENSG00000211604
ENSG00000211626	ENSG00000211605
ENSG00000211626	ENSG00000211606
ENSG00000211626	ENSG00000211607
ENSG00000211626	ENSG00000211608
ENSG00000211626	ENSG00000211609
ENSG00000211626	ENSG00000211610
ENSG00000211626	ENSG00000211611
ENSG00000211626	ENSG00000211613
ENSG00000211626	ENSG00000211616
ENSG00000211626	ENSG00000211617
ENSG00000211626	ENSG00000211618
ENSG00000211626	ENSG00000211620
ENSG00000211626	ENSG00000211625
ENSG00000211626	ENSG00000211627
ENSG00000211626	ENSG00000211628
ENSG00000211626	ENSG00000211629
ENSG00000211626	ENSG00000211630
ENSG00000211626	ENSG00000211631
ENSG00000211626	ENSG00000211632
ENSG00000211626	ENSG00000211633
ENSG00000211626	ENSG00000211634
ENSG00000211626	ENSG00000211635
ENSG00000211626	ENSG00000211636
ENSG00000211627	ENSG00000136700
ENSG00000211627	ENSG00000156755
ENSG00000211627	ENSG00000183719
ENSG00000211627	ENSG00000197794
ENSG00000211627	ENSG00000204670
ENSG00000211627	ENSG00000204776
ENSG00000211627	ENSG00000204780
ENSG00000211627	ENSG00000211599
ENSG00000211627	ENSG00000211600
ENSG00000211627	ENSG00000211601
ENSG00000211627	ENSG00000211602
ENSG00000211627	ENSG00000211603
ENSG00000211627	ENSG00000211604
ENSG00000211627	ENSG00000211605
ENSG00000211627	ENSG00000211606
ENSG00000211627	ENSG00000211607

ENSG00000211627	ENSG00000211608
ENSG00000211627	ENSG00000211609
ENSG00000211627	ENSG00000211610
ENSG00000211627	ENSG00000211611
ENSG00000211627	ENSG00000211613
ENSG00000211627	ENSG00000211616
ENSG00000211627	ENSG00000211617
ENSG00000211627	ENSG00000211618
ENSG00000211627	ENSG00000211620
ENSG00000211627	ENSG00000211625
ENSG00000211627	ENSG00000211626
ENSG00000211627	ENSG00000211628
ENSG00000211627	ENSG00000211629
ENSG00000211627	ENSG00000211630
ENSG00000211627	ENSG00000211631
ENSG00000211627	ENSG00000211632
ENSG00000211627	ENSG00000211633
ENSG00000211627	ENSG00000211634
ENSG00000211627	ENSG00000211635
ENSG00000211627	ENSG00000211636
ENSG00000211628	ENSG00000136700
ENSG00000211628	ENSG00000156755
ENSG00000211628	ENSG00000183719
ENSG00000211628	ENSG00000197794
ENSG00000211628	ENSG00000204670
ENSG00000211628	ENSG00000204776
ENSG00000211628	ENSG00000204780
ENSG00000211628	ENSG00000211599
ENSG00000211628	ENSG00000211600
ENSG00000211628	ENSG00000211601
ENSG00000211628	ENSG00000211602
ENSG00000211628	ENSG00000211603
ENSG00000211628	ENSG00000211604
ENSG00000211628	ENSG00000211605
ENSG00000211628	ENSG00000211606
ENSG00000211628	ENSG00000211607
ENSG00000211628	ENSG00000211608
ENSG00000211628	ENSG00000211609
ENSG00000211628	ENSG00000211610
ENSG00000211628	ENSG00000211611
ENSG00000211628	ENSG00000211613
ENSG00000211628	ENSG00000211616
ENSG00000211628	ENSG00000211617
ENSG00000211628	ENSG00000211618
ENSG00000211628	ENSG00000211620
ENSG00000211628	ENSG00000211625
ENSG00000211628	ENSG00000211626
ENSG00000211628	ENSG00000211627
ENSG00000211628	ENSG00000211629
ENSG00000211628	ENSG00000211630
ENSG00000211628	ENSG00000211631
ENSG00000211628	ENSG00000211632
ENSG00000211628	ENSG00000211633
ENSG00000211628	ENSG00000211634
ENSG00000211628	ENSG00000211635
ENSG00000211628	ENSG00000211636
ENSG00000211629	ENSG00000136700
ENSG00000211629	ENSG00000156755
ENSG00000211629	ENSG00000183719

ENSG00000211629	ENSG00000197794
ENSG00000211629	ENSG00000204670
ENSG00000211629	ENSG00000204776
ENSG00000211629	ENSG00000204780
ENSG00000211629	ENSG00000211599
ENSG00000211629	ENSG00000211600
ENSG00000211629	ENSG00000211601
ENSG00000211629	ENSG00000211602
ENSG00000211629	ENSG00000211603
ENSG00000211629	ENSG00000211604
ENSG00000211629	ENSG00000211605
ENSG00000211629	ENSG00000211606
ENSG00000211629	ENSG00000211607
ENSG00000211629	ENSG00000211608
ENSG00000211629	ENSG00000211609
ENSG00000211629	ENSG00000211610
ENSG00000211629	ENSG00000211611
ENSG00000211629	ENSG00000211613
ENSG00000211629	ENSG00000211616
ENSG00000211629	ENSG00000211617
ENSG00000211629	ENSG00000211618
ENSG00000211629	ENSG00000211620
ENSG00000211629	ENSG00000211625
ENSG00000211629	ENSG00000211626
ENSG00000211629	ENSG00000211627
ENSG00000211629	ENSG00000211628
ENSG00000211629	ENSG00000211630
ENSG00000211629	ENSG00000211631
ENSG00000211629	ENSG00000211632
ENSG00000211629	ENSG00000211633
ENSG00000211629	ENSG00000211634
ENSG00000211629	ENSG00000211635
ENSG00000211629	ENSG00000211636
ENSG00000211630	ENSG00000136700
ENSG00000211630	ENSG00000156755
ENSG00000211630	ENSG00000183719
ENSG00000211630	ENSG00000197794
ENSG00000211630	ENSG00000204670
ENSG00000211630	ENSG00000204776
ENSG00000211630	ENSG00000204780
ENSG00000211630	ENSG00000211599
ENSG00000211630	ENSG00000211600
ENSG00000211630	ENSG00000211601
ENSG00000211630	ENSG00000211602
ENSG00000211630	ENSG00000211603
ENSG00000211630	ENSG00000211604
ENSG00000211630	ENSG00000211605
ENSG00000211630	ENSG00000211606
ENSG00000211630	ENSG00000211607
ENSG00000211630	ENSG00000211608
ENSG00000211630	ENSG00000211609
ENSG00000211630	ENSG00000211610
ENSG00000211630	ENSG00000211611
ENSG00000211630	ENSG00000211613
ENSG00000211630	ENSG00000211616
ENSG00000211630	ENSG00000211617
ENSG00000211630	ENSG00000211618
ENSG00000211630	ENSG00000211620
ENSG00000211630	ENSG00000211625

ENSG00000211630	ENSG00000211626
ENSG00000211630	ENSG00000211627
ENSG00000211630	ENSG00000211628
ENSG00000211630	ENSG00000211629
ENSG00000211630	ENSG00000211631
ENSG00000211630	ENSG00000211632
ENSG00000211630	ENSG00000211633
ENSG00000211630	ENSG00000211634
ENSG00000211630	ENSG00000211635
ENSG00000211630	ENSG00000211636
ENSG00000211631	ENSG00000136700
ENSG00000211631	ENSG00000156755
ENSG00000211631	ENSG00000183719
ENSG00000211631	ENSG00000197794
ENSG00000211631	ENSG00000204670
ENSG00000211631	ENSG00000204776
ENSG00000211631	ENSG00000204780
ENSG00000211631	ENSG00000211599
ENSG00000211631	ENSG00000211600
ENSG00000211631	ENSG00000211601
ENSG00000211631	ENSG00000211602
ENSG00000211631	ENSG00000211603
ENSG00000211631	ENSG00000211604
ENSG00000211631	ENSG00000211605
ENSG00000211631	ENSG00000211606
ENSG00000211631	ENSG00000211607
ENSG00000211631	ENSG00000211608
ENSG00000211631	ENSG00000211609
ENSG00000211631	ENSG00000211610
ENSG00000211631	ENSG00000211611
ENSG00000211631	ENSG00000211613
ENSG00000211631	ENSG00000211616
ENSG00000211631	ENSG00000211617
ENSG00000211631	ENSG00000211618
ENSG00000211631	ENSG00000211620
ENSG00000211631	ENSG00000211625
ENSG00000211631	ENSG00000211626
ENSG00000211631	ENSG00000211627
ENSG00000211631	ENSG00000211628
ENSG00000211631	ENSG00000211629
ENSG00000211631	ENSG00000211630
ENSG00000211631	ENSG00000211632
ENSG00000211631	ENSG00000211633
ENSG00000211631	ENSG00000211634
ENSG00000211631	ENSG00000211635
ENSG00000211631	ENSG00000211636
ENSG00000211632	ENSG00000136700
ENSG00000211632	ENSG00000156755
ENSG00000211632	ENSG00000183719
ENSG00000211632	ENSG00000197794
ENSG00000211632	ENSG00000204670
ENSG00000211632	ENSG00000204776
ENSG00000211632	ENSG00000204780
ENSG00000211632	ENSG00000211599
ENSG00000211632	ENSG00000211600
ENSG00000211632	ENSG00000211601
ENSG00000211632	ENSG00000211602
ENSG00000211632	ENSG00000211603
ENSG00000211632	ENSG00000211604

ENSG00000211632	ENSG00000211605
ENSG00000211632	ENSG00000211606
ENSG00000211632	ENSG00000211607
ENSG00000211632	ENSG00000211608
ENSG00000211632	ENSG00000211609
ENSG00000211632	ENSG00000211610
ENSG00000211632	ENSG00000211611
ENSG00000211632	ENSG00000211613
ENSG00000211632	ENSG00000211616
ENSG00000211632	ENSG00000211617
ENSG00000211632	ENSG00000211618
ENSG00000211632	ENSG00000211620
ENSG00000211632	ENSG00000211625
ENSG00000211632	ENSG00000211626
ENSG00000211632	ENSG00000211627
ENSG00000211632	ENSG00000211628
ENSG00000211632	ENSG00000211629
ENSG00000211632	ENSG00000211630
ENSG00000211632	ENSG00000211631
ENSG00000211632	ENSG00000211633
ENSG00000211632	ENSG00000211634
ENSG00000211632	ENSG00000211635
ENSG00000211632	ENSG00000211636
ENSG00000211633	ENSG00000136700
ENSG00000211633	ENSG00000156755
ENSG00000211633	ENSG00000183719
ENSG00000211633	ENSG00000197794
ENSG00000211633	ENSG00000204670
ENSG00000211633	ENSG00000204776
ENSG00000211633	ENSG00000204780
ENSG00000211633	ENSG00000211599
ENSG00000211633	ENSG00000211600
ENSG00000211633	ENSG00000211601
ENSG00000211633	ENSG00000211602
ENSG00000211633	ENSG00000211603
ENSG00000211633	ENSG00000211604
ENSG00000211633	ENSG00000211605
ENSG00000211633	ENSG00000211606
ENSG00000211633	ENSG00000211607
ENSG00000211633	ENSG00000211608
ENSG00000211633	ENSG00000211609
ENSG00000211633	ENSG00000211610
ENSG00000211633	ENSG00000211611
ENSG00000211633	ENSG00000211613
ENSG00000211633	ENSG00000211616
ENSG00000211633	ENSG00000211617
ENSG00000211633	ENSG00000211618
ENSG00000211633	ENSG00000211620
ENSG00000211633	ENSG00000211625
ENSG00000211633	ENSG00000211626
ENSG00000211633	ENSG00000211627
ENSG00000211633	ENSG00000211628
ENSG00000211633	ENSG00000211629
ENSG00000211633	ENSG00000211630
ENSG00000211633	ENSG00000211631
ENSG00000211633	ENSG00000211632
ENSG00000211633	ENSG00000211634
ENSG00000211633	ENSG00000211635
ENSG00000211633	ENSG00000211636

ENSG00000211634	ENSG00000136700
ENSG00000211634	ENSG00000156755
ENSG00000211634	ENSG00000183719
ENSG00000211634	ENSG00000197794
ENSG00000211634	ENSG00000204670
ENSG00000211634	ENSG00000204776
ENSG00000211634	ENSG00000204780
ENSG00000211634	ENSG00000211599
ENSG00000211634	ENSG00000211600
ENSG00000211634	ENSG00000211601
ENSG00000211634	ENSG00000211602
ENSG00000211634	ENSG00000211603
ENSG00000211634	ENSG00000211604
ENSG00000211634	ENSG00000211605
ENSG00000211634	ENSG00000211606
ENSG00000211634	ENSG00000211607
ENSG00000211634	ENSG00000211608
ENSG00000211634	ENSG00000211609
ENSG00000211634	ENSG00000211610
ENSG00000211634	ENSG00000211611
ENSG00000211634	ENSG00000211613
ENSG00000211634	ENSG00000211616
ENSG00000211634	ENSG00000211617
ENSG00000211634	ENSG00000211618
ENSG00000211634	ENSG00000211620
ENSG00000211634	ENSG00000211625
ENSG00000211634	ENSG00000211626
ENSG00000211634	ENSG00000211627
ENSG00000211634	ENSG00000211628
ENSG00000211634	ENSG00000211629
ENSG00000211634	ENSG00000211630
ENSG00000211634	ENSG00000211631
ENSG00000211634	ENSG00000211632
ENSG00000211634	ENSG00000211633
ENSG00000211634	ENSG00000211635
ENSG00000211634	ENSG00000211636
ENSG00000211635	ENSG00000136700
ENSG00000211635	ENSG00000156755
ENSG00000211635	ENSG00000183719
ENSG00000211635	ENSG00000197794
ENSG00000211635	ENSG00000204670
ENSG00000211635	ENSG00000204776
ENSG00000211635	ENSG00000204780
ENSG00000211635	ENSG00000211599
ENSG00000211635	ENSG00000211600
ENSG00000211635	ENSG00000211601
ENSG00000211635	ENSG00000211602
ENSG00000211635	ENSG00000211603
ENSG00000211635	ENSG00000211604
ENSG00000211635	ENSG00000211605
ENSG00000211635	ENSG00000211606
ENSG00000211635	ENSG00000211607
ENSG00000211635	ENSG00000211608
ENSG00000211635	ENSG00000211609
ENSG00000211635	ENSG00000211610
ENSG00000211635	ENSG00000211611
ENSG00000211635	ENSG00000211613
ENSG00000211635	ENSG00000211616
ENSG00000211635	ENSG00000211617

ENSG00000211635	ENSG00000211618
ENSG00000211635	ENSG00000211620
ENSG00000211635	ENSG00000211625
ENSG00000211635	ENSG00000211626
ENSG00000211635	ENSG00000211627
ENSG00000211635	ENSG00000211628
ENSG00000211635	ENSG00000211629
ENSG00000211635	ENSG00000211630
ENSG00000211635	ENSG00000211631
ENSG00000211635	ENSG00000211632
ENSG00000211635	ENSG00000211633
ENSG00000211635	ENSG00000211634
ENSG00000211635	ENSG00000211636
ENSG00000211636	ENSG00000136700
ENSG00000211636	ENSG00000156755
ENSG00000211636	ENSG00000183719
ENSG00000211636	ENSG00000197794
ENSG00000211636	ENSG00000204670
ENSG00000211636	ENSG00000204776
ENSG00000211636	ENSG00000204780
ENSG00000211636	ENSG00000211599
ENSG00000211636	ENSG00000211600
ENSG00000211636	ENSG00000211601
ENSG00000211636	ENSG00000211602
ENSG00000211636	ENSG00000211603
ENSG00000211636	ENSG00000211604
ENSG00000211636	ENSG00000211605
ENSG00000211636	ENSG00000211606
ENSG00000211636	ENSG00000211607
ENSG00000211636	ENSG00000211608
ENSG00000211636	ENSG00000211609
ENSG00000211636	ENSG00000211610
ENSG00000211636	ENSG00000211611
ENSG00000211636	ENSG00000211613
ENSG00000211636	ENSG00000211616
ENSG00000211636	ENSG00000211617
ENSG00000211636	ENSG00000211618
ENSG00000211636	ENSG00000211620
ENSG00000211636	ENSG00000211625
ENSG00000211636	ENSG00000211626
ENSG00000211636	ENSG00000211627
ENSG00000211636	ENSG00000211628
ENSG00000211636	ENSG00000211629
ENSG00000211636	ENSG00000211630
ENSG00000211636	ENSG00000211631
ENSG00000211636	ENSG00000211632
ENSG00000211636	ENSG00000211633
ENSG00000211636	ENSG00000211634
ENSG00000211636	ENSG00000211635
ENSG00000211675	ENSG00000128322
ENSG00000211675	ENSG00000206066
ENSG00000211675	ENSG00000211592
ENSG00000211675	ENSG00000211677
ENSG00000211675	ENSG00000211679
ENSG00000211675	ENSG00000211683
ENSG00000211675	ENSG00000211685
ENSG00000211675	ENSG00000211898
ENSG00000211677	ENSG00000128322
ENSG00000211677	ENSG00000206066

ENSG00000211677	ENSG00000211592
ENSG00000211677	ENSG00000211675
ENSG00000211677	ENSG00000211679
ENSG00000211677	ENSG00000211683
ENSG00000211677	ENSG00000211685
ENSG00000211677	ENSG00000211898
ENSG00000211679	ENSG00000128322
ENSG00000211679	ENSG00000206066
ENSG00000211679	ENSG00000211592
ENSG00000211679	ENSG00000211675
ENSG00000211679	ENSG00000211677
ENSG00000211679	ENSG00000211683
ENSG00000211679	ENSG00000211685
ENSG00000211679	ENSG00000211898
ENSG00000211683	ENSG00000128322
ENSG00000211683	ENSG00000206066
ENSG00000211683	ENSG00000211592
ENSG00000211683	ENSG00000211675
ENSG00000211683	ENSG00000211677
ENSG00000211683	ENSG00000211679
ENSG00000211683	ENSG00000211685
ENSG00000211683	ENSG00000211898
ENSG00000211685	ENSG00000128322
ENSG00000211685	ENSG00000206066
ENSG00000211685	ENSG00000211592
ENSG00000211685	ENSG00000211675
ENSG00000211685	ENSG00000211677
ENSG00000211685	ENSG00000211679
ENSG00000211685	ENSG00000211683
ENSG00000211685	ENSG00000211898
ENSG00000211695	ENSG00000211774
ENSG00000211695	ENSG00000211775
ENSG00000211695	ENSG00000211776
ENSG00000211695	ENSG00000211777
ENSG00000211695	ENSG00000211782
ENSG00000211695	ENSG00000211783
ENSG00000211695	ENSG00000211786
ENSG00000211695	ENSG00000211787
ENSG00000211695	ENSG00000211790
ENSG00000211695	ENSG00000211792
ENSG00000211695	ENSG00000211793
ENSG00000211695	ENSG00000211795
ENSG00000211695	ENSG00000211796
ENSG00000211695	ENSG00000211798
ENSG00000211695	ENSG00000211799
ENSG00000211695	ENSG00000211804
ENSG00000211695	ENSG00000211808
ENSG00000211695	ENSG00000211816
ENSG00000211695	ENSG00000211817
ENSG00000211695	ENSG00000211819
ENSG00000211695	ENSG00000211830
ENSG00000211774	ENSG00000211695
ENSG00000211774	ENSG00000211775
ENSG00000211774	ENSG00000211776
ENSG00000211774	ENSG00000211777
ENSG00000211774	ENSG00000211782
ENSG00000211774	ENSG00000211783
ENSG00000211774	ENSG00000211786
ENSG00000211774	ENSG00000211787

ENSG00000211774	ENSG00000211790
ENSG00000211774	ENSG00000211792
ENSG00000211774	ENSG00000211793
ENSG00000211774	ENSG00000211795
ENSG00000211774	ENSG00000211796
ENSG00000211774	ENSG00000211798
ENSG00000211774	ENSG00000211799
ENSG00000211774	ENSG00000211804
ENSG00000211774	ENSG00000211808
ENSG00000211774	ENSG00000211816
ENSG00000211774	ENSG00000211817
ENSG00000211774	ENSG00000211819
ENSG00000211774	ENSG00000211830
ENSG00000211775	ENSG00000211695
ENSG00000211775	ENSG00000211774
ENSG00000211775	ENSG00000211776
ENSG00000211775	ENSG00000211777
ENSG00000211775	ENSG00000211782
ENSG00000211775	ENSG00000211783
ENSG00000211775	ENSG00000211786
ENSG00000211775	ENSG00000211787
ENSG00000211775	ENSG00000211790
ENSG00000211775	ENSG00000211792
ENSG00000211775	ENSG00000211793
ENSG00000211775	ENSG00000211795
ENSG00000211775	ENSG00000211796
ENSG00000211775	ENSG00000211798
ENSG00000211775	ENSG00000211799
ENSG00000211775	ENSG00000211804
ENSG00000211775	ENSG00000211808
ENSG00000211775	ENSG00000211816
ENSG00000211775	ENSG00000211817
ENSG00000211775	ENSG00000211819
ENSG00000211775	ENSG00000211830
ENSG00000211776	ENSG00000211695
ENSG00000211776	ENSG00000211774
ENSG00000211776	ENSG00000211775
ENSG00000211776	ENSG00000211777
ENSG00000211776	ENSG00000211782
ENSG00000211776	ENSG00000211783
ENSG00000211776	ENSG00000211786
ENSG00000211776	ENSG00000211787
ENSG00000211776	ENSG00000211790
ENSG00000211776	ENSG00000211792
ENSG00000211776	ENSG00000211793
ENSG00000211776	ENSG00000211795
ENSG00000211776	ENSG00000211796
ENSG00000211776	ENSG00000211798
ENSG00000211776	ENSG00000211799
ENSG00000211776	ENSG00000211804
ENSG00000211776	ENSG00000211808
ENSG00000211776	ENSG00000211816
ENSG00000211776	ENSG00000211817
ENSG00000211776	ENSG00000211819
ENSG00000211776	ENSG00000211830
ENSG00000211777	ENSG00000211695
ENSG00000211777	ENSG00000211774
ENSG00000211777	ENSG00000211775
ENSG00000211777	ENSG00000211776

ENSG00000211777	ENSG00000211782
ENSG00000211777	ENSG00000211783
ENSG00000211777	ENSG00000211786
ENSG00000211777	ENSG00000211787
ENSG00000211777	ENSG00000211790
ENSG00000211777	ENSG00000211792
ENSG00000211777	ENSG00000211793
ENSG00000211777	ENSG00000211795
ENSG00000211777	ENSG00000211796
ENSG00000211777	ENSG00000211798
ENSG00000211777	ENSG00000211799
ENSG00000211777	ENSG00000211804
ENSG00000211777	ENSG00000211808
ENSG00000211777	ENSG00000211816
ENSG00000211777	ENSG00000211817
ENSG00000211777	ENSG00000211819
ENSG00000211777	ENSG00000211830
ENSG00000211782	ENSG00000211695
ENSG00000211782	ENSG00000211774
ENSG00000211782	ENSG00000211775
[truncated: 146,227 more chars]
